# Unified Access to Biaryl-Bridged Linkages Unlocks Structural Diversification of Noncanonical Cyclic Peptides

**DOI:** 10.1021/jacs.6c05294

**Published:** 2026-06-29

**Authors:** Longhui Yu, Jie Zhang, Xinwei Zhang, Xilun Wu, Ruoyu Liu, Chak Hin Au, Hiroshige Ogawa, Rongbiao Tong, Hugh Nakamura

**Affiliations:** Department of Chemistry, 162635 The Hong Kong University of Science and Technology, Clear Water Bay, 999077 Kowloon, Hong Kong SAR, China

## Abstract

Among ribosomally synthesized and post-translationally modified peptides (RiPPs), there exist cyclic peptides featuring rigid and highly linear biaryl linkages. These biaryl cyclic peptides often exhibit unique structural characteristics absent in conventional cyclic peptides, and many display valuable biological activities. Nevertheless, the supply of such biaryl-bridged cyclic peptides and their analogs has been constrained by their intrinsic rigidity. In this study, we report a systematic and comprehensive synthetic strategy for incorporating ten distinct natural and artificial biaryl/triaryl linkages into arbitrary peptide linkers, inspired by RiPP architectures. This unified synthetic platform, which combines electrochemical decarboxylative C–C bond formation with Larock macrocyclization, enables (i) systematic access to RiPP derivatives from readily available building blocks and (ii) facile preparation of fluorine-containing RiPPs, which are of particular importance in medicinal chemistry, as well as biaryl-bridged cyclic peptides spanning diverse ring sizes. In addition, several of the cihunamide analogs synthesized in this study exhibited antibacterial activity.

## Graphical abstract

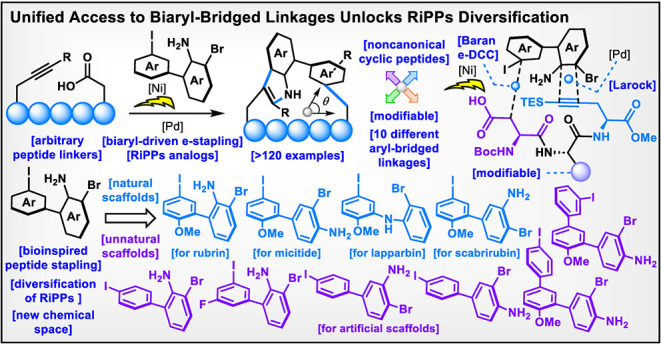

## Introduction

In recent years, peptide-based therapeutics have established themselves as a new drug discovery modality, with their importance and market size as pharmaceuticals continuing to expand annually.^1–3^ Peptides offer several advantages, including the relatively low cost of raw materials and synthetic accessibility at the medium-molecule scale. Moreover, by varying the length of amino acid residues, the modes of connectivity between residues, and the introduction of three-dimensional structures, diverse biological activities can potentially be achieved from inexpensive amino acid building blocks, making peptides a valuable modality for drug development.^1–3^ On the other hand, the principal challenges for peptides as pharmaceuticals lie in maintaining structural stability and enhancing membrane permeability.^4–7^ For example, linear peptides often suffer from (i) excessive flexibility, which reduces specificity toward target proteins, (ii) rapid degradation by proteases, and (iii) high hydrophilicity, which limits membrane permeability. In contrast, stapled peptides, in which amino acid side chains are cross-linked to form a ring, are designed to stabilize three-dimensional structures such as protein α-helices and to impart rigidity to otherwise flexible linear peptides, thereby yielding more stable and robust derivatives (Figure 1A).^8–10^ The introduction of such cross-linking structures into peptides facilitates the retention of helical conformations in solution and enhances properties such as membrane permeability when linear peptides are converted into cyclic architectures.^8–12^

**Figure 1:**
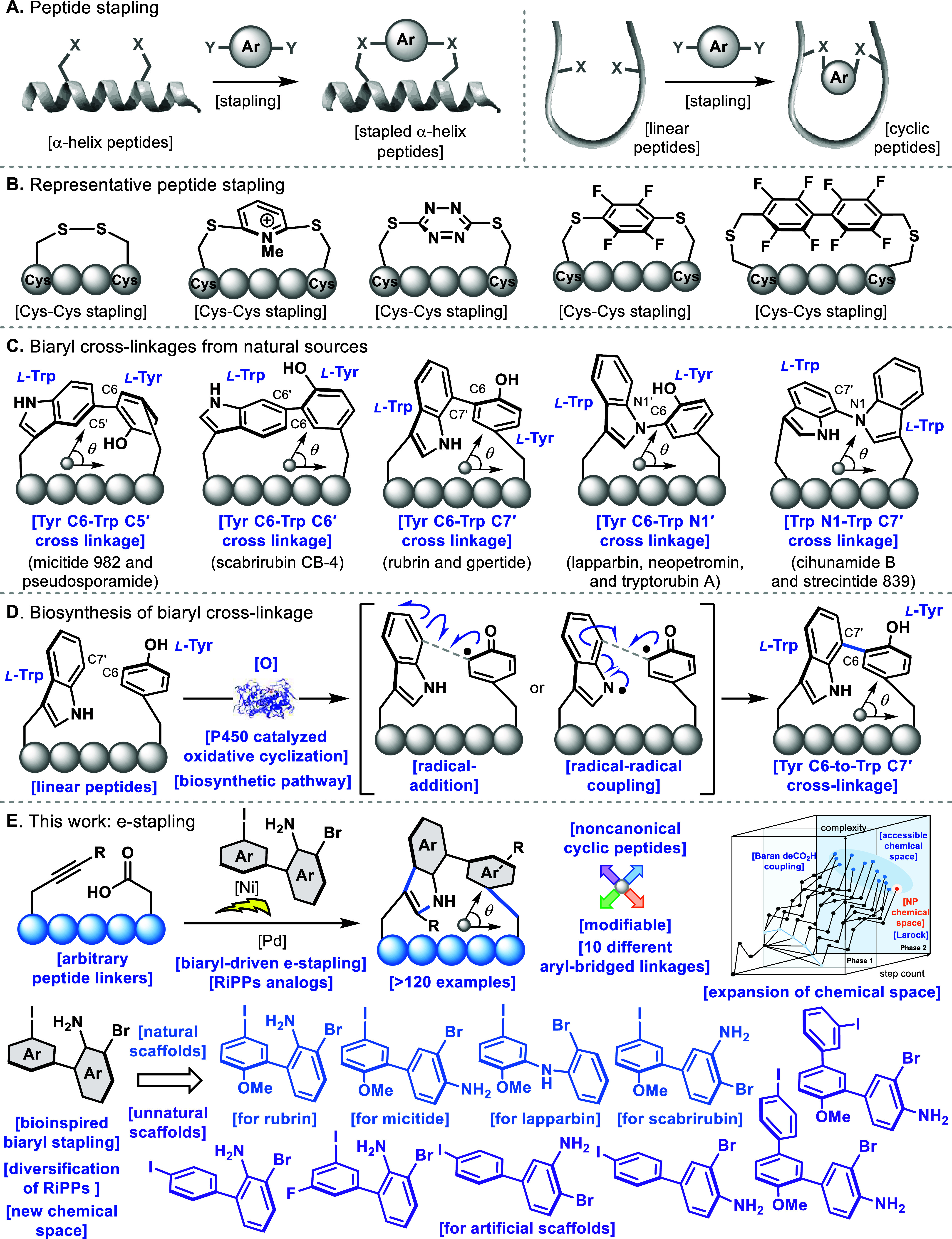
(A) Peptide stapling. (B) Representative peptide stapling. (C) Biaryl cross-linkages from natural sources. (D) Biosynthesis of biaryl cross-linkage. (E) This work: e-stapling.

Cyclic and linear peptides are known to exhibit distinct physicochemical and biological properties.^12^ With respect to general physicochemical differences, cyclic peptides are conformationally more rigid than their linear counterparts due to their ring structures, rendering them less susceptible to proteolytic degradation. This confers markedly improved stability in vivo, thereby enhancing their potential as pharmaceuticals.^13,14^ Furthermore, the formation of cyclic structures often positions hydrophobic groups on the outer surface of the ring, reducing the apparent polarity of the molecule and improving its ability to permeate lipid bilayer membranes compared to linear peptides.^15^ Regarding biological properties, cyclic peptides typically display higher stereospecificity toward target proteins as a result of ring formation.^13,14^

This reduces undesired interactions with off-target proteins or biomolecules, thereby mitigating side effects. Collectively, cyclic peptides possess several physicochemical and biological advantages over linear peptides.^12^

Various strategies for peptide stapling involving different amino acid residues have been reported to date.^16–22^ Among these, cysteine-based stapling represents one of the most widely employed approaches (Figure 1B). The thiol functionality of cysteine residues, owing to its strong nucleophilicity, has been exploited in numerous cross-linking methods. For example, cysteine residues can be oxidatively coupled to form disulfide (S–S) cross-linkages,^23^ or the thiol group can be linked to aromatic units via nucleophilic aromatic substitution (S_N_Ar) reactions, yielding stapled peptides cross-linked through motifs such as pyridinium,^24^ tetrazine,^25^ tetrafluorobenzene,^19^ and octafluorobiphenyl.^26^

In parallel, advances in genome mining have recently uncovered RiPPs bearing diverse biaryl-bridged linkages (Figure 1C).^27–34^ These naturally occurring cyclic peptides are characterized by the presence of cross-links between two aromatic rings. Biaryl-bridged cyclic peptides generally exhibit several advantages not observed in canonical cyclic peptides:^35–37^ (i) The C–C bonds formed between two aromatic rings are relatively resistant to hydrolysis and oxidation, thereby conferring enhanced stability against proteolytic degradation and improving in vivo stability; (ii) the presence of biaryl-bridged linkages increases conformational rigidity and enables conformational control, thereby enhancing selectivity and binding affinity toward target proteins; (iii) the incorporation of hydrophobic motifs increases overall hydrophobicity, improving cell membrane permeability; (iv) the combined improvements in stability and permeability contribute to favorable oral bioavailability; and (v) the synthetic versatility of biaryl-bridged cyclic peptides allows fine-tuning of physicochemical properties by varying the nature and connectivity of the aromatic rings, thereby expanding accessible chemical space.

The biosynthetic pathways of biaryl-bridged linkages are thought to involve oxidative cyclization catalyzed by cytochrome P450 enzymes (Figure 1D).^27–34,38–42^ Electron-rich tyrosine and tryptophan residues undergo single-electron oxidation mediated by cytochrome P450, generating radical species on the aromatic rings. These radicals subsequently couple with another aromatic ring (via radical addition or radical–radical coupling), thereby constructing the biaryl-bridged linkage.^43^ While this enzymatic pathway represents an efficient biosynthetic route to biaryl cross-linkages in vivo, it suffers from several limitations:^30^ (i) The cyclization reaction is restricted to naturally electron-rich aromatic residues (primarily tyrosine and tryptophan); (ii) enzyme specificity confines the reaction to particular peptide linkers, limiting the ability to introduce biaryl-bridged linkages into arbitrary peptide sequences; and (iii) biosynthetic approaches typically require identification of the relevant enzymes through genome mining, followed by heterologous expression of the corresponding gene clusters in *Escherichia coli*, which is not amenable to large-scale production. Indeed, isolation or biosynthesis of RiPPs and their analogs bearing biaryl-bridged linkages generally yields only milligram-scale quantities (e.g., micitide 982: 5 mg; pseudosporamide: 16 mg; scabrirubin CB-4: 1.3 mg; rubrin: 8.8 mg; gpertide: 3 mg; lapparbin: 9.3 mg; neopetromin: 2.8 mg; cihunamide B: 1.2 mg; strecintide 839: 5.0 mg).^27–34^

As noted above, biaryl-bridged cyclic peptides possess physicochemical and biological advantages not shared by canonical cyclic peptides. Chen and co-workers have previously reported pioneering peptide stapling methodologies targeting various amino acid residues, including proline,^44^ lysine,^45–50^ tyrosine,^50,51^ tryptophan,^52^ histidine,^52–54^ and cysteine.^55^ These approaches enable modular synthesis of diverse cyclic peptides from linear native or artificial peptides under mild conditions. Building upon this foundation, we herein report a systematic and comprehensive strategy for introducing diverse natural and artificial biaryl-bridged linkages into arbitrary peptide linkers with the aim of analog synthesis (Figure 1E). Specifically, modular incorporation of naturally inspired biaryl-bridged linkages derived from rubrin, micitide, lapparbin, and scabrirubin CB-4, as well as construction of artificial biaryl-bridged linkages, was achieved.

Recent advances in electrochemistry^56–58^ have highlighted cross-electrophile coupling mediated by nickel catalysis. Baran and co-workers demonstrated that *N*-hydroxyphthalimide (NHPI) esters are effective in nickel-catalyzed electrochemical decarboxylative cross-electrophile coupling.^59,60^ This reaction enables formation of C­(sp^3^)-C­(sp^2^) bonds under mild conditions, thereby allowing introduction of diverse aromatic rings into sp^3^ carbons of complex substrates. Indeed, a number of elegant applications of this reaction toward complex-molecule synthesis have been reported in recent years.^61^ In parallel, our group has recently reported the synthesis of biaryl-bridged cyclic peptides including cihunamide B, lapparbin, neopetromin, strecintide 839, micitide 982, and scabrirubin CB-4.^62–67^ These noncanonical cyclic peptides are characterized by significant ring strain arising from the rigidity of the biaryl units, rendering them inaccessible via conventional intramolecular amide coupling or intramolecular Ullmann coupling. Through extensive investigation, we identified palladium-catalyzed Larock macrocyclization as a suitable method for constructing these biaryl-bridged cyclic peptides.^68–79^

Against this backdrop, this study demonstrates structural diversification of noncanonical cyclic peptides via biaryl-driven electrochemical stapling, achieved by combining nickel-catalyzed electrochemical decarboxylative cross-electrophile coupling with palladium-catalyzed Larock macrocyclization (Figure 2). This methodology enables systematic incorporation of ten distinct biaryl- and triaryl-bridged linkages, comprising four naturally inspired motifs (rubrin, micitide, lapparbin, scabrirubin CB-4) and six artificial variants. Furthermore, the approach is extendable to diverse peptide linkers, thereby facilitating the supply of various noncanonical cyclic peptides. This systematic strategy for accessing biaryl-bridged cyclic peptides effectively broadens the accessible chemical space, thereby facilitating the development of novel cyclic peptide scaffolds.^80–86^

**Figure 2:**
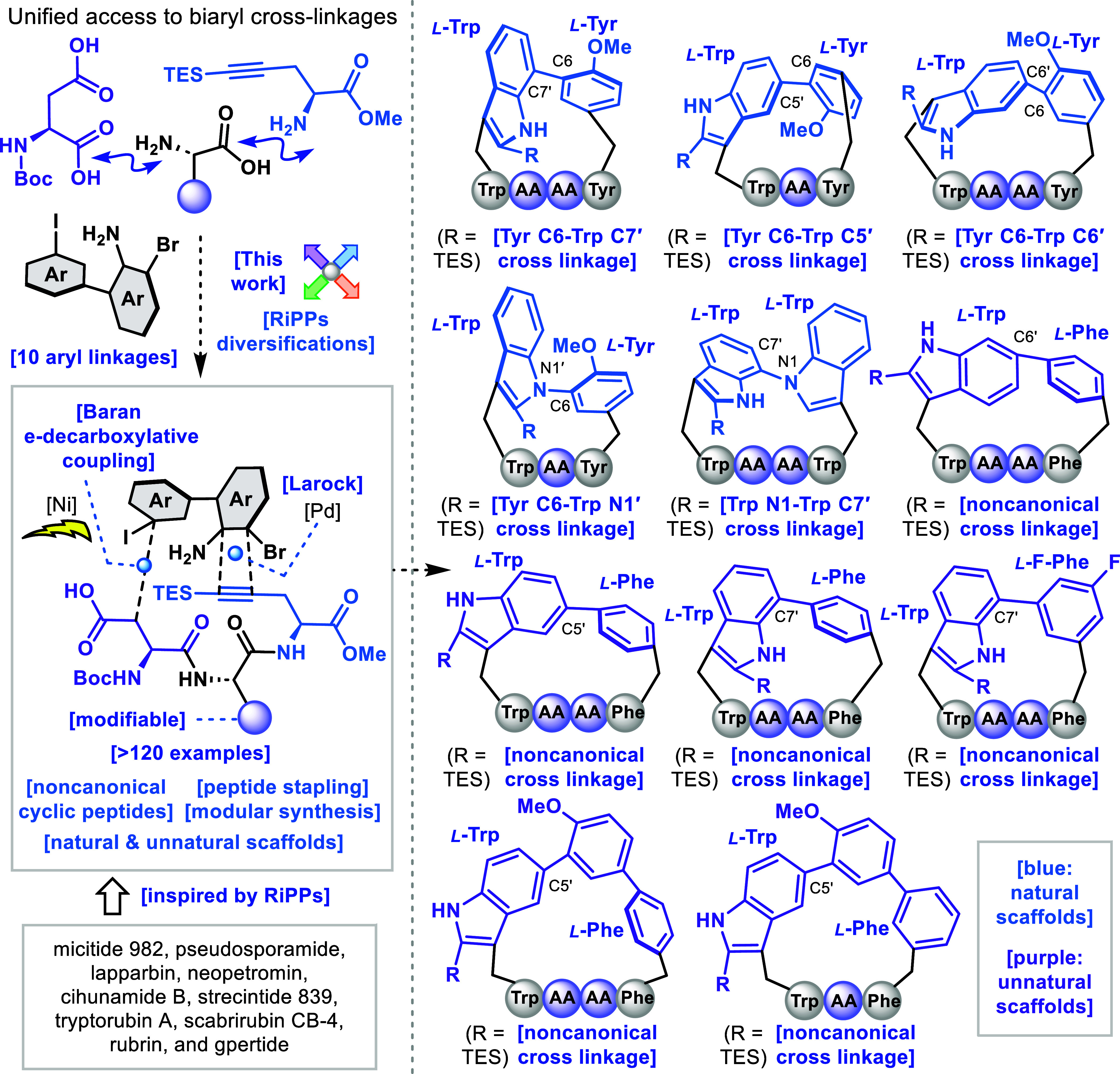
Synthetic plan: Unified access to biaryl cross-linkages.

## Results and Discussion

Initially, a series of biaryl units was synthesized (Scheme 1). The comprehensive and divergent preparation of diverse biaryl units from readily available building blocks represented a key objective of this study, as it enables the synthesis of a wide variety of cyclic peptides cross-linked through different aromatic motifs. Following cyclization, four biaryl units (**3a–d**) corresponding to naturally derived cyclic peptides such as rubrin, micitide,^66^ scabrirubin, and lapparbin,^63^ together with six biaryl/triaryl units (**7a–d**, **12a–b**) corresponding to artificial cyclic peptide structures not found in nature, were successfully prepared.

**Scheme 1:**
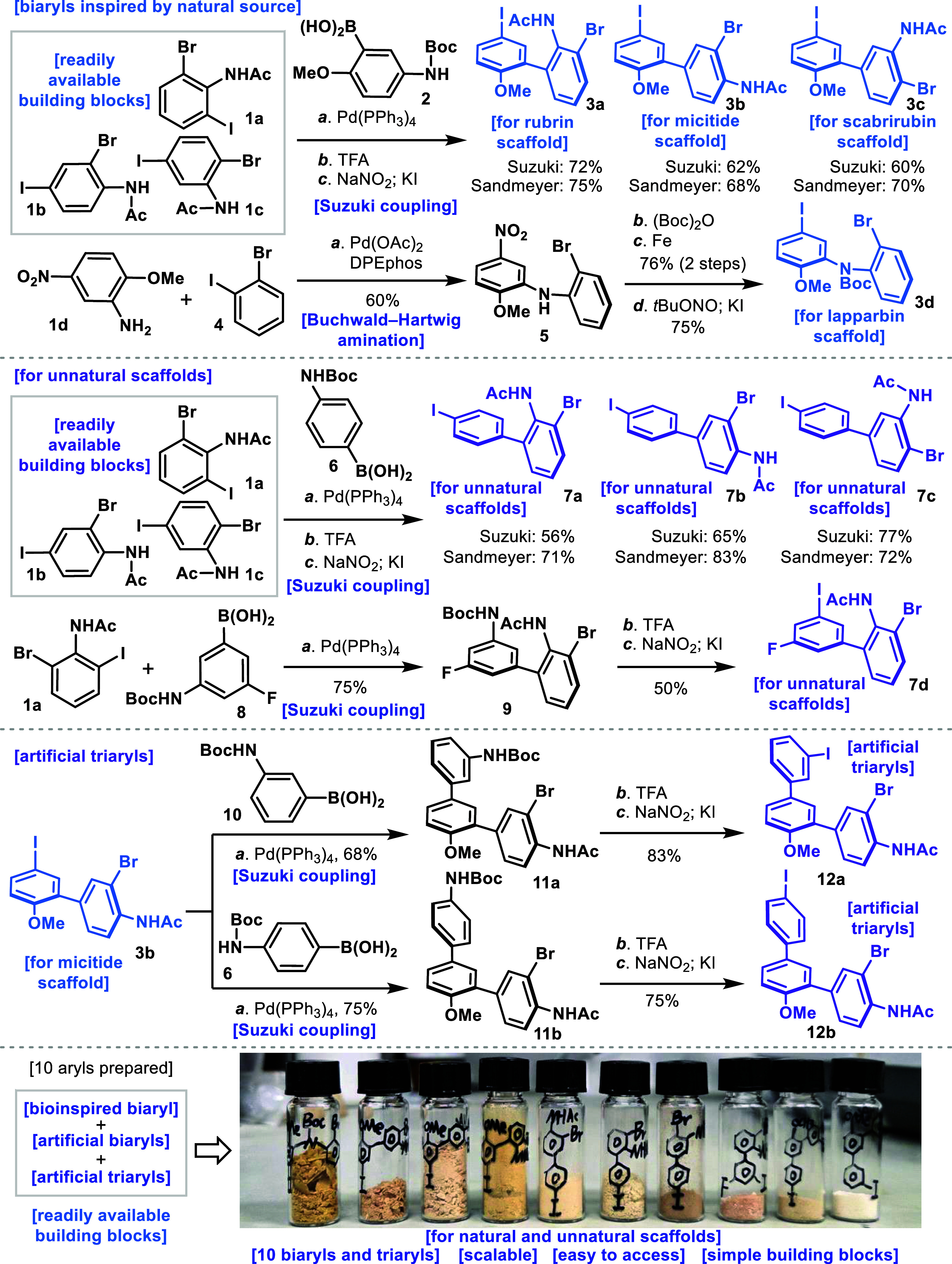
Preparation of the Biaryls and Triaryls^a^

a For detailed reagents and conditions, see the Supporting Information.

Iodoaniline derivatives **1a–c** were coupled with boronic acid **2** via Suzuki coupling to afford the corresponding biaryls. Subsequent *tert*-butyloxycarbonyl (Boc) group deprotection followed by a Sandmeyer reaction introduced iodine substituents, yielding the desired biaryl units **3a–c**.^66^ In addition, aniline derivative **1d** was coupled with 1-bromo-2-iodobenzene (**4**) through Buchwald–Hartwig amination to give compound **5**. Boc protection, nitro group reduction, and a subsequent Sandmeyer reaction converted compound **5** into biaryl unit **3d**.^63^

Next, four biaryl units (**7a–d**) corresponding to artificial cyclic peptide structures not found in nature were synthesized. Following a similar sequence, iodoaniline derivatives **1a–c** were coupled with boronic acid **6** via Suzuki coupling. Boc deprotection with trifluoroacetic acid (TFA) and subsequent Sandmeyer reaction furnished biaryl units **7a–c**. Biaryl unit **7d** was obtained through Suzuki coupling of iodoaniline derivative **1a** with boronic acid **8**, followed by Boc deprotection and a Sandmeyer reaction.

In addition to the biaryl derivatives (**3a–d**, **7a–d**), triaryl derivatives (**12a–b**) were also prepared. Biaryl unit **3b** was subjected to Suzuki coupling with boronic acids **10** and **6**, affording triaryl intermediates **11a–b**. Subsequent transformations following the same sequence of steps as described above furnished the triaryl units **12a–b**. Through these procedures, a total of ten aromatic cross-linking units was synthesized from readily available building blocks: four biaryl units (**3a–d**) corresponding to naturally derived cyclic peptides, and six biaryl/triaryl units (**7a–d**, **12a–b**) corresponding to artificial cyclic peptide structures not found in nature.

As an initial application, construction of the rubrin scaffold was attempted (Figure 3A). Rubrin, a RiPP isolated in 2023, features a Tyr C6–Trp C7′ cross-linkage.^31^ More recently, gpertide, which shares this structural motif, was reported in 2025.^30^ For both RiPPs, biosynthetic supply is limited to only a few milligrams (rubrin: 8.8 mg; gpertide: 3 mg).^30,31^ To address this limitation, C­(sp^3^)-C­(sp^2^) bond cross-coupling and subsequent cyclization of biaryl unit **3a** with tetrapeptide linker **13** were investigated (method A). Direct incorporation of aromatic units into polypeptides requires high functional group tolerance and mild reaction conditions. Guided by Baran’s report on nickel-catalyzed electrochemical decarboxylative cross-electrophile coupling,^59,60^ introduction of biaryl unit **3a** into tetrapeptide linker **13** was achieved, affording cyclization precursor **19a** in 53% yield. The reaction proceeded smoothly even on gram scale, enabling quantitative supply of precursor **19a**. Subsequent Larock macrocyclization converted precursor **19a** into cyclized compound **20a** in 51% isolated yield. The configuration of the biaryl moiety within the rubrin scaffold was assigned as *R* based on ROESY analysis (**20 m**), which is the same configuration as the gpertide core structure.^30^

**Figure 3:**
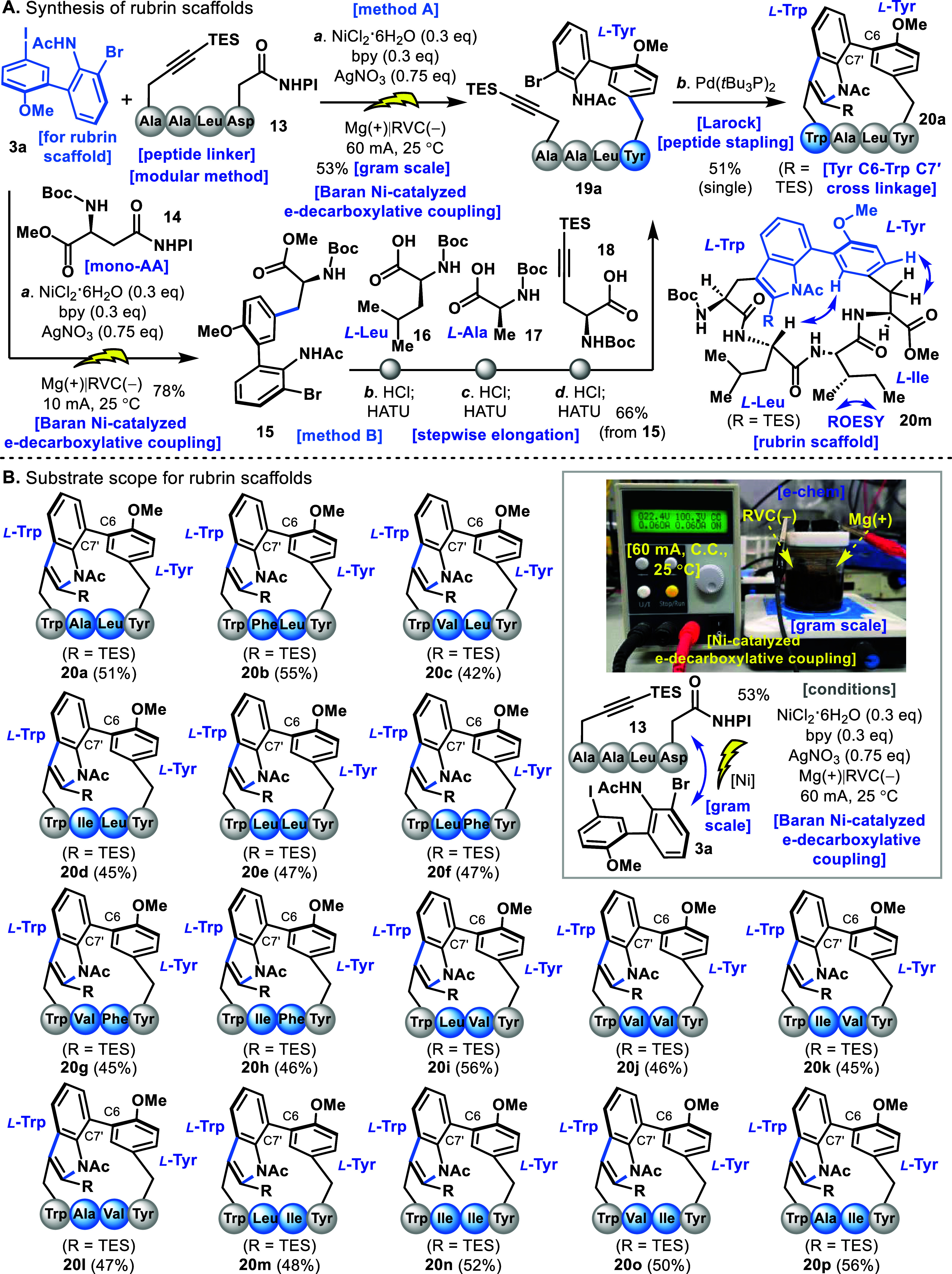
^*a*^(A) Synthesis of rubrin scaffolds. (B) Substrate scope for rubrin scaffolds. ^*a*^For detailed reagents and conditions, see the Supporting Information.

In parallel, an alternative approach (method B) employing the mono amino acid fragment **14** was examined. Electrochemical decarboxylative cross-electrophile coupling of biaryl **3a** with redox-active ester **14** proceeded efficiently, affording coupling product **15** in 78% yield. After Boc deprotection, sequential introduction of leucine derivative **16**, alanine derivative **17**, and alkyne fragment **18** furnished cyclization precursor **19a**. Notably, precursor **19a** could be prepared by both method A, which offers high modularity, and method B, which provides divergent access through sequential incorporation of different amino acids. Thus, for analog synthesis, method A is advantageous when diverse biaryl units are to be rapidly introduced into peptide linkers, whereas method B is preferable when diverse peptide linkers are to be introduced into a single biaryl unit, enabling efficient analog preparation.

The generality of method B was further evaluated by preparing precursors in which amino acids within the ring were replaced with various lipophilic residues (Figure 3B). In all cases, Larock macrocyclization proceeded smoothly, affording 16 corresponding cyclized products (**20a–20p**) in 42–56% yield. Remarkably, the reaction was insensitive to steric bulk of the amino acid side chains: for example, cyclization of **20b**, bearing the bulkiest amino acid combination, proceeded in 55% yield, while **20l**, bearing the least bulky combination, was obtained in 47% yield. These results demonstrate that the developed methodology is broadly applicable and independent of steric hindrance from amino acid side chains.

Next, application of the developed strategy to the micitide scaffold was investigated (Figure 4A). Micitide 982, a RiPP isolated in 2023, features a Tyr C6–Trp C5′ cross-linkage.^27^ We previously reported the synthesis of micitide 982 using cross-electrophile coupling between a serine-derived alkyl bromide and an aryl iodide, followed by Larock macrocyclization.^66^

**Figure 4:**
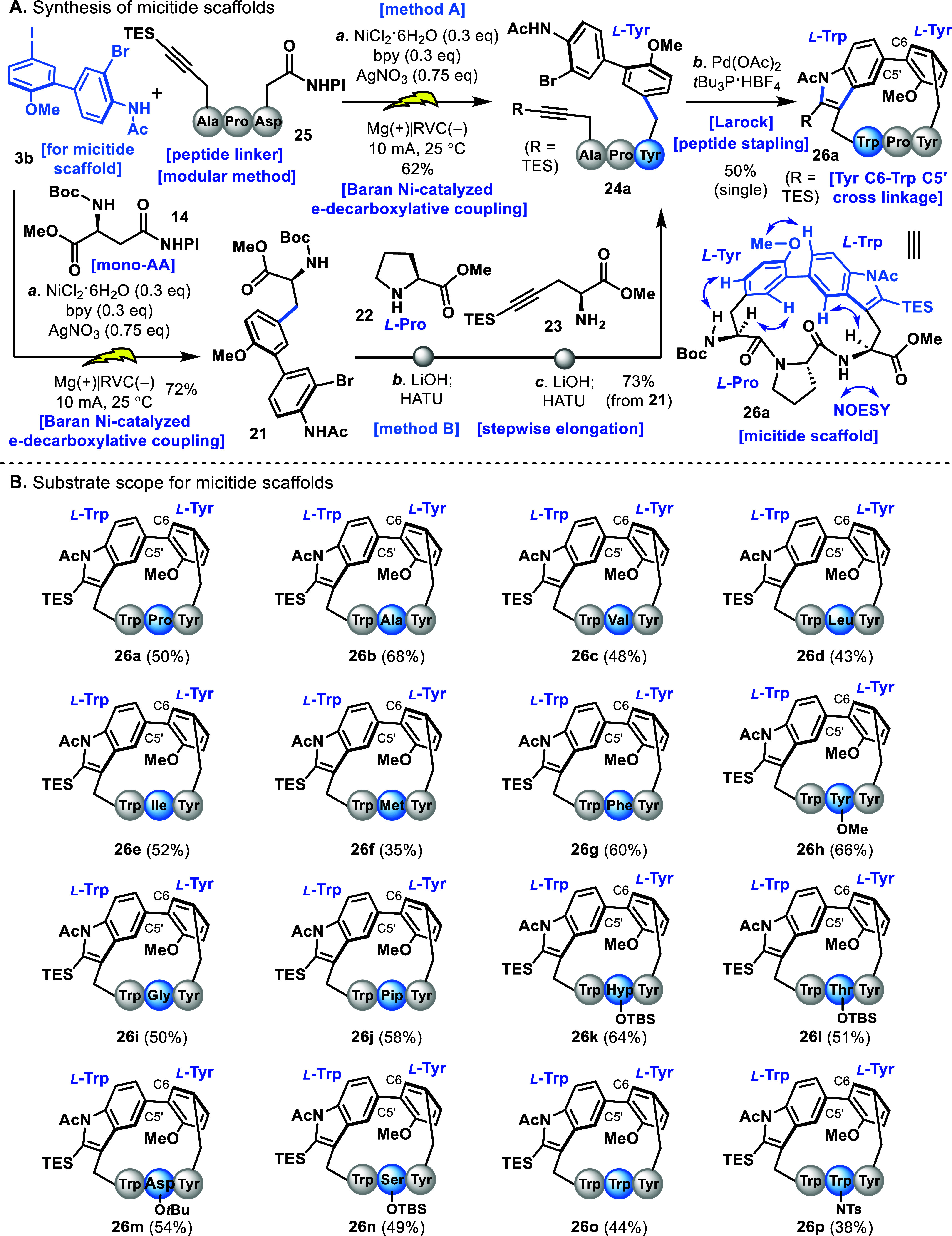
^*a*^(A) Synthesis of micitide scaffolds. (B) Substrate scope for micitide scaffolds. *^a^* For detailed reagents and conditions, see the Supporting Information.

While this approach allowed direct incorporation of aromatic units into tripeptides with high modularity, the cross-electrophile coupling proceeded in only moderate yield, with challenges including dehalogenation of the serine-derived alkyl bromide and over-reduction. To overcome these limitations, decarboxylative coupling using an aspartic acid unit was employed to improve yields and establish a more practical methodology for complex substrates. Using method A, decarboxylative coupling of biaryl **3b** with tripeptide linker **25** afforded cyclization precursor **24a** in 62% yield. Subsequent Larock macrocyclization yielded cyclized product **26a**, bearing the micitide core, in 50% yield. The ^1^H NMR spectrum of **26a** matched our previously reported data, and NOESY analysis confirmed the *S* configuration of the biaryl moiety.^66^ In parallel, method B was applied to synthesize precursor **24a**. Decarboxylative coupling of biaryl **3b** with aspartic acid derivative **14** proceeded efficiently, and the resulting amino acid fragment **21** was converted to precursor **24a** via amide coupling in 73% overall yield. Evaluation of the generality of method B (Figure 4B) demonstrated that Larock macrocyclization proceeded across a range of neutral amino acid substrates, affording 16 cyclized products (**26a–26p**) in 35–68% yield. Notably, substrates containing methionine gave lower yields, with the cyclized product obtained in 35% yield.

Finally, the developed methodology was extended to scaffolds featuring a Tyr C6–Trp C6′ linkage (Figure 5A). This structural motif was first reported in 2025 in scabrirubin CB-4, a RiPP discovered through combinatorial biosynthesis.^29^ To date, no natural products bearing a Tyr C6–Trp C6′ cross-linkage have been isolated, making scabrirubin CB-4 a rare example of a biaryl-bridged cyclic peptide. Electrochemical decarboxylative cross-electrophile coupling of biaryl **3c** with tetrapeptide linker **13** (method A) proceeded smoothly, affording cyclization precursor **28a** in 48% yield. Subsequent Larock macrocyclization converted precursor **28a** into cyclized product **29a**, bearing the Tyr C6–Trp C6′ linkage, in 55% yield. The protecting groups of cyclized compound **29a** were removed by treatment with BBr_3_ followed by LiOH, affording the deprotected product **29a′**. The configuration of the biaryl moiety in **29a′** was determined to be *R* through ROESY experiments.^67^ Subsequently, decarboxylative coupling of biaryl **3c** with aspartic acid derivative **14** followed by introduction of amino acid units (method B) proceeded in good yield, affording cyclization precursor **28a**. The generality of the developed methodology was then evaluated for the scabrirubin scaffold using method B (Figure 5B).

**Figure 5:**
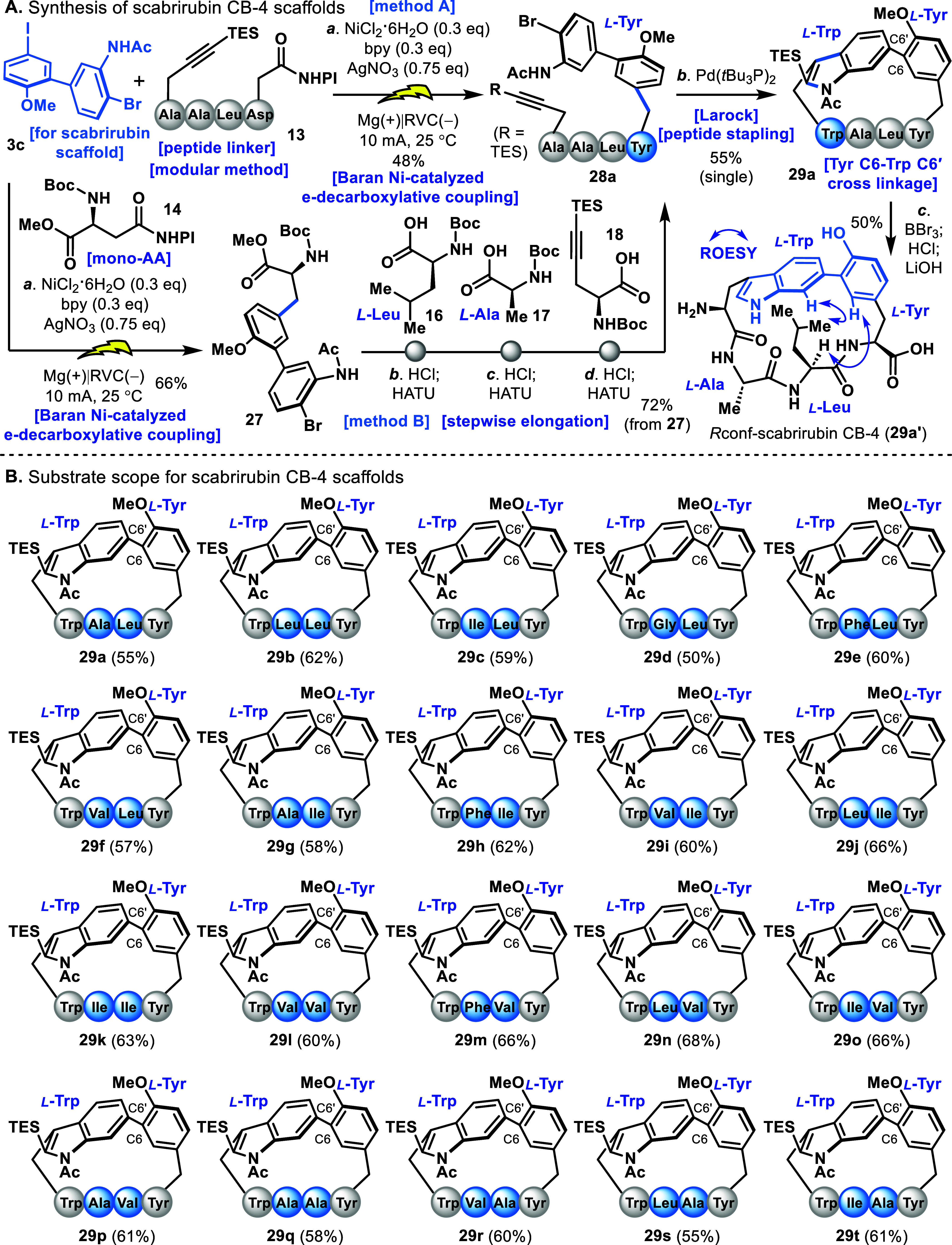
^*a*^(A) Synthesis of scabrirubin CB-4 scaffolds. (B) Substrate scope for scabrirubin CB-4 scaffolds. *^a^* For detailed reagents and conditions, see the Supporting Information.

Cyclization was attempted with precursors in which amino acids within the ring were replaced with various hydrophobic residues. Regardless of sequence, cyclization proceeded smoothly to afford 20 corresponding cyclic products (**29a–29t**) in 50–68% yield.

The biaryl-driven electrochemical stapling strategy was next applied to the lapparbin scaffold (Figure 6A). Lapparbin, a RiPP isolated in 2023, features a characteristic Tyr C6–Trp N1′ cross-linkage.^31^ Other cyclic peptides bearing this motif include tryptorubin A^33^ and nocapeptin A.^68^ Our group previously reported the synthesis of lapparbin^63^ and neopetromin^64^ via C–H activation. While these methods enabled gram-scale supply, they required introduction of the aromatic cross-linking unit through mono amino acids, limiting modularity. In the present study, application of the newly developed methodology was investigated to establish a more general and divergent synthetic approach. Electrochemical decarboxylative cross-electrophile coupling of aromatic unit **3d** with tripeptide linker **25** proceeded in 33% yield, and subsequent treatment with TFA and Boc_2_O furnished cyclization precursor **31a**. The relatively low yield compared to other aromatic units is likely attributable to electronic effects arising from nitrogen bridging between the two aromatic rings in **3d**. Indeed, substrates lacking Boc protection on the aniline moiety of **3d** failed to undergo coupling with **25**.

**Figure 6:**
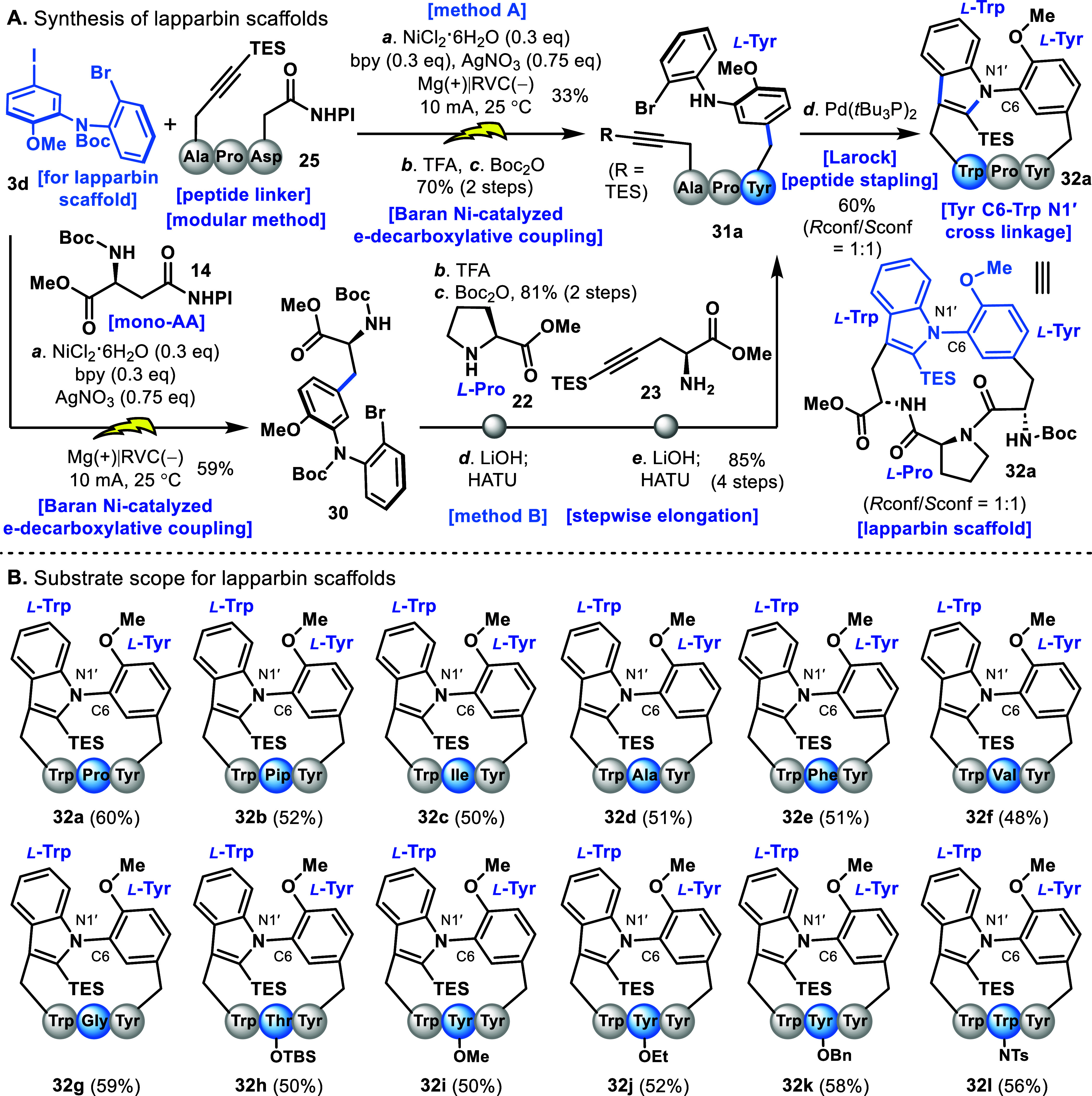
^*a*^(A) Synthesis of lapparbin scaffolds. (B) Substrate scope for lapparbin scaffolds. ^*a*^For detailed reagents and conditions, see the Supporting Information.

Larock macrocyclization of precursor **31a** afforded cyclized product **32a**, bearing the lapparbin scaffold, as a 1:1 atropisomeric mixture (method A).^63^ In contrast, method B involving decarboxylative coupling of **3d** with aspartic acid derivative **14** proceeded in 59% yield, affording amino acid fragment **30**. Sequential introduction of proline derivative **22** and alkyne fragment **23** converted **30** into precursor **31a**. Evaluation of the generality of method B for the lapparbin scaffold (Figure 6B) demonstrated that cyclization proceeded smoothly with substrates bearing various aliphatic amino acids (**32a–32g**), affording the corresponding cyclic products in 48–60% yield. Cyclization also proceeded with substrates containing threonine (**32h**), tyrosine (**32i–32k**), and tryptophan (**32l**) derivatives, yielding the desired products in moderate yields.

Traditionally, chemical synthesis of RiPPs has relied on building blocks derived from natural amino acids, making incorporation of non-natural biaryl motifs challenging.^87,88^ The methodology developed herein enables direct incorporation of arbitrary non-natural biaryl and triaryl units into cyclic peptides. To demonstrate the high modularity and versatility of biaryl-driven electrochemical stapling, systematic introduction of non-natural biaryl and triaryl-bridged linkages into peptide linkers was undertaken (Figure 7). This included incorporation of fluorine-containing aromatic units, which are frequently encountered in pharmaceuticals.^89–92^ Representative examples of fluorine-containing peptide drugs include enlicitide, voxilaprevir, and ulimorelin. These cyclic peptides have been designed to incorporate fluorine in order to enhance metabolic stability and lipophilicity, while maintaining appropriate membrane permeability.^93^

**Figure 7:**
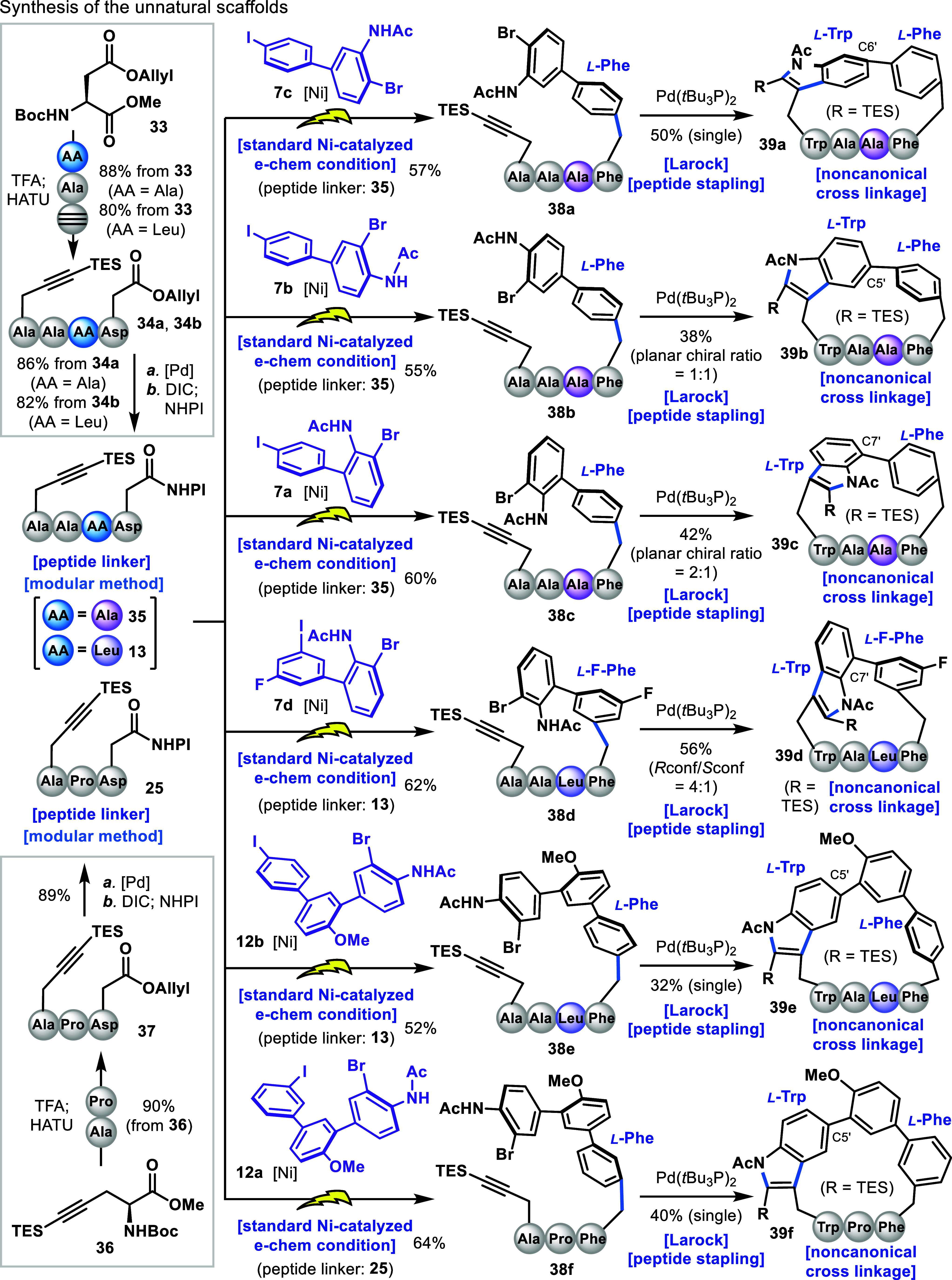
^*a*^Synthesis of the unnatural scaffolds. *^a^* For detailed reagents and conditions, see the Supporting Information.

Tetrapeptide linkers (**34a–b**) were synthesized by sequential introduction of two amino acids and an alkyne fragment into aspartic acid derivative **33** using Boc chemistry, affording tetrapeptides **34a–b**. Subsequent Pd(0)-mediated allyl ester removal and esterification with NHPI converted **34a–b** into redox-active esters (**13**, **35**). In parallel, tripeptide linker **25** was prepared by elongation of alkyne fragment **36** toward the *N*-terminus with proline and aspartic acid derivatives, yielding tripeptide **37**, which was converted into redox-active ester **25** by Pd(0)-mediated allyl ester removal and NHPI esterification. Electrochemical stapling was then attempted using oligopeptide linker **35** with biaryl derivatives **7c**, **7b**, and **7a**, each differing in indole substitution pattern. Decarboxylative coupling proceeded in moderate yields, affording cyclization precursors **38a–38c** in 55–60% yield. Subsequent Pd-catalyzed peptide stapling furnished cyclized products **39a–39c** in 38–50% yield, successfully incorporating artificial biaryl-bridged linkages.

Similarly, tetrapeptide linker **13** was coupled with fluorinated biaryl **7d**. Incorporation of fluorine into bioactive molecules is known to enhance metabolic stability and lipophilicity, among other properties. Indeed, 20–25% of approved pharmaceuticals contain at least one fluorine atom.^94^ Application of the developed methodology to fluorine-containing aromatic units proceeded smoothly, affording cyclization precursor **38d** in 62% yield. Subsequent peptide stapling converted **38d** into cyclized product **39d** in 56% yield, demonstrating the feasibility of synthesizing fluorine-containing biaryl-bridged cyclic peptides.

Finally, the methodology was applied to triaryl units **12a** and **12b**. Incorporation of triaryl structures into peptides offers significant advantages in medicinal chemistry. Aromatic motifs can stabilize three-dimensional conformations and reduce entropic penalties upon binding to target proteins, thereby enhancing specificity and affinity. To date, synthetic access to triaryl motifs has been limited to copper-catalyzed methods reported by Chen and co-workers.^52^ In this study, decarboxylative coupling of triaryl unit **12b** with tetrapeptide linker **13** and triaryl unit **12a** with tripeptide linker **25** proceeded smoothly, affording cyclization precursors **38e** and **38f**. Application of Larock macrocyclization conditions yielded the desired triaryl-bridged cyclic peptides in 32% and 40% yield, respectively, demonstrating successful synthesis of artificial triaryl-containing noncanonical cyclic peptides.

Construction of biaryl-bridged linkages by enzymatic methods is inherently limited by the size of the enzyme pocket and the specific amino acid residues involved in catalysis, restricting applicability to substrates with defined sequences and residue numbers.^30^ Consequently, biosynthetic approaches impose constraints on the synthesis of cyclic peptides with varying ring sizes. To address this limitation, the generality of the developed methodology was evaluated with respect to cyclic structures of different residue counts (Figure 8).

**Figure 8:**
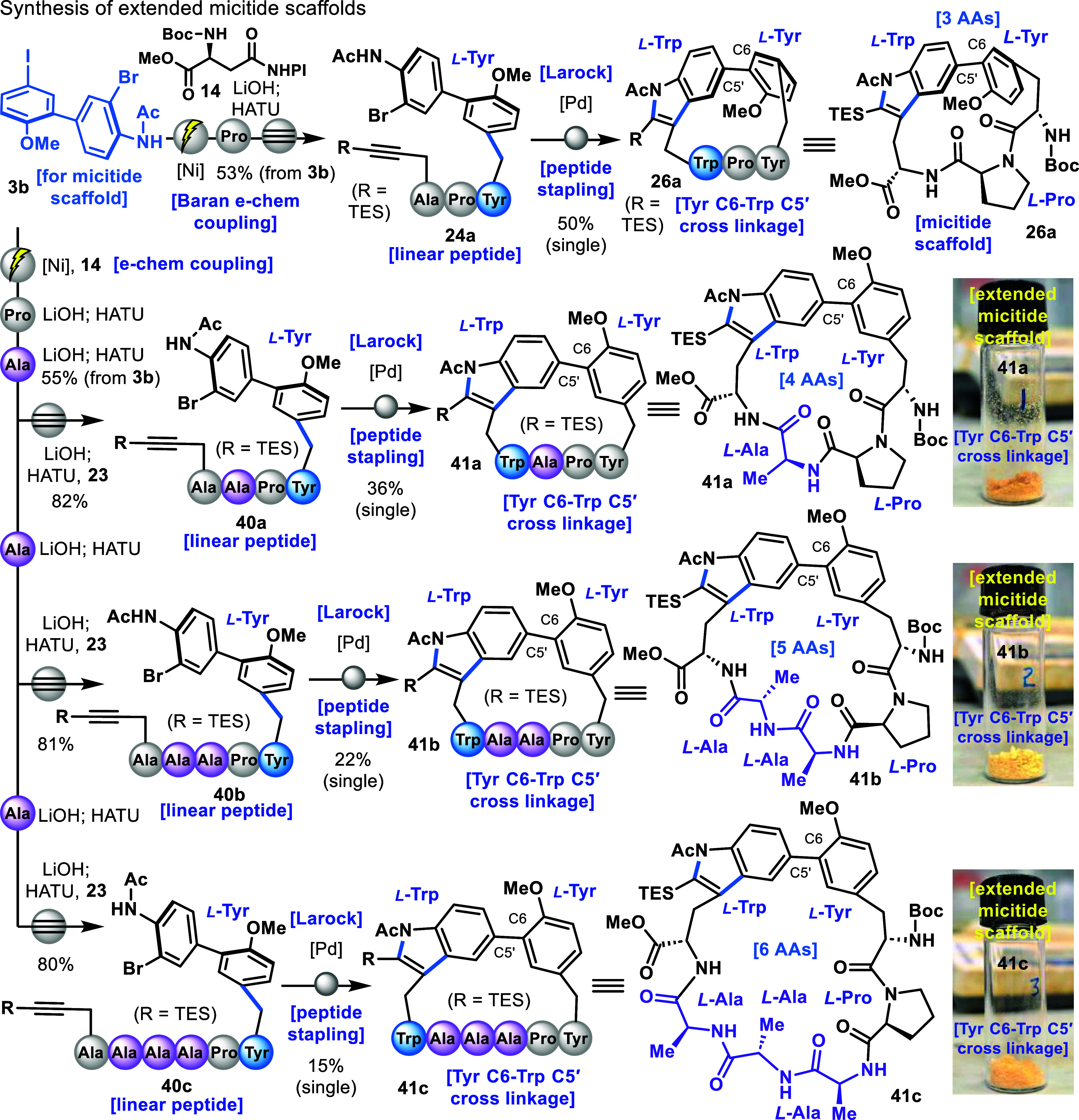
^*a*^Synthesis of extended micitide scaffolds. ^*a*^For detailed reagents and conditions, see the Supporting Information.

Decarboxylative coupling of biaryl unit **3b** with aspartic acid derivative **14** afforded amino acid fragment **21**. Hydrolysis of **21** followed by sequential introduction of proline, alanine, and an alkyne fragment furnished cyclization precursor **40a**. In a similar manner, cyclization precursors **40b** and **40c**, containing two and three alanine residues, respectively, were prepared. Application of the cyclization reaction to these precursors revealed that, relative to the natural micitide 982 core (**26a**), the yield of Larock macrocyclization decreased with increasing ring size. Regardless of the ring size, no isomer formation was observed during the macrocyclization, and the corresponding cyclic products were obtained as single isomers, consistent with the case of the micitide 982 core **26a**. Nevertheless, these results demonstrate that the methodology enables access to biaryl-bridged cyclic peptides of diverse ring sizes, which are difficult to obtain through biosynthetic supply.

The generality of the developed methodology was next evaluated using the lapparbin scaffold as a model with cyclization precursors of varying residue numbers (Figure 9). Decarboxylative coupling of aspartic acid derivative **14** with biaryl **3d** afforded amino acid fragment **30**. Hydrolysis of **30** followed by sequential introduction of two alanine residues and an alkyne fragment furnished cyclization precursor **42a**. Similarly, introduction of three alanine residues and an alkyne fragment yielded precursor **42b**. Peptide stapling of these precursors (**42a**, **42b**) was then attempted. As observed for the micitide scaffold, cyclization of the natural precursor (**31d**) proceeded in 51% yield, whereas increasing ring size led to a gradual decrease in yield. Consistent with the case of the natural product-corresponding lapparbin core **32d**, the macrocyclization gave the desired products as inseparable mixtures of atropisomers, regardless of the amino acid residue length of the precursors. The cyclized products were obtained with an isomer ratio of 3:2 for **43a**, and a 1:1 ratio for **43b**. Nevertheless, these results demonstrate that peptide stapling can be achieved across a range of artificial ring sizes that are difficult to access by enzymatic methods.

**Figure 9:**
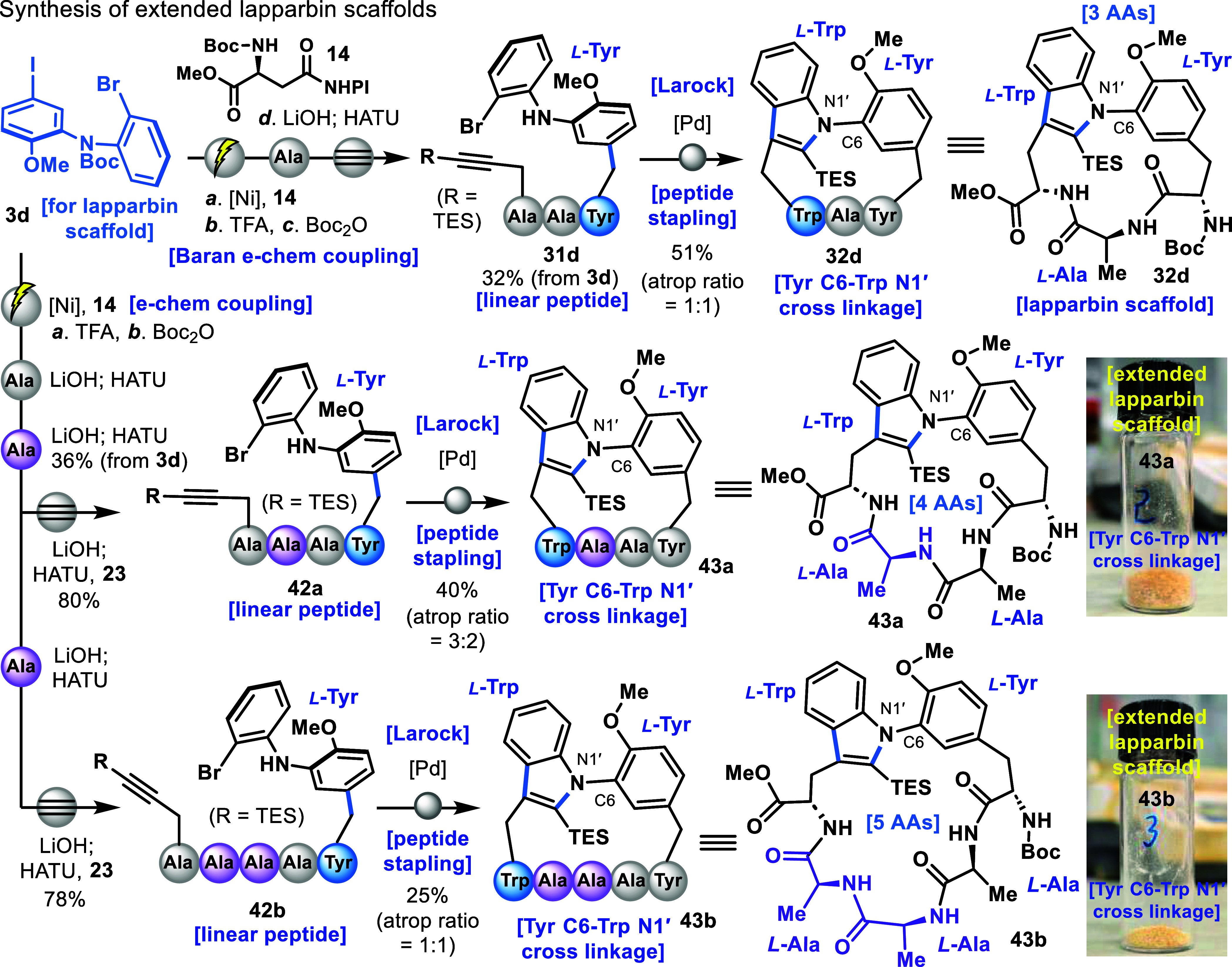
^*a*^Synthesis of extended lapparbin scaffolds. ^*a*^For detailed reagents and conditions, see the Supporting Information.

In parallel, our group reported the synthesis of cihunamide B in 2024.^62^ Cihunamide B, a RiPP isolated in 2023, is a noncanonical biaryl cyclic peptide featuring a unique Trp N1–Trp C7′ linkage not found in conventional cyclic peptides.^34^ The emergence of drug-resistant bacteria represents a major global public health challenge, and chemical synthesis and modification of natural antibiotics provide a promising approach toward the discovery of new agents.^95–105^ For example, chemical modification of the natural antibiotic erythromycin improved pharmacokinetics and reduced side effects, leading to the development of clarithromycin.^106^ Although cihunamide congeners have been reported to exhibit potent antibacterial activity, systematic analog synthesis has not been undertaken due to the specificity and rigidity imparted by the two tryptophan units. Against this backdrop, structure–activity relationship (SAR) studies of cihunamide congeners were conducted (Figure 10). We previously demonstrated that the characteristic Trp N1–Trp C7′ linkage of cihunamide congeners can be constructed from tryptophan derivative **44** through sequential incorporation of amino acids followed by Larock macrocyclization (Figure 10A).^62^ Using this methodology, a series of analogs bearing different amino acids within the ring was successfully synthesized (Figure 10B).

**Figure 10:**
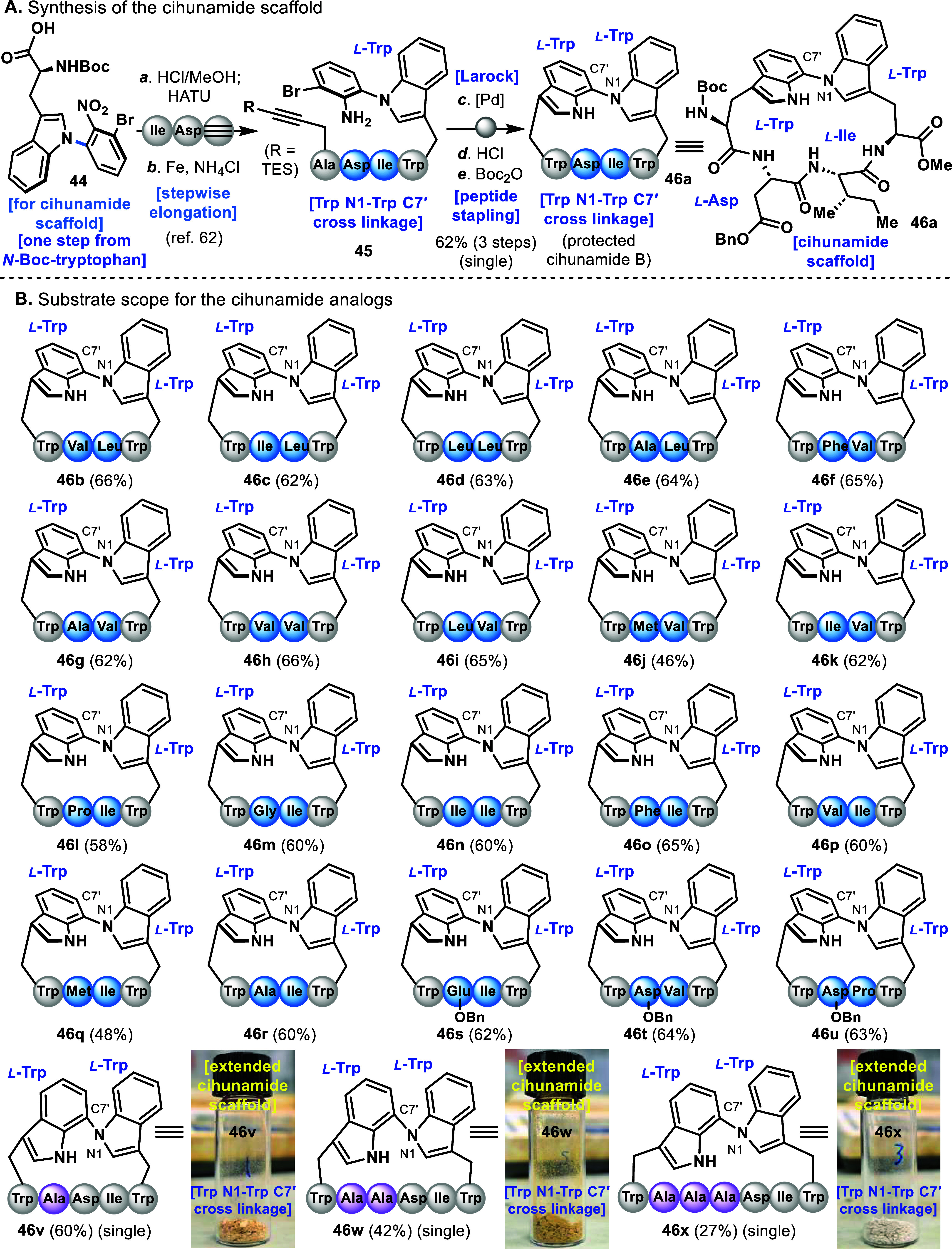
^*a*^(A) Synthesis of the cihunamide scaffold. (B) Substrate scope for the cihunamide analogs. ^*a*^For detailed reagents and conditions, see the Supporting Information.

Specifically, amino acid fragment **44**, which can be synthesized from commercially available L-tryptophan derivatives via S_N_Ar reaction, was employed as the starting material. Sequential introduction of various amino acid residues was carried out using Boc chemistry. Following Boc deprotection, an alkyne fragment was introduced to furnish a tetrapeptide. The nitro group of the resulting tetrapeptide was reduced with Fe(0), affording the cyclization precursor. Subsequent Larock macrocyclization under palladium catalysis, followed by removal of the triethylsilyl (TES) group on the indole, yielded cyclized products **46b–46u**, each incorporating different amino acids within the ring, in isolated yields of 46–66%. In addition, analogous to the micitide and lapparbin scaffolds, synthesis of cihunamide B congeners with varying ring sizes was attempted, confirming the versatility of this synthetic strategy (Figure 10B, bottom). Beyond the natural ring structure, cyclization precursors containing one, two, or three additional alanine residues were prepared and subjected to Larock cyclization. For precursor of **46v**, bearing one additional alanine, cyclization proceeded with a yield comparable to that of the natural product (60%), affording the desired cyclic peptide. In contrast, precursors of **46w** and **46x**, containing two and three additional alanine residues, respectively, afforded the desired cyclic products in reduced yields of 42% and 27%, reflecting the impact of increased ring size. Consistent with the case of the cihunamide B-corresponding cyclic core **46a**, the desired cyclized products were obtained as single isomers, regardless of the ring size.

In parallel, analogs of cihunamide B modified at the carboxyl group of the aspartic acid residue within the ring were synthesized (Figure 11A). The cihunamide core (**47**) was prepared on gram scale, and subsequent hydrogenation removed the benzyl ester to yield the corresponding carboxylic acid. This acid was then coupled with a variety of primary and secondary amines using HATU, affording cihunamide analogs (**48a–48x**) bearing modifications at the aspartic acid residue. In total, 24 analogs with diverse steric and electronic environments were synthesized. All protecting groups of the prepared analogs (**46a–46x**, **48a–48x**) were removed by treatment with LiOH and HCl to give the protecting group free cihunamide analogs.

**Figure 11:**
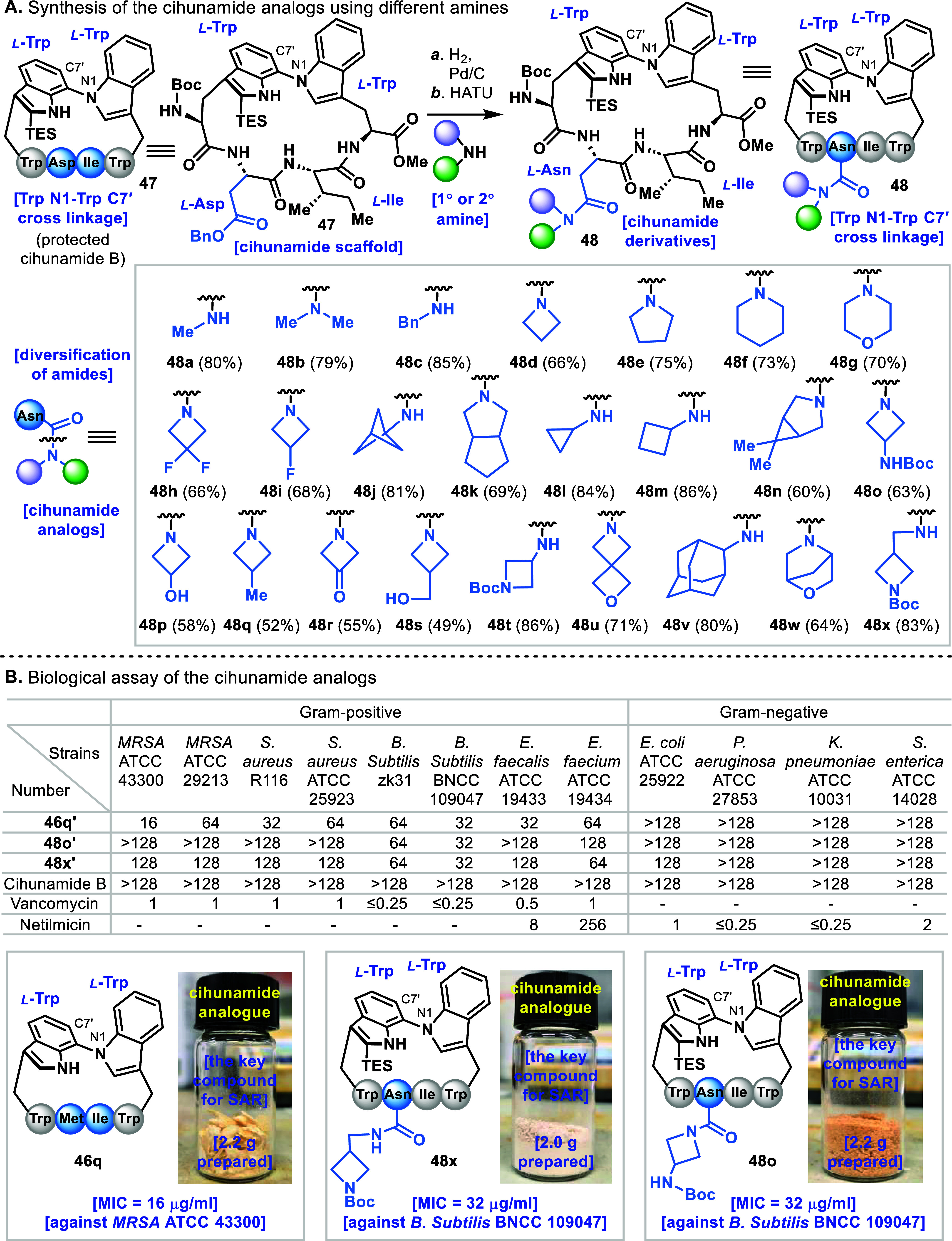
^*a*^(A) Synthesis of the cihunamide analogs using different amines. (B) Biological assay of the cihunamide analogs. ^*a*^For detailed reagents and conditions, see the Supporting Information.

Figure 11B summarizes the antibacterial activity of ci-hunamide B and its analogs. The potency of the synthesized analogs was systematically assessed against eight Gram-positive and four Gram-negative bacterial strains. Based on the structural elucidation by 2D NMR, the cihunamide B analogs synthesized herein share the same *Sa* configuration as cihunamide B. In this study, the impact of the amino acid residues within the ring systems and ester substituents on antibacterial activity was systematically investigated.

The cihunamide analog **46q′** (deprotected from **46q**), containing a methionine residue exhibited antibacterial activity against methicillin-resistant *Staphylococcus aureus* (MRSA ATCC 43300) with an MIC of 16 μg/mL. Furthermore, analogs **48x′** (deprotected from **48x**) and **48o′** (deprotected from **48o**), bearing azetidine units at the aspartic acid position, displayed antibacterial activity against *Bacillus subtilis* (BNCC 109047) with MIC values of 32 μg/mL. Although the antibacterial activity of cihunamide B was reported in the original isolation study (MIC = 8–16 μg/mL),^34^ no significant activity was observed under the assay conditions employed in this work. This discrepancy is speculated to be due to a force majeure event, such as the presence of impurities. The amount of cihunamide B obtained in the reported paper was extremely limited at 1.2 mg,^34^ meaning that even a trace amount of impurities could significantly impact the results.

## Conclusion

Recent advances in genome mining have uncovered a wide array of biaryl cyclic peptides with diverse biological activities. Nonetheless, among these RiPPs, certain members exhibit structural complexity that complicates their supply, and broadly applicable, systematic synthetic methodologies aimed at analog generation remain underdeveloped.

In this study, integration of electrochemical decarboxylative cross-electrophile coupling with Larock macrocyclization enabled systematic and modular incorporation of ten distinct biaryl/triaryl-bridged linkages, centered on Tyr–Trp motifs, into RiPPs from readily available building blocks. The principal strengths of this strategy are (i) its modularity, divergent nature, and broad substrate applicability. Using this platform, a library of more than 120 RiPP derivatives was constructed, encompassing four naturally inspired scaffolds (rubrin, micitide, scabrirubin, lapparbin) and six artificial biaryl/triaryl-bridged motifs, thereby systematically expanding chemical space and achieving structural diversity in RiPPs. (ii) The methodology also enabled synthesis of RiPP derivatives with non-natural ring sizes and frameworks inaccessible by enzymatic methods, including fluorine-substituted and triaryl architectures. (iii) Furthermore, structure–activity relationship (SAR) studies were conducted on cihunamide B, a noncanonical biaryl cyclic peptide featuring a unique Trp N1–Trp C7′ linkage. More than 40 analogs were synthesized and evaluated, with several compounds exhibiting antibacterial activity.

Collectively, this modular biaryl-driven electrochemical stapling strategy establishes a systematic supply platform for RiPPs and provides a versatile synthetic framework for both natural and non-natural biaryl cyclic peptides. The methodology is expected to facilitate exploration of previously inaccessible RiPP chemical space and enable synthesis of noncanonical cyclic peptides that have not yet been reported.

## Supplementary Material





## Notes

Experimental procedures (PDF)
Analytical data (^1^H and ^13^C NMR, MS) for all new compounds, as well as optimization tables (PDF)
