# Supplementary material for: Unified Access to Biaryl-Bridged Linkages Unlocks Structural Diversification of Noncanonical Cyclic Peptides
Source: J Am Chem Soc. 2026 Jun 29;148(27):28553–72. doi: 10.1021/jacs.6c05294 (PMC13383641; doi:10.1021/jacs.6c05294)

SUPPORTING INFORMATION
-NMR and LCMS spectrums-

Unified Access to Biaryl-Bridged Linkages Unlocks Structural Diversification of Noncanonical Cyclic Peptides

Longhui Yu, [§] Jie Zhang, [§] Xinwei Zhang, [§] Xilun Wu, [§] Ruoyu Liu, Chak Hin Au, Hiroshige Ogawa, Rongbiao Tong, * Hugh Nakamura*

Department of Chemistry, The Hong Kong University of Science and Technology,
Clear Water Bay, 999077, Hong Kong SAR, China

General information:

All the reagents were purchased from commercial sources (Sigma-Aldrich, TCI, Bide, Energy and Leyan) and were used without further purification unless otherwise stated. The yield refers to column chromatography separation yield unless otherwise stated. All the reactions were monitored by thin layer chromatography (TLC) and LC/MS. TLC was performed using 0.2-0.25 mm silica plates. ^{1}H NMR and ^{13}C NMR spectra were recorded on Bruker AVII 400, and JEOL 600 instruments. The following abbreviations were used to explain multiplicities: s = singlet, d = doublet, t = triplet, q = quartet, m = multiplet, br = broad. Column chromatography was performed using 230-400 mesh silica gel, and PTLC was performed using 0.2-0.25 mm silica plates. High-resolution mass spectra (HRMS) were recorded on an Agilent LC/MSD TOF mass spectrometer by electrospray ionization time of flight reflectron experiments.

Abbreviations

Ac	Acetyl
aq.	Aqueous
Boc	*tert*-Butoxycarbonyl
Bu	Butyl
DCM	Dichloromethane
DIC	*N,N′*-Diisopropylcarbodiimide
DIPEA	*N,N*-Diisopropylethylamine
DMA	*N,N*-Dimethylacetamide
DMF	*N,N*-Dimethylformamide
DMSO	Dimethyl sulfoxide
Et	Ethyl
HATU	Hexafluorophosphate Azabenzotriazole Tetramethyl Uronium
HRMS	High Resolution Mass Spectrometry
LCMS	Liquid Chromatograph Mass Spectrometer
Me	Methyl
NHPI	*N*-Hydroxyphthalimide
NMP	*N*-Methyl-2-pyrrolidone
NMR	Nuclear Magnetic Resonance Spectroscopy
Ph	Phenyl
PPh_3	Triphenylphosphine
PTLC	Preparative Thin Layer Chromatography
RVC	Reticulated Vitreous Carbon
TES	Triethylsilyl
TFA	Trifluoroacetic acid
THF	Tetrahydrofuran
TLC	Thin Layer Chromatography

The NMR spectrums:

Compound S1a ^{1}H NMR (600 MHz, $CDCl_3$)

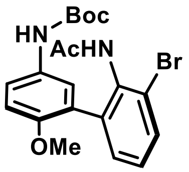

S1a

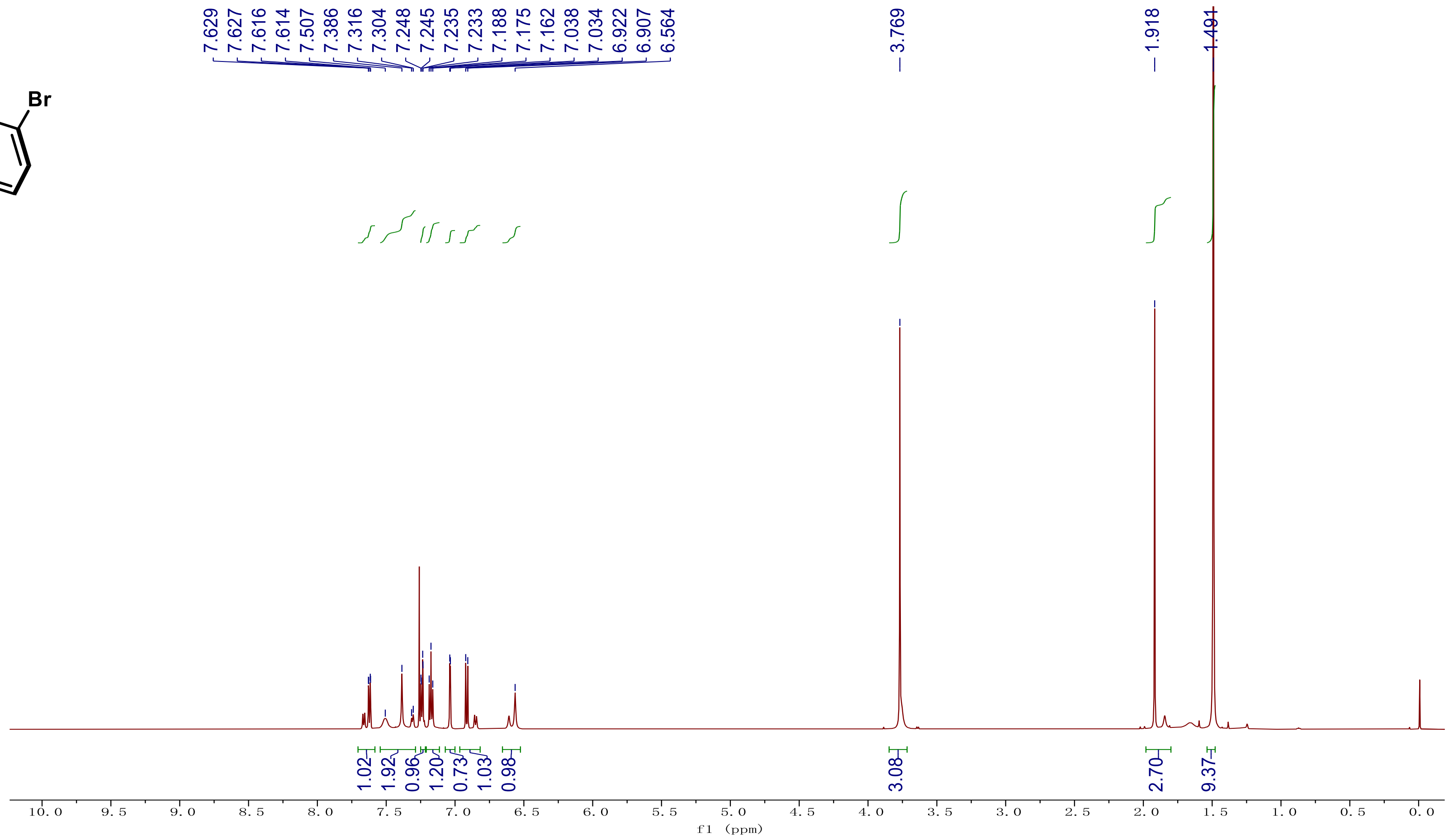

Compound S1a ^{13}C NMR (151 MHz, $CDCl_3$)

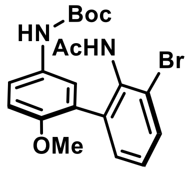

S1a

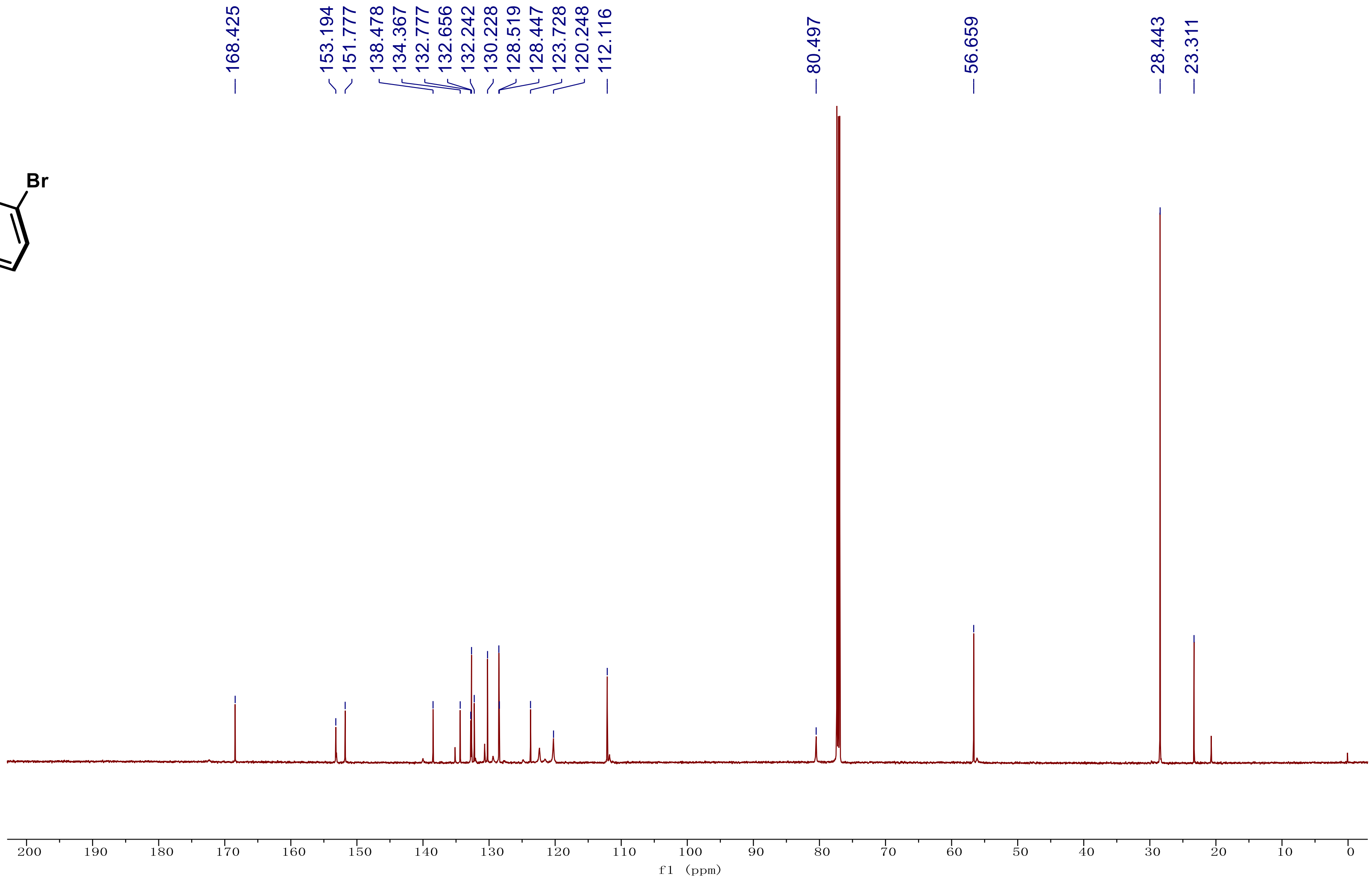

Compound 3a ^{1}H NMR (600 MHz, $CDCl_3$)

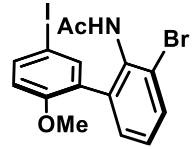

3a

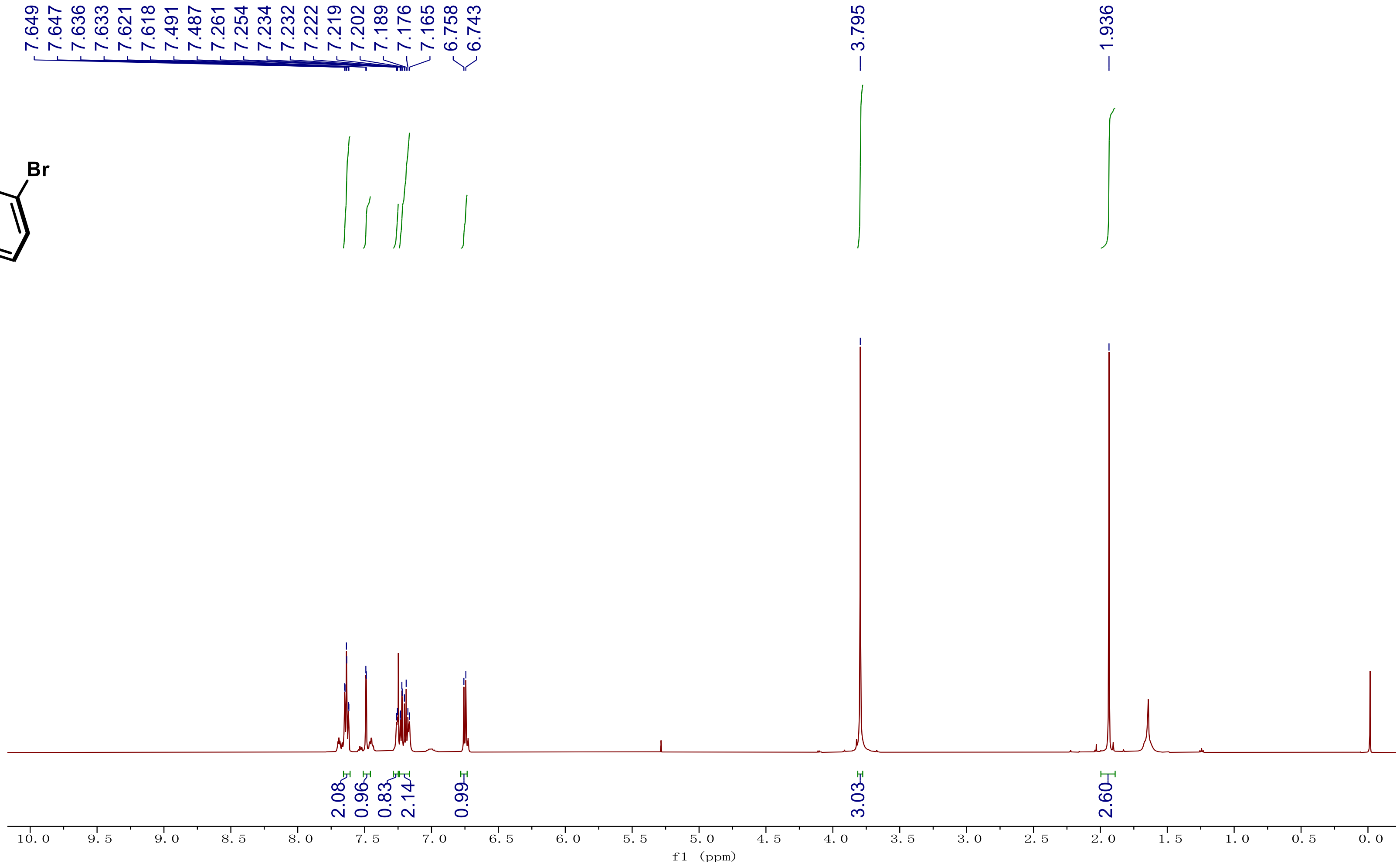

Compound 3a ^{13}C NMR (151 MHz, $CDCl_3$)

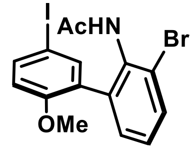

3a

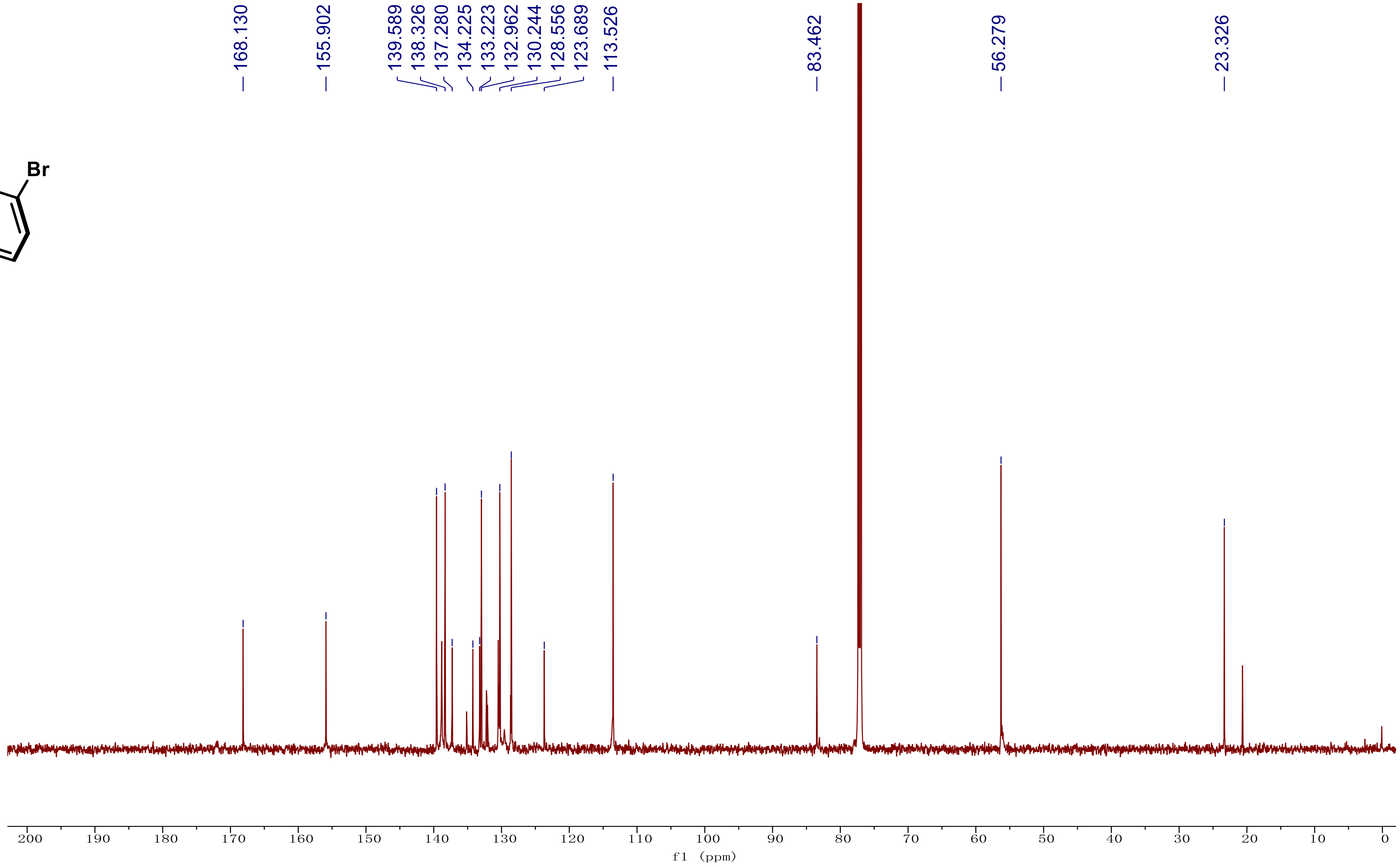

Compound S1c ¹H NMR (600 MHz, $CDCl_3$)

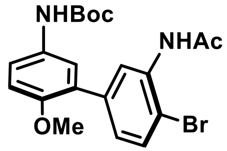

S1c

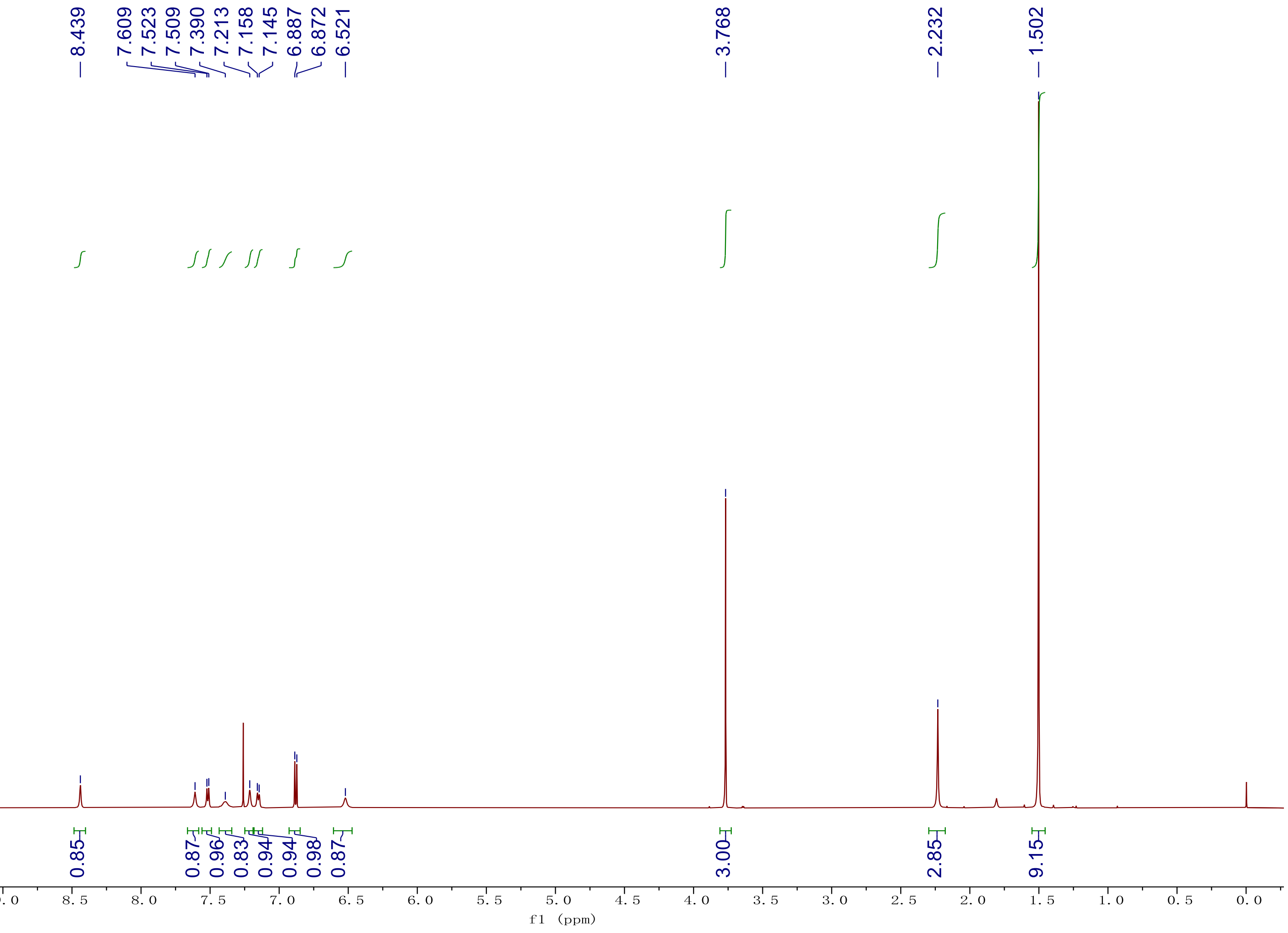

Compound S1c ^{13}C NMR (151 MHz, $CDCl_3$)

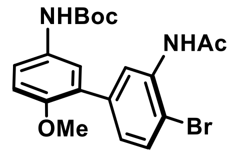

S1c

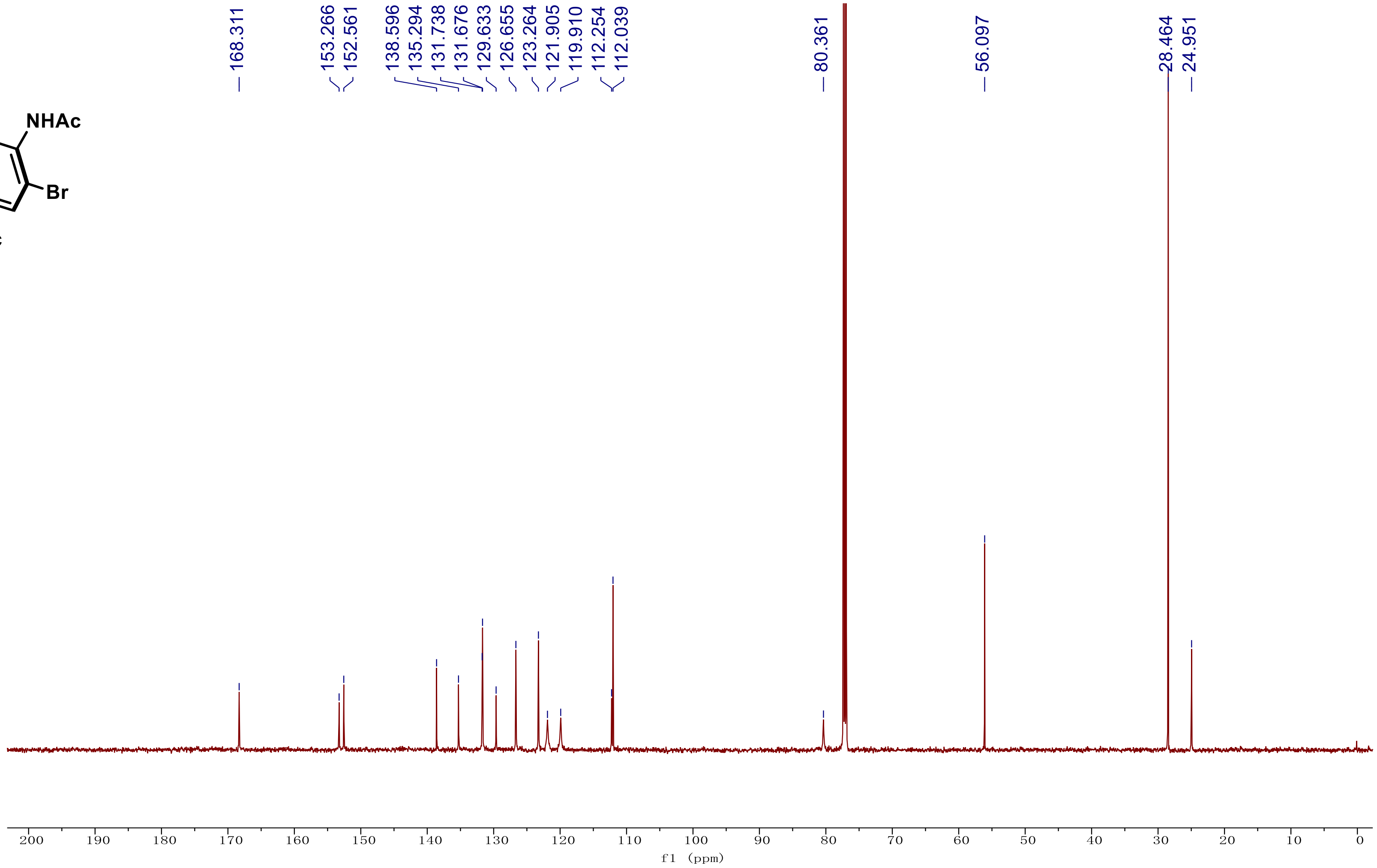

Compound 3c ¹H NMR (600 MHz, CDCl₃)

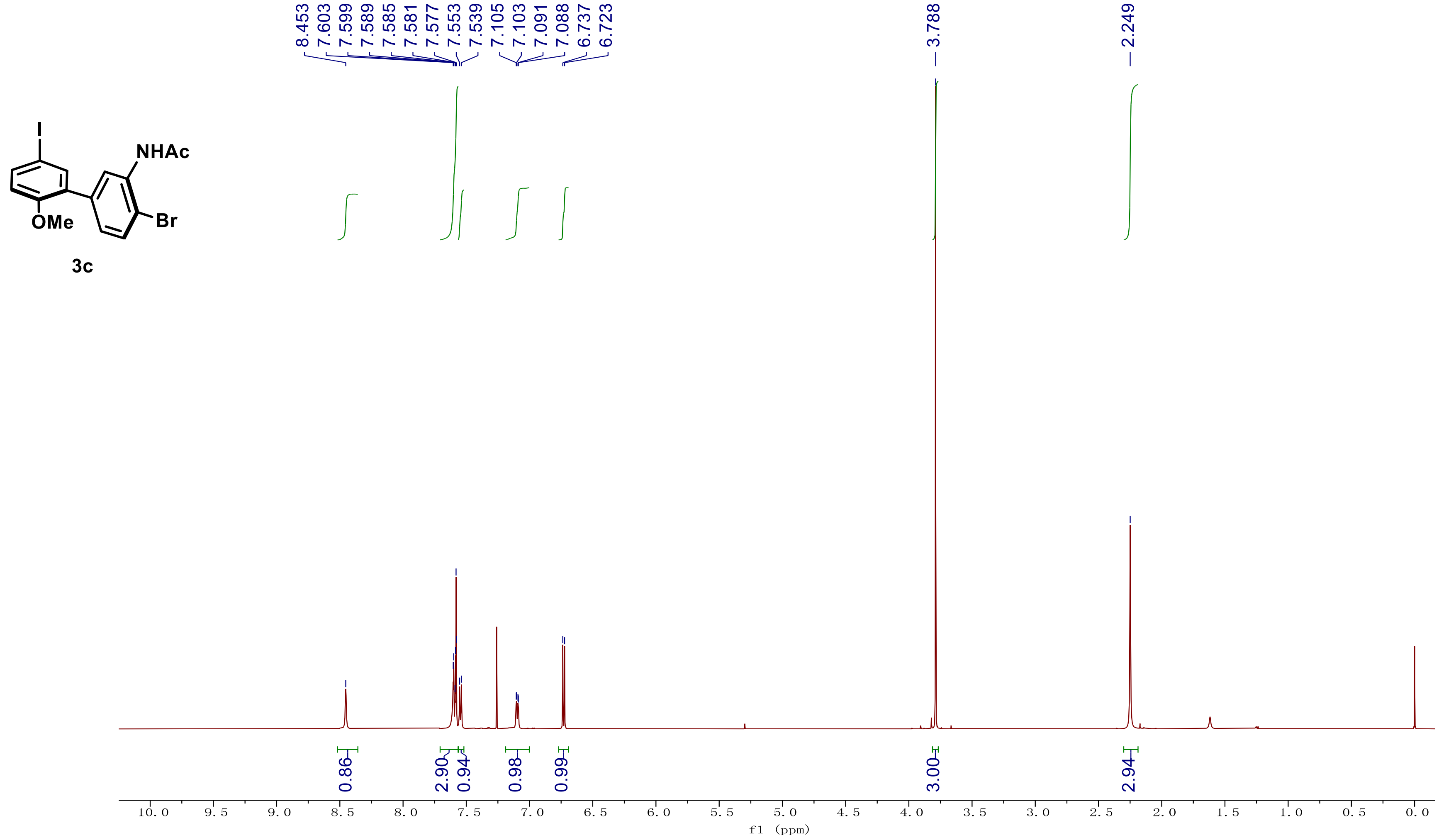

Compound 3c ^{13}C NMR (151 MHz, $CDCl_3$)

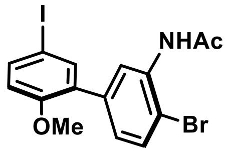

3c

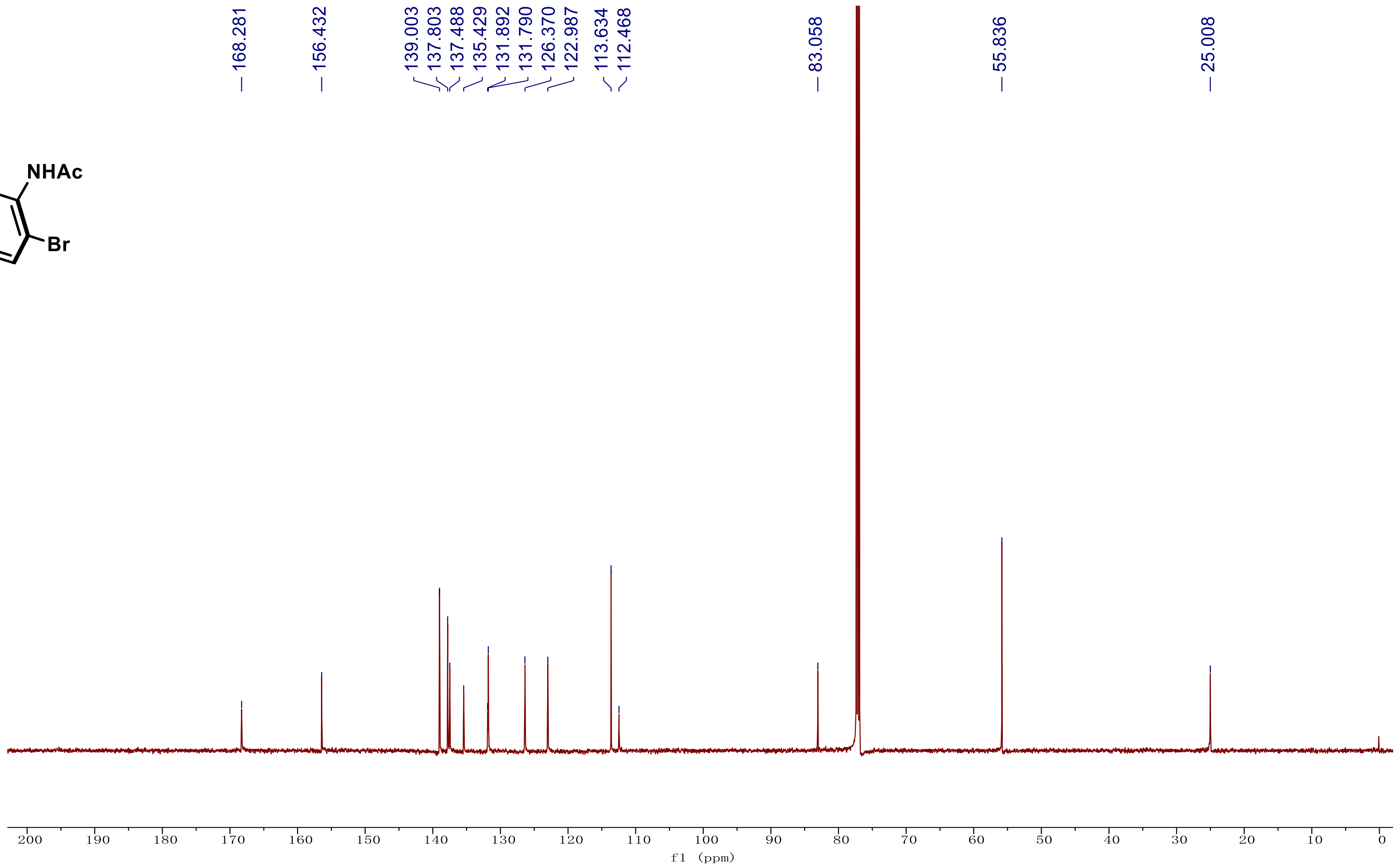

Compound S2a ¹H NMR (600 MHz, $CDCl_3$)

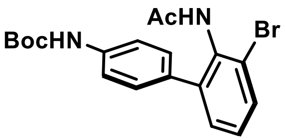

S2a

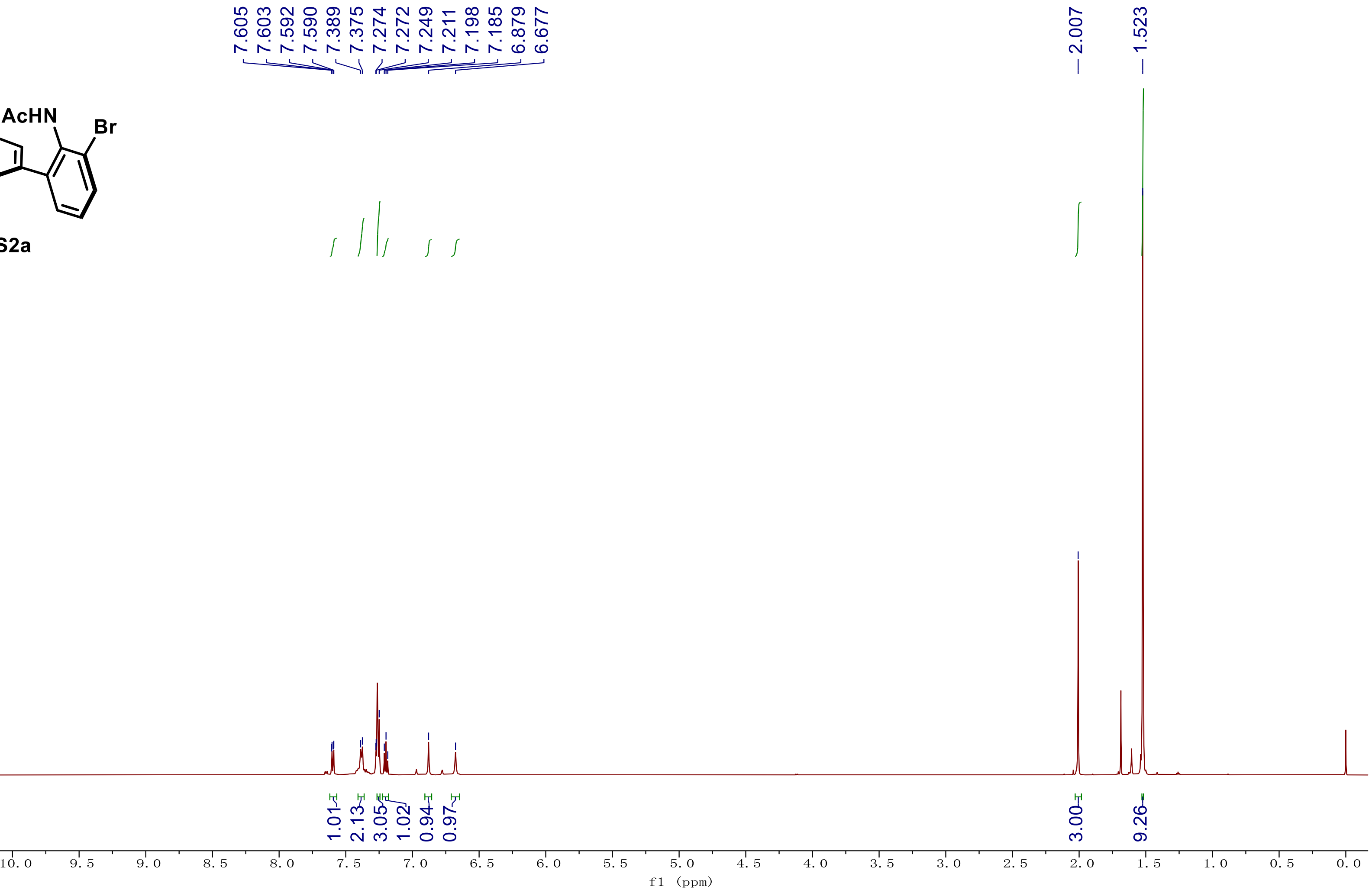

Compound S2a ^{13}C NMR (151 MHz, $CDCl_3$)

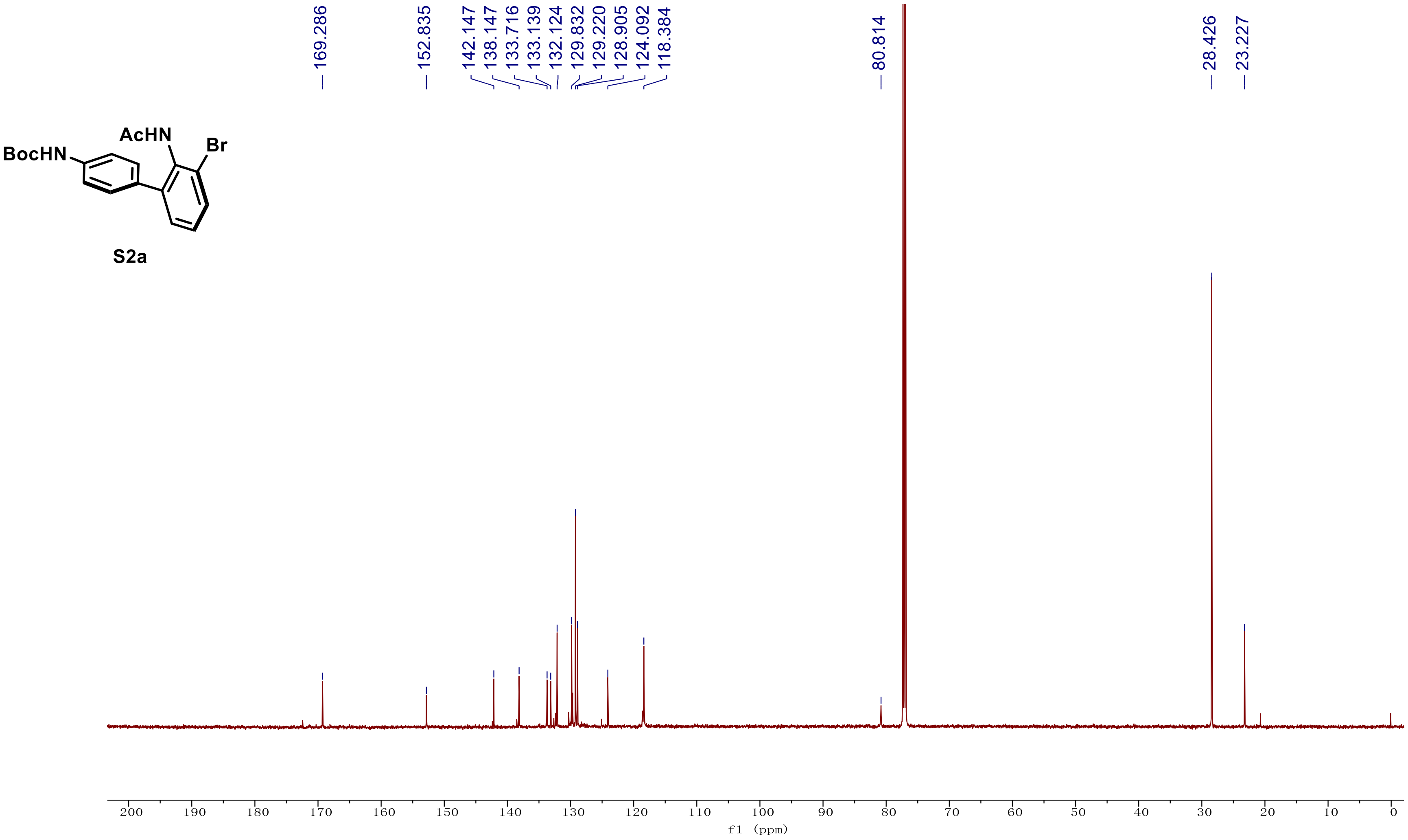

Compound 7a ^{1}H NMR (600 MHz, $CDCl_3$)

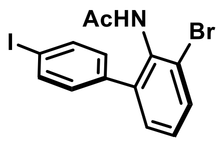

7a

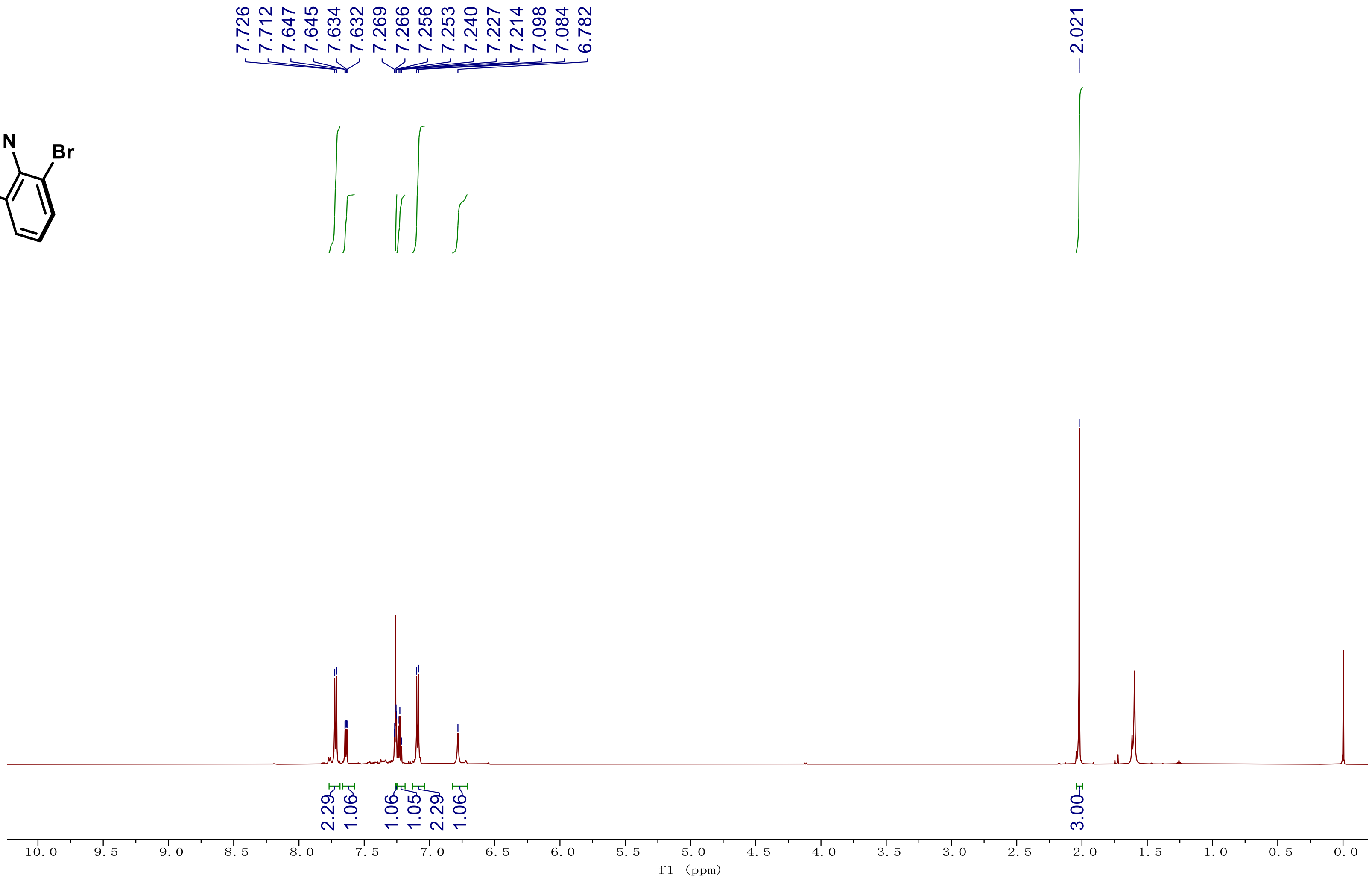

Compound 7a ^{13}C NMR (151 MHz, $CDCl_3$)

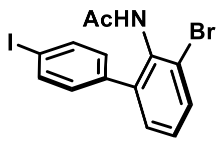

7a

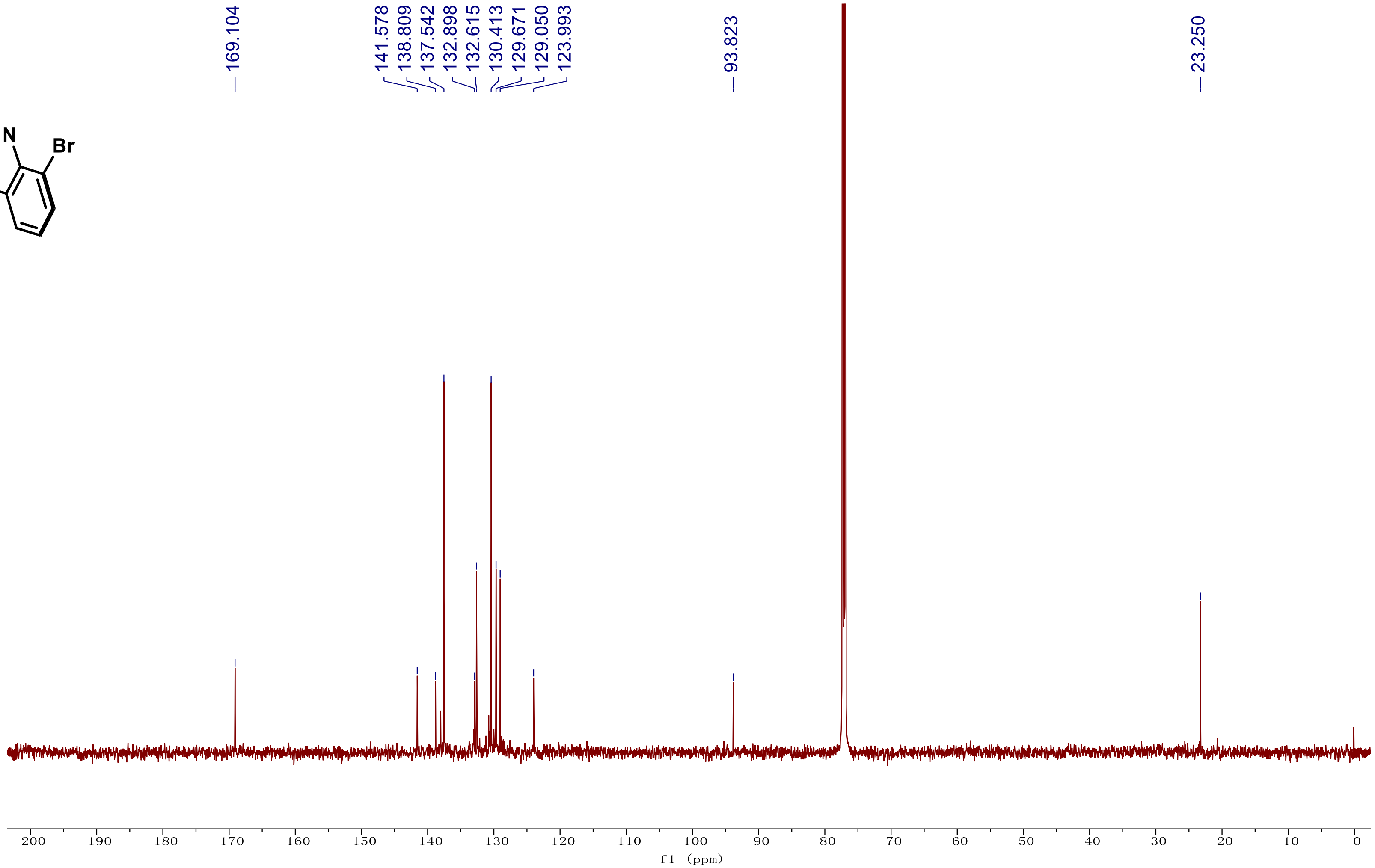

Compound S2b ^{1}H NMR (600 MHz, $CDCl_3$)

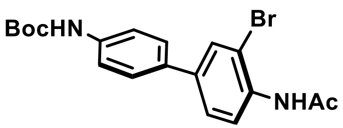

S2b

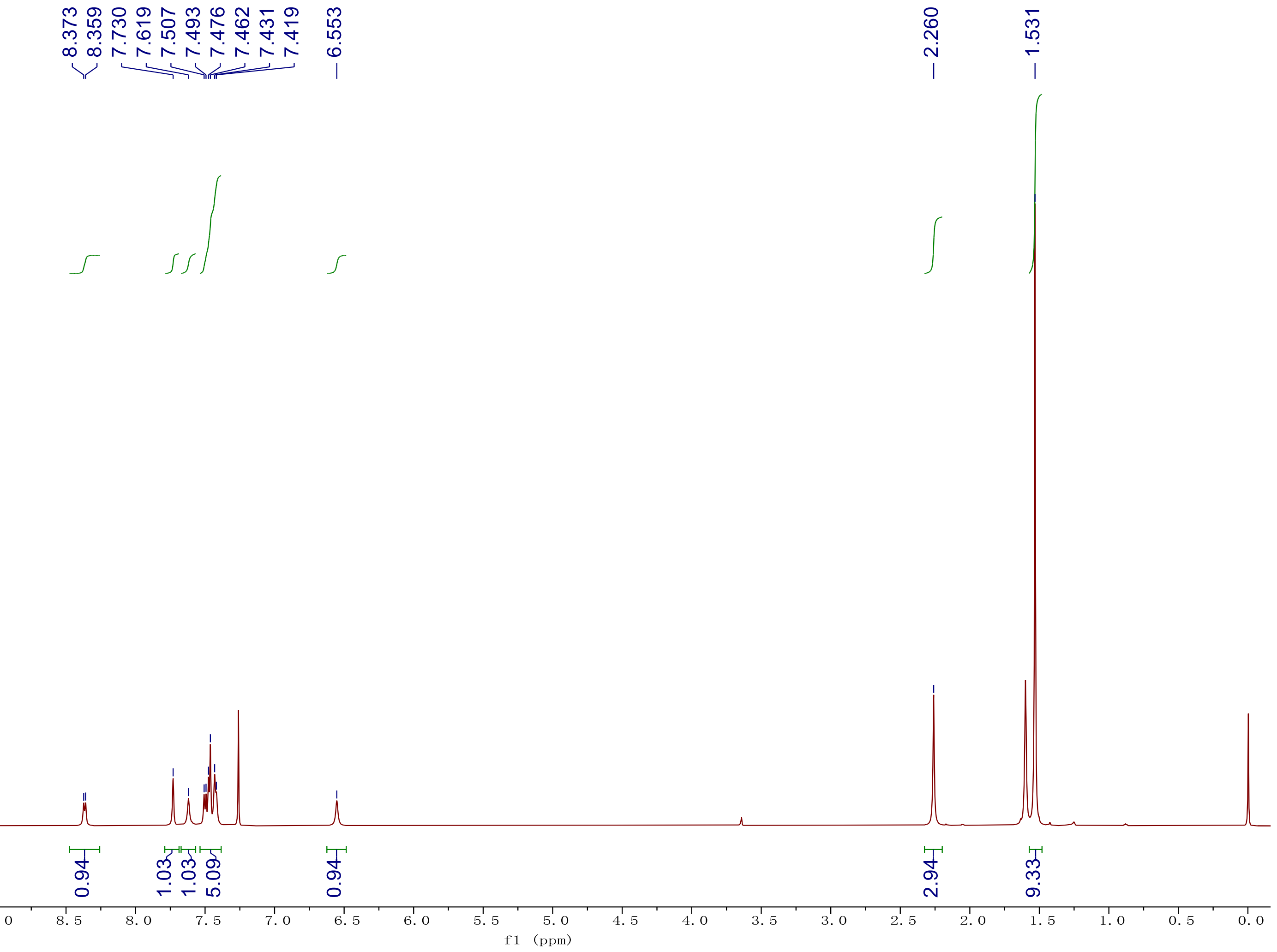

Compound S2b ^{13}C NMR (151 MHz, $CDCl_3$)

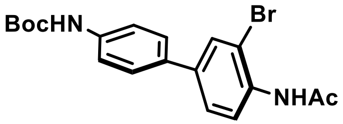
S2b

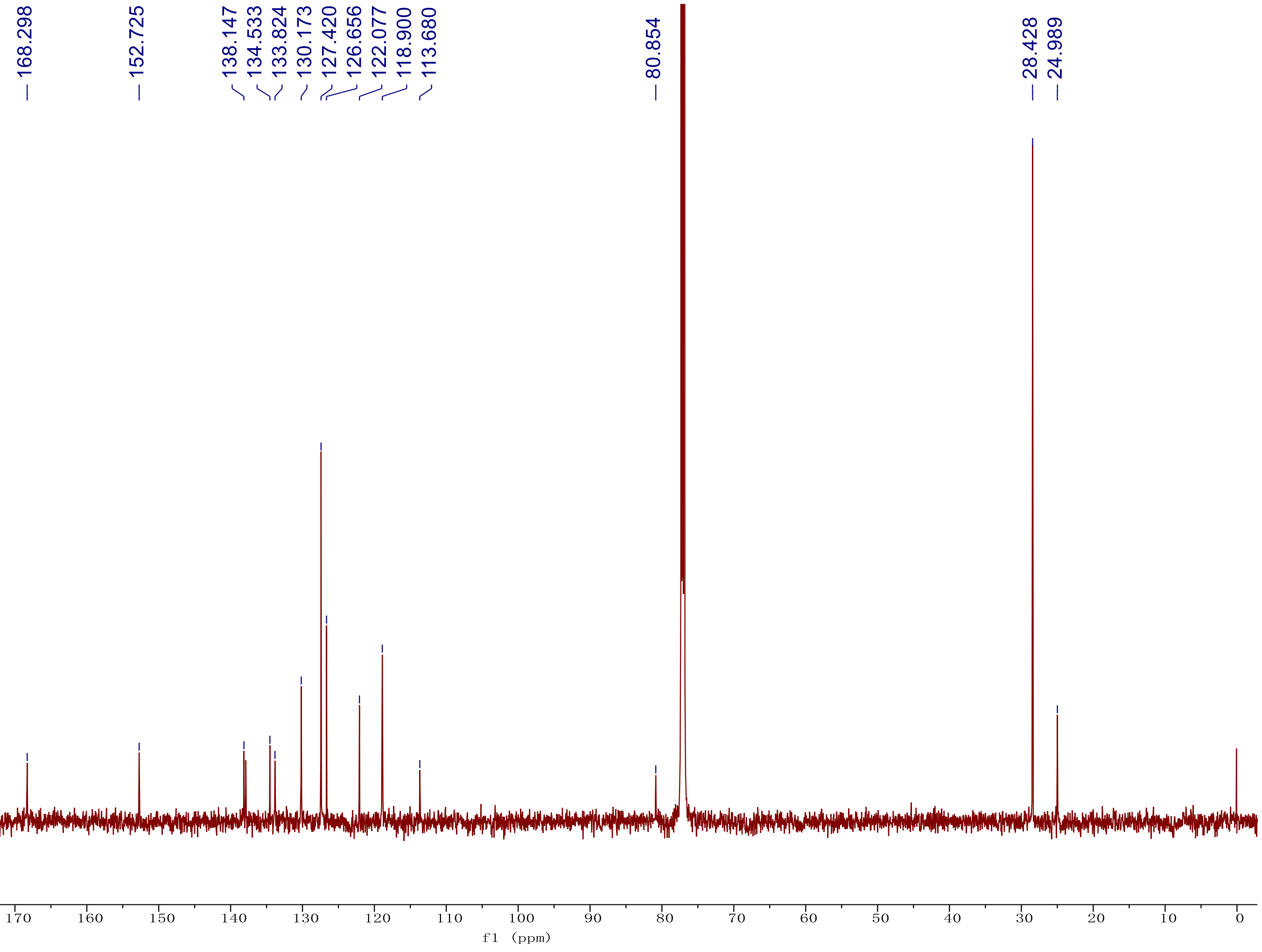

Compound 7b ¹H NMR (600 MHz, $CDCl_3$)

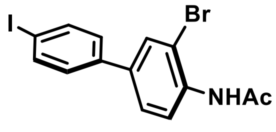

7b

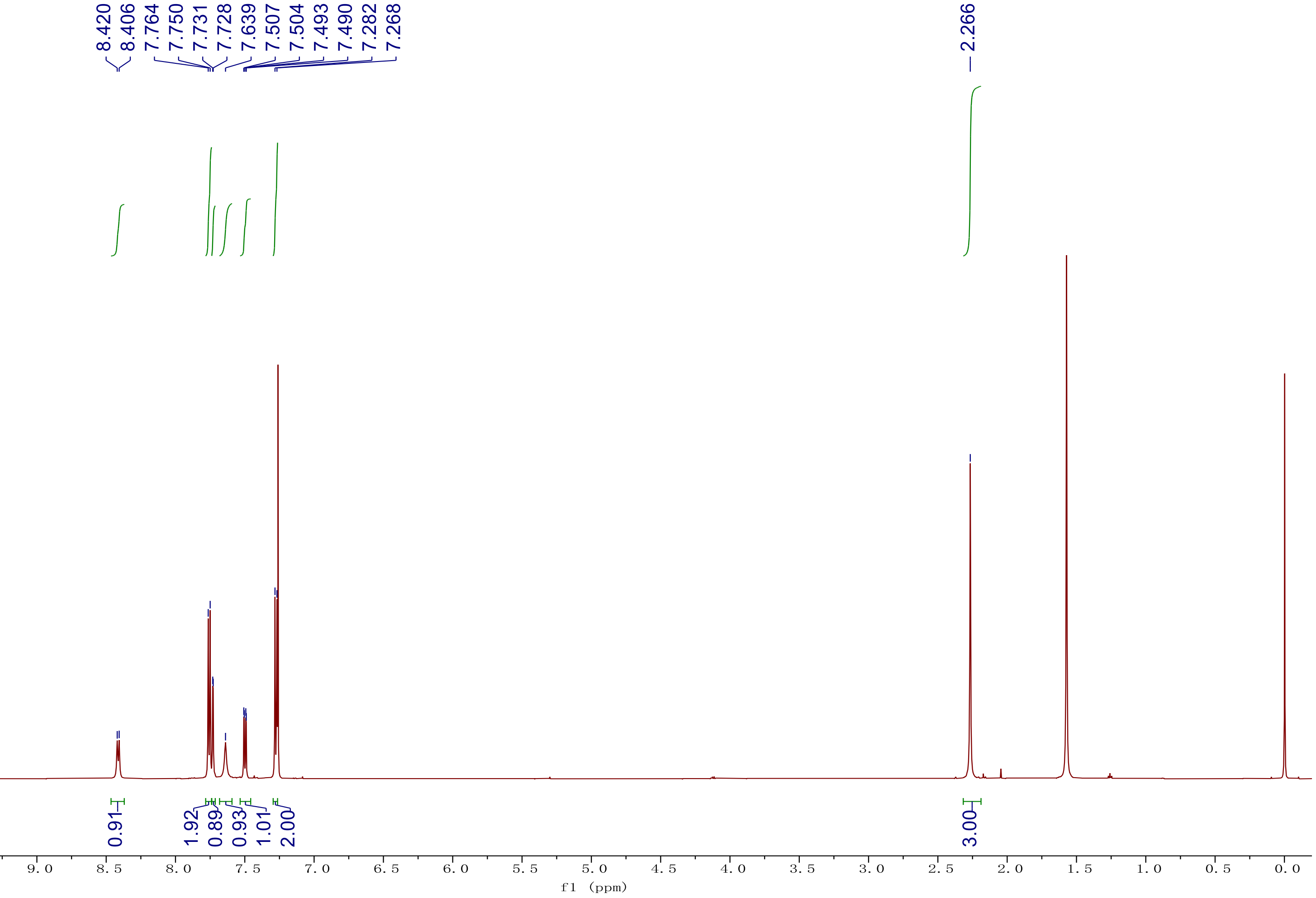

Compound 7b ^{13}C NMR (151 MHz, $CDCl_3$)

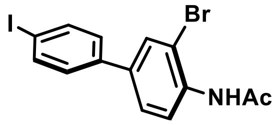

7b

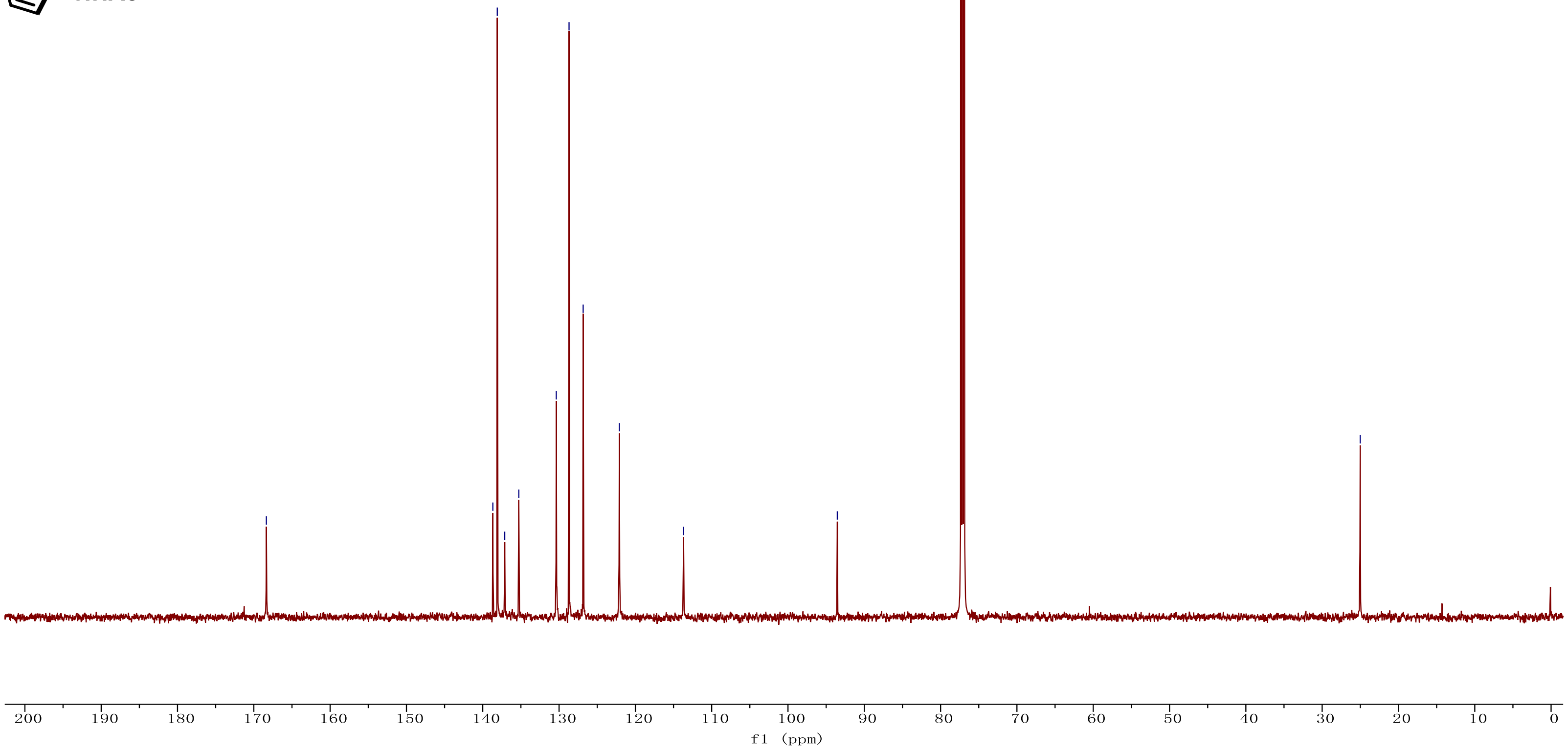

Compound S2c 1H NMR (600 MHz, $CDCl_3$)

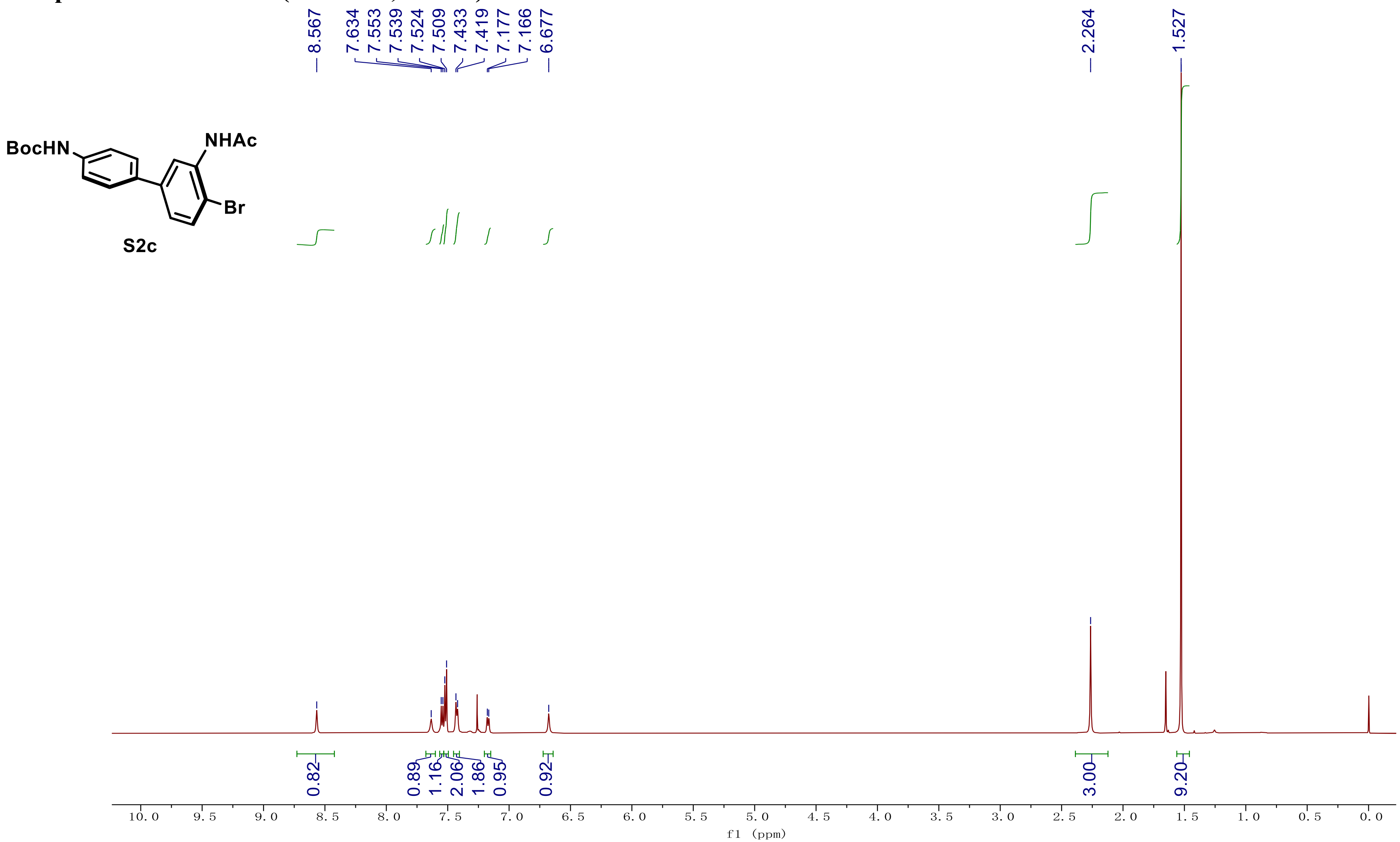

Compound S2c ^{13}C NMR (151 MHz, CDCl$_3$)

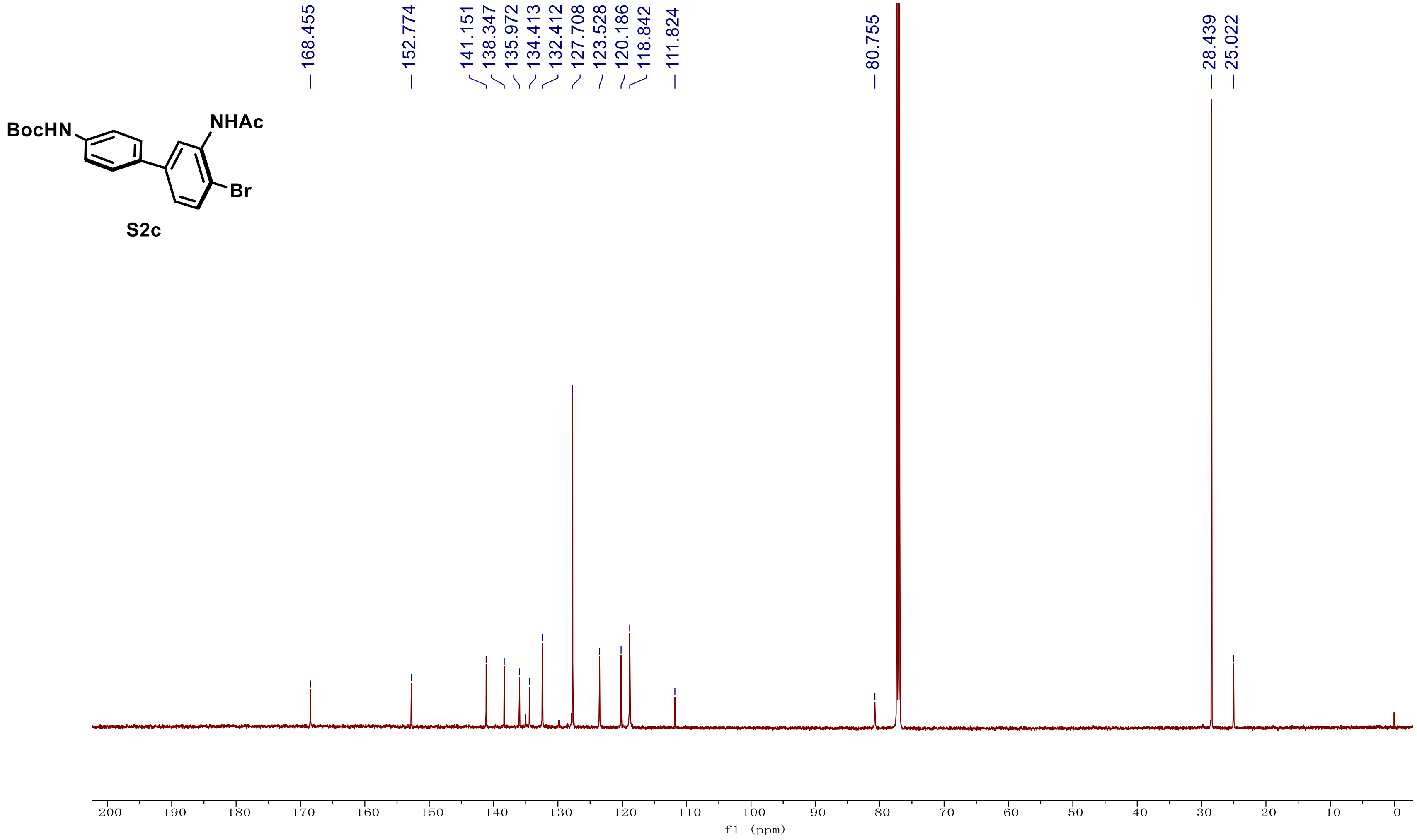

Compound 7c ^{1}H NMR (600 MHz, $CDCl_3$)

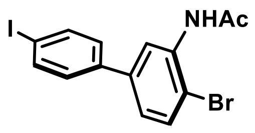

7c

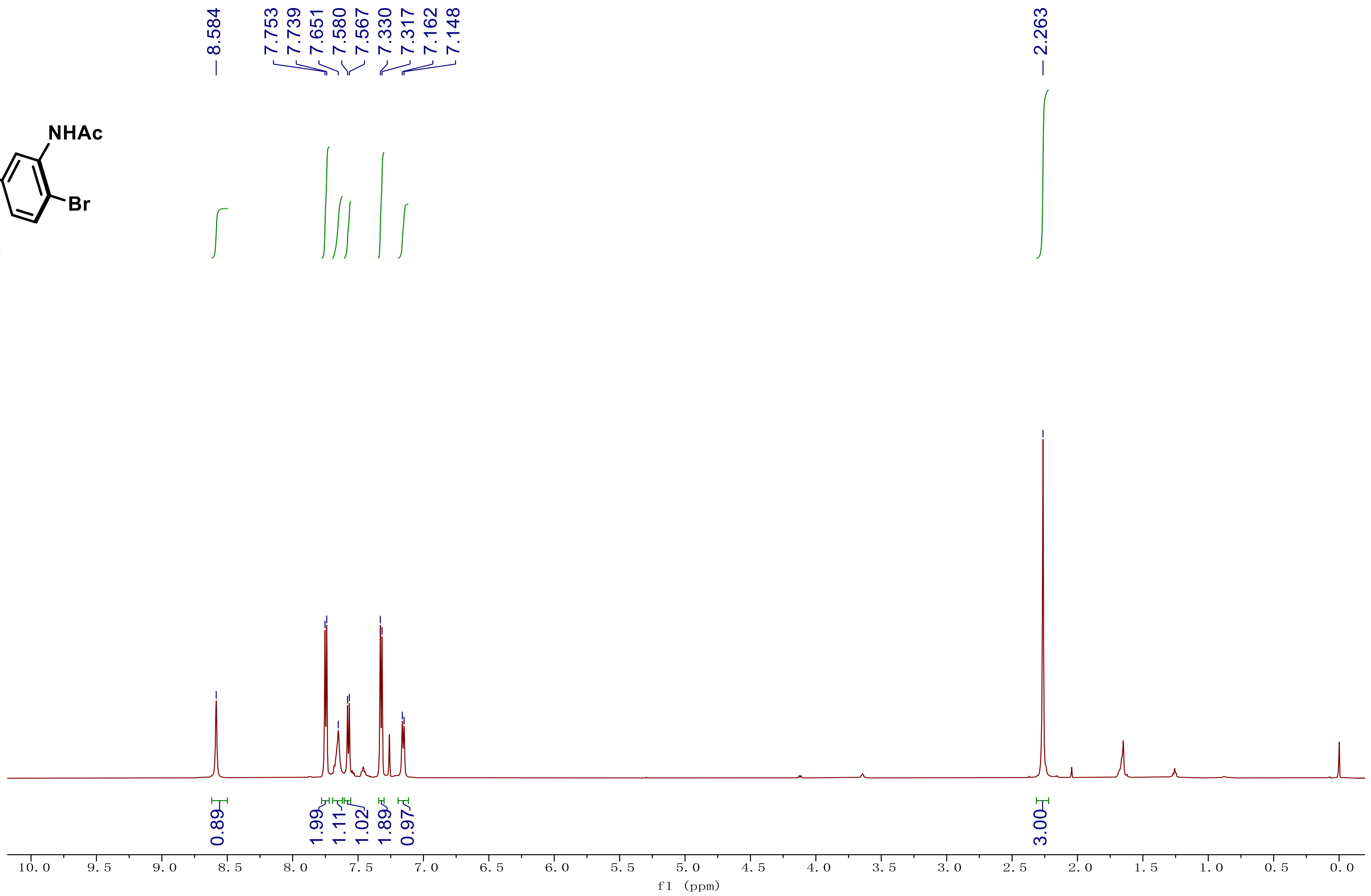

Compound 7c ^{13}C NMR (151 MHz, $CDCl_3$)

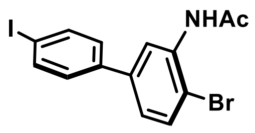

7c

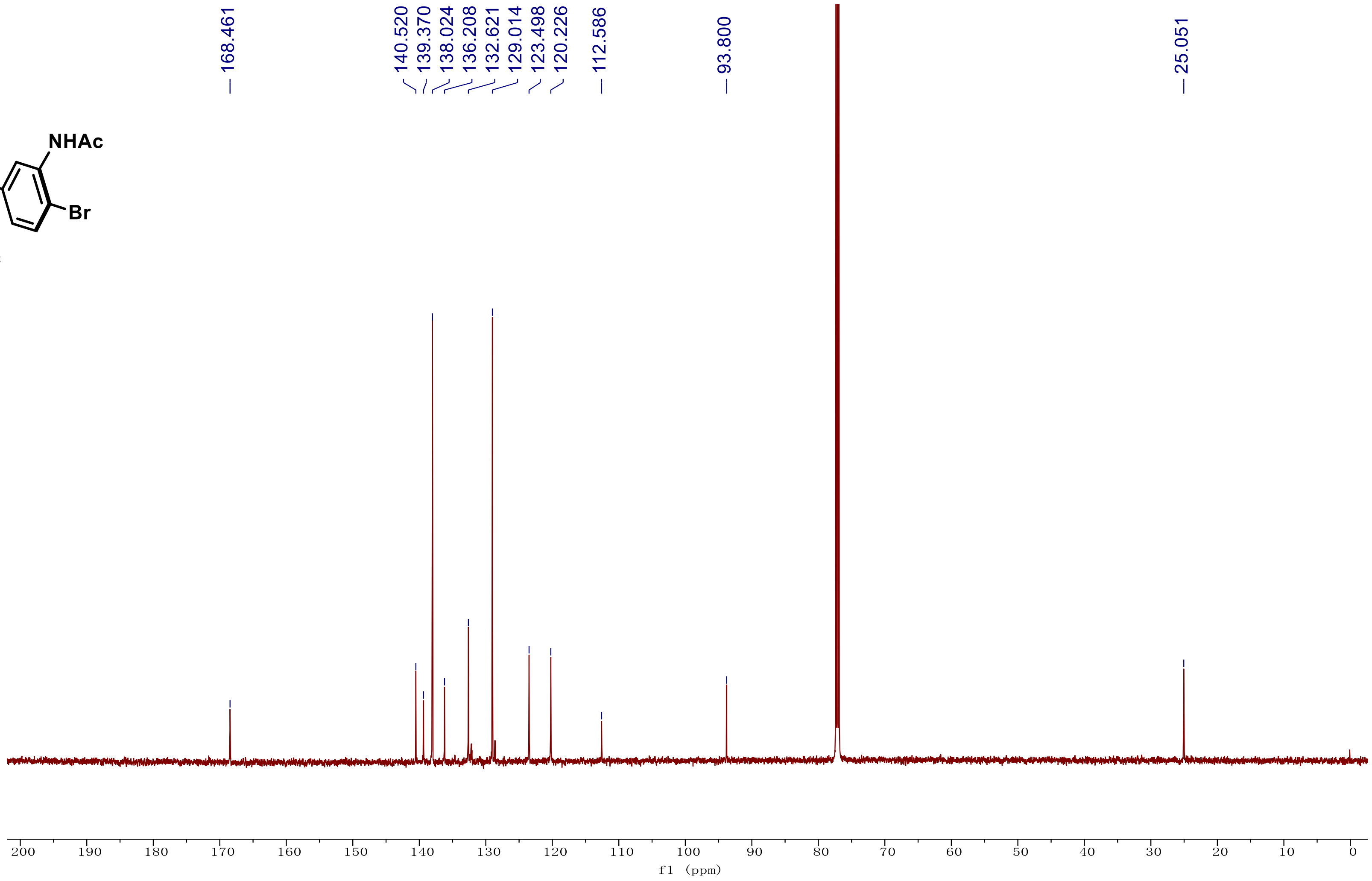

Compound 9 ^{1}H NMR (600 MHz, $CDCl_3$)

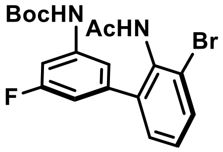

9

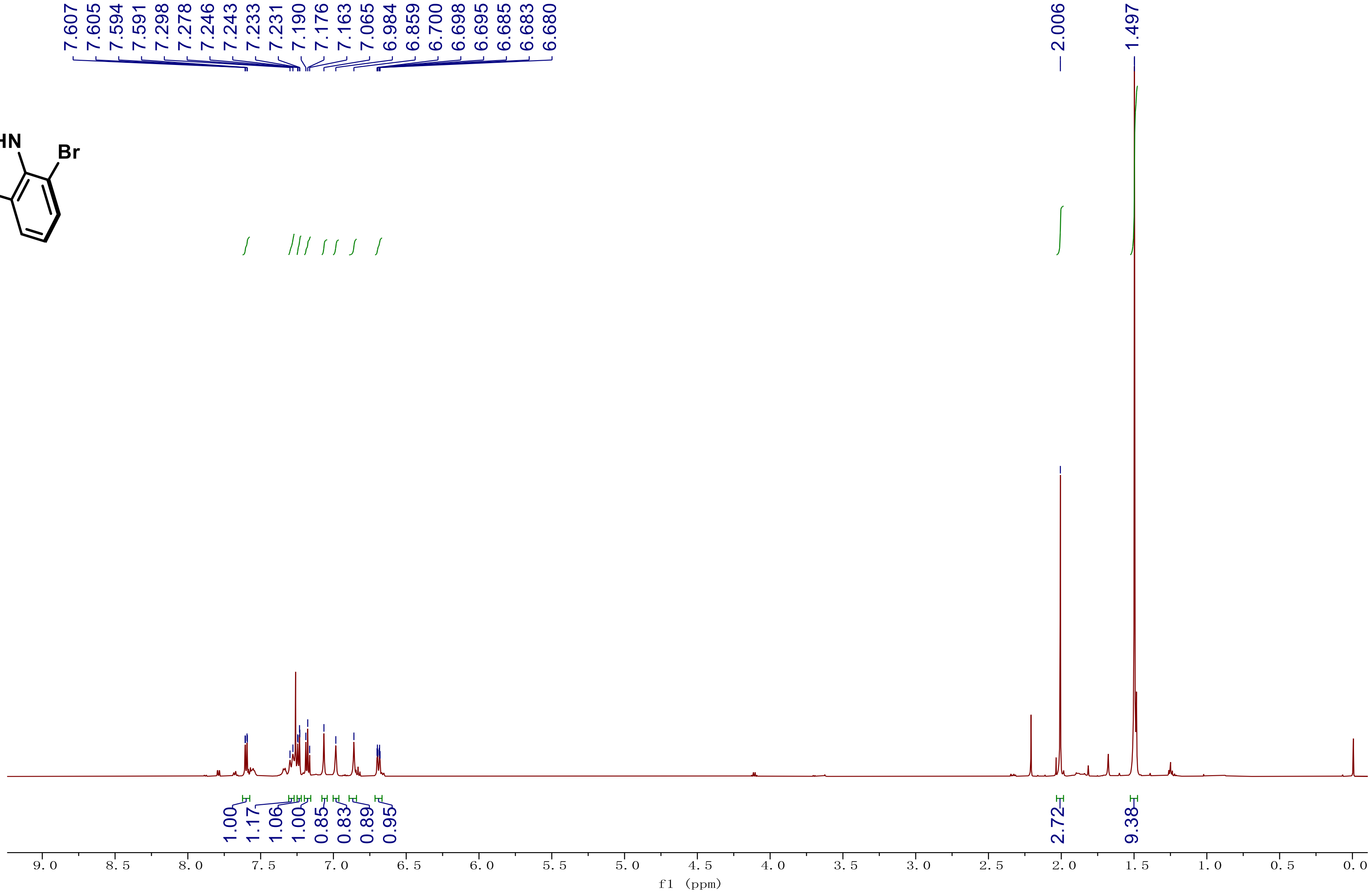

Compound 9 ^{13}C NMR (151 MHz, $CDCl_3$)

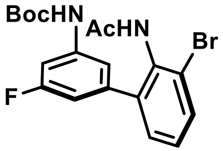

9

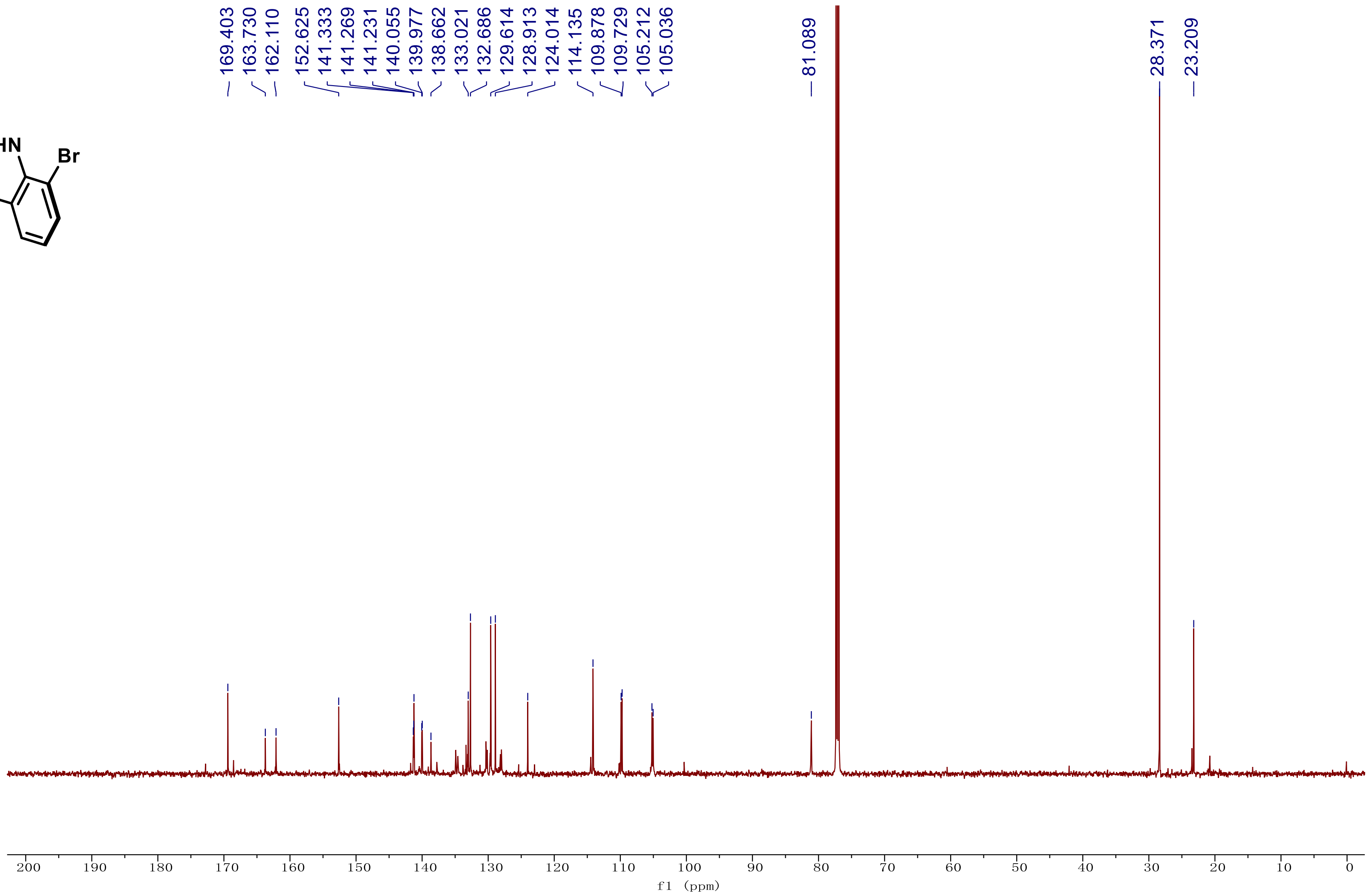

Compound 9 ^{19}F NMR (565 MHz, $CDCl_3$)

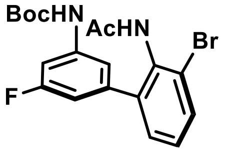

9

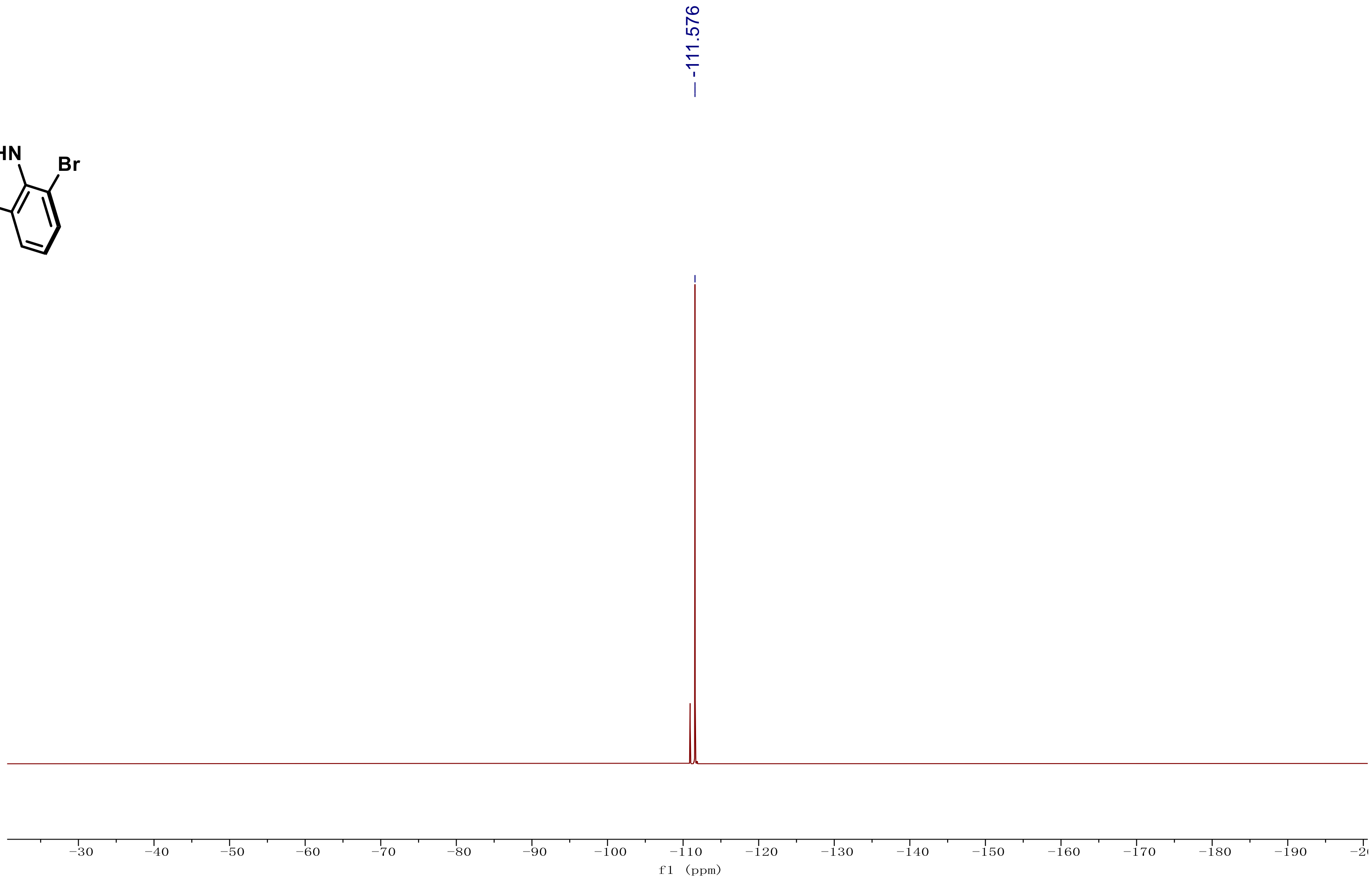

Compound 7d ^{1}H NMR (600 MHz, CDCl$_3$)

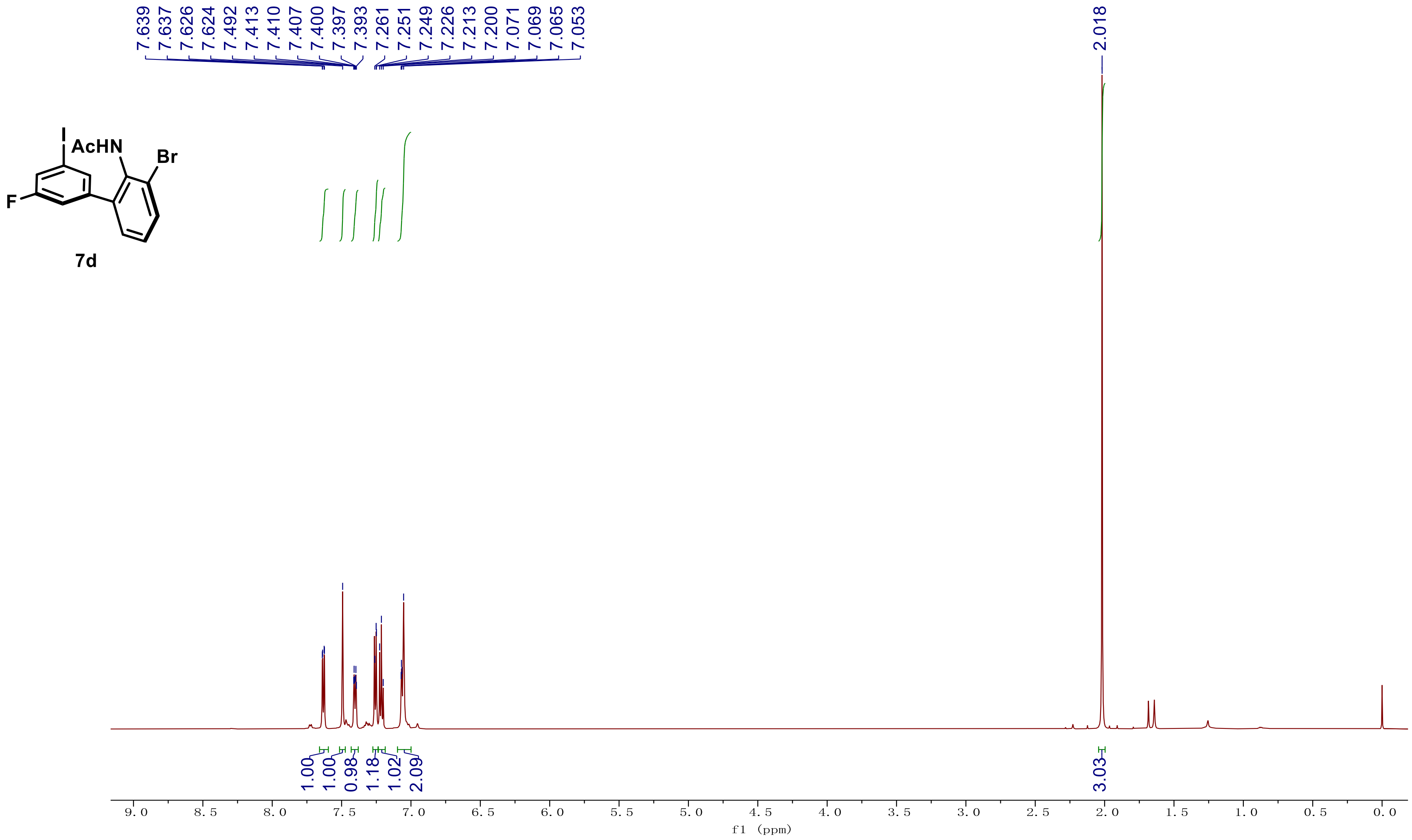

Compound 7d ^{13}C NMR (151 MHz, $CDCl_3$)

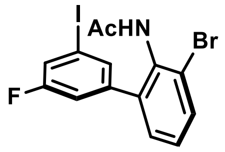

7d

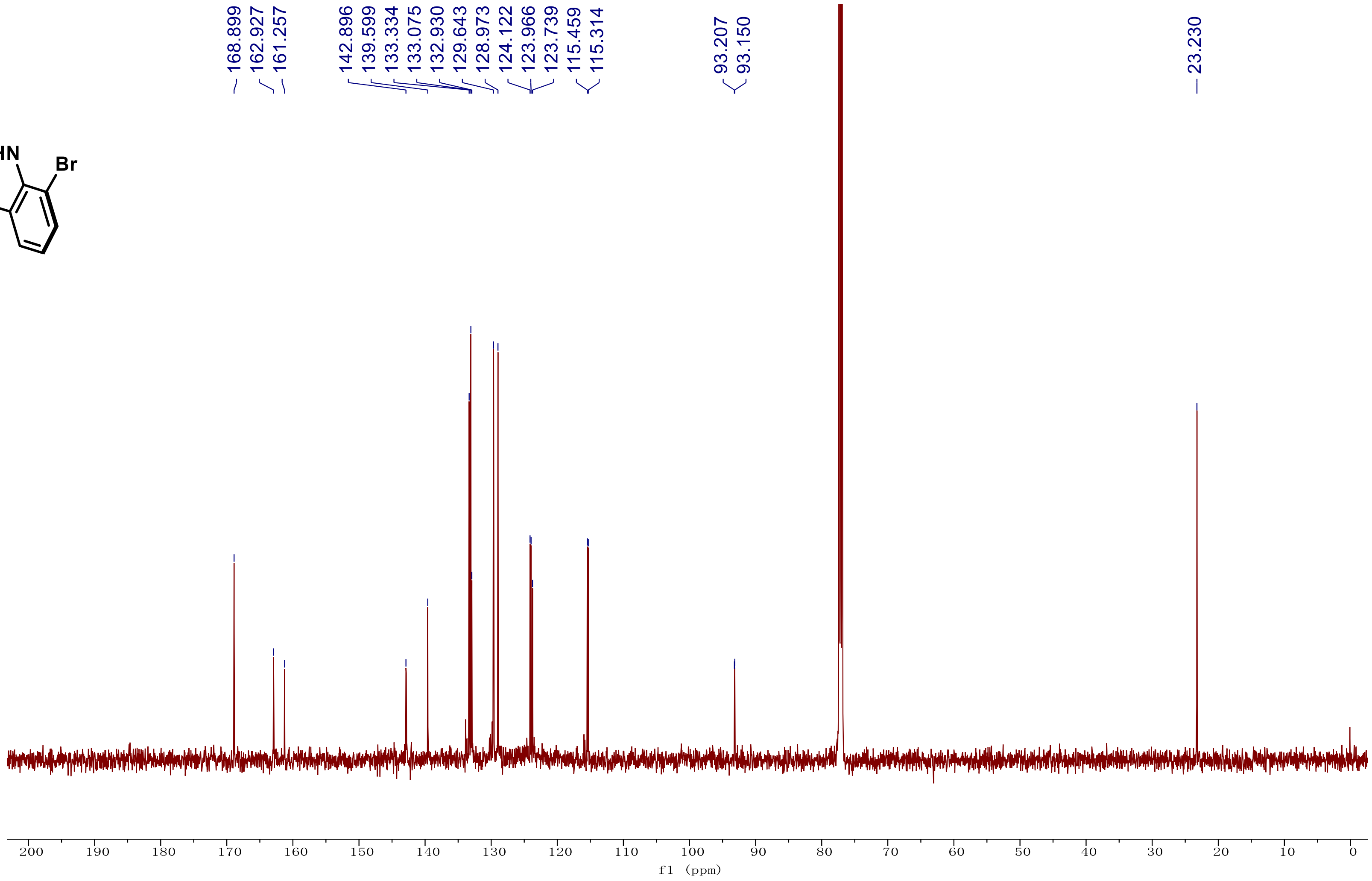

Compound 7d ^{19}F NMR (565 MHz, $CDCl_3$)

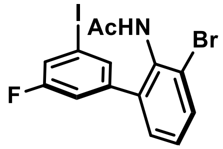

7d

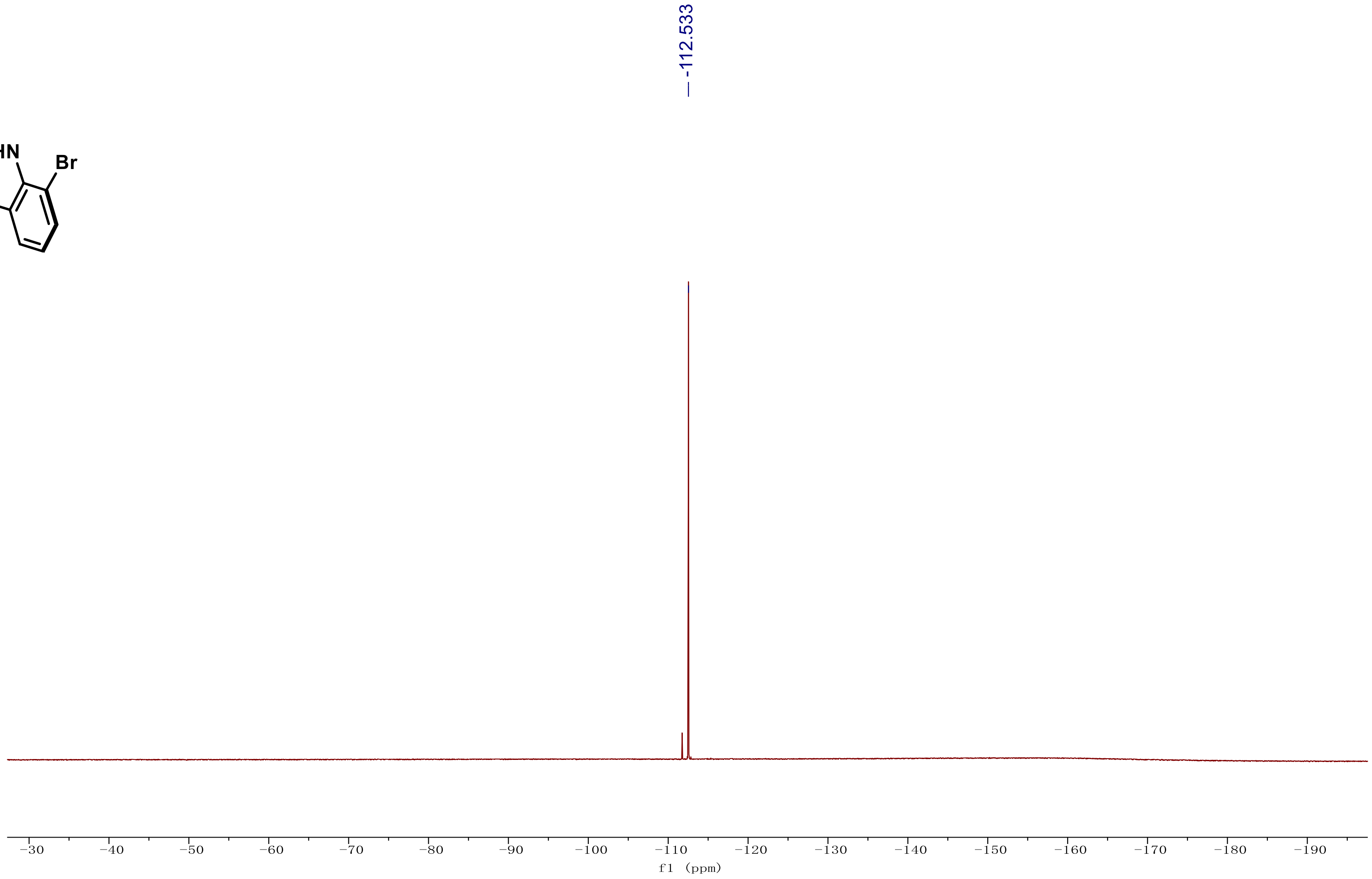

Compound 11a ¹H NMR (600 MHz, CDCl₃)

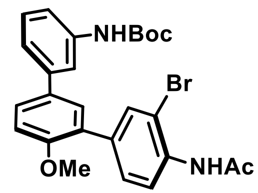

11a

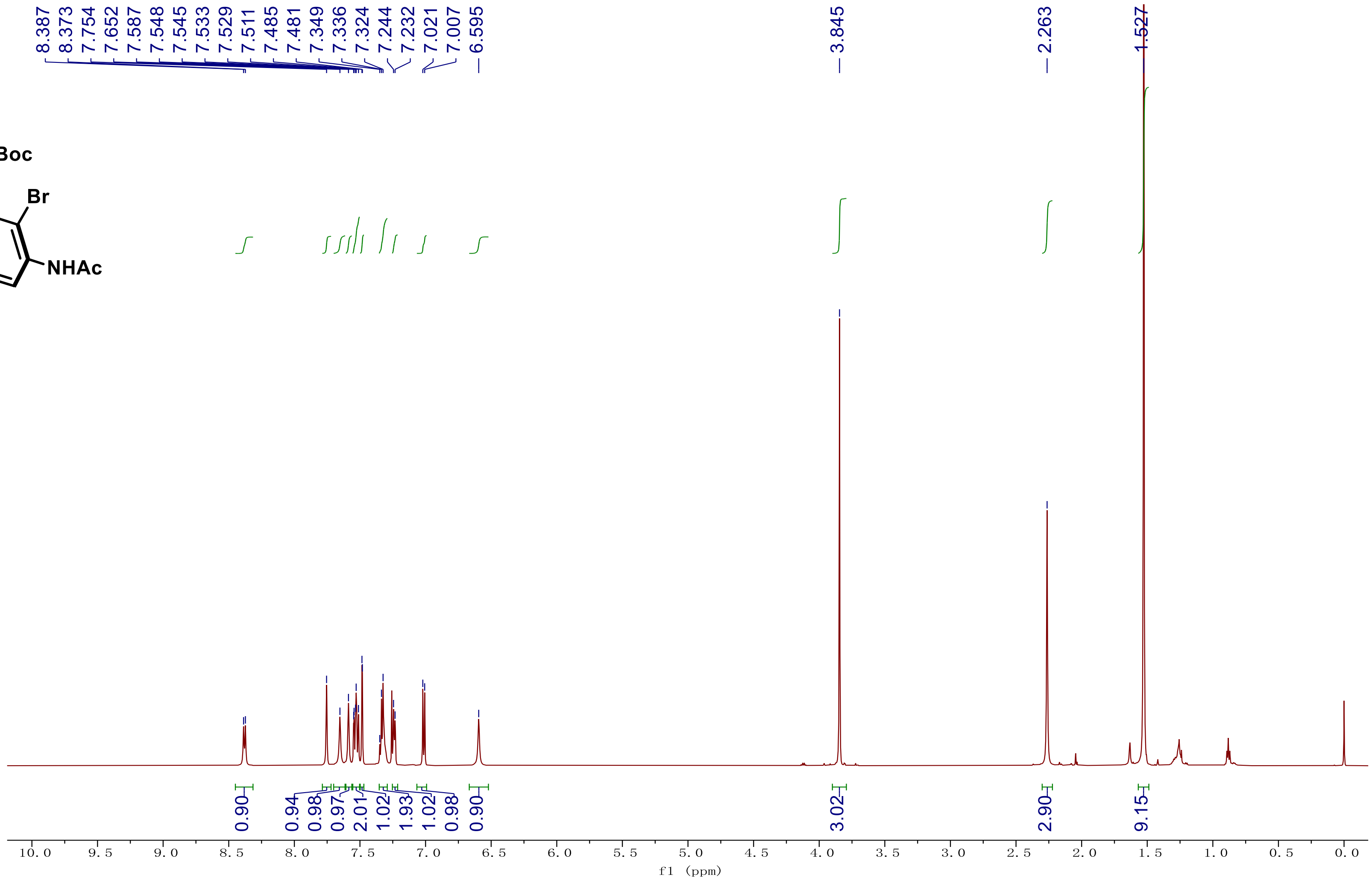

Compound 11a ^{13}C NMR (151 MHz, $CDCl_3$)

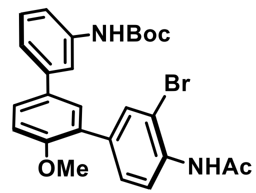

11a

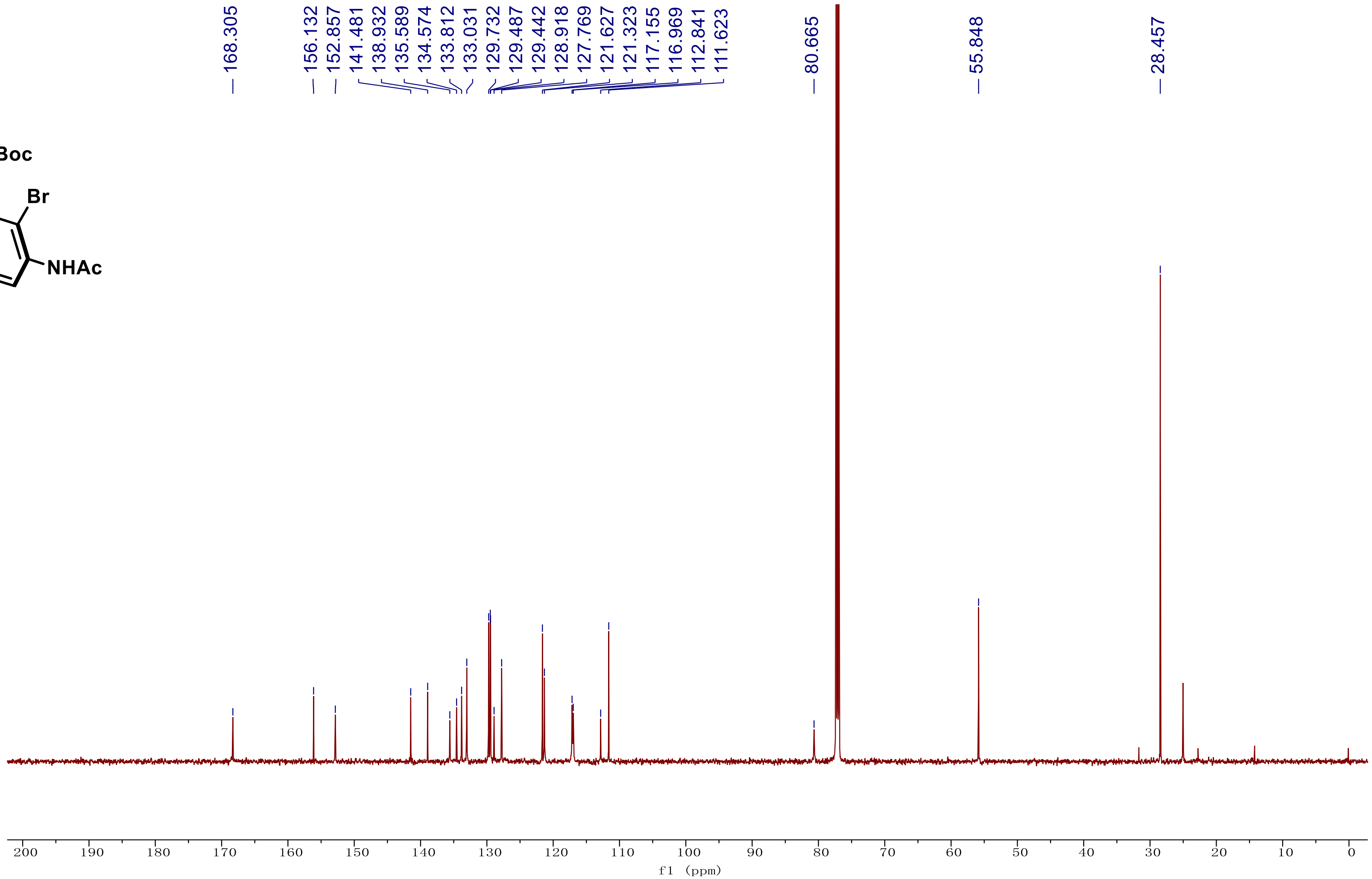

Compound 12a ¹H NMR (600 MHz, $CDCl_3$)

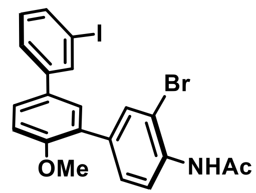

12a

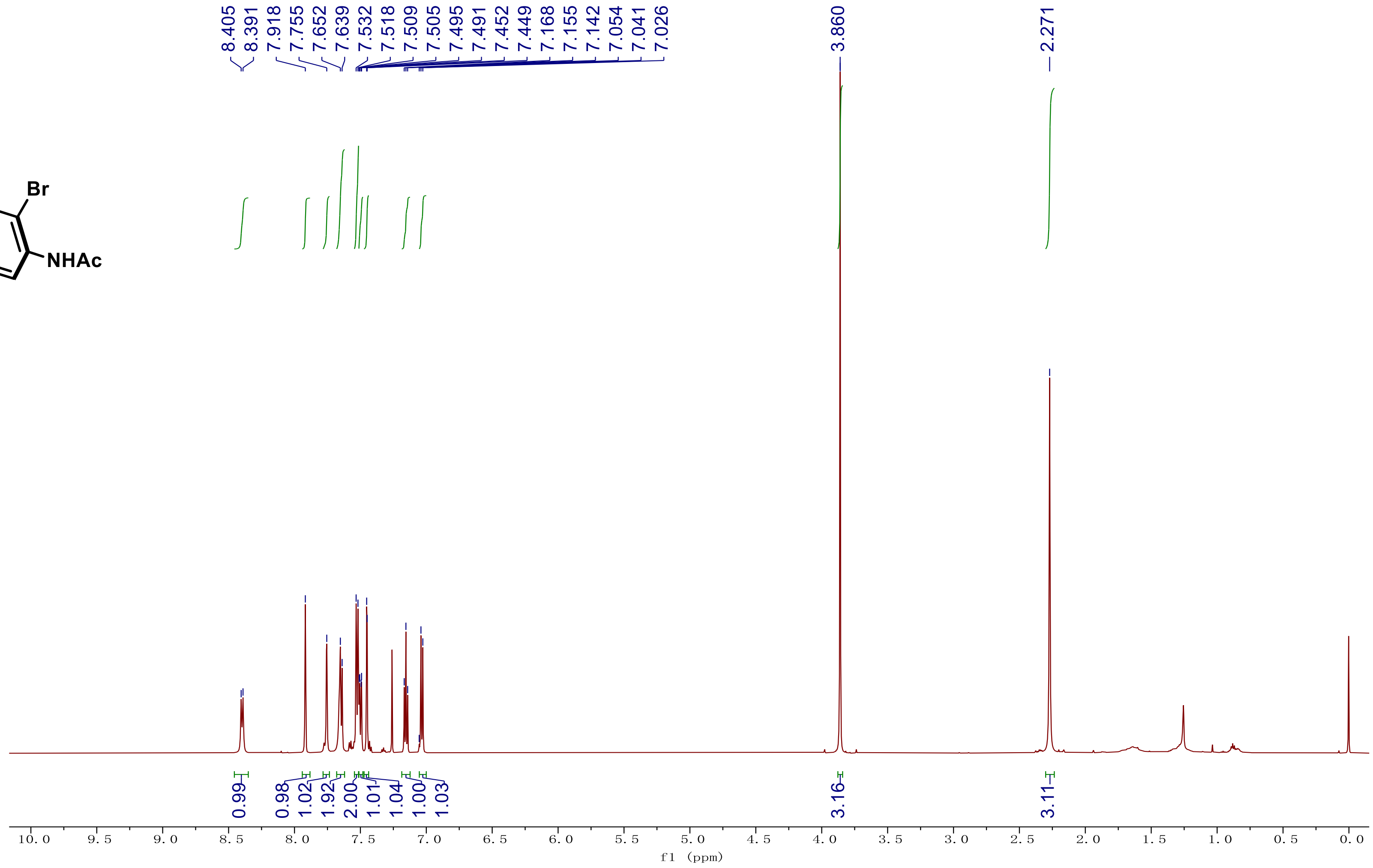

Compound 12a ^{13}C NMR (151 MHz, CDCl$_3$)

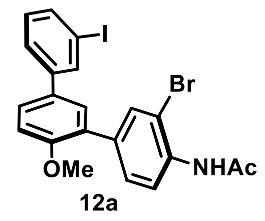

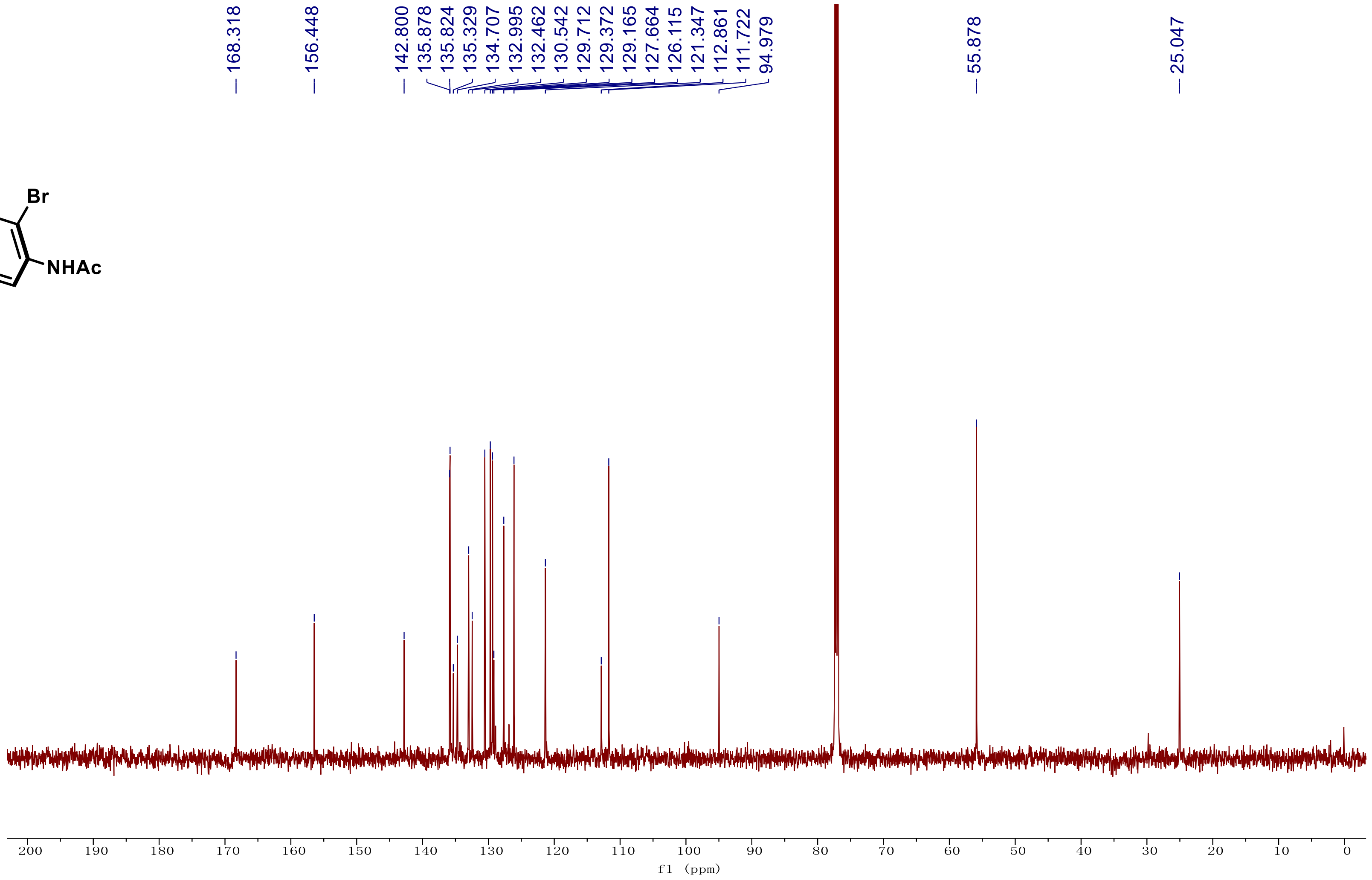

Compound 11b ^{1}H NMR (600 MHz, $CDCl_3$)

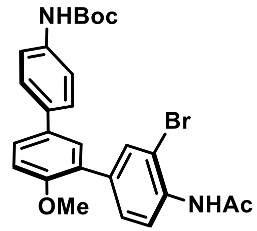

11b

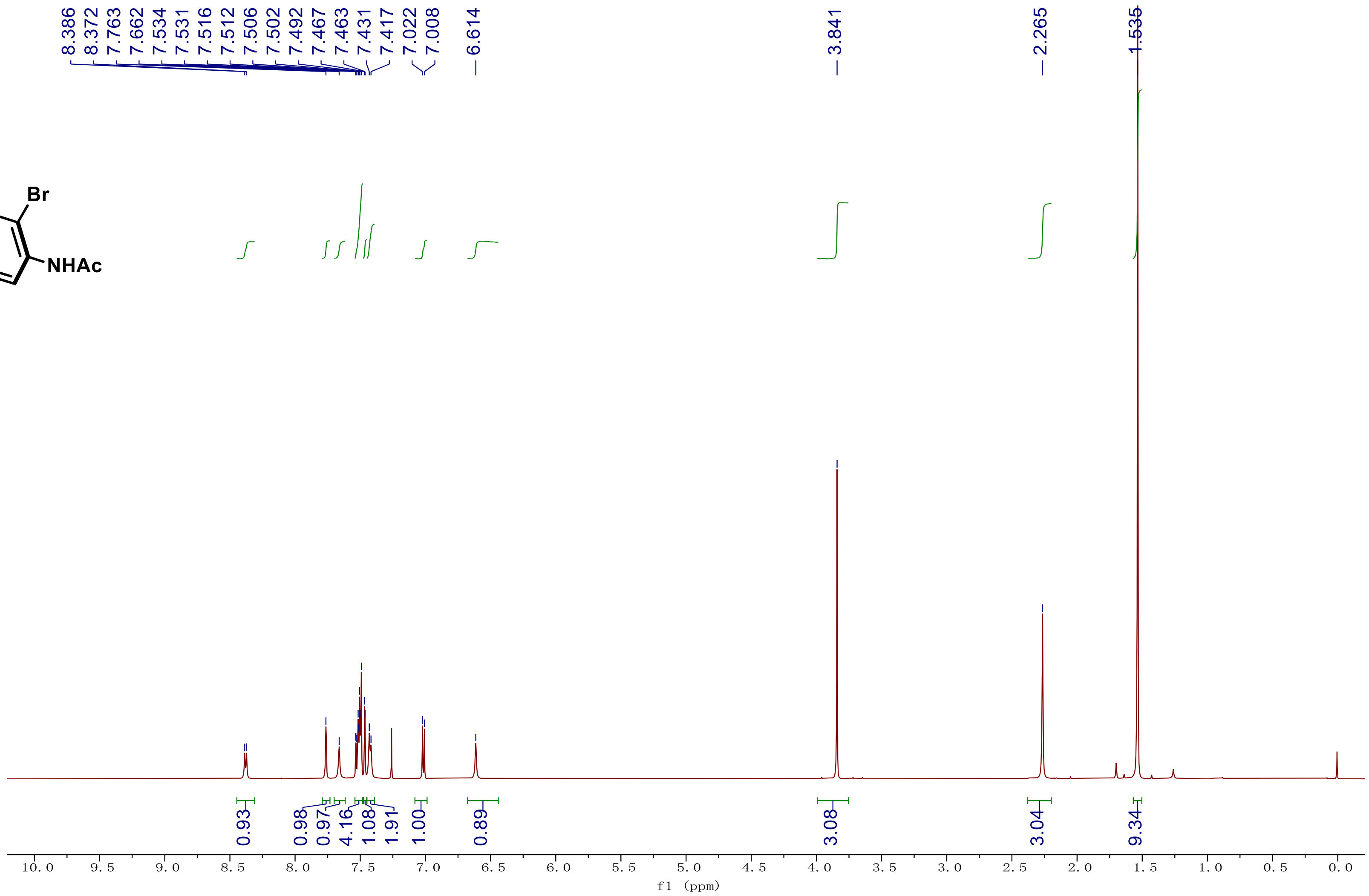

Compound 11b ^{13}C NMR (151 MHz, CDCl$_3$)

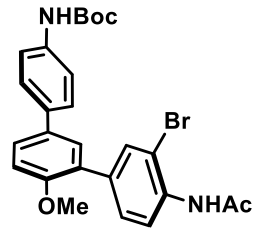

11b

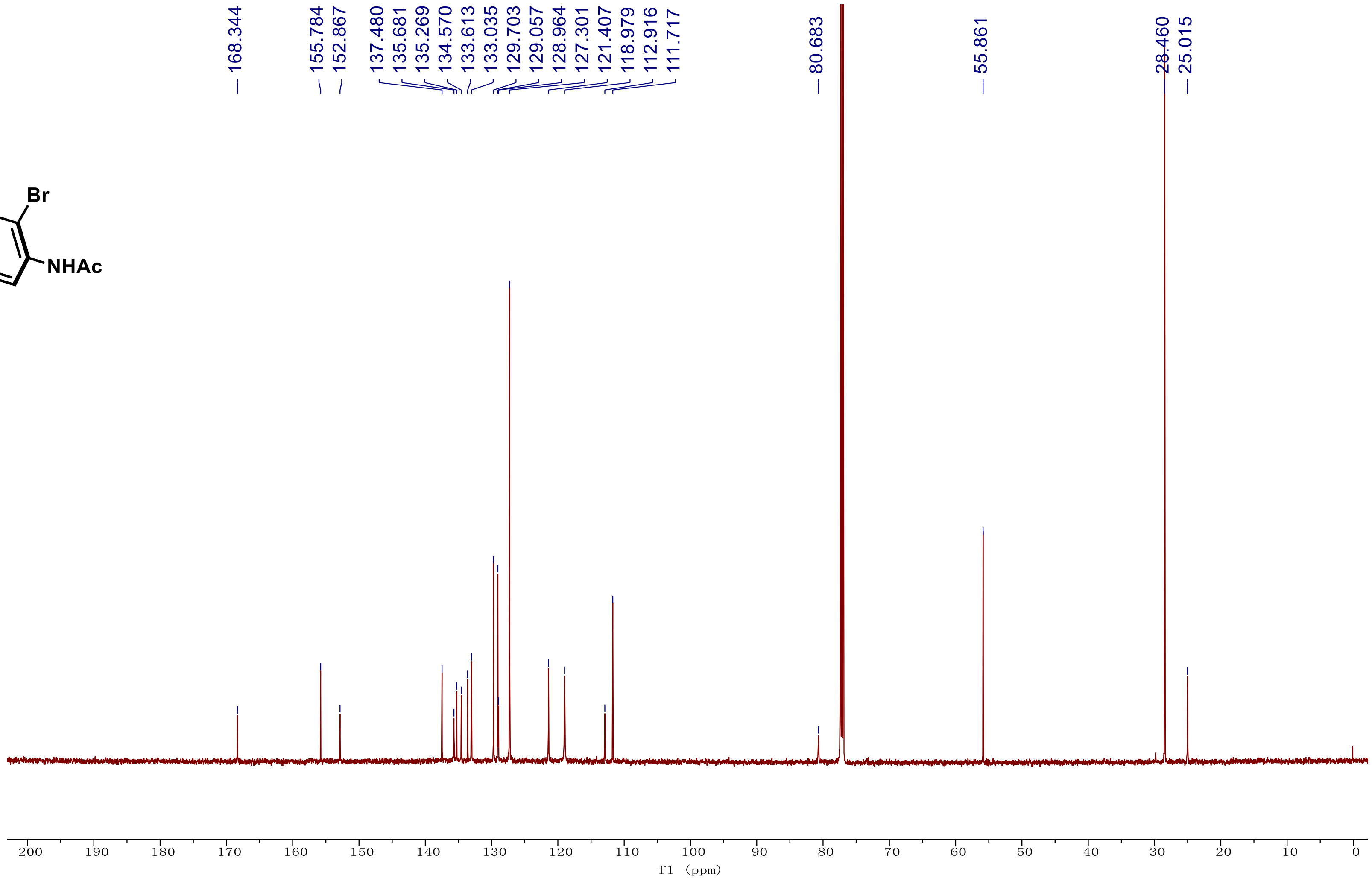

Compound 12b ^{1}H NMR (600 MHz, CDCl₃)

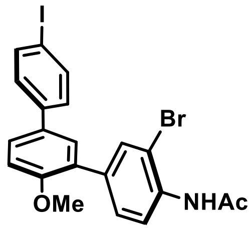

12b

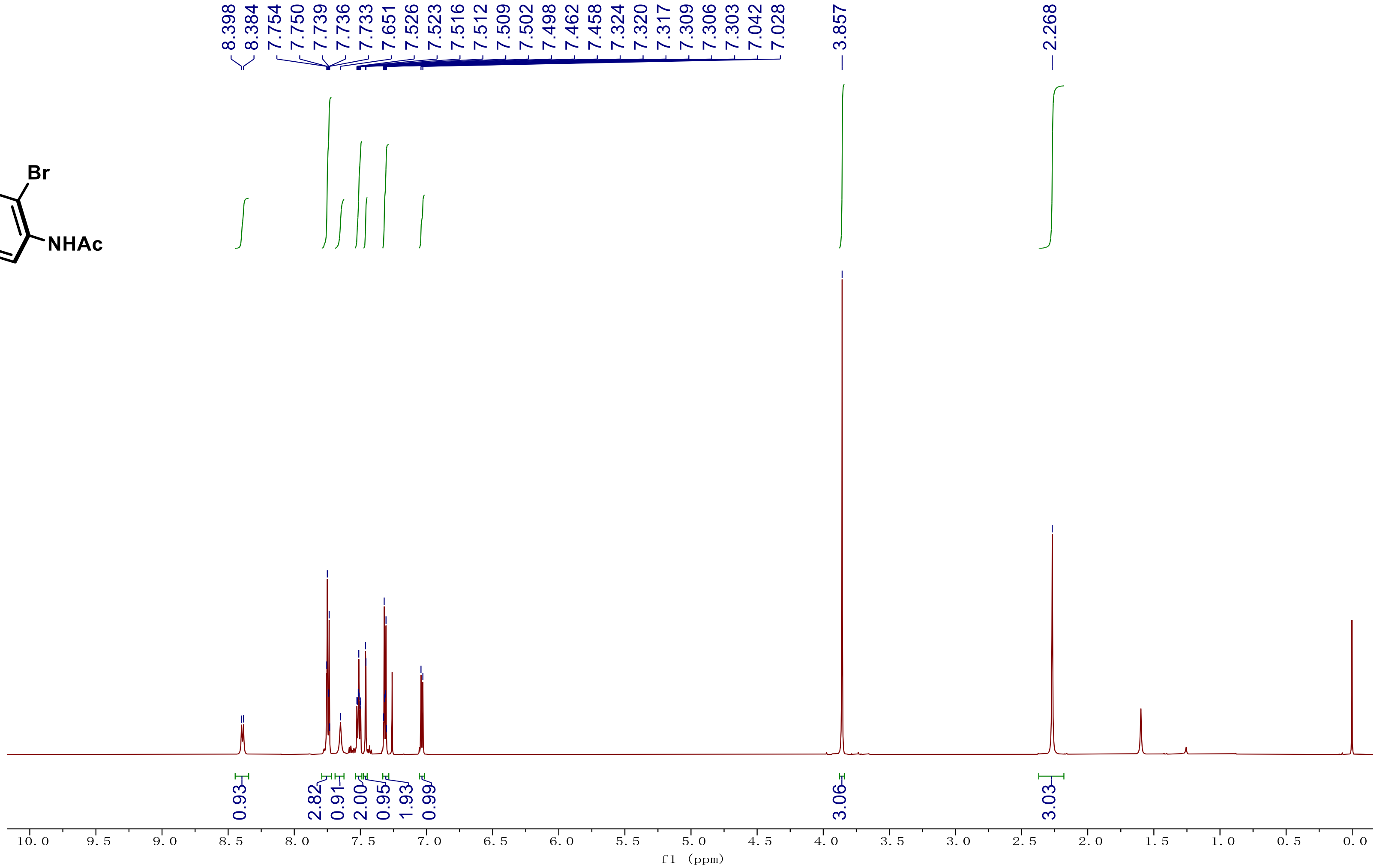

Compound 12b ^{13}C NMR (151 MHz, $CDCl_3$)

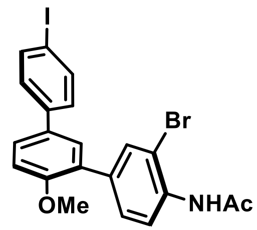

12b

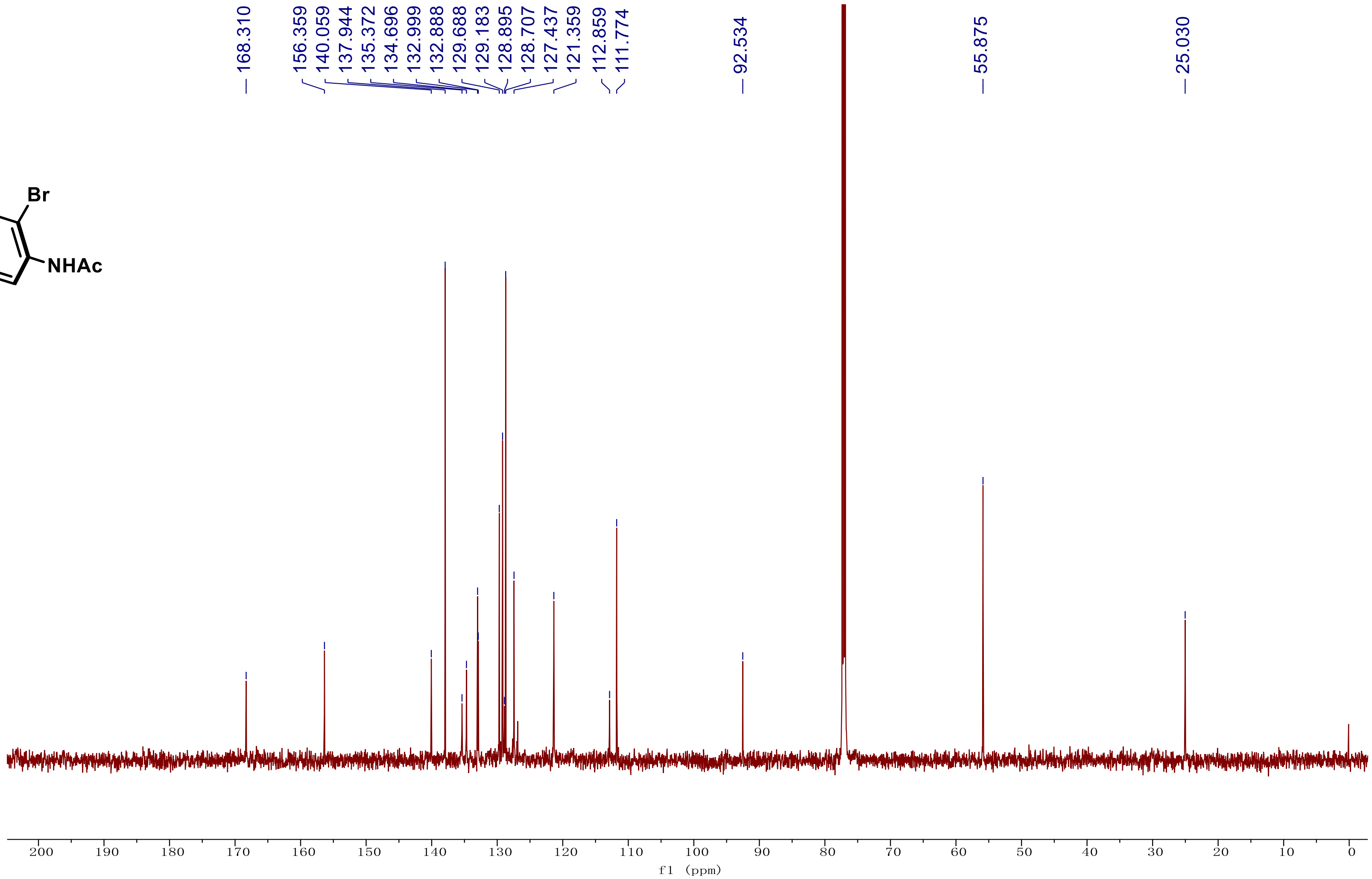

Compound 34a ^{1}H NMR (600 MHz, $CDCl_3$)

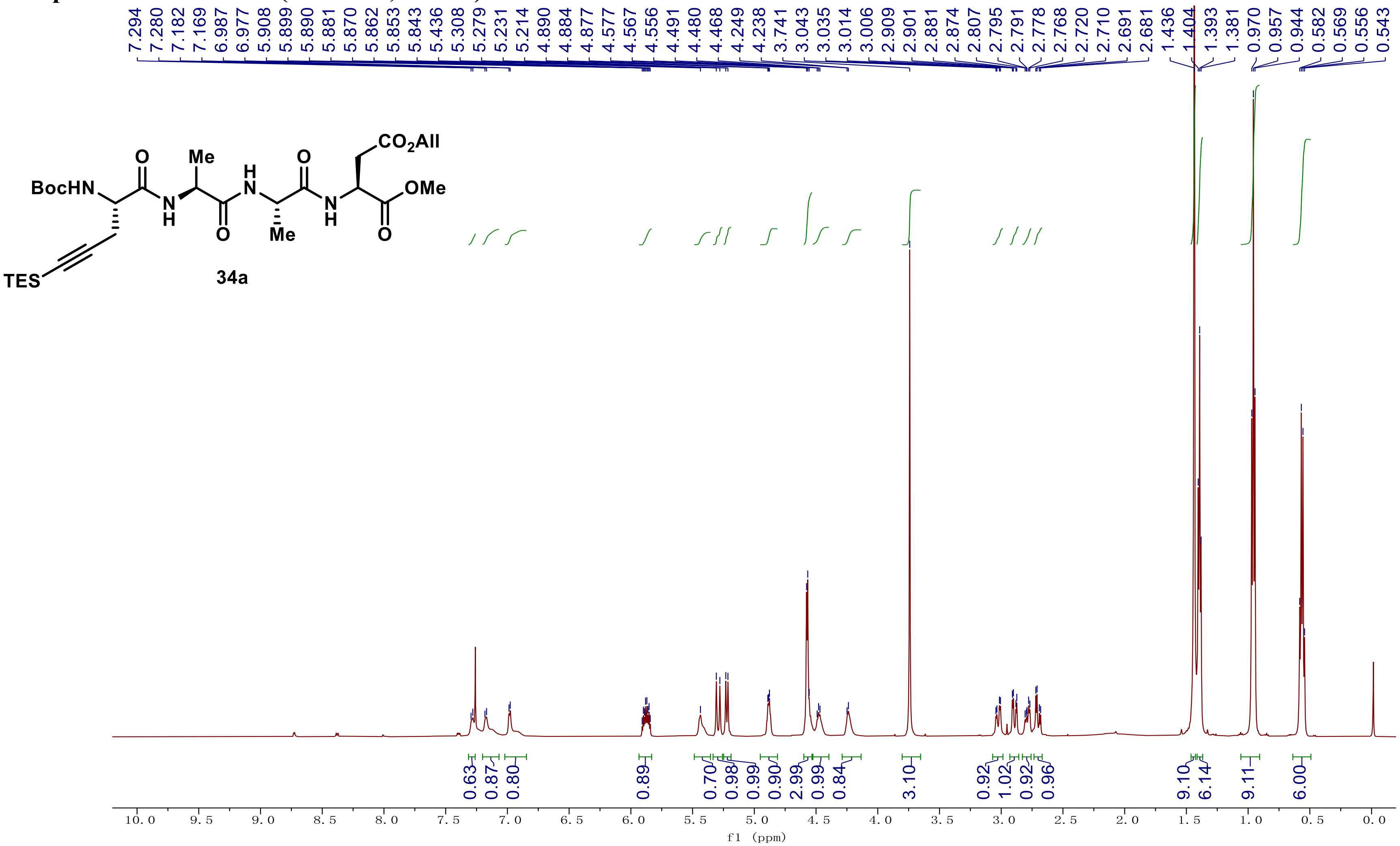

Compound 34a ^{13}C NMR (151 MHz, $CDCl_3$)

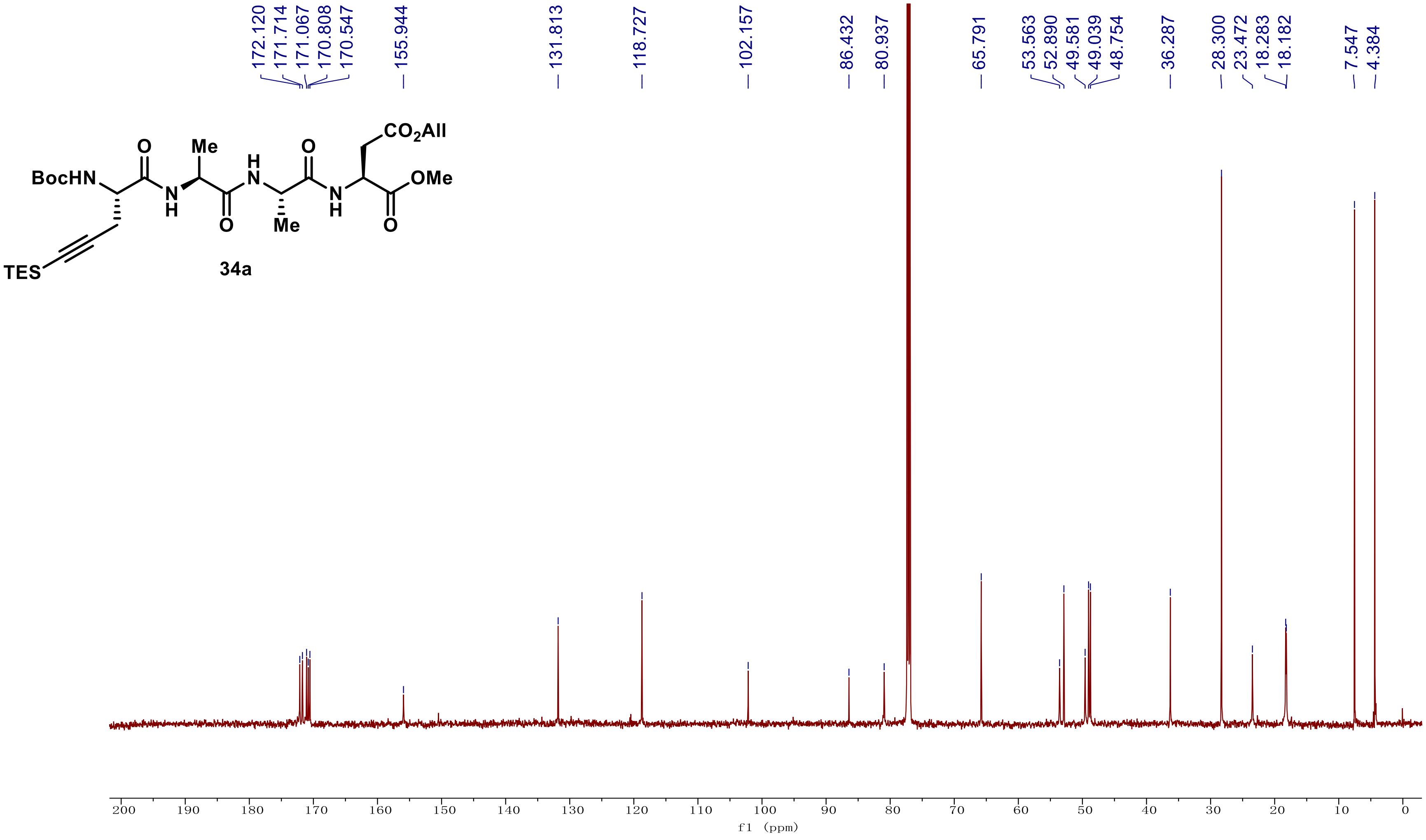

Compound 34b ^{1}H NMR (600 MHz, CDCl$_3$)

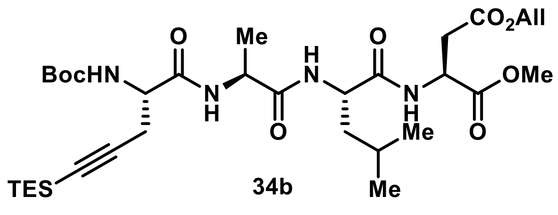

34b

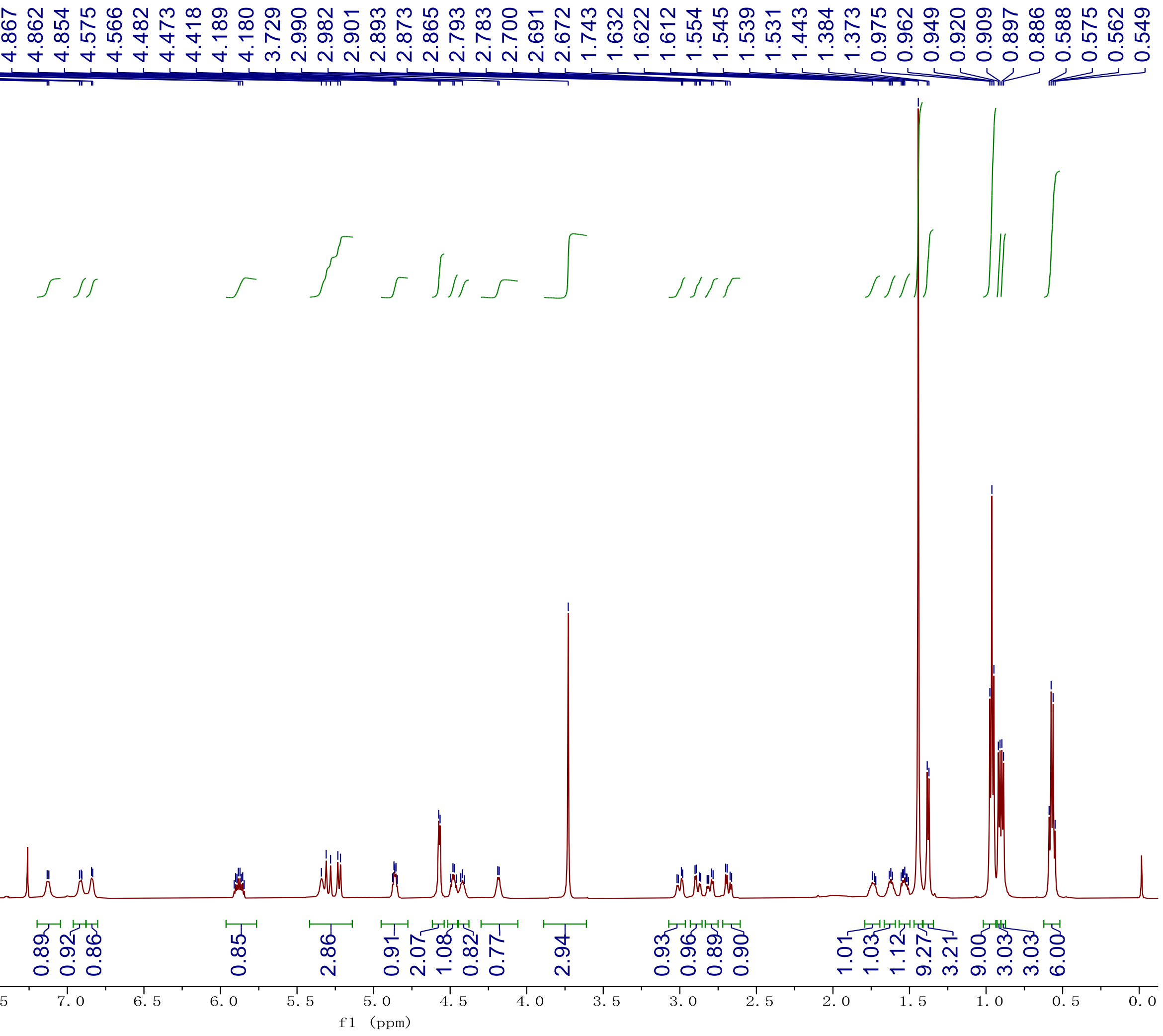

Compound 34b ^{13}C NMR (151 MHz, $CDCl_3$)

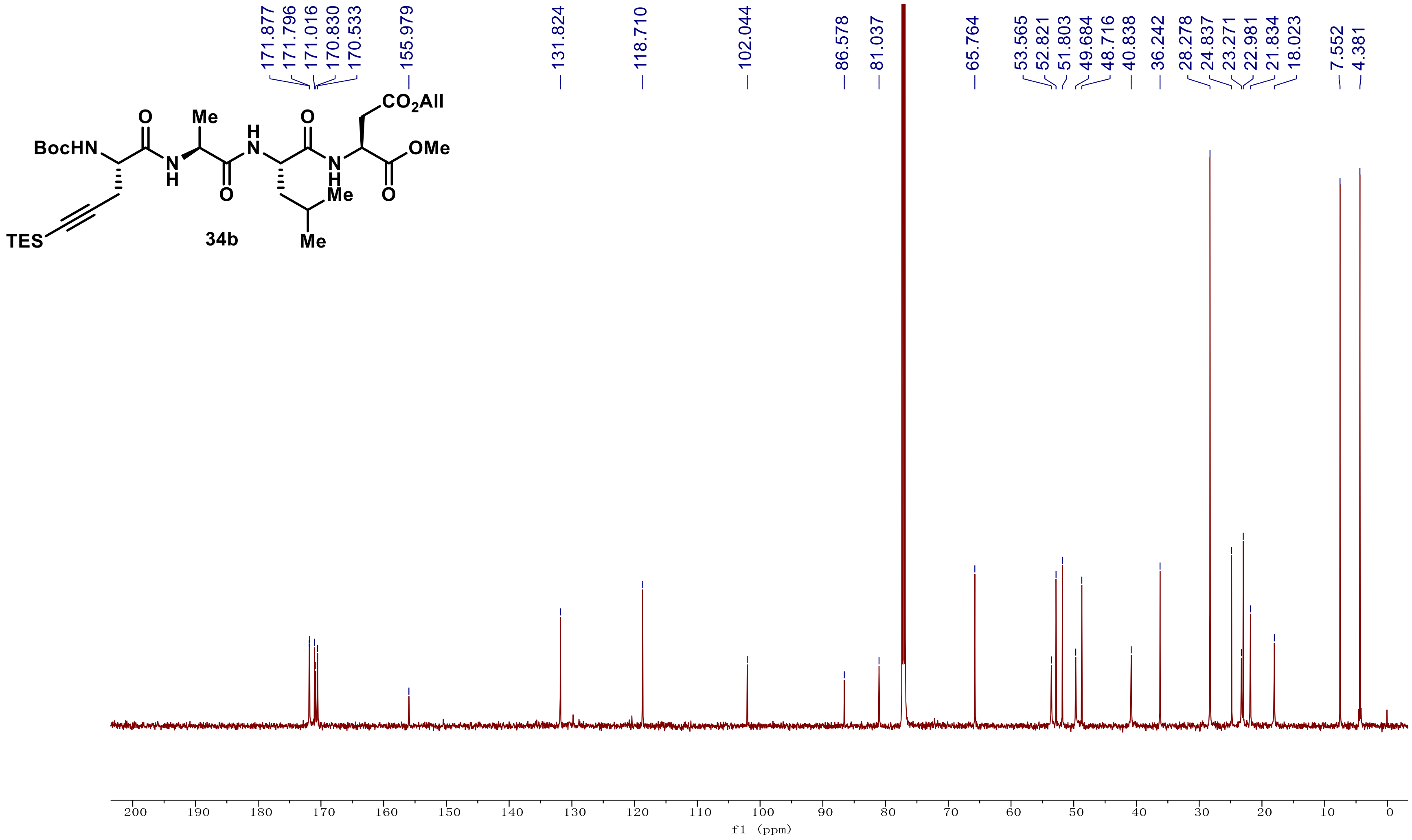

Compound 35 ^{1}H NMR (600 MHz, $CDCl_3$)

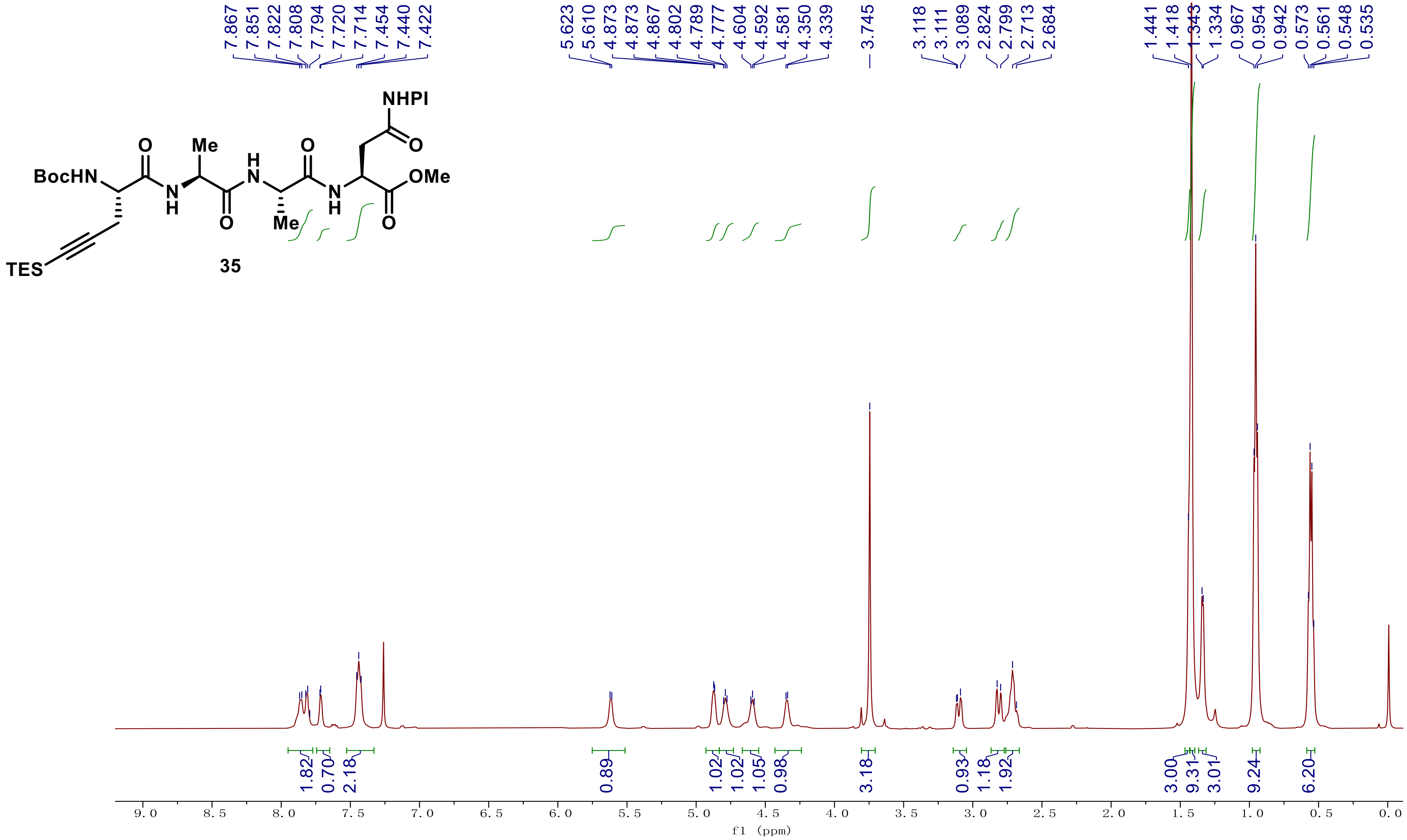

Compound 35 ^{13}C NMR (151 MHz, $CDCl_3$)

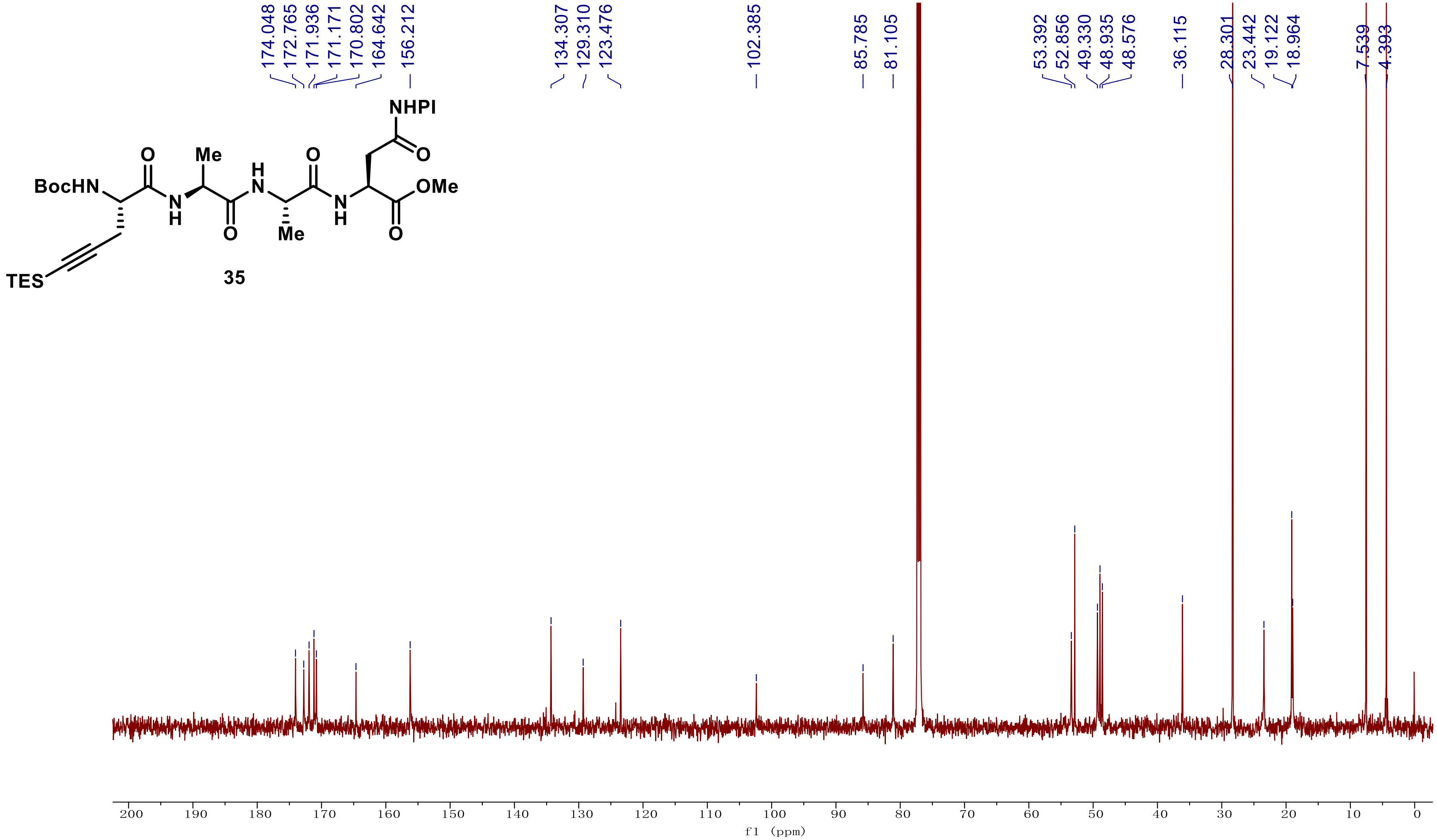

Compound 13 ^{1}H NMR (600 MHz, $CDCl_3$)

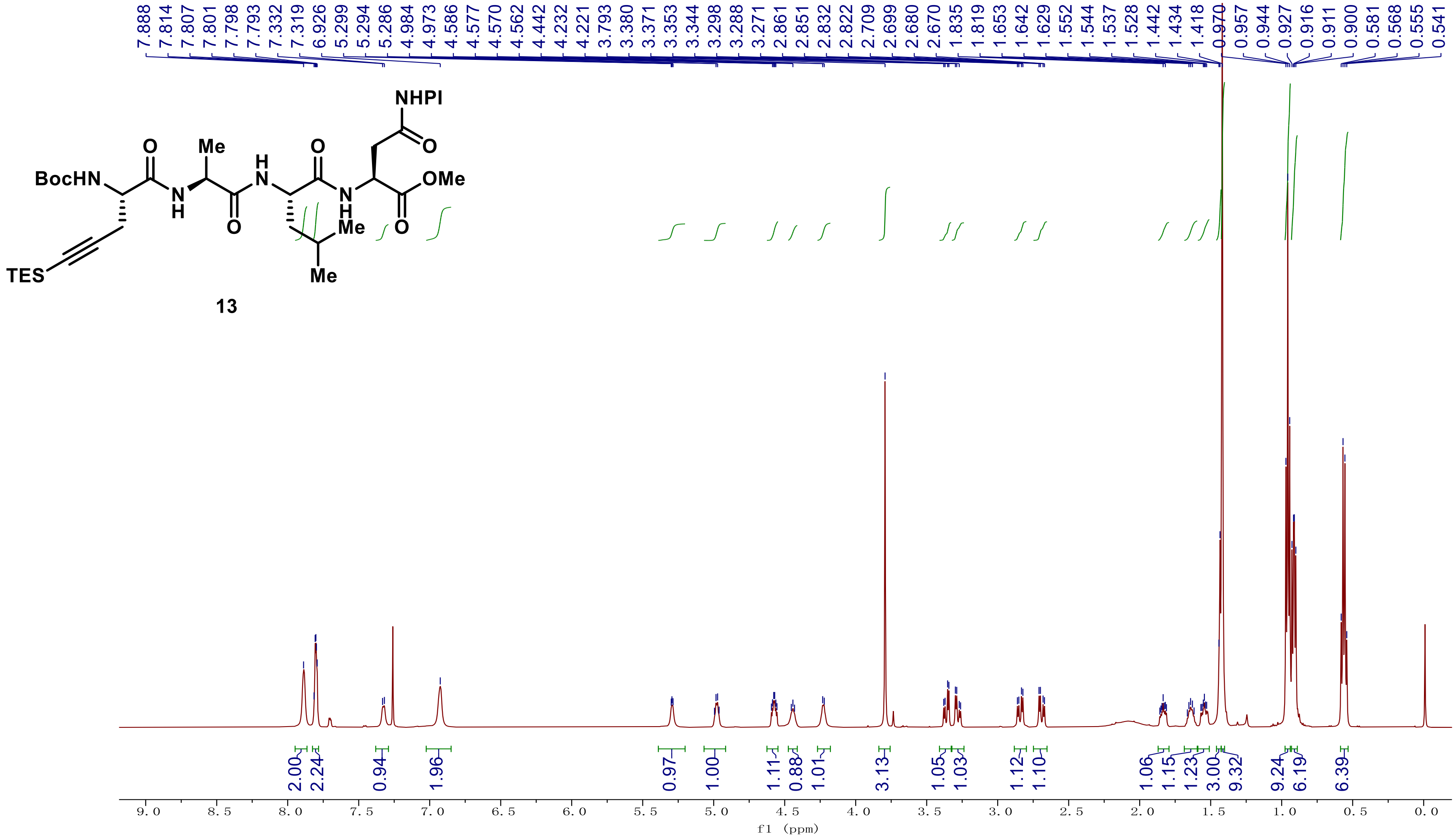

Compound 13 ^{13}C NMR (151 MHz, $CDCl_3$)

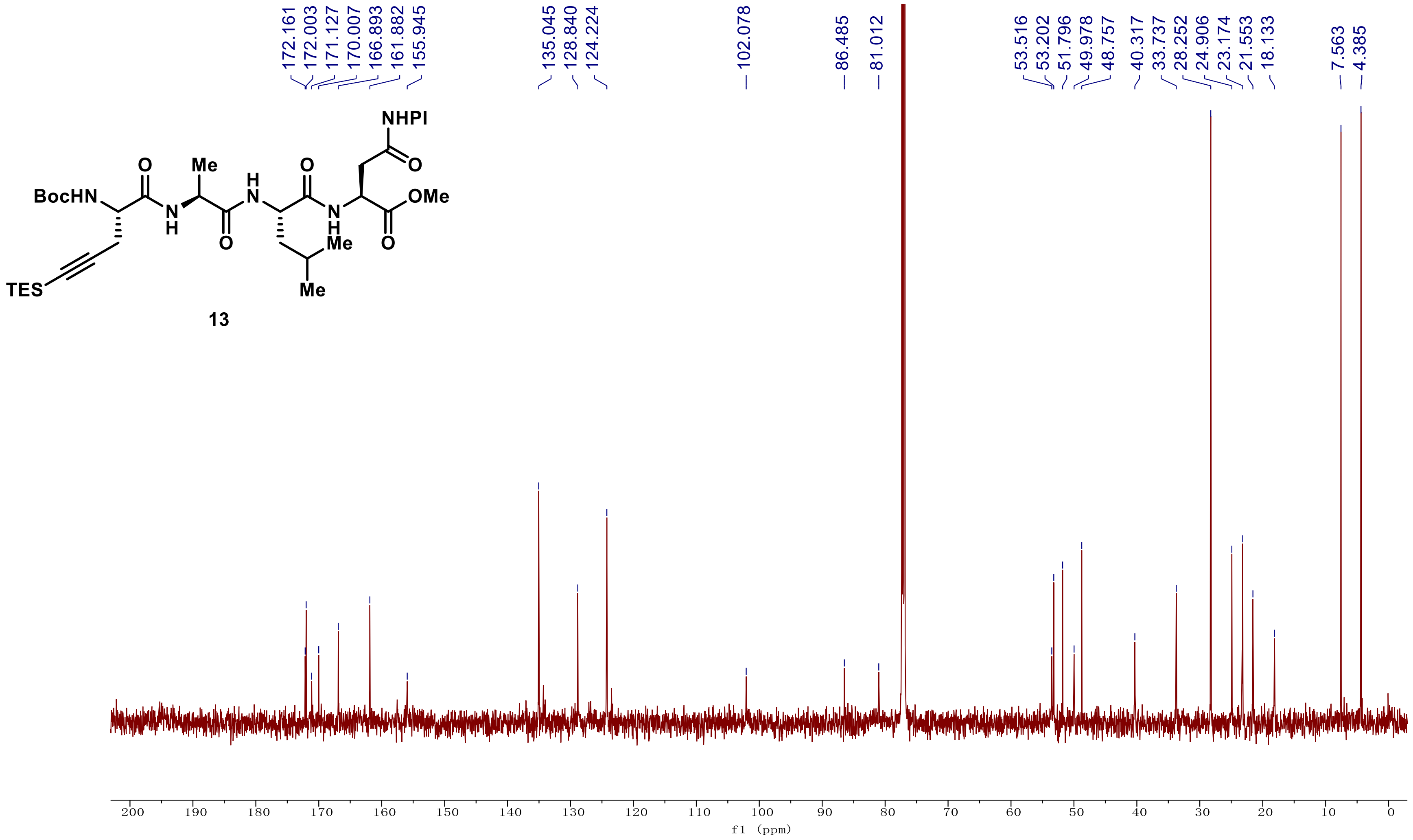

Compound 37 ^{1}H NMR (600 MHz, CDCl$_3$)

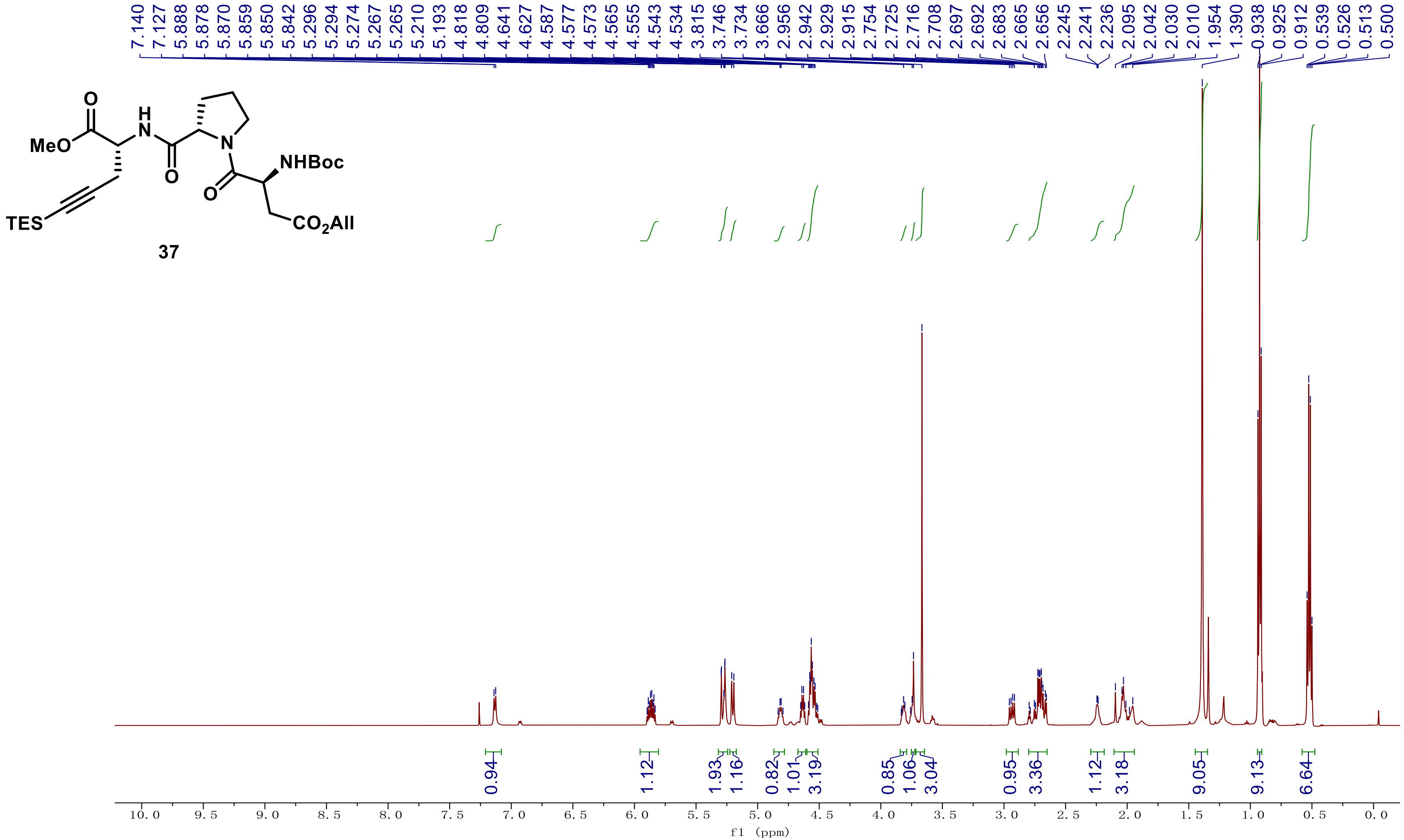

Compound 37 ^{13}C NMR (151 MHz, $CDCl_3$)

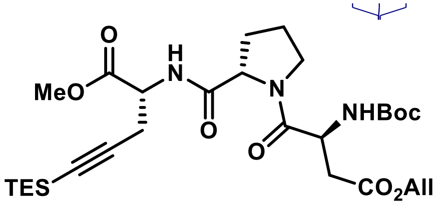

37

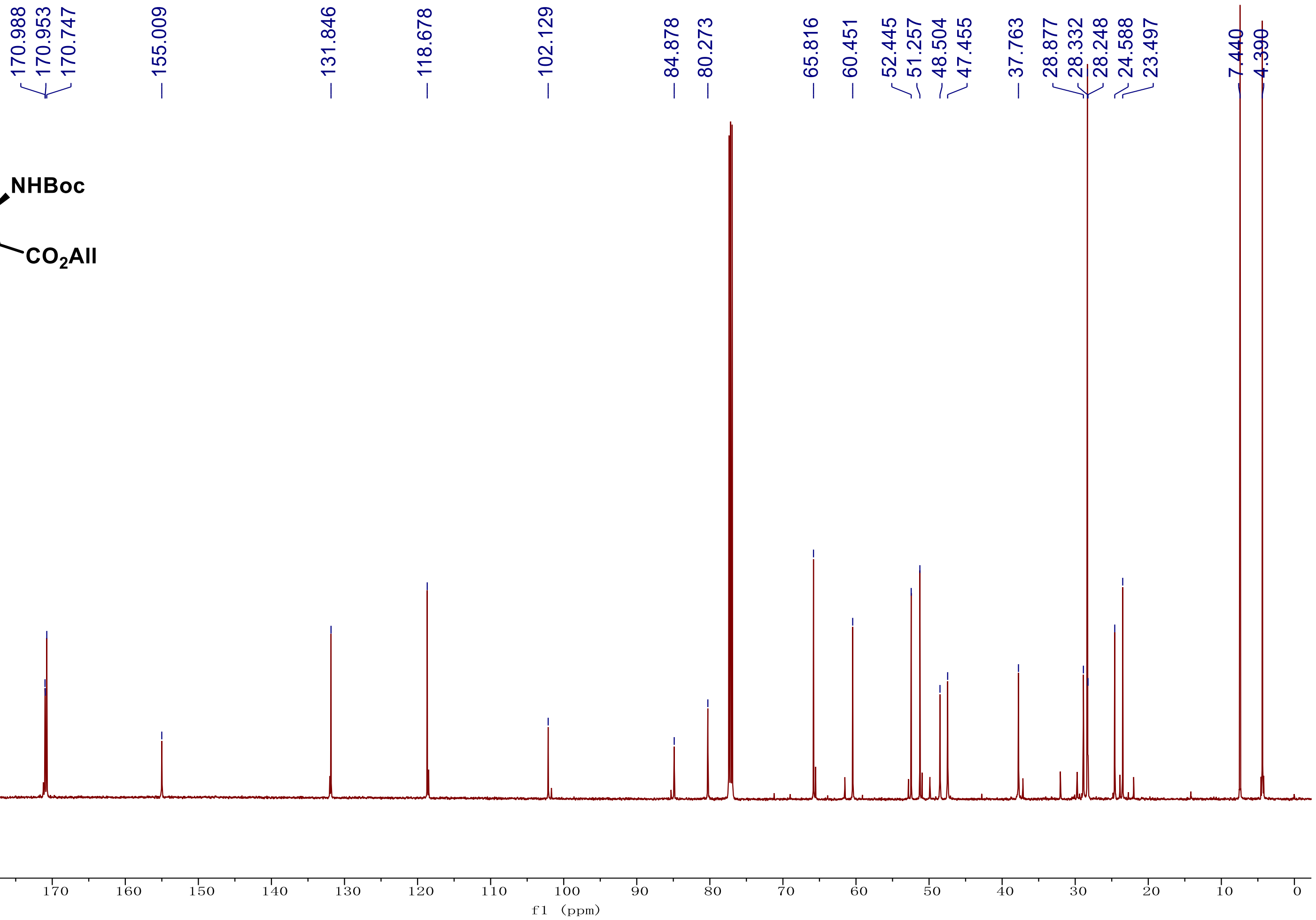

Compound 25 ^{1}H NMR (600 MHz, $CDCl_3$)

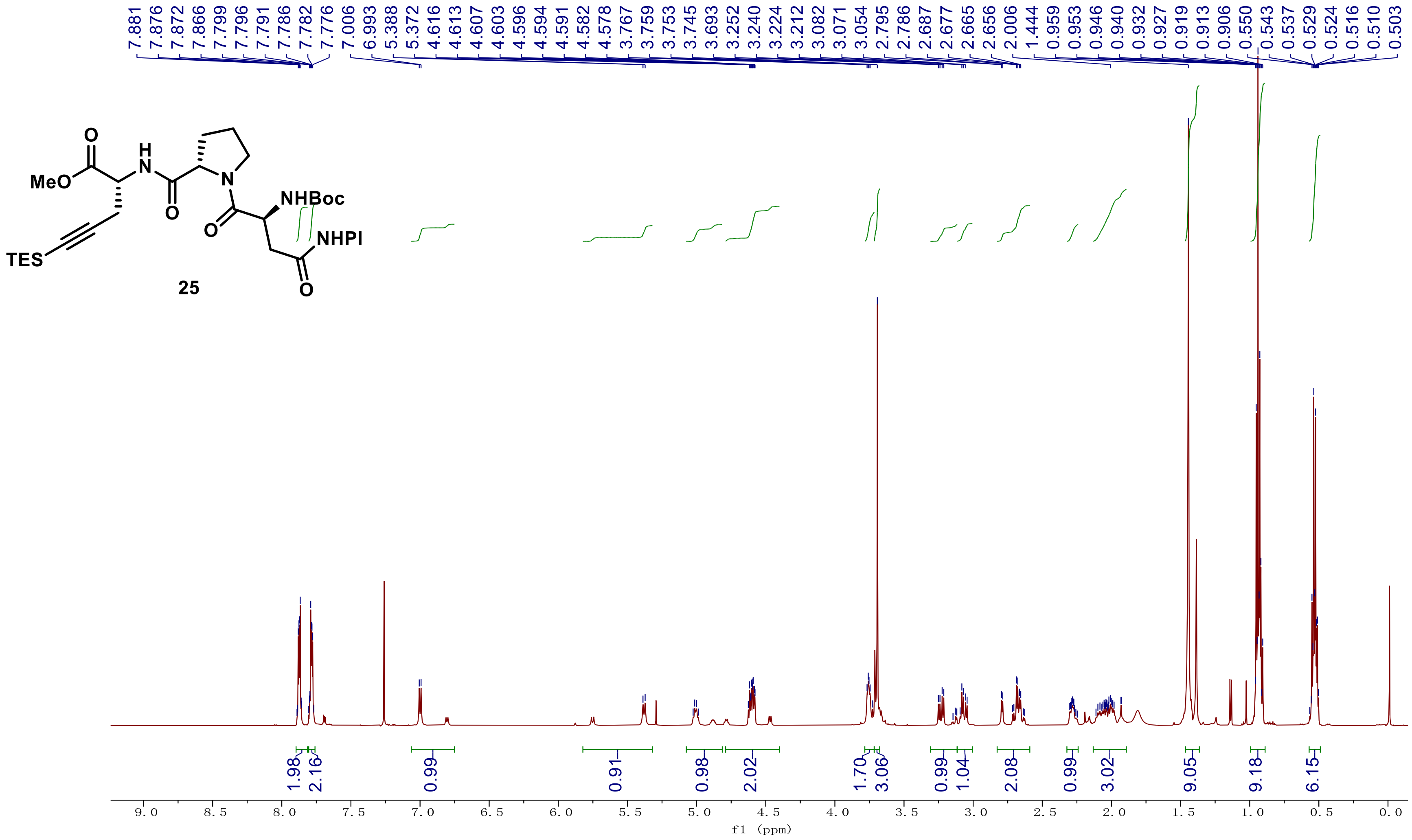

Compound 25 ^{13}C NMR (151 MHz, $CDCl_3$)

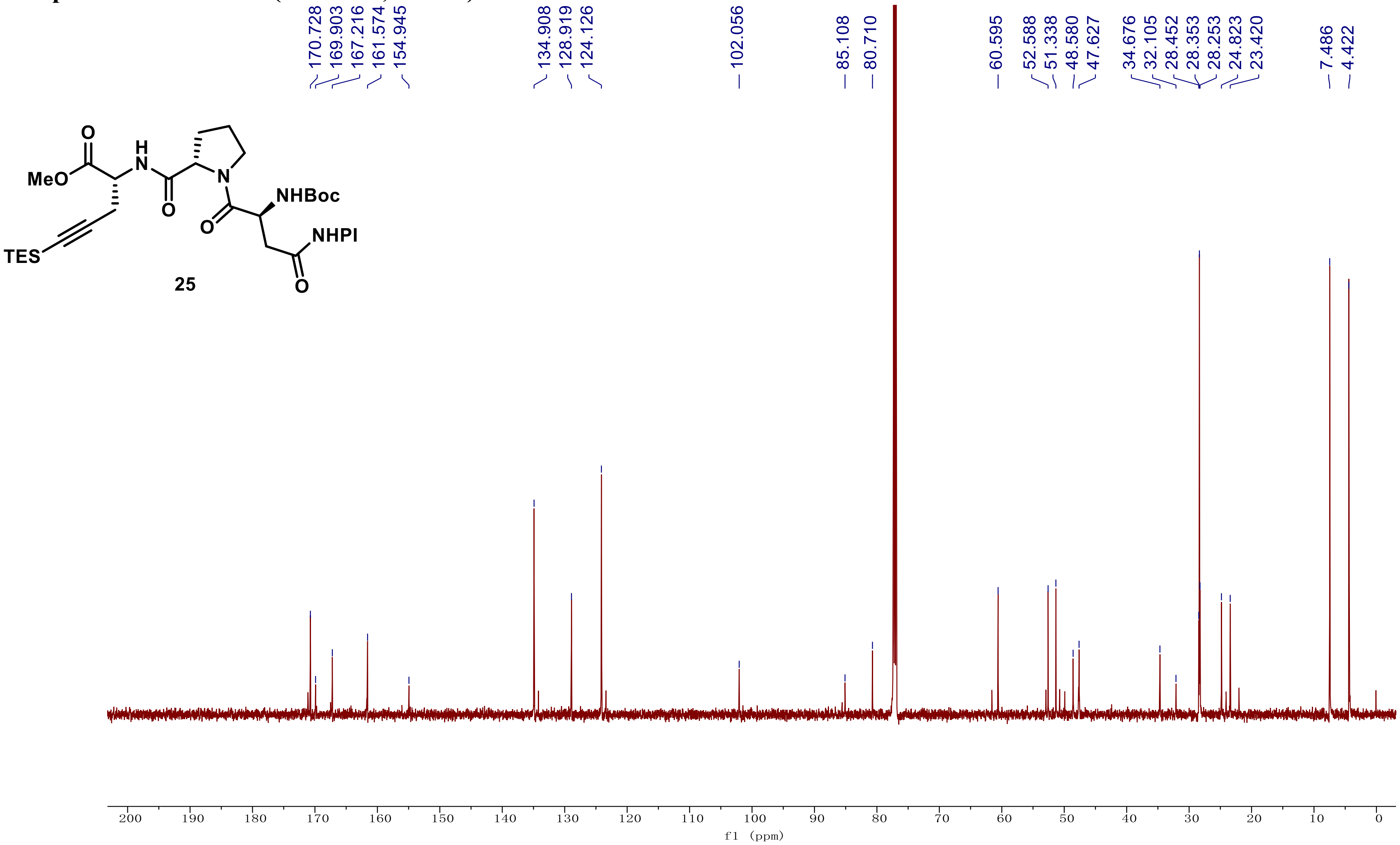

Compound 15 ^{1}H NMR (600 MHz, $CDCl_3$)

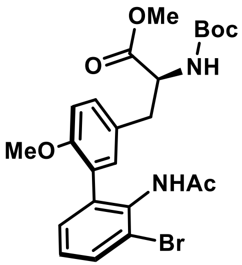

15

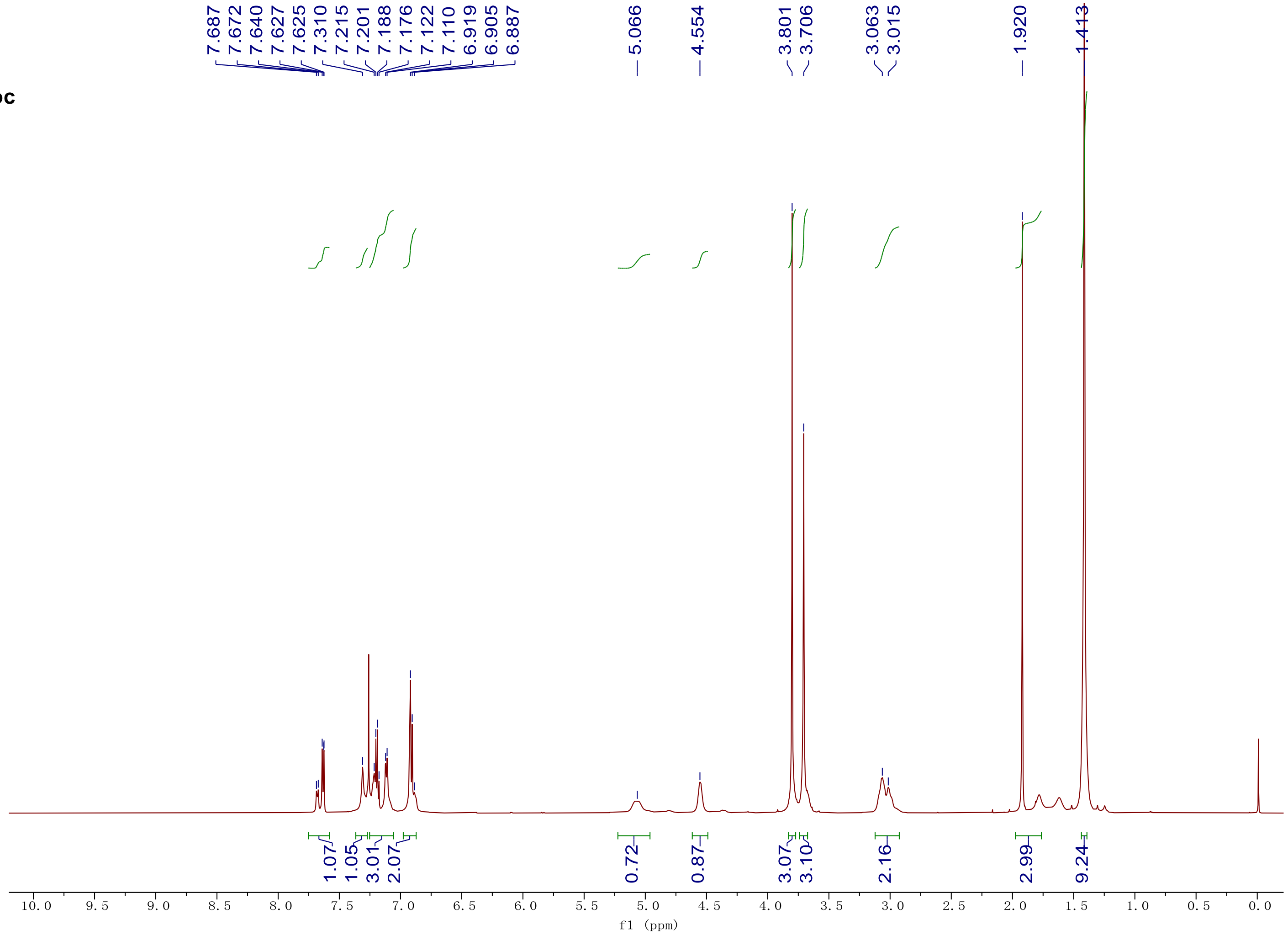

Compound 15 ^{13}C NMR (151 MHz, $CDCl_3$)

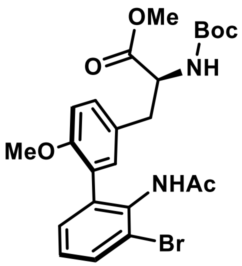

15

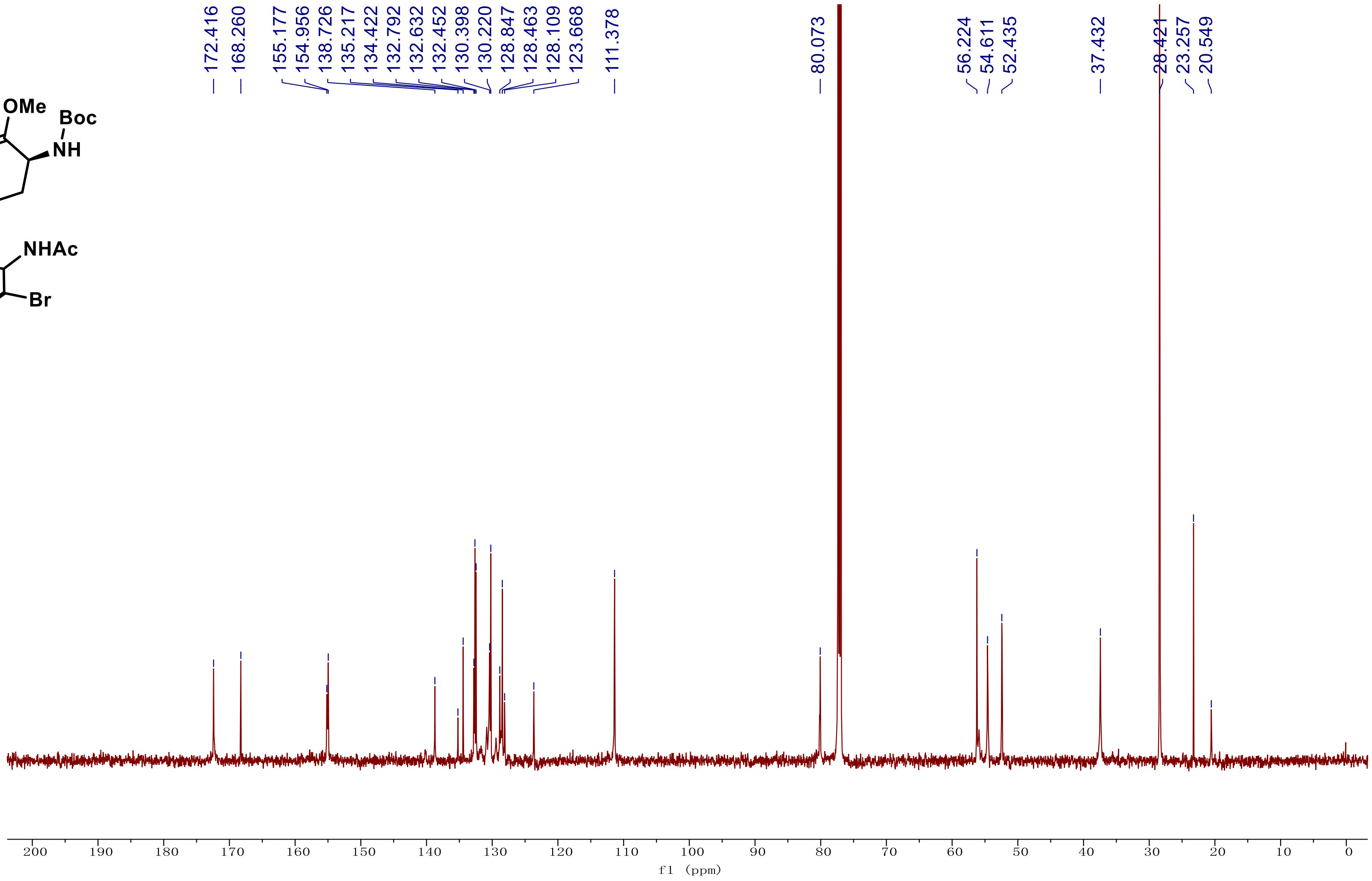

Compound 21 ^{1}H NMR (600 MHz, $CDCl_3$)

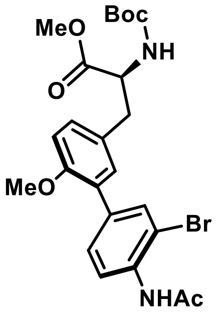

21

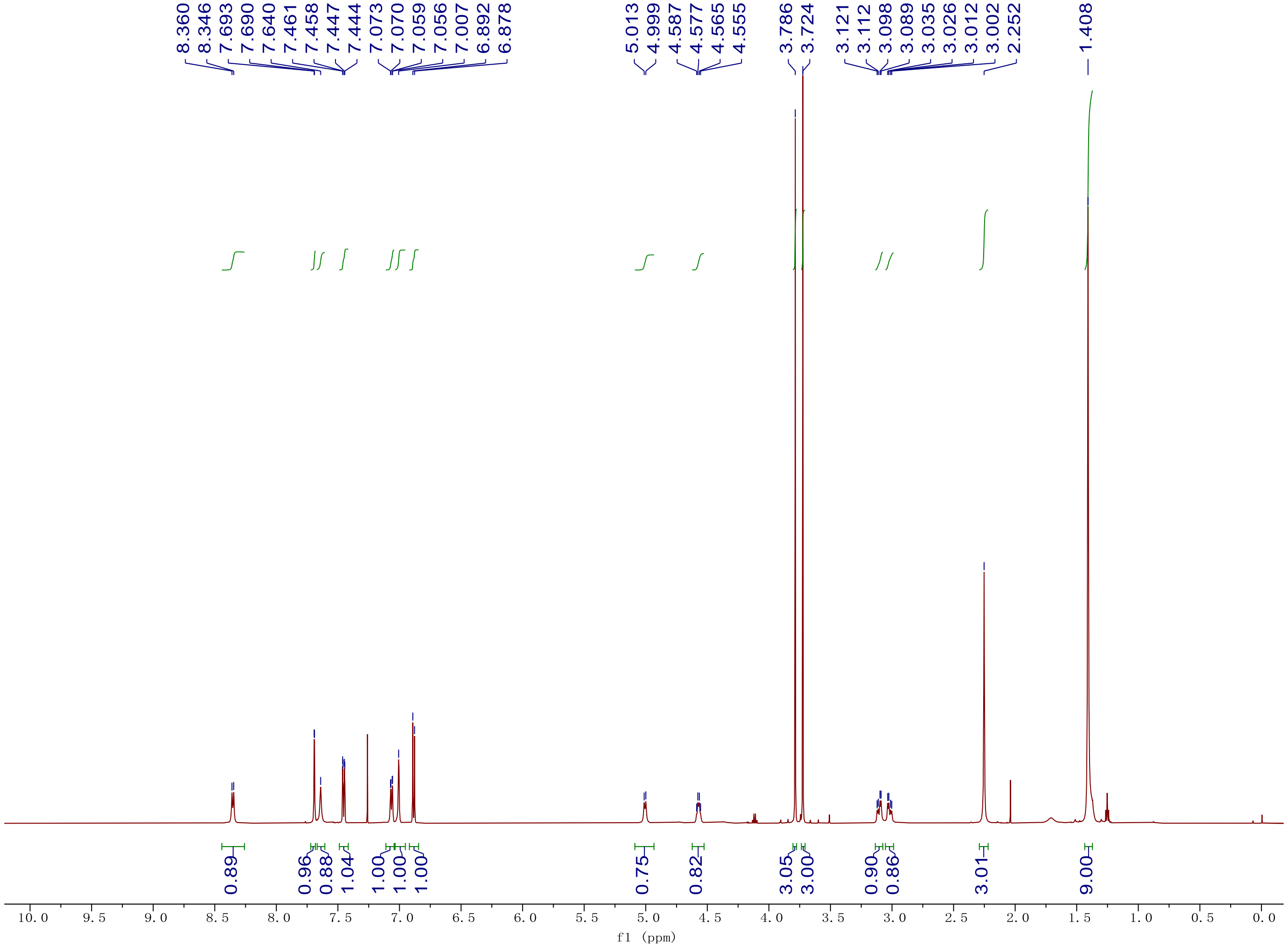

Compound 21 ^{13}C NMR (151 MHz, $CDCl_3$)

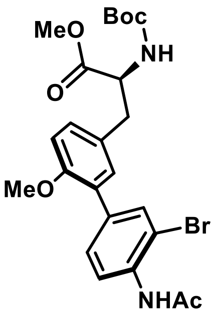

21

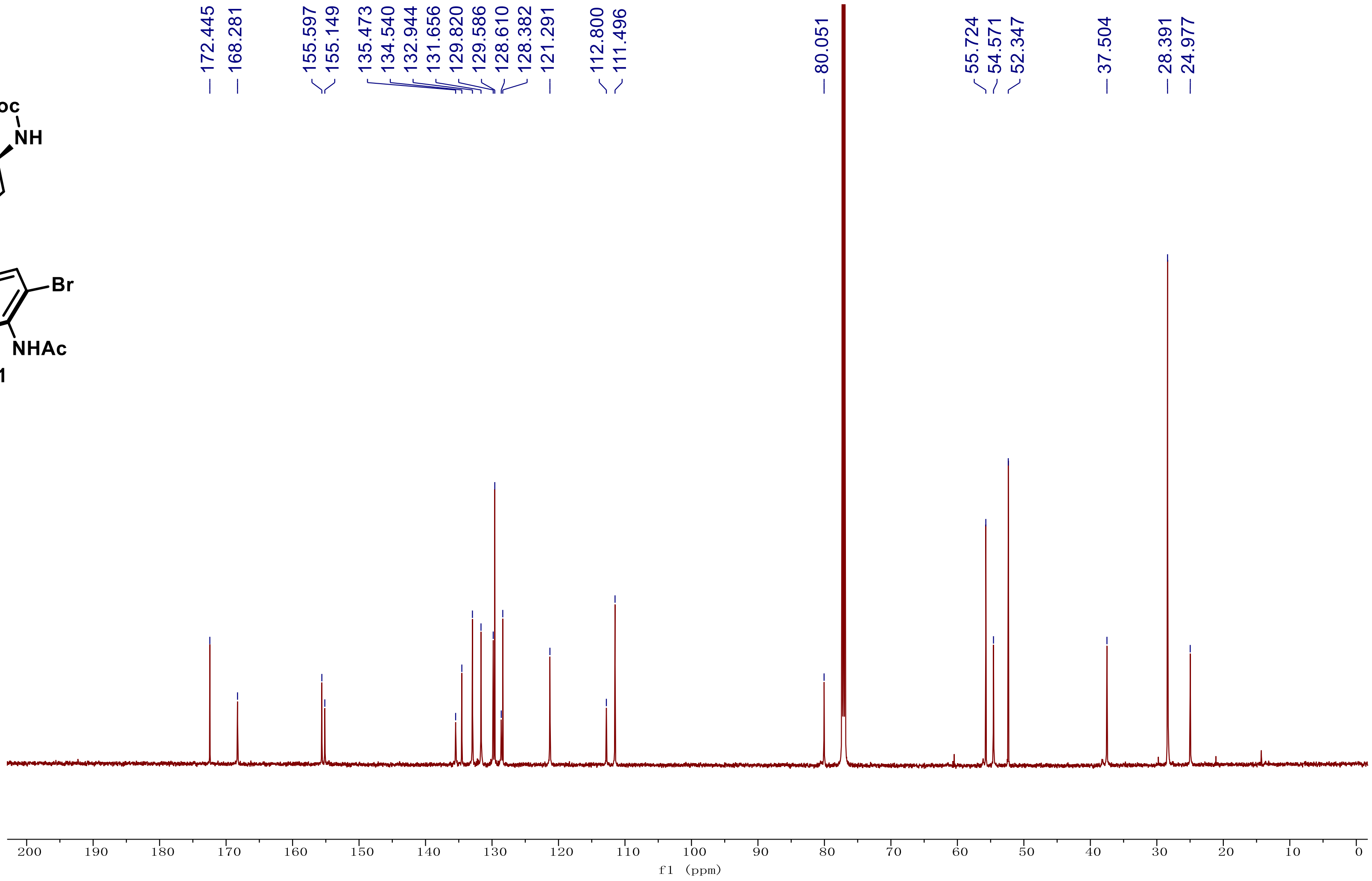

Compound 27 ^{1}H NMR (600 MHz, $CDCl_3$)

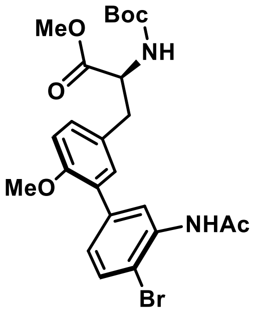

27

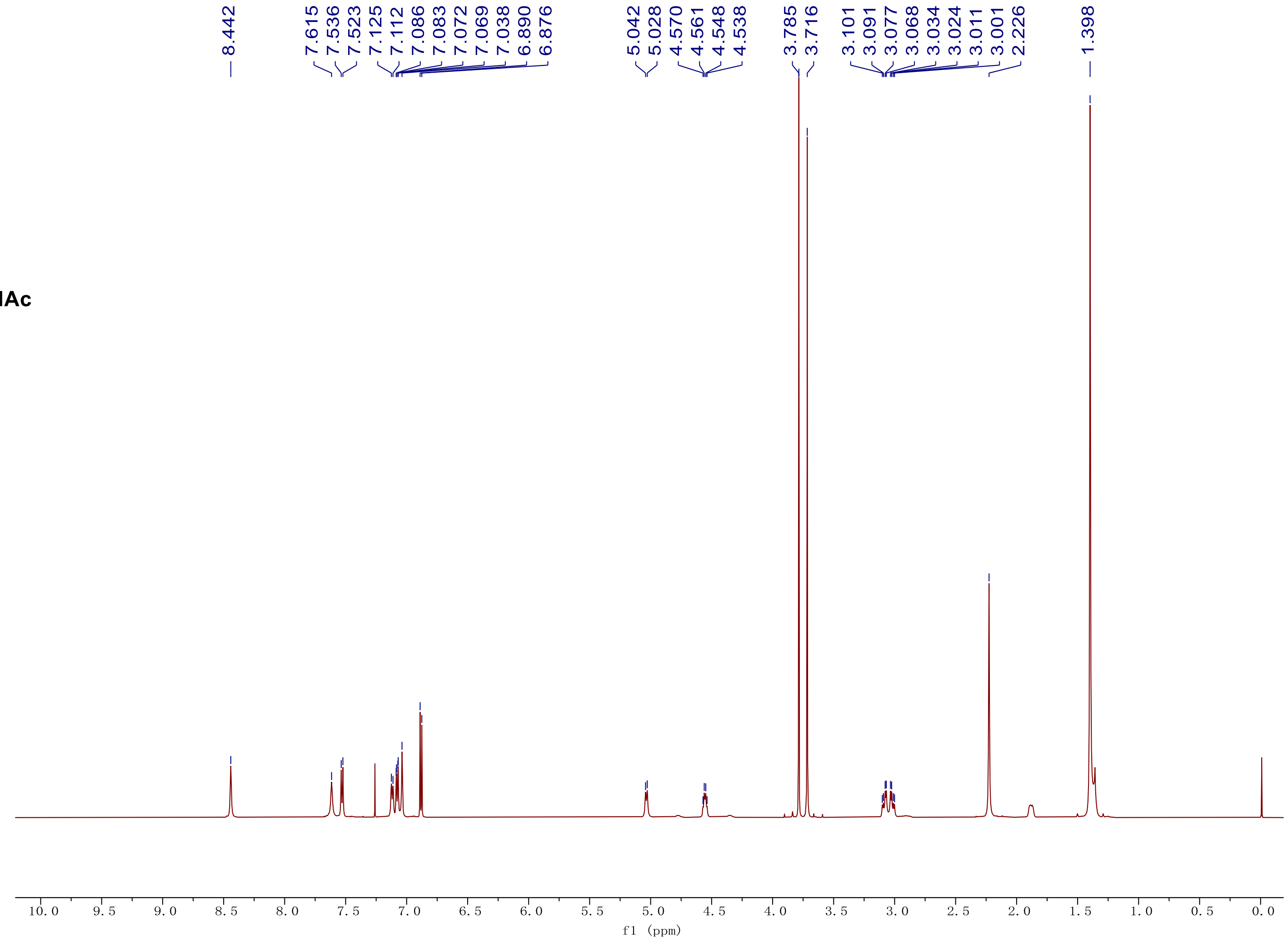

Compound 27 ^{13}C NMR (151 MHz, CDCl$_3$)

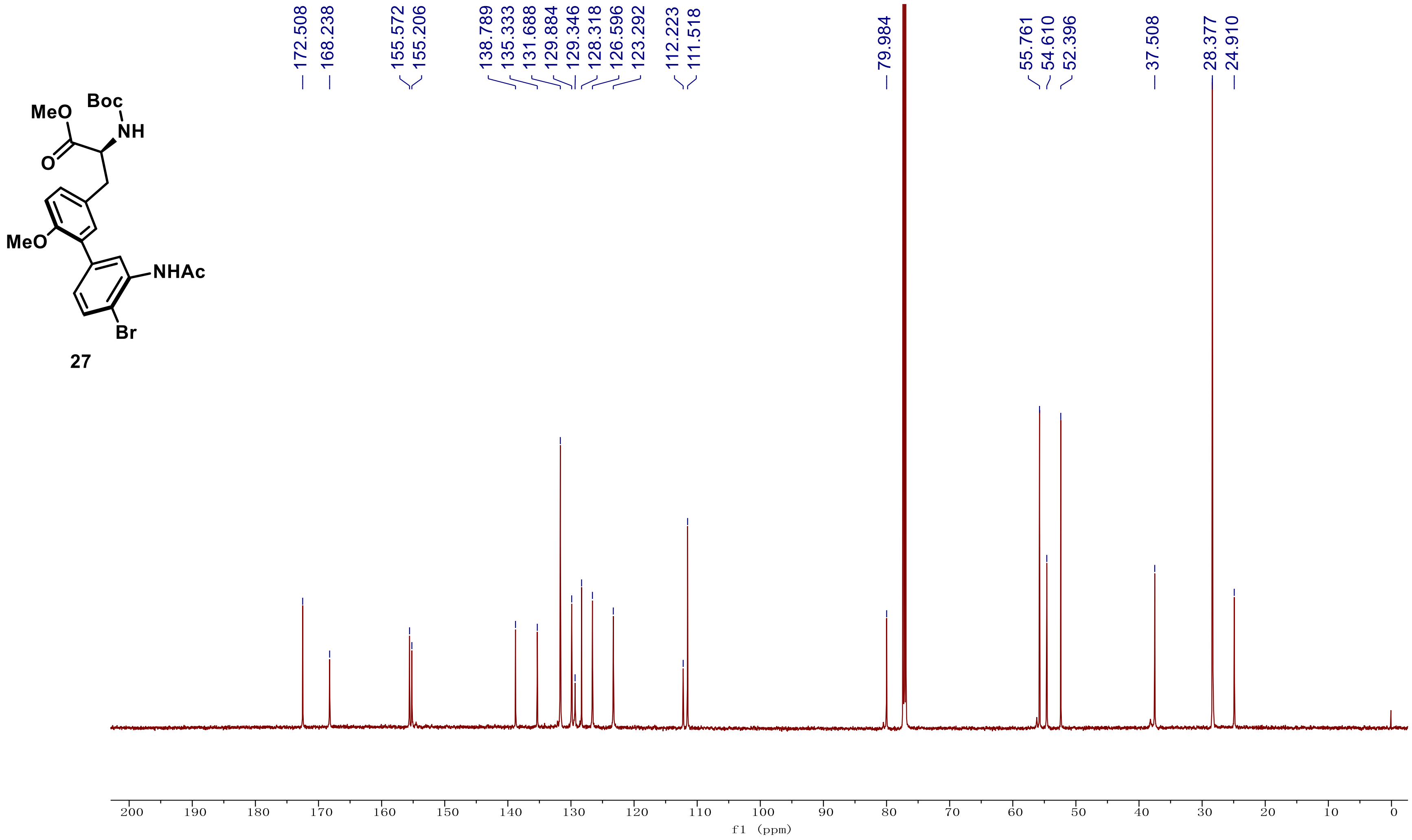

Compound 30 ¹H NMR (600 MHz, $CDCl_3$)

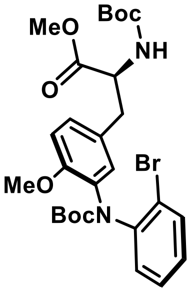

30

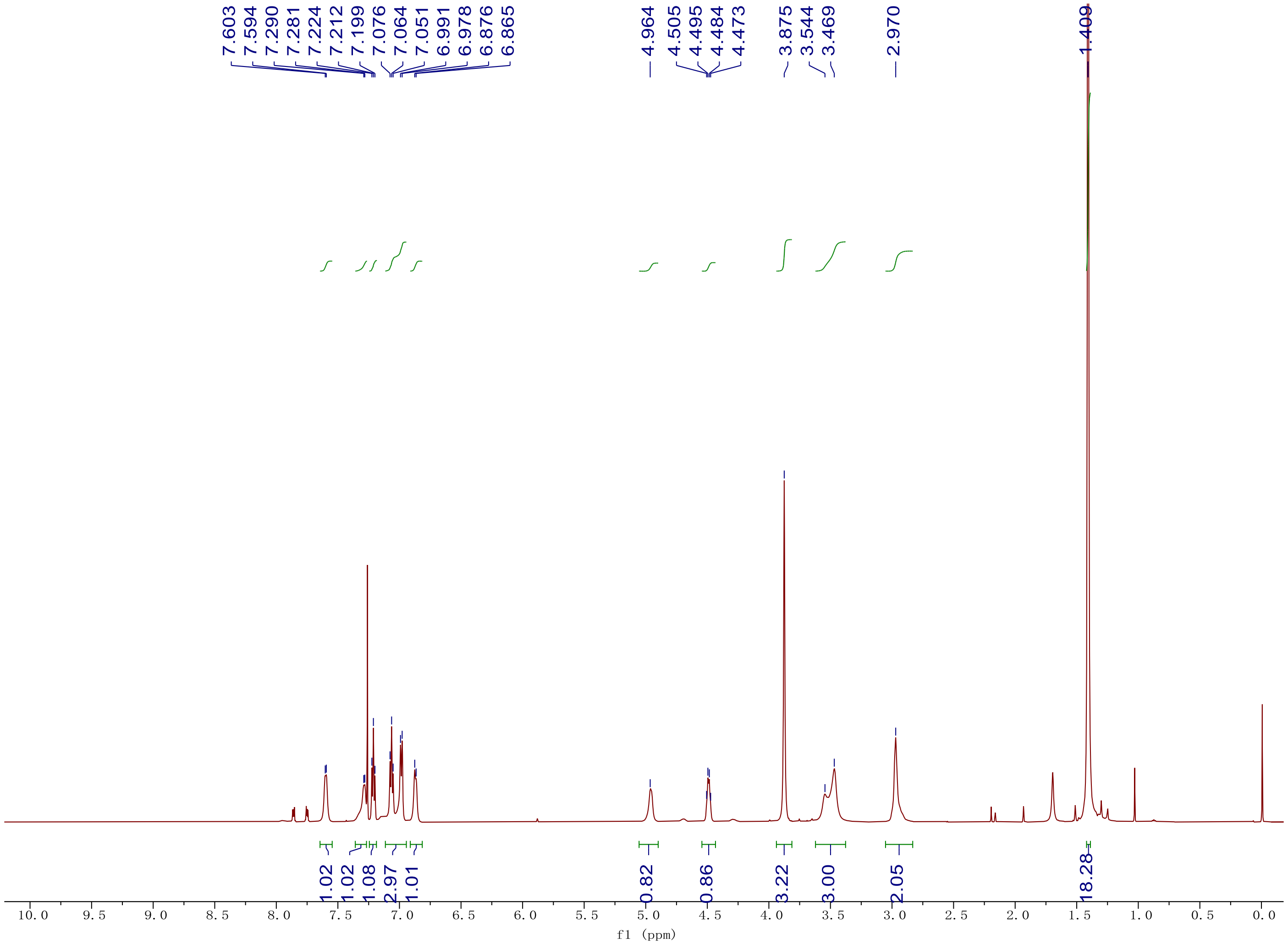

Compound 30 ^{13}C NMR (151 MHz, $CDCl_3$)

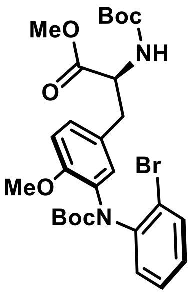

30

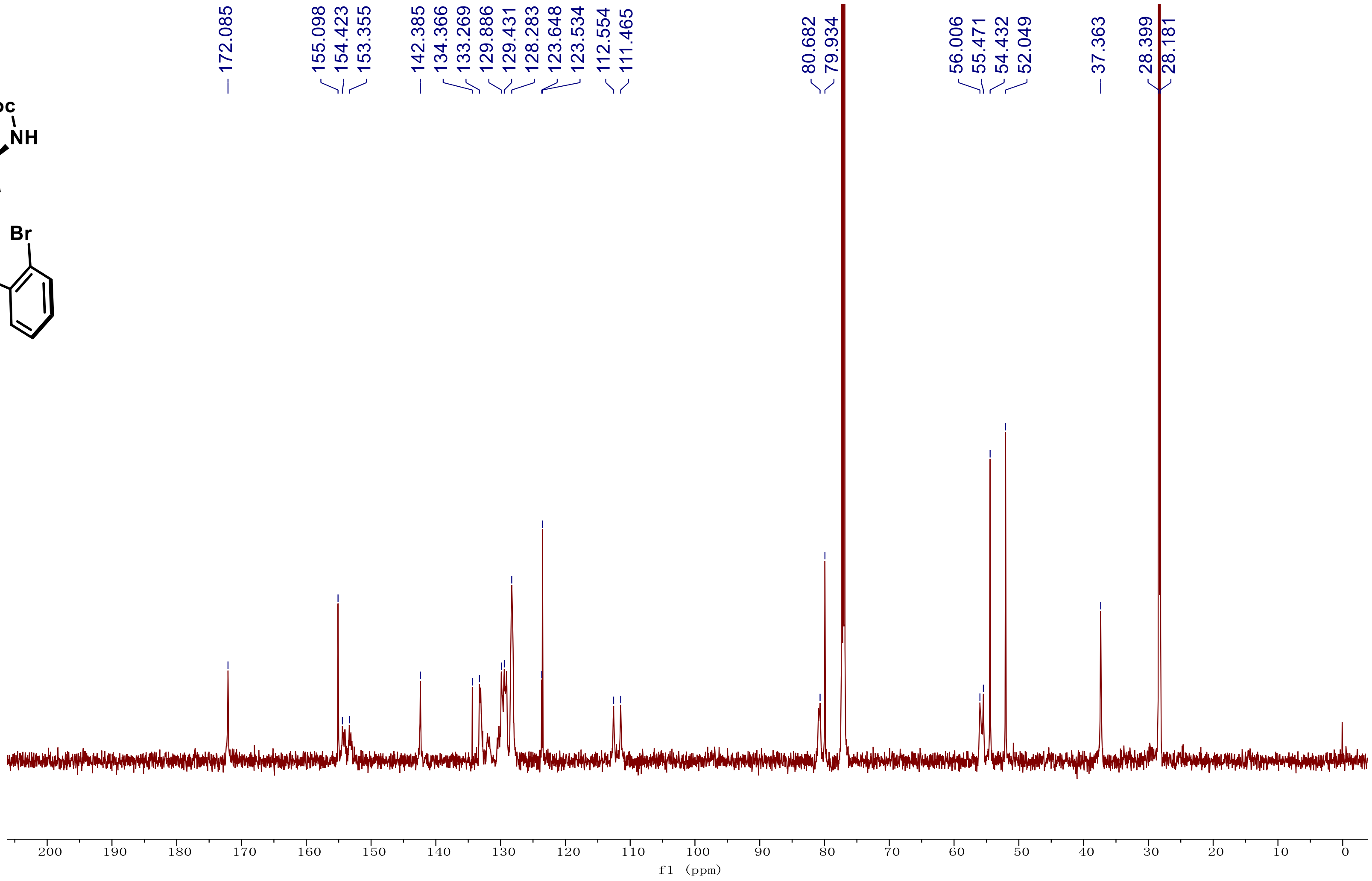

Compound S6 ^{1}H NMR (600 MHz, $CDCl_3$)

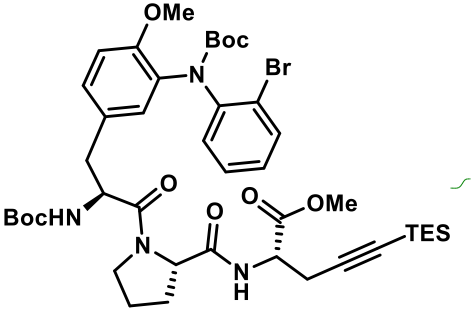

S6

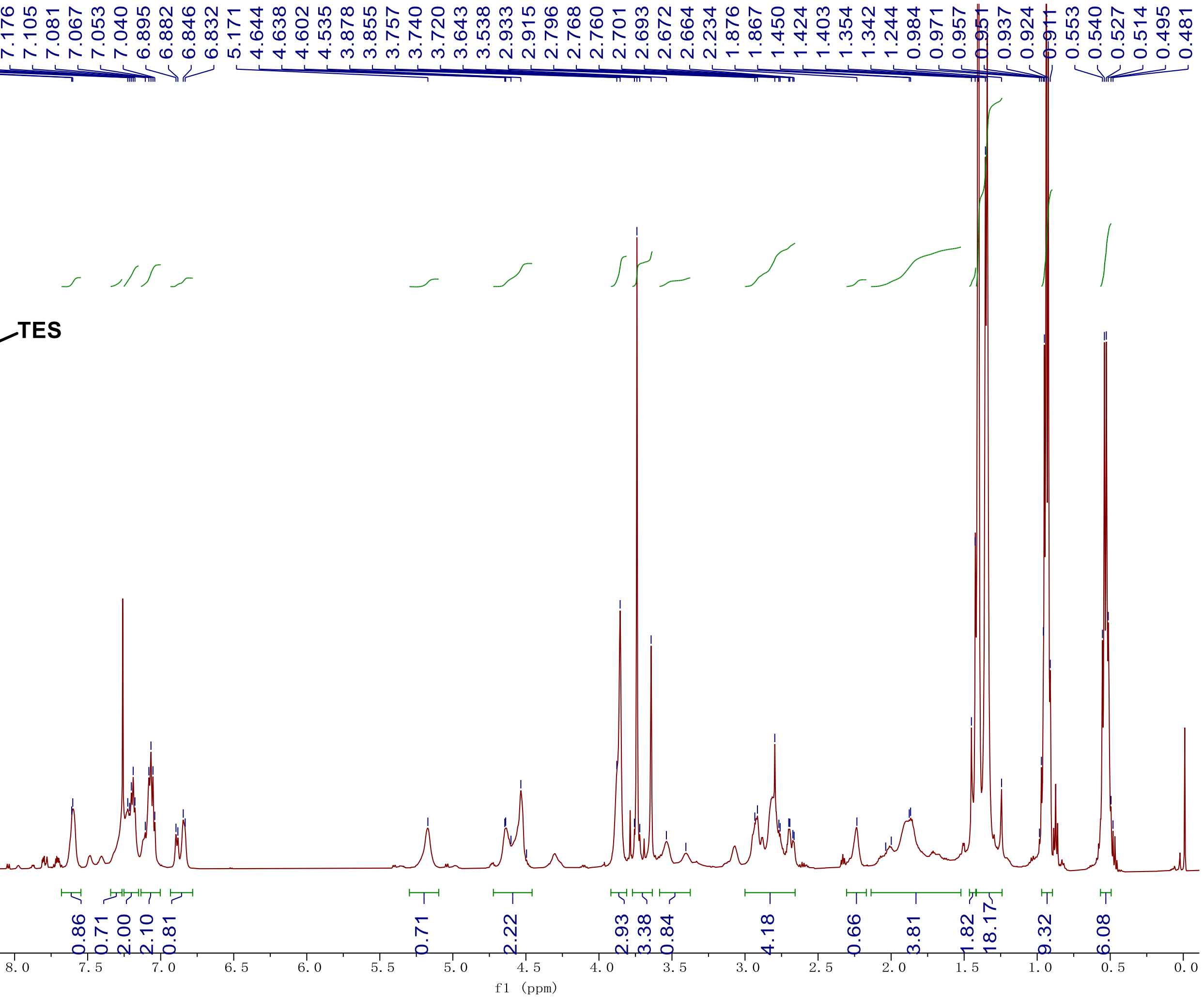

Compound S6 ^{13}C NMR (151 MHz, $CDCl_3$)

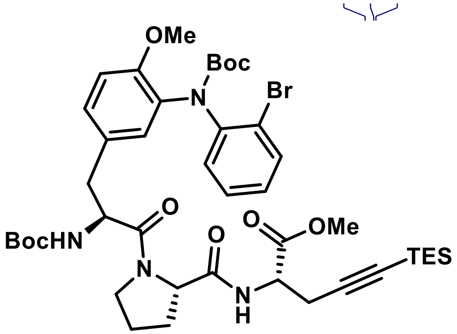

S6

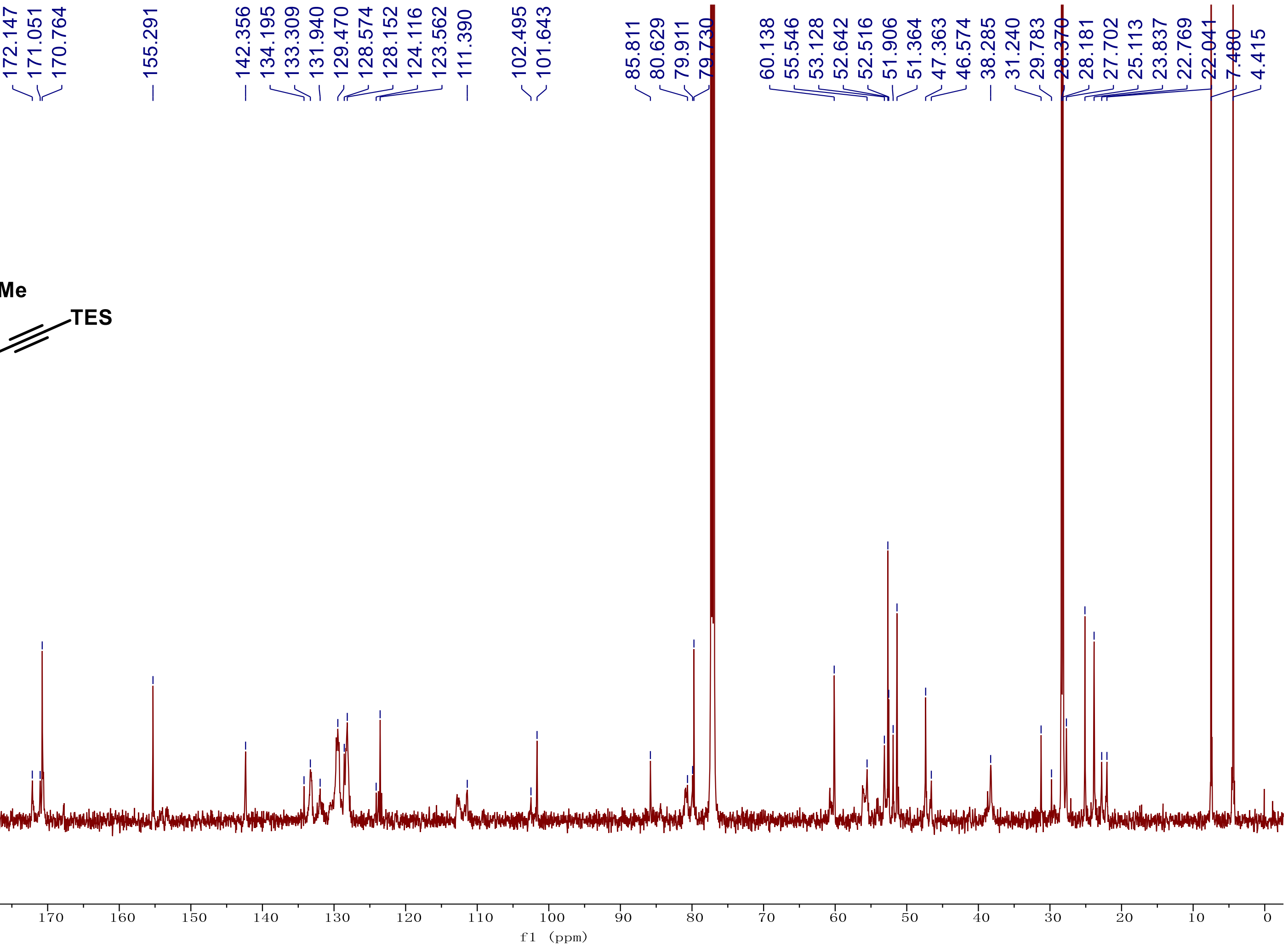

Compound S7 ¹H NMR (600 MHz, $CDCl_3$)

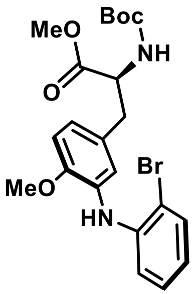

S7

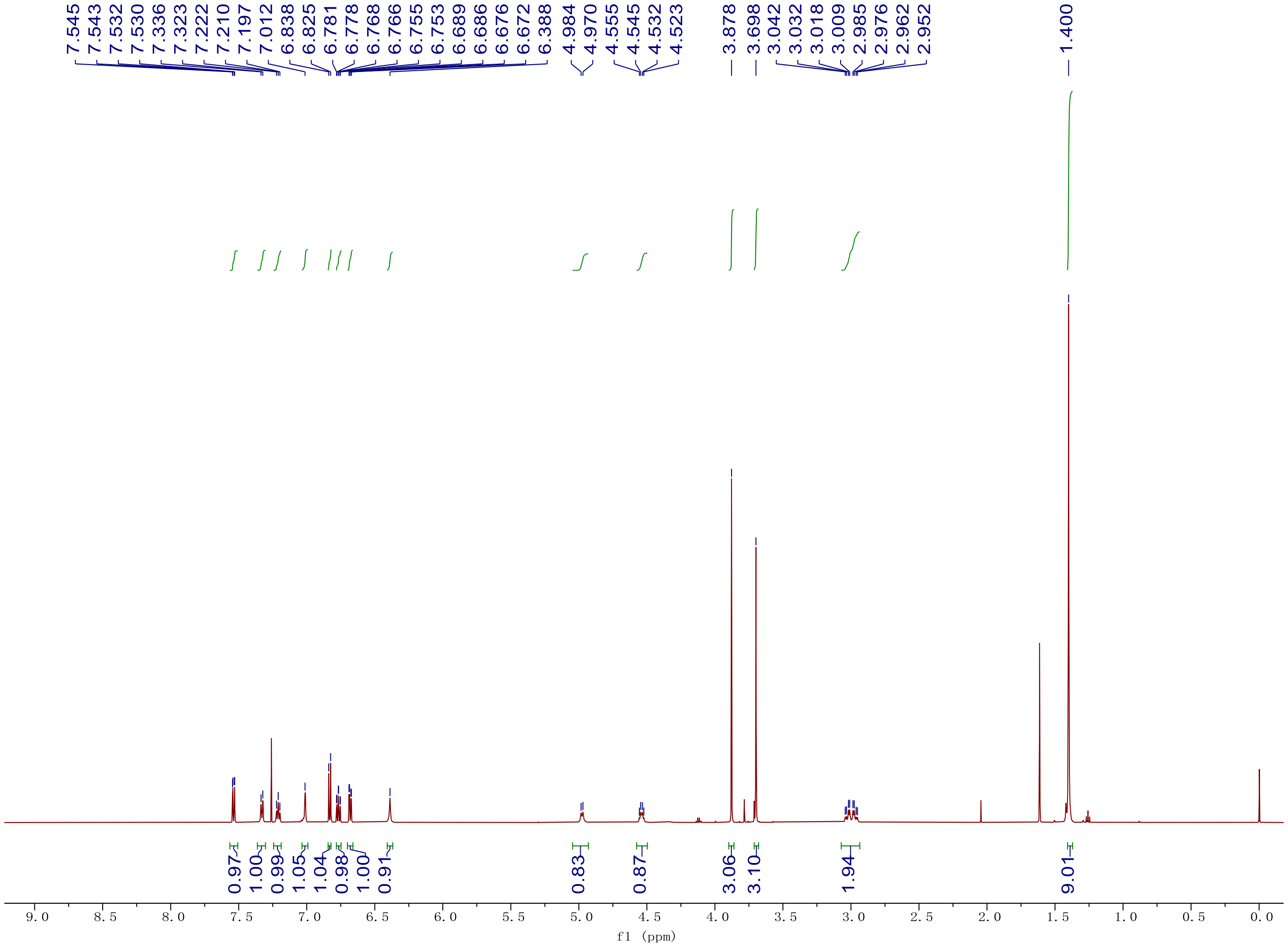

Compound S7 ^{13}C NMR (151 MHz, $CDCl_3$)

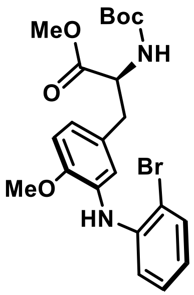

S7

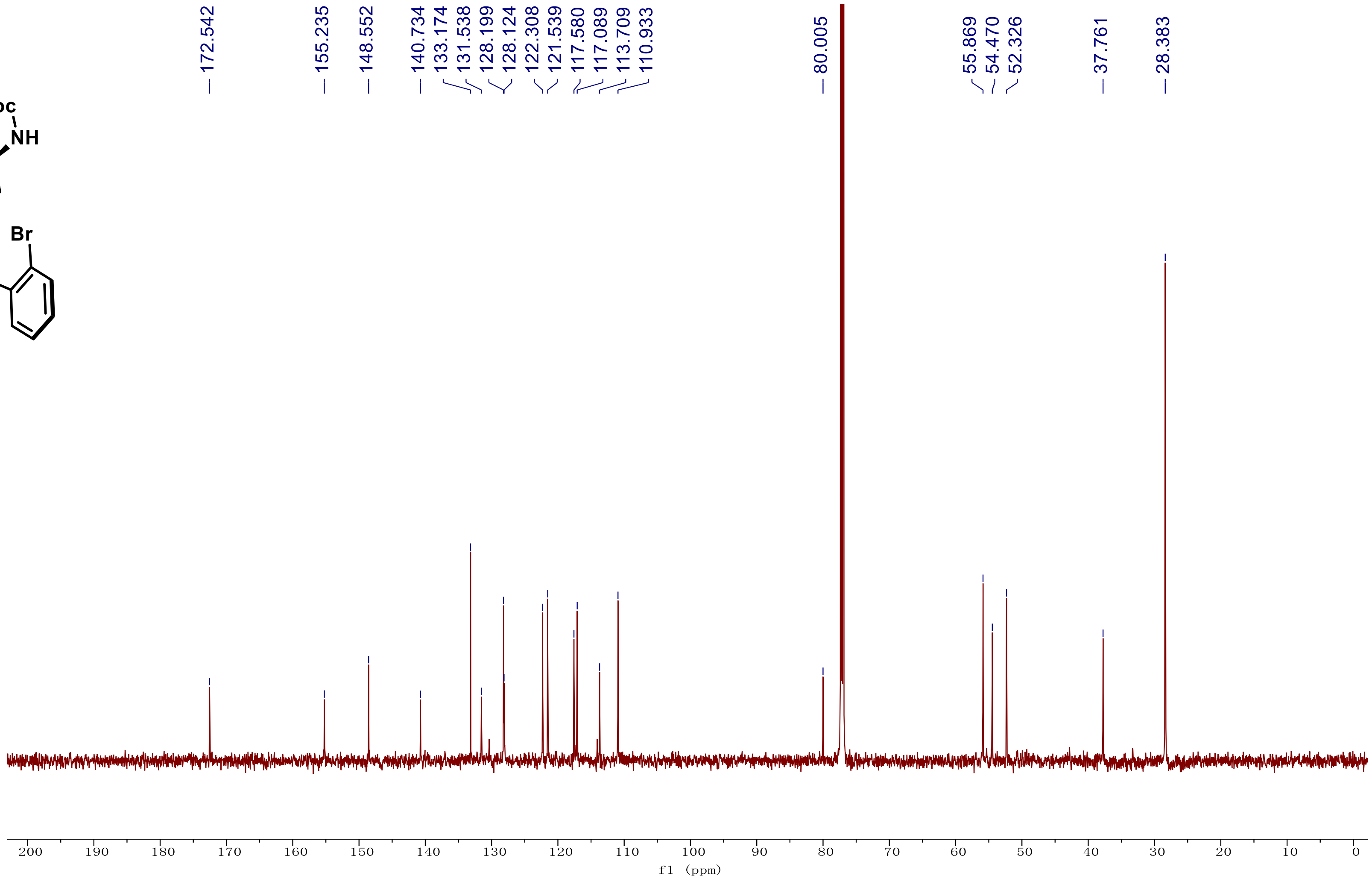

Compound 38a ^{1}H NMR (600 MHz, DMSO-*d6*)

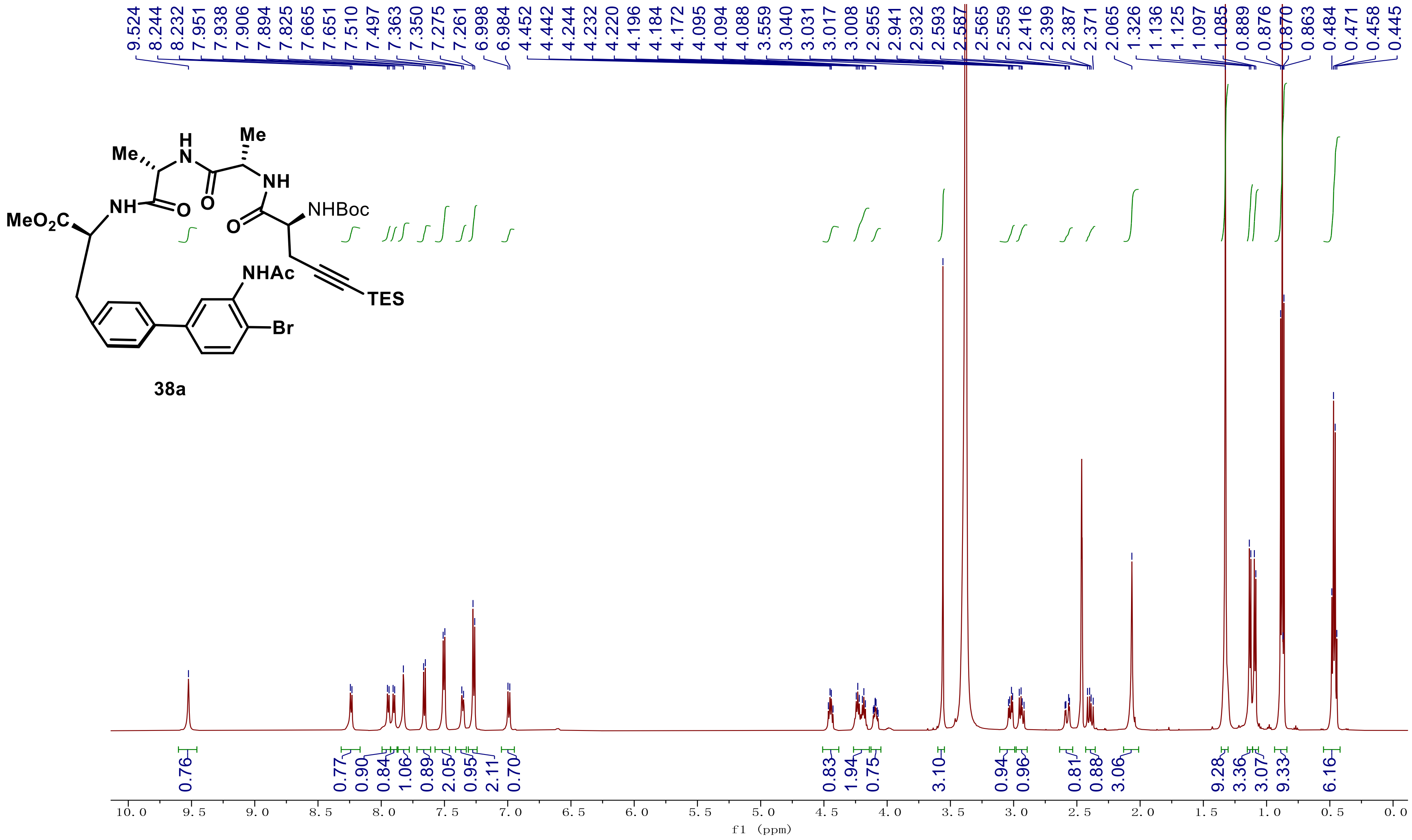

Compound 38a ^{13}C NMR (151 MHz, DMSO-*d6*)

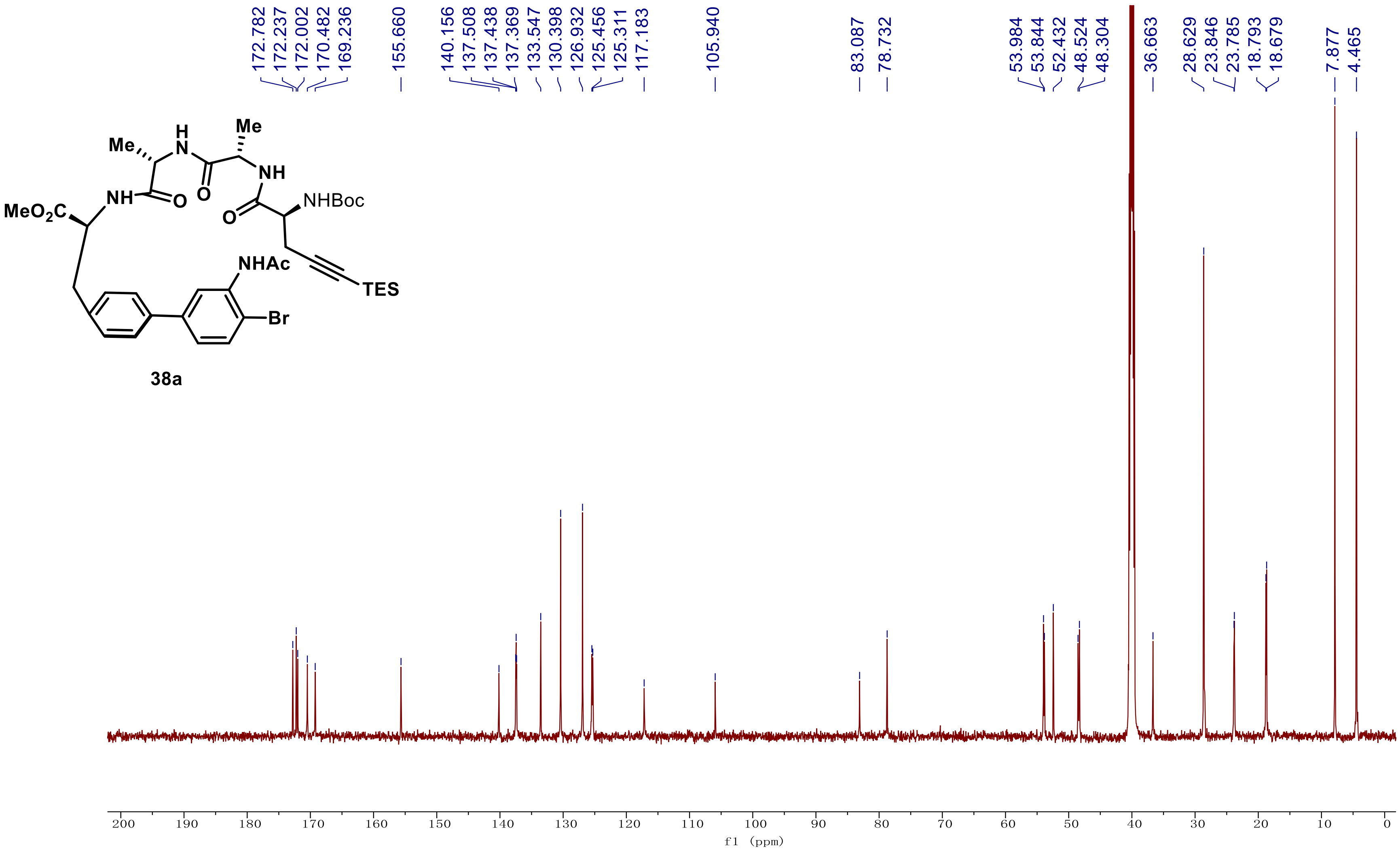

Compound 38b ^{1}H NMR (600 MHz, METHANOL-D_4)

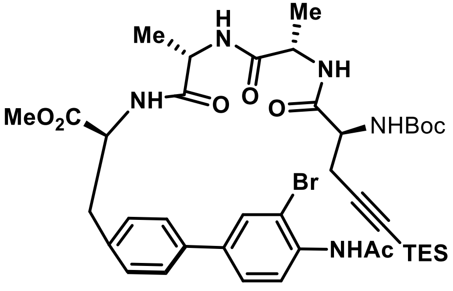

38b

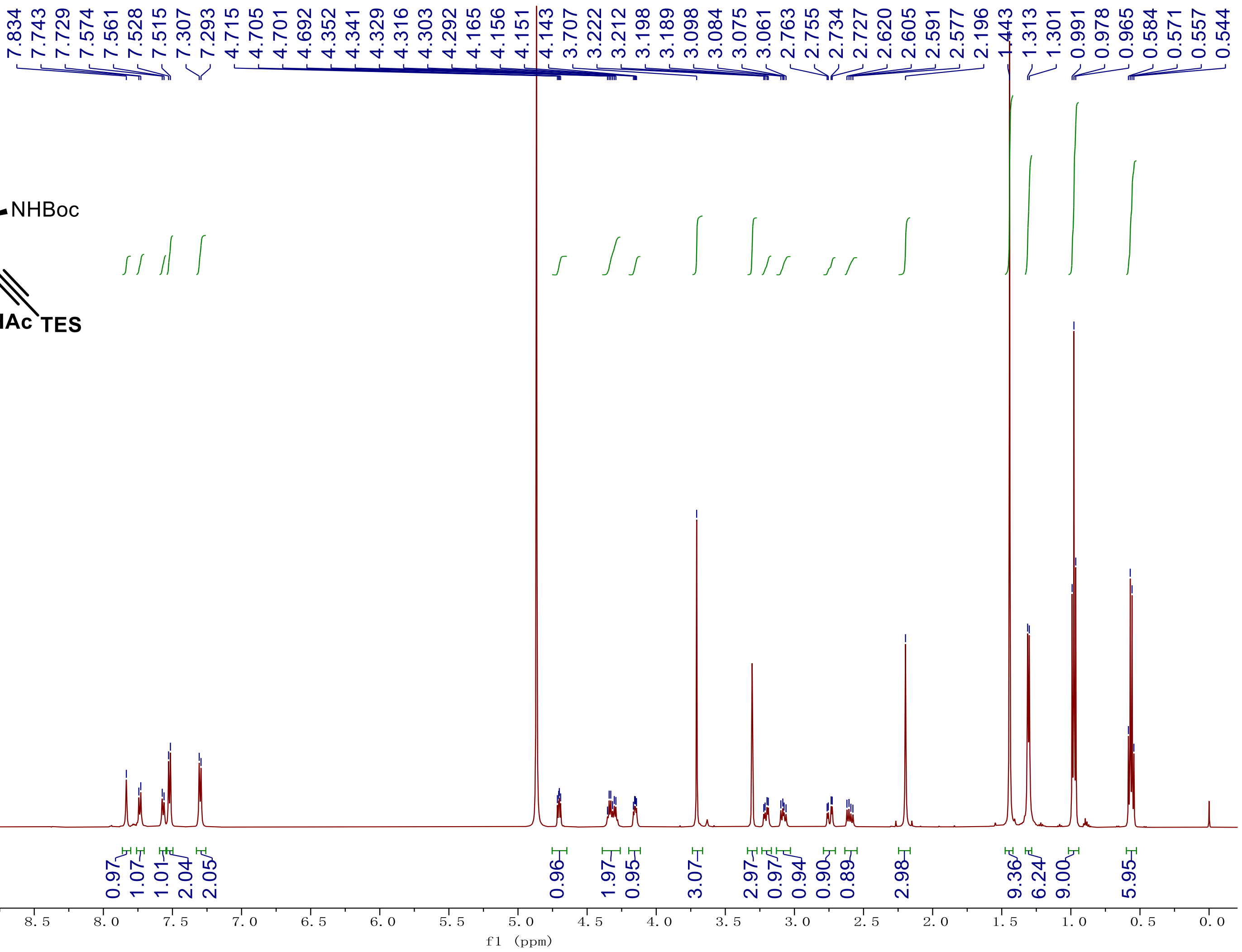

Compound 38b ^{13}C NMR (151 MHz, METHANOL-*D*4)

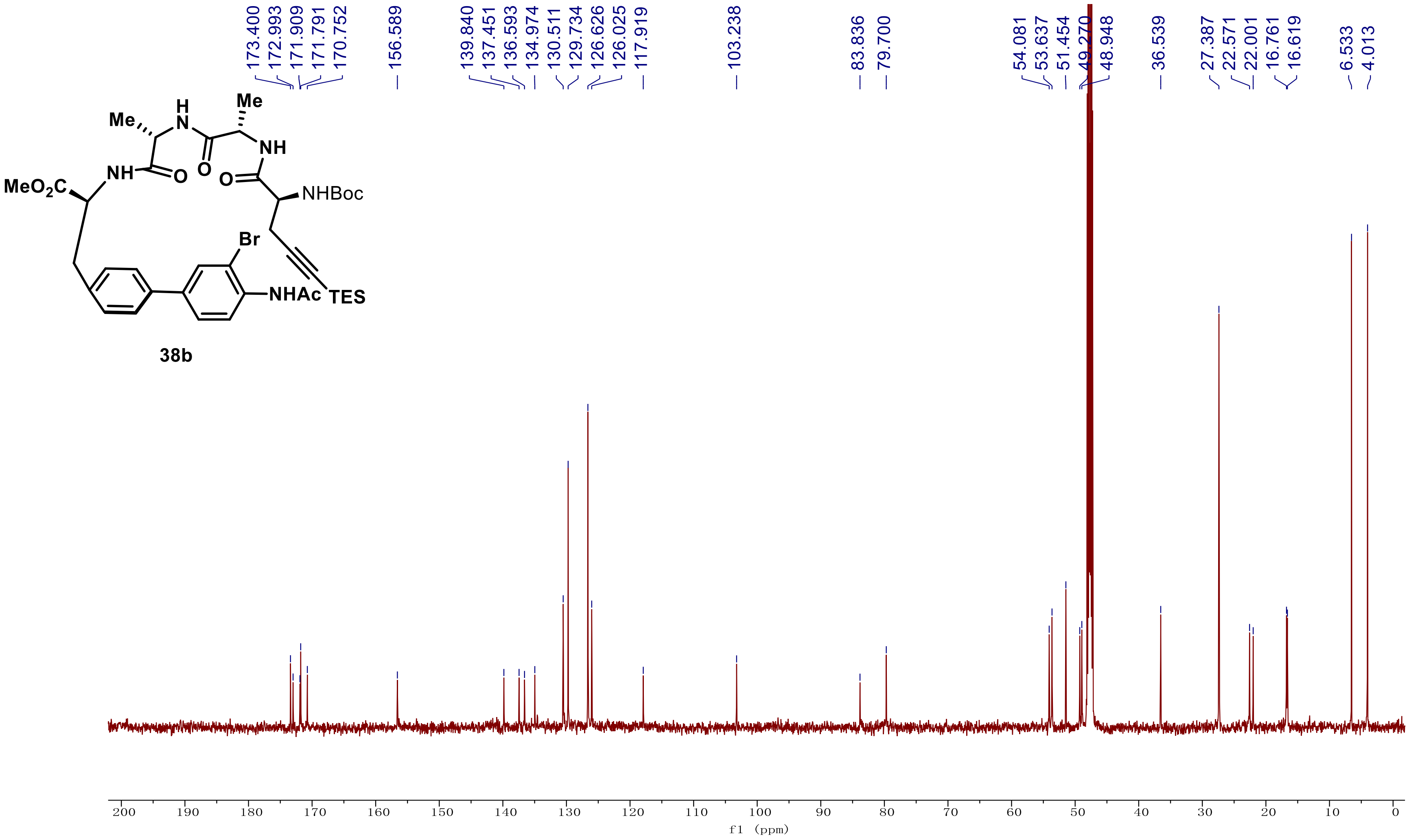

Compound 38c ^{1}H NMR (600 MHz, METHANOL-D_4)

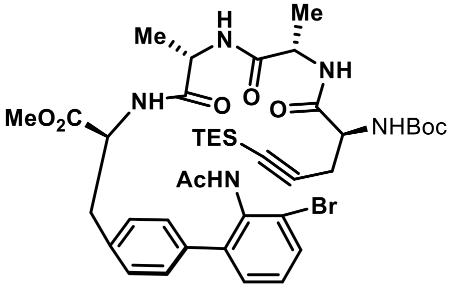

38c

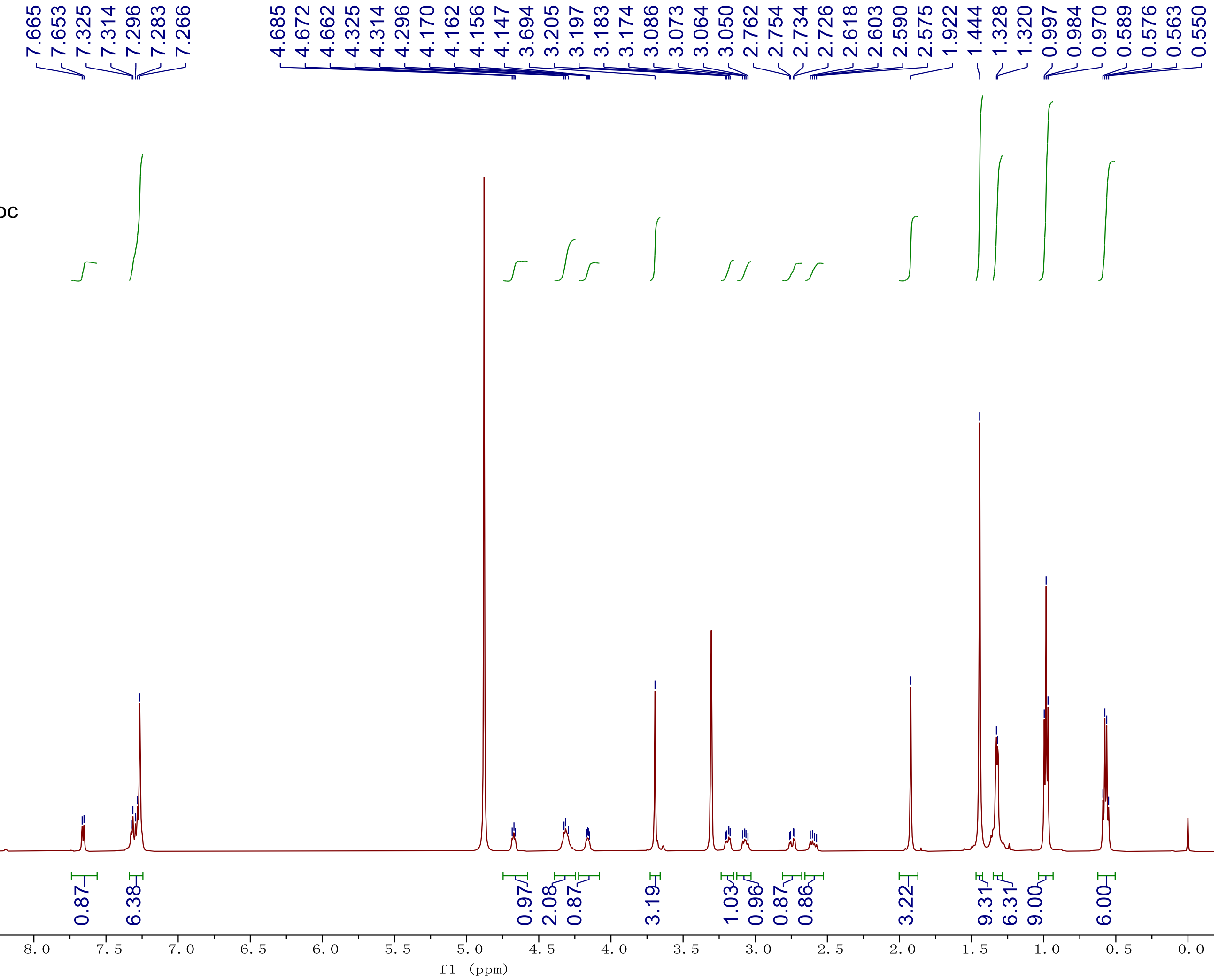

Compound 38c ^{13}C NMR (151 MHz, METHANOL-D_4)

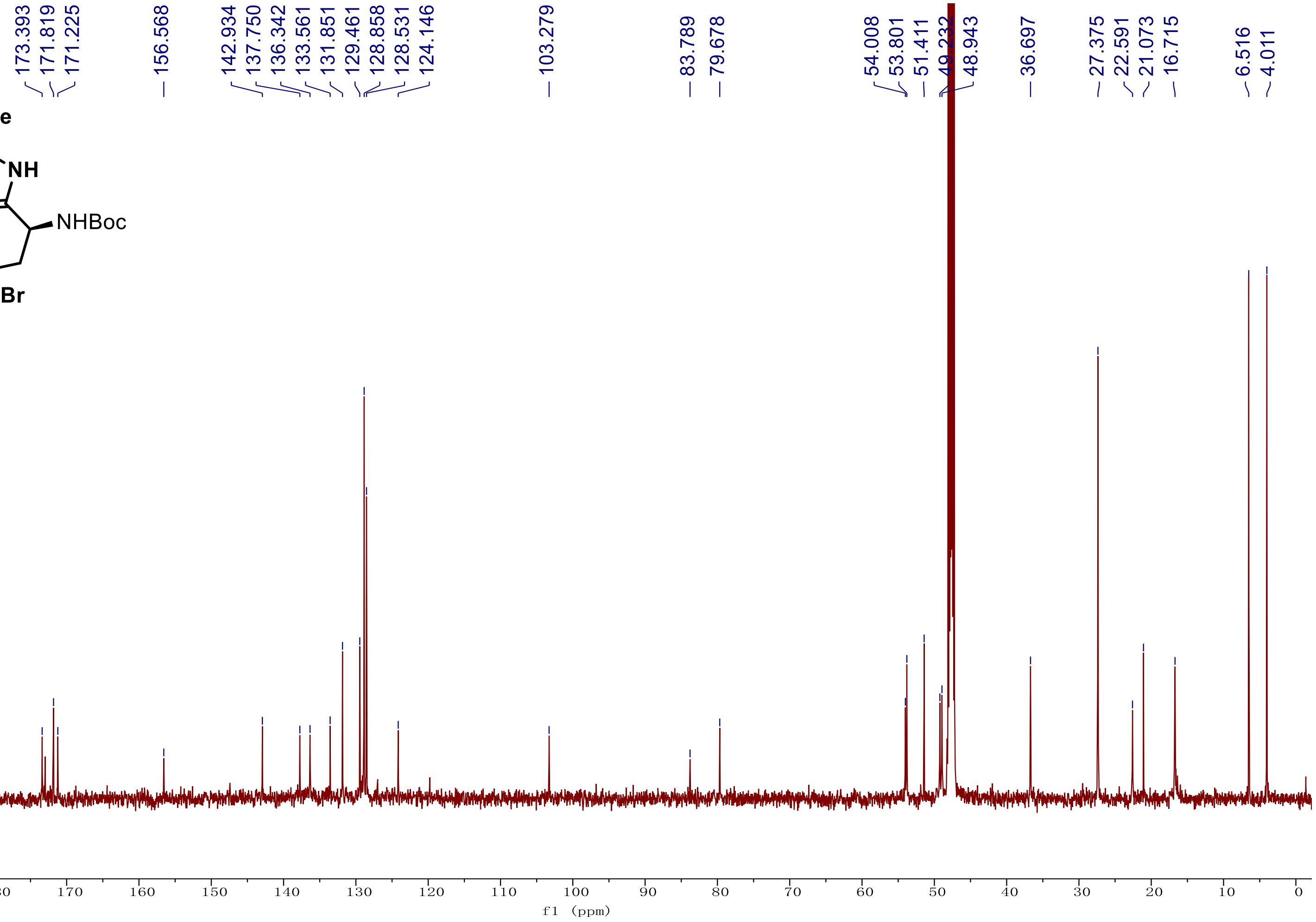

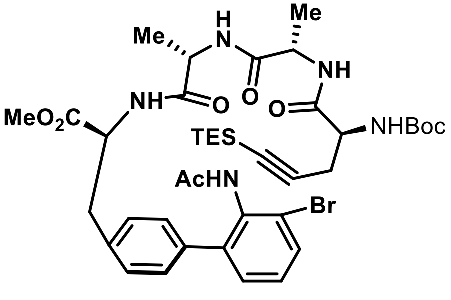

38c

Compound 38d ^{1}H NMR (600 MHz, METHANOL-D_4)

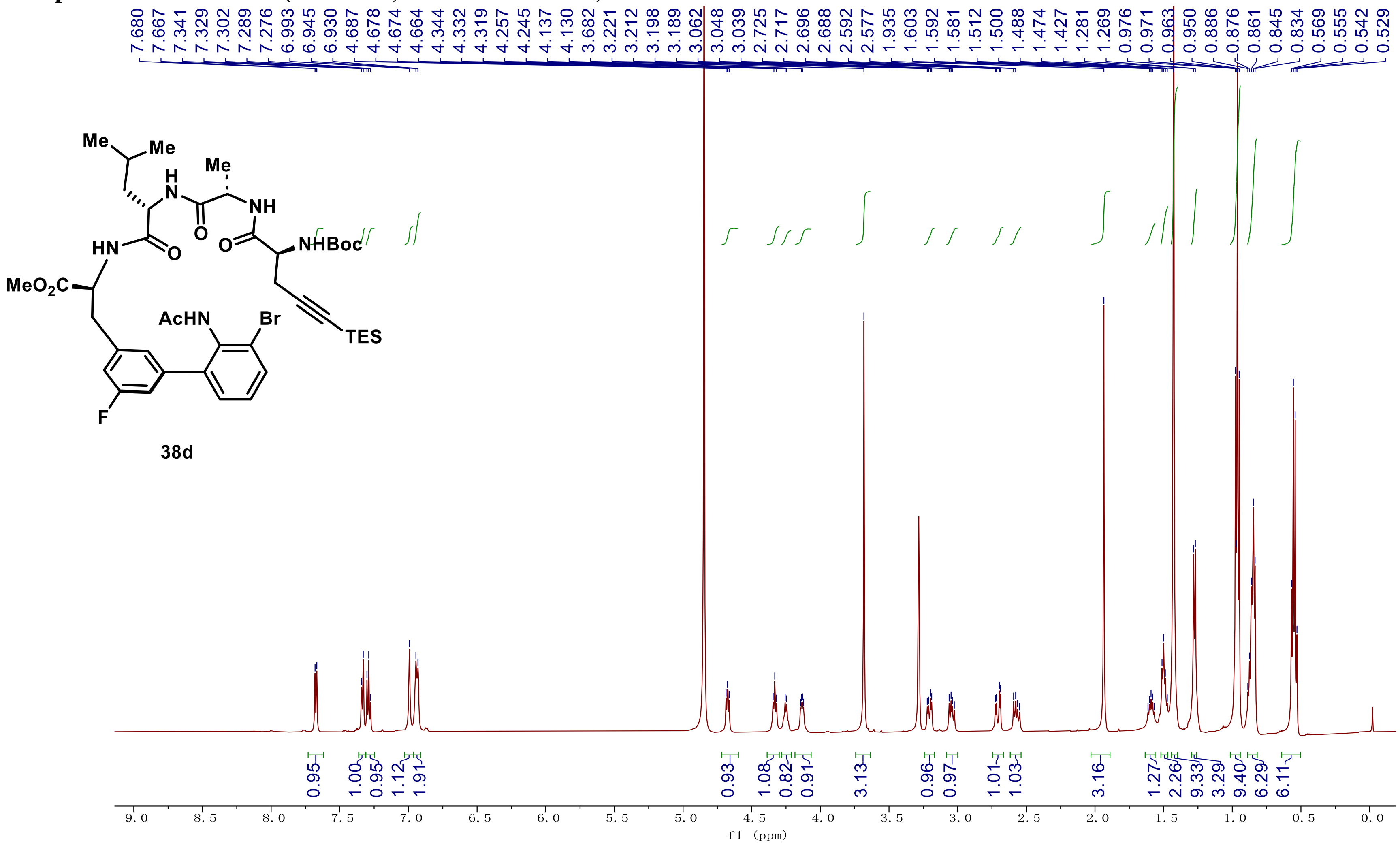

Compound 38d ^{13}C NMR (151 MHz, METHANOL-D_4)

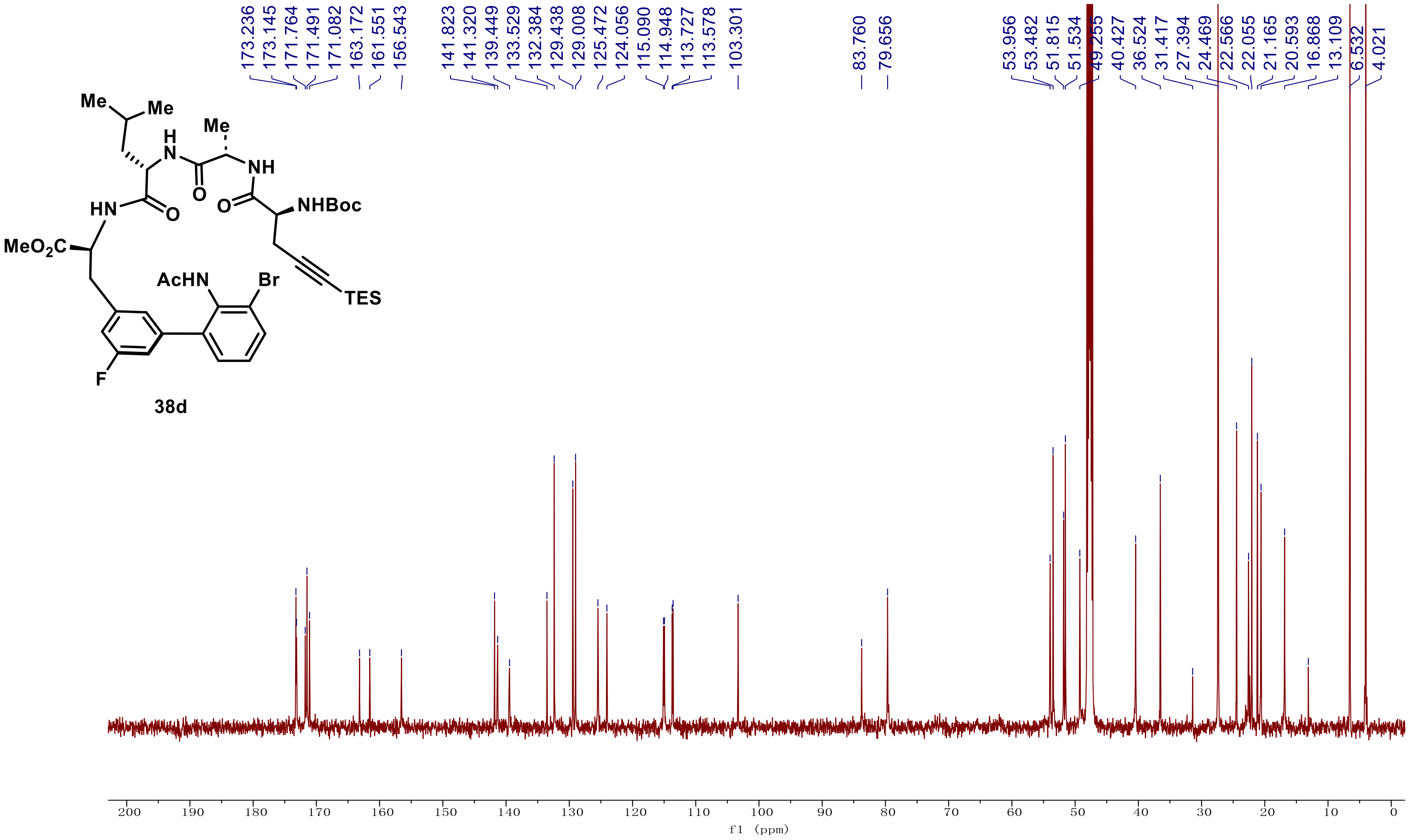

Compound 38d ^{19}F NMR (565 MHz, METHANOL-D_4)

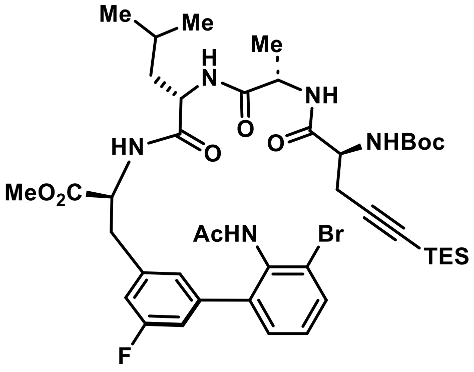

38d

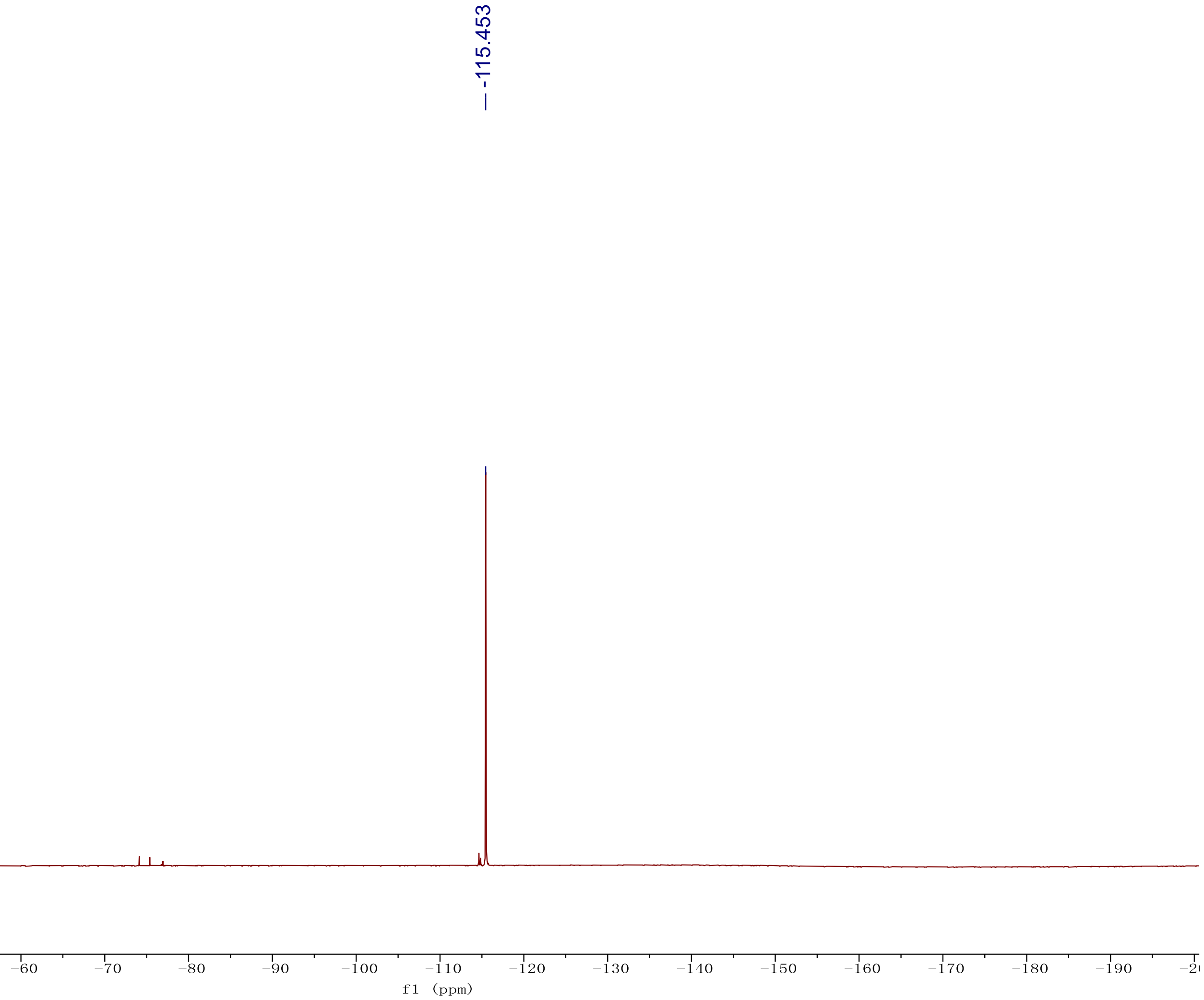

Compound 38e ^{1}H NMR (600 MHz, METHANOL-D_4)

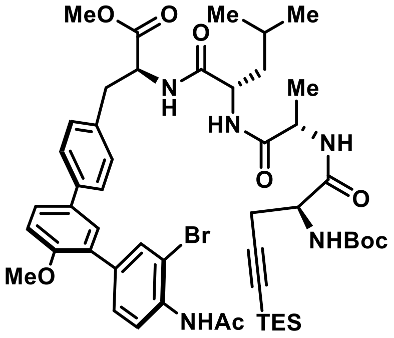

38e

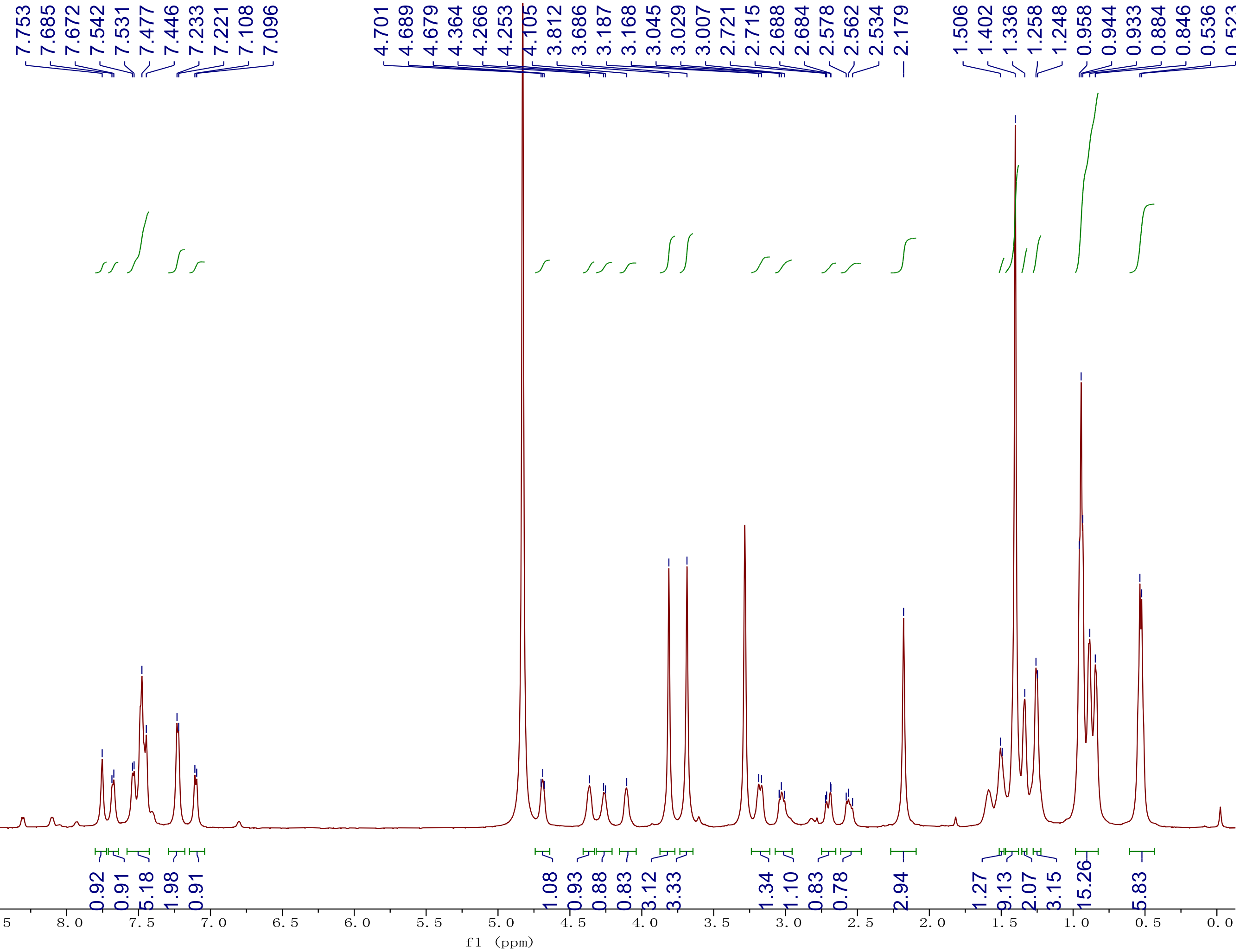

Compound 38e ^{13}C NMR (151 MHz, METHANOL-D_4)

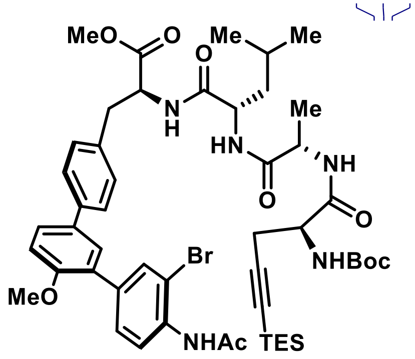

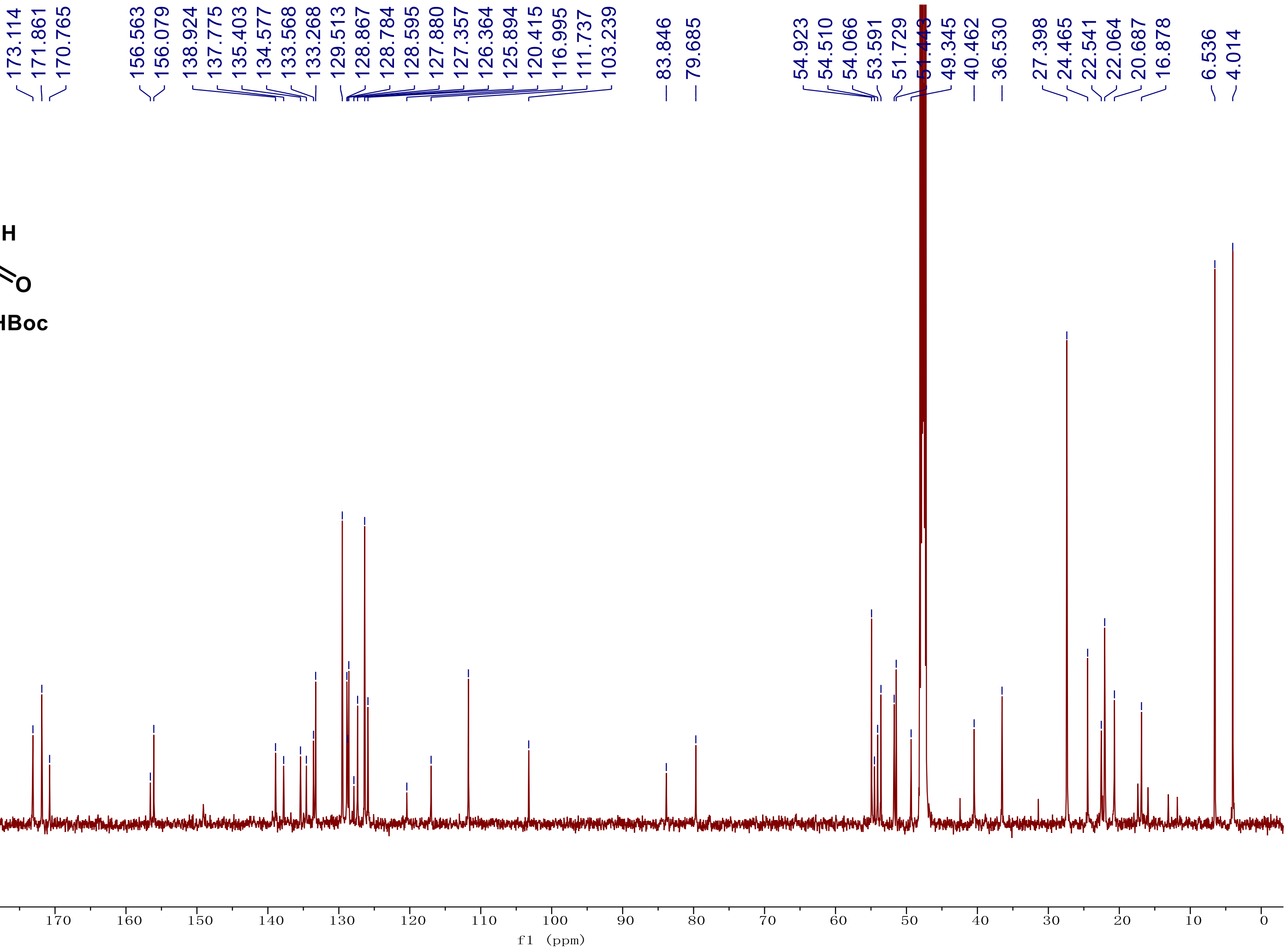

Compound 38f ^{1}H NMR (600 MHz, METHANOL-D_4)

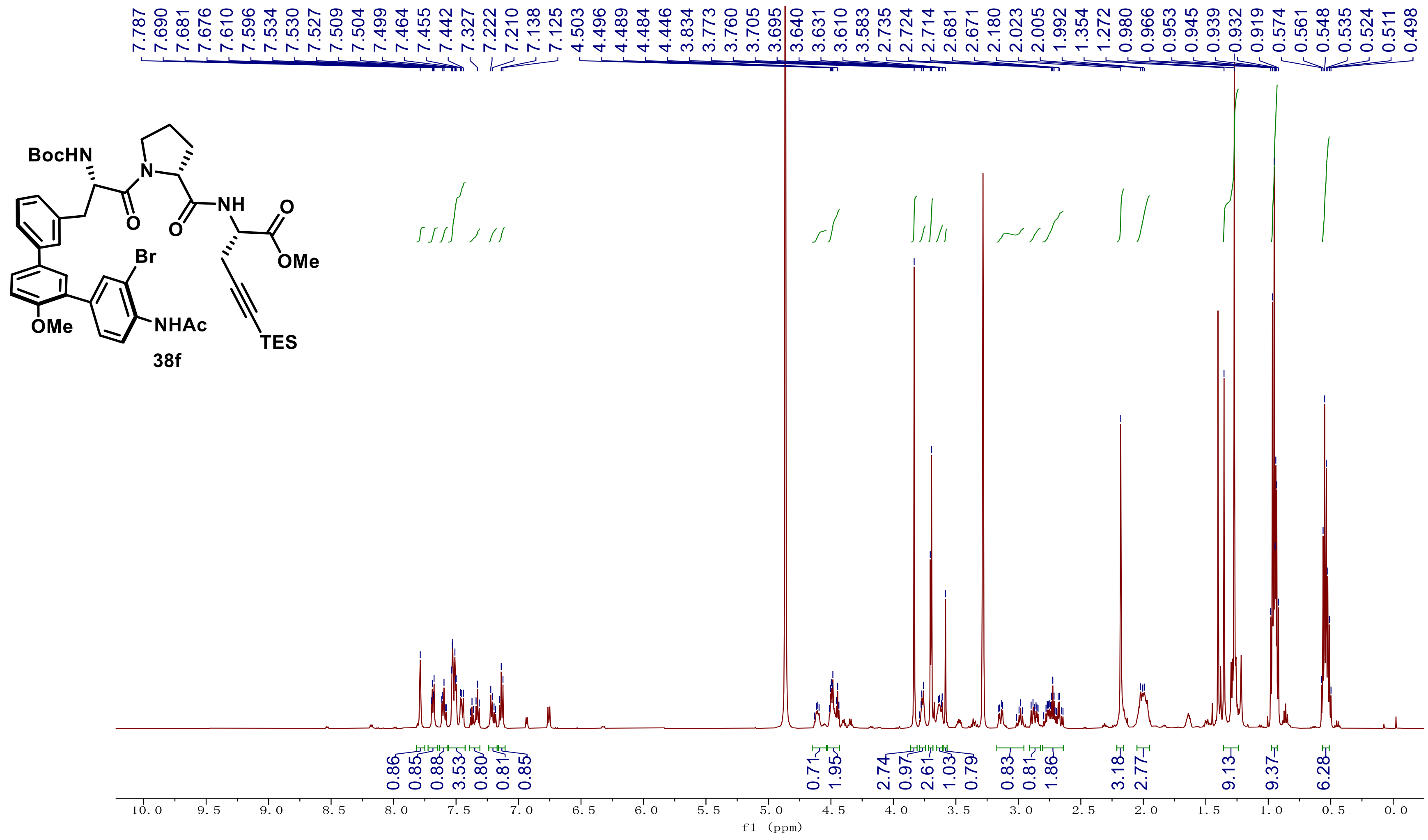

Compound 38f ^{13}C NMR (151 MHz, METHANOL-D_4)

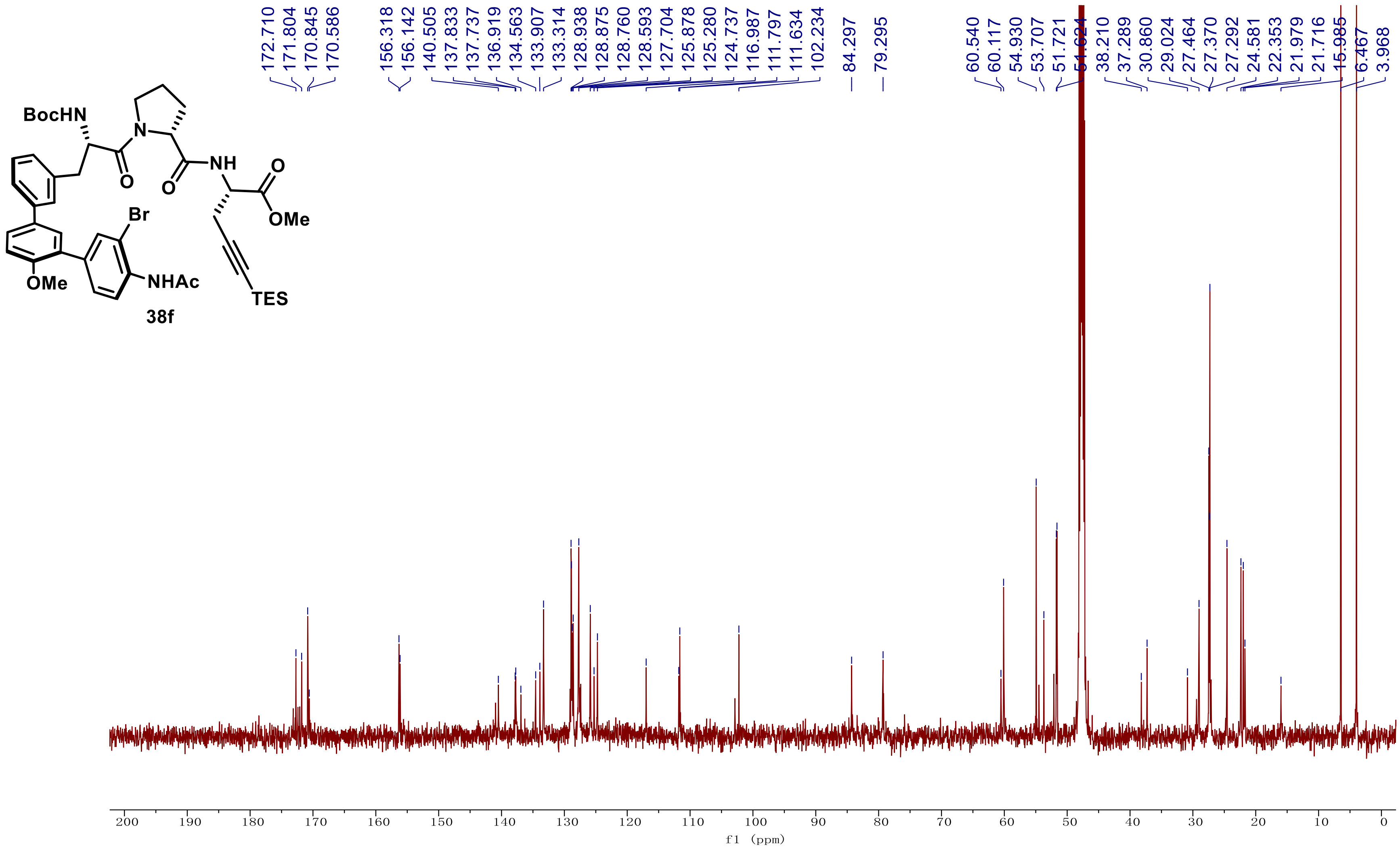

Compound 39a ¹H NMR (600 MHz, CDCl₃)

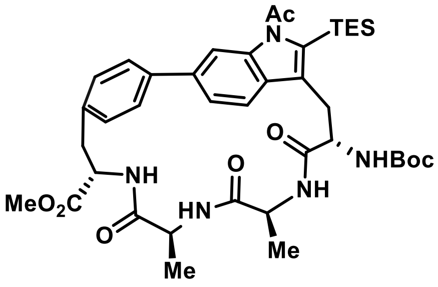

39a

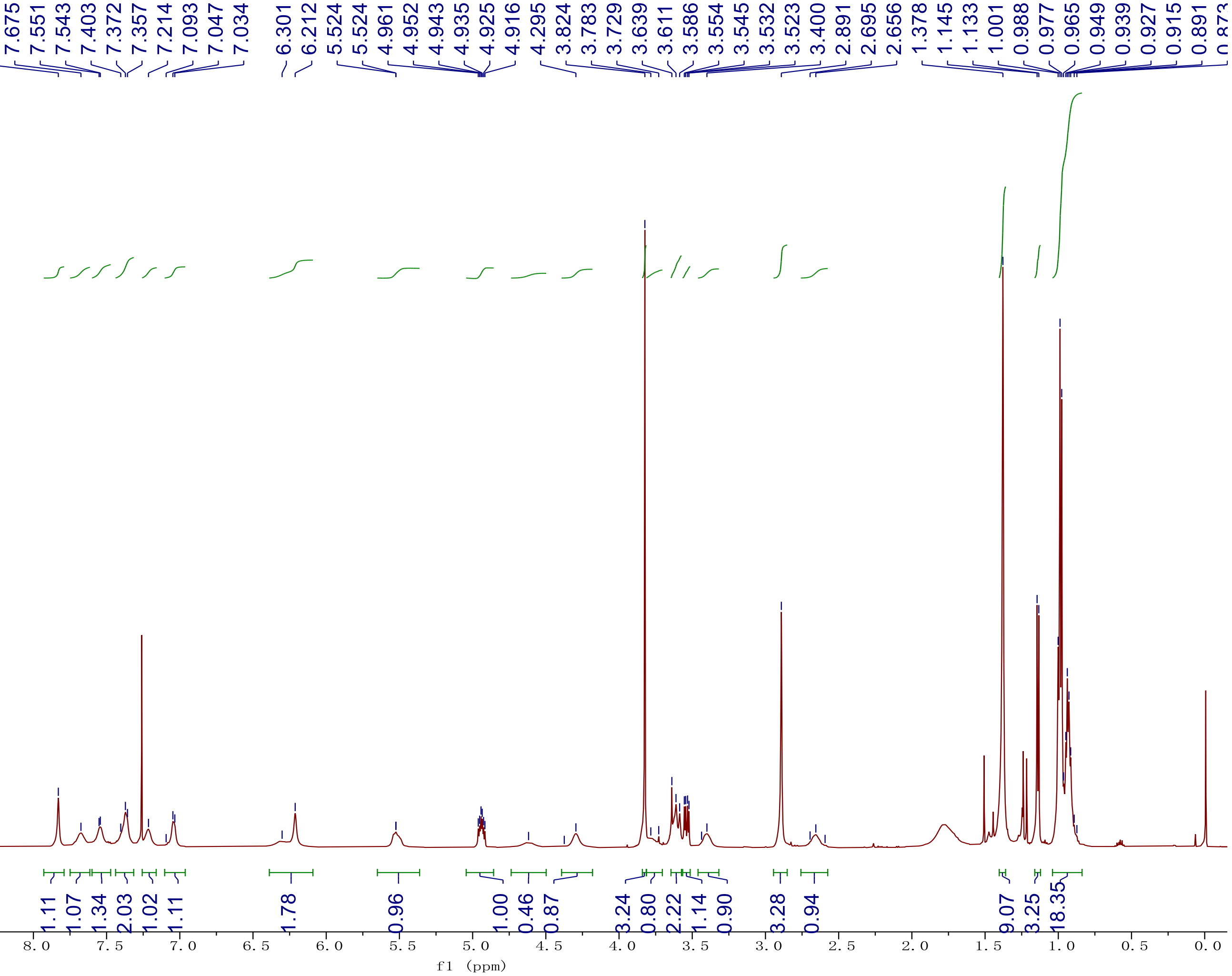

Compound 39a ^{1}H NMR (600 MHz, DMSO-d_6)

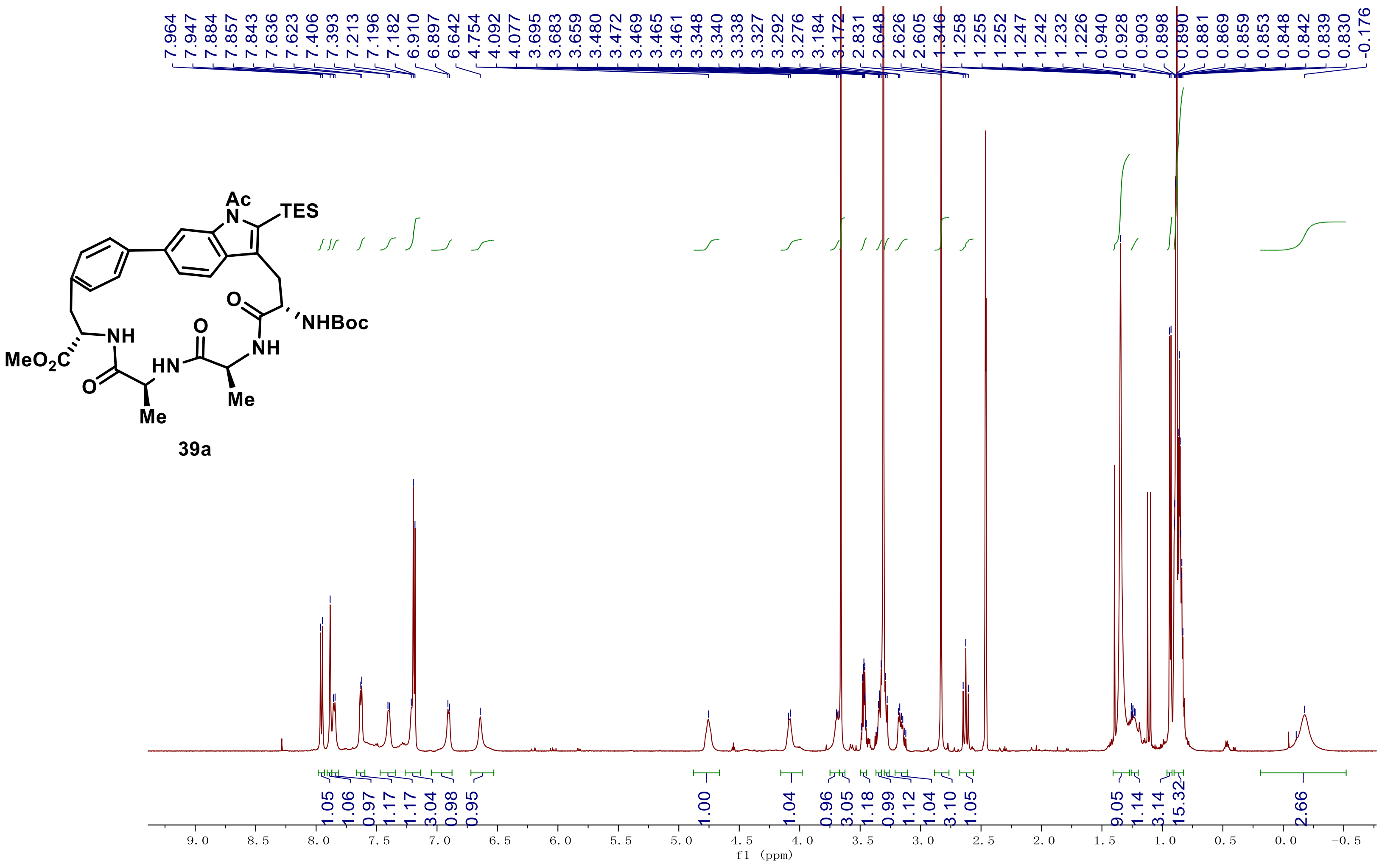

Compound 39a ^{13}C NMR (151 MHz, $CDCl_3$)

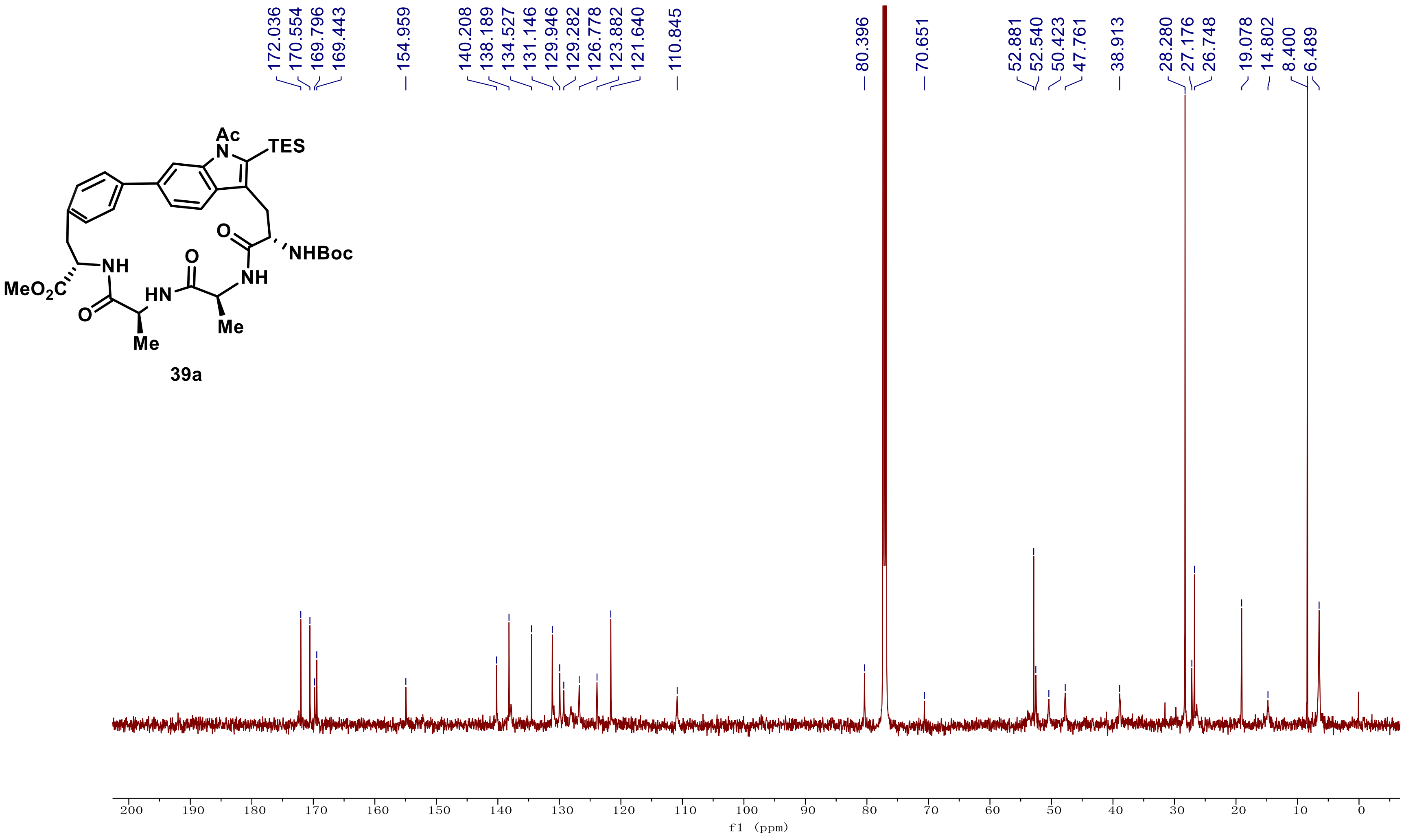

Compound 39a ^{13}C NMR (151 MHz, DMSO-d_6)

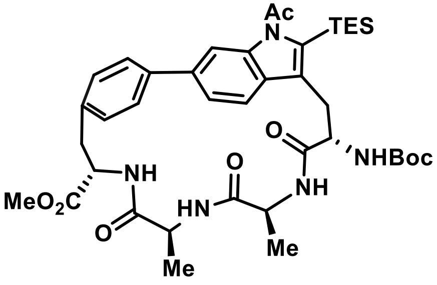

39a

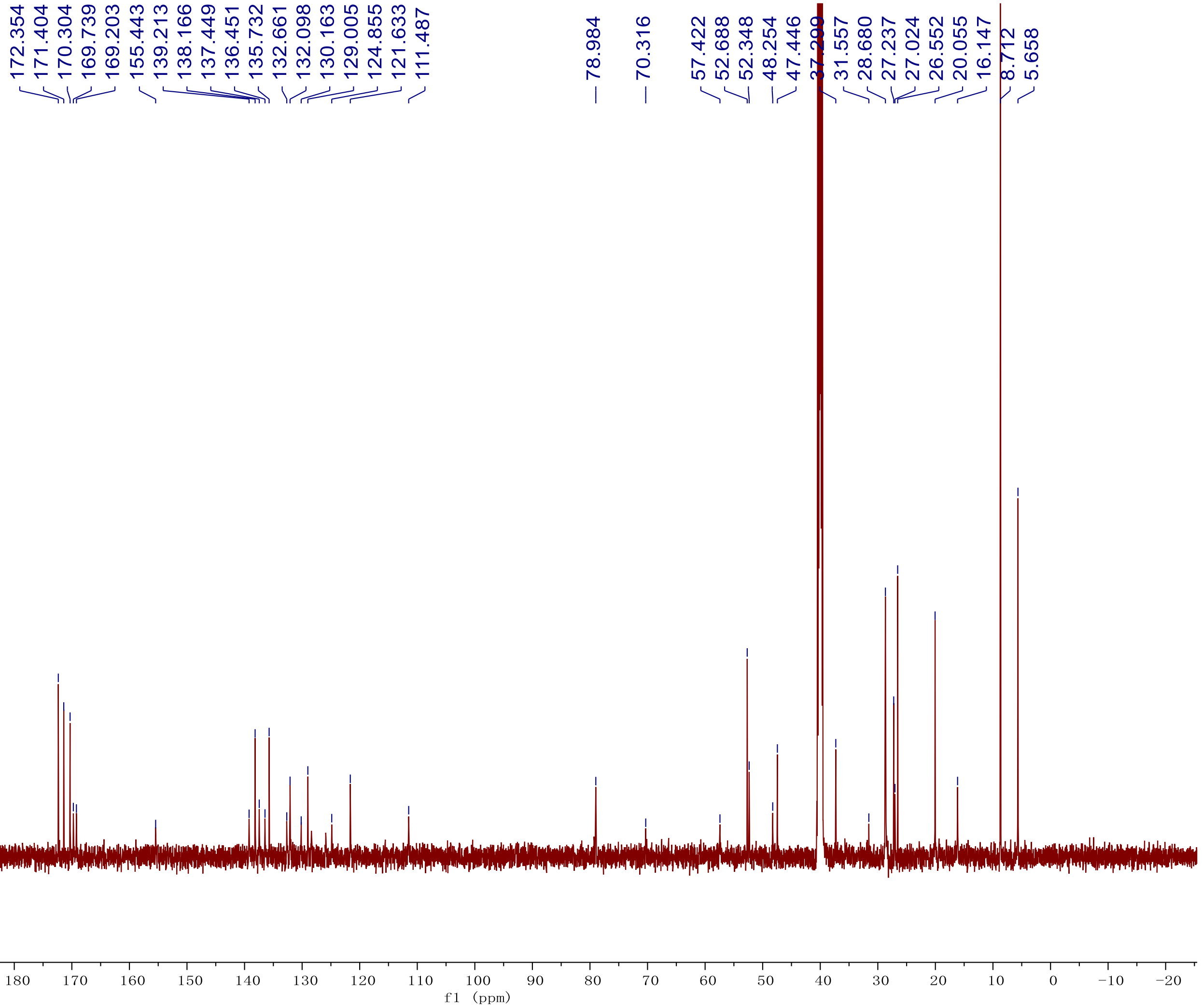

Compound 39a ROESY (400 MHz, DMSO-d_6)

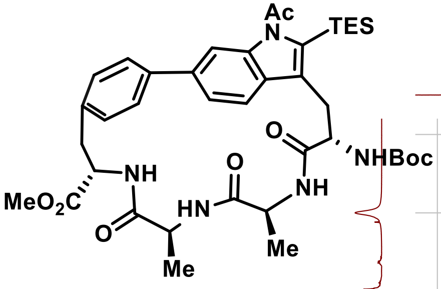

39a

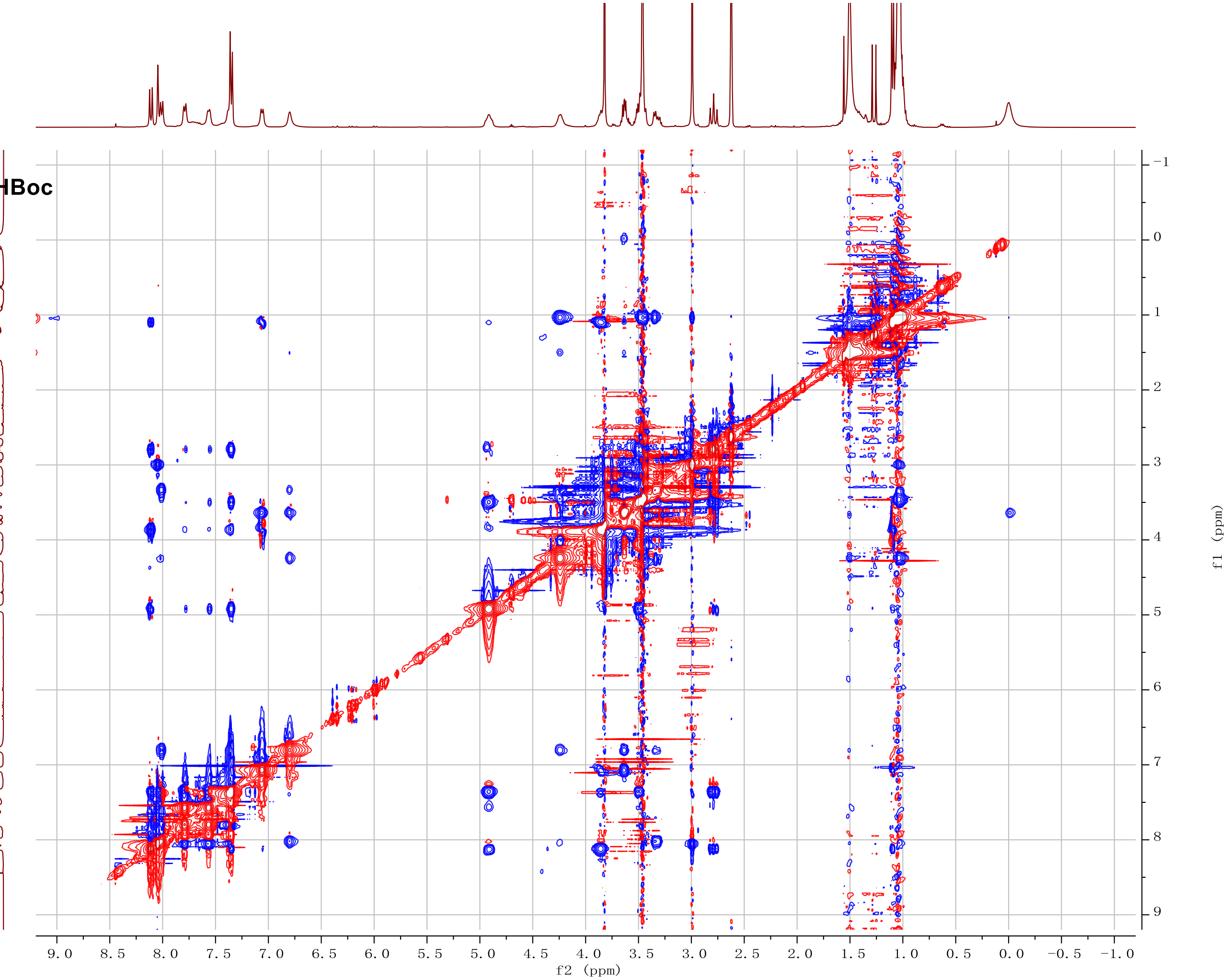

LCMS trace of Compound 39a

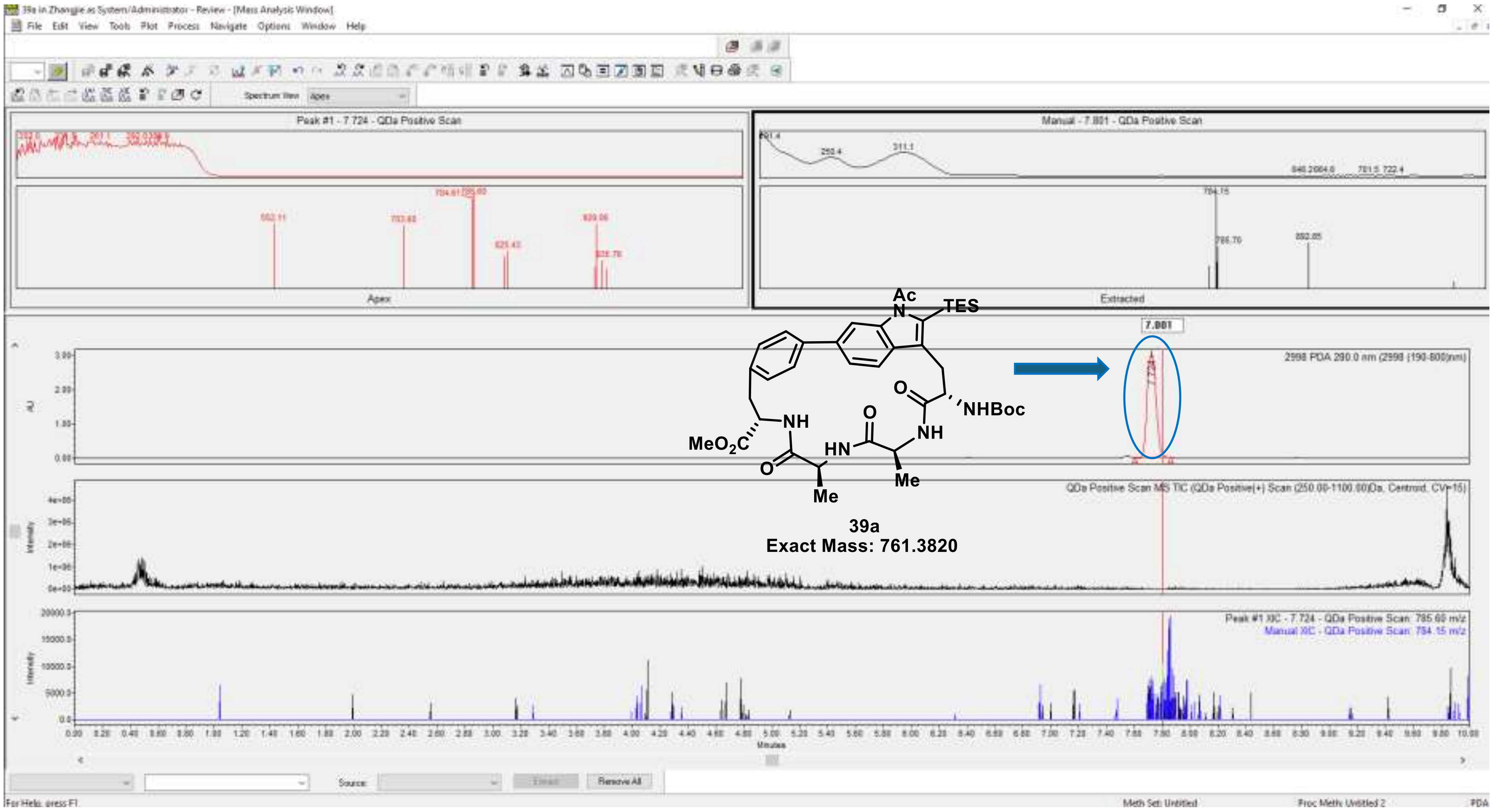

Compound 39b ^{1}H NMR (600 MHz, $CDCl_3$)

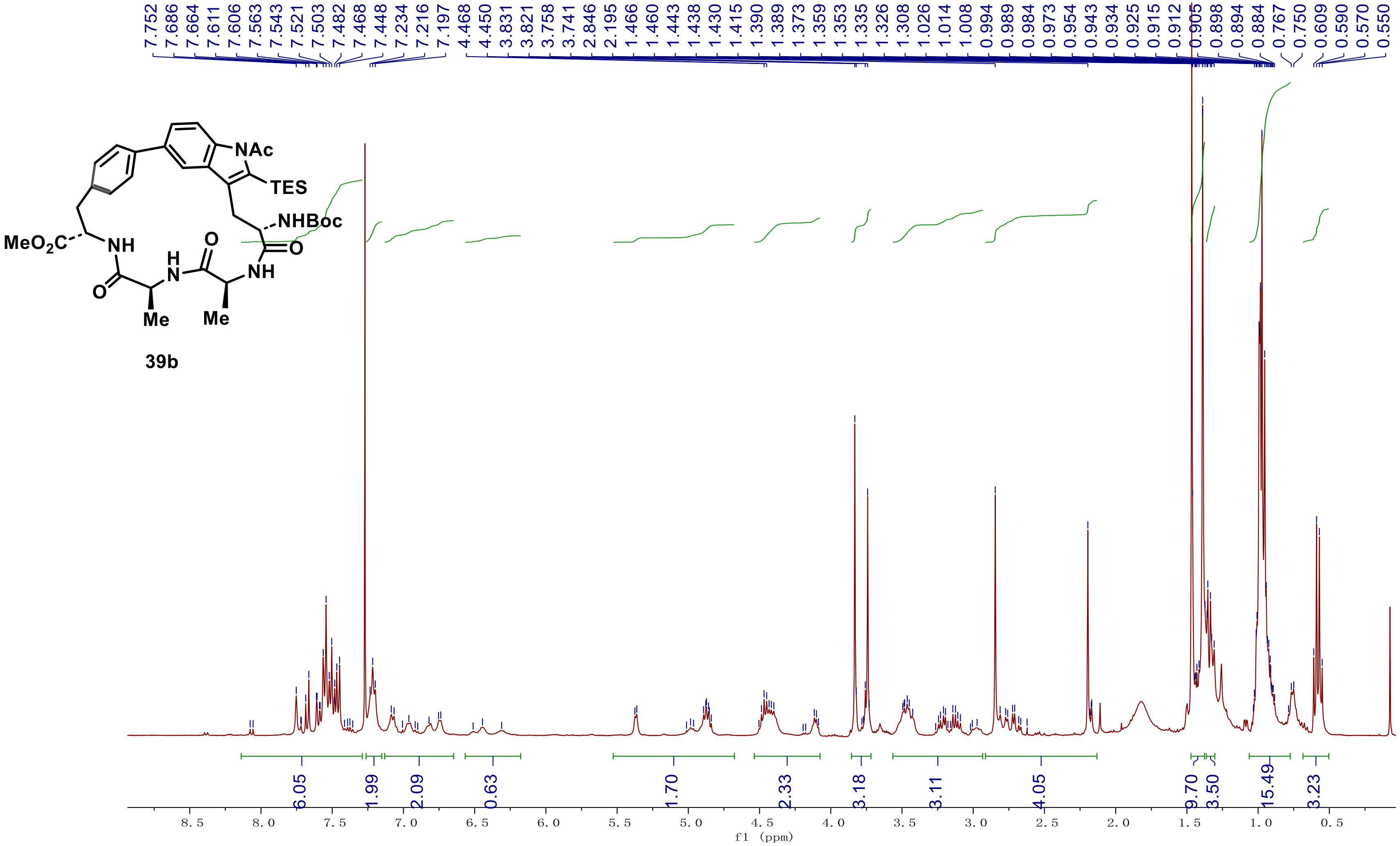

Compound 39b ^{13}C NMR (151 MHz, $CDCl_3$)

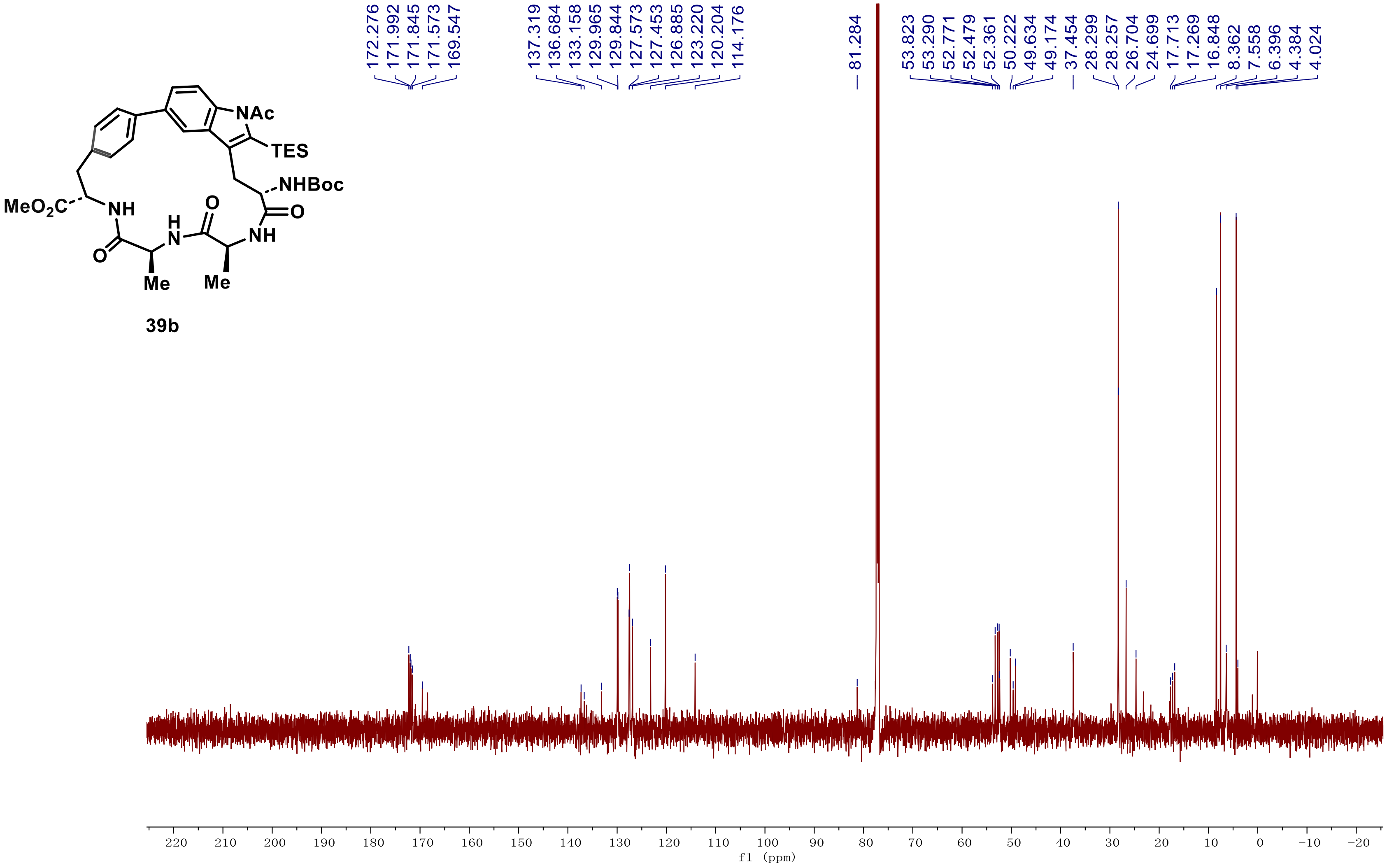

LCMS trace of Compound 39b

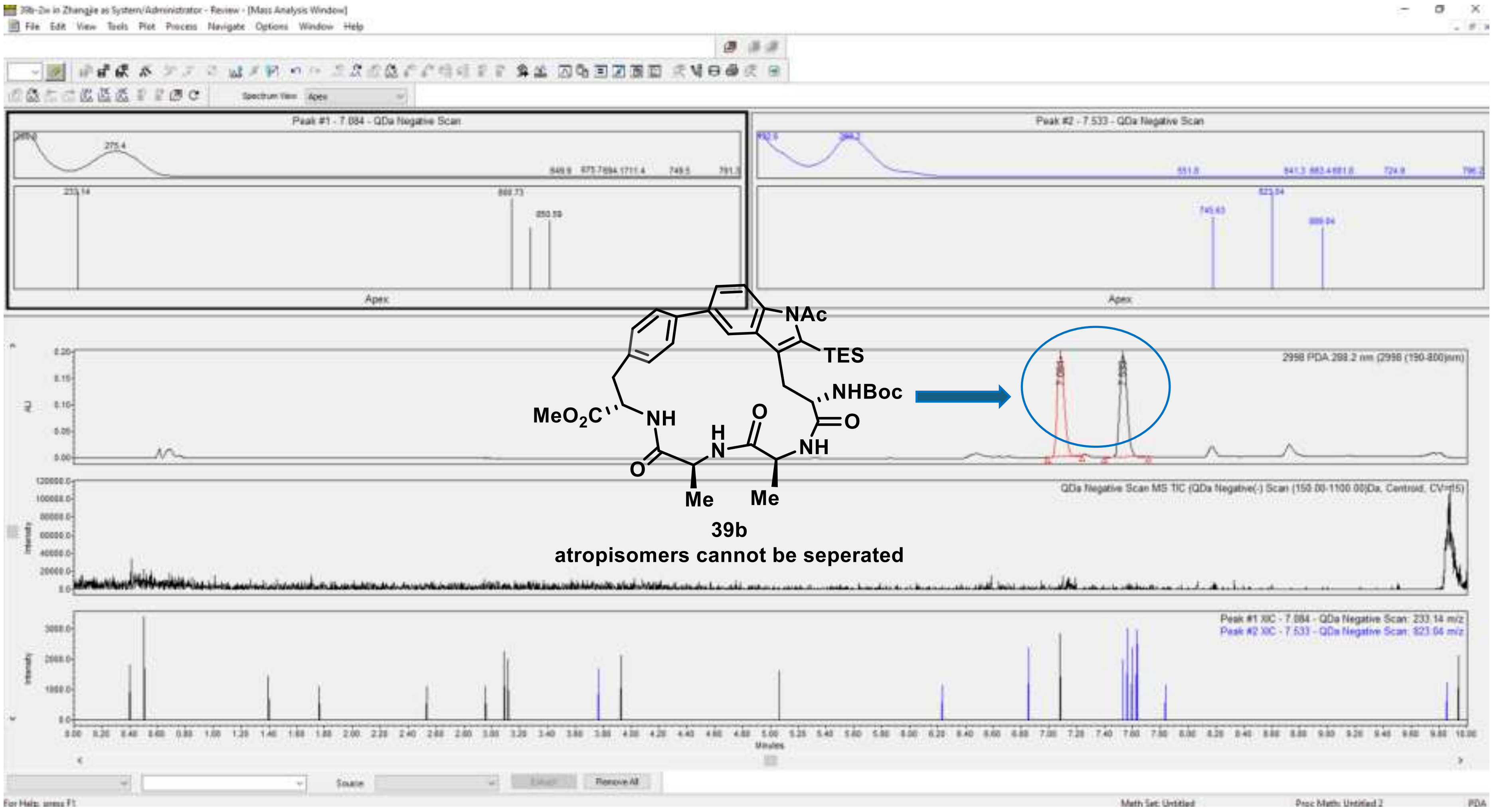

Compound 39c-major ^{1}H NMR (600 MHz, CDCl₃)

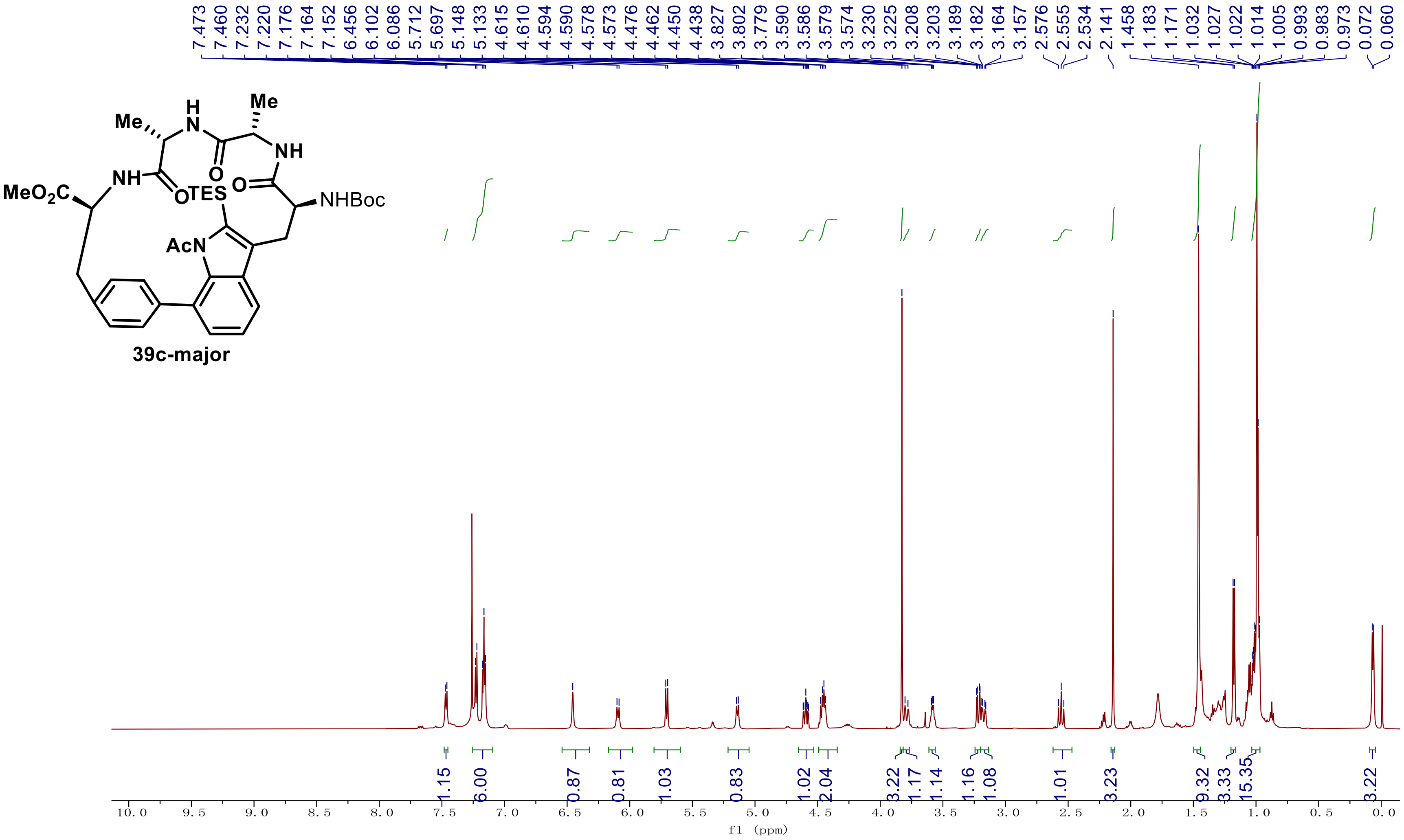

39c-major

Compound 39c-major ^{1}H NMR (600 MHz, DMSO-d_6)

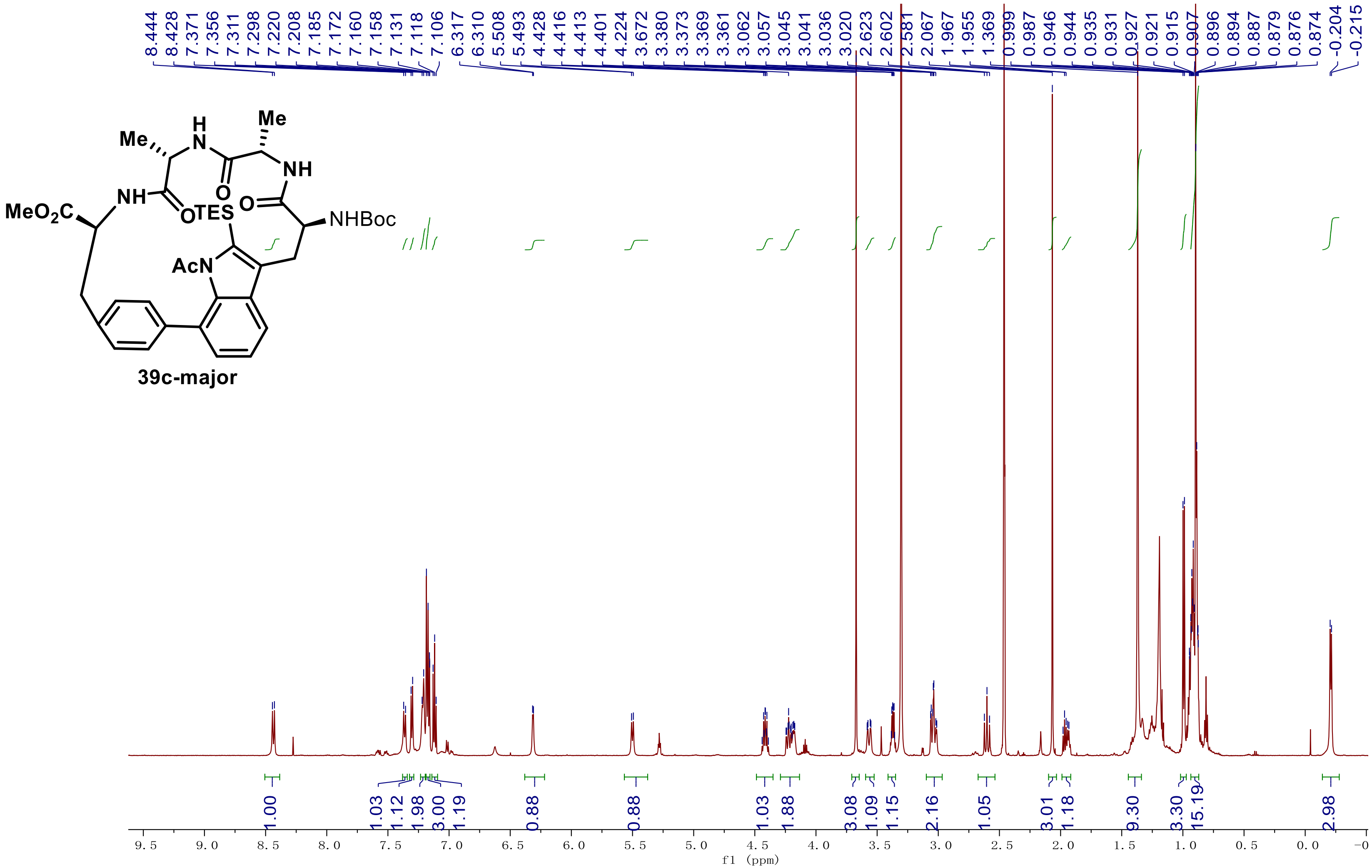

Compound 39c-major ^{13}C NMR (151 MHz, $CDCl_3$)

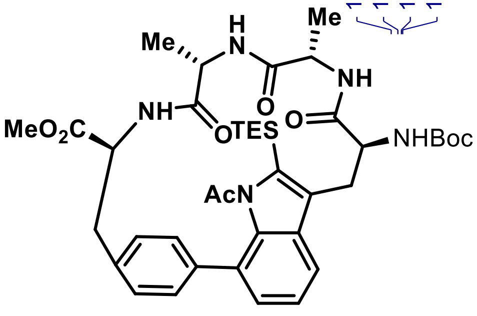

39c-major

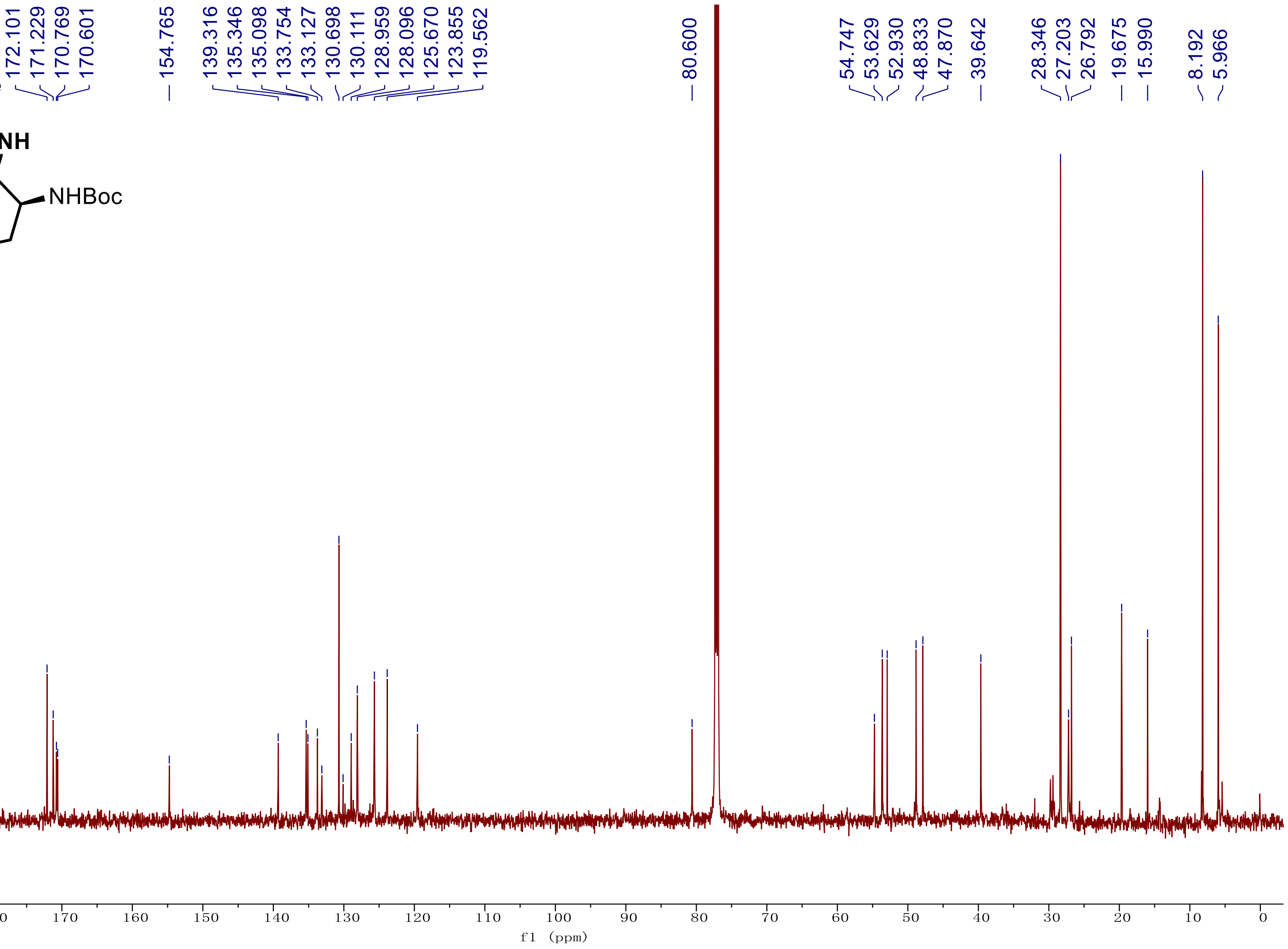

Compound 39c-major ^{13}C NMR (151 MHz, DMSO-d_6)

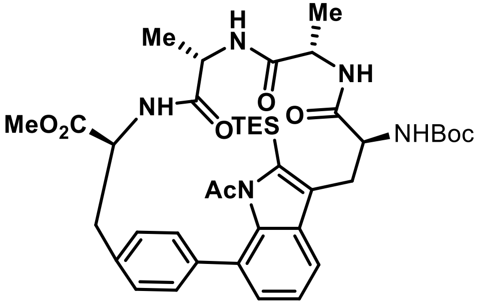

39c-major

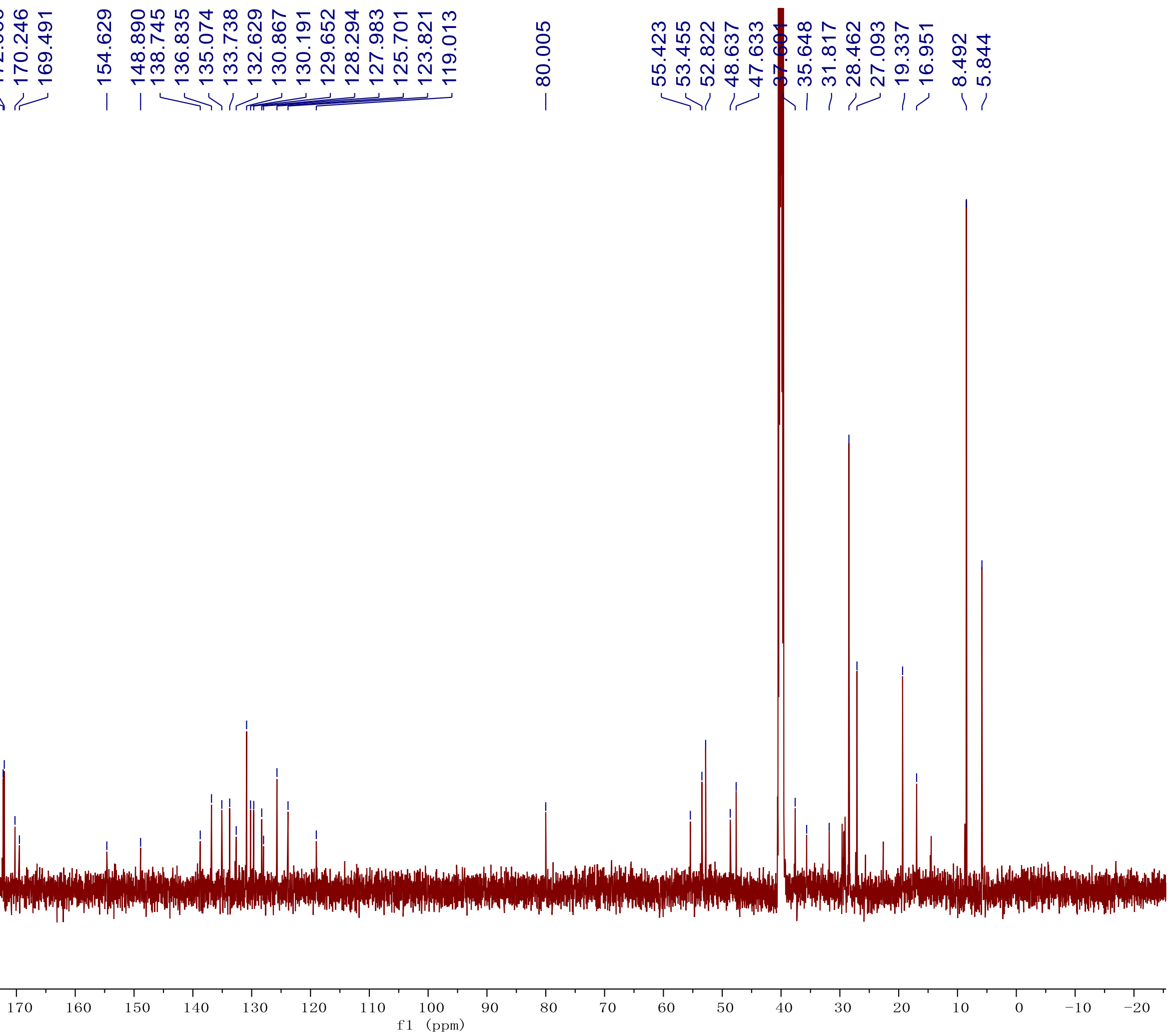

Compound 39c-major ROESY (400 MHz, DMSO-d_6)

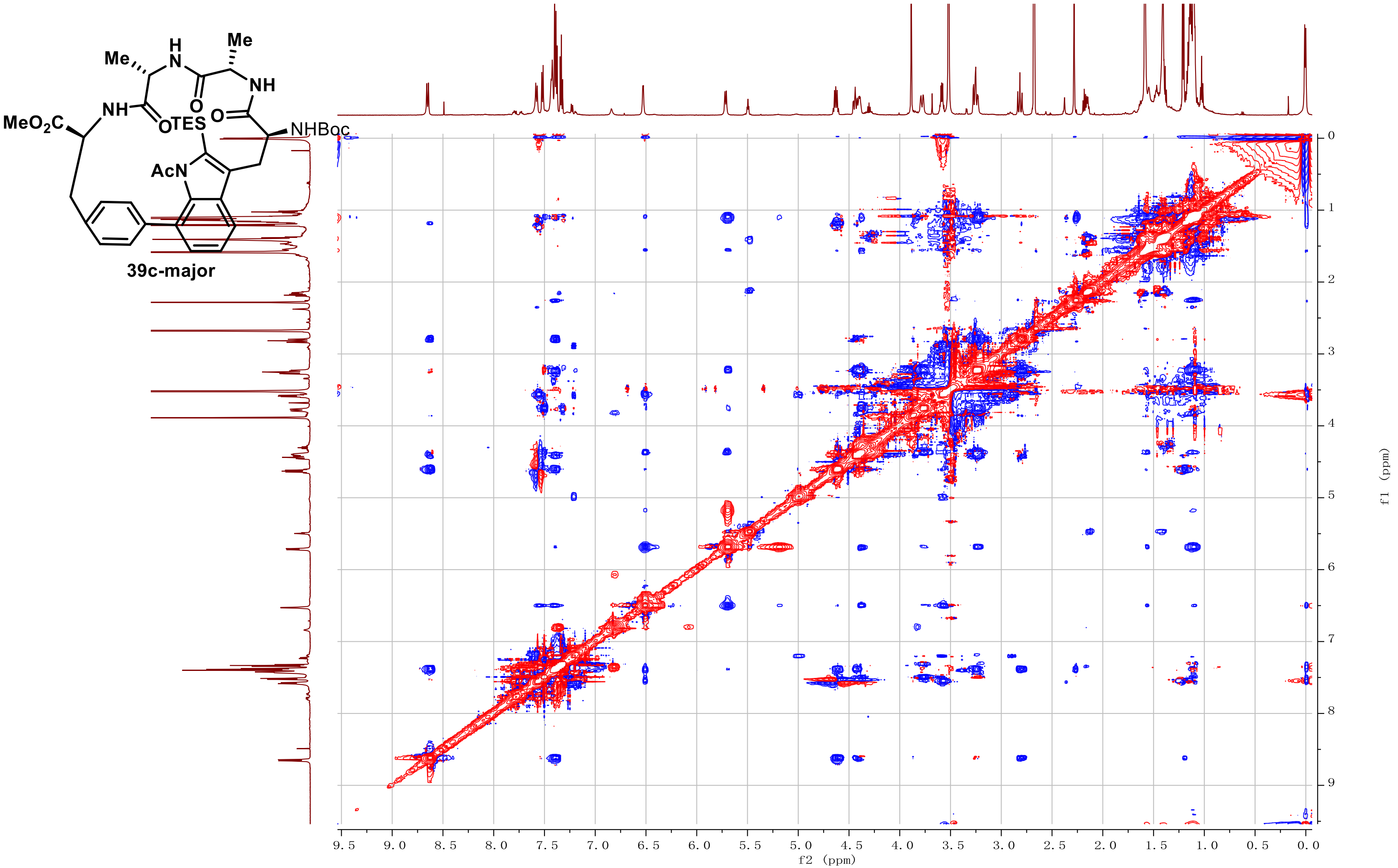

LCMS trace of Compound 39c-major

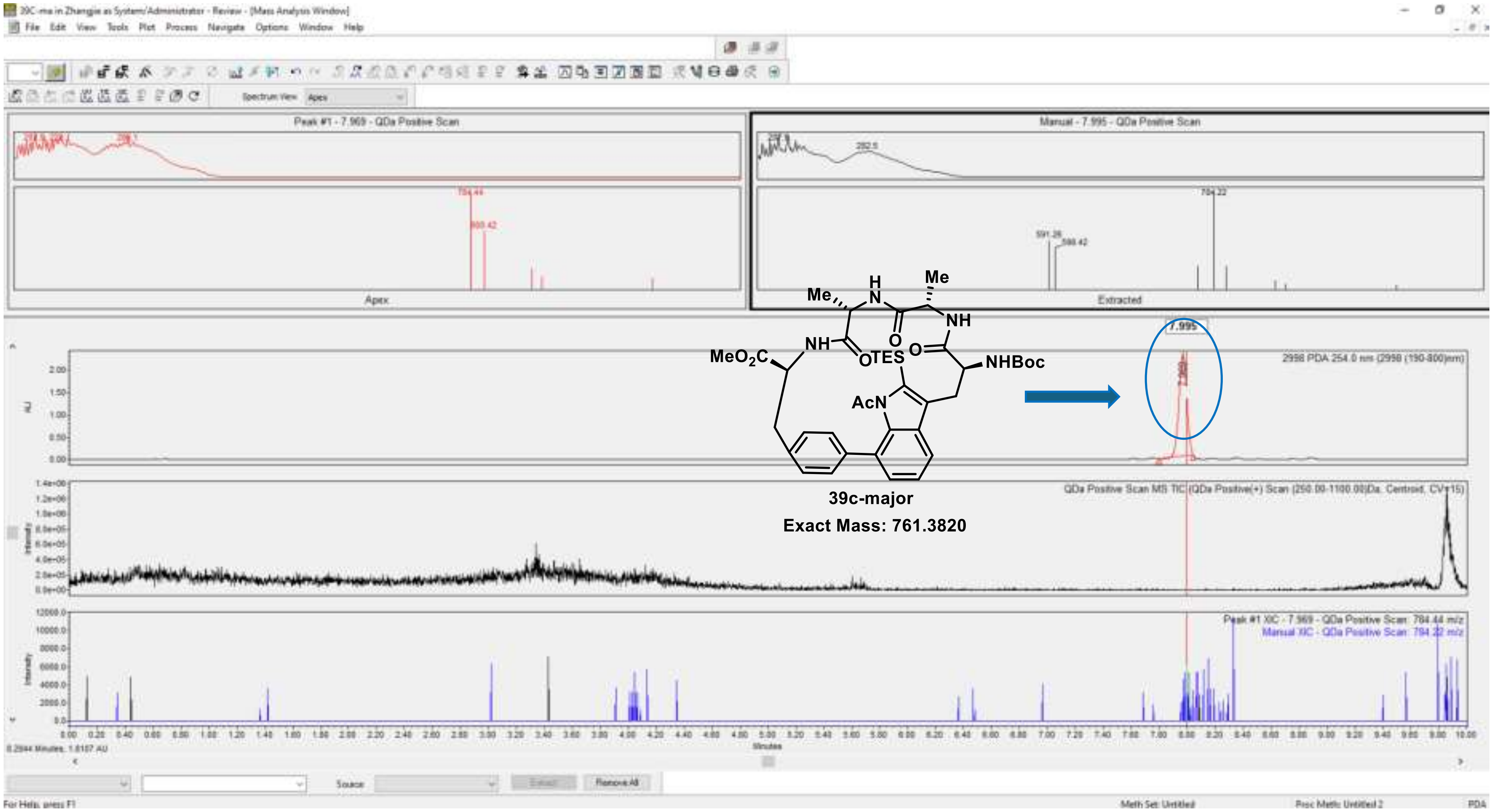

Compound 39c-minor ¹H NMR (600 MHz, CDCl₃)

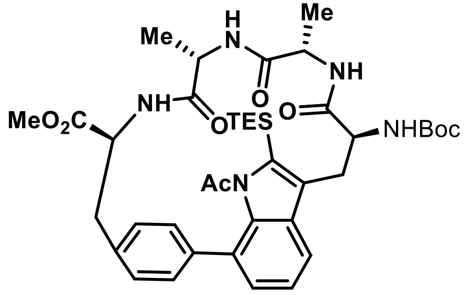

39c-minor

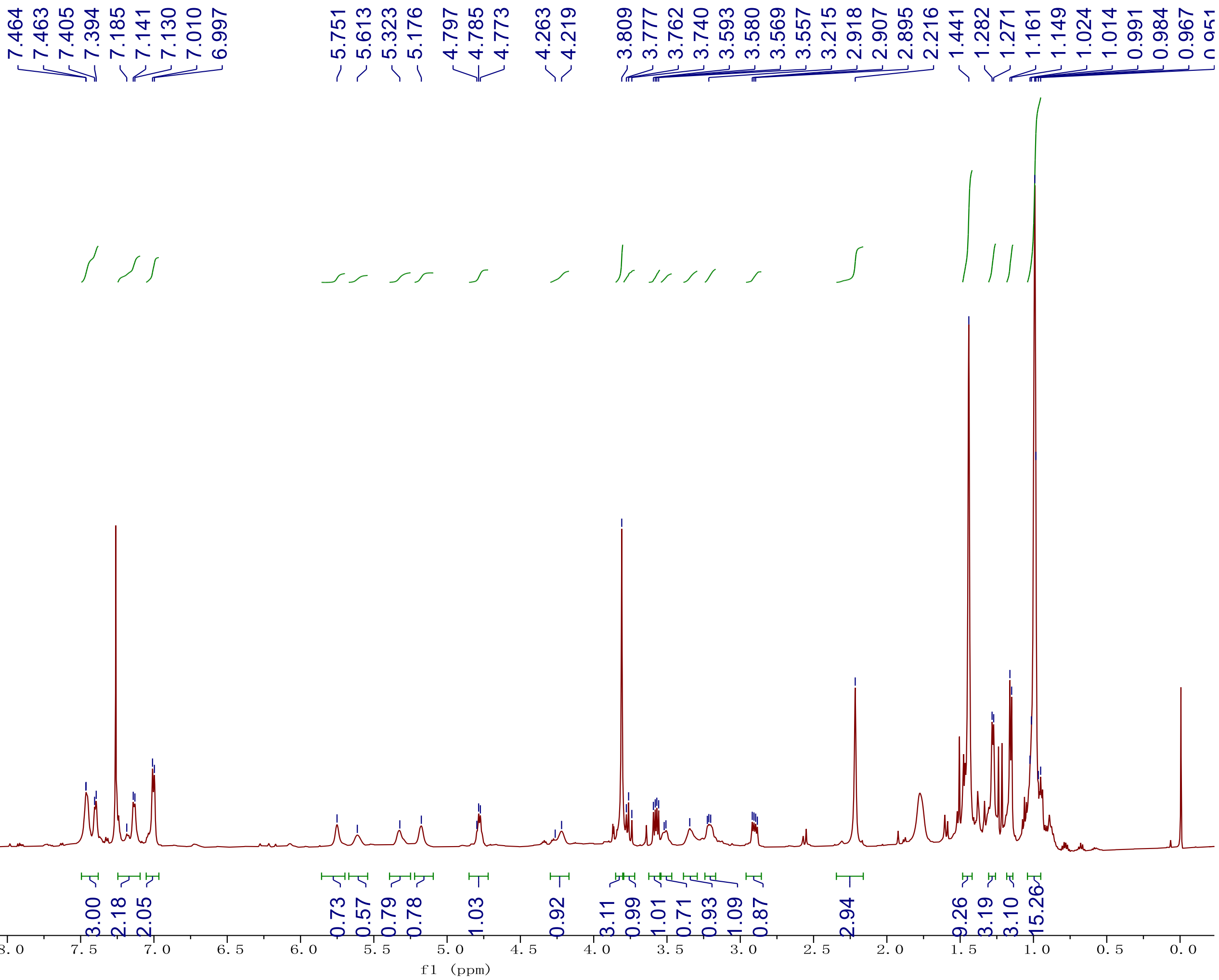

Compound 39c-minor ^{13}C NMR (151 MHz, $CDCl_3$)

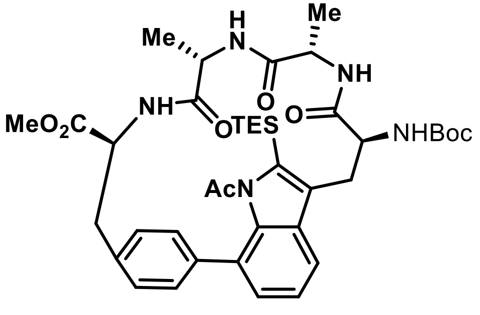

39c-minor

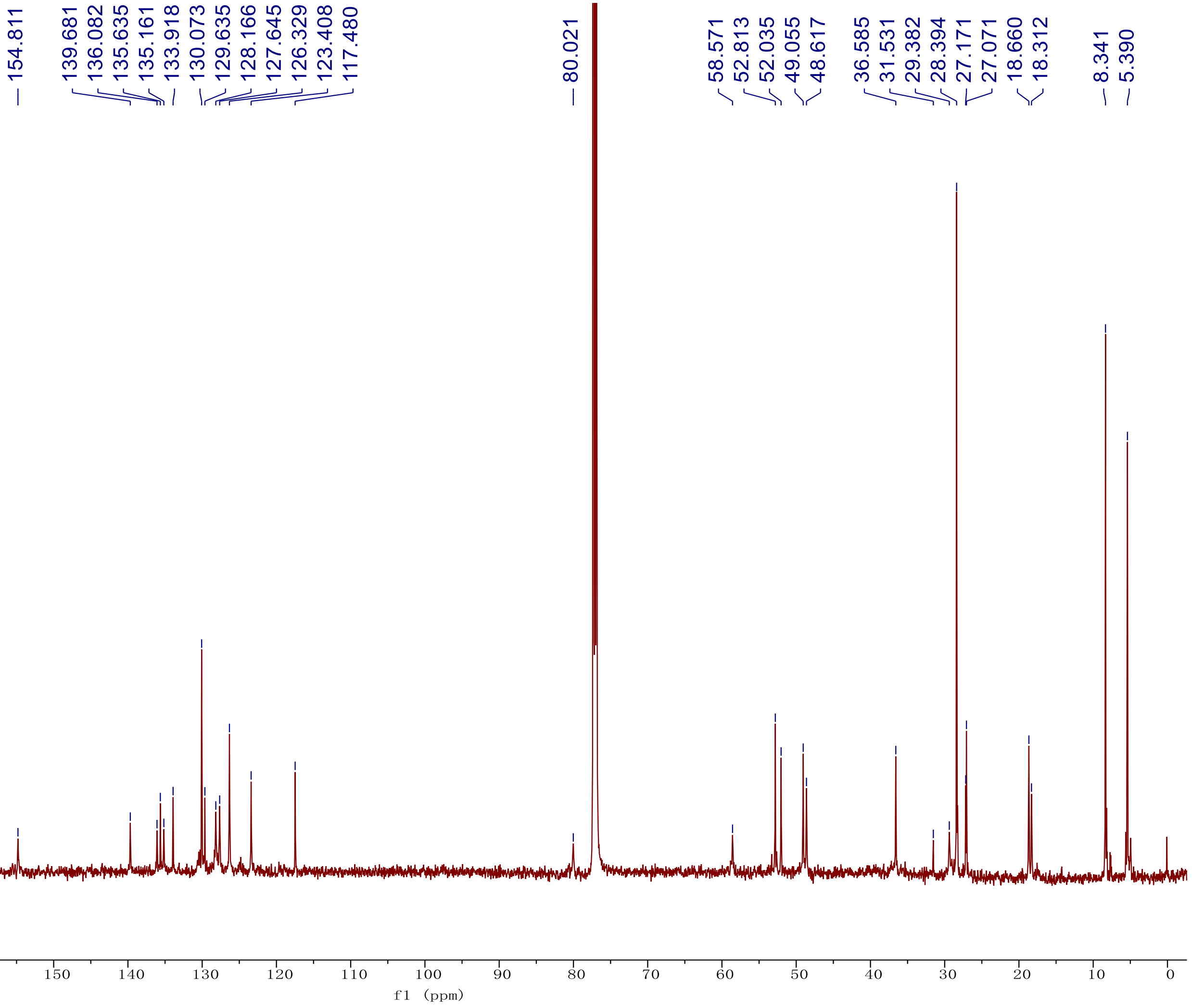

LCMS trace of Compound 39c-minor

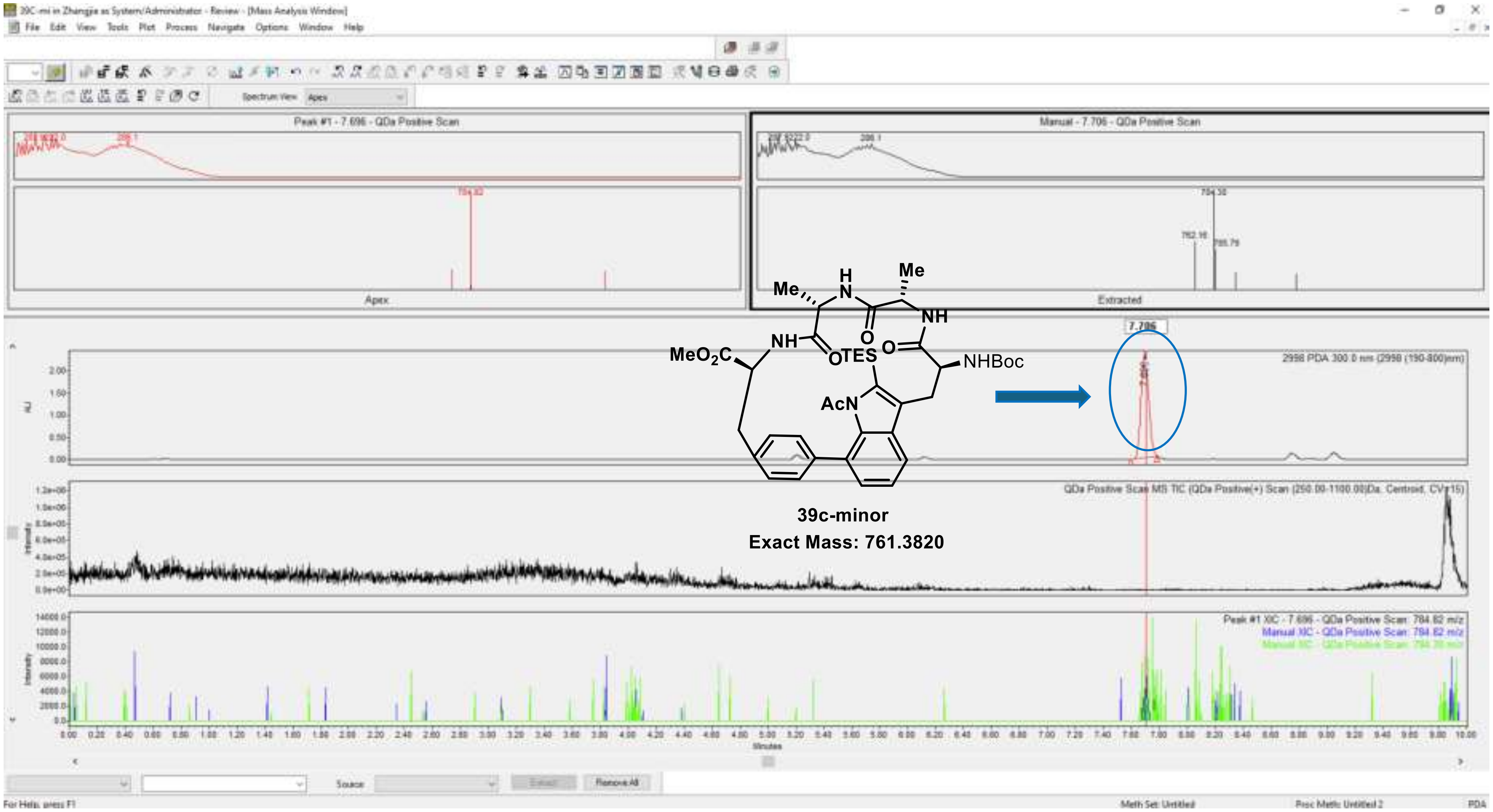

Compound 39d-major ^{1}H NMR (600 MHz, $CDCl_3$)

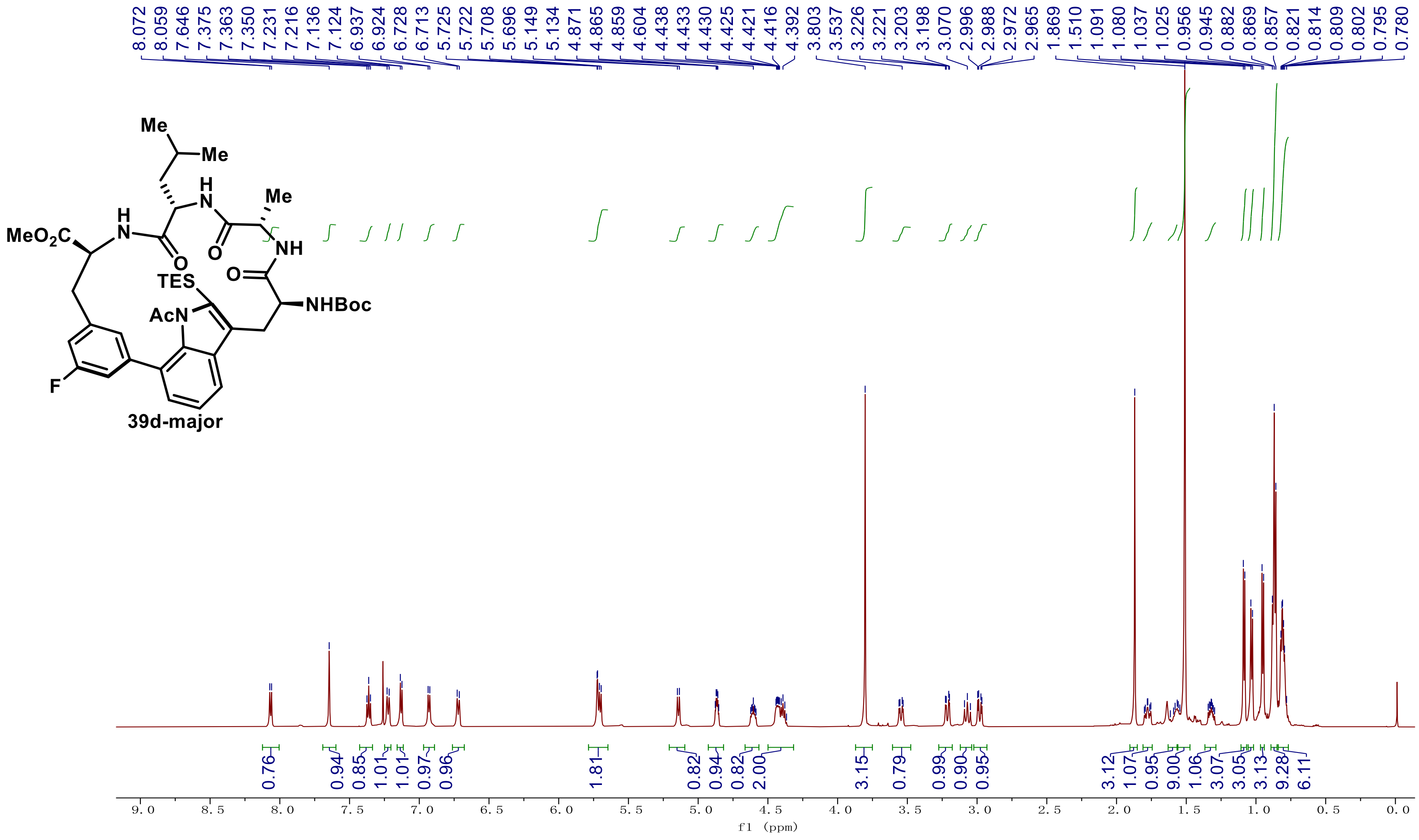

Compound 39d-major ^{13}C NMR (151 MHz, $CDCl_3$)

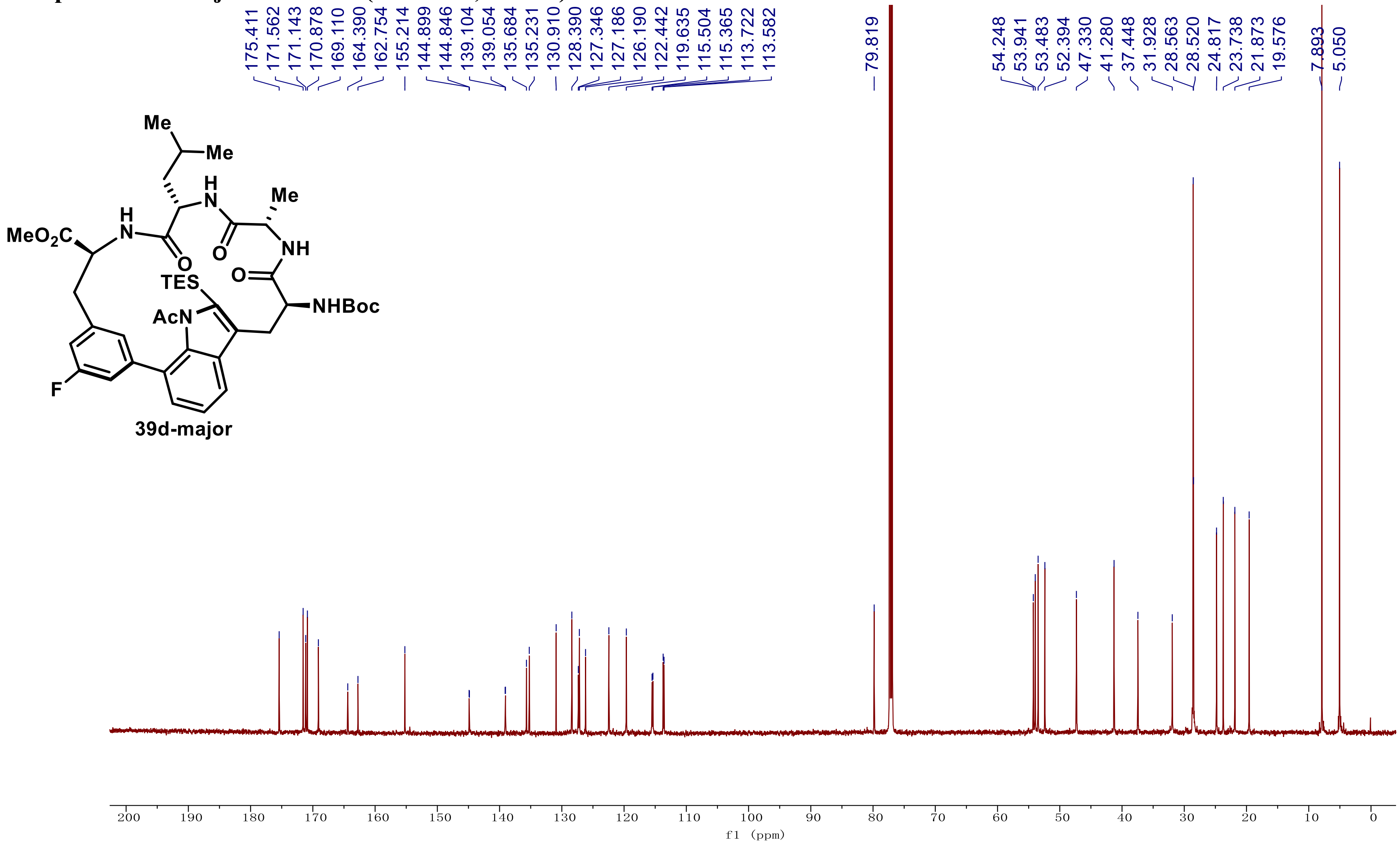

Compound 39d-major ^{19}F NMR (565 MHz, $CDCl_3$)

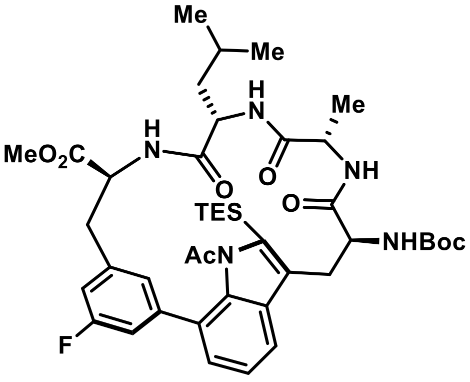

39d-major

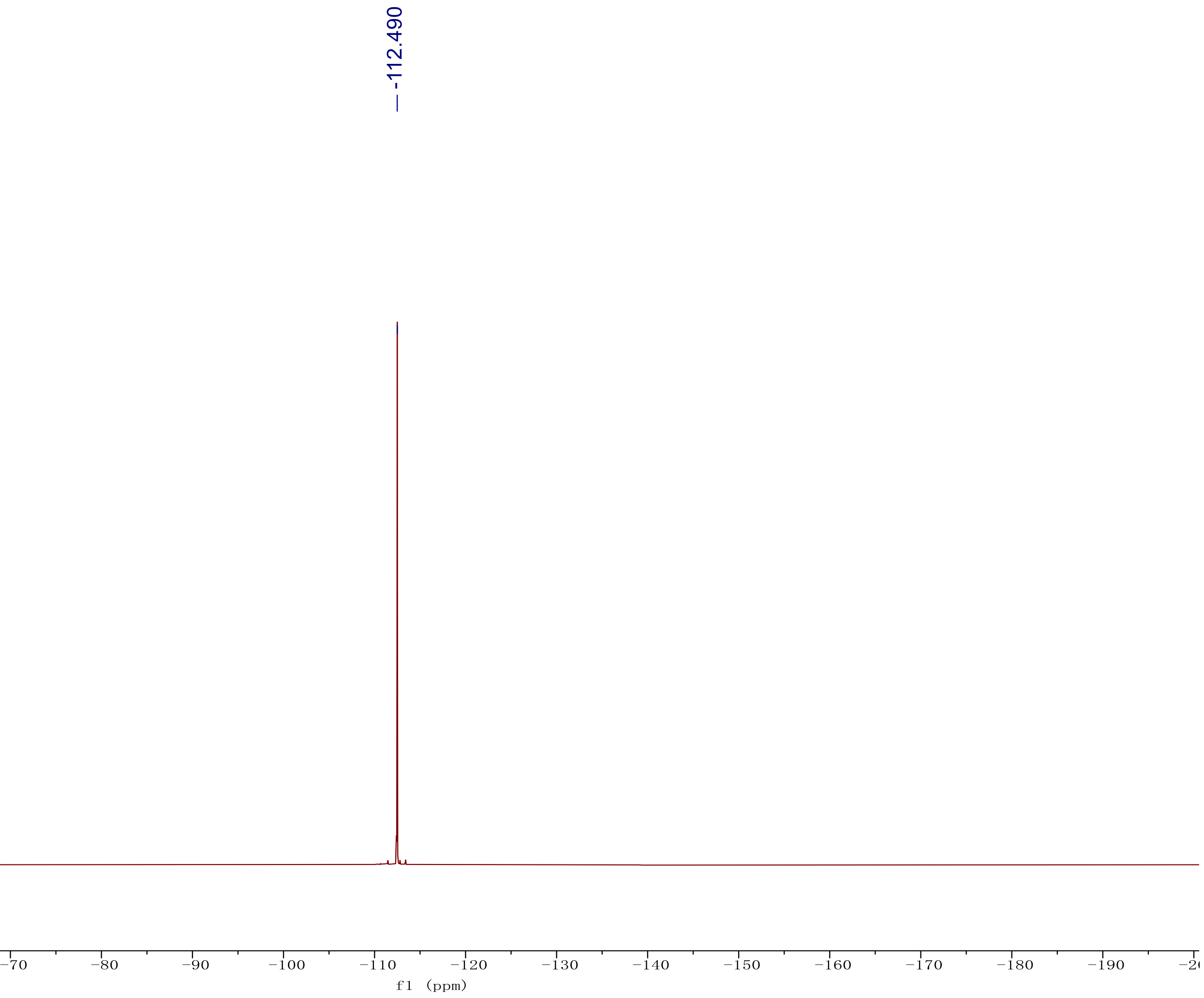

Compound 39d-major ROESY (400 MHz, CDCl3)

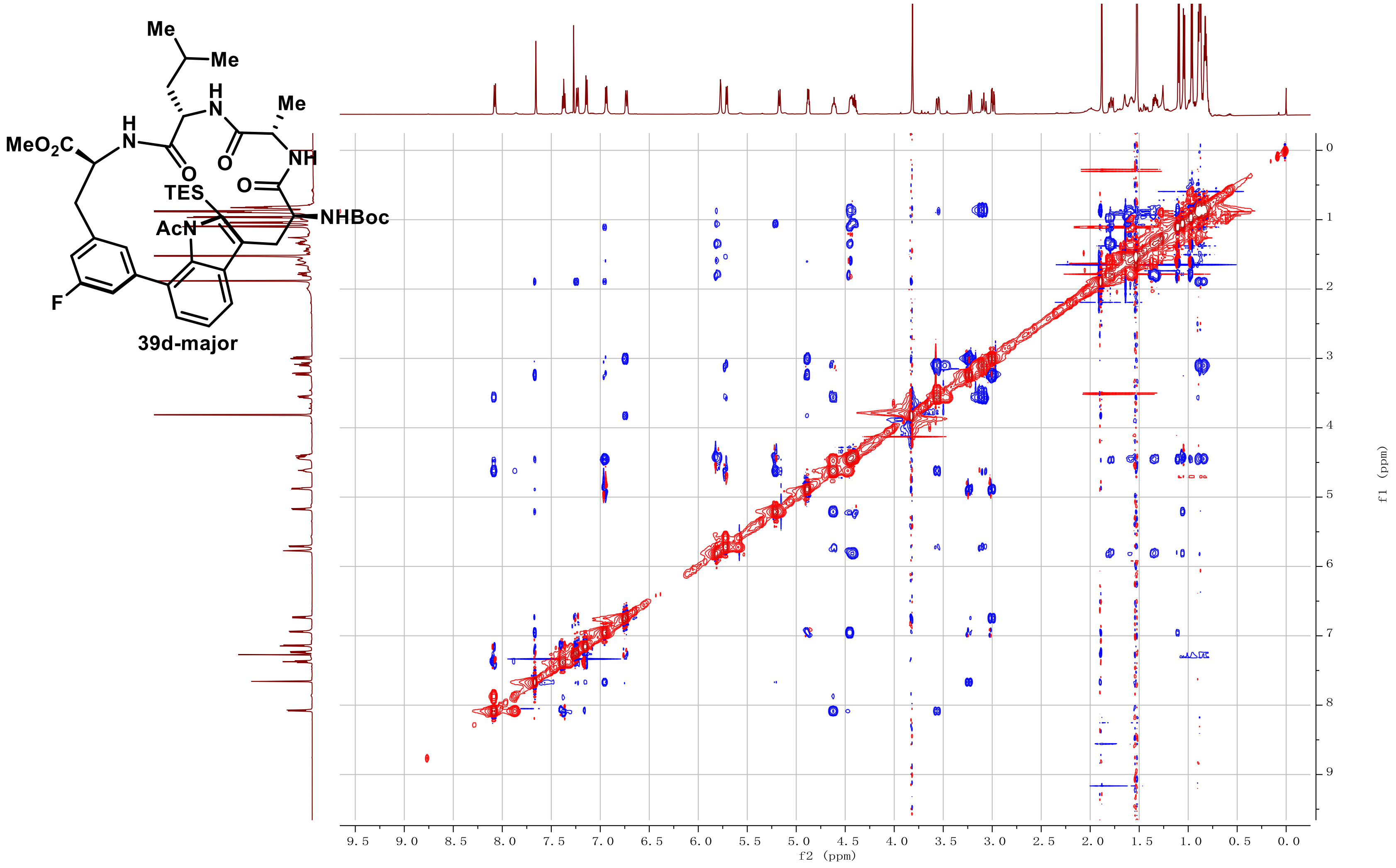

LCMS trace of Compound 39d-major

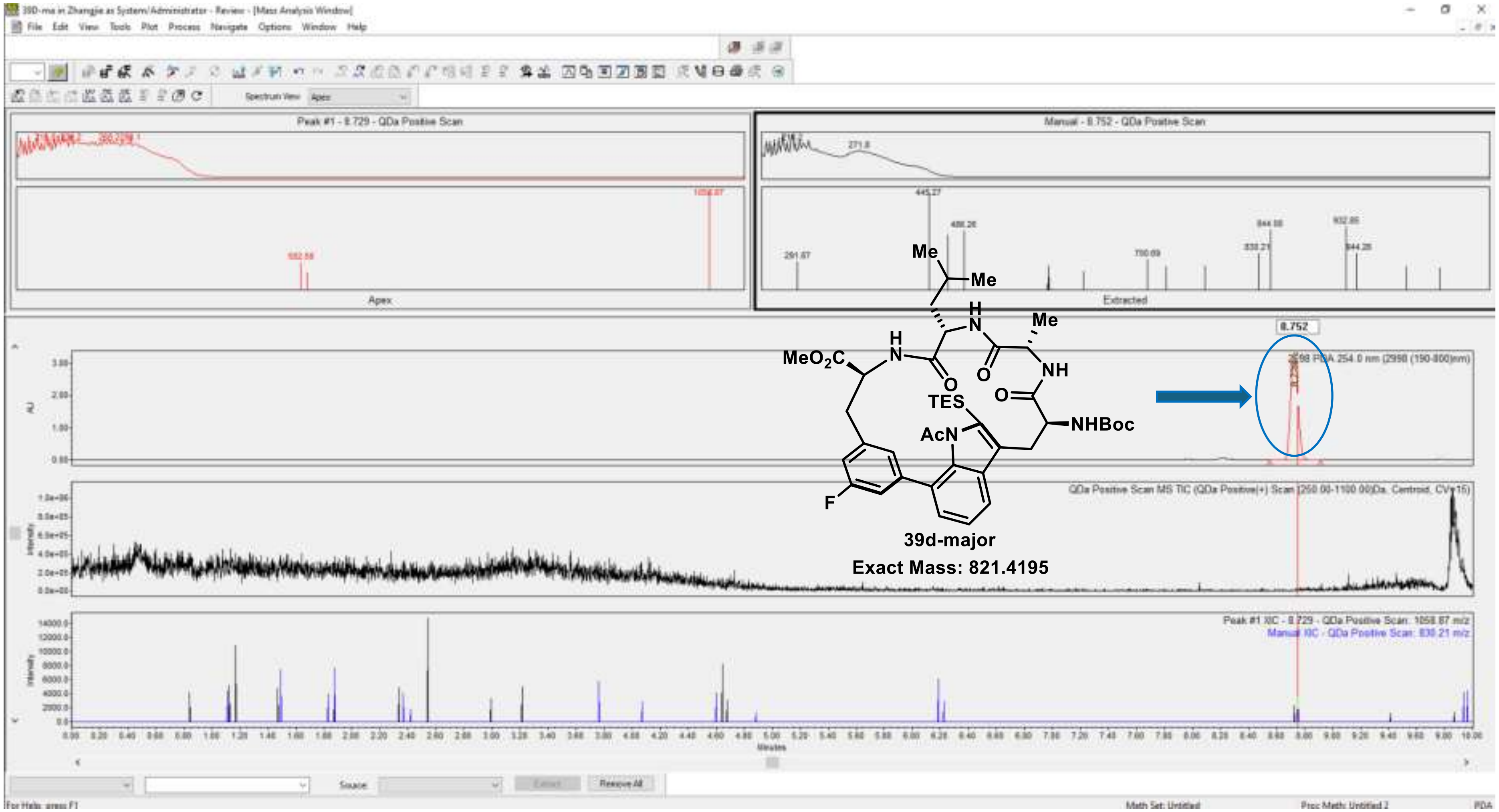

Compound 39d-minor ^{1}H NMR (600 MHz, $CDCl_3$)

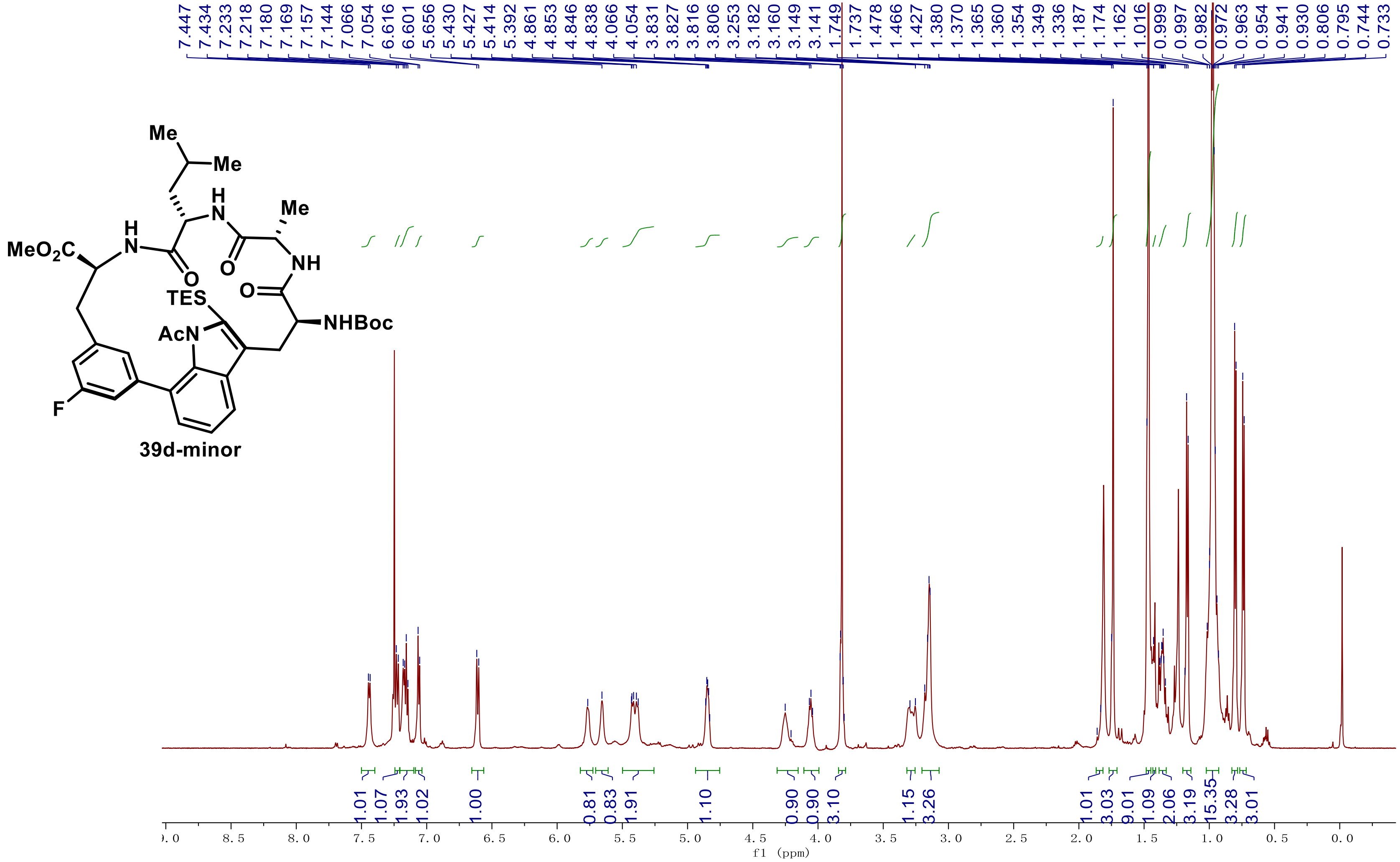

Compound 39d-minor ¹³C NMR (151 MHz, $CDCl_3$)

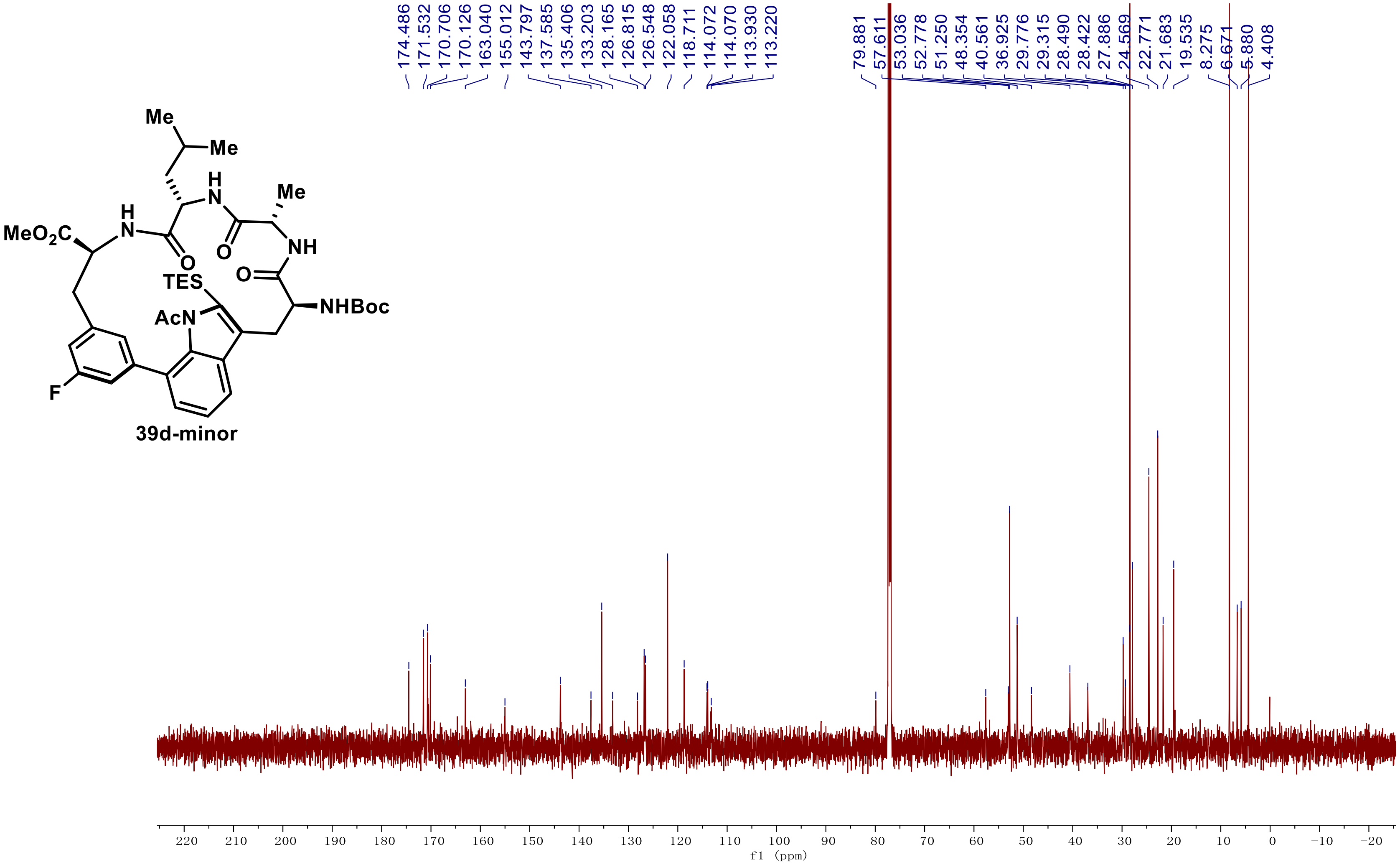

39d-minor

LCMS trace of Compound 39d-minor

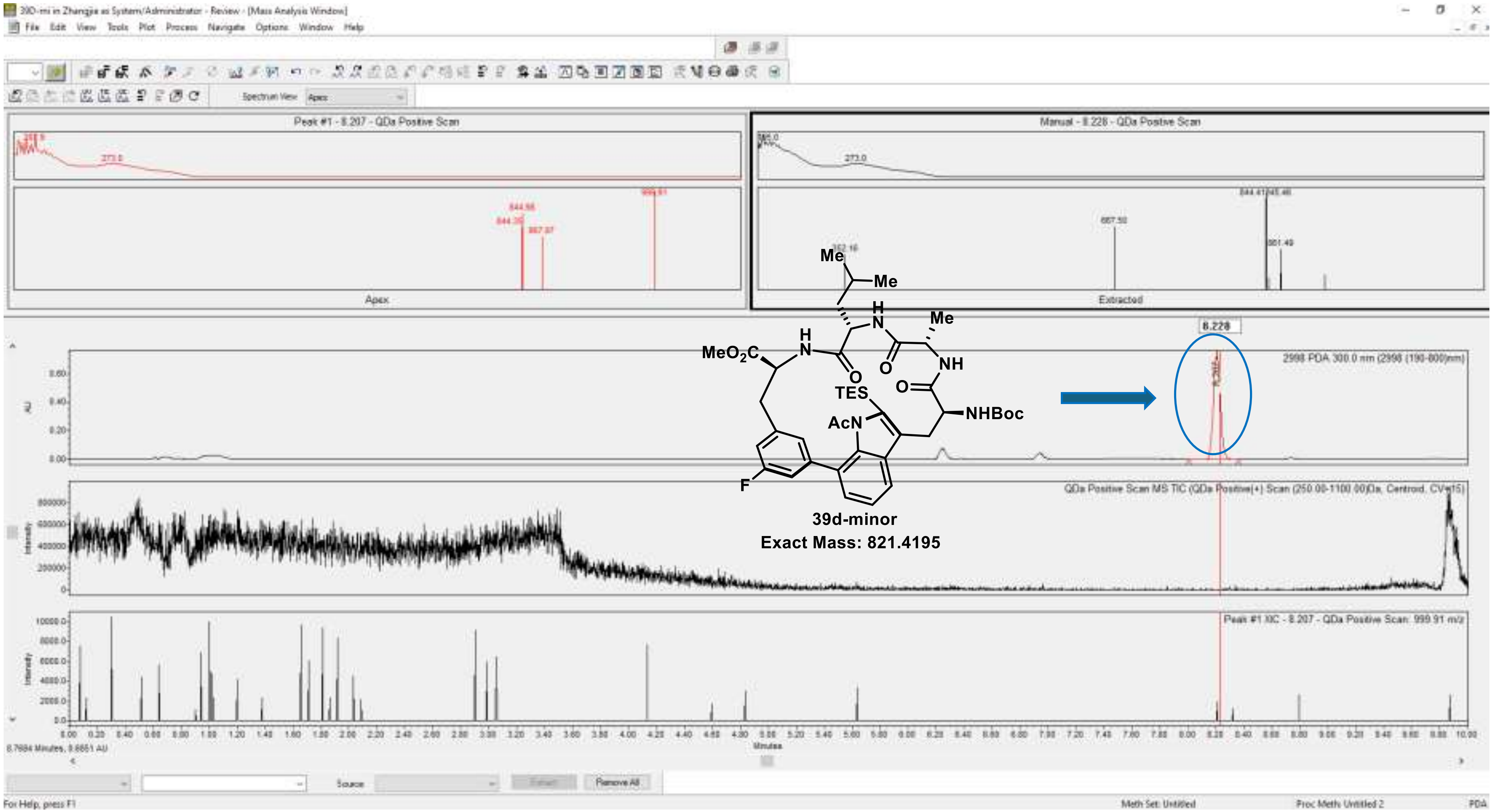

Compound 39e ^{1}H NMR (600 MHz, $CDCl_3$)

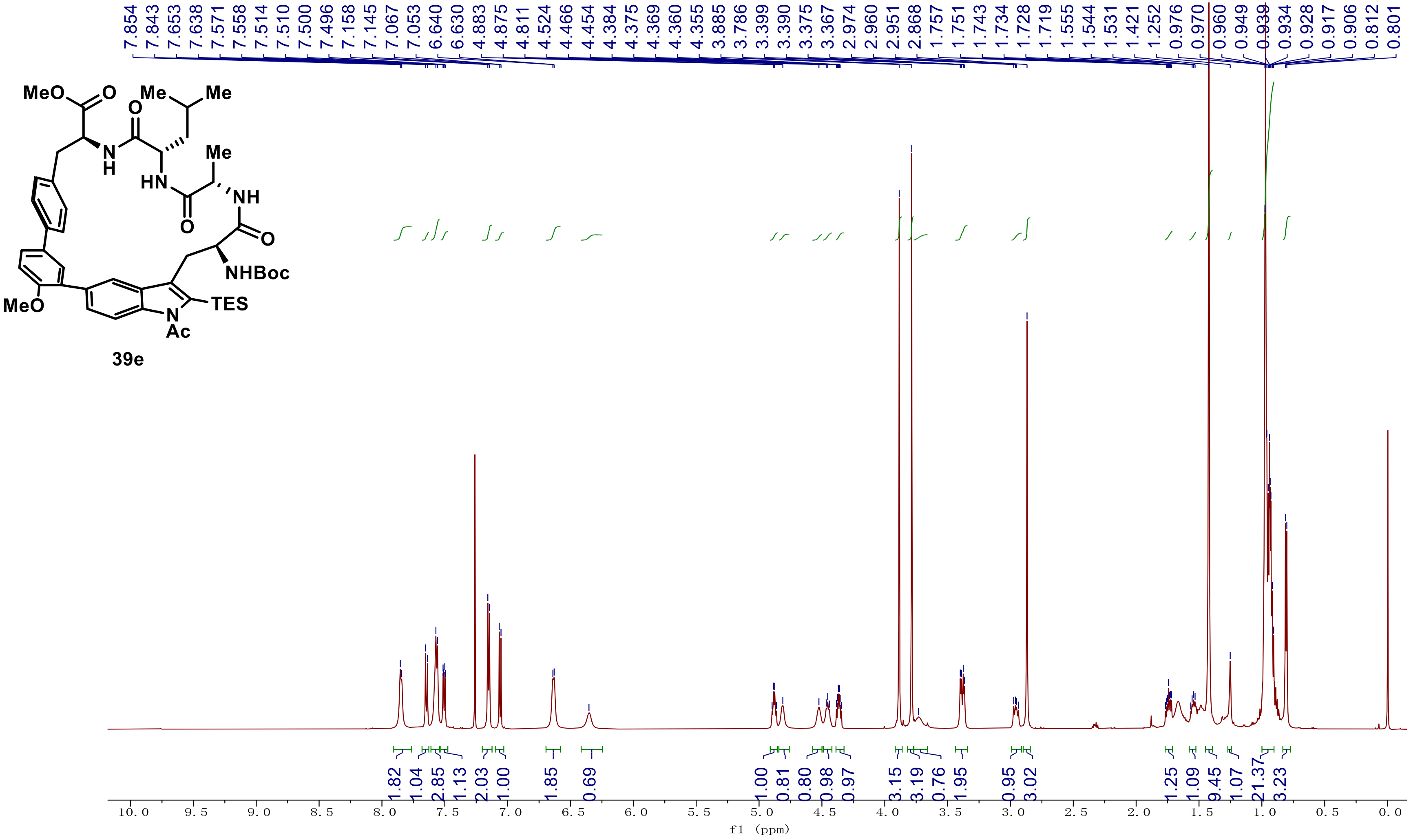

Compound 39e ^{13}C NMR (151 MHz, $CDCl_3$)

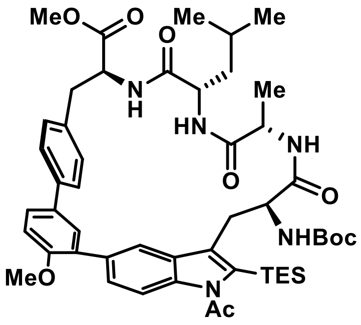

39e

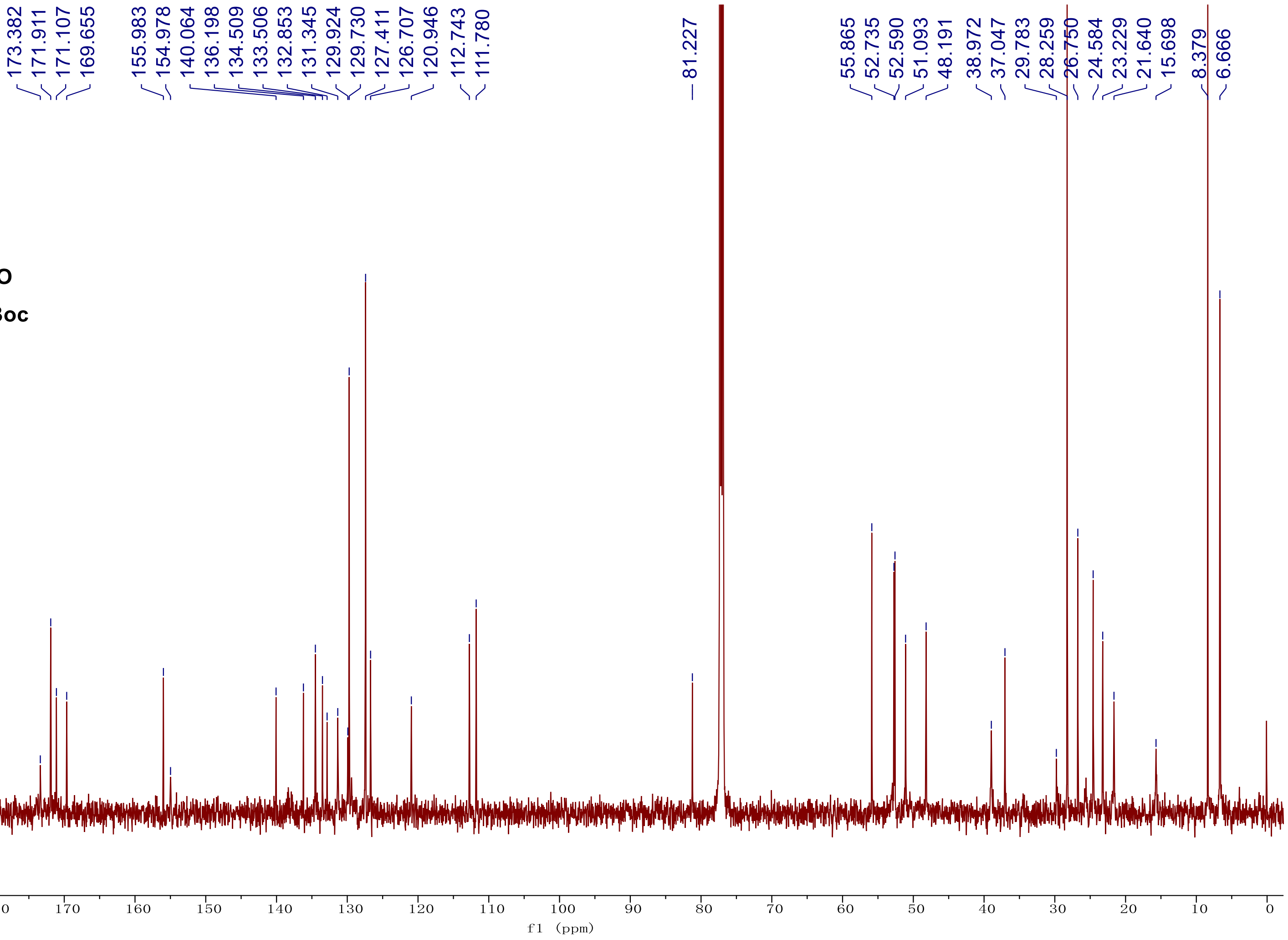

Compound 39e NOESY (400 MHz, $CDCl_3$)

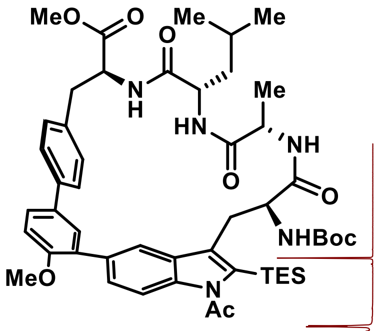

39e

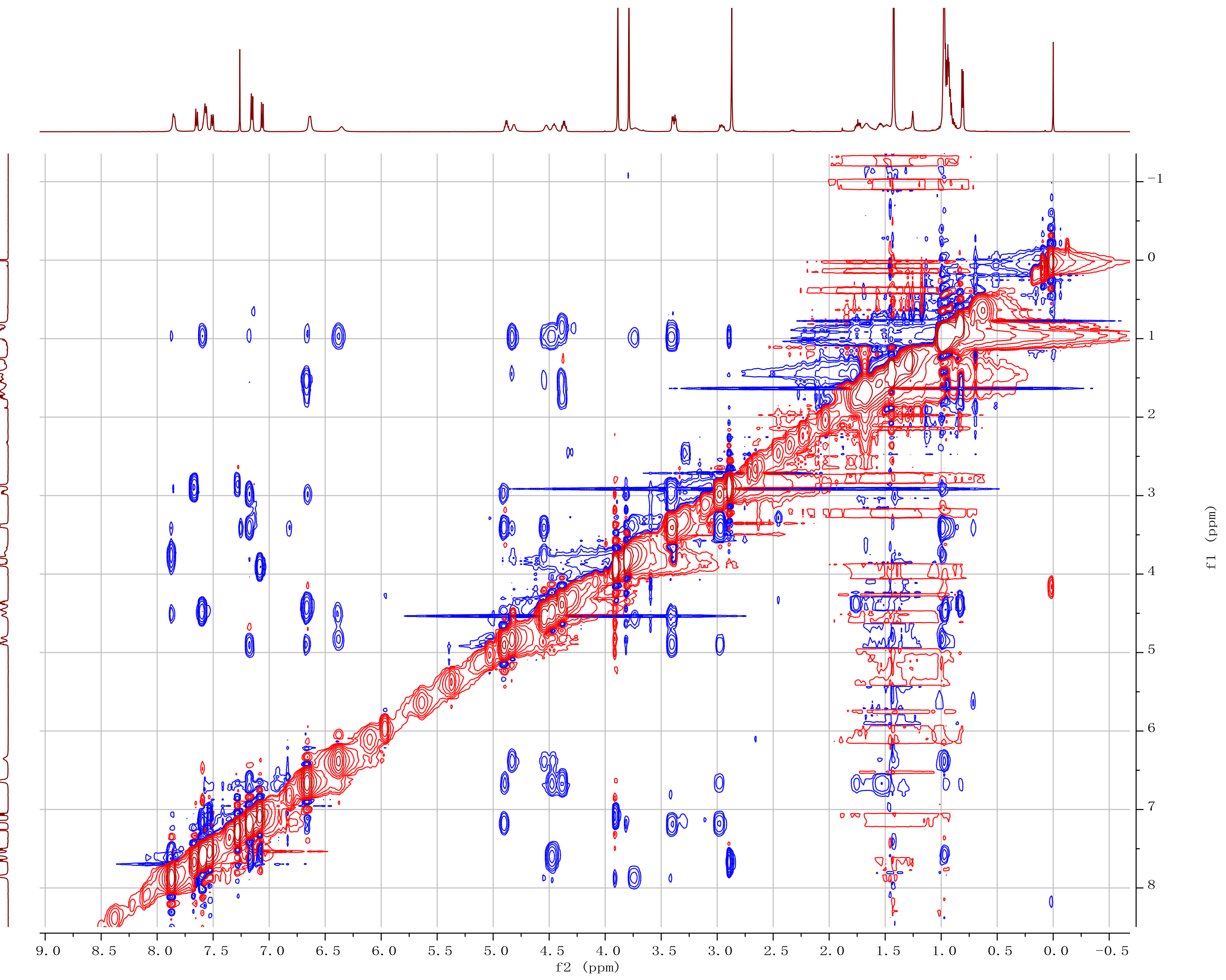

LCMS trace of Compound 39e

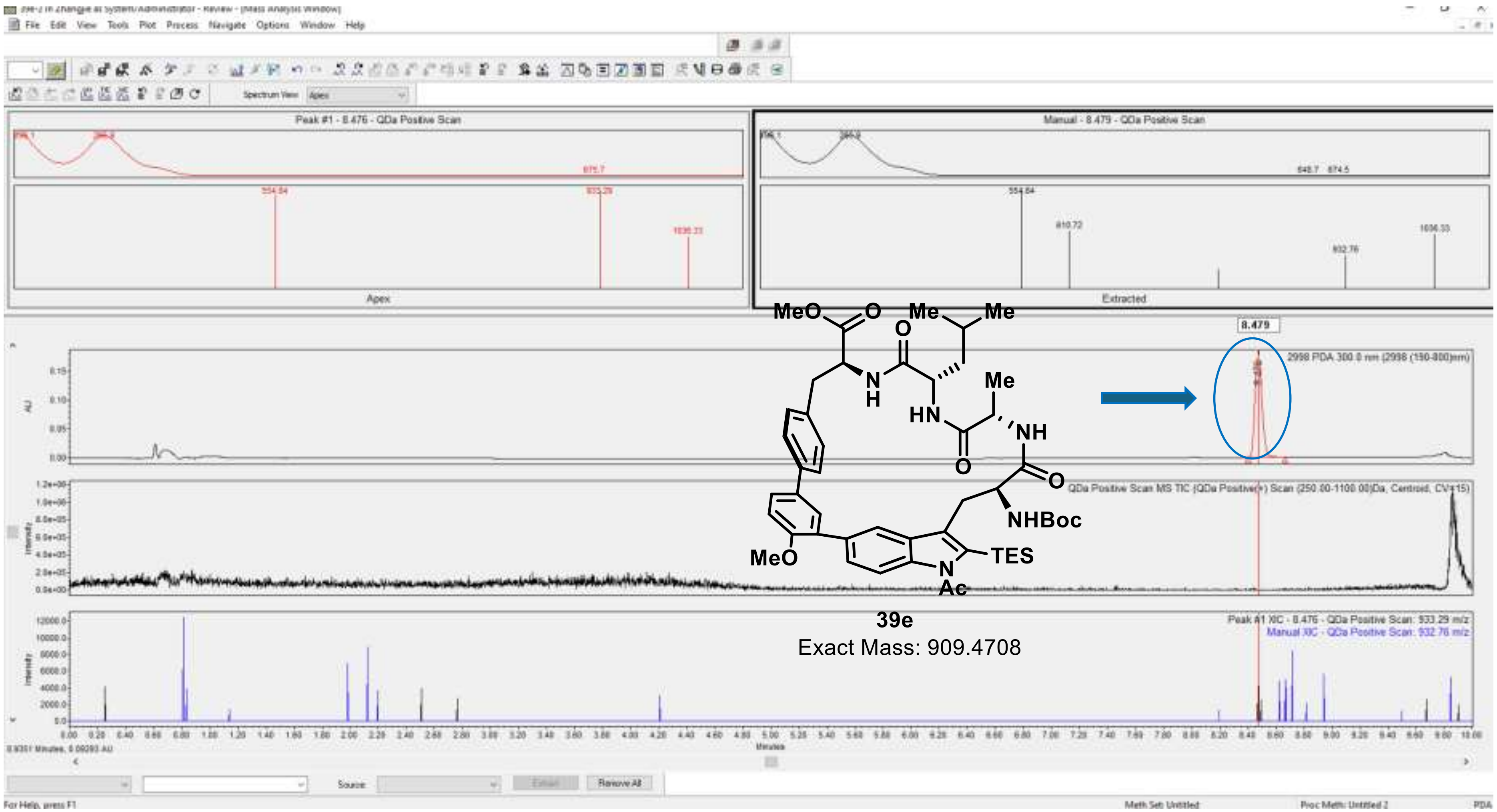

Compound 39f ^{1}H NMR (600 MHz, $CDCl_3$)

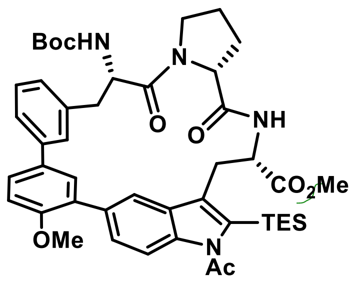

39f

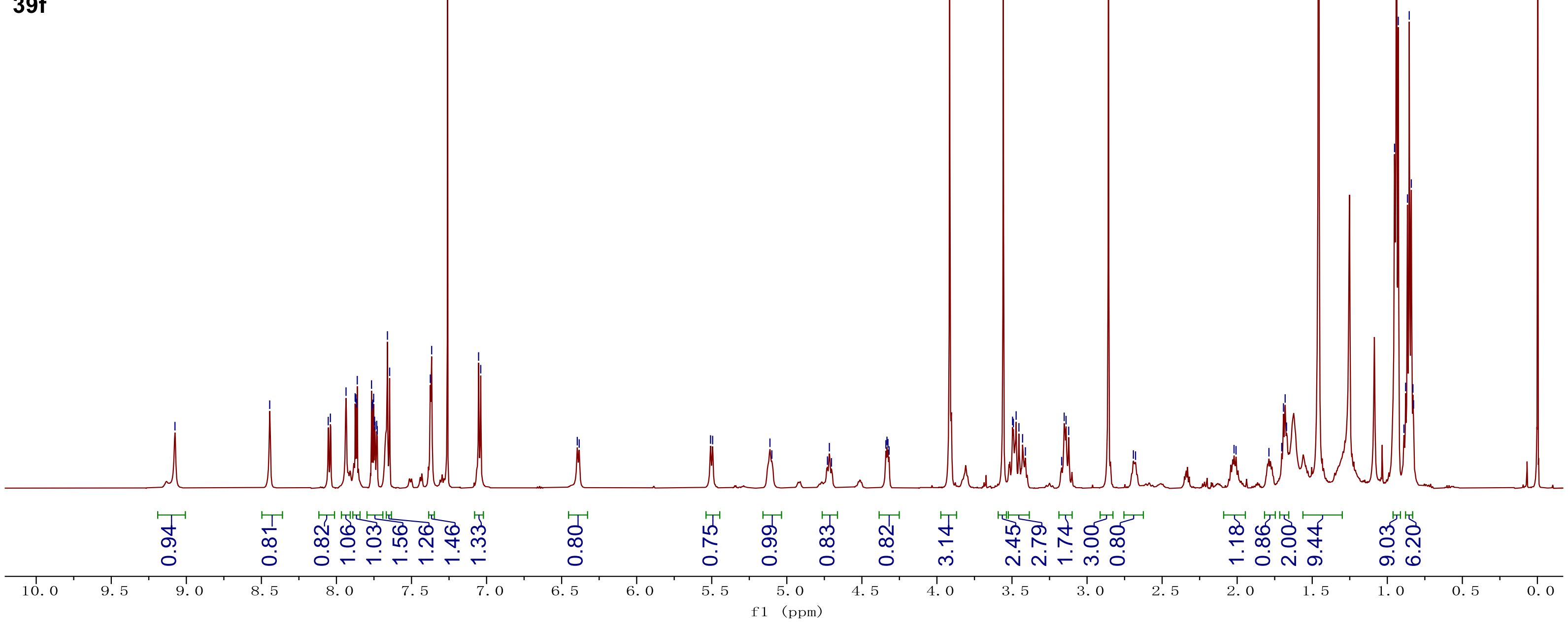

Compound 39f ^{13}C NMR (151 MHz, $CDCl_3$)

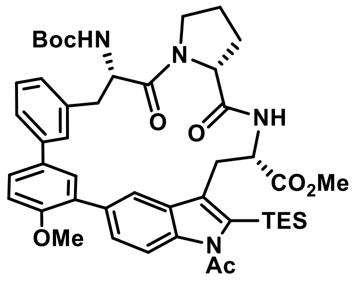

39f

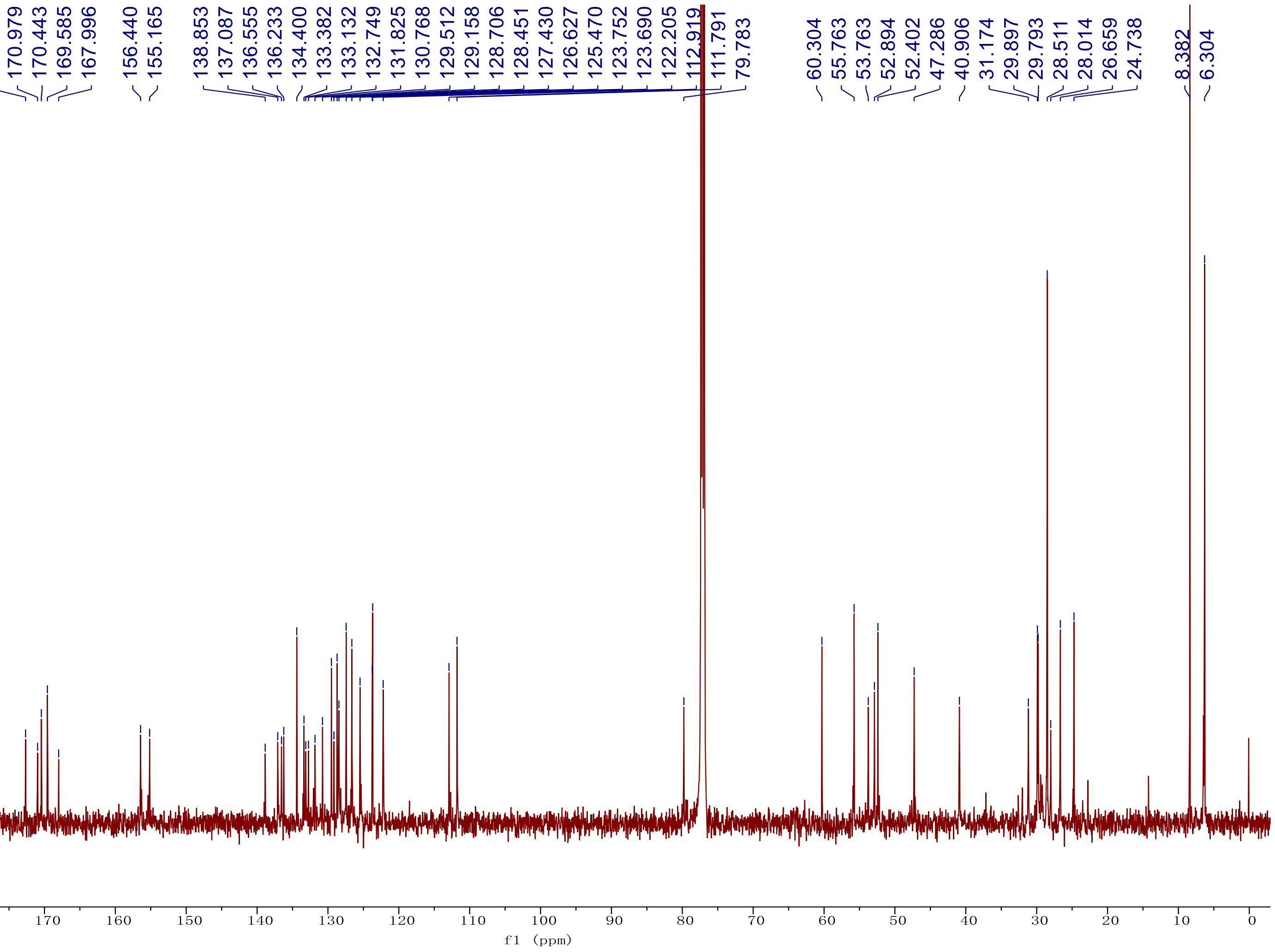

Compound 39f ROESY (400 MHz, CDCl3)

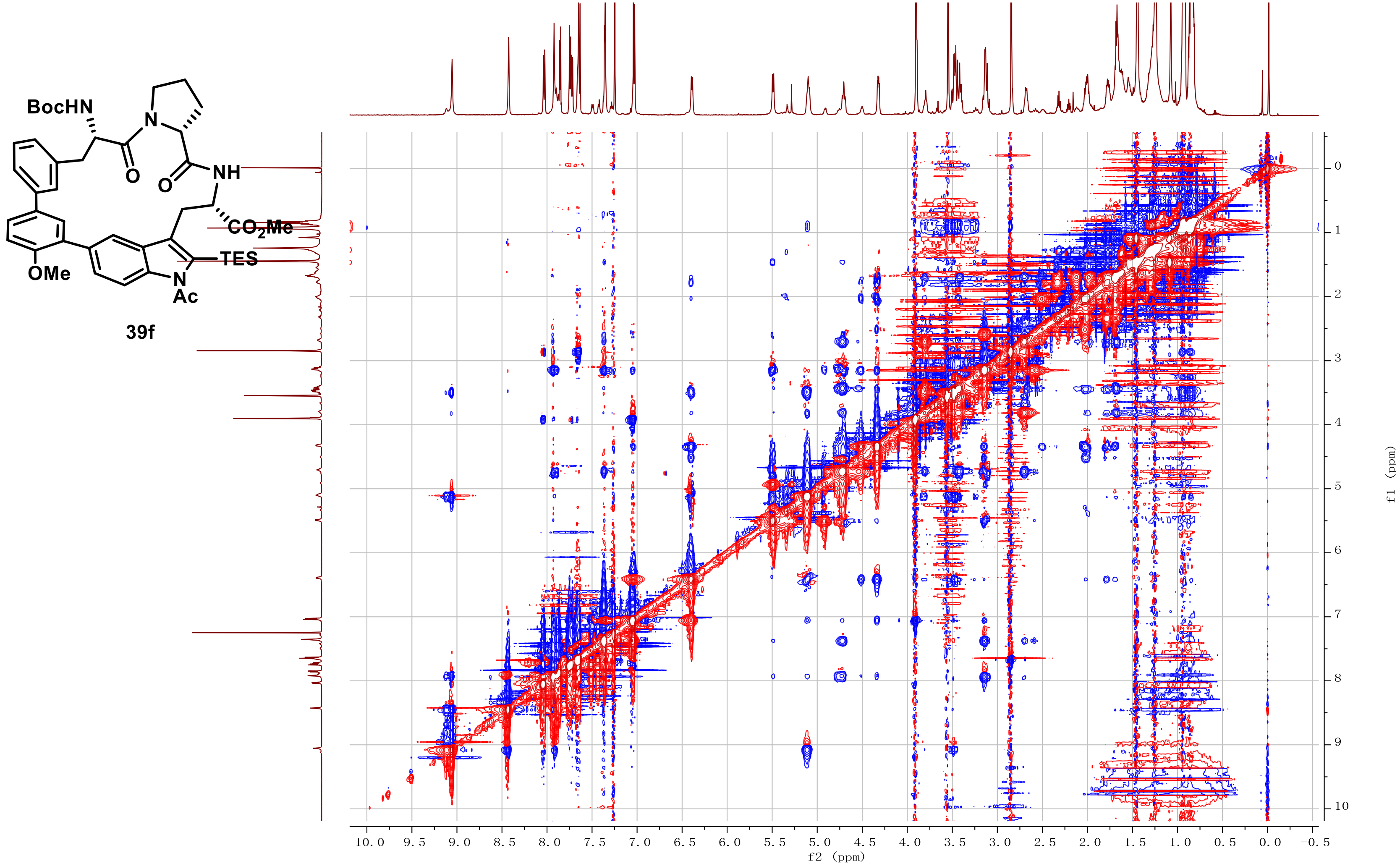

LCMS trace of Compound 39f

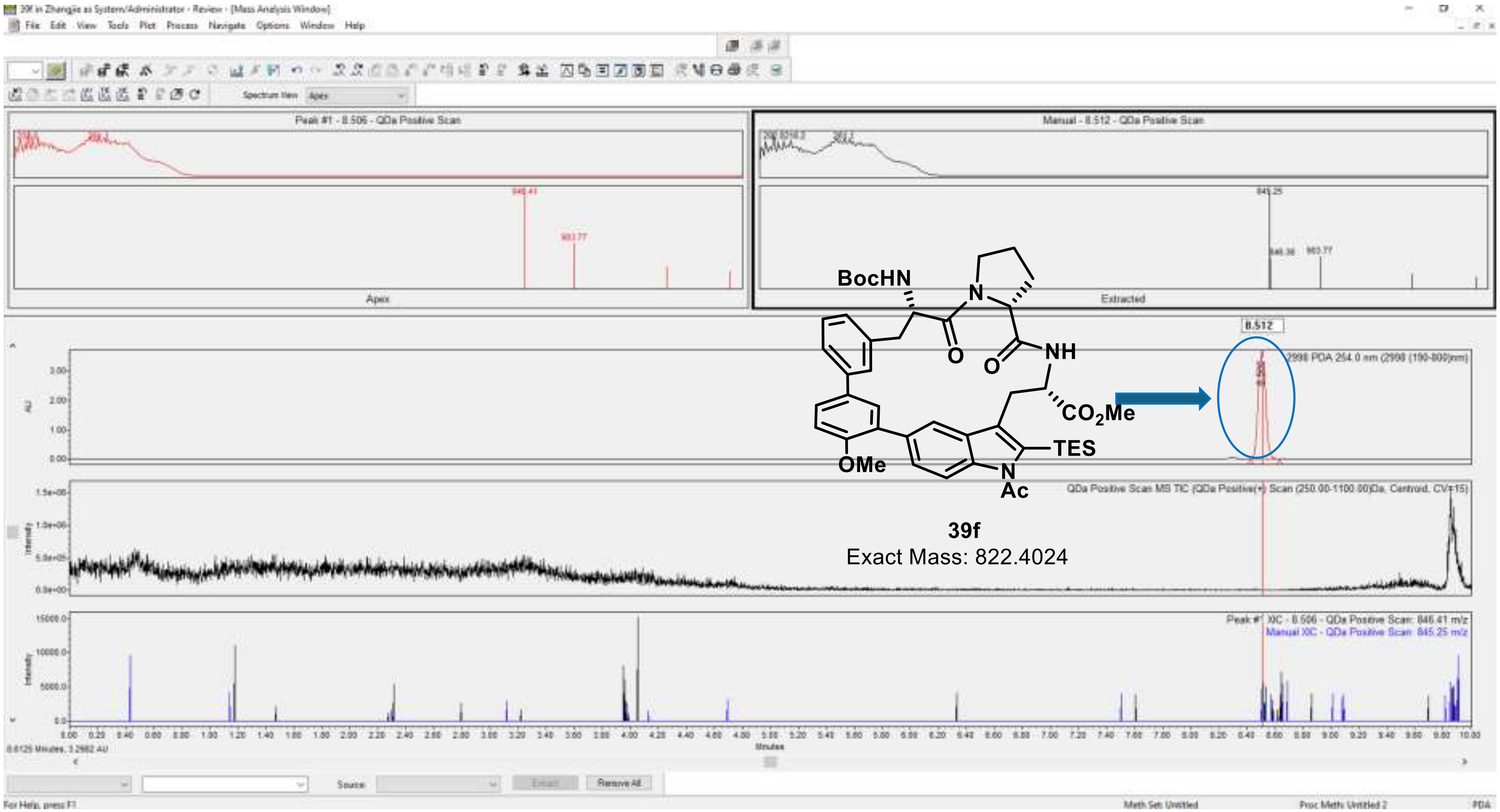

Compound 19a ^{13}C NMR (151 MHz, $CDCl_3$)

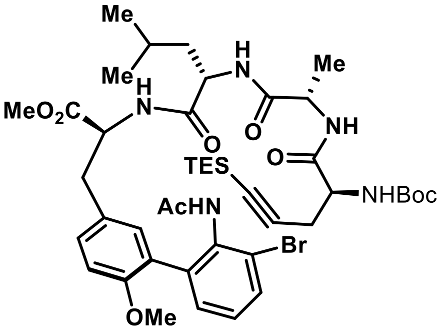

19a

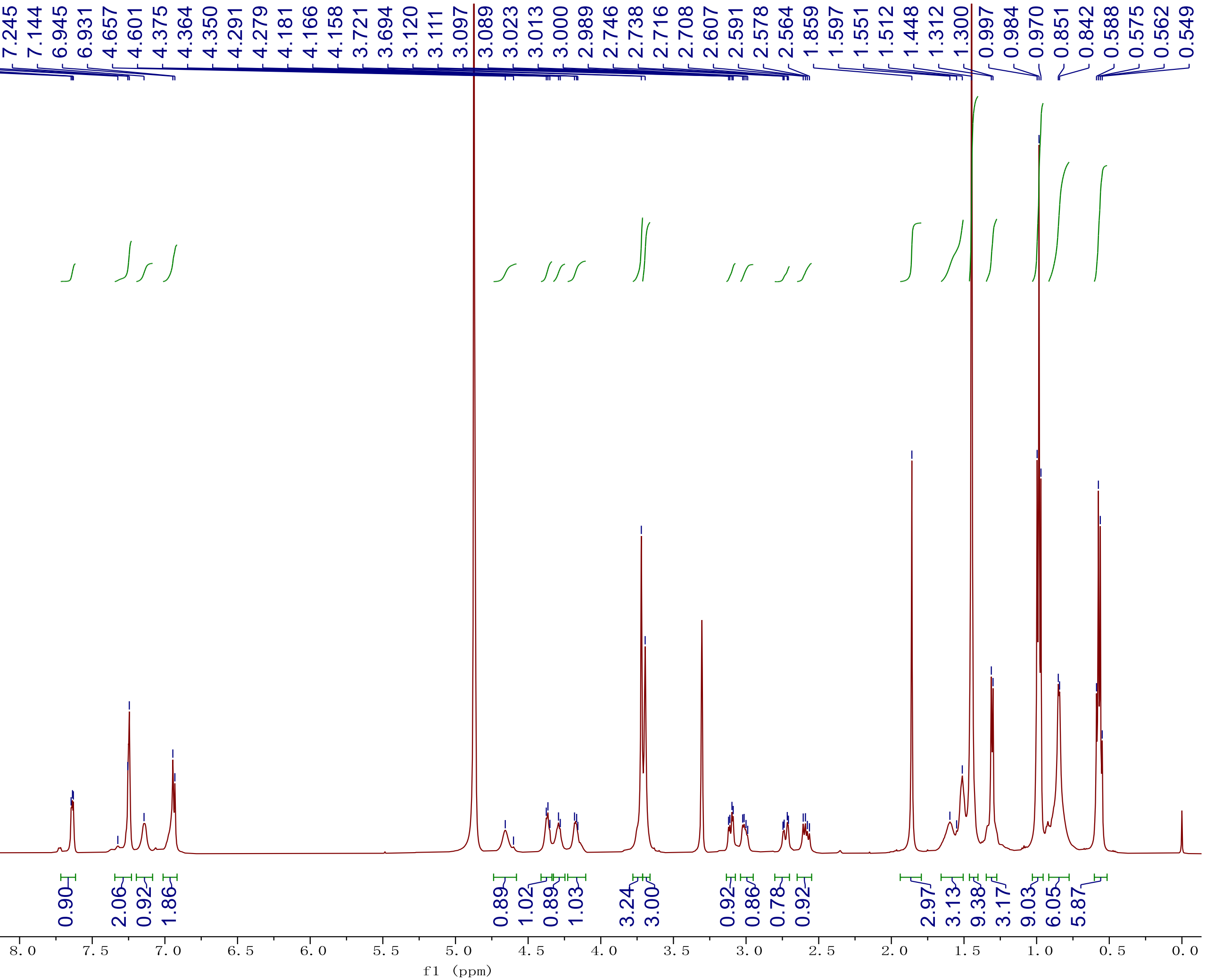

Compound 19a ^{13}C NMR (151 MHz, METHANOL-D_4)

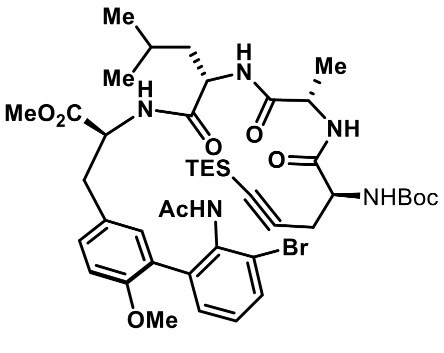

19a

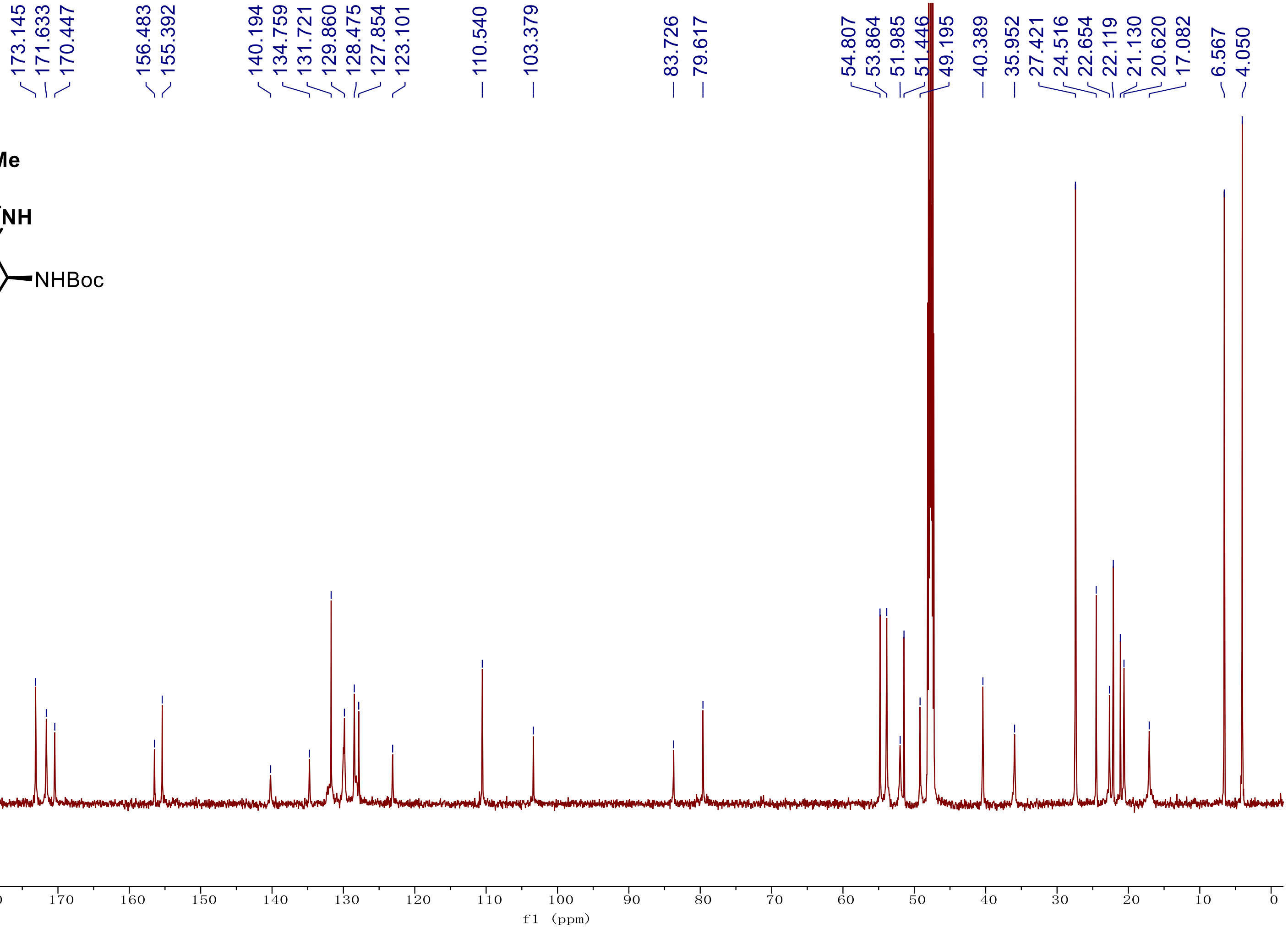

Compound 19b ¹H NMR (600 MHz, METHANOL-*D*₄)

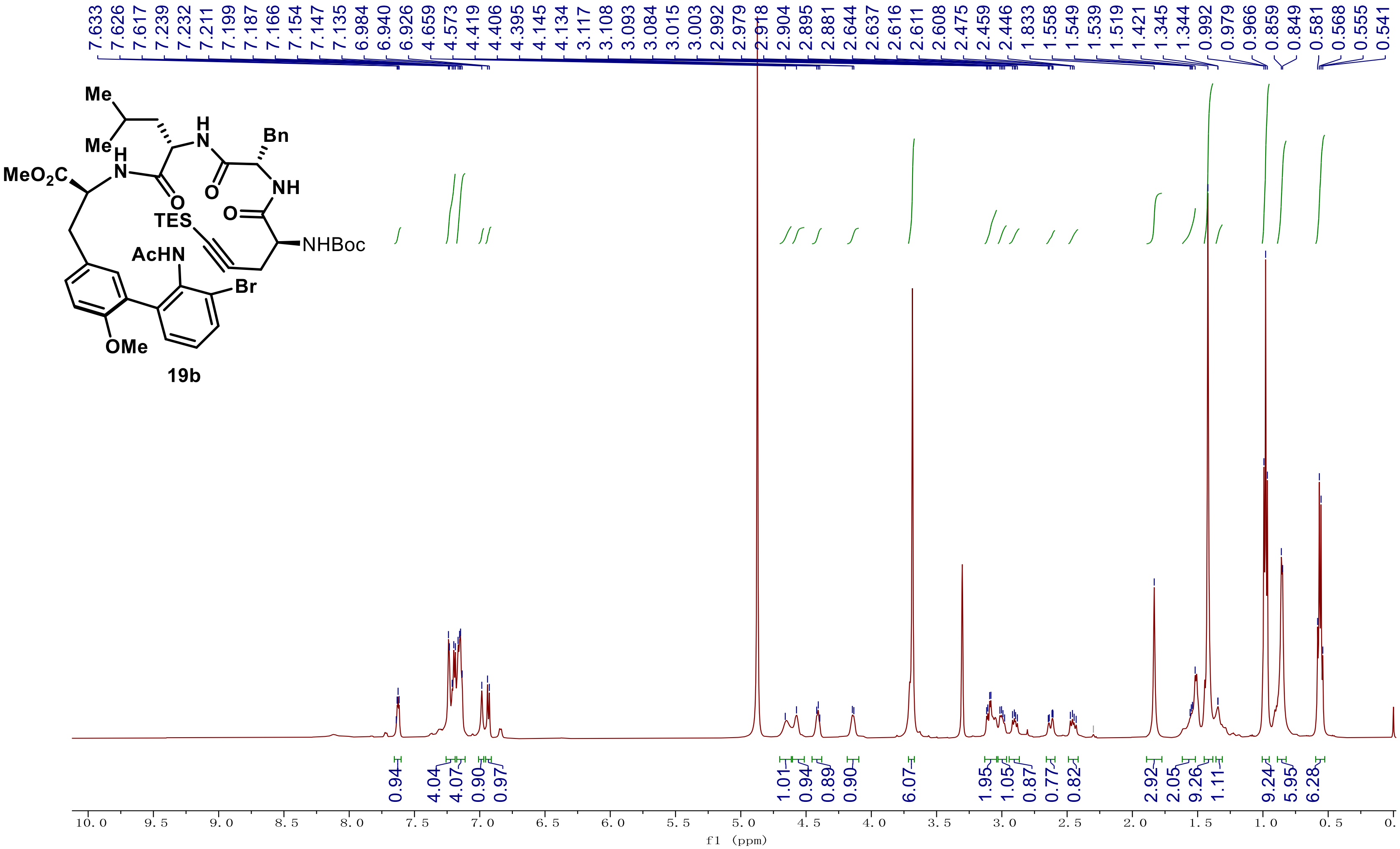

Compound 19b ^{13}C NMR (151 MHz, METHANOL-D_4)

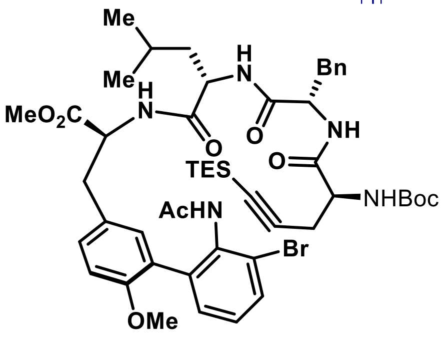

19b

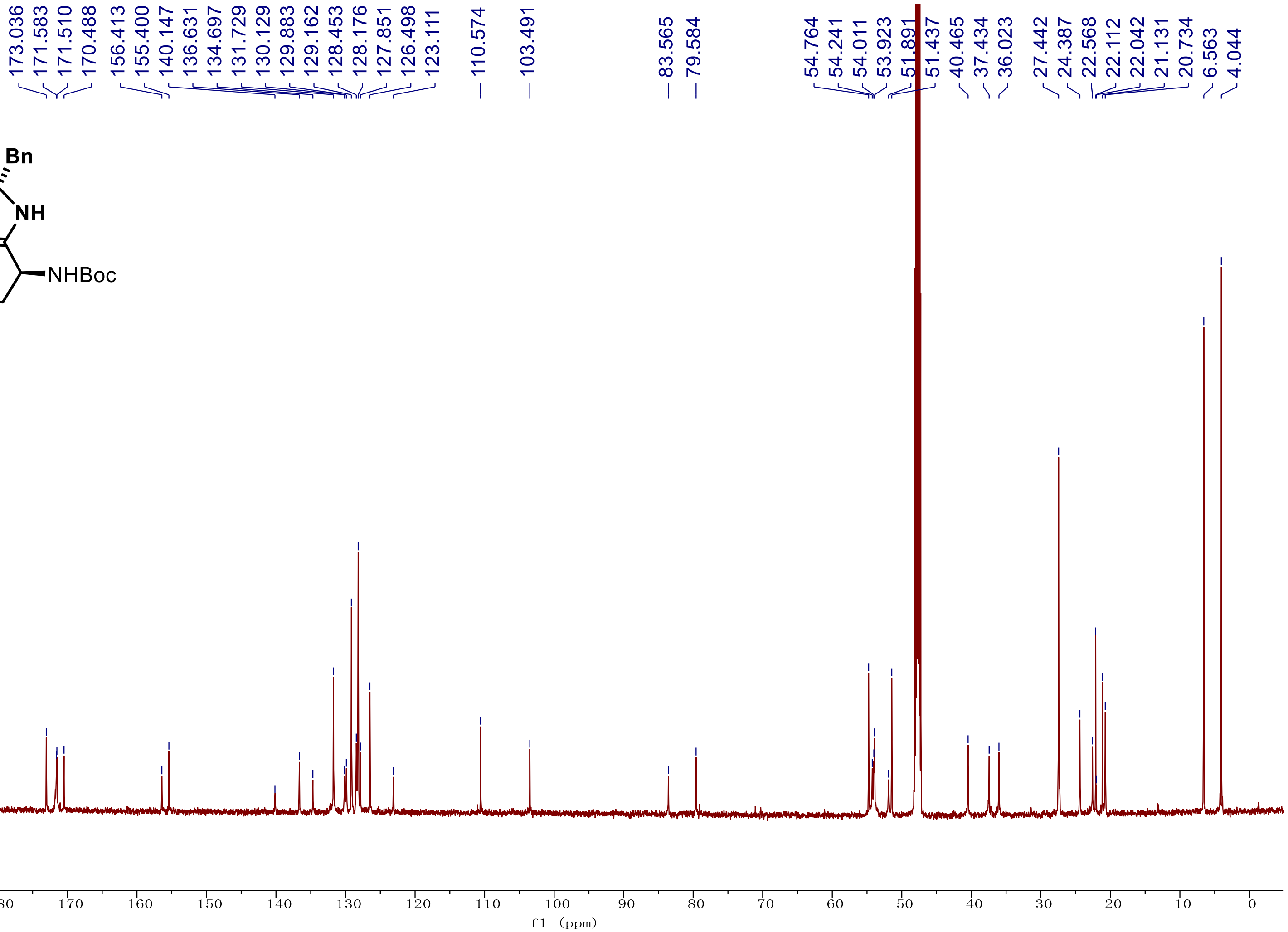

Compound 19c ^{1}H NMR (600 MHz, METHANOL-D_4)

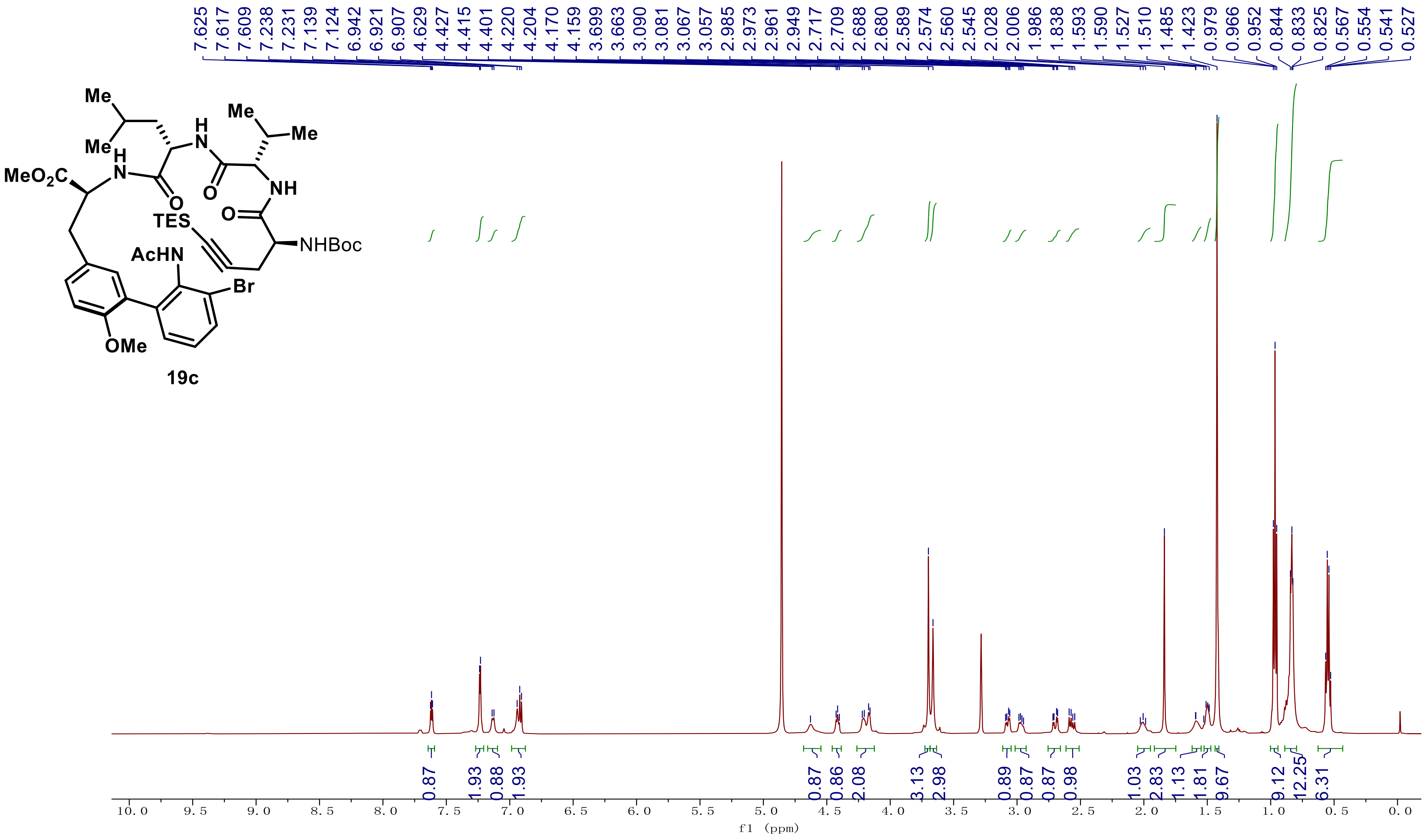

Compound 19c ^{13}C NMR (151 MHz, METHANOL-D_4)

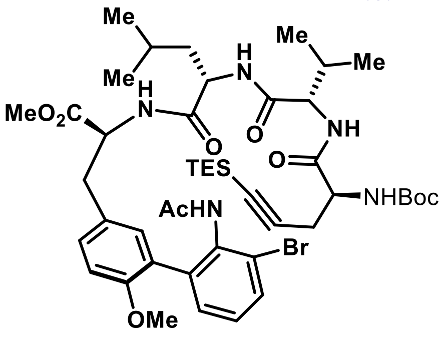

19c

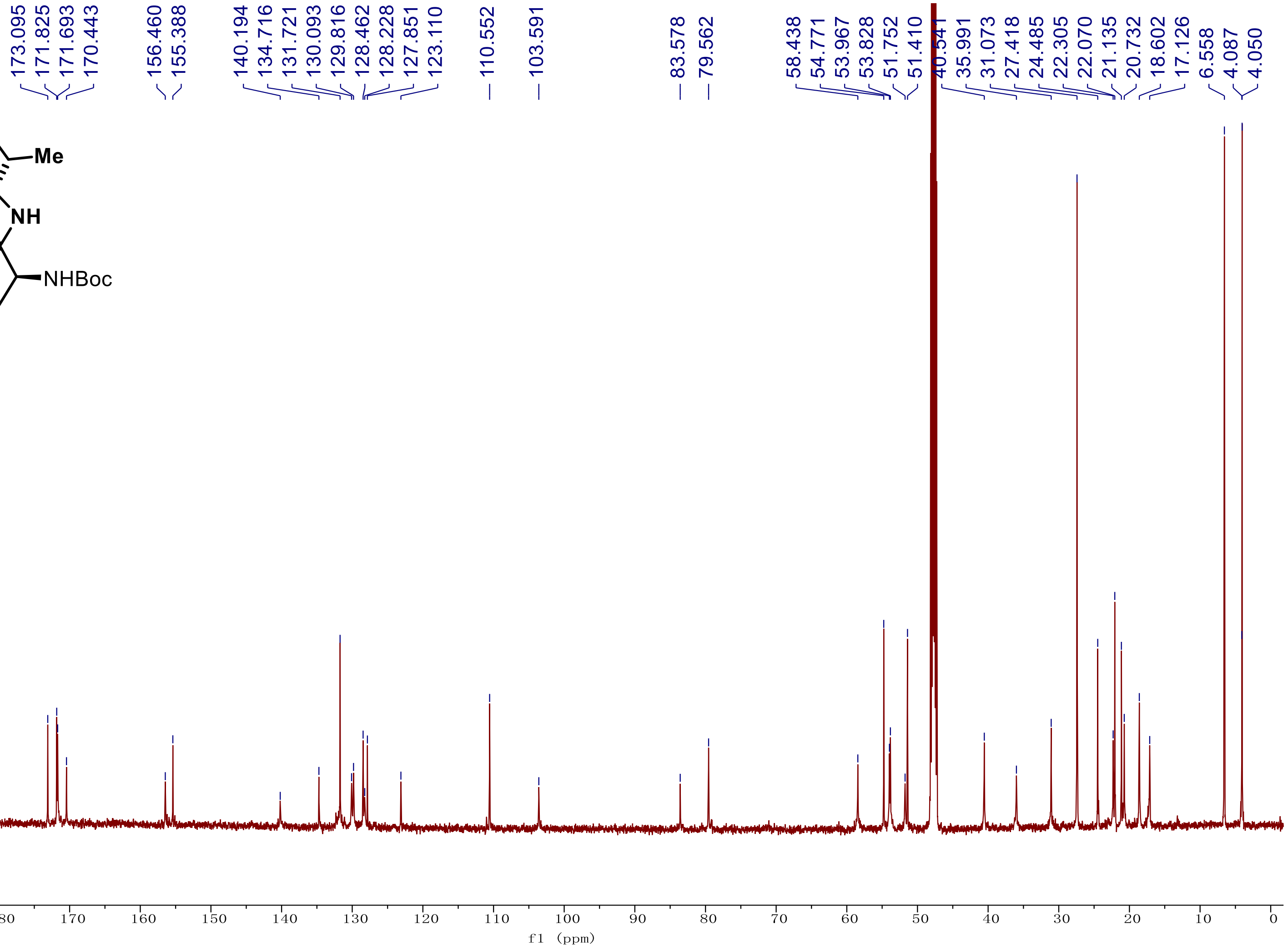

Compound 19d ^{1}H NMR (600 MHz, METHANOL-D_4)

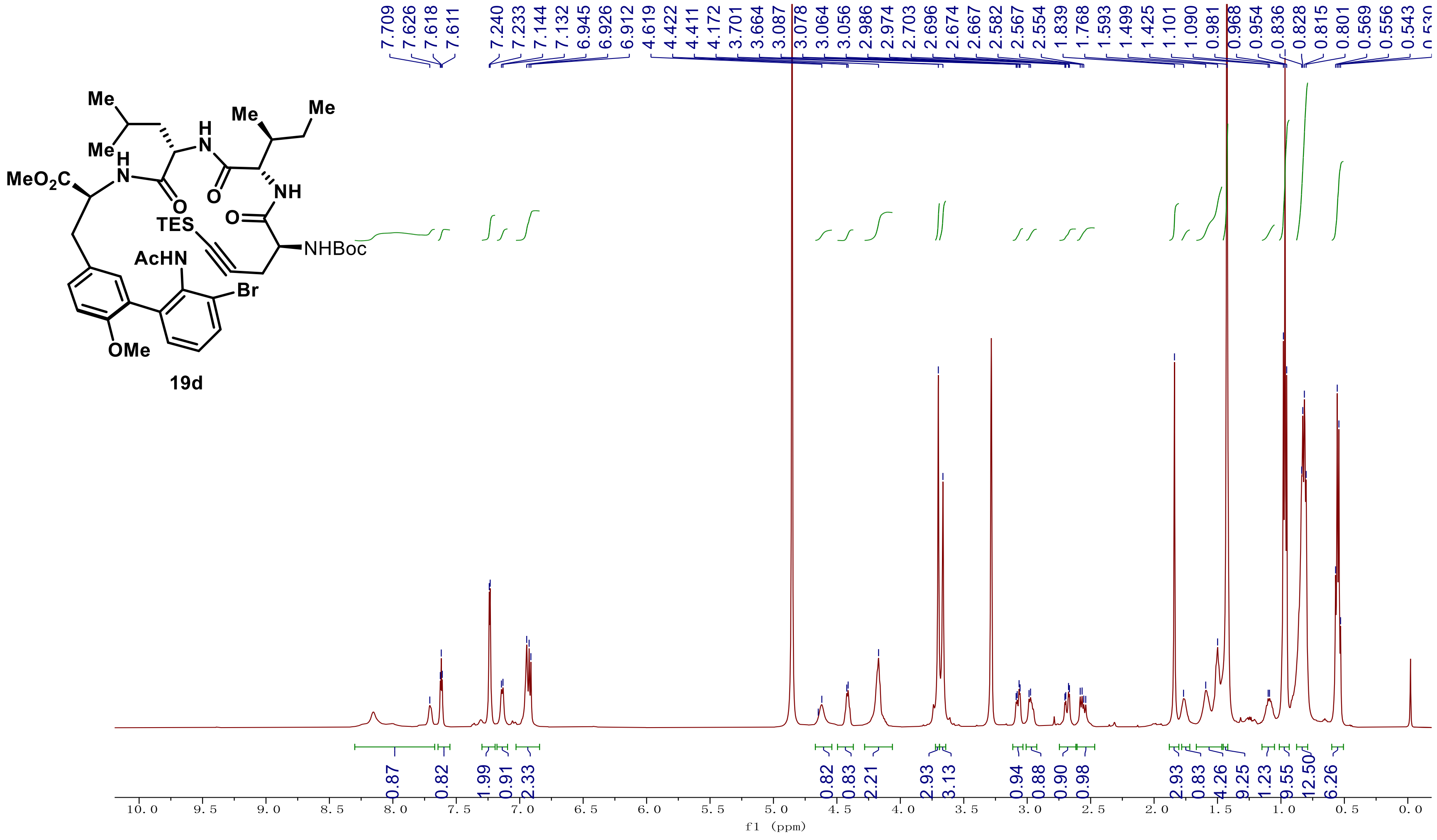

Compound 19d ^{13}C NMR (151 MHz, METHANOL-D_4)

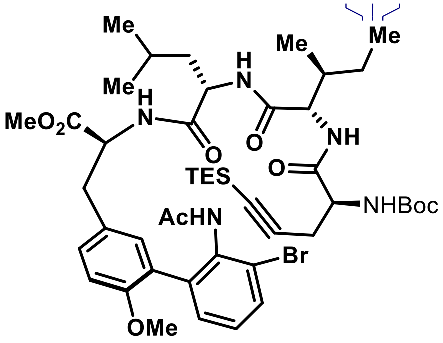

19d

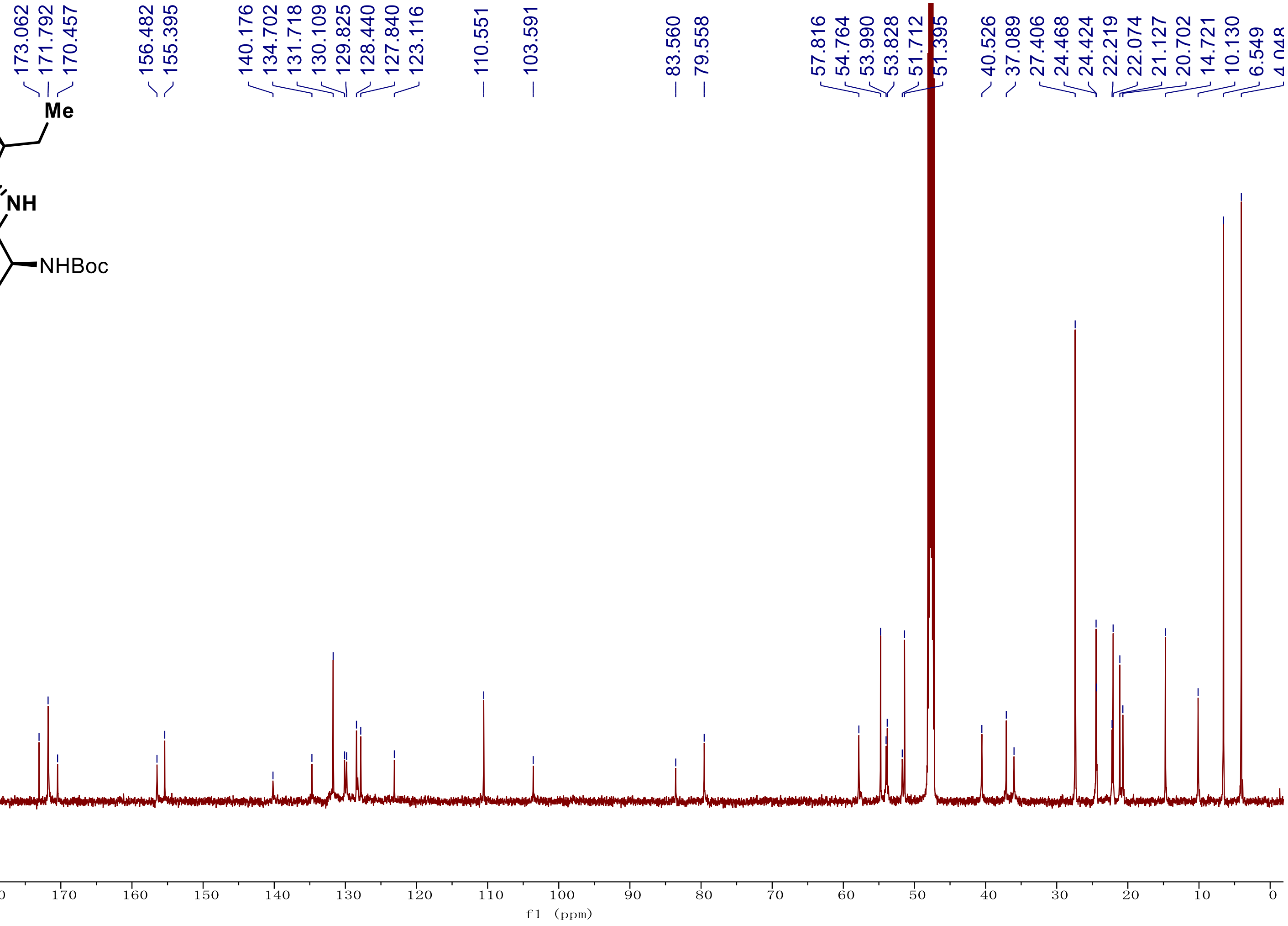

Compound 19e ^{1}H NMR (600 MHz, METHANOL-D_4)

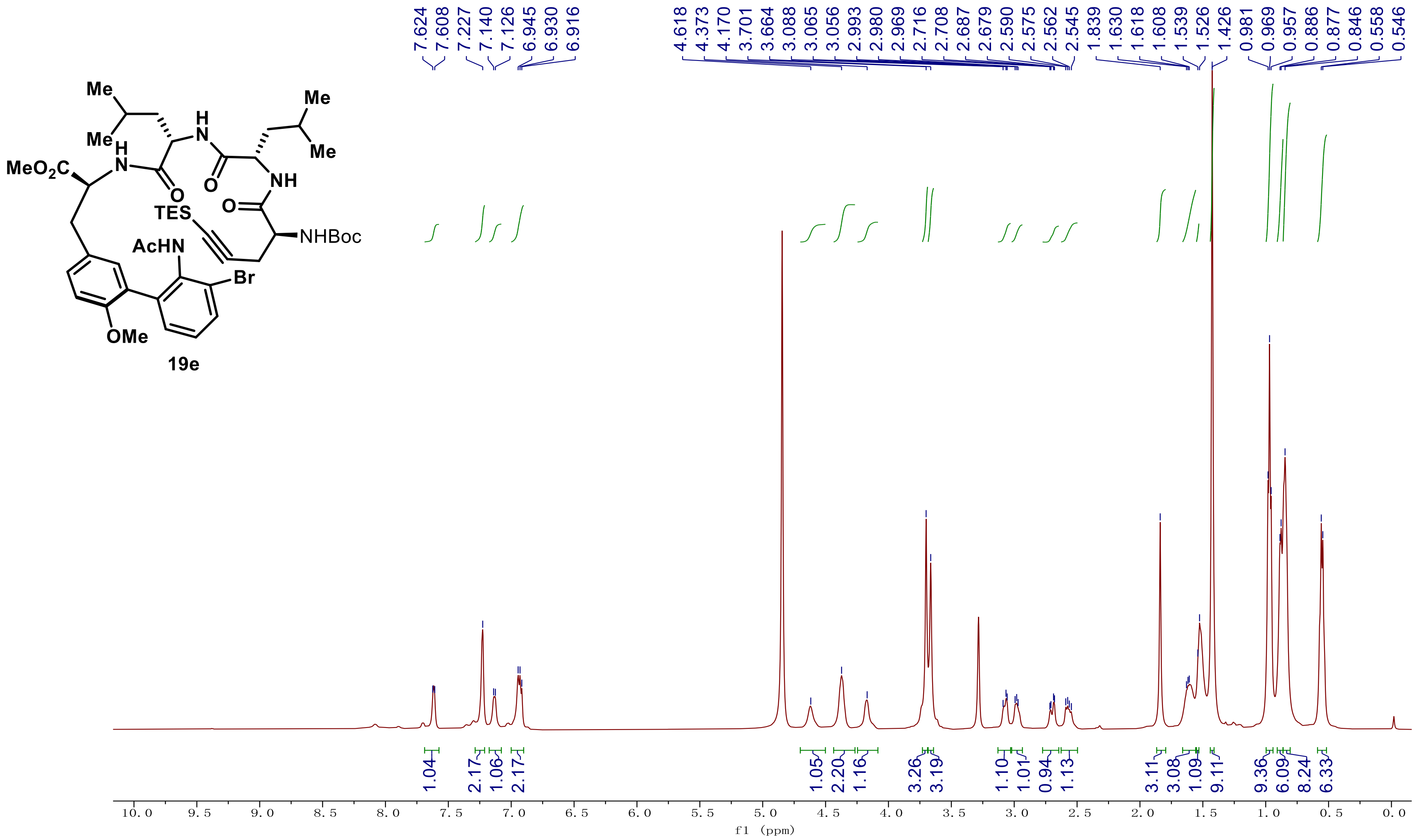

Compound 19e ^{13}C NMR (151 MHz, METHANOL-D_4)

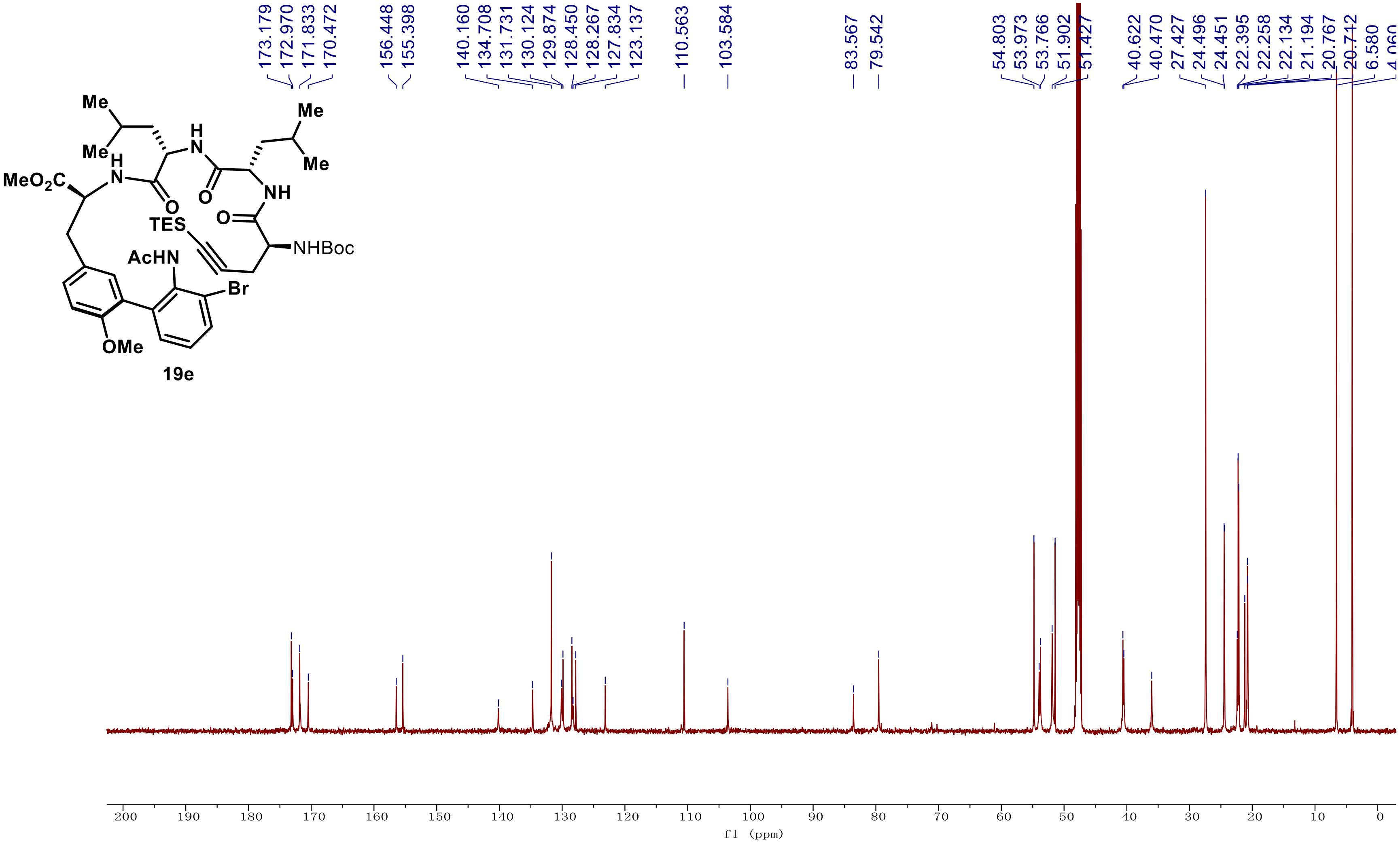

Compound 19f ^{1}H NMR (600 MHz, METHANOL-D_4)

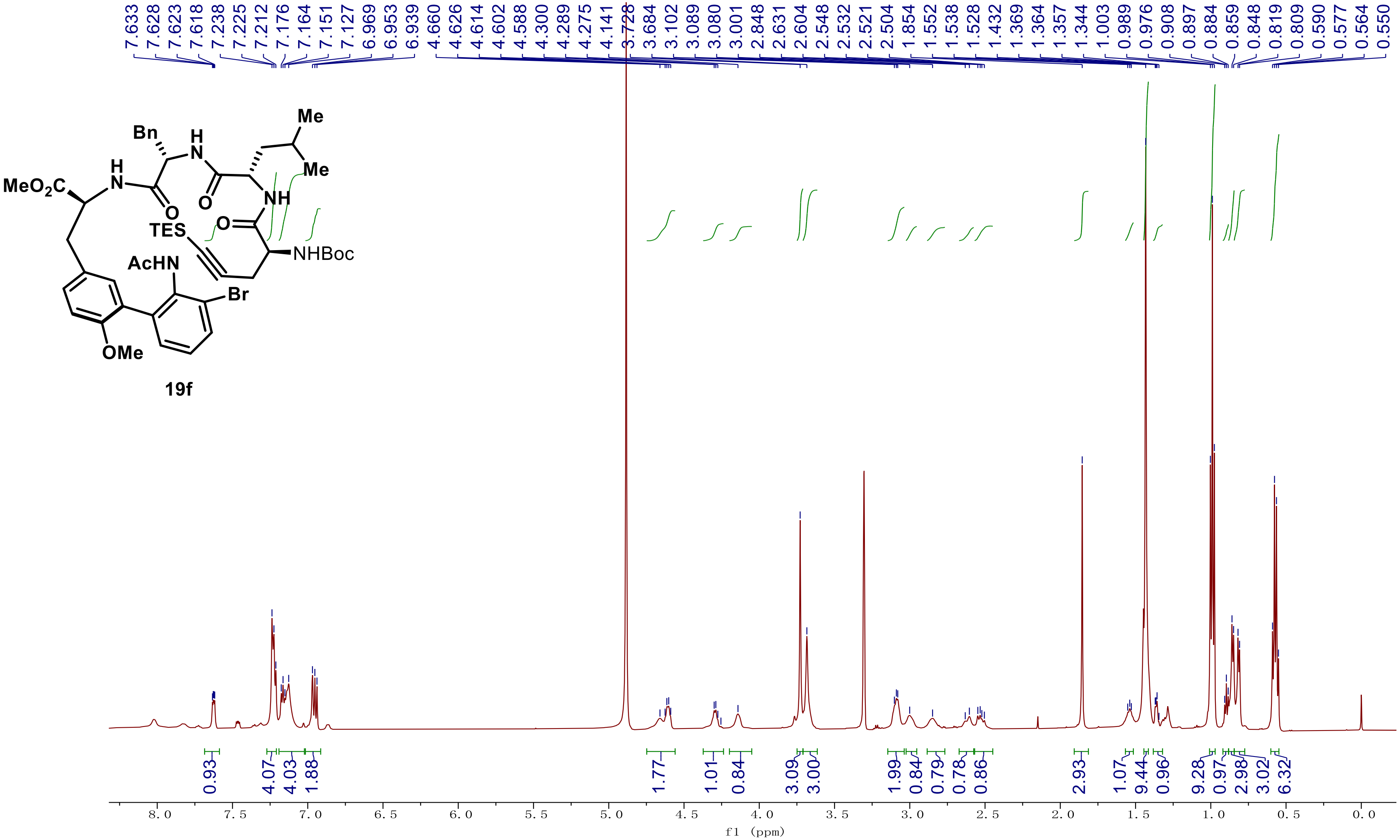

Compound 19f ^{13}C NMR (151 MHz, METHANOL-D_4)

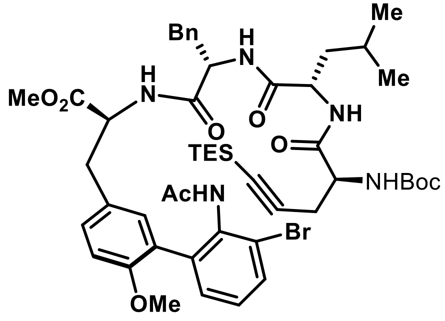

19f

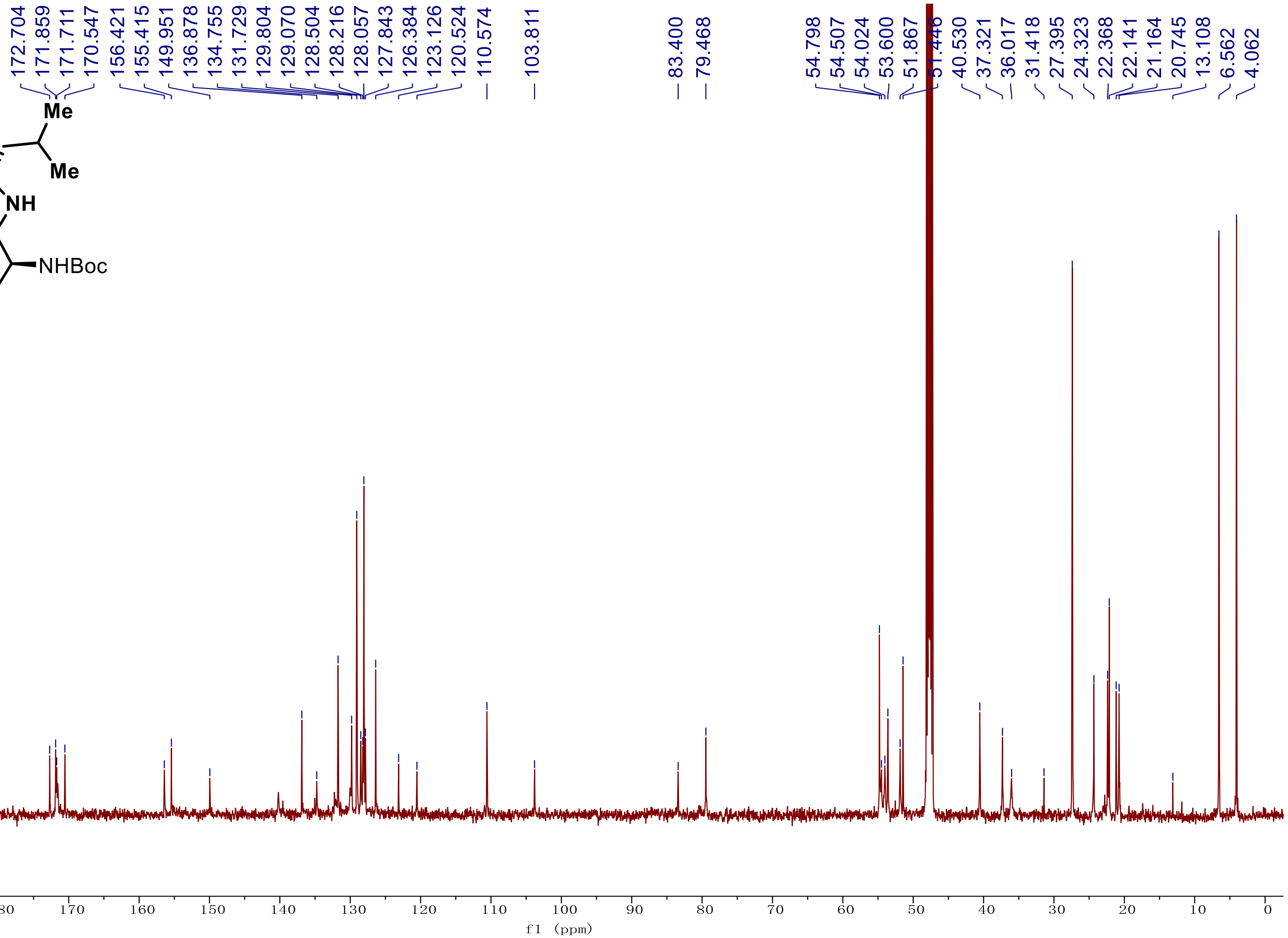

Compound 19g ^{1}H NMR (600 MHz, METHANOL-D_4)

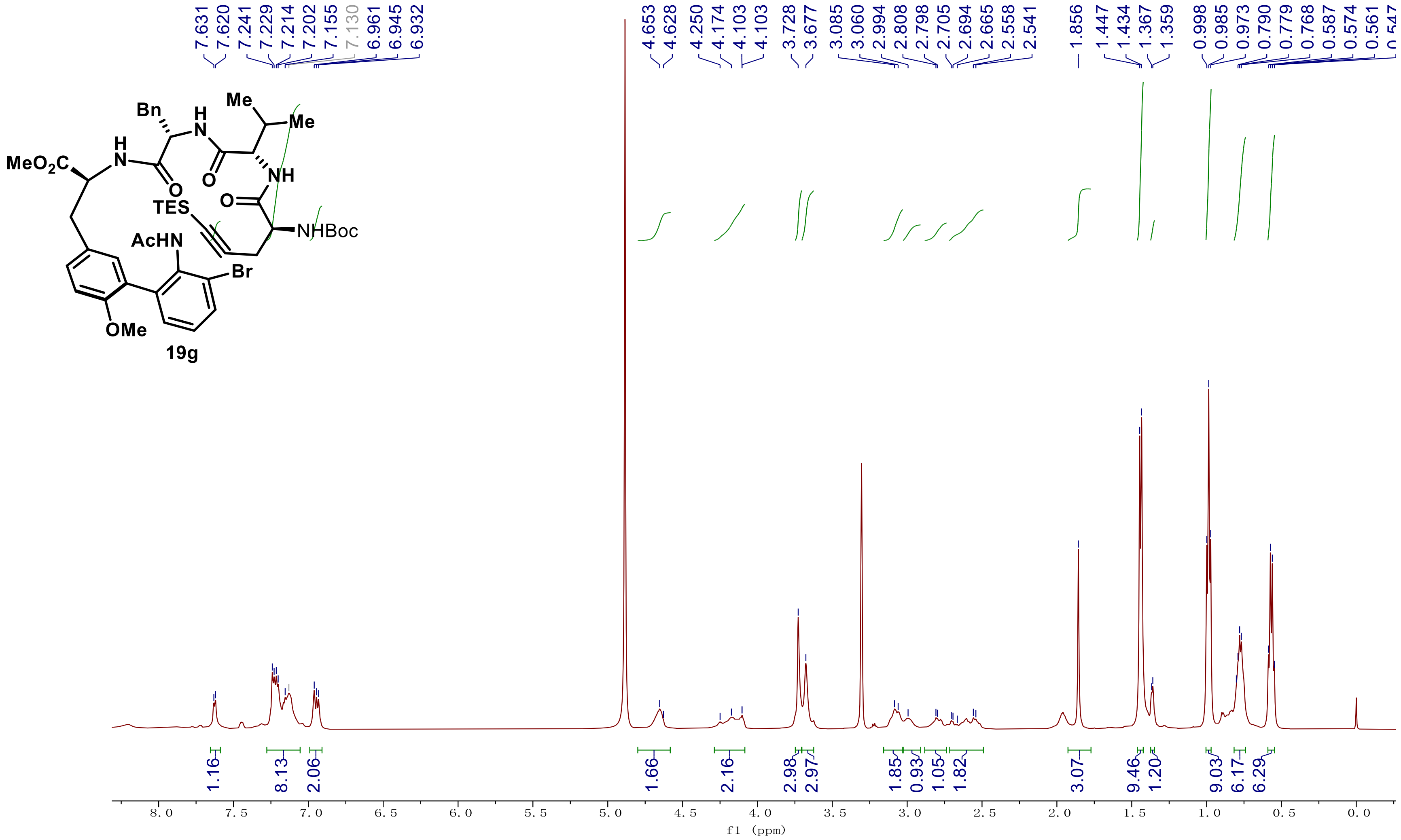

Compound 19g ^{13}C NMR (151 MHz, METHANOL-D_4)

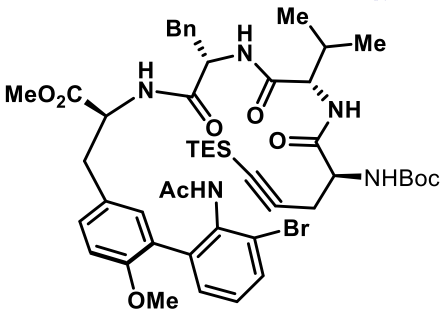

19g

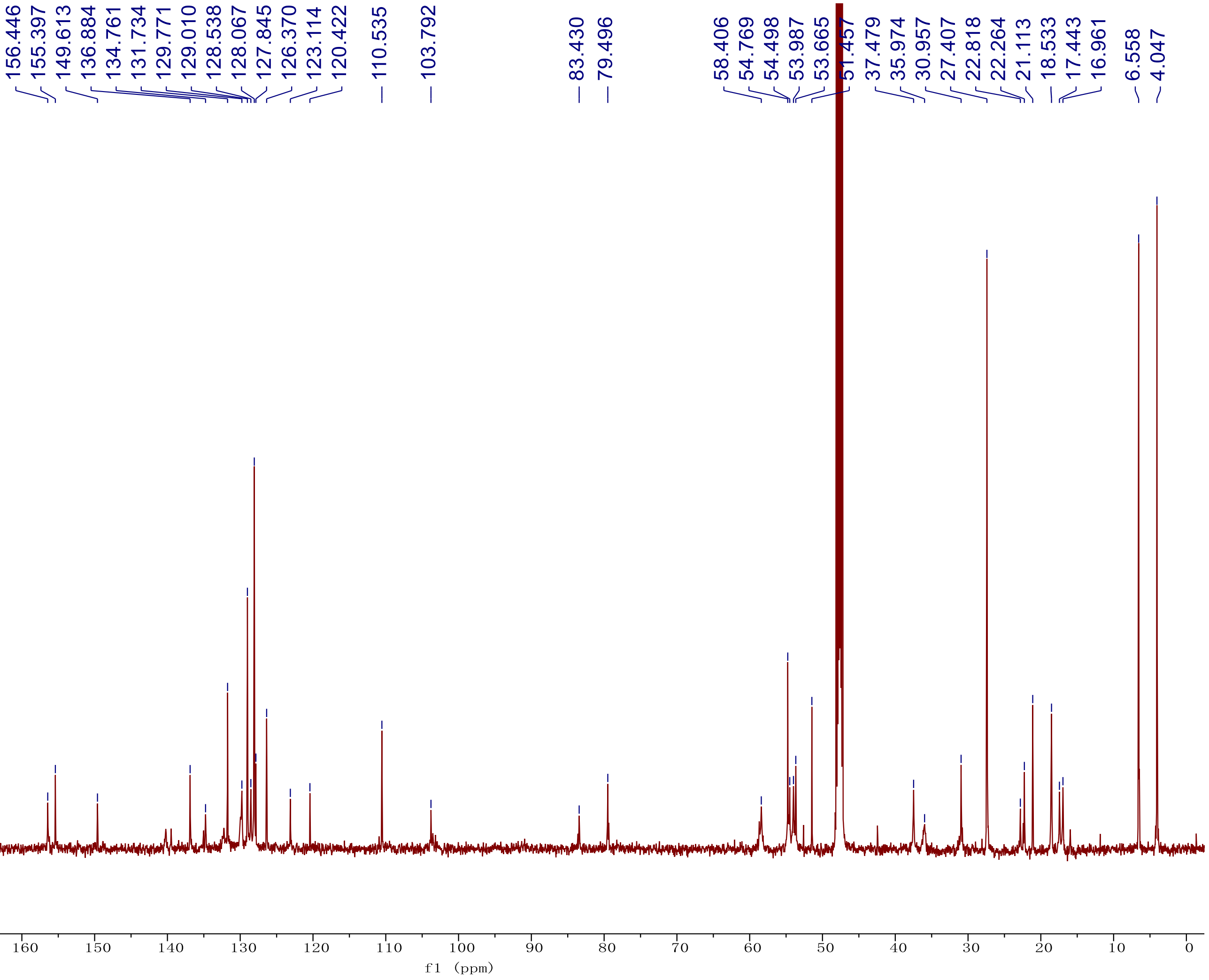

Compound 19h ^{1}H NMR (600 MHz, METHANOL-D_4)

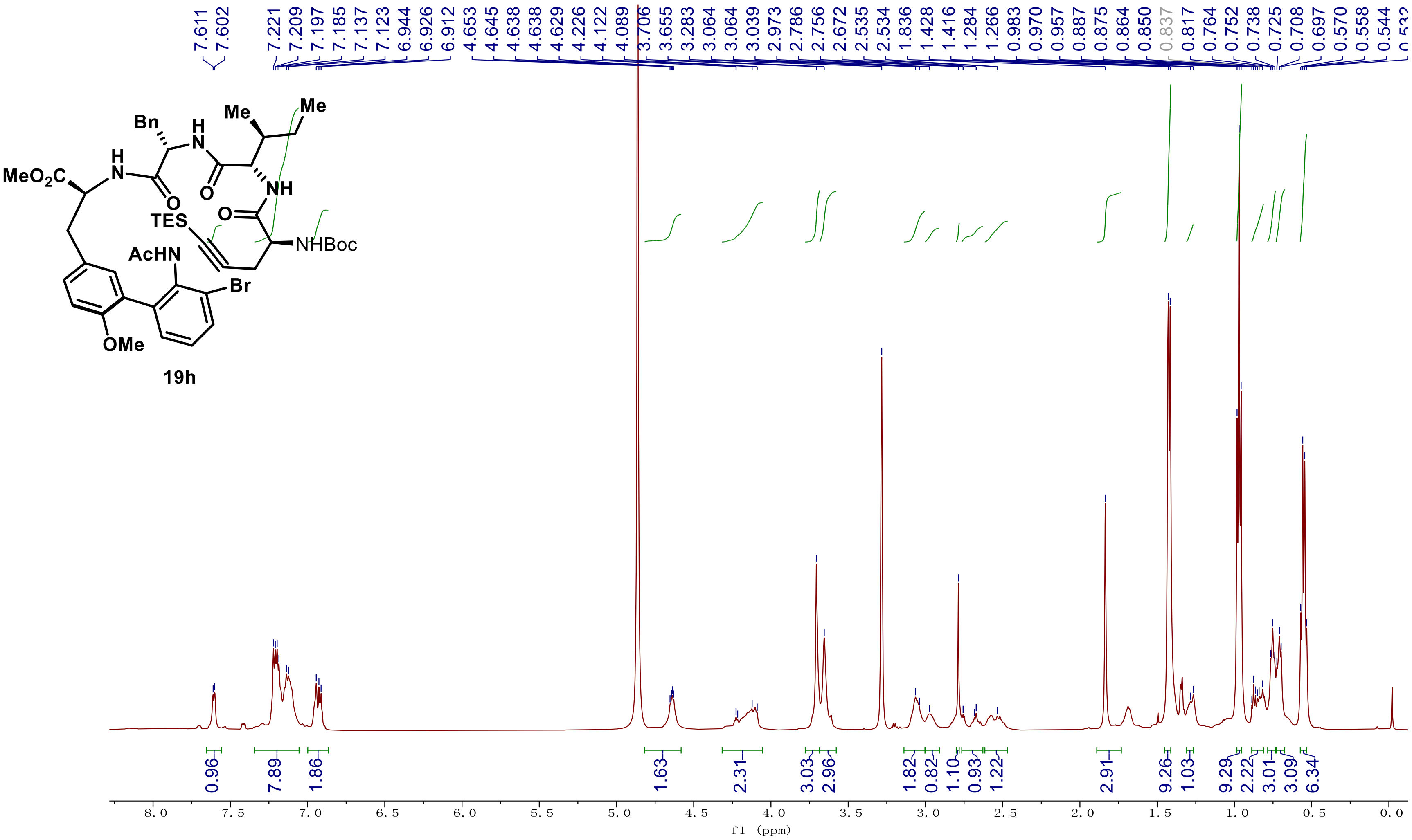

Compound 19h ^{13}C NMR (151 MHz, DMSO-d_6)

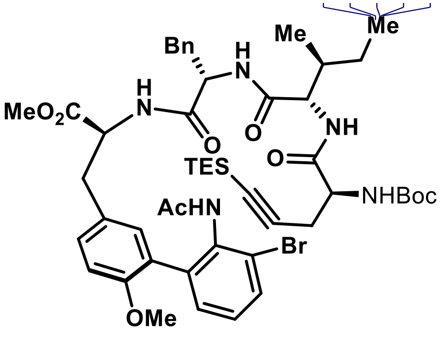

19h

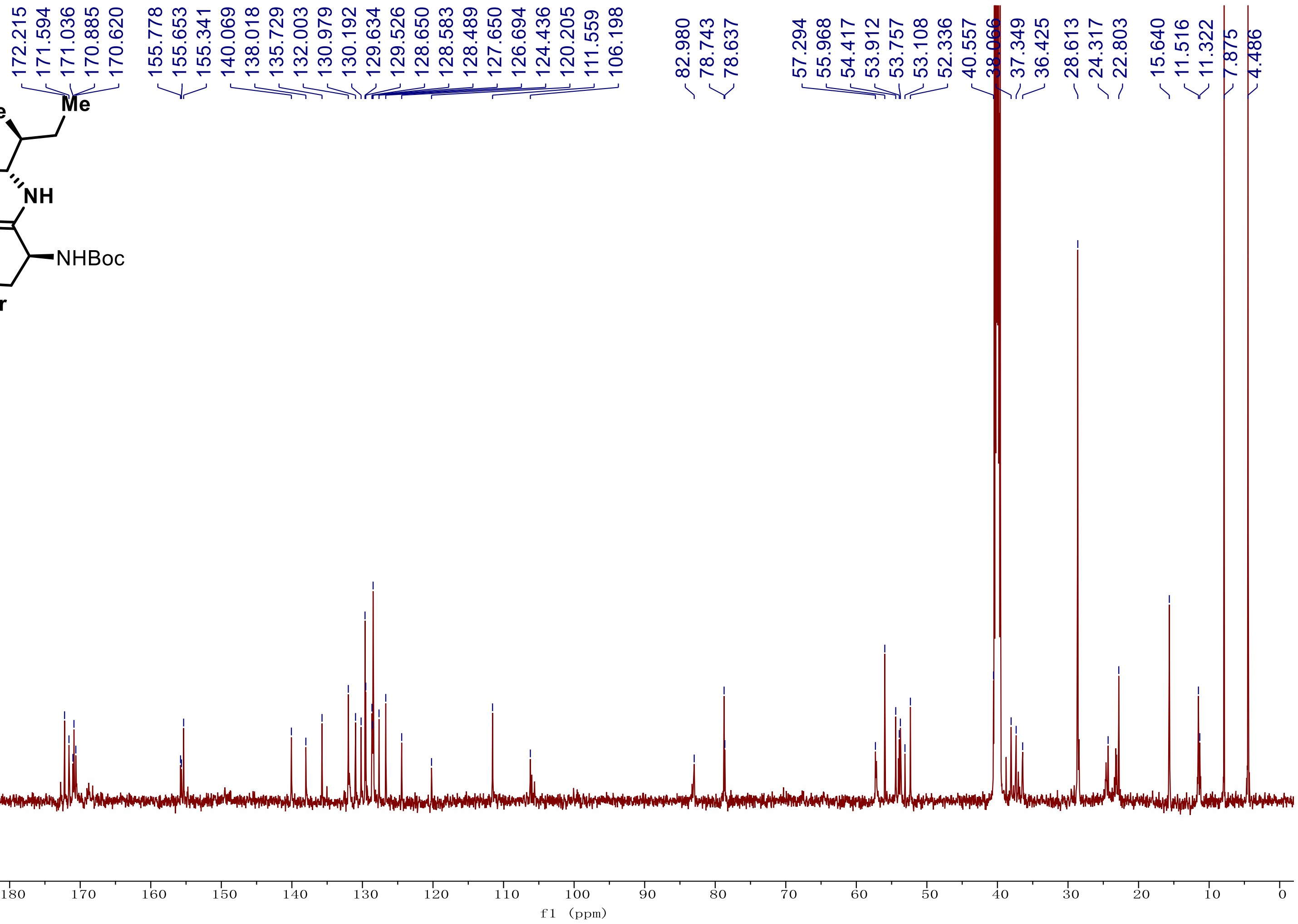

Compound 19i ^{1}H NMR (600 MHz, METHANOL-D_4)

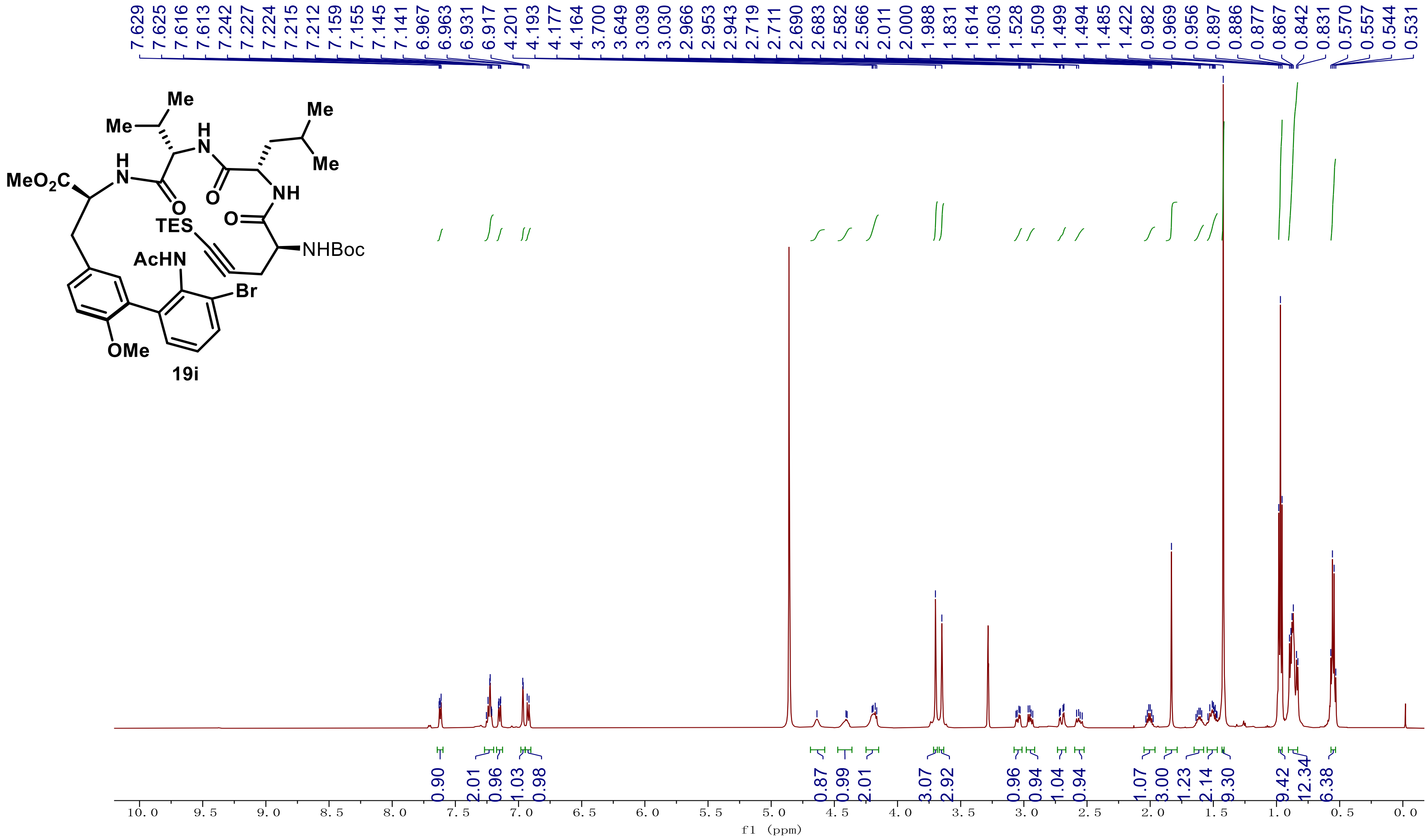

Compound 19i ^{13}C NMR (151 MHz, METHANOL-D_4)

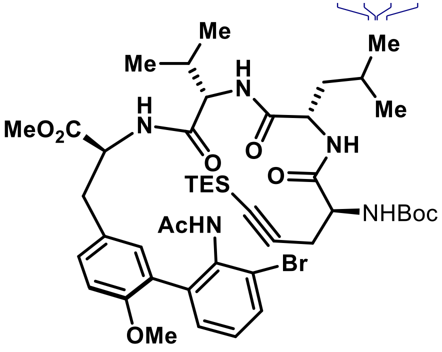

19i

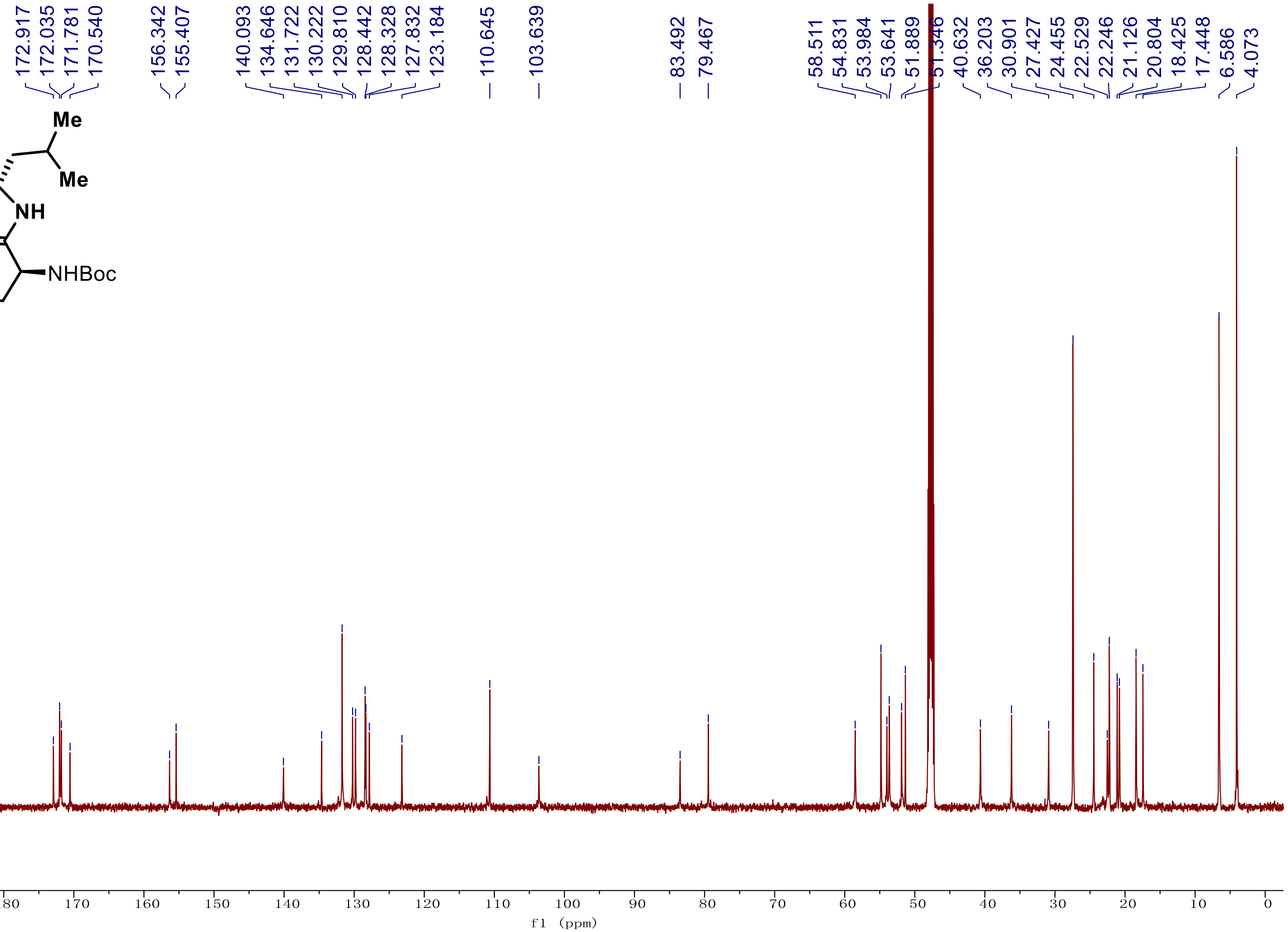

Compound 19j ^{1}H NMR (600 MHz, METHANOL-D_4)

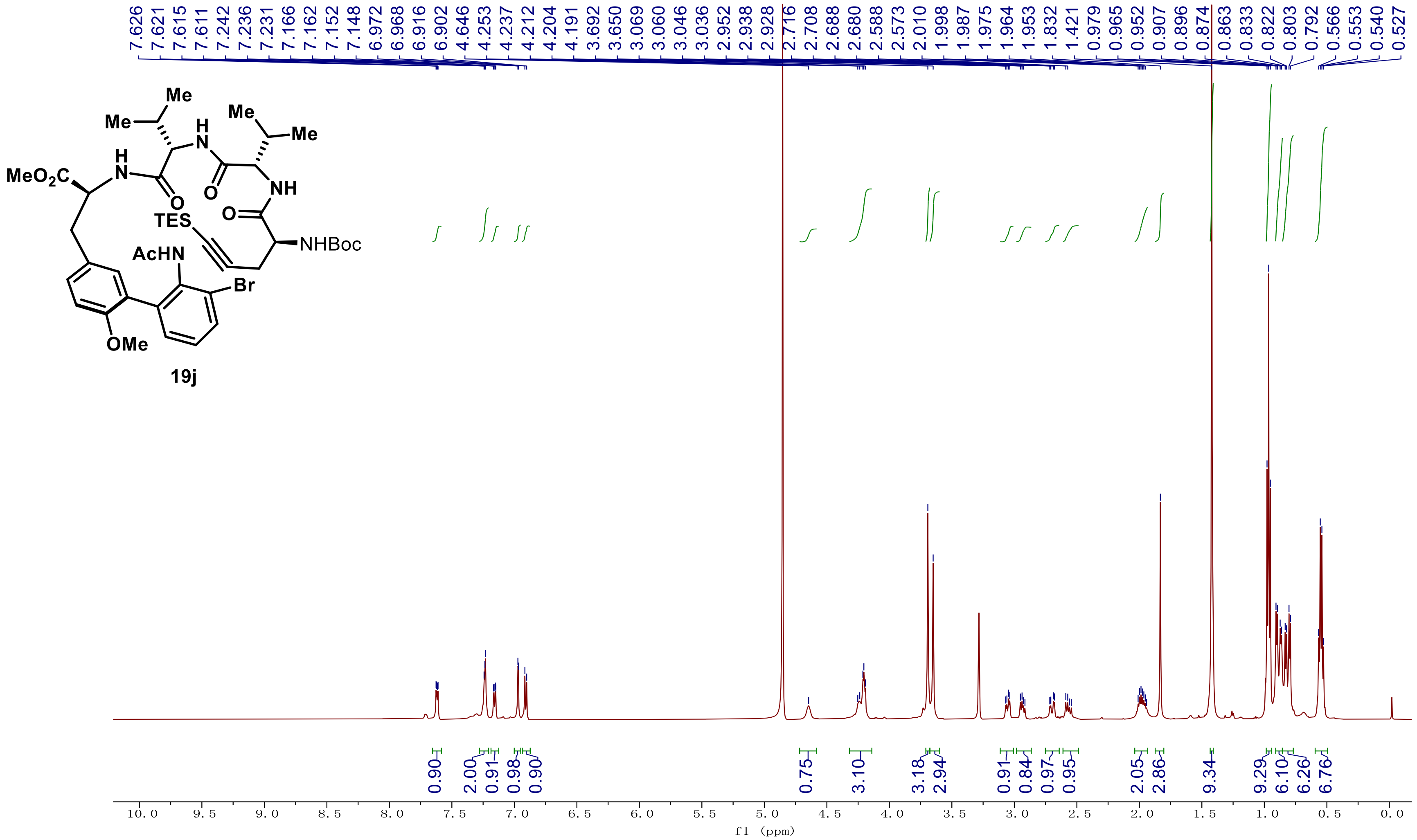

Compound 19j ^{13}C NMR (151 MHz, METHANOL-D_4)

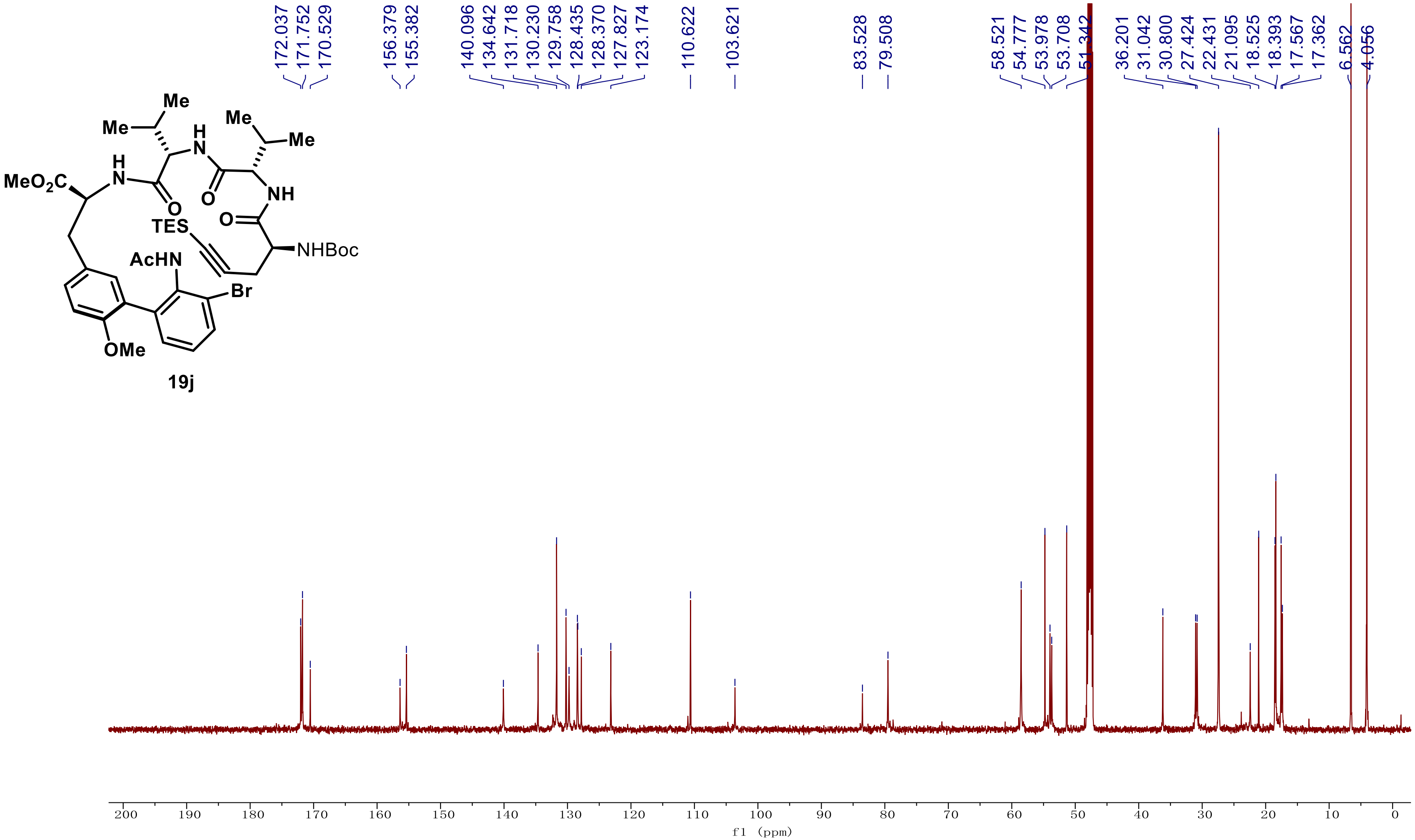

19j

Compound 19k ^{1}H NMR (600 MHz, METHANOL-D_4)

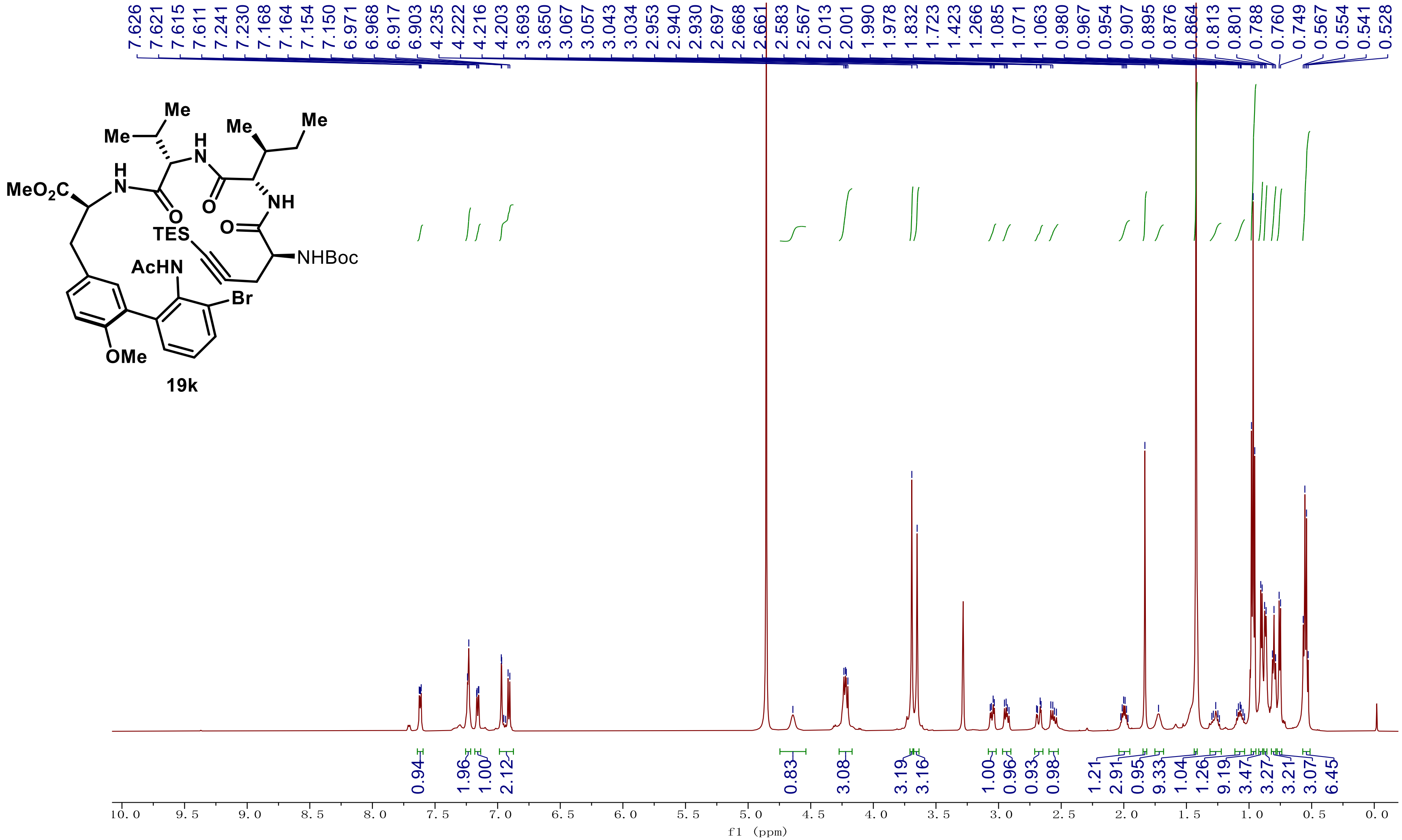

Compound 19k ^{13}C NMR (151 MHz, METHANOL-D_4)

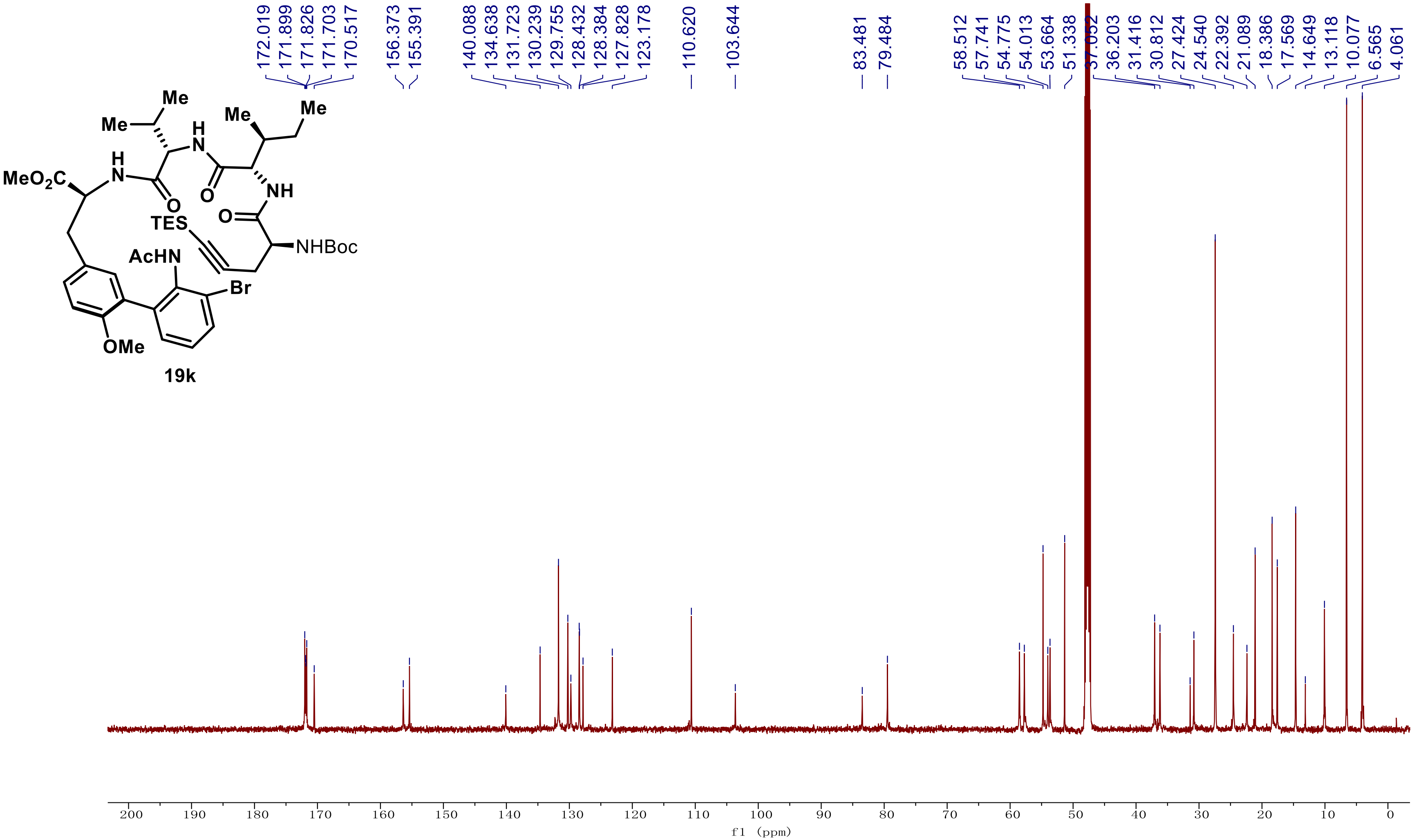

19k

Compound 19l ^{1}H NMR (600 MHz, METHANOL-D_4)

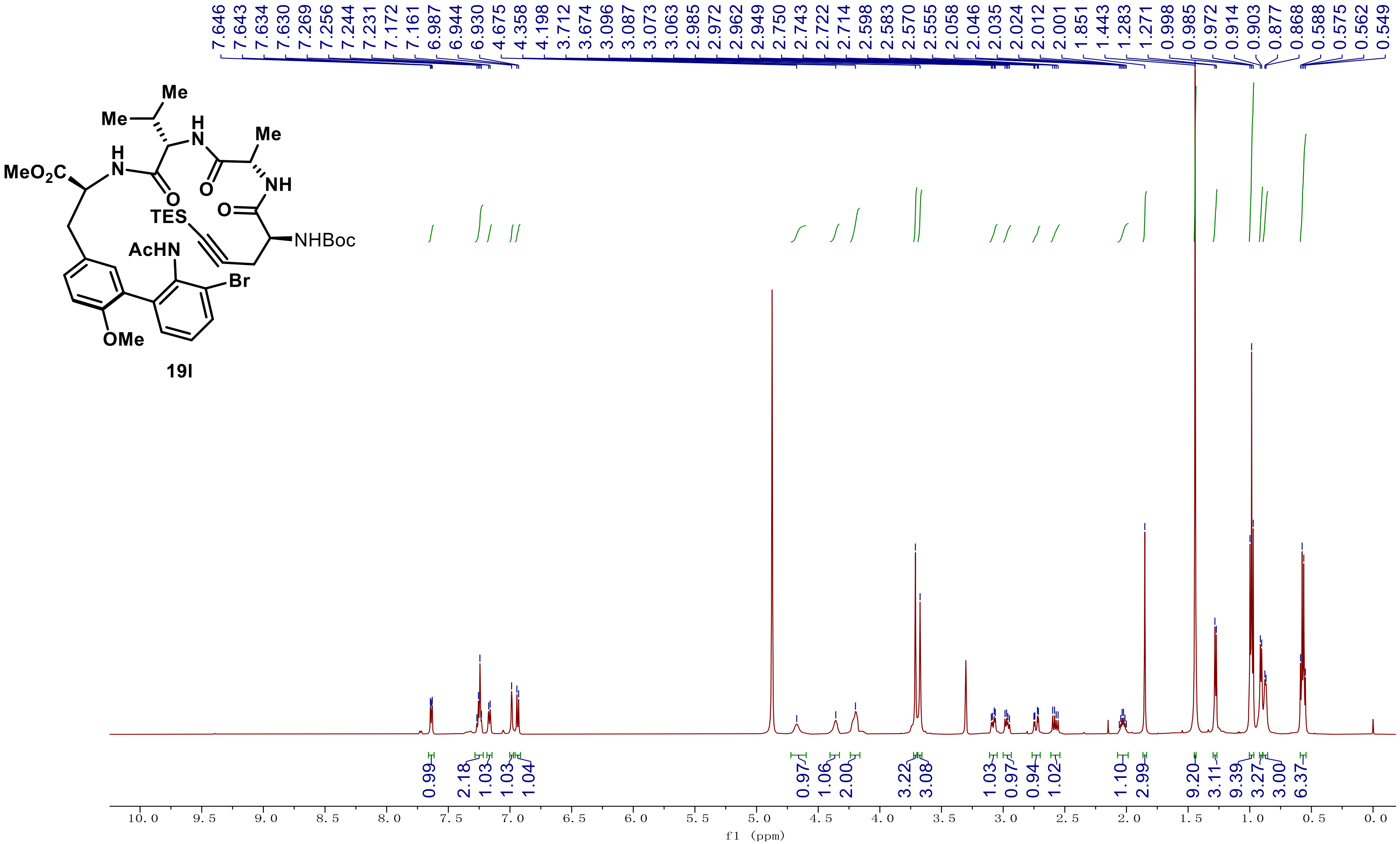

Compound 19l ^{13}C NMR (151 MHz, METHANOL-D_4)

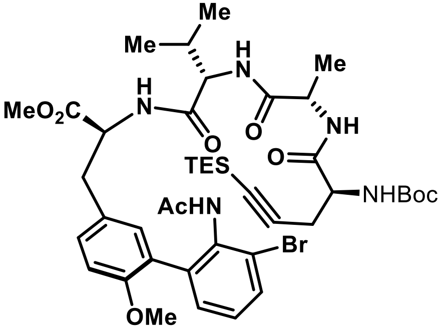

19l

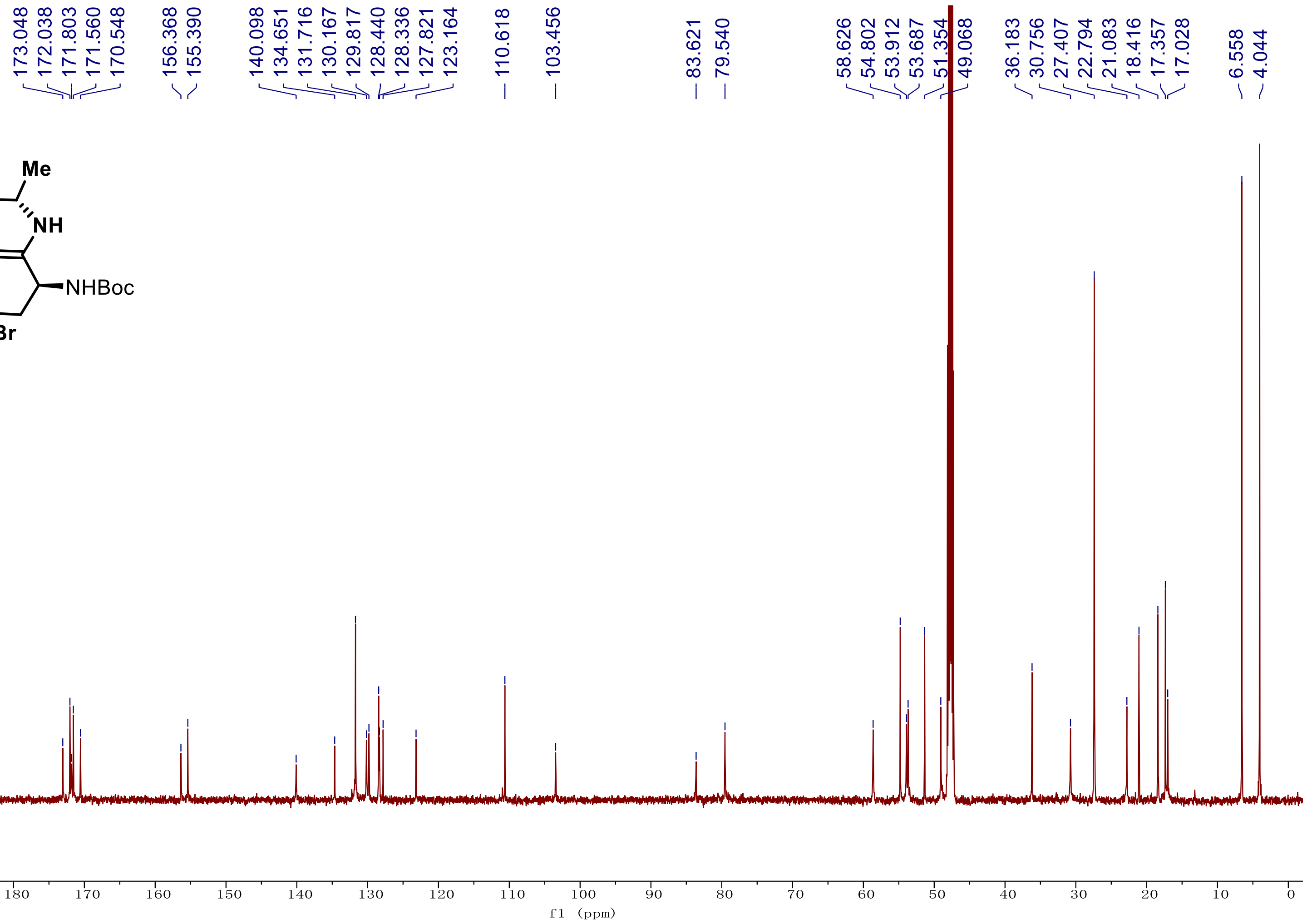

Compound 19m ^{1}H NMR (600 MHz, METHANOL-D_4)

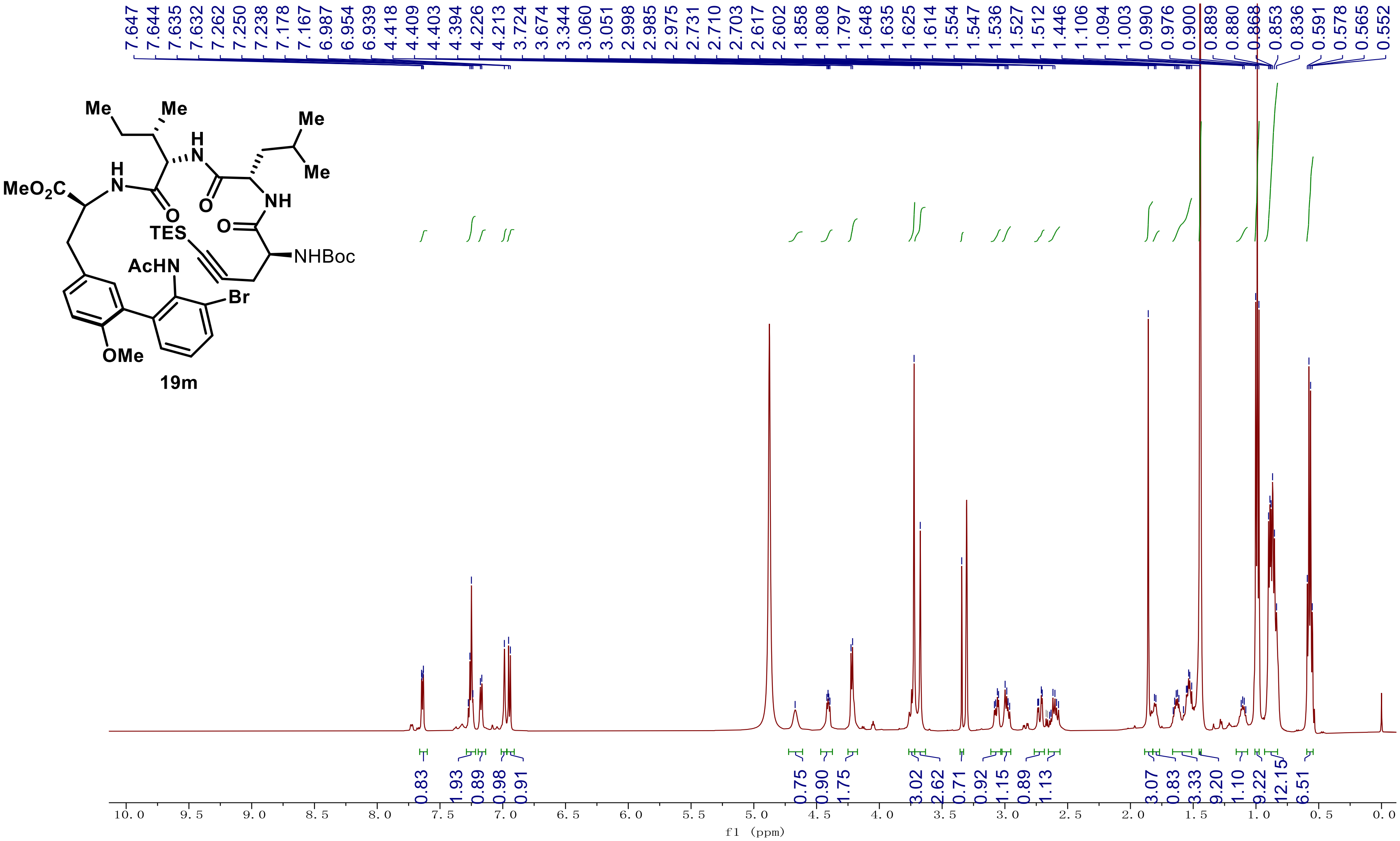

Compound 19m ^{13}C NMR (151 MHz, METHANOL-D_4)

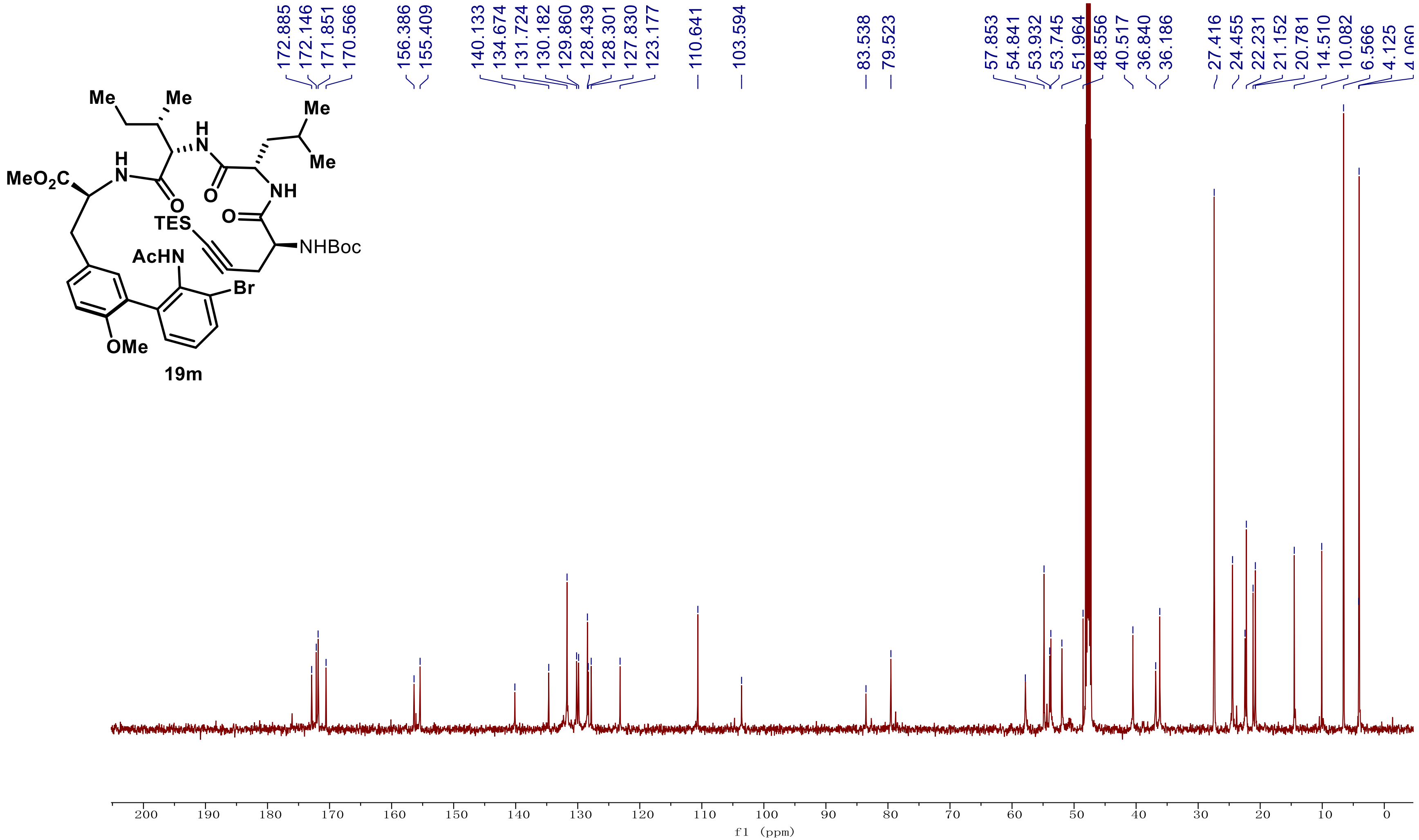

Compound 19n ^{1}H NMR (600 MHz, METHANOL-D_4)

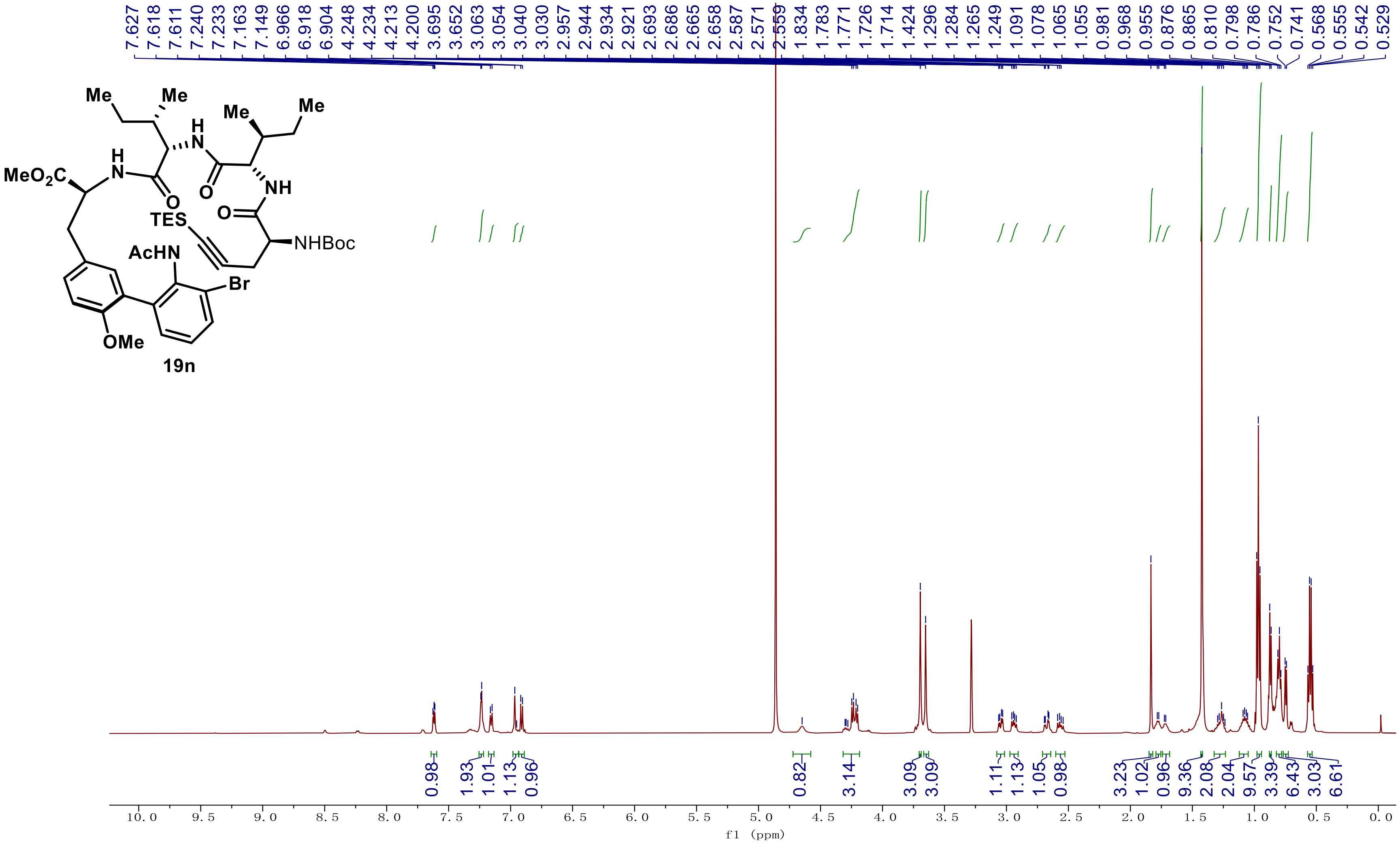

Compound 19n ^{13}C NMR (151 MHz, METHANOL-*D*$_4$)

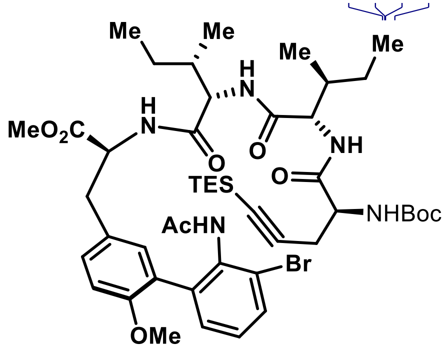

19n

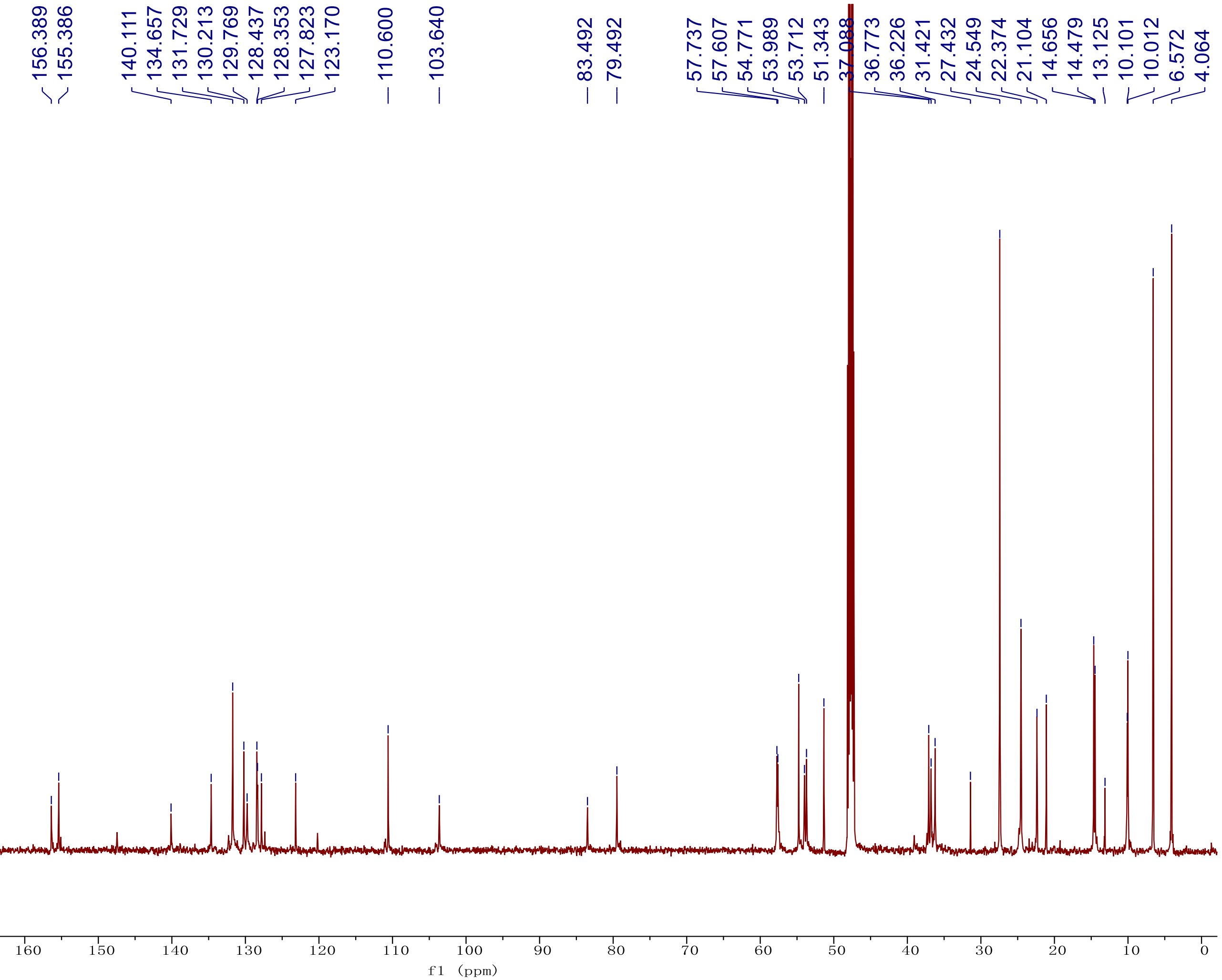

Compound 19o ^{1}H NMR (600 MHz, METHANOL-D_4)

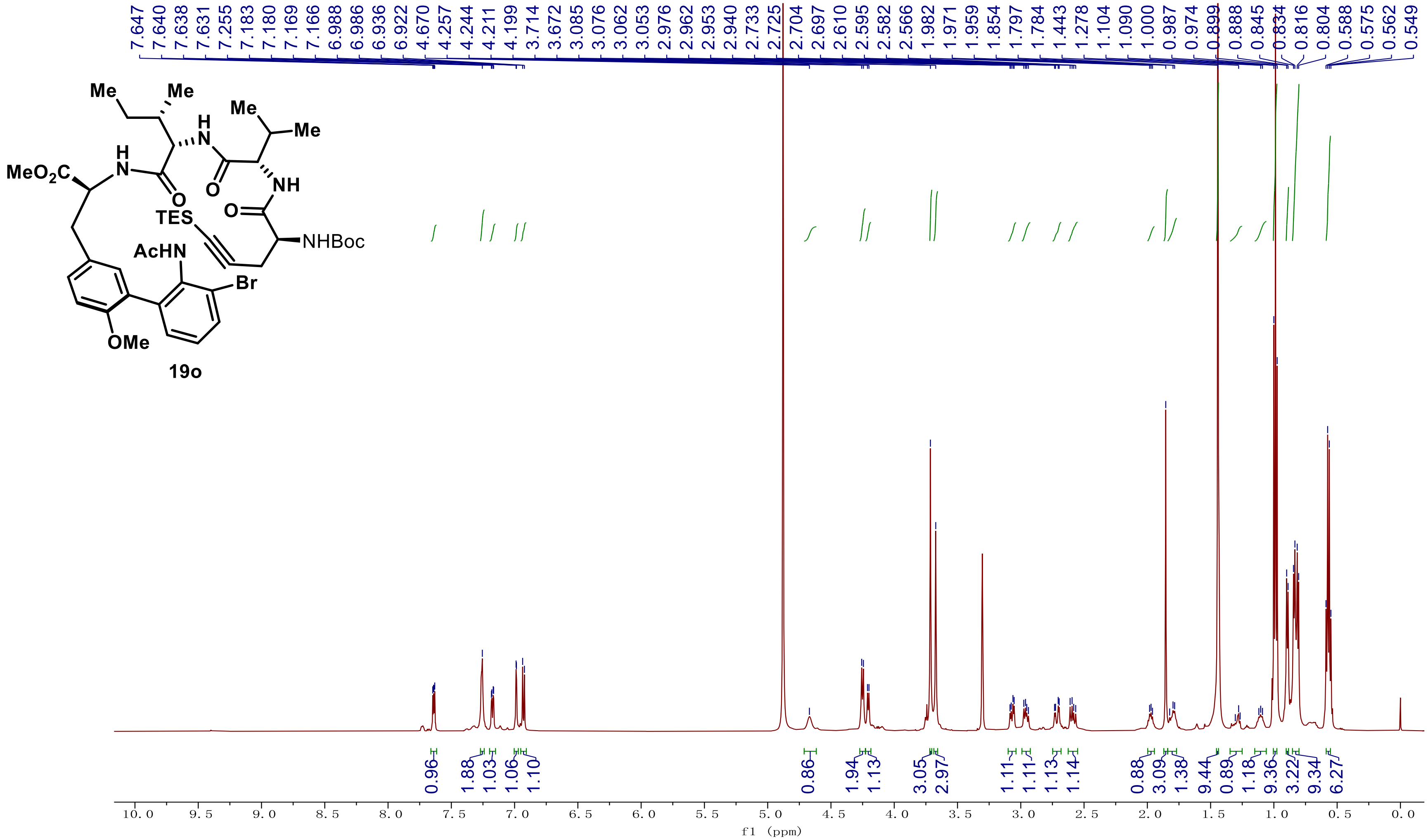

Compound 19o ^{13}C NMR (151 MHz, METHANOL-D_4)

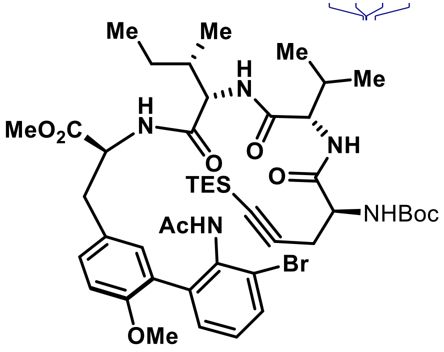

19o

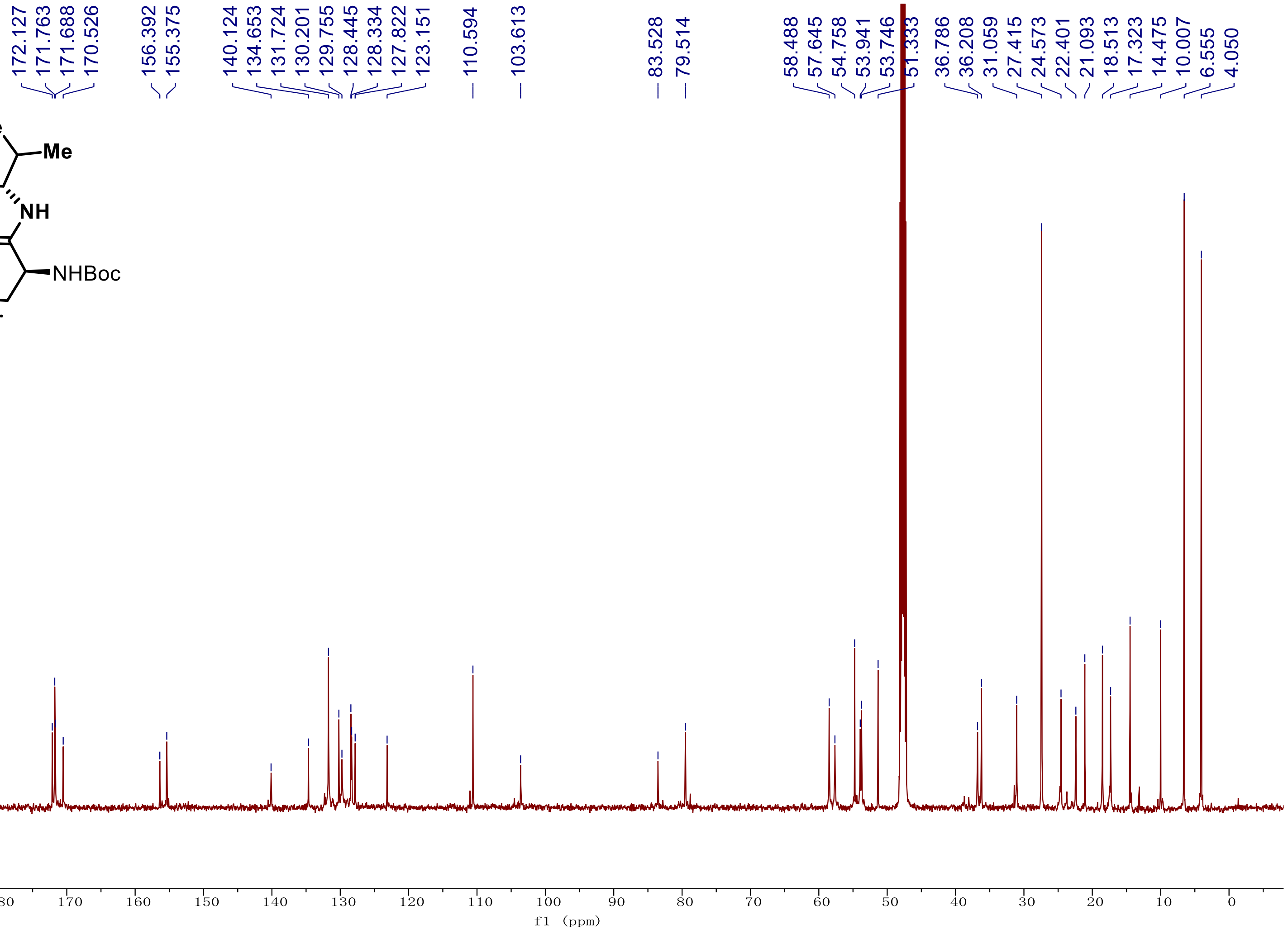

Compound 19p ^{1}H NMR (600 MHz, METHANOL-D_4)

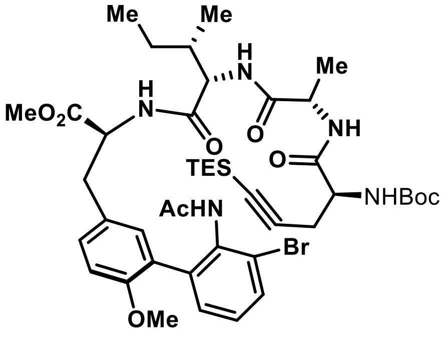

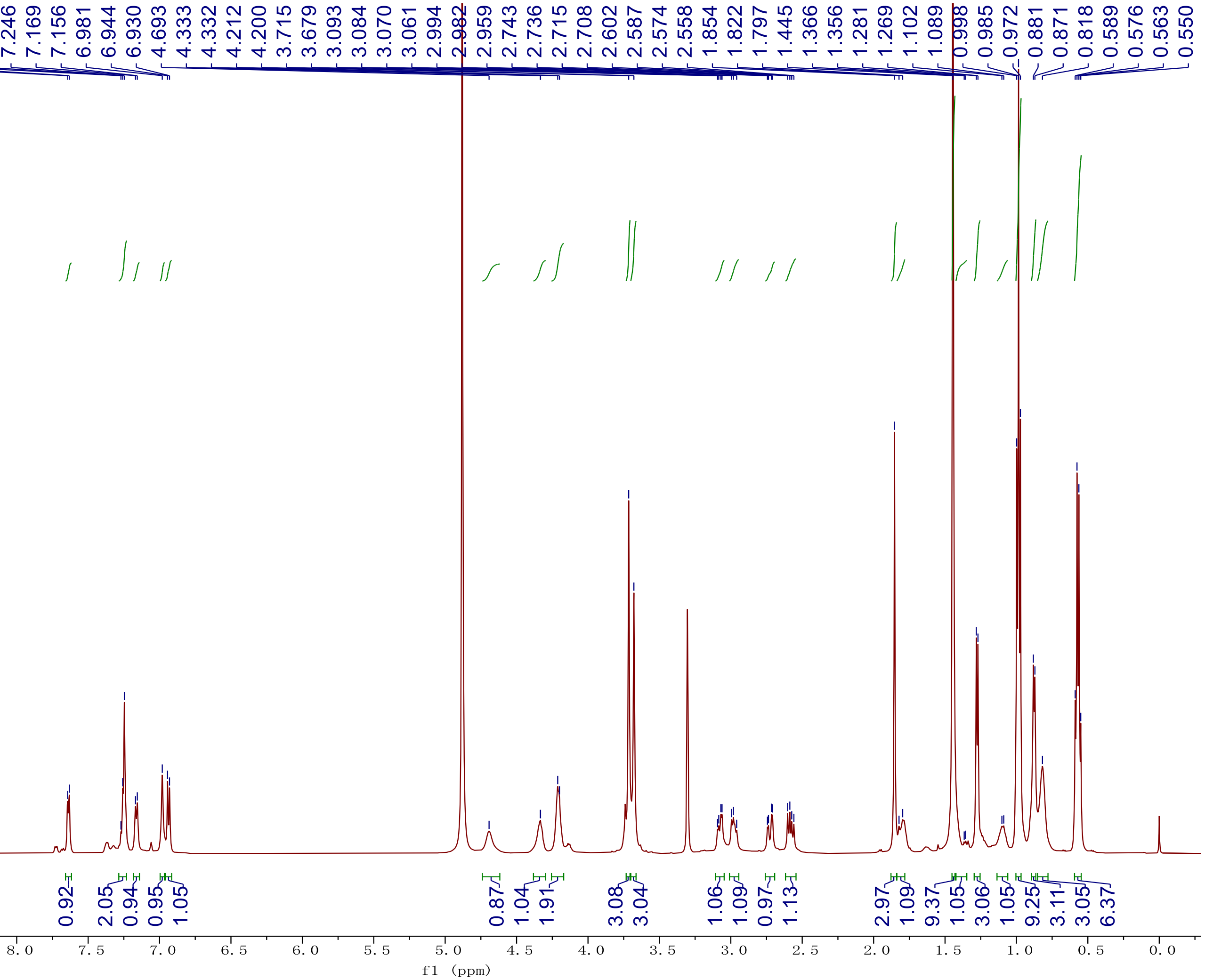

Compound 19p ^{13}C NMR (151 MHz, METHANOL-D_4)

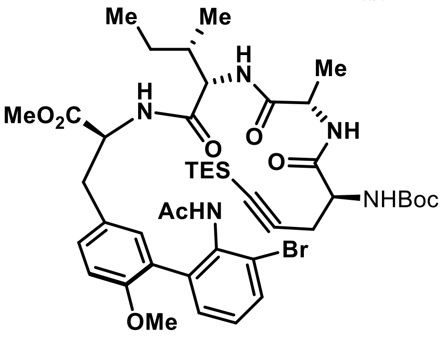

19p

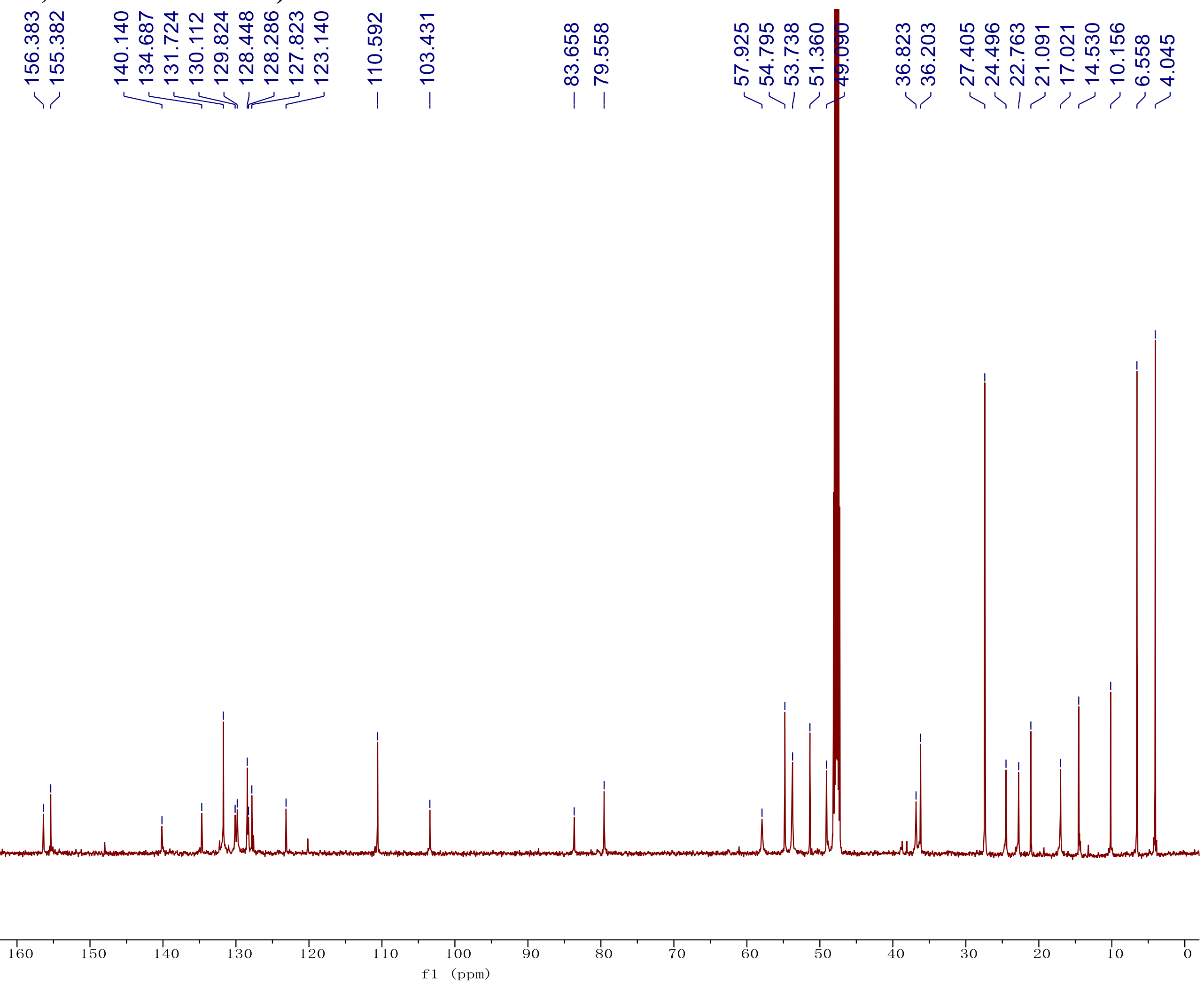

Compound 20a ^{1}H NMR (600 MHz, $CDCl_3$)

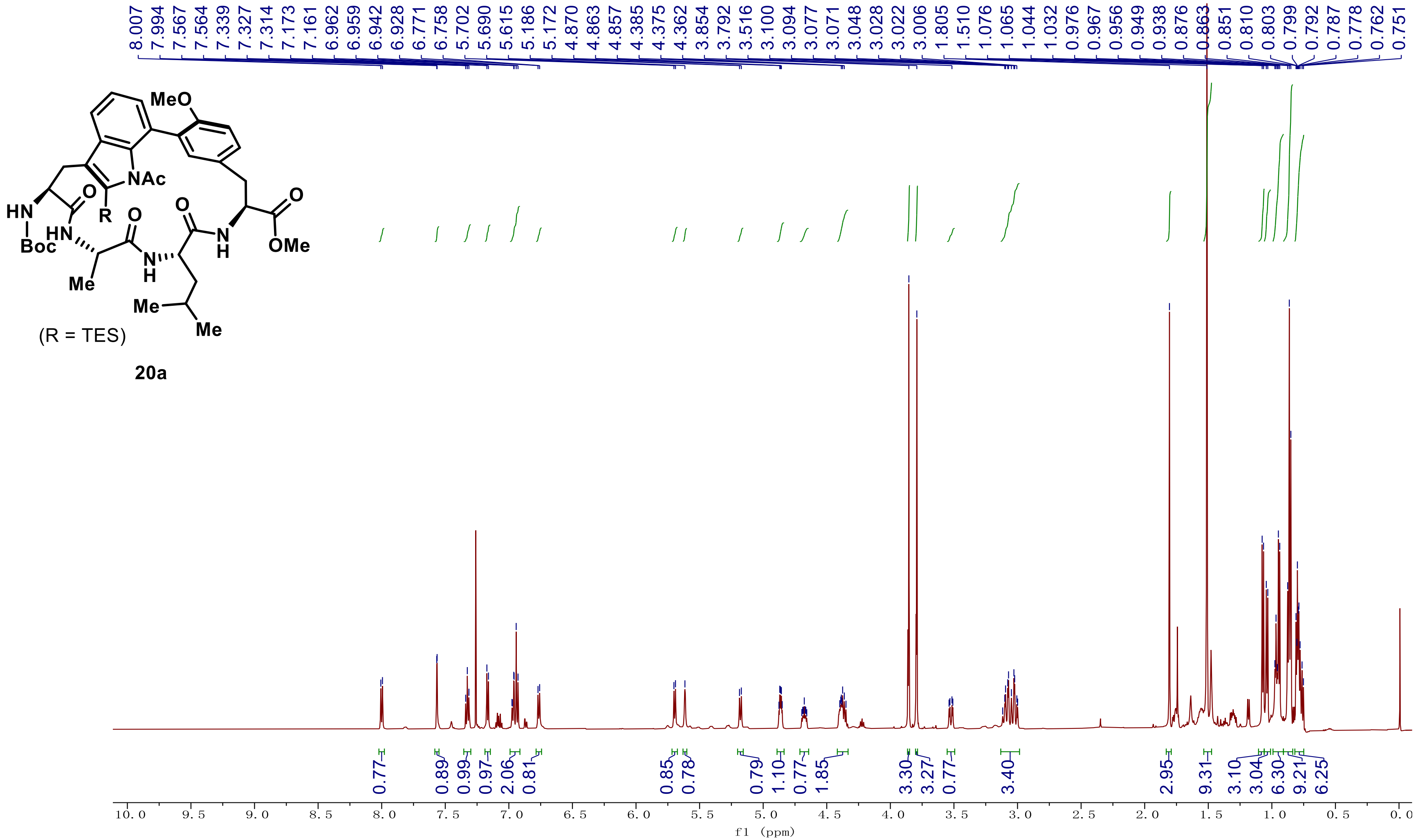

Compound 20a ^{13}C NMR (151 MHz, $CDCl_3$)

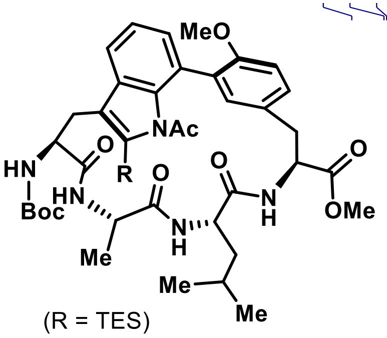

20a

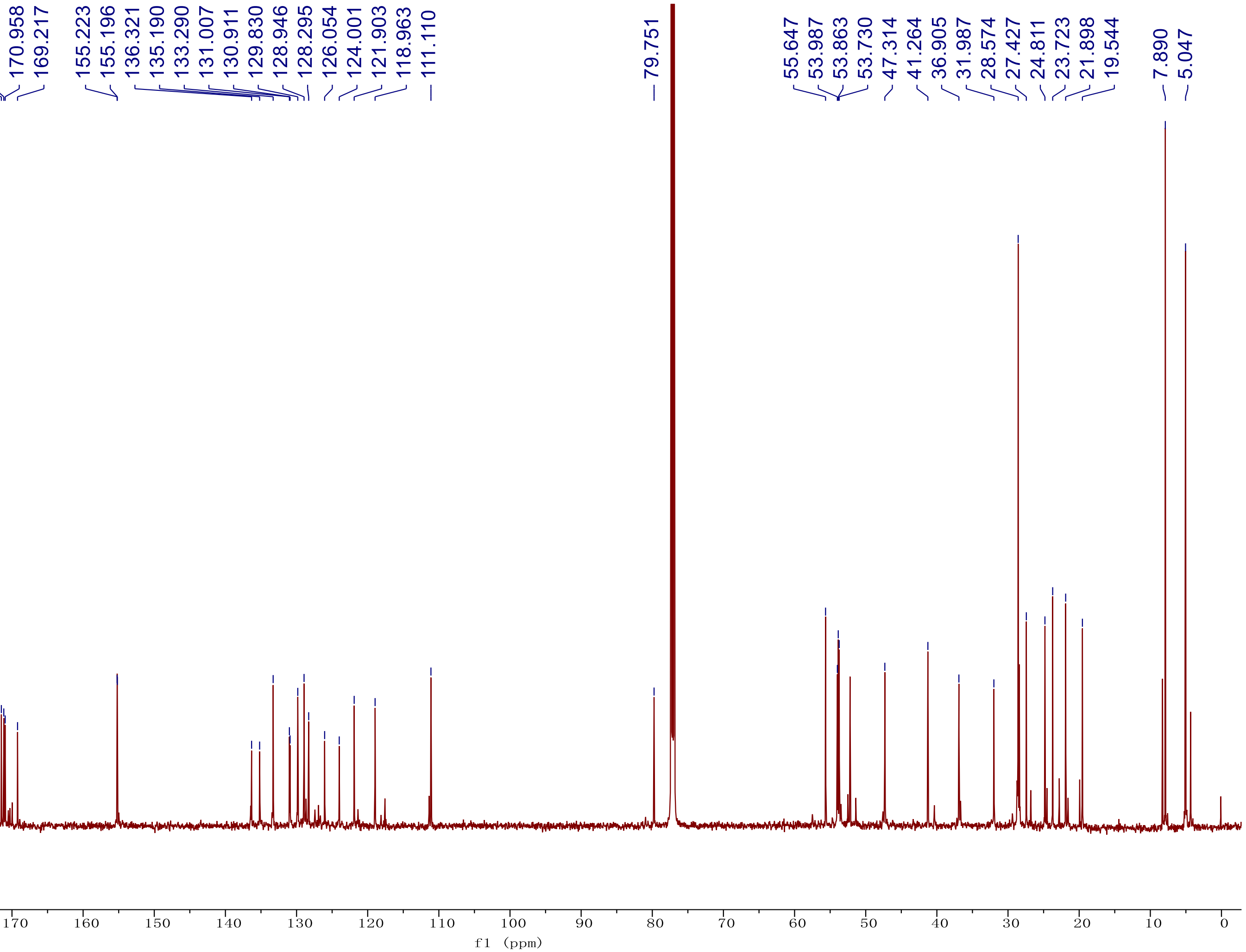

Compound 20b ^{1}H NMR (600 MHz, CDCl$_3$)

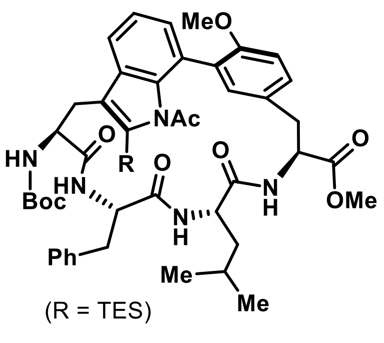

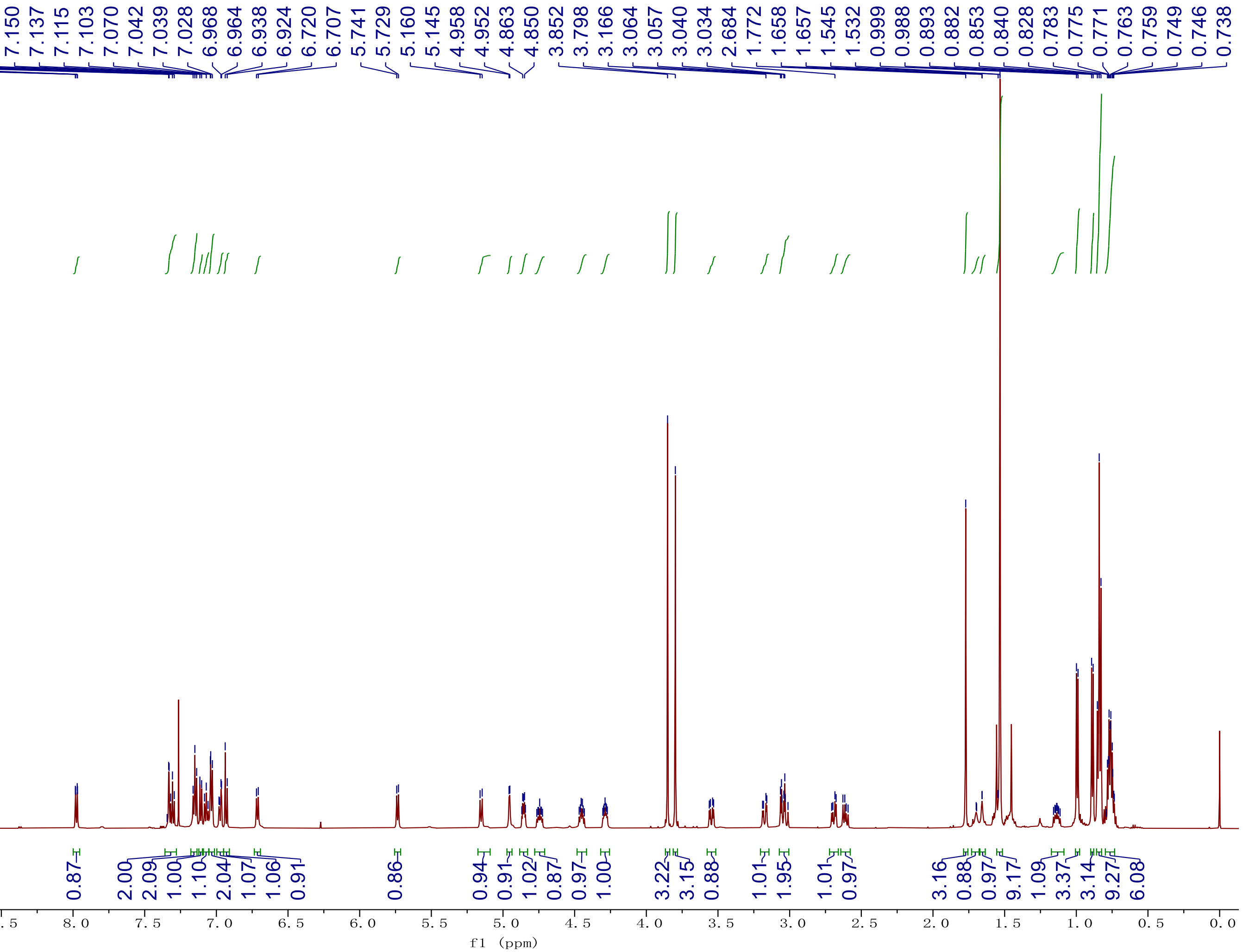

Compound 20b ^{13}C NMR (151 MHz, $CDCl_3$)

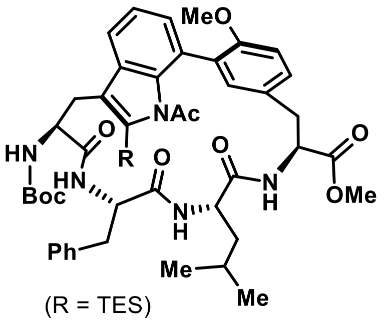

20b

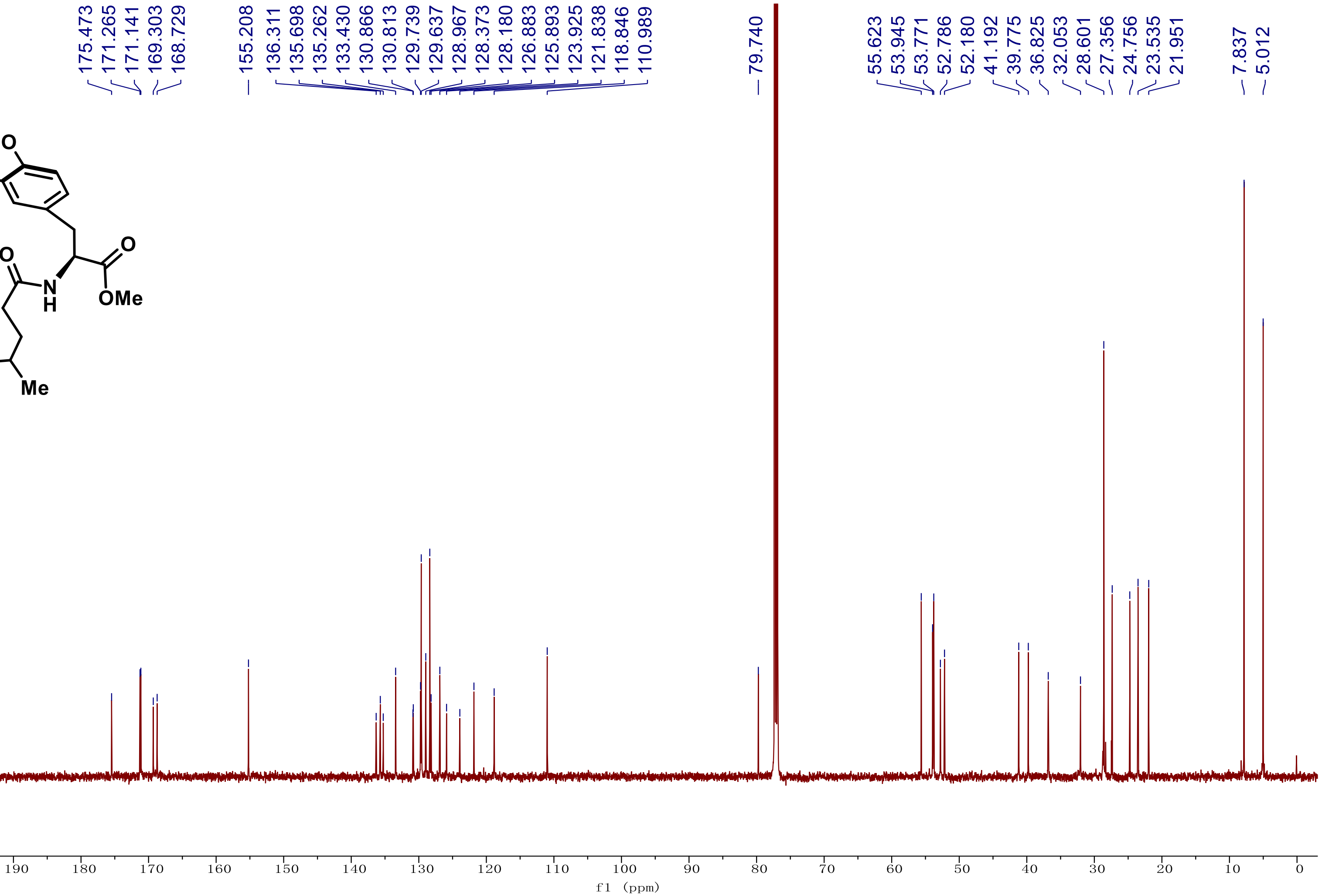

Compound 20c ^1H NMR (600 MHz, $CDCl_3$)

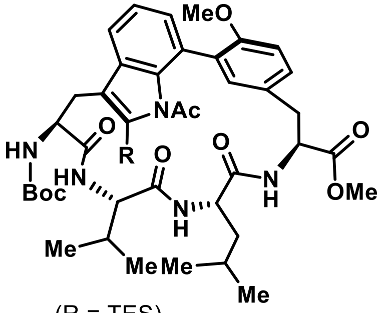

(R = TES)

20c

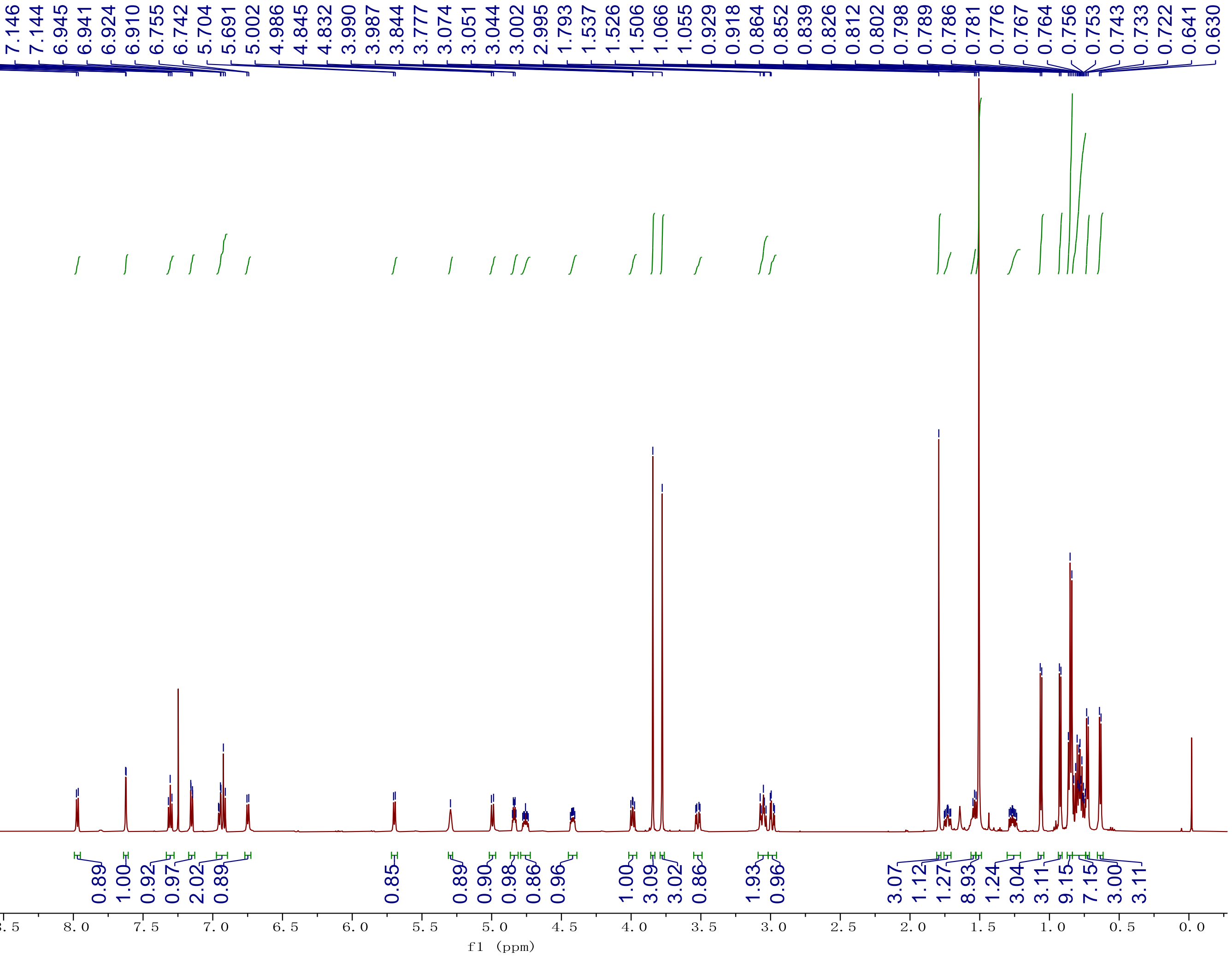

Compound 20c ^{13}C NMR (151 MHz, $CDCl_3$)

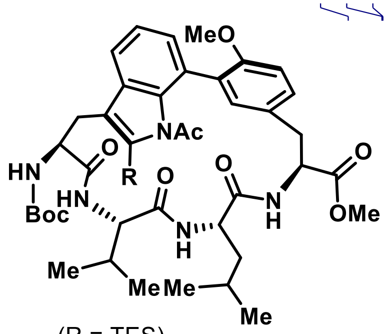

(R = TES)

20c

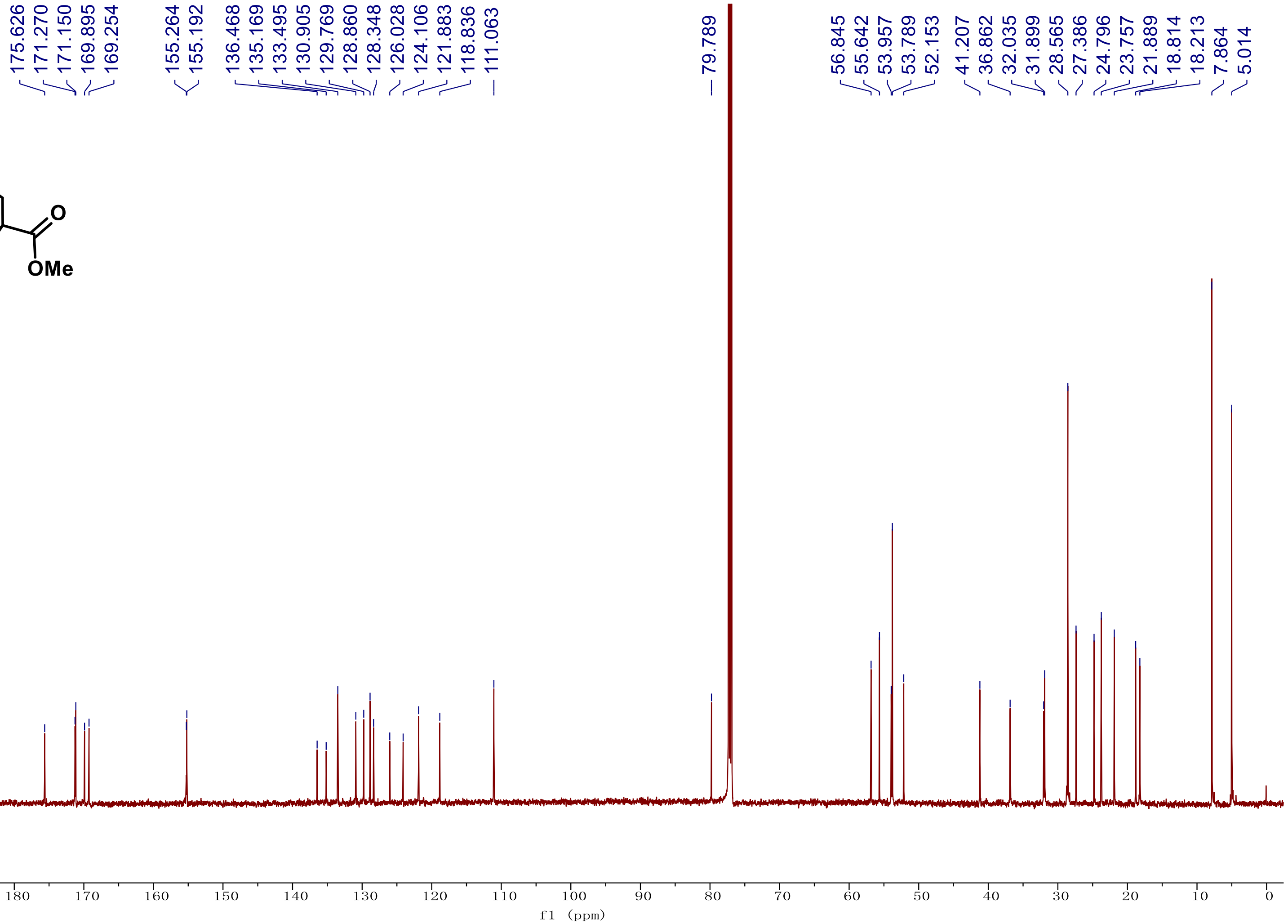

Compound 20d ^{1}H NMR (600 MHz, $CDCl_3$)

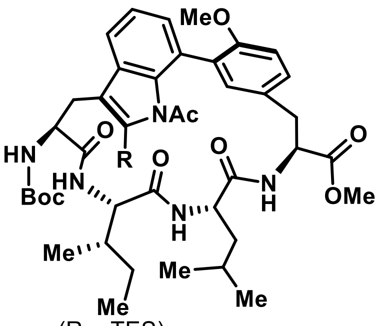

(R = TES)

20d

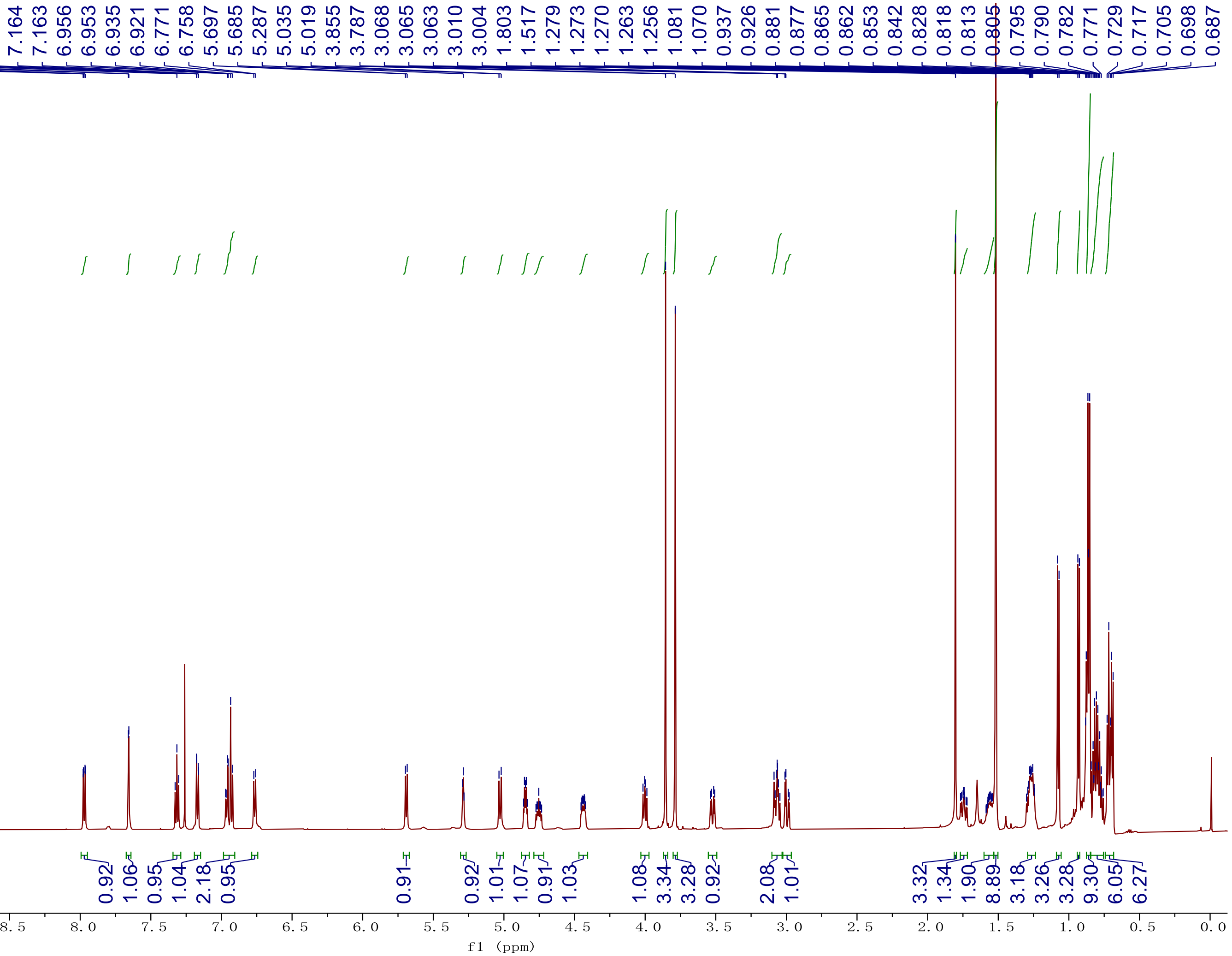

Compound 20b ^{13}C NMR (151 MHz, CDCl$_3$)

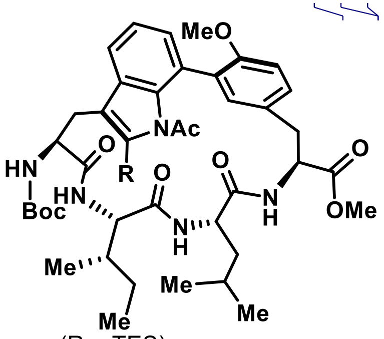

(R = TES)
20d

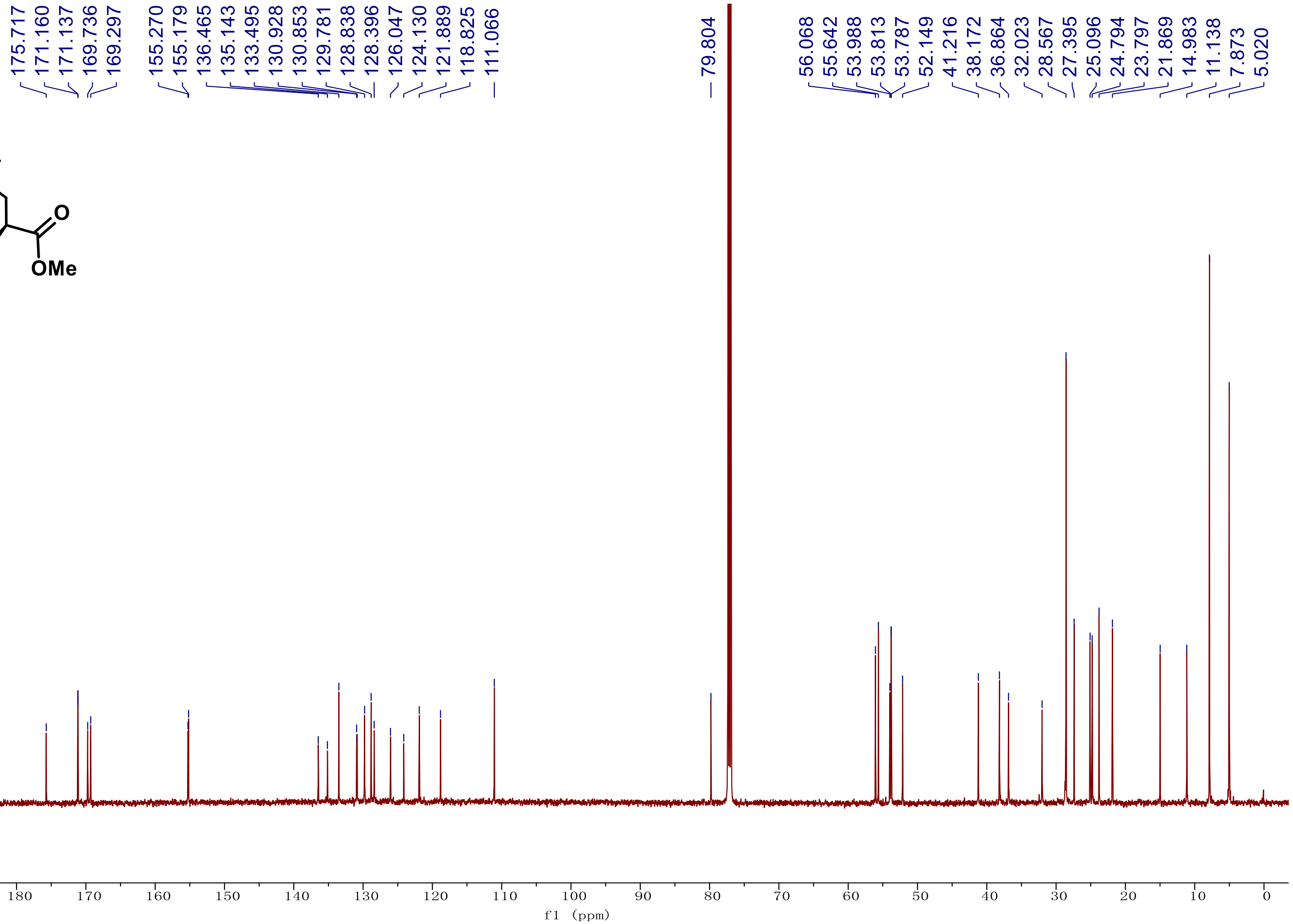

Compound 20e ^{1}H NMR (600 MHz, CDCl₃)

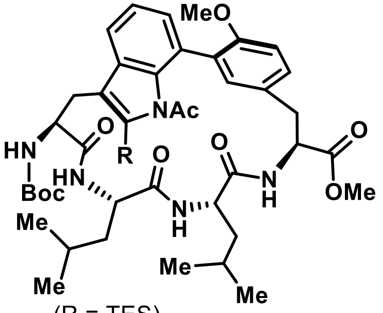

(R = TES)

20e

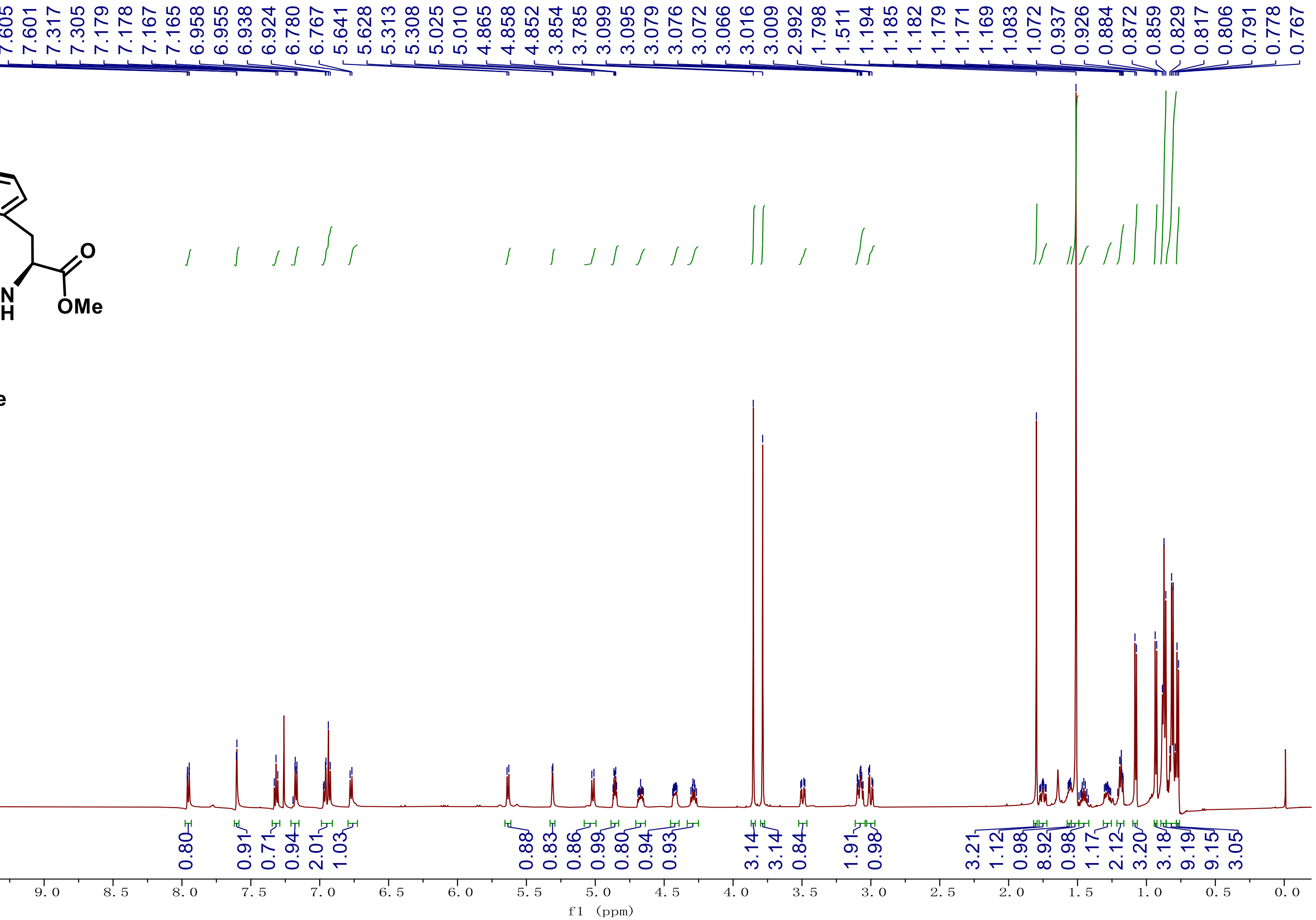

Compound 20e ^{13}C NMR (151 MHz, $CDCl_3$)

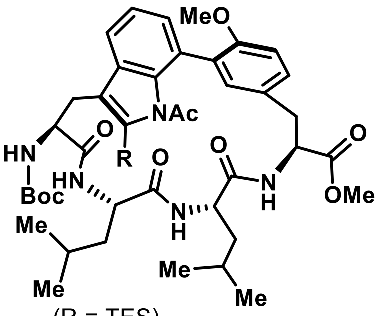

(R = TES)

20e

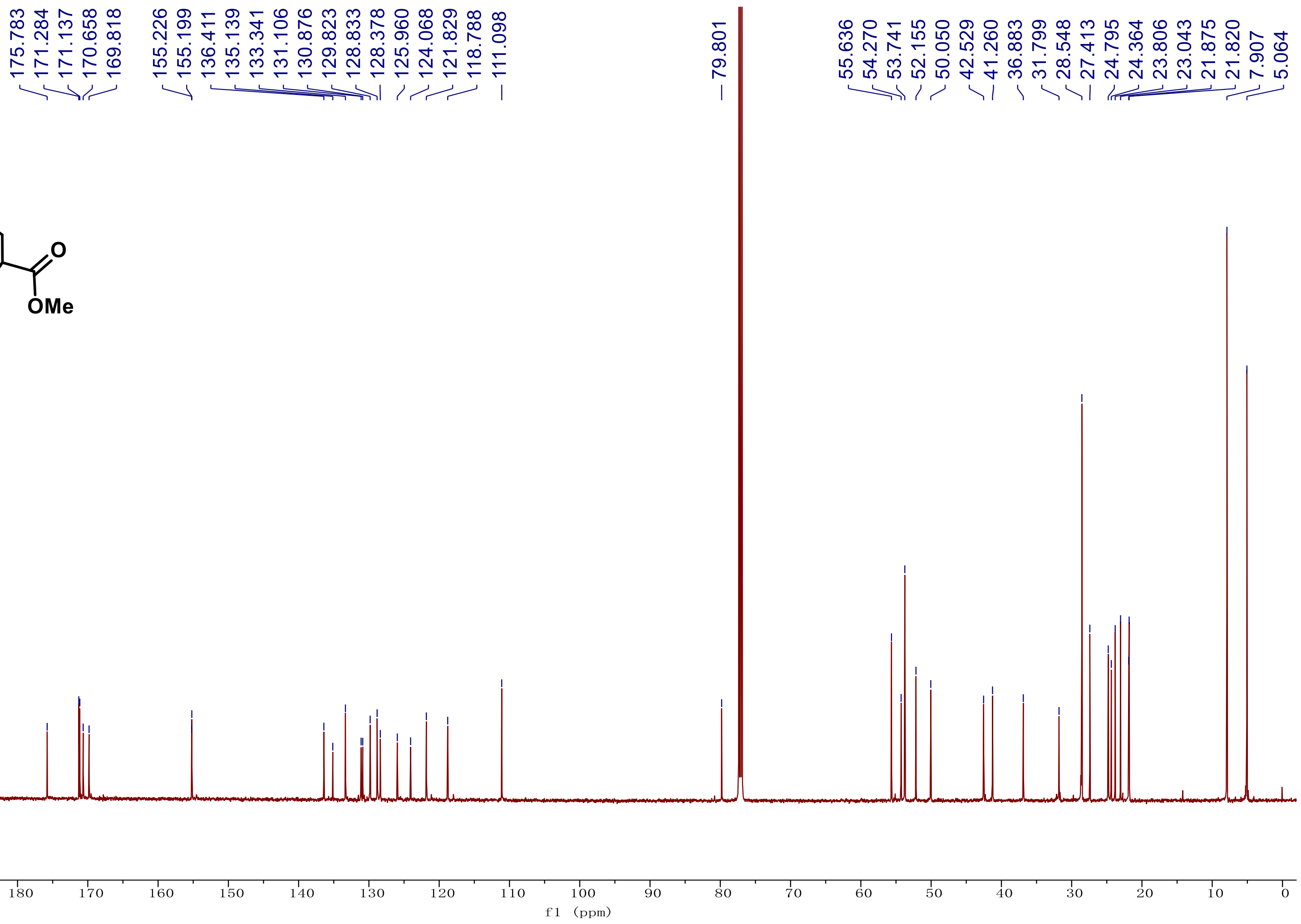

Compound 20f ¹H NMR (600 MHz, $CDCl_3$)

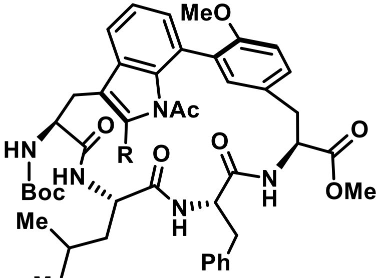

(R = TES)

20f

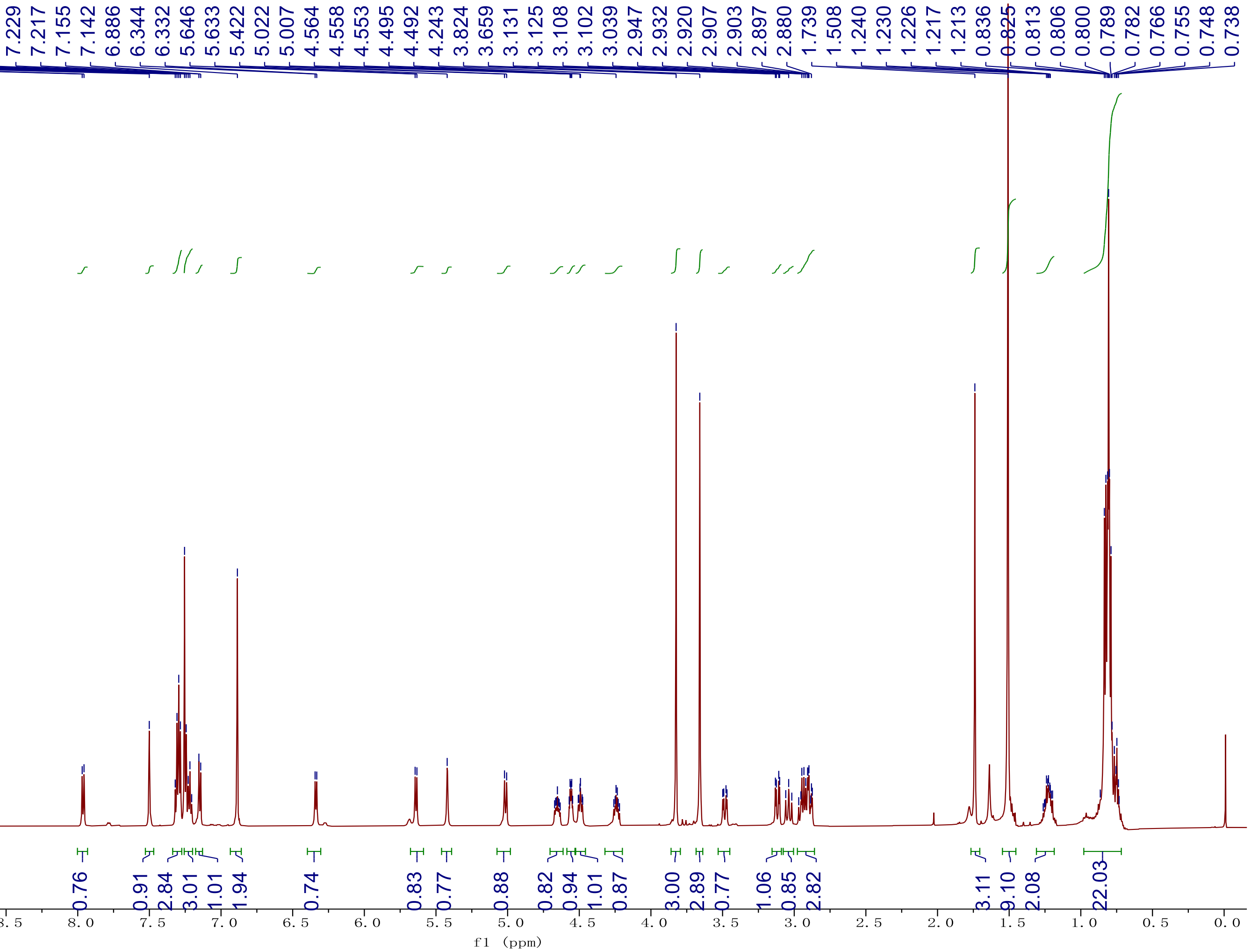

Compound 20f ^{13}C NMR (151 MHz, $CDCl_3$)

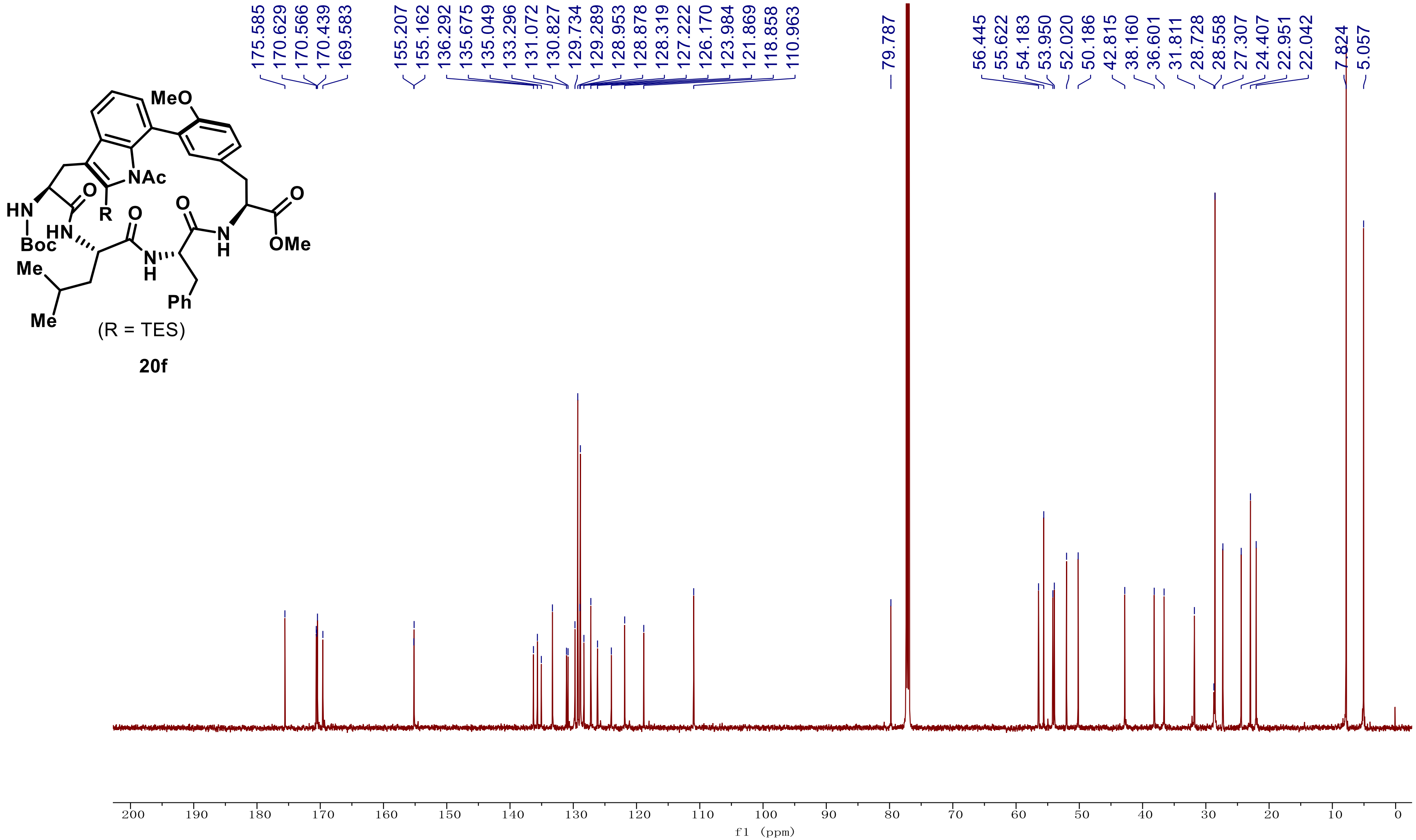

Compound 20g ^{1}H NMR (600 MHz, $CDCl_3$)

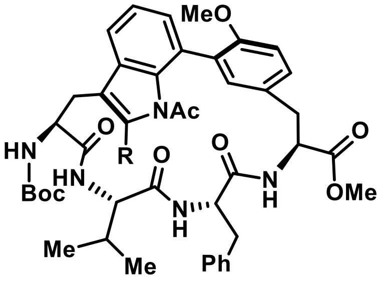

(R = TES)

20g

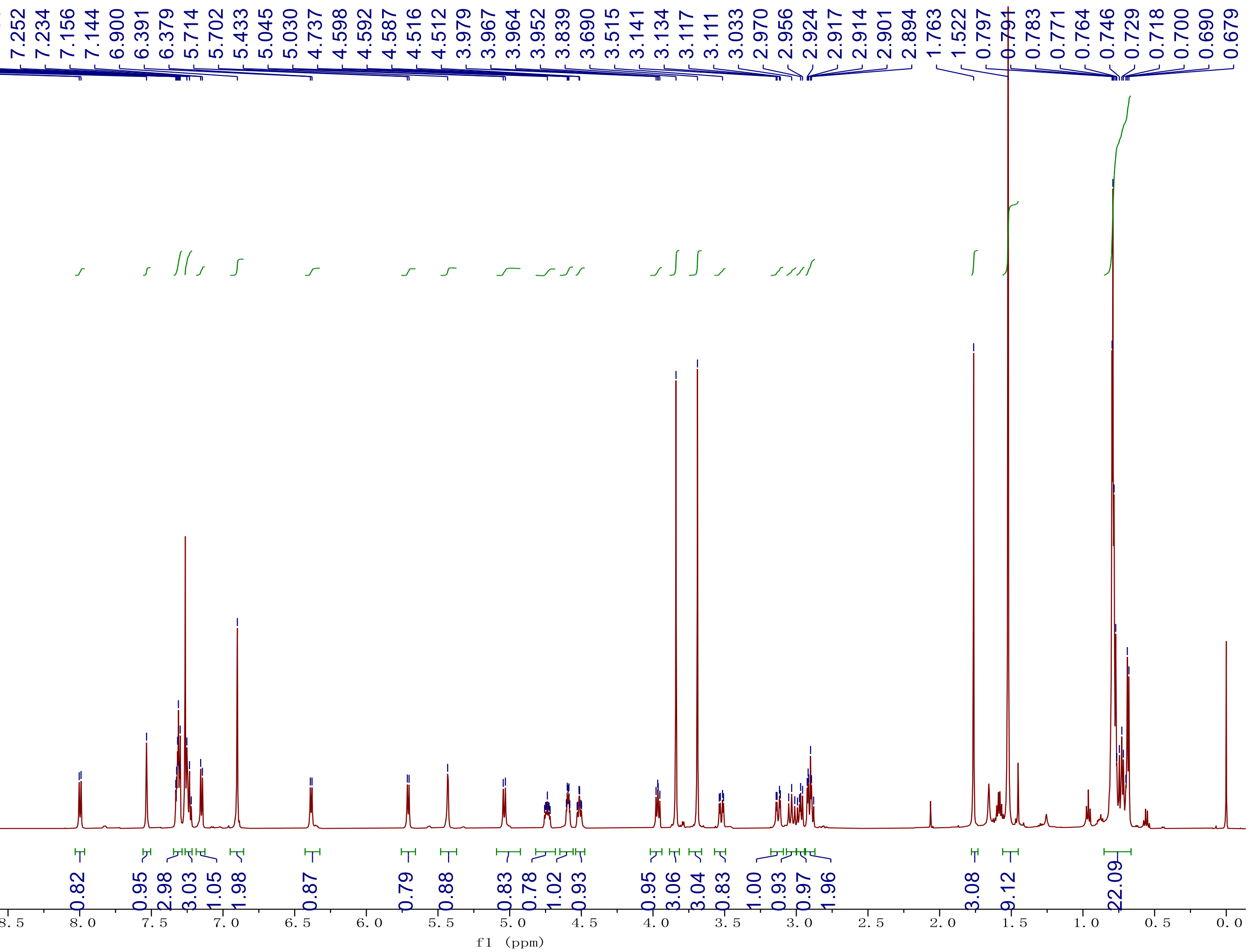

Compound 20g ^{13}C NMR (151 MHz, $CDCl_3$)

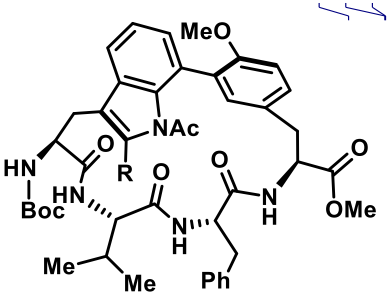

(R = TES)

20g

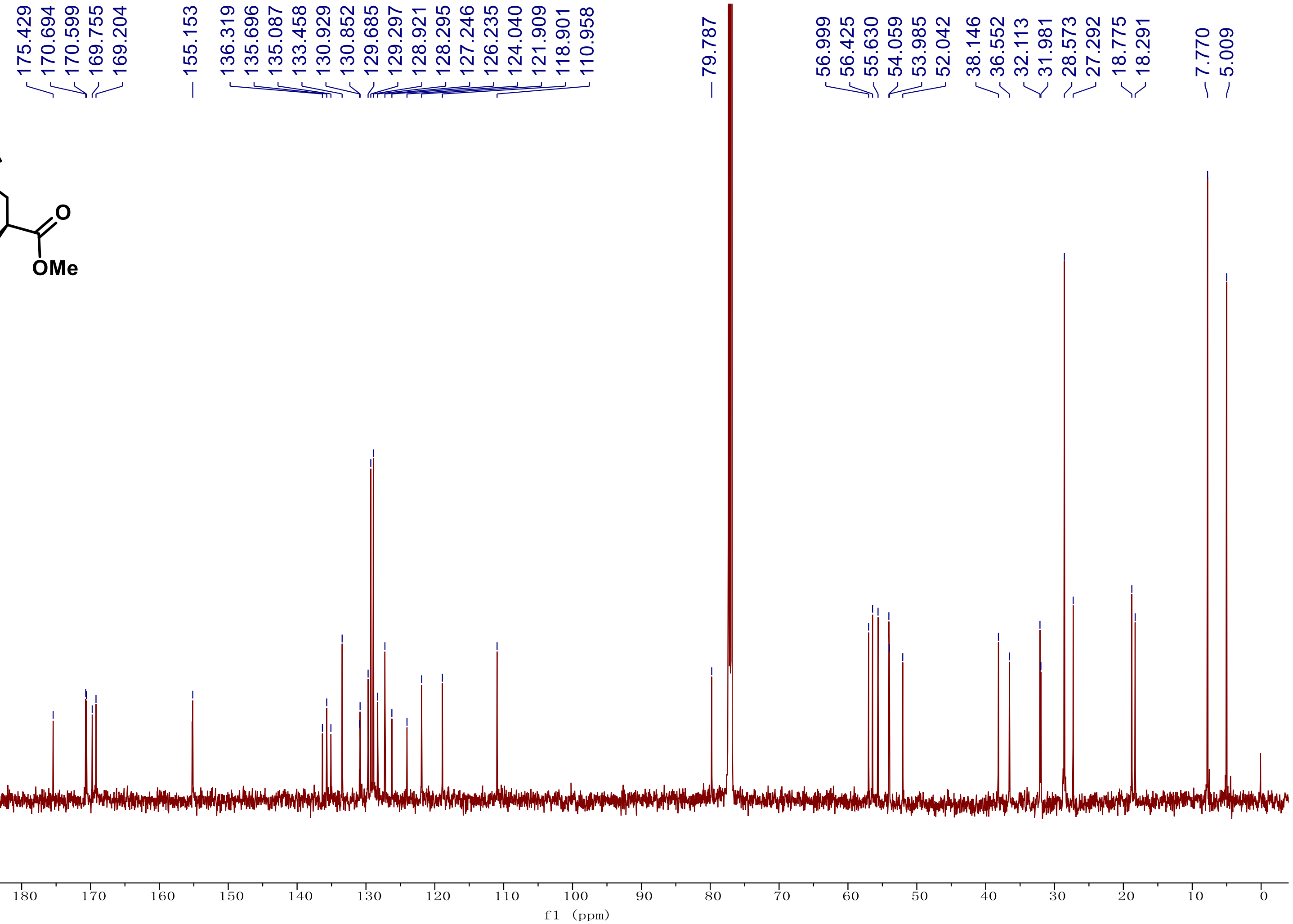

Compound 20g ROESY (400 MHz, $CDCl_3$)

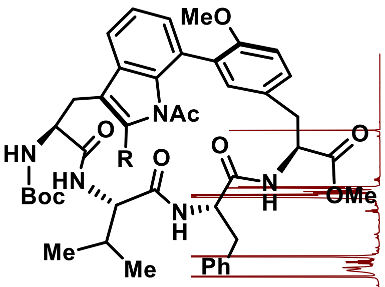

(R = TES)

20g

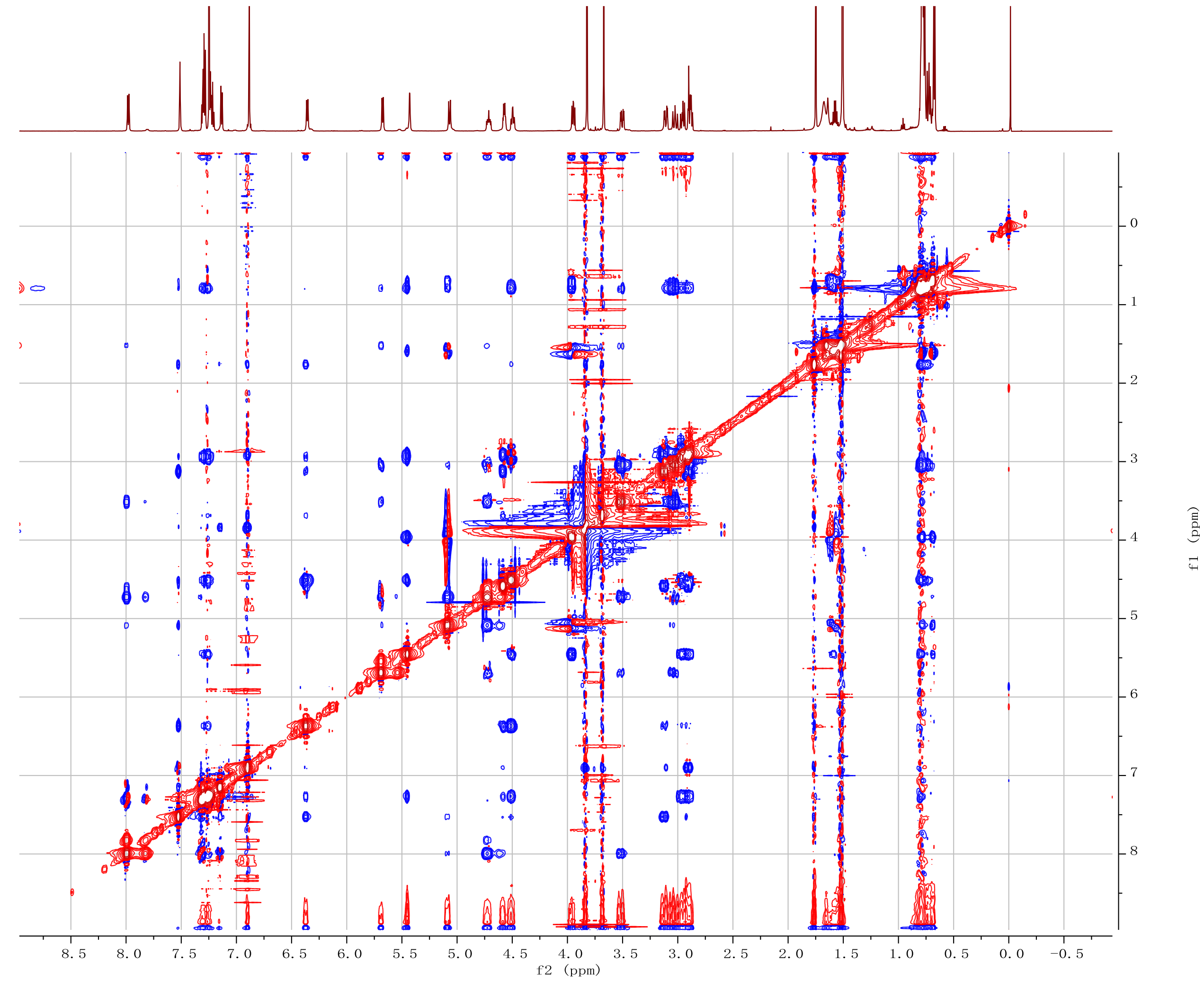

LCMS trace of compound 20g

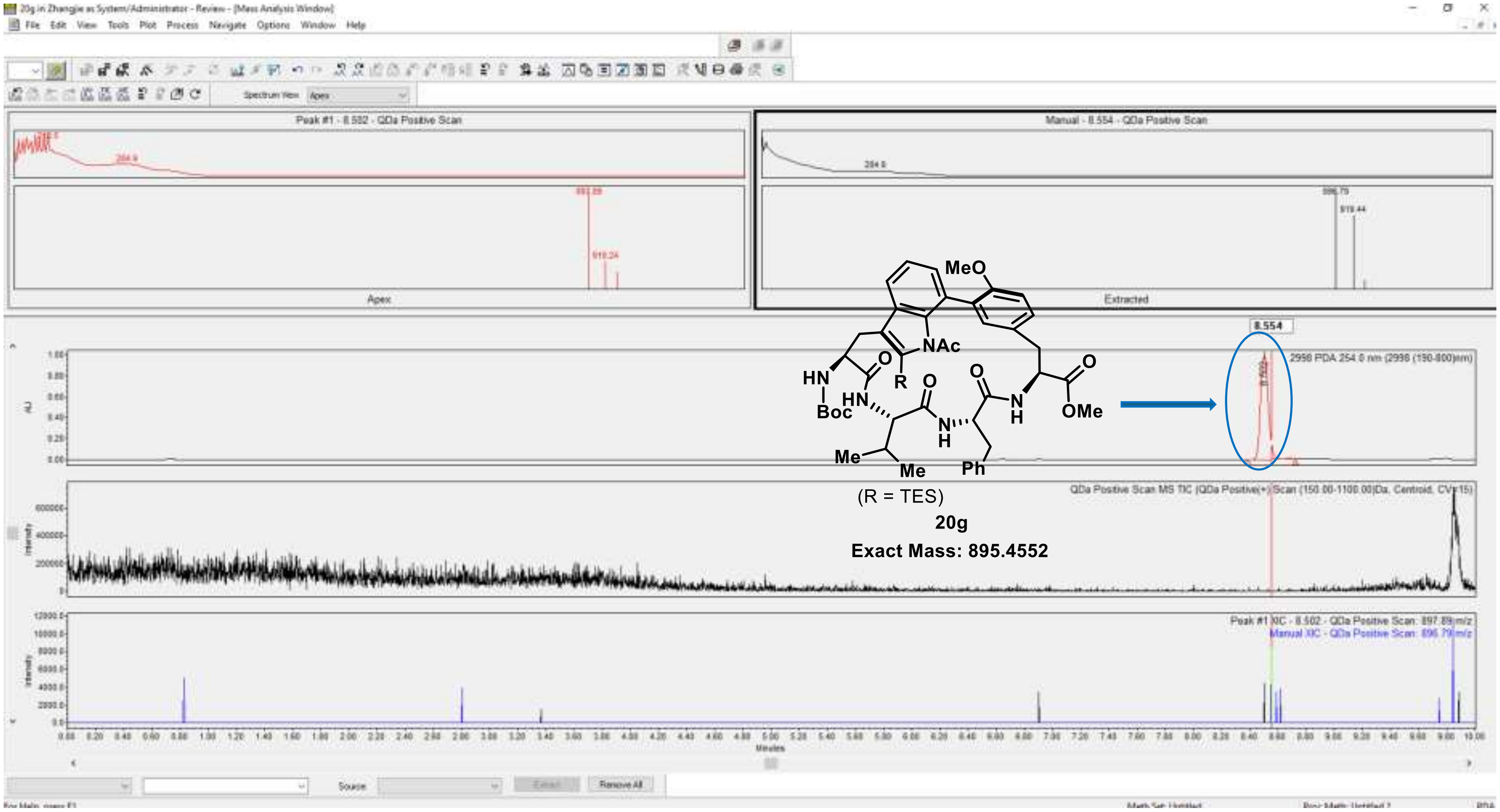

Compound 20h ^{1}H NMR (600 MHz, $CDCl_3$)

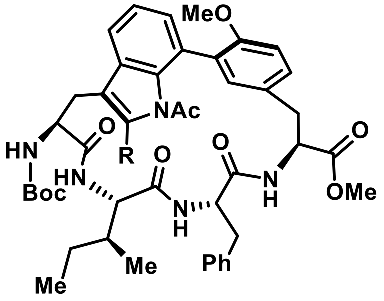

(R = TES)

20h

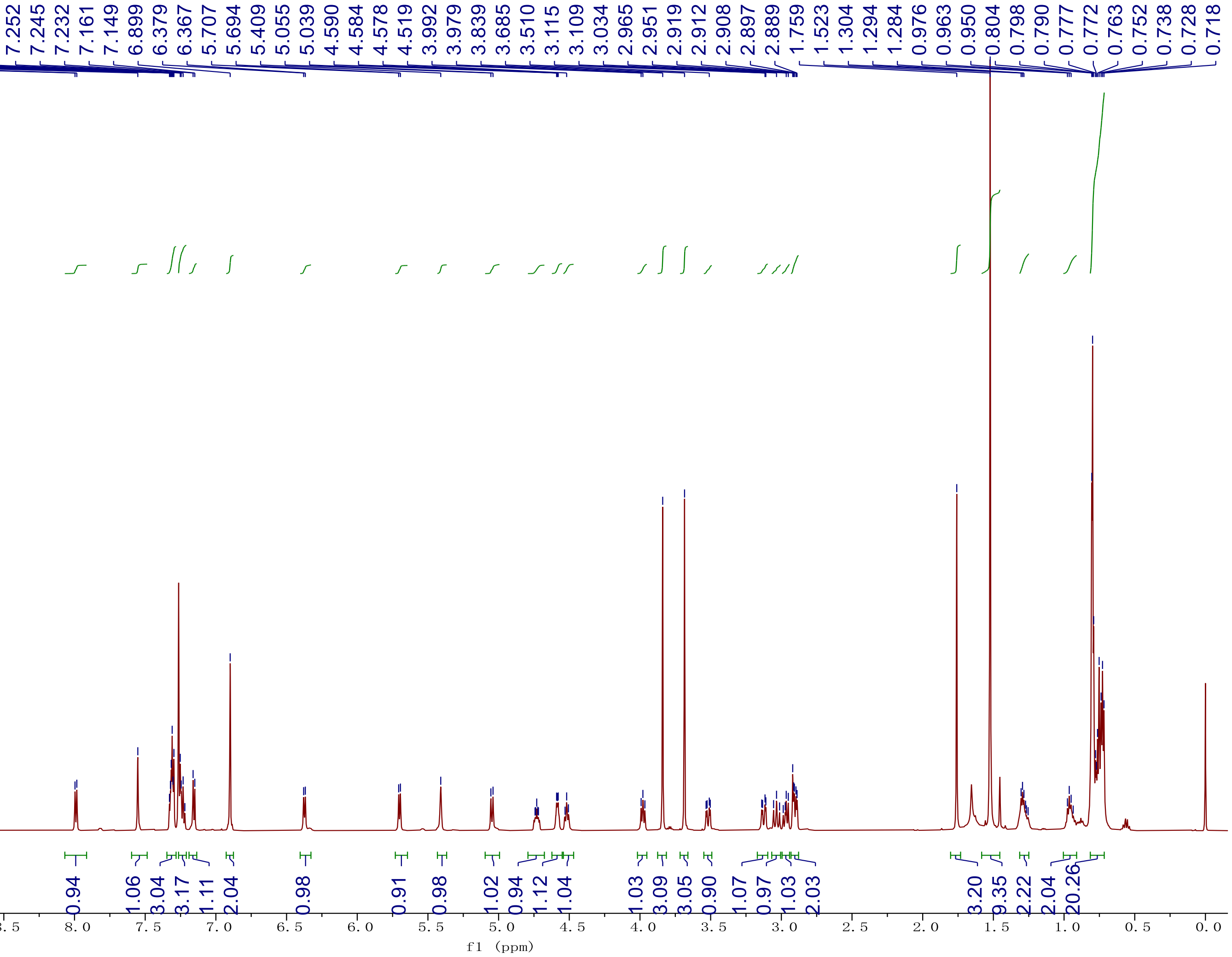

Compound 20h ^{13}C NMR (151 MHz, $CDCl_3$)

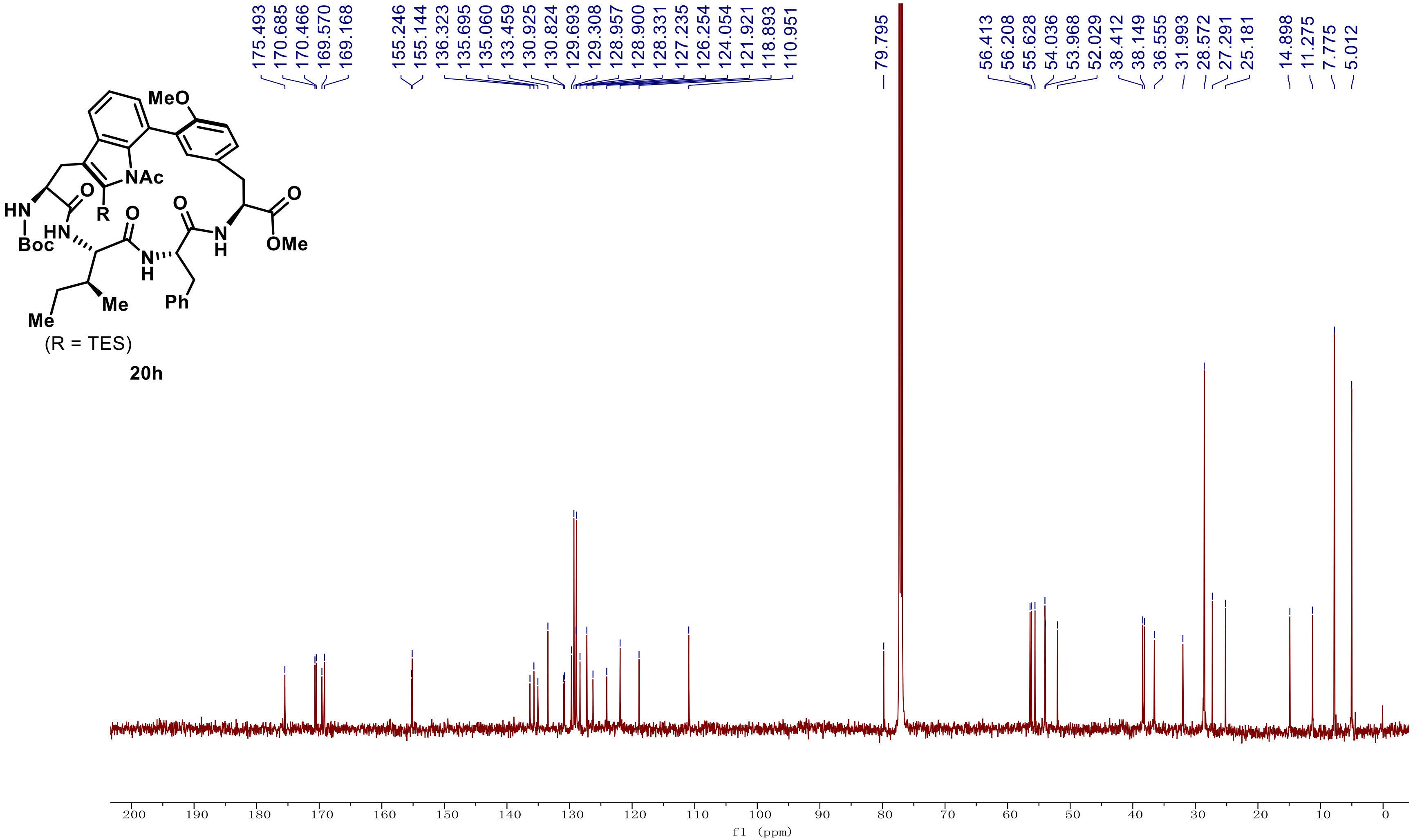

Compound 20h ROESY (400 MHz, $CDCl_3$)

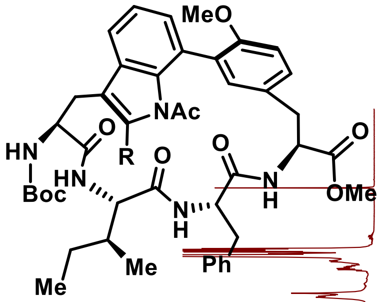

(R = TES)

20h

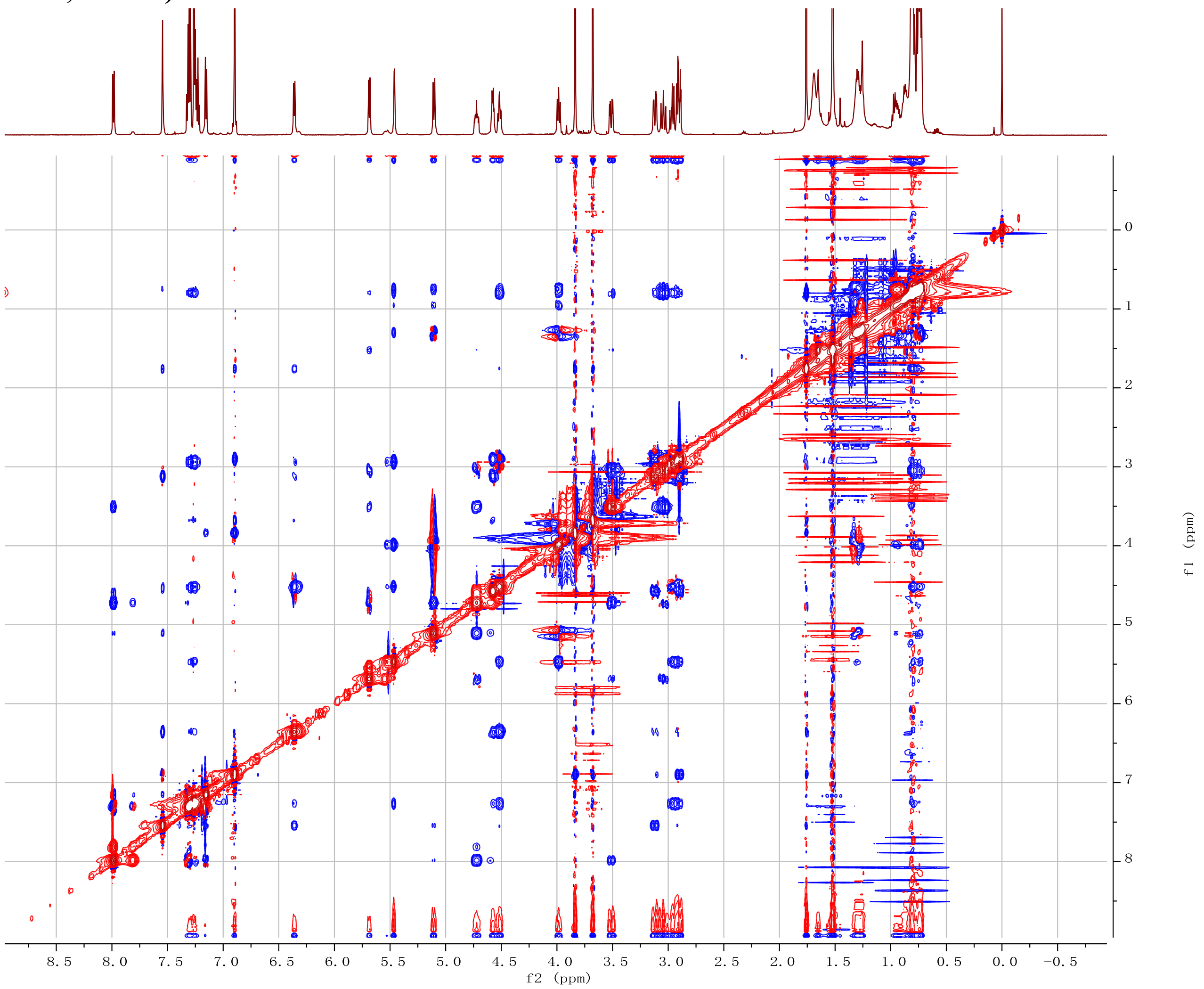

LCMS trace of compound 20h

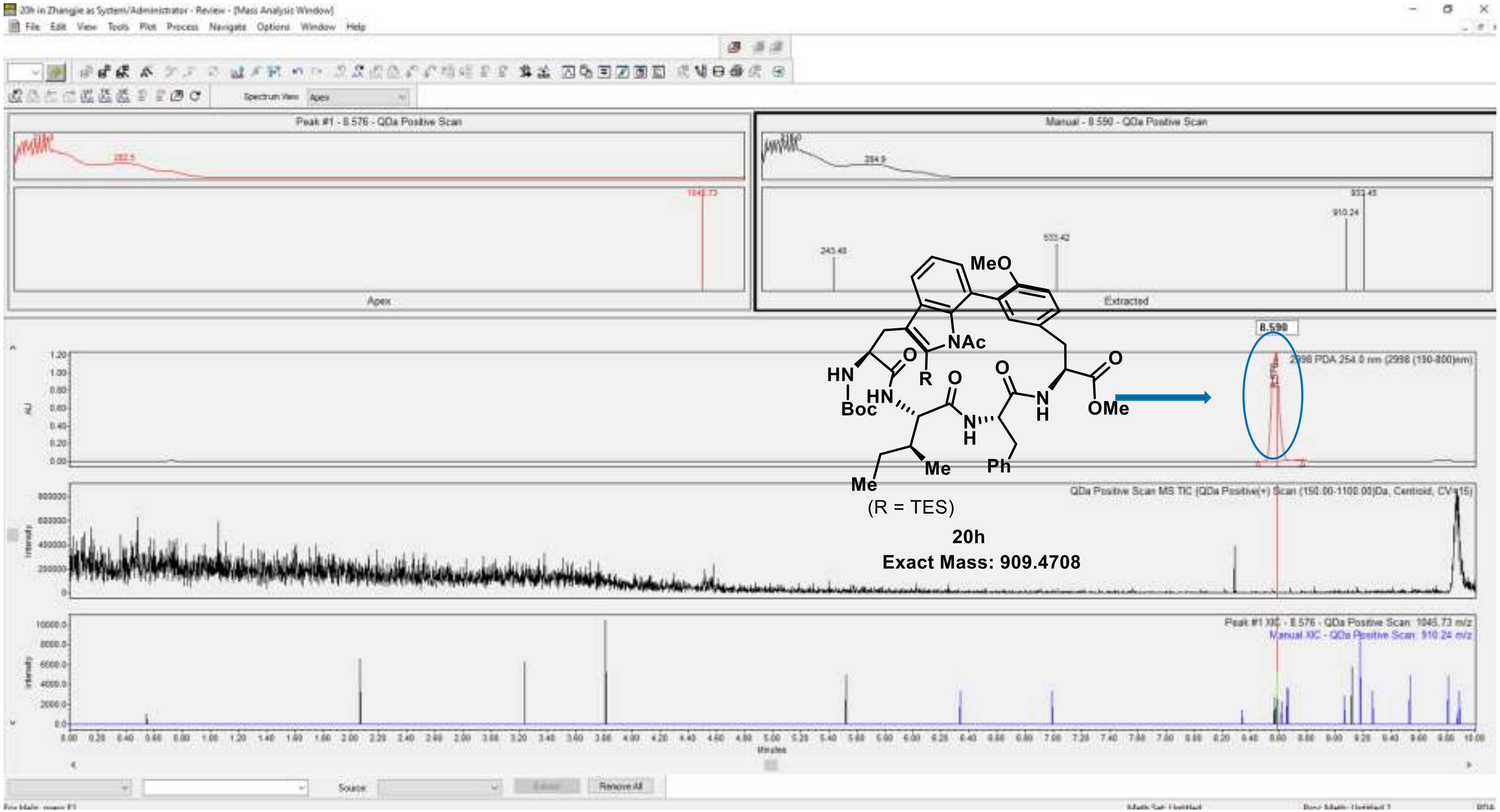

Compound 20i ^{1}H NMR (600 MHz, $CDCl_3$)

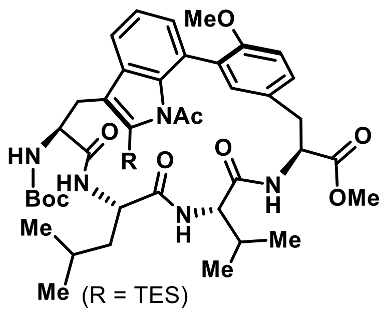

20i

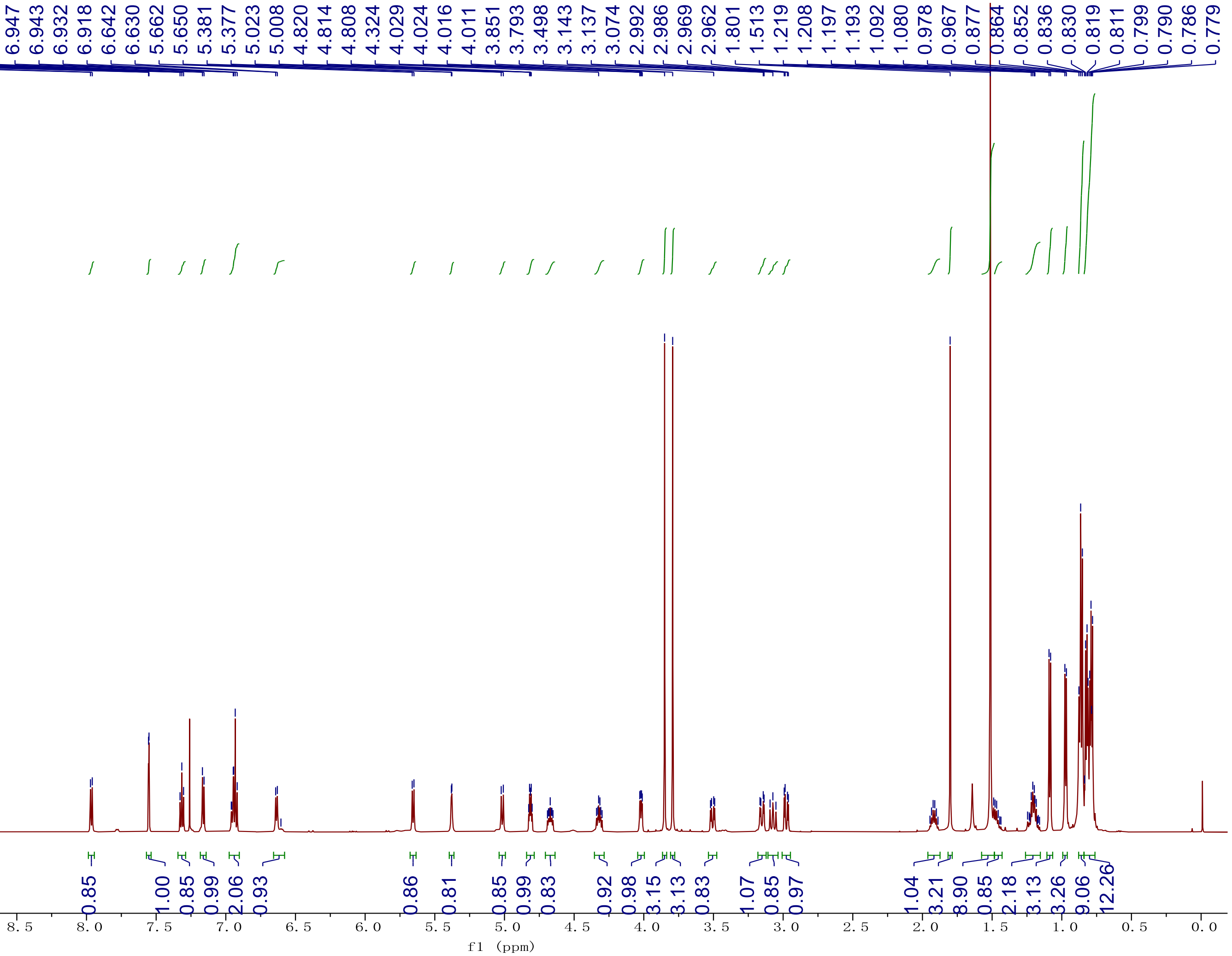

Compound 20i ^{13}C NMR (151 MHz, $CDCl_3$)

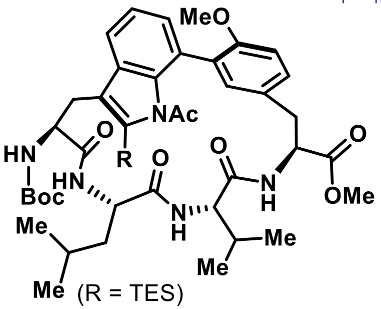

20i

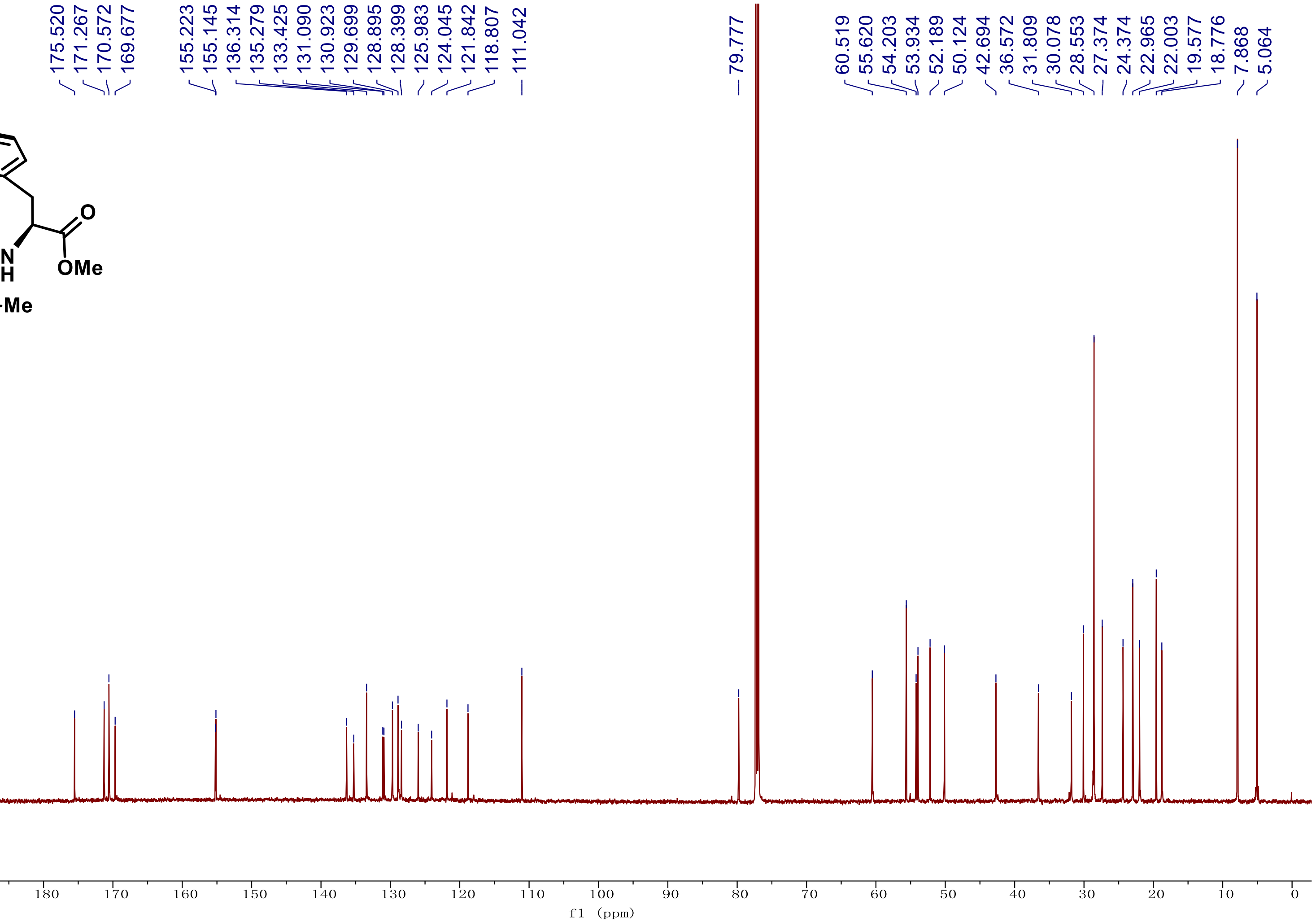

Compound 20j ^{1}H NMR (600 MHz, CDCl$_3$)

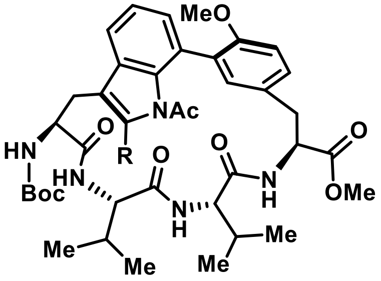

(R = TES)

20j

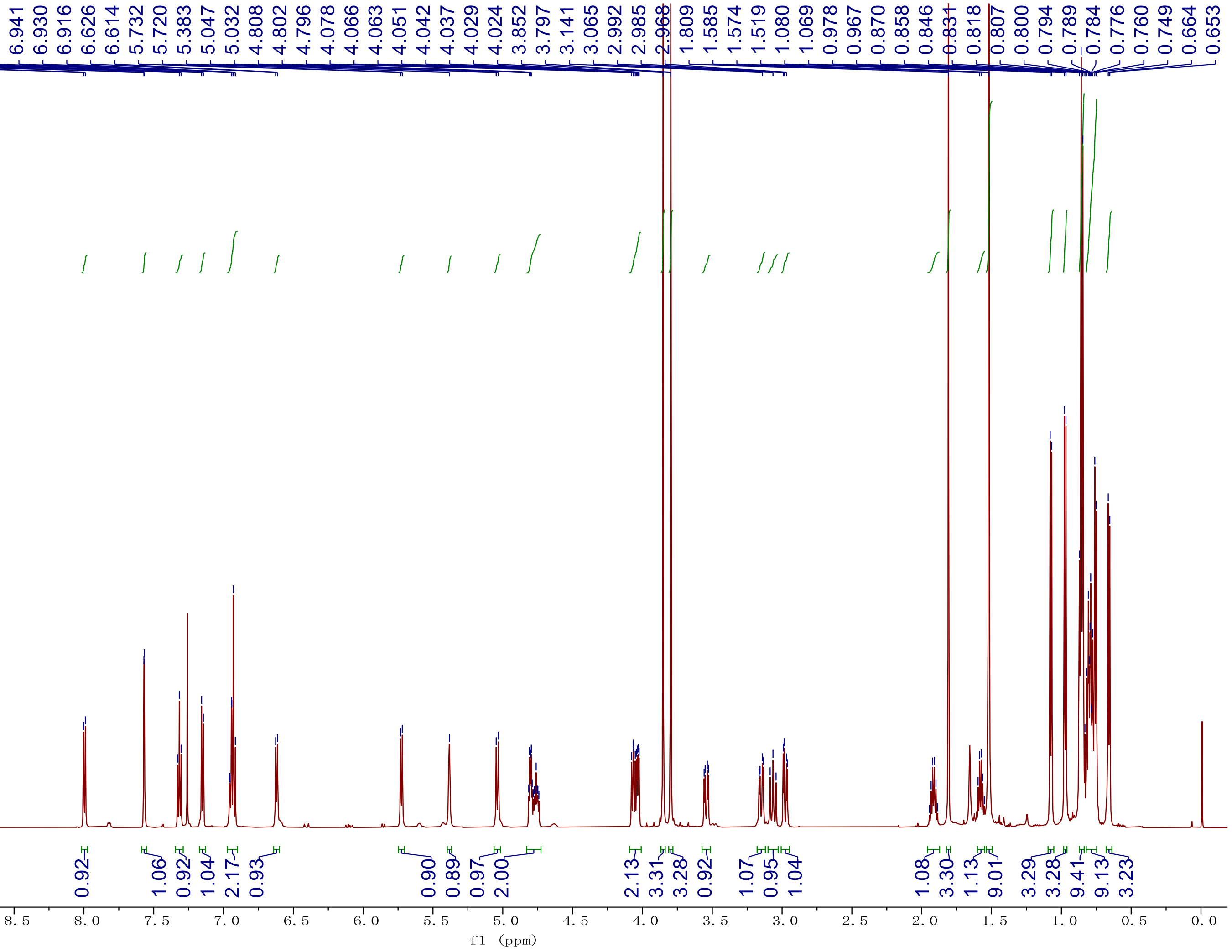

Compound 20j ^{13}C NMR (151 MHz, CDCl$_3$)

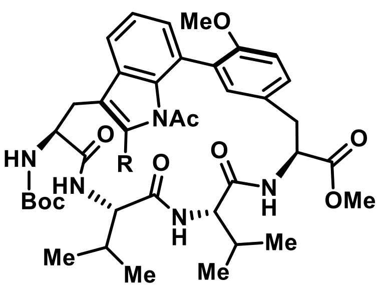

(R = TES)

20j

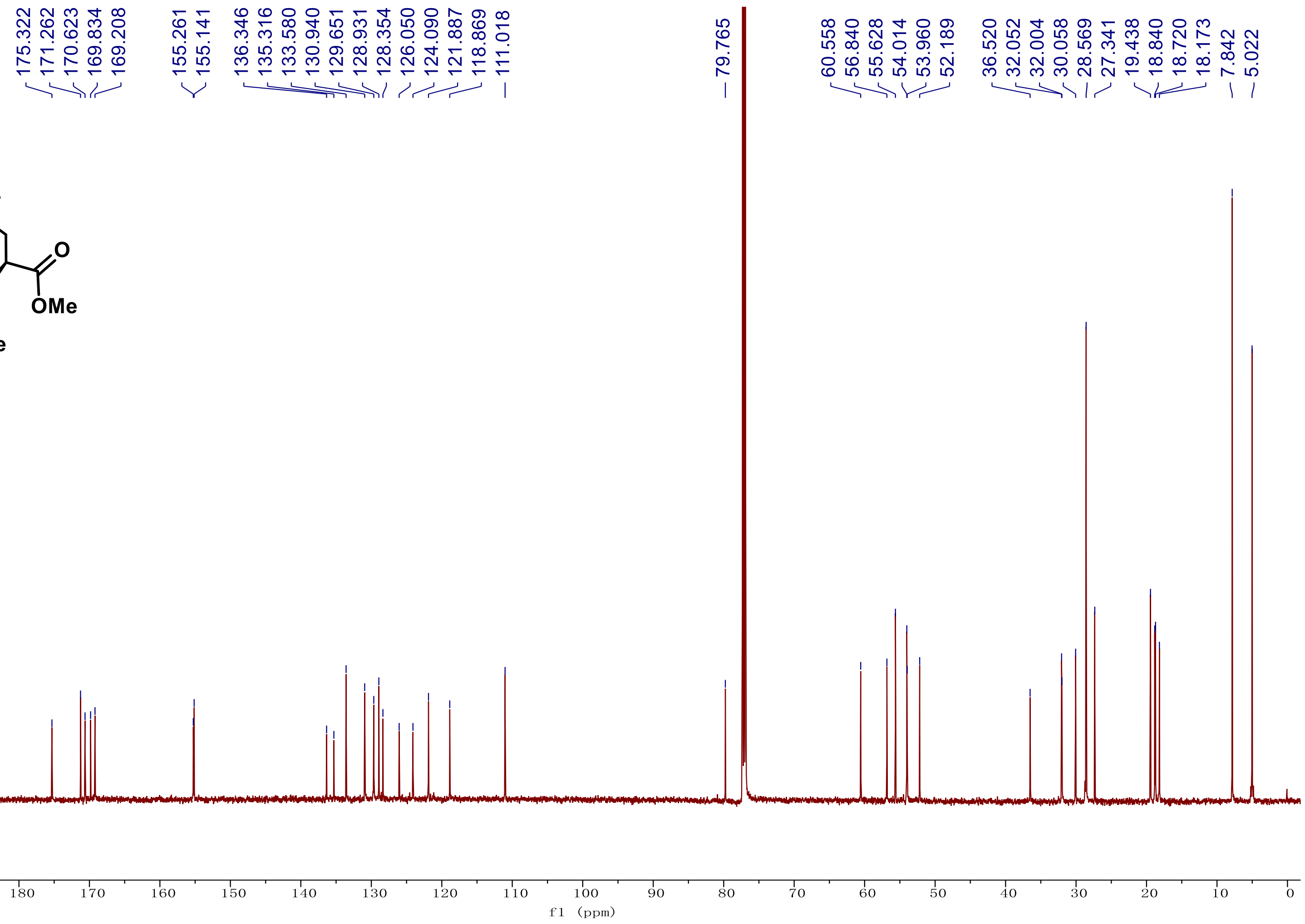

Compound 20k ^{1}H NMR (600 MHz, $CDCl_3$)

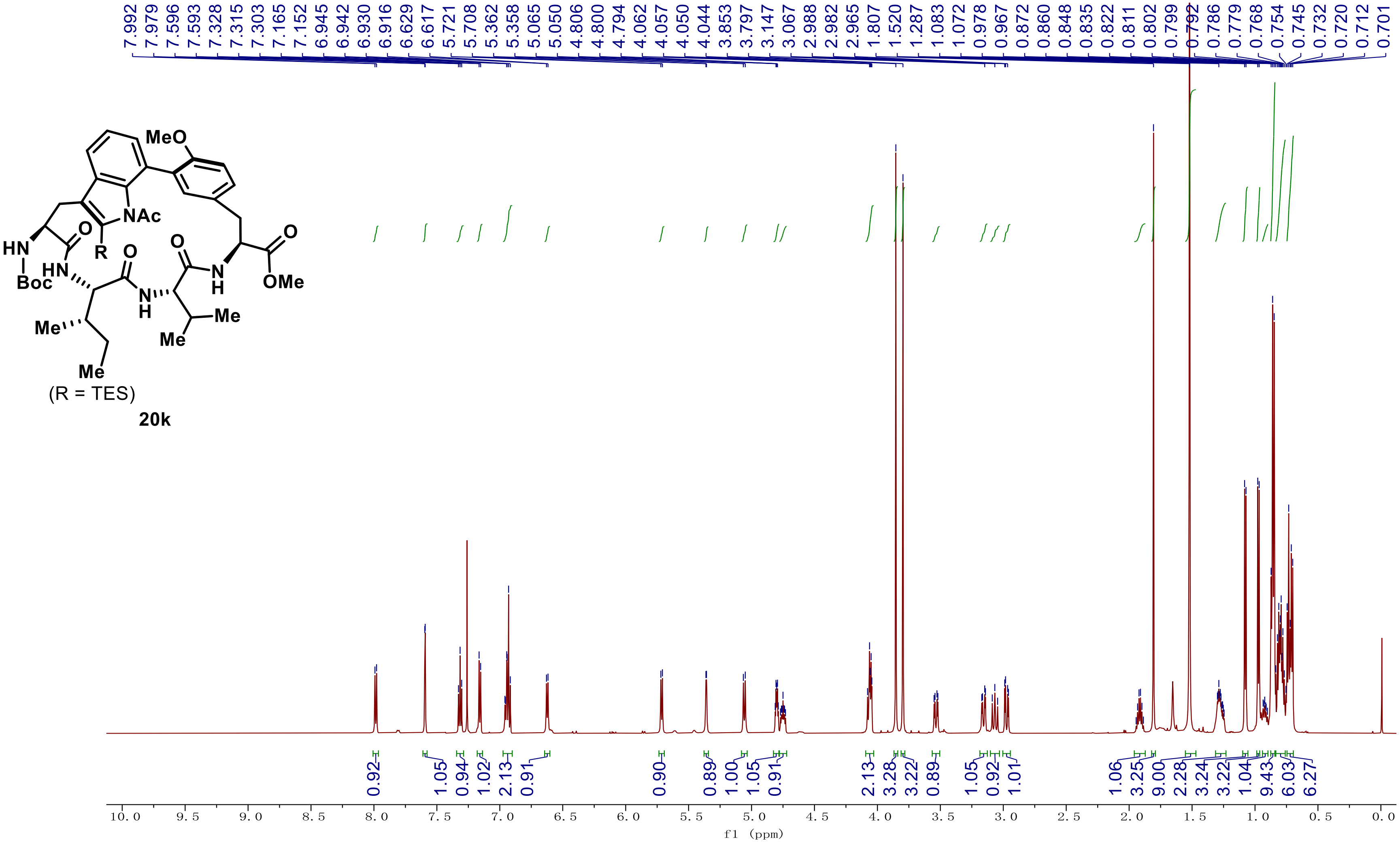

Compound 20k ^{13}C NMR (151 MHz, $CDCl_3$)

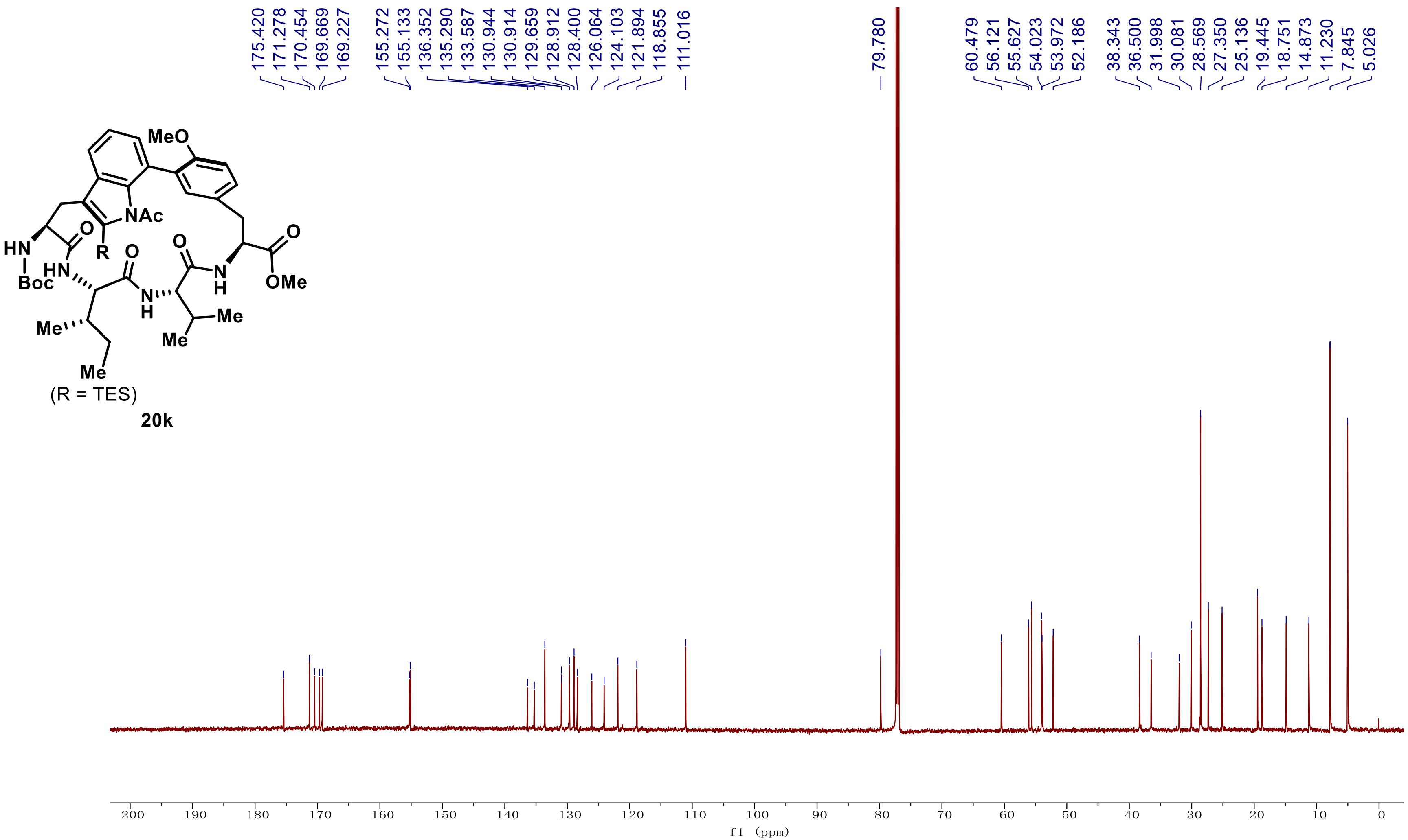

Compound 20l ^{1}H NMR (600 MHz, CDCl$_3$)

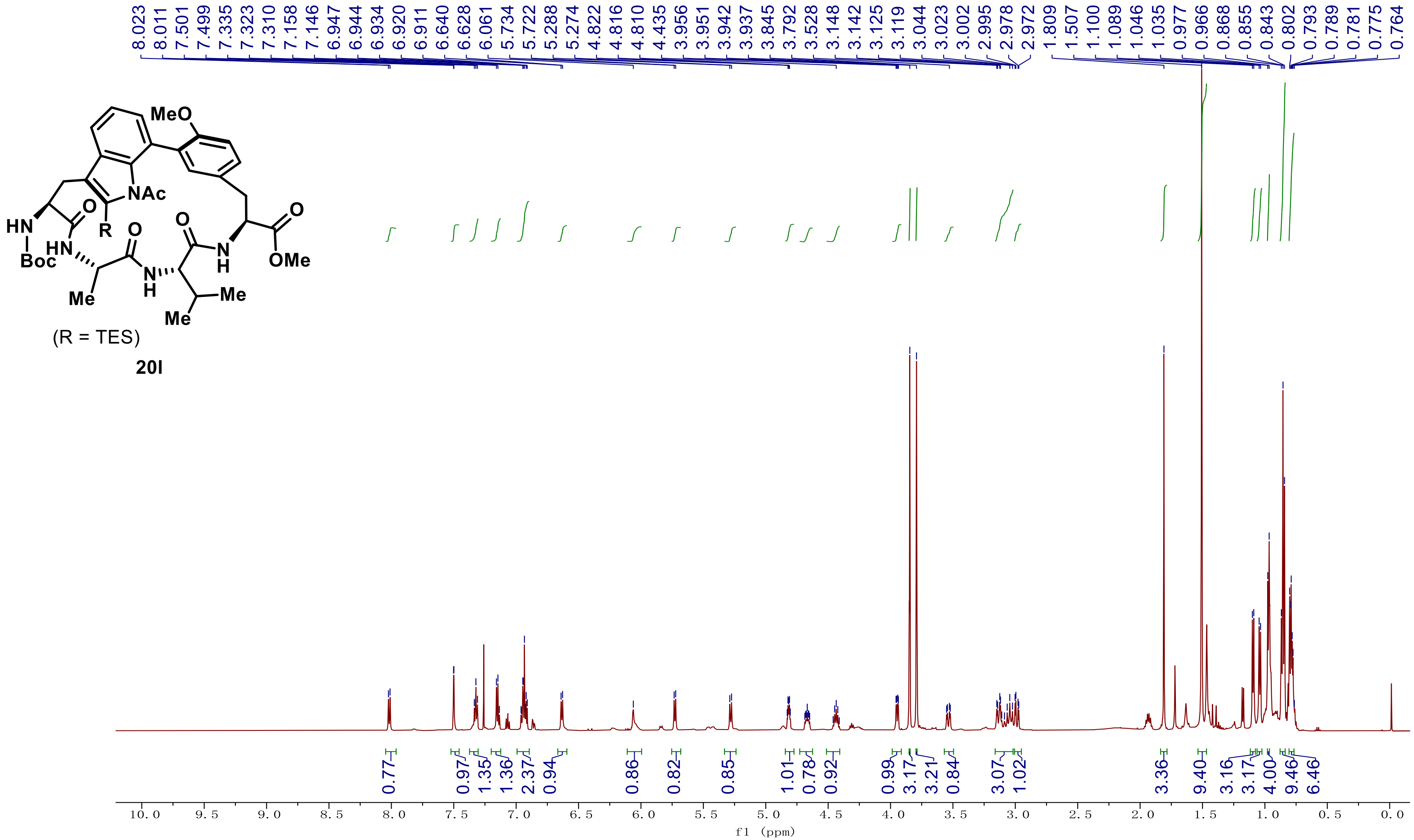

(R = TES)

20l

Compound 20l ^{13}C NMR (151 MHz, CDCl$_3$)

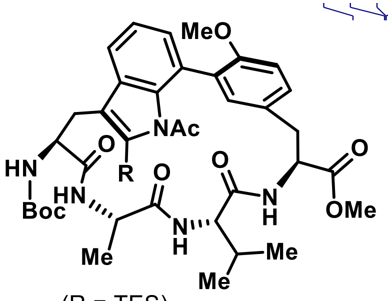

(R = TES)

20l

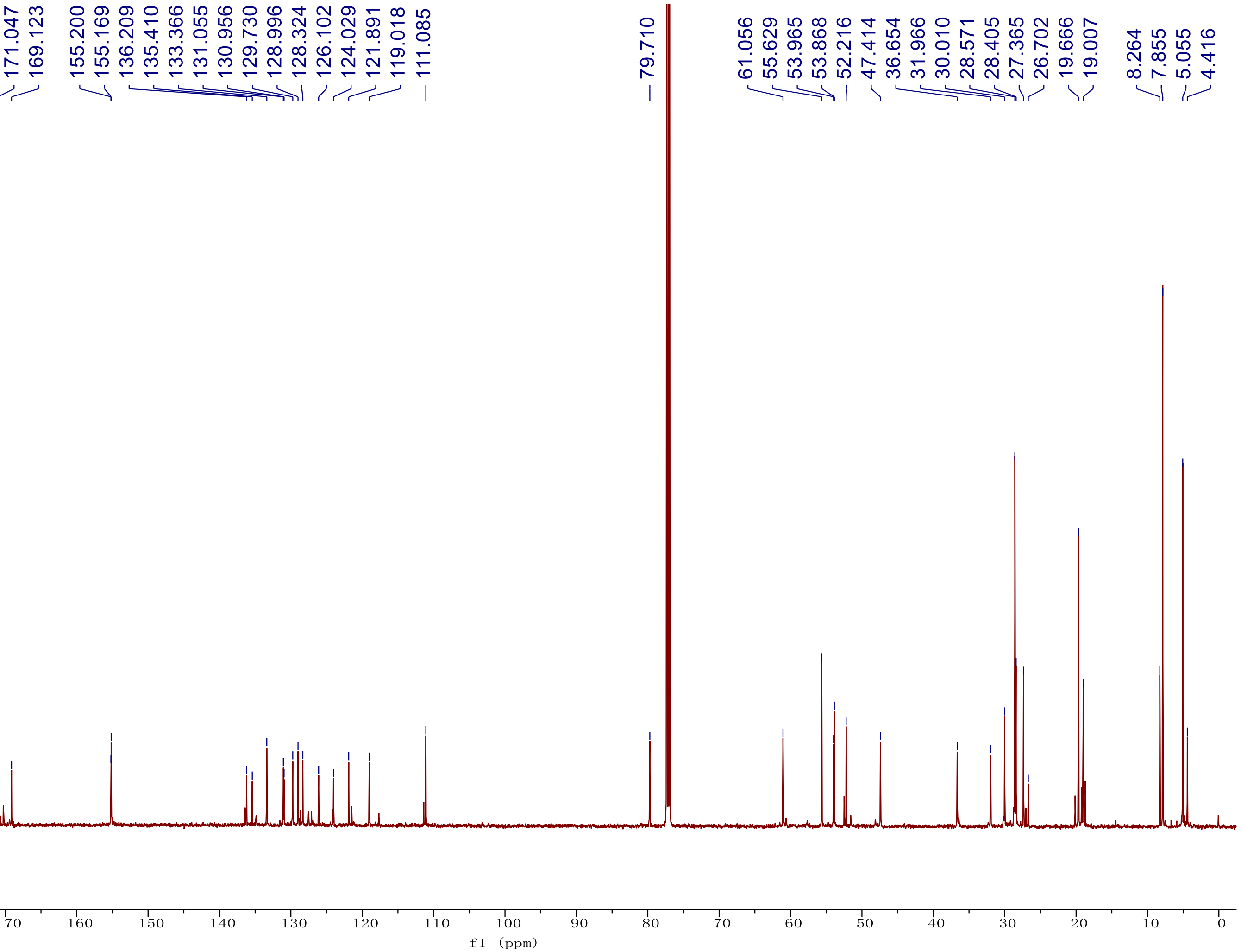

Compound 20m ^{1}H NMR (600 MHz, CDCl3)

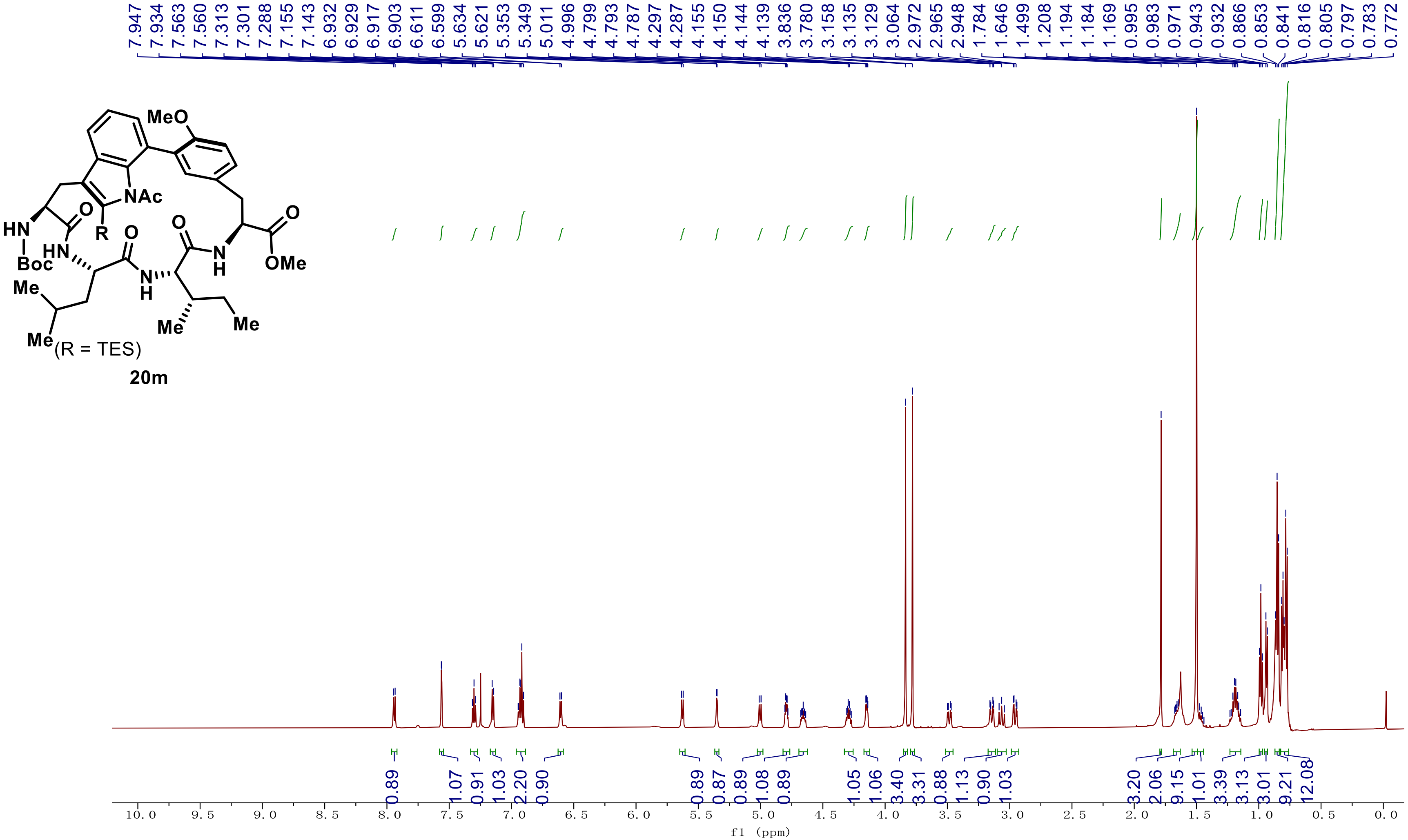

Compound 20m ^{13}C NMR (151 MHz, CDCl$_3$)

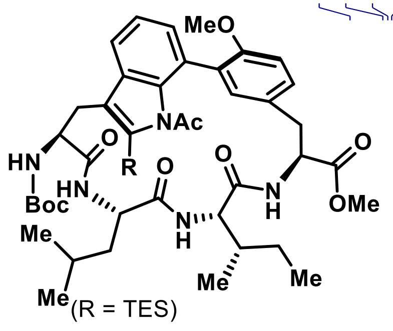

20m

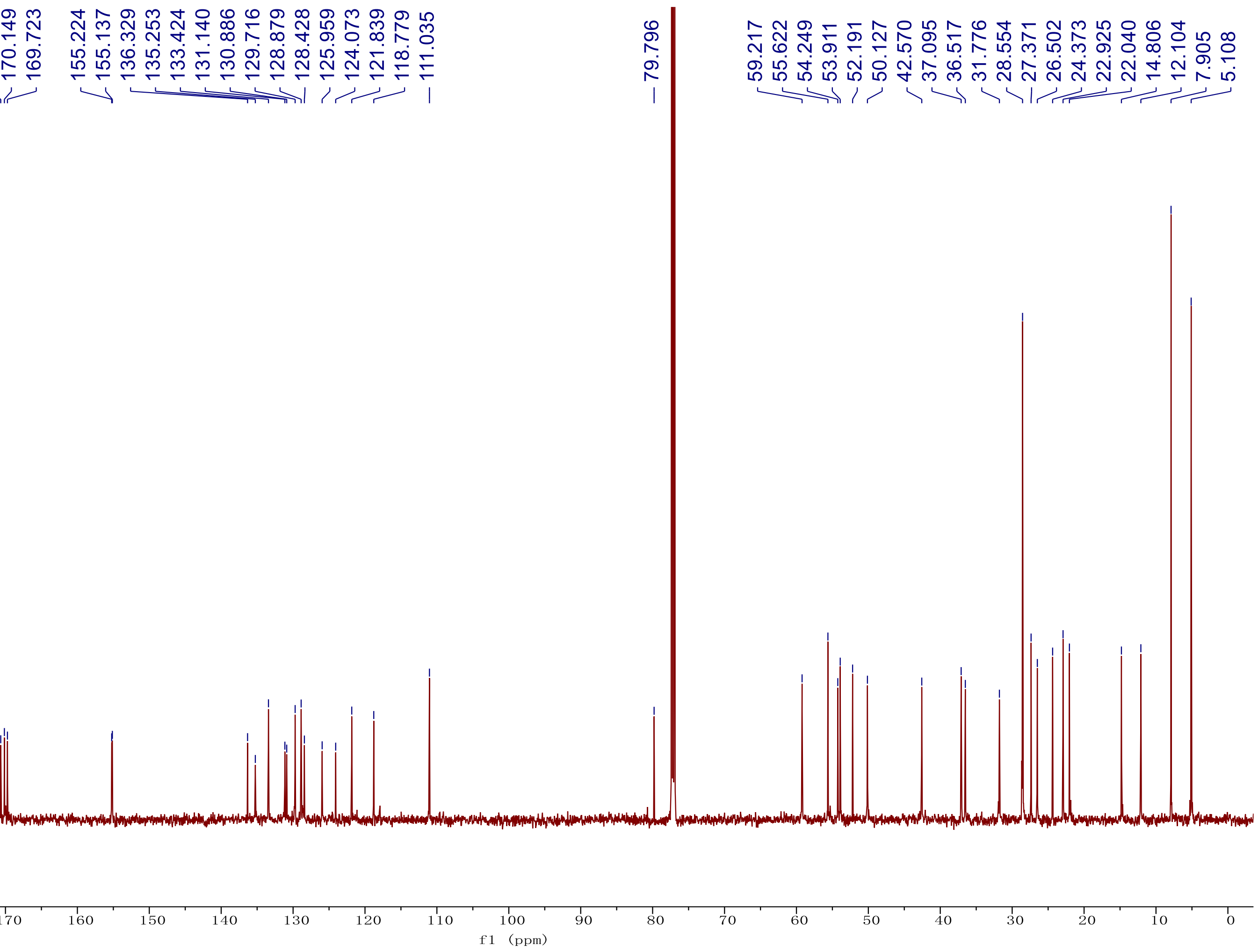

Compound 20m HSQC (600 MHz, CDCl3)

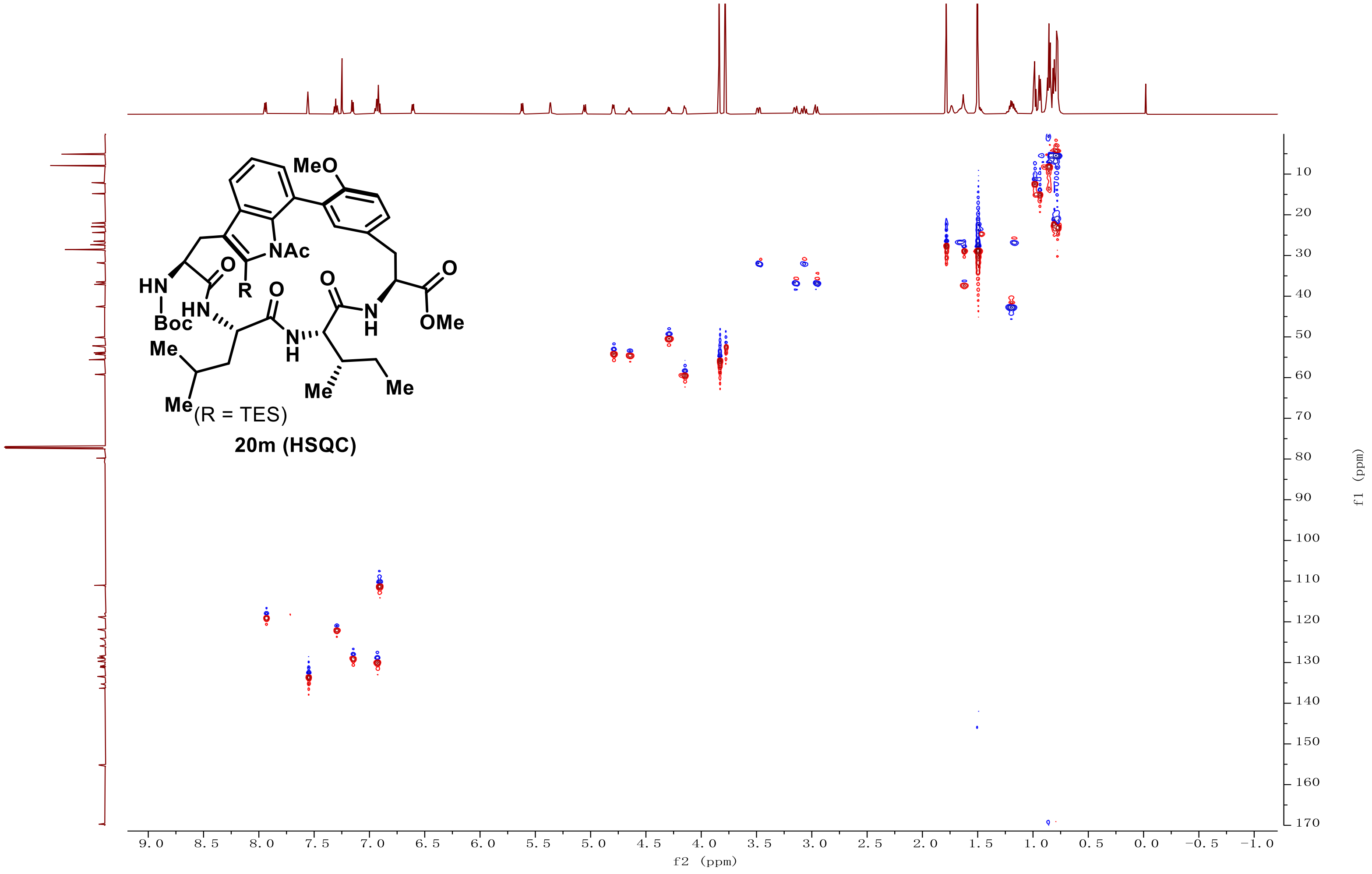

Compound 20m COSY (600 MHz, CDCl₃)

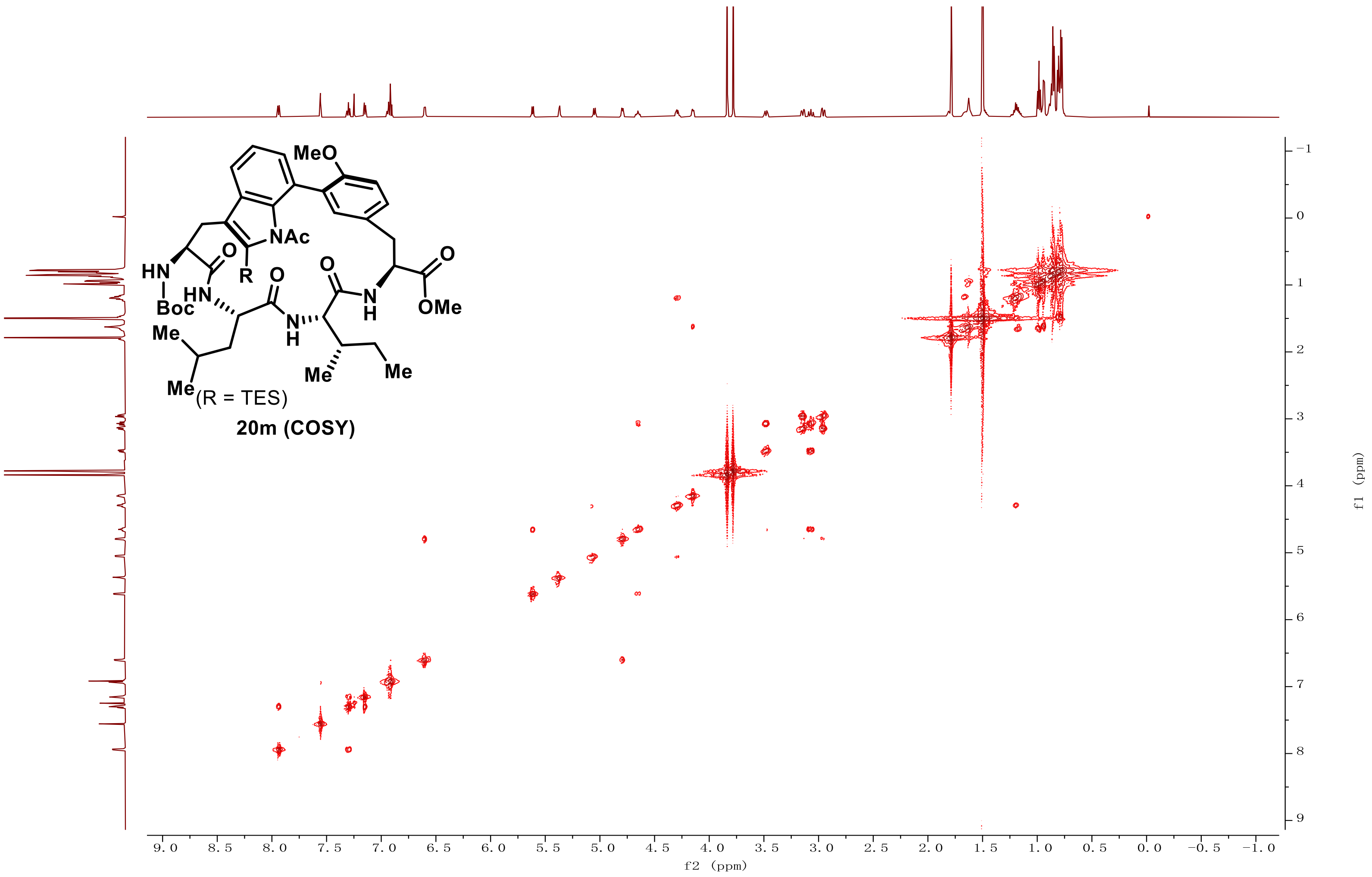

Compound 20m ROESY (600 MHz, $CDCl_3$)

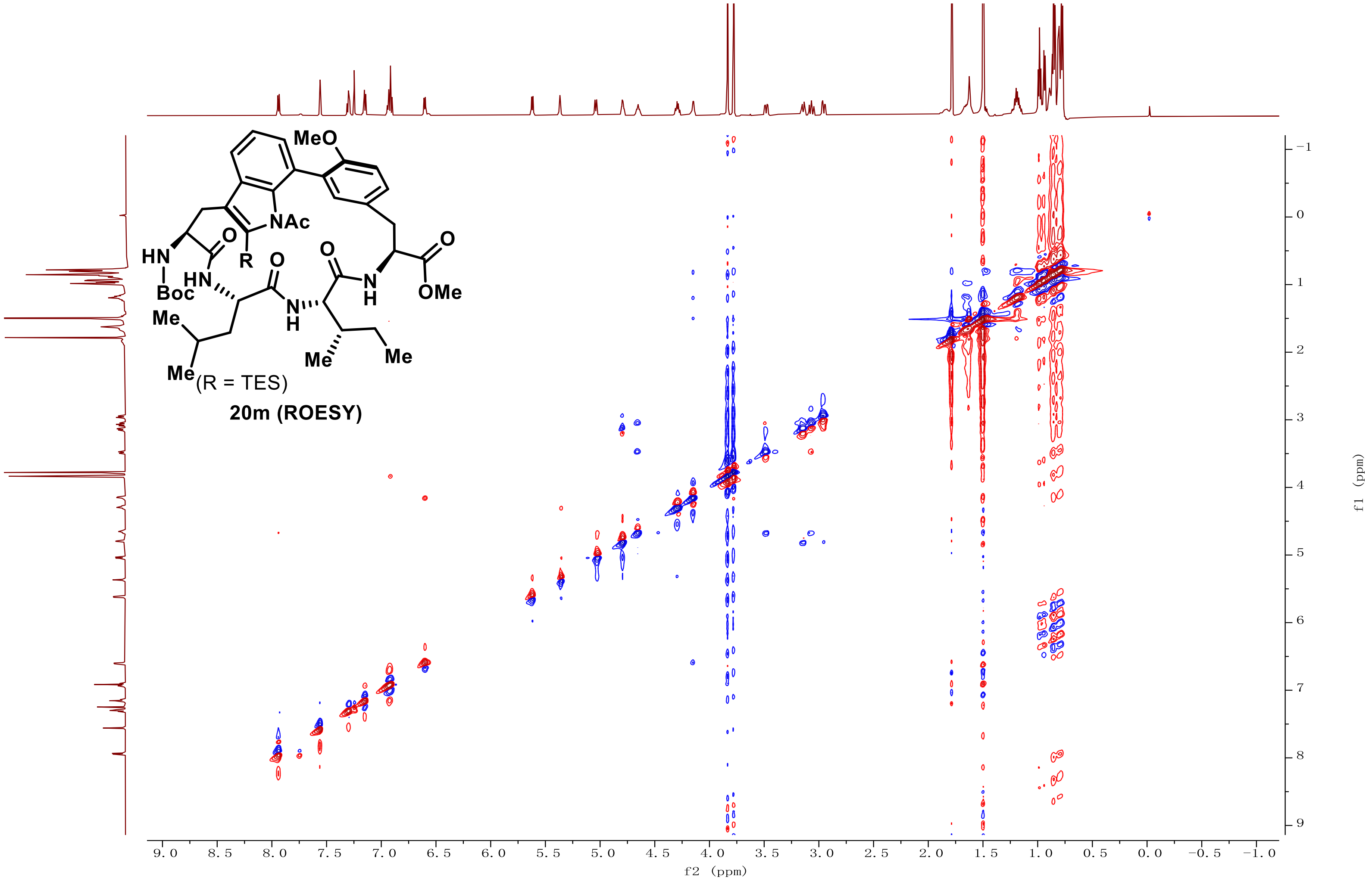

Compound 20m NOESY (600 MHz, $CDCl_3$)

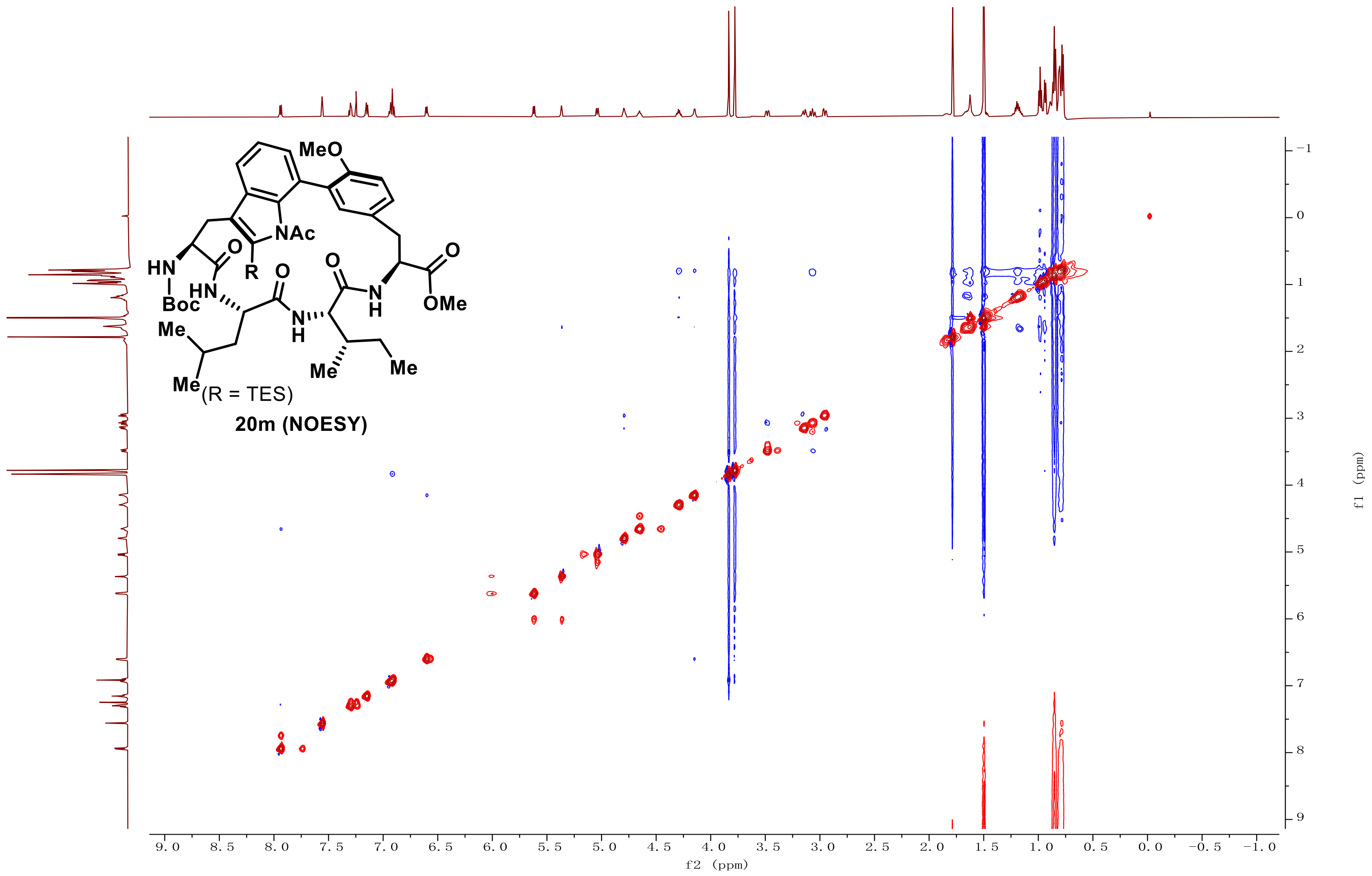

Compound 20m HMBC (600 MHz, CDCl3)

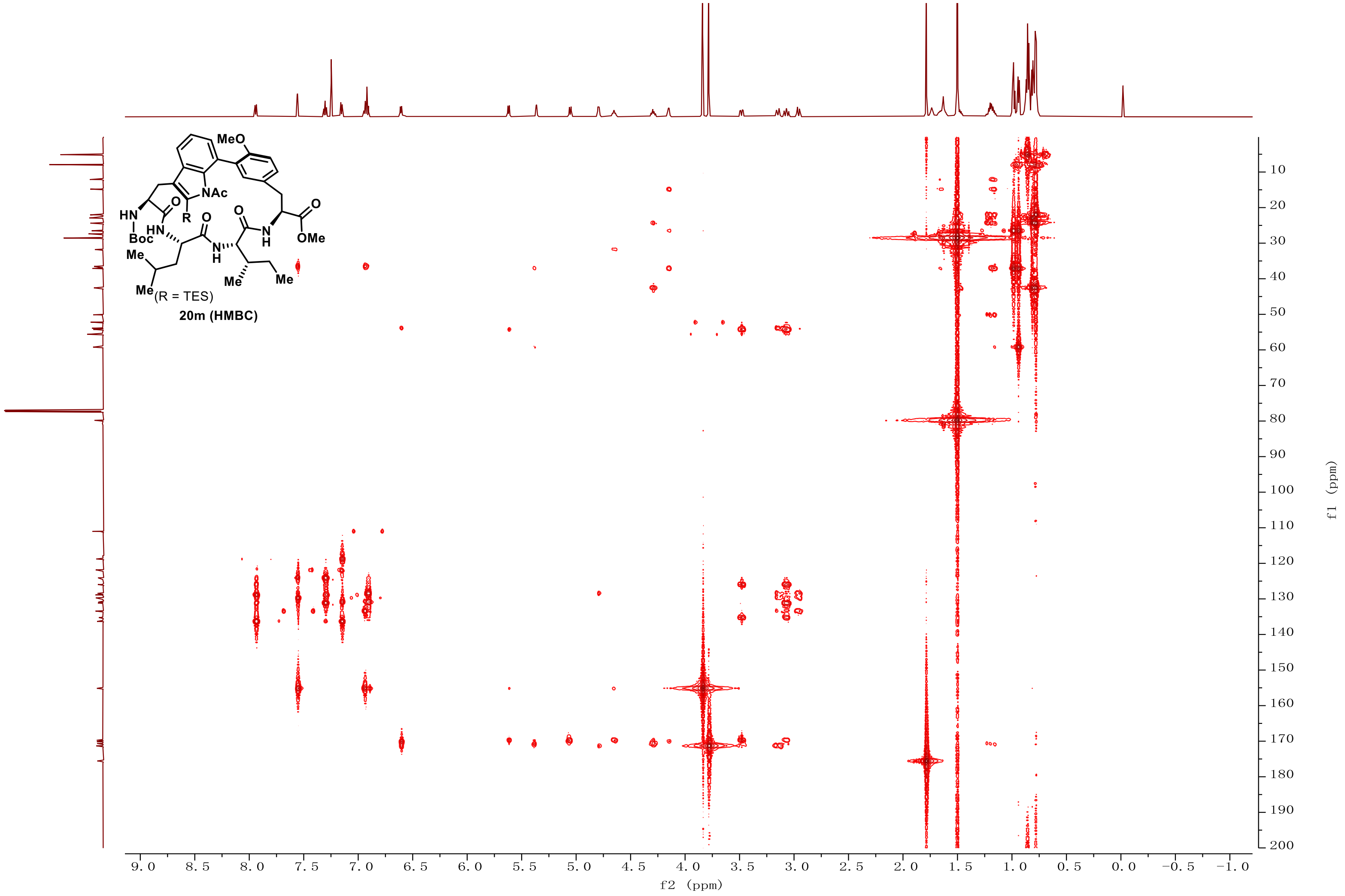

Compound 20n ¹H NMR (600 MHz, CDCl₃)

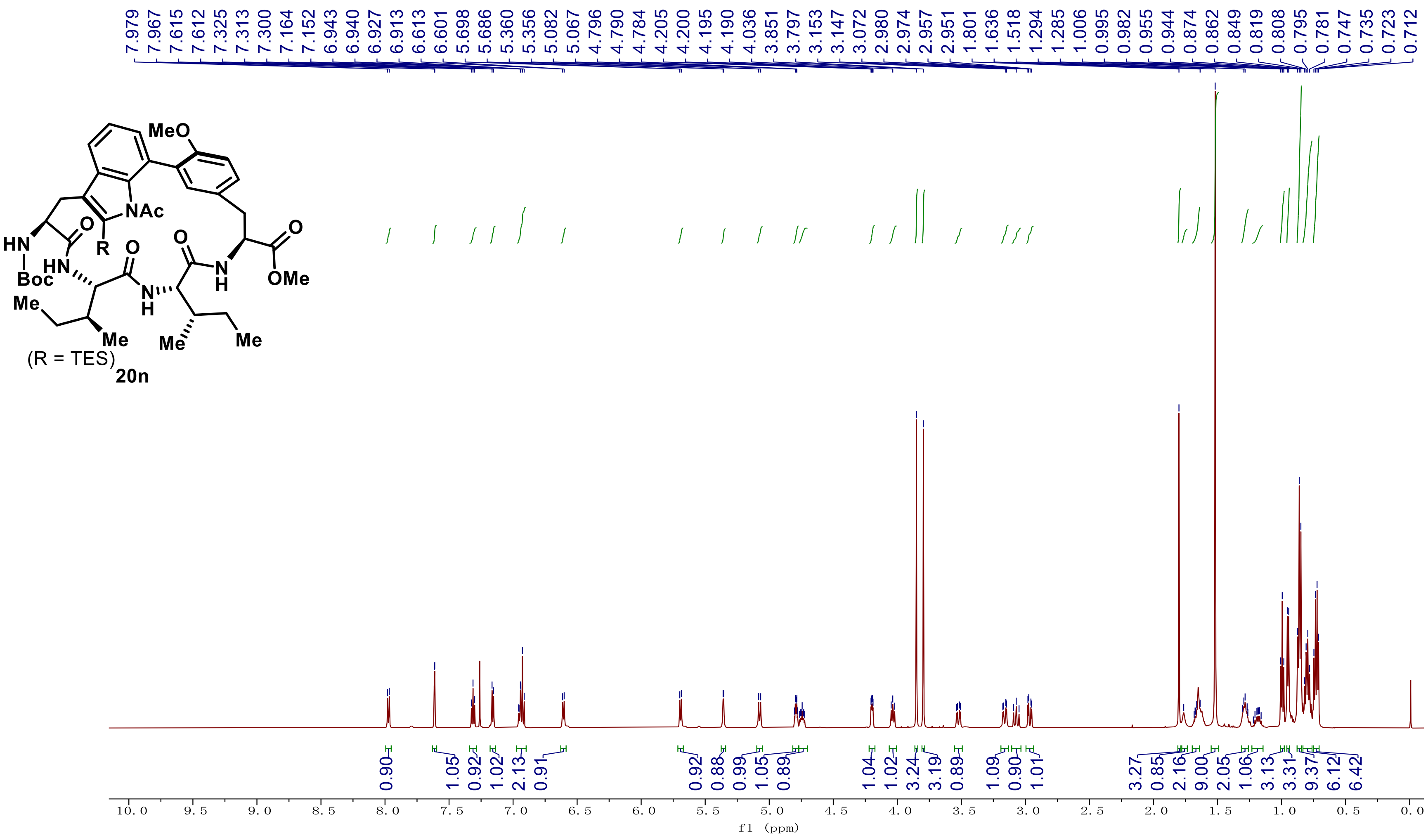

Compound 20n ^{13}C NMR (151 MHz, CDCl$_3$)

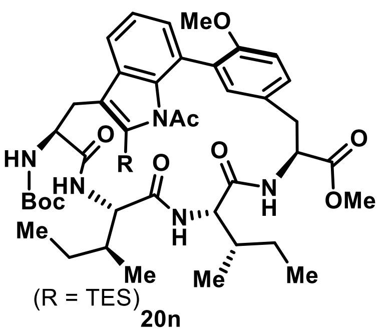

20n

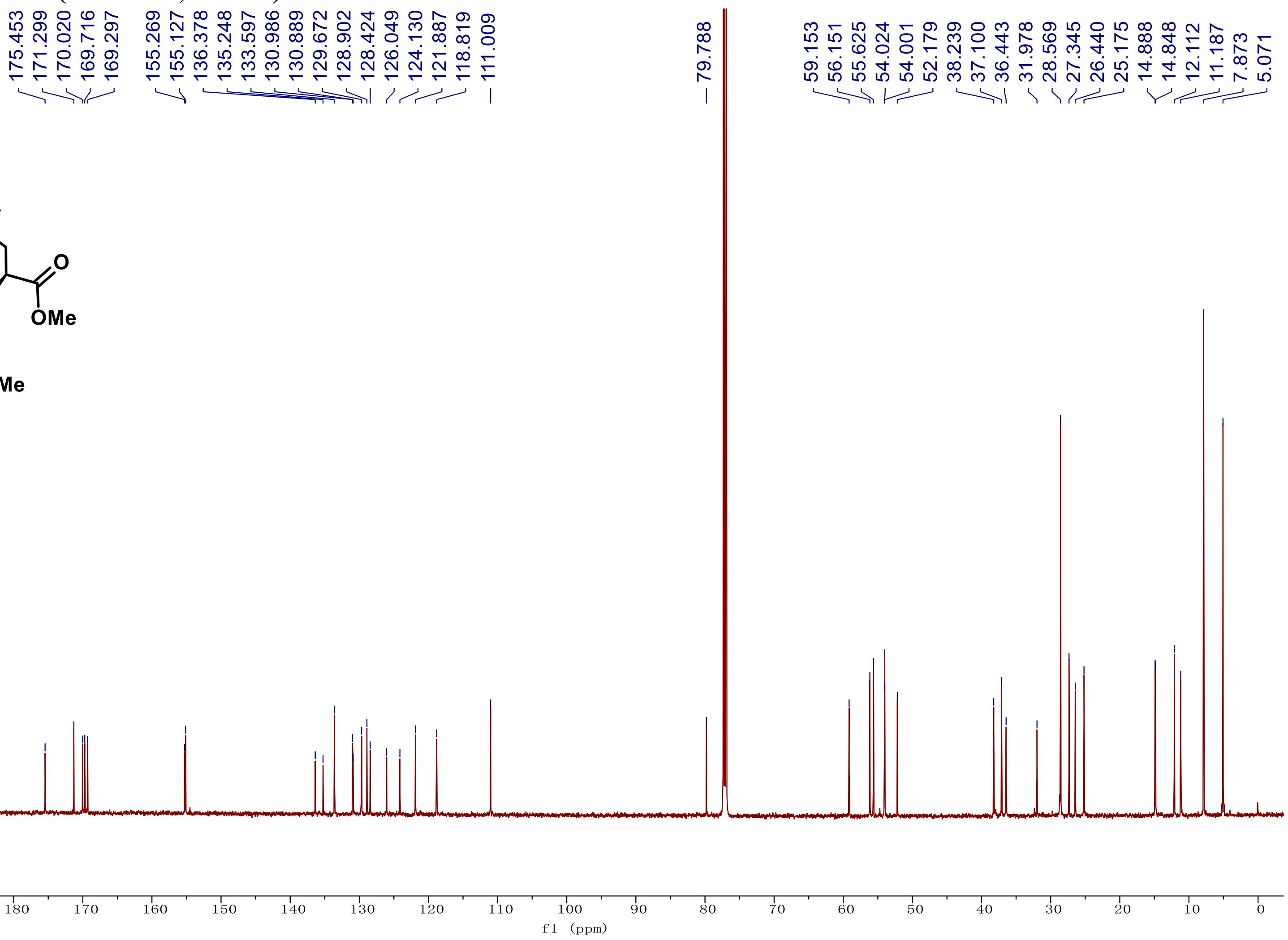

Compound 20o ^{1}H NMR (600 MHz, CDCl₃)

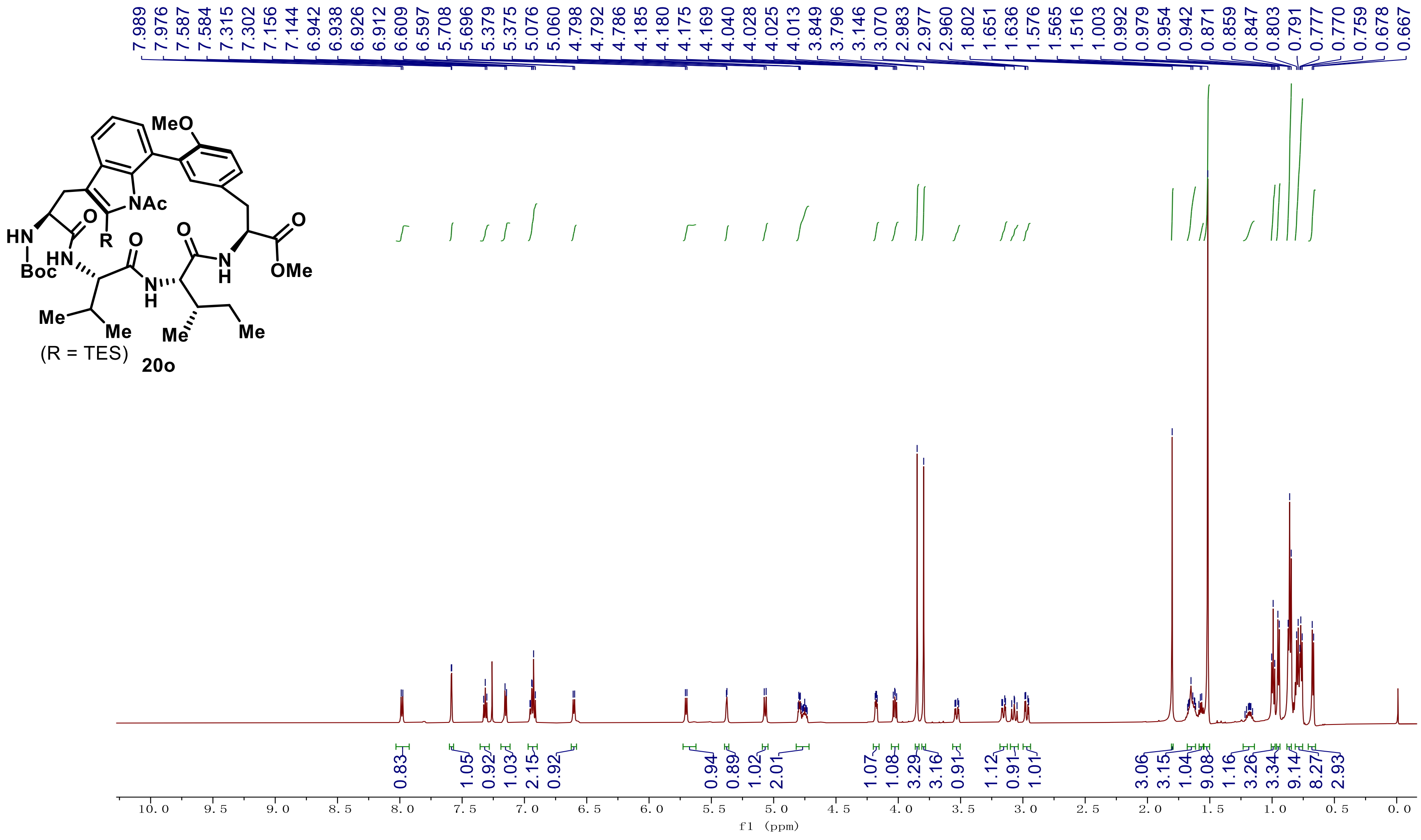

Compound 20o ^{13}C NMR (151 MHz, CDCl$_3$)

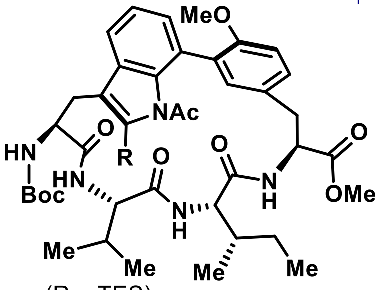

(R = TES) **20o**

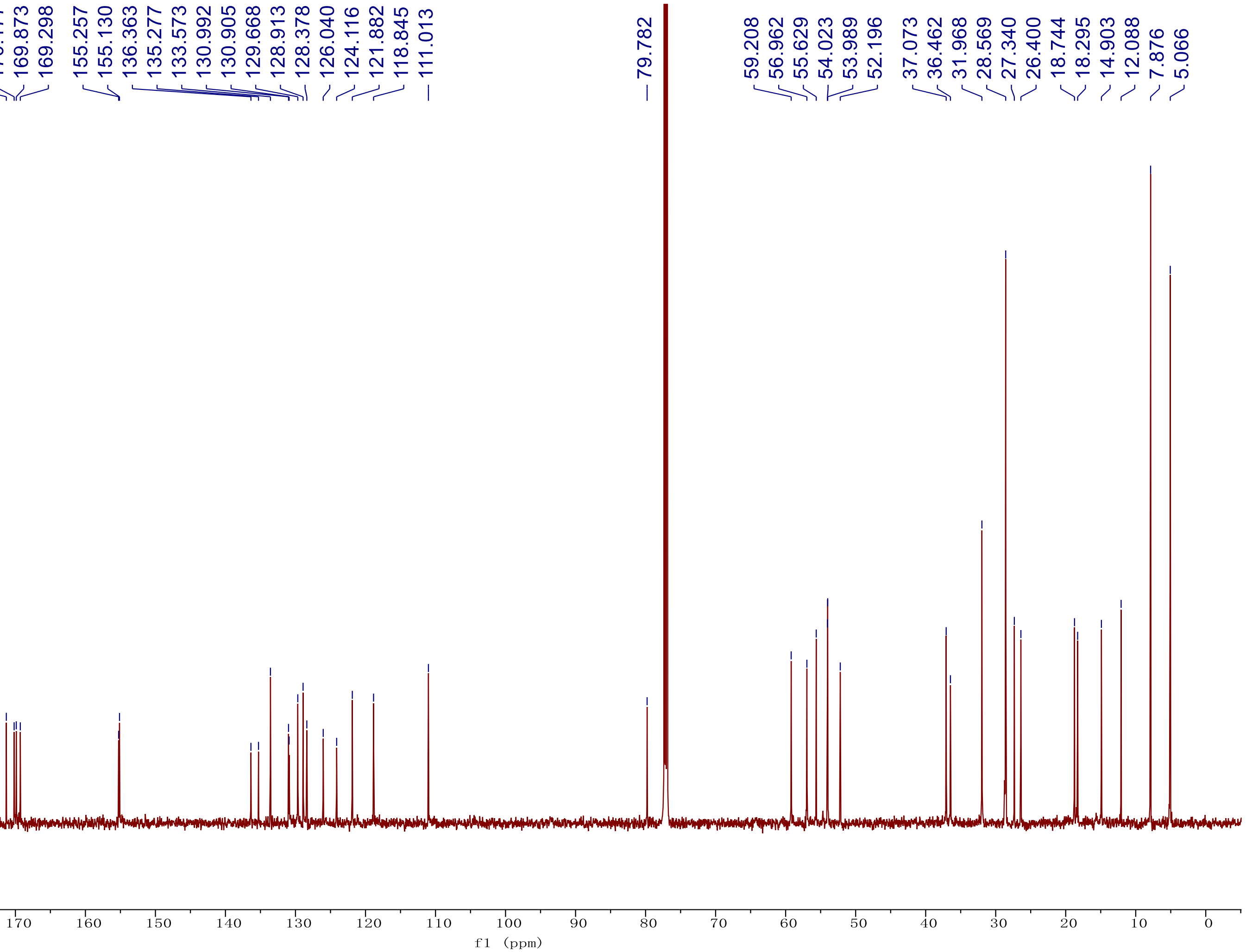

Compound 20p ^1H NMR (600 MHz, $CDCl_3$)

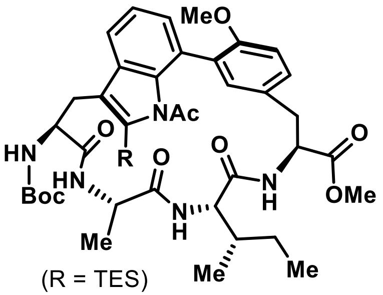

20p

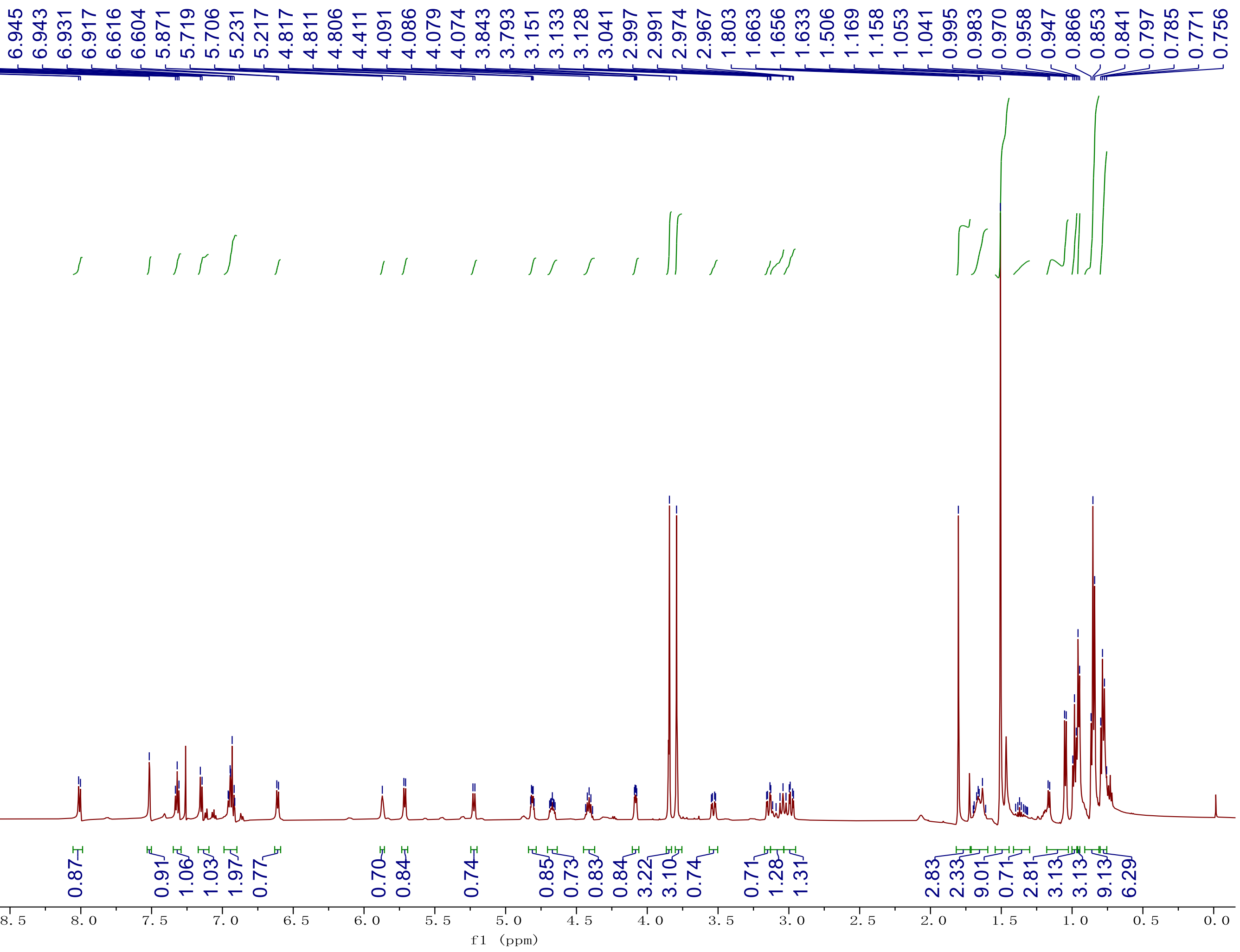

Compound 20p ^{13}C NMR (151 MHz, CDCl$_3$)

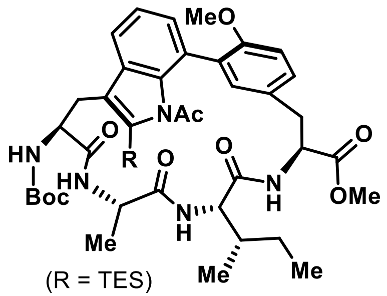

(R = TES)

20p

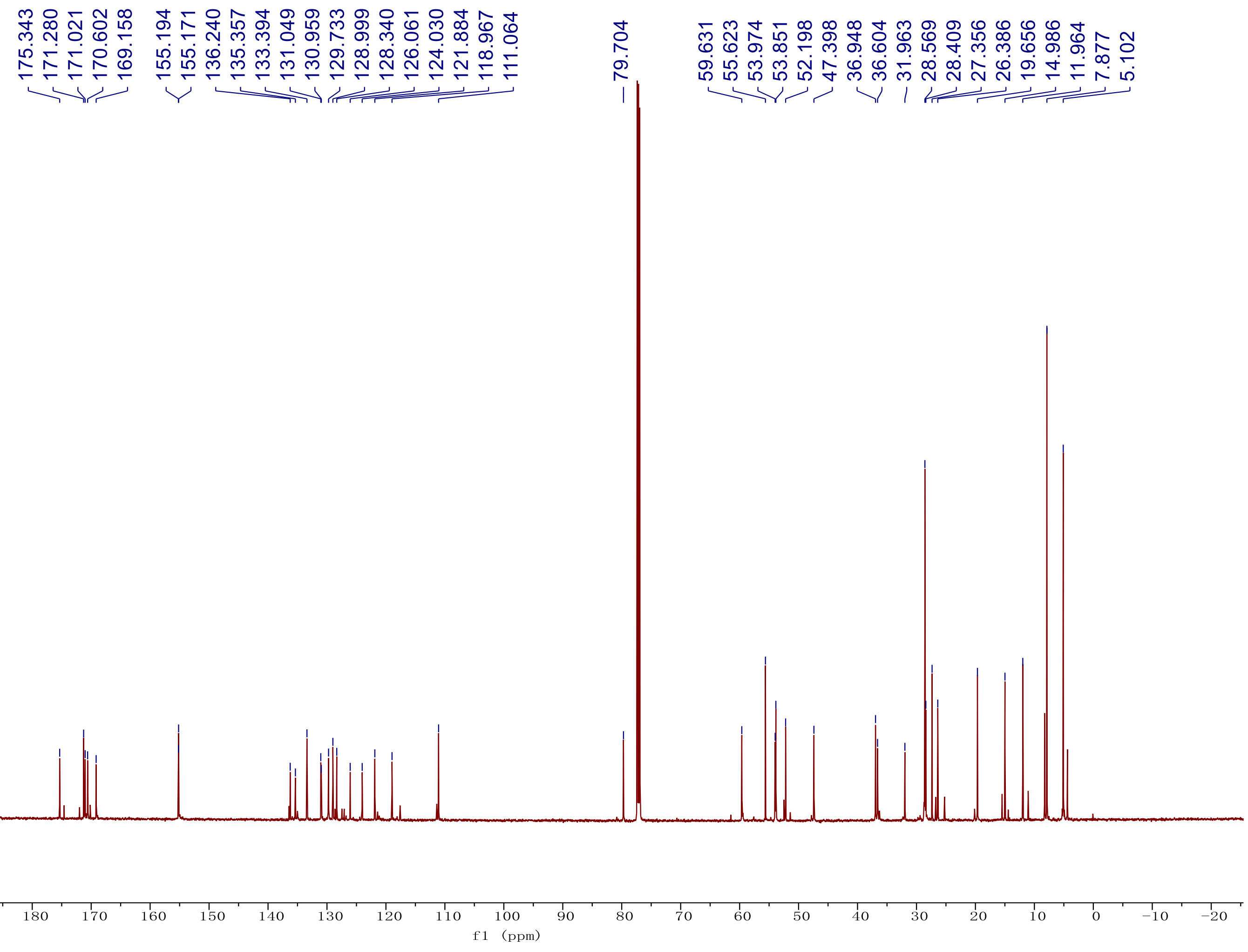

Compound 24a ^{1}H NMR (600 MHz, $CDCl_3$)

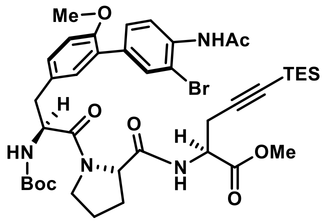

24a

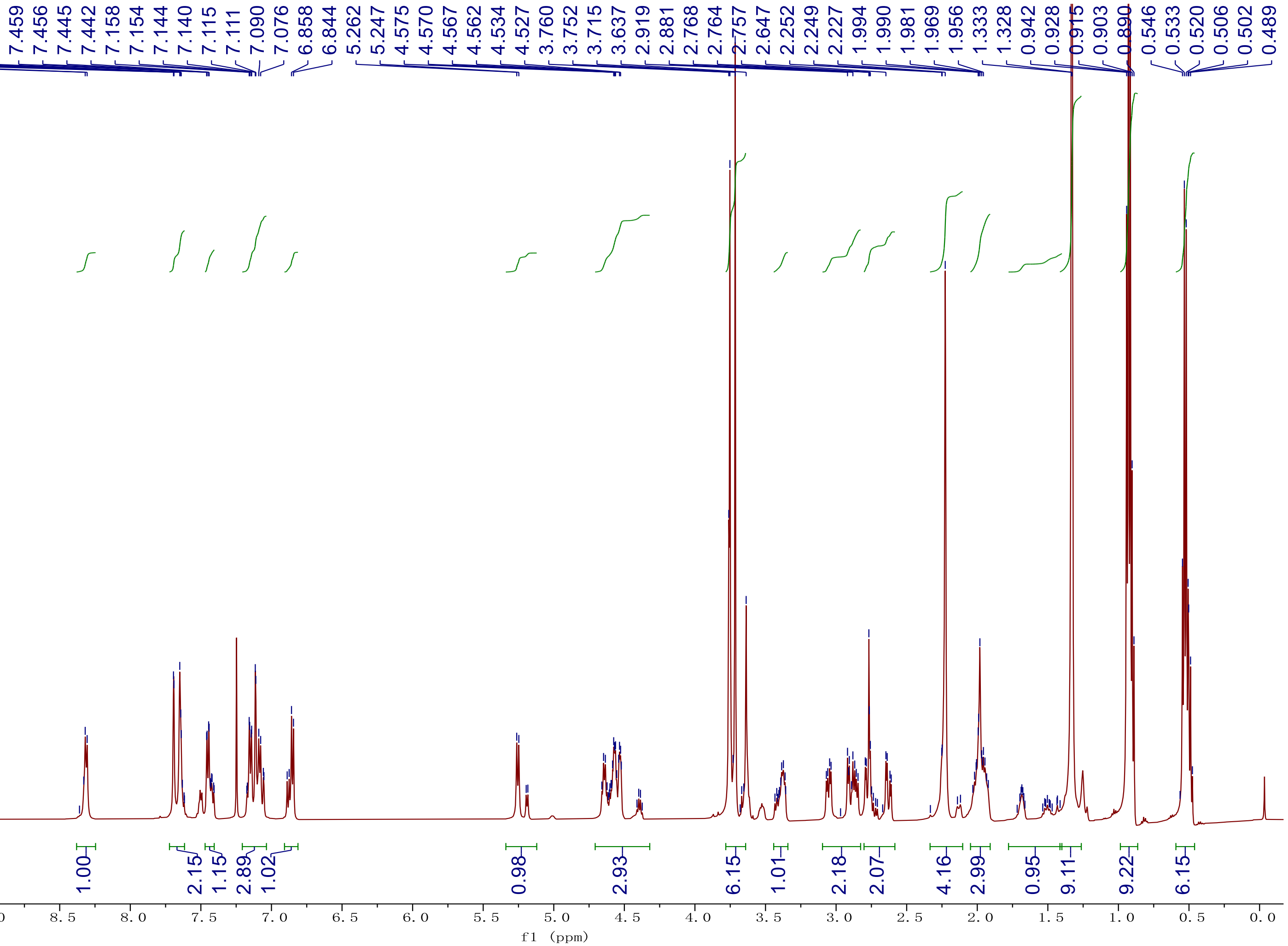

Compound 24a ^{13}C NMR (151 MHz, $CDCl_3$)

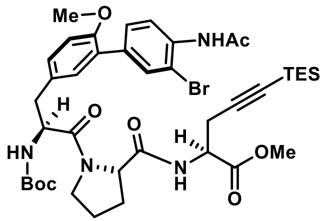

24a

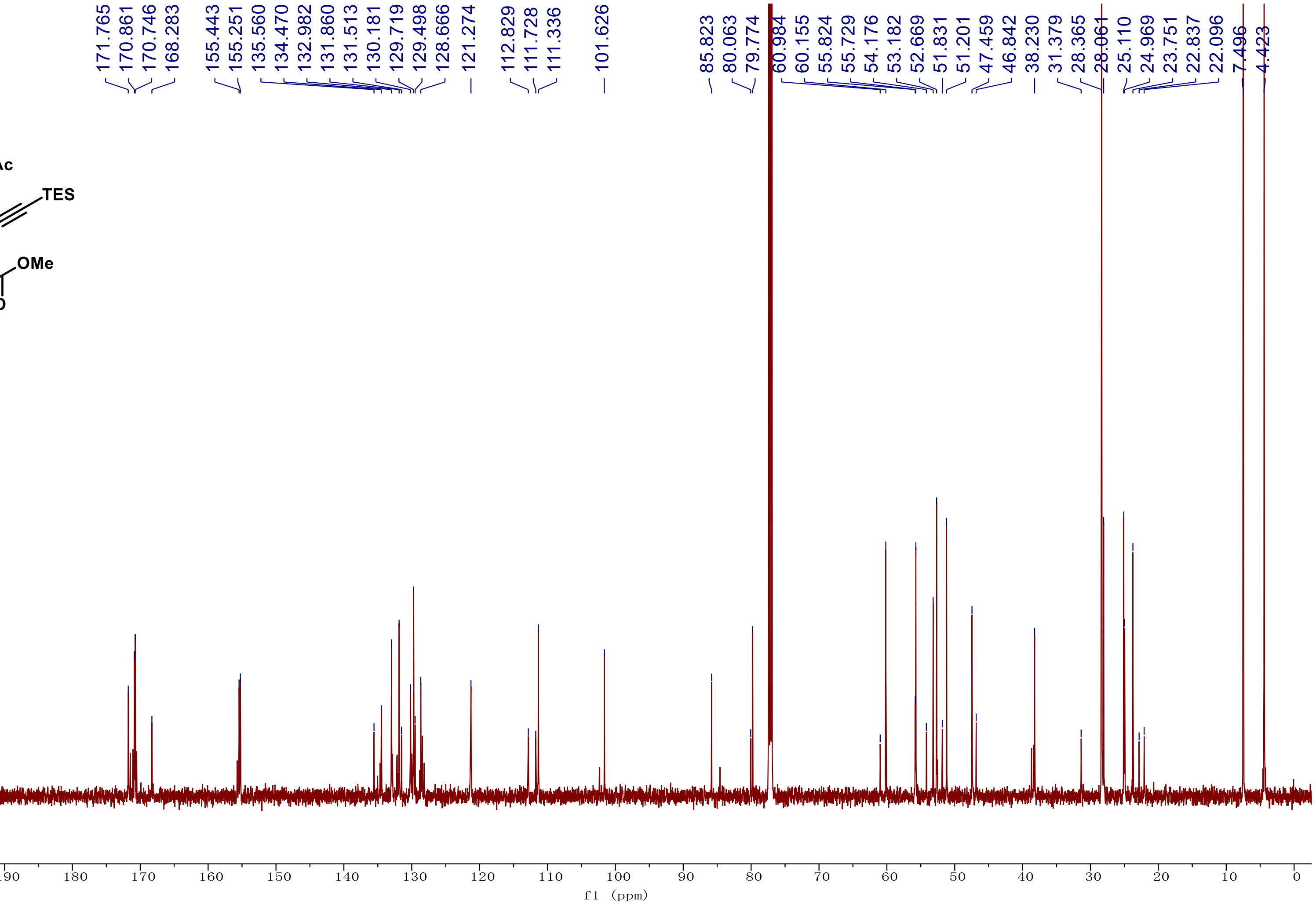

Compound 24b ^{1}H NMR (600 MHz, $CDCl_3$)

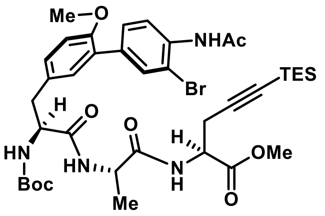

24b

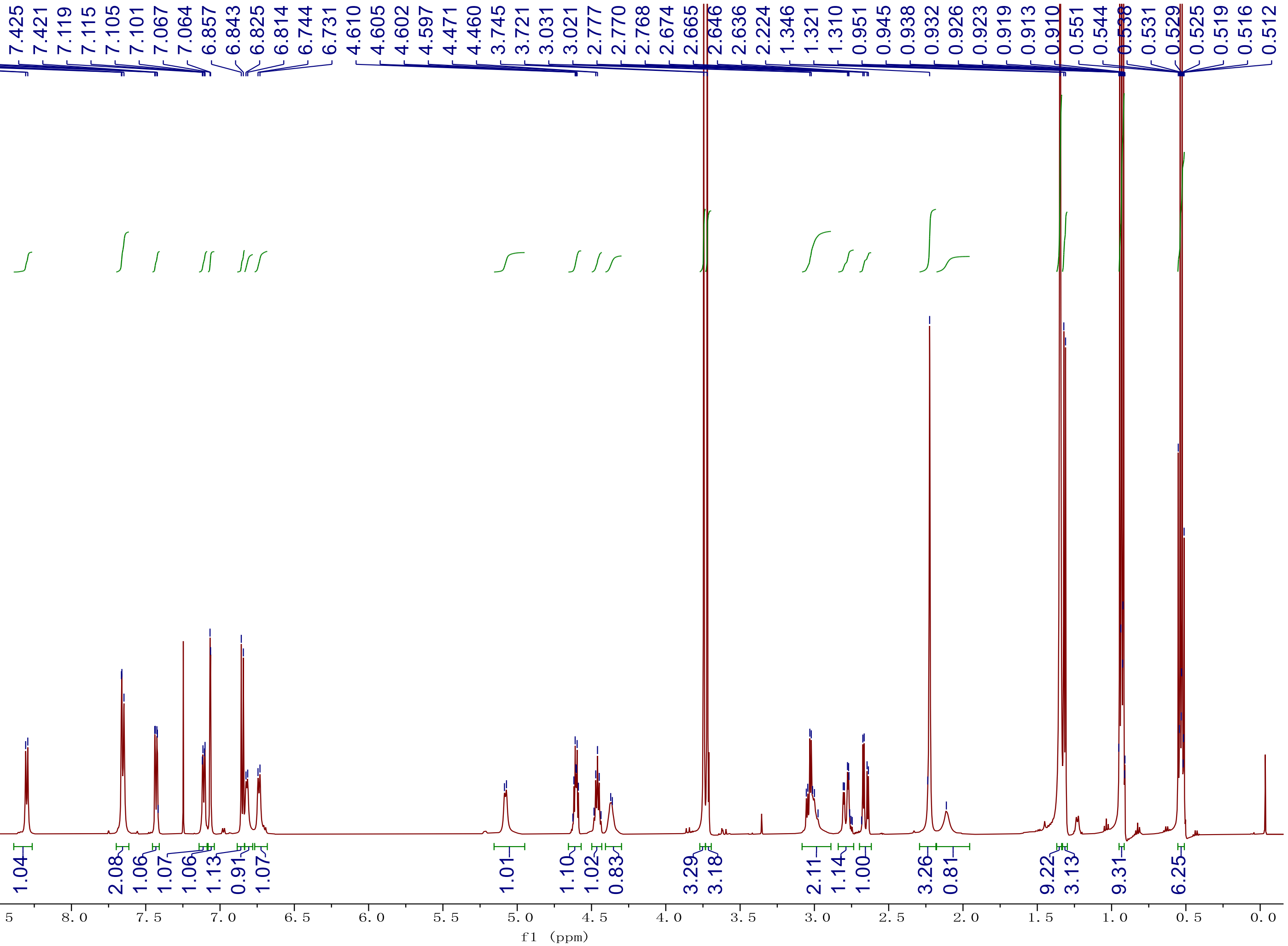

Compound 24b ^{13}C NMR (151 MHz, $CDCl_3$)

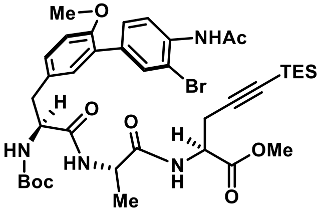

24b

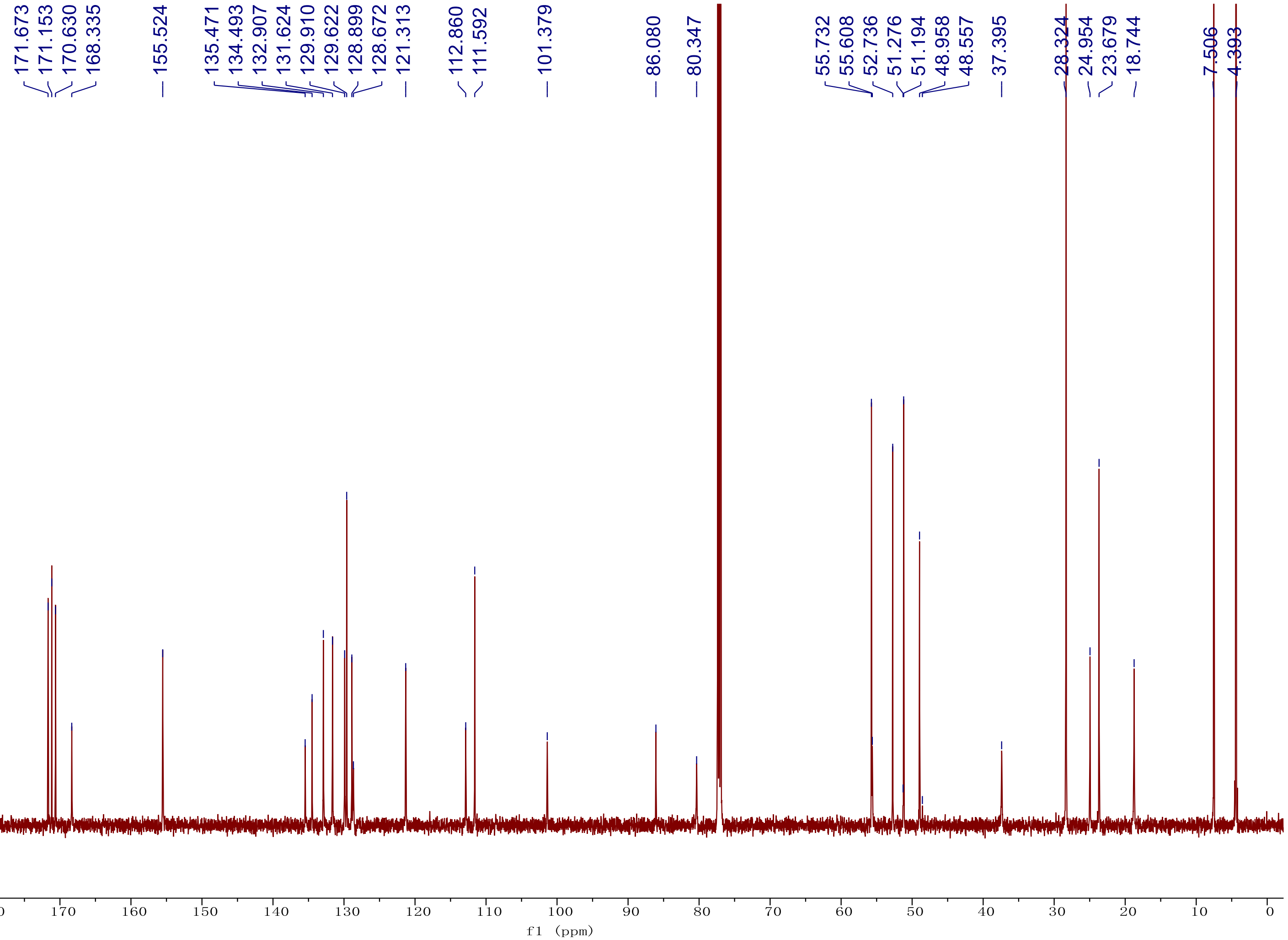

Compound 24c ¹H NMR (600 MHz, CDCl₃)

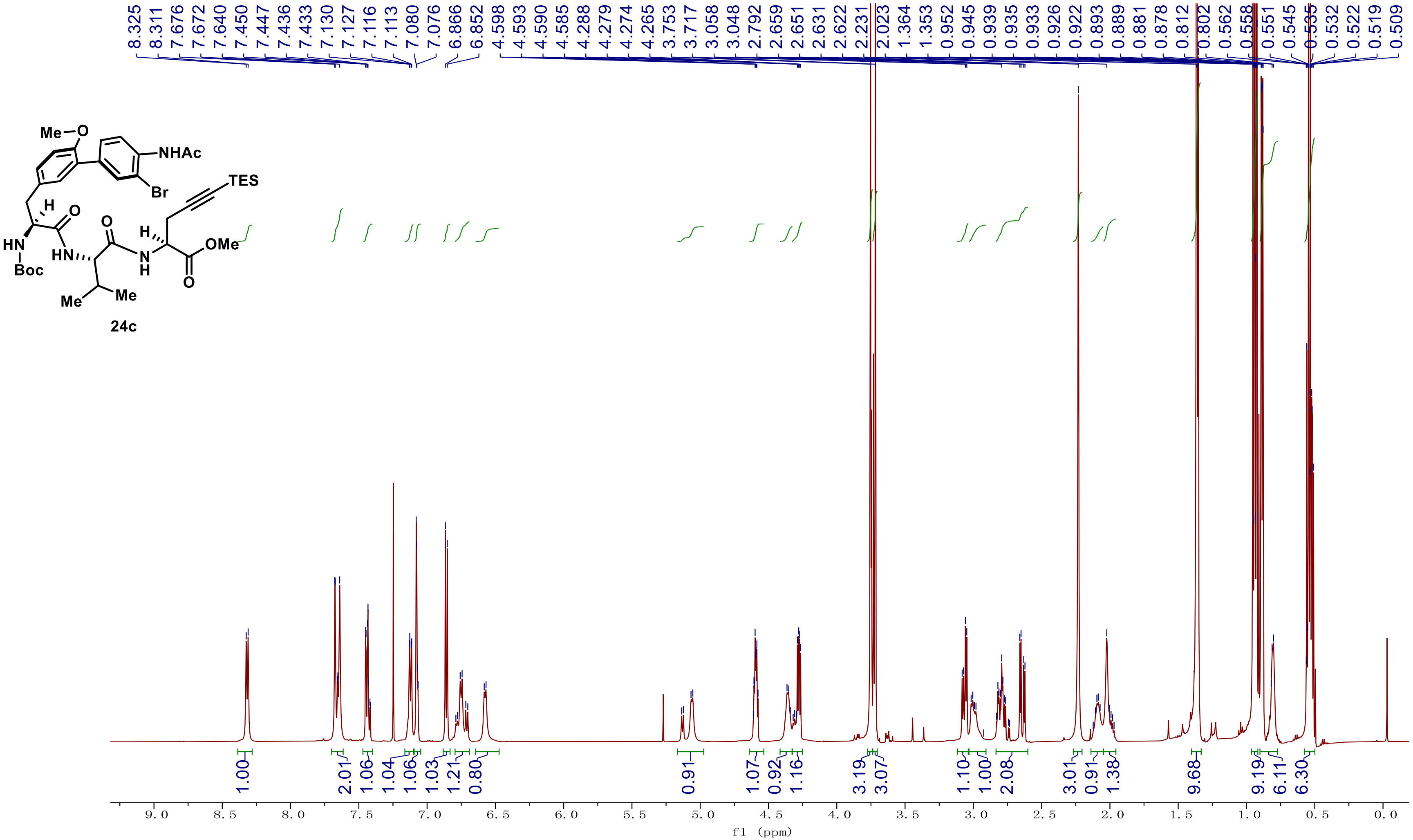

24c

Compound 24c ^{13}C NMR (151 MHz, $CDCl_3$)

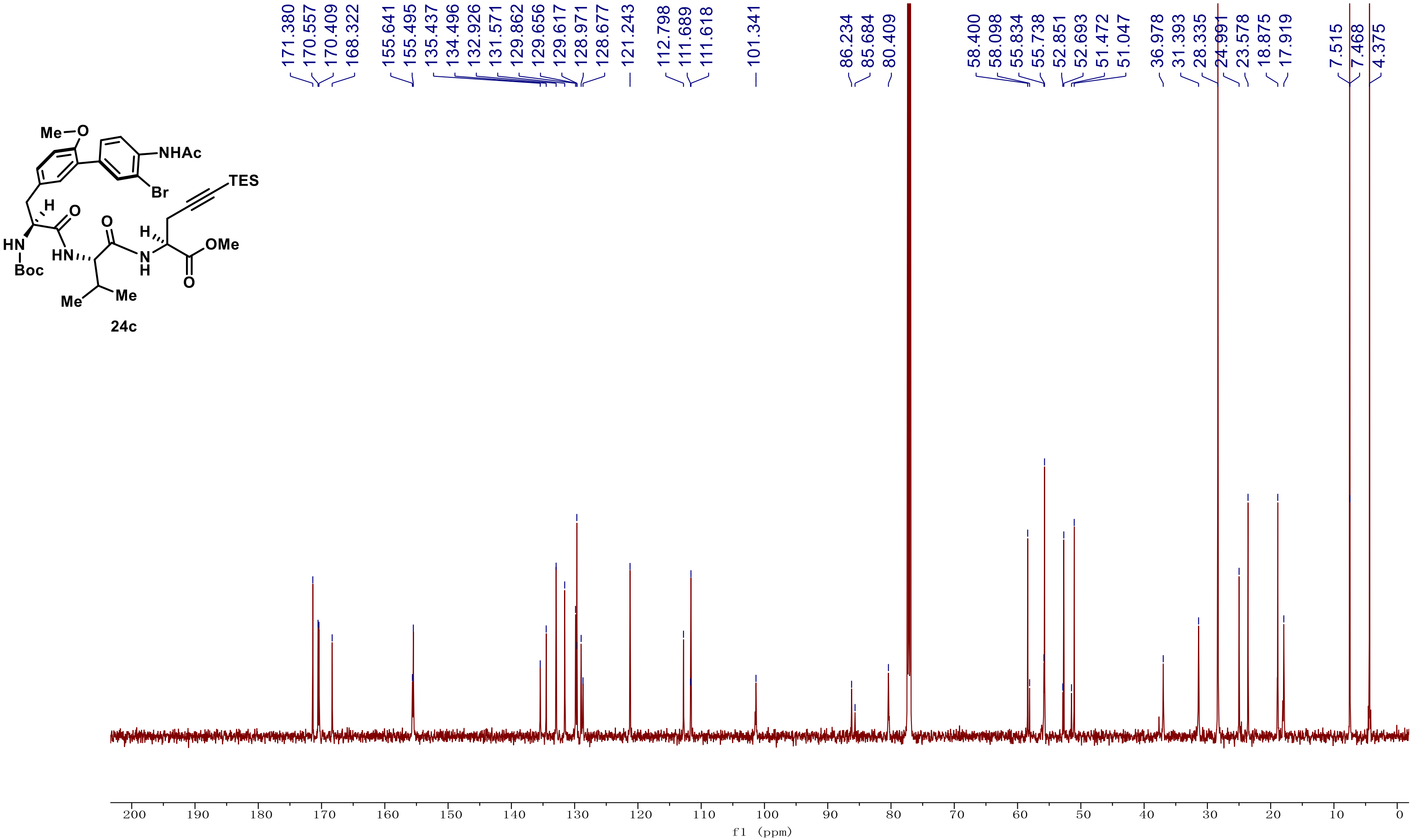

Compound 24d ^{1}H NMR (600 MHz, $CDCl_3$)

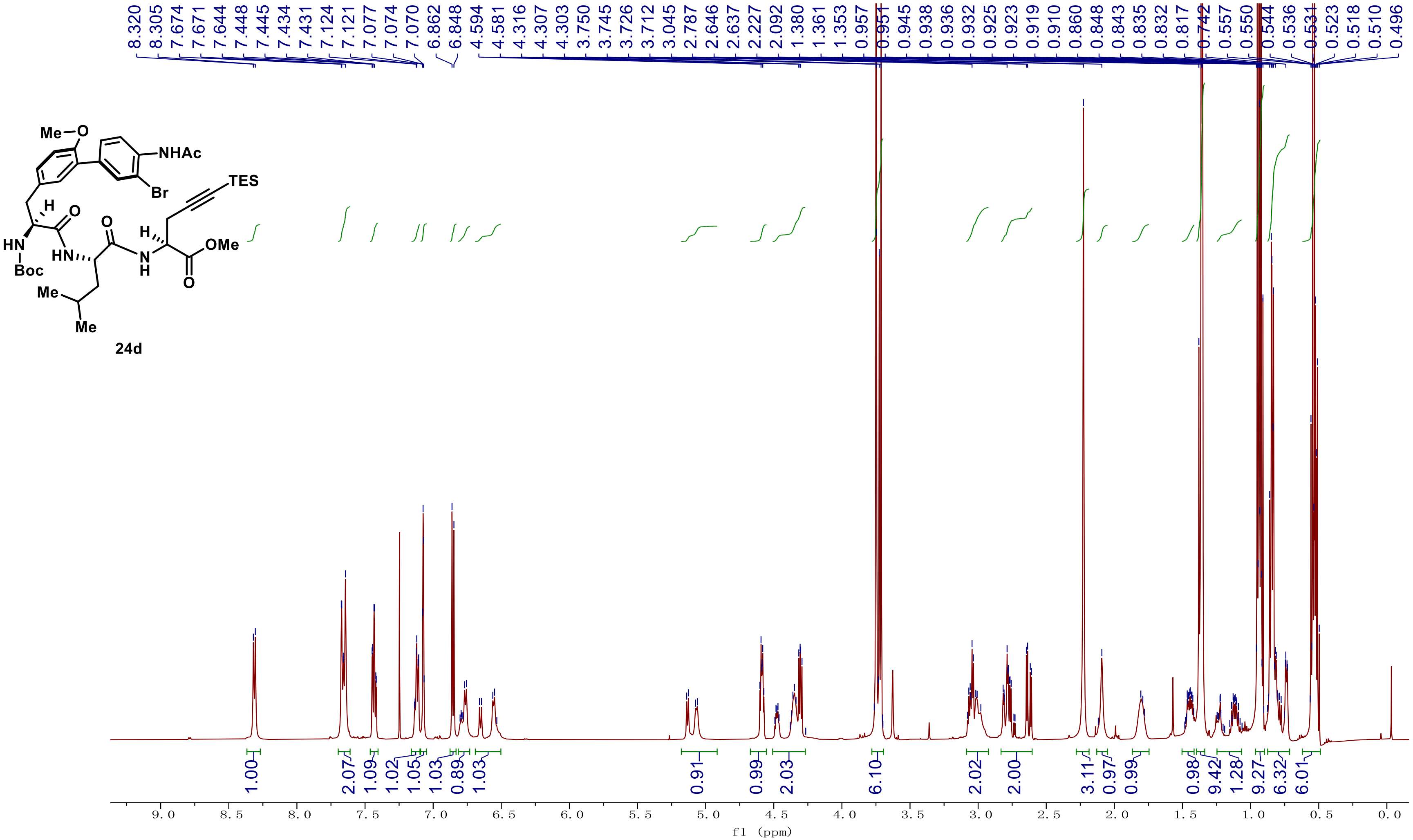

Compound 24d ^{13}C NMR (151 MHz, CDCl$_3$)

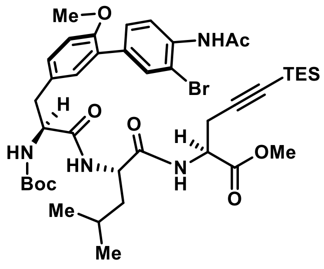

24d

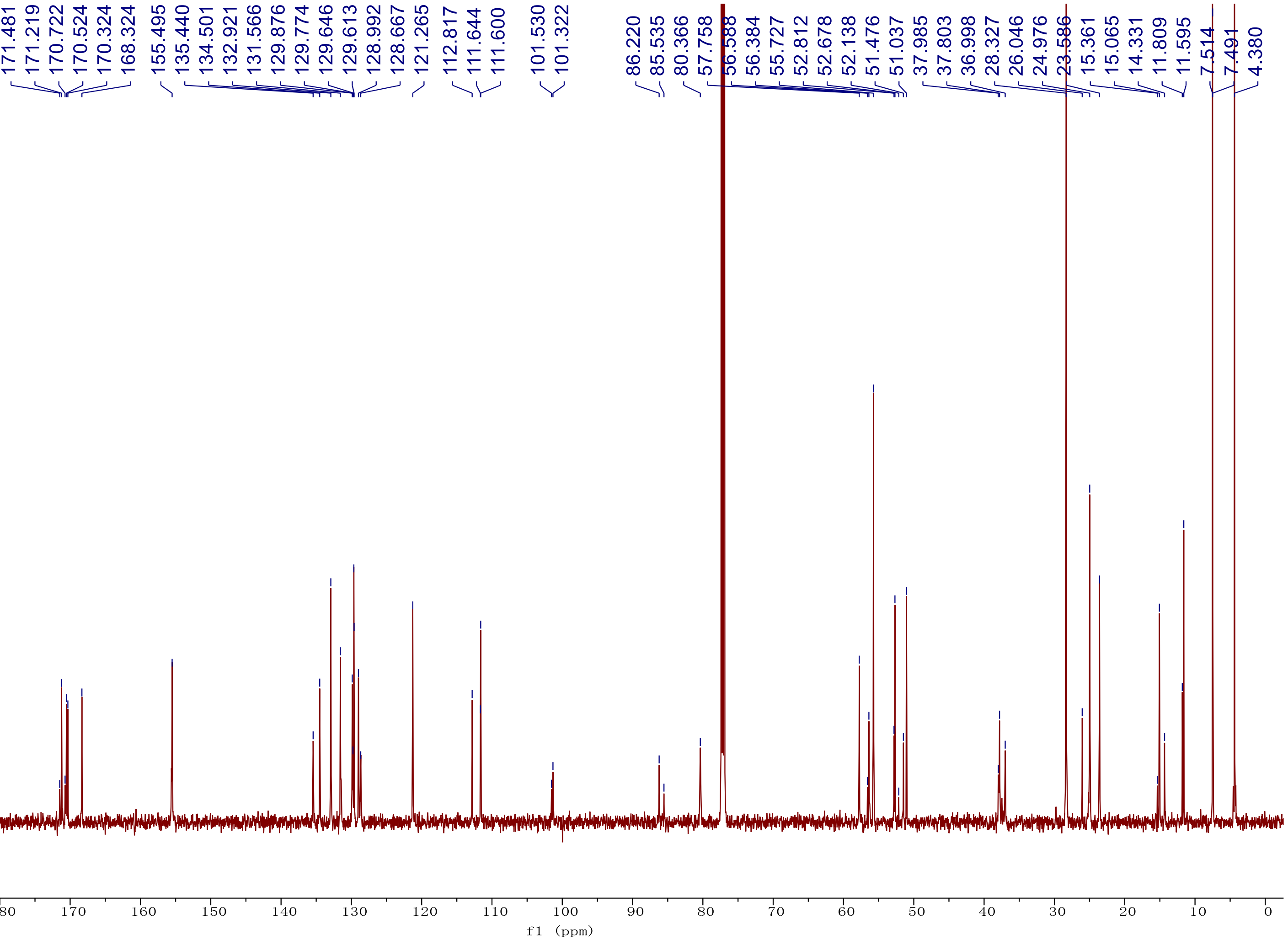

Compound 24e ^{1}H NMR (600 MHz, $CDCl_3$)

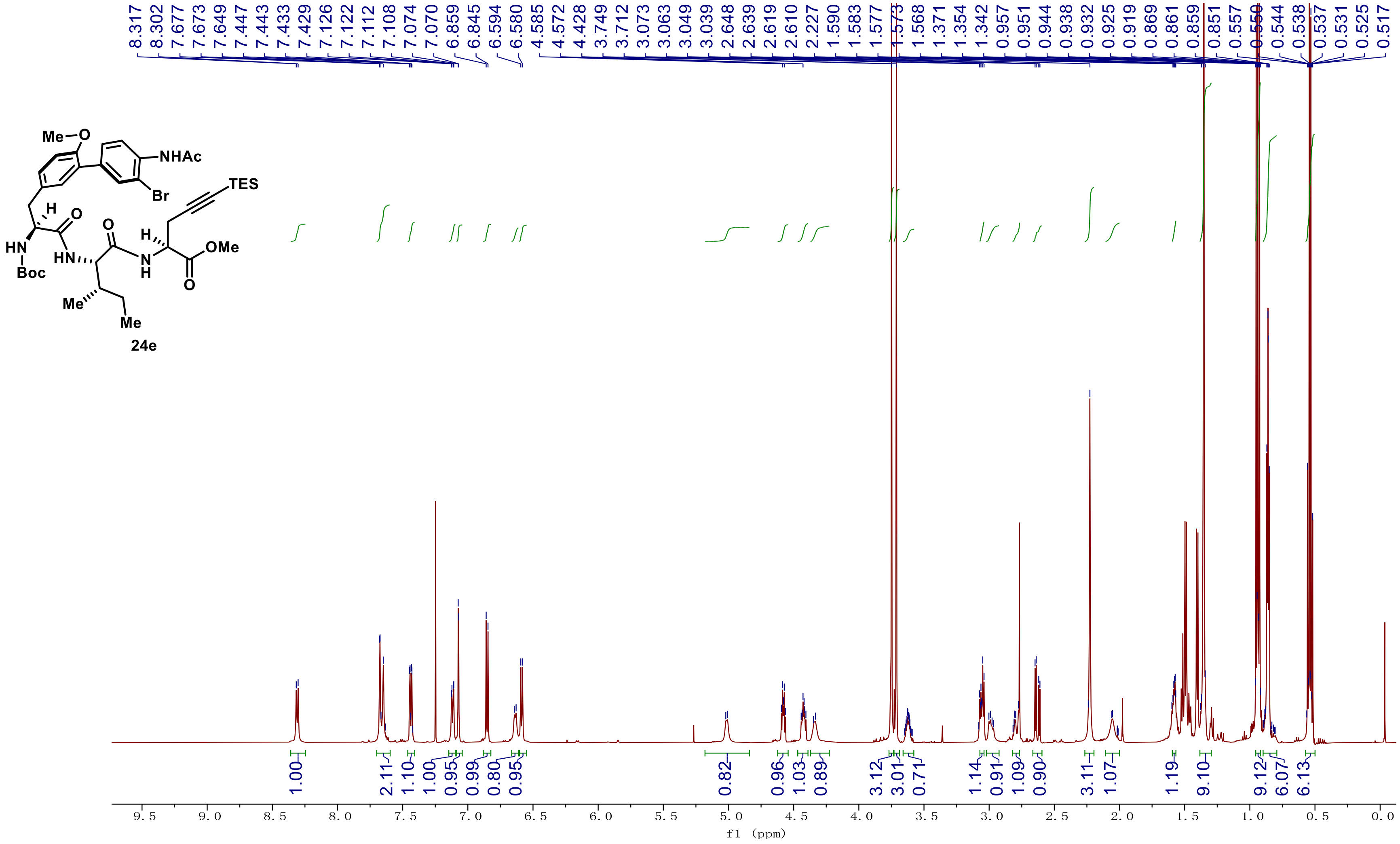

Compound 24e ^{13}C NMR (151 MHz, $CDCl_3$)

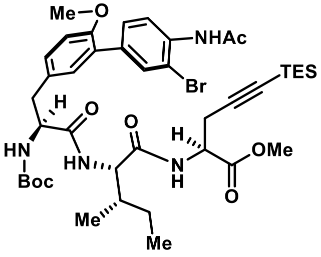

24e

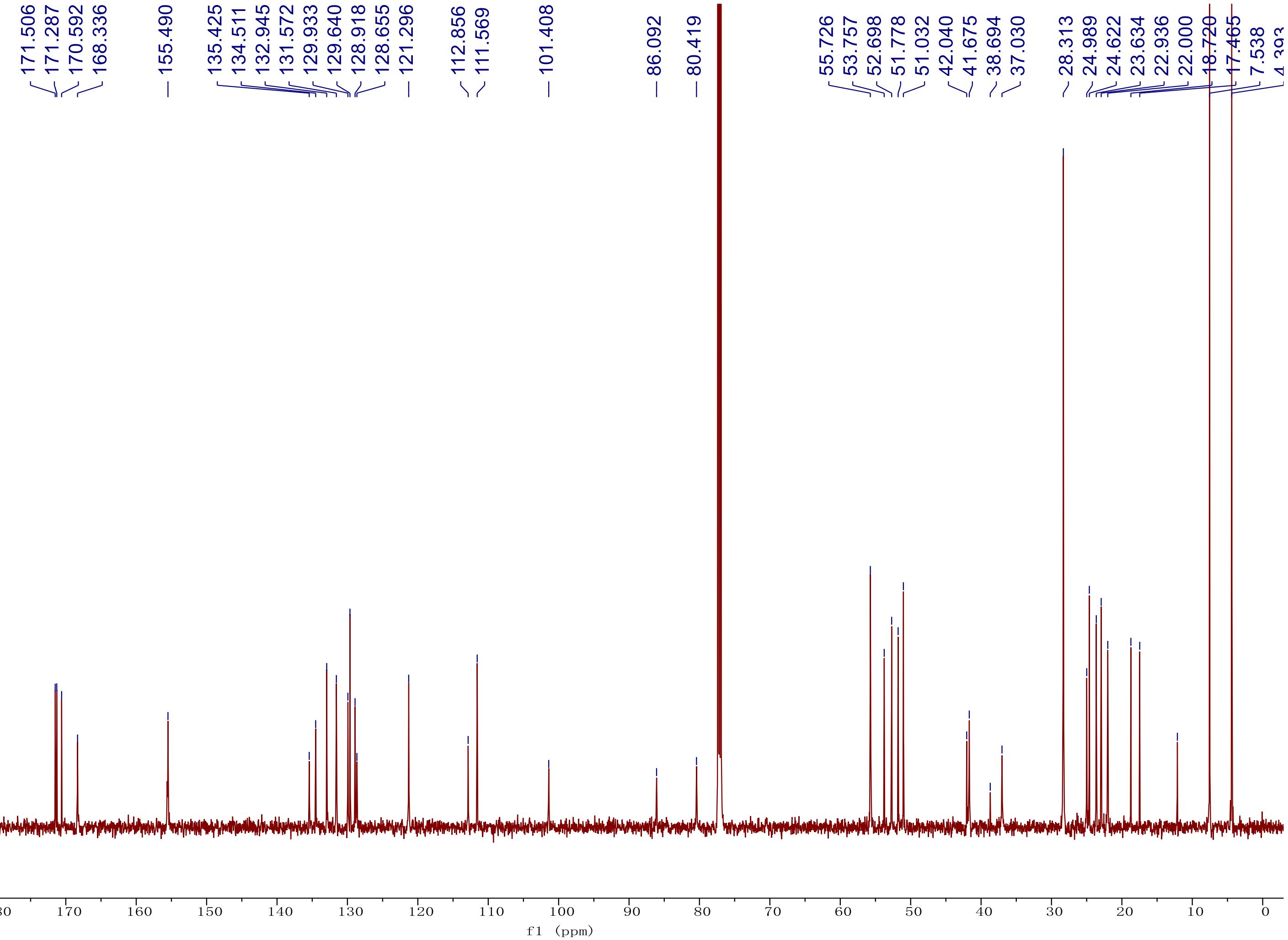

Compound 24f ¹H NMR (400 MHz, CDCl₃)

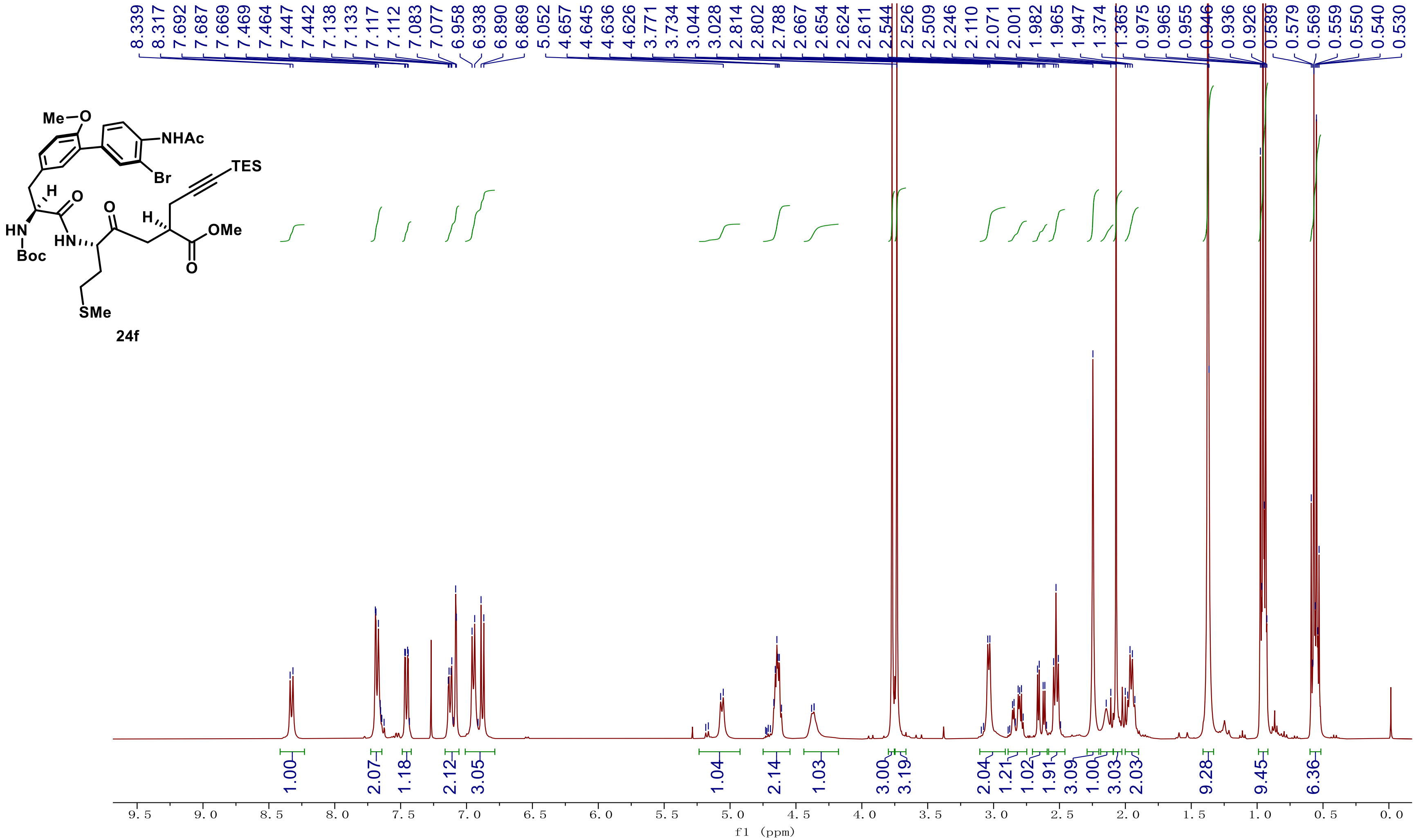

Compound 24f ^{13}C NMR (101 MHz, $CDCl_3$)

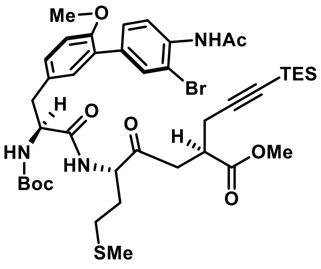

24f

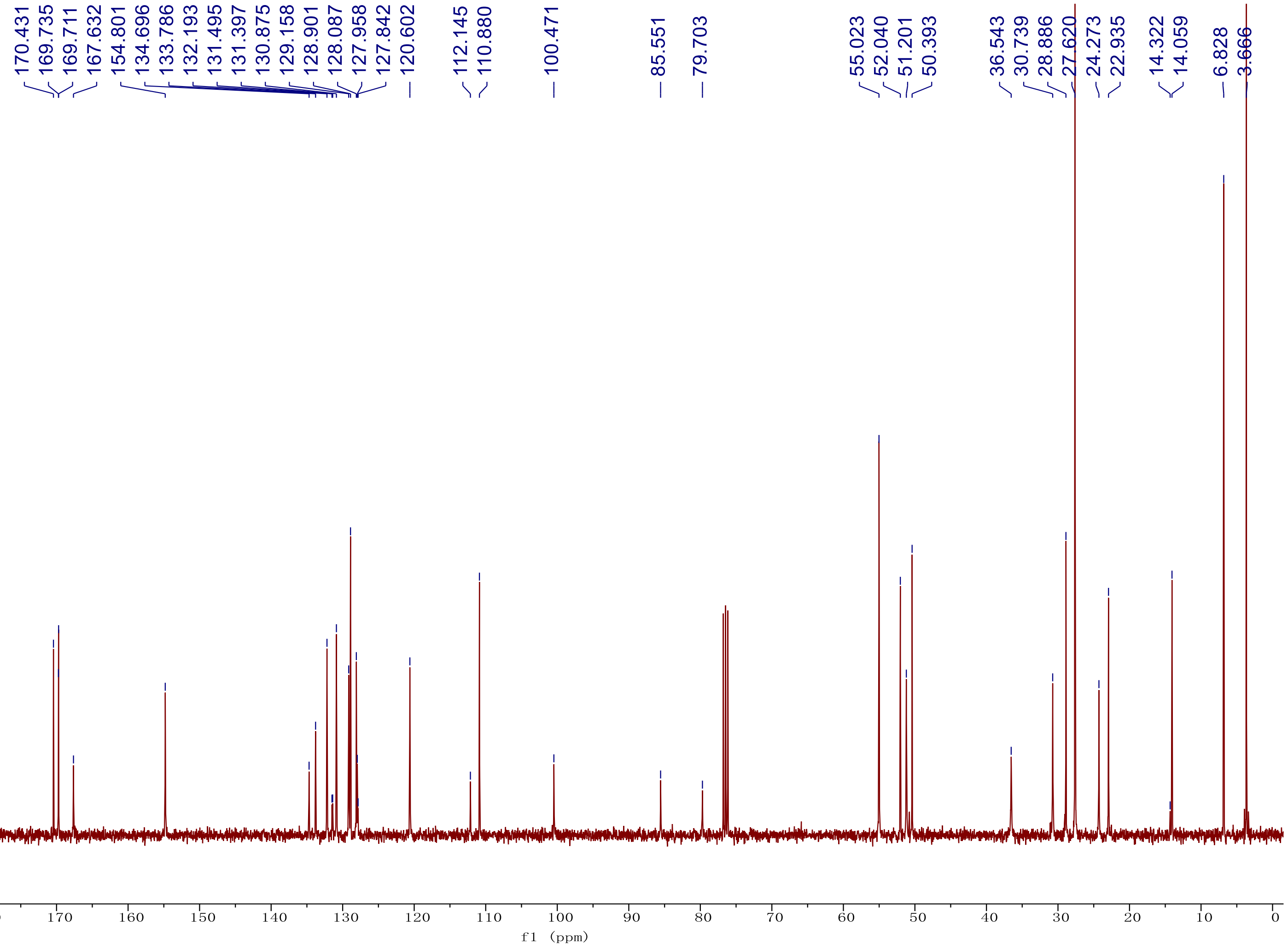

Compound 24g ^{1}H NMR (600 MHz, $CDCl_3$)

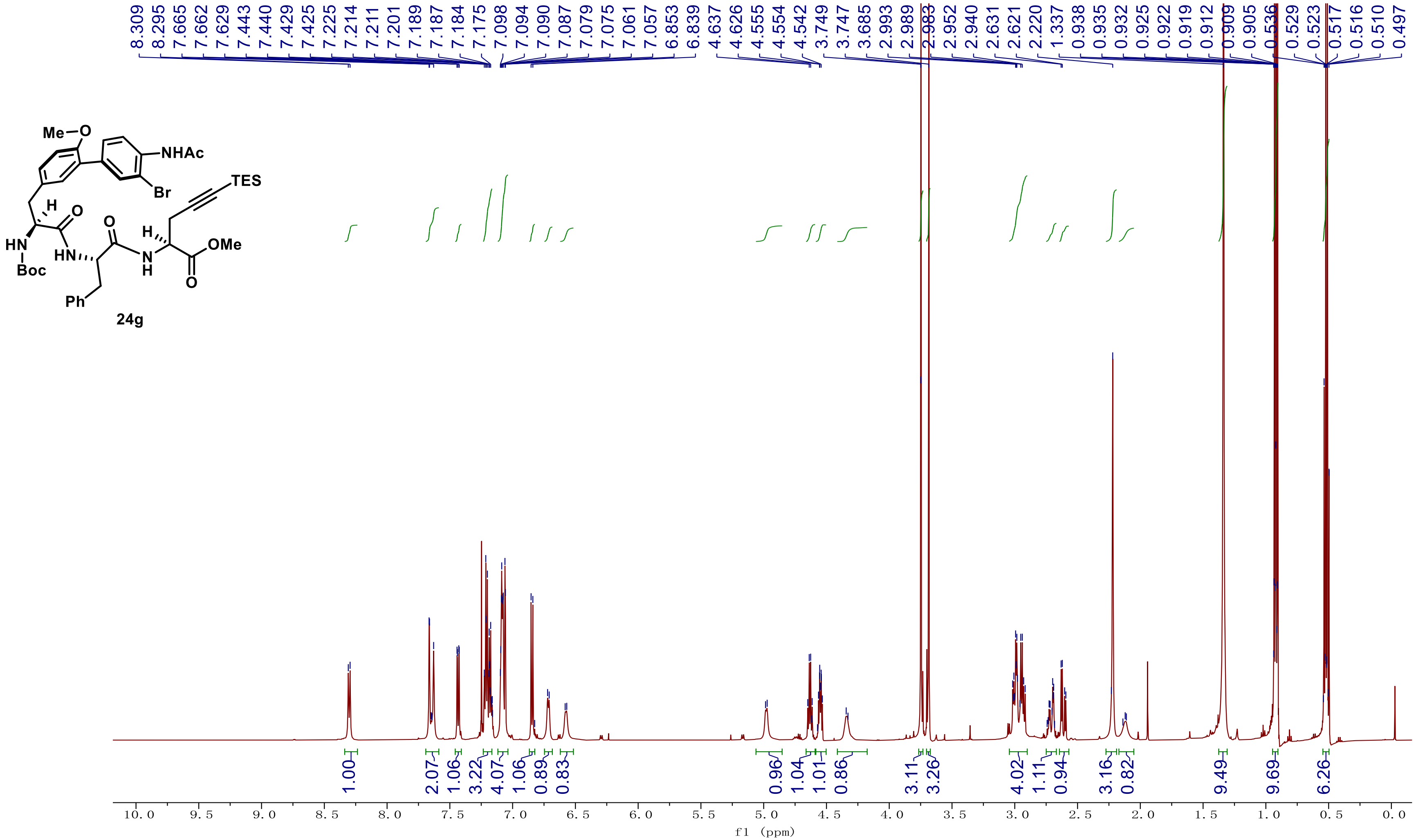

24g

Compound 24g ^{13}C NMR (151 MHz, CDCl$_3$)

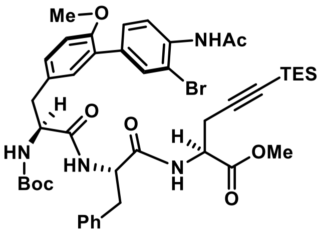

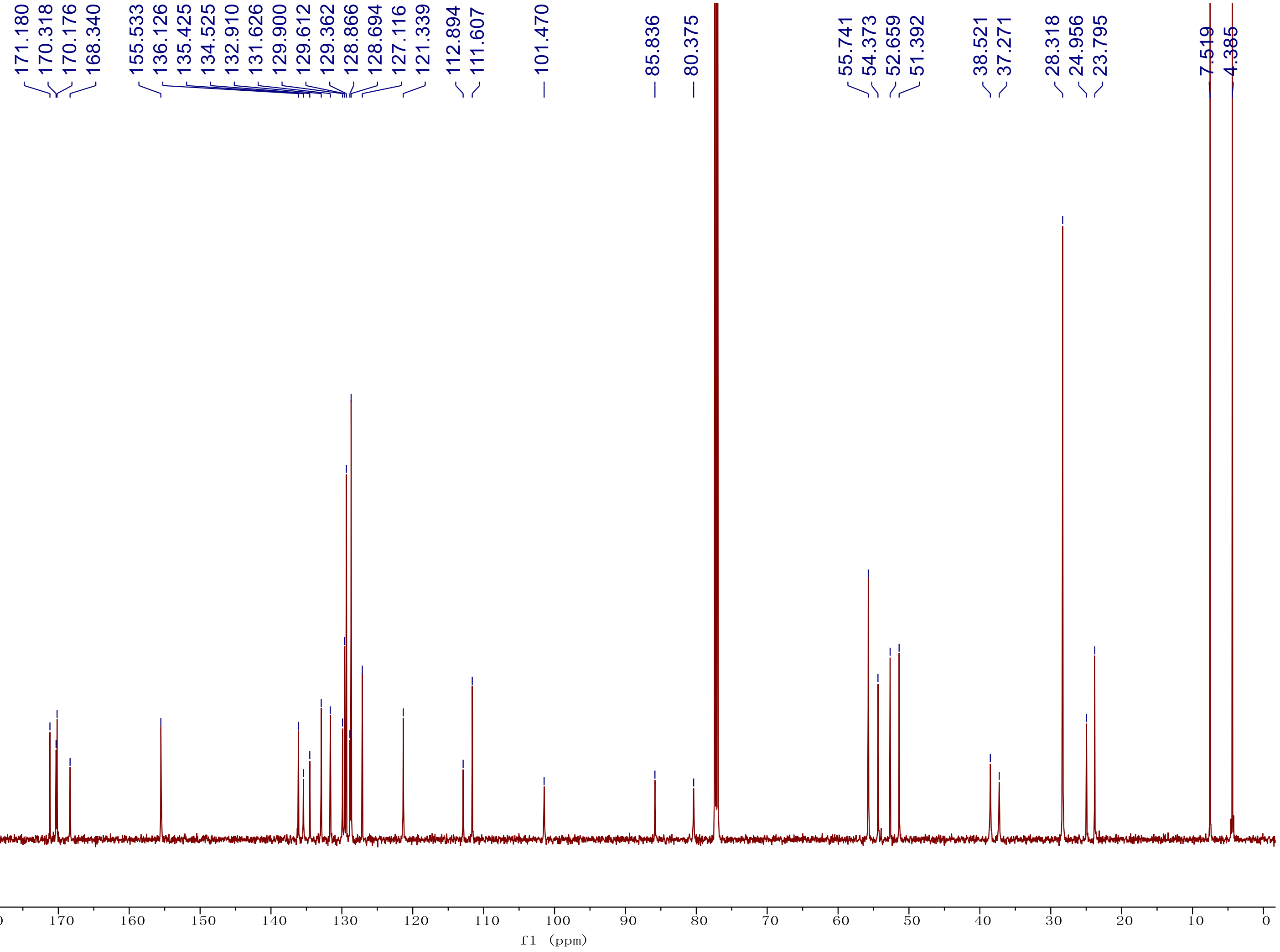

Compound 24h ^{1}H NMR (600 MHz, $CDCl_3$)

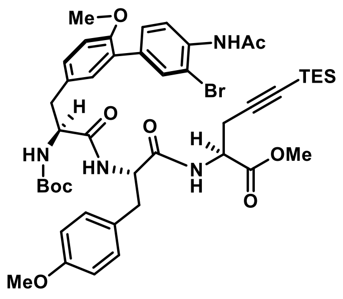

24h

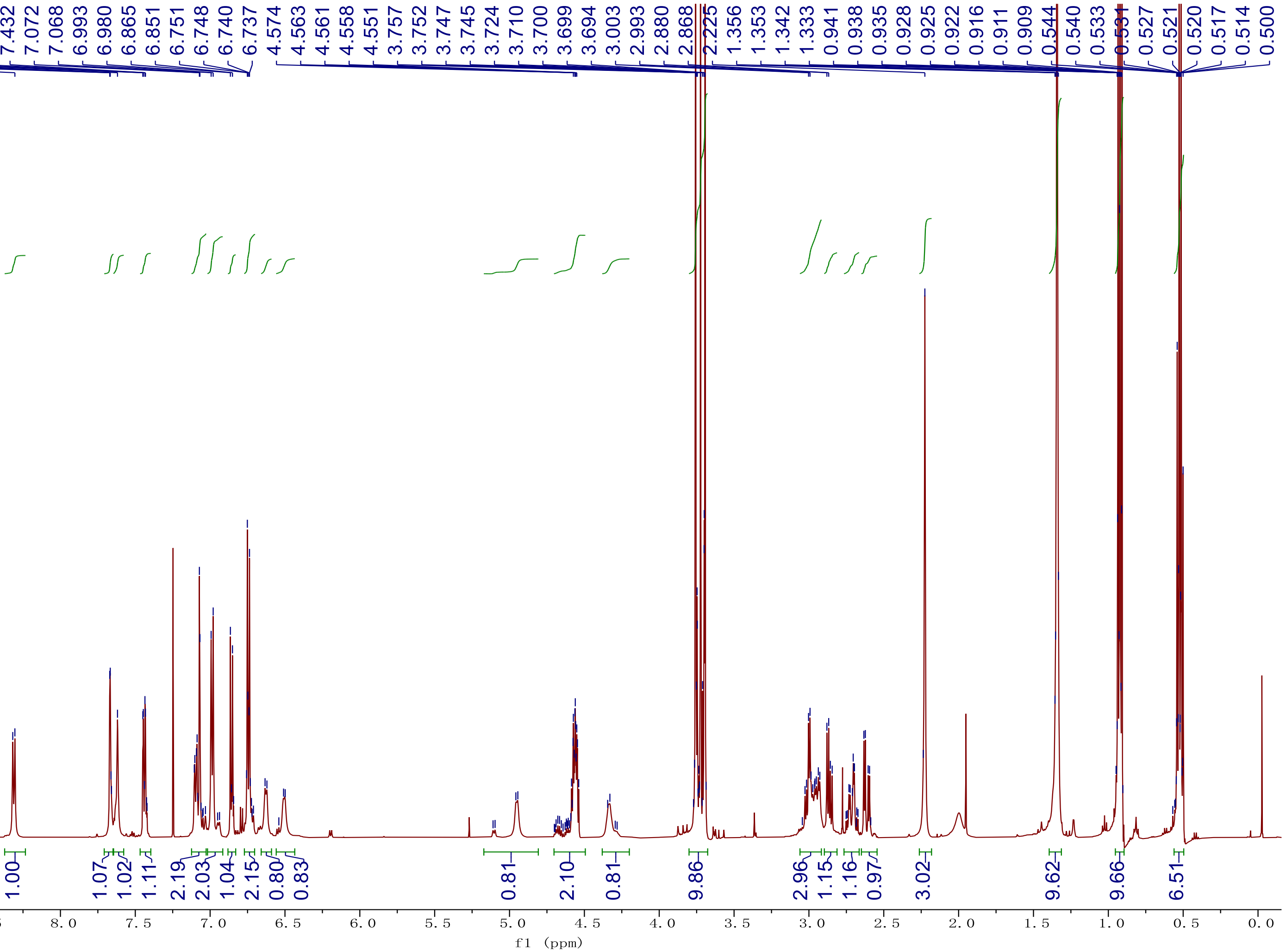

Compound 24h ^{13}C NMR (151 MHz, $CDCl_3$)

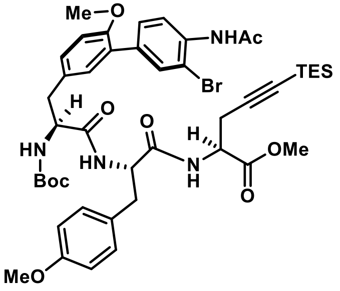

24h

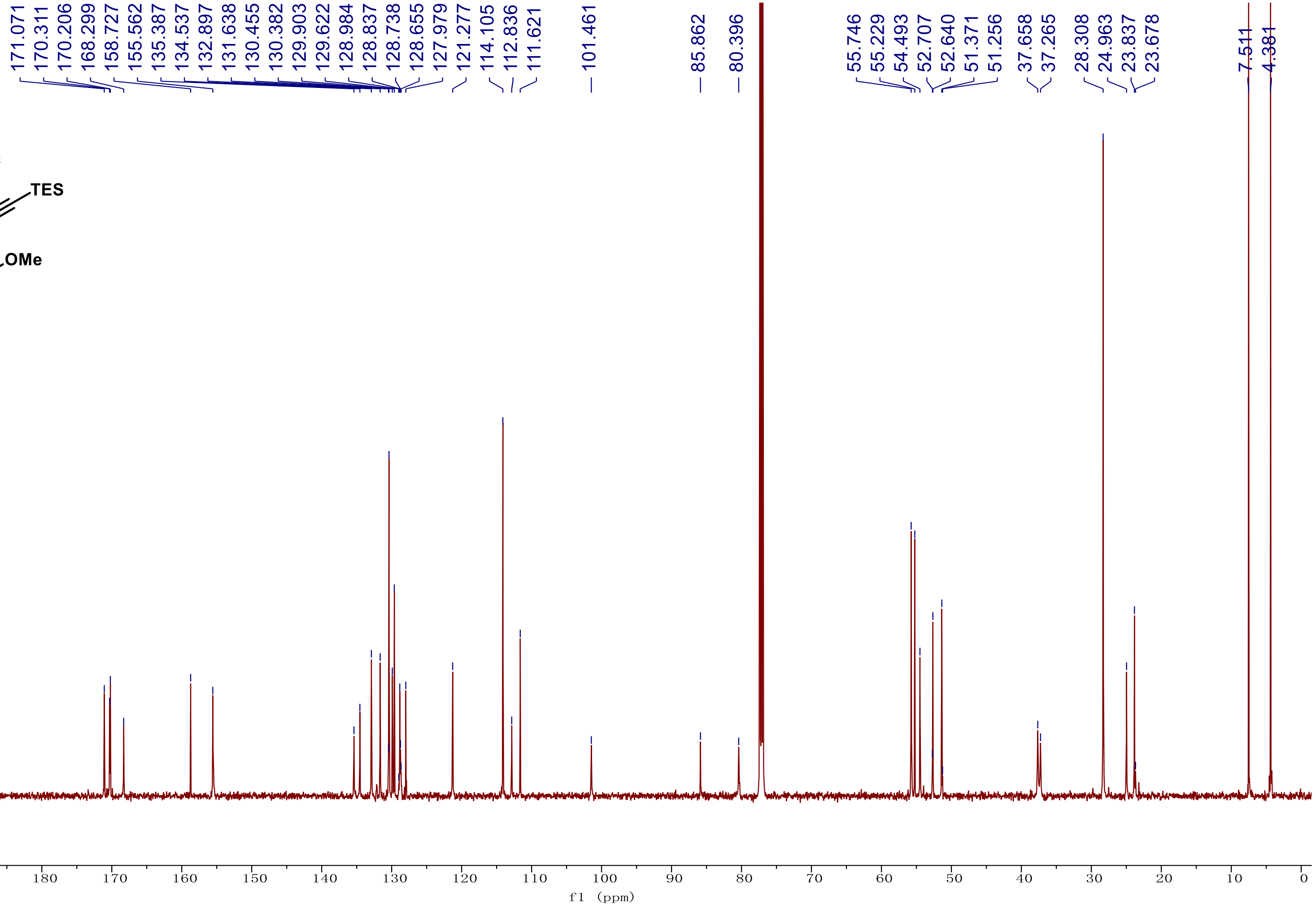

Compound 24i ¹H NMR (600 MHz, $CDCl_3$)

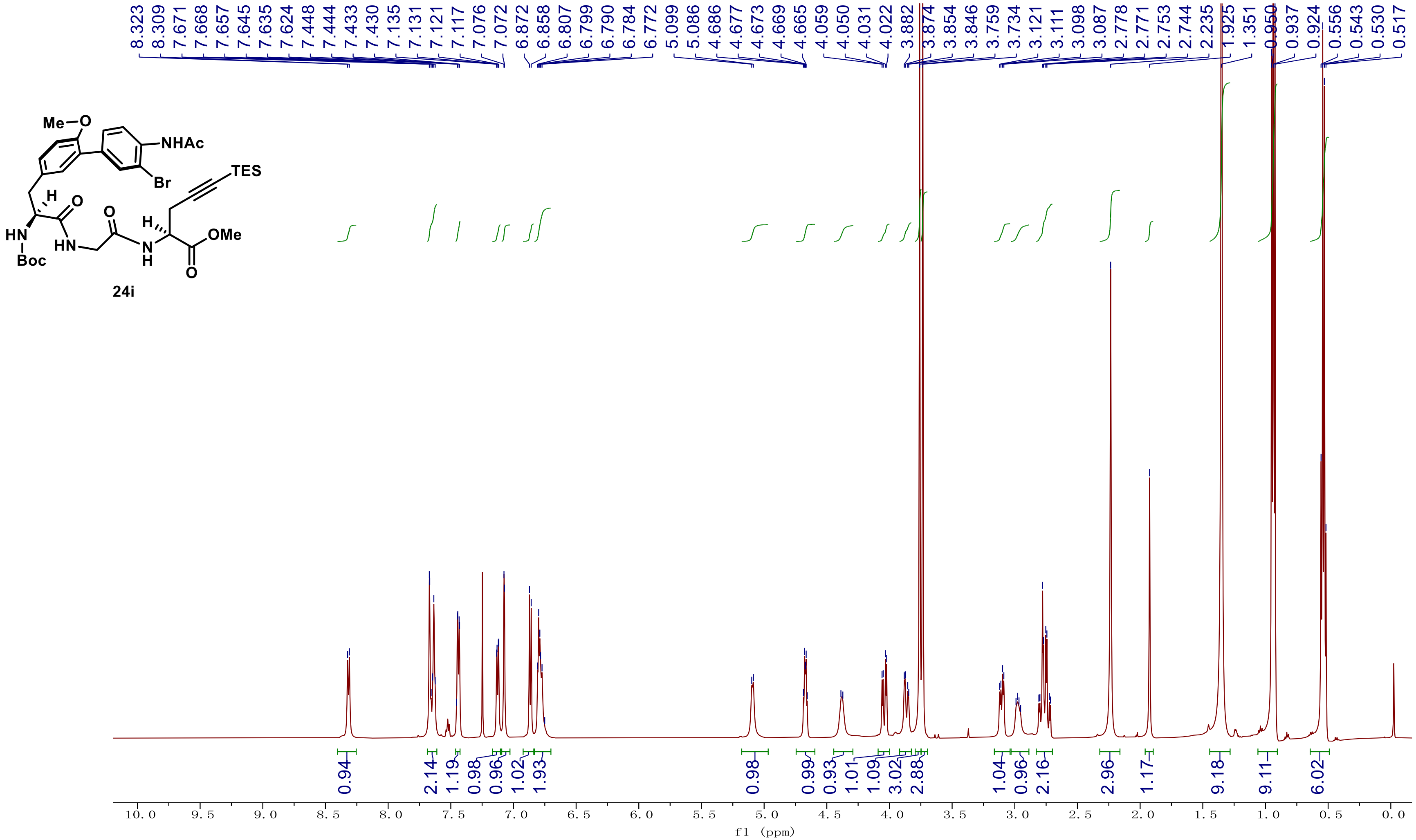

Compound 24i ^{13}C NMR (151 MHz, $CDCl_3$)

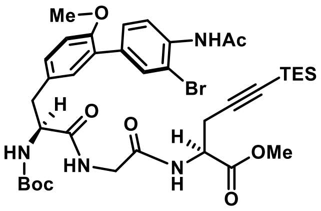

24i

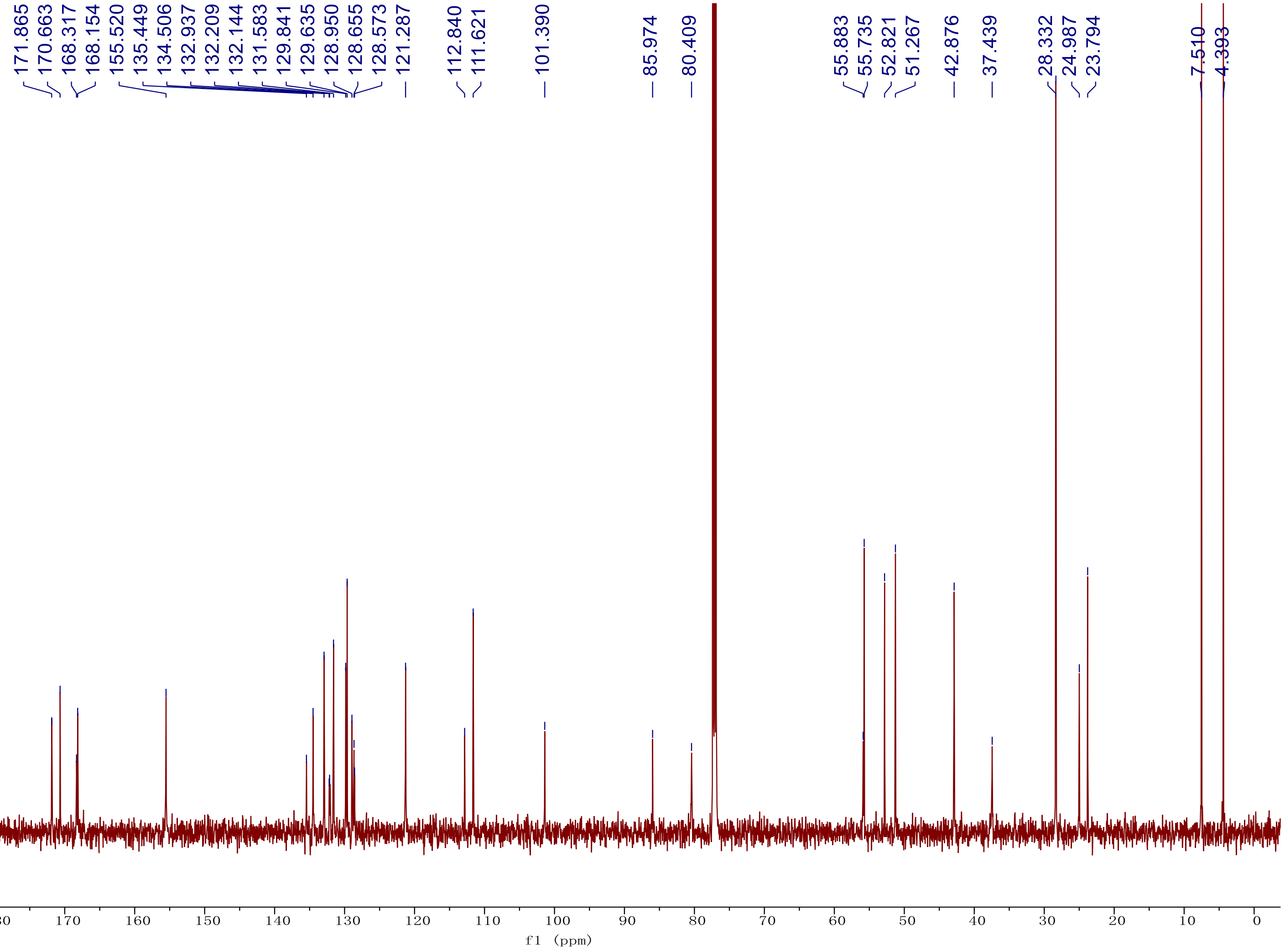

Compound 24j ^{1}H NMR (600 MHz, $CDCl_3$)

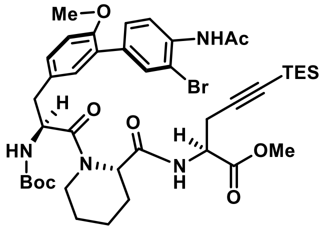

24j

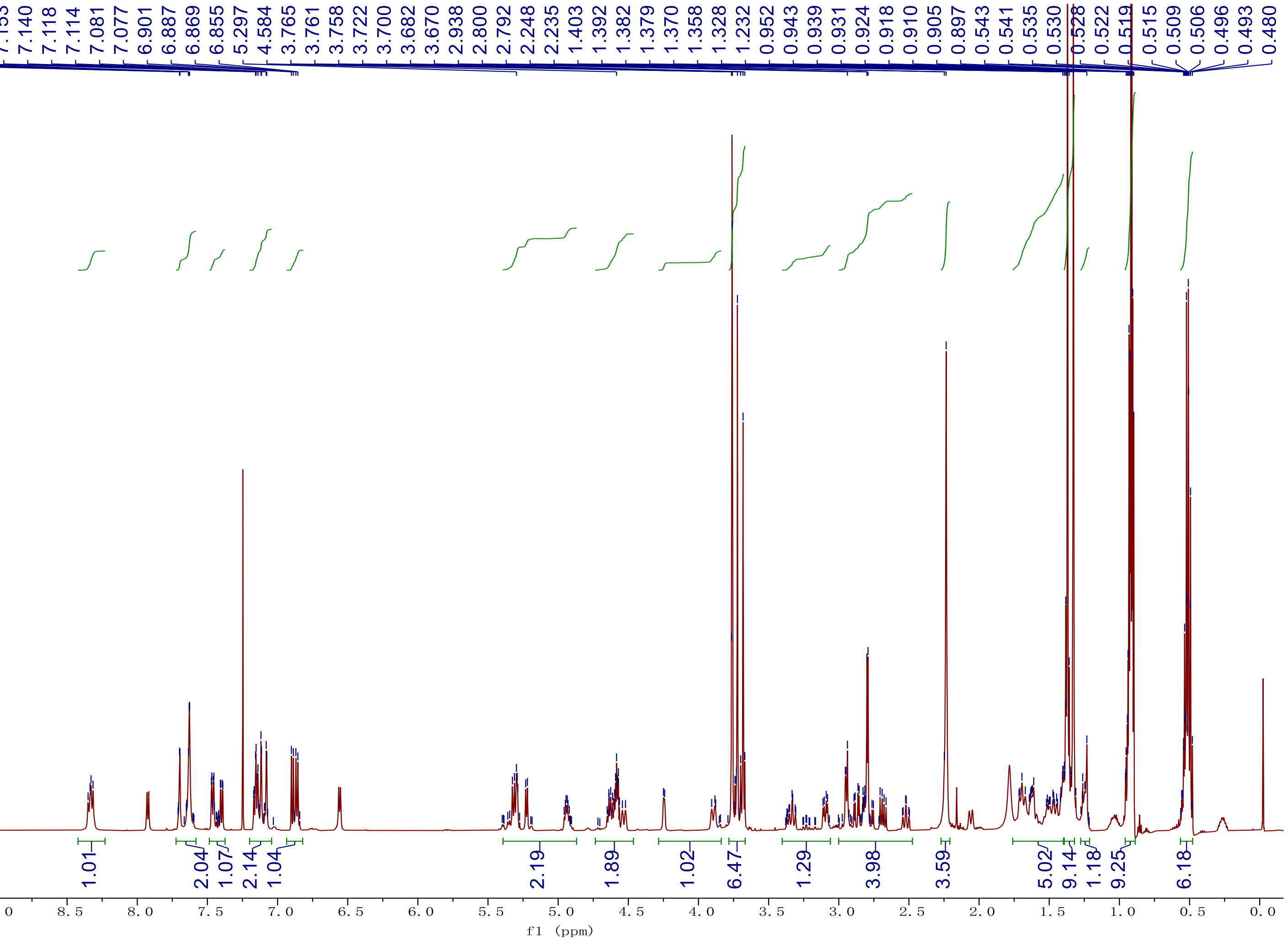

Compound 24j ^{13}C NMR (151 MHz, $CDCl_3$)

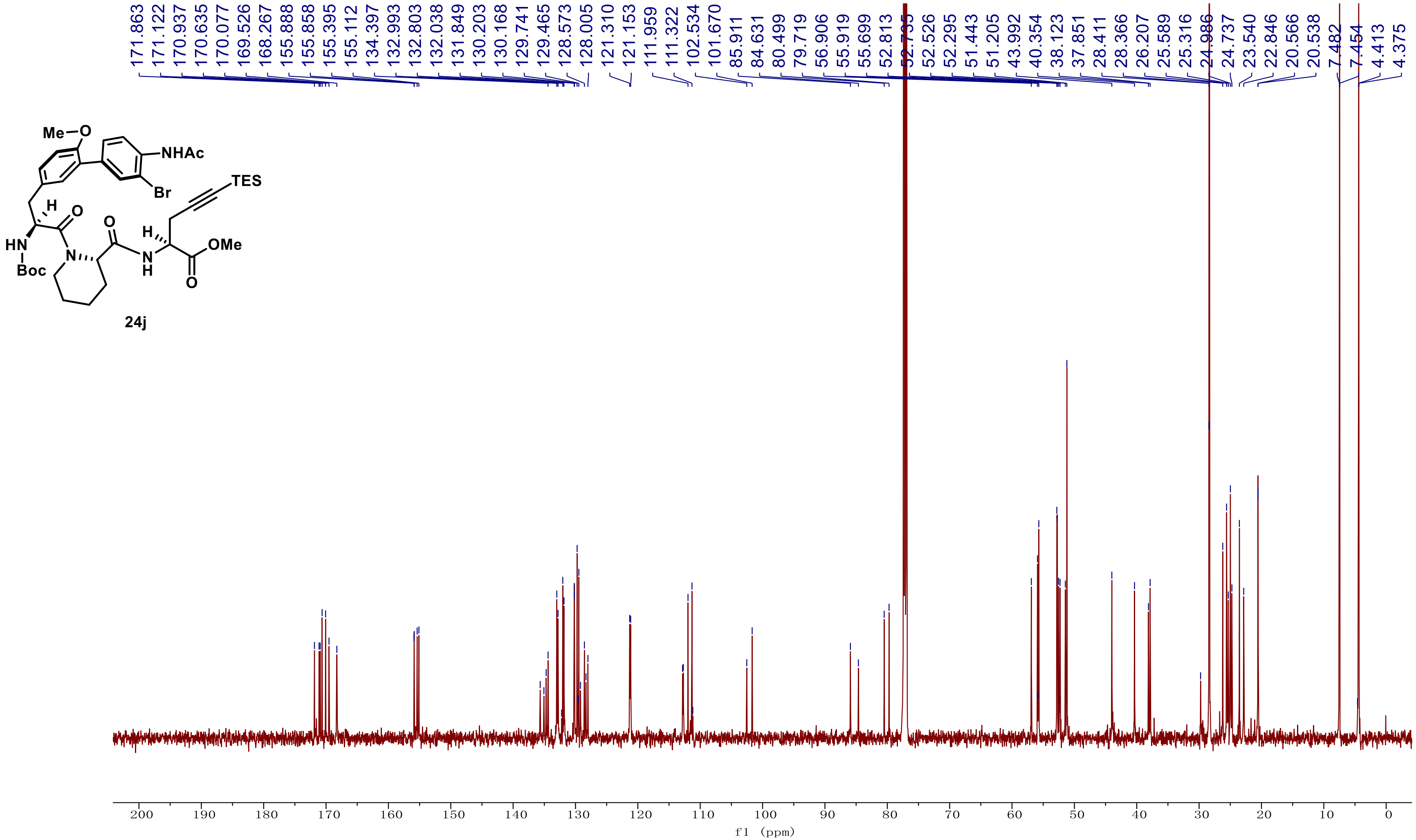

Compound 24k ^{1}H NMR (600 MHz, CDCl$_3$)

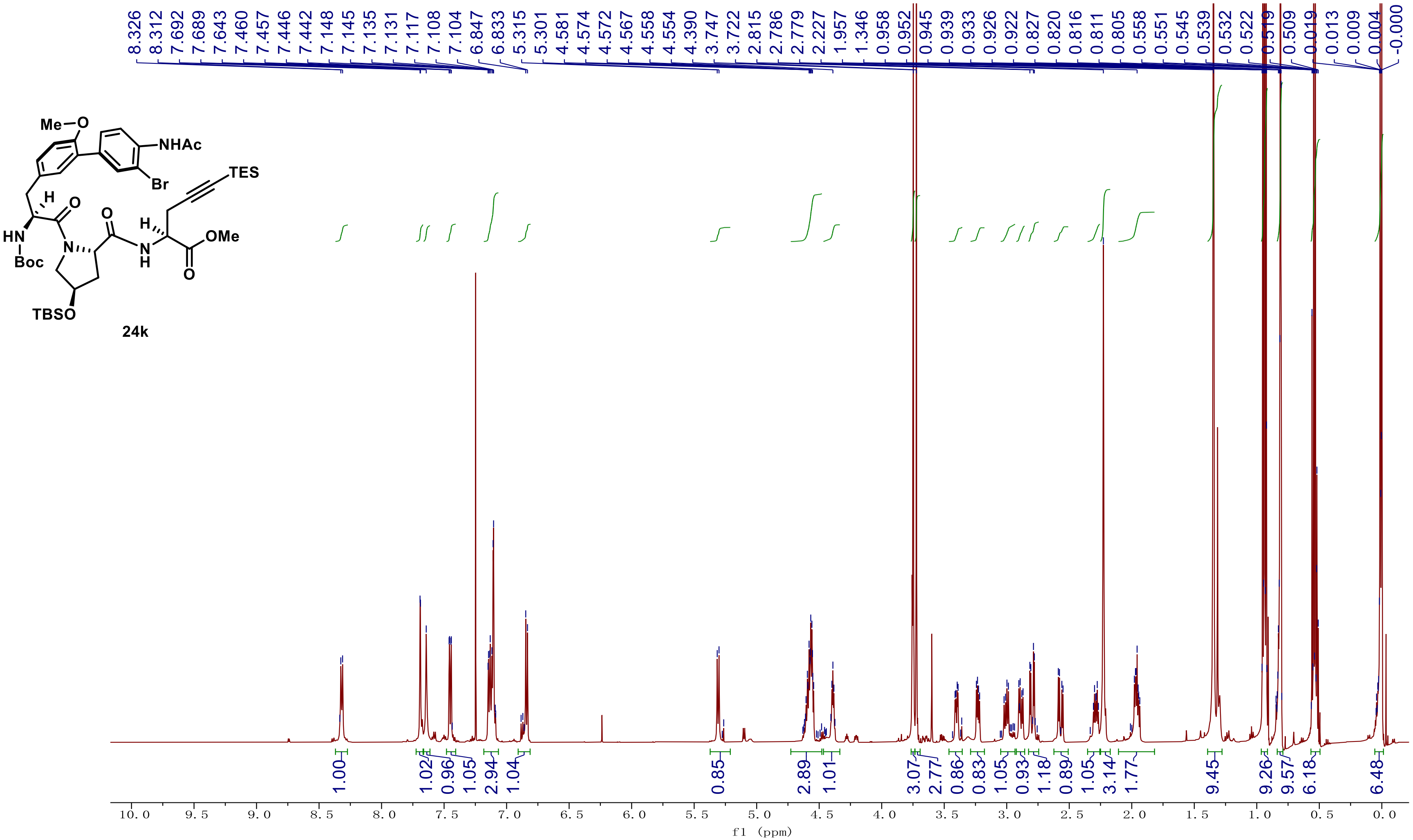

Compound 24k ^{13}C NMR (151 MHz, $CDCl_3$)

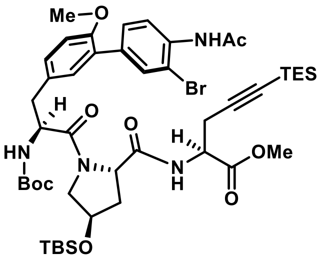

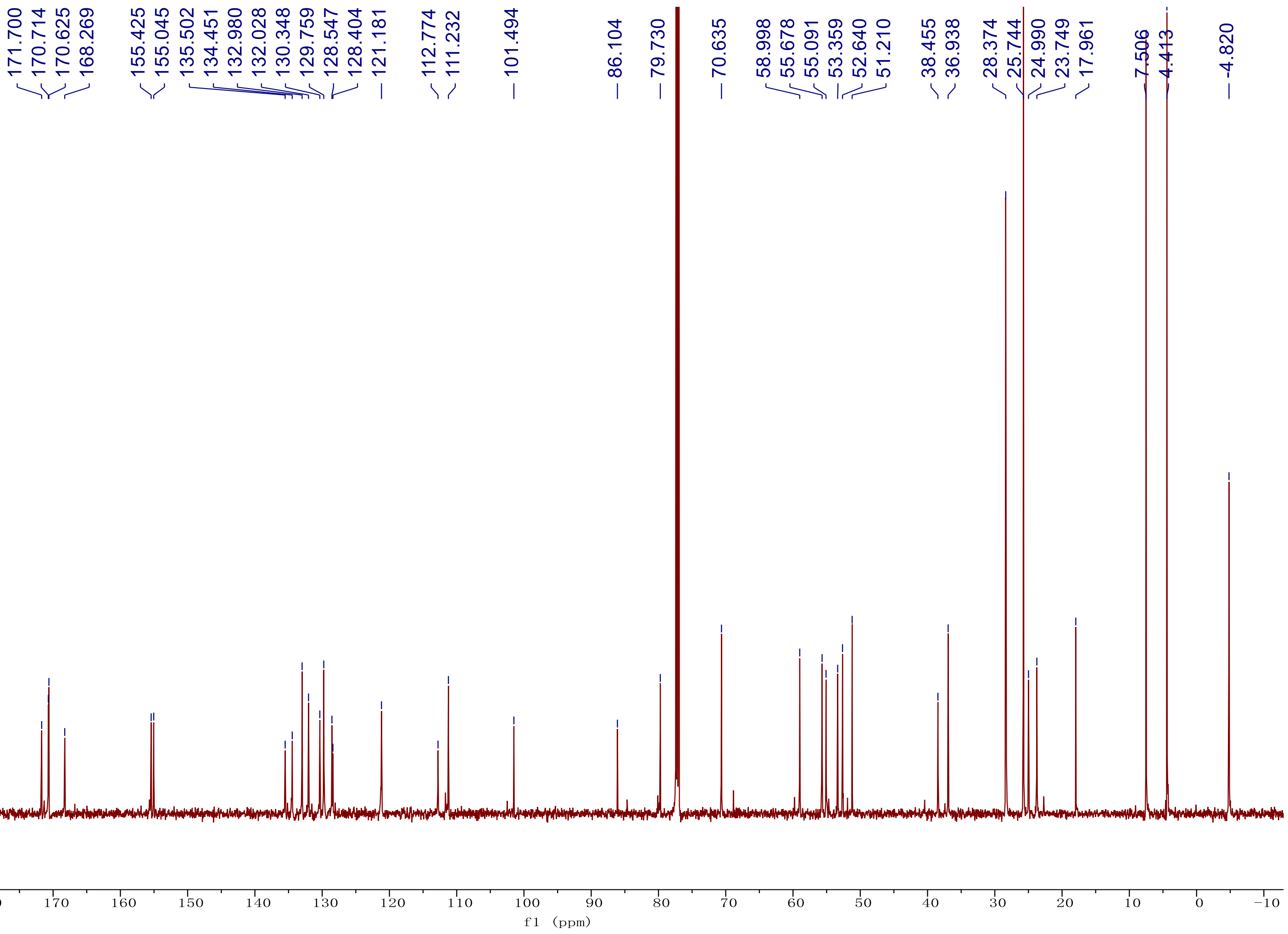

Compound 24l ¹H NMR (600 MHz, CDCl₃)

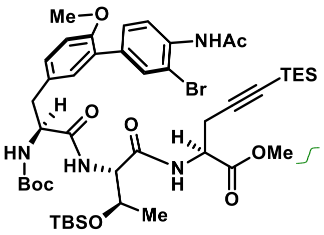

24l

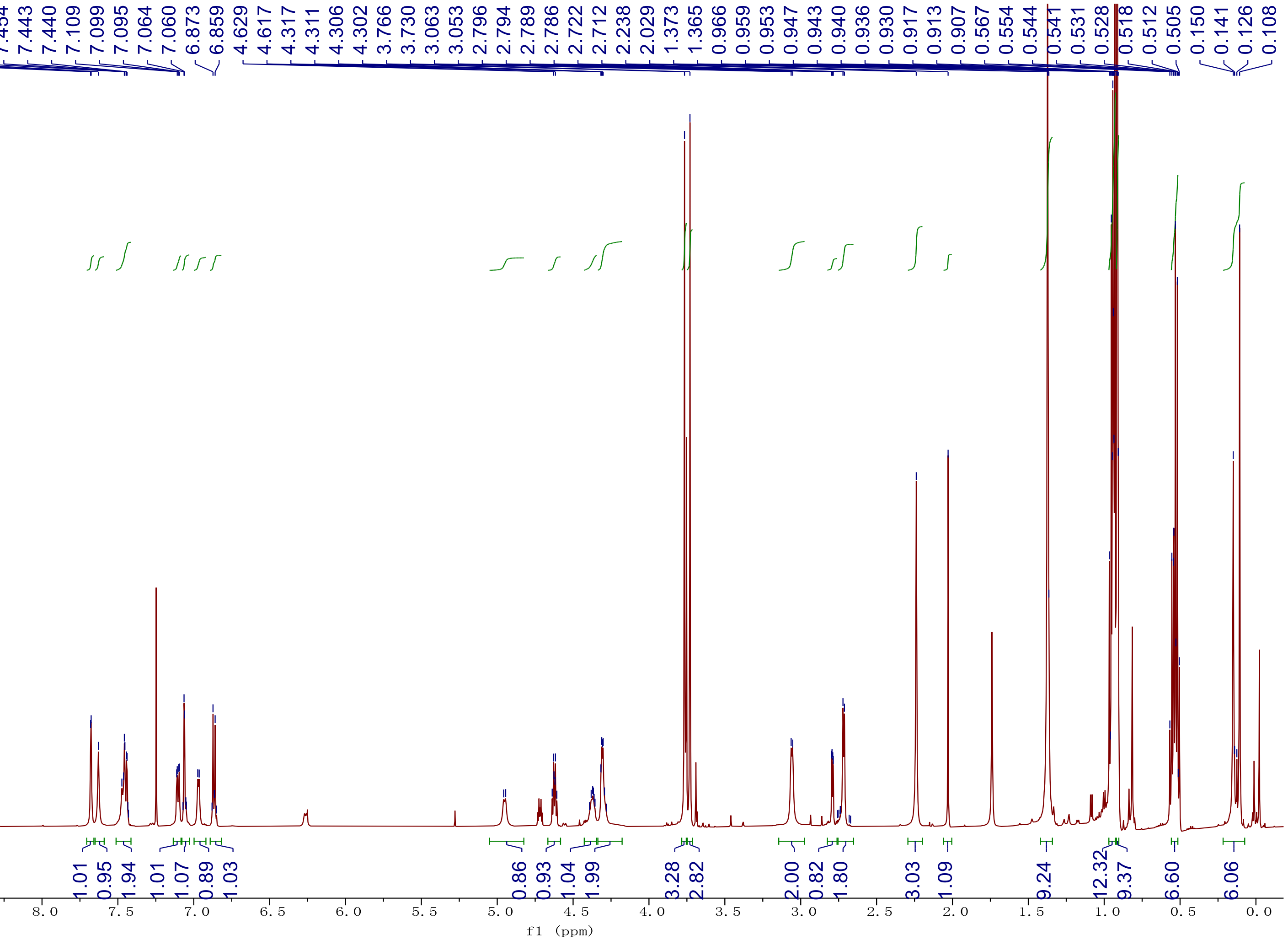

Compound 24l ^{13}C NMR (151 MHz, $CDCl_3$)

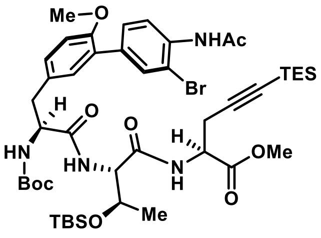

24l

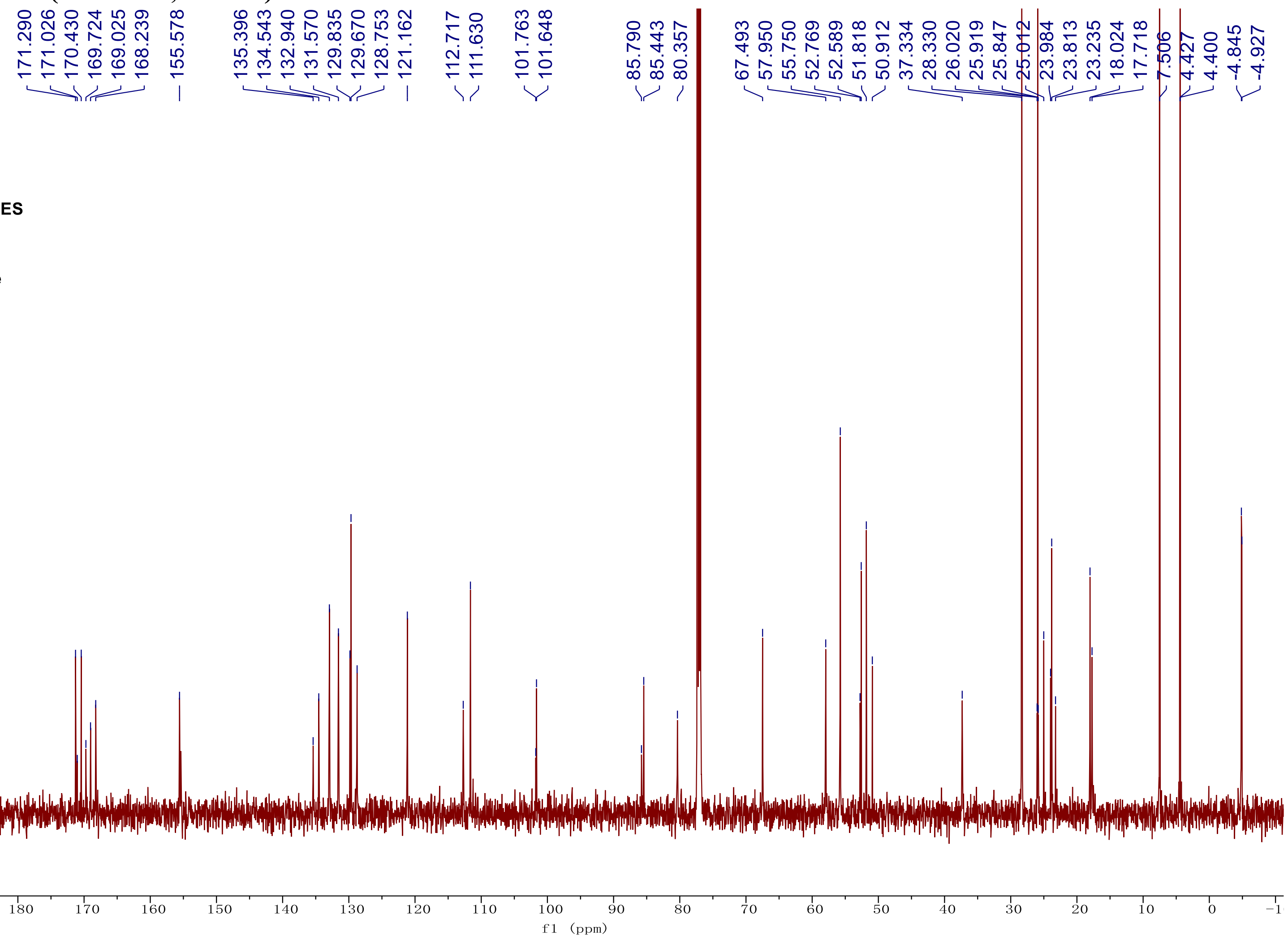

Compound 24m ^{1}H NMR (600 MHz, $CDCl_3$)

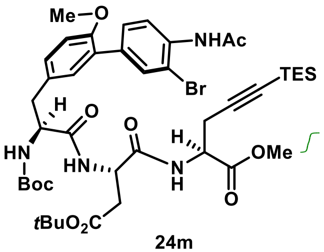

24m

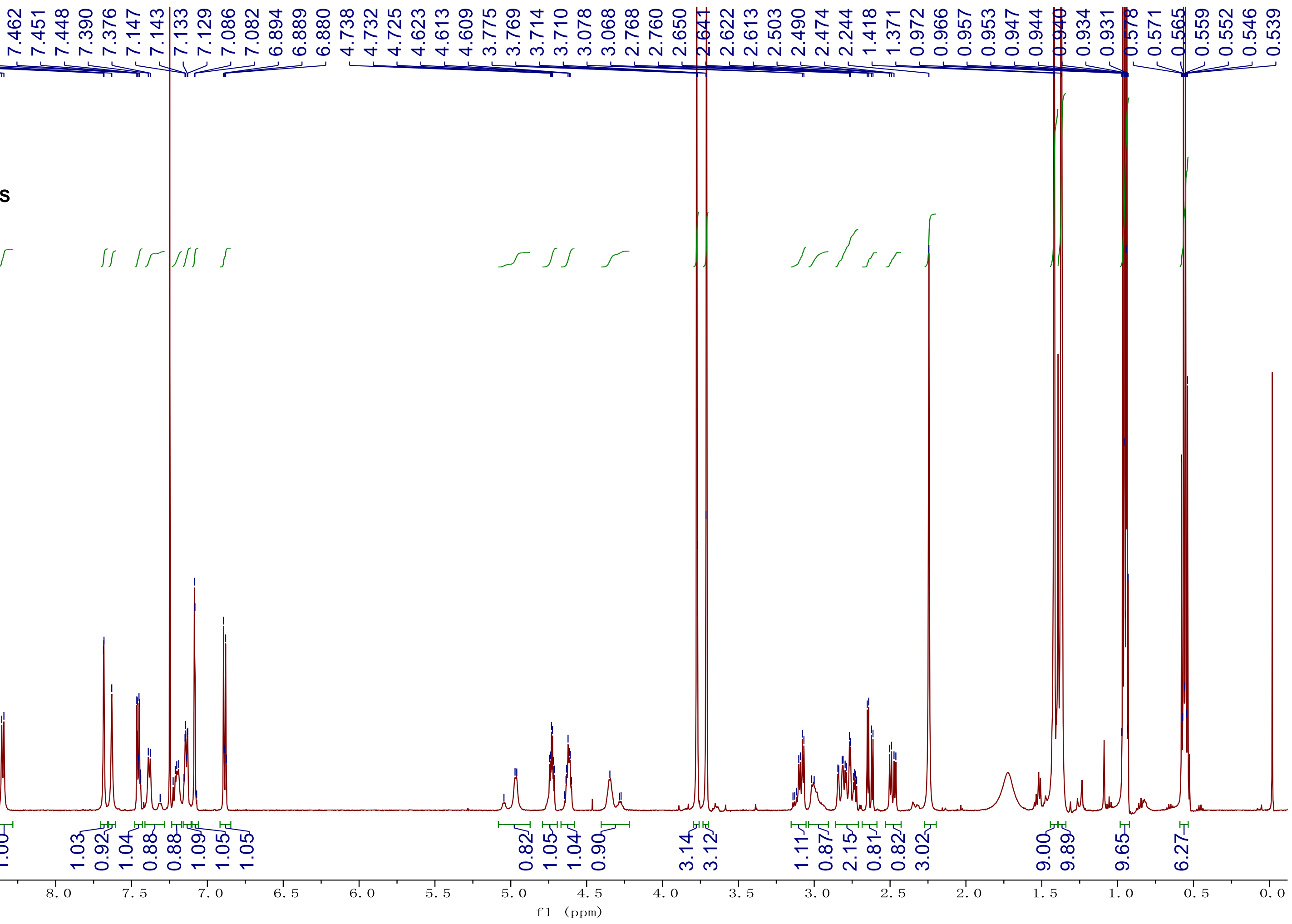

Compound 24m ^{13}C NMR (151 MHz, $CDCl_3$)

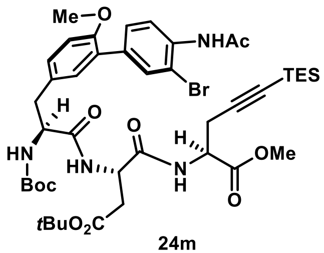

24m

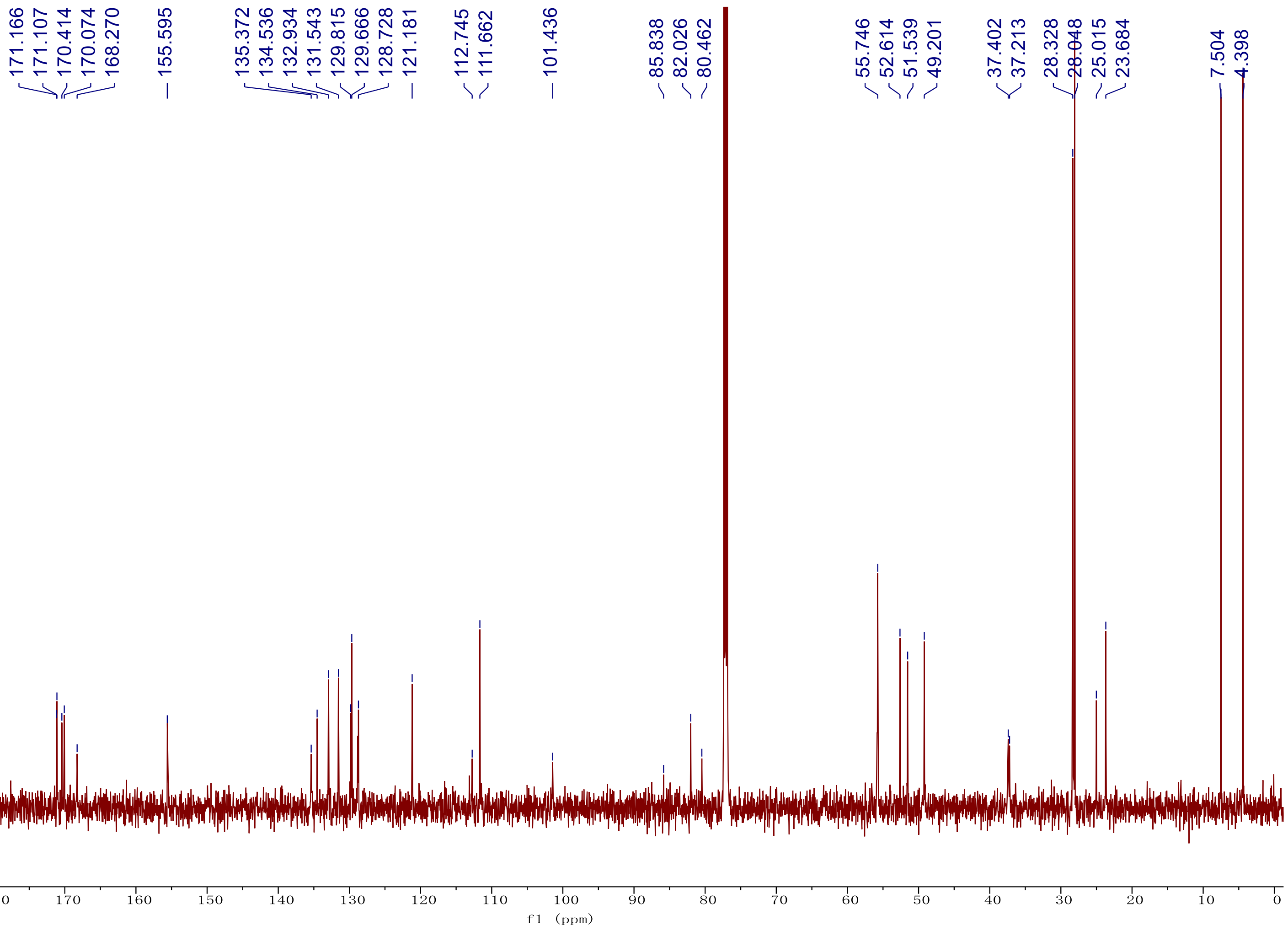

Compound 24n ¹H NMR (600 MHz, $CDCl_3$)

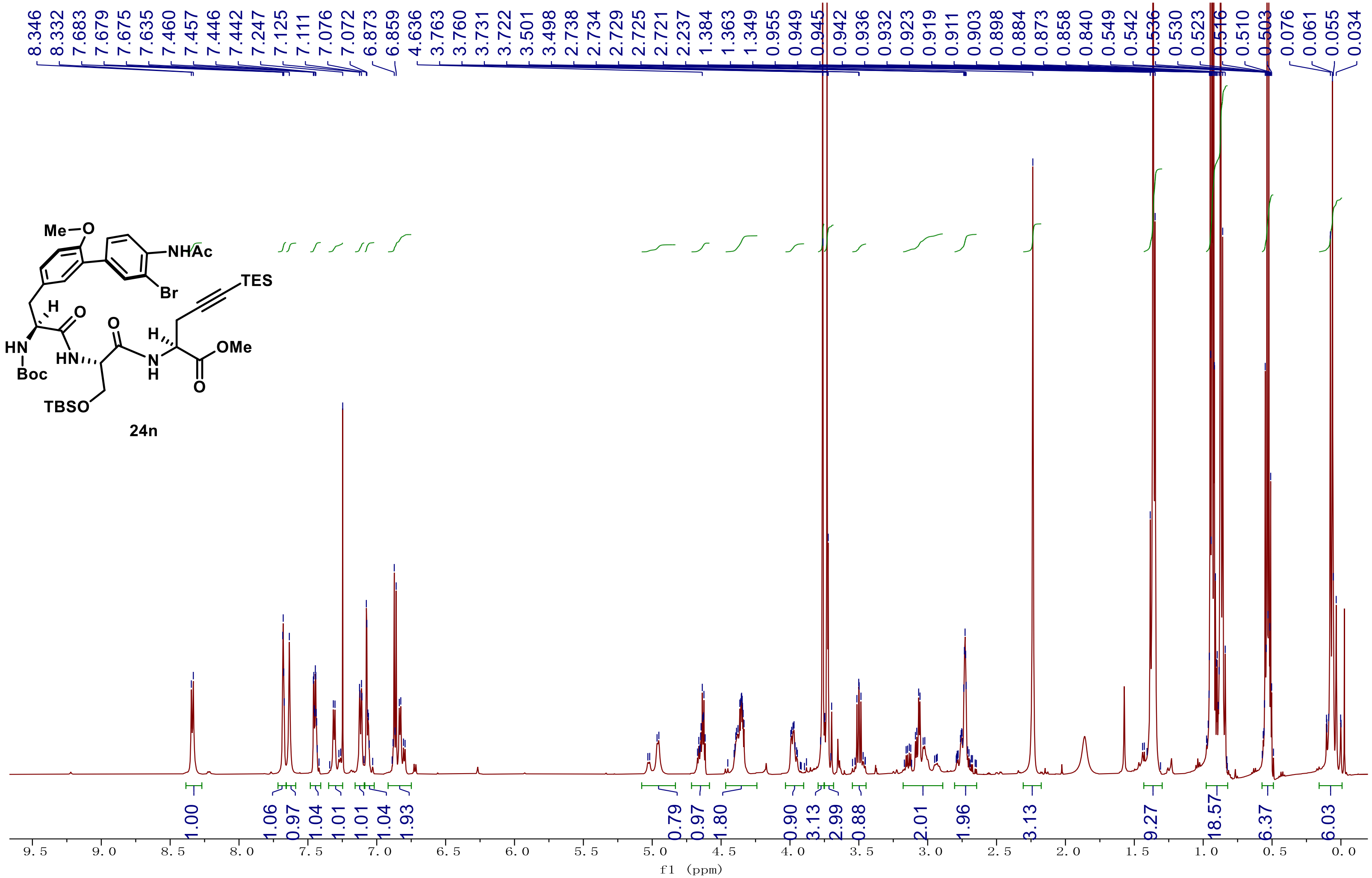

Compound 24n ^{13}C NMR (151 MHz, $CDCl_3$)

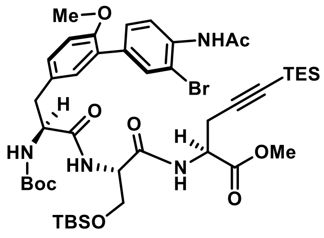

24n

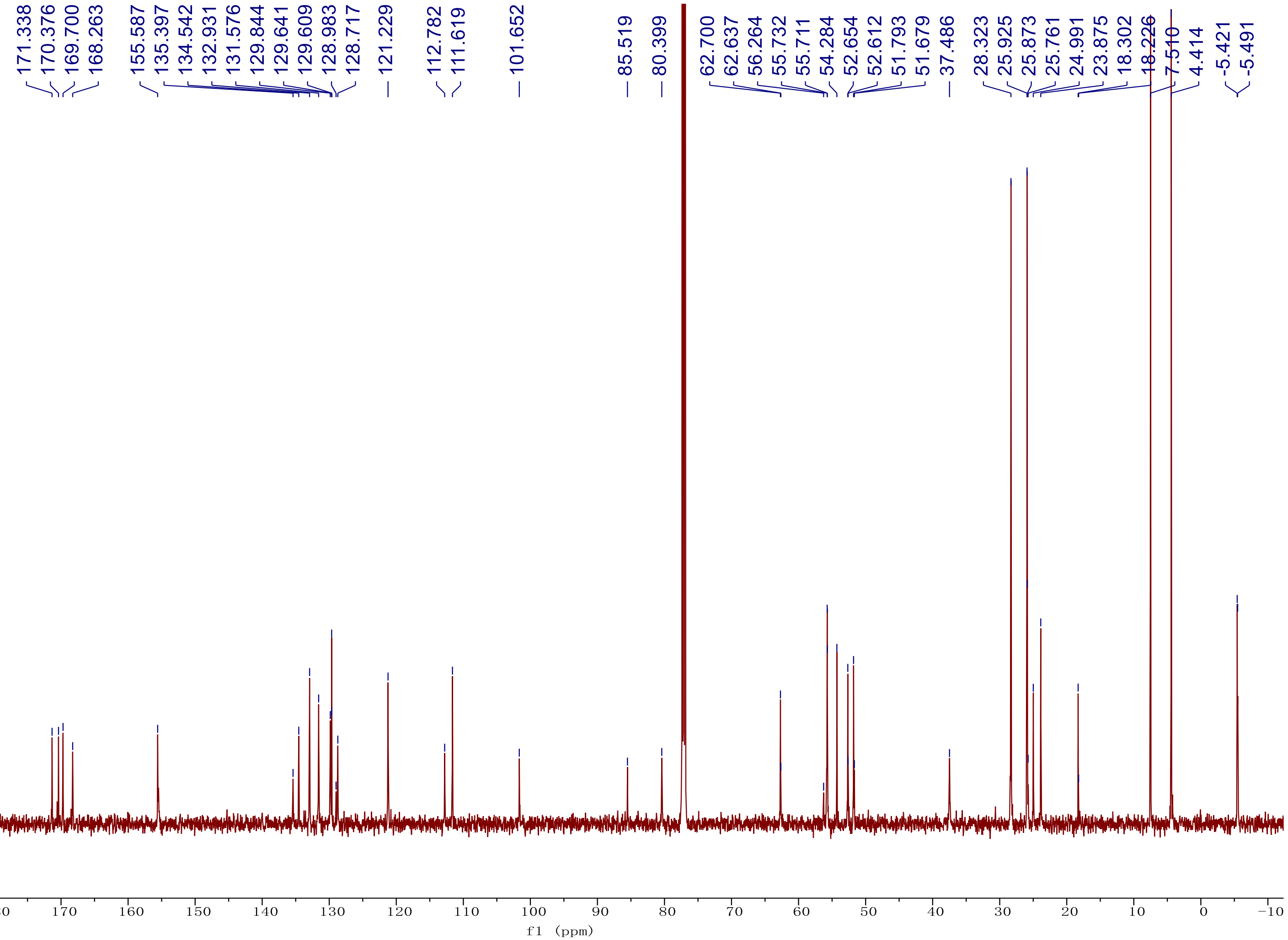

Compound 24o ¹H NMR (600 MHz, CDCl₃)

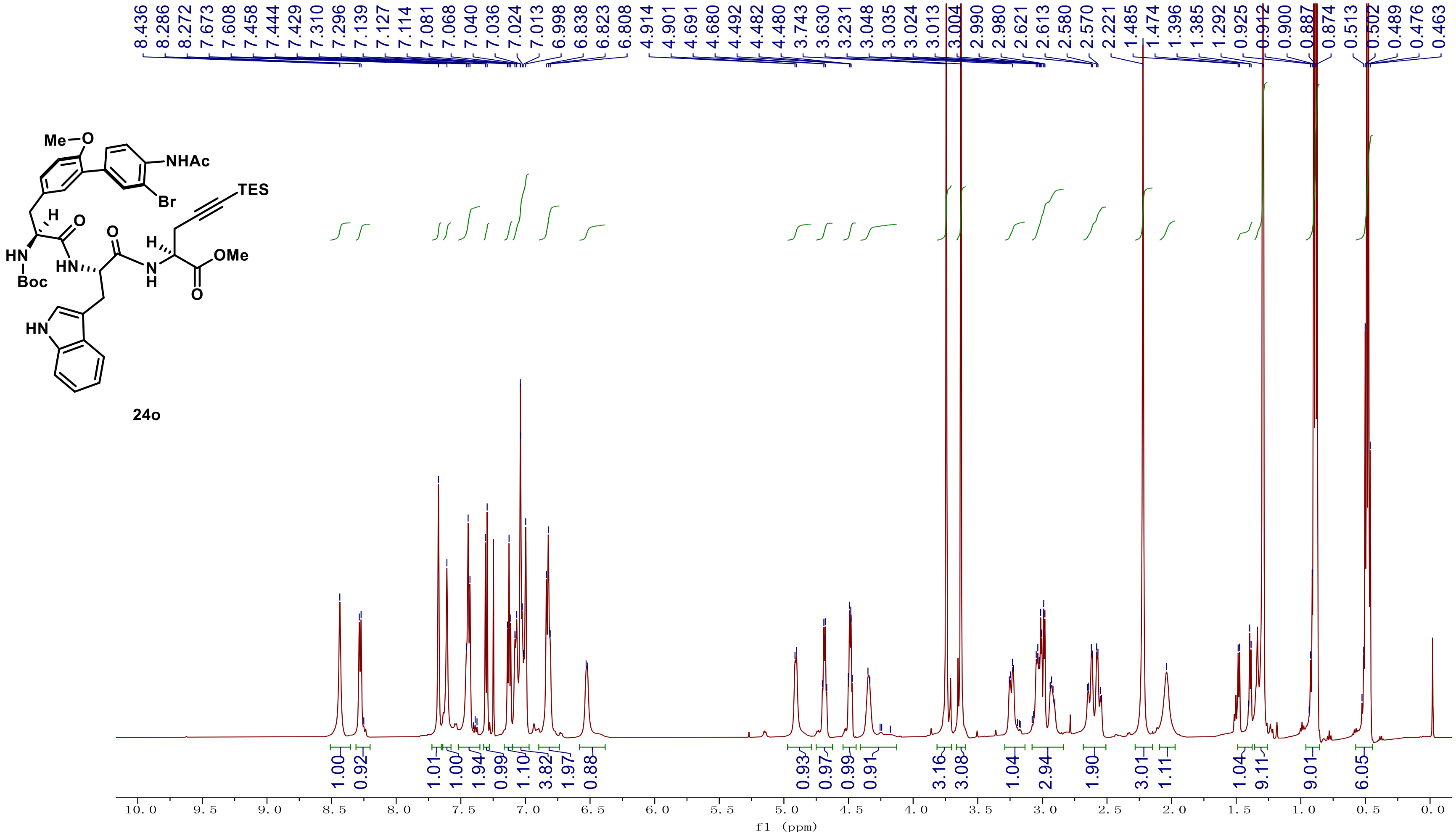

Compound 24o ^{13}C NMR (151 MHz, $CDCl_3$)

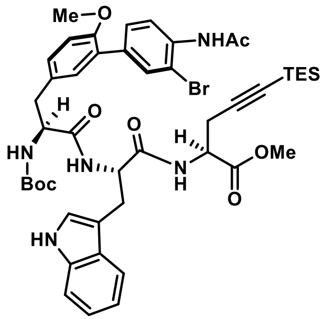

24o

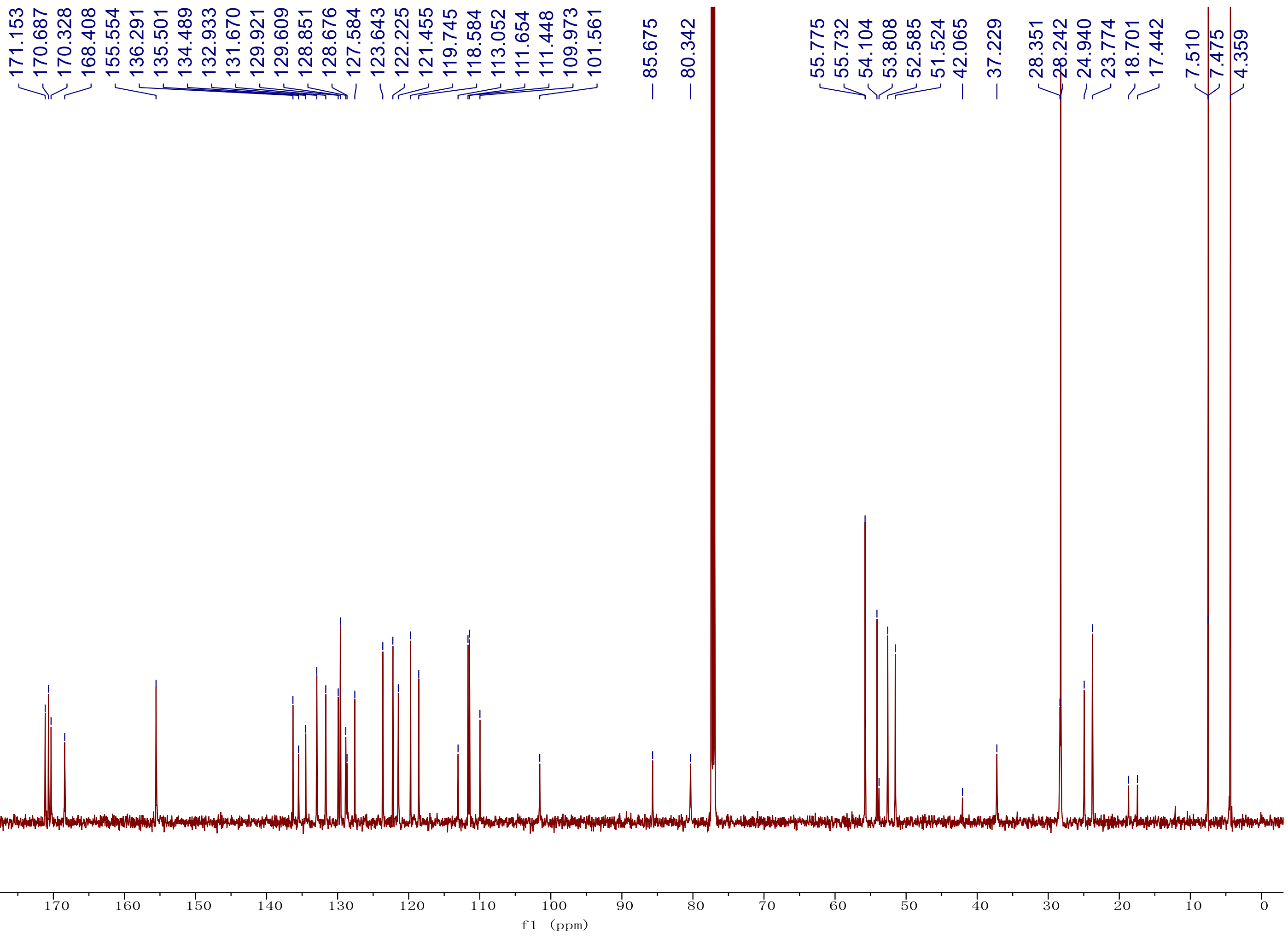

Compound 24p ^{1}H NMR (600 MHz, $CDCl_3$)

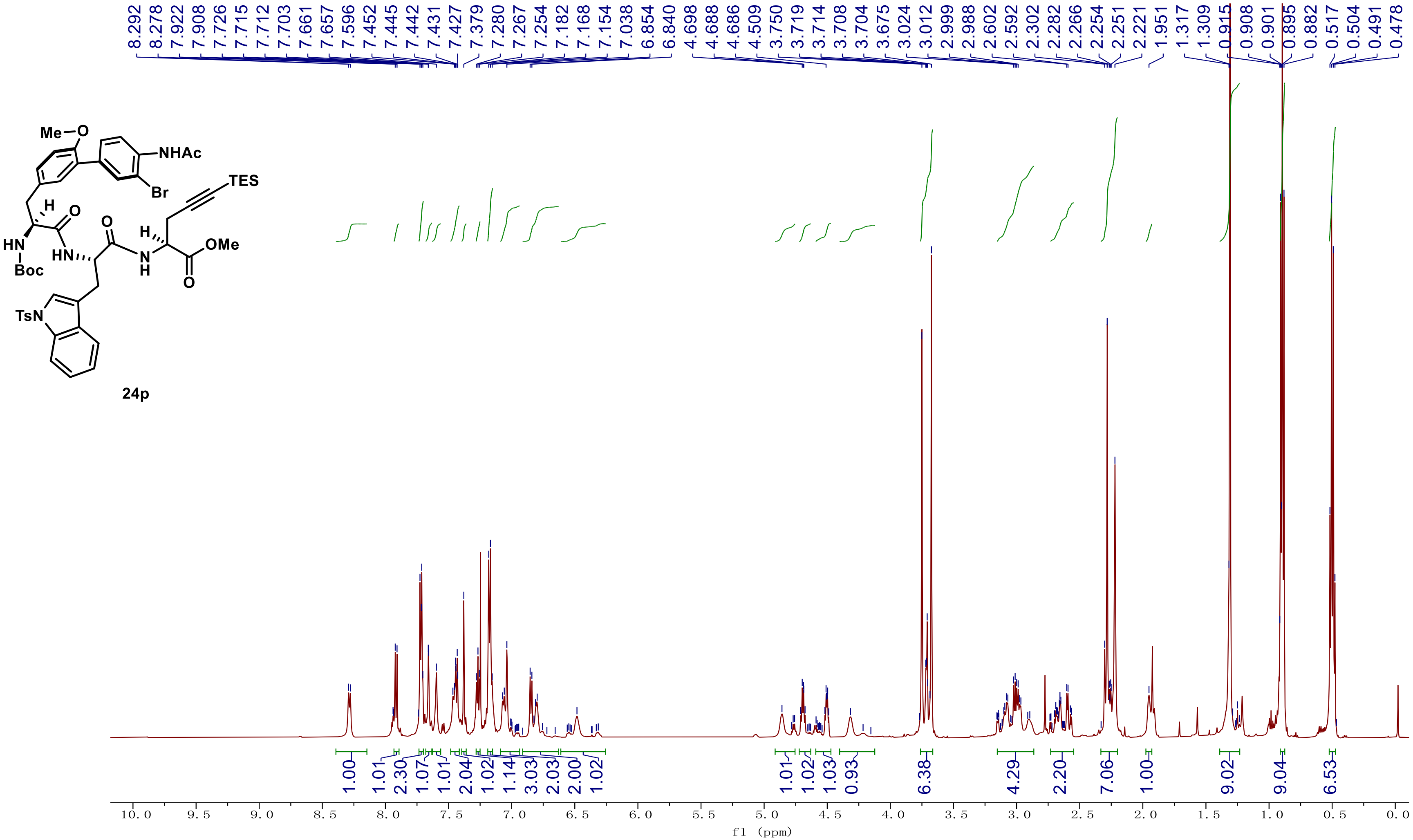

Compound 24p ^{13}C NMR (151 MHz, $CDCl_3$)

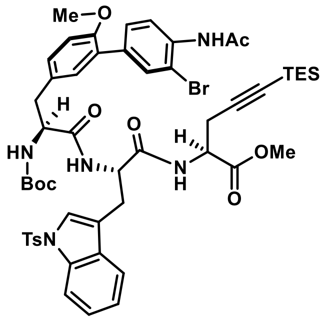

24p

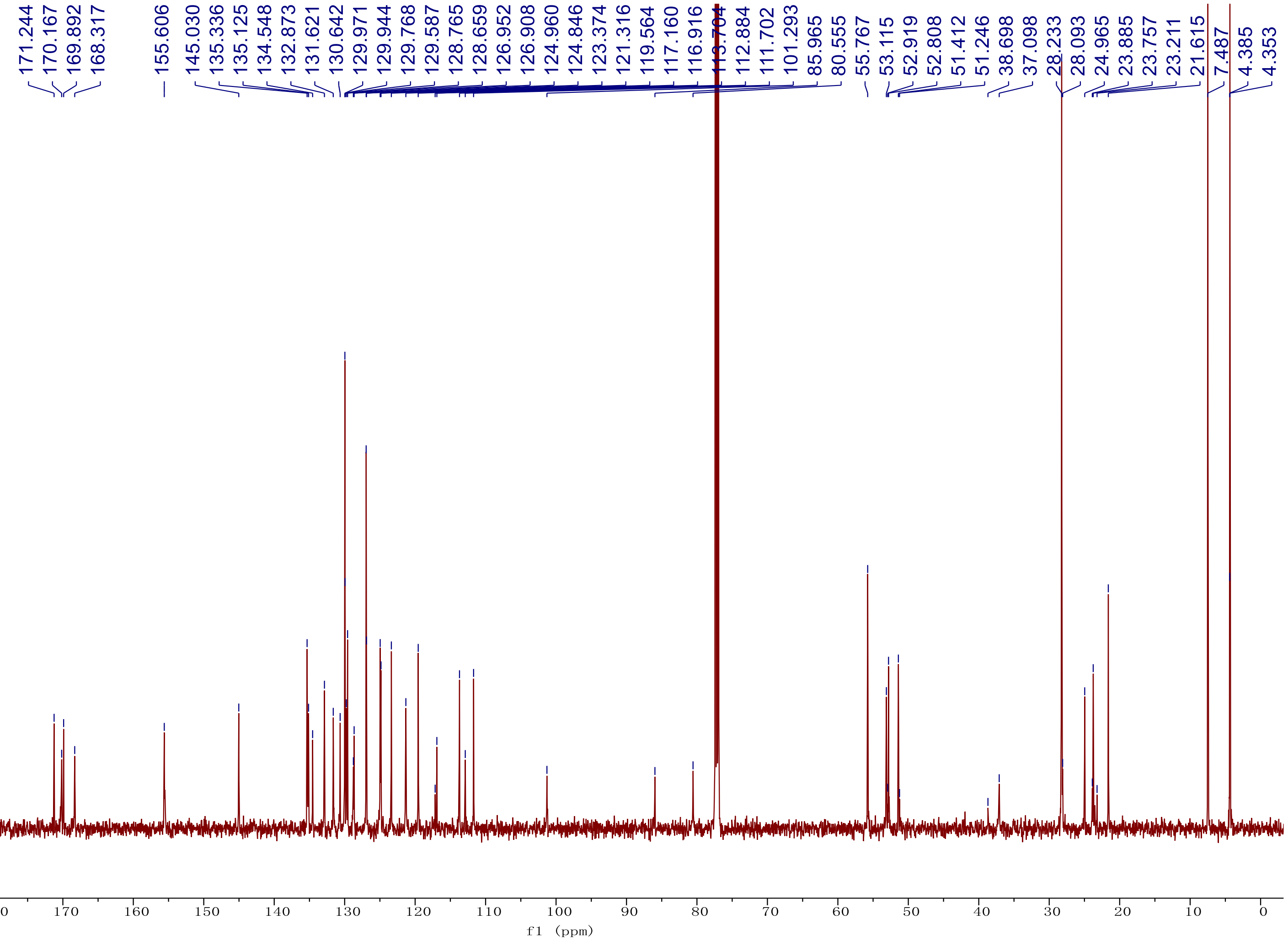

Compound 26a ^{1}H NMR (400 MHz, $CDCl_3$)

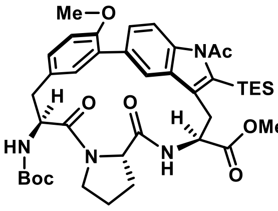

26a

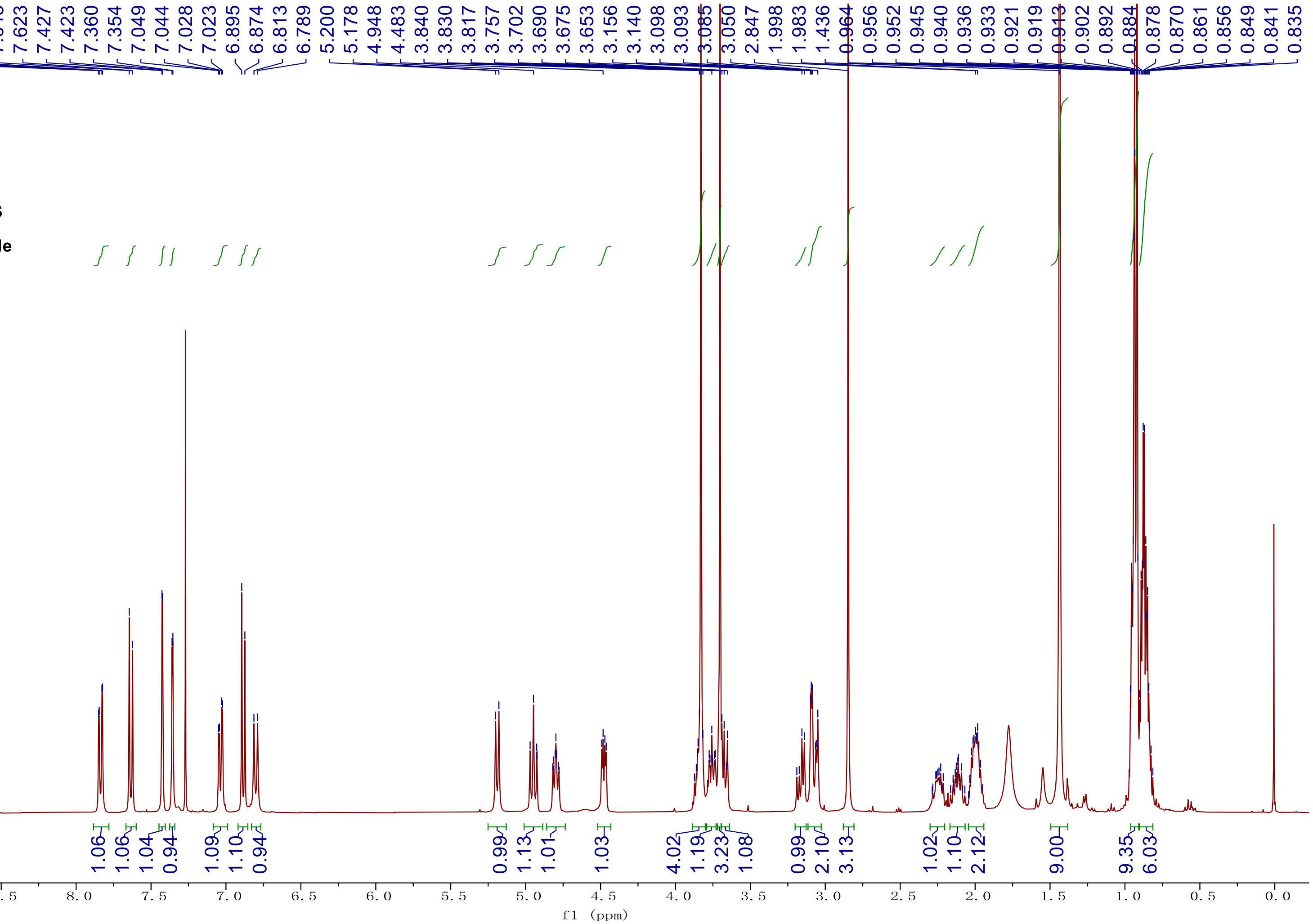

Compound 26a ^{13}C NMR (101 MHz, $CDCl_3$)

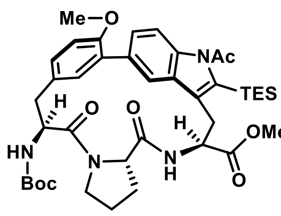

26a

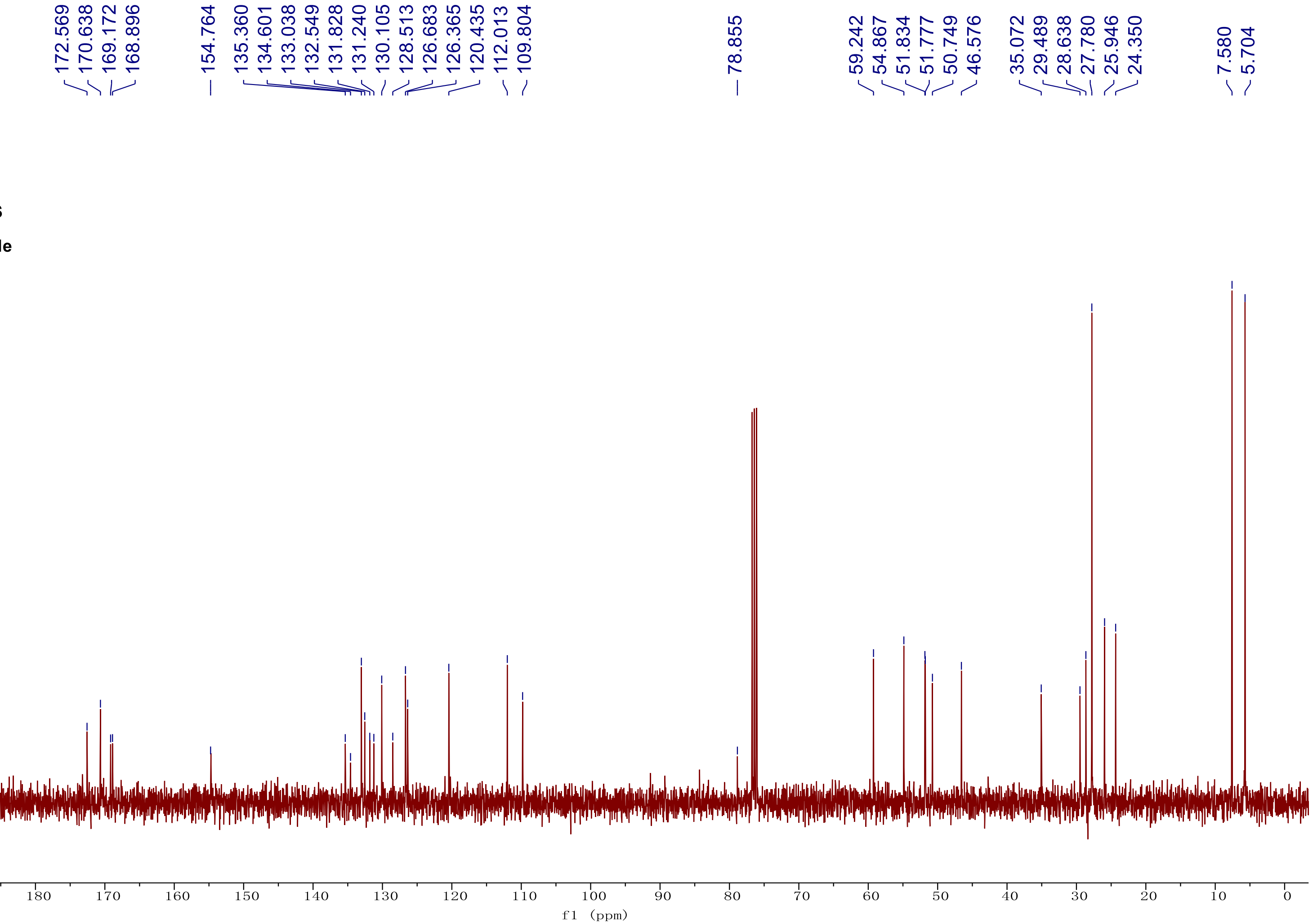

Compound 26b ^{1}H NMR (600 MHz, $CDCl_3$)

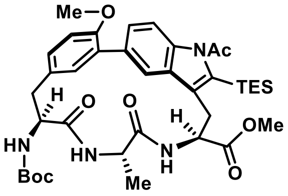

26b

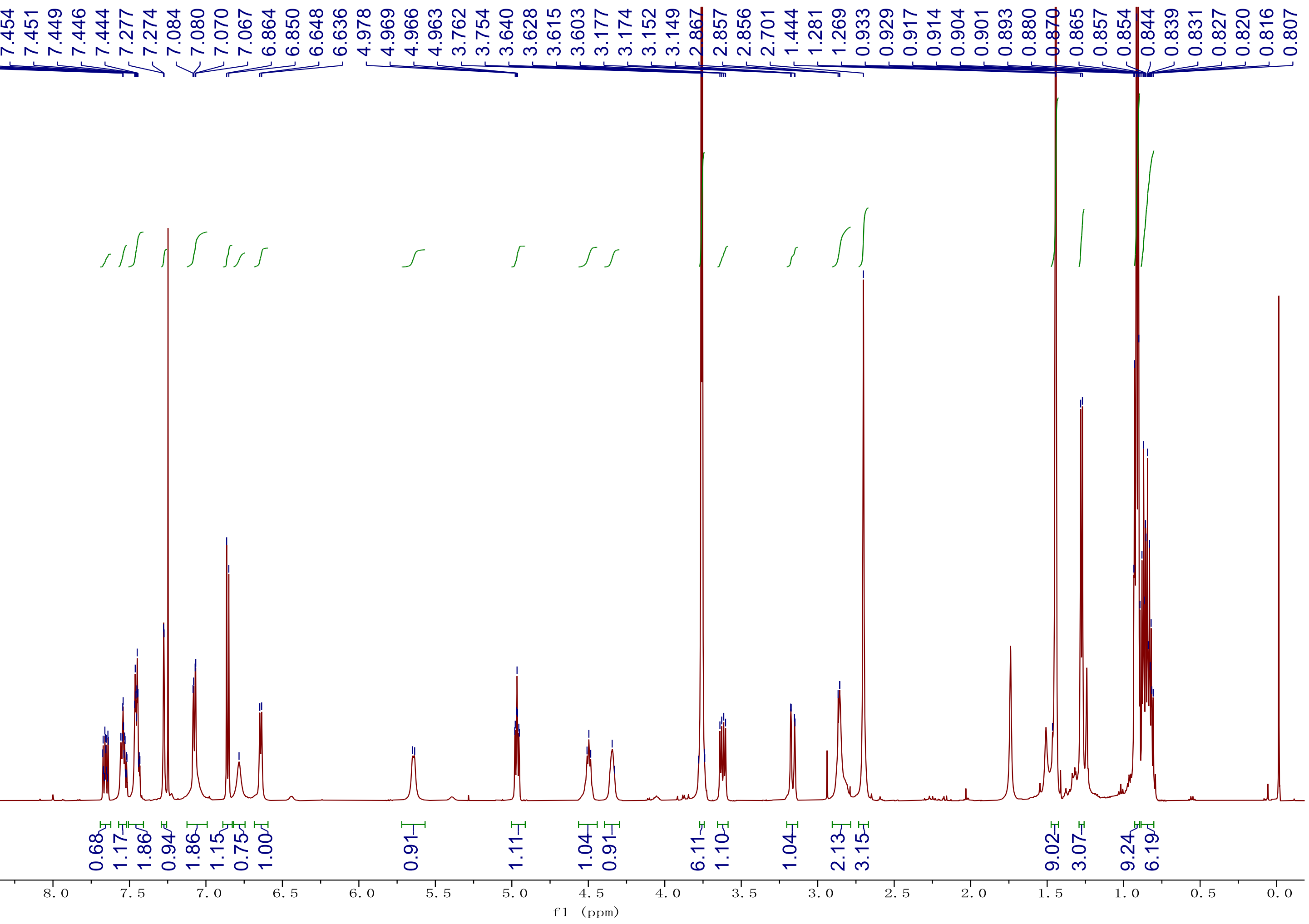

Compound 26b ^{13}C NMR (151 MHz, $CDCl_3$)

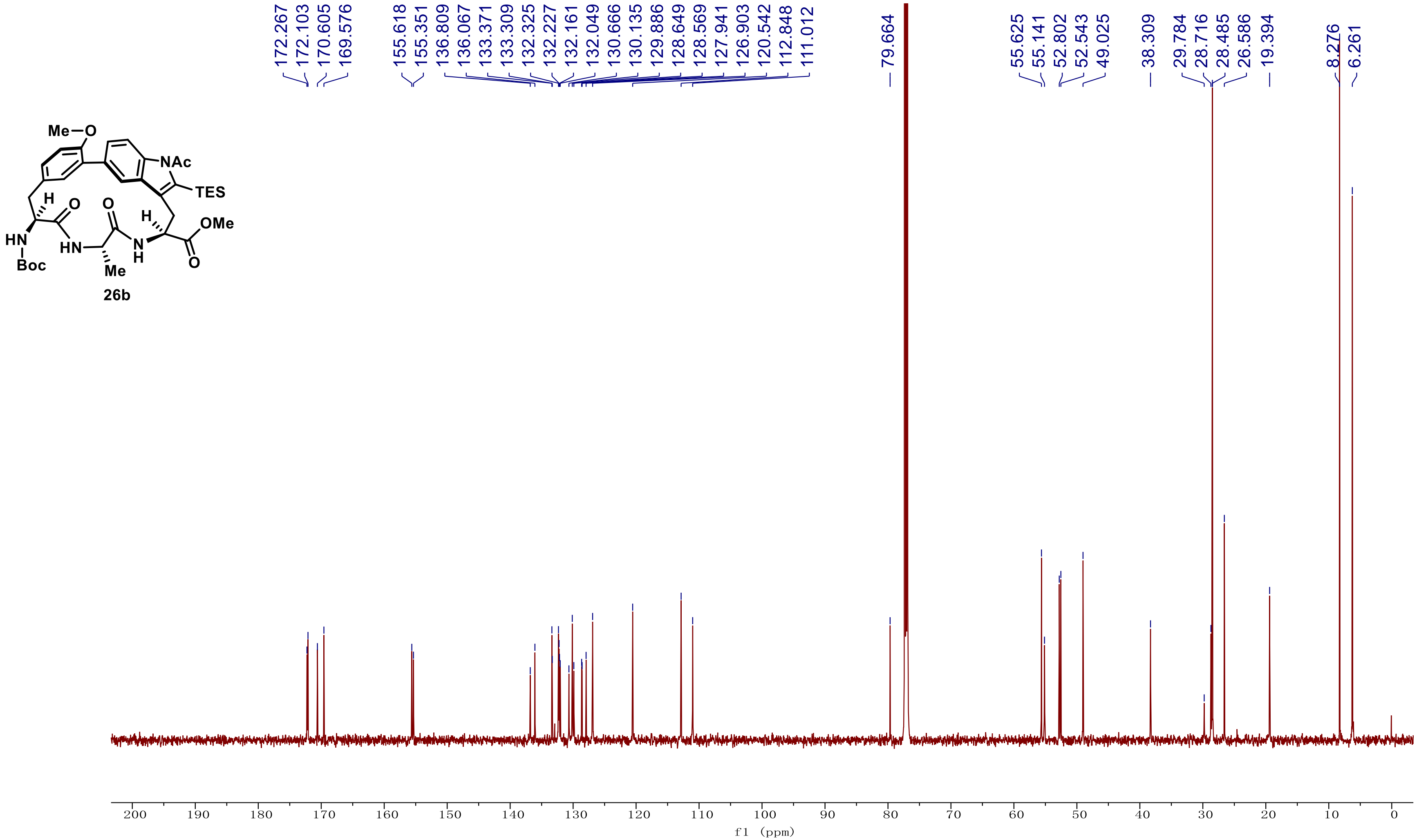

Compound 26c ¹H NMR (600 MHz, CDCl₃)

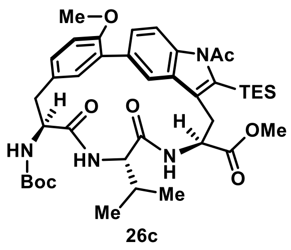

26c

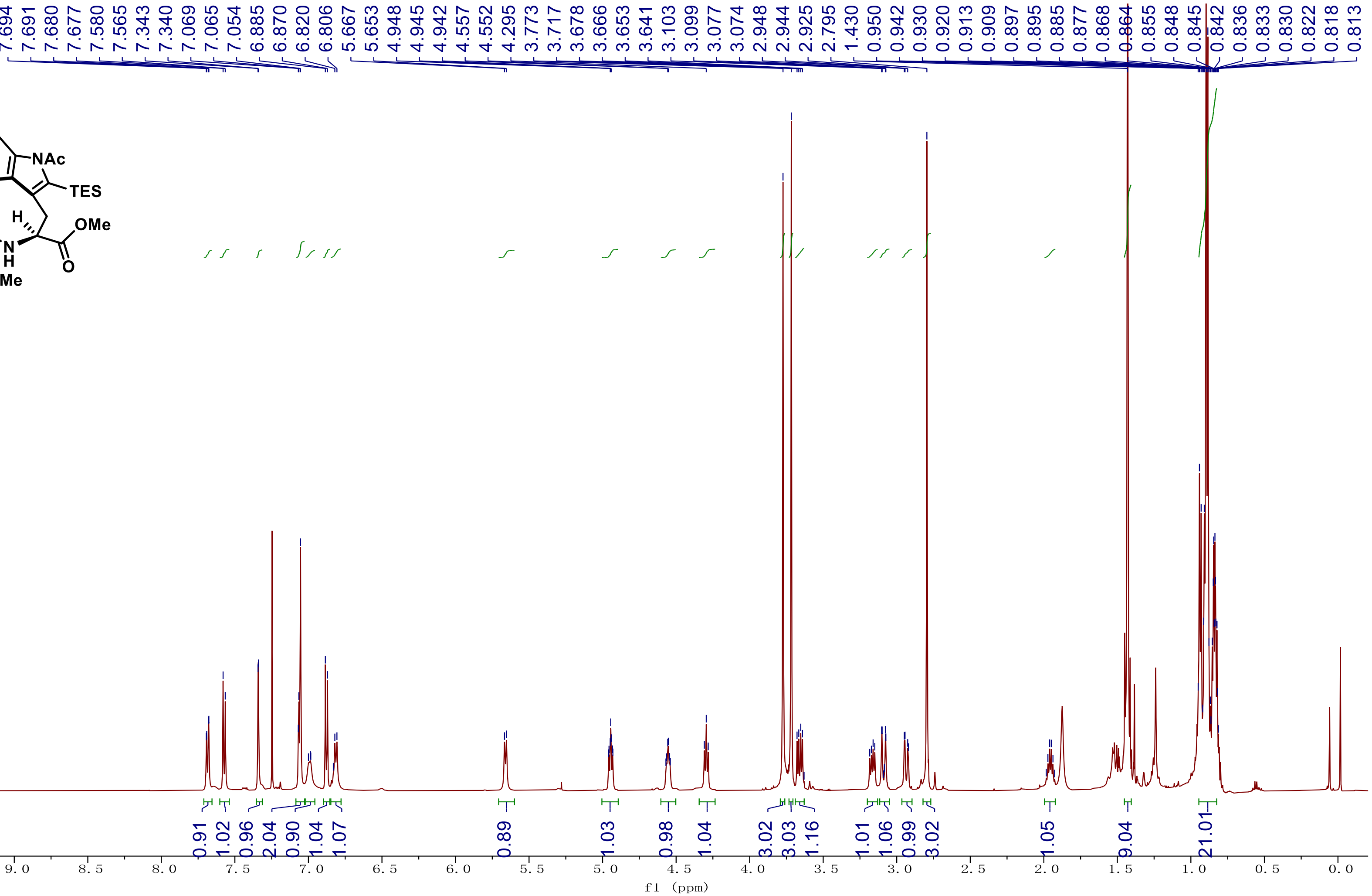

Compound 26c ^{13}C NMR (151 MHz, CDCl$_3$)

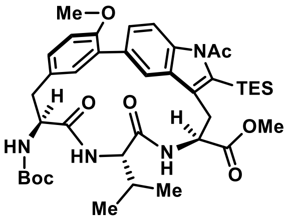

26c

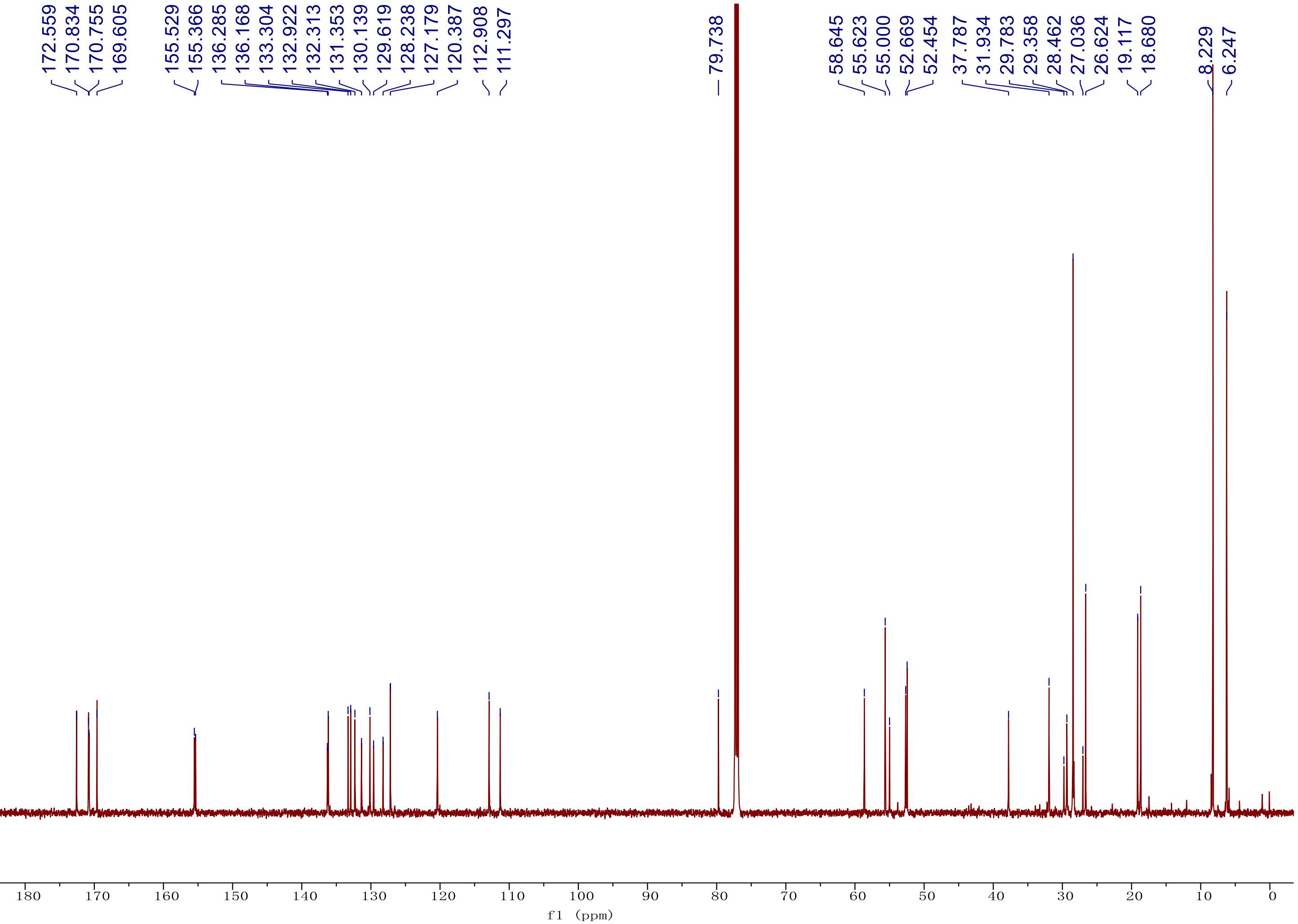

Compound 26c NOESY (400 MHz, $CDCl_3$)

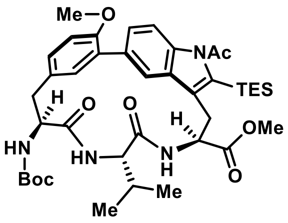

26c

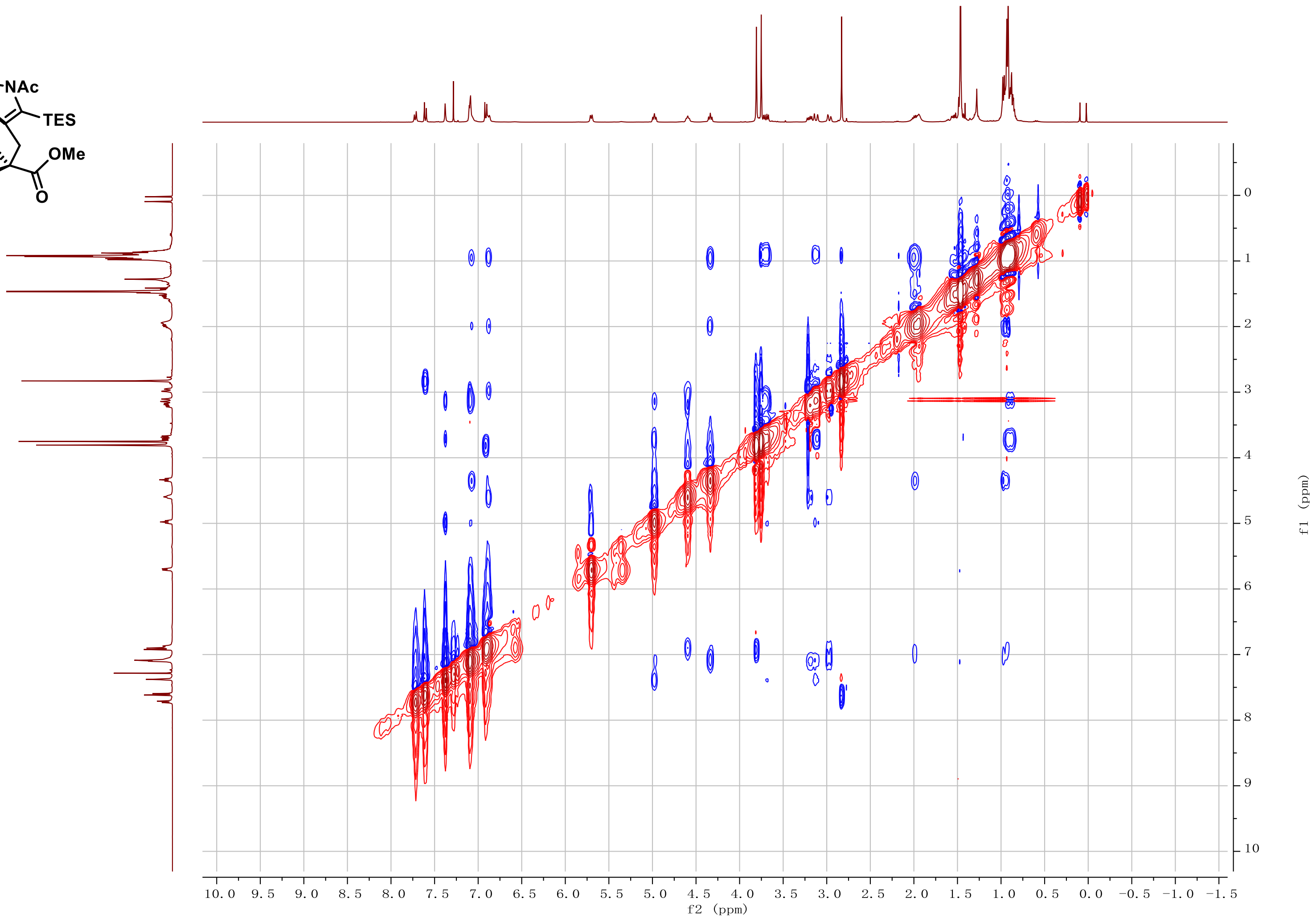

LCMS trace of compound 26c

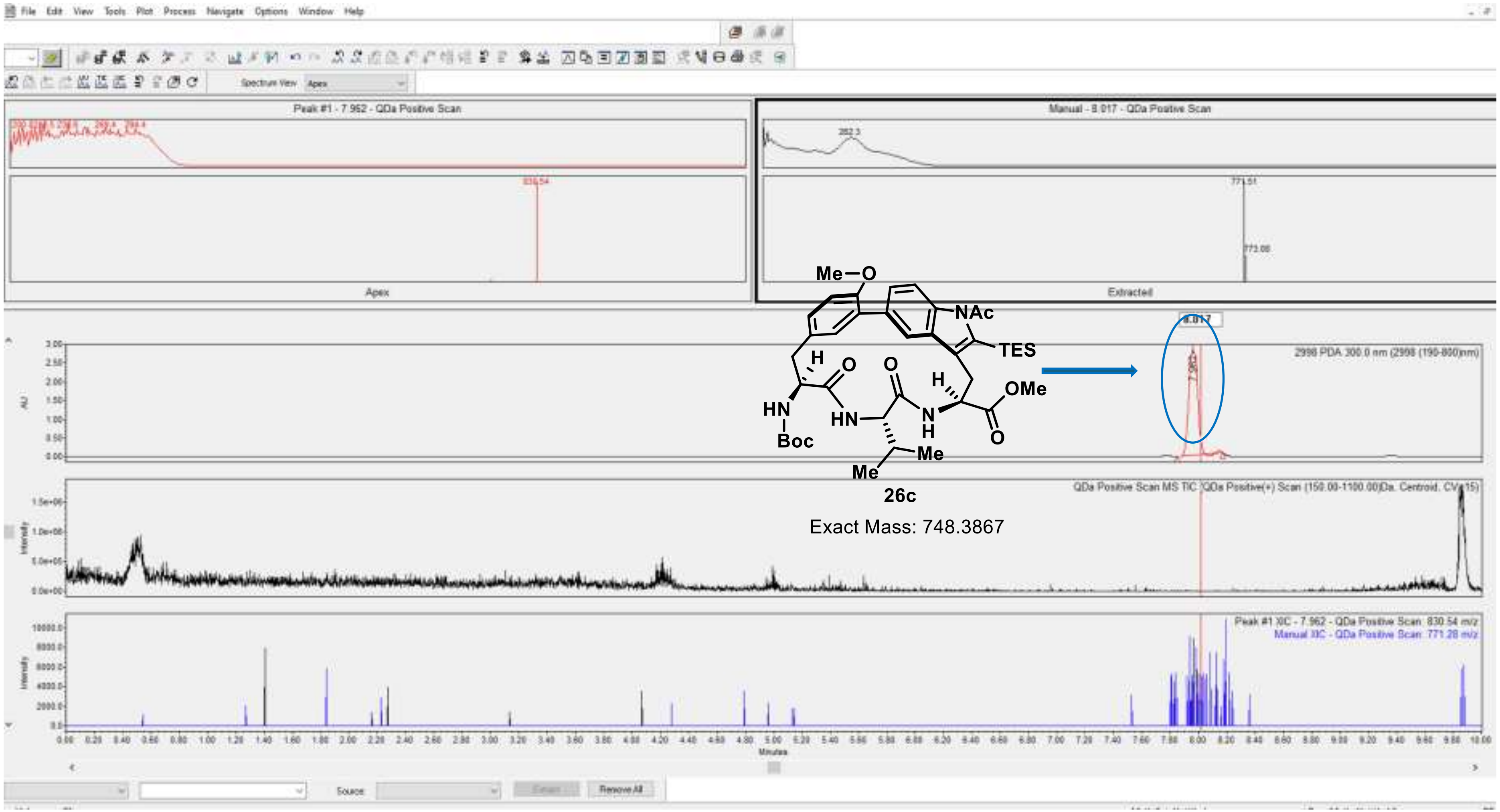

Compound 26d ^1H NMR (600 MHz, $CDCl_3$)

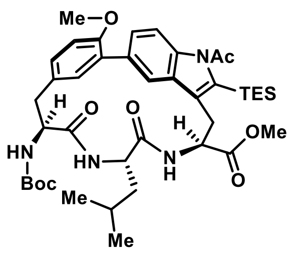

26d

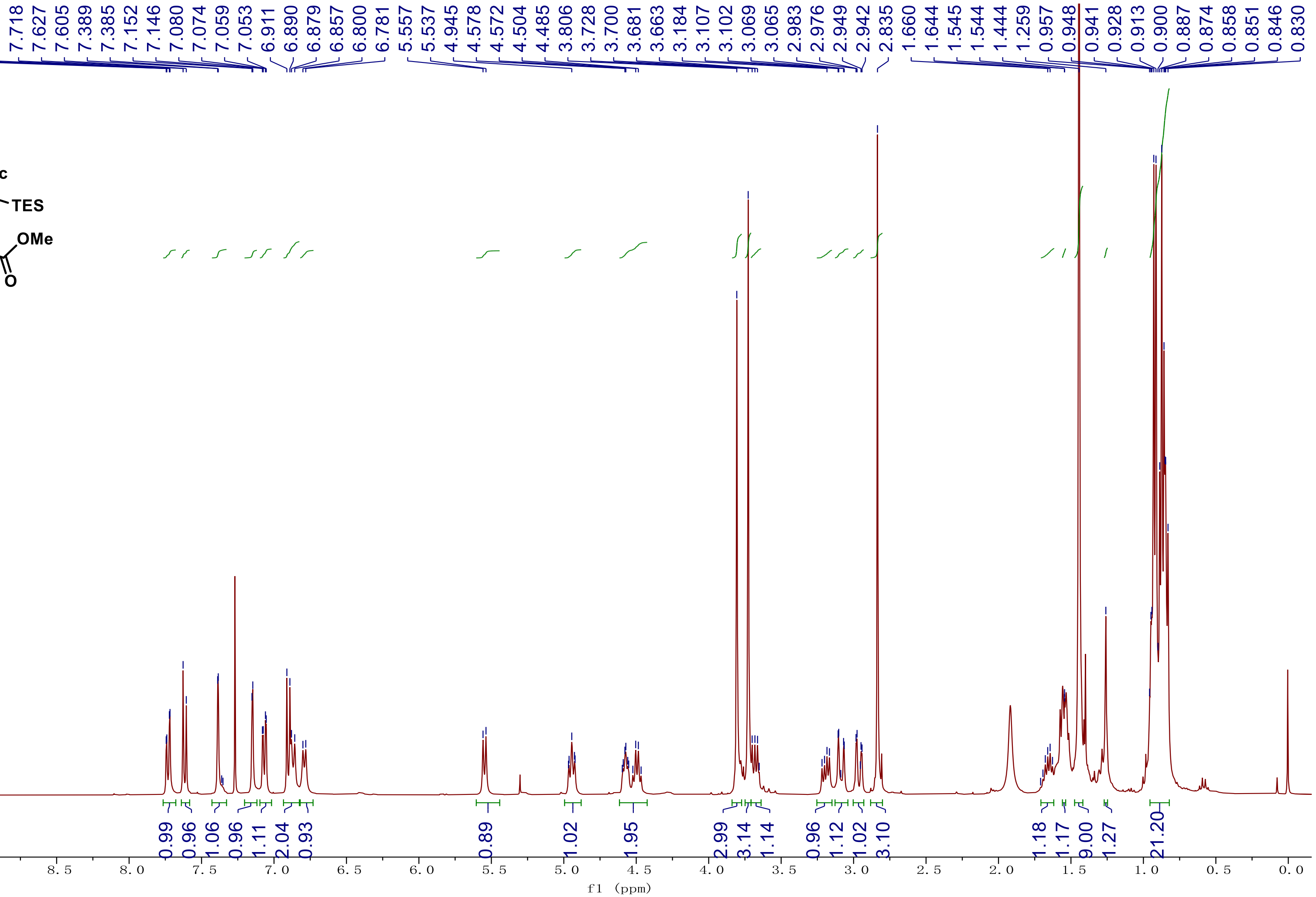

Compound 26d ^{13}C NMR (151 MHz, $CDCl_3$)

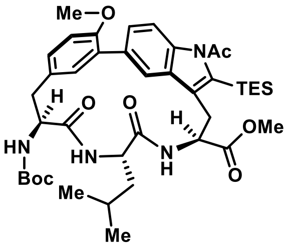

26d

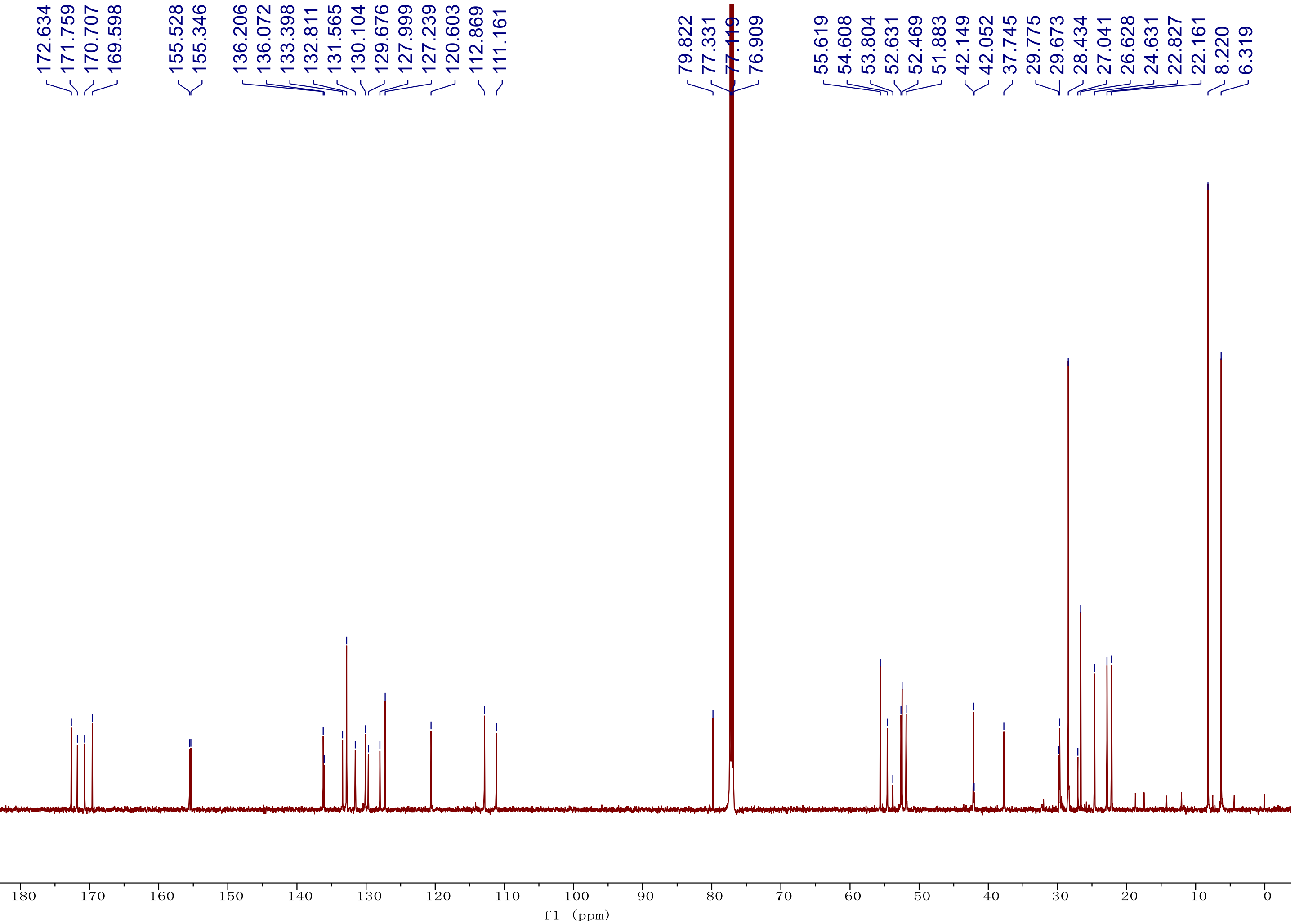

Compound 26d NOESY (400 MHz, $CDCl_3$)

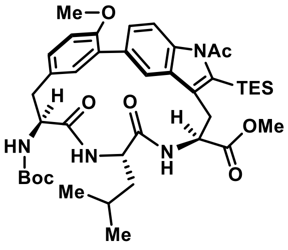

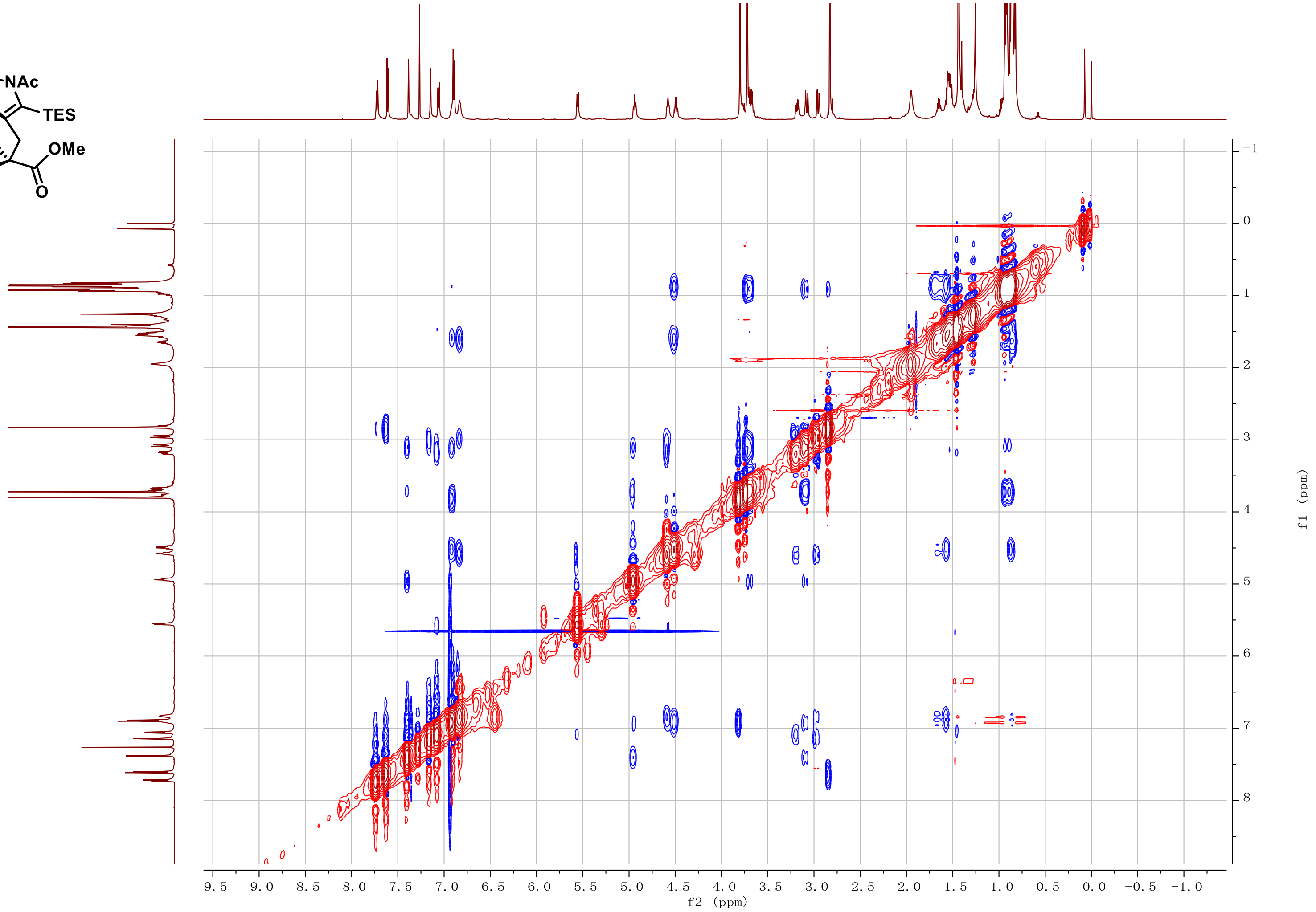

LCMS trace of compound 26d

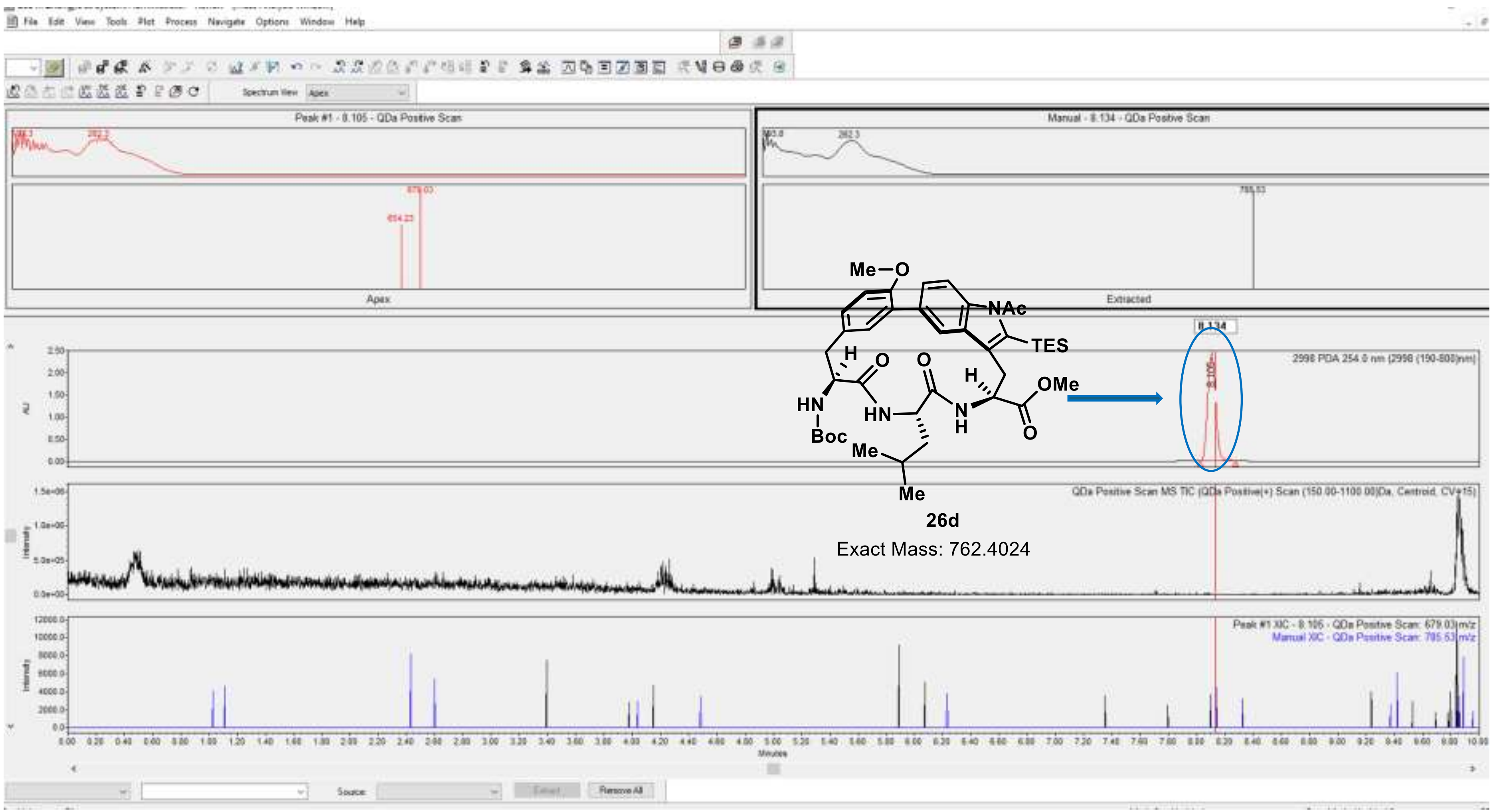

Compound 26e ^{1}H NMR (600 MHz, CDCl₃)

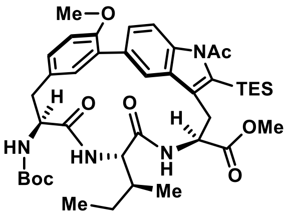

26e

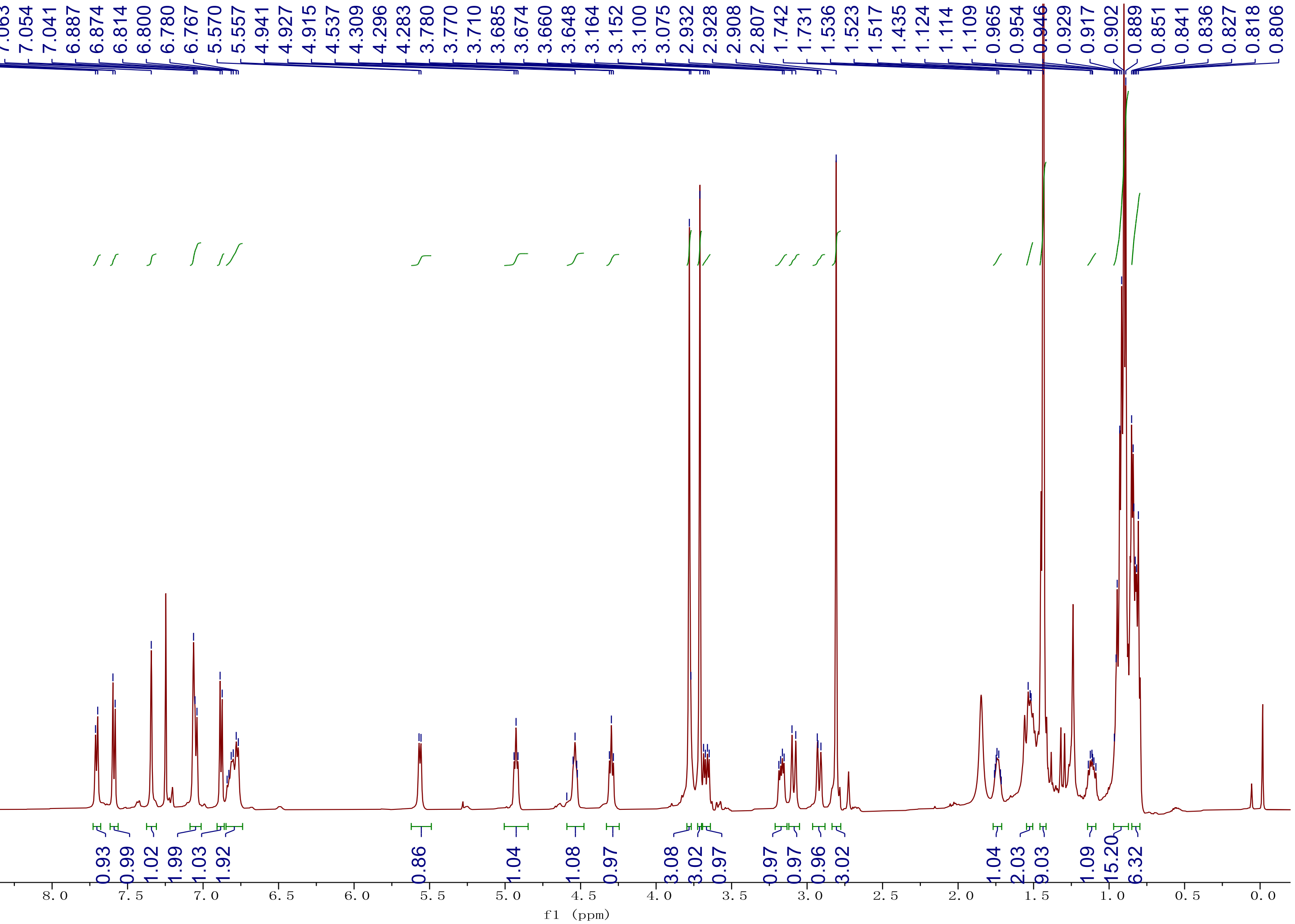

Compound 26e ^{13}C NMR (151 MHz, $CDCl_3$)

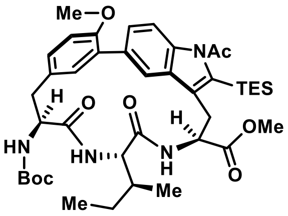

26e

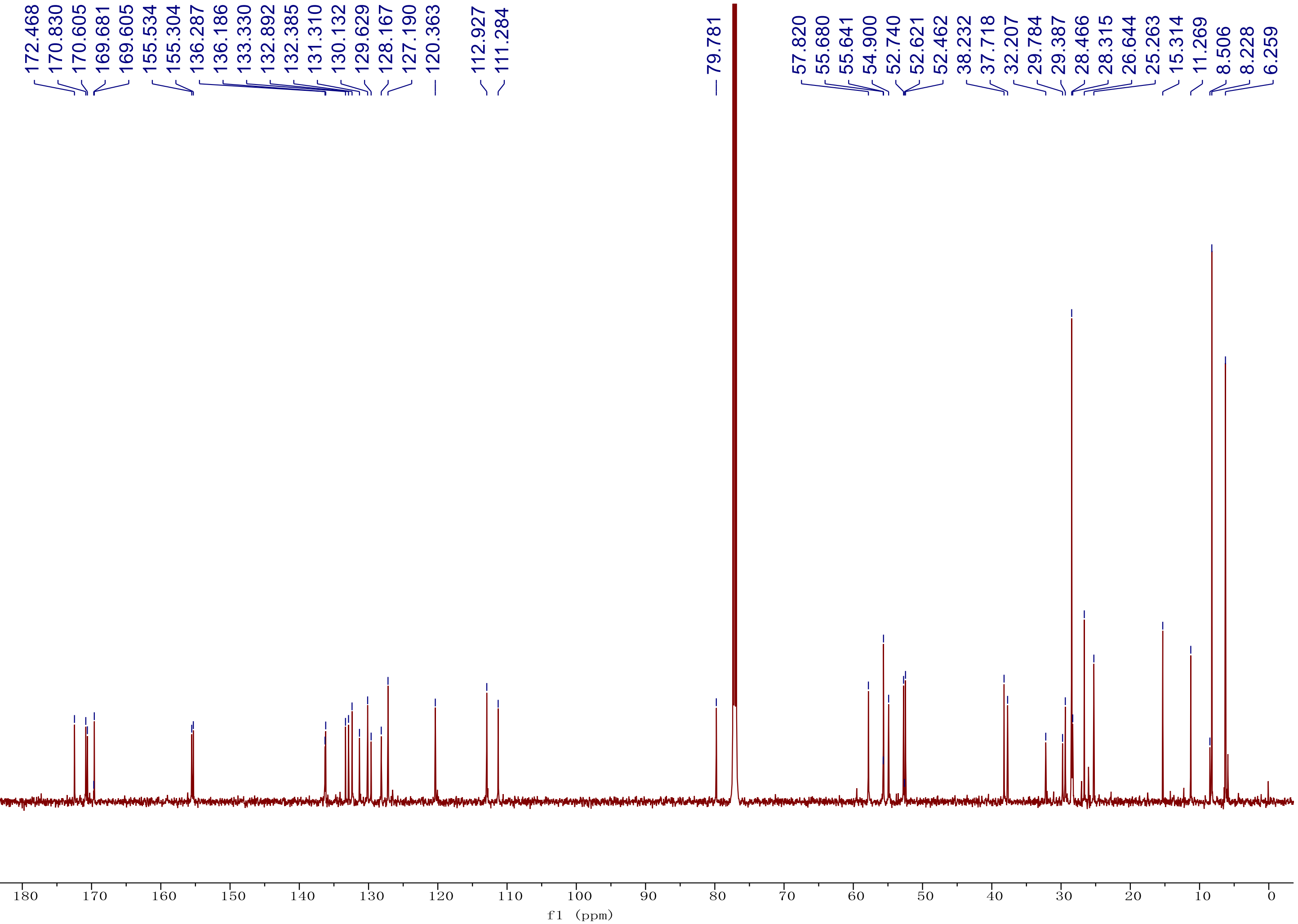

Compound 26f ^{1}H NMR (600 MHz, $CDCl_3$)

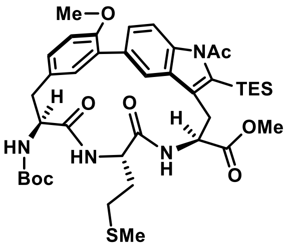

26f

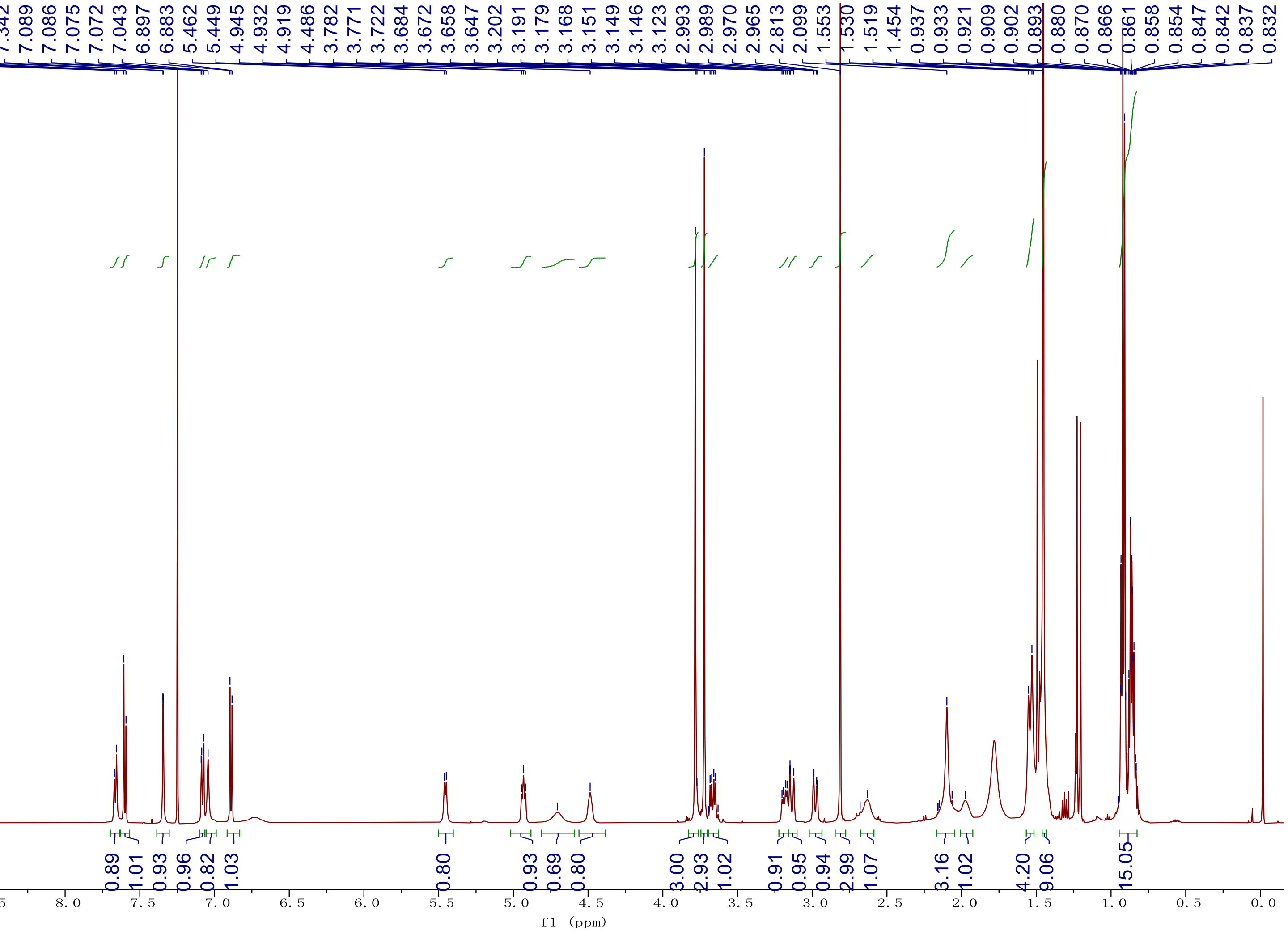

Compound 26f ^{13}C NMR (151 MHz, $CDCl_3$)

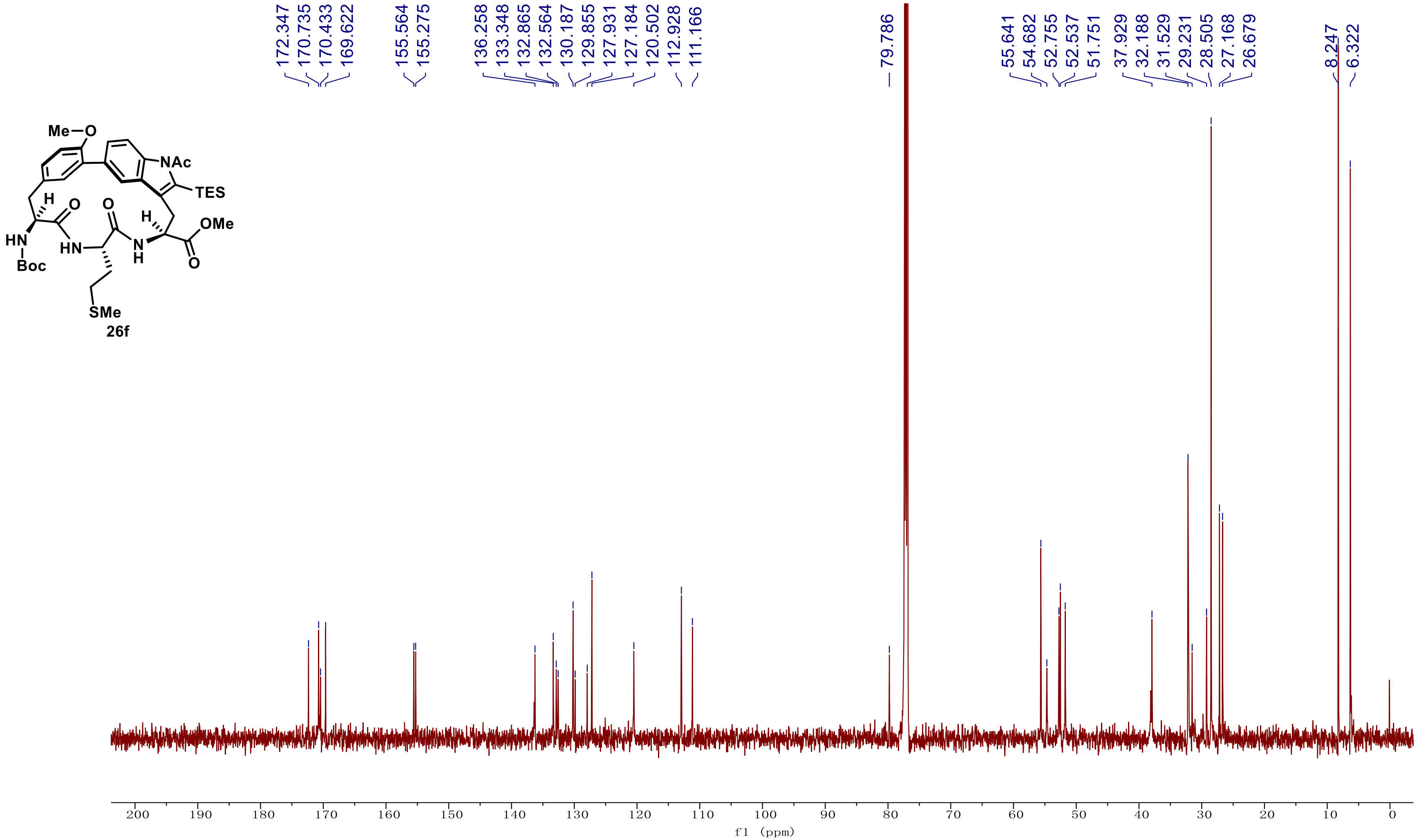

Compound 26g ^{1}H NMR (600 MHz, $CDCl_3$)

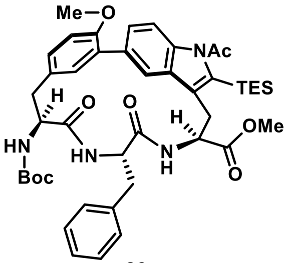

26g

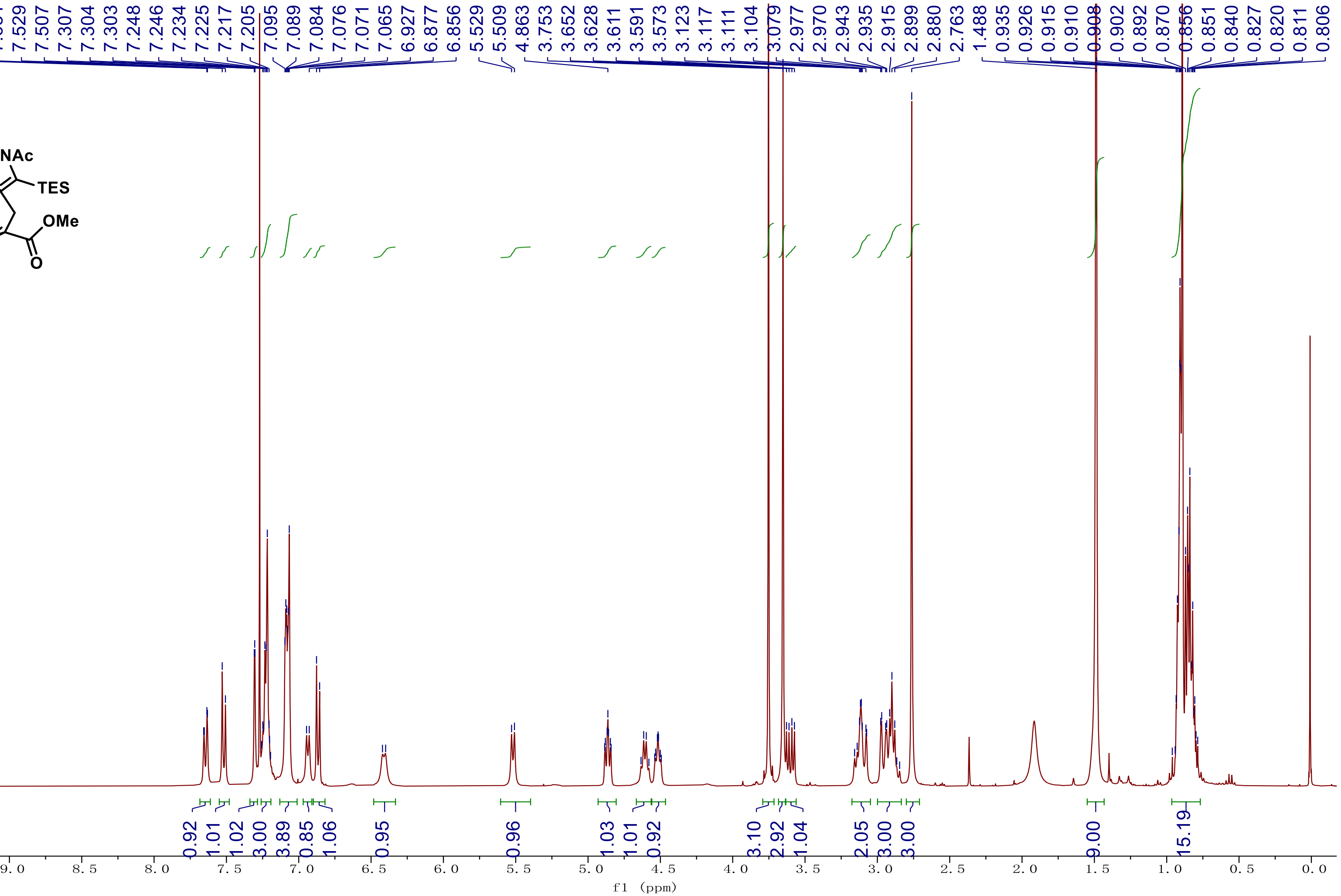

Compound 26g ^{13}C NMR (151 MHz, CDCl$_3$)

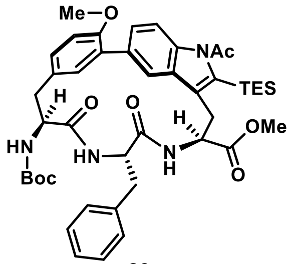

26g

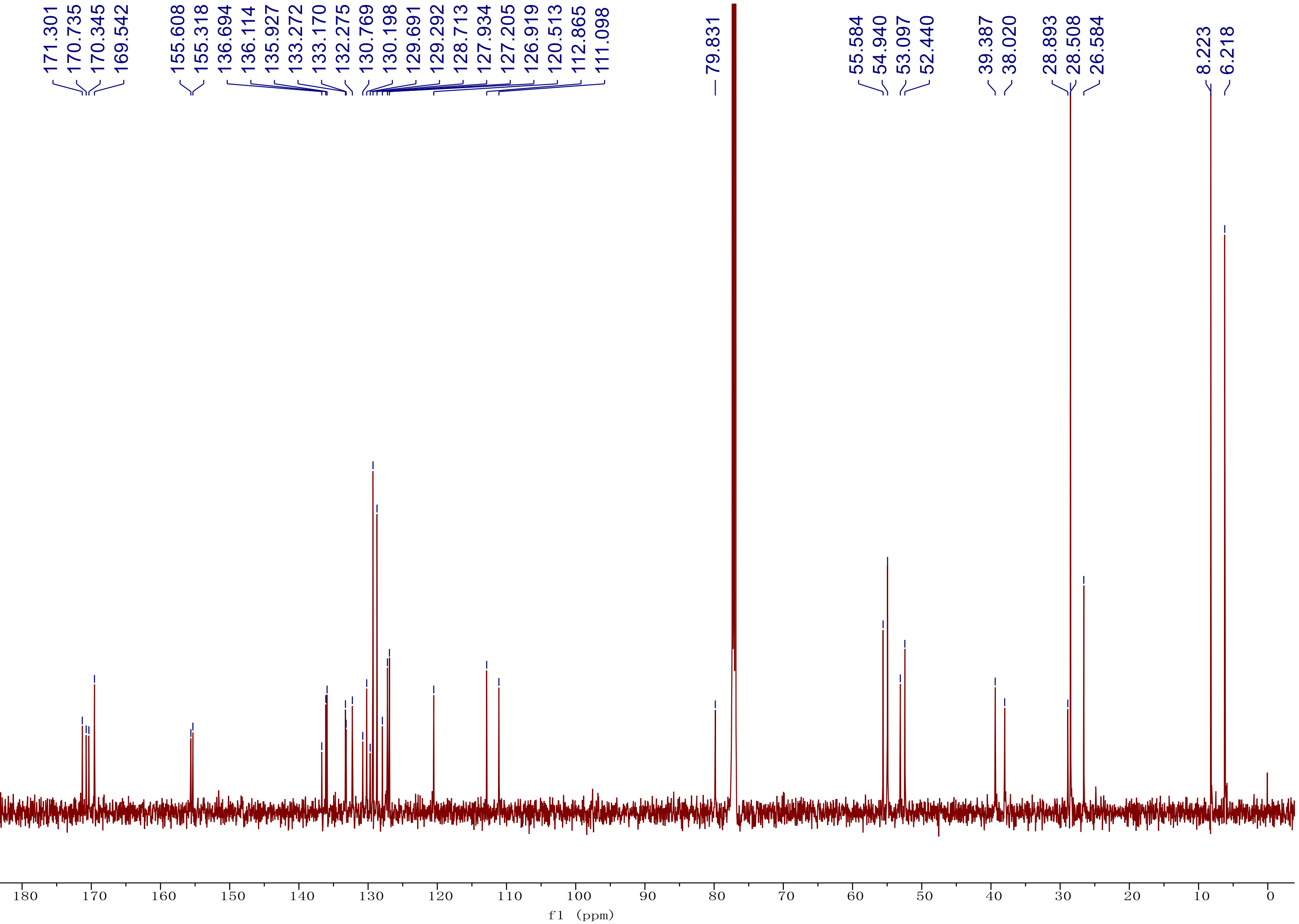

Compound 26h ^{1}H NMR (400 MHz, $CDCl_3$)

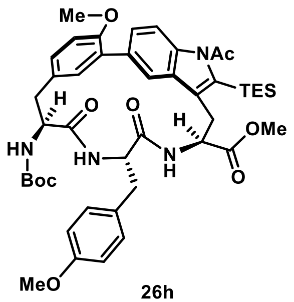

26h

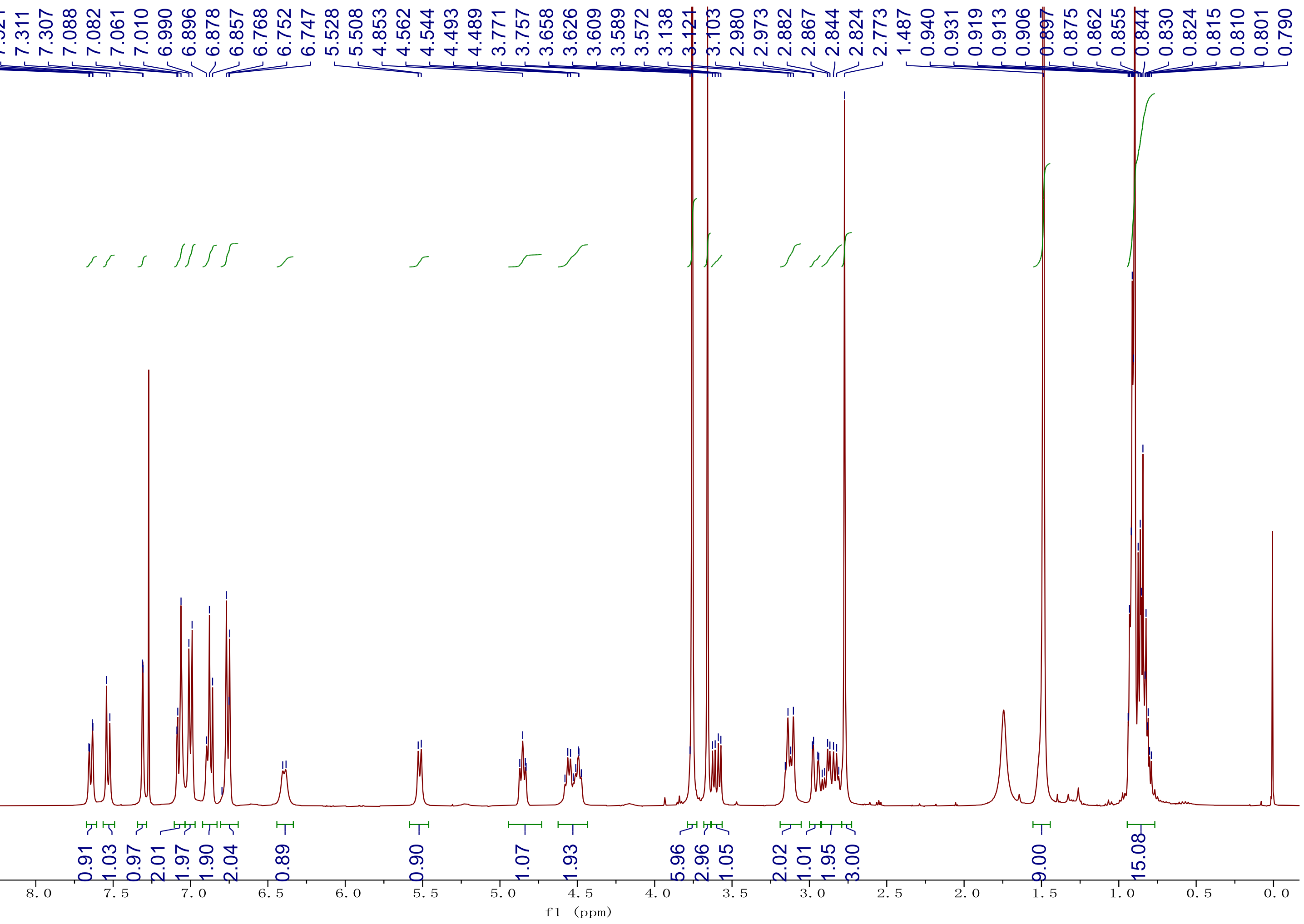

Compound 26h ^{13}C NMR (101 MHz, $CDCl_3$)

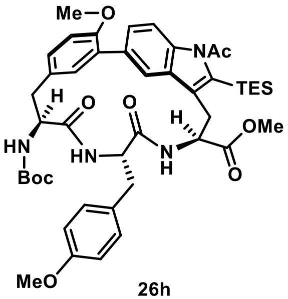

26h

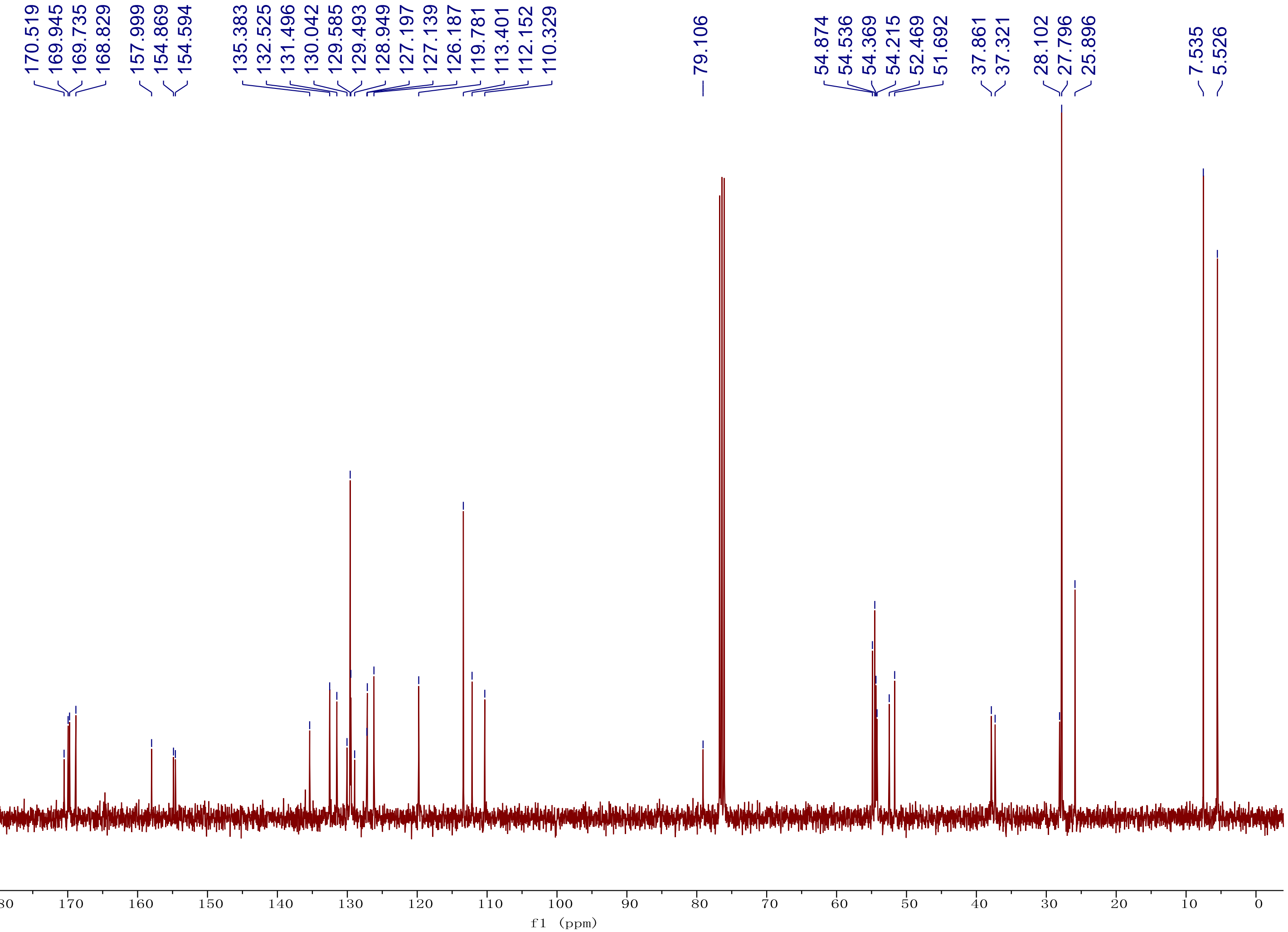

Compound 26i ^{1}H NMR (600 MHz, $CDCl_3$)

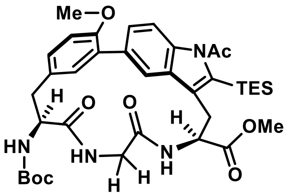

26i

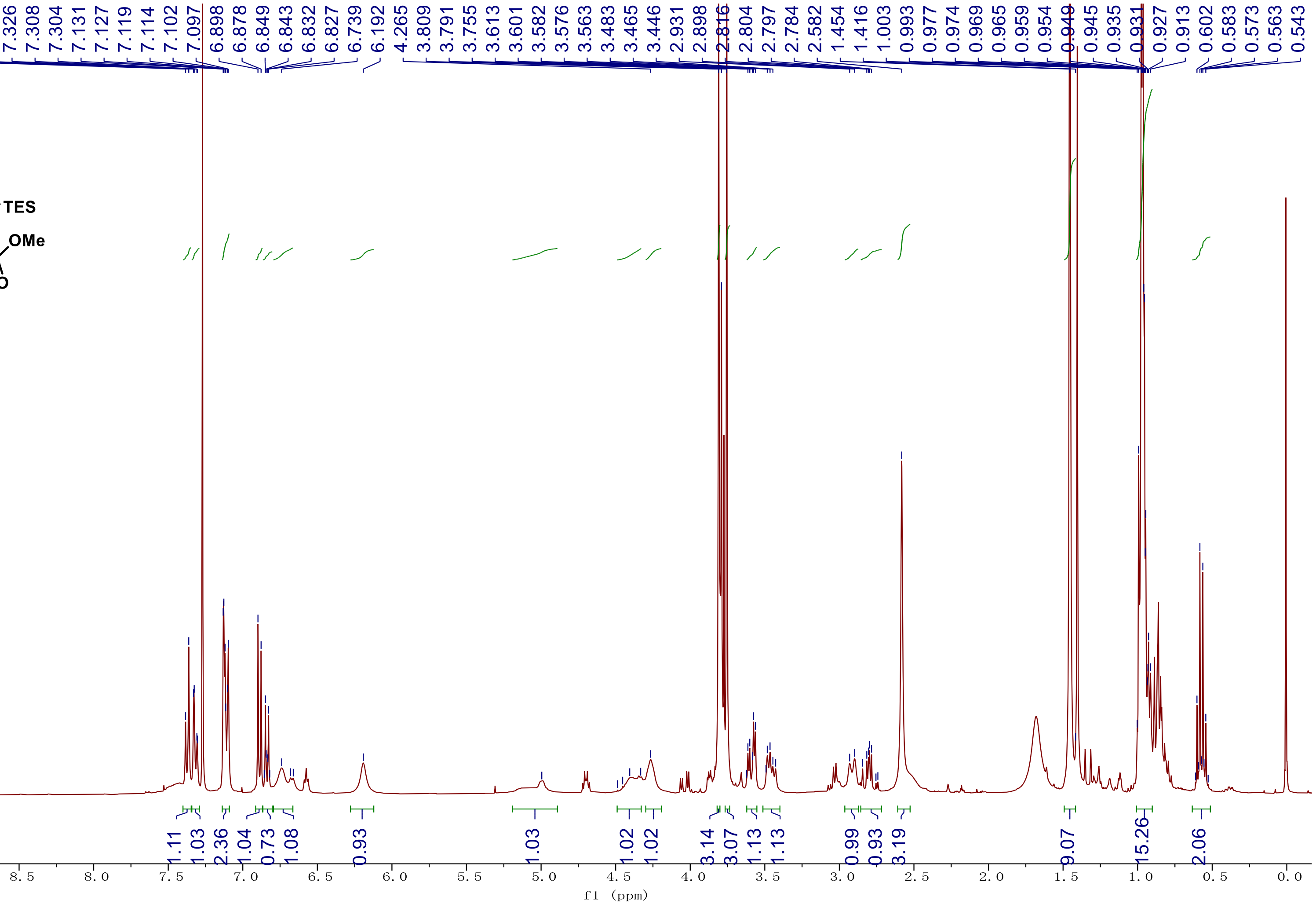

Compound 26i ^{13}C NMR (151 MHz, $CDCl_3$)

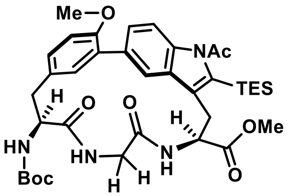

26i

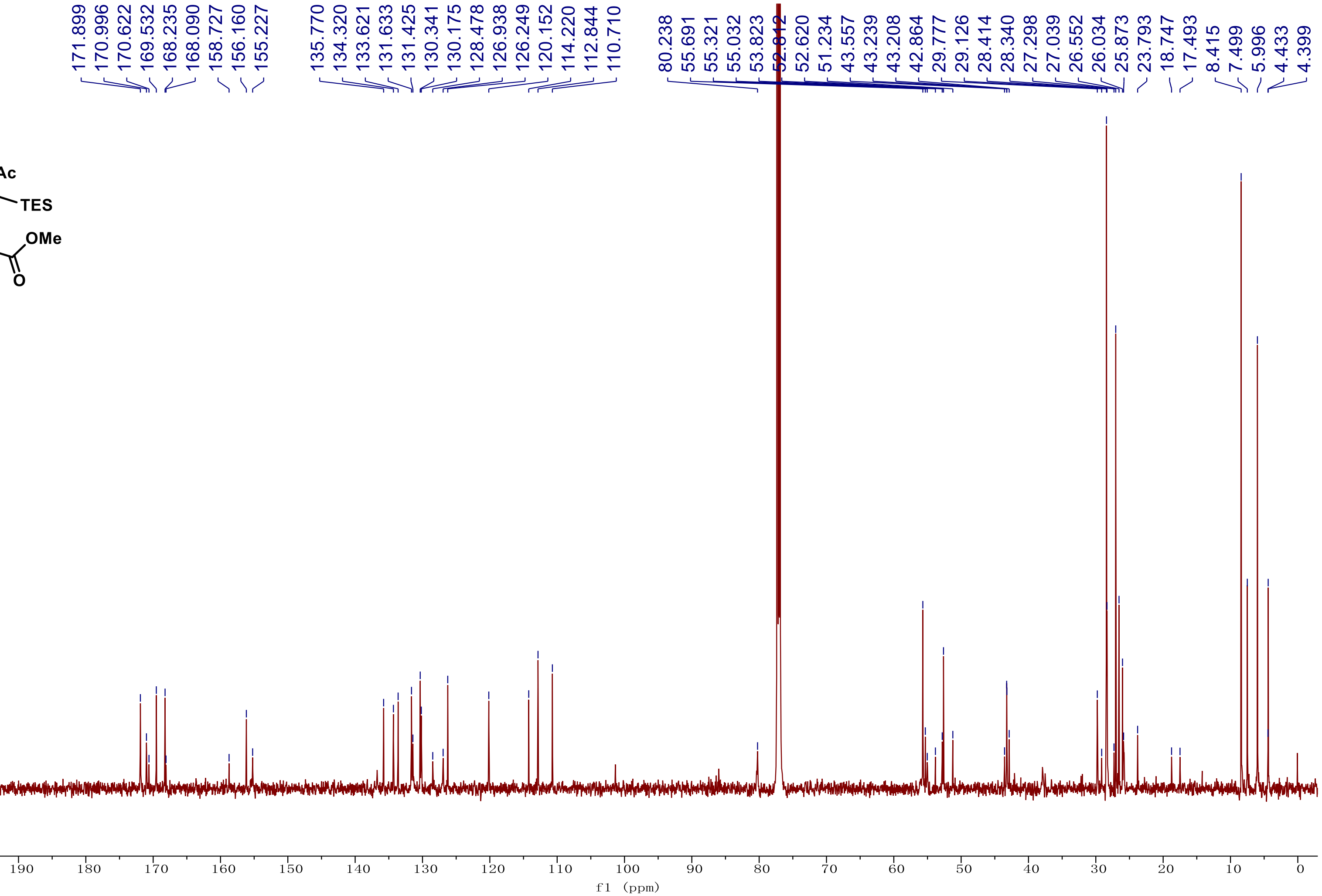

Compound 26j ^{1}H NMR (600 MHz, CDCl3)

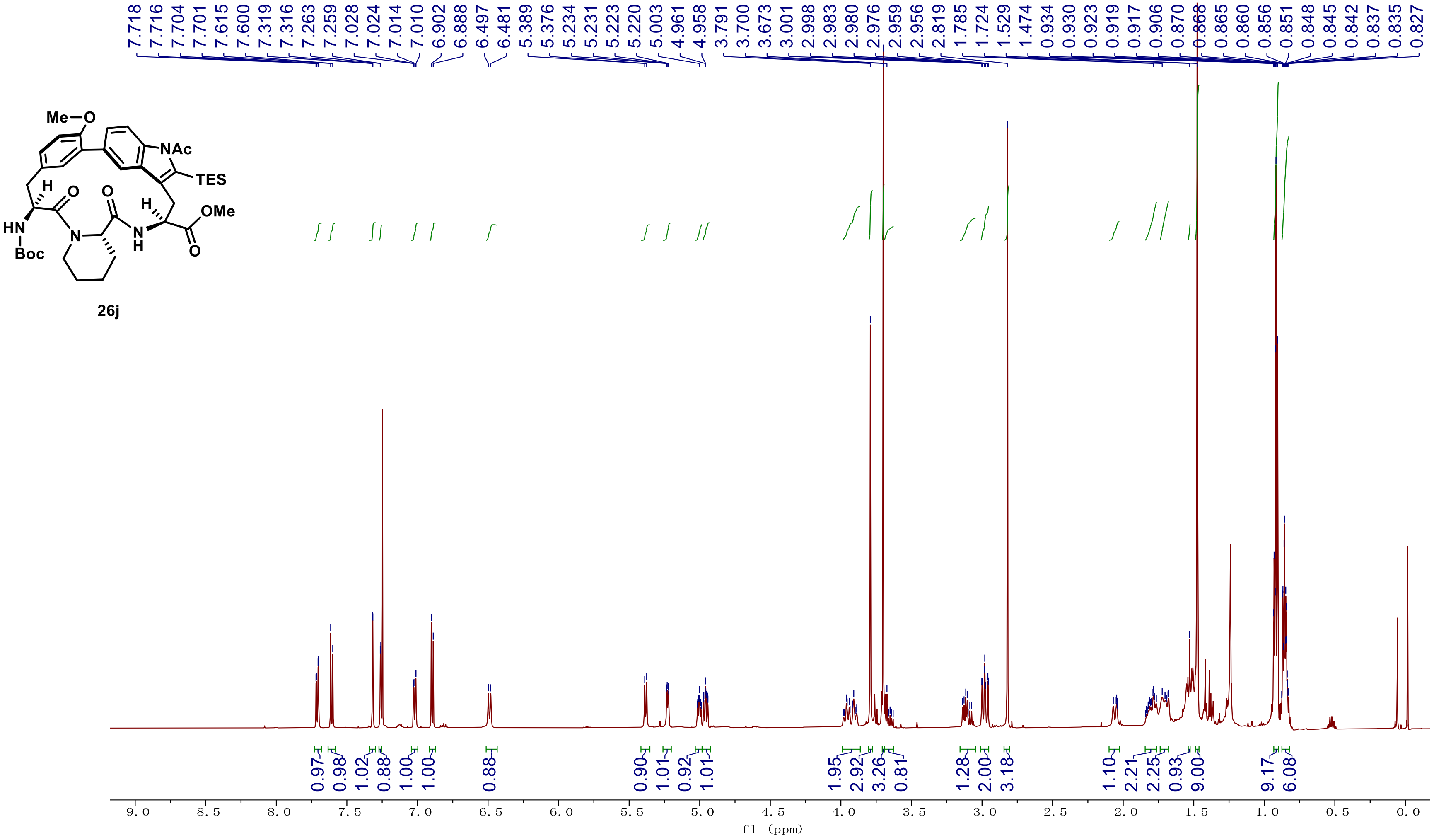

26j

Compound 26j ^{13}C NMR (151 MHz, $CDCl_3$)

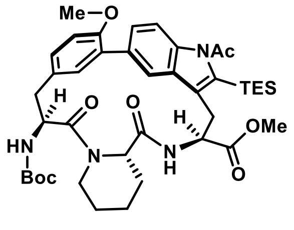

26j

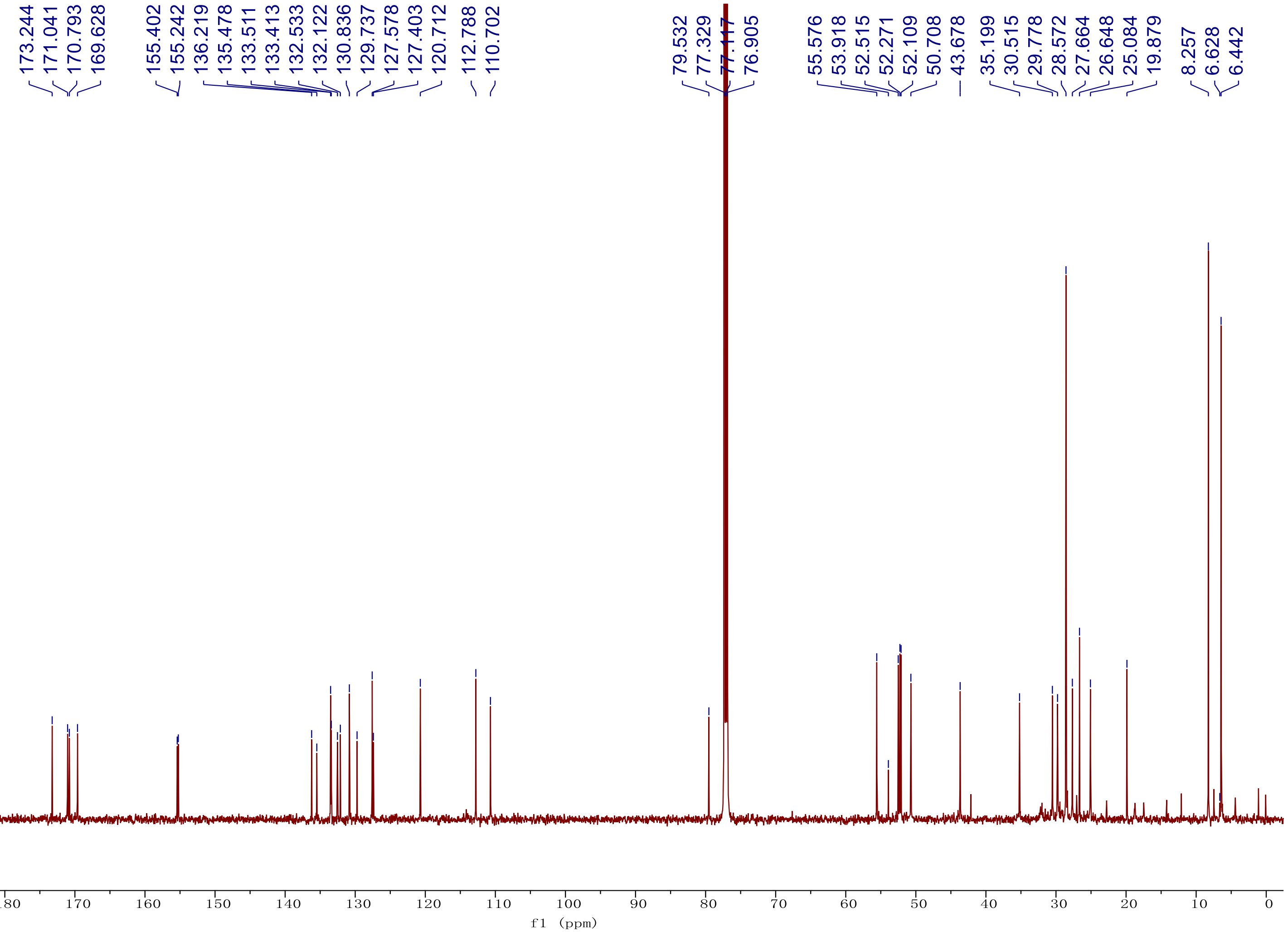

Compound 26k ¹H NMR (600 MHz, CDCl₃)

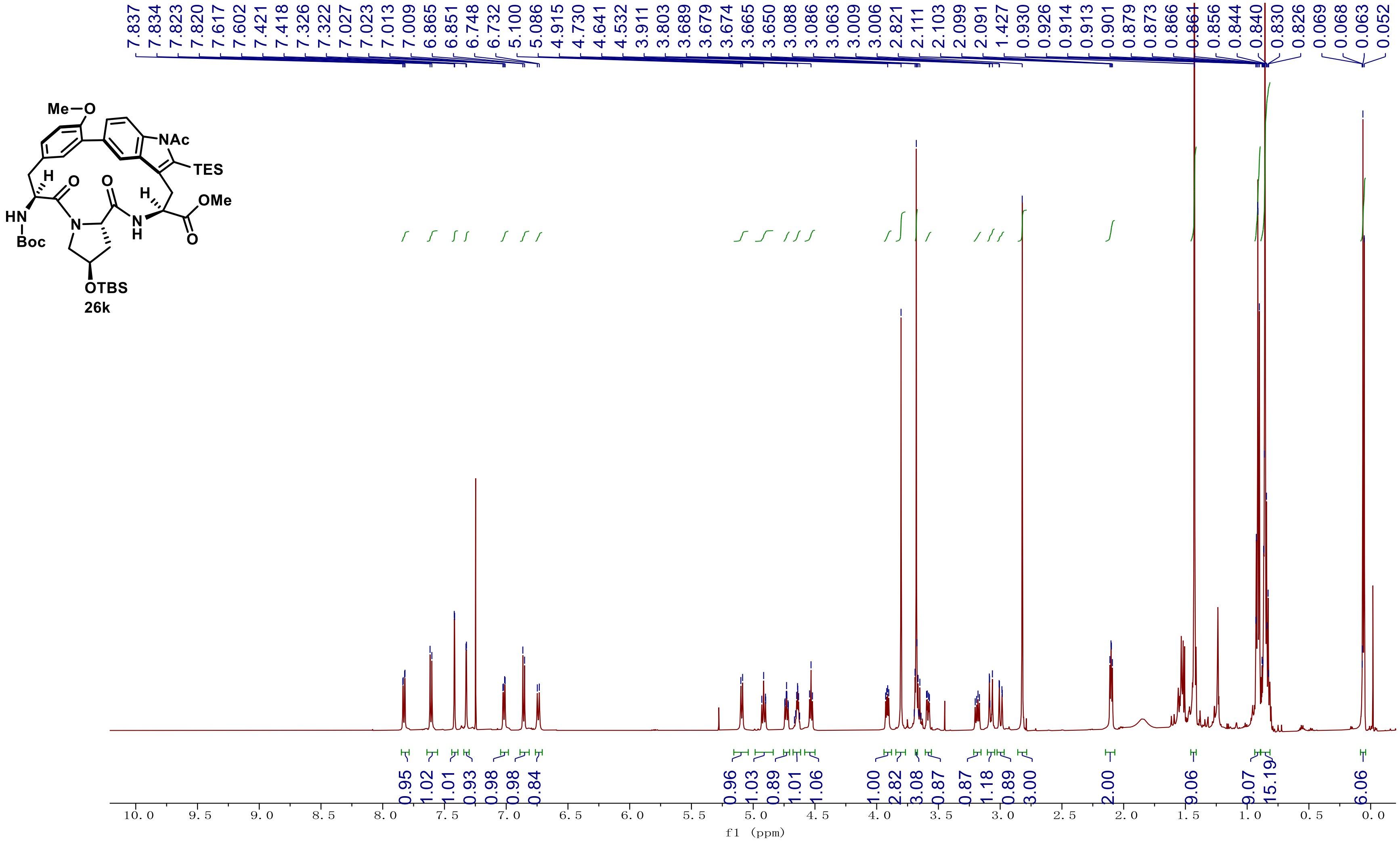

Compound 26k ^{13}C NMR (151 MHz, CDCl$_3$)

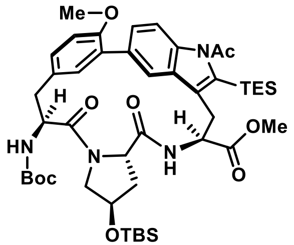

26k

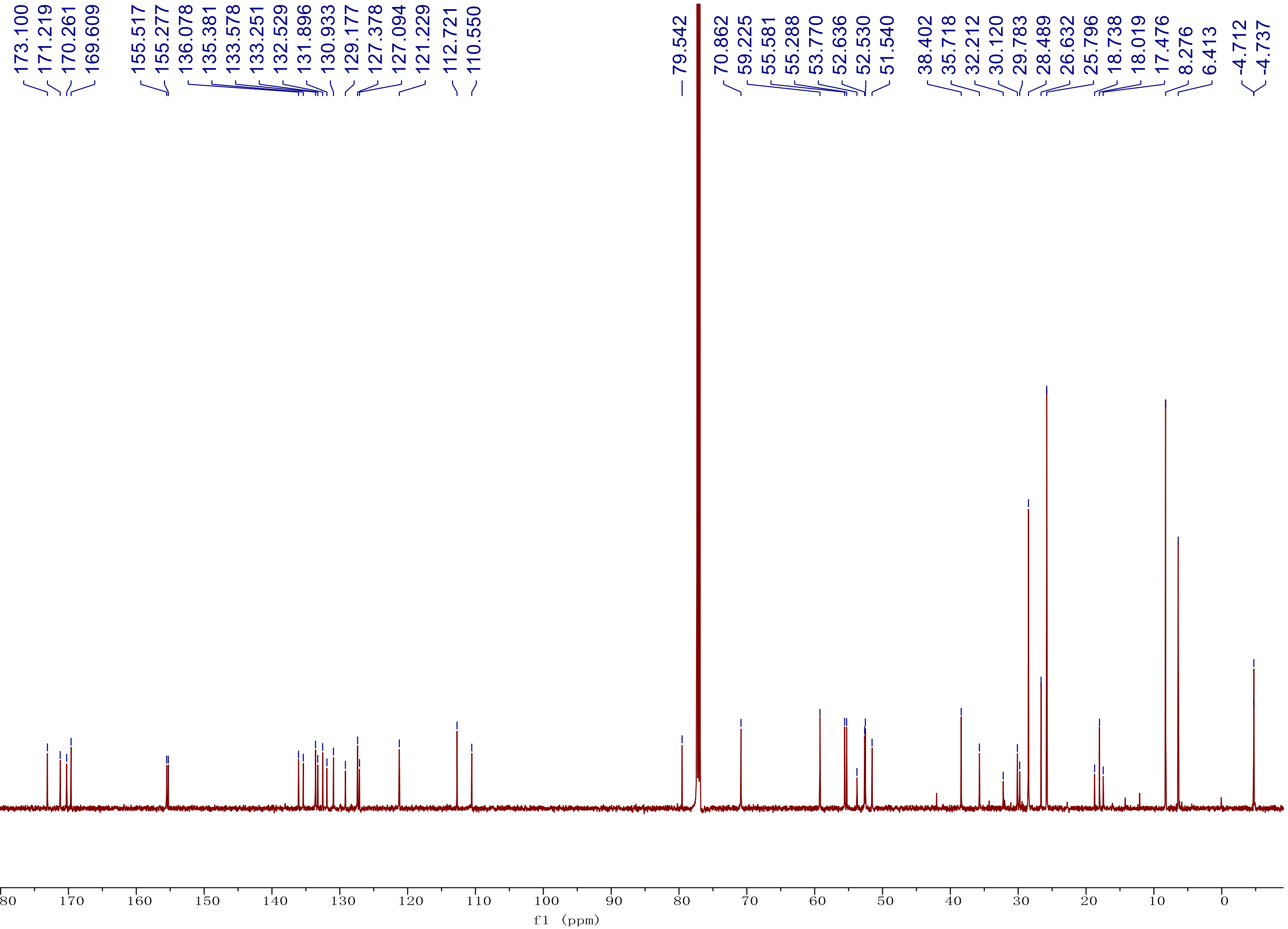

Compound 26l ^{1}H NMR (600 MHz, $CDCl_3$)

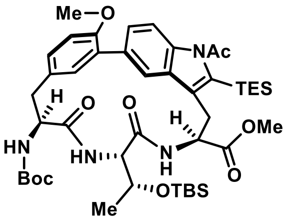

26l

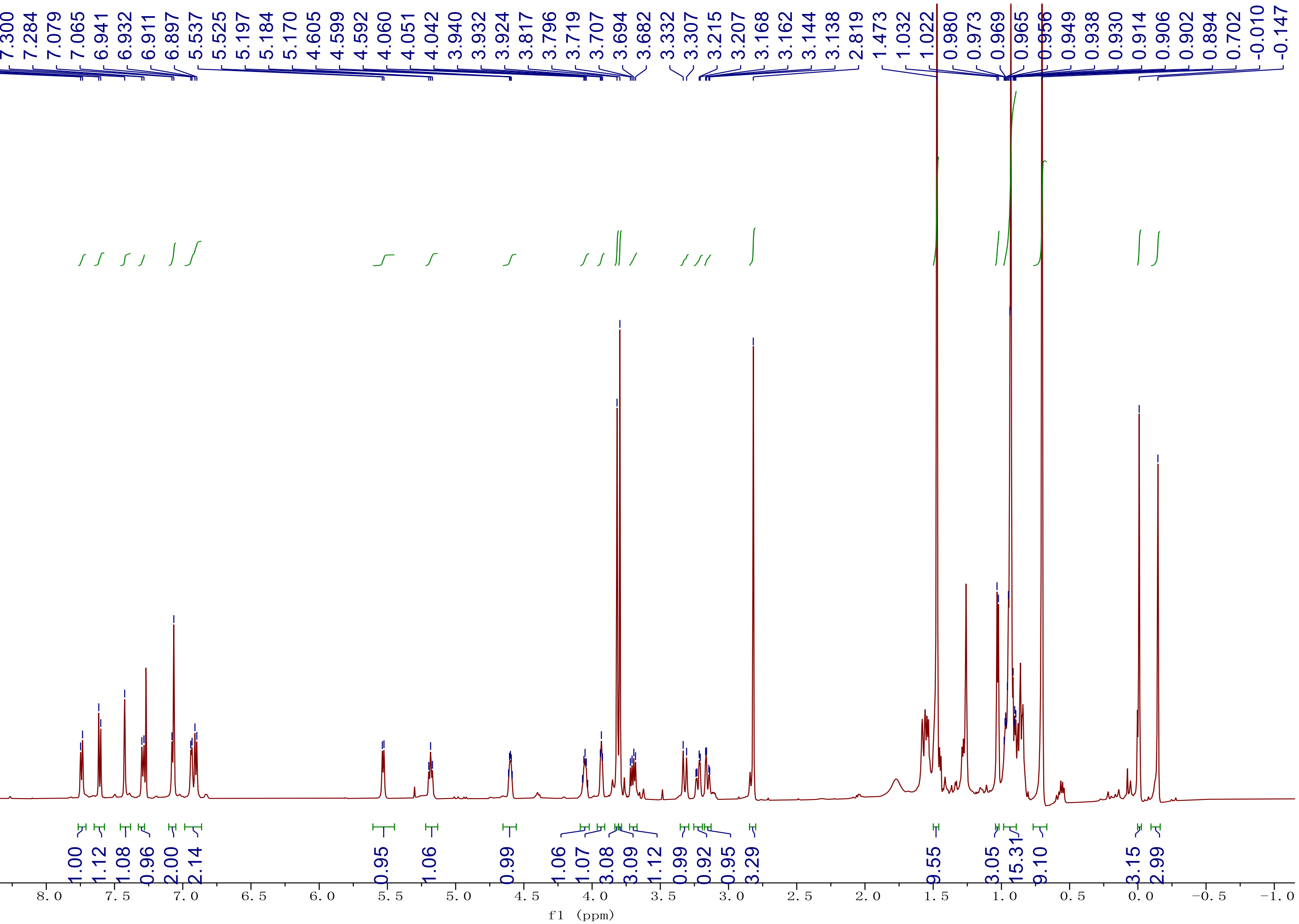

Compound 26l ^{13}C NMR (151 MHz, $CDCl_3$)

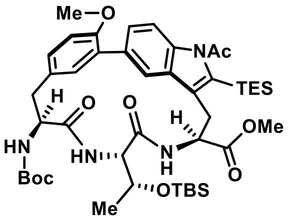

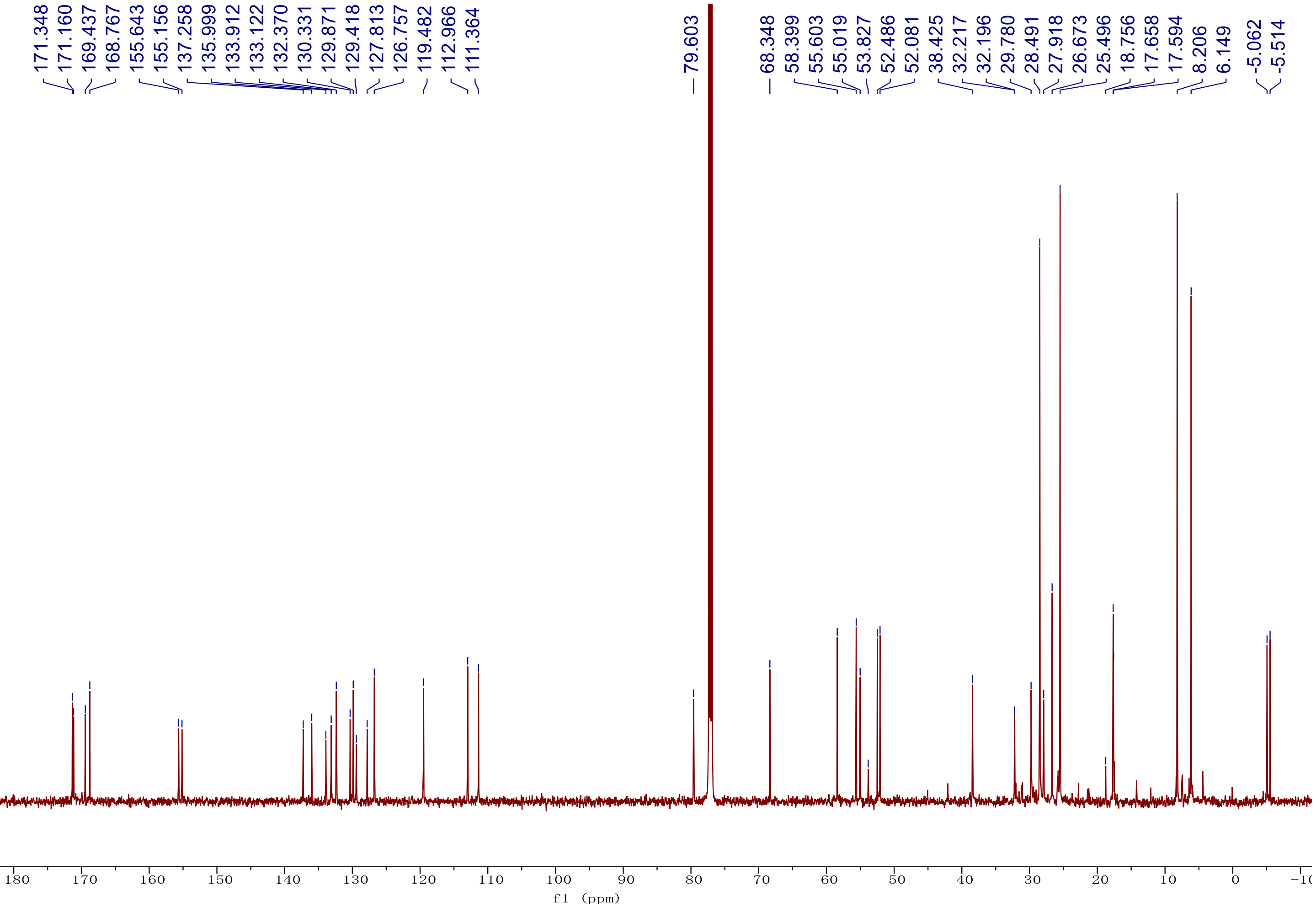

26l

Compound 26m ¹H NMR (600 MHz, CDCl₃)

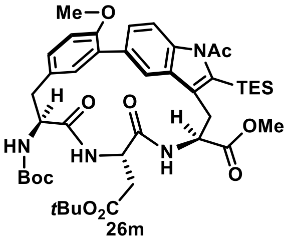

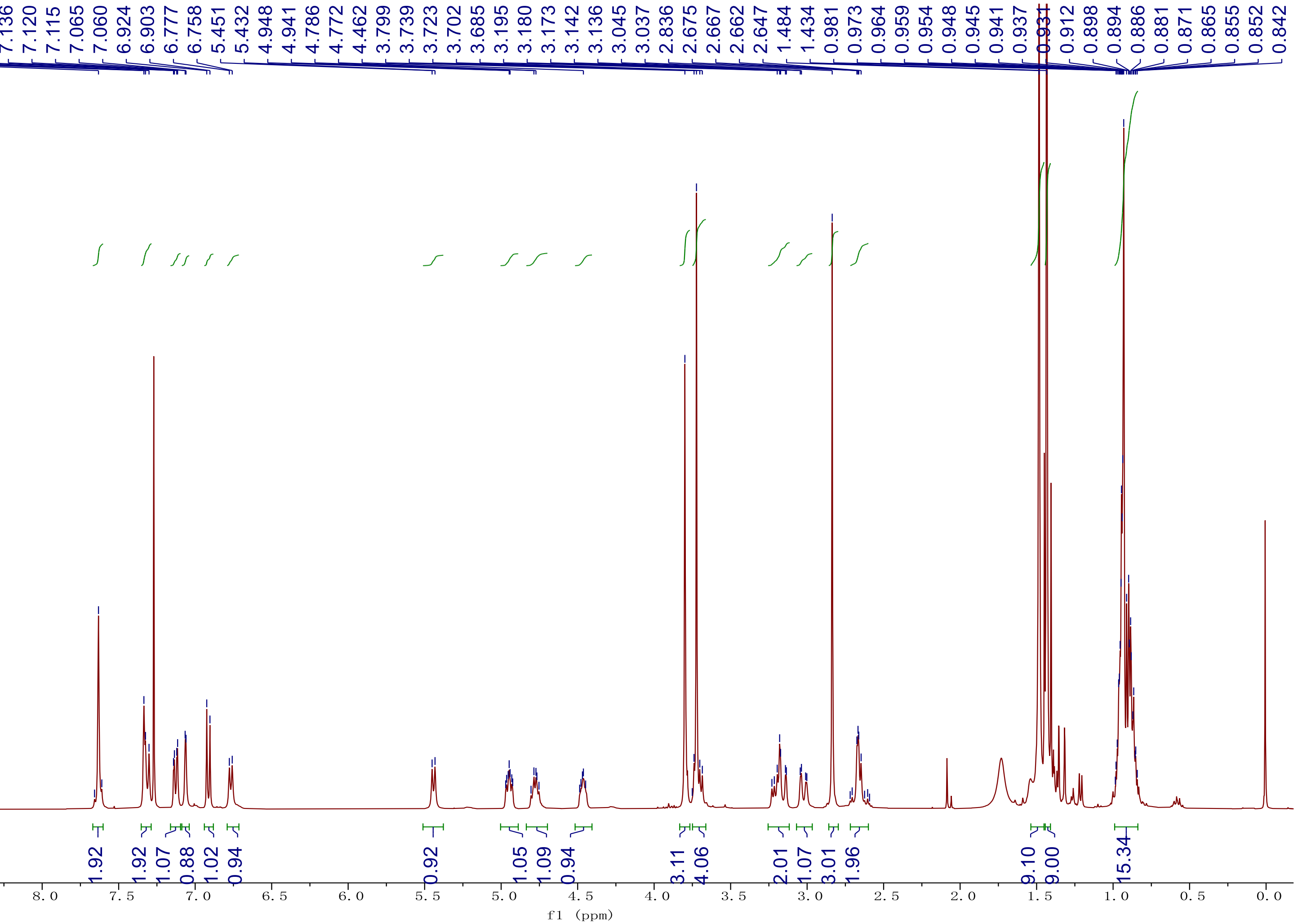

Compound 26m ^{13}C NMR (151 MHz, $CDCl_3$)

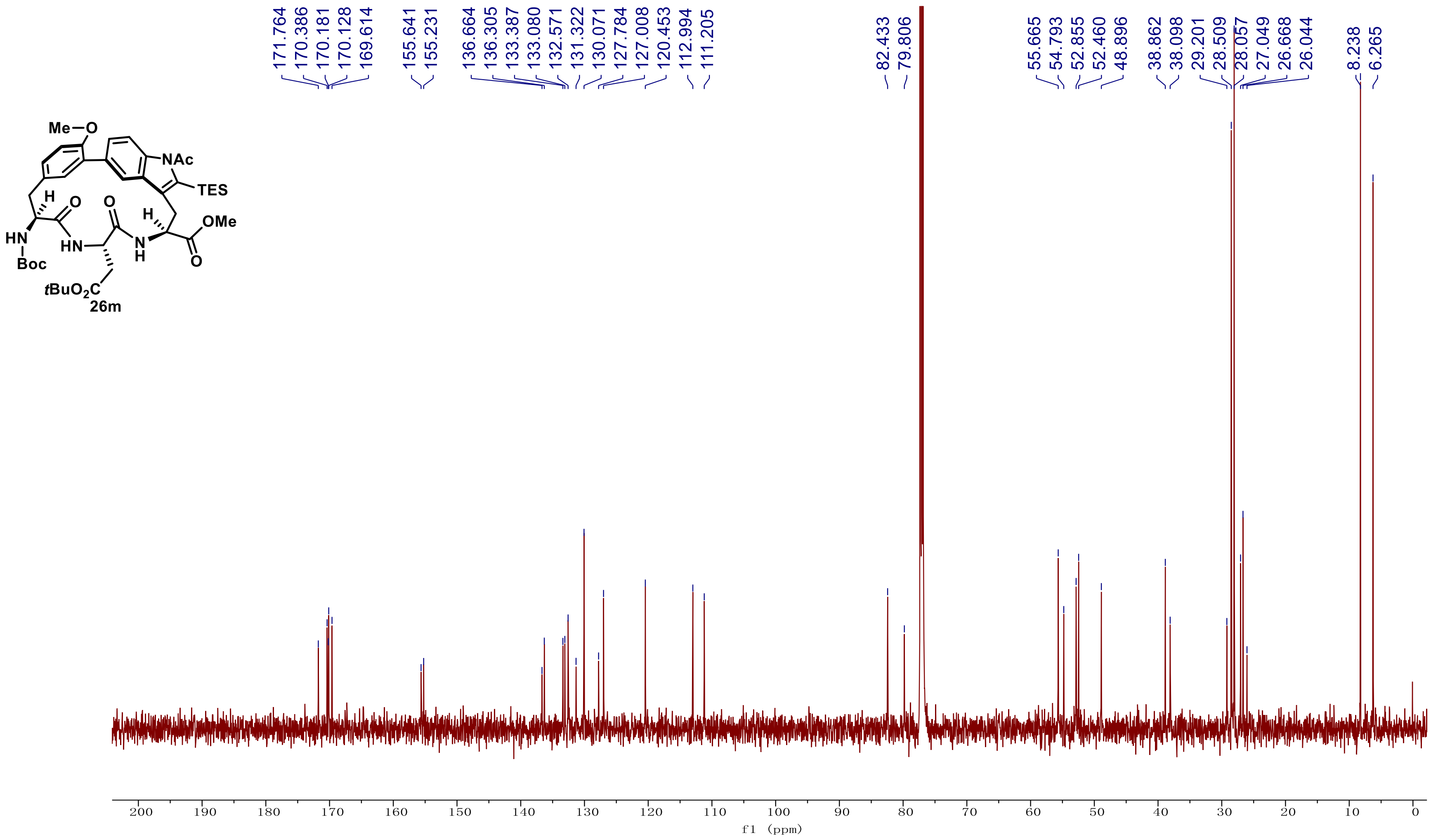

Compound 26n ^{1}H NMR (600 MHz, $CDCl_3$)

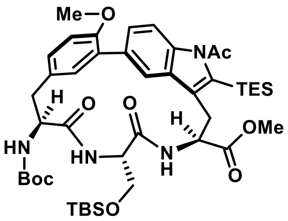

26n

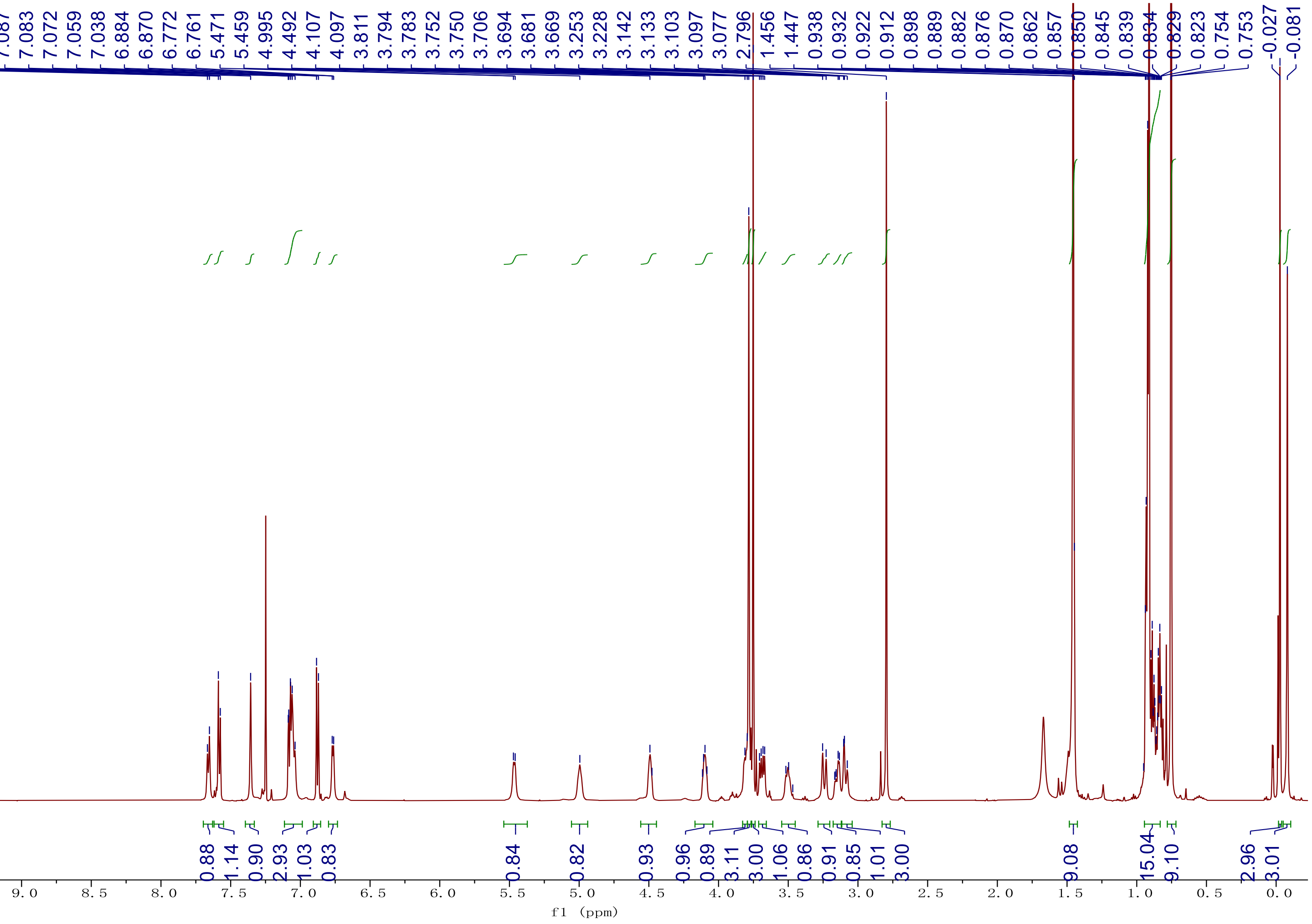

Compound 26n ^{13}C NMR (151 MHz, $CDCl_3$)

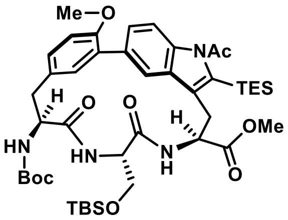

26n

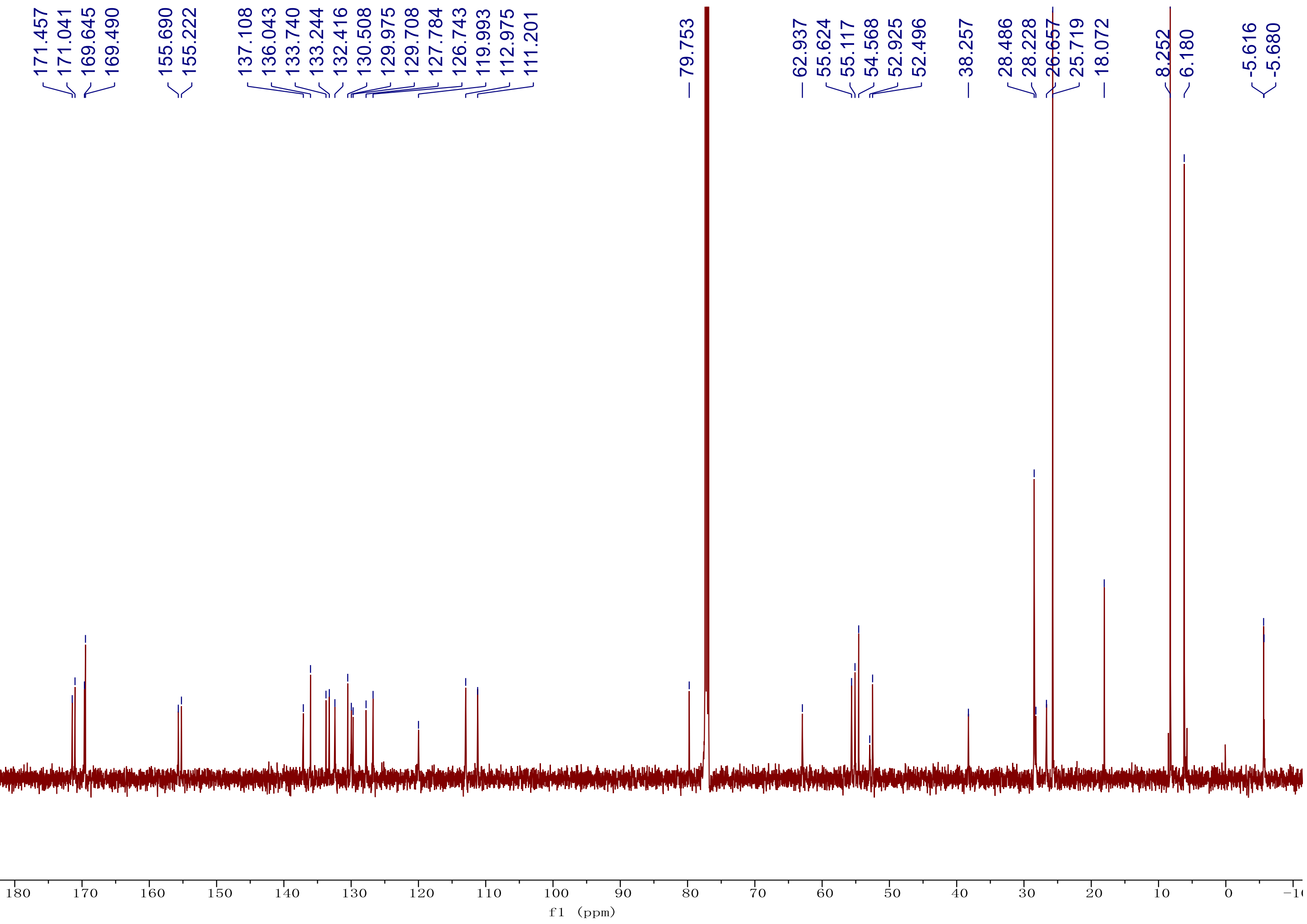

Compound 26o ^{1}H NMR (600 MHz, CDCl₃)

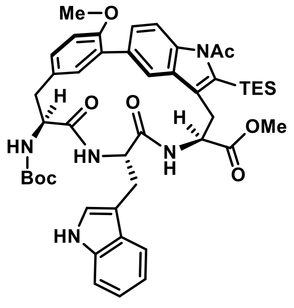

26o

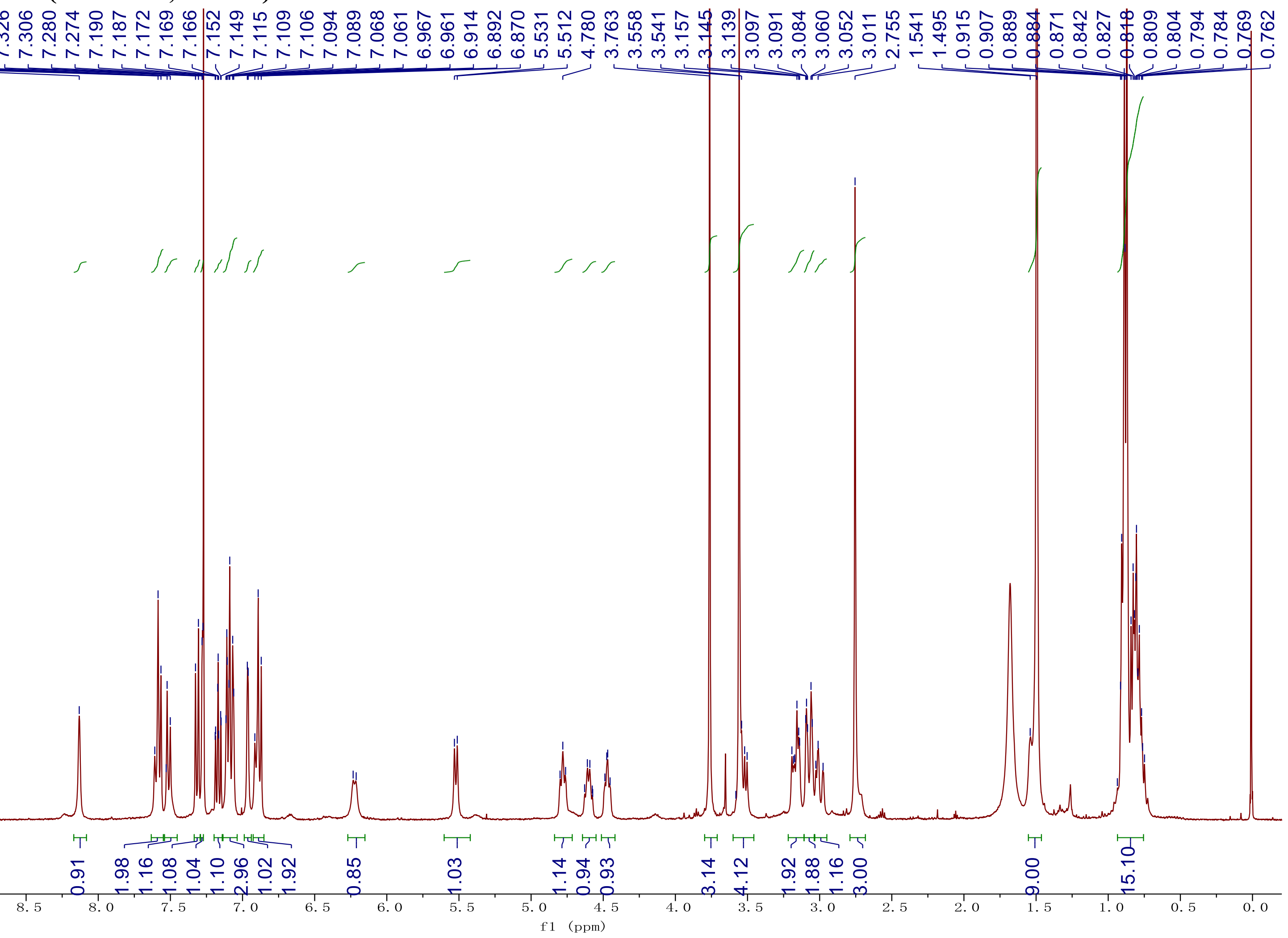

Compound 26o ^{13}C NMR (151 MHz, $CDCl_3$)

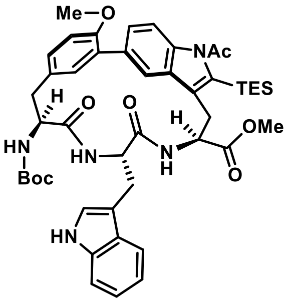

26o

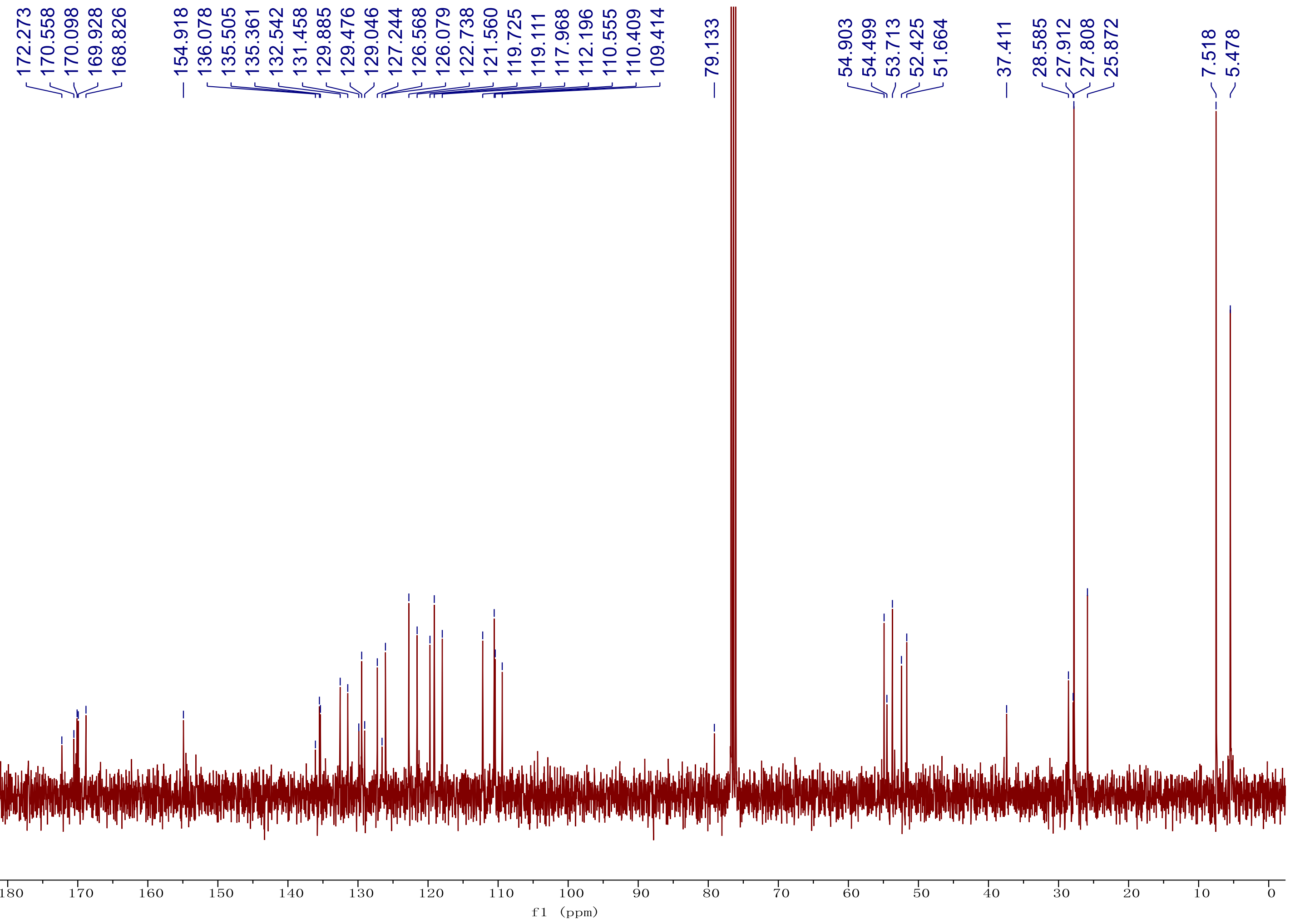

Compound 26p ¹H NMR (600 MHz, CDCl₃)

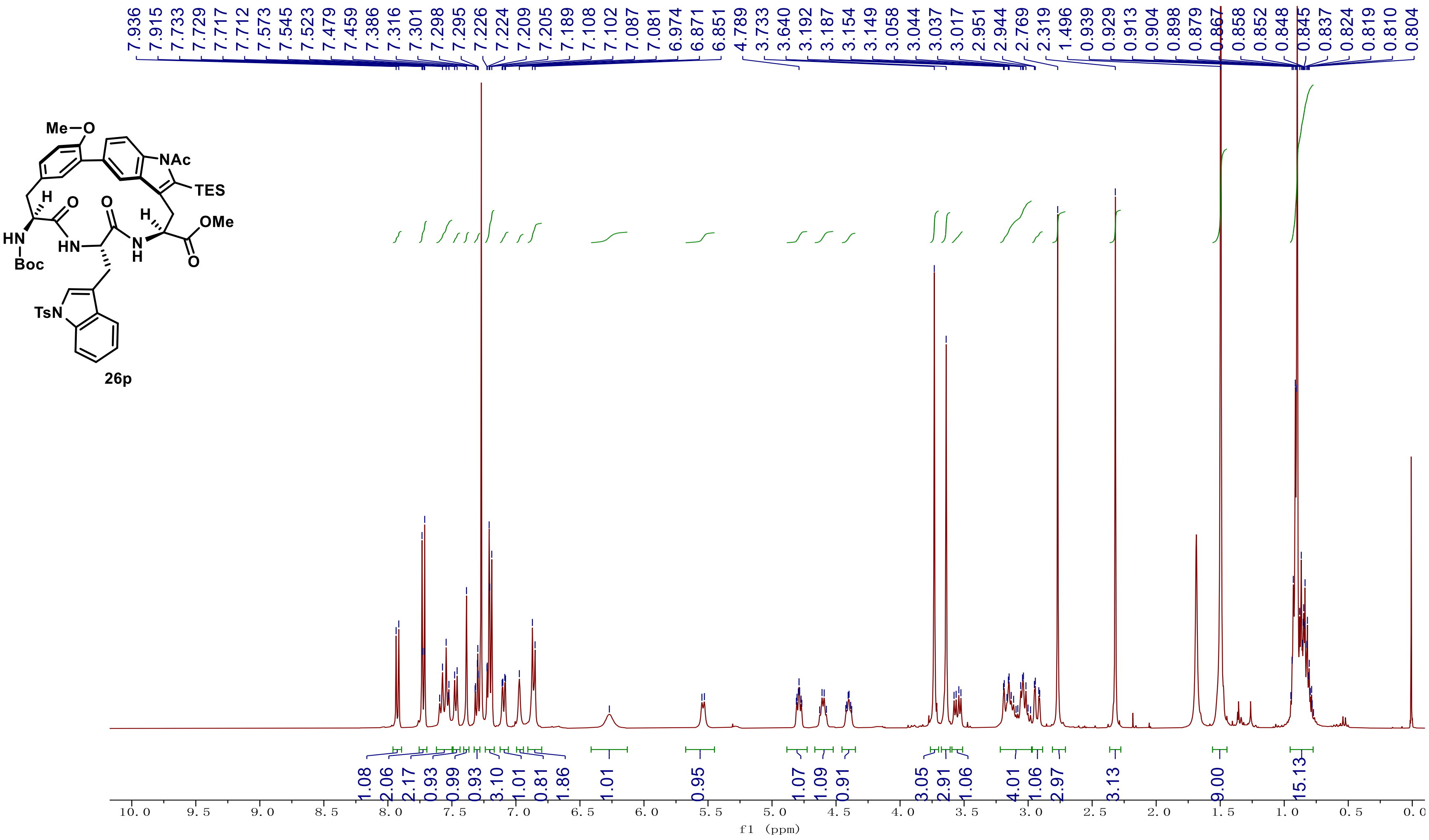

Compound 26p ^{13}C NMR (151 MHz, $CDCl_3$)

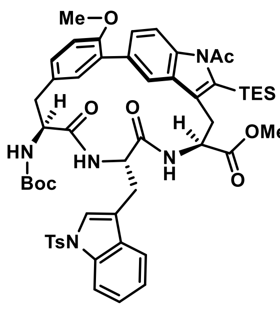

26p

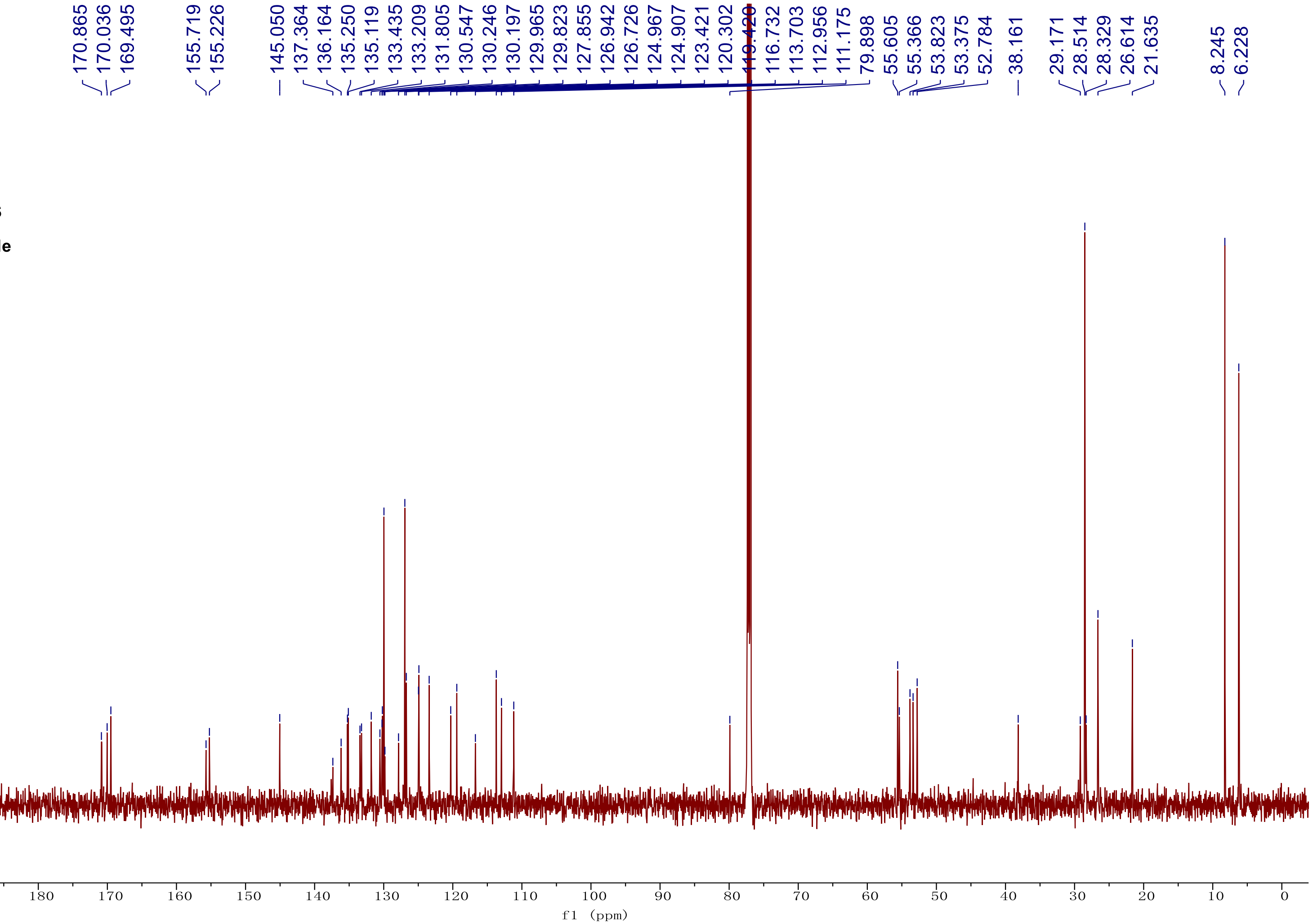

Compound 28a ¹H NMR (600 MHz, CDCl₃)

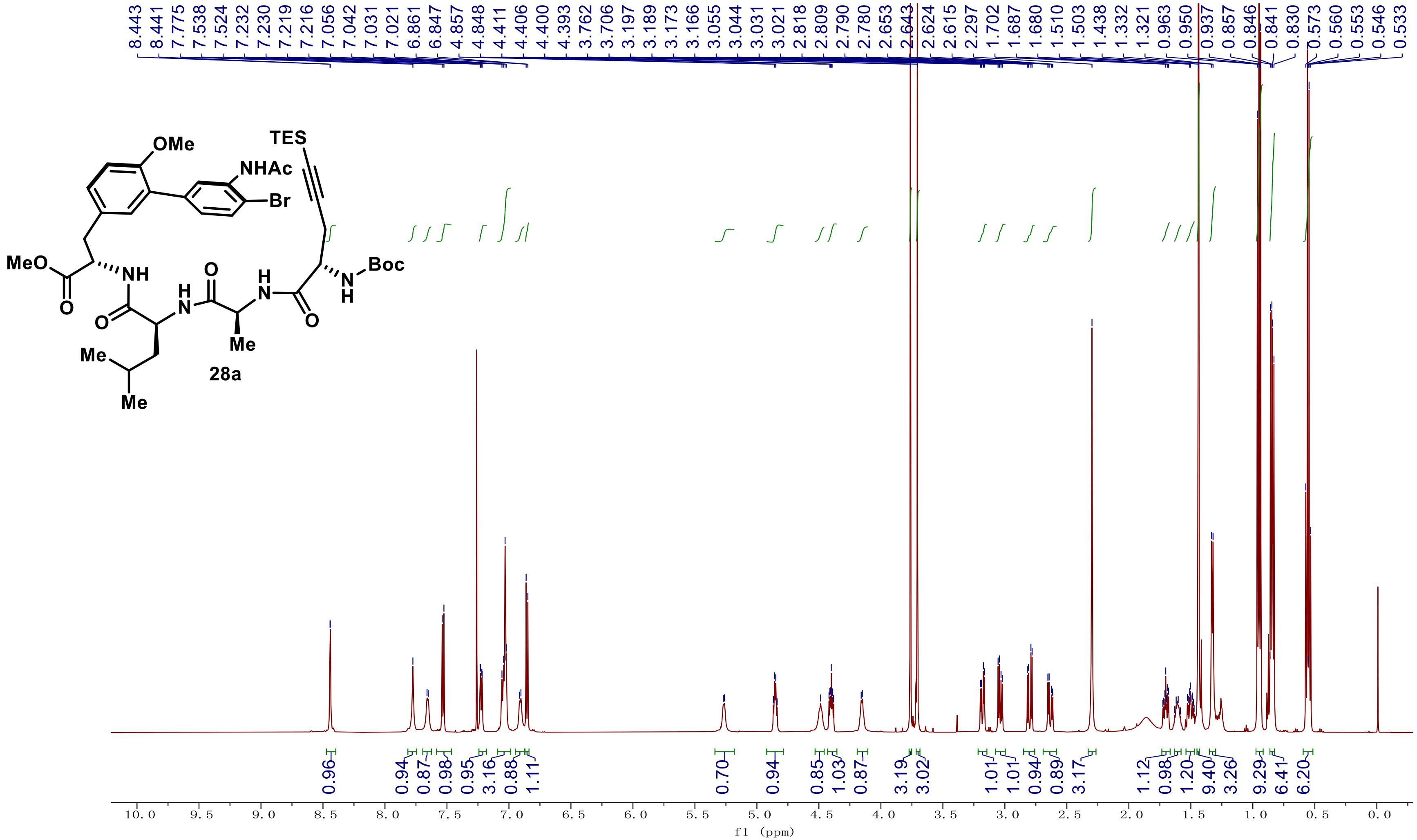

Compound 28a ^{13}C NMR (151 MHz, $CDCl_3$)

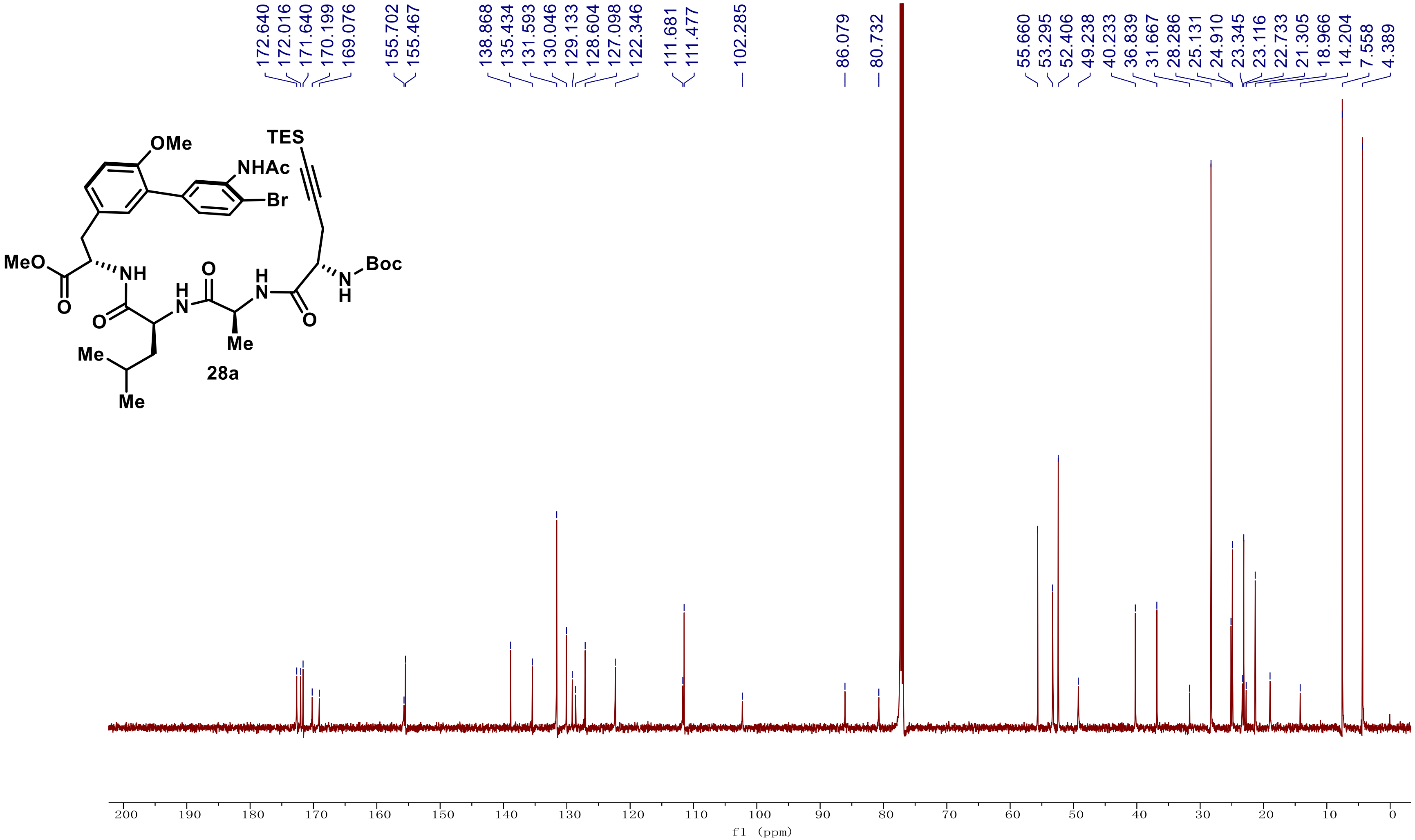

Compound 28b ^{1}H NMR (600 MHz, $CDCl_3$)

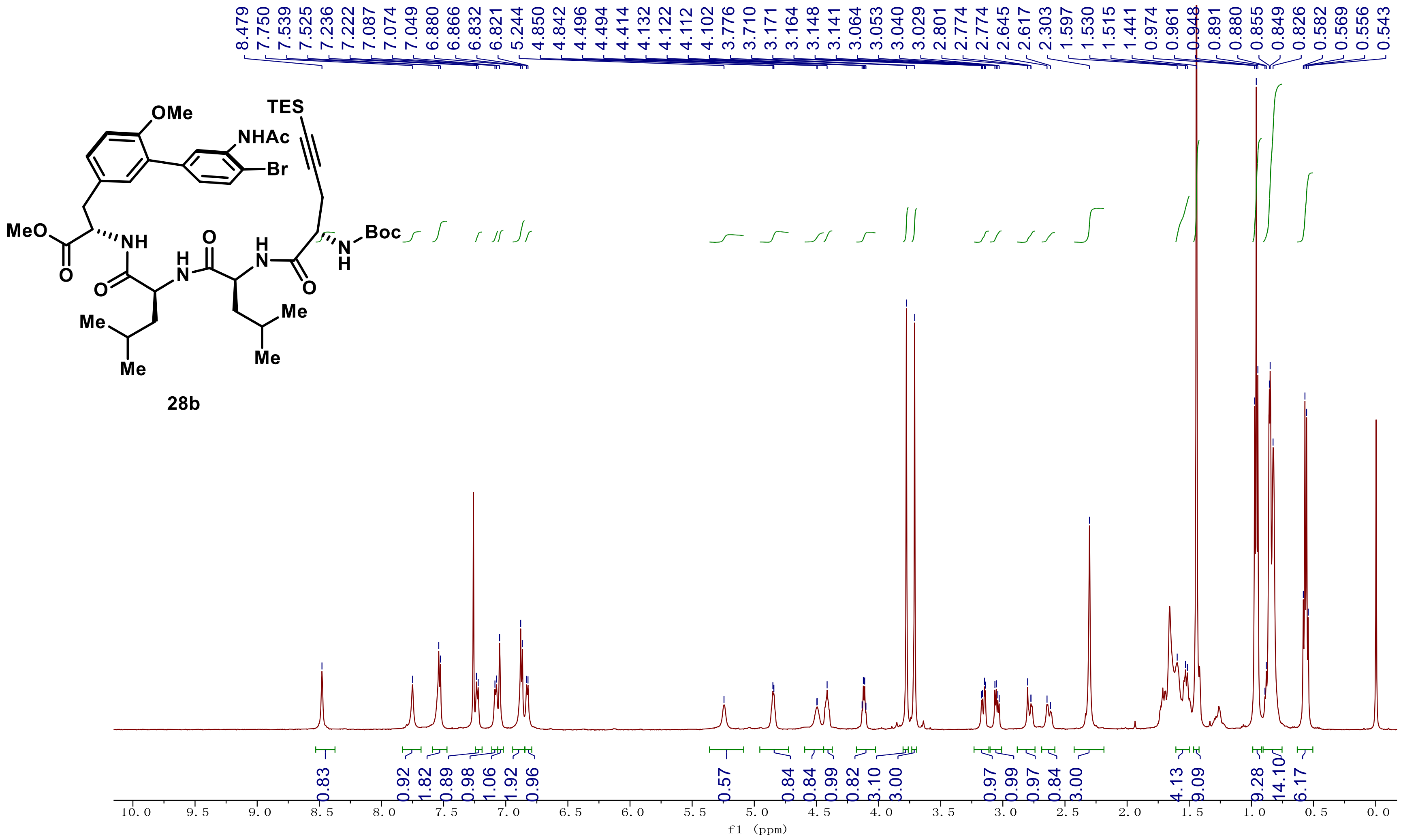

Compound 28b ^{13}C NMR (151 MHz, CDCl$_3$)

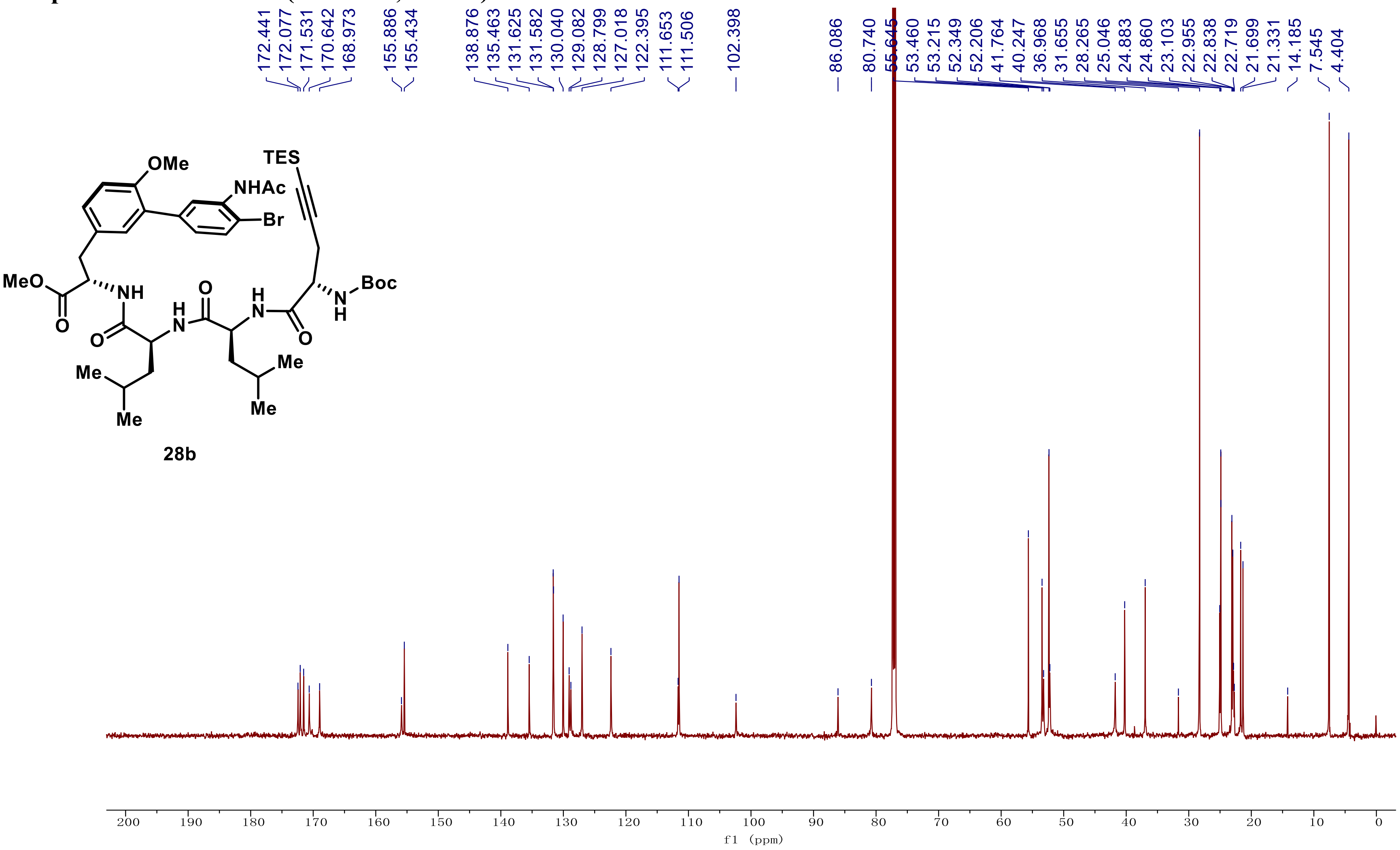

Compound 28c ^{1}H NMR (600 MHz, $CDCl_3$)

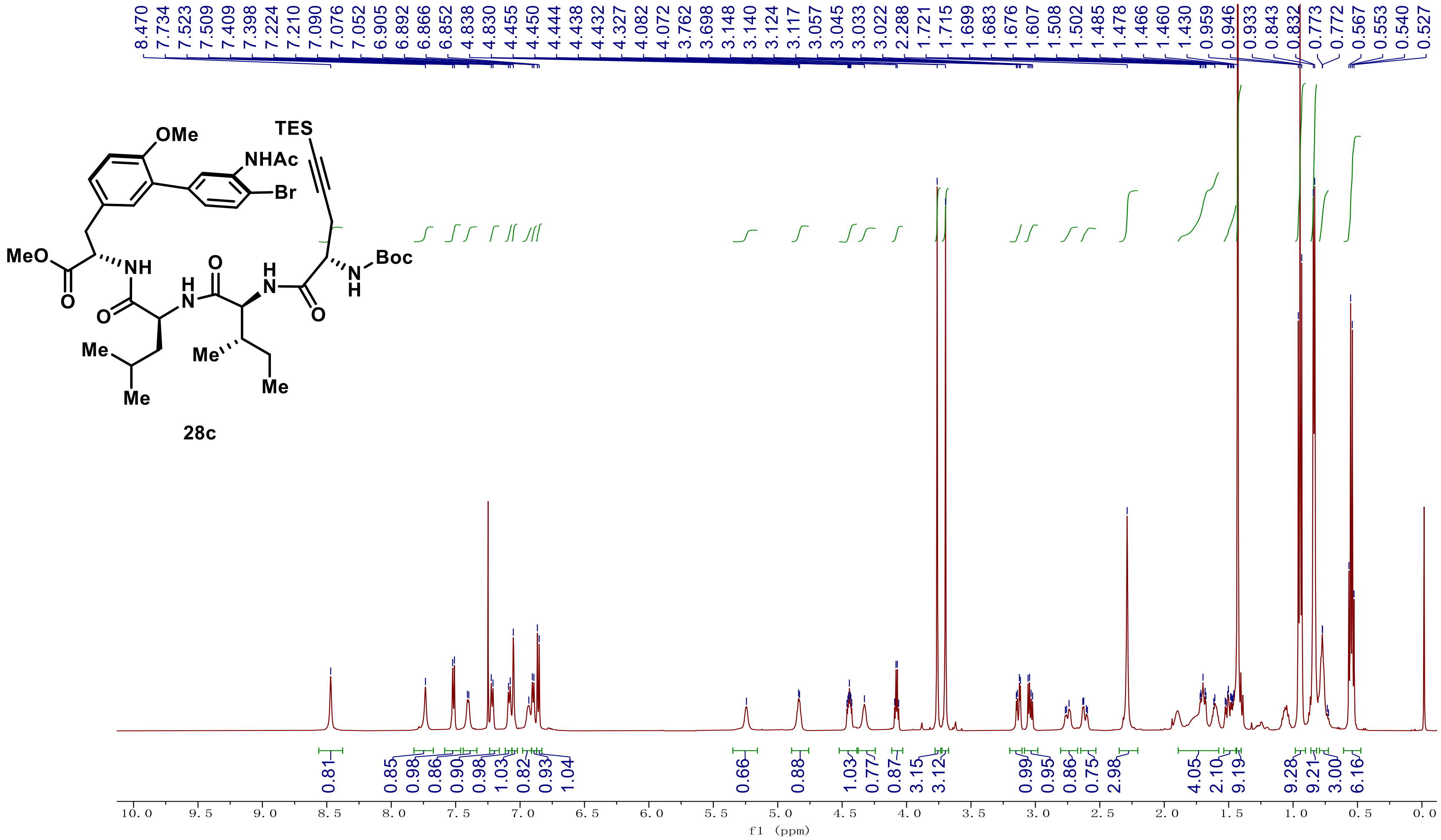

Compound 28c ^{13}C NMR (151 MHz, CDCl$_3$)

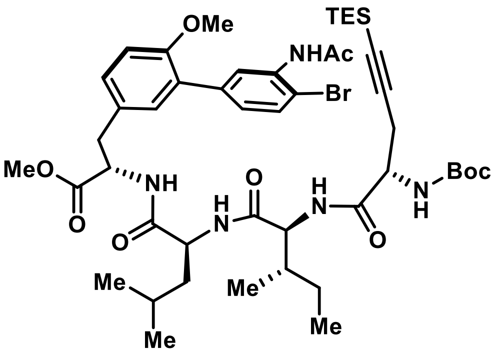

28c

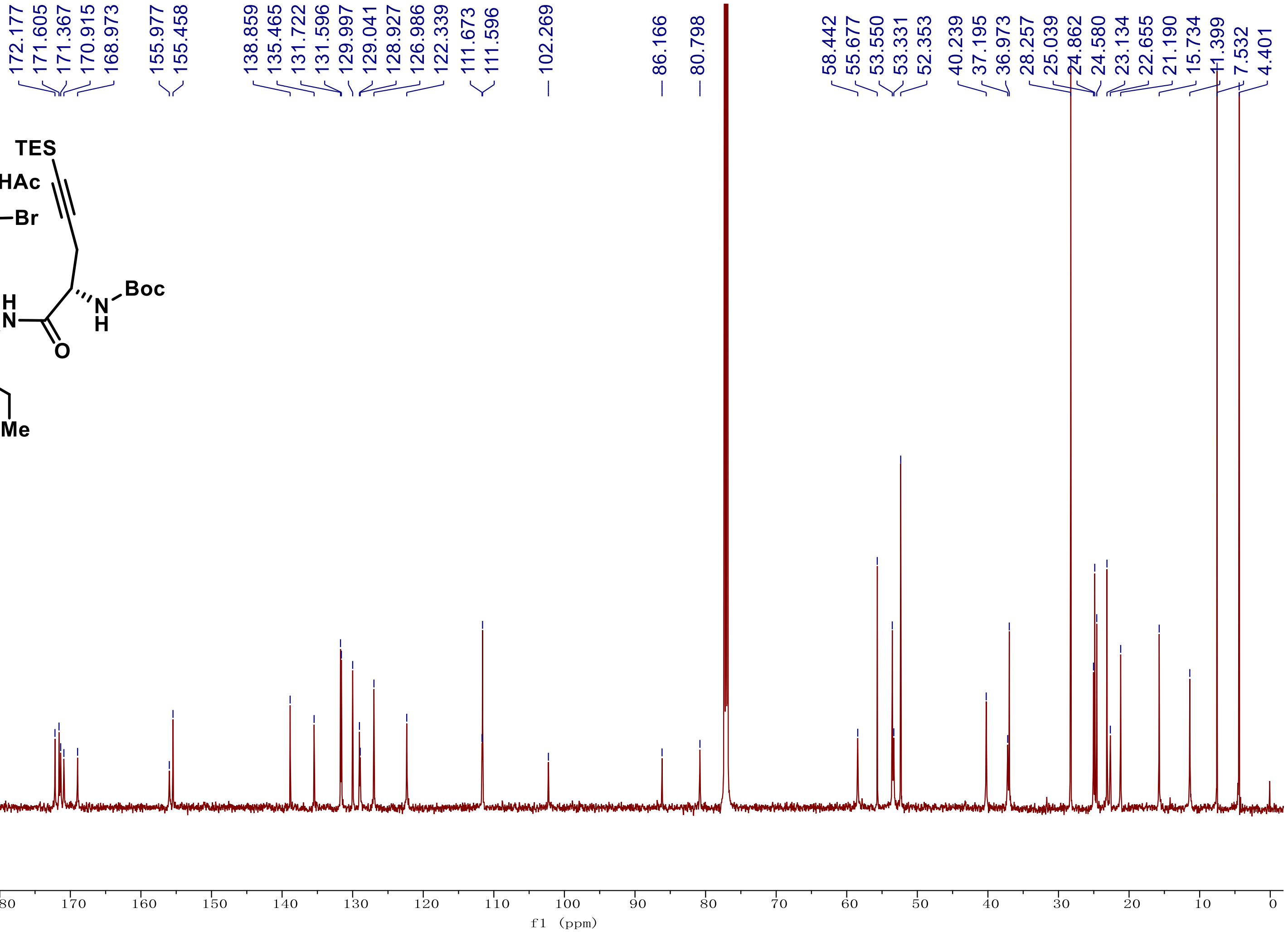

Compound 28d ¹H NMR (600 MHz, CDCl₃)

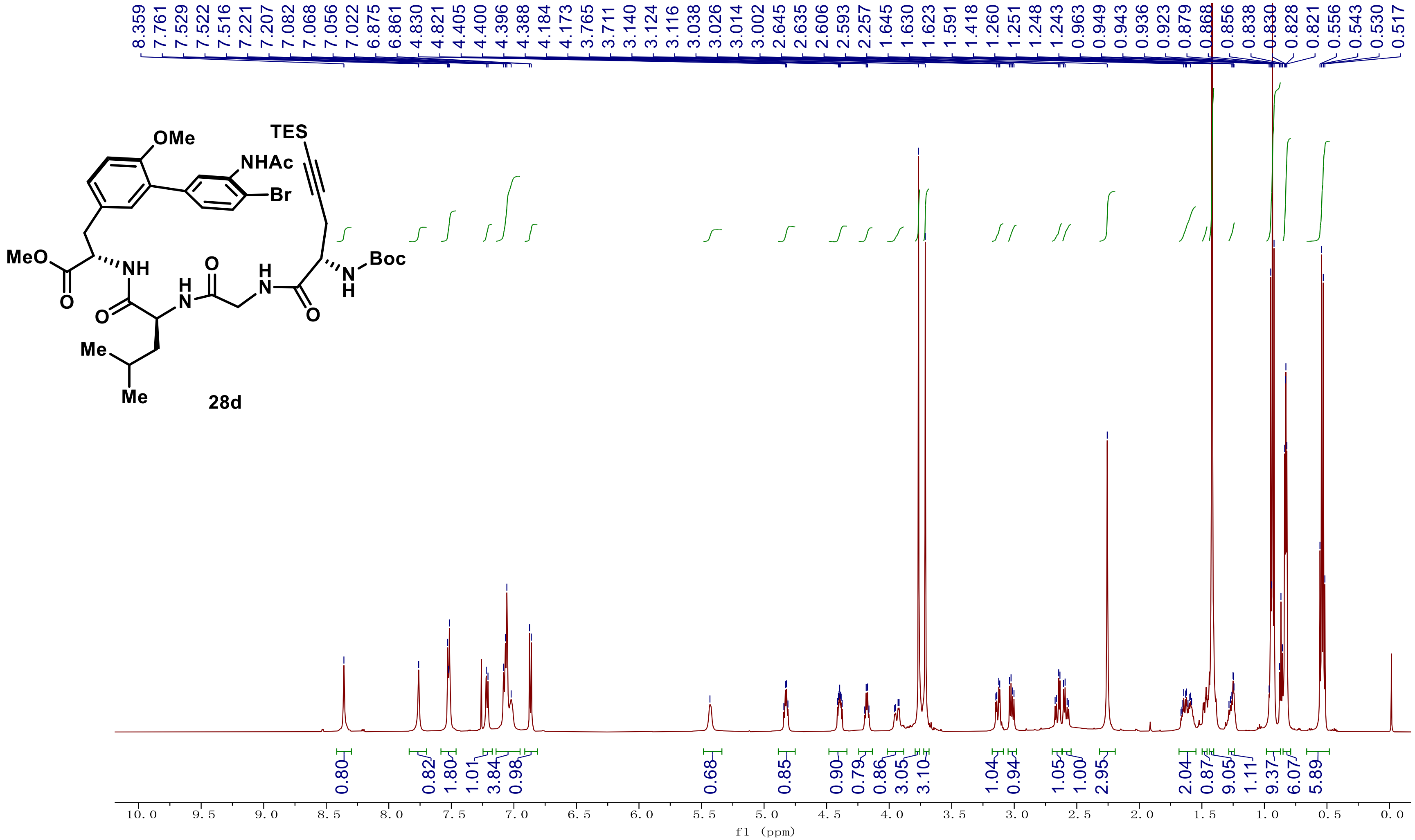

Compound 28d ^{13}C NMR (151 MHz, CDCl$_3$)

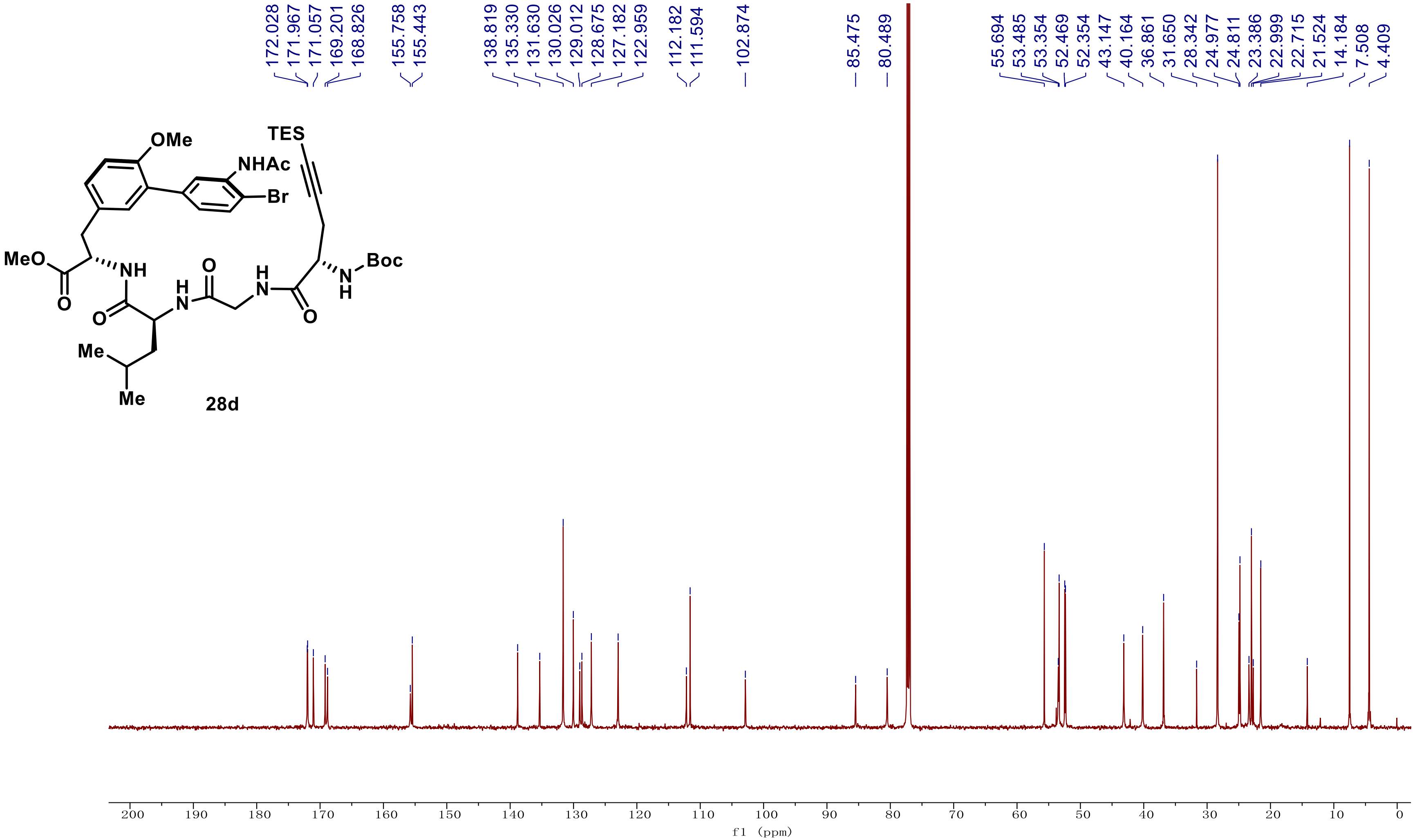

Compound 28e ^{1}H NMR (600 MHz, CDCl₃)

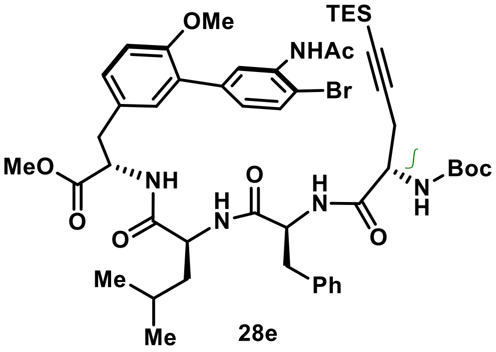

28e

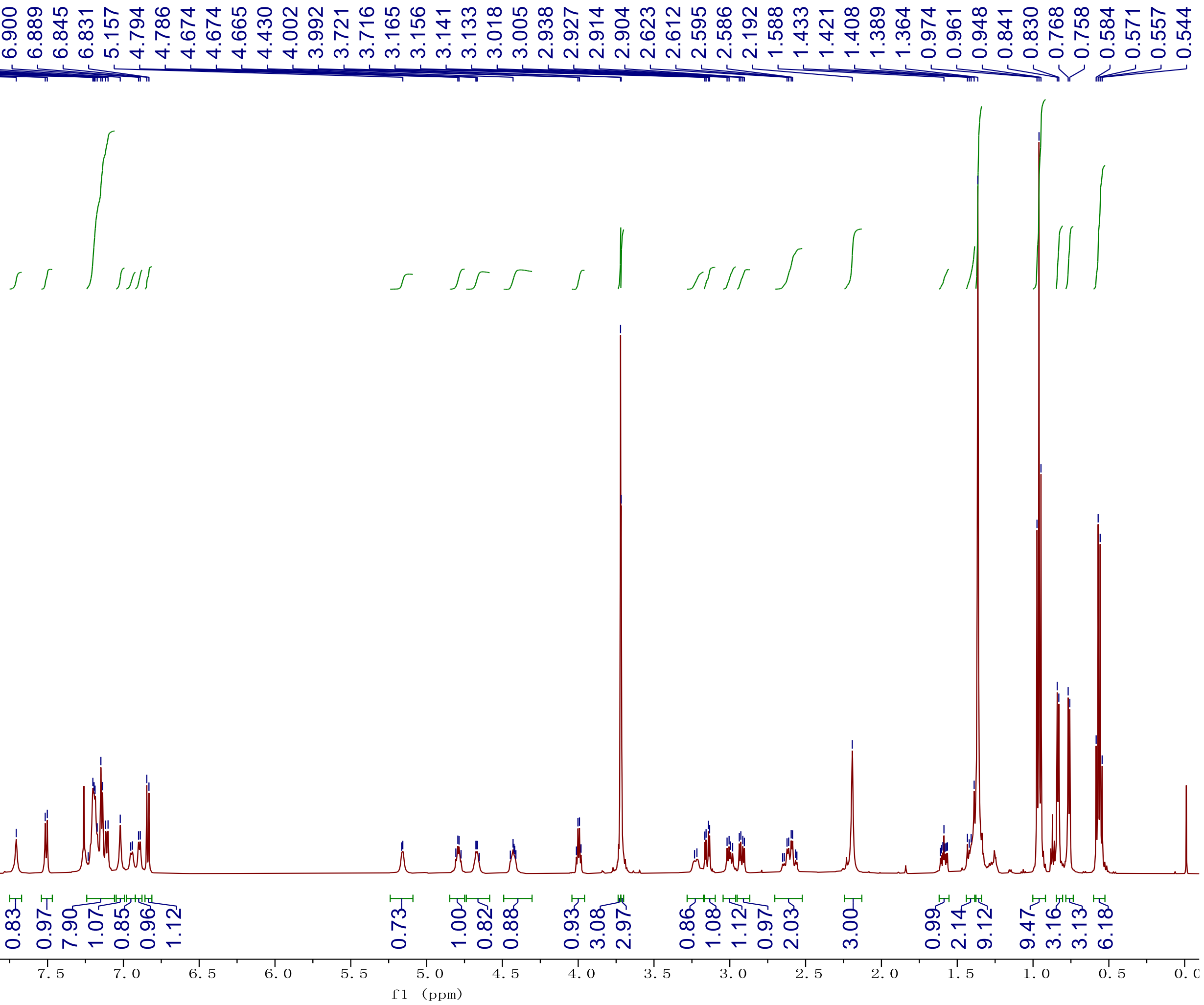

Compound 28e ^{13}C NMR (151 MHz, $CDCl_3$)

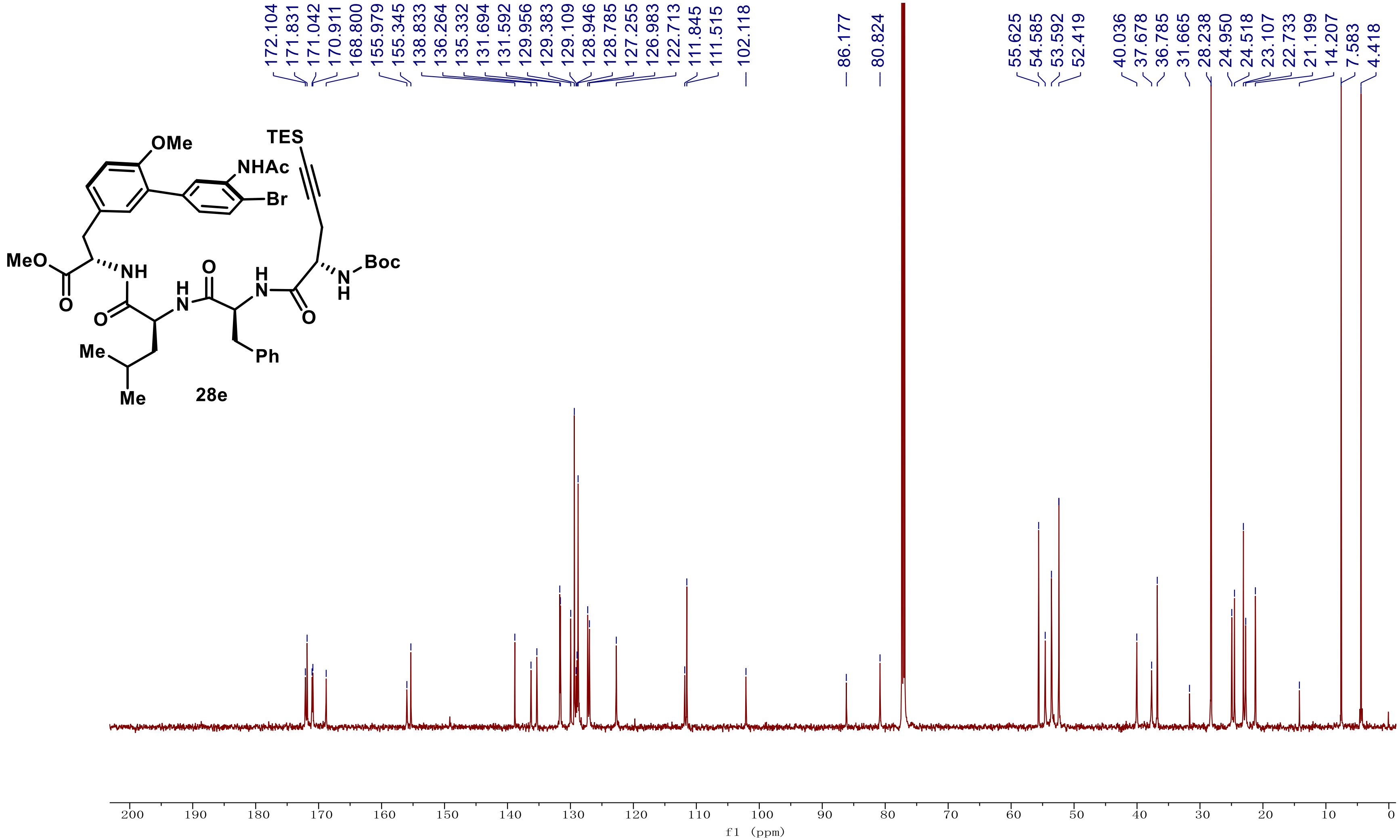

Compound 28f ¹H NMR (600 MHz, $CDCl_3$)

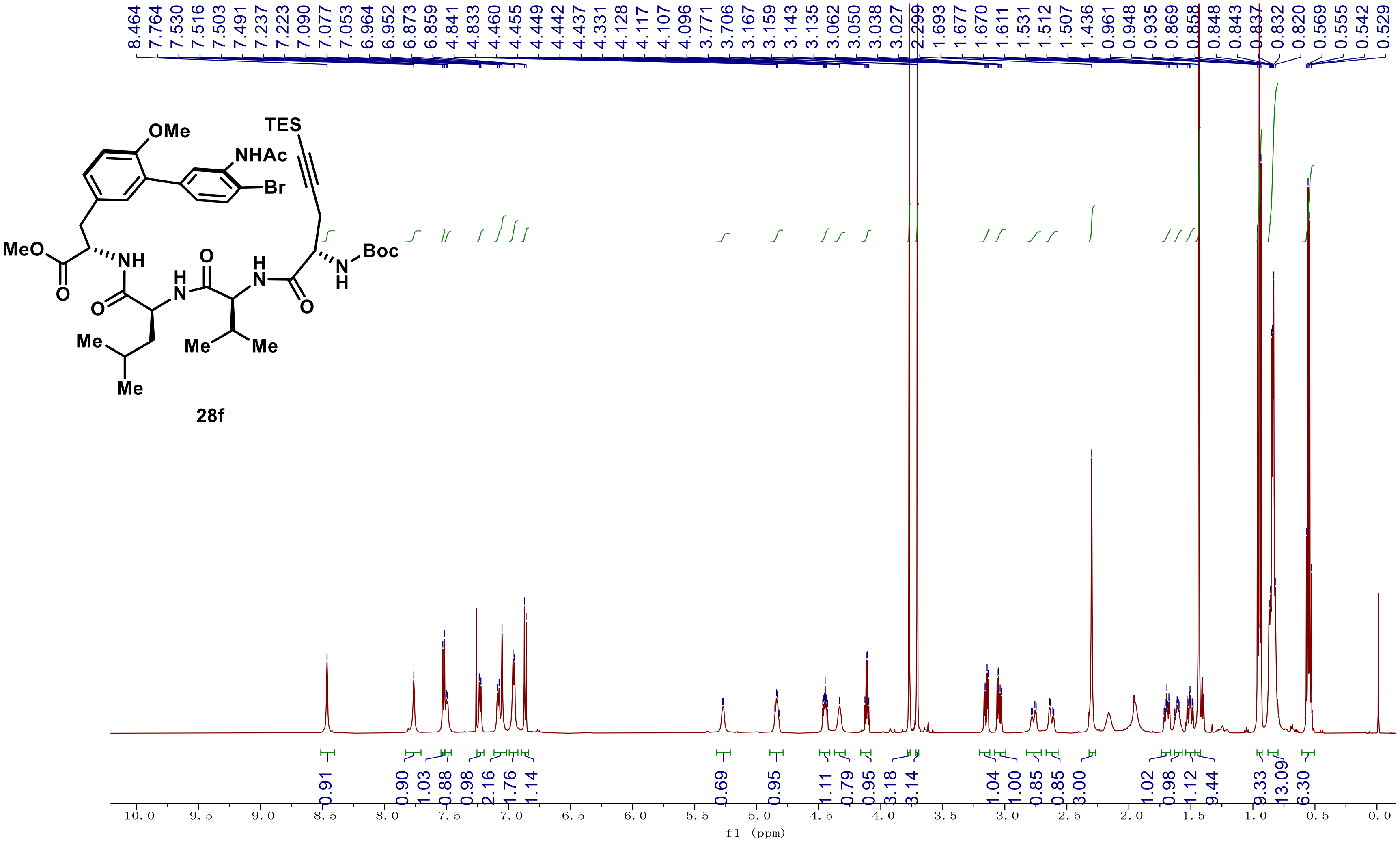

Compound 28a ^{13}C NMR (151 MHz, $CDCl_3$)

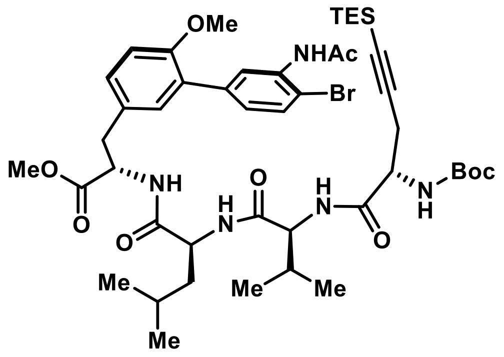

28f

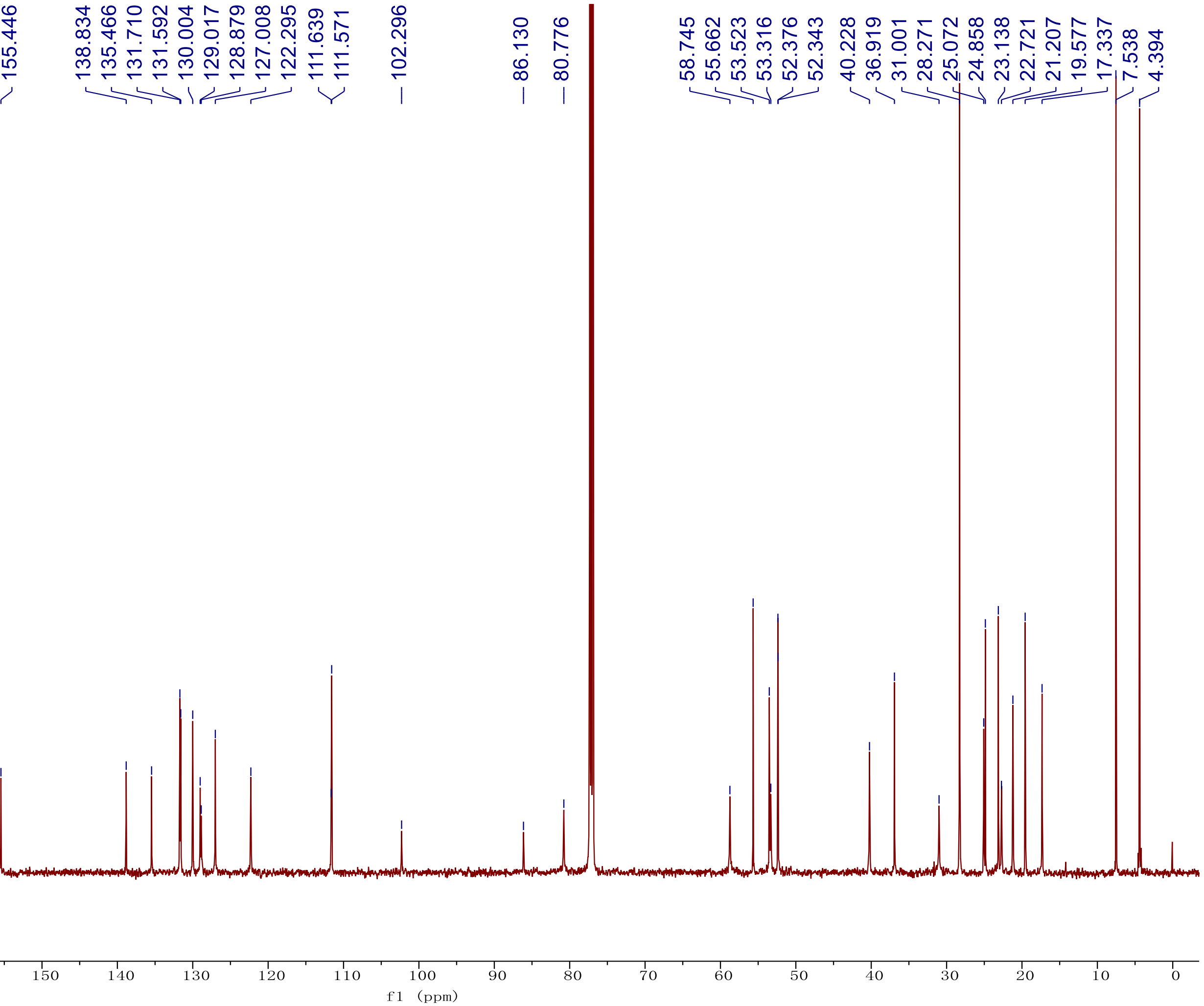

Compound 28g ^{1}H NMR (600 MHz, $CDCl_3$)

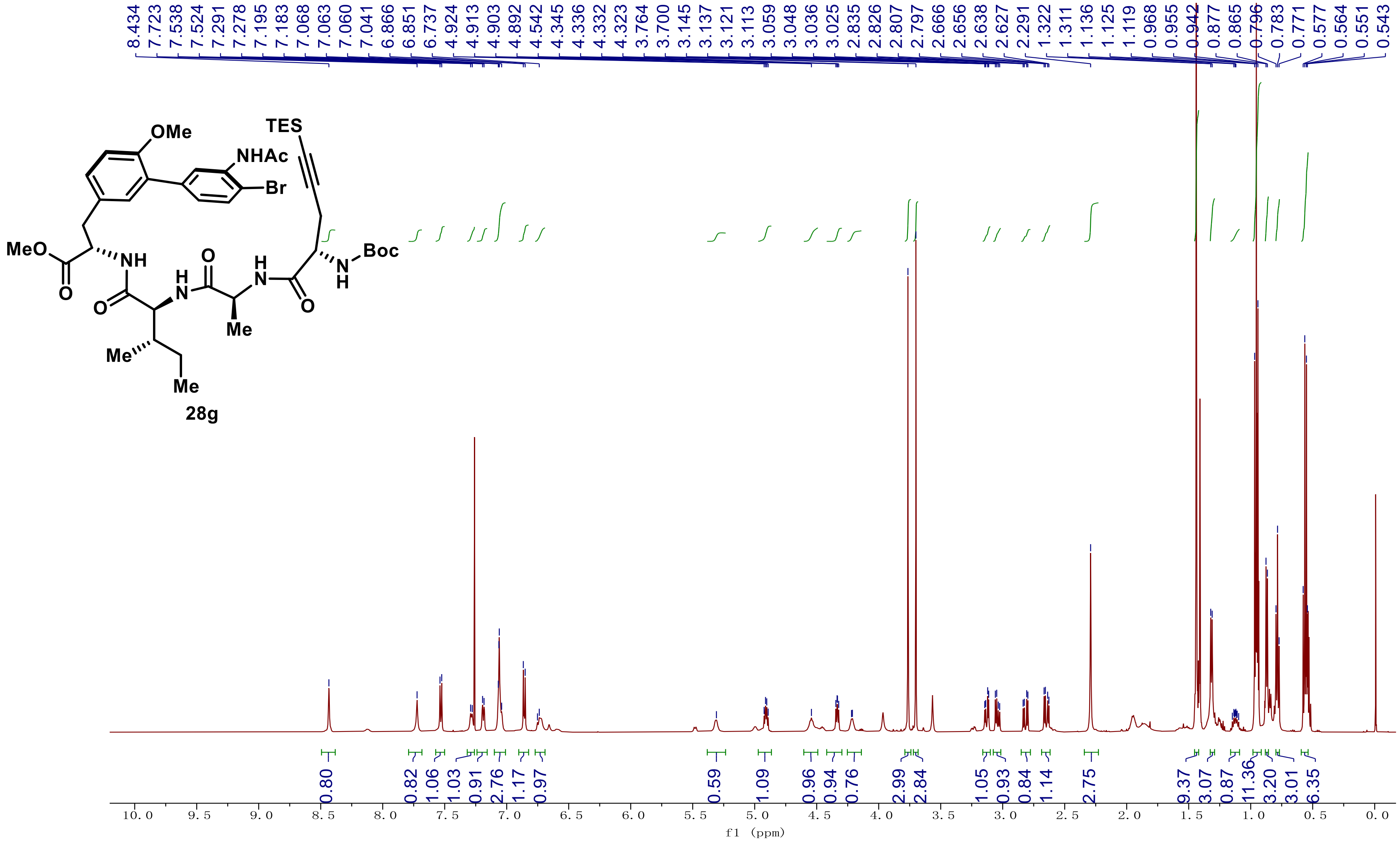

Compound 28g ^{13}C NMR (151 MHz, $CDCl_3$)

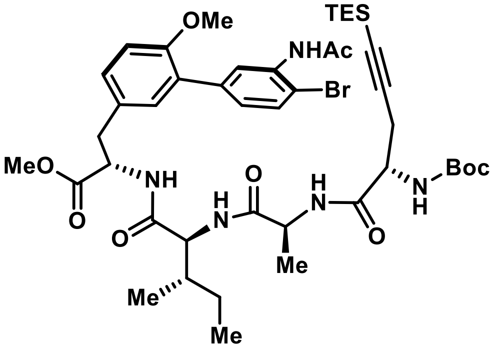

28g

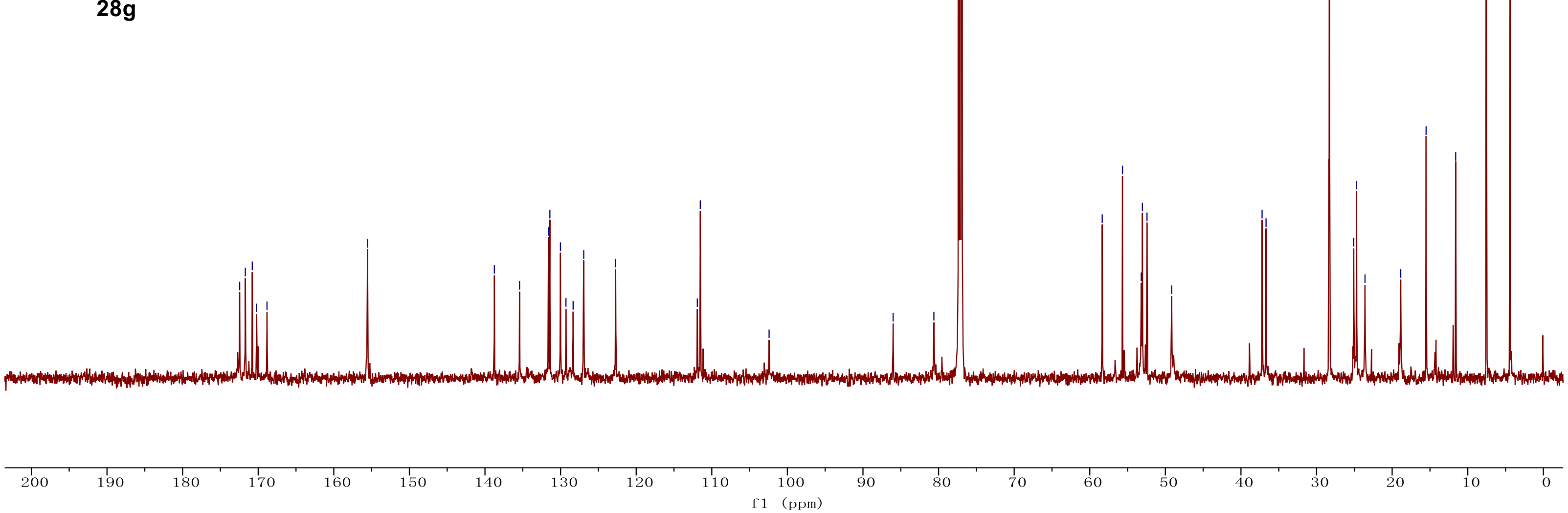

Compound 28h ^{1}H NMR (600 MHz, CDCl$_3$)

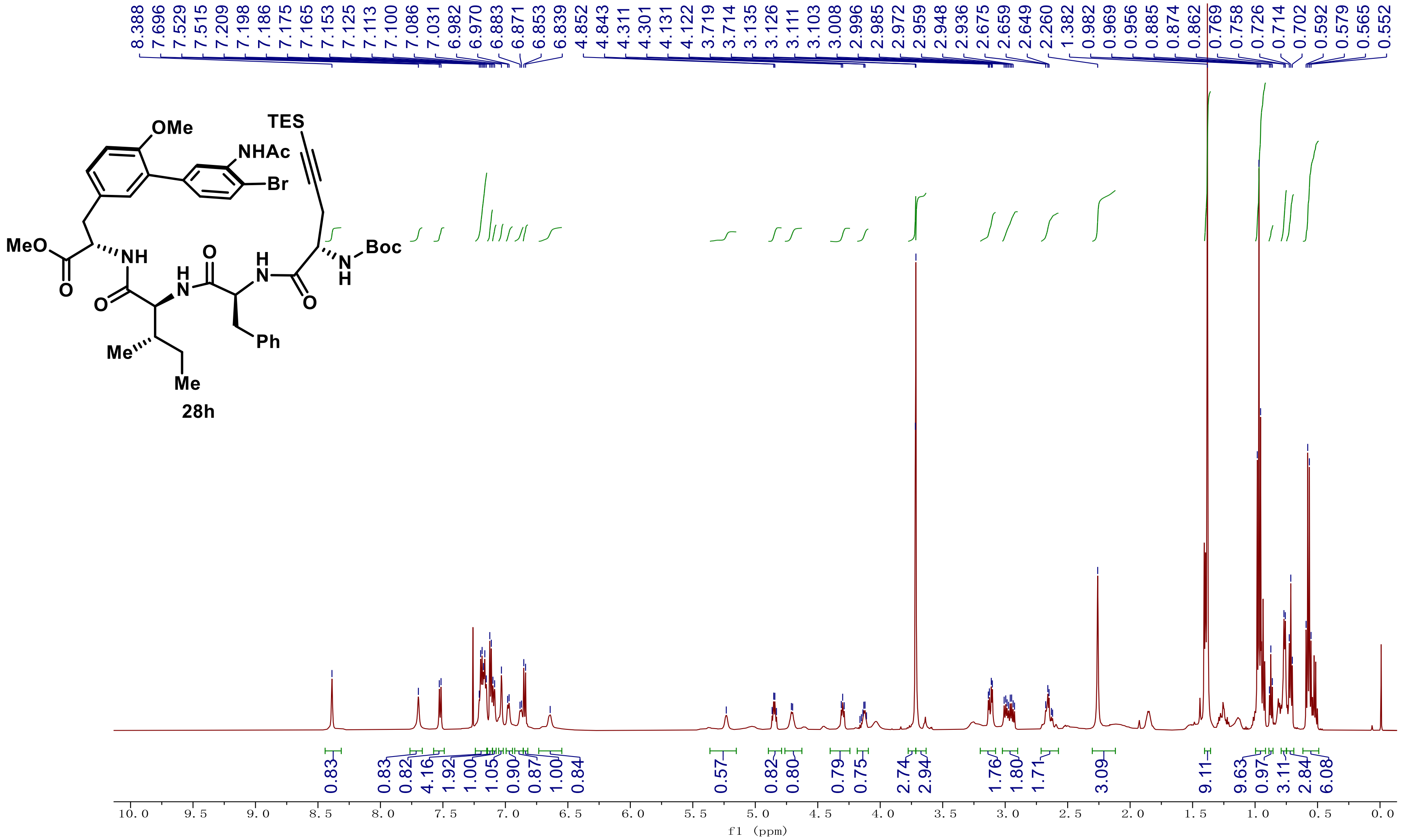

Compound 28h ^{13}C NMR (151 MHz, $CDCl_3$)

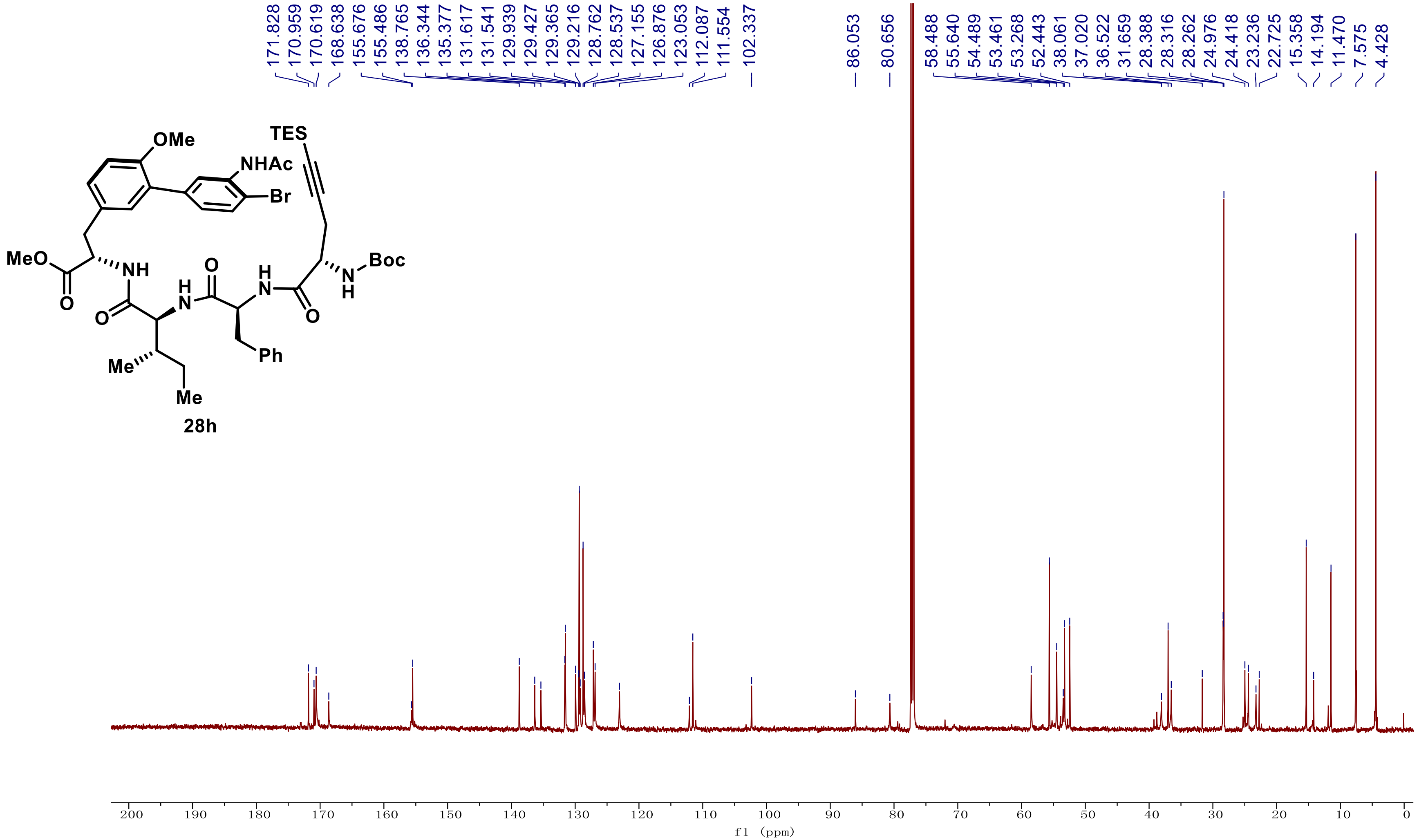

Compound 28i ¹H NMR (600 MHz, $CDCl_3$)

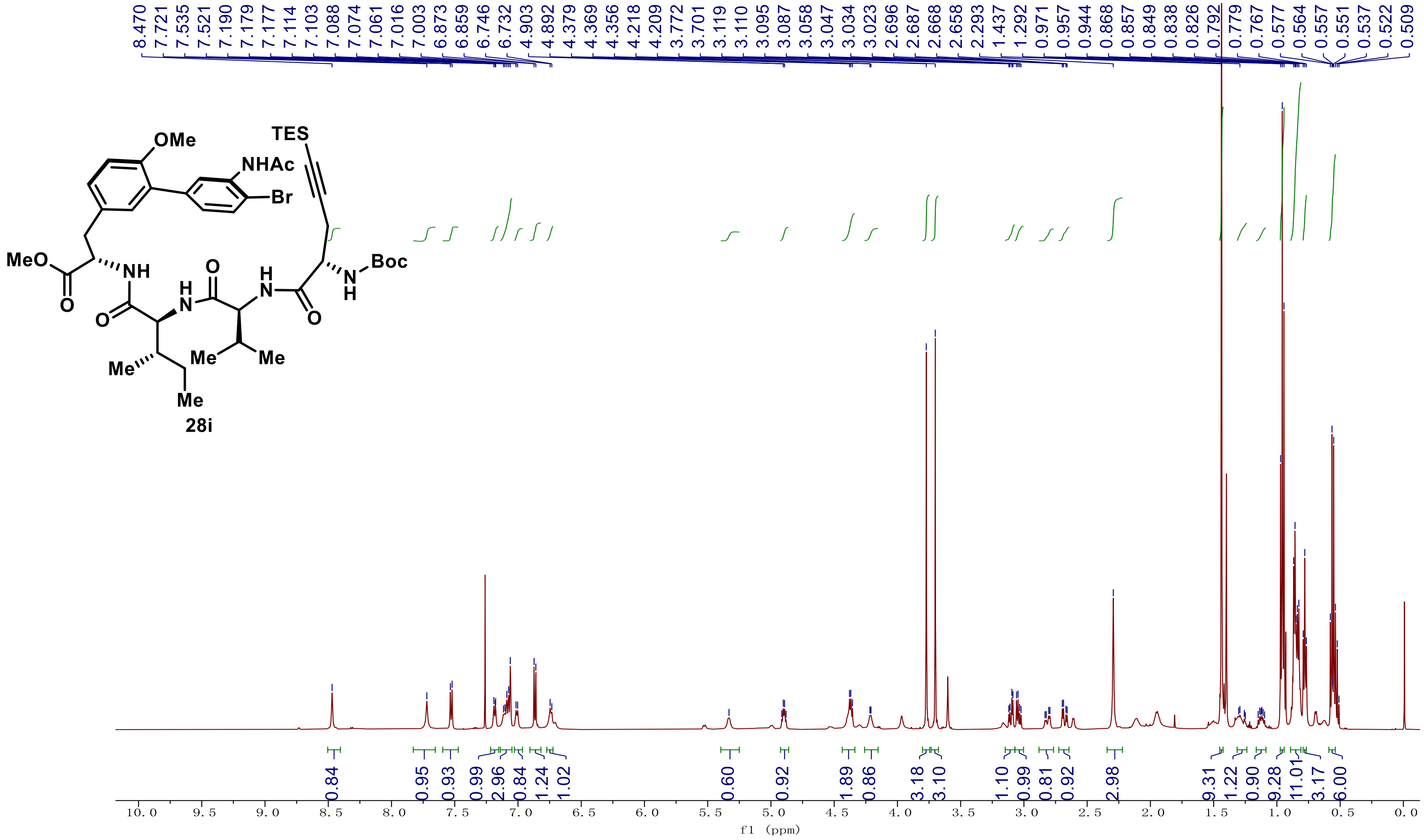

Compound 28i ^{13}C NMR (151 MHz, $CDCl_3$)

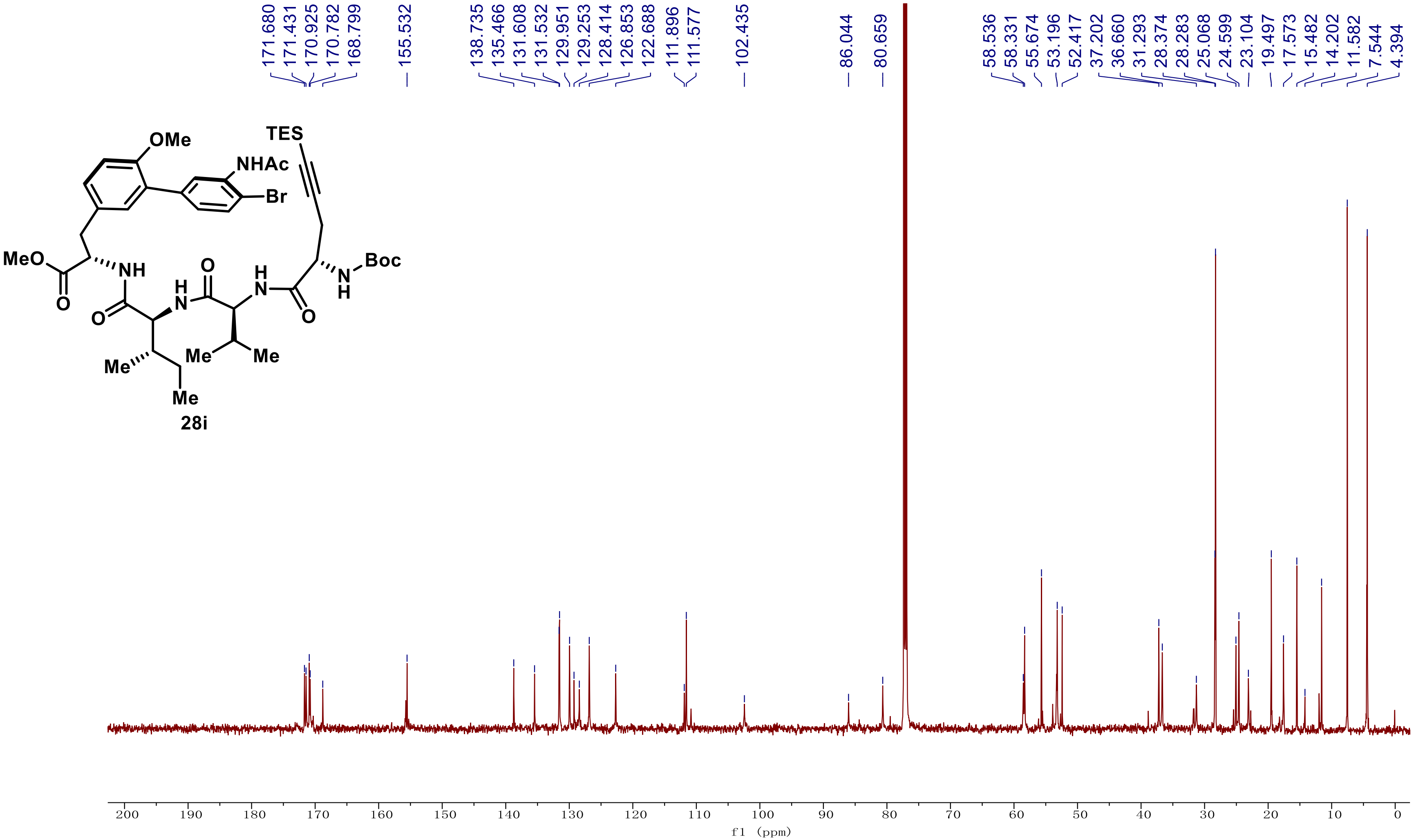

Compound 28j ¹H NMR (600 MHz, CDCl₃)

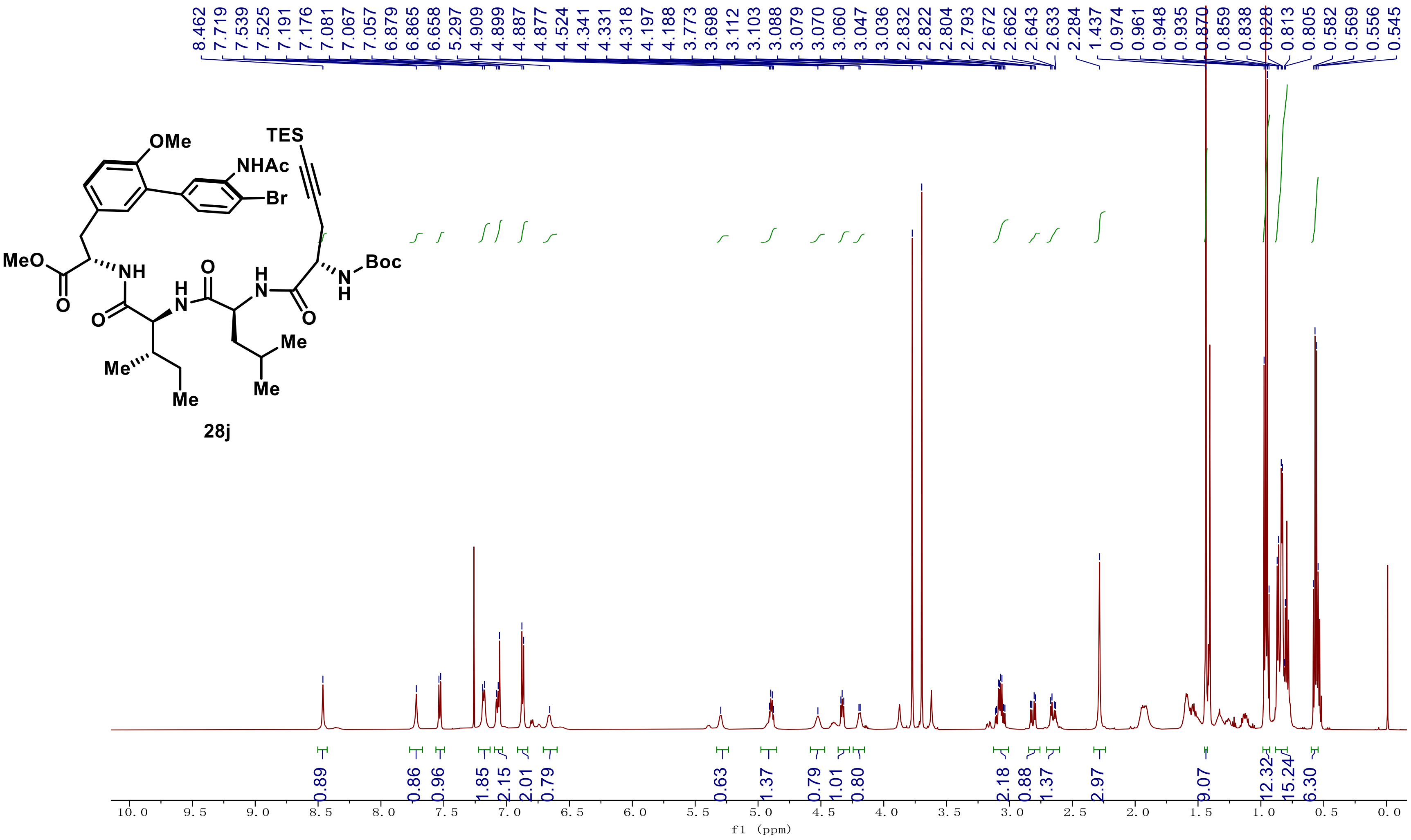

28j

Compound 28j ^{13}C NMR (151 MHz, CDCl$_3$)

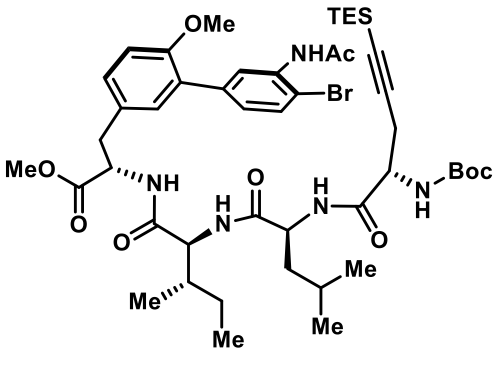

28j

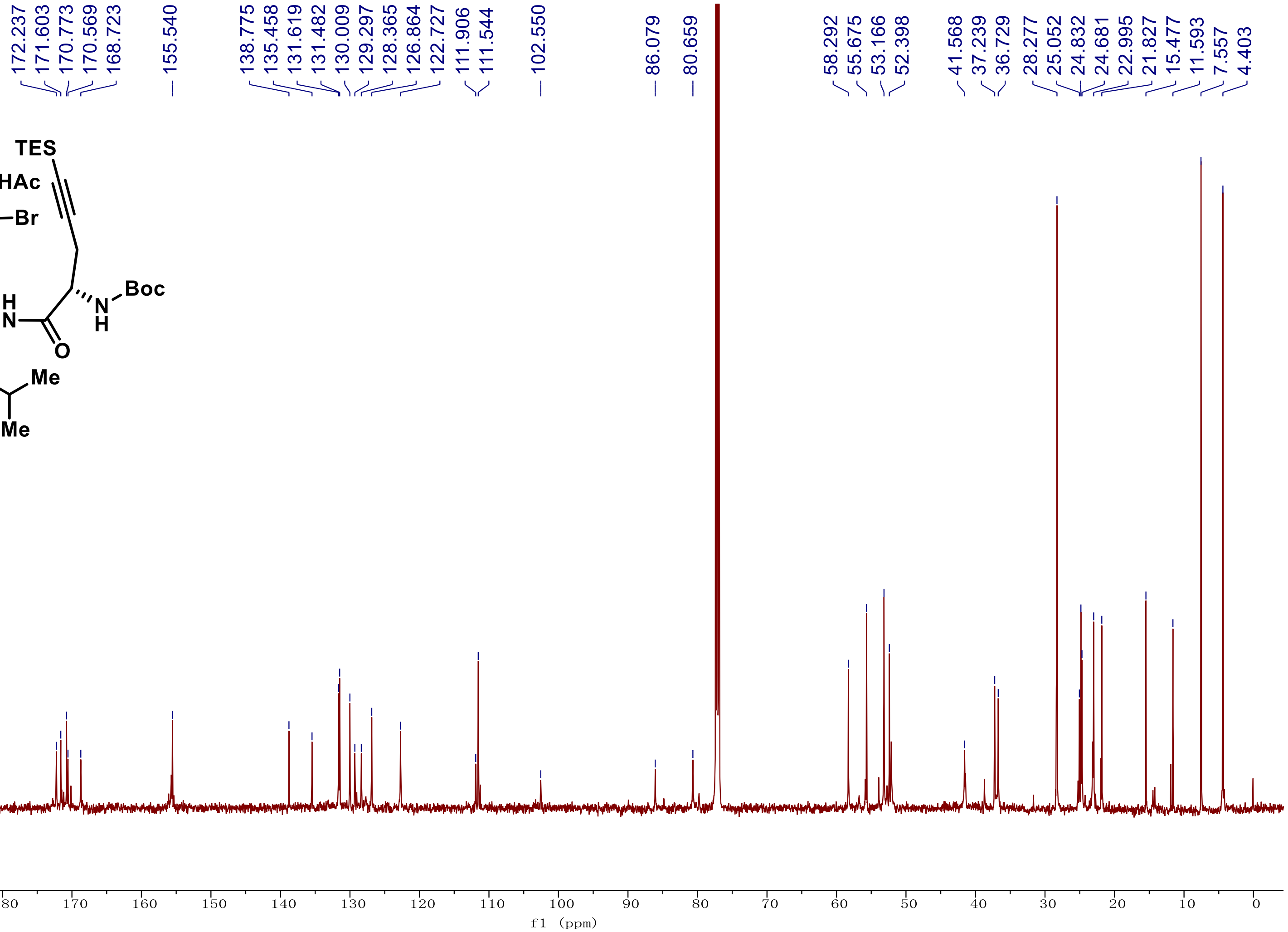

Compound 28k ^{1}H NMR (600 MHz, $CDCl_3$)

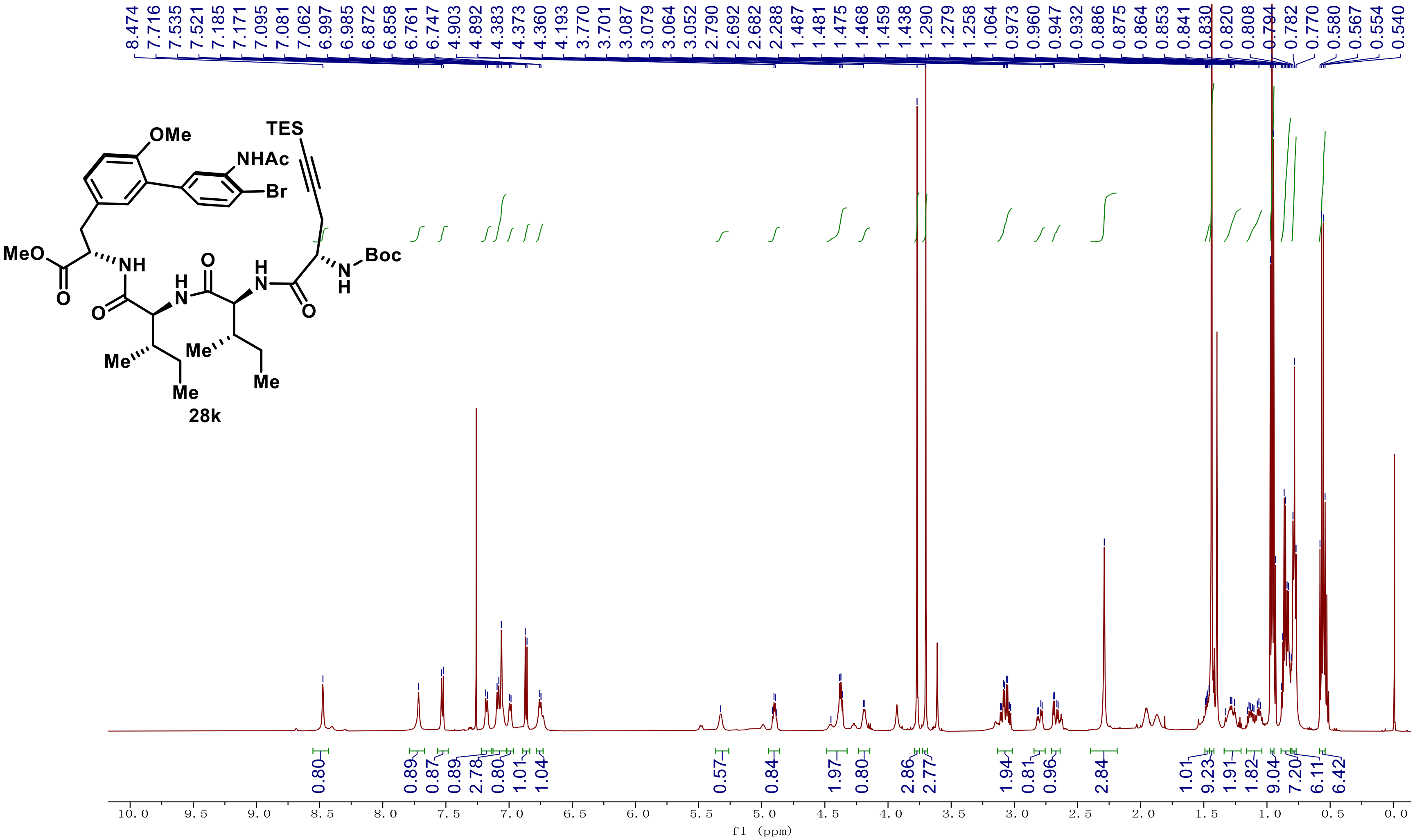

Compound 28k ^{13}C NMR (151 MHz, $CDCl_3$)

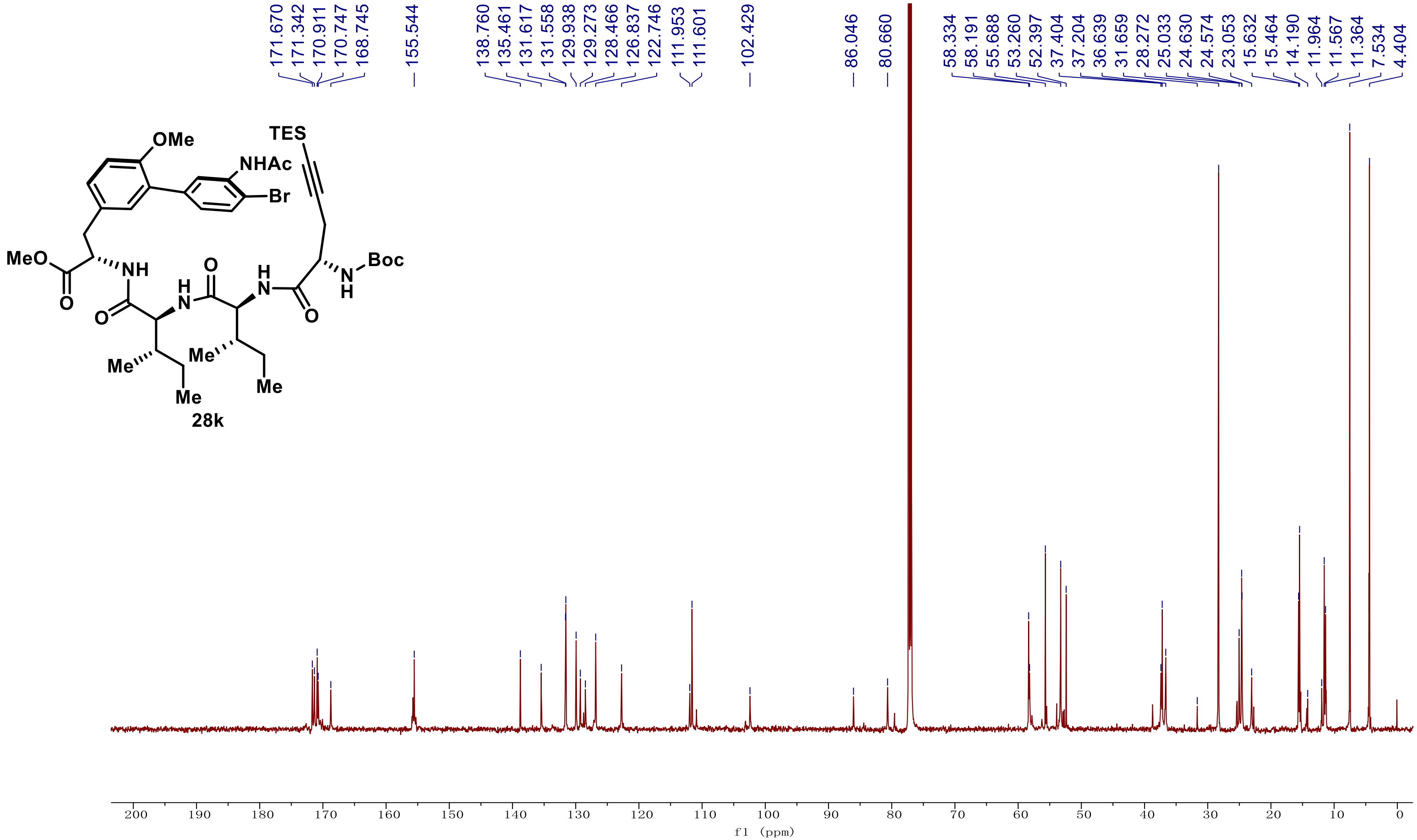

Compound 28l ^{13}C NMR (151 MHz, CDCl$_3$)

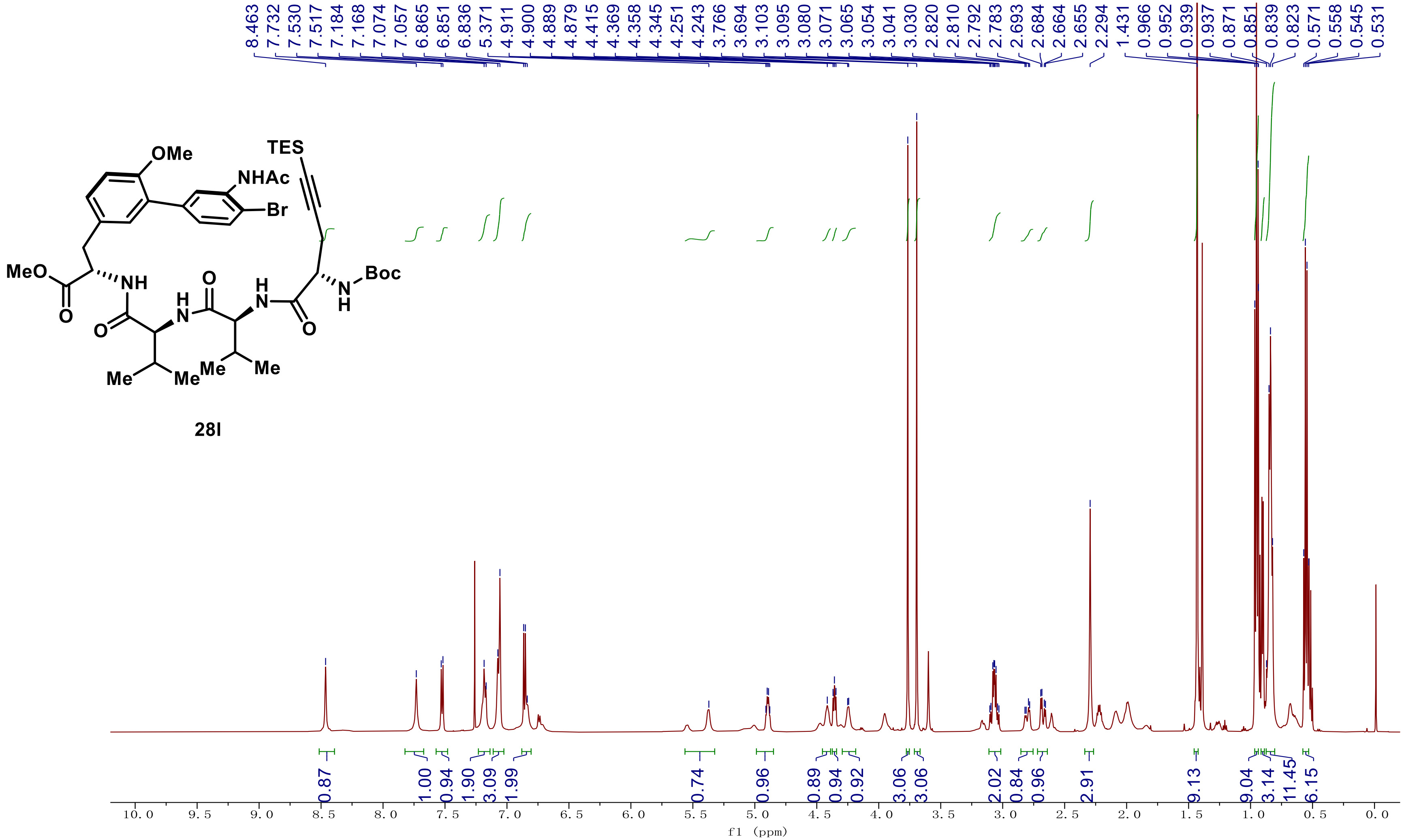

Compound 28l ^{13}C NMR (151 MHz, $CDCl_3$)

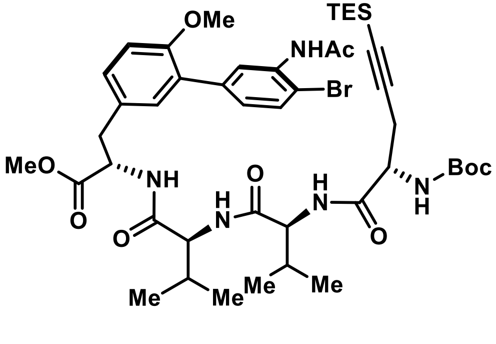

28l

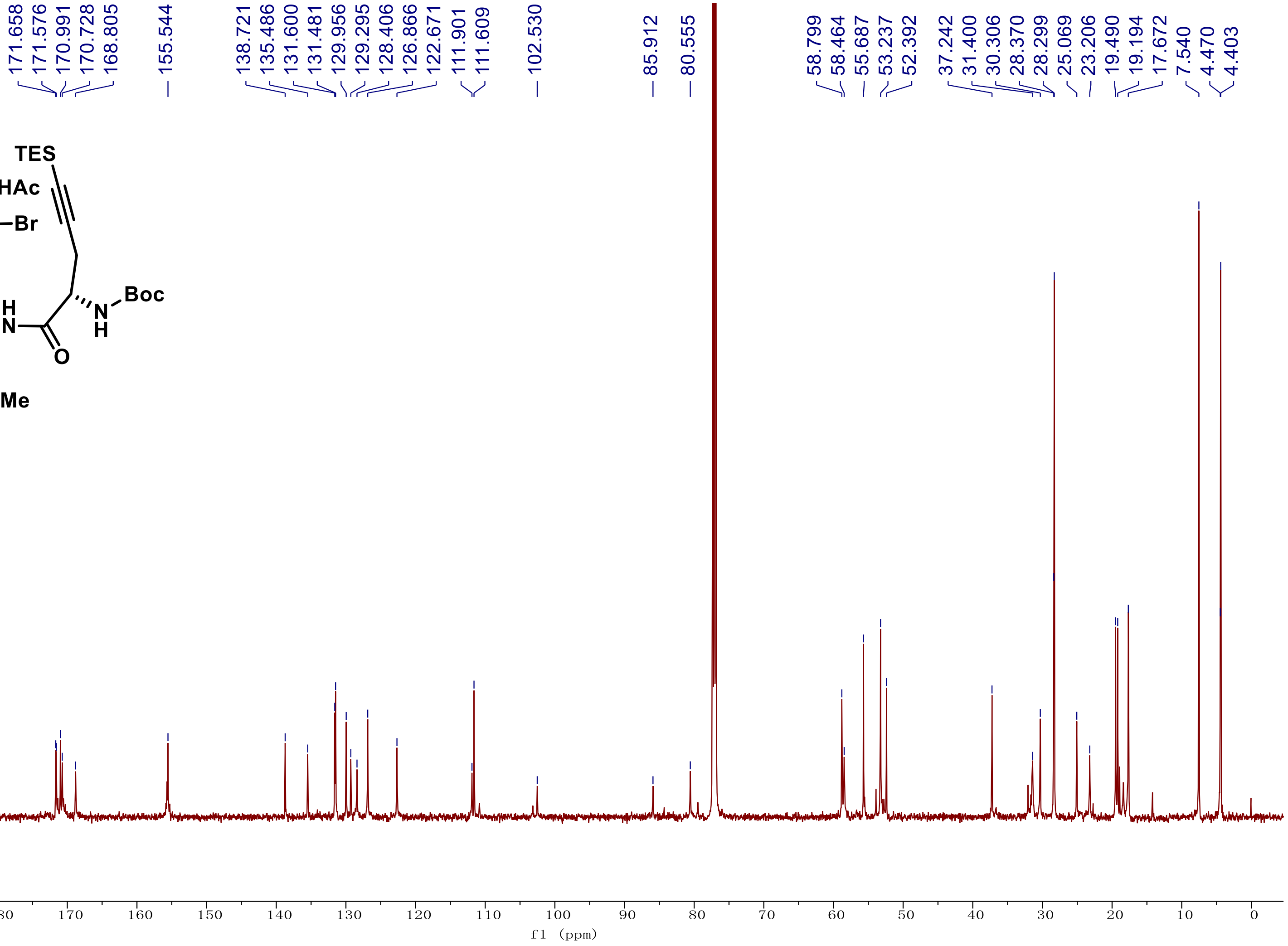

Compound 28m ^{1}H NMR (600 MHz, $CDCl_3$)

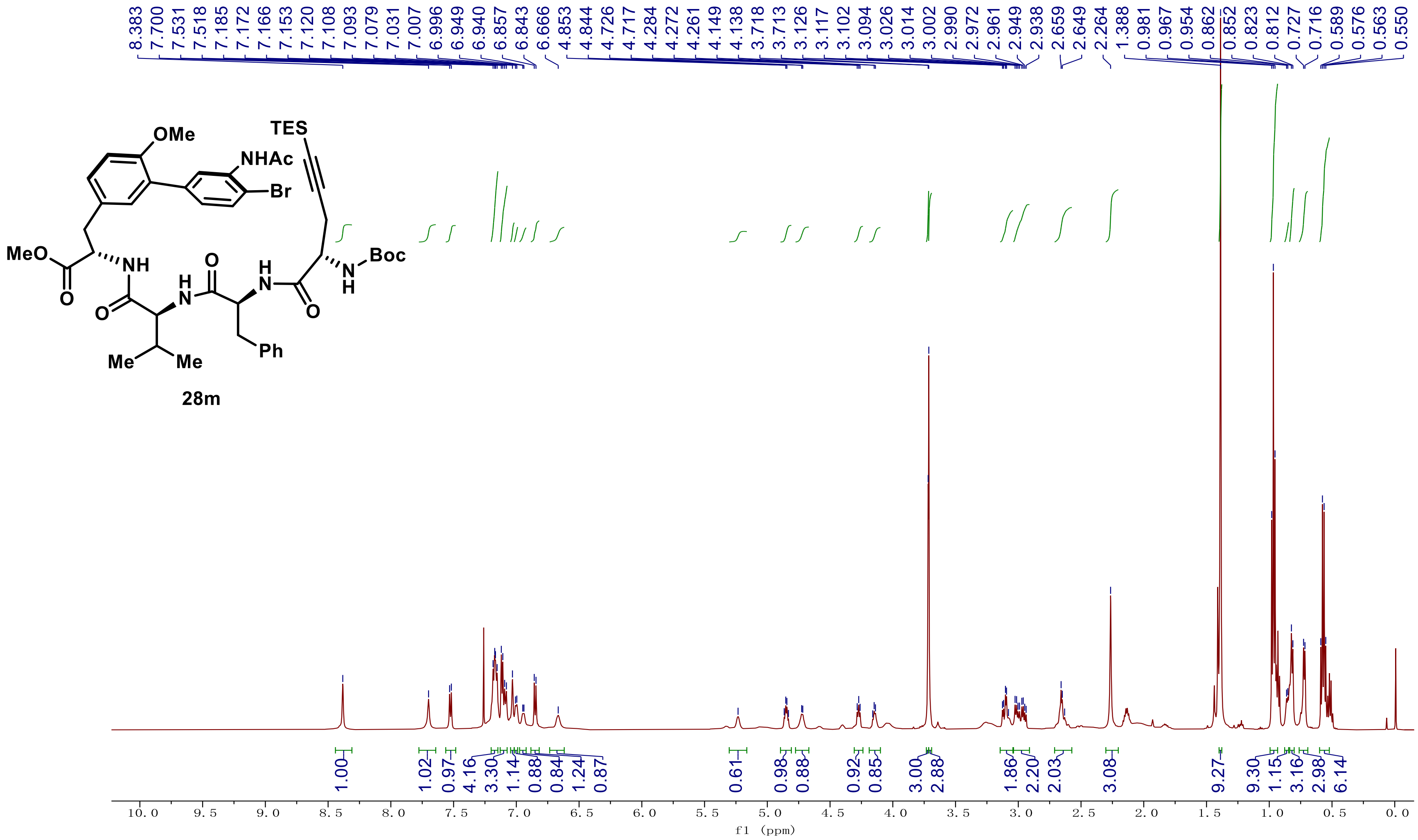

Compound 28m ^{13}C NMR (151 MHz, $CDCl_3$)

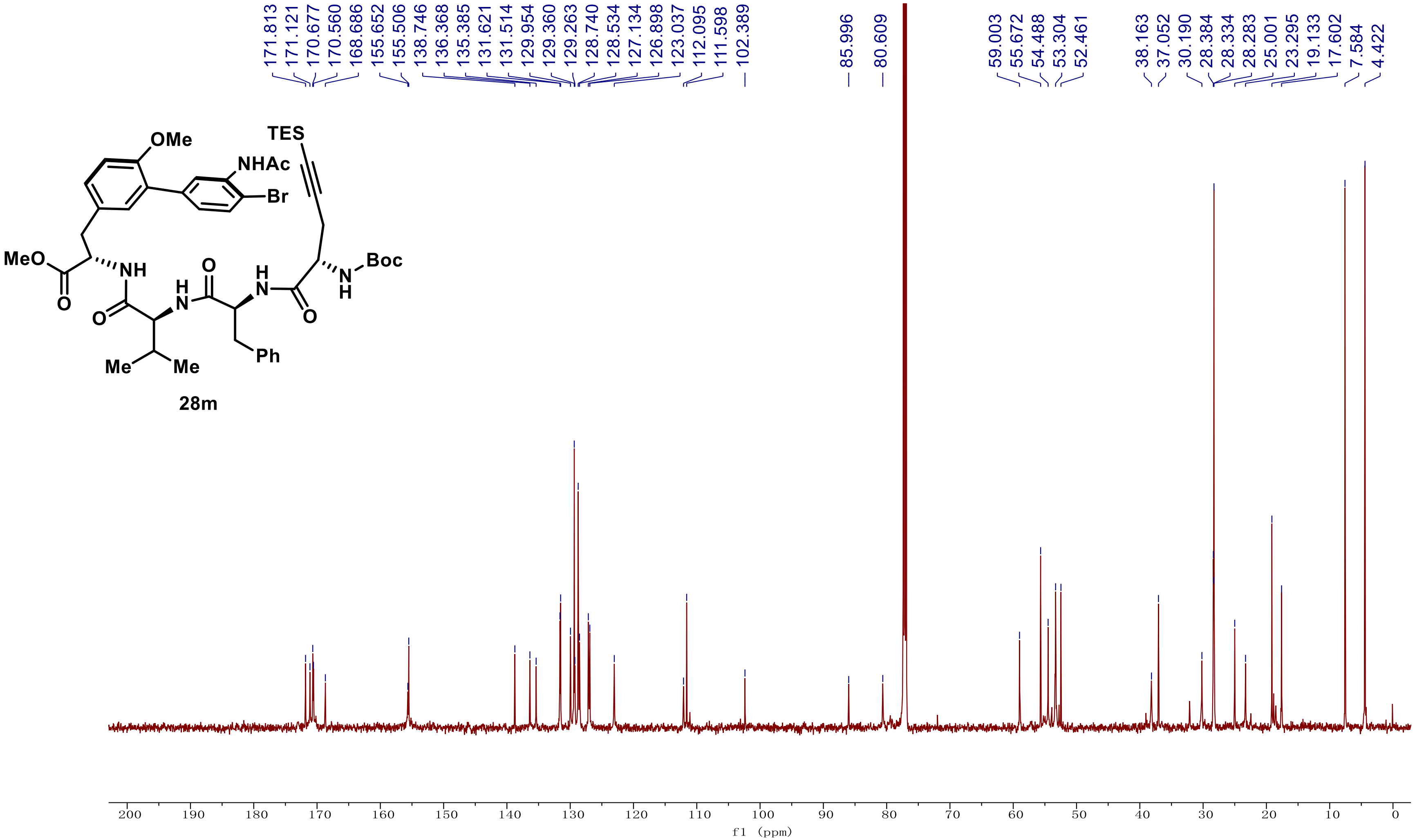

Compound 28n ^{1}H NMR (600 MHz, CDCl₃)

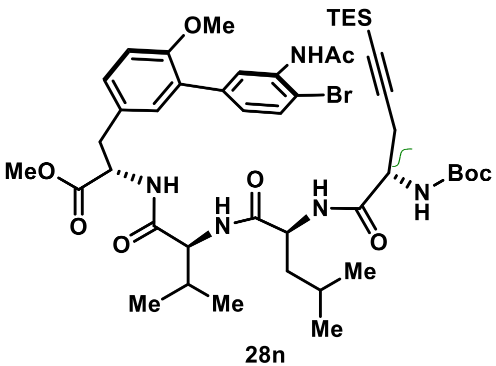

28n

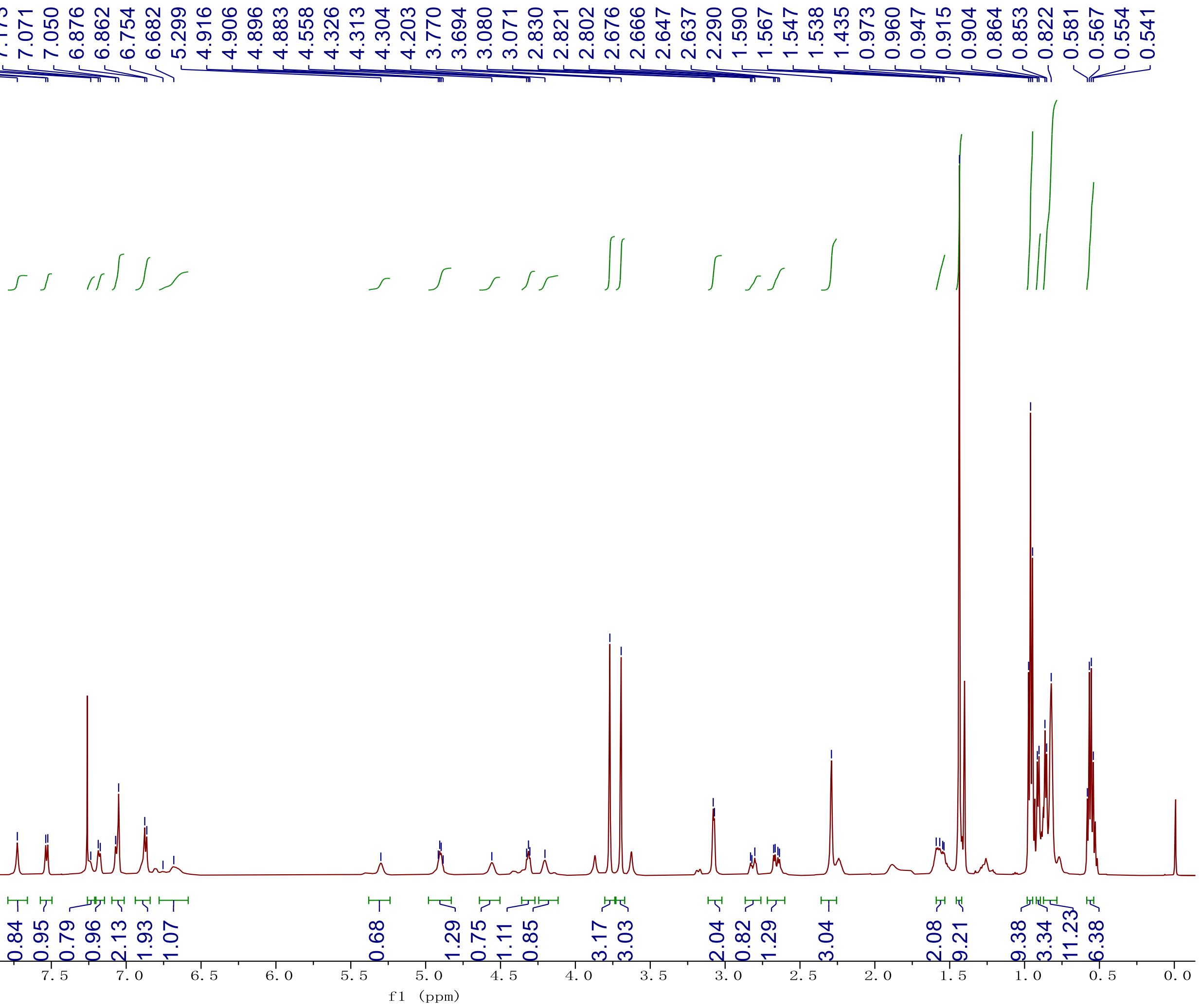

Compound 28n ^{13}C NMR (151 MHz, $CDCl_3$)

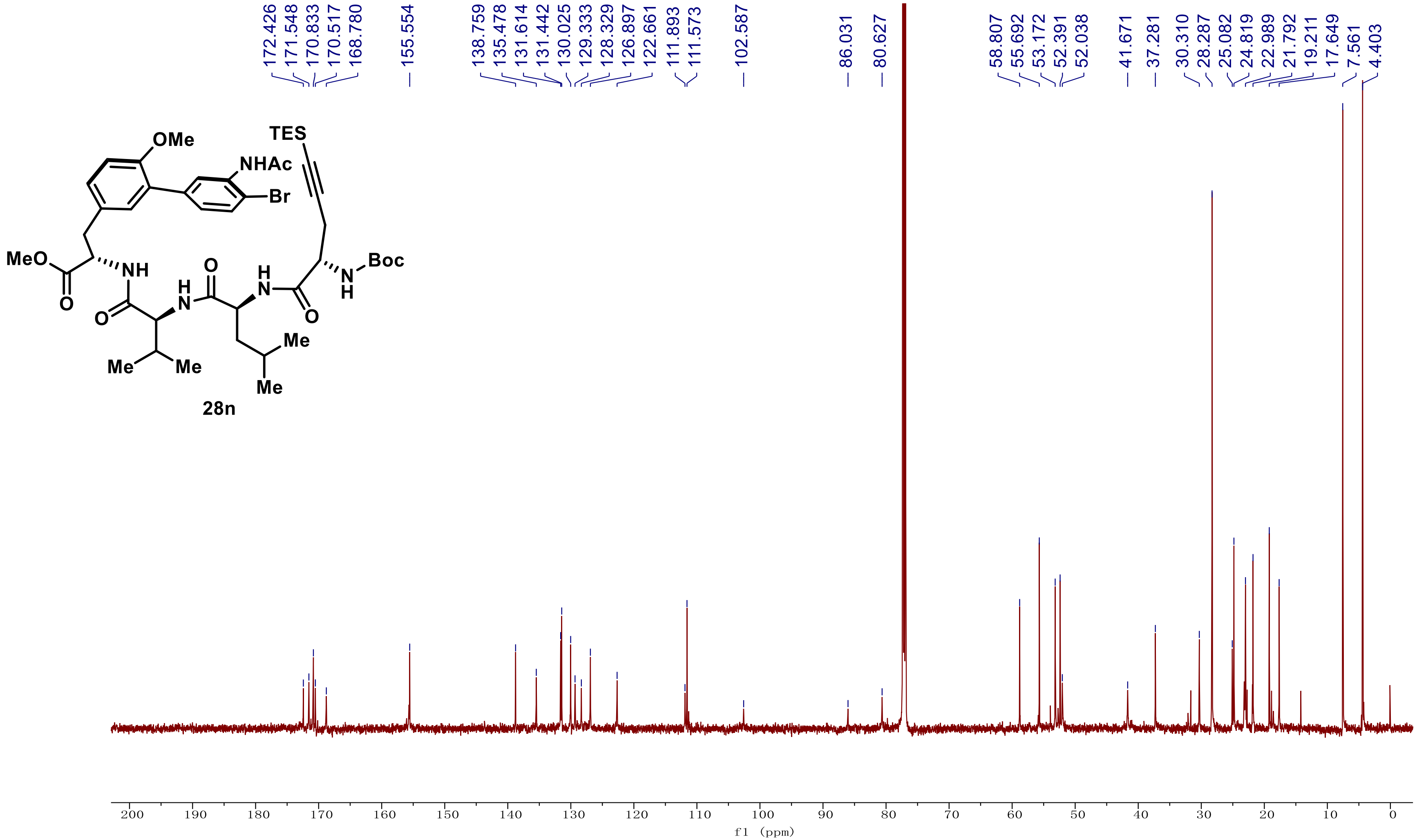

Compound 28o ^{1}H NMR (600 MHz, $CDCl_3$)

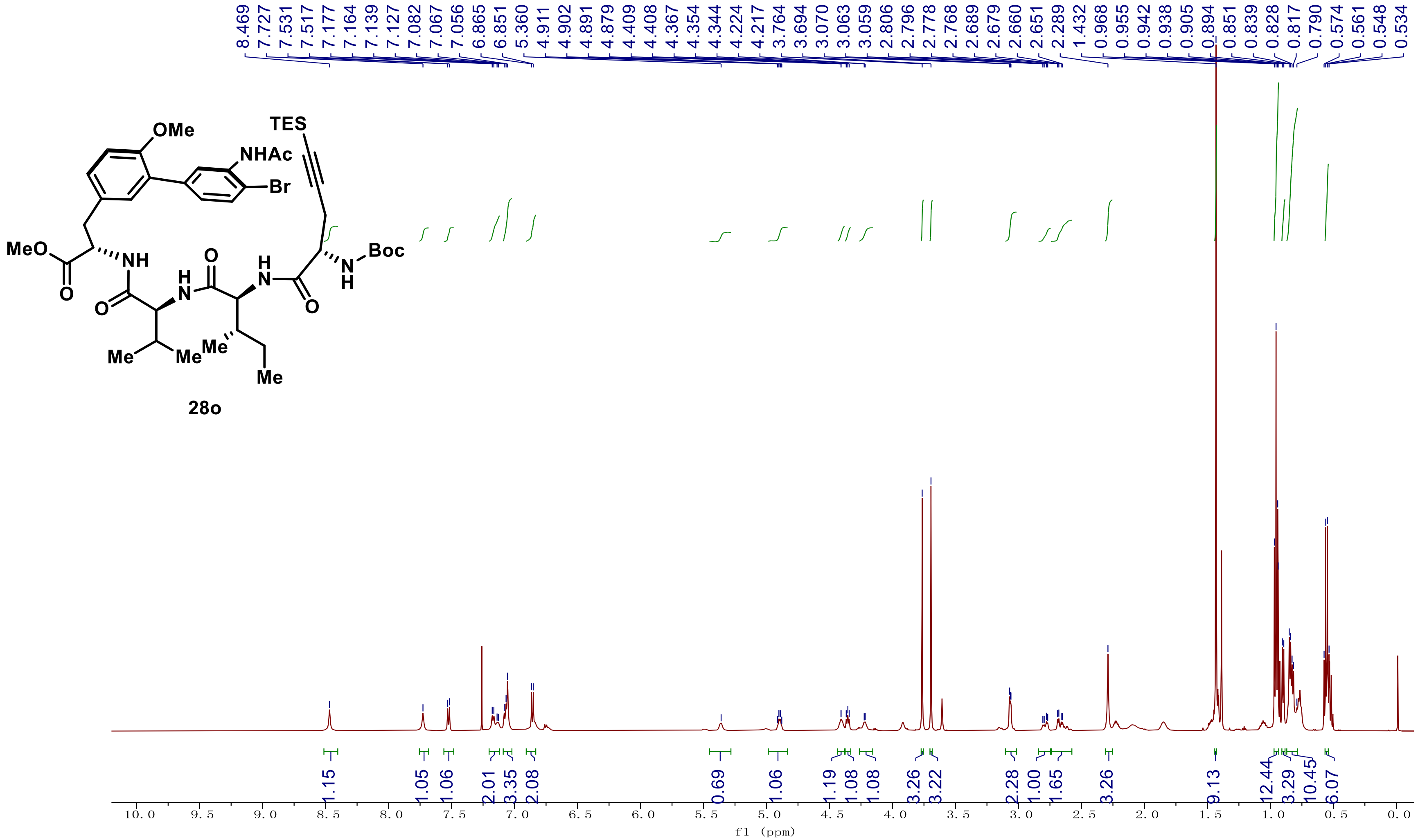

Compound 28o ^{13}C NMR (151 MHz, $CDCl_3$)

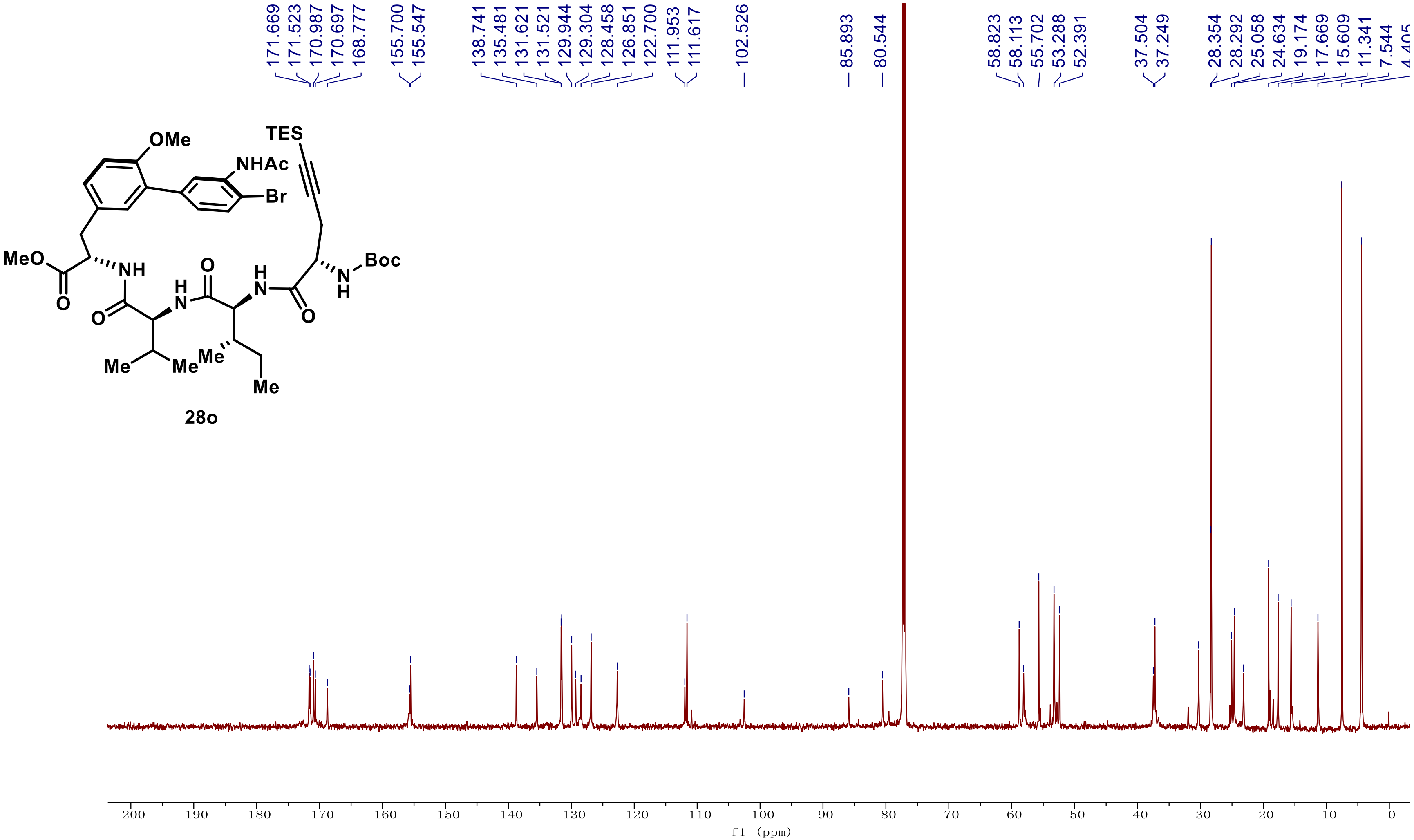

Compound 28p ^{1}H NMR (600 MHz, $CDCl_3$)

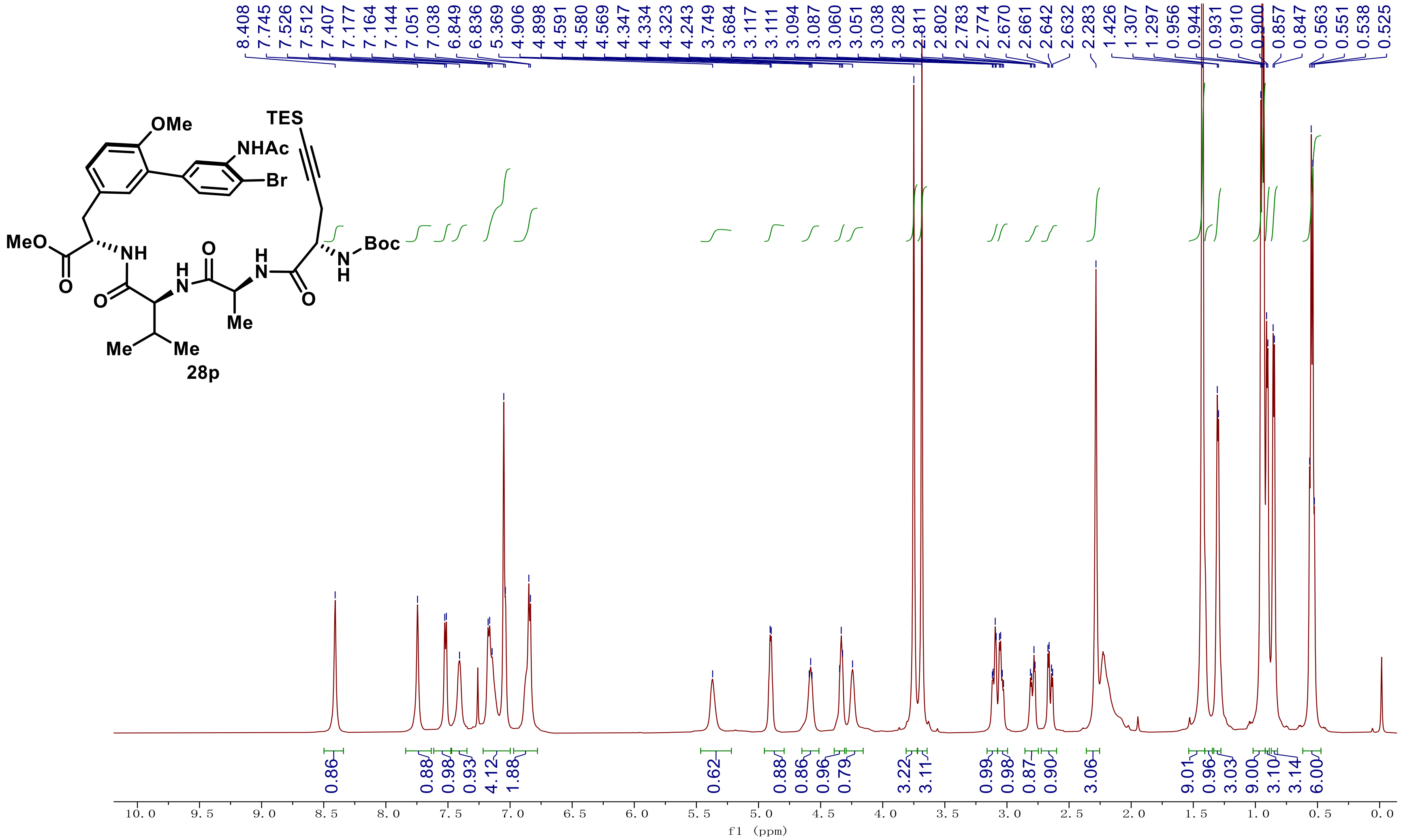

Compound 28p ^{13}C NMR (151 MHz, $CDCl_3$)

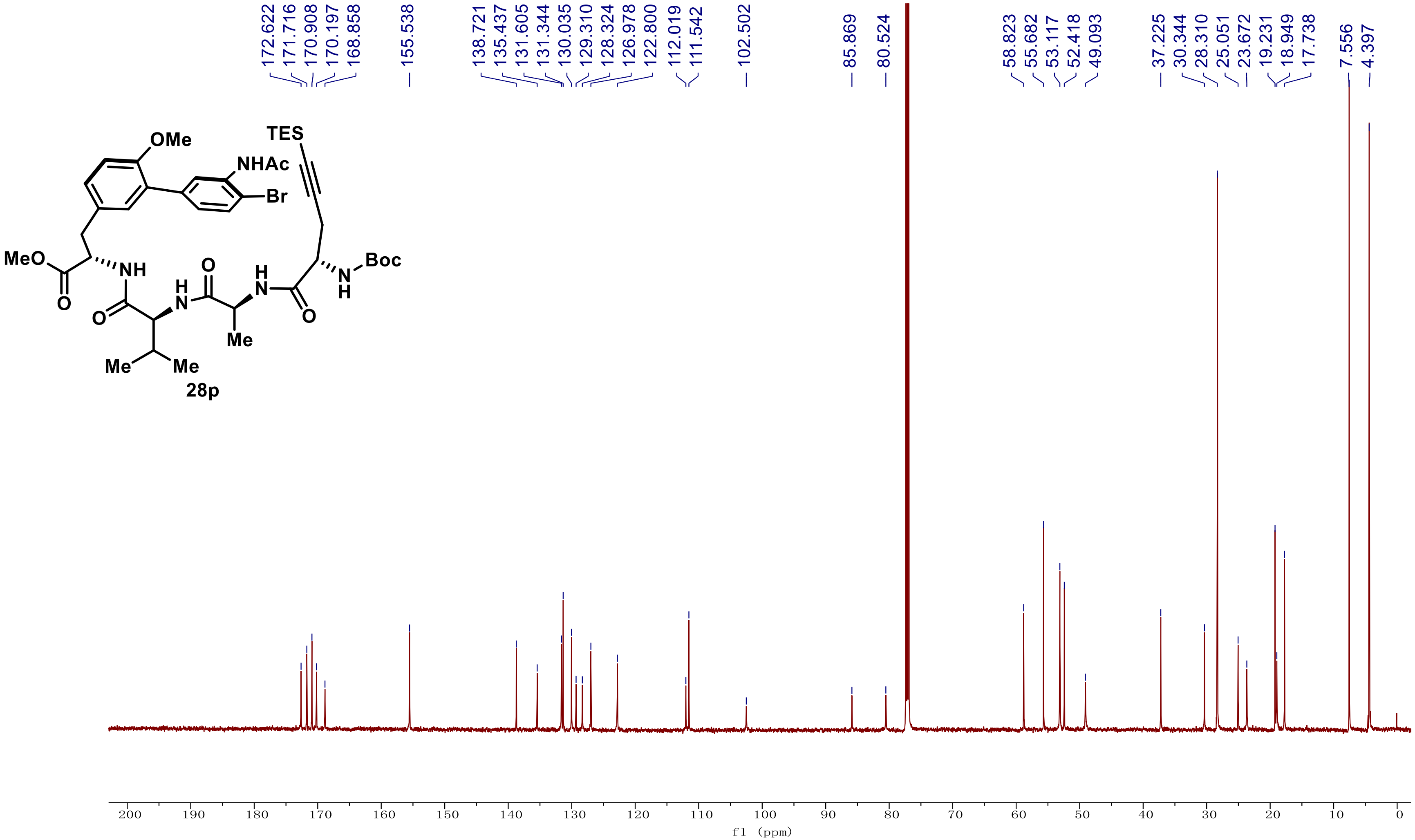

Compound 28q ^{1}H NMR (600 MHz, $CDCl_3$)

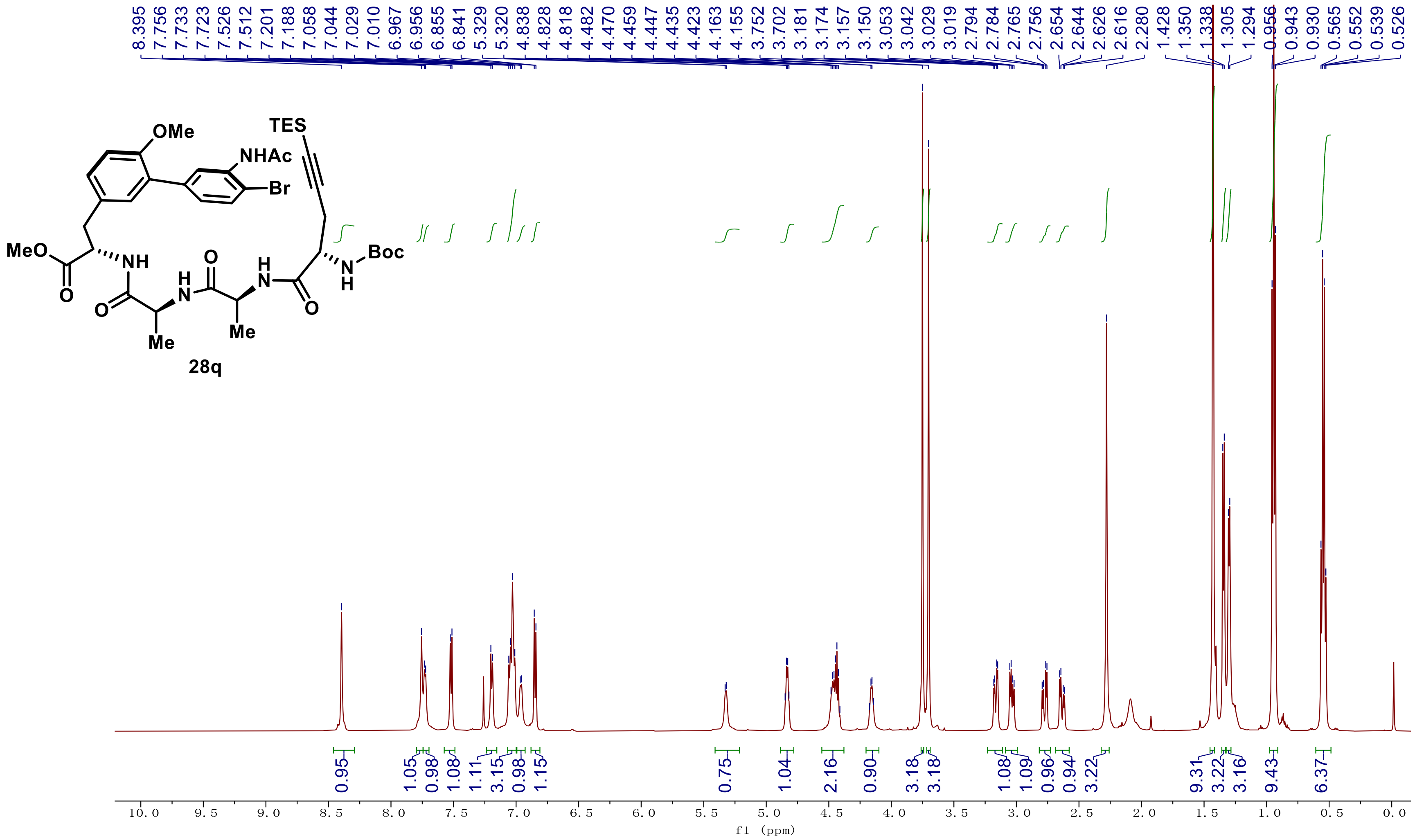

Compound 28q ^{13}C NMR (151 MHz, $CDCl_3$)

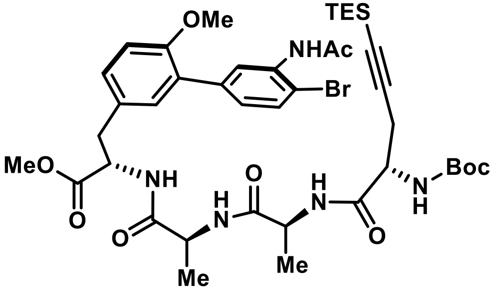

28q

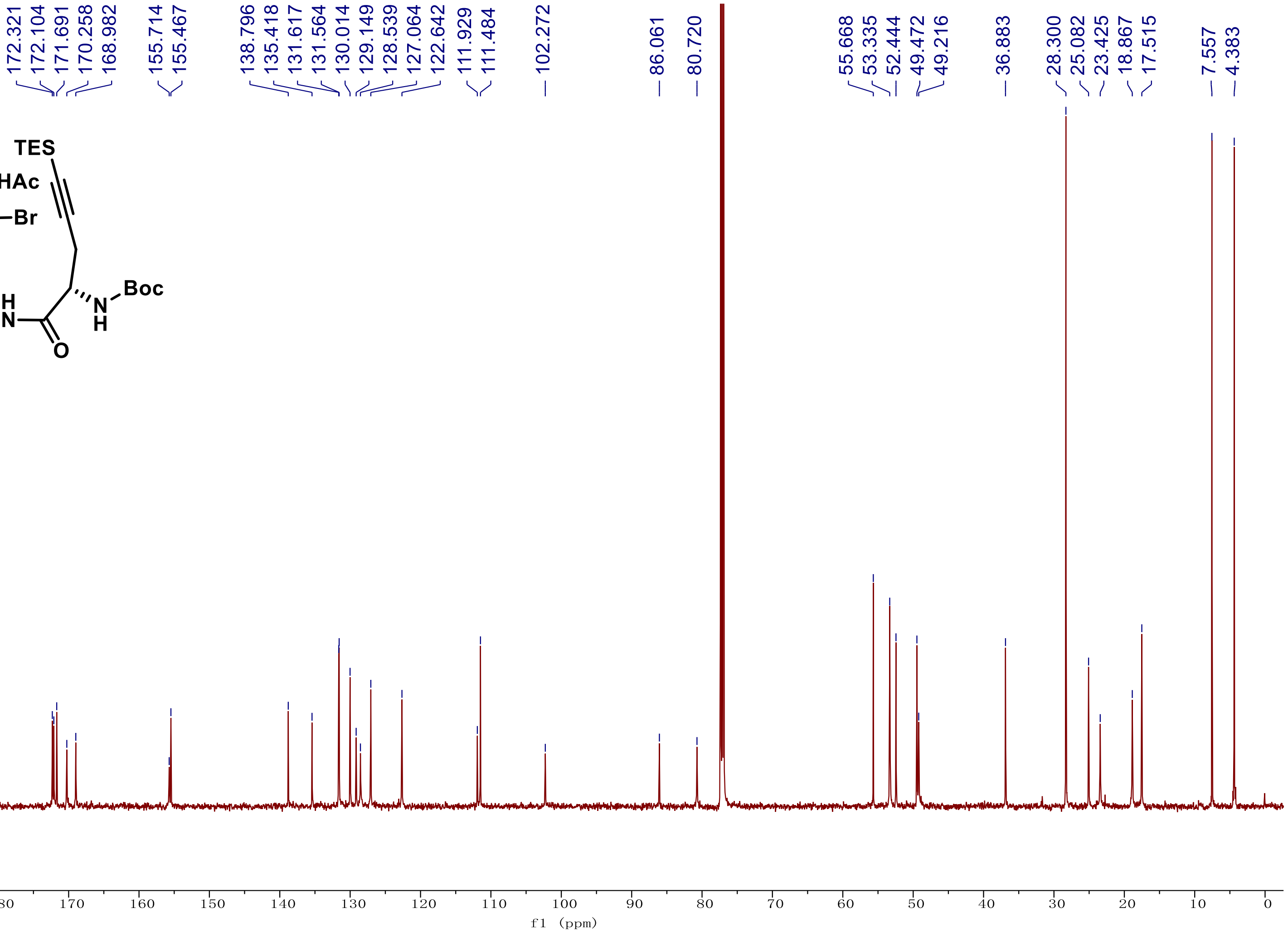

Compound 28r ^{1}H NMR (600 MHz, $CDCl_3$)

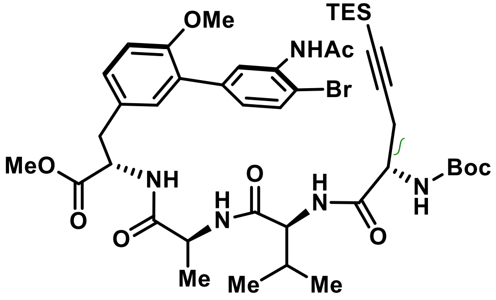

28r

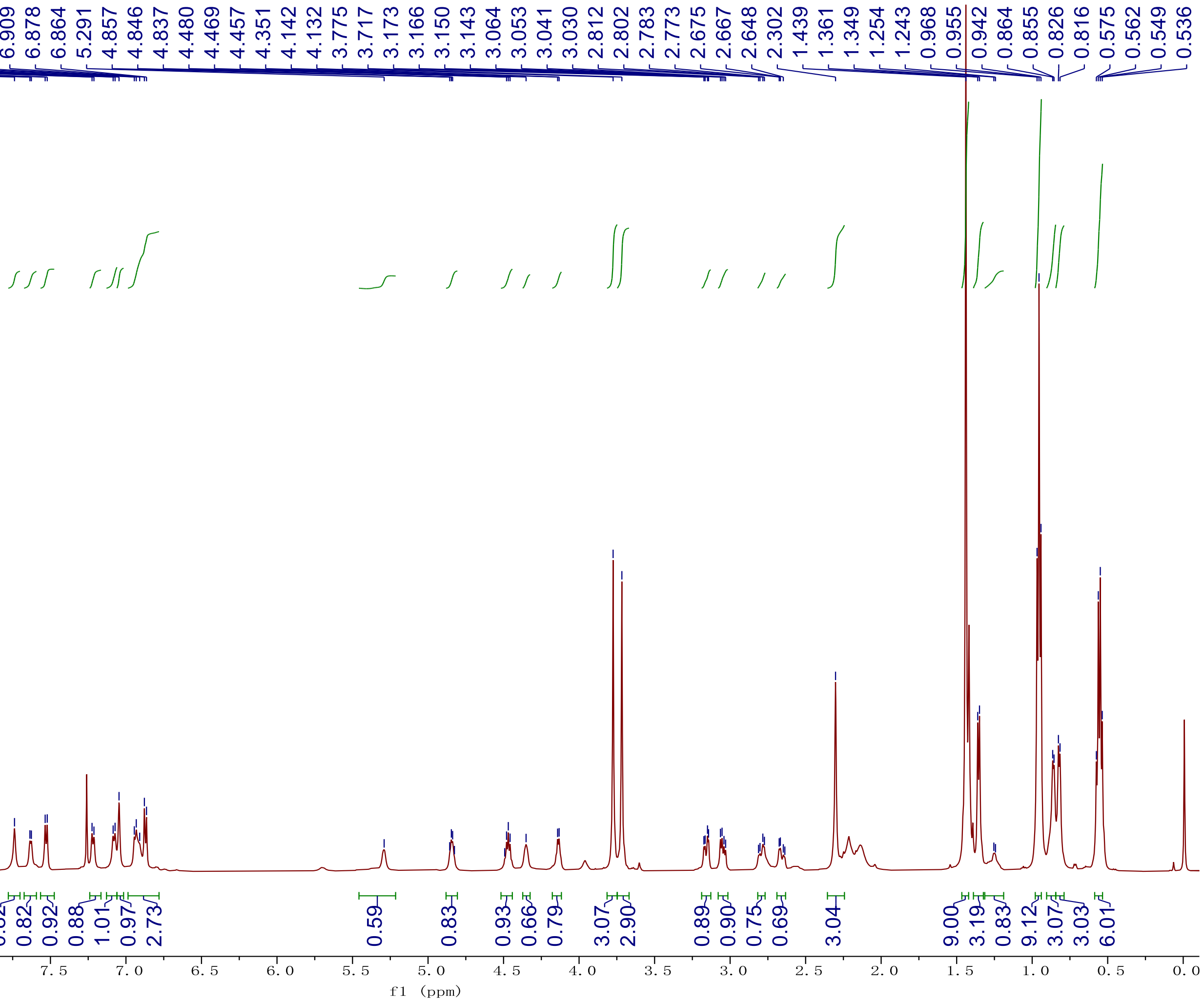

Compound 28r ^{13}C NMR (151 MHz, $CDCl_3$)

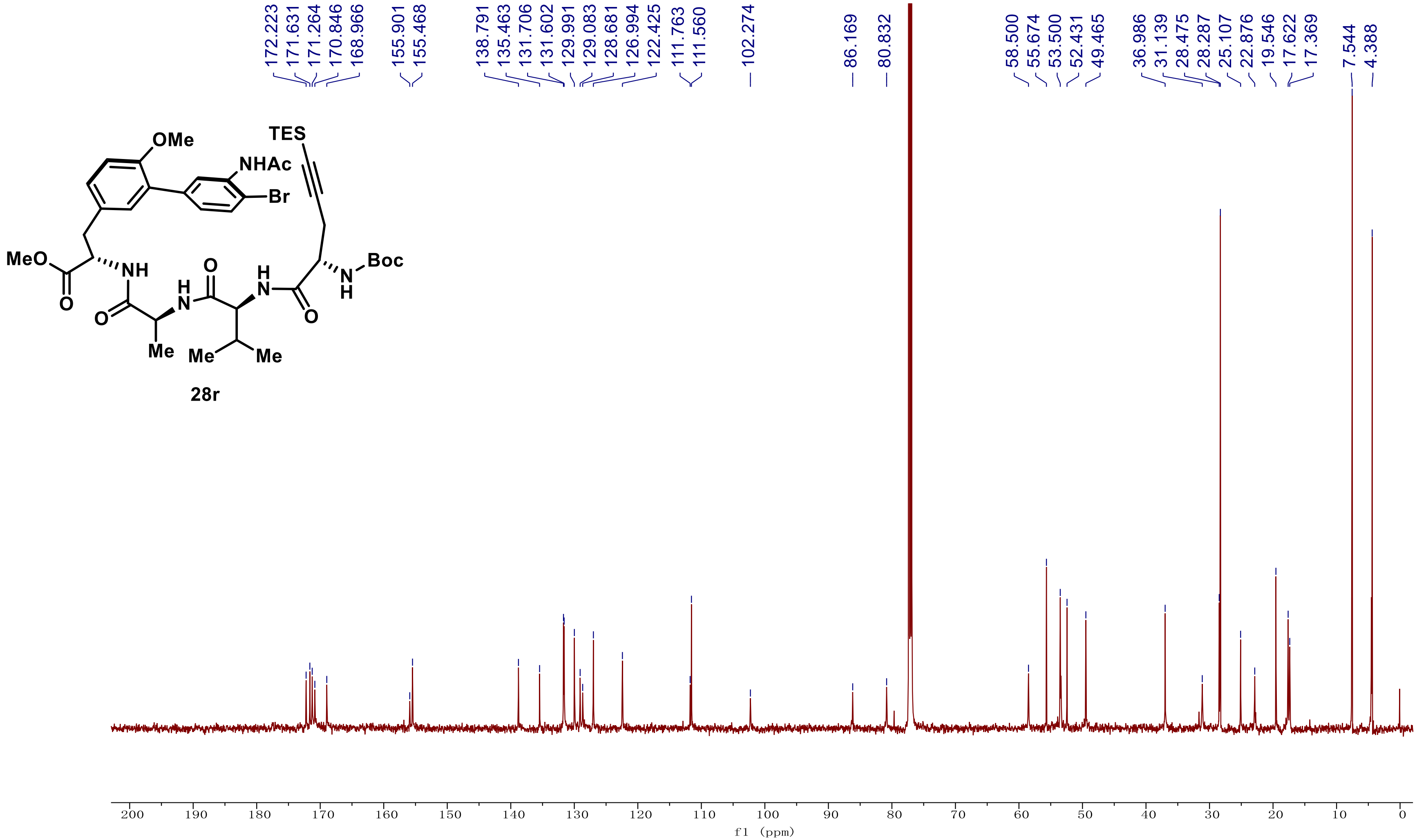

Compound 28s ^{1}H NMR (600 MHz, CDCl$_3$)

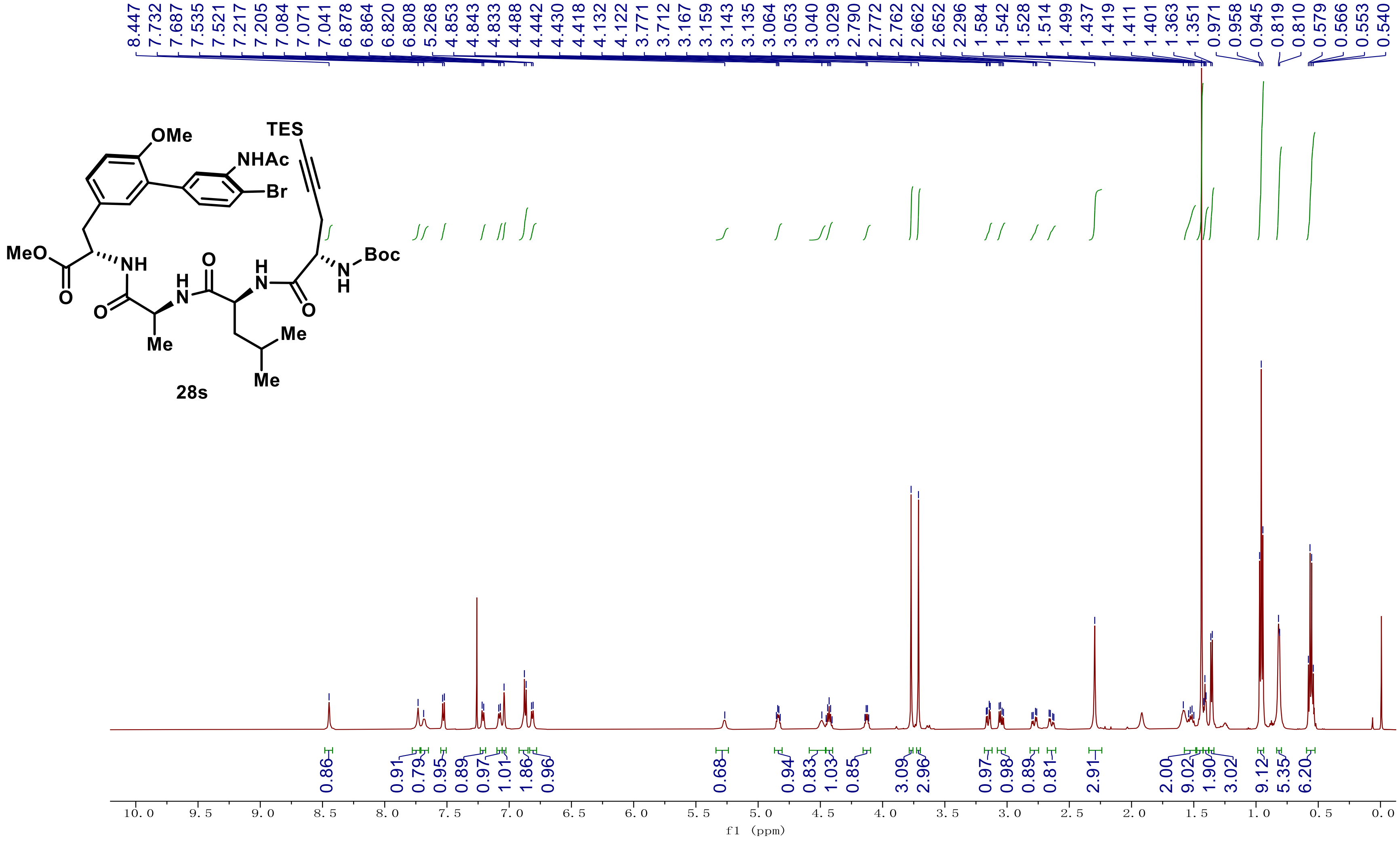

Compound 28s ^{13}C NMR (151 MHz, $CDCl_3$)

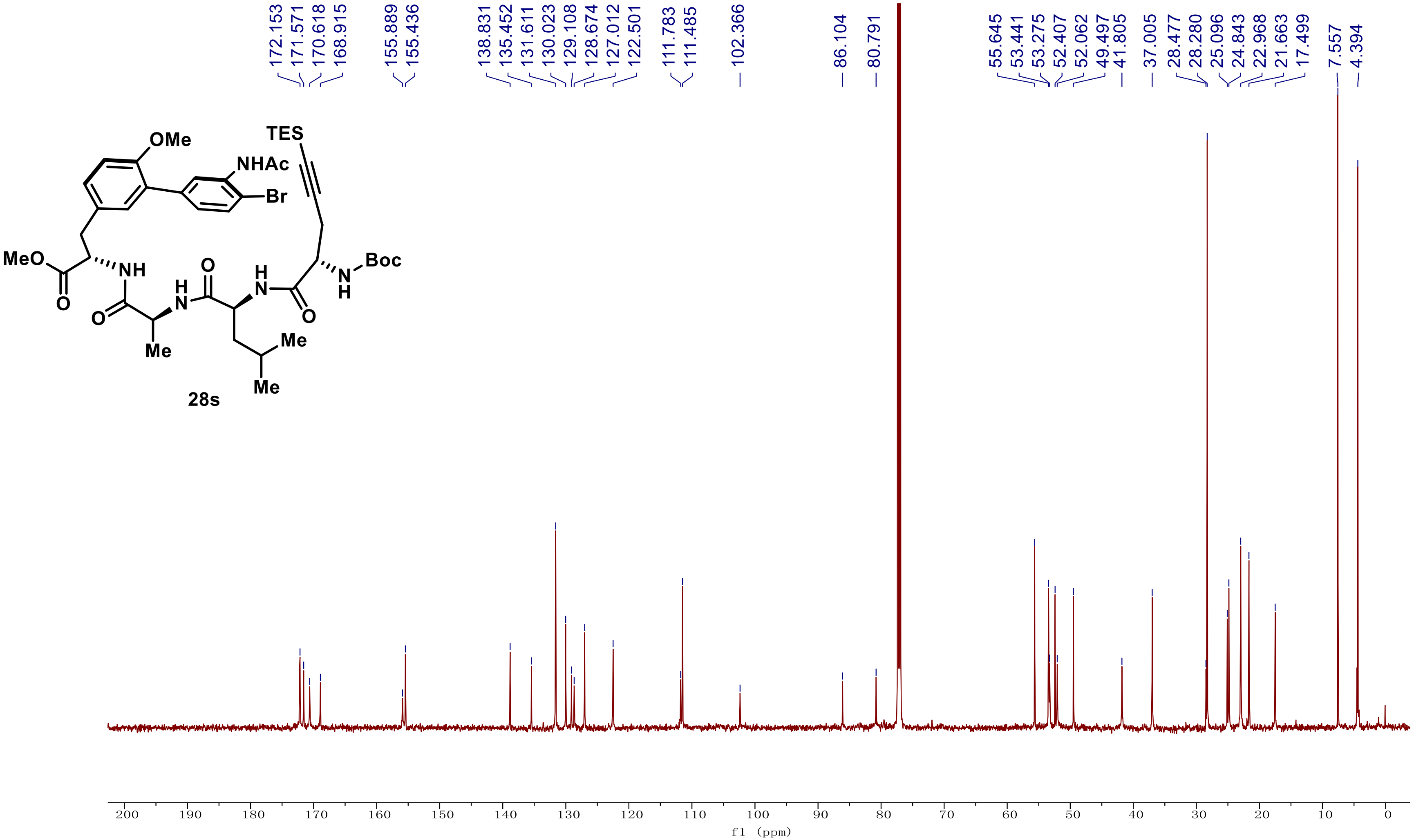

Compound 28t ^{1}H NMR (600 MHz, $CDCl_3$)

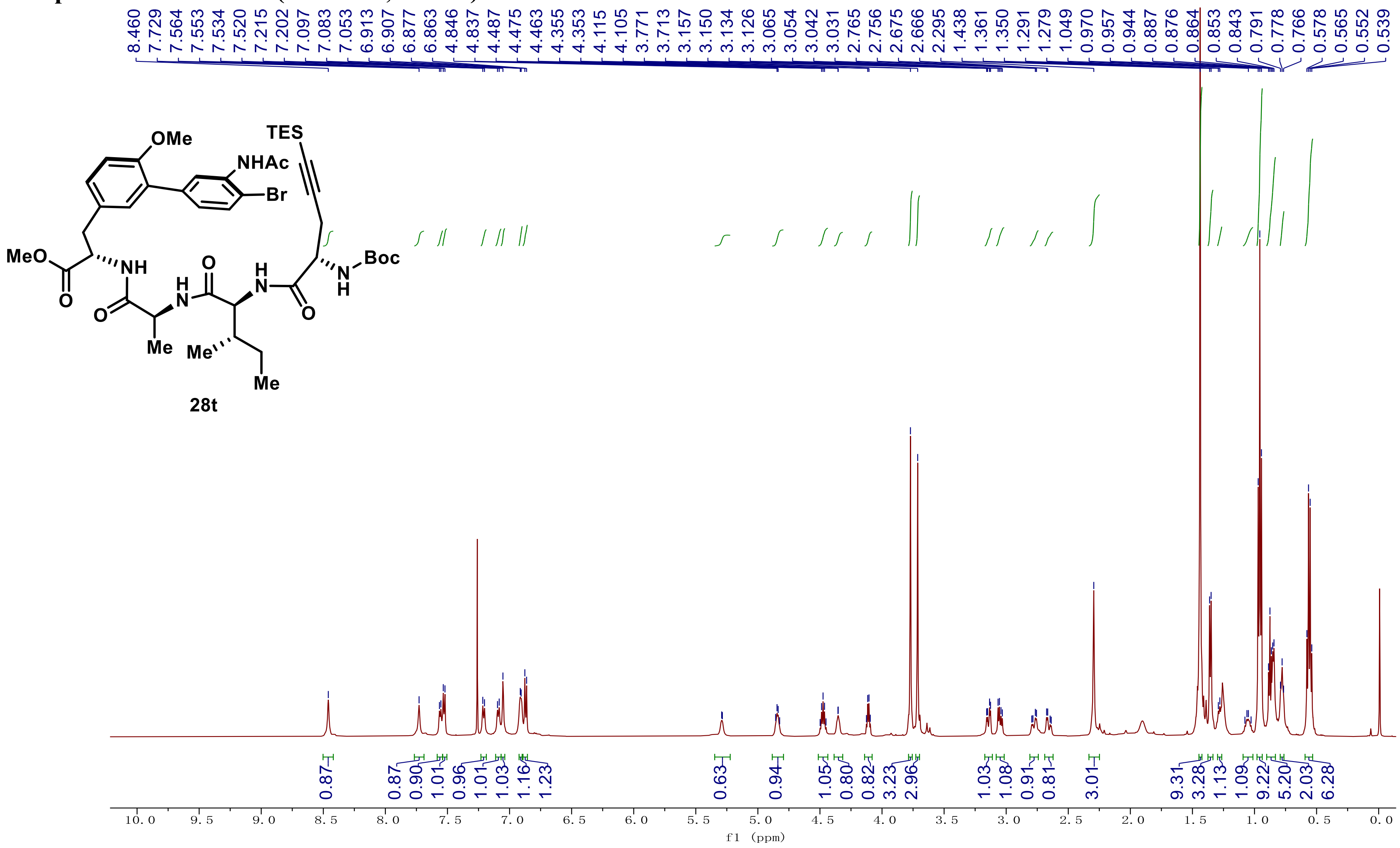

Compound 28t ^{13}C NMR (151 MHz, $CDCl_3$)

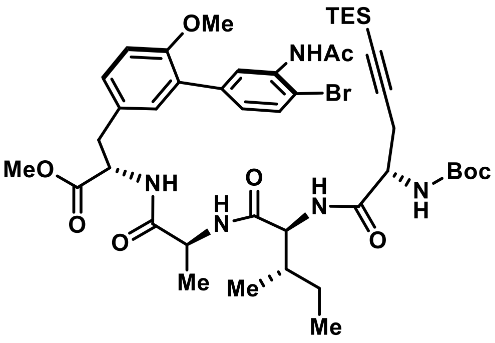

28t

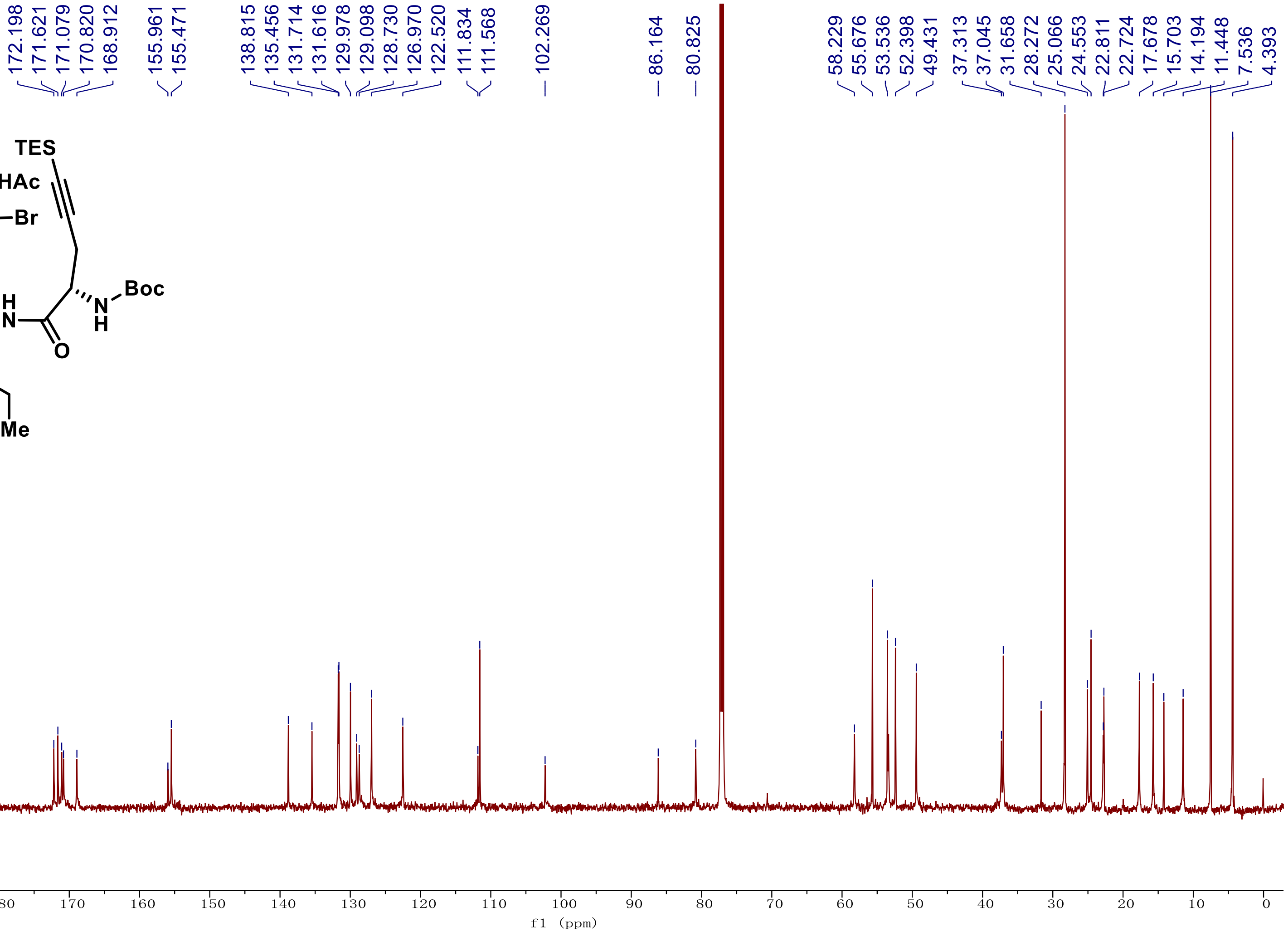

Compound 29a ^{1}H NMR (600 MHz, $CDCl_3$)

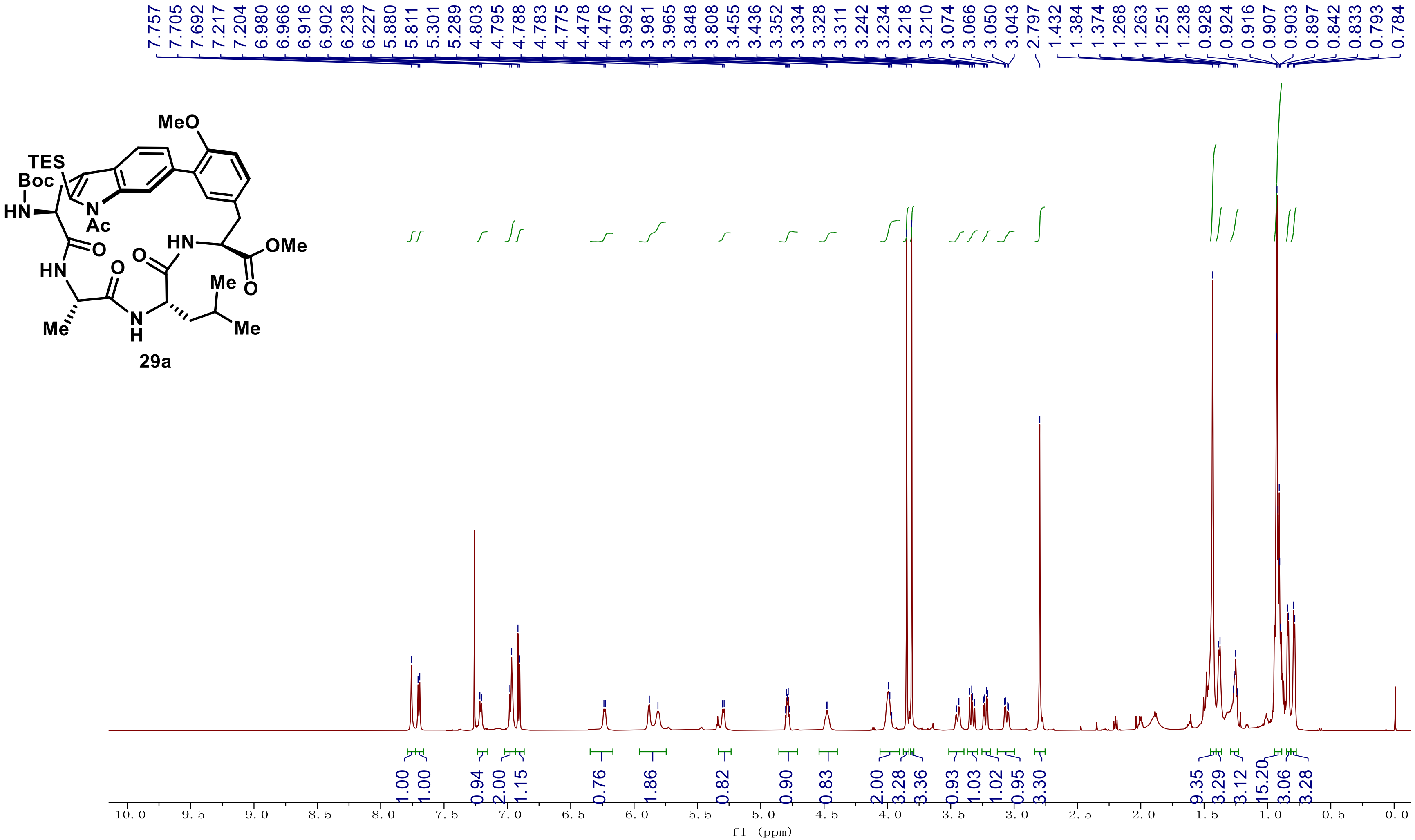

29a

Compound 29a ^{13}C NMR (151 MHz, CDCl$_3$)

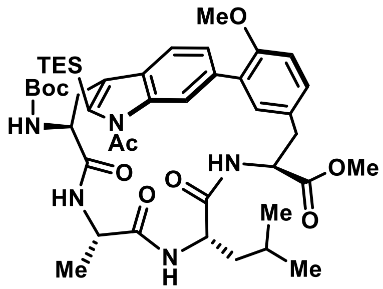

29a

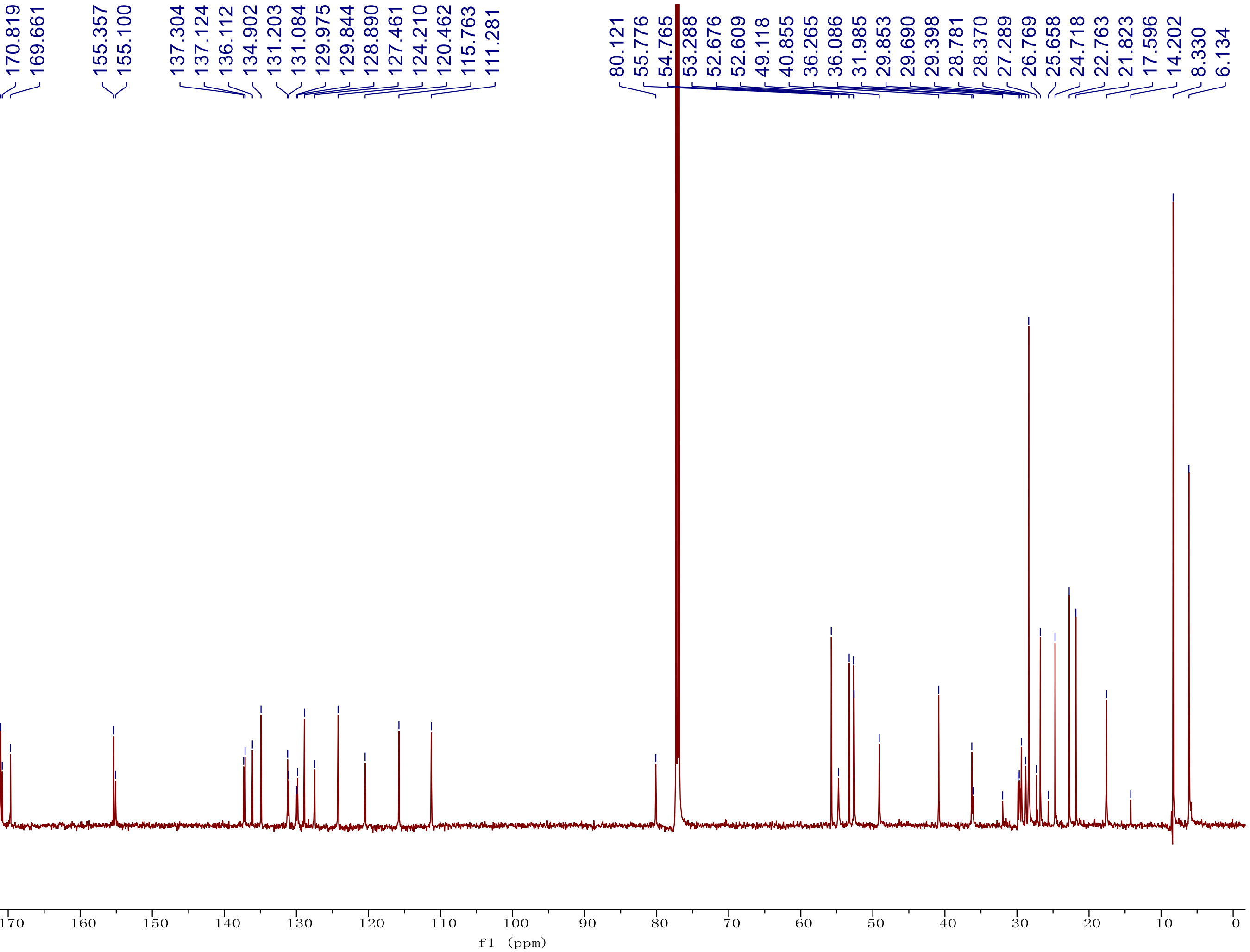

Compound 29b ^{1}H NMR (600 MHz, $CDCl_3$)

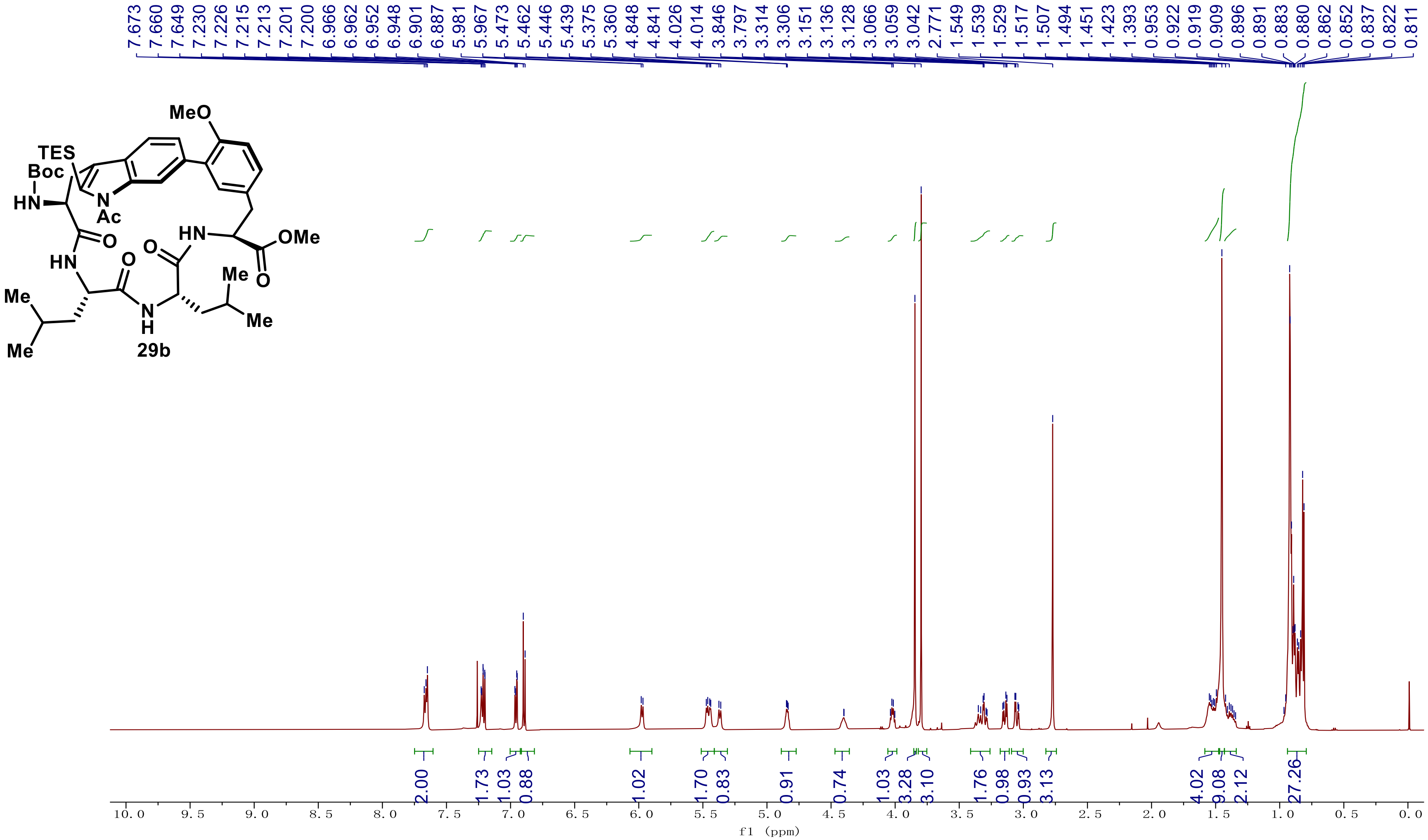

Compound 29b ^{13}C NMR (151 MHz, $CDCl_3$)

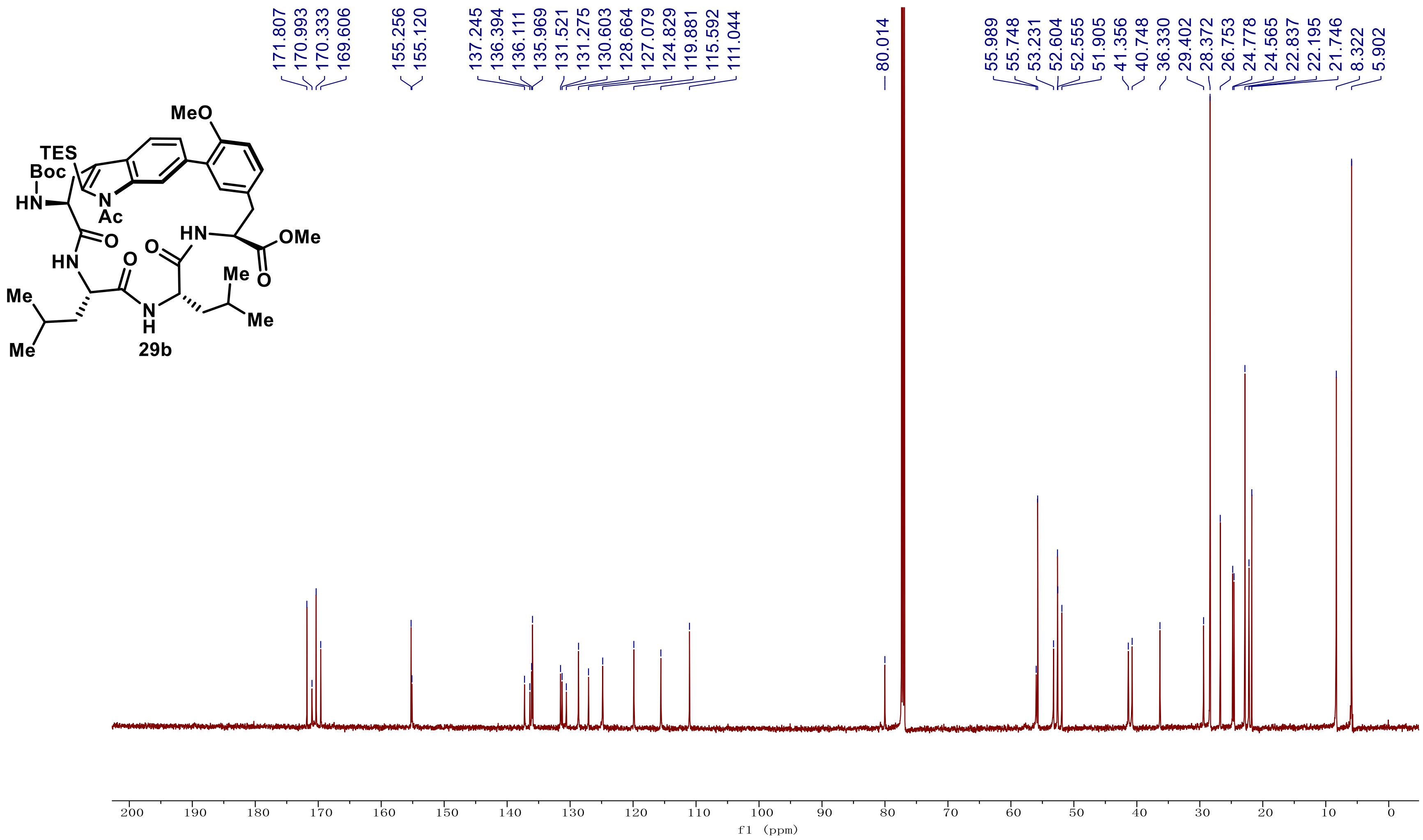

Compound 29c ¹H NMR (600 MHz, CDCl₃)

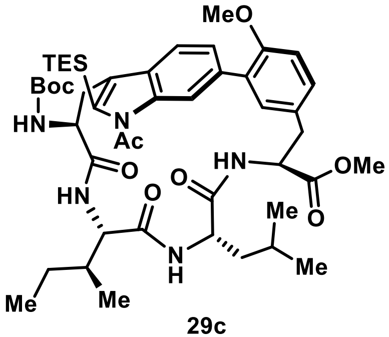

29c

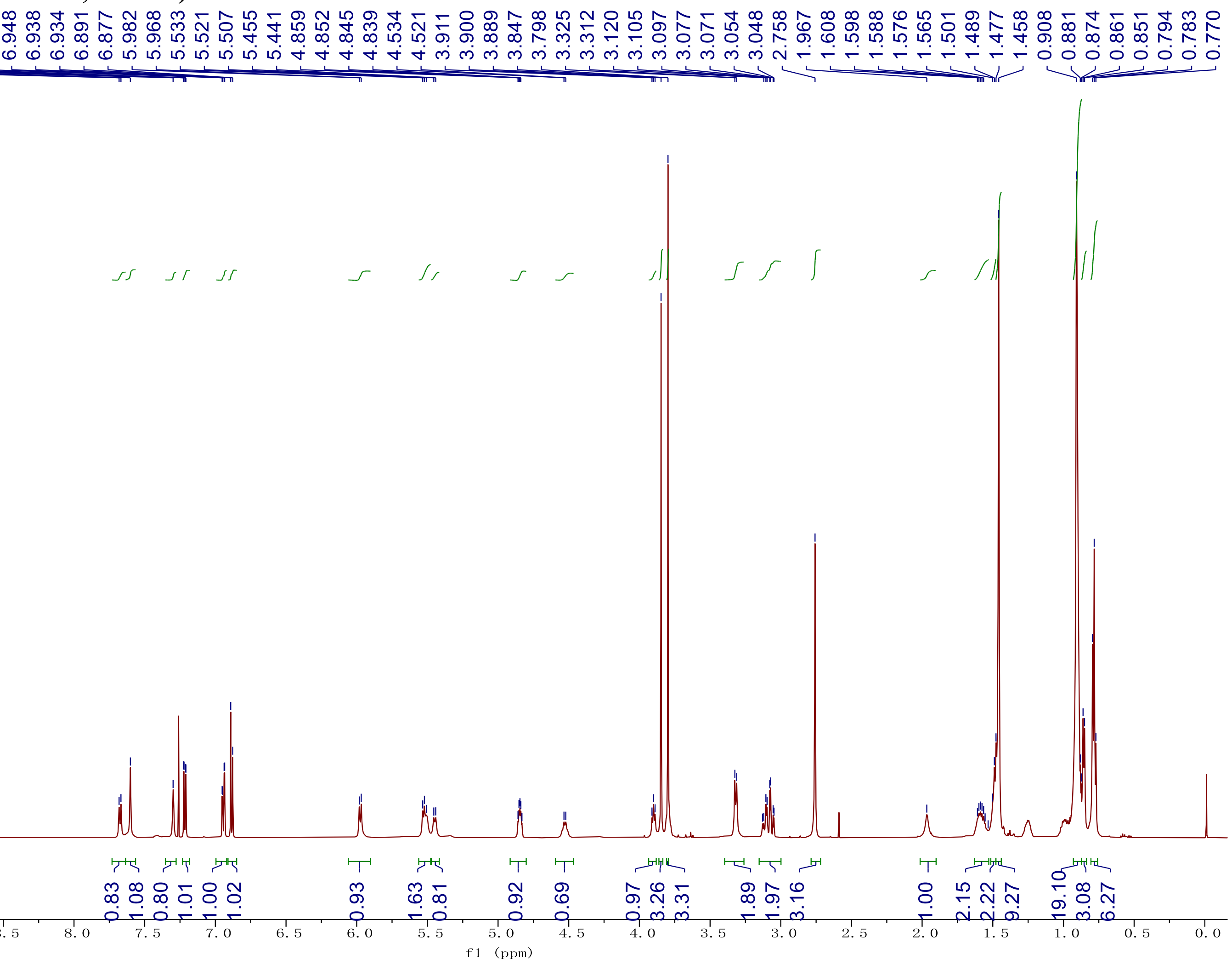

Compound 29c ^{13}C NMR (151 MHz, $CDCl_3$)

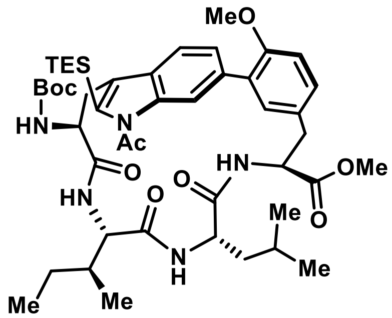

29c

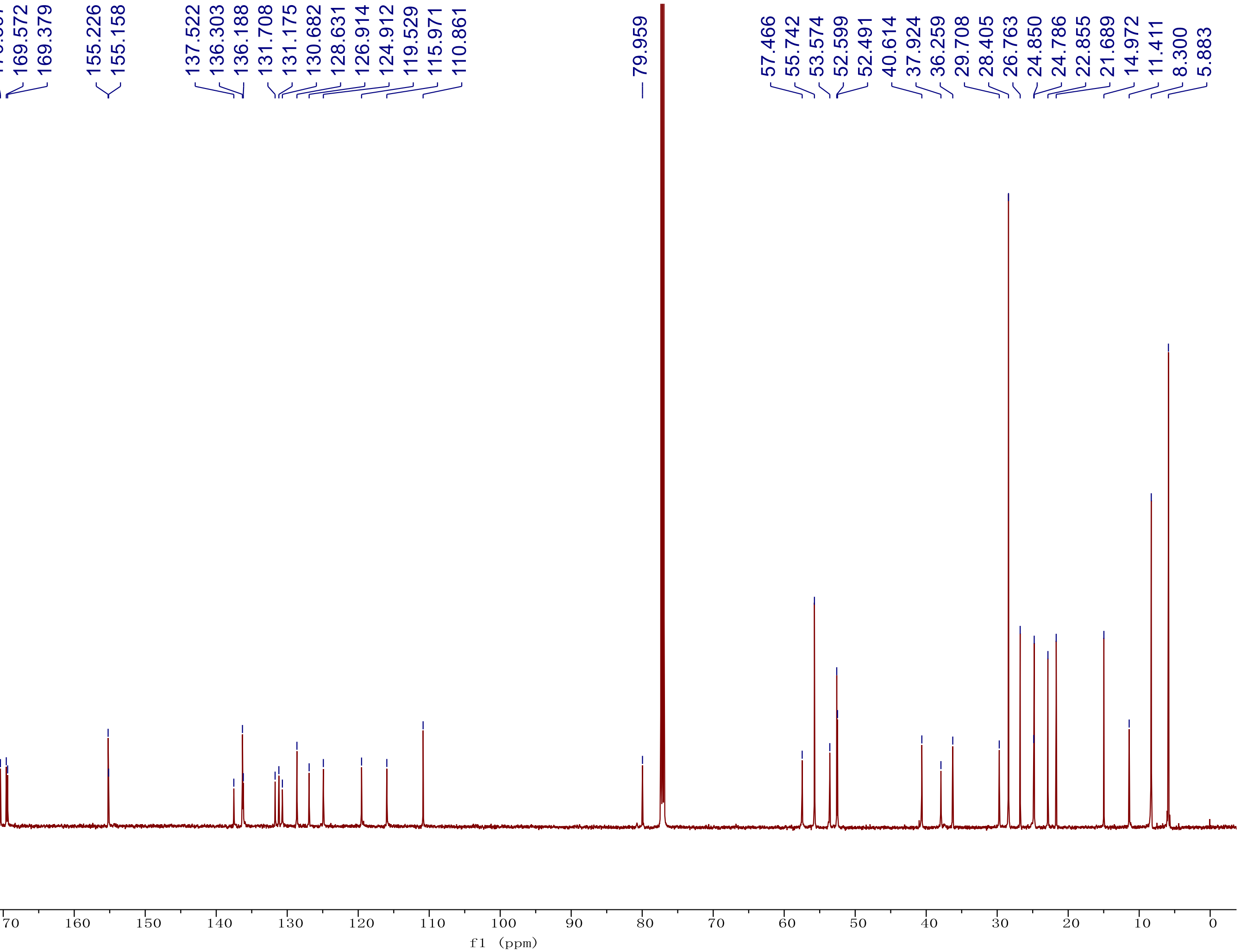

Compound 29d ^1H NMR (600 MHz, $CDCl_3$)

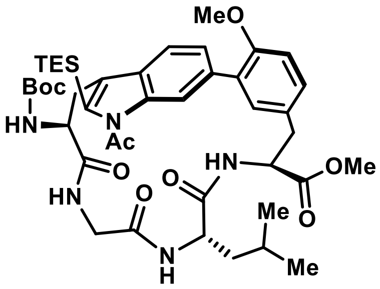

29d

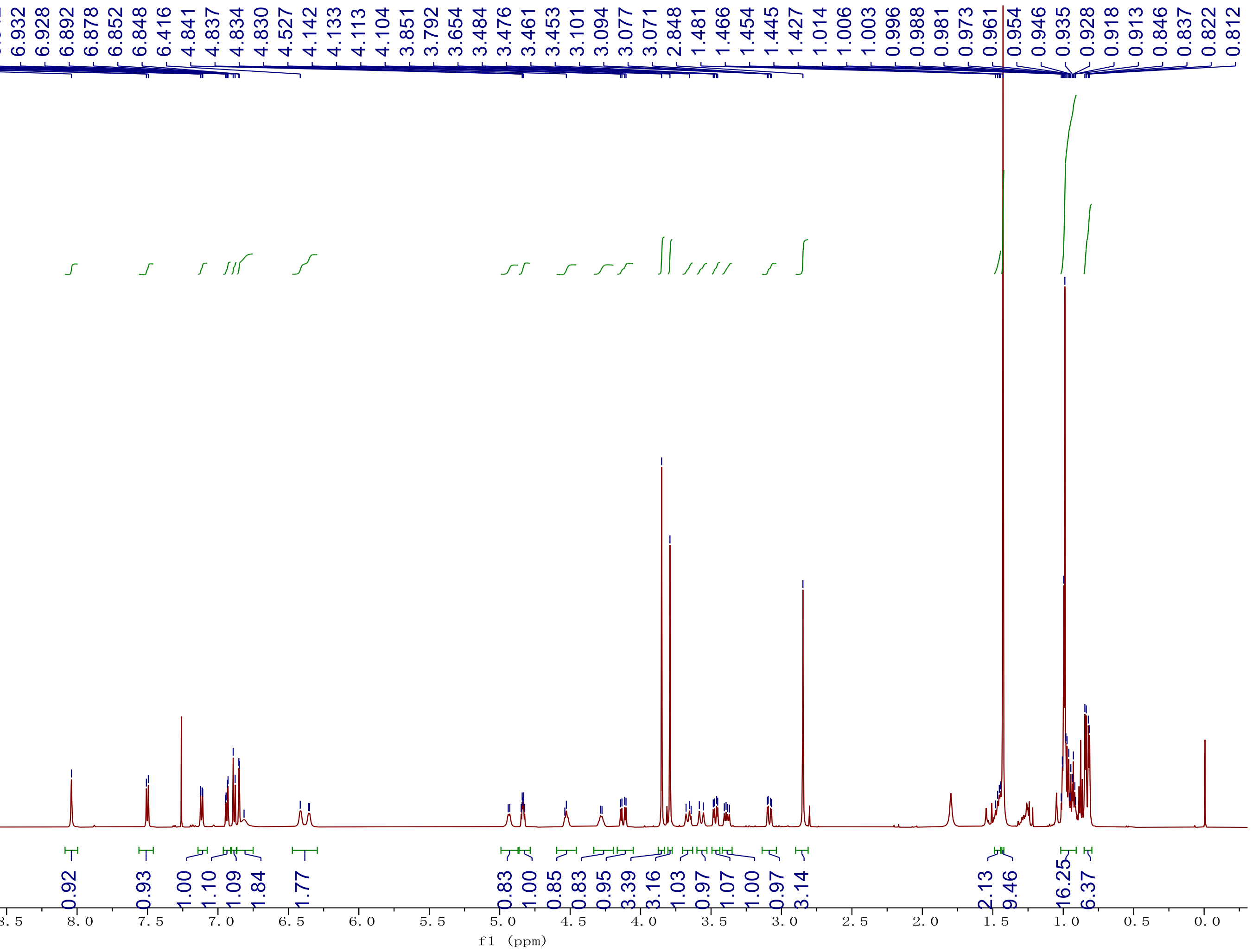

Compound 29d ^{13}C NMR (151 MHz, $CDCl_3$)

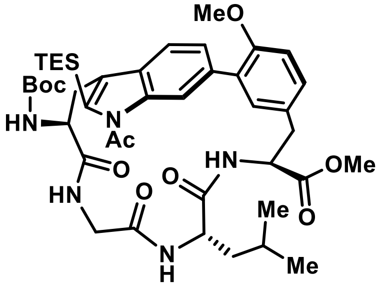

29d

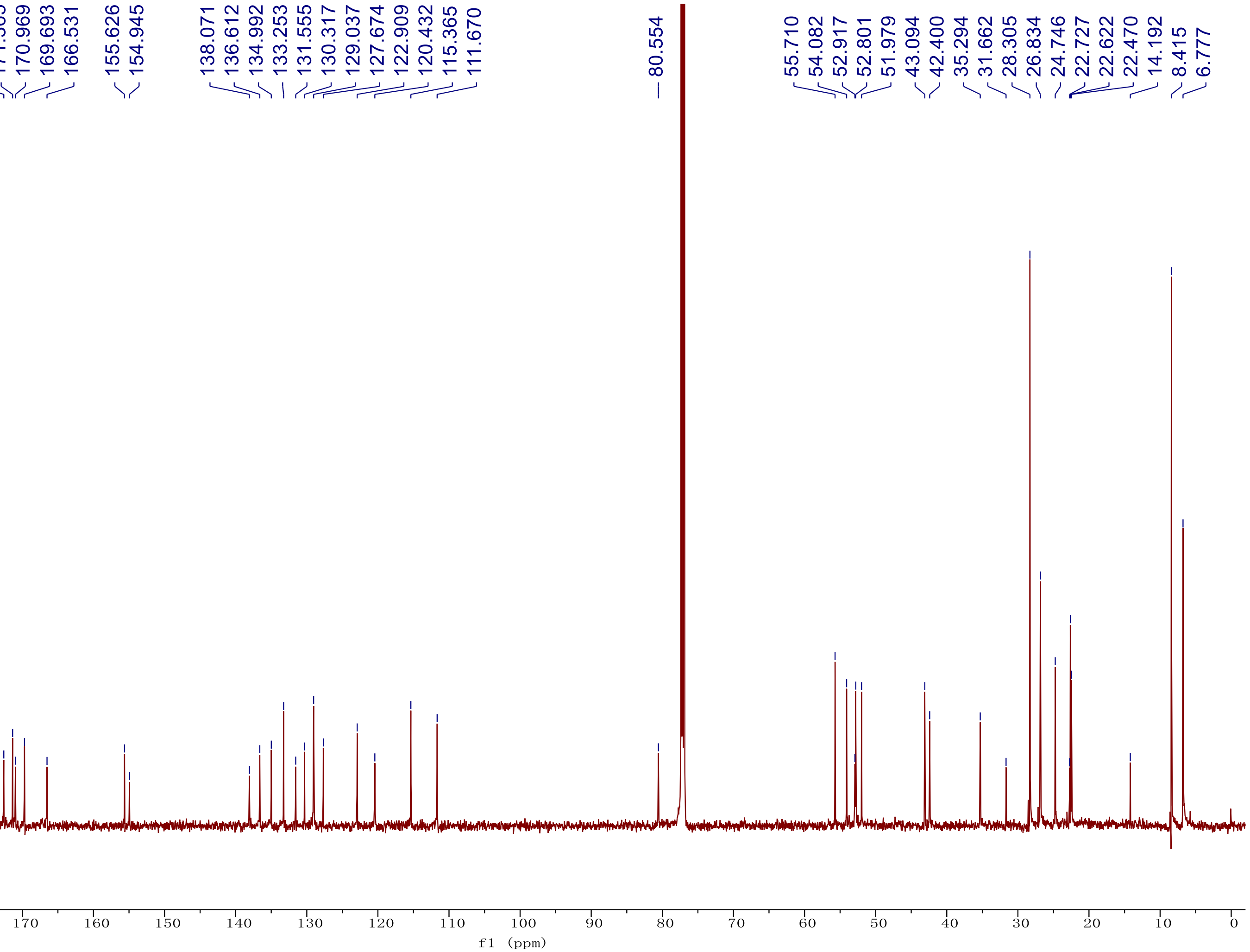

Compound 29e ¹H NMR (600 MHz, CDCl₃)

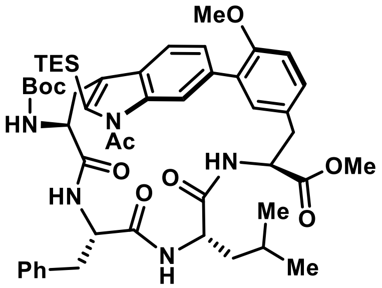

29e

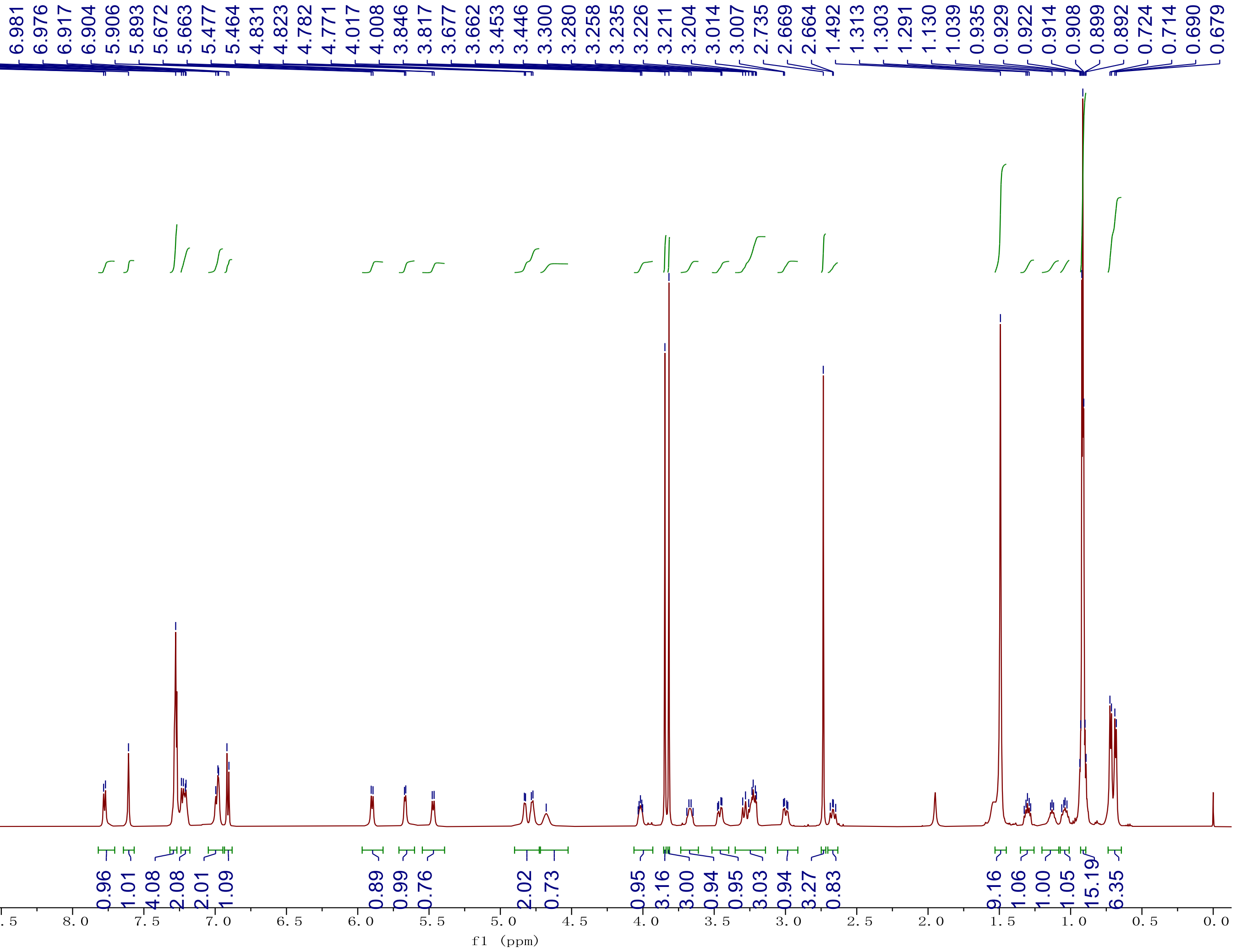

Compound 29e ^{13}C NMR (151 MHz, $CDCl_3$)

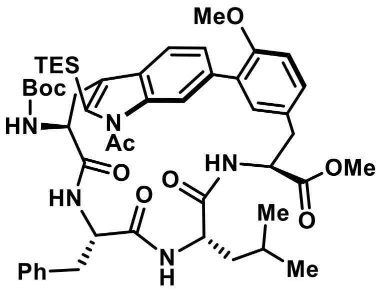

29e

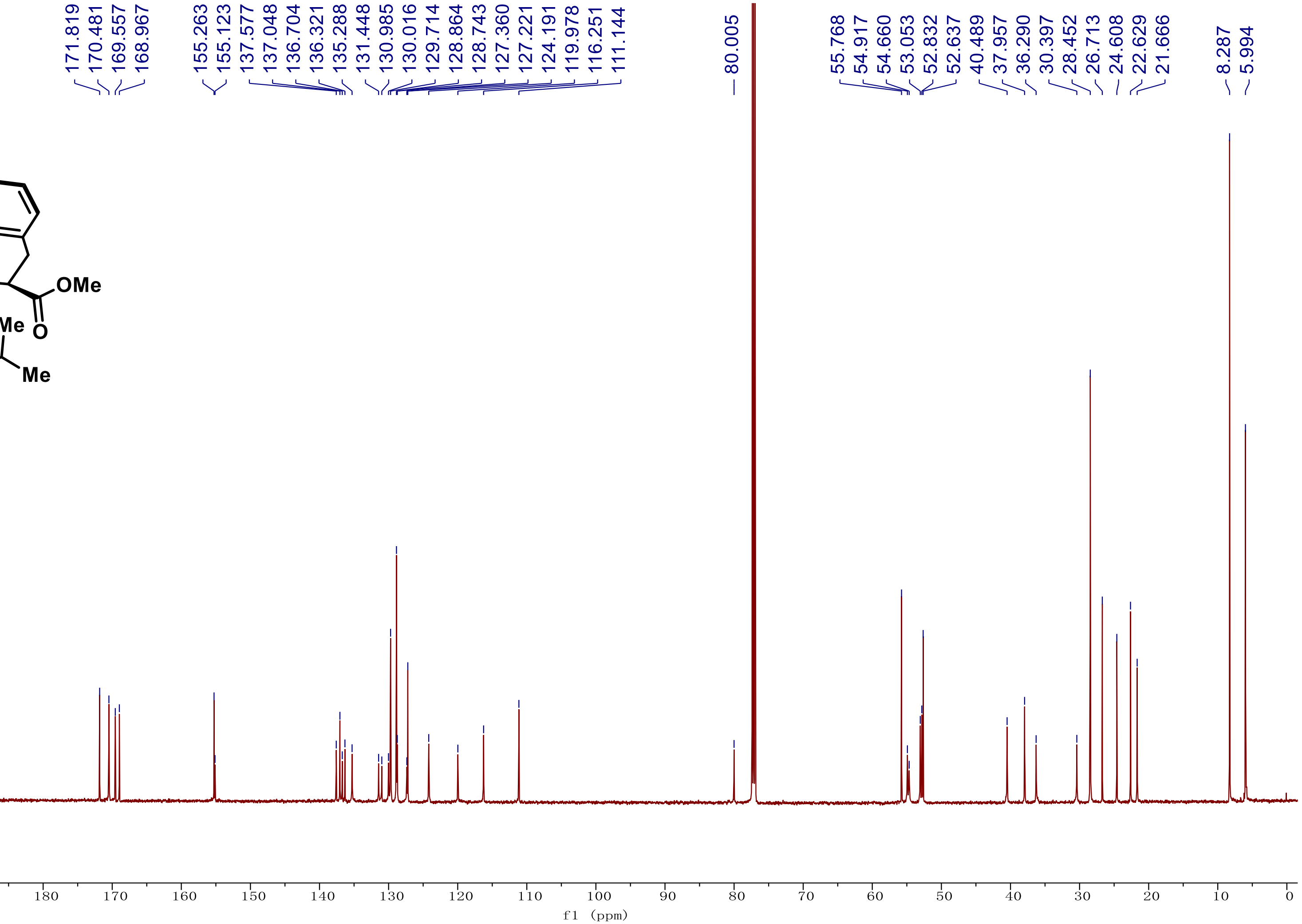

Compound 29f ¹H NMR (600 MHz, CDCl₃)

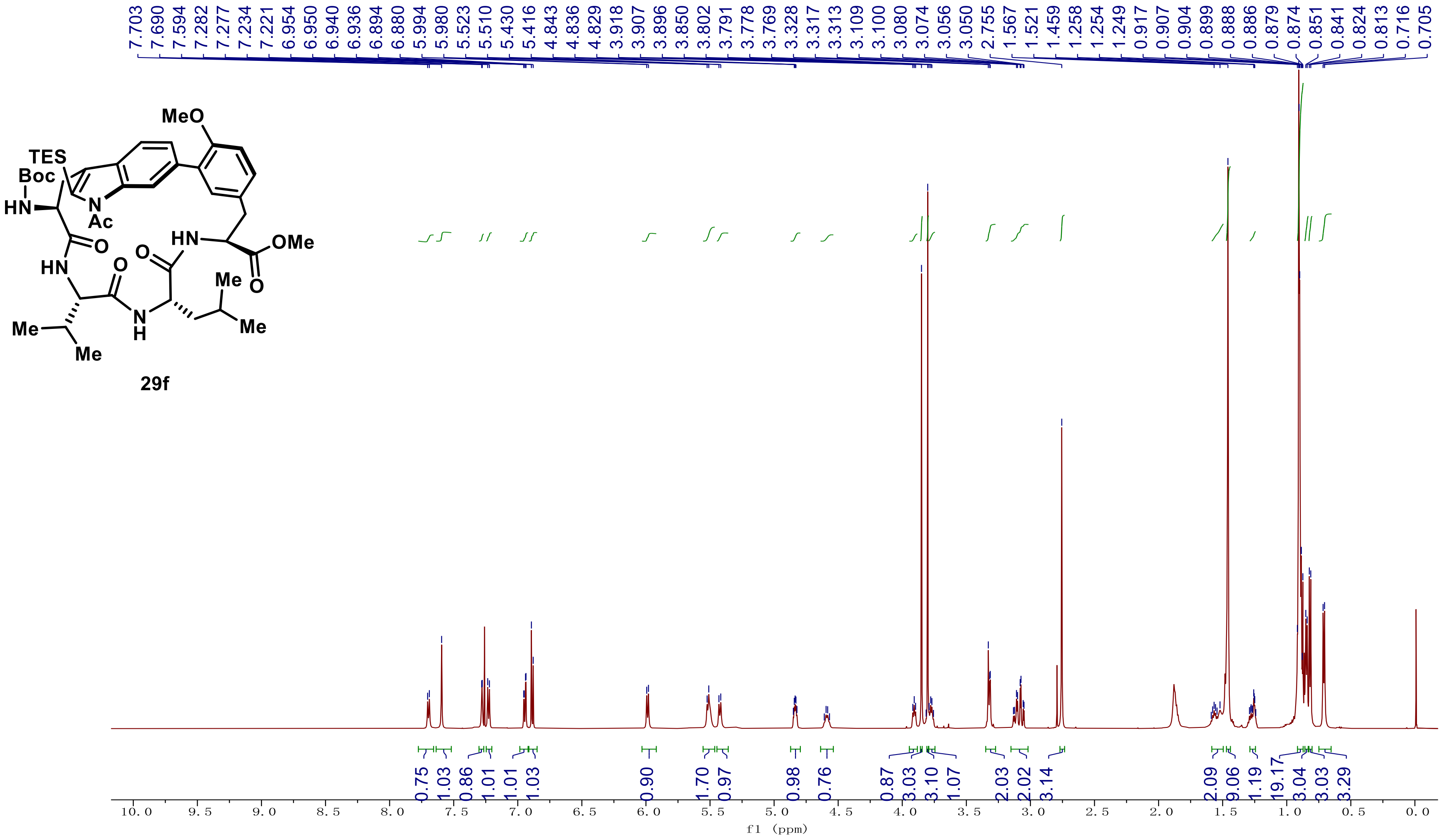

Compound 29f ^{13}C NMR (151 MHz, CDCl$_3$)

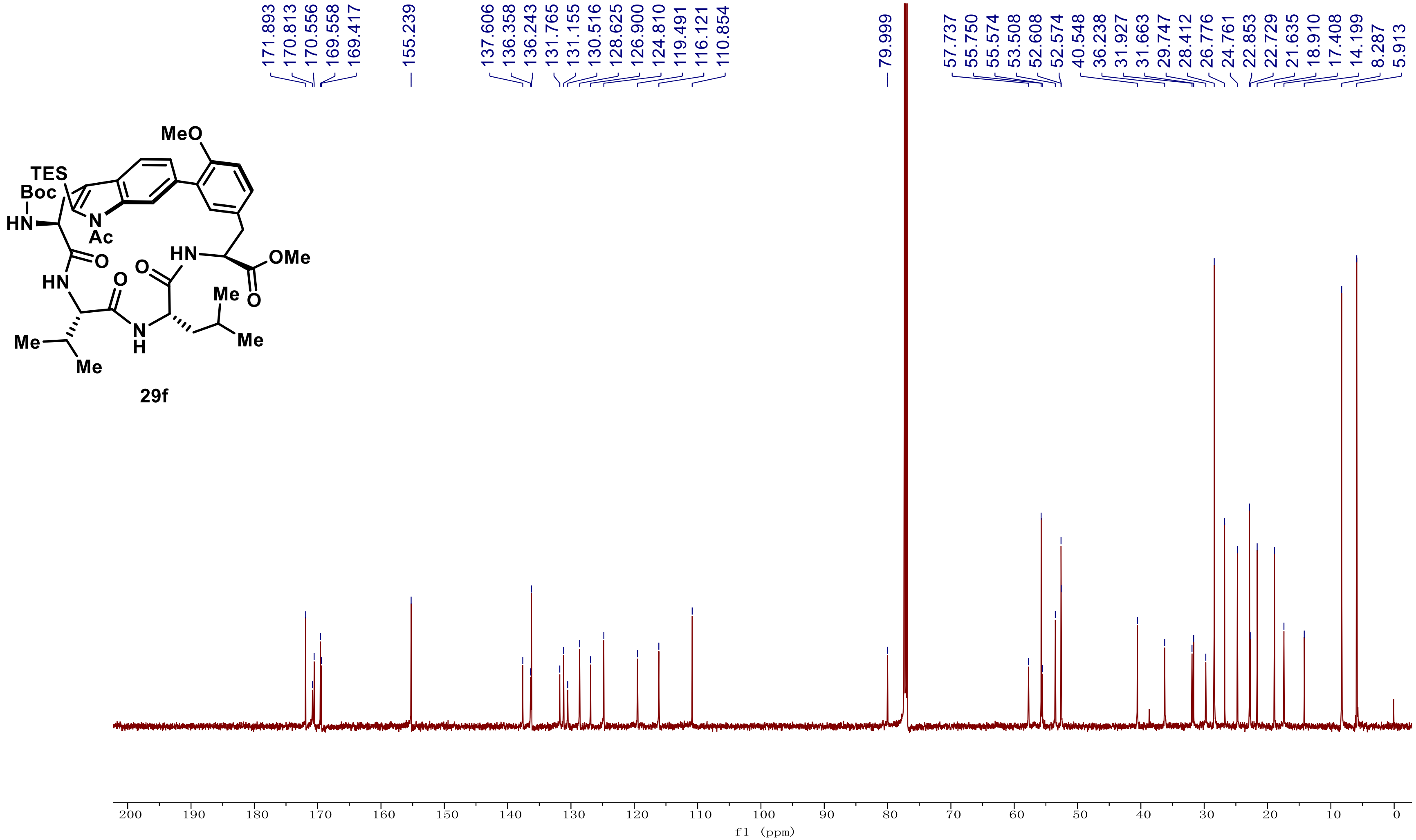

Compound 29f NOESY (400 MHz, CDCl₃)

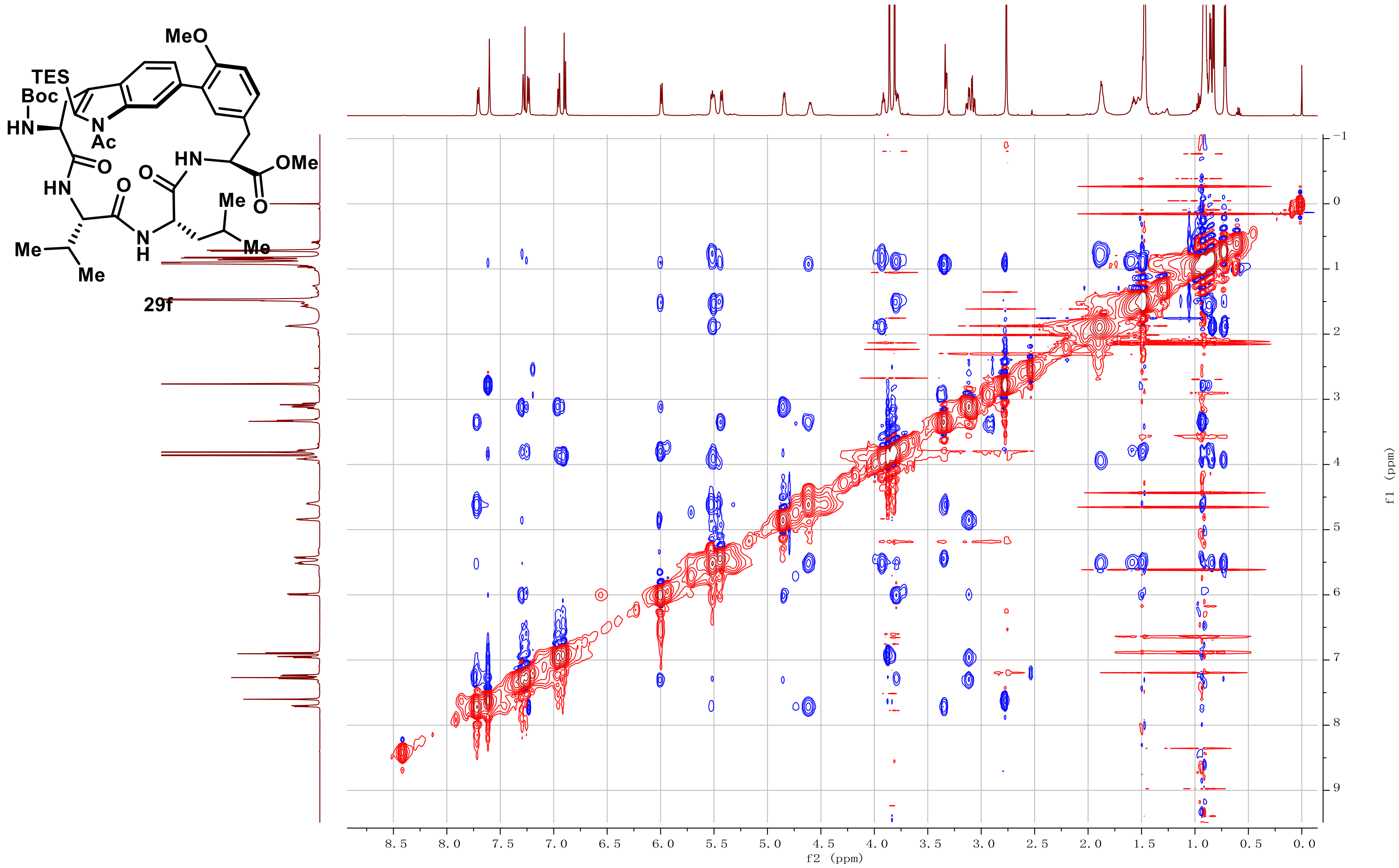

LCMS trace of compound 29f

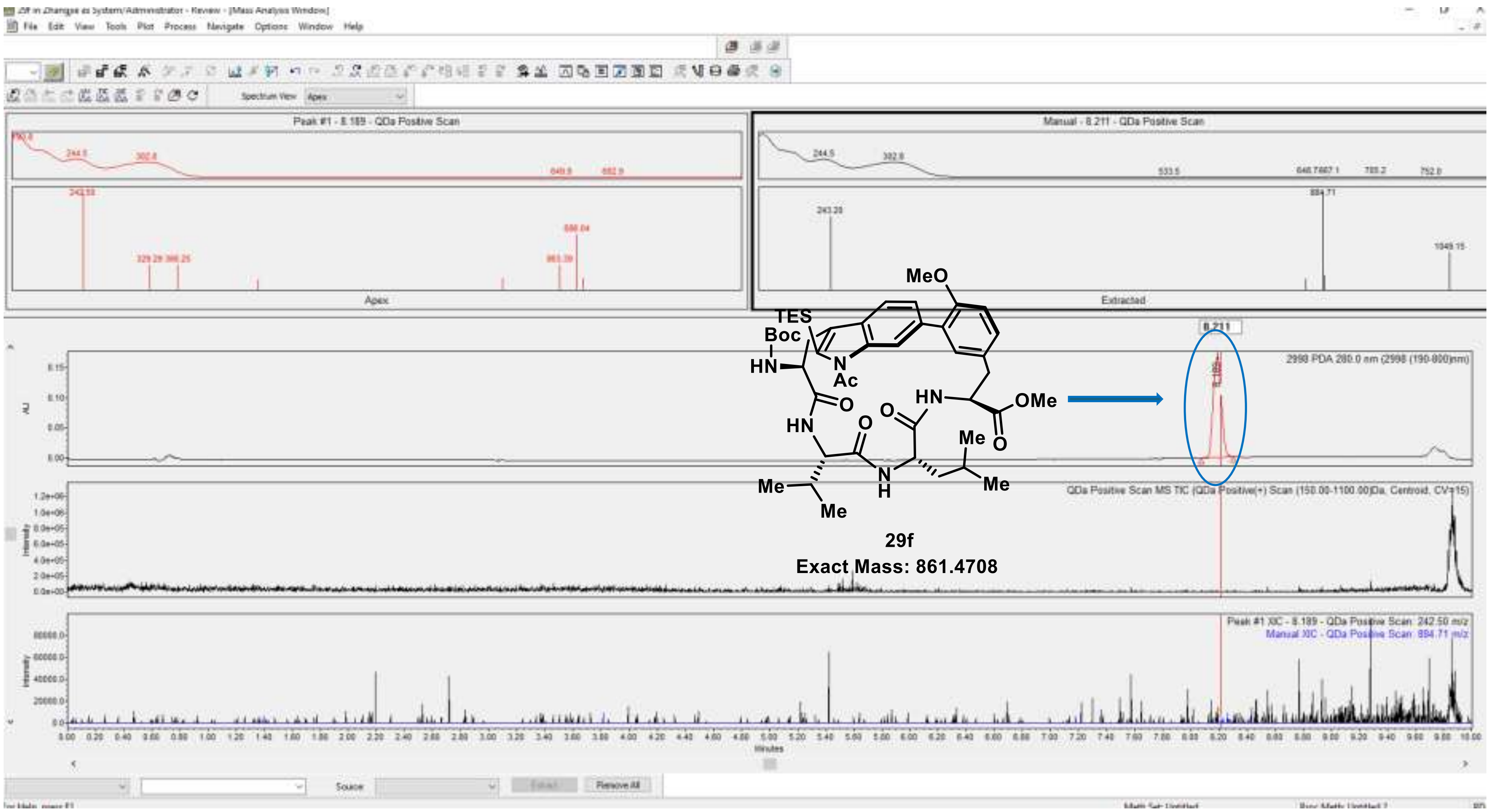

Compound 29g ^{1}H NMR (600 MHz, $CDCl_3$)

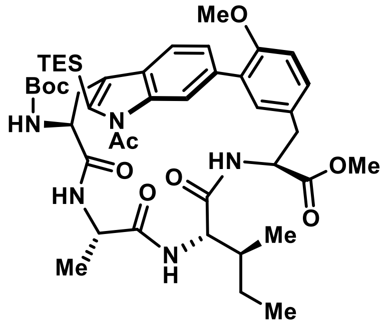

29g

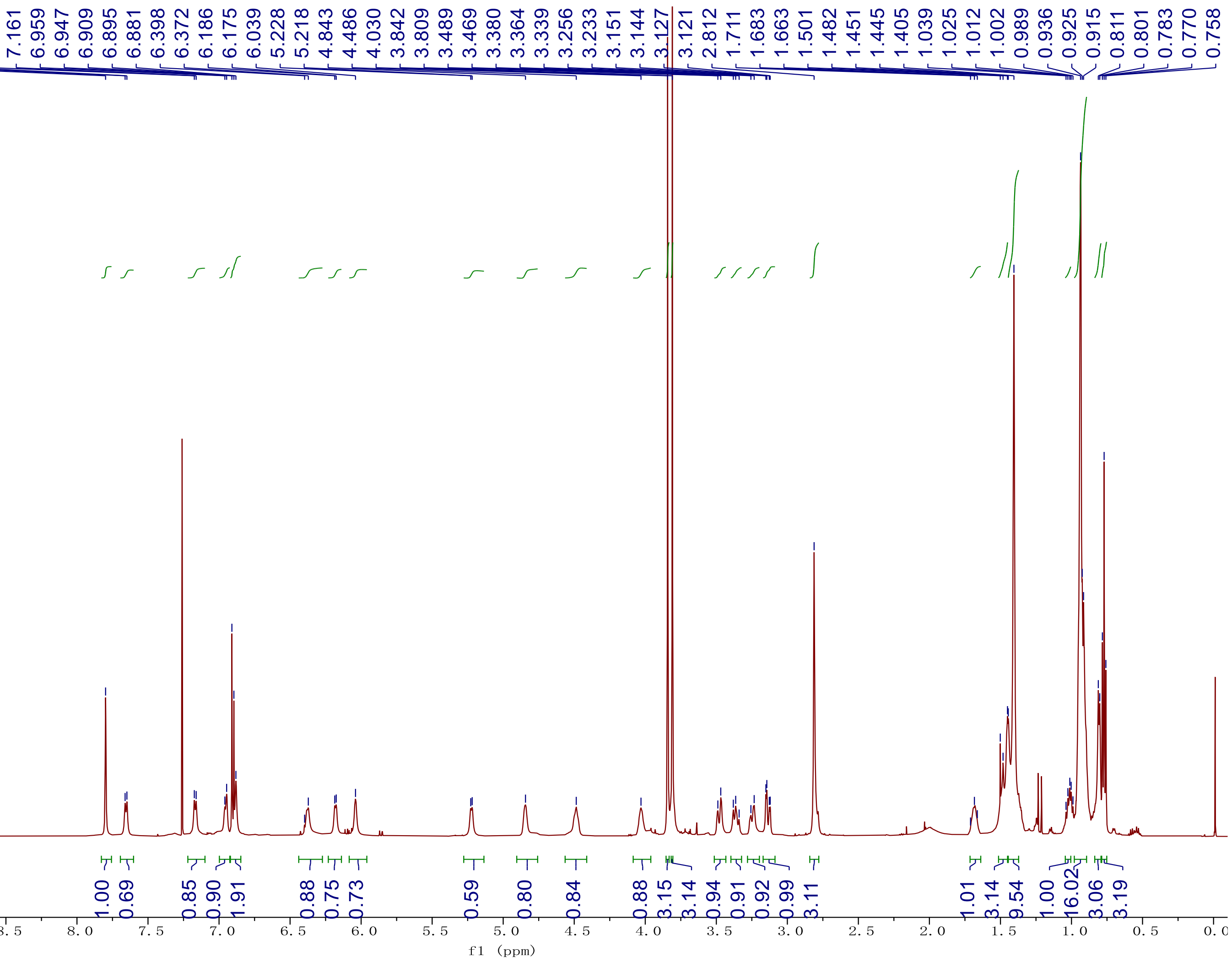

Compound 29g ^{13}C NMR (151 MHz, $CDCl_3$)

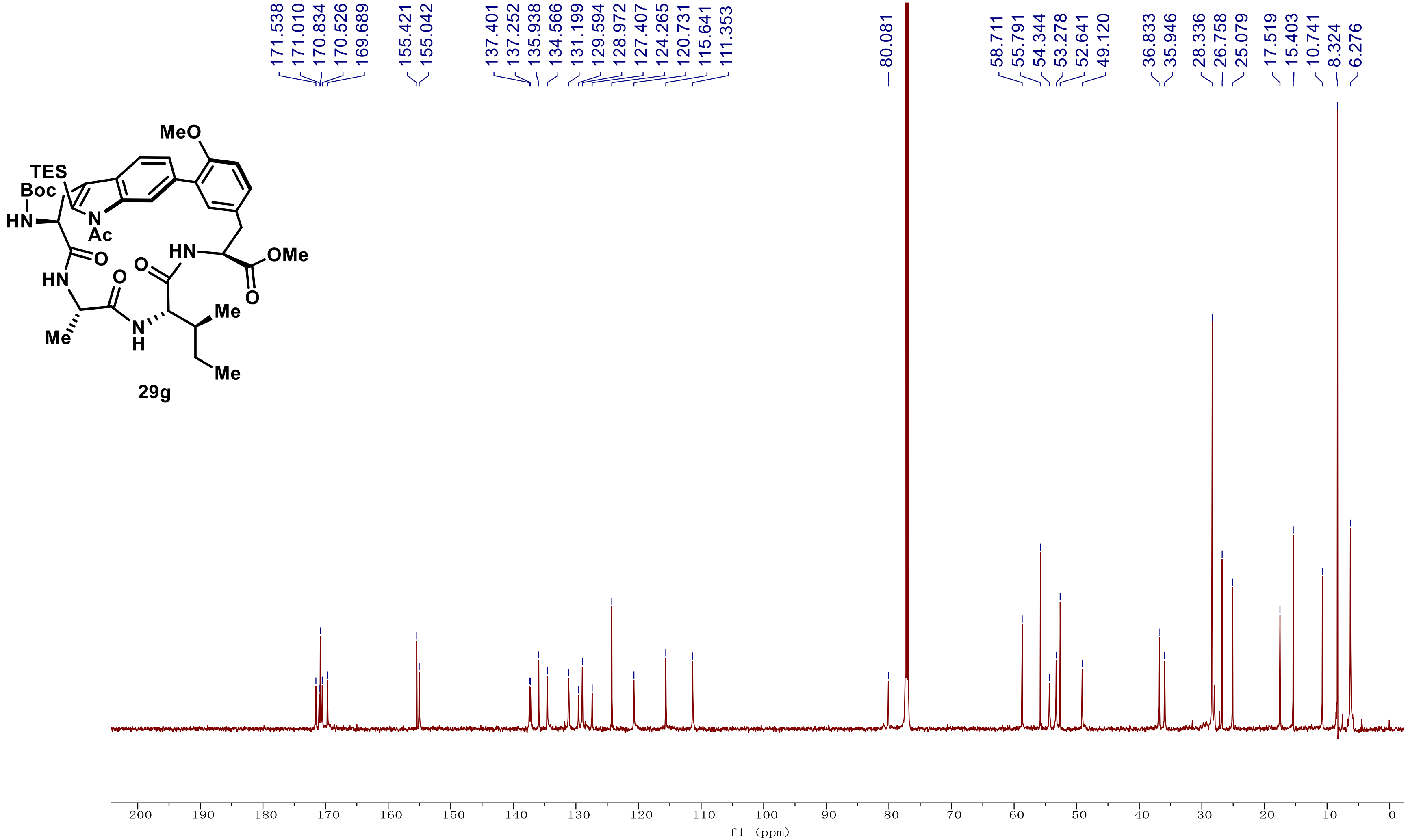

Compound 29h ^{1}H NMR (600 MHz, $CDCl_3$)

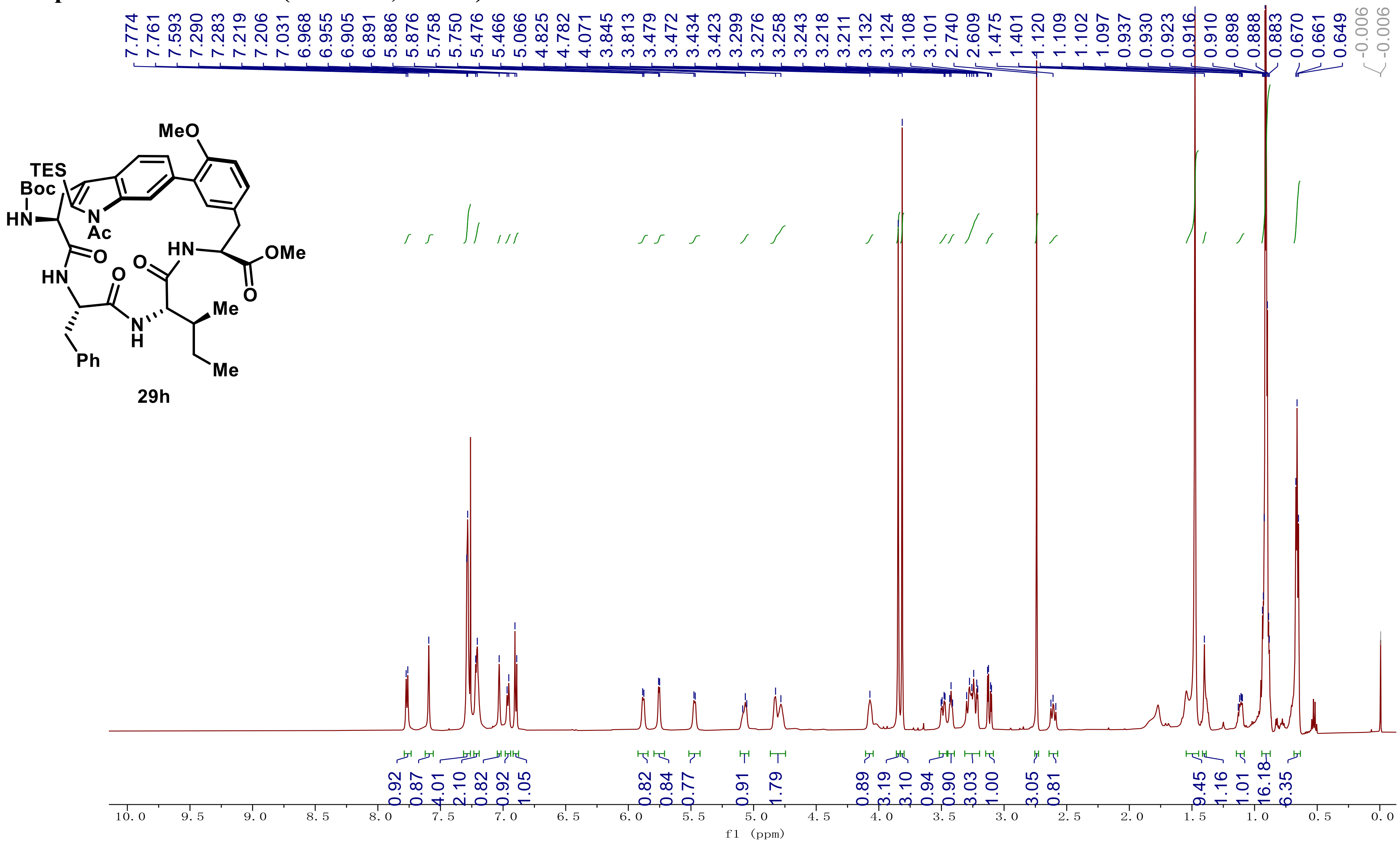

Compound 29h ^{13}C NMR (151 MHz, $CDCl_3$)

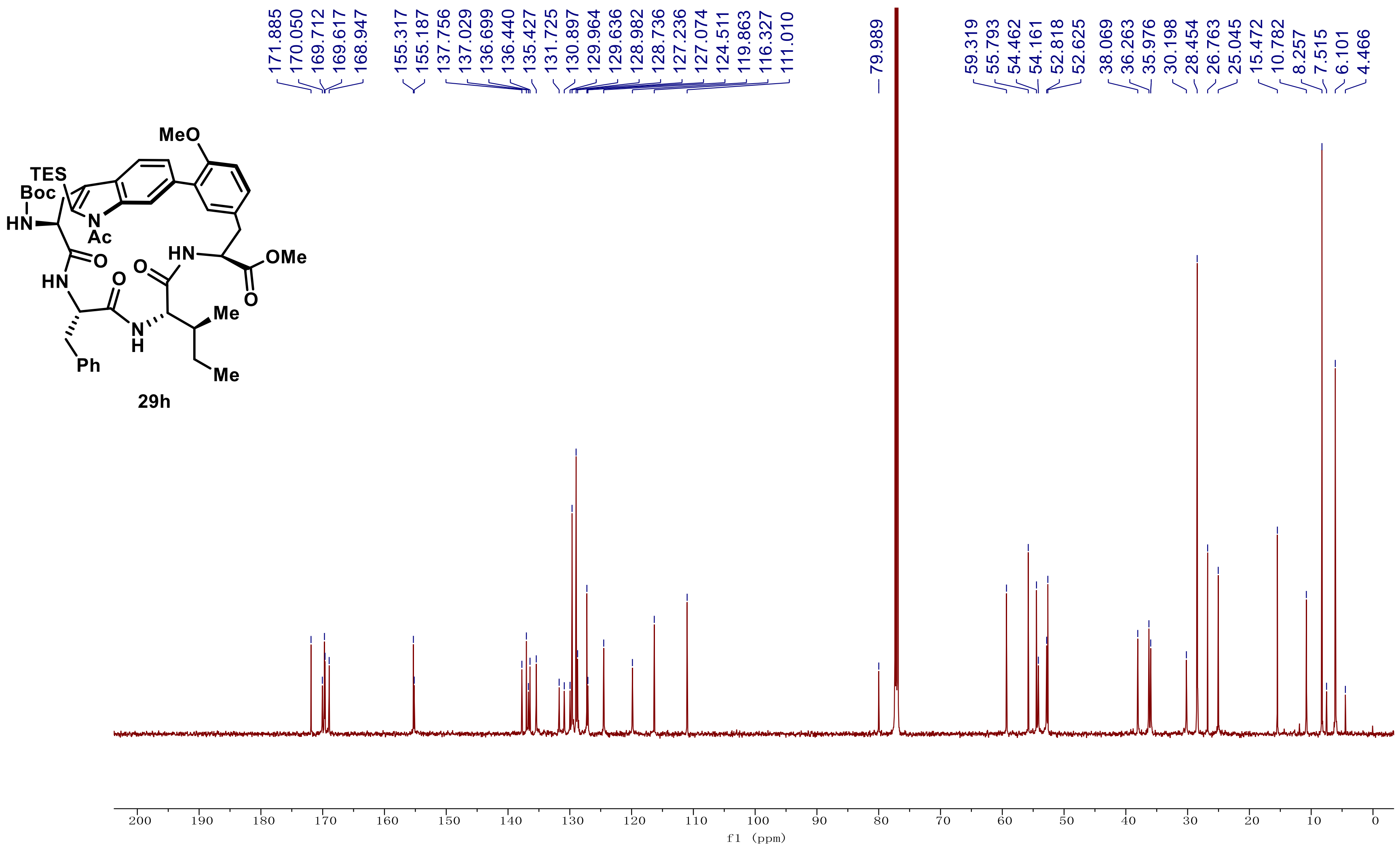

29h

Compound 29i 1H NMR (600 MHz, CDCl3)

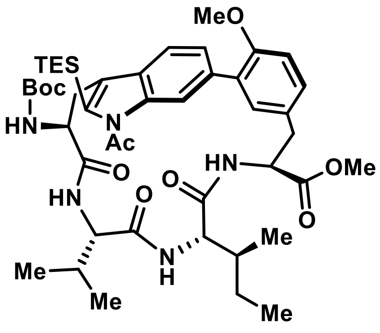

29i

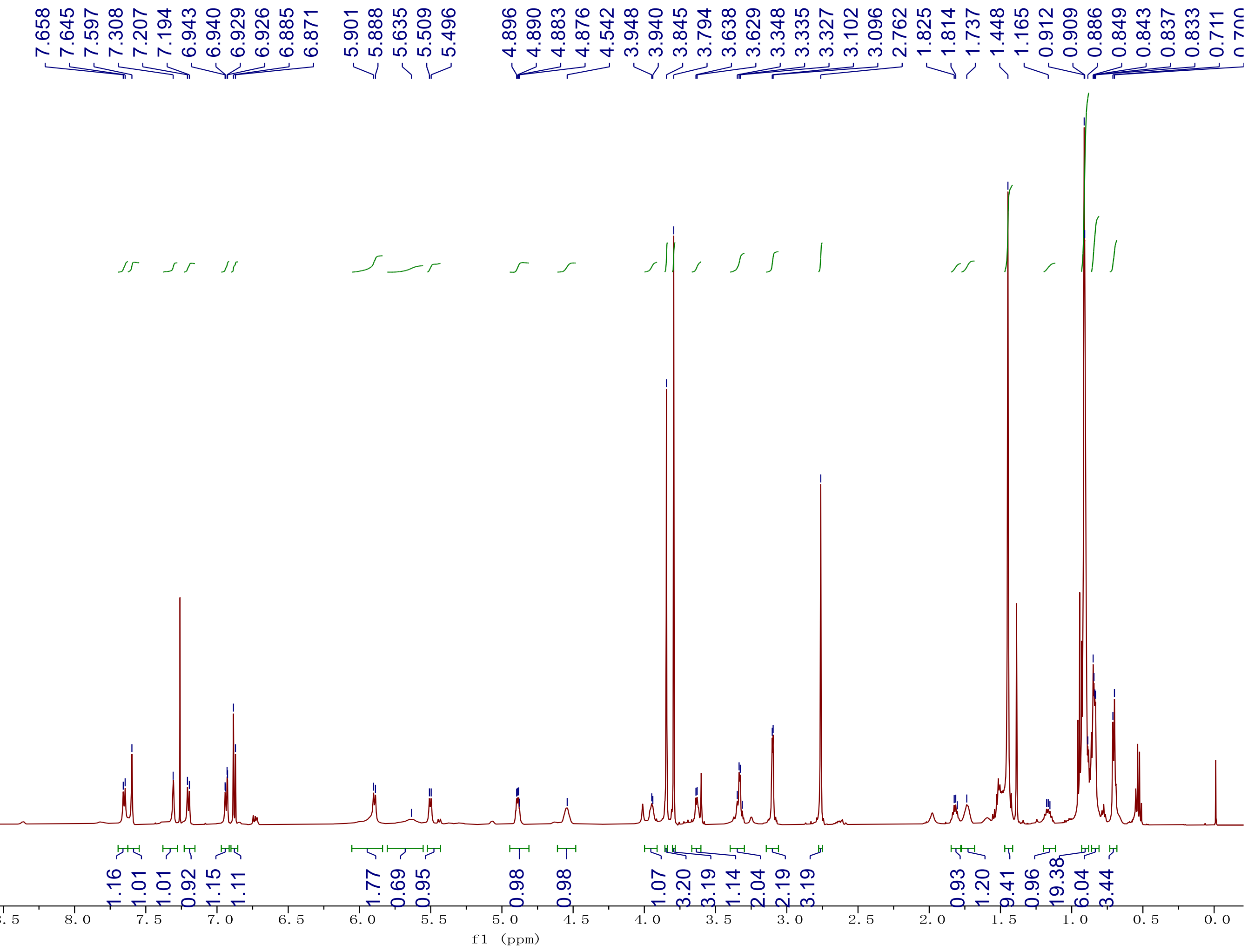

Compound 29i ^{13}C NMR (151 MHz, CDCl$_3$)

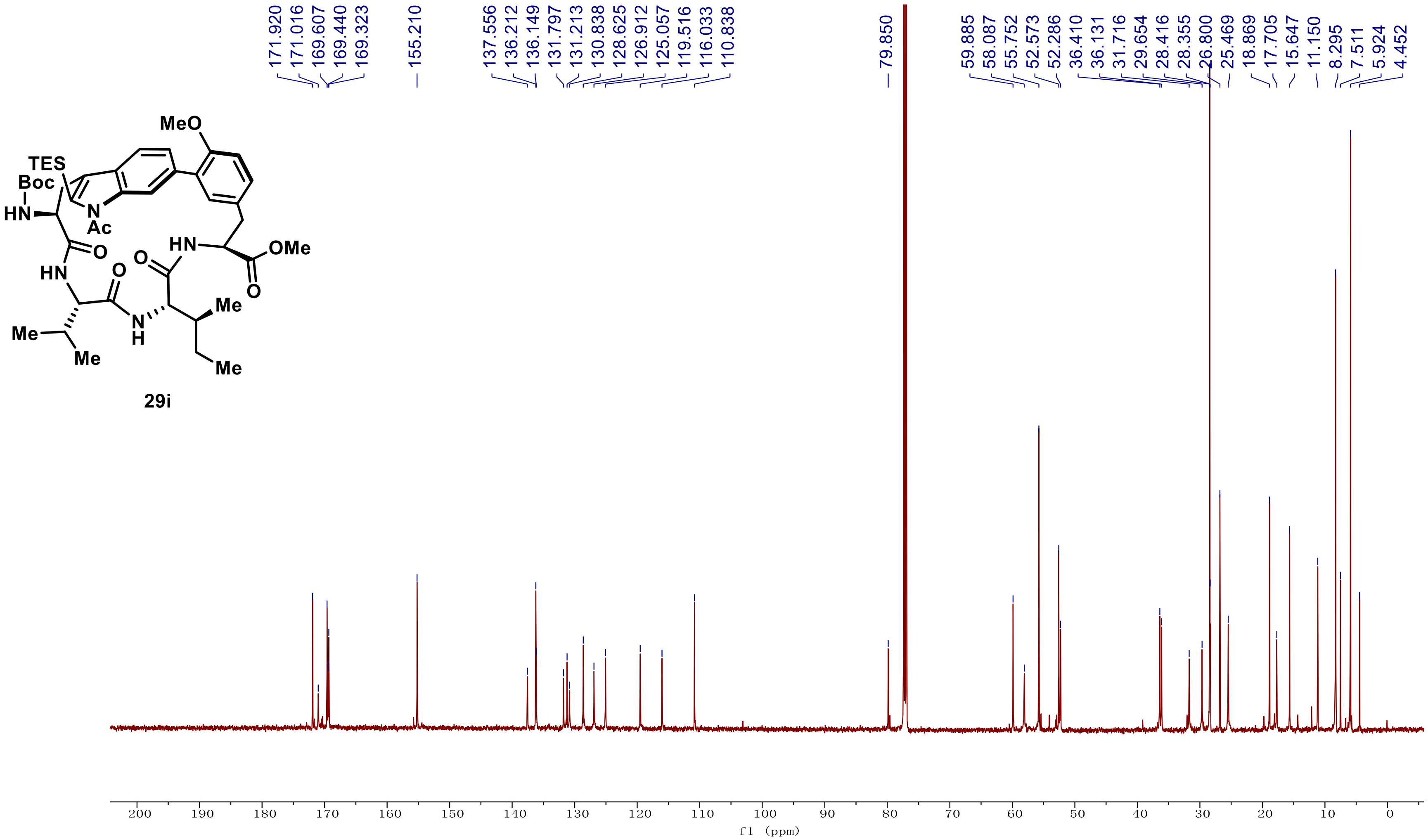

Compound 29j ^{1}H NMR (600 MHz, $CDCl_3$)

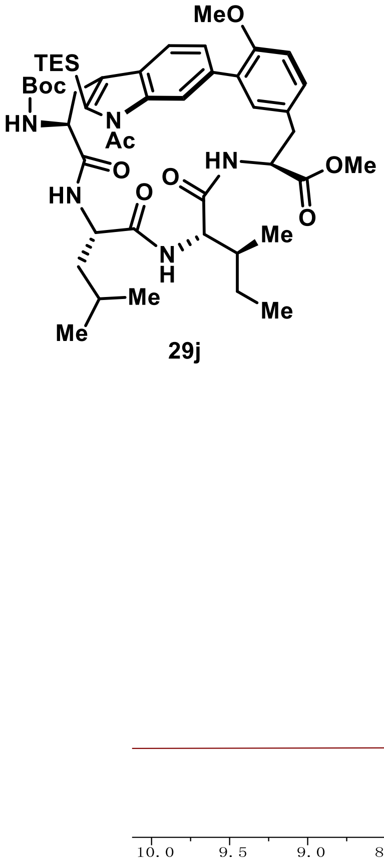

29j

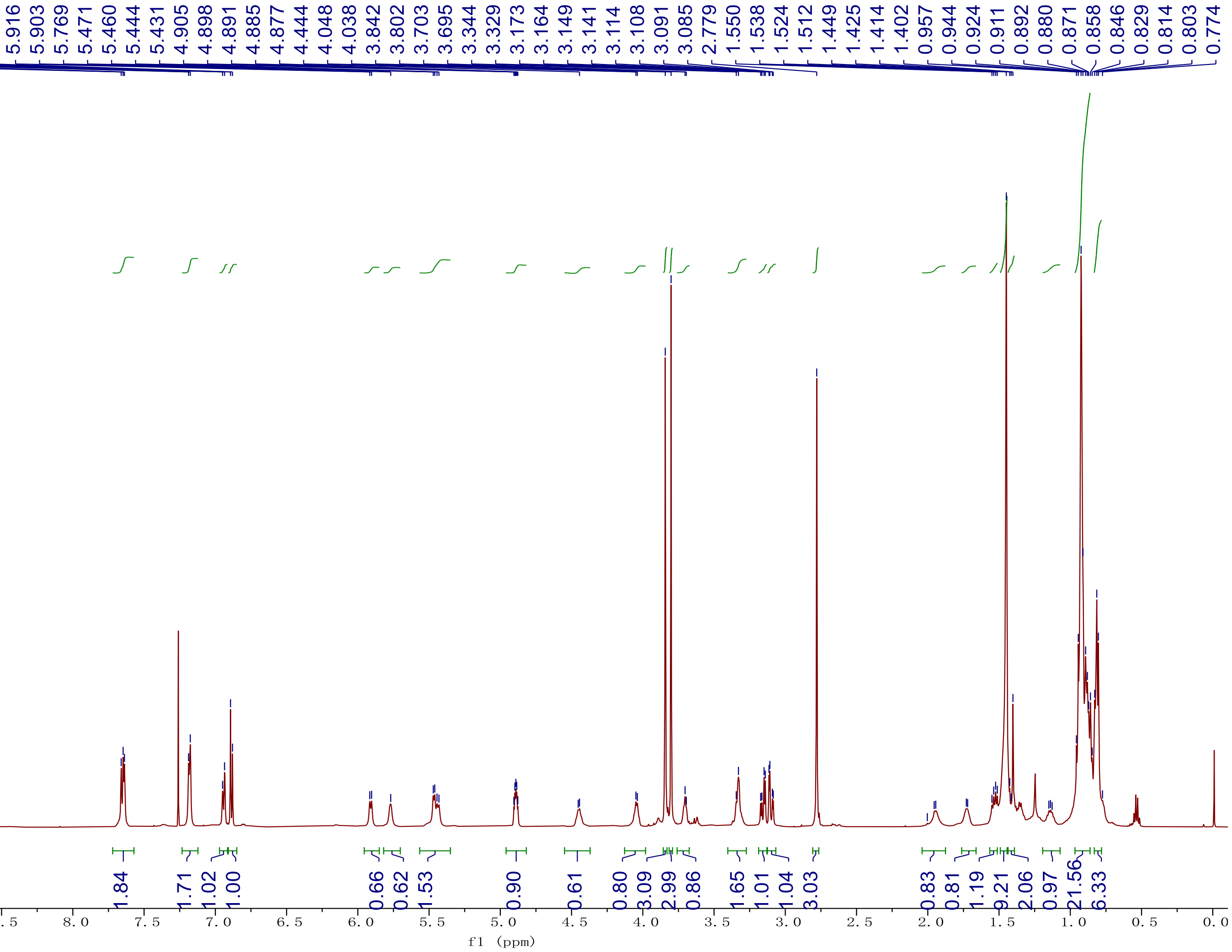

Compound 29j ^{13}C NMR (151 MHz, $CDCl_3$)

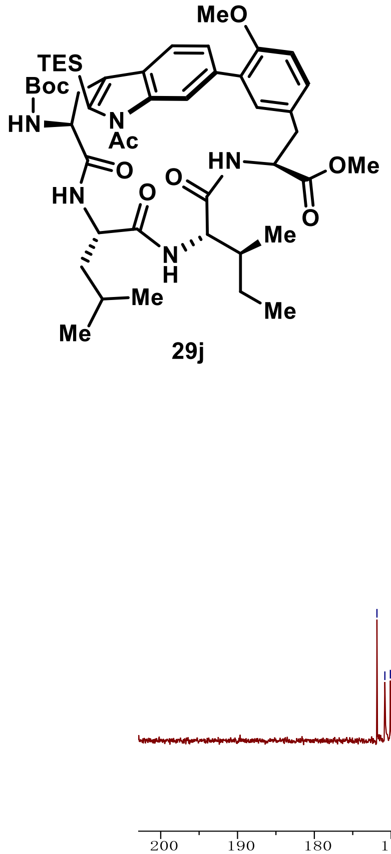

29j

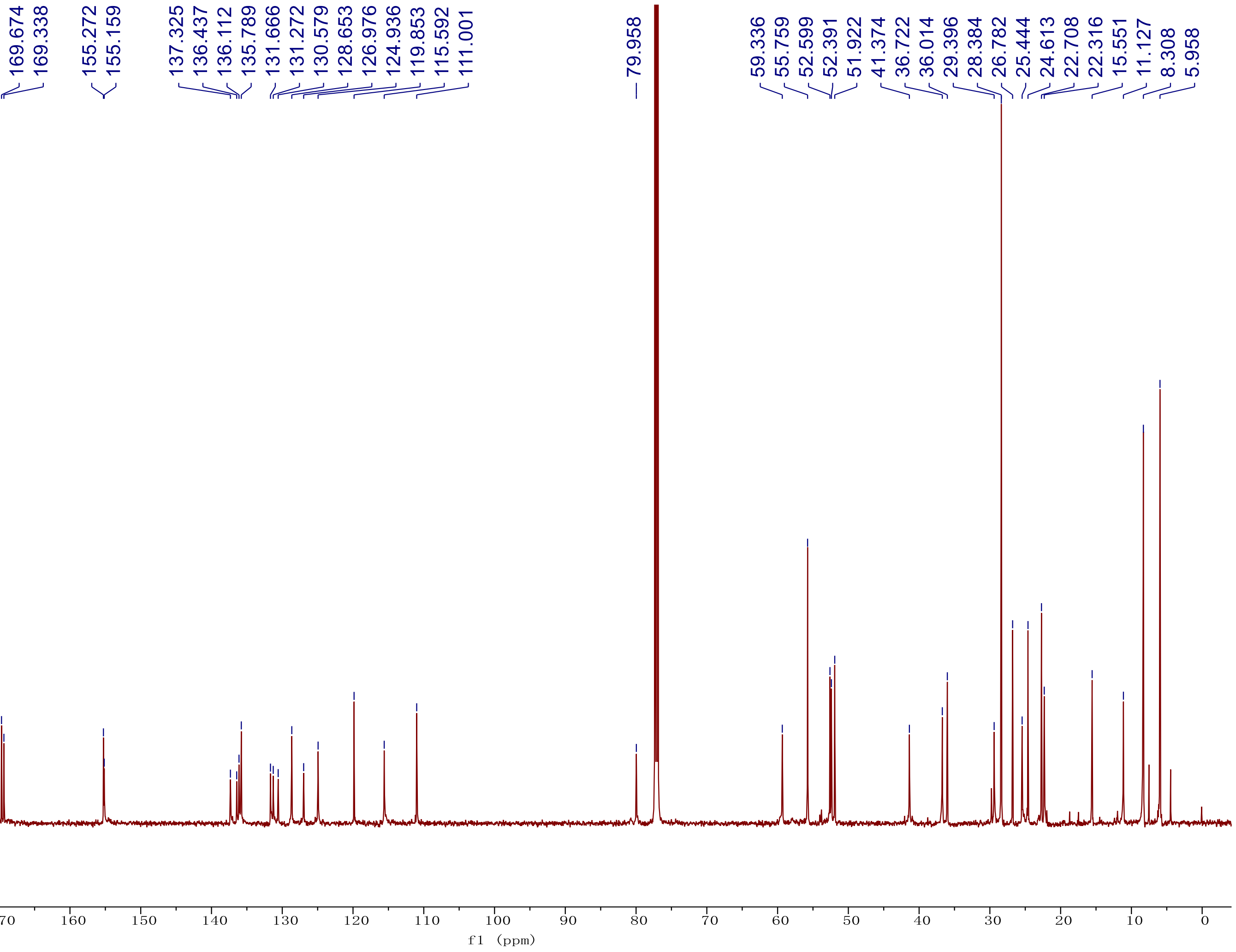

Compound 29k ^{1}H NMR (600 MHz, $CDCl_3$)

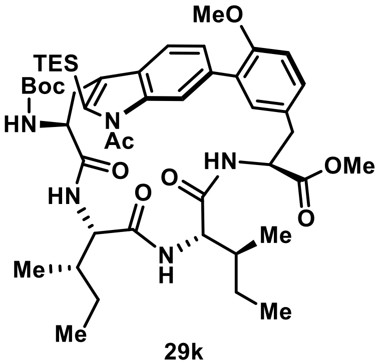

29k

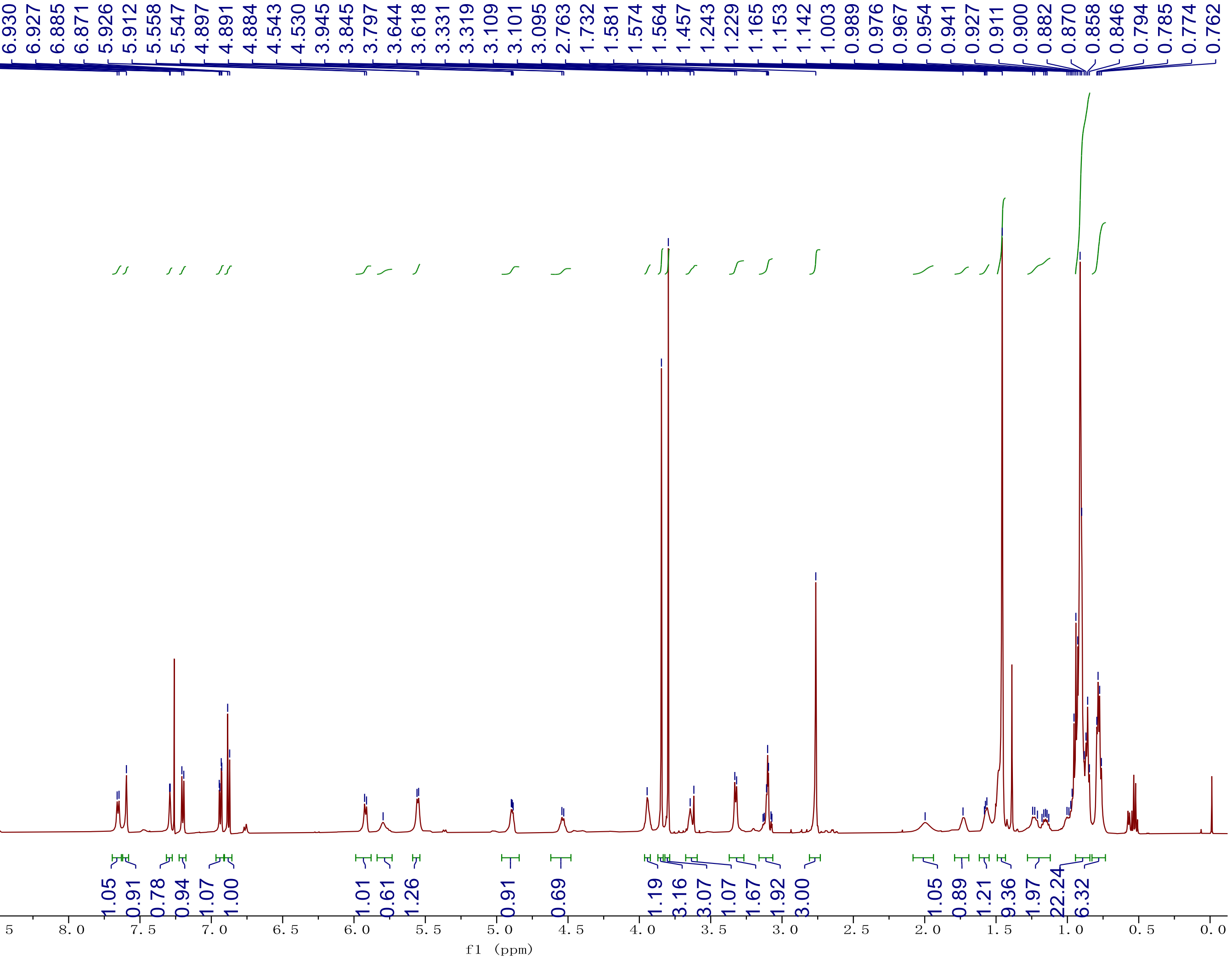

Compound 29k ^{13}C NMR (151 MHz, $CDCl_3$)

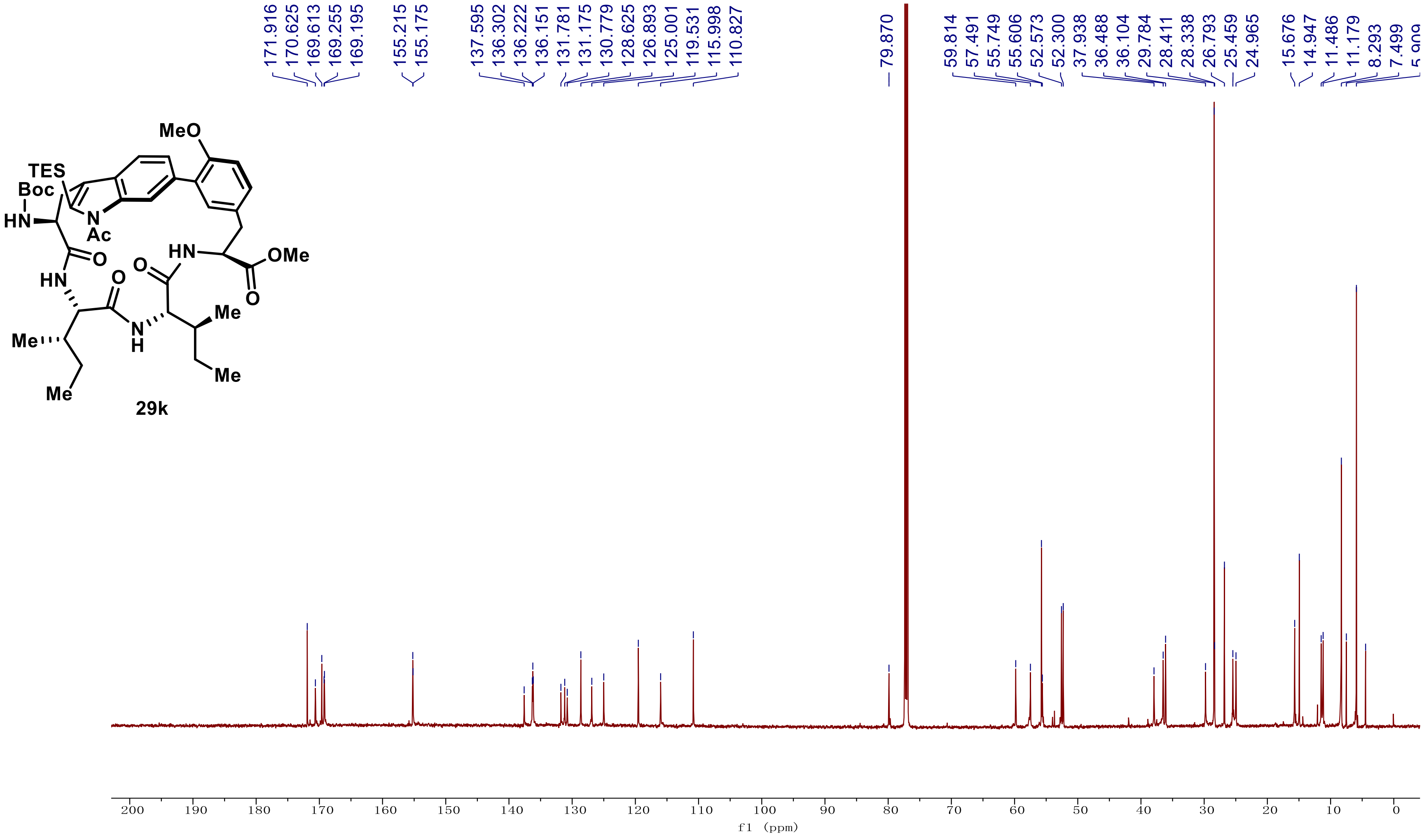

Compound 29l ^{1}H NMR (600 MHz, $CDCl_3$)

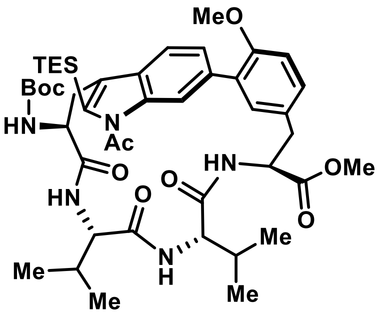

29l

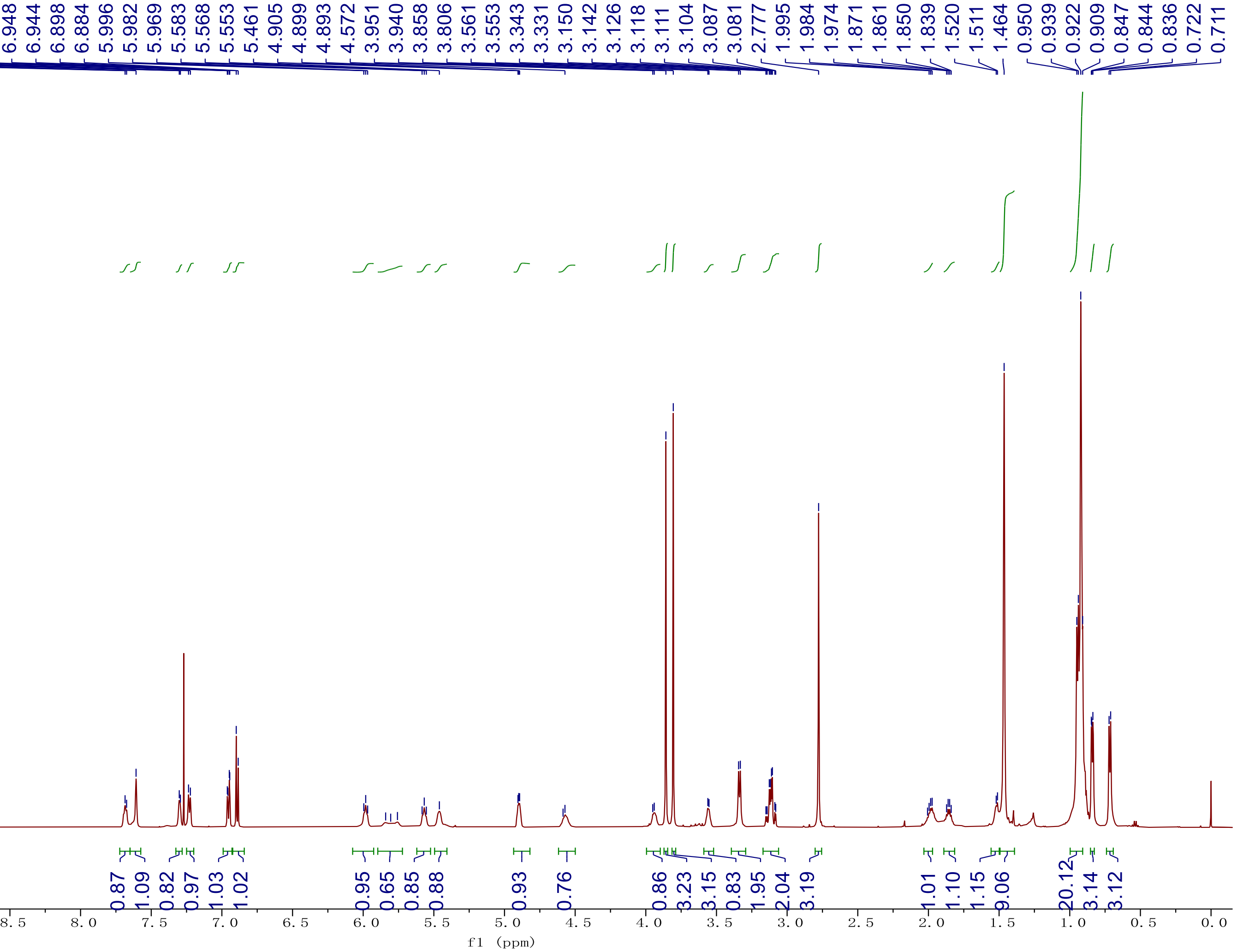

Compound 29l ^{13}C NMR (151 MHz, $CDCl_3$)

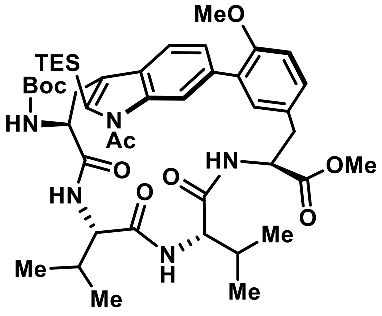

29l

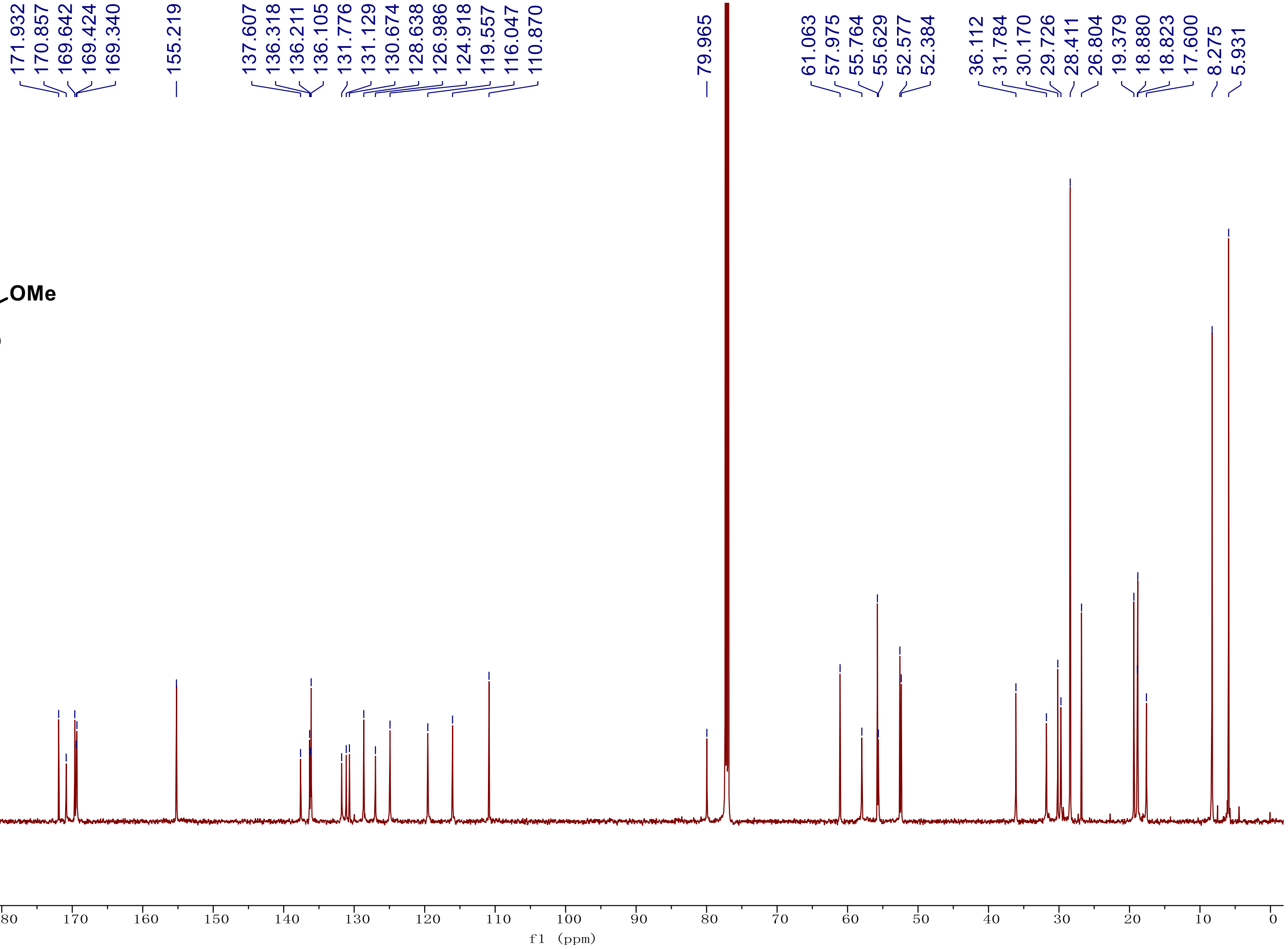

Compound 29m ^{1}H NMR (600 MHz, CDCl$_3$)

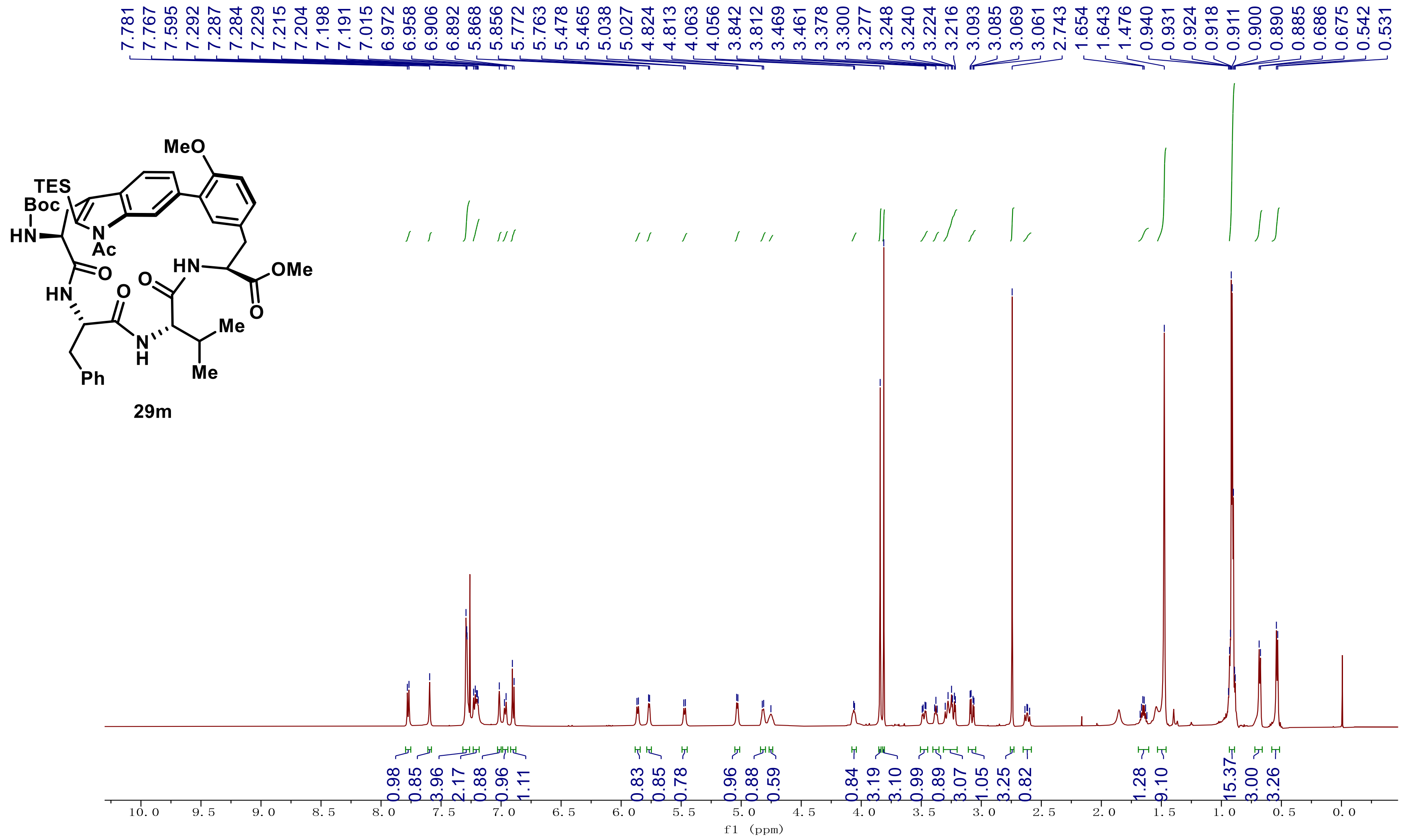

29m

Compound 29m ^{13}C NMR (151 MHz, CDCl$_3$)

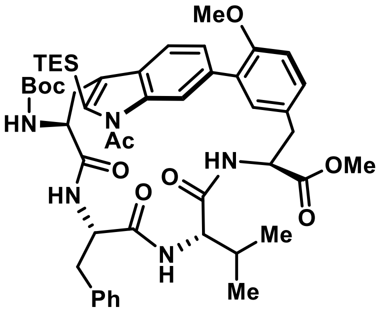

29m

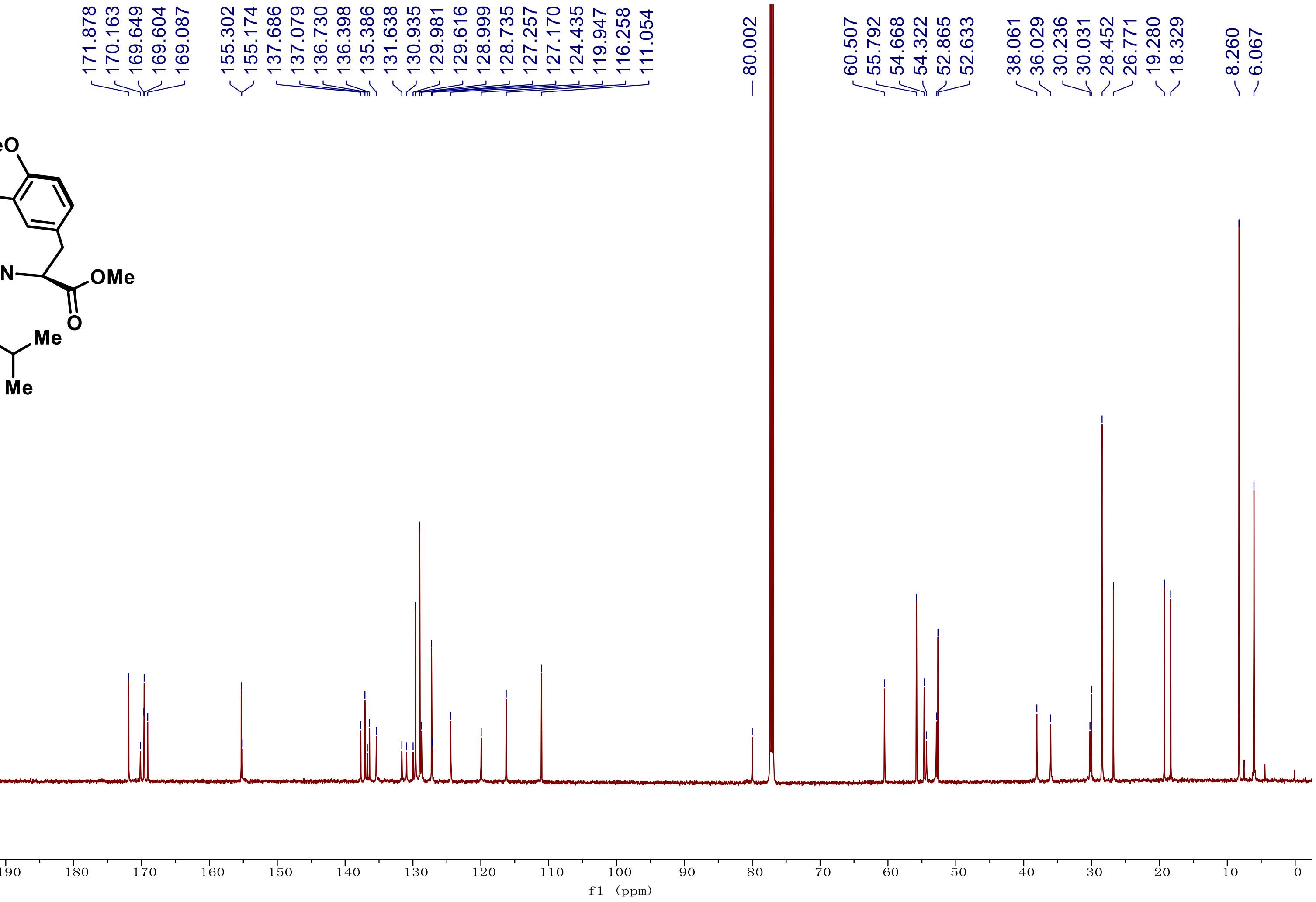

Compound 29n ¹H NMR (600 MHz, CDCl₃)

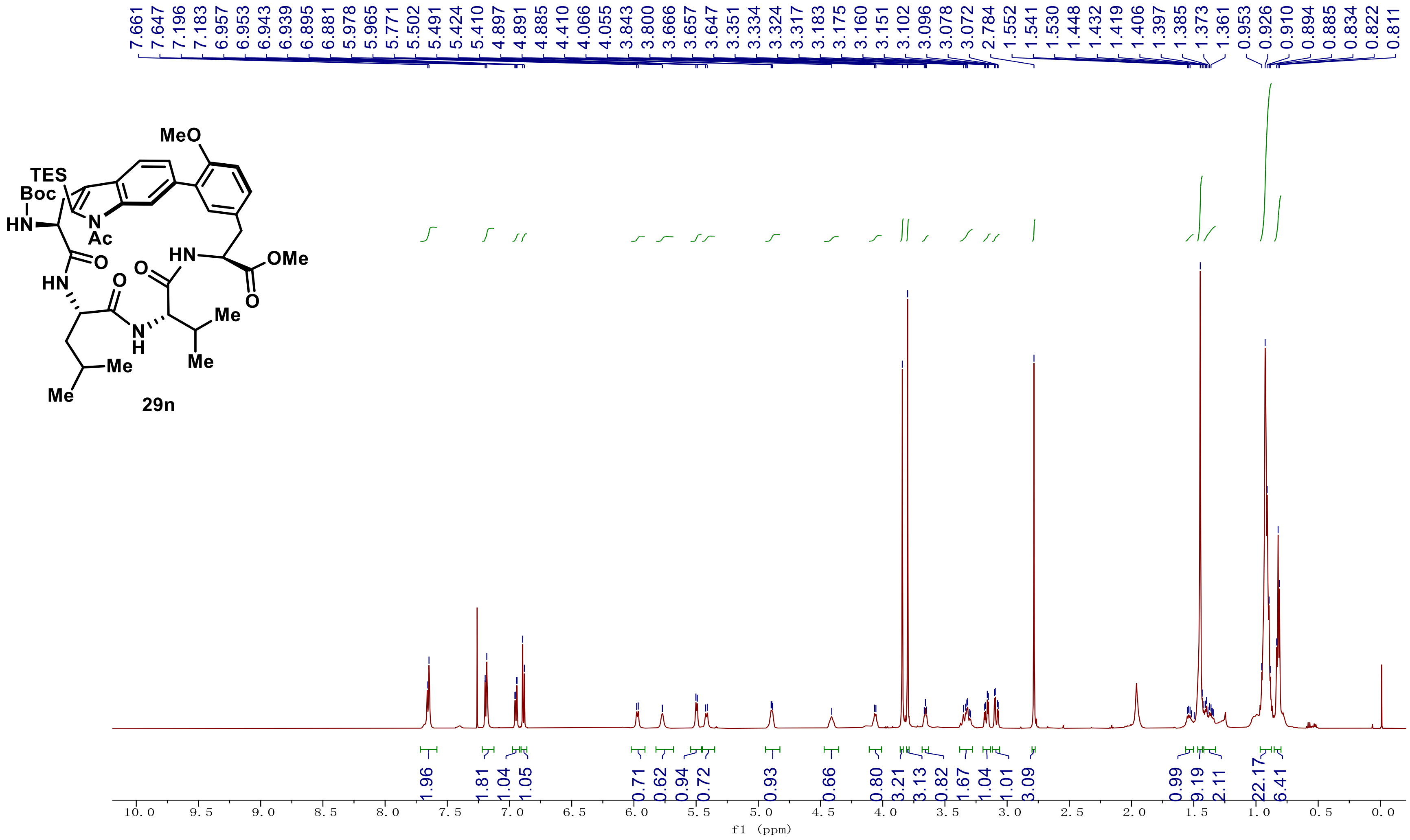

Compound 29n ^{13}C NMR (151 MHz, $CDCl_3$)

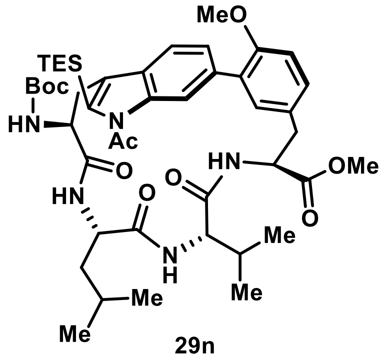

29n

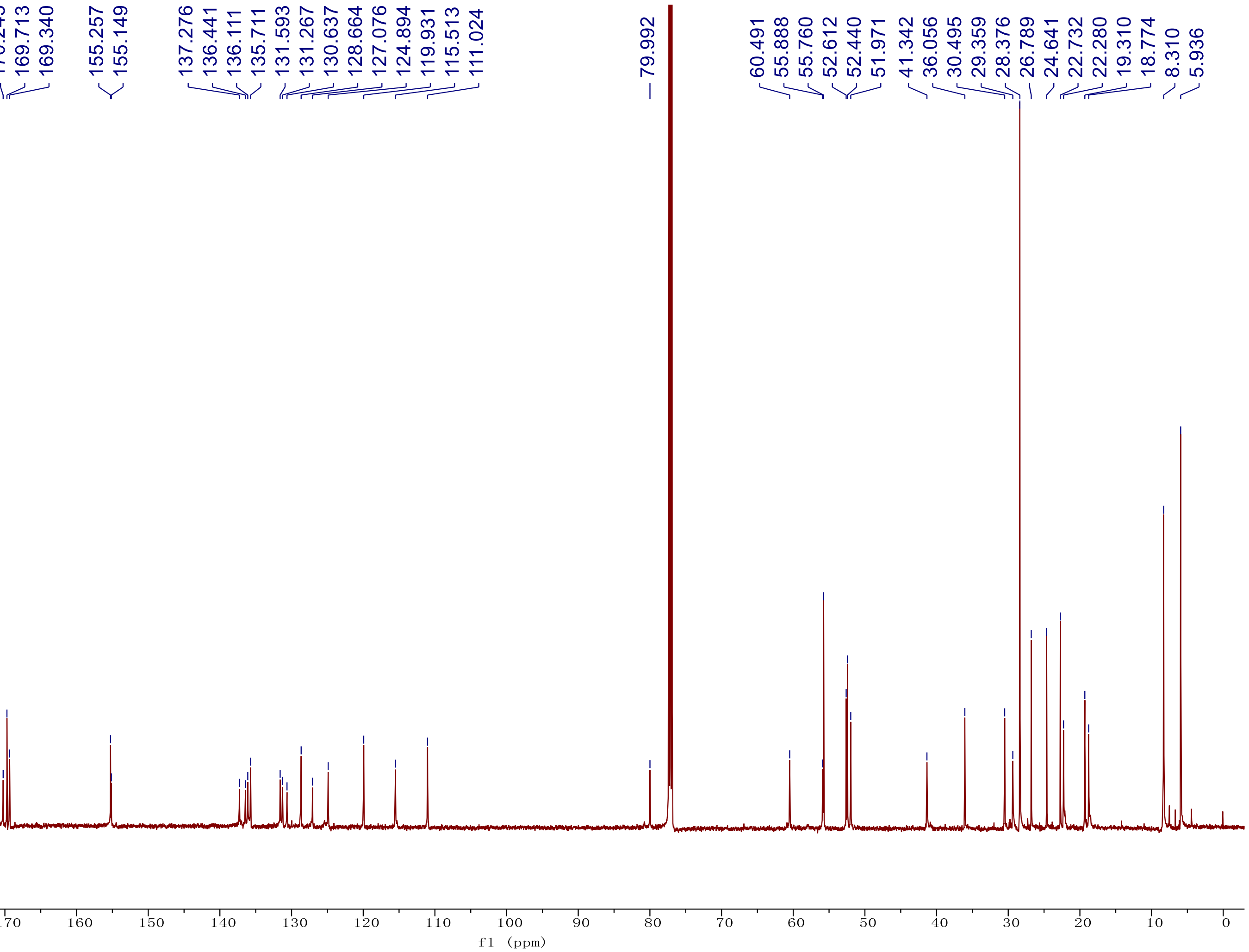

Compound 29o ^{1}H NMR (600 MHz, $CDCl_3$)

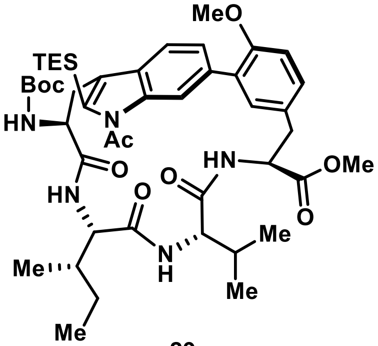

29o

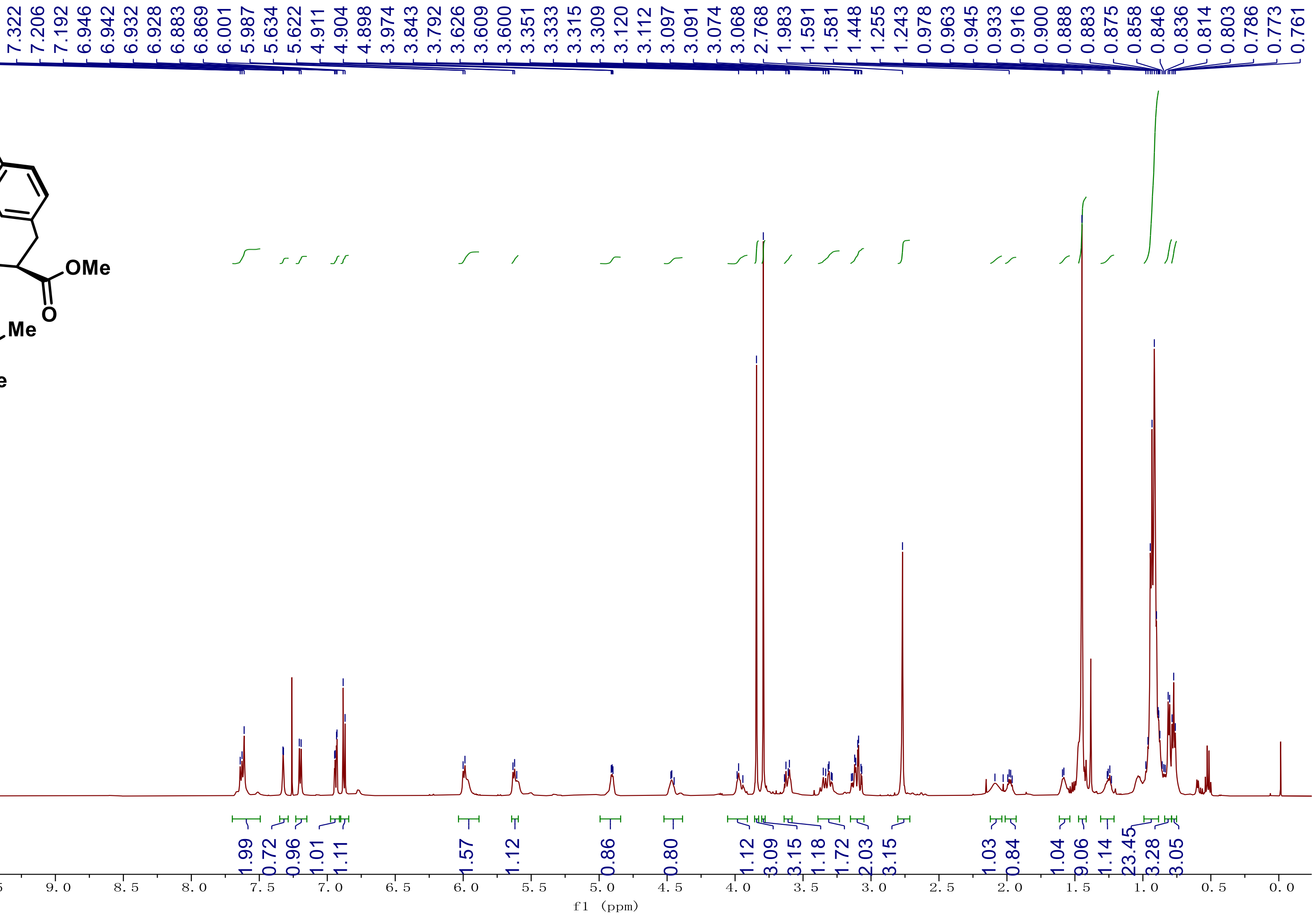

Compound 29o ^{13}C NMR (151 MHz, $CDCl_3$)

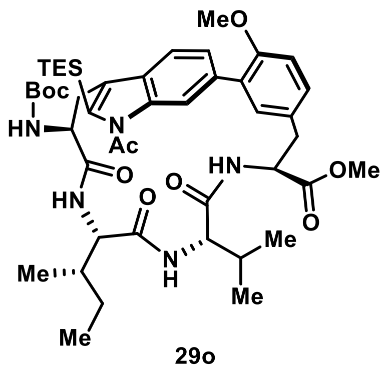

29o

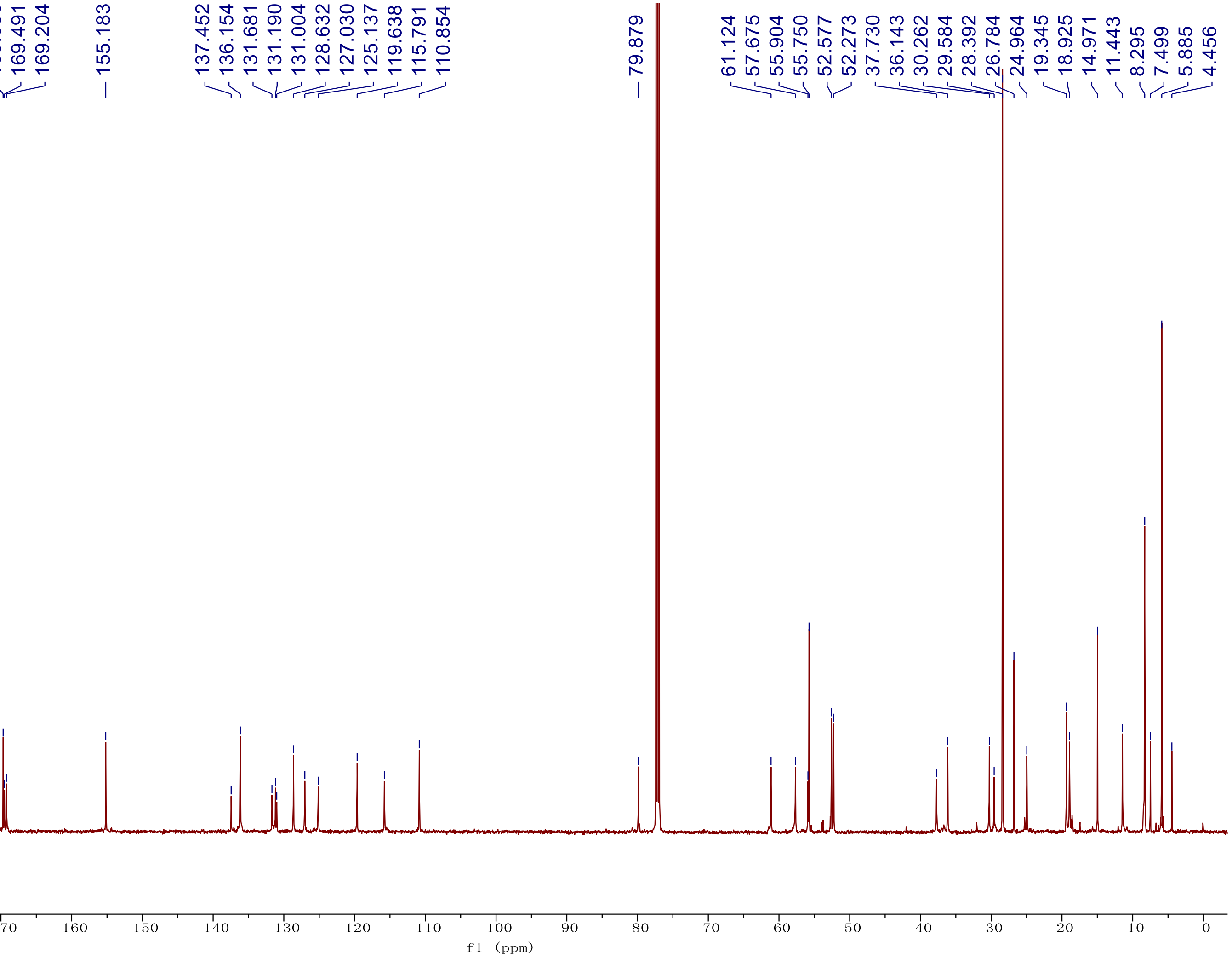

Compound 29p ^{1}H NMR (600 MHz, $CDCl_3$)

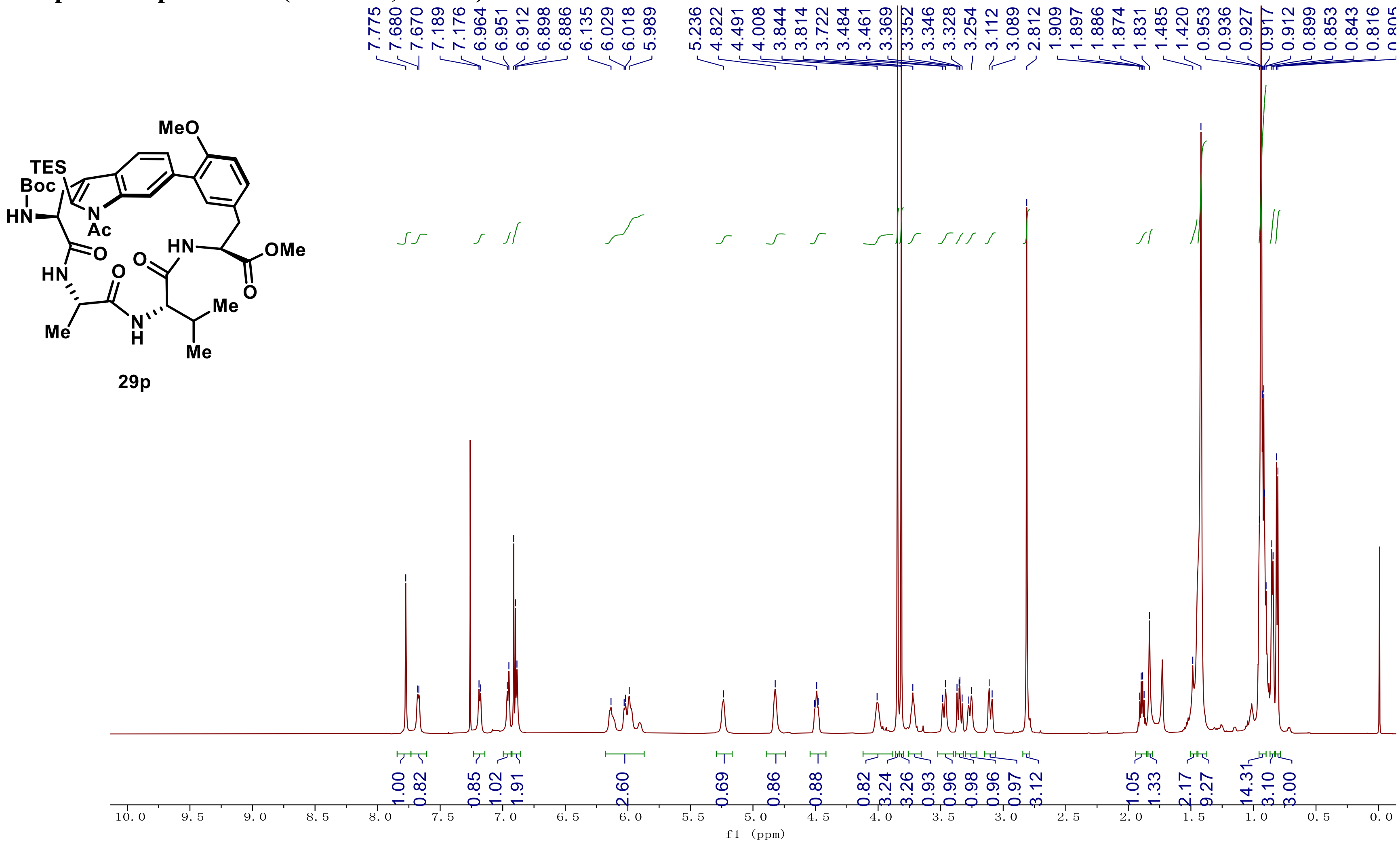

Compound 29p ^{13}C NMR (151 MHz, $CDCl_3$)

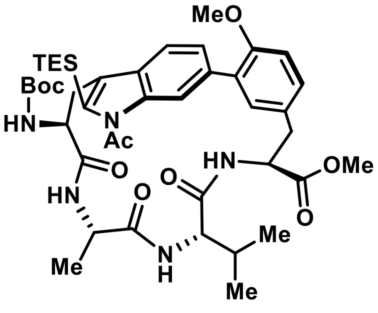

29p

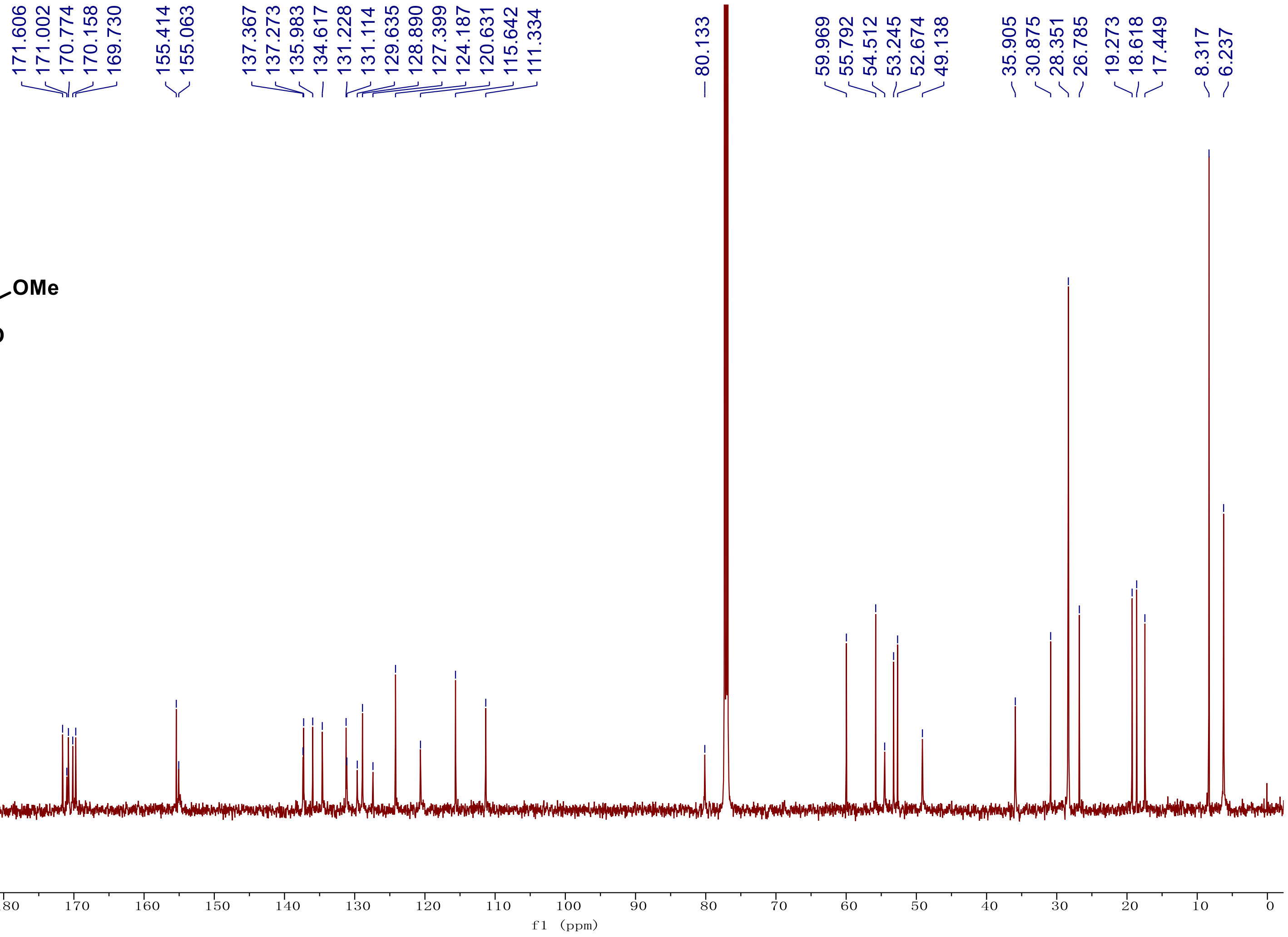

Compound 29q ^{1}H NMR (600 MHz, $CDCl_3$)

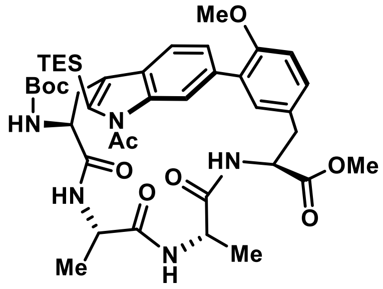

29q

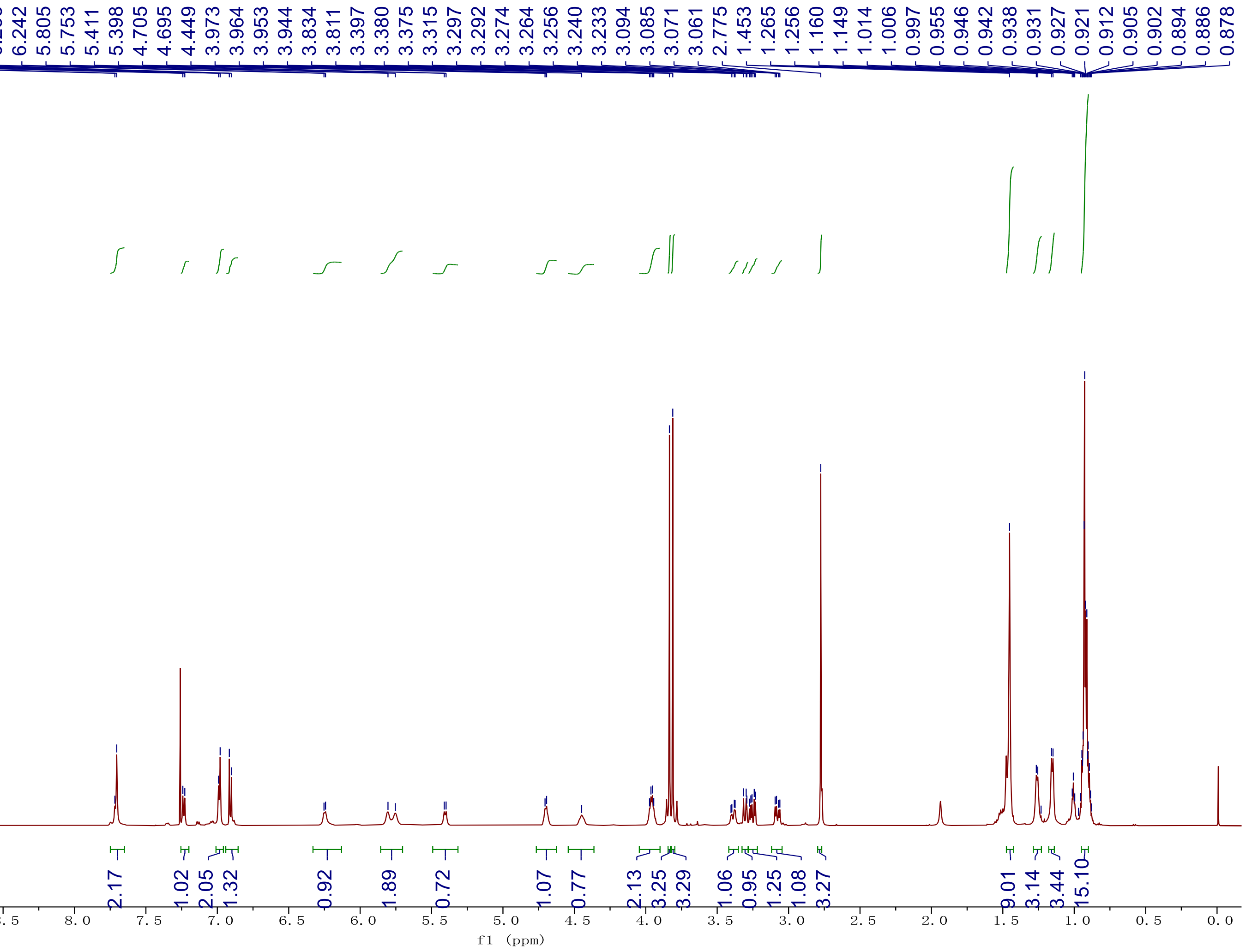

Compound 29q ^{13}C NMR (151 MHz, $CDCl_3$)

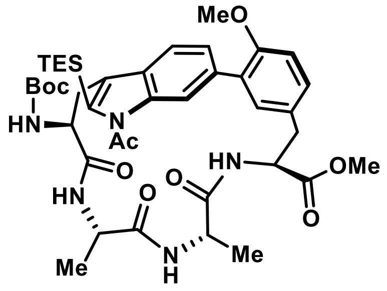

29q

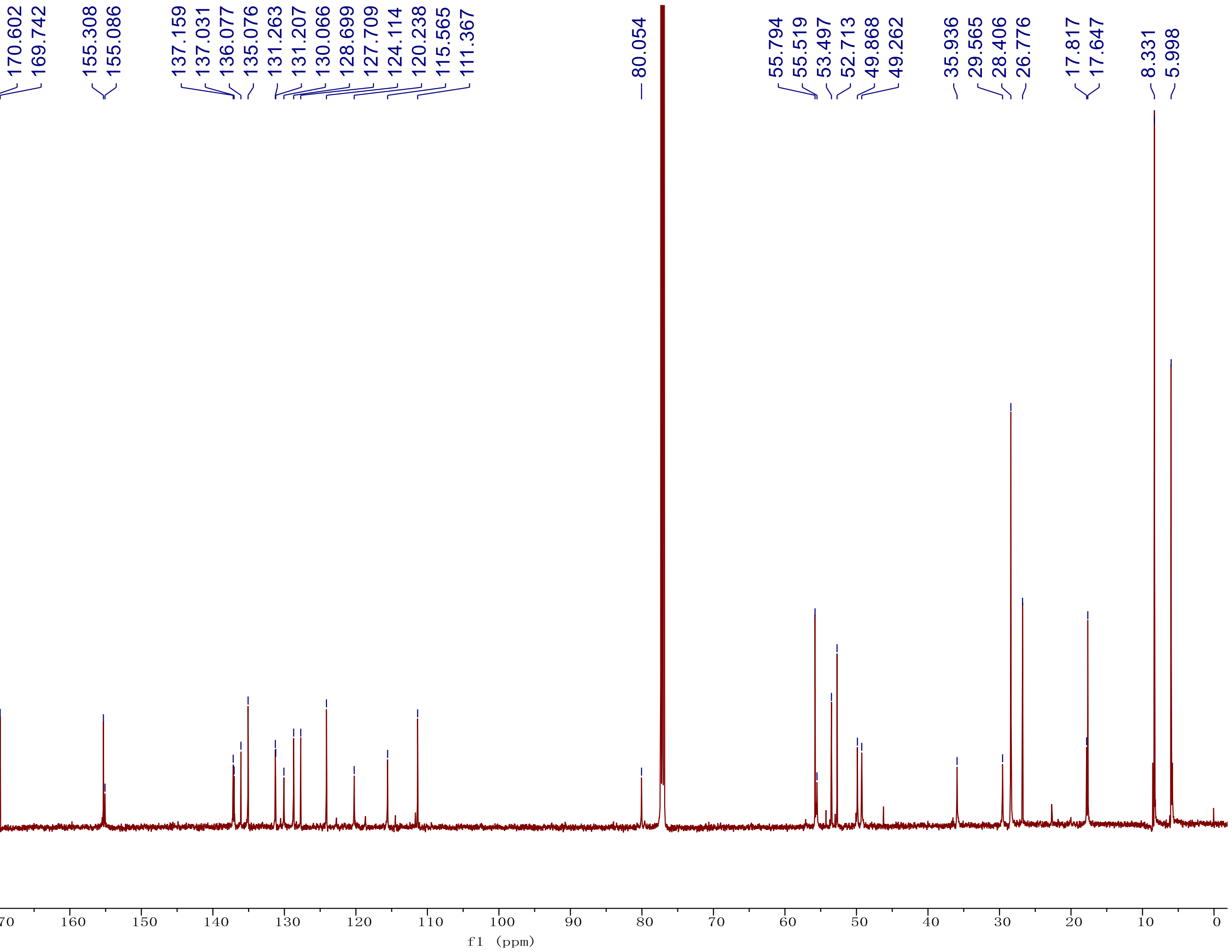

Compound 29r ^{1}H NMR (600 MHz, $CDCl_3$)

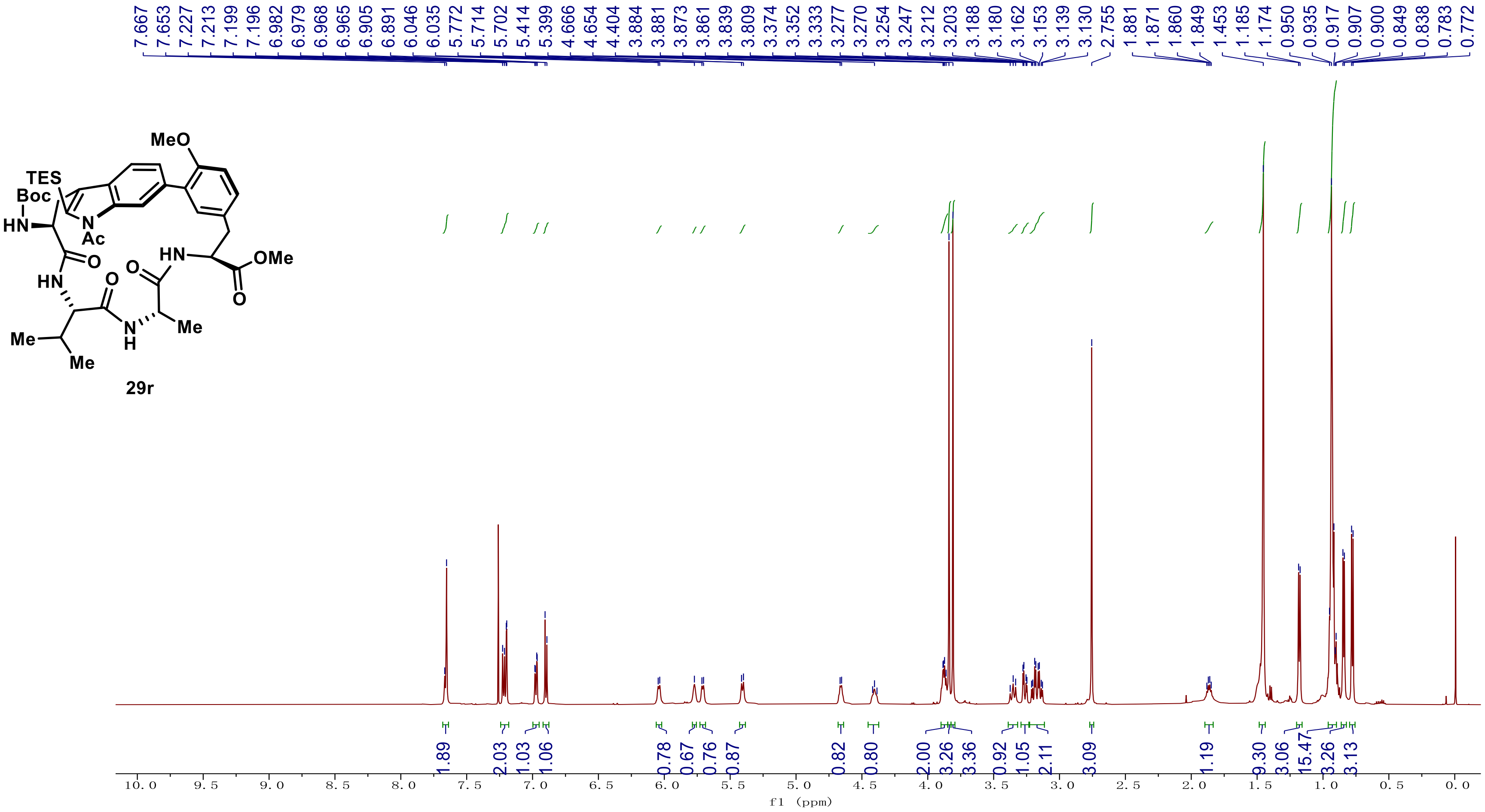

Compound 29r ^{13}C NMR (151 MHz, $CDCl_3$)

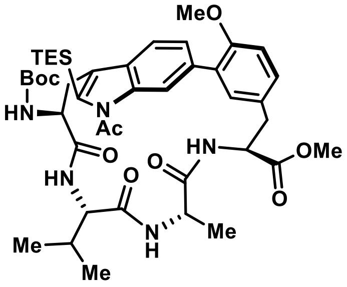

29r

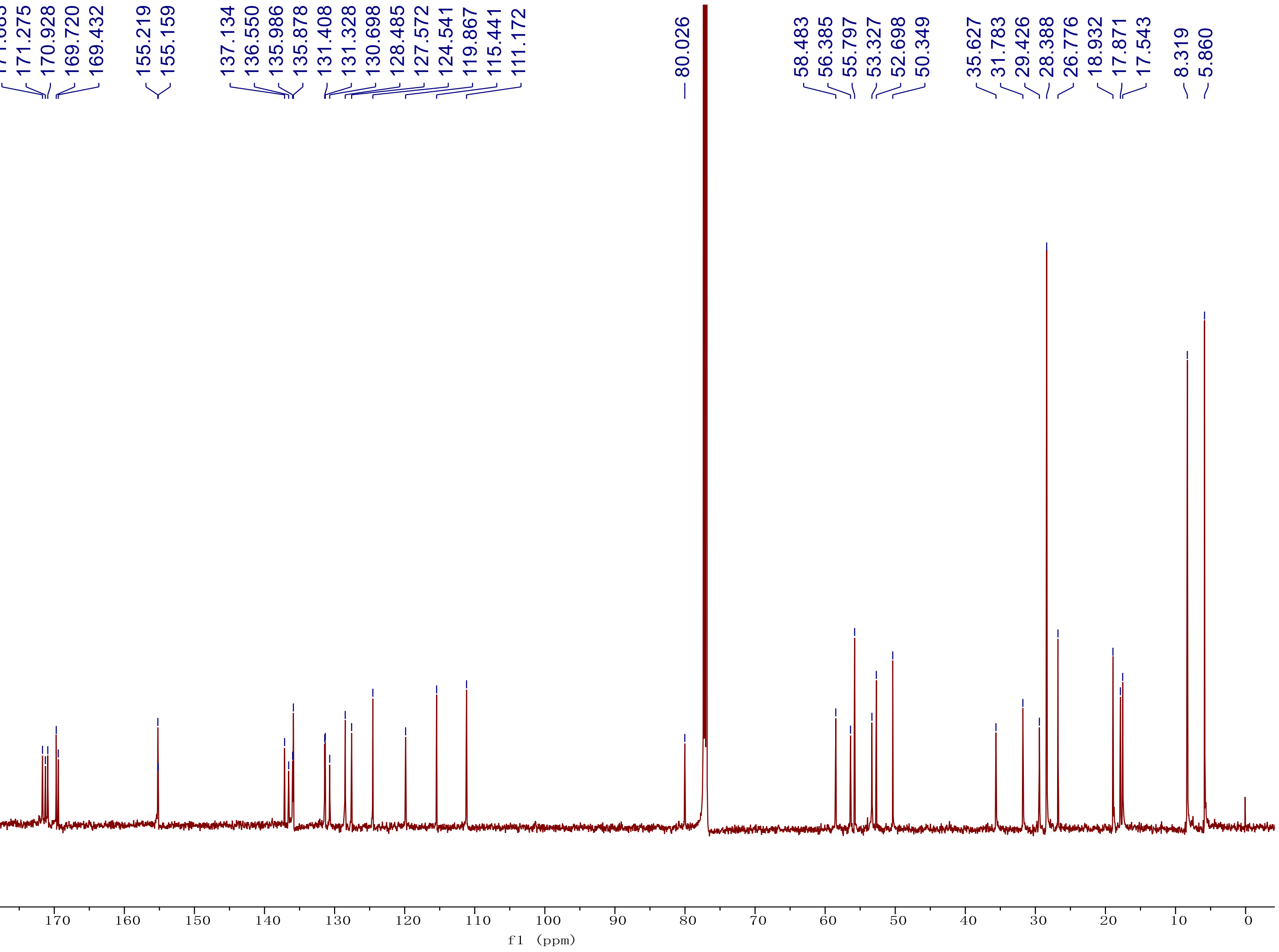

Compound 29s ^{1}H NMR (600 MHz, $CDCl_3$)

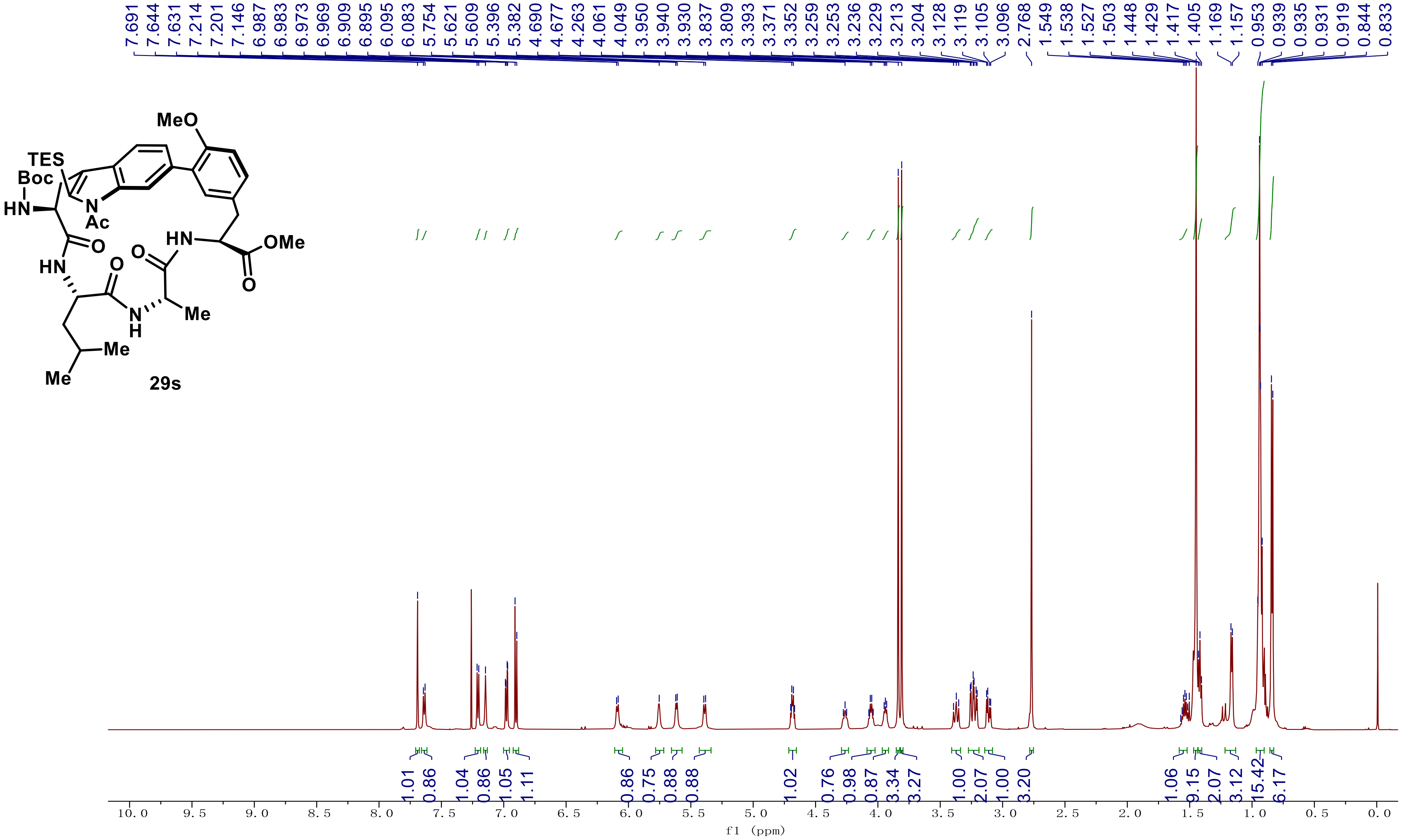

29s

Compound 29s ^{13}C NMR (151 MHz, CDCl$_3$)

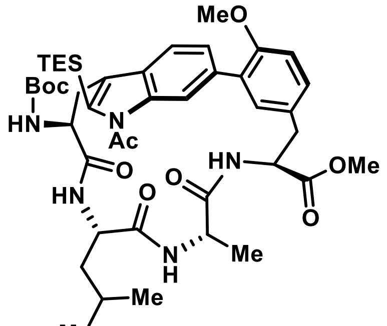

29s

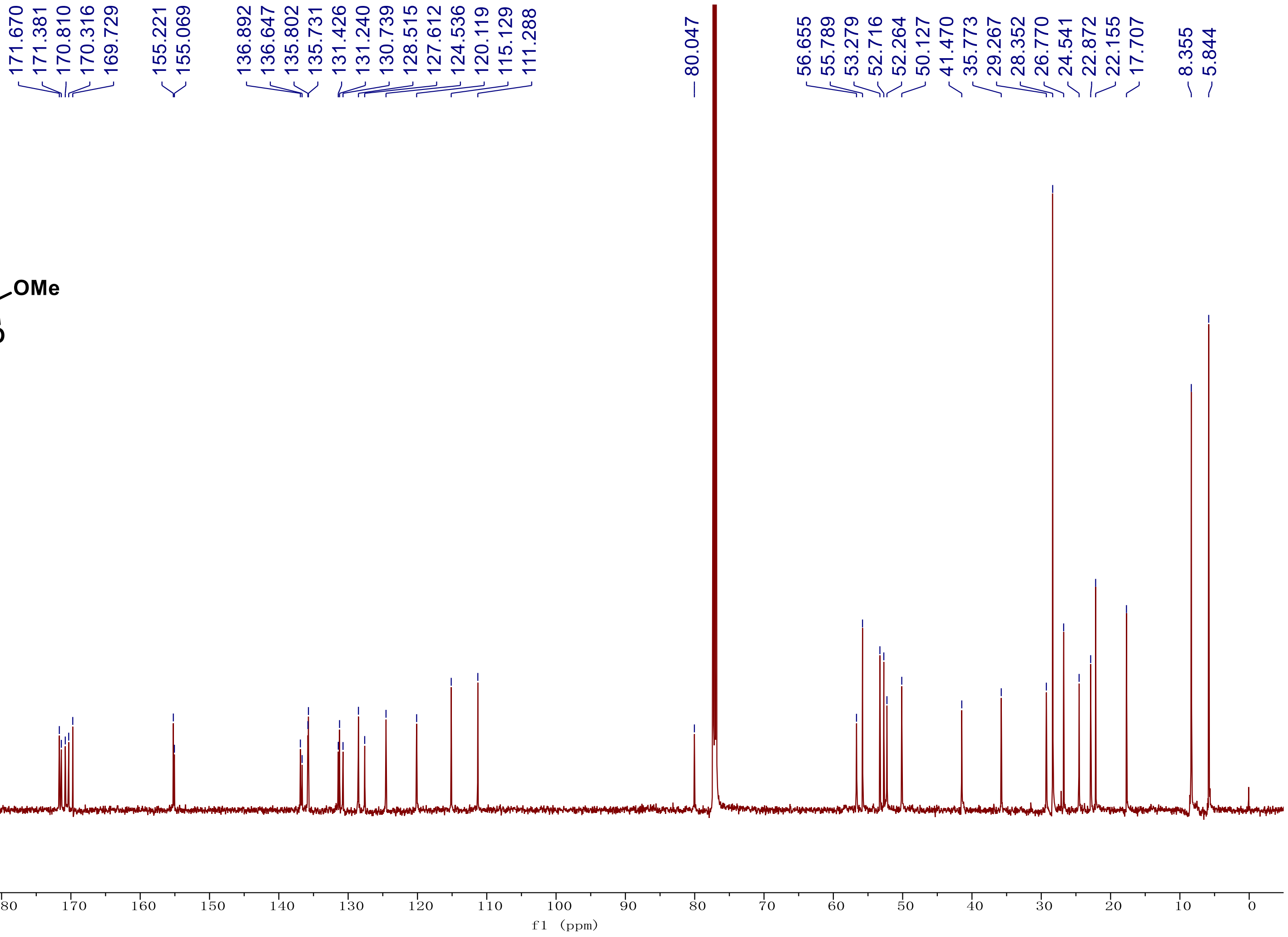

Compound 29s NOESY (400 MHz, $CDCl_3$)

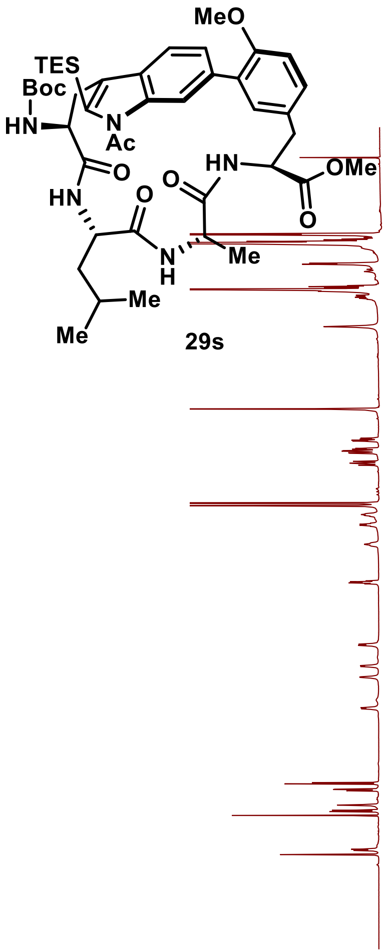

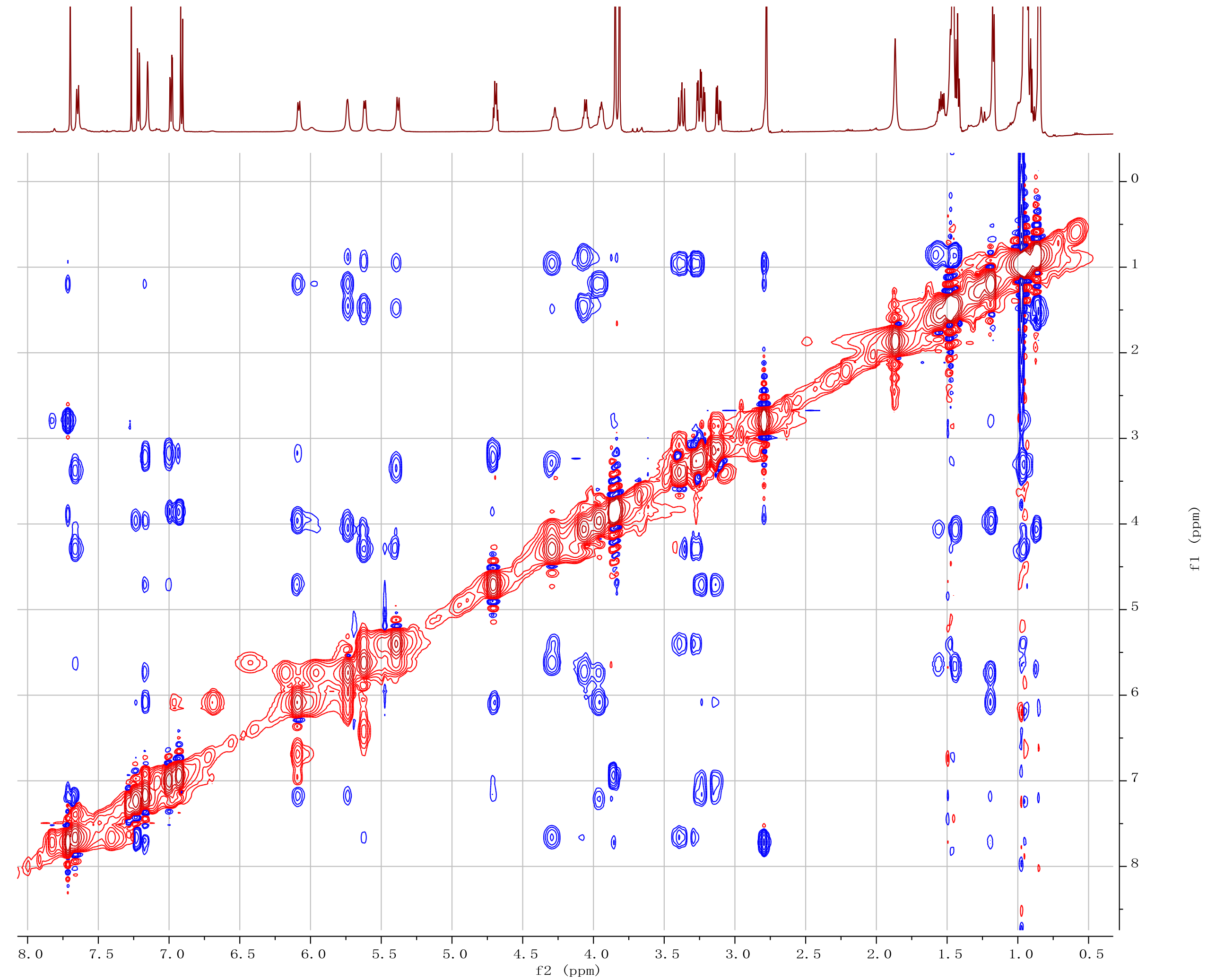

LCMS trace of compound 29s

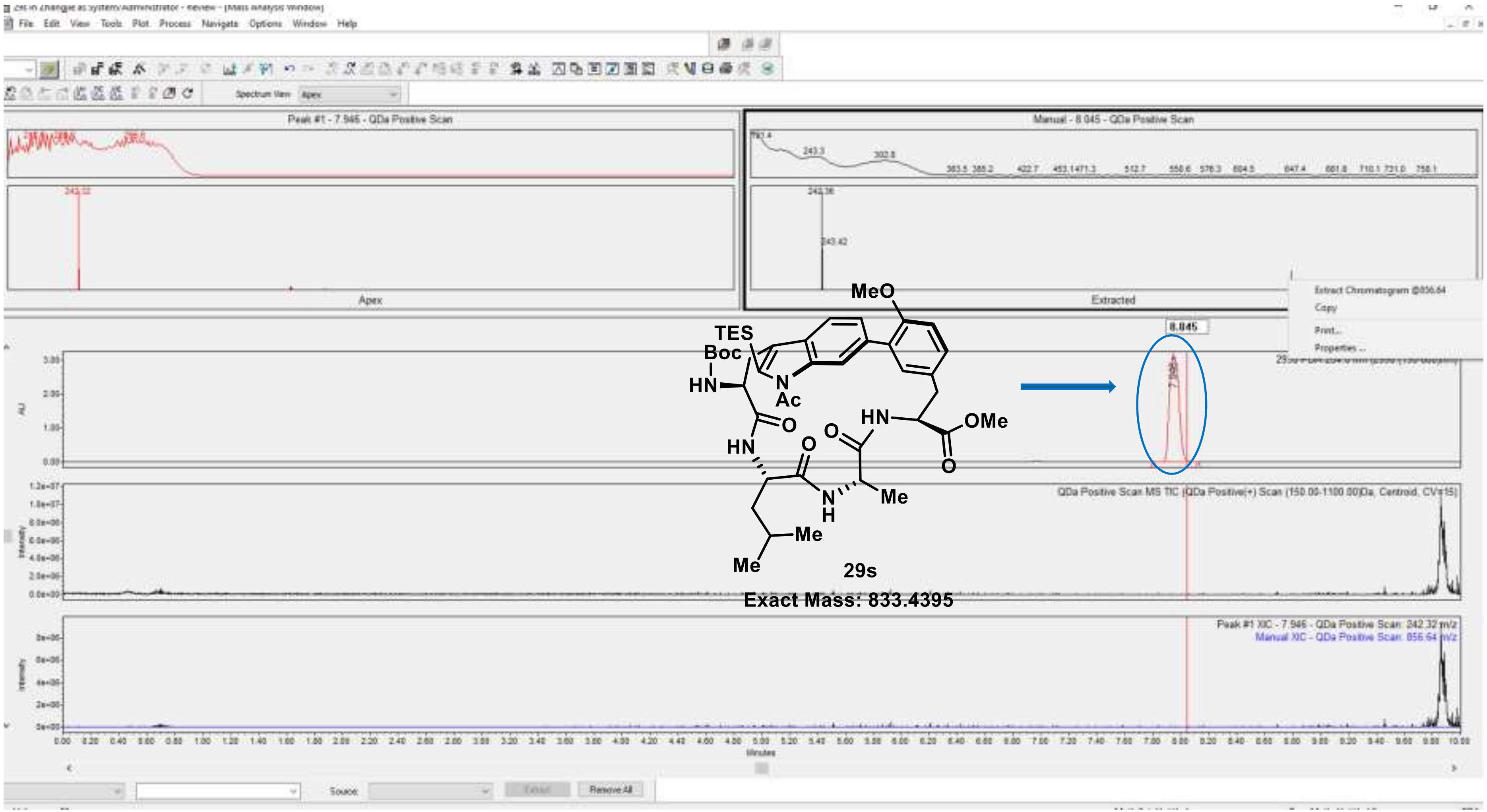

Compound 29t ^{1}H NMR (600 MHz, $CDCl_3$)

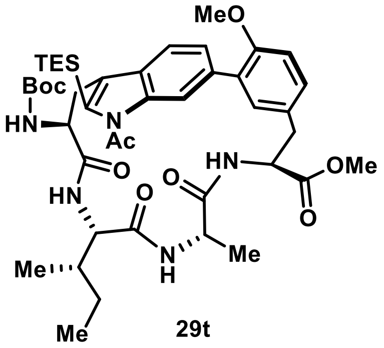

29t

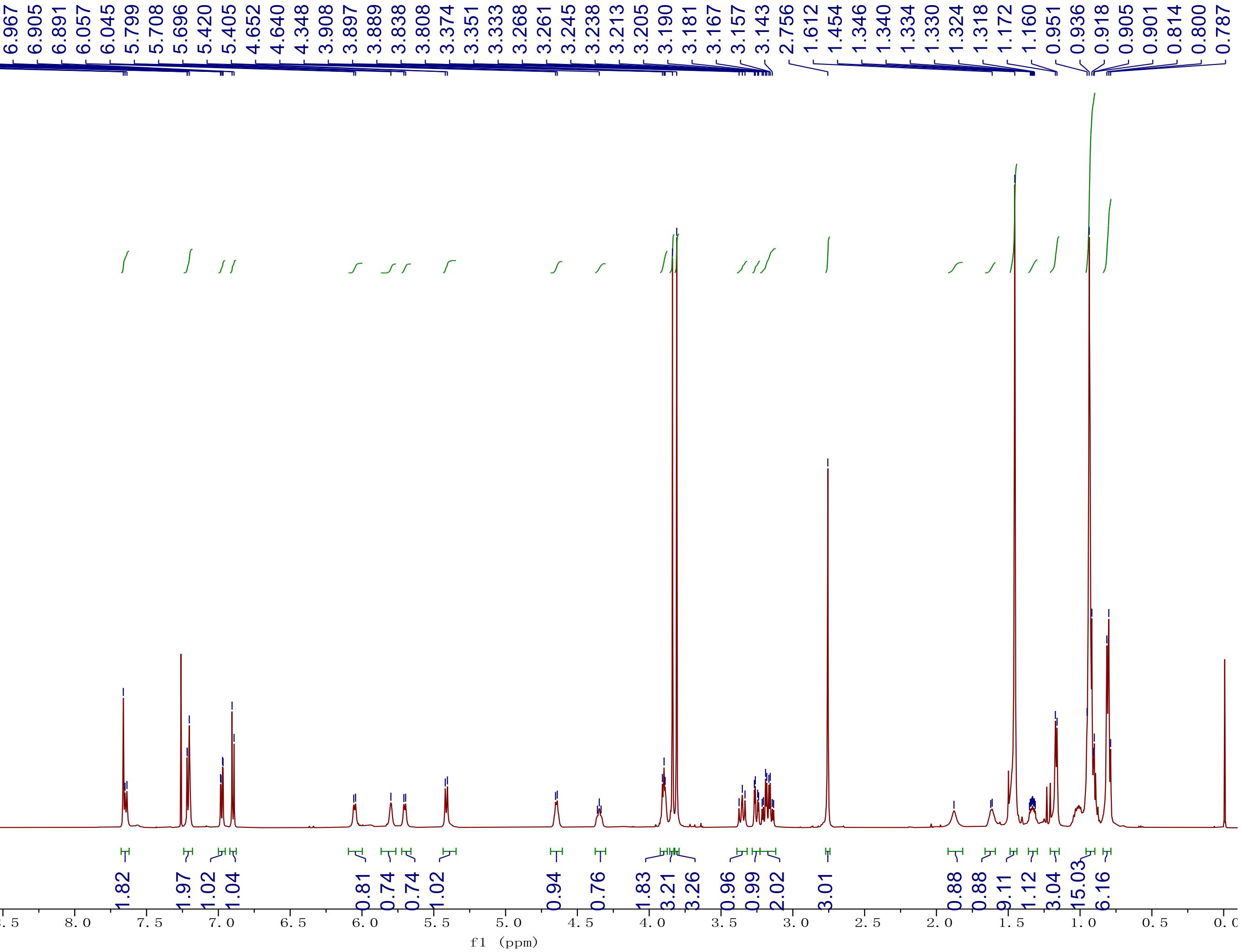

Compound 29t ^{13}C NMR (151 MHz, $CDCl_3$)

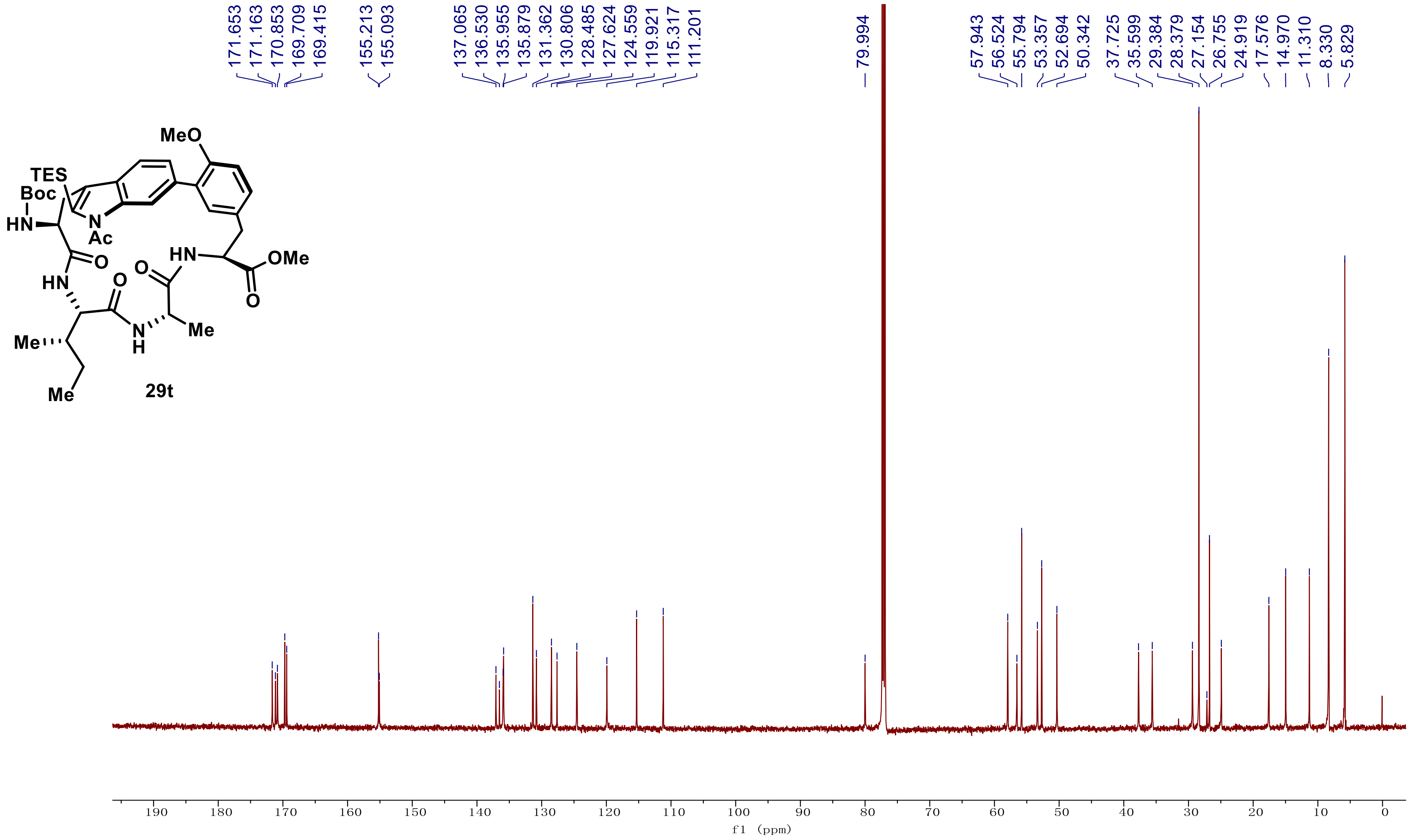

Compound 31a ¹H NMR (600 MHz, METHANOL-D_4)

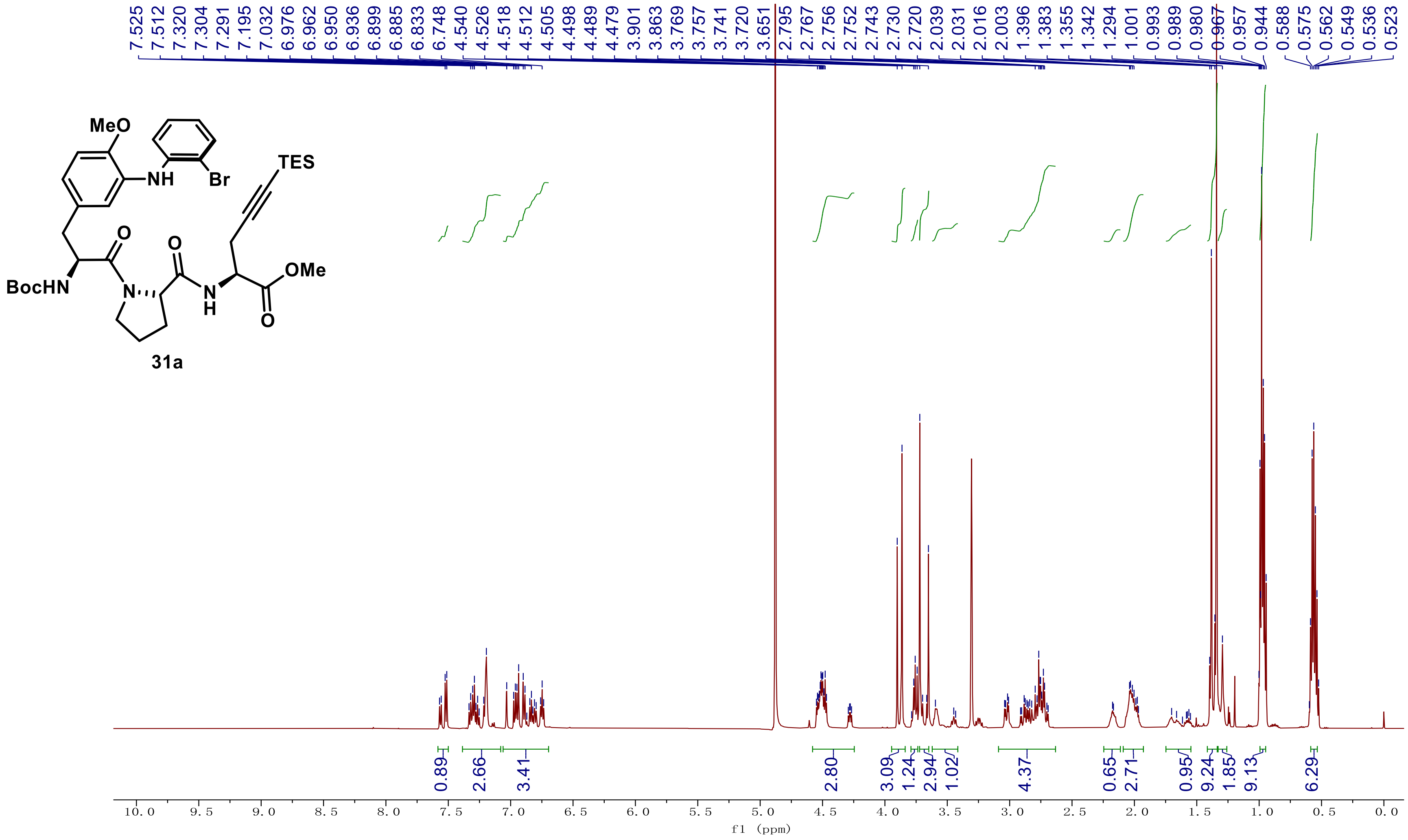

Compound 31a ^{13}C NMR (151 MHz, METHANOL-D_4)

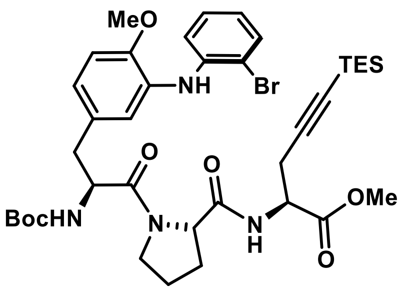

31a

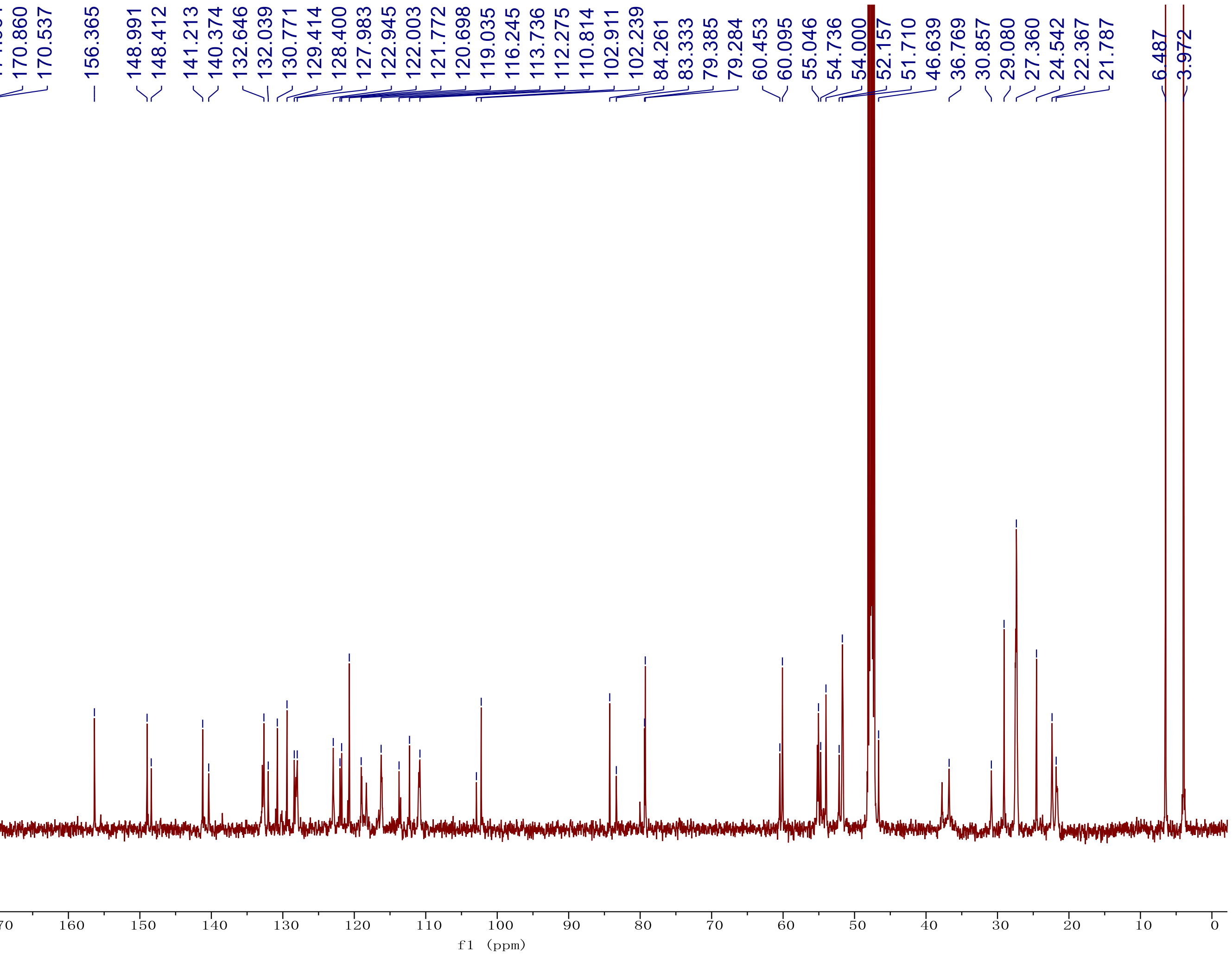

Compound 31b ^{1}H NMR (600 MHz, $CDCl_3$)

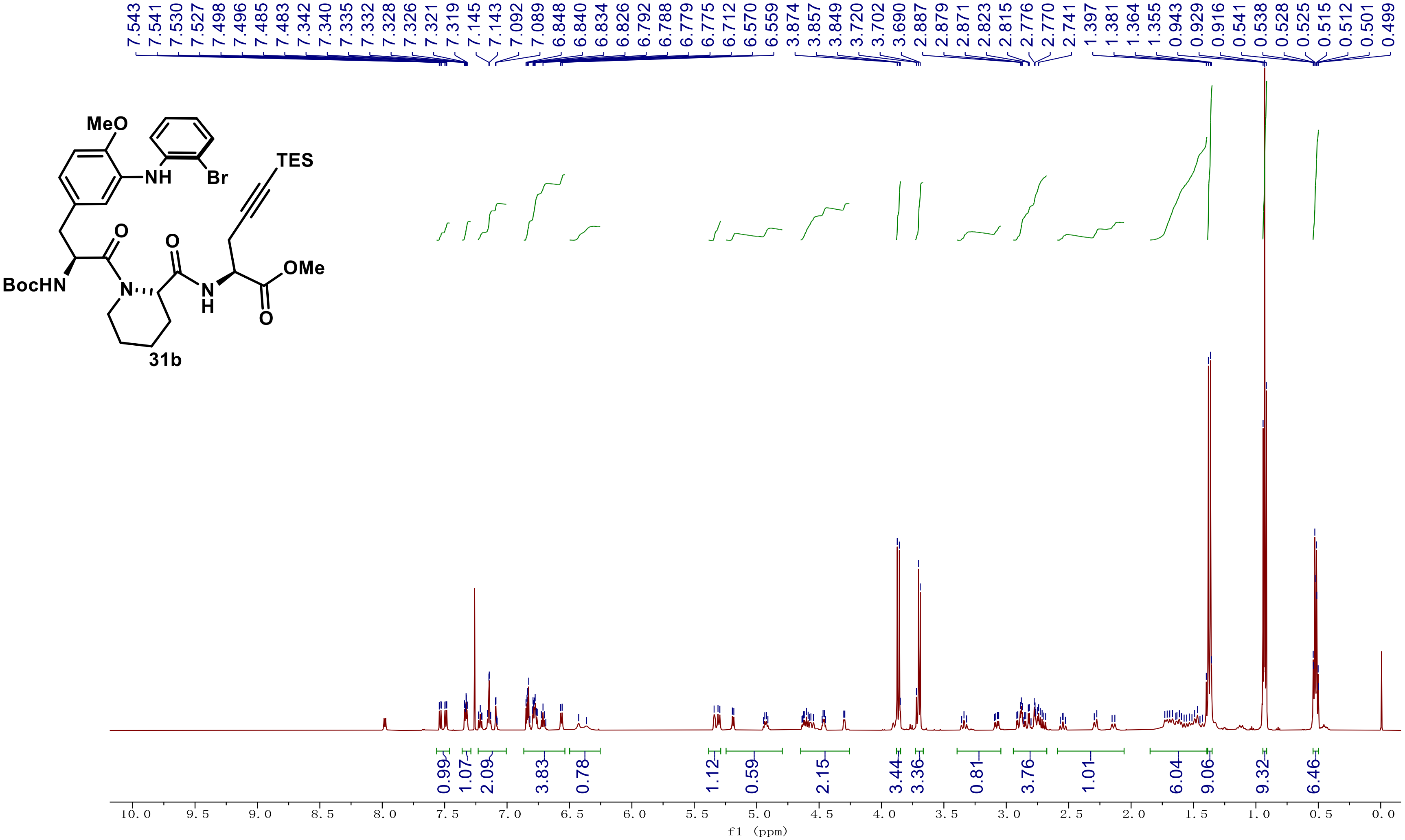

Compound 31b ^{13}C NMR (151 MHz, $CDCl_3$)

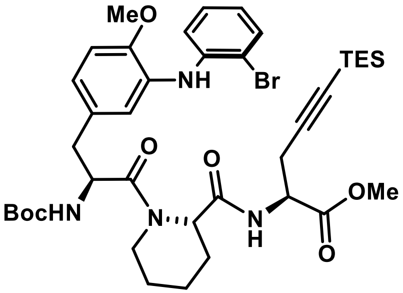

31b

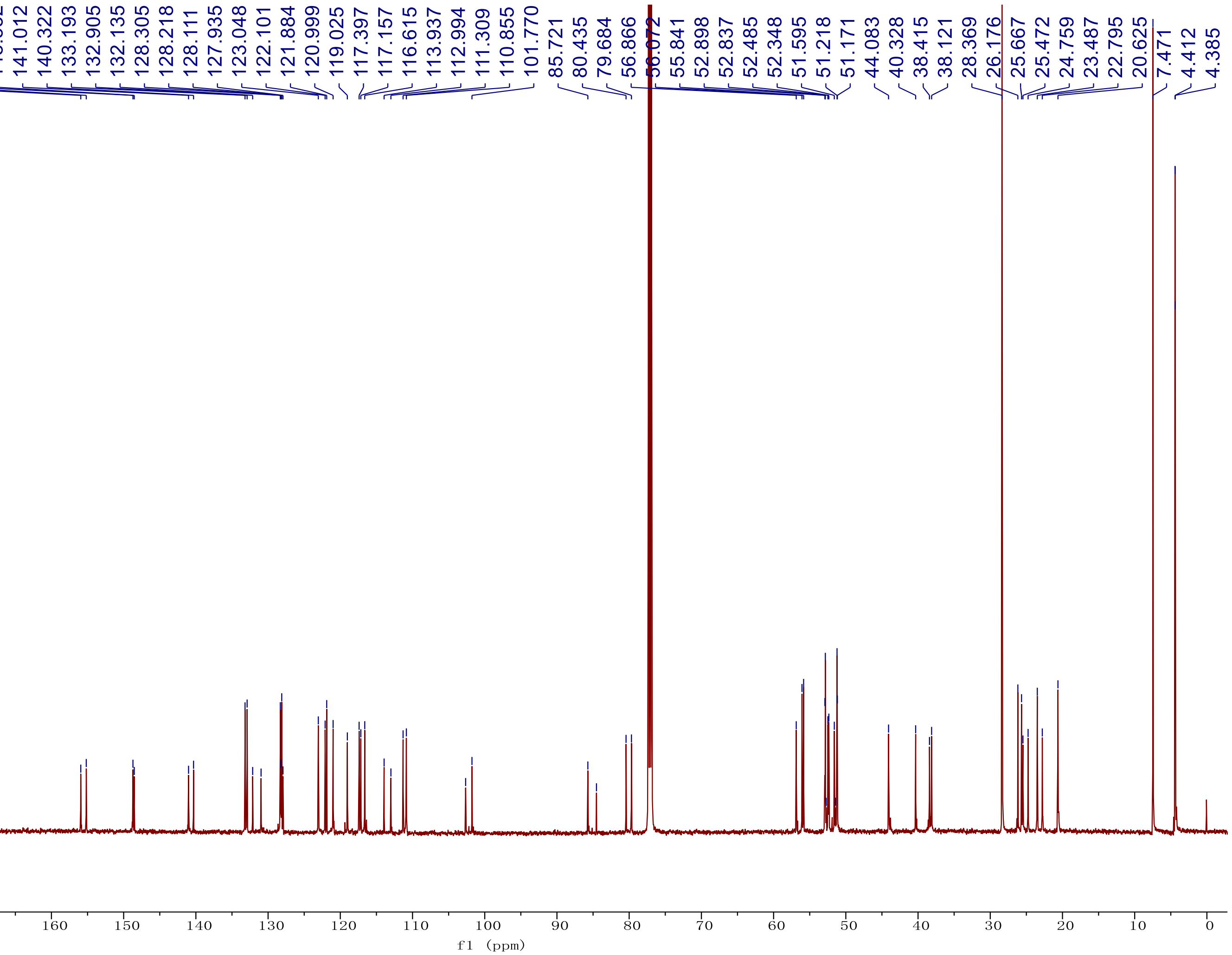

Compound 31c ^{1}H NMR (600 MHz, METHANOL-D_4)

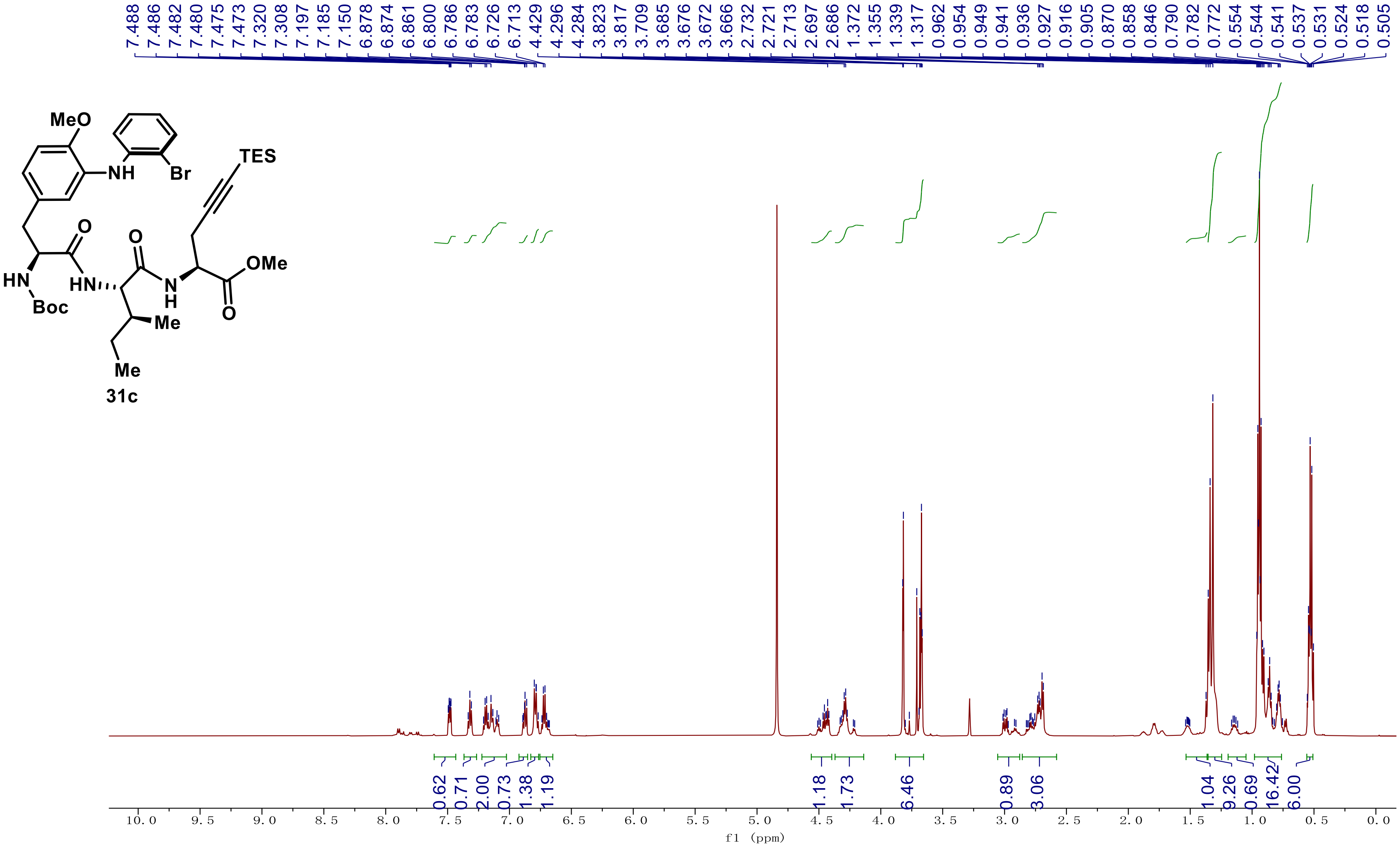

Compound 31c ^{13}C NMR (151 MHz, METHANOL-D_4)

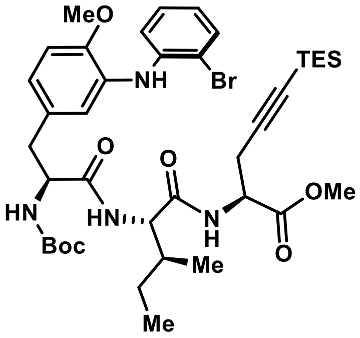

31c

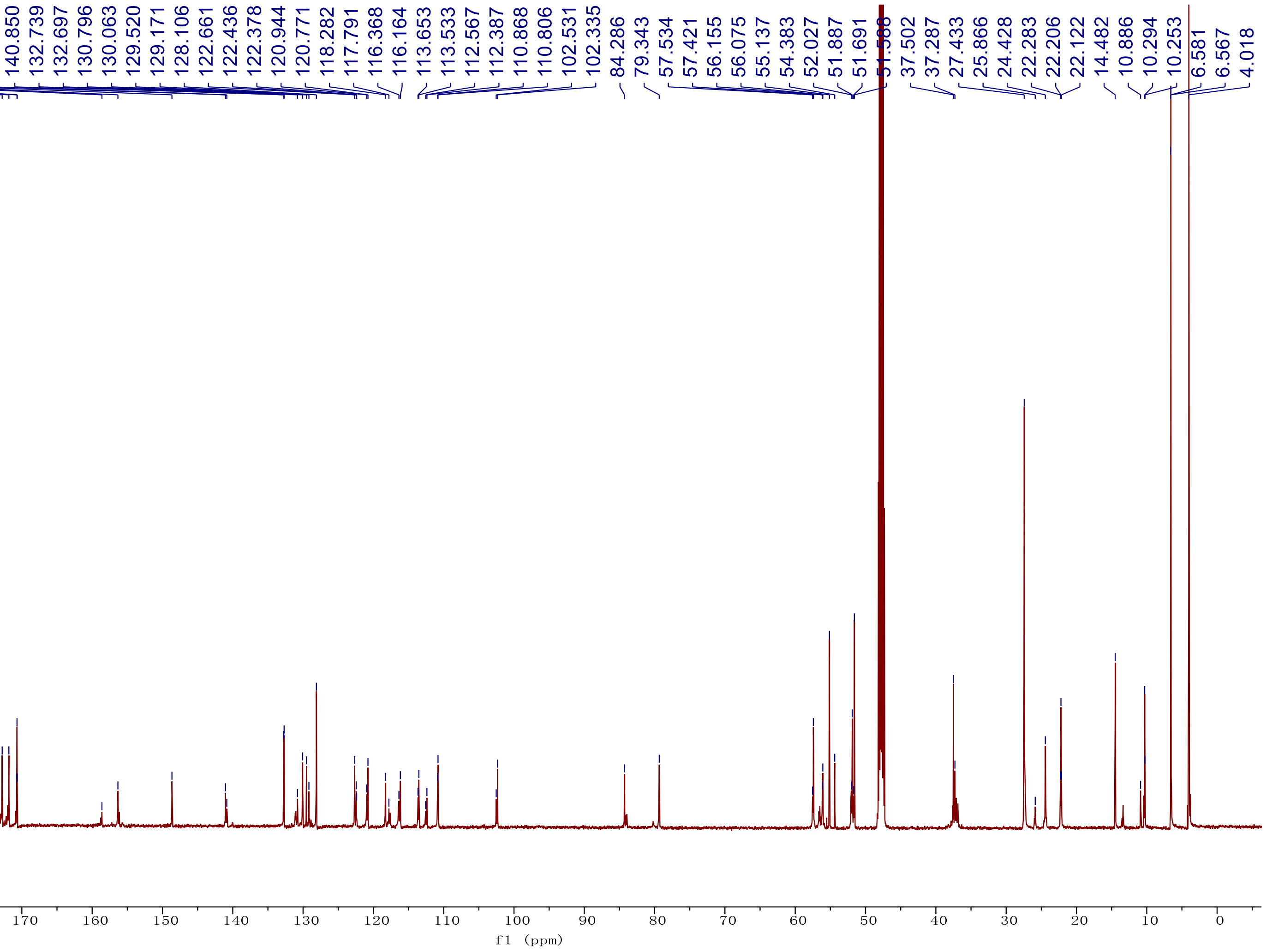

Compound 31d ^{1}H NMR (600 MHz, METHANOL-*D*$_4$)

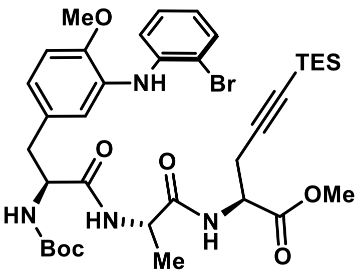

31d

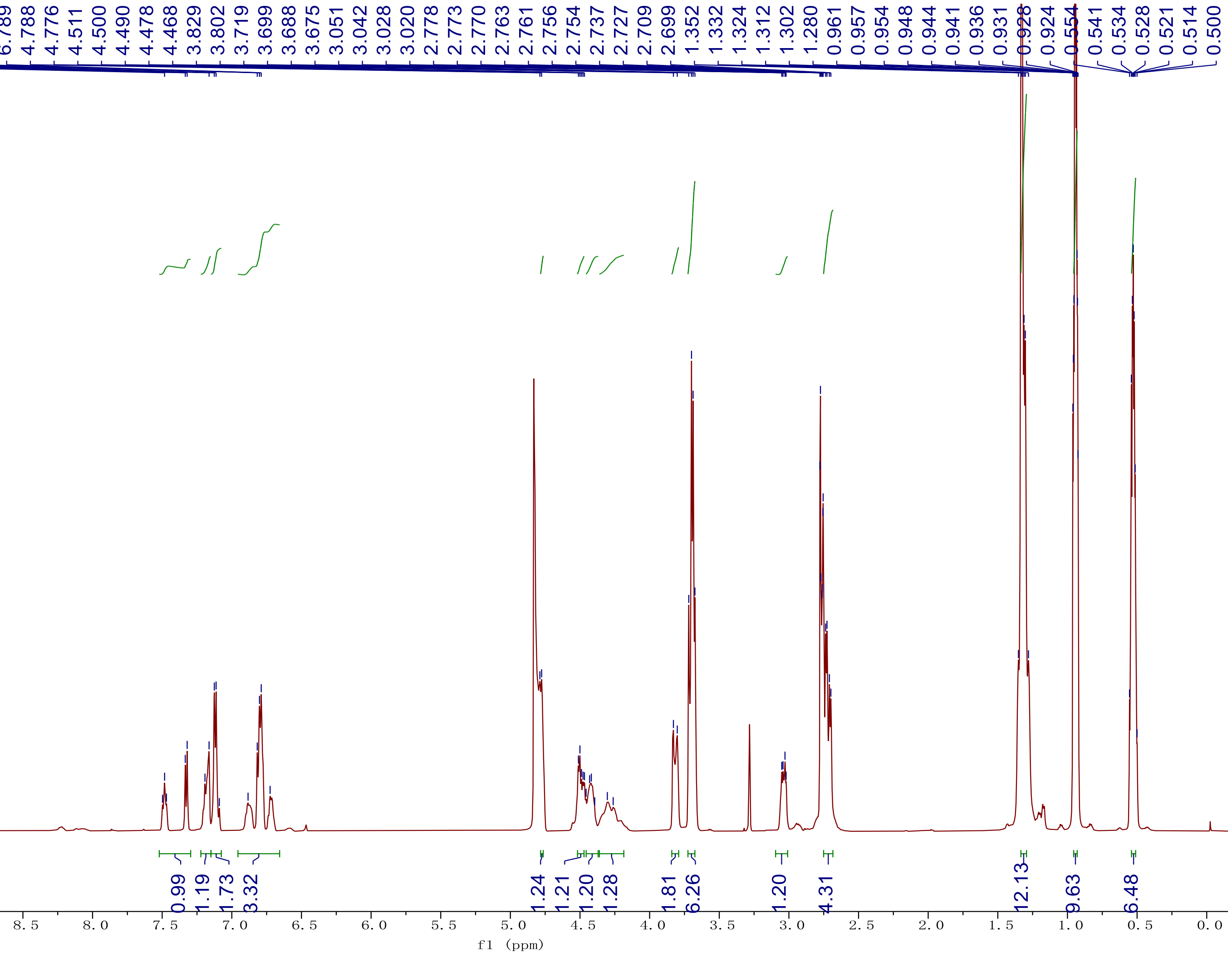

Compound 31d ^{13}C NMR (151 MHz, METHANOL-D_4)

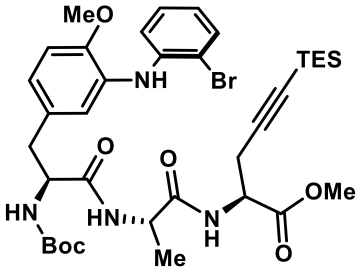

31d

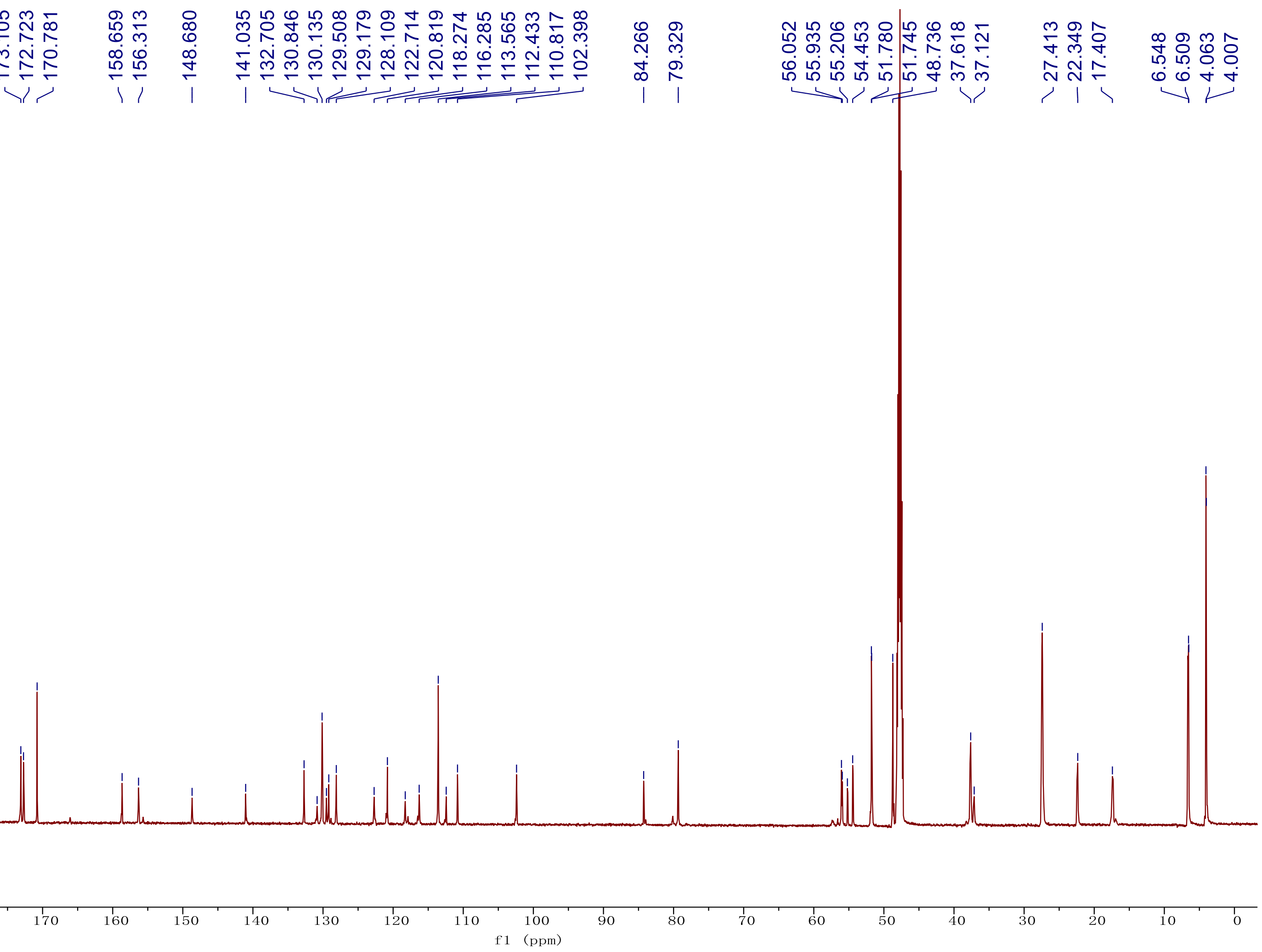

Compound 31e ^{1}H NMR (600 MHz, METHANOL-D_4)

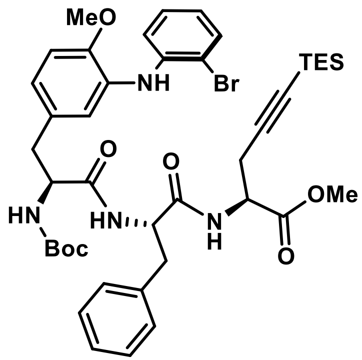

31e

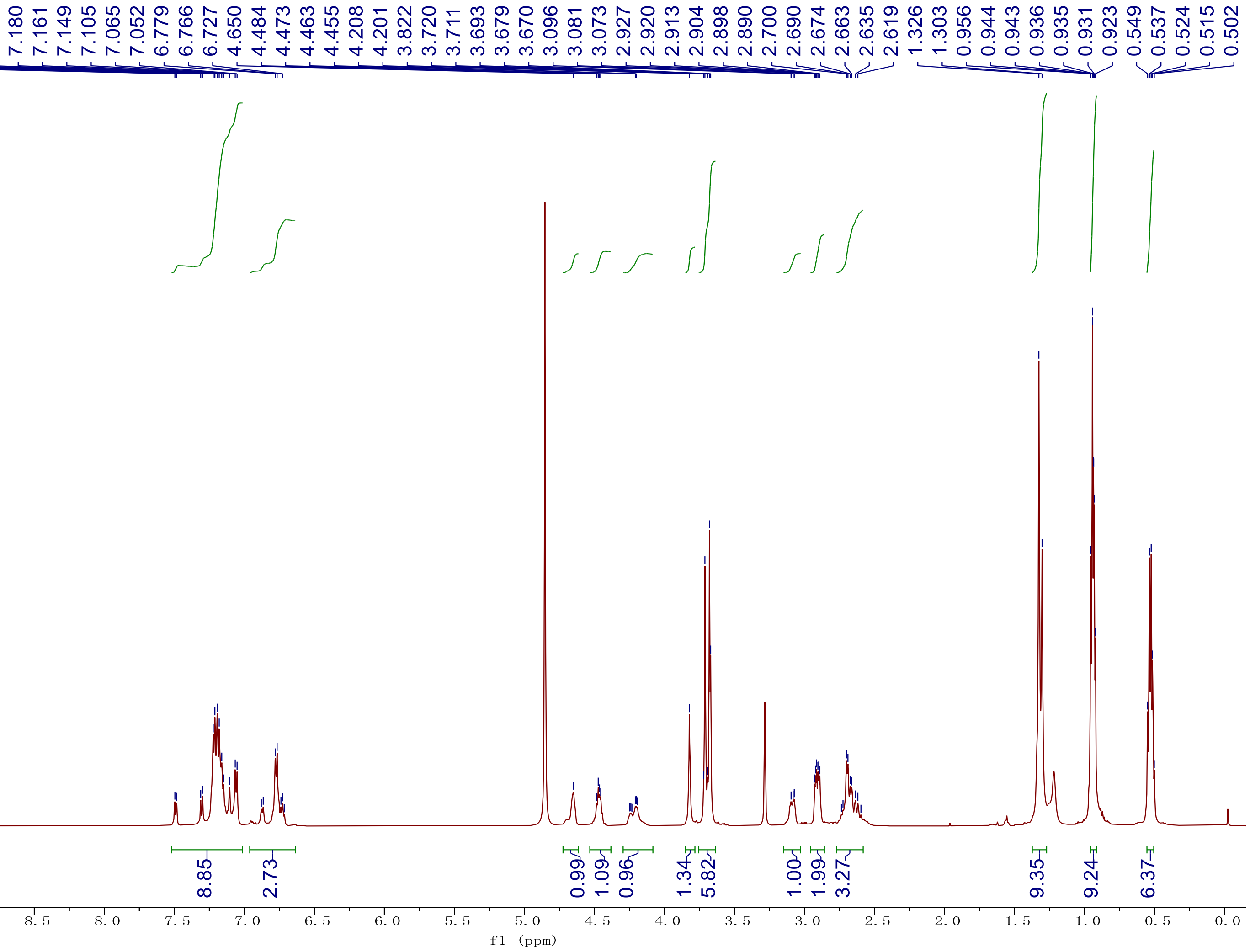

Compound 31e ^{13}C NMR (151 MHz, METHANOL-D_4)

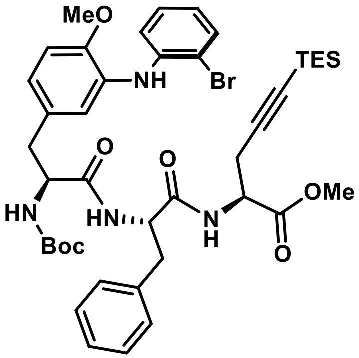

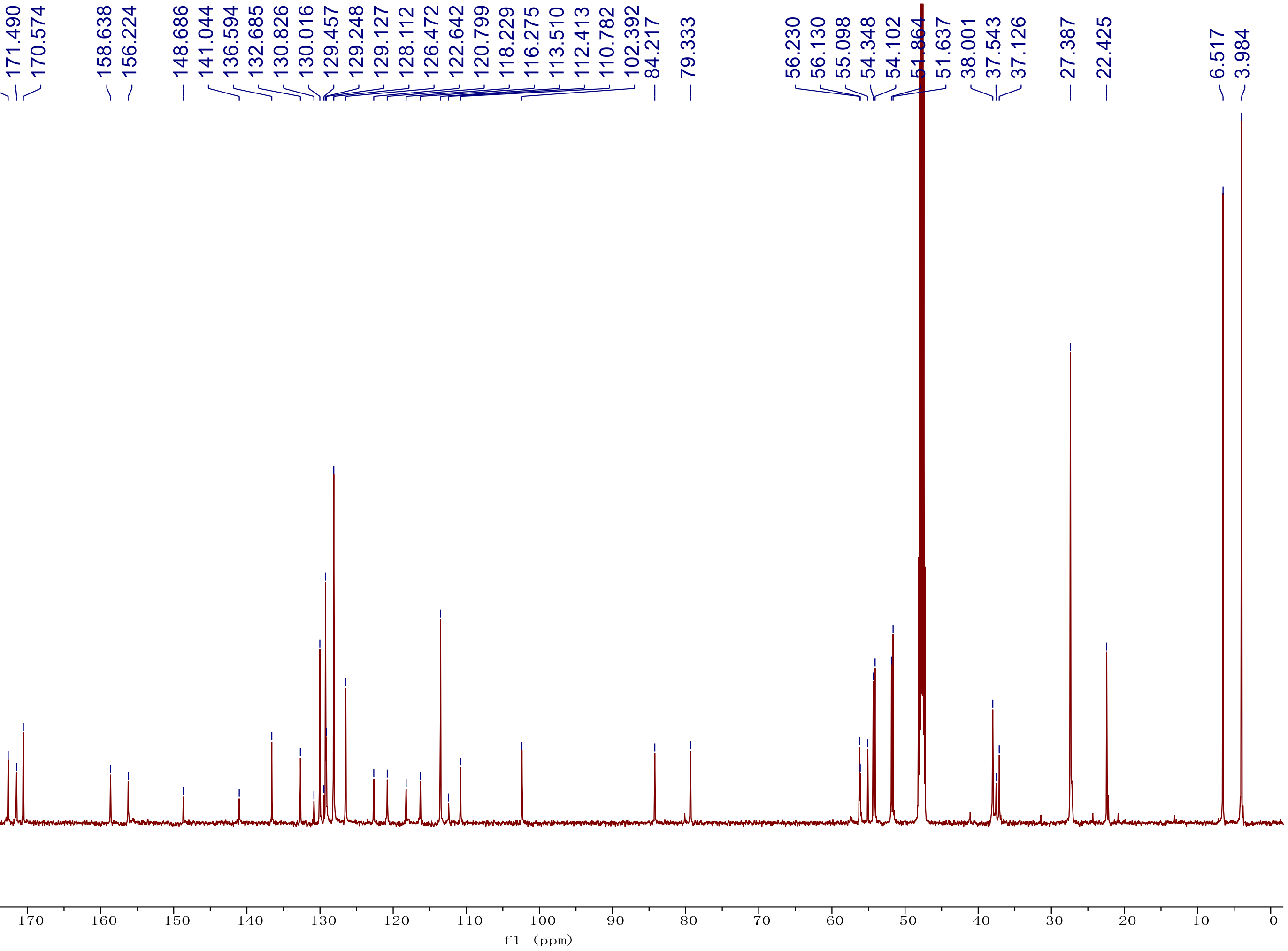

Compound 31f ^{1}H NMR (600 MHz, CDCl3)

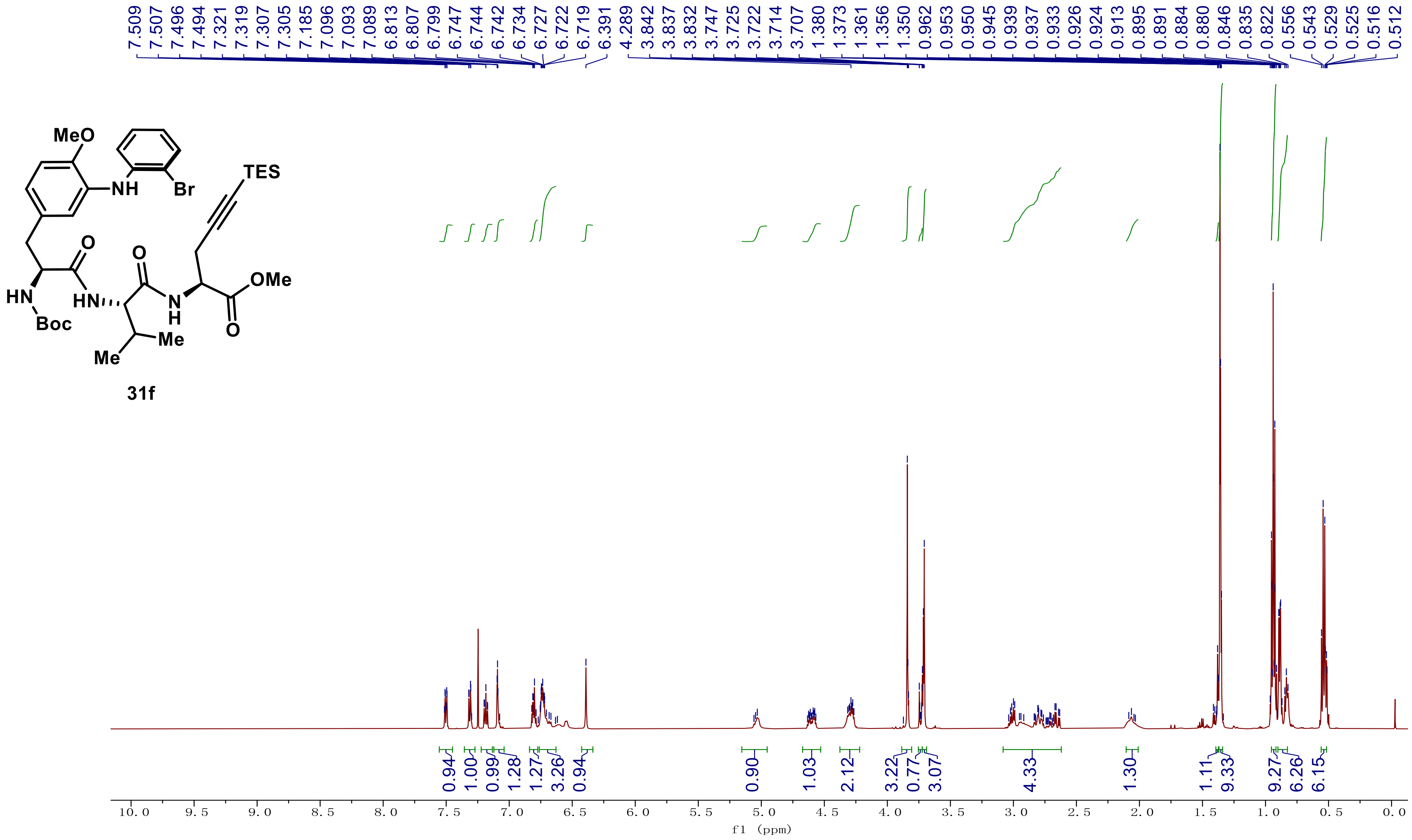

Compound 31f ^{13}C NMR (151 MHz, $CDCl_3$)

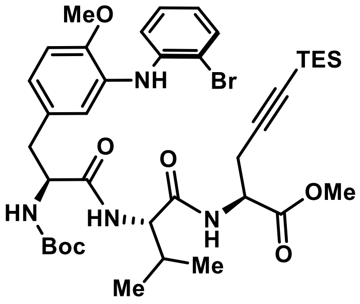

31f

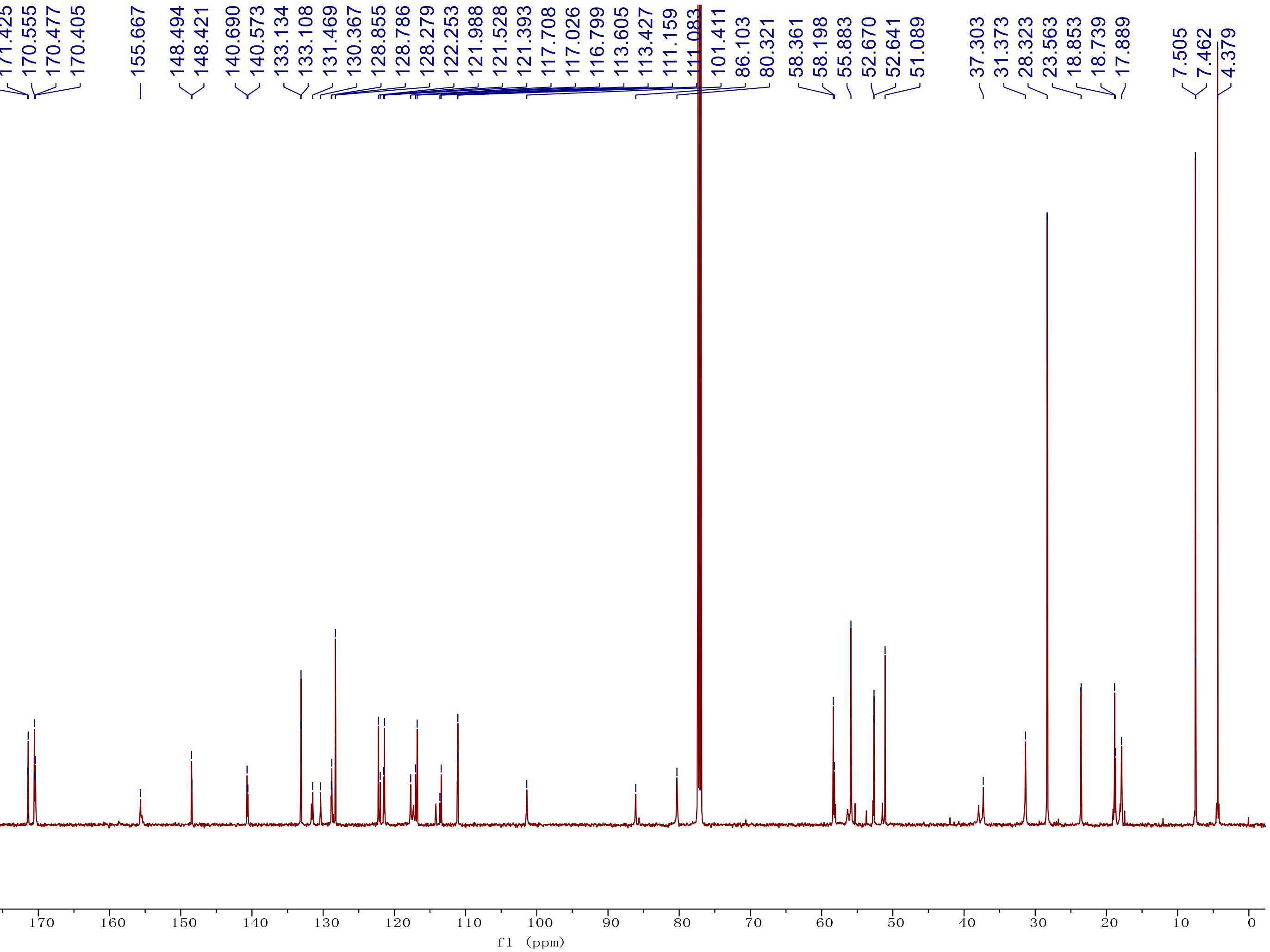

Compound 31g ¹H NMR (600 MHz, METHANOL-D_4)

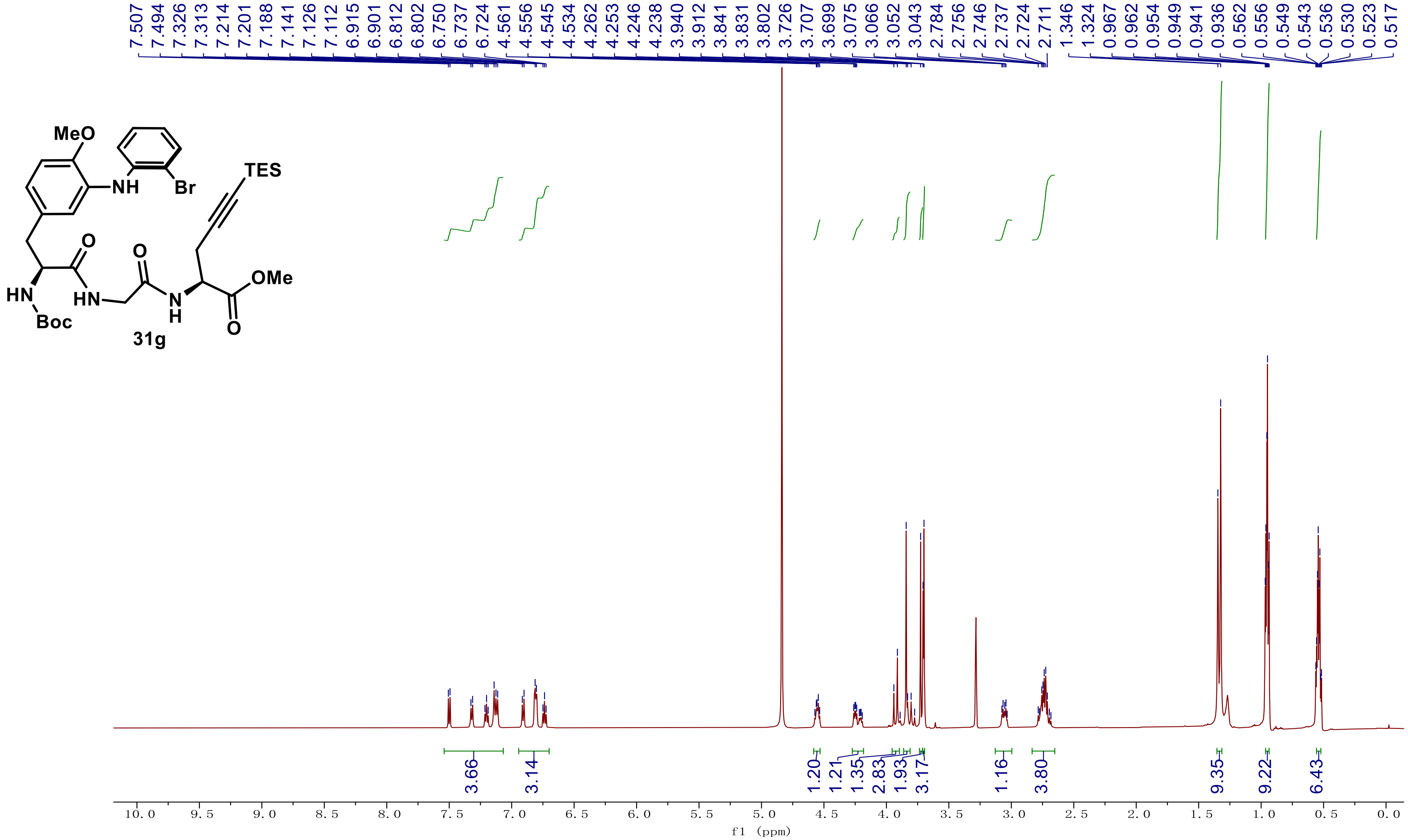

Compound 31g ^{13}C NMR (151 MHz, METHANOL-D_4)

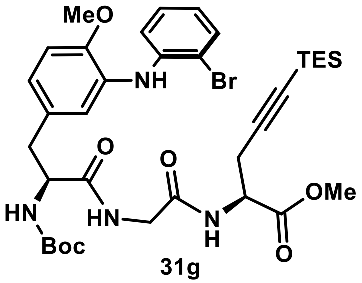

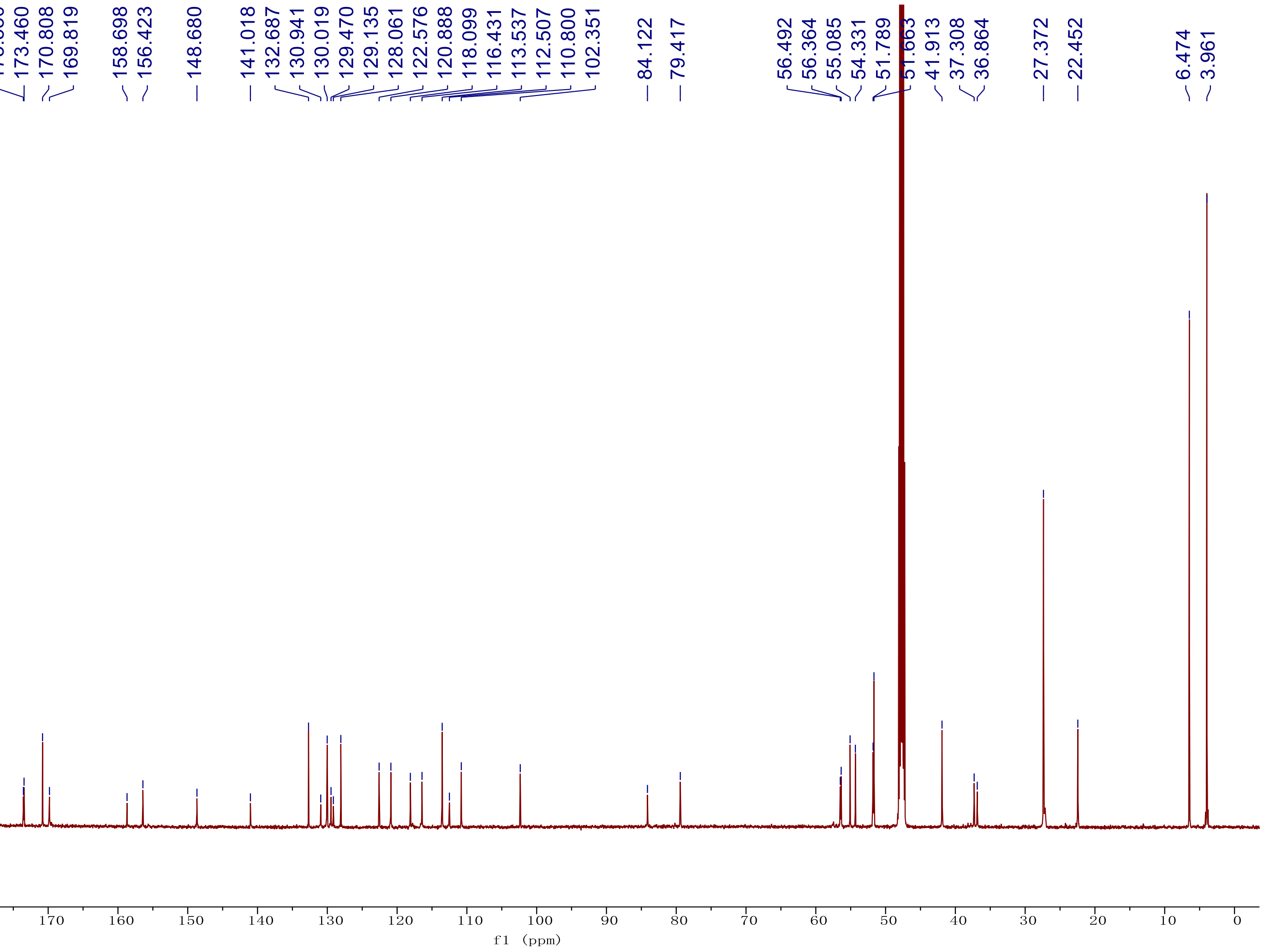

Compound 31h ^{1}H NMR (600 MHz, METHANOL-D_4)

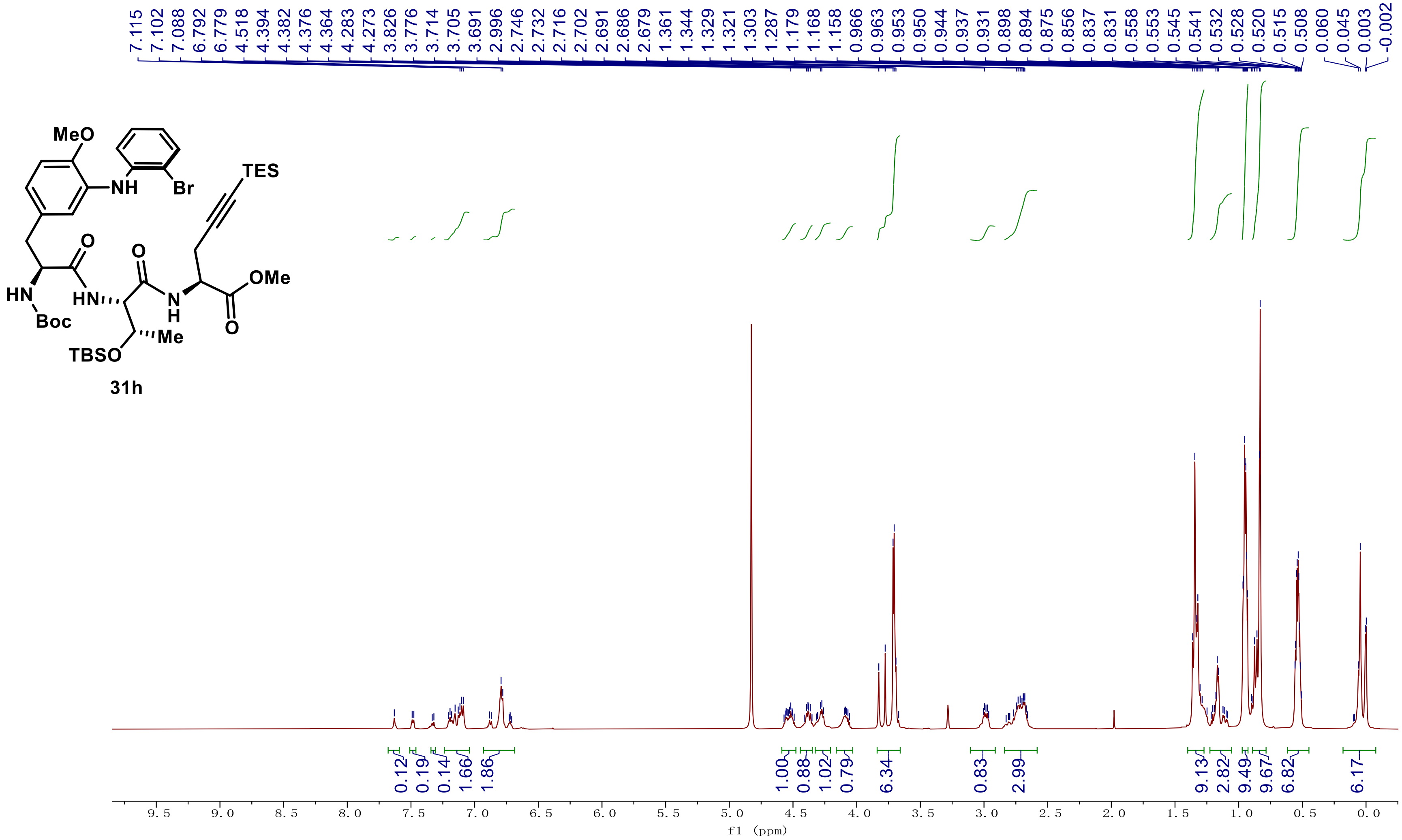

Compound 31h ^{13}C NMR (151 MHz, METHANOL-D_4)

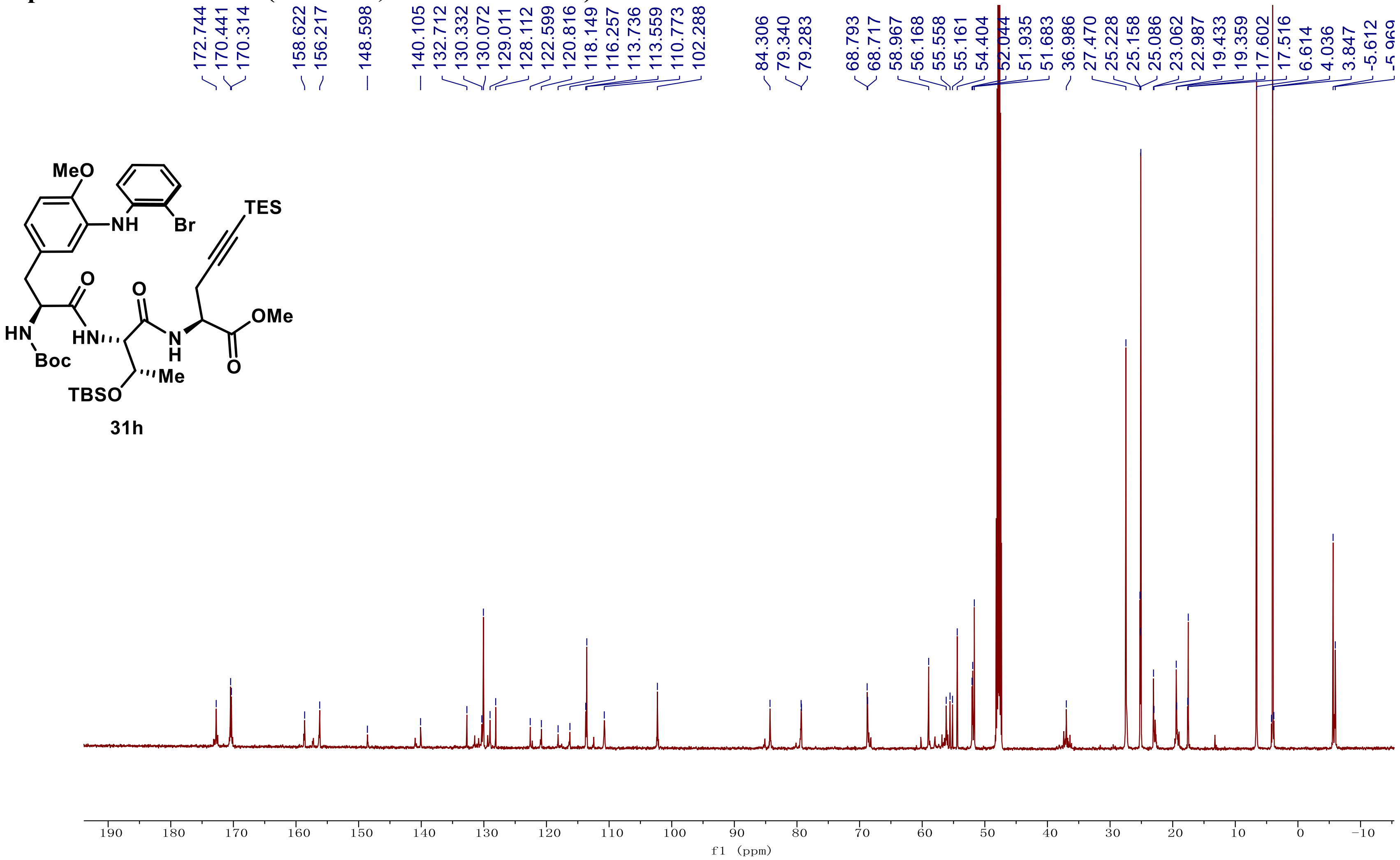

Compound 31i ¹H NMR (600 MHz, METHANOL-D_4)

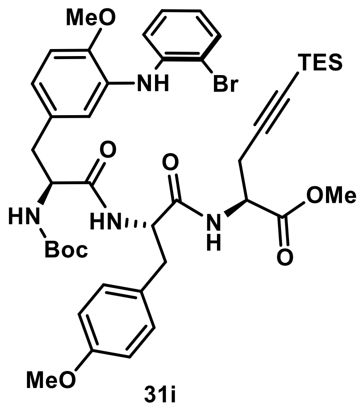

31i

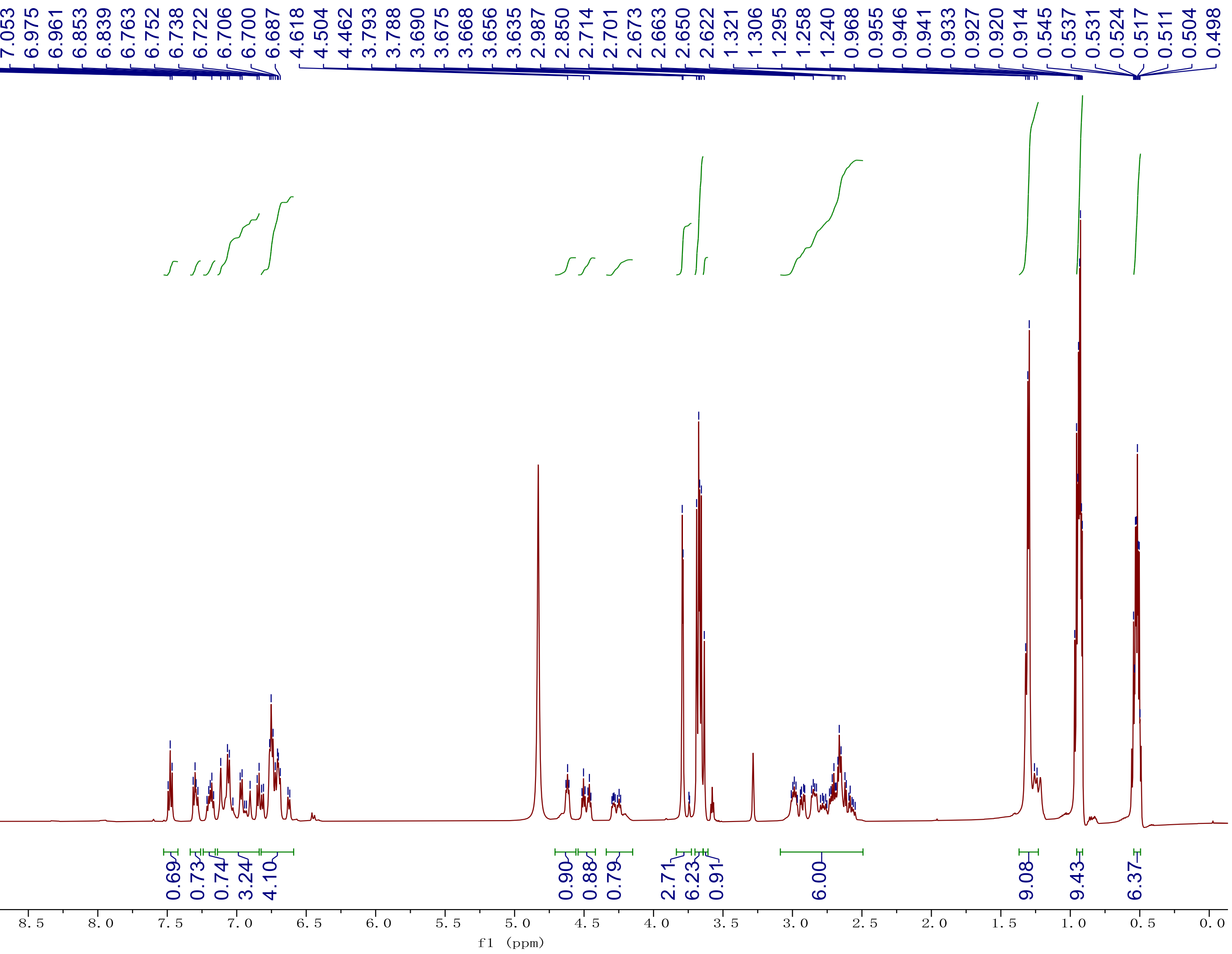

Compound 31i ^{13}C NMR (151 MHz, METHANOL-*D*$_4$)

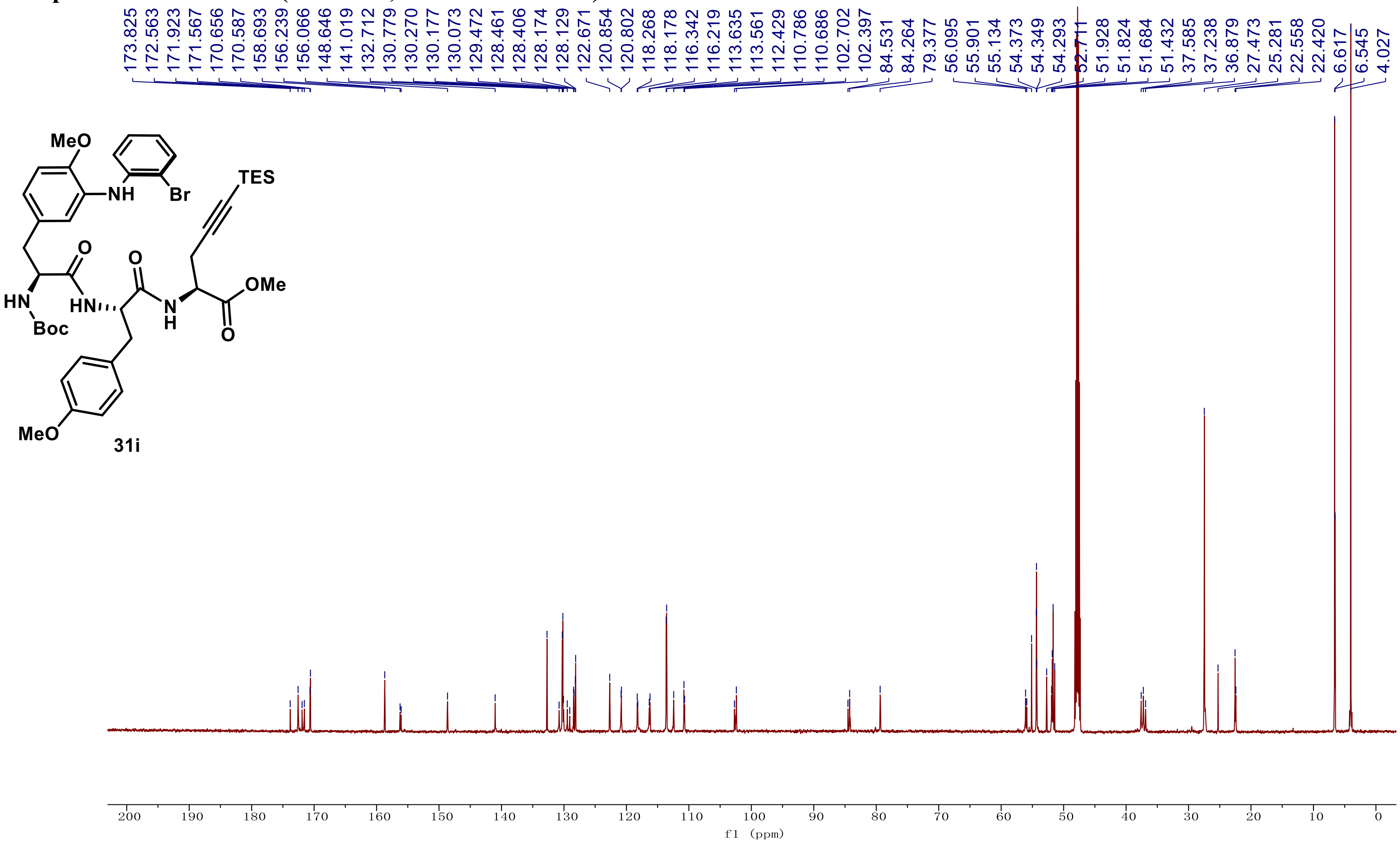

Compound 31j ^{1}H NMR (600 MHz, METHANOL-D_4)

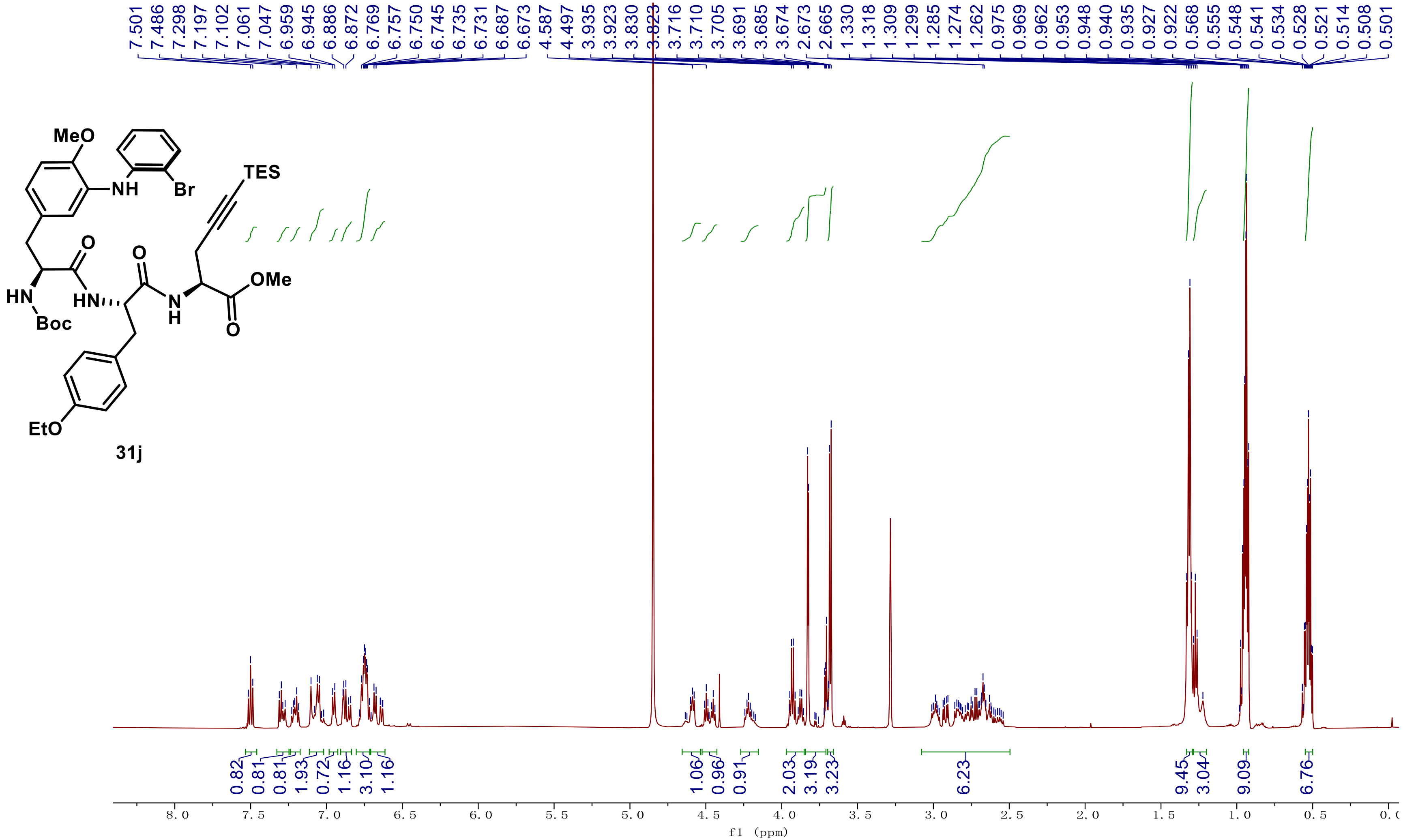

Compound 31j ^{13}C NMR (151 MHz, METHANOL-D_4)

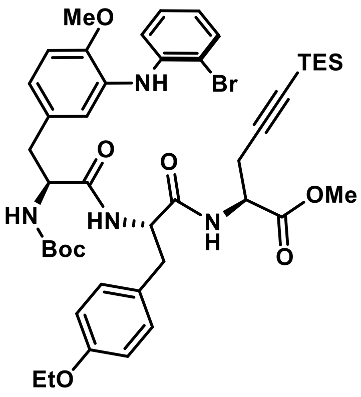

31j

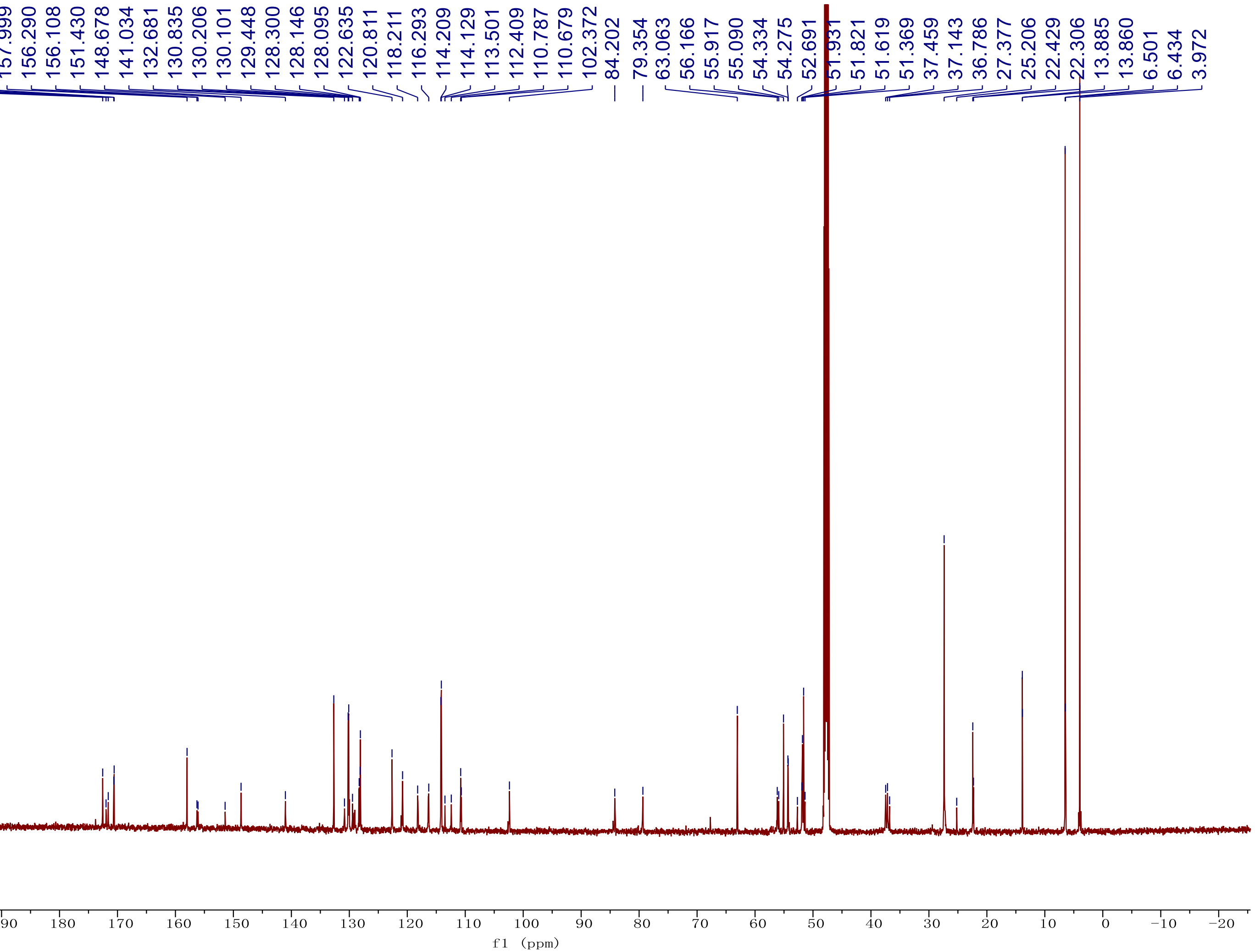

Compound 31k ^{1}H NMR (600 MHz, METHANOL-D_4)

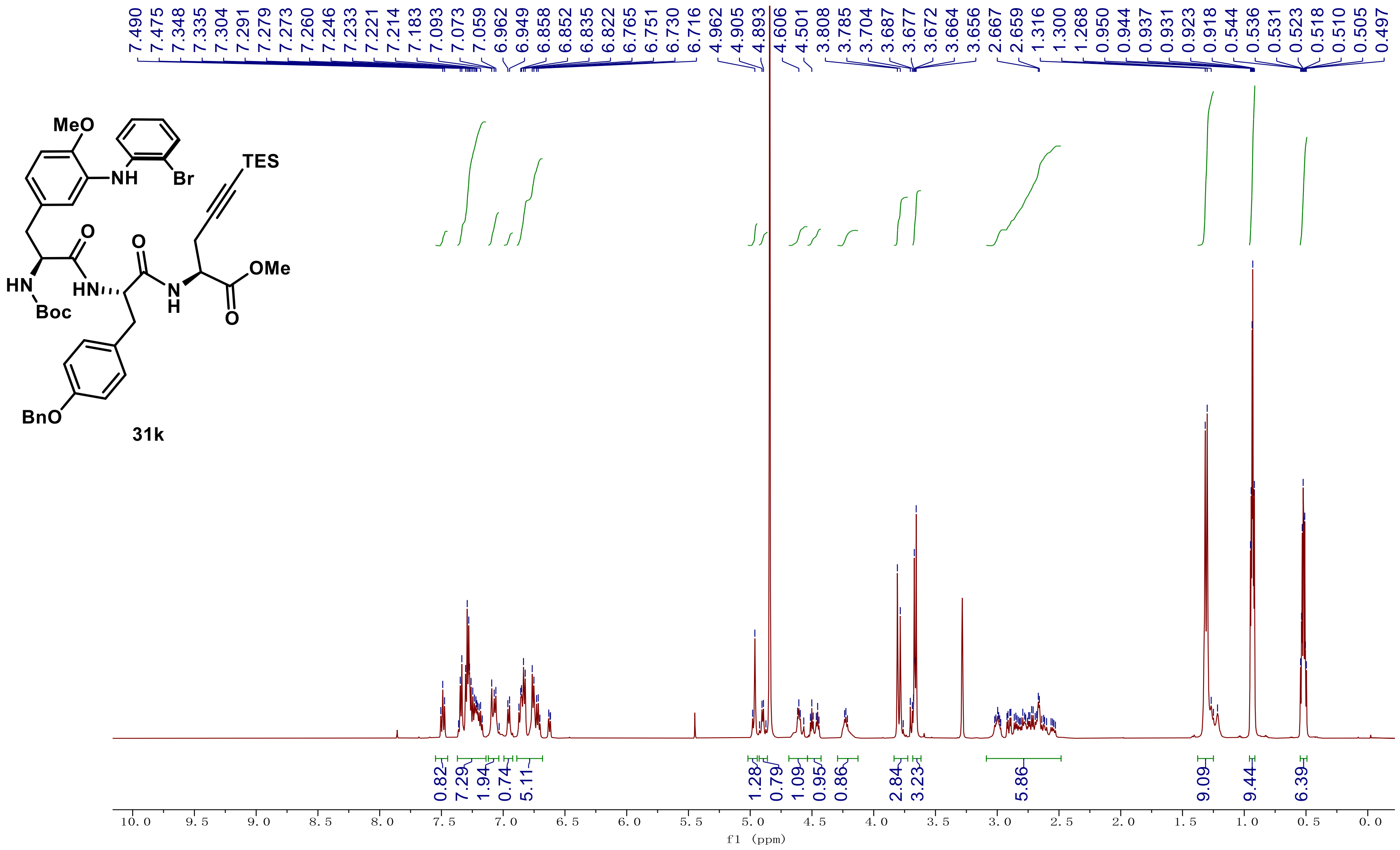

Compound 31k ^{13}C NMR (151 MHz, METHANOL-D_4)

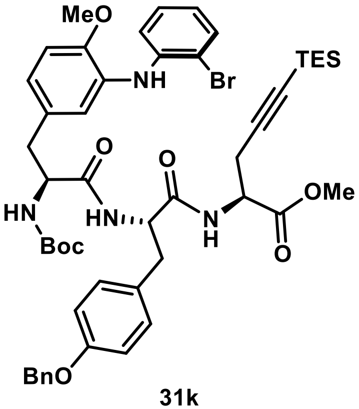

31k

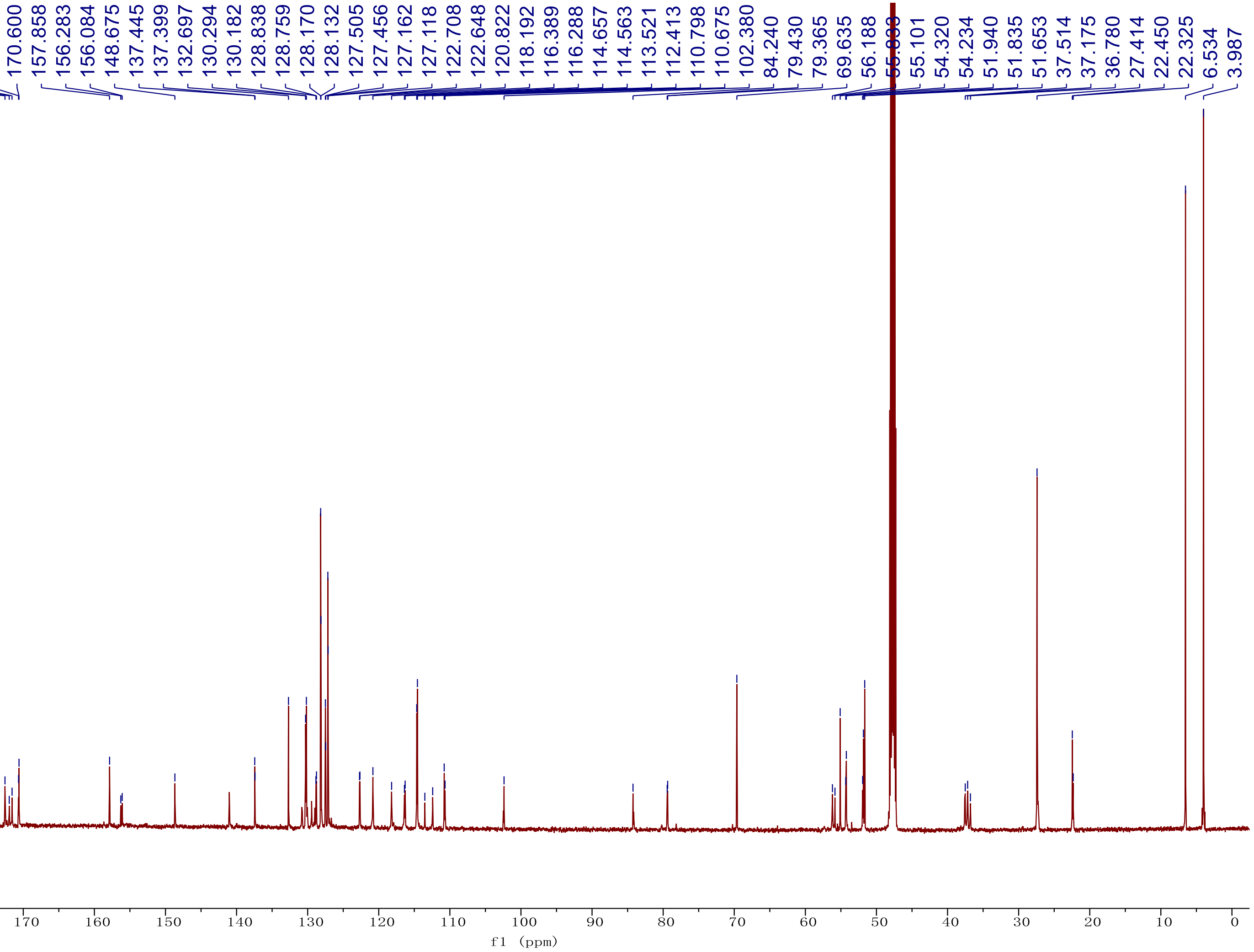

Compound 31l ^{1}H NMR (600 MHz, $CDCl_3$)

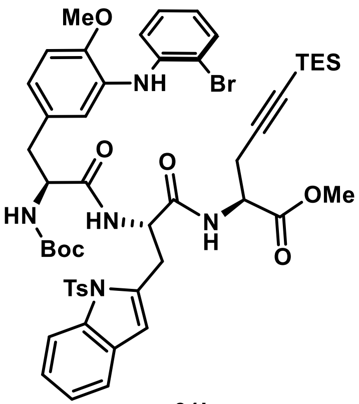

31l

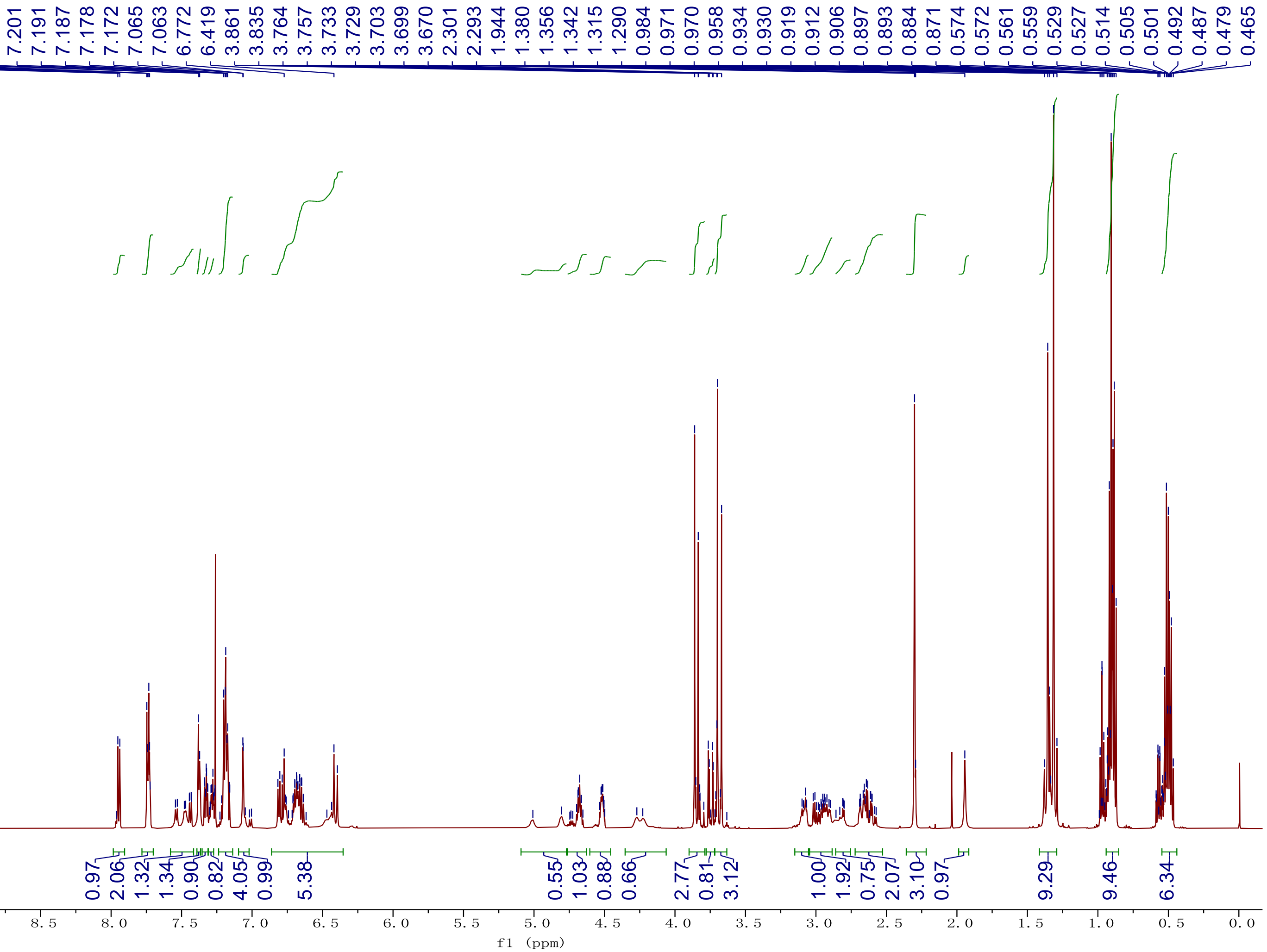

Compound 31l ^{13}C NMR (151 MHz, CDCl$_3$)

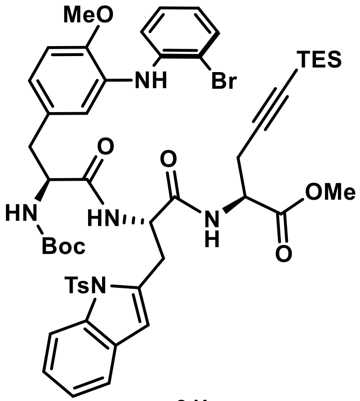

31l

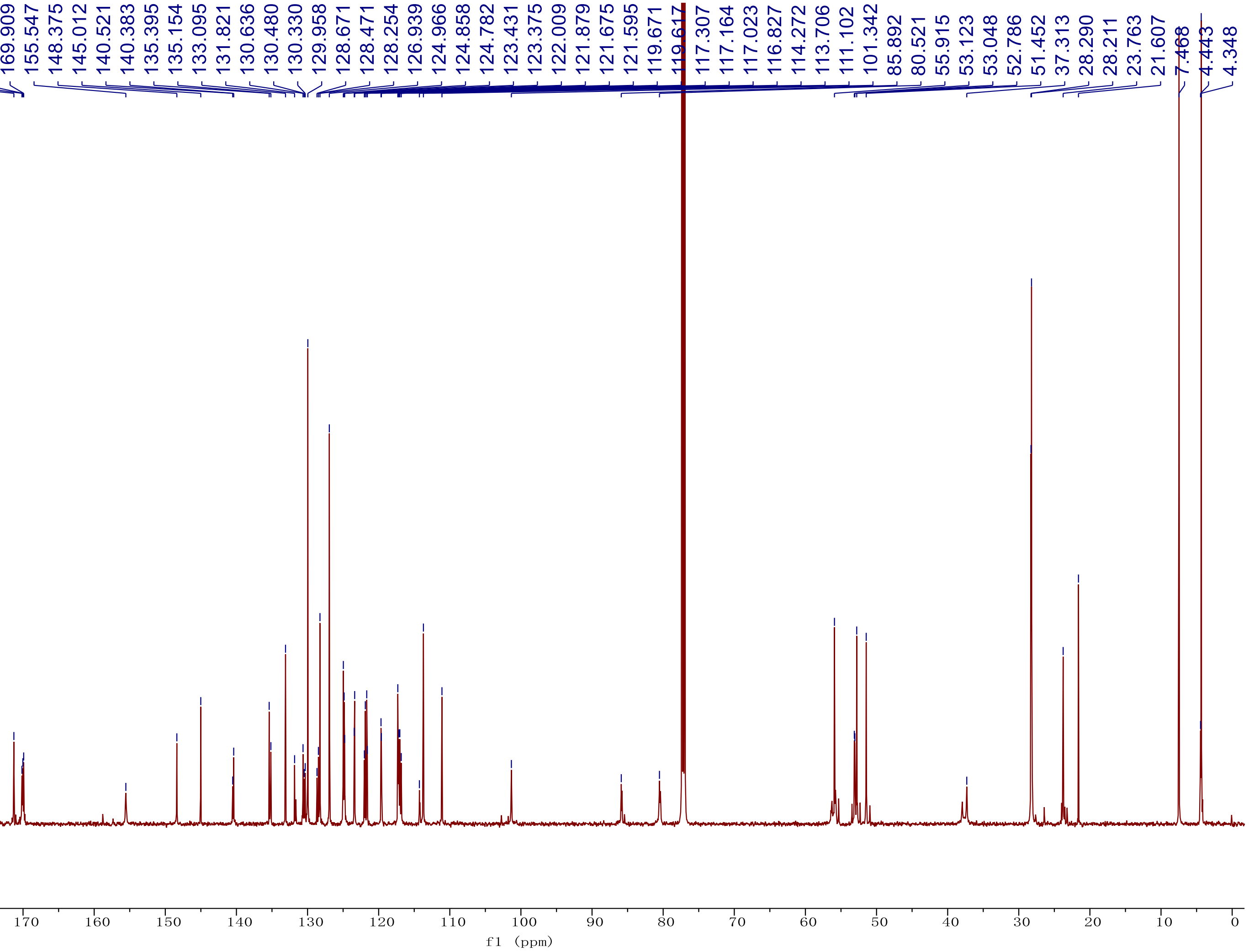

Compound 32a ^{1}H NMR (600 MHz, CDCl₃)

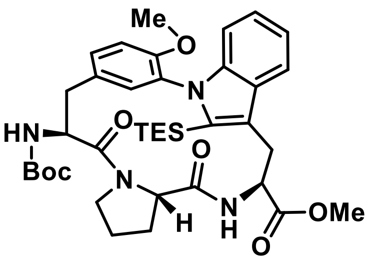

32a

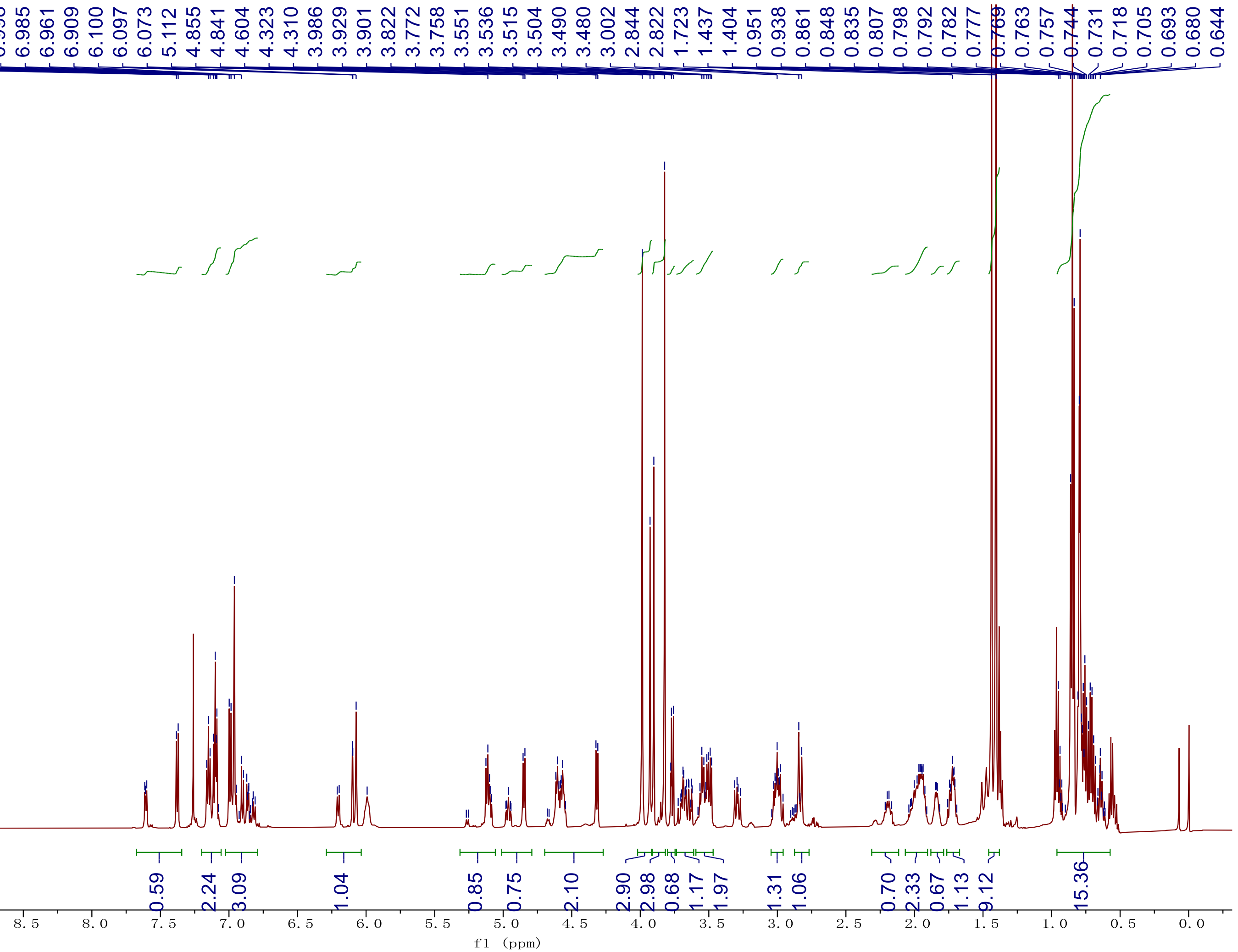

Compound 32a ^{13}C NMR (151 MHz, $CDCl_3$)

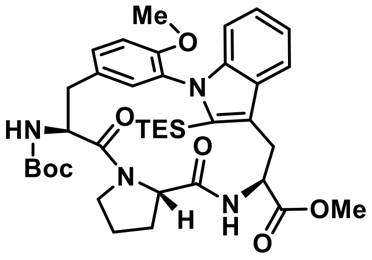

32a

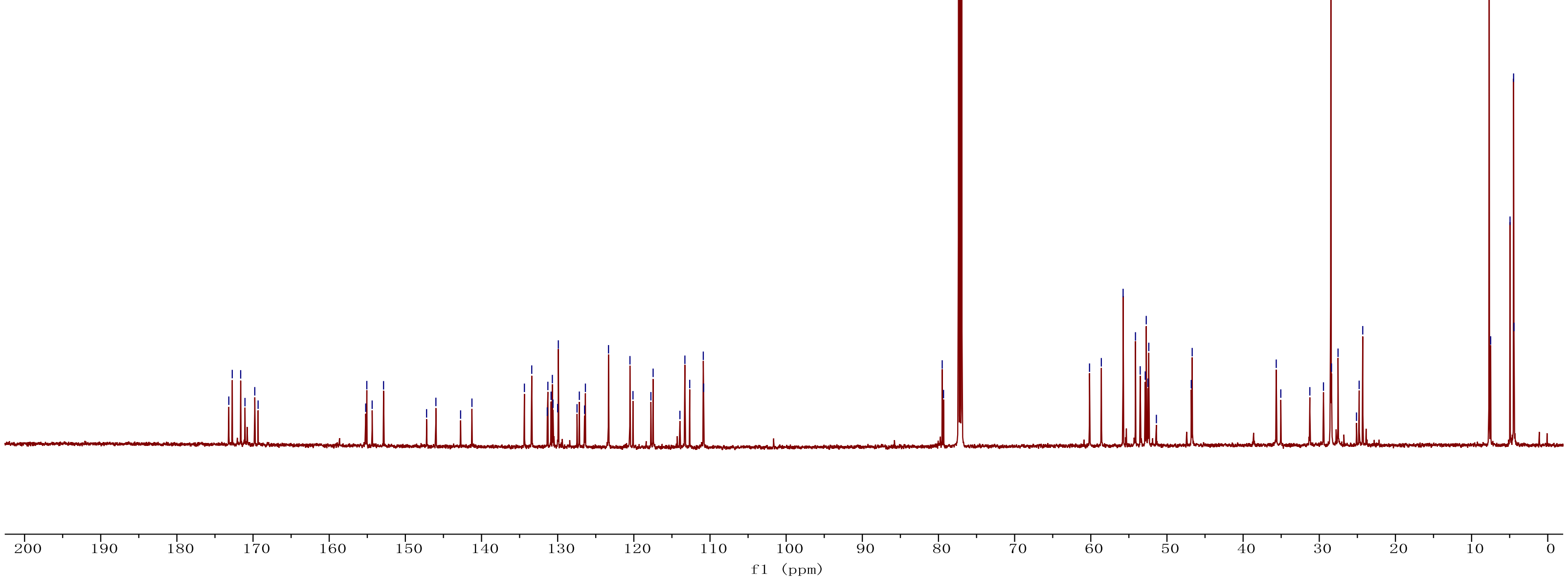

Compound 32b ¹H NMR (600 MHz, CDCl₃)

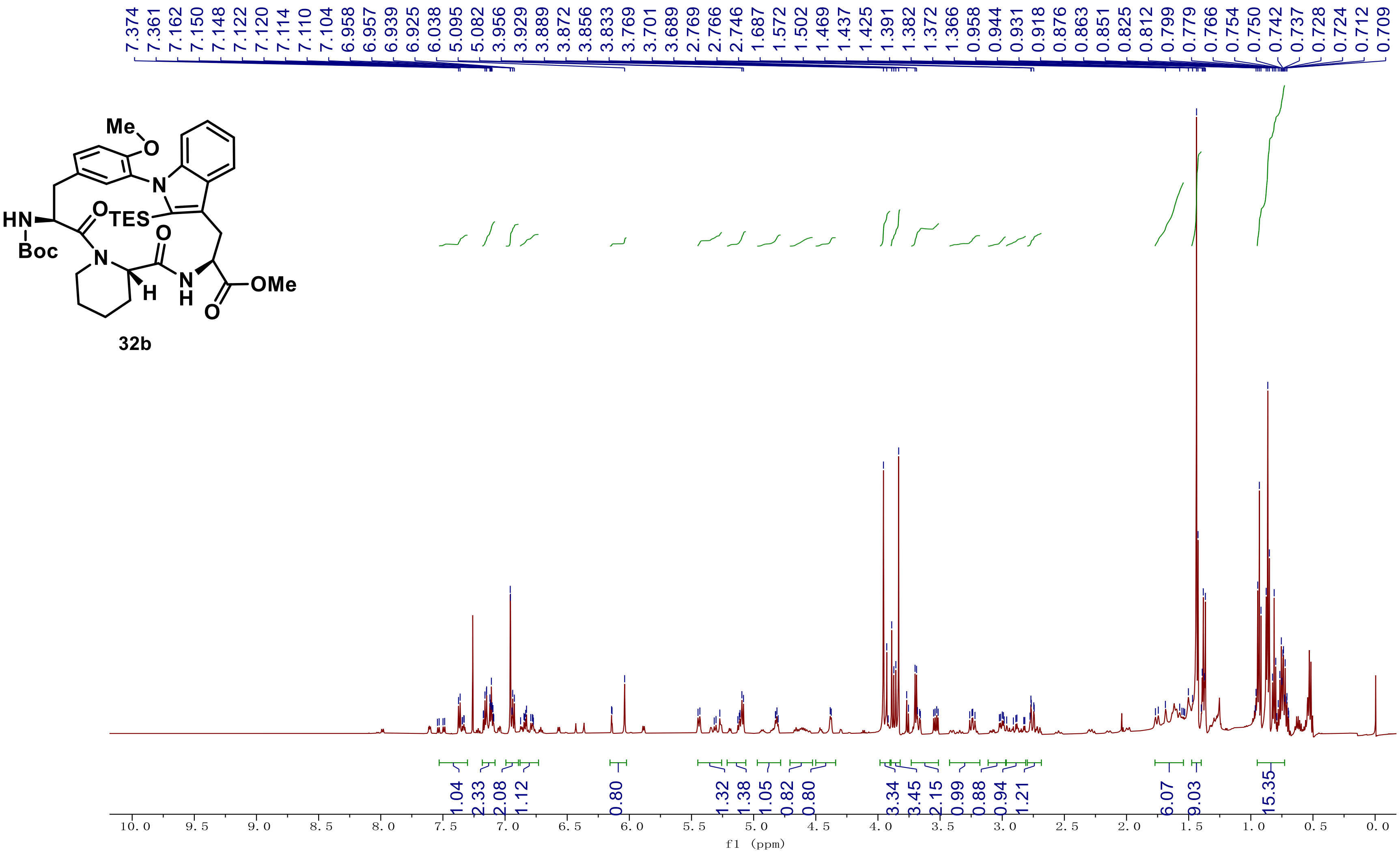

Compound 32b ^{13}C NMR (151 MHz, CDCl$_3$)

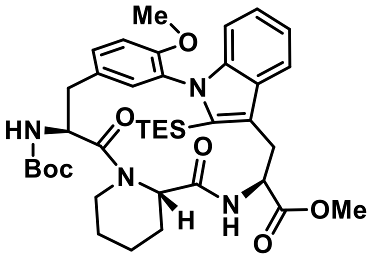

32b

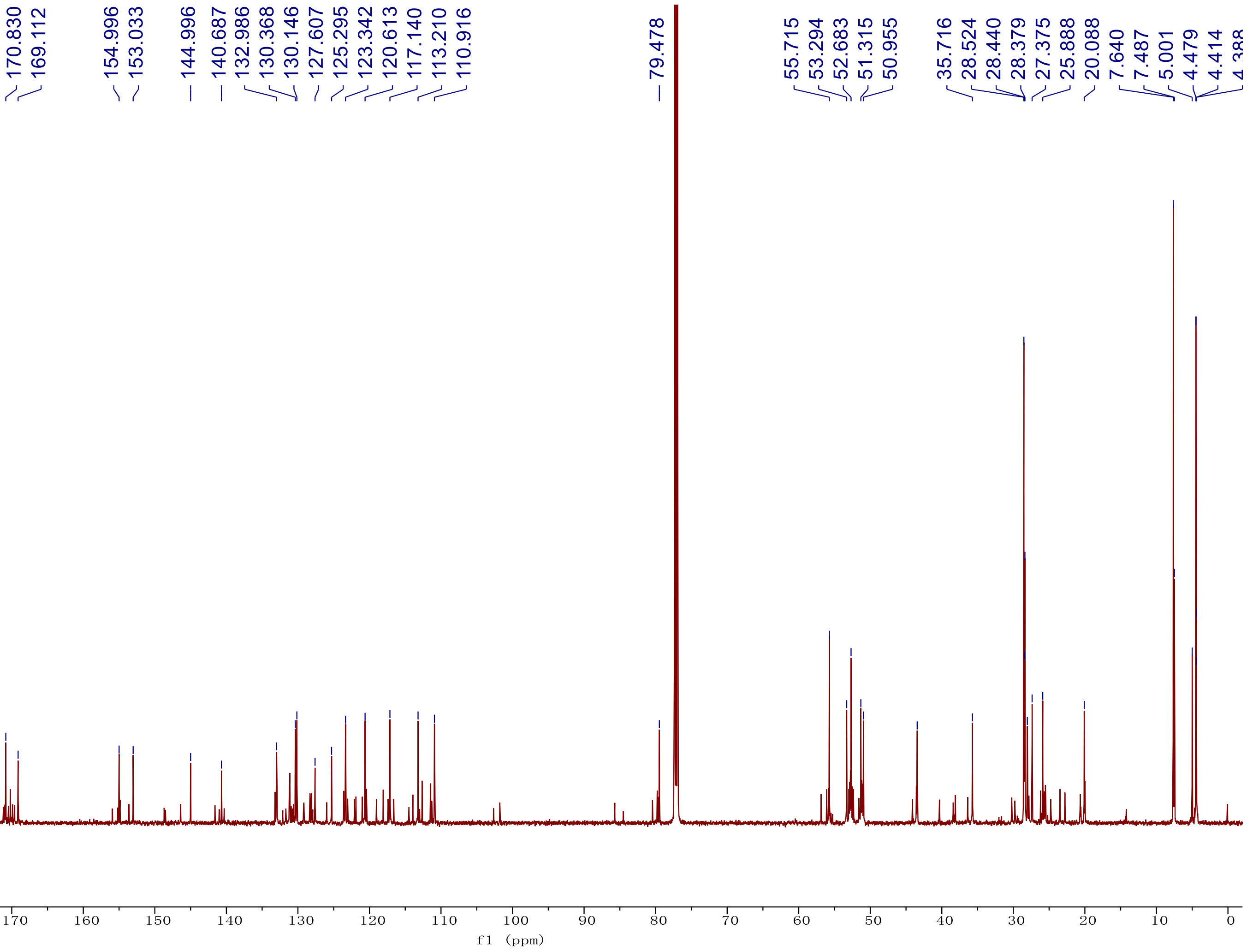

Compound 32c ^{1}H NMR (600 MHz, CDCl₃)

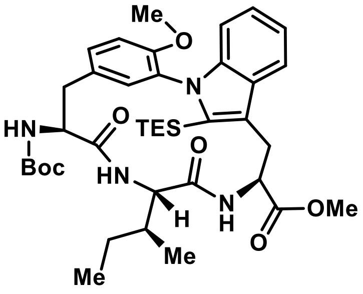

32c

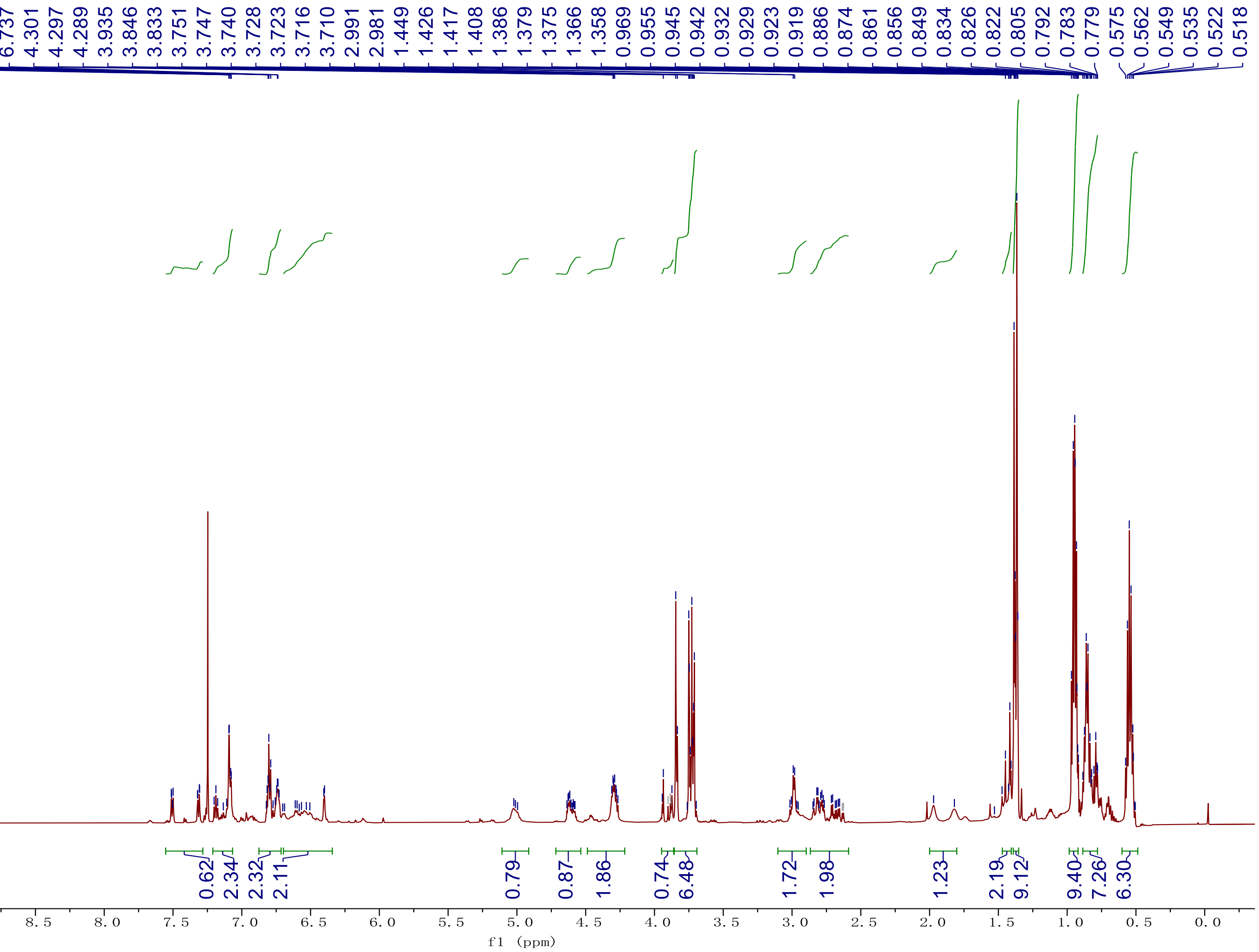

Compound 32c ^{13}C NMR (151 MHz, $CDCl_3$)

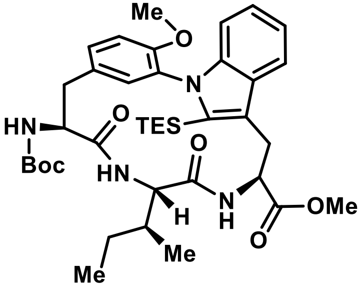

32c

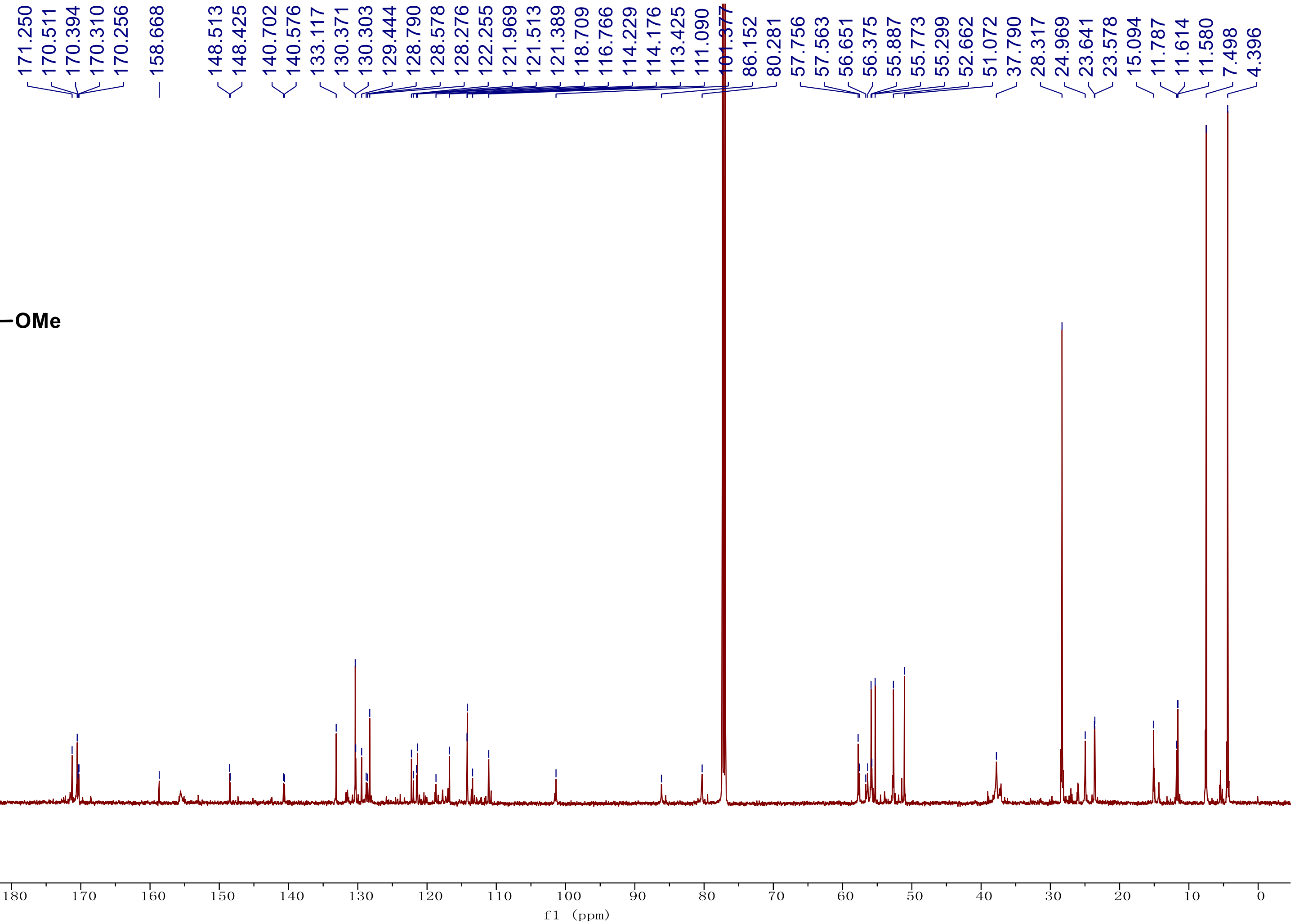

Compound 32d ^{1}H NMR (600 MHz, CDCl₃)

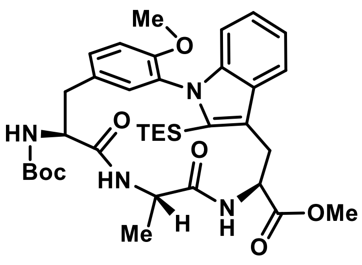

32d

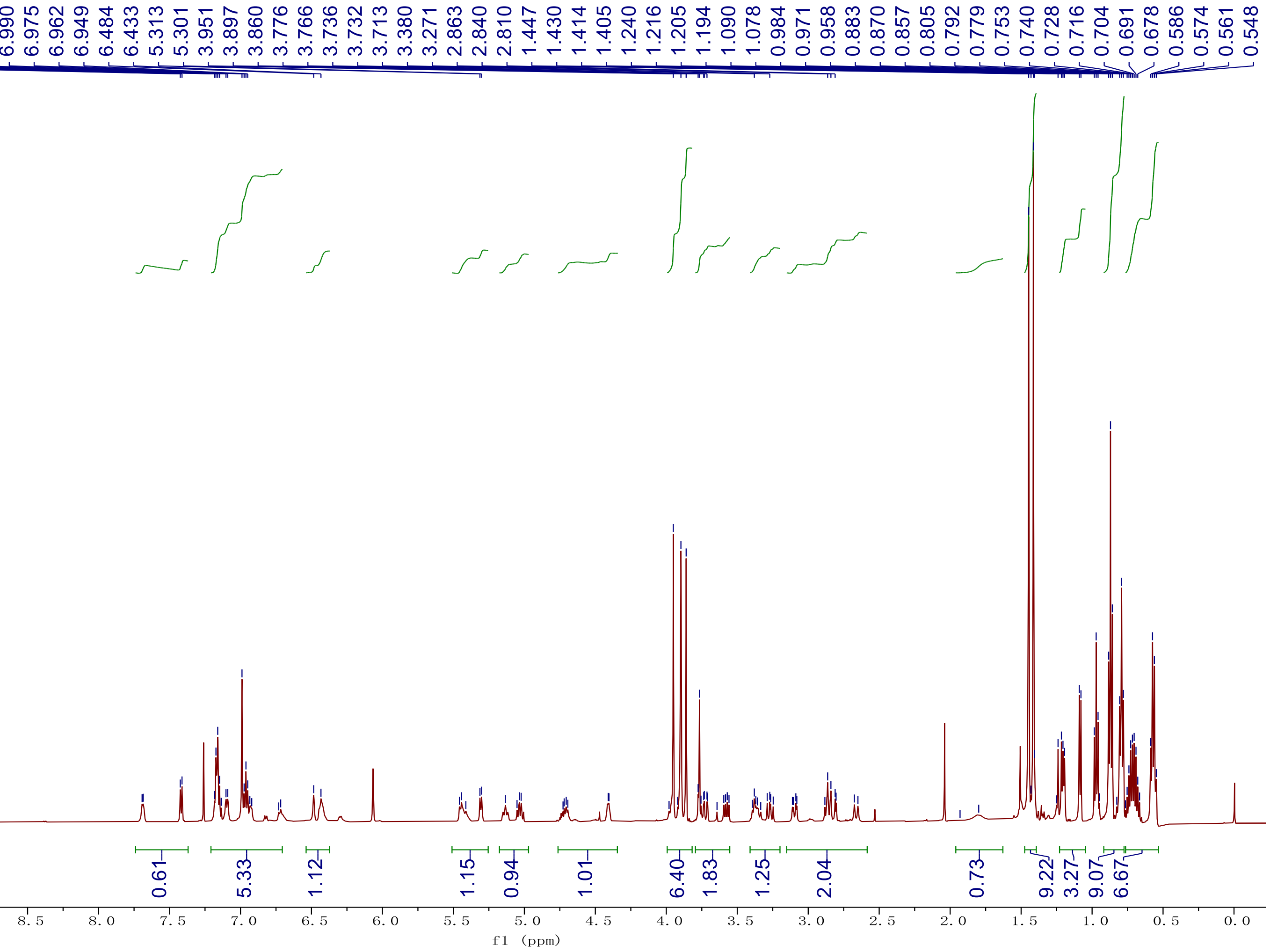

Compound 32d ^{13}C NMR (151 MHz, $CDCl_3$)

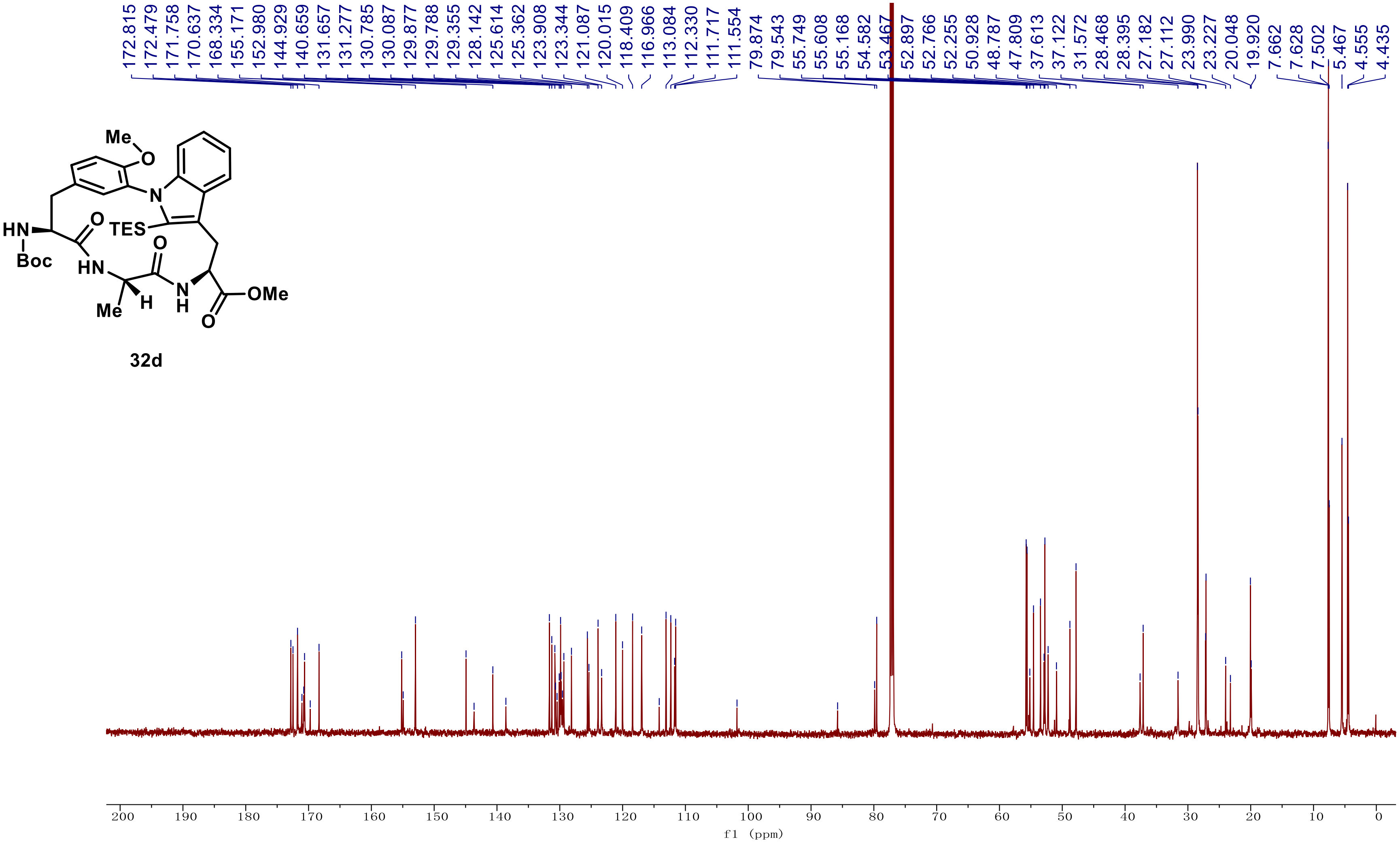

Compound 32e ^{1}H NMR (600 MHz, CDCl$_3$)

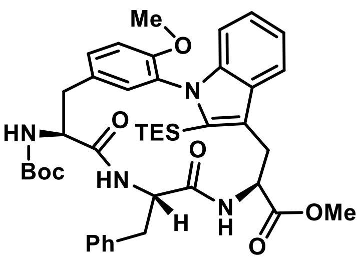

32e

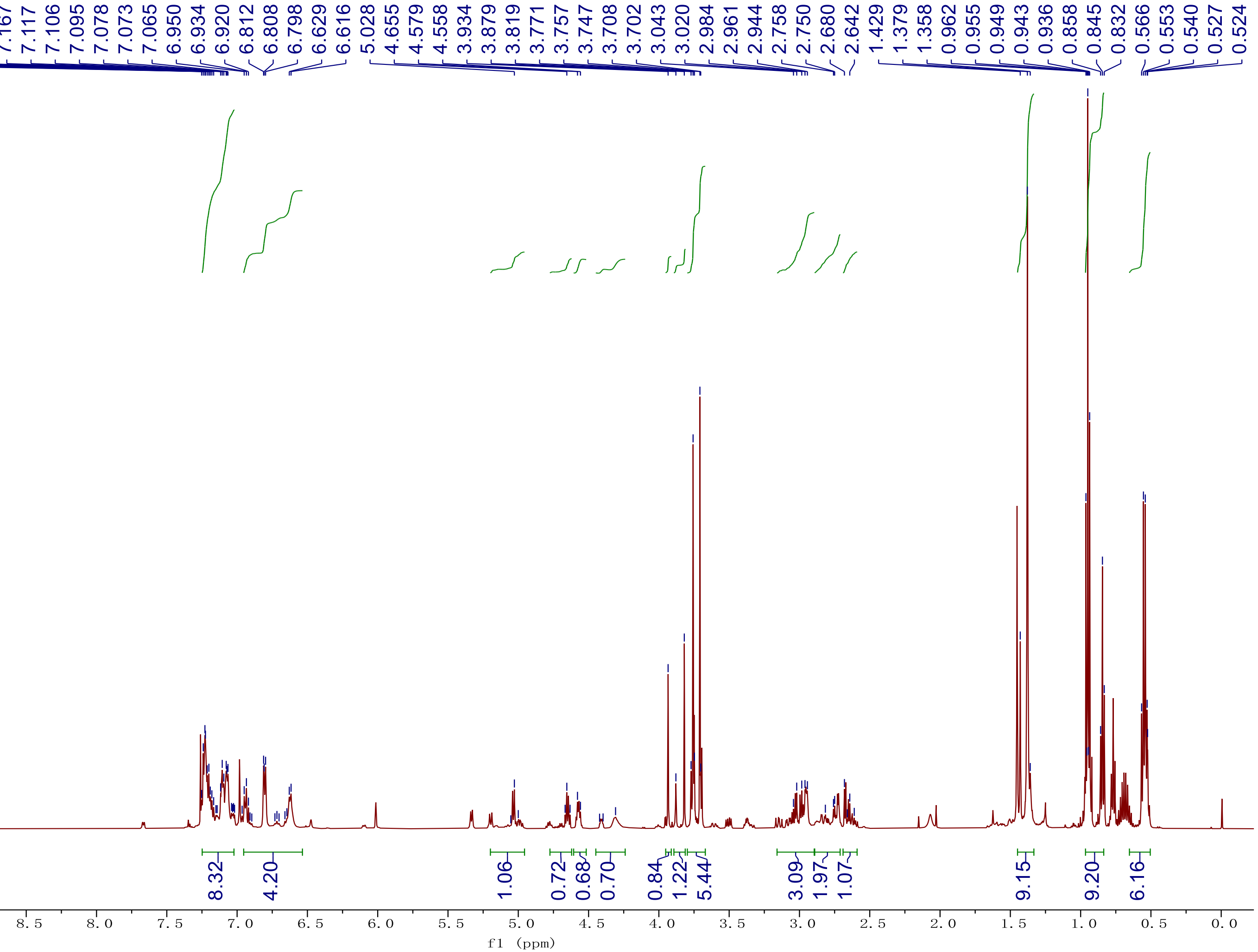

Compound 32e ^{13}C NMR (151 MHz, CDCl$_3$)

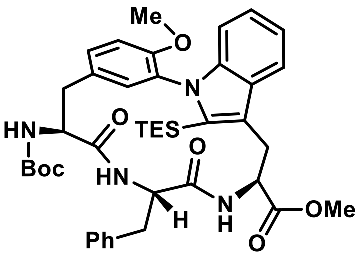

32e

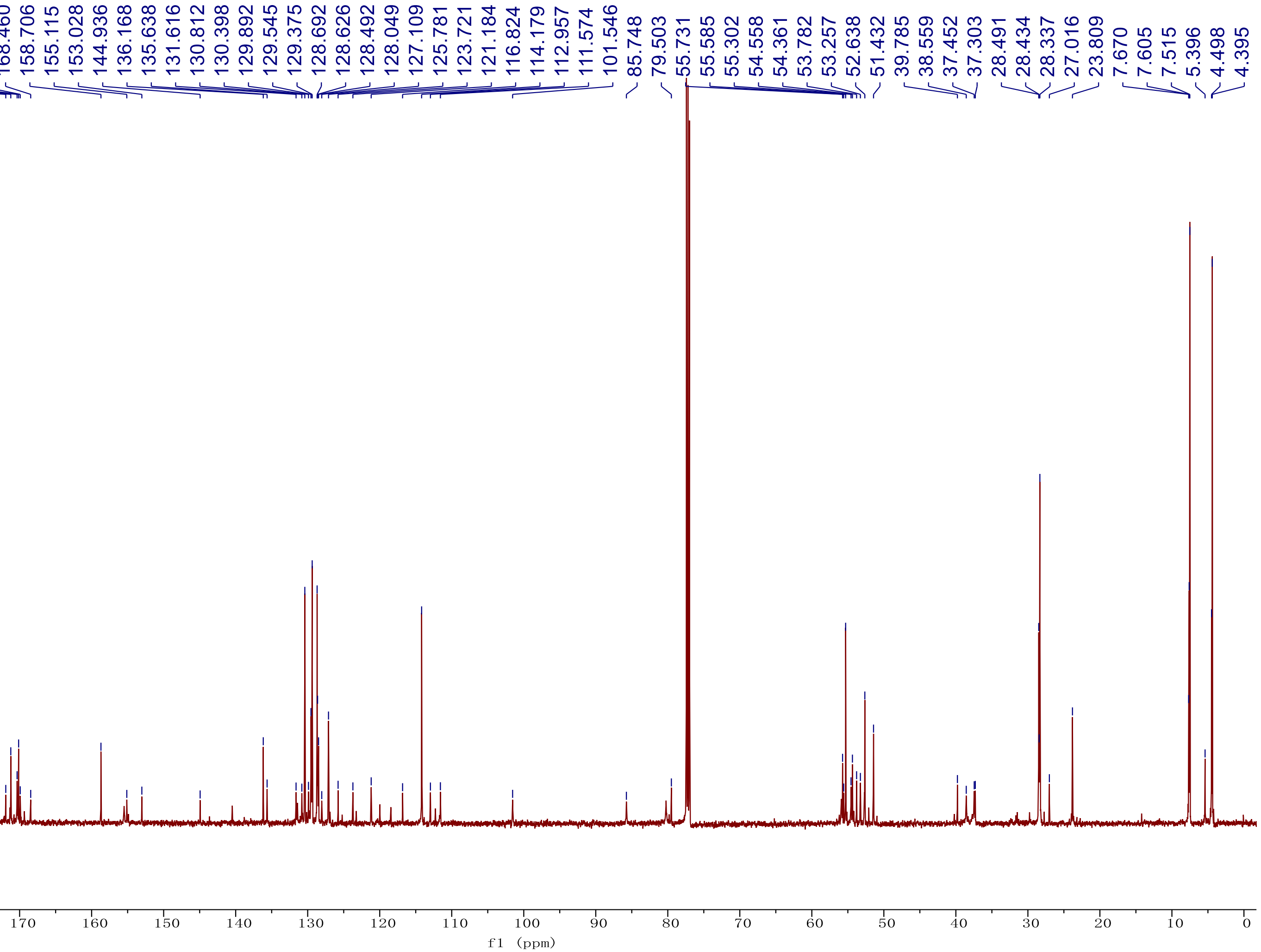

Compound 32f ^{1}H NMR (600 MHz, CDCl₃)

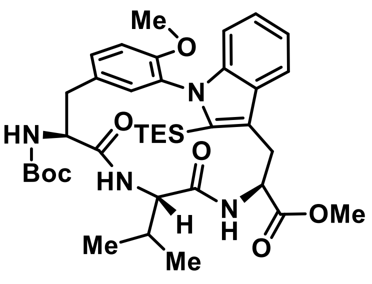

32f

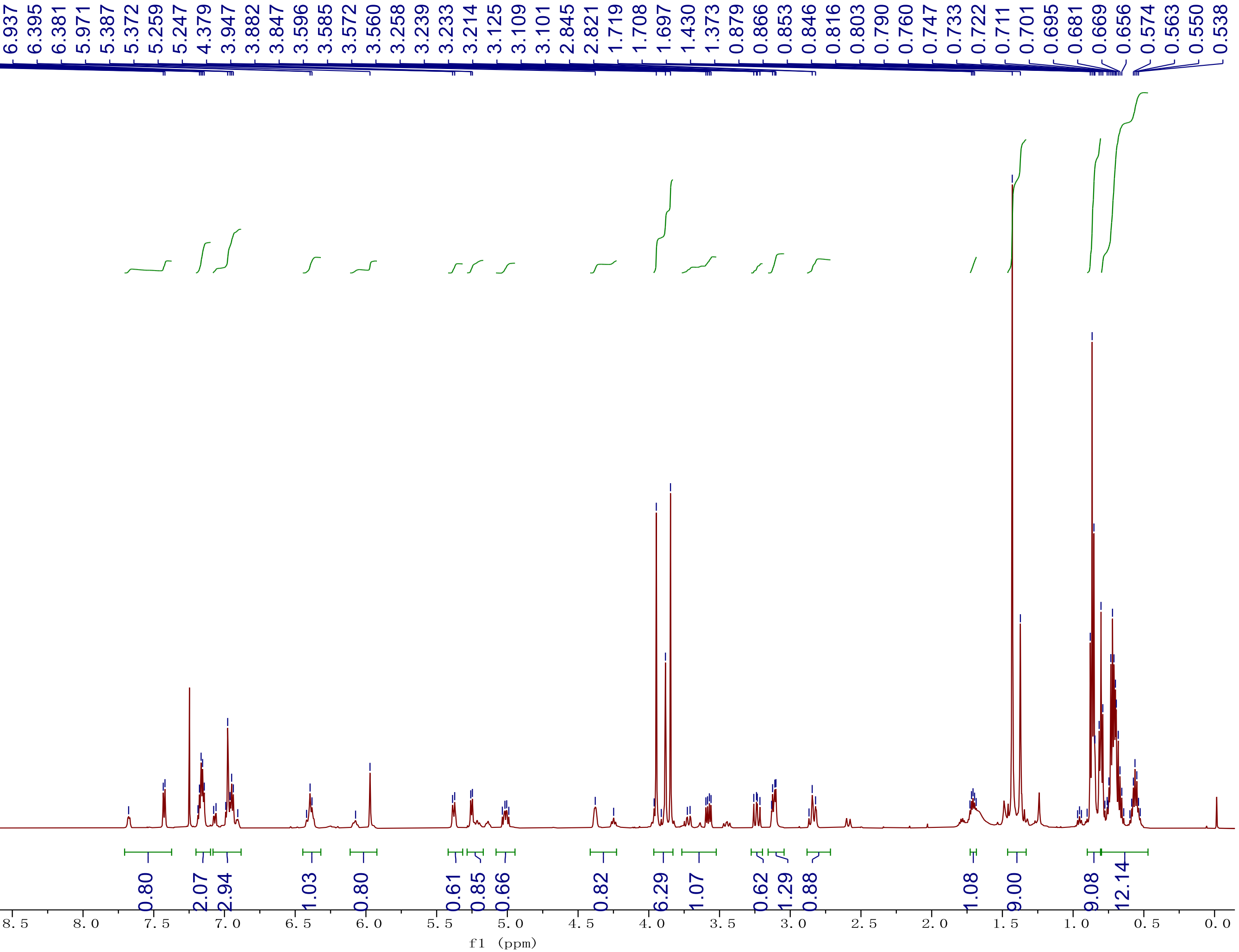

Compound 32f ^{13}C NMR (151 MHz, CDCl$_3$)

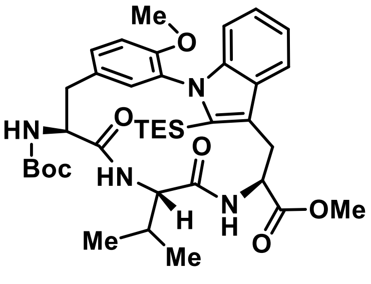

32f

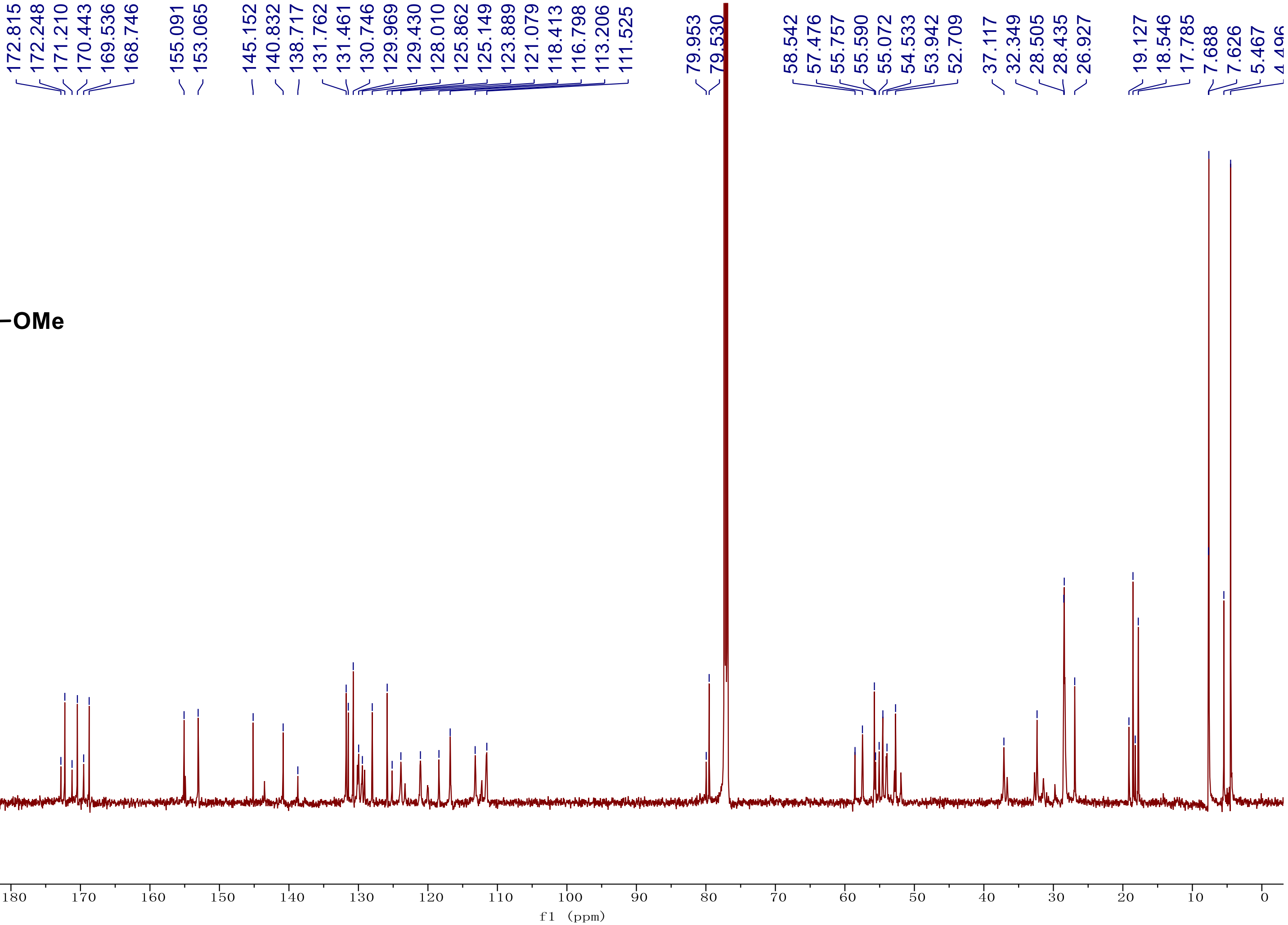

Compound 32g ^1H NMR (600 MHz, $CDCl_3$)

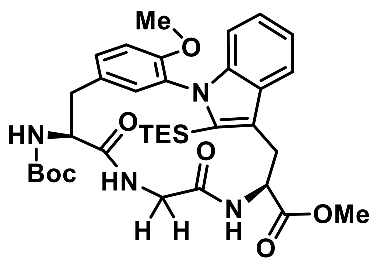

32g

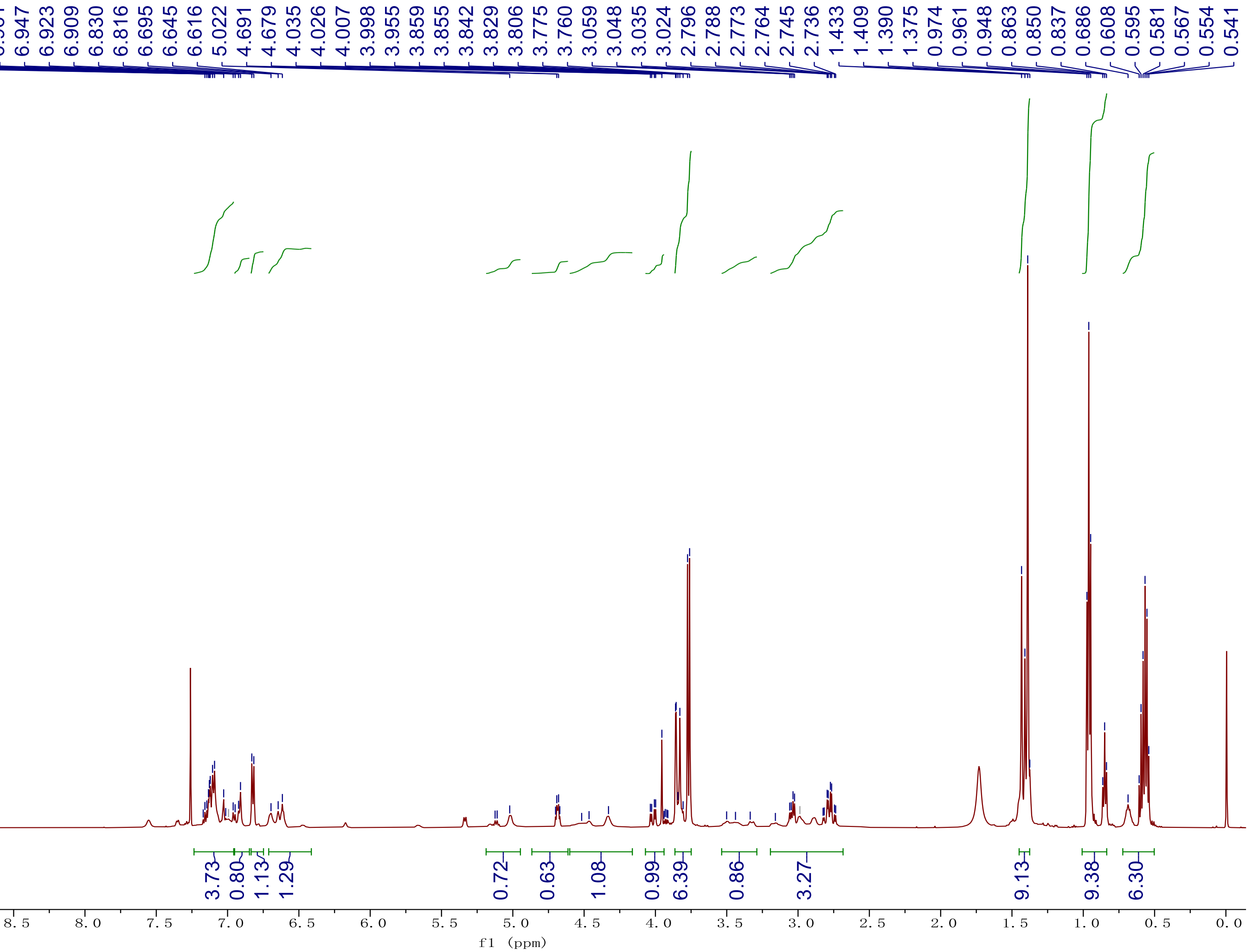

Compound 32g ^{13}C NMR (151 MHz, $CDCl_3$)

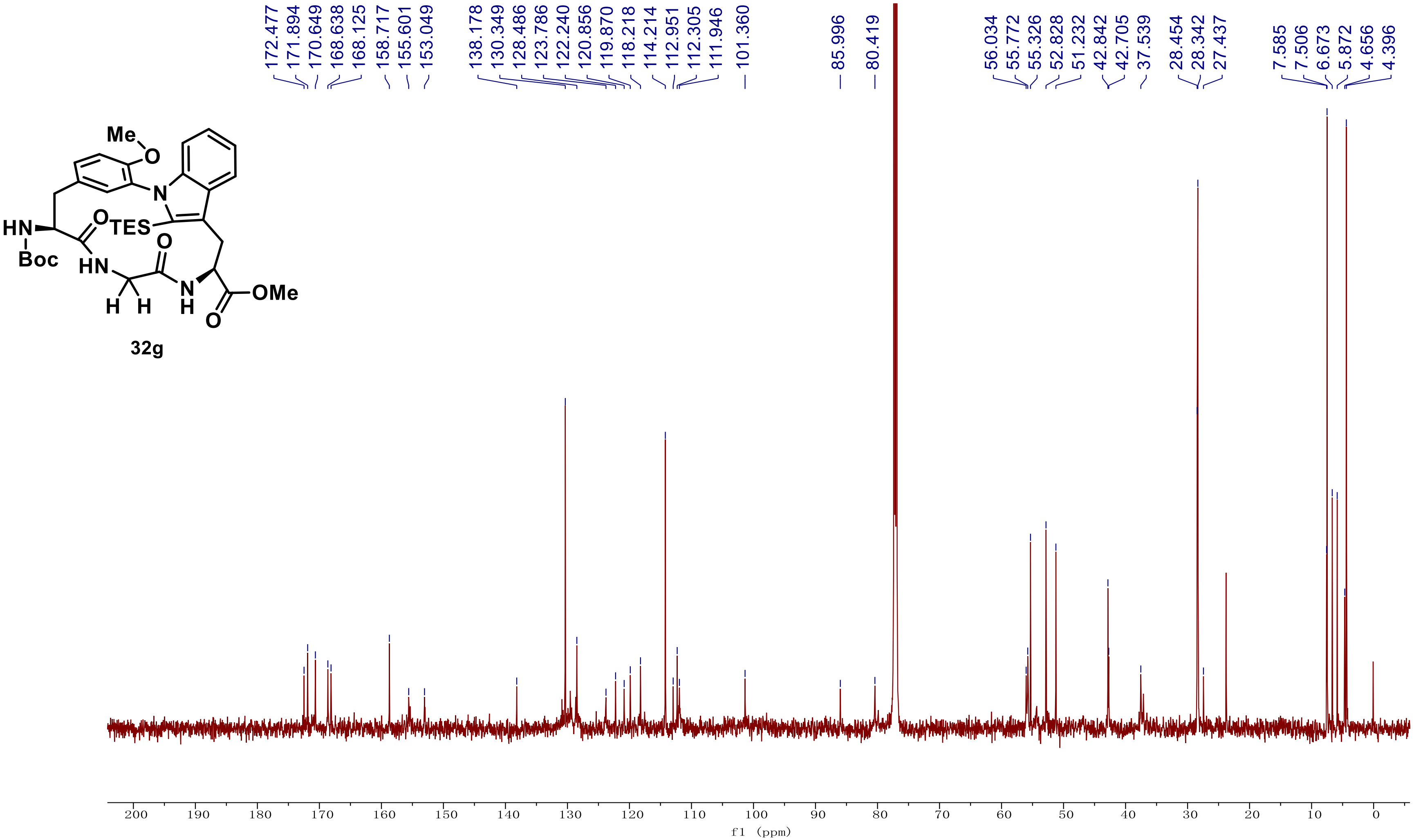

32g

Compound 32h ¹H NMR (600 MHz, CDCl₃)

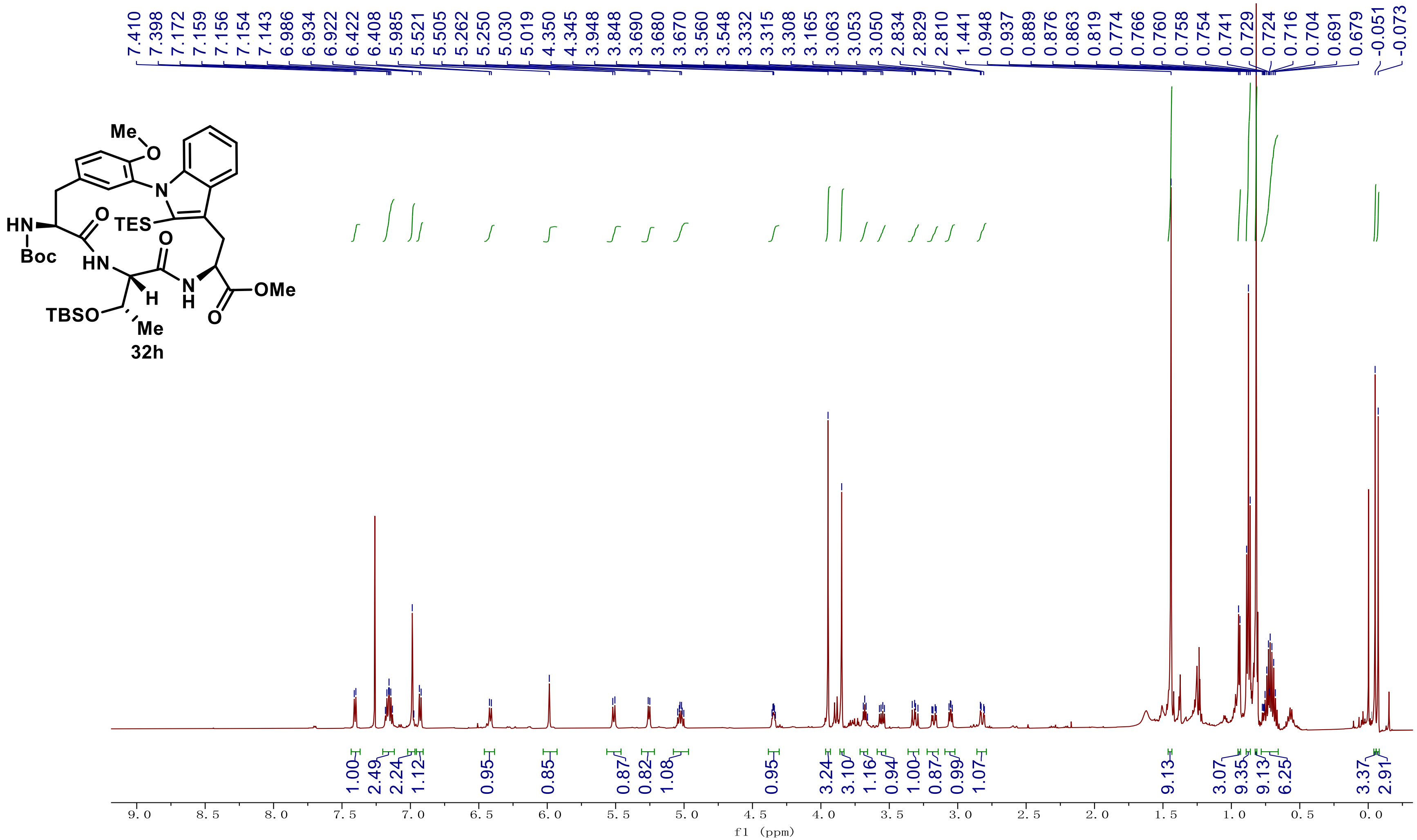

Compound 32h ^{13}C NMR (151 MHz, $CDCl_3$)

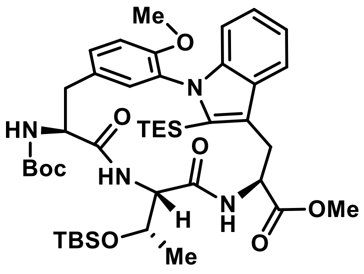

32h

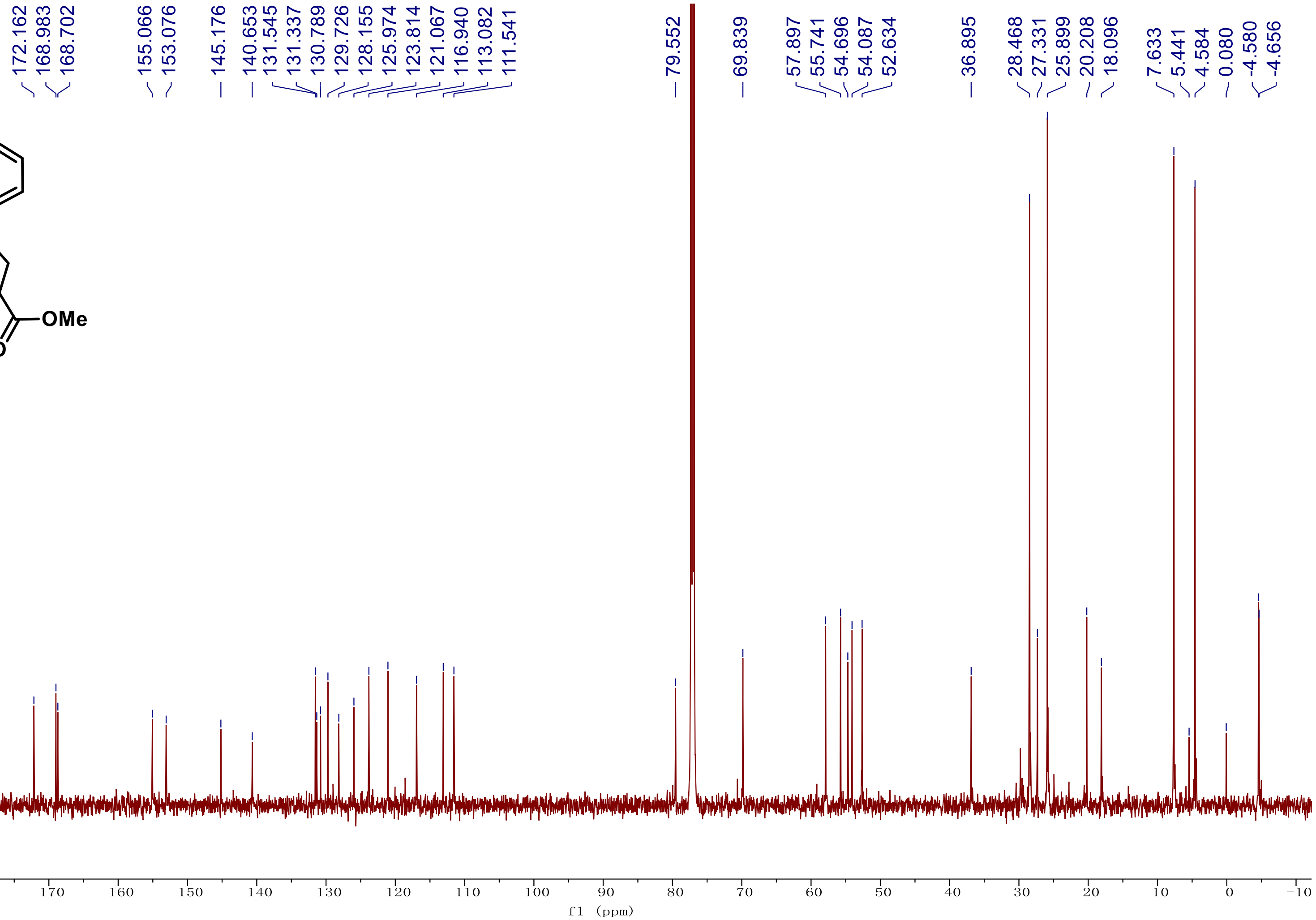

Compound 32i ^{1}H NMR (600 MHz, CDCl3)

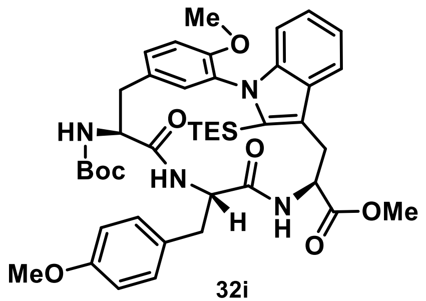

32i

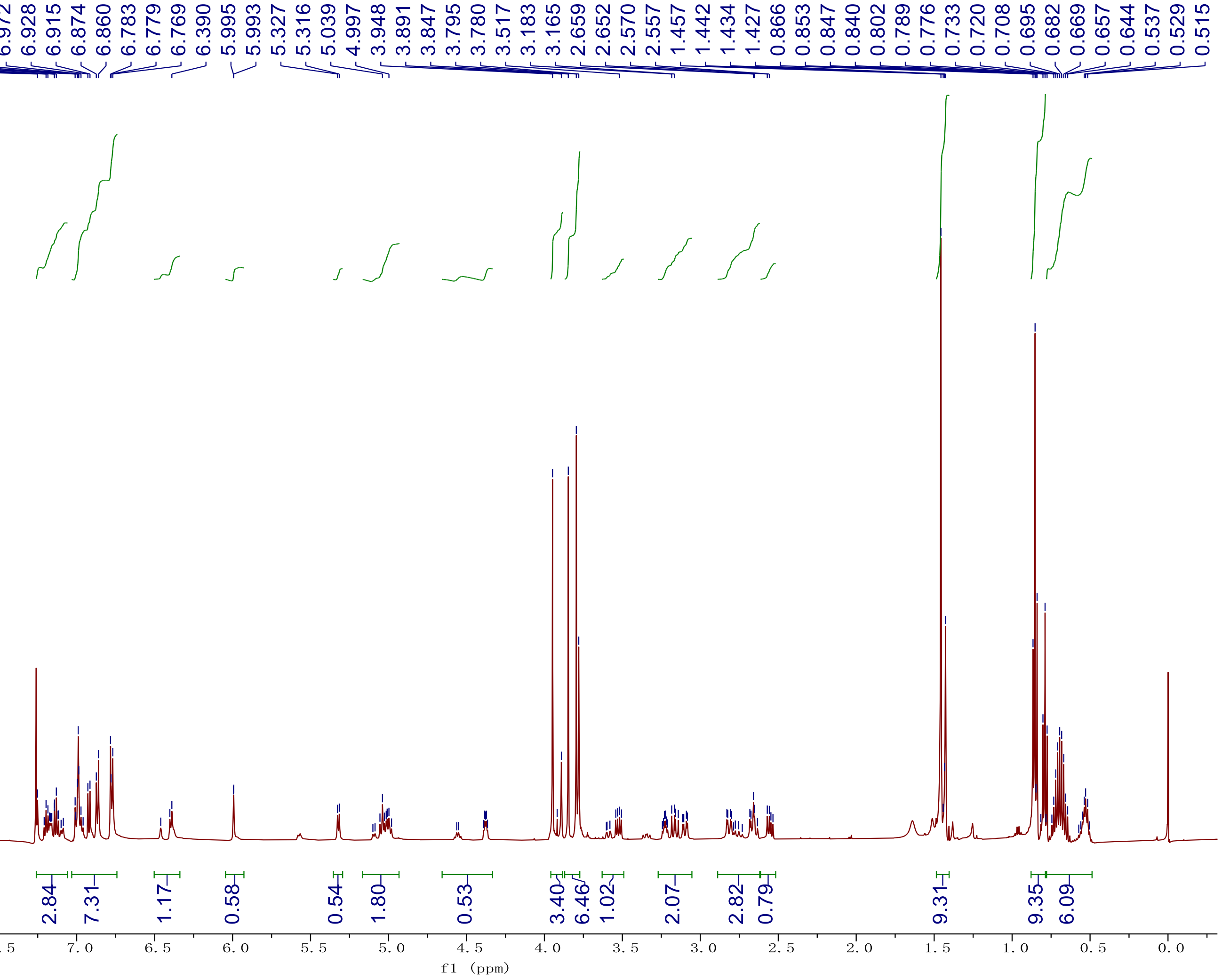

Compound 32i ^{13}C NMR (151 MHz, $CDCl_3$)

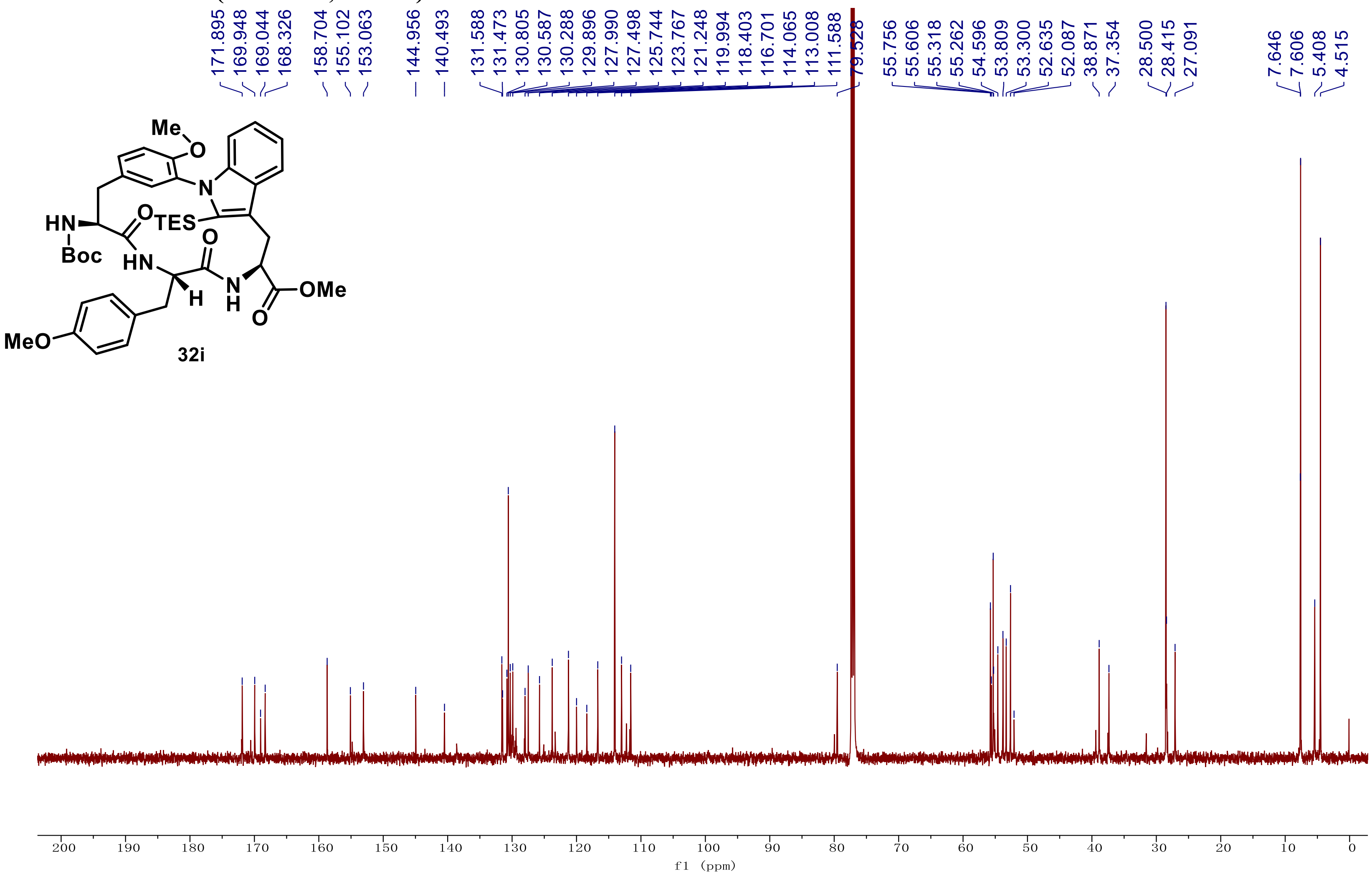

Compound 32j ¹H NMR (600 MHz, $CDCl_3$)

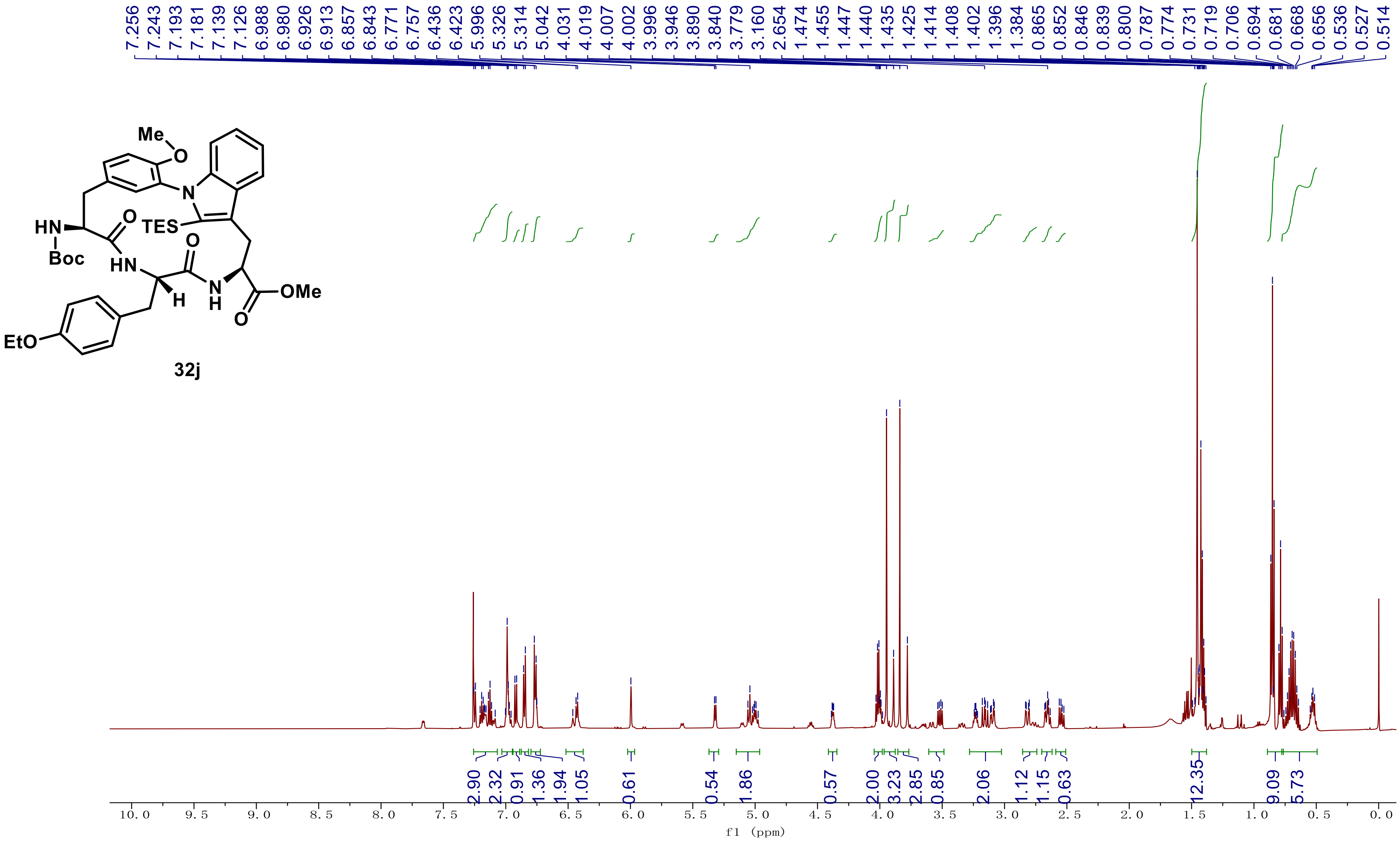

32j

Compound 32j ^{13}C NMR (151 MHz, $CDCl_3$)

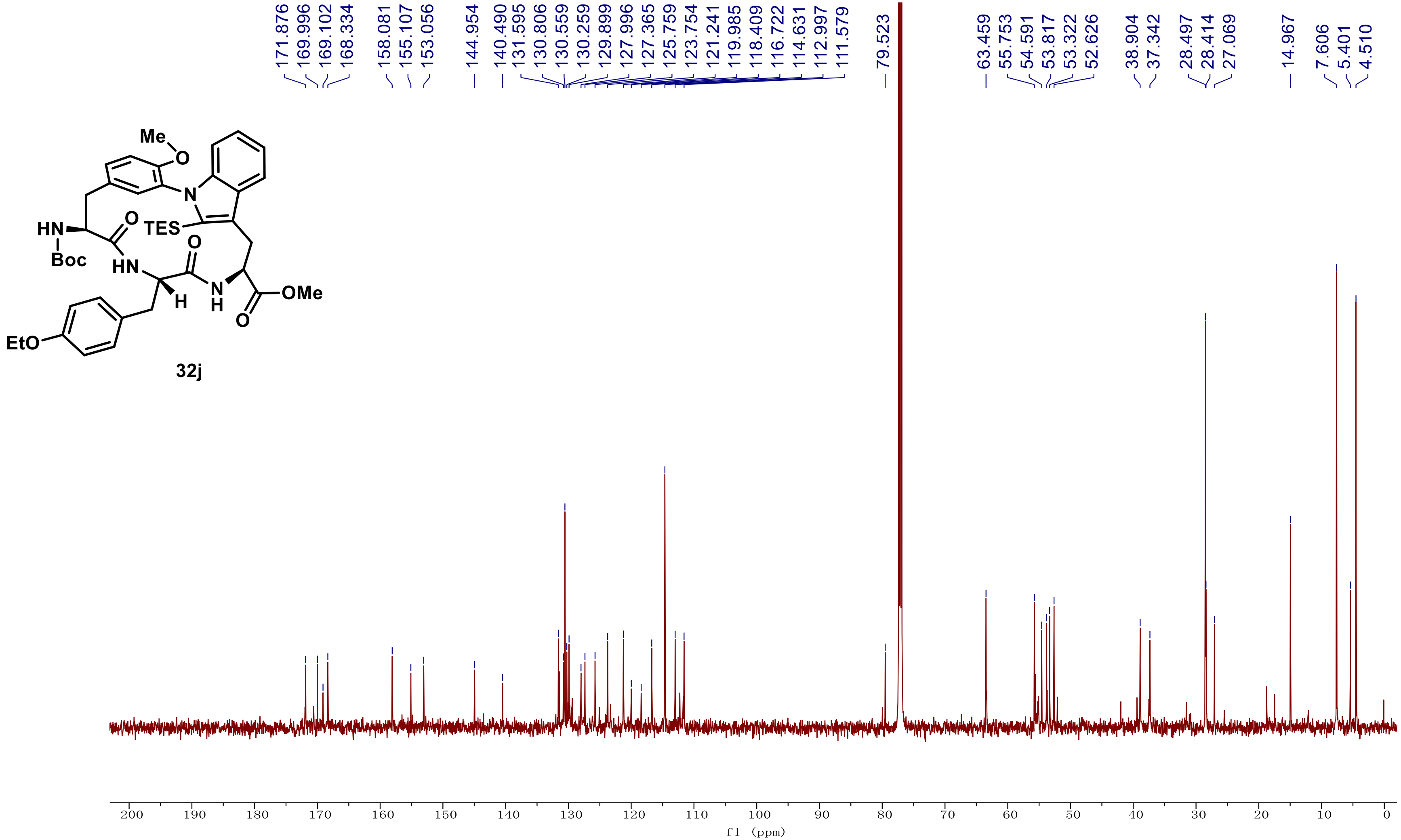

Compound 32k ^{1}H NMR (600 MHz, $CDCl_3$)

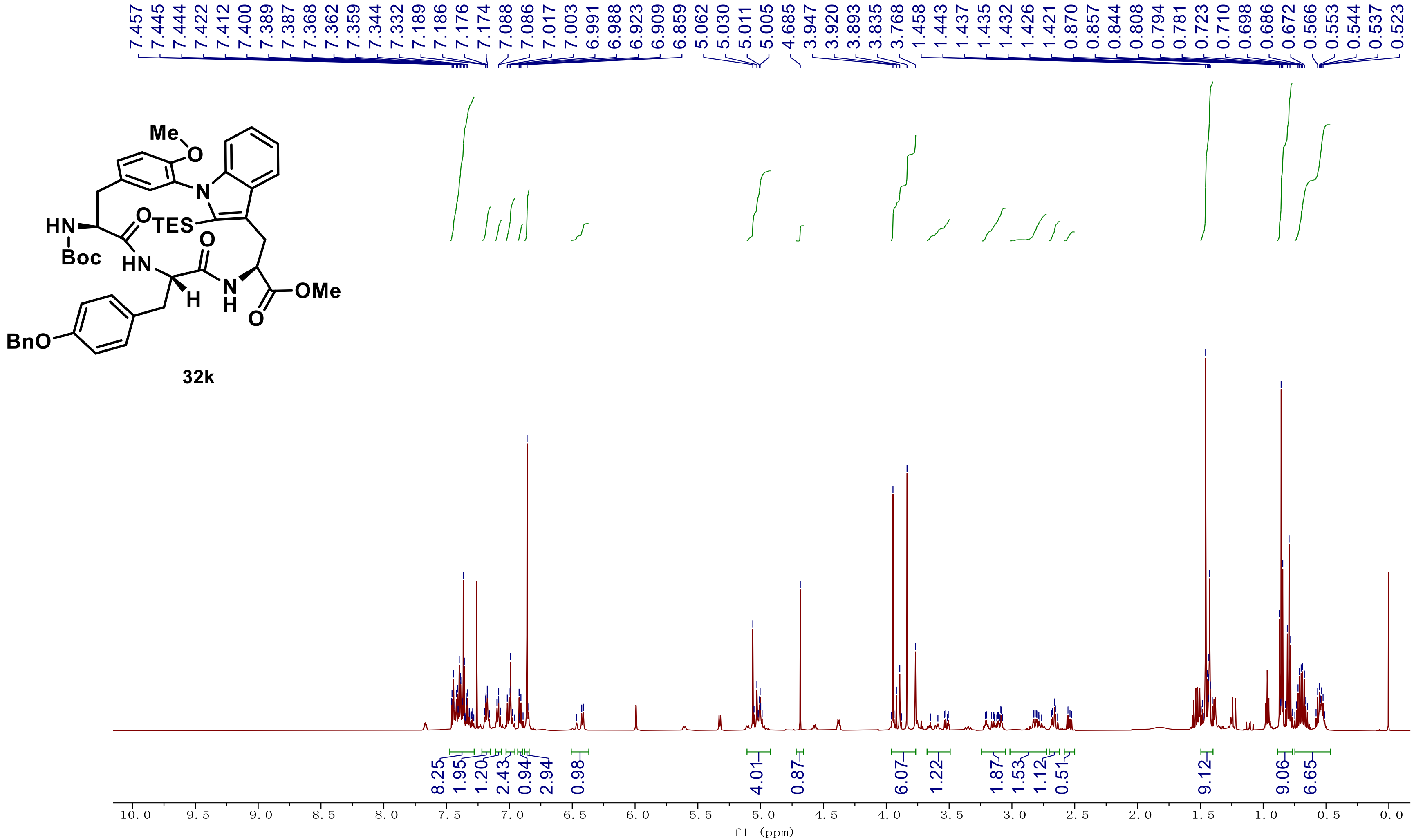

Compound 32k ^{13}C NMR (151 MHz, CDCl$_3$)

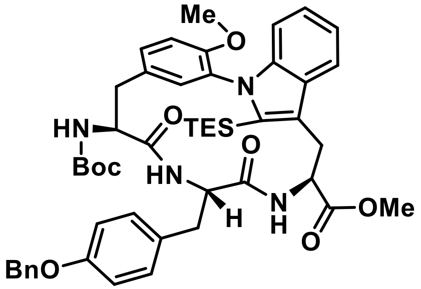

32k

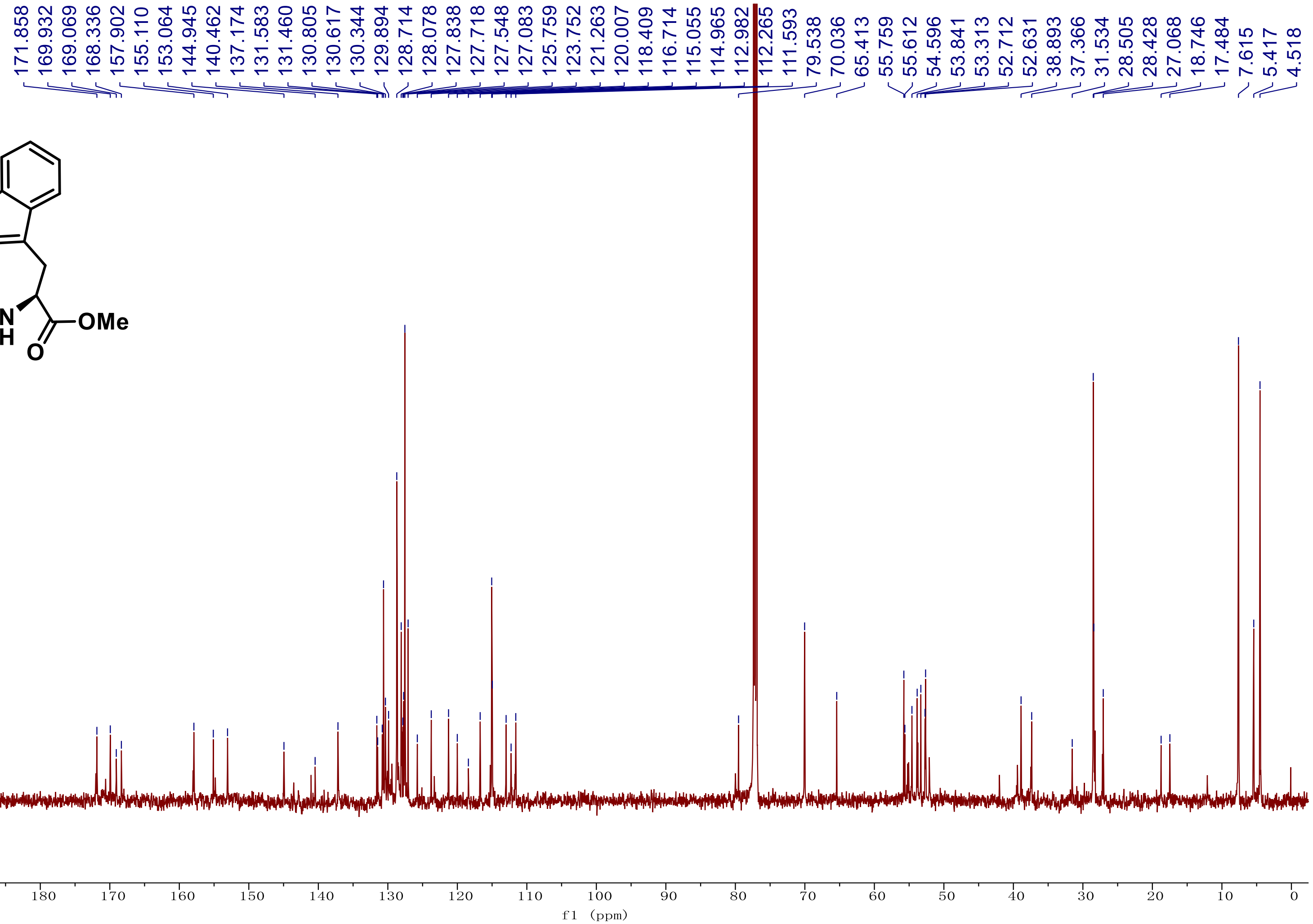

Compound 32l ¹H NMR (600 MHz, $CDCl_3$)

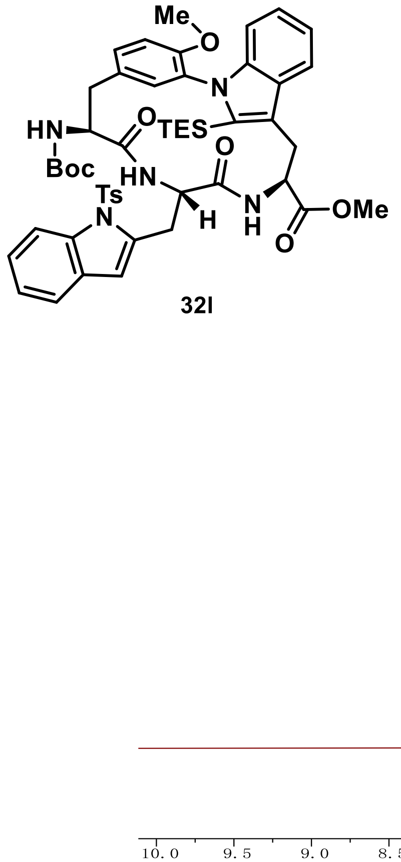

32l

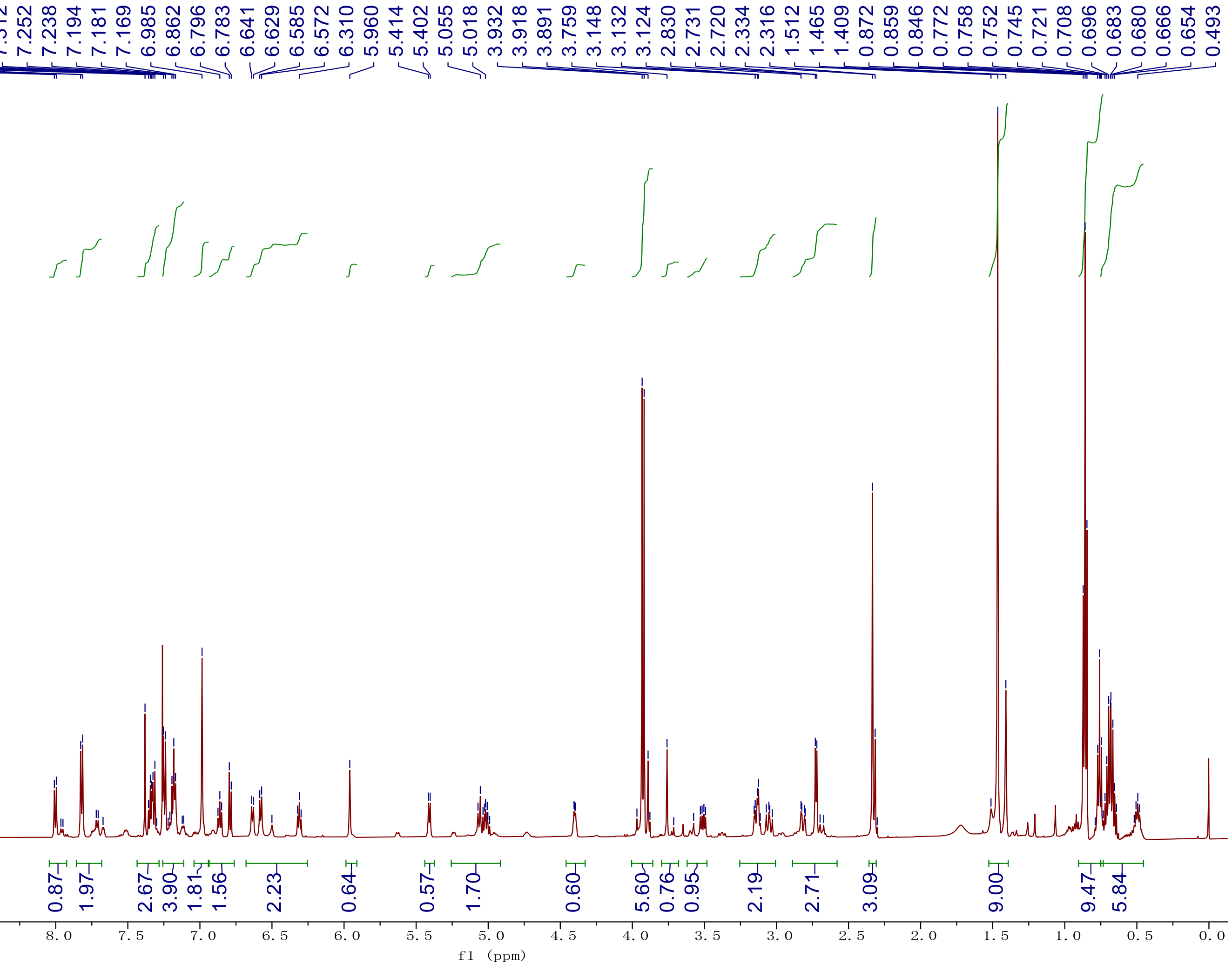

Compound 32l ^{13}C NMR (151 MHz, $CDCl_3$)

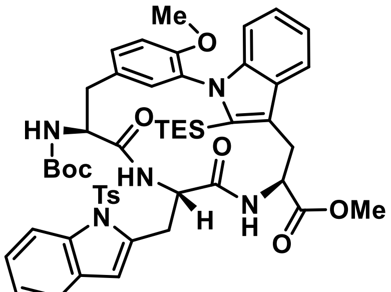

32l

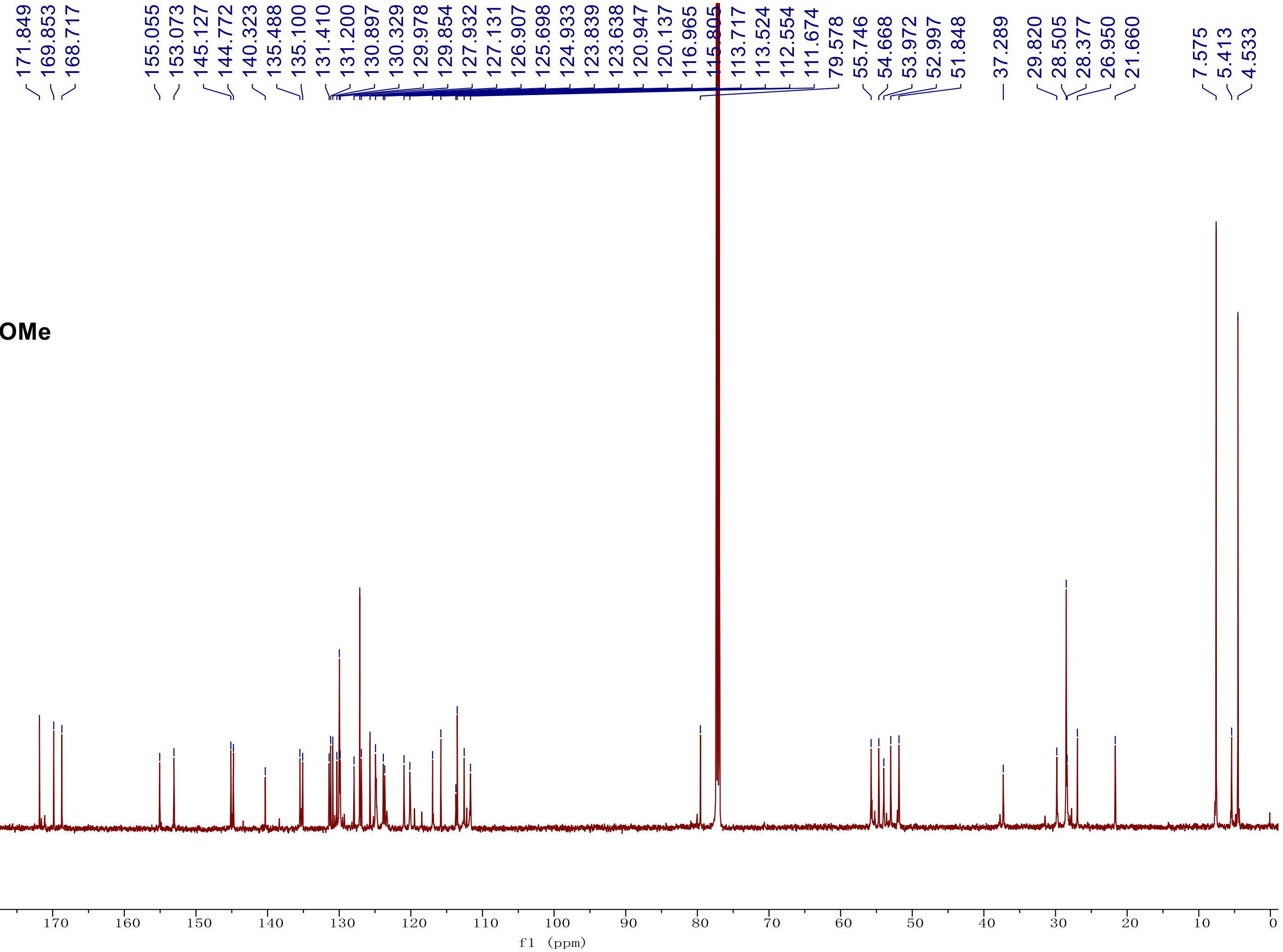

Compound S8 ¹H NMR (600 MHz, CDCl₃)

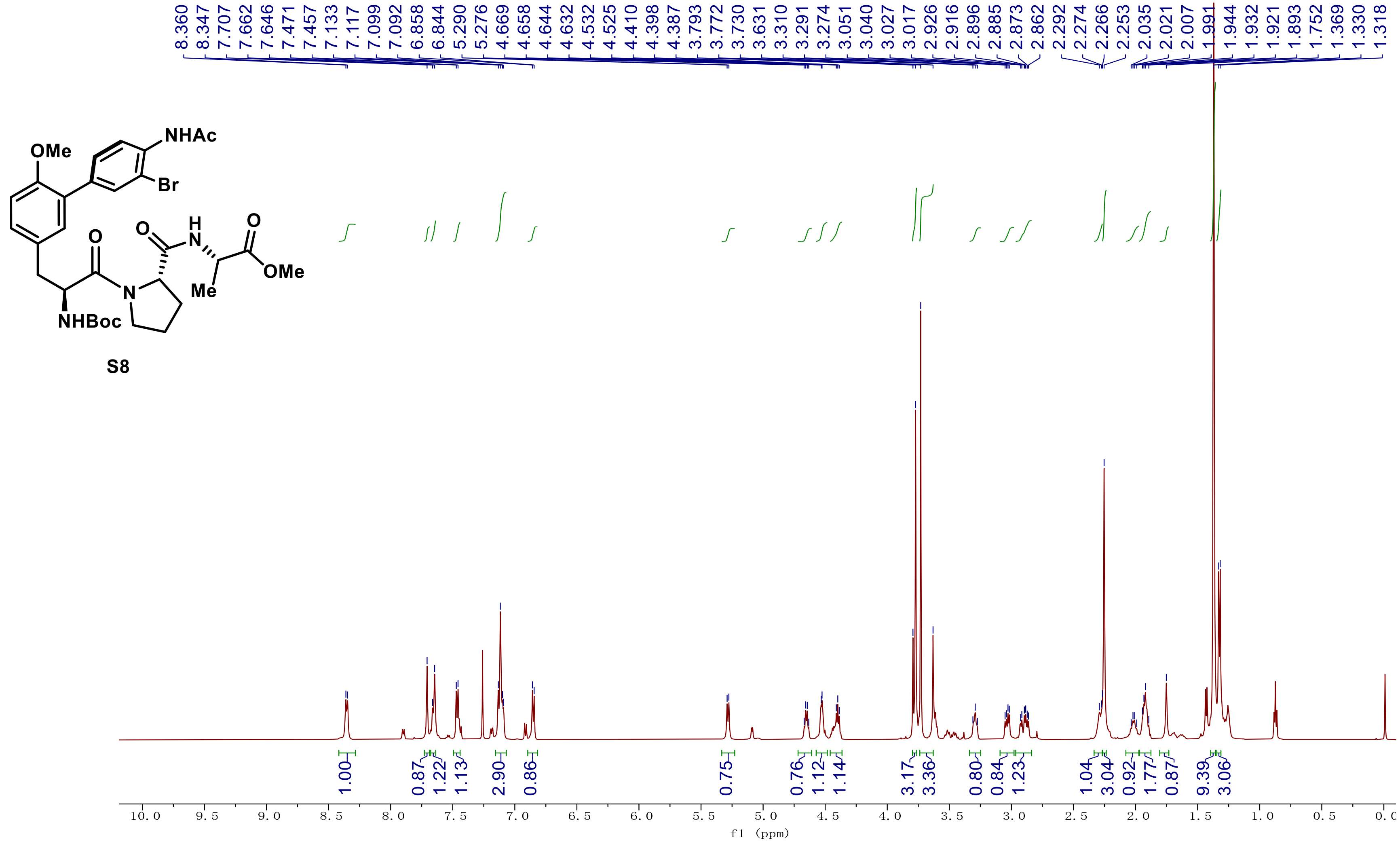

Compound S8 ^{13}C NMR (151 MHz, $CDCl_3$)

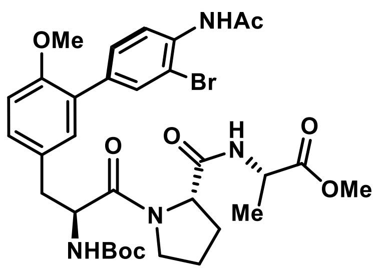

S8

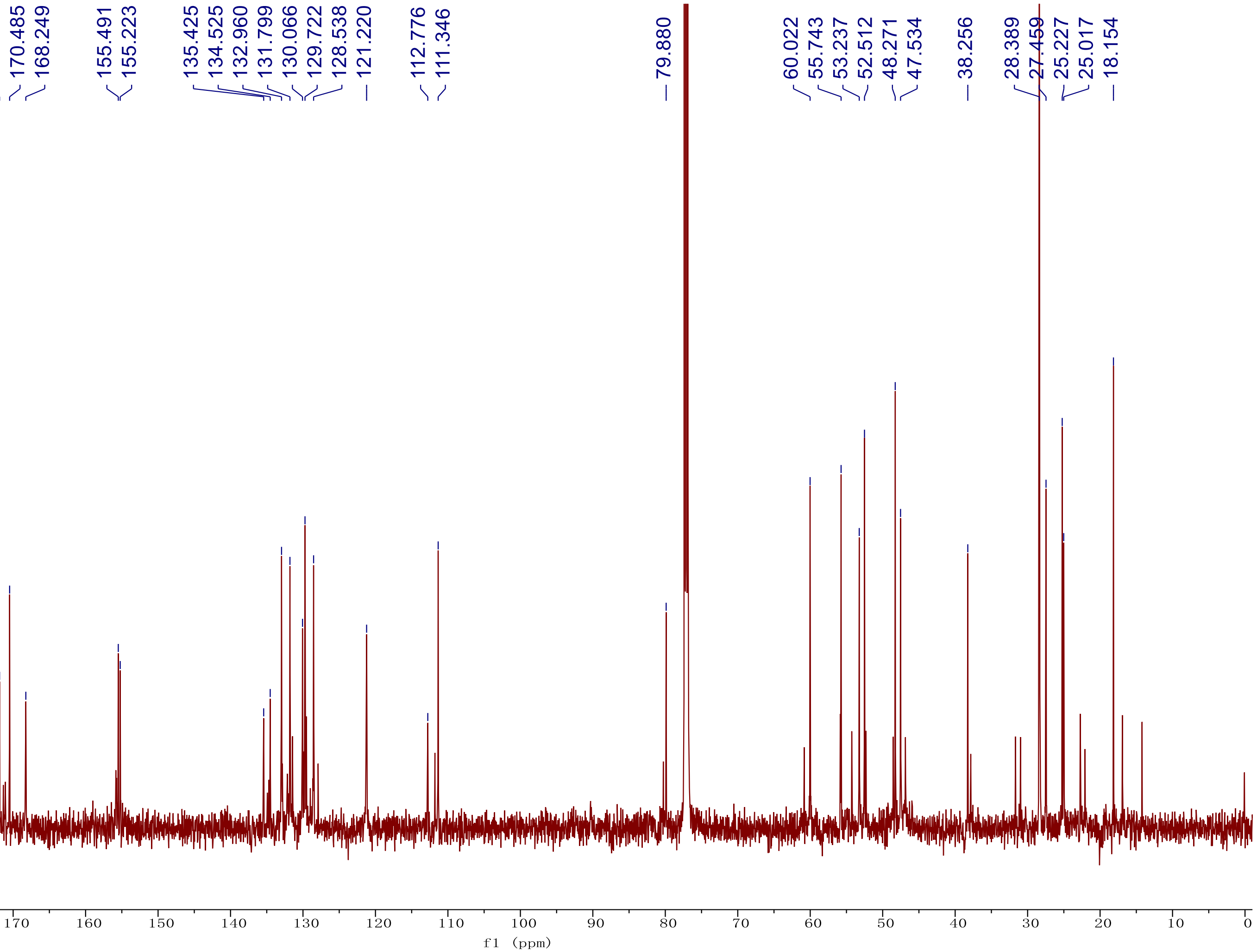

Compound 40a ^{1}H NMR (600 MHz, $CDCl_3$)

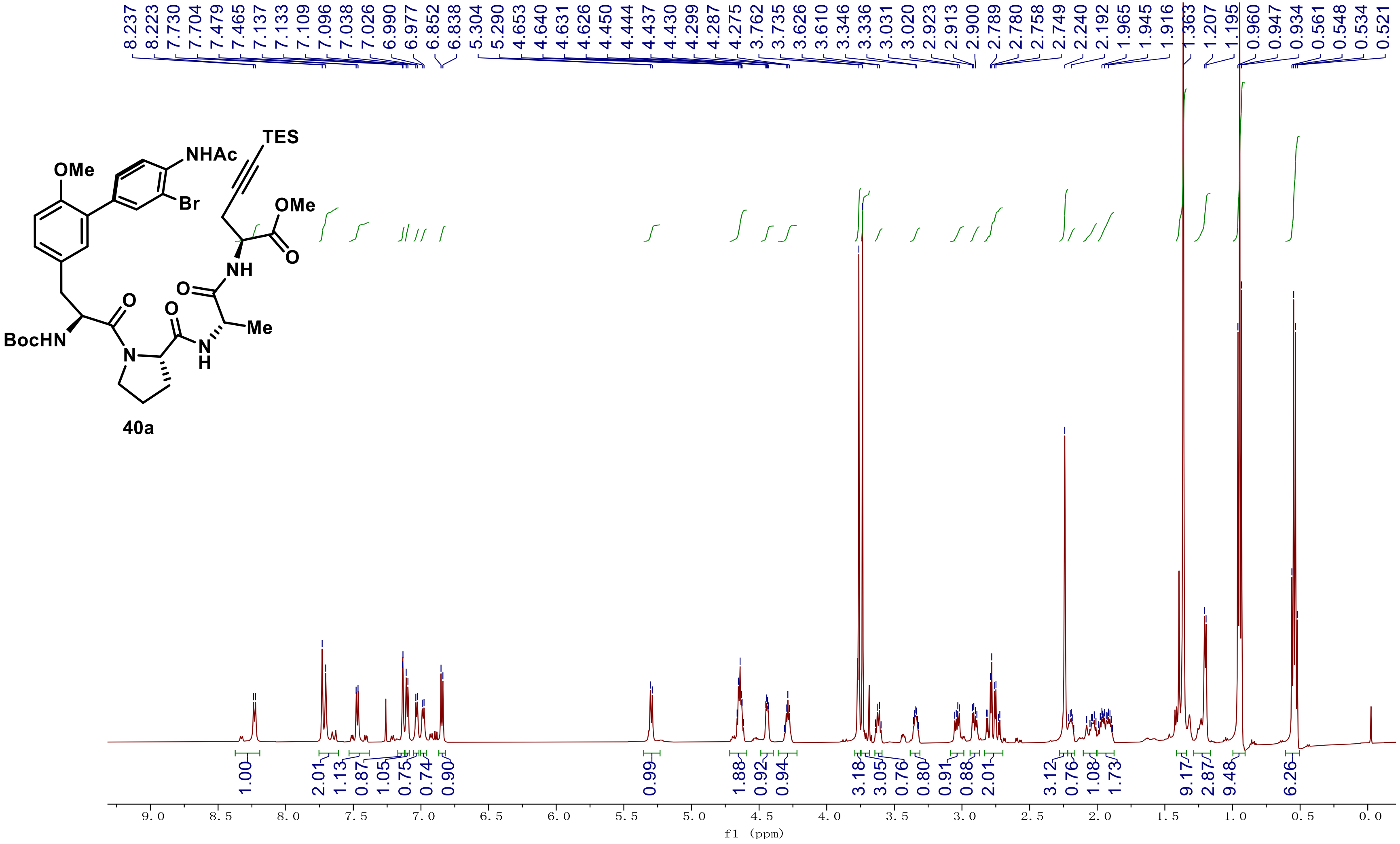

Compound 40a ^{13}C NMR (151 MHz, $CDCl_3$)

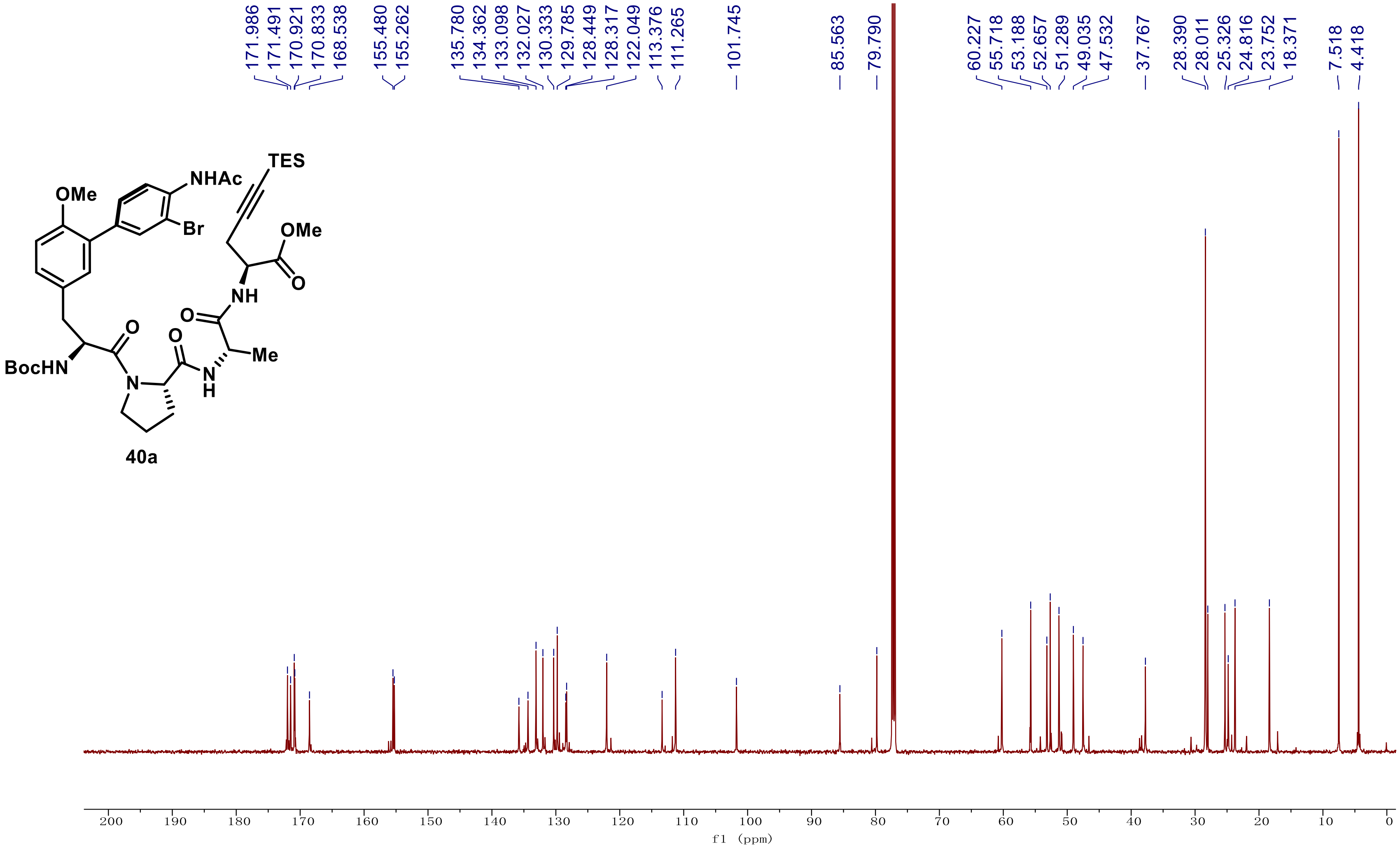

40a

Compound 41a ¹H NMR (600 MHz, CDCl₃)

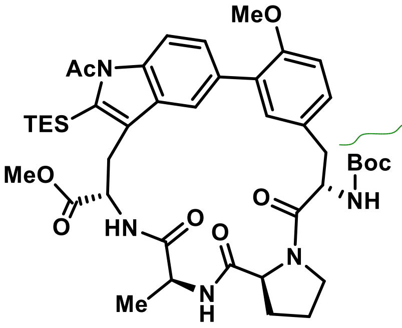

41a

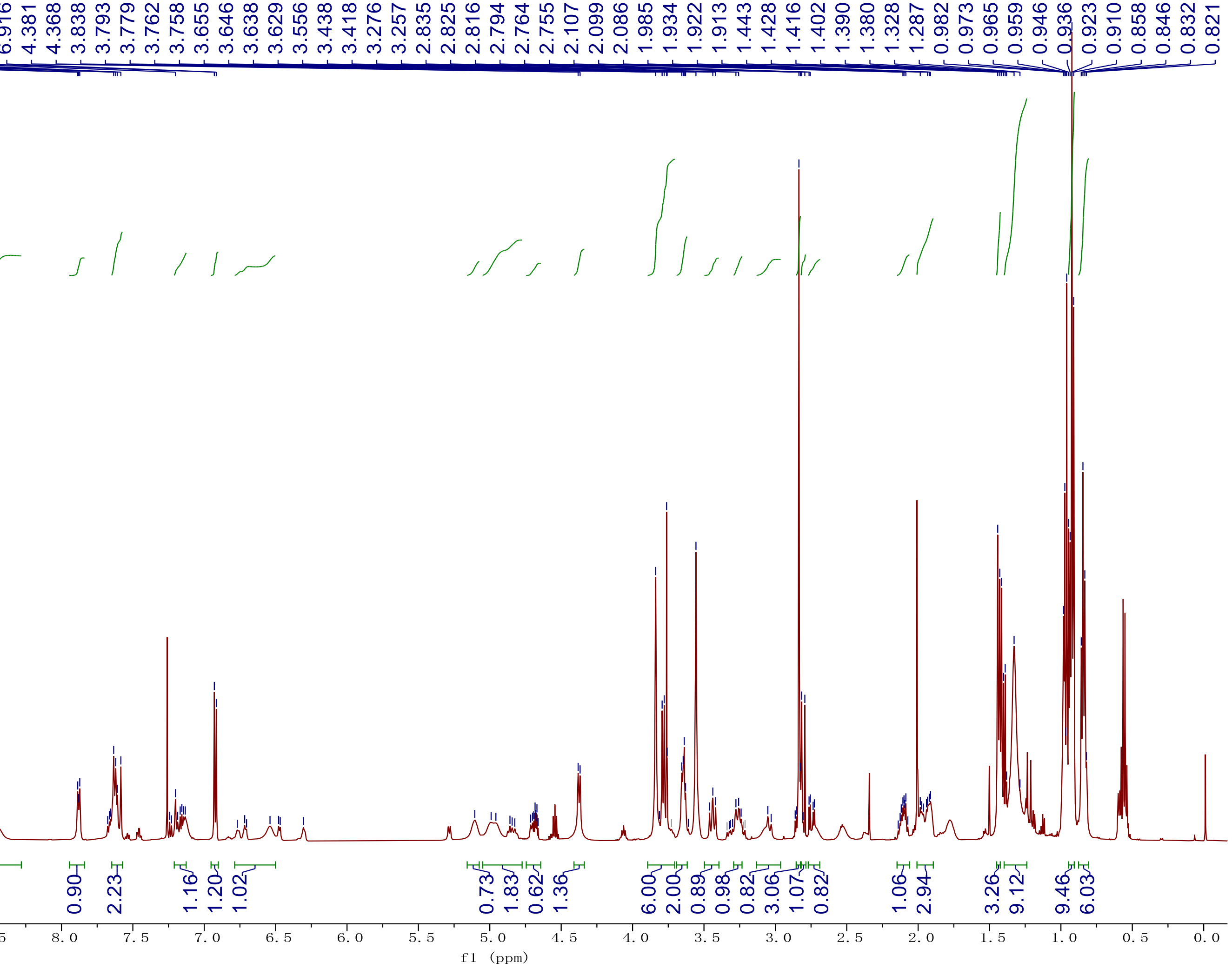

Compound 41a ^{13}C NMR (151 MHz, CDCl$_3$)

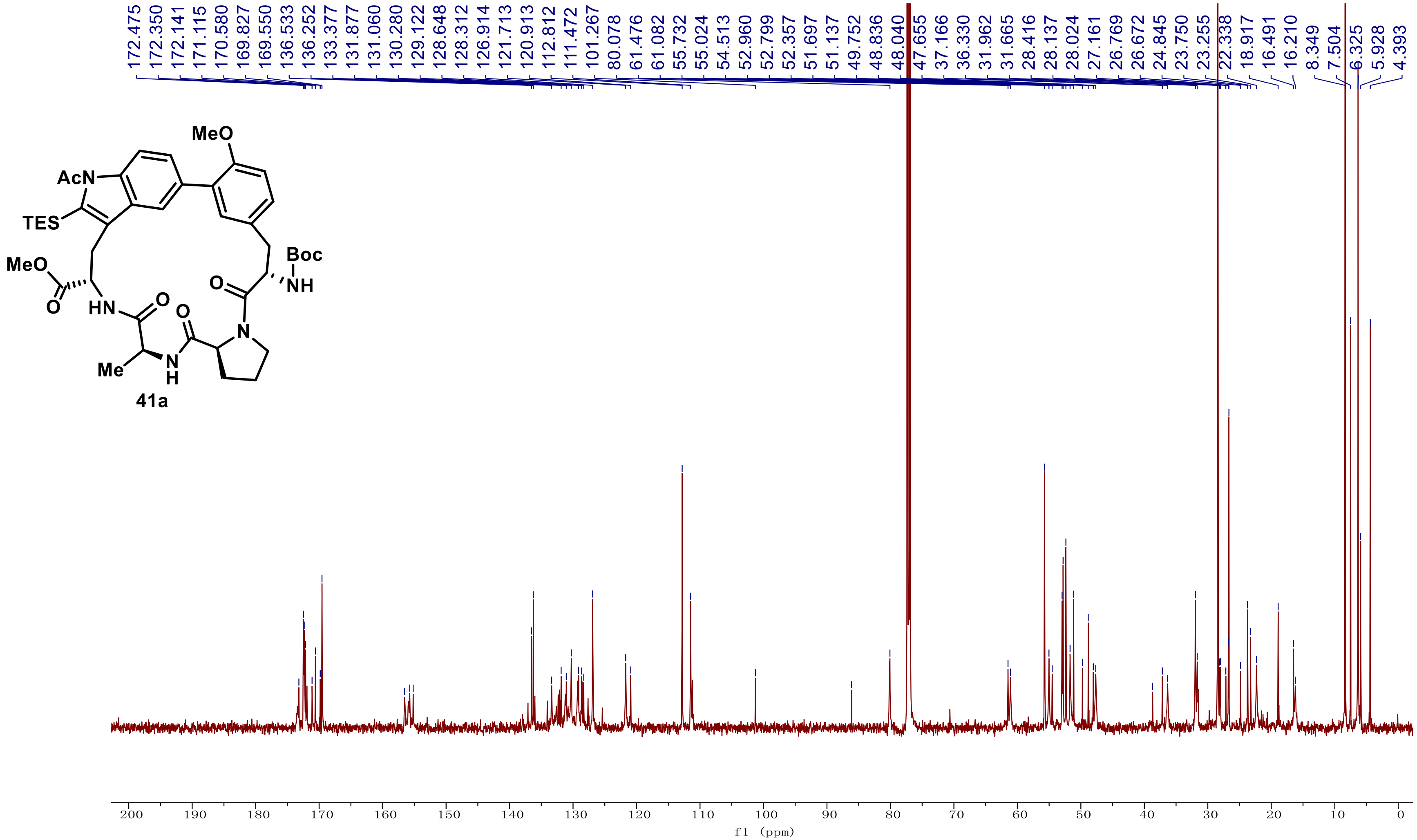

41a

Compound 40b ^{1}H NMR (600 MHz, $CDCl_3$)

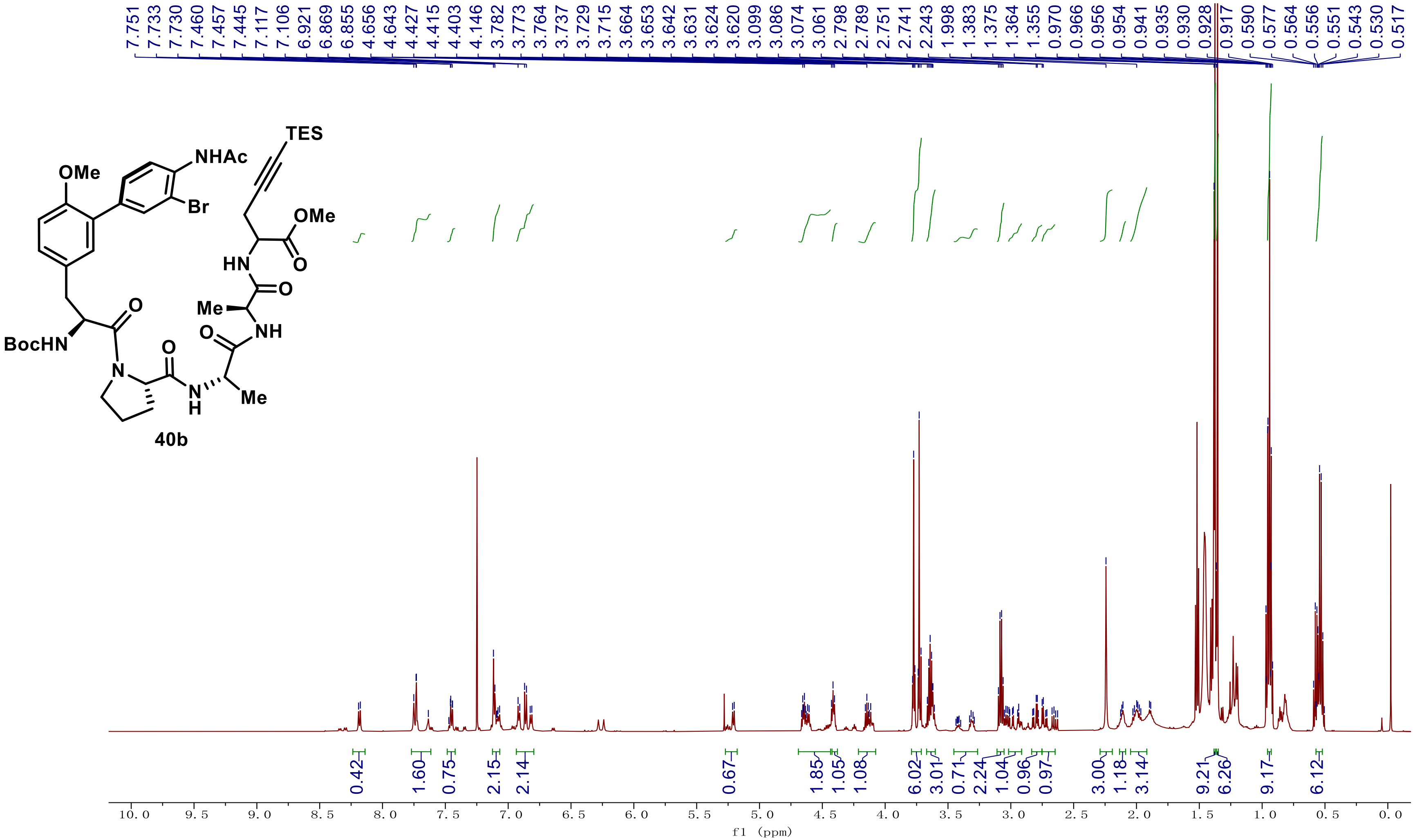

Compound 40b ^{13}C NMR (151 MHz, CDCl$_3$)

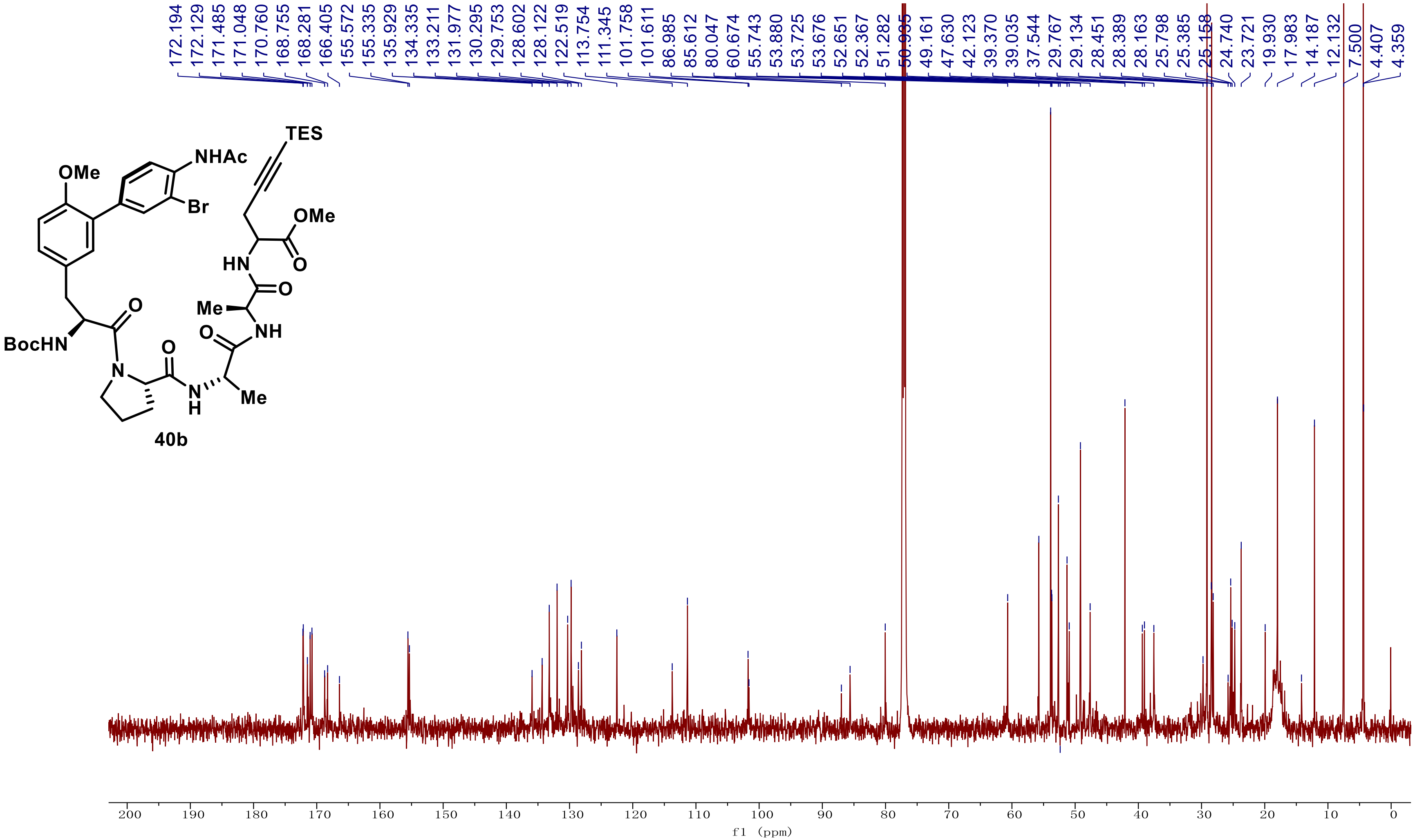

Compound 41b ^{1}H NMR (600 MHz, $CDCl_3$)

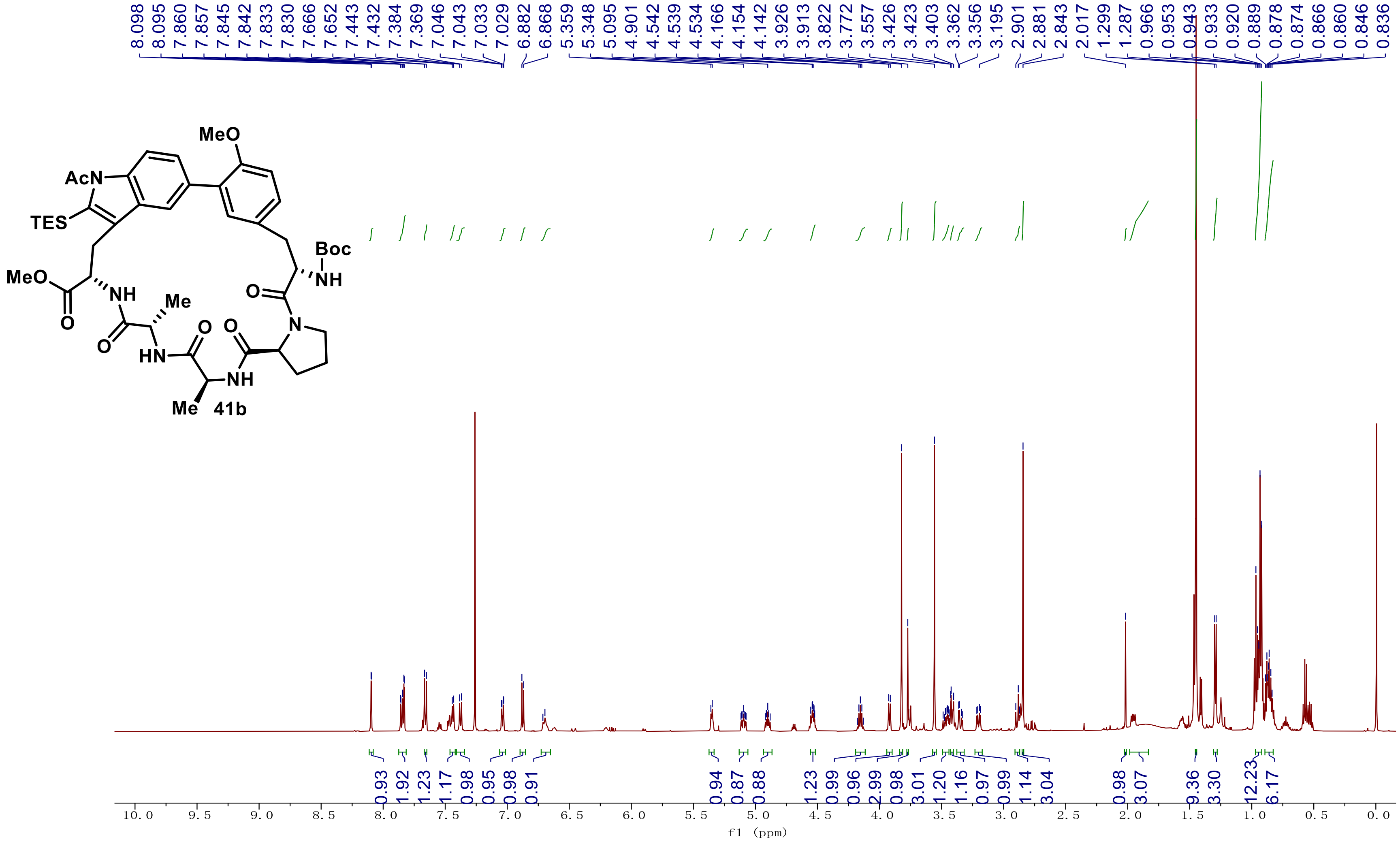

Compound 41b ^{13}C NMR (151 MHz, $CDCl_3$)

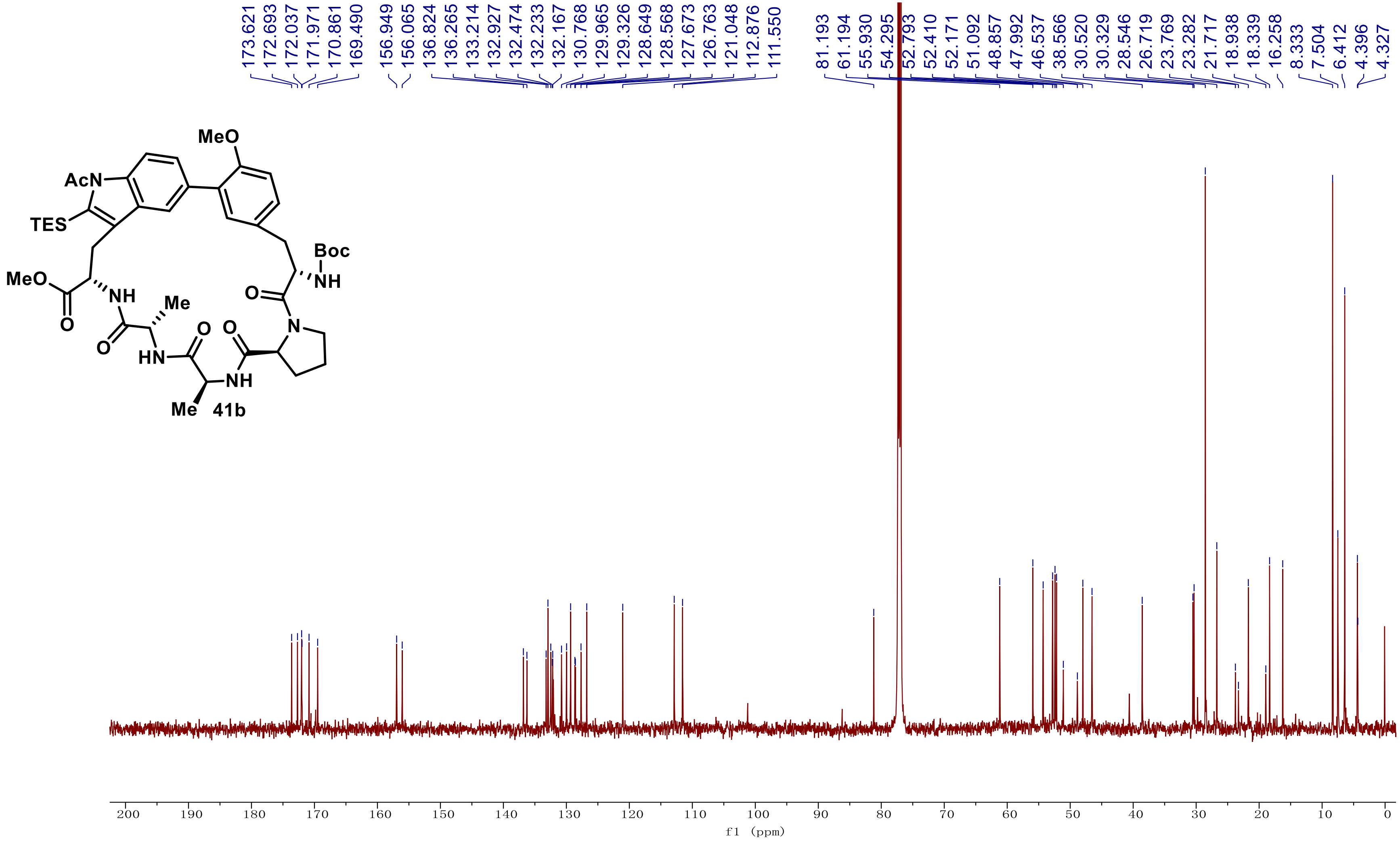

Compound 40c ^{1}H NMR (600 MHz, DMSO-*d6*)

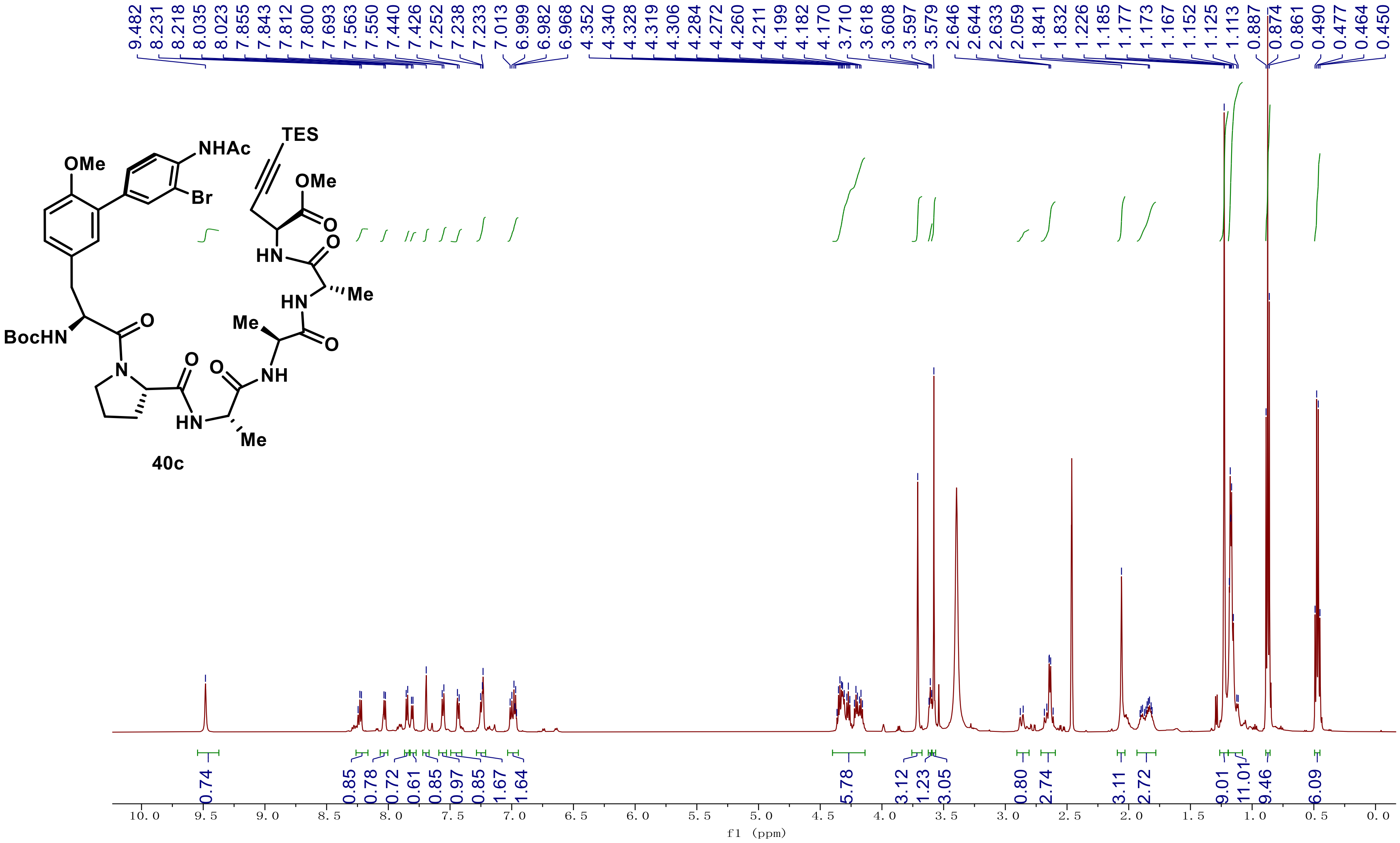

40c

Compound 40c ^{13}C NMR (151 MHz, DMSO-*d6*)

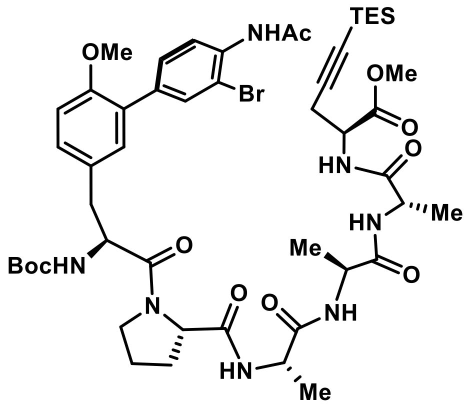

40c

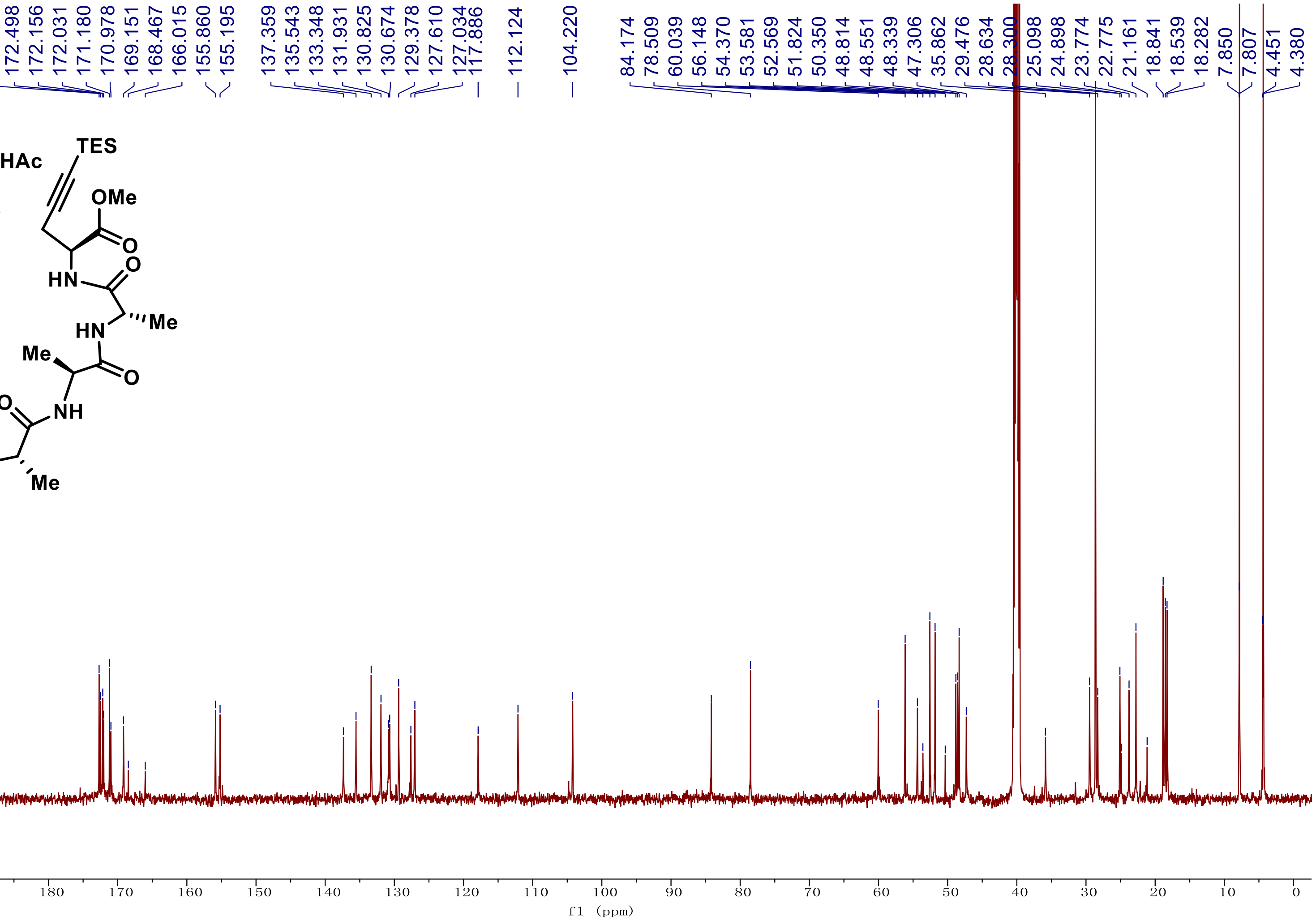

Compound 41c ^{1}H NMR (600 MHz, CDCl$_3$)

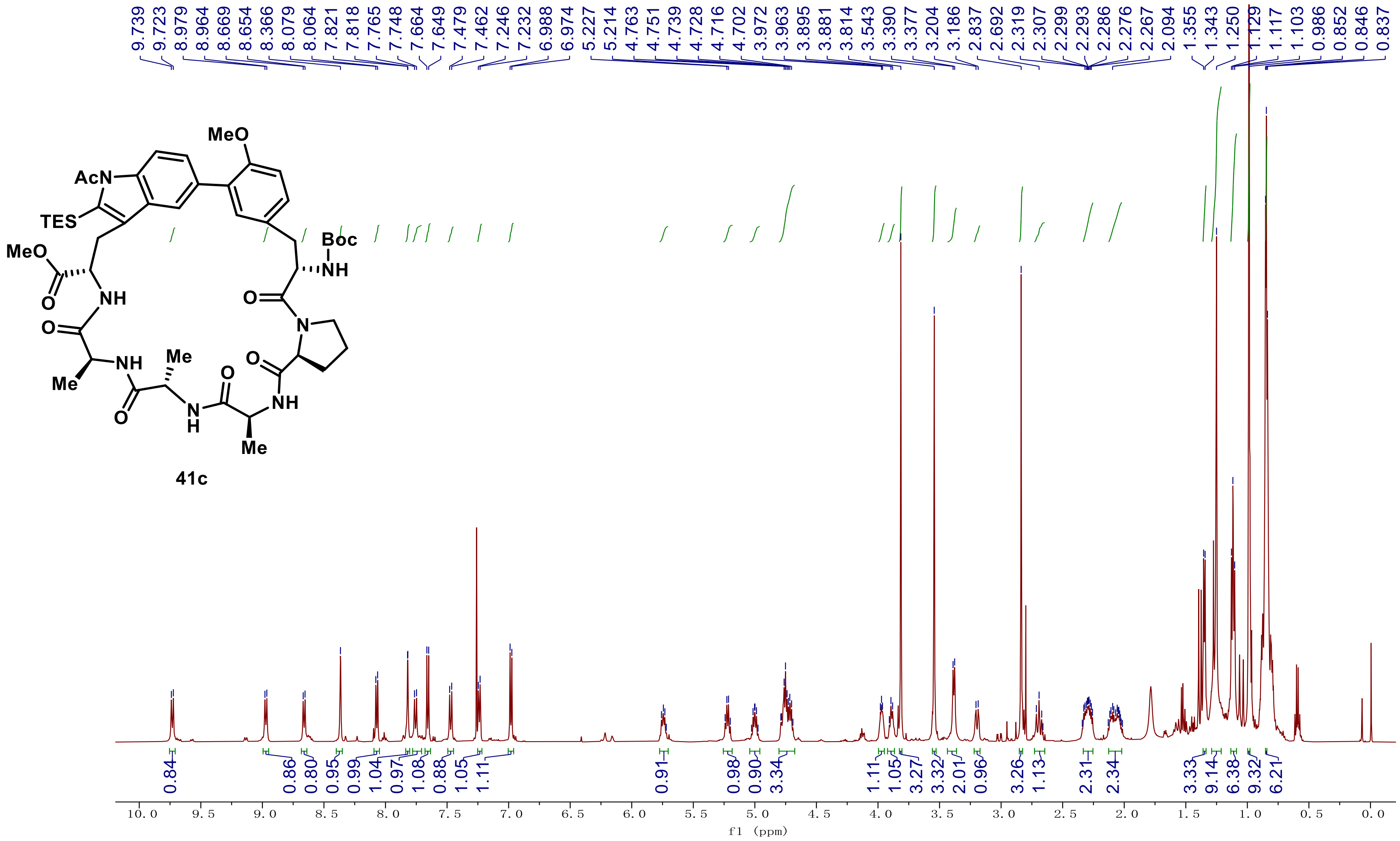

Compound 41cC NMR (151 MHz, CDCl₃)

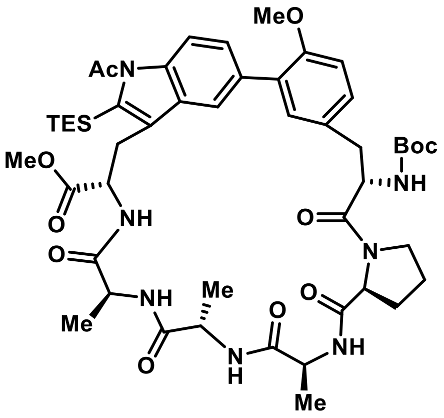

41c

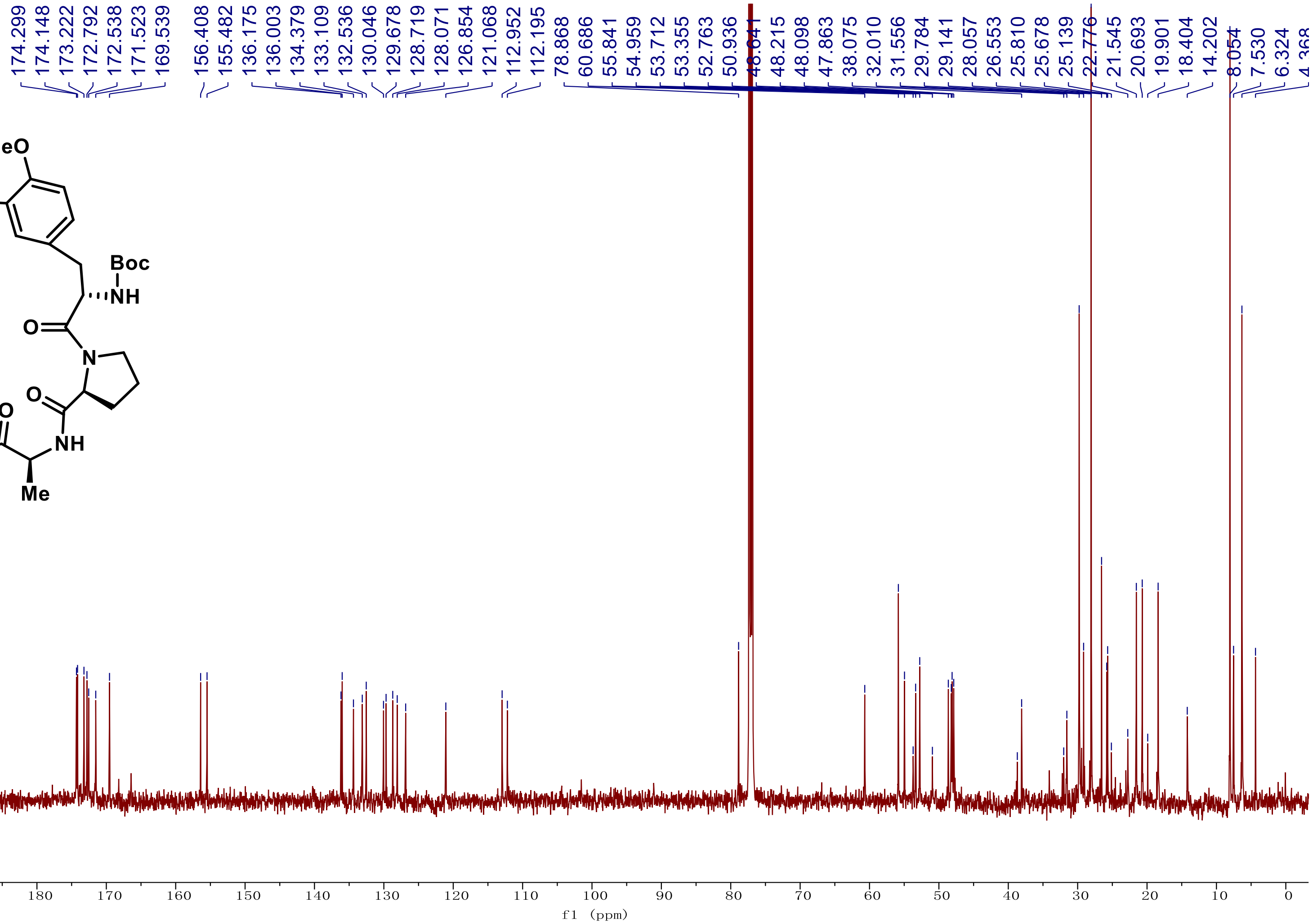

Compound S9 ^{1}H NMR (600 MHz, $CDCl_3$)

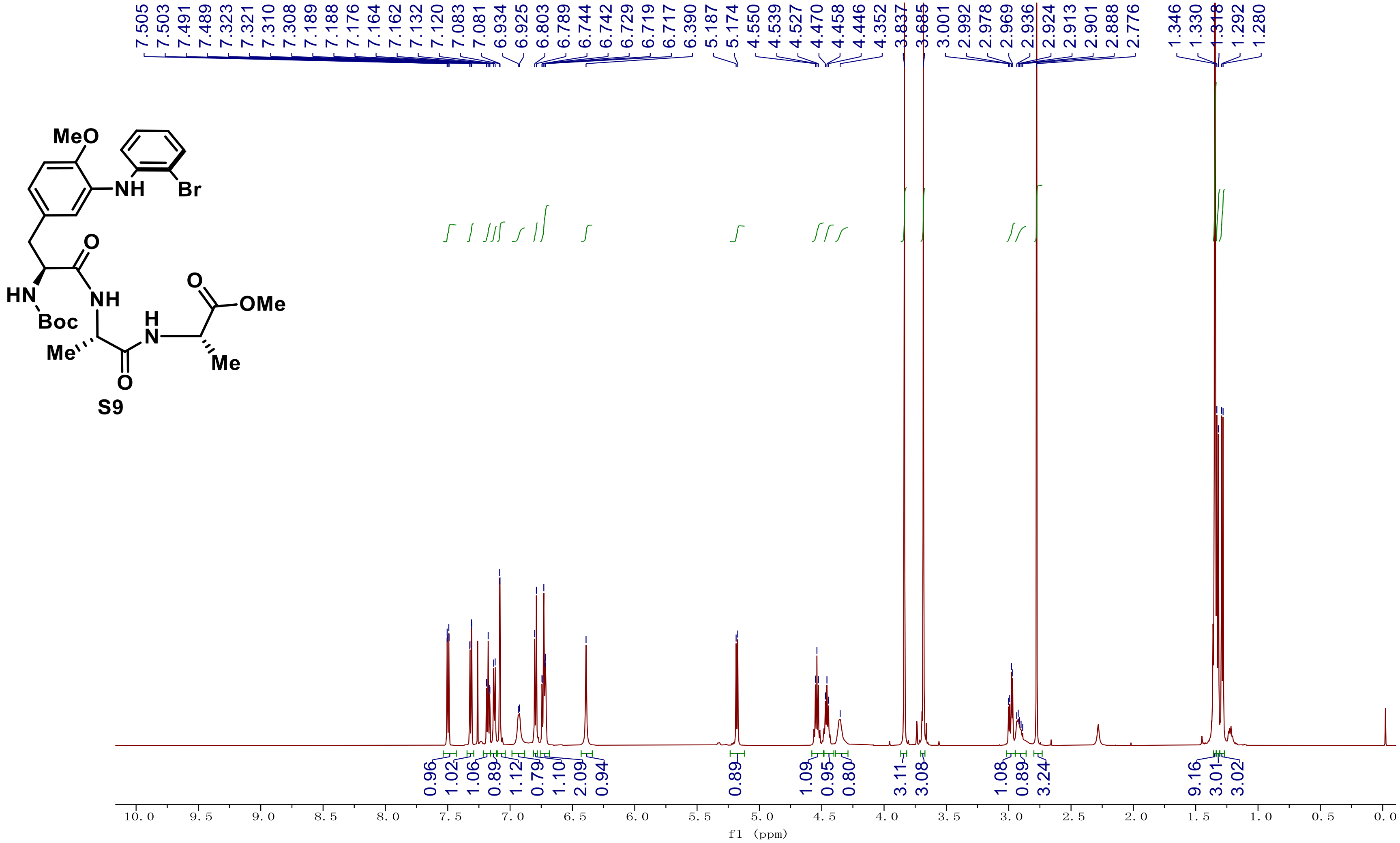

Compound S9 ^{13}C NMR (151 MHz, CDCl$_3$)

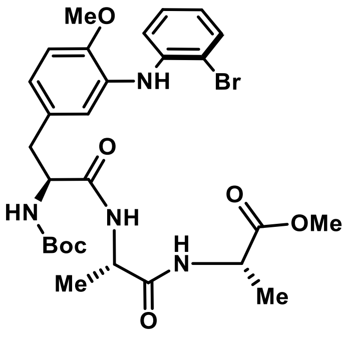

S9

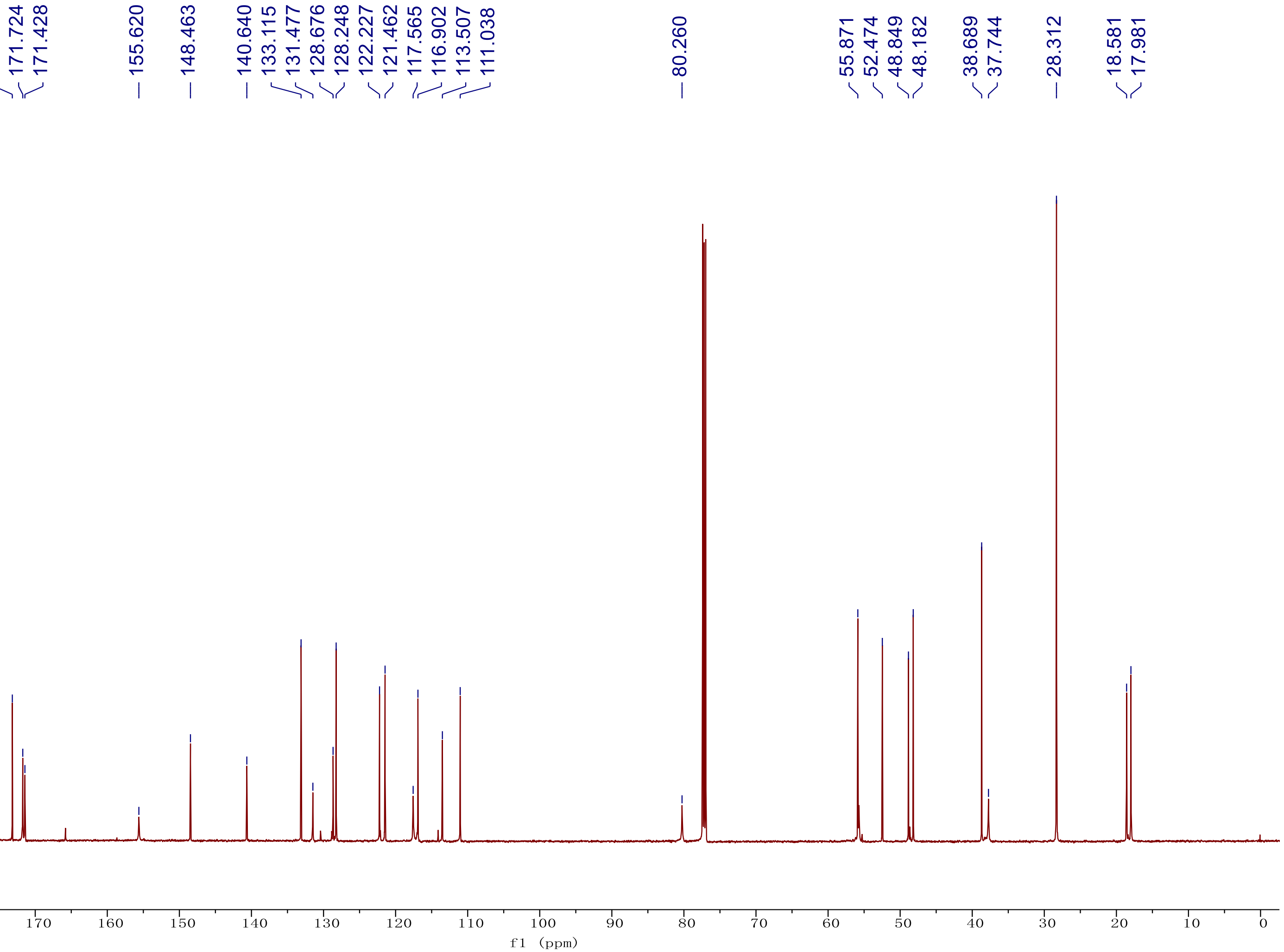

Compound 42a ^{1}H NMR (600 MHz, CDCl$_3$)

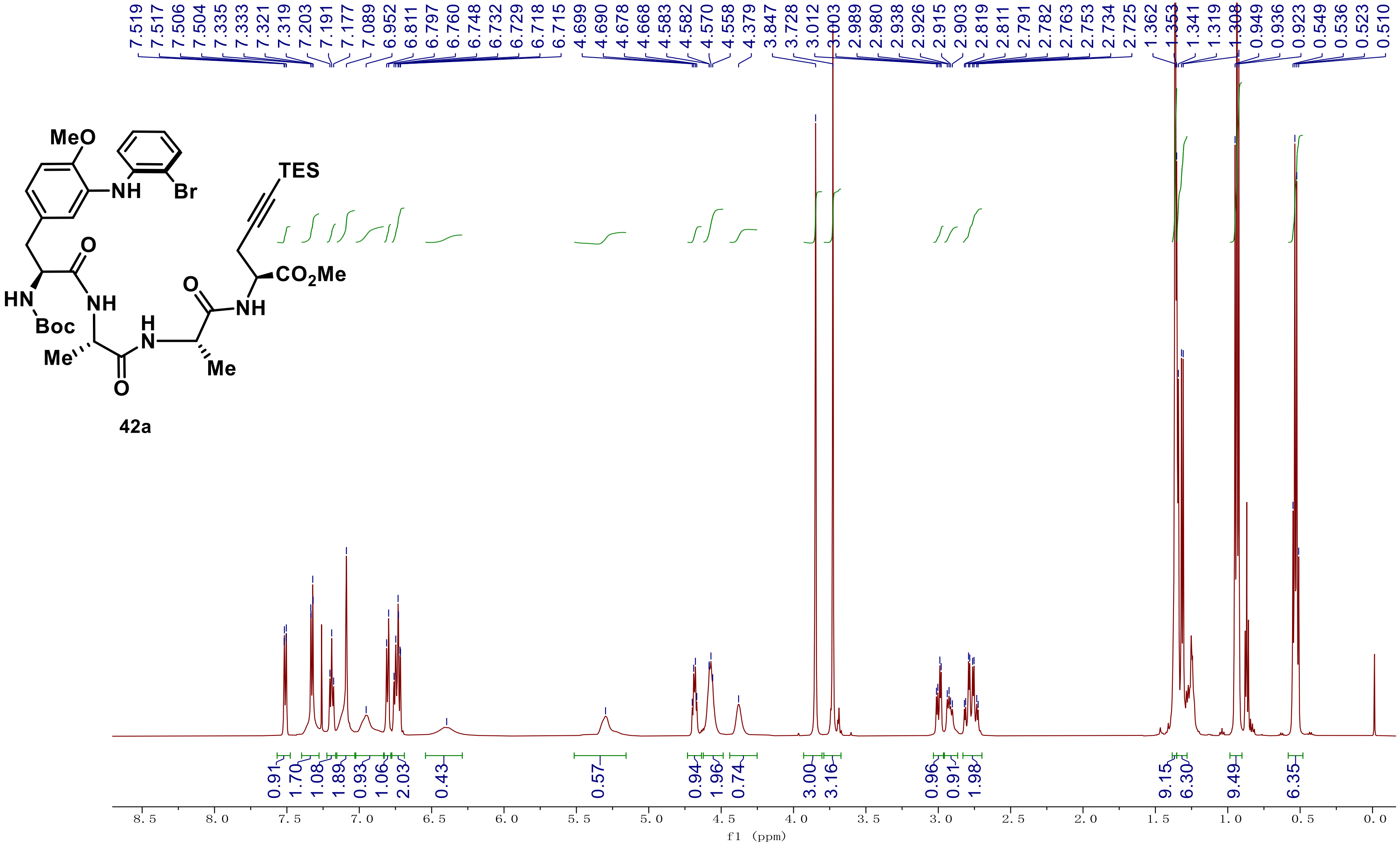

Compound 42a ^{13}C NMR (151 MHz, $CDCl_3$)

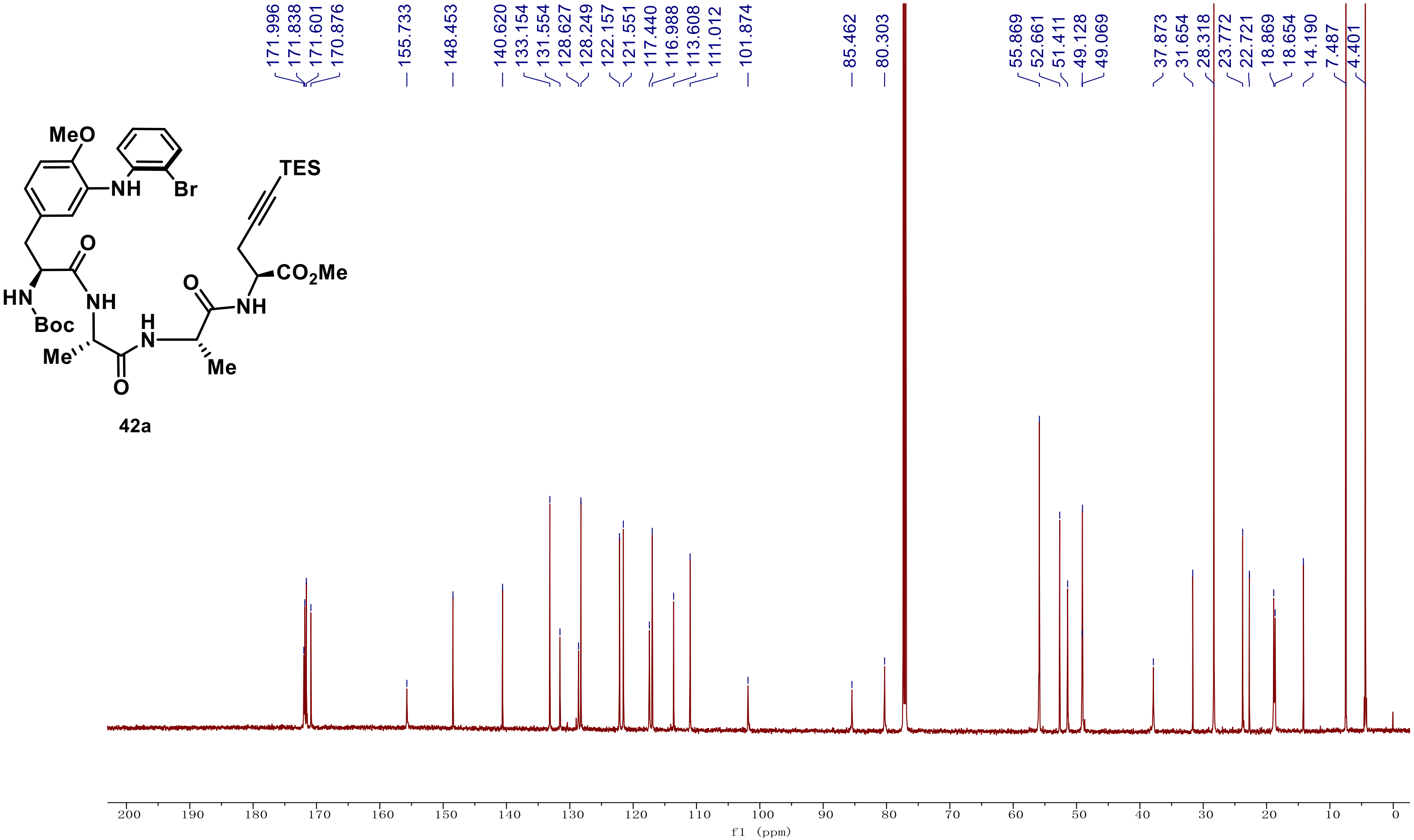

Compound 43a ^{1}H NMR (600 MHz, $CDCl_3$)

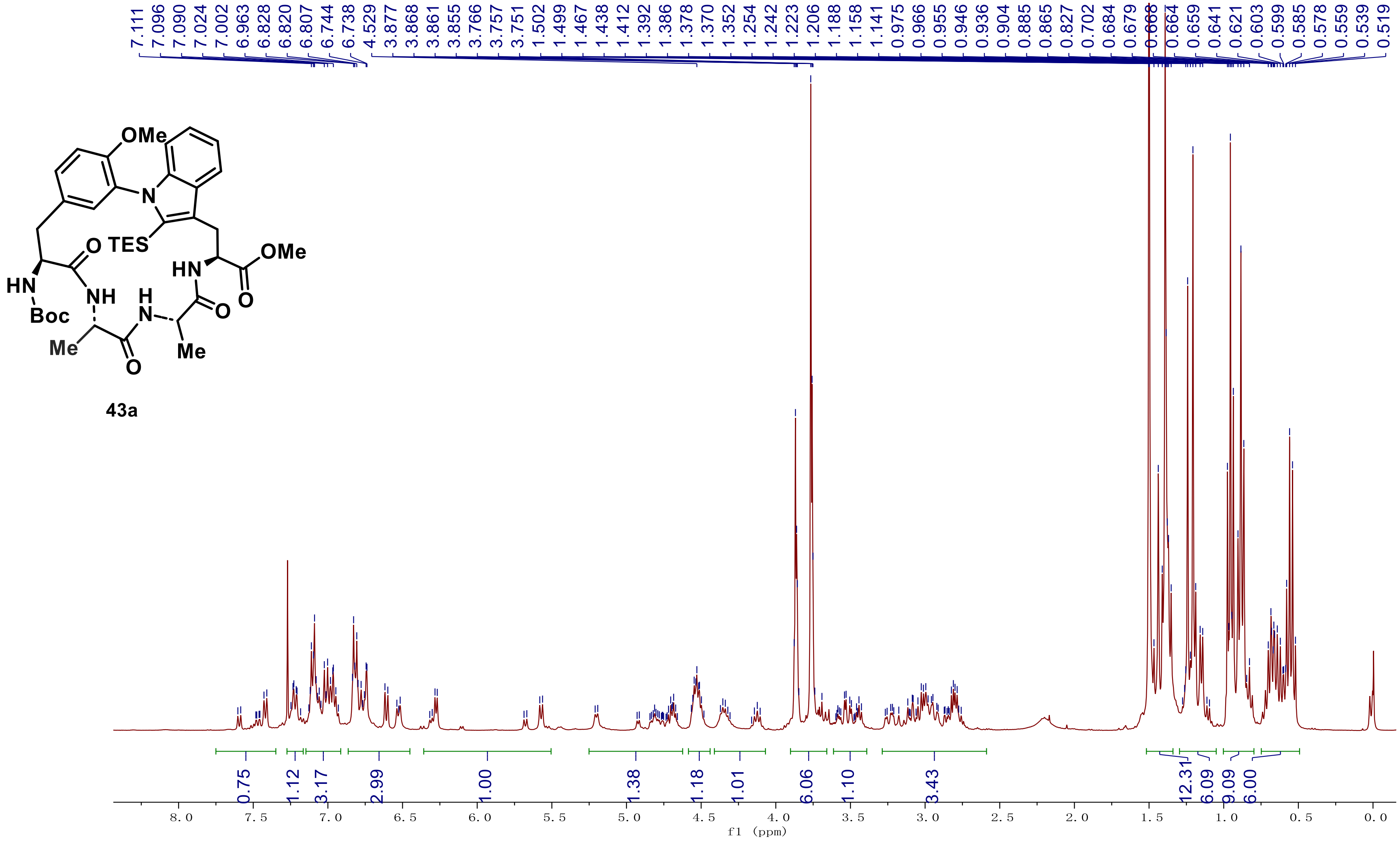

Compound 43a ^{13}C NMR (151 MHz, $CDCl_3$)

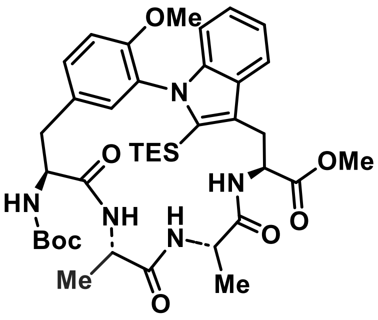

43a

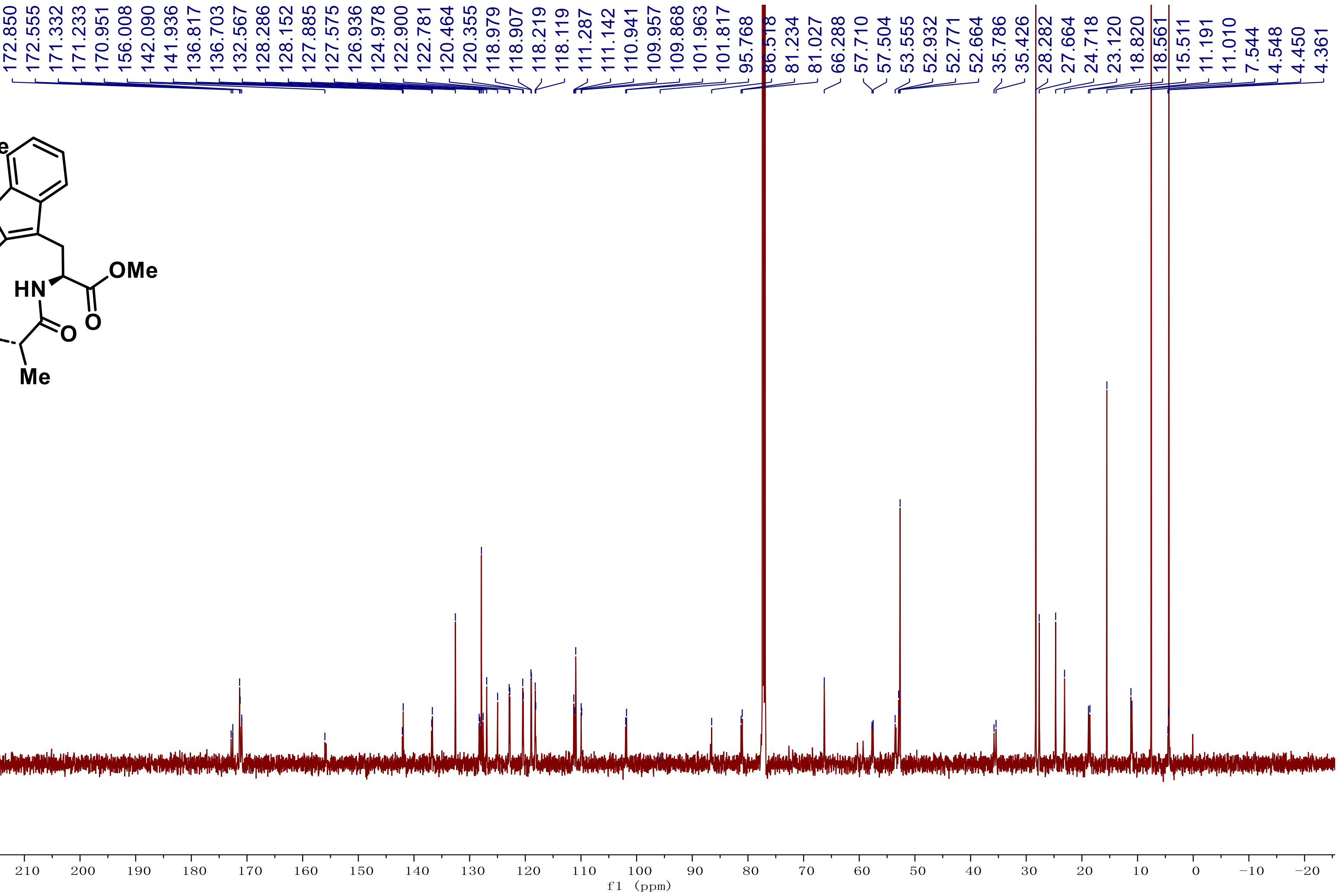

Compound 42b ^{1}H NMR (600 MHz, $CDCl_3$)

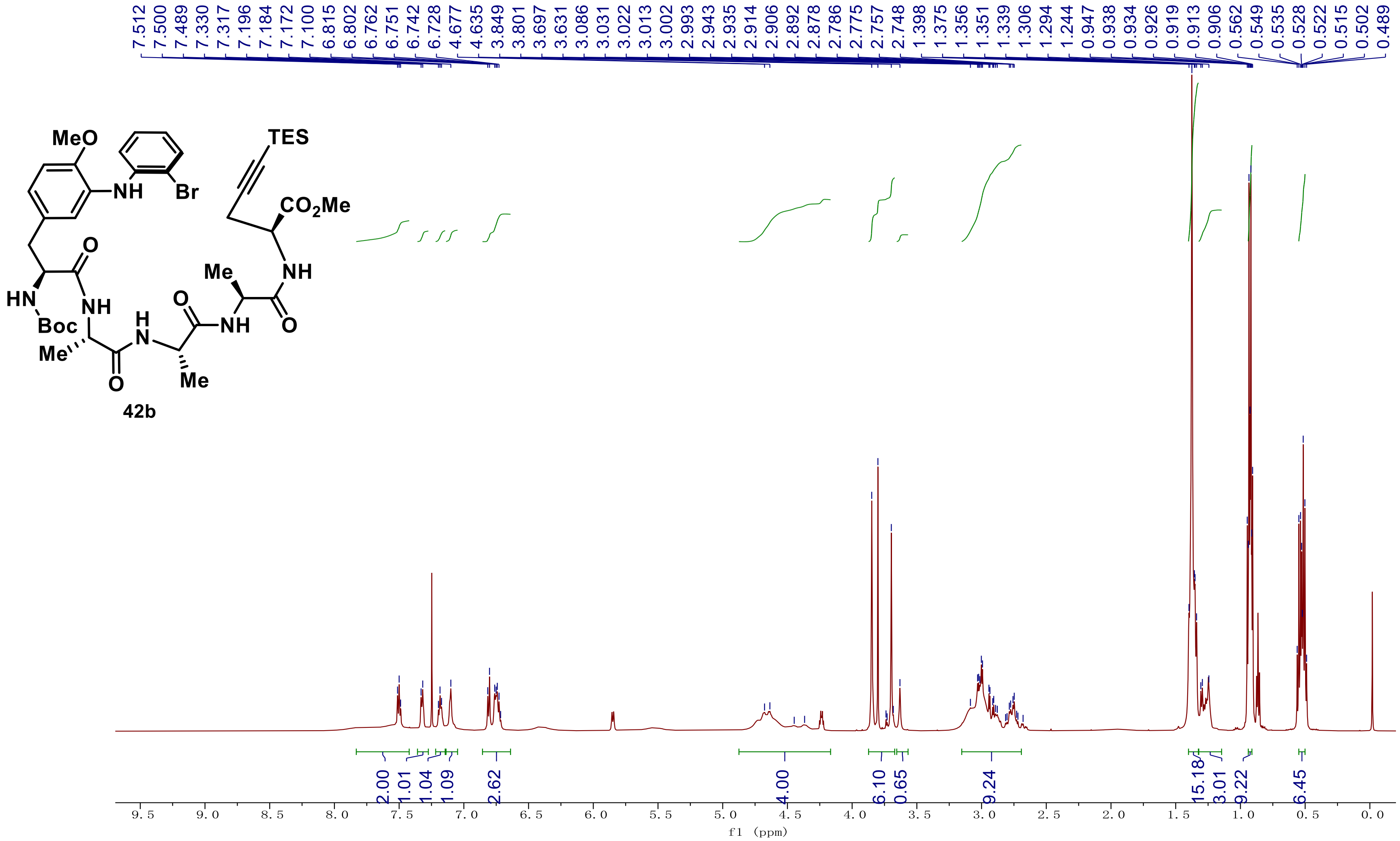

Compound 42b ^{13}C NMR (151 MHz, $CDCl_3$)

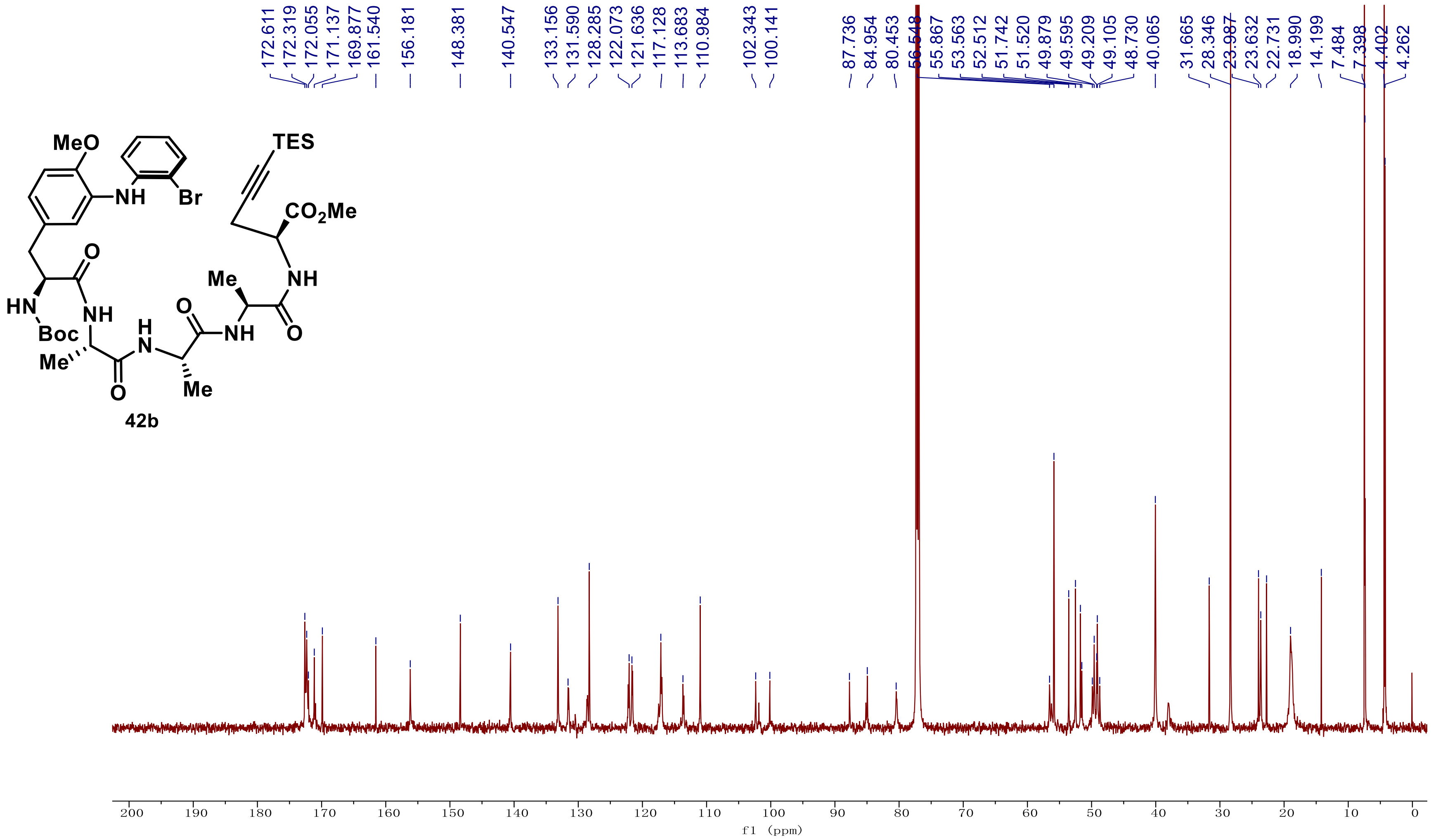

Compound 43b ¹H NMR (600 MHz, CDCl₃)

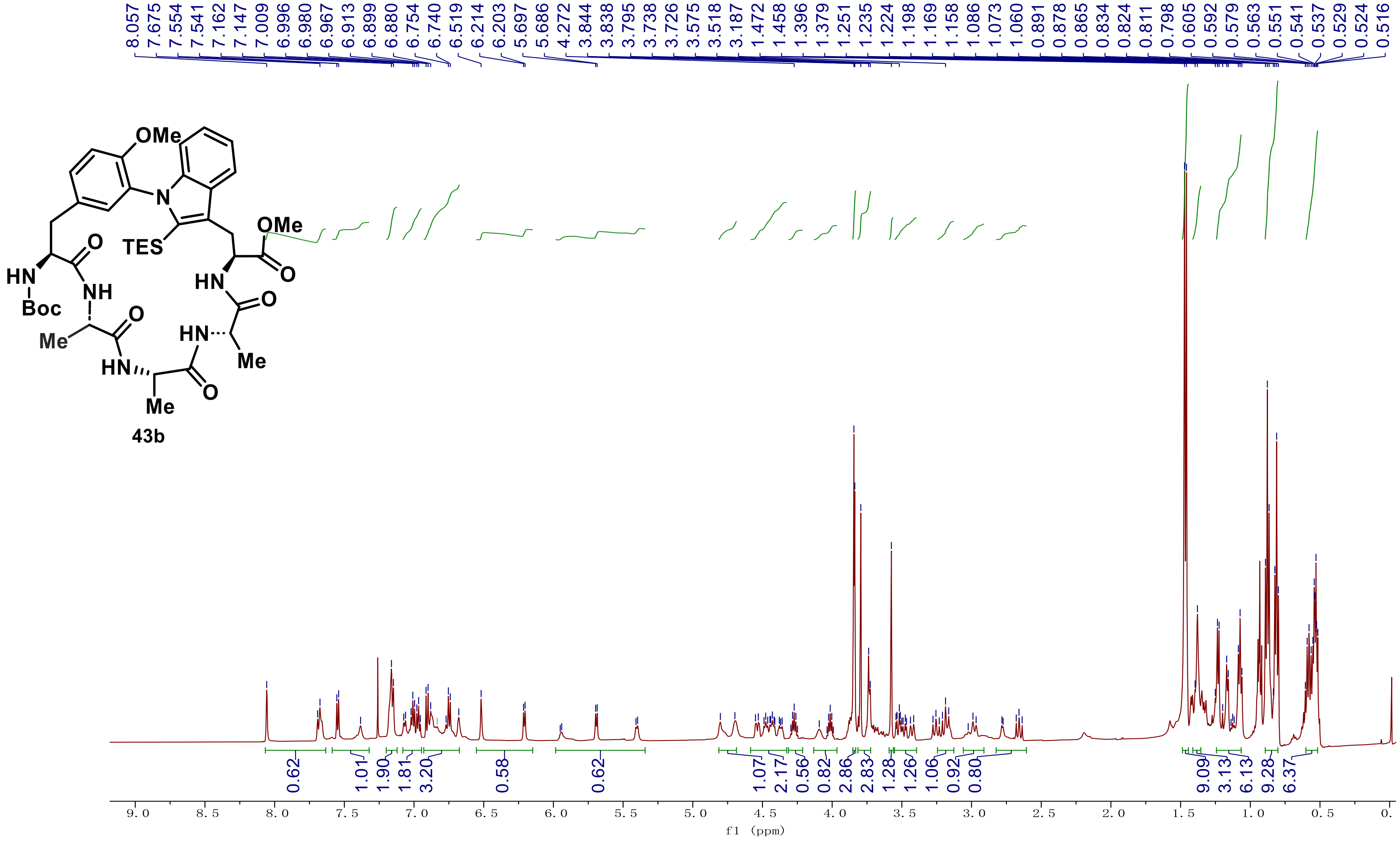

Compound 43b ^{13}C NMR (151 MHz, $CDCl_3$)

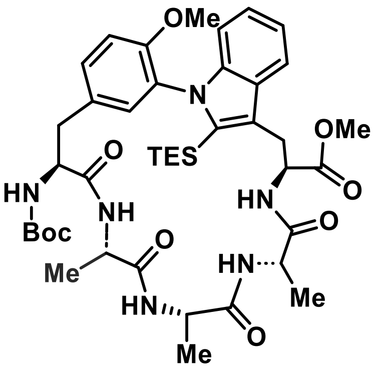

43b

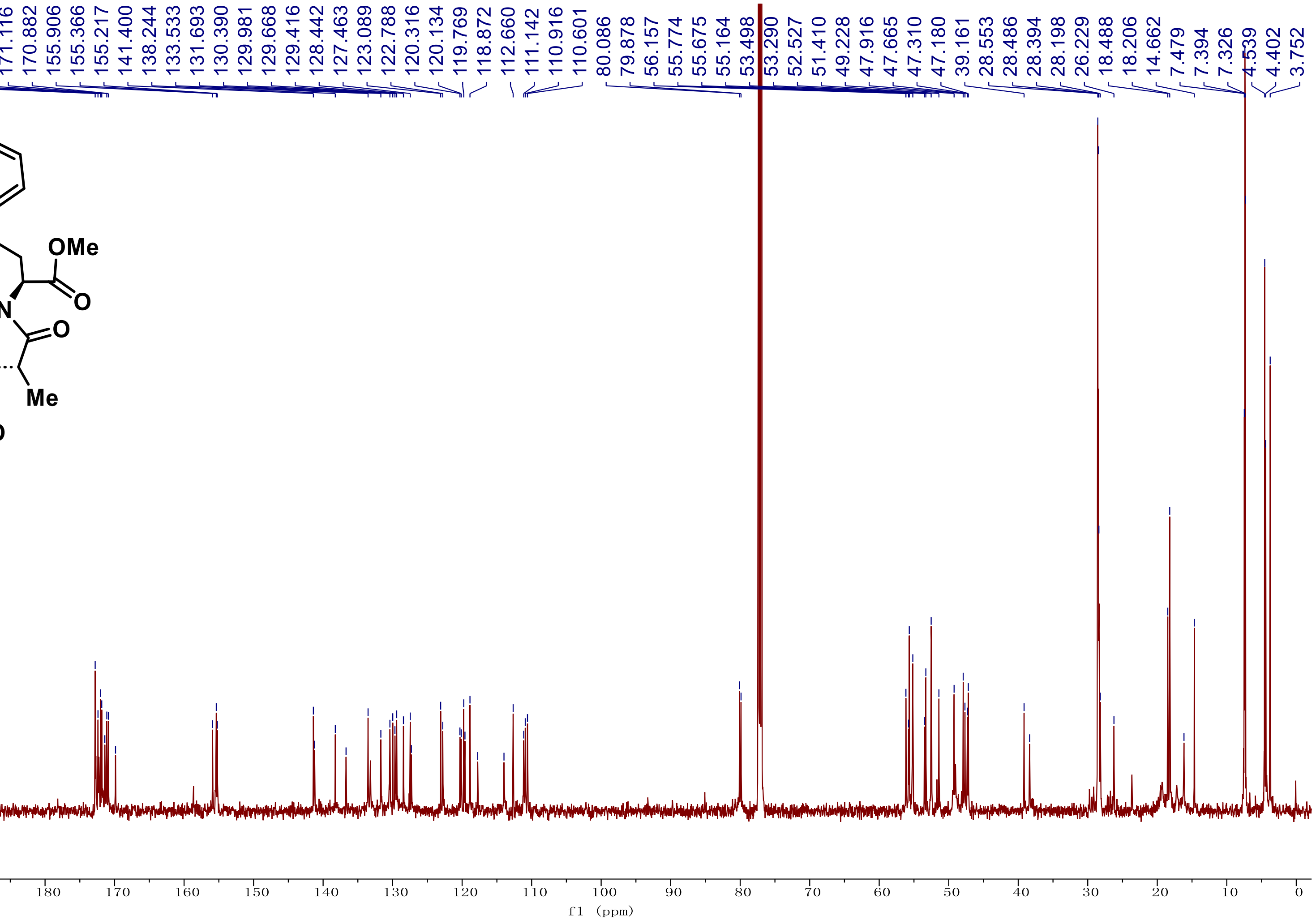

Compound S11a ¹H NMR (600 MHz, CDCl₃)

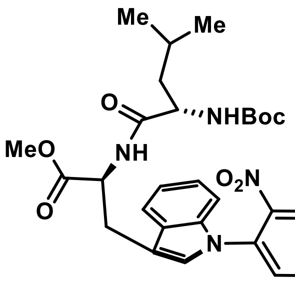

S11a

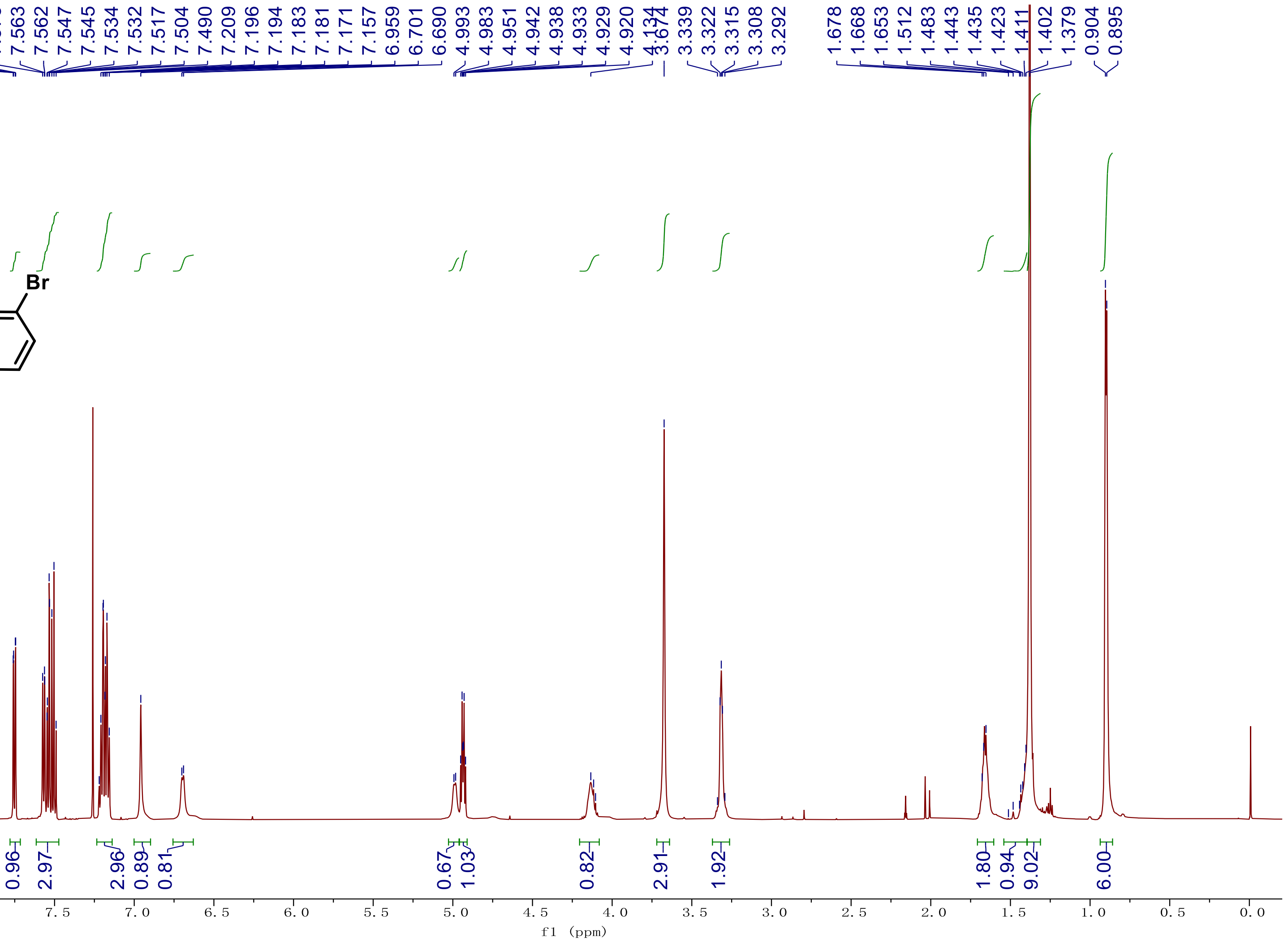

Compound S11a ^{13}C NMR (151 MHz, CDCl$_3$)

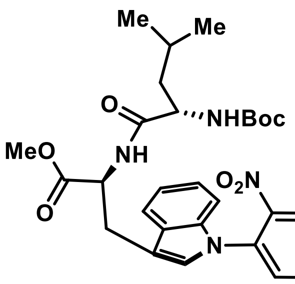

S11a

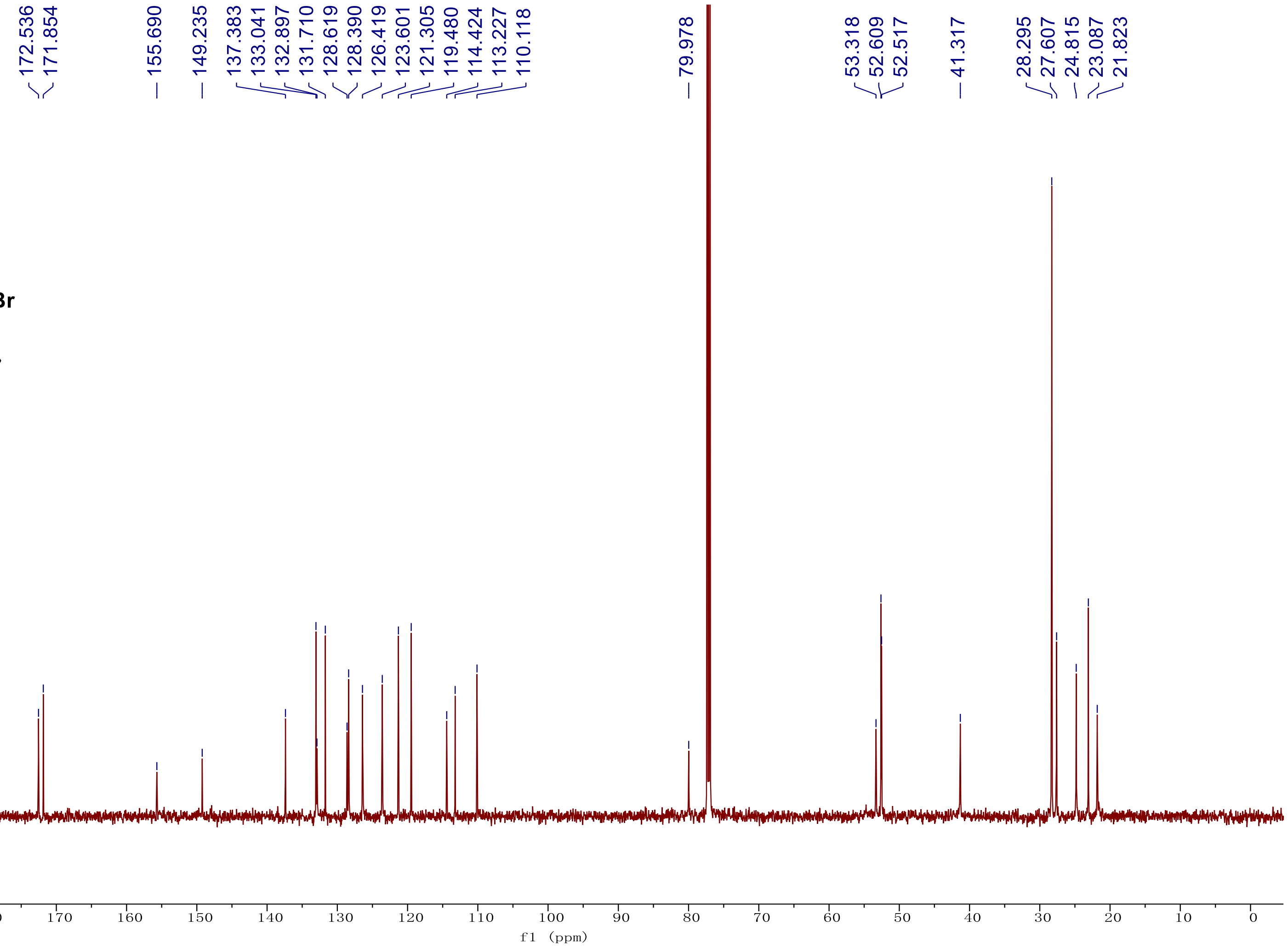

Compound S11b ^{1}H NMR (600 MHz, $CDCl_3$)

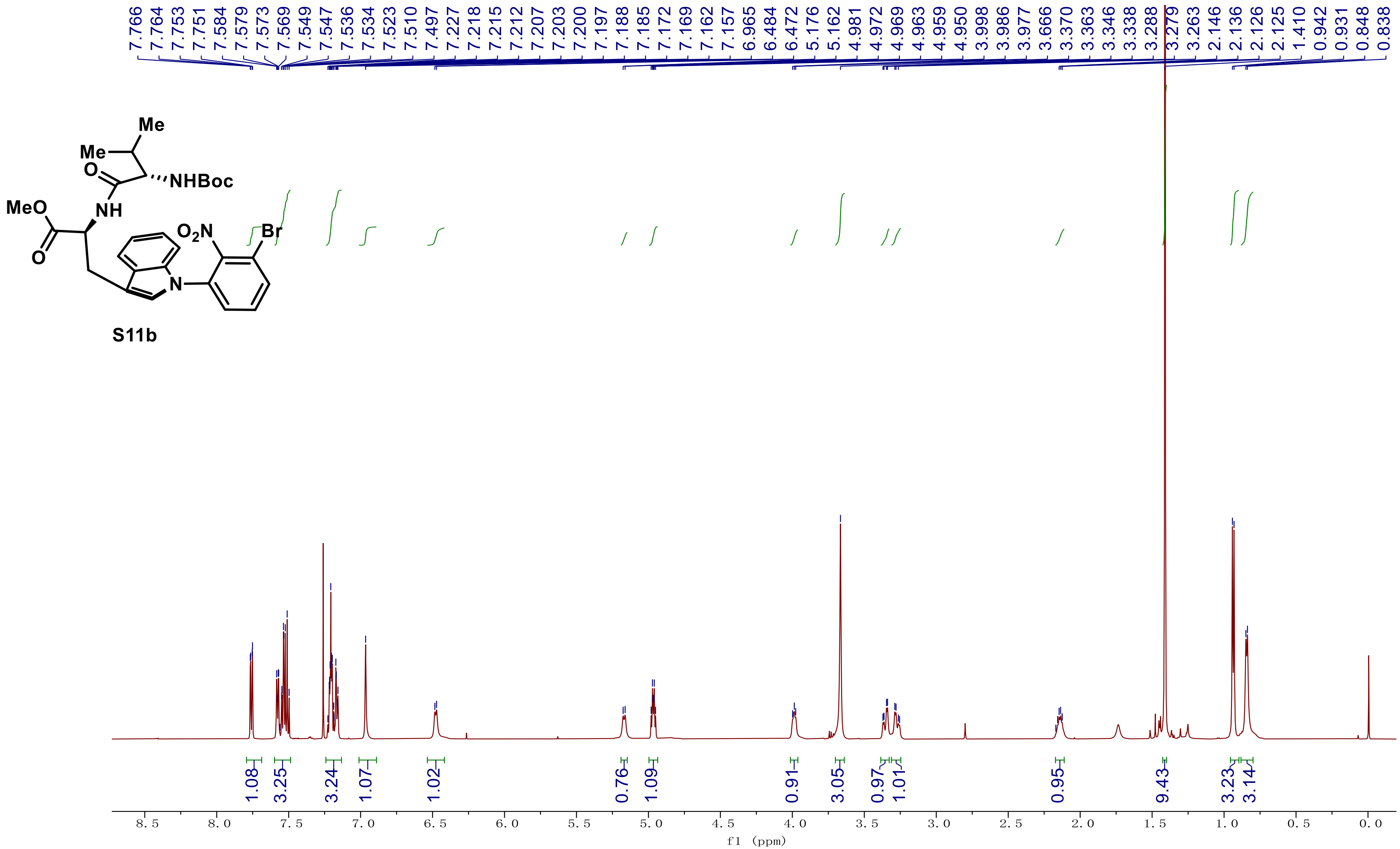

S11b

Compound S11b ^{13}C NMR (151 MHz, $CDCl_3$)

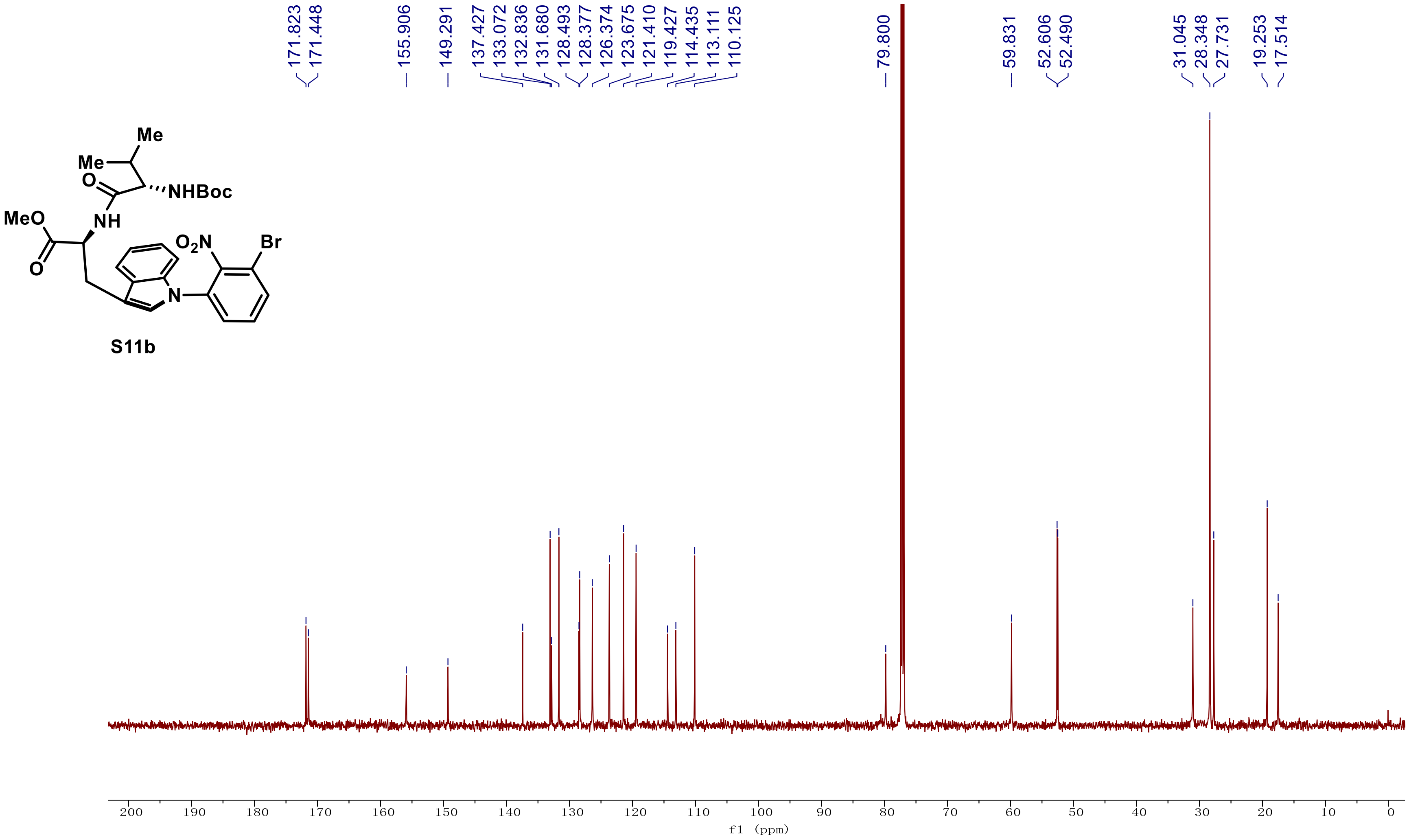

S11b

Compound S11c ^{1}H NMR (600 MHz, $CDCl_3$)

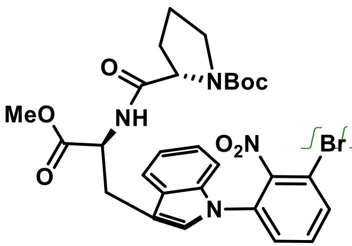

S11c

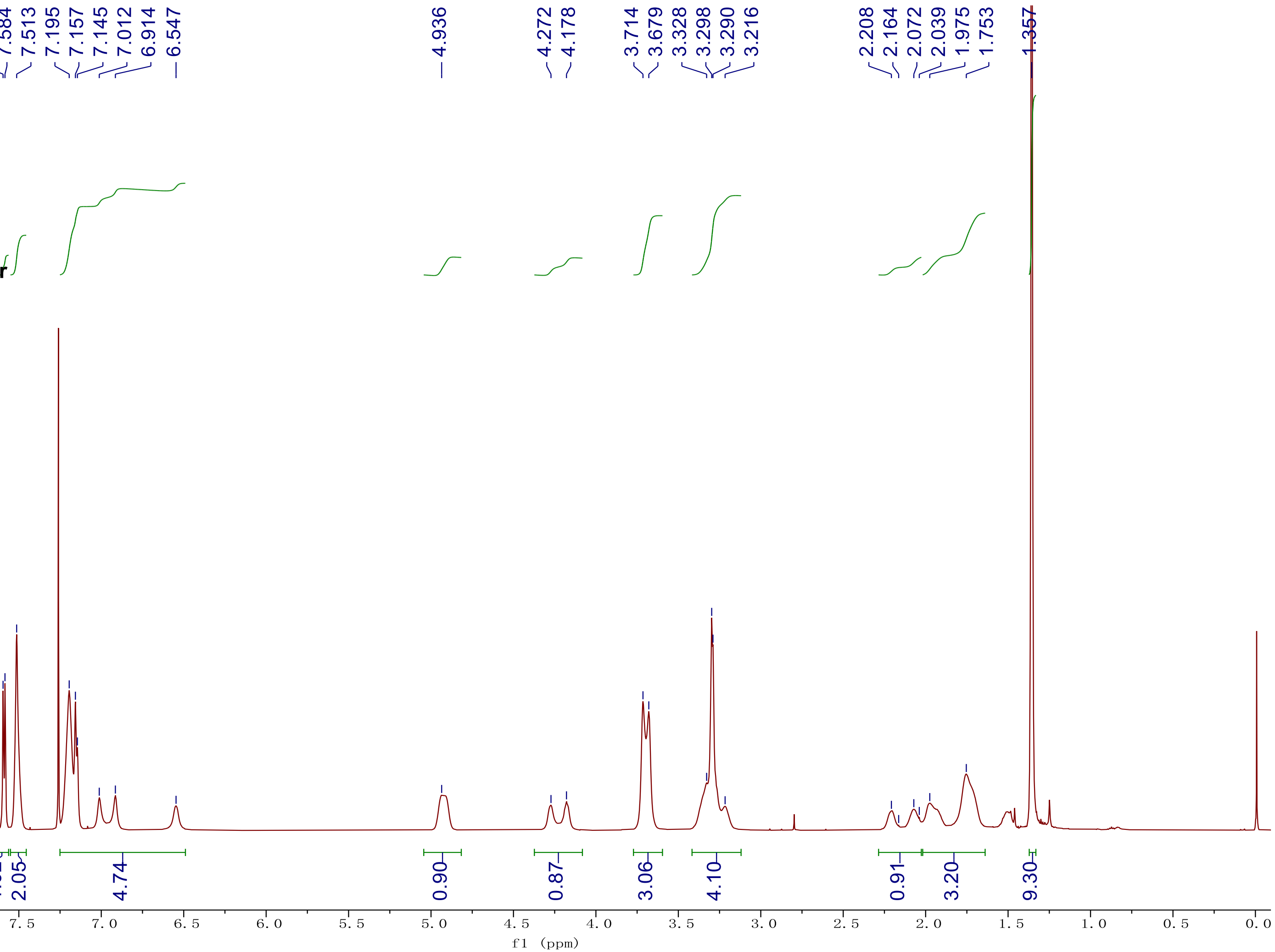

Compound S11c ^{13}C NMR (151 MHz, CDCl$_3$)

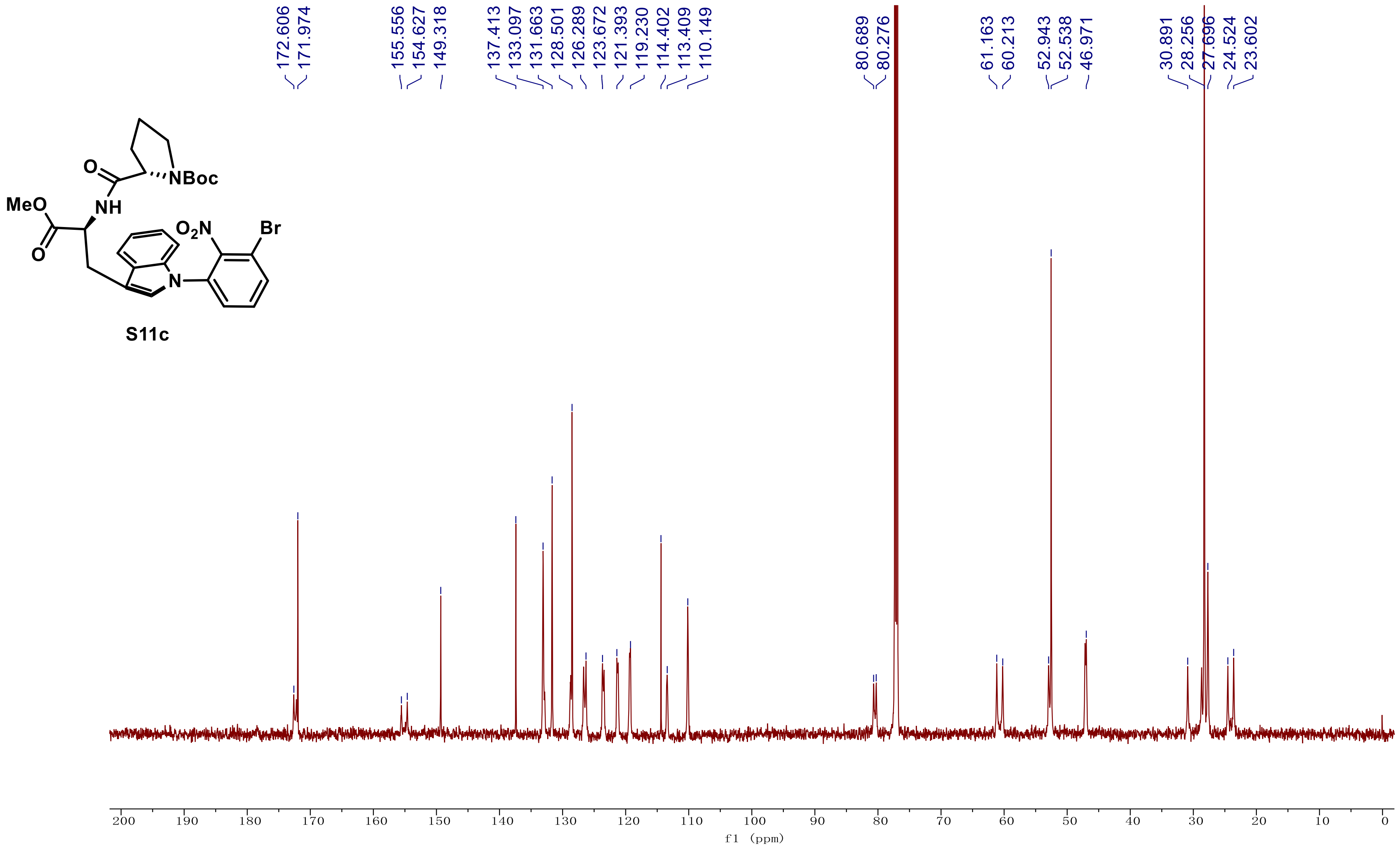

Compound 45b ^{1}H NMR (600 MHz, $CDCl_3$)

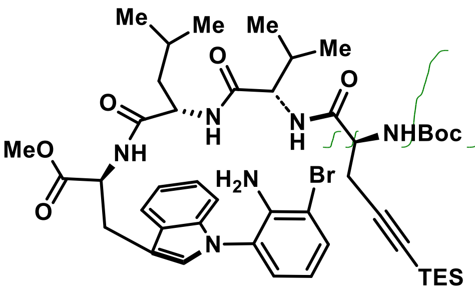

45b

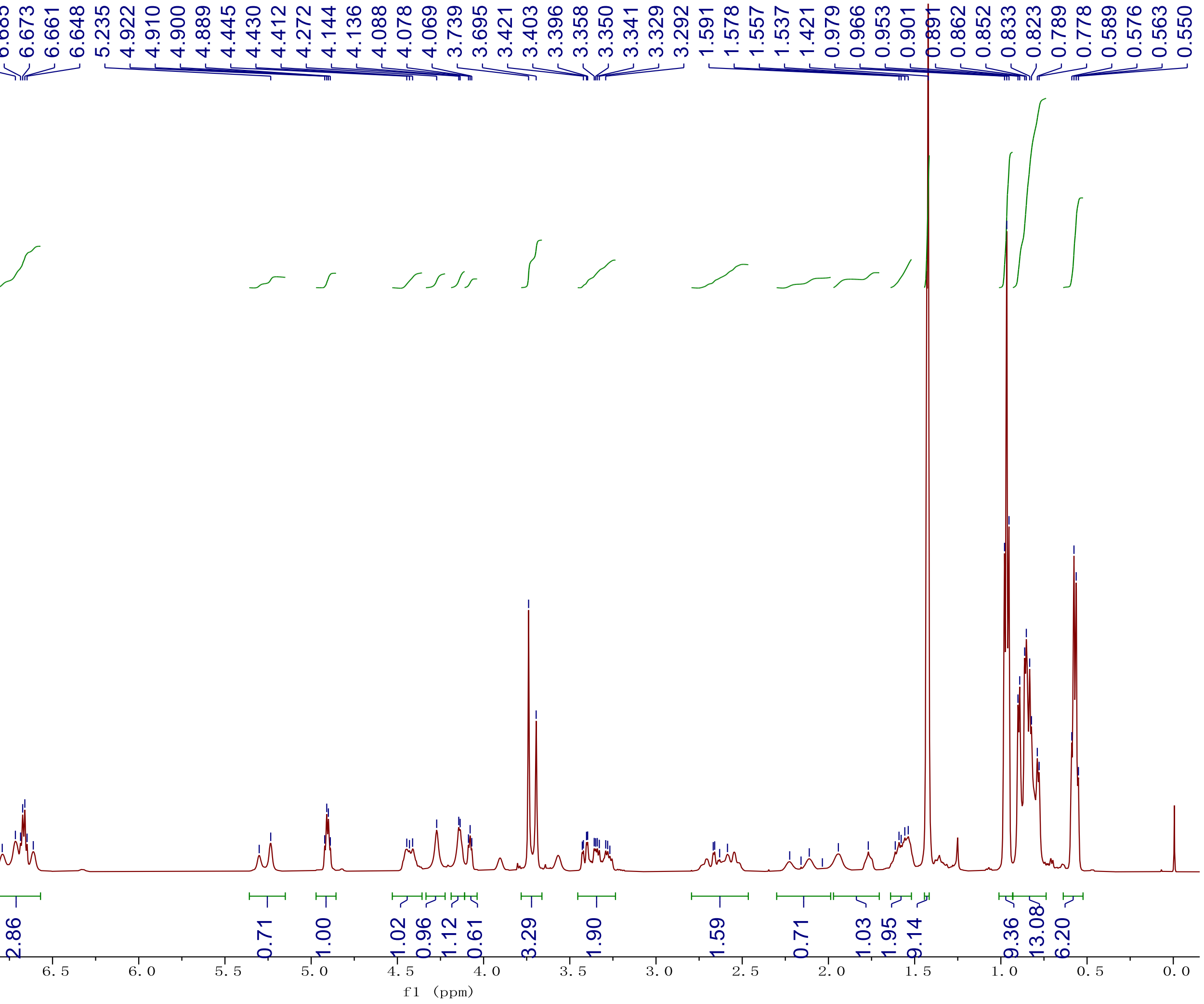

Compound 45b ^{13}C NMR (151 MHz, CDCl$_3$)

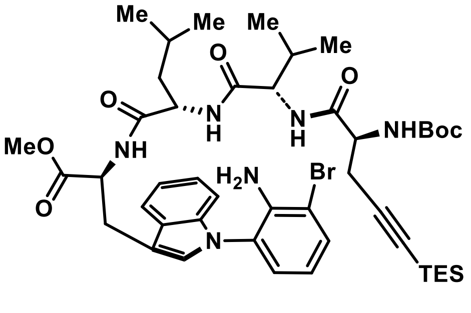

45b

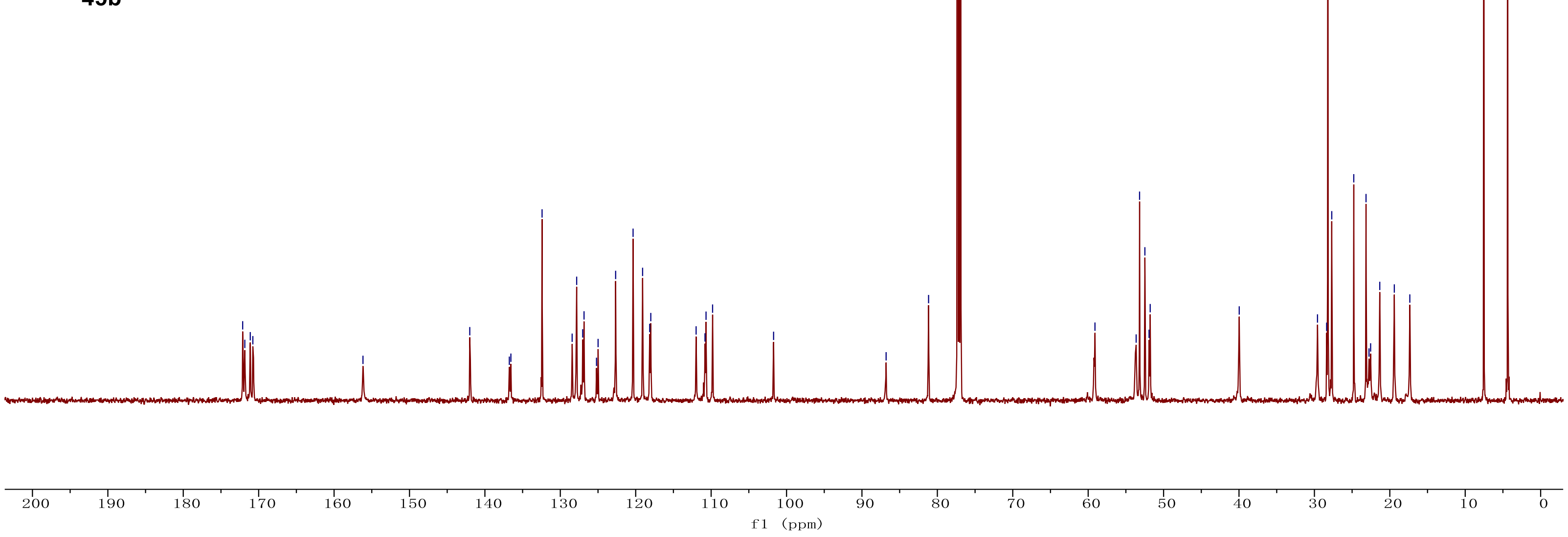

Compound 45c ^{1}H NMR (600 MHz, $CDCl_3$)

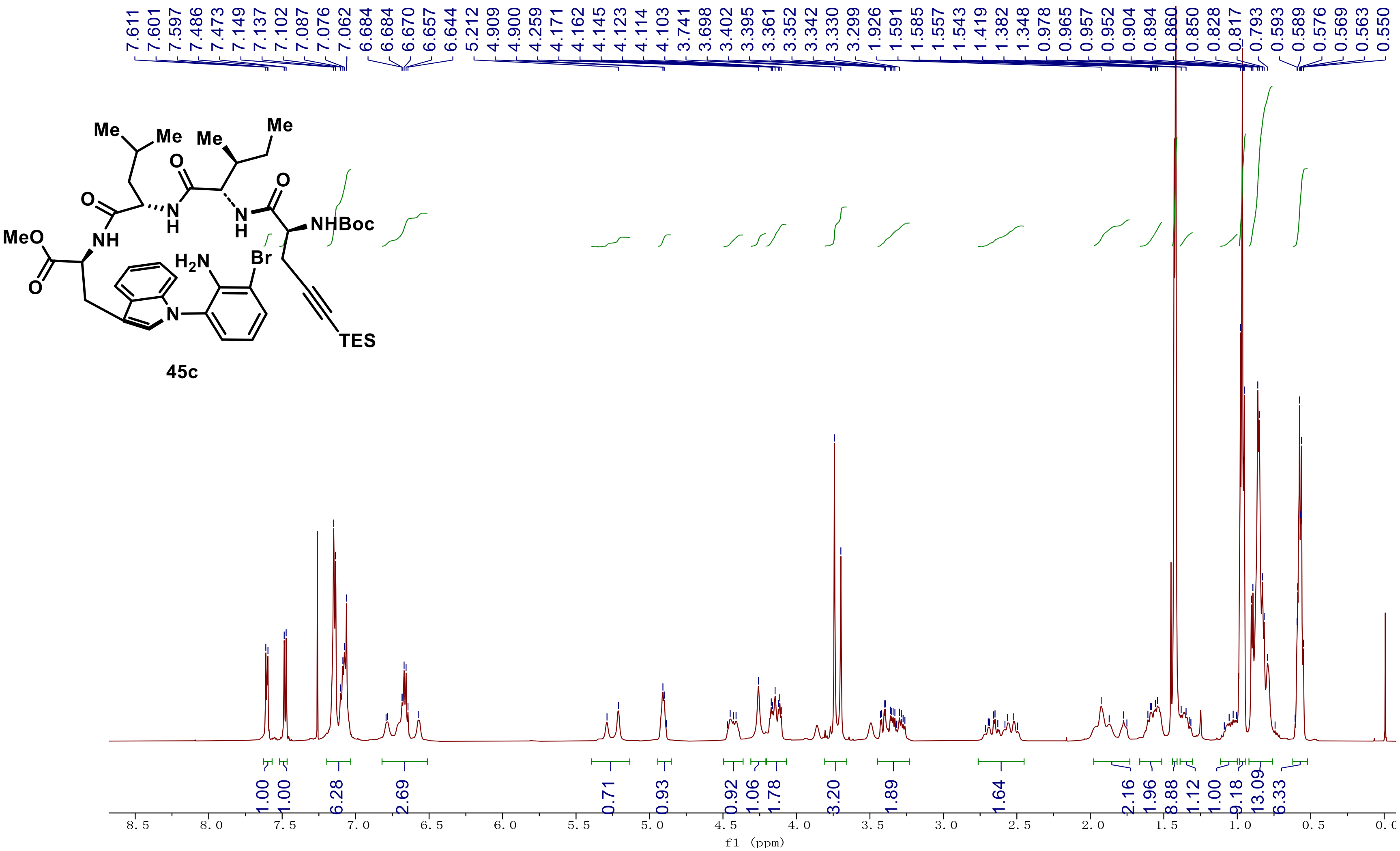

Compound 45c ^{13}C NMR (151 MHz, $CDCl_3$)

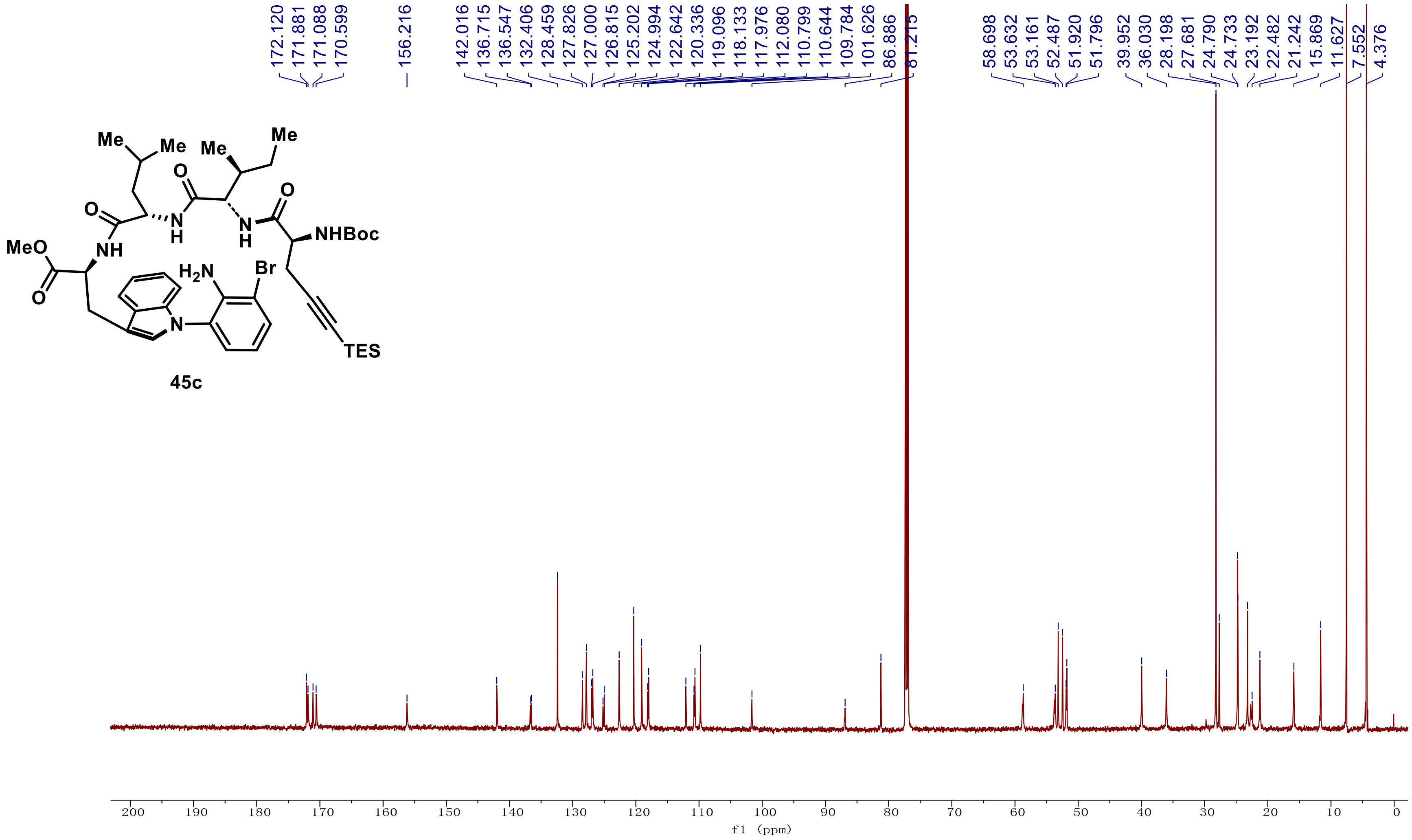

Compound 45d ¹H NMR (600 MHz, CDCl₃)

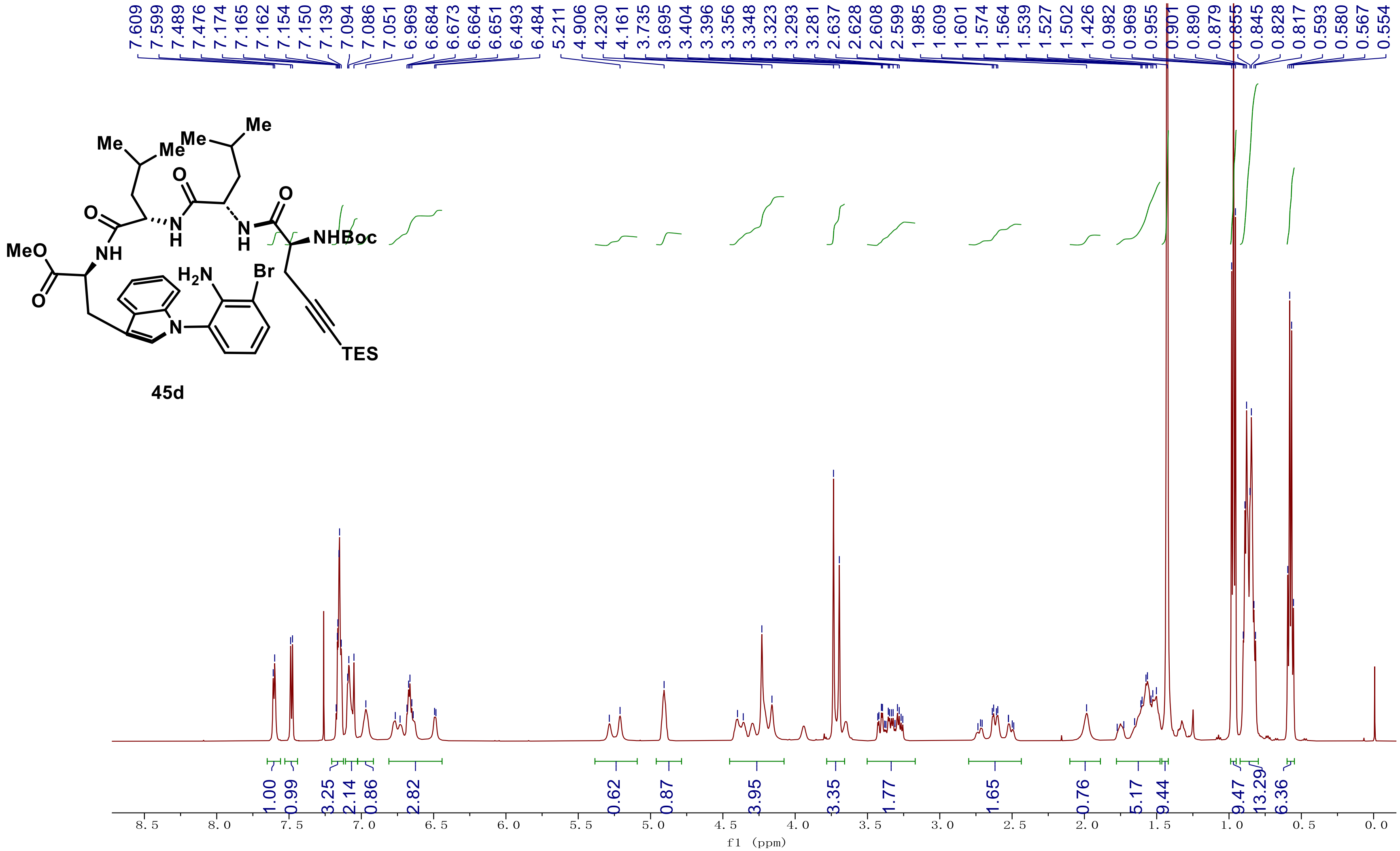

Compound 45d ^{13}C NMR (151 MHz, $CDCl_3$)

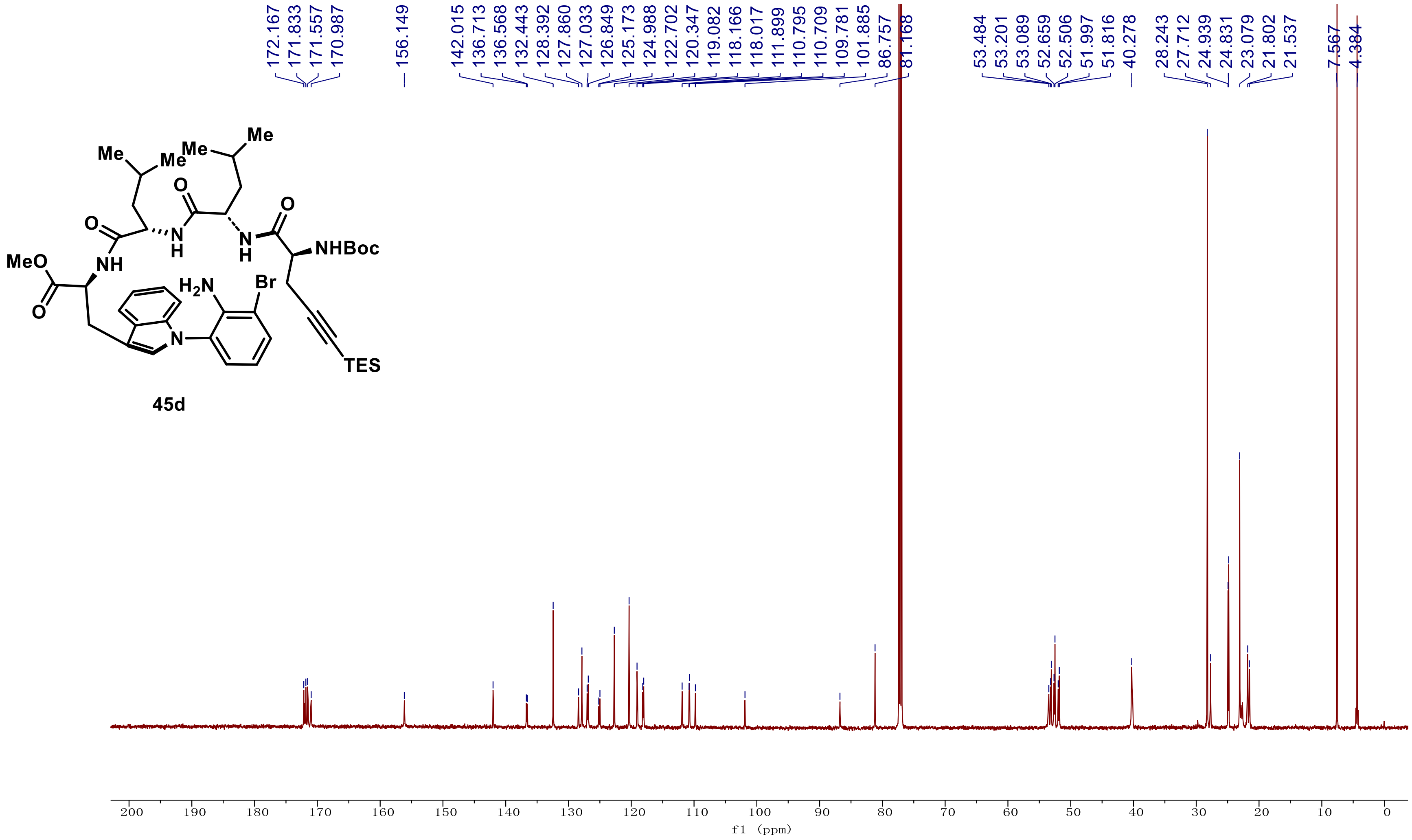

Compound 45e ^{1}H NMR (600 MHz, $CDCl_3$)

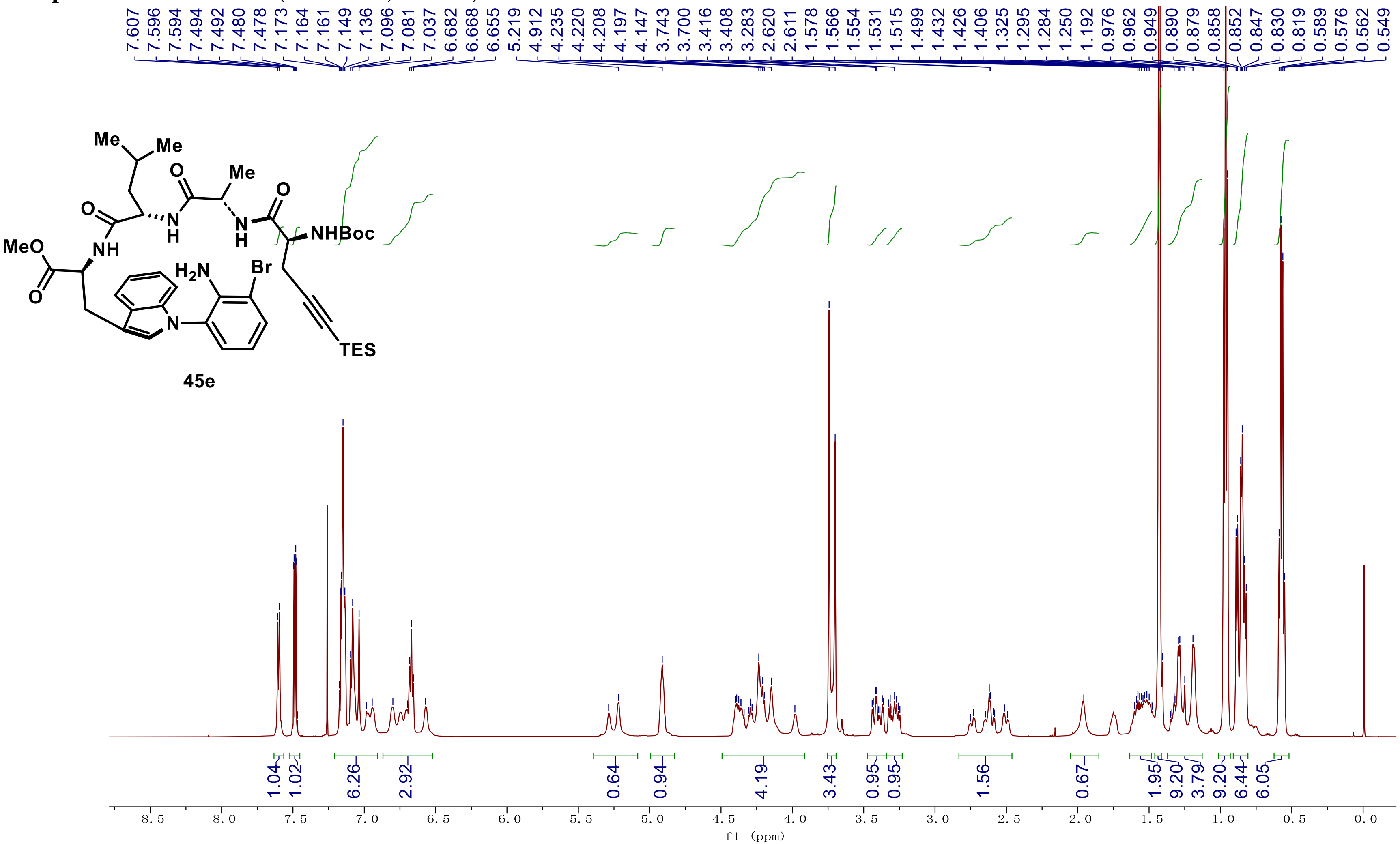

Compound 45e ^{13}C NMR (151 MHz, $CDCl_3$)

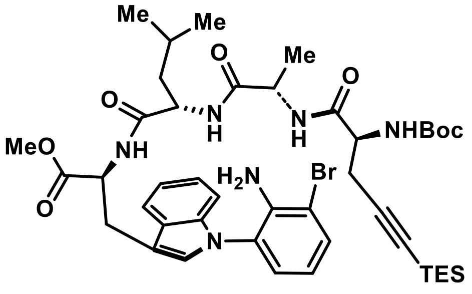

45e

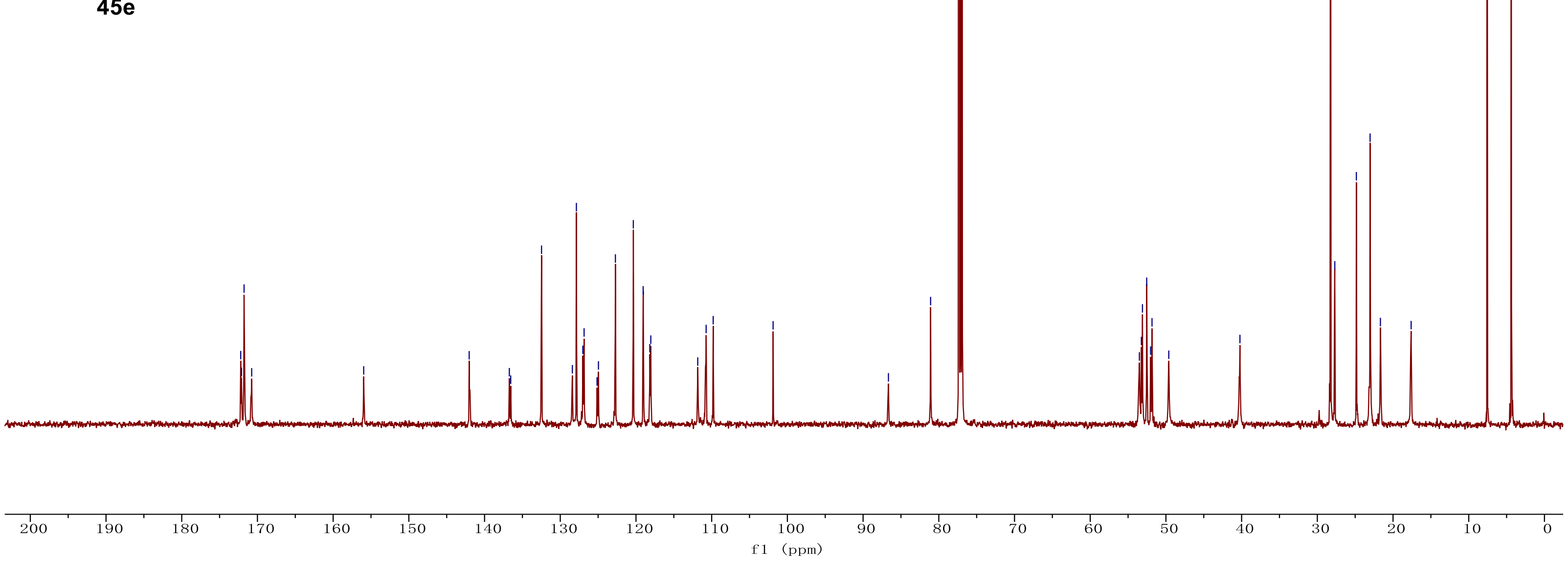

Compound 45f ^{1}H NMR (600 MHz, $CDCl_3$)

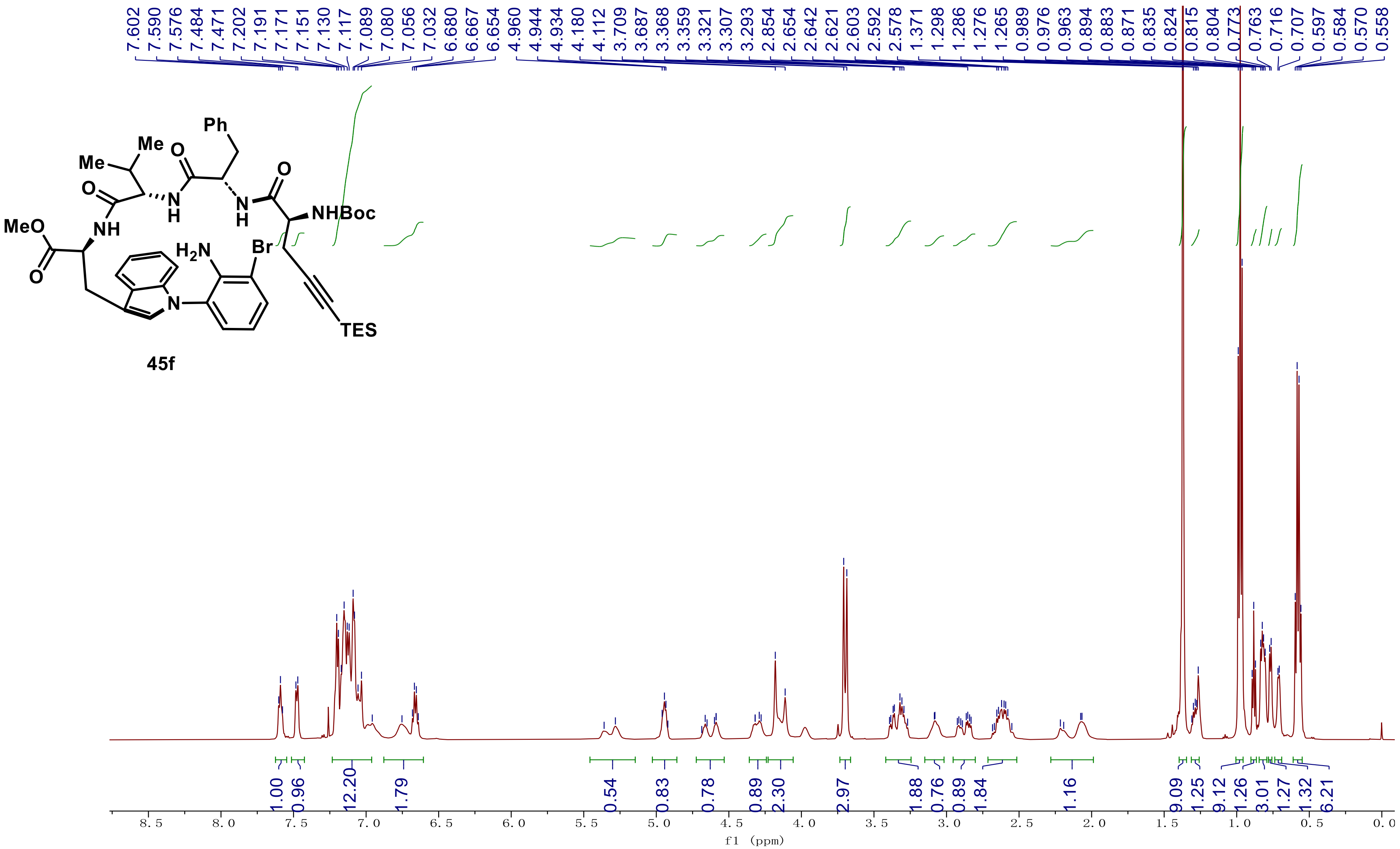

Compound 45f ^{13}C NMR (151 MHz, CDCl$_3$)

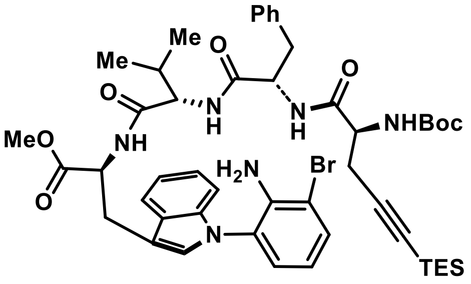

45f

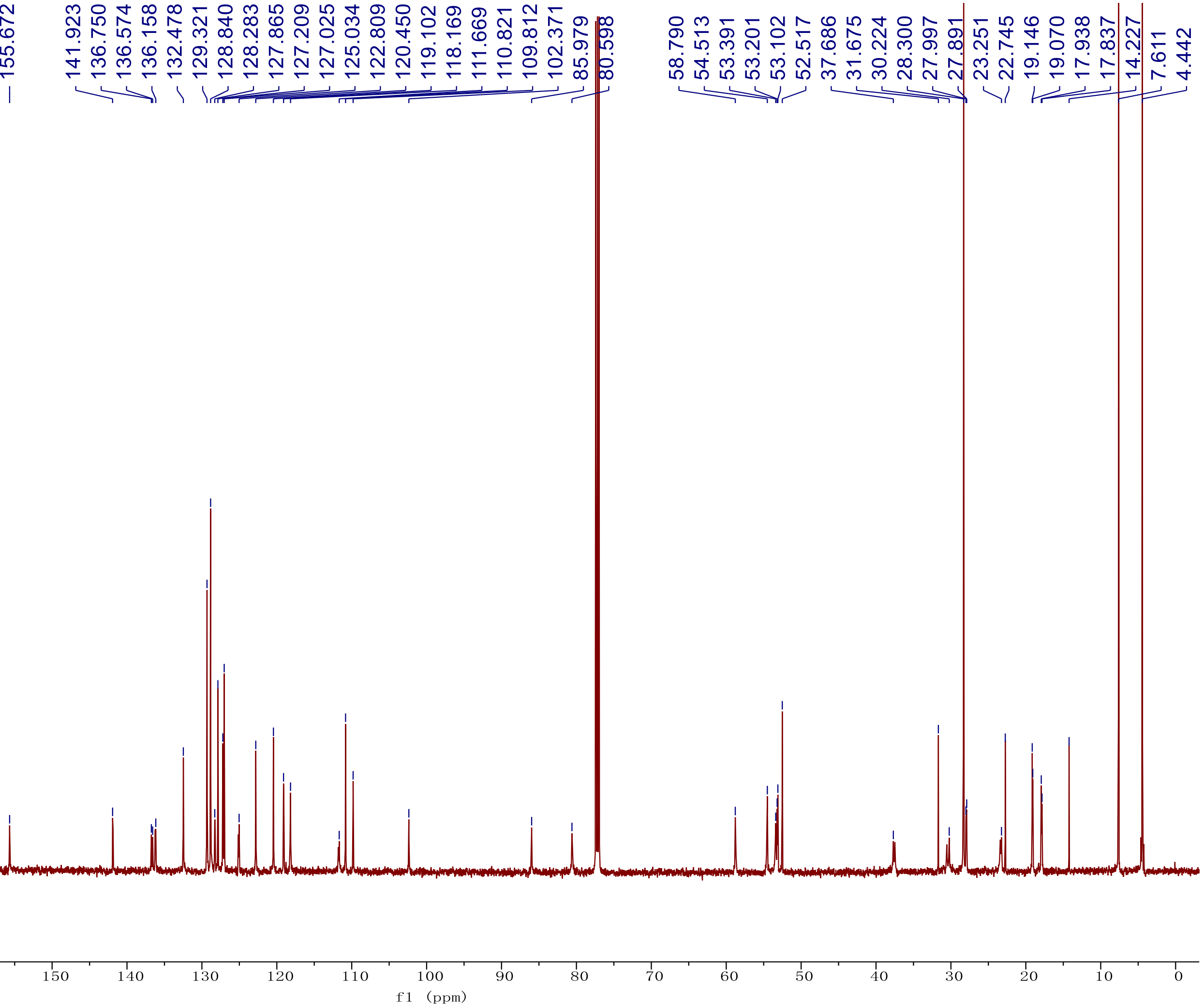

Compound 45g ¹H NMR (600 MHz, $CDCl_3$)

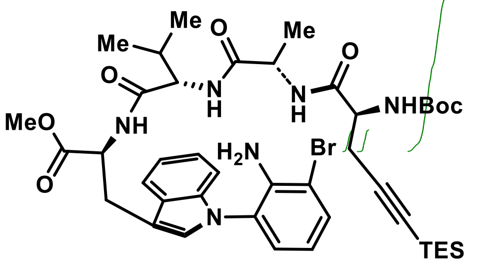

45g

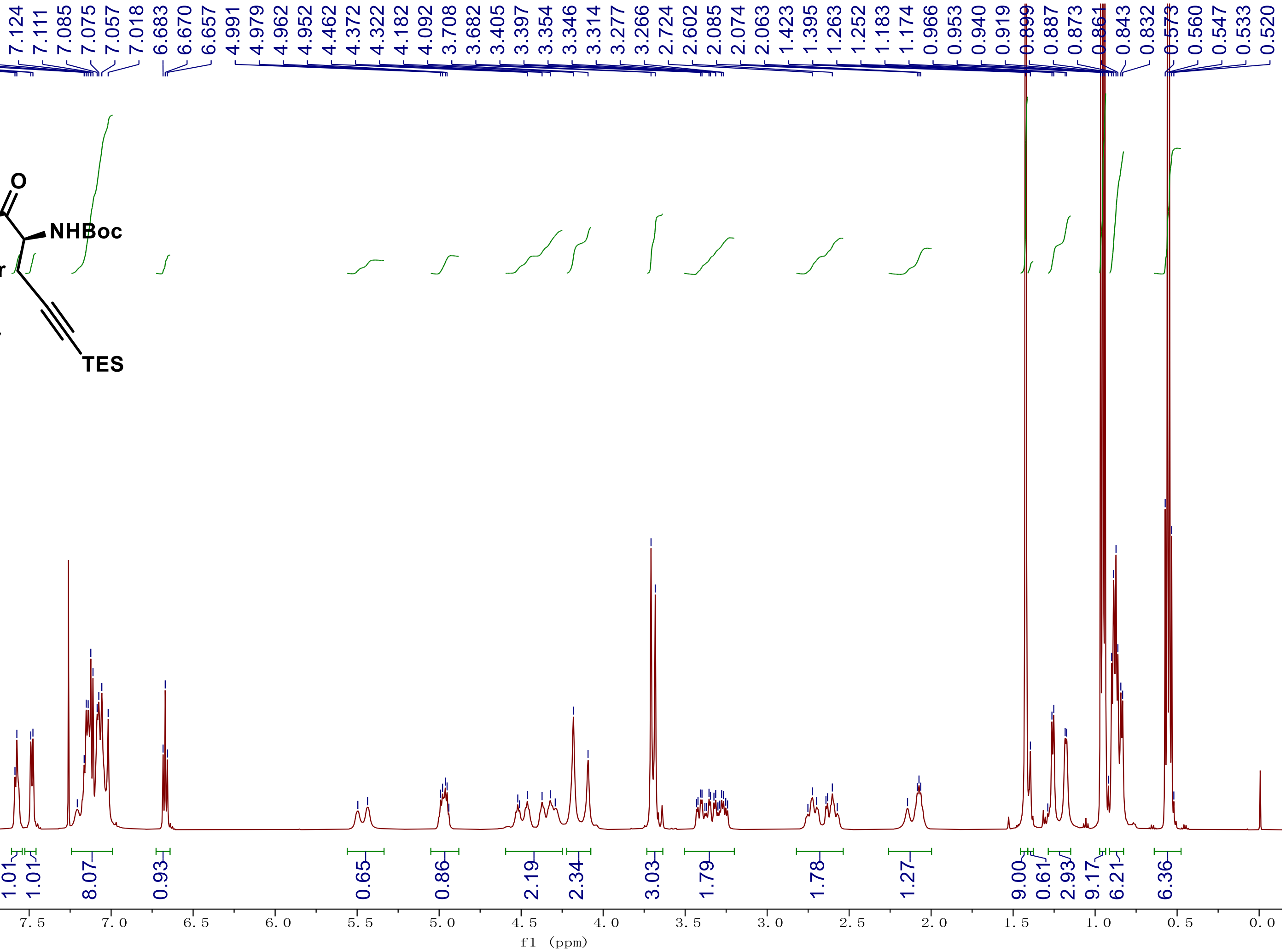

Compound 45g ^{13}C NMR (151 MHz, CDCl$_3$)

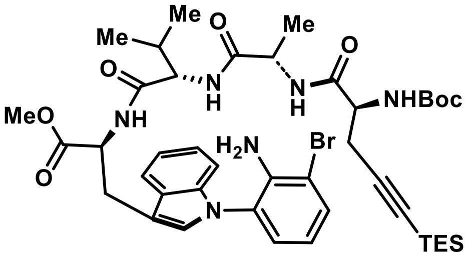

45g

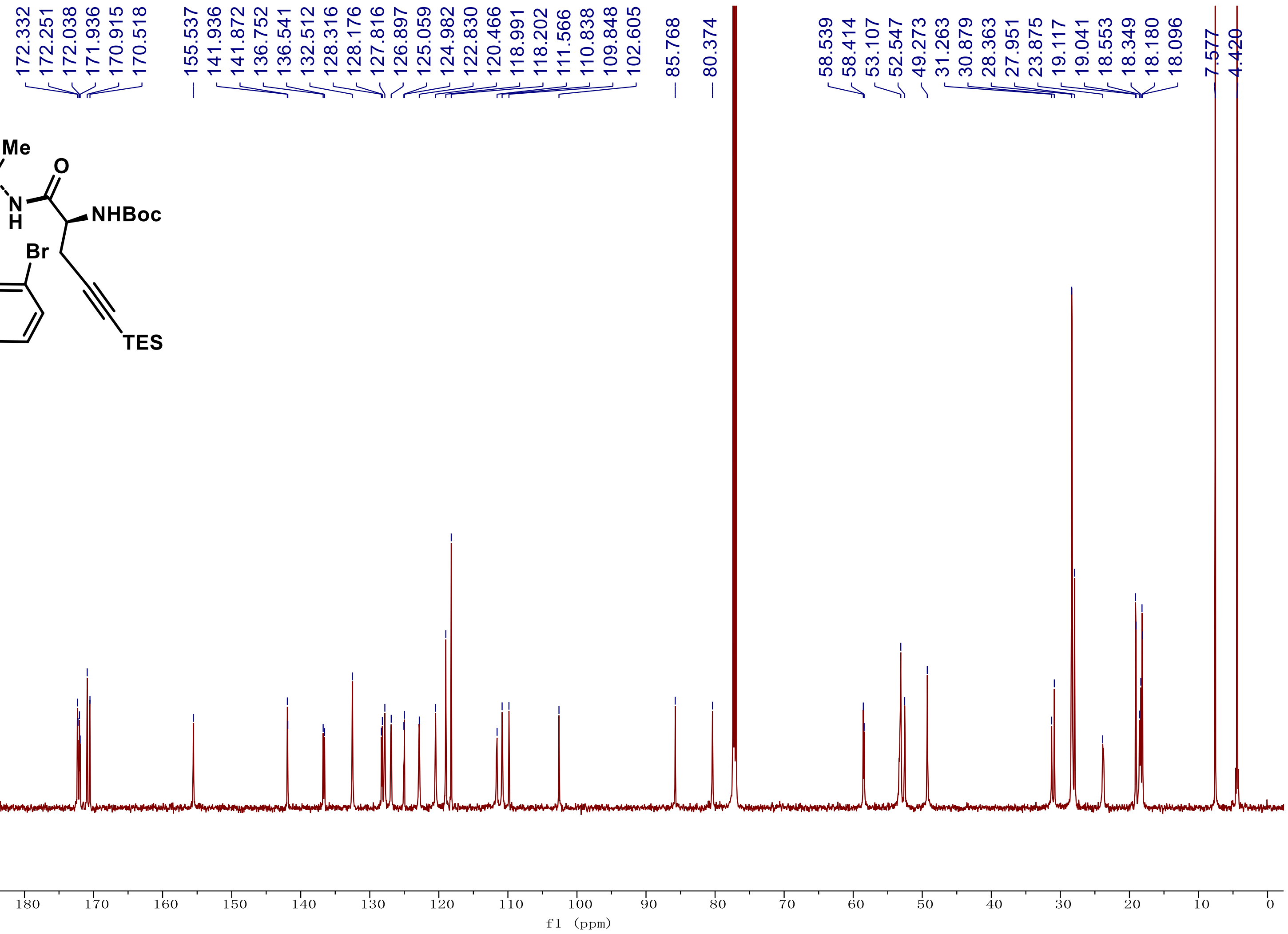

Compound 45h ^{1}H NMR (600 MHz, CDCl$_3$)

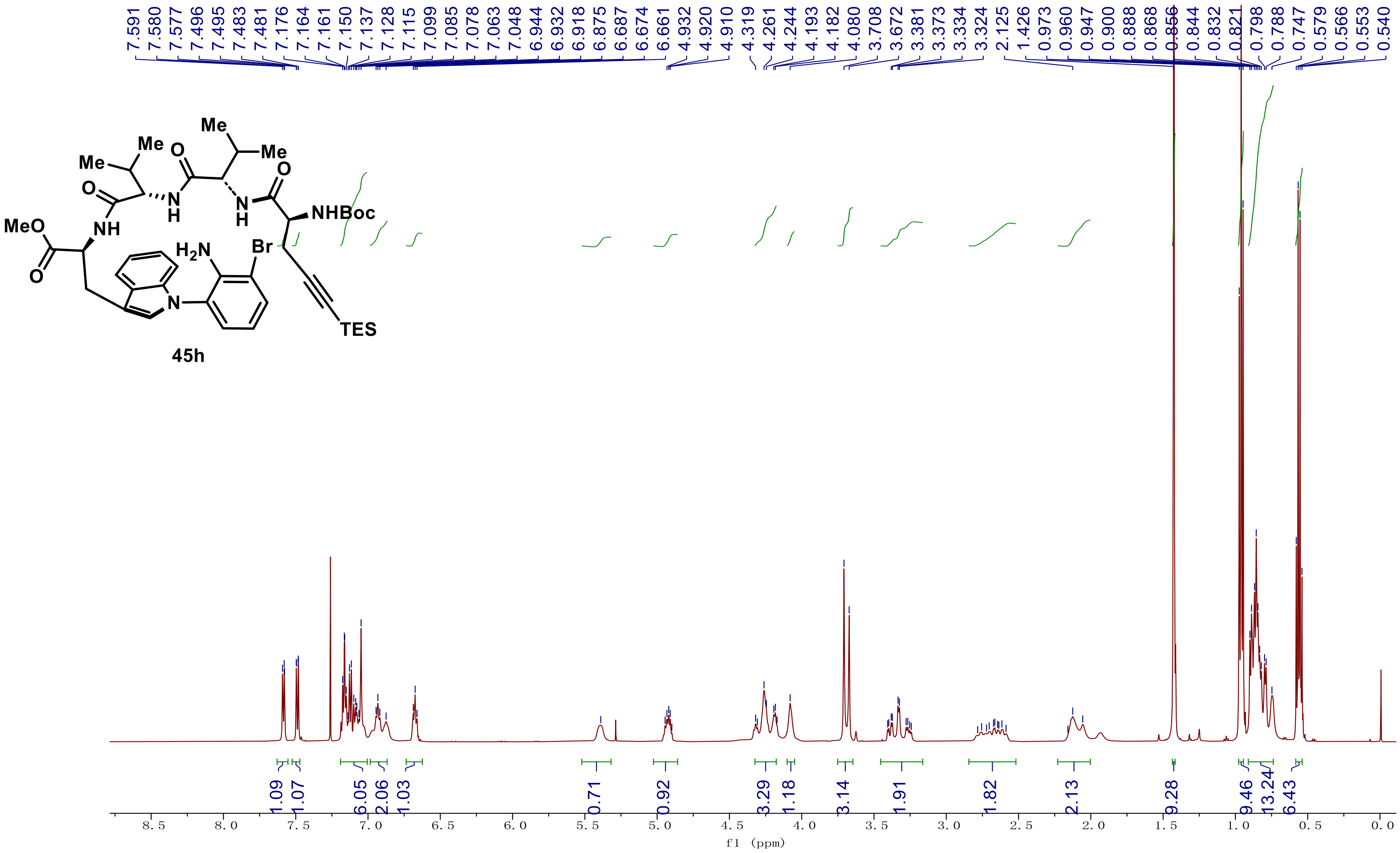

45h

Compound 45h ^{13}C NMR (151 MHz, CDCl$_3$)

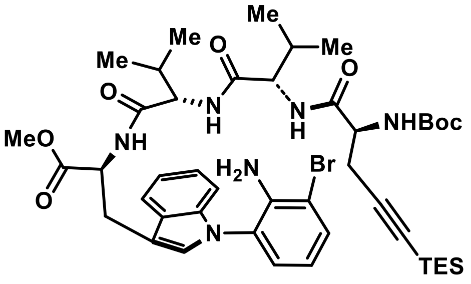

45h

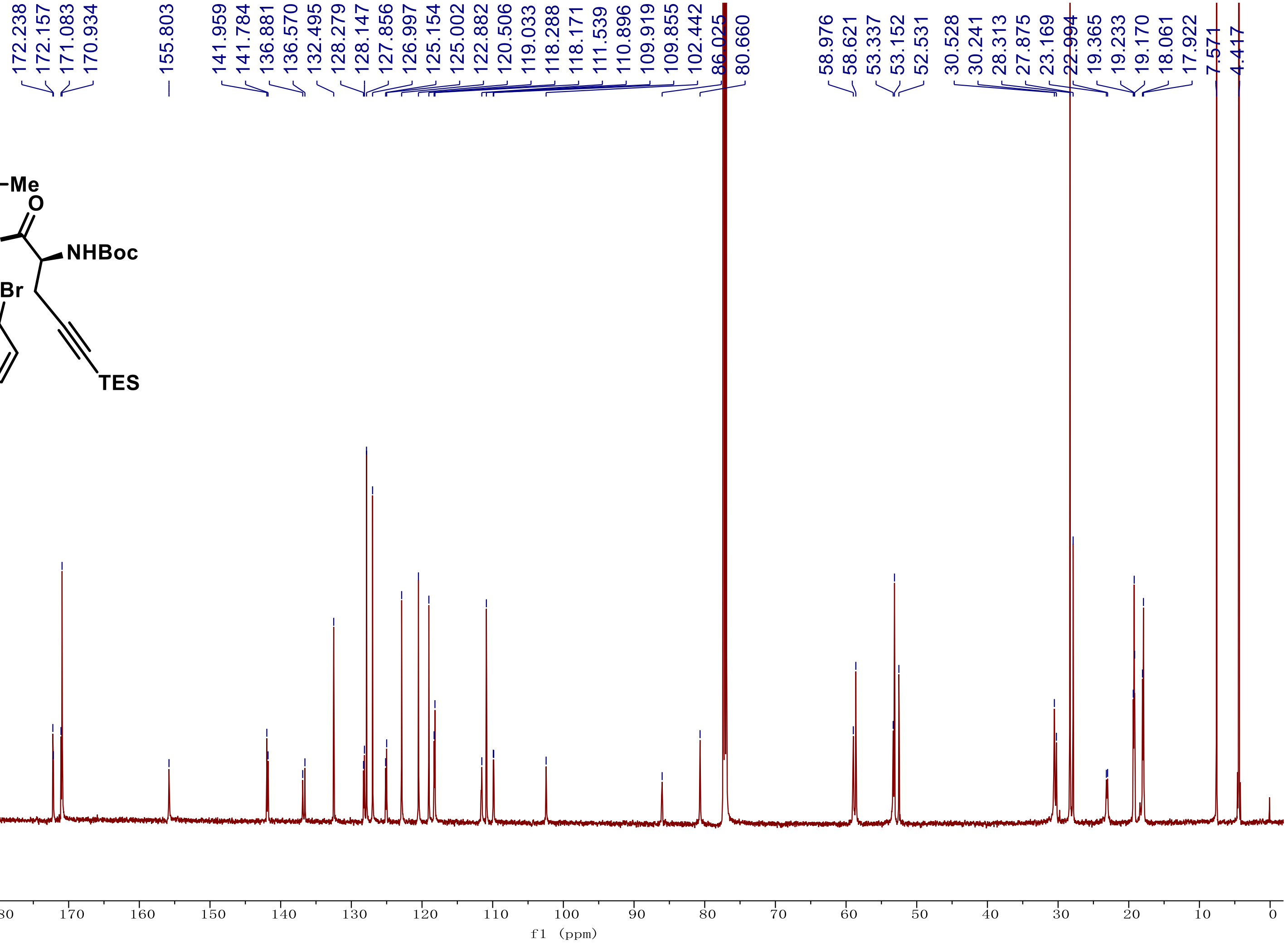

Compound 45i ^{1}H NMR (600 MHz, $CDCl_3$)

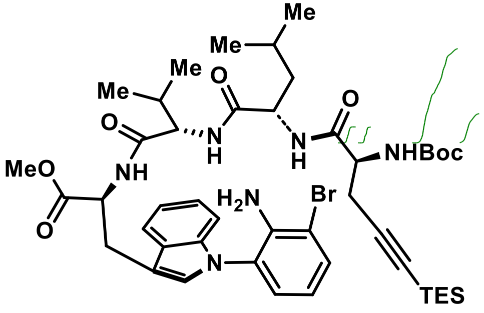

45i

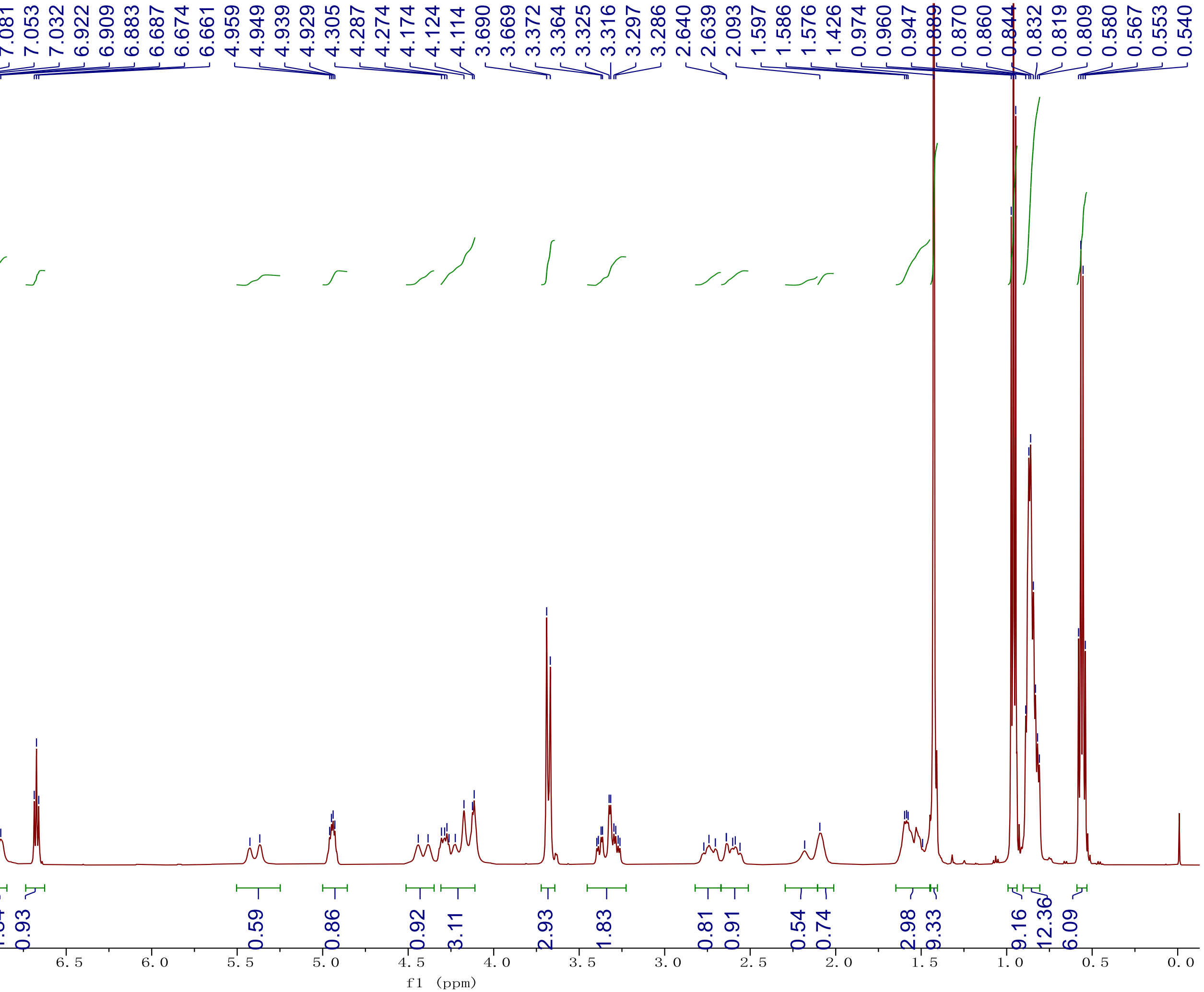

Compound 45i ^{13}C NMR (151 MHz, CDCl$_3$)

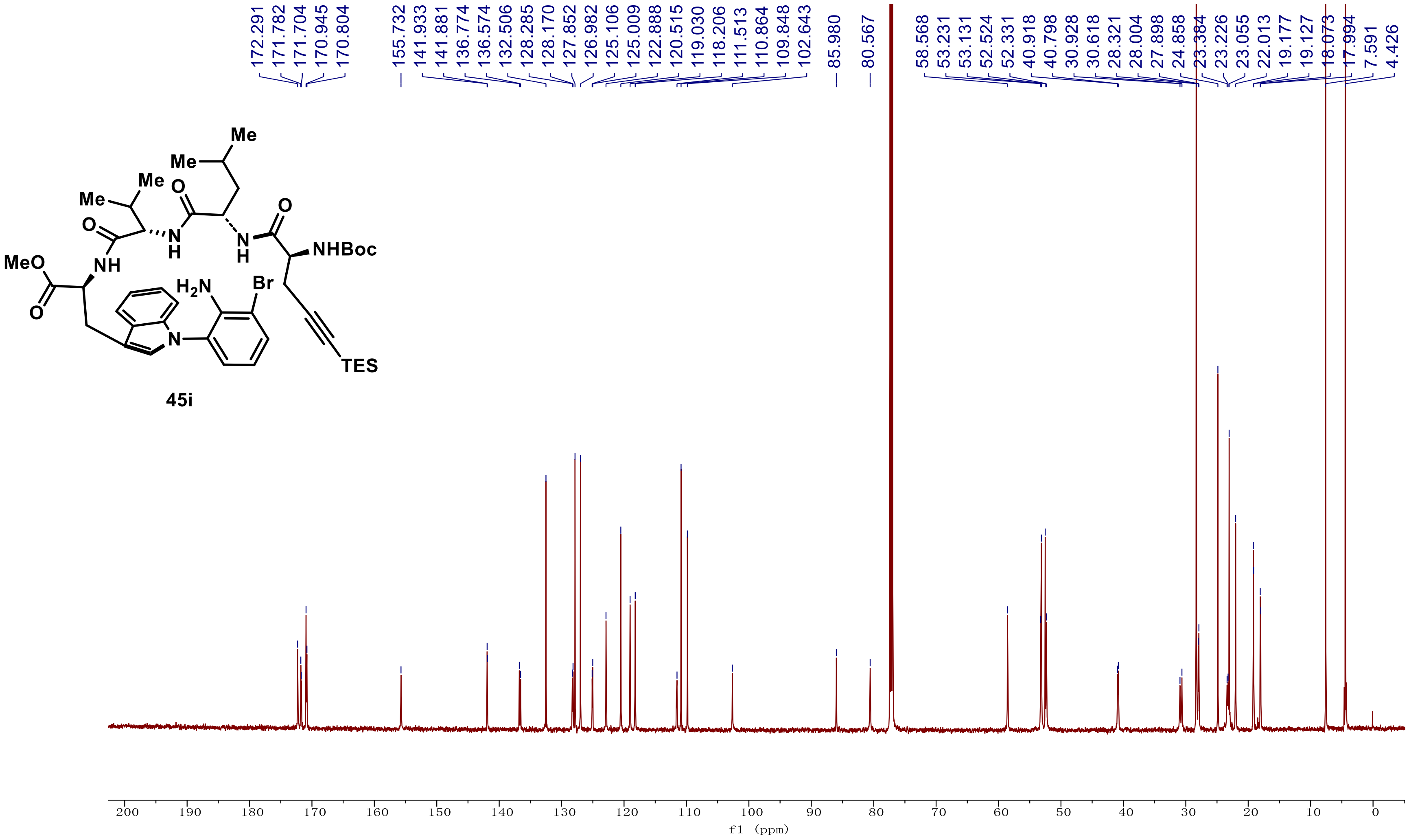

Compound 45j ^{1}H NMR (600 MHz, $CDCl_3$)

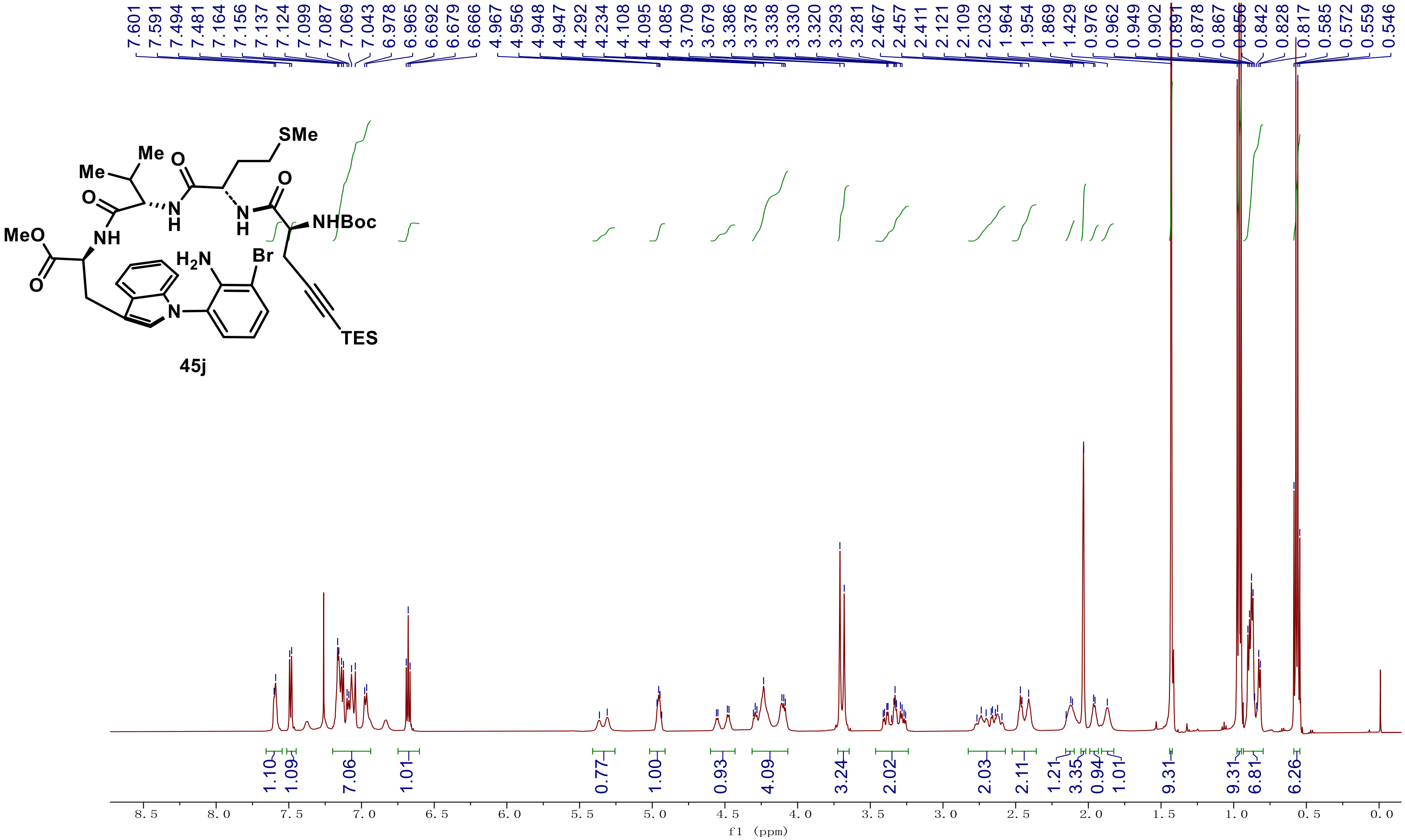

Compound 45j ^{13}C NMR (151 MHz, $CDCl_3$)

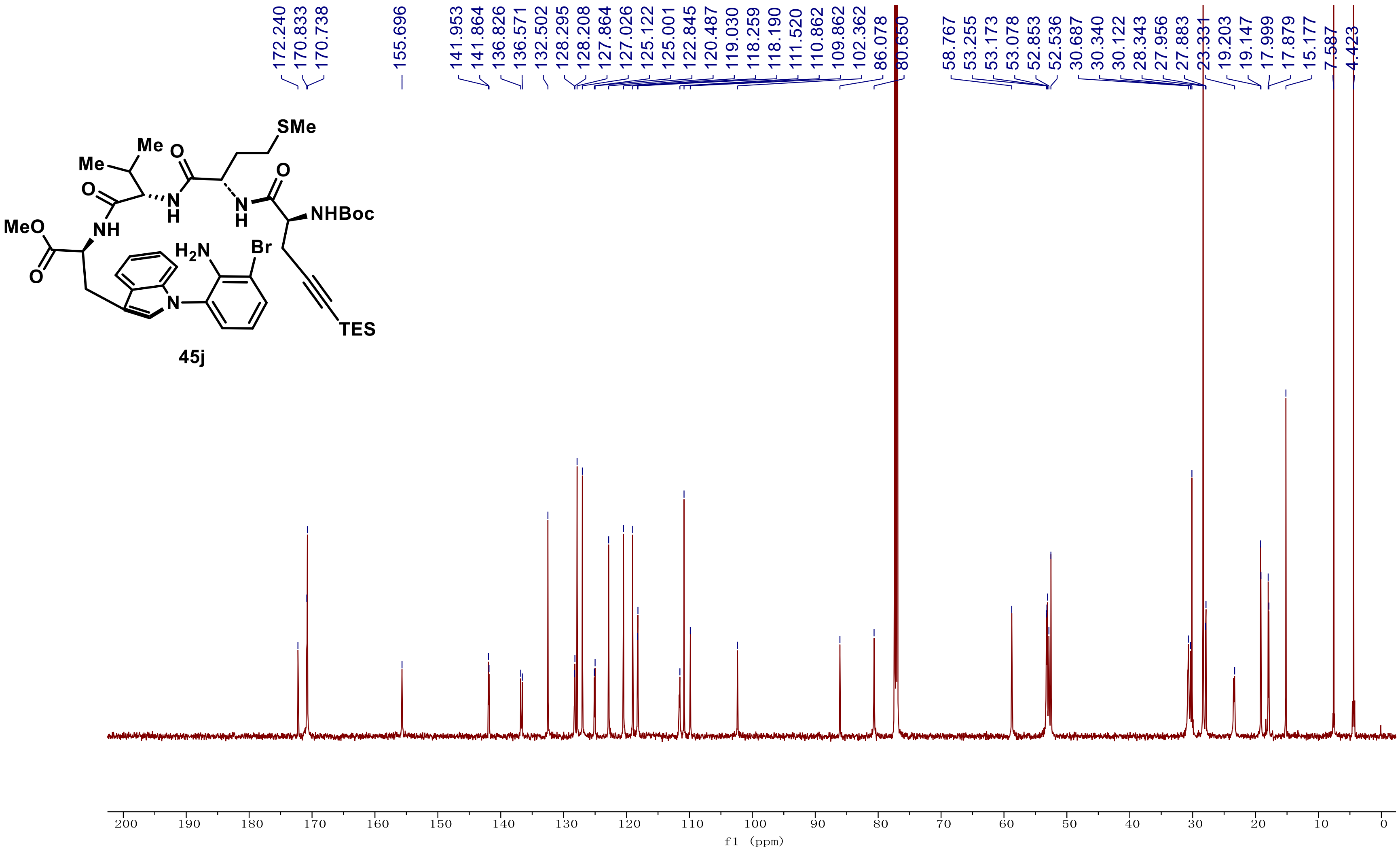

Compound 45k ^{1}H NMR (600 MHz, $CDCl_3$)

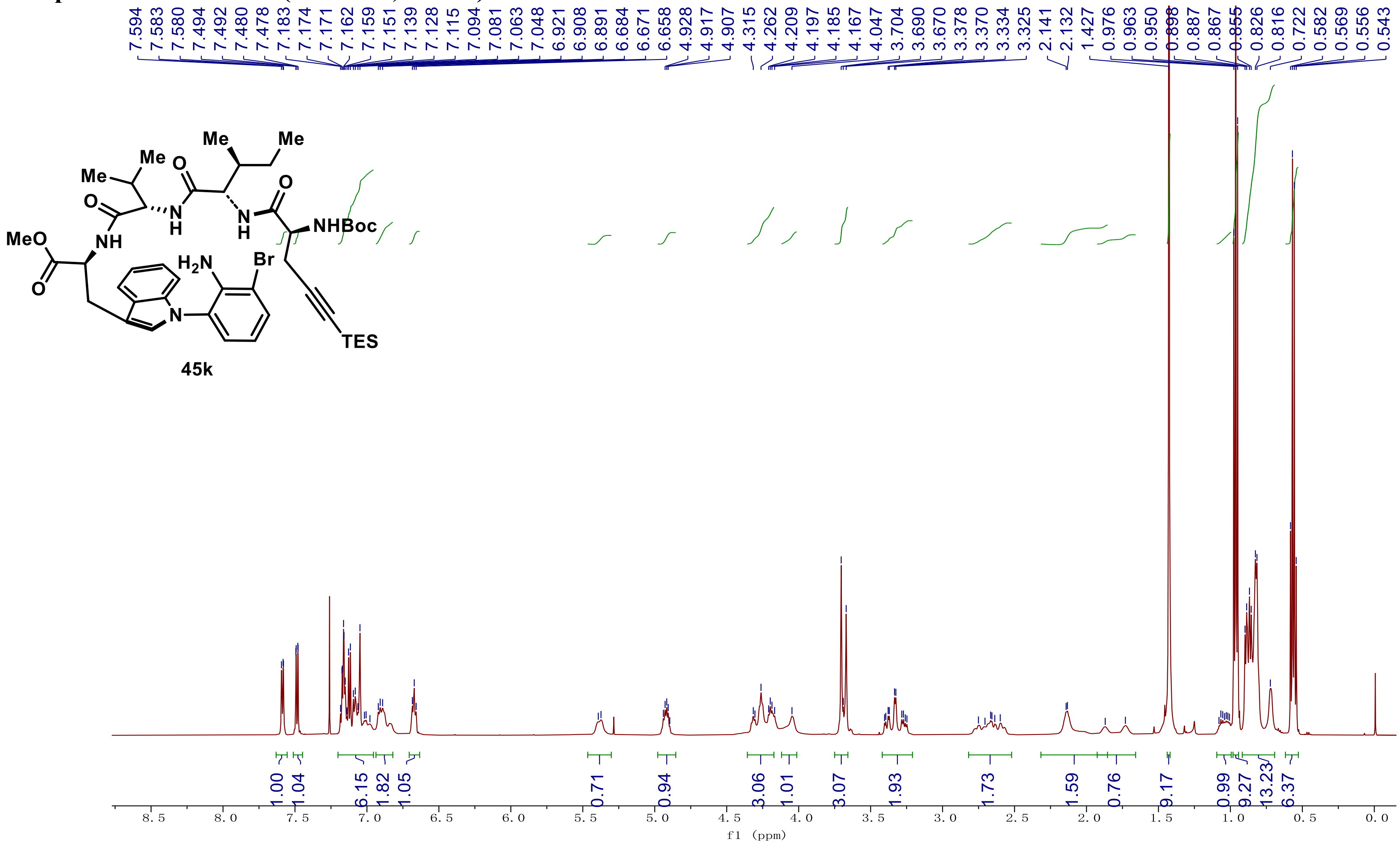

Compound 45k ^{13}C NMR (151 MHz, $CDCl_3$)

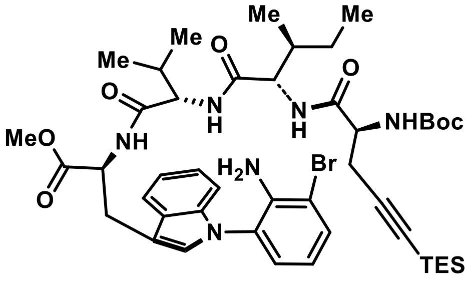

45k

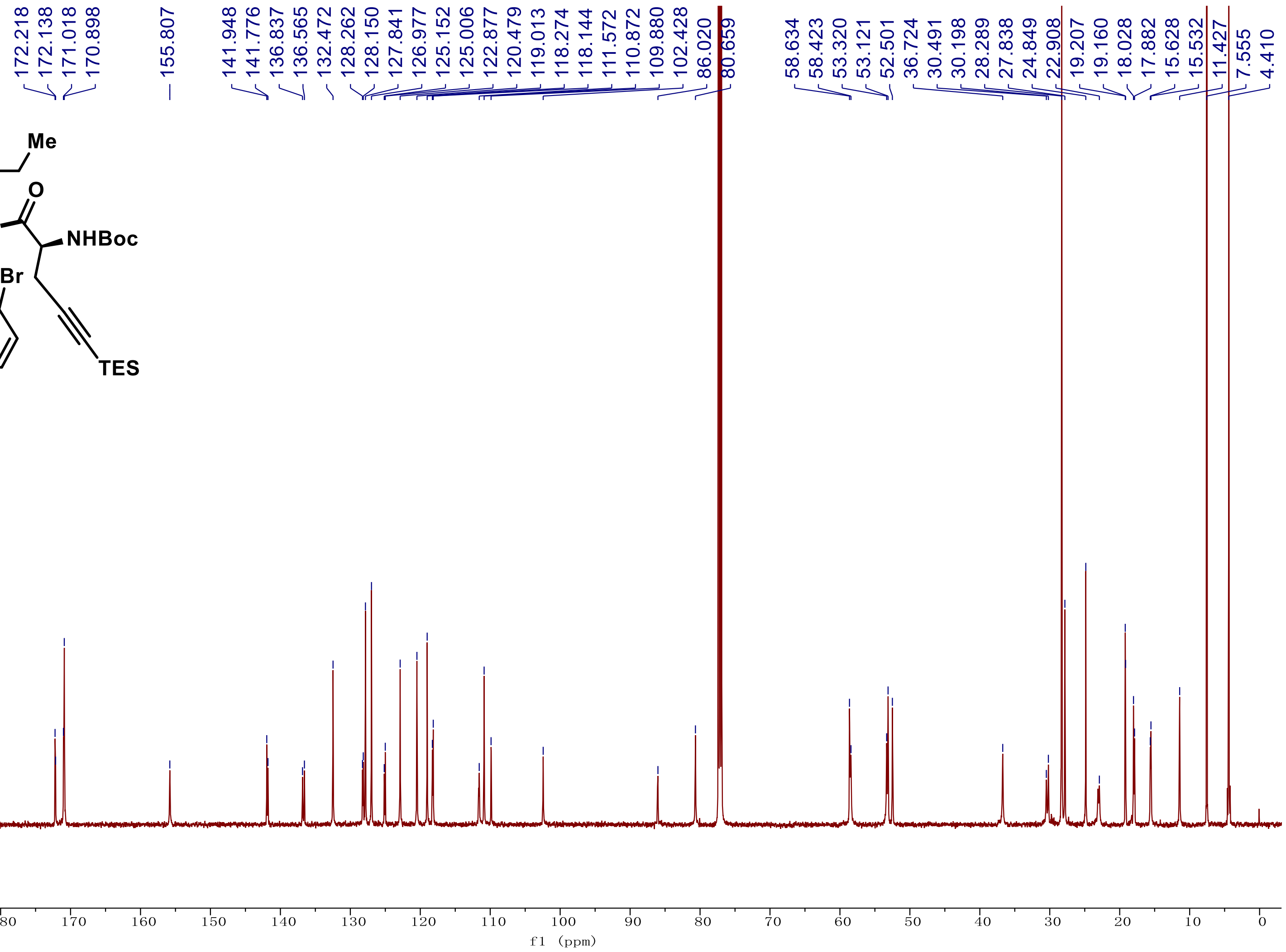

Compound 45l ^{1}H NMR (600 MHz, $CDCl_3$)

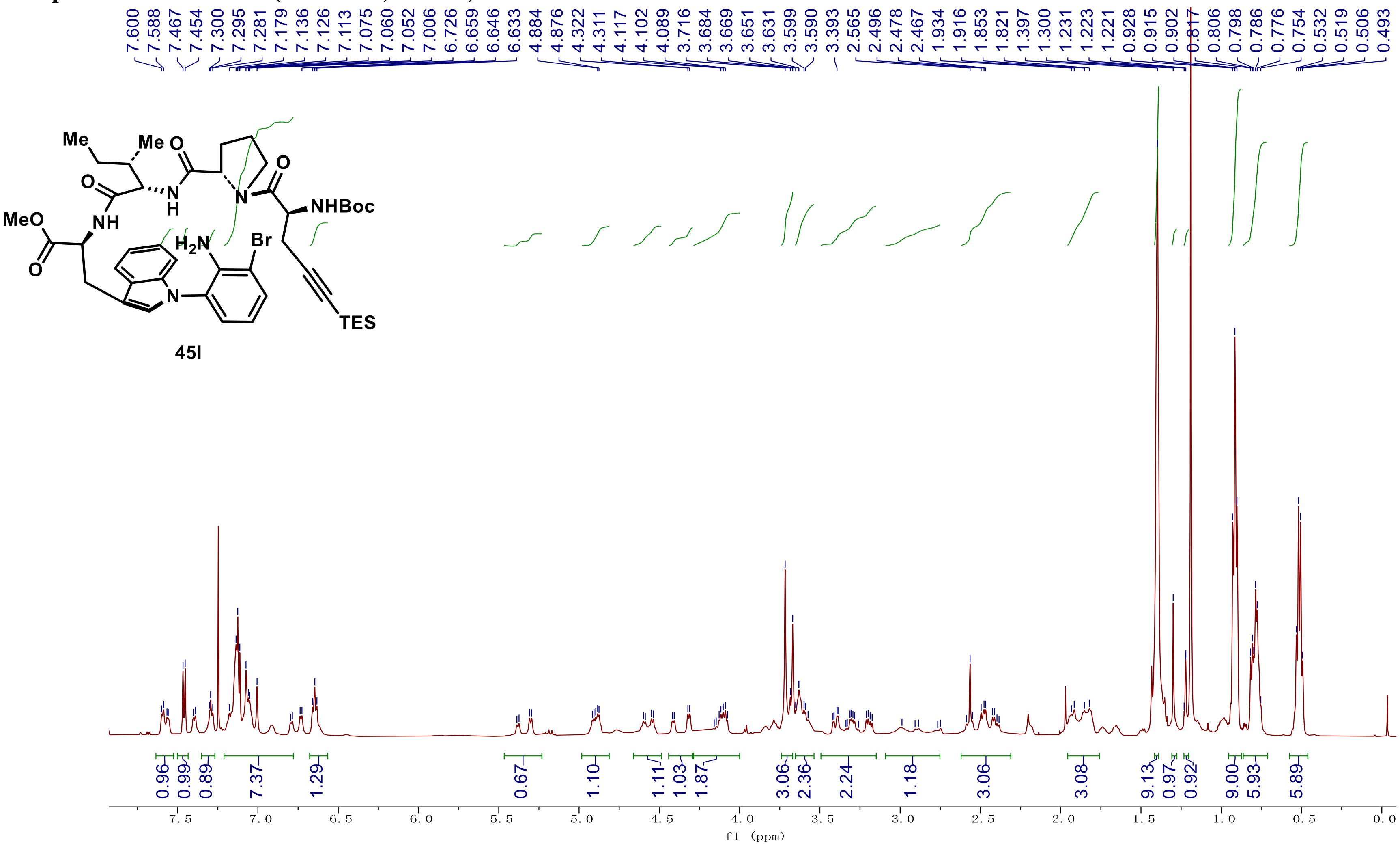

Compound 45l ^{13}C NMR (151 MHz, $CDCl_3$)

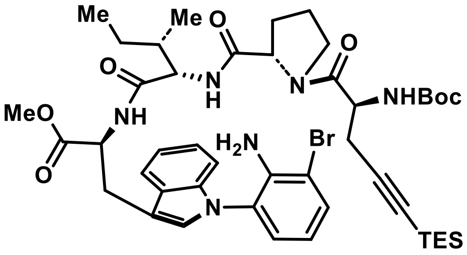

45l

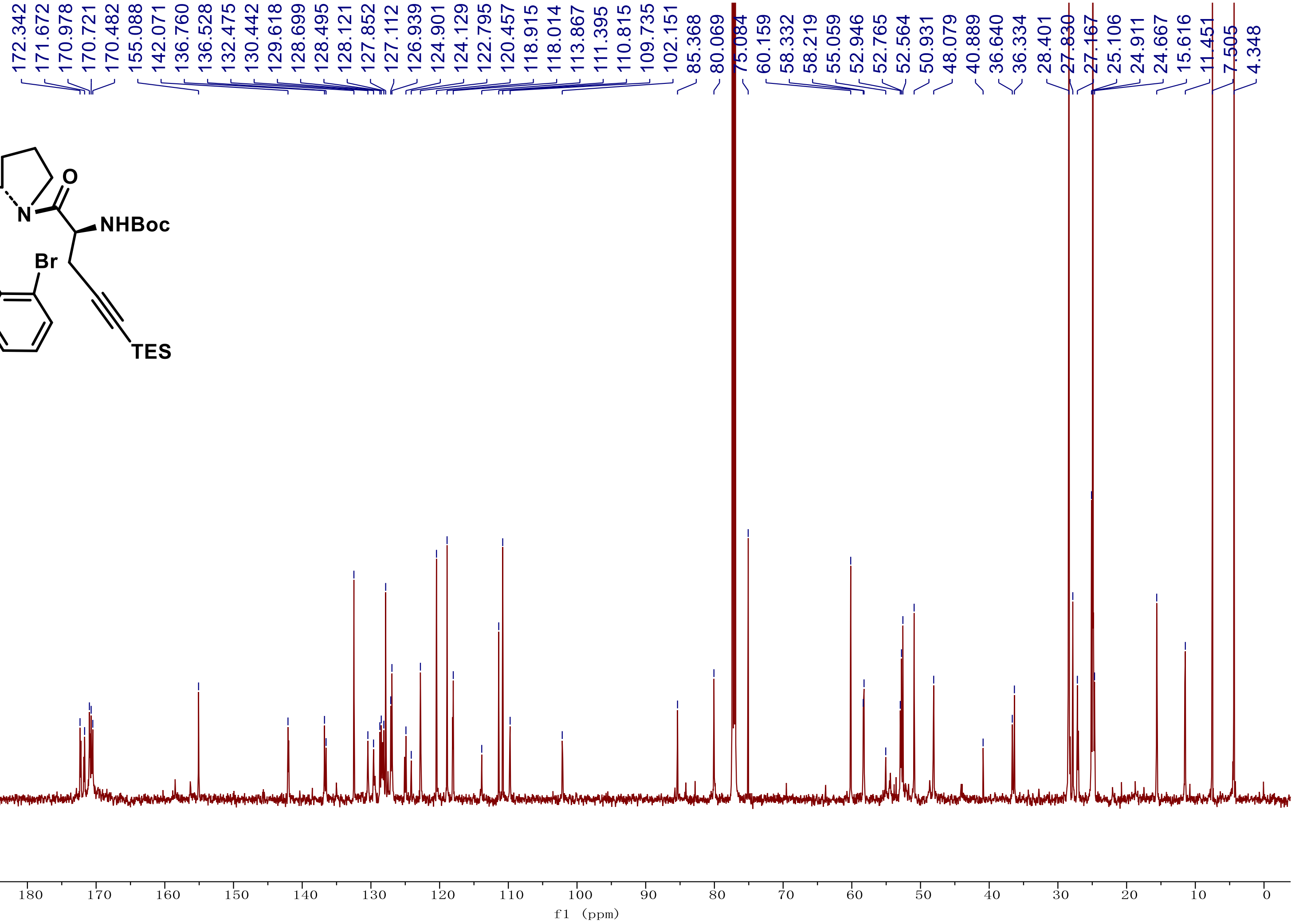

Compound 45m ^{1}H NMR (600 MHz, $CDCl_3$)

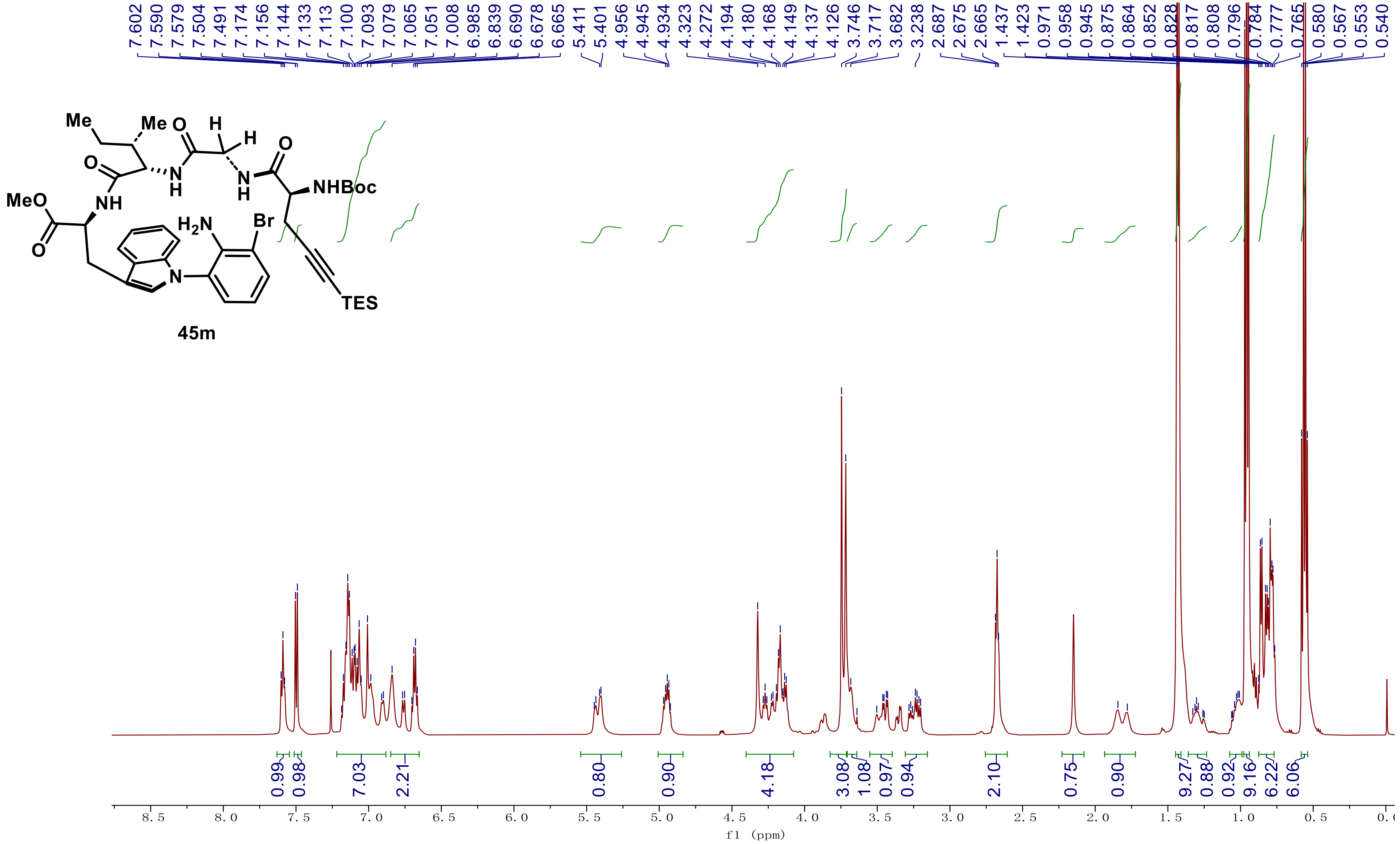

Compound 45m ^{13}C NMR (151 MHz, $CDCl_3$)

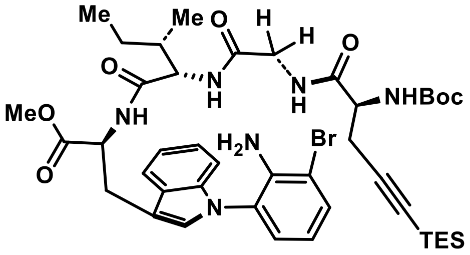

45m

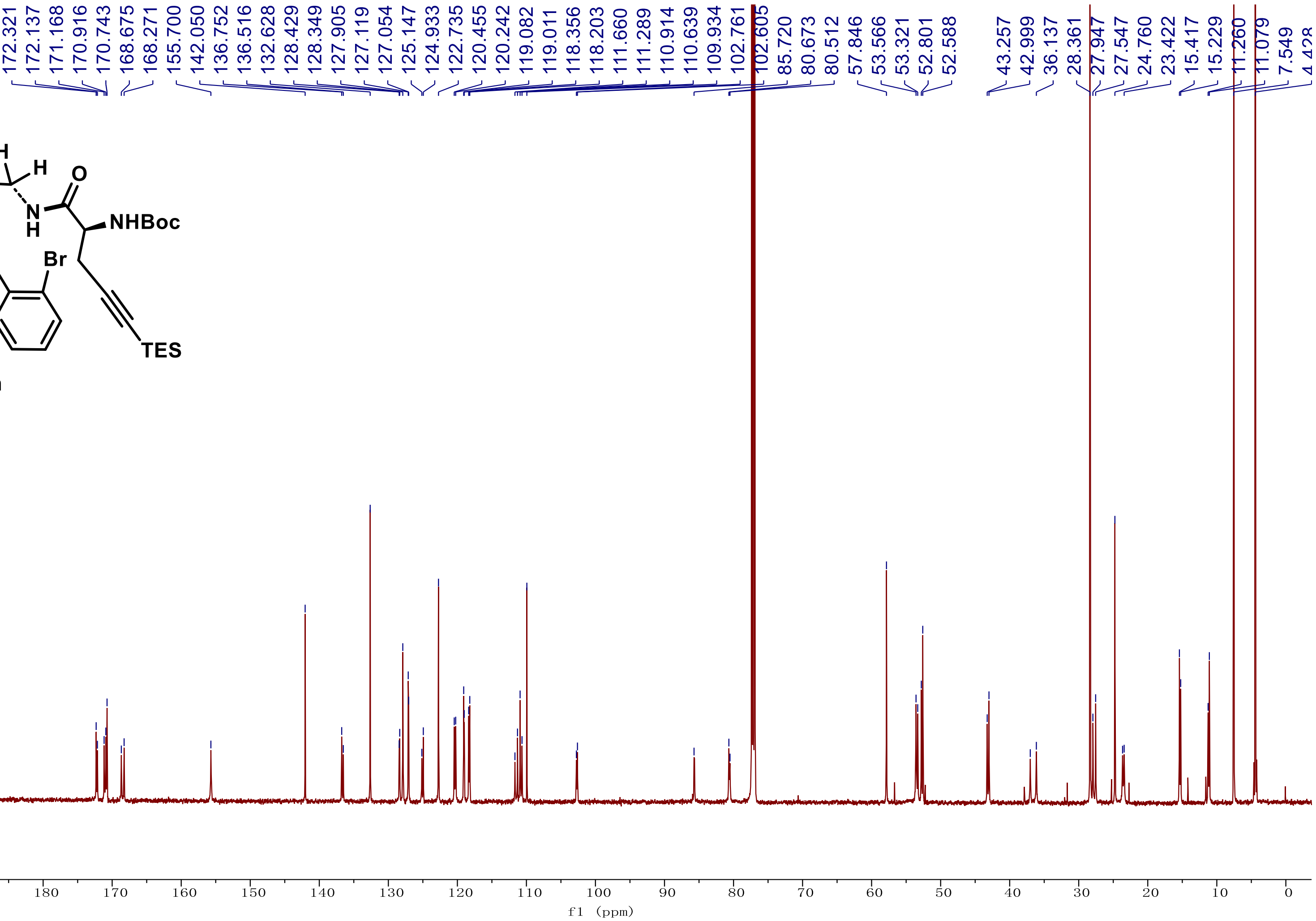

Compound 45n ¹H NMR (600 MHz, CDCl₃)

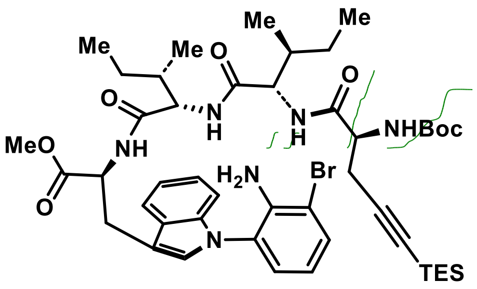

45n

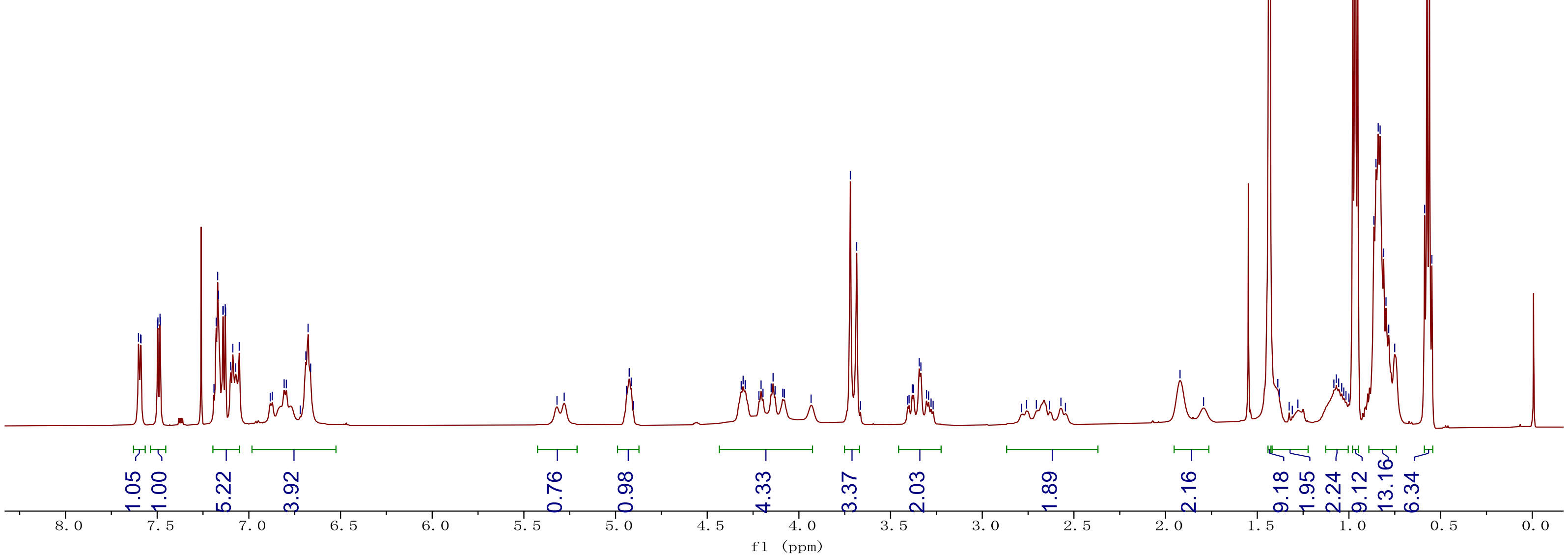

Compound 45n ^{13}C NMR (151 MHz, CDCl$_3$)

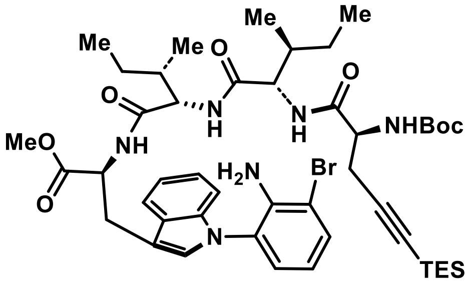

45n

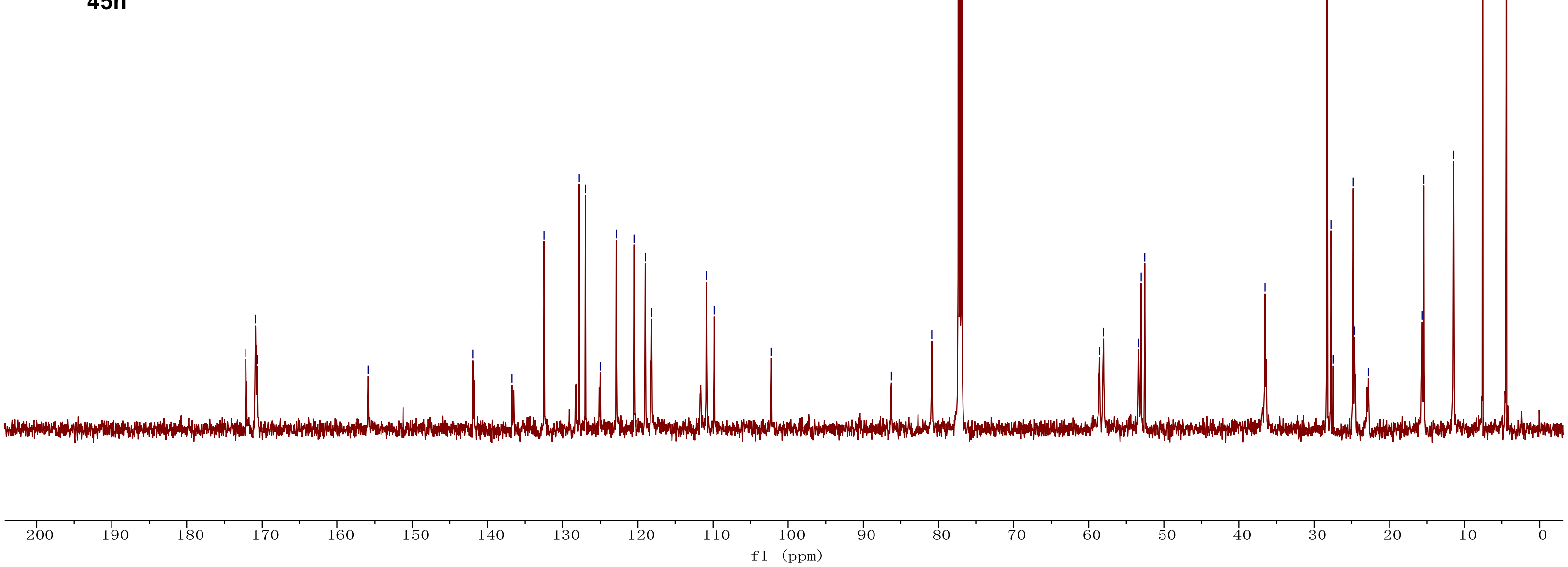

Compound 45o ^{1}H NMR (600 MHz, $CDCl_3$)

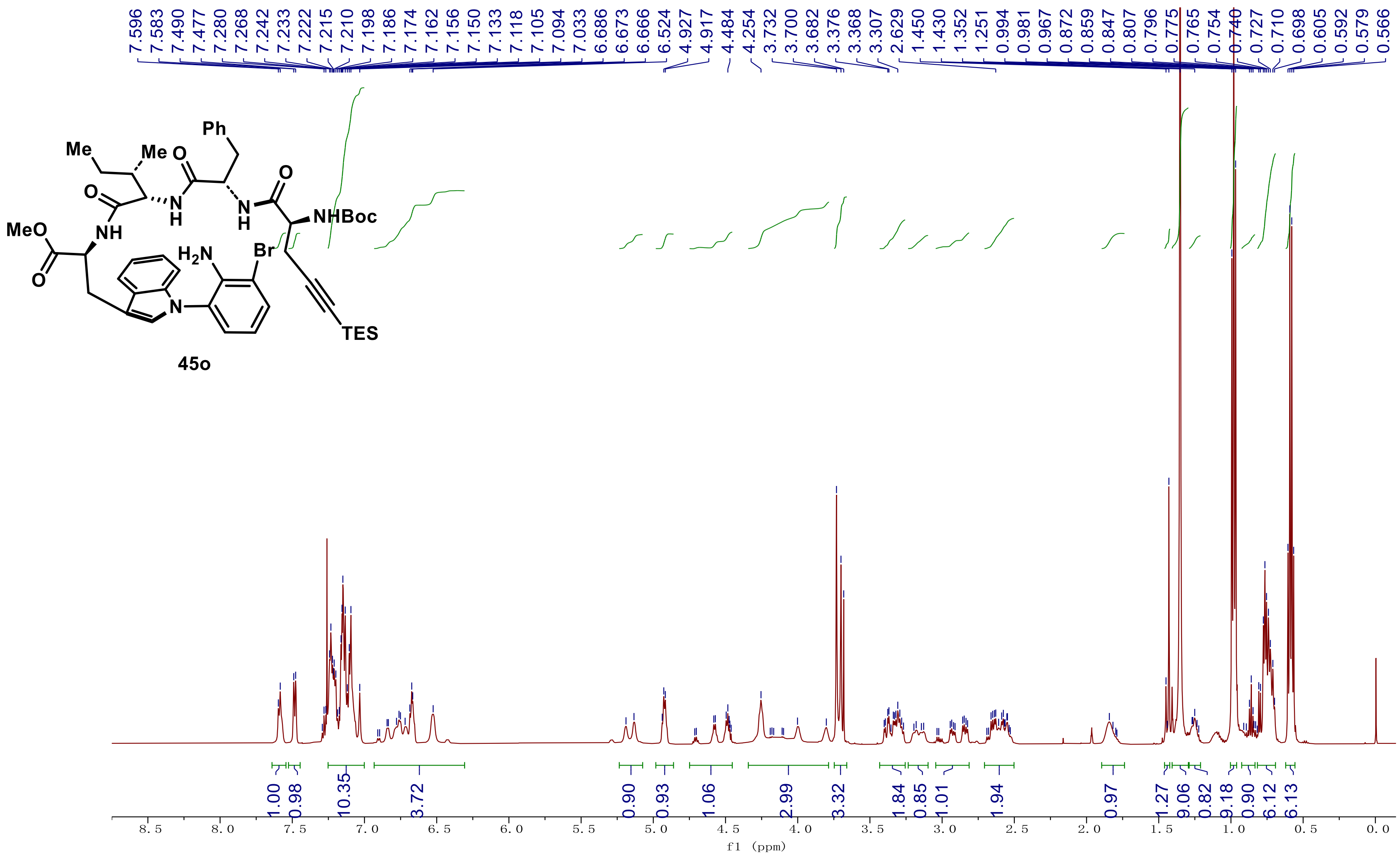

Compound 45o ^{13}C NMR (151 MHz, $CDCl_3$)

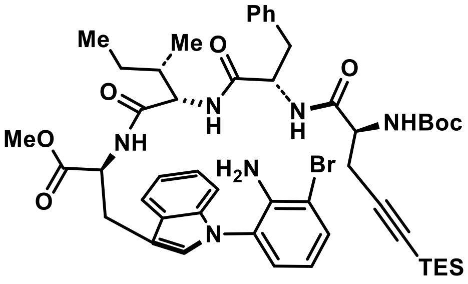

45o

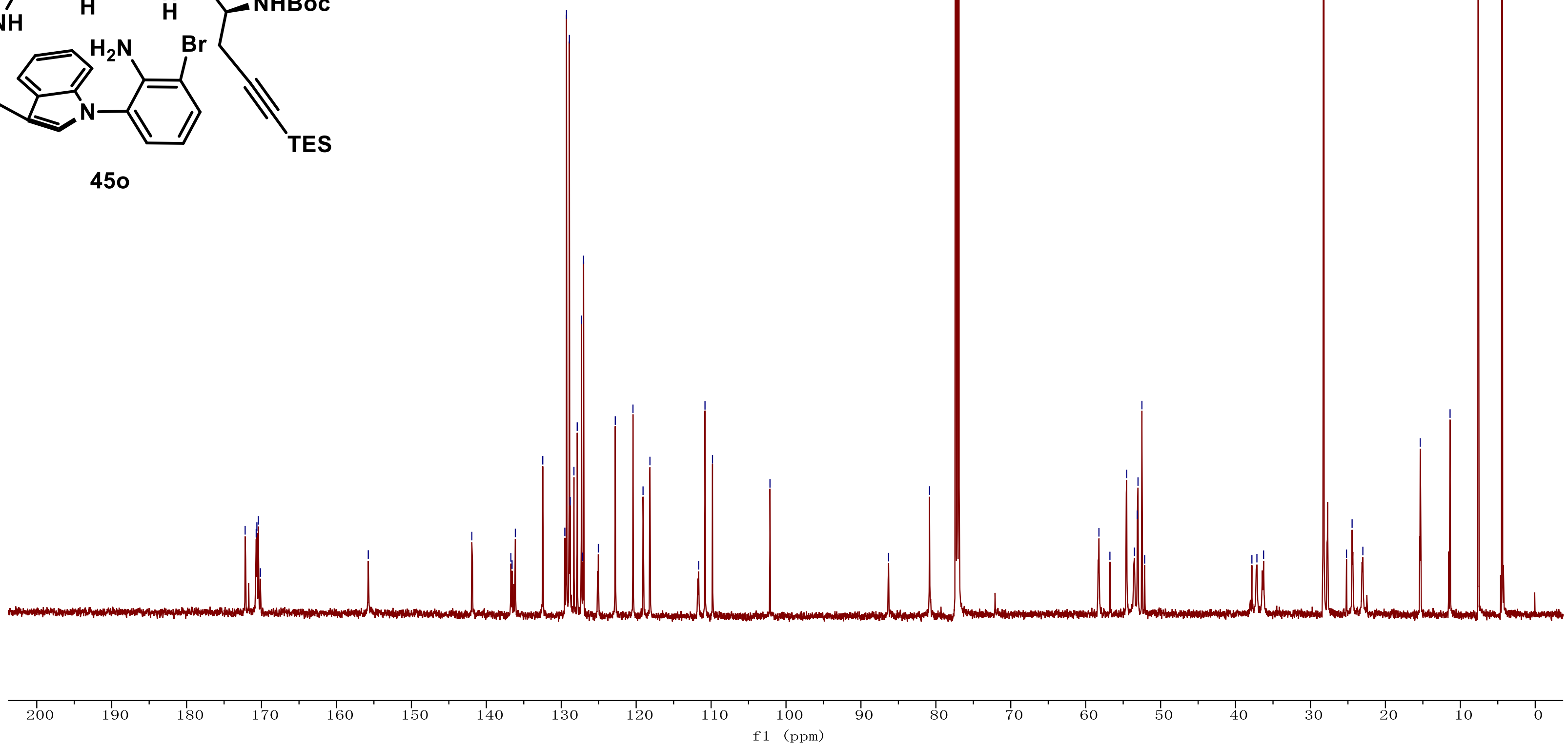

Compound 45p ^{1}H NMR (600 MHz, $CDCl_3$)

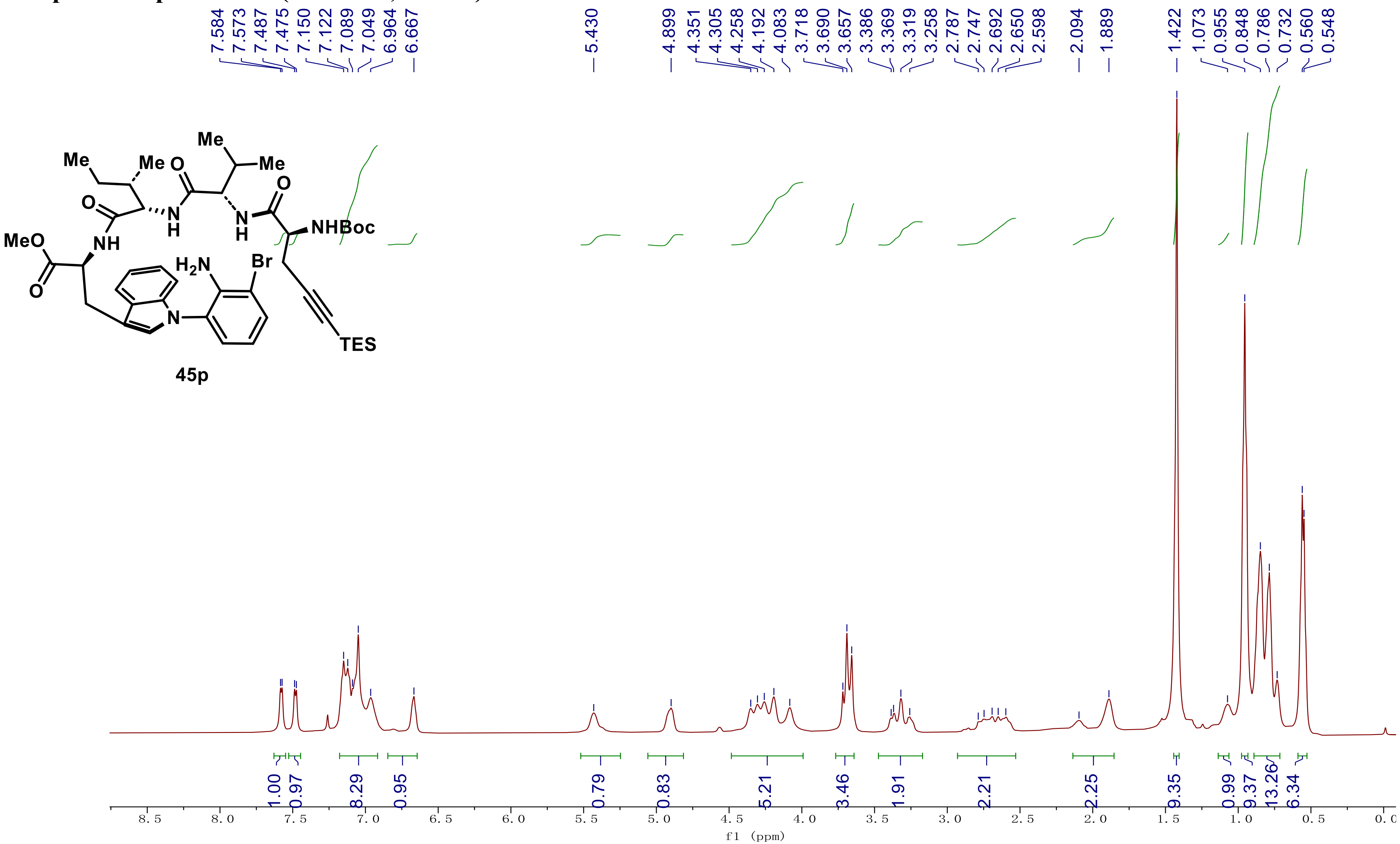

Compound 45p ^{13}C NMR (151 MHz, CDCl$_3$)

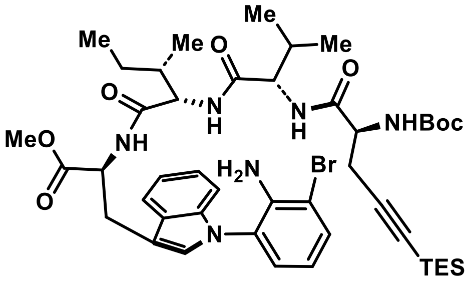

45p

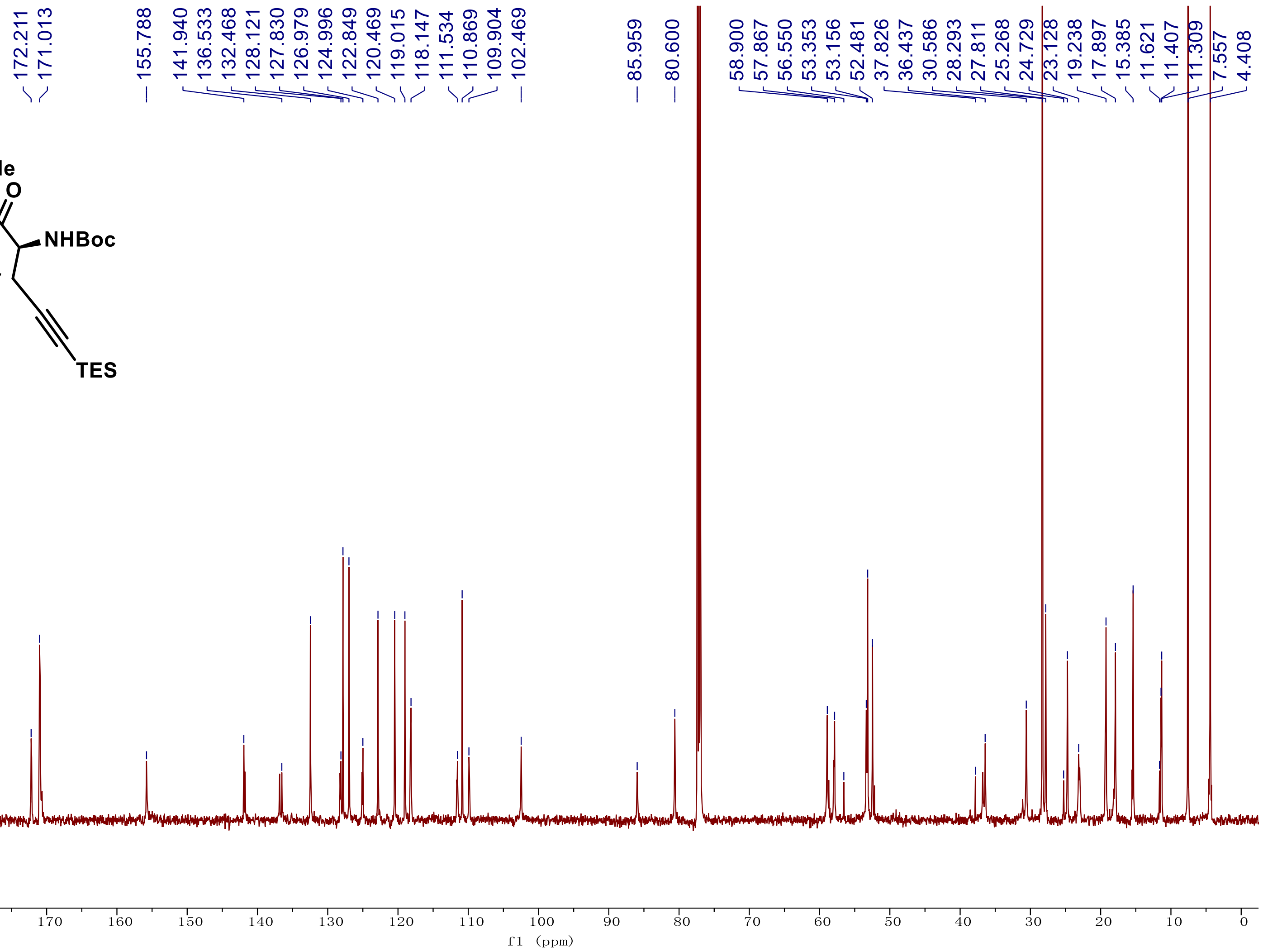

Compound 45q ^{1}H NMR (600 MHz, $CDCl_3$)

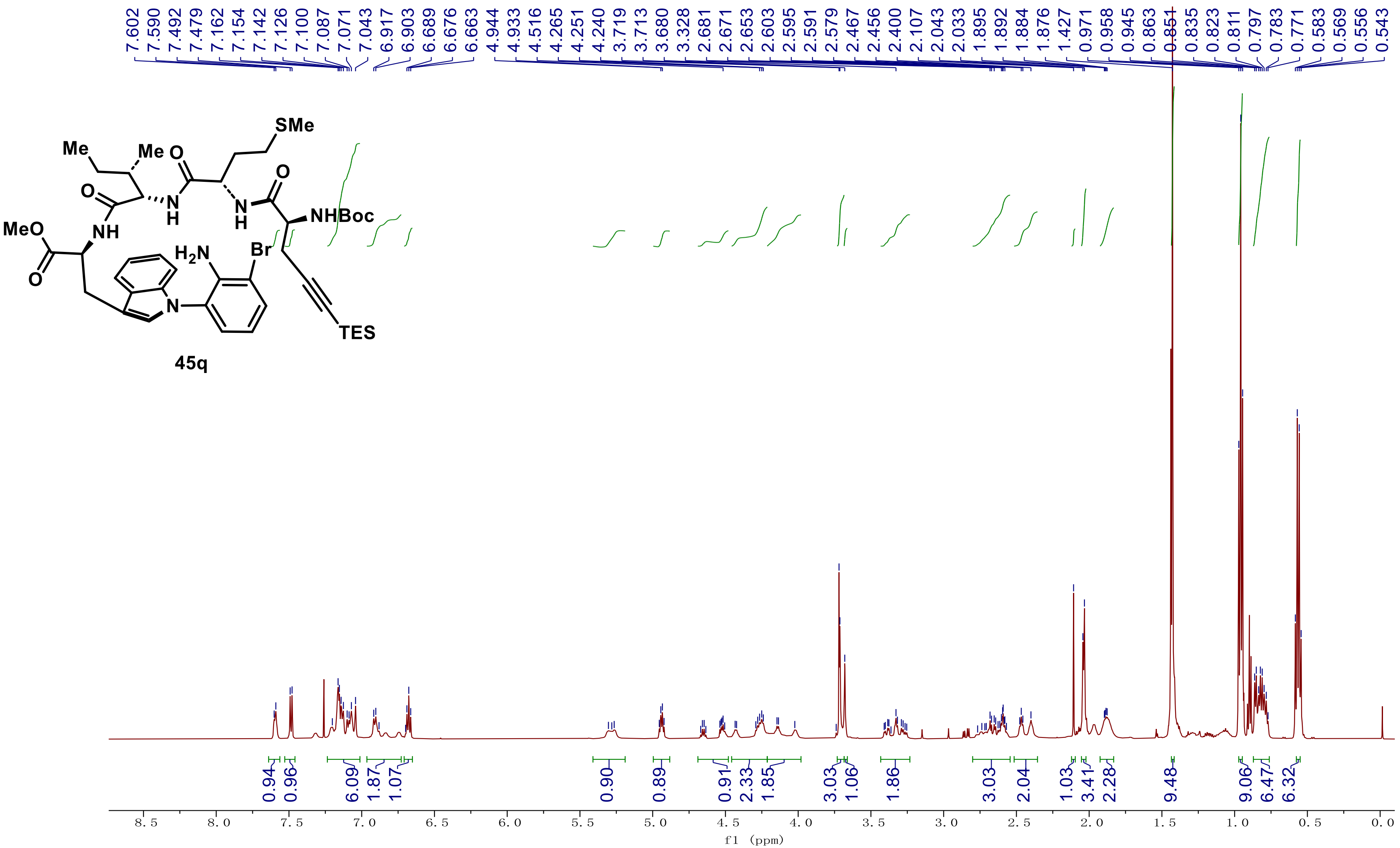

Compound 45q ^{13}C NMR (151 MHz, $CDCl_3$)

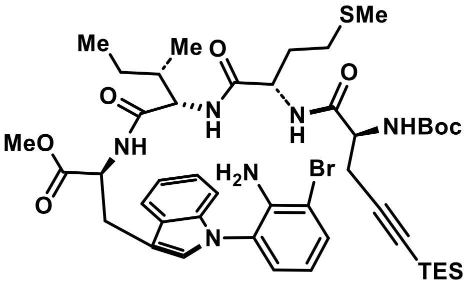

45q

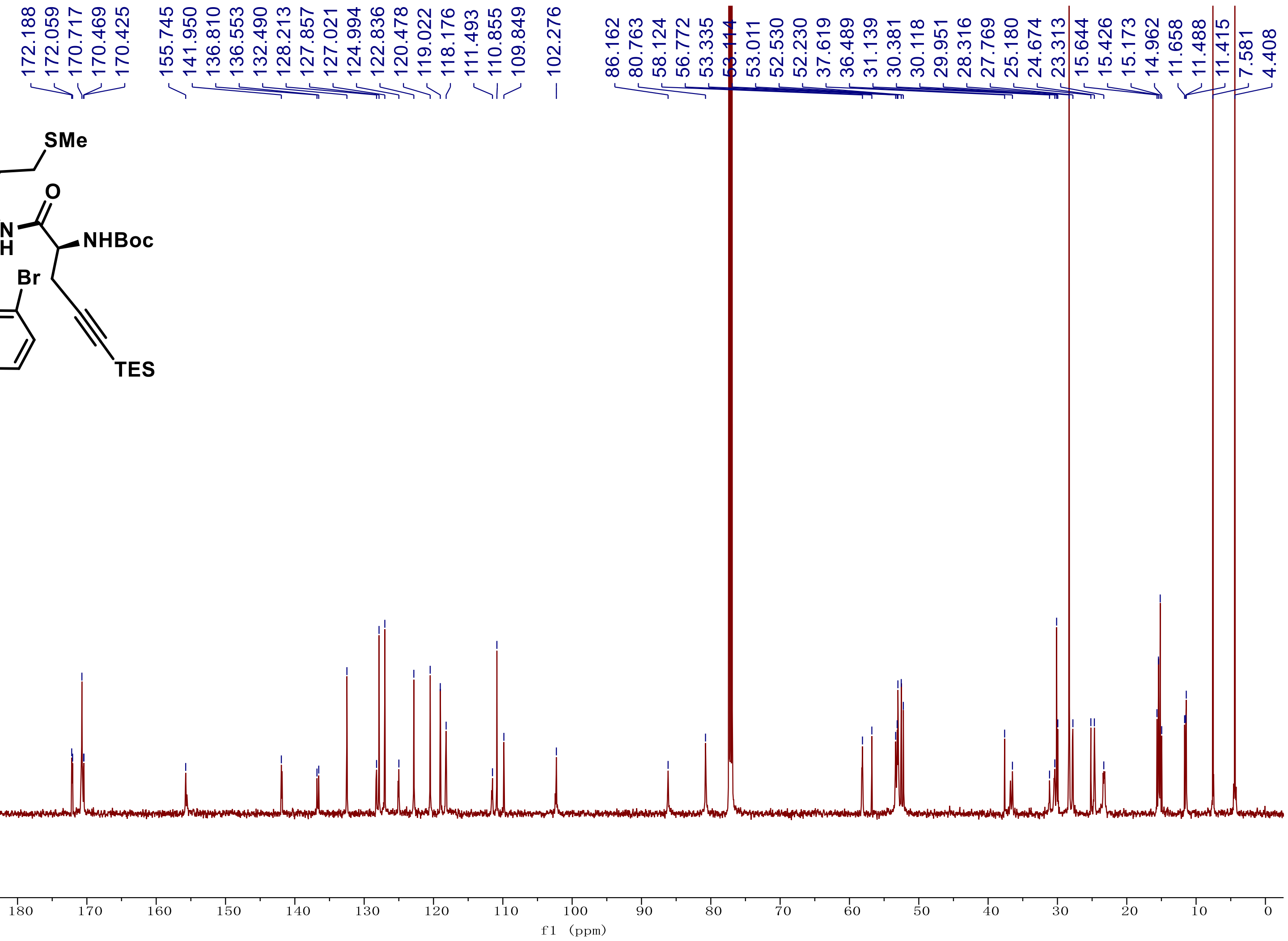

Compound 45r ^{1}H NMR (600 MHz, $CDCl_3$)

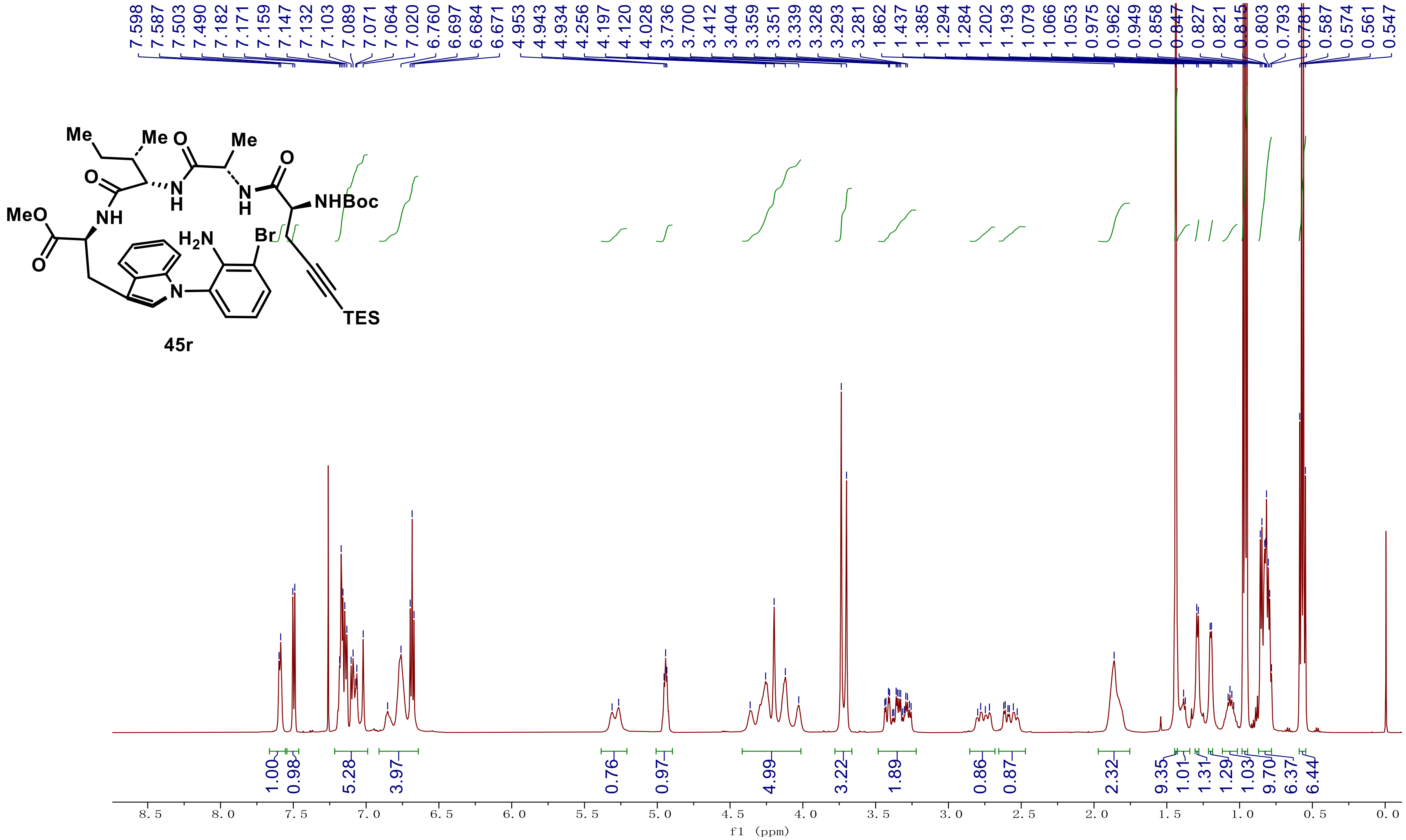

Compound 45r ^{13}C NMR (151 MHz, CDCl$_3$)

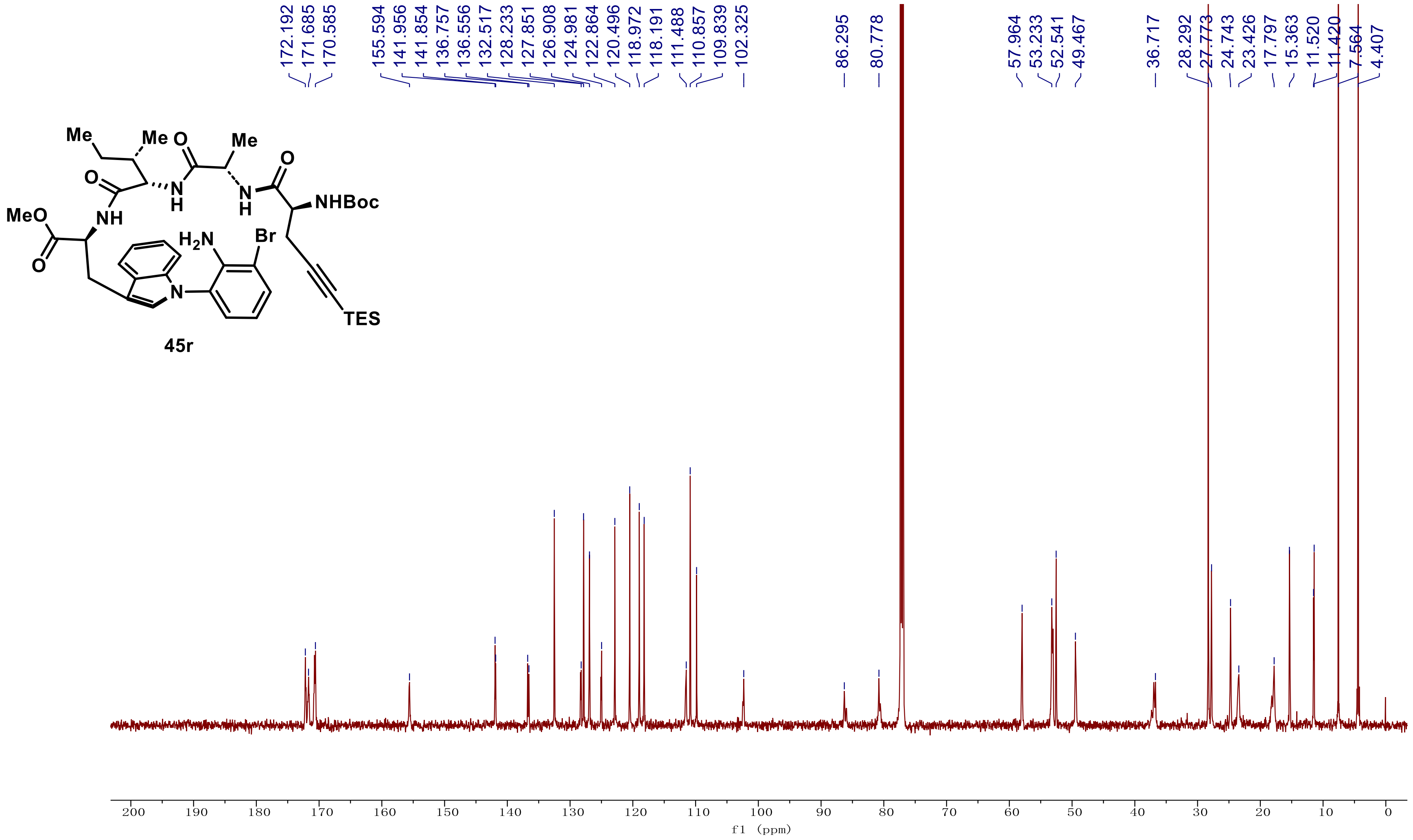

Compound 45s ¹H NMR (600 MHz, CDCl₃)

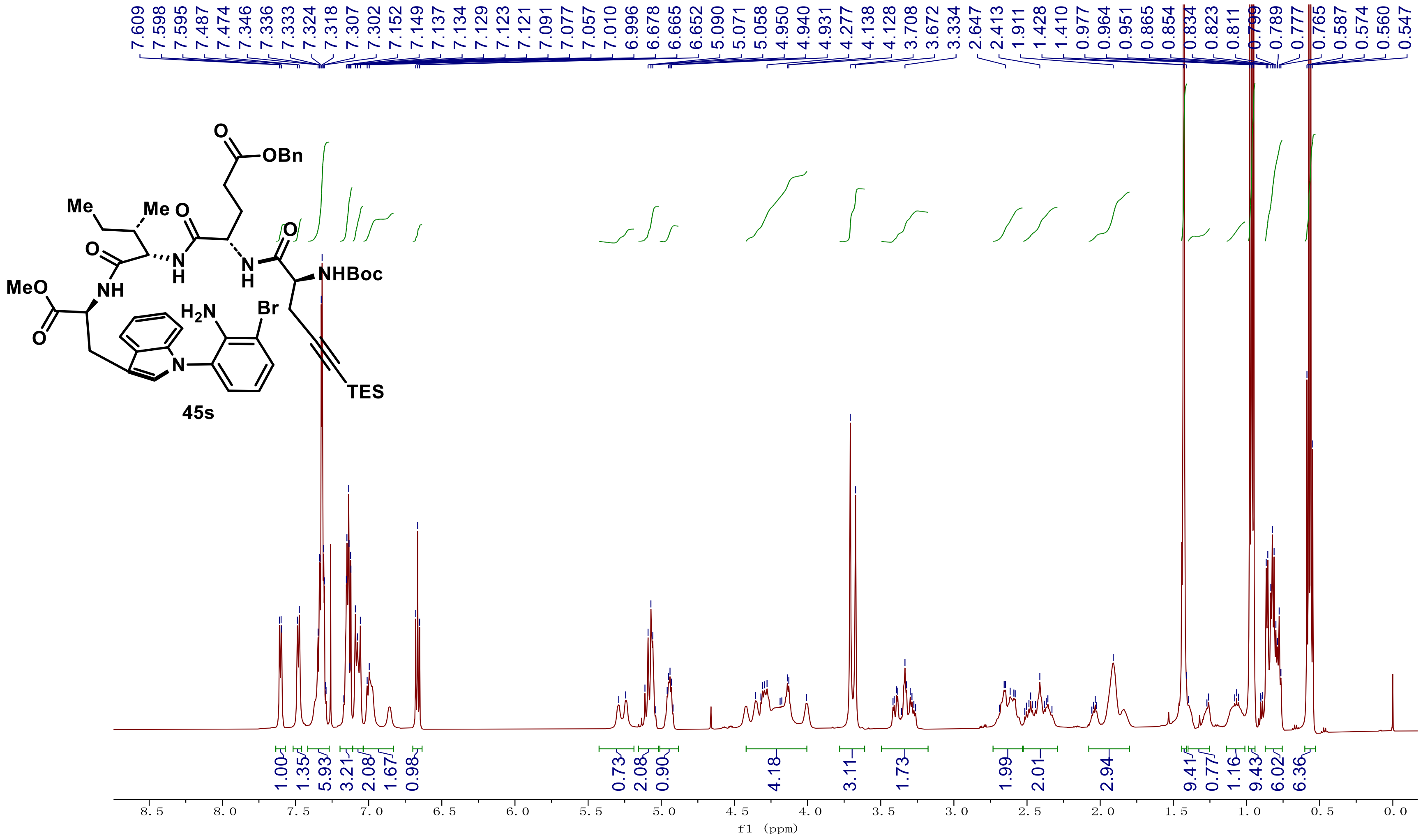

Compound 45s ^{13}C NMR (151 MHz, $CDCl_3$)

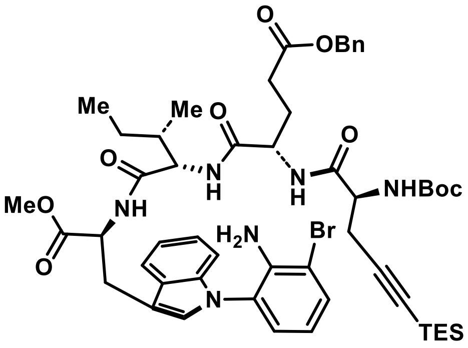

45s

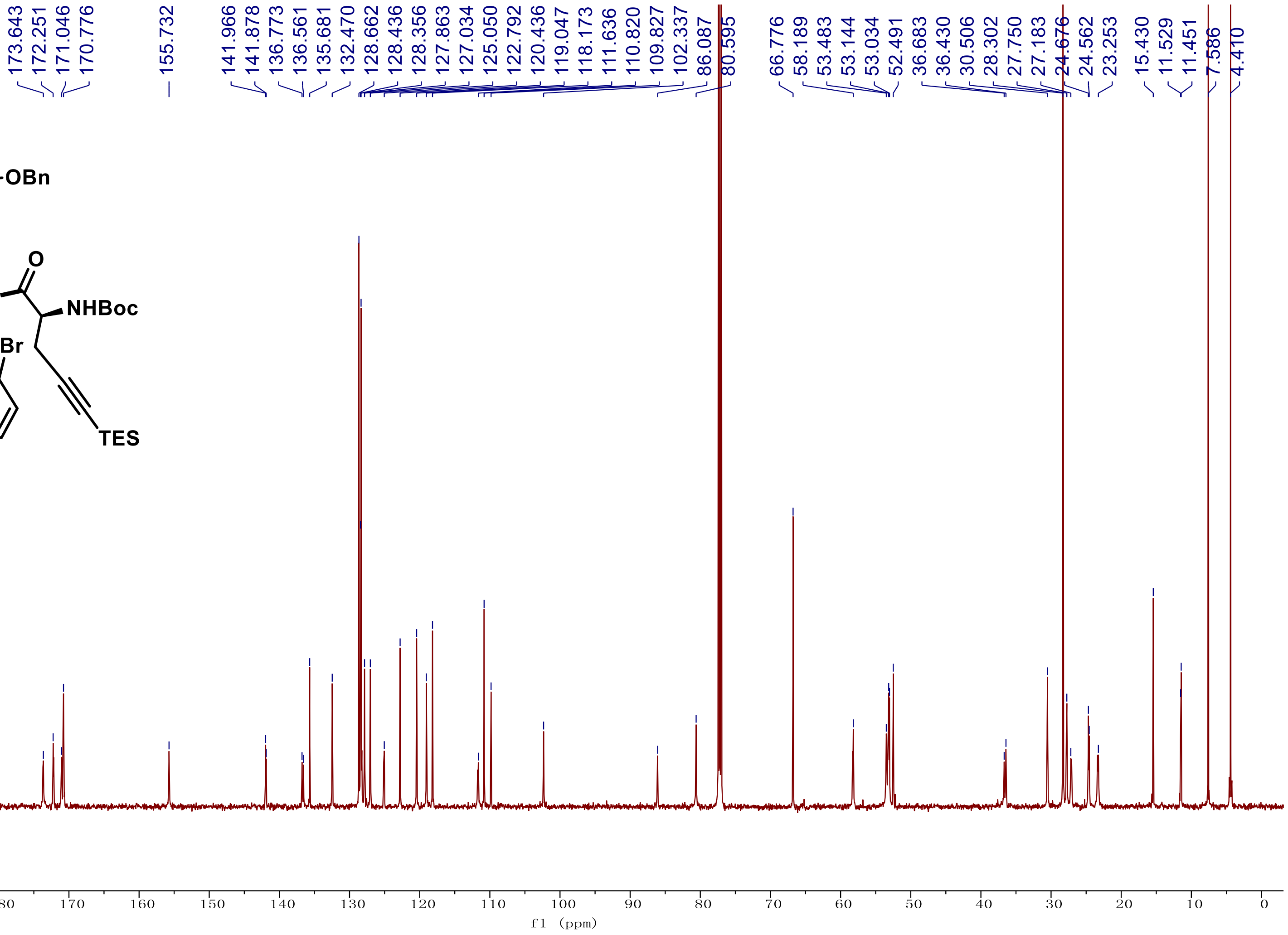

Compound 45t ^{1}H NMR (600 MHz, $CDCl_3$)

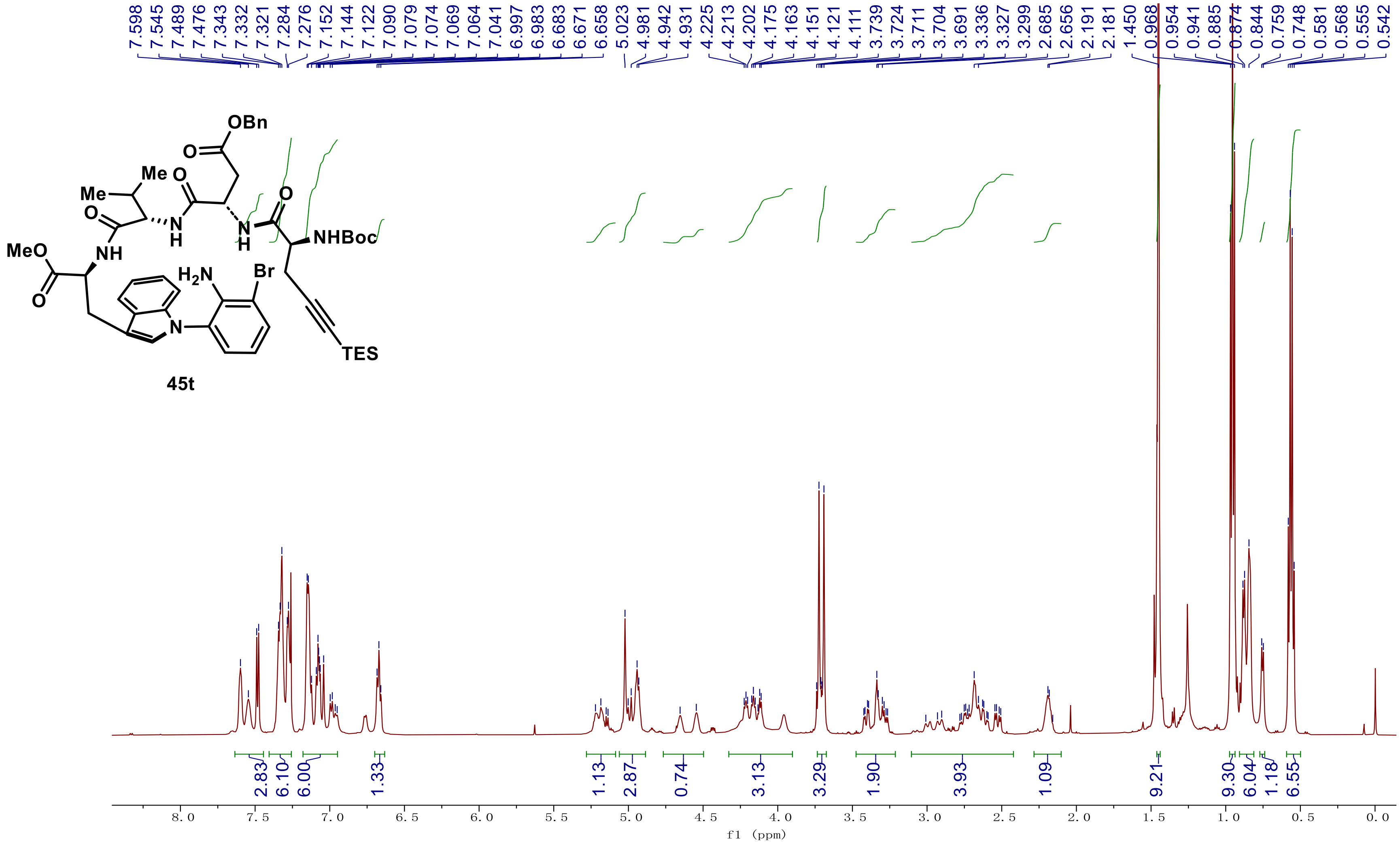

Compound 45t ^{13}C NMR (151 MHz, CDCl$_3$)

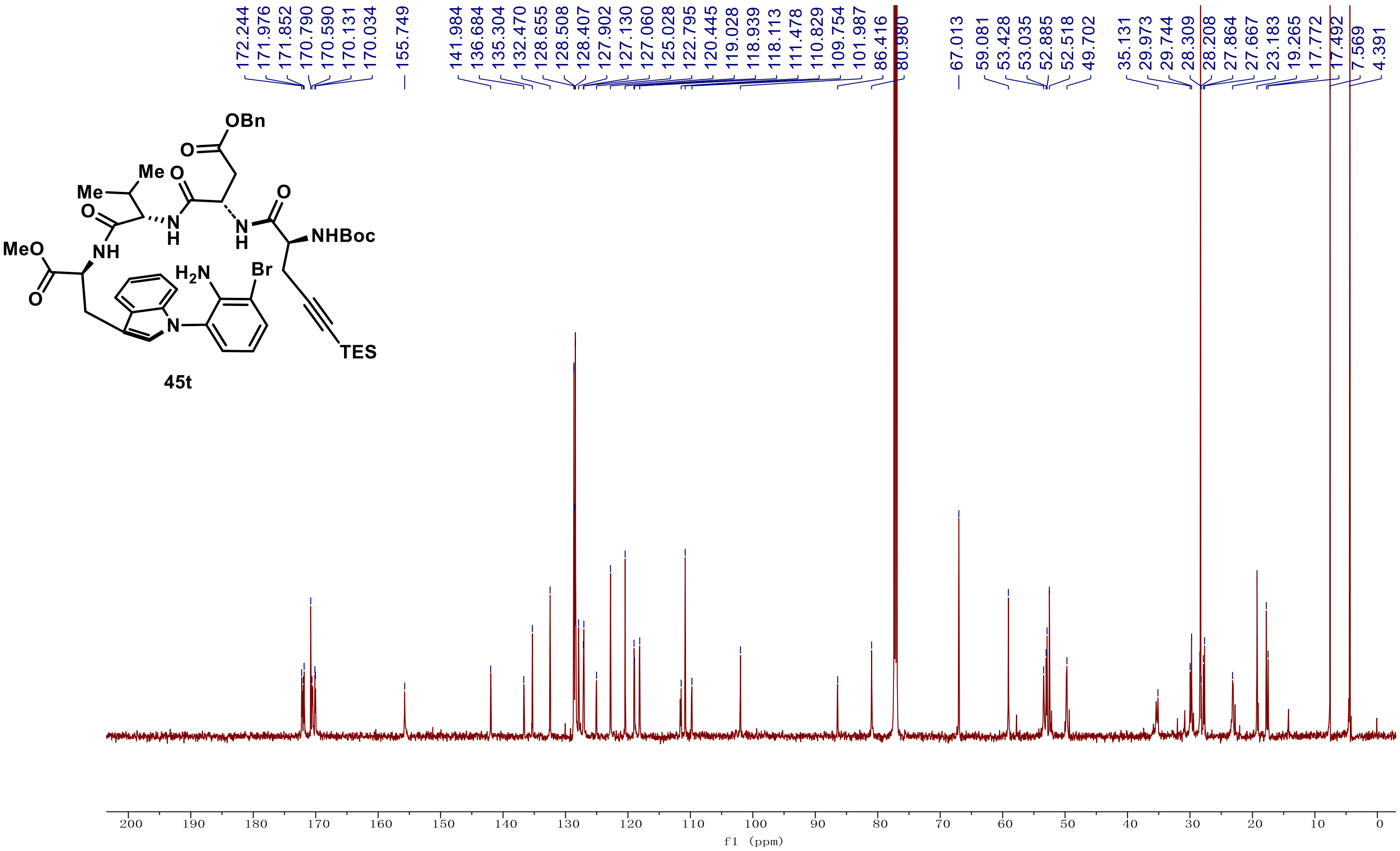

Compound 45u ^{1}H NMR (600 MHz, CDCl₃)

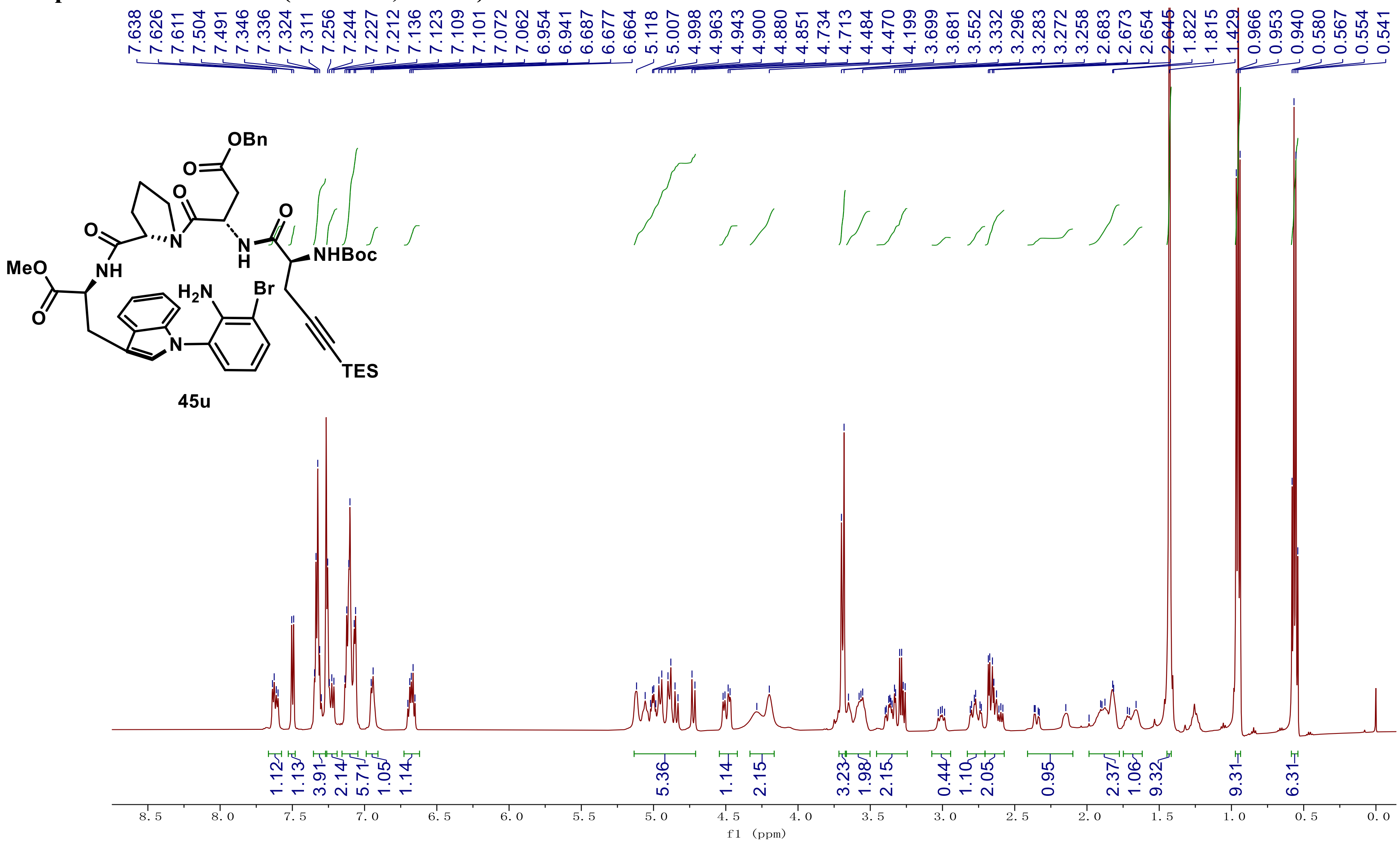

Compound 45u ^{13}C NMR (151 MHz, $CDCl_3$)

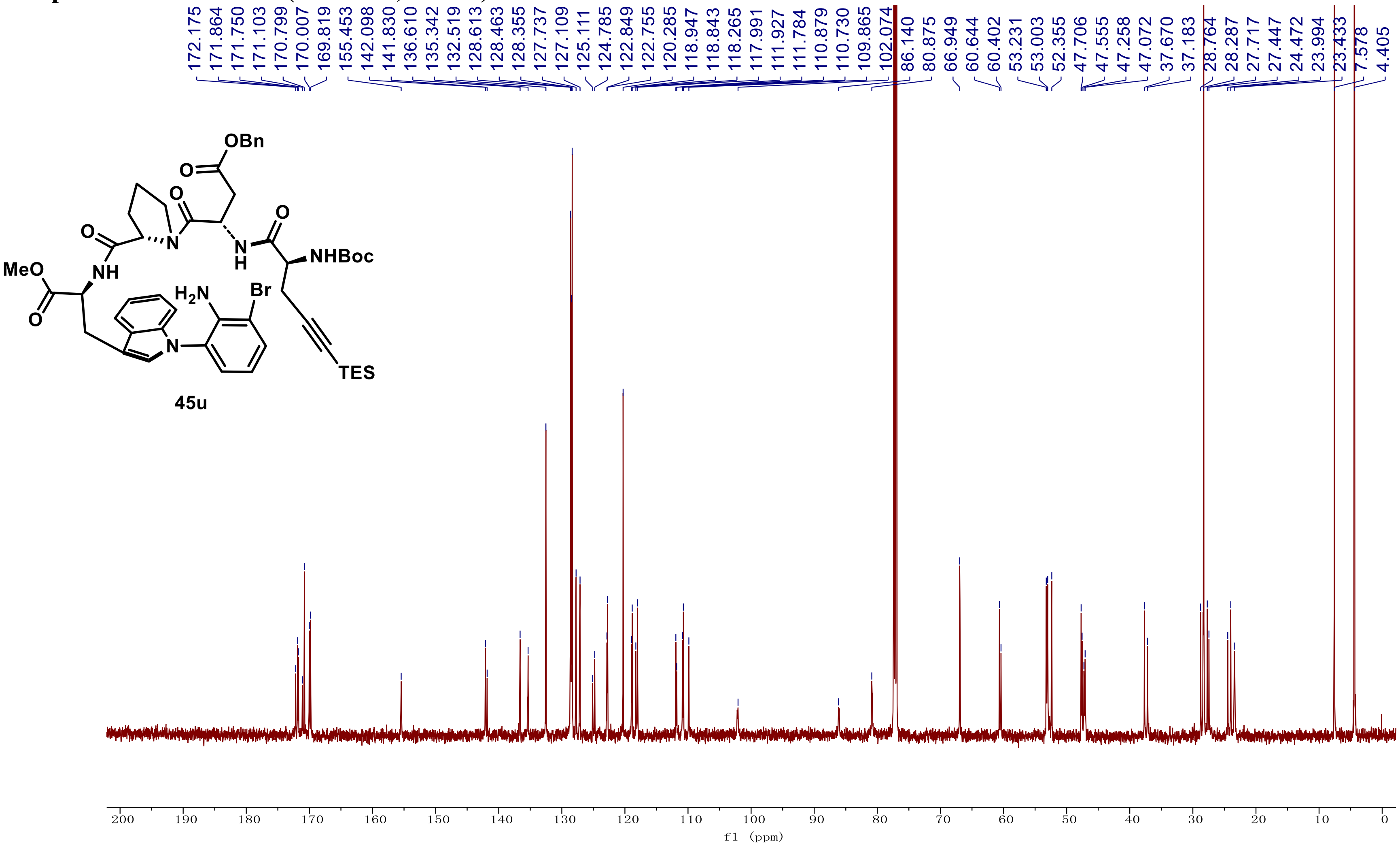

Compound 45y ¹H NMR (600 MHz, CDCl₃)

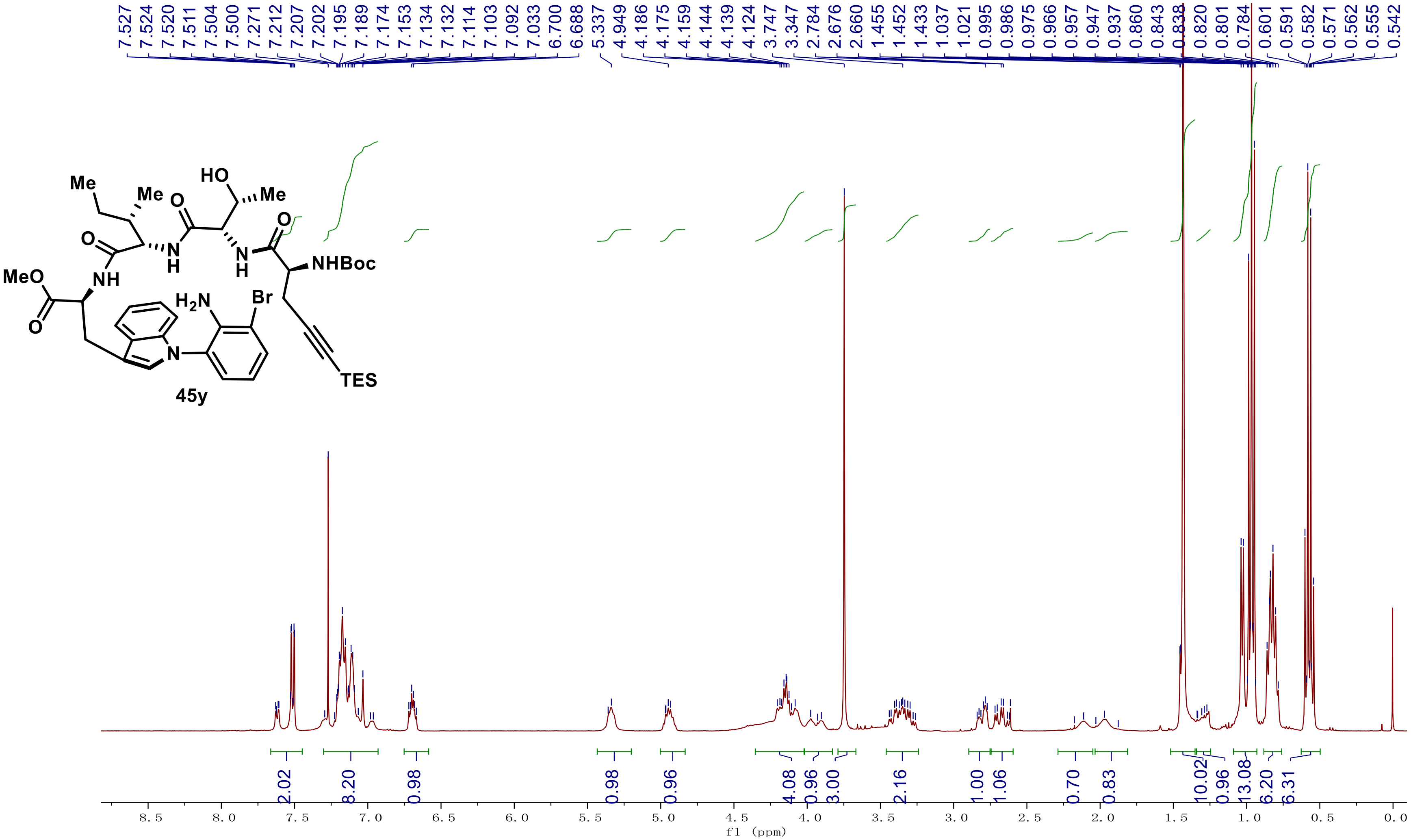

Compound 45y ^{13}C NMR (151 MHz, CDCl$_3$)

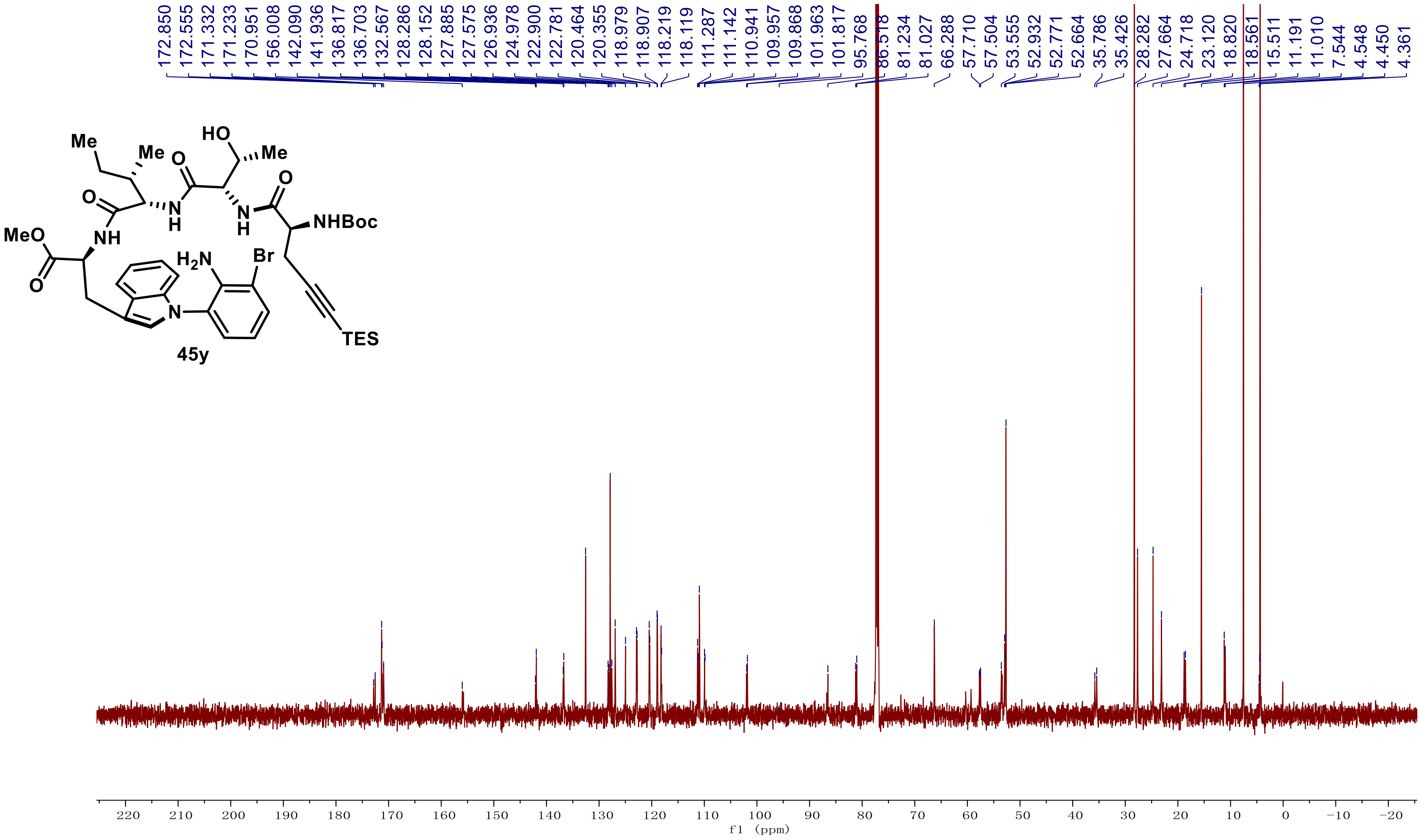

Compound 46a ^{1}H NMR (600 MHz, $CDCl_3$)

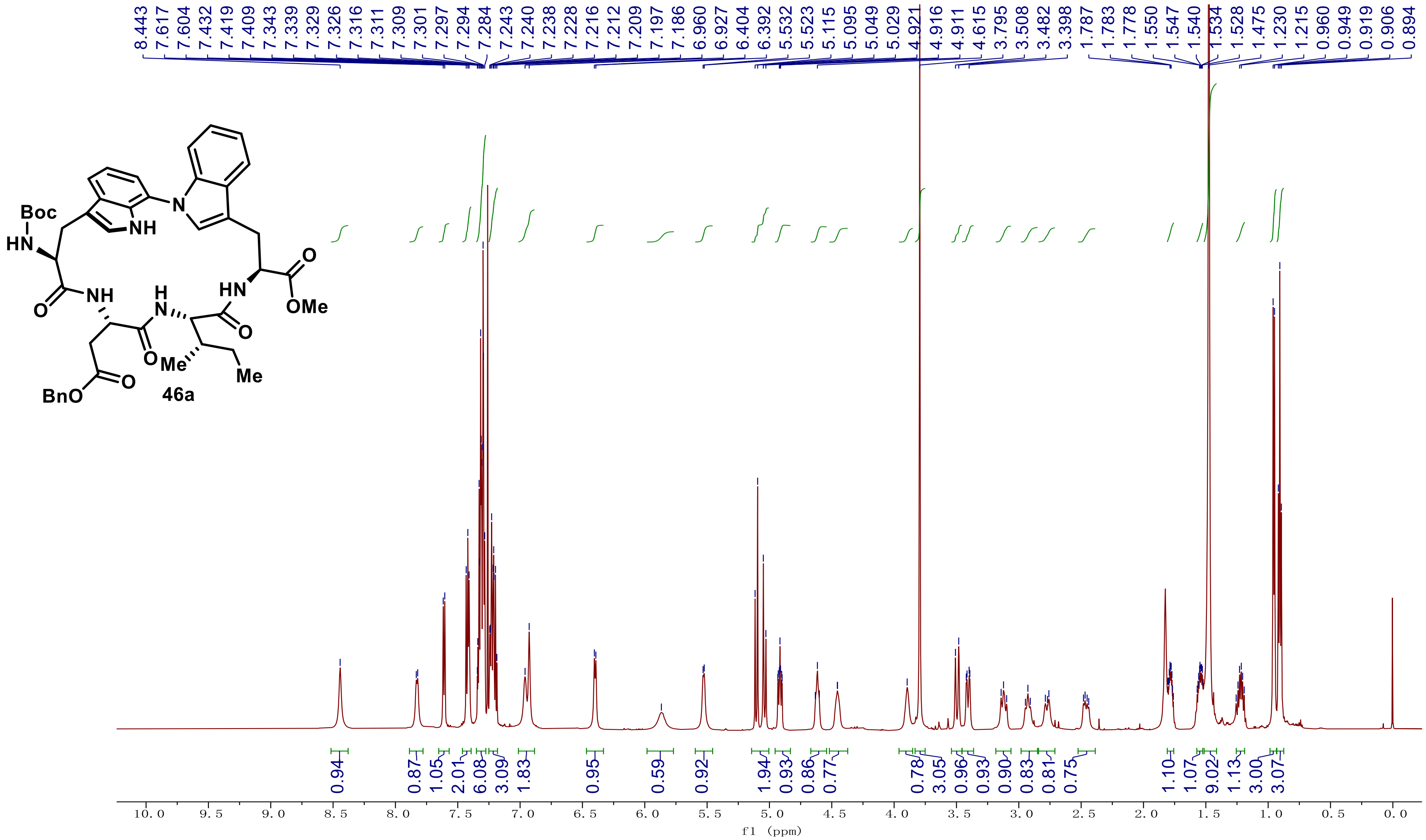

Compound 46a ^{13}C NMR (151 MHz, CDCl$_3$)

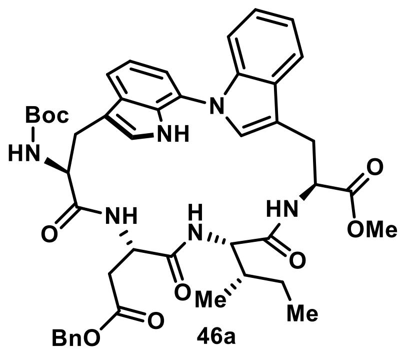

46a

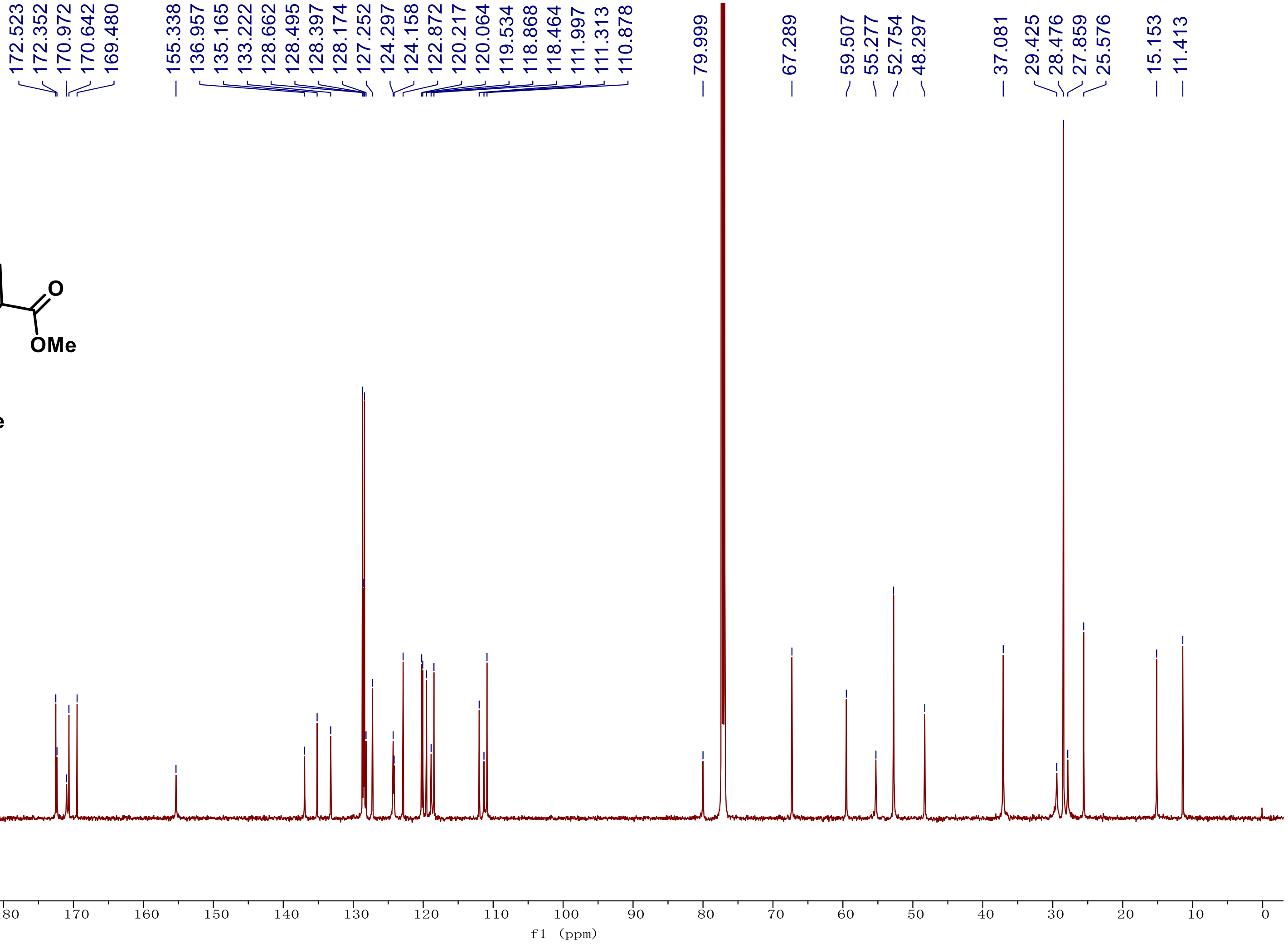

Compound 46b ¹H NMR (600 MHz, CDCl₃)

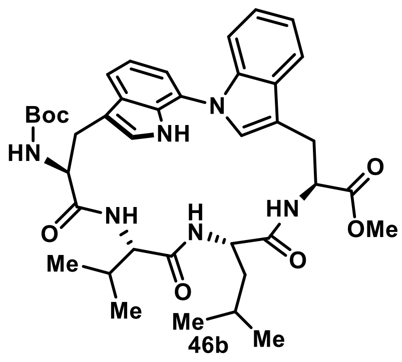

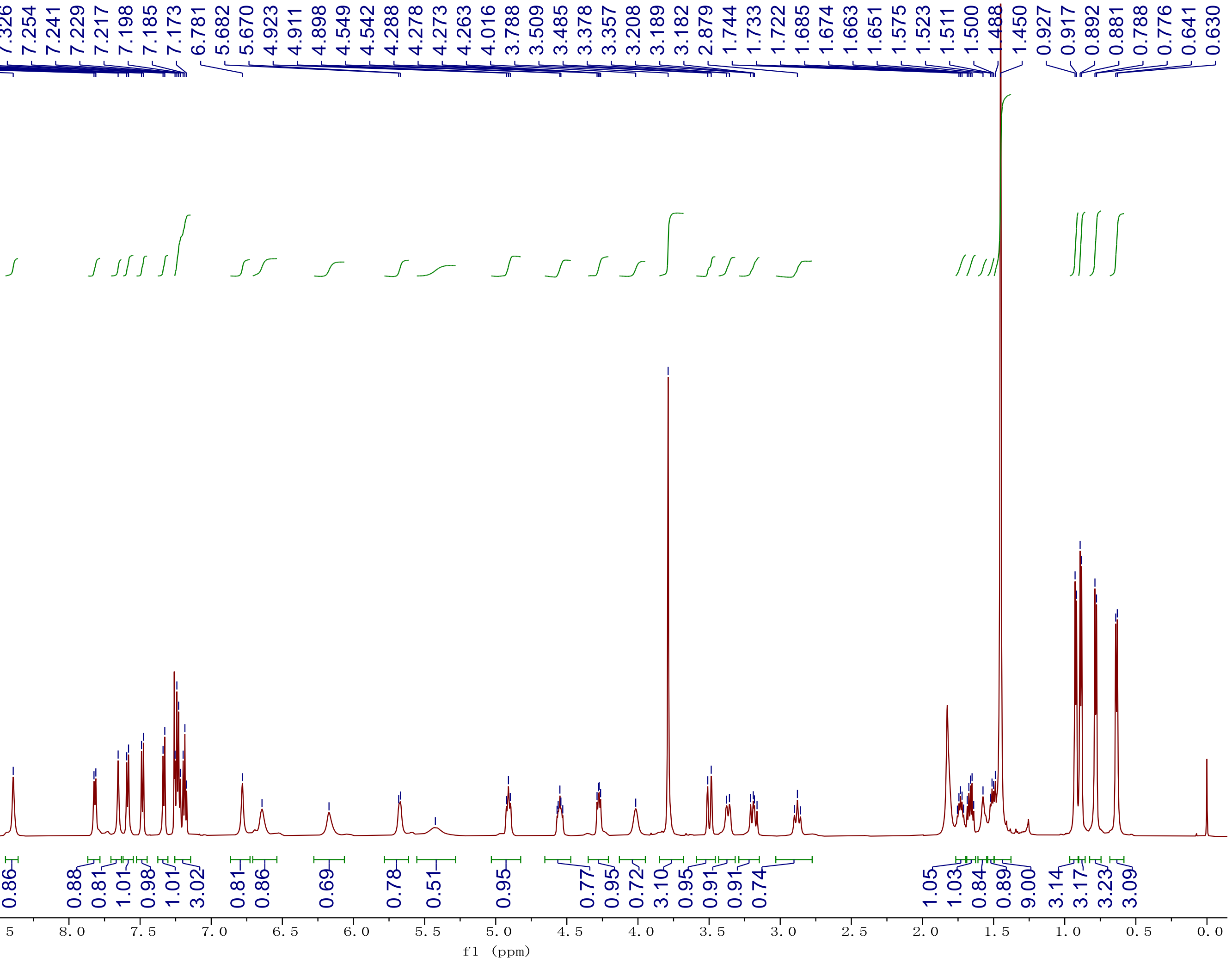

Compound 46b ^{13}C NMR (151 MHz, $CDCl_3$)

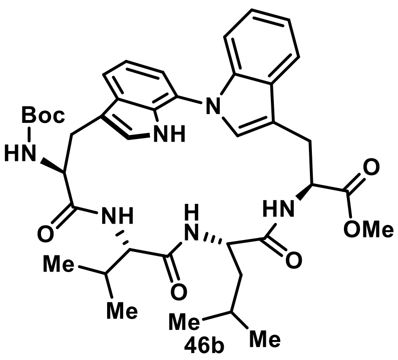

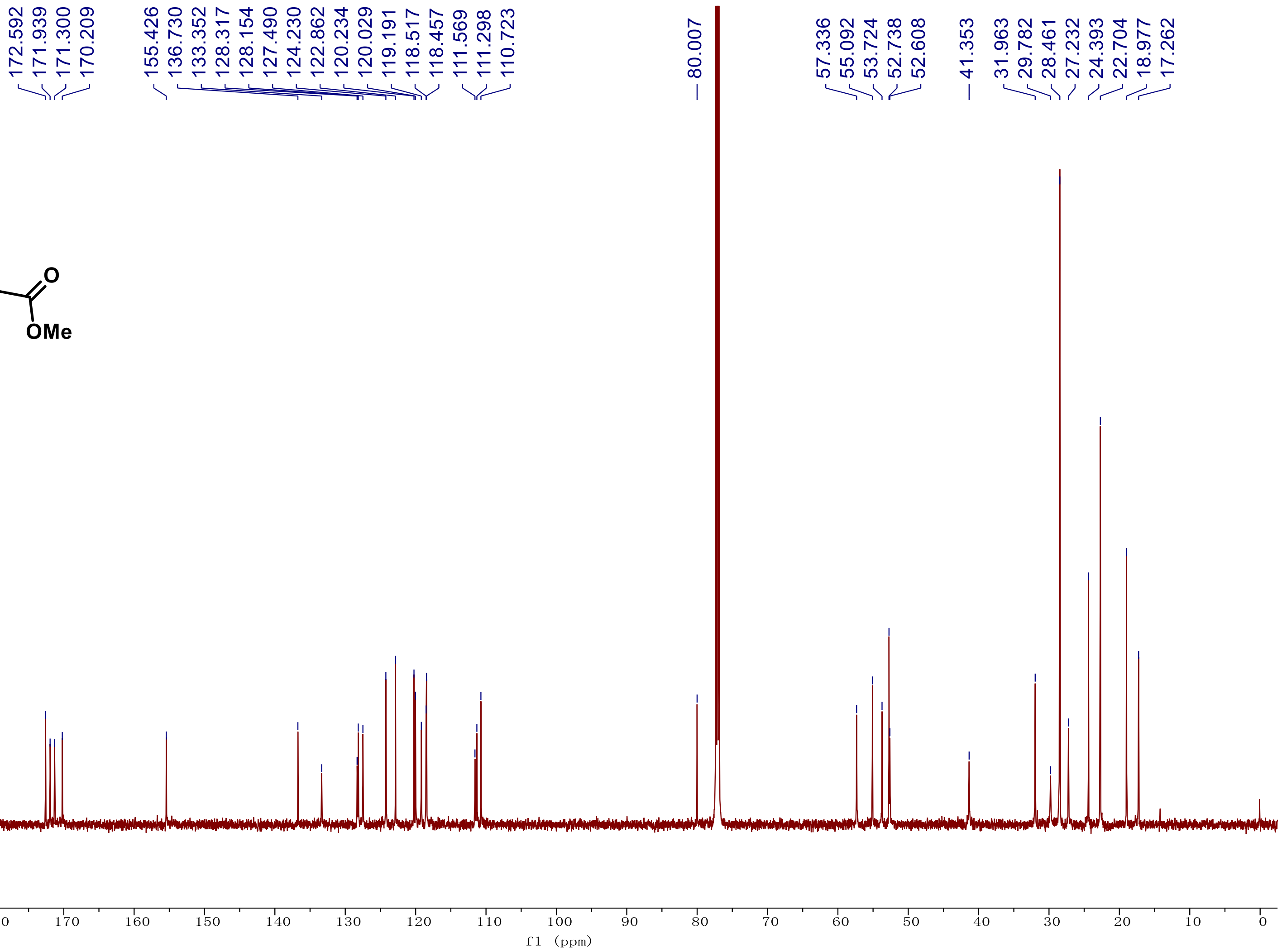

Compound 46c ¹H NMR (600 MHz, CDCl₃)

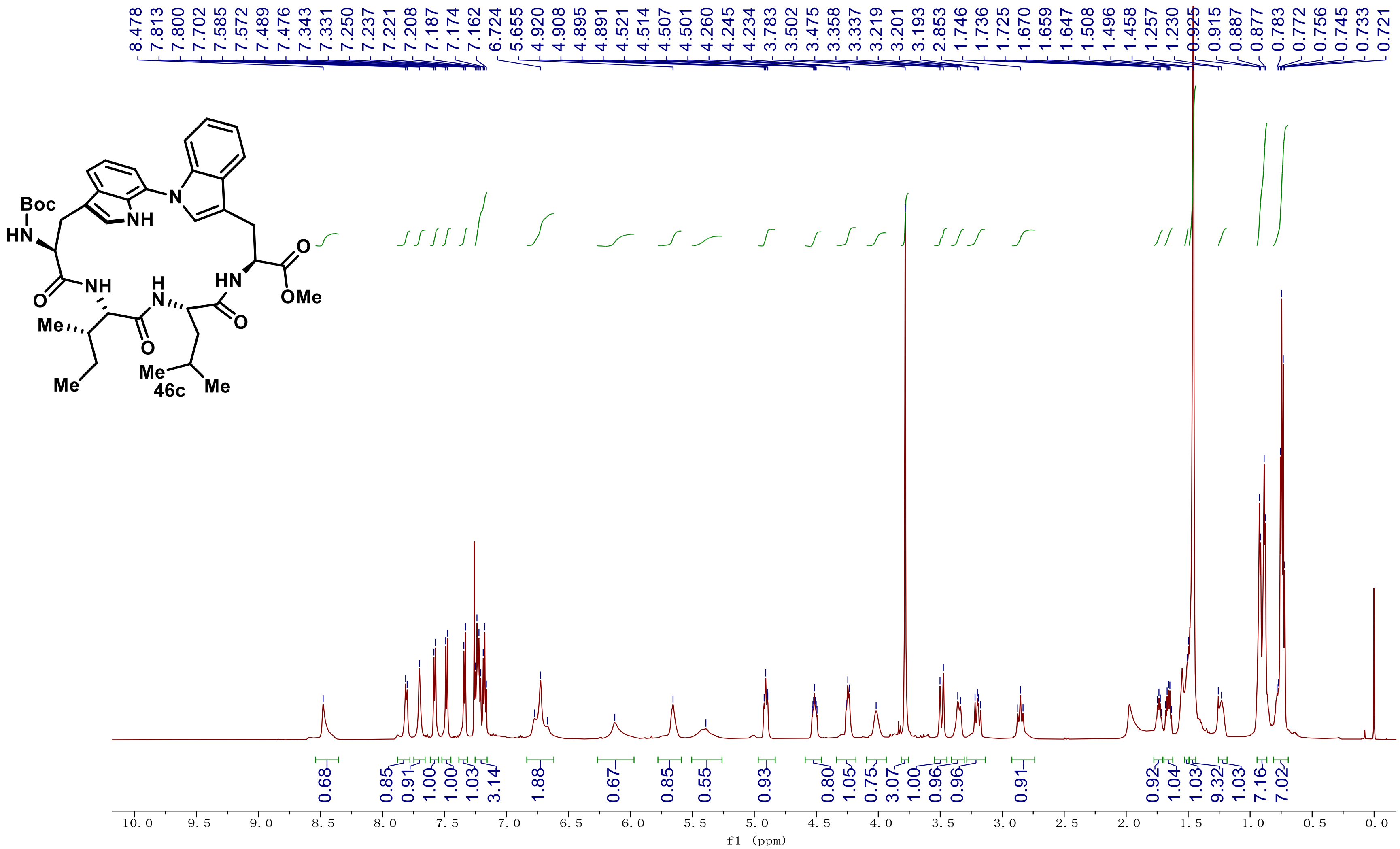

Compound 46c ^{13}C NMR (151 MHz, $CDCl_3$)

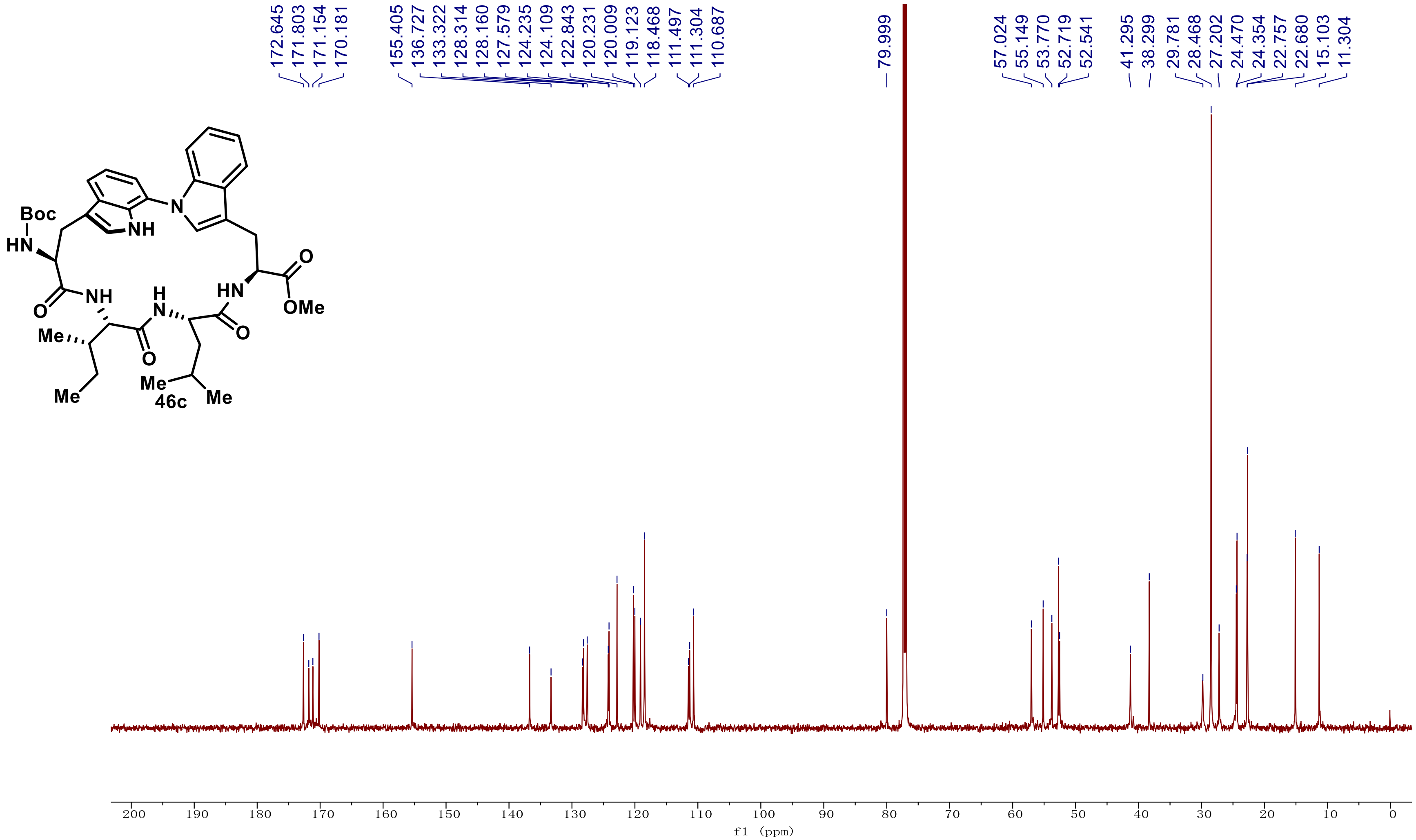

Compound 46d ^{1}H NMR (600 MHz, CDCl₃)

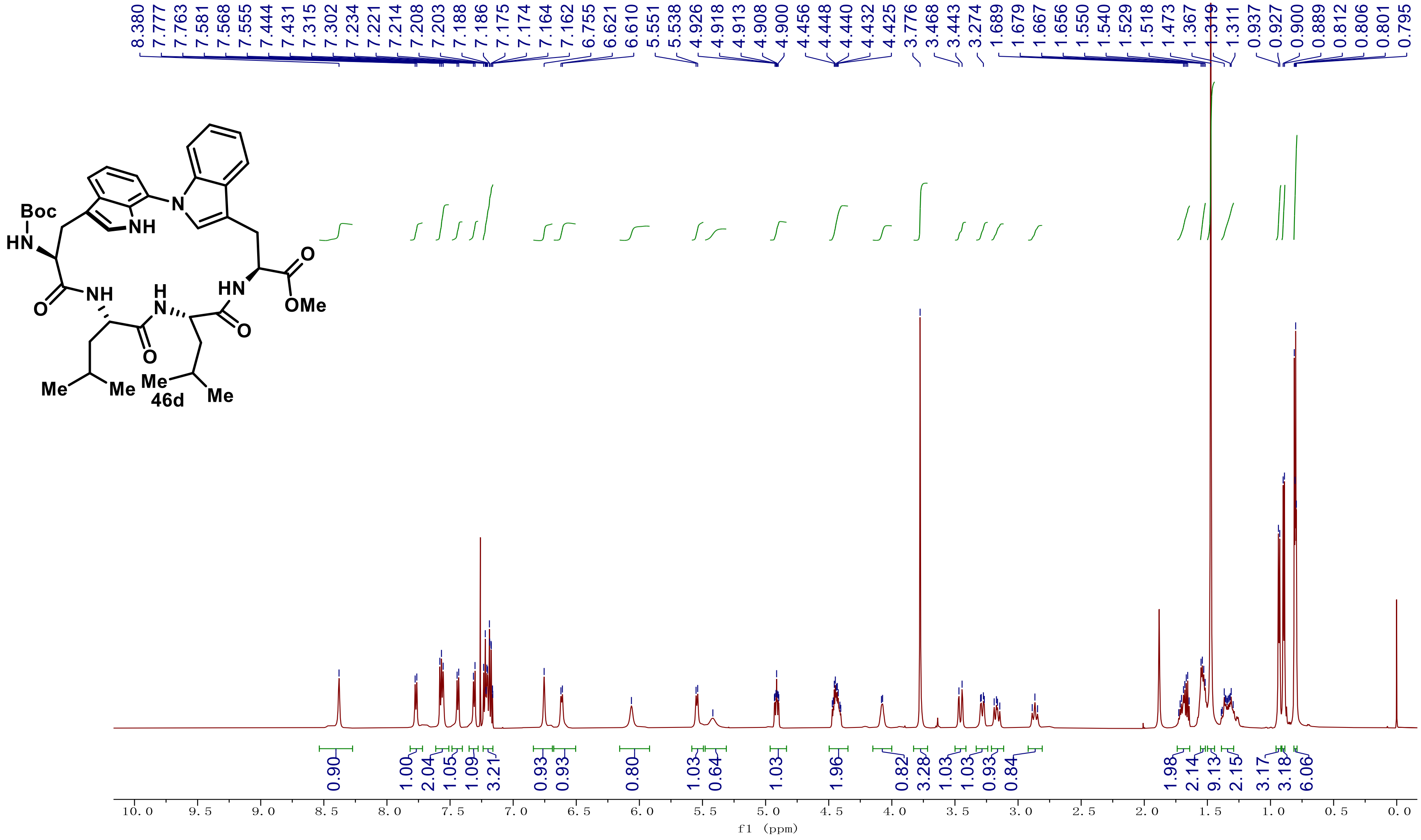

Compound 46d ^{13}C NMR (151 MHz, $CDCl_3$)

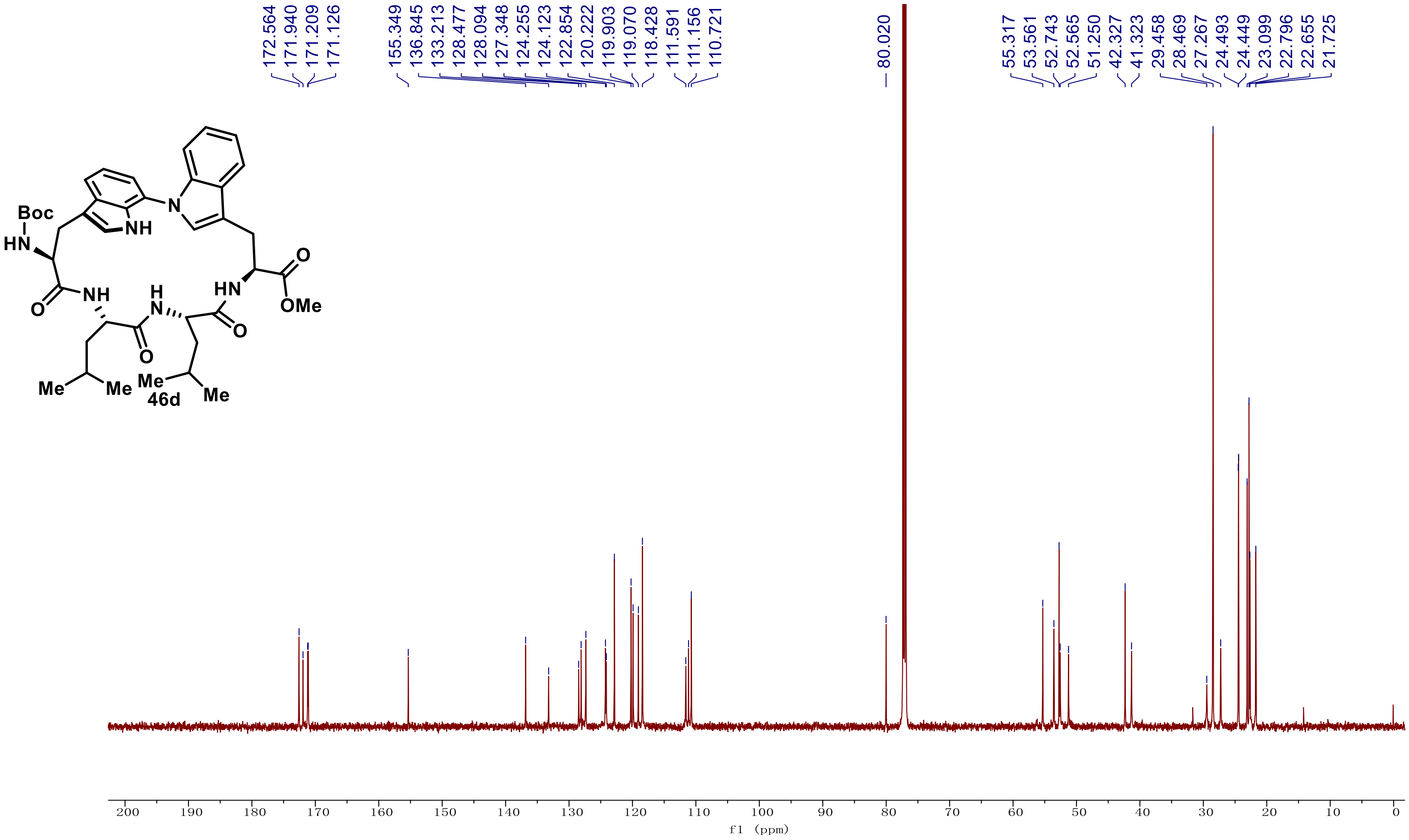

Compound 46e ^{1}H NMR (600 MHz, $CDCl_3$)

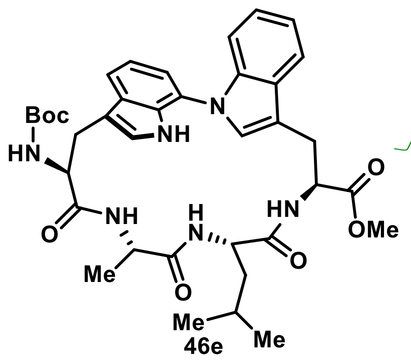

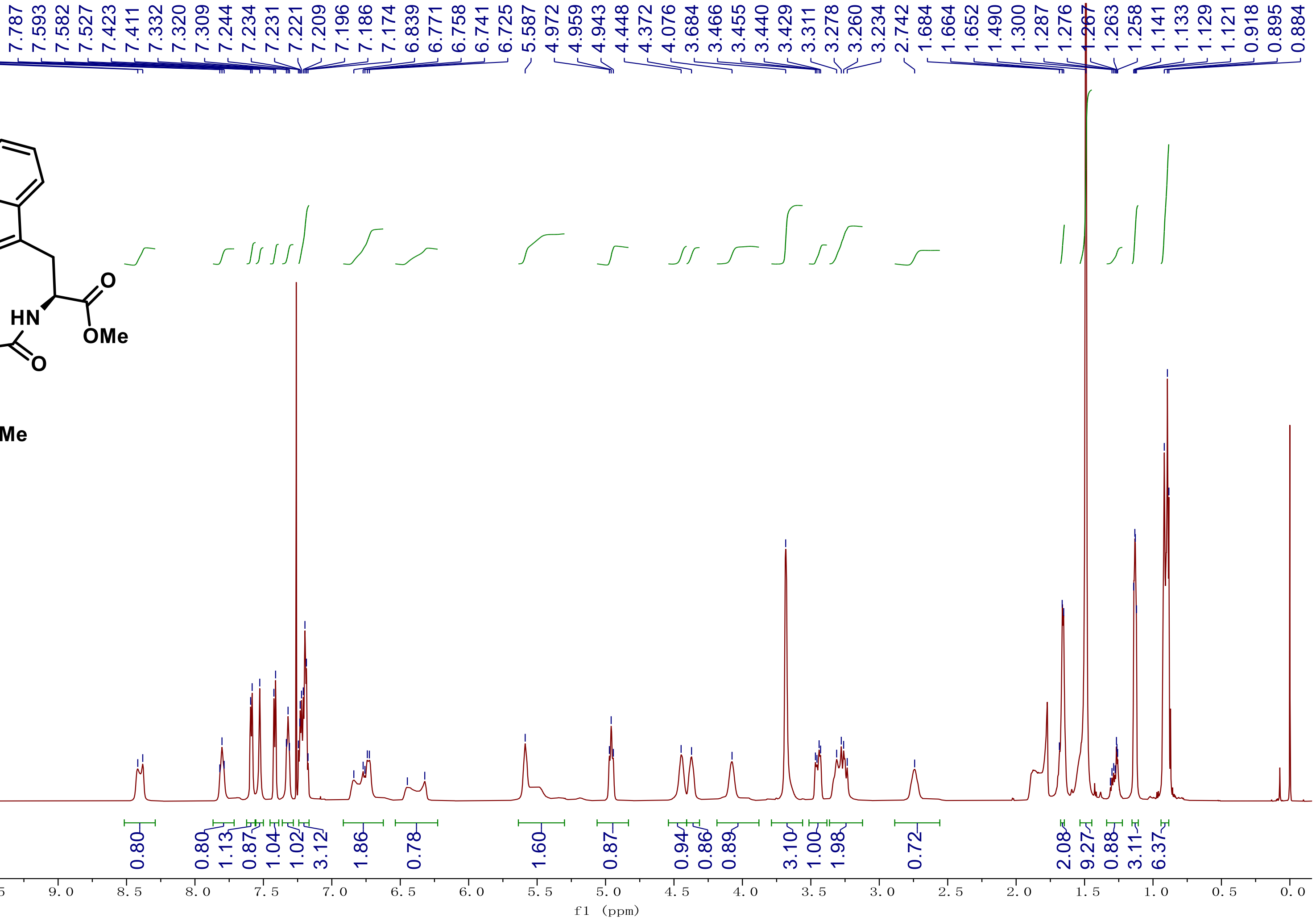

Compound 46e ^{13}C NMR (151 MHz, $CDCl_3$)

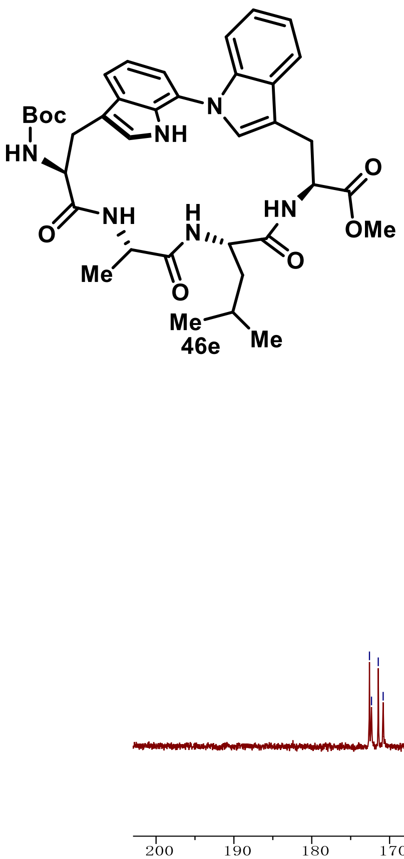

46e

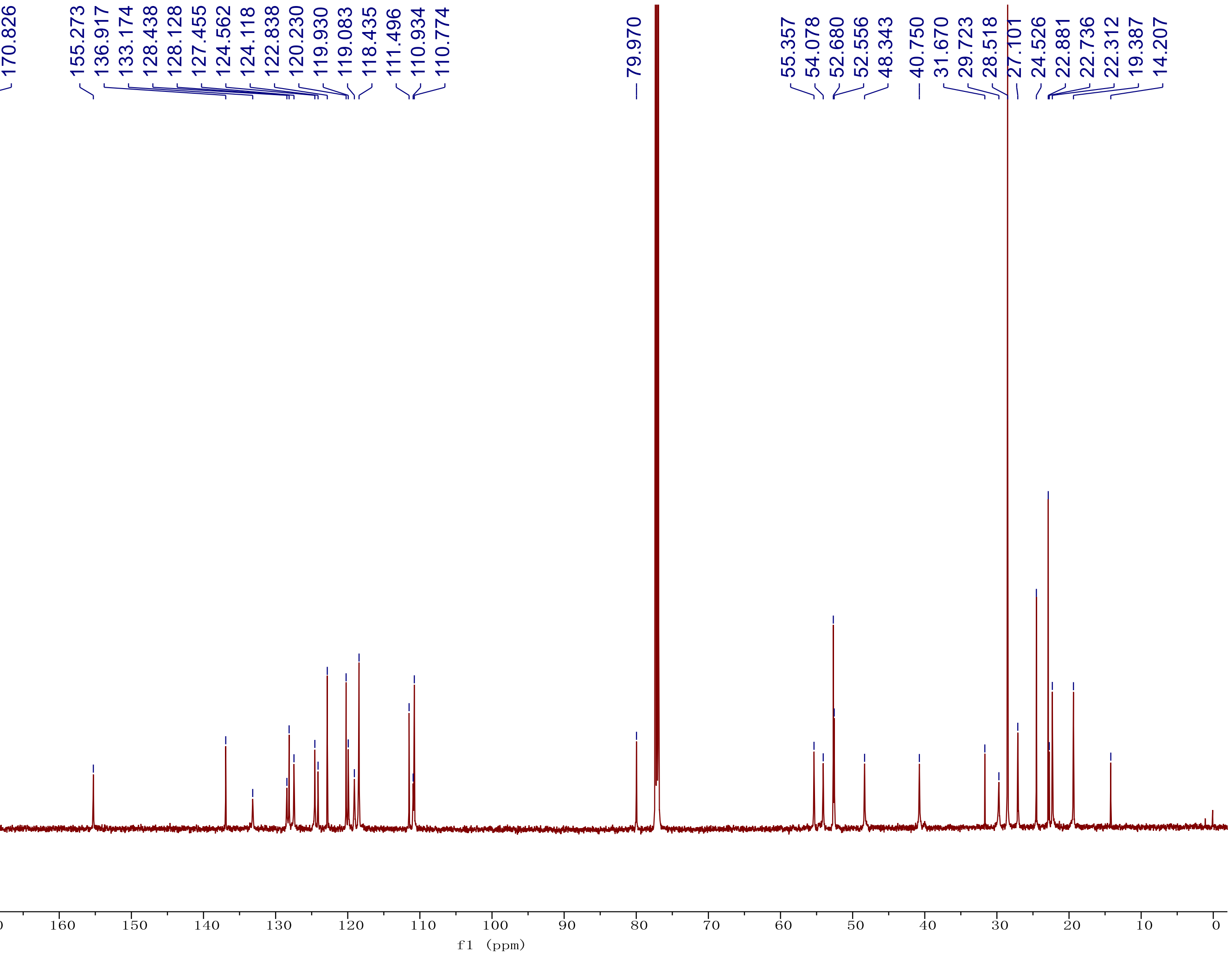

Compound 46e ROESY (600 MHz, CDCl3)

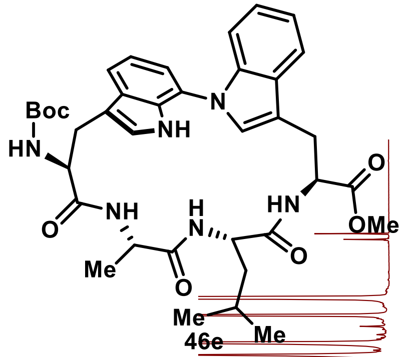

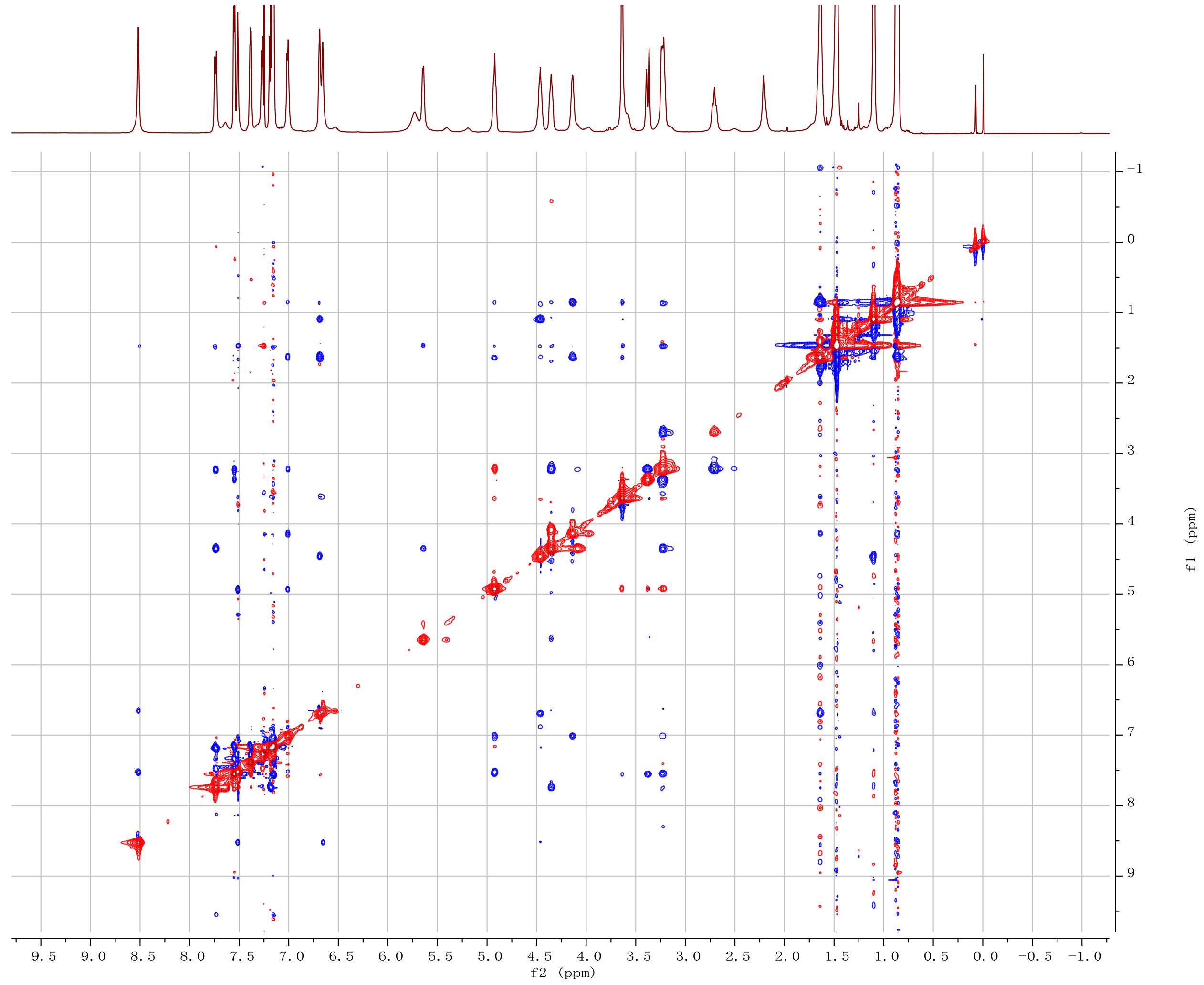

LCMS trace of compound 46e

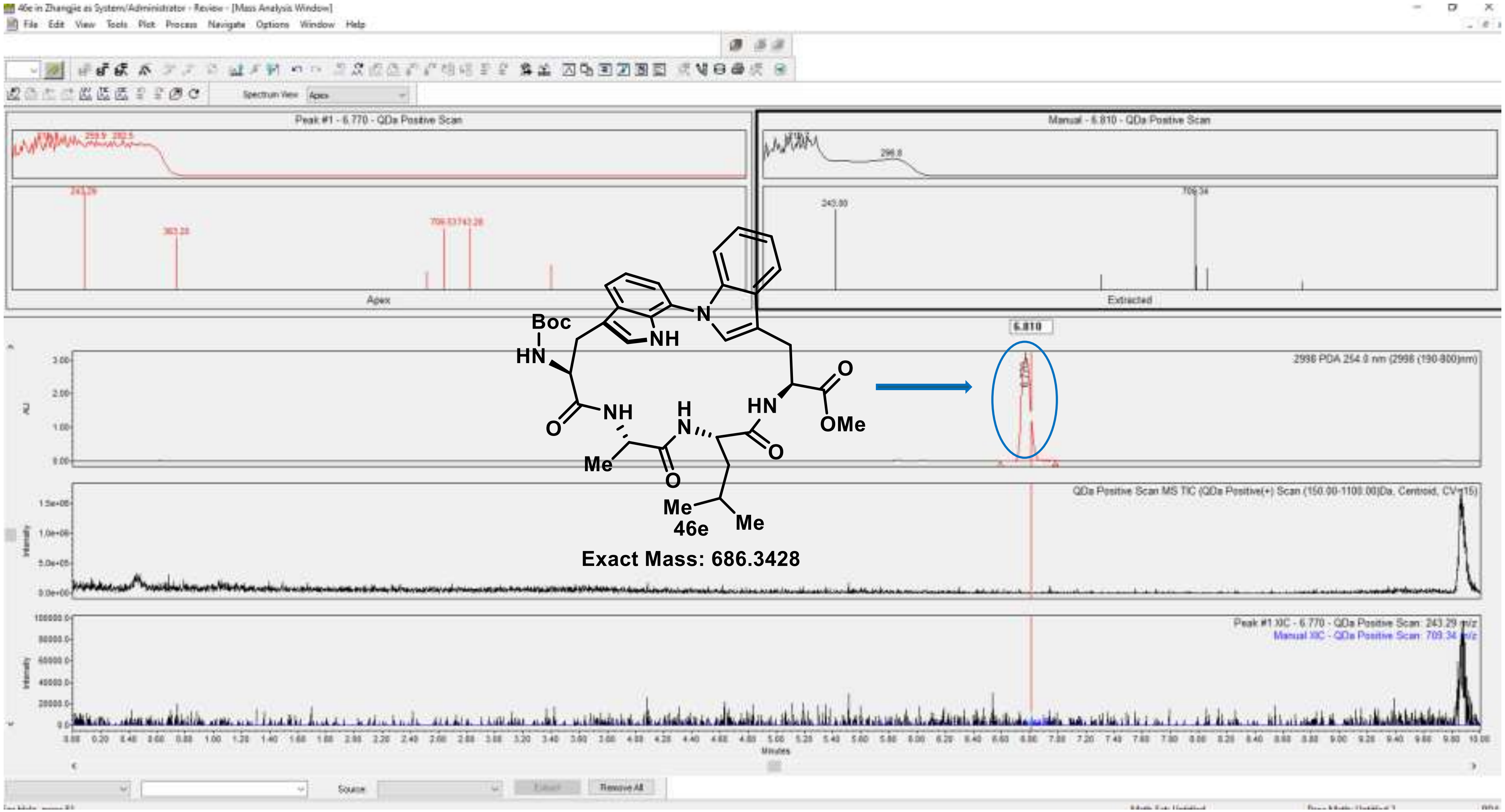

Compound 46f ¹H NMR (600 MHz, $CDCl_3$)

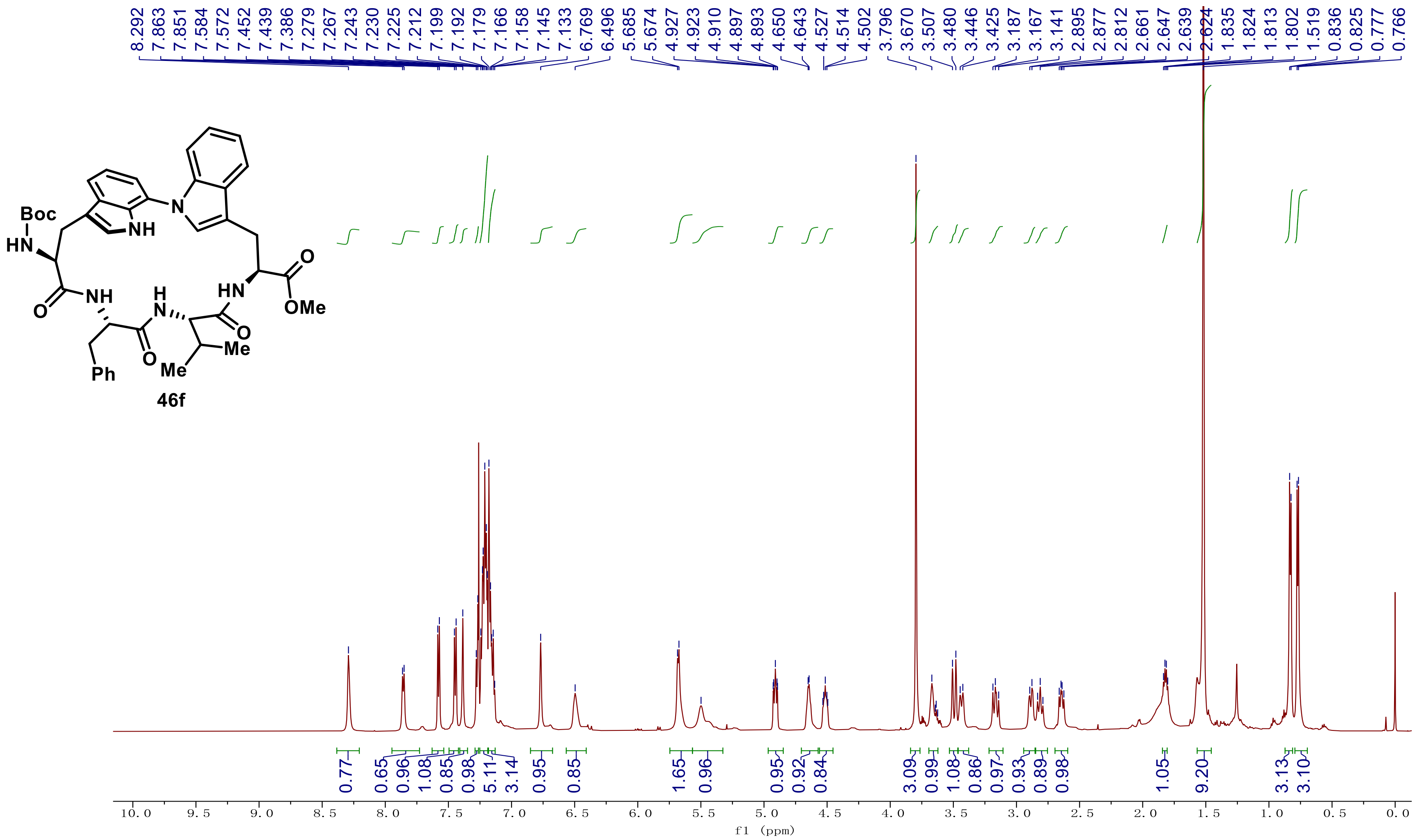

Compound 46f ^{13}C NMR (151 MHz, CDCl$_3$)

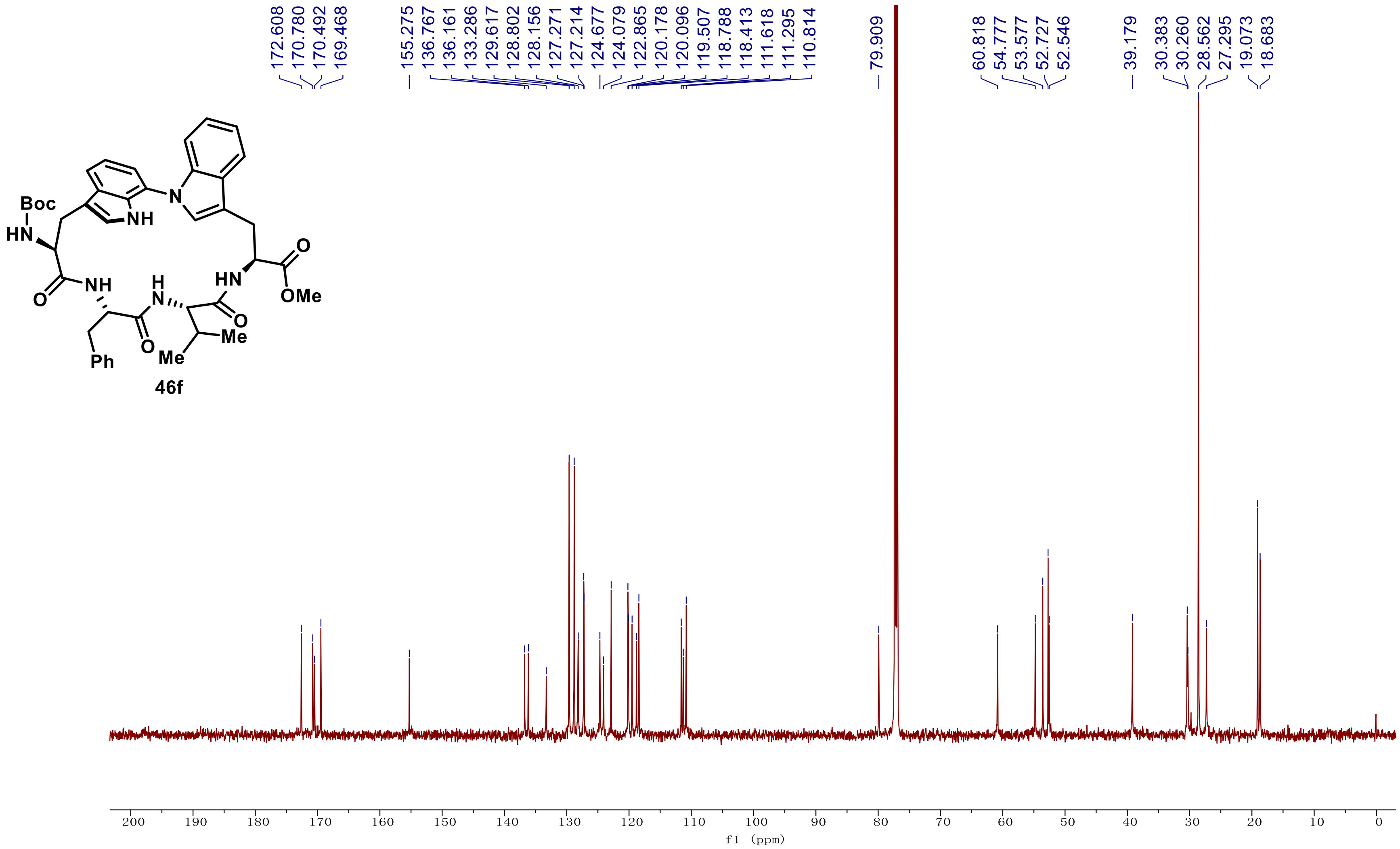

Compound 46g ^{1}H NMR (600 MHz, $CDCl_3$)

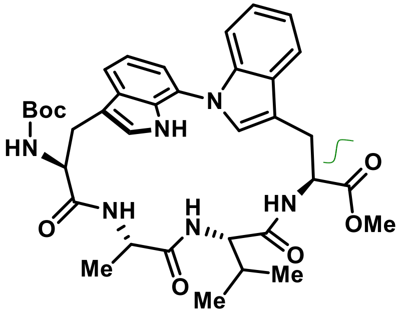

46g

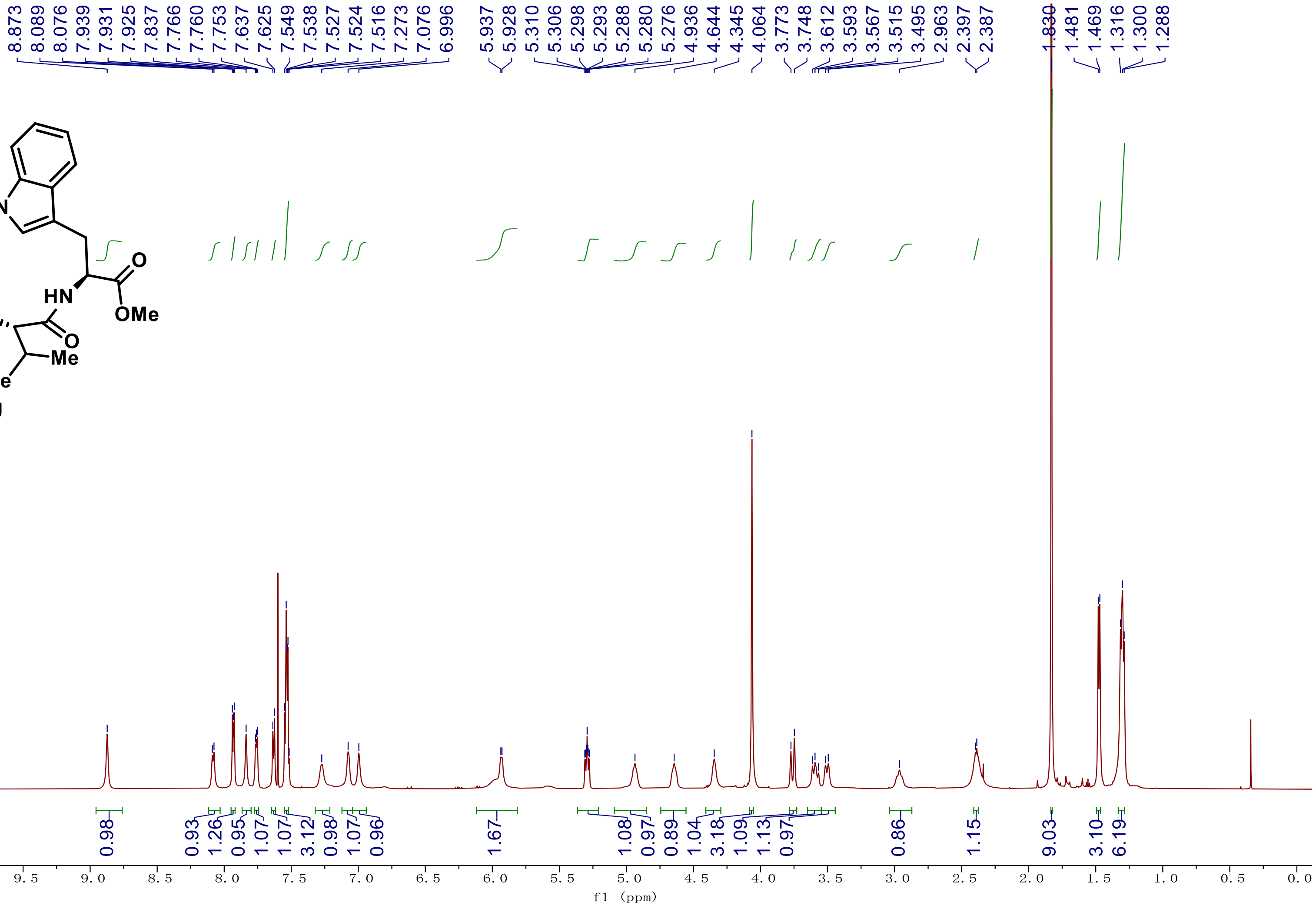

Compound 46g ^{13}C NMR (151 MHz, CDCl$_3$)

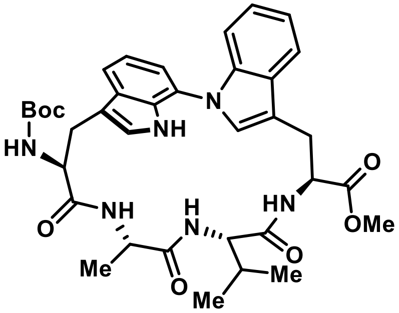

46g

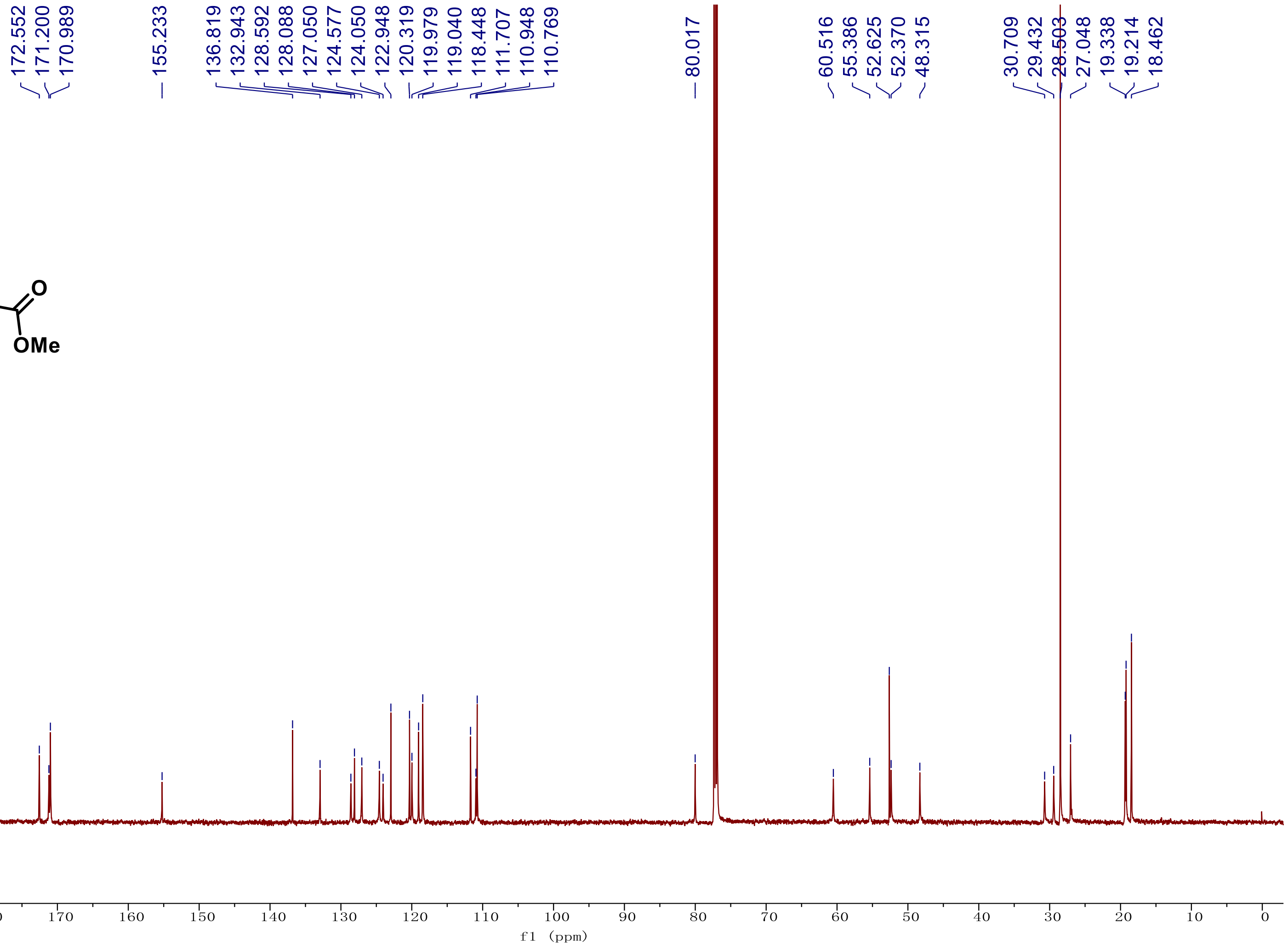

Compound 46h ¹H NMR (600 MHz, CDCl₃)

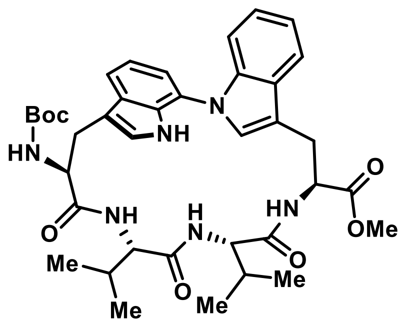

46h

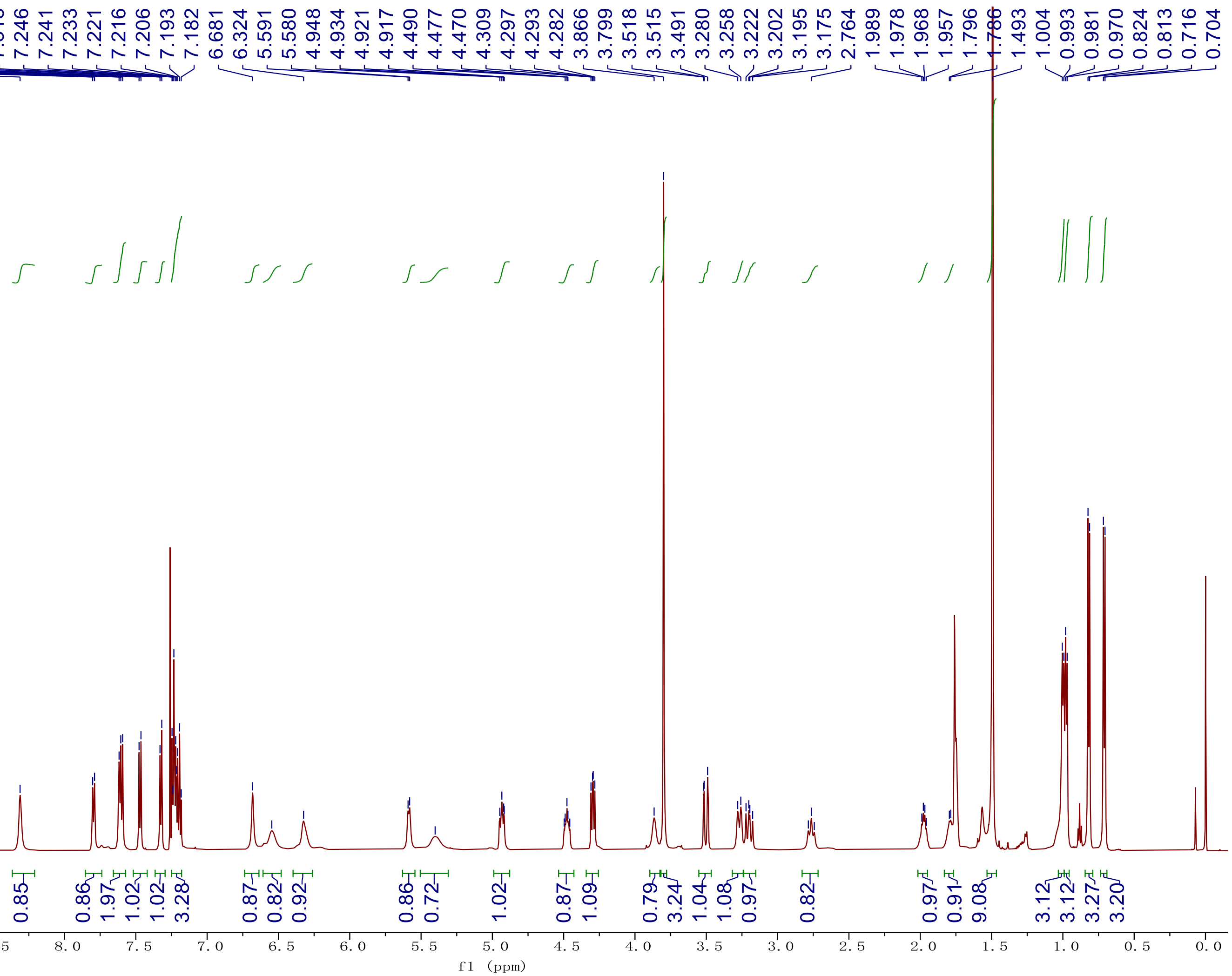

Compound 46h ^{13}C NMR (151 MHz, CDCl$_3$)

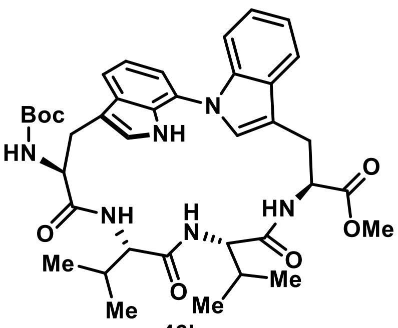

46h

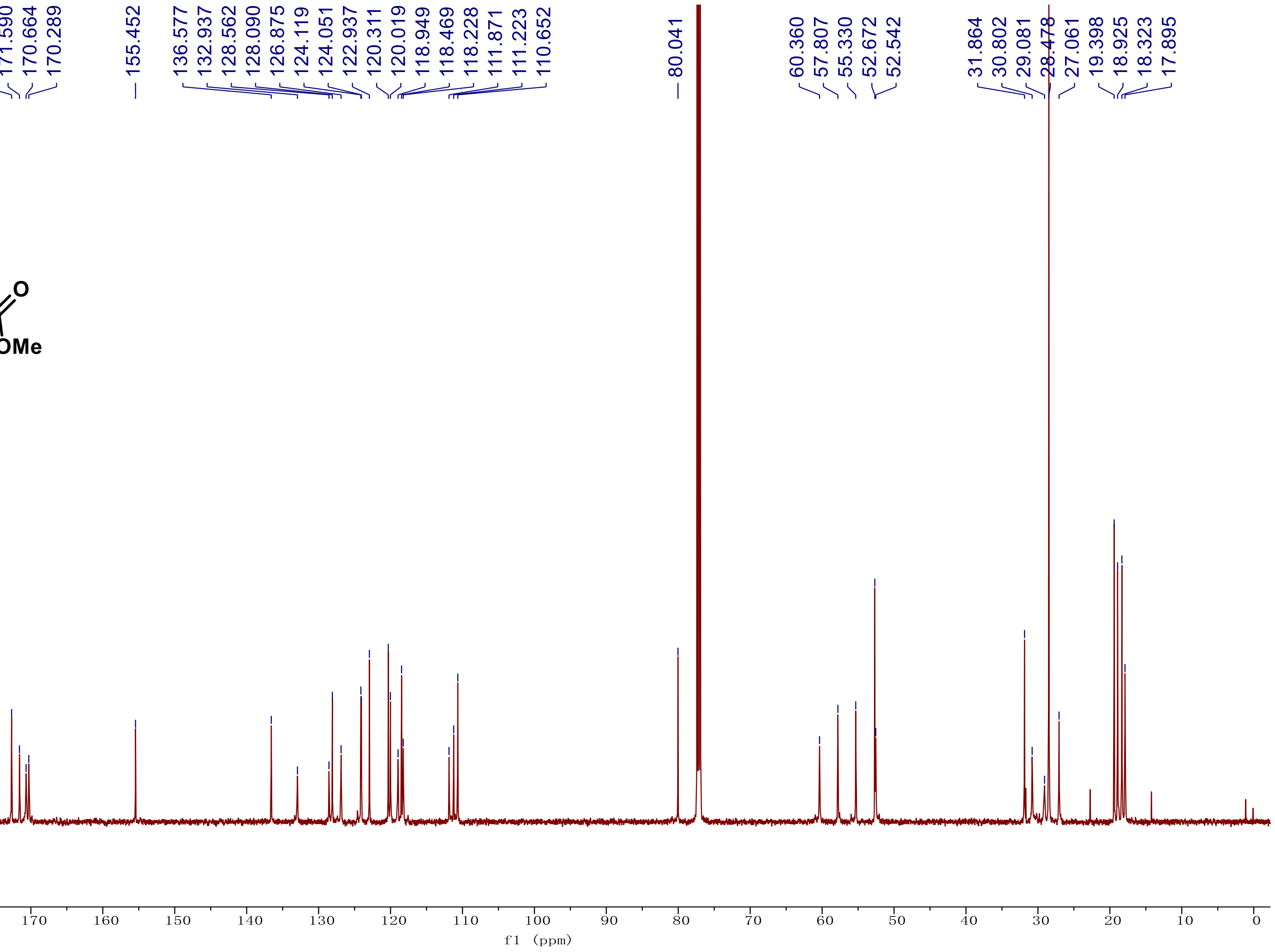

Compound 46i ^{1}H NMR (600 MHz, $CDCl_3$)

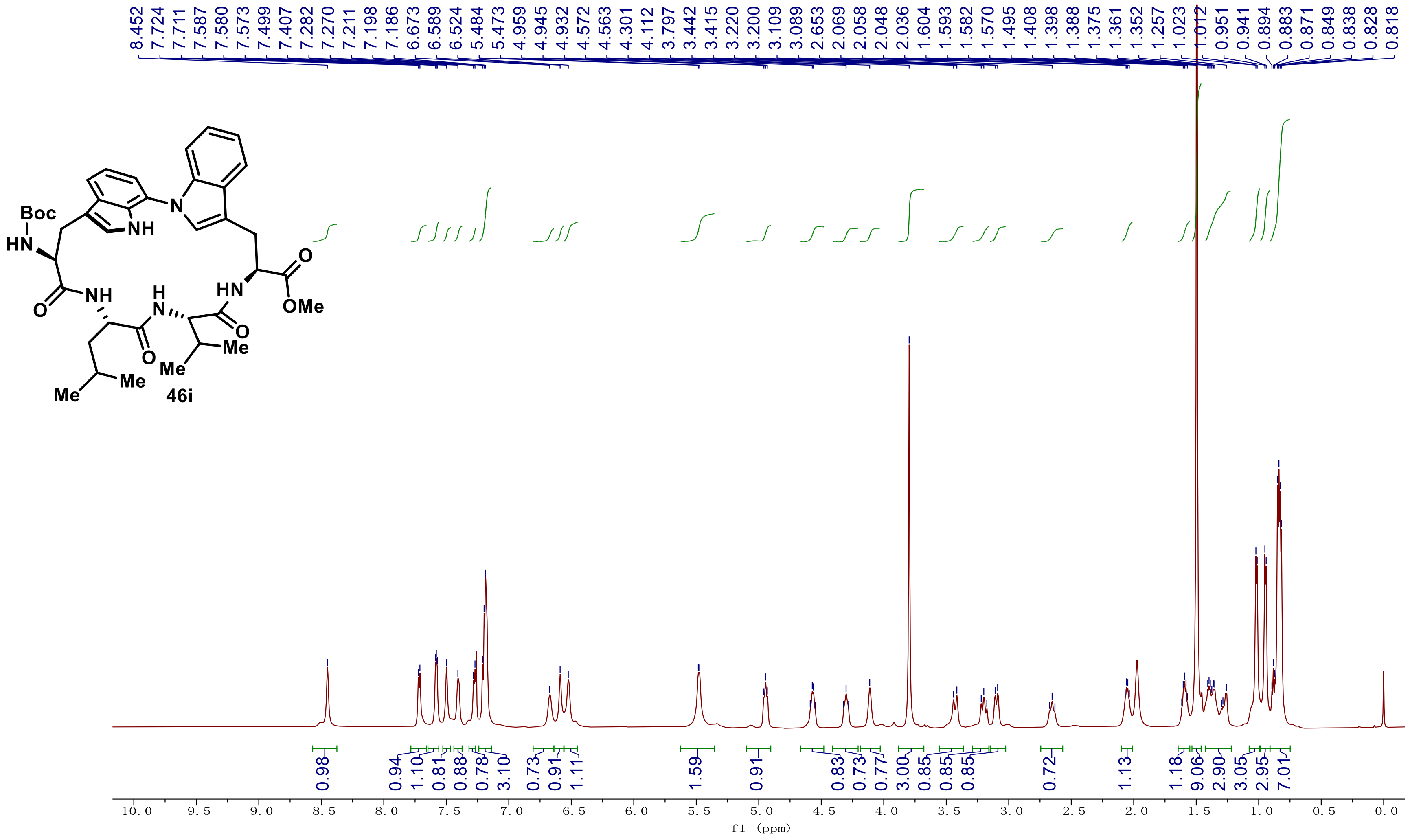

Compound 46i ^{13}C NMR (151 MHz, CDCl$_3$)

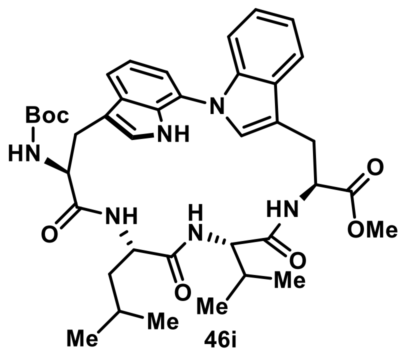

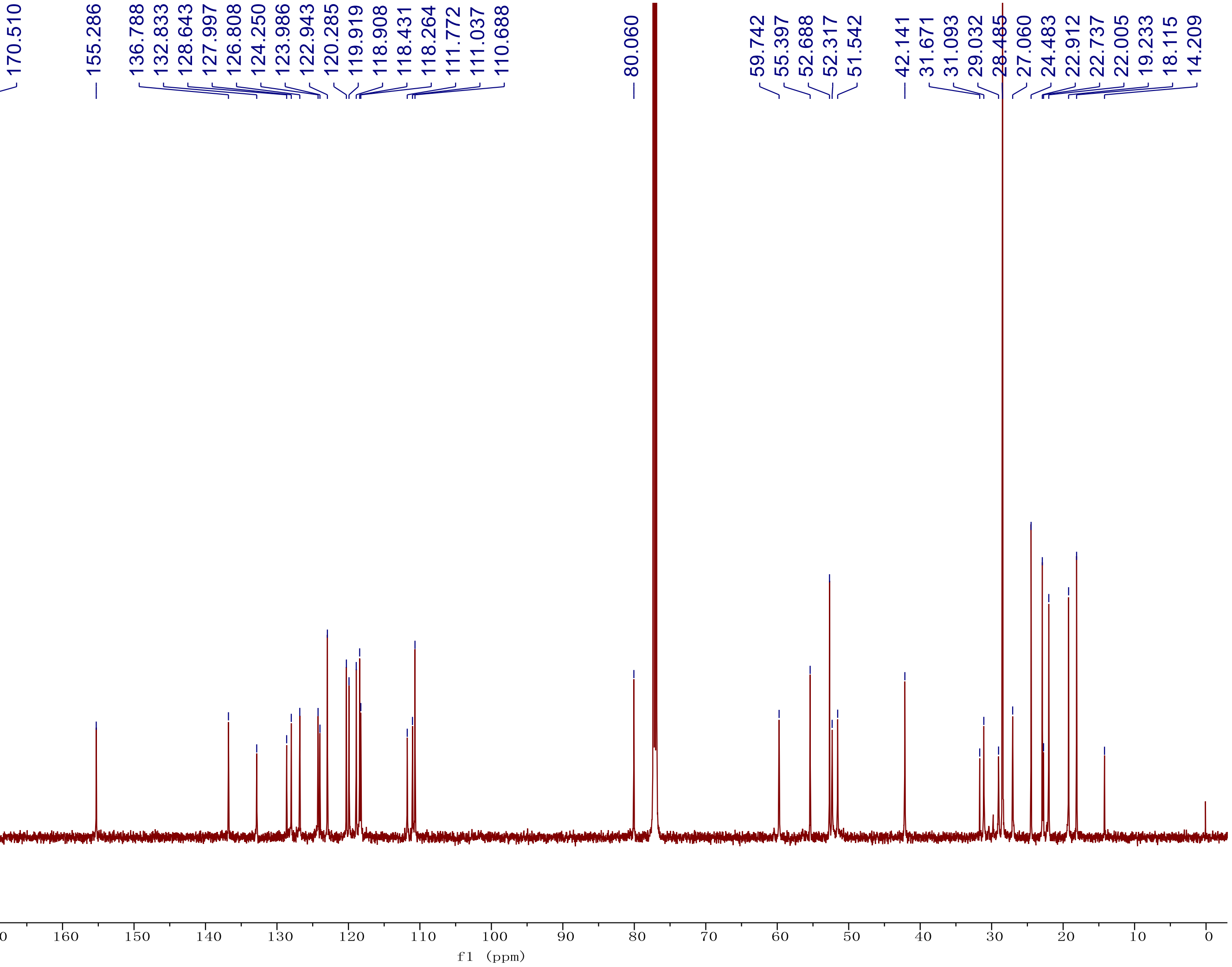

Compound 46i ROESY (400 MHz, $CDCl_3$)

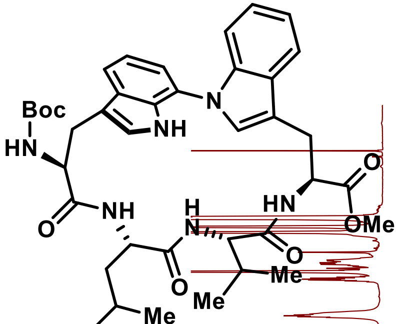

46i

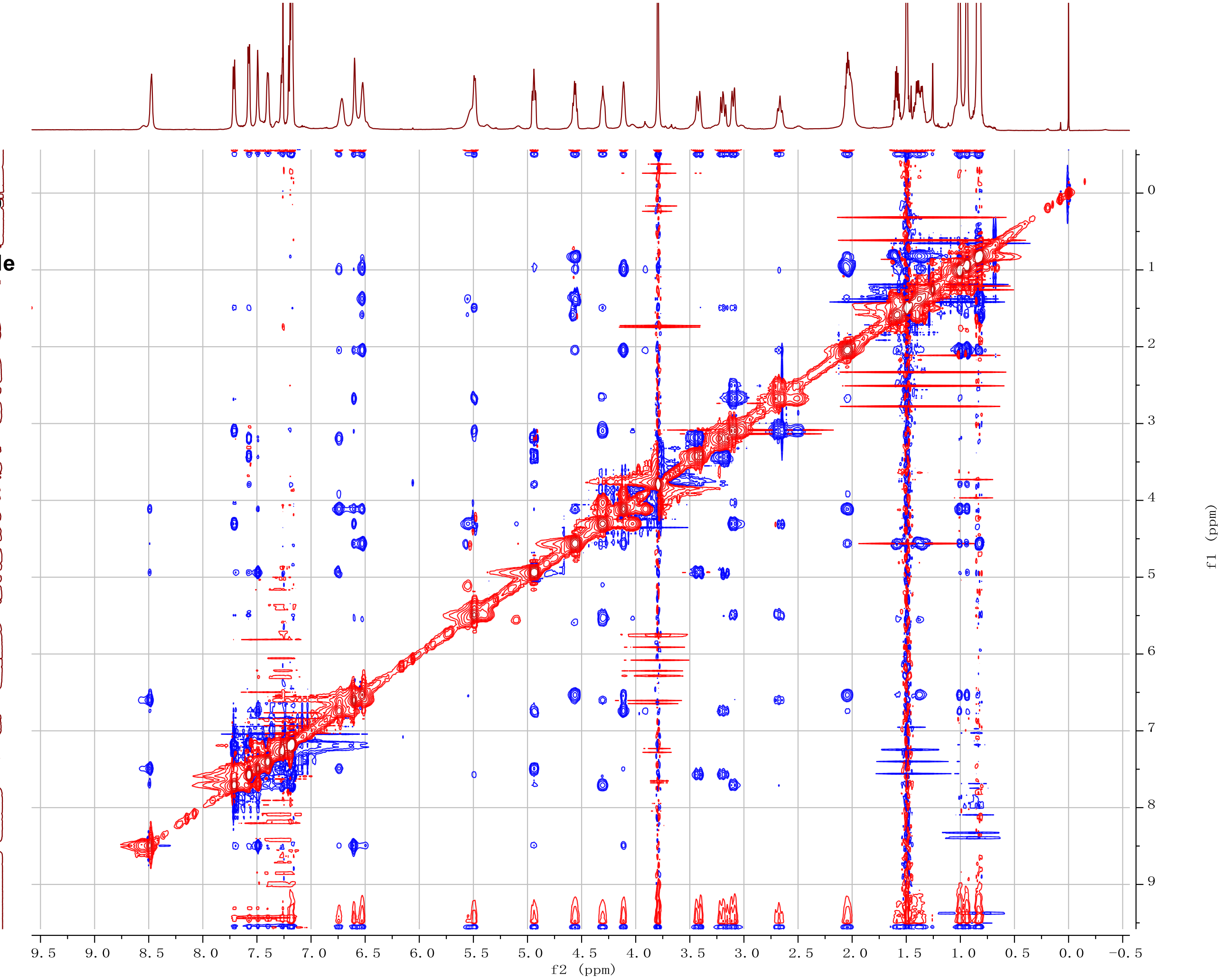

LCMS trace of compound 46i

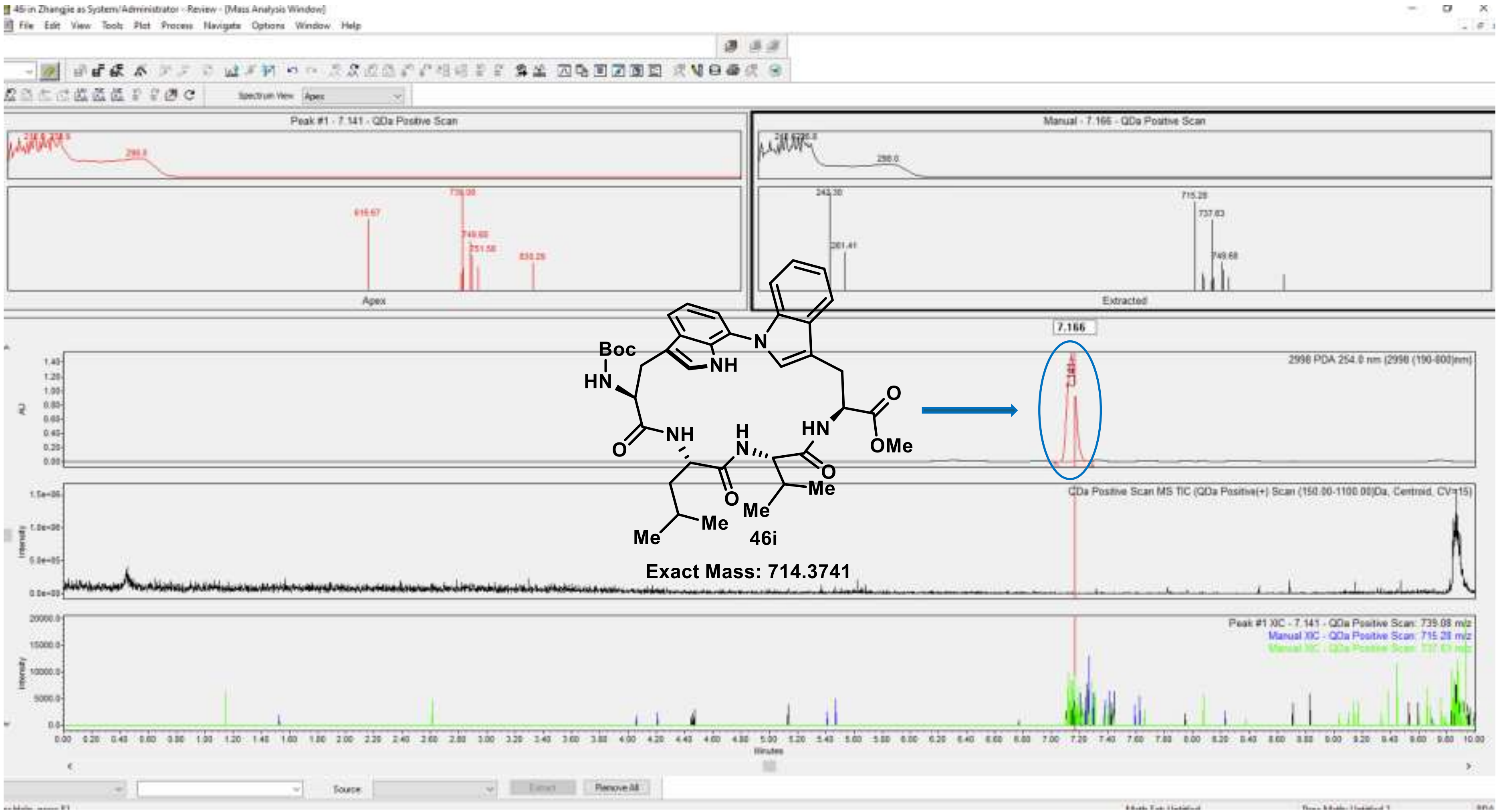

Compound 46j ^{1}H NMR (600 MHz, $CDCl_3$)

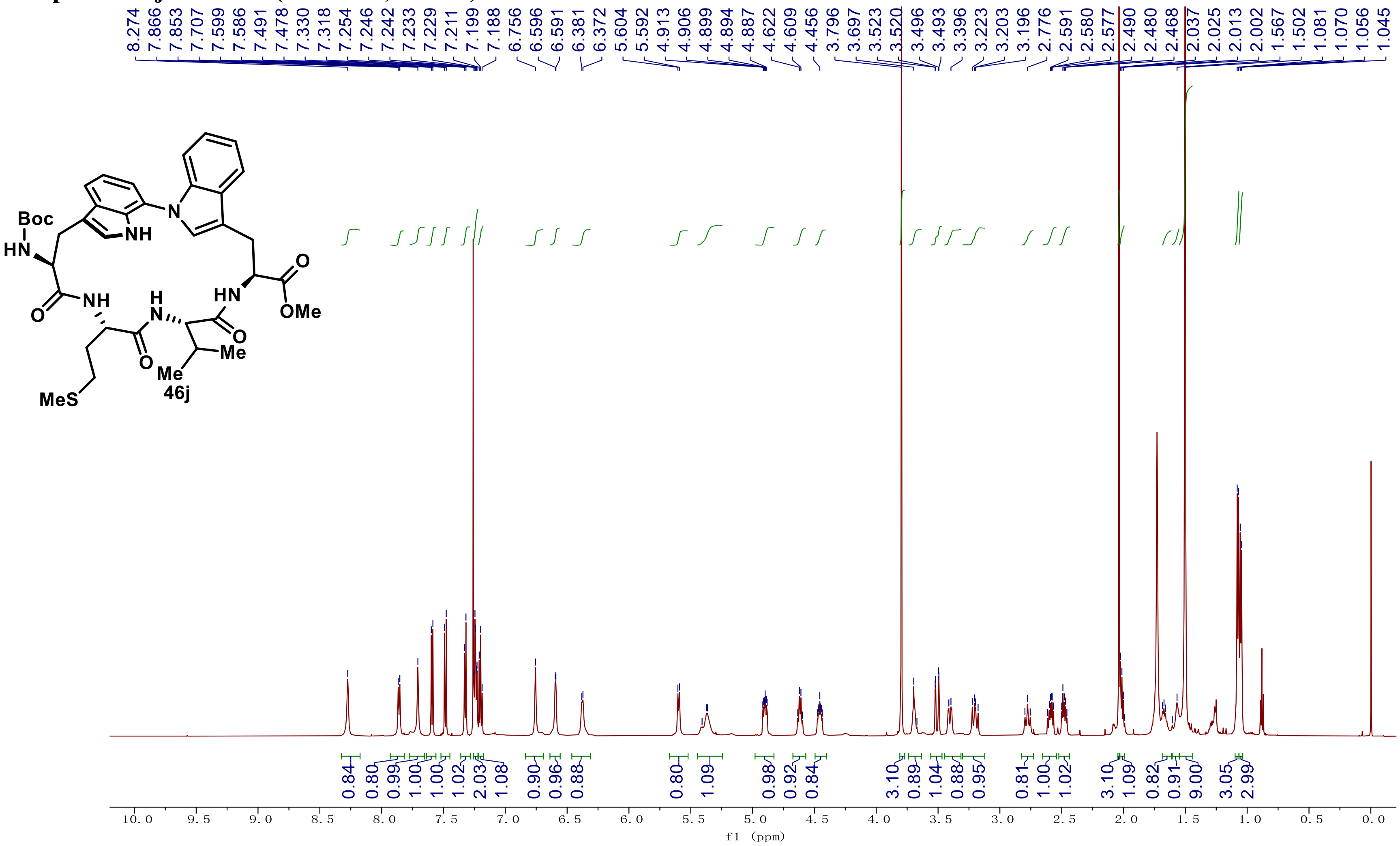

Compound 46j ^{13}C NMR (151 MHz, CDCl$_3$)

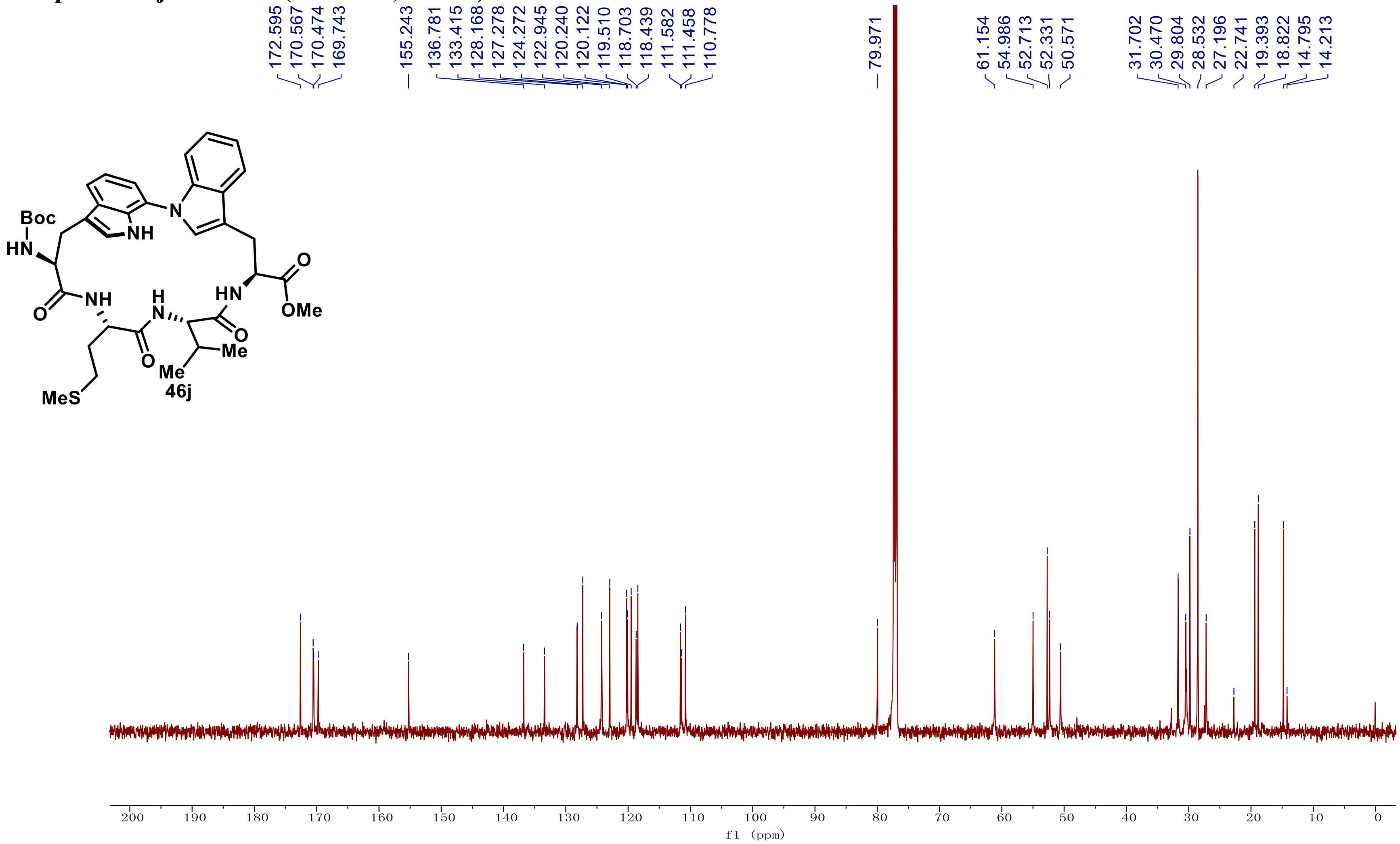

Compound 46k ^{1}H NMR (600 MHz, $CDCl_3$)

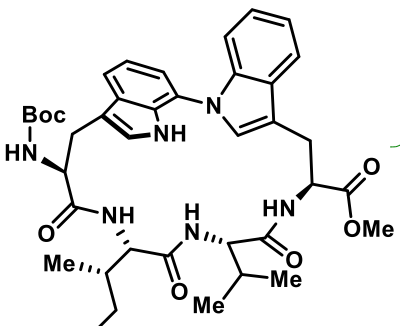

46k

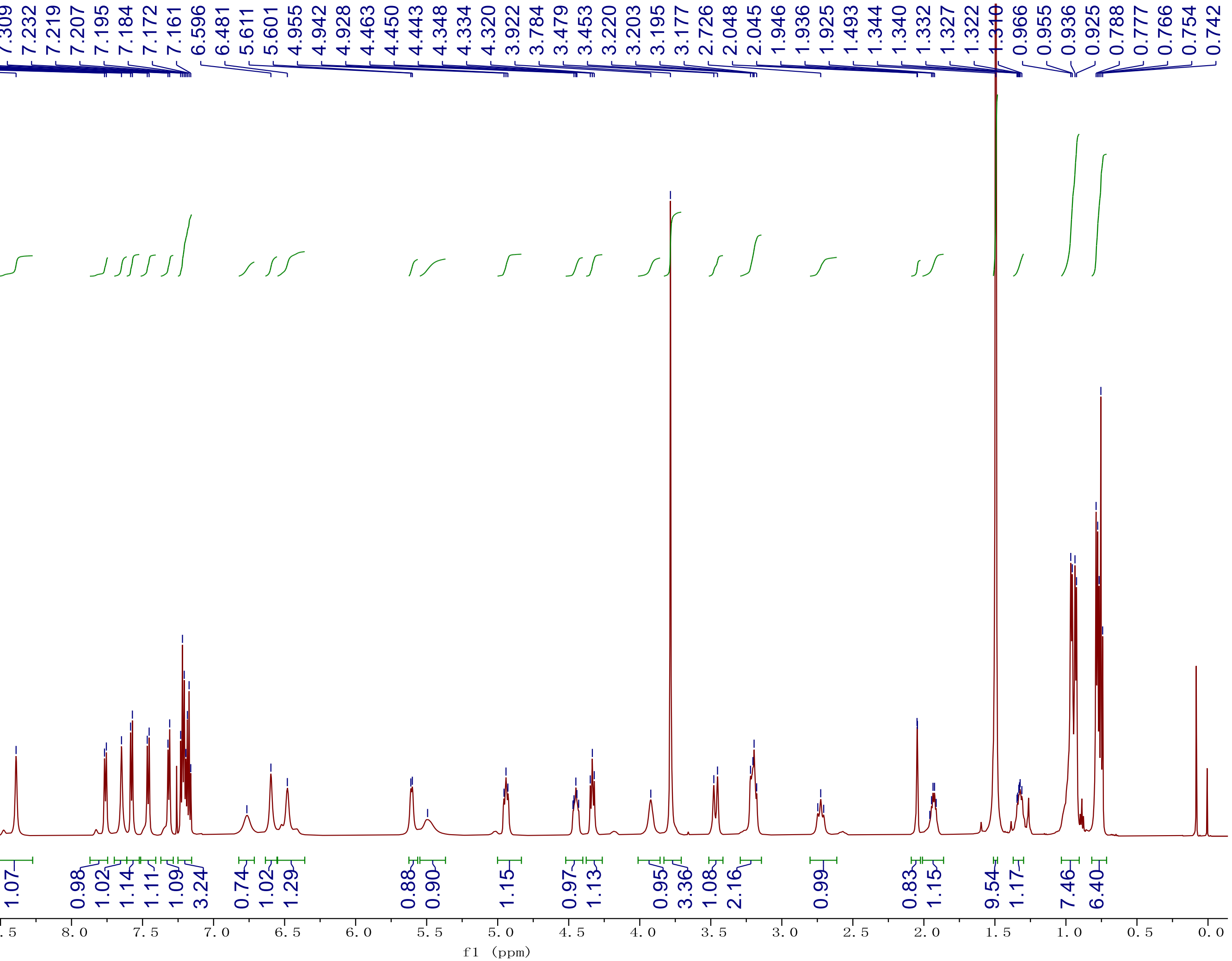

Compound 46k ^{13}C NMR (151 MHz, $CDCl_3$)

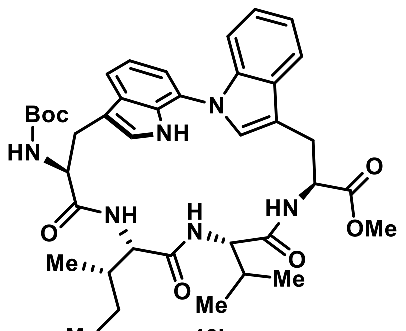

46k

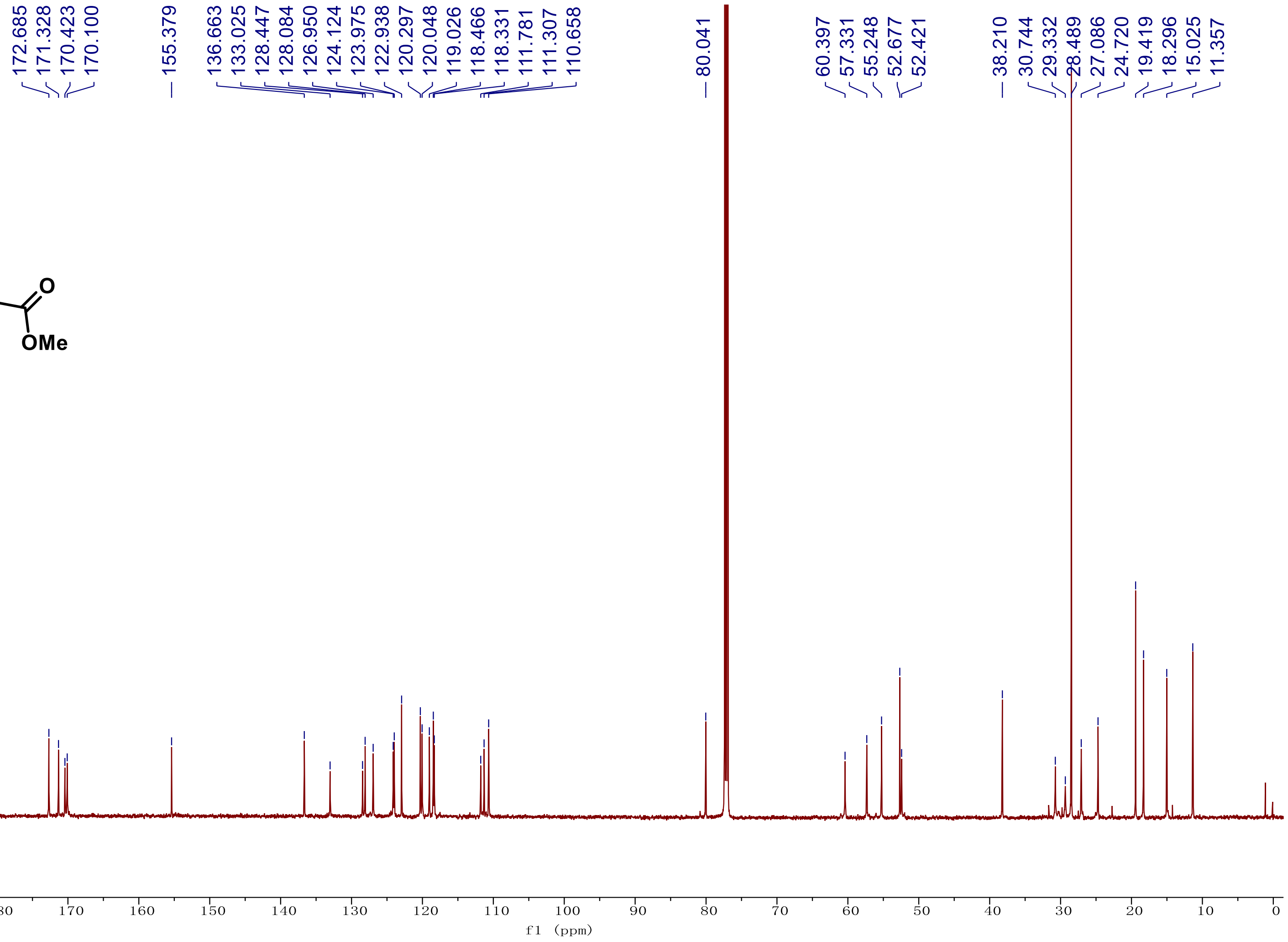

Compound 46l ^{1}H NMR (600 MHz, $CDCl_3$)

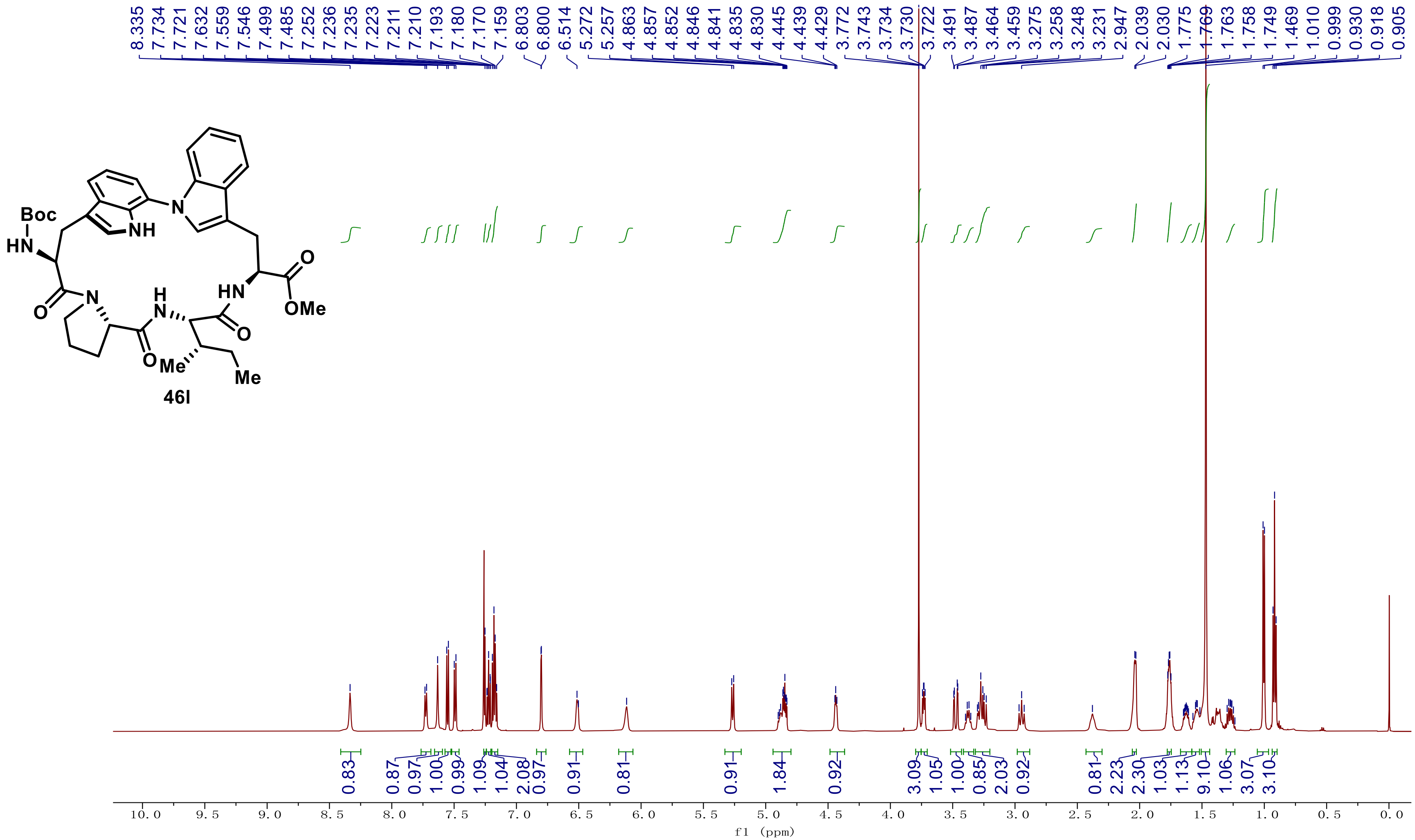

Compound 46l ^{13}C NMR (151 MHz, $CDCl_3$)

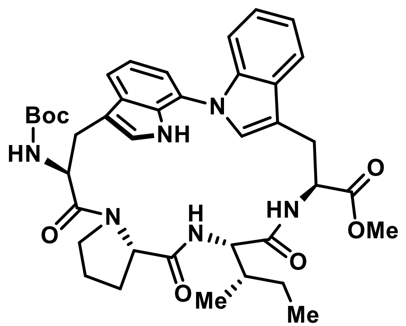

46l

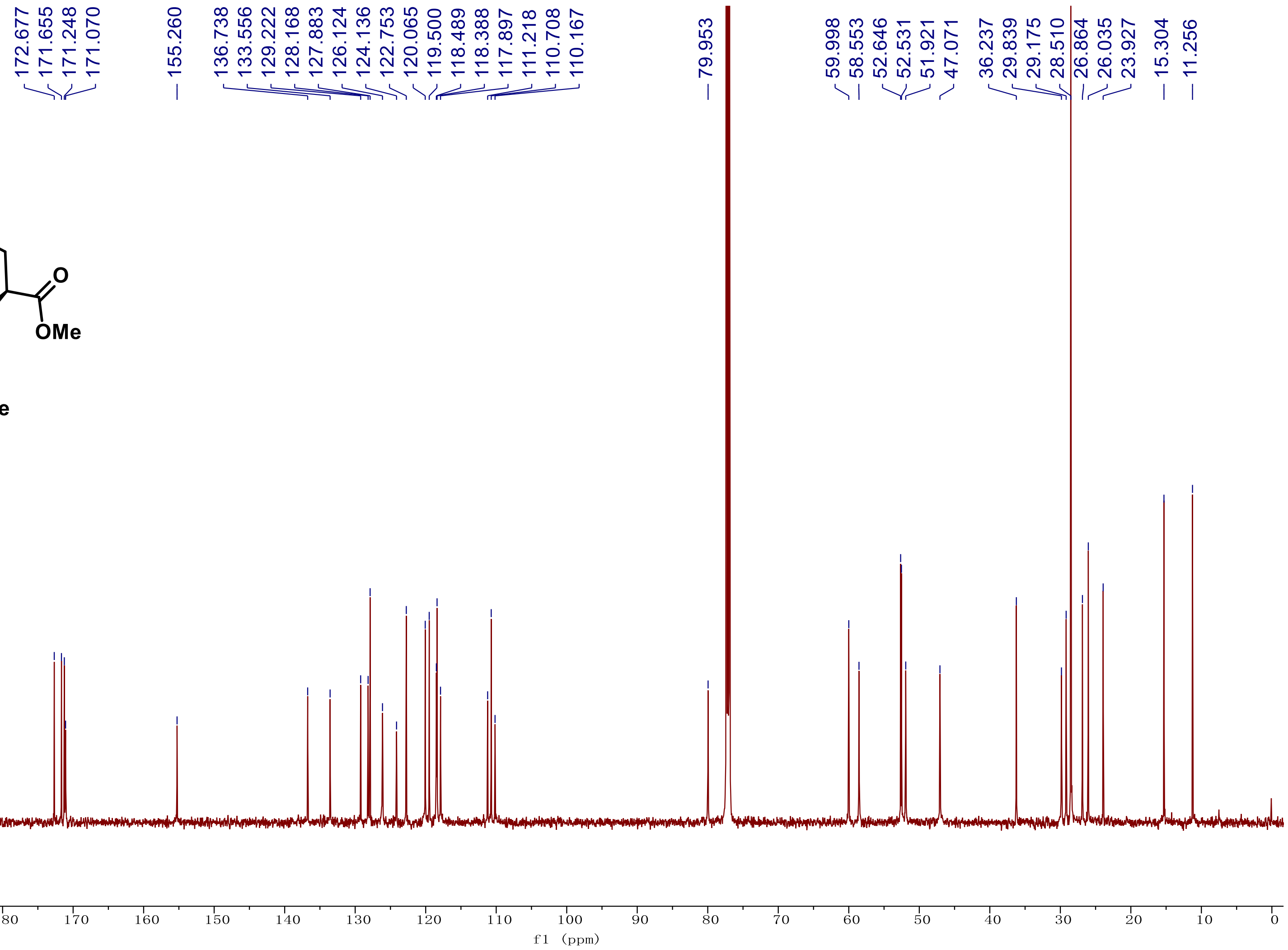

Compound 46m ^{1}H NMR (600 MHz, DMSO-*d6*)

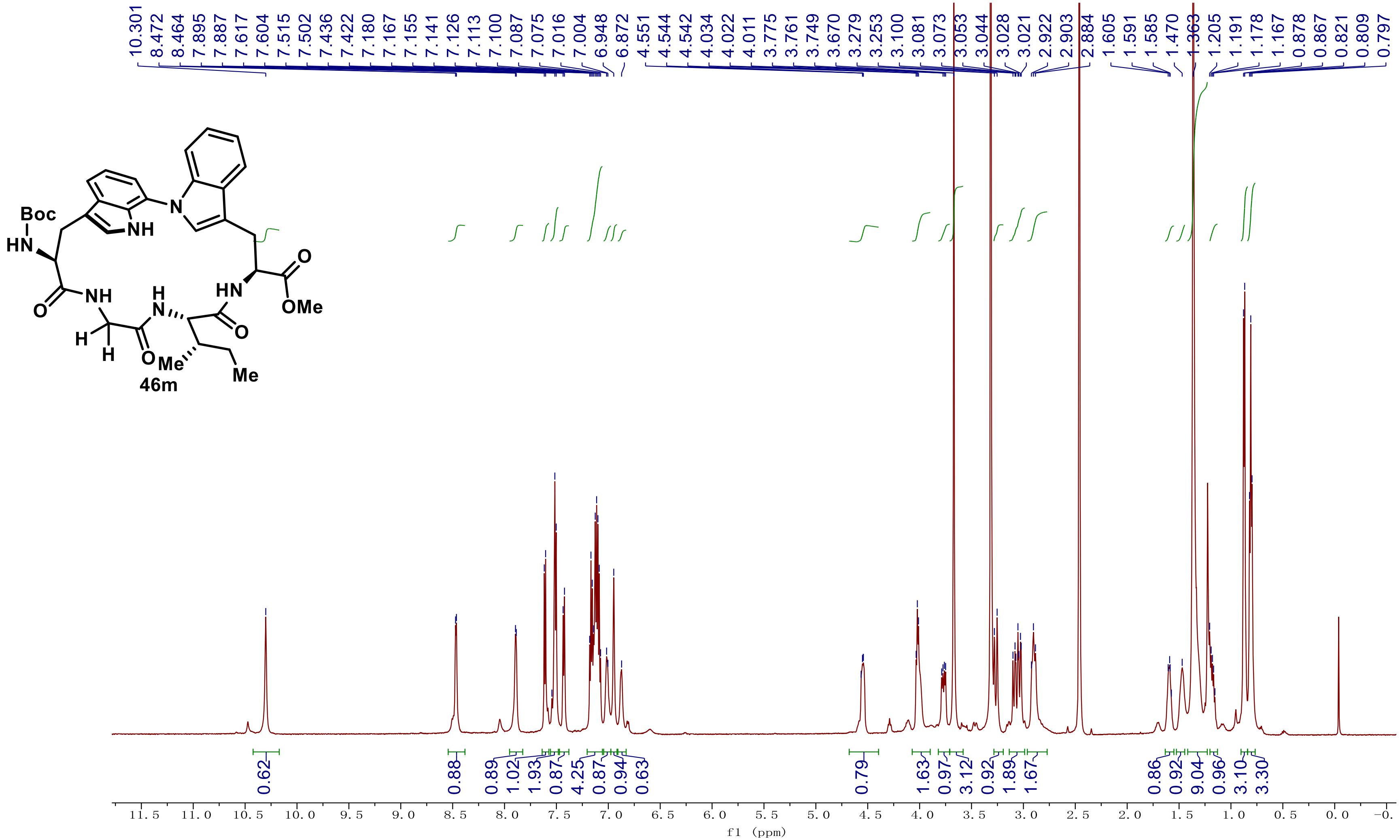

46m

Compound 46m ^{13}C NMR (151 MHz, DMSO-*d6*)

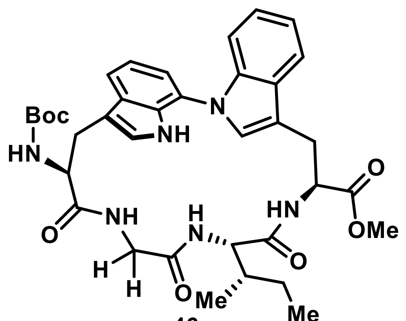

46m

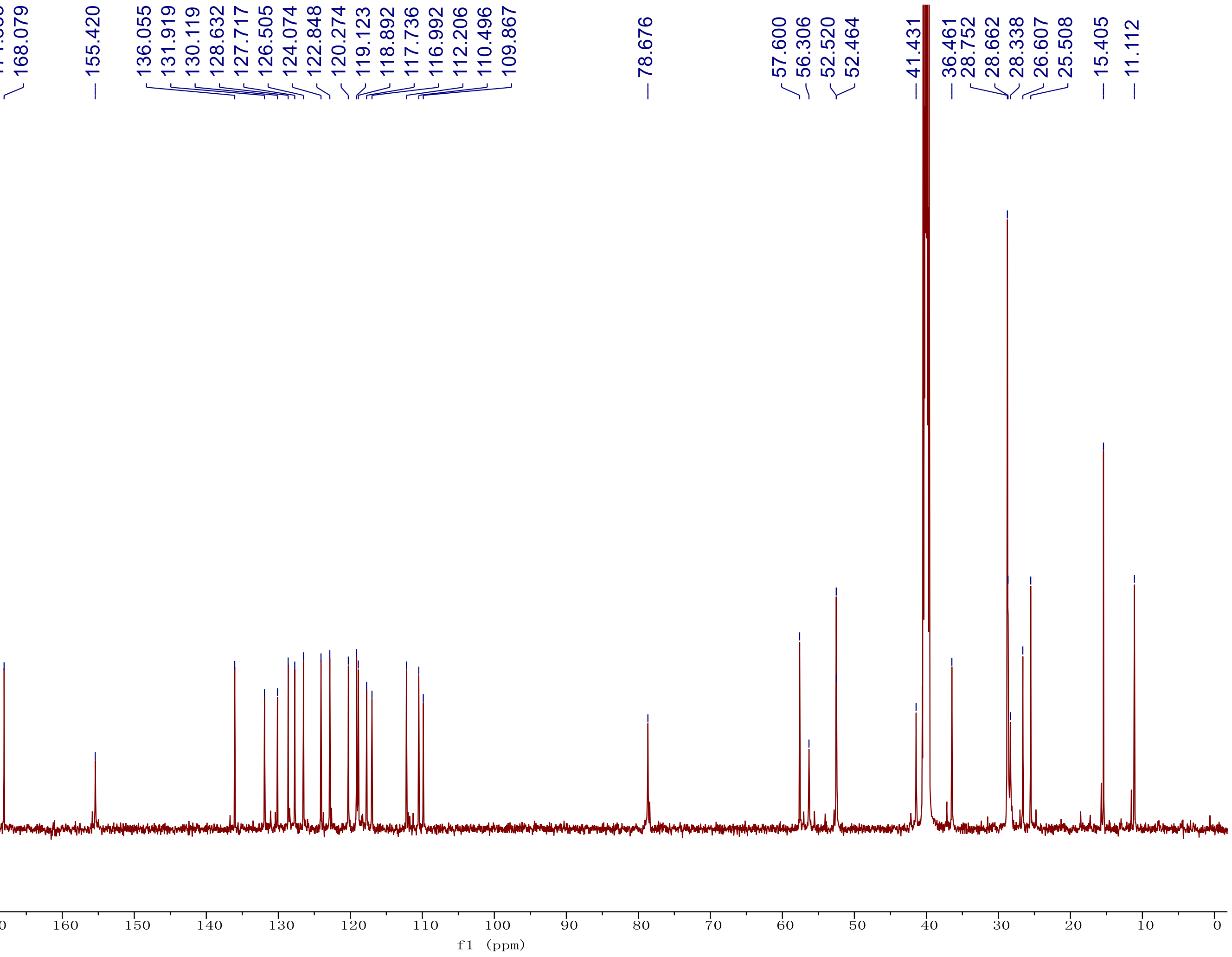

Compound 46n ¹H NMR (600 MHz, CDCl₃)

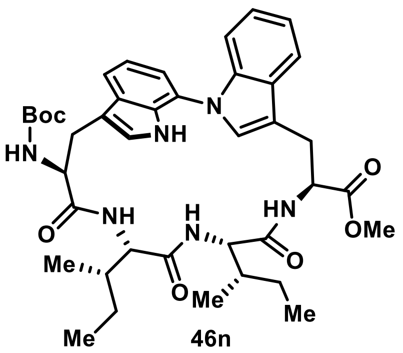

46n

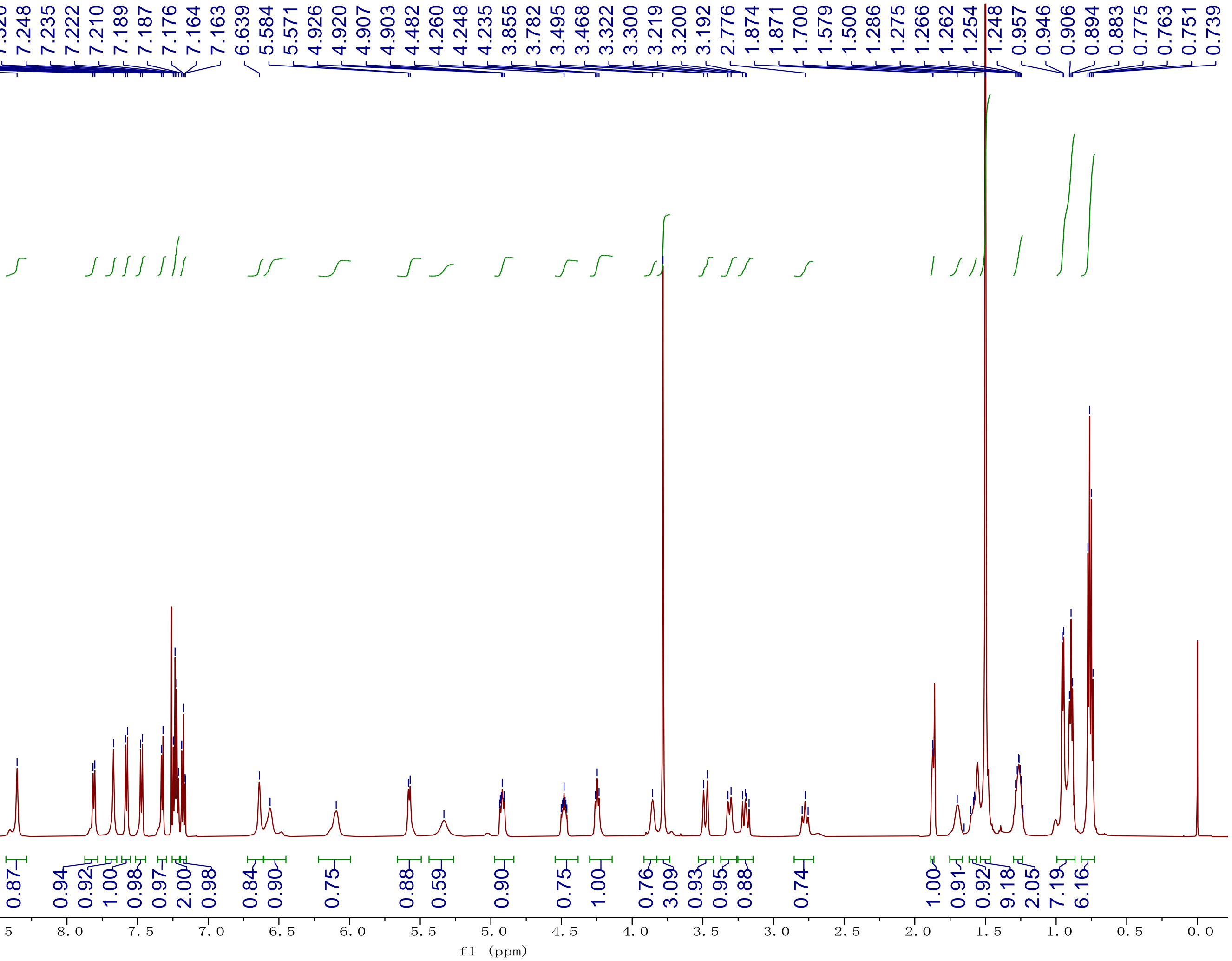

Compound 46n ^{13}C NMR (151 MHz, $CDCl_3$)

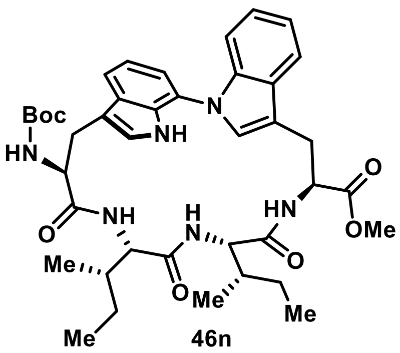

46n

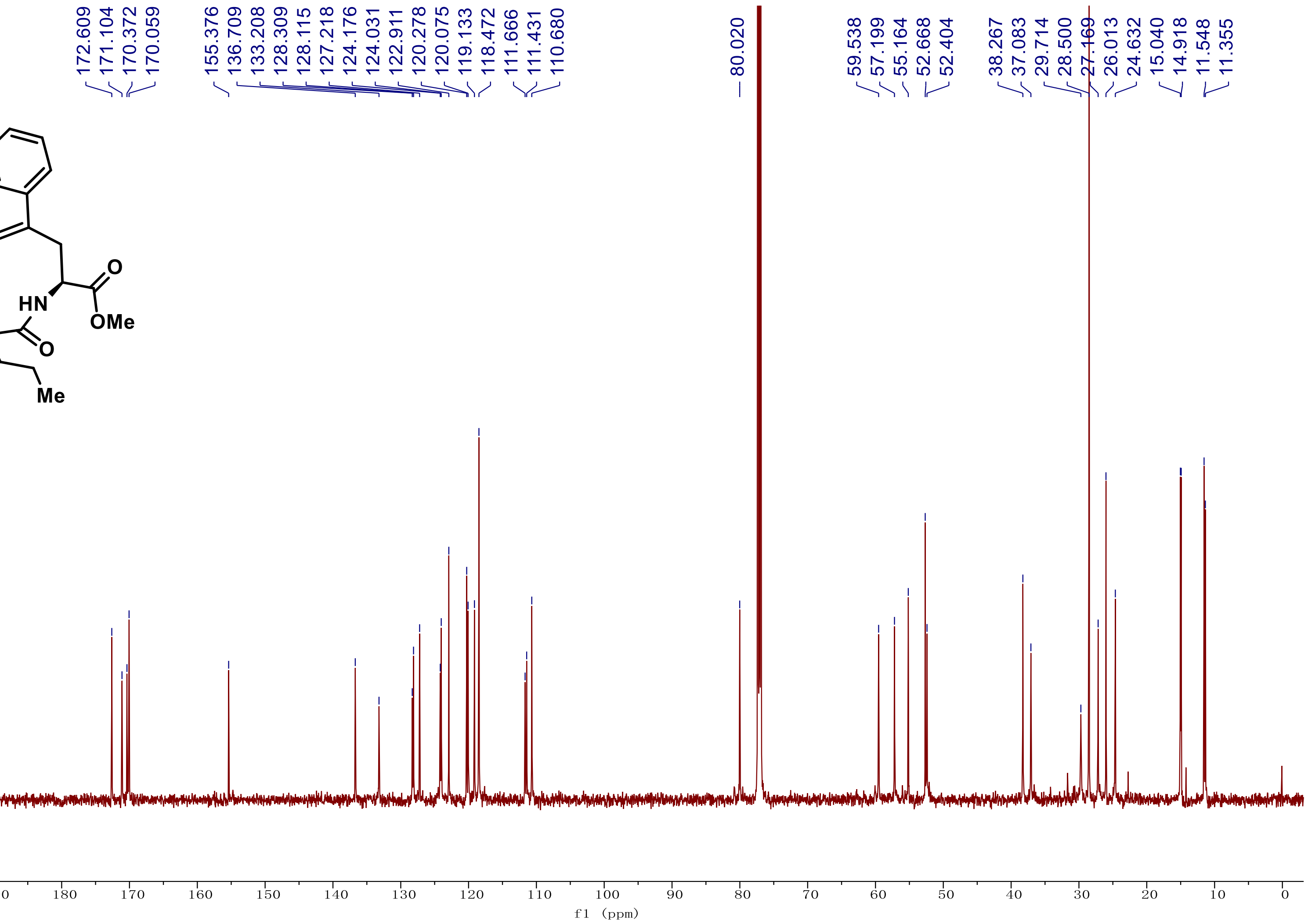

Compound 46o ¹H NMR (600 MHz, CDCl₃)

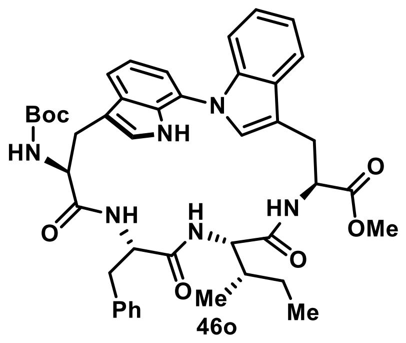

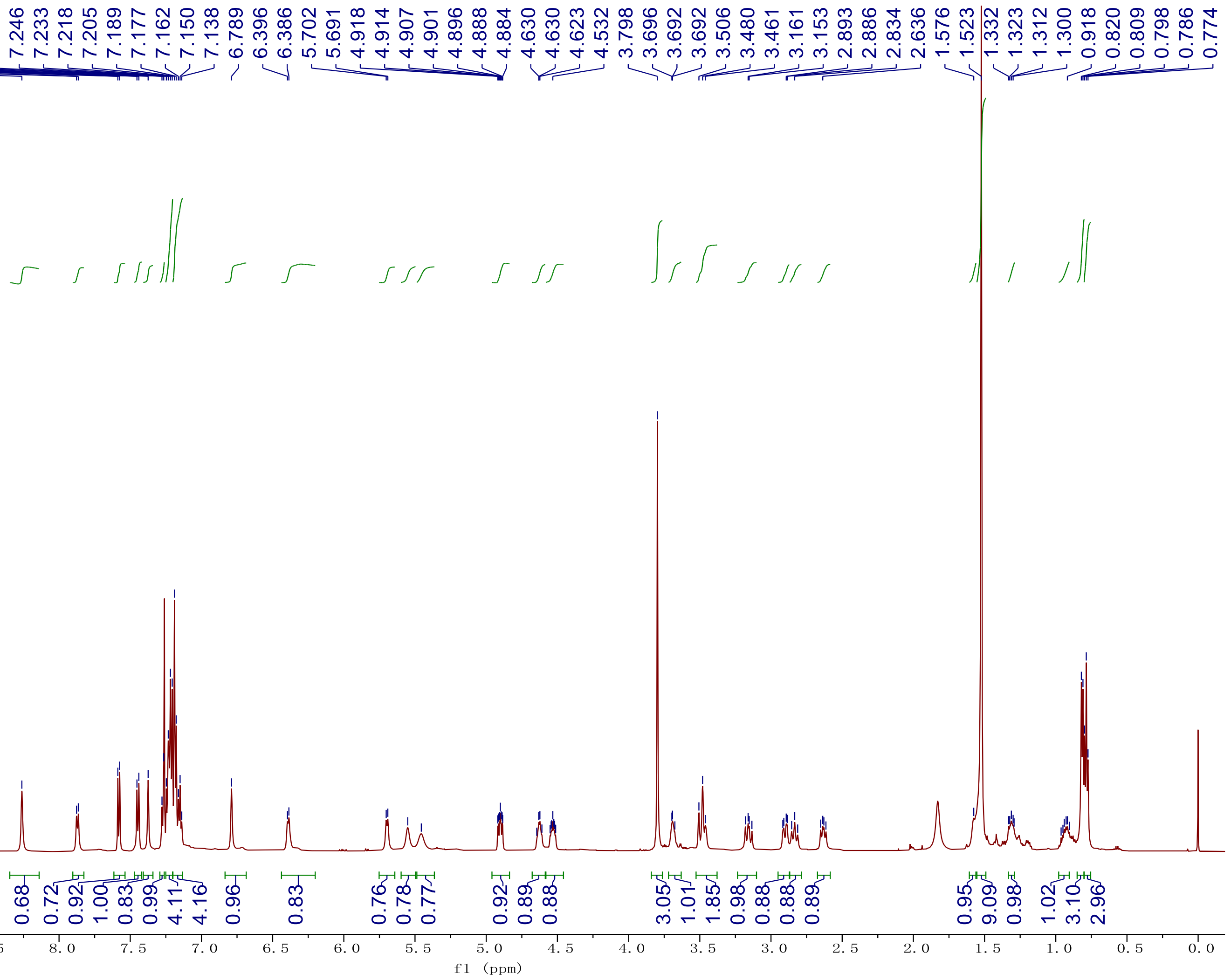

Compound 46o ^{13}C NMR (151 MHz, CDCl$_3$)

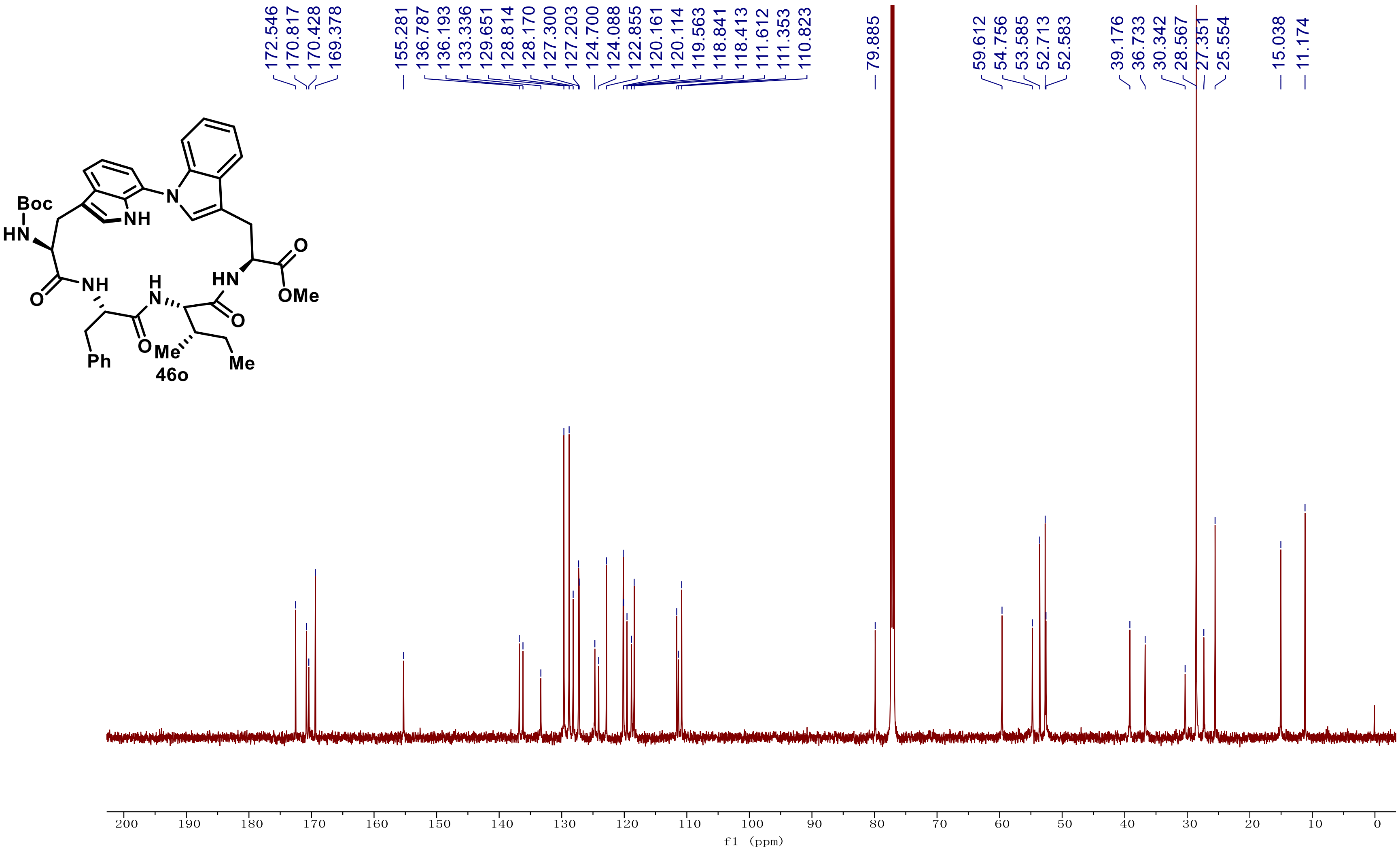

Compound 46p ^{1}H NMR (600 MHz, $CDCl_3$)

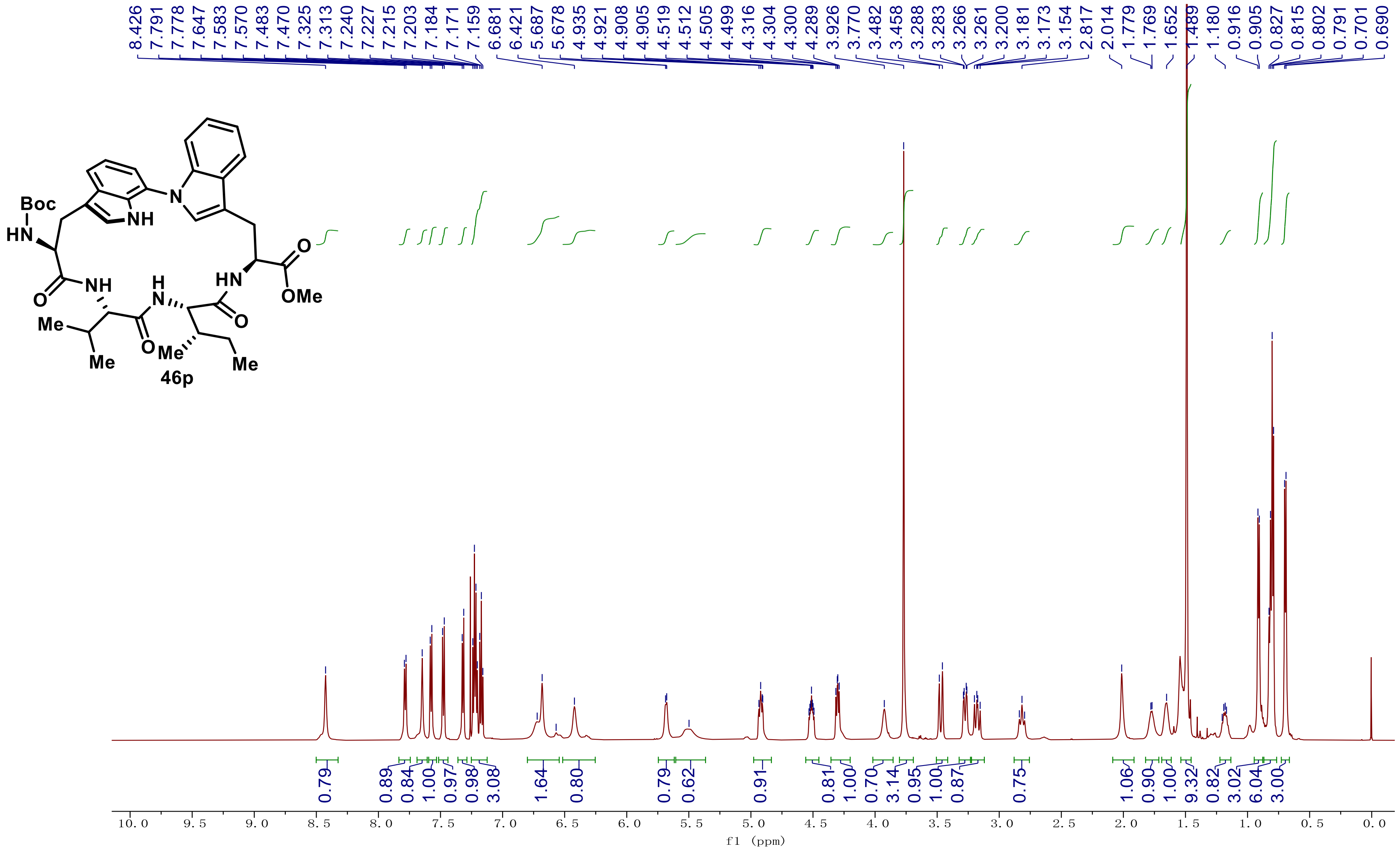

Compound 46p ^{13}C NMR (151 MHz, $CDCl_3$)

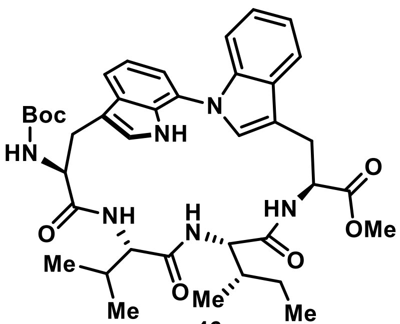

46p

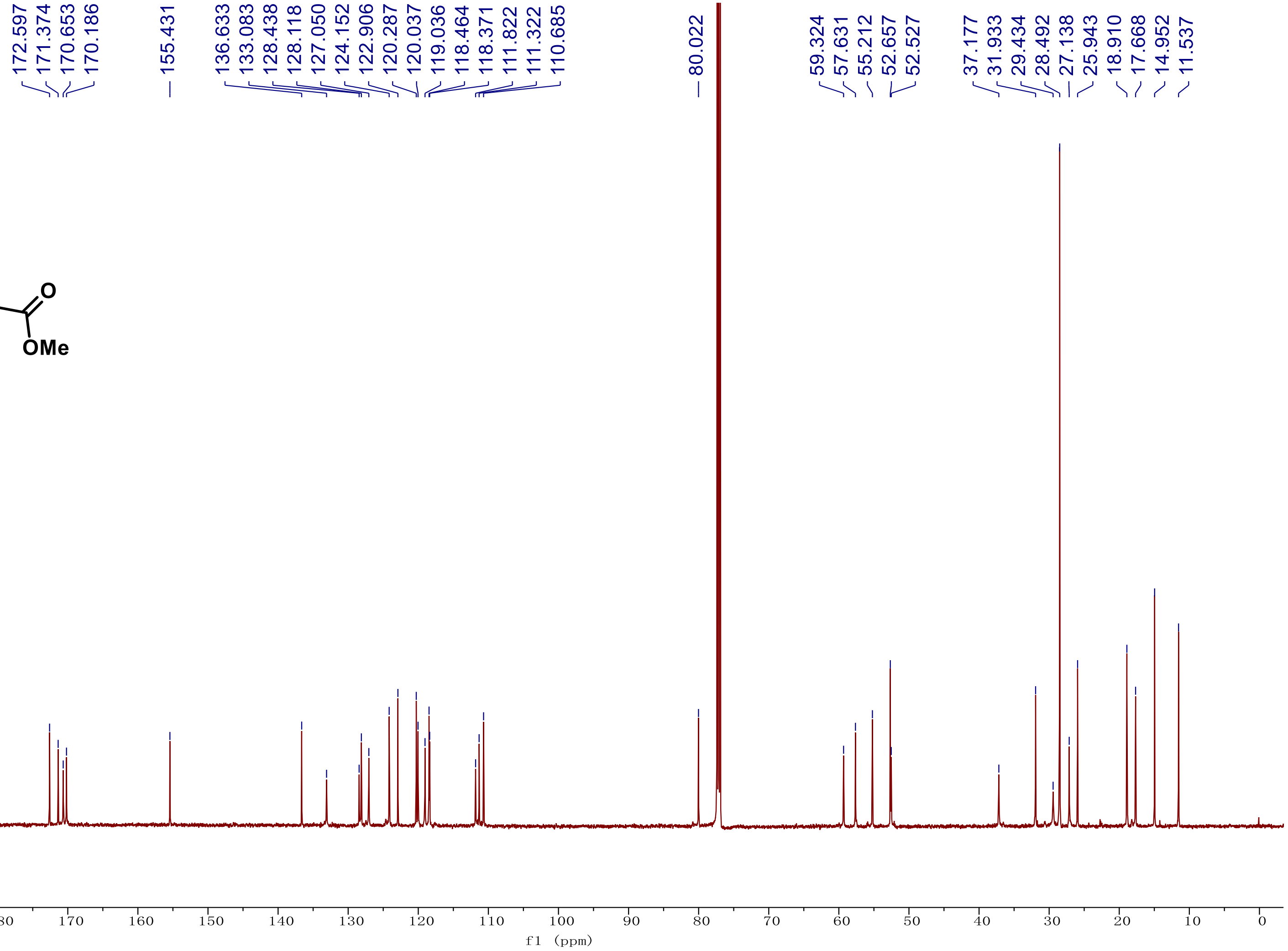

Compound 46q ^{1}H NMR (600 MHz, $CDCl_3$)

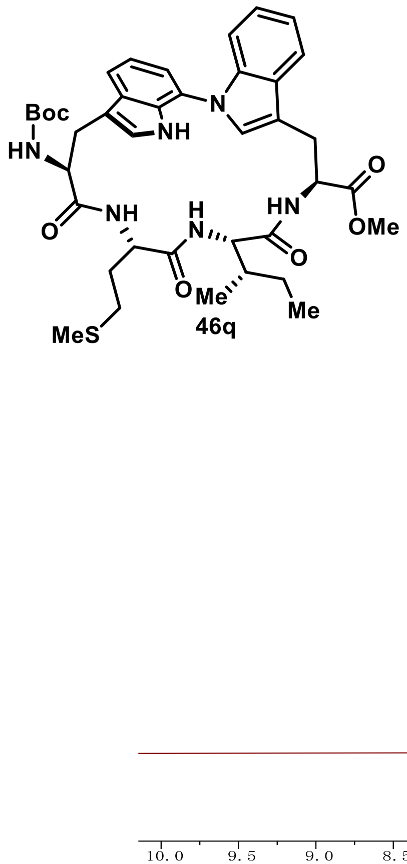

46q

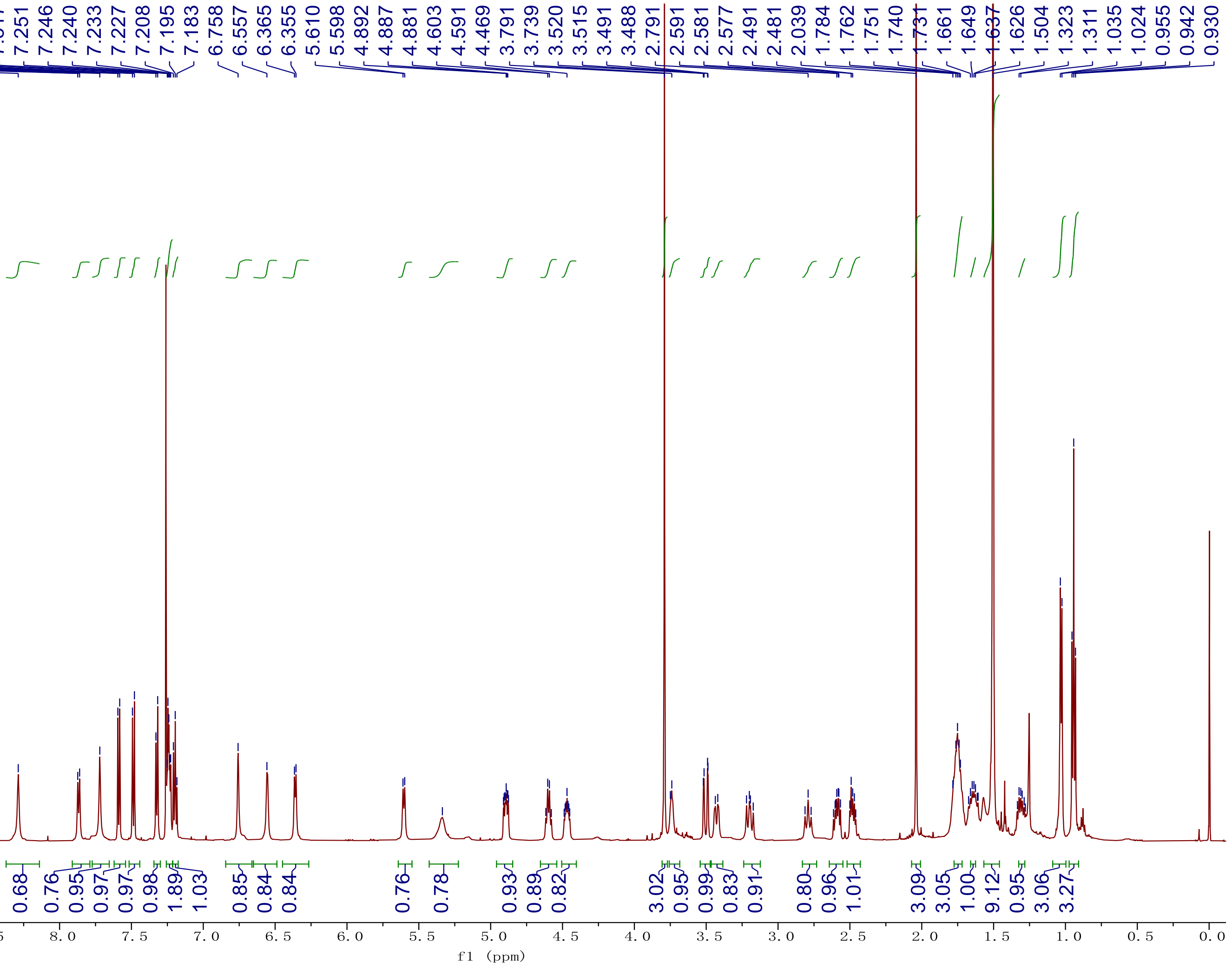

Compound 46q ^{13}C NMR (151 MHz, $CDCl_3$)

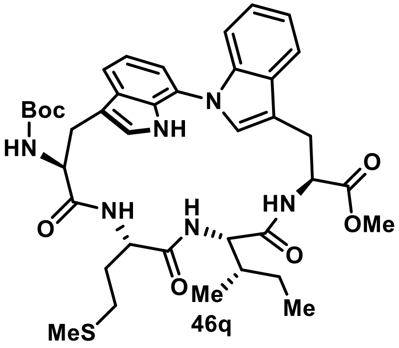

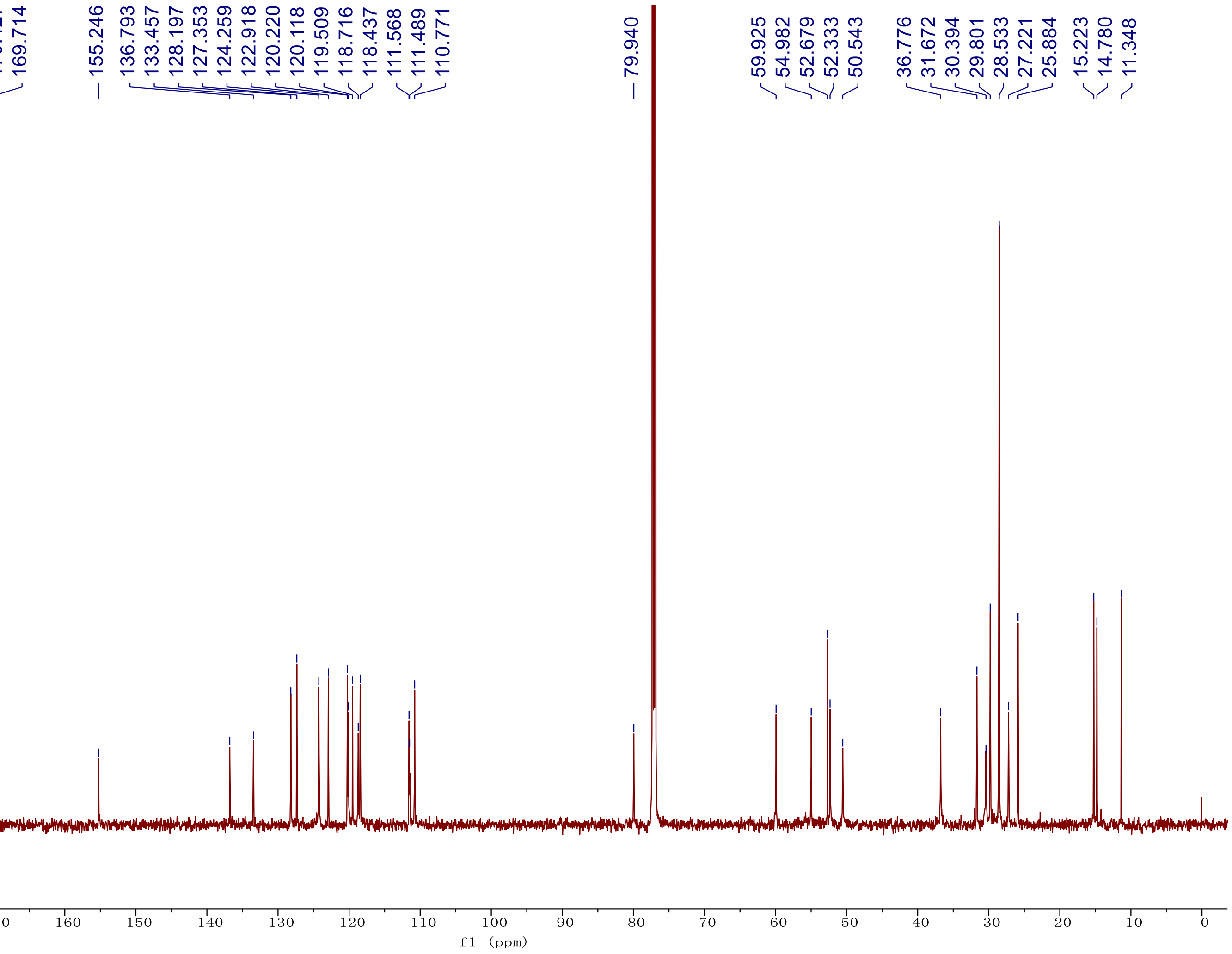

Compound 46r ^{1}H NMR (600 MHz, $CDCl_3$)

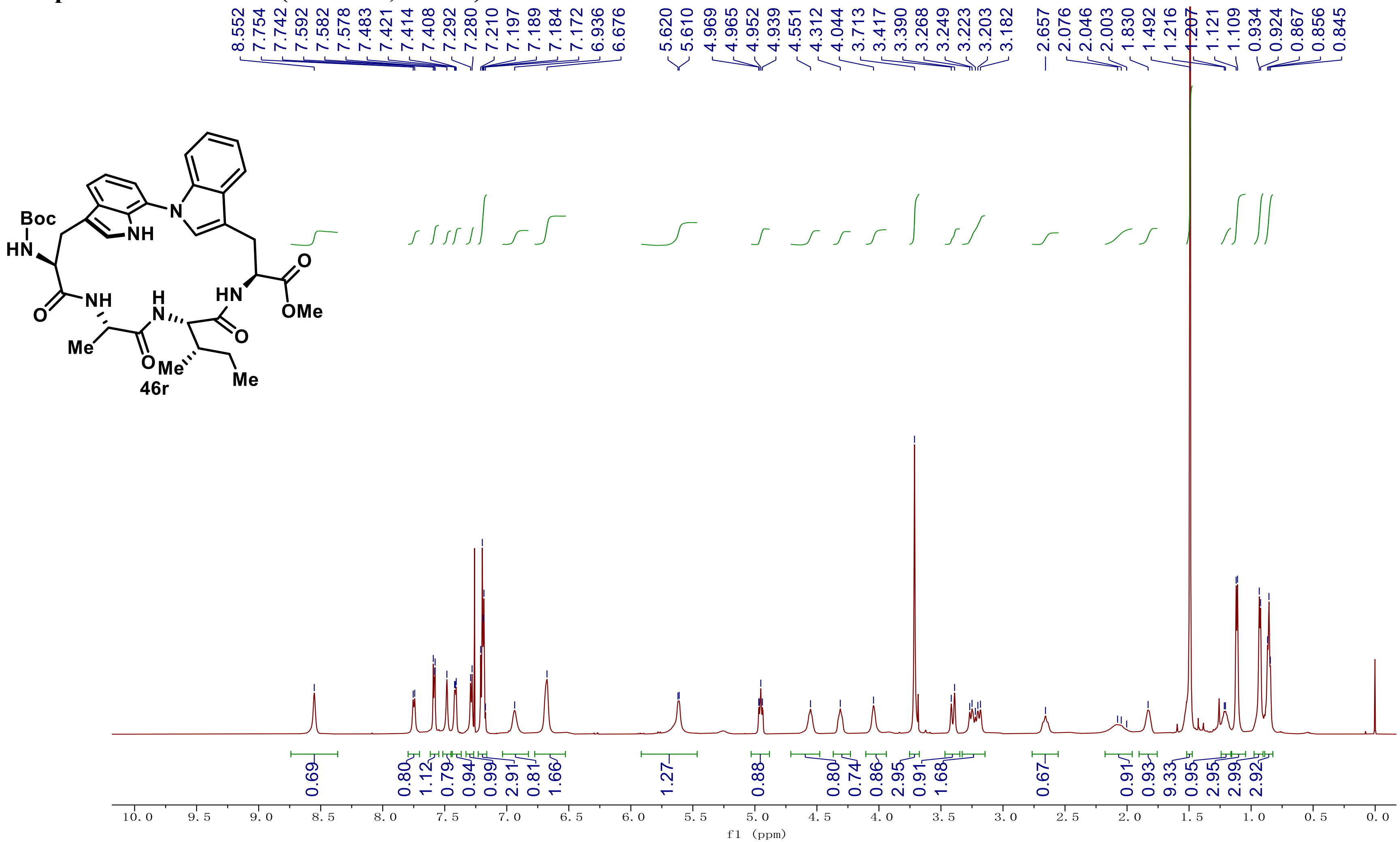

Compound 46r ^{13}C NMR (151 MHz, $CDCl_3$)

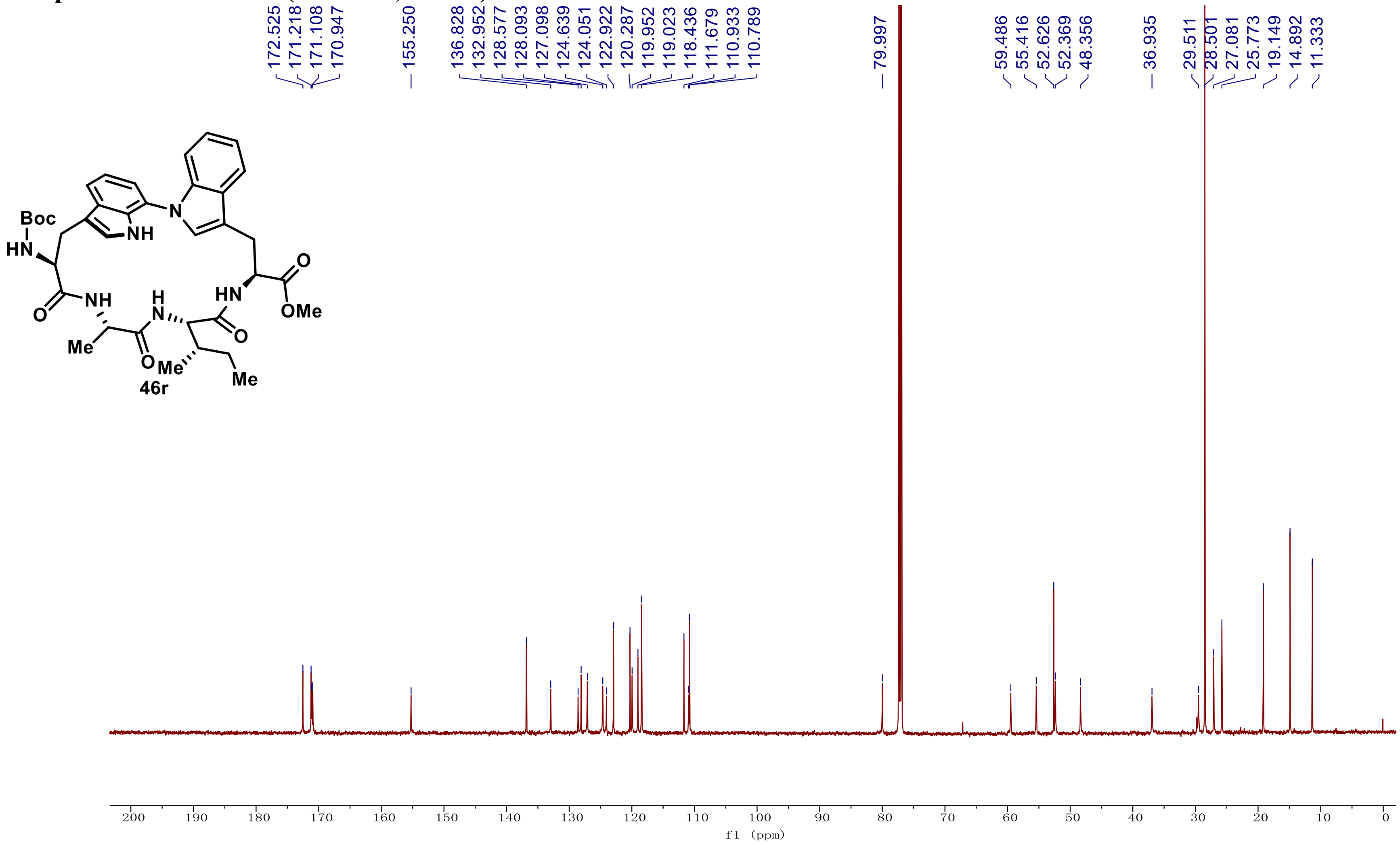

Compound 46s ¹H NMR (600 MHz, CDCl₃)

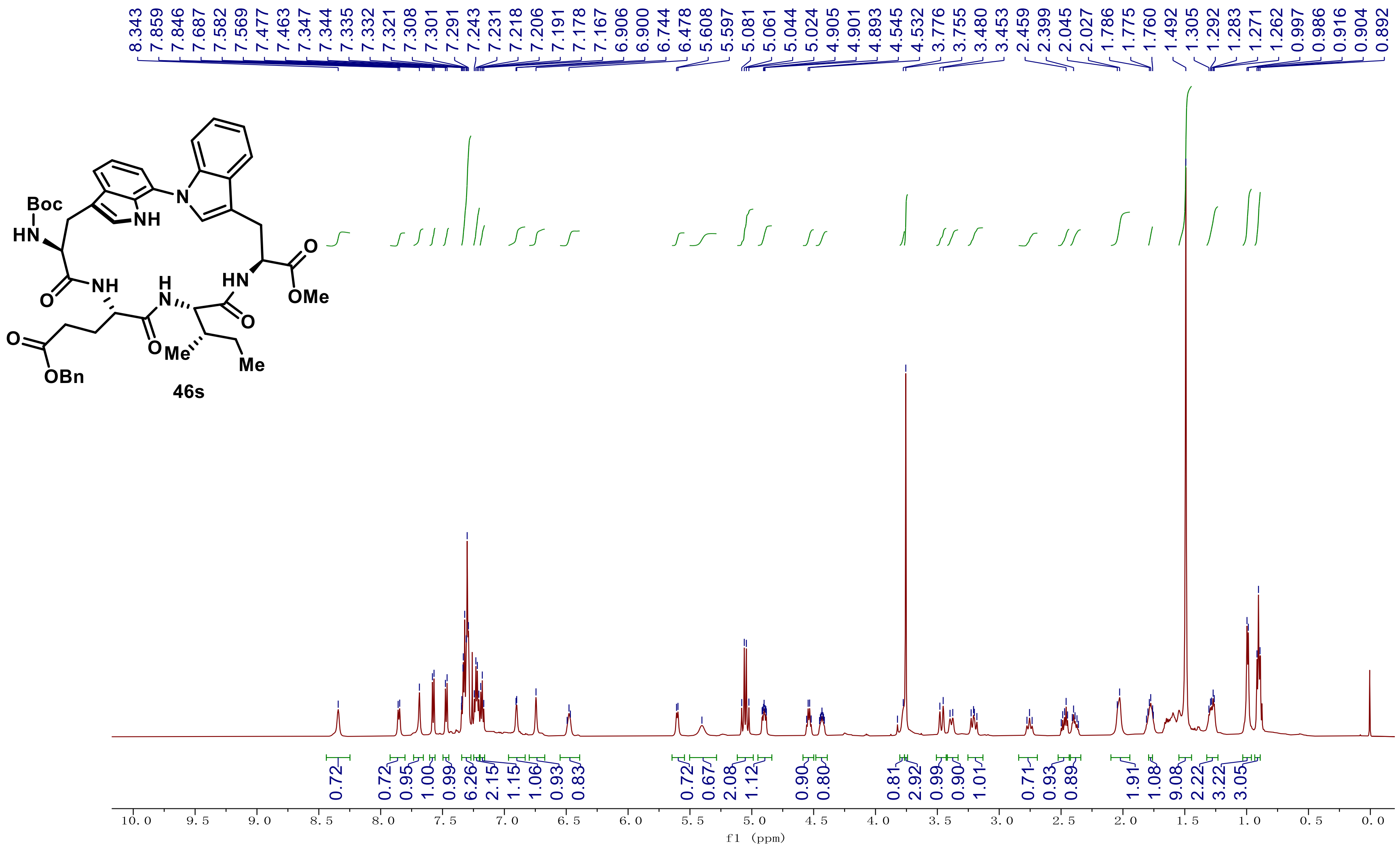

Compound 46s ^{13}C NMR (151 MHz, CDCl$_3$)

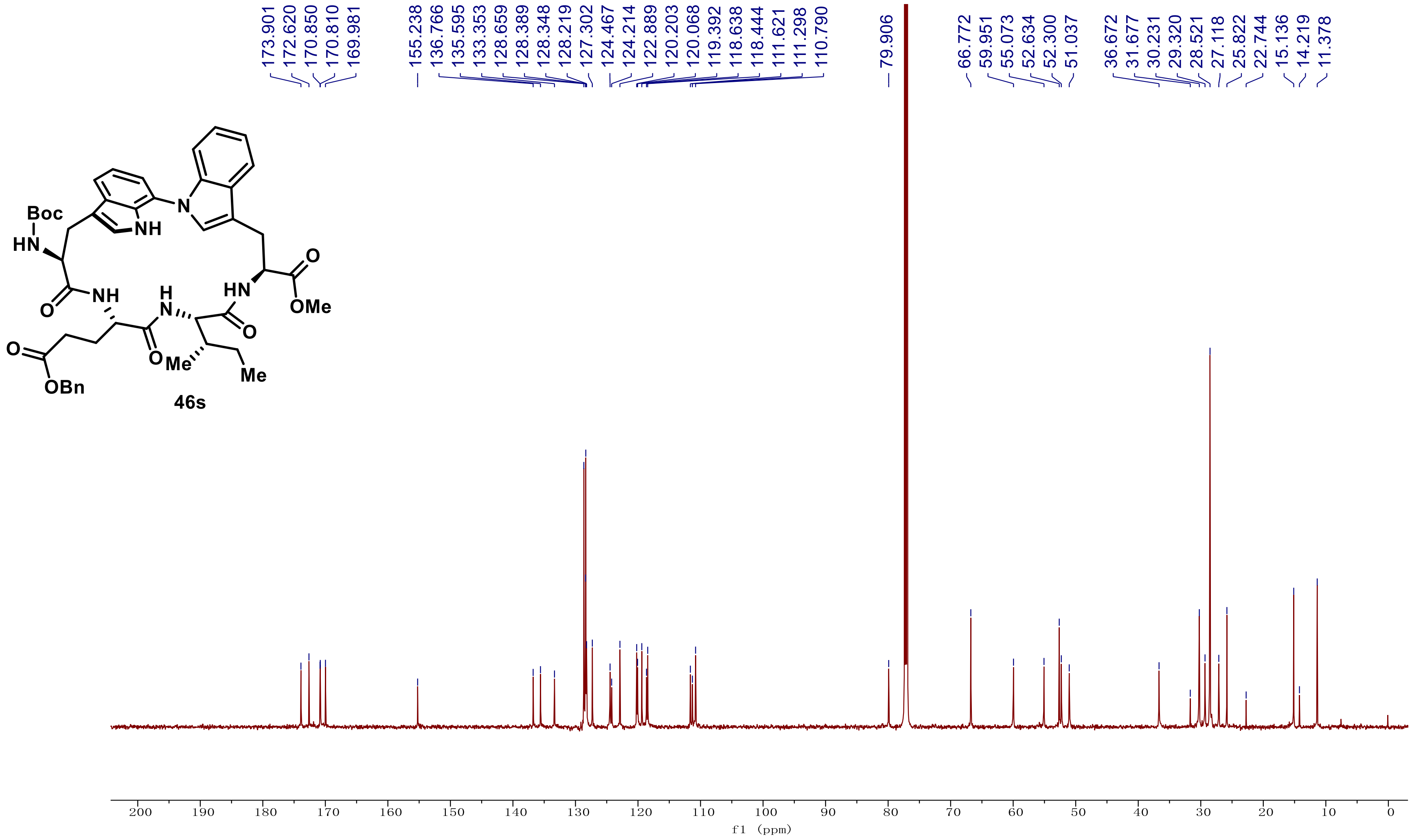

Compound 46t ^{1}H NMR (600 MHz, $CDCl_3$)

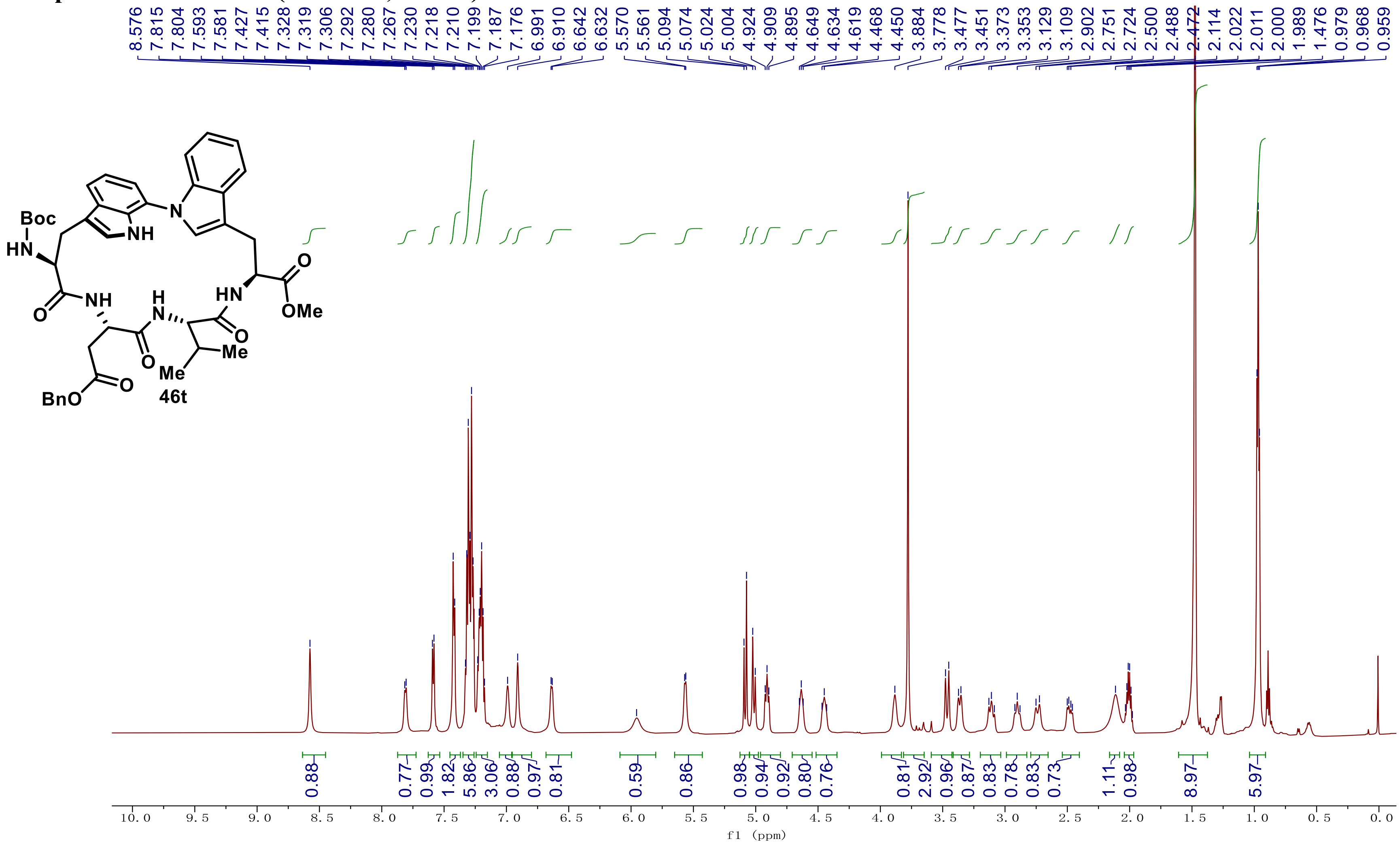

Compound 46t ^{13}C NMR (151 MHz, $CDCl_3$)

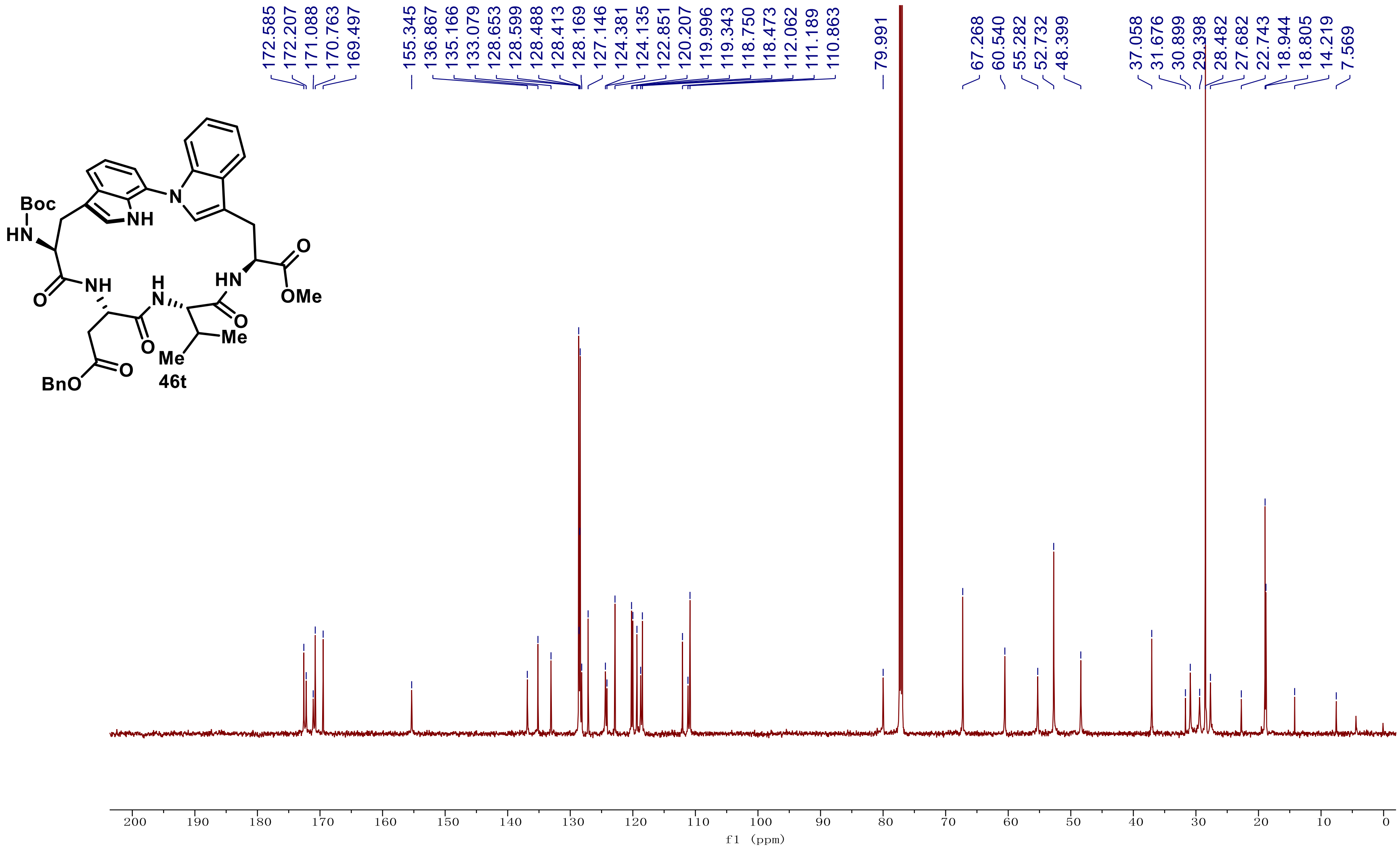

Compound 46u ¹H NMR (600 MHz, CDCl₃)

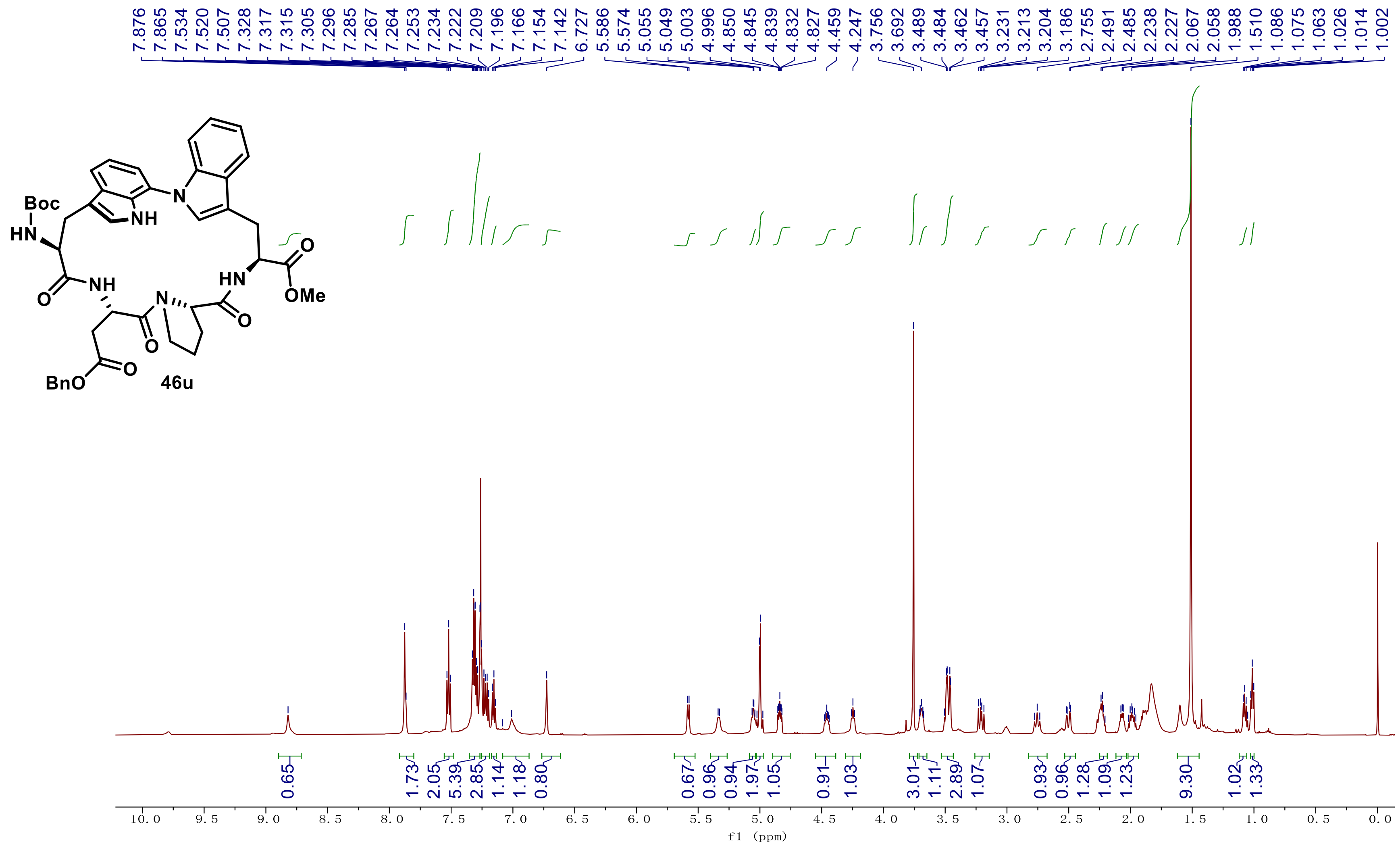

Compound 46u ^{13}C NMR (151 MHz, CDCl$_3$)

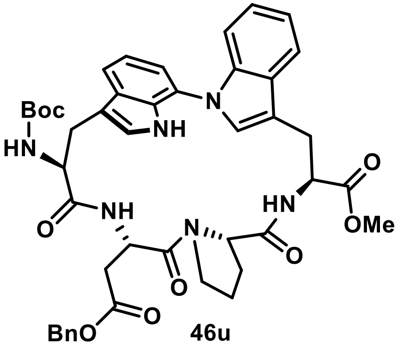

46u

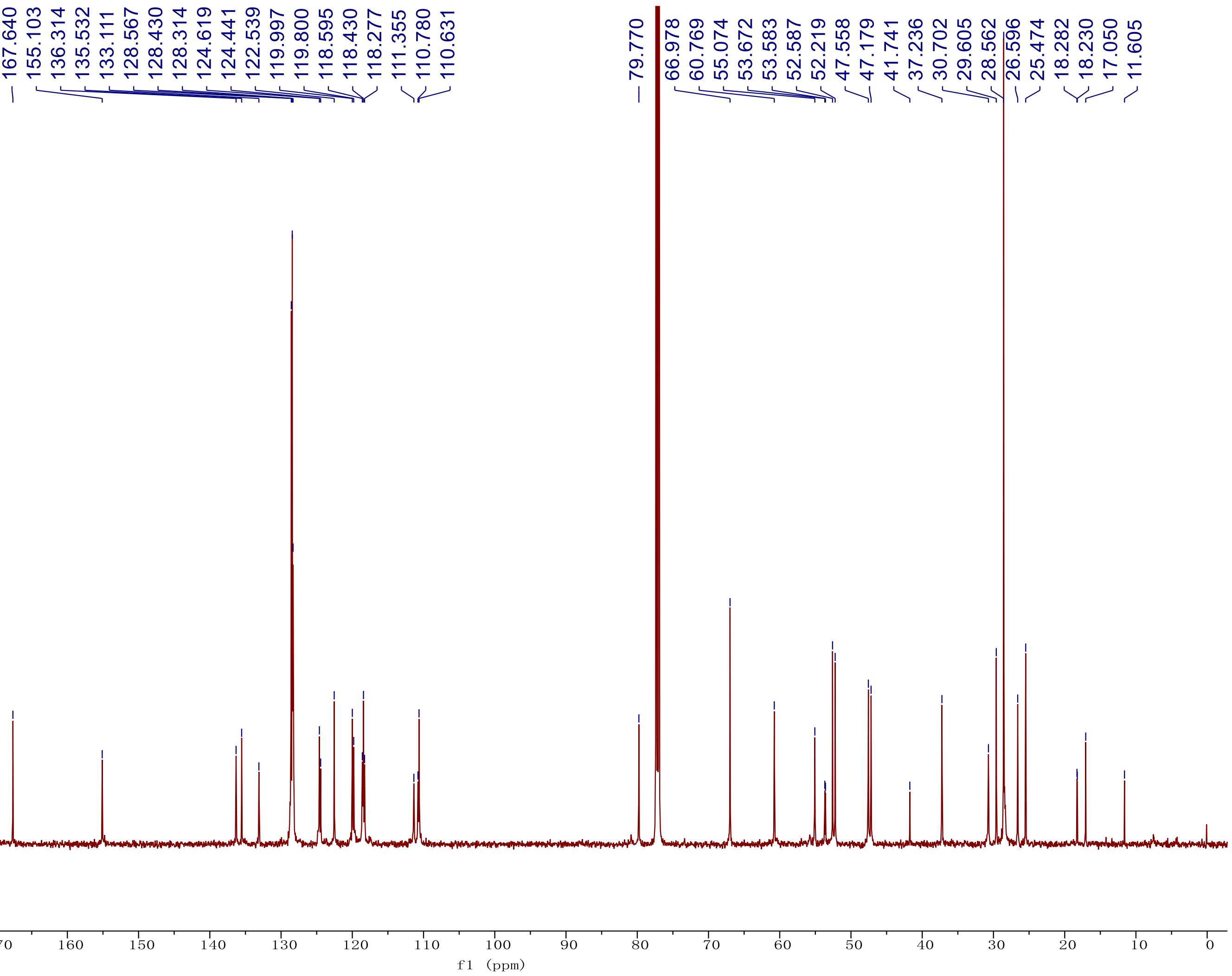

Compound 46y ^1H NMR (600 MHz, $CDCl_3$)

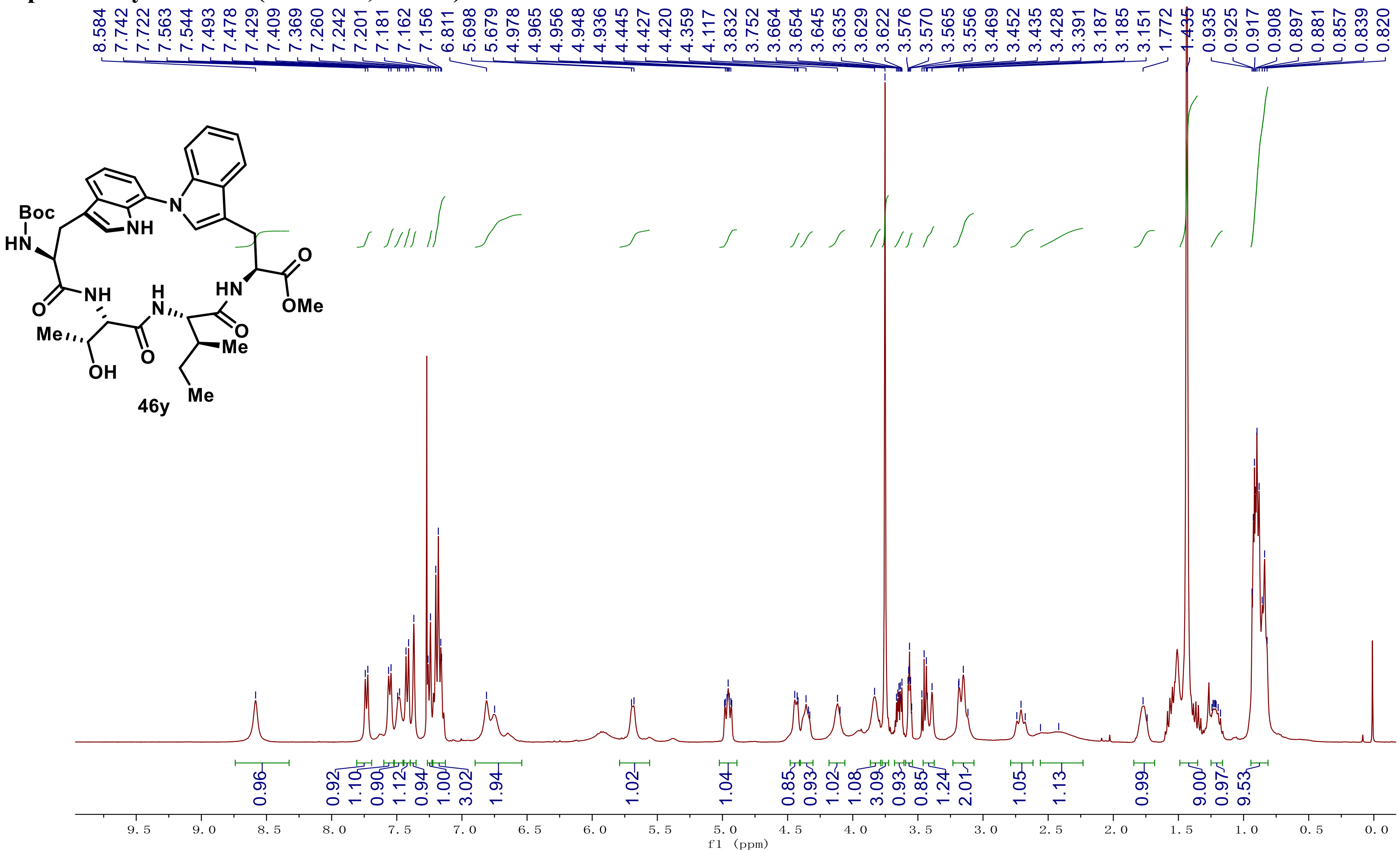

Compound 46y ^{13}C NMR (151 MHz, $CDCl_3$)

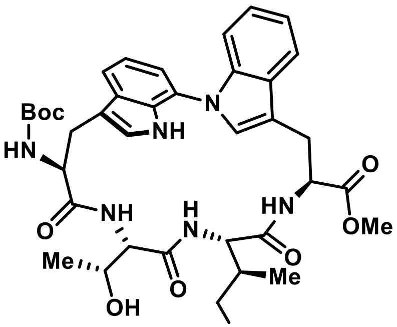

46y

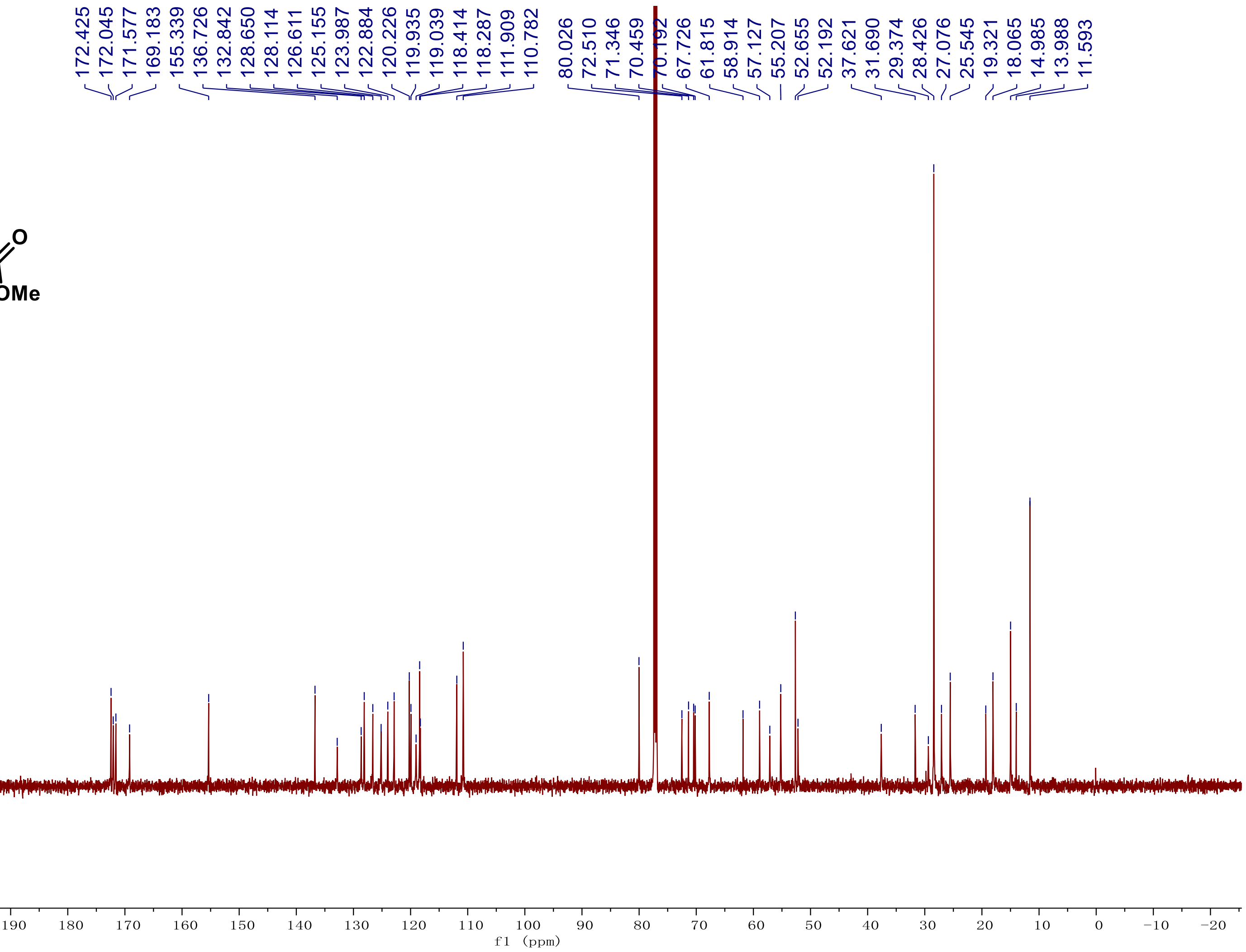

Compound 46z ^{1}H NMR (600 MHz, CD3OD)

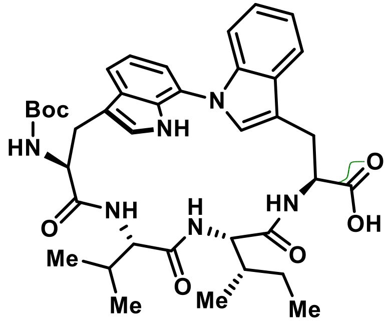

46z

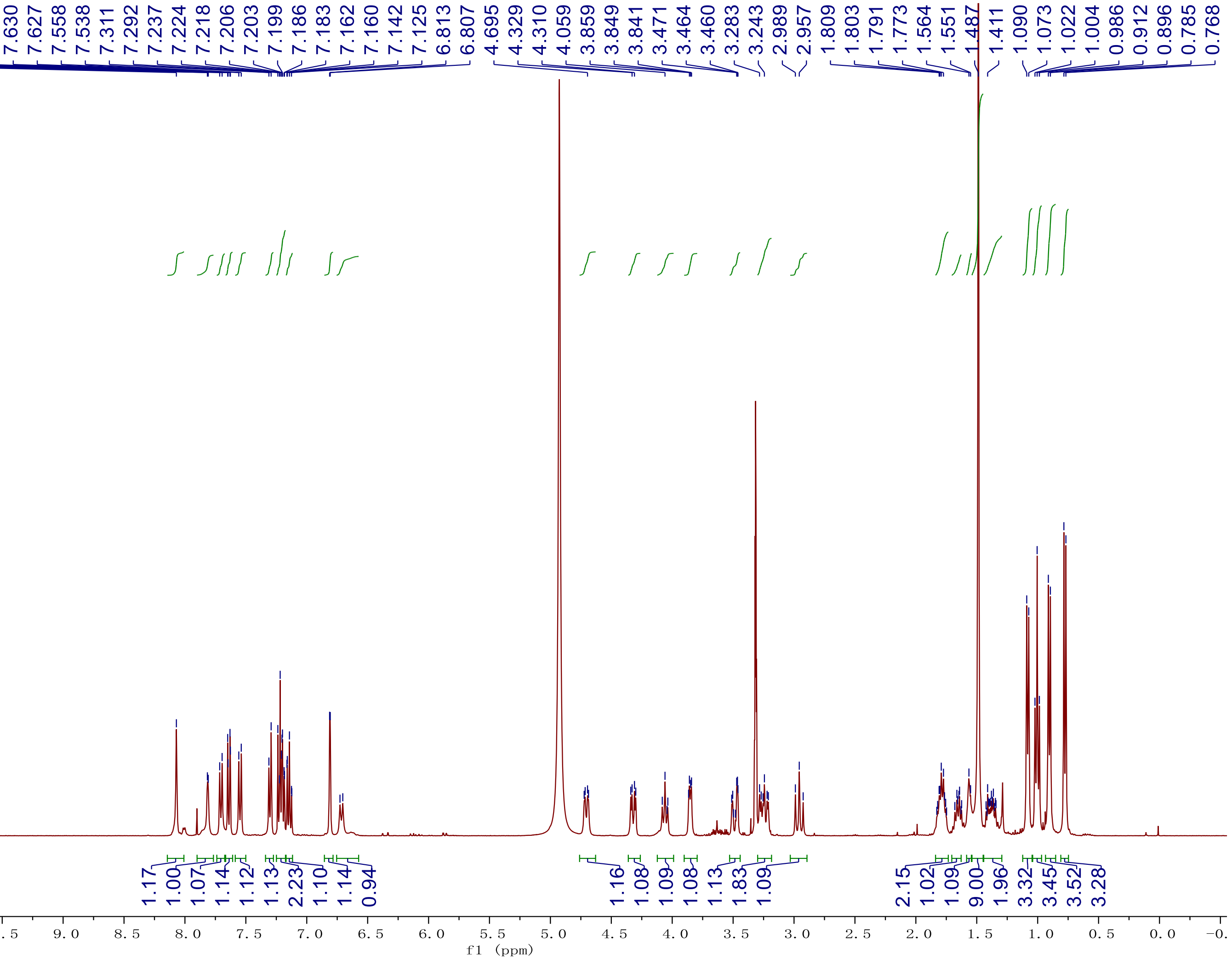

Compound 46z ^{13}C NMR (151 MHz, CD_3OD)

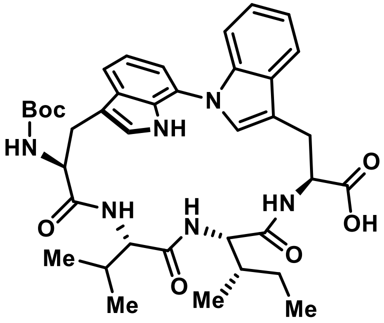

46z

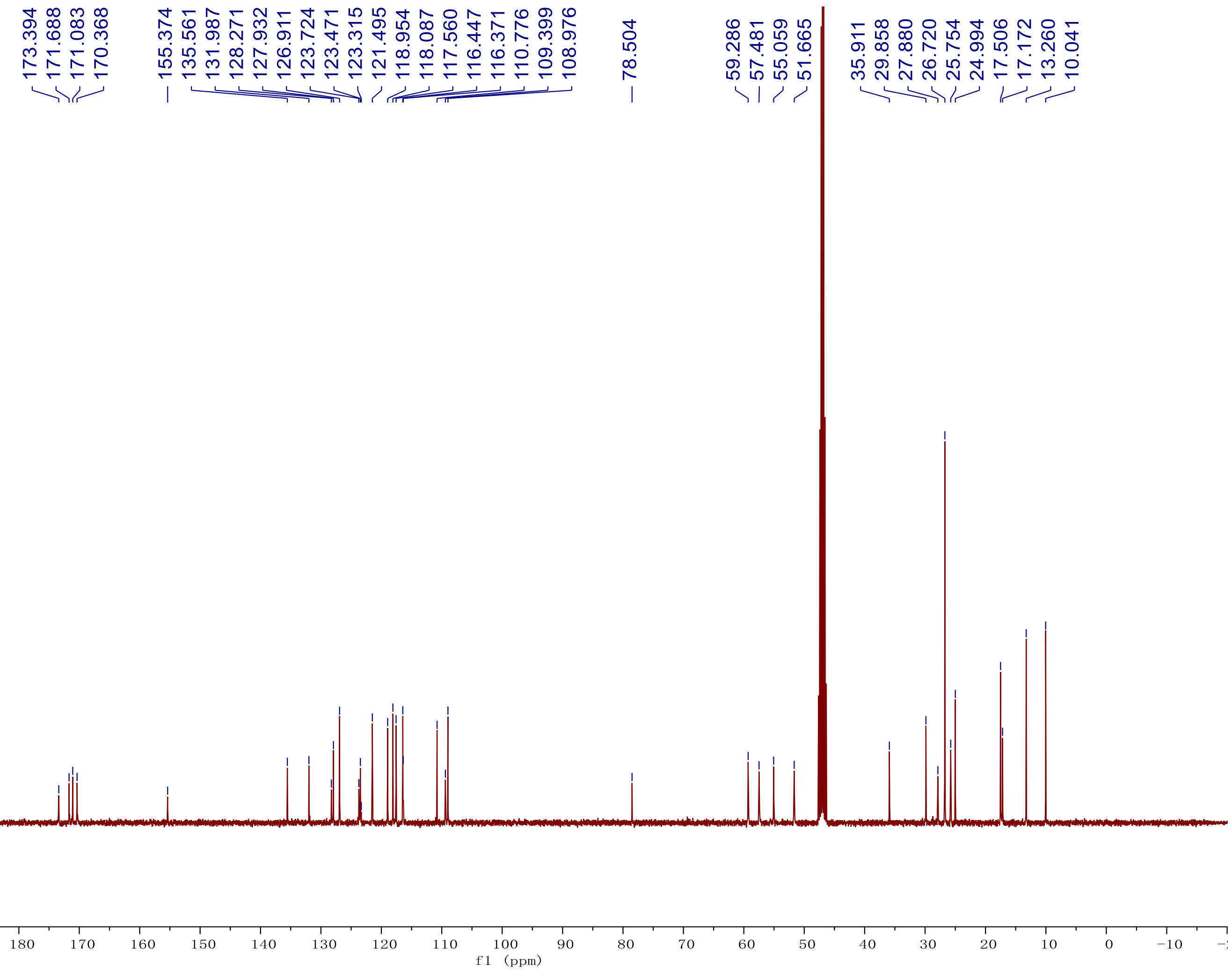

Compound S13 ^{1}H NMR (600 MHz, CDCl$_3$)

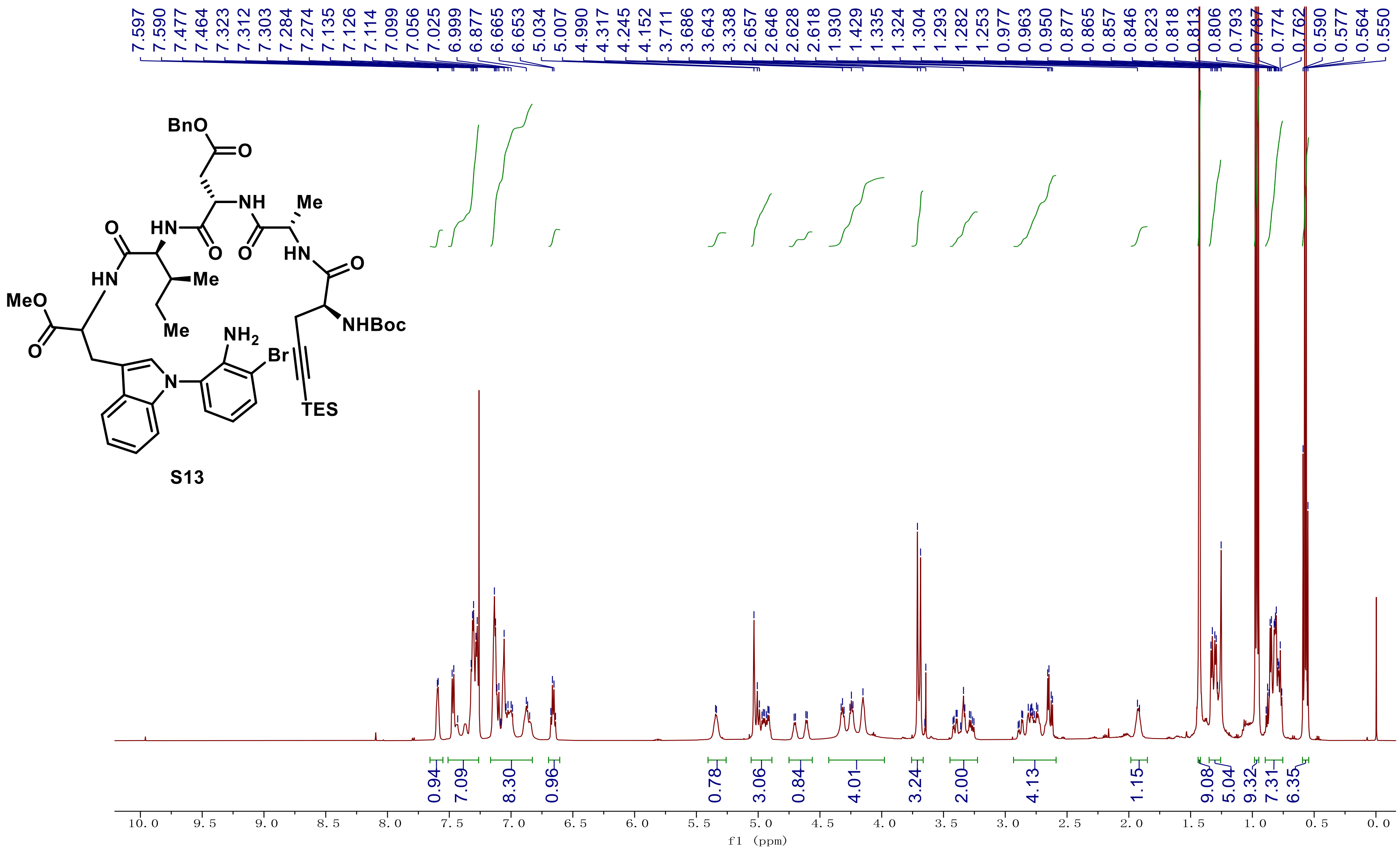

Compound S13 ^{13}C NMR (151 MHz, $CDCl_3$)

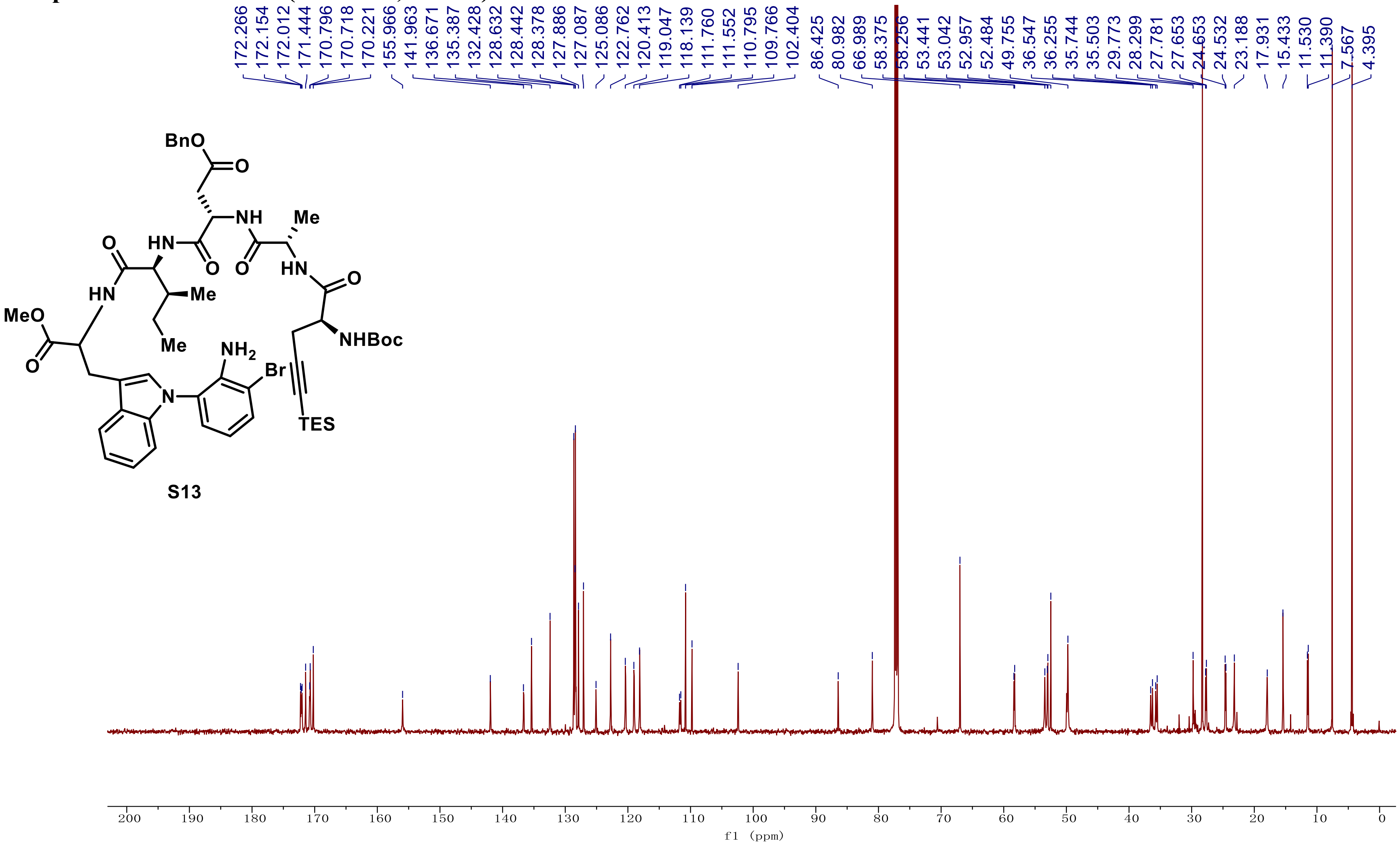

Compound 46v ¹H NMR (600 MHz, CDCl₃)

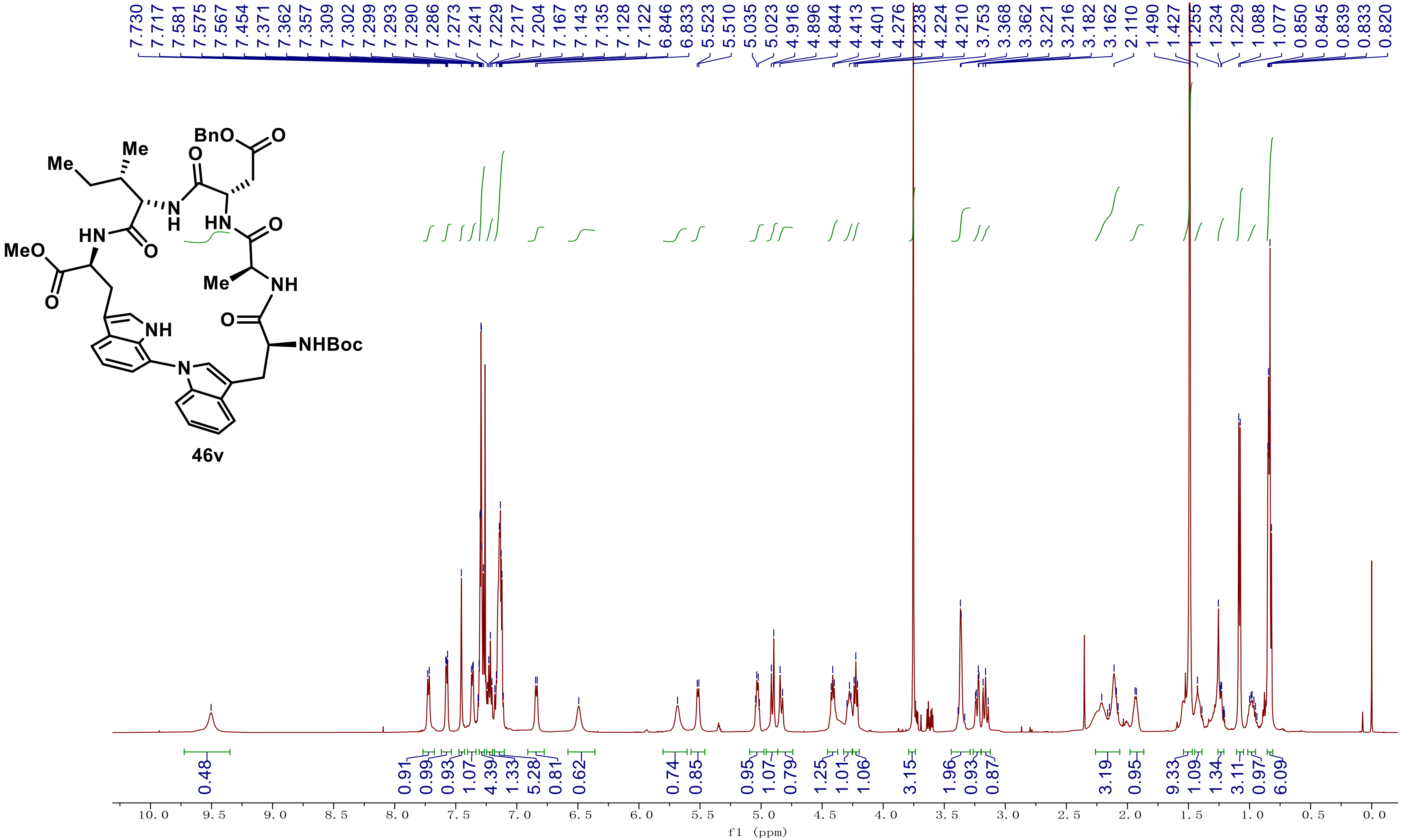

46v

Compound 46v ^{13}C NMR (151 MHz, $CDCl_3$)

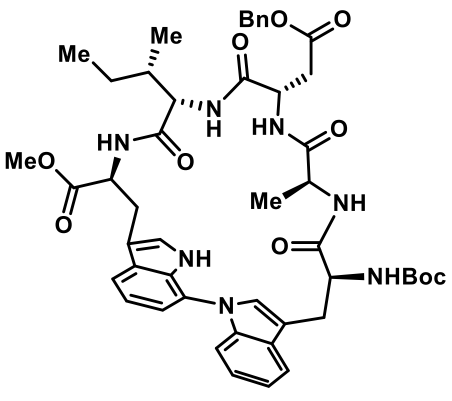

46v

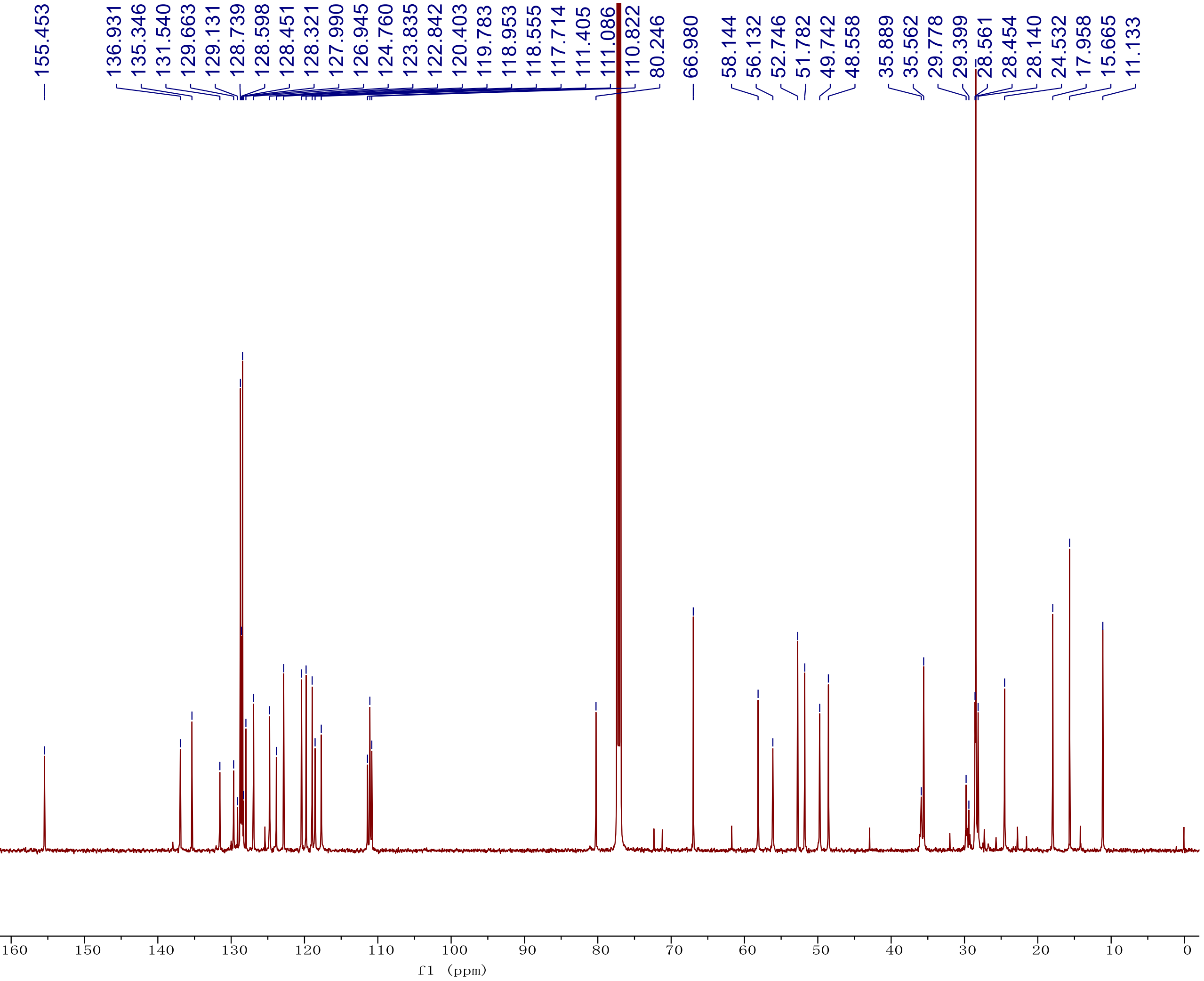

Compound S14 ^{1}H NMR (600 MHz, DMSO-d_6)

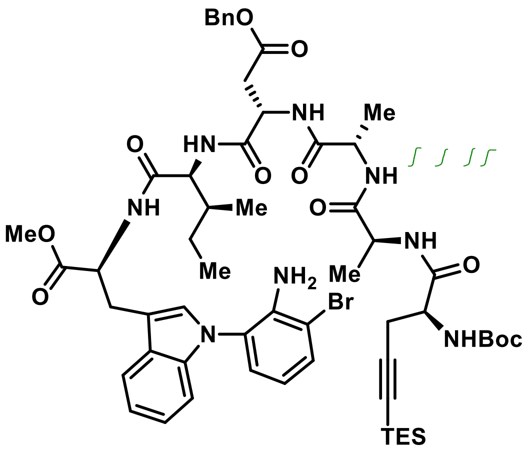

S14

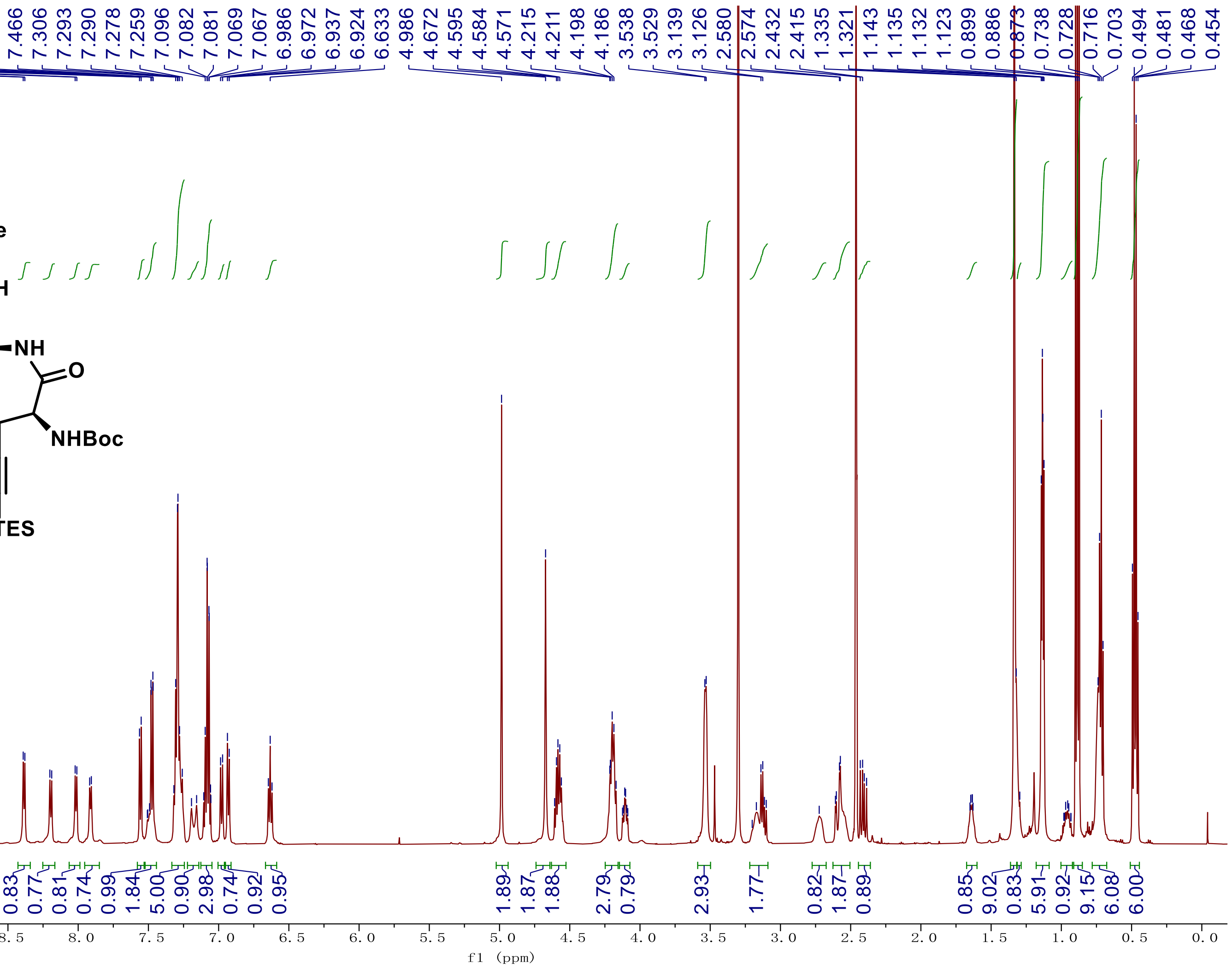

Compound S14 ^{13}C NMR (151 MHz, DMSO-d_6)

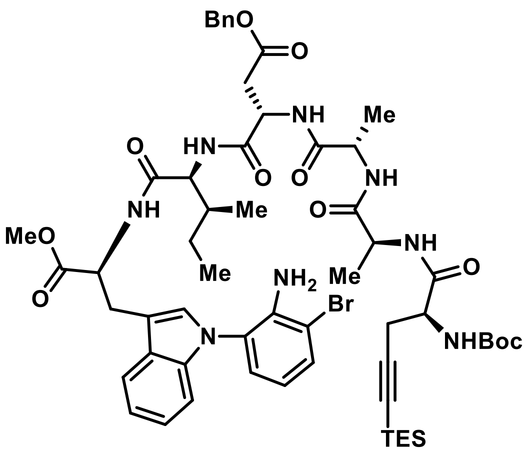

S14

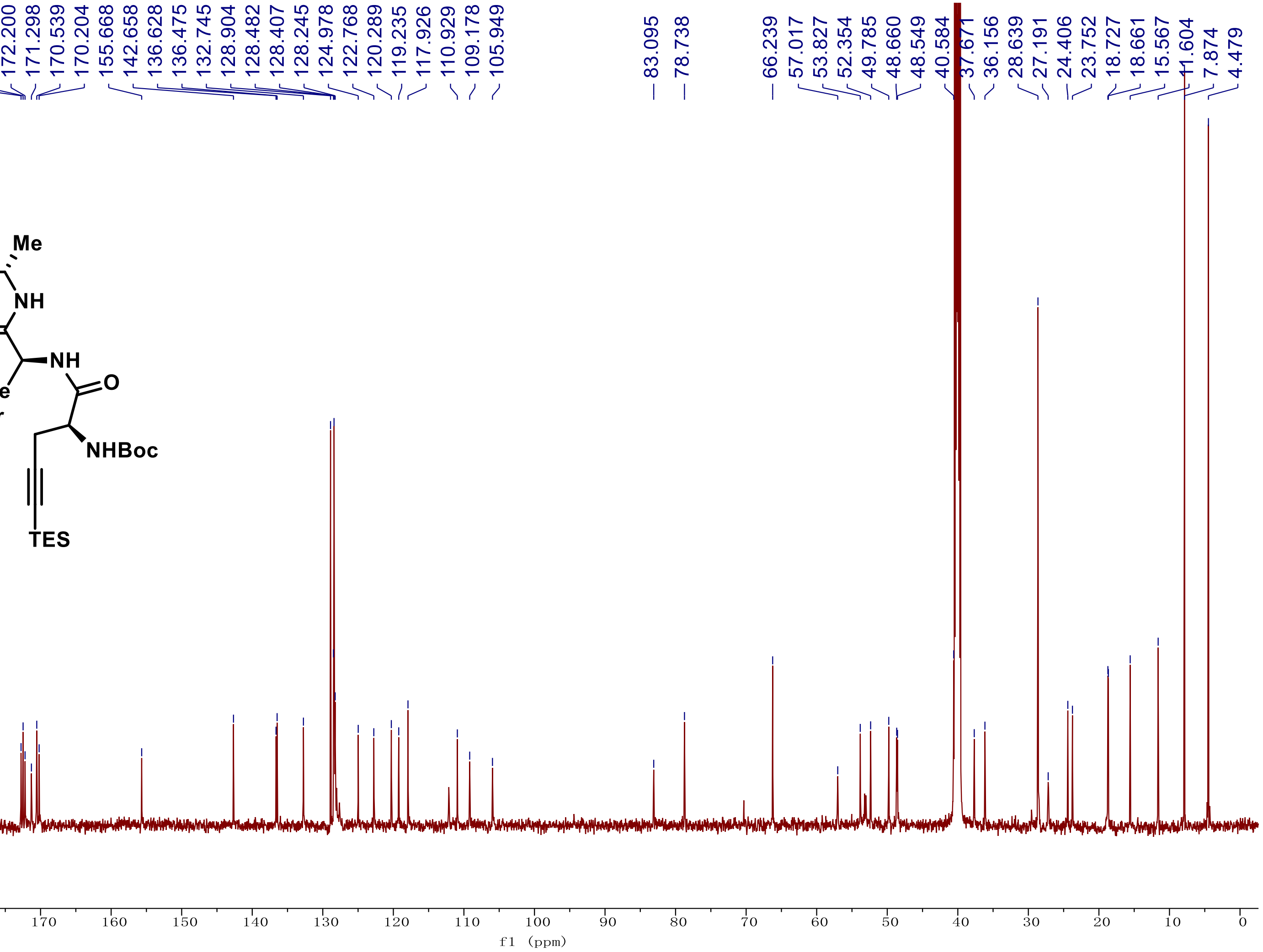

Compound 46w ^{1}H NMR (600 MHz, $CDCl_3$)

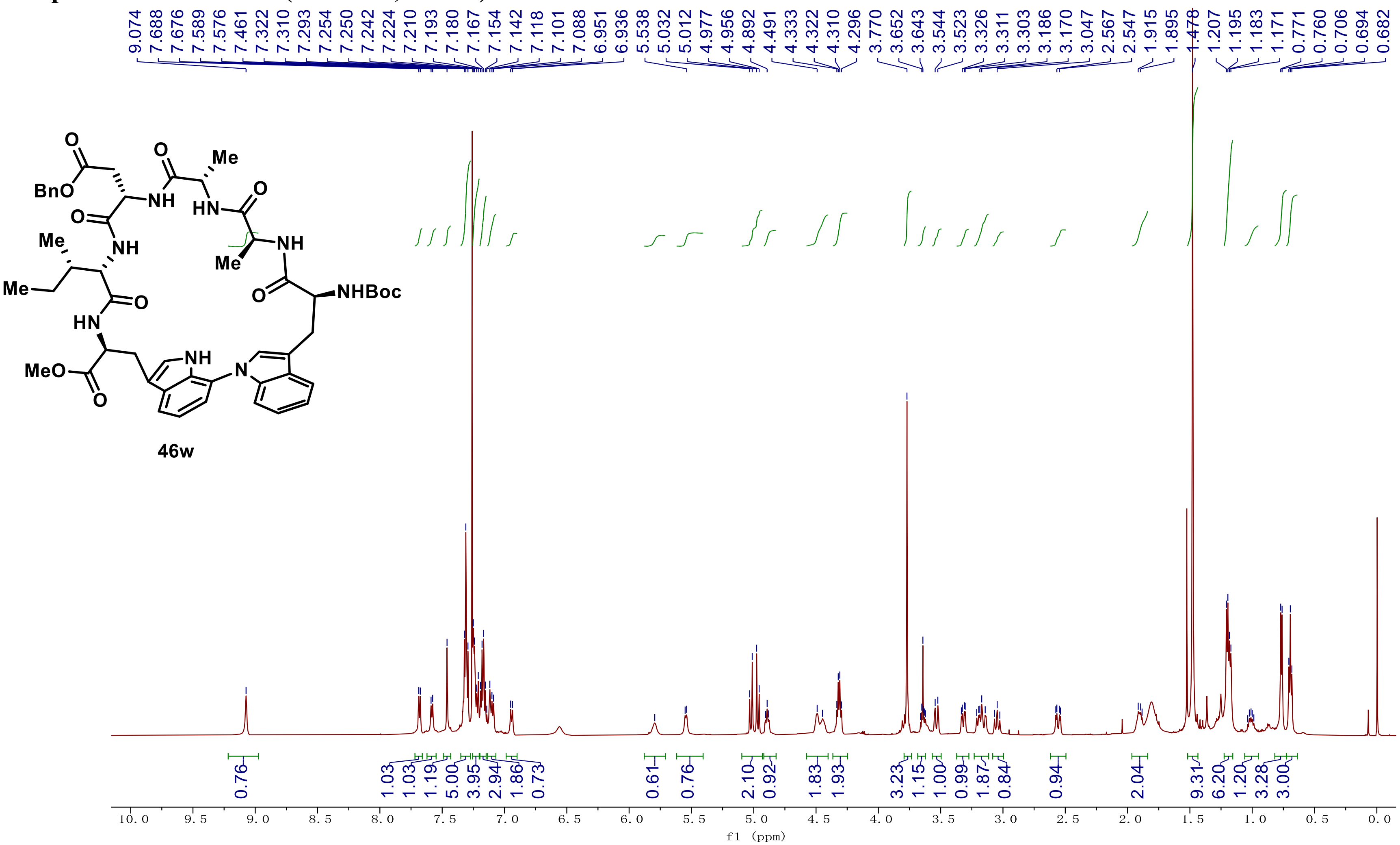

Compound 46w ^{13}C NMR (151 MHz, $CDCl_3$)

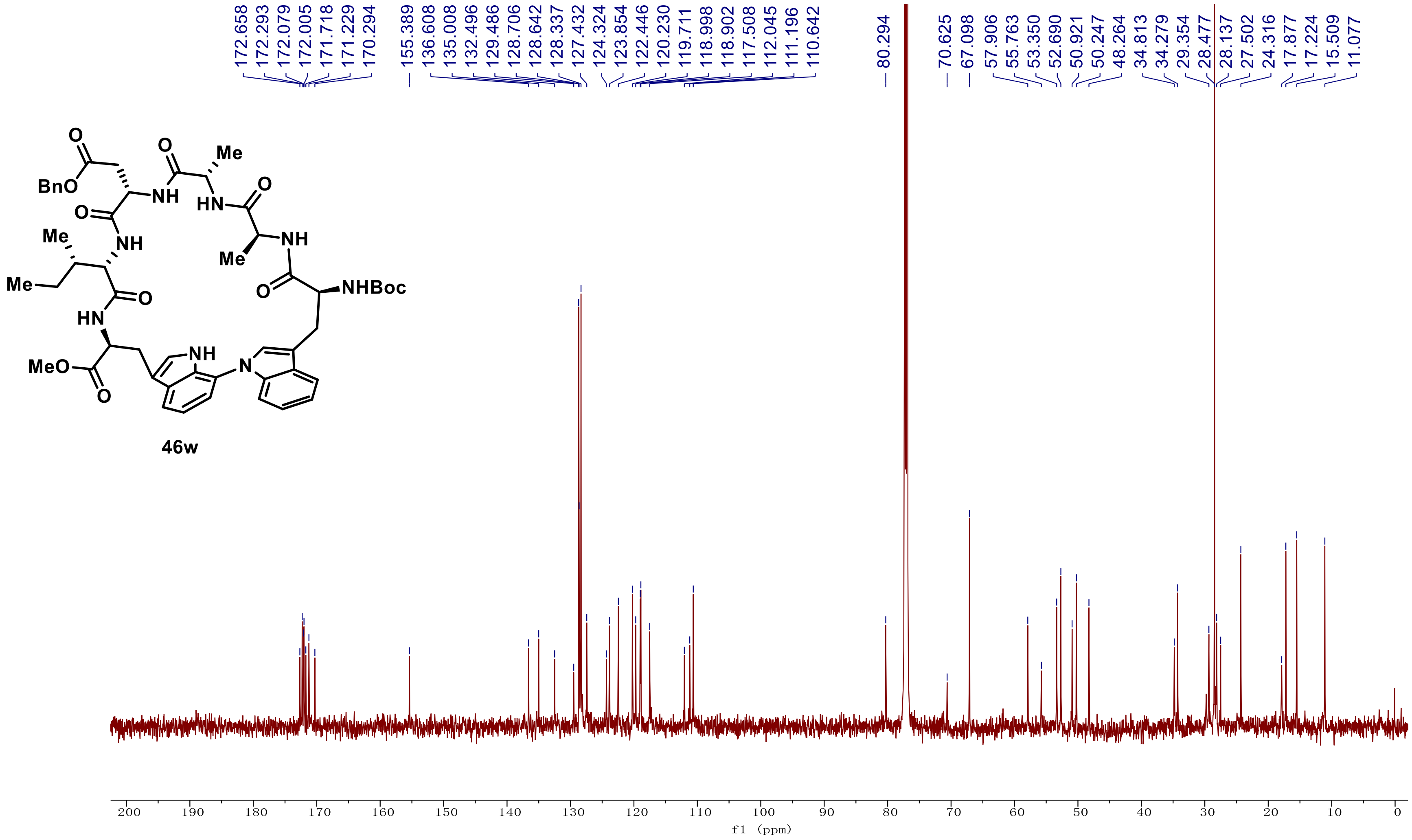

Compound S15 ¹H NMR (600 MHz, DMSO-*d6*)

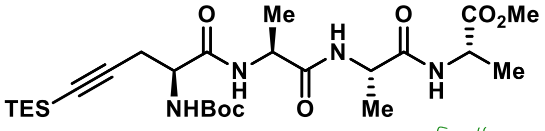

S15

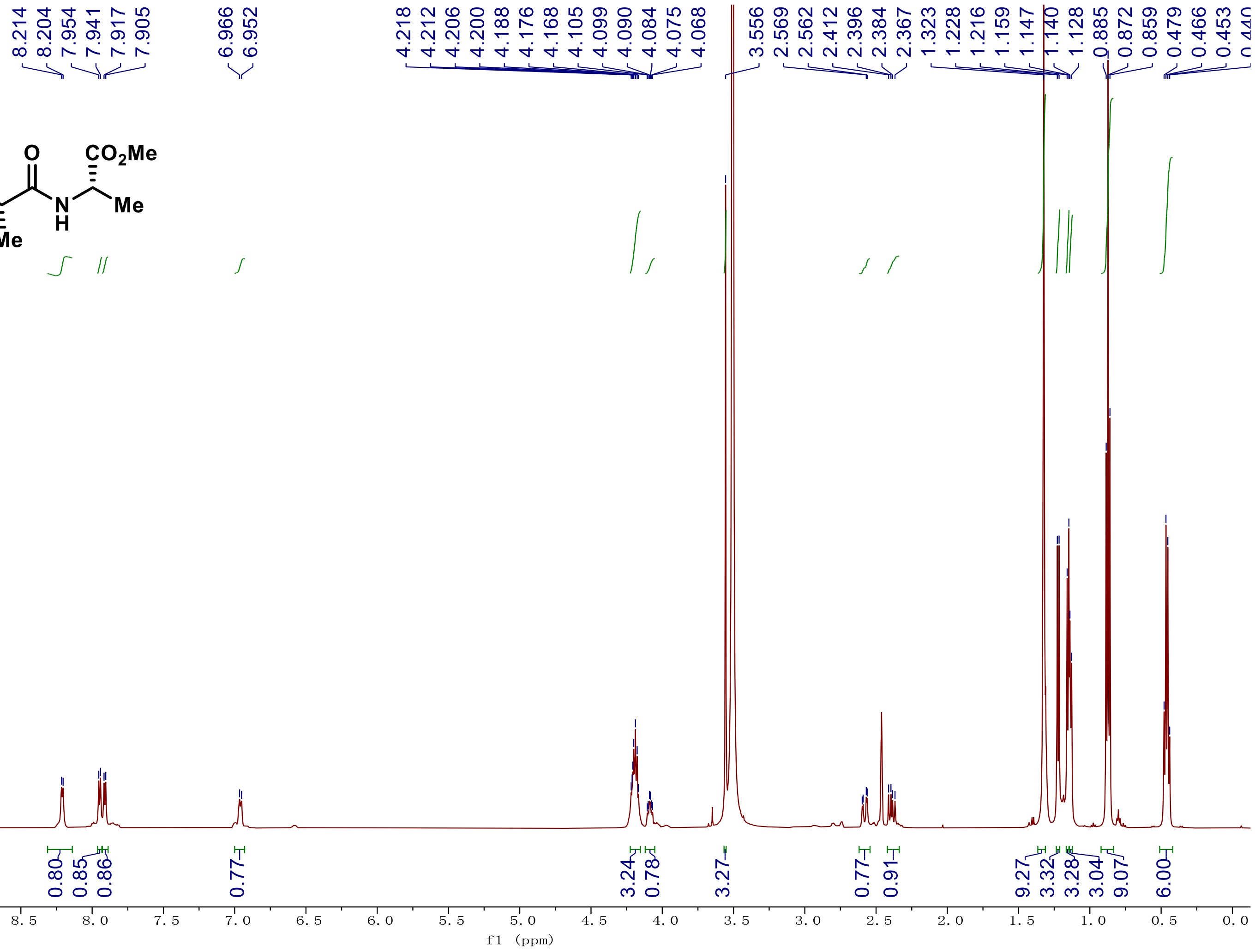

Compound S15 ^{13}C NMR (151 MHz, DMSO-*d6*)

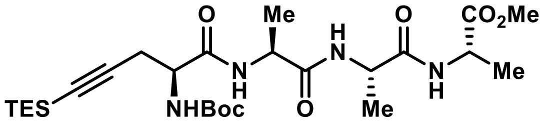

S15

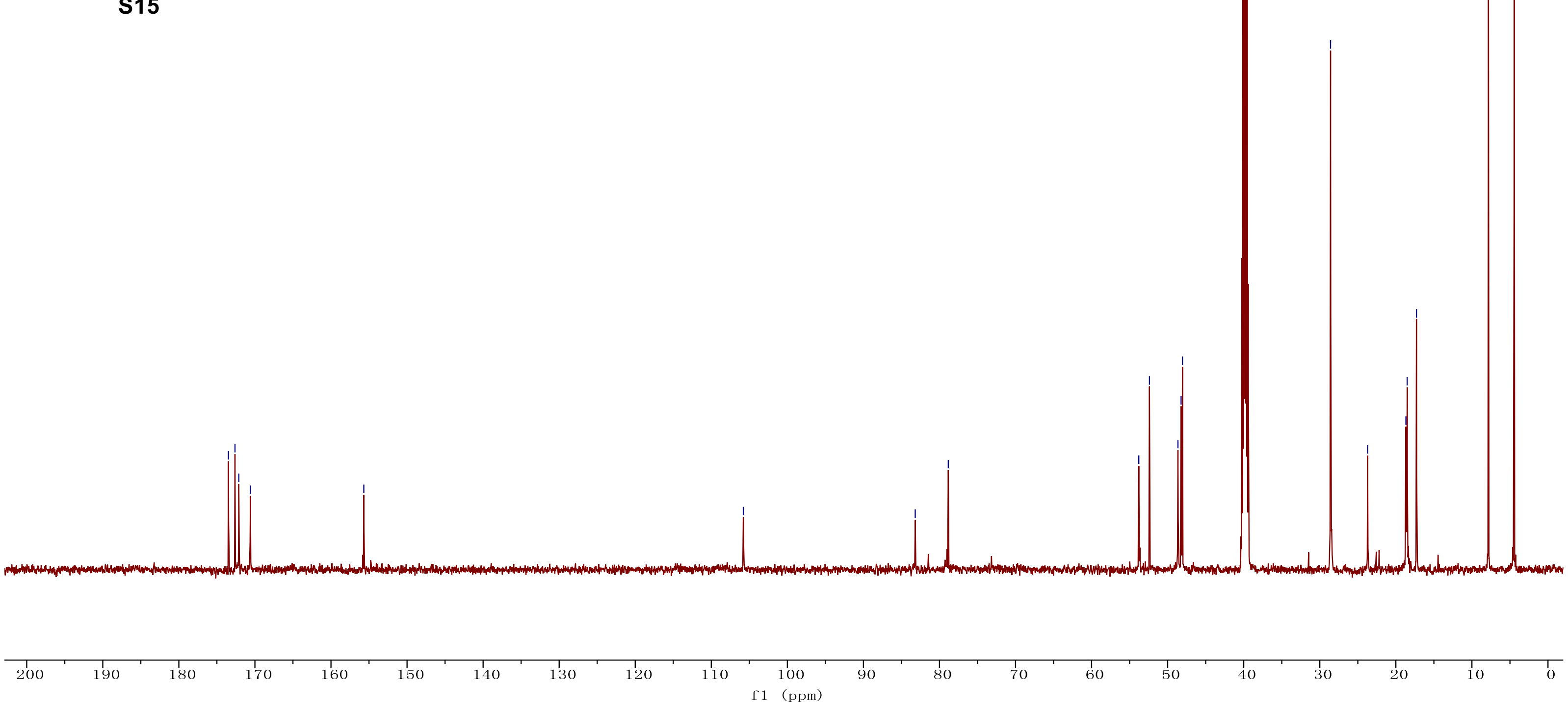

Compound S17 ^{1}H NMR (600 MHz, DMSO-d_6)

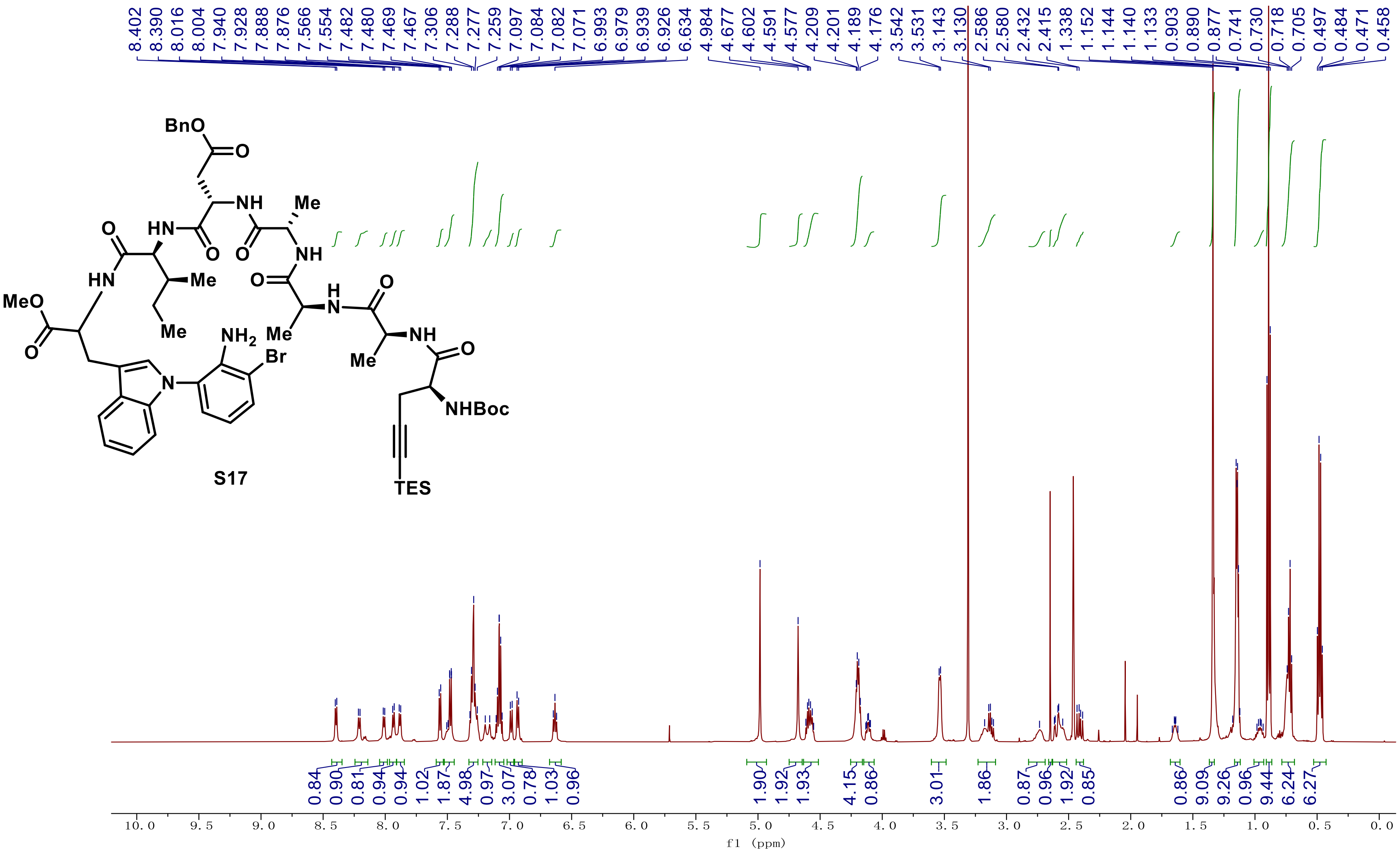

Compound S17 ^{13}C NMR (151 MHz, DMSO-d_6)

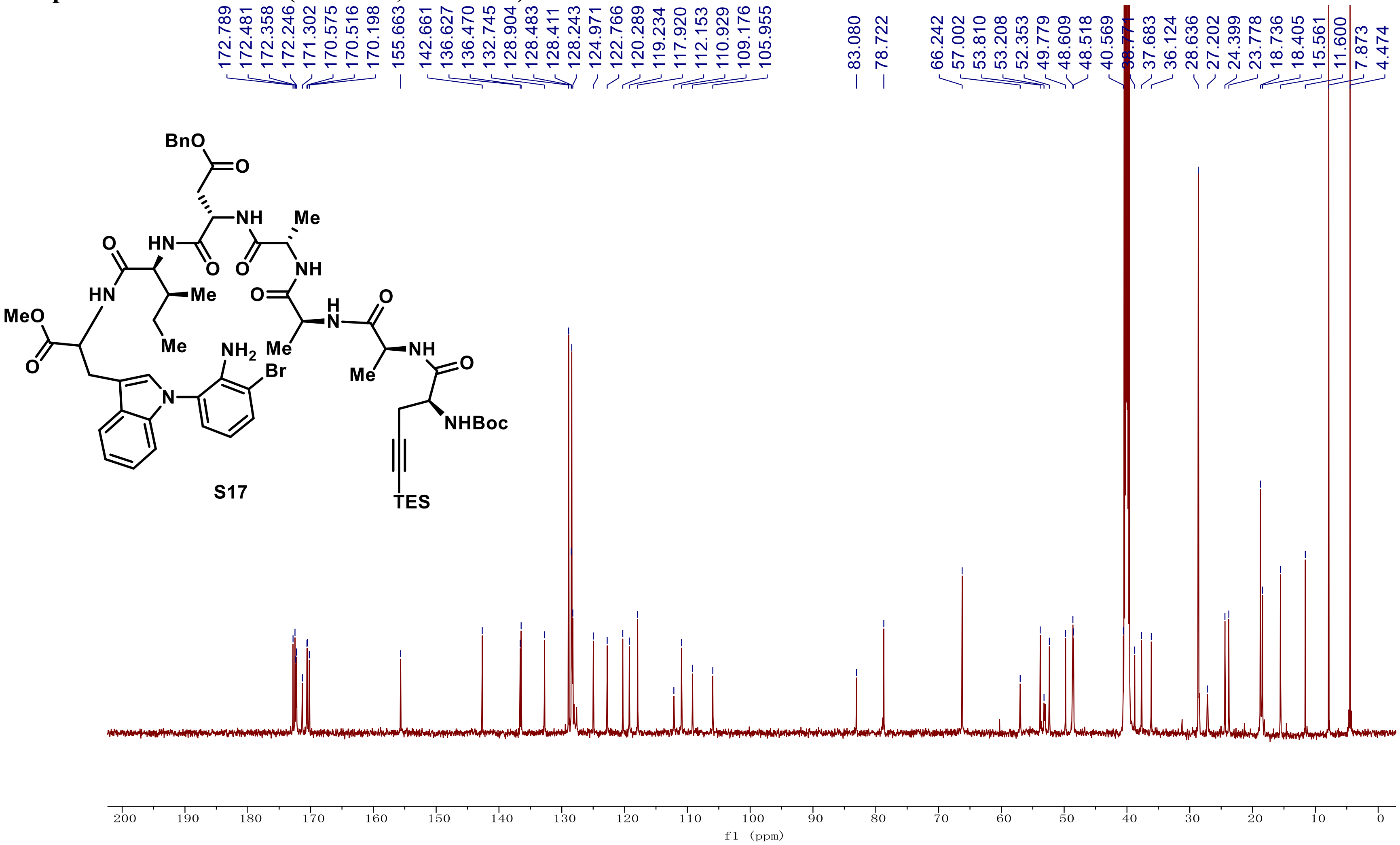

Compound 46x ^{1}H NMR (600 MHz, DMSO-d_6)

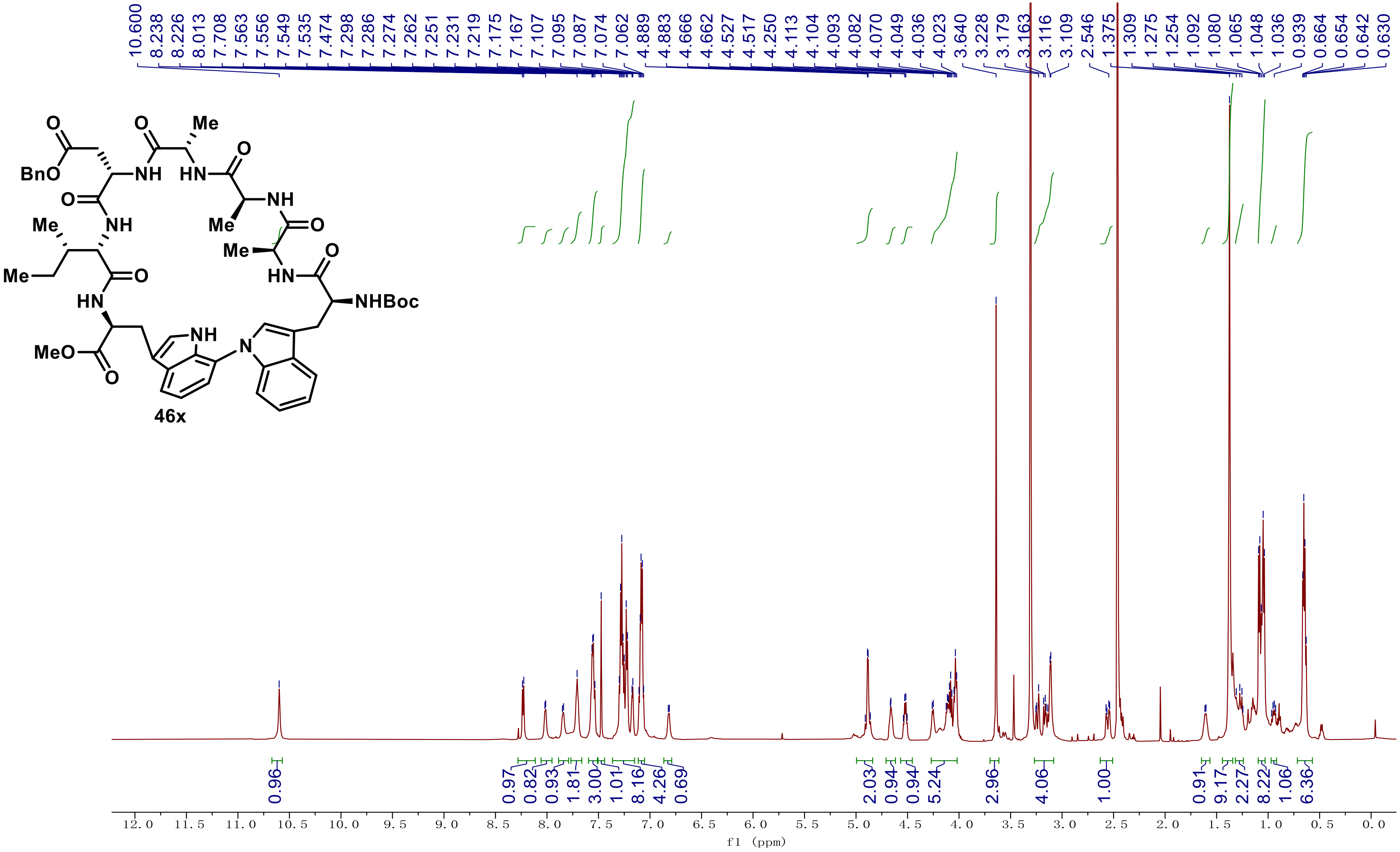

46x

Compound 46x ^{13}C NMR (151 MHz, DMSO-d_6)

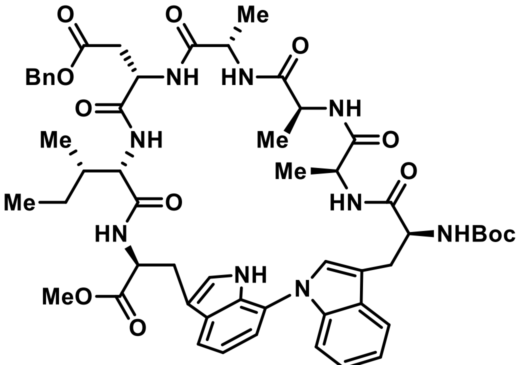

46x

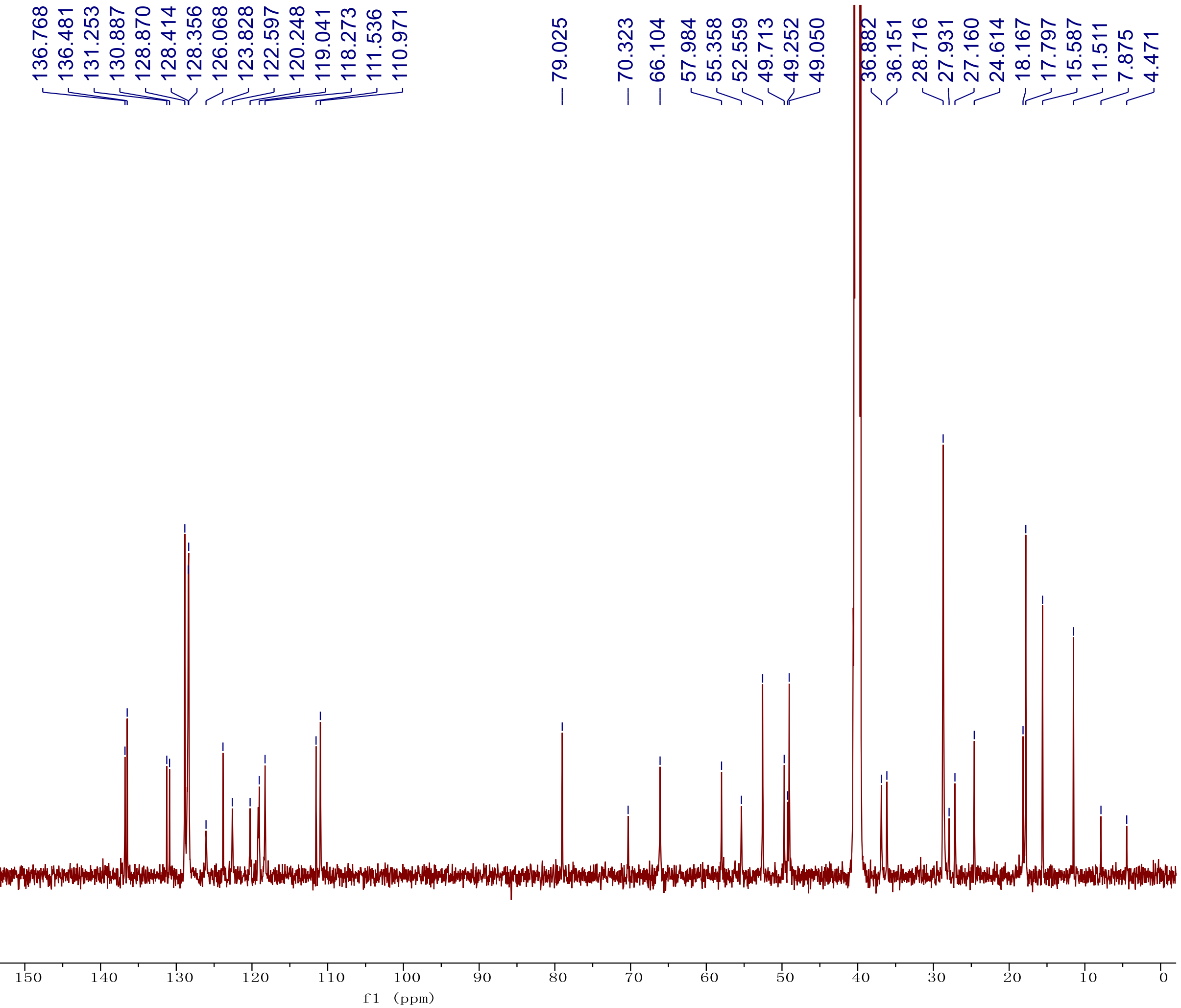

Compound S18a ¹H NMR (600 MHz, CDCl₃)

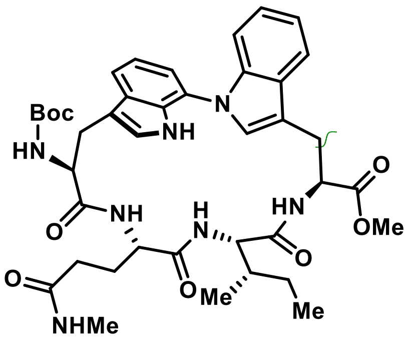

S18a

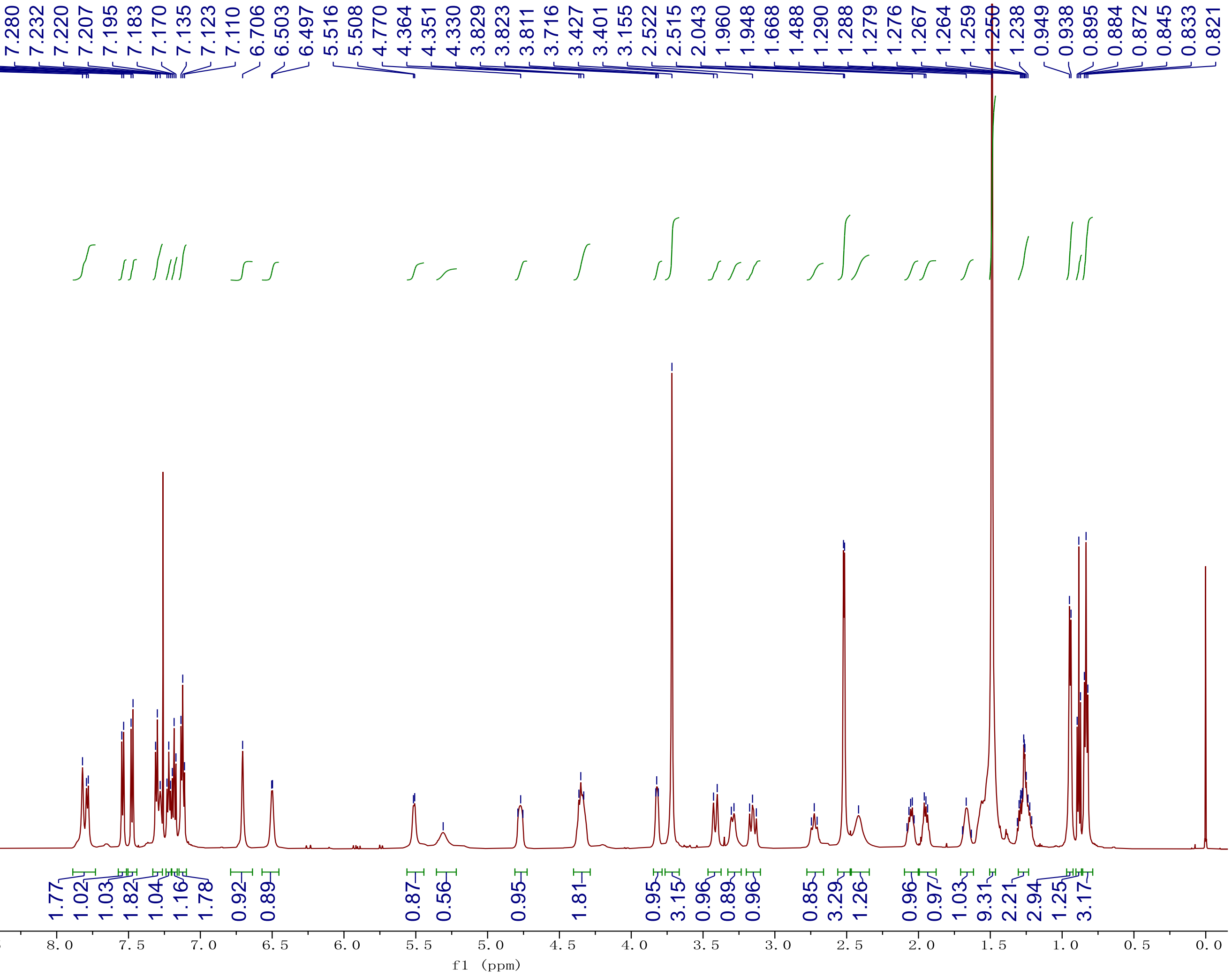

Compound S18a ^{13}C NMR (151 MHz, $CDCl_3$)

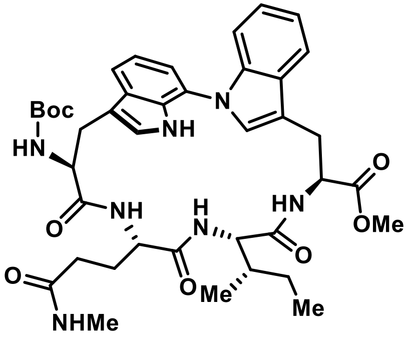

S18a

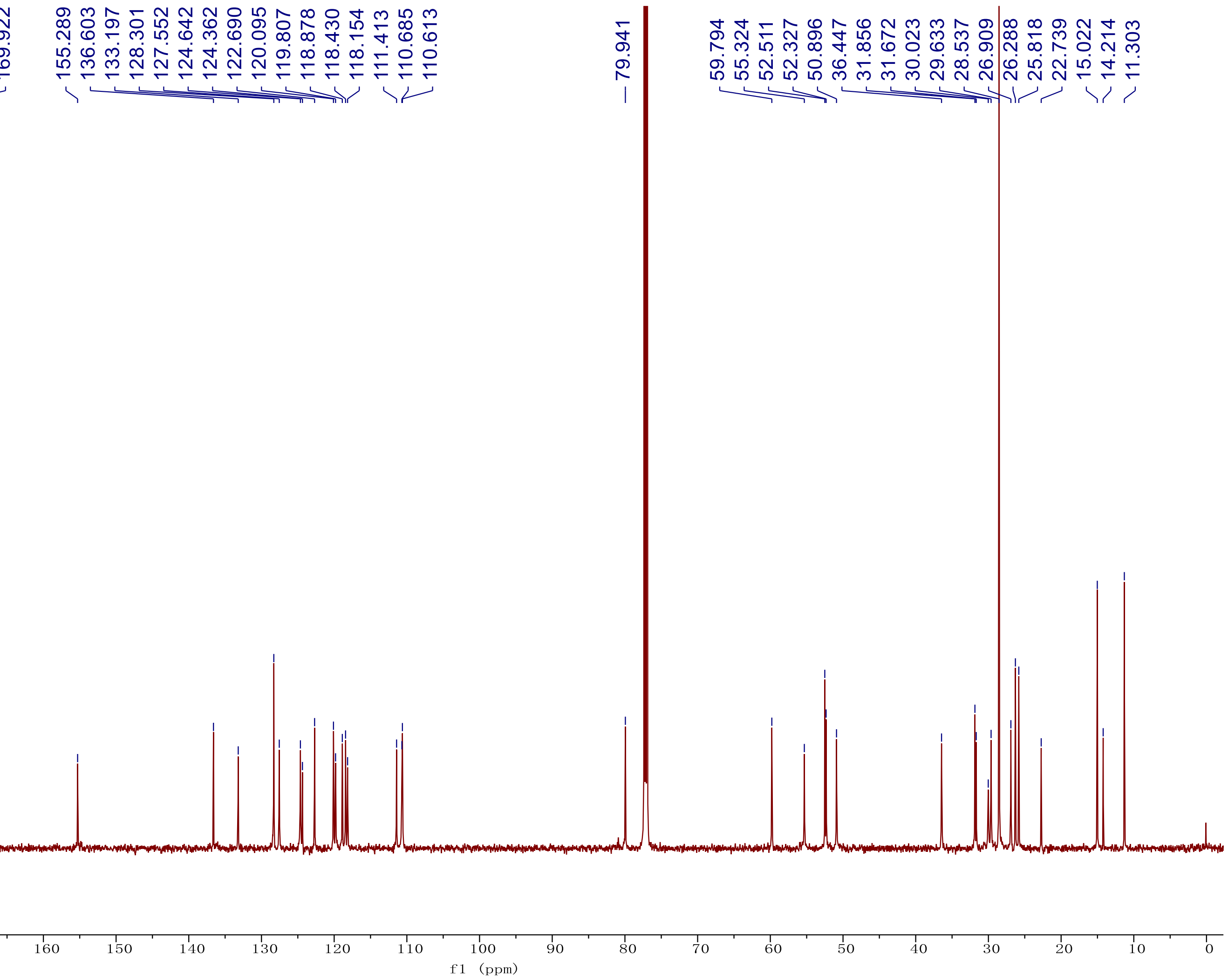

Compound S18b ^{1}H NMR (600 MHz, METHANOL-D_4)

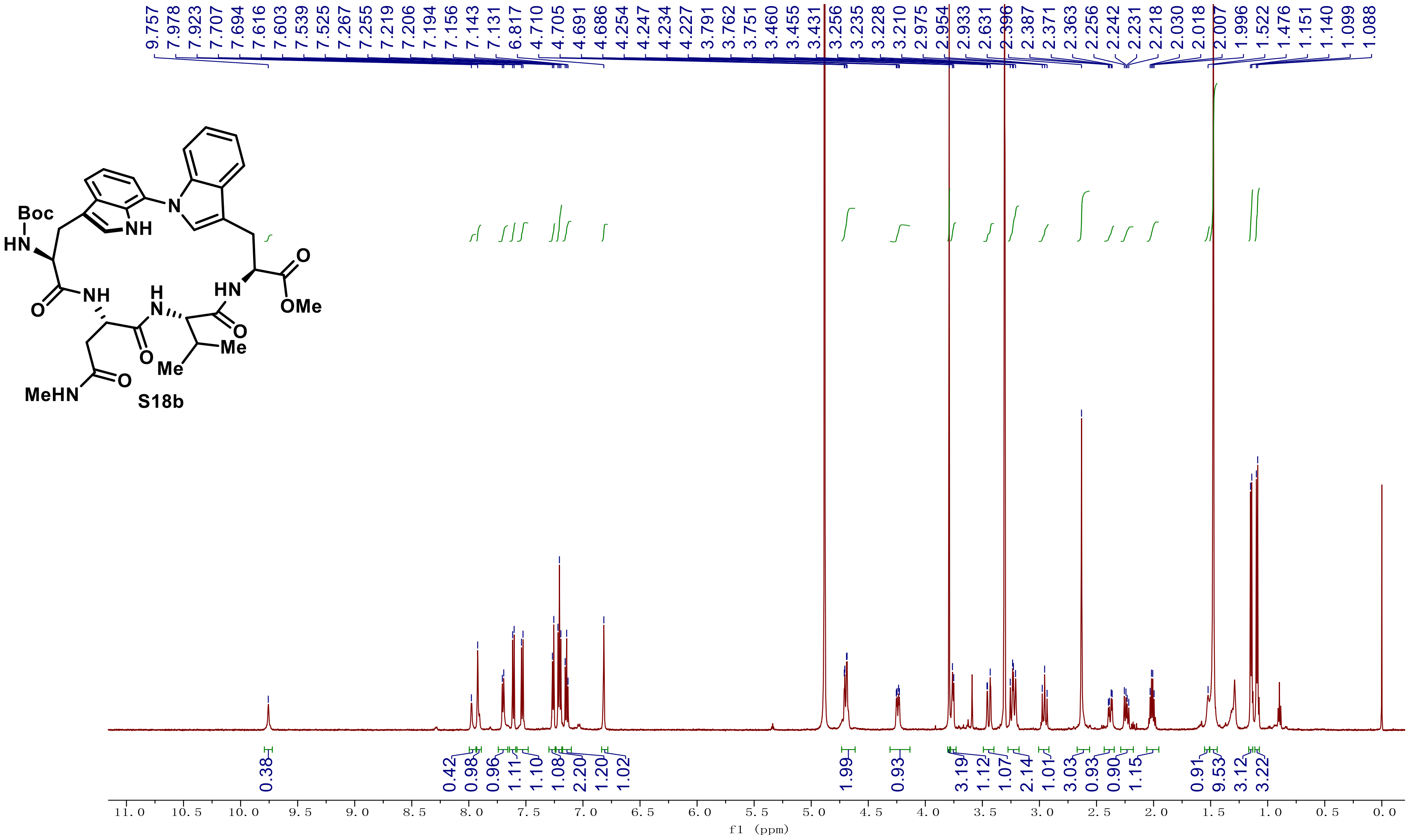

Compound S18b ^{13}C NMR (151 MHz, METHANOL-D_4)

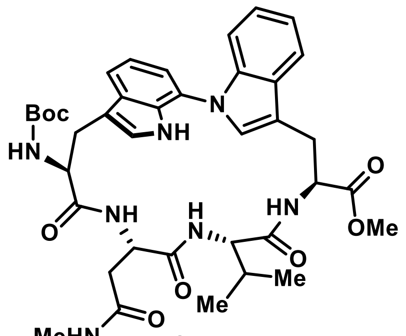

S18b

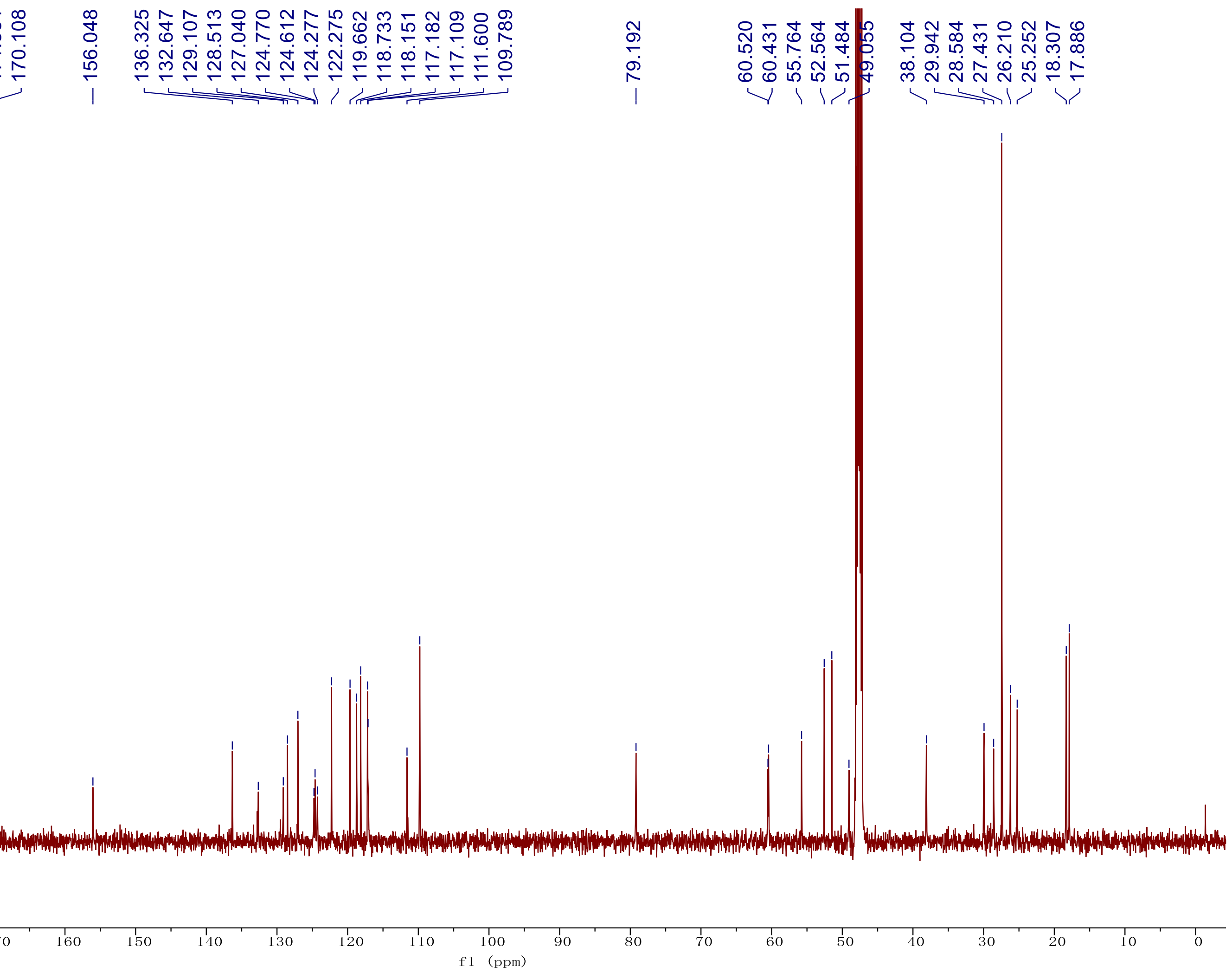

Compound S18c ¹H NMR (600 MHz, CDCl₃)

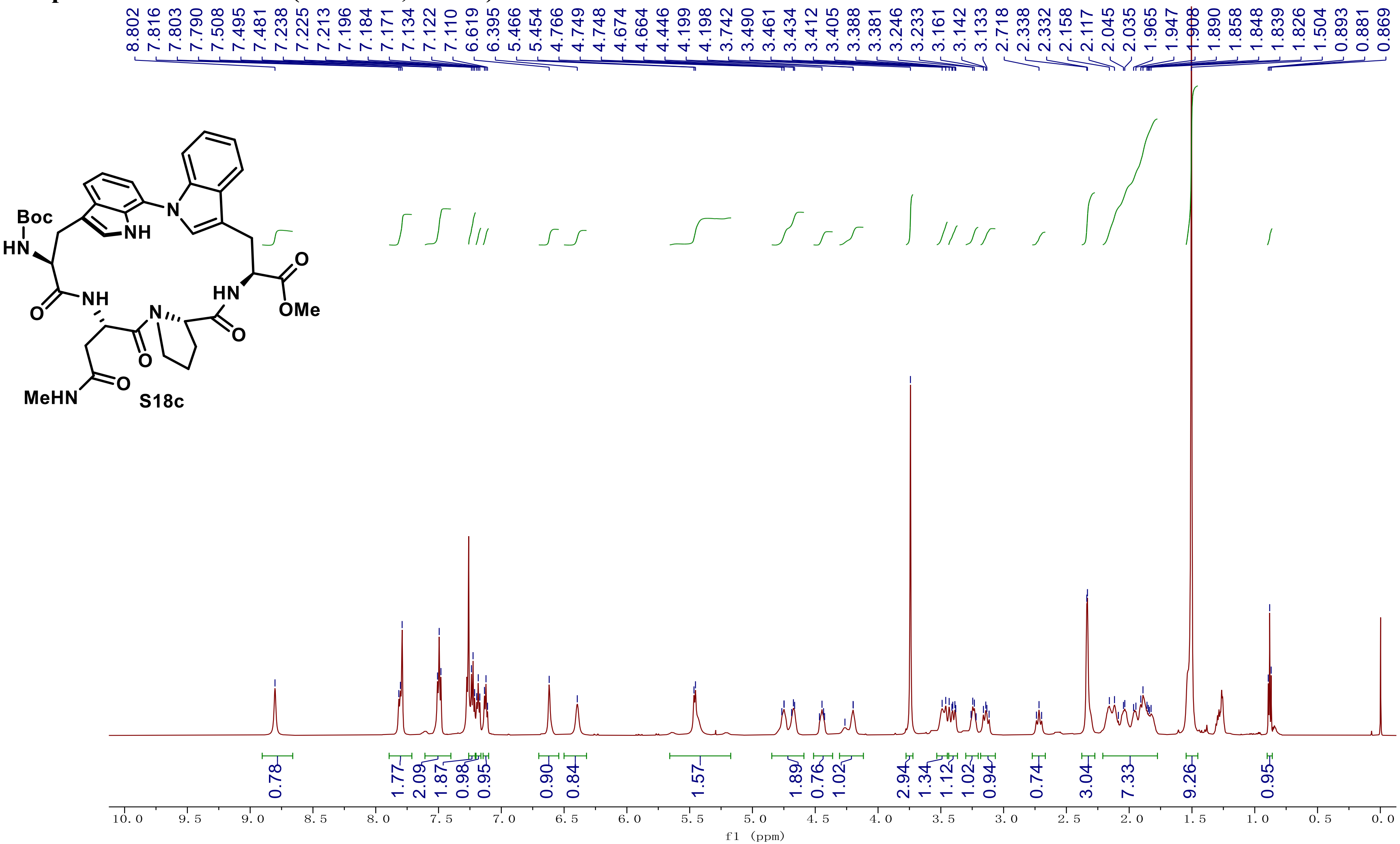

Compound S18c ^{13}C NMR (151 MHz, $CDCl_3$)

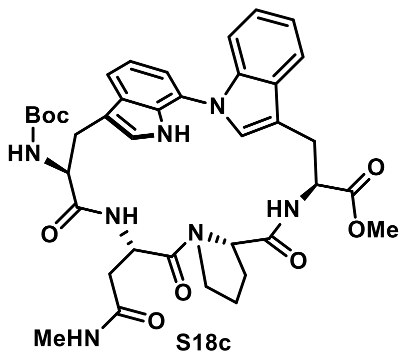

S18c

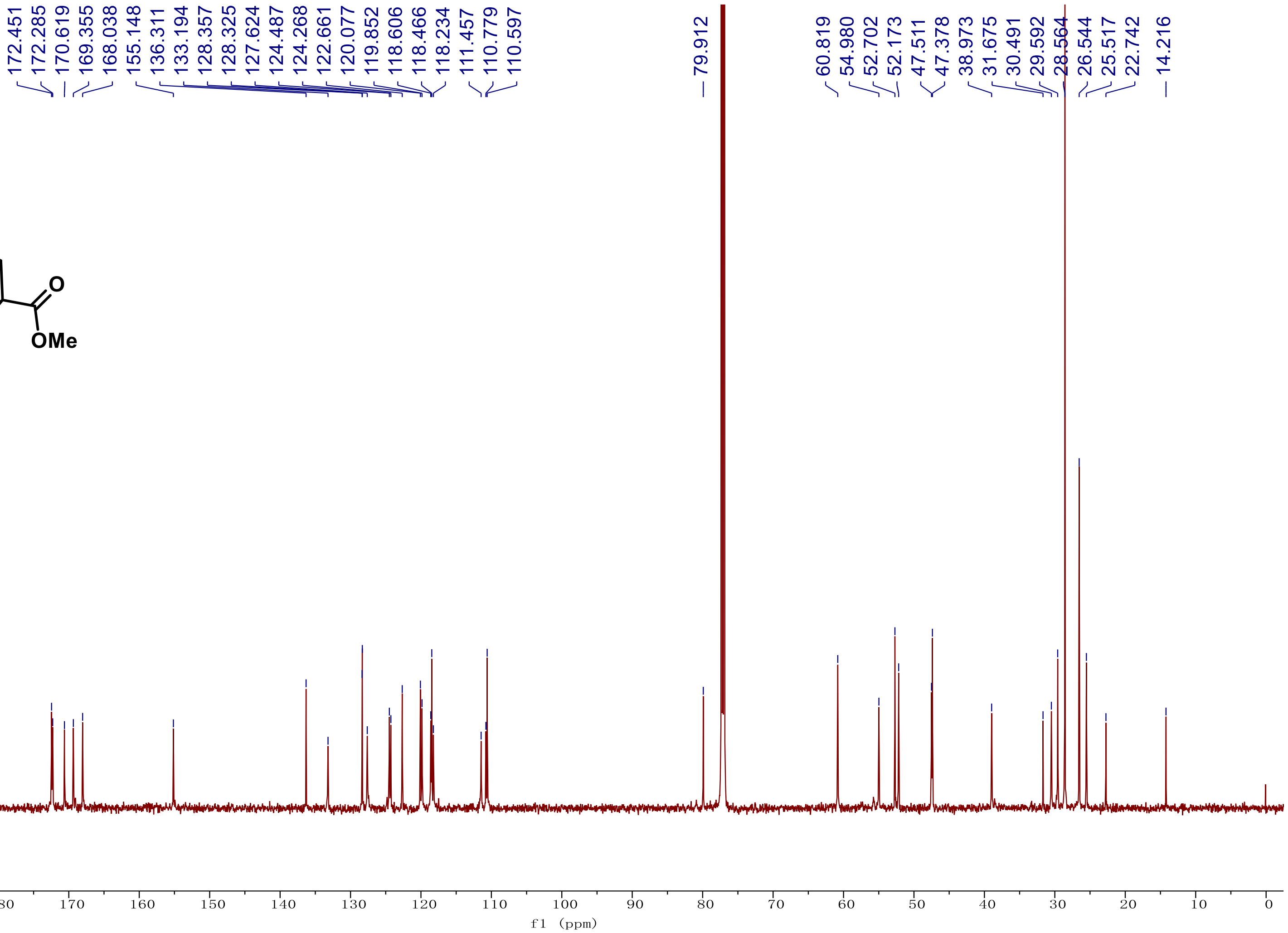

Compound 48x′ ^{1}H NMR (600 MHz, DMSO-d_6)

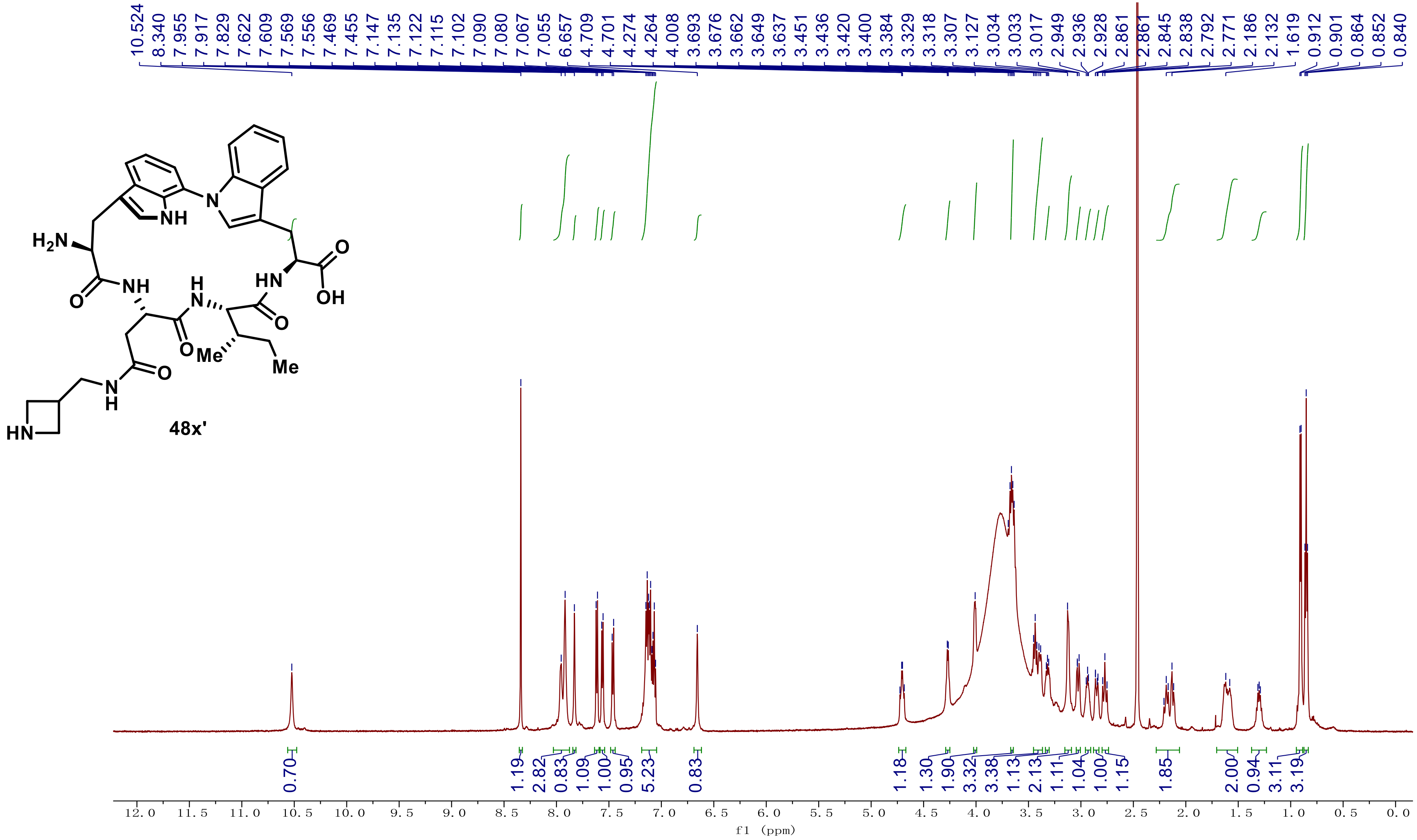

Compound 48x′ ^{13}C NMR (151 MHz, DMSO-d_6)

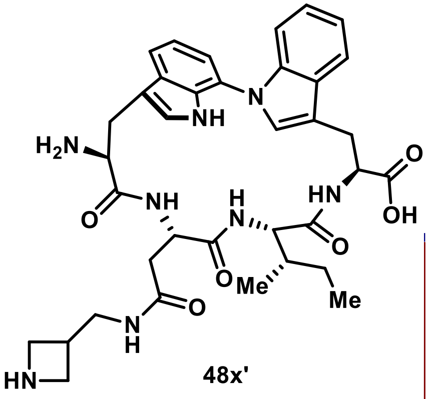

48x'

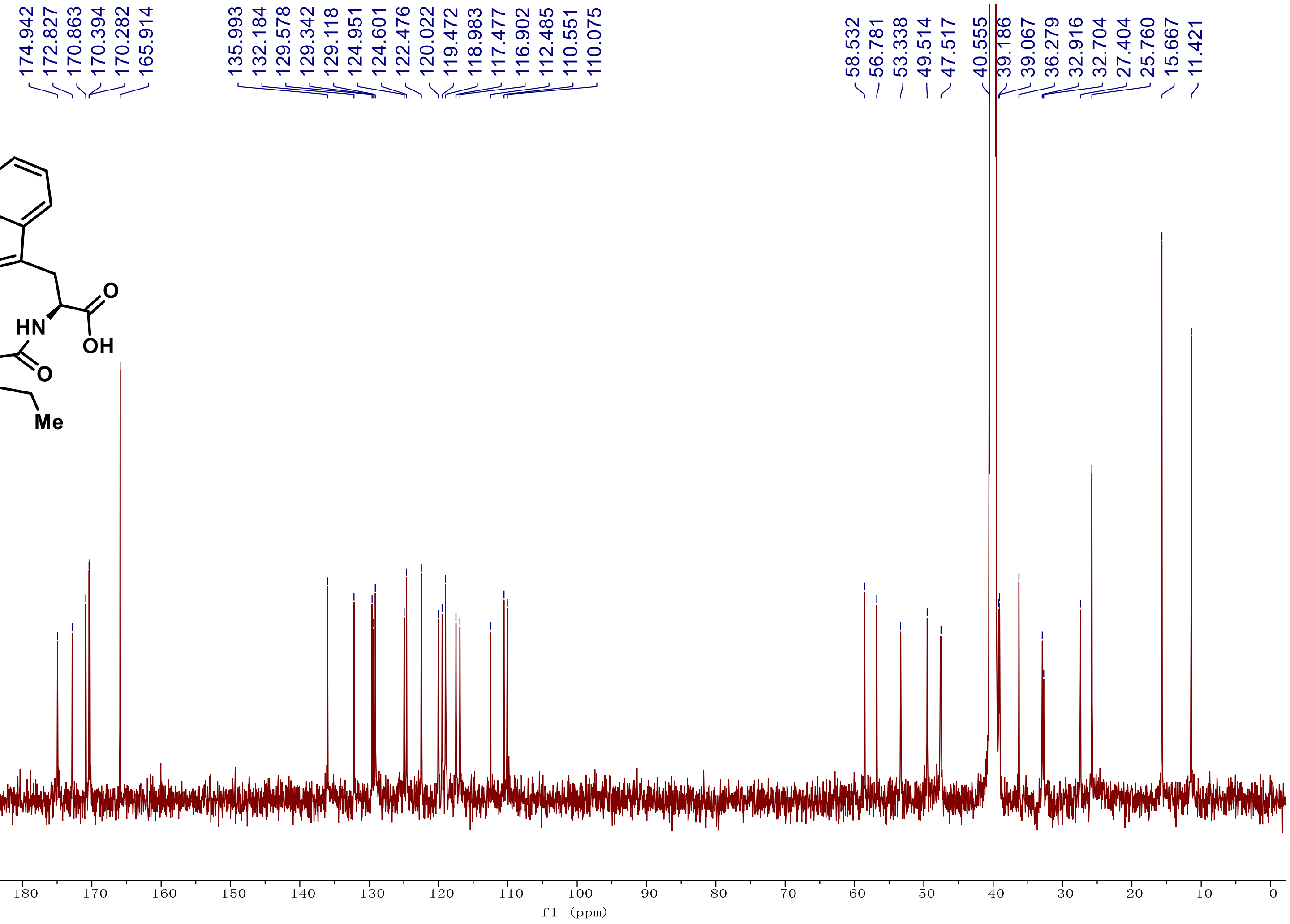

Compound 48o′ ^{1}H NMR (600 MHz, DMSO-d_6)

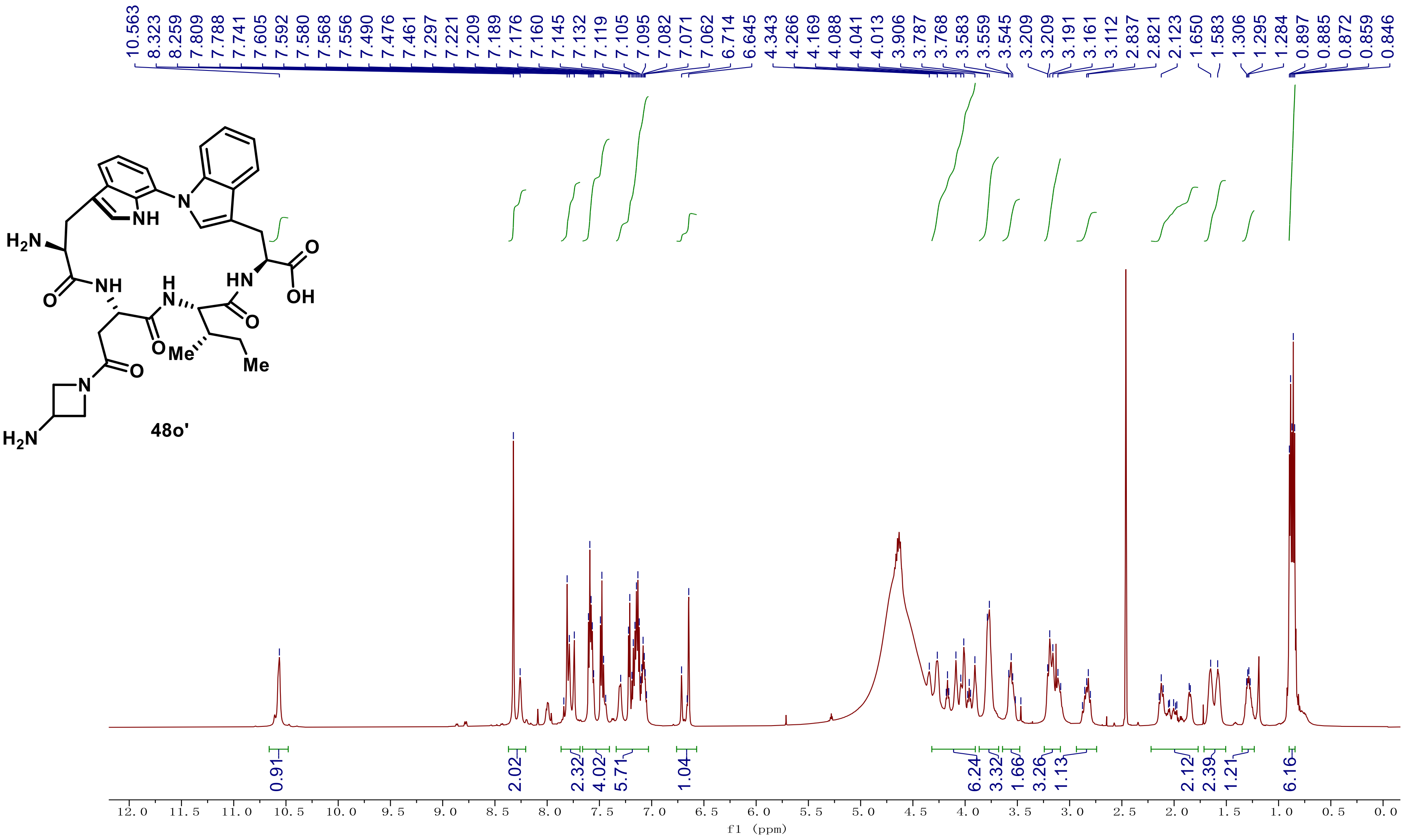

Compound 48o′ ^{13}C NMR (151 MHz, DMSO-d_6)

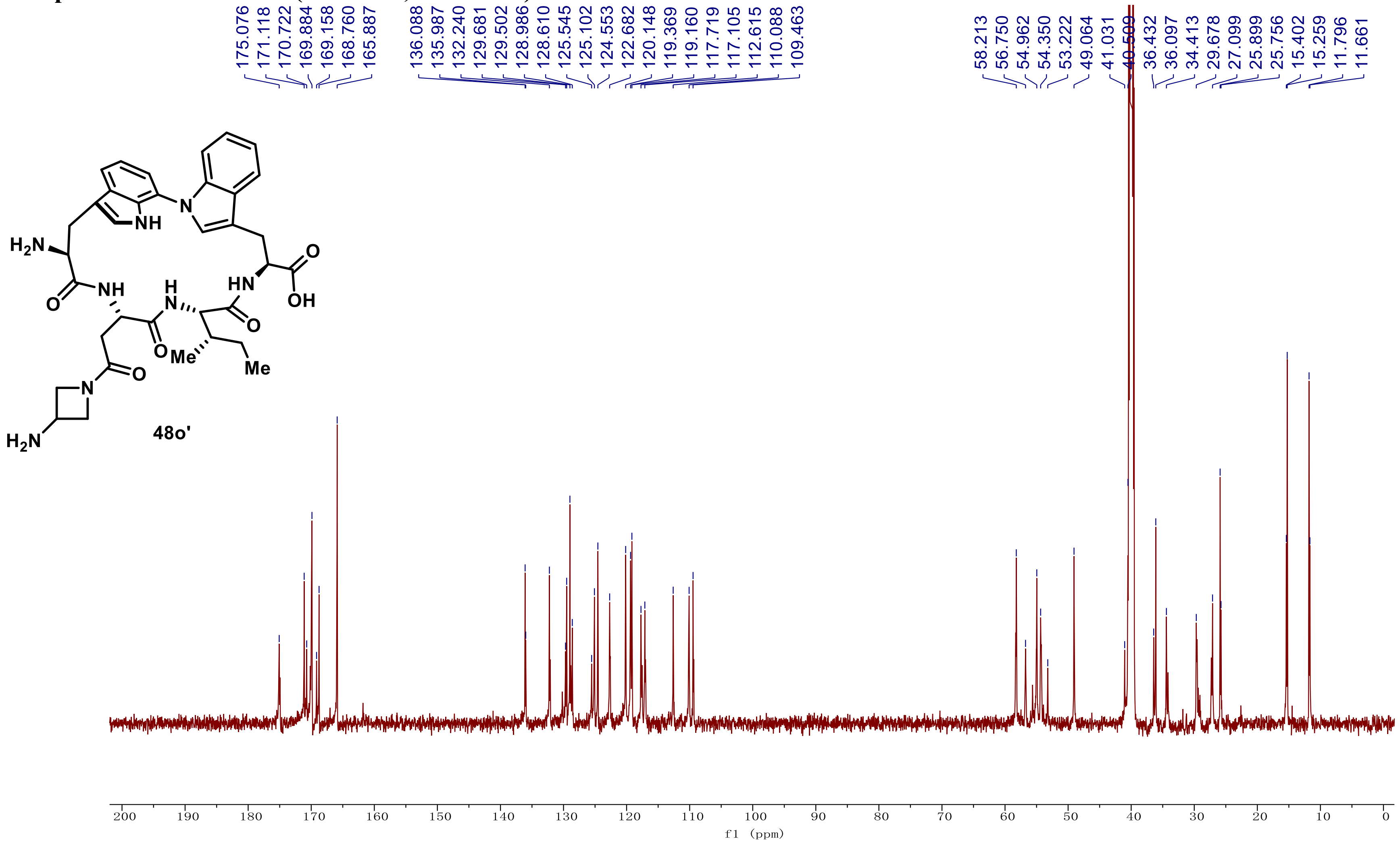

Compound 46q′ ^{1}H NMR (600 MHz, DMSO-d_6)

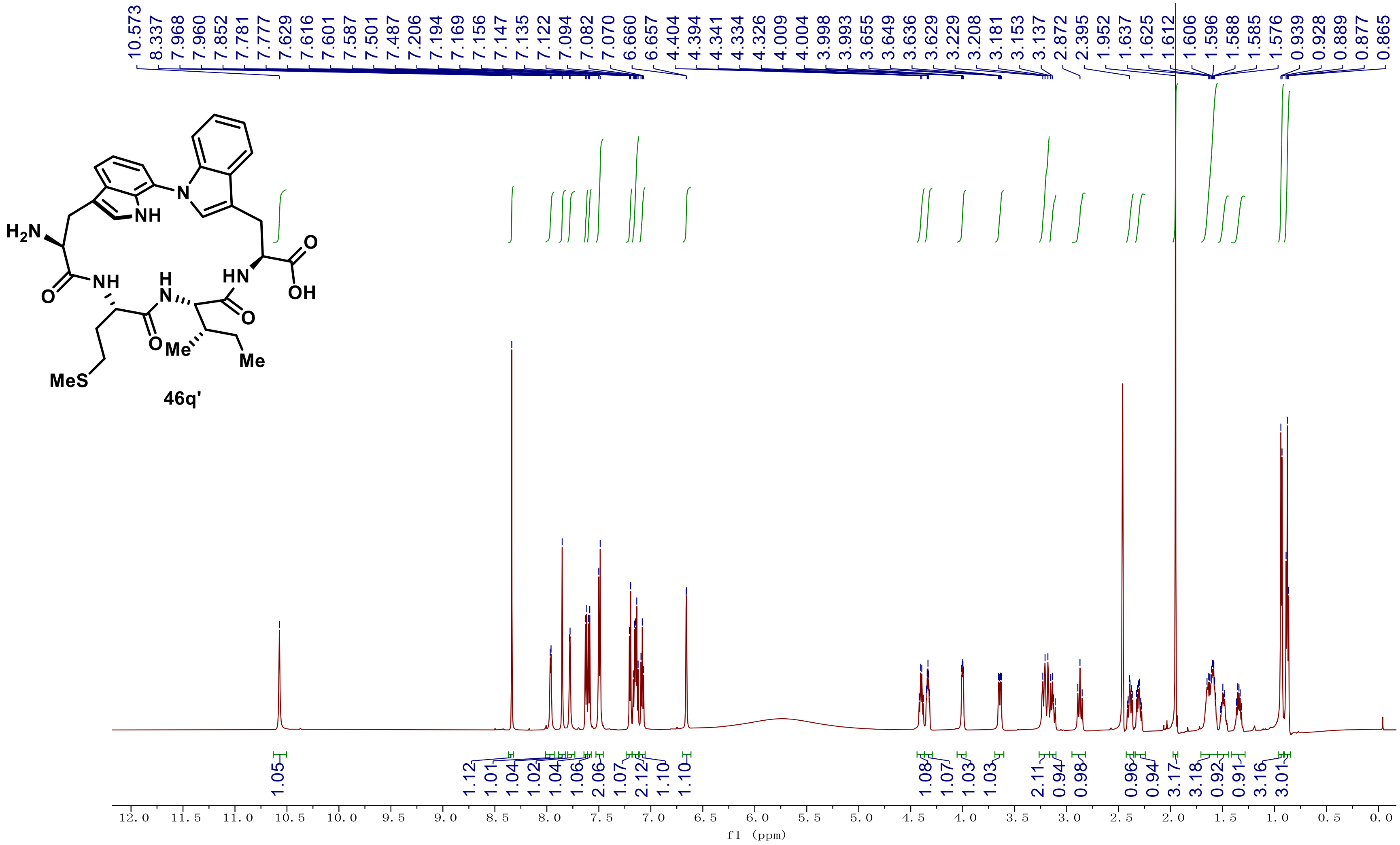

Compound 46q′ ^{13}C NMR (151 MHz, DMSO-d_6)

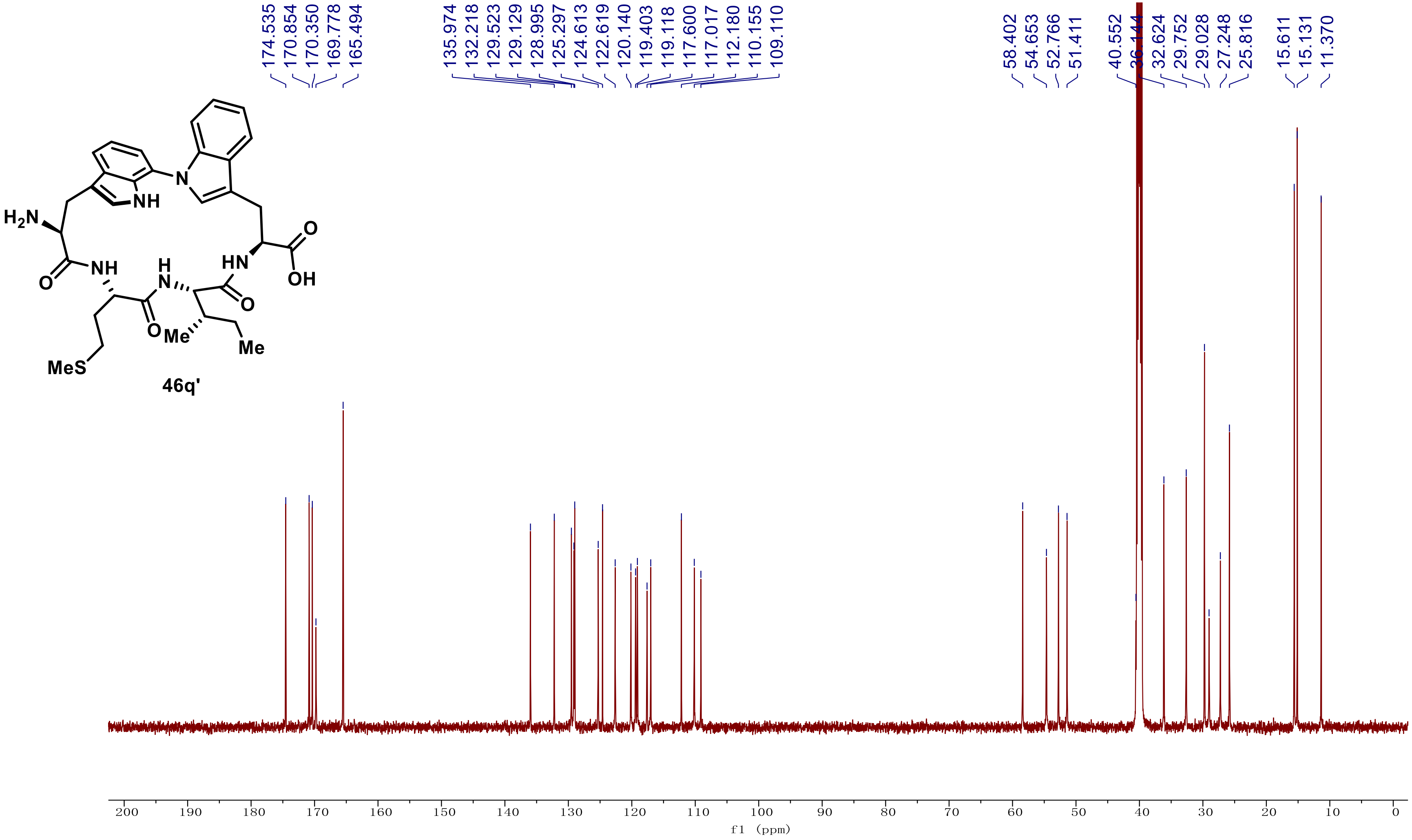

Compound 48a ¹H NMR (600 MHz, CDCl₃)

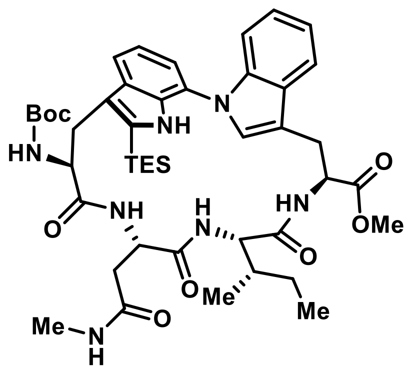

48a

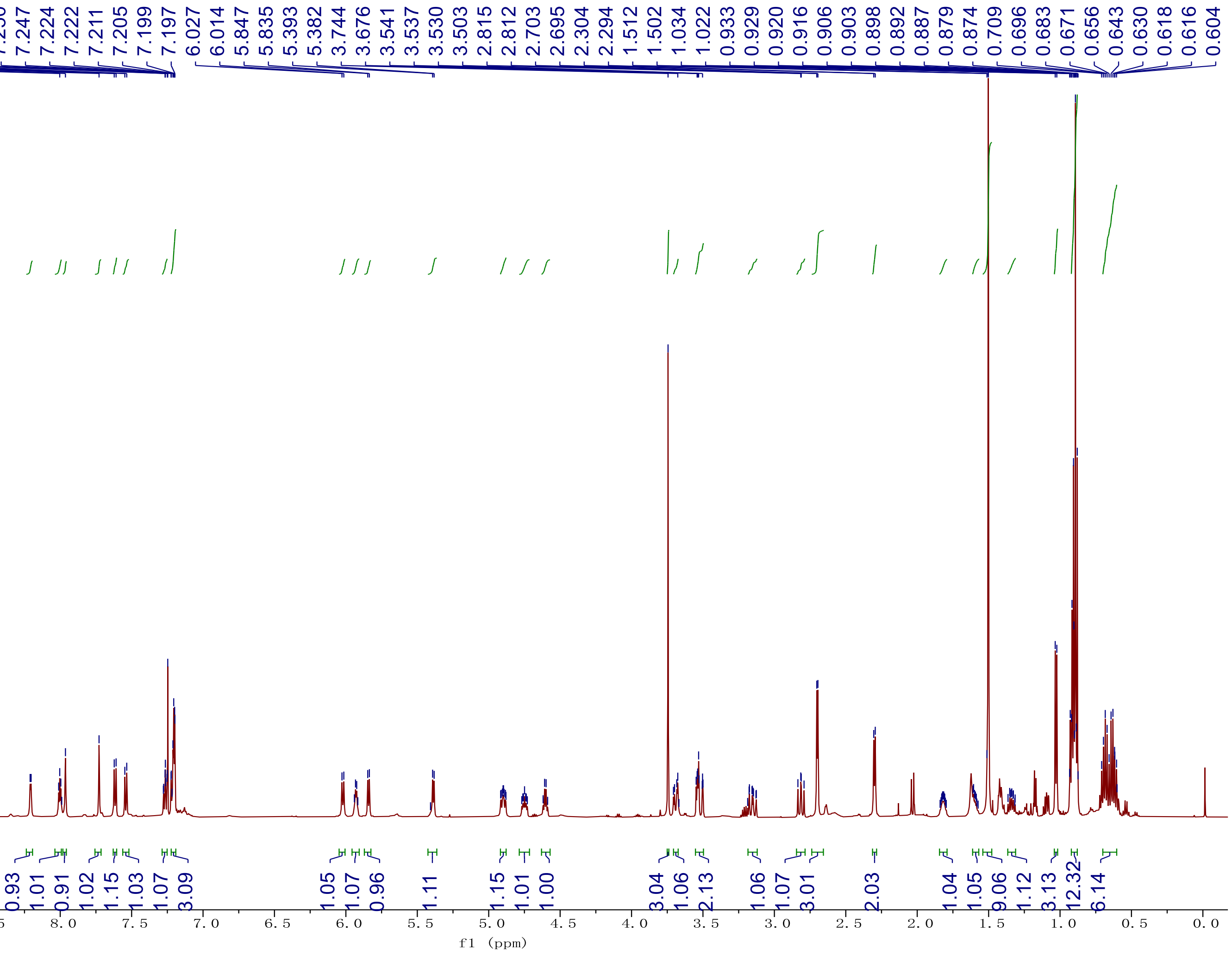

Compound 48a ^{13}C NMR (151 MHz, $CDCl_3$)

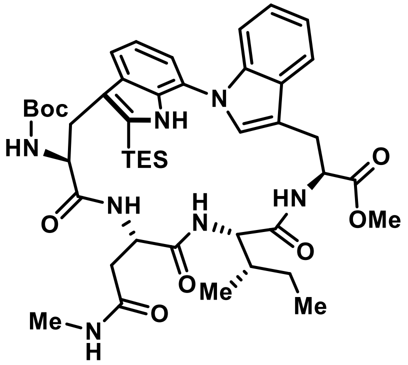

48a

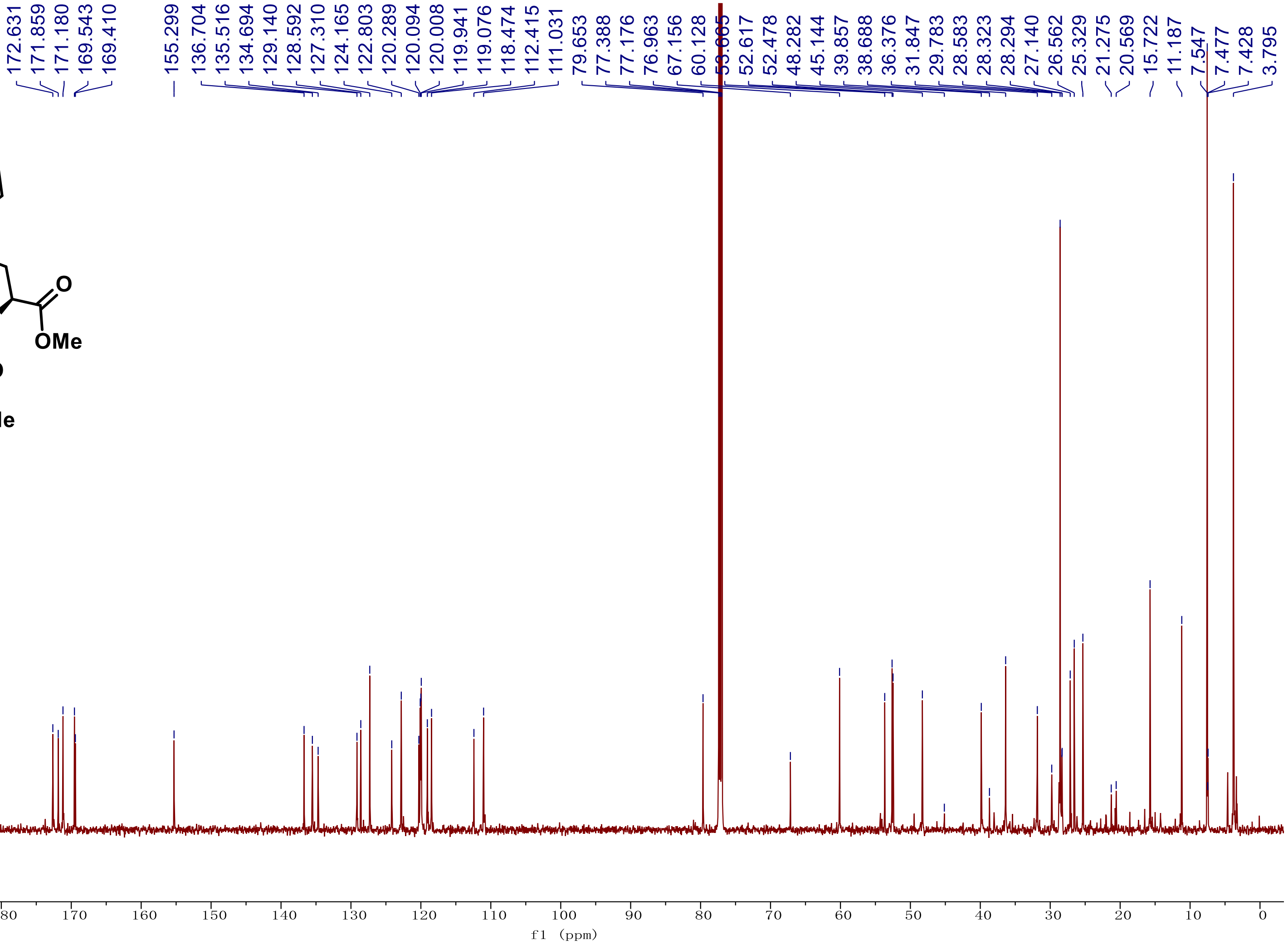

Compound 48b ^{1}H NMR (600 MHz, $CDCl_3$)

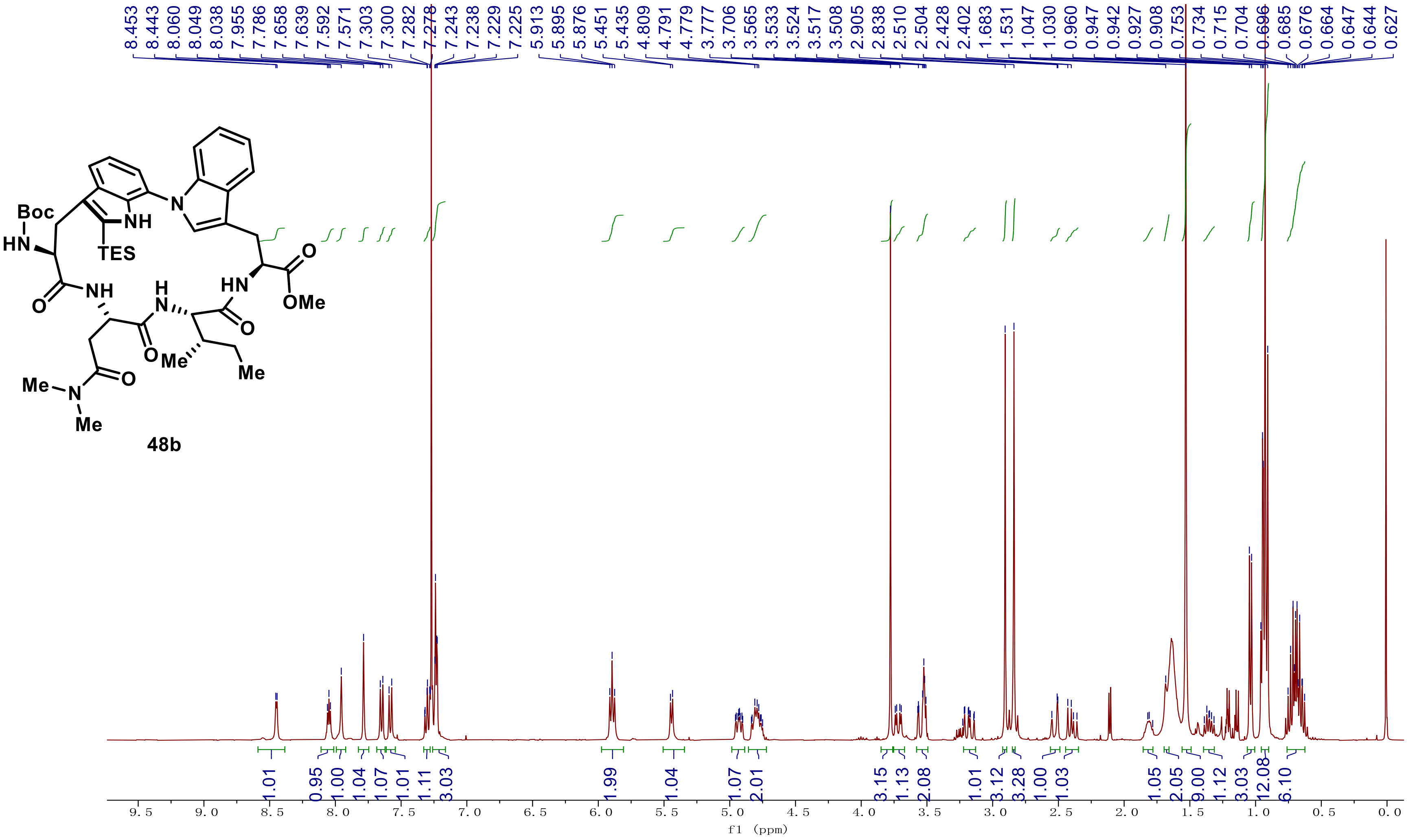

48b

Compound 48b ^{13}C NMR (151 MHz, CDCl$_3$)

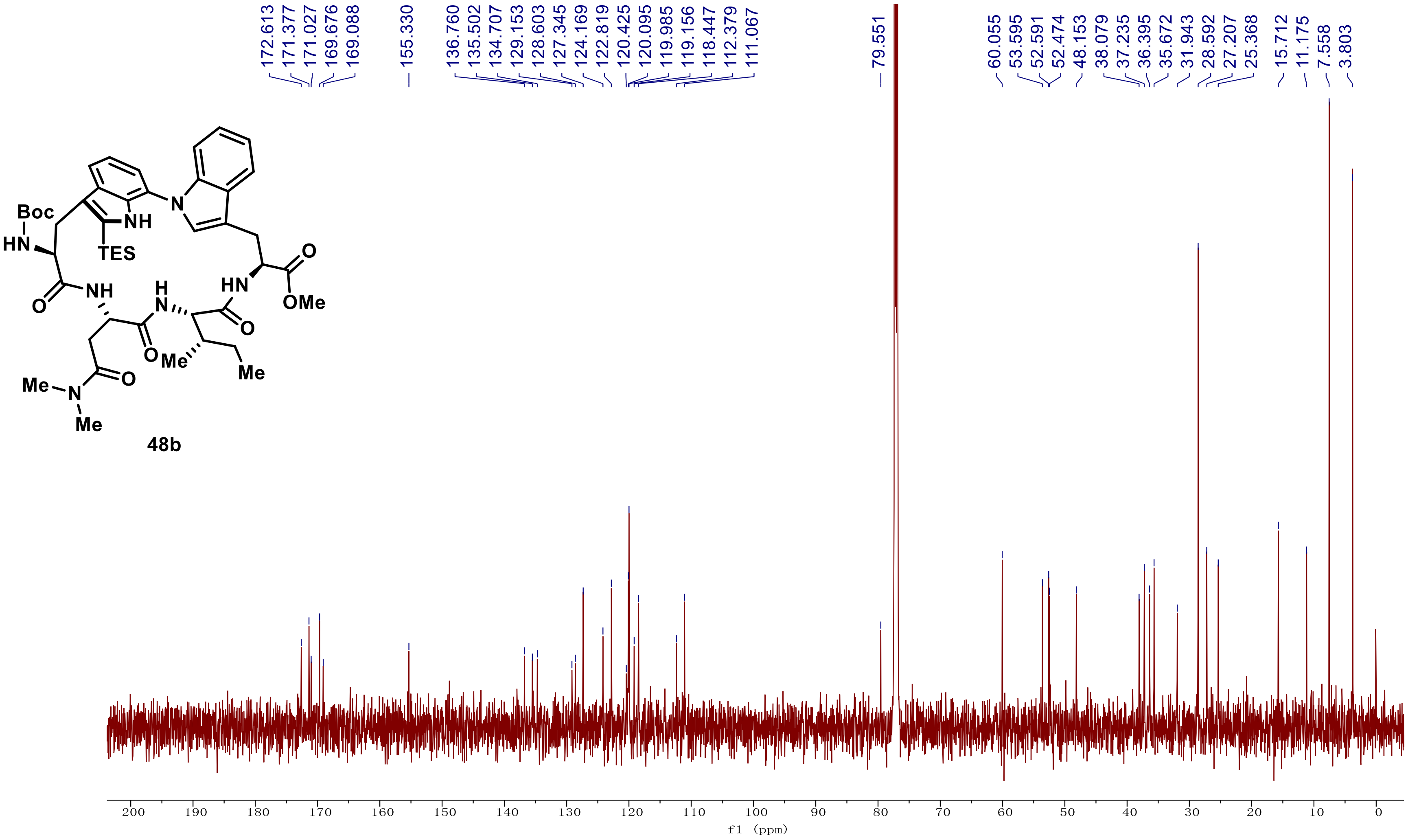

Compound 48c ^{1}H NMR (600 MHz, $CDCl_3$)

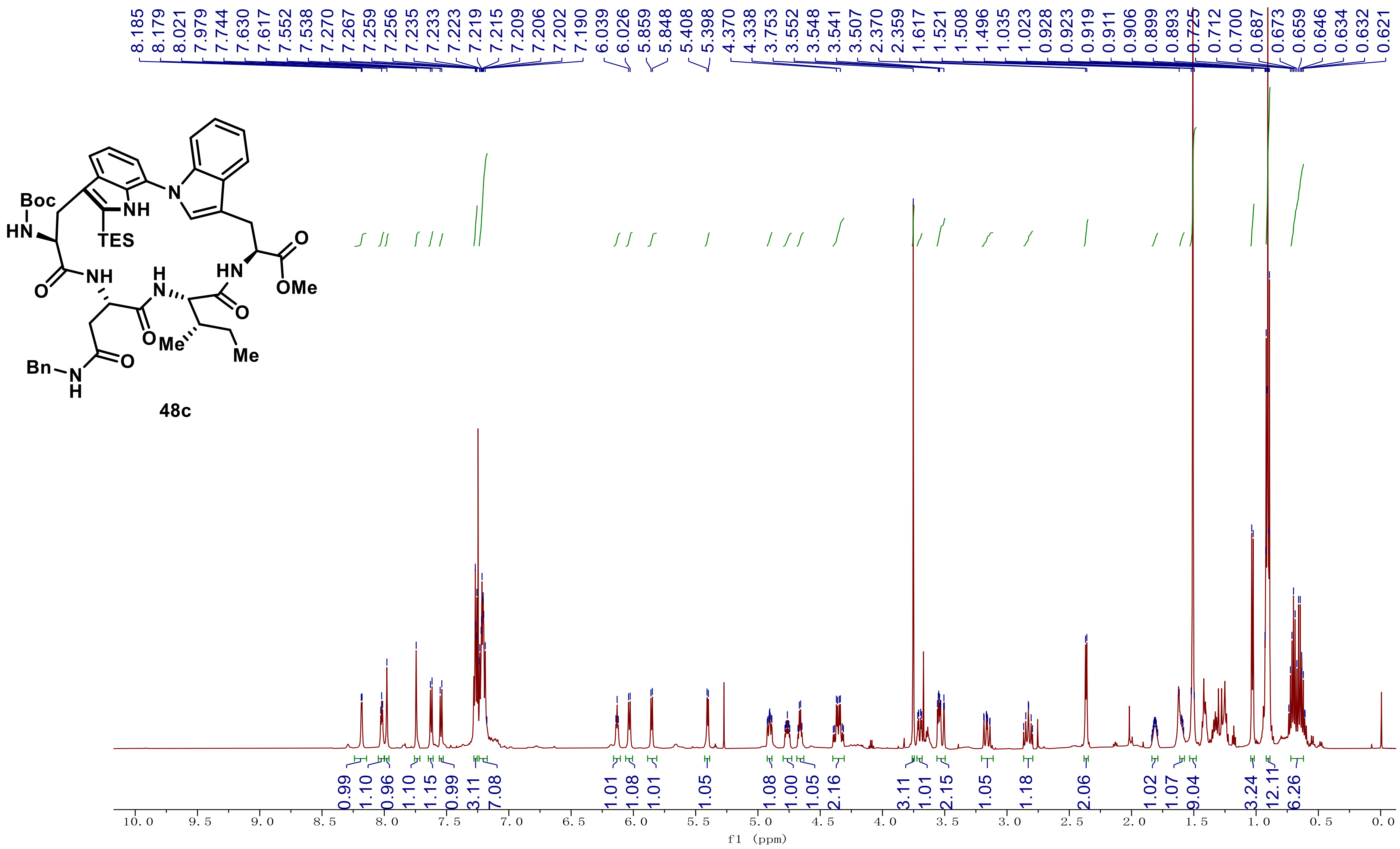

Compound 48c ${}^{13}C$ NMR (151 MHz, $CDCl_3$)

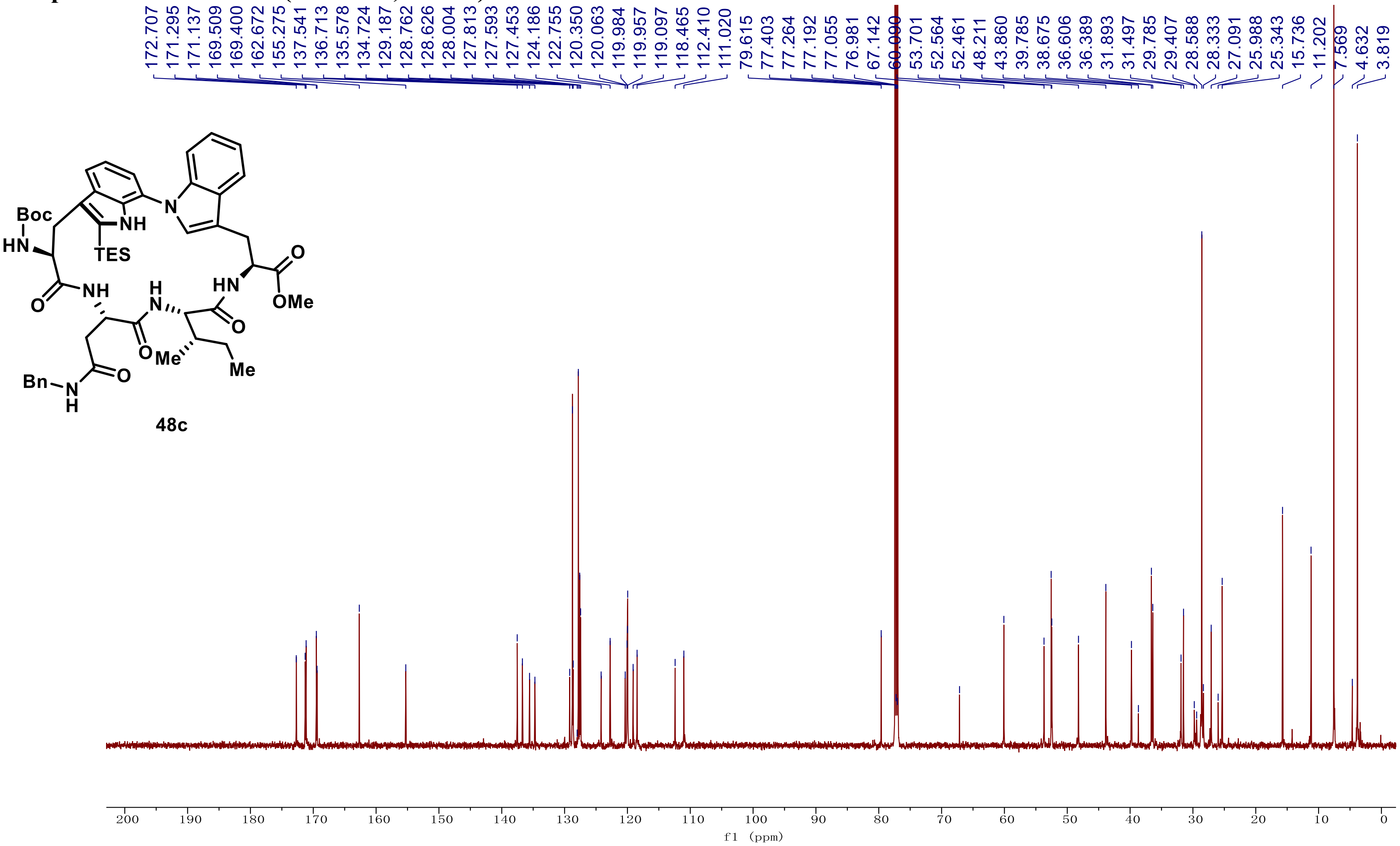

Compound 48d ¹H NMR (400 MHz, CDCl₃)

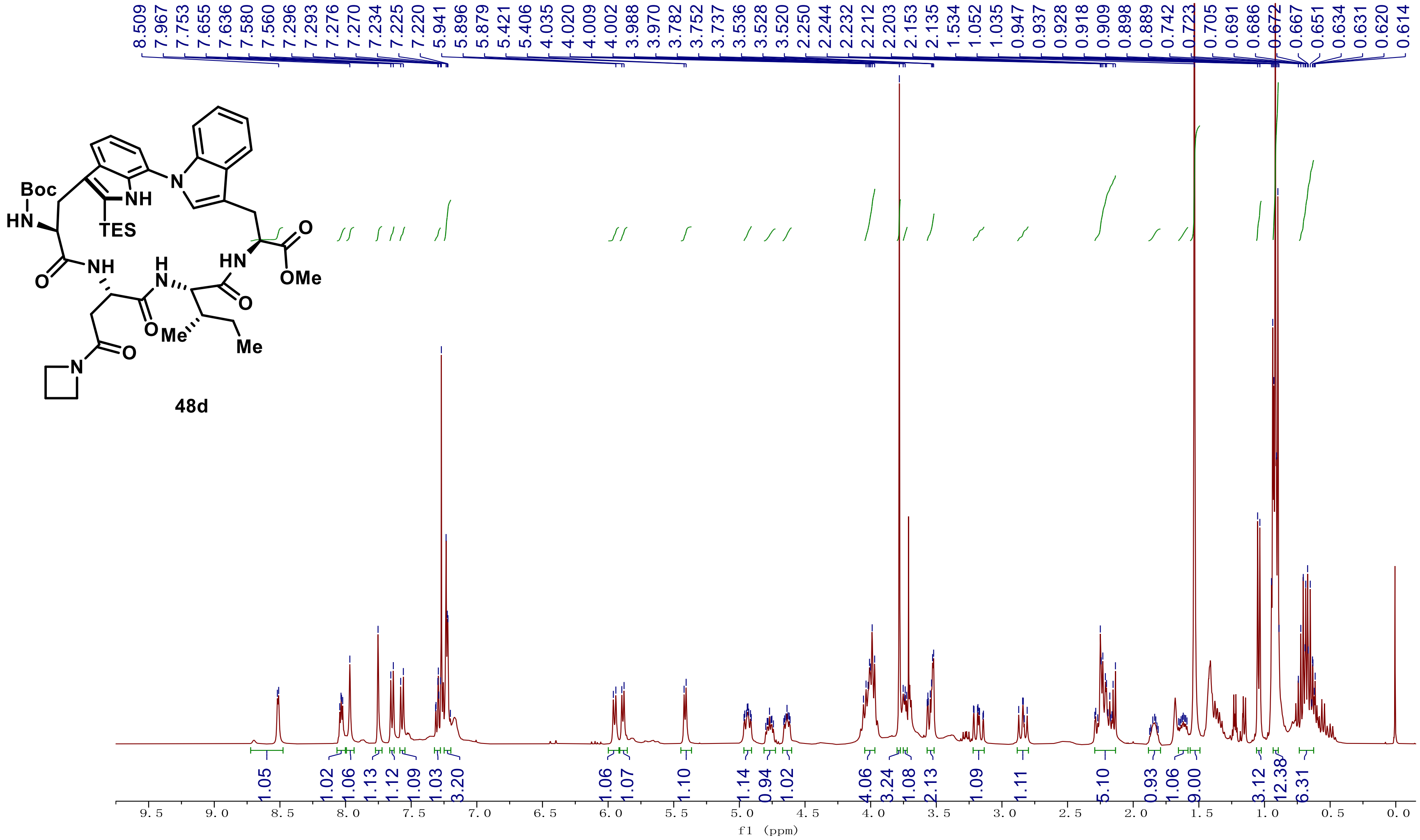

Compound 48d ^{13}C NMR (151 MHz, CDCl$_3$)

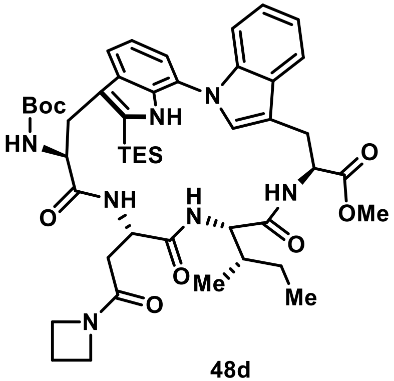

48d

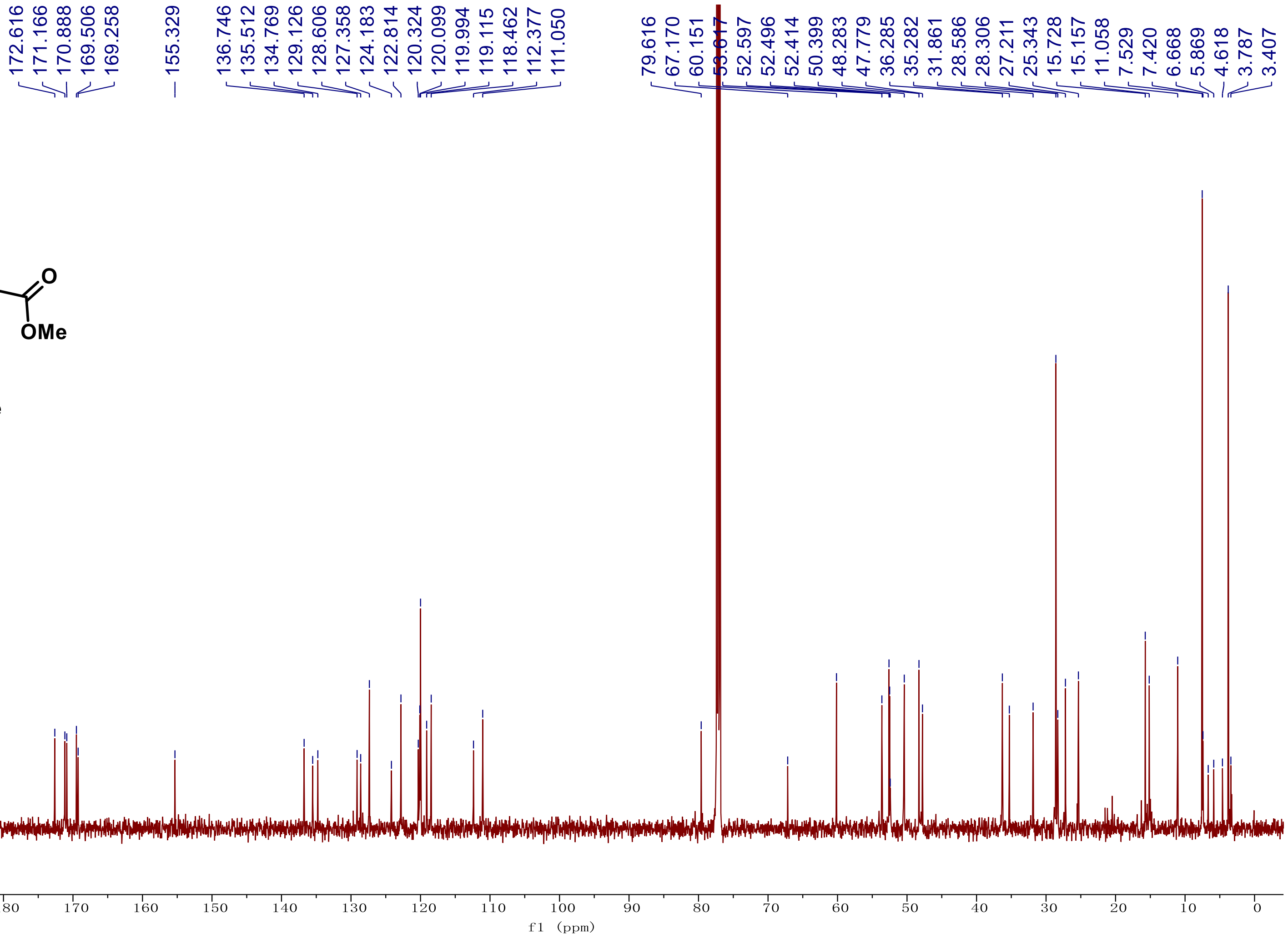

Compound 48e ¹H NMR (600 MHz, CDCl₃)

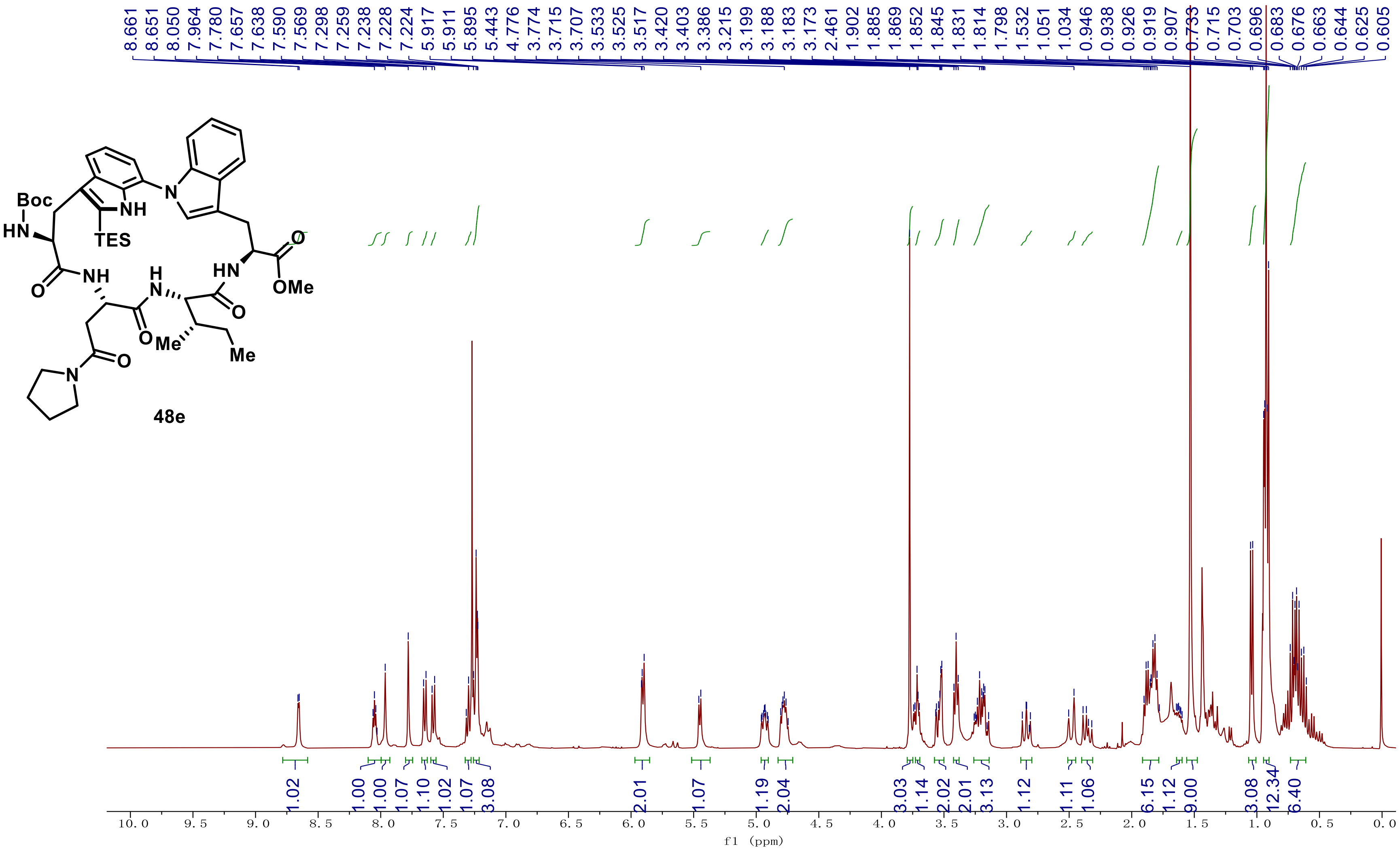

Compound 48e ^{13}C NMR (151 MHz, $CDCl_3$)

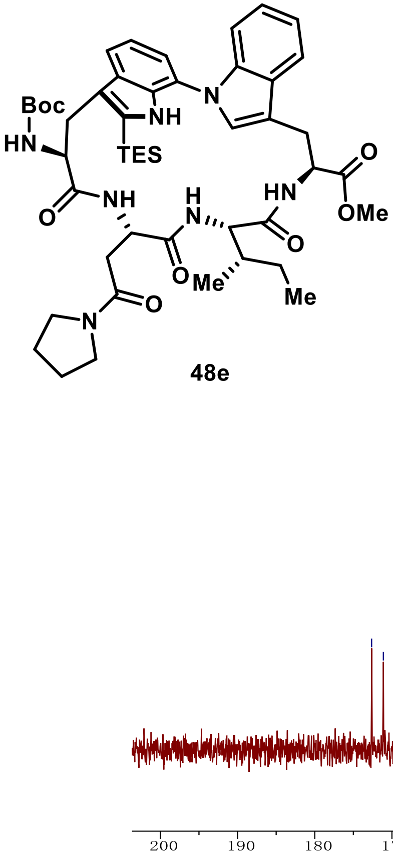

48e

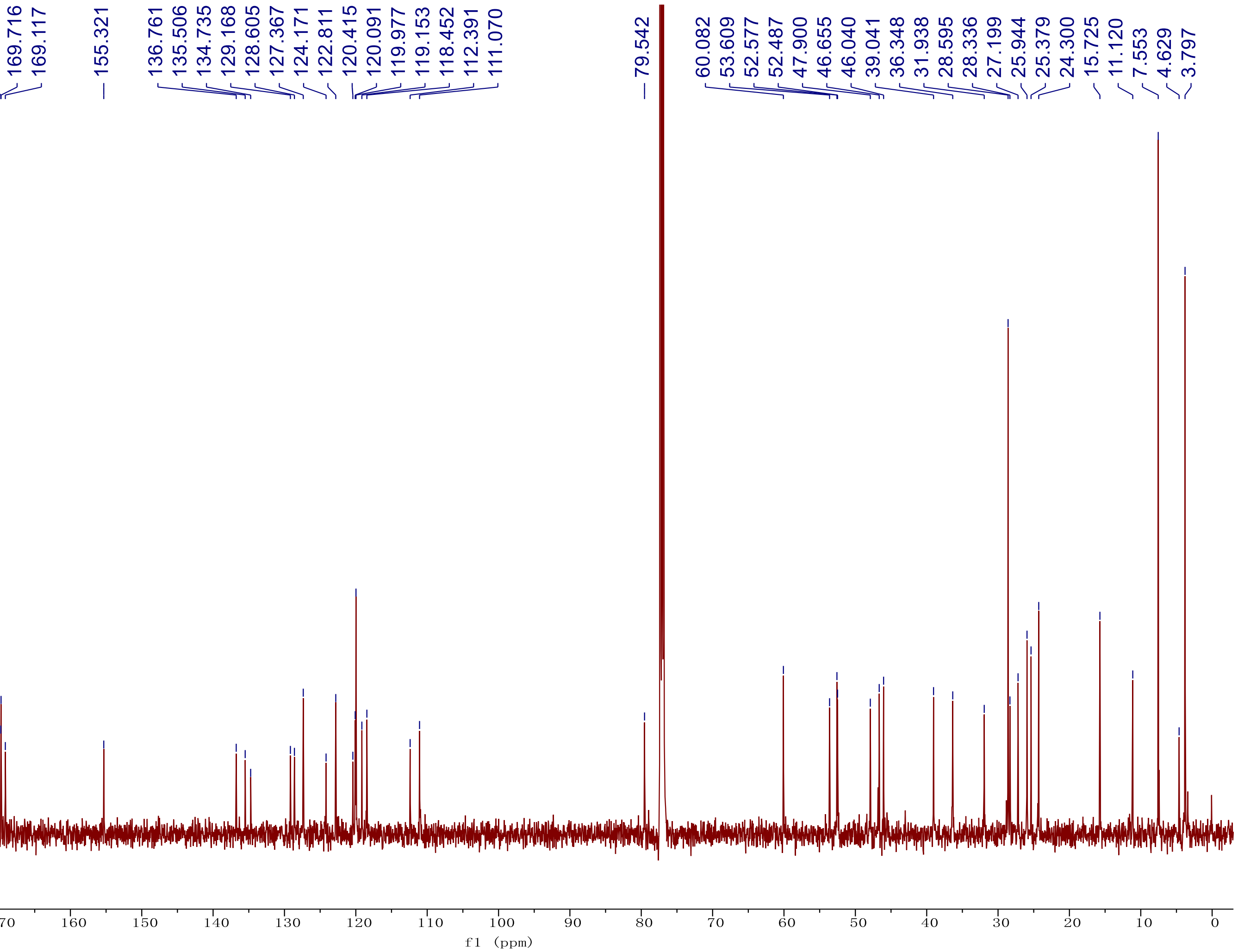

Compound 48f ^{1}H NMR (600 MHz, $CDCl_3$)

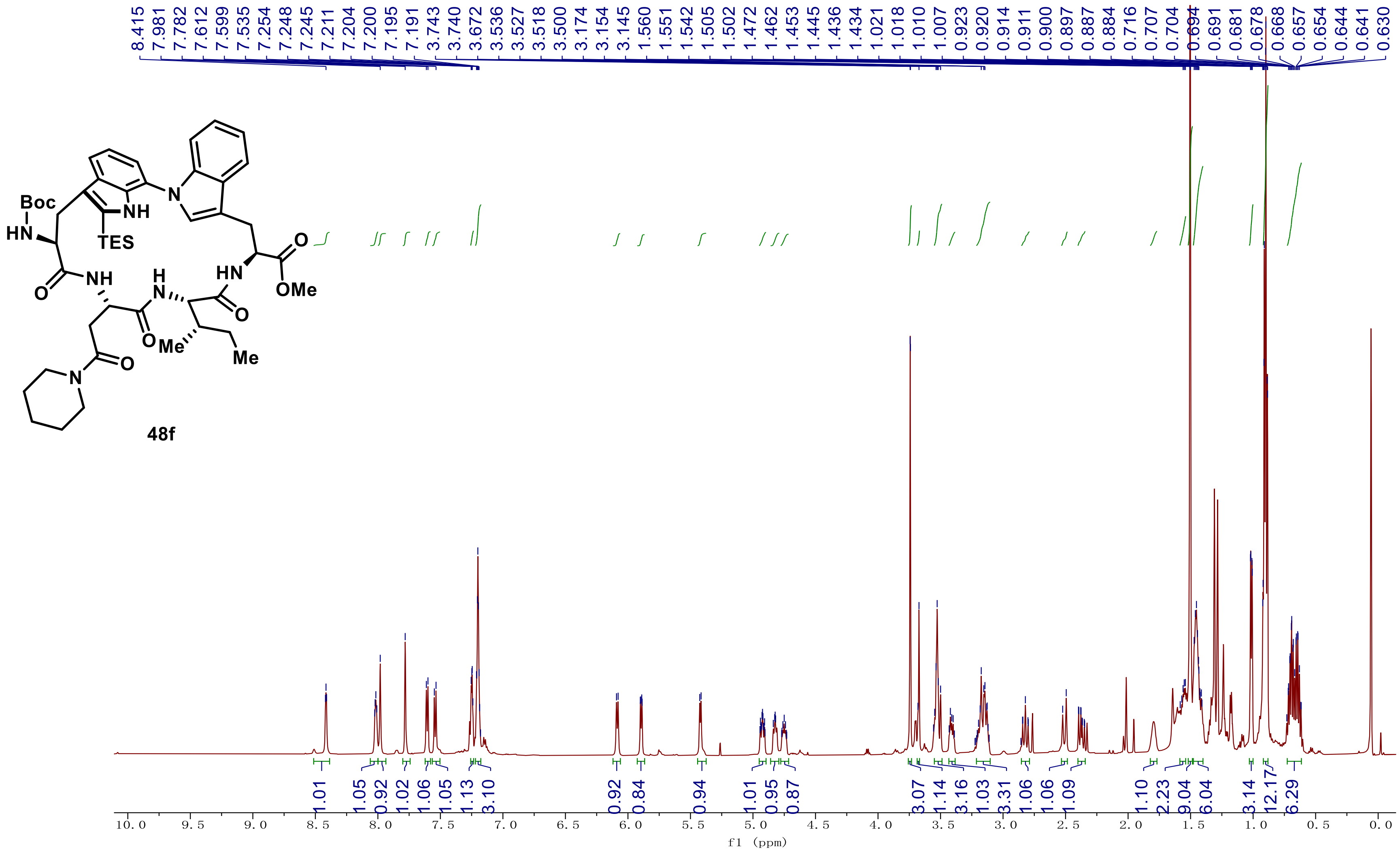

Compound 48f ^{13}C NMR (151 MHz, $CDCl_3$)

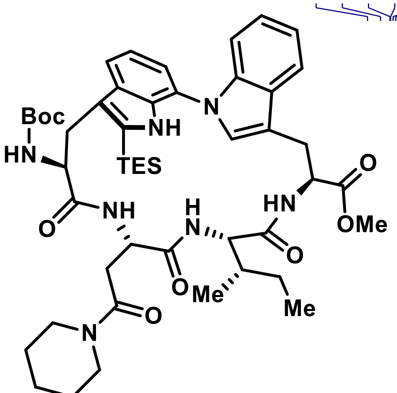

48f

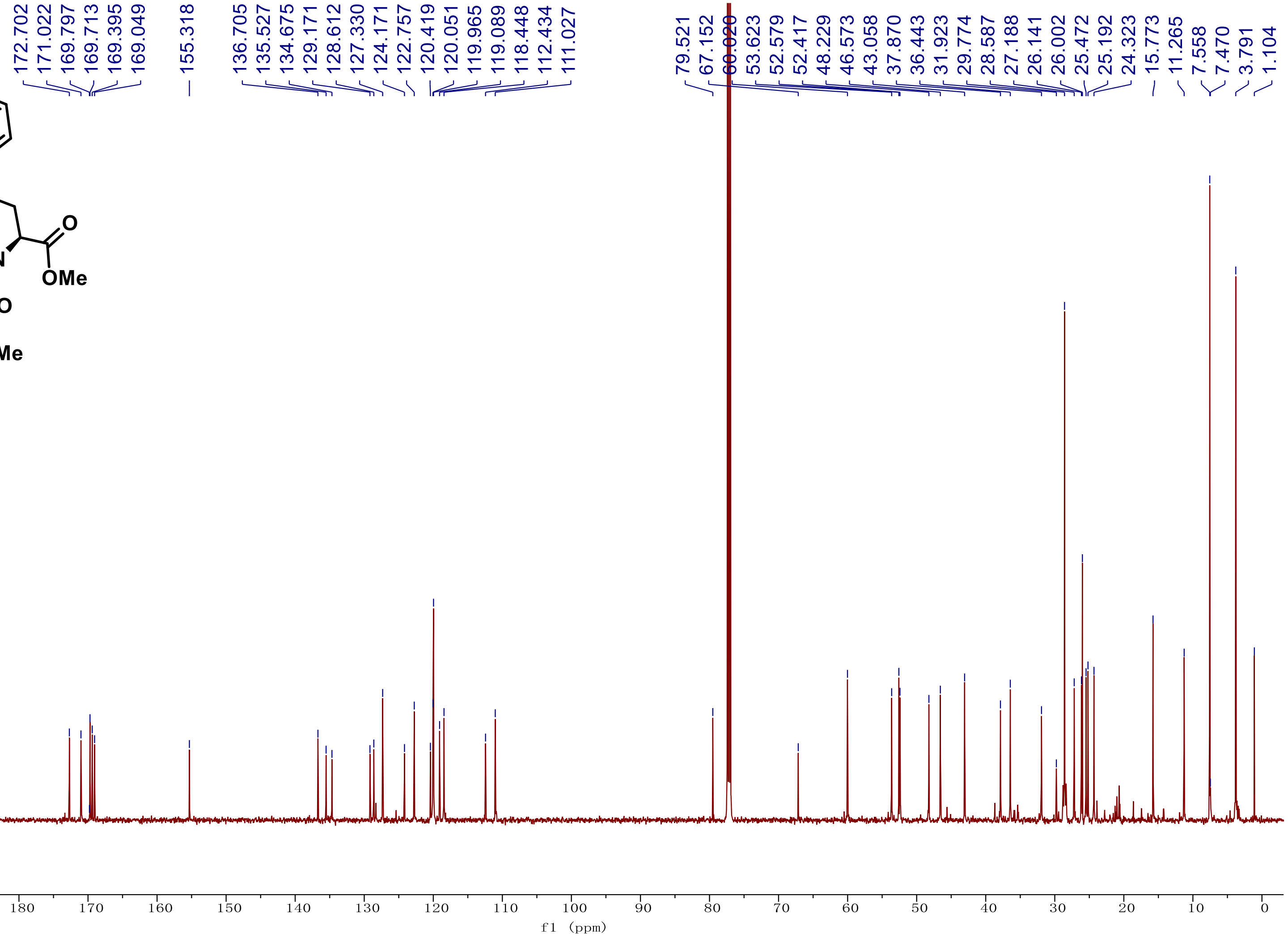

Compound 48g ¹H NMR (600 MHz, CDCl₃)

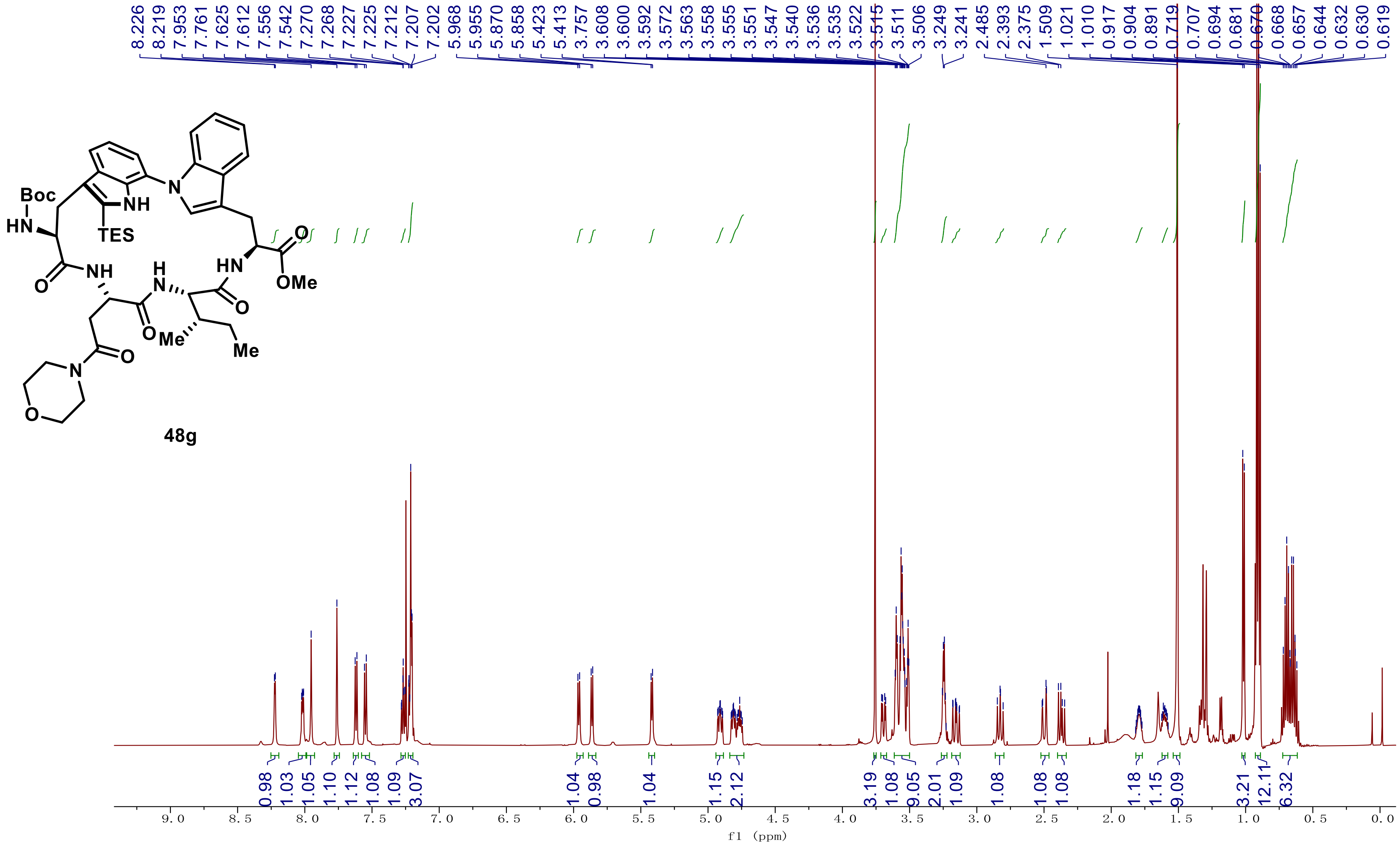

48g

Compound 48g ^{13}C NMR (151 MHz, $CDCl_3$)

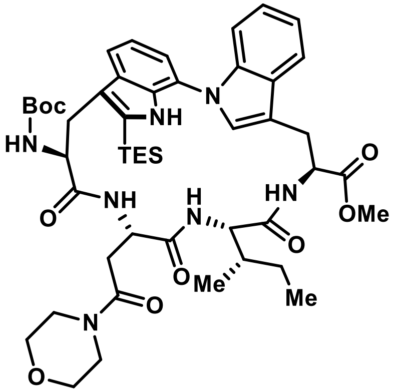

48g

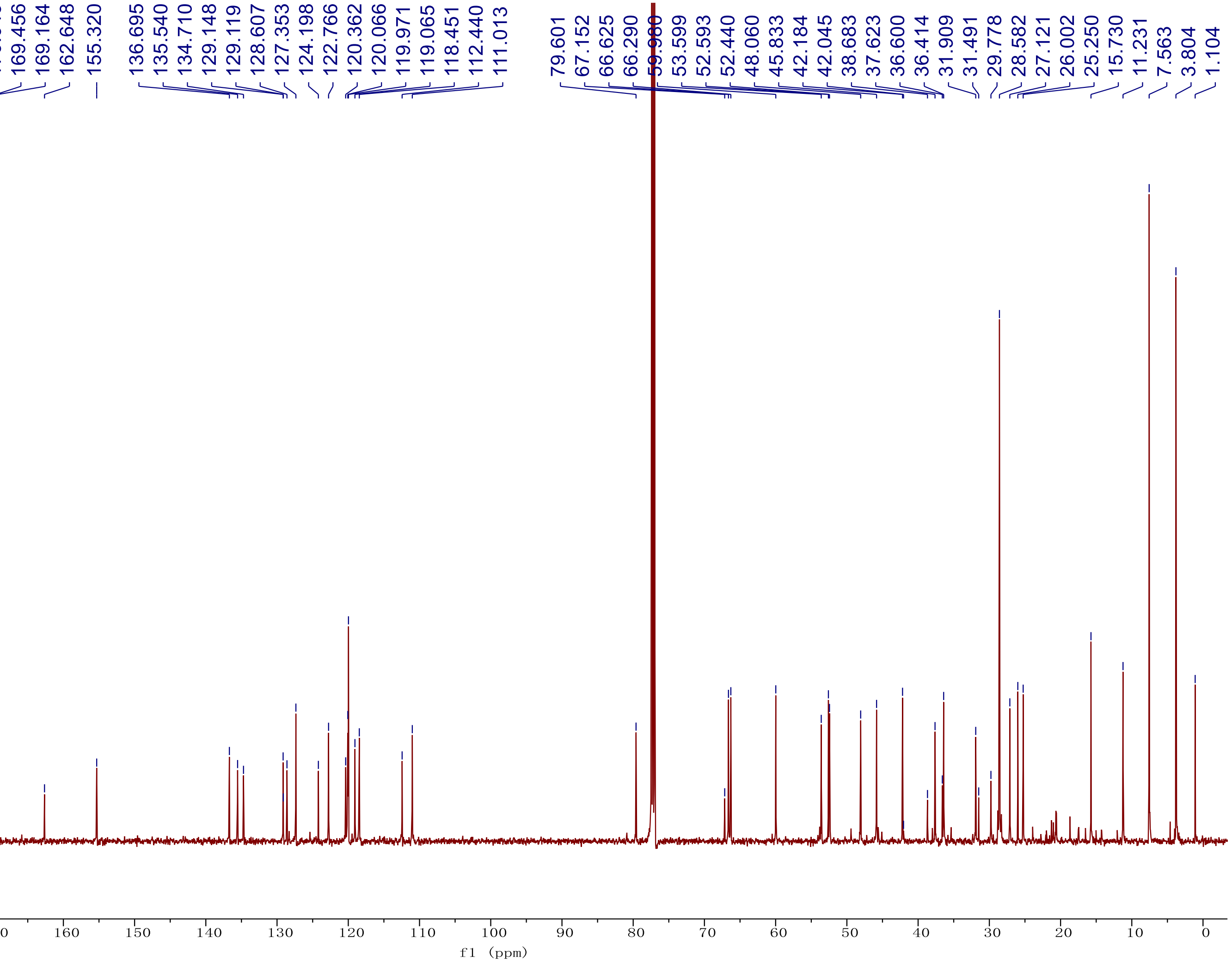

Compound 48h ^{1}H NMR (400 MHz, $CDCl_3$)

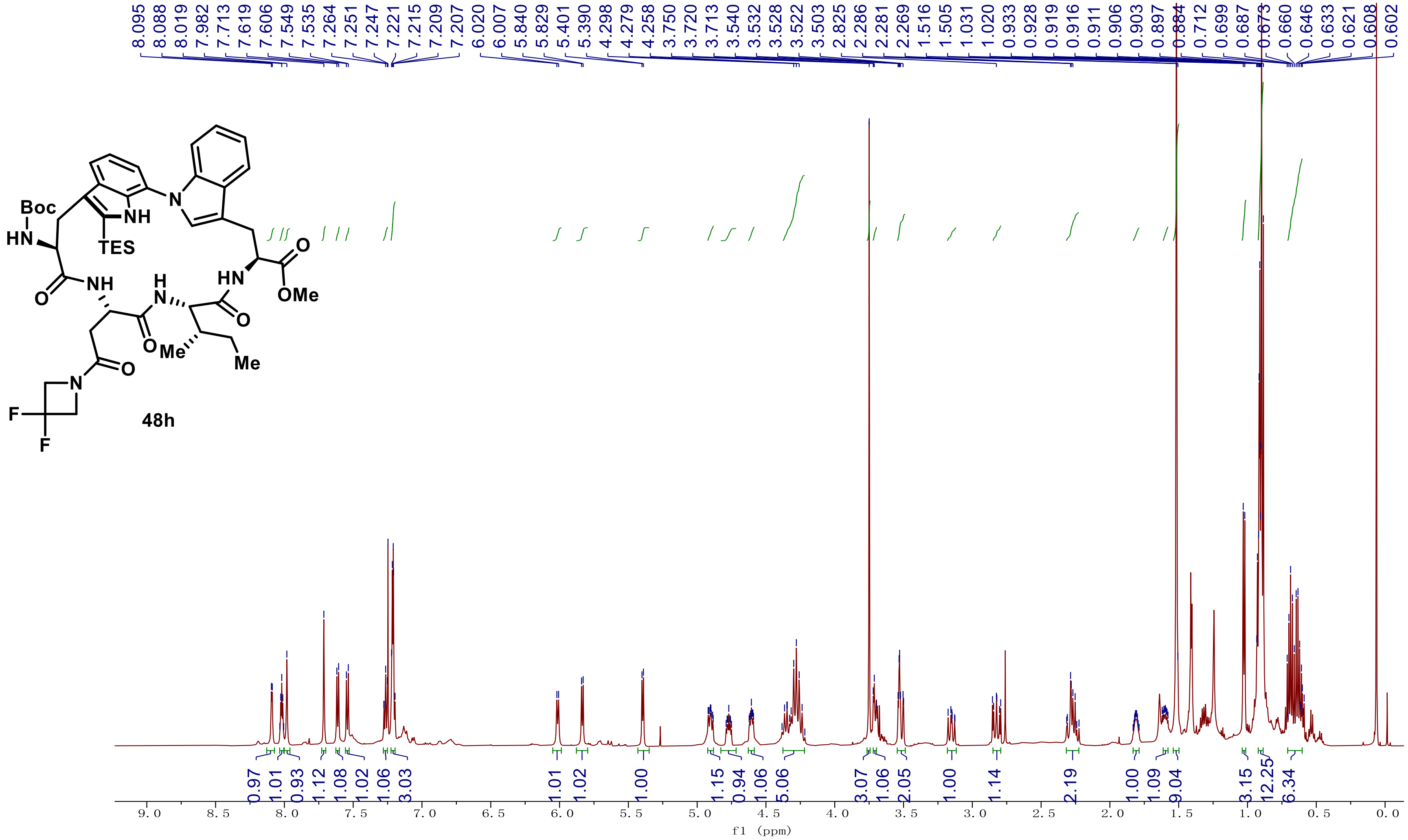

Compound 48h ^{13}C NMR (151 MHz, $CDCl_3$)

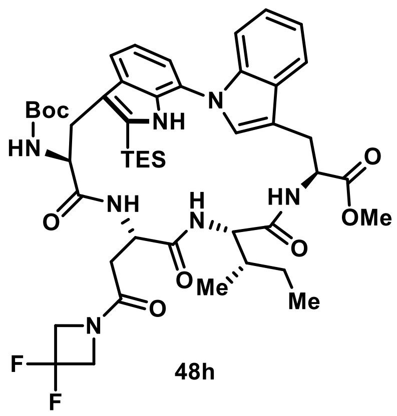

48h

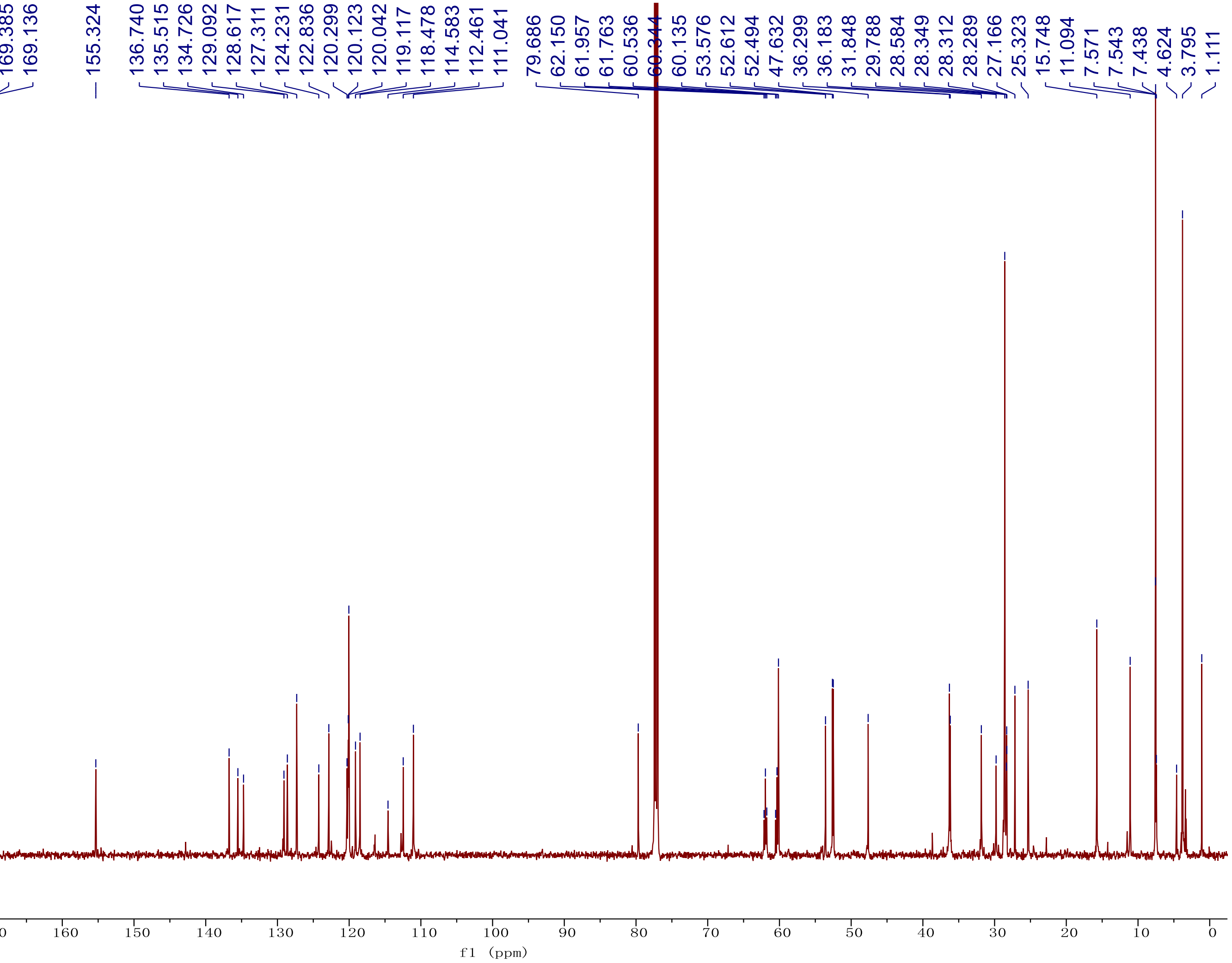

Compound 48h ^{19}F NMR (565 MHz, CDCl$_3$)

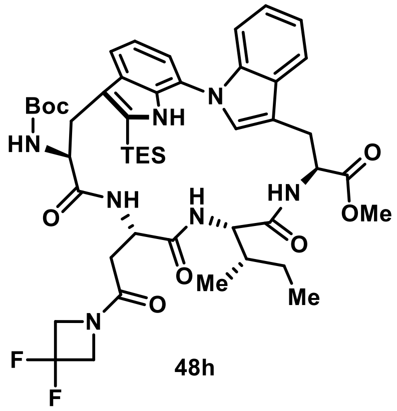

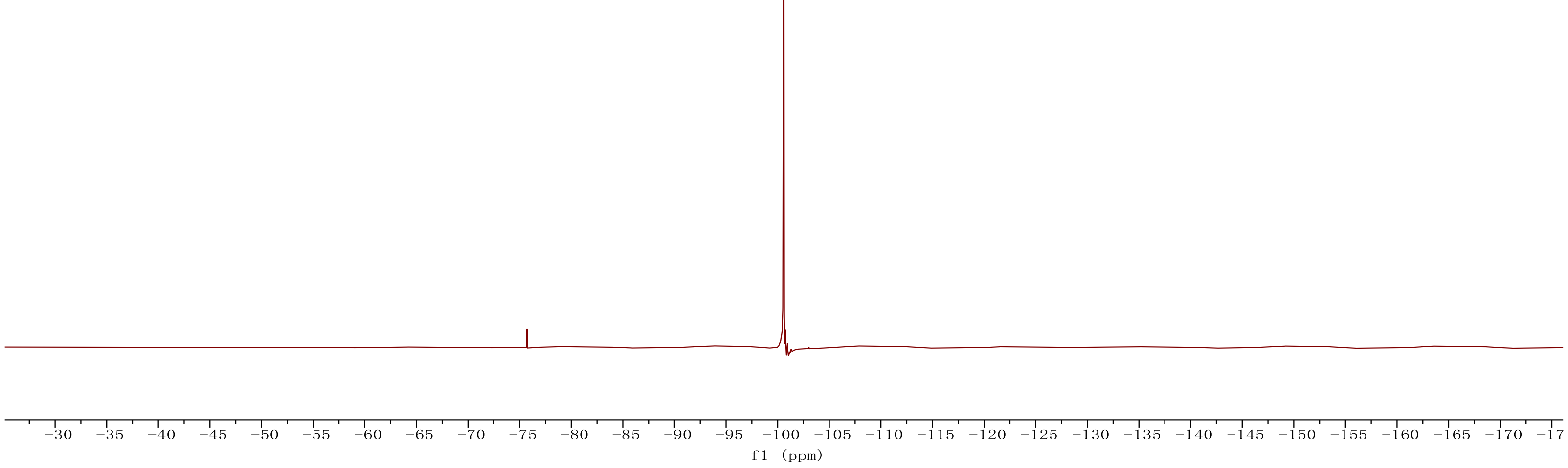

Compound 48i ¹H NMR (600 MHz, $CDCl_3$)

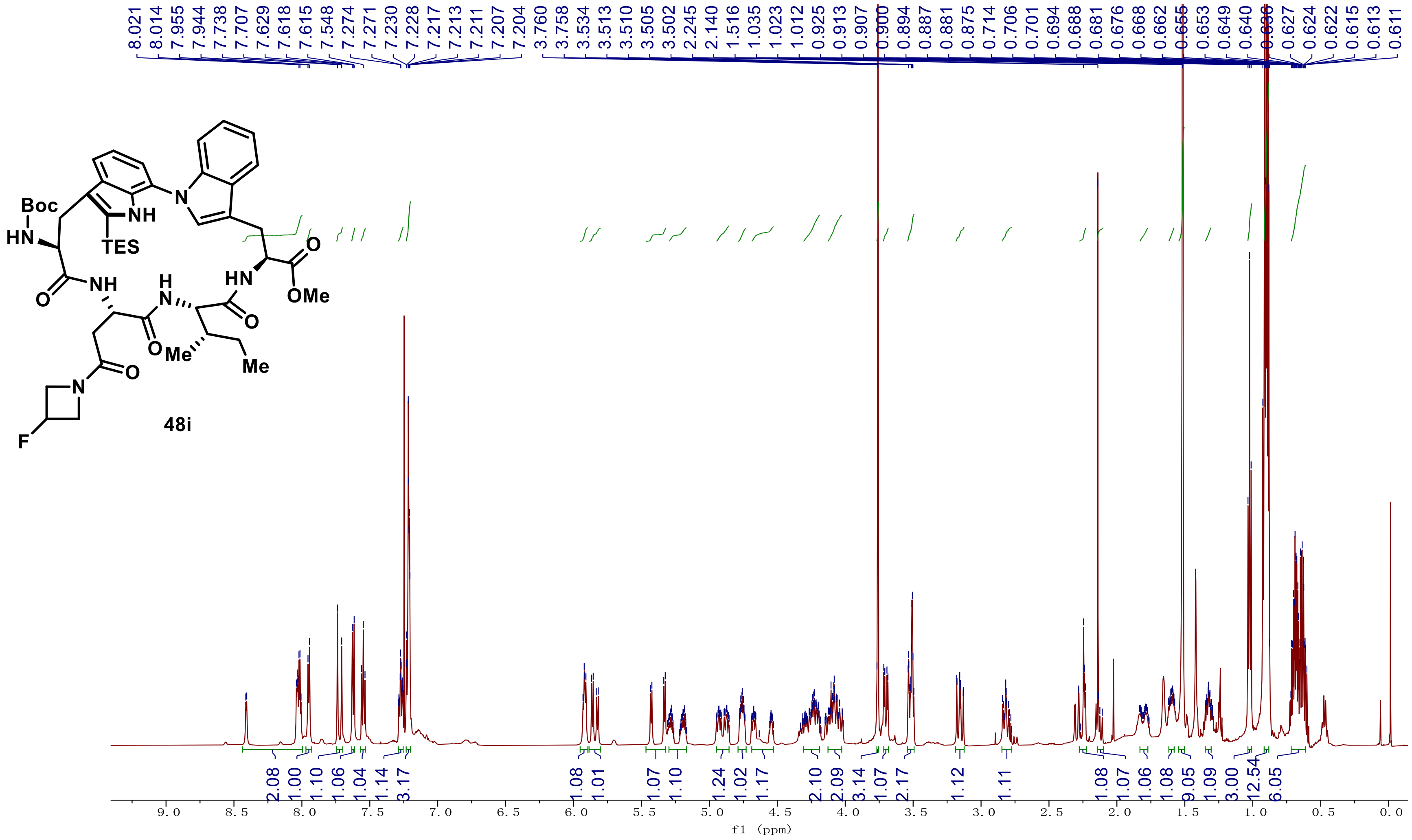

Compound 48i ^{13}C NMR (151 MHz, $CDCl_3$)

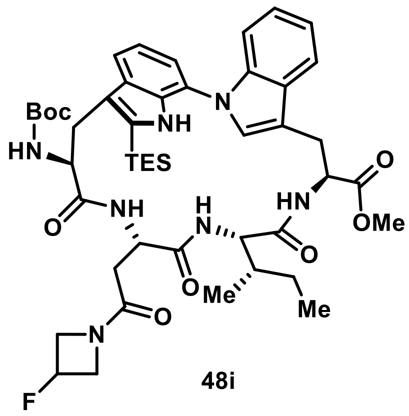

48i

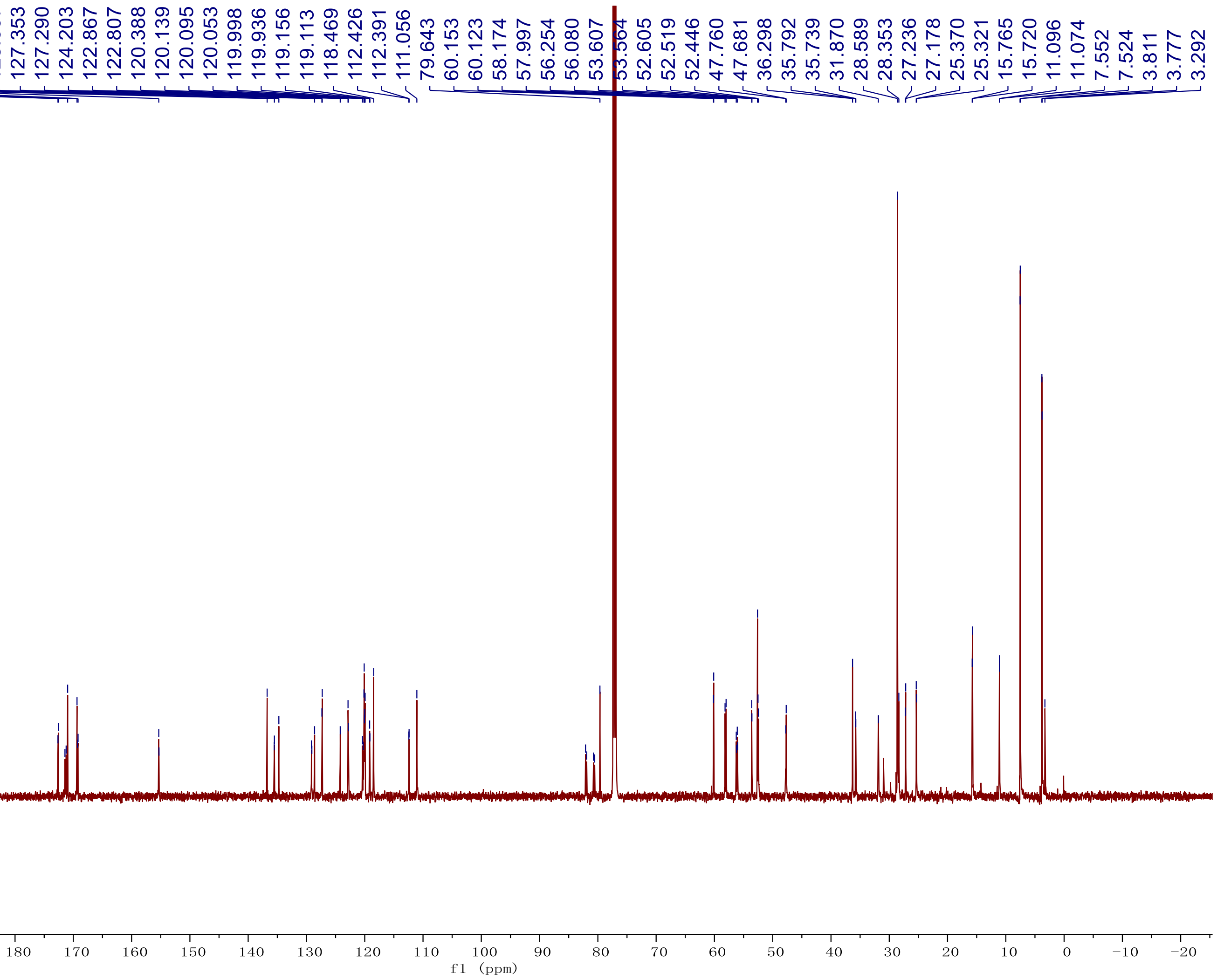

Compound 48i ^{19}F NMR (565 MHz, $CDCl_3$)

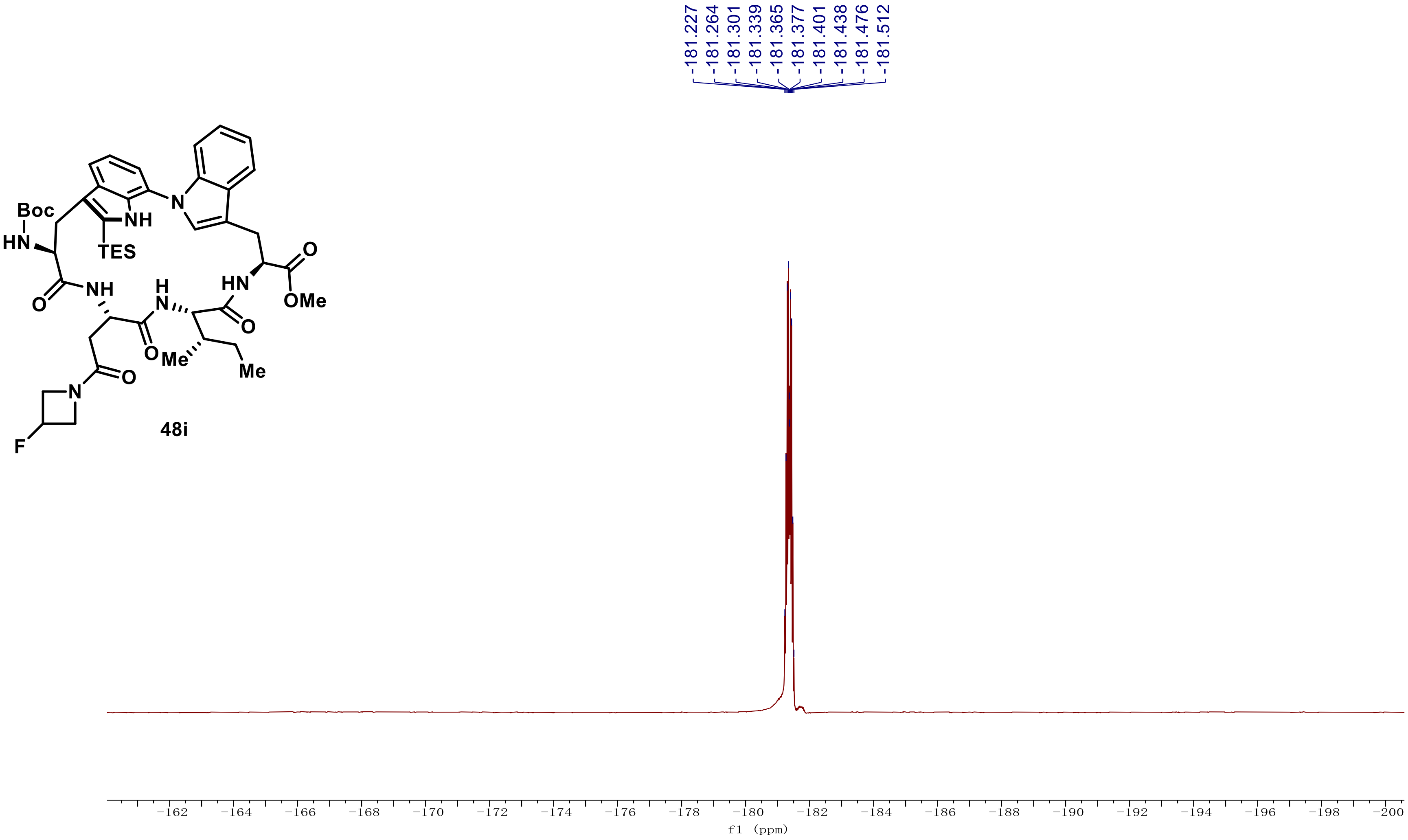

Compound 48j ¹H NMR (600 MHz, $CDCl_3$)

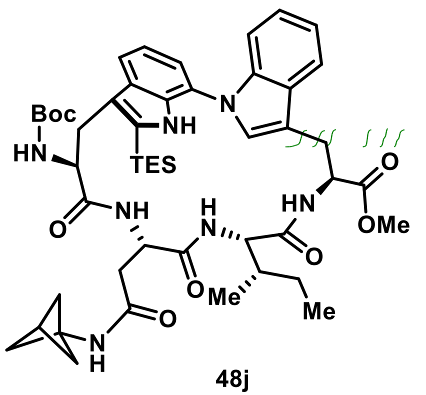

48j

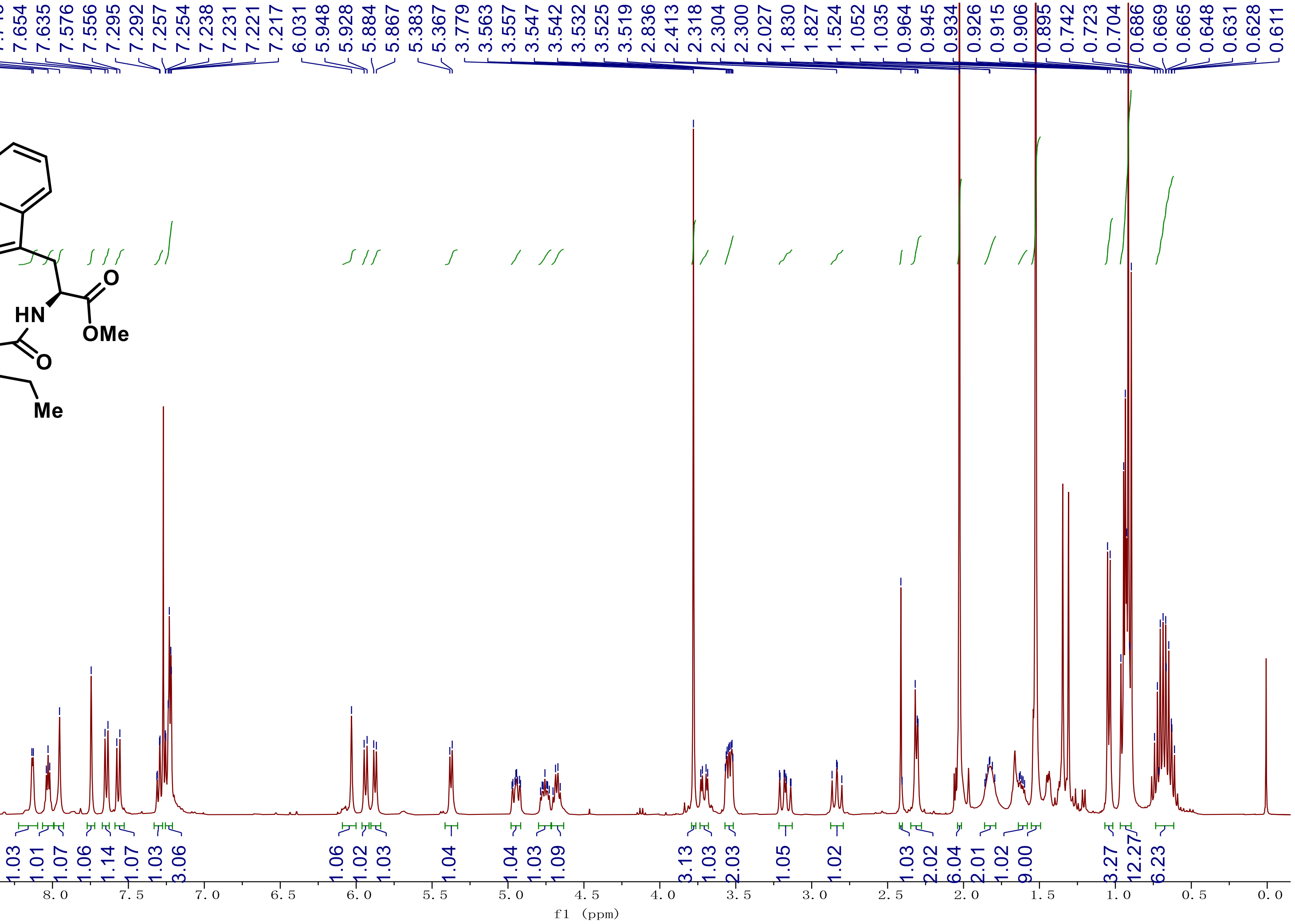

Compound 48j ^{13}C NMR (151 MHz, CDCl$_3$)

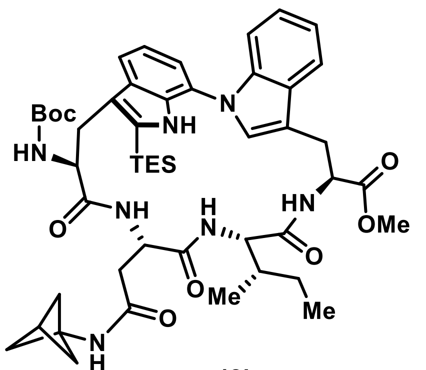

48j

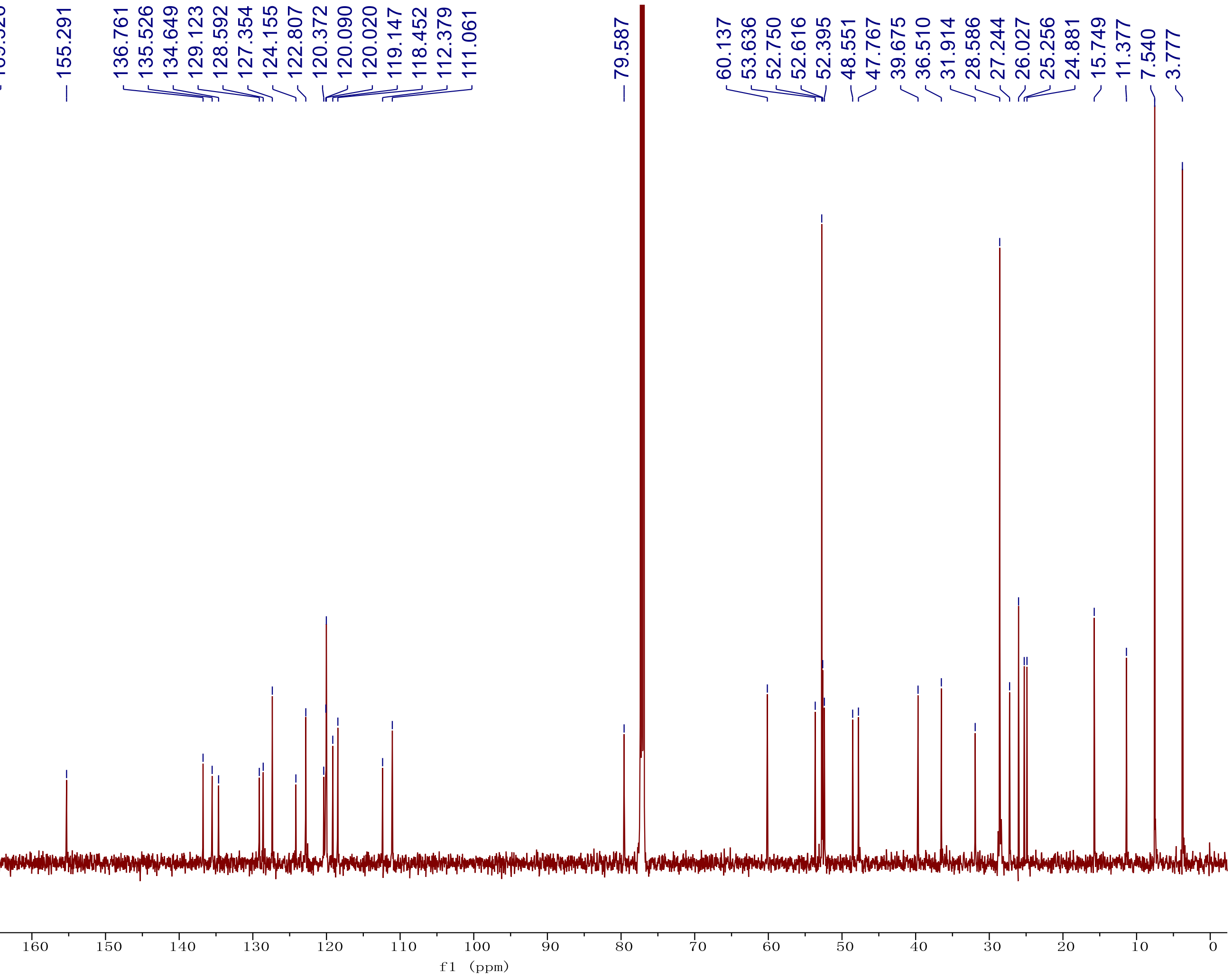

Compound 48k ^{1}H NMR (600 MHz, $CDCl_3$)

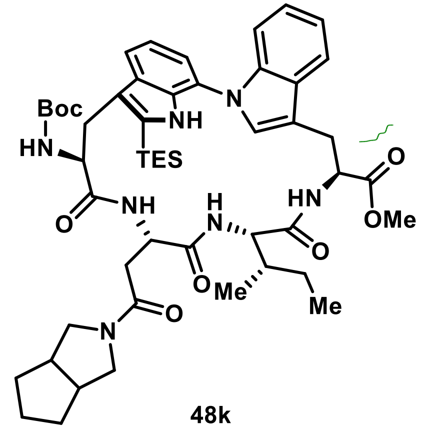

48k

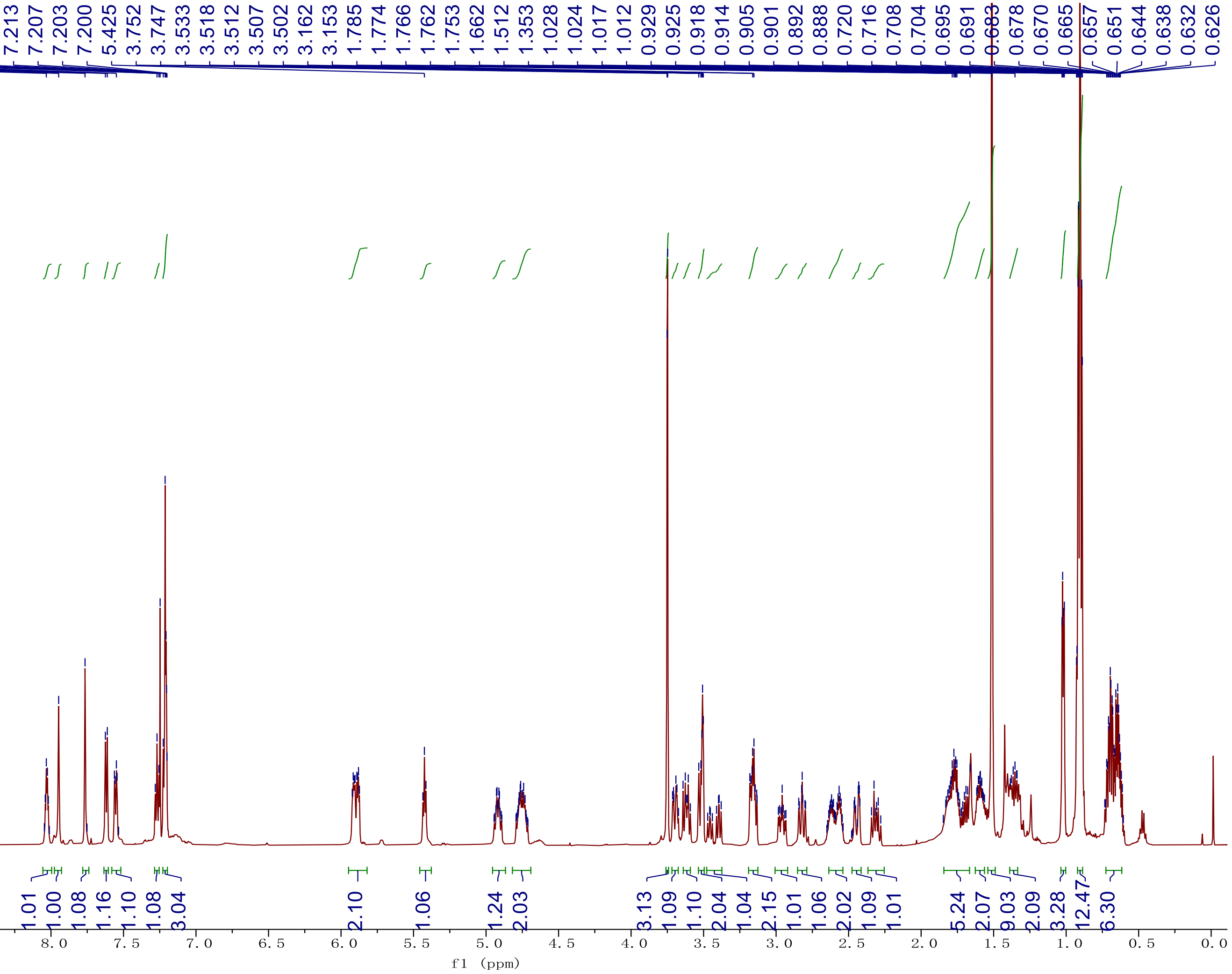

Compound 48k ^{13}C NMR (151 MHz, $CDCl_3$)

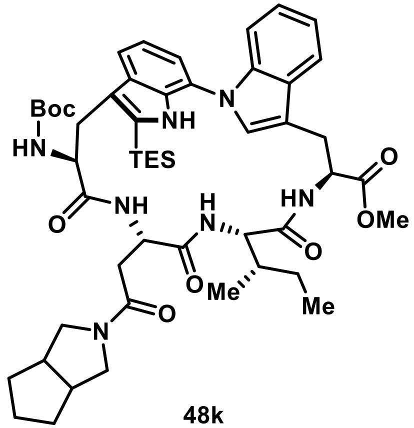

48k

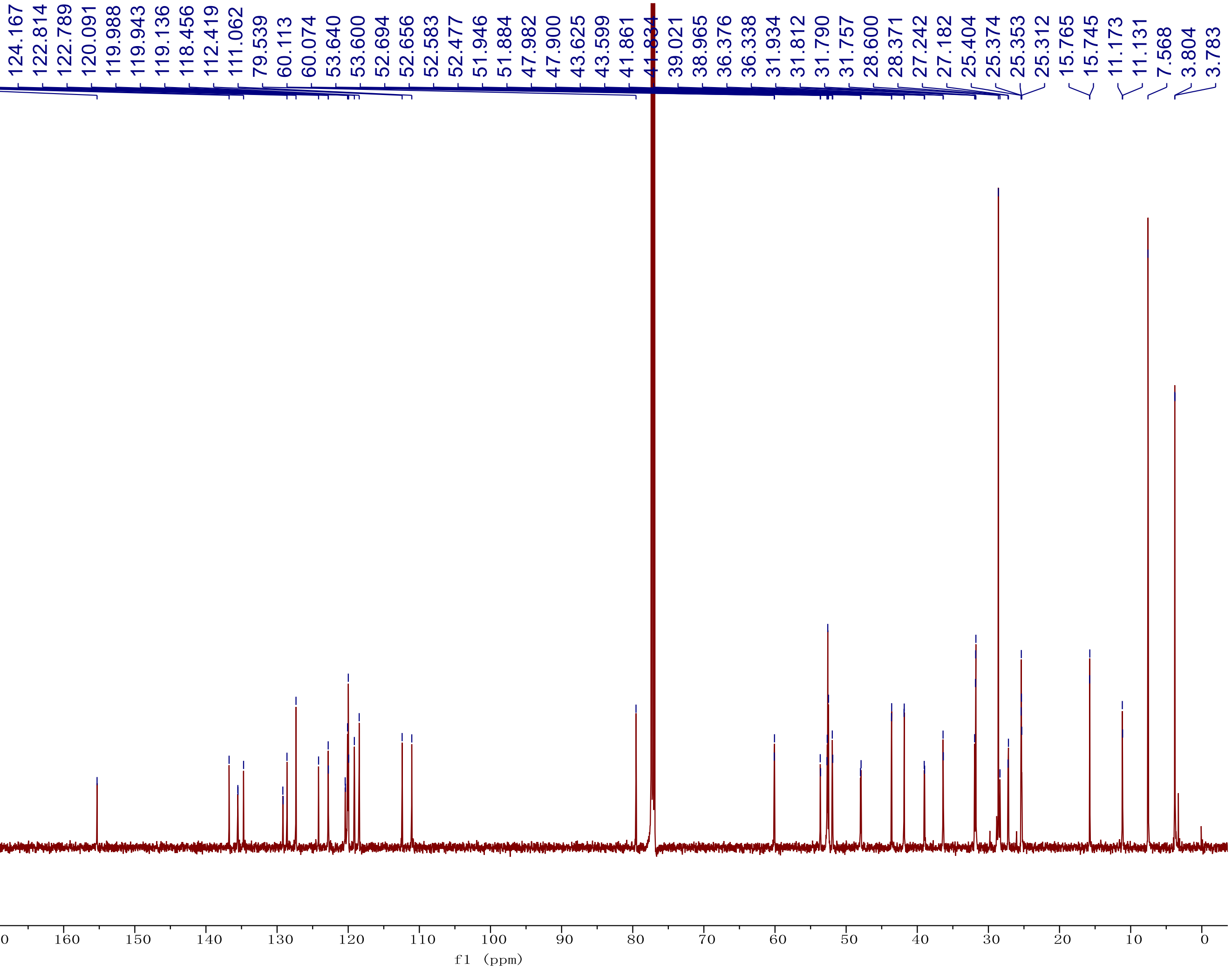

Compound 48l ¹H NMR (600 MHz, CDCl₃)

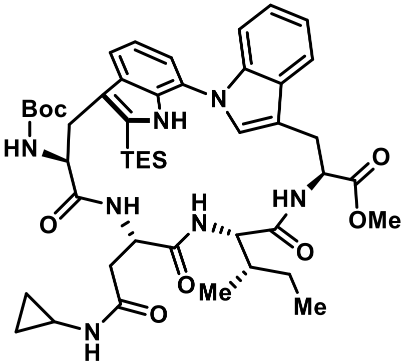

48l

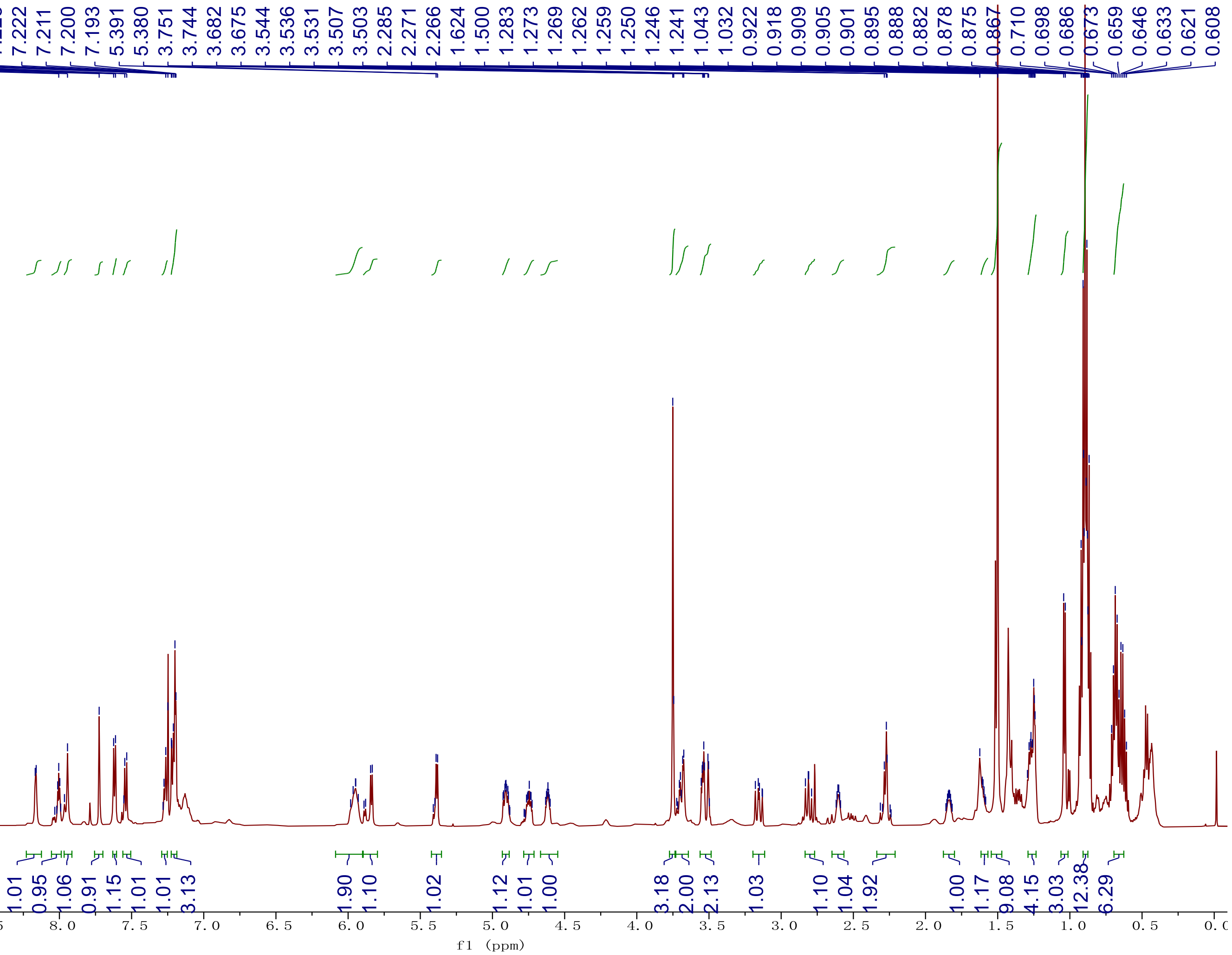

Compound 48l ^{13}C NMR (151 MHz, $CDCl_3$)

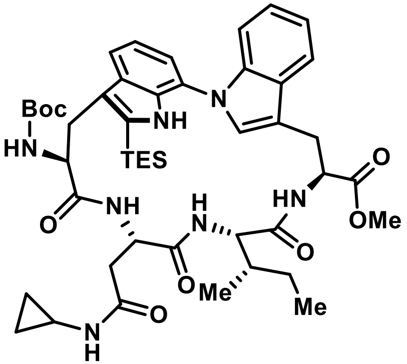

48l

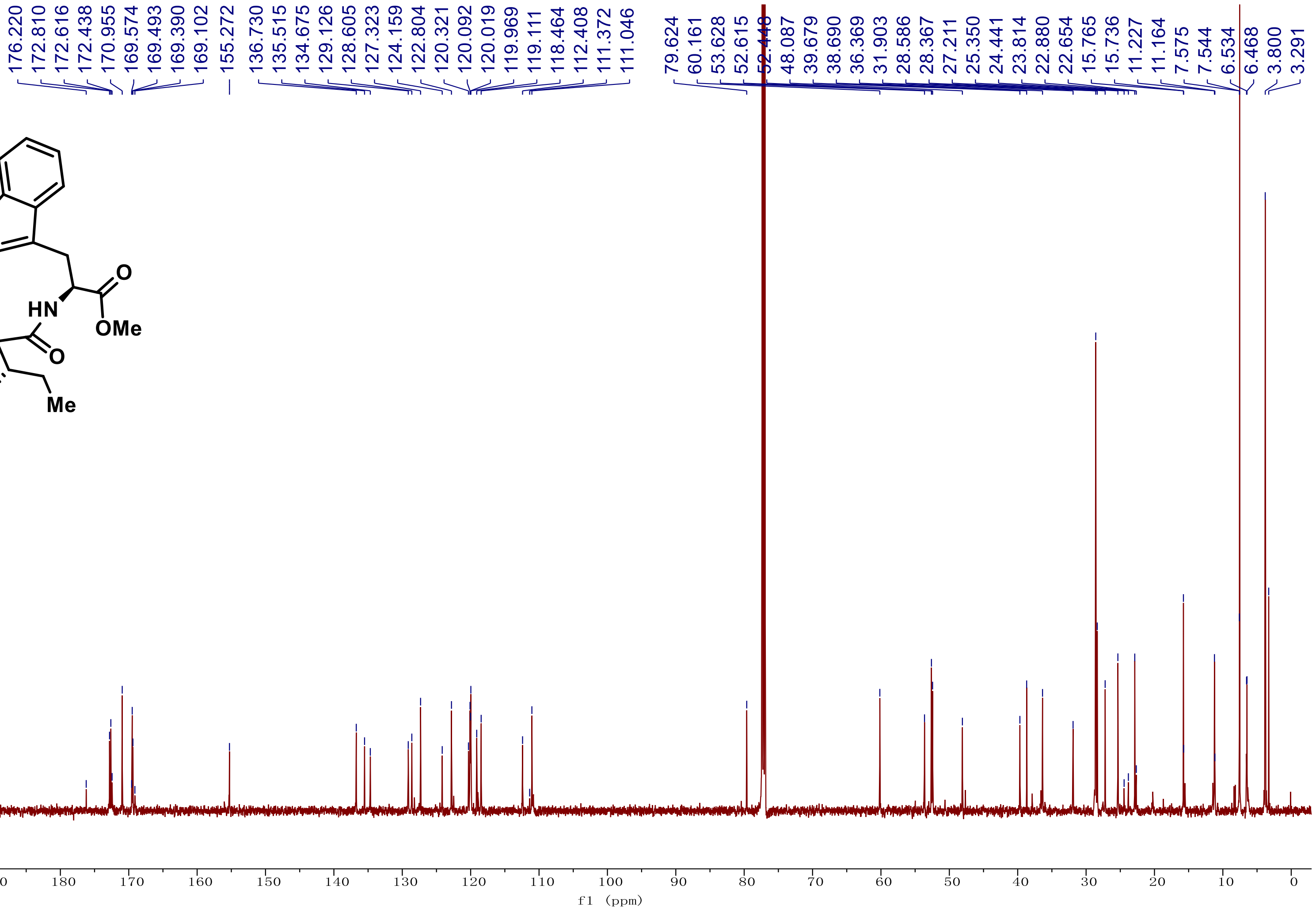

Compound 48m ^{1}H NMR (600 MHz, $CDCl_3$)

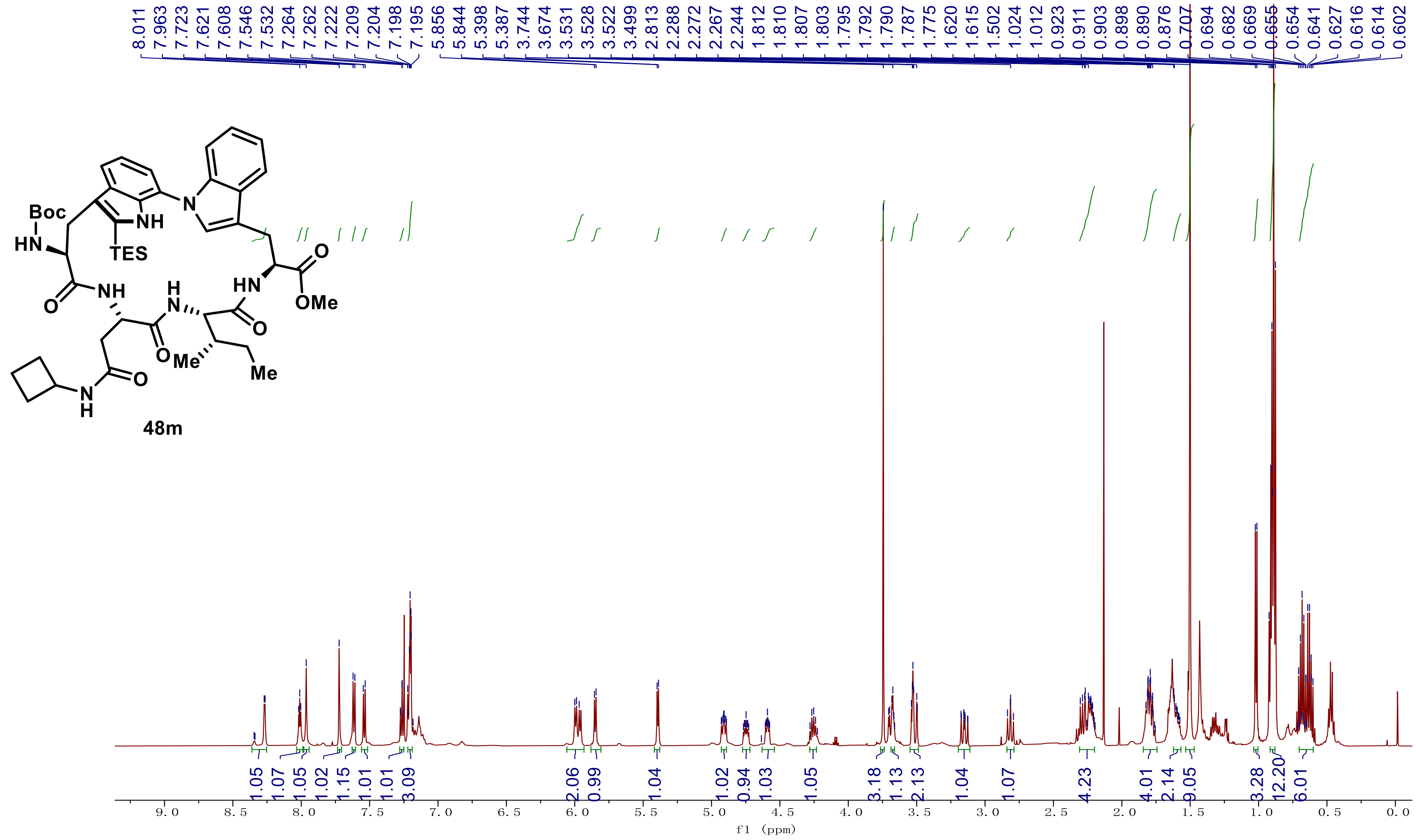

Compound 48m ^{13}C NMR (151 MHz, $CDCl_3$)

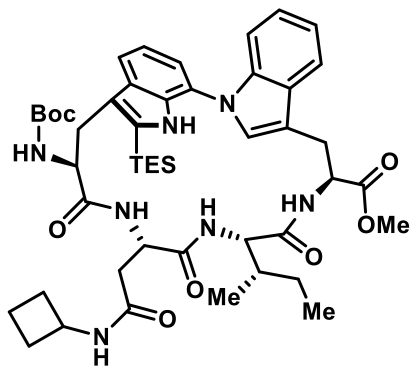

48m

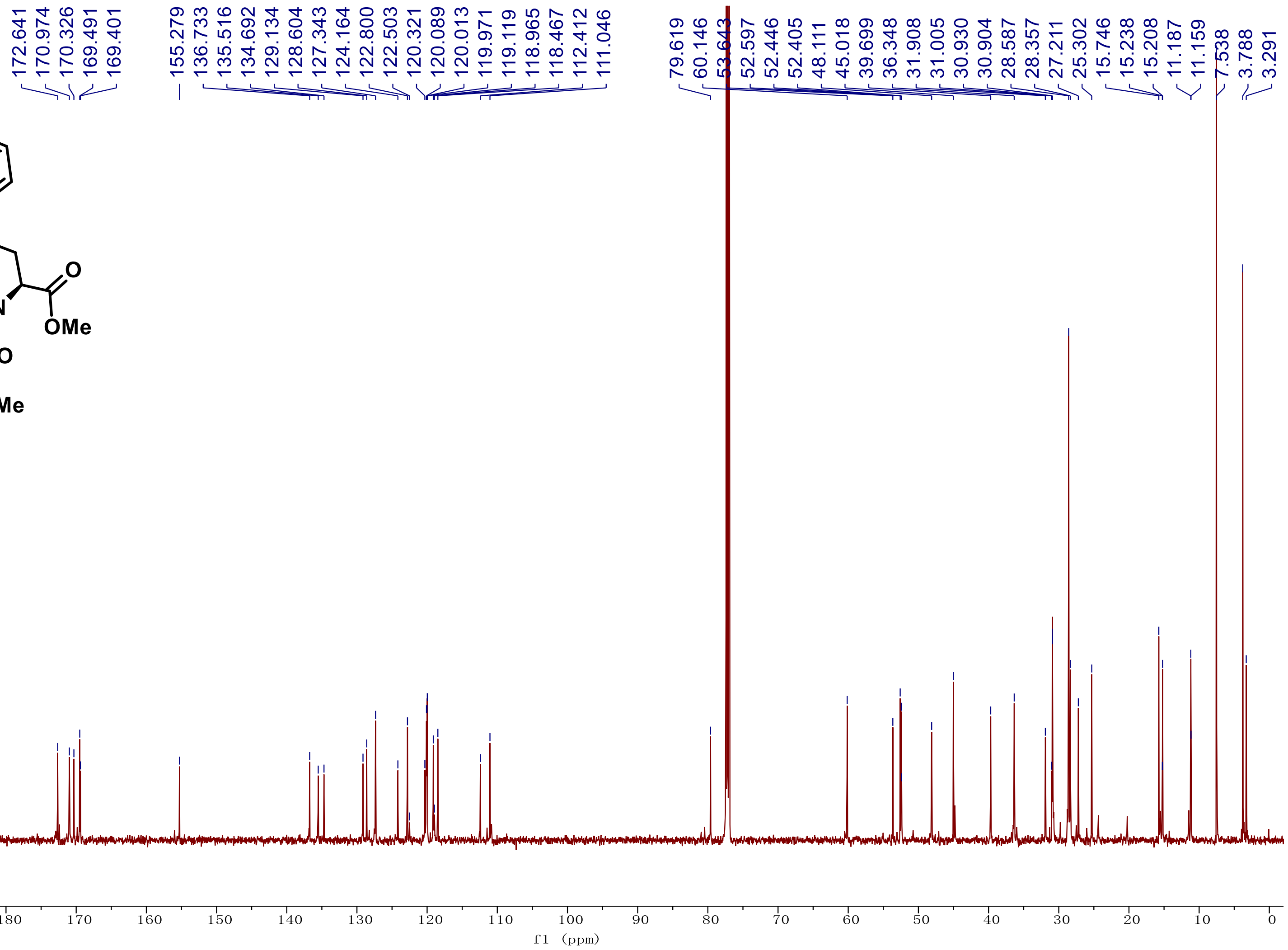

Compound 48n ^{1}H NMR (600 MHz, CDCl₃, rt)

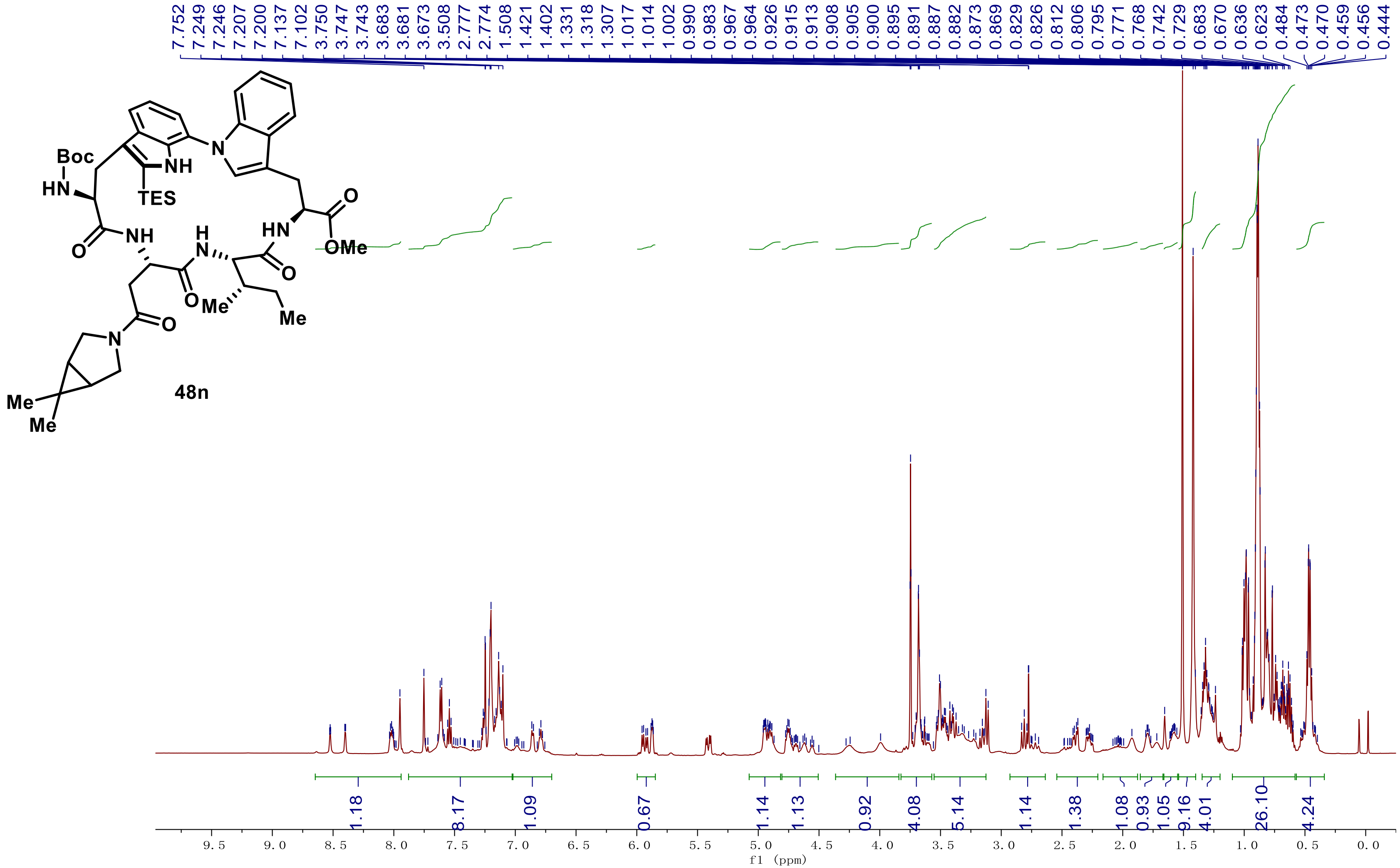

Compound 48n ^{13}C NMR (151 MHz, CDCl$_3$, rt)

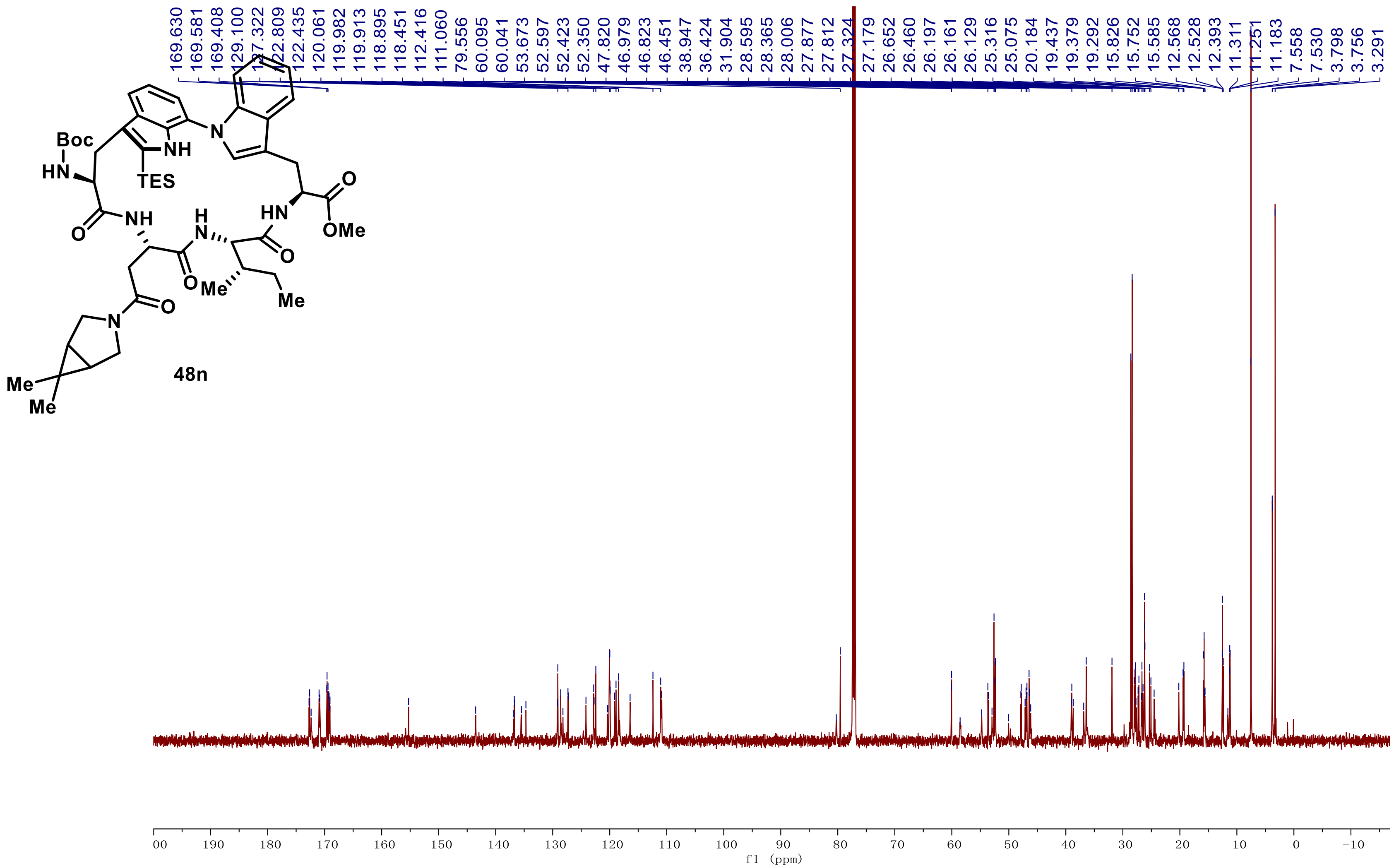

48n

Compound 48n ^{1}H NMR (400 MHz, DMSO-d_6, rt)

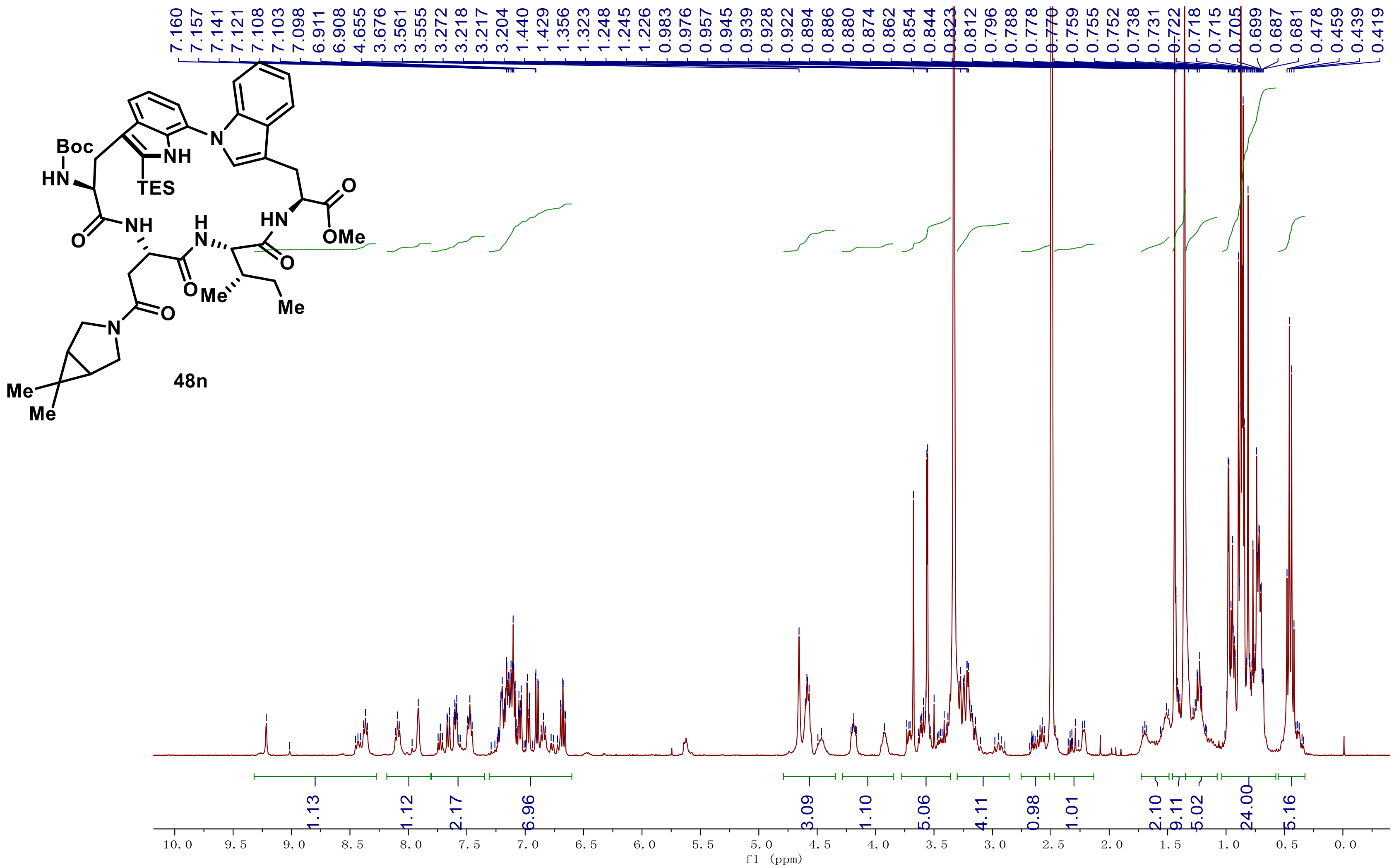

Compound 48n ¹H NMR (400 MHz, DMSO-d_6, 60 °C)

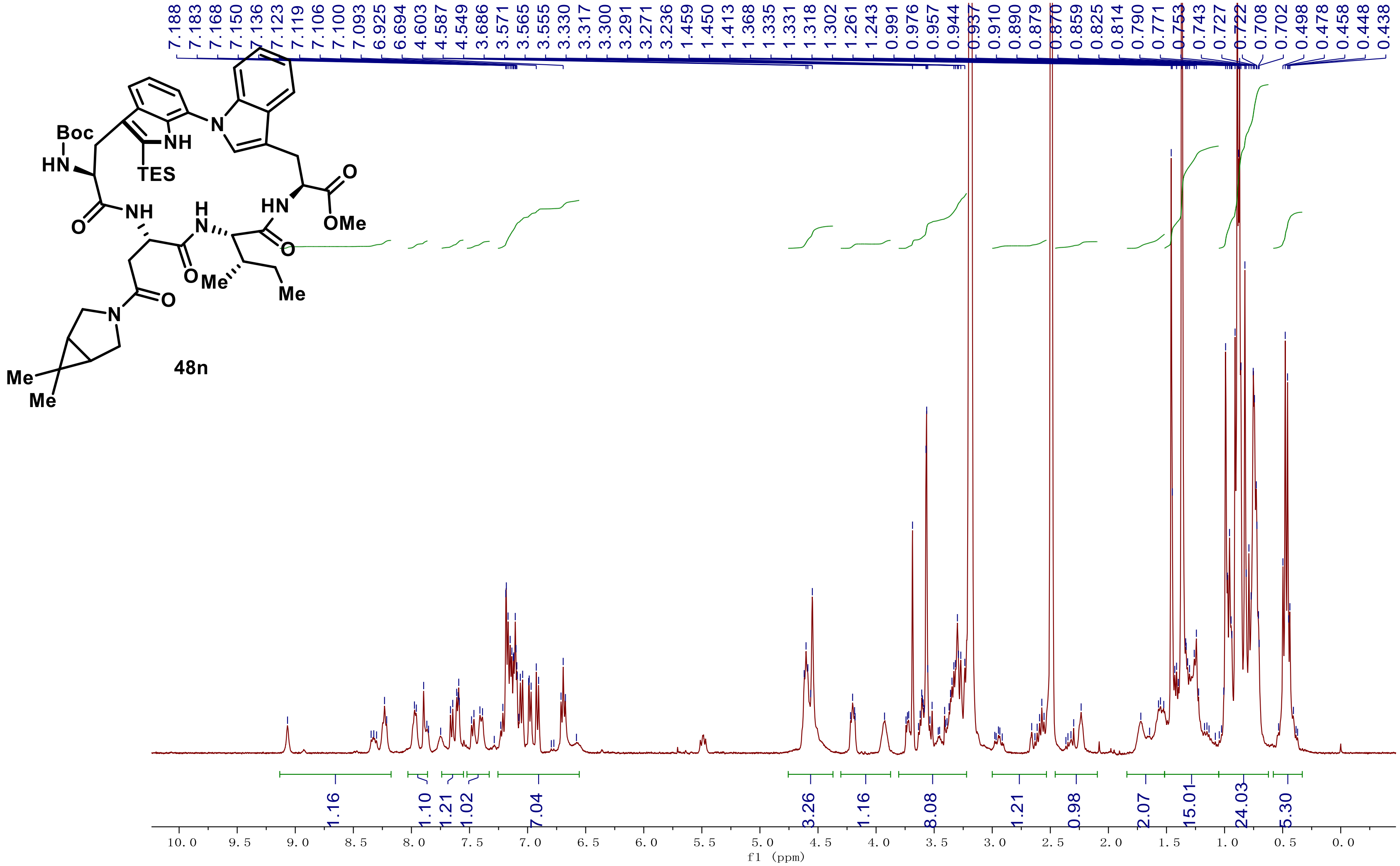

Compound 48n ^{13}C NMR (101 MHz, DMSO-d_6, rt)

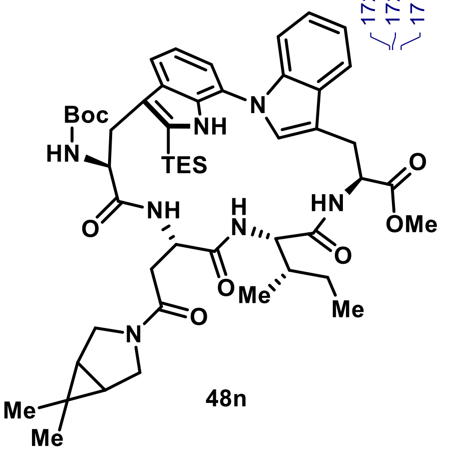

48n

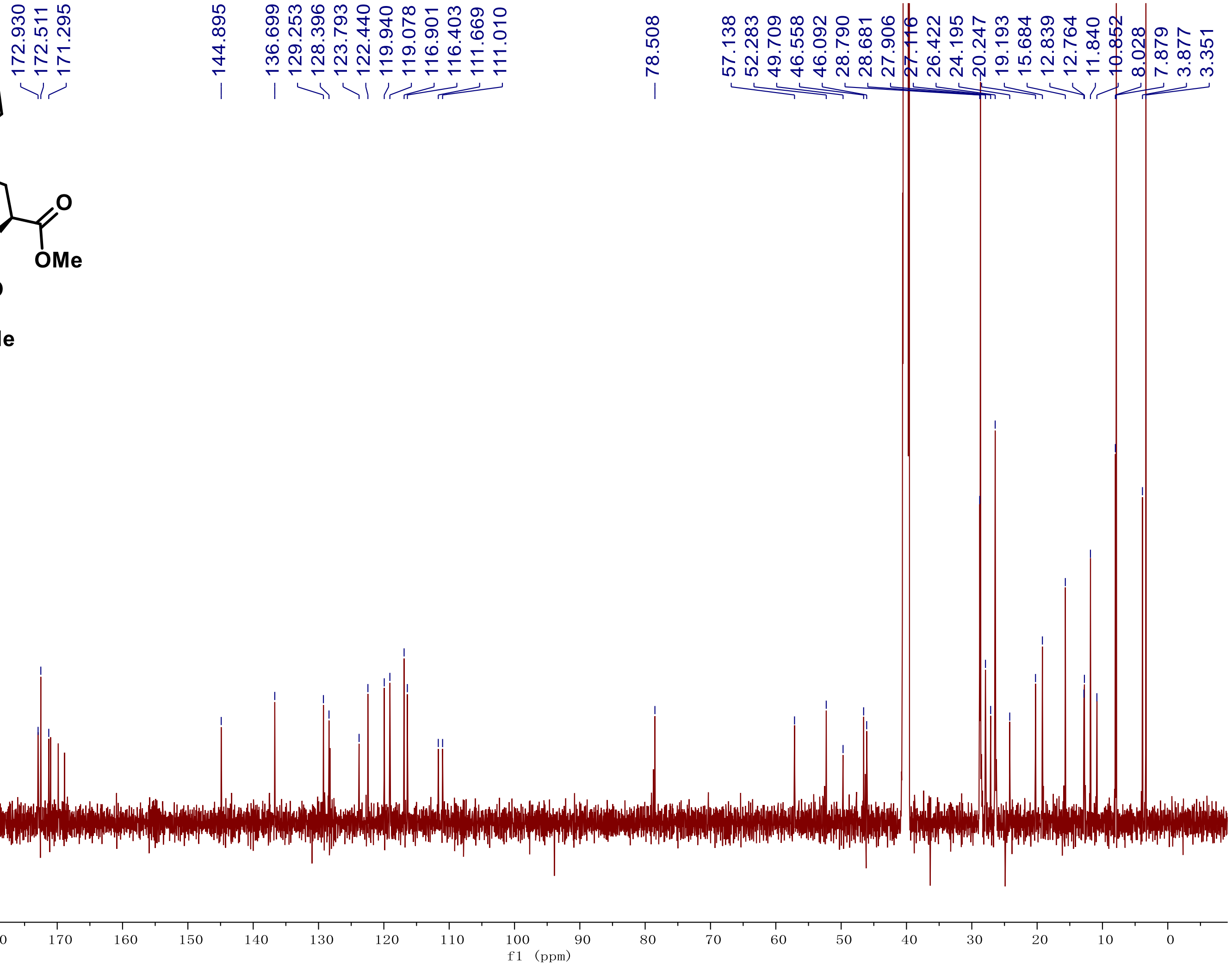

Compound 48o ^{1}H NMR (600 MHz, CDCl₃, rt)

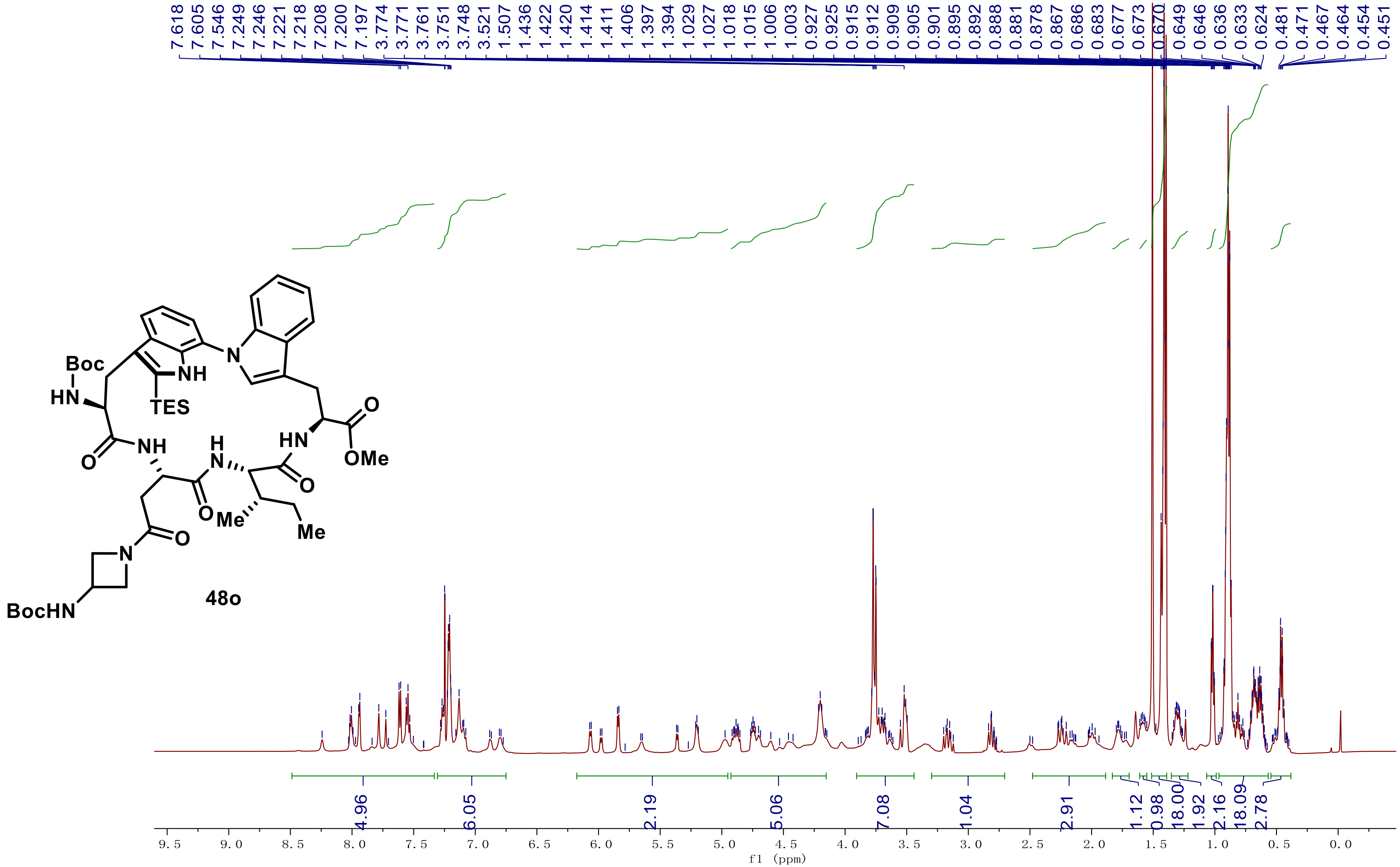

Compound 48o ^{13}C NMR (151 MHz, CDCl$_3$, rt)

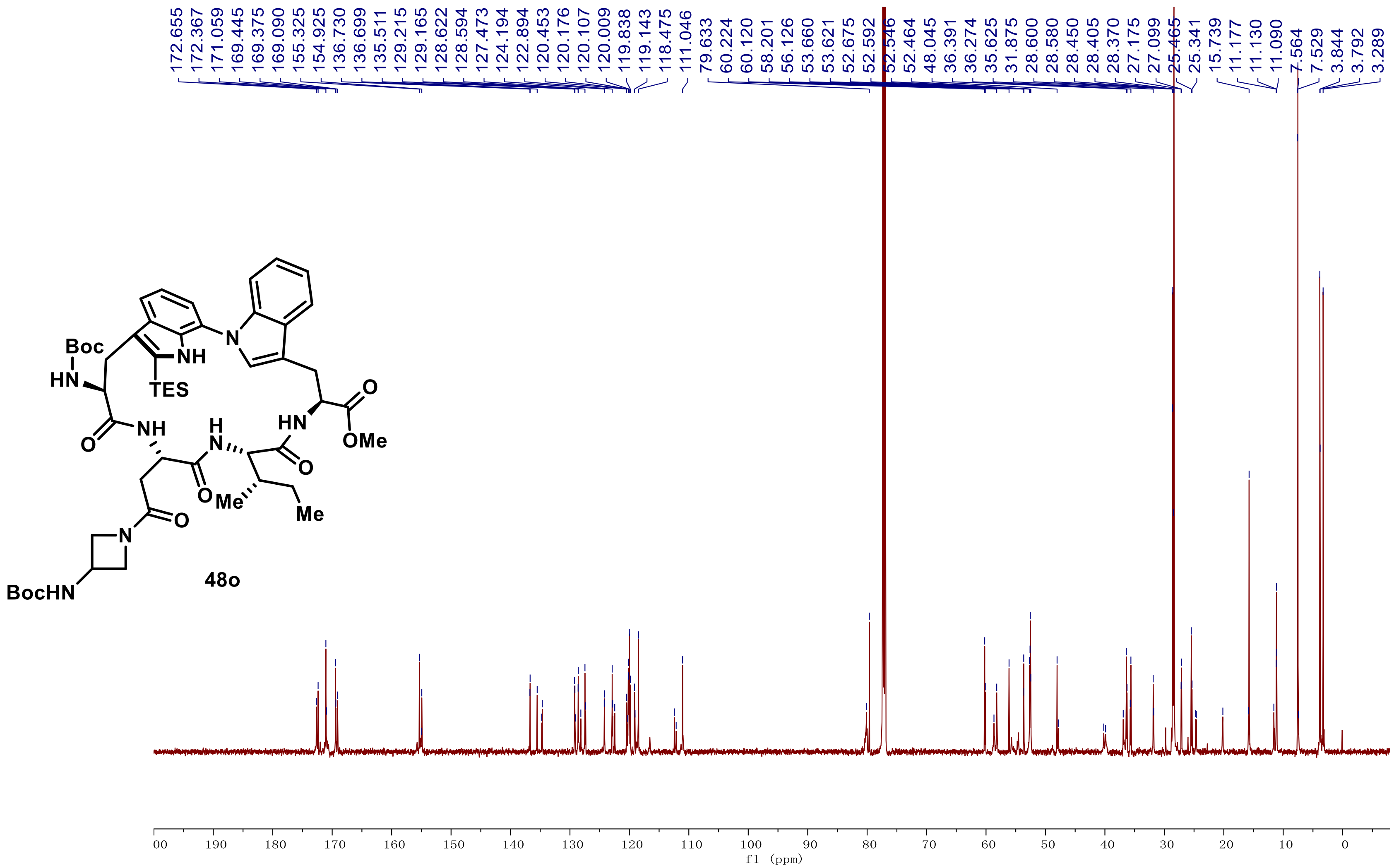

Compound 48o ^{1}H NMR (400 MHz, DMSO-d_6, rt)

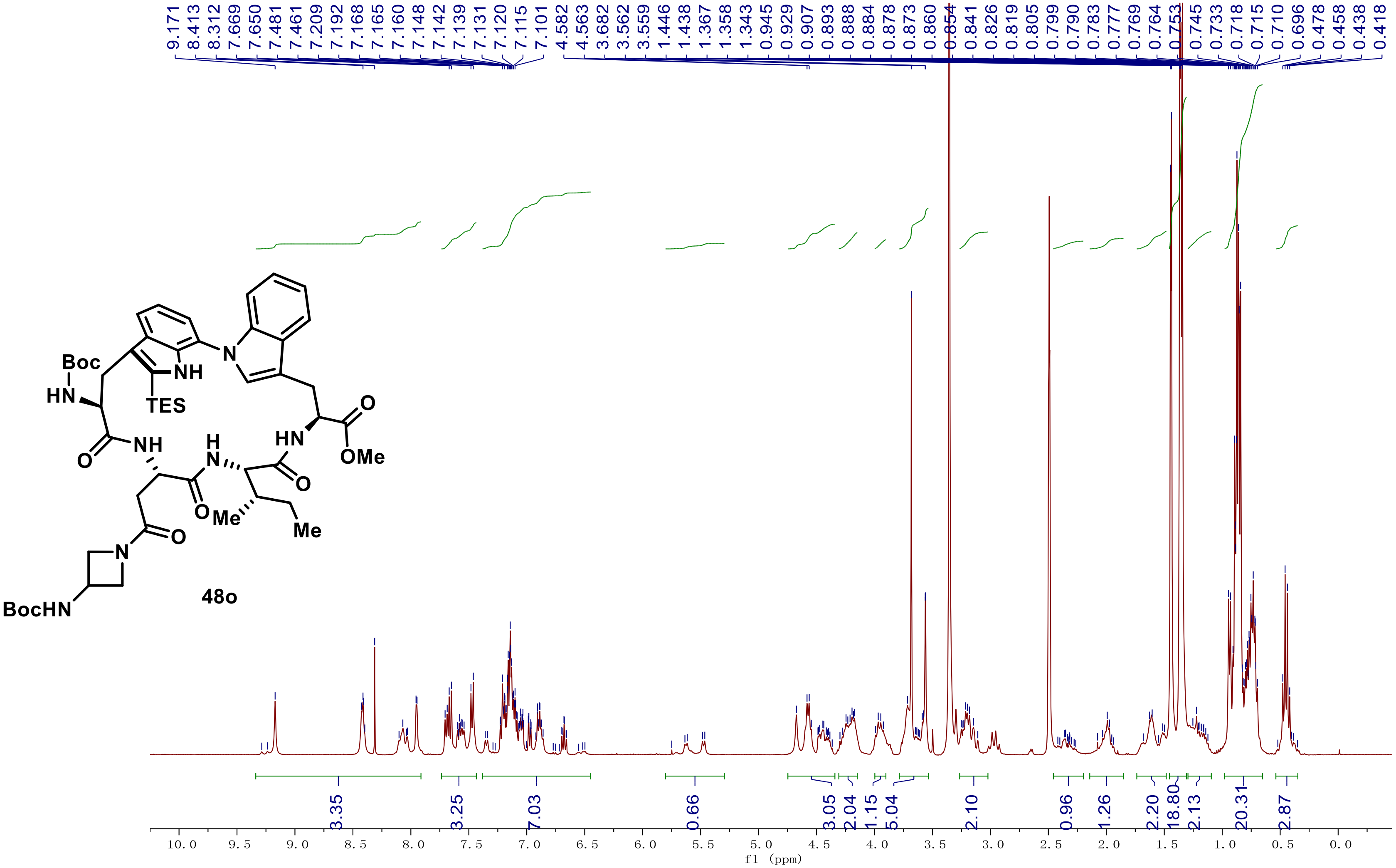

48o

Compound 48o ¹H NMR (400 MHz, DMSO-d_6, 60 °C)

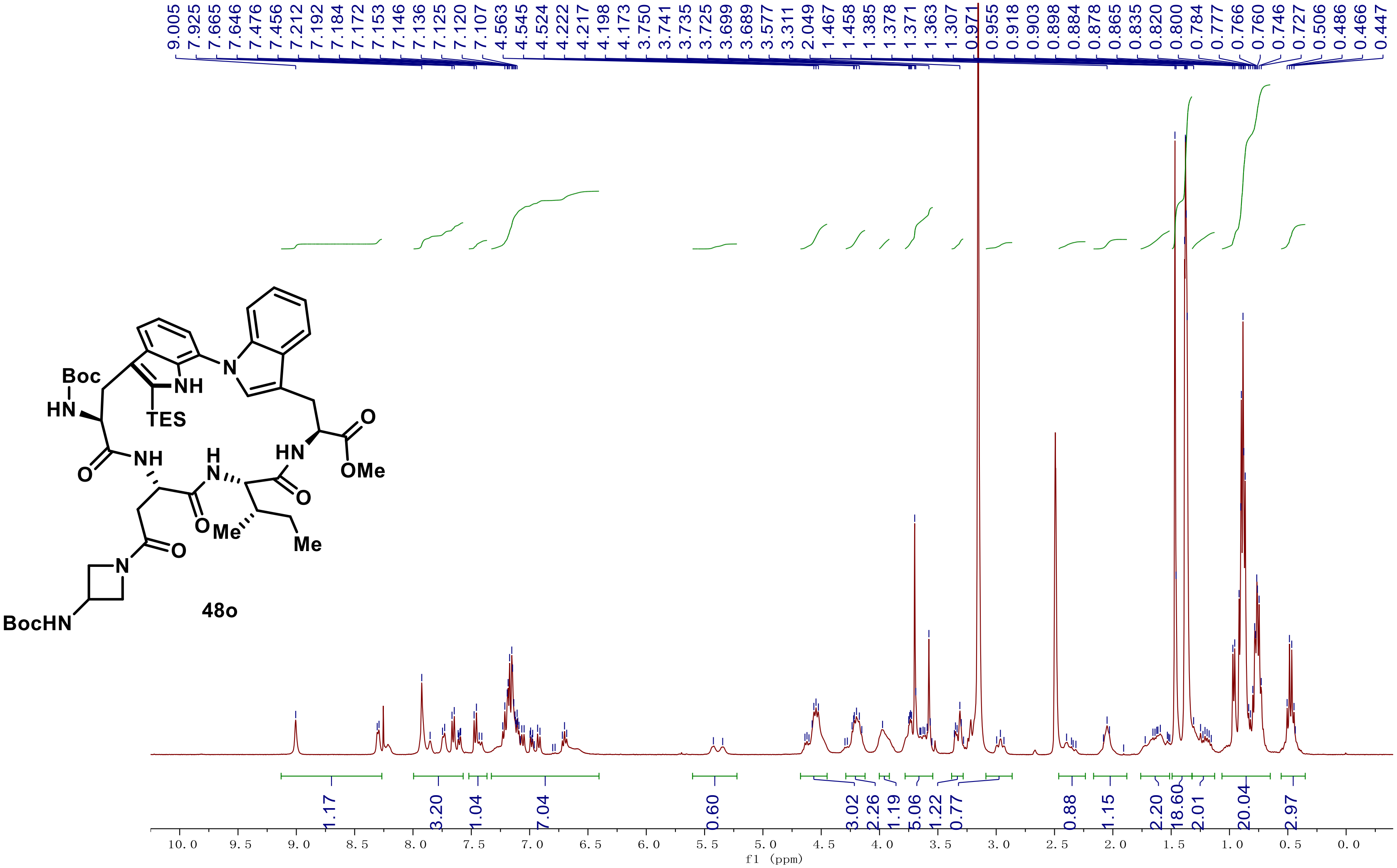

48o

Compound 48o ^{13}C NMR (101 MHz, DMSO-d_6, rt)

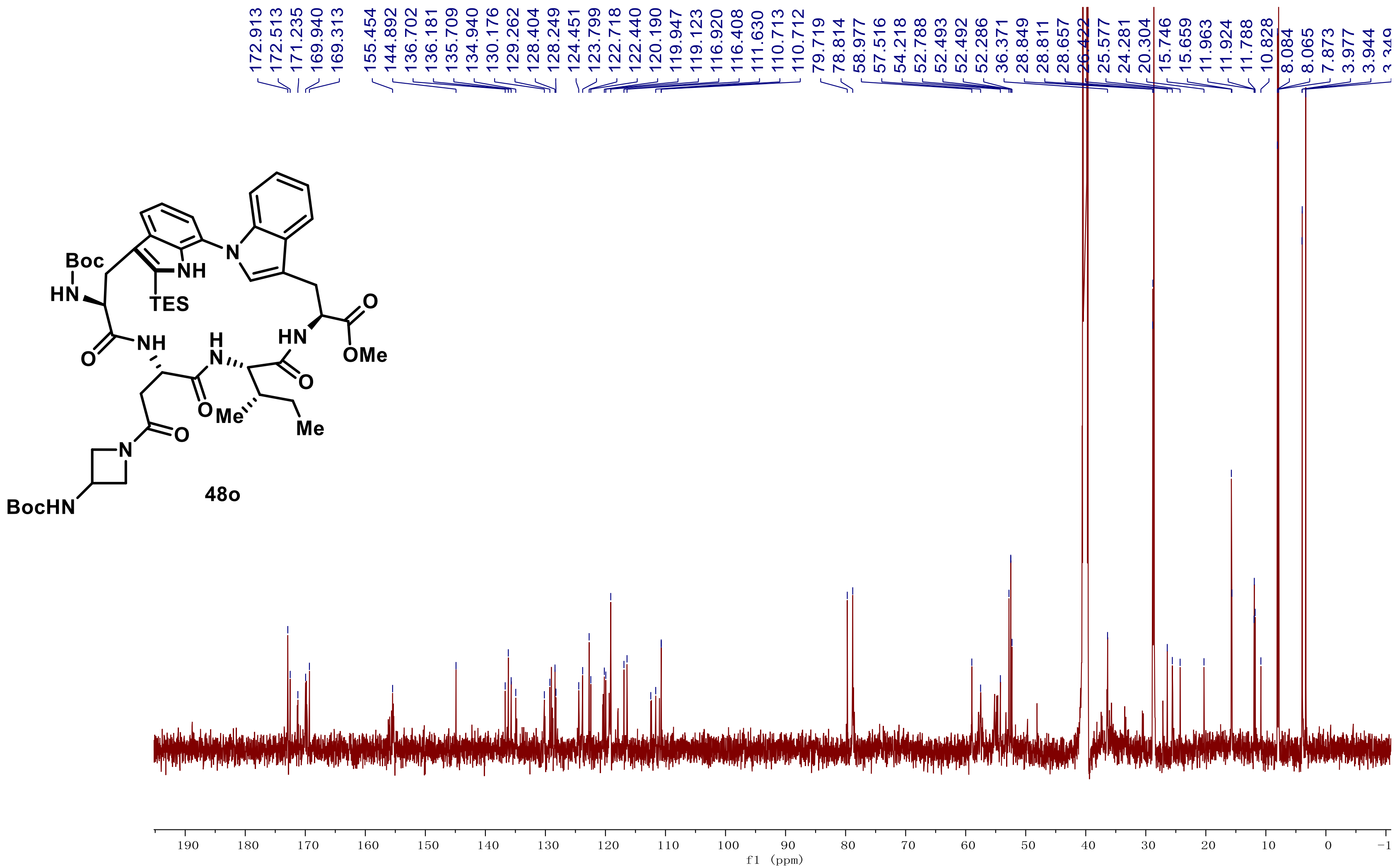

Compound 48p ^{1}H NMR (600 MHz, $CDCl_3$)

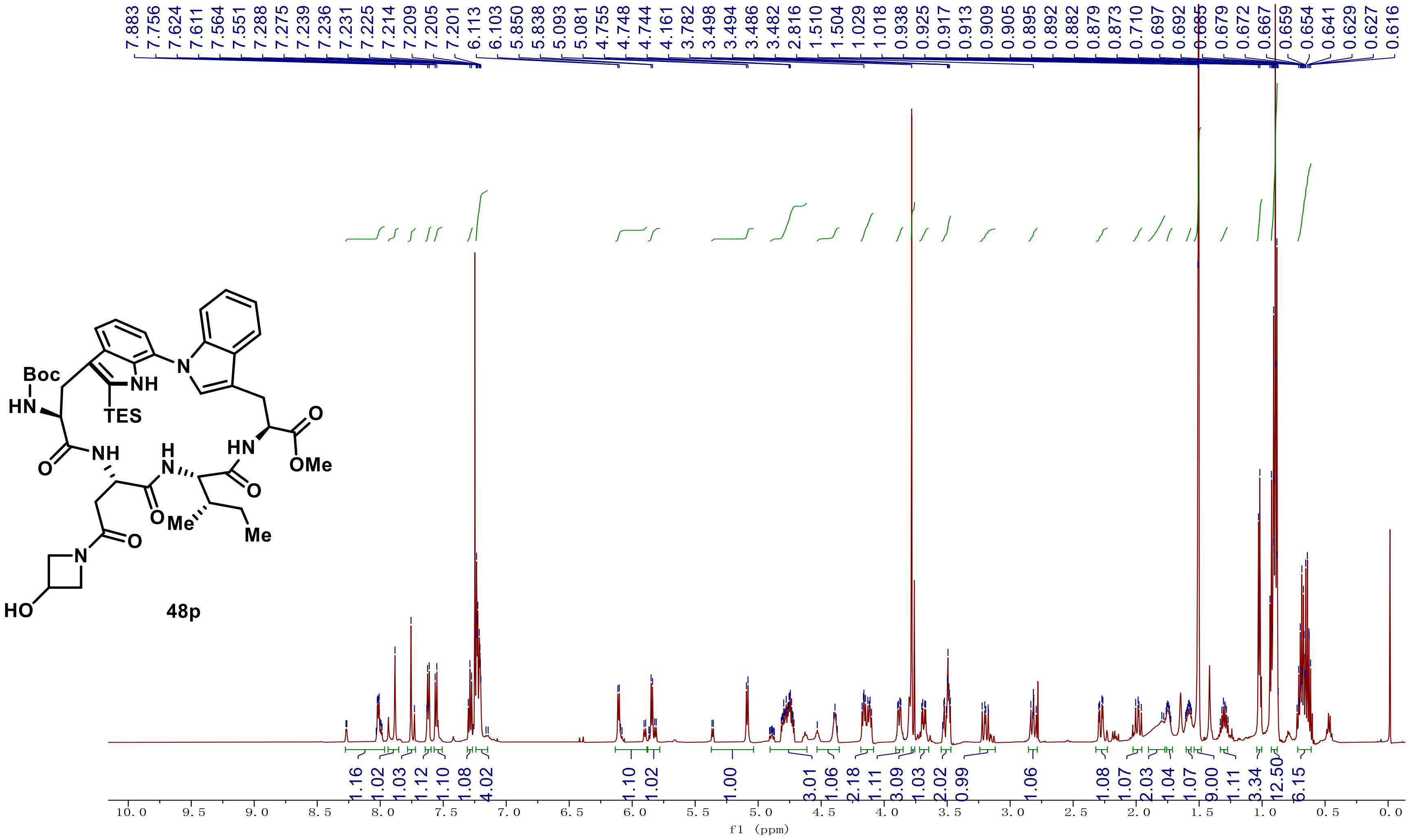

48p

Compound 48p ^{13}C NMR (151 MHz, $CDCl_3$)

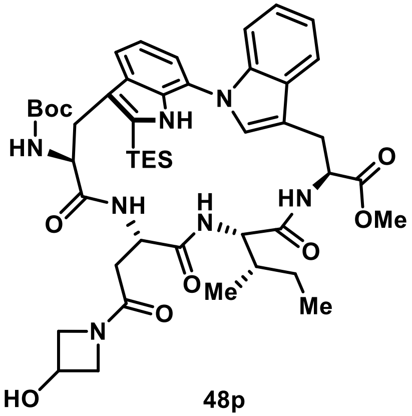

48p

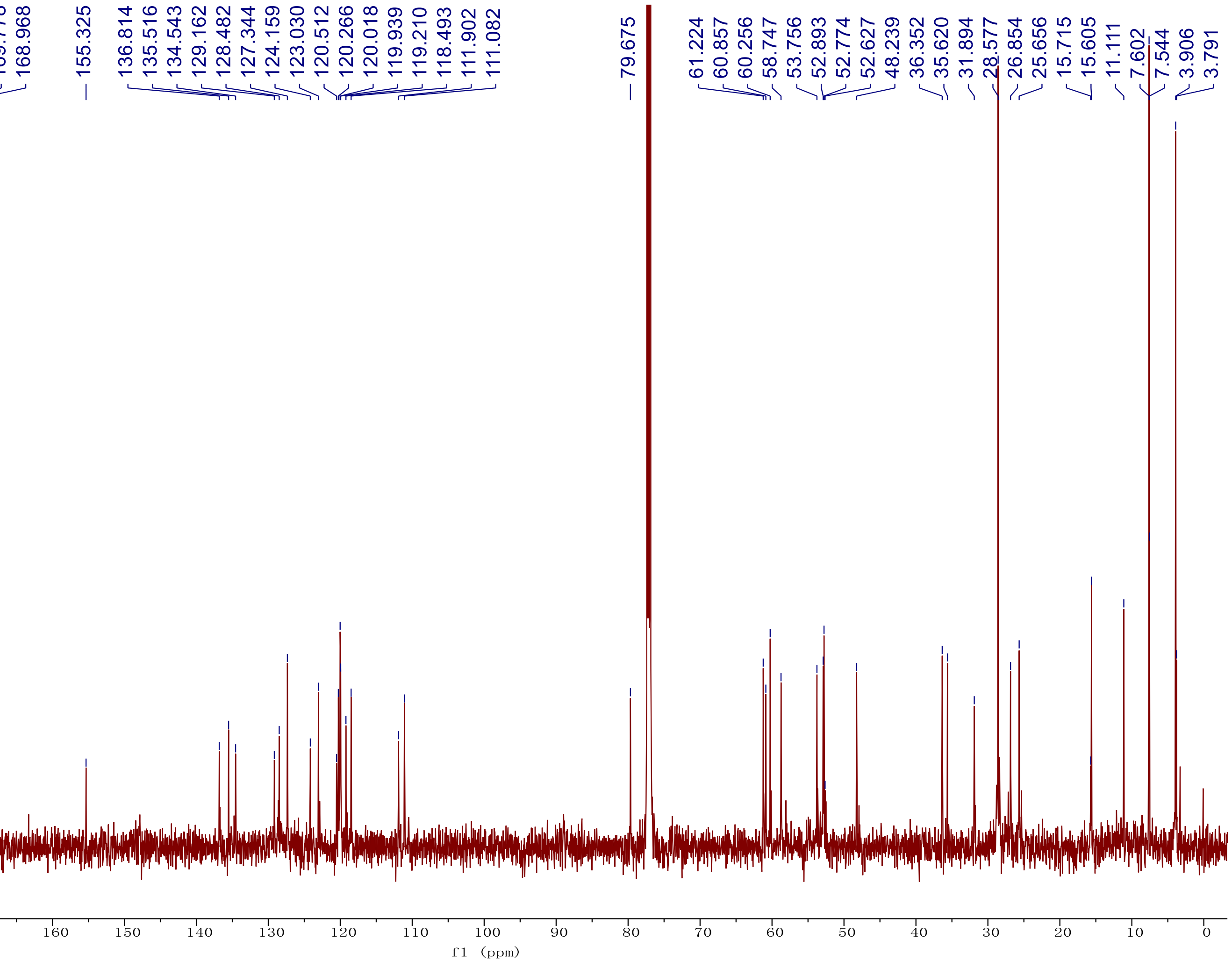

Compound 48q ¹H NMR (600 MHz, CDCl₃, rt)

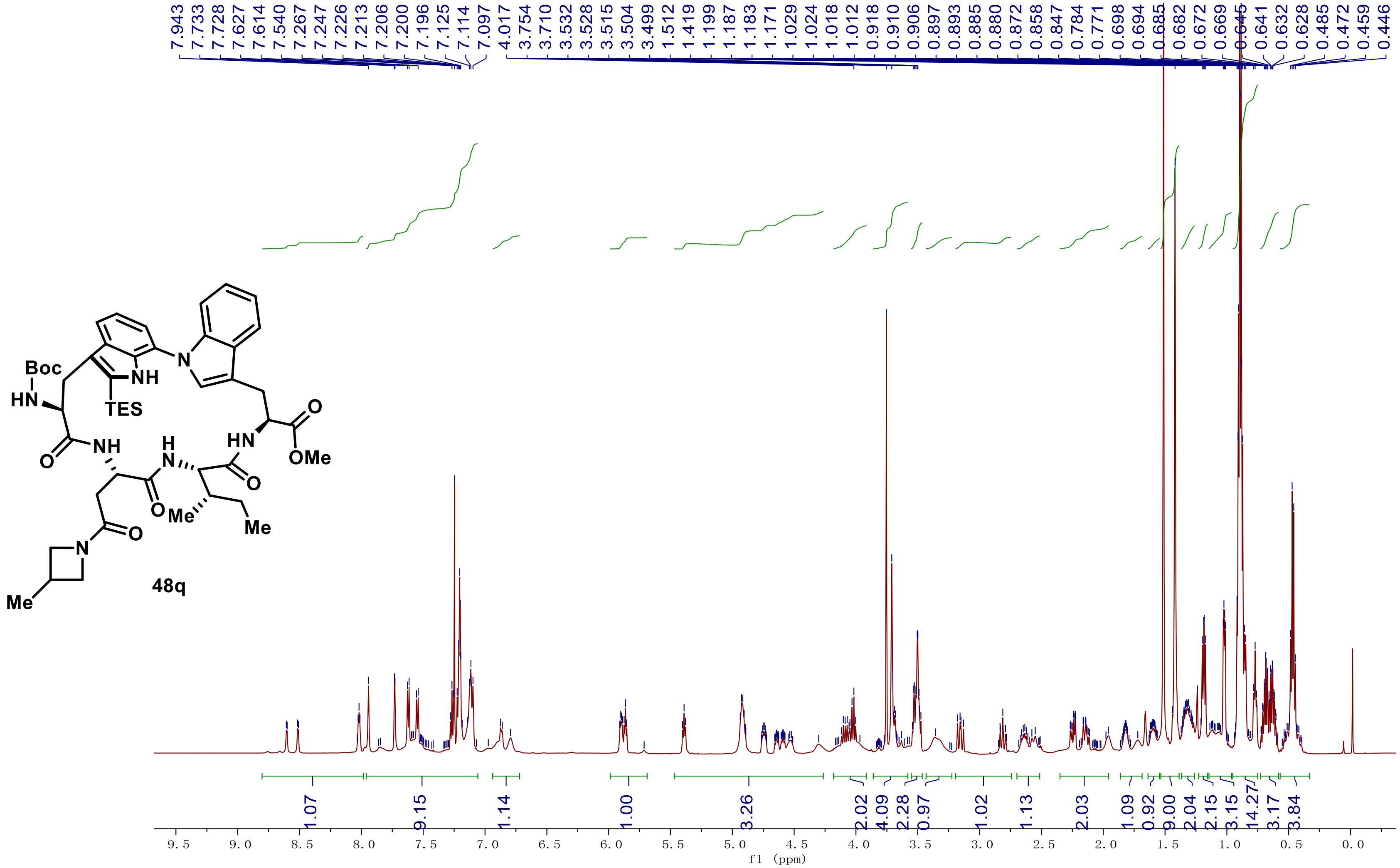

Compound 48q ^{13}C NMR (151 MHz, $CDCl_3$, rt)

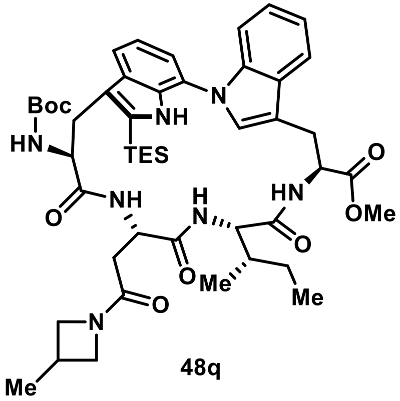

48q

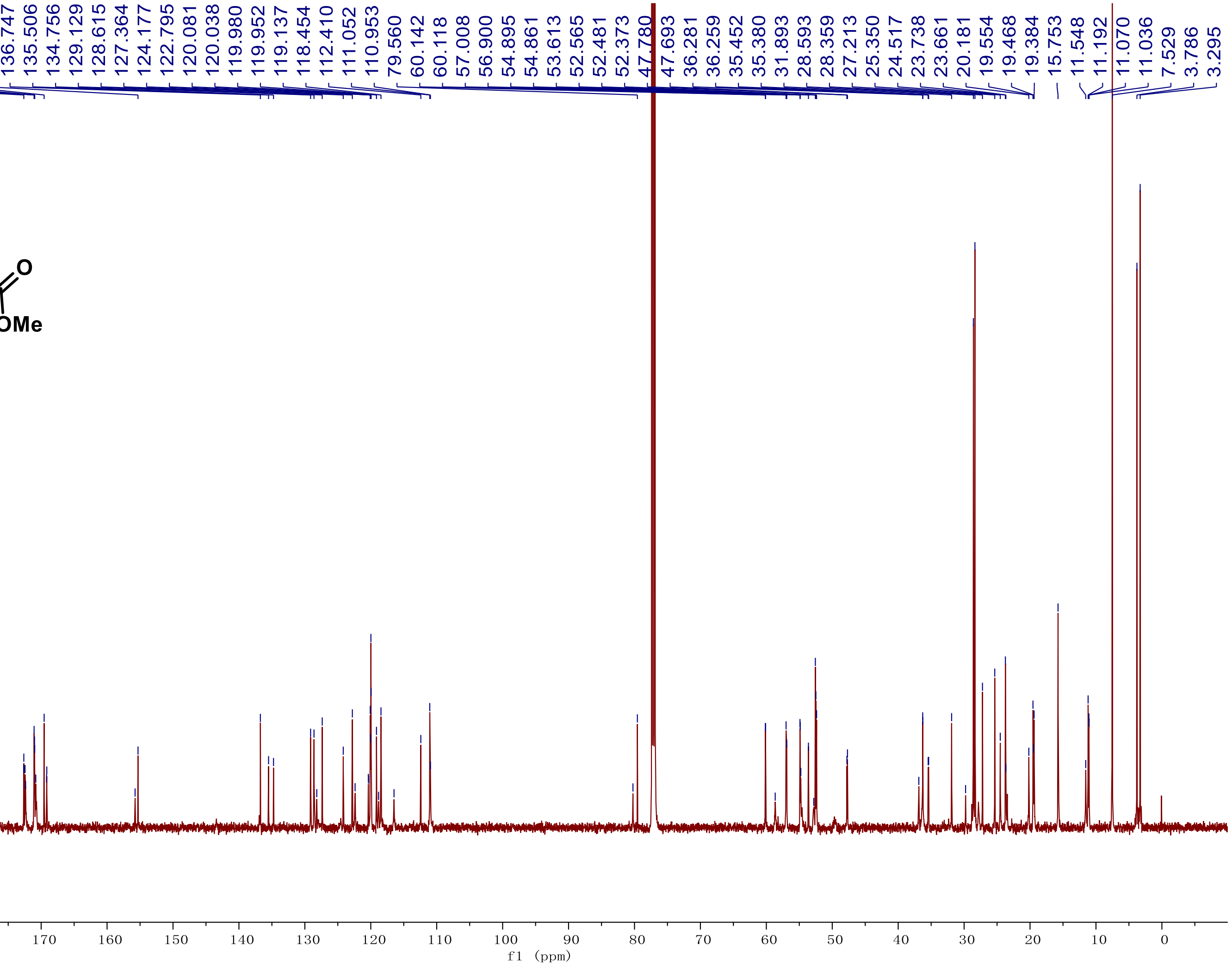

Compound 48q ^{1}H NMR (400 MHz, DMSO-d_6, rt)

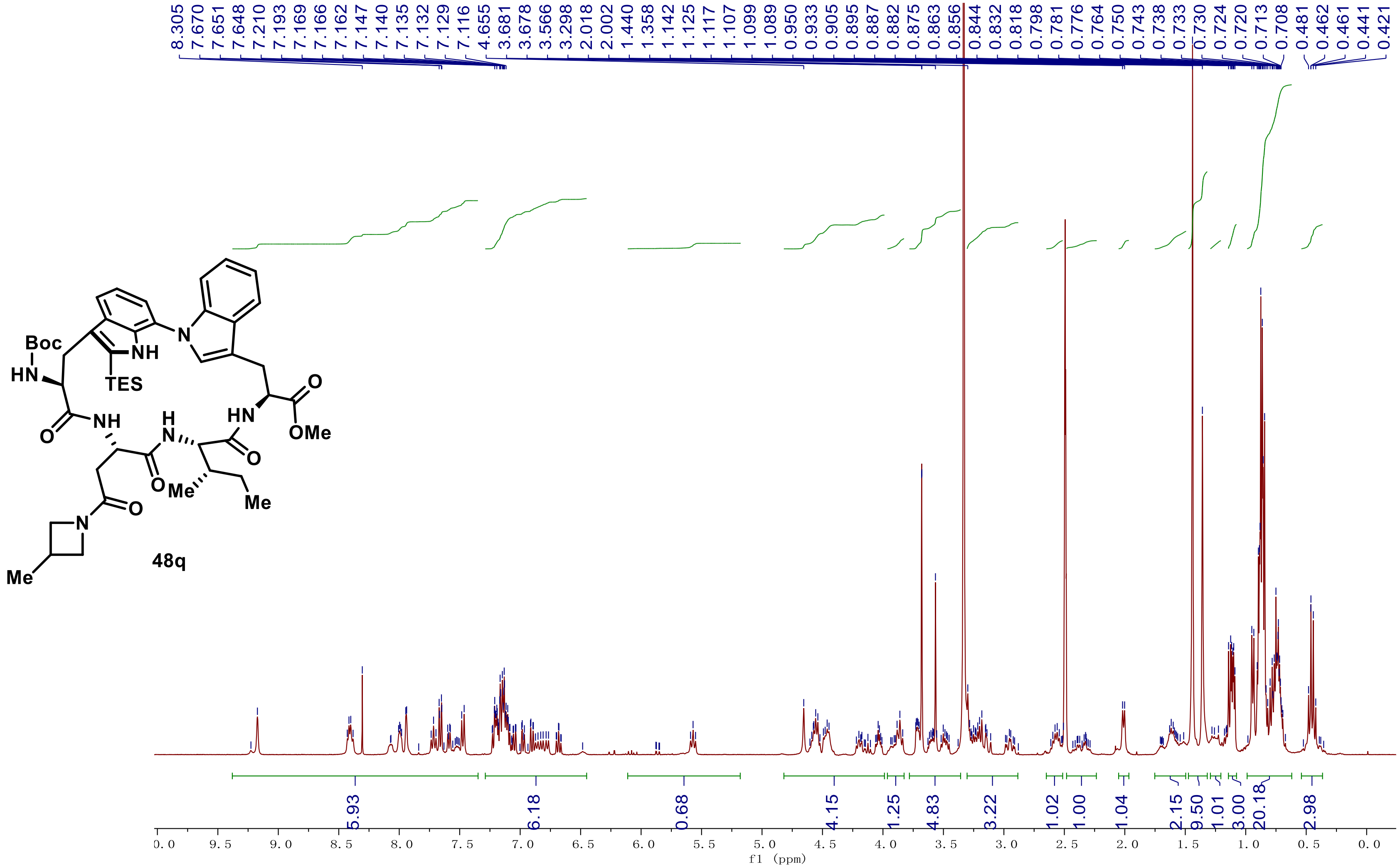

48q

Compound 48q ^{1}H NMR (400 MHz, DMSO-d_6, 60 °C)

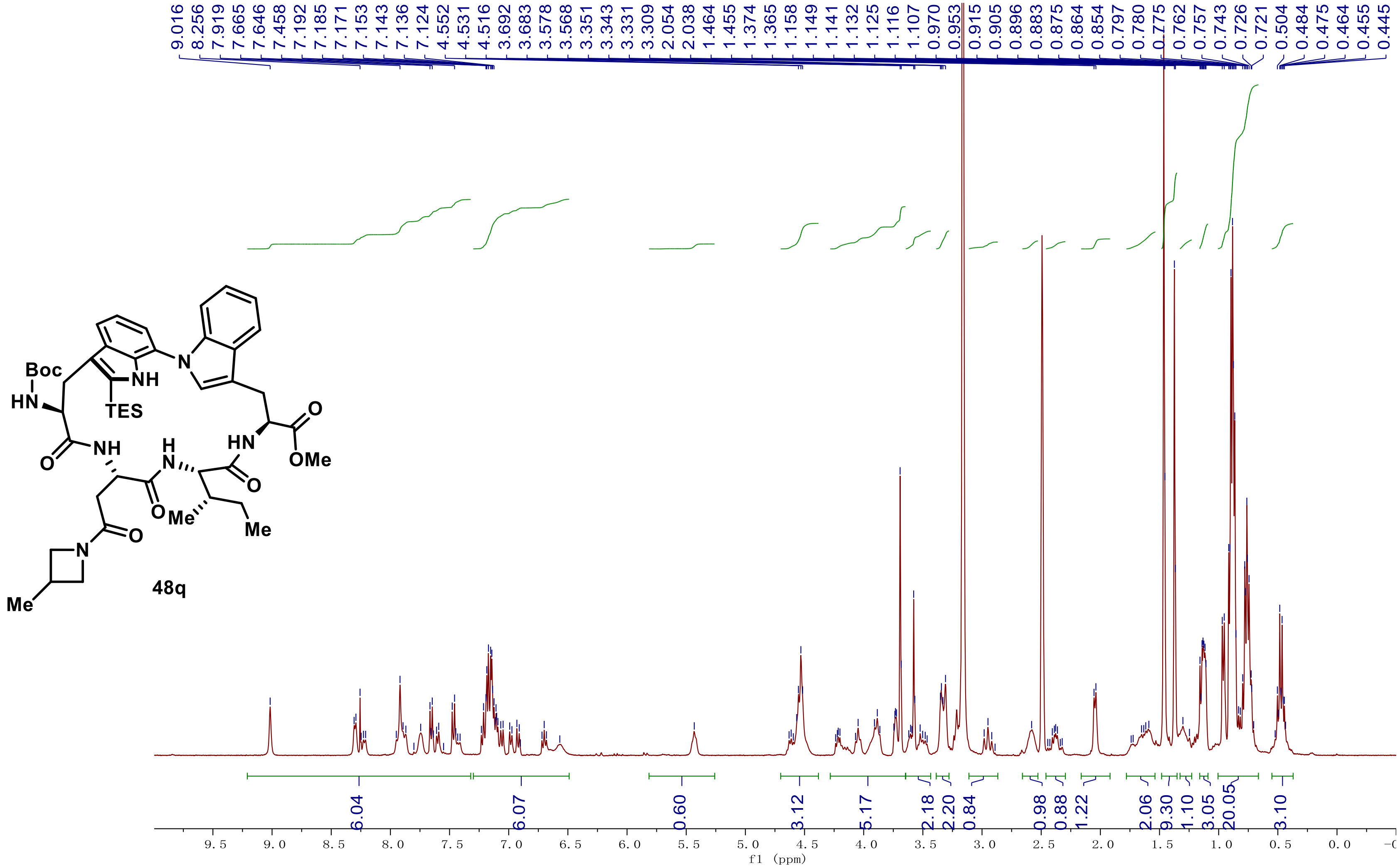

Compound 48q ^{13}C NMR (101 MHz, DMSO-d_6, rt)

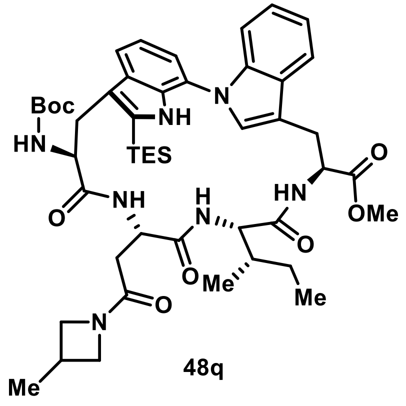

48q

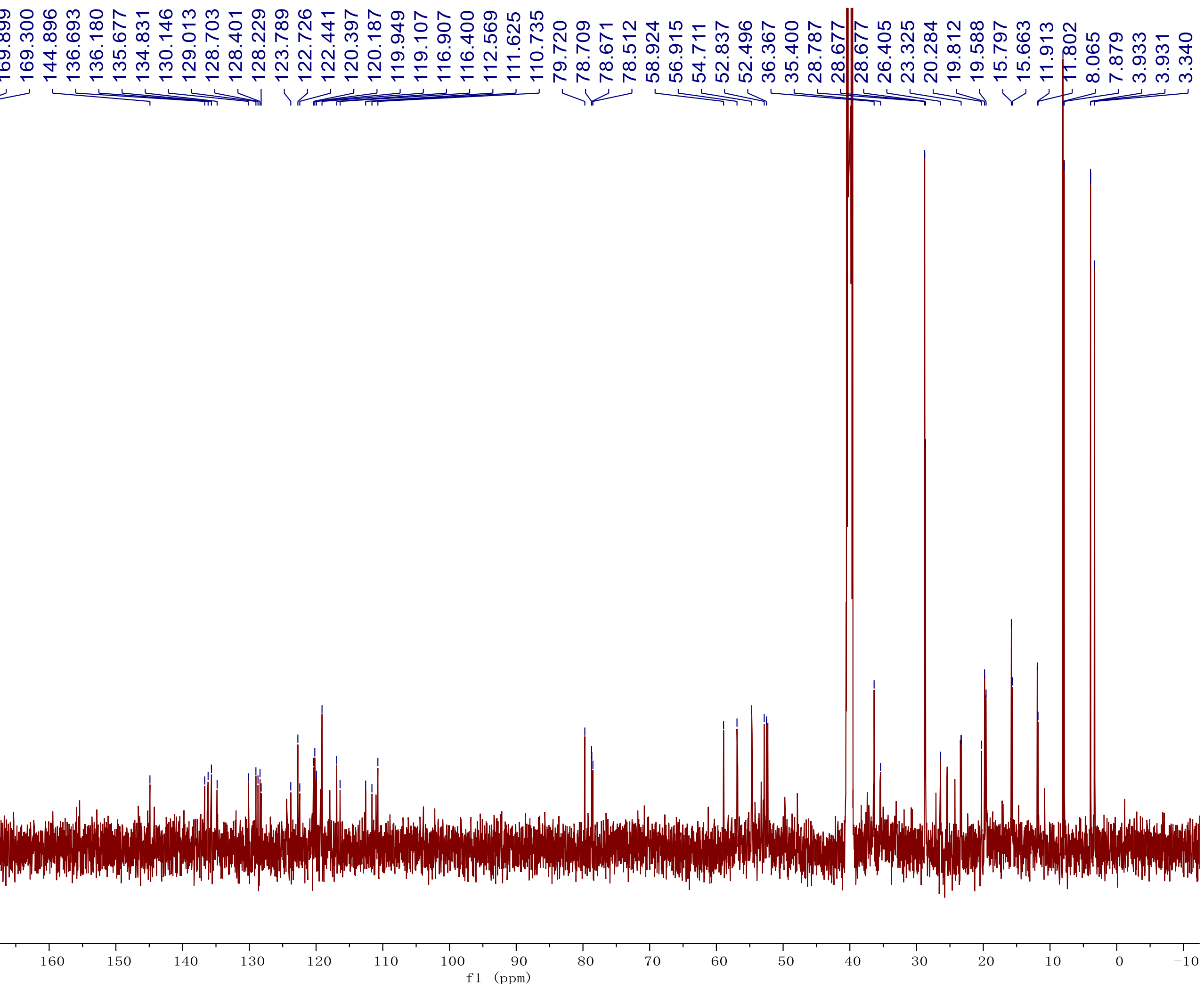

Compound 48r ^{1}H NMR (600 MHz, $CDCl_3$)

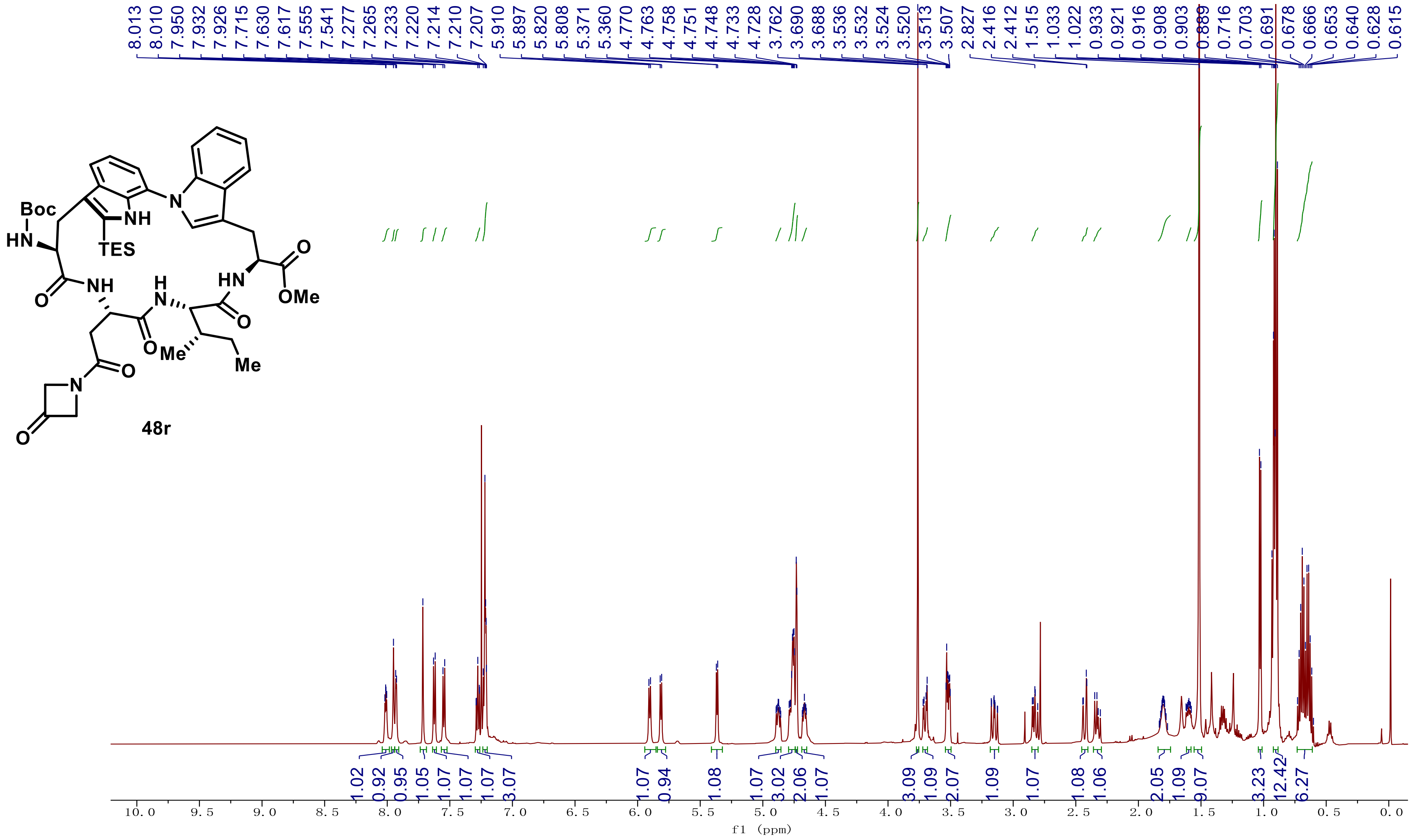

Compound 48r ^{13}C NMR (151 MHz, $CDCl_3$)

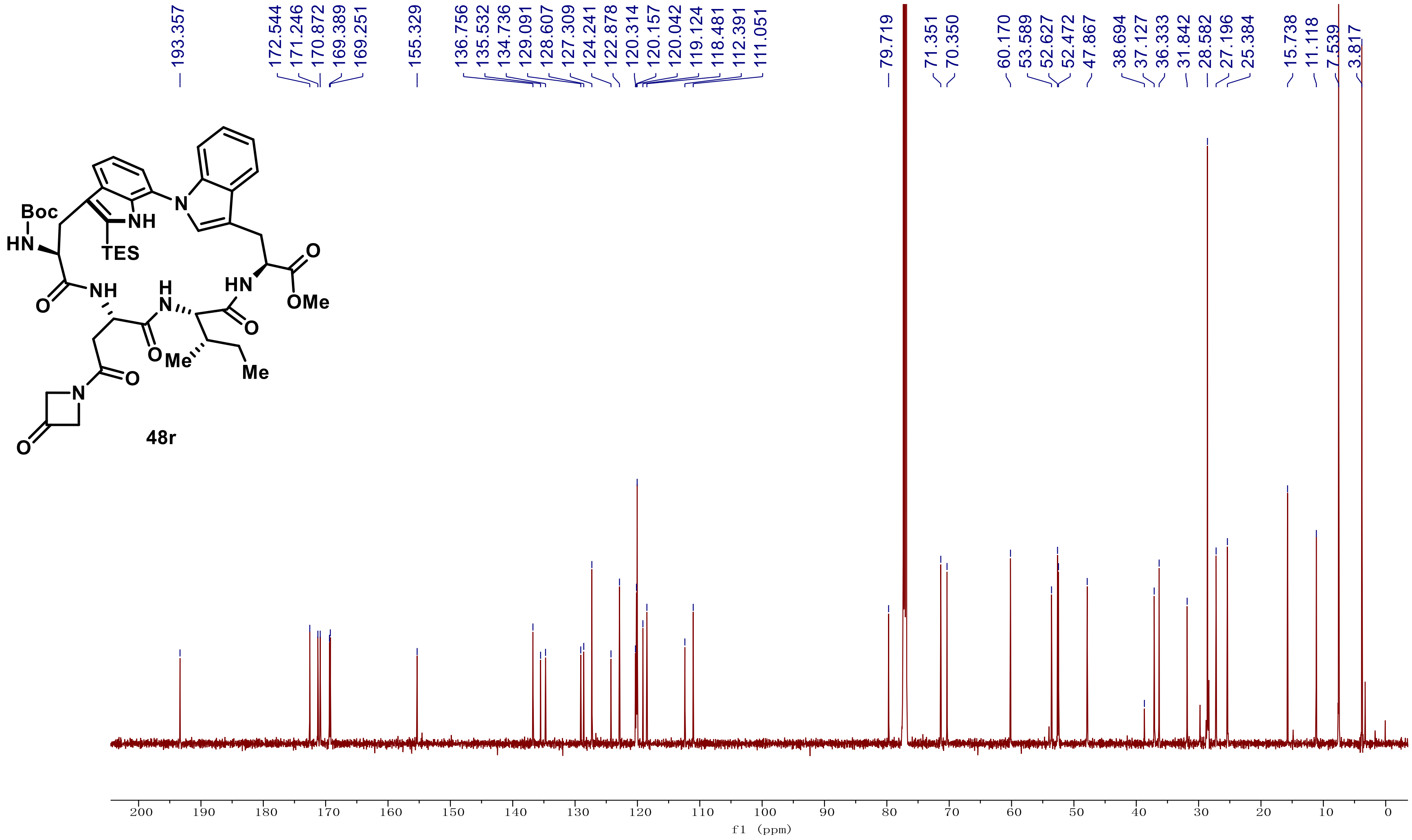

Compound 48s ^{1}H NMR (600 MHz, CDCl₃, rt)

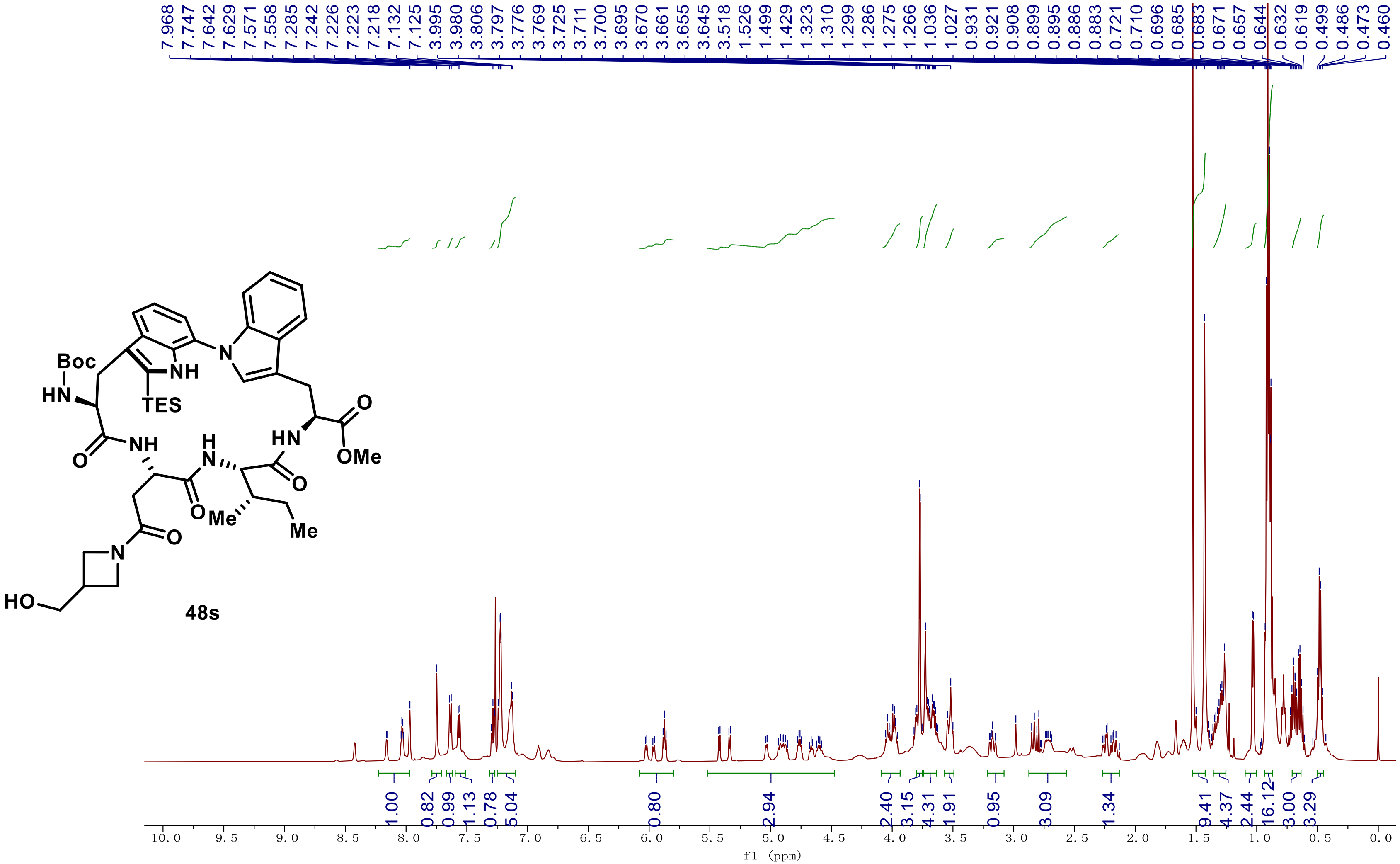

Compound 48s ^{13}C NMR (151 MHz, CDCl$_3$, rt)

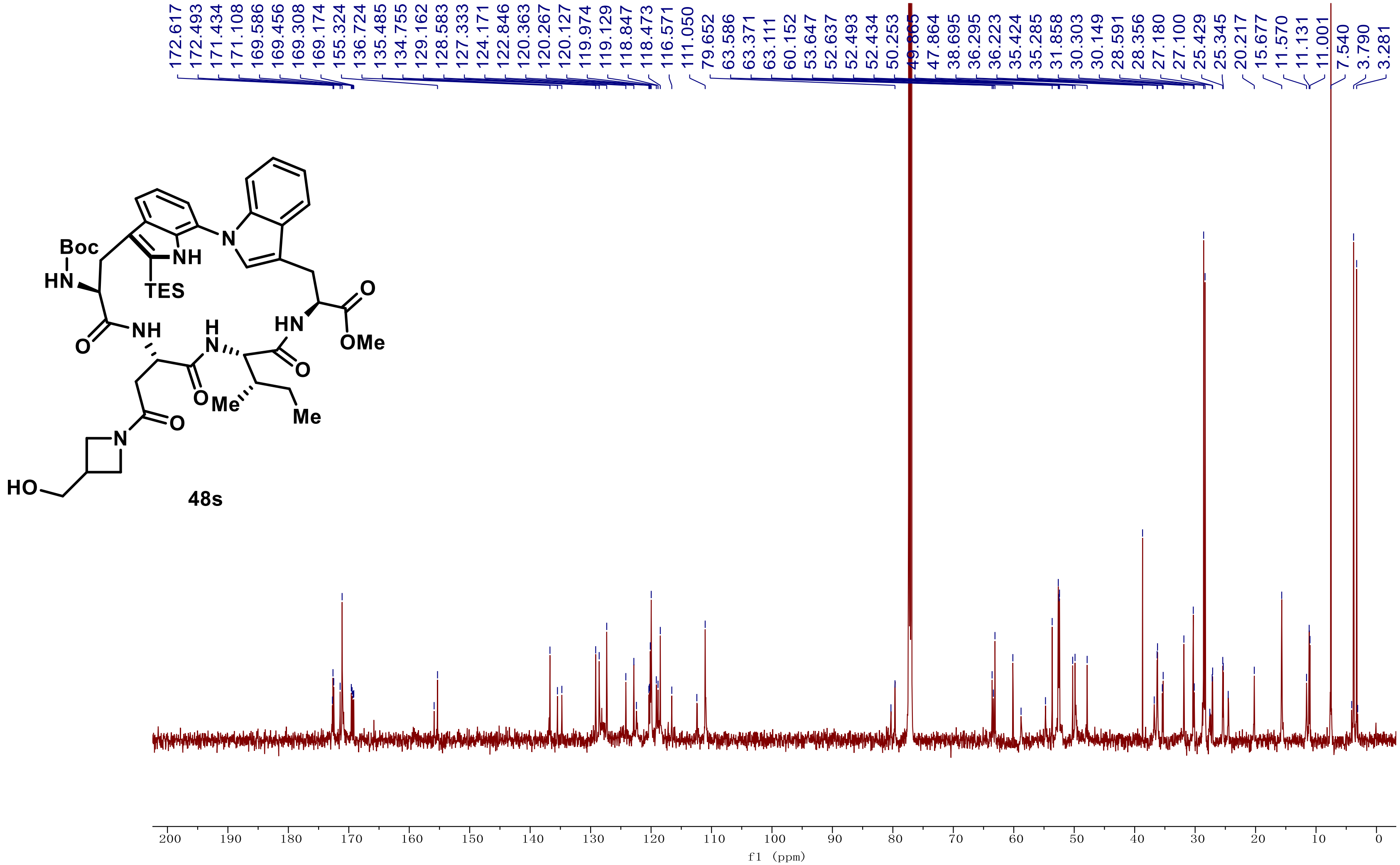

48s

Compound 48s ^{1}H NMR (400 MHz, DMSO-d_6, rt)

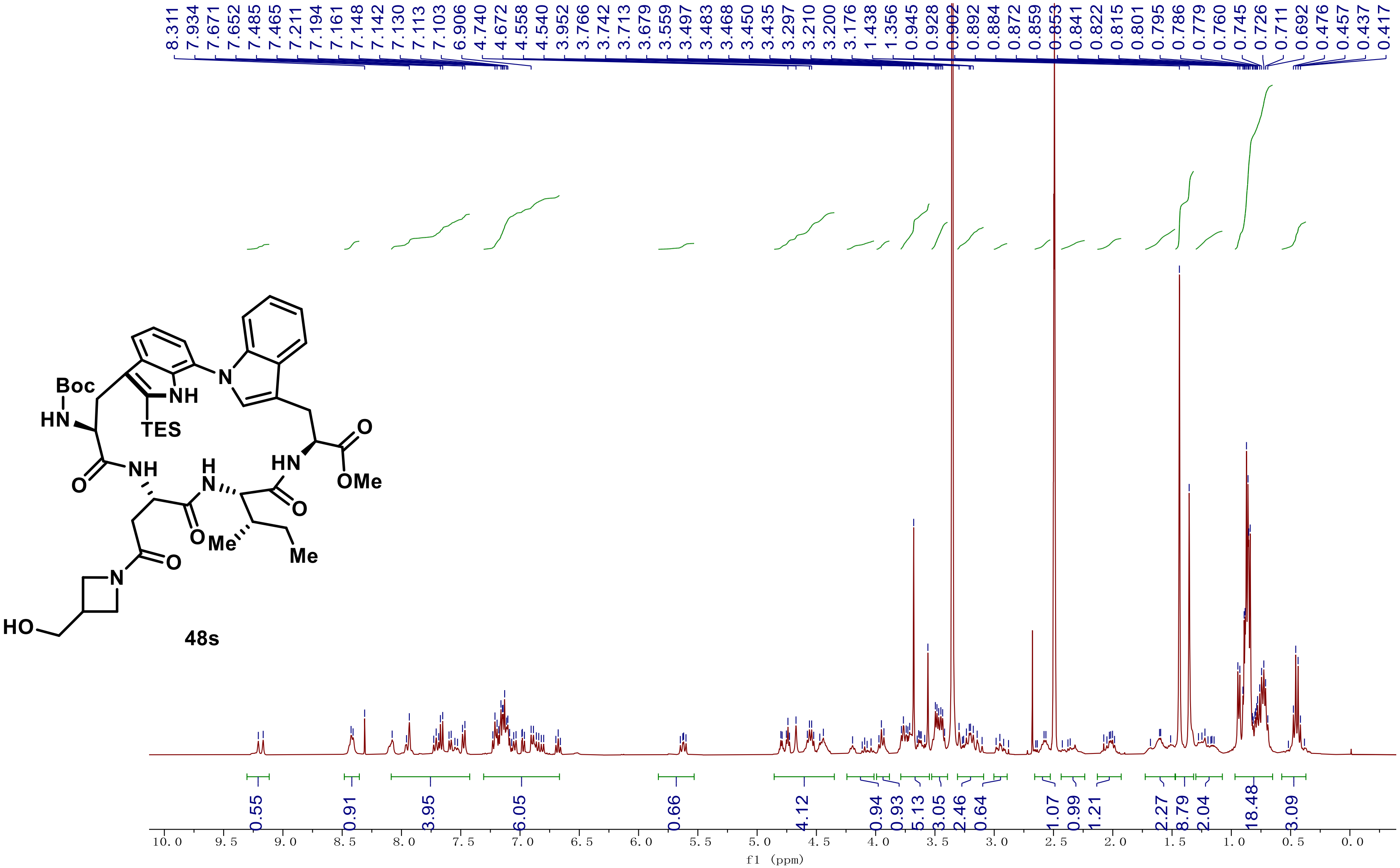

Compound 48s ^{1}H NMR (400 MHz, DMSO-d_6, 60 °C)

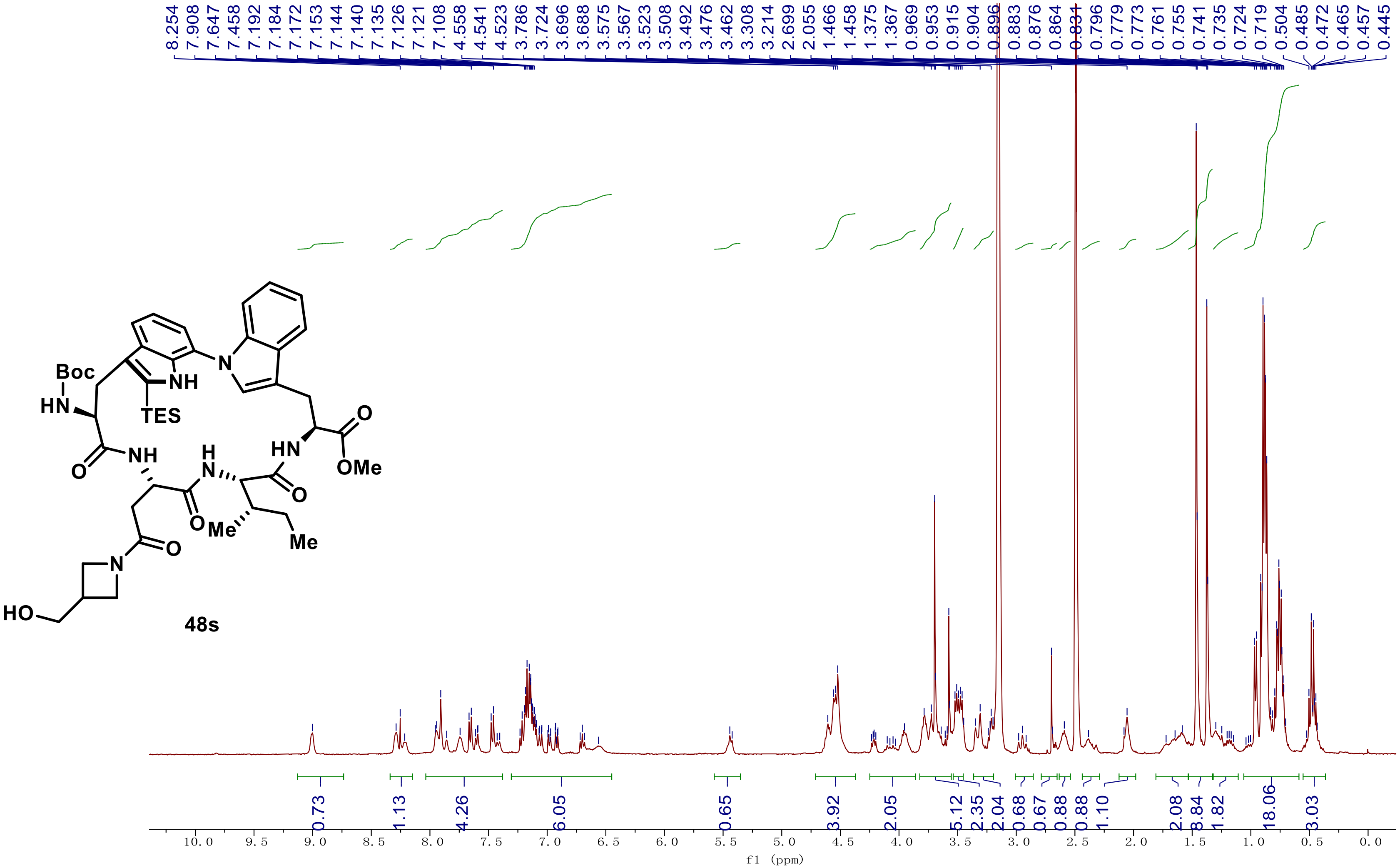

Compound 48s ^{13}C NMR (101 MHz, DMSO-d_6, rt)

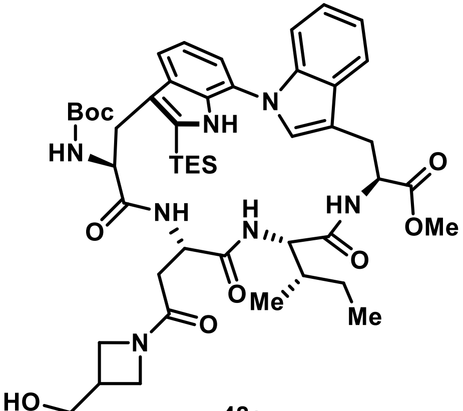

48s

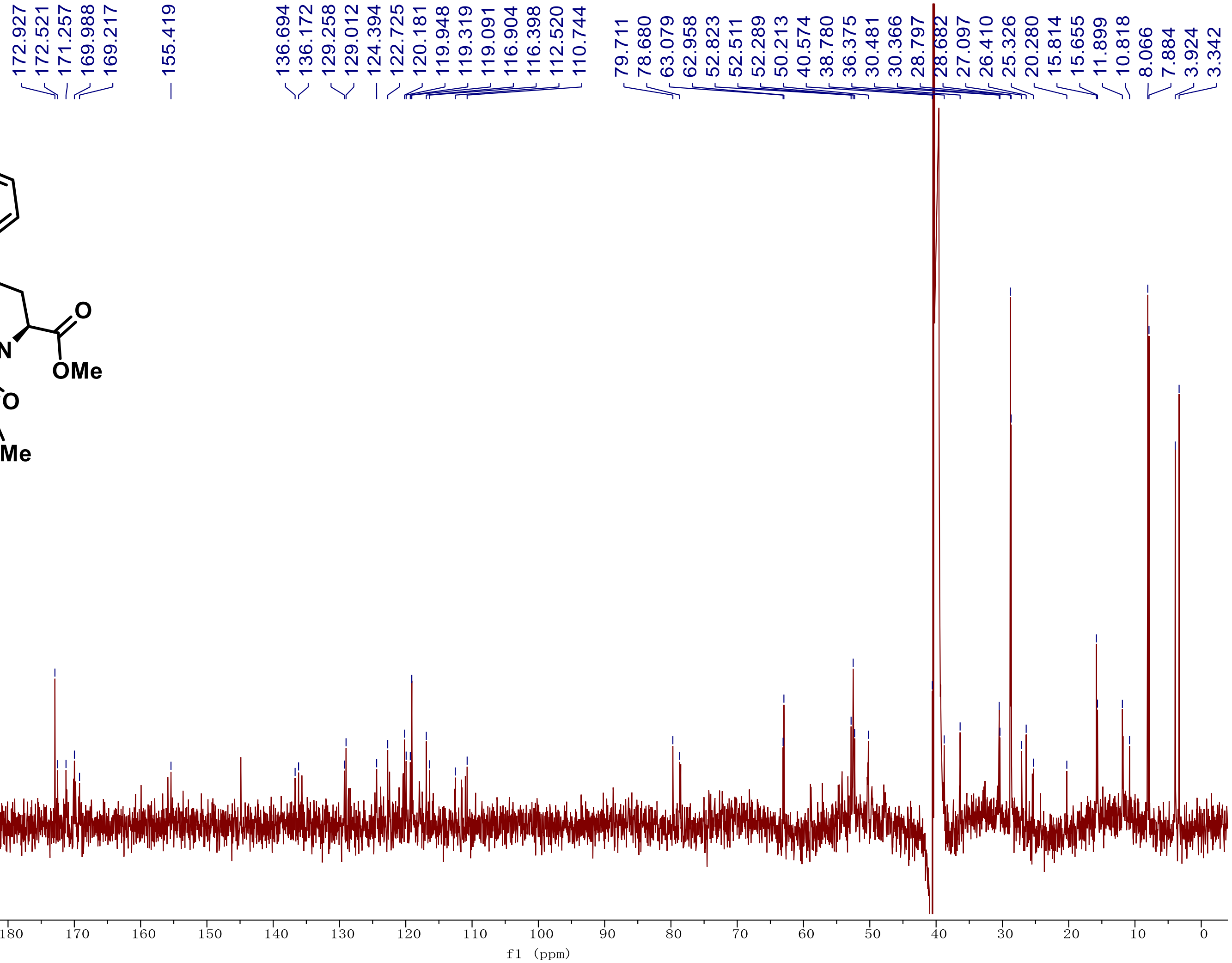

Compound 48t ^{1}H NMR (600 MHz, $CDCl_3$)

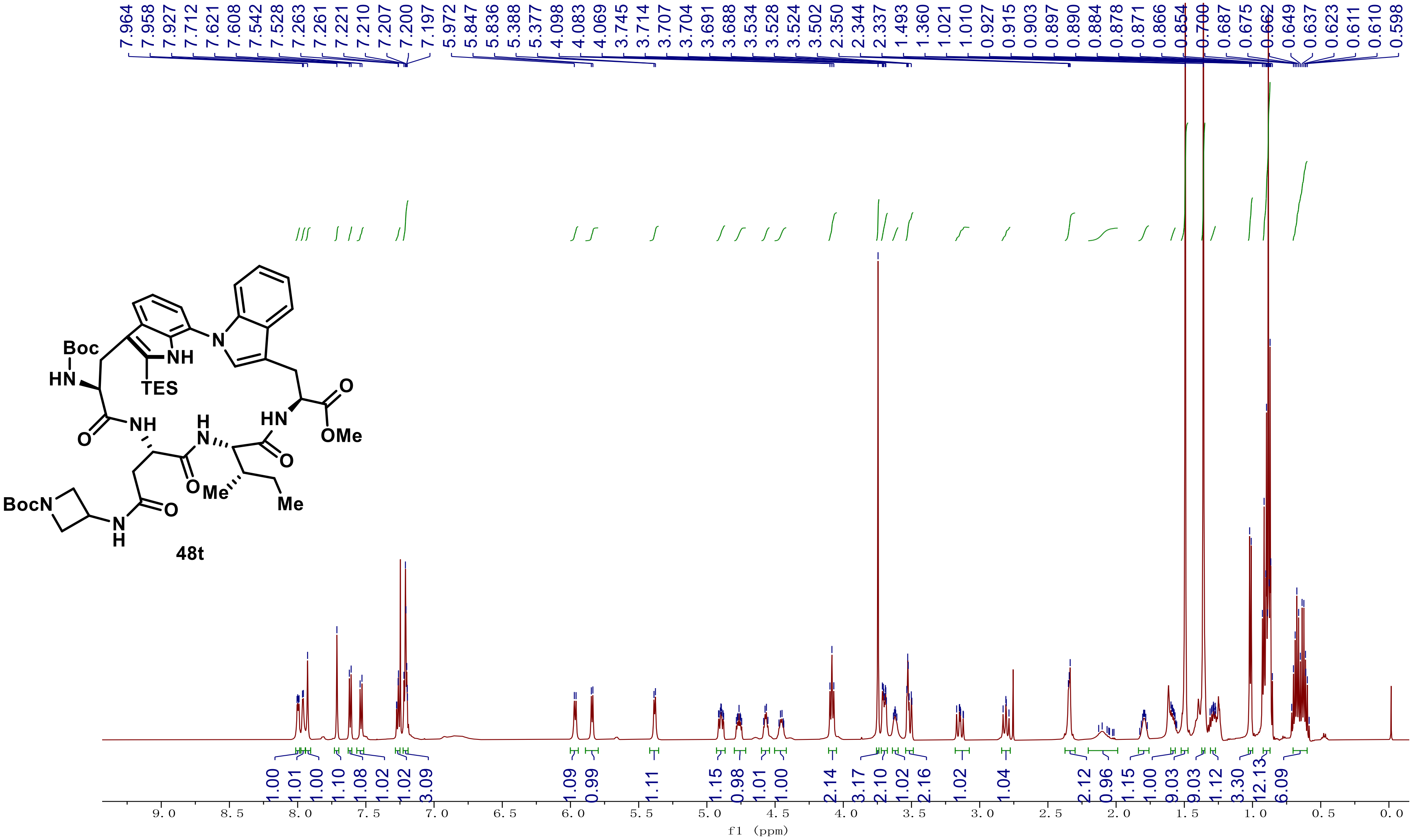

Compound 48t ^{13}C NMR (151 MHz, CDCl$_3$)

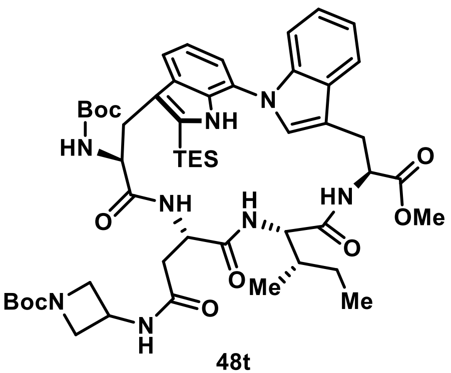

48t

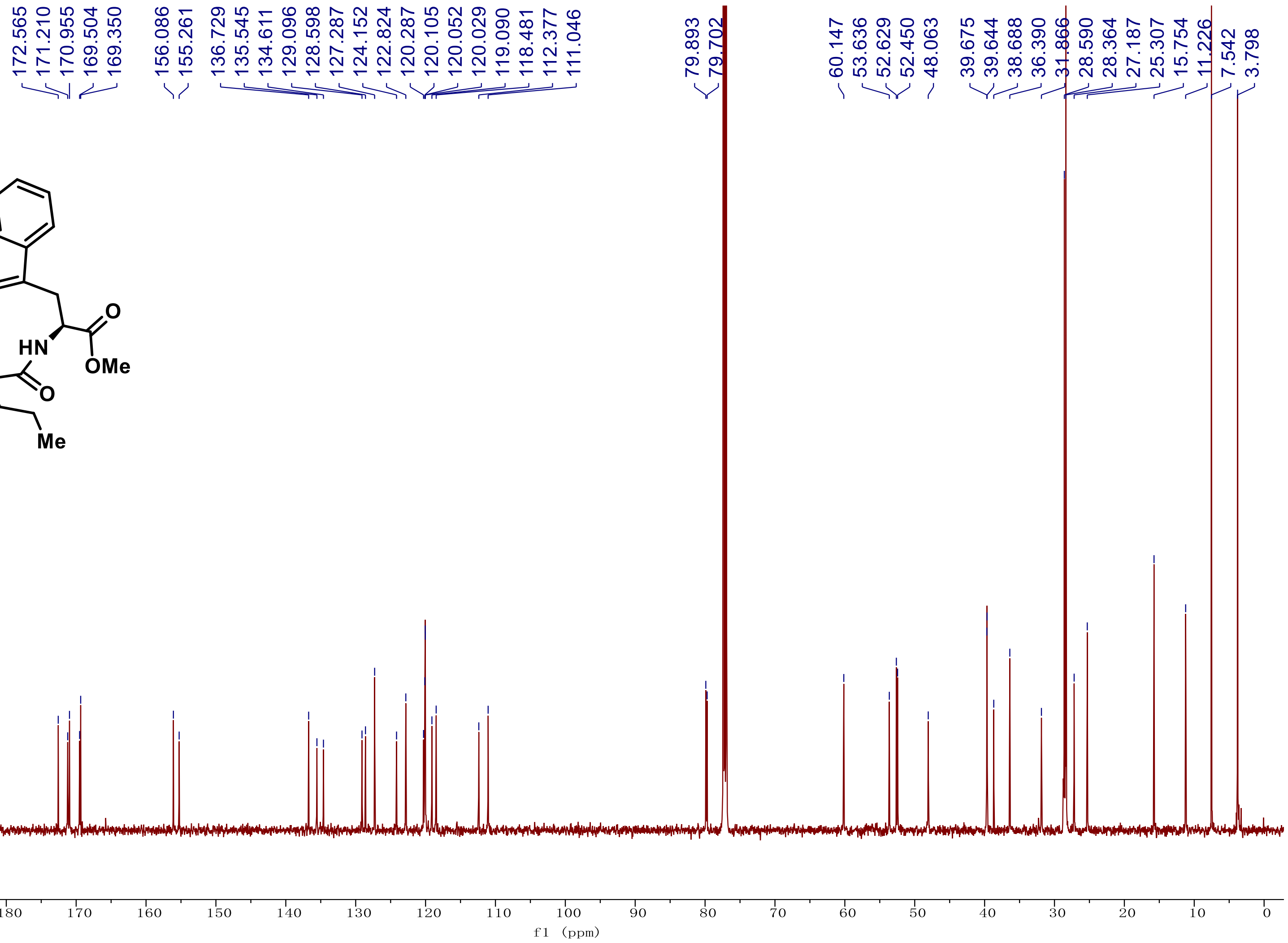

Compound 48u ^{1}H NMR (600 MHz, CDCl₃, rt)

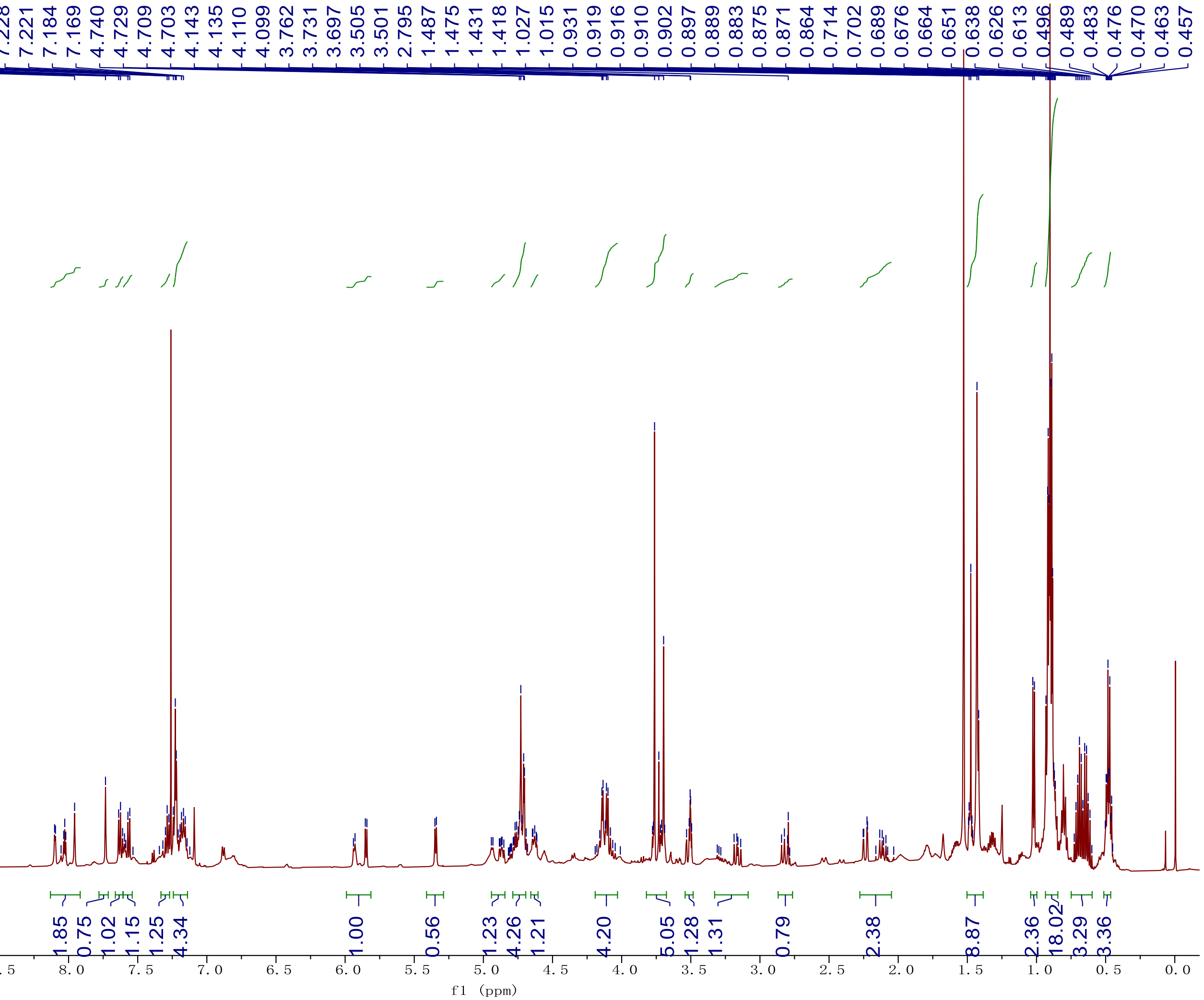

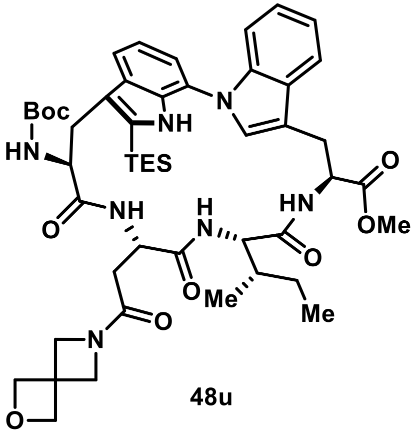

48u

Compound 48u ^{13}C NMR (151 MHz, $CDCl_3$, rt)

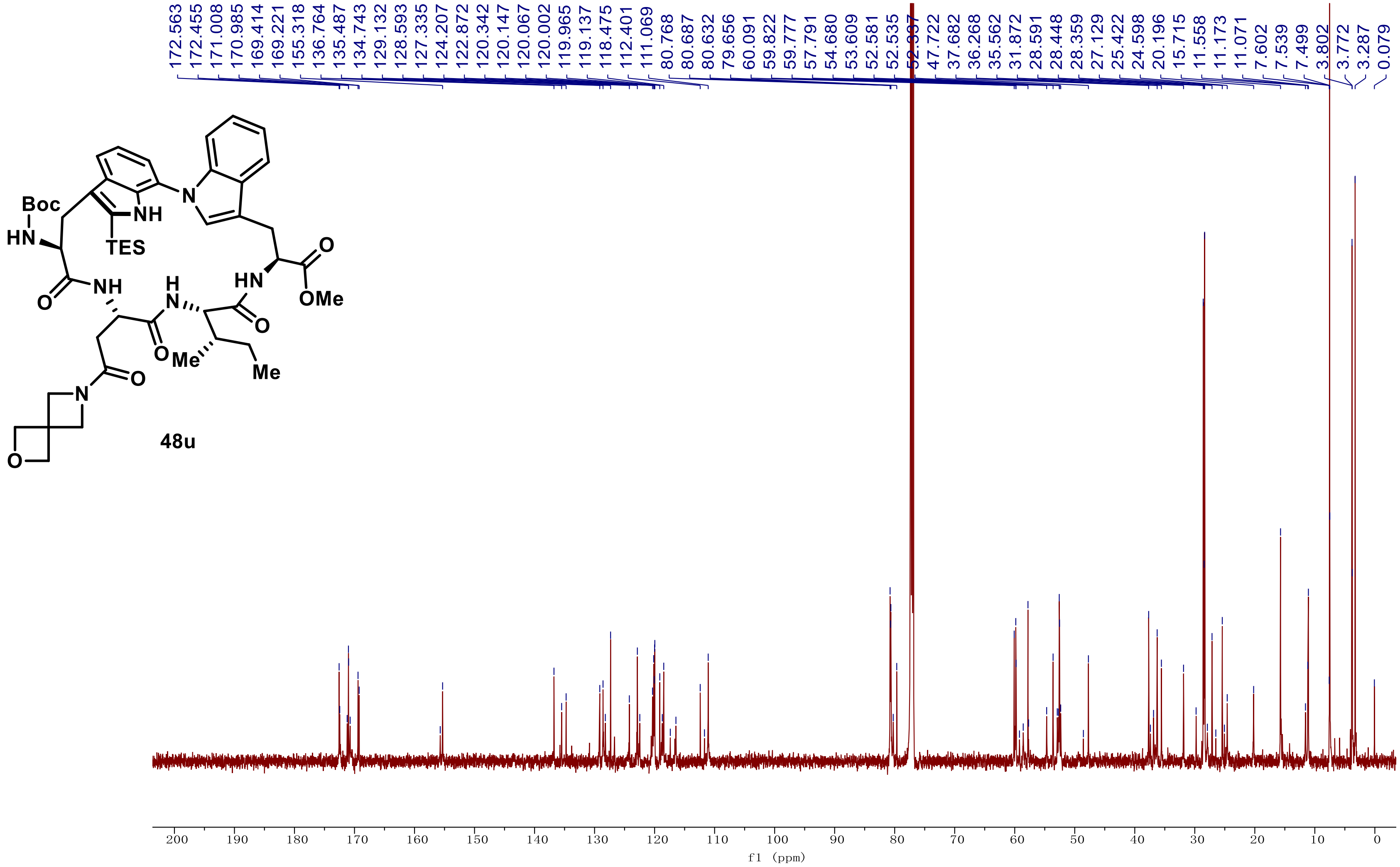

Compound 48u ^{1}H NMR (400 MHz, DMSO-d_6, rt)

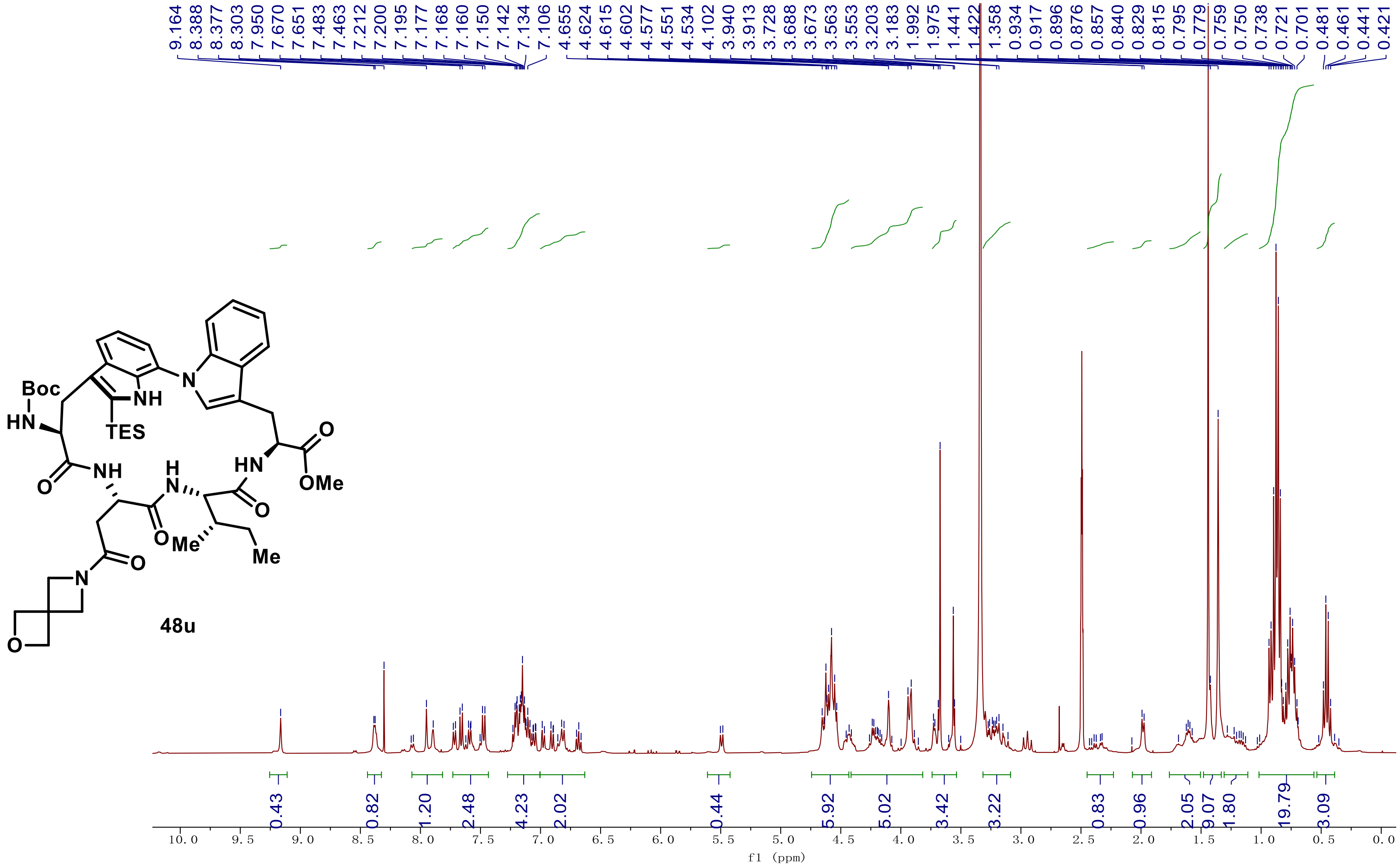

Compound 48u ^{1}H NMR (400 MHz, DMSO-d_6, 60 °C)

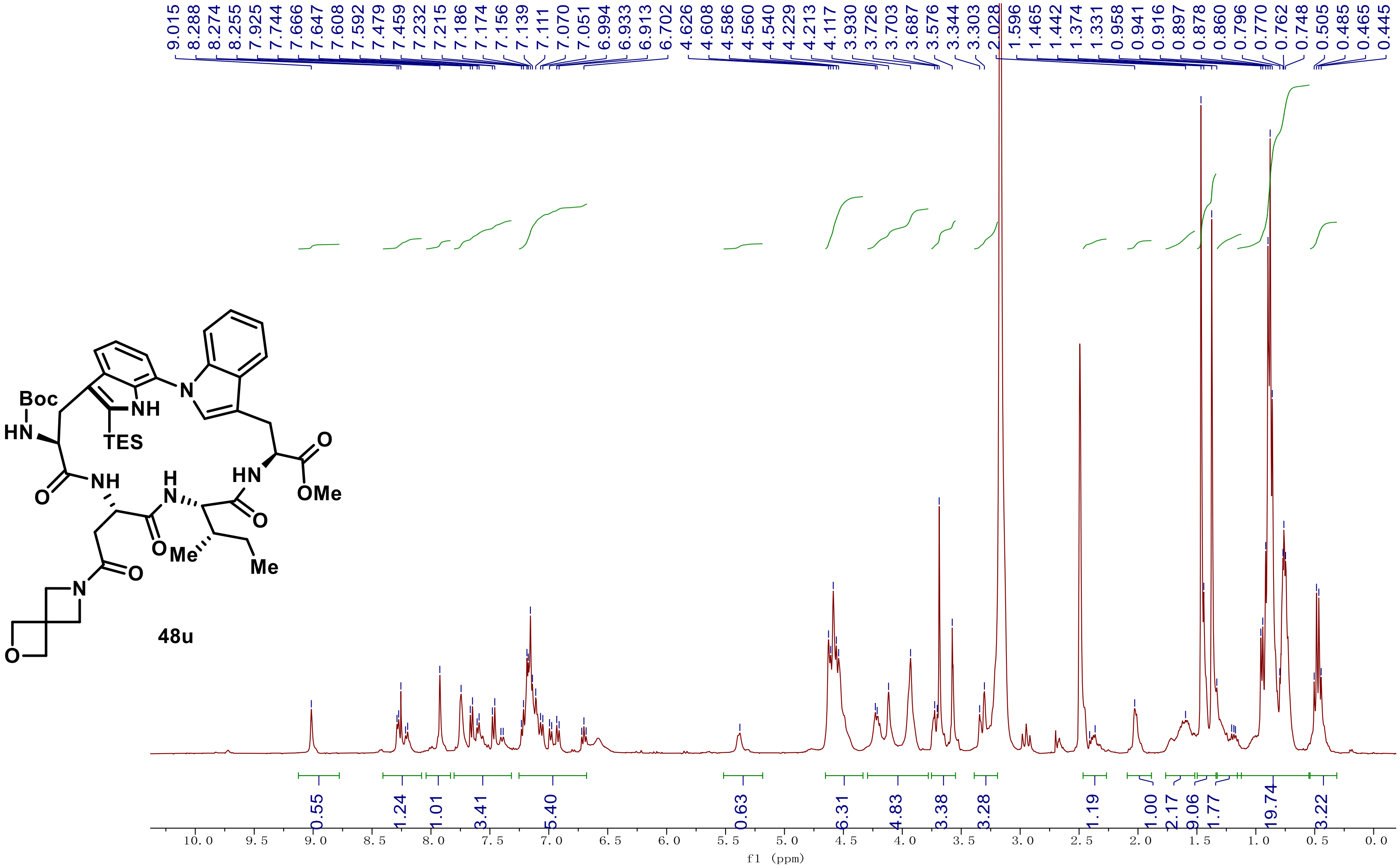

48u

Compound 48u ^{13}C NMR (101 MHz, DMSO-d_6, rt)

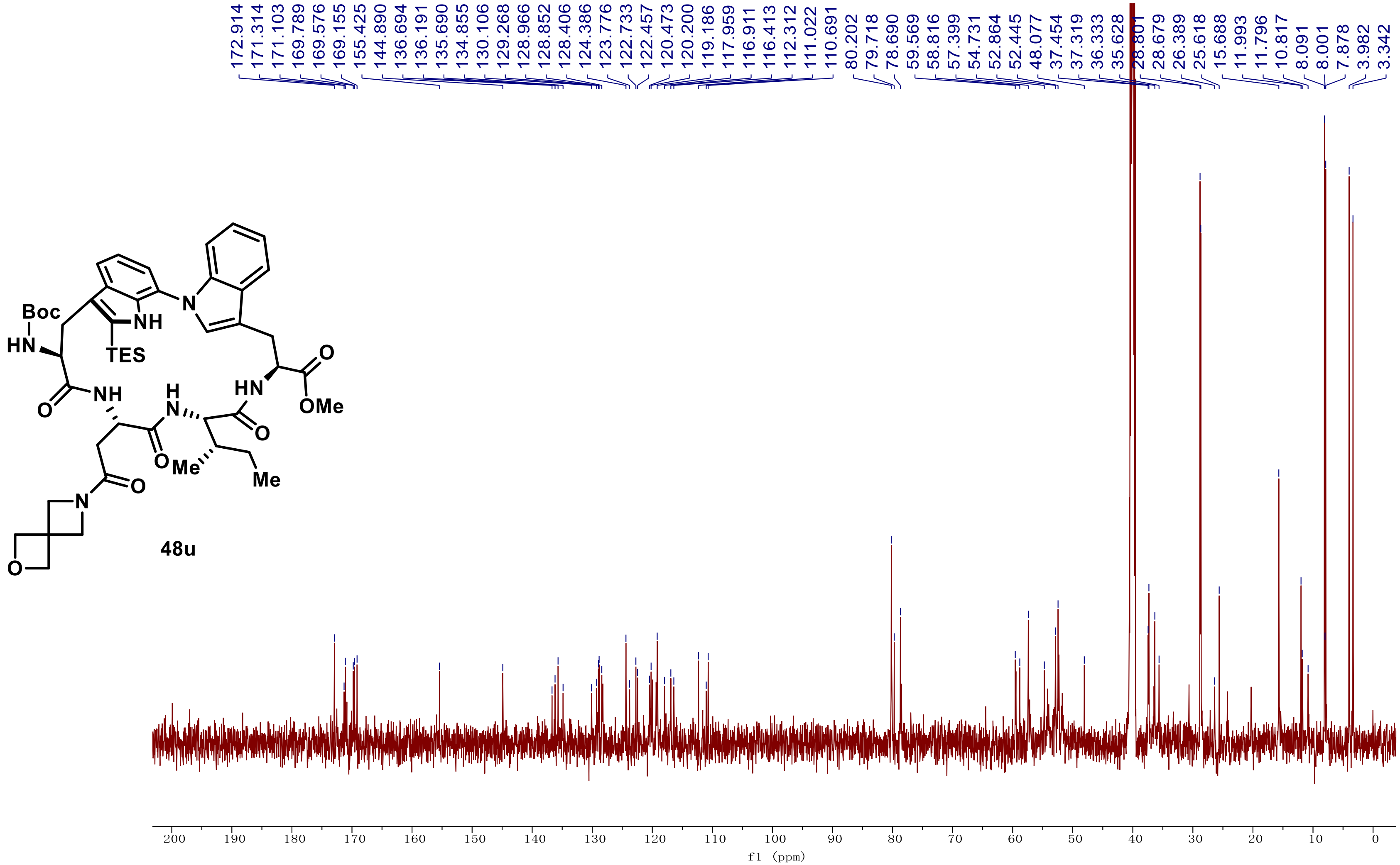

Compound 48v ^{1}H NMR (400 MHz, CDCl₃)

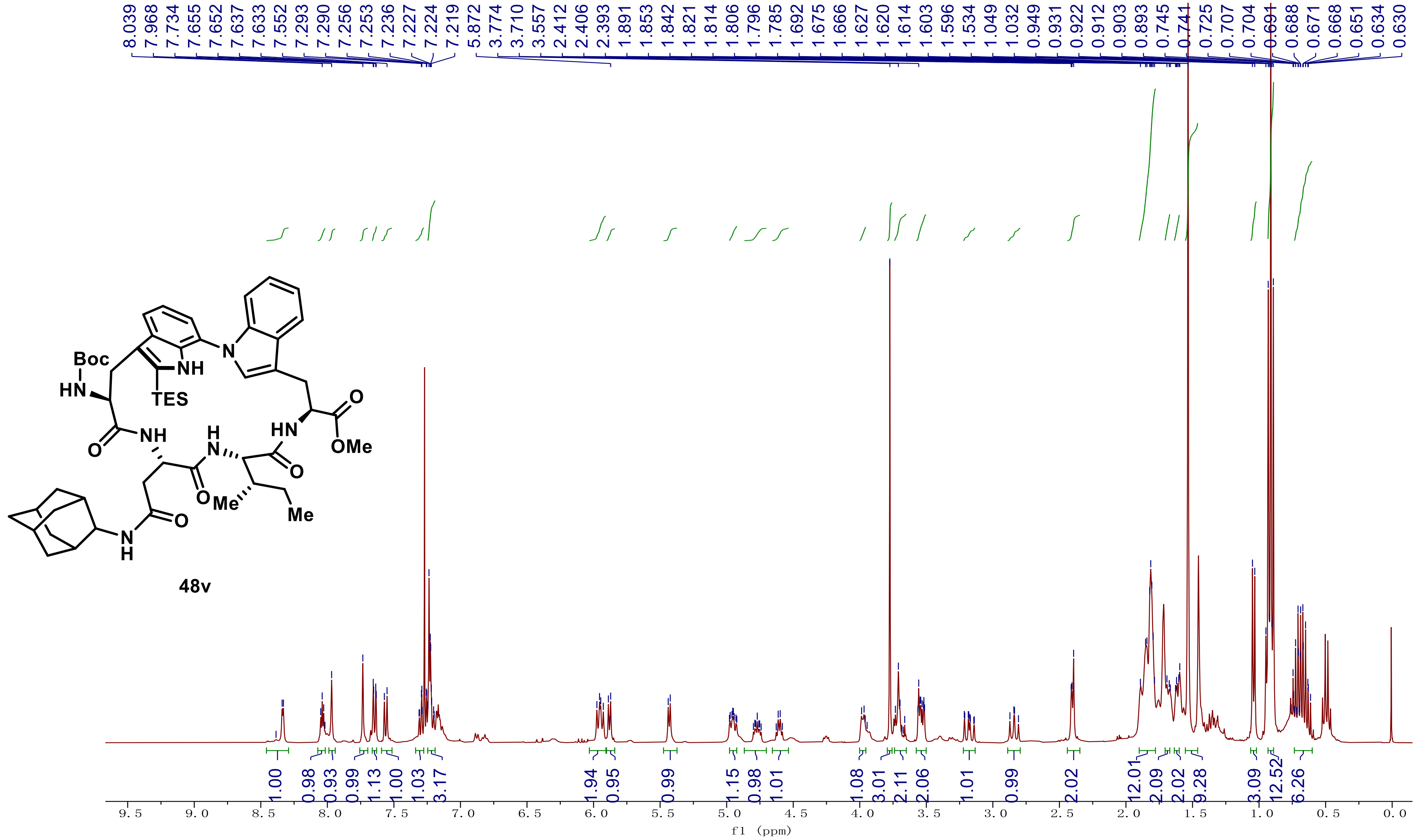

Compound 48v ^{13}C NMR (151 MHz, $CDCl_3$)

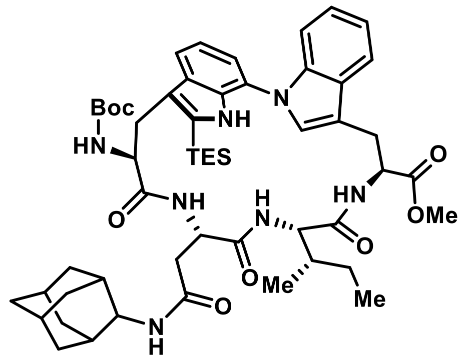

48v

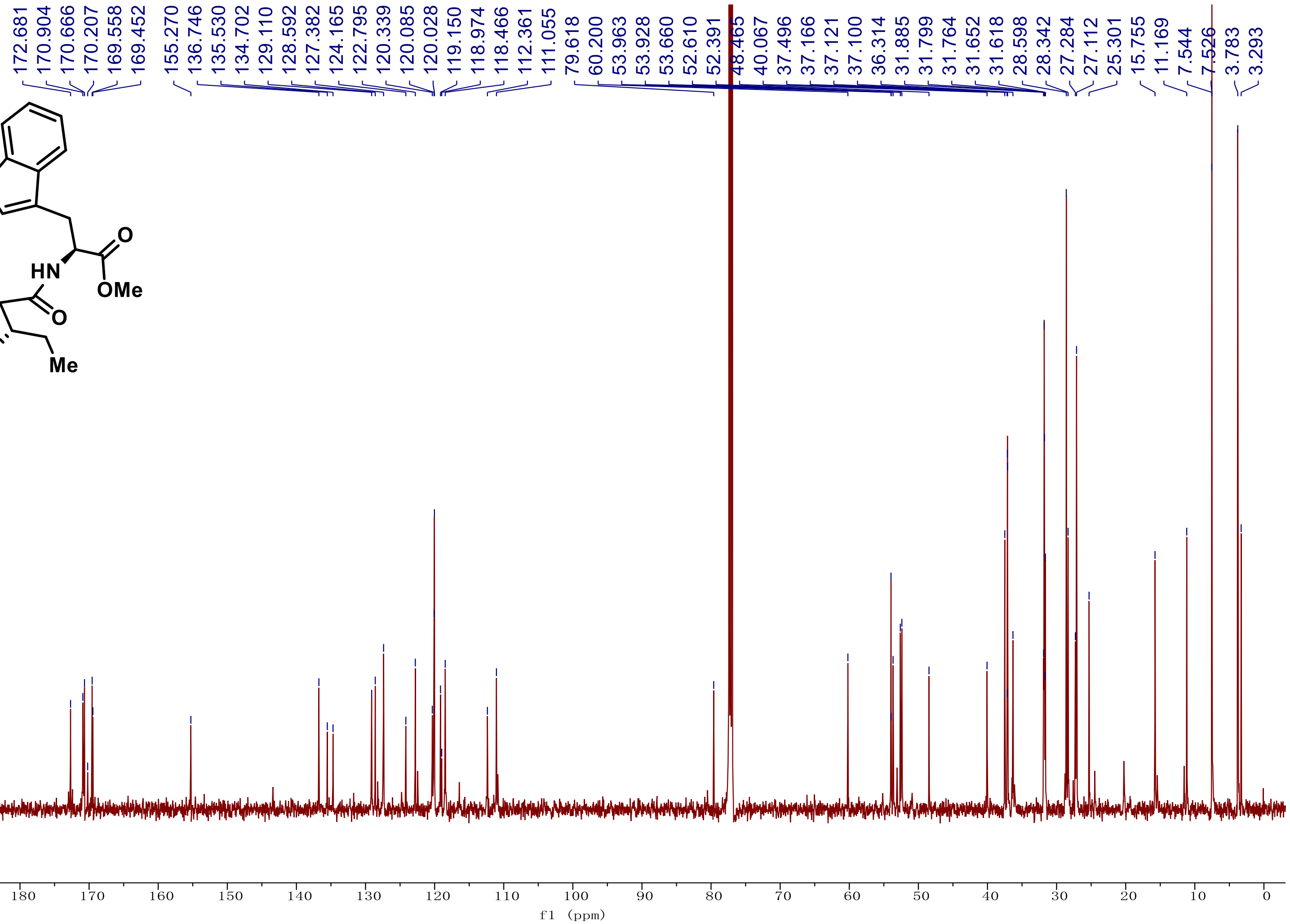

Compound 48w ^{1}H NMR (600 MHz, $CDCl_3$, rt)

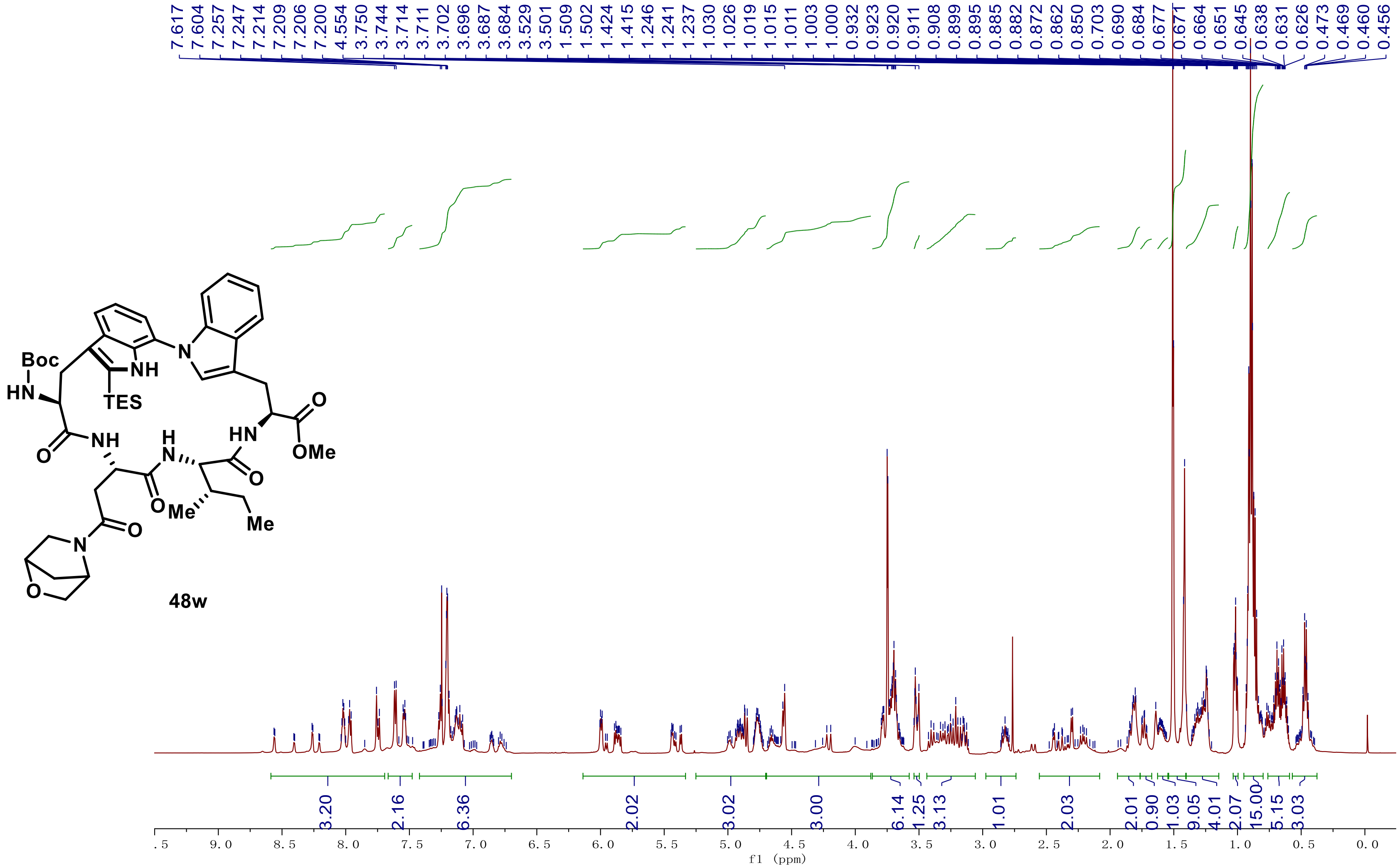

Compound 48w ^{13}C NMR (151 MHz, $CDCl_3$, rt)

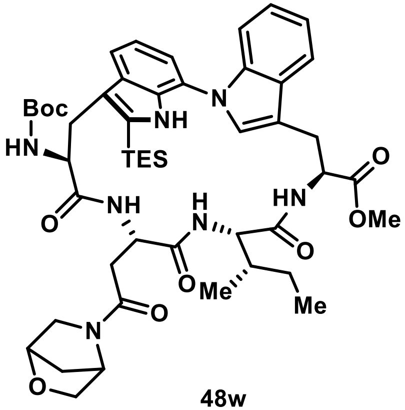

48w

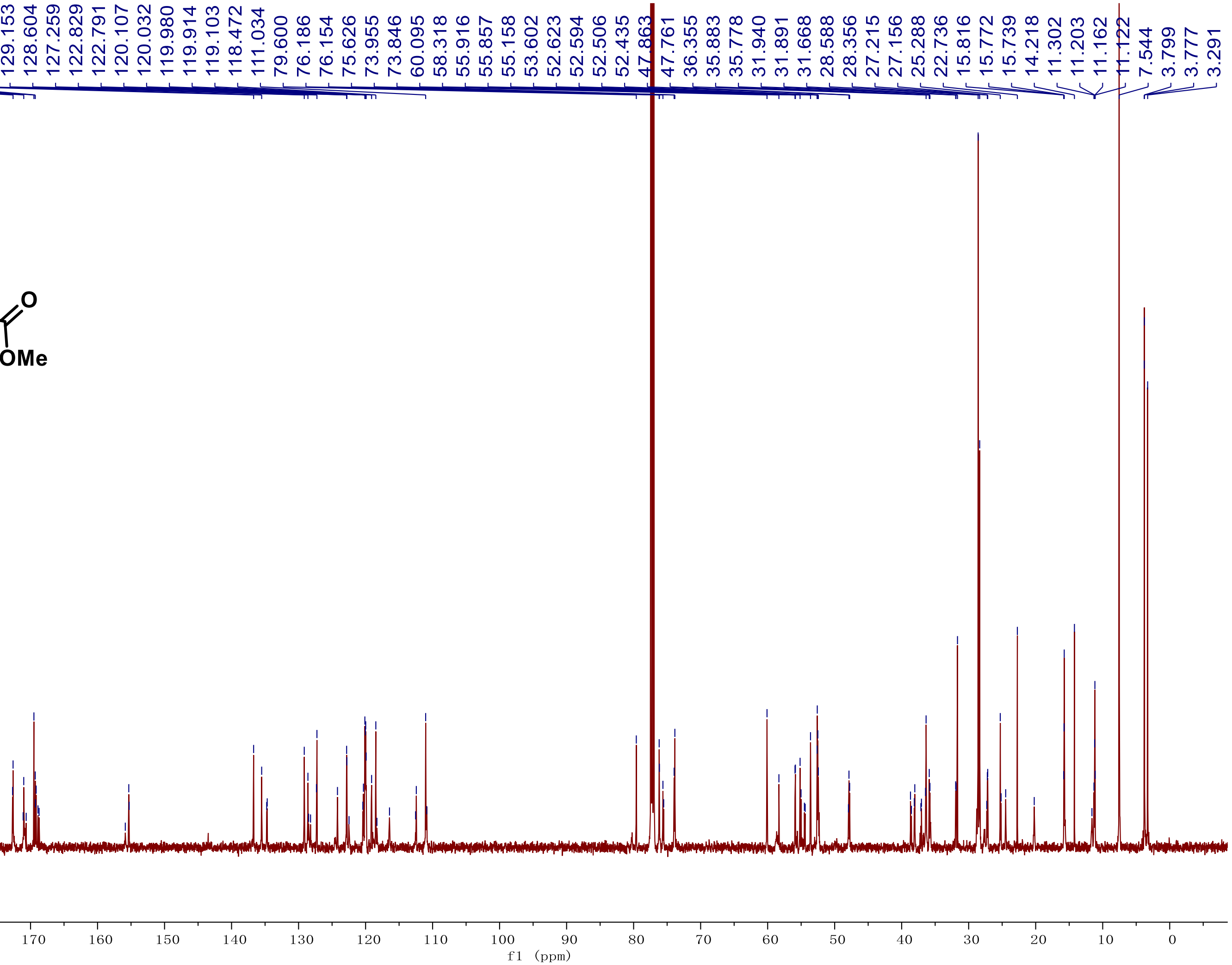

Compound 48w ^{1}H NMR (400 MHz, DMSO-d_6, rt)

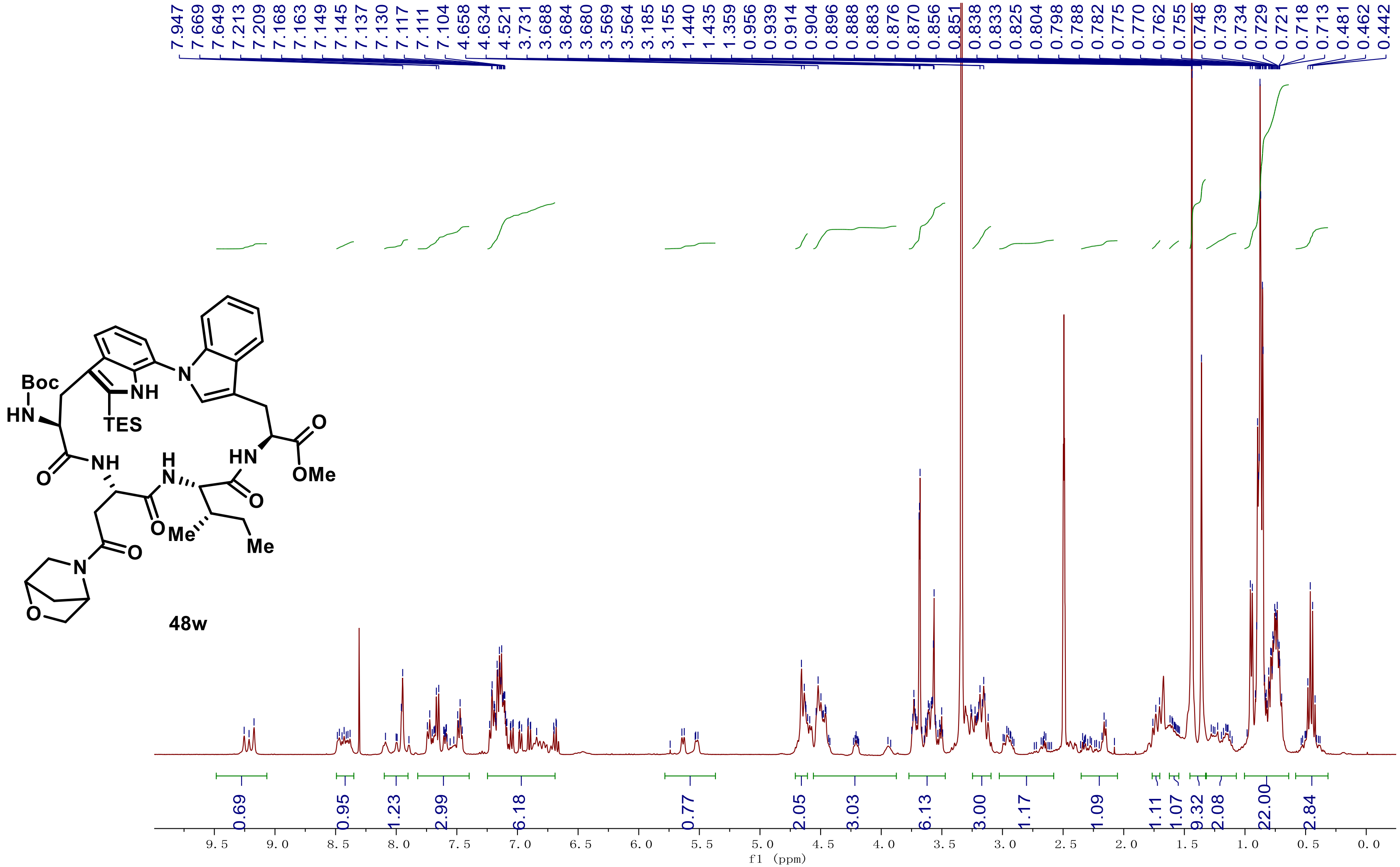

48w

Compound 48w ^{1}H NMR (400 MHz, DMSO-d_6, 60 °C)

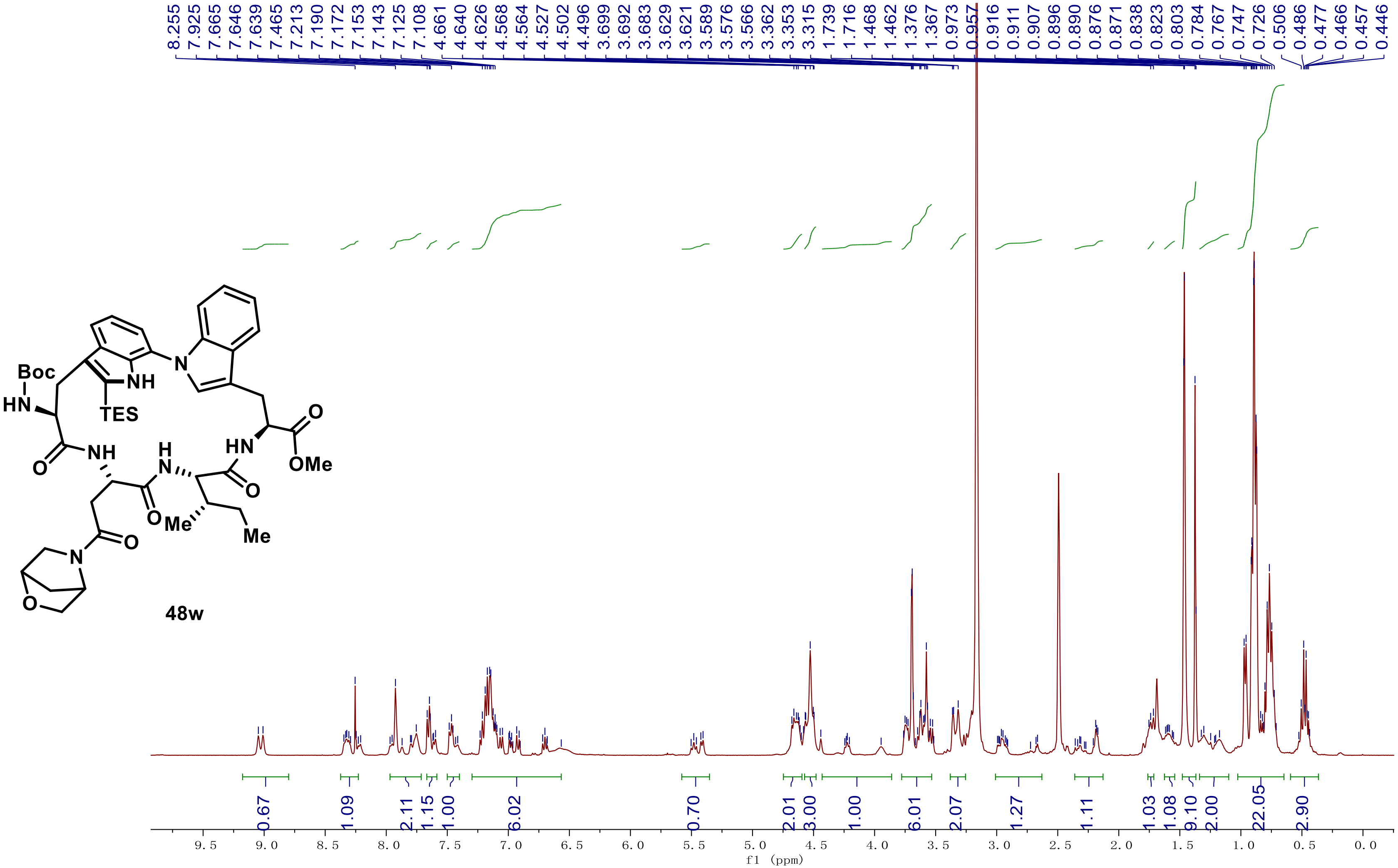

48w

Compound 48w ^{13}C NMR (101 MHz, DMSO-d_6, rt)

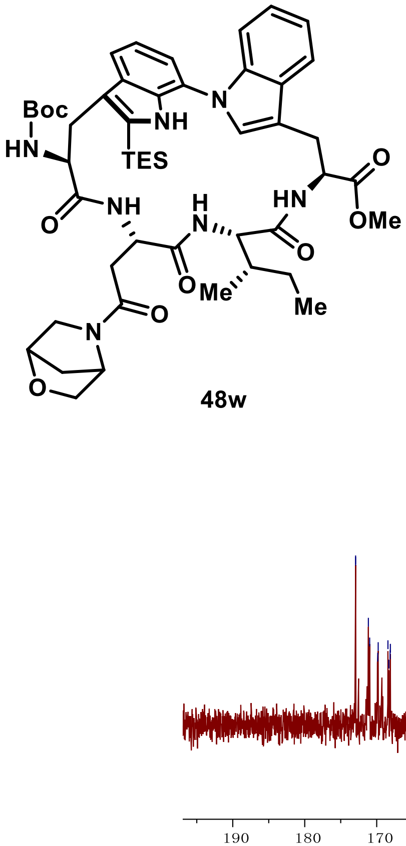

48w

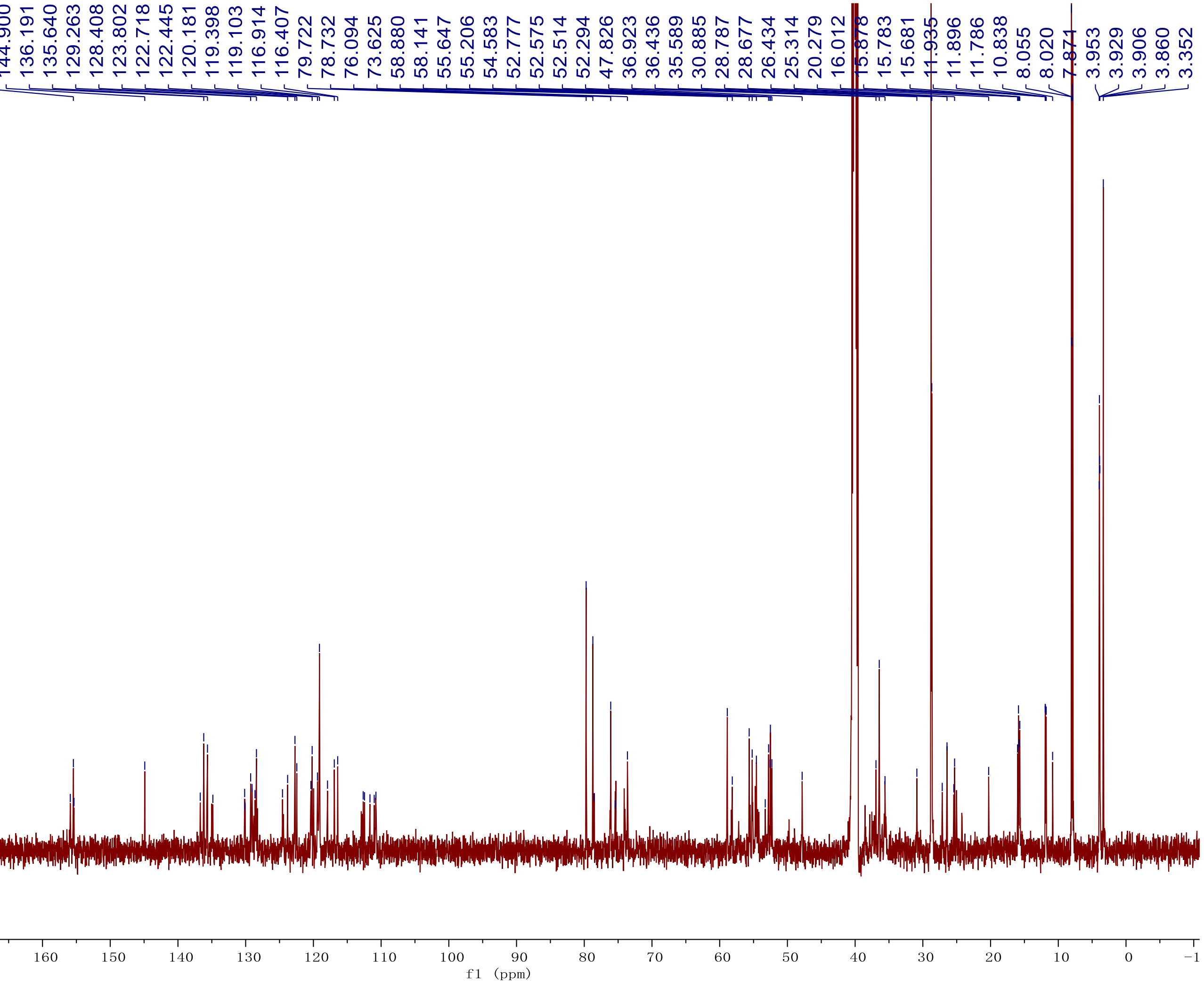

Compound 48x ^{1}H NMR (400 MHz, $CDCl_3$)

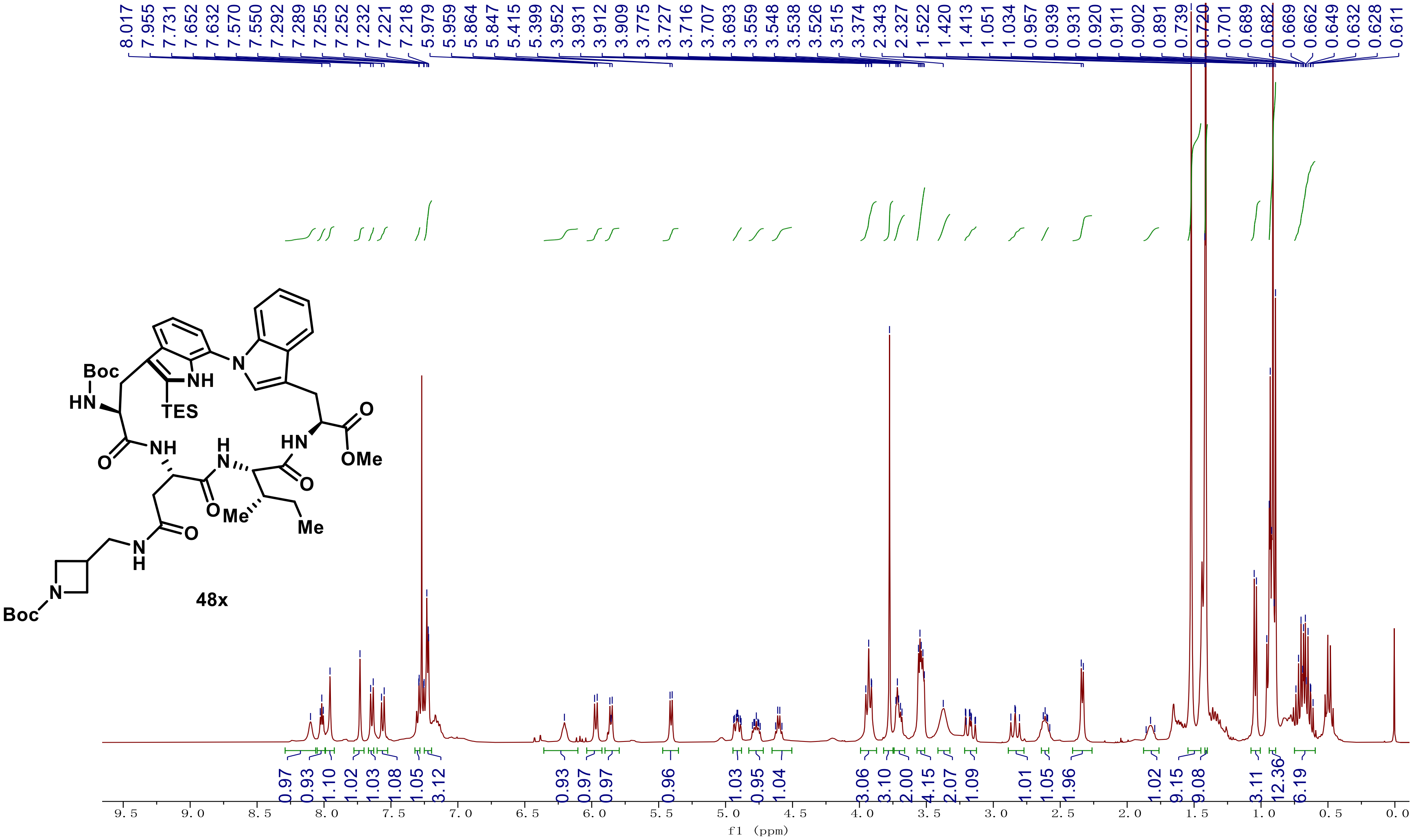

Compound 48x ^{13}C NMR (151 MHz, CDCl$_3$)

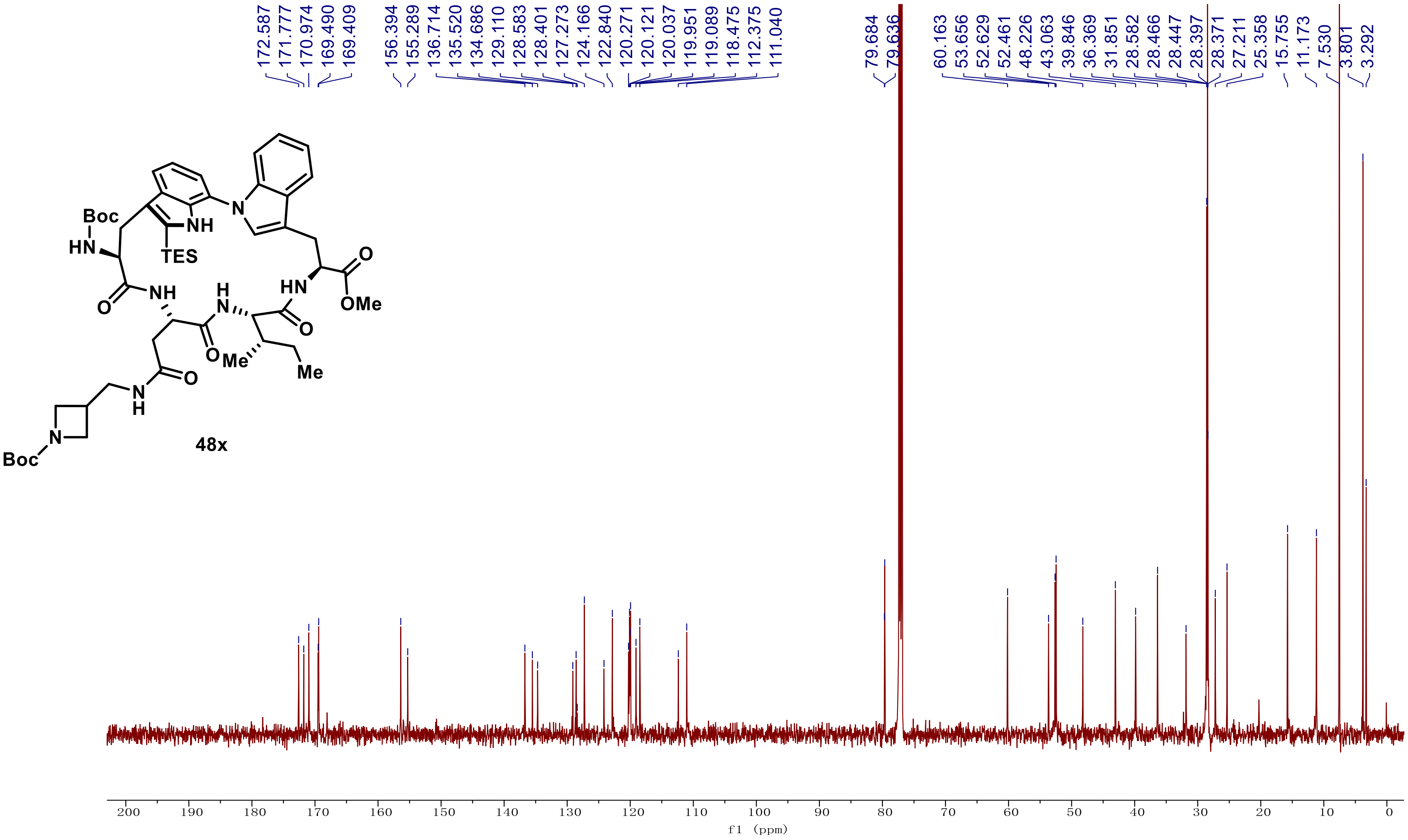

Supplement: Supplementary file 1 [file ja6c05294_si_001.pdf]
