# Supplementary material for: Unified Access to Biaryl-Bridged Linkages Unlocks Structural Diversification of Noncanonical Cyclic Peptides
Source: J Am Chem Soc. 2026 Jun 29;148(27):28553–72. doi: 10.1021/jacs.6c05294 (PMC13383641; doi:10.1021/jacs.6c05294)
Supplement: Supplementary file 2 [file ja6c05294_si_002.pdf]

SUPPORTING INFORMATION

Unified Access to Biaryl-Bridged Linkages Unlocks Structural Diversification of Noncanonical Cyclic Peptides

Longhui Yu, [§] Jie Zhang, [§] Xinwei Zhang, [§] Xilun Wu, [§] Ruoyu Liu, Chak Hin Au, Hiroshige Ogawa, Rongbiao Tong, * Hugh Nakamura*

Department of Chemistry, The Hong Kong University of Science and Technology,
Clear Water Bay, 999077, Hong Kong SAR, China

Content

General information: 3
Abbreviations 4
Substrate Scope Overview 5
Procedure for the synthesis of 35, 13: 19
Procedure for the synthesis of 25: 23
The screening of the e-chem reaction condition: 25
The scope of the e-chem reaction: 26
Larock macrocyclization of unnatural scaffold： 37
General procedure E for the synthesis of 19： 43
General procedure F for the synthesis of 20 via Larock macrocyclization： 53
General procedure G for the synthesis of 24: 64
General procedure H for the synthesis of 26 via Larock macrocyclization: 74
General procedure I for the synthesis of 28: 84
General procedure J for the synthesis of 29 via Larock macrocyclization： 96
General procedure K for the synthesis of 31: 108
General procedure L for the synthesis of 32 via Larock macrocyclization: 115
Synthesis of extended micitide scaffolds: 122
Synthesis of extended lapparbin scaffolds: 128
General procedure M for the synthesis of S11: 132
General procedure N for the synthesis of 45: 134
General procedure O for the synthesis of 46 via Larock macrocyclization: 146
Synthesis of extended Cihunamide scaffolds: 160
General procedure P for the synthesis of S18: 167
General procedure Q for the hydrolysis and deprotection, the synthesis of 46’, 48’: 170
General procedure R for the synthesis of 48: 173
Biological assay of the cihunamide analogs: 190
References: 196

General information:

All the reagents were purchased from commercial sources (Sigma-Aldrich, TCI, Bide, Energy and Leyan) and were used without further purification unless otherwise stated. The yield refers to column chromatography separation yield unless otherwise stated. All the reactions were monitored by thin layer chromatography (TLC) and LC/MS. TLC was performed using 0.2-0.25 mm silica plates. ^{1}H NMR and ^{13}C NMR spectra were recorded on Bruker AVII 400, and JEOL 600 instruments. The following abbreviations were used to explain multiplicities: s = singlet, d = doublet, t = triplet, q = quartet, m = multiplet, br = broad. Column chromatography was performed using 230-400 mesh silica gel, and PTLC was performed using 0.2-0.25 mm silica plates. High-resolution mass spectra (HRMS) were recorded on an Agilent LC/MSD TOF mass spectrometer by electrospray ionization time of flight reflectron experiments.

Abbreviations

Ac	Acetyl
aq.	Aqueous
Boc	*tert*-Butoxycarbonyl
Bu	Butyl
DCM	Dichloromethane
DIC	*N,N'*-Diisopropylcarbodiimide
DIPEA	*N,N*-Diisopropylethylamine
DMA	*N,N*-Dimethylacetamide
DMF	*N,N*-Dimethylformamide
DMSO	Dimethyl sulfoxide
Et	Ethyl
HATU	Hexafluorophosphate Azabenzotriazole Tetramethyl Uronium
HRMS	High Resolution Mass Spectrometry
LCMS	Liquid Chromatograph Mass Spectrometer
Me	Methyl
NHPI	*N*-Hydroxyphthalimide
NMP	*N*-Methyl-2-pyrrolidone
NMR	Nuclear Magnetic Resonance Spectroscopy
Ph	Phenyl
PPh_3	Triphenylphosphine
PTLC	Preparative Thin Layer Chromatography
RVC	Reticulated Vitreous Carbon
TES	Triethylsilyl
TFA	Trifluoroacetic acid
THF	Tetrahydrofuran
TLC	Thin Layer Chromatography

Substrate Scope Overview

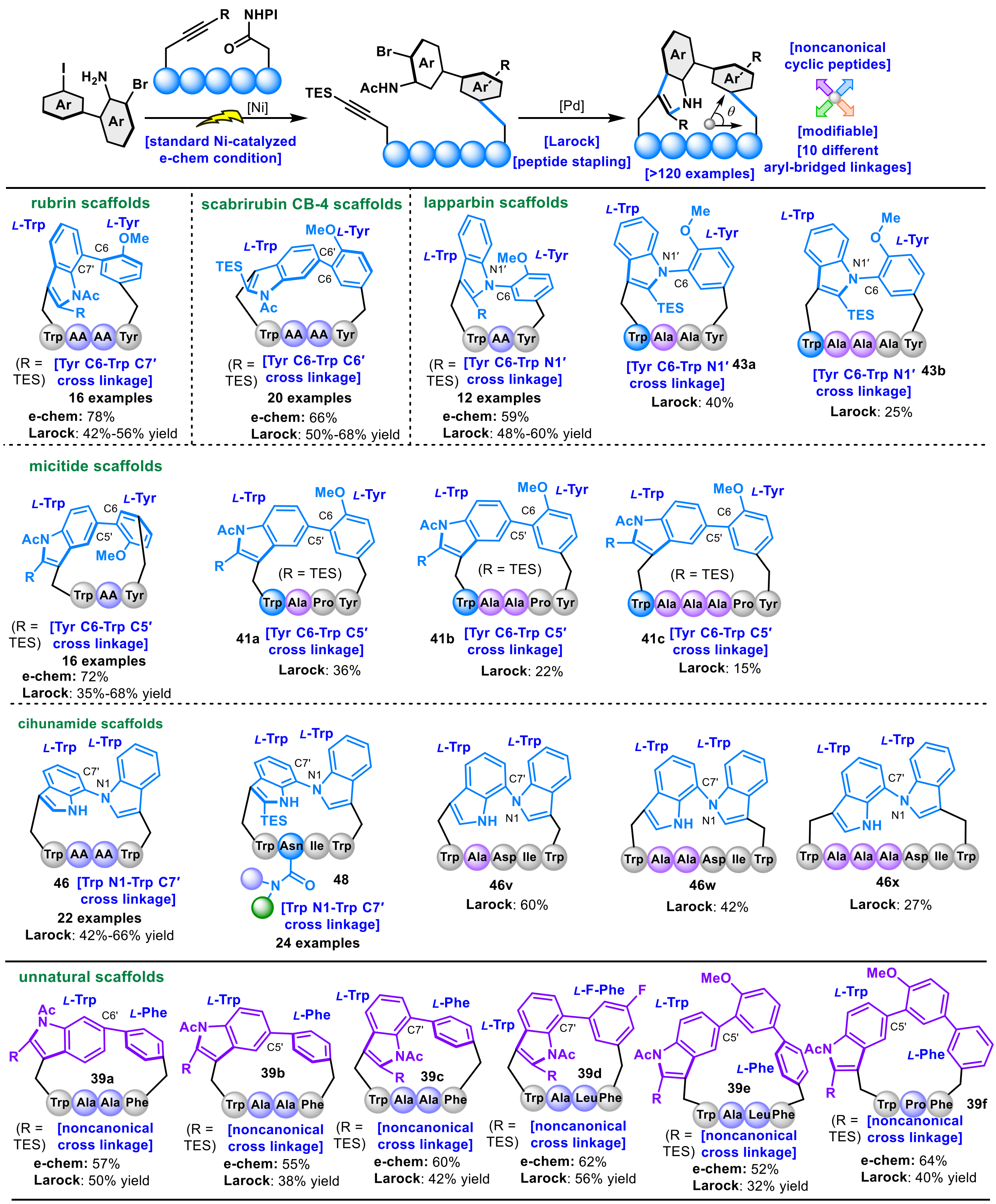

Figure S1. Ultimate summary of the substrate scope.

Functional Group Compatibility of the Larock Cyclization

A Additionally assessed functional group compatibility.

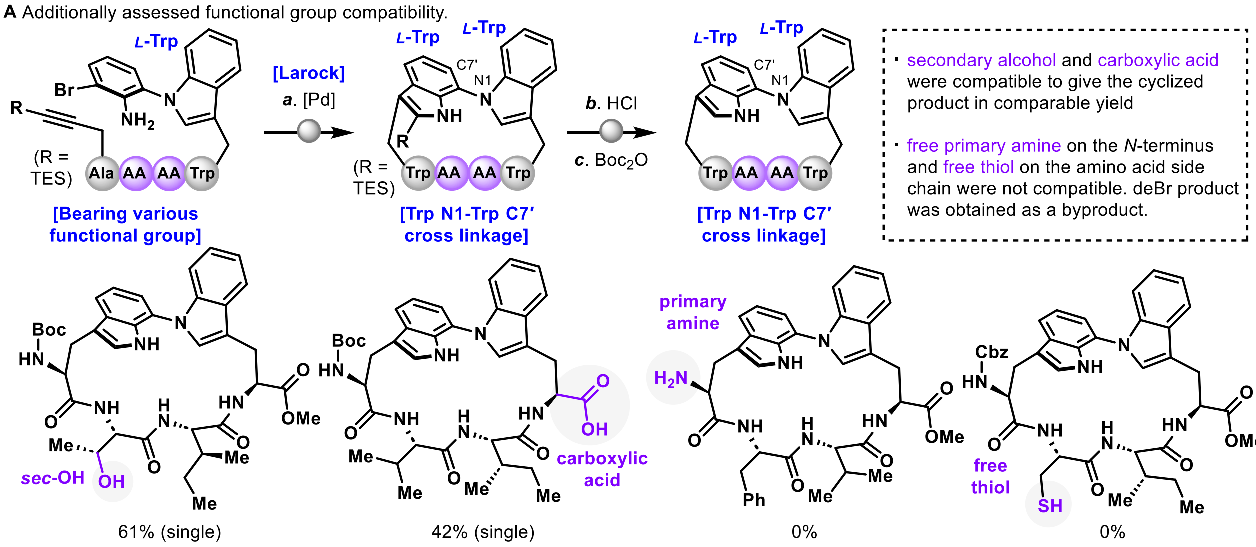

B Functional groups tolerance of Larock macrocyclization.

functional groups \ scaffolds	[Tyr C6-Trp C7′ cross linkage] rubrin scaffolds	[Tyr C6-Trp C5′ cross linkage] micitide scaffolds	[Tyr C6-Trp C6′ cross linkage] Scabrirubin CB-4 scaffolds	[Tyr C6-Trp N1′ cross linkage] lapparbin scaffolds	[Trp N1-Trp C7′ cross linkage] cihunamide scaffolds	[Trp-Phe cross linkages] unnatural scaffolds	[triaryl cross linkages] unnatural scaffolds
ester	42%-56%	15%-68%	50%-68%	25%-60%	27%-66%	38%-56%	32%-40%
carboxylic acid					42%		
carbamate	42%-56%	15%-68%	50%-68%	25%-60%	27%-66%	38%-56%	32%-40%
tosyl indole		38%		56%			
free indole		44%					
anisole	42%-56%	15%-68%	50%-68%	25%-60%			32%-40%
sec-OH					61%		
silyl ether		49-64%					
thio ether		35%			46%		
free thiol					0% (incompatible)		
primary amine					0% (incompatible		

Figure S2. Summary of the functional group compatibility.

High-level Summary and Comparison with the Reported Strategies

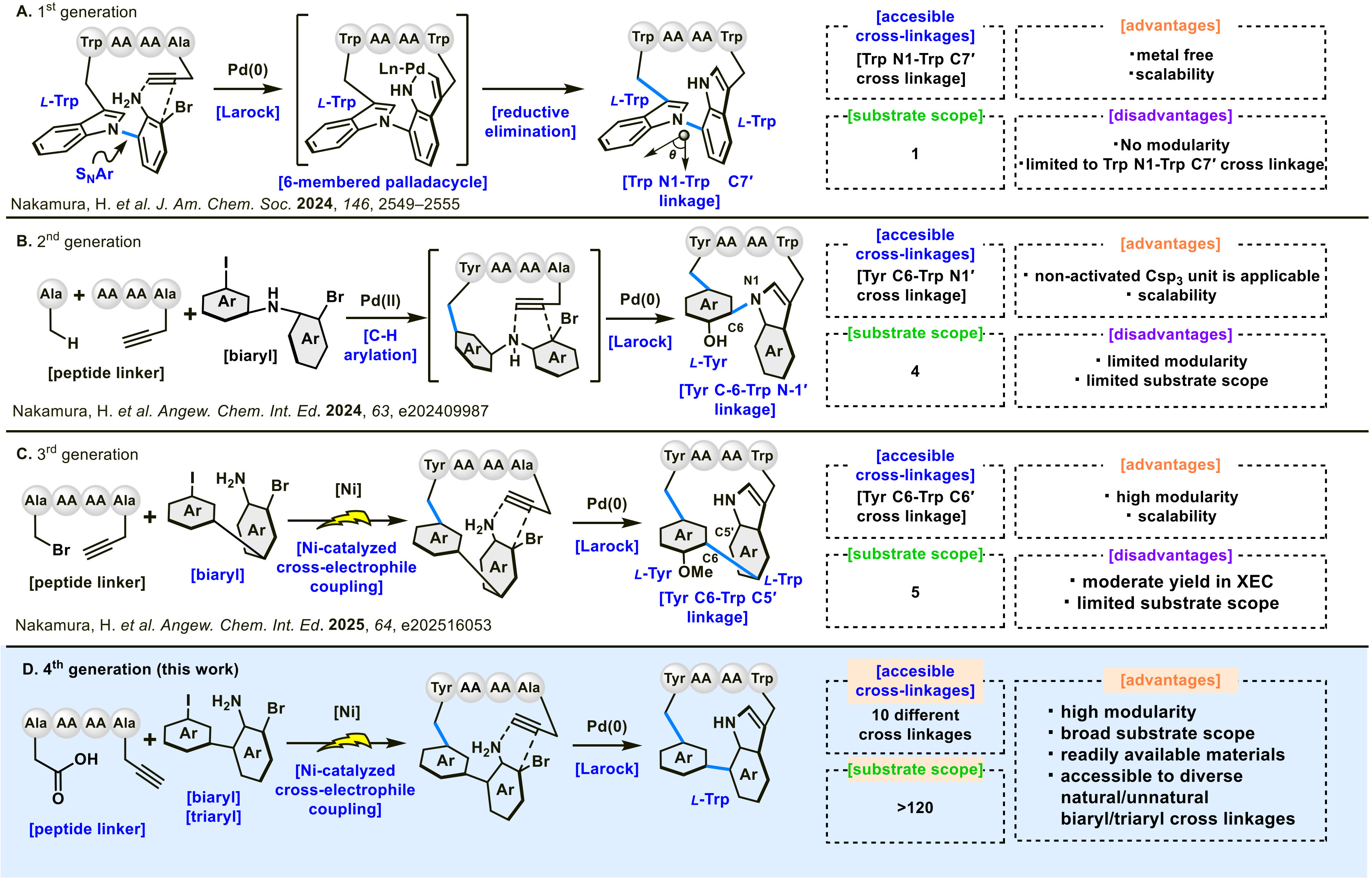

Figure S3. High level summary of the reported strategies. (A) 1st generation. (B) 2nd generation. (C) 3rd generation. (D) 4th generation (this work).

Summary of the atropisomeric outcomes

Featuring Linkage	Picked substrate	Conformational Isomer detected? [Y/N]	Separable? [Y/N]	Configuration was assigned?			NMR	LCMS
				Configuration assigned? [Y/N]	Method to assign its configuration	Configuration		
Tyr C6–Trp C7′ **(Rubrin Scaffold)**	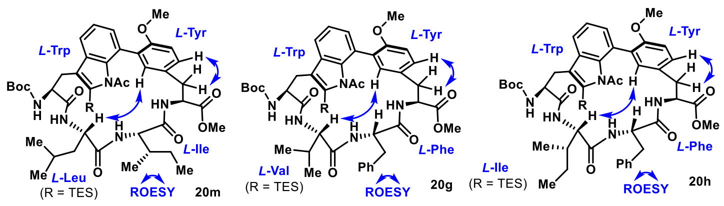	No	Single isomer	Yes	ROESY	***Ra***	Single	Single
Tyr C6–Trp C5′ **(Micitide Scaffold)**	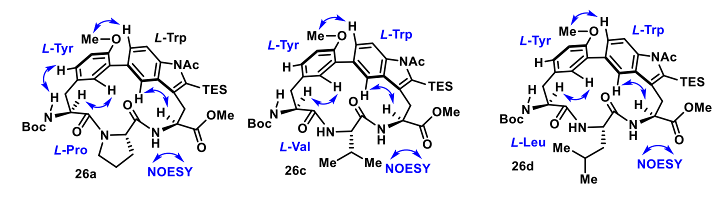	No	Single isomer	Yes	NOESY	***Sa***	Single	Single
Tyr C6-Trp C6′ **(Scabrirubin Scaffold)**	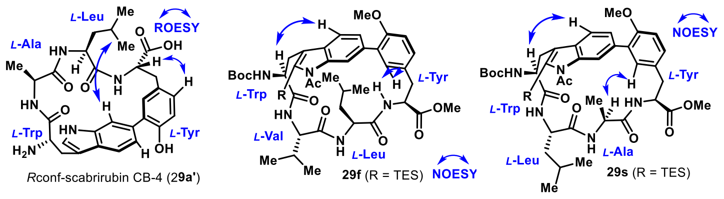	No	Single isomer	Yes	ROESY	***Ra***	Single	Single
Trp N1-Trp C7′ **(Cihunamide Scaffold)**	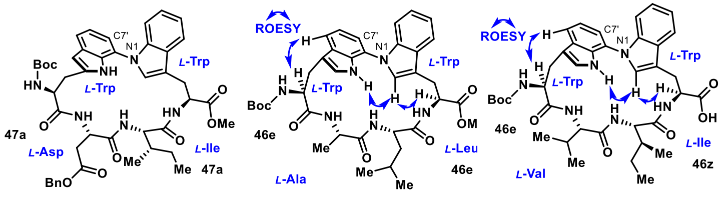	No	Single isomer	Yes	NMR comparison with the isolation report	***Sa***	Single	Single
Tyr C6-Trp N1′ **(lapparbin Scaffold)**	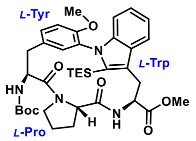 (*R*conf/*S*conf = 1:1) [lapparbin scaffold] 32a	Yes (*R* : *S* 1:1)	No	No (*R/S* mixture)	*R/S* mixture	***R/S* mixture**	*R/S* mixture	Single (Not separated)

Table S1. Summary of atropisomerism. (natural product-derived scaffolds).

substrate	Conformational Isomer detected? [Y/N]	Separable? [Y/N]	Configuration was assigned?			NMR [single/Mixture]	LCMS [single/Mixture]
			Configuration assigned? [Y/N]	Method to assign its configuration	Configuration [R/S]		
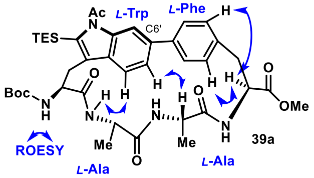 39a	No	Single isomer	Yes	ROESY	as shown in the structure (planar chiral)	Single	Single
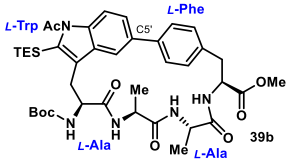 39b	Yes (ratio 1:1)	No	mixture	mixture	mixture (Planar Chiral)	mixture	mixture of two isomers
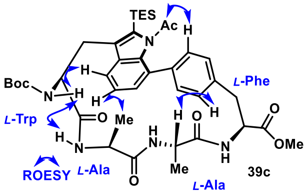 39c	Yes (ratio 2:1)	Yes	Yes	ROESY	as shown in the structure (planar chiral)	Single in each isolated isomer	Single in each isolated isomer
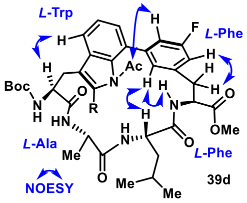 39d	Yes (ratio 4:1)	Yes	Yes	NOESY	***Ra***	Single in each isolated isomer	Single in each isolated isomer
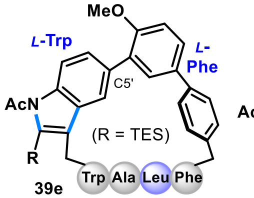 39e 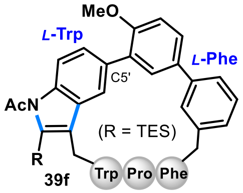 39f	No	Single isomer	No	Not assigned	Not assigned	Single	Single

Table S2. Summary of atropisomerism. (artificial scaffolds).

Featuring Linkage	substrate	Number of alanine	Conformational Isomer detected? [Y/N]	Separable? [Y/N]	NMR [single/Mixture]	LCMS [single/Mixture]
Tyr C6–Trp C5′ **(Micitide Scaffold)**	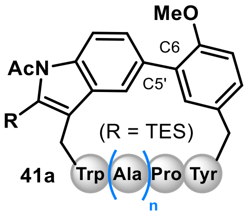	0	No	Single isomer	Single	Single
		1	No	Single isomer	Single	Single
		2	No	Single isomer	Single	Single
		3	No	Single isomer	Single	Single
Tyr C6-Trp N1' **(lapparbin Scaffold)**	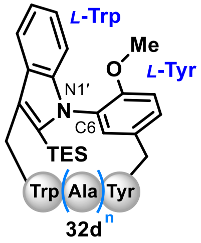	1	Yes (ratio=1:1)	No	mixture	mixture
		2	Yes (ratio=3:2)	No	mixture	mixture
		3	Yes (ratio=1:1)	No	mixture	mixture
Trp N1-to-Trp C7′ **(Cihunamide Scaffold)**	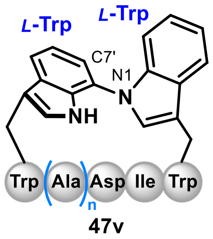	1	No	Single isomer	Single	Single
		2	No	Single isomer	Single	Single
		3	No	Single isomer	Single	Single

Table S3. Summary of atropisomerism. (different ring size).

General procedure A for the synthesis of 3a-3c:

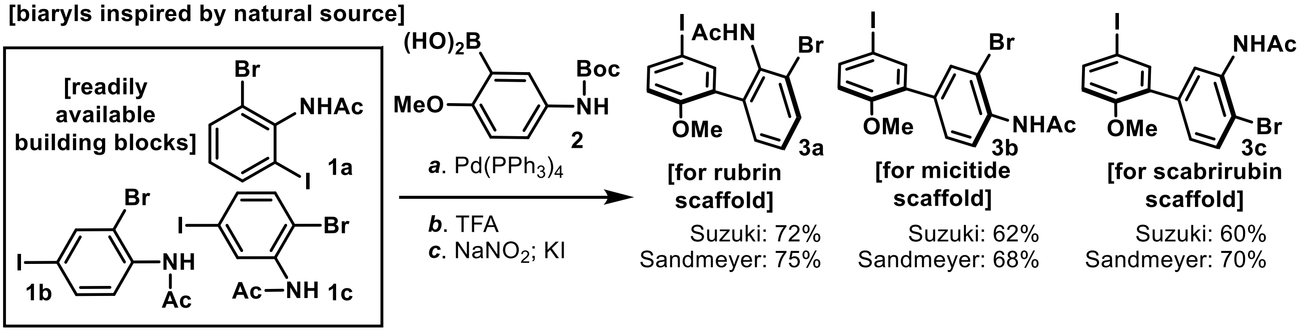

Suzuki: To a solution of iodide compound **1a-1c** (1.0 eq.) in toluene:H_2O:EtOH 4:2:1 (0.2 M) was added boronic acid **2** (1.1 eq.), K_2CO_3 (5.0 eq.) and $Pd(PPh_3)_4$ (0.05 eq.). The reaction was stirred at 80 °C under nitrogen atmosphere for 4 h, the reaction mixture was allowed to be cooled to rt and diluted with water, extracted with EtOAc for three times, the organic layers were combined, washed with saturated aq. NaCl, dried over Na_2SO_4, and removed under reduced pressure to give the crude, the crude was purified by silica gel chromatography to give compound **S1a-S1c**.

Compound **S1a-S1c** (1.0 eq.) was dissolved in DCM/TFA 3:1 (0.2 M), the reaction was stirred at rt for 1 h. The solvent was removed under reduced pressure, saturated aq. $NaHCO_3$ was added, extracted with DCM for three times. The organic layers were combined, washed with saturated aq. NaCl, dried over Na_2SO_4. The solvent was removed under reduced pressure to give the amine (quant.).

Sandmeyer: To a solution of the amine (1.0 eq.) and *p*-TsOH·H_2O (4.0 eq.) in MeCN (0.2 M) at 0 °C was added $NaNO_2$ (1.3 eq.) in H_2O dropwise. The reaction was stirred at rt for 3 h, then the KI (6.5 eq.) in H_2O was added dropwise at 0 °C, the reaction was stirred at rt for 6 h. The reaction was quenched by saturated aq. $NaHCO_3$ and saturated aq. $Na_2S_2O_3$, extracted with EtOAc for three times. The organic layers were combined and washed with saturated aq. NaCl. The solvent was removed under reduced pressure to give the crude, the crude was purified by silica gel chromatography to give iodide compound **3a-3c**.

*Note: Compound **3b, 3d** was reported in our previous work.[1-2]*

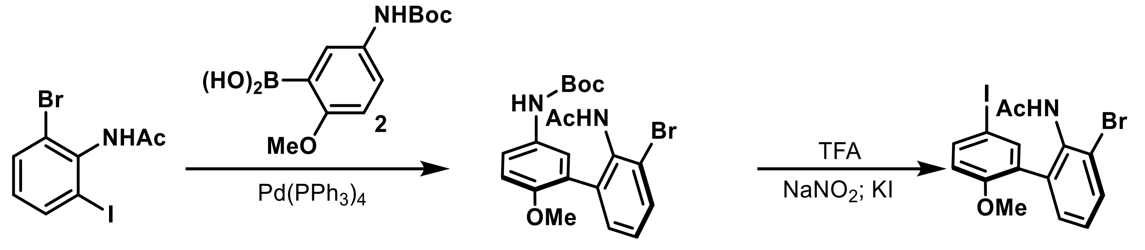

1a S1a 3a

On 16.6 mmol scale, **General Procedure A** was followed with aryl iodide **1a** and boronic acid **2** via Suzuki. Purification by silica gel column chromatography gave the title compound **S1a** (5.2 g, 72% yield).

Compound S1a

Physical State: amorphous solid

^{1}H NMR (600 MHz, $CDCl_3$): δ 7.62 (dd, J = 8.0, 1.2 Hz, 1H), 7.50 – 7.30 (m, 2H), 7.24 (dd, J = 7.6, 1.3 Hz, 1H), 7.17 (t, J = 7.8 Hz, 1H), 7.04 (d, J = 2.6 Hz, 1H), 6.91 (d, J = 8.9 Hz, 1H), 6.56 (s, 1H), 3.77 (s, 3H), 1.92 (s, 3H), 1.49 (s, 9H).

^{13}C NMR (151 MHz, $CDCl_3$): δ 168.43, 153.19, 151.78, 138.48, 134.37, 132.78, 132.66, 132.24, 130.23, 128.52, 128.45, 123.73, 120.25, 112.12, 80.50, 56.66, 28.44, 23.31.

HRMS (ESI-TOF): calculated for $C_{20}H_{23}BrN_2NaO_4^+$ $[M+Na]^+$: 457.0733, found: 457.0746.

On 17.3 mmol scale, **General Procedure A** was followed with **S1a** via Boc deprotection and Sandmeyer.

Purification by silica gel column chromatography gave the title compound **3a**.

Compound 3a

Physical State: amorphous solid

(5.8 g, 75% yield).

^{1}H NMR (600 MHz, CDCl$_3$): δ 7.67 – 7.62 (m, 2H), 7.50 (d, *J* = 2.2 Hz, 1H), 7.27 (d, *J* = 4.0 Hz, 1H), 7.25 – 7.18 (m, 2H), 6.76 (d, *J* = 8.7 Hz, 1H), 3.81 (s, 3H), 1.95 (s, 3H).

^{13}C NMR (151 MHz, CDCl$_3$): δ 168.13, 155.90, 139.59, 138.33, 137.28, 134.22, 133.22, 132.96, 130.24, 128.56, 123.69, 113.53, 83.46, 56.28, 23.33.

HRMS (ESI-TOF): calculated for $C_{15}H_{13}BrINNaO_2^+$ [M+Na]$^+$: 467.9067, found: 467.9081.

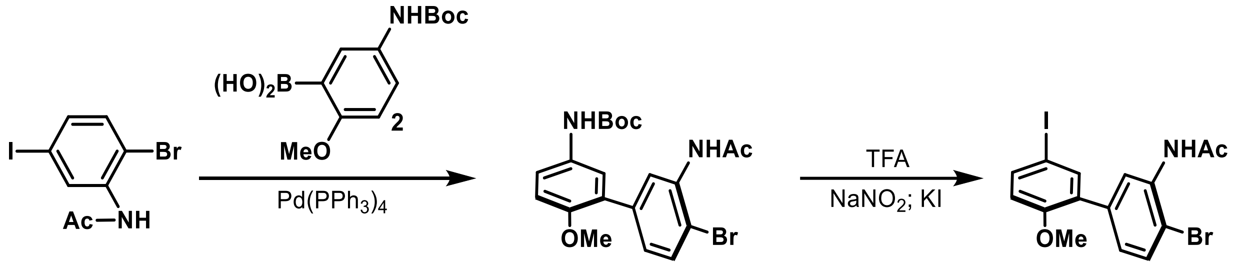

1c S1c 3c

On 4.6 mmol scale, **General Procedure A** was followed with aryl iodide **1c** and boronic acid **2** via Suzuki. Purification by silica gel column chromatography gave the title compound **S1c** (1.2 g, 60% yield).

Compound S1c

Physical State: colorless oil

^{1}H NMR (600 MHz, CDCl$_3$): δ 8.44 (s, 1H), 7.61 (s, 1H), 7.52 (d, *J* = 8.3 Hz, 1H), 7.39 (s, 1H), 7.21 (s, 1H), 7.15 (d, *J* = 7.9 Hz, 1H), 6.88 (d, *J* = 8.9 Hz, 1H), 6.52 (s, 1H), 3.77 (s, 3H), 2.23 (s, 3H), 1.50 (s, 9H).

^{13}C NMR (151 MHz, CDCl$_3$): δ 168.31, 153.27, 152.56, 138.60, 135.29, 131.74, 131.68, 129.63, 126.65, 123.26, 121.90, 119.91, 112.25, 112.04, 80.36, 56.10, 28.46, 24.95.

HRMS (ESI-TOF): calculated for $C_{20}H_{23}BrN_2NaO_4^+$ [M+Na]$^+$: 457.0733, found: 457.0746.

On 2.56 mmol scale, **General Procedure A** was followed with **S1c** via Boc deprotection and Sandmeyer. Purification by silica gel column chromatography gave the title compound **3c** (800 mg, 70% yield).

Compound 3c

Physical State: amorphous solid

^{1}H NMR (600 MHz, CDCl$_3$): δ 8.45 (s, 1H), 7.71 – 7.57 (m, 3H), 7.55 (d, *J* = 8.3 Hz, 1H), 7.19 – 7.00 (m, 1H), 6.73 (d, *J* = 8.5 Hz, 1H), 3.79 (s, 3H), 2.25 (s, 3H).

^{13}C NMR (151 MHz, CDCl$_3$): δ 168.28, 156.43, 139.00, 137.80, 137.49, 135.43, 131.89, 131.79, 126.37, 122.99, 113.63, 112.47, 83.06, 55.84, 25.01.

HRMS (ESI-TOF): calculated for $C_{15}H_{13}BrINNaO_2^+$ [M+Na]$^+$: 467.9067, found: 467.9078.

General Procedure B for the synthesis of 7a-7c:

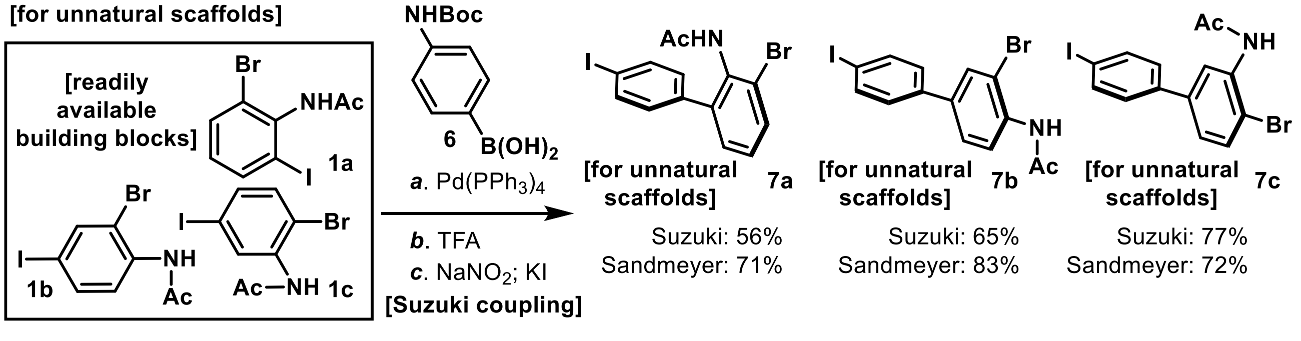

Suzuki: To a solution of iodide compound **1a-1c** (1.0 eq.) in toluene:H_2O:EtOH 4:2:1 (0.2 M) was added boric

acid **6** (1.1 eq.), K_2CO_3 (5.0 eq.) and $Pd(PPh_3)_4$ (0.05 eq.). The reaction was stirred at 80 °C under nitrogen atmosphere for 4 h, the reaction mixture was allowed to be cooled to rt and diluted with water, extracted with EtOAc for three times, the organic layers were combined, washed with saturated aq. NaCl, dried over Na_2SO_4, and removed under reduced pressure to give the crude, the crude was purified by silica gel chromatography to give compound **S2a-S2c**.

Compound **S2a-S2c** (1.0 eq.) was dissolved in DCM/TFA 3:1 (0.2 M), the reaction was stirred at rt for 1 h. The solvent was removed under reduced pressure, saturated aq. $NaHCO_3$ was added, extracted with DCM for three times. The organic layers were combined, washed with saturated aq. NaCl, dried over Na_2SO_4. The solvent was removed under reduced pressure to give the amine (quant.).

Sandmeyer: To a solution of the amine (1.0 eq.) and *p*-TsOH·H_2O (4.0 eq.) in MeCN (0.2 M) at 0 °C was added $NaNO_2$ (1.3 eq.) in H_2O dropwise. The reaction was stirred at rt for 3 h, then the KI (6.5 eq.) in H_2O was added dropwise at 0 °C, the reaction was stirred at rt for 6 h. The reaction was quenched by saturated aq. $NaHCO_3$ and saturated aq. $Na_2S_2O_3$, extracted with EtOAc for three times. The organic layers were combined and washed with saturated aq. NaCl. The solvent was removed under reduced pressure to give the crude, the crude was purified by silica gel chromatography to give iodide compound **7a-7c**.

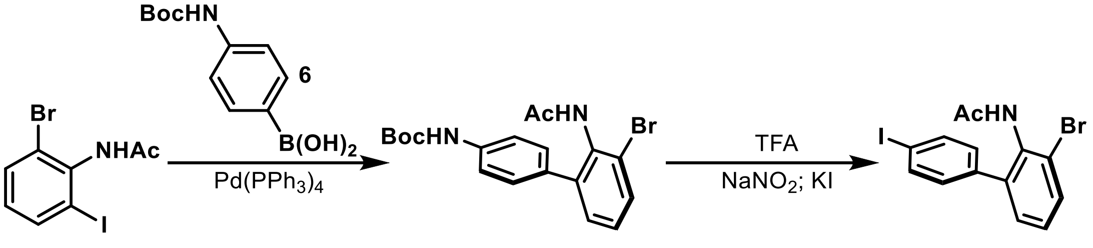

1a **S2a** **7a**

On 3.14 mmol scale, **General Procedure B** was followed with aryl iodide **1a** and boronic acid **6** via Suzuki. Purification by silica gel column chromatography gave the title compound **S2a** (712 mg, 56% yield).

Compound S2a

Physical State: amorphous solid

^{1}H NMR (600 MHz, CDCl$_3$): δ 7.60 (dd, J = 8.0, 1.3 Hz, 1H), 7.38 (d, J = 8.0 Hz, 2H), 7.26 – 7.24 (m, 3H), 7.20 (t, J = 7.8 Hz, 1H), 6.88 (s, 1H), 6.68 (s, 1H), 2.01 (s, 3H), 1.52 (s, 9H).

^{13}C NMR (151 MHz, CDCl$_3$): δ 169.29, 152.83, 142.15, 138.15, 133.72, 133.14, 132.12, 129.83, 129.22, 128.90, 124.09, 118.38, 80.81, 28.43, 23.23.

HRMS (ESI-TOF): calculated for $C_{19}H_{21}BrN_2NaO_3^+$ $[M+Na]^+$: 427.0628, found: 427.0653.

On 1.04 mmol scale, **General Procedure B** was followed with **S2a** via Boc deprotection and Sandmeyer. Purification by silica gel column chromatography gave the title compound **7a** (306 mg, 71% yield).

Compound 7a

Physical State: amorphous solid

^{1}H NMR (600 MHz, CDCl$_3$): δ 7.72 (d, J = 8.3 Hz, 2H), 7.64 (dd, J = 7.8, 1.5 Hz, 1H), 7.26 – 7.25 (m, 1H), 7.23 (t, J = 7.7 Hz, 1H), 7.09 (d, J = 8.3 Hz, 2H), 6.78 (s, 1H), 2.02 (s, 3H).

^{13}C NMR (151 MHz, CDCl$_3$): δ 169.10, 141.58, 138.81, 137.54, 132.90, 132.61, 130.41, 129.67, 129.05, 123.99, 93.82, 23.25.

HRMS (ESI-TOF): calculated for $C_{14}H_{11}BrINNaO^+$ $[M+Na]^+$: 437.8961, found: 437.8951.

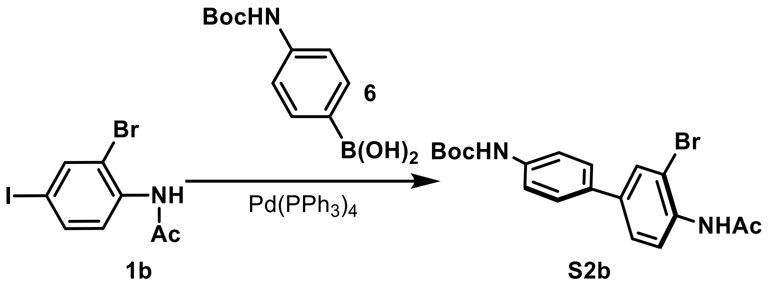

On 4.18 mmol scale, **General Procedure B** was followed with aryl iodide **1b** and boronic acid **6** via Suzuki. Purification by silica gel column chromatography gave the title compound **S2b** (1.1 g, 65% yield).

Compound S2b

Physical State: amorphous solid

^{1}H NMR (600 MHz, CDCl$_3$): δ 8.37 (d, *J* = 8.2 Hz, 1H), 7.73 (s, 1H), 7.62 (s, 1H), 7.50 – 7.41 (m, 5H), 6.55 (s, 1H), 2.26 (s, 3H), 1.53 (s, 9H).

^{13}C NMR (151 MHz, CDCl$_3$): δ 168.30, 152.72, 138.15, 134.53, 133.82, 130.17, 127.42, 126.66, 122.08, 118.90, 113.68, 80.85, 28.43, 24.99.

HRMS (ESI-TOF): calculated for $C_{19}H_{21}BrN_2NaO_3^+$ [M+Na]$^+$: 427.0628, found: 427.0634.

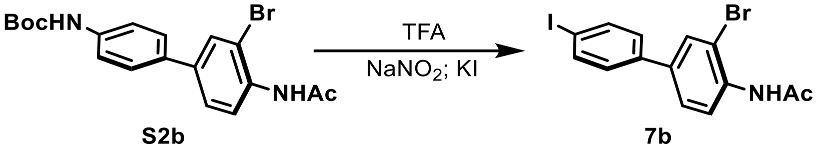

On 1.91 mmol scale, **General Procedure B** was followed with **S2b** via Boc deprotection and Sandmeyer. Purification by silica gel column chromatography gave the title compound **7b** (660 mg, 83% yield).

Compound 7b

Physical State: amorphous solid

^{1}H NMR (600 MHz, CDCl$_3$): δ 8.41 (d, *J* = 8.4 Hz, 1H), 7.76 (d, *J* = 8.5 Hz, 2H), 7.73 (d, *J* = 1.9 Hz, 1H), 7.64 (s, 1H), 7.50 (dd, *J* = 8.6, 2.0 Hz, 1H), 7.28 (d, *J* = 8.5 Hz, 2H), 2.27 (s, 3H).

^{13}C NMR (151 MHz, CDCl$_3$): δ 168.36, 138.68, 138.10, 137.12, 135.28, 130.37, 128.69, 126.84, 122.10, 113.68, 93.54, 25.02.

HRMS (ESI-TOF): calculated for $C_{14}H_{11}BrINNaO^+$ [M+Na]$^+$: 437.8961, found: 437.8983.

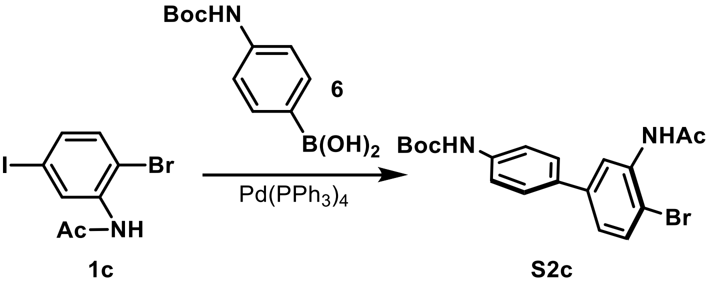

On 3.2 mmol scale, **General Procedure B** was followed with aryl iodide **1c** and boronic acid **6** via Suzuki. Purification by silica gel column chromatography gave the title compound **S2c** (1.0 g, 77% yield).

Compound S2c

Physical State: amorphous solid

^{1}H NMR (600 MHz, CDCl$_3$): δ 8.57 (s, 1H), 7.63 (s, 1H), 7.55 (d, *J* = 8.3 Hz, 1H), 7.52 (d, *J* = 8.6 Hz, 2H), 7.43 (d, *J* = 8.0 Hz, 2H), 7.17 (d, *J* = 6.9 Hz, 1H), 6.68 (s, 1H), 2.26 (s, 3H), 1.53 (s, 9H).

^{13}C NMR (151 MHz, CDCl$_3$): δ 168.45, 152.77, 141.15, 138.35, 135.97, 134.41, 132.41, 127.71, 123.53, 120.19, 118.84, 111.82, 80.75, 28.44, 25.02.

HRMS (ESI-TOF): calculated for $C_{19}H_{21}BrN_2NaO_3^+$ [M+Na]$^+$: 427.0628, found: 427.0630.

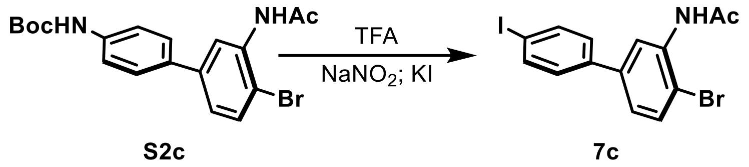

On 1.84 mmol scale, **General Procedure B** was followed with **S2c** via Boc deprotection and Sandmeyer. Purification by silica gel column chromatography gave the title compound **7c** (550 mg, 72% yield).

Compound 7c

Physical State: amorphous solid

^{1}H NMR (600 MHz, $CDCl_3$): δ 8.58 (s, 1H), 7.75 (d, J = 8.1 Hz, 2H), 7.65 (s, 1H), 7.57 (d, J = 8.3 Hz, 1H), 7.32 (d, J = 8.0 Hz, 2H), 7.15 (d, J = 8.1 Hz, 1H), 2.26 (s, 3H).

^{13}C NMR (151 MHz, $CDCl_3$): δ 168.46, 140.52, 139.37, 138.02, 136.21, 132.62, 129.01, 123.50, 120.23, 112.59, 93.80, 25.05.

HRMS (ESI-TOF): calculated for $C_{14}H_{11}BrINNaO^+$ $[M+Na]^+$: 437.8961, found: 437.8974.

Procedure for the synthesis of 7d:

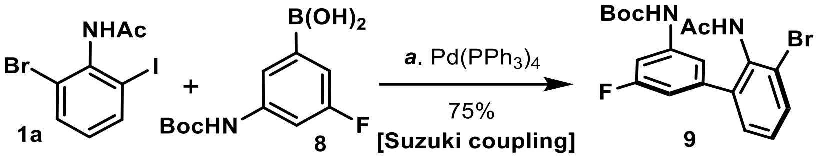

Suzuki: To a solution of iodide compound **1a** (996 mg, 2.93 mmol, 1.0 eq.) in toluene:H_2O:EtOH 4:2:1 (15 mL) was added boric acid **8** (822 mg, 3.2 mmol, 1.1 eq.), K_2CO_3 (2.0 g, 14.66 mmol, 5.0 eq.) and $Pd(PPh_3)_4$ (170 mg, 0.15 mmol, 0.05 eq.). The reaction was stirred at 80 °C under nitrogen atmosphere for 4 h, the reaction mixture was allowed to be cooled to rt and diluted with water, extracted with EtOAc for three times, the organic layers were combined, washed with saturated aq. NaCl, dried over Na_2SO_4, and removed under reduced pressure to give the crude, the crude was purified by silica gel chromatography to give compound **9** (930 mg, 75% yield).

Compound 9

Physical State: amorphous solid

^{1}H NMR (600 MHz, $CDCl_3$): δ 7.60 (dd, J = 8.0, 1.4 Hz, 1H), 7.29 (d, J = 12.1 Hz, 1H), 7.24 (dd, J = 7.7, 1.4 Hz, 1H), 7.18 (t, J = 7.8 Hz, 1H), 7.07 (s, 1H), 6.98 (s, 1H), 6.86 (s, 1H), 6.69 (dt, J = 9.0, 1.7 Hz, 1H), 2.01 (s, 3H), 1.50 (s, 9H).

^{13}C NMR (151 MHz, $CDCl_3$) δ 169.40, 162.92 (d, J = 244.4 Hz), 152.62, 141.30 (d, J = 9.7 Hz), 141.23, 140.02 (d, J = 11.7 Hz), 138.66, 133.02, 132.69, 129.61, 128.91, 124.01, 114.13, 109.80 (d, J = 22.6 Hz), 105.12 (d, J = 26.6 Hz), 81.09, 28.37, 23.21.

HRMS (ESI-TOF): calculated for $C_{19}H_{20}BrFN_2NaO_3^+$ $[M+Na]^+$: 445.0534, found: 445.0545.

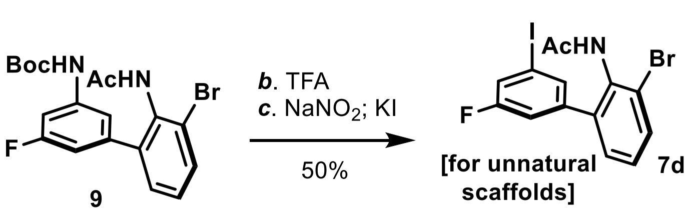

Compound **9** (786 mg, 1.86 mmol, 1.0 eq.) was dissolved in DCM/TFA 3:1 (10 mL), the reaction was stirred at rt for 1 h. The solvent was removed under reduced pressure, saturated aq. $NaHCO_3$ was added, extracted with DCM for three times. The organic layers were combined, washed with saturated aq. NaCl, dried over Na_2SO_4. The solvent was removed under reduced pressure to give the amine.

Sandmeyer: To a solution of the amine (600 mg, 1.86 mmol, 1.0 eq.) and p-TsOH·H_2O (1.41 g, 7.43 mmol, 4.0 eq.) in MeCN (10 mL) at 0 °C was added $NaNO_2$ (166.3 mg, 2.41 mmol, 1.3 eq.) in H_2O dropwise. The

reaction was stirred at rt for 3 h, then the KI (2 g, 12.07 mmol, 6.5 eq.) in H_2O was added dropwise at 0 °C, the reaction was stirred at rt for 6 h. The reaction was quenched by saturated aq. $NaHCO_3$ and saturated aq. $Na_2S_2O_3$, extracted with EtOAc for three times. The organic layers were combined and washed with saturated aq. NaCl. The solvent was removed under reduced pressure to give the crude, the crude was purified by silica gel chromatography to give iodide compound **7d** (403 mg, 50% yield).

Compound 7d

Physical State: amorphous solid

^{1}H NMR (600 MHz, CDCl$_3$): δ 7.63 (dd, *J* = 7.9, 1.4 Hz, 1H), 7.49 (s, 1H), 7.43 – 7.38 (m, 1H), 7.27 – 7.24 (m, 1H), 7.21 (t, *J* = 7.8 Hz, 1H), 7.10 – 7.00 (m, 2H), 2.02 (s, 3H).

^{13}C NMR (151 MHz, CDCl$_3$): δ 168.90, 162.09 (d, *J* = 252.0 Hz), 142.87(d, *J* = 22.2 Hz), 139.60, 133.33, 133.07, 132.93, 129.64, 128.97, 124.04 (d, *J* = 23.5 Hz), 123.74, 115.39 (d, *J* = 22.0 Hz), 93.18 (d, *J* = 8.6 Hz), 23.23.

HRMS (ESI-TOF): calculated for $C_{14}H_{10}BrFINNaO^+$ $[M+Na]^+$: 455.8867, found: 455.8870.

General procedure C for the synthesis of 12a-12b:

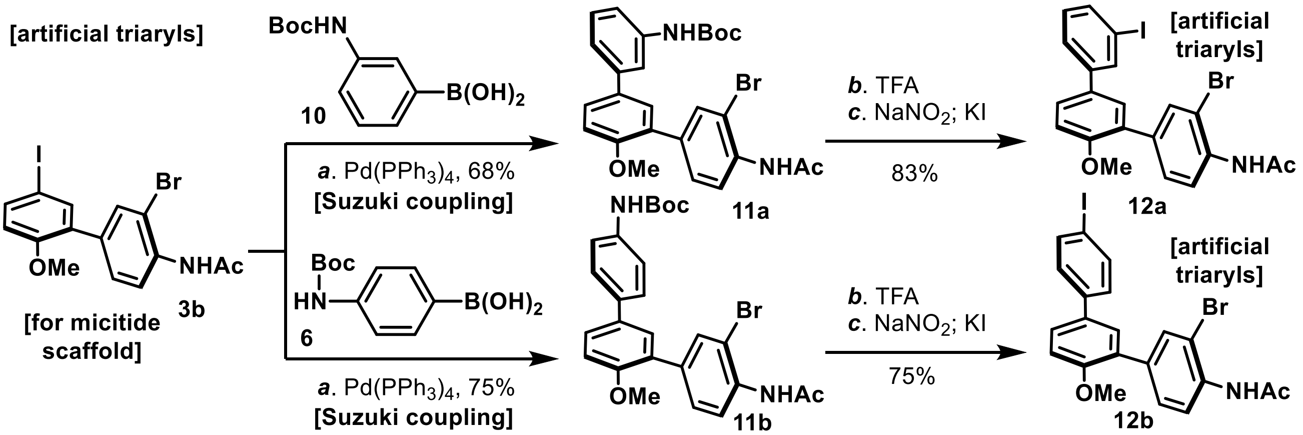

Suzuki: To a solution of iodide compound **3b** (1.0 eq.) in toluene:H_2O:EtOH 4:2:1 (0.2 M) was added boric acid **10** or **6** (1.1 eq.), K_2CO_3 (5.0 eq.) and $Pd(PPh_3)_4$ (0.05 eq.). The reaction was stirred at 80 °C under nitrogen atmosphere for 4 h, the reaction mixture was allowed to be cooled to rt and diluted with water, extracted with EtOAc for three times, the organic layers were combined, washed with saturated aq. NaCl, dried over Na_2SO_4, and removed under reduced pressure to give the crude, the crude was purified by silica gel chromatography to give compound **11a-11b**.

Compound **11a-11b** (1.0 eq.) was dissolved in DCM/TFA 3:1 (0.2 M), the reaction was stirred at rt for 1 h. The solvent was removed under reduced pressure, saturated aq. $NaHCO_3$ was added, extracted with DCM for three times. The organic layers were combined, washed with saturated aq. NaCl, dried over Na_2SO_4. The solvent was removed under reduced pressure to give the amine (quant.).

Sandmeyer: To a solution of the amine (1.0 eq.) and *p*-TsOH·H_2O (4.0 eq.) in MeCN (0.2 M) at 0 °C was added $NaNO_2$ (1.3 eq.) in H_2O dropwise. The reaction was stirred at rt for 3 h, then the KI (6.5 eq.) in H_2O was added dropwise at 0 °C, the reaction was stirred at rt for 6 h. The reaction was quenched by saturated aq. $NaHCO_3$ and saturated aq. $Na_2S_2O_3$, extracted with EtOAc for three times. The organic layers were combined and washed with saturated aq. NaCl. The solvent was removed under reduced pressure to give the crude, the crude was purified by silica gel chromatography to give iodide compound **12a-12b**.

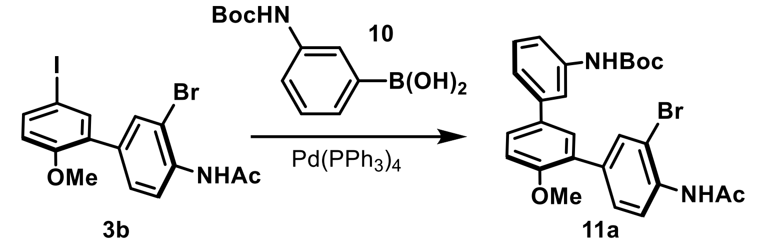

On 2.36 mmol scale, **General Procedure C** was followed with aryl iodide **3b** and boronic acid **10** via Suzuki. Purification by silica gel column chromatography gave the title compound **11a** (820 mg, 68% yield).

Compound 11a

Physical State: amorphous solid

^{1}H NMR (600 MHz, CDCl$_3$): δ 8.38 (d, *J* = 8.4 Hz, 1H), 7.75 (s, 1H), 7.65 (s, 1H), 7.59 (s, 1H), 7.55 – 7.50 (m, 2H), 7.48 (d, *J* = 1.9 Hz, 1H), 7.35 – 7.30 (m, 2H), 7.24 (d, *J* = 7.1 Hz, 1H), 7.01 (d, *J* = 8.5 Hz, 1H), 6.59 (s, 1H), 3.85 (s, 3H), 2.26 (s, 3H), 1.53 (s, 9H).

^{13}C NMR (151 MHz, CDCl$_3$): δ 168.30, 156.13, 152.86, 141.48, 138.93, 135.59, 134.57, 133.81, 133.03, 129.73, 129.49, 129.44, 128.92, 127.77, 121.63, 121.32, 117.15, 116.97, 112.84, 111.62, 80.67, 55.85, 28.46.

HRMS (ESI-TOF): calculated for $C_{26}H_{27}BrN_2NaO_4^+$ [M+Na]$^+$: 533.1046, found: 533.1053.

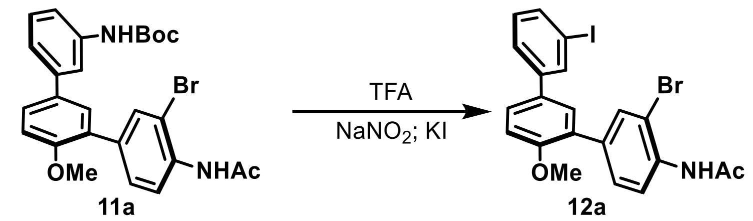

On 1.06 mmol scale, **General Procedure C** was followed with **11a** via Boc deprotection and Sandmeyer. Purification by silica gel column chromatography gave the title compound **12a** (460 mg, 83% yield).

Compound 12a

Physical State: amorphous solid

^{1}H NMR (600 MHz, CDCl$_3$): δ 8.40 (d, *J* = 8.4 Hz, 1H), 7.92 (s, 1H), 7.76 (s, 1H), 7.66 – 7.73 (m, 2H), 7.53 (d, *J* = 8.4 Hz, 2H), 7.50 (dd, *J* = 8.5, 2.2 Hz, 1H), 7.45 (d, *J* = 2.2 Hz, 1H), 7.15 (t, *J* = 7.8 Hz, 1H), 7.03 (d, *J* = 8.5 Hz, 1H), 3.86 (s, 3H), 2.27 (s, 3H).

^{13}C NMR (151 MHz, CDCl$_3$): δ 168.32, 156.45, 142.80, 135.88, 135.82, 135.33, 134.71, 133.00, 132.46, 130.54, 129.71, 129.37, 129.16, 127.66, 126.12, 121.35, 112.86, 111.72, 94.98, 55.88, 25.05.

HRMS (ESI-TOF): calculated for $C_{21}H_{17}BrINNaO_2^+$ [M+Na]$^+$: 543.9380, found: 543.9385.

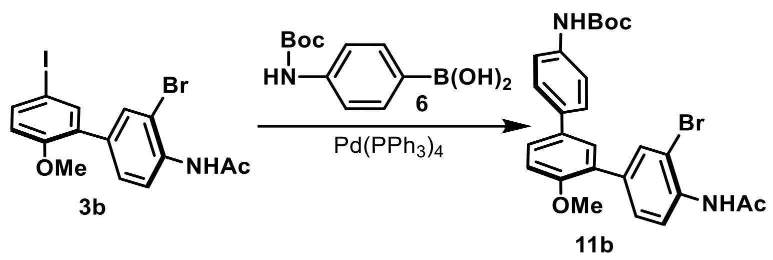

On 1.47 mmol scale, **General Procedure C** was followed with aryl iodide **3b** and boronic acid **6** via Suzuki. Purification by silica gel column chromatography gave the title compound **11b** (563 mg, 75% yield).

Compound 11b

Physical State: amorphous solid

^{1}H NMR (600 MHz, CDCl$_3$): δ 8.38 (d, *J* = 8.4 Hz, 1H), 7.76 (s, 1H), 7.66 (s, 1H), 7.54 – 7.48 (m, 4H), 7.46 (d, *J* = 2.2 Hz, 1H), 7.42 (d, *J* = 7.9 Hz, 2H), 7.01 (d, *J* = 8.5 Hz, 1H), 6.61 (s, 1H), 3.84 (s, 3H), 2.26 (s, 3H), 1.53 (s, 9H).

^{13}C NMR (151 MHz, CDCl$_3$): δ 168.34, 155.78, 152.87, 137.48, 135.68, 135.27, 134.57, 133.61, 133.03, 129.70, 129.06, 128.96, 127.30, 121.41, 118.98, 112.92, 111.72, 80.68, 55.86, 28.46, 25.02.
HRMS (ESI-TOF): calculated for $C_{26}H_{27}BrN_2NaO_4^+$ [M+Na]$^+$: 533.1046, found: 533.1052.

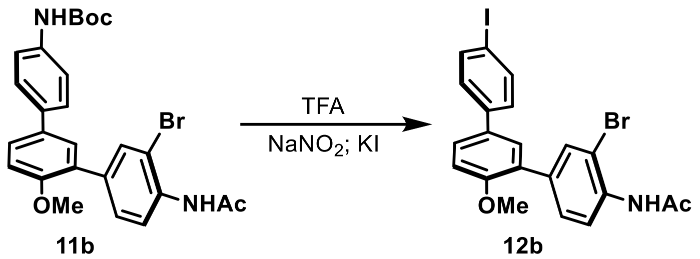

On 1.18 mmol scale, **General Procedure C** was followed with **11b** via Boc deprotection and Sandmeyer. Purification by silica gel column chromatography gave the title compound **12b** (463 mg, 75% yield).
Compound 12b
Physical State: amorphous solid
^{1}H NMR (600 MHz, CDCl$_3$): δ 8.39 (d, *J* = 8.4 Hz, 1H), 7.75 – 7.73 (m, 3H), 7.65 (s, 1H), 7.51 (ddd, *J* = 8.5, 6.5, 2.2 Hz, 2H), 7.46 (d, *J* = 2.4 Hz, 1H), 7.33 – 7.29 (m, 2H), 7.03 (d, *J* = 8.5 Hz, 1H), 3.86 (s, 3H), 2.27 (s, 3H).
^{13}C NMR (151 MHz, CDCl$_3$): δ 168.31, 156.36, 140.06, 137.94, 135.37, 134.70, 133.00, 132.89, 129.69, 129.18, 128.89, 128.71, 127.44, 121.36, 112.86, 111.77, 92.53, 55.87, 25.03.
HRMS (ESI-TOF): calculated for $C_{21}H_{17}BrINNaO_2^+$ [M+Na]$^+$: 543.9380, found: 543.9386.

Procedure for the synthesis of 35, 13:

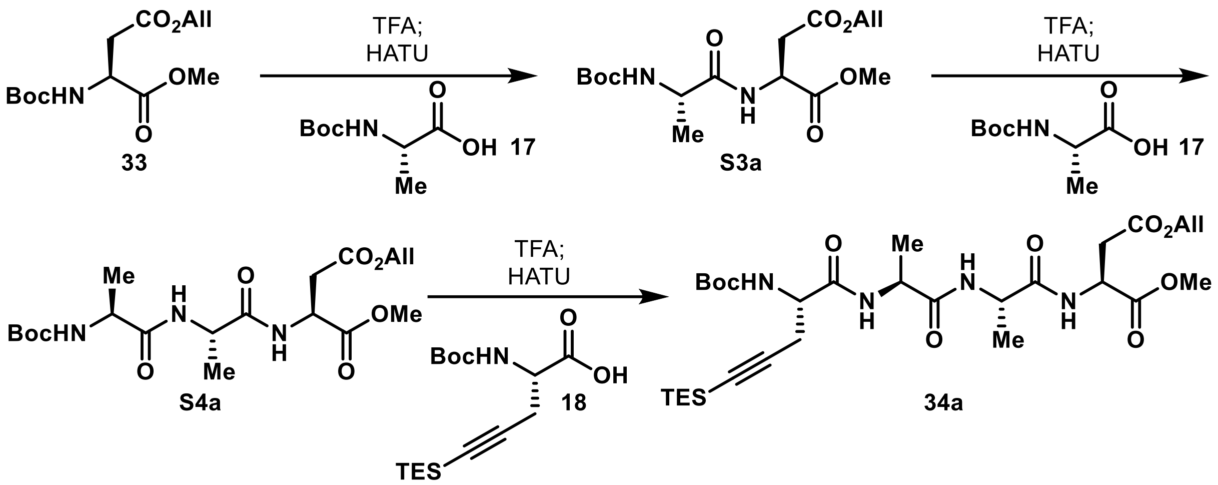

Compound **33** (2.87 g, 10.0 mmol, 1.0 eq.) was dissolved in DCM (30 mL), TFA (15 mL) was added dropwise, and stirred at rt for 1 h. The mixture was co-evaporated with toluene (40 mL × 3) *in vacuo* to afford the residue, the residue was used directly in the next step without further purification.

To a solution of the crude residue in DMF (10 mL) was added Boc-*L*-alanine (2.27 g, 12.0 mmol, 1.2 eq.), HATU (4.56 g, 12.0 mmol, 1.2 eq.) and DIPEA (7.0 mL, 40.0 mmol, 4.0 eq.). After stirring at rt for 1 h, the reaction was quenched with 0.5 M HCl (100 mL) and extracted with EtOAc (100 mL × 3). The combined organic layers were washed with saturated aq. NaCl, dried over Na_2SO_4, and concentrated *in vacuo* to give the residue **S3a**, the residue was used directly in the subsequent step.

This residue **S3a** was dissolved in DCM (30 mL), TFA (15 mL) was added dropwise, and stirred at rt for 1 h. The mixture was co-evaporated with toluene (40 mL × 3) *in vacuo* to afford the residue, the residue was used directly in the next step without further purification.

A mixture of the residue and Boc-*L*-alanine (2.27 g, 12.0 mmol, 1.2 eq.) in DMF (10 mL) was added sequentially with DIPEA (7.0 mL, 40.0 mmol, 4.0 eq.) and HATU (4.56 g, 12.0 mmol, 1.2 eq.). The reaction was stirred for 1 h, quenched with 0.5 M HCl (100 mL), and extracted with EtOAc (100 mL × 3). The combined organic layers were washed with saturated aq. NaCl, dried over Na_2SO_4, and concentrated *in vacuo* to give the residue **S4a**, the residue was used directly in the next step.

The residue **S4a** was dissolved in DCM (30 mL), TFA (15 mL) was added dropwise, and stirred at rt for 1 h. The mixture was co-evaporated with toluene (40 mL × 3) *in vacuo* to afford the residue, the residue was used directly in the next step without further purification.

To a solution of this crude residue in DMF (10 mL), (*S*)-2-((*tert*-butoxycarbonyl)amino)-5-(triethylsilyl)pent-4-ynoic acid (3.92 g, 12.0 mmol, 1.2 eq.), HATU (4.56 g, 12.0 mmol, 1.2 eq.) and DIPEA (7.0 mL, 40.0 mmol, 4.0 eq.) was added. After stirring at rt for 1 h, the reaction was quenched with 0.5 M HCl (100 mL) and extracted with EtOAc (100 mL × 3). The combined organic layers were washed with saturated aq. NaCl, dried over Na_2SO_4, and concentrated *in vacuo*. The resulting residue was purified by silica gel chromatography to afford the desired compound **34a** (5.6 g, 88% yield)**.**

Compound 34a

Physical State: amorphous white solid

^{1}H NMR (600 MHz, CDCl$_3$): δ 7.29 (d, *J* = 8.7 Hz, 1H), 7.18 (d, *J* = 7.8 Hz, 1H), 6.98 (d, *J* = 6.0 Hz, 1H), 5.91 – 5.84 (m, 1H), 5.44 – 5.41 (m, 1H), 5.29 (d, *J* = 17.2 Hz, 1H), 5.22 (d, *J* = 10.4 Hz, 1H), 4.95 – 4.81 (m, 1H), 4.57 – 4.54 (m, 3H), 4.50 – 4.46 (m, 1H), 4.26 – 4.22 (m, 1H), 3.74 (s, 3H), 3.02 (dd, *J* = 17.1, 4.8 Hz, 1H), 2.89 (dd, *J* = 16.8, 4.4 Hz, 1H), 2.83 – 2.76 (m, 1H), 2.70 (dd, *J* = 17.2, 6.0 Hz, 1H), 1.44 (s, 9H), 1.39 (t, *J* = 7.0 Hz, 6H), 0.96 (t, *J* = 7.9 Hz, 9H), 0.56 (q, *J* = 7.9 Hz, 6H).

^{13}C NMR (151 MHz, CDCl$_3$): δ 172.12, 171.71, 171.07, 170.81, 170.55, 155.94, 131.81, 118.73, 102.16,

86.43, 80.94, 65.79, 53.56, 52.89, 49.58, 49.04, 48.75, 36.29, 28.30, 23.47, 18.28, 18.18, 7.55, 4.38.
HRMS (ESI-TOF): calculated for $C_{30}H_{50}N_4NaO_9Si^+$ [M+Na]$^+$: 661.3239, found: 661.3254.
[α]$^{25}_{D}$: –3.4 (*c* = 0.5, $CHCl_3$)

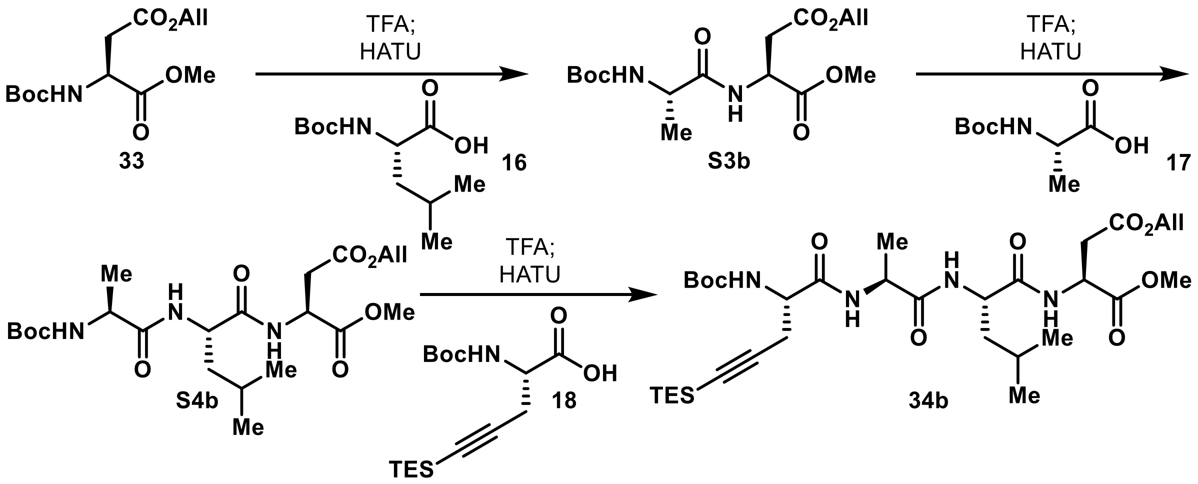

Compound **33** (2.87 g, 10.0 mmol, 1.0 eq.) was dissolved in DCM (30 mL), TFA (15 mL) was added dropwise, and stirred at rt for 1 h. The mixture was co-evaporated with toluene (40 mL × 3) *in vacuo* to afford the residue, the residue was used directly in the next step without further purification.

To a solution of the crude residue in DMF (10 mL) was added Boc-*L*-leucine (2.78 g, 12.0 mmol, 1.2 eq.), HATU (4.56 g, 12.0 mmol, 1.2 eq.) and DIPEA (7.0 mL, 40.0 mmol, 4.0 eq.). After stirring at rt for 1 h, the reaction was quenched with 0.5 M HCl (100 mL) and extracted with EtOAc (100 mL × 3). The combined organic layers were washed with saturated aq. NaCl, dried over Na_2SO_4, and concentrated *in vacuo* to give the residue **S3b**, the residue was used directly in the subsequent step.

This residue **S3b** was dissolved in DCM (30 mL), TFA (15 mL) was added dropwise, and stirred at rt for 1 h. The mixture was co-evaporated with toluene (40 mL × 3) *in vacuo* to afford the residue, the residue was used directly in the next step without further purification.

A mixture of the residue and Boc-*L*-alanine (2.27 g, 12.0 mmol, 1.2 eq.) in DMF (10 mL) was added sequentially with DIPEA (7.0 mL, 40.0 mmol, 4.0 eq.) and HATU (4.56 g, 12.0 mmol, 1.2 eq.). The reaction was stirred for 1 h, quenched with 0.5 M HCl (100 mL), and extracted with EtOAc (100 mL × 3). The combined organic layers were washed with saturated aq. NaCl, dried over Na_2SO_4, and concentrated *in vacuo* to give the residue **S4b**, the residue was used directly in the next step.

The residue **S4b** was dissolved in DCM (30 mL), TFA (15 mL) was added dropwise, and stirred at rt for 1 h. The mixture was co-evaporated with toluene (40 mL × 3) *in vacuo* to afford the residue, the residue was used directly in the next step without further purification.

To a solution of this crude residue in DMF (10 mL), (*S*)-2-((*tert*-butoxycarbonyl)amino)-5-(triethylsilyl)pent-4-ynoic acid (3.92 g, 12.0 mmol, 1.2 eq.), HATU (4.56 g, 12.0 mmol, 1.2 eq.) and DIPEA (7.0 mL, 40.0 mmol, 4.0 eq.) was added. After stirring at rt for 1 h, the reaction was quenched with 0.5 M HCl (100 mL) and extracted with EtOAc (100 mL × 3). The combined organic layers were washed with saturated aq. NaCl, dried over Na_2SO_4, and concentrated *in vacuo*. The resulting residue was purified by silica gel chromatography to afford the desired compound **34b** (5.4 g, 80% yield).

Compound 34b

Physical State: amorphous white solid

^{1}H NMR (600 MHz, $CDCl_3$): δ 7.13 (d, *J* = 6.7 Hz, 1H), 6.96 – 6.88 (m, 1H), 6.84 (d, *J* = 4.7 Hz, 1H), 5.91 – 5.84 (m, 1H), 5.42 – 5.14 (m, 3H), 4.87 – 4.84 (m, 1H), 4.57 (d, *J* = 5.4 Hz, 2H), 4.52 – 4.45 (m, 1H), 4.43 – 4.41 (m, 1H), 4.30 – 4.06 (m, 1H), 3.73 (s, 3H), 3.00 (dd, *J* = 16.8, 4.8 Hz, 1H), 2.88 (dd, *J* = 16.9, 4.7 Hz, 1H), 2.80 (dd, *J* = 17.1, 6.0 Hz, 1H), 2.68 (dd, *J* = 17.2, 5.9 Hz, 1H), 1.79 – 1.69 (m, 1H), 1.67 – 1.59 (m, 1H),

1.55 – 1.50 (m, 1H), 1.44 (s, 9H), 1.38 (d, *J* = 7.0 Hz, 3H), 0.96 (t, *J* = 7.9 Hz, 9H), 0.91 (d, *J* = 6.5 Hz, 3H), 0.89 (d, *J* = 6.5 Hz, 3H), 0.57 (q, *J* = 7.9 Hz, 6H).
^{13}C NMR (151 MHz, CDCl$_3$): δ 171.88, 171.80, 171.02, 170.83, 170.53, 155.98, 131.82, 118.71, 102.04, 86.58, 81.04, 65.76, 53.57, 52.82, 51.80, 49.68, 48.72, 40.84, 36.24, 28.28, 24.84, 23.27, 22.98, 21.83, 18.02, 7.55, 4.38.
HRMS (ESI-TOF): calculated for $C_{33}H_{56}N_4NaO_9Si^+$ [M+Na]$^+$: 703.3709, found: 703.3713.
[α]$^{25}_D$: –9.9 (*c* = 0.5, CHCl$_3$)

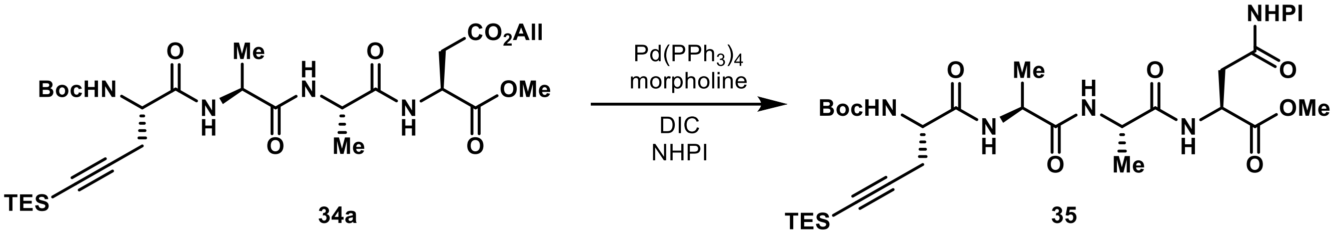

To a solution of compound **34a** (5.09 g, 7.97 mmol, 1.0 eq.) in THF (40 mL) was added Pd(PPh$_3$)$_4$ (460.5 mg, 0.4 mmol, 5 mol%) and morpholine (4.17 g, 47.8 mmol, 6.0 eq.). The reaction mixture was allowed to stir at rt for 2 h under nitrogen atmosphere. The reaction was quenched with 0.5 M HCl and extracted with DCM for three times. The combined organic layers were washed with saturated aq. NaCl, dried over Na$_2$SO$_4$, and concentrated *in vacuo* to give the residue, the residue was used directly in the next step.
The residue and NHPI (1.43 g, 8.77 mmol, 1.1 eq.) were dissolved in DCM (40 mL). To this solution was added DIC (1.21 mL, 7.97 mmol, 1.0 eq.) dropwise, the reaction mixture was stirred at rt overnight. The solvent was removed under reduced pressure, the resulting residue was purified by silica gel chromatography to afford the desired compound **35** (5.1 g, 86% yield).
Compound 35
Physical State: amorphous white solid
^{1}H NMR (600 MHz, CDCl$_3$): δ 7.94 – 7.76 (m, 2H), 7.72 – 7.68 (m, 1H), 7.44 – 7.40 (m, 2H), 5.62 – 5.58 (m, 1H), 4.89 – 4.84 (m, 1H), 4.80 – 4.74 (m, 1H), 4.61 – 4.57 (m, 1H), 4.36 – 4.32 (m, 1H), 3.73 (s, 3H), 3.09 (dd, *J* = 17.5, 4.5 Hz, 1H), 2.86 – 2.76 (m, 1H), 2.74 – 2.66 (m, 2H), 1.43 (d, *J* = 7.2 Hz, 3H), 1.41 (s, 9H), 1.33 (d, *J* = 7.2 Hz, 3H), 0.94 (t, *J* = 7.4 Hz, 9H), 0.54 (q, *J* = 7.7 Hz, 6H).
^{13}C NMR (151 MHz, CDCl$_3$): δ 174.05, 172.76, 171.94, 171.17, 170.80, 164.64, 156.21, 134.31, 129.31, 123.48, 102.38, 85.78, 81.11, 53.39, 52.86, 49.33, 48.94, 48.58, 36.11, 28.30, 23.44, 19.12, 18.96, 7.54, 4.39.
HRMS (ESI-TOF): calculated for $C_{35}H_{49}N_5NaO_{11}Si^+$ [M+Na]$^+$: 766.3090, found: 766.3079.
[α]$^{25}_D$: +3.1 (*c* = 0.2, CHCl$_3$)

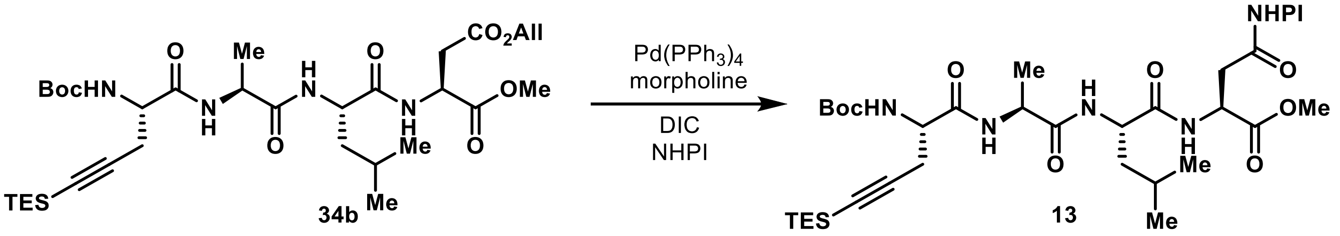

To a solution of compound **34b** (6.87 g, 10.09 mmol, 1.0 eq.) in THF (50 mL) was added Pd(PPh$_3$)$_4$ (583 mg, 0.51 mmol, 5 mol%) and morpholine (5.27 g, 60.5 mmol, 6.0 eq.). The reaction mixture was allowed to stir at rt for 2 h under nitrogen atmosphere. The reaction was quenched with 0.5 M HCl and extracted with DCM for three times. The combined organic layers were washed with saturated aq. NaCl, dried over Na$_2$SO$_4$, and concentrated *in vacuo* to give the residue, the residue was used directly in the next step.
The residue and NHPI (1.81 g, 11.1 mmol, 1.1 eq.) were dissolved in DCM (50 mL). To this solution was added DIC (1.53 mL, 10.09 mmol, 1.0 eq.) dropwise, the reaction mixture was stirred at rt overnight. The

solvent was removed under reduced pressure, the resulting residue was purified by silica gel chromatography to afford the desired compound **13** (6.5 g, 82% yield).

Compound 13

Physical State: amorphous solid

^{1}H NMR (600 MHz, $CDCl_3$): δ 7.89 (s, 2H), 7.81 – 7.79 (m, 2H), 7.33 (d, *J* = 7.7 Hz, 1H), 6.93 (s, 2H), 5.39 – 5.20 (m, 1H), 4.98 (q, *J* = 6.3 Hz, 1H), 4.59 – 4.54 (m, 1H), 4.47 – 4.41 (m, 1H), 4.23 (d, *J* = 6.6 Hz, 1H), 3.79 (s, 3H), 3.36 (dd, *J* = 16.2, 5.5 Hz, 1H), 3.28 (dd, *J* = 16.3, 5.6 Hz, 1H), 2.84 (dd, *J* = 17.2, 6.0 Hz, 1H), 2.69 (dd, *J* = 17.2, 5.9 Hz, 1H), 1.86 – 1.81 (m, 1H), 1.67 – 1.60 (m, 1H), 1.57 – 1.52 (m, 1H), 1.45 – 1.43 (m, 3H), 1.42 (s, 9H), 0.96 (t, *J* = 7.9 Hz, 9H), 0.91 (dd, *J* = 9.8, 6.5 Hz, 6H), 0.56 (q, *J* = 7.9 Hz, 6H).

^{13}C NMR (151 MHz, $CDCl_3$): δ 172.16, 172.00, 171.13, 170.01, 166.89, 161.88, 155.95, 135.04, 128.84, 124.22, 102.08, 86.48, 81.01, 53.52, 53.20, 51.80, 49.98, 48.76, 40.32, 33.74, 28.25, 24.91, 23.17, 21.55, 18.13, 7.56, 4.39.

HRMS (ESI-TOF): calculated for $C_{38}H_{55}N_5NaO_{11}Si^+$ $[M+Na]^+$: 808.3560, found: 808.3562.

$[\alpha]^{25}_{D}$: +2.4 (*c* = 0.5, $CHCl_3$)

Procedure for the synthesis of 25:

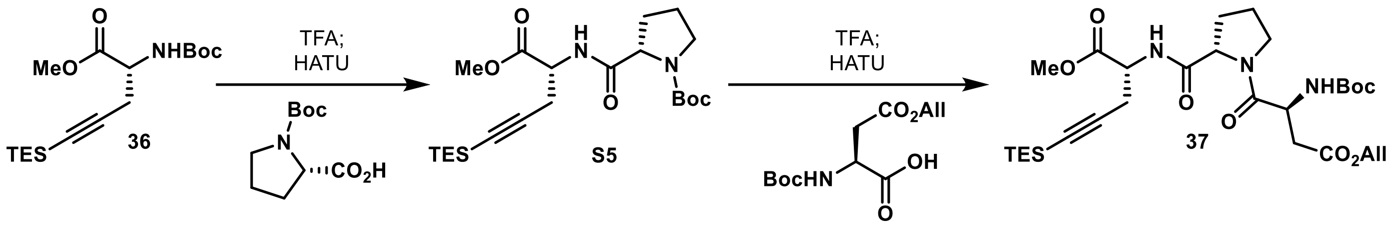

Compound **36** (1.71 g, 5.0 mmol, 1.0 eq.) was dissolved in DCM (30 mL), TFA (7.5 mL) was added dropwise, and stirred at rt for 1 h. The mixture was co-evaporated with toluene (30 mL × 3) *in vacuo* to afford the residue, the residue was used directly in the next step without further purification.

To a solution of the residue and Boc-*L*-proline (1.29 g, 6.0 mmol, 1.2 eq.) in DMF (50 mL) was added sequentially with DIPEA (3.5 mL, 20.0 mmol, 4.0 eq.) and HATU (2.28 g, 6.0 mmol, 1.2 eq.). The reaction was stirred at rt for 1 h, quenched with 0.5 M HCl (50 mL), and extracted with EtOAc (50 mL × 3). The combined organic layers were washed with saturated aq. NaCl, dried over Na_2SO_4, and concentrated *in vacuo* to give the residue **S5**, the residue **S5** was used directly in the next step.

The residue **S5** was dissolved in DCM (30 mL), TFA (7.5 mL) was added dropwise, and stirred at rt for 1 h. The mixture was co-evaporated with toluene (30 mL × 3) *in vacuo* to afford the residue, the residue was used directly in the next step without further purification.

To a solution of this residue in DMF (50 mL) was added Boc-amino acid (1.64 g, 6.0 mmol, 1.2 eq.), DIPEA (3.5 mL, 20.0 mmol, 4.0 eq.) and HATU (2.28 g, 6.0 mmol, 1.2 eq.). After stirring at rt for 1 h, the reaction was quenched with 0.5 M HCl (50 mL) and extracted with EtOAc (50 mL × 3). The combined organic layers were washed with saturated aq. NaCl, dried over Na_2SO_4, and concentrated *in vacuo*. The resulting residue was purified by silica gel chromatography to afford the desired compound **37** (2.7 g, 90% yield).

Compound 37

Physical State: colorless oil

^{1}H NMR (600 MHz, CDCl$_3$): δ 7.13 (d, *J* = 8.0 Hz, 1H), 5.90 – 5.83 (m, 1H), 5.32 – 5.24 (m, 2H), 5.20 (d, *J* = 10.4 Hz, 1H), 4.83 – 4.79 (m, 1H), 4.65 – 4.61 (m, 1H), 4.59 – 4.51 (m, 3H), 3.83 – 3.80 (m, 1H), 3.76 – 3.73 (m, 1H), 3.67 (s, 3H), 2.94 (dd, *J* = 16.4, 8.3 Hz, 1H), 2.80 – 2.65 (m, 3H), 2.30 – 2.19 (m, 1H), 2.11 – 1.94 (m, 3H), 1.39 (s, 9H), 0.92 (t, *J* = 7.9 Hz, 9H), 0.52 (q, *J* = 7.9 Hz, 6H).

^{13}C NMR (151 MHz, CDCl$_3$): δ 170.99, 170.95, 170.75, 155.01, 131.85, 118.68, 102.13, 84.88, 80.27, 65.82, 60.45, 52.44, 51.26, 48.50, 47.46, 37.76, 28.88, 28.33, 28.25, 24.59, 23.50, 7.44, 4.39.

HRMS (ESI-TOF): calculated for $C_{29}H_{47}N_3NaO_8Si^+$ [M+Na]$^+$: 616.3025, found: 616.3029.

$[\alpha]^{25}_D$: –79.7 (*c* = 0.5, $CHCl_3$)

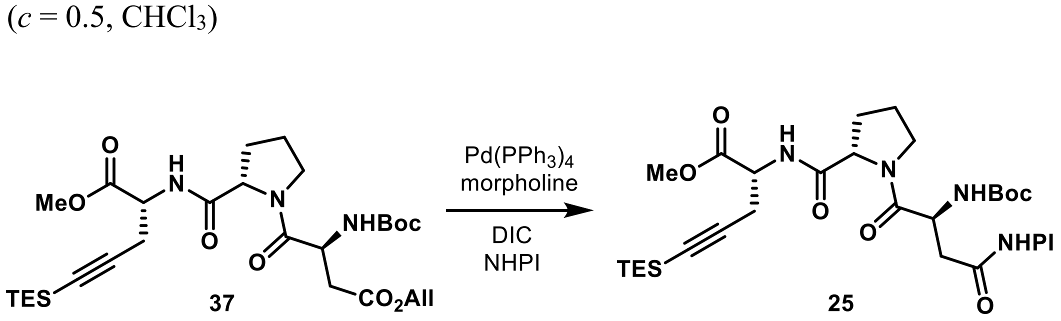

To a solution of compound **37** (2. 68 g, 4.5 mmol, 1.0 eq.) in THF (24 mL) was added $Pd(PPh_3)_4$ (259 mg, 0.23 mmol, 5 mol%) and morpholine (2.4 mL, 27.0 mmol, 6.0 eq.). The reaction mixture was allowed to stir at rt for 2 h under nitrogen atmosphere. The reaction was quenched with 0.5 M HCl 30 mL and extracted with DCM (30 mL × 3). The combined organic layers were washed with saturated aq. NaCl, dried over Na_2SO_4, and concentrated *in vacuo* to give the residue, the residue was used directly in the next step.

The residue and NHPI (790 mg, 4.95 mmol, 1.1 eq.) were dissolved in DCM (24 mL). To this solution was

added DIC (0.75 mL, 4.95 mmol, 1.0 eq.) dropwise, and the mixture was stirred at rt overnight. The solvent was removed under reduced pressure, the resulting residue was purified by silica gel chromatography to afford the desired compound **25** (2.80 g, 89% yield).

Compound 25

Physical State: amorphous solid

^{1}H NMR (600 MHz, $CDCl_3$): δ 7.90 – 7.81 (m, 2H), 7.81 – 7.76 (m, 2H), 7.00 (d, *J* = 8.0 Hz, 1H), 5.38 (d, *J* = 9.4 Hz, 1H), 5.01 (q, *J* = 7.4 Hz, 1H), 4.79 – 4.40 (m, 2H), 3.78 – 3.72 (m, 2H), 3.69 (s, 3H), 3.31 – 3.12 (m, 1H), 3.06 (dd, *J* = 16.4, 6.3 Hz, 1H), 2.83 – 2.59 (m, 2H), 2.31 – 2.24 (m, 1H), 2.13 – 1.89 (m, 3H), 1.44 (s, 9H), 0.96 – 0.90 (m, 9H), 0.57 – 0.50 (m, 6H).

^{13}C NMR (151 MHz, $CDCl_3$): δ 170.73, 169.90, 167.22, 161.57, 154.94, 134.91, 128.92, 124.13, 102.06, 85.11, 80.71, 60.60, 52.59, 51.34, 48.58, 47.63, 34.68, 32.11, 28.45, 28.35, 28.25, 24.82, 23.42, 7.49, 4.42.

HRMS (ESI-TOF): calculated for $C_{34}H_{46}N_4NaO_{10}Si^+$ $[M+Na]^+$: 721.2875, found: 721.2883.

$[\alpha]^{25}_D$: –31.6 (*c* = 0.5, $CHCl_3$)

The screening of the e-chem reaction condition:

The reaction has been carried out according to the procedure reported by Baran et al. [3]
To an oven dried 10 mL vial, $NiCl_2 \cdot 6H_2O$ (14.3 mg, 0.06 mmol, 0.3 eq.), and 2,2′-bipyridine (9.4 mg, 0.06 mmol, 0.3 eq.) were all directly added and dissolved in anhydrous solvent (3 mL). The Ni catalytic mixture was allowed to stir for 5 minutes. Then, redox active ester (RAE) **14** (0.5 mmol, 2.5 eq.) and aryl halide **3a** (0.2 mmol, 1.0 eq.) were added to the catalytic solution. The solution of the vial was stirred until all solids were dissolved (roughly 10 minutes). $AgNO_3$ (25.5 mg, 0.15 mmol, 0.75 eq.) was then added to the reaction mixture directly as a solid. The vial was closed with a cap with a magnesium sacrificial anode and RVC cathode. The vial was then placed on a stir plate and electrolysis was set to 10 mA. The reaction underwent the programmed electrolysis open to air. After completion of the reaction, the reaction mass was transferred to a separatory funnel, the electrodes were rinsed with EtOAc (5 mL) and saturated aq. $NaHCO_3$ (10 mL) was slowly added. The aqueous layer was extracted with EtOAc for three times. The combined organics were washed with water then brine, then dried over Na_2SO_4 before being filtered and concentrated via rotary evaporation. The crude oily solid was purified via flash chromatography to afford the desired product **15**.

Table S1. Optimization of the electrochemical decarboxylative coupling

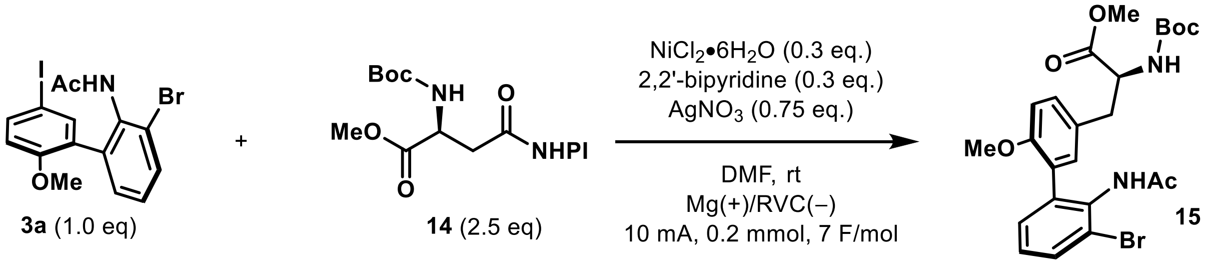

Entry[a]	Modification from standard condition	Yield (%)[b]
	Ligand screening(0.3 eq.)	
1	5,5'-dimethyl-2,2'-bipyridine	56%
2	4,4'-dimethyl-2,2'-bipyridine	trace
3	4,4'-di-*tert*-butyl-2,2'-bipyridine	41%
4	o-Phenanthroline	11%
5	BINAP	trace
6	1,3-bis(diphenylphosphaneyl)propane	trace
	Solvents screening	
**7**	**NMP**	**78%**
8	DMA	46%
9	MeCN	tarce
10	THF	trace
	Electrodes screening (from Entry 7)	
11	Zn(+)/RVC(–)	trace
12	Mg(+)/C(–)	12%
13	Ni_{foam}(+)/RVC(–)	N.D.
14	Zn(+)/Ni_{foam}(–)	trace
15	Al(+)/RVC(–)	N.D.
16	Mg(+)/Ni_{foam}(–)	27%

[a]The reactions were carried out on a 0.2 mmol scale in 3 mL solvent open to air. [b]Isolated yields.

The scope of the e-chem reaction:

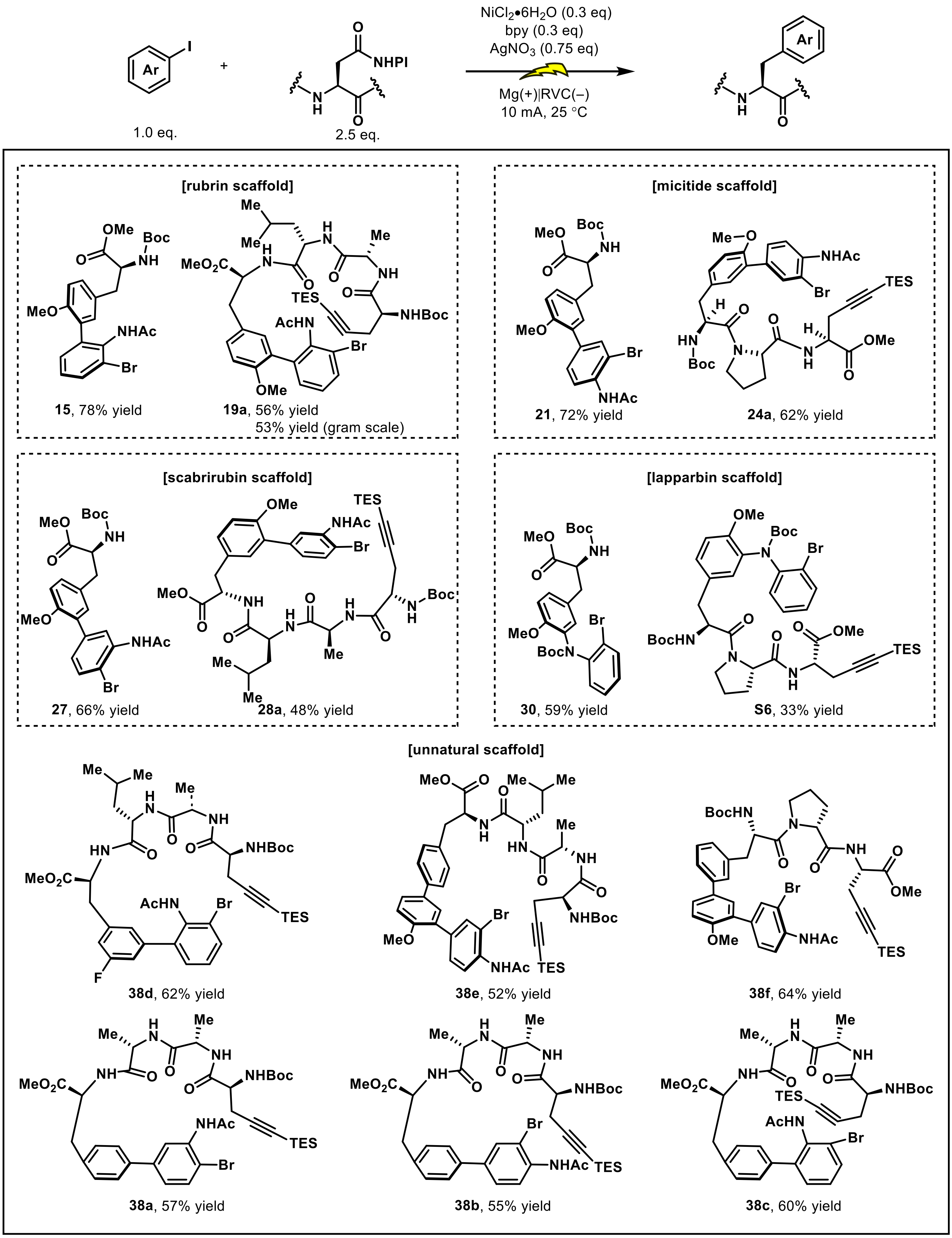

Figure S1. High-level summary of the electrochemical decarboxylative coupling

General procedure D: Electrochemical decarboxylative cross-coupling

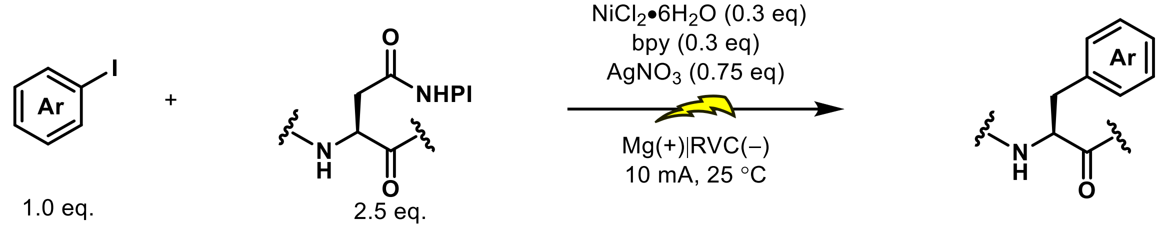

To an oven dried 10 mL vial, $NiCl_2 \cdot 6H_2O$ (14.3 mg, 0.06 mmol, 0.3 eq.), and 2,2′-bipyridine (9.4 mg, 0.06 mmol, 0.3 eq.) were all directly added and dissolved in anhydrous solvent (3 mL). The Ni catalytic mixture was allowed to stir for 5 minutes. Then, redox active ester (RAE) (0.5 mmol, 2.5 eq.) and aryl halide (0.2 mmol, 1.0 eq.) were added to the catalytic solution. The solution of the vial was stirred until all solids were dissolved (roughly 10 minutes). $AgNO_3$ (25.5 mg, 0.15 mmol, 0.75 eq.) was then added to the reaction mixture directly as a solid. The vial was closed with a cap with a magnesium sacrificial anode and RVC cathode. The vial was then placed on a stir plate and electrolysis was set to 10 mA. The reaction underwent the programmed electrolysis open to air. After completion of the reaction, the reaction mass was transferred to a separatory funnel, the electrodes were rinsed with EtOAc (5 mL) and saturated aq. $NaHCO_3$ (10 mL) was slowly added. The aqueous layer was extracted with EtOAc for three times. The combined organics were washed with water then brine, then dried over Na_2SO_4 before being filtered and concentrated via rotary evaporation. The crude oily solid was purified via flash chromatography to afford the desired product.

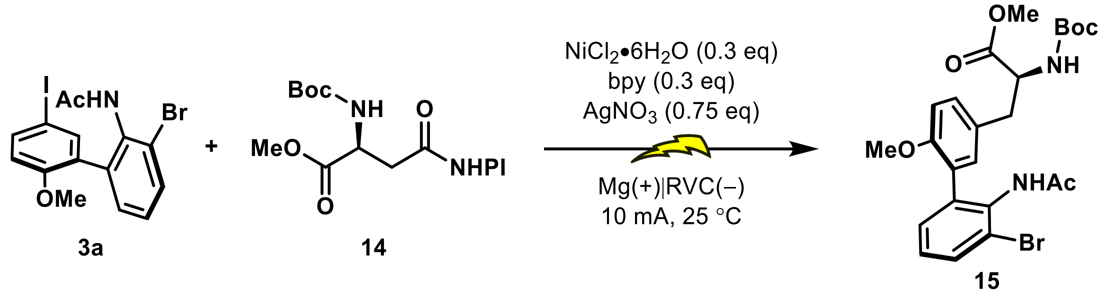

On 0.2 mmol scale, **General Procedure D** was followed with aryl iodide **3a** and redox active ester (RAE) **14**. Purification by silica gel column chromatography gave the title compound **15** (81.3 mg, 78% yield).

Compound 15

Physical State: sticky oil

^{1}H NMR (600 MHz, CDCl$_3$): δ 7.75 – 7.58 (m, 1H), 7.31 (s, 1H), 7.25 – 7.06 (m, 3H), 6.92 – 6.88 (m, 2H), 5.10 – 5.05 (m, 1H), 4.55 (s, 1H), 3.80 (s, 3H), 3.71 (s, 3H), 3.10 – 3.00 (m, 2H), 1.92 (s, 3H), 1.41 (s, 9H).

^{13}C NMR (151 MHz, CDCl$_3$): δ 172.42, 168.26, 155.18, 154.96, 138.73, 135.22, 134.42, 132.79, 132.63, 132.45, 130.40, 130.22, 128.85, 128.46, 128.11, 123.67, 111.38, 80.07, 56.22, 54.61, 52.44, 37.43, 28.42, 23.26, 20.55.

HRMS (ESI-TOF): calculated for $C_{24}H_{29}BrN_2NaO_6^+$ [M+Na]$^+$: 543.1101, found: 543.1119.

$[\alpha]^{25}_D$: +5.9 (c = 0.5, CHCl$_3$)

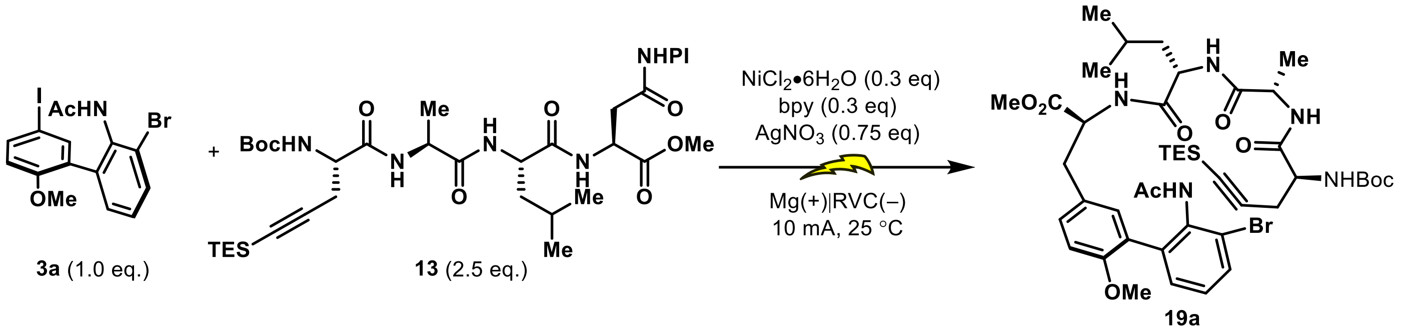

On 0.2 mmol scale, **General Procedure D** was followed with aryl iodide **3a** and redox active ester (RAE) **13**. Purification by silica gel column chromatography gave the title compound **19a** (102.5 mg, 56% yield).
On 3.0 mmol scale, synthesis for the **19a**:
To an oven dried 100 mL vial, $NiCl_2\cdot 6H_2O$ (214.2 mg, 0.9 mmol, 0.3 eq.), and 2,2′-bipyridine (141.3 mg, 0.9 mmol, 0.3 eq.) were all directly added and dissolved in anhydrous NMP (45 mL). The Ni catalytic mixture was allowed to stir for 5 minutes. Then, redox active ester (RAE) **13** (5.9 g, 7.5 mmol, 2.5 eq.) and aryl halide **3a** (1.34 g, 3.0 mmol, 1.0 eq.) were added to the catalytic solution. The solution of the vial was stirred until all solids were dissolved (roughly 15 minutes). $AgNO_3$ (382.5 mg, 2.25 mmol, 0.75 eq.) was then added to the reaction mixture directly as a solid. The vial was closed with a cap with a magnesium sacrificial anode and RVC cathode. The vial was then placed on a stir plate and electrolysis was set to 60 mA. The reaction underwent the programmed electrolysis open to air for 8h. After completion of the reaction, the reaction mass was transferred to a separatory funnel, the electrodes were rinsed with EtOAc (50 mL) and saturated. aq. $NaHCO_3$ (100 mL) was slowly added. The aqueous layer was extracted with EtOAc for three times. The combined organics were washed with water then brine, then dried over Na_2SO_4 before being filtered and concentrated via rotary evaporation. The crude oily solid was purified via flash chromatography (33% Hexane/ EtOAc) to afford the desired product **19a** (1.45 g, 53% yield).
Compound 19a
Physical State: amorphous solid
^{1}H NMR (600 MHz, METHANOL-*D*$_4$): δ 7.72 – 7.62 (m, 1H), 7.32 – 7.24 (m, 2H), 7.14 (s, 1H), 6.95 – 6.93 (m, 2H), 4.66 – 4.60 (m, 1H), 4.37 – 4.35 (m, 1H), 4.29 – 4.26 (m, 1H), 4.23 – 4.10 (m, 1H), 3.72 (s, 3H), 3.69 (s, 3H), 3.10 (dd, *J* = 13.9, 5.2 Hz, 1H), 3.01 (dd, *J* = 14.4, 6.4 Hz, 1H), 2.80 – 2.70 (m, 1H), 2.58 (dd, *J* = 16.9, 9.0 Hz, 1H), 1.86 (s, 3H), 1.59 – 1.51 (m, 3H), 1.45 (s, 9H), 1.31 (d, *J* = 7.0 Hz, 3H), 0.98 (t, *J* = 7.9 Hz, 9H), 0.92 – 0.78 (m, 6H), 0.57 (q, *J* = 7.9 Hz, 6H).
^{13}C NMR (151 MHz, METHANOL-*D*$_4$): δ 173.14, 171.63, 170.45, 156.48, 155.39, 140.19, 134.76, 131.72, 129.86, 128.48, 127.85, 123.10, 110.54, 103.38, 83.73, 79.62, 54.81, 53.86, 51.99, 51.45, 49.20, 40.39, 35.95, 27.42, 24.52, 22.65, 22.12, 21.13, 20.62, 17.08, 6.57, 4.05.
HRMS (ESI-TOF): calculated for $C_{44}H_{64}BrN_5NaO_9Si^+$ $[M+Na]^+$: 936.3549, found: 936.3559.
$[\alpha]^{25}_D$: –11.4 (*c* = 0.2, $CHCl_3$)

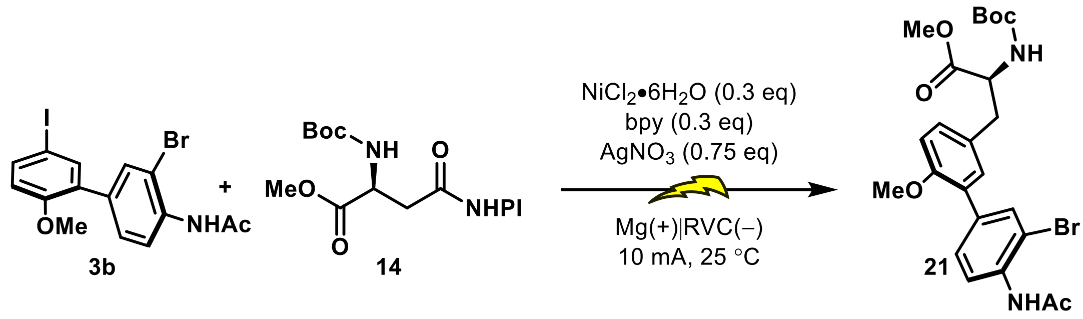

On 0.2 mmol scale, **General Procedure D** was followed with aryl iodide **3b** and redox active ester (RAE) **14**. Purification by silica gel column chromatography gave the title compound **21** (75.0 mg, 72% yield).

Compound 21

Physical State: sticky oil

^{1}H NMR (600 MHz, CDCl$_3$): δ 8.35 (d, *J* = 8.4 Hz, 1H), 7.69 (d, *J* = 1.7 Hz, 1H), 7.64 (s, 1H), 7.45 (dd, *J* = 8.5, 1.9 Hz, 1H), 7.11 – 7.04 (m, 1H), 7.01 (s, 1H), 6.88 (d, *J* = 8.4 Hz, 1H), 5.01 (d, *J* = 8.0 Hz, 1H), 4.57 (q, *J* = 5.9 Hz, 1H), 3.79 (s, 3H), 3.72 (s, 3H), 3.10 (dd, *J* = 13.9, 5.5 Hz, 1H), 3.02 (dd, *J* = 13.9, 5.8 Hz, 1H), 2.25 (s, 3H), 1.41 (s, 9H).

^{13}C NMR (151 MHz, CDCl$_3$): δ 172.45, 168.28, 155.60, 155.15, 135.47, 134.54, 132.94, 131.66, 129.82, 129.59, 128.61, 128.38, 121.29, 112.80, 111.50, 80.05, 55.72, 54.57, 52.35, 37.50, 28.39, 24.98.

HRMS (ESI-TOF): calculated for $C_{24}H_{29}BrN_2NaO_6^+$ [M+Na]$^+$: 543.1101, found: 543.1119.

[α]$^{25}_{D}$: +10.5 (*c* = 0.5, CHCl$_3$)

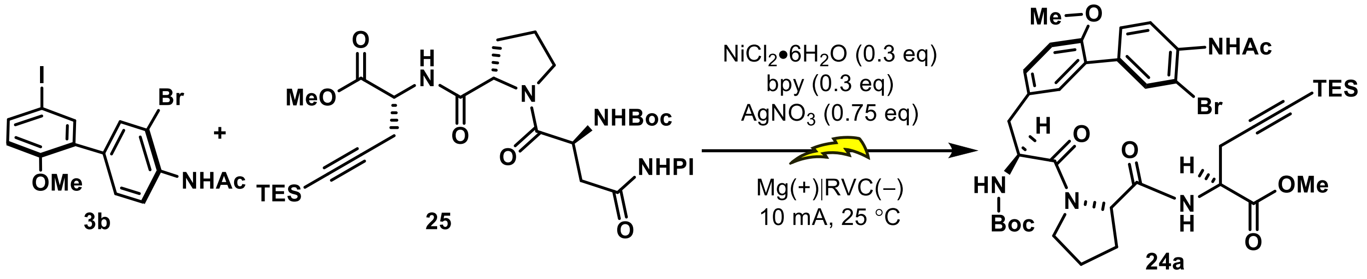

On 0.2 mmol scale, **General Procedure D** was followed with aryl iodide **3b** and redox active ester (RAE) **25**. Purification by silica gel column chromatography gave the title compound **24a** (102.6 mg, 62% yield).The characterization data were identical to the data reported by our group previously.[1]

Compound 24a

Physical State: white solid

^{1}H NMR (600 MHz, CDCl$_3$): δ 8.32 (t, *J* = 7.2 Hz, 1H), 7.72 – 7.61 (m, 2H), 7.48 – 7.40 (m, 1H), 7.21 – 7.01 (m, 3H), 6.87 (dd, *J* = 18.4, 8.4 Hz, 1H), 5.22 (dd, *J* = 39.3, 8.0 Hz, 1H), 4.74 – 4.32 (m, 3H), 3.80 – 3.64 (m, 6H), 3.39 (ddt, *J* = 21.1, 9.8, 5.2 Hz, 1H), 3.12 – 2.83 (m, 2H), 2.81 – 2.58 (m, 2H), 2.23 (s, 4H), 2.06 – 1.90 (m, 3H), 1.78 – 1.41 (m, 1H), 1.39 – 1.30 (m, 9H), 1.00 – 0.86 (m, 9H), 0.60 – 0.45 (m, 6H).

^{13}C NMR (151 MHz, CDCl$_3$): δ 171.76, 170.86, 170.75, 168.28, 155.44, 155.25, 135.56, 134.47, 132.98, 131.86, 131.51, 130.18, 129.72, 129.50, 128.67, 121.27, 112.83, 111.73, 111.34, 101.63, 85.82, 80.06, 79.77, 60.98, 60.15, 55.82, 55.73, 54.18, 53.18, 52.67, 51.83, 51.20, 47.46, 46.84, 38.23, 31.38, 28.37, 28.06, 25.11, 24.97, 23.75, 22.84, 22.10, 7.50, 4.42.

HRMS (ESI-TOF): calculated for $C_{40}H_{55}BrN_4NaO_8Si^+$ [M+Na]$^+$: 849.2865, found: 849.2855.

[α]$^{25}_{D}$: +7.9 (*c* = 0.5, CHCl$_3$)

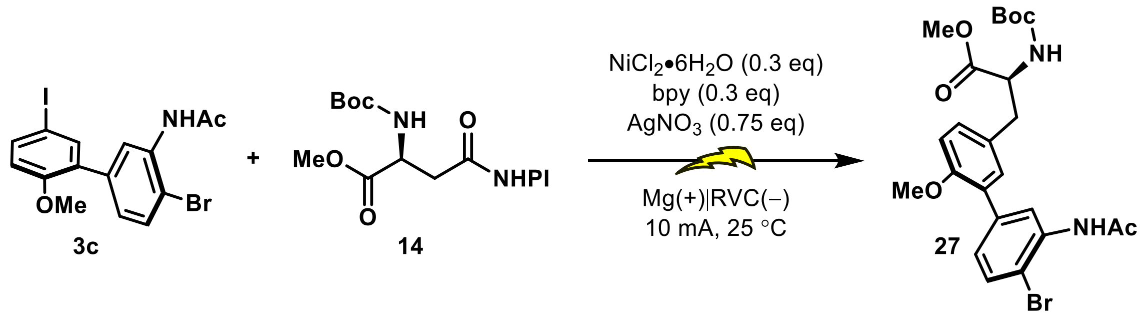

On 0.2 mmol scale, **General Procedure D** was followed with aryl iodide **3c** and redox active ester (RAE) **14**. Purification by silica gel column chromatography gave the title compound **27** (68.8 mg, 66% yield).

Compound 27

Physical State: sticky oil

^{1}H NMR (600 MHz, CDCl$_3$): δ 8.44 (s, 1H), 7.62 (s, 1H), 7.53 (d, *J* = 8.3 Hz, 1H), 7.12 (d, *J* = 7.9 Hz, 1H), 7.09 – 7.06 (m, 1H), 7.04 (s, 1H), 6.88 (d, *J* = 8.4 Hz, 1H), 5.03 (d, *J* = 8.1 Hz, 1H), 4.55 (q, *J* = 6.0 Hz, 1H), 3.79 (s, 3H), 3.72 (s, 3H), 3.08 (dd, *J* = 13.9, 5.6 Hz, 1H), 3.02 (dd, *J* = 14.0, 6.0 Hz, 1H), 2.23 (s, 3H), 1.40 (s, 9H).

^{13}C NMR (151 MHz, CDCl$_3$): δ 172.51, 168.24, 155.57, 155.21, 138.79, 135.33, 131.69, 129.88, 129.35, 128.32, 126.60, 123.29, 112.22, 111.52, 79.98, 55.76, 54.61, 52.40, 37.51, 28.38, 24.91.

HRMS (ESI-TOF): calculated for $C_{24}H_{29}BrN_2NaO_6^+$ [M+Na]$^+$: 543.1101, found: 543.1119.

[α]$^{25}_D$: +8.6 (*c* = 0.5, CHCl$_3$)

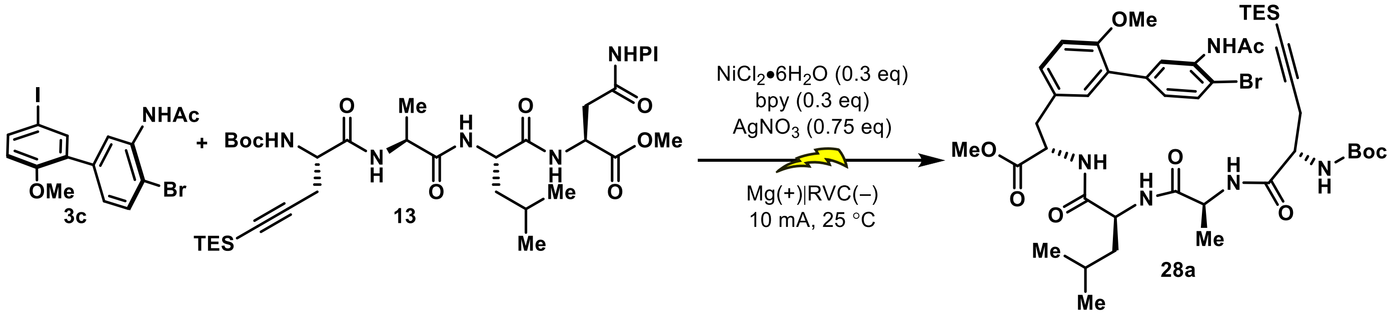

On 0.2 mmol scale, **General Procedure D** was followed with aryl iodide **3c** and redox active ester (RAE) **13**. Purification by silica gel column chromatography gave the title compound **28a** (87.8 mg, 48% yield).

Compound 28a

Physical State: amorphous solid

^{1}H NMR (600 MHz, CDCl$_3$): δ 8.47 – 8.40 (m, 1H), 7.77 (s, 1H), 7.66 (d, *J* = 5.8 Hz, 1H), 7.53 (d, *J* = 8.3 Hz, 1H), 7.24 – 7.18 (m, 1H), 7.06 – 7.02 (m, 3H), 6.91 (d, *J* = 6.4 Hz, 1H), 6.85 (d, *J* = 8.4 Hz, 1H), 5.27 (d, *J* = 5.5 Hz, 1H), 4.85 (q, *J* = 6.5 Hz, 1H), 4.49 – 4.46 (m, 1H), 4.41 – 4.38 (m, 1H), 4.19 – 4.11 (m, 1H), 3.76 (s, 3H), 3.71 (s, 3H), 3.18 (dd, *J* = 14.2, 4.5 Hz, 1H), 3.04 (dd, *J* = 14.2, 6.3 Hz, 1H), 2.80 (dd, *J* = 17.1, 5.8 Hz, 1H), 2.63 (dd, *J* = 17.1, 5.9 Hz, 1H), 2.30 (s, 3H), 1.73 – 1.68 (m, 1H), 1.63 – 1.58 (m, 1H), 1.53 – 1.47 (m, 1H), 1.44 (s, 9H), 1.33 (d, *J* = 6.8 Hz, 3H), 0.95 (t, *J* = 7.9 Hz, 9H), 0.86 – 0.83 (m, 6H), 0.55 (q, *J* = 7.9 Hz, 6H).

^{13}C NMR (151 MHz, CDCl$_3$): δ 172.64, 172.02, 171.64, 170.20, 169.08, 155.70, 155.47, 138.87, 135.43, 131.59, 130.05, 129.13, 128.60, 127.10, 122.35, 111.68, 111.48, 102.29, 86.08, 80.73, 55.66, 53.30, 52.41, 49.24, 40.23, 36.84, 31.67, 28.29, 25.13, 24.91, 23.34, 23.12, 22.73, 21.31, 18.97, 14.20, 7.56, 4.39.

HRMS (ESI-TOF): calculated for $C_{44}H_{64}BrN_5NaO_9Si$+ [M+Na]$^+$: 936.3549, found: 936.3550.

[α]$^{25}_D$: +23.7 (*c* = 0.2, CHCl$_3$)

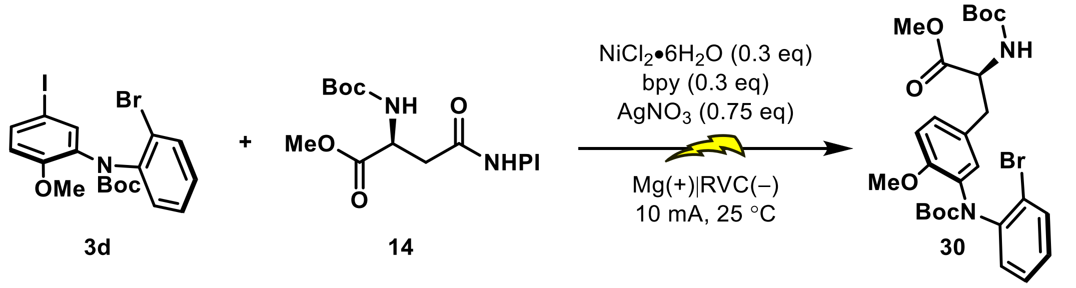

On 0.2 mmol scale, **General Procedure D** was followed with aryl iodide **3d** and redox active ester (RAE) **14**. Purification by silica gel column chromatography gave the title compound **30** (68.4 mg, 59% yield).

Compound 30

Physical State: amorphous solid

^{1}H NMR (600 MHz, CDCl$_3$): δ 7.60 (d, *J* = 5.2 Hz, 1H), 7.32 – 7.28 (m, 1H), 7.21 (t, *J* = 7.5 Hz, 1H), 7.11 – 6.94 (m, 3H), 6.87 (d, *J* = 6.7 Hz, 1H), 4.96 (s, 1H), 4.51 – 4.47 (m, 1H), 3.88 (s, 3H), 3.55 – 3.45 (m, 3H), 2.97 (s, 2H), 1.41 (s, 18H).

^{13}C NMR (151 MHz, CDCl$_3$): δ 172.09, 155.10, 154.42, 153.36, 142.38, 134.37, 133.27, 129.89, 129.43, 128.28, 123.65, 123.53, 112.55, 111.47, 80.68, 79.93, 56.01, 55.47, 54.43, 52.05, 37.36, 28.40, 28.18.

HRMS (ESI-TOF): calculated for $C_{27}H_{35}BrN_2NaO_7^+$ [M+Na]$^+$: 601.1520, found: 601.1530.

[α]^{25}$_D$: +56.5 (*c* = 0.5, CHCl$_3$)

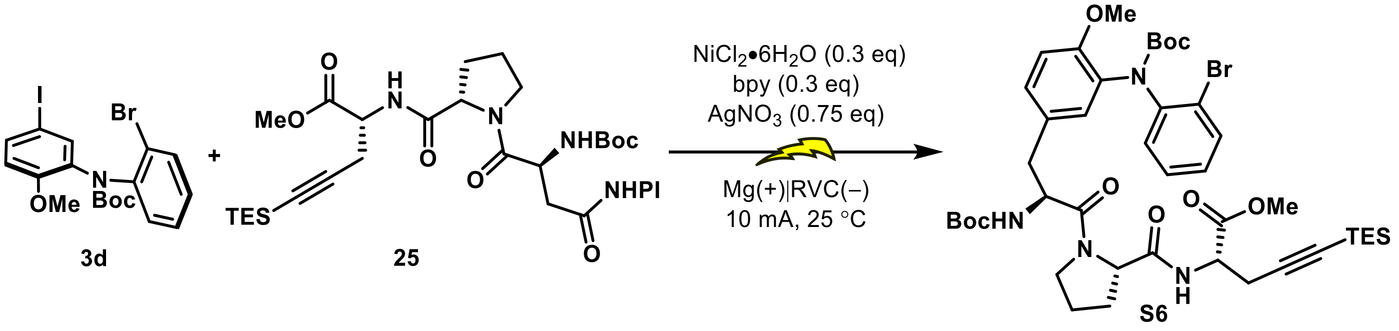

On 0.2 mmol scale, **General Procedure D** was followed with aryl iodide **3d** and redox active ester (RAE) **25**. Purification by silica gel column chromatography gave the title compound **S6** (58.5 mg, 33% yield).

Compound S6

Physical State: colorless oil

^{1}H NMR (600 MHz, CDCl$_3$): δ 7.62 – 7.59 (m, 1H), 7.28 – 7.27 (m, 1H), 7.25 – 7.15 (m, 2H), 7.13 – 7.00 (m, 2H), 6.90 – 6.83 (m, 1H), 5.17 (s, 1H), 4.64 – 4.49 (s, 2H), 3.89 – 3.82 (m, 3H), 3.76 – 3.64 (m, 3H), 3.54 – 3.40 (m, 1H), 3.00 – 2.66 (m, 4H), 2.23 (s, 1H), 2.14 – 1.52 (m, 4H), 1.45 – 1.42 (m, 2H), 1.42 – 1.24 (m, 18H), 0.97 – 0.90 (m, 9H), 0.53 (q, *J* = 7.9 Hz, 6H).

^{13}C NMR (151 MHz, CDCl$_3$): δ 172.15, 171.05, 170.76, 155.29, 142.36, 134.19, 133.31, 131.94, 129.47, 128.57, 128.15, 124.12, 123.56, 111.39, 102.49, 101.64, 85.81, 80.63, 79.91, 79.73, 60.14, 55.55, 53.13, 52.64, 52.52, 51.91, 51.36, 47.36, 46.57, 38.29, 31.24, 29.78, 28.37, 28.18, 27.70, 25.11, 23.84, 22.77, 22.04, 7.48, 4.41.

HRMS (ESI-TOF): calculated for $C_{43}H_{61}BrN_4NaO_9Si^+$ [M+Na]$^+$: 907.3283, found: 907.3288.

[α]^{25}$_D$: +2.4 (*c* = 0.5, CHCl$_3$)

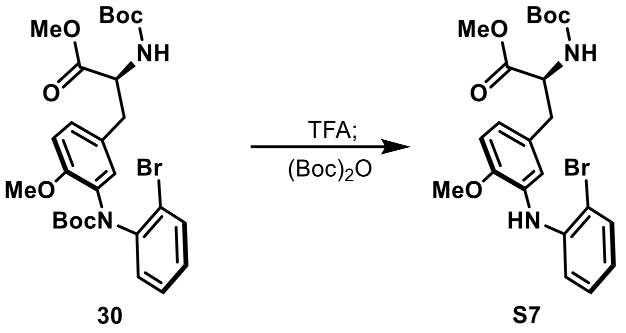

The compound **59** (0.1 mmol, 58.0 mg, 1.0 eq.) was dissolved in DCM (2 mL), TFA (0.5 mL) was added dropwise, the reaction mixture was stirred at rt for 1 h, the solvents were removed under reduced pressure, and then quenched by saturated aq. $NaHCO_3$, extracted with DCM for three times, the organic layers were concentrated under reduced pressure to give the crude amine, the crude was then dissolved in DCM (2 mL), DIPEA (0.3 mmol, 3.0 eq.), $(Boc)_2O$ (0.15 mmol, 1.5 eq.) was added, the reaction was stirred at rt for 4 h. The solvents were removed under reduced pressure to give the residue, the residue was purified by silica gel chromatography to give the product **S7** (38.8 mg, 81% yield).[2]

Compound S7

Physical State: amorphous solid

(38.8 mg, 81% yield).

^{1}H NMR (600 MHz, $CDCl_3$): δ 7.54 (dd, *J* = 8.0, 1.4 Hz, 1H), 7.33 (d, *J* = 7.7 Hz, 1H), 7.21 (t, *J* = 7.4 Hz, 1H), 7.01 (s, 1H), 6.83 (d, *J* = 8.2 Hz, 1H), 6.77 (td, *J* = 7.9, 1.5 Hz, 1H), 6.68 (dd, *J* = 8.2, 2.1 Hz, 1H), 6.39 (s, 1H), 4.98 (d, *J* = 8.2 Hz, 1H), 4.54 (q, *J* = 5.8 Hz, 1H), 3.88 (s, 3H), 3.70 (s, 3H), 3.07 – 2.93 (m, 2H), 1.40 (s, 9H).

^{13}C NMR (151 MHz, $CDCl_3$): δ 172.54, 155.24, 148.55, 140.73, 133.17, 131.54, 128.20, 128.12, 122.31, 121.54, 117.58, 117.09, 113.71, 110.93, 80.00, 55.87, 54.47, 52.33, 37.76, 28.38.

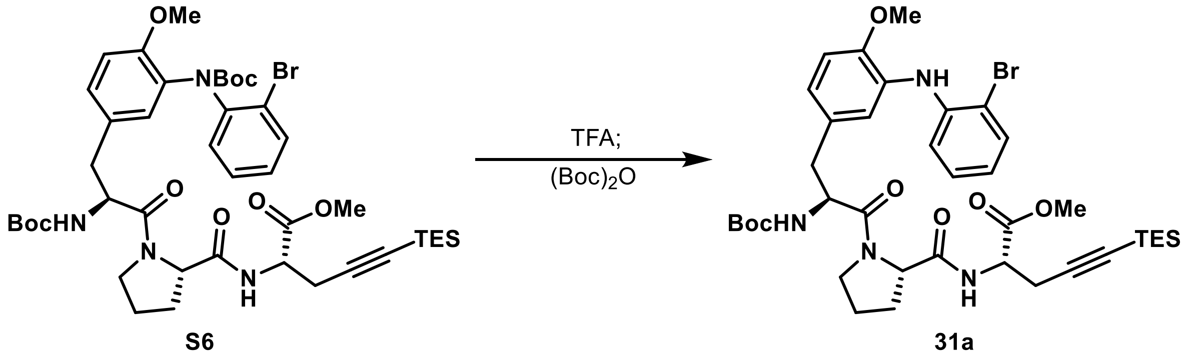

The compound **S6** (0.1 mmol, 88.6 mg, 1.0 eq.) was dissolved in DCM (2 mL), TFA (0.5 mL) was added dropwise, the reaction mixture was stirred at rt for 1 h, the solvents were removed under reduced pressure, and then quenched by saturated aq. $NaHCO_3$, extracted with DCM for three times, the organic layers were concentrated under reduced pressure to give the crude amine, the crude was then dissolved in DCM (2 mL), DIPEA (0.3 mmol, 3.0 eq.), $(Boc)_2O$ (0.15 mmol, 1.5 eq.) was added, the reaction was stirred at rt for 4 h. The solvents were removed under reduced pressure to give the residue, the residue was purified by silica gel chromatography to give the product **31a** (55.0 mg, 70% yield). The characterization data were identical to the data reported by our group previously.[2]

Compound 31a

Physical State: amorphous solid

^{1}H NMR (600 MHz, $CDCl_3$): δ 7.57 – 7.51 (m, 1H), 7.39 – 7.08 (m, 3H), 7.06 – 6.70 (m, 3H), 4.58 – 4.25 (m, 3H), 3.90 – 3.86 (m, 3H), 3.79 – 3.74 (m, 1H), 3.72 – 3.65 (m, 3H), 3.62 – 3.42 (m, 1H), 3.09 – 2.63 (m, 4H), 2.18 – 2.15 (m, 1H), 2.09 – 1.93 (m, 3H), 1.75 – 1.55 (m, 1H), 1.39 – 1.34 (m, 9H), 1.31 – 1.26 (m, 2H),

0.99 – 0.95 (m, 9H), 0.60 – 0.52 (m, 6H).
^{13}C NMR (151 MHz, CDCl$_3$): δ 172.69, 172.56, 172.17, 171.96, 170.86, 170.54, 156.36, 148.99, 148.41, 141.21, 140.37, 132.65, 132.04, 130.77, 129.41, 128.40, 127.98, 122.94, 122.00, 121.77, 120.70, 119.04, 116.24, 113.74, 112.27, 110.81, 102.91, 102.24, 84.26, 83.33, 79.38, 79.28, 60.45, 60.09, 55.05, 54.74, 54.00, 52.16, 51.71, 46.64, 36.77, 30.86, 29.08, 27.36, 24.54, 22.37, 21.79, 6.49, 3.97.[2]

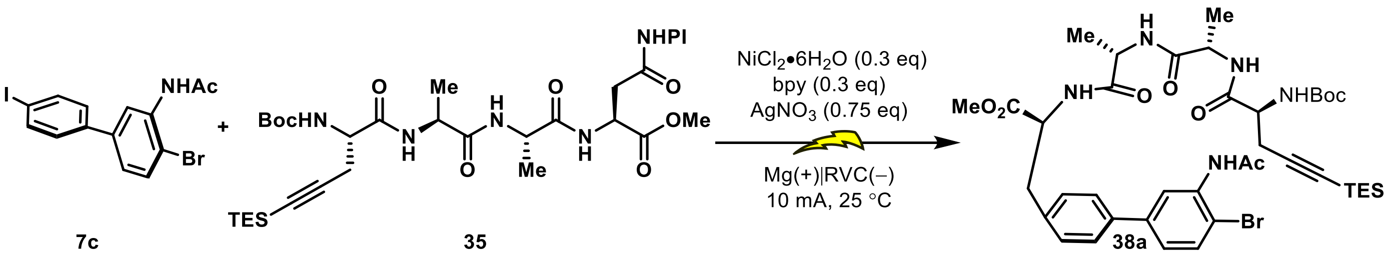

On 0.2 mmol scale, **General Procedure D** was followed with aryl iodide **7c** and redox active ester (RAE) **35**. Purification by silica gel column chromatography gave the title compound **38a** (96.7 mg, 57% yield).

Compound 38a

Physical State: amorphous solid

^{1}H NMR (600 MHz, DMSO-*d*$_6$): δ 9.52 (s, 1H), 8.24 (d, *J* = 7.5 Hz, 1H), 7.94 (d, *J* = 7.5 Hz, 1H), 7.90 (d, *J* = 7.4 Hz, 1H), 7.82 (s, 1H), 7.66 (d, *J* = 8.3 Hz, 1H), 7.50 (d, *J* = 8.1 Hz, 2H), 7.36 (d, *J* = 8.1 Hz, 1H), 7.27 (d, *J* = 8.2 Hz, 2H), 6.99 (d, *J* = 8.7 Hz, 1H), 4.46 – 4.42 (m, 1H), 4.24 – 4.17 (m, 2H), 4.11 – 4.07 (m, 1H), 3.56 (s, 3H), 3.02 (dd, *J* = 13.8, 5.6 Hz, 1H), 2.94 (dd, *J* = 13.9, 8.7 Hz, 1H), 2.58 (dd, *J* = 17.0, 3.9 Hz, 1H), 2.39 (dd, *J* = 17.0, 10.0 Hz, 1H), 2.07 (s, 3H), 1.33 (s, 9H), 1.13 (d, *J* = 7.1 Hz, 3H), 1.09 (d, *J* = 7.0 Hz, 3H), 0.88 (t, *J* = 7.9 Hz, 9H), 0.46 (q, *J* = 7.9 Hz, 6H).

^{13}C NMR (151 MHz, DMSO-*d*$_6$): δ 172.78, 172.24, 172.00, 170.48, 169.24, 155.66, 140.16, 137.51, 137.44, 137.37, 133.55, 130.40, 126.93, 125.46, 125.31, 117.18, 105.94, 83.09, 78.73, 53.98, 53.84, 52.43, 48.52, 48.30, 36.66, 28.63, 23.85, 23.78, 18.79, 18.68, 7.88, 4.46.

HRMS (ESI-TOF): calculated for $C_{40}H_{56}BrN_5NaO_8Si^+$ [M+Na]$^+$: 864.2974, found: 864.2976.

[α]$^{25}_{D}$: +20.7 (*c* = 0.2, CHCl$_3$)

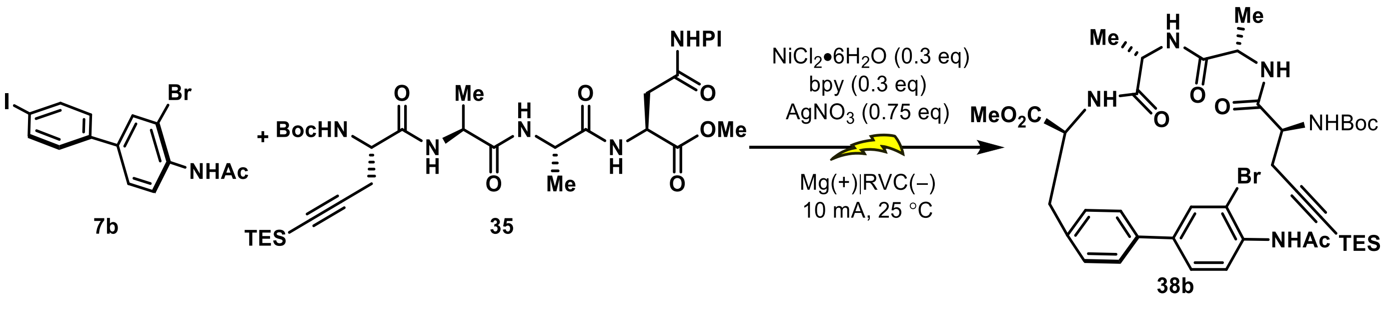

On 0.2 mmol scale, **General Procedure D** was followed with aryl iodide **7b** and redox active ester (RAE) **35**. Purification by silica gel column chromatography gave the title compound **38b** (92.7 mg, 55% yield).

Compound 38b

Physical State: amorphous solid

^{1}H NMR (600 MHz, METHANOL-*D*$_4$): δ 7.83 (s, 1H), 7.74 (d, *J* = 8.4 Hz, 1H), 7.57 (d, *J* = 7.8 Hz, 1H), 7.52 (d, *J* = 8.0 Hz, 2H), 7.30 (d, *J* = 7.9 Hz, 2H), 4.70 (dd, *J* = 8.3, 5.6 Hz, 1H), 4.35 – 4.28 (m, 2H), 4.15 (dd, *J* = 8.3, 5.0 Hz, 1H), 3.71 (s, 3H), 3.21 (dd, *J* = 14.0, 5.5 Hz, 1H), 3.08 (dd, *J* = 13.9, 8.5 Hz, 1H), 2.74 (dd, *J* = 17.2, 4.8 Hz, 1H), 2.60 (dd, *J* = 17.1, 8.8 Hz, 1H), 2.20 (s, 3H), 1.44 (s, 9H), 1.31 (d, *J* = 7.1 Hz, 6H), 0.98 (t, *J* = 7.9 Hz, 9H), 0.56 (q, *J* = 7.9 Hz, 6H).

^{13}C NMR (151 MHz, METHANOL-*D*$_4$): δ 173.40, 172.99, 171.91, 171.79, 170.75, 156.59, 139.84, 137.45,

136.59, 134.97, 130.51, 129.73, 126.63, 126.03, 117.92, 103.24, 83.84, 79.70, 54.08, 53.64, 51.45, 49.27, 48.95, 36.54, 27.39, 22.57, 22.00, 16.76, 16.62, 6.53, 4.01.
HRMS (ESI-TOF): calculated for $C_{40}H_{56}BrN_5NaO_8Si^+$ $[M+Na]^+$: 864.2974, found: 864.2975.
$[\alpha]^{25}_D$: +17.9 (c = 0.2, $CHCl_3$)

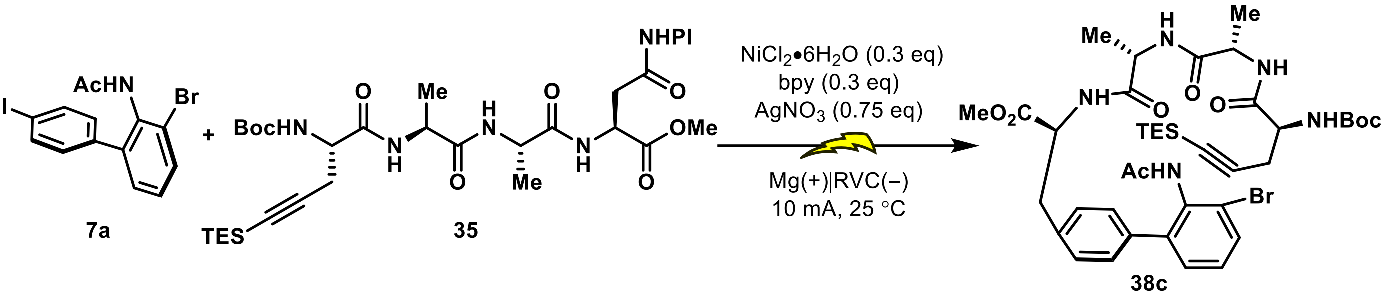

On 0.2 mmol scale, **General Procedure D** was followed with aryl iodide **7a** and redox active ester (RAE) **35**. Purification by silica gel column chromatography gave the title compound **38c** (101.2 mg, 60% yield).
Compound 38c
Physical State: amorphous solid
^{1}H NMR (600 MHz, METHANOL-*D*$_4$): δ 7.66 (d, J = 7.7 Hz, 1H), 7.34 – 7.24 (m, 6H), 4.75 – 4.58 (m, 1H), 4.33 – 4.29 (m, 2H), 4.17 – 4.14 (m, 1H), 3.69 (s, 3H), 3.19 (dd, J = 13.5, 5.3 Hz, 1H), 3.07 (dd, J = 13.5, 8.3 Hz, 1H), 2.74 (dd, J = 17.1, 4.8 Hz, 1H), 2.60 (dd, J = 17.1, 8.9 Hz, 1H), 1.92 (s, 3H), 1.44 (s, 9H), 1.32 (d, J = 5.0 Hz, 6H), 0.98 (t, J = 7.9 Hz, 9H), 0.57 (q, J = 7.9 Hz, 6H).
^{13}C NMR (151 MHz, METHANOL-*D*$_4$): δ 173.39, 171.82, 171.22, 156.57, 142.93, 137.75, 136.34, 133.56, 131.85, 129.46, 128.86, 128.53, 124.15, 103.28, 83.79, 79.68, 54.01, 53.80, 51.41, 49.23, 48.94, 36.70, 27.37, 22.59, 21.07, 16.71, 6.52, 4.01.
HRMS (ESI-TOF): calculated for $C_{40}H_{56}BrN_5NaO_8Si^+$ $[M+Na]^+$: 864.2974, found: 864.2949.
$[\alpha]^{25}_D$: –1.2 (c = 0.5, $CHCl_3$)

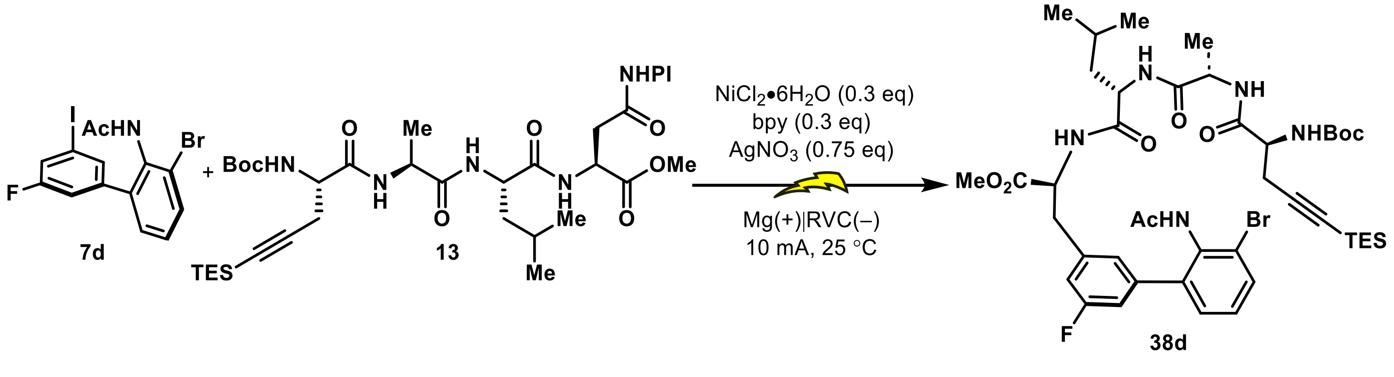

On 0.2 mmol scale, **General Procedure D** was followed with aryl iodide **7d** and redox active ester (RAE) **13**. Purification by silica gel column chromatography gave the title compound **28d** (112.0 mg, 62% yield).
Compound 38d
Physical State: amorphous solid
^{1}H NMR (600 MHz, METHANOL-*D*$_4$): δ 7.67 (d, J = 7.7 Hz, 1H), 7.34 (d, J = 7.0 Hz, 1H), 7.29 (t, J = 7.8 Hz, 1H), 6.99 (s, 1H), 6.94 (d, J = 8.9 Hz, 2H), 4.68 (dd, J = 8.0, 5.6 Hz, 1H), 4.33 (t, J = 7.5 Hz, 1H), 4.27 – 4.22 (m, 1H), 4.13 (dd, J = 8.2, 4.4 Hz, 1H), 3.68 (s, 3H), 3.21 (dd, J = 14.0, 5.3 Hz, 1H), 3.04 (dd, J = 14.0, 8.4 Hz, 1H), 2.71 (dd, J = 17.1, 4.7 Hz, 1H), 2.57 (dd, J = 17.0, 8.9 Hz, 1H), 1.94 (s, 3H), 1.61 – 1.56 (m, 1H), 1.52 – 1.47 (m, 2H), 1.43 (s, 9H), 1.28 (d, J = 7.1 Hz, 3H), 0.96 (t, J = 7.9 Hz, 9H), 0.88 – 0.83 (m, 6H), 0.55 (q, J = 7.9 Hz, 6H).
^{13}C NMR (151 MHz, METHANOL-*D*$_4$): δ 173.24, 173.14, 171.76, 171.49, 171.08, 162.36 (d, J = 244.6 Hz),

156.54, 141.82, 141.32, 139.45, 133.53, 132.38, 129.44, 129.01, 125.47, 124.06, 115.02 (d, J = 21.5 Hz), 113.65 (d, J = 22.5 Hz), 103.30, 83.76, 79.66, 53.96, 53.48, 51.82, 51.53, 49.25, 40.43, 36.52, 31.42, 27.39, 24.47, 22.57, 22.05, 21.16, 20.59, 16.87, 13.11, 6.53, 4.02.
HRMS (ESI-TOF): calculated for $C_{43}H_{61}BrFN_5NaO_8Si^+$ $[M+Na]^+$: 924.3349, found: 924.3361.
$[\alpha]^{25}_D$: -4.6 (c = 0.2, $CHCl_3$)

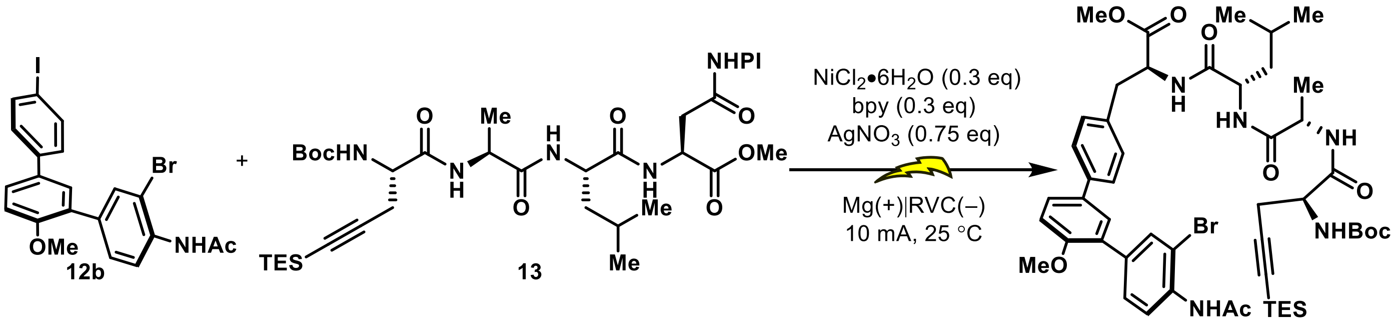

On 0.2 mmol scale, **General Procedure D** was followed with aryl iodide **12b** and redox active ester (RAE) **13**. Purification by silica gel column chromatography gave the title compound **38e** (103.1 mg, 52% yield).
Compound 38e
Physical State: amorphous solid
^{1}H NMR (600 MHz, METHANOL-D_4): δ 7.75 (s, 1H), 7.68 (d, J = 7.9 Hz, 1H), 7.58 – 7.37 (m, 5H), 7.23 (d, J = 7.2 Hz, 2H), 7.10 (d, J = 7.6 Hz, 1H), 4.69 (t, J = 6.5 Hz, 1H), 4.36 (s, 1H), 4.26 (d, J = 7.8 Hz, 1H), 4.10 (s, 1H), 3.81 (s, 3H), 3.69 (s, 3H), 3.18 (d, J = 11.8 Hz, 1H), 3.03 (t, J = 11.3 Hz, 1H), 2.75 – 2.65 (m, 1H), 2.59 – 2.51 (m, 1H), 2.18 (s, 3H), 1.51 – 1.48 (m, 1H), 1.40 (s, 9H), 1.36 – 1.33 (m, 2H), 1.25 (d, J = 6.4 Hz, 3H), 0.98 – 0.83 (m, 15H), 0.61 – 0.43 (m, 6H).
^{13}C NMR (151 MHz, METHANOL-D_4): δ 173.11, 171.86, 170.76, 156.56, 156.08, 138.92, 137.77, 135.40, 134.58, 133.57, 133.27, 129.51, 128.87, 128.78, 128.60, 127.88, 127.36, 126.36, 125.89, 120.42, 116.99, 111.74, 103.24, 83.85, 79.69, 54.92, 54.51, 54.07, 53.59, 51.73, 51.44, 49.35, 40.46, 36.53, 27.40, 24.47, 22.54, 22.06, 20.69, 16.88, 6.54, 4.01.
HRMS (ESI-TOF): calculated for $C_{50}H_{68}BrN_5NaO_9Si^+$ $[M+Na]^+$: 1012.3862, found: 1012.3870.
$[\alpha]^{25}_D$: +6.2 (c = 0.2, $CHCl_3$)

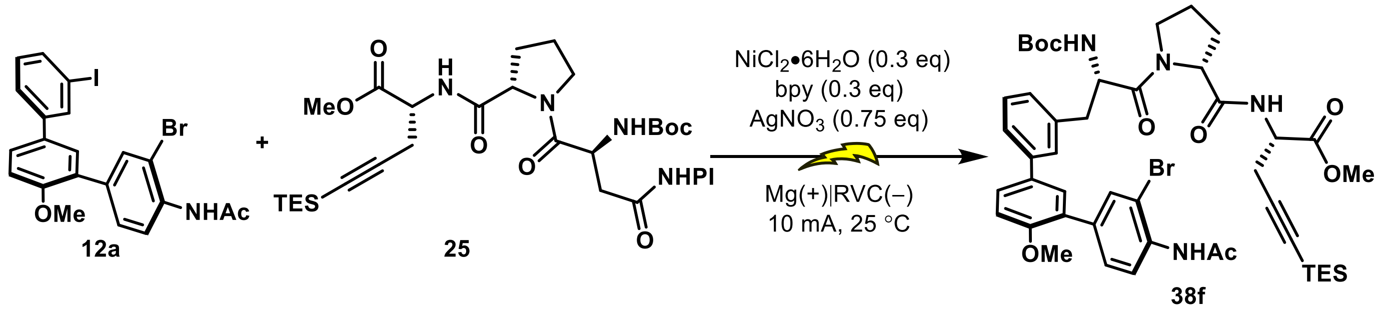

On 0.2 mmol scale, **General Procedure D** was followed with aryl iodide **12a** and redox active ester (RAE) **25**. Purification by silica gel column chromatography gave the title compound **38f** (115.7 mg, 64% yield).
Compound 38f
Physical State: amorphous solid
^{1}H NMR (600 MHz, METHANOL-D_4): δ 7.79 (s, 1H), 7.69 (dd, J = 8.3, 3.1 Hz, 1H), 7.63 – 7.57 (m, 1H), 7.56 – 7.43 (m, 4H), 7.38 – 7.31 (m, 1H), 7.20 (dd, J = 15.5, 7.6 Hz, 1H), 7.14 (t, J = 7.9 Hz, 1H), 4.65 – 4.54 (m, 1H), 4.53 – 4.43 (m, 2H), 3.83 (s, 3H), 3.79 – 3.74 (m, 1H), 3.71 – 3.69 (m, 3H), 3.66 – 3.61 (m, 1H), 3.58 (s, 1H), 3.17 – 2.96 (m, 1H), 2.91 – 2.82 (m, 1H), 2.81 – 2.64 (m, 2H), 2.18 (s, 3H), 2.05 – 1.95 (m, 3H),

1.35 – 1.27 (m, 9H), 0.97 – 0.93 (m, 9H), 0.57 – 0.51 (m, 6H).

^{13}C NMR (151 MHz, METHANOL-*D*$_4$): δ 172.71, 171.80, 170.84, 170.59, 156.32, 156.14, 140.50, 137.83, 137.74, 136.92, 134.56, 133.91, 133.31, 128.94, 128.88, 128.76, 128.59, 127.70, 125.88, 125.28, 124.74, 116.99, 111.80, 111.63, 102.23, 84.30, 79.29, 60.54, 60.12, 54.93, 53.71, 51.72, 51.62, 38.21, 37.29, 30.86, 29.02, 27.46, 27.37, 27.29, 24.58, 22.35, 21.98, 21.72, 15.98, 6.47, 3.97.

HRMS (ESI-TOF): calculated for $C_{46}H_{59}BrN_4NaO_8Si^+$ [M+Na]$^+$: 925.3178, found: 925.3182.

[α]$^{25}_{D}$: +3.3 (*c* = 0.5, $CHCl_3$)

Larock macrocyclization of unnatural scaffold：

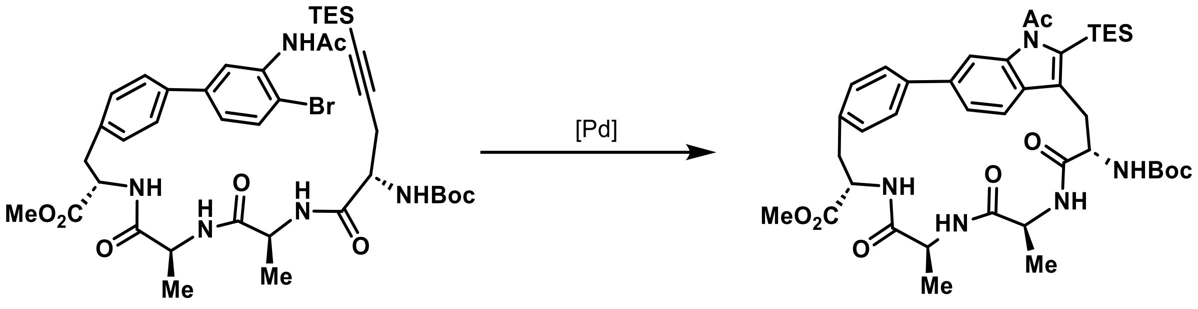

38a 39a

The precursor **38a** (84.3 mg, 0.1 mmol, 1.0 eq.) was dissolved in acetonitrile (1.5 mL). Pd(tBu$_3$P)$_2$ (10.2 mg, 0.02 mmol, 0.2 eq.) and DIPEA (32.3 mg, 0.25 mmol, 2.5 eq.) were added. The reaction mixture was stirred at 110 °C under nitrogen atmosphere for 12 h. The reaction mixture was concentrated under reduced pressure and purified by silica gel chromatography to give the cyclized compound **39a** (38.1 mg, 50% yield).

Compound 39a

Physical State: amorphous solid

^{1}H NMR (600 MHz, CDCl$_3$): δ 7.83 (s, 1H), 7.68 (s, 1H), 7.57 – 7.52 (m, 1H), 7.40 – 7.35 (m, 2H), 7.21 (s, 1H), 7.09 – 7.01 (m, 1H), 6.31 – 6.20 (m, 2H), 5.65 – 5.36 (m, 1H), 4.97 – 4.91 (m, 1H), 4.61 (s, 1H), 4.37 – 4.29 (m, 1H), 3.82 (s, 3H), 3.78 – 3.72 (m, 1H), 3.65 – 3.57 (m, 2H), 3.54 (dd, J = 13.4, 5.6 Hz, 1H), 3.43 – 3.41 (m, 1H), 2.89 (s, 3H), 2.69 – 2.59 (m, 1H), 1.38 (s, 9H), 1.14 (d, J = 7.1 Hz, 3H), 1.00 – 0.87 (m, 18H).

^{1}H NMR (600 MHz, DMSO-*d*$_6$) δ 7.96 (d, J = 9.9 Hz, 1H), 7.88 (s, 1H), 7.85 (d, J = 8.4 Hz, 1H), 7.63 (d, J = 7.8 Hz, 1H), 7.40 (d, J = 7.9 Hz, 1H), 7.19 (d, J = 8.1 Hz, 3H), 6.90 (d, J = 8.0 Hz, 1H), 6.64 (s, 1H), 4.75 (s, 1H), 4.08 (d, J = 8.9 Hz, 1H), 3.75 – 3.67 (m, 1H), 3.66 (s, 3H), 3.50 – 3.44 (m, 1H), 3.37 – 3.32 (m, 1H), 3.28 (d, J = 9.1 Hz, 1H), 3.21 – 3.11 (m, 1H), 2.83 (s, 3H), 2.63 (t, J = 12.8 Hz, 1H), 1.35 (s, 9H), 1.26 – 1.20 (m, 1H), 0.93 (d, J = 7.1 Hz, 3H), 0.91 – 0.83 (m, 15H), -0.18 (s, 3H).

^{13}C NMR (151 MHz, CDCl$_3$): δ 172.04, 170.55, 169.80, 169.44, 154.96, 140.21, 138.19, 134.53, 131.15, 129.95, 129.28, 126.78, 123.88, 121.64, 110.84, 80.40, 70.65, 52.88, 52.54, 50.42, 47.76, 38.91, 28.28, 27.18, 26.75, 19.08, 14.80, 8.40, 6.49.

^{13}C NMR (151 MHz, DMSO-*d*$_6$) δ 172.35, 171.40, 170.30, 169.74, 169.20, 155.44, 139.21, 138.17, 137.45, 136.45, 135.73, 132.66, 132.10, 130.16, 129.01, 124.86, 121.63, 111.49, 78.98, 70.32, 57.42, 52.69, 52.35, 48.25, 47.45, 37.30, 31.56, 28.68, 27.24, 27.02, 26.55, 20.06, 16.15, 8.71, 5.66.

HRMS (ESI-TOF): calculated for $C_{40}H_{55}N_5NaO_8Si^+$ [M+Na]$^+$: 784.3712, found: 784.3719.

[α]$^{25}_D$: +48.1 (c = 0.5, CHCl$_3$)

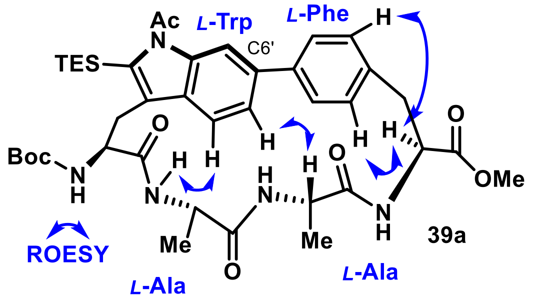

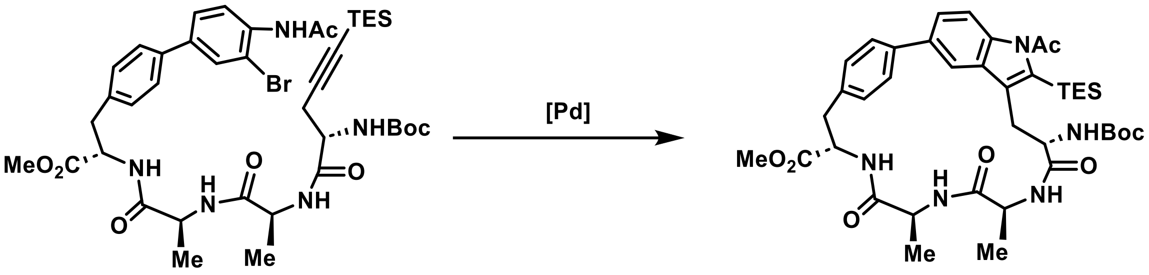

38b **39b**

The precursor **38b** (84.3 mg, 0.1 mmol, 1.0 eq.) was dissolved in acetonitrile (1.5 mL). Pd(*t*Bu3P)2 (10.2 mg, 0.02 mmol, 0.2 eq.) and DIPEA (32.3 mg, 0.25 mmol, 2.5 eq.) were added. The reaction mixture was stirred at 110 °C under nitrogen atmosphere for 12 h. The reaction mixture was concentrated under reduced pressure and purified by silica gel chromatography to give the cyclized compound **39b** (28.9 mg, 38% yield).

Compound 39b

Physical State: amorphous solid

^{1}H NMR (600 MHz, CDCl$_3$): (1/1 atropisomers cannot be separated) δ 8.13 – 7.29 (m, 6H), 7.26 – 7.16 (m, 2H), 7.13 – 6.64 (m, 2H), 6.56 – 6.18 (m, 1H), 5.52 – 4.68 (m, 2H), 4.54 – 4.04 (m, 2H), 3.86 – 3.70 (m, 3H), 3.56 – 2.92 (m, 3H), 2.88 – 2.14 (m, 4H), 1.49 – 1.37 (m, 9H), 1.37 – 1.30 (m, 3H), 1.06 – 0.75 (m, 15H), 0.69 – 0.49 (m, 3H).

^{13}C NMR (151 MHz, CDCl$_3$): (1/1 atropisomers cannot be separated) δ 172.28, 171.99, 171.85, 171.57, 169.55, 137.32, 136.68, 133.16, 129.97, 129.84, 127.57, 127.45, 126.89, 123.22, 120.20, 114.18, 81.28, 53.82, 53.29, 52.77, 52.48, 52.36, 50.22, 49.63, 49.17, 37.45, 28.30, 28.26, 26.70, 24.70, 17.71, 17.27, 16.85, 8.36, 7.56, 6.40, 4.38, 4.02.

HRMS (ESI-TOF): calculated for $C_{40}H_{55}N_5NaO_8Si^+$ [M+Na]$^+$: 784.3712, found: 784.3719.

[α]$^{25}_D$: + 4.7 (*c* = 0.5, CHCl$_3$)

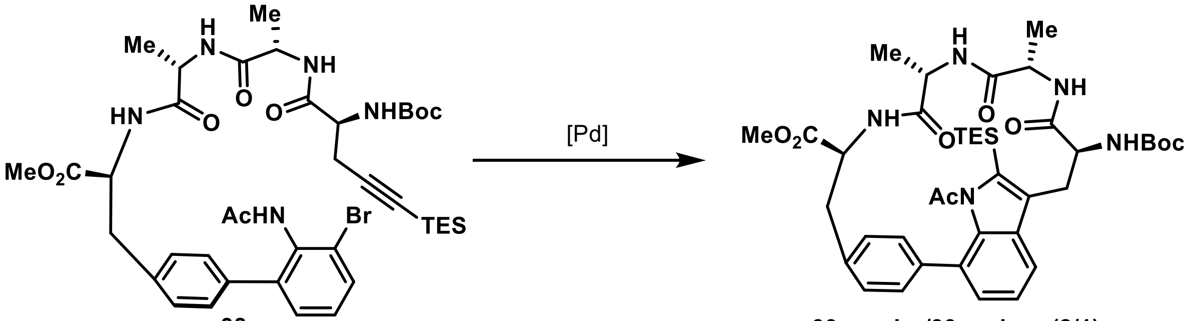

38c **39c-major/39c-minor (2/1)**

The precursor **38c** (84.3 mg, 0.1 mmol, 1.0 eq.) was dissolved in acetonitrile (1.5 mL). Pd(*t*Bu3P)2 (10.2 mg, 0.02 mmol, 0.2 eq.) and DIPEA (32.3 mg, 0.25 mmol, 2.5 eq.) were added. The reaction mixture was stirred at 110 °C under nitrogen atmosphere for 12 h. The reaction mixture was concentrated under reduced pressure and purified by silica gel chromatography to give the cyclized compound **39c-major** (21.5 mg, 28% yield) and **39c-minor** (10.7 mg, 14% yield)

Compound 39c-major

Physical State: amorphous solid

^{1}H NMR (600 MHz, CDCl$_3$): δ 7.47 (d, *J* = 7.5 Hz, 1H), 7.25 – 7.09 (m, 6H), 6.46 (s, 1H), 6.09 (d, *J* = 9.8 Hz, 1H), 5.70 (d, *J* = 9.0 Hz, 1H), 5.14 (d, *J* = 8.5 Hz, 1H), 4.59 (td, *J* = 11.3, 3.1 Hz, 1H), 4.49 – 4.34 (m, 2H), 3.83 (s, 3H), 3.80 – 3.77 (m, 1H), 3.59 – 3.57 (m, 1H), 3.22 (dd, *J* = 13.2, 3.1 Hz, 1H), 3.17 (dd, *J* = 14.6, 4.2 Hz, 1H), 2.56 (t, *J* = 12.7 Hz, 1H), 2.14 (s, 3H), 1.46 (s, 9H), 1.18 (d, *J* = 7.2 Hz, 3H), 1.03 – 0.97 (m, 15H), 0.07 (d, *J* = 6.9 Hz, 3H).

^{1}H NMR (600 MHz, DMSO-*d*$_6$) δ 8.44 (d, *J* = 9.9 Hz, 1H), 7.36 (d, *J* = 9.2 Hz, 1H), 7.30 (d, *J* = 7.6 Hz, 1H),

7.21 (d, J = 7.7 Hz, 2H), 7.19 – 7.16 (m, 3H), 7.12 (t, J = 7.5 Hz, 1H), 6.31 (d, J = 4.3 Hz, 1H), 5.50 (d, J = 8.9 Hz, 1H), 4.41 (dq, J = 9.1, 7.2 Hz, 1H), 4.26 – 4.14 (m, 2H), 3.67 (s, 3H), 3.59 – 3.52 (m, 1H), 3.37 (dt, J = 6.8, 3.4 Hz, 1H), 3.09 – 2.98 (m, 2H), 2.60 (t, J = 12.6 Hz, 1H), 2.07 (s, 3H), 1.96 (dt, J = 13.8, 6.9 Hz, 1H), 1.37 (s, 8H), 0.99 (d, J = 7.2 Hz, 3H), 0.95 – 0.87 (m, 13H), -0.21 (d, J = 6.8 Hz, 3H).

^{13}C NMR (151 MHz, CDCl$_3$): δ 172.10, 171.23, 170.77, 170.60, 154.76, 139.32, 135.35, 135.10, 133.75, 133.13, 130.70, 130.11, 128.96, 128.10, 125.67, 123.86, 119.56, 80.60, 54.75, 53.63, 52.93, 48.83, 47.87, 39.64, 28.35, 27.20, 26.79, 19.67, 15.99, 8.19, 5.97.

^{13}C NMR (151 MHz, DMSO-*d*$_6$) δ 172.26, 172.07, 170.25, 169.49, 154.63, 148.89, 138.74, 136.84, 135.07, 133.74, 132.63, 130.87, 130.19, 129.65, 128.29, 127.98, 125.70, 123.82, 119.01, 80.00, 55.42, 53.46, 52.82, 48.64, 47.63, 37.60, 35.65, 31.82, 28.46, 27.09, 19.34, 16.95, 8.49, 5.84.

HRMS (ESI-TOF): calculated for $C_{40}H_{55}N_5NaO_8Si$+ [M+Na]$^+$: 784.3712, found: 784.3719.

[α]^{25}$_D$: +109.0 (c = 0.5, CHCl$_3$)

Compound 39c-minor

Physical State: amorphous solid

^{1}H NMR (600 MHz, CDCl$_3$): δ 7.46 – 7.39 (m, 3H), 7.25 – 7.09 (m, 2H), 7.00 (d, J = 7.6 Hz, 2H), 5.77 – 5.74 (m, 1H), 5.63 – 5.59 (m, 1H), 5.34 – 5.30 (m, 1H), 5.21 – 5.14 (m, 1H), 4.80 – 4.75 (m, 1H), 4.26 – 4.21 (m, 1H), 3.81 (s, 3H), 3.80 – 3.72 (m, 1H), 3.57 (dd, J = 14.0, 7.6 Hz, 1H), 3.53 – 3.50 (m, 1H), 3.36 – 3.33 (m, 1H), 3.24 – 3.17 (m, 1H), 2.90 (dd, J = 13.9, 7.0 Hz, 1H), 2.22 (s, 3H), 1.44 (s, 9H), 1.28 (d, J = 6.5 Hz, 3H), 1.15 (d, J = 7.1 Hz, 3H), 1.02 – 0.95 (m, 15H).

^{13}C NMR (151 MHz, CDCl$_3$): δ 172.10, 171.18, 170.33, 169.39, 154.81, 139.68, 136.08, 135.64, 135.16, 133.92, 130.07, 129.63, 128.17, 127.64, 126.33, 123.41, 117.48, 80.02, 58.57, 52.81, 52.03, 49.06, 48.62, 36.58, 31.53, 29.38, 28.39, 27.17, 27.07, 18.66, 18.31, 8.34, 5.39.

HRMS (ESI-TOF): calculated for $C_{40}H_{55}N_5NaO_8Si$+ [M+Na]$^+$: 784.3712, found: 784.3714.

[α]^{25}$_D$: -49.5 (c = 0.5, CHCl$_3$)

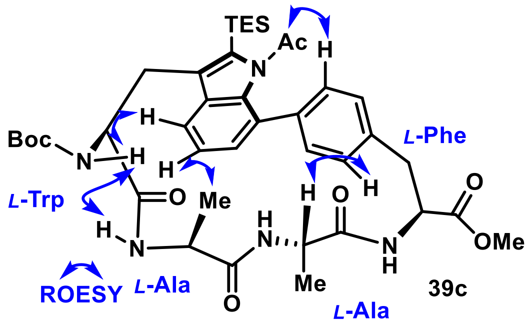

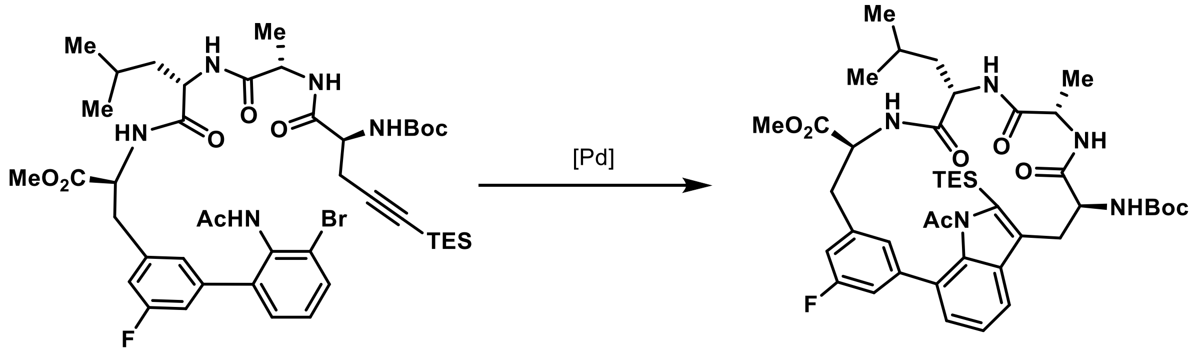

38d

39d-major/39d-minor (4/1)

The precursor **38d** (86.1 mg, 0.1 mmol, 1.0 eq.) was dissolved in acetonitrile (1.5 mL). Pd(tBu$_3$P)$_2$ (10.2 mg, 0.02 mmol, 0.2 eq.) and DIPEA (32.3 mg, 0.25 mmol, 2.5 eq.) were added. The reaction mixture was stirred at 110 °C under nitrogen atmosphere for 12 h. The reaction mixture was concentrated under reduced pressure and purified by silica gel chromatography to give the cyclized compound **39d-major** (36.4 mg, 45% yield) and **39d-minor** (9.1 mg, 11% yield)

Compound 39d-major

Physical State: amorphous solid

^{1}H NMR (600 MHz, CDCl$_3$): δ 8.07 (d, *J* = 7.7 Hz, 1H), 7.65 (s, 1H), 7.36 (t, *J* = 7.5 Hz, 1H), 7.22 (d, *J* = 8.9 Hz, 1H), 7.13 (d, *J* = 7.1 Hz, 1H), 6.93 (d, *J* = 7.4 Hz, 1H), 6.72 (d, *J* = 9.2 Hz, 1H), 5.73 – 5.69 (m, 2H), 5.14 (d, *J* = 8.6 Hz, 1H), 4.88 – 4.85 (m, 1H), 4.62 – 4.58 (m, 1H), 4.50 – 4.32 (m, 2H), 3.80 (s, 3H), 3.55 (dd, *J* = 13.6, 4.0 Hz, 1H), 3.21 (dd, *J* = 13.9, 3.2 Hz, 1H), 3.07 (t, *J* = 12.7 Hz, 1H), 2.98 (dd, *J* = 13.9, 4.2 Hz, 1H), 1.87 (s, 3H), 1.80 – 1.75 (m, 1H), 1.63 – 1.57 (m, 1H), 1.51 (s, 9H), 1.34 – 1.29 (m, 1H), 1.09 (d, *J* = 6.5 Hz, 3H), 1.03 (d, *J* = 7.0 Hz, 3H), 0.95 (d, *J* = 6.5 Hz, 3H), 0.87 (t, *J* = 7.4 Hz, 9H), 0.82 – 0.78 (m, 6H).

^{13}C NMR (151 MHz, CDCl$_3$): δ 175.41, 171.56, 171.14, 170.88, 169.11, 163.57 (d, *J* = 246.9 Hz), 155.21, 144.87 (d, *J* = 7.9 Hz), 139.08 (d, *J* = 7.6 Hz), 135.68, 135.23, 130.91, 128.39, 127.35, 127.19, 126.19, 122.44, 119.63, 115.43 (d, *J* = 20.9 Hz), 113.65 (d, *J* = 21.1 Hz), 79.82, 54.25, 53.94, 53.48, 52.39, 47.33, 41.28, 37.45, 31.93, 28.56, 28.52, 24.82, 23.74, 21.87, 19.58, 7.89, 5.05.

HRMS (ESI-TOF): calculated for $C_{43}H_{60}FN_5NaO_8Si^+$ $[M+Na]^+$: 844.4087, found: 844.4092.

$[\alpha]^{25}_D$: +81.0 (*c* = 0.5, CHCl$_3$)

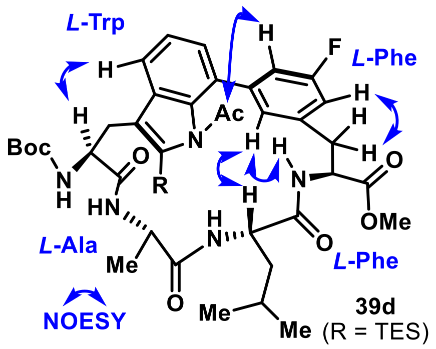

39d (R = TES)

The configuration of the ring system is deduced to be *Rconf*.

Compound 39d-minor

Physical State: amorphous solid

^{1}H NMR (600 MHz, CDCl$_3$): δ 7.44 (d, *J* = 8.0 Hz, 1H), 7.23 (d, *J* = 9.1 Hz, 1H), 7.16 (dd, *J* = 14.7, 7.2 Hz, 2H), 7.06 (d, *J* = 7.3 Hz, 1H), 6.61 (d, *J* = 9.1 Hz, 1H), 5.77 (s, 1H), 5.66 (s, 1H), 5.51 – 5.27 (m, 2H), 4.85 (dt, *J* = 9.3, 4.9 Hz, 1H), 4.25 (s, 1H), 4.11 – 4.01 (m, 1H), 3.82 (s, 3H), 3.27 (d, *J* = 25.1 Hz, 1H), 3.21 – 3.07 (m, 3H), 1.85 (d, *J* = 16.3 Hz, 1H), 1.74 (s, 3H), 1.47 (s, 9H), 1.43 – 1.41 (m, 1H), 1.38 – 1.33 (m, 2H), 1.17 (d, *J* = 7.1 Hz, 3H), 1.03 – 0.93 (m, 15H), 0.80 (d, *J* = 6.6 Hz, 3H), 0.74 (d, *J* = 6.6 Hz, 3H).

^{13}C NMR (151 MHz, CDCl$_3$): δ 174.49, 171.53, 170.71, 170.13, 163.04, 155.01, 143.80, 137.58, 135.41, 133.20, 128.17, 126.82, 126.55, 122.06, 118.71, 114.07, 114.07, 113.93, 113.22, 79.88, 57.61, 53.04, 52.78, 51.25, 48.35, 40.56, 36.93, 29.78, 29.31, 28.49, 28.42, 27.89, 24.57, 22.77, 21.68, 19.53, 8.27, 6.67, 5.88, 4.41.

HRMS (ESI-TOF): calculated for $C_{43}H_{60}FN_5NaO_8Si^+$ $[M+Na]^+$: 844.4087, found: 844.4091.

$[\alpha]^{25}_D$: +56.2 (*c* = 0.5, CHCl$_3$)

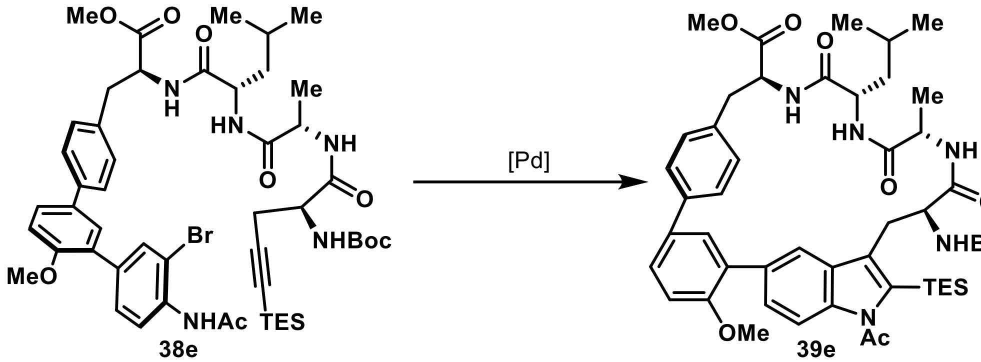

The precursor **38e** (99.1 mg, 0.1 mmol, 1.0 eq.) was dissolved in DMF (1.5 mL). Pd(*t*Bu$_3$P)$_2$ (10.2 mg, 0.02 mmol, 0.2 eq.) and DIPEA (32.3 mg, 0.25 mmol, 2.5 eq.) were added. The reaction mixture was stirred at 110 °C under nitrogen atmosphere for 12 h. The reaction was quenched by 0.5 M HCl and extracted by EtOAc for three times, the combined organic layers were removed under reduced pressure to give the residue, the residue was purified by silica gel chromatography to give the cyclized compound **39e** (29.1 mg, 32% yield).

Compound 39e

Physical State: amorphous solid

^{1}H NMR (600 MHz, CDCl$_3$): δ 7.85 (d, *J* = 6.6 Hz, 2H), 7.65 (d, *J* = 9.1 Hz, 1H), 7.56 (d, *J* = 7.6 Hz, 3H), 7.51 (dd, *J* = 8.4, 2.0 Hz, 1H), 7.15 (d, *J* = 7.9 Hz, 2H), 7.06 (d, *J* = 8.5 Hz, 1H), 6.64 (d, *J* = 6.0 Hz, 2H), 6.35 (s, 1H), 4.91 – 4.85 (m, 1H), 4.81 (s, 1H), 4.52 (s, 1H), 4.45 (t, *J* = 7.4 Hz, 1H), 4.36 (td, *J* = 9.1, 5.7 Hz, 1H), 3.88 (s, 3H), 3.79 (s, 3H), 3.73 (s, 1H), 3.38 (dd, *J* = 13.9, 5.3 Hz, 2H), 2.96 (dd, *J* = 13.7, 8.4 Hz, 1H), 2.87 (s, 3H), 1.77 – 1.71 (m, 1H), 1.56 – 1.53 (m, 1H), 1.42 (s, 9H), 1.27 – 1.23 (m, 1H), 1.00 – 0.90 (m, 21H), 0.81 (d, *J* = 6.4 Hz, 3H).

^{13}C NMR (151 MHz, CDCl$_3$): δ 173.38, 171.91, 171.11, 169.65, 155.98, 154.98, 140.06, 136.20, 134.51, 133.51, 132.85, 131.34, 129.92, 129.73, 127.41, 126.71, 120.95, 112.74, 111.78, 81.23, 55.86, 52.73, 52.59, 51.09, 48.19, 38.97, 37.05, 29.78, 28.26, 26.75, 24.58, 23.23, 21.64, 15.70, 8.38, 6.67, 0.08.

HRMS (ESI-TOF): calculated for $C_{50}H_{67}N_5NaO_9Si^+$ [M+Na]$^+$: 932.4600, found: 932.4580.

[α]^{25}$_D$: -64.8 (*c* = 0.5, CHCl$_3$)

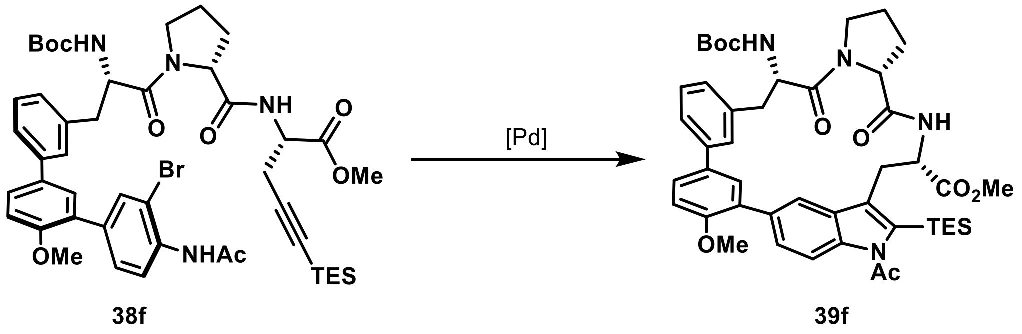

The precursor **38f** (90.4 mg, 0.1 mmol, 1.0 eq.) was dissolved in DMF (1.5 mL). Pd(*t*Bu$_3$P)$_2$ (10.2 mg, 0.02 mmol, 0.2 eq.) and DIPEA (32.3 mg, 0.25 mmol, 2.5 eq.) were added. The reaction mixture was stirred at 110 °C under nitrogen atmosphere for 12 h. The reaction was quenched by 0.5 M HCl and extracted by EtOAc for three times, the combined organic layers were removed under reduced pressure to give the residue, the residue was purified by silica gel chromatography to give the cyclized compound **39f** (32.9 mg, 40% yield).

Compound 39f

Physical State: amorphous solid

^{1}H NMR (600 MHz, CDCl$_3$): δ 9.08 (s, 1H), 8.44 (s, 1H), 8.05 (d, *J* = 8.8 Hz, 1H), 7.94 (s, 1H), 7.87 (dd, *J* = 5.5, 3.0 Hz, 1H), 7.76 – 7.72 (m, 2H), 7.65 (d, *J* = 8.9 Hz, 1H), 7.37 (d, *J* = 5.4 Hz, 1H), 7.05 (d, *J* = 8.6 Hz, 1H), 6.39 (d, *J* = 7.6 Hz, 1H), 5.50 (d, *J* = 8.4 Hz, 1H), 5.13 – 5.10 (m, 1H), 4.72 (t, *J* = 8.0 Hz, 1H), 4.33 (dd, *J* = 8.3, 4.4 Hz, 1H), 3.92 (s, 3H), 3.56 (s, 2H), 3.52 – 3.38 (m, 3H), 3.18 – 3.12 (m, 2H), 2.86 (s, 3H), 2.68

(d, J = 8.6 Hz, 1H), 2.03 – 2.00 (m, 1H), 1.80 – 1.76 (m, 1H), 1.72 – 1.66 (m, 2H), 1.46 (s, 9H), 0.94 (t, J = 7.6 Hz, 9H), 0.86 (q, J = 7.8 Hz, 6H).

^{13}C NMR (151 MHz, CDCl$_3$): δ 172.66, 170.98, 170.44, 169.59, 168.00, 156.44, 155.16, 138.85, 137.09, 136.55, 136.23, 134.40, 133.38, 133.13, 132.75, 131.82, 130.77, 129.51, 129.16, 128.71, 128.45, 127.43, 126.63, 125.47, 123.75, 123.69, 122.20, 112.92, 111.79, 79.78, 60.30, 55.76, 53.76, 52.89, 52.40, 47.29, 40.91, 31.17, 29.90, 29.79, 28.51, 28.01, 26.66, 24.74, 8.38, 6.30.

HRMS (ESI-TOF): calculated for $C_{46}H_{58}N_4NaO_8Si^+$ [M+Na]$^+$: 845.3916, found: 845.3923.

[α]$^{25}_D$: +8.0 (c = 0.5, CHCl$_3$)

General procedure E for the synthesis of 19：

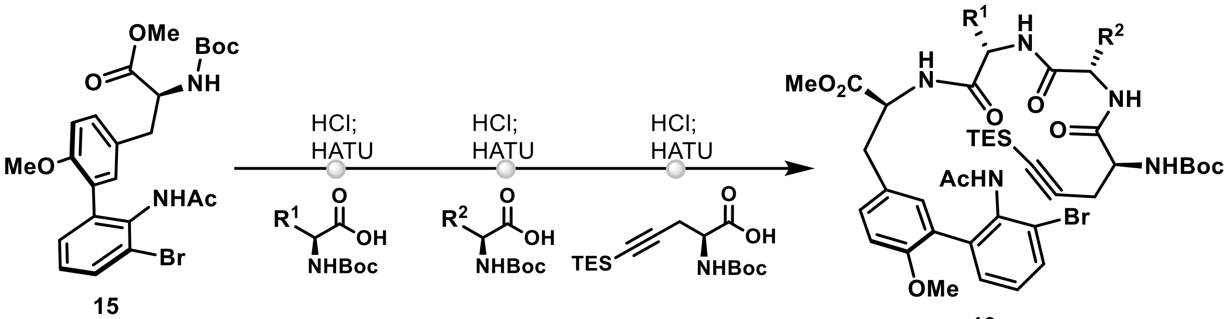

The compound **15** was dissolved in DCM/4M HCl = 3:1 (0.2 M), The reaction mixture was stirred at rt for 1 h, the solvents were removed under reduced pressure, and then quenched by saturated aq. $NaHCO_3$, extracted with DCM for three times, The organic layers were concentrated under reduced pressure to give the crude amine. To the amine was added DCM (0.2 M), Boc-amino acid (1.1 eq.), DIPEA (3.0 eq.) and HATU (1.5 eq.) were added sequentially, the reaction mixture was stirred at rt for 1 h, quenched by *aq.* 0.5 M HCl, the organic layers were concentrated under reduced pressure to give the dipeptide.
The dipeptide was dissolved in DCM/4M HCl = 3:1 (0.2 M), The reaction mixture was stirred at rt for 1 h, the solvents were removed under reduced pressure, and then quenched by saturated aq. $NaHCO_3$, extracted with DCM for three times, The organic layers were concentrated under reduced pressure to give the crude amine. To the amine was added DCM (0.2 M), Boc-amino acid (1.1 eq.), DIPEA (3.0 eq.) and HATU (1.5 eq.) were added sequentially, the reaction mixture was stirred at rt for 1 h, quenched by *aq.* 0.5 M HCl, the organic layers were concentrated under reduced pressure to give the tripeptide.
The tripeptide was dissolved in DCM/4M HCl = 3:1 (0.2 M), The reaction mixture was stirred at rt for 1 h, the solvents were removed under reduced pressure, and then quenched by saturated aq. $NaHCO_3$, extracted with DCM for three times, The organic layers were concentrated under reduced pressure to give the crude amine. To the amine was added DCM (0.1 M), (*S*)-2-((tert-butoxycarbonyl)amino)-5-(triethylsilyl)pent-4-ynoic acid (1.1 eq.), DIPEA (3.0 eq.) and HATU (1.5 eq.) were added sequentially, the reaction mixture was stirred at rt for 1 h, quenched by aq. 0.5 M HCl, the organic layers were concentrated under reduced pressure to give crude, the crude was precipitated in hexane to give the tetrapeptide **19a**-**19p**.

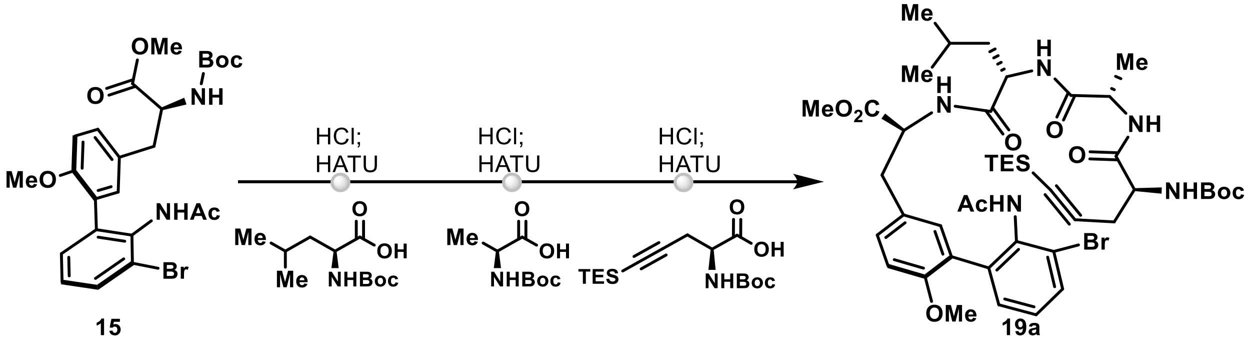

On 0.30 mmol scale, **General Procedure E** was followed from compound **15** as an intermediate. Purification by precipitation in hexane gave the title compound **19a** (178 mg, 66% yield from **15**).

Compound 19a

Physical State: amorphous solid

^{1}H NMR (600 MHz, METHANOL-D_4): δ 7.72 – 7.62 (m, 1H), 7.32 – 7.24 (m, 2H), 7.14 (s, 1H), 6.95 – 6.93 (m, 2H), 4.66 – 4.60 (m, 1H), 4.37 – 4.35 (m, 1H), 4.29 – 4.26 (m, 1H), 4.23 – 4.10 (m, 1H), 3.72 (s, 3H), 3.69 (s, 3H), 3.10 (dd, J = 13.9, 5.2 Hz, 1H), 3.01 (dd, J = 14.4, 6.4 Hz, 1H), 2.80 – 2.70 (m, 1H), 2.58 (dd, J

= 16.9, 9.0 Hz, 1H), 1.86 (s, 3H), 1.59 – 1.51 (m, 3H), 1.45 (s, 9H), 1.31 (d, *J* = 7.0 Hz, 3H), 0.98 (t, *J* = 7.9 Hz, 9H), 0.92 – 0.78 (m, 6H), 0.57 (q, *J* = 7.9 Hz, 6H).
^{13}C NMR (151 MHz, METHANOL-*D*$_4$): δ 173.14, 171.63, 170.45, 156.48, 155.39, 140.19, 134.76, 131.72, 129.86, 128.48, 127.85, 123.10, 110.54, 103.38, 83.73, 79.62, 54.81, 53.86, 51.99, 51.45, 49.20, 40.39, 35.95, 27.42, 24.52, 22.65, 22.12, 21.13, 20.62, 17.08, 6.57, 4.05.
HRMS (ESI-TOF): calculated for $C_{44}H_{64}BrN_5NaO_9Si^+$ [M+Na]$^+$: 936.3549, found: 936.3559.
[α]$^{25}_{D}$: –11.4 (*c* = 0.2, $CHCl_3$)

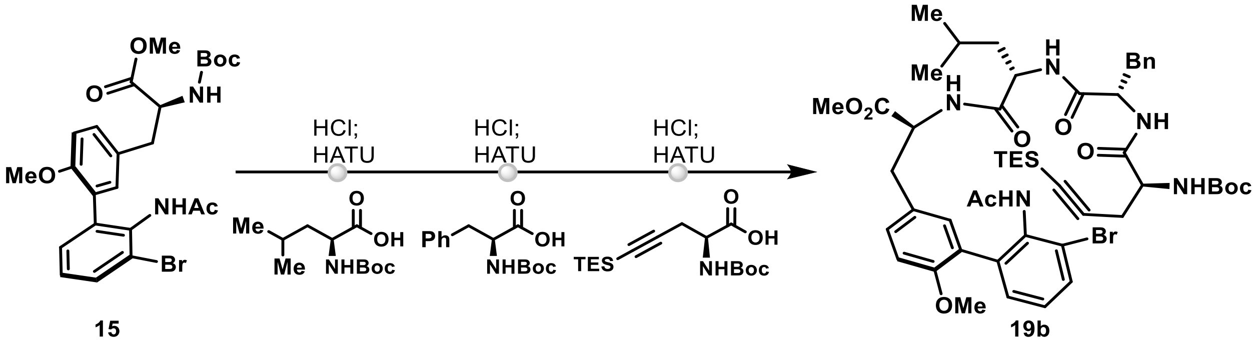

On 0.40 mmol scale, **General Procedure E** was followed from compound **15** as an intermediate. Purification by precipitation in hexane gave the title compound **19b** (191 mg, 48% yield from **15**).

Compound 19b

Physical State: amorphous solid
^{1}H NMR (600 MHz, METHANOL-*D*$_4$): δ 7.63 (q, *J* = 4.5 Hz, 1H), 7.25 – 7.12 (m, 8H), 6.98 (s, 1H), 6.93 (d, *J* = 8.5 Hz, 1H), 4.66 (s, 1H), 4.57 (s, 1H), 4.43 – 4.38 (m, 1H), 4.14 (d, *J* = 6.8 Hz, 1H), 3.68 (s, 6H), 3.12 – 3.08 (m, 2H), 3.00 (dd, *J* = 14.0, 7.4 Hz, 1H), 2.90 (dd, *J* = 14.0, 8.3 Hz, 1H), 2.69 – 2.58 (m, 1H), 2.45 (dd, *J* = 17.1, 9.5 Hz, 1H), 1.83 (s, 3H), 1.56-1.52 (d, *J* = 23.2 Hz, 2H), 1.42 (s, 9H), 1.36 – 1.33 (m, 1H), 0.98 (t, *J* = 7.9 Hz, 9H), 0.85 (d, *J* = 5.9 Hz, 7H), 0.56 (q, *J* = 7.9 Hz, 6H).
^{13}C NMR (151 MHz, METHANOL-*D*$_4$): δ 173.04, 171.58, 171.51, 170.49, 156.41, 155.40, 140.15, 136.63, 134.70, 131.73, 130.13, 129.88, 129.16, 128.45, 128.18, 127.85, 126.50, 123.11, 110.57, 103.49, 83.57, 79.58, 54.76, 54.24, 54.01, 53.92, 51.89, 51.44, 40.46, 37.43, 36.02, 27.44, 24.39, 22.57, 22.11, 21.13, 20.73, 6.56, 4.04.
HRMS (ESI-TOF): calculated for $C_{50}H_{68}BrN_5NaO_9Si^+$ [M+Na]$^+$: 1012.3862, found: 1012.3867.
[α]$^{25}_{D}$: –20.6 (*c* = 0.2, $CHCl_3$)

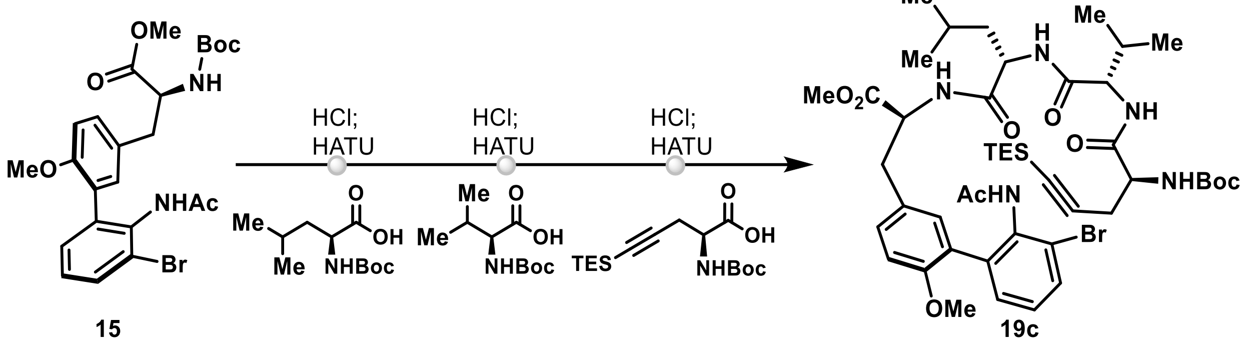

On 0.30 mmol scale, **General Procedure E** was followed from compound **15** as an intermediate. Purification by precipitation in hexane gave the title compound **19c** (166 mg, 58% yield from **15**).

Compound 19c

Physical State: amorphous solid
^{1}H NMR (600 MHz, METHANOL-*D*$_4$): δ 7.62 (t, *J* = 4.7 Hz, 1H), 7.23 (d, *J* = 4.3 Hz, 2H), 7.13 (d, *J* = 8.7

Hz, 1H), 6.99 – 6.84 (m, 2H), 4.63 (s, 1H), 4.41 (t, J = 7.7 Hz, 1H), 4.22-4.16 (m, 2H), 3.70 (s, 3H), 3.66 (s, 2H), 3.07 (dd, J = 14.1, 5.6 Hz, 1H), 2.97 (dd, J = 14.3, 7.4 Hz, 1H), 2.70 (dd, J = 17.2, 4.9 Hz, 1H), 2.57 (dd, J = 17.2, 9.3 Hz, 1H), 2.03 – 1.99 (m, 1H), 1.84 (s, 3H), 1.61 – 1.58 (m, 1H), 1.53 - 1.49 (m, 2H), 1.42 (s, 9H), 0.97 (t, J = 7.9 Hz, 9H), 0.91 – 0.81 (m, 12H), 0.55 (q, J = 7.9 Hz, 6H).
^{13}C NMR (151 MHz, METHANOL-*D*$_4$): δ 173.10, 171.83, 171.69, 170.44, 156.46, 155.39, 140.19, 134.72, 131.72, 130.09, 129.82, 128.46, 128.23, 127.85, 123.11, 110.55, 103.59, 83.58, 79.56, 58.44, 54.77, 53.97, 53.83, 51.75, 51.41, 40.54, 35.99, 31.07, 27.42, 24.48, 22.30, 22.07, 21.13, 20.73, 18.60, 17.13, 6.56, 4.09, 4.05.
HRMS (ESI-TOF): calculated for $C_{46}H_{68}BrN_5NaO_9Si^+$ [M+Na]$^+$: 964.3862, found: 964.3864.
[α]$^{25}_D$: –8.1 (c = 0.2, $CHCl_3$)

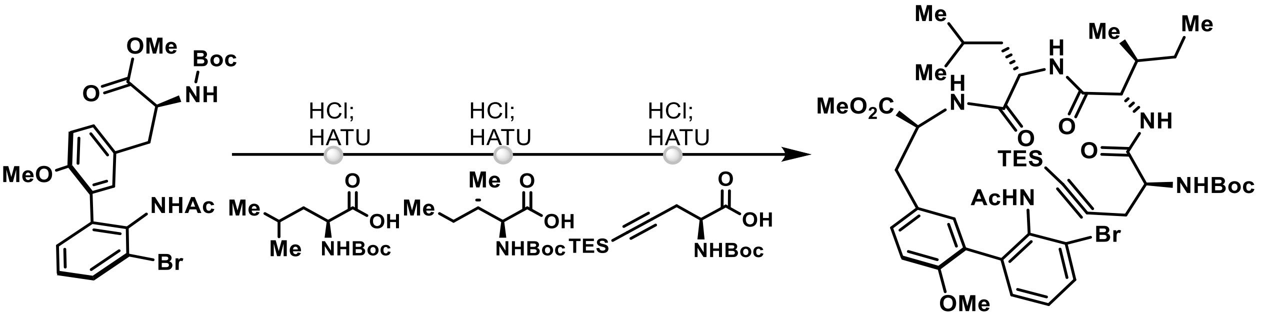

15 19d

On 0.37 mmol scale, **General Procedure E** was followed from compound **15** as an intermediate. Purification by precipitation in hexane gave the title compound **19d** (186 mg, 53% yield from **15**).

Compound 19d

Physical State: amorphous solid

^{1}H NMR (600 MHz, METHANOL-*D*$_4$): δ 7.71 (s, 1H), 7.65 – 7.59 (m, 1H), 7.24 (d, J = 4.5 Hz, 2H), 7.14 (d, J = 7.3 Hz, 1H), 6.99 – 6.81 (m, 2H), 4.65 – 4.62 (m, 1H), 4.42 (d, J = 6.5 Hz, 1H), 4.18 – 4.16 (m, 2H), 3.70 (s, 3H), 3.66 (s, 3H), 3.07 (dd, J = 13.6, 4.9 Hz, 1H), 2.99 – 2.97 (m, 1H), 2.69 (dd, J = 17.0, 4.2 Hz, 1H), 2.56 (dd, J = 17.0, 9.2 Hz, 1H), 1.84 (s, 3H), 1.78 - 1.76 (m, 1H), 1.59 – 1.50 (m, 4H), 1.43 (s, 9H), 1.10 – 1.09 (m, 1H), 0.97 (t, J = 7.8 Hz, 9H), 0.84 – 0.80 (m, 12H), 0.55 (q, J = 7.8 Hz, 6H).
^{13}C NMR (151 MHz, METHANOL-*D*$_4$): δ 173.06, 171.79, 170.46, 156.48, 155.39, 140.18, 134.70, 131.72, 130.11, 129.82, 128.44, 127.84, 123.12, 110.55, 103.59, 83.56, 79.56, 57.82, 54.76, 53.99, 53.83, 51.71, 51.40, 40.53, 37.09, 36.00, 27.41, 24.47, 24.42, 22.22, 22.07, 21.13, 20.70, 14.72, 10.13, 6.55, 4.05.
HRMS (ESI-TOF): calculated for $C_{47}H_{70}BrN_5NaO_9Si^+$ [M+Na]$^+$: 978.4018, found: 978.4025.
[α]$^{25}_D$: –4.2 (c = 0.2, $CHCl_3$)

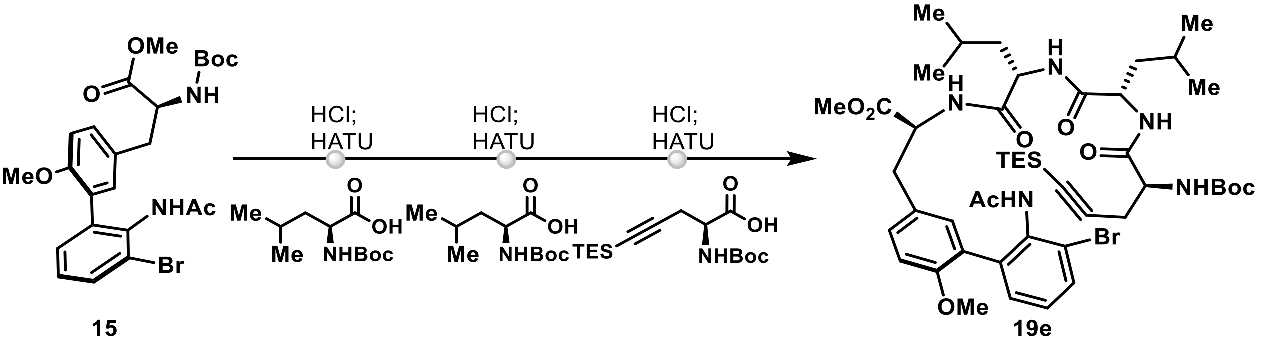

15 19e

On 0.32 mmol scale, **General Procedure E** was followed from compound **15** as an intermediate. Purification by precipitation in hexane gave the title compound **19e** (196 mg, 65% yield from **15**).

Compound 19e

Physical State: amorphous solid
^{1}H NMR (600 MHz, METHANOL-*D*$_4$): δ 7.62 (d, *J* = 9.6 Hz, 1H), 7.24 – 7.22 (m, 2H), 7.16 – 7.11 (m, 1H), 6.96 – 6.90 (m, 2H), 4.64 – 4.60 (m, 1H), 4.39 – 4.35 (m, 2H), 4.17 (s, 1H), 3.70 (s, 4H), 3.66 (s, 4H), 3.10 – 3.04 (m, 1H), 3.01 – 2.94 (m, 1H), 2.70 (dd, *J* = 17.2, 4.9 Hz, 1H), 2.57 (dd, *J* = 17.4, 9.5 Hz, 1H), 1.84 (s, 3H), 1.67 – 1.58 (m, 3H), 1.54 - 1.53 (m, 1H), 1.43 (s, 9H), 0.97 (t, *J* = 7.4 Hz, 9H), 0.90 – 0.82 (m, 14H), 0.55 (d, *J* = 7.5 Hz, 6H).
^{13}C NMR (151 MHz, METHANOL-*D*$_4$): δ 173.18, 172.97, 171.83, 170.47, 156.45, 155.40, 140.16, 134.71, 131.73, 130.12, 129.87, 128.45, 128.27, 127.83, 123.14, 110.56, 103.58, 83.57, 79.54, 54.80, 53.97, 53.77, 51.90, 51.43, 40.62, 40.47, 36.02, 27.43, 24.50, 24.45, 22.39, 22.26, 22.13, 21.19, 20.77, 20.71, 6.58, 4.06.
HRMS (ESI-TOF): calculated for $C_{47}H_{70}BrN_5NaO_9Si^+$ [M+Na]$^+$: 978.4018, found: 978.4019.
[α]^{25}$_D$: –8.3 (*c* = 0.2, $CHCl_3$)

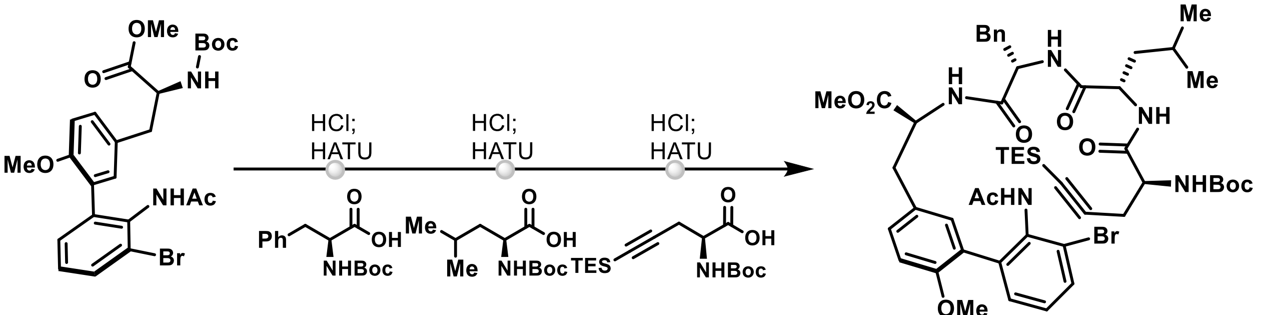

15
19f

On 0.34 mmol scale, **General Procedure E** was followed from compound **15** as an intermediate. Purification by precipitation in hexane gave the title compound **19f** (202 mg, 60% yield from **15**).

Compound 19f

Physical State: amorphous solid
^{1}H NMR (600 MHz, METHANOL-*D*$_4$): δ 7.63 (dd, *J* = 5.9, 3.1 Hz, 1H), 7.27 – 7.21 (m, 4H), 7.17 – 7.12 (m, 4H), 7.02 – 6.91 (m, 2H), 4.75 – 4.56 (m, 2H), 4.30 – 4.25 (m, 1H), 4.14 (s, 1H), 3.73 (s, 3H), 3.68 (s, 3H), 3.15 – 3.04 (m, 2H), 3.02 – 2.99 (m, 1H), 2.86 – 2.83 (s, 1H), 2.64 – 2.60 (m, 1H), 2.53 (dd, *J* = 16.4, 9.7 Hz, 1H), 1.85 (s, 3H), 1.56 – 1.51 (m, 1H), 1.43 (s, 9H), 1.38 – 1.32 (m, 1H), 0.99 (t, *J* = 7.9 Hz, 9H), 0.90 (t, *J* = 7.0 Hz, 1H), 0.85 (d, *J* = 6.4 Hz, 3H), 0.81 (d, *J* = 6.3 Hz, 3H), 0.57 (q, *J* = 7.9 Hz, 6H).
^{13}C NMR (151 MHz, METHANOL-*D*$_4$): δ 172.70, 171.86, 171.71, 170.55, 156.42, 155.42, 149.95, 136.88, 134.75, 131.73, 129.80, 129.07, 128.50, 128.22, 128.06, 127.84, 126.38, 123.13, 120.52, 110.57, 103.81, 83.40, 79.47, 54.80, 54.51, 54.02, 53.60, 51.87, 51.45, 40.53, 37.32, 36.02, 31.42, 27.40, 24.32, 22.37, 22.14, 21.16, 20.75, 13.11, 6.56, 4.06.
HRMS (ESI-TOF): calculated for $C_{50}H_{68}BrN_5NaO_9Si^+$ [M+Na]$^+$: 1012.3862, found: 1012.3868.
[α]^{25}$_D$: –25.5 (*c* = 0.2, $CHCl_3$)

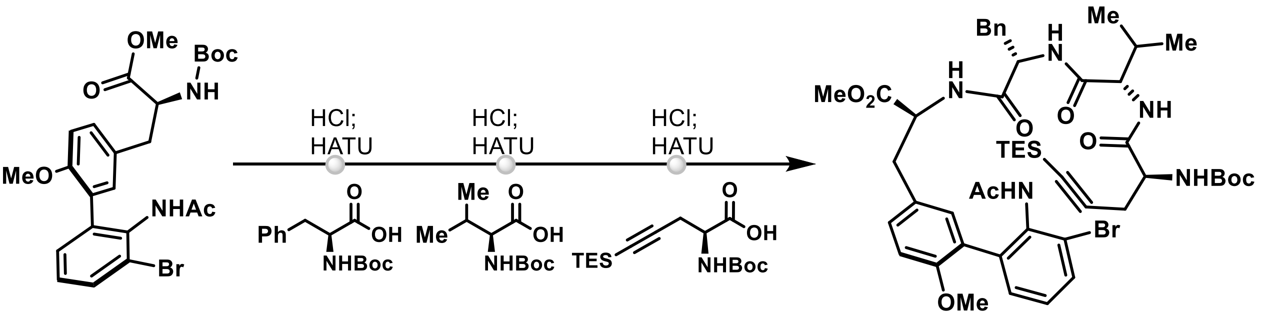

15
19g

On 0.39 mmol scale, **General Procedure E** was followed from compound **15** as an intermediate. Purification

by precipitation in hexane gave the title compound **19g** (222 mg, 58% yield from **15**).

Compound 19g

Physical State: amorphous solid

^{1}H NMR (600 MHz, METHANOL-*D*$_4$): δ 7.63 (d, *J* = 6.7 Hz, 1H), 7.28 – 7.05 (m, 8H), 6.99 – 6.91 (m, 2H), 4.70 – 4.61 (m, 2H), 4.29 – 4.08 (m, 2H), 3.73 (s, 3H), 3.68 (s, 3H), 3.13 – 3.04 (m, 2H), 2.99 (s, 1H), 2.85 – 2.75 (m, 1H), 2.70 – 2.54 (m, 2H), 1.86 (s, 3H), 1.44 (s, 9H), 1.37 – 1.35 (m, 1H), 0.99 (t, *J* = 7.8 Hz, 9H), 0.82 – 0.74 (m, 6H), 0.57 (q, *J* = 8.0 Hz, 6H).

^{13}C NMR (151 MHz, METHANOL-*D*$_4$): δ 171.87, 171.71, 171.53, 170.50, 156.45, 155.40, 149.61, 136.88, 134.76, 131.73, 129.77, 129.01, 128.54, 128.07, 127.85, 126.37, 123.11, 120.42, 110.53, 103.79, 83.43, 79.50, 58.41, 54.77, 54.50, 53.99, 53.67, 51.46, 37.48, 35.97, 30.96, 27.41, 22.82, 22.26, 21.11, 18.53, 17.44, 16.96, 6.56, 4.05.

HRMS (ESI-TOF): calculated for $C_{49}H_{66}BrN_5NaO_9Si^+$ [M+Na]$^+$: 998.3705, found: 998.3687.

[α]$^{25}_D$: –35.8 (*c* = 0.2, CHCl$_3$)

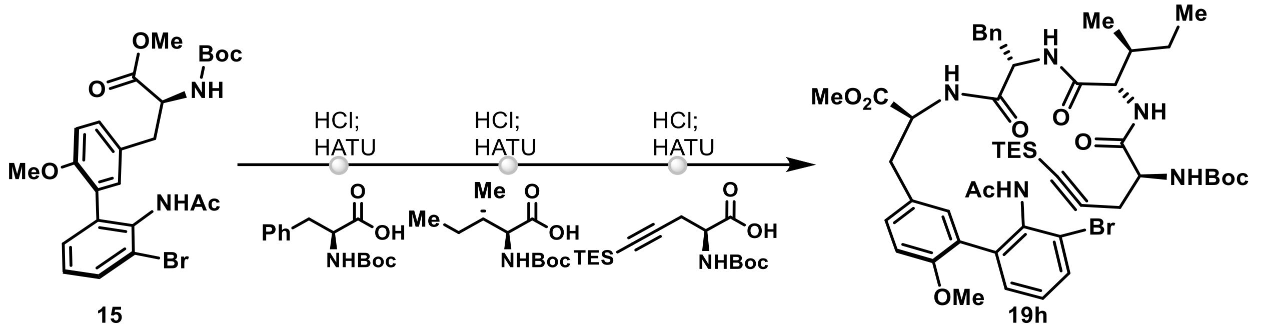

On 0.30 mmol scale, **General Procedure E** was followed from compound **15** as an intermediate. Purification by precipitation in hexane gave the title compound **19h** (182 mg, 62% yield from **15**).

Compound 19h

Physical State: amorphous solid

^{1}H NMR (600 MHz, METHANOL-*D*$_4$): δ 7.61 (d, *J* = 5.6 Hz, 1H), 7.34 – 7.05 (m, 8H), 7.00 – 6.87 (m, 2H), 4.82 – 4.58 (m, 2H), 4.32 – 4.05 (m, 2H), 3.71 (s, 3H), 3.66 (s, 3H), 3.14 – 3.00 (m, 2H), 2.99 – 2.96 (m, 1H), 2.79 (s, 1H), 2.76 – 2.63 (m, 1H), 2.62 – 2.47 (m, 1H), 1.84 (s, 3H), 1.42 (s, 9H), 1.29 – 1.26 (m, 1H), 0.97 (t, *J* = 7.8 Hz, 9H), 0.89 – 0.81 (m, 2H), 0.76 (t, *J* = 7.3 Hz, 3H), 0.71 (t, *J* = 7.3 Hz, 3H), 0.57 – 0.53 (q, *J* = 7.6 Hz, 6H).

^{13}C NMR (151 MHz, DMSO-*d*$_6$): δ 172.22, 171.59, 171.04, 170.88, 170.62, 155.78, 155.65, 155.34, 140.07, 138.02, 135.73, 132.00, 130.98, 130.19, 129.63, 129.53, 128.65, 128.58, 128.49, 127.65, 126.69, 124.44, 120.20, 111.56, 106.20, 82.98, 78.74, 78.64, 57.29, 55.97, 54.42, 53.91, 53.76, 53.11, 52.34, 40.56, 38.07, 37.35, 36.43, 28.61, 24.32, 22.80, 15.64, 11.52, 11.32, 7.87, 4.49.

HRMS (ESI-TOF): calculated for $C_{50}H_{68}BrN_5NaO_9Si^+$ [M+Na]$^+$: 1012.3862, found: 1012.3868.

[α]$^{25}_D$: –15.0 (*c* = 0.2, CHCl$_3$)

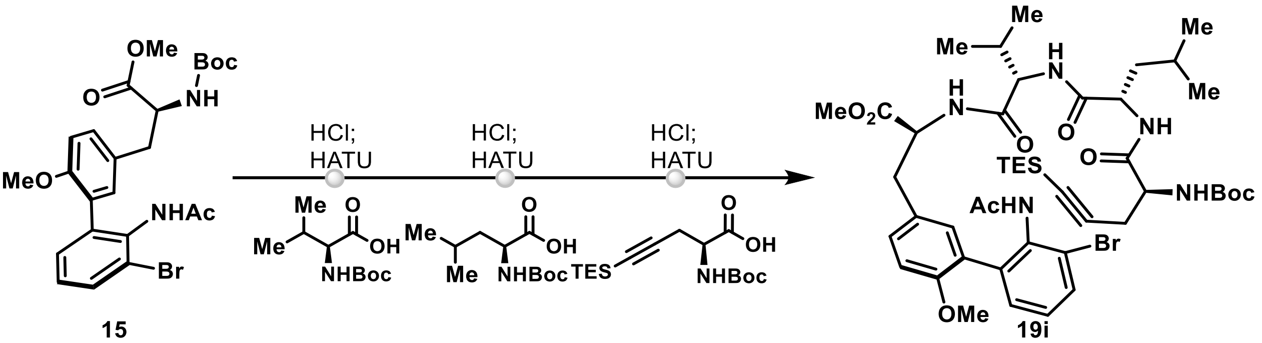

On 0.34 mmol scale, **General Procedure E** was followed from compound **15** as an intermediate. Purification by precipitation in hexane gave the title compound **19i** (166 mg, 52% yield from **15**).

Compound 19i

Physical State: amorphous solid

^{1}H NMR (600 MHz, METHANOL-*D*$_4$): δ 7.72 – 7.51 (m, 1H), 7.27 – 7.20 (m, 2H), 7.15 (dd, *J* = 8.4, 2.0 Hz, 1H), 6.97 – 6.96 (m, 1H), 6.93 – 6.92 (m, 1H), 4.64 (s, 1H), 4.41 - 4.40 (m, 1H), 4.20 – 4.16 (m, 2H), 3.70 (s, 3H), 3.65 (s, 3H), 3.05 (dd, *J* = 14.0, 5.7 Hz, 1H), 2.95 (dd, *J* = 14.0, 7.8 Hz, 1H), 2.70 (dd, *J* = 17.1, 4.4 Hz, 1H), 2.56 (dd, *J* = 17.1, 9.5 Hz, 1H), 2.03 – 1.98 (m, 1H), 1.83 (s, 3H), 1.64 – 1.59 (m, 1H), 1.54 – 1.47 (m, 2H), 1.42 (s, 9H), 0.97 (t, *J* = 7.9 Hz, 9H), 0.92 – 0.78 (m, 12H), 0.55 (q, *J* = 7.9 Hz, 6H).

^{13}C NMR (151 MHz, METHANOL-*D*$_4$): δ 172.92, 172.04, 171.78, 170.54, 156.34, 155.41, 140.09, 134.65, 131.72, 130.22, 129.81, 128.44, 128.33, 127.83, 123.18, 110.65, 103.64, 83.49, 79.47, 58.51, 54.83, 53.98, 53.64, 51.89, 51.35, 40.63, 36.20, 30.90, 27.43, 24.46, 22.53, 22.25, 21.13, 20.80, 18.43, 17.45, 6.59, 4.07.

HRMS (ESI-TOF): calculated for $C_{46}H_{68}BrN_5NaO_9Si^+$ [M+Na]$^+$: 964.3862, found: 964.3871.

[α]$^{25}_{D}$: –4.1 (*c* = 0.2, $CHCl_3$)

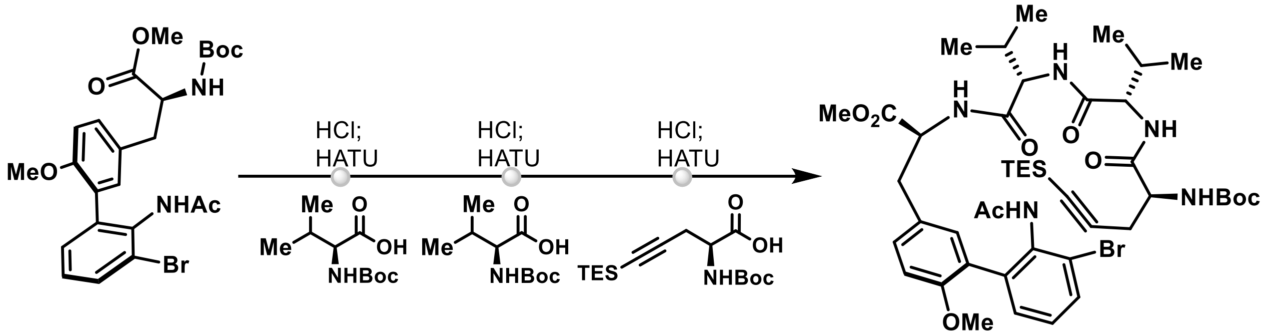

15 19j

On 0.30 mmol scale, **General Procedure E** was followed from compound **15** as an intermediate. Purification by precipitation in hexane gave the title compound **19j** (181 mg, 66% yield from **15**).

Compound 19j

Physical State: amorphous solid

^{1}H NMR (600 MHz, METHANOL-*D*$_4$): δ 7.62 (dd, *J* = 6.5, 2.9 Hz, 1H), 7.27 – 7.20 (m, 2H), 7.16 (dd, *J* = 8.4, 2.4 Hz, 1H), 6.97 (d, *J* = 2.3 Hz, 1H), 6.91 (d, *J* = 8.5 Hz, 1H), 4.65 (s, 1H), 4.32 – 4.11 (m, 3H), 3.69 (s, 3H), 3.65 (s, 3H), 3.05 (dd, *J* = 14.0, 5.8 Hz, 1H), 2.93 (dd, *J* = 14.0, 8.0 Hz, 1H), 2.70 (dd, *J* = 17.2, 4.8 Hz, 1H), 2.57 (dd, *J* = 17.2, 9.3 Hz, 1H), 2.01 - 1.94 (m, 1H), 1.83 (s, 3H), 1.42 (s, 9H), 0.97 (t, *J* = 7.9 Hz, 9H), 0.90 (d, *J* = 6.8 Hz, 3H), 0.87 (d, *J* = 6.6 Hz, 3H), 0.83 (d, *J* = 6.8 Hz, 3H), 0.80 (d, *J* = 6.8 Hz, 3H), 0.55 (q, *J* = 7.8 Hz, 6H).

^{13}C NMR (151 MHz, METHANOL-*D*$_4$): δ 172.04, 171.75, 170.53, 156.38, 155.38, 140.10, 134.64, 131.72, 130.23, 129.76, 128.44, 128.37, 127.83, 123.17, 110.62, 103.62, 83.53, 79.51, 58.52, 54.78, 53.98, 53.71, 51.34, 36.20, 31.04, 30.80, 27.42, 22.43, 21.10, 18.52, 18.39, 17.57, 17.36, 6.56, 4.06.

HRMS (ESI-TOF): calculated for $C_{45}H_{66}BrN_5NaO_9Si^+$ [M+Na]$^+$: 950.3705, found: 950.3709.

[α]$^{25}_{D}$: –3.3 (*c* = 0.2, $CHCl_3$)

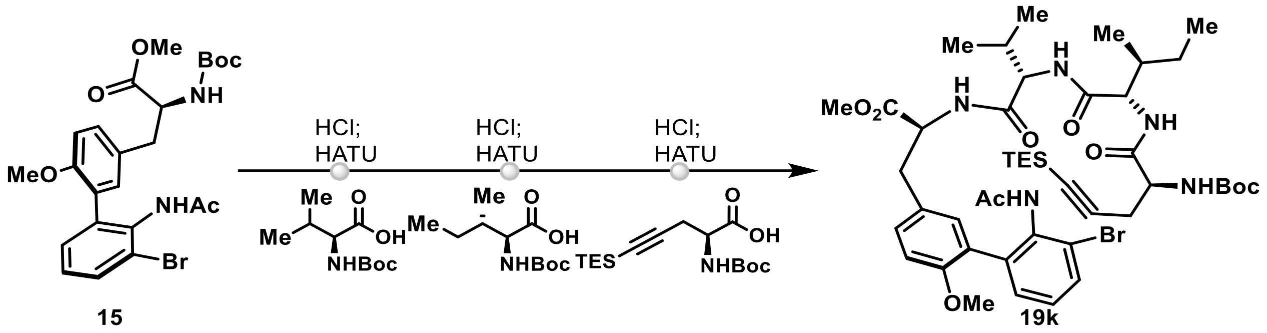

On 0.33 mmol scale, **General Procedure E** was followed from compound **15** as an intermediate. Purification by precipitation in hexane gave the title compound **19k** (188 mg, 60% yield from **15**).

Compound 19k

Physical State: amorphous solid

^{1}H NMR (600 MHz, METHANOL-*D*$_4$): δ 7.63 – 7.61 (m, 1H), 7.24 – 7.23 (m, 2H), 7.16 (dd, *J* = 8.4, 1.9 Hz, 1H), 7.01 – 6.86 (m, 2H), 4.64 (s, 1H), 4.23 4.20 (m, 3H), 3.69 (s, 3H), 3.65 (s, 3H), 3.05 (dd, *J* = 13.9, 5.6 Hz, 1H), 2.93 (dd, *J* = 13.9, 8.1 Hz, 1H), 2.68 (dd, *J* = 17.1, 4.4 Hz, 1H), 2.56 (dd, *J* = 17.1, 9.4 Hz, 1H), 2.02 – 1.96 (m, 1H), 1.83 (s, 3H), 1.73 – 1.71 (m, 1H), 1.42 (s, 9H), 1.30 - 1.24 (m, 1H), 1.10 – 1.04 (m, 1H), 0.97 (t, *J* = 7.9 Hz, 9H), 0.90 (d, *J* = 6.7 Hz, 3H), 0.87 (d, *J* = 7.1 Hz, 3H), 0.80 (t, *J* = 7.4 Hz, 3H), 0.75 (d, *J* = 6.7 Hz, 3H), 0.55 (q, *J* = 7.9 Hz, 6H).

^{13}C NMR (151 MHz, METHANOL-*D*$_4$): δ 172.02, 171.90, 171.83, 171.70, 170.52, 156.37, 155.39, 140.09, 134.64, 131.72, 130.24, 129.76, 128.43, 128.38, 127.83, 123.18, 110.62, 103.64, 83.48, 79.48, 58.51, 57.74, 54.78, 54.01, 53.66, 51.34, 37.05, 36.20, 31.42, 30.81, 27.42, 24.54, 22.39, 21.09, 18.39, 17.57, 14.65, 13.12, 10.08, 6.56, 4.06.

HRMS (ESI-TOF): calculated for $C_{46}H_{68}BrN_5NaO_9Si^+$ [M+Na]$^+$: 964.3862, found: 964.3867.

[α]$^{25}_{D}$: +3.6 (*c* = 0.2, $CHCl_3$)

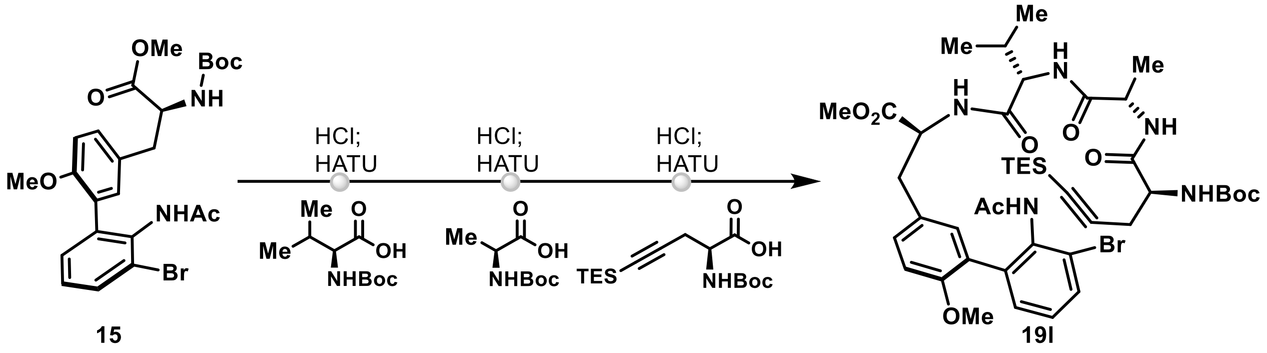

On 0.39 mmol scale, **General Procedure E** was followed from compound **15** as an intermediate. Purification by precipitation in hexane gave the title compound **19l** (176 mg, 50% yield from **15**).

Compound 19l

Physical State: amorphous solid

^{1}H NMR (600 MHz, METHANOL-*D*$_4$): δ 7.64 (dd, *J* = 7.3, 1.9 Hz, 1H), 7.27 – 7.23 (m, 2H), 7.17 (d, *J* = 6.8 Hz, 1H), 6.99 (s, 1H), 6.94 (d, *J* = 8.5 Hz, 1H), 4.67 (s, 1H), 4.37 – 4.35 (m, 1H), 4.21 – 4.18 (m, 2H), 3.71 (s, 3H), 3.67 (s, 3H), 3.08 (dd, *J* = 13.9, 5.6 Hz, 1H), 2.97 (dd, *J* = 13.9, 7.9 Hz, 1H), 2.73 (dd, *J* = 17.1, 4.6 Hz, 1H), 2.58 (dd, *J* = 17.1, 9.2 Hz, 1H), 2.08 – 1.96 (m, 1H), 1.85 (s, 3H), 1.44 (s, 9H), 1.28 (d, *J* = 7.0 Hz, 2H), 0.98 (t, *J* = 7.9 Hz, 9H), 0.91 (d, *J* = 6.7 Hz, 3H), 0.87 (d, *J* = 5.6 Hz, 3H), 0.57 (q, *J* = 7.9 Hz, 6H).

^{13}C NMR (151 MHz, METHANOL-*D*$_4$): δ 173.05, 172.04, 171.80, 171.56, 170.55, 156.37, 155.39, 140.10, 134.65, 131.72, 130.17, 129.82, 128.44, 128.34, 127.82, 123.16, 110.62, 103.46, 83.62, 79.54, 58.63, 54.80, 53.91, 53.69, 51.35, 49.07, 36.18, 30.76, 27.41, 22.79, 21.08, 18.42, 17.36, 17.03, 6.56, 4.04.

HRMS (ESI-TOF): calculated for $C_{43}H_{62}BrN_5NaO_9Si^+$ [M+Na]$^+$: 922.3392, found: 922.3399.

[α]^{25}D: –9.8 (*c* = 0.2, $CHCl_3$)

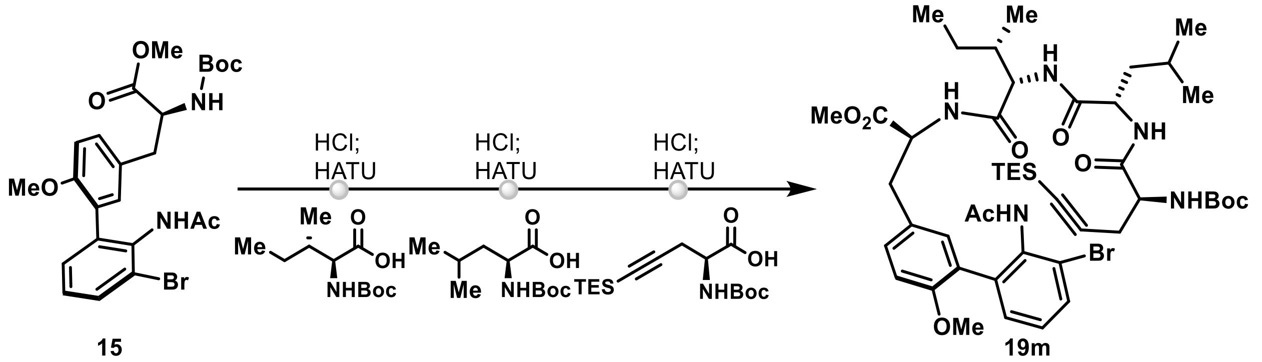

On 0.37 mmol scale, **General Procedure E** was followed from compound **15** as an intermediate. Purification by precipitation in hexane gave the title compound **19m** (216 mg, 61% yield from **15**).

Compound 19m

Physical State: amorphous solid

^{1}H NMR (600 MHz, METHANOL-*D*4): δ 7.64 (dd, *J* = 7.1, 1.8 Hz, 1H), 7.26 (q, *J* = 7.2 Hz, 2H), 7.17 (d, *J* = 6.8 Hz, 1H), 6.99 (s, 1H), 6.95 (d, *J* = 8.5 Hz, 1H), 4.67 (s, 1H), 4.41 (dd, *J* = 9.2, 5.5 Hz, 1H), 4.22 (d, *J* = 7.6 Hz, 2H), 3.72 (s, 3H), 3.67 (s, 3H), 3.34 (s, 1H), 3.07 (dd, *J* = 14.0, 5.7 Hz, 1H), 2.98 (dd, *J* = 14.0, 7.8 Hz, 1H), 2.72 (dd, *J* = 17.1, 4.3 Hz, 1H), 2.60 (dd, *J* = 17.1, 9.5 Hz, 1H), 1.86 (s, 3H), 1.81 - 1.80 (m, 1H), 1.66 – 1.51 (m, 3H), 1.45 (s, 9H), 1.12 – 1.08 (m, 1H), 0.99 (t, *J* = 7.8 Hz, 9H), 0.90 – 0.84 (m, 12H), 0.57 (q, *J* = 7.9 Hz, 6H).

^{13}C NMR (151 MHz, METHANOL-*D*4): δ 172.89, 172.15, 171.85, 170.57, 156.39, 155.41, 140.13, 134.67, 131.72, 130.18, 129.86, 128.44, 128.30, 127.83, 123.18, 110.64, 103.59, 83.54, 79.52, 57.85, 54.84, 53.93, 53.74, 51.96, 48.56, 40.52, 36.84, 36.19, 27.42, 24.45, 22.45, 22.23, 21.15, 20.78, 14.51, 10.08, 6.57, 4.13, 4.06.

HRMS (ESI-TOF): calculated for $C_{47}H_{70}BrN_5NaO_9Si^+$ $[M+Na]^+$: 978.4018, found: 978.4030.

[α]^{25}D: –2.7 (*c* = 0.2, $CHCl_3$)

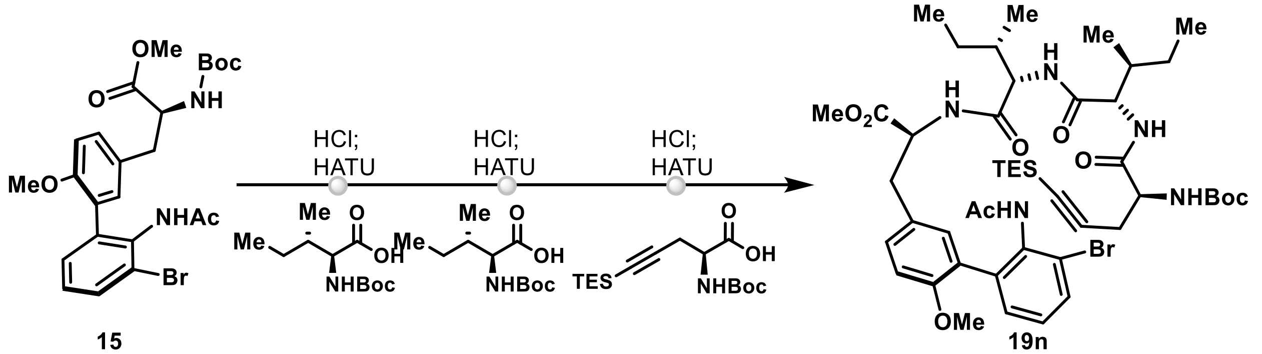

On 0.49 mmol scale, **General Procedure E** was followed from compound **15** as an intermediate. Purification by precipitation in hexane gave the title compound **19n** (225 mg, 48% yield from **15**).

Compound 19n

Physical State: amorphous solid

^{1}H NMR (600 MHz, METHANOL-*D*4): δ 7.66 – 7.57 (m, 1H), 7.24 – 7.23 (m, 2H), 7.16 (d, *J* = 8.4 Hz, 1H), 6.97 (s, 1H), 6.91 (d, *J* = 8.5 Hz, 1H), 4.70 – 4.58 (m, 1H), 4.22 (dd, *J* = 20.6, 8.0 Hz, 3H), 3.69 (s, 3H), 3.65 (s, 3H), 3.05 (dd, *J* = 13.9, 5.5 Hz, 1H), 2.94 (dd, *J* = 13.9, 8.0 Hz, 1H), 2.68 (dd, *J* = 17.2, 4.4 Hz, 1H), 2.56 (dd, *J* = 17.0, 9.4 Hz, 1H), 1.83 (s, 3H), 1.78 – 1.76 (m, 1H), 1.74 – 1.71 (m, 1H), 1.42 (s, 9H), 1.32 – 1.22 (m, 2H), 1.09 – 1.05 (m, 2H), 0.97 (t, *J* = 7.8 Hz, 9H), 0.87 (d, *J* = 6.8 Hz, 3H), 0.82 – 0.78 (m, 6H), 0.75 (d, *J* = 6.7 Hz, 3H), 0.55 (q, *J* = 7.8 Hz, 6H).

^{13}C NMR (151 MHz, METHANOL-*D*$_4$): δ 172.11, 171.82, 171.72, 170.52, 156.39, 155.39, 140.11, 134.66, 131.73, 130.21, 129.77, 128.44, 128.35, 127.82, 123.17, 110.60, 103.64, 83.49, 79.49, 57.74, 57.61, 54.77, 53.99, 53.71, 51.34, 37.09, 36.77, 36.23, 31.42, 27.43, 24.55, 22.37, 21.10, 14.66, 14.48, 13.12, 10.10, 10.01, 6.57, 4.06.
HRMS (ESI-TOF): calculated for $C_{47}H_{70}BrN_5NaO_9Si^+$ $[M+Na]^+$: 978.4018, found: 978.4017.
[α]^{25}$_D$: –11.6 (c = 0.2, CHCl$_3$)

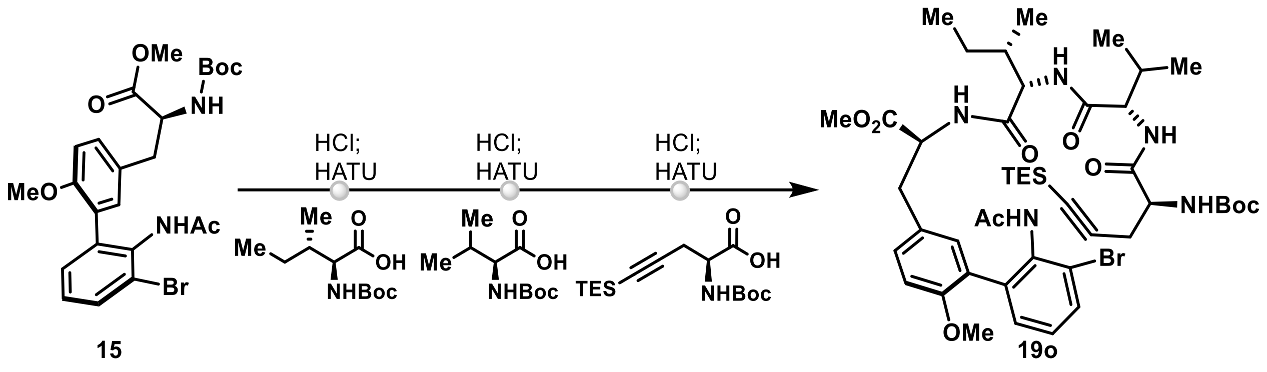

On 0.29 mmol scale, **General Procedure E** was followed from compound **15** as an intermediate. Purification by precipitation in hexane gave the title compound **19o** (169 mg, 62% yield from **15**).
Compound 19o
Physical State: amorphous solid
^{1}H NMR (600 MHz, METHANOL-*D*$_4$): δ 7.64 (dd, J = 5.5, 3.9 Hz, 1H), 7.27 – 7.23 (m, 2H), 7.17 (dd, J = 8.4, 1.9 Hz, 1H), 7.01 – 6.97 (m, 1H), 6.93 (d, J = 8.5 Hz, 1H), 4.67 (s, 1H), 4.25 (d, J = 8.2 Hz, 2H), 4.21 (d, J = 7.0 Hz, 1H), 3.71 (s, 3H), 3.67 (s, 3H), 3.07 (dd, J = 13.9, 5.6 Hz, 1H), 2.96 (dd, J = 13.8, 7.9 Hz, 1H), 2.71 (dd, J = 17.1, 4.5 Hz, 1H), 2.59 (dd, J = 17.2, 9.5 Hz, 1H), 2.02 – 1.93 (m, 1H), 1.85 (s, 3H), 1.83 – 1.75 (m, 1H), 1.44 (s, 9H), 1.35 – 1.24 (m, 1H), 1.15 – 1.06 (m, 1H), 0.99 (t, J = 7.9 Hz, 9H), 0.89 (d, J = 6.6 Hz, 3H), 0.85 – 0.80 (m, 9H), 0.57 (q, J = 7.7 Hz, 6H).
^{13}C NMR (151 MHz, METHANOL-*D*$_4$): δ 172.13, 171.76, 171.69, 170.53, 156.39, 155.38, 140.12, 134.65, 131.72, 130.20, 129.76, 128.45, 128.33, 127.82, 123.15, 110.59, 103.61, 83.53, 79.51, 58.49, 57.64, 54.76, 53.94, 53.75, 51.33, 36.79, 36.21, 31.06, 27.42, 24.57, 22.40, 21.09, 18.51, 17.32, 14.47, 10.01, 6.55, 4.05.
HRMS (ESI-TOF): calculated for $C_{46}H_{68}BrN_5NaO_9Si^+$ $[M+Na]^+$: 964.3862, found: 964.3864.
[α]^{25}$_D$: –3.7 (c = 0.2, CHCl$_3$)

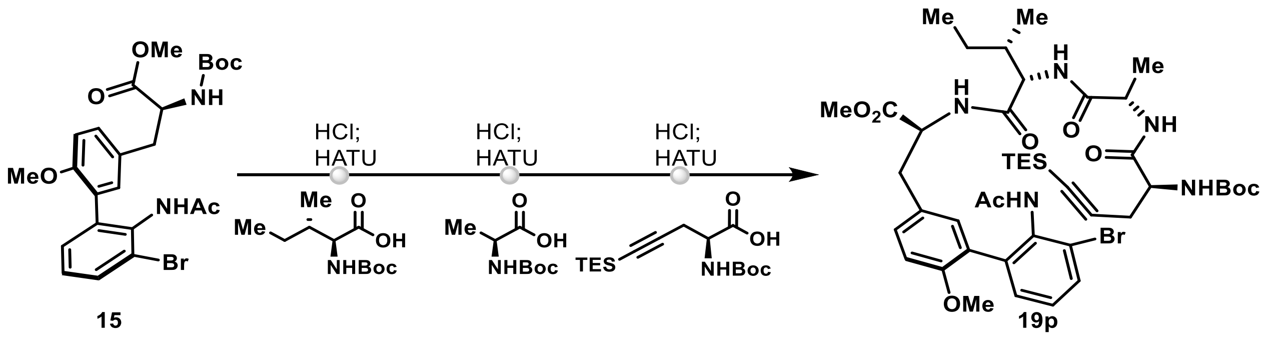

On 0.36 mmol scale, **General Procedure E** was followed from compound **15** as an intermediate. Purification by precipitation in hexane gave the title compound **19p** (183 mg, 55% yield from **15**).
Compound 19p
Physical State: amorphous solid
^{1}H NMR (600 MHz, METHANOL-*D*$_4$): δ 7.64 (d, J = 6.9 Hz, 1H), 7.27 – 7.24 (m, 2H), 7.16 (d, J = 7.6 Hz, 1H), 6.98 (s, 1H), 6.94 (d, J = 8.4 Hz, 1H), 4.69 (s, 1H), 4.44 – 4.29 (m, 1H), 4.22 – 4.19 (m, 2H), 3.72 (s, 3H), 3.68 (s, 3H), 3.08 (dd, J = 13.7, 5.0 Hz, 1H), 3.01 – 2.91 (m, 1H), 2.73 (dd, J = 17.1, 4.2 Hz, 1H), 2.58

(dd, J = 17.1, 9.1 Hz, 1H), 1.85 (s, 3H), 1.82 – 1.80 (m, 1H), 1.45 (s, 9H), 1.38 – 1.35 (m, 1H), 1.28 (d, J = 7.0 Hz, 3H), 1.14 – 1.07 (m, 1H), 0.99 (t, J = 7.9 Hz, 9H), 0.88 (d, J = 6.4 Hz, 3H), 0.85 – 0.82 (m, 3H), 0.57 (q, J = 7.8 Hz, 6H).

^{13}C NMR (151 MHz, METHANOL-D_4): δ 173.00, 172.10, 171.73, 171.58, 170.54, 156.38, 155.38, 140.14, 134.69, 131.72, 130.11, 129.82, 128.45, 128.29, 127.82, 123.14, 110.59, 103.43, 83.66, 79.56, 57.93, 54.79, 53.74, 51.36, 49.09, 36.82, 36.20, 27.41, 24.50, 22.76, 21.09, 17.02, 14.53, 10.16, 6.56, 4.04.

HRMS (ESI-TOF): calculated for $C_{44}H_{64}BrN_5NaO_9Si^+$ [M+Na]$^+$: 936.3549, found: 936.3552.

$[\alpha]^{25}_D$: +6.2 (c = 0.2, $CHCl_3$)

General procedure F for the synthesis of 20 via Larock macrocyclization：

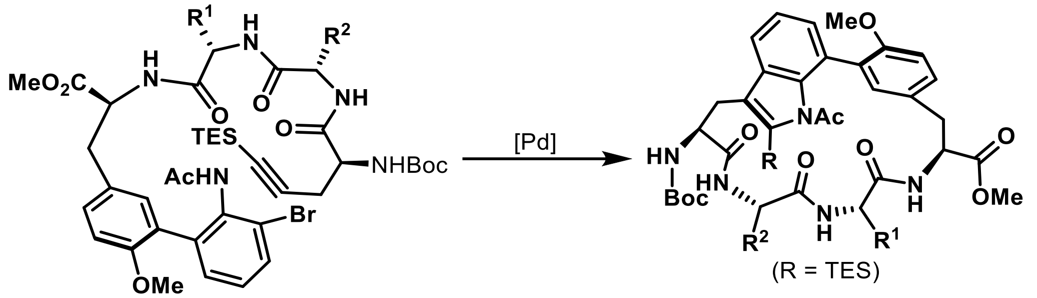

19 20

The tetrapeptide **19** (0.1 mmol, 1.0 eq.) was dissolved in acetonitrile (1.5 mL). Pd(tBu$_3$P)$_2$ (0.02 mmol, 0.2 eq.) and DIPEA (0.25 mmol, 2.5 eq.) were added. The reaction mixture was stirred at 110 ºC under nitrogen atmosphere for 12 h. The reaction mixture was concentrated under reduced pressure and purified by silica gel chromatography to give the cyclized compound **20**.

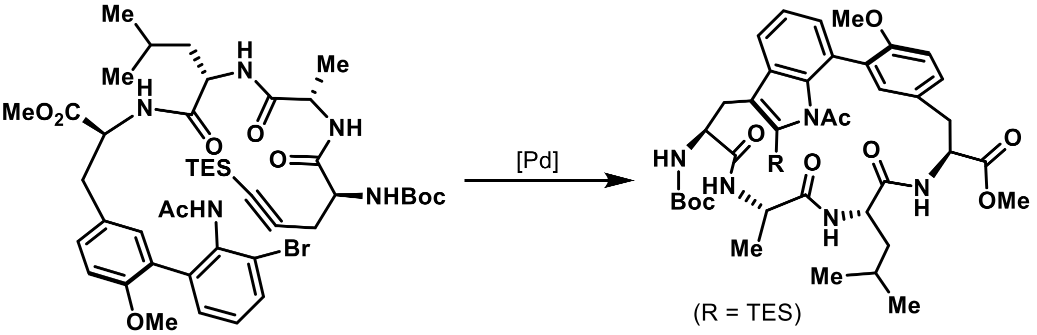

19a 20a

On 0.2 mmol scale, **General Procedure F** was followed from compound **19a** via Larock macrocyclization. Purification by silica gel column chromatography gave the title compound **20a** (42.4 mg, 51% yield).

Compound 20a

Physical State: amorphous solid

^{1}H NMR (600 MHz, CDCl$_3$): δ 8.00 (d, J = 7.8 Hz, 1H), 7.57 (d, J = 1.7 Hz, 1H), 7.33 (t, J = 7.6 Hz, 1H), 7.17 (d, J = 7.3 Hz, 1H), 6.99 – 6.91 (m, 2H), 6.76 (d, J = 7.8 Hz, 1H), 5.70 (d, J = 7.4 Hz, 1H), 5.62 (s, 1H), 5.18 (d, J = 8.6 Hz, 1H), 4.88 – 4.85 (m, 1H), 4.70 – 4.65 (m, 1H), 4.42 – 4.33 (m, 2H), 3.85 (s, 3H), 3.79 (s, 3H), 3.52 (dd, J = 13.6, 4.2 Hz, 1H), 3.13 – 2.98 (m, 3H), 1.81 (s, 3H), 1.51 (s, 9H), 1.07 (d, J = 6.5 Hz, 3H), 1.04 (d, J = 7.0 Hz, 3H), 0.98 – 0.93 (m, 6H), 0.86 (t, J = 7.5 Hz, 9H), 0.82 – 0.75 (m, 6H).

^{13}C NMR (151 MHz, CDCl$_3$): δ 175.68, 171.49, 171.15, 170.96, 169.22, 155.22, 155.20, 136.32, 135.19, 133.29, 131.01, 130.91, 129.83, 128.95, 128.30, 126.05, 124.00, 121.90, 118.96, 111.11, 79.75, 55.65, 53.99, 53.86, 53.73, 47.31, 41.26, 36.90, 31.99, 28.57, 27.43, 24.81, 23.72, 21.90, 19.54, 7.89, 5.05.

HRMS (ESI-TOF): calculated for $C_{44}H_{63}N_5NaO_9Si^+$ [M+Na]$^+$: 856.4287, found: 856.4282.

[α]$^{25}_D$: +47.6 (c = 0.5, CHCl$_3$)

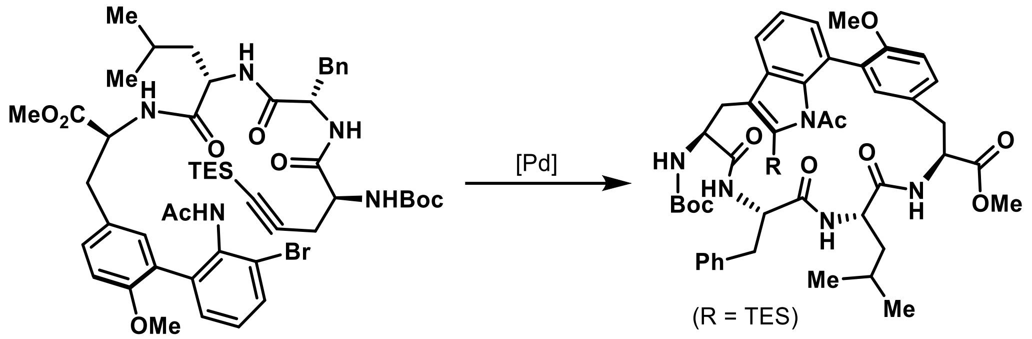

19b **20b**

On 0.2 mmol scale, **General Procedure F** was followed from compound **19b** via Larock macrocyclization. Purification by silica gel column chromatography gave the title compound **20b** (49.9 mg, 55% yield).

Compound 20b

Physical State: amorphous solid

^{1}H NMR (600 MHz, CDCl$_3$): δ 7.98 (dd, *J* = 7.8, 1.1 Hz, 1H), 7.43 – 7.28 (m, 2H), 7.15 (t, *J* = 7.4 Hz, 2H), 7.11 (d, *J* = 7.3 Hz, 1H), 7.07 (t, *J* = 7.3 Hz, 1H), 7.05 – 7.02 (m, 2H), 6.97 (dd, *J* = 8.4, 2.3 Hz, 1H), 6.93 (d, *J* = 8.4 Hz, 1H), 6.71 (d, *J* = 7.6 Hz, 1H), 5.74 (d, *J* = 7.3 Hz, 1H), 5.15 (d, *J* = 8.8 Hz, 1H), 4.95 (d, *J* = 3.3 Hz, 1H), 4.87 – 4.84 (m, 1H), 4.76 – 4.72 (m, 1H), 4.46 – 4.43 (m, 1H), 4.30 – 4.27 (m, 1H), 3.85 (s, 3H), 3.80 (s, 3H), 3.55 (dd, *J* = 13.7, 4.4 Hz, 1H), 3.17 (dd, *J* = 13.9, 3.7 Hz, 1H), 3.07 – 2.99 (m, 2H), 2.69 (dd, *J* = 13.6, 5.0 Hz, 1H), 2.61 (dd, *J* = 13.6, 7.9 Hz, 1H), 1.77 (s, 3H), 1.73 – 1.68 (m, 1H), 1.67 – 1.63 (m, 1H), 1.53 (s, 9H), 1.16 – 1.11 (m, 1H), 0.99 (d, *J* = 6.5 Hz, 3H), 0.89 (d, *J* = 6.5 Hz, 3H), 0.84 (t, *J* = 7.6 Hz, 9H), 0.79 – 0.73 (m, 6H).

^{13}C NMR (151 MHz, CDCl$_3$): δ 175.47, 171.26, 171.14, 169.30, 168.73, 155.21, 136.31, 135.70, 135.26, 133.43, 130.87, 130.81, 129.74, 129.64, 128.97, 128.37, 128.18, 126.88, 125.89, 123.92, 121.84, 118.85, 110.99, 79.74, 55.62, 53.95, 53.77, 52.79, 52.18, 41.19, 39.78, 36.83, 32.05, 28.60, 27.36, 24.76, 23.53, 21.95, 7.84, 5.01.

HRMS (ESI-TOF): calculated for $C_{50}H_{67}N_5NaO_9Si^+$ $[M+Na]^+$: 932.4600, found: 932.4608.

$[\alpha]^{25}_D$: +41.7 (*c* = 0.5, CHCl$_3$)

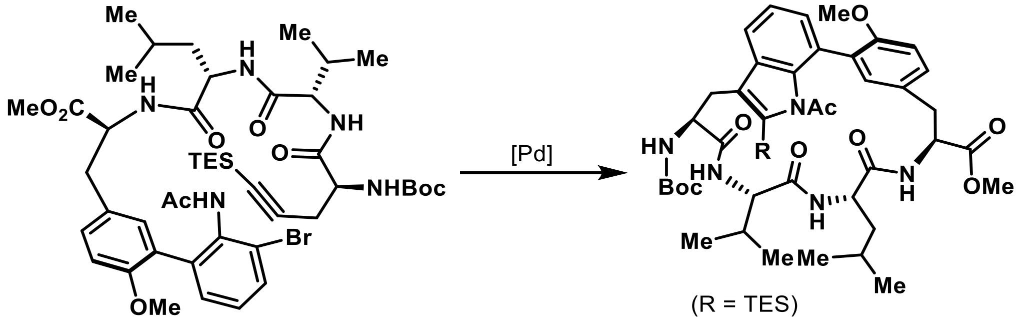

19c **20c**

On 0.2 mmol scale, **General Procedure F** was followed from compound **19c** via Larock macrocyclization. Purification by silica gel column chromatography gave the title compound **20c** (36.2 mg, 42% yield).

Compound 20c

Physical State: amorphous solid

^{1}H NMR (600 MHz, CDCl$_3$): δ 7.97 (d, *J* = 7.8 Hz, 1H), 7.62 (d, *J* = 2.0 Hz, 1H), 7.31 (t, *J* = 7.6 Hz, 1H), 7.17 – 7.13 (m, 1H), 6.97 – 6.89 (m, 2H), 6.75 (d, *J* = 7.8 Hz, 1H), 5.70 (d, *J* = 7.5 Hz, 1H), 5.30 (s, 1H), 4.99 (d, *J* = 9.3 Hz, 1H), 4.85 – 4.82 (m, 1H), 4.77 – 4.73 (m, 1H), 4.45 – 4.39 (m, 1H), 3.99 (dd, *J* = 9.2, 6.9 Hz, 1H), 3.84 (s, 3H), 3.78 (s, 3H), 3.52 (dd, *J* = 13.6, 4.4 Hz, 1H), 3.12 – 3.02 (m, 2H), 2.99 (dd, *J* = 14.0, 3.9 Hz, 1H), 1.79 (s, 3H), 1.75 – 1.69 (m, 1H), 1.56 – 1.52 (m, 1H), 1.51 (s, 9H), 1.30 – 1.21 (m, 1H), 1.06 (d, *J* = 6.5 Hz, 3H), 0.92 (d, *J* = 6.6 Hz, 3H), 0.85 (t, *J* = 7.5 Hz, 9H), 0.82 – 0.75 (m, 7H), 0.73 (d, *J* = 6.7 Hz, 3H),

0.64 (d, J = 6.7 Hz, 3H).
^{13}C NMR (151 MHz, CDCl$_3$): δ 175.63, 171.27, 171.15, 169.90, 169.25, 155.26, 155.19, 136.47, 135.17, 133.49, 130.91, 129.77, 128.86, 128.35, 126.03, 124.11, 121.88, 118.84, 111.06, 79.79, 56.84, 55.64, 53.96, 53.79, 52.15, 41.21, 36.86, 32.03, 31.90, 28.56, 27.39, 24.80, 23.76, 21.89, 18.81, 18.21, 7.86, 5.01.
HRMS (ESI-TOF): calculated for $C_{46}H_{67}N_5NaO_9Si^+$ $[M+Na]^+$: 884.4600, found: 884.4600.
[α]$^{25}_{D}$: +68.3 (c = 0.5, CHCl$_3$)

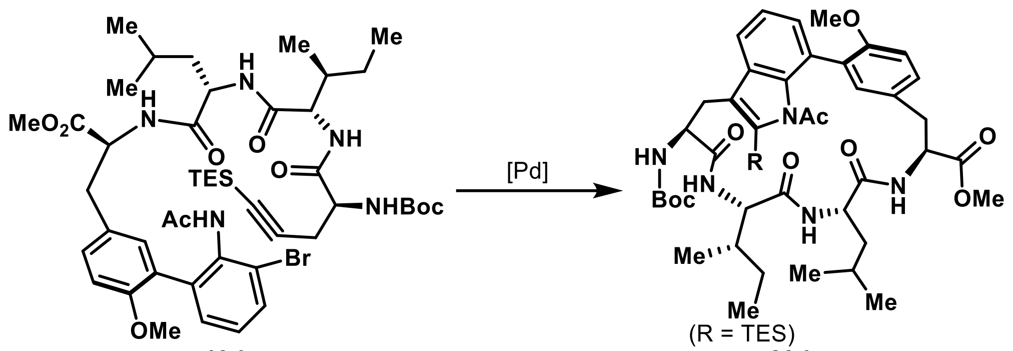

19d 20d

On 0.2 mmol scale, **General Procedure F** was followed from compound **19d** via Larock macrocyclization. Purification by silica gel column chromatography gave the title compound **20d** (39.8 mg, 45% yield).

Compound 20d

Physical State: amorphous solid
^{1}H NMR (600 MHz, CDCl$_3$): δ 7.97 (dd, J = 7.8, 1.2 Hz, 1H), 7.66 (d, J = 2.2 Hz, 1H), 7.32 (t, J = 7.6 Hz, 1H), 7.17 (dd, J = 7.3, 1.1 Hz, 1H), 6.99 – 6.91 (m, 2H), 6.76 (d, J = 7.8 Hz, 1H), 5.69 (d, J = 7.5 Hz, 1H), 5.30 – 5.28 (m, 1H), 5.03 (d, J = 9.3 Hz, 1H), 4.85 (dt, J = 7.7, 3.8 Hz, 1H), 4.78 – 4.73 (m, 1H), 4.44 (ddd, J = 10.8, 5.1, 3.2 Hz, 1H), 4.00 (dd, J = 9.3, 7.3 Hz, 1H), 3.86 (s, 3H), 3.79 (s, 3H), 3.52 (dd, J = 13.6, 4.4 Hz, 1H), 3.10 – 3.03 (m, 2H), 3.00 (dd, J = 13.9, 3.9 Hz, 1H), 1.80 (s, 3H), 1.74 (ddd, J = 12.5, 10.7, 4.3 Hz, 1H), 1.59 – 1.55 (m, 2H), 1.52 (s, 9H), 1.31-1.25 (m, 3H), 1.08 (d, J = 6.5 Hz, 3H), 0.93 (d, J = 6.6 Hz, 3H), 0.88 – 0.84 (m, 9H), 0.83 – 0.76 (m, 6H), 0.74 – 0.67 (m, 6H).
^{13}C NMR (151 MHz, CDCl$_3$): δ 175.72, 171.16, 171.14, 169.74, 169.30, 155.27, 155.18, 136.47, 135.14, 133.50, 130.93, 130.85, 129.78, 128.84, 128.40, 126.05, 124.13, 121.89, 118.83, 111.07, 79.80, 56.07, 55.64, 53.99, 53.81, 53.79, 52.15, 41.22, 38.17, 36.86, 32.02, 28.57, 27.39, 25.10, 24.79, 23.80, 21.87, 14.98, 11.14, 7.87, 5.02.
HRMS (ESI-TOF): calculated for $C_{47}H_{69}N_5NaO_9Si^+$ $[M+Na]^+$: 898.4757, found: 898.4759.
[α]$^{25}_{D}$: +57.1 (c = 0.5, CHCl$_3$)

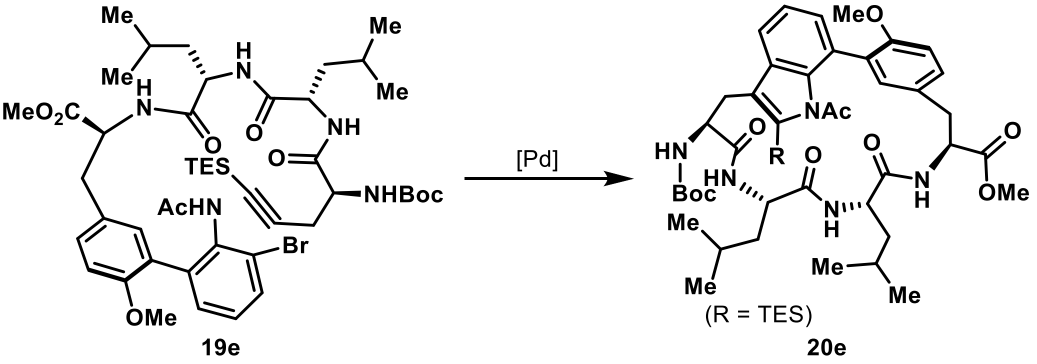

19e 20e

On 0.2 mmol scale, **General Procedure F** was followed from compound **19e** via Larock macrocyclization. Purification by silica gel column chromatography gave the title compound **20e** (41.1 mg, 47% yield).

Compound 20e

Physical State: amorphous solid

^{1}H NMR (600 MHz, CDCl$_3$): δ 7.95 (dd, *J* = 7.8, 1.2 Hz, 1H), 7.60 (d, *J* = 2.2 Hz, 1H), 7.32 (t, *J* = 7.6 Hz, 1H), 7.17 (dd, *J* = 7.3, 1.1 Hz, 1H), 6.99 – 6.91 (m, 2H), 6.77 (d, *J* = 7.8 Hz, 1H), 5.63 (d, *J* = 7.7 Hz, 1H), 5.31 (d, *J* = 2.9 Hz, 1H), 5.02 (d, *J* = 9.3 Hz, 1H), 4.87 – 4.84 (dt, *J* = 7.7, 3.8 Hz, 1H), 4.69 – 4.65 (m, 1H), 4.44 – 4.40 (m, 1H), 4.31 – 4.23 (m, 1H), 3.85 (s, 3H), 3.79 (s, 3H), 3.49 (dd, *J* = 13.5, 4.4 Hz, 1H), 3.08 (ddd, *J* = 13.5, 7.5, 3.8 Hz, 2H), 3.00 (dd, *J* = 14.0, 3.9 Hz, 1H), 1.80 (s, 3H), 1.75 (ddd, *J* = 12.5, 10.7, 4.3 Hz, 1H), 1.60 – 1.53 (m, 1H), 1.51 (s, 9H), 1.48 – 1.42 (m, 1H), 1.31 – 1.26 (m, 1H), 1.22 – 1.14 (m, 2H), 1.08 (d, *J* = 6.5 Hz, 3H), 0.93 (d, *J* = 6.6 Hz, 3H), 0.87 (t, *J* = 7.8 Hz, 9H), 0.83 – 0.79 (d, *J* = 6.8 Hz, 9H), 0.77 (d, *J* = 6.7 Hz, 3H).
^{13}C NMR (151 MHz, CDCl$_3$): δ 175.78, 171.28, 171.14, 170.66, 169.82, 155.23, 155.20, 136.41, 135.14, 133.34, 131.11, 130.88, 129.82, 128.83, 128.38, 125.96, 124.07, 121.83, 118.79, 111.10, 79.80, 55.64, 54.27, 53.74, 52.15, 50.05, 42.53, 41.26, 36.88, 31.80, 28.55, 27.41, 24.79, 24.36, 23.81, 23.04, 21.87, 21.82, 7.91, 5.06.
HRMS (ESI-TOF): calculated for $C_{47}H_{69}N_5NaO_9Si^+$ [M+Na]$^+$: 898.4757, found: 898.4767.
[α]$^{25}_D$: +7.3 (*c* = 0.5, CHCl$_3$)

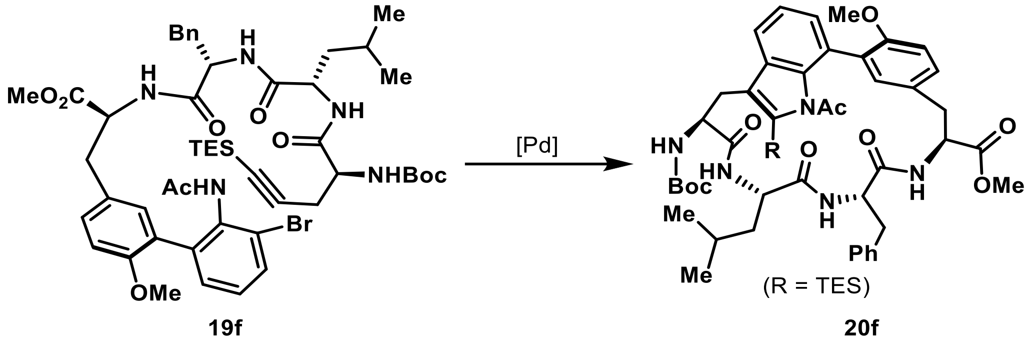

On 0.2 mmol scale, **General Procedure F** was followed from compound **19f** via Larock macrocyclization. Purification by silica gel column chromatography gave the title compound **20f** (42.3 mg, 47% yield).
Compound 20f
Physical State: amorphous solid
^{1}H NMR (600 MHz, CDCl$_3$): δ 7.96 (d, *J* = 7.6 Hz, 1H), 7.50 (s, 1H), 7.32 – 7.28 (m, 3H), 7.26 – 7.20 (m, 3H), 7.15 (d, *J* = 7.6 Hz, 1H), 6.89 (s, 2H), 6.34 (d, *J* = 7.1 Hz, 1H), 5.64 (d, *J* = 7.6 Hz, 1H), 5.42 (s, 1H), 5.01 (d, *J* = 9.1 Hz, 1H), 4.67 – 4.63 (m, 1H), 4.57 – 4.54 (m, 1H), 4.53 – 4.46 (m, 1H), 4.24 (td, *J* = 9.2, 5.4 Hz, 1H), 3.82 (s, 3H), 3.66 (s, 3H), 3.48 (dd, *J* = 13.5, 4.2 Hz, 1H), 3.12 (dd, *J* = 14.0, 3.8 Hz, 1H), 3.04 (t, *J* = 12.6 Hz, 1H), 2.98 – 2.86 (m, 3H), 1.74 (s, 3H), 1.51 (s, 9H), 1.31 – 1.19 (m, 2H), 0.98 – 0.72 (m, 22H).
^{13}C NMR (151 MHz, CDCl$_3$): δ 175.58, 170.63, 170.57, 170.44, 169.58, 155.21, 155.16, 136.29, 135.67, 135.05, 133.30, 131.07, 130.83, 129.73, 129.29, 128.95, 128.88, 128.32, 127.22, 126.17, 123.98, 121.87, 118.86, 110.96, 79.79, 56.44, 55.62, 54.18, 53.95, 52.02, 50.19, 42.81, 38.16, 36.60, 31.81, 28.73, 28.56, 27.31, 24.41, 22.95, 22.04, 7.82, 5.06.
HRMS (ESI-TOF): calculated for $C_{50}H_{67}N_5NaO_9Si^+$ [M+Na]$^+$: 932.4600, found: 932.4608.
[α]$^{25}_D$: +22.2 (*c* = 0.5, CHCl$_3$)

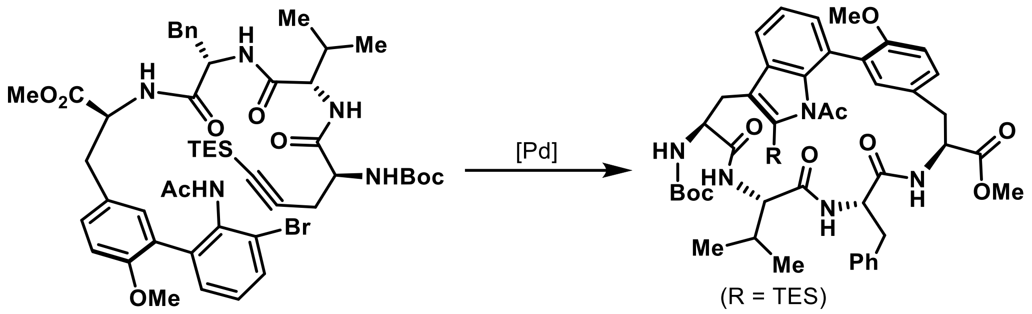

On 0.2 mmol scale, **General Procedure F** was followed from compound **19g** via Larock macrocyclization. Purification by silica gel column chromatography gave the title compound **20g** (40.4 mg, 45% yield).

Compound 20g

Physical State: amorphous solid

^{1}H NMR (600 MHz, CDCl$_3$): δ 8.00 (d, *J* = 7.7 Hz, 1H), 7.53 (s, 1H), 7.33 – 7.29 (m, 3H), 7.25 - 722 (m, 3H), 7.15 (d, *J* = 7.2 Hz, 1H), 6.90 (s, 2H), 6.38 (d, *J* = 7.0 Hz, 1H), 5.71 (d, *J* = 7.4 Hz, 1H), 5.43 (s, 1H), 5.04 (d, *J* = 9.1 Hz, 1H), 4.76 – 4.71 (m, 1H), 4.60 – 4.58 (m, 1H), 4.51 (td, *J* = 8.1, 2.3 Hz, 1H), 3.97 (dd, *J* = 8.9, 7.0 Hz, 1H). 3.84 (s, 3H), 3.69 (s, 3H), 3.52 (dd, *J* = 13.5, 4.2 Hz, 1H), 3.13 (dd, *J* = 14.0, 3.7 Hz, 1H), 3.07 – 3.00 (m, 1H), 2.97 (dd, *J* = 12.9, 8.5 Hz, 1H), 2.94 – 2.87 (m, 2H), 1.76 (s, 3H), 1.52 (s, 9H), 0.85 – 0.66 (m, 22H).

^{13}C NMR (151 MHz, CDCl$_3$): δ 175.43, 170.69, 170.60, 169.76, 169.20, 155.15, 136.32, 135.70, 135.09, 133.46, 130.93, 130.85, 129.69, 129.30, 128.92, 128.29, 127.25, 126.23, 124.04, 121.91, 118.90, 110.96, 79.79, 57.00, 56.42, 55.63, 54.06, 53.98, 52.04, 38.15, 36.55, 32.11, 31.98, 28.57, 27.29, 18.77, 18.29, 7.77, 5.01.

HRMS (ESI-TOF): calculated for $C_{49}H_{65}N_5NaO_9Si^+$ [M+Na]$^+$: 918.4444, found: 932.4452.

[α]$^{25}_{D}$: +49.6 (*c* = 0.5, CHCl$_3$)

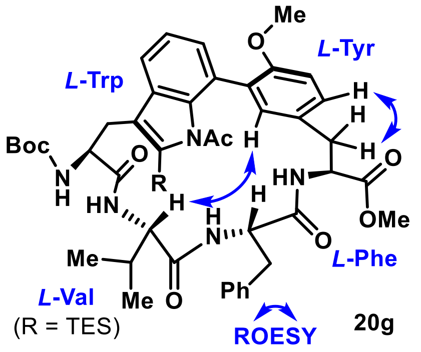

The configuration of the ring system is deduced to be Rconf.

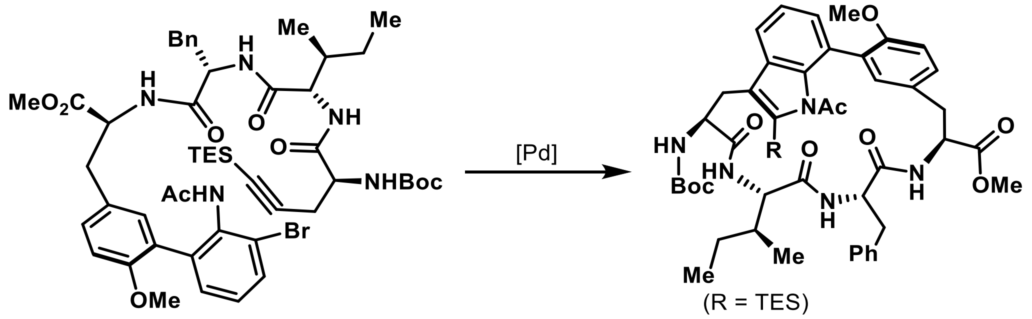

On 0.2 mmol scale, **General Procedure F** was followed from compound **19h** via Larock macrocyclization.

Purification by silica gel column chromatography gave the title compound **20h** (41.9 mg, 46% yield).

Compound 20h

Physical State: amorphous solid

^{1}H NMR (600 MHz, CDCl$_3$): δ 7.99 (d, *J* = 7.7 Hz, 1H), 7.55 (s, 1H), 7.31 (td, *J* = 7.5, 4.0 Hz, 3H), 7.25 – 7.21 (m, 3H), 7.16 (d, *J* = 7.3 Hz, 1H), 6.90 (s, 2H), 6.37 (d, *J* = 7.0 Hz, 1H), 5.70 (d, *J* = 7.4 Hz, 1H), 5.41 (s, 1H), 5.05 (d, *J* = 9.1 Hz, 1H), 4.79 – 4.68 (m, 1H), 4.62 – 4.55 (m, 1H), 4.52 (t, *J* = 7.1 Hz, 1H), 4.02 – 3.95 (m, 1H), 3.84 (s, 3H), 3.68 (s, 3H), 3.52 (dd, *J* = 13.5, 4.2 Hz, 1H), 3.12 (dd, *J* = 13.9, 3.7 Hz, 1H), 3.03 (t, *J* = 12.7 Hz, 1H), 2.97 (dd, *J* = 12.8, 8.7 Hz, 1H), 2.93 – 2.88 (m, 2H), 1.76 (s, 3H), 1.52 (s, 9H), 1.30 – 1.25 (m, 2H), 1.00 – 0.91 (m, 2H), 0.81 – 0.72 (m, 20H).

^{13}C NMR (151 MHz, CDCl$_3$): δ 175.49, 170.69, 170.47, 169.57, 169.17, 155.25, 155.14, 136.32, 135.70, 135.06, 133.46, 130.92, 130.82, 129.69, 129.31, 128.96, 128.90, 128.33, 127.24, 126.25, 124.05, 121.92, 118.89, 110.95, 79.80, 56.41, 56.21, 55.63, 54.04, 53.97, 52.03, 38.41, 38.15, 36.56, 31.99, 28.57, 27.29, 25.18, 14.90, 11.27, 7.77, 5.01.

HRMS (ESI-TOF): calculated for $C_{50}H_{67}N_5NaO_9Si^+$ [M+Na]$^+$: 932.4600, found: 932.4606.

[α]^{25}D: +35.1 (*c* = 0.5, CHCl$_3$)

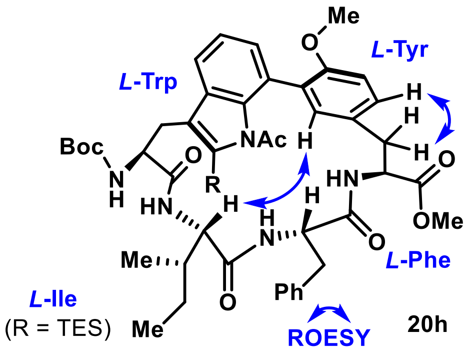

The configuration of the ring system is deduced to be Rconf.

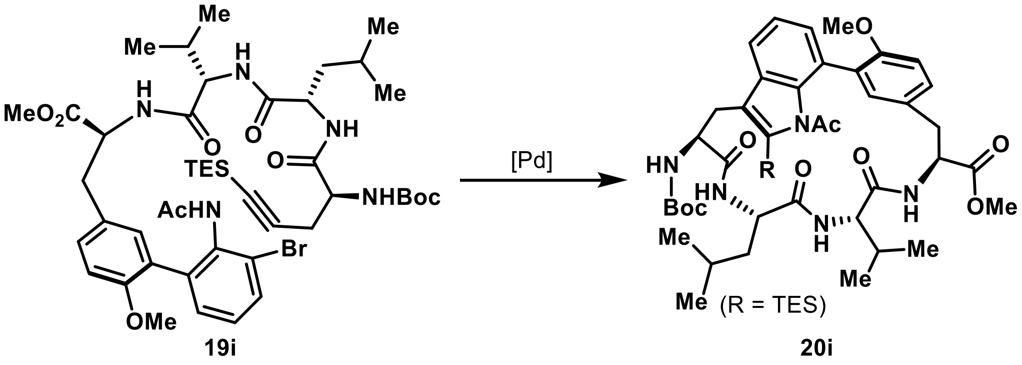

On 0.2 mmol scale, **General Procedure F** was followed from compound **19i** via Larock macrocyclization. Purification by silica gel column chromatography gave the title compound **20i** (48.1 mg, 56% yield).

Compound 20i

Physical State: amorphous solid

^{1}H NMR (600 MHz, CDCl$_3$): δ 7.96 (d, *J* = 7.7 Hz, 1H), 7.55 (d, *J* = 2.0 Hz, 1H), 7.32 (t, *J* = 7.6 Hz, 1H), 7.16 (d, *J* = 6.9 Hz, 1H), 6.98 – 6.90 (m, 2H), 6.64 (d, *J* = 7.3 Hz, 1H), 5.66 (d, *J* = 7.6 Hz, 1H), 5.38 (d, *J* = 2.2 Hz, 1H), 5.02 (d, *J* = 9.1 Hz, 1H), 4.83 – 4.80 (m, 1H), 4.69 – 4.65 (m, 1H), 4.34 – 4.30 (m, 1H), 4.02 (dd, *J* = 7.8, 3.0 Hz, 1H), 3.85 (s, 3H), 3.79 (s, 3H), 3.51 (dd, *J* = 13.5, 4.3 Hz, 1H), 3.15 (dd, *J* = 14.0, 3.6 Hz, 1H), 3.11 – 3.04 (m, 1H), 2.98 (dd, *J* = 14.0, 3.9 Hz, 1H), 1.95 – 1.89 (m, 1H), 1.80 (s, 3H), 1.51 (s, 9H), 1.49 – 1.42 (m, 1H), 1.25 – 1.16 (m, 2H), 1.09 (d, *J* = 6.8 Hz, 3H), 0.97 (d, *J* = 6.7 Hz, 3H), 0.86 (t, *J* = 7.4 Hz, 9H), 0.84 – 0.74 (m, 12H).

^{13}C NMR (151 MHz, CDCl$_3$): δ 175.52, 171.27, 170.57, 169.68, 155.22, 155.14, 136.31, 135.28, 133.42,

131.09, 130.92, 129.70, 128.90, 128.40, 125.98, 124.05, 121.84, 118.81, 111.04, 79.78, 60.52, 55.62, 54.20, 53.93, 52.19, 50.12, 42.69, 36.57, 31.81, 30.08, 28.55, 27.37, 24.37, 22.97, 22.00, 19.58, 18.78, 7.87, 5.06.
HRMS (ESI-TOF): calculated for $C_{46}H_{67}N_5NaO_9Si^+$ [M+Na]$^+$: 884.4600, found: 884.4603.
[α]^{25}D: +66.3 (c = 0.5, $CHCl_3$)

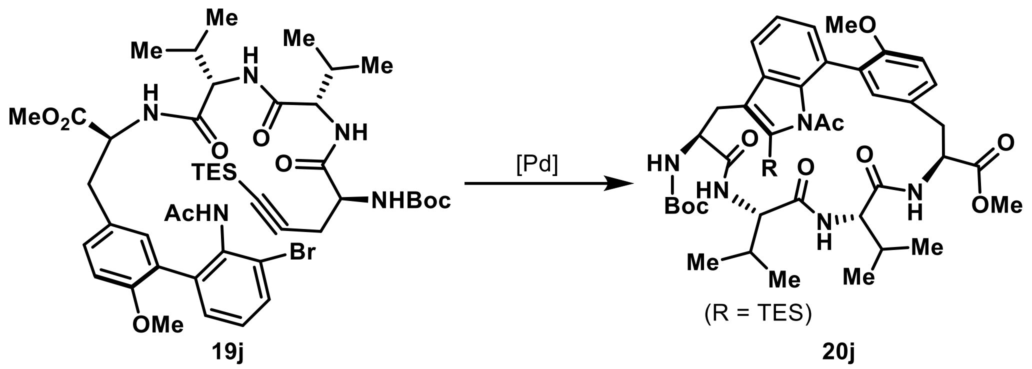

On 0.2 mmol scale, **General Procedure F** was followed from compound **19j** via Larock macrocyclization. Purification by silica gel column chromatography gave the title compound **20j** (38.8 mg, 46% yield).
Compound 20j
Physical State: amorphous solid
^{1}H NMR (600 MHz, $CDCl_3$): δ 8.00 (d, J = 7.7 Hz, 1H), 7.57 (d, J = 1.8 Hz, 1H), 7.32 (t, J = 7.6 Hz, 1H), 7.15 (d, J = 7.3 Hz, 1H), 6.94 – 6.87 (m, 2H), 6.62 (d, J = 7.2 Hz, 1H), 5.73 (d, J = 7.4 Hz, 1H), 5.38 (s, 1H), 5.04 (d, J = 9.1 Hz, 1H), 4.81 – 4.74 (m, 2H), 4.09 – 4.01 (m, 2H), 3.85 (s, 3H), 3.80 (s, 3H), 3.54 (dd, J = 13.6, 4.3 Hz, 1H), 3.15 (dd, J = 13.9, 3.6 Hz, 1H), 3.10 – 3.03 (m, 1H), 2.98 (dd, J = 14.0, 3.9 Hz, 1H), 1.94 – 1.88 (m, 1H), 1.81 (s, 3H), 1.60 – 1.55 (m, 1H), 1.52 (s, 9H), 1.07 (d, J = 6.8 Hz, 3H), 0.97 (d, J = 6.7 Hz, 3H), 0.86 (t, J = 7.4 Hz, 9H), 0.82 – 0.75 (m, 9H), 0.66 (d, J = 6.7 Hz, 3H).
^{13}C NMR (151 MHz, $CDCl_3$): δ 175.32, 171.26, 170.62, 169.83, 169.21, 155.26, 155.14, 136.35, 135.32, 133.58, 130.94, 129.65, 128.93, 128.35, 126.05, 124.09, 121.89, 118.87, 111.02, 79.76, 60.56, 56.84, 55.63, 54.01, 53.96, 52.19, 36.52, 32.05, 32.00, 30.06, 28.57, 27.34, 19.44, 18.84, 18.72, 18.17, 7.84, 5.02.
HRMS (ESI-TOF): calculated for $C_{45}H_{65}N_5NaO_9Si^+$ [M+Na]$^+$: 870.4444, found: 870.4448.
[α]^{25}D: +53.7 (c = 0.5, $CHCl_3$)

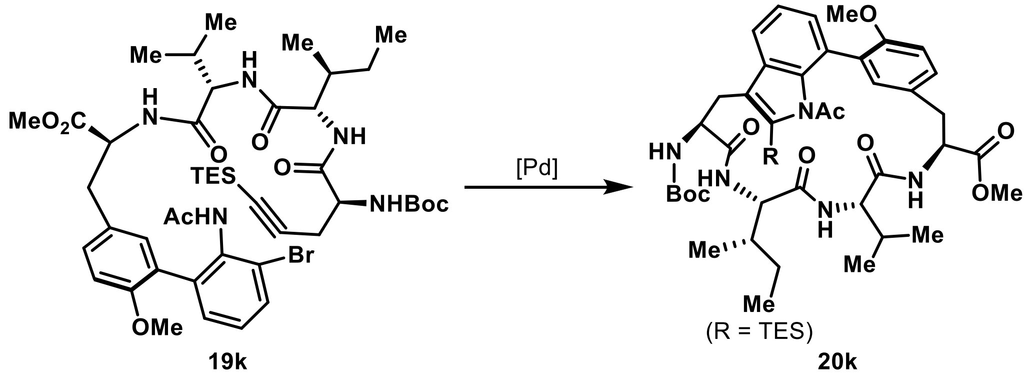

On 0.2 mmol scale, **General Procedure F** was followed from compound **19k** via Larock macrocyclization. Purification by silica gel column chromatography gave the title compound **20k** (39.1 mg, 45% yield).
Compound 20k
Physical State: amorphous solid
^{1}H NMR (600 MHz, $CDCl_3$): δ 7.99 (d, J = 7.7 Hz, 1H), 7.59 (d, J = 1.8 Hz, 1H), 7.32 (t, J = 7.6 Hz, 1H), 7.16 (d, J = 7.3 Hz, 1H), 6.97 – 6.90 (m, 2H), 6.62 (d, J = 7.2 Hz, 1H), 5.71 (d, J = 7.4 Hz, 1H), 5.36 (d, J = 2.7 Hz, 1H), 5.06 (d, J = 9.1 Hz, 1H), 4.81 – 4.79 (m, 1H), 4.77 – 4.73 (m, 1H), 4.09 – 4.03 (m, 2H), 3.85 (s, 3H), 3.80 (s, 3H), 3.53 (dd, J = 13.6, 4.3 Hz, 1H), 3.16 (dd, J = 13.9, 3.6 Hz, 1H), 3.10 – 3.03 (m, 1H), 2.97

(dd, J = 13.9, 3.9 Hz, 1H), 1.94 – 1.88 (m, 1H), 1.81 (s, 3H), 1.52 (s, 9H), 1.30 – 1.24 (m, 2H), 1.08 (d, J = 6.8 Hz, 3H), 0.97 (d, J = 6.8 Hz, 3H), 0.94 – 0.90 (m, 1H), 0.86 (t, J = 7.3 Hz, 9H), 0.84 – 0.76 (m, 6H), 0.76 – 0.69 (m, 6H).
^{13}C NMR (151 MHz, CDCl$_3$): δ 175.42, 171.28, 170.45, 169.67, 169.23, 155.27, 155.13, 136.35, 135.29, 133.59, 130.94, 130.91, 129.66, 128.91, 128.40, 126.06, 124.10, 121.89, 118.85, 111.02, 79.78, 60.48, 56.12, 55.63, 54.02, 53.97, 52.19, 38.34, 36.50, 32.00, 30.08, 28.57, 27.35, 25.14, 19.45, 18.75, 14.87, 11.23, 7.84, 5.03.
HRMS (ESI-TOF): calculated for $C_{46}H_{67}N_5NaO_9Si^+$ [M+Na]$^+$: 884.4600, found: 884.4598.
[α]$^{25}_{D}$: +69.8 (c = 0.5, CHCl$_3$)

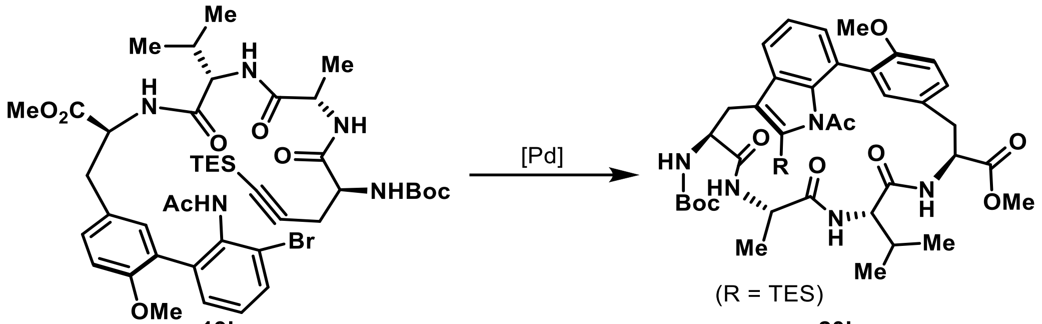

19l 20l

On 0.2 mmol scale, **General Procedure F** was followed from compound **19l** via Larock macrocyclization. Purification by silica gel column chromatography gave the title compound **20l** (38.4 mg, 47% yield).

Compound 20l

Physical State: amorphous solid
^{1}H NMR (600 MHz, CDCl$_3$): δ 8.02 (d, J = 7.7 Hz, 1H), 7.50 (d, J = 1.6 Hz, 1H), 7.32 (t, J = 7.5 Hz, 1H), 7.15 (t, J = 7.1 Hz, 1H), 6.99 – 6.90 (m, 2H), 6.63 (d, J = 7.3 Hz, 1H), 6.06 (s, 1H), 5.73 (d, J = 7.3 Hz, 1H), 5.28 (d, J = 8.4 Hz, 1H), 4.83 – 4.80 (m, 1H), 4.68 – 4.64 (m, 1H), 4.46 – 4.41 (m, 1H), 3.95 (dd, J = 8.3, 2.9 Hz, 1H), 3.85 (s, 3H), 3.79 (s, 3H), 3.54 (dd, J = 13.6, 4.1 Hz, 1H), 3.16 – 3.02 (m, 3H), 2.99 (dd, J = 14.0, 3.9 Hz, 1H), 1.81 (s, 3H), 1.51 (s, 9H), 1.09 (d, J = 6.7 Hz, 3H), 1.04 (d, J = 7.0 Hz, 3H), 0.98 – 0.95 (m, 4H), 0.86 (t, J = 7.6 Hz, 9H), 0.81 – 0.77 (m, 6H).
^{13}C NMR (151 MHz, CDCl$_3$): δ 175.35, 171.26, 171.08, 171.05, 169.12, 155.20, 155.17, 136.21, 135.41, 133.37, 131.06, 130.96, 129.73, 129.00, 128.32, 126.10, 124.03, 121.89, 119.02, 111.08, 79.71, 61.06, 55.63, 53.97, 53.87, 52.22, 47.41, 36.65, 31.97, 30.01, 28.57, 28.40, 27.36, 26.70, 19.67, 19.01, 8.26, 7.85, 5.06, 4.42.
HRMS (ESI-TOF): calculated for $C_{43}H_{61}N_5NaO_9Si^+$ [M+Na]$^+$: 842.4131, found: 842.4153.
[α]$^{25}_{D}$: +67.1 (c = 0.5, CHCl$_3$)

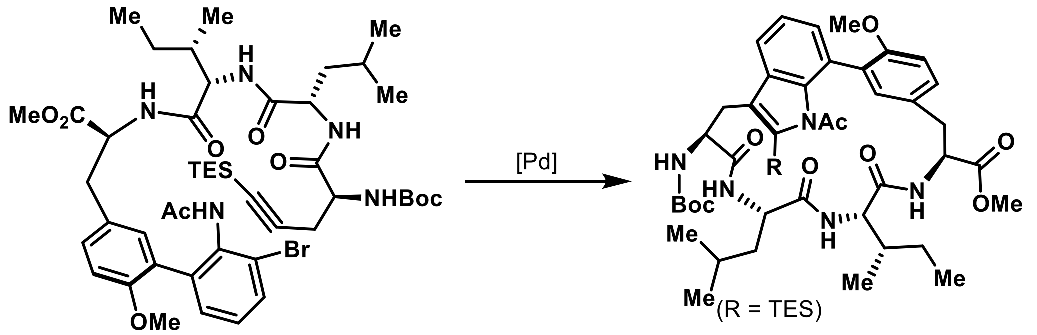

19m 20m

On 0.2 mmol scale, **General Procedure F** was followed from compound **19m** via Larock macrocyclization. Purification by silica gel column chromatography gave the title compound **20m** (42.1 mg, 48% yield).

Compound 20m

Physical State: amorphous solid

^{1}H NMR (600 MHz, CDCl$_3$): δ 7.94 (d, *J* = 7.7 Hz, 1H), 7.56 (d, *J* = 1.7 Hz, 1H), 7.30 (t, *J* = 7.6 Hz, 1H), 7.15 (d, *J* = 7.2 Hz, 1H), 7.00 – 6.84 (m, 2H), 6.60 (d, *J* = 7.2 Hz, 1H), 5.63 (d, *J* = 7.6 Hz, 1H), 5.39 – 5.32 (m, 1H), 5.00 (d, *J* = 9.1 Hz, 1H), 4.79 (dt, *J* = 7.3, 3.7 Hz, 1H), 4.66 (ddd, *J* = 11.8, 7.6, 4.4 Hz, 1H), 4.31 – 4.27 (m, 1H), 4.15 (dd, *J* = 6.5, 2.9 Hz, 1H), 3.84 (s, 3H), 3.78 (s, 3H), 3.48 (dd, *J* = 13.5, 4.3 Hz, 1H), 3.14 (dd, *J* = 13.9, 3.6 Hz, 1H), 3.06 (t, *J* = 12.7 Hz, 1H), 2.96 (dd, *J* = 13.9, 3.8 Hz, 1H), 1.78 (s, 3H), 1.71 – 1.59 (m, 2H), 1.50 (s, 9H), 1.49 – 1.43 (m, 1H), 1.23 – 1.14 (m, 3H), 0.98 (t, *J* = 7.3 Hz, 3H), 0.94 (d, *J* = 6.7 Hz, 3H), 0.85 (t, *J* = 7.5 Hz, 9H), 0.83 – 0.71 (m, 12H).

^{13}C NMR (151 MHz, CDCl$_3$): δ 175.56, 171.30, 170.65, 170.15, 169.72, 155.22, 155.14, 136.33, 135.25, 133.42, 131.14, 130.89, 129.72, 128.88, 128.43, 125.96, 124.07, 121.84, 118.78, 111.04, 79.80, 59.22, 55.62, 54.25, 53.91, 52.19, 50.13, 42.57, 37.09, 36.52, 31.78, 28.55, 27.37, 26.50, 24.37, 22.92, 22.04, 14.81, 12.10, 7.91, 5.11.

HRMS (ESI-TOF): calculated for $C_{47}H_{69}N_5NaO_9Si^+$ [M+Na]$^+$: 898.4757, found: 898.4764.

[α]$^{25}_D$: +69.2 (*c* = 0.5, CHCl$_3$)

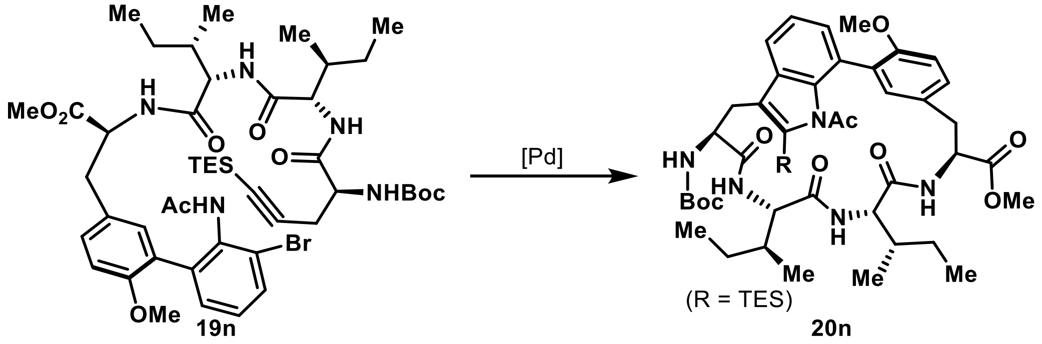

On 0.2 mmol scale, **General Procedure F** was followed from compound **19n** via Larock macrocyclization. Purification by silica gel column chromatography gave the title compound **20n** (45.3 mg,52% yield).

Compound 20n

Physical State: amorphous solid

^{1}H NMR (600 MHz, CDCl$_3$): δ 7.97 (d, *J* = 7.7 Hz, 1H), 7.61 (d, *J* = 1.8 Hz, 1H), 7.31 (t, *J* = 7.6 Hz, 1H), 7.16 (d, *J* = 7.2 Hz, 1H), 6.97 – 6.90 (m, 2H), 6.61 (d, *J* = 7.2 Hz, 1H), 5.69 (d, *J* = 7.5 Hz, 1H), 5.36 (d, *J* = 2.7 Hz, 1H), 5.07 (d, *J* = 9.2 Hz, 1H), 4.79 (dt, *J* = 7.3, 3.7 Hz, 1H), 4.74 (ddd, *J* = 11.8, 7.4, 4.5 Hz, 1H), 4.20 (dd, *J* = 6.2, 3.1 Hz, 1H), 4.10 – 4.01 (m, 1H), 3.85 (s, 3H), 3.80 (s, 3H), 3.52 (dd, *J* = 13.6, 4.3 Hz, 1H), 3.16 (dd, *J* = 13.9, 3.6 Hz, 1H), 3.11 – 3.03 (m, 1H), 2.97 (dd, *J* = 13.9, 3.8 Hz, 1H), 1.80 (s, 3H), 1.76 (s, 1H), 1.70 – 1.60 (m, 1H), 1.52 (s, 9H), 1.38 – 1.23 (m, 2H), 1.22 – 1.15 (m, 1H), 0.99 (t, *J* = 7.3 Hz, 3H), 0.95 (d, *J* = 6.7 Hz, 3H), 0.86 (t, *J* = 7.4 Hz, 9H), 0.80 (q, *J* = 7.4 Hz, 6H), 0.76 – 0.69 (m, 6H).

^{13}C NMR (151 MHz, CDCl$_3$): δ 175.45, 171.30, 170.02, 169.72, 169.30, 155.27, 155.13, 136.38, 135.25, 133.60, 130.99, 130.89, 129.67, 128.90, 128.42, 126.05, 124.13, 121.89, 118.82, 111.01, 79.79, 59.15, 56.15, 55.62, 54.02, 54.00, 52.18, 38.24, 37.10, 36.44, 31.98, 28.57, 27.35, 26.44, 25.17, 14.89, 14.85, 12.11, 11.19, 7.87, 5.07.

HRMS (ESI-TOF): calculated for $C_{47}H_{69}N_5NaO_9Si^+$ [M+Na]$^+$: 898.4757, found: 898.4766.

[α]$^{25}_D$: +70.2 (*c* = 0.5, CHCl$_3$)

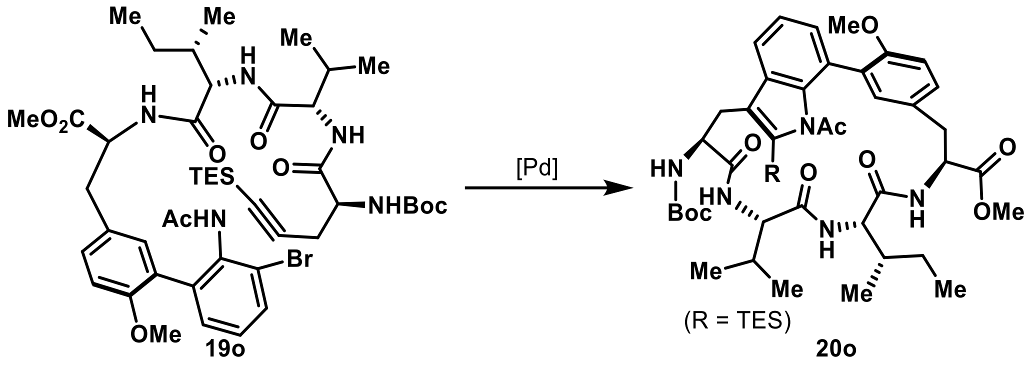

On 0.2 mmol scale, **General Procedure F** was followed from compound **19o** via Larock macrocyclization. Purification by silica gel column chromatography gave the title compound **20o** (43.4 mg, 50% yield).

Compound 20o

Physical State: amorphous solid

^{1}H NMR (600 MHz, CDCl$_3$): δ 7.98 (d, *J* = 7.8 Hz, 1H), 7.59 (d, *J* = 1.9 Hz, 1H), 7.31 (t, *J* = 7.6 Hz, 1H), 7.15 (d, *J* = 7.3 Hz, 1H), 6.98 – 6.89 (m, 2H), 6.60 (d, *J* = 7.2 Hz, 1H), 5.70 (d, *J* = 7.5 Hz, 1H), 5.38 (d, *J* = 2.4 Hz, 1H), 5.07 (d, *J* = 9.2 Hz, 1H), 4.81 – 4.73 (m, 2H), 4.18 (dd, *J* = 6.4, 3.2 Hz, 1H), 4.03 (dd, *J* = 9.1, 7.0 Hz, 1H), 3.85 (s, 3H), 3.80 (s, 3H), 3.53 (dd, *J* = 13.6, 4.4 Hz, 1H), 3.15 (dd, *J* = 13.9, 3.6 Hz, 1H), 3.07 (dd, *J* = 13.7, 11.8 Hz, 1H), 2.97 (dd, *J* = 13.9, 3.8 Hz, 1H), 1.80 (s, 3H), 1.71 – 1.61 (m, 3H), 1.57 (d, *J* = 6.7 Hz, 1H), 1.52 (s, 9H), 1.25 – 1.13 (m, 1H), 0.99 (t, *J* = 7.3 Hz, 3H), 0.95 (d, *J* = 6.7 Hz, 3H), 0.86 (t, *J* = 7.4 Hz, 9H), 0.82 – 0.74 (m, 8H), 0.67 (d, *J* = 6.7 Hz, 3H).

^{13}C NMR (151 MHz, CDCl$_3$): δ 175.37, 171.29, 170.18, 169.87, 169.30, 155.26, 155.13, 136.36, 135.28, 133.57, 130.99, 130.90, 129.67, 128.91, 128.38, 126.04, 124.12, 121.88, 118.85, 111.01, 79.78, 59.21, 56.96, 55.63, 54.02, 53.99, 52.20, 37.07, 36.46, 31.97, 28.57, 27.34, 26.40, 18.74, 18.30, 14.90, 12.09, 7.88, 5.07.

HRMS (ESI-TOF): calculated for $C_{46}H_{67}N_5NaO_9Si^+$ $[M+Na]^+$: 884.4600, found: 884.4609.

$[\alpha]^{25}_D$: +70.9 (*c* = 0.2, CHCl$_3$)

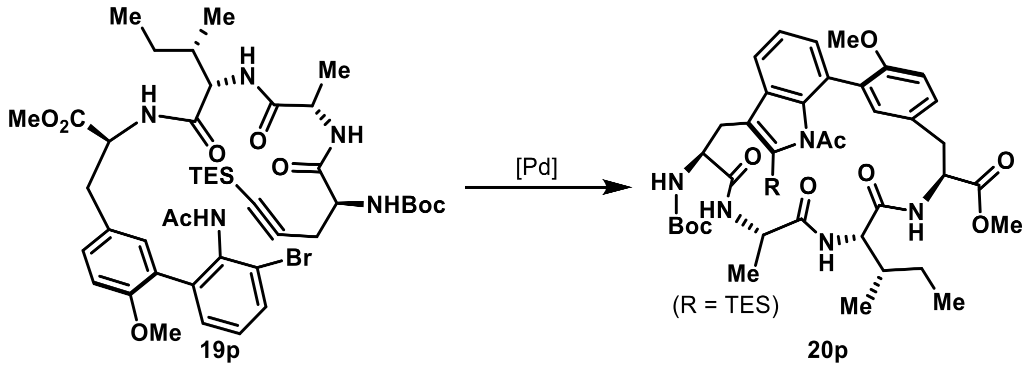

On 0.2 mmol scale, **General Procedure F** was followed from compound **19p** via Larock macrocyclization. Purification by silica gel column chromatography gave the title compound **20p** (55.1 mg, 56% yield).

Compound 20p

Physical State: amorphous solid

^{1}H NMR (600 MHz, CDCl$_3$): δ 8.01 (d, *J* = 7.7 Hz, 1H), 7.52 (s, 1H), 7.32 (t, *J* = 7.6 Hz, 1H), 7.15 (d, *J* = 7.3 Hz, 1H), 7.00 – 6.90 (m, 2H), 6.61 (d, *J* = 7.3 Hz, 1H), 5.87 (s, 1H), 5.71 (d, *J* = 7.3 Hz, 1H), 5.22 (d, *J* = 8.4 Hz, 1H), 4.81 (dt, *J* = 7.3, 3.7 Hz, 1H), 4.69 – 4.65 (m, 1H), 4.44 – 4.38 (m, 1H), 4.08 (dd, *J* = 7.0, 2.8 Hz, 1H), 3.84 (s, 3H), 3.79 (s, 3H), 3.53 (dd, *J* = 13.5, 4.1 Hz, 1H), 3.14 (dd, *J* = 14.0, 3.5 Hz, 1H), 3.12 – 3.01 (m, 1H), 2.98 (dd, *J* = 14.0, 3.9 Hz, 1H), 1.80 (s, 3H), 1.71 – 1.61 (m, 2H), 1.51 (s, 9H), 1.41 – 1.31 (m, 1H), 1.05 (d, *J* = 7.0 Hz, 3H), 0.98 (t, *J* = 7.4 Hz, 3H), 0.95 (d, *J* = 6.7 Hz, 3H), 0.85 (t, *J* = 7.6 Hz, 9H), 0.78 (q, *J* = 8.1 Hz, 6H).

^{13}C NMR (151 MHz, CDCl$_3$): δ 175.34, 171.28, 171.02, 170.60, 169.16, 155.19, 155.17, 136.24, 135.36,

133.39, 131.05, 130.96, 129.73, 129.00, 128.34, 126.06, 124.03, 121.88, 118.97, 111.06, 79.70, 59.63, 55.62, 53.97, 53.85, 52.20, 47.40, 36.95, 36.60, 31.96, 28.57, 28.41, 27.36, 26.39, 19.66, 14.99, 11.96, 7.88, 5.10.
HRMS (ESI-TOF): calculated for $C_{44}H_{63}N_5NaO_9Si^+$ $[M+Na]^+$: 856.4287, found: 856.4299.
$[\alpha]^{25}_D$: +57.4 (c = 0.5, $CHCl_3$)

General procedure G for the synthesis of 24:

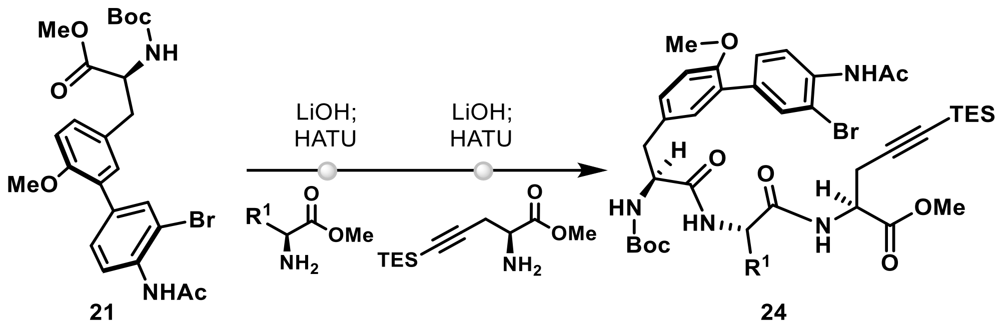

A solution of compound **21** (1.0 eq.) in THF/H_2O (2:1, 0.1 M) was treated with LiOH·H_2O (3.0 eq.). The reaction mixture was stirred at rt for 30 minutes. Upon completion, the reaction was quenched by adjusting the pH to 3 with 0.5 M aq. HCl and then extracted with DCM for three times. The combined organic layers were washed with saturated aq. NaCl, dried over Na_2SO_4, and concentrated *in vacuo* to give the residue, the residue was used directly in the subsequent step.
To a solution of the residue in DMF (0.1 M) was added amino acid methylester (1.2 eq.), DIPEA (4.0 eq.) HATU (1.2 eq.) sequentially. After stirring at rt for 1 h, the reaction was quenched with 0.5 M HCl and extracted with EtOAc for three times. The combined organic layers were washed with saturated aq. NaCl, dried over Na_2SO_4, and concentrated *in vacuo* to give the residue, the residue was used directly in the subsequent step.
The residue was dissolved in THF/H_2O (2:1, 0.1 M), treated with LiOH·H_2O (3.0 eq.), and stirred at rt for 30 minutes. Upon completion, the reaction was quenched by adjusting the pH to 3 with 0.5 M aq. HCl and then extracted with DCM for three times. The combined organic layers were washed with saturated aq. NaCl, dried over Na_2SO_4, and concentrated *in vacuo* to give the residue, the residue was used directly in the subsequent step.
A mixture of the residue in DMF (0.1 M) was added DIPEA (4.0 eq.), HATU (1.2 eq.), methyl (*S*)-2-amino-5-(triethylsilyl)pent-4-ynoate (1.2 eq.) sequentially. The reaction was stirred for 1 h, quenched with 0.5 M HCl and extracted with EtOAc for three times. The combined organic layers were washed with saturated aq. NaCl, dried over Na_2SO_4, and concentrated *in vacuo* to give the residue, the residue was purified by silica gel chromatography to afford the compound **24**.

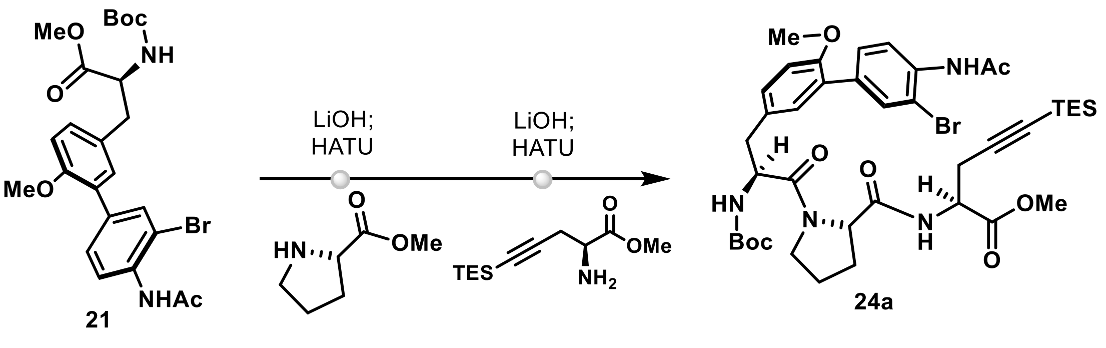

On 1.0 mmol scale, **General Procedure G** was followed. Purification by silica gel column chromatography gave the title compound **24a** (604.4 mg, 73% yield from **21**). The characterization data were identical to the data reported by our group previously.[1]

Compound 24a

Physical State: white solid

^{1}H NMR (600 MHz, CDCl$_3$): δ 8.32 (t, *J* = 7.2 Hz, 1H), 7.72 – 7.61 (m, 2H), 7.48 – 7.40 (m, 1H), 7.21 – 7.01 (m, 3H), 6.87 (dd, *J* = 18.4, 8.4 Hz, 1H), 5.22 (dd, *J* = 39.3, 8.0 Hz, 1H), 4.74 – 4.32 (m, 3H), 3.80 – 3.64 (m, 6H), 3.39 (ddt, *J* = 21.1, 9.8, 5.2 Hz, 1H), 3.12 – 2.83 (m, 2H), 2.81 – 2.58 (m, 2H), 2.23 (s, 4H), 2.06 – 1.90

(m, 3H), 1.78 – 1.41 (m, 1H), 1.39 – 1.30 (m, 9H), 1.00 – 0.86 (m, 9H), 0.60 – 0.45 (m, 6H).
^{13}C NMR (151 MHz, CDCl$_3$): δ 171.76, 170.86, 170.75, 168.28, 155.44, 155.25, 135.56, 134.47, 132.98, 131.86, 131.51, 130.18, 129.72, 129.50, 128.67, 121.27, 112.83, 111.73, 111.34, 101.63, 85.82, 80.06, 79.77, 60.98, 60.15, 55.82, 55.73, 54.18, 53.18, 52.67, 51.83, 51.20, 47.46, 46.84, 38.23, 31.38, 28.37, 28.06, 25.11, 24.97, 23.75, 22.84, 22.10, 7.50, 4.42.
HRMS (ESI-TOF): calculated for $C_{40}H_{55}BrN_4NaO_8Si^+$ [M+Na]$^+$: 849.2865, found: 849.2855.
[α]$^{25}_{D}$: +7.9 (c = 0.5, $CHCl_3$)

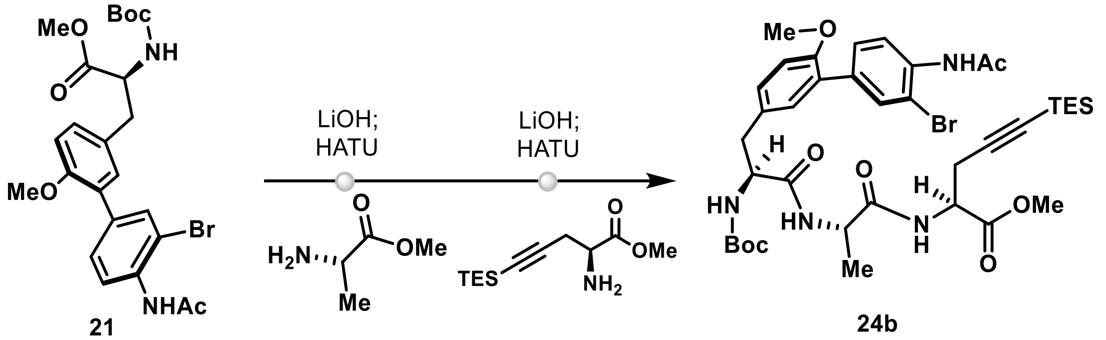

21 24b

On 0.5 mmol scale, **General Procedure G** was followed. Purification by silica gel column chromatography gave the title compound **24b** (268.6 mg, 67% yield from **21**).

Compound 24b

Physical State: white solid

^{1}H NMR (600 MHz, CDCl$_3$): δ 8.30 (d, J = 8.5 Hz, 1H), 7.74 – 7.57 (m, 2H), 7.43 (dd, J = 8.6, 2.0 Hz, 1H), 7.11 (dd, J = 8.4, 2.3 Hz, 1H), 7.07 (d, J = 2.3 Hz, 1H), 6.85 (d, J = 8.4 Hz, 1H), 6.82 (d, J = 7.0 Hz, 1H), 6.74 (d, J = 8.0 Hz, 1H), 5.08 (d, J = 8.2 Hz, 1H), 4.60 (dt, J = 8.0, 5.1 Hz, 1H), 4.46 (p, J = 7.0 Hz, 1H), 4.41 – 4.29 (m, 1H), 3.74 (s, 3H), 3.72 (s, 3H), 3.03 (td, J = 13.4, 12.7, 6.7 Hz, 2H), 2.85 – 2.74 (m, 1H), 2.66 (dd, J = 17.1, 5.4 Hz, 1H), 2.22 (s, 3H), 2.11 (s, 1H), 1.35 (s, 9H), 1.32 (d, J = 7.0 Hz, 3H), 0.93 (t, J = 7.9 Hz, 9H), 0.53 (q, J = 7.9 Hz, 6H).
^{13}C NMR (151 MHz, CDCl$_3$): δ 171.67, 171.15, 170.63, 168.33, 155.52, 135.47, 134.49, 132.91, 131.62, 129.91, 129.62, 128.90, 128.67, 121.31, 112.86, 111.59, 101.38, 86.08, 80.35, 55.73, 55.61, 52.74, 51.28, 51.19, 48.96, 48.56, 37.40, 28.32, 24.95, 23.68, 18.74, 7.51, 4.39.
HRMS (ESI-TOF): calculated for $C_{38}H_{53}BrN_4NaO_8Si^+$ [M+Na]$^+$: 823.2708, found: 823.2714.
[α]$^{25}_{D}$: +25.1 (c = 0.5, $CHCl_3$)

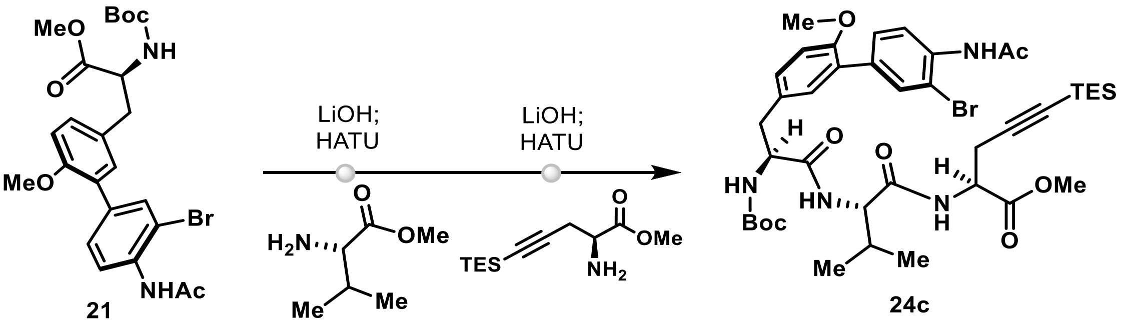

21 24c

On 0.4 mmol scale, **General Procedure G** was followed. Purification by silica gel column chromatography gave the title compound **24c** (209 mg, 63% yield from **21**).

Compound 24c

Physical State: white solid

^{1}H NMR (600 MHz, CDCl$_3$): δ 8.32 (d, J = 8.5 Hz, 1H), 7.70 – 7.61 (m, 2H), 7.43 (ddd, J = 10.3, 8.5, 2.0 Hz, 1H), 7.12 (dd, J = 8.4, 2.2 Hz, 1H), 7.07 (dd, J = 5.8, 2.3 Hz, 1H), 6.86 (d, J = 8.4 Hz, 1H), 6.79 – 6.69

(m, 1H), 6.58 (d, J = 8.0 Hz, 1H), 5.15 – 4.98 (m, 1H), 4.59 (dt, J = 8.2, 4.9 Hz, 1H), 4.41 – 4.33 (m, 1H), 4.33 – 4.25 (m, 1H), 3.75 (s, 3H), 3.72 (s, 3H), 3.06 (dd, J = 14.1, 6.2 Hz, 1H), 3.04 – 2.91 (m, 1H), 2.84 – 2.60 (m, 2H), 2.23 (s, 3H), 2.14 – 2.04 (m, 1H), 2.05 – 1.96 (m, 1H), 1.36 (s, 9H), 0.96 – 0.92 (m, 9H), 0.90 – 0.77 (m, 6H) 0.58 – 0.50 (m, 6H).
^{13}C NMR (151 MHz, CDCl$_3$): δ 171.38, 170.56, 170.41, 168.32, 155.64, 155.50, 135.44, 134.50, 132.93, 131.57, 129.86, 129.66, 129.62, 128.97, 128.68, 121.24, 112.80, 111.69, 111.62, 101.34, 86.23, 85.68, 80.41, 58.40, 58.10, 55.83, 55.74, 52.85, 52.69, 51.47, 51.05, 36.98, 31.39, 28.33, 24.99, 23.58, 18.88, 17.92, 7.52, 7.47, 4.37.
HRMS (ESI-TOF): calculated for $C_{40}H_{57}BrN_4NaO_8Si^+$ [M+Na]$^+$: 851.3021, found: 851.3023.
[α]$^{25}_{D}$: +27.4 (c = 0.5, CHCl$_3$)

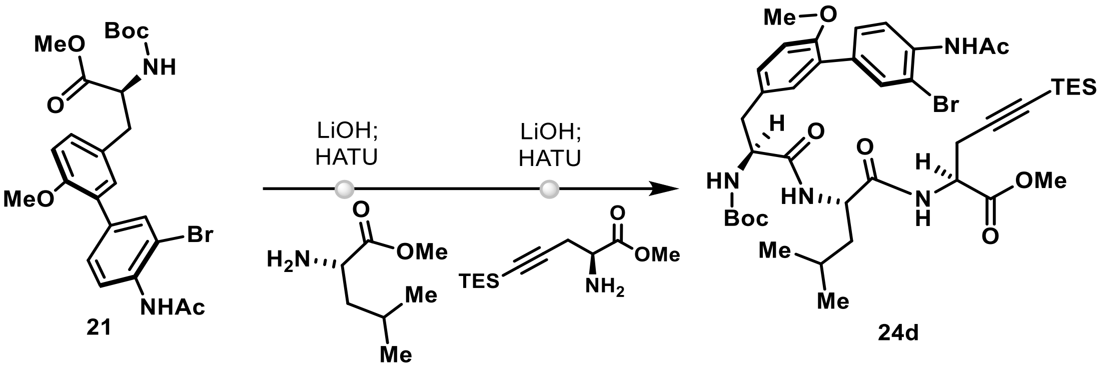

On 0.4 mmol scale, **General Procedure G** was followed. Purification by silica gel column chromatography gave the title compound **24d** (219.4 mg, 65% yield from **21**).

Compound 24d

Physical State: white solid
^{1}H NMR (600 MHz, CDCl$_3$): δ 8.31 (d, J = 8.5 Hz, 1H), 7.70 – 7.61 (m, 2H), 7.43 (td, J = 8.2, 2.0 Hz, 1H), 7.16 – 7.09 (m, 1H), 7.07 (t, J = 1.9 Hz, 1H), 6.85 (d, J = 8.5 Hz, 1H), 6.78 (dd, J = 21.5, 6.4 Hz, 1H), 6.70 – 6.49 (m, 1H), 5.17 – 4.91 (m, 1H), 4.59 (dt, J = 7.6, 4.9 Hz, 1H), 4.52 – 4.26 (m, 2H), 3.79 – 3.68 (m, 6H), 3.09 – 2.92 (m, 2H), 2.85 – 2.59 (m, 2H), 2.23 (s, 3H), 2.09 (s, 1H), 1.79 (d, J = 15.4 Hz, 1H), 1.45 (ddt, J = 11.3, 7.5, 5.7 Hz, 1H), 1.40 – 1.34 (m, 9H), 1.25 – 1.06 (m, 1H), 0.93 (dt, J = 9.4, 7.9 Hz, 9H), 0.88 – 0.71 (m, 6H), 0.53 (dq, J = 12.7, 7.9 Hz, 6H).
^{13}C NMR (151 MHz, CDCl$_3$): δ 171.48, 171.22, 170.72, 170.52, 170.32, 168.32, 155.50, 135.44, 134.50, 132.92, 131.57, 129.88, 129.77, 129.65, 129.61, 128.99, 128.67, 121.27, 112.82, 111.64, 111.60, 101.53, 101.32, 86.22, 85.54, 80.37, 57.76, 56.59, 56.38, 55.73, 52.81, 52.68, 52.14, 51.48, 51.04, 37.99, 37.80, 37.00, 28.33, 26.05, 24.98, 23.59, 15.36, 15.07, 14.33, 11.81, 11.60, 7.51, 7.49, 4.38.
HRMS (ESI-TOF): calculated for $C_{41}H_{59}BrN_4NaO_8Si^+$ [M+Na]$^+$: 865.3178, found: 865.3177.
[α]$^{25}_{D}$: +16.0 (c = 0.5, CHCl$_3$)

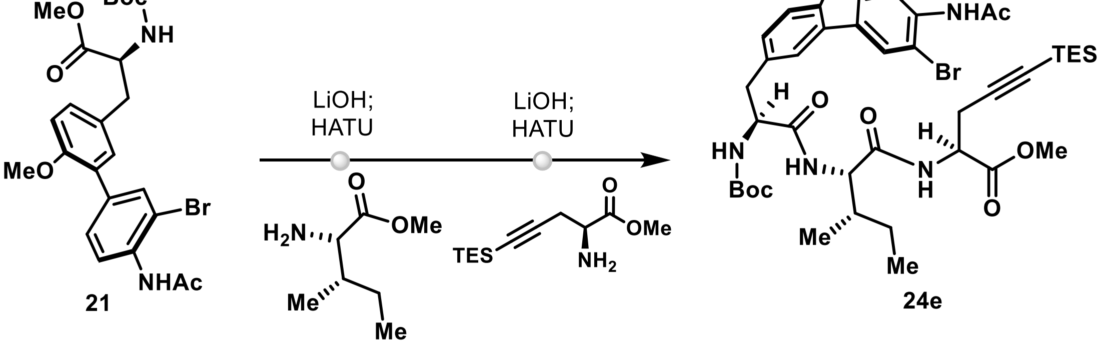

On 0.4 mmol scale, **General Procedure G** was followed. Purification by silica gel column chromatography

gave the title compound **24e** (216 mg, 64% yield from **21**).

Compound 24e

Physical State: white solid

^{1}H NMR (600 MHz, CDCl$_3$): δ 8.31 (d, *J* = 8.5 Hz, 1H), 7.72 – 7.60 (m, 2H), 7.44 (dd, *J* = 8.6, 2.1 Hz, 1H), 7.12 (dd, *J* = 8.5, 2.2 Hz, 1H), 7.07 (d, *J* = 2.3 Hz, 1H), 6.85 (d, *J* = 8.4 Hz, 1H), 6.64 (d, *J* = 8.0 Hz, 1H), 6.59 (d, *J* = 8.0 Hz, 1H), 5.01 (d, *J* = 8.0 Hz, 1H), 4.58 (dt, *J* = 8.0, 5.0 Hz, 1H), 4.42 (td, *J* = 8.4, 4.7 Hz, 1H), 4.34 (d, *J* = 9.1 Hz, 1H), 3.75 (s, 3H), 3.71 (s, 3H), 3.66 – 3.57 (m, 1H), 3.10 – 3.02 (m, 1H), 2.98 (dd, *J* = 14.7, 7.1 Hz, 1H), 2.80 (dd, *J* = 14.8, 10.2 Hz, 1H), 2.63 (dd, *J* = 17.1, 5.4 Hz, 1H), 2.23 (s, 3H), 2.11 – 2.00 (m, 1H), 1.60 – 1.56 (m, 1H), 1.35 (s, 9H), 0.94 (t, *J* = 7.9 Hz, 9H), 0.90 – 0.79 (m, 6H), 0.54 (q, *J* = 7.9 Hz, 6H).

^{13}C NMR (151 MHz, CDCl$_3$): δ 171.51, 171.29, 170.59, 168.34, 155.49, 135.43, 134.51, 132.94, 131.57, 129.93, 129.64, 128.92, 128.66, 121.30, 112.86, 111.57, 101.41, 86.09, 80.42, 55.73, 53.76, 52.70, 51.78, 51.03, 42.04, 41.68, 38.69, 37.03, 28.31, 24.99, 24.62, 23.63, 22.94, 22.00, 18.72, 17.46, 12.12, 7.54, 4.39.

HRMS (ESI-TOF): calculated for $C_{41}H_{59}BrN_4NaO_8Si^+$ [M+Na]$^+$: 865.3178, found: 865.3184.

[α]$^{25}_D$: +23.3 (*c* = 0.5, CHCl$_3$)

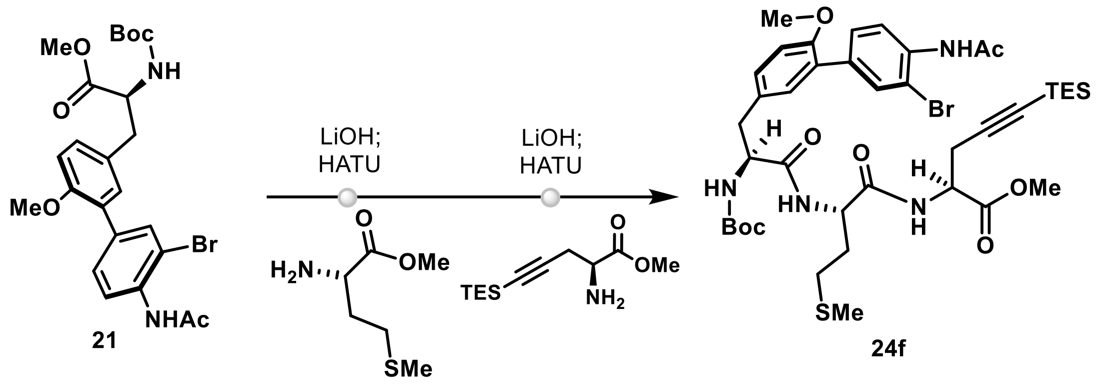

On 0.6 mmol scale, **General Procedure G** was followed. Purification by silica gel column chromatography gave the title compound **24f** (243 mg, 47% yield from **21**).

Compound 24f

Physical State: yellow solid

^{1}H NMR (400 MHz, CDCl$_3$): δ 8.33 (d, *J* = 8.6 Hz, 1H), 7.72 – 7.60 (m, 2H), 7.46 (dd, *J* = 8.6, 2.0 Hz, 1H), 7.18 – 7.04 (m, 2H), 6.91 (dd, *J* = 27.5, 8.2 Hz, 3H), 5.24 – 4.91 (m, 1H), 4.64 (dq, *J* = 10.6, 6.5, 5.4 Hz, 2H), 4.44 – 4.16 (m, 1H), 3.77 (s, 3H), 3.73 (s, 3H), 3.04 (d, *J* = 6.5 Hz, 2H), 2.90 – 2.75 (m, 1H), 2.64 (dd, *J* = 17.1, 5.3 Hz, 1H), 2.53 (t, *J* = 7.1 Hz, 2H), 2.25 (s, 3H), 2.13 (d, *J* = 13.9 Hz, 1H), 2.07 (s, 3H), 1.96 (p, *J* = 7.4 Hz, 2H), 1.37 (s, 9H), 0.96 (t, *J* = 7.9 Hz, 9H), 0.56 (q, *J* = 7.9 Hz, 6H).

^{13}C NMR (101 MHz, CDCl$_3$): δ 170.43, 169.73, 169.71, 167.63, 154.80, 134.70, 133.79, 132.19, 131.50, 131.40, 130.88, 129.16, 128.90, 128.09, 127.96, 127.84, 120.60, 112.14, 110.88, 100.47, 85.55, 79.70, 55.02, 52.04, 51.20, 50.39, 36.54, 30.74, 28.89, 27.62, 24.27, 22.94, 14.32, 14.06, 6.83, 3.67.

HRMS (ESI-TOF): calculated for $C_{40}H_{57}BrN_4NaO_8SSi^+$ [M+Na]$^+$: 883.2742, found: 883.2731.

[α]$^{25}_D$: +20.2 (*c* = 0.5, CHCl$_3$)

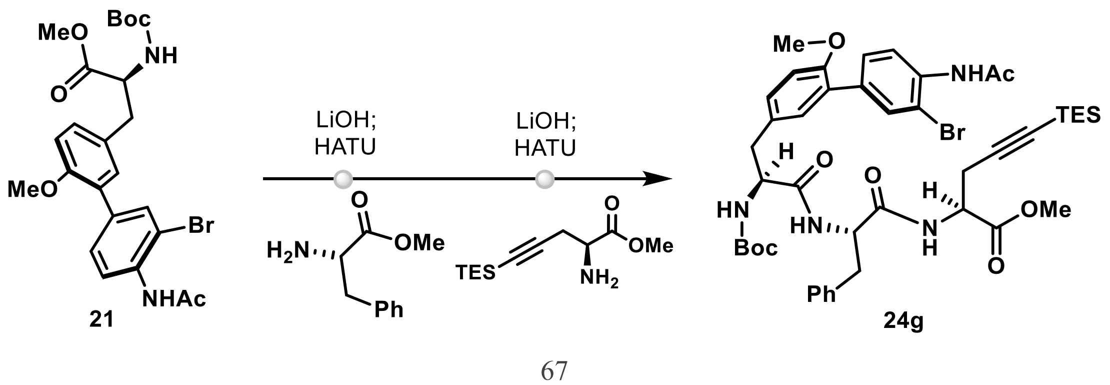

On 0.5 mmol scale, **General Procedure G** was followed. Purification by silica gel column chromatography gave the title compound **24g** (267.8 mg, 61% yield from **21**).

Compound 24g.

Physical State: white solid

^{1}H NMR (600 MHz, CDCl$_3$): δ 8.30 (d, *J* = 8.5 Hz, 1H), 7.69 – 7.58 (m, 2H), 7.43 (dd, *J* = 8.5, 2.0 Hz, 1H), 7.23 – 7.16 (m, 3H), 7.12 – 7.03 (m, 4H), 6.85 (d, *J* = 8.4 Hz, 1H), 6.72 (d, *J* = 7.6 Hz, 1H), 6.58 (d, *J* = 7.6 Hz, 1H), 4.98 (d, *J* = 7.8 Hz, 1H), 4.63 (q, *J* = 7.1 Hz, 1H), 4.59 – 4.51 (m, 1H), 4.42 – 4.18 (m, 1H), 3.75 (s, 3H), 3.68 (s, 3H), 3.02 – 2.90 (m, 4H), 2.75 – 2.67 (m, 1H), 2.61 (dd, *J* = 17.0, 5.8 Hz, 1H), 2.22 (s, 3H), 2.12 (s, 1H), 1.34 (s, 9H), 0.92 (t, *J* = 7.9 Hz, 9H), 0.52 (q, *J* = 7.9 Hz, 6H).

^{13}C NMR (151 MHz, CDCl$_3$): δ 171.18, 170.32, 170.18, 168.34, 155.53, 136.13, 135.43, 134.52, 132.91, 131.63, 129.90, 129.61, 129.36, 128.87, 128.69, 127.12, 121.34, 112.89, 111.61, 101.47, 85.84, 80.38, 55.74, 54.37, 52.66, 51.39, 38.52, 37.27, 28.32, 24.96, 23.79, 7.52, 4.38.

HRMS (ESI-TOF): calculated for $C_{44}H_{57}BrN_4NaO_8Si^+$ [M+Na]$^+$: 899.3021, found: 899.3027.

$[\alpha]^{25}_D$: +21.3 (*c* = 0.5, CHCl$_3$)

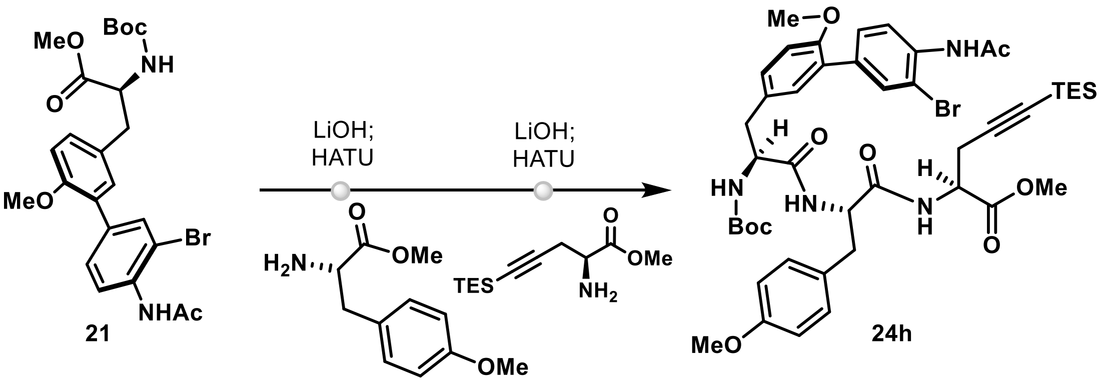

On 0.5 mmol scale, **General Procedure G** was followed. Purification by silica gel column chromatography gave the title compound **24h** (245.2 mg, 54% yield from **21**).

Compound 24h

Physical State: white solid

^{1}H NMR (600 MHz, CDCl$_3$): δ 8.31 (d, *J* = 8.5 Hz, 1H), 7.70 – 7.64 (m, 1H), 7.62 (s, 1H), 7.44 (ddd, *J* = 8.5, 6.4, 2.0 Hz, 1H), 7.12 – 7.03 (m, 2H), 6.99 (d, *J* = 8.2 Hz, 2H), 6.88 – 6.83 (m, 1H), 6.77 – 6.70 (m, 2H), 6.63 (d, *J* = 7.4 Hz, 1H), 6.50 (d, *J* = 6.5 Hz, 1H), 5.15 – 4.81 (m, 1H), 4.74 – 4.51 (m, 2H), 4.39 – 4.21 (m, 1H), 3.80 – 3.68 (m, 9H), 3.06 – 2.90 (m, 3H), 2.86 (dd, *J* = 13.9, 7.2 Hz, 1H), 2.76 – 2.66 (m, 1H), 2.61 (dd, *J* = 17.0, 5.8 Hz, 1H), 2.23 (s, 3H), 1.34 (s, 9H), 0.92 (t, *J* = 7.9 Hz, 9H), 0.52 (q, *J* = 7.9 Hz, 6H).

^{13}C NMR (151 MHz, CDCl$_3$): δ 171.07, 170.31, 170.21, 168.30, 158.73, 155.56, 135.39, 134.54, 132.90, 131.64, 130.45, 130.38, 129.90, 129.62, 128.98, 128.84, 128.74, 128.65, 127.98, 121.28, 114.10, 112.84, 111.62, 101.46, 85.86, 80.40, 55.75, 55.23, 54.49, 52.71, 52.64, 51.37, 51.26, 37.66, 37.27, 28.31, 24.96, 23.84, 23.68, 7.51, 4.38.

HRMS (ESI-TOF): calculated for $C_{45}H_{59}BrN_4NaO_9Si^+$ [M+Na]$^+$: 929.3127, found: 929.3127.

$[\alpha]^{25}_D$: +10.4 (*c* = 0.5, CHCl$_3$)

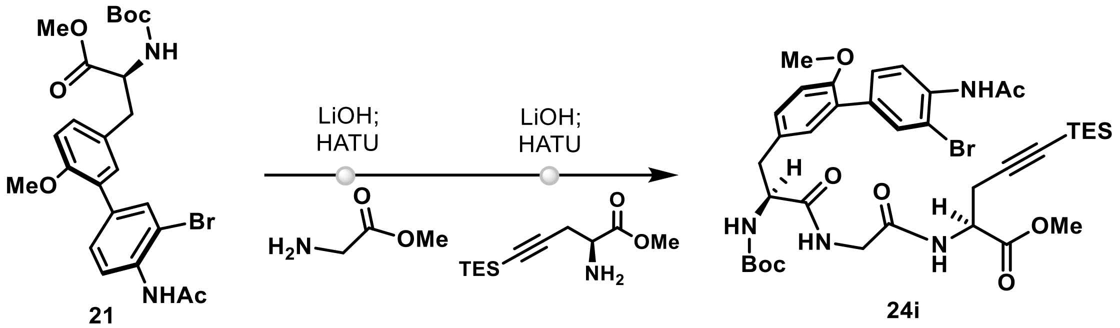

On 0.5 mmol scale, **General Procedure G** was followed. Purification by silica gel column chromatography gave the title compound **24i** (244.2 mg, 62% yield from **21**).

Compound 24i

Physical State: white solid

^{1}H NMR (600 MHz, CDCl$_3$): δ 8.32 (d, *J* = 8.5 Hz, 1H), 7.70 – 7.61 (m, 2H), 7.44 (dd, *J* = 8.4, 2.2 Hz, 1H), 7.13 (dd, *J* = 8.4, 2.2 Hz, 1H), 7.07 (d, *J* = 2.3 Hz, 1H), 6.87 (d, *J* = 8.4 Hz, 1H), 6.79 (dt, *J* = 10.5, 6.1 Hz, 2H), 5.09 (d, *J* = 7.9 Hz, 1H), 4.67 (dt, *J* = 7.7, 5.1 Hz, 1H), 4.38 (d, *J* = 9.1 Hz, 1H), 4.04 (dd, *J* = 16.8, 5.5 Hz, 1H), 3.86 (dd, *J* = 16.8, 4.8 Hz, 1H), 3.76 (s, 3H), 3.73 (s, 3H), 3.10 (dd, *J* = 14.1, 6.1 Hz, 1H), 2.97 (dd, *J* = 14.5, 7.5 Hz, 1H), 2.76 (qd, *J* = 17.4, 5.2 Hz, 2H), 2.24 (s, 3H), 1.92 (s, 1H), 1.35 (s, 9H), 0.94 (t, *J* = 7.9 Hz, 9H), 0.54 (q, *J* = 7.9 Hz, 6H).

^{13}C NMR (151 MHz, CDCl$_3$): δ 171.86, 170.66, 168.32, 168.15, 155.52, 135.45, 134.51, 132.94, 132.21, 132.14, 131.58, 129.84, 129.64, 128.95, 128.66, 128.57, 121.29, 112.84, 111.62, 101.39, 85.97, 80.41, 55.88, 55.73, 52.82, 51.27, 42.88, 37.44, 28.33, 24.99, 23.79, 7.51, 4.39.

HRMS (ESI-TOF): calculated for $C_{37}H_{51}BrN_4NaO_8Si^+$ [M+Na]$^+$: 809.2552, found: 809.2558.

[α]$^{25}_{D}$: +21.5 (*c* = 0.5, CHCl$_3$)

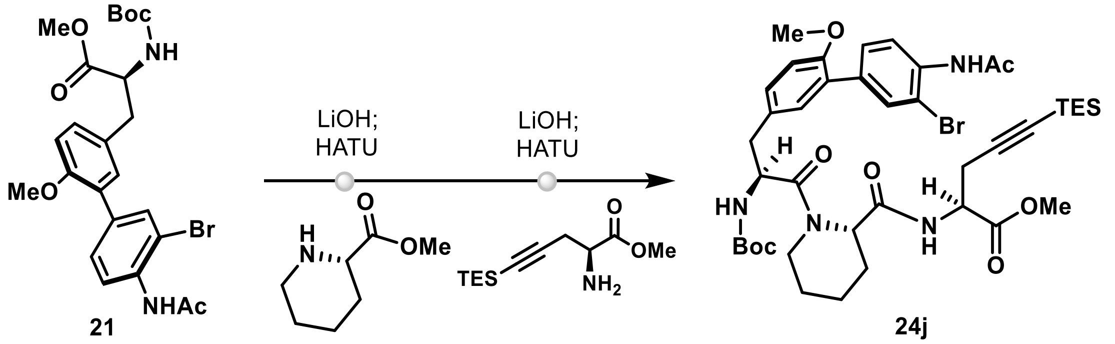

On 0.5 mmol scale, **General Procedure G** was followed. Purification by silica gel column chromatography gave the title compound **24j** (210.5 mg, 50% yield from **21**).

Compound 24j

Physical State: white solid

^{1}H NMR (600 MHz, CDCl$_3$): δ 8.33 (dd, *J* = 12.6, 8.5 Hz, 1H), 7.73 – 7.57 (m, 2H), 7.50 – 7.37 (m, 1H), 7.20 – 7.03 (m, 2H), 6.94 – 6.81 (m, 1H), 5.40 – 4.86 (m, 2H), 4.75 – 4.44 (m, 2H), 4.29 – 3.83 (m, 1H), 3.79 – 3.66 (m, 6H), 3.41 – 3.05 (m, 1H), 3.01 – 2.47 (m, 4H), 2.23 (s, 3H), 1.76 – 1.39 (m, 5H), 1.39 – 1.31 (m, 9H), 1.28 – 1.21 (m, 1H), 0.96 – 0.88 (m, 9H), 0.56 – 0.48 (m, 6H).

^{13}C NMR (151 MHz, CDCl$_3$): δ 171.86, 171.12, 170.94, 170.63, 170.08, 169.53, 168.27, 155.89, 155.86, 155.39, 155.11, 135.66, 135.06, 134.70, 134.40, 132.99, 132.80, 132.23, 132.04, 131.85, 130.20, 130.17, 129.74, 129.53, 129.47, 129.20, 128.57, 128.32, 128.01, 121.31, 121.15, 112.83, 112.71, 111.96, 111.32, 111.19, 102.53, 101.67, 85.91, 84.63, 80.50, 79.72, 56.91, 55.92, 55.85, 55.70, 52.81, 52.73, 52.53, 52.39, 52.30, 51.44, 51.20, 43.99, 40.35, 38.12, 37.85, 29.76, 28.41, 28.37, 26.21, 25.59, 25.32, 24.99, 24.74, 23.54, 22.85, 20.57, 20.54, 7.48, 7.45, 4.60, 4.41, 4.37.

HRMS (ESI-TOF): calculated for $C_{41}H_{57}BrN_4NaO_8Si^+$ $[M+Na]^+$: 863.3021, found: 863.3024.
$[\alpha]^{25}_D$: +8.2 (c = 0.5, $CHCl_3$)

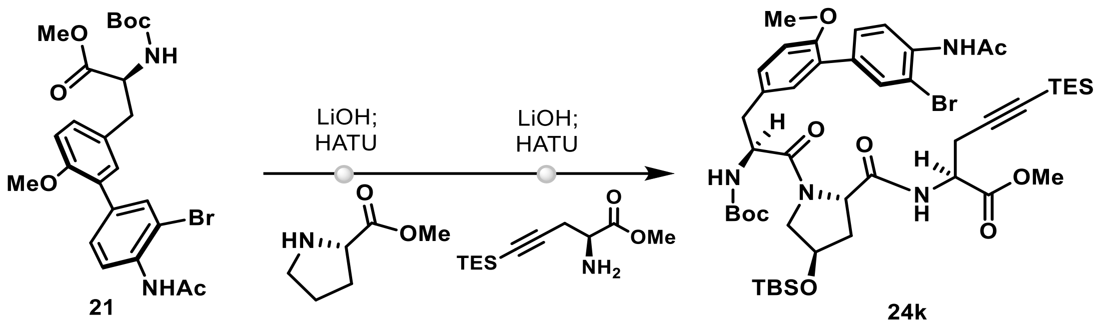

On 0.5 mmol scale, **General Procedure G** was followed. Purification by silica gel column chromatography gave the title compound **24k** (249.1 mg, 52% yield from **21**).

Compound 24k

Physical State: colorless oil

^{1}H NMR (600 MHz, $CDCl_3$): δ 8.32 (d, J = 8.4 Hz, 1H), 7.69 (d, J = 2.0 Hz, 1H), 7.64 (s, 1H), 7.45 (dd, J = 8.5, 2.0 Hz, 1H), 7.18 – 7.06 (m, 3H), 6.91 – 6.81 (m, 1H), 5.40 – 5.21 (m, 1H), 4.72 – 4.48 (m, 3H), 4.46 – 4.34 (m, 1H), 3.75 (s, 3H), 3.72 (s, 3H), 3.47 – 3.35 (m, 1H), 3.23 (dd, J = 10.2, 5.1 Hz, 1H), 3.06 – 2.93 (m, 1H), 2.89 (dd, J = 14.0, 6.0 Hz, 1H), 2.80 (dd, J = 17.1, 4.4 Hz, 1H), 2.57 (dd, J = 17.1, 5.6 Hz, 1H), 2.29 (dt, J = 12.7, 5.4 Hz, 1H), 2.23 (s, 3H), 2.11 – 1.82 (m, 2H), 1.35 (s, 9H), 0.94 (t, J = 7.9 Hz, 9H), 0.87 – 0.77 (m, 9H), 0.58 – 0.50 (m, 6H), 0.06 – -0.01 (m, 6H).

^{13}C NMR (151 MHz, $CDCl_3$): δ 171.70, 170.71, 170.63, 168.27, 155.43, 155.05, 135.50, 134.45, 132.98, 132.03, 130.35, 129.76, 128.55, 128.40, 121.18, 112.77, 111.23, 101.49, 86.10, 79.73, 70.63, 59.00, 55.68, 55.09, 53.36, 52.64, 51.21, 38.45, 36.94, 28.37, 25.74, 24.99, 23.75, 17.96, 7.51, 4.41, -4.82.

HRMS (ESI-TOF): calculated for $C_{46}H_{69}BrN_4NaO_9Si_2^+$ $[M+Na]^+$: 979.3679, found: 979.3770.
$[\alpha]^{25}_D$: +10.7 (c = 0.5, $CHCl_3$)

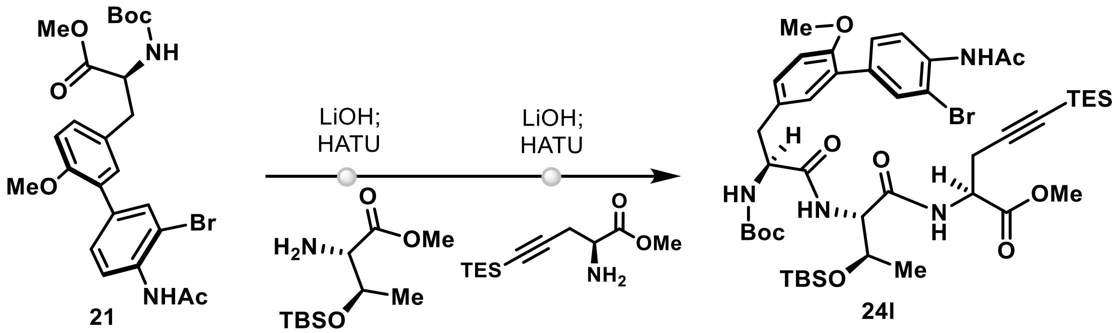

On 0.5 mmol scale, **General Procedure G** was followed. Purification by silica gel column chromatography gave the title compound **24l** (194 mg, 41% yield from **21**).

Compound 24l

Physical State: colorless oil

^{1}H NMR (600 MHz, $CDCl_3$): δ 8.35 (d, J = 8.6 Hz, 1H), 7.68 (d, J = 1.9 Hz, 1H), 7.63 (s, 1H), 7.45 (td, J = 9.2, 8.5, 4.4 Hz, 2H), 7.10 (dd, J = 8.3, 2.3 Hz, 1H), 7.06 (d, J = 2.3 Hz, 1H), 6.97 (d, J = 5.6 Hz, 1H), 6.89 – 6.83 (m, 1H), 4.95 (d, J = 7.9 Hz, 1H), 4.62 (dt, J = 7.4, 5.6 Hz, 1H), 4.44 – 4.34 (m, 1H), 4.31 (dt, J = 7.9, 4.2 Hz, 2H), 3.77 (s, 3H), 3.73 (s, 3H), 3.06 (d, J = 6.4 Hz, 2H), 2.79 (dd, J = 4.7, 1.8 Hz, 1H), 2.72 (d, J = 5.7 Hz, 2H), 2.24 (s, 3H), 2.03 (s, 1H), 1.37 (s, 9H), 0.97 – 0.93 (m, 12H), 0.92 – 0.90 (m, 9H), 0.58 – 0.50 (m, 6H), 0.19 – 0.10 (m, 6H).

^{13}C NMR (151 MHz, $CDCl_3$): δ 171.29, 171.03, 170.43, 169.72, 169.03, 168.24, 155.58, 135.40, 134.54, 132.94, 131.57, 129.83, 129.67, 128.75, 121.16, 112.72, 111.63, 101.76, 101.65, 85.79, 85.44, 80.36, 67.49,

57.95, 55.75, 52.77, 52.59, 51.82, 50.91, 37.33, 28.33, 26.02, 25.92, 25.85, 25.01, 23.98, 23.81, 23.23, 18.02, 17.72, 7.51, 4.43, 4.40, -4.85, -4.93.
HRMS (ESI-TOF): calculated for $C_{45}H_{69}BrN_4NaO_9Si_2^+$ [M+Na]$^+$: 967.3679, found: 967.3688.
[α]^{25}D: +8.8 (c = 0.5, CHCl$_3$)

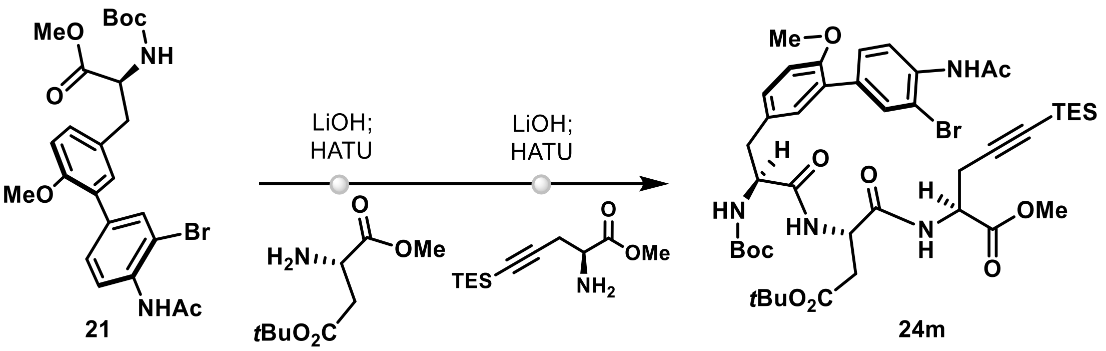

On 0.4 mmol scale, **General Procedure G** was followed. Purification by silica gel column chromatography gave the title compound **24m** (220.1 mg, 61% yield from **21**).
Compound 24m
Physical State: white solid
^{1}H NMR (600 MHz, CDCl$_3$): δ 8.35 (d, J = 8.6 Hz, 1H), 7.68 (d, J = 2.0 Hz, 1H), 7.63 (s, 1H), 7.45 (ddd, J = 8.7, 4.9, 2.0 Hz, 1H), 7.40 – 7.28 (m, 1H), 7.23 – 7.17 (m, 1H), 7.14 (dt, J = 8.3, 3.2 Hz, 1H), 7.08 (d, J = 2.3 Hz, 1H), 6.88 (dd, J = 8.5, 2.6 Hz, 1H), 5.07 – 4.89 (m, 1H), 4.73 (td, J = 7.4, 3.8 Hz, 1H), 4.62 (td, J = 8.0, 4.0 Hz, 1H), 4.40 – 4.22 (m, 1H), 3.77 (s, 3H), 3.71 (s, 3H), 3.16 – 3.05 (m, 1H), 3.04 – 2.92 (m, 1H), 2.86 – 2.71 (m, 2H), 2.63 (dd, J = 17.0, 5.8 Hz, 1H), 2.48 (dd, J = 17.2, 7.4 Hz, 1H), 2.24 (s, 3H), 1.42 (s, 9H), 1.37 (s, 9H), 0.95 (td, J = 7.9, 5.4 Hz, 9H), 0.56 (q, J = 7.8 Hz, 6H).
^{13}C NMR (151 MHz, CDCl$_3$): δ 171.17, 171.11, 170.41, 170.07, 168.27, 155.59, 135.37, 134.54, 132.93, 131.54, 129.81, 129.67, 128.73, 121.18, 112.75, 111.66, 101.44, 85.84, 82.03, 80.46, 55.75, 52.61, 51.54, 49.20, 37.40, 37.21, 28.33, 28.05, 25.01, 23.68, 7.50, 4.40.
HRMS (ESI-TOF): calculated for $C_{43}H_{61}BrN_4NaO_{10}Si^+$ [M+Na]$^+$: 923.3233, found: 923.3263.
[α]^{25}D: +10.4 (c = 0.5, CHCl$_3$)

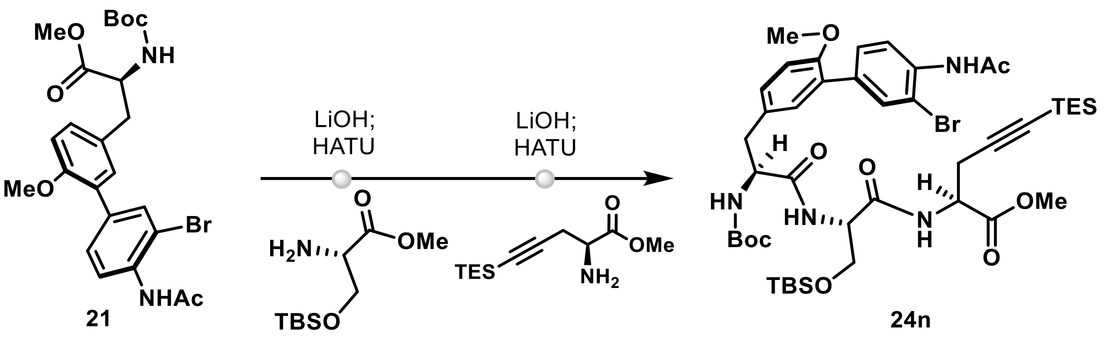

On 0.5 mmol scale, **General Procedure G** was followed. Purification by silica gel column chromatography gave the title compound **24n** (191.1 mg, 41% yield from **21**).
Compound 24n
Physical State: colorless oil
^{1}H NMR (600 MHz, CDCl$_3$): δ 8.34 (d, J = 8.6 Hz, 1H), 7.68 (dd, J = 4.5, 2.1 Hz, 1H), 7.63 (s, 1H), 7.45 (ddd, J = 8.6, 3.7, 2.1 Hz, 1H), 7.36 – 7.25 (m, 1H), 7.12 (dd, J = 8.4, 2.1 Hz, 1H), 7.07 (dd, J = 6.8, 2.4 Hz, 1H), 6.92 – 6.74 (m, 2H), 4.99 (dd, J = 40.0, 7.9 Hz, 1H), 4.75 – 4.57 (m, 1H), 4.46 – 4.24 (m, 2H), 3.97 (ddd, J = 14.5, 9.8, 3.6 Hz, 1H), 3.76 (s, 3H), 3.75 – 3.69 (m, 3H), 3.55 – 3.44 (m, 1H), 3.17 – 2.90 (m, 2H), 2.80 – 2.64 (m, 2H), 2.24 (s, 3H), 1.44 – 1.29 (m, 9H), 0.98 – 0.82 (m, 18H), 0.58 – 0.49 (m, 6H), 0.14 – -0.01 (m,

6H).
^{13}C NMR (151 MHz, CDCl$_3$): δ 171.34, 170.38, 169.70, 168.26, 155.59, 135.40, 134.54, 132.93, 131.58, 129.84, 129.64, 129.61, 128.98, 128.72, 121.23, 112.78, 111.62, 101.65, 85.52, 80.40, 62.70, 62.64, 56.26, 55.73, 55.71, 54.28, 52.65, 52.61, 51.79, 51.68, 37.49, 28.32, 25.93, 25.87, 25.76, 24.99, 23.88, 18.30, 18.23, 7.51, 4.41, -5.42, -5.49.
HRMS (ESI-TOF): calculated for $C_{44}H_{67}BrN_4NaO_9Si_2^+$ [M+Na]$^+$: 953.3522, found: 953.3527.
[α]$^{25}_{D}$: +18.9 (*c* = 0.5, CHCl$_3$)

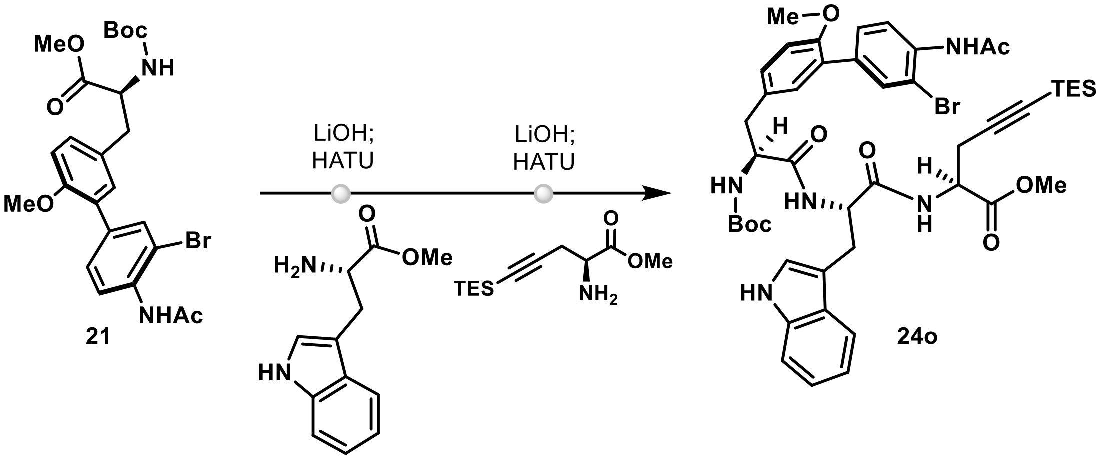

On 0.4 mmol scale, **General Procedure G** was followed. Purification by silica gel column chromatography gave the title compound **24o** (216.4 mg, 59% yield from **21**).

Compound 24o

Physical State: yellow solid

^{1}H NMR (600 MHz, CDCl$_3$): δ 8.44 (s, 1H), 8.28 (d, *J* = 8.5 Hz, 1H), 7.67 (s, 1H), 7.61 (s, 1H), 7.51 – 7.35 (m, 2H), 7.30 (d, *J* = 8.2 Hz, 1H), 7.13 (t, *J* = 7.6 Hz, 1H), 7.10 – 6.98 (m, 4H), 6.82 (t, *J* = 8.8 Hz, 2H), 6.52 (d, *J* = 7.1 Hz, 1H), 4.91 (d, *J* = 7.8 Hz, 1H), 4.69 (q, *J* = 6.8 Hz, 1H), 4.53 – 4.44 (m, 1H), 4.40 – 4.14 (m, 1H), 3.74 (s, 3H), 3.63 (s, 3H), 3.29 – 3.14 (m, 1H), 3.09 – 2.83 (m, 3H), 2.69 – 2.51 (m, 2H), 2.22 (s, 3H), 2.04 (s, 1H), 1.49 – 1.37 (m, 1H), 1.29 (s, 9H), 0.89 (t, *J* = 7.9 Hz, 9H), 0.48 (q, *J* = 7.9 Hz, 6H).

^{13}C NMR (151 MHz, CDCl$_3$): δ 171.15, 170.69, 170.33, 168.41, 155.55, 136.29, 135.50, 134.49, 132.93, 131.67, 129.92, 129.61, 128.85, 128.68, 127.58, 123.64, 122.23, 121.46, 119.74, 118.58, 113.05, 111.65, 111.45, 109.97, 101.56, 85.68, 80.34, 55.77, 55.73, 54.10, 53.81, 52.59, 51.52, 42.06, 37.23, 28.35, 28.24, 24.94, 23.77, 18.70, 17.44, 7.51, 7.48, 4.36.

HRMS (ESI-TOF): calculated for $C_{46}H_{58}BrN_5NaO_8Si^+$ [M+Na]$^+$: 938.3130, found: 938.3132.

[α]$^{25}_{D}$: +18.4 (*c* = 0.5, CHCl$_3$)

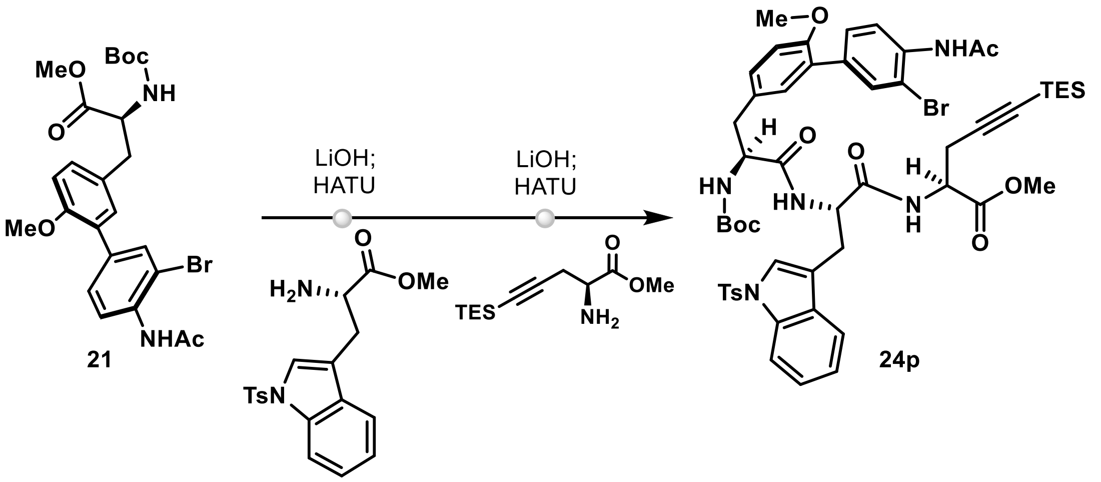

On 0.4 mmol scale, **General Procedure G** was followed. Purification by silica gel column chromatography gave the title compound **24p** (240 mg, 56% yield from **21**).

Compound 24p

Physical State: light yellow oil

^{1}H NMR (600 MHz, CDCl$_3$): δ 8.28 (d, *J* = 8.4 Hz, 1H), 7.92 (d, *J* = 8.5 Hz, 1H), 7.74 – 7.70 (m, 2H), 7.68 – 7.64 (m, 1H), 7.60 (s, 1H), 7.48 – 7.42 (m, 2H), 7.38 (s, 1H), 7.27 (t, *J* = 7.8 Hz, 1H), 7.20 – 7.15 (m, 3H), 7.09 – 6.94 (m, 2H), 6.91 – 6.64 (m, 2H), 6.62 – 6.27 (m, 1H), 4.91 – 4.75 (m, 1H), 4.73 – 4.63 (m, 1H), 4.59 – 4.46 (m, 1H), 4.27 (d, *J* = 59.9 Hz, 1H), 3.78 – 3.65 (m, 6H), 3.16 – 2.87 (m, 4H), 2.74 – 2.54 (m, 2H), 2.34 – 2.19 (m, 7H), 1.97 – 1.92 (m, 1H), 1.31 (s, 9H), 0.89 (t, *J* = 7.9 Hz, 9H), 0.50 (q, *J* = 7.9 Hz, 6H).

^{13}C NMR (151 MHz, CDCl$_3$): δ 171.24, 170.17, 169.89, 168.32, 155.61, 145.03, 135.34, 135.12, 134.55, 132.87, 131.62, 130.64, 129.97, 129.94, 129.77, 129.59, 128.77, 128.66, 126.95, 126.91, 124.96, 124.85, 123.37, 121.32, 119.56, 117.16, 116.92, 113.70, 112.88, 111.70, 101.29, 85.97, 80.56, 55.77, 53.12, 52.92, 52.81, 51.41, 51.25, 38.70, 37.10, 28.23, 28.09, 24.96, 23.89, 23.76, 23.21, 21.62, 7.49, 4.38, 4.35.

HRMS (ESI-TOF): calculated for $C_{40}H_{57}BrN_4NaO_8SSi^+$ $[M+Na]^+$: 883.2742, found: 883.2731.

$[\alpha]^{25}_D$: +14.6 (*c* = 0.5, CHCl$_3$)

General procedure H for the synthesis of 26 via Larock macrocyclization:

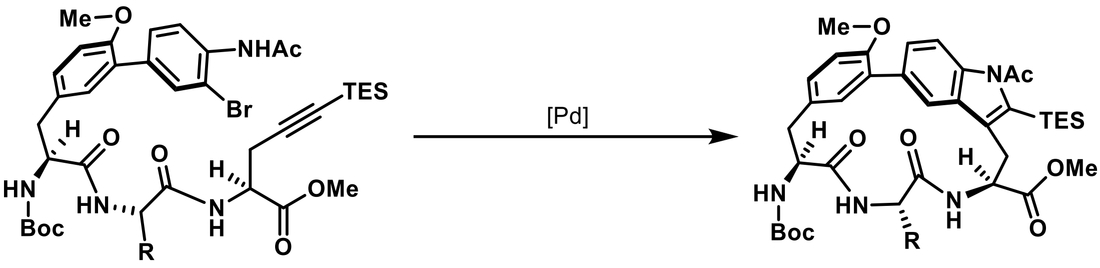

24 **26**

The tripeptide **24** (0.1 mmol, 1.0 eq.) was dissolved toluene (1.5 mL). $Pd(OAc)_2$ (30 mol%), *t*$Bu_3P{\cdot}HBF_4$ (60 mol%) and DIPEA (0.3 mmol, 3.0 eq.) were added. The reaction mixture was stirred at 110 °C under nitrogen atmosphere for 16 h. The reaction mixture was concentrated under reduced pressure and purified by silica gel chromatography to give the cyclized compound **26**.

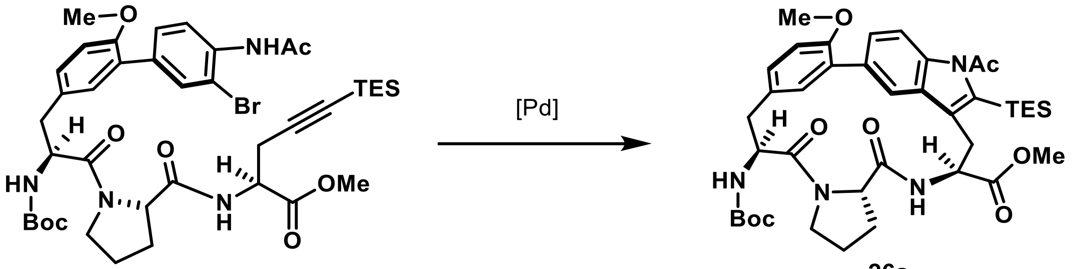

24a **26a**

On 0.1 mmol scale, **General Procedure H** was followed from compound **24a** via Larock macrocyclization. Purification by silica gel column chromatography gave the title compound **26a** (37.3 mg, 50% yield). The characterization data were identical to the data previously reported by our group.[1]

Compound 26a

Physical State: amorphous solid

^{1}H NMR (400 MHz, $CDCl_3$): δ 7.84 (dd, *J* = 8.8, 1.7 Hz, 1H), 7.63 (d, *J* = 8.9 Hz, 1H), 7.43 (d, *J* = 1.7 Hz, 1H), 7.36 (d, *J* = 2.3 Hz, 1H), 7.04 (dd, *J* = 8.4, 2.2 Hz, 1H), 6.88 (d, *J* = 8.3 Hz, 1H), 6.80 (d, *J* = 9.7 Hz, 1H), 5.19 (d, *J* = 8.7 Hz, 1H), 4.95 (t, *J* = 8.8 Hz, 1H), 4.80 (ddd, *J* = 8.6, 6.5, 1.8 Hz, 1H), 4.48 (dd, *J* = 8.4, 3.7 Hz, 1H), 3.88 – 3.80 (m, 4H), 3.75 (td, *J* = 7.0, 2.5 Hz, 1H), 3.70 (s, 3H), 3.69 – 3.64 (m, 1H), 3.16 (dd, *J* = 13.6, 6.6 Hz, 1H), 3.12 – 3.03 (m, 2H), 2.85 (s, 3H), 2.30 – 2.20 (m, 1H), 2.18 – 2.07 (m, 1H), 2.05 – 1.95 (m, 2H), 1.44 (s, 9H), 0.97 – 0.91 (m, 9H), 0.90 – 0.81 (m, 6H).

^{13}C NMR (101 MHz, $CDCl_3$): δ 172.57, 170.64, 169.17, 168.90, 154.76, 135.36, 134.60, 133.04, 132.55, 131.83, 131.24, 130.11, 128.51, 126.68, 126.36, 120.44, 112.01, 109.80, 78.86, 59.24, 54.87, 51.83, 51.78, 50.75, 46.58, 35.07, 29.49, 28.64, 27.78, 25.95, 24.35, 7.58, 5.70.

HRMS (ESI-TOF): calculated for $C_{40}H_{54}BrN_4NaO_8Si^+$ $[M+Na]^+$: 769.3603, found: 769.3604.

$[\alpha]^{25}_{D}$: –33.7 (*c* = 0.5, $CHCl_3$)

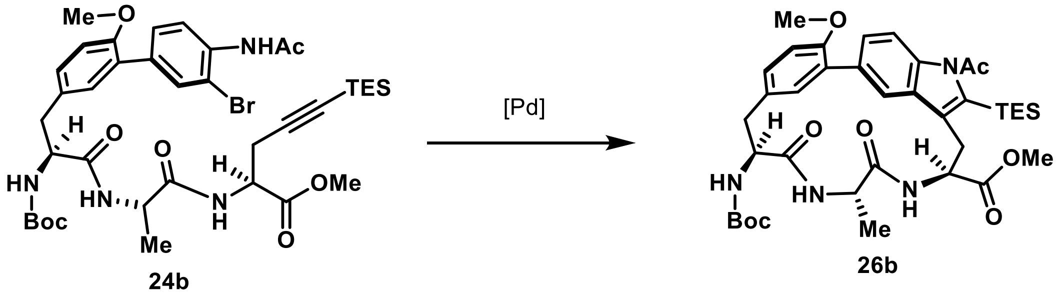

On 0.1 mmol scale, **General Procedure H** was followed from compound **24b** via Larock macrocyclization. Purification by silica gel column chromatography gave the title compound **26b** (49.0 mg, 68% yield).

Compound 26b

Physical State: amorphous solid

^{1}H NMR (600 MHz, CDCl$_3$): δ 7.66 (ddt, J = 12.0, 6.8, 1.4 Hz, 1H), 7.57 – 7.51 (m, 1H), 7.50 – 7.41 (m, 2H), 7.28 (d, J = 1.8 Hz, 1H), 7.08 (dd, J = 8.3, 2.3 Hz, 2H), 6.86 (d, J = 8.5 Hz, 1H), 6.78 (s, 1H), 6.64 (d, J = 7.4 Hz, 1H), 5.72 – 5.57 (m, 1H), 4.97 (ddd, J = 9.0, 7.1, 1.9 Hz, 1H), 4.57 – 4.44 (m, 1H), 4.34 (d, J = 9.7 Hz, 1H), 3.79 – 3.72 (m, 6H), 3.62 (dd, J = 15.1, 7.1 Hz, 1H), 3.16 (dd, J = 15.1, 2.0 Hz, 1H), 2.91 – 2.79 (m, 2H), 2.70 (s, 3H), 1.44 (s, 9H), 1.28 (d, J = 7.0 Hz, 3H), 0.94 – 0.89 (m, 9H), 0.89 – 0.80 (m, 6H).

^{13}C NMR (151 MHz, CDCl$_3$): δ 172.27, 172.10, 170.61, 169.58, 155.62, 155.35, 136.81, 136.07, 133.37, 133.31, 132.33, 132.23, 132.16, 132.05, 130.67, 130.13, 129.89, 128.65, 128.57, 127.94, 126.90, 120.54, 112.85, 111.01, 79.66, 55.62, 55.14, 52.80, 52.54, 49.03, 38.31, 29.78, 28.72, 28.49, 26.59, 19.39, 8.28, 6.26.

HRMS (ESI-TOF): calculated for $C_{38}H_{52}BrN_4NaO_8Si^+$ [M+Na]$^+$: 743.3447, found: 743.3456.

[α]$^{25}_D$: –17.5 (c = 0.5, CHCl$_3$)

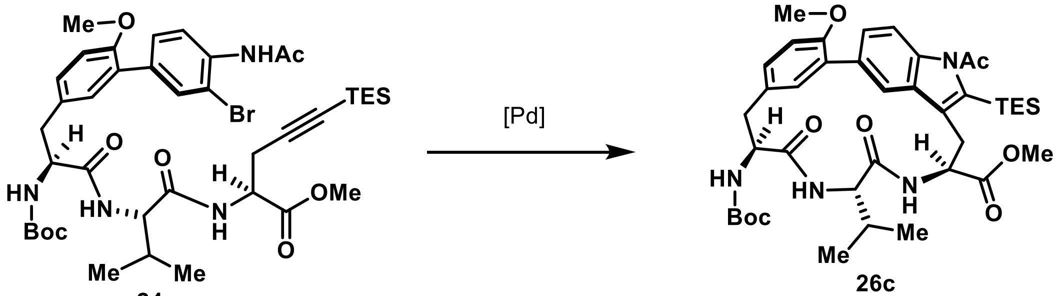

On 0.1 mmol scale, **General Procedure H** was followed from compound **24c** via Larock macrocyclization. Purification by silica gel column chromatography gave the title compound **26c** (36.0 mg, 48% yield).

Compound 26c

Physical State: amorphous solid

^{1}H NMR (600 MHz, CDCl$_3$): δ 7.69 (dd, J = 8.8, 1.7 Hz, 1H), 7.57 (d, J = 8.8 Hz, 1H), 7.34 (d, J = 1.9 Hz, 1H), 7.06 (d, J = 6.7 Hz, 2H), 6.99 (d, J = 9.4 Hz, 1H), 6.88 (d, J = 8.8 Hz, 1H), 6.81 (d, J = 8.6 Hz, 1H), 5.66 (d, J = 8.0 Hz, 1H), 4.94 (ddd, J = 9.0, 6.9, 1.8 Hz, 1H), 4.55 (td, J = 7.5, 2.8 Hz, 1H), 4.30 (t, J = 7.9 Hz, 1H), 3.77 (s, 3H), 3.72 (s, 3H), 3.66 (dd, J = 15.1, 7.0 Hz, 1H), 3.17 (dd, J = 13.8, 6.9 Hz, 1H), 3.09 (dd, J = 15.3, 2.0 Hz, 1H), 2.93 (dd, J = 13.8, 2.9 Hz, 1H), 2.79 (s, 3H), 1.95 (h, J = 6.8 Hz, 1H), 1.43 (s, 9H), 0.96 – 0.81 (m, 21H).

^{13}C NMR (151 MHz, CDCl$_3$): δ 172.56, 170.83, 170.76, 169.60, 155.53, 155.37, 136.29, 136.17, 133.30, 132.92, 132.31, 131.35, 130.14, 129.62, 128.24, 127.18, 120.39, 112.91, 111.30, 79.74, 58.65, 55.62, 55.00, 52.67, 52.45, 37.79, 31.93, 29.78, 29.36, 28.46, 27.04, 26.62, 19.12, 18.68, 8.23, 6.25.

HRMS (ESI-TOF): calculated for $C_{40}H_{56}BrN_4NaO_8Si^+$ [M+Na]$^+$: 771.3760, found: 771.3763.

[α]$^{25}_D$: –2.2 (c = 0.5, CHCl$_3$)

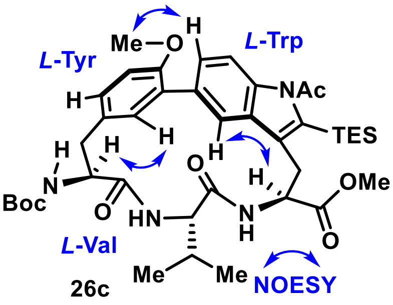

The configuration of the ring system is deduced to be *Sconf*.

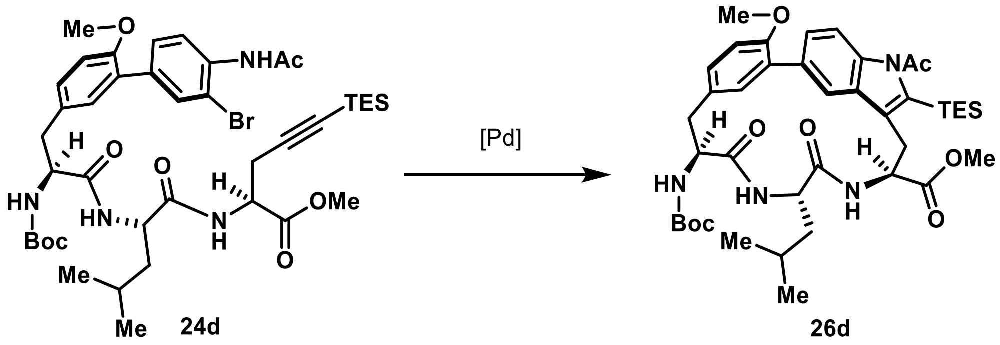

On 0.1 mmol scale, **General Procedure H** was followed from compound **24d** via Larock macrocyclization. Purification by silica gel column chromatography gave the title compound **26d** (32.8 mg, 43% yield).

Compound 26d

Physical State: amorphous solid

^{1}H NMR (600 MHz, CDCl$_3$): δ 7.73 (dd, *J* = 8.8, 1.7 Hz, 1H), 7.62 (d, *J* = 8.8 Hz, 1H), 7.39 (d, *J* = 1.7 Hz, 1H), 7.15 (d, *J* = 2.2 Hz, 1H), 7.07 (dd, *J* = 8.3, 2.2 Hz, 1H), 6.88 (dd, *J* = 13.0, 8.7 Hz, 2H), 6.79 (d, *J* = 7.9 Hz, 1H), 5.55 (d, *J* = 8.1 Hz, 1H), 4.99 – 4.88 (m, 1H), 4.61 – 4.42 (m, 2H), 3.81 (s, 3H), 3.73 (s, 3H), 3.71 – 3.63 (m, 1H), 3.19 (dd, *J* = 13.7, 6.8 Hz, 1H), 3.09 (dd, *J* = 15.1, 1.8 Hz, 1H), 2.96 (dd, *J* = 13.7, 2.8 Hz, 1H), 2.84 (s, 3H), 1.71 – 1.62 (m, 1H), 1.54 (s, 1H), 1.44 (s, 9H), 1.26 (s, 1H), 0.96 – 0.81 (m, 21H).

^{13}C NMR (151 MHz, CDCl$_3$): δ 172.63, 171.76, 170.71, 169.60, 155.53, 155.35, 136.21, 136.07, 133.40, 132.81, 131.56, 130.10, 129.68, 128.00, 127.24, 120.60, 112.87, 111.16, 79.82, 77.33, 77.12, 76.91, 55.62, 54.61, 53.80, 52.63, 52.47, 51.88, 42.15, 42.05, 37.74, 29.78, 29.67, 28.43, 27.04, 26.63, 24.63, 22.83, 22.16, 8.22, 6.32.

HRMS (ESI-TOF): calculated for $C_{41}H_{58}BrN_4NaO_8Si^+$ [M+Na]$^+$: 785.3916, found: 785.3922.

$[\alpha]^{25}_{D}$: +1.9 (*c* = 0.5, CHCl$_3$)

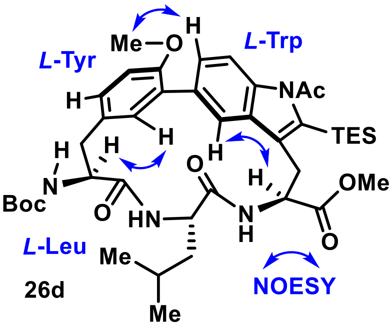

The configuration of the ring system is deduced to be *Sconf*.

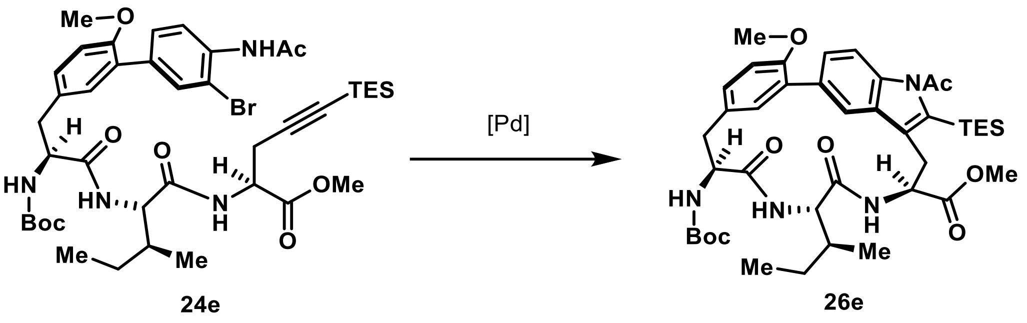

On 0.1 mmol scale, **General Procedure H** was followed from compound **24e** via Larock macrocyclization. Purification by silica gel column chromatography gave the title compound **26e** (39.7 mg, 52% yield).

Compound 26e

Physical State: amorphous solid

^{1}H NMR (600 MHz, CDCl$_3$): δ 7.71 (d, J = 8.7 Hz, 1H), 7.59 (d, J = 8.8 Hz, 1H), 7.34 (s, 1H), 7.09 – 7.02 (m, 2H), 6.88 (d, J = 8.3 Hz, 1H), 6.85 – 6.74 (m, 2H), 5.56 (d, J = 7.9 Hz, 1H), 4.93 (t, J = 7.8 Hz, 1H), 4.60 – 4.47 (m, 1H), 4.30 (t, J = 7.9 Hz, 1H), 3.78 (s, 3H), 3.71 (s, 3H), 3.67 (dd, J = 15.2, 7.0 Hz, 1H), 3.17 (dd, J = 13.9, 6.9 Hz, 1H), 3.09 (d, J = 15.0 Hz, 1H), 2.95 – 2.91 (m, 1H), 2.81 (s, 3H), 1.77 – 1.70 (m, 1H), 1.55 – 1.50 (m, 2H), 1.44 (s, 9H), 1.15 – 1.08 (m, 1H), 0.97 – 0.88 (m, 15H), 0.85 – 0.79 (m, 6H).

^{13}C NMR (151 MHz, CDCl$_3$): δ 172.47, 170.83, 170.61, 169.68, 169.60, 155.53, 155.30, 136.29, 136.19, 133.33, 132.89, 132.38, 131.31, 130.13, 129.63, 128.17, 127.19, 120.36, 112.93, 111.28, 79.78, 57.82, 55.68, 55.64, 54.90, 52.74, 52.62, 52.46, 38.23, 37.72, 32.21, 29.78, 29.39, 28.47, 28.31, 26.64, 25.26, 15.31, 11.27, 8.51, 8.23, 6.26.

HRMS (ESI-TOF): calculated for $C_{41}H_{58}BrN_4NaO_8Si^+$ [M+Na]$^+$: 785.3916, found: 785.3923.

[α]$^{25}_{D}$: –1.4 (c = 0.5, CHCl$_3$)

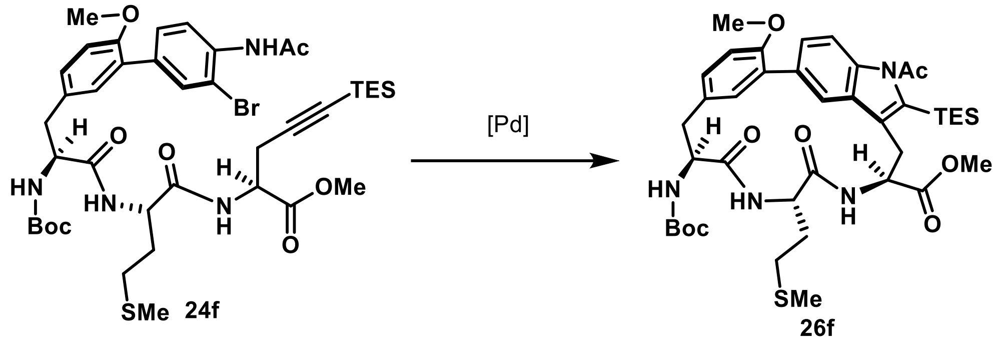

On 0.1 mmol scale, **General Procedure H** was followed from compound **24f** via Larock macrocyclization. Purification by silica gel column chromatography gave the title compound **26f** (27.3 mg, 35% yield).

Compound 26f

Physical State: amorphous solid

^{1}H NMR (600 MHz, CDCl$_3$): δ 7.66 (d, J = 8.8 Hz, 1H), 7.60 (d, J = 8.7 Hz, 1H), 7.38 – 7.30 (m, 1H), 7.08 (dd, J = 8.4, 2.3 Hz, 1H), 7.04 (s, 1H), 6.89 (d, J = 8.4 Hz, 1H), 5.46 (d, J = 7.7 Hz, 1H), 4.93 (t, J = 7.8 Hz, 1H), 4.70 (s, 1H), 4.49 (s, 1H), 3.78 (s, 3H), 3.72 (s, 3H), 3.66 (dt, J = 16.8, 8.4 Hz, 1H), 3.19 (dd, J = 13.9, 6.4 Hz, 1H), 3.16 – 3.10 (m, 1H), 2.98 (dd, J = 14.1, 2.9 Hz, 1H), 2.81 (s, 3H), 2.66 (d, J = 28.8 Hz, 1H), 2.10 (s, 3H), 1.97 (s, 1H), 1.54 (d, J = 13.8 Hz, 4H), 1.45 (s, 9H), 0.95 – 0.82 (m, 15H).

^{13}C NMR (151 MHz, CDCl$_3$): δ 172.35, 170.73, 170.43, 169.62, 155.56, 155.28, 136.26, 133.35, 132.86, 132.56, 130.19, 129.86, 127.93, 127.18, 120.50, 112.93, 111.17, 79.79, 55.64, 54.68, 52.75, 52.54, 51.75, 37.93, 32.19, 31.53, 29.23, 28.50, 27.17, 26.68, 8.25, 6.32.

HRMS (ESI-TOF): calculated for $C_{40}H_{56}BrN_4NaO_8SSi^+$ [M+Na]$^+$: 803.3480, found: 803.3447.

[α]$^{25}_{D}$: +10.4 (c = 0.5, CHCl$_3$)

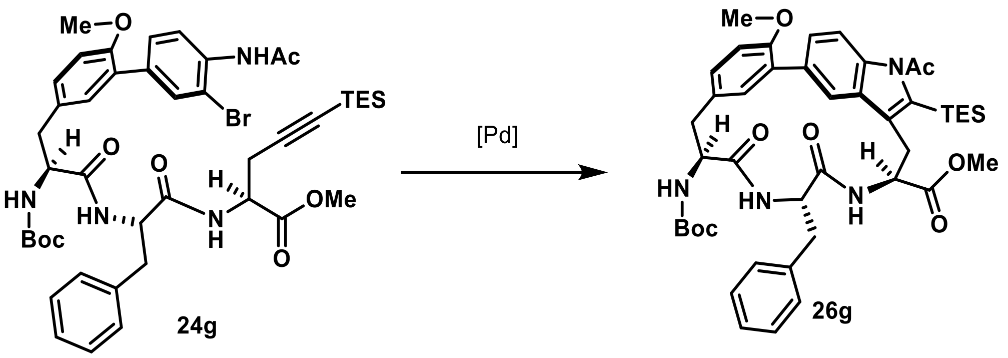

On 0.1 mmol scale, **General Procedure H** was followed from compound **24g** via Larock macrocyclization. Purification by silica gel column chromatography gave the title compound **26g** (47.8 mg, 60% yield).

Compound 26g

Physical State: amorphous solid

^{1}H NMR (600 MHz, CDCl$_3$): δ 7.64 (dd, J = 8.8, 1.6 Hz, 1H), 7.52 (d, J = 8.8 Hz, 1H), 7.33 – 7.29 (m, 1H), 7.26 – 7.18 (m, 3H), 7.14 – 7.01 (m, 4H), 6.94 (d, J = 7.5 Hz, 1H), 6.87 (d, J = 8.6 Hz, 1H), 6.41 (d, J = 8.7 Hz, 1H), 5.52 (d, J = 8.0 Hz, 1H), 4.86 (ddd, J = 8.8, 6.9, 2.0 Hz, 1H), 4.61 (q, J = 7.3 Hz, 1H), 4.56 – 4.46 (m, 1H), 3.75 (s, 3H), 3.65 (s, 3H), 3.60 (dd, J = 14.9, 7.1 Hz, 1H), 3.18 – 3.05 (m, 2H), 2.99 – 2.84 (m, 3H), 2.76 (s, 3H), 1.49 (s, 9H), 0.96 – 0.77 (m, 15H).

^{13}C NMR (151 MHz, CDCl$_3$): δ 171.30, 170.73, 170.34, 169.54, 155.61, 155.32, 136.69, 136.11, 135.93, 133.27, 133.17, 132.27, 130.77, 130.20, 129.69, 129.29, 128.71, 127.93, 127.20, 126.92, 120.51, 112.87, 111.10, 79.83, 55.58, 54.94, 53.10, 52.44, 39.39, 38.02, 28.89, 28.51, 26.58, 8.22, 6.22.

HRMS (ESI-TOF): calculated for $C_{44}H_{56}BrN_4NaO_8Si^+$ [M+Na]$^+$: 819.3760, found: 819.3760.

[α]$^{25}_{D}$: +34.6 (c = 0.5, CHCl$_3$)

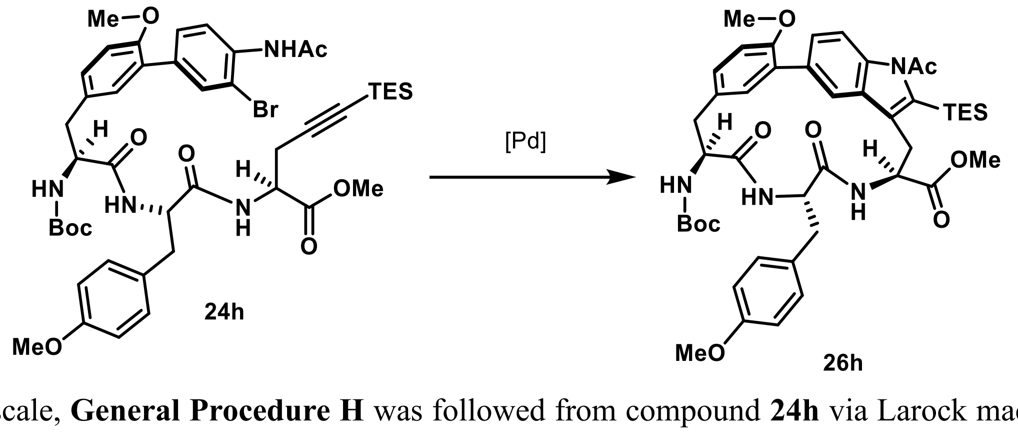

On 0.1 mmol scale, **General Procedure H** was followed from compound **24h** via Larock macrocyclization. Purification by silica gel column chromatography gave the title compound **26h** (54.6 mg, 66% yield).

Compound 26h

Physical State: amorphous solid

^{1}H NMR (400 MHz, CDCl$_3$): δ 7.64 (dd, J = 8.8, 1.6 Hz, 1H), 7.53 (d, J = 8.7 Hz, 1H), 7.31 (d, J = 1.7 Hz, 1H), 7.07 (d, J = 8.5 Hz, 2H), 7.00 (d, J = 8.3 Hz, 2H), 6.88 (t, J = 7.8 Hz, 2H), 6.76 (d, J = 8.5 Hz, 2H), 6.39 (d, J = 8.5 Hz, 1H), 5.52 (d, J = 8.0 Hz, 1H), 4.85 (t, J = 7.1 Hz, 1H), 4.53 (dt, J = 28.0, 7.5 Hz, 2H), 3.76 (s, 6H), 3.66 (s, 3H), 3.60 (dd, J = 14.8, 6.8 Hz, 1H), 3.13 (dd, J = 14.1, 7.0 Hz, 2H), 2.96 (dd, J = 13.8, 2.7 Hz, 1H), 2.92 – 2.80 (m, 2H), 2.77 (s, 3H), 1.49 (s, 9H), 0.95 – 0.77 (m, 15H).

^{13}C NMR (101 MHz, CDCl$_3$): δ 170.52, 169.95, 169.73, 168.83, 158.00, 154.87, 154.59, 135.38, 132.52, 131.50, 130.04, 129.58, 129.49, 128.95, 127.20, 127.14, 126.19, 119.78, 113.40, 112.15, 110.33, 79.11, 54.87, 54.54, 54.37, 54.22, 52.47, 51.69, 37.86, 37.32, 28.10, 27.80, 25.90, 7.53, 5.53.

HRMS (ESI-TOF): calculated for $C_{45}H_{58}BrN_4NaO_9Si^+$ [M+Na]$^+$: 849.3865, found: 849.3870.

[α]$^{25}_{D}$: +32.0 (c = 0.5, CHCl$_3$)

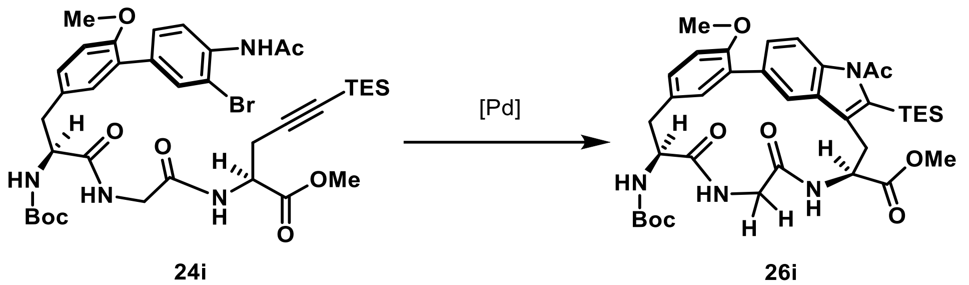

On 0.1 mmol scale, **General Procedure H** was followed from compound **24i** via Larock macrocyclization. Purification by silica gel column chromatography gave the title compound **26i** (35.3 mg, 50% yield).

Compound 26i

Physical State: amorphous solid

^{1}H NMR (600 MHz, CDCl$_3$): δ 7.37 (d, *J* = 8.6 Hz, 1H), 7.32 (dd, *J* = 8.8, 1.5 Hz, 1H), 7.14 – 7.09 (m, 2H), 6.89 (d, *J* = 8.4 Hz, 1H), 6.86 – 6.81 (m, 1H), 6.80 – 6.63 (m, 1H), 6.19 (s, 1H), 5.00 (s, 1H), 4.42 (dd, *J* = 40.6, 21.5 Hz, 1H), 4.27 (s, 1H), 3.81 (s, 3H), 3.75 (s, 3H), 3.59 (dd, *J* = 15.0, 5.0 Hz, 1H), 3.46 (dd, *J* = 15.0, 7.4 Hz, 1H), 2.91 (d, *J* = 13.4 Hz, 1H), 2.85 – 2.73 (m, 1H), 2.58 (s, 3H), 1.45 (s, 9H), 1.01 – 0.90 (m, 15H), 0.57 (q, *J* = 7.9 Hz, 2H).

^{13}C NMR (151 MHz, CDCl$_3$): δ 171.90, 171.00, 170.62, 169.53, 168.23, 168.09, 158.73, 156.16, 155.23, 135.77, 134.32, 133.62, 131.63, 131.43, 130.34, 130.18, 128.48, 126.94, 126.25, 120.15, 114.22, 112.84, 110.71, 80.24, 55.69, 55.32, 55.03, 53.82, 52.81, 52.62, 51.23, 43.56, 43.24, 43.21, 42.86, 29.78, 29.13, 28.41, 28.34, 27.30, 27.04, 26.55, 26.03, 25.87, 23.79, 18.75, 17.49, 8.42, 7.50, 6.00, 4.43, 4.40.

HRMS (ESI-TOF): calculated for $C_{37}H_{50}BrN_4NaO_8Si^+$ [M+Na]$^+$: 729.3290, found: 729.3297.

[α]$^{25}_{D}$: –10.1 (*c* = 0.5, CHCl$_3$)

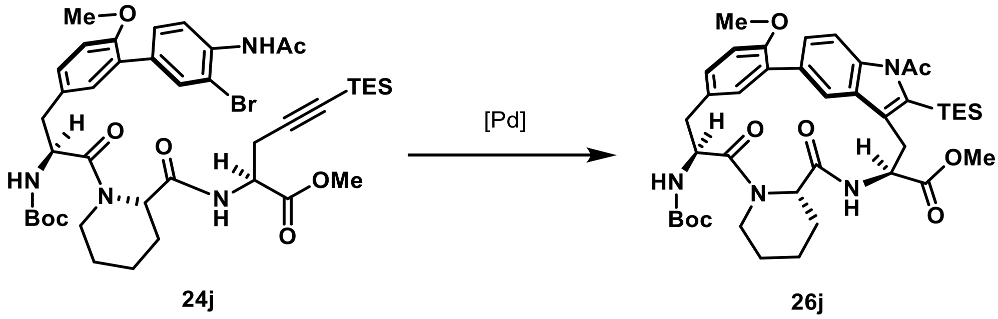

On 0.1 mmol scale, **General Procedure H** was followed from compound **24j** via Larock macrocyclization. Purification by silica gel column chromatography gave the title compound **26j** (44.1 mg, 58% yield).

Compound 26j

Physical State: amorphous solid

^{1}H NMR (600 MHz, CDCl$_3$): δ 7.71 (dd, *J* = 8.8, 1.7 Hz, 1H), 7.61 (d, *J* = 8.8 Hz, 1H), 7.32 (d, *J* = 1.7 Hz, 1H), 7.26 (d, *J* = 2.3 Hz, 1H), 7.02 (dd, *J* = 8.4, 2.3 Hz, 1H), 6.90 (d, *J* = 8.3 Hz, 1H), 6.49 (d, *J* = 9.8 Hz, 1H), 5.38 (d, *J* = 8.2 Hz, 1H), 5.23 (dd, *J* = 6.7, 2.1 Hz, 1H), 5.00 (ddd, *J* = 8.5, 6.3, 1.8 Hz, 1H), 4.96 (ddd, *J* = 9.5, 7.6, 1.6 Hz, 1H), 3.96 (td, *J* = 12.5, 3.3 Hz, 1H), 3.92 – 3.88 (m, 2H), 3.79 (s, 3H), 3.70 (s, 3H), 3.69 – 3.62 (m, 1H), 3.15 – 3.06 (m, 1H), 3.02 – 2.95 (m, 2H), 2.82 (s, 3H), 2.09 – 2.02 (m, 1H), 1.84 – 1.76 (m, 2H), 1.73 – 1.67 (m, 2H), 1.53 (s, 1H), 1.47 (s, 9H), 0.92 (dd, *J* = 8.0, 6.8 Hz, 9H), 0.88 – 0.82 (m, 6H).

^{13}C NMR (151 MHz, CDCl$_3$): δ 173.24, 171.04, 170.79, 169.63, 155.40, 155.24, 136.22, 135.48, 133.51, 133.41, 132.53, 132.12, 130.84, 129.74, 127.58, 127.40, 120.71, 112.79, 110.70, 79.53, 77.33, 77.12, 76.91, 55.58, 53.92, 52.51, 52.27, 52.11, 50.71, 43.68, 35.20, 30.51, 29.78, 28.57, 27.66, 26.65, 25.08, 19.88, 8.26, 6.63, 6.44.

HRMS (ESI-TOF): calculated for $C_{41}H_{56}BrN_4NaO_8Si^+$ [M+Na]$^+$: 783.3760, found: 783.3757.

[α]$^{25}_{D}$: –38.3 (*c* = 0.5, CHCl$_3$)

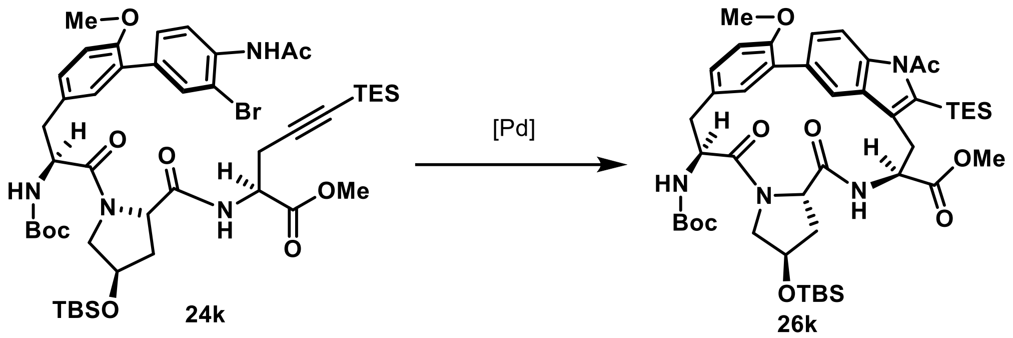

On 0.1 mmol scale, **General Procedure H** was followed from compound **24k** via Larock macrocyclization. Purification by silica gel column chromatography gave the title compound **26k** (56.1 mg, 64% yield).

Compound 26k

Physical State: amorphous solid

^{1}H NMR (600 MHz, CDCl$_3$): δ 7.83 (dd, *J* = 8.8, 1.7 Hz, 1H), 7.61 (d, *J* = 8.8 Hz, 1H), 7.42 (d, *J* = 1.8 Hz, 1H), 7.32 (d, *J* = 2.3 Hz, 1H), 7.02 (dd, *J* = 8.3, 2.3 Hz, 1H), 6.86 (d, *J* = 8.3 Hz, 1H), 6.74 (d, *J* = 9.6 Hz, 1H), 5.09 (d, *J* = 8.5 Hz, 1H), 4.92 (t, *J* = 8.9 Hz, 1H), 4.73 (ddd, *J* = 8.6, 7.0, 1.5 Hz, 1H), 4.68 – 4.61 (m, 1H), 4.53 (t, *J* = 7.1 Hz, 1H), 3.91 (dd, *J* = 10.1, 5.2 Hz, 1H), 3.80 (s, 3H), 3.68 (s, 3H), 3.58 (dd, *J* = 10.1, 3.8 Hz, 1H), 3.19 (dd, *J* = 13.6, 7.0 Hz, 1H), 3.10 – 3.05 (m, 1H), 3.02 – 2.97 (m, 1H), 2.82 (s, 3H), 2.10 (dd, *J* = 7.1, 5.1 Hz, 2H), 1.43 (s, 9H), 0.95 – 0.89 (m, 9H), 0.89 – 0.82 (m, 15H), 0.06 (d, *J* = 6.5 Hz, 6H).

^{13}C NMR (151 MHz, CDCl$_3$): δ 173.10, 171.22, 170.26, 169.61, 155.52, 155.28, 136.08, 135.38, 133.58, 133.25, 132.53, 131.90, 130.93, 129.18, 127.38, 127.09, 121.23, 112.72, 110.55, 79.54, 70.86, 59.22, 55.58, 55.29, 53.77, 52.64, 52.53, 51.54, 38.40, 35.72, 32.21, 30.12, 29.78, 28.49, 26.63, 25.80, 18.74, 18.02, 17.48, 8.28, 6.41, -4.71, -4.74.

HRMS (ESI-TOF): calculated for $C_{46}H_{68}BrN_4NaO_9Si_2^+$ [M+Na]$^+$: 899.4417, found: 899.4451.

[α]$^{25}_{D}$: -15.2 (*c* = 0.5, CHCl$_3$)

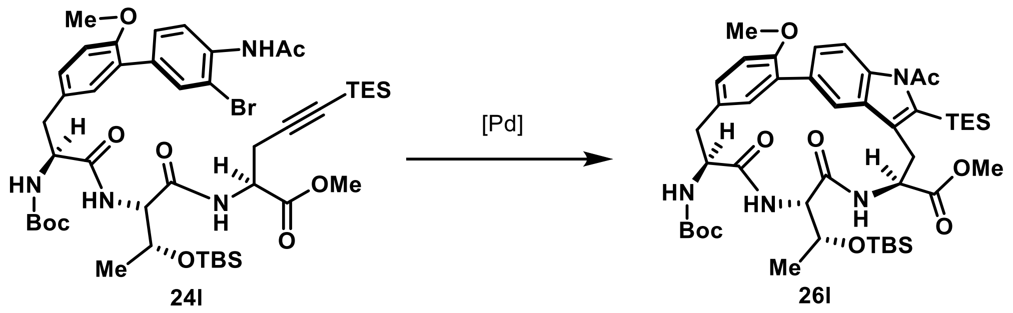

On 0.1 mmol scale, **General Procedure H** was followed from compound **24l** via Larock macrocyclization. Purification by silica gel column chromatography gave the title compound **26l** (44.1 mg, 51% yield).

Compound 26l

Physical State: amorphous solid

^{1}H NMR (600 MHz, CDCl$_3$): δ 7.74 (d, *J* = 8.7 Hz, 1H), 7.61 (d, *J* = 8.8 Hz, 1H), 7.43 (s, 1H), 7.29 (d, *J* = 9.1 Hz, 1H), 7.10 – 7.04 (m, 2H), 6.98 – 6.86 (m, 2H), 5.53 (d, *J* = 7.3 Hz, 1H), 5.18 (t, *J* = 8.2 Hz, 1H), 4.60 (dt, *J* = 7.9, 4.1 Hz, 1H), 4.08 – 4.02 (m, 1H), 3.93 (t, *J* = 4.8 Hz, 1H), 3.82 (s, 3H), 3.80 (s, 3H), 3.70 (dd, *J* = 15.0, 7.3 Hz, 1H), 3.32 (d, *J* = 15.1 Hz, 1H), 3.22 (dd, *J* = 14.2, 4.7 Hz, 1H), 3.15 (dd, *J* = 14.1, 3.7 Hz, 1H), 2.82 (s, 3H), 1.47 (s, 9H), 1.03 (d, *J* = 6.3 Hz, 3H), 0.98 – 0.89 (m, 15H), 0.70 (s, 9H), -0.01 (s, 3H), -0.15 (s, 3H).

^{13}C NMR (151 MHz, CDCl$_3$): δ 171.35, 171.16, 169.44, 168.77, 155.64, 155.16, 137.26, 136.00, 133.91, 133.12, 132.37, 130.33, 129.87, 129.42, 127.81, 126.76, 119.48, 112.97, 111.36, 79.60, 68.35, 58.40, 55.60, 55.02, 53.83, 52.49, 52.08, 38.43, 32.22, 32.20, 29.78, 28.49, 27.92, 26.67, 25.50, 18.76, 17.66, 17.59, 8.21, 6.15, -5.06, -5.51.

HRMS (ESI-TOF): calculated for $C_{45}H_{68}BrN_4NaO_9Si_2^+$ $[M+Na]^+$: 887.4417, found: 887.4410.
$[\alpha]^{25}_D$: +32.3 (*c* = 0.5, $CHCl_3$)

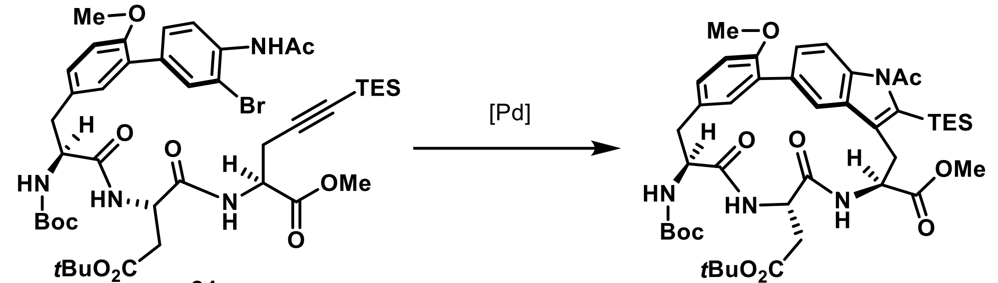

24m 26m

On 0.1 mmol scale, **General Procedure H** was followed from compound **24m** via Larock macrocyclization. Purification by silica gel column chromatography gave the title compound **26m** (44.3 mg, 54% yield).

Compound 26m

Physical State: amorphous solid

^{1}H NMR (400 MHz, $CDCl_3$): δ 7.67 – 7.59 (m, 2H), 7.36 – 7.28 (m, 2H), 7.13 (dd, *J* = 8.4, 2.3 Hz, 1H), 7.06 (d, *J* = 2.3 Hz, 1H), 6.91 (d, *J* = 8.4 Hz, 1H), 6.77 (d, *J* = 7.6 Hz, 1H), 5.44 (d, *J* = 7.7 Hz, 1H), 4.95 (ddd, *J* = 9.3, 6.7, 2.4 Hz, 1H), 4.85 – 4.68 (m, 1H), 4.52 – 4.41 (m, 1H), 3.80 (s, 3H), 3.72 (s, 4H), 3.18 (ddd, *J* = 17.6, 14.5, 4.3 Hz, 2H), 3.02 (dd, *J* = 14.1, 3.2 Hz, 1H), 2.84 (s, 3H), 2.72 – 2.59 (m, 2H), 1.48 (s, 9H), 1.43 (s, 9H), 0.99 – 0.83 (m, 15H).

^{13}C NMR (151 MHz, $CDCl_3$): δ 171.76, 170.39, 170.18, 170.13, 169.61, 155.64, 155.23, 136.66, 136.30, 133.39, 133.08, 132.57, 131.32, 130.07, 127.78, 127.01, 120.45, 112.99, 111.20, 82.43, 79.81, 55.66, 54.79, 52.86, 52.46, 48.90, 38.86, 38.10, 29.20, 28.51, 28.06, 27.05, 26.67, 26.04, 8.24, 6.27.

HRMS (ESI-TOF): calculated for $C_{43}H_{60}BrN_4NaO_{10}Si^+$ $[M+Na]^+$: 843.3971, found: 843.3979.
$[\alpha]^{25}_D$: +27.9 (*c* = 0.5, $CHCl_3$)

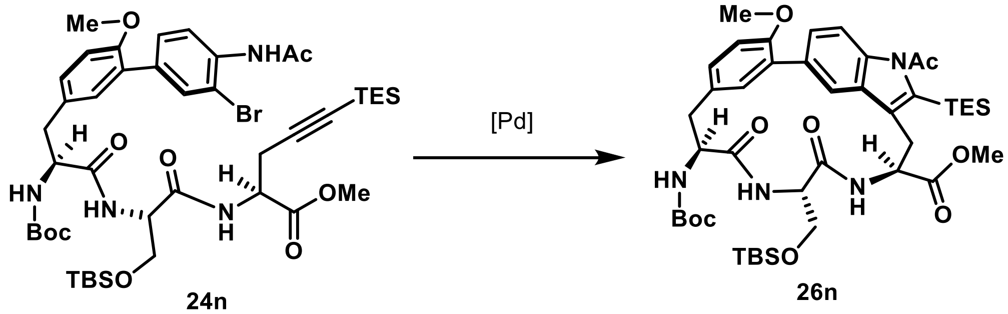

24n 26n

On 0.1 mmol scale, **General Procedure H** was followed from compound **24n** via Larock macrocyclization. Purification by silica gel column chromatography gave the title compound **26n** (41.7 mg, 49% yield).

Compound 26n

Physical State: amorphous solid

^{1}H NMR (600 MHz, $CDCl_3$): δ 7.66 (d, *J* = 8.7 Hz, 1H), 7.58 (d, *J* = 8.5 Hz, 1H), 7.36 (s, 1H), 7.11 – 6.98 (m, 3H), 6.88 (d, *J* = 8.3 Hz, 1H), 6.77 (d, *J* = 6.2 Hz, 1H), 5.47 (d, *J* = 7.5 Hz, 1H), 5.00 (s, 1H), 4.48 (d, *J* = 8.8 Hz, 1H), 4.10 (q, *J* = 8.5, 6.5 Hz, 1H), 3.81 (s, 1H), 3.78 (s, 3H), 3.75 (d, *J* = 1.0 Hz, 3H), 3.69 (dd, *J* = 15.1, 7.1 Hz, 1H), 3.51 (d, *J* = 11.9 Hz, 1H), 3.24 (d, *J* = 15.1 Hz, 1H), 3.15 (dd, *J* = 13.6, 5.1 Hz, 1H), 3.12 – 3.06 (m, 1H), 2.80 (s, 3H), 1.46 (s, 9H), 0.95 – 0.82 (m, 15H), 0.75 (d, *J* = 1.1 Hz, 9H), -0.03 (s, 3H), -0.08 (s, 3H).

^{13}C NMR (151 MHz, $CDCl_3$): δ 171.46, 171.04, 169.64, 169.49, 155.69, 155.22, 137.11, 136.04, 133.74, 133.24, 132.42, 130.51, 129.98, 129.71, 127.78, 126.74, 119.99, 112.98, 111.20, 79.75, 62.94, 55.62, 55.12, 54.57, 52.93, 52.50, 38.26, 28.49, 28.23, 26.66, 25.72, 18.07, 8.25, 6.18, -5.62, -5.68.

HRMS (ESI-TOF): calculated for $C_{44}H_{66}N_4NaO_9Si_2^+$ $[M+Na]^+$: 873.4261, found: 873.4271.
$[\alpha]^{25}{}_D$: +10.4 (*c* = 0.5, $CHCl_3$)

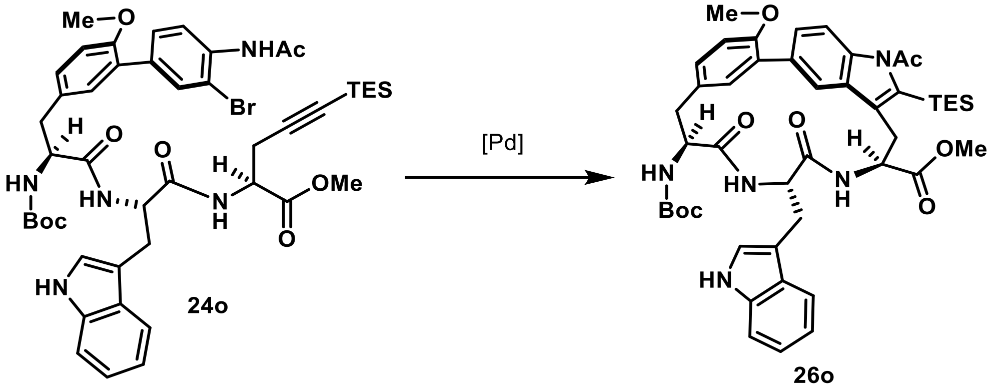

24o

26o

On 0.1 mmol scale, **General Procedure H** was followed from compound **24o** via Larock macrocyclization. Purification by silica gel column chromatography gave the title compound **26o** (36.8 mg, 44% yield).
Compound 26o
Physical State: amorphous solid
^{1}H NMR (600 MHz, $CDCl_3$): δ 8.13 (s, 1H), 7.59 (t, *J* = 8.6 Hz, 2H), 7.51 (d, *J* = 8.8 Hz, 1H), 7.32 (d, *J* = 8.1 Hz, 1H), 7.28 (d, *J* = 2.5 Hz, 1H), 7.17 (ddd, *J* = 8.1, 7.0, 1.2 Hz, 1H), 7.14 – 7.04 (m, 3H), 6.96 (d, *J* = 2.3 Hz, 1H), 6.89 (t, *J* = 8.8 Hz, 2H), 6.22 (d, *J* = 8.4 Hz, 1H), 5.52 (d, *J* = 7.9 Hz, 1H), 4.78 (t, *J* = 7.7 Hz, 1H), 4.60 (q, *J* = 7.1, 6.6 Hz, 1H), 4.53 – 4.41 (m, 1H), 3.76 (s, 3H), 3.56 (s, 4H), 3.23 – 3.12 (m, 2H), 3.11 – 3.04 (m, 2H), 3.04 – 2.96 (m, 1H), 2.75 (s, 3H), 1.49 (s, 9H), 0.94 – 0.75 (m, 15H).
^{13}C NMR (151 MHz, $CDCl_3$): δ 172.27, 170.56, 170.10, 169.93, 168.83, 154.92, 136.08, 135.51, 135.36, 132.54, 131.46, 129.88, 129.48, 129.05, 127.24, 126.57, 126.08, 122.74, 121.56, 119.73, 119.11, 117.97, 112.20, 110.56, 110.41, 109.41, 79.13, 54.90, 54.50, 53.71, 52.43, 51.66, 37.41, 28.59, 27.91, 27.81, 25.87, 7.52, 5.48.
HRMS (ESI-TOF): calculated for $C_{46}H_{57}BrN_5NaO_8Si^+$ $[M+Na]^+$: 858.3869, found: 858.3866.
$[\alpha]^{25}{}_D$: +38.4 (*c* = 0.5, $CHCl_3$)

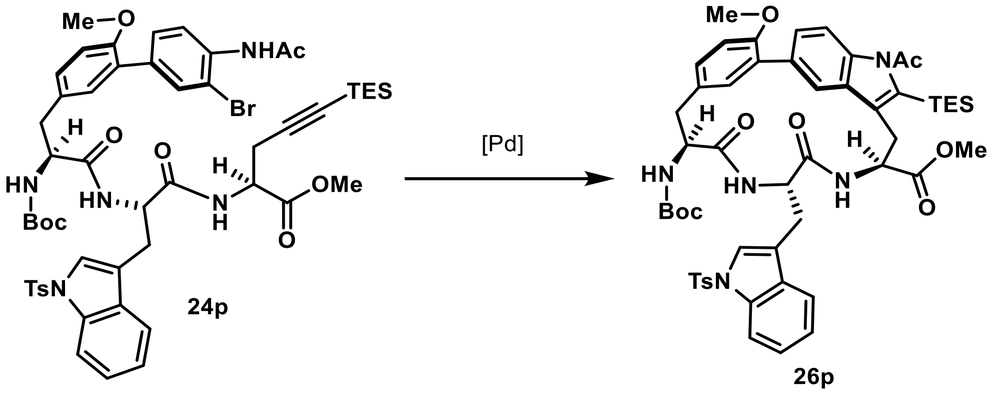

24p

26p

On 0.1 mmol scale, **General Procedure H** was followed from compound **24p** via Larock macrocyclization. Purification by silica gel column chromatography gave the title compound **26p** (37.6 mg, 38% yield).
Compound 26p
Physical State: amorphous solid
^{1}H NMR (600 MHz, $CDCl_3$): δ 7.93 (d, *J* = 8.3 Hz, 1H), 7.76 – 7.68 (m, 2H), 7.56 (q, *J* = 8.8 Hz, 2H), 7.47 (d, *J* = 7.8 Hz, 1H), 7.39 (s, 1H), 7.33 – 7.29 (m, 1H), 7.21 (dd, *J* = 8.4, 6.6 Hz, 3H), 7.09 (dd, *J* = 8.4, 2.2 Hz, 1H), 6.97 (s, 1H), 6.86 (d, *J* = 8.3 Hz, 2H), 6.27 (s, 1H), 5.54 (d, *J* = 7.8 Hz, 1H), 4.79 (ddd, *J* = 8.6, 6.4, 2.1 Hz, 1H), 4.60 (q, *J* = 7.1 Hz, 1H), 4.40 (td, *J* = 8.2, 7.8, 2.9 Hz, 1H), 3.73 (s, 3H), 3.64 (s, 3H), 3.55 (dd, *J* =

15.0, 6.5 Hz, 1H), 3.22 – 2.98 (m, 4H), 2.93 (dd, J = 13.7, 2.9 Hz, 1H), 2.77 (s, 3H), 2.32 (s, 3H), 1.50 (s, 9H), 0.95 – 0.77 (m, 15H).
^{13}C NMR (151 MHz, $CDCl_3$): δ 170.86, 170.04, 169.49, 155.72, 155.23, 145.05, 137.36, 136.16, 135.25, 135.12, 133.44, 133.21, 131.80, 130.55, 130.25, 130.20, 129.96, 129.82, 127.86, 126.94, 126.73, 124.97, 124.91, 123.42, 120.30, 119.42, 116.73, 113.70, 112.96, 111.17, 79.90, 55.61, 55.37, 53.82, 53.37, 52.78, 38.16, 29.17, 28.51, 28.33, 26.61, 21.63, 8.25, 6.23.
HRMS (ESI-TOF): calculated for $C_{53}H_{63}BrN_5NaO_{10}SSi^+$ [M+Na]$^+$: 1012.3957, found: 1012.3961.
$[\alpha]^{25}_D$: +25.7 (c = 0.5, $CHCl_3$)

General procedure I for the synthesis of 28:

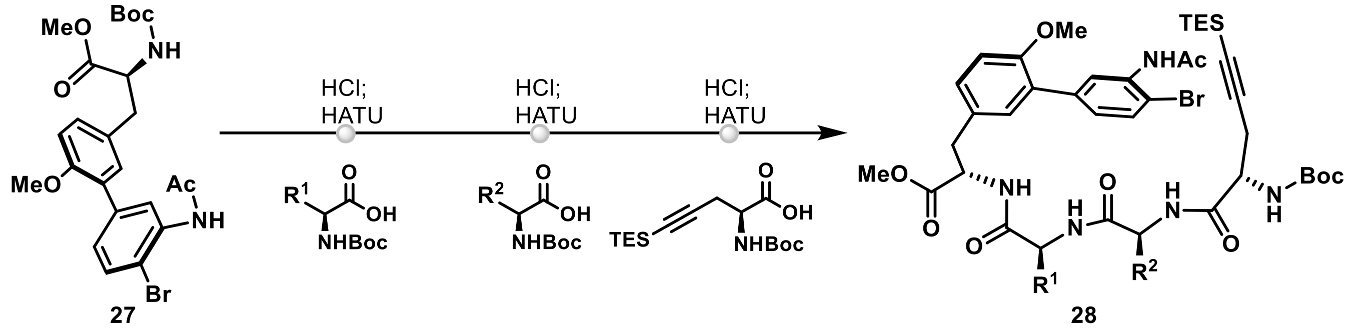

The compound **27** was dissolved in DCM/4M HCl = 3:1 (0.2 M), The reaction mixture was stirred at rt for 1 h, the solvents were removed under reduced pressure, and then quenched by saturated aq. $NaHCO_3$, extracted with DCM for three times, The organic layers were concentrated under reduced pressure to give the crude amine. To the amine was added DCM (0.2 M), Boc-amino acid (1.1 eq.), DIPEA (3.0 eq.) and HATU (1.5 eq.) were added sequentially, the reaction mixture was stirred at rt for 1 h, quenched by *aq.* 0.5 M HCl, the organic layers were concentrated under reduced pressure to give the dipeptide.
The dipeptide was dissolved in DCM/4M HCl = 3:1 (0.2 M), The reaction mixture was stirred at rt for 1 h, the solvents were removed under reduced pressure, and then quenched by saturated aq. $NaHCO_3$, extracted with DCM for three times, The organic layers were concentrated under reduced pressure to give the crude amine. To the amine was added DCM (0.2 M), Boc-amino acid (1.1 eq.), DIPEA (3.0 eq.) and HATU (1.5 eq.) were added sequentially, the reaction mixture was stirred at rt for 1 h, quenched by *aq.* 0.5 M HCl, the organic layers were concentrated under reduced pressure to give the tripeptide.
The tripeptide was dissolved in DCM/4M HCl = 3:1 (0.2 M), The reaction mixture was stirred at rt for 1 h, the solvents were removed under reduced pressure, and then quenched by saturated aq. $NaHCO_3$, extracted with DCM for three times, The organic layers were concentrated under reduced pressure to give the crude amine. To the amine was added DCM (0.1 M), (*S*)-2-((tert-butoxycarbonyl)amino)-5-(triethylsilyl)pent-4-ynoic acid (1.1 eq.), DIPEA (3.0 eq.) and HATU (1.5 eq.) were added sequentially, the reaction mixture was stirred at rt for 1 h, quenched by aq. 0.5 M HCl, the organic layers were concentrated under reduced pressure to give the crude, the crude was precipitated in hexane to give the tetrapeptide **28**.

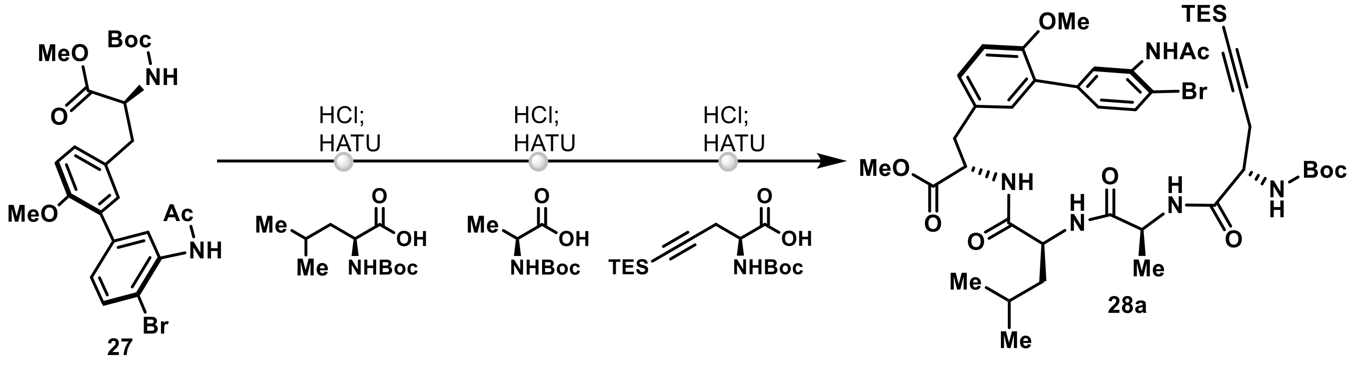

On 0.30 mmol scale, **General Procedure I** was followed. Purification by precipitation in hexane gave the title compound **28a** (198 mg, 72% yield from **27**).

Compound 28a

Physical State: amorphous solid

^{1}H NMR (600 MHz, $CDCl_3$): δ 8.47 – 8.40 (m, 1H), 7.77 (s, 1H), 7.66 (d, *J* = 5.8 Hz, 1H), 7.53 (d, *J* = 8.3 Hz, 1H), 7.24 – 7.18 (m, 1H), 7.06 – 7.02 (m, 3H), 6.91 (d, *J* = 6.4 Hz, 1H), 6.85 (d, *J* = 8.4 Hz, 1H), 5.27 (d, *J* = 5.5 Hz, 1H), 4.85 (q, *J* = 6.5 Hz, 1H), 4.49 – 4.46 (m, 1H), 4.41 – 4.38 (m, 1H), 4.19 – 4.11 (m, 1H), 3.76 (s, 3H), 3.71 (s, 3H), 3.18 (dd, *J* = 14.2, 4.5 Hz, 1H), 3.04 (dd, *J* = 14.2, 6.3 Hz, 1H), 2.80 (dd, *J* = 17.1, 5.8

Hz, 1H), 2.63 (dd, $J = 17.1, 5.9$ Hz, 1H), 2.30 (s, 3H), 1.73 – 1.68 (m, 1H), 1.63 – 1.58 (m, 1H), 1.53 – 1.47 (m, 1H), 1.44 (s, 9H), 1.33 (d, $J = 6.8$ Hz, 3H), 0.95 (t, $J = 7.9$ Hz, 9H), 0.86 – 0.83 (m, 6H), 0.55 (q, $J = 7.9$ Hz, 6H).

^{13}C NMR (151 MHz, $CDCl_3$): δ 172.64, 172.02, 171.64, 170.20, 169.08, 155.70, 155.47, 138.87, 135.43, 131.59, 130.05, 129.13, 128.60, 127.10, 122.35, 111.68, 111.48, 102.29, 86.08, 80.73, 55.66, 53.30, 52.41, 49.24, 40.23, 36.84, 31.67, 28.29, 25.13, 24.91, 23.34, 23.12, 22.73, 21.31, 18.97, 14.20, 7.56, 4.39.

HRMS (ESI-TOF): calculated for $C_{44}H_{64}BrN_5NaO_9Si+$ $[M+Na]^+$: 936.3549, found: 936.3550.

$[\alpha]^{25}_D$: +23.7 ($c = 0.2$, $CHCl_3$)

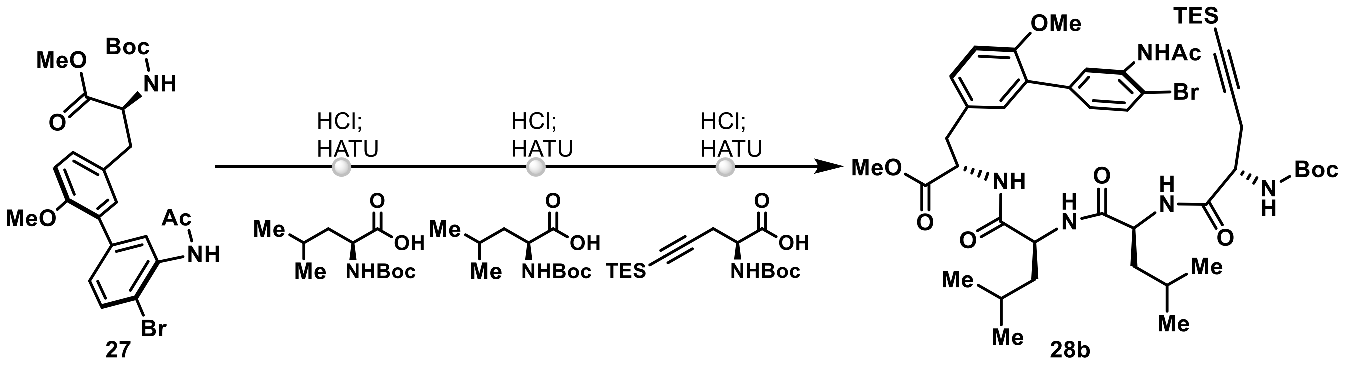

On 0.48 mmol scale, **General Procedure I** was followed. Purification by precipitation in hexane gave the title compound **28b** (268 mg, 58% yield from **27**).

Compound 28b

Physical State: amorphous solid

^{1}H NMR (600 MHz, $CDCl_3$): δ 8.48 (s, 1H), 7.75 (s, 1H), 7.53 (d, $J = 8.2$ Hz, 2H), 7.23 (d, $J = 8.4$ Hz, 1H), 7.08 (d, $J = 7.5$ Hz, 1H), 7.05 (s, 1H), 6.87 (d, $J = 8.3$ Hz, 2H), 6.83 (d, $J = 6.9$ Hz, 1H), 5.24 (s, 1H), 4.86 – 4.84 (m, 1H), 4.50 (s, 1H), 4.43 – 4.40 (m, 1H), 4.12 (q, $J = 6.1$ Hz, 1H), 3.78 (s, 3H), 3.71 (s, 3H), 3.16 (dd, $J = 14.1, 4.2$ Hz, 1H), 3.05 (dd, $J = 14.0, 6.6$ Hz, 1H), 2.80 – 2.76 (m, 1H), 2.65 – 2.60 (m, 1H), 2.30 (s, 3H), 1.60 – 1.51 (m, 4H), 1.44 (s, 9H), 0.96 (t, $J = 7.9$ Hz, 9H), 0.91 – 0.75 (m, 14H), 0.56 (q, $J = 7.9$ Hz, 6H).

^{13}C NMR (151 MHz, $CDCl_3$): δ 172.44, 172.08, 171.53, 170.64, 168.97, 155.89, 155.43, 138.88, 135.46, 131.63, 131.58, 130.04, 129.08, 128.80, 127.02, 122.39, 111.65, 111.51, 102.40, 86.09, 80.74, 55.64, 53.46, 53.22, 52.35, 52.21, 41.76, 40.25, 36.97, 31.65, 28.27, 25.05, 24.88, 24.86, 23.10, 22.95, 22.84, 22.72, 21.70, 21.33, 14.19, 7.54, 4.40.

HRMS (ESI-TOF): calculated for $C_{47}H_{70}BrN_5NaO_9Si^+$ $[M+Na]^+$: 978.4018, found: 978.4025.

$[\alpha]^{25}_D$: –165.8 ($c = 0.2$, $CHCl_3$)

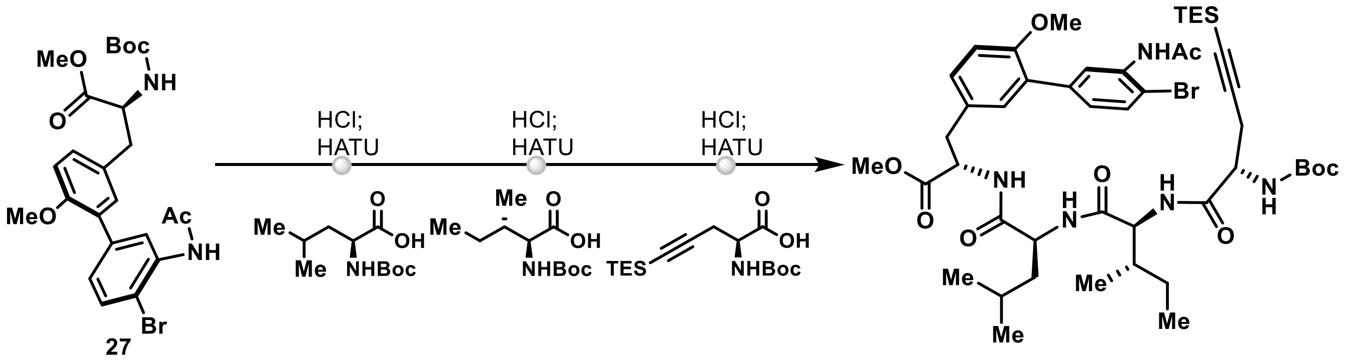

28c

On 0.39 mmol scale, **General Procedure I** was followed. Purification by precipitation in hexane gave the title compound **28c** (186 mg, 50% yield from **27**).

Compound 28c

Physical State: amorphous solid
^{1}H NMR (600 MHz, CDCl$_3$): δ 8.47 (s, 1H), 7.73 (s, 1H), 7.52 (d, *J* = 8.3 Hz, 1H), 7.40 (d, *J* = 6.6 Hz, 1H), 7.22 (d, *J* = 8.2 Hz, 1H), 7.08 (d, *J* = 8.2 Hz, 1H), 7.05 (s, 1H), 6.94 – 6.92 (m, 1H), 6.90 (d, *J* = 7.5 Hz, 1H), 6.86 (d, *J* = 8.4 Hz, 1H), 5.24 (s, 1H), 4.84 – 4.82 (m, 1H), 4.47 – 4.42 (m, 1H), 4.33 – 4.31(m, 1H), 4.08 (q, *J* = 6.2 Hz, 1H), 3.76 (s, 3H), 3.70 (s, 3H), 3.13 (dd, *J* = 14.1, 4.6 Hz, 1H), 3.04 (dd, *J* = 14.1, 6.9 Hz, 1H), 2.76 – 2.72 (m, 1H), 2.61 (dd, *J* = 16.9, 4.7 Hz, 1H), 2.29 (s, 3H), 1.89 – 1.58 (m, 4H), 1.54 – 1.44 (m, 2H), 1.43 (s, 9H), 0.95 (t, *J* = 7.9 Hz, 9H), 0.84 (d, *J* = 6.4 Hz, 9H), 0.77 – 0.72 (m, 3H), 0.55 (q, *J* = 7.9 Hz, 6H).
^{13}C NMR (151 MHz, CDCl$_3$): δ 172.18, 171.60, 171.37, 170.91, 168.97, 155.98, 155.46, 138.86, 135.46, 131.72, 131.60, 130.00, 129.04, 128.93, 126.99, 122.34, 111.67, 111.60, 102.27, 86.17, 80.80, 58.44, 55.68, 53.55, 53.33, 52.35, 40.24, 37.20, 36.97, 28.26, 25.04, 24.86, 24.58, 23.13, 22.66, 21.19, 15.73, 11.40, 7.53, 4.40.
HRMS (ESI-TOF): calculated for $C_{47}H_{70}BrN_5NaO_9Si^+$ [M+Na]$^+$: 978.4018, found: 978.4026.
[α]$^{25}_D$: +12.8 (*c* = 0.2, CHCl$_3$)

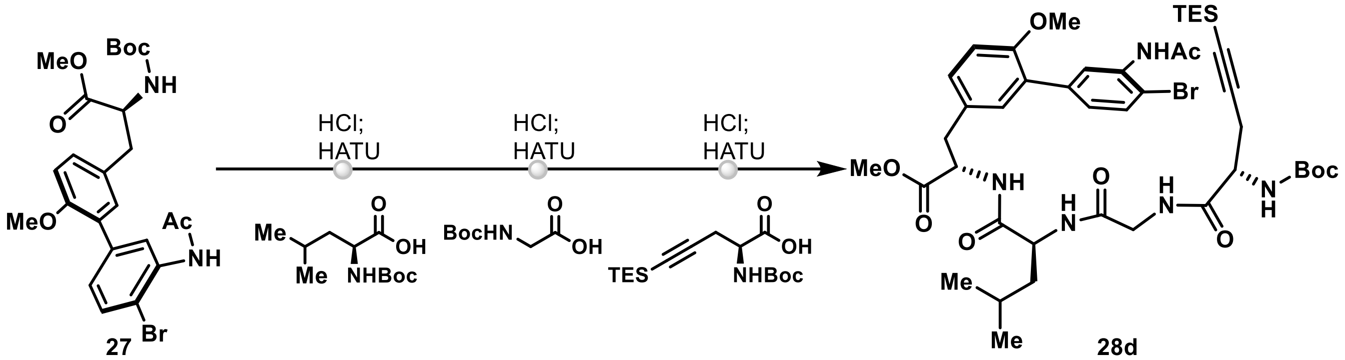

27 28d

On 0.32 mmol scale, **General Procedure I** was followed. Purification by precipitation in hexane gave the title compound **28d** (168 mg, 59% yield from **27**).

Compound 28d
Physical State: amorphous solid
^{1}H NMR (600 MHz, CDCl$_3$): δ 8.36 (s, 1H), 7.76 (s, 1H), 7.58 – 7.46 (m, 2H), 7.21 (d, *J* = 8.3 Hz, 1H), 7.14 – 6.95 (m, 4H), 6.87 (d, *J* = 8.3 Hz, 1H), 5.43 (s, 1H), 4.83 (q, *J* = 7.3 Hz, 1H), 4.41 – 4.37 (m, 1H), 4.18 (q, *J* = 6.8 Hz, 1H), 3.94 (dd, *J* = 16.4, 4.7 Hz, 1H), 3.77 (s, 3H), 3.71 (s, 3H), 3.13 (dd, *J* = 14.1, 4.6 Hz, 1H), 3.02 (dd, *J* = 14.1, 7.3 Hz, 1H), 2.65 (dd, *J* = 17.2, 6.0 Hz, 1H), 2.59 (dd, *J* = 17.1, 7.4 Hz, 1H), 2.26 (s, 3H), 1.68 – 1.55 (m, 2H), 1.50 – 1.46 (m, 1H), 1.42 (s, 9H), 1.28 – 1.24 (m, 1H), 0.94 (t, *J* = 7.9 Hz, 9H), 0.85 – 0.79 (m, 6H), 0.54 (q, *J* = 7.9 Hz, 6H).
^{13}C NMR (151 MHz, CDCl$_3$): δ 172.03, 171.97, 171.06, 169.20, 168.83, 155.76, 155.44, 138.82, 135.33, 131.63, 130.03, 129.01, 128.68, 127.18, 122.96, 112.18, 111.59, 102.87, 85.48, 80.49, 55.69, 53.49, 53.35, 52.47, 52.35, 43.15, 40.16, 36.86, 31.65, 28.34, 24.98, 24.81, 23.39, 23.00, 22.72, 21.52, 14.18, 7.51, 4.41.
HRMS (ESI-TOF): calculated for $C_{43}H_{62}BrN_5NaO_9Si^+$ [M+Na]$^+$: 922.3392, found: 922.3403.
[α]$^{25}_D$: +7.4 (*c* = 0.2, CHCl$_3$)

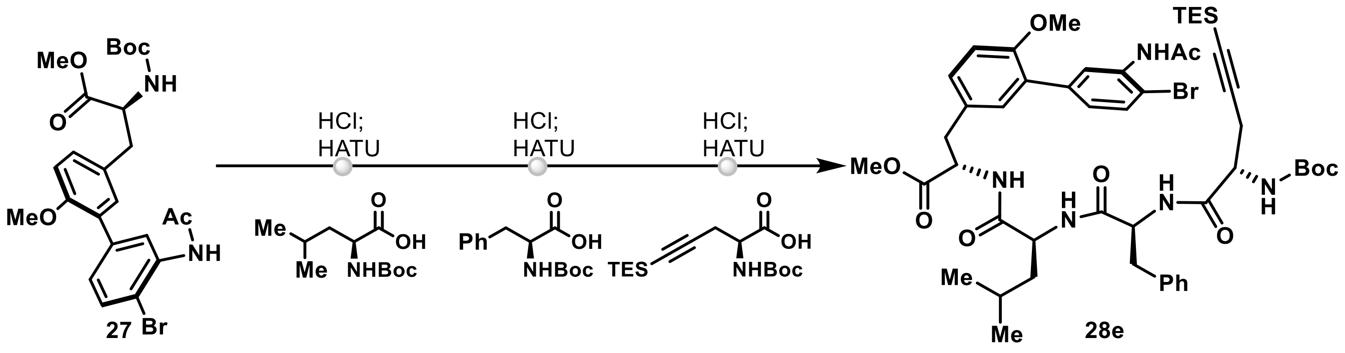

On 0.60 mmol scale, **General Procedure I** was followed. Purification by precipitation in hexane gave the title compound **28e** (266 mg, 45% yield from **27**).

Compound 28e

Physical State: amorphous solid

^{1}H NMR (600 MHz, CDCl$_3$): δ 8.34 (s, 1H), 7.71 (s, 1H), 7.51 (d, *J* = 8.3 Hz, 1H), 7.24 – 7.06 (m, 8H), 7.02 (s, 1H), 6.95 (d, *J* = 7.1 Hz, 1H), 6.89 (d, *J* = 6.8 Hz, 1H), 6.84 (d, *J* = 8.4 Hz, 1H), 5.16 (d, *J* = 4.5 Hz, 1H), 4.79 (q, *J* = 7.8 Hz, 1H), 4.74 – 4.58 (m, 1H), 4.49 – 4.30 (m, 1H), 4.00 (q, *J* = 6.1 Hz, 1H), 3.72 (s, 3H), 3.72 (s, 3H), 3.23 – 3.21 (m, 1H), 3.15 (dd, *J* = 14.1, 4.8 Hz, 1H), 3.00 (dd, *J* = 14.1, 8.0 Hz, 1H), 2.92 (dd, *J* = 13.9, 6.1 Hz, 1H), 2.65 – 2.55 (m, 2H), 2.19 (s, 3H), 1.61-1.56 (m, 1H), 1.44 – 1.38 (m, 2H), 1.36 (s, 9H), 0.96 (t, *J* = 7.9 Hz, 9H), 0.84 (d, *J* = 6.4 Hz, 3H), 0.76 (d, *J* = 6.5 Hz, 3H), 0.56 (q, *J* = 7.9 Hz, 6H).

^{13}C NMR (151 MHz, CDCl$_3$): δ 172.10, 171.83, 171.04, 170.91, 168.80, 155.98, 155.35, 138.83, 136.26, 135.33, 131.69, 131.59, 129.96, 129.38, 129.11, 128.95, 128.78, 127.25, 126.98, 122.71, 111.85, 111.51, 102.12, 86.18, 80.82, 55.62, 54.58, 53.59, 52.42, 40.04, 37.68, 36.78, 31.66, 28.24, 24.95, 24.52, 23.11, 22.73, 21.20, 14.21, 7.58, 4.42.

HRMS (ESI-TOF): calculated for $C_{50}H_{68}BrN_5NaO_9Si^+$ [M+Na]$^+$: 1012.3862, found: 1012.3868.

[α]$^{25}_{D}$: +8.6 (*c* = 0.2, CHCl$_3$)

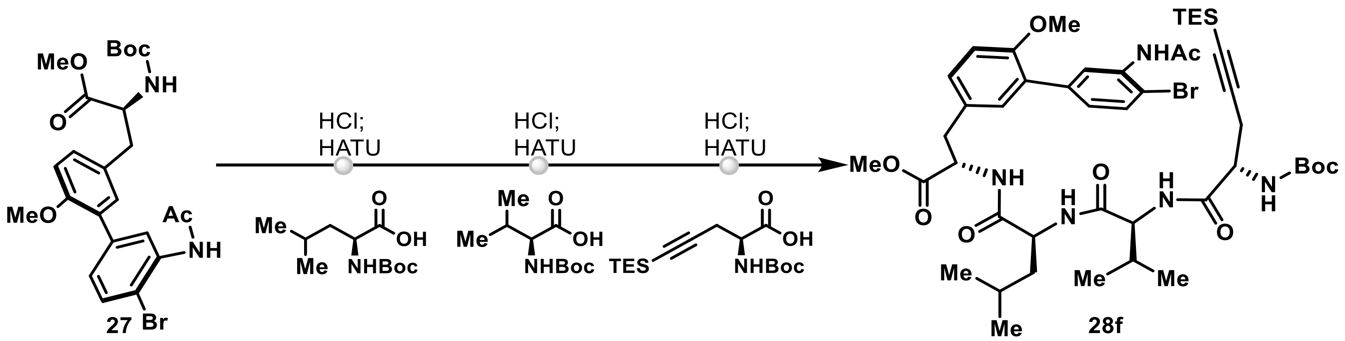

On 0.40 mmol scale, **General Procedure I** was followed. Purification by precipitation in hexane gave the title compound **28f** (226 mg, 60% yield from **27**).

Compound 28f

Physical State: amorphous solid

^{1}H NMR (600 MHz, CDCl$_3$): δ 8.46 (s, 1H), 7.76 (s, 1H), 7.52 (d, *J* = 8.3 Hz, 1H), 7.50 (d, *J* = 6.6 Hz, 1H), 7.23 (d, *J* = 8.2 Hz, 1H), 7.12 – 7.02 (m, 2H), 6.96 (d, *J* = 7.6 Hz, 2H), 6.87 (d, *J* = 8.4 Hz, 1H), 5.27 (d, *J* = 4.0 Hz, 1H), 4.84 (q, *J* = 7.4 Hz, 1H), 4.47 – 4.42 (m, 1H), 4.35 – 4.32 (m, 1H), 4.11 (q, *J* = 6.2 Hz, 1H), 3.77 (s, 3H), 3.71 (s, 3H), 3.15 (dd, *J* = 14.1, 4.6 Hz, 1H), 3.04 (dd, *J* = 14.1, 6.9 Hz, 1H), 2.77 (dd, *J* = 17.2, 5.8 Hz, 1H), 2.62 (dd, *J* = 17.1, 4.6 Hz, 1H), 2.30 (s, 3H), 1.72 – 1.66 (m, 1H), 1.64 – 1.59 (m, 1H), 1.53 – 1.48 (m, 1H), 1.44 (s, 9H), 0.95 (t, *J* = 7.9 Hz, 9H), 0.88 – 0.80 (m, 13H), 0.55 (q, *J* = 7.9 Hz, 6H).

^{13}C NMR (151 MHz, CDCl$_3$): δ 172.21, 171.61, 171.51, 170.98, 169.02, 155.95, 155.45, 138.83, 135.47, 131.71, 131.59, 130.00, 129.02, 128.88, 127.01, 122.30, 111.64, 111.57, 102.30, 86.13, 80.78, 58.74, 55.66, 53.52, 53.32, 52.38, 52.34, 40.23, 36.92, 31.00, 28.27, 25.07, 24.86, 23.14, 22.72, 21.21, 19.58, 17.34, 7.54,

4.39.
HRMS (ESI-TOF): calculated for $C_{46}H_{68}BrN_5NaO_9Si^+$ $[M+Na]^+$: 964.3862, found: 964.3867.
$[\alpha]^{25}_D$: +10.3 (c = 0.5, $CHCl_3$)

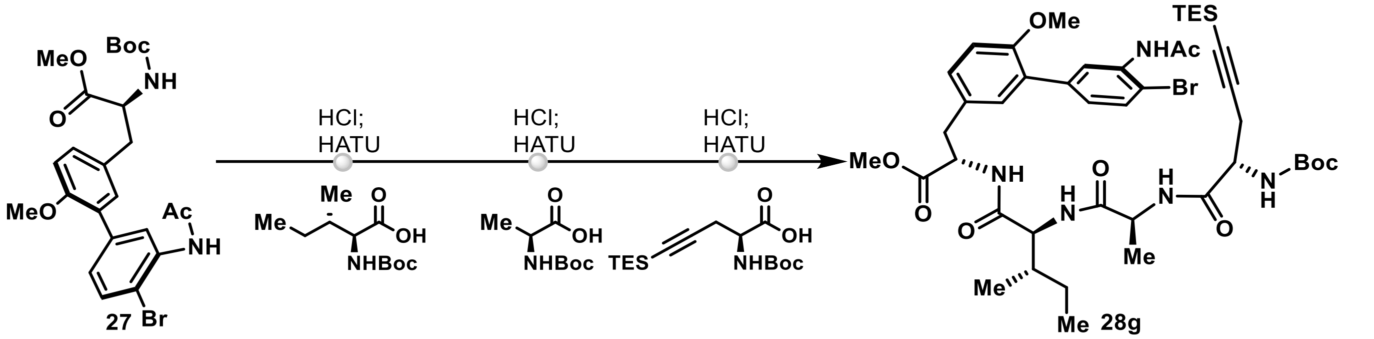

On 0.54 mmol scale, **General Procedure I** was followed. Purification by precipitation in hexane gave the title compound **28g** (268 mg, 54% yield from **27**).

Compound 28g

Physical State: amorphous solid

^{1}H NMR (600 MHz, $CDCl_3$): δ 8.43 (s, 1H), 7.72 (s, 1H), 7.53 (d, J = 8.3 Hz, 1H), 7.28 (d, J = 7.9 Hz, 1H), 7.19 (d, J = 7.1 Hz, 1H), 7.10 – 7.01 (m, 3H), 6.86 (d, J = 9.0 Hz, 1H), 6.75 – 6.71 (m, 1H), 5.31 (s, 1H), 4.92 – 4.89 (m, 1H), 4.55 – 4.53 (m, 1H), 4.33 (dd, J = 7.8, 5.9 Hz, 1H), 4.24 – 4.20 (m, 1H), 3.76 (s, 3H), 3.70 (s, 3H), 3.13 (dd, J = 14.2, 4.9 Hz, 1H), 3.04 (dd, J = 14.2, 6.6 Hz, 1H), 2.82 (dd, J = 17.1, 5.8 Hz, 1H), 2.65 (dd, J = 17.1, 6.1 Hz, 1H), 2.29 (s, 3H), 1.44 (s, 9H), 1.32 (d, J = 6.9 Hz, 3H), 1.15 – 1.09 (m, 1H), 0.97 – 0.94 (m, 11H), 0.87 (d, J = 6.8 Hz, 3H), 0.78 (t, J = 7.4 Hz, 3H), 0.59 – 0.54 (m, 6H).

^{13}C NMR (151 MHz, $CDCl_3$): δ 172.45, 171.70, 170.78, 170.20, 168.83, 155.53, 138.76, 135.43, 131.60, 131.42, 130.02, 129.29, 128.34, 126.94, 122.72, 111.91, 111.52, 102.42, 86.01, 80.62, 58.35, 55.67, 53.19, 53.04, 52.43, 49.17, 37.21, 36.68, 28.30, 25.08, 24.71, 23.59, 18.87, 15.51, 11.60, 7.56, 4.40.

HRMS (ESI-TOF): calculated for $C_{44}H_{64}BrN_5NaO_9Si+$ $[M+Na]^+$: 936.3549, found: 936.3558.
$[\alpha]^{25}_D$: +48.7 (c = 0.2, $CHCl_3$)

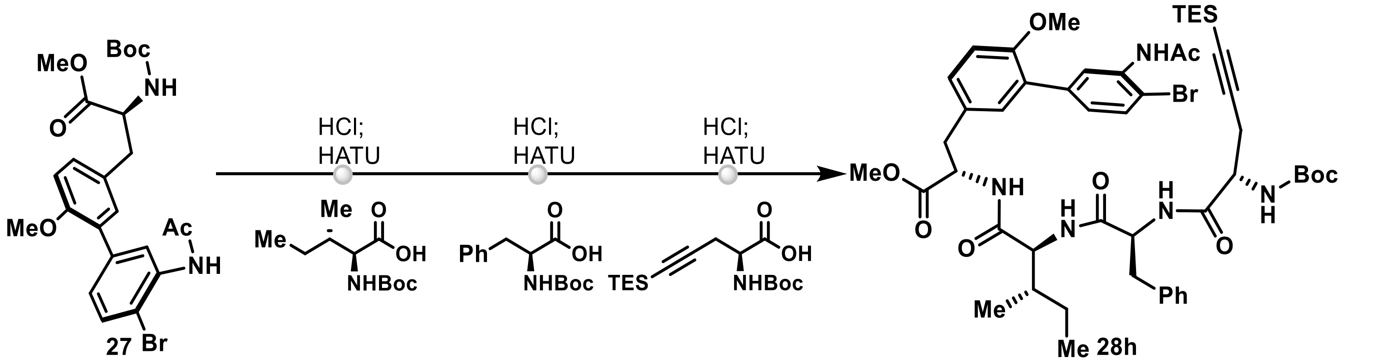

On 0.47 mmol scale, **General Procedure I** was followed. Purification by precipitation in hexane gave the title compound **28h** (246 mg, 53% yield from **27**).

Compound 28h

Physical State: amorphous solid

^{1}H NMR (600 MHz, $CDCl_3$): δ 8.39 (s, 1H), 7.70 (s, 1H), 7.52 (d, J = 8.3 Hz, 1H), 7.21 – 7.15 (m, 4H), 7.12 (d, J = 7.2 Hz, 2H), 7.09 (d, J = 8.3 Hz, 1H), 7.03 (s, 1H), 6.98 (d, J = 7.0 Hz, 1H), 6.88 (d, J = 7.1 Hz, 1H), 6.85 (d, J = 8.4 Hz, 1H), 6.66 – 6.63 (m, 1H), 5.23 (s, 1H), 4.85 (q, J = 7.7 Hz, 1H), 4.76 – 4.63 (m, 1H), 4.40 – 4.24 (m, 1H), 4.16 – 4.11 (m, 1H), 3.72 (s, 3H), 3.71 (s, 3H), 3.12 (dd, J = 14.1, 5.1 Hz, 2H), 3.00 - 2.92 (m, 2H), 2.68 – 2.62 (m, 2H), 2.26 (s, 3H), 1.38 (s, 9H), 0.97 (t, J = 7.9 Hz, 10H), 0.87 (t, J = 6.9 Hz, 1H), 0.76 (d, J = 6.5 Hz, 3H), 0.71 (t, J = 7.4 Hz, 3H), 0.57 (q, J = 7.9 Hz, 6H).

^{13}C NMR (151 MHz, CDCl$_3$): δ 171.83, 170.96, 170.62, 168.64, 155.68, 155.49, 138.77, 136.34, 135.38, 131.62, 131.54, 129.94, 129.43, 129.37, 129.22, 128.76, 128.54, 127.15, 126.88, 123.05, 112.09, 111.55, 102.34, 86.05, 80.66, 58.49, 55.64, 54.49, 53.46, 53.27, 52.44, 38.06, 37.02, 36.52, 31.66, 28.39, 28.32, 28.26, 24.98, 24.42, 23.24, 22.72, 15.36, 14.19, 11.47, 7.58, 4.43.
HRMS (ESI-TOF): calculated for $C_{50}H_{68}BrN_5NaO_9Si^+$ [M+Na]$^+$: 1012.3862, found: 1012.3867.
[α]^{25}$_D$: +18.2 (c = 0.2, CHCl$_3$)

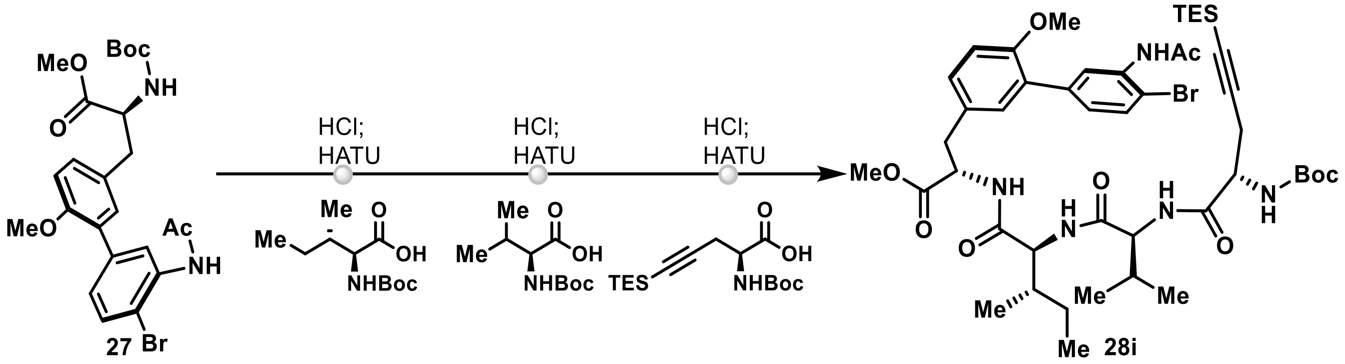

On 0.50 mmol scale, **General Procedure I** was followed. Purification by precipitation in hexane gave the title compound **28i** (235 mg, 50% yield from **27**).

Compound 28i

Physical State: amorphous solid

^{1}H NMR (600 MHz, CDCl$_3$): δ 8.47 (s, 1H), 7.72 (s, 1H), 7.53 (d, J = 8.3 Hz, 1H), 7.18 (d, J = 8.2 Hz, 1H), 7.14 – 7.05 (m, 3H), 7.01 (d, J = 7.7 Hz, 1H), 6.87 (d, J = 8.4 Hz, 1H), 6.74 (d, J = 8.0 Hz, 1H), 5.33 (s, 1H), 4.90 (q, J = 6.8 Hz, 1H), 4.45 – 4.34 (m, 2H), 4.21 (d, J = 5.0 Hz, 1H), 3.77 (s, 3H), 3.70 (s, 3H), 3.10 (dd, J = 14.1, 5.1 Hz, 1H), 3.04 (dd, J = 14.1, 6.8 Hz, 1H), 2.81 (dd, J = 17.3, 5.8 Hz, 1H), 2.68 (dd, J = 17.2, 5.7 Hz, 1H), 2.29 (s, 3H), 1.44 (s, 9H), 1.30 – 1.24 (m, 1H), 1.16 – 1.09 (m, 1H), 0.96 (t, J = 8.0 Hz, 9H), 0.89 – 0.81 (m, 11H), 0.78 (t, J = 7.4 Hz, 3H), 0.59 – 0.54 (m, 6H).

^{13}C NMR (151 MHz, CDCl$_3$): δ 171.68, 171.43, 170.93, 170.78, 168.80, 155.53, 138.74, 135.47, 131.61, 131.53, 129.95, 129.25, 128.41, 126.85, 122.69, 111.90, 111.58, 102.43, 86.04, 80.66, 58.54, 58.33, 55.67, 53.20, 52.42, 37.20, 36.66, 31.29, 28.37, 28.28, 25.07, 24.60, 23.10, 19.50, 17.57, 15.48, 14.20, 11.58, 7.54, 4.39.

HRMS (ESI-TOF): calculated for $C_{46}H_{68}BrN_5NaO_9Si^+$ [M+Na]$^+$: 964.3862, found: 964.3867.

[α]^{25}$_D$: +18.8 (c = 0.2, CHCl$_3$)

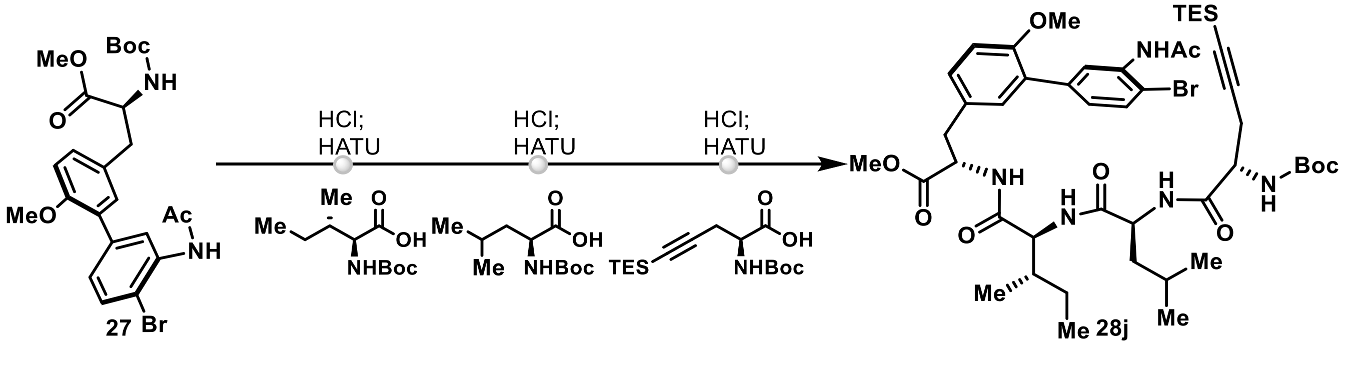

On 0.49 mmol scale, **General Procedure I** was followed. Purification by precipitation in hexane gave the title compound **28j** (227 mg, 48% yield from **27**).

Compound 28j

Physical State: amorphous solid

^{1}H NMR (600 MHz, CDCl$_3$): δ 8.46 (s, 1H), 7.72 (s, 1H), 7.53 (d, J = 8.3 Hz, 1H), 7.18 (d, J = 8.9 Hz, 2H),

7.10 – 7.03 (m, 2H), 6.87 (d, *J* = 8.3 Hz, 2H), 6.68 – 6.66 (m, 1H), 5.30 – 5.28 (m, 1H), 4.91 – 4.87 (m, 1H), 4.52 (s, 1H), 4.36 – 4.27 (m, 1H), 4.24 – 4.15 (m, 1H), 3.77 (s, 3H), 3.70 (s, 3H), 3.10 – 3.03 (m, 2H), 2.81 (dd, *J* = 17.2, 6.2 Hz, 1H), 2.65 (dd, *J* = 17.2, 6.2 Hz, 1H), 2.28 (s, 3H), 1.44 (s, 9H), 0.95 (q, *J* = 7.9 Hz, 12H), 0.88 – 0.79 (m, 15H), 0.57 (t, *J* = 7.9 Hz, 6H).
^{13}C NMR (151 MHz, $CDCl_3$): δ 172.24, 171.60, 170.77, 170.57, 168.72, 155.54, 138.77, 135.46, 131.62, 131.48, 130.01, 129.30, 128.37, 126.86, 122.73, 111.91, 111.54, 102.55, 86.08, 80.66, 58.29, 55.68, 53.17, 52.40, 41.57, 37.24, 36.73, 28.28, 25.05, 24.83, 24.68, 22.99, 21.83, 15.48, 11.59, 7.56, 4.40.
HRMS (ESI-TOF): calculated for $C_{47}H_{70}BrN_5NaO_9Si^+$ [M+Na]$^+$: 978.4018, found: 978.4022.
$[\alpha]^{25}_D$: +3.6 (*c* = 0.2, $CHCl_3$)

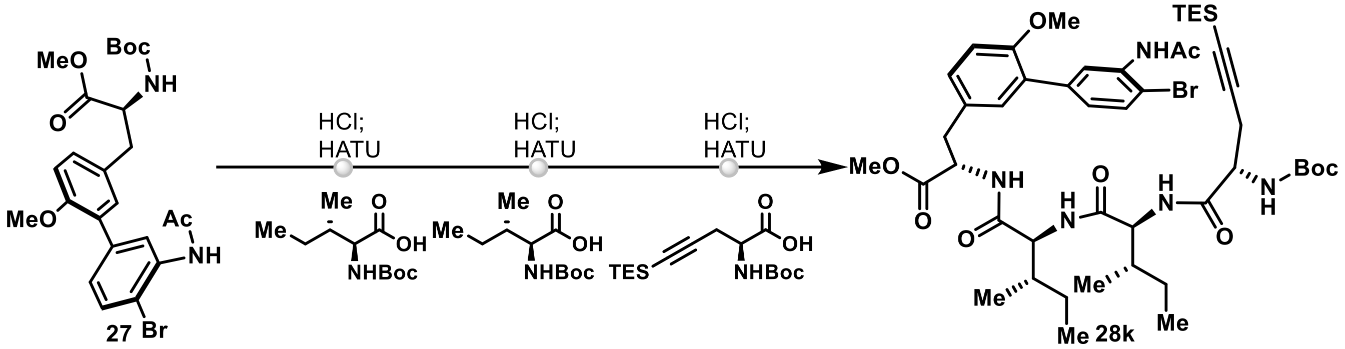

On 0.47 mmol scale, **General Procedure I** was followed. Purification by precipitation in hexane gave the title compound **28k** (252 mg, 56% yield from **27**).

Compound 28k

Physical State: amorphous solid

^{1}H NMR (600 MHz, $CDCl_3$): δ 8.47 (s, 1H), 7.72 (s, 1H), 7.53 (d, *J* = 8.3 Hz, 1H), 7.18 (d, *J* = 8.1 Hz, 1H), 7.13 – 7.02 (m, 3H), 6.99 (d, *J* = 7.6 Hz, 1H), 6.87 (d, *J* = 8.4 Hz, 1H), 6.76 – 6.73 (m, 1H), 5.32 (s, 1H), 4.90 (q, *J* = 6.8 Hz, 1H), 4.49 – 4.32 (m, 2H), 4.19 (d, *J* = 5.5 Hz, 1H), 3.77 (s, 3H), 3.70 (s, 3H), 3.11 – 3.02 (m, 2H), 2.80 (dd, *J* = 17.2, 6.2 Hz, 1H), 2.67 (dd, *J* = 17.3, 5.7 Hz, 1H), 2.29 (s, 3H), 1.49 – 1.46 (m, 1H), 1.44 (s, 9H), 1.34 – 1.21 (m, 2H), 1.16 – 1.04 (m, 2H), 0.96 (t, *J* = 7.9 Hz, 9H), 0.88 – 0.80 (m, 7H), 0.78 (t, *J* = 7.3 Hz, 6H), 0.56 (q, *J* = 8.0 Hz, 6H).
^{13}C NMR (151 MHz, $CDCl_3$): δ 171.67, 171.34, 170.91, 170.75, 168.75, 155.54, 138.76, 135.46, 131.62, 131.56, 129.94, 129.27, 128.47, 126.84, 122.75, 111.95, 111.60, 102.43, 86.05, 80.66, 58.33, 58.19, 55.69, 53.26, 52.40, 37.40, 37.20, 36.64, 31.66, 28.27, 25.03, 24.63, 24.57, 23.05, 15.63, 15.46, 14.19, 11.96, 11.57, 11.36, 7.53, 4.40.
HRMS (ESI-TOF): calculated for $C_{47}H_{70}BrN_5NaO_9Si^+$ [M+Na]$^+$: 978.4018, found: 978.4013.
$[\alpha]^{25}_D$: –65.4 (*c* = 0.2, $CHCl_3$)

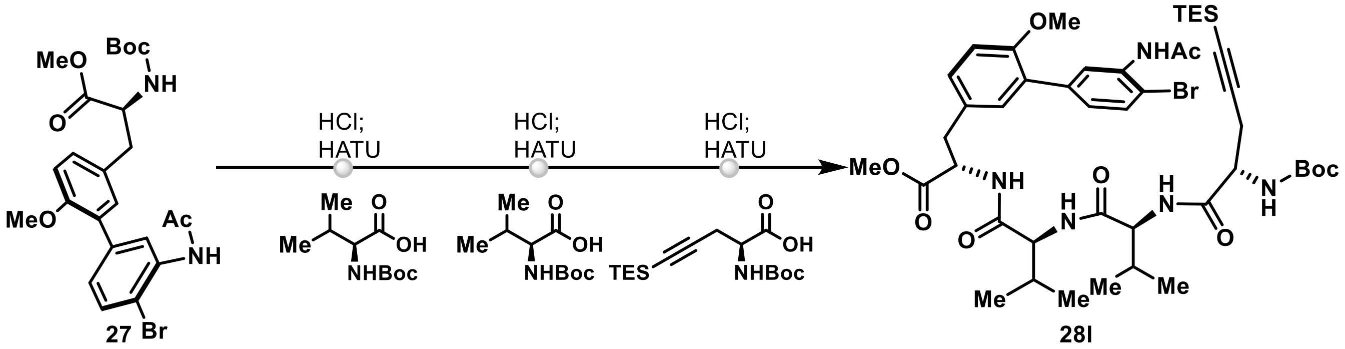

On 0.42 mmol scale, **General Procedure I** was followed. Purification by precipitation in hexane gave the title compound **28l** (237 mg, 61% yield from **27**).

Compound 28l

Physical State: amorphous solid

^{1}H NMR (600 MHz, CDCl$_3$): δ 8.46 (s, 1H), 7.73 (s, 1H), 7.52 (d, *J* = 8.3 Hz, 1H), 7.19 – 7.16 (m, 2H), 7.08 – 7.05 (m, 3H), 6.86 – 6.82 (m, 2H), 5.37 (s, 1H), 4.89 (q, *J* = 6.2 Hz, 1H), 4.43 - 4.40 (m, 1H), 4.38 – 4.34 (m, 1H), 4.29 – 4.19 (m, 1H), 3.77 (s, 3H), 3.69 (s, 3H), 3.10 – 3.03 (m, 2H), 2.80 (dd, *J* = 16.8, 5.8 Hz, 1H), 2.67 (dd, *J* = 17.2, 5.6 Hz, 1H), 2.29 (s, 3H), 1.43 (s, 9H), 0.95 (t, *J* = 8.0 Hz, 9H), 0.90 (d, *J* = 6.8 Hz, 3H), 0.87 – 0.81 (m, 11H), 0.55 (q, *J* = 7.9 Hz, 6H).

^{13}C NMR (151 MHz, CDCl$_3$): δ 171.66, 171.58, 170.99, 170.73, 168.81, 155.54, 138.72, 135.49, 131.60, 131.48, 129.96, 129.29, 128.41, 126.87, 122.67, 111.90, 111.61, 102.53, 85.91, 80.56, 58.80, 58.46, 55.69, 53.24, 52.39, 37.24, 31.40, 30.31, 28.37, 28.30, 25.07, 23.21, 19.49, 19.19, 17.67, 7.54, 4.47, 4.40.

HRMS (ESI-TOF): calculated for $C_{45}H_{66}BrN_5NaO_9Si^+$ [M+Na]$^+$: 950.3705, found: 950.3715.

[α]$^{25}_{D}$: –26.1 (*c* = 0.2, CHCl$_3$)

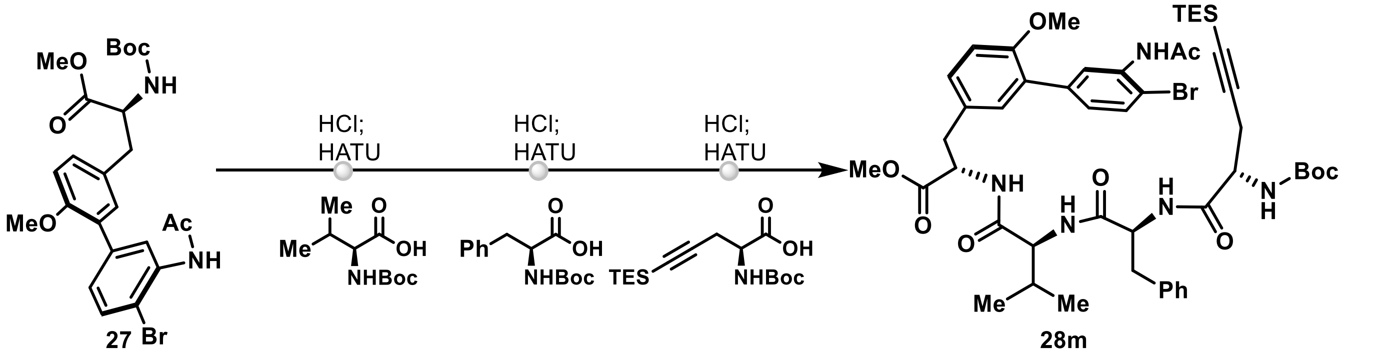

On 0.60 mmol scale, **General Procedure I** was followed. Purification by precipitation in hexane gave the title compound **28m** (248 mg, 42% yield from **27**).

Compound 28m

Physical State: amorphous solid

^{1}H NMR (600 MHz, CDCl$_3$): δ 8.38 (s, 1H), 7.70 (s, 1H), 7.52 (d, *J* = 8.3 Hz, 1H), 7.20 – 7.15 (m, 4H), 7.12 – 7.07 (m, 3H), 7.03 (s, 1H), 7.00 (d, *J* = 6.4 Hz, 1H), 6.94 (d, *J* = 5.7 Hz, 1H), 6.85 (d, *J* = 8.4 Hz, 1H), 6.67 (s, 1H), 5.23 (s, 1H), 4.85 (q, *J* = 7.4 Hz, 1H), 4.72 (d, *J* = 5.3 Hz, 1H), 4.31 – 4.24 (m, 1H), 4.19 – 4.10 (m, 1H), 3.72 (s, 3H), 3.71 (s, 3H), 3.11 (dd, *J* = 14.1, 5.1 Hz, 2H), 3.03 - 2.93 (m, 2H), 2.66 – 2.63 (m, 2H), 2.26 (s, 3H), 1.39 (s, 9H), 0.97 (t, *J* = 7.9 Hz, 9H), 0.86 – 0.84 (m, 1H), 0.82 (d, *J* = 6.9 Hz, 3H), 0.72 (d, *J* = 6.7 Hz, 3H), 0.57 (q, *J* = 7.9 Hz, 6H).

^{13}C NMR (151 MHz, CDCl$_3$): δ 171.81, 171.12, 170.68, 170.56, 168.69, 155.65, 155.51, 138.75, 136.37, 135.39, 131.62, 131.51, 129.95, 129.36, 129.26, 128.74, 128.53, 127.13, 126.90, 123.04, 112.10, 111.60, 102.39, 86.00, 80.61, 59.00, 55.67, 54.49, 53.30, 52.46, 38.16, 37.05, 30.19, 28.38, 28.33, 28.28, 25.00, 23.30, 19.13, 17.60, 7.58, 4.42.

HRMS (ESI-TOF): calculated for $C_{49}H_{66}BrN_5NaO_9Si^+$ [M+Na]$^+$: 998.3705, found: 998.3730.

[α]$^{25}_{D}$: +23.2 (*c* = 0.2, CHCl$_3$)

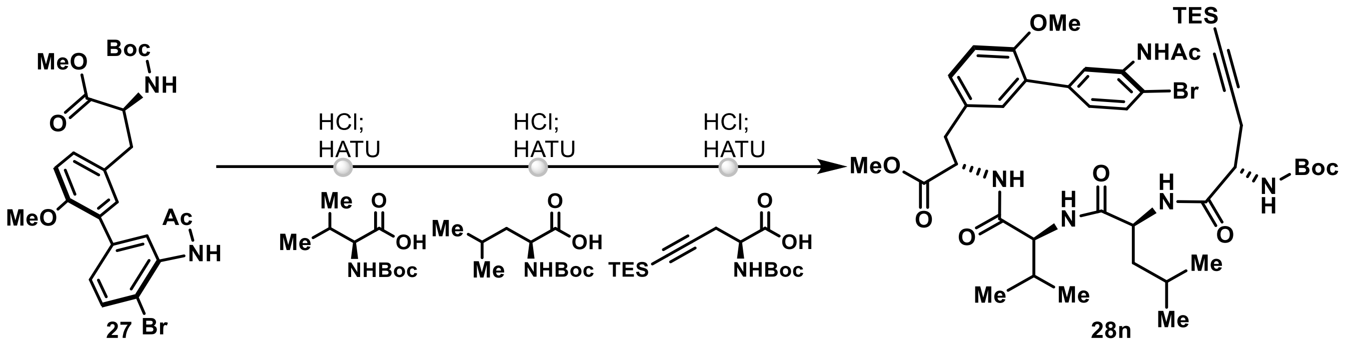

On 0.39 mmol scale, **General Procedure I** was followed. Purification by precipitation in hexane gave the title compound **28n** (170 mg, 46% yield from **27**).

Compound 28n

Physical State: amorphous solid

^{1}H NMR (600 MHz, CDCl$_3$): δ 8.47 (s, 1H), 7.73 (s, 1H), 7.53 (d, *J* = 8.3 Hz, 1H), 7.25 – 7.23 (m, 1H), 7.18 (d, *J* = 8.2 Hz, 1H), 7.08 – 7.04 (m, 2H), 6.89 – 6.86 (m, 2H), 6.75 – 6.68 (m, 1H), 5.30 (s, 1H), 4.91 (t, *J* = 5.8 Hz, 1H), 4.57 – 4.54 (m, 1H), 4.36 – 4.27 (m, 1H), 4.20 (s, 1H), 3.77 (s, 3H), 3.69 (s, 3H), 3.08 (d, *J* = 4.9 Hz, 2H), 2.87 – 2.76 (m, 1H), 2.66 (dd, *J* = 17.1, 6.1 Hz, 1H), 2.29 (s, 3H), 1.59 – 1.53 (m, 2H), 1.43 (s, 9H), 0.96 (t, *J* = 7.9 Hz, 9H), 0.91 (d, *J* = 6.7 Hz, 3H), 0.87 – 0.78 (m, 11H), 0.56 (q, *J* = 7.9 Hz, 6H).

^{13}C NMR (151 MHz, CDCl$_3$): δ 172.43, 171.55, 170.83, 170.52, 168.78, 155.55, 138.76, 135.48, 131.61, 131.44, 130.03, 129.33, 128.33, 126.90, 122.66, 111.89, 111.57, 102.59, 86.03, 80.63, 58.81, 55.69, 53.17, 52.39, 52.04, 41.67, 37.28, 30.31, 28.29, 25.08, 24.82, 22.99, 21.79, 19.21, 17.65, 7.56, 4.40.

HRMS (ESI-TOF): calculated for $C_{46}H_{68}BrN_5NaO_9Si^+$ $[M+Na]^+$: 964.3862, found: 964.3877.

$[\alpha]^{25}_D$: +20.6 (*c* = 0.2, CHCl$_3$)

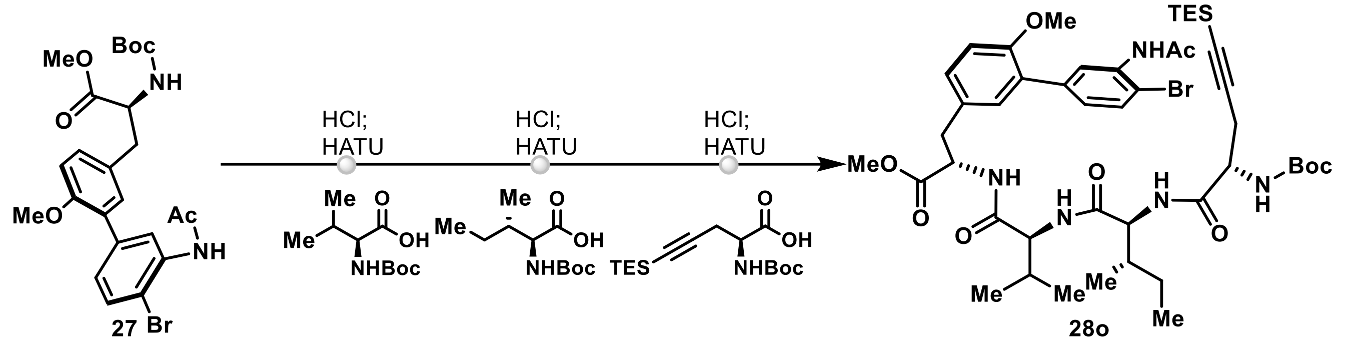

On 0.46 mmol scale, **General Procedure I** was followed. Purification by precipitation in hexane gave the title compound **28o** (238 mg, 55% yield from **27**).

Compound 28o

Physical State: amorphous solid

^{1}H NMR (600 MHz, CDCl$_3$): δ 8.47 (s, 1H), 7.73 (s, 1H), 7.52 (d, *J* = 8.3 Hz, 1H), 7.15 (dd, *J* = 22.7, 7.4 Hz, 2H), 7.09 – 7.02 (m, 3H), 6.86 (d, *J* = 8.4 Hz, 2H), 5.36 (s, 1H), 4.90 (q, *J* = 6.5 Hz, 1H), 4.43 – 4.38 (m, 1H), 4.38 – 4.33 (m, 1H), 4.29 – 4.16 (m, 1H), 3.76 (s, 3H), 3.69 (s, 3H), 3.10 – 3.02 (m, 2H), 2.79 (dd, *J* = 16.9, 6.2 Hz, 1H), 2.67 (dd, *J* = 17.2, 5.8 Hz, 2H), 2.29 (s, 3H), 1.43 (s, 9H), 0.97 – 0.93 (m, 12H), 0.90 (d, *J* = 6.8 Hz, 3H), 0.86 – 0.79 (m, 10H), 0.55 (q, *J* = 7.9 Hz, 6H).

^{13}C NMR (151 MHz, CDCl$_3$): δ 171.67, 171.52, 170.99, 170.70, 168.78, 155.70, 155.55, 138.74, 135.48, 131.62, 131.52, 129.94, 129.30, 128.46, 126.85, 122.70, 111.95, 111.62, 102.53, 85.89, 80.54, 58.82, 58.11, 55.70, 53.29, 52.39, 37.50, 37.25, 30.30, 28.35, 28.29, 25.06, 24.63, 23.17, 19.17, 17.67, 15.61, 11.34, 7.54, 4.41.

HRMS (ESI-TOF): calculated for $C_{46}H_{68}BrN_5NaO_9Si^+$ $[M+Na]^+$: 964.3862, found: 964.3873.

[α]^{25}D: +21.0 (c = 0.2, $CHCl_3$)

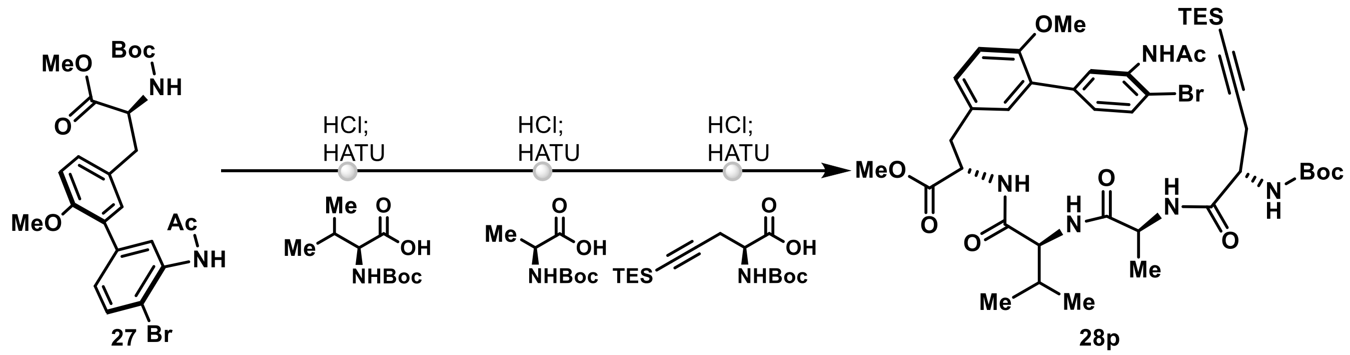

On 0.40 mmol scale, **General Procedure I** was followed. Purification by precipitation in hexane gave the title compound **28p** (236 mg, 66% yield from **27**).

Compound 28p

Physical State: amorphous solid

^{1}H NMR (600 MHz, $CDCl_3$): δ 8.41 (s, 1H), 7.74 (s, 1H), 7.52 (d, J = 7.9 Hz, 1H), 7.41 (s, 1H), 7.22 – 7.00 (m, 4H), 6.86 – 6.83 (m, 2H), 5.37 (s, 1H), 4.95 – 4.79 (m, 1H), 4.65 – 4.51 (m, 1H), 4.35 – 4.32 (m, 1H), 4.24 (s, 1H), 3.75 (s, 3H), 3.68 (s, 3H), 3.12 – 3.08 (m, 1H), 3.06 – 3.02 (m, 1H), 2.79 (dd, J = 16.8, 5.2 Hz, 1H), 2.65 (dd, J = 17.0, 5.6 Hz, 1H), 2.28 (s, 3H), 1.43 (s, 9H), 1.35 (s, 1H), 1.30 (d, J = 6.0 Hz, 3H), 0.94 (t, J = 7.7 Hz, 9H), 0.90 (d, J = 6.1 Hz, 3H), 0.85 (d, J = 6.3 Hz, 3H), 0.54 (q, J = 7.5 Hz, 6H).

^{13}C NMR (151 MHz, $CDCl_3$): δ 172.62, 171.72, 170.91, 170.20, 168.86, 155.54, 138.72, 135.44, 131.61, 131.34, 130.04, 129.31, 128.32, 126.98, 122.80, 112.02, 111.54, 102.50, 85.87, 80.52, 58.82, 55.68, 53.12, 52.42, 49.09, 37.23, 30.34, 28.31, 25.05, 23.67, 19.23, 18.95, 17.74, 7.56, 4.40.

HRMS (ESI-TOF): calculated for $C_{43}H_{62}BrN_5NaO_9Si^+$ [M+Na]$^+$: 922.3392, found: 922.3420.

[α]^{25}D: +18.6 (c = 0.2, $CHCl_3$)

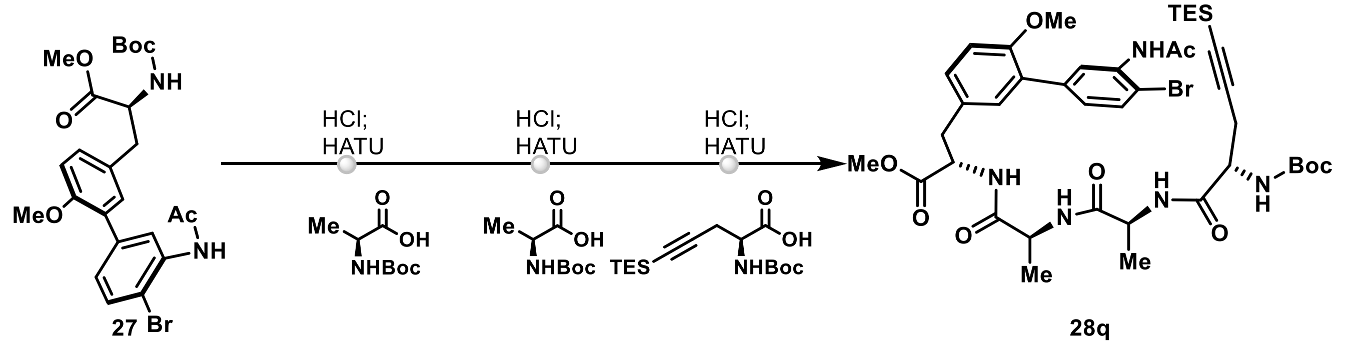

On 0.42 mmol scale, **General Procedure I** was followed. Purification by precipitation in hexane gave the title compound **28q** (225 mg, 62% yield from **27**).

Compound 28q

Physical State: amorphous solid

^{1}H NMR (600 MHz, $CDCl_3$): δ 8.40 (s, 1H), 7.76 (s, 1H), 7.73 (d, J = 5.7 Hz, 1H), 7.52 (d, J = 8.3 Hz, 1H), 7.19 (d, J = 8.1 Hz, 1H), 7.06 – 7.01 (m, 3H), 6.96 (d, J = 6.2 Hz, 1H), 6.85 (d, J = 8.3 Hz, 1H), 5.32 (d, J = 5.4 Hz, 1H), 4.83 (q, J = 6.4 Hz, 1H), 4.48 – 4.41 (m, 2H), 4.18 – 4.14 (m, 1H), 3.75 (s, 3H), 3.70 (s, 3H), 3.17 (dd, J = 14.1, 4.3 Hz, 1H), 3.04 (dd, J = 14.1, 6.3 Hz, 1H), 2.77 (dd, J = 17.1, 5.7 Hz, 1H), 2.64 (dd, J = 17.1, 5.9 Hz, 1H), 2.28 (s, 3H), 1.43 (s, 9H), 1.34 (d, J = 7.1 Hz, 3H), 1.30 (d, J = 6.5 Hz, 3H), 0.94 (t, J = 7.9 Hz, 9H), 0.55 (q, J = 7.8 Hz, 6H).

^{13}C NMR (151 MHz, $CDCl_3$): δ 172.32, 172.10, 171.69, 170.26, 168.98, 155.71, 155.47, 138.80, 135.42, 131.62, 131.56, 130.01, 129.15, 128.54, 127.06, 122.64, 111.93, 111.48, 102.27, 86.06, 80.72, 55.67, 53.33,

52.44, 49.47, 49.22, 36.88, 28.30, 25.08, 23.42, 18.87, 17.51, 7.56, 4.38.
HRMS (ESI-TOF): calculated for $C_{41}H_{58}BrN_5NaO_9Si^+$ [M+Na]$^+$: 894.3079, found: 894.3099.
[α]$^{25}_{D}$: +22.0 (*c* = 0.2, CHCl$_3$)

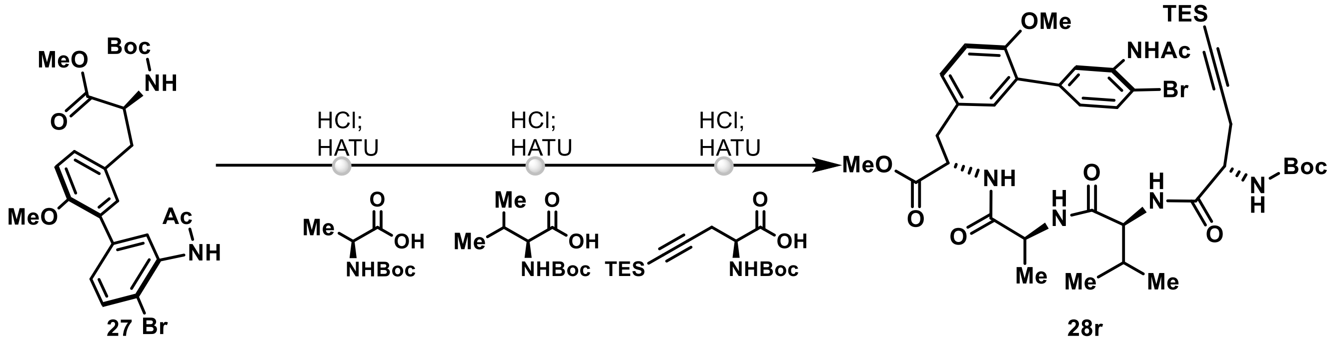

On 0.52 mmol scale, **General Procedure I** was followed. Purification by precipitation in hexane gave the title compound **28r** (226 mg, 48% yield from **27**).

Compound 28r

Physical State: amorphous solid

^{1}H NMR (600 MHz, CDCl$_3$): δ 8.46 (s, 1H), 7.74 (s, 1H), 7.63 (d, *J* = 5.8 Hz, 1H), 7.53 (d, *J* = 8.2 Hz, 1H), 7.22 (d, *J* = 7.6 Hz, 1H), 7.08 (d, *J* = 7.7 Hz, 1H), 7.05 (s, 1H), 6.95 – 6.86 (m, 3H), 5.29 (s, 1H), 4.84 (q, *J* = 6.9 Hz, 1H), 4.49 – 4.45 (m, 1H), 4.35 (s, 1H), 4.15 – 4.13 (m, 1H), 3.77 (s, 3H), 3.72 (s, 3H), 3.16 (dd, *J* = 13.9, 4.4 Hz, 1H), 3.05 (dd, *J* = 14.1, 6.5 Hz, 1H), 2.82 – 2.77 (m, 1H), 2.66 (dd, *J* = 17.2, 5.3 Hz, 1H), 2.30 (s, 3H), 1.44 (s, 9H), 1.35 (d, *J* = 7.1 Hz, 3H), 1.26 – 1.23 (m, 1H), 0.95 (t, *J* = 7.8 Hz, 9H), 0.86 (d, *J* = 6.3 Hz, 3H), 0.82 (d, *J* = 6.3 Hz, 3H), 0.56 (q, *J* = 7.8 Hz, 6H).

^{13}C NMR (151 MHz, CDCl$_3$): δ 172.22, 171.63, 171.26, 170.85, 168.97, 155.90, 155.47, 138.79, 135.46, 131.71, 131.60, 129.99, 129.08, 128.68, 126.99, 122.42, 111.76, 111.56, 102.27, 86.17, 80.83, 58.50, 55.67, 53.50, 52.43, 49.46, 36.99, 31.14, 28.47, 28.29, 25.11, 22.88, 19.55, 17.62, 17.37, 7.54, 4.39.

HRMS (ESI-TOF): calculated for $C_{43}H_{62}BrN_5NaO_9Si^+$ [M+Na]$^+$: 922.3392, found: 922.3397.

[α]$^{25}_{D}$: +16.7 (*c* = 0.2, CHCl$_3$)

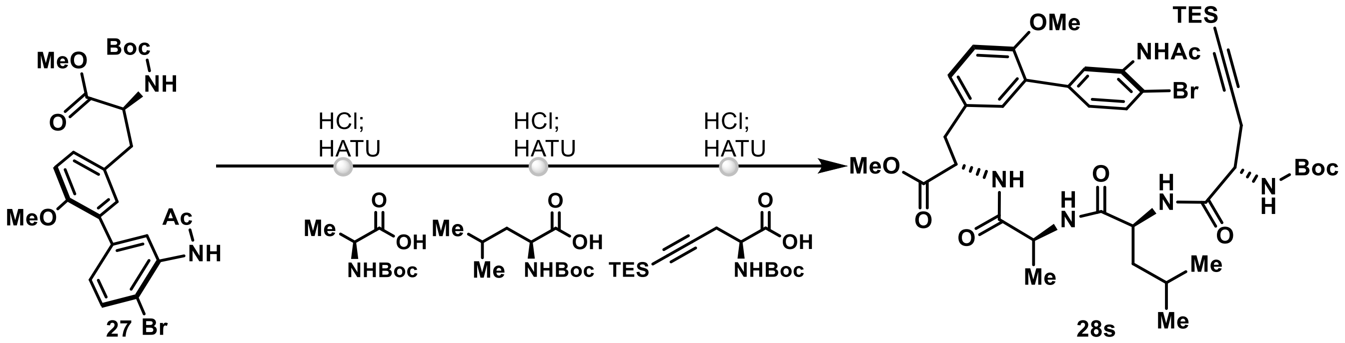

On 0.49 mmol scale, **General Procedure I** was followed. Purification by precipitation in hexane gave the title compound **28s** (265 mg, 59% yield from **27**).

Compound 28s

Physical State: amorphous solid

^{1}H NMR (600 MHz, CDCl$_3$): δ 8.45 (s, 1H), 7.73 (s, 1H), 7.69 - 7.66 (m, 1H), 7.53 (d, *J* = 8.3 Hz, 1H), 7.21 (d, *J* = 7.5 Hz, 1H), 7.08 (d, *J* = 7.8 Hz, 1H), 7.04 (s, 1H), 6.87 (d, *J* = 8.4 Hz, 2H), 6.81 (d, *J* = 7.6 Hz, 1H), 5.28 – 5.25 (m, 1H), 4.84 (q, *J* = 6.6 Hz, 1H), 4.48 – 4.44 (m, 1H), 4.43 – 4.40 (m, 1H), 4.13 (q, *J* = 6.0 Hz, 1H), 3.77 (s, 3H), 3.71 (s, 3H), 3.15 (dd, *J* = 14.1, 4.7 Hz, 1H), 3.05 (dd, *J* = 14.1, 6.5 Hz, 1H), 2.78 (dd, *J* = 17.2, 6.0 Hz, 1H), 2.64 (dd, *J* = 17.1, 5.8 Hz, 1H), 2.30 (s, 3H), 1.58 – 1.48 (m, 2H), 1.44 (s, 9H), 1.41 (t, *J* =

5.4 Hz, 2H), 1.36 (d, J = 7.2 Hz, 3H), 0.96 (t, J = 7.9 Hz, 9H), 0.82 – 0.80 (m, 5H), 0.56 (q, J = 7.9 Hz, 6H).
^{13}C NMR (151 MHz, CDCl$_3$): δ 172.15, 171.57, 170.62, 168.92, 155.89, 155.44, 138.83, 135.45, 131.61, 130.02, 129.11, 128.67, 127.01, 122.50, 111.78, 111.49, 102.37, 86.10, 80.79, 55.64, 53.44, 53.28, 52.41, 52.06, 49.50, 41.81, 37.01, 28.48, 28.28, 25.10, 24.84, 22.97, 21.66, 17.50, 7.56, 4.39.
HRMS (ESI-TOF): calculated for $C_{44}H_{64}BrN_5NaO_9Si+$ [M+Na]$^+$: 936.3549, found: 936.3558.
[α]^{25}$_D$: –17.7 (c = 0.2, CHCl$_3$)

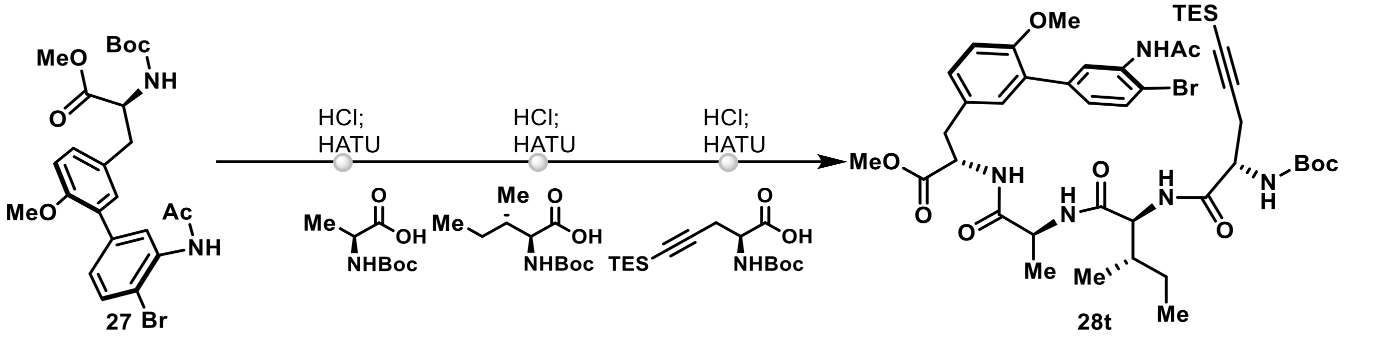

On 0.43 mmol scale, **General Procedure I** was followed. Purification by precipitation in hexane gave the title compound **28t** (178 mg, 45% yield from **27**).

Compound 28t

Physical State: amorphous solid

^{1}H NMR (600 MHz, CDCl$_3$): δ 8.46 (s, 1H), 7.73 (s, 1H), 7.56 (d, J = 6.8 Hz, 1H), 7.53 (d, J = 8.2 Hz, 1H), 7.21 (d, J = 8.2 Hz, 1H), 7.09 (d, J = 8.2 Hz, 1H), 7.05 (s, 1H), 6.92 – 6.90 (m, 1H), 6.87 (d, J = 8.4 Hz, 1H), 5.35 – 5.22 (m, 1H), 4.84 (q, J = 7.0 Hz, 1H), 4.50 – 4.45 (m, 1H), 4.39 – 4.32 (m, 1H), 4.11 (q, J = 6.1 Hz, 1H), 3.77 (s, 3H), 3.71 (s, 3H), 3.14 (dd, J = 14.1, 4.5 Hz, 1H), 3.05 (dd, J = 14.0, 6.7 Hz, 1H), 2.77 (dd, J = 17.2, 5.5 Hz, 1H), 2.66 (dd, J = 17.0, 5.4 Hz, 1H), 2.29 (s, 3H), 1.44 (s, 9H), 1.36 (d, J = 7.2 Hz, 3H), 1.29 – 1.26 (m, 1H), 1.09 – 1.01 (m, 1H), 0.96 (t, J = 7.9 Hz, 9H), 0.88 – 0.84 (m, 5H), 0.79 – 0.76 (m, 2H), 0.56 (q, J = 7.8 Hz, 6H).
^{13}C NMR (151 MHz, CDCl$_3$): δ 172.20, 171.62, 171.08, 170.82, 168.91, 155.96, 155.47, 138.81, 135.46, 131.71, 131.62, 129.98, 129.10, 128.73, 126.97, 122.52, 111.83, 111.57, 102.27, 86.16, 80.82, 58.23, 55.68, 53.54, 52.40, 49.43, 37.31, 37.04, 31.66, 28.27, 25.07, 24.55, 22.81, 22.72, 17.68, 15.70, 14.19, 11.45, 7.54, 4.39.
HRMS (ESI-TOF): calculated for $C_{44}H_{64}BrN_5NaO_9Si+$ [M+Na]$^+$: 936.3549, found: 936.3564.
[α]^{25}$_D$: +12.8 (c = 0.2, CHCl$_3$)

General procedure J for the synthesis of 29 via Larock macrocyclization：

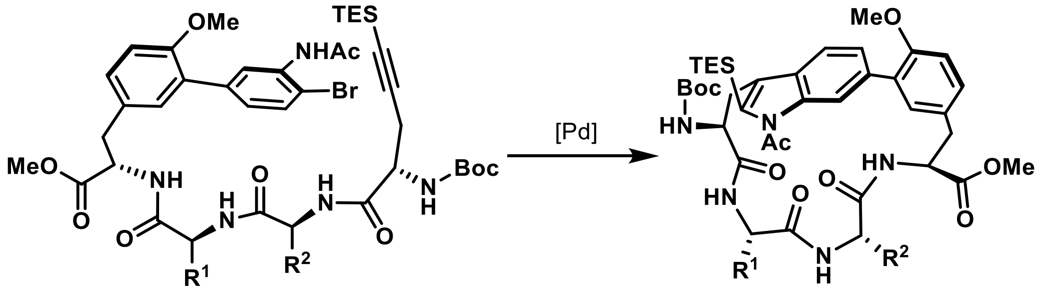

28 29

The tetrapeptide **28** (0.1 mmol, 1.0 eq.) was dissolved in acetonitrile (1.5 mL). Pd(tBu$_3$P)$_2$ (0.01 mmol, 0.1 eq.) and DIPEA (0.25 mmol, 2.5 eq.) were added. The reaction mixture was stirred at 110 ºC under nitrogen atmosphere for 12 h. The reaction mixture was concentrated under reduced pressure and purified by silica gel chromatography to give the cyclized compound **29**.

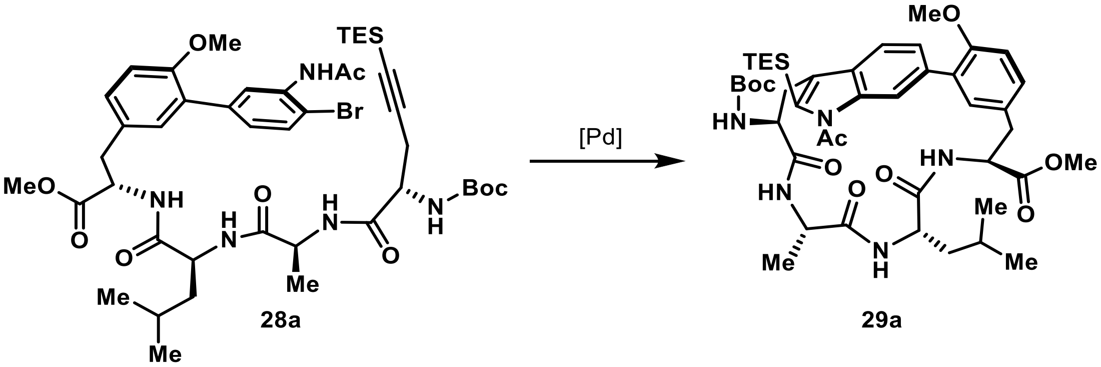

28a 29a

On 0.1 mmol scale, **General Procedure J** was followed from compound **28a** via Larock macrocyclization. Purification by silica gel column chromatography gave the title compound **29a** (45.8 mg, 55% yield).

Compound 29a

Physical State: amorphous solid

^{1}H NMR (600 MHz, CDCl$_3$): δ 7.76 (s, 1H), 7.70 (d, J = 8.0 Hz, 1H), 7.21 (d, J = 7.7 Hz, 1H), 6.99 – 6.93 (m, 2H), 6.91 (d, J = 8.4 Hz, 1H), 6.23 (d, J = 6.6 Hz, 1H), 5.88 – 5.81 (m, 2H), 5.29 (d, J = 7.1 Hz, 1H), 4.86 – 4.71 (m, 1H), 4.49 – 4.46 (m, 1H), 3.99 – 3.96 (m, 2H), 3.85 (s, 3H), 3.81 (s, 3H), 3.45 (d, J = 11.5 Hz, 1H), 3.33 (dd, J = 14.1, 10.6 Hz, 1H), 3.23 (dd, J = 14.2, 4.5 Hz, 1H), 3.06 (dd, J = 14.2, 4.5 Hz, 1H), 2.80 (s, 3H), 1.43 (s, 9H), 1.38 (d, J = 6.1 Hz, 3H), 1.29 – 1.23 (m, 3H), 0.93 – 0.89 (m, 15H), 0.84 (d, J = 5.5 Hz, 3H), 0.79 (d, J = 5.5 Hz, 3H).

^{13}C NMR (151 MHz, CDCl$_3$): δ 171.67, 171.03, 170.82, 169.66, 155.36, 155.10, 137.30, 137.12, 136.11, 134.90, 131.20, 131.08, 129.98, 129.84, 128.89, 127.46, 124.21, 120.46, 115.76, 111.28, 80.12, 55.78, 54.77, 53.29, 52.68, 52.61, 49.12, 40.85, 36.27, 36.09, 31.99, 29.85, 29.69, 29.40, 28.78, 28.37, 27.29, 26.77, 25.66, 24.72, 22.76, 21.82, 17.60, 14.20, 8.33, 6.13.

HRMS (ESI-TOF): calculated for $C_{44}H_{63}N_5NaO_9Si$+ $[M+Na]^+$: 856.4287, found: 856.4297.

$[\alpha]^{25}_D$: +8.6 (c = 0.5, CHCl$_3$)

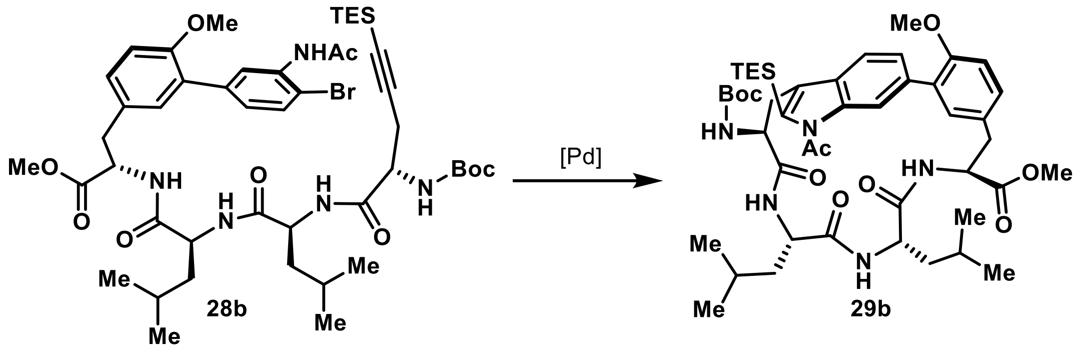

On 0.1 mmol scale, **General Procedure J** was followed from compound **28b** via Larock macrocyclization. Purification by silica gel column chromatography gave the title compound **29b** (54.3 mg, 62% yield).

Compound 29b

Physical State: amorphous solid

^{1}H NMR (600 MHz, CDCl$_3$): δ 7.75 – 7.60 (m, 2H), 7.24 – 7.19 (m, 2H), 6.96 (dd, *J* = 8.4, 2.3 Hz, 1H), 6.89 (d, *J* = 8.4 Hz, 1H), 5.97 (d, *J* = 8.2 Hz, 1H), 5.46 (dd, *J* = 15.0, 5.6 Hz, 2H), 5.37 (d, *J* = 8.7 Hz, 1H), 4.89 – 4.77 (m, 1H), 4.42 – 4.38 (m, 1H), 4.02 (q, *J* = 6.8 Hz, 1H), 3.85 (s, 3H), 3.80 (s, 3H), 3.41 – 3.26 (m, 2H), 3.14 (dd, *J* = 14.1, 5.1 Hz, 1H), 3.05 (dd, *J* = 14.1, 4.1 Hz, 1H), 2.77 (s, 3H), 1.55 – 1.49 (m, 4H), 1.45 (s, 9H), 1.43 – 1.34 (m, 2H), 0.94 – 0.79 (m, 27H).

^{13}C NMR (151 MHz, CDCl$_3$): δ 171.81, 170.99, 170.33, 169.61, 155.26, 155.12, 137.24, 136.39, 136.11, 135.97, 131.52, 131.27, 130.60, 128.66, 127.08, 124.83, 119.88, 115.59, 111.04, 80.01, 55.99, 55.75, 53.23, 52.60, 52.55, 51.91, 41.36, 40.75, 36.33, 29.40, 28.37, 26.75, 24.78, 24.57, 22.84, 22.20, 21.75, 8.32, 5.90.

HRMS (ESI-TOF): calculated for $C_{47}H_{69}N_5NaO_9Si^+$ [M+Na]$^+$: 898.4757, found: 898.4809.

[α]$^{25}_{D}$: +25.6 (*c* = 0.2, CHCl$_3$)

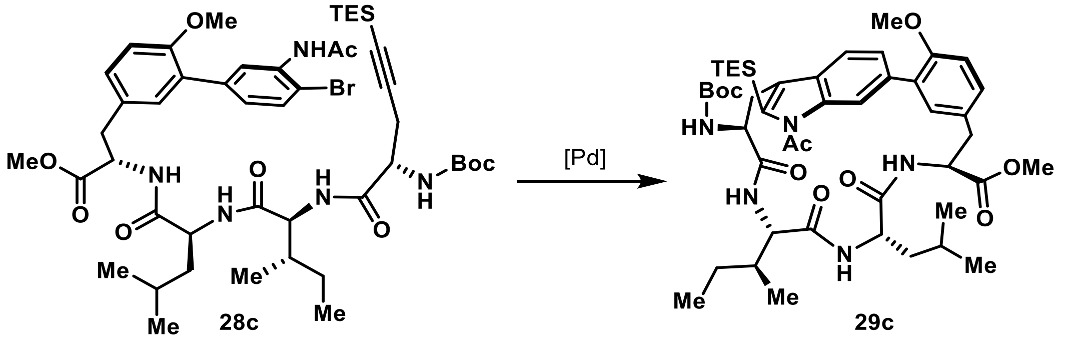

On 0.1 mmol scale, **General Procedure J** was followed from compound **28c** via Larock macrocyclization. Purification by silica gel column chromatography gave the title compound **29c** (51.6 mg, 59% yield).

Compound 29c

Physical State: amorphous solid

^{1}H NMR (600 MHz, CDCl$_3$): δ 7.67 (d, *J* = 8.0 Hz, 1H), 7.60 (s, 1H), 7.30 (s, 1H), 7.23 – 7.18 (m, 1H), 6.94 (dd, *J* = 8.4, 2.3 Hz, 1H), 6.88 (d, *J* = 8.4 Hz, 1H), 5.97 (d, *J* = 8.3 Hz, 1H), 5.56 – 5.48 (m, 2H), 5.45 (d, *J* = 8.3 Hz, 1H), 4.85 (dt, *J* = 8.4, 4.4 Hz, 1H), 4.56- 4.51 (m, 1H), 3.90 (t, *J* = 6.5 Hz, 1H), 3.85 (s, 3H), 3.80 (s, 3H), 3.32 (d, *J* = 7.8 Hz, 2H), 3.14 – 3.04 (m, 2H), 2.76 (s, 3H), 1.97 (s, 1H), 1.61 – 1.56 (m, 2H), 1.50 – 1.46 (m, 2H), 1.46 (s, 9H), 0.92 – 0.87 (m, 19H), 0.86 (d, *J* = 6.4 Hz, 3H), 0.78 (t, *J* = 7.1 Hz, 6H).

^{13}C NMR (151 MHz, CDCl$_3$): δ 171.88, 170.69, 170.40, 169.57, 169.38, 155.23, 155.16, 137.52, 136.30, 136.19, 131.71, 131.18, 130.68, 128.63, 126.91, 124.91, 119.53, 115.97, 110.86, 79.96, 57.47, 55.74, 53.57, 52.60, 52.49, 40.61, 37.92, 36.26, 29.71, 28.40, 26.76, 24.85, 24.79, 22.86, 21.69, 14.97, 11.41, 8.30, 5.88.

HRMS (ESI-TOF): calculated for $C_{47}H_{69}N_5NaO_9Si^+$ [M+Na]$^+$: 898.4757, found: 898.4768.

[α]$^{25}_{D}$: +54.4 (*c* = 0.5, CHCl$_3$)

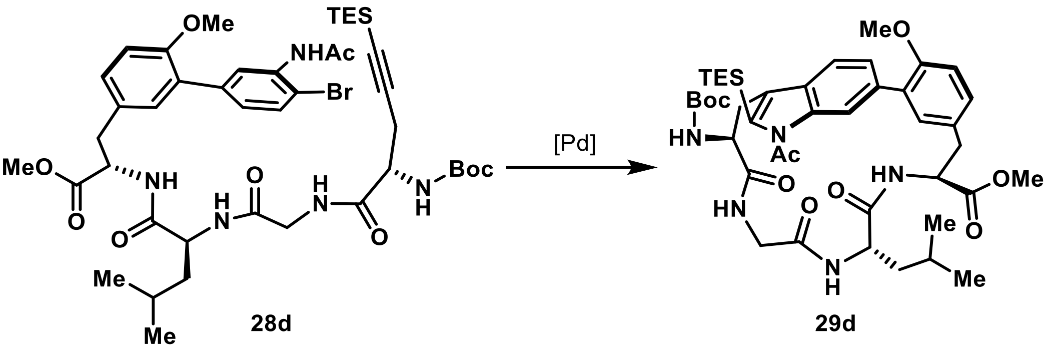

On 0.1 mmol scale, **General Procedure J** was followed from compound **28d** via Larock macrocyclization. Purification by silica gel column chromatography gave the title compound **29d** (41.0 mg, 50% yield).

Compound 29d

Physical State: amorphous solid

^{1}H NMR (600 MHz, CDCl$_3$): δ 8.04 (s, 1H), 7.50 (d, *J* = 8.2 Hz, 1H), 7.12 (dd, *J* = 8.2, 1.3 Hz, 1H), 6.94 (dd, *J* = 8.4, 2.1 Hz, 1H), 6.89 (d, *J* = 8.4 Hz, 1H), 6.85 (d, *J* = 2.0 Hz, 2H), 6.47 – 6.30 (m, 2H), 4.93 (d, *J* = 6.5 Hz, 1H), 4.84 (dt, *J* = 6.4, 4.3 Hz, 1H), 4.53 (d, *J* = 6.6 Hz, 1H), 4.33 – 4.19 (m, 1H), 4.12 (dd, *J* = 17.5, 5.8 Hz, 1H), 3.85 (s, 3H), 3.79 (s, 3H), 3.70 – 3.63 (m, 1H), 3.57 (d, *J* = 17.4 Hz, 1H), 3.47 (dd, *J* = 14.1, 4.6 Hz, 1H), 3.39 (dd, *J* = 14.5, 7.5 Hz, 1H), 3.09 (dd, *J* = 14.1, 3.6 Hz, 1H), 2.85 (s, 3H), 1.48 – 1.44 (m, 2H), 1.43 (s, 9H), 1.02 – 0.91 (m, 16H), 0.85 – 0.80 (m, 6H).

^{13}C NMR (151 MHz, CDCl$_3$): δ 172.61, 171.36, 170.97, 169.69, 166.53, 155.63, 154.94, 138.07, 136.61, 134.99, 133.25, 131.56, 130.32, 129.04, 127.67, 122.91, 120.43, 115.36, 111.67, 80.55, 55.71, 54.08, 52.92, 52.80, 51.98, 43.09, 42.40, 35.29, 31.66, 28.31, 26.83, 24.75, 22.73, 22.62, 22.47, 14.19, 8.42, 6.78.

HRMS (ESI-TOF): calculated for $C_{43}H_{61}N_5NaO_9Si^+$ [M+Na]$^+$: 842.4131, found: 842.4132.

[α]$^{25}_{D}$: –19.7 (*c* = 0.5, CHCl$_3$)

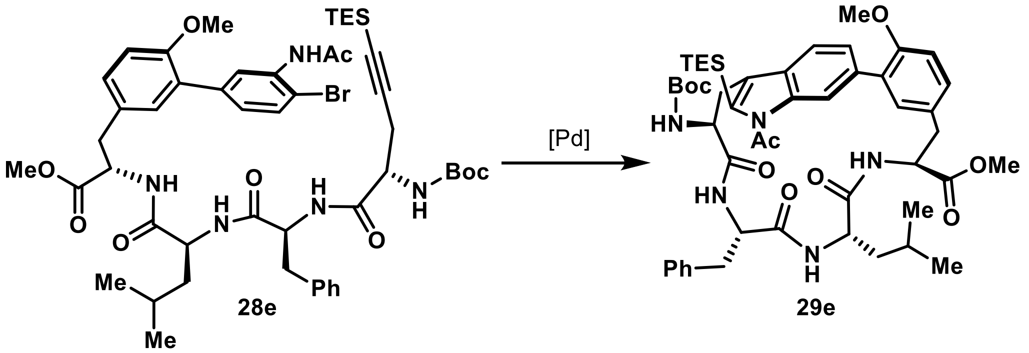

On 0.1 mmol scale, **General Procedure J** was followed from compound **28e** via Larock macrocyclization. Purification by silica gel column chromatography gave the title compound **29e** (54.6 mg, 60% yield).

Compound 29e

Physical State: amorphous solid

^{1}H NMR (600 MHz, CDCl$_3$): δ 7.78 (d, *J* = 7.9 Hz, 1H), 7.61 (s, 1H), 7.31 – 7.27 (m, 4H), 7.24 – 7.19 (m, 2H), 7.05 – 6.95 (m, 2H), 6.91 (d, *J* = 8.3 Hz, 1H), 5.90 (d, *J* = 7.7 Hz, 1H), 5.67 (d, *J* = 5.3 Hz, 1H), 5.47 (d, *J* = 7.9 Hz, 1H), 4.83 – 4.77 (m, 2H), 4.68 (s, 1H), 4.05 – 3.99 (m, 1H), 3.85 (s, 3H), 3.82 (s, 3H), 3.67 (q, *J* = 8.5 Hz, 1H), 3.46 (dd, *J* = 13.8, 4.1 Hz, 1H), 3.35 – 3.14 (m, 3H), 3.00 (dd, *J* = 13.8, 4.4 Hz, 1H), 2.73 (s, 3H), 2.70 – 2.63 (m, 1H), 1.49 (s, 9H), 1.33 – 1.28 (m, 1H), 1.20 – 1.08 (m, 1H), 1.06 – 1.01 (m, 1H), 0.94 – 0.89 (m, 15H), 0.72 – 0.67 (m, 6H).

^{13}C NMR (151 MHz, CDCl$_3$): δ 171.82, 170.48, 169.56, 168.97, 155.26, 155.12, 137.58, 137.05, 136.70, 136.32, 135.29, 131.45, 130.99, 130.02, 129.71, 128.86, 128.74, 127.36, 127.22, 124.19, 119.98, 116.25, 111.14, 80.01, 55.77, 54.92, 54.66, 53.05, 52.83, 52.64, 40.49, 37.96, 36.29, 30.40, 28.45, 26.71, 24.61, 22.63,

21.67, 8.29, 5.99.

HRMS (ESI-TOF): calculated for $C_{50}H_{67}N_5NaO_9Si^+$ [M+Na]$^+$: 932.4600, found: 932.4601.

[α]^{25}D: +69.5 (*c* = 0.5, CHCl$_3$)

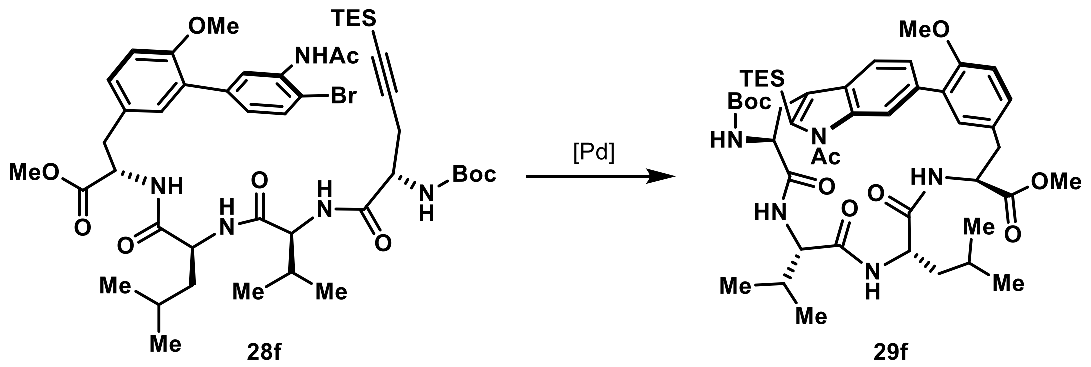

On 0.1 mmol scale, **General Procedure J** was followed from compound **28f** via Larock macrocyclization. Purification by silica gel column chromatography gave the title compound **29f** (49.1 mg, 57% yield).

Compound 29f

Physical State: amorphous solid

^{1}H NMR (600 MHz, CDCl$_3$): δ 7.70 (d, *J* = 8.0 Hz, 1H), 7.59 (s, 1H), 7.28 (d, *J* = 2.6 Hz, 1H), 7.23 (d, *J* = 8.0 Hz, 1H), 6.94 (dd, *J* = 8.4, 2.2 Hz, 1H), 6.89 (d, *J* = 8.4 Hz, 1H), 5.99 (d, *J* = 8.2 Hz, 1H), 5.52 (d, *J* = 7.8 Hz, 2H), 5.42 (d, *J* = 8.6 Hz, 1H), 4.84 (dt, *J* = 8.4, 4.5 Hz, 1H), 4.59 (q, *J* = 8.6 Hz, 1H), 3.94 – 3.88 (m, 1H), 3.85 (s, 3H), 3.80 (s, 3H), 3.77 (q, *J* = 7.4 Hz, 1H), 3.35 – 3.27 (m, 2H), 3.15 – 3.02 (m, 2H), 2.76 (s, 3H), 1.57 – 1.53 (m, 2H), 1.46 (s, 9H), 1.29 – 1.24 (m, 1H), 0.92 – 0.87 (m, 19H), 0.85 (d, *J* = 6.4 Hz, 3H), 0.82 (d, *J* = 6.7 Hz, 3H), 0.71 (d, *J* = 6.7 Hz, 3H).

^{13}C NMR (151 MHz, CDCl$_3$): δ 171.89, 170.81, 170.56, 169.56, 169.42, 155.24, 137.61, 136.36, 136.24, 131.77, 131.16, 130.52, 128.62, 126.90, 124.81, 119.49, 116.12, 110.85, 80.00, 57.74, 55.75, 55.57, 53.51, 52.61, 52.57, 40.55, 36.24, 31.93, 31.66, 29.75, 28.41, 26.78, 24.76, 22.85, 22.73, 21.63, 18.91, 17.41, 14.20, 8.29, 5.91.

HRMS (ESI-TOF): calculated for $C_{46}H_{67}N_5NaO_9Si^+$ [M+Na]$^+$: 884.4600, found: 884.4610.

[α]^{25}D: +58.8 (*c* = 0.5, CHCl$_3$)

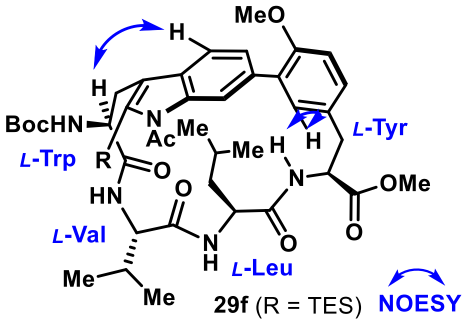

29f (R = TES)

The configuration of the ring system is deduced to be *Rconf*.

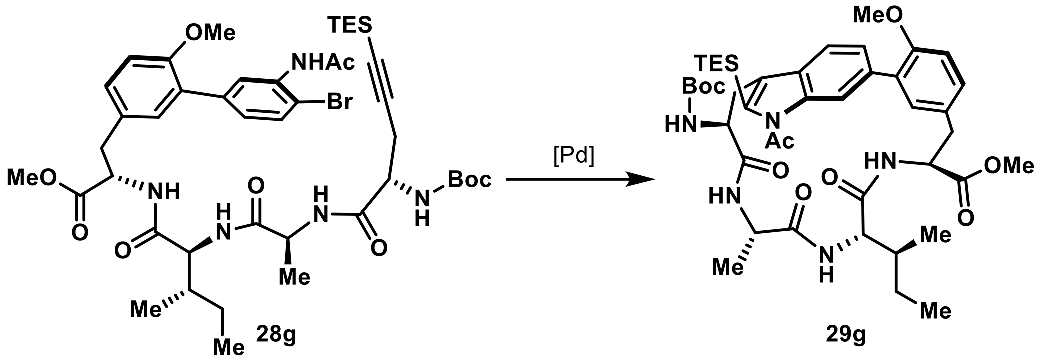

On 0.1 mmol scale, **General Procedure J** was followed from compound **28g** via Larock macrocyclization. Purification by silica gel column chromatography gave the title compound **29g** (48.3 mg, 58% yield).

Compound 29g

Physical State: amorphous solid

^{1}H NMR (600 MHz, CDCl$_3$): δ 7.80 (s, 1H), 7.65 (d, *J* = 7.7 Hz, 1H), 7.17 (d, *J* = 7.8 Hz, 1H), 6.95 (d, *J* = 7.4 Hz, 1H), 6.92 – 6.87 (m, 2H), 6.38 (d, *J* = 15.6 Hz, 1H), 6.18 (d, *J* = 6.2 Hz, 1H), 6.04 (s, 1H), 5.22 (d, *J* = 6.0 Hz, 1H), 4.84 (s, 1H), 4.49 (s, 1H), 4.03 (s, 1H), 3.84 (s, 3H), 3.81 (s, 4H), 3.48 (d, *J* = 12.3 Hz, 1H), 3.36 (t, *J* = 12.3 Hz, 1H), 3.24 (d, *J* = 13.8 Hz, 1H), 3.14 (dd, *J* = 14.4, 3.9 Hz, 1H), 2.81 (s, 3H), 1.70 (d, *J* = 16.3 Hz, 1H), 1.47 (dd, *J* = 26.2, 7.5 Hz, 4H), 1.41 (s, 7H), 1.01 (dt, *J* = 13.4, 7.8 Hz, 2H), 0.93 (d, *J* = 7.0 Hz, 17H), 0.81 (d, *J* = 6.2 Hz, 3H), 0.77 (t, *J* = 7.4 Hz, 3H).

^{13}C NMR (151 MHz, CDCl$_3$): δ 171.54, 171.01, 170.83, 170.53, 169.69, 155.42, 155.04, 137.40, 137.25, 135.94, 134.57, 131.20, 129.59, 128.97, 127.41, 124.27, 120.73, 115.64, 111.35, 80.08, 58.71, 55.79, 54.34, 53.28, 52.64, 49.12, 36.83, 35.95, 28.34, 26.76, 25.08, 17.52, 15.40, 10.74, 8.32, 6.28.

HRMS (ESI-TOF): calculated for $C_{44}H_{63}N_5NaO_9Si$+ $[M+Na]^+$: 856.4287, found: 856.4283.

$[\alpha]^{25}_D$: +2.6 (*c* = 0.5, CHCl$_3$)

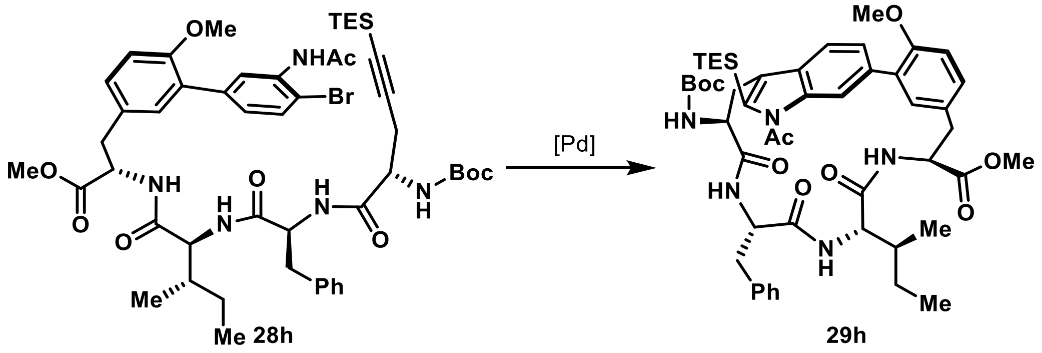

On 0.1 mmol scale, **General Procedure J** was followed from compound **28h** via Larock macrocyclization. Purification by silica gel column chromatography gave the title compound **29h** (56.4 mg, 62% yield).

Compound 29h

Physical State: amorphous solid

^{1}H NMR (600 MHz, CDCl$_3$): δ 7.77 (d, *J* = 8.0 Hz, 1H), 7.59 (s, 1H), 7.30 – 7.26 (m, 4H), 7.23 – 7.17 (m, 2H), 7.03 (s, 1H), 6.96 (d, *J* = 8.1 Hz, 1H), 6.90 (d, *J* = 8.4 Hz, 1H), 5.88 (d, *J* = 5.8 Hz, 1H), 5.75 (d, *J* = 4.9 Hz, 1H), 5.47 (d, *J* = 6.2 Hz, 1H), 5.07 (t, *J* = 9.8 Hz, 1H), 4.83 – 4.76 (m, 1H), 4.10 – 4.05 (m, 1H), 3.85 (s, 3H), 3.81 (s, 3H), 3.49 (dd, *J* = 14.0, 4.6 Hz, 1H), 3.42 (t, *J* = 6.8 Hz, 1H), 3.31 – 3.19 (m, 3H), 3.12 (dd, *J* = 14.4, 4.4 Hz, 1H), 2.74 (s, 3H), 2.64 – 2.57 (m, 1H), 1.48 (s, 9H), 1.42 – 1.38 (m, 1H), 1.16 – 1.07 (m, 1H), 0.94 – 0.87 (m, 16H), 0.73 – 0.62 (m, 6H).

^{13}C NMR (151 MHz, CDCl$_3$): δ 171.88, 170.05, 169.71, 169.62, 168.95, 155.32, 155.19, 137.76, 137.03, 136.70, 136.44, 135.43, 131.73, 130.90, 129.96, 129.64, 128.98, 128.74, 127.24, 127.07, 124.51, 119.86, 116.33, 111.01, 79.99, 59.32, 55.79, 54.46, 54.16, 52.82, 52.63, 38.07, 36.26, 35.98, 30.20, 28.45, 26.76, 25.05,

15.47, 10.78, 8.26, 7.51, 6.10, 4.47.
HRMS (ESI-TOF): calculated for $C_{50}H_{67}N_5NaO_9Si^+$ $[M+Na]^+$: 932.4600, found: 932.4601.
[α]^{25}D: +34.8 (*c* = 0.5, CHCl$_3$)

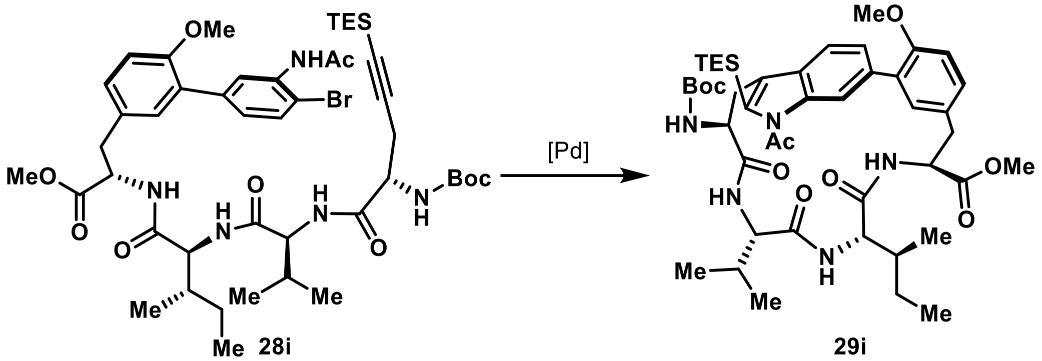

On 0.1 mmol scale, **General Procedure J** was followed from compound **28i** via Larock macrocyclization. Purification by silica gel column chromatography gave the title compound **29i** (51.7 mg, 60% yield).

Compound 29i

Physical State: amorphous solid

^{1}H NMR (600 MHz, CDCl$_3$): δ 7.65 (d, *J* = 7.9 Hz, 1H), 7.60 (s, 1H), 7.31 (s, 1H), 7.20 (d, *J* = 8.0 Hz, 1H), 6.93 (dd, *J* = 8.3, 1.9 Hz, 1H), 6.88 (d, *J* = 8.4 Hz, 1H), 5.89 (d, *J* = 8.1 Hz, 2H), 5.63 (s, 1H), 5.50 (d, *J* = 7.3 Hz, 1H), 4.89 (dd, *J* = 8.0, 4.2 Hz, 1H), 4.54 (s, 1H), 3.99 – 3.91 (m, 1H), 3.84 (s, 3H), 3.79 (s, 3H), 3.63 (d, *J* = 5.0 Hz, 1H), 3.33 (q, *J* = 8.2 Hz, 2H), 3.10 (d, *J* = 3.8 Hz, 2H), 2.76 (s, 3H), 1.86 – 1.78 (m, 1H), 1.77 – 1.71 (m, 1H), 1.45 (s, 9H), 1.21 – 1.12 (m, 1H), 0.97 – 0.88 (m, 19H), 0.86 – 0.82 (m, 6H), 0.71 (d, *J* = 6.5 Hz, 3H).

^{13}C NMR (151 MHz, CDCl$_3$): δ 171.92, 171.02, 169.61, 169.44, 169.32, 155.21, 137.56, 136.21, 136.15, 131.80, 131.21, 130.84, 128.63, 126.91, 125.06, 119.52, 116.03, 110.84, 79.85, 59.89, 58.09, 55.75, 52.57, 52.29, 36.41, 36.13, 31.72, 29.65, 28.42, 28.35, 26.80, 25.47, 18.87, 17.70, 15.65, 11.15, 8.29, 7.51, 5.92, 4.45.

HRMS (ESI-TOF): calculated for $C_{46}H_{67}N_5NaO_9Si^+$ $[M+Na]^+$: 884.4600, found: 884.4605.

[α]^{25}D: +72.4 (*c* = 0.5, CHCl$_3$)

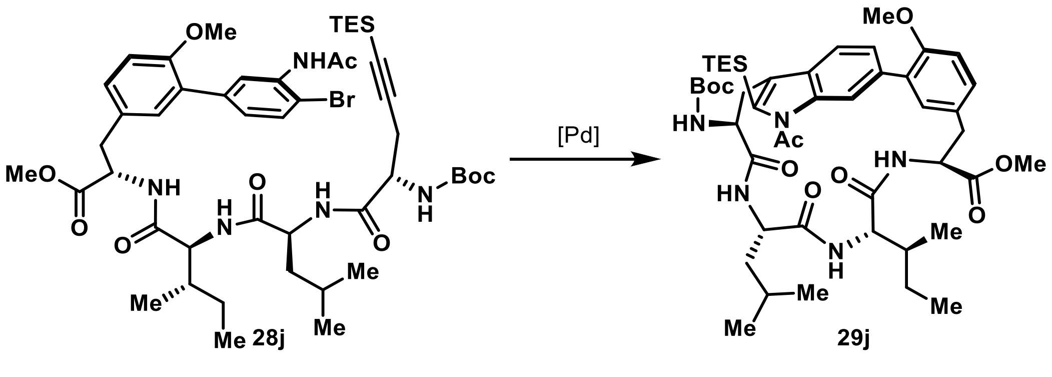

On 0.1 mmol scale, **General Procedure J** was followed from compound **28j** via Larock macrocyclization. Purification by silica gel column chromatography gave the title compound **29j** (57.8 mg, 66% yield).

Compound 29j

Physical State: amorphous solid

^{1}H NMR (600 MHz, CDCl$_3$): δ 7.69 – 7.60 (m, 2H), 7.18 (d, *J* = 7.3 Hz, 2H), 6.94 (d, *J* = 8.3 Hz, 1H), 6.89 (d, *J* = 8.4 Hz, 1H), 5.91 (d, *J* = 7.7 Hz, 1H), 5.77 (s, 1H), 5.45 (dd, *J* = 16.7, 7.2 Hz, 2H), 4.89 (dt, *J* = 8.4, 4.5 Hz, 1H), 4.51 – 4.38 (m, 1H), 4.04 (d, *J* = 6.2 Hz, 1H), 3.84 (s, 3H), 3.80 (s, 3H), 3.74 – 3.67 (m, 1H), 3.36 – 3.34 (m, 2H), 3.16 (dd, *J* = 14.2, 5.2 Hz, 1H), 3.10 (dd, *J* = 14.2, 3.6 Hz, 1H), 2.78 (s, 3H), 1.99 – 1.91 (m, 1H), 1.78 – 1.66 (m, 1H), 1.55 – 1.50 (m, 1H), 1.45 (s, 9H), 1.43 – 1.39 (m, 2H), 1.20 – 1.09 (m, 1H),

0.98 – 0.84 (m, 21H), 0.83 – 0.77 (m, 6H).

^{13}C NMR (151 MHz, CDCl$_3$): δ 171.85, 170.82, 170.10, 169.67, 169.34, 155.27, 155.16, 137.33, 136.44, 136.11, 135.79, 131.67, 131.27, 130.58, 128.65, 126.98, 124.94, 119.85, 115.59, 111.00, 79.96, 59.34, 55.76, 52.60, 52.39, 51.92, 41.37, 36.72, 36.01, 29.40, 28.38, 26.78, 25.44, 24.61, 22.71, 22.32, 15.55, 11.13, 8.31, 5.96.

HRMS (ESI-TOF): calculated for $C_{47}H_{69}N_5NaO_9Si^+$ [M+Na]$^+$: 898.4757, found: 898.4756.

[α]$^{25}_D$: +47.6 (c = 0.5, CHCl$_3$)

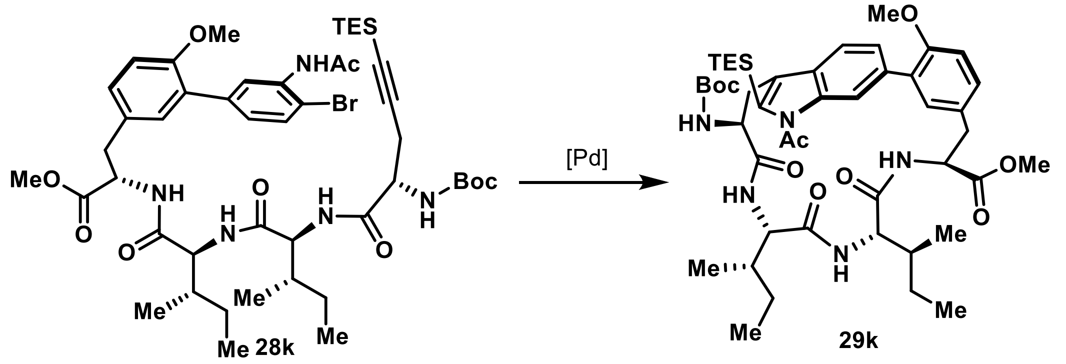

On 0.1 mmol scale, **General Procedure J** was followed from compound **28k** via Larock macrocyclization. Purification by silica gel column chromatography gave the title compound **29k** (55.2 mg, 63% yield).

Compound 29k

Physical State: amorphous solid

^{1}H NMR (600 MHz, CDCl$_3$): δ 7.65 (d, J = 7.9 Hz, 1H), 7.60 (s, 1H), 7.31 – 7.27 (m, 1H), 7.20 (d, J = 8.0 Hz, 1H), 6.94 (dd, J = 8.4, 2.3 Hz, 1H), 6.88 (d, J = 8.4 Hz, 1H), 5.92 (d, J = 8.0 Hz, 1H), 5.80 (s, 1H), 5.55 (d, J = 6.8 Hz, 1H), 4.92 – 4.86 (m, 1H), 4.55 – 4.52 (m, 1H), 3.96 – 3.93 (m, 1H), 3.85 (s, 3H), 3.80 (s, 3H), 3.65 – 3.61 (m, 1H), 3.32 (d, J = 7.6 Hz, 2H), 3.17 – 3.03 (m, 2H), 2.76 (s, 3H), 2.01 – 1.98 (m, 1H), 1.75 – 1.71 (m, 1H), 1.59 – 1.55 (m, 1H), 1.46 (s, 9H), 1.29 – 1.10 (m, 2H), 0.99 – 0.84 (m, 22H), 0.78 (q, J = 6.2 Hz, 6H).

^{13}C NMR (151 MHz, CDCl$_3$): δ 171.92, 170.63, 169.61, 169.25, 169.20, 155.21, 155.18, 137.59, 136.30, 136.22, 136.15, 131.78, 131.18, 130.78, 128.63, 126.89, 125.00, 119.53, 116.00, 110.83, 79.87, 59.81, 57.49, 55.75, 55.61, 52.57, 52.30, 37.94, 36.49, 36.10, 29.78, 28.41, 28.34, 26.79, 25.46, 24.97, 15.68, 14.95, 11.49, 11.18, 8.29, 7.50, 5.91, 4.46.

HRMS (ESI-TOF): calculated for $C_{47}H_{69}N_5NaO_9Si^+$ [M+Na]$^+$: 898.4757, found: 898.4760.

[α]$^{25}_D$: +65.0 (c = 0.5, CHCl$_3$)

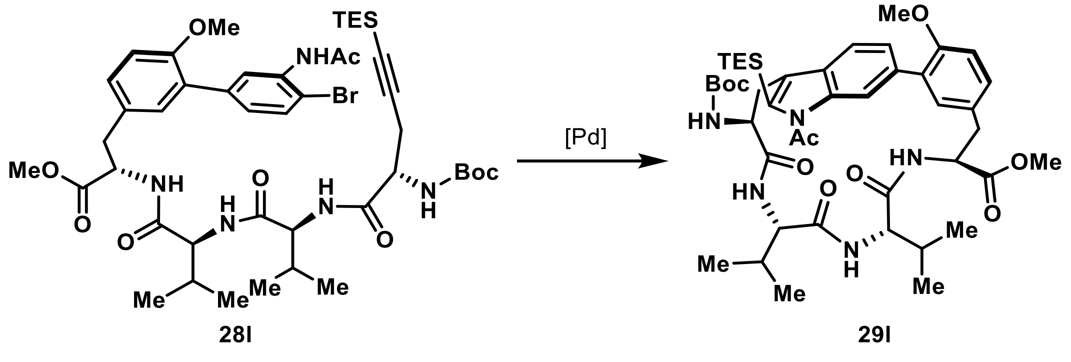

On 0.1 mmol scale, **General Procedure J** was followed from compound **28l** via Larock macrocyclization. Purification by silica gel column chromatography gave the title compound **29l** (50.8 mg, 60% yield).

Compound 29l

Physical State: amorphous solid

^{1}H NMR (600 MHz, CDCl$_3$): δ 7.68 (d, *J* = 6.5 Hz, 1H), 7.61 (s, 1H), 7.30 (d, *J* = 4.8 Hz, 1H), 7.23 (d, *J* = 7.9 Hz, 1H), 6.95 (dd, *J* = 8.4, 2.1 Hz, 1H), 6.89 (d, *J* = 8.4 Hz, 1H), 5.98 (t, *J* = 8.1 Hz, 1H), 5.80 (d, *J* = 50.2 Hz, 1H), 5.56 (d, *J* = 8.8 Hz, 1H), 5.48 – 5.44 (m, 1H), 4.94 – 4.82 (m, 1H), 4.64 – 4.49 (m, 1H), 3.97 – 3.92 (m, 1H), 3.86 (s, 3H), 3.81 (s, 3H), 3.59 – 3.52 (m, 1H), 3.34 (d, *J* = 7.3 Hz, 2H), 3.17 – 3.06 (m, 2H), 2.78 (s, 3H), 1.99 (dd, *J* = 13.1, 6.4 Hz, 1H), 1.89 – 1.81 (m, 1H), 1.56 – 1.50 (m, 1H), 1.46 (s, 9H), 1.00 – 0.91 (m, 20H), 0.85 – 0.83 (m, 3H), 0.72 (d, *J* = 6.7 Hz, 3H).
^{13}C NMR (151 MHz, CDCl$_3$): δ 171.93, 170.86, 169.64, 169.42, 169.34, 155.22, 137.61, 136.32, 136.21, 136.11, 131.78, 131.13, 130.67, 128.64, 126.99, 124.92, 119.56, 116.05, 110.87, 79.96, 61.06, 57.98, 55.76, 55.63, 52.58, 52.38, 36.11, 31.78, 30.17, 29.73, 28.41, 26.80, 19.38, 18.88, 18.82, 17.60, 8.28, 5.93.
HRMS (ESI-TOF): calculated for $C_{45}H_{65}N_5NaO_9Si^+$ [M+Na]$^+$: 870.4444, found: 870.4448.
[α]$^{25}_{D}$: +0.8 (*c* = 0.5, CHCl$_3$)

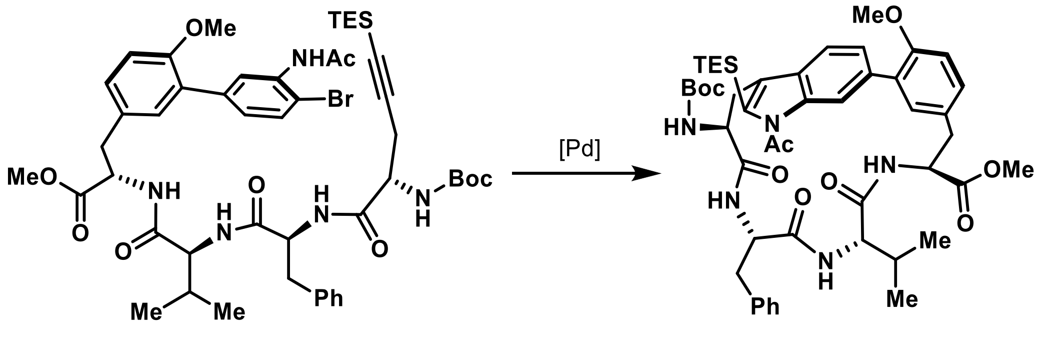

28m **29m**

On 0.1 mmol scale, **General Procedure J** was followed from compound **28m** via Larock macrocyclization. Purification by silica gel column chromatography gave the title compound **29m** (59.1 mg, 66% yield).

Compound 29m

Physical State: amorphous solid
^{1}H NMR (600 MHz, CDCl$_3$): δ 7.77 (d, *J* = 8.0 Hz, 1H), 7.60 (s, 1H), 7.32 – 7.26 (m, 4H), 7.23 – 7.18 (m, 2H), 7.01 (s, 1H), 6.97 (d, *J* = 8.2 Hz, 1H), 6.90 (d, *J* = 8.4 Hz, 1H), 5.86 (d, *J* = 7.1 Hz, 1H), 5.77 (d, *J* = 5.1 Hz, 1H), 5.47 (d, *J* = 7.8 Hz, 1H), 5.03 (d, *J* = 6.1 Hz, 1H), 4.82 (d, *J* = 6.8 Hz, 1H), 4.78 – 4.72 (m, 1H), 4.08 – 4.04 (m, 1H), 3.84 (s, 3H), 3.81 (s, 3H), 3.48 (dd, *J* = 13.9, 4.6 Hz, 1H), 3.38 (t, *J* = 6.4 Hz, 1H), 3.32 – 3.20 (m, 3H), 3.08 (dd, *J* = 14.4, 4.7 Hz, 1H), 2.74 (s, 3H), 2.62 (dd, *J* = 13.3, 10.0 Hz, 1H), 1.68 – 1.62 (m, 1H), 1.48 (s, 9H), 0.94 – 0.88 (m, 15H), 0.68 (d, *J* = 6.6 Hz, 3H), 0.54 (d, *J* = 6.7 Hz, 3H).
^{13}C NMR (151 MHz, CDCl$_3$): δ 171.88, 170.16, 169.65, 169.60, 169.09, 155.30, 155.17, 137.69, 137.08, 136.73, 136.40, 135.39, 131.64, 130.93, 129.98, 129.62, 129.00, 128.73, 127.26, 127.17, 124.44, 119.95, 116.26, 111.05, 80.00, 60.51, 55.79, 54.67, 54.32, 52.86, 52.63, 38.06, 36.03, 30.24, 30.03, 28.45, 26.77, 19.28, 18.33, 8.26, 6.07.
HRMS (ESI-TOF): calculated for $C_{49}H_{65}N_5NaO_9Si^+$ [M+Na]$^+$: 918.4444, found: 918.4448.
[α]$^{25}_{D}$: +52.2 (*c* = 0.5, CHCl$_3$)

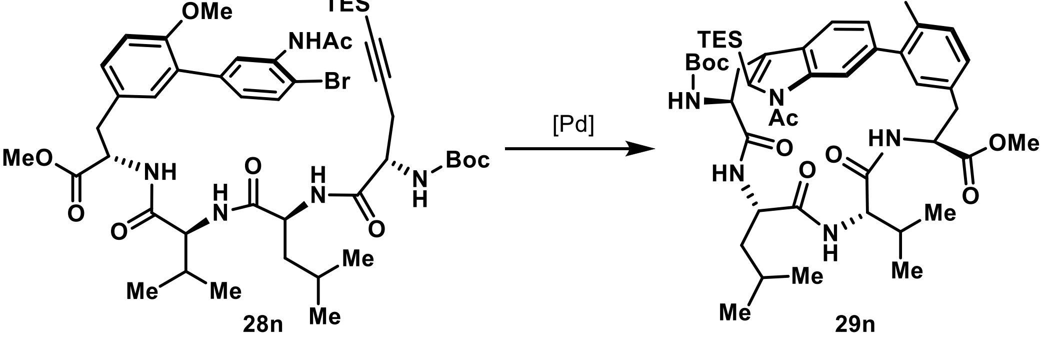

28n **29n**

On 0.1 mmol scale, **General Procedure J** was followed from compound **28n** via Larock macrocyclization. Purification by silica gel column chromatography gave the title compound **29n** (58.6 mg, 68% yield).

Compound 29n

Physical State: amorphous solid

^{1}H NMR (600 MHz, CDCl$_3$): δ 7.65 (d, *J* = 8.6 Hz, 2H), 7.19 (d, *J* = 7.9 Hz, 2H), 6.95 (dd, *J* = 8.4, 2.2 Hz, 1H), 6.89 (d, *J* = 8.4 Hz, 1H), 5.97 (d, *J* = 7.7 Hz, 1H), 5.77 (s, 1H), 5.50 (d, *J* = 6.5 Hz, 1H), 5.42 (d, *J* = 8.2 Hz, 1H), 4.94 – 4.83 (m, 1H), 4.43 – 4.38 (m, 1H), 4.06 (d, *J* = 6.5 Hz, 1H), 3.84 (s, 3H), 3.80 (s, 3H), 3.66 (t, *J* = 5.7 Hz, 1H), 3.36 – 3.28 (m, 2H), 3.17 (dd, *J* = 14.2, 5.1 Hz, 1H), 3.09 (dd, *J* = 14.2, 3.8 Hz, 1H), 2.78 (s, 3H), 1.54 (dd, *J* = 13.1, 6.5 Hz, 1H), 1.45 (s, 9H), 1.42 – 1.33 (m, 2H), 0.95 – 0.85 (m, 22H), 0.82 (t, *J* = 6.9 Hz, 6H).

^{13}C NMR (151 MHz, CDCl$_3$): δ 171.87, 170.94, 170.24, 169.71, 169.34, 155.26, 155.15, 137.28, 136.44, 136.11, 135.71, 131.59, 131.27, 130.64, 128.66, 127.08, 124.89, 119.93, 115.51, 111.02, 79.99, 60.49, 55.89, 55.76, 52.61, 52.44, 51.97, 41.34, 36.06, 30.49, 29.36, 28.38, 26.79, 24.64, 22.73, 22.28, 19.31, 18.77, 8.31, 5.94.

HRMS (ESI-TOF): calculated for $C_{46}H_{67}N_5NaO_9Si^+$ [M+Na]$^+$: 884.4600, found: 884.4608.

[α]$^{25}_D$: +52.7 (*c* = 0.5, CHCl$_3$)

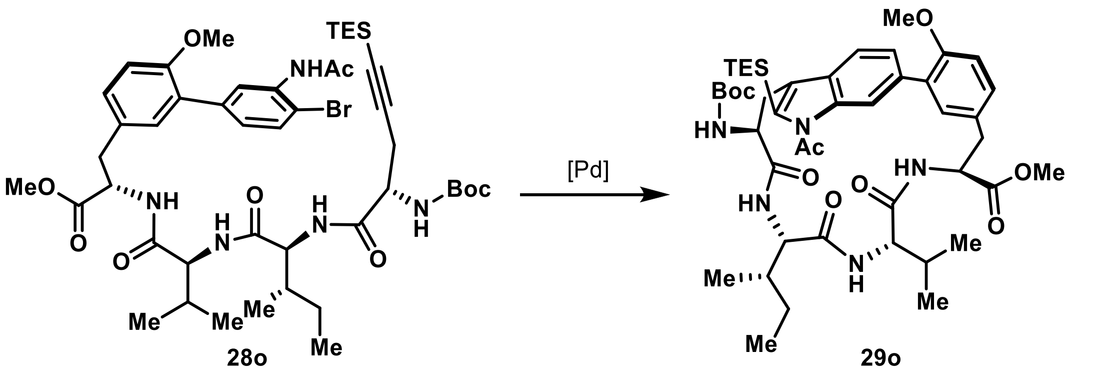

On 0.1 mmol scale, **General Procedure J** was followed from compound **28o** via Larock macrocyclization. Purification by silica gel column chromatography gave the title compound **29o** (56.8 mg, 66% yield).

Compound 29o

Physical State: amorphous solid

^{1}H NMR (600 MHz, CDCl$_3$): δ 7.70 – 7.49 (m, 2H), 7.32 (d, *J* = 2.3 Hz, 1H), 7.20 (d, *J* = 8.0 Hz, 1H), 6.94 (dd, *J* = 8.4, 2.2 Hz, 1H), 6.88 (d, *J* = 8.4 Hz, 1H), 6.01 - 5.96 (m, 2H), 5.64 – 5.60 (m, 1H), 4.99 – 4.84 (m, 1H), 4.48 – 4.44 (m, 1H), 3.99 – 3.93 (m, 1H), 3.84 (s, 3H), 3.79 (s, 3H), 3.65- 3.60 (m, 1H), 3.39 – 3.23 (m, 2H), 3.15 – 3.06 (m, 2H), 2.77 (s, 3H), 2.01 – 1.93 (m, 1H), 1.61 – 1.54 (m, 1H), 1.45 (s, 9H), 1.31 – 1.21 (m, 1H), 0.98 – 0.83 (m, 23H), 0.81 (d, *J* = 6.5 Hz, 3H), 0.77 (t, *J* = 7.3 Hz, 3H).

^{13}C NMR (151 MHz, CDCl$_3$): δ 171.96, 170.94, 169.69, 169.49, 169.20, 155.18, 137.45, 136.15, 131.68, 131.19, 131.00, 128.63, 127.03, 125.14, 119.64, 115.79, 110.85, 79.88, 61.12, 57.67, 55.90, 55.75, 52.58, 52.27, 37.73, 36.14, 30.26, 29.58, 28.39, 26.78, 24.96, 19.34, 18.92, 14.97, 11.44, 8.29, 7.50, 5.88, 4.46.

HRMS (ESI-TOF): calculated for $C_{46}H_{67}N_5NaO_9Si^+$ [M+Na]$^+$: 884.4600, found: 884.4608.

[α]$^{25}_D$: +55.0 (*c* = 0.5, CHCl$_3$)

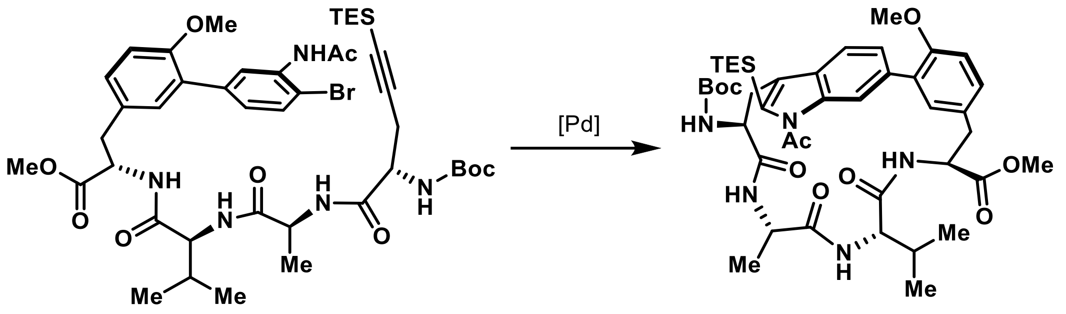

28p 29p

On 0.1 mmol scale, **General Procedure J** was followed from compound **28p** via Larock macrocyclization. Purification by silica gel column chromatography gave the title compound **29p** (50.0 mg, 61% yield).

Compound 29p

Physical State: amorphous solid

^{1}H NMR (600 MHz, CDCl$_3$): δ 7.78 (s, 1H), 7.67 (d, *J* = 5.6 Hz, 1H), 7.18 (d, *J* = 7.8 Hz, 1H), 6.96 (d, *J* = 7.7 Hz, 1H), 6.93 – 6.86 (m, 2H), 6.23 – 5.82 (m, 3H), 5.24 (s, 1H), 4.82 (s, 1H), 4.49 (t, *J* = 8.5 Hz, 1H), 4.00 (s, 1H), 3.84 (s, 3H), 3.81 (s, 3H), 3.72 (s, 1H), 3.47 (d, *J* = 13.7 Hz, 1H), 3.37 – 3.27 (m, 1H), 3.26 (d, *J* = 12.6 Hz, 1H), 3.10 (d, *J* = 14.2 Hz, 1H), 2.81 (s, 3H), 1.94 – 1.85 (m, 1H), 1.83 (s, 1H), 1.49 – 1.46 (m, 2H), 1.42 (s, 9H), 0.96 – 0.90 (m, 14H), 0.85 (d, *J* = 6.2 Hz, 3H), 0.81 (d, *J* = 6.7 Hz, 3H).

^{13}C NMR (151 MHz, CDCl$_3$): δ 171.61, 171.00, 170.77, 170.16, 169.73, 155.41, 155.06, 137.37, 137.27, 135.98, 134.62, 131.23, 131.11, 129.63, 128.89, 127.40, 124.19, 120.63, 115.64, 111.33, 80.13, 59.97, 55.79, 54.51, 53.24, 52.67, 49.14, 35.90, 30.88, 28.35, 26.79, 19.27, 18.62, 17.45, 8.32, 6.24.

HRMS (ESI-TOF): calculated for $C_{43}H_{61}N_5NaO_9Si^+$ [M+Na]$^+$: 842.4131, found: 842.4131.

$[\alpha]^{25}_D$: +33.6 (*c* = 0.2, CHCl$_3$)

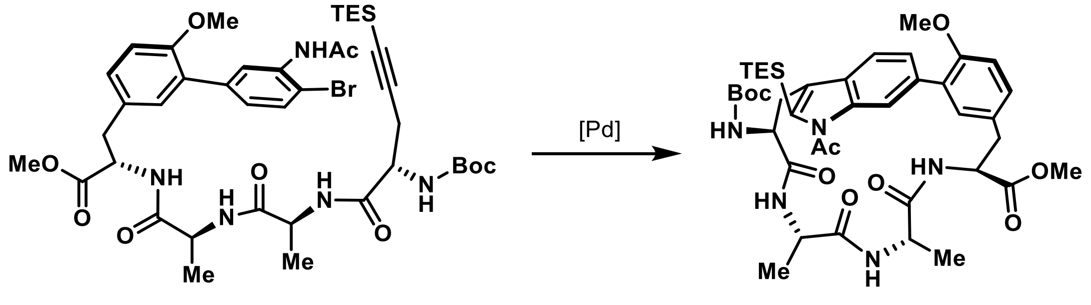

28q 29q

On 0.1 mmol scale, **General Procedure J** was followed from compound **28q** via Larock macrocyclization. Purification by silica gel column chromatography gave the title compound **29q** (45.9 mg, 58% yield).

Compound 29q

Physical State: amorphous solid

^{1}H NMR (600 MHz, CDCl$_3$): δ 7.72 – 7.69 (m, 2H), 7.23 (d, *J* = 8.1 Hz, 1H), 7.00 – 6.97 (m, 2H), 6.91 (d, *J* = 8.8 Hz, 1H), 6.25 (d, *J* = 6.5 Hz, 1H), 5.81 – 5.76 (m, 2H), 5.40 (d, *J* = 7.7 Hz, 1H), 4.72 – 4.68 (m, 1H), 4.46 – 4.43 (m, 1H), 3.98 – 3.94 (m, 2H), 3.83 (s, 3H), 3.81 (s, 3H), 3.39 (dd, *J* = 13.7, 3.4 Hz, 1H), 3.33 – 3.28 (m, 1H), 3.28 – 3.22 (m, 1H), 3.08 (dd, *J* = 14.2, 5.7 Hz, 1H), 2.77 (s, 3H), 1.45 (s, 9H), 1.26 (d, *J* = 5.9 Hz, 3H), 1.15 (d, *J* = 6.7 Hz, 3H), 0.96 – 0.91 (m, 15H).

^{13}C NMR (151 MHz, CDCl$_3$): δ 171.73, 171.12, 170.91, 170.60, 169.74, 155.31, 155.09, 137.16, 137.03, 136.08, 135.08, 131.26, 131.21, 130.07, 128.70, 127.71, 124.11, 120.24, 115.56, 111.37, 80.05, 55.79, 55.52, 53.50, 52.71, 49.87, 49.26, 35.94, 29.56, 28.41, 26.78, 17.82, 17.65, 8.33, 6.00.

HRMS (ESI-TOF): calculated for $C_{41}H_{57}N_5NaO_9Si^+$ [M+Na]$^+$: 814.3818, found: 814.3824.

$[\alpha]^{25}_D$: +57.5 (*c* = 0.5, CHCl$_3$)

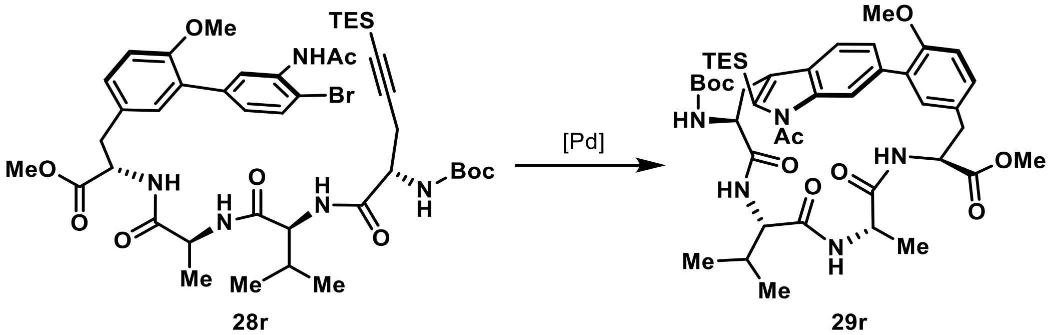

On 0.1 mmol scale, **General Procedure J** was followed from compound **28r** via Larock macrocyclization. Purification by silica gel column chromatography gave the title compound **29r** (49.2 mg, 60% yield).

Compound 29r

Physical State: amorphous solid

^{1}H NMR (600 MHz, CDCl$_3$): δ 7.66 (d, J = 8.2 Hz, 2H), 7.24 – 7.18 (m, 2H), 6.97 (dd, J = 8.3, 2.1 Hz, 1H), 6.90 (d, J = 8.4 Hz, 1H), 6.04 (d, J = 7.0 Hz, 1H), 5.77 (s, 1H), 5.71 (d, J = 7.3 Hz, 1H), 5.41 (d, J = 8.6 Hz, 1H), 4.68 – 4.64 (m, 1H), 4.42 – 4.38 (m, 1H), 3.89 – 3.85 (m, 2H), 3.84 (s, 3H), 3.81 (s, 3H), 3.39 – 3.32 (m, 1H), 3.26 (dd, J = 13.8, 4.1 Hz, 1H), 3.21 – 3.13 (m, 2H), 2.76 (s, 3H), 1.90 – 1.83 (m, 1H), 1.45 (s, 9H), 1.18 (d, J = 7.0 Hz, 3H), 0.95 – 0.90 (m, 15H), 0.84 (d, J = 6.7 Hz, 3H), 0.78 (d, J = 6.8 Hz, 3H).

^{13}C NMR (151 MHz, CDCl$_3$): δ 171.68, 171.28, 170.93, 169.72, 169.43, 155.22, 155.16, 137.13, 136.55, 135.99, 135.88, 131.41, 131.33, 130.70, 128.48, 127.57, 124.54, 119.87, 115.44, 111.17, 80.03, 58.48, 56.39, 55.80, 53.33, 52.70, 50.35, 35.63, 31.78, 29.43, 28.39, 26.78, 18.93, 17.87, 17.54, 8.32, 5.86.

HRMS (ESI-TOF): calculated for $C_{43}H_{61}N_5NaO_9Si^+$ [M+Na]$^+$: 842.4131, found: 842.4142.

[α]$^{25}_D$: +33.4 (c = 0.5, CHCl$_3$)

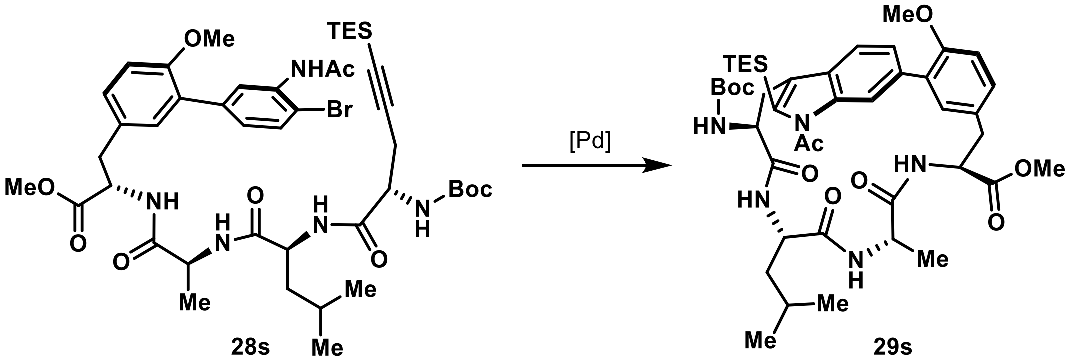

On 0.1 mmol scale, **General Procedure J** was followed from compound **28s** via Larock macrocyclization. Purification by silica gel column chromatography gave the title compound **29s** (45.8 mg, 55% yield).

Compound 29s

Physical State: amorphous solid

^{1}H NMR (600 MHz, CDCl$_3$): δ 7.69 (s, 1H), 7.64 (d, J = 8.0 Hz, 1H), 7.21 (d, J = 8.1 Hz, 1H), 7.15 (s, 1H), 6.98 (dd, J = 8.4, 2.2 Hz, 1H), 6.90 (d, J = 8.4 Hz, 1H), 6.09 (d, J = 7.7 Hz, 1H), 5.75 (s, 1H), 5.62 (d, J = 6.9 Hz, 1H), 5.39 (d, J = 8.7 Hz, 1H), 4.71 – 4.65 (m, 1H), 4.26 (t, J = 7.9 Hz, 1H), 4.06 (q, J = 6.9 Hz, 1H), 3.96 – 3.91 (m, 1H), 3.84 (s, 3H), 3.81 (s, 3H), 3.41 – 3.34 (m, 1H), 3.23 (td, J = 14.3, 4.4 Hz, 2H), 3.11 (dd, J = 14.0, 5.5 Hz, 1H), 2.77 (s, 3H), 1.58 – 1.50 (m, 1H), 1.45 (s, 9H), 1.42 (t, J = 7.2 Hz, 2H), 1.16 (d, J = 6.9 Hz, 3H), 0.97 – 0.90 (m, 15H), 0.84 (d, J = 6.5 Hz, 6H).

^{13}C NMR (151 MHz, CDCl$_3$): δ 171.67, 171.38, 170.81, 170.32, 169.73, 155.22, 155.07, 136.89, 136.65, 135.80, 135.73, 131.43, 131.24, 130.74, 128.52, 127.61, 124.54, 120.12, 115.13, 111.29, 80.05, 56.65, 55.79, 53.28, 52.72, 52.26, 50.13, 41.47, 35.77, 29.27, 28.35, 26.77, 24.54, 22.87, 22.16, 17.71, 8.36, 5.84.

HRMS (ESI-TOF): calculated for $C_{44}H_{63}N_5NaO_9Si+$ [M+Na]$^+$: 856.4287, found: 856.4295.

[α]$^{25}_D$: +31.8 (c = 0.5, CHCl$_3$)

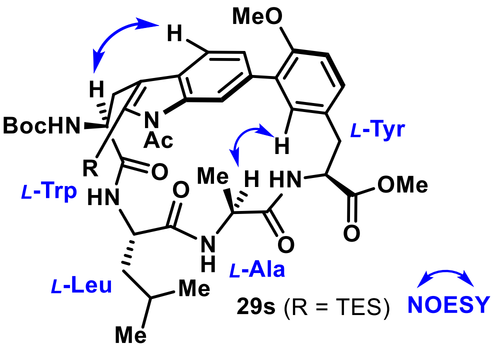

The configuration of the ring system is deduced to be *Rconf*.

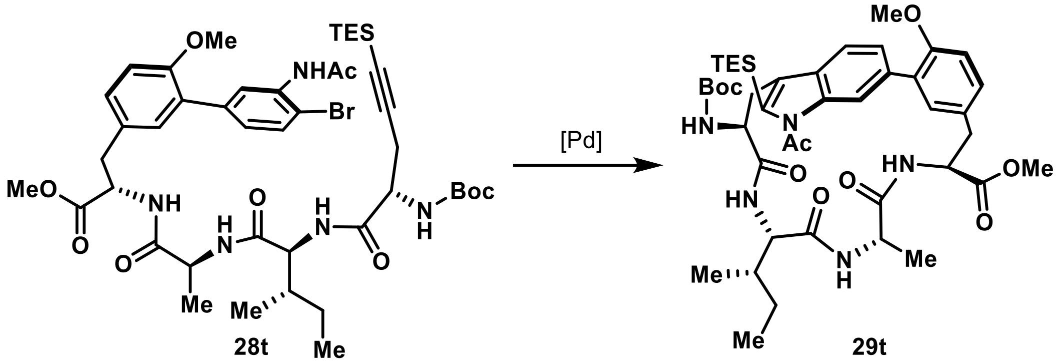

On 0.1 mmol scale, **General Procedure J** was followed from compound **28t** via Larock macrocyclization. Purification by silica gel column chromatography gave the title compound **29t** (50.8 mg, 61% yield).

Compound 29t

Physical State: amorphous solid

^{1}H NMR (600 MHz, CDCl$_3$): δ 7.66 – 7.63 (m, 2H), 7.24 – 7.18 (m, 2H), 6.98 (dd, *J* = 8.4, 2.2 Hz, 1H), 6.90 (d, *J* = 8.4 Hz, 1H), 6.05 (d, *J* = 7.7 Hz, 1H), 5.80 (s, 1H), 5.70 (d, *J* = 7.5 Hz, 1H), 5.41 (d, *J* = 8.9 Hz, 1H), 4.66 – 4.63 (m, 1H), 4.35 (t, *J* = 7.9 Hz, 1H), 3.92 – 3.87 (m, 2H), 3.84 (s, 3H), 3.81 (s, 3H), 3.39 – 3.32 (m, 1H), 3.25 (dd, *J* = 13.9, 3.9 Hz, 1H), 3.21 – 3.13 (m, 2H), 2.76 (s, 3H), 1.88 (s, 1H), 1.66 – 1.59 (m, 1H), 1.45 (s, 9H), 1.35 – 1.31 (m, 1H), 1.17 (d, *J* = 6.9 Hz, 3H), 0.95 – 0.90 (m, 15H), 0.80 (t, *J* = 7.9 Hz, 6H).

^{13}C NMR (151 MHz, CDCl$_3$): δ 171.65, 171.16, 170.85, 169.71, 169.42, 155.21, 155.09, 137.07, 136.53, 135.95, 135.88, 131.36, 130.81, 128.49, 127.62, 124.56, 119.92, 115.32, 111.20, 79.99, 57.94, 56.52, 55.79, 53.36, 52.69, 50.34, 37.72, 35.60, 29.38, 28.38, 27.15, 26.76, 24.92, 17.58, 14.97, 11.31, 8.33, 5.83.

HRMS (ESI-TOF): calculated for $C_{44}H_{63}N_5NaO_9Si+$ $[M+Na]^+$: 856.4287, found: 856.4294.

$[\alpha]^{25}_D$: +30.1 (*c* = 0.5, CHCl$_3$)

General procedure K for the synthesis of 31:

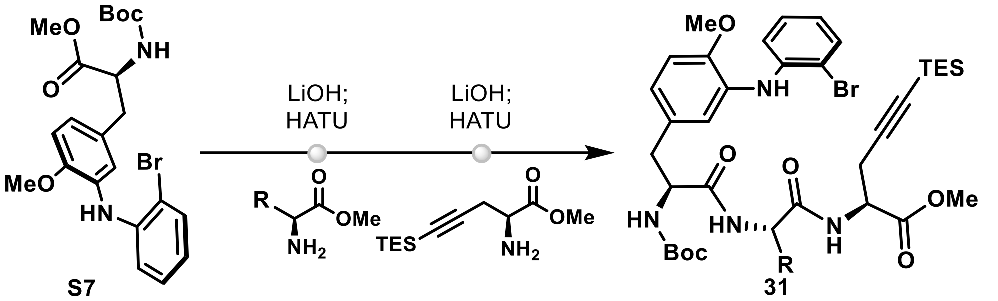

A solution of compound **S7**(1.0 eq.) in MeOH/H_2O (1:1, 0.4 M) was treated with LiOH·H_2O (2.0 eq.). The reaction mixture was stirred at rt for 30 minutes. Upon completion, the reaction was quenched by adjusting the pH to 3 with 0.5 M aq. HCl and then extracted with DCM for three times. The combined organic layers were washed with saturated aq. NaCl, dried over Na_2SO_4, and concentrated *in vacuo* to give the residue, the residue was used directly in the subsequent step.
To a solution of the residue in DCM (0.2 M) was added amino acid methylester (1.2 eq.), DIPEA (4.0 eq.) HATU (1.2 eq.) sequentially. After stirring at rt for 1 h, the reaction was quenched with 0.5 M HCl and extracted with DCM for three times. The combined organic layers were washed with saturated aq. NaCl, dried over Na_2SO_4, and concentrated *in vacuo* to give the residue, the residue was used directly in the subsequent step.
The residue was dissolved in MeOH/H_2O (1:1, 0.4 M), treated with LiOH·H_2O (2.0 eq.), and stirred at rt for 30 minutes. Upon completion, the reaction was quenched by adjusting the pH to 3 with 0.5 M aq. HCl and then extracted with DCM for three times. The combined organic layers were washed with saturated aq. NaCl, dried over Na_2SO_4, and concentrated *in vacuo* to give the residue, the residue was used directly in the subsequent step.
A mixture of the residue in DCM (0.2 M) was added DIPEA (4.0 eq.), HATU (1.2 eq.), methyl (*S*)-2-amino-5-(triethylsilyl)pent-4-ynoate (1.2 eq.) sequentially. The reaction was stirred for 1 h, quenched with 0.5 M HCl and extracted with DCM for three times. The combined organic layers were washed with saturated aq. NaCl, dried over Na_2SO_4, and concentrated *in vacuo* to give the residue, the residue was purified by silica gel chromatography to afford the compound **31**. A mixture of rotamers was observed in **31**.[2]

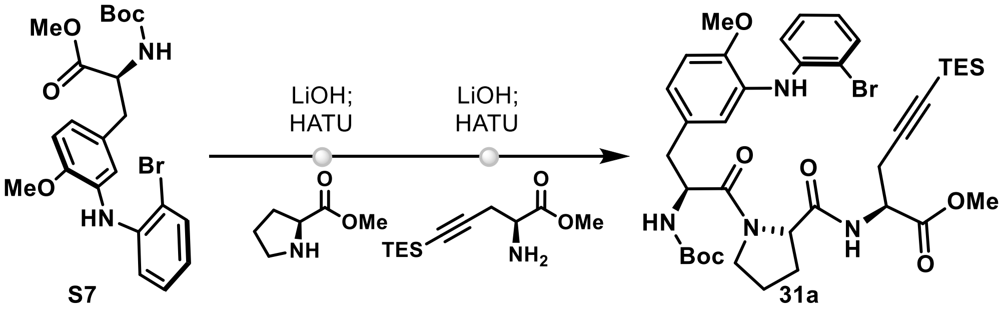

On 0.3 mmol scale, **General Procedure K** was followed. Purification by silica gel column chromatography gave the title compound **31a** (200.4 mg, 85% yield from **S7**).

Compound 31a

Physical State: amorphous solid

^{1}H NMR (600 MHz, CDCl$_3$): δ 7.57 – 7.51 (m, 1H), 7.39 – 7.08 (m, 3H), 7.06 – 6.70 (m, 3H), 4.58 – 4.25 (m, 3H), 3.90 – 3.86 (m, 3H), 3.79 – 3.74 (m, 1H), 3.72 – 3.65 (m, 3H), 3.62 – 3.42 (m, 1H), 3.09 – 2.63 (m, 4H), 2.18 – 2.15 (m, 1H), 2.09 – 1.93 (m, 3H), 1.75 – 1.55 (m, 1H), 1.39 – 1.34 (m, 9H), 1.31 – 1.26 (m, 2H), 0.99 – 0.95 (m, 9H), 0.60 – 0.52 (m, 6H).

^{13}C NMR (151 MHz, CDCl$_3$): δ 172.69, 172.56, 172.17, 171.96, 170.86, 170.54, 156.36, 148.99, 148.41, 141.21, 140.37, 132.65, 132.04, 130.77, 129.41, 128.40, 127.98, 122.94, 122.00, 121.77, 120.70, 119.04, 116.24, 113.74, 112.27, 110.81, 102.91, 102.24, 84.26, 83.33, 79.38, 79.28, 60.45, 60.09, 55.05, 54.74, 54.00, 52.16, 51.71, 46.64, 36.77, 30.86, 29.08, 27.36, 24.54, 22.37, 21.79, 6.49, 3.97.[2]

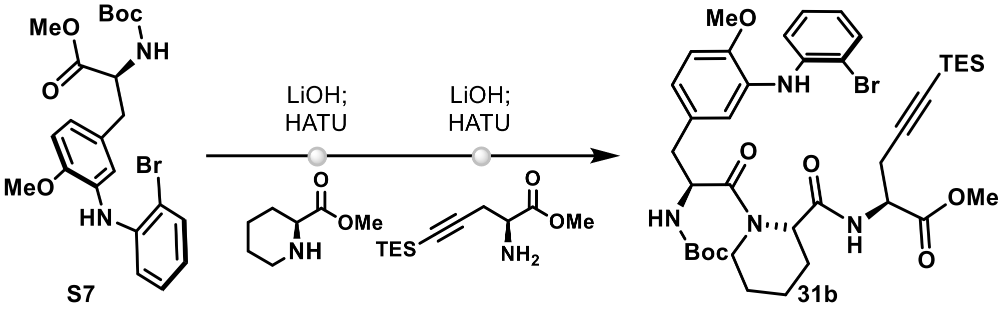

On 0.4 mmol scale, **General Procedure K** was followed. Purification by silica gel column chromatography gave the title compound **31b** (224 mg, 70% yield from **S7**).

Compound 31b

Physical State: amorphous solid

^{1}H NMR (600 MHz, CDCl$_3$): δ 7.51 (ddd, J = 26.6, 7.9, 1.2 Hz, 1H), 7.33 (ddd, J = 8.1, 4.3, 1.3 Hz, 1H), 7.23 – 7.01 (m, 2H), 6.87 – 6.54 (m, 4H), 6.45 – 6.36 (m, 1H), 5.38 – 5.29 (m, 1H), 5.25 – 4.79 (m, 1H), 4.65 – 4.26 (m, 2H), 3.87 – 3.84 (m, 3H), 3.73 – 3.67 (m, 3H), 3.39 – 3.04 (m, 1H), 2.94 – 2.68 (m, 4H), 2.59 – 2.06 (m, 1H), 1.85 – 1.39 (m, 6H), 1.40 – 1.35 (m, 9H), 0.93 (t, J = 7.9 Hz, 9H), 0.54 – 0.49 (m, 6H).

^{13}C NMR (151 MHz, CDCl$_3$): δ 171.96, 171.19, 170.94, 170.46, 169.91, 169.61, 155.94, 155.18, 148.71, 148.53, 141.01, 140.32, 133.19, 132.90, 132.14, 130.97, 128.31, 128.22, 128.11, 127.94, 123.05, 122.10, 121.88, 121.00, 119.03, 117.40, 117.16, 116.62, 113.94, 112.99, 111.31, 110.85, 102.64, 101.77, 85.72, 84.53, 80.44, 79.68, 56.87, 56.07, 55.84, 52.90, 52.84, 52.72, 52.48, 52.35, 51.59, 51.44, 51.22, 51.17, 44.08, 40.33, 38.41, 38.12, 28.37, 26.18, 25.67, 25.47, 24.76, 23.49, 22.79, 20.62, 7.47, 4.41, 4.39.

HRMS (ESI-TOF): calculated for $C_{44}H_{59}BrN_4NaO_8Si^+$ [M+Na]$^+$: 901.3178, found: 901.3188.

[α]$^{25}_{D}$: +39.5 (c = 0.5, CHCl$_3$)

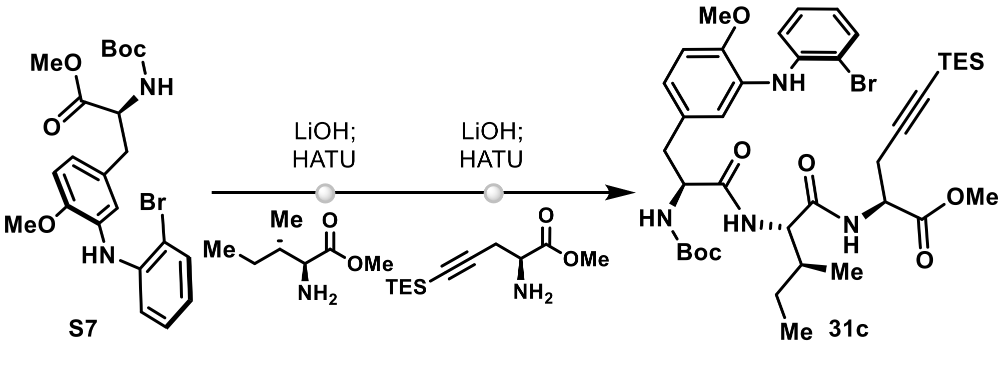

On 0.4 mmol scale, **General Procedure K** was followed. Purification by silica gel column chromatography gave the title compound **31c** (211.7 mg, 66% yield from **S7**).

Compound 31c

Physical State: sticky oil

^{1}H NMR (600 MHz, METHANOL-D_4): δ 7.48 (ddd, J = 7.9, 4.1, 1.1 Hz, 1H), 7.32 (t, J = 7.7 Hz, 1H), 7.22 – 7.02 (m, 2H), 6.92 – 6.85 (m, 1H), 6.83 – 6.76 (m, 1H), 6.74 – 6.66 (m, 1H), 4.56 – 4.40 (m, 1H), 4.37 – 4.14 (m, 2H), 3.88 – 3.65 (m, 6H), 3.06 – 2.88 (m, 1H), 2.86 – 2.58 (m, 3H), 1.53 – 1.36 (m, 1H), 1.36 – 1.25 (m, 9H), 1.17 – 1.11 (m, 1H), 0.98 – 0.76 (m, 16H), 0.56 – 0.51 (m, 6H).

^{13}C NMR (151 MHz, METHANOL-D_4): δ 172.80, 171.84, 170.69, 170.65, 158.61, 156.34, 148.65, 141.03,

140.85, 132.74, 132.70, 130.80, 130.06, 129.52, 129.17, 128.11, 122.66, 122.44, 122.38, 120.94, 120.77, 118.28, 117.79, 116.37, 116.16, 113.65, 113.53, 112.57, 112.39, 110.87, 110.81, 102.53, 102.33, 84.29, 79.34, 57.53, 57.42, 56.15, 56.08, 55.14, 54.38, 52.03, 51.89, 51.69, 51.59, 37.50, 37.29, 27.43, 25.87, 24.43, 22.28, 22.21, 22.12, 14.48, 10.89, 10.29, 10.25, 6.58, 6.57, 4.02.
HRMS (ESI-TOF): calculated for $C_{39}H_{57}BrN_4NaO_7Si^+$ $[M+Na]^+$: 823.3072, found: 823.3076.
$[\alpha]^{25}_D$: +8.4 (c = 0.5, $CHCl_3$)

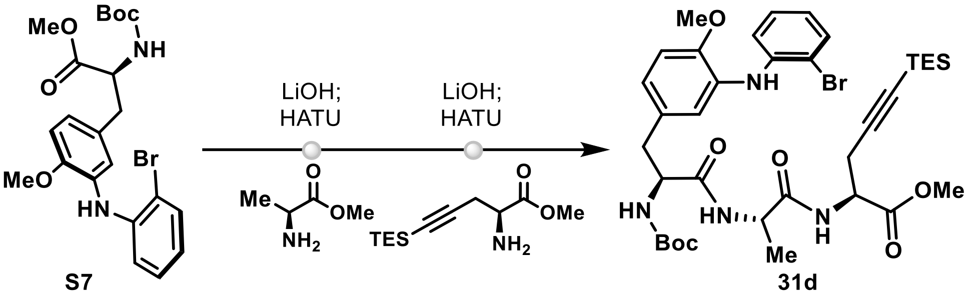

On 0.4 mmol scale, **General Procedure K** was followed. Purification by silica gel column chromatography gave the title compound **31d** (206.7 mg, 68% yield from **S7**).

Compound 31d

Physical State: sticky oil
^{1}H NMR (600 MHz, METHANOL-D_4): δ 7.49 – 7.32 (m, 1H), 7.19 – 7.12 (m, 1H), 7.19 – 7.09 (m, 2H), 6.96 – 6.66 (m, 3H), 4.78 – 4.77 (m, 1H), 4.51 – 4.45 (m, 1H), 4.43 – 4.39 (m, 1H), 4.30 – 4.26 (m, 1H), 3.83 – 3.81 (m, 2H), 3.72 – 3.68 (m, 6H), 3.06 – 3.02 (m, 1H), 2.78 – 2.69 (m, 4H), 1.35 – 1.28 (m, 12H), 0.96 – 0.92 (m, 9H), 0.55 – 0.50 (m, 6H).
^{13}C NMR (151 MHz, METHANOL-D_4): δ 173.11, 172.72, 170.78, 158.66, 156.31, 148.68, 141.04, 132.70, 130.85, 130.14, 129.51, 129.18, 128.11, 122.71, 120.82, 118.27, 116.29, 113.57, 112.43, 110.82, 102.40, 84.27, 79.33, 56.05, 55.93, 55.21, 54.45, 51.78, 51.75, 48.74, 37.62, 37.12, 27.41, 22.35, 17.41, 6.55, 6.51, 4.06, 4.01.
HRMS (ESI-TOF): calculated for $C_{36}H_{51}BrN_4NaO_7Si^+$ $[M+Na]^+$: 781.2603, found: 781.2604.
$[\alpha]^{25}_D$: +14.2 (c = 0.5, $CHCl_3$)

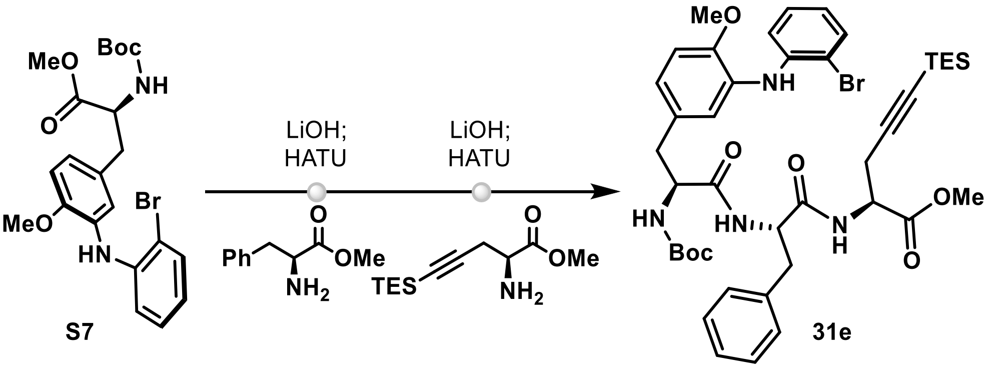

On 0.4 mmol scale, **General Procedure K** was followed. Purification by silica gel column chromatography gave the title compound **31e** (230.7 mg, 69% yield from **S7**).

Compound 31e

Physical State: sticky oil
^{1}H NMR (600 MHz, METHANOL-D_4): δ 7.52 – 7.01 (m, 9H), 6.96 – 6.64 (m, 3H), 4.65 (s, 1H), 4.48 – 4.45 (m, 1H), 4.30 – 4.08 (m, 1H), 3.82 (s, 1H), 3.76 – 3.64 (m, 6H), 3.15 – 3.03 (m, 1H), 2.96 – 2.86 (m, 2H), 2.73 – 2.59 (m, 3H), 1.32 – 1.30 (m, 9H), 0.96 – 0.91 (m, 9H), 0.55 – 0.50 (m, 6H).
^{13}C NMR (151 MHz, METHANOL-D_4): δ 172.64, 171.49, 170.57, 158.64, 156.22, 148.69, 141.04, 136.59, 132.68, 130.83, 130.02, 129.46, 129.25, 129.13, 128.11, 126.47, 122.64, 120.80, 118.23, 116.28, 113.51,

112.41, 110.78, 102.39, 84.22, 79.33, 56.23, 56.13, 55.10, 54.35, 54.10, 51.86, 51.64, 38.00, 37.54, 37.13, 27.39, 22.43, 6.52, 3.98.
HRMS (ESI-TOF): calculated for $C_{42}H_{55}BrN_4NaO_7Si^+$ [M+Na]$^+$: 857.2916, found: 857.2922.
[α]^{25}$_D$: +35.9 (c = 0.5, $CHCl_3$)

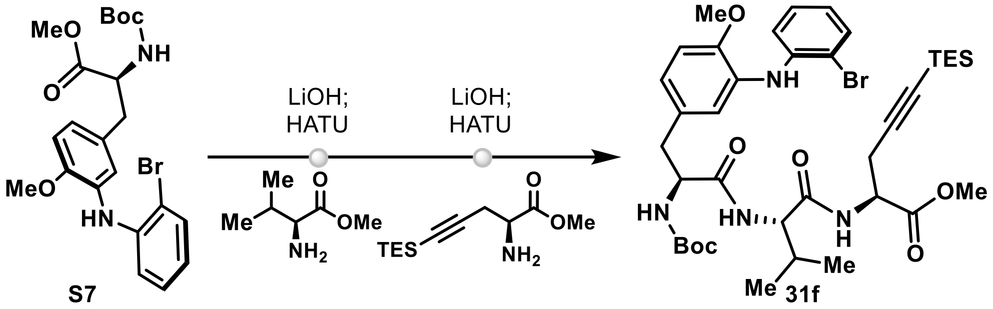

On 0.5 mmol scale, **General Procedure K** was followed. Purification by silica gel column chromatography gave the title compound **31f** (236.4 mg, 60% yield from **S7**).

Compound 31f

Physical State: sticky oil

^{1}H NMR (600 MHz, CDCl$_3$): δ 7.51 – 7.49 (m, 1H), 7.32 – 7.29 (m, 1H), 7.22 – 7.13 (m, 1H), 7.09 – 7.07 (m, 1H), 6.82 – 6.75 (m, 1H), 6.76 – 6.63 (m, 3H), 6.39 (s, 1H), 5.15 – 4.95 (m, 1H), 4.63 – 4.56 (m, 1H), 4.38 – 4.22 (m, 2H), 3.87 – 3.83 (m, 3H), 3.75 – 3.72 (m, 1H), 3.72 – 3.69 (m, 3H), 3.08 – 2.62 (m, 4H), 2.08 – 2.03 (m, 1H), 1.41 – 1.37 (m, 1H), 1.39 – 1.33 (m, 9H), 0.96 – 0.92 (m, 9H), 0.90 – 0.83 (m, 6H), 0.56 – 0.51 (m, 6H).

^{13}C NMR (151 MHz, CDCl$_3$): δ 171.47, 171.43, 170.56, 170.48, 170.41, 155.67, 148.49, 148.42, 140.69, 140.57, 133.13, 133.11, 131.47, 130.37, 128.86, 128.79, 128.28, 122.25, 121.99, 121.53, 121.39, 117.71, 117.03, 116.80, 113.61, 113.43, 111.16, 111.08, 101.41, 86.10, 80.32, 58.36, 58.20, 55.88, 52.67, 52.64, 51.09, 37.30, 31.37, 28.32, 23.56, 18.85, 18.74, 17.89, 7.51, 7.46, 4.38.

HRMS (ESI-TOF): calculated for $C_{38}H_{55}BrN_4NaO_7Si^+$ [M+Na]$^+$: 809.2916, found: 809.2921.
[α]^{25}$_D$: +5.5 (c = 0.5, $CHCl_3$)

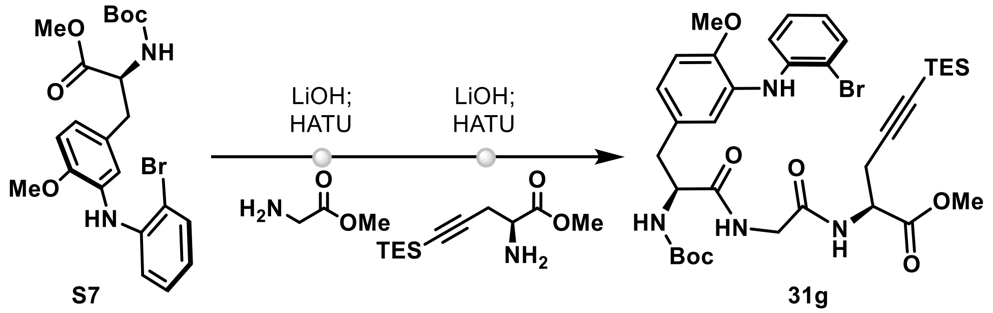

On 0.5 mmol scale, **General Procedure K** was followed. Purification by silica gel column chromatography gave the title compound **31g** (220 mg, 59% yield from **S7**).

Compound 31g

Physical State: sticky oil

^{1}H NMR (600 MHz, METHANOL-D_4): δ 7.54 – 7.07 (m, 4H), 6.94 – 6.70 (m, 3H), 4.57 – 4.53 (m, 2H), 4.26 – 4.19 (m, 1H), 3.94 – 3.89 (m, 1H), 3.84 – 3.80 (m, 3H), 3.73 (s, 2H), 3.72 – 3.68 (m, 3H), 3.07 – 3.03 (m, 1H), 2.83 – 2.65 (m, 4H), 1.34 – 1.32 (m, 9H), 0.97 – 0.93 (m, 9H), 0.56 – 0.51 (m, 6H).

^{13}C NMR (151 MHz, METHANOL-D_4): δ 173.55, 173.46, 170.81, 169.82, 158.70, 156.42, 148.68, 141.02, 132.69, 130.94, 130.02, 129.47, 129.13, 128.06, 122.58, 120.89, 118.10, 116.43, 113.54, 112.51, 110.80, 102.35, 84.12, 79.42, 56.49, 56.36, 55.08, 54.33, 51.79, 51.66, 41.91, 37.31, 36.86, 27.37, 22.45, 6.47, 3.96.

HRMS (ESI-TOF): calculated for $C_{35}H_{49}BrN_4NaO_7Si^+$ $[M+Na]^+$: 767.2446, found: 767.2455.
$[\alpha]^{25}_D$: +18.9 (c = 0.5, $CHCl_3$)

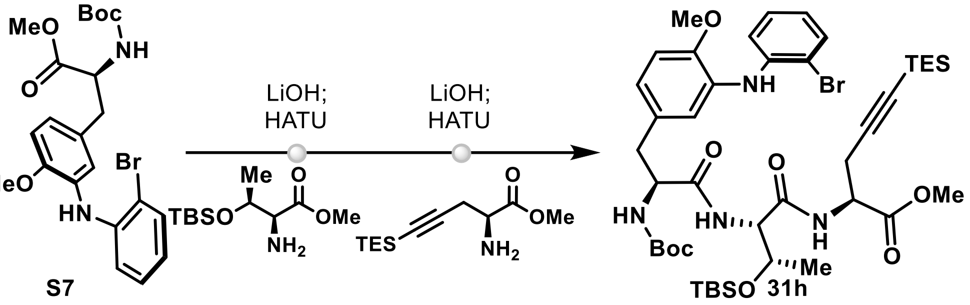

On 0.4 mmol scale, **General Procedure K** was followed. Purification by silica gel column chromatography gave the title compound **31h** (231.5 mg, 64% yield from **S7**).

Compound 31h

Physical State: sticky oil

^{1}H NMR (600 MHz, METHANOL-*D*$_4$): δ 7.63 – 7.61 (m, 1H), 7.47 – 7.50 (m, 1H), 7.35 – 7.30 (m, 1H), 7.24 – 7.04 (m, 2H), 6.93 – 6.69 (m, 2H), 4.57 – 4.50 (m, 1H), 4.42 – 4.34 (m, 1H), 4.33 – 4.21 (m, 1H), 4.15 – 4.04 (m, 1H), 3.84 – 3.66 (m, 6H), 3.00 - 2.96 (m, 1H), 2.84 – 2.58 (m, 3H), 1.36 – 1.25 (m, 9H), 1.23 – 1.06 (m, 3H), 0.98 – 0.92 (m, 9H), 0.89 – 0.78 (m, 9H), 0.56 – 0.50 (m, 6H), 0.09 – 0.02 (m, 6H).

^{13}C NMR (151 MHz, METHANOL-*D*$_4$): δ 172.74, 170.44, 170.31, 158.62, 156.22, 148.60, 140.11, 132.71, 130.33, 130.07, 129.01, 128.11, 122.60, 120.82, 118.15, 116.26, 113.74, 113.56, 110.77, 102.29, 84.31, 79.34, 79.28, 68.79, 68.72, 58.97, 56.17, 55.56, 55.16, 54.40, 52.04, 51.94, 51.68, 36.99, 27.47, 25.23, 25.16, 25.09, 23.06, 22.99, 19.43, 19.36, 17.60, 17.52, 6.61, 4.22, 4.04, 3.85, -5.61, -5.97.

HRMS (ESI-TOF): calculated for $C_{44}H_{59}BrN_4NaO_8Si_2^+$ $[M+Na]^+$: 941.3178, found: 941.3188.
$[\alpha]^{25}_D$: +20.9 (c = 0.5, $CHCl_3$)

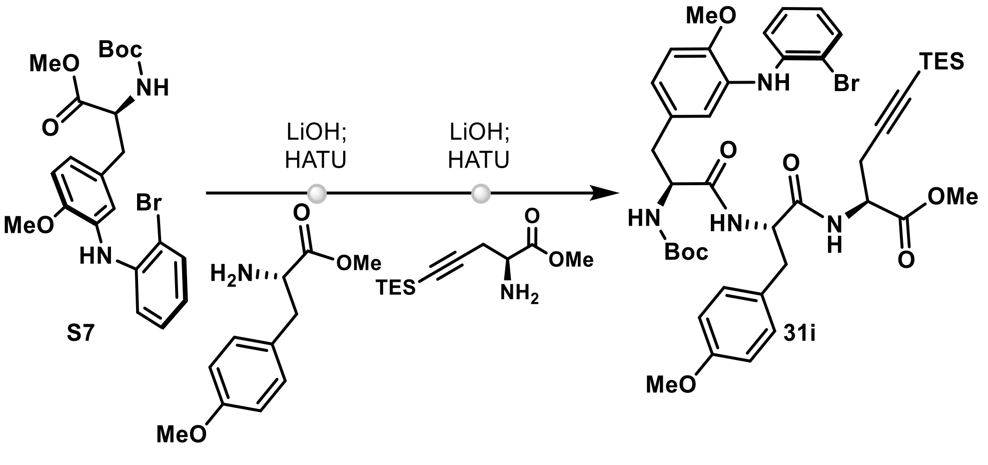

On 0.4 mmol scale, **General Procedure K** was followed. Purification by silica gel column chromatography gave the title compound **31i** (218.2 mg, 63% yield from **S7**).

Compound 31i

Physical State: amorphous solid

^{1}H NMR (600 MHz, METHANOL-*D*$_4$): δ 7.48 (t, J = 8.5 Hz, 1H), 7.30 (dd, J = 12.4, 8.0 Hz, 1H), 7.22 – 7.16 (m, 1H), 7.14 – 6.84 (m, 3H), 6.82 – 6.59 (m, 4H), 4.65 – 4.58 (m, 1H), 4.53 – 4.43 (m, 1H), 4.34 – 4.15 (m, 1H), 3.79 – 3.74 (m, 3H), 3.69 – 3.65 (m, 6H), 3.63 (s, 1H), 3.09 – 2.49 (m, 6H), 1.37 – 1.23 (m, 9H), 0.97 – 0.91 (m, 9H), 0.55 – 0.49 (m, 6H).

^{13}C NMR (151 MHz, METHANOL-*D*$_4$): δ 173.83, 172.56, 171.92, 171.57, 170.66, 170.59, 158.69, 156.24, 156.07, 148.65, 141.02, 132.71, 130.78, 130.27, 130.18, 130.07, 129.47, 129.07, 128.46, 128.41, 128.17,

128.13, 122.67, 120.85, 120.80, 118.27, 118.18, 116.34, 116.22, 113.63, 113.56, 112.43, 110.79, 110.69, 102.70, 102.40, 84.53, 84.26, 79.38, 56.10, 55.90, 55.13, 54.37, 54.35, 54.29, 52.71, 51.93, 51.82, 51.68, 51.43, 37.59, 37.24, 36.88, 27.47, 25.28, 22.56, 22.42, 6.62, 6.54, 4.03.
[α]^{25}$_D$: +201 (c = 0.5, $CHCl_3$)

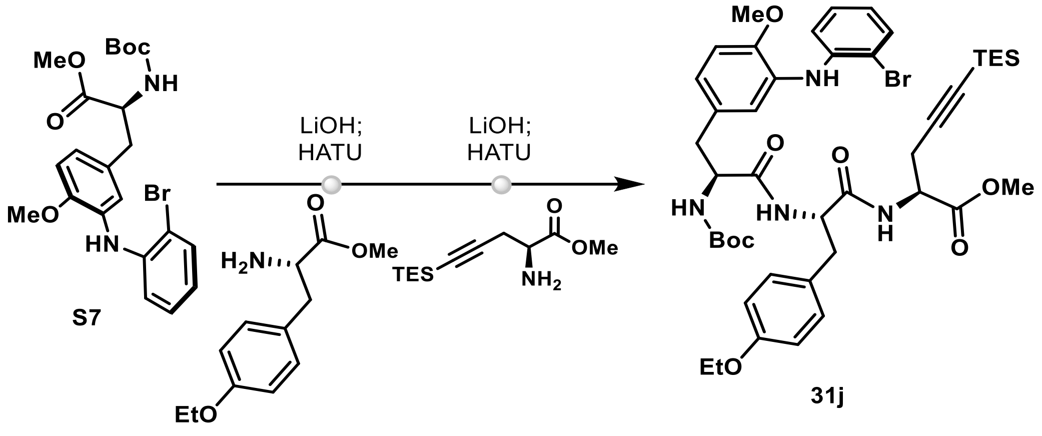

On 0.4 mmol scale, **General Procedure K** was followed. Purification by silica gel column chromatography gave the title compound **31j** (239.4 mg, 68% yield from **S7**).

Compound 31j

Physical State: sticky oil

^{1}H NMR (600 MHz, METHANOL-*D*$_4$): δ 7.50 (t, J = 8.7 Hz, 1H), 7.31 – 7.27 (m, 1H), 7.23 – 7.18 (m, 1H), 7.11 – 7.02 (m, 2H), 6.95 (d, J = 8.3 Hz, 1H), 6.91 – 6.84 (m, 1H), 6.80 – 6.71 (m, 3H), 6.71 – 6.61 (m, 1H), 4.66 – 4.53 (m, 1H), 4.50 – 4.44 (m, 1H), 4.24 – 4.17 (m, 1H), 3.95 – 3.91 (m, 2H), 3.83 – 3.71 (m, 3H), 3.71 – 3.67 (m, 3H), 3.08 – 2.50 (m, 6H), 1.33 – 1.28 (m, 9H), 1.29 – 1.20 (m, 3H), 0.96 – 0.92 (m, 9H), 0.56 – 0.50 (m, 6H).

^{13}C NMR (151 MHz, METHANOL-*D*$_4$): δ 172.55, 171.97, 171.60, 170.64, 170.57, 158.00, 156.29, 156.11, 151.43, 148.68, 141.03, 132.68, 130.84, 130.21, 130.10, 129.45, 128.30, 128.15, 128.09, 122.63, 120.81, 118.21, 116.29, 114.21, 114.13, 113.50, 112.41, 110.79, 110.68, 102.37, 84.20, 79.35, 63.06, 56.17, 55.92, 55.09, 54.33, 54.28, 52.69, 51.93, 51.82, 51.62, 51.37, 37.46, 37.14, 36.79, 27.38, 25.21, 22.43, 22.31, 13.88, 13.86, 6.50, 6.43, 3.97.

HRMS (ESI-TOF): calculated for $C_{44}H_{59}BrN_4NaO_8Si^+$ $[M+Na]^+$: 901.3178, found: 901.3188.

[α]^{25}$_D$: +17.7 (c = 0.5, $CHCl_3$)

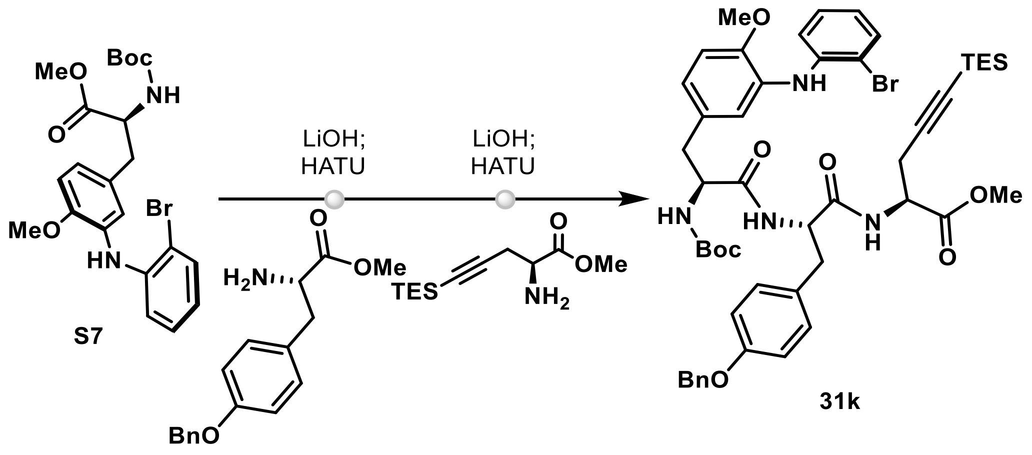

On 0.3 mmol scale, **General Procedure K** was followed. Purification by silica gel column chromatography gave the title compound **31k** (189.4 mg, 67% yield from **S7**).

Compound 31k

Physical State: amorphous solid

^{1}H NMR (600 MHz, METHANOL-*D*$_4$): δ 7.49 (t, *J* = 9.1 Hz, 1H), 7.37 – 7.14 (m, 7H), 7.12 – 7.04 (m, 2H), 6.96 (d, *J* = 8.2 Hz, 1H), 6.89 – 6.68 (m, 5H), 4.98 – 4.96 (m, 1H), 4.93 – 4.86 (m, 1H), 4.69 – 4.54 (m, 1H), 4.51 – 4.44 (m, 1H), 4.29 – 4.13 (m, 1H), 3.81 – 3.76 (m, 3H), 3.70 – 3.65 (m, 3H), 3.09 – 2.48 (m, 6H), 1.31 – 1.21 (m, 9H), 0.95 – 0.91 (m, 9H), 0.54 – 0.49 (m, 6H).
^{13}C NMR (151 MHz, METHANOL-*D*$_4$): δ 172.57, 171.98, 171.58, 170.66, 170.60, 157.86, 156.28, 156.08, 148.67, 137.44, 137.40, 132.70, 130.29, 130.18, 128.84, 128.76, 128.17, 128.13, 127.51, 127.46, 127.16, 127.12, 122.71, 122.65, 120.82, 118.19, 116.39, 116.29, 114.66, 114.56, 113.52, 112.41, 110.80, 110.68, 102.38, 84.24, 79.43, 79.36, 69.63, 56.19, 55.83, 55.10, 54.32, 54.23, 51.94, 51.84, 51.65, 37.51, 37.17, 36.78, 27.41, 22.45, 22.33, 6.53, 3.99.
HRMS (ESI-TOF): calculated for $C_{49}H_{61}BrN_4NaO_8Si^+$ [M+Na]$^+$: 963.3334, found: 963.3333.
[α]^{25}$_D$: +13.0 (*c* = 0.5, $CHCl_3$)

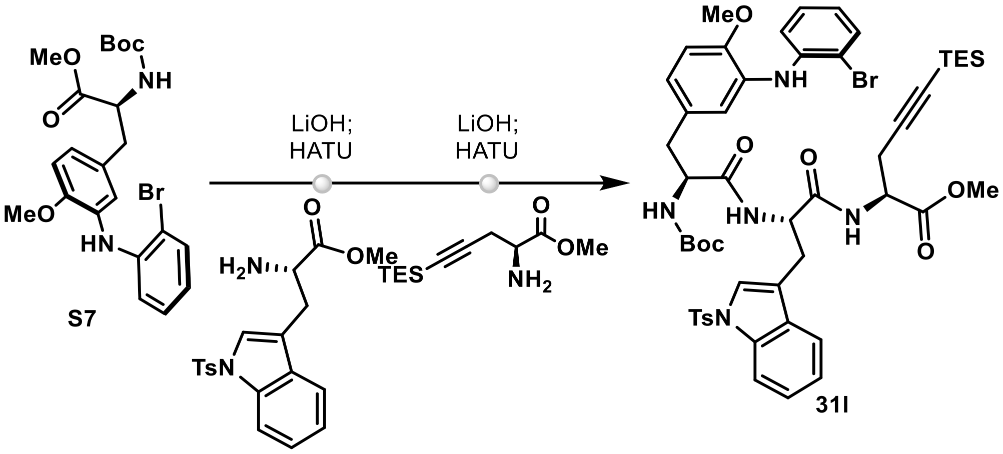

On 0.3 mmol scale, **General Procedure K** was followed. Purification by silica gel column chromatography gave the title compound **31l** (194.5 mg, 63% yield from **S7**).

Compound 31l

Physical State: amorphous solid

^{1}H NMR (600 MHz, $CDCl_3$): δ 7.95 (d, *J* = 8.5 Hz, 1H), 7.75 – 7.72 (m, 2H), 7.58 – 7.41 (m, 1H), 7.38 – 7.36 (m, 1H), 7.34 – 7.31 (m, 1H), 7.31 – 7.27 (m, 1H), 7.24 – 7.14 (m, 4H), 7.10 – 7.02 (m, 1H), 6.86 – 6.35 (m, 5H), 5.01 - 4.91 (m, 1H), 4.76 – 4.63 (m, 1H), 4.60 – 4.46 (m, 1H), 4.27 – 4.22 (m, 1H), 3.90 – 3.79 (m, 3H), 3.78 – 3.72 (m, 1H), 3.72 – 3.63 (m, 3H), 3.09 – 3.06 (m, 1H), 3.05 – 2.89 (m, 2H), 2.86 – 2.76 (m, 1H), 2.72 – 2.53 (m, 2H), 2.30 – 2.28 (m, 3H), 1.94 (s, 1H), 1.42 – 1.29 (m, 9H), 0.99 – 0.87 (m, 9H), 0.55 – 0.44 (m, 6H).
^{13}C NMR (151 MHz, $CDCl_3$): δ 171.29, 170.16, 170.03, 169.91, 155.55, 148.38, 145.01, 140.52, 140.38, 135.39, 135.15, 133.10, 131.82, 130.64, 130.48, 130.33, 129.96, 128.67, 128.47, 128.25, 126.94, 124.97, 124.86, 124.78, 123.43, 123.37, 122.01, 121.88, 121.68, 121.59, 119.67, 119.62, 117.31, 117.16, 117.02, 116.83, 114.27, 113.71, 111.10, 101.34, 85.89, 80.52, 55.92, 53.12, 53.05, 52.79, 51.45, 37.31, 28.29, 28.21, 23.76, 21.61, 7.47, 4.44, 4.35.
HRMS (ESI-TOF): calculated for $C_{51}H_{62}BrN_5NaO_9SSi^+$ [M+Na]$^+$: 1050.3113, found: 1050.3123.
[α]^{25}$_D$: +26.0 (*c* = 0.5, $CHCl_3$)

General procedure L for the synthesis of 32 via Larock macrocyclization:

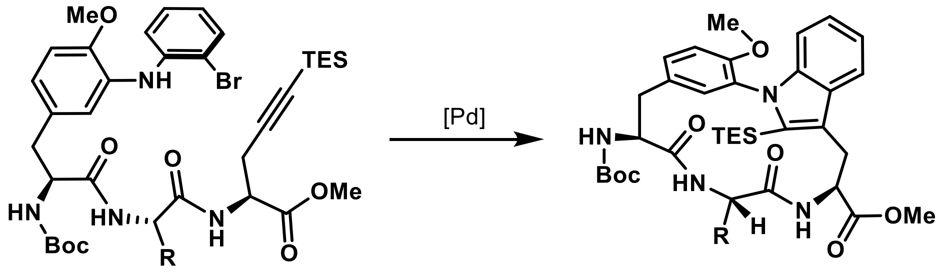

31 32

The precursor **31** (0.1 mmol, 1.0 eq.) was dissolved in toluene (1.5 mL). Pd(tBu$_3$P)$_2$ (0.02 mmol, 0.2 eq.) and DIPEA (0.25 mmol, 2.5 eq.) were added. The reaction mixture was stirred at 110 °C under nitrogen atmosphere for 16 h. The solvents were removed under reduced pressure to give the residue, the residue was purified by silica gel chromatography to give the product **32**. A mixture of conformational isomers as well as rotamers were observed in **32**, which aligns with our previous work.[2]

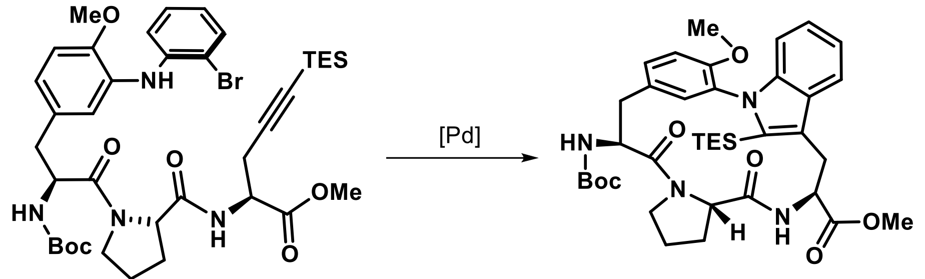

31a 32a

On 0.1 mmol scale, **General Procedure L** was followed from compound **31a** via Larock macrocyclization. Purification by silica gel column chromatography gave the title compound **32a** (42.3 mg, 60% yield). The characterization data were identical to the data previously reported by our group.[2]

Compound 32a

Physical State: amorphous solid

^{1}H NMR (600 MHz, CDCl$_3$): δ 7.67 – 7.34 (m, 1H), 7.20 – 7.06 (m, 2H), 7.02 – 6.79 (m, 3H), 6.29 – 6.04 (m, 1H), 5.31 – 5.06 (m, 1H), 5.01 – 4.79 (m, 1H), 4.70 – 4.27 (m, 2H), 3.99 – 3.92 (m, 3H), 3.90 – 3.82 (m, 3H), 3.80 – 3.75 (m, 1H), 3.74 – 3.61 (m, 1H), 3.57 – 3.47 (m, 2H), 3.05 – 2.96 (m, 1H), 2.87 – 2.77 (m, 1H), 2.31 – 2.12 (m, 1H), 2.07 – 1.90 (m, 2H), 1.88 – 1.79 (m, 1H), 1.76 – 1.67 (m, 1H), 1.48 – 1.36 (m, 9H), 0.96 – 0.57 (m, 15H).

^{13}C NMR (151 MHz, CDCl$_3$): δ 173.18, 172.73, 171.63, 171.06, 169.77, 169.34, 155.21, 155.06, 154.36, 152.86, 147.21, 145.99, 142.75, 141.26, 134.37, 133.40, 131.37, 131.29, 130.87, 130.70, 130.61, 130.01, 129.92, 127.46, 127.16, 126.47, 126.36, 123.33, 120.51, 120.11, 117.76, 117.47, 113.95, 113.29, 112.66, 110.88, 110.83, 79.51, 79.33, 60.17, 58.62, 55.76, 54.13, 53.51, 52.85, 52.72, 52.50, 52.38, 51.39, 46.81, 46.68, 35.65, 35.05, 31.24, 29.45, 28.47, 28.40, 27.54, 25.11, 24.76, 24.29, 7.69, 7.50, 4.95, 4.48, 4.43.

$[\alpha]^{25}_D$: +60.1 (c = 0.5, CHCl$_3$)

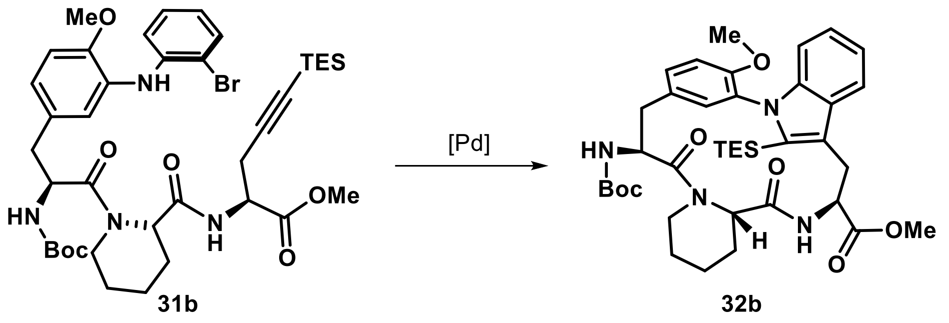

On 0.1 mmol scale, **General Procedure L** was followed from compound **31b** via Larock macrocyclization. Purification by silica gel column chromatography gave the title compound **32b** (37.4 mg, 52% yield).

Compound 32b

Physical State: sticky oil

^{1}H NMR (600 MHz, CDCl$_3$): δ 7.53 – 7.30 (m, 1H), 7.18 – 7.08 (m, 2H), 6.99 – 6.89 (m, 2H), 6.88 – 6.73 (m, 1H), 6.16 – 6.02 (m, 1H), 5.45 – 5.26 (m, 1H), 5.21 – 5.06 (m, 1H), 4.97 – 4.78 (m, 1H), 4.71 – 4.53 (m, 1H), 4.47 – 4.35 (m, 1H), 3.97 – 3.90 (m, 3H), 3.89 – 3.82 (m, 3H), 3.73 – 3.51 (m, 2H), 3.42 – 3.18 (m, 1H), 3.11 – 2.97 (m, 1H), 2.97 – 2.81 (m, 1H), 2.80 – 2.68 (m, 1H), 1.77 – 1.54 (m, 6H), 1.47 – 1.40 (m, 9H), 0.95 – 0.73 (m, 15H).

^{13}C NMR (151 MHz, CDCl$_3$): δ 173.01, 172.81, 170.83, 169.11, 155.00, 153.03, 145.00, 140.69, 132.99, 130.37, 130.15, 127.61, 125.29, 123.34, 120.61, 117.14, 113.21, 110.92, 79.48, 55.71, 53.29, 52.68, 51.32, 50.96, 43.44, 35.72, 28.52, 28.44, 28.38, 28.04, 27.38, 25.89, 20.09, 7.64, 7.49, 5.00, 4.48, 4.41, 4.39.

$[\alpha]^{25}_D$: +22.8 (c = 0.5, CHCl$_3$)

HRMS (ESI-TOF): calculated for $C_{39}H_{54}N_4NaO_7Si^+$ $[M+Na]^+$: 741.3654, found: 741.3658.

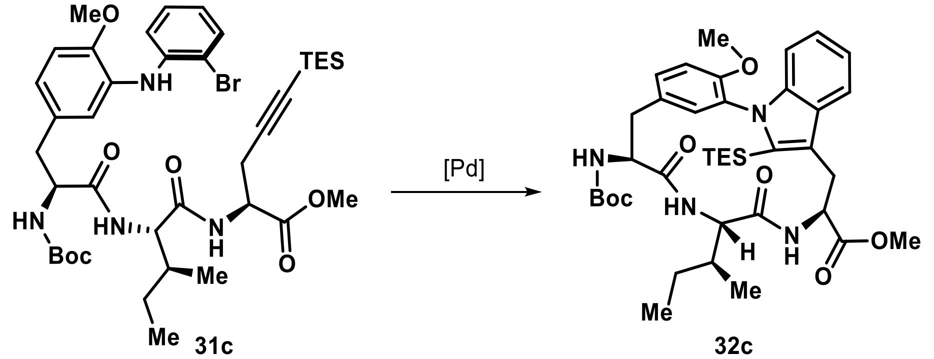

On 0.1 mmol scale, **General Procedure L** was followed from compound **31c** via Larock macrocyclization. Purification by silica gel column chromatography gave the title compound **32c** (36.0 mg, 50% yield).

Compound 32c

Physical State: amorphous solid

^{1}H NMR (600 MHz, CDCl$_3$): δ 7.55 – 7.28 (m, 1H), 7.21 – 7.07 (m, 2H), 6.88 – 6.71 (m, 2H), 6.70 – 6.34 (m, 2H), 5.11 – 4.91 (m, 1H), 4.64 – 4.57 (m, 1H), 4.49 – 4.22 (m, 2H), 3.95 – 3.86 (m, 1H), 3.86 – 3.69 (m, 6H), 3.10 – 2.90 (m, 2H), 2.87 – 2.59 (m, 2H), 1.97 – 1.82 (m, 1H), 1.47 – 1.41 (m, 2H), 1.47 – 1.35 (m, 9H), 0.99 – 0.91 (m, 9H), 0.89 – 0.78 (m, 7H), 0.60 – 0.49 (m, 6H).

^{13}C NMR (151 MHz, CDCl$_3$): δ 171.25, 170.51, 170.39, 170.31, 170.26, 158.67, 148.51, 148.42, 140.70, 140.58, 133.12, 130.37, 130.30, 129.44, 128.79, 128.58, 128.28, 122.25, 121.97, 121.51, 121.39, 118.71, 116.77, 114.23, 114.18, 113.42, 111.09, 101.38, 86.15, 80.28, 57.76, 57.56, 56.65, 56.37, 55.89, 55.77, 55.30, 52.66, 51.07, 37.79, 28.32, 24.97, 23.64, 23.58, 15.09, 11.79, 11.61, 11.58, 7.50, 4.40.

HRMS (ESI-TOF): calculated for $C_{39}H_{56}N_4NaO_7Si^+$ $[M+Na]^+$: 743.3810, found: 743.3818.

$[\alpha]^{25}_D$: +8.4 (c = 0.5, CHCl$_3$)

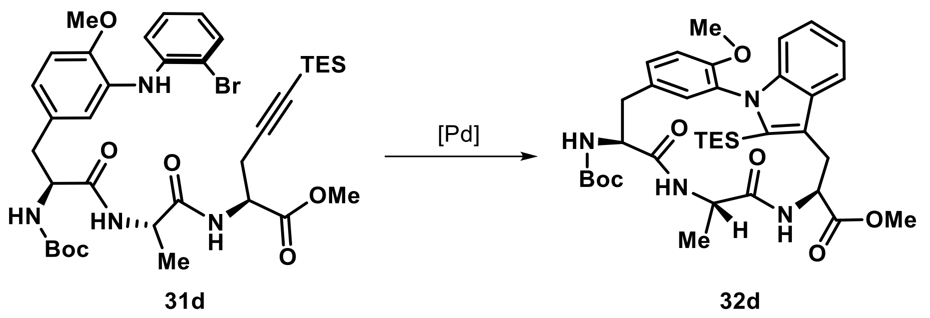

On 0.1 mmol scale, **General Procedure L** was followed from compound **31d** via Larock macrocyclization. Purification by silica gel column chromatography gave the title compound **32d** (34.6 mg, 51% yield).

Compound 32d

Physical State: amorphous solid

^{1}H NMR (600 MHz, CDCl$_3$): δ 7.69 – 7.41 (m, 1H), 7.21 – 6.71 (m, 5H), 6.49 – 6.42 (m, 1H), 5.51 – 5.25 (m, 1H), 5.18 – 4.97 (m, 1H), 4.76 – 4.34 (m, 1H), 3.99 – 3.82 (m, 6H), 3.79 – 3.55 (m, 2H), 3.41 – 3.20 (m, 1H), 3.15 – 2.58 (m, 2H), 1.89 – 1.79 (m, 1H), 1.45 – 1.40 (m, 9H), 1.23 – 1.05 (m, 3H), 0.88 – 0.77 (m, 9H), 0.76 – 0.53 (m, 6H).

^{13}C NMR (151 MHz, CDCl$_3$): δ 172.82, 172.48, 171.76, 171.05, 170.76, 170.64, 169.73, 168.33, 155.17, 154.93, 152.98, 144.93, 143.65, 140.66, 138.58, 131.66, 131.28, 130.79, 130.68, 130.44, 130.09, 129.88, 129.79, 129.57, 129.36, 128.14, 125.61, 125.36, 123.91, 123.34, 121.09, 120.02, 118.41, 116.97, 114.17, 113.08, 112.33, 111.72, 111.55, 101.78, 85.77, 79.87, 79.54, 55.75, 55.61, 55.17, 54.58, 53.47, 52.90, 52.77, 52.26, 50.93, 48.79, 47.81, 37.61, 37.12, 31.57, 28.47, 28.39, 27.18, 27.11, 23.99, 23.23, 20.05, 19.92, 7.66, 7.63, 7.50, 5.47, 4.55, 4.44.

HRMS (ESI-TOF): calculated for $C_{36}H_{50}N_4NaO_7Si^+$ [M+Na]$^+$: 701.3341, found: 701.3345.

[α]$^{25}_D$: +8.6 (c = 0.5, CHCl$_3$)

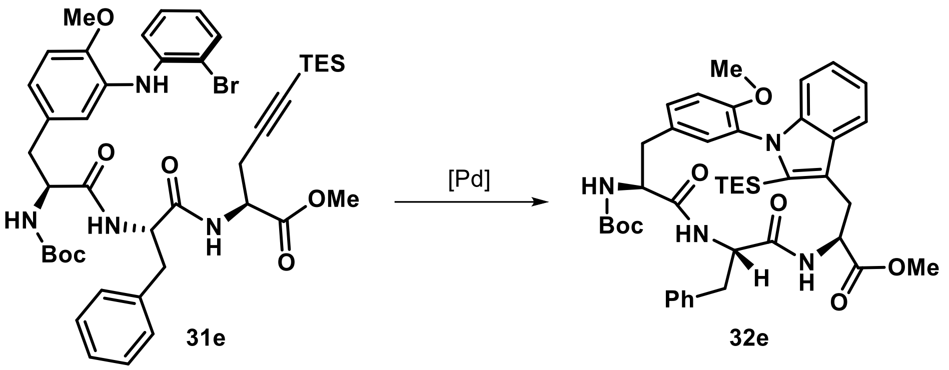

On 0.1 mmol scale, **General Procedure L** was followed from compound **31e** via Larock macrocyclization. Purification by silica gel column chromatography gave the title compound **32e** (38.5 mg, 51% yield).

Compound 32e

Physical State: amorphous solid

^{1}H NMR (600 MHz, CDCl$_3$): δ 7.25 – 7.02 (m, 8H), 6.95 – 6.54 (m, 4H), 5.05 – 5.00 (m, 1H), 4.77 – 4.62 (m, 1H), 4.58 – 4.55 (m, 1H), 4.42 – 4.30 (m, 1H), 3.94 – 3.93 (m, 1H), 3.88 – 3.82 (m, 1H), 3.80 – 3.67 (m, 6H), 3.16 – 2.90 (m, 3H), 2.89 – 2.71 (m, 2H), 2.69 – 2.59 (m, 1H), 1.43 – 1.35 (m, 9H), 0.97 – 0.84 (m, 9H), 0.57 – 0.52 (m, 6H).

^{13}C NMR (151 MHz, CDCl$_3$): δ 171.92, 171.23, 170.35, 170.14, 169.91, 168.46, 158.71, 155.12, 153.03, 144.94, 136.17, 135.64, 131.62, 130.81, 130.40, 129.89, 129.55, 129.38, 128.69, 128.63, 128.49, 128.05, 127.11, 125.78, 123.72, 121.18, 116.82, 114.18, 112.96, 111.57, 101.55, 85.75, 79.50, 55.73, 55.59, 55.30, 54.56, 54.36, 53.78, 53.26, 52.64, 51.43, 39.79, 38.56, 37.45, 37.30, 28.49, 28.43, 28.34, 27.02, 23.81, 7.67, 7.60, 7.51, 5.40, 4.50, 4.40.

HRMS (ESI-TOF): calculated for $C_{42}H_{54}N_4NaO_7Si$+ $[M+Na]^+$: 777.3654, found: 777.3661.
$[\alpha]^{25}{}_D$: +29.2 (c = 0.5, $CHCl_3$)

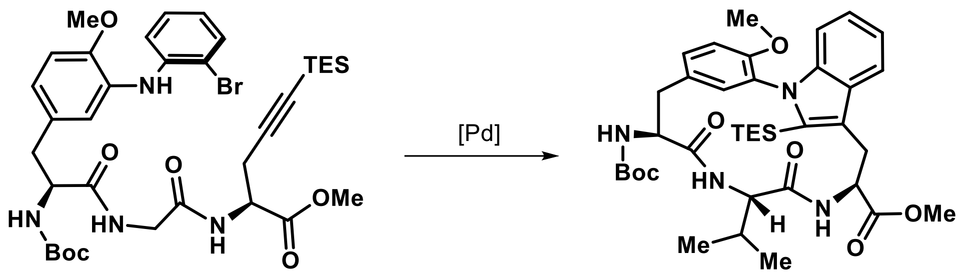

31f 32f

On 0.1 mmol scale, **General Procedure L** was followed from compound **31f** via Larock macrocyclization. Purification by silica gel column chromatography gave the title compound **32f** (33.9 mg, 48% yield).

Compound 32f

Physical State: amorphous solid

^{1}H NMR (600 MHz, $CDCl_3$): δ 7.67 – 7.43 (m, 1H), 7.19 – 7.14 (m, 2H), 7.08 – 6.88 (m, 3H), 6.42 – 6.39 (m, 1H), 6.07 - 5.97 (m, 1H), 5.39 – 5.37 (m, 1H), 5.26 – 5.23 (m, 1H), 5.08 – 4.95 (m, 1H), 4.38 – 4.24 (m, 1H), 3.96 – 3.83 (m, 6H), 3.77 – 3.52 (m, 1H), 3.26 – 3.21 (m, 1H), 3.13 – 3.10 (m, 1H), 2.87 – 2.82 (m, 1H), 1.73 – 1.68 (m, 1H), 1.43 – 1.37 (m, 9H), 0.90 – 0.81 (m, 9H), 0.80 – 0.47 (m, 12H).

^{13}C NMR (151 MHz, $CDCl_3$): δ 172.82, 172.25, 171.21, 170.44, 169.54, 168.75, 155.09, 153.07, 145.15, 140.83, 138.72, 131.76, 131.46, 130.75, 129.97, 129.43, 128.01, 125.86, 125.15, 123.89, 121.08, 118.41, 116.80, 113.21, 111.52, 79.95, 79.53, 58.54, 57.48, 55.76, 55.59, 55.07, 54.53, 53.94, 52.71, 37.12, 32.35, 28.50, 28.44, 26.93, 19.13, 18.55, 18.22, 17.78, 7.69, 7.63, 5.47, 4.50.

HRMS (ESI-TOF): calculated for $C_{51}H_{71}N_7NaO_8Si^+$ $[M+Na]^+$: 960.5026, found: 960.5035.
$[\alpha]^{25}{}_D$: +22.9 (c = 0.5, $CHCl_3$)

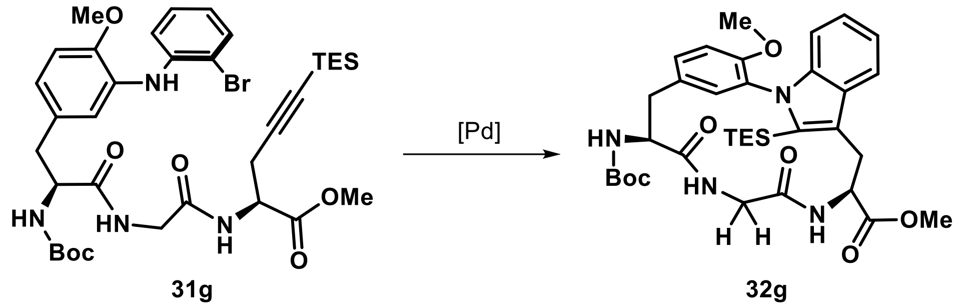

31g 32g

On 0.1 mmol scale, **General Procedure L** was followed from compound **31g** via Larock macrocyclization. Purification by silica gel column chromatography gave the title compound **32g** (39.2 mg, 59% yield).

Compound 32g

Physical State: amorphous solid

^{1}H NMR (600 MHz, $CDCl_3$): δ 7.24 – 6.96 (m, 4H), 6.95 – 6.85 (m, 1H), 6.83 – 6.81 (m, 1H), 6.71 – 6.41 (m, 1H), 5.12 – 5.02 (m, 1H), 4.87 – 4.61 (m, 1H), 4.60 – 4.16 (m, 1H), 4.07 – 3.94 (m, 1H), 3.87 – 3.75 (m, 6H), 3.54 – 3.29 (m, 1H), 3.19 – 2.68 (m, 3H), 1.44 – 1.37 (m, 9H), 0.97 – 0.83 (m, 9H), 0.69 – 0.54 (m, 6H).

^{13}C NMR (151 MHz, $CDCl_3$): δ 172.48, 171.89, 170.65, 168.64, 168.13, 158.72, 155.60, 153.05, 138.18, 130.35, 128.49, 123.79, 122.24, 120.86, 119.87, 118.22, 114.21, 112.95, 112.31, 111.95, 101.36, 86.00, 80.42, 56.03, 55.77, 55.33, 52.83, 51.23, 42.84, 42.71, 37.54, 28.45, 28.34, 27.44, 7.58, 7.51, 6.67, 5.87, 4.66, 4.40.

HRMS (ESI-TOF): calculated for $C_{35}H_{48}N_4NaO_7Si^+$ $[M+Na]^+$: 687.3184, found: 687.3190.
$[\alpha]^{25}{}_D$: +3.5 (c = 0.5, $CHCl_3$)

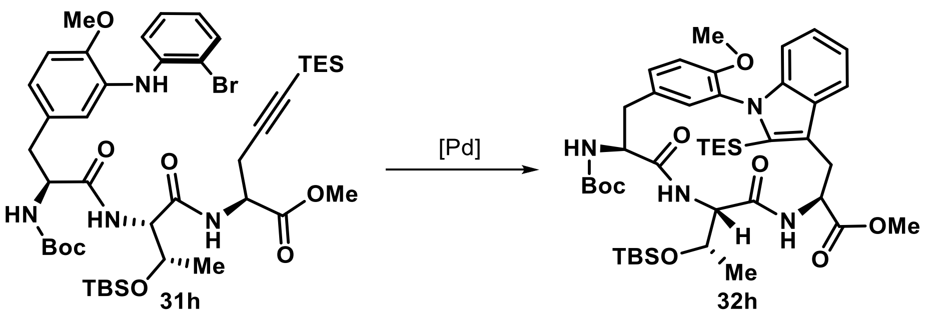

On 0.1 mmol scale, **General Procedure L** was followed from compound **31h** via Larock macrocyclization. Purification by silica gel column chromatography gave the title compound **32h** (41.1 mg, 50% yield).

Compound 32h

Physical State: amorphous solid

^{1}H NMR (600 MHz, CDCl$_3$): δ 7.40 (d, J = 7.2 Hz, 1H), 7.20 – 7.12 (m, 2H), 6.99 (s, 2H), 6.93 (d, J = 7.7 Hz, 1H), 6.42 (d, J = 8.4 Hz, 1H), 5.98 (s, 1H), 5.51 (d, J = 9.3 Hz, 1H), 5.26 (d, J = 7.1 Hz, 1H), 5.02 (td, J = 10.0, 7.1 Hz, 1H), 4.34 (dt, J = 7.5, 4.2 Hz, 1H), 3.95 (s, 3H), 3.85 (s, 3H), 3.67 (q, J = 6.2 Hz, 1H), 3.55 (dd, J = 14.7, 7.0 Hz, 1H), 3.31 (dd, J = 14.6, 10.5 Hz, 1H), 3.17 (dd, J = 14.3, 4.7 Hz, 1H), 3.05 (dd, J = 8.2, 6.1 Hz, 1H), 2.82 (dd, J = 14.3, 3.2 Hz, 1H), 1.44 (s, 9H), 0.94 (d, J = 6.3 Hz, 3H), 0.88 (t, J = 7.8 Hz, 9H), 0.82 (s, 9H), 0.78 – 0.66 (m, 6H), -0.05 (s, 3H), -0.07 (s, 3H).

^{13}C NMR (151 MHz, CDCl$_3$): δ 172.16, 168.98, 168.70, 155.07, 153.08, 145.18, 140.65, 131.55, 131.34, 130.79, 129.73, 128.15, 125.97, 123.81, 121.07, 116.94, 113.08, 111.54, 79.55, 69.84, 57.90, 55.74, 54.70, 54.09, 52.63, 36.89, 28.47, 27.33, 25.90, 20.21, 18.10, 7.63, 5.44, 4.58, 0.08, -4.58, -4.66.

HRMS (ESI-TOF): calculated for $C_{43}H_{66}N_4NaO_8Si_2^+$ [M+Na]$^+$: 845.4311, found: 845.4316.

[α]$^{25}_D$: +8.4 (c = 0.5, CHCl$_3$)

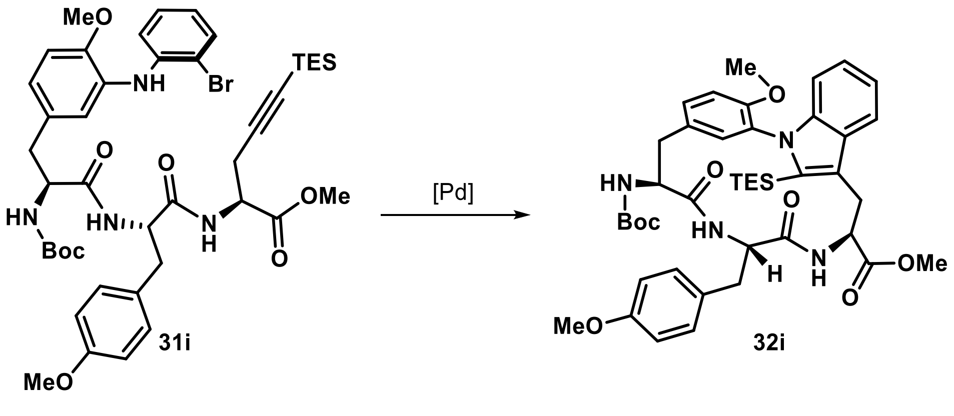

On 0.1 mmol scale, **General Procedure L** was followed from compound **31i** via Larock macrocyclization. Purification by silica gel column chromatography gave the title compound **32i** (39.2 mg, 50% yield).

Compound 32i

Physical State: amorphous solid

^{1}H NMR (600 MHz, CDCl$_3$): δ 7.26 – 7.06 (m, 3H), 7.03 – 6.74 (m, 7H), 6.50 – 6.34 (m, 1H), 6.05 – 5.93 (m, 1H), 5.32 (d, J = 7.0 Hz, 1H), 5.17 – 4.93 (m, 2H), 4.66 – 4.33 (m, 1H), 3.95 – 3.89 (m, 3H), 3.87 – 3.77 (m, 6H), 3.63 – 3.49 (m, 1H), 3.27 – 3.05 (m, 2H), 2.89 – 2.62 (m, 3H), 2.57 – 2.53 (m, 1H), 1.45 – 1.42 (m, 9H), 0.88 – 0.79 (m, 9H), 0.78 – 0.49 (m, 6H).

^{13}C NMR (151 MHz, CDCl$_3$): δ 171.89, 169.95, 169.04, 168.33, 158.70, 155.10, 153.06, 144.96, 140.49, 131.59, 131.47, 130.80, 130.59, 130.29, 129.90, 127.99, 127.50, 125.74, 123.77, 121.25, 119.99, 118.40, 116.70, 114.06, 113.01, 111.59, 79.53, 55.76, 55.61, 55.32, 55.26, 54.60, 53.81, 53.30, 52.64, 52.09, 38.87, 37.35, 28.50, 28.41, 27.09, 7.65, 7.61, 5.41, 4.52.

HRMS (ESI-TOF): calculated for $C_{49}H_{60}N_4NaO_8Si^+$ [M+Na]$^+$: 883.4073, found: 883.4075.

[α]$^{25}_{D}$: +7.8 (c = 0.5, $CHCl_3$)

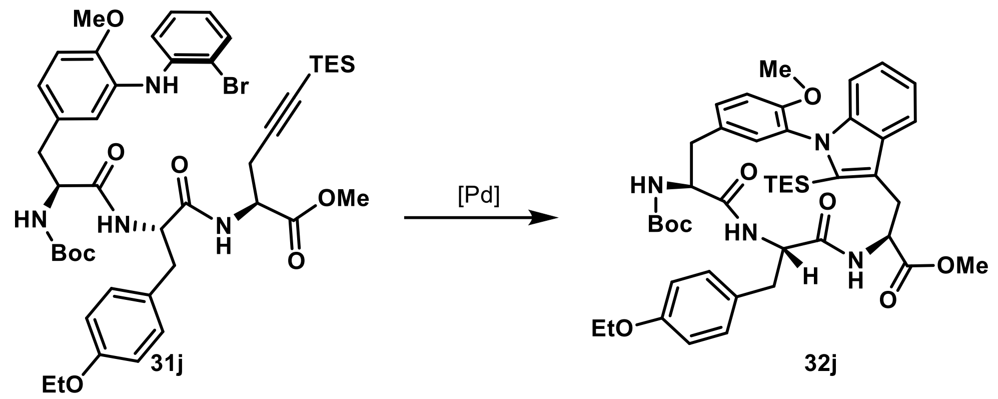

31j 32j

On 0.1 mmol scale, **General Procedure L** was followed from compound **31j** via Larock macrocyclization. Purification by silica gel column chromatography gave the title compound **32j** (41.5 mg, 52% yield).

Compound 32j

Physical State: amorphous solid

^{1}H NMR (600 MHz, $CDCl_3$): δ 7.26 – 7.07 (m, 3H), 6.99 (q, J = 6.7 Hz, 2H), 6.92 (d, J = 8.2 Hz, 1H), 6.85 (d, J = 8.5 Hz, 1H), 6.76 (d, J = 8.5 Hz, 2H), 6.52 – 6.38 (m, 1H), 6.00 (s, 1H), 5.32 (d, J = 6.9 Hz, 1H), 5.15 – 4.96 (m, 2H), 4.39 – 4.37 (m, 1H), 4.05 – 3.98 (m, 2H), 3.95 – 3.89 (m, 3H), 3.84 – 3.77 (m, 3H), 3.54 – 3.49 (m, 1H), 3.28 – 3.02 (m, 2H), 2.83 – 2.80 (m, 1H), 2.70 – 2.62 (m, 1H), 2.56 – 2.52 (m, 1H), 1.50 – 1.38 (m, 12H), 0.82 (dt, J = 39.0, 7.9 Hz, 9H), 0.78 – 0.49 (m, 6H).

^{13}C NMR (151 MHz, $CDCl_3$): δ 171.88, 170.00, 169.10, 168.33, 158.08, 155.11, 153.06, 144.95, 140.49, 131.60, 130.81, 130.56, 130.26, 129.90, 128.00, 127.37, 125.76, 123.75, 121.24, 119.98, 118.41, 116.72, 114.63, 113.00, 111.58, 79.52, 63.46, 55.75, 54.59, 53.82, 53.32, 52.63, 38.90, 37.34, 28.50, 28.41, 27.07, 14.97, 7.61, 5.40, 4.51.

HRMS (ESI-TOF): calculated for $C_{44}H_{58}N_4NaO_8Si^+$ $[M+Na]^+$: 821.3916, found: 821.3922.

[α]$^{25}_{D}$: +5.6 (c = 0.5, $CHCl_3$)

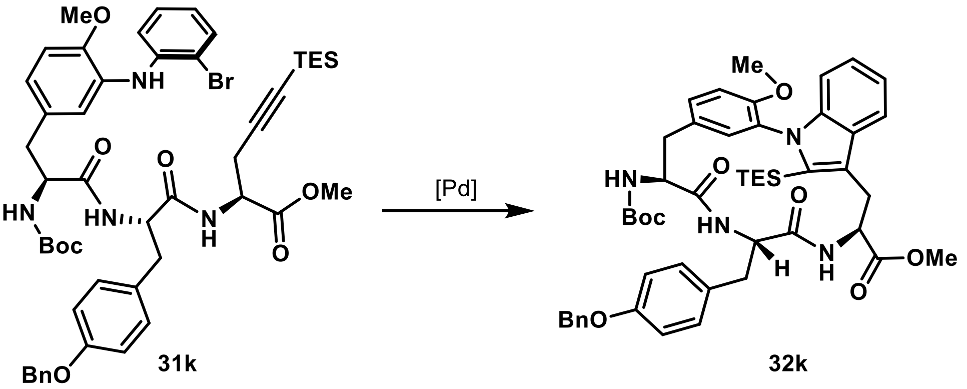

31k 32k

On 0.1 mmol scale, **General Procedure L** was followed from compound **31k** via Larock macrocyclization. Purification by silica gel column chromatography gave the title compound **32k** (49.9 mg, 58% yield).

Compound 32k

Physical State: amorphous solid

^{1}H NMR (600 MHz, $CDCl_3$): δ 7.47 – 7.28 (m, 8H), 7.22 – 7.15 (m, 2H), 7.11 – 7.06 (m, 1H), 7.03 – 6.96 (m, 2H), 6.92 – 6.89 (m, 1H), 6.86 – 6.84 (m, 3H), 6.51 – 6.37 (m, 1H), 5.11 – 4.92 (m, 4H), 4.68 (s, 1H), 3.96 – 3.76 (m, 6H), 3.68 – 3.49 (m, 1H), 3.24 – 3.05 (m, 2H), 3.02 – 2.72 (m, 2H), 2.70 – 2.62 (m, 1H), 2.54 (dd, J = 13.9, 8.1 Hz, 1H), 1.50 – 1.40 (m, 9H), 0.87 – 0.64 (m, 9H), 0.75 – 0.47 (m, 6H).

^{13}C NMR (151 MHz, $CDCl_3$): δ 171.86, 169.93, 169.07, 168.34, 157.90, 155.11, 153.06, 144.95, 140.46, 137.17, 131.58, 131.46, 130.80, 130.62, 130.34, 129.89, 128.71, 128.08, 127.84, 127.72, 127.55, 127.08,

125.76, 123.75, 121.26, 120.01, 118.41, 116.71, 115.06, 114.96, 112.98, 112.26, 111.59, 79.54, 70.04, 65.41, 55.76, 55.61, 54.60, 53.84, 53.31, 52.71, 52.63, 38.89, 37.37, 31.53, 28.50, 28.43, 27.07, 18.75, 17.48, 7.62, 5.42, 4.52.

HRMS (ESI-TOF): calculated for $C_{49}H_{60}N_4NaO_8Si^+$ $[M+Na]^+$: 883.4073, found: 883.4075.

[α]^{25}$_D$: +6.2 (c = 0.5, $CHCl_3$)

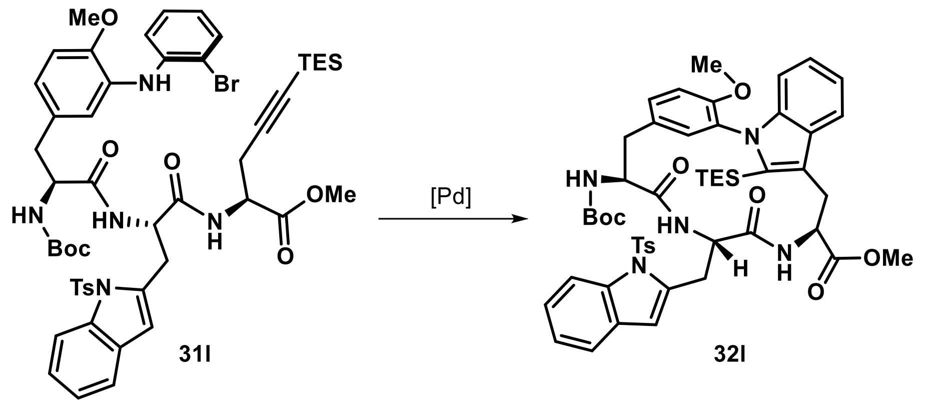

On 0.1 mmol scale, **General Procedure L** was followed from compound **31l** via Larock macrocyclization. Purification by silica gel column chromatography gave the title compound **32l** (53.0 mg, 56% yield).

Compound 32l

Physical State: amorphous solid

^{1}H NMR (600 MHz, CDCl$_3$): δ 8.01 - 7.95 (m, 1H), 7.82 – 7.67 (m, 2H), 7.44 – 7.28 (m, 3H), 7.26 – 7.11 (m, 4H), 6.99 (s, 2H), 6.94 – 6.76 (m, 2H), 6.68 – 6.25 (m, 2H), 5.96 (s, 1H), 5.41 (d, J = 6.9 Hz, 1H), 5.26 – 4.92 (m, 2H), 4.46 – 4.33 (m, 1H), 3.97 - 3.87 (m, 6H), 3.76 (s, 1H), 3.62 – 3.48 (m, 1H), 3.25 – 3.01 (m, 2H), 2.89 – 2.58 (m, 3H), 2.33 – 2.32 (m, 3H), 1.51 – 1.41 (m, 9H), 0.87 – 0.87 (m, 9H), 0.75 – 0.45 (m, 6H).

^{13}C NMR (151 MHz, CDCl$_3$): δ 171.85, 169.85, 168.72, 155.05, 153.07, 145.13, 144.77, 140.32, 135.49, 135.10, 131.41, 131.20, 130.90, 130.33, 129.98, 129.85, 127.93, 127.13, 126.91, 125.70, 124.93, 123.84, 123.64, 120.95, 120.14, 116.96, 115.80, 113.72, 113.52, 112.55, 111.67, 79.58, 55.75, 54.67, 53.97, 53.00, 51.85, 37.29, 29.82, 28.51, 28.38, 26.95, 21.66, 7.58, 5.41, 4.53.

HRMS (ESI-TOF): calculated for $C_{51}H_{61}N_5NaO_9SSi^+$ $[M+Na]^+$: 970.3851, found: 970.3853.

[α]^{25}$_D$: +53.9 (c = 0.5, $CHCl_3$)

Synthesis of extended micitide scaffolds:

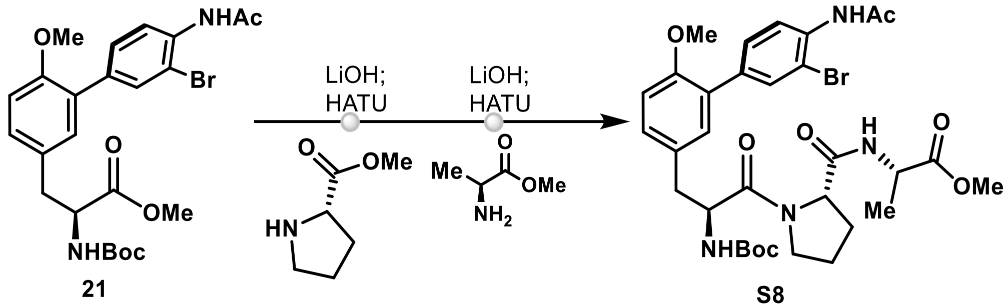

The compound **21** (3.18 g, 6.11 mmol, 1.0 eq.) was dissolved in MeOH/H_2O (1:1, 15 mL), treated with LiOH·H_2O (513 mg, 12.22 mmol, 2.0 eq.), and stirred at rt for 30 minutes. Upon completion, the reaction was quenched by adjusting the pH to 3 with 0.5 M aq. HCl and then extracted with DCM for three times. The combined organic layers were washed with saturated aq. NaCl, dried over Na_2SO_4, and concentrated *in vacuo* to give the residue, the residue was used directly in the subsequent step.
To a solution of the residue in DCM (30 mL) was added *L*-proline methyl ester hydrochloride (1.21 g, 7.33 mmol, 1.2 eq.), DIPEA (3.16 g, 24.44 mmol, 4.0 eq.) HATU (2.79 g, 7.33 mmol, 1.2 eq.) sequentially. After stirring at rt for 1 h, the reaction was quenched with 0.5 M HCl and extracted with DCM for three times. The combined organic layers were washed with saturated aq. NaCl, dried over Na_2SO_4, and concentrated *in vacuo* to give the residue, the residue was used directly in the subsequent step.
The residue was dissolved in MeOH/H_2O (1:1, 15 mL), treated with LiOH·H_2O (513 mg, 12.22 mmol, 2.0 eq.), and stirred at rt for 30 minutes. Upon completion, the reaction was quenched by adjusting the pH to 3 with 0.5 M aq. HCl and then extracted with DCM for three times. The combined organic layers were washed with saturated aq. NaCl, dried over Na_2SO_4, and concentrated *in vacuo* to give the residue, the residue was used directly in the subsequent step.
To a solution of the residue in DCM (30 mL) was added *L*-alanine methyl ester hydrochloride (1.02 g, 7.33 mmol, 1.2 eq.), DIPEA (3.16 g, 24.44 mmol, 4.0 eq.) HATU (2.79 g, 7.33 mmol, 1.2 eq.) sequentially. After stirring at rt for 1 h, the reaction was quenched with 0.5 M HCl and extracted with DCM for three times. The combined organic layers were washed with saturated aq. NaCl, dried over Na_2SO_4, and concentrated *in vacuo* to give the residue, the residue was purified by silica gel chromatography to afford the compound **S8** (3.20 g, 76% yield).

Compound S8

Physical State: amorphous solid

^{1}H NMR (600 MHz, $CDCl_3$): δ 8.35 (d, *J* = 8.1 Hz, 1H), 7.71 (s, 1H), 7.66 – 7.63 (m, 1H), 7.47 – 7.44 (m, 1H), 7.16 – 7.07 (m, 3H), 6.85 (d, *J* = 8.1 Hz, 1H), 5.28 (d, *J* = 8.5 Hz, 1H), 4.65 (q, *J* = 7.9 Hz, 1H), 4.57 – 4.48 (m, 1H), 4.46 – 4.36 (m, 1H), 3.79 – 3.77 (m, 3H), 3.73 – 3.63 (m, 3H), 3.31 – 3.27 (m, 1H), 3.03 (dd, *J* = 13.8, 6.5 Hz, 1H), 2.93 – 2.85 (m, 1H), 2.32 – 2.26 (m, 1H), 2.25 (s, 3H), 2.04 – 1.97 (m, 1H), 1.96 – 1.88 (m, 2H), 1.75 (s, 1H), 1.37 (s, 9H), 1.32 (d, *J* = 7.2 Hz, 3H).

^{13}C NMR (151 MHz, $CDCl_3$): δ 173.27, 171.87, 170.48, 168.25, 155.49, 155.22, 135.42, 134.52, 132.96, 131.80, 130.07, 129.72, 128.54, 121.22, 112.78, 111.35, 79.88, 60.02, 55.74, 53.24, 52.51, 48.27, 47.53, 38.26, 28.39, 27.46, 25.23, 25.02, 18.15.

HRMS (ESI-TOF): calculated for $C_{32}H_{41}BrN_4NaO_8^+$ $[M+Na]^+$: 711.2000, found: 711.2005.

$[\alpha]^{25}_D$: +11.6 (*c* = 0.5, $CHCl_3$)

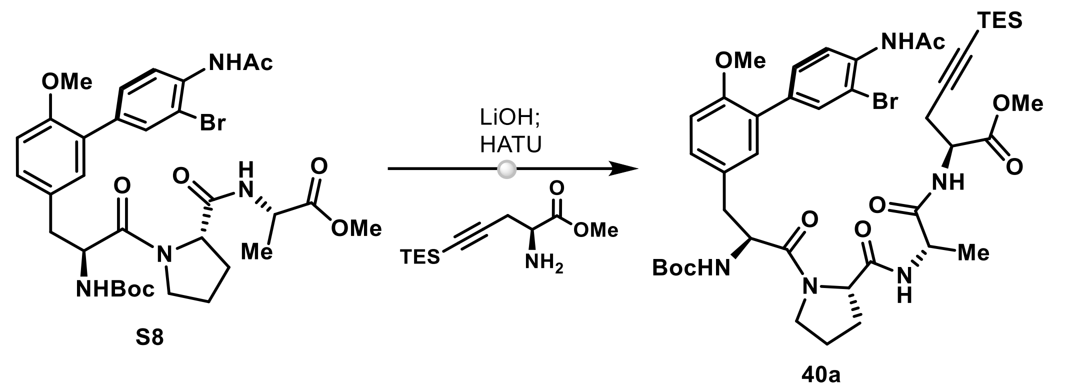

The compound **S8** (750 mg, 1.09 mmol, 1.0 eq.) was dissolved in $MeOH/H_2O$ (1:1, 3 mL), treated with $LiOH{\cdot}H_2O$ (91.5 mg, 2.18 mmol, 2.0 eq.), and stirred at rt for 30 minutes. Upon completion, the reaction was quenched by adjusting the pH to 3 with 0.5 M aq. HCl and then extracted with DCM for three times. The combined organic layers were washed with saturated aq. NaCl, dried over Na_2SO_4, and concentrated *in vacuo* to give the residue, the residue was used directly in the subsequent step.

A mixture of the residue in DCM (6 mL) was added DIPEA (563.5 mg, 4.36 mmol, 4.0 eq.), HATU (497.4 mg, 1.31 mmol, 1.2 eq.), methyl (*S*)-2-amino-5-(triethylsilyl)pent-4-ynoate (315.8 mg, 1.31 mmol, 1.2 eq.) sequentially. The reaction was stirred for 1 h, quenched with 0.5 M HCl and extracted with DCM for three times. The combined organic layers were washed with saturated aq. NaCl, dried over Na_2SO_4, and concentrated *in vacuo* to give the residue, the residue was purified by silica gel chromatography to afford the compound **40a** (802 mg, 82% yield from **S8**).

Compound 40a.

Physical State: amorphous solid

^{1}H NMR (600 MHz, $CDCl_3$): δ 8.23 (d, *J* = 8.5 Hz, 1H), 7.72 (d, *J* = 15.7 Hz, 2H), 7.47 (d, *J* = 8.5 Hz, 1H), 7.13 (d, *J* = 2.3 Hz, 1H), 7.10 (d, *J* = 7.8 Hz, 1H), 7.03 (d, *J* = 7.3 Hz, 1H), 6.98 (d, *J* = 7.8 Hz, 1H), 6.84 (d, *J* = 8.3 Hz, 1H), 5.30 (d, *J* = 8.7 Hz, 1H), 4.72 – 4.59 (m, 2H), 4.44 (dd, *J* = 7.9, 4.0 Hz, 1H), 4.31 – 4.26 (m, 1H), 3.76 (s, 3H), 3.73 (s, 3H), 3.62 (q, *J* = 7.4 Hz, 1H), 3.36 – 3.32 (m, 1H), 3.04 (dd, *J* = 13.9, 6.8 Hz, 1H), 2.91 (dd, *J* = 13.8, 5.9 Hz, 1H), 2.82 – 2.71 (m, 2H), 2.24 (s, 3H), 2.21 – 2.17 (m, 1H), 2.10 – 2.00 (m, 1H), 2.00 – 1.88 (m, 2H), 1.36 (s, 9H), 1.20 (d, *J* = 6.8 Hz, 3H), 0.95 (t, *J* = 7.9 Hz, 9H), 0.54 (q, *J* = 7.9 Hz, 6H).

^{13}C NMR (151 MHz, $CDCl_3$): δ 171.99, 171.49, 170.92, 170.83, 168.54, 155.48, 155.26, 135.78, 134.36, 133.10, 132.03, 130.33, 129.79, 128.45, 128.32, 122.05, 113.38, 111.27, 101.75, 85.56, 79.79, 60.23, 55.72, 53.19, 52.66, 51.29, 49.04, 47.53, 37.77, 28.39, 28.01, 25.33, 24.82, 23.75, 18.37, 7.52, 4.42.

HRMS (ESI-TOF): calculated for $C_{43}H_{60}BrN_5NaO_9Si^+$ $[M+Na]^+$: 920.3236, found: 920.3256.

$[\alpha]^{25}_D$: +14.4 (*c* = 0.5, $CHCl_3$)

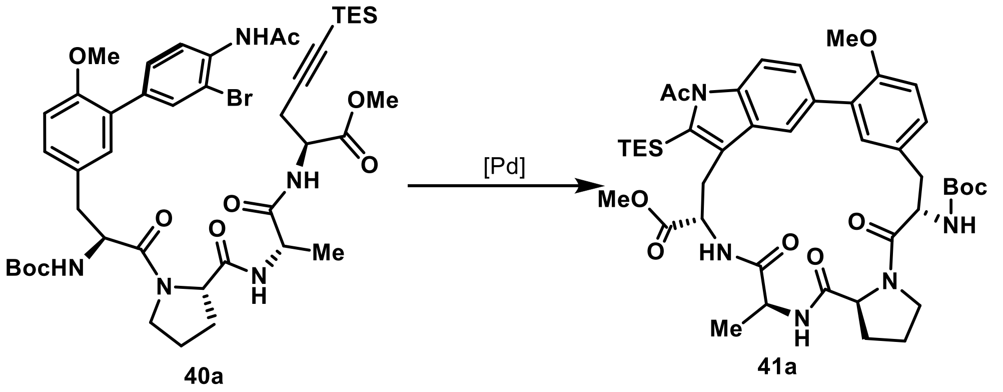

The compound **40a** (671 mg, 0.75 mmol, 1.0 eq.) was dissolved toluene (10.0 mL). $Pd(OAc)_2$ (30 mol%), *t*$Bu_3P{\cdot}HBF_4$ (60 mol%) and DIPEA (2.25 mmol, 3.0 eq.) were added. The reaction mixture was stirred at 110 °C under nitrogen atmosphere for 16 h. The reaction mixture was concentrated under reduced pressure and

purified by silica gel chromatography to give the cyclized compound **41a** (220 mg, 36% yield).

Compound 41a

Physical State: amorphous solid

^{1}H NMR (600 MHz, CDCl$_3$): δ 8.70 – 8.42 (m, 1H), 7.93 – 7.83 (m, 1H), 7.64 – 7.56 (m, 2H), 7.20 – 7.11 (m, 1H), 6.91 (d, *J* = 8.3 Hz, 1H), 6.77 – 6.49 (m, 1H), 5.09 (s, 1H), 5.04 – 4.76 (m, 2H), 4.73 – 4.63 (m, 1H), 4.36 (d, *J* = 8.3 Hz, 1H), 3.88 – 3.69 (m, 6H), 3.68 – 3.61 (m, 2H), 3.48 – 3.38 (m, 1H), 3.28 – 3.22 (m, 1H), 3.06 – 3.00 (m, 1H), 2.82 (s, 3H), 2.83 – 2.78 (m, 1H), 2.77 - 2.70 (m, 1H), 2.14 – 2.05 (m, 1H), 2.00 – 1.88 (m, 3H), 1.42 (d, *J* = 8.6 Hz, 3H), 1.39 – 1.23 (m, 9H), 0.96 – 0.90 (m, 9H), 0.86 – 0.80 (m, 6H).

^{13}C NMR (151 MHz, CDCl$_3$): δ 173.19, 172.48, 172.35, 172.14, 171.11, 170.58, 169.83, 169.55, 156.54, 155.72, 155.19, 136.53, 136.25, 133.38, 131.88, 131.06, 130.28, 129.12, 128.65, 128.31, 126.91, 121.71, 120.91, 112.81, 111.47, 101.27, 86.11, 80.08, 61.48, 61.08, 55.73, 55.02, 54.51, 52.96, 52.80, 52.36, 51.70, 51.14, 49.75, 48.84, 48.04, 47.66, 38.70, 37.17, 36.33, 31.96, 31.67, 28.42, 28.14, 28.02, 27.16, 26.77, 26.67, 24.84, 23.75, 23.26, 22.34, 18.92, 16.49, 16.21, 8.35, 7.50, 6.32, 5.93, 4.39.

HRMS (ESI-TOF): calculated for $C_{43}H_{59}N_5NaO_9Si^+$ $[M+Na]^+$: 840.3974, found: 840.3997.

$[\alpha]^{25}_D$: –11.6 (*c* = 0.2, CHCl$_3$)

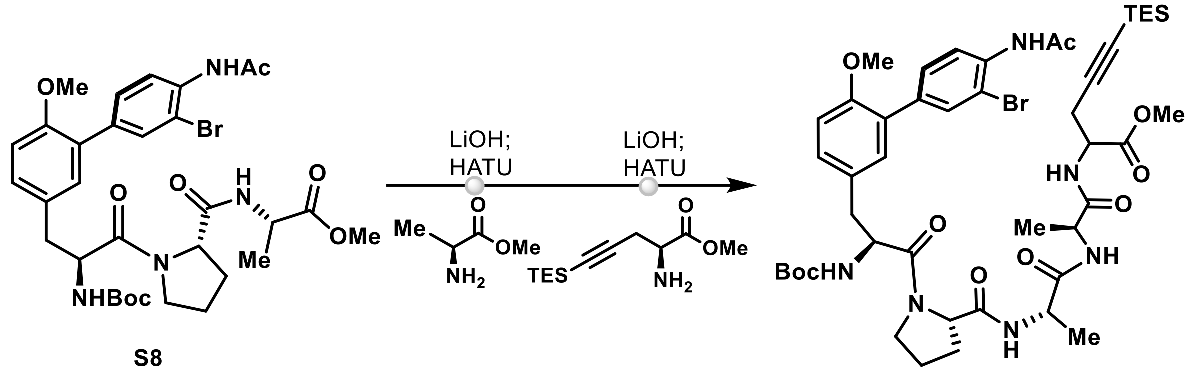

S8 — 40b

The compound **S8** (623.1 mg, 0.9 mmol, 1.0 eq.) was dissolved in MeOH/H_2O (1:1, 3 mL), treated with LiOH·H_2O (76 mg, 1.81 mmol, 2.0 eq.), and stirred at rt for 30 minutes. Upon completion, the reaction was quenched by adjusting the pH to 3 with 0.5 M aq. HCl and then extracted with DCM for three times. The combined organic layers were washed with saturated aq. NaCl, dried over Na_2SO_4, and concentrated *in vacuo* to give the residue, the residue was used directly in the subsequent step.

To a solution of the residue in DCM (5 mL) was added *L*-alanine methyl ester hydrochloride (151 mg, 1.08 mmol, 1.2 eq.), DIPEA (467 mg, 3.61 mmol, 4.0 eq.) and HATU (411 mg, 1.08 mmol, 1.2 eq.) sequentially. After stirring at rt for 1 h, the reaction was quenched with 0.5 M HCl and extracted with DCM for three times. The combined organic layers were washed with saturated aq. NaCl, dried over Na_2SO_4, and concentrated *in vacuo* to give the residue, the residue was directly in the subsequent step.

The residue was dissolved in MeOH/H_2O (1:1, 3 mL), treated with LiOH·H_2O (76 mg, 1.81 mmol, 2.0 eq.), and stirred at rt for 30 minutes. Upon completion, the reaction was quenched by adjusting the pH to 3 with 0.5 M aq. HCl and then extracted with DCM for three times. The combined organic layers were washed with saturated aq. NaCl, dried over Na_2SO_4, and concentrated *in vacuo* to give the residue, the residue was used directly in the subsequent step.

A mixture of the residue in DCM (5 mL) was added DIPEA (467 mg, 3.61 mmol, 4.0 eq.), HATU (411 mg, 1.08 mmol, 1.2 eq.), methyl (*S*)-2-amino-5-(triethylsilyl)pent-4-ynoate (260.7 mg, 1.08 mmol, 1.2 eq.) sequentially. The reaction was stirred for 1 h, quenched with 0.5 M HCl and extracted with DCM for three times. The combined organic layers were washed with saturated aq. NaCl, dried over Na_2SO_4, and concentrated *in vacuo* to give the residue, the residue was purified by silica gel chromatography to afford the compound **40b** (710 mg, 81% yield from **S8**).

Compound 40b

Physical State: amorphous solid

^{1}H NMR (600 MHz, CDCl$_3$): δ 8.18 (d, *J* = 8.4 Hz, 1H), 7.77 – 7.62 (m, 2H), 7.45 (dd, *J* = 8.6, 1.7 Hz, 1H), 7.12 – 7.07 (m, 2H), 6.93 – 6.80 (m, 2H), 5.21 (d, *J* = 7.9 Hz, 1H), 4.69 – 4.43 (m, 2H), 4.43 – 4.40 (m, 1H), 4.21 – 4.08 (m, 1H), 3.79 – 3.71 (m, 6H), 3.67 – 3.60 (m, 3H), 3.45 – 3.26 (m, 1H), 3.08 (q, *J* = 7.4 Hz, 2H), 3.02 – 2.91 (m, 1H), 2.83 – 2.75 (m, 1H), 2.75 – 2.65 (m, 1H), 2.24 (s, 3H), 2.13 – 2.09 (m, 1H), 2.05 – 1.92 (m, 3H), 1.40 – 1.37 (m, 9H), 1.36 – 1.33 (m, 6H), 0.96 – 0.93 (m, 9H), 0.57 – 0.52 (m, 6H).

^{13}C NMR (151 MHz, CDCl$_3$): δ 172.19, 172.13, 171.49, 171.05, 170.76, 168.75, 168.28, 166.41, 155.57, 155.34, 135.93, 134.34, 133.21, 131.98, 130.30, 129.75, 128.60, 128.12, 122.52, 113.75, 111.35, 101.76, 101.61, 86.98, 85.61, 80.05, 60.67, 55.74, 53.88, 53.73, 53.68, 52.65, 52.37, 51.28, 50.93, 49.16, 47.63, 42.12, 39.37, 39.04, 37.54, 29.77, 29.13, 28.45, 28.39, 28.16, 25.80, 25.38, 25.16, 24.74, 23.72, 19.93, 17.98, 14.19, 12.13, 7.50, 4.41, 4.36.

HRMS (ESI-TOF): calculated for $C_{46}H_{65}BrN_6NaO_{10}Si^+$ [M+Na]$^+$: 991.3607, found: 991.3610.

$[\alpha]^{25}_D$: +2.8 (*c* = 0.5, CHCl$_3$)

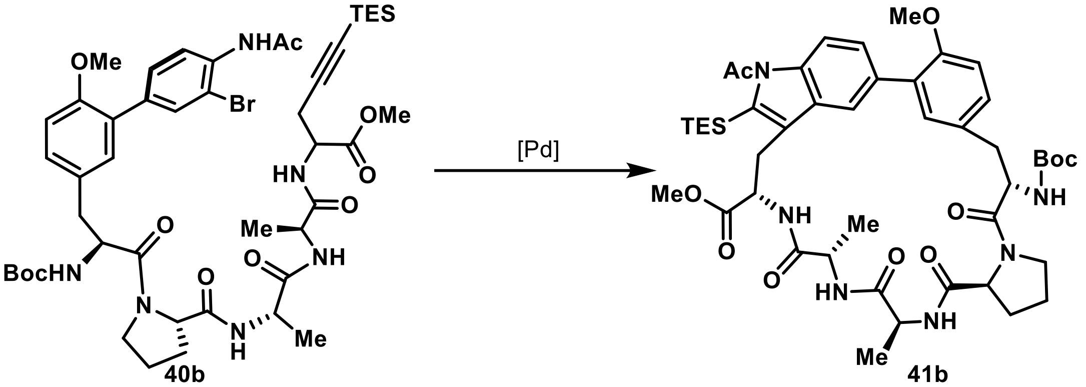

40b 41b

The compound **40b** (604 mg, 0.62 mmol, 1.0 eq.) was dissolved toluene (8 mL). Pd(OAc)$_2$ (30 mol%), *t*Bu$_3$P·HBF$_4$ (60 mol%) and DIPEA (1.86 mmol, 3.0 eq.) were added. The reaction mixture was stirred at 110 °C under nitrogen atmosphere for 16 h. The reaction mixture was concentrated under reduced pressure and purified by silica gel chromatography to give the cyclized compound **41b** (120 mg, 22% yield).

Compound 41b

Physical State: amorphous solid

^{1}H NMR (600 MHz, CDCl$_3$): δ 8.10 (d, *J* = 1.5 Hz, 1H), 7.87 – 7.81 (m, 2H), 7.66 (d, *J* = 8.9 Hz, 1H), 7.44 (d, *J* = 6.8 Hz, 1H), 7.38 (d, *J* = 9.1 Hz, 1H), 7.04 (dd, *J* = 8.3, 2.2 Hz, 1H), 6.88 (d, *J* = 8.4 Hz, 1H), 6.72 – 6.69 (m, 1H), 5.35 (d, *J* = 6.5 Hz, 1H), 5.10 (ddd, *J* = 11.9, 9.2, 3.6 Hz, 1H), 4.90 (dt, *J* = 11.7, 5.8 Hz, 1H), 4.56 – 4.52 (m, 1H), 4.18 – 4.12 (m, 1H), 3.92 (d, *J* = 7.9 Hz, 1H), 3.82 (s, 3H), 3.77 (s, 1H), 3.56 (s, 3H), 3.49 – 3.44 (m, 1H), 3.42 (d, *J* = 1.9 Hz, 1H), 3.35 (dd, *J* = 14.0, 3.5 Hz, 1H), 3.20 (dd, *J* = 12.3, 4.7 Hz, 1H), 2.91 – 2.87 (m, 1H), 2.84 (s, 3H), 2.02 (s, 1H), 1.98 – 1.83 (m, 3H), 1.45 (s, 9H), 1.29 (d, *J* = 7.0 Hz, 3H), 0.97 – 0.92 (m, 12H), 0.89 – 0.83 (m, 6H).

^{13}C NMR (151 MHz CDCl$_3$): δ 173.62, 172.69, 172.04, 171.97, 170.86, 169.49, 156.95, 156.07, 136.82, 136.26, 133.21, 132.93, 132.47, 132.23, 132.17, 130.77, 129.97, 129.33, 128.65, 128.57, 127.67, 126.76, 121.05, 112.88, 111.55, 81.19, 61.19, 55.93, 54.29, 52.79, 52.41, 52.17, 51.09, 48.86, 47.99, 46.54, 38.57, 30.52, 30.33, 28.55, 26.72, 23.77, 23.28, 21.72, 18.94, 18.34, 16.26, 8.33, 7.50, 6.41, 4.40, 4.33.

HRMS (ESI-TOF): calculated for $C_{46}H_{64}N_6NaO_{10}Si^+$ [M+Na]$^+$: 911.4345, found: 911.4357.

$[\alpha]^{25}_D$: –14.8 (*c* = 0.2, CHCl$_3$)

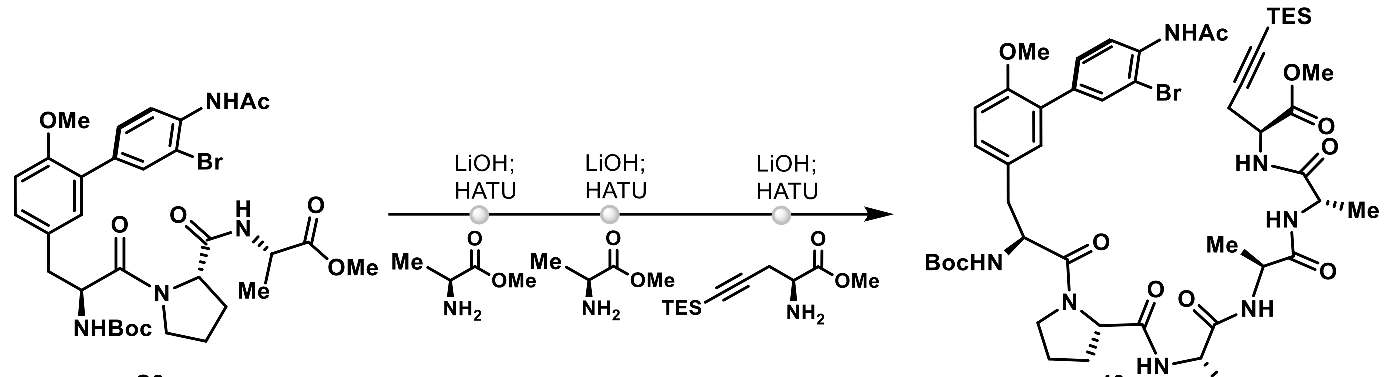

The compound **S8** (1.16 g, 1.68 mmol, 1.0 eq.) was dissolved in MeOH/H_2O (1:1, 5 mL), treated with LiOH·H_2O (141 mg, 3.36 mmol, 2.0 eq.), and stirred at rt for 30 minutes. Upon completion, the reaction was quenched by adjusting the pH to 3 with 0.5 M aq. HCl and then extracted with DCM for three times. The combined organic layers were washed with saturated aq. NaCl, dried over Na_2SO_4, and concentrated *in vacuo* to give the residue, the residue was used directly in the subsequent step.

To a solution of the residue in DCM (8 mL) was added *L*-alanine methyl ester hydrochloride (281.4 mg, 2.02 mmol, 1.2 eq.), DIPEA (869 mg, 6.72 mmol, 4.0 eq.) HATU (767 mg, 2.02 mmol, 1.2 eq.) sequentially. After stirring at rt for 1 h, the reaction was quenched with 0.5 M HCl and extracted with DCM for three times. The combined organic layers were washed with saturated aq. NaCl, dried over Na_2SO_4, and concentrated *in vacuo* to give the residue, the residue was used directly in the subsequent step.

The residue was dissolved in MeOH/H_2O (1:1, 5 mL), treated with LiOH·H_2O (141 mg, 3.36 mmol, 2.0 eq.), and stirred at rt for 30 minutes. Upon completion, the reaction was quenched by adjusting the pH to 3 with 0.5 M aq. HCl and then extracted with DCM for three times. The combined organic layers were washed with saturated aq. NaCl, dried over Na_2SO_4, and concentrated *in vacuo* to give the residue, the residue was used directly in the subsequent step.

To a solution of the residue in DCM (8 mL) was added *L*-alanine methyl ester hydrochloride (281.4 mg, 2.02 mmol, 1.2 eq.), DIPEA (869 mg, 6.72 mmol, 4.0 eq.) HATU (767 mg, 2.02 mmol, 1.2 eq.) sequentially. After stirring at rt for 1 h, the reaction was quenched with 0.5 M HCl and extracted with DCM for three times. The combined organic layers were washed with saturated aq. NaCl, dried over Na_2SO_4, and concentrated *in vacuo* to give the residue, the residue was directly in the subsequent step.

The residue was dissolved in MeOH/H_2O (1:1, 5 mL), treated with LiOH·H_2O (141 mg, 3.36 mmol, 2.0 eq.), and stirred at rt for 30 minutes. Upon completion, the reaction was quenched by adjusting the pH to 3 with 0.5 M aq. HCl and then extracted with DCM for three times. The combined organic layers were washed with saturated aq. NaCl, dried over Na_2SO_4, and concentrated *in vacuo* to give the residue, the residue was used directly in the subsequent step.

A mixture of the residue in DCM (8 mL) was added DIPEA (869 mg, 6.72 mmol, 4.0 eq.), HATU (767 mg, 2.02 mmol, 1.2 eq.), methyl (*S*)-2-amino-5-(triethylsilyl)pent-4-ynoate (488 mg, 2.02 mmol, 1.2 eq.) sequentially. The reaction was stirred for 1 h, quenched with 0.5 M HCl and extracted with DCM for three times. The combined organic layers were washed with saturated aq. NaCl, dried over Na_2SO_4, and concentrated *in vacuo* to give the residue, the residue was purified by silica gel chromatography to afford the compound **40c** (1.4 g, 80% yield from **S8**).

Compound 40c

Physical State: amorphous solid

^{1}H NMR (600 MHz, DMSO-*d*$_6$): δ 9.48 (s, 1H), 8.26 – 8.17 (m, 1H), 8.03 (d, *J* = 7.1 Hz, 1H), 7.85 (d, *J* = 7.5 Hz, 1H), 7.81 (d, *J* = 7.2 Hz, 1H), 7.69 (s, 1H), 7.56 (d, *J* = 8.2 Hz, 1H), 7.43 (d, *J* = 8.4 Hz, 1H), 7.29 – 7.21 (m, 2H), 7.03 – 6.95 (m, 2H), 4.40 – 4.14 (m, 6H), 3.71 (s, 3H), 3.61 (t, *J* = 6.4 Hz, 1H), 3.58 (s, 3H), 2.89 – 2.85 (m, 1H), 2.71 – 2.59 (m, 3H), 2.06 (s, 3H), 1.91 – 1.81 (m, 3H), 1.23 (s, 9H), 1.19 – 1.08 (m,

11H), 0.87 (t, J = 7.9 Hz, 9H), 0.48 (t, J = 7.9 Hz, 6H).
^{13}C NMR (151 MHz, DMSO-d_6): δ 172.69, 172.50, 172.16, 172.03, 171.18, 170.98, 169.15, 168.47, 166.01, 155.86, 155.19, 137.36, 135.54, 133.35, 131.93, 130.82, 130.67, 129.38, 127.61, 127.03, 117.89, 112.12, 104.22, 84.17, 78.51, 60.04, 56.15, 54.37, 53.58, 52.57, 51.82, 50.35, 48.81, 48.55, 48.34, 47.31, 35.86, 29.48, 28.63, 28.30, 25.10, 24.90, 23.77, 22.77, 21.16, 18.84, 18.54, 18.28, 7.85, 7.81, 4.45, 4.38.
HRMS (ESI-TOF): calculated for $C_{49}H_{70}BrN_7NaO_{11}Si^+$ [M+Na]$^+$: 1062.3978, found: 1062.3990.
[α]$^{25}_{D}$: –0.4 (c = 0.2, $CHCl_3$)

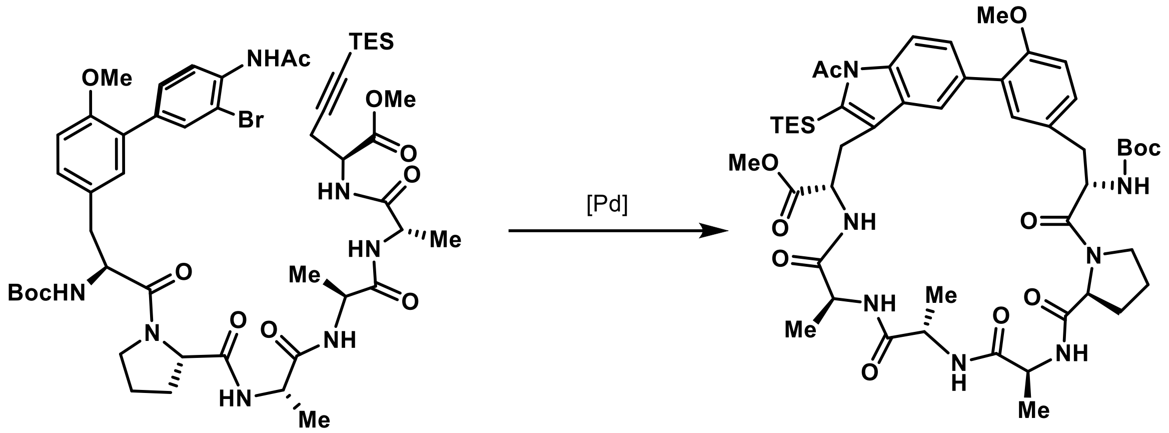

40c 41c

The compound **40c** (1.3 g, 1.25 mmol, 1.0 eq.) was dissolved toluene (15 mL). $Pd(OAc)_2$ (30 mol%), $t$$Bu_3P·HBF_4$ (60 mol%) and DIPEA (3.75 mmol, 3.0 eq.) were added. The reaction mixture was stirred at 110 °C under nitrogen atmosphere for 16 h. The reaction mixture was concentrated under reduced pressure and purified by silica gel chromatography to give the cyclized compound **41c** (180 mg, 15% yield).

Compound 41c

Physical State: amorphous solid

^{1}H NMR (600 MHz, $CDCl_3$): δ 9.73 (d, J = 9.6 Hz, 1H), 8.97 (d, J = 9.2 Hz, 1H), 8.66 (d, J = 9.0 Hz, 1H), 8.37 (s, 1H), 8.07 (d, J = 8.7 Hz, 1H), 7.84 – 7.80 (m, 1H), 7.76 (d, J = 9.9 Hz, 1H), 7.66 (d, J = 8.8 Hz, 1H), 7.47 (d, J = 10.0 Hz, 1H), 7.24 (d, J = 8.5 Hz, 1H), 6.98 (d, J = 8.6 Hz, 1H), 5.76 – 5.70 (m, 1H), 5.22 (q, J = 8.5 Hz, 1H), 5.03 – 4.96 (m, 1H), 4.80 – 4.68 (m, 3H), 4.00 – 3.95 (m, 1H), 3.91 – 3.86 (m, 1H), 3.81 (s, 3H), 3.54 (s, 3H), 3.38 (d, J = 7.8 Hz, 2H), 3.20 (d, J = 10.6 Hz, 1H), 2.84 (s, 3H), 2.69 (t, J = 13.0 Hz, 1H), 2.35 – 2.23 (m, 2H), 2.13 – 2.02 (m, 2H), 1.35 (d, J = 7.0 Hz, 3H), 1.25 (s, 9H), 1.13 – 1.09 (m, 6H), 0.99 – 0.96 (m, 9H), 0.87 – 0.82 (m, 6H).
^{13}C NMR (151 MHz, $CDCl_3$): δ 174.30, 174.15, 173.22, 172.79, 172.54, 171.52, 169.54, 156.41, 155.48, 136.18, 136.00, 134.38, 133.11, 132.54, 130.05, 129.68, 128.72, 128.07, 126.85, 121.07, 112.95, 112.19, 78.87, 60.69, 55.84, 54.96, 53.71, 53.35, 52.76, 50.94, 48.64, 48.22, 48.10, 47.86, 38.70, 38.07, 32.01, 31.56, 29.78, 29.14, 28.06, 26.55, 25.81, 25.68, 25.14, 22.78, 21.55, 20.69, 19.90, 18.40, 14.20, 8.05, 7.53, 6.32, 4.37.
HRMS (ESI-TOF): calculated for $C_{49}H_{69}N_7NaO_{11}Si^+$ [M+Na]$^+$: 982.4717, found: 982.4724.
[α]$^{25}_{D}$: +28.8 (c = 0.5, $CHCl_3$)

Synthesis of extended lapparbin scaffolds:

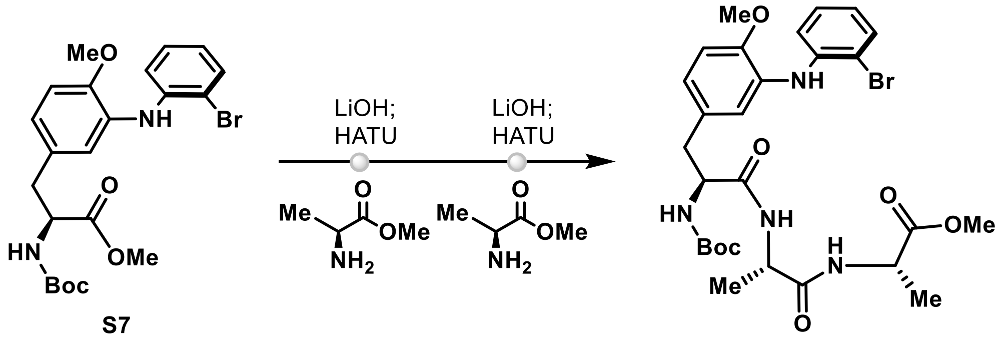

A solution of compound **S7** (3.55 g, 7.41 mmol, 1.0 eq.) in MeOH/H_2O (1:1, 20 mL) was treated with LiOH·H_2O (622 mg, 14.82 mmol, 2.0 eq.). The reaction mixture was stirred at rt for 30 minutes. Upon completion, the reaction was quenched by adjusting the pH to 3 with 0.5 M aq. HCl and then extracted with DCM for three times. The combined organic layers were washed with saturated aq. NaCl, dried over Na_2SO_4, and concentrated *in vacuo* to give the residue, the residue was used directly in the subsequent step.

To a solution of the residue in DCM (20 mL) was added *L*-alanine methyl ester hydrochloride (1.24 g, 8.89 mmol, 1.2 eq.), DIPEA (3.83 g, 29.6 mmol, 4.0 eq.) HATU (3.38 g, 8.89 mmol, 1.2 eq.) sequentially. After stirring at rt for 1 h, the reaction was quenched with 0.5 M HCl and extracted with DCM for three times. The combined organic layers were washed with saturated aq. NaCl, dried over Na_2SO_4, and concentrated *in vacuo* to give the residue, the residue was used directly in the subsequent step.

The residue was dissolved in MeOH/H_2O (1:1, 20 mL), treated with LiOH·H_2O (622 mg, 14.82 mmol, 2.0 eq.), and stirred at rt for 30 minutes. Upon completion, the reaction was quenched by adjusting the pH to 3 with 0.5 M aq. HCl and then extracted with DCM for three times. The combined organic layers were washed with saturated aq. NaCl, dried over Na_2SO_4, and concentrated *in vacuo* to give the residue, the residue was used directly in the subsequent step.

To a solution of the residue in DCM (20 mL) was added *L*-alanine methyl ester hydrochloride (1.24 g, 8.89 mmol, 1.2 eq.), DIPEA (3.83 g, 29.6 mmol, 4.0 eq.) HATU (3.38 g, 8.89 mmol, 1.2 eq.) sequentially. After stirring at rt for 1 h, the reaction was quenched with 0.5 M HCl and extracted with DCM for three times. The combined organic layers were washed with saturated aq. NaCl, dried over Na_2SO_4, and concentrated *in vacuo* to give the residue, the residue was purified by silica gel chromatography to afford the compound **S9** (3.5 g, 76% yield from **S7**).

Compound S9

Physical State: amorphous solid

^{1}H NMR (600 MHz, $CDCl_3$): δ 7.50 (dd, *J* = 8.0, 1.1 Hz, 1H), 7.35 – 7.29 (m, 1H), 7.21 – 7.16 (m, 1H), 7.13 (d, *J* = 7.3 Hz, 1H), 7.08 (d, *J* = 1.7 Hz, 1H), 6.98 – 6.88 (m, 1H), 6.80 (d, *J* = 8.3 Hz, 1H), 6.76 – 6.68 (m, 2H), 6.39 (s, 1H), 5.18 (d, *J* = 7.8 Hz, 1H), 4.58 – 4.49 (m, 1H), 4.48 – 4.40 (m, 1H), 4.35 (s, 1H), 3.84 (s, 3H), 3.68 (s, 3H), 2.98 (dd, *J* = 14.0, 5.6 Hz, 1H), 2.95 – 2.87 (m, 1H), 2.78 (s, 3H), 1.35 (s, 9H), 1.32 (d, *J* = 7.2 Hz, 3H), 1.29 (d, *J* = 7.0 Hz, 3H).

^{13}C NMR (151 MHz, $CDCl_3$): δ 173.18, 171.72, 171.43, 155.62, 148.46, 140.64, 133.11, 131.48, 128.68, 128.25, 122.23, 121.46, 117.57, 116.90, 113.51, 111.04, 80.26, 55.87, 52.47, 48.85, 48.18, 38.69, 37.74, 28.31, 18.58, 17.98.

HRMS (ESI-TOF): calculated for $C_{28}H_{37}BrN_4NaO_7^+$ [M+Na]$^+$: 643.1738, found: 643.1744.

$[\alpha]^{25}_D$: –8.2 (*c* = 0.5, $CHCl_3$)

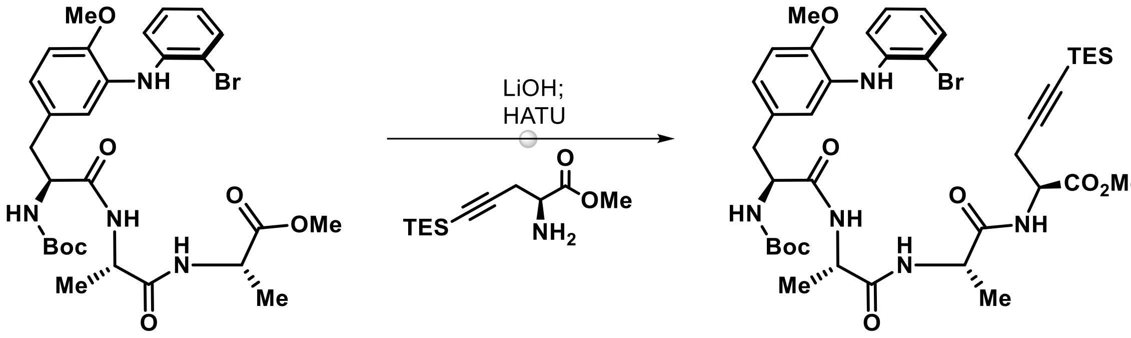

S9 **42a**

The compound **S9** (570.4 mg, 0.92 mmol, 1.0 eq.) was dissolved in MeOH/H_2O (1:1, 3 mL), treated with LiOH·H_2O (77 mg, 1.84 mmol, 2.0 eq.), and stirred at rt for 30 minutes. Upon completion, the reaction was quenched by adjusting the pH to 3 with 0.5 M aq. HCl and then extracted with DCM for three times. The combined organic layers were washed with saturated aq. NaCl, dried over Na_2SO_4, and concentrated *in vacuo* to give the residue, the residue was used directly in the subsequent step.

A mixture of the residue in DCM (5 mL) was added DIPEA (475.6 mg, 3.68 mmol, 4.0 eq.), HATU (420 mg, 1.1 mmol, 1.2 eq.), methyl (*S*)-2-amino-5-(triethylsilyl)pent-4-ynoate (266 mg, 1.1 mmol, 1.2 eq.) sequentially. The reaction was stirred for 1 h, quenched with 0.5 M HCl and extracted with DCM for three times. The combined organic layers were washed with saturated aq. NaCl, dried over Na_2SO_4, and concentrated *in vacuo* to give the residue, the residue was purified by silica gel chromatography to afford the compound **42a** (610 mg, 80% yield from **S9**).

Compound 42a

Physical State: amorphous solid

^{1}H NMR (600 MHz, CDCl$_3$): δ 7.57 – 7.48 (m, 1H), 7.40 – 7.28 (m, 2H), 7.19 (t, J = 7.7 Hz, 1H), 7.15 – 7.04 (m, 2H), 6.99 – 6.85 (m, 1H), 6.80 (d, J = 8.3 Hz, 1H), 6.78 – 6.69 (m, 2H), 6.52 – 6.26 (m, 1H), 5.33 – 5.27 (m, 1H), 4.68 (q, J = 5.7 Hz, 1H), 4.62 – 4.49 (m, 2H), 4.38 (s, 1H), 3.85 (s, 3H), 3.73 (s, 3H), 3.00 (dd, J = 14.0, 5.5 Hz, 1H), 2.92 (dd, J = 13.7, 7.4 Hz, 1H), 2.82 – 2.72 (m, 2H), 1.36 (s, 9H), 1.35 – 1.28 (m, 6H), 0.94 (t, J = 7.9 Hz, 9H), 0.53 (q, J = 7.9 Hz, 6H).

^{13}C NMR (151 MHz, CDCl$_3$): δ 172.00, 171.84, 171.60, 170.88, 155.73, 148.45, 140.62, 133.15, 131.55, 128.63, 128.25, 122.16, 121.55, 117.44, 116.99, 113.61, 111.01, 101.87, 85.46, 80.30, 55.87, 52.66, 51.41, 49.13, 49.07, 37.87, 31.65, 28.32, 23.77, 22.72, 18.87, 18.65, 14.19, 7.49, 4.40.

HRMS (ESI-TOF): calculated for $C_{39}H_{56}BrN_5NaO_8Si^+$ $[M+Na]^+$: 852.2974, found: 852.2979.

$[\alpha]^{25}_D$: –17.6 (c = 0.2, $CHCl_3$)

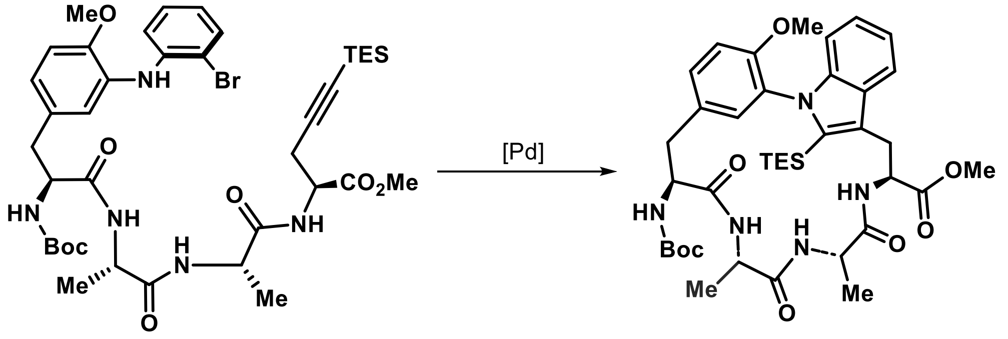

42a **43a**

The precursor **42a** (582 mg, 0.7 mmol, 1.0 eq.) was dissolved in toluene (10.0 mL). Pd(tBu$_3$P)$_2$ (0.175 mmol, 0.25 eq.) and DIPEA (1.75 mmol, 2.5 eq.) were added. The reaction mixture was stirred at 110 ºC under nitrogen atmosphere for 16 h. The solvents were removed under reduced pressure to give the residue, the residue was purified by silica gel chromatography to give the product **43a** (210 mg, 40% yield).

Compound 43a

Physical State: amorphous solid

^{1}H NMR (600 MHz, CDCl$_3$): δ 7.73 – 7.36 (m, 1H), 7.26 – 7.17 (m, 1H), 7.15 – 6.92 (m, 3H), 6.87 – 6.45 (m, 3H), 6.37 – 5.51 (m, 1H), 5.25 – 4.63 (m, 1H), 4.60 – 4.44 (m, 1H), 4.42 – 4.07 (m, 1H), 3.91 – 3.67 (m, 6H), 3.60 – 3.39 (m, 1H), 3.30 – 2.60 (m, 3H), 1.52 – 1.34 (m, 12H), 1.29 – 1.05 (m, 6H), 1.01 – 0.81 (m, 9H), 0.76 – 0.50 (m, 6H).

^{13}C NMR (151 MHz, CDCl$_3$): δ 172.94, 172.38, 172.34, 172.02, 171.96, 171.78, 171.60, 171.20, 170.99, 170.81, 170.59, 170.19, 158.76, 155.73, 155.28, 155.16, 154.04, 142.34, 142.13, 138.88, 137.87, 133.47, 133.24, 130.47, 130.38, 129.80, 129.58, 129.18, 128.62, 128.27, 128.09, 127.75, 122.90, 122.52, 122.08, 120.97, 119.71, 119.60, 119.08, 118.31, 117.65, 114.18, 112.13, 111.54, 111.32, 101.86, 85.49, 80.46, 80.04, 79.85, 56.60, 56.06, 55.78, 55.63, 55.37, 55.32, 55.30, 54.35, 53.54, 52.72, 52.66, 52.45, 51.40, 49.21, 49.14, 47.78, 45.93, 39.80, 37.42, 37.21, 36.43, 35.86, 31.53, 31.51, 28.98, 28.62, 28.55, 28.49, 28.45, 28.36, 28.32, 27.16, 23.72, 19.22, 18.69, 18.61, 18.39, 16.34, 14.64, 7.49, 7.45, 7.41, 7.37, 6.69, 5.89, 4.93, 4.40, 3.90.

HRMS (ESI-TOF): calculated for $C_{39}H_{55}N_5NaO_8Si^+$ [M+Na]$^+$: 772.3712, found: 772.3719.

$[\alpha]^{25}_D$: –32.5 (*c* = 0.2, CHCl$_3$)

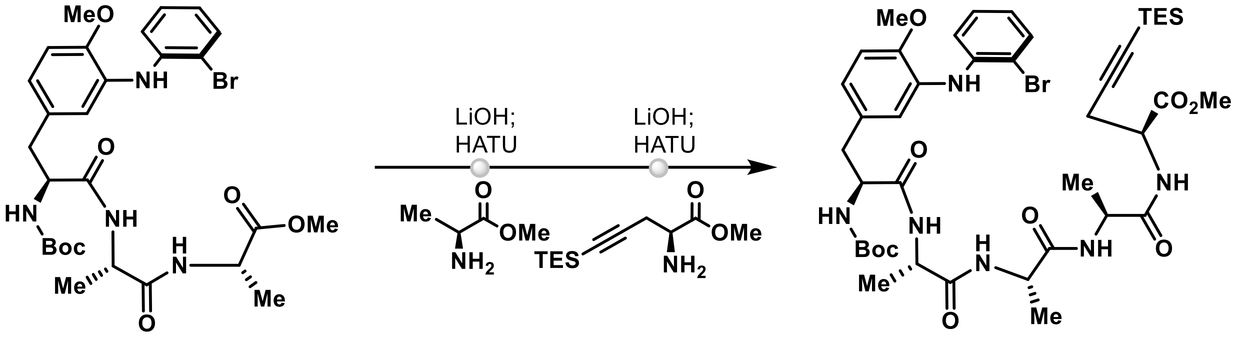

A solution of compound **S9** (715.6 mg, 1.15 mmol, 1.0 eq.) in MeOH/H_2O (1:1, 3 mL) was treated with LiOH·H_2O (96.5 mg, 2.3 mmol, 2.0 eq.). The reaction mixture was stirred at rt for 30 minutes. Upon completion, the reaction was quenched by adjusting the pH to 3 with 0.5 M aq. HCl and then extracted with DCM for three times. The combined organic layers were washed with saturated aq. NaCl, dried over Na_2SO_4, and concentrated *in vacuo* to give the residue, the residue was used directly in the subsequent step.

To a solution of the residue in DCM (6 mL) was added *L*-alanine methyl ester hydrochloride (192.6 mg, 1.38 mmol, 1.2 eq.), DIPEA (594.5 mg, 4.6 mmol, 4.0 eq.) HATU (524.7 mg, 1.38 mmol, 1.2 eq.) sequentially. After stirring at rt for 1 h, the reaction was quenched with 0.5 M HCl and extracted with DCM for three times. The combined organic layers were washed with saturated aq. NaCl, dried over Na_2SO_4, and concentrated *in vacuo* to give the residue, the residue was used directly in the subsequent step.

The residue was dissolved in MeOH/H_2O (1:1, 3 mL), treated with LiOH·H_2O (96.5 mg, 2.3 mmol, 2.0 eq.), and stirred at rt for 30 minutes. Upon completion, the reaction was quenched by adjusting the pH to 3 with 0.5 M aq. HCl and then extracted with DCM for three times. The combined organic layers were washed with saturated aq. NaCl, dried over Na_2SO_4, and concentrated *in vacuo* to give the residue, the residue was used directly in the subsequent step.

A mixture of the residue in DCM (6 mL) was added DIPEA (594.5 mg, 4.6 mmol, 4.0 eq.), HATU (524.7 mg, 1.38 mmol, 1.2 eq.), methyl (*S*)-2-amino-5-(triethylsilyl)pent-4-ynoate (333 mg, 1.38 mmol, 1.2 eq.) sequentially. The reaction was stirred for 1 h, quenched with 0.5 M HCl and extracted with DCM for three times. The combined organic layers were washed with saturated aq. NaCl, dried over Na_2SO_4, and concentrated *in vacuo* to give the residue, the residue was purified by silica gel chromatography to afford the compound **42b** (810 mg, 78% yield from **S9**).

Compound 42b

Physical State: amorphous solid

^{1}H NMR (600 MHz, CDCl$_3$): δ 7.50 (t, *J* = 7.0 Hz, 2H), 7.32 (d, *J* = 7.8 Hz, 1H), 7.18 (t, *J* = 7.2 Hz, 1H), 7.12 – 7.09 (m, 1H), 6.86 – 6.67 (m, 3H), 4.87 – 4.26 (m, 4H), 4.01 – 3.67 (m, 6H), 3.63 (s, 1H), 3.17 – 2.67 (m, 9H), 1.45 – 1.30 (m, 15H), 1.30 – 1.24 (m, 3H), 0.93 (q, *J* = 7.7, 9H), 0.56 – 0.48 (m, 6H).
^{13}C NMR (151 MHz, CDCl$_3$): δ 172.61, 172.32, 172.06, 171.14, 169.88, 161.54, 156.18, 148.38, 140.55, 133.16, 131.59, 128.29, 122.07, 121.64, 117.13, 113.68, 110.98, 102.34, 100.14, 87.74, 84.95, 80.45, 56.55, 55.87, 53.56, 52.51, 51.74, 51.52, 49.88, 49.59, 49.21, 49.10, 48.73, 40.07, 31.67, 28.35, 23.99, 23.63, 22.73, 18.99, 14.20, 7.48, 7.40, 4.40, 4.26.
HRMS (ESI-TOF): calculated for $C_{42}H_{61}BrN_6NaO_9Si^+$ [M+Na]$^+$: 923.3345, found: 923.3353.
[α]$^{25}_D$: –23.3 (*c* = 0.2, CHCl$_3$)

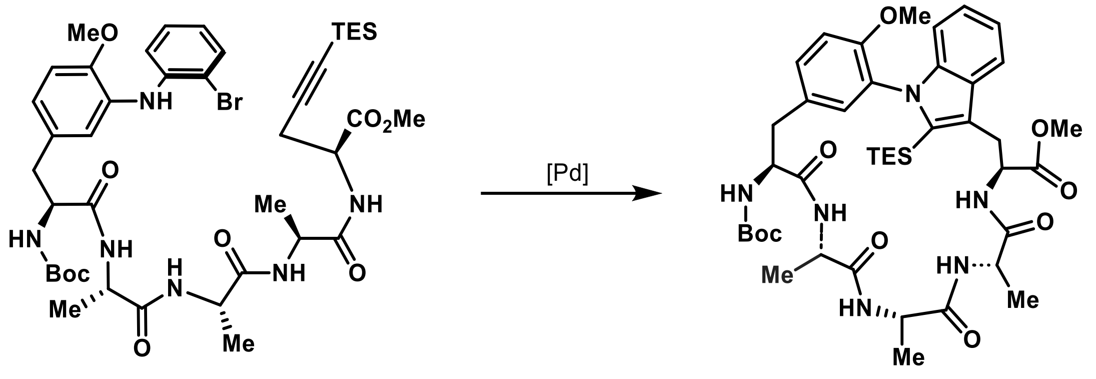

The precursor **42b** (729 mg, 0.81 mmol, 1.0 eq.) was dissolved in toluene (10 mL). Pd(*t*Bu$_3$P)$_2$ (0.2 mmol, 0.25 eq.) and DIPEA (2.0 mmol, 2.5 eq.) were added. The reaction mixture was stirred at 110 °C under nitrogen atmosphere for 16 h. The solvents were removed under reduced pressure to give the residue, the residue was purified by silica gel chromatography to give the product **43b** (166 mg, 25% yield).

Compound 43b

Physical State: amorphous solid
^{1}H NMR (600 MHz, CDCl$_3$): δ 8.07 – 7.63 (m, 1H), 7.59 – 7.32 (m, 1H), 7.15 (d, *J* = 8.8 Hz, 2H), 7.08 – 6.95 (m, 2H), 6.93 – 6.67 (m, 3H), 6.55 – 6.15 (m, 1H), 5.98 – 5.34 (m, 1H), 4.80 – 4.68 (m, 1H), 4.59 – 4.33 (m, 2H), 4.30 – 4.24 (m, 1H), 4.13 – 3.97 (m, 1H), 3.86 – 3.82 (m, 3H), 3.79 – 3.72 (m, 3H), 3.58 (s, 1H), 3.55 – 3.39 (m, 1H), 3.24 – 3.13 (m, 1H), 3.02 – 2.97 (m, 1H), 2.82 – 2.61 (m, 1H), 1.48 – 1.44 (m, 9H), 1.41 – 1.36 (m, 3H), 1.24 – 1.07 (m, 6H), 0.90 – 0.78 (m, 9H), 0.60 – 0.52 (m, 6H).
^{13}C NMR (151 MHz, CDCl$_3$): δ 172.79, 172.39, 172.01, 171.84, 171.40, 171.12, 170.88, 169.86, 155.91, 155.37, 155.22, 141.40, 141.23, 138.24, 136.70, 133.53, 131.69, 130.39, 129.98, 129.67, 129.42, 128.44, 127.46, 127.30, 123.09, 122.79, 120.32, 120.13, 119.77, 119.59, 118.87, 117.76, 113.98, 112.66, 111.14, 110.92, 110.60, 80.09, 79.88, 56.16, 55.77, 55.68, 55.16, 53.50, 53.29, 52.53, 51.41, 49.23, 47.92, 47.67, 47.31, 47.18, 39.16, 38.36, 28.55, 28.49, 28.39, 28.20, 26.23, 18.49, 18.21, 16.16, 14.66, 7.48, 7.39, 7.33, 4.54, 4.40, 3.75.
HRMS (ESI-TOF): calculated for $C_{42}H_{60}N_6NaO_9Si^+$ [M+Na]$^+$: 843.4083, found: 843.4088.
[α]$^{25}_D$: –15.7 (*c* = 0.2, CHCl$_3$)

General procedure M for the synthesis of S11:

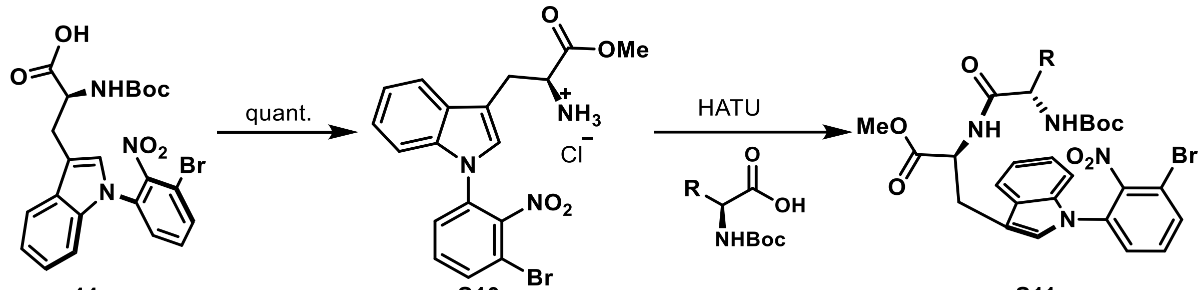

44 S10 S11

Step 1: Compound **44** (201.7 g, 400 mmol, 1.0 eq.) was dissolved in methanol (3000 mL), $SOCl_2$ (400 mL) was added dropwise at 0 ºC, the reaction was stirred at rt for 24 h. The solvent was removed under reduced pressure to give a yellow solid **S10** in quantitative yield.

Step 2: Compound **S10** (1.0 eq.) was dissolved in DMF (0.2 M), Boc-amino acid (1.1 eq.), DIPEA (3.0 eq.) and HATU (1.5 eq.) was added sequentially, the reaction was stirred at rt for 4 h, quenched by aq. NH_4Cl and extracted by Hexane/Ethyl acetate = 1:1 for three times, the solvent was removed under reduced pressure to give the residue, the residue was purified by silica gel chromatography to give the dipeptide **S11a-S11d**. **S11d** was reported in our previous work.[4]

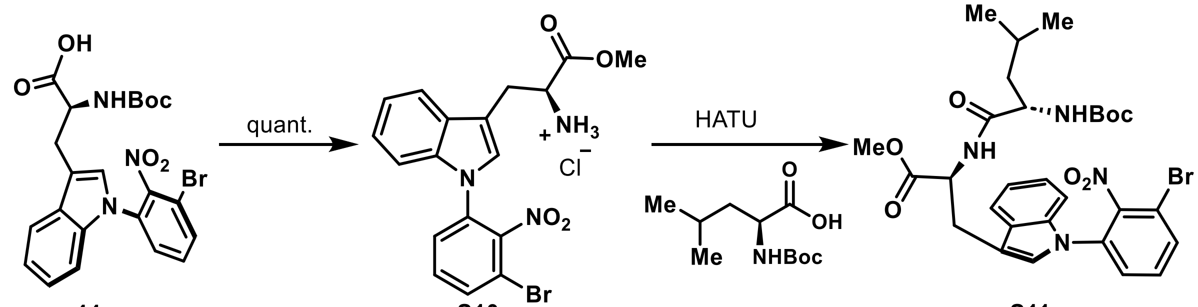

44 S10 S11a

On 100 mmol scale, **General Procedure M** was followed with **S10** and Boc-*L*-leucine via amide coupling. Purification by silica gel column chromatography gave the title compound **S11a** (60.6 g, 96% yield from **44**).

Compound S11a

Physical State: amorphous solid

^{1}H NMR (600 MHz, $CDCl_3$): δ 7.75 (dd, J = 7.9, 1.4 Hz, 1H), 7.62 – 7.47 (m, 3H), 7.23 – 7.14 (m, 3H), 6.96 (s, 1H), 6.70 (d, J = 6.4 Hz, 1H), 4.99 (d, J = 6.4 Hz, 1H), 4.94 (dt, J = 7.6, 5.4 Hz, 1H), 4.20 – 4.08 (m, 1H), 3.67 (s, 3H), 3.37 – 3.26 (m, 2H), 1.71 – 1.60 (m, 2H), 1.54 – 1.40 (m, 1H), 1.38 (s, 9H), 0.90 (d, J = 5.1 Hz, 6H).

^{13}C NMR (151 MHz, $CDCl_3$): δ 172.54, 171.85, 155.69, 149.23, 137.38, 133.04, 132.90, 131.71, 128.62, 128.39, 126.42, 123.60, 121.30, 119.48, 114.42, 113.23, 110.12, 79.98, 53.32, 52.61, 52.52, 41.32, 28.29, 27.61, 24.81, 23.09, 21.82.

HRMS (ESI-TOF): calculated for $C_{29}H_{35}BrN_4NaO_7^+$ $[M+Na]^+$: 653.1581, found: 653.1594.

$[\alpha]^{25}_D$: +21.4 (c = 0.5, $CHCl_3$)

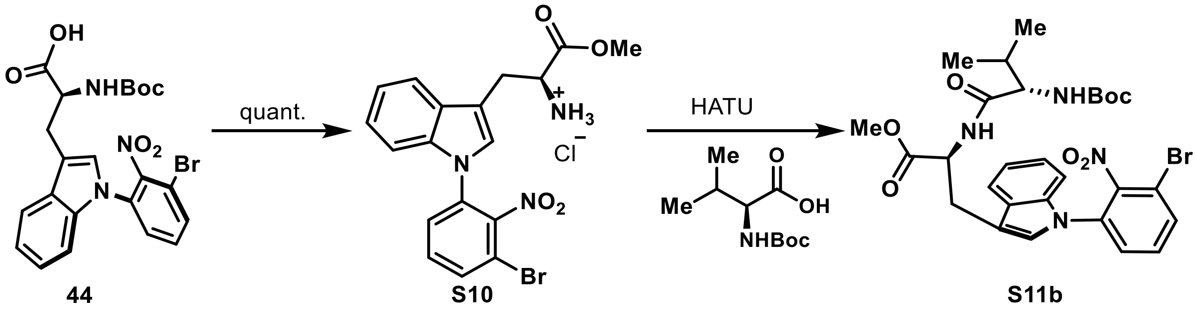

On 100 mmol scale, **General Procedure M** was followed with **S10** and Boc-*L*-valine via amide coupling. Purification by silica gel column chromatography gave the title compound **S11b** (60.1 g, 97% yield from **44**).

Compound S11b

Physical State: amorphous solid

^{1}H NMR (600 MHz, CDCl$_3$): δ 7.76 (dd, *J* = 7.8, 1.4 Hz, 1H), 7.60 – 7.49 (m, 3H), 7.24 – 7.13 (m, 3H), 6.97 (s, 1H), 6.48 (d, *J* = 7.0 Hz, 1H), 5.17 (d, *J* = 8.2 Hz, 1H), 4.97 (dt, *J* = 7.5, 5.4 Hz, 1H), 4.01 – 3.96 (m, 1H), 3.67 (s, 3H), 3.35 (dd, *J* = 14.8, 4.4 Hz, 1H), 3.27 (dd, *J* = 14.8, 5.0 Hz, 1H), 2.17 – 2.11 (m, 1H), 1.41 (s, 9H), 0.94 (d, *J* = 6.8 Hz, 3H), 0.84 (d, *J* = 6.3 Hz, 3H).

^{13}C NMR (151 MHz, CDCl$_3$): δ 171.82, 171.45, 155.91, 149.29, 137.43, 133.07, 132.84, 131.68, 128.49, 128.38, 126.37, 123.67, 121.41, 119.43, 114.44, 113.11, 110.13, 79.80, 59.83, 52.61, 52.49, 31.04, 28.35, 27.73, 19.25, 17.51.

HRMS (ESI-TOF): calculated for $C_{28}H_{33}BrN_4NaO_7^+$ [M+Na]$^+$: 639.1425, found: 639.1432.

[α]^{25}$_D$: +20.1. (*c* = 0.5, CHCl$_3$)

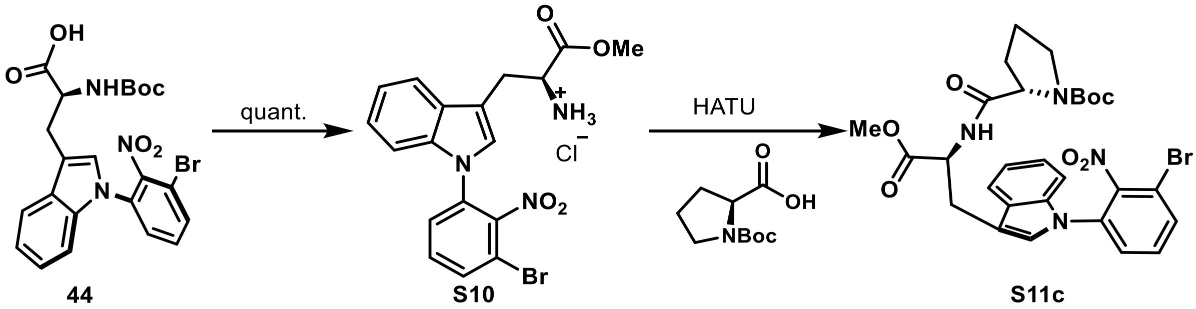

On 100 mmol scale, **General Procedure M** was followed with **S10** and Boc-*L*-proline via amide coupling. Purification by silica gel column chromatography gave the title compound **S11c** (56.6 g, 92% yield from **44**).

Compound S11c

Physical State: amorphous solid

^{1}H NMR (600 MHz, CDCl$_3$): δ 7.75 (d, *J* = 6.9 Hz, 1H), 7.59 (d, *J* = 7.0 Hz, 1H), 7.51 (s, 2H), 7.25 – 6.49 (m, 5H), 4.94 (s, 1H), 4.28 – 4.18 (m, 1H), 3.72 – 3.67 (m, 3H), 3.42 – 3.12 (m, 4H), 2.21 – 2.03 (m, 1H), 1.97 – 1.75 (m, 3H), 1.36 (s, 9H).

^{13}C NMR (151 MHz, CDCl$_3$): δ 171.97, 155.56, 154.63, 149.32, 137.41, 133.10, 131.66, 128.50, 126.29, 123.67, 121.39, 119.23, 114.40, 113.41, 110.15, 80.69, 80.28, 61.16, 60.21, 52.94, 52.54, 46.97, 30.89, 28.26, 27.70, 24.52, 23.60.

HRMS (ESI-TOF): calculated for $C_{28}H_{31}BrN_4NaO_7^+$ [M+Na]$^+$: 637.1268, found: 637.1277.

[α]^{25}$_D$: –11.4 (*c* = 0.5, CHCl$_3$)

General procedure N for the synthesis of 45:

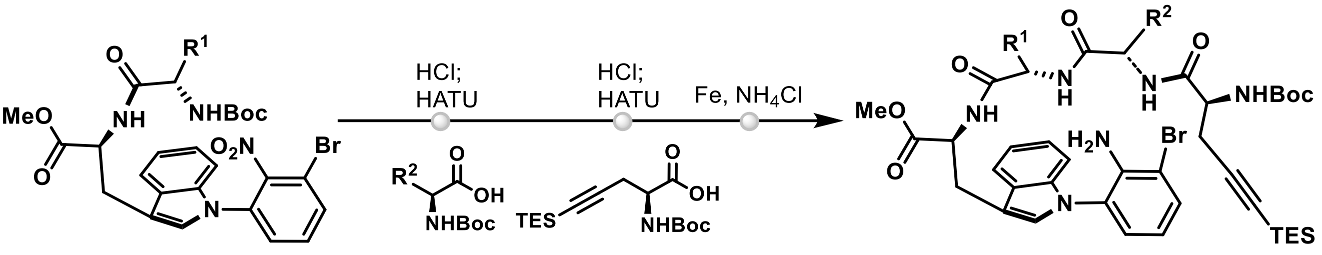

S11 45

The dipeptide compound **S11** was dissolved in DCM/4M HCl (3:1, 0.2 M) was added portion wise, the reaction was stirred at rt for 2 h. The solvent was removed under reduced pressure to give the crude, the crude was dissolved in DMF (0.2 M), Boc-amino acid (1.1 eq.), DIPEA (3.0 eq.) and HATU (1.5 eq.) was added sequentially, the reaction was stirred at rt for 4 h, quenched by aq. NH_4Cl and extracted by Hexane/Ethyl acetate = 1:1 for three times, the solvent was removed under reduced pressure to give the tripeptide.
The tripeptide compound was dissolved in DCM/4M HCl (3:1, 0.2 M) was added portion wise, the reaction was stirred at rt for 2 h. The solvent was removed under reduced pressure to give the crude, the crude was dissolved in DMF (0.2 M), Boc-amino acid (1.1 eq.), DIPEA (3.0 eq.) and HATU (1.5 eq.) was added sequentially, the reaction was stirred at rt for 4 h, quenched by aq. NH_4Cl and extracted by Hexane/Ethyl acetate = 1:1 for three times, the solvent was removed under reduced pressure to give the tetrapeptide.
The tetrapeptide was dissolved in EtOH/H_2O/EtOAc (3:1:1, 0.1 M), Fe (15.0 eq.) and NH_4Cl (4.0 eq.) was added, the reaction was stirred at 60 ºC for 4 h, the solvent was removed under reduced pressure and water was added, extracted by EtOAc for three times, the combined organic layers were removed under reduced pressure to give the residue, the residue was purified by silica gel chromatography to give the precursor **45**.
45a was reported in our previous work.[4]

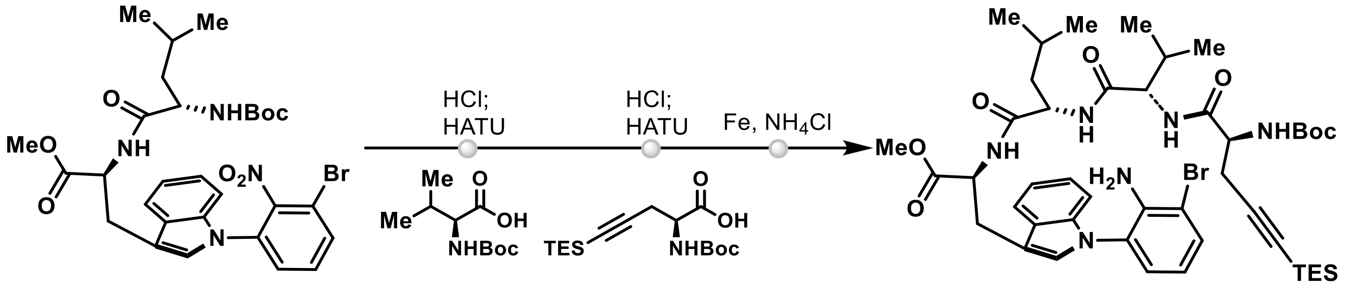

S11a 45b

On 3.82 mmol scale, **General Procedure N** was followed from compound **S11a** as an intermediate. Purification by silica gel column chromatography gave the title compound **45b** (1.6 g, 46% yield from **S11a**).

Compound 45b

Physical State: amorphous solid

^{1}H NMR (600 MHz, $CDCl_3$): δ 7.66 – 7.55 (m, 1H), 7.48 (d, J = 8.0 Hz, 1H), 7.22 – 6.96 (m, 6H), 6.79 – 6.61 (m, 3H), 5.23 – 5.23 (m, 1H), 4.91 (q, J = 6.9 Hz, 1H), 4.44 – 4.41 (m, 1H), 4.27 (s, 1H), 4.14 (d, J = 4.9 Hz, 1H), 4.11 – 4.04 (m, 1H), 3.74 – 3.69 (m, 3H), 3.45 – 3.24 (m, 2H), 2.66 – 2.58 (m, 2H), 2.22 – 2.03 (m, 1H), 1.94 – 1.77 (m, 1H), 1.61 – 1.53 (m, 2H), 1.42 (s, 9H), 0.97 (t, J = 7.8 Hz, 9H), 0.90 – 0.77 (m, 13H), 0.57 (q, J = 7.7 Hz, 6H).

^{13}C NMR (151 MHz, $CDCl_3$): δ 172.12, 171.84, 171.11, 170.77, 156.17, 142.01, 136.76, 136.55, 132.42, 128.43, 127.83, 127.03, 126.85, 125.20, 124.99, 122.66, 120.35, 119.09, 118.15, 118.00, 111.99, 110.81, 110.68, 109.81, 101.72, 86.80, 81.16, 59.10, 53.64, 53.18, 52.48, 51.93, 51.78, 39.97, 29.59, 28.38, 28.22, 27.70, 24.78, 23.15, 22.76, 22.55, 21.33, 19.40, 17.35, 7.54, 4.38.

HRMS (ESI-TOF): calculated for $C_{45}H_{65}BrN_6NaO_7Si^+$ [M+Na]$^+$: 931.3760, found: 931.3766.

[α]^{25}D: -5.2 (c = 0.5, $CHCl_3$)

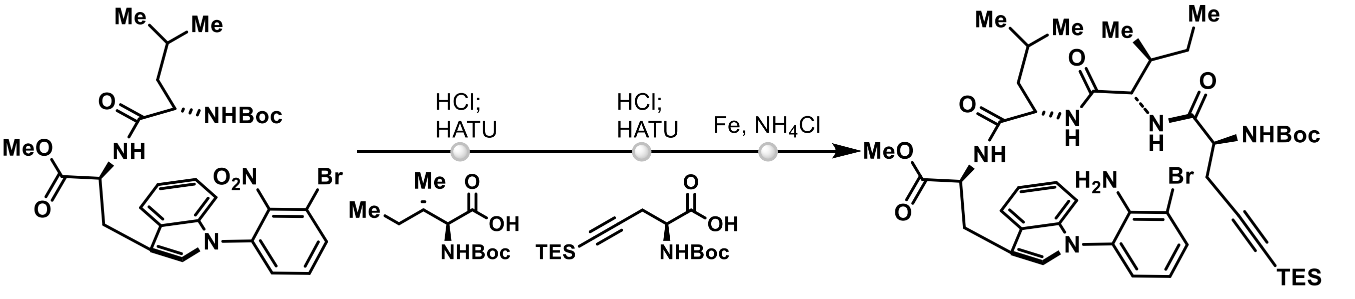

On 4.81 mmol scale, **General Procedure N** was followed from compound **S11a** as an intermediate. Purification by silica gel column chromatography gave the title compound **45c** (2.0 g, 45% yield from **S11a**).

Compound 45c

Physical State: amorphous solid

^{1}H NMR (600 MHz, CDCl$_3$): δ 7.63 – 7.57 (m, 1H), 7.48 (d, J = 8.0 Hz, 1H), 7.20 – 7.03 (m, 6H), 6.82 – 6.51 (m, 3H), 5.29 – 5.21 (m, 1H), 4.91 – 4.88 (m, 1H), 4.47 – 4.41 (m, 1H), 4.26 (s, 1H), 4.20 – 4.07 (m, 2H), 3.74 – 3.69 (m, 3H), 3.45 – 3.23 (m, 2H), 2.76 – 2.45 (m, 2H), 1.98 – 1.73 (m, 2H), 1.66 – 1.51 (m, 2H), 1.42 (s, 9H), 1.38 – 1.31 (m, 1H), 1.08 – 1.00 (m, 1H), 0.99 – 0.95 (m, 9H), 0.90 – 0.74 (m, 13H), 0.60 – 0.55 (m, 6H).

^{13}C NMR (151 MHz, CDCl$_3$): δ 172.12, 171.88, 171.09, 170.60, 156.22, 142.02, 136.71, 136.55, 132.41, 128.46, 127.83, 127.00, 126.81, 125.20, 124.99, 122.64, 120.34, 119.10, 118.13, 117.98, 112.08, 110.80, 110.64, 109.78, 101.63, 86.89, 81.22, 58.70, 53.63, 53.16, 52.49, 51.92, 51.80, 39.95, 36.03, 28.20, 27.68, 24.79, 24.73, 23.19, 22.48, 21.24, 15.87, 11.63, 7.55, 4.38.

HRMS (ESI-TOF): calculated for $C_{46}H_{67}BrN_6NaO_7Si^+$ [M+Na]$^+$: 945.3916, found: 945.3921.

[α]^{25}D: –4.4 (c = 0.5, $CHCl_3$)

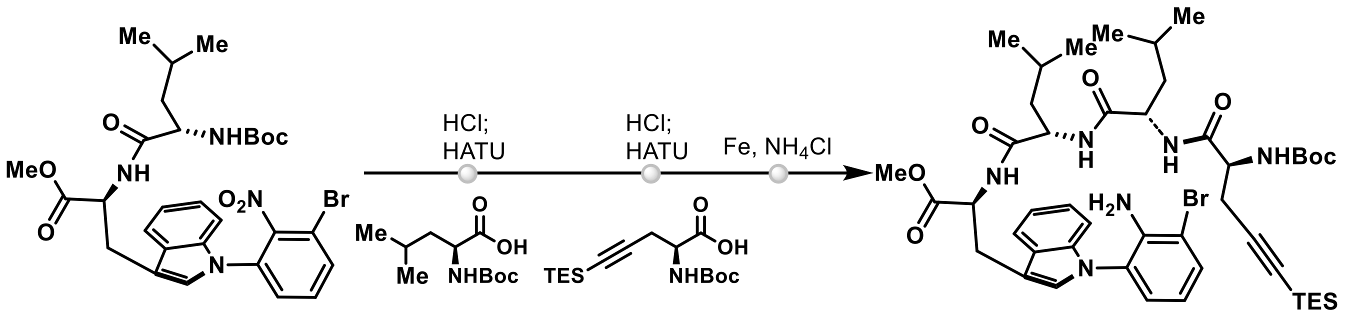

On 5.86 mmol scale, **General Procedure N** was followed from compound **S11a** as an intermediate. Purification by silica gel column chromatography gave the title compound **45d** (2.6 g, 48% yield from **S11a**).

Compound 45d

Physical State: amorphous solid

^{1}H NMR (600 MHz, CDCl$_3$): δ 7.60 (d, J = 6.0 Hz, 1H), 7.48 (d, J = 8.0 Hz, 1H), 7.17 – 7.14 (m, 3H), 7.11 – 7.03 (m, 2H), 6.97 (s, 1H), 6.81 – 6.44 (m, 3H), 5.25 (d, J = 44.5 Hz, 1H), 4.91 (s, 1H), 4.45 – 4.08 (m, 4H), 3.73 – 3.69 (m, 3H), 3.50 – 3.17 (m, 2H), 2.80 – 2.44 (m, 2H), 1.98 (s, 1H), 1.78 – 1.48 (m, 5H), 1.43 (s, 9H), 0.97 (t, J = 7.9 Hz, 9H), 0.92 – 0.80 (m, 13H), 0.57 (q, J = 7.9 Hz, 6H).

^{13}C NMR (151 MHz, CDCl$_3$): δ 172.17, 171.83, 171.56, 170.99, 156.15, 142.01, 136.71, 136.57, 132.44, 128.39, 127.86, 127.03, 126.85, 125.17, 124.99, 122.70, 120.35, 119.08, 118.17, 118.02, 111.90, 110.79, 110.71, 109.78, 101.88, 86.76, 81.17, 53.48, 53.20, 53.09, 52.66, 52.51, 52.00, 51.82, 40.28, 28.24, 27.71, 24.94, 24.83, 23.08, 21.80, 21.54, 7.57, 4.38.

HRMS (ESI-TOF): calculated for $C_{46}H_{67}BrN_6NaO_7Si^+$ [M+Na]$^+$: 945.3916, found: 945.3920.
[α]^{25}D: –7.0 (c = 0.5, $CHCl_3$)

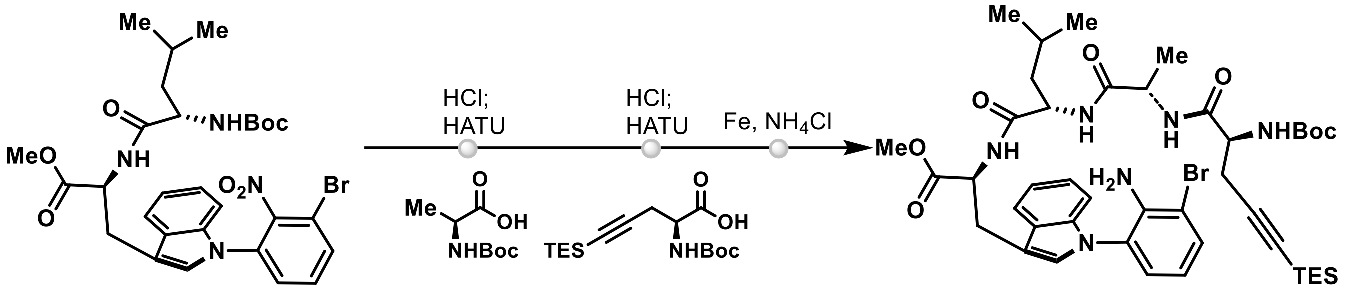

S11a 45e

On 7.37 mmol scale, **General Procedure N** was followed from compound **S11a** as an intermediate. Purification by silica gel column chromatography gave the title compound **45e** (2.6 g, 40% yield from **S11a**).

Compound 45e

Physical State: amorphous solid

^{1}H NMR (600 MHz, CDCl$_3$): δ 7.63 – 7.56 (m, 1H), 7.49 (dd, J = 8.1, 1.3 Hz, 1H), 7.21 – 6.91 (m, 6H), 6.87 – 6.52 (m, 3H), 5.29 – 5.21 (m, 1H), 4.93 – 4.88 (m, 1H), 4.49 – 3.91 (m, 4H), 3.74 – 3.70 (m, 3H), 3.44 – 3.36 (m, 1H), 3.32 – 3.24 (m, 1H), 2.83 – 2.46 (m, 2H), 1.96 (s, 1H), 1.60 – 1.48 (m, 2H), 1.43 (d, J = 3.9 Hz, 9H), 1.37 – 1.13 (m, 4H), 0.96 (t, J = 7.9 Hz, 9H), 0.91 – 0.81 (m, 6H), 0.57 (q, J = 7.8 Hz, 6H).

^{13}C NMR (151 MHz, CDCl$_3$): δ 172.21, 172.11, 171.77, 170.76, 155.96, 142.03, 136.73, 136.54, 132.47, 128.40, 127.87, 127.02, 126.85, 125.14, 124.96, 122.72, 120.35, 119.05, 118.17, 118.04, 111.84, 110.73, 109.80, 101.89, 86.65, 81.08, 53.50, 53.24, 53.10, 52.54, 52.02, 51.84, 49.62, 40.22, 28.26, 27.69, 24.84, 23.03, 21.67, 17.61, 7.57, 4.39.

HRMS (ESI-TOF): calculated for $C_{43}H_{61}BrN_6NaO_7Si^+$ [M+Na]$^+$: 903.3447, found: 903.3453.
[α]^{25}D: +3.3 (c = 0.5, $CHCl_3$)

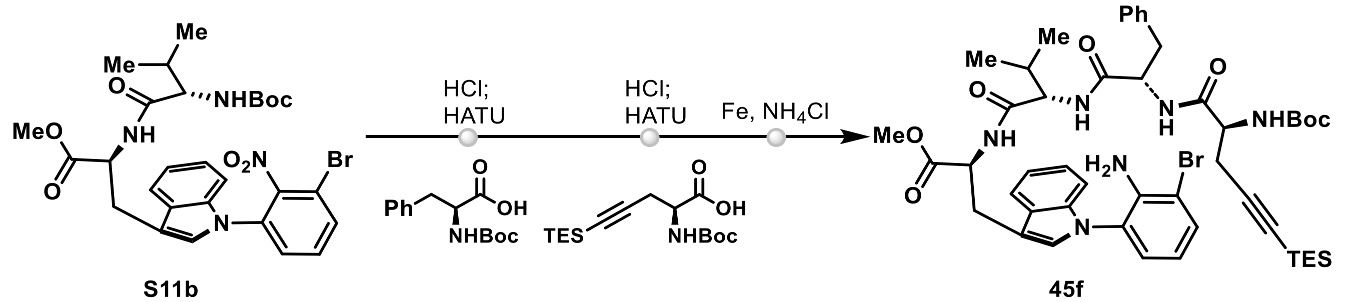

S11b 45f

On 7.63 mmol scale, **General Procedure N** was followed from compound **S11a** as an intermediate. Purification by silica gel column chromatography gave the title compound **45f** (3.6 g, 50% yield from **S11b**).

Compound 45f

Physical State: amorphous solid

^{1}H NMR (600 MHz, CDCl$_3$): δ 7.60 – 7.57 (m, 1H), 7.48 (d, J = 7.7 Hz, 1H), 7.20 – 6.96 (m, 12H), 6.75 – 6.64 (m, 2H), 5.36 – 5.28 (m, 1H), 4.96 – 4.92 (m, 1H), 4.72 – 4.53 (m, 1H), 4.36 – 4.24 (m, 1H), 4.19 – 4.10 (m, 2H), 3.71 – 3.68 (m, 3H), 3.42 – 3.24 (m, 2H), 3.11 – 3.04 (m, 1H), 2.92 – 2.83 (m, 1H), 2.71 – 2.52 (m, 2H), 2.28 – 1.99 (m, 1H), 1.37 (s, 9H), 1.31 – 1.26 (m, 1H), 0.98 (t, J = 7.9 Hz, 9H), 0.88 (t, J = 6.9 Hz, 1H), 0.82 (dd, J = 11.9, 6.5 Hz, 3H), 0.77 (d, J = 6.3 Hz, 1H), 0.71 (d, J = 5.4 Hz, 1H), 0.58 (q, J = 7.8 Hz, 6H).

^{13}C NMR (151 MHz, CDCl$_3$): δ 172.29, 170.72, 170.62, 155.67, 141.92, 136.75, 136.57, 136.16, 132.48, 129.32, 128.84, 128.28, 127.86, 127.21, 127.03, 125.03, 122.81, 120.45, 119.10, 118.17, 111.67, 110.82, 109.81, 102.37, 85.98, 80.60, 58.79, 54.51, 53.39, 53.20, 53.10, 52.52, 37.69, 31.68, 30.22, 28.30, 28.00, 27.89, 23.25, 22.75, 19.15, 19.07, 17.94, 17.84, 14.23, 7.61, 4.44.

HRMS (ESI-TOF): calculated for $C_{48}H_{63}BrN_6NaO_7Si^+$ $[M+Na]^+$: 965.3603, found: 965.3608.
$[\alpha]^{25}_D$: +2.2 (c = 0.5, $CHCl_3$)

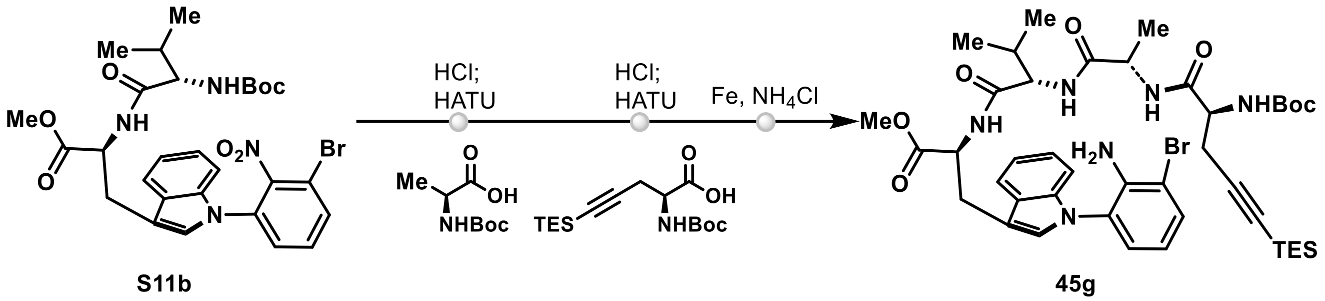

On 8.30 mmol scale, **General Procedure N** was followed from compound **S11b** as an intermediate. Purification by silica gel column chromatography gave the title compound **45g** (3.6 g, 50% yield from **S11b**).

Compound 45g

Physical State: amorphous solid

^{1}H NMR (600 MHz, CDCl$_3$): δ 7.58 (d, J = 7.0 Hz, 1H), 7.48 (d, J = 7.9 Hz, 1H), 7.24 – 6.99 (m, 8H), 6.67 (t, J = 7.9 Hz, 1H), 5.50 – 5.43 (m, 1H), 4.99 – 4.94 (m, 1H), 4.59 – 4.25 (m, 2H), 4.18 – 4.09 (m, 2H), 3.70 – 3.68 (m, 3H), 3.43 – 3.24 (m, 2H), 2.82 – 2.54 (m, 2H), 2.26 – 2.00 (m, 1H), 1.42 (s, 9H), 1.39 (s, 1H), 1.29 – 1.17 (m, 3H), 0.95 (t, J = 7.9 Hz, 9H), 0.91 – 0.83 (m, 6H), 0.55 (q, J = 7.9 Hz, 6H).

^{13}C NMR (151 MHz, CDCl$_3$): δ 172.33, 172.25, 172.04, 171.94, 170.91, 170.52, 155.54, 141.94, 141.87, 136.75, 136.54, 132.51, 128.32, 128.18, 127.82, 126.90, 125.06, 124.98, 122.83, 120.47, 118.99, 118.20, 111.57, 110.84, 109.85, 102.61, 85.77, 80.37, 58.54, 58.41, 53.11, 52.55, 49.27, 31.26, 30.88, 28.36, 27.95, 23.88, 19.12, 19.04, 18.55, 18.35, 18.18, 18.10, 7.58, 4.42.

HRMS (ESI-TOF): calculated for $C_{42}H_{59}BrN_6NaO_7Si^+$ $[M+Na]^+$: 889.3290, found: 889.3298.
$[\alpha]^{25}_D$: +10.7 (c = 0.5, $CHCl_3$)

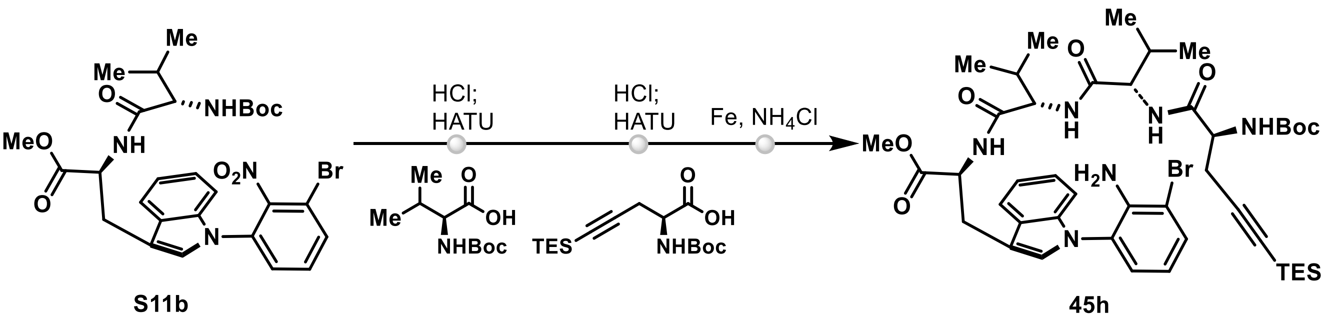

On 4.25 mmol scale, **General Procedure N** was followed from compound **S11b** as an intermediate. Purification by silica gel column chromatography gave the title compound **45h** (1.6 g, 42% yield from **S11b**).

Compound 45h

Physical State: amorphous solid

^{1}H NMR (600 MHz, CDCl$_3$): δ 7.63 – 7.55 (m, 1H), 7.52 – 7.47 (m, 1H), 7.19 – 7.01 (m, 6H), 6.98 – 6.87 (m, 2H), 6.69 – 6.65 (m, 1H), 5.41 – 5.36 (m, 1H), 4.94 – 4.89 (m, 1H), 4.32 – 4.17 (m, 3H), 4.08 (s, 1H), 3.71 – 3.67 (m, 3H), 3.45 – 3.16 (m, 2H), 2.84 – 2.52 (m, 2H), 2.18 – 2.02 (m, 2H), 1.43 (s, 9H), 0.96 (t, J = 7.9 Hz, 9H), 0.91 – 0.74 (m, 13H), 0.56 (q, J = 7.9 Hz, 6H).

^{13}C NMR (151 MHz, CDCl$_3$): δ 172.24, 172.16, 171.08, 170.93, 155.80, 141.96, 141.78, 136.88, 136.57, 132.49, 128.28, 128.15, 127.86, 127.00, 125.15, 125.00, 122.88, 120.51, 119.03, 118.29, 118.17, 111.54, 110.90, 109.92, 109.85, 102.44, 86.02, 80.66, 58.98, 58.62, 53.34, 53.15, 52.53, 30.53, 30.24, 28.31, 27.88, 23.17, 22.99, 19.37, 19.23, 19.17, 18.06, 17.92, 7.57, 4.42.

HRMS (ESI-TOF): calculated for $C_{44}H_{63}BrN_6NaO_7Si^+$ $[M+Na]^+$: 917.3603, found: 917.3610.

[α]^{25}D: +4.4 (c = 0.5, $CHCl_3$)

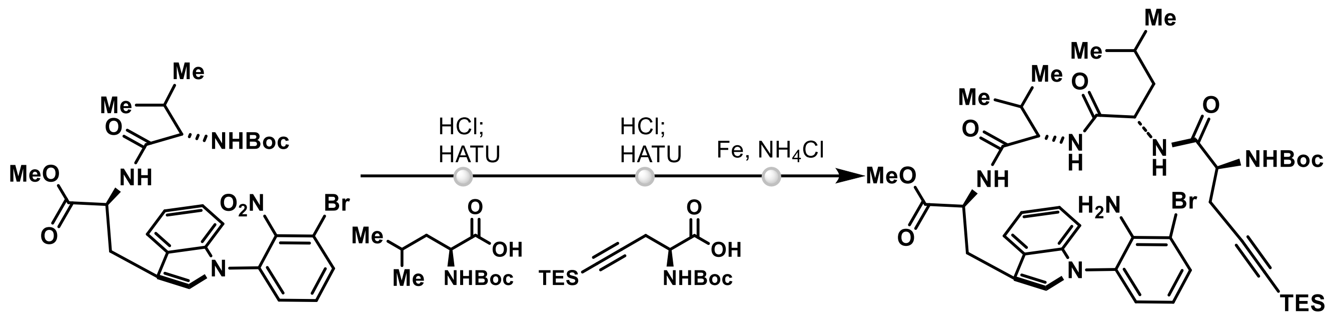

S11b 45i

On 5.62 mmol scale, **General Procedure N** was followed from compound **S11b** as an intermediate. Purification by silica gel column chromatography gave the title compound **45i** (2.2 g, 43% yield from **S11b**).

Compound 45i

Physical State: amorphous solid

^{1}H NMR (600 MHz, CDCl$_3$): δ 7.58 (d, J = 6.4 Hz, 1H), 7.53 – 7.45 (m, 1H), 7.22 – 6.97 (m, 6H), 6.96 – 6.85 (m, 2H), 6.67 (t, J = 7.9 Hz, 1H), 5.43 – 5.36 (m, 1H), 4.96 – 4.92 (m, 1H), 4.44 – 4.38 (m, 1H), 4.31 – 4.11 (m, 3H), 3.69 – 3.66 (m, 3H), 3.45 – 3.23 (m, 2H), 2.77 – 2.70 (m, 1H), 2.67 – 2.51 (m, 1H), 2.20 – 2.16 (m, 1H), 2.11 – 2.06 (m, 1H),1.65 – 1.45 (m, 3H), 1.43 (s, 9H), 0.96 (t, J = 7.9 Hz, 9H), 0.89 – 0.80 (m, 12H), 0.56 (q, J = 7.9 Hz, 6H).

^{13}C NMR (151 MHz, CDCl$_3$): δ 172.29, 171.78, 171.70, 170.94, 170.80, 155.73, 141.93, 141.88, 136.77, 136.57, 132.51, 128.29, 128.17, 127.85, 126.98, 125.11, 125.01, 122.89, 120.52, 119.03, 118.21, 111.51, 110.86, 109.85, 102.64, 85.98, 80.57, 58.57, 53.23, 53.13, 52.52, 52.33, 40.92, 40.80, 30.93, 30.62, 28.32, 28.00, 27.90, 24.86, 23.38, 23.23, 23.06, 22.01, 19.18, 19.13, 18.07, 17.99, 7.59, 4.43.

HRMS (ESI-TOF): calculated for $C_{45}H_{65}BrN_6NaO_7Si^+$ $[M+Na]^+$: 931.3760, found: 931.3764.

[α]^{25}D: +2.8 (c = 0.5, $CHCl_3$)

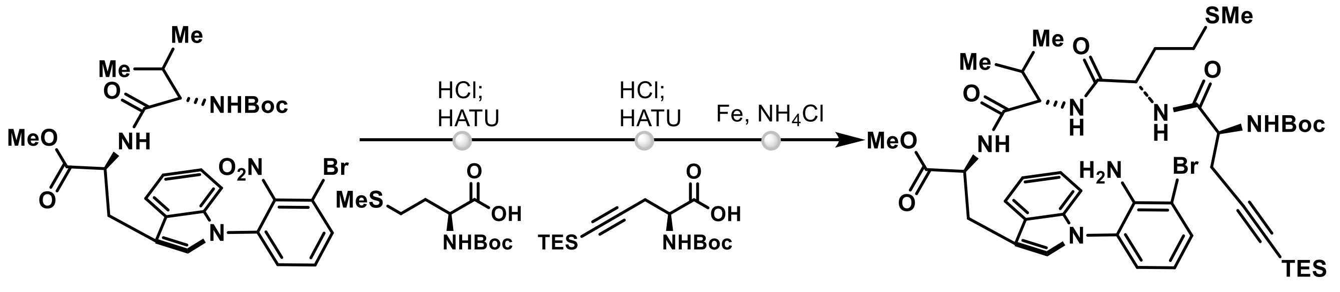

S11b 45j

On 5.72 mmol scale, **General Procedure N** was followed from compound **S11b** as an intermediate. Purification by silica gel column chromatography gave the title compound **45j** (2.6 g, 49% yield from **S11b**).

Compound 45j

Physical State: amorphous solid

^{1}H NMR (600 MHz, CDCl$_3$): δ 7.60 – 7.58 (m, 1H), 7.49 (d, J = 8.0 Hz, 1H), 7.20 – 6.94 (m, 7H), 6.68 (t, J = 7.9 Hz, 1H), 5.36 – 5.30 (m, 1H), 5.02 – 4.91 (m, 1H), 4.56 – 4.47 (m, 1H), 4.31 – 4.07 (m, 4H), 3.70 – 3.67 (m, 3H), 3.46 – 3.24 (m, 2H), 2.83 – 2.57 (m, 2H), 2.52 – 2.36 (m, 2H), 2.16 – 2.10 (m, 1H), 2.03 (s, 3H), 1.99 – 1.93 (m, 1H), 1.89 – 1.83 (m, 1H) 1.43 (s, 9H), 0.96 (t, J = 7.9 Hz, 9H), 0.90 – 0.81 (m, 7H), 0.57 (q, J = 7.9 Hz, 6H).

^{13}C NMR (151 MHz, CDCl$_3$): δ 172.24, 170.83, 170.74, 155.70, 141.95, 141.86, 136.83, 136.57, 132.50, 128.30, 128.21, 127.86, 127.03, 125.12, 125.00, 122.84, 120.49, 119.03, 118.26, 118.19, 111.52, 110.86, 109.86, 102.36, 86.08, 80.65, 58.77, 53.26, 53.17, 53.08, 52.85, 52.54, 30.69, 30.34, 30.12, 28.34, 27.96,

27.88, 23.33, 19.20, 19.15, 18.00, 17.88, 15.18, 7.59, 4.42.
HRMS (ESI-TOF): calculated for $C_{44}H_{63}BrN_6NaO_7SSi^+$ [M+Na]$^+$: 949.3324, found: 949.3325.
[α]^{25}D: +6.6 (c = 0.5, $CHCl_3$)

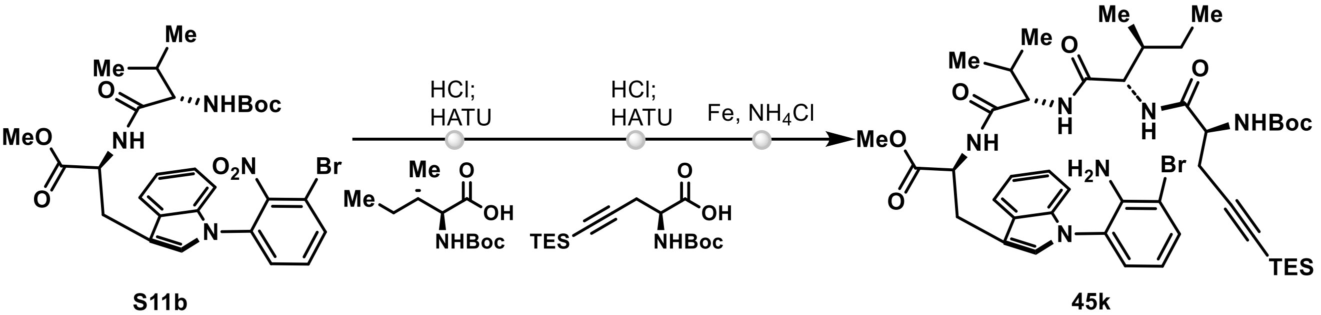

S11b 45k

On 6.50 mmol scale, **General Procedure N** was followed from compound **S11b** as an intermediate. Purification by silica gel column chromatography gave the title compound **45k** (2.9 g, 49% yield from **S11b**).

Compound 45k

Physical State: amorphous solid

^{1}H NMR (600 MHz, CDCl$_3$): δ 7.63 – 7.55 (m, 1H), 7.49 (dd, J = 8.1, 1.2 Hz, 1H), 7.20 – 6.95 (m, 6H), 6.94 – 6.82 (m, 2H), 6.67 (t, J = 7.9 Hz, 1H), 5.39 – 5.37 (m, 1H), 4.94 – 4.89 (m, 1H), 4.36 – 4.17 (m, 3H), 4.09 – 4.03 (m, 1H), 3.70 – 3.67 (m, 3H), 3.42 – 3.21 (m, 2H), 2.82 – 2.52 (m, 2H), 2.32 – 1.85 (m, 2H), 1.87 - 1.73 (m, 1H), 1.43 (s, 9H), 1.09 – 1.00 (m, 1H), 0.96 (t, J = 7.9 Hz, 9H), 0.92 – 0.69 (m, 13H), 0.56 (q, J = 7.9 Hz, 6H).

^{13}C NMR (151 MHz, CDCl$_3$): δ 172.22, 172.14, 171.02, 170.90, 155.81, 141.95, 141.78, 136.84, 136.57, 132.47, 128.26, 128.15, 127.84, 126.98, 125.15, 125.01, 122.88, 120.48, 119.01, 118.27, 118.14, 111.57, 110.87, 109.88, 102.43, 86.02, 80.66, 58.63, 58.42, 53.32, 53.12, 52.50, 36.72, 30.49, 30.20, 28.29, 27.84, 24.85, 22.91, 19.21, 19.16, 18.03, 17.88, 15.63, 15.53, 11.43, 7.56, 4.41.

HRMS (ESI-TOF): calculated for $C_{45}H_{65}BrN_6NaO_7Si^+$ [M+Na]$^+$: 931.3760, found: 931.3762.
[α]^{25}D: +4.8 (c = 0.5, $CHCl_3$)

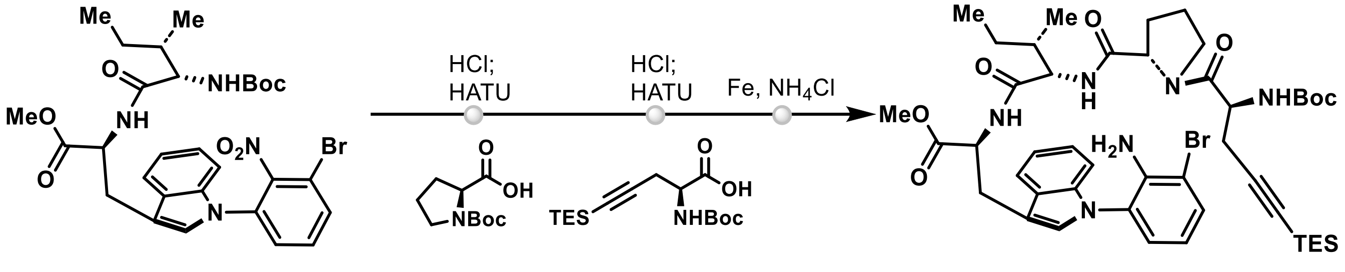

S11d 45l

On 4.10 mmol scale, **General Procedure N** was followed from compound **S11d** as an intermediate. Purification by silica gel column chromatography gave the title compound **45l** (1.6 g, 43% yield from **S11d**).

Compound 45l

Physical State: amorphous solid

^{1}H NMR (600 MHz, CDCl$_3$): δ 7.58 (dd, J = 18.9, 6.0 Hz, 1H), 7.46 (d, J = 7.9 Hz, 1H), 7.35 – 7.27 (m, 1H), 7.21 – 6.78 (m, 7H), 6.65 (t, J = 7.8 Hz, 1H), 5.34 (dd, J = 47.3, 8.1 Hz, 1H), 4.98 – 4.81 (m, 1H), 4.60 – 4.53 (m, 1H), 4.41 – 4.31 (m, 1H), 4.16 – 4.07 (m, 2H), 3.73 – 3.66 (m, 3H), 3.65 – 3.54 (m, 2H), 3.49 – 3.15 (m, 2H), 2.91 (s, 1H), 2.62 – 2.31 (m, 3H), 1.93 – 1.82 (m, 3H), 1.40 (s, 9H), 1.30 (s, 1H), 1.23 – 1.20 (m, 1H), 0.92 (t, J = 7.8 Hz, 9H), 0.86 – 0.71 (m, 6H), 0.51 (q, J = 7.7 Hz, 6H).

^{13}C NMR (151 MHz, CDCl$_3$): δ 172.34, 171.67, 170.98, 170.72, 170.48, 155.09, 142.07, 136.76, 136.53, 132.48, 130.44, 129.62, 128.70, 128.49, 128.12, 127.85, 127.11, 126.94, 124.90, 124.13, 122.80, 120.46,

118.92, 118.01, 113.87, 111.40, 110.82, 109.73, 102.15, 85.37, 80.07, 75.08, 60.16, 58.33, 58.22, 55.06, 52.95, 52.76, 52.56, 50.93, 48.08, 40.89, 36.64, 36.33, 28.40, 27.83, 27.17, 25.11, 24.91, 24.67, 15.62, 11.45, 7.50, 4.35.
HRMS (ESI-TOF): calculated for $C_{45}H_{63}BrN_6NaO_7Si^+$ [M+Na]$^+$: 929.3603, found: 929.3608.
[α]^{25}D: –14.7 (*c* = 0.5, CHCl$_3$)

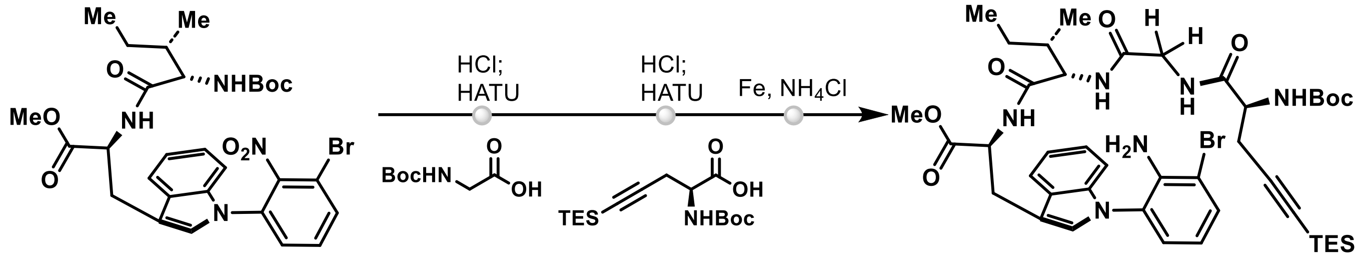

S11d **45m**

On 5.06 mmol scale, **General Procedure N** was followed from compound **S11d** as an intermediate. Purification by silica gel column chromatography gave the title compound **45m** (1.8 g, 41% yield from **S11d**).
Compound 45m
Physical State: amorphous solid
^{1}H NMR (600 MHz, CDCl$_3$): δ 7.59 (t, *J* = 7.1 Hz, 1H), 7.50 (d, *J* = 8.0 Hz, 1H), 7.22 – 6.88 (m, 7H), 6.85 – 6.65 (m, 2H), 5.45 – 5.40 (m, 1H), 4.97 – 4.92 (m, 1H), 4.40 – 4.08 (m, 4H), 3.75 – 3.71 (m, 3H), 3.68 – 3.64 (m, 1H), 3.55 – 3.40 (m, 1H), 3.28 – 3.20 (m, 1H), 2.68 (t, *J* = 6.4 Hz, 2H), 2.15 (s, 1H), 1.84 – 1.78 (m, 1H), 1.44 (s, 9H), 1.36 – 1.23 (m, 1H), 1.07 – 0.99 (m, 1H), 0.96 (t, *J* = 7.9 Hz, 9H), 0.87 – 0.77 (m, 6H), 0.56 (q, *J* = 7.9 Hz, 6H).
^{13}C NMR (151 MHz, CDCl$_3$): δ 172.32, 172.14, 171.17, 170.92, 170.74, 168.67, 168.27, 155.70, 142.05, 136.75, 136.52, 132.63, 128.43, 128.35, 127.90, 127.12, 127.05, 125.15, 124.93, 122.73, 120.46, 120.24, 119.08, 119.01, 118.36, 118.20, 111.66, 111.29, 110.91, 110.64, 109.93, 102.76, 102.61, 85.72, 80.67, 80.51, 57.85, 53.57, 53.32, 52.80, 52.59, 43.26, 43.00, 37.02, 36.14, 28.36, 27.95, 27.55, 24.76, 23.65, 23.42, 15.42, 15.23, 11.26, 11.08, 7.55, 4.43.
HRMS (ESI-TOF): calculated for $C_{42}H_{59}BrN_6NaO_7Si^+$ [M+Na]$^+$: 889.3290, found: 889.3290.
[α]^{25}D: +19.6 (*c* = 0.5, CHCl$_3$)

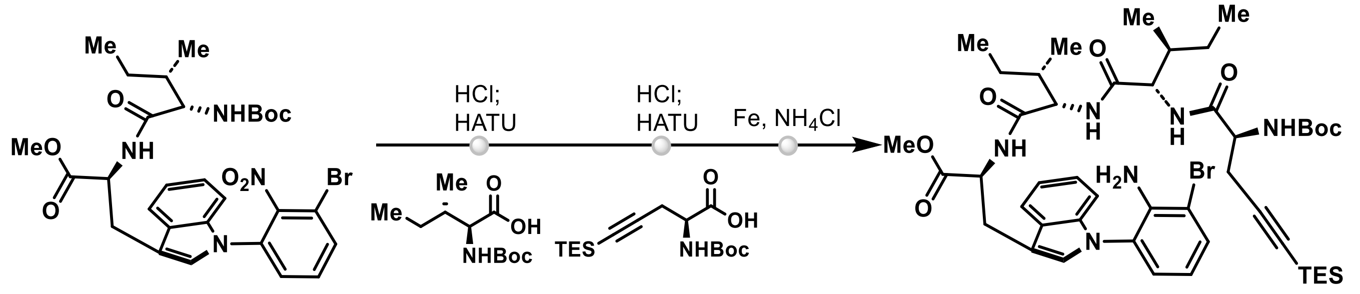

S11d **45n**

On 4.33 mmol scale, **General Procedure N** was followed from compound **S11d** as an intermediate. Purification by silica gel column chromatography gave the title compound **45n** (1.6 g, 40% yield from **S11d**).
Compound 45n
Physical State: amorphous solid
^{1}H NMR (600 MHz, CDCl$_3$): δ 7.63 – 7.57 (m, 1H), 7.54 – 7.45 (m, 1H), 7.20 – 7.05 (m, 5H), 6.98 – 6.52 (m, 4H), 5.32 – 5.27 (m, 1H), 4.94 – 4.90 (m, 1H), 4.43 – 3.93 (m, 4H), 3.72 – 3.66 (m, 3H), 3.46 – 3.22 (m, 2H), 2.78 – 2.54 (m, 2H), 1.92 – 1.79 (m, 2H), 1.43 (s, 9H), 1.38 – 1.27 (m, 2H), 1.09 – 1.00 (m, 2H), 0.97 (t, *J* = 7.9 Hz, 9H), 0.89 – 0.74 (m, 13H), 0.57 (q, *J* = 7.9 Hz, 6H).

^{13}C NMR (151 MHz, CDCl$_3$): δ 172.15, 170.85, 170.64, 155.88, 141.92, 136.78, 132.46, 127.84, 126.94, 125.01, 122.86, 120.47, 119.02, 118.16, 110.86, 109.86, 102.24, 86.30, 80.87, 58.55, 58.00, 53.40, 53.06, 52.51, 36.54, 28.26, 27.74, 27.47, 24.81, 24.64, 22.77, 15.63, 15.42, 11.48, 7.56, 4.40.
HRMS (ESI-TOF): calculated for $C_{46}H_{67}BrN_6NaO_7Si^+$ [M+Na]$^+$: 945.3916, found: 945.3922.
[α]^{25}$_D$: +3.8 (c = 0.5, CHCl$_3$)

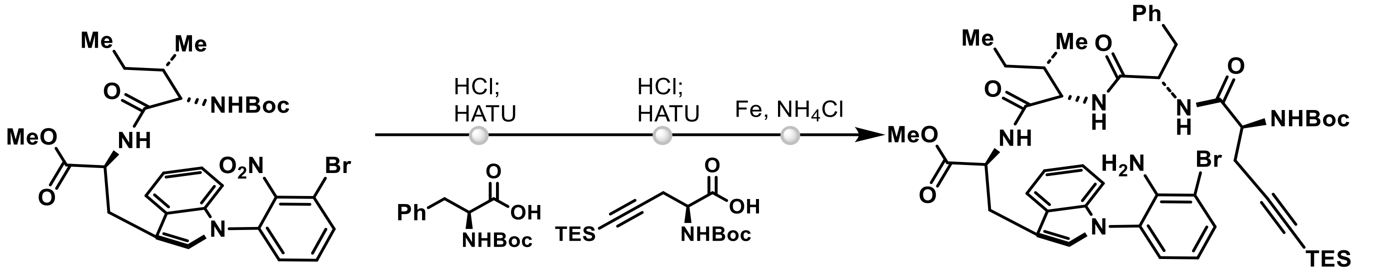

S11d 45o

On 5.33 mmol scale, **General Procedure N** was followed from compound **S11d** as an intermediate. Purification by silica gel column chromatography gave the title compound **45o** (2.3 g, 45% yield from **S11d**).
Compound 45o
Physical State: amorphous solid
^{1}H NMR (600 MHz, CDCl$_3$): δ 7.60 – 7.56 (m, 1H), 7.48 (d, J = 7.9 Hz, 1H), 7.25 – 7.00 (m, 10H), 6.93 – 6.31 (m, 4H), 5.19 – 5.13 (m, 1H), 4.98 – 4.86 (m, 1H), 4.75 – 4.45 (m, 1H), 4.34 – 3.79 (m, 3H), 3.75 – 3.66 (m, 3H), 3.40 – 3.26 (m, 2H), 3.19 – 3.12 (m, 1H), 3.03 – 2.82 (m, 1H), 2.71 – 2.50 (m, 2H), 1.84 – 1.79 (m, 1H), 1.45 – 1.43 (m, 1H), 1.35 (s, 9H), 1.29 – 1.21 (m, 1H), 0.98 (t, J = 7.9 Hz, 9H), 0.93 – 0.83 (m, 1H), 0.80 – 0.69 (m, 6H), 0.59 (q, J = 7.9 Hz, 6H).
^{13}C NMR (151 MHz, CDCl$_3$): δ 172.19, 170.71, 170.62, 170.43, 170.16, 155.76, 141.93, 136.73, 136.56, 136.13, 132.46, 129.52, 129.30, 128.92, 128.80, 128.30, 127.87, 127.31, 127.14, 127.01, 125.05, 122.79, 120.42, 119.08, 118.16, 111.65, 110.81, 109.81, 102.14, 86.31, 80.84, 58.23, 56.77, 54.53, 53.50, 53.12, 53.02, 52.50, 52.13, 37.82, 37.15, 36.26, 28.23, 25.19, 24.45, 23.01, 15.35, 11.37, 7.59, 4.43.
HRMS (ESI-TOF): calculated for $C_{49}H_{65}BrN_6NaO_7Si^+$ [M+Na]$^+$: 979.3760, found: 979.3764.
[α]^{25}$_D$: +1.4 (c = 0.5, CHCl$_3$)

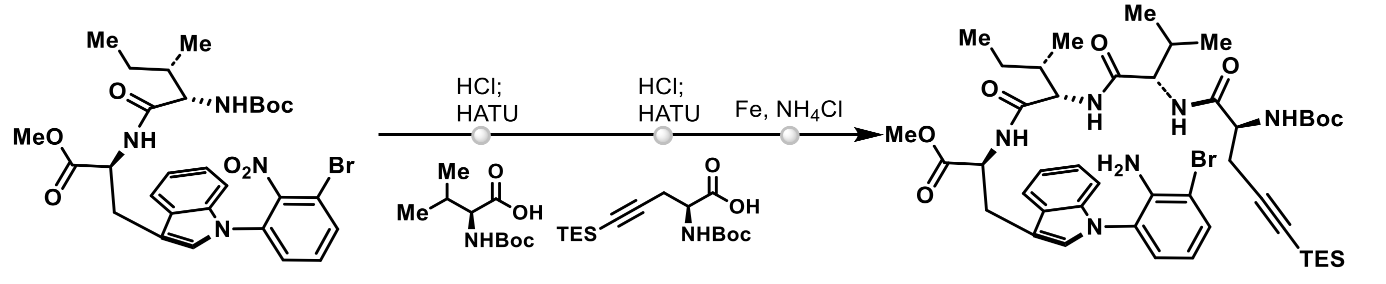

S11d 45p

On 3.30 mmol scale, **General Procedure N** was followed from compound **S11d** as an intermediate. Purification by silica gel column chromatography gave the title compound **45p** (1.5 g, 50% yield from **S11d**).
Compound 45p
Physical State: amorphous solid
^{1}H NMR (600 MHz, CDCl$_3$): δ 7.58 (d, J = 6.4 Hz, 1H), 7.48 (d, J = 7.2 Hz, 1H), 7.18 – 6.92 (m, 8H), 6.70 – 6.65 (m, 1H), 5.45 – 5.41 (m, 1H), 4.92 – 4.88 (m, 1H), 4.48 – 3.99 (m, 5H), 3.73 – 3.63 (m, 3H), 3.47 – 3.17 (m, 2H), 2.93 – 2.53 (m, 2H), 2.09 – 1.88 (m, 2H), 1.42 (s, 9H), 1.09 – 1.05 (m, 1H), 0.98 – 0.93 (m, 9H), 0.89 – 0.71 (m, 13H), 0.58 – 0.52 (m, 6H).
^{13}C NMR (151 MHz, CDCl$_3$): δ 172.21, 171.01, 155.79, 141.94, 136.53, 132.47, 128.12, 127.83, 126.98,

125.00, 122.85, 120.47, 119.02, 118.15, 111.53, 110.87, 109.90, 102.47, 85.96, 80.60, 58.90, 57.87, 56.55, 53.35, 53.16, 52.48, 37.83, 36.44, 30.59, 28.29, 27.81, 25.27, 24.73, 23.13, 19.24, 17.90, 15.38, 11.62, 11.41, 11.31, 7.56, 4.41.

HRMS (ESI-TOF): calculated for $C_{45}H_{65}BrN_6NaO_7Si^+$ $[M+Na]^+$: 931.3760, found: 931.3770.

[α]^{25}$_D$: +5.3 (c = 0.5, $CHCl_3$)

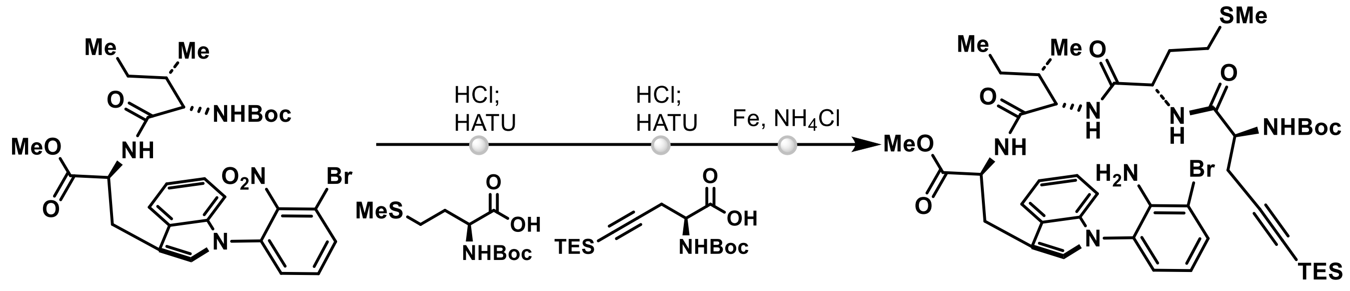

S11d 45q

On 3.11 mmol scale, **General Procedure N** was followed from compound **S11d** as an intermediate. Purification by silica gel column chromatography gave the title compound **45q** (1.2 g, 41% yield from **S11d**).

Compound 45q

Physical State: amorphous solid

^{1}H NMR (600 MHz, $CDCl_3$): δ 7.61 – 7.58 (m, 1H), 7.49 (d, J = 7.9 Hz, 1H), 7.24 – 7.01 (m, 6H), 6.91 – 6.87 (m, 2H), 6.68 (t, J = 7.9 Hz, 1H), 5.41 – 5.19 (m, 1H), 4.94 (q, J = 6.5 Hz, 1H), 4.69 – 4.48 (m, 1H), 4.46 – 4.21 (m, 2H), 4.21 – 3.98 (m, 2H), 3.74 – 3.70 (m, 3H), 3.68 (s, 1H), 3.43 – 3.23 (m, 2H), 2.80 – 2.54 (m, 3H), 2.52 – 2.36 (m, 2H), 2.11 (s, 1H), 2.04 – 2.03 (m, 3H), 1.92 – 1.83 (m, 2H), 1.43 (s, 9H), 0.96 (t, J = 8.0 Hz, 9H), 0.86 – 0.77 (m, 6H), 0.56 (q, J = 7.9 Hz, 6H).

^{13}C NMR (151 MHz, $CDCl_3$): δ 172.19, 172.06, 170.72, 170.47, 170.42, 155.74, 141.95, 136.81, 136.55, 132.49, 128.21, 127.86, 127.02, 124.99, 122.84, 120.48, 119.02, 118.18, 111.49, 110.85, 109.85, 102.28, 86.16, 80.76, 58.12, 56.77, 53.34, 53.11, 53.01, 52.53, 52.23, 37.62, 36.49, 31.14, 30.38, 30.12, 29.95, 28.32, 27.77, 25.18, 24.67, 23.31, 15.64, 15.43, 15.17, 14.96, 11.66, 11.49, 11.42, 7.58, 4.41.

HRMS (ESI-TOF): calculated for $C_{45}H_{65}BrN_6NaO_7SSi^+$ $[M+Na]^+$: 963.3480, found: 963.3486.

[α]^{25}$_D$: +13.5 (c = 0.5, $CHCl_3$)

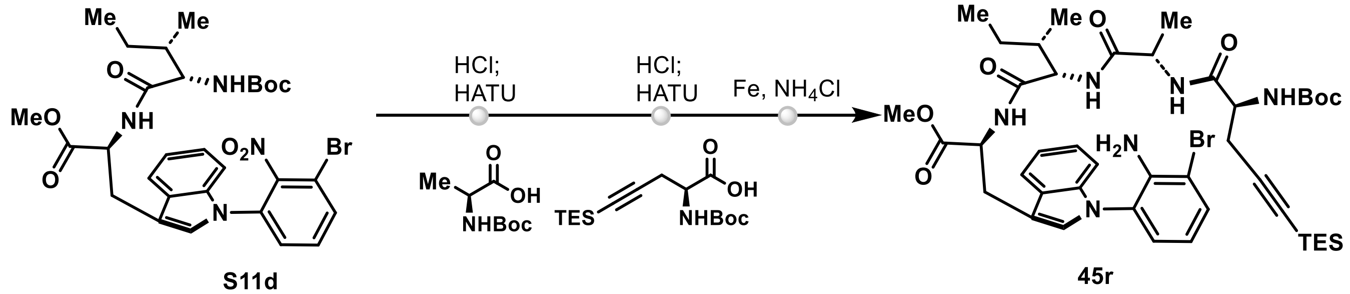

S11d 45r

On 3.70 mmol scale, **General Procedure N** was followed from compound **S11d** as an intermediate. Purification by silica gel column chromatography gave the title compound **45r** (1.5 g, 46% yield from **S11d**).

Compound 45r

Physical State: amorphous solid

^{1}H NMR (600 MHz, $CDCl_3$): δ 7.60 – 7.57 (m, 1H), 7.50 (d, J = 8.0 Hz, 1H), 7.22 – 6.99 (m, 5H), 6.89 – 6.64 (m, 4H), 5.32 – 5.26 (m, 1H), 5.01 – 4.90 (m, 1H), 4.42 – 4.01 (m, 5H), 3.74 – 3.70 (m, 3H), 3.43 – 3.25 (m, 2H), 2.80 – 2.71 (m, 1H), 2.62 – 2.52 (m, 1H), 1.86 – 1.79 (m, 2H), 1.44 (s, 9H), 1.40 – 1.36 (m, 1H), 1.29 (d, J = 6.5 Hz, 1H), 1.20 (d, J = 5.6 Hz, 1H), 1.12 – 1.01 (m, 1H), 0.96 (t, J = 7.9 Hz, 10H), 0.86 – 0.78 (m, 6H), 0.57 (q, J = 7.9 Hz, 6H).

^{13}C NMR (151 MHz, CDCl$_3$): δ 172.19, 171.68, 170.59, 155.59, 141.96, 141.85, 136.76, 136.56, 132.52, 128.23, 127.85, 126.91, 124.98, 122.86, 120.50, 118.97, 118.19, 111.49, 110.86, 109.84, 102.32, 86.30, 80.78, 57.96, 53.23, 52.54, 49.47, 36.72, 28.29, 27.77, 24.74, 23.43, 17.80, 15.36, 11.52, 11.42, 7.56, 4.41.
HRMS (ESI-TOF): calculated for $C_{43}H_{61}BrN_6NaO_7Si^+$ [M+Na]$^+$: 903.3447, found: 903.3455.
[α]^{25}$_D$: +13.7 (c = 0.5, CHCl$_3$)

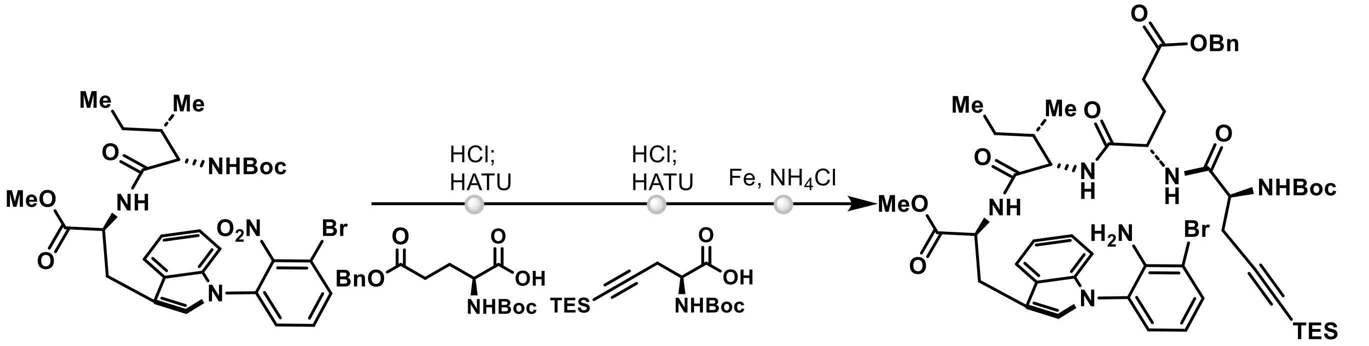

S11d 45s

On 2.81 mmol scale, **General Procedure N** was followed from compound **S11d** as an intermediate. Purification by silica gel column chromatography gave the title compound **45s** (1.1 g, 38% yield from **S11d**).
Compound 45s
Physical State: amorphous solid
^{1}H NMR (600 MHz, CDCl$_3$): δ 7.63 – 7.57 (m, 1H), 7.48 (d, J = 7.8 Hz, 1H), 7.35 – 7.29 (m, 6H), 7.20 – 7.11 (m, 3H), 7.11 – 7.04 (m, 2H), 7.01 – 6.88 (m, 2H), 6.67 (t, J = 7.9 Hz, 1H), 5.30 – 5.24 (m, 1H), 5.16 – 5.02 (m, 2H), 4.96 – 4.92 (m, 1H), 4.42 – 4.00 (m, 4H), 3.71 – 3.67 (m, 3H), 3.50 – 3.18 (m, 2H), 2.73 – 2.53 (m, 2H), 2.53 – 2.29 (m, 2H), 2.07 – 1.80 (m, 3H), 1.43 (s, 9H), 1.40 – 1.25 (m, 1H), 1.14 – 1.01 (m, 1H), 0.96 (t, J = 7.9 Hz, 9H), 0.87 – 0.76 (m, 6H), 0.57 (q, J = 7.9 Hz, 6H).
^{13}C NMR (151 MHz, CDCl$_3$): δ 173.64, 172.25, 171.05, 170.78, 155.73, 141.97, 141.88, 136.77, 136.56, 135.68, 132.47, 128.66, 128.44, 128.36, 127.86, 127.03, 125.05, 122.79, 120.44, 119.05, 118.17, 111.64, 110.82, 109.83, 102.34, 86.09, 80.59, 66.78, 58.19, 53.48, 53.14, 53.03, 52.49, 36.68, 36.43, 30.51, 28.30, 27.75, 27.18, 24.68, 24.56, 23.25, 15.43, 11.53, 11.45, 7.59, 4.41.
HRMS (ESI-TOF): calculated for $C_{52}H_{69}BrN_6NaO_9Si^+$ [M+Na]$^+$: 1051.3971, found: 1051.3970.
[α]^{25}$_D$: +5.8 (c = 0.5, CHCl$_3$)

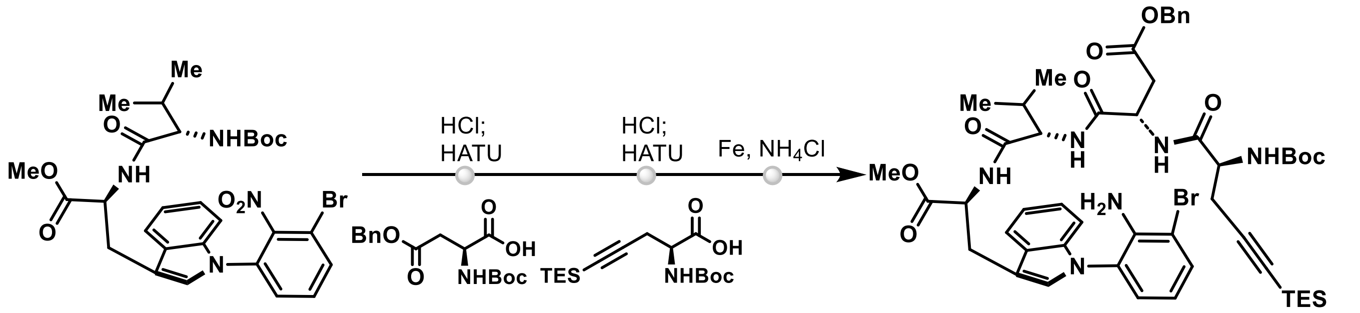

S11b 45t

On 3.24 mmol scale, **General Procedure N** was followed from compound **S11b** as an intermediate. Purification by silica gel column chromatography gave the title compound **45t** (1.3 g, 40% yield from **S11b**).
Compound 45t
Physical State: amorphous solid
^{1}H NMR (600 MHz, CDCl$_3$): δ 7.64 – 7.44 (m, 3H), 7.41 – 7.26 (m, 6H), 7.18 – 6.95 (m, 6H), 6.67 (t, J = 7.6 Hz, 1H), 5.28 – 5.09 (m, 1H), 5.06 – 4.89 (m, 3H), 4.65 – 4.54 (m, 1H), 4.33 – 3.90 (m, 3H), 3.73 – 3.67 (m, 3H), 3.48 – 3.21 (m, 2H), 3.10 – 2.42 (m, 4H), 2.24 – 2.15 (m, 1H), 1.45 (s, 9H), 0.95 (t, J = 7.9 Hz, 9H),

0.91 – 0.81 (m, 6H), 0.75 (d, J = 6.5 Hz, 1H), 0.56 (q, J = 7.9 Hz, 6H).

^{13}C NMR (151 MHz, CDCL3) δ 172.24, 171.98, 171.85, 170.79, 170.59, 170.13, 170.03, 155.75, 141.98, 136.68, 135.30, 132.47, 128.65, 128.51, 128.41, 127.90, 127.13, 127.06, 125.03, 122.79, 120.45, 119.03, 118.94, 118.11, 111.48, 110.83, 109.75, 101.99, 86.42, 80.98, 67.01, 59.08, 53.43, 53.03, 52.88, 52.52, 49.70, 35.13, 29.97, 29.74, 28.31, 28.21, 27.86, 27.67, 23.18, 19.26, 17.77, 17.49, 7.57, 4.39.

HRMS (ESI-TOF): calculated for $C_{50}H_{65}BrN_6NaO_9Si^+$ [M+Na]$^+$: 1023.3658, found: 1023.3647.

[α]^{25}D: +5.6 (c = 0.5, $CHCl_3$)

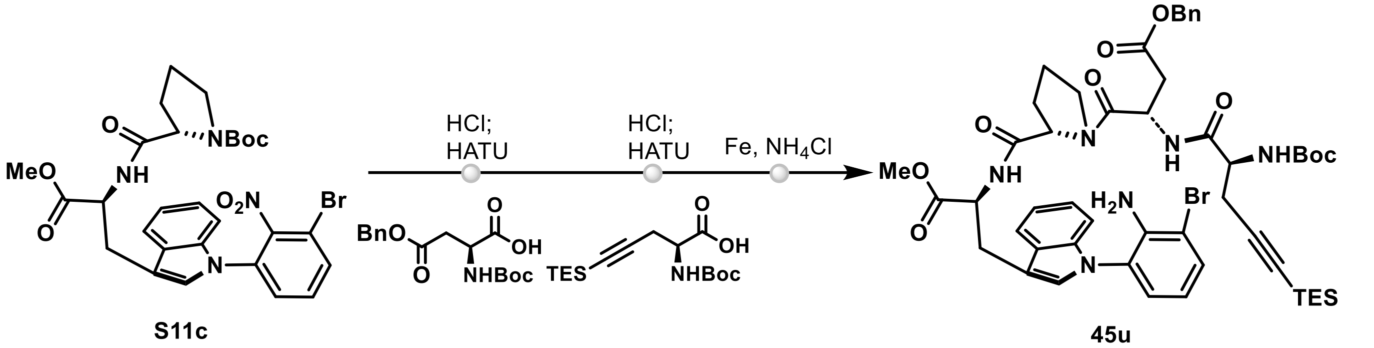

On 2.82 mmol scale, **General Procedure N** was followed from compound **S11c** as an intermediate. Purification by silica gel column chromatography gave the title compound **45u** (1.1 g, 39% yield from **S11c**).

Compound 45u

Physical State: amorphous solid

^{1}H NMR (600 MHz, CDCl$_3$): δ 7.62 (dd, J = 16.0, 7.2 Hz, 1H), 7.50 (d, J = 8.0 Hz, 1H), 7.35 – 7.30 (m, 4H), 7.26 – 7.19 (m, 2H), 7.16 – 7.05 (m, 6H), 6.96 – 6.92 (m, 1H), 6.70 – 6.65 (m, 1H), 5.13 – 4.71 (m, 5H), 4.49 (dd, J = 21.1, 7.8 Hz, 1H), 4.28 – 4.19 (m, 2H), 3.70 – 3.65 (m, 3H), 3.67 – 3.50 (m, 2H), 3.46 – 3.24 (m, 2H), 3.03 – 2.98 (m, 1H), 2.83 – 2.71 (m, 1H), 2.68 – 2.58 (m, 2H), 2.41 – 2.10 (m, 1H), 1.99 – 1.78 (m, 2H), 1.75 – 1.62 (m, 1H), 1.43 (s, 9H), 0.95 (t, J = 7.9 Hz, 9H), 0.56 (q, J = 7.9 Hz, 6H).

^{13}C NMR (151 MHz, CDCl$_3$): δ 172.17, 171.86, 171.75, 171.10, 170.80, 170.01, 169.82, 155.45, 142.10, 141.83, 136.61, 135.34, 132.52, 128.61, 128.46, 128.35, 127.74, 127.11, 125.11, 124.79, 122.85, 122.75, 120.28, 118.95, 118.84, 118.26, 117.99, 111.93, 111.78, 110.88, 110.73, 109.87, 102.07, 86.14, 80.88, 66.95, 60.64, 60.40, 53.23, 53.00, 52.36, 47.71, 47.55, 47.26, 47.07, 37.67, 37.18, 28.76, 28.29, 27.72, 27.45, 24.47, 23.99, 23.43, 7.58, 4.41.

HRMS (ESI-TOF): calculated for $C_{50}H_{63}BrN_6NaO_9Si^+$ [M+Na]$^+$: 1021.3501, found: 1021.3506.

[α]^{25}D: –24.5 (c = 0.5, $CHCl_3$)

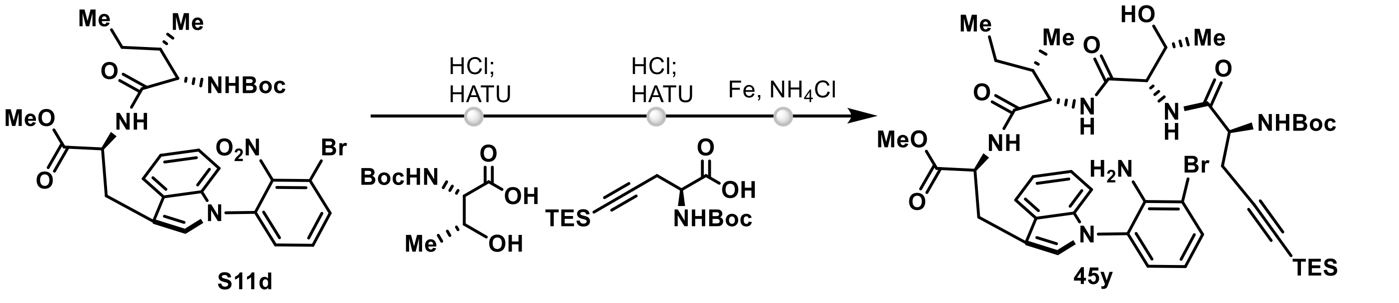

On 1.0 mmol scale, **General Procedure N** was followed from compound **S11d** as an intermediate. Purification by silica gel column chromatography gave the title compound **45y** (582 mg, 63% yield from **S11d**).

Compound 45y

Physical State: amorphous solid

^{1}H NMR (600 MHz, CDCl$_3$): δ 7.64 – 7.44 (m, 2H), 7.30 – 6.93 (m, 8H), 6.69 (td, J = 7.8, 4.5 Hz, 1H), 5.35 (d, J = 8.7 Hz, 1H), 5.03 – 4.78 (m, 1H), 4.35 – 4.02 (m, 4H), 4.02 – 3.84 (m, 1H), 3.75 (s, 3H), 3.46 – 3.21

(m, 2H), 2.81 (dt, J = 17.1, 5.2 Hz, 1H), 2.74 – 2.60 (m, 1H), 2.14 (d, J = 25.2 Hz, 1H), 1.97 (s, 1H), 1.43 (s, 10H), 1.35 – 1.26 (m, 1H), 1.08 – 0.92 (m, 13H), 0.82 (dq, J = 14.4, 6.8 Hz, 6H), 0.57 (q, J = 7.9 Hz, 6H).
^{13}C NMR (151 MHz, $CDCl_3$): δ 172.85, 172.56, 171.33, 171.23, 170.95, 156.01, 142.09, 141.94, 136.82, 136.70, 132.57, 128.29, 128.15, 127.89, 127.58, 126.94, 124.98, 122.90, 122.78, 120.46, 120.36, 118.98, 118.91, 118.22, 118.12, 111.29, 111.14, 110.94, 109.96, 109.87, 101.96, 101.82, 95.77, 86.52, 81.23, 81.03, 66.29, 57.71, 57.50, 53.56, 52.93, 52.77, 52.66, 35.79, 35.43, 28.28, 27.66, 24.72, 23.12, 18.82, 18.56, 15.51, 11.19, 11.01, 7.54, 4.55, 4.45, 4.36.
HRMS (ESI-TOF): calculated for $C_{44}H_{63}BrN_6NaO_8Si^+$ [M+Na]$^+$: 933.3552, found: 933.3552.
$[\alpha]^{25}_D$: +59.8 (c = 0.5, $CHCl_3$)

General procedure O for the synthesis of 46 via Larock macrocyclization:

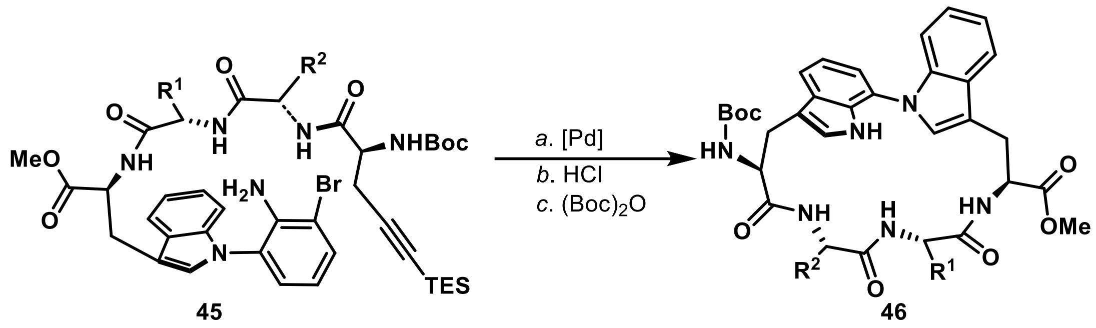

The precursor **45** (0.2 mmol, 1.0 eq.) was dissolved in toluene (3 mL). Pd($t$$Bu_3P)_2$ (0.02 mmol, 0.1 eq.) and DIPEA (0.5 mmol, 2.5 eq.) were added. The reaction mixture was stirred at 110 °C under nitrogen atmosphere for 4 h. After Celite filtration followed by concentration, the residue was dissolved in DCM/4M HCl = 1 mL /1 mL, the reaction was stirred at rt for 2 h, The solvents were removed under reduced pressure to give the crude, the crude was dissolved in DCM (1 mL), DIPEA (0.6 mmol, 3.0 eq.), $(Boc)_2O$ (0.3 mmol, 1.5 eq.) was added, the reaction was stirred at rt for 4 h. The solvents were removed under reduced pressure to give the residue, the residue was purified by silica gel chromatography to give the product **46**.
Unless otherwise noted, the reactions are conducted in 0.2 mmol scale.

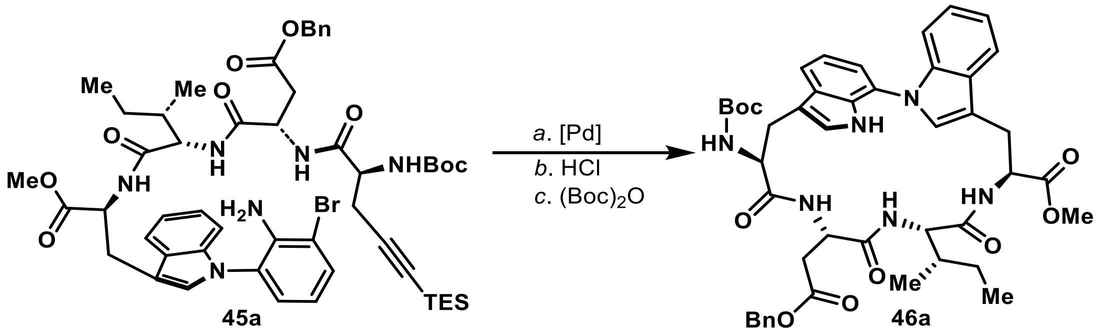

On 0.41 mmol scale, **General Procedure O** was followed with compound **45a** via Larock macrocyclization. Purification by silica gel column chromatography gave the title compound **46a** (210 mg, 62% yield).

Compound 46a

Physical State: amorphous solid

^{1}H NMR (600 MHz, CDCl$_3$): δ 8.44 (s, 1H), 7.83 (d, J = 6.7 Hz, 1H), 7.61 (d, J = 7.6 Hz, 1H), 7.46 – 7.39 (m, 2H), 7.35 – 7.27 (m, 6H), 7.25 – 7.18 (m, 3H), 6.98 – 6.91 (m, 2H), 6.40 (d, J = 7.2 Hz, 1H), 5.94 – 5.78 (m, 1H), 5.53 (d, J = 5.5 Hz, 1H), 5.14 – 5.00 (m, 2H), 4.94 – 4.88 (m, 1H), 4.64 – 4.59 (m, 1H), 4.52 – 4.37 (m, 1H), 3.90 (s, 1H), 3.79 (s, 3H), 3.49 (d, J = 15.5 Hz, 1H), 3.41 (dd, J = 13.6, 3.5 Hz, 1H), 3.18 – 3.06 (m, 1H), 2.93 (t, J = 11.8 Hz, 1H), 2.84 – 2.71 (m, 1H), 2.46 (dd, J = 17.4, 7.7 Hz, 1H), 1.80 – 1.76 (m, 1H), 1.57 – 1.52 (m, 1H), 1.48 (s, 9H), 1.26 – 1.19 (m, 1H), 0.95 (d, J = 6.8 Hz, 3H), 0.91 (t, J = 7.4 Hz, 3H).

^{13}C NMR (151 MHz, CDCl$_3$): δ 172.52, 172.35, 170.97, 170.64, 169.48, 155.34, 136.96, 135.16, 133.22, 128.66, 128.49, 128.40, 128.17, 127.25, 124.30, 124.16, 122.87, 120.22, 120.06, 119.53, 118.87, 118.46, 112.00, 111.31, 110.88, 80.00, 67.29, 59.51, 55.28, 52.75, 48.30, 37.08, 29.42, 28.48, 27.86, 25.58, 15.15, 11.41.

HRMS (ESI-TOF): calculated for $C_{45}H_{52}N_6NaO_9^+$ $[M+Na]^+$: 843.3688, found: 843.3687.

$[\alpha]^{25}_D$: +13.4 (c = 0.2, $CHCl_3$)

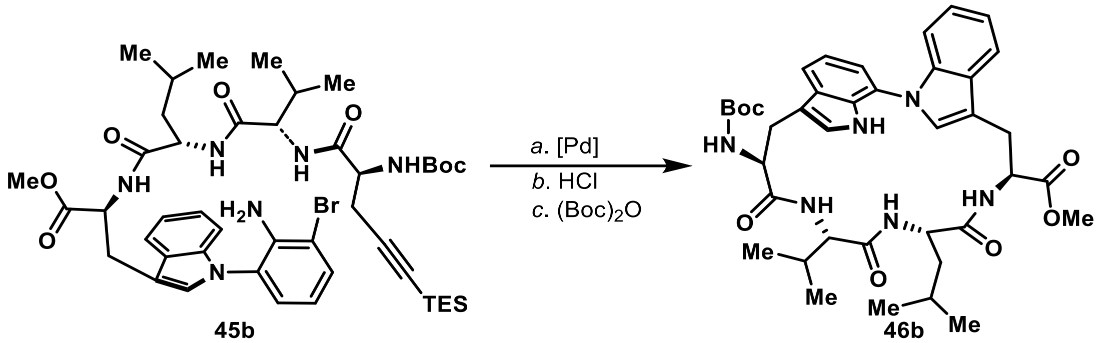

On 0.2 mmol scale, **General Procedure O** was followed with compound **45b** via Larock macrocyclization. Purification by silica gel column chromatography gave the title compound **46b** (94.4 mg, 66% yield).

Compound 46b

Physical State: amorphous solid

^{1}H NMR (600 MHz, CDCl$_3$): δ 8.39 (s, 1H), 7.82 (d, *J* = 7.8 Hz, 1H), 7.65 (s, 1H), 7.59 (d, *J* = 7.8 Hz, 1H), 7.48 (d, *J* = 8.1 Hz, 1H), 7.33 (d, *J* = 7.4 Hz, 1H), 7.26 – 7.14 (m, 3H), 6.78 (s, 1H), 6.64 (s, 1H), 6.17 (s, 1H), 5.68 (d, *J* = 7.5 Hz, 1H), 5.42 (s, 1H), 4.92 – 4.89 (m, 1H), 4.57 – 4.52 (m, 1H), 4.28 (dd, *J* = 9.0, 6.0 Hz, 1H), 4.01 (s, 1H), 3.79 (s, 3H), 3.50 (d, *J* = 14.5 Hz, 1H), 3.37 (d, *J* = 12.6 Hz, 1H), 3.19 (dd, *J* = 15.8, 11.1 Hz, 1H), 2.88 (t, *J* = 12.8 Hz, 1H), 1.75 – 1.71 (m, 1H), 1.69 – 1.64 (m, 1H), 1.57 (s, 1H), 1.54 – 1.50 (m, 1H), 1.45 (s, 9H), 0.92 (d, *J* = 6.4 Hz, 3H), 0.89 (d, *J* = 6.4 Hz, 3H), 0.78 (d, *J* = 6.6 Hz, 3H), 0.64 (d, *J* = 6.6 Hz, 3H).

^{13}C NMR (151 MHz, CDCl$_3$): δ 172.59, 171.94, 171.30, 170.21, 155.43, 136.73, 133.35, 128.32, 128.15, 127.49, 124.23, 122.86, 120.23, 120.03, 119.19, 118.52, 118.46, 111.57, 111.30, 110.72, 80.01, 57.34, 55.09, 53.72, 52.74, 52.61, 41.35, 31.96, 29.78, 28.46, 27.23, 24.39, 22.70, 18.98, 17.26.

HRMS (ESI-TOF): calculated for $C_{39}H_{50}N_6NaO_7^+$ [M+Na]$^+$: 737.3633, found: 737.3633.

[α]$^{25}_D$: +39.4 (*c* = 0.5, CHCl$_3$)

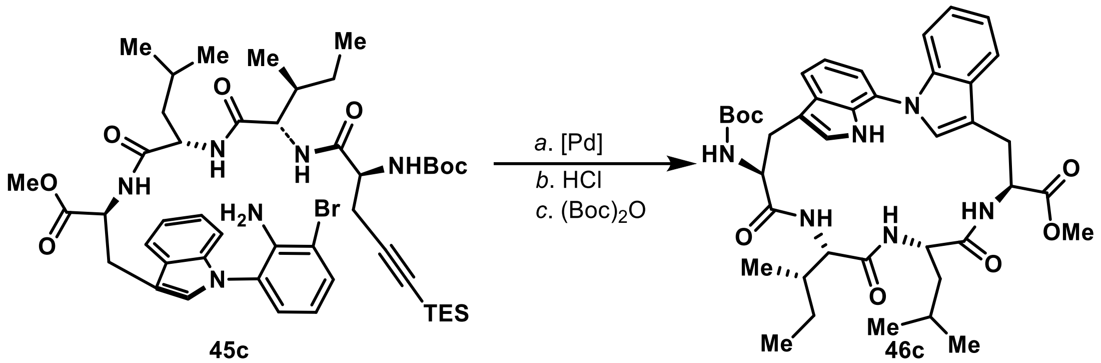

On 0.2 mmol scale, **General Procedure O** was followed with compound **45c** via Larock macrocyclization. Purification by silica gel column chromatography gave the title compound **46c** (90.4 mg, 62% yield).

Compound 46c

Physical State: amorphous solid

^{1}H NMR (600 MHz, CDCl$_3$): δ 8.48 (s, 1H), 7.81 (d, *J* = 7.3 Hz, 1H), 7.70 (s, 1H), 7.58 (d, *J* = 7.7 Hz, 1H), 7.48 (d, *J* = 8.1 Hz, 1H), 7.34 (d, *J* = 7.3 Hz, 1H), 7.25 – 7.16 (m, 3H), 6.77 – 6.66 (m, 2H), 6.13 (s, 1H), 5.65 (s, 1H), 5.39 (s, 1H), 4.97 – 4.83 (m, 1H), 4.53 – 4.49 (m, 1H), 4.34 – 4.18 (m, 1H), 4.02 (s, 1H), 3.78 (s, 3H), 3.49 (d, *J* = 16.1 Hz, 1H), 3.35 (d, *J* = 12.8 Hz, 1H), 3.20 (dd, *J* = 15.9, 11.1 Hz, 1H), 2.85 (t, *J* = 12.7 Hz, 1H), 1.75 – 1.71 (m, 1H), 1.68 – 1.63 (m, 1H), 1.51 – 1.49 (m, 1H), 1.46 (s, 9H), 1.25 – 1.23 (m, 1H), 0.93 – 0.86 (m, 7H), 0.78 – 0.72 (m, 7H).

^{13}C NMR (151 MHz, CDCl$_3$): δ 172.64, 171.80, 171.15, 170.18, 155.41, 136.73, 133.32, 128.31, 128.16, 127.58, 124.24, 124.11, 122.84, 120.23, 120.01, 119.12, 118.47, 111.50, 111.30, 110.69, 80.00, 57.02, 55.15,

53.77, 52.72, 52.54, 41.29, 38.30, 29.78, 28.47, 27.20, 24.47, 24.35, 22.76, 22.68, 15.10, 11.30.
HRMS (ESI-TOF): calculated for $C_{40}H_{52}N_6NaO_7^+$ $[M+Na]^+$: 751.3790, found: 751.3797.
$[\alpha]^{25}_D$: +49.3 (c = 0.5, $CHCl_3$)

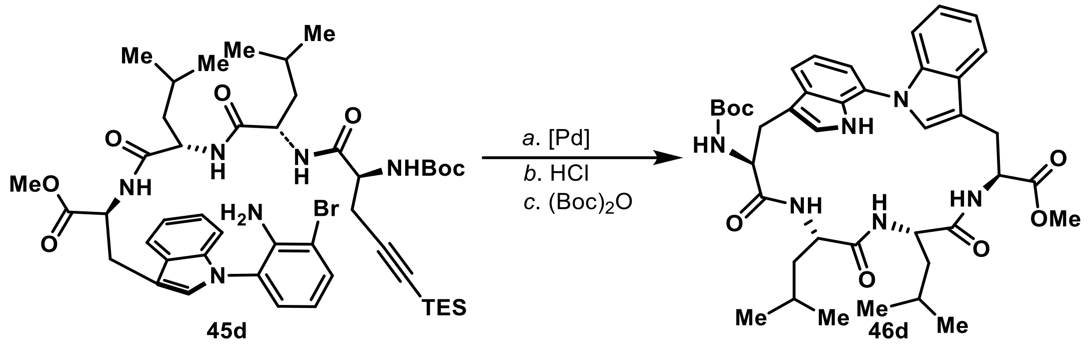

On 0.2 mmol scale, **General Procedure O** was followed with compound **45d** via Larock macrocyclization. Purification by silica gel column chromatography gave the title compound **46d** (91.8 mg, 63% yield).

Compound 46d

Physical State: amorphous solid

^{1}H NMR (600 MHz, $CDCl_3$): δ 8.38 (s, 1H), 7.77 (d, J = 7.9 Hz, 1H), 7.61 – 7.51 (m, 2H), 7.44 (d, J = 7.7 Hz, 1H), 7.31 (d, J = 7.3 Hz, 1H), 7.24 – 7.16 (m, 3H), 6.75 (s, 1H), 6.62 (d, J = 6.9 Hz, 1H), 6.06 (s, 1H), 5.54 (d, J = 7.8 Hz, 1H), 5.42 (s, 1H), 4.91 (ddd, J = 10.4, 7.4, 2.7 Hz, 1H), 4.47 – 4.40 (m, 2H), 4.09 – 4.05 (m, 1H), 3.78 (s, 3H), 3.46 (d, J = 14.9 Hz, 1H), 3.28 (dd, J = 13.9, 3.7 Hz, 1H), 3.17 (dd, J = 15.8, 10.9 Hz, 1H), 2.87 (t, J = 12.6 Hz, 1H), 1.73 – 1.64 (m, 2H), 1.55 – 1.51 (m, 2H), 1.47 (s, 9H), 1.39 – 1.29 (m, 2H), 0.93 (d, J = 6.3 Hz, 3H), 0.89 (d, J = 6.3 Hz, 3H), 0.82 – 0.78 (m, 6H).

^{13}C NMR (151 MHz, $CDCl_3$): δ 172.56, 171.94, 171.21, 171.13, 155.35, 136.84, 133.21, 128.48, 128.09, 127.35, 124.26, 124.12, 122.85, 120.22, 119.90, 119.07, 118.43, 111.59, 111.16, 110.72, 80.02, 55.32, 53.56, 52.74, 52.57, 51.25, 42.33, 41.32, 29.46, 28.47, 27.27, 24.49, 24.45, 23.10, 22.80, 22.65, 21.72.

HRMS (ESI-TOF): calculated for $C_{40}H_{52}N_6NaO_7^+$ $[M+Na]^+$: 751.3790, found: 751.3793.

$[\alpha]^{25}_D$: +41.2 (c = 0.5, $CHCl_3$)

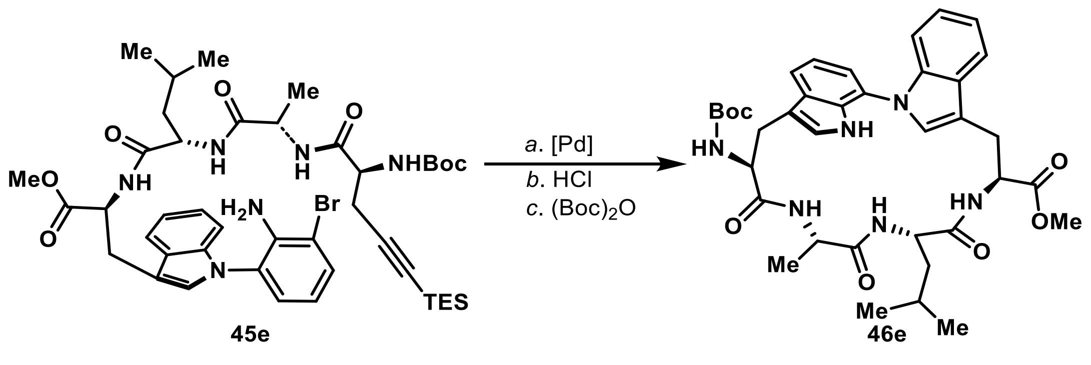

On 0.2 mmol scale, **General Procedure O** was followed with compound **45e** via Larock macrocyclization. Purification by silica gel column chromatography gave the title compound **46e** (87.9 mg, 64% yield).

Compound 46e

Physical State: amorphous solid

^{1}H NMR (600 MHz, $CDCl_3$): δ 8.41 – 8.37 (m, 1H), 7.82 – 7.78 (m, 1H), 7.59 (d, J = 6.6 Hz, 1H), 7.53 (s, 1H), 7.42 (d, J = 7.4 Hz, 1H), 7.32 (t, J = 6.8 Hz, 1H), 7.24 – 7.17 (m, 3H), 6.92 – 6.62 (m, 2H), 6.45 – 6.32 (m, 1H), 5.59 (s, 2H), 4.97 (t, J = 7.7 Hz, 1H), 4.45 (s, 1H), 4.37 (s, 1H), 4.08 (s, 1H), 3.68 (s, 3H), 3.45 (dd, J = 16.0, 6.6 Hz, 1H), 3.36 – 3.12 (m, 2H), 2.74 (s, 1H), 1.67 – 1.63 (m, 2H), 1.49 (s, 9H), 1.34 – 1.22 (m, 1H), 1.15 – 1.11 (m, 3H), 0.92 – 0.87 (m, 6H).

^{13}C NMR (151 MHz, CDCl$_3$): δ 172.58, 172.33, 171.46, 170.83, 155.27, 136.92, 133.17, 128.44, 128.13, 127.45, 124.56, 124.12, 122.84, 120.23, 119.93, 119.08, 118.43, 111.50, 110.93, 110.77, 79.97, 55.36, 54.08, 52.68, 52.56, 48.34, 40.75, 31.67, 29.72, 28.52, 27.10, 24.53, 22.88, 22.74, 22.31, 19.39, 14.21.
HRMS (ESI-TOF): calculated for $C_{37}H_{46}N_6NaO_7^+$ [M+Na]$^+$: 709.3320, found: 709.3322.
[α]$^{25}_D$: +114.2 (c = 0.5, CHCl$_3$)

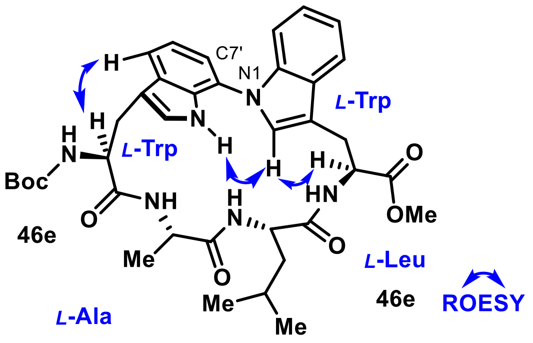

The configuration of the ring system is deduced to be *Sconf*.

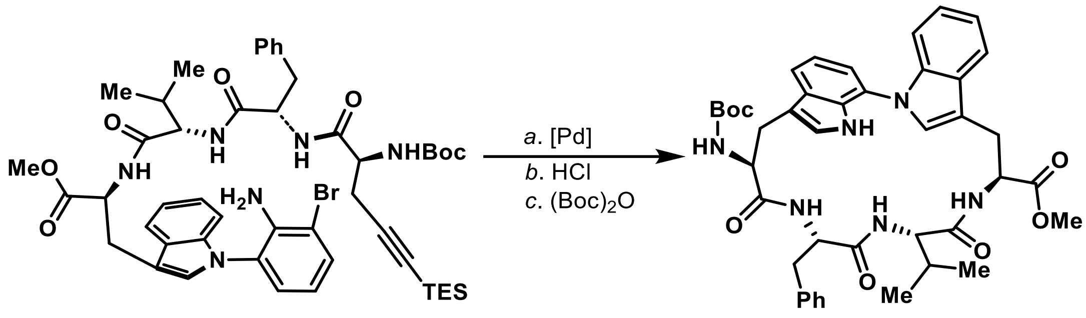

45f 46f

On 0.2 mmol scale, **General Procedure O** was followed with compound **45f** via Larock macrocyclization. Purification by silica gel column chromatography gave the title compound **46f** (97.4 mg, 65% yield).

Compound 46f

Physical State: amorphous solid

^{1}H NMR (600 MHz, CDCl$_3$): δ 8.29 (s, 1H), 7.86 (d, J = 7.4 Hz, 1H), 7.58 (d, J = 7.7 Hz, 1H), 7.45 (d, J = 8.0 Hz, 1H), 7.39 (s, 1H), 7.27 (d, J = 7.4 Hz, 1H), 7.25 – 7.19 (m, 5H), 7.18 – 7.13 (m, 3H), 6.77 (s, 1H), 6.50 (s, 1H), 5.68 (d, J = 6.7 Hz, 2H), 5.50 (s, 1H), 4.97 – 4.85 (m, 1H), 4.70 – 4.57 (m, 1H), 4.54 – 4.49 (m, 1H), 3.80 (s, 3H), 3.69 – 3.62 (m, 1H), 3.49 (d, J = 15.9 Hz, 1H), 3.44 (d, J = 12.6 Hz, 1H), 3.22 – 3.11 (m, 1H), 2.89 (d, J = 10.4 Hz, 1H), 2.81 (t, J = 12.6 Hz, 1H), 2.64 (dd, J = 13.6, 8.5 Hz, 1H), 1.84 – 1.81 (m, 1H), 1.52 (s, 9H), 0.83 (d, J = 6.5 Hz, 3H), 0.77 (d, J = 6.6 Hz, 3H).

^{13}C NMR (151 MHz, CDCl$_3$): δ 172.61, 170.78, 170.49, 169.47, 155.27, 136.77, 136.16, 133.29, 129.62, 128.80, 128.16, 127.27, 127.21, 124.68, 124.08, 122.86, 120.18, 120.10, 119.51, 118.79, 118.41, 111.62, 111.29, 110.81, 79.91, 60.82, 54.78, 53.58, 52.73, 52.55, 39.18, 30.38, 30.26, 28.56, 27.29, 19.07, 18.68.
HRMS (ESI-TOF): calculated for $C_{42}H_{48}N_6NaO_7^+$ [M+Na]$^+$: 771.3477, found: 771.3483.
[α]$^{25}_D$: +43.2 (c = 0.5, CHCl$_3$)

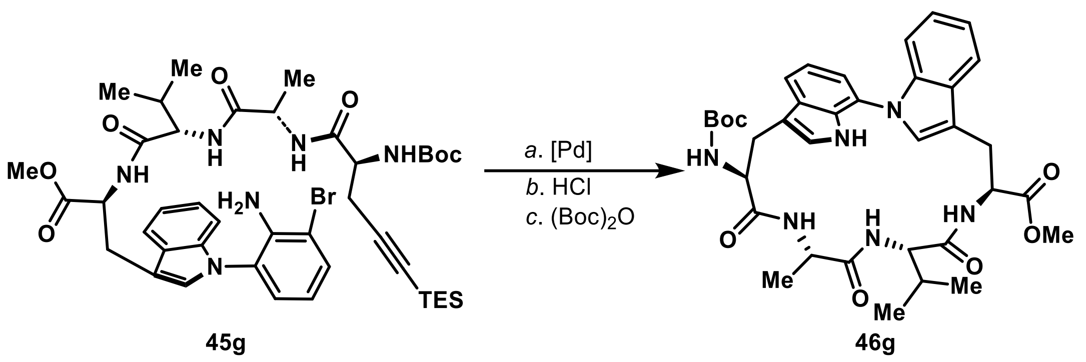

45g **46g**

On 0.2 mmol scale, **General Procedure O** was followed with compound **45g** via Larock macrocyclization. Purification by silica gel column chromatography gave the title compound **46g** (83.4 mg, 62% yield).

Compound 46g

Physical State: amorphous solid

^{1}H NMR (600 MHz, CDCl$_3$): δ 8.87 (s, 1H), 8.08 (d, *J* = 7.5 Hz, 1H), 7.95 – 7.92 (m, 1H), 7.84 (s, 1H), 7.77 – 7.74 (m, 1H), 7.63 (d, *J* = 7.3 Hz, 1H), 7.55 – 7.52 (m, 3H), 7.27 (s, 1H), 7.08 (s, 1H), 7.00 (s, 1H), 6.04 - 5.90 (m, 2H), 5.31 – 5.27 (m, 1H), 4.98 – 4.90 (m, 1H), 4.64 (s, 1H), 4.35 (s, 1H), 4.06 (s, 3H), 3.76 (d, *J* = 15.3 Hz, 1H), 3.65 – 3.55 (m, 1H), 3.50 (d, *J* = 11.9 Hz, 1H), 2.96 (s, 1H), 2.41 – 2.37 (m, 1H), 1.83 (s, 9H), 1.48 (d, *J* = 6.9 Hz, 3H), 1.33 – 1.28 (m, 6H).

^{13}C NMR (151 MHz, CDCl$_3$): δ 172.55, 171.20, 170.99, 155.23, 136.82, 132.94, 128.59, 128.09, 127.05, 124.58, 124.05, 122.95, 120.32, 119.98, 119.04, 118.45, 111.71, 110.95, 110.77, 80.02, 60.52, 55.39, 52.63, 52.37, 48.32, 30.71, 29.43, 28.50, 27.05, 19.34, 19.21, 18.46.

HRMS (ESI-TOF): calculated for $C_{36}H_{44}N_6NaO_7^+$ [M+Na]$^+$: 695.3164, found: 695.3166.

[α]$^{25}_{D}$: +98.2 (*c* = 0.5, CHCl$_3$)

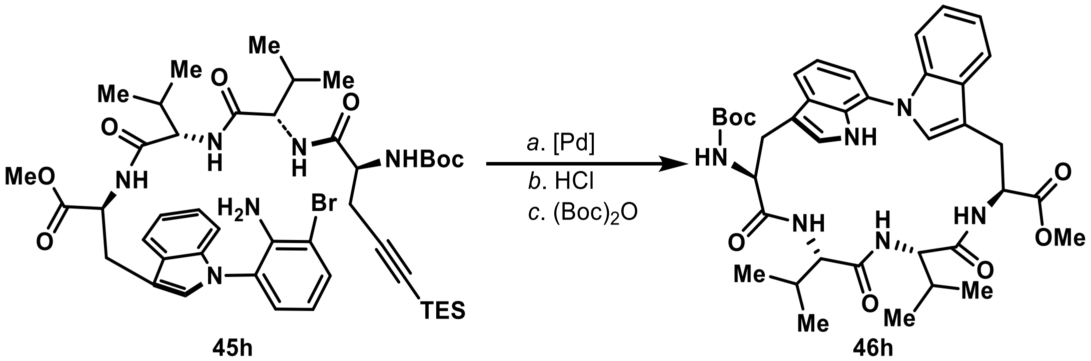

45h **46h**

On 0.2 mmol scale, **General Procedure O** was followed with compound **45h** via Larock macrocyclization. Purification by silica gel column chromatography gave the title compound **46h** (92.5 mg, 66% yield).

Compound 46h

Physical State: amorphous solid

^{1}H NMR (600 MHz, CDCl$_3$): δ 8.31 (s, 1H), 7.80 (d, *J* = 7.8 Hz, 1H), 7.66 – 7.57 (m, 2H), 7.47 (d, *J* = 8.0 Hz, 1H), 7.32 (d, *J* = 7.4 Hz, 1H), 7.25 – 7.18 (m, 3H), 6.68 (s, 1H), 6.55 (s, 1H), 6.32 (s, 1H), 5.59 (d, *J* = 6.7 Hz, 1H), 5.40 (s, 1H), 4.99 – 4.88 (m, 1H), 4.50 – 4.45 (m, 1H), 4.30 (dd, *J* = 9.5, 6.7 Hz, 1H), 3.87 (s, 1H), 3.80 (s, 3H), 3.55 – 3.47 (m, 1H), 3.27 (d, *J* = 13.1 Hz, 1H), 3.20 (dd, *J* = 16.2, 11.7 Hz, 1H), 2.76 (t, *J* = 12.5 Hz, 1H), 2.02 – 1.95 (m, 1H), 1.80 – 1.78 (m, 1H), 1.49 (s, 9H), 1.00 (d, *J* = 6.5 Hz, 3H), 0.98 (d, *J* = 6.5 Hz, 3H), 0.82 (d, *J* = 6.7 Hz, 3H), 0.71 (d, *J* = 6.7 Hz, 3H).

^{13}C NMR (151 MHz, CDCl$_3$): δ 172.68, 171.59, 170.66, 170.29, 155.45, 136.58, 132.94, 128.56, 128.09, 126.87, 124.12, 124.05, 122.94, 120.31, 120.02, 118.95, 118.47, 118.23, 111.87, 111.22, 110.65, 80.04, 60.36, 57.81, 55.33, 52.67, 52.54, 31.86, 30.80, 29.08, 28.48, 27.06, 19.40, 18.93, 18.32, 17.90.

HRMS (ESI-TOF): calculated for $C_{38}H_{48}N_6NaO_7^+$ [M+Na]$^+$: 723.3477, found: 723.3481.

[α]^{25}D: +43.6 (c = 0.5, $CHCl_3$)

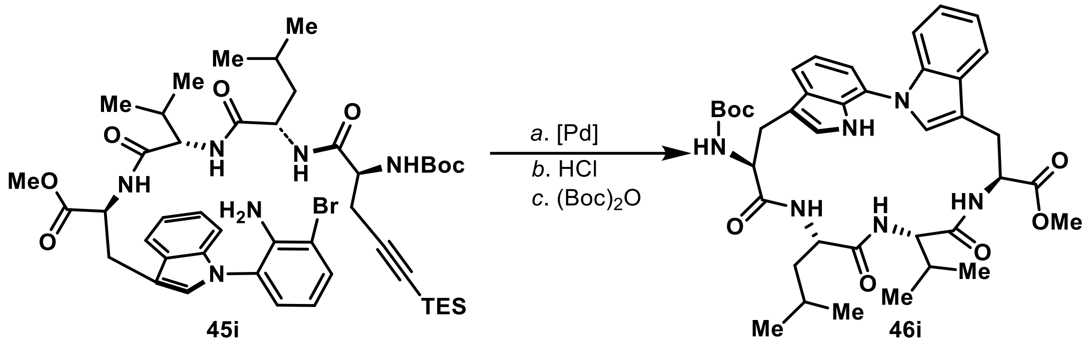

On 0.2 mmol scale, **General Procedure O** was followed with compound **45i** via Larock macrocyclization. Purification by silica gel column chromatography gave the title compound **46i** (92.9 mg, 65% yield).

Compound 46i

Physical State: amorphous solid

^{1}H NMR (600 MHz, $CDCl_3$): δ 8.45 (s, 1H), 7.72 (d, J = 7.5 Hz, 1H), 7.65 – 7.56 (m, 1H), 7.50 (s, 1H), 7.41 (s, 1H), 7.28 (d, J = 6.7 Hz, 1H), 7.24 – 7.14 (m, 3H), 6.67 (s, 1H), 6.59 (s, 1H), 6.52 (s, 1H), 5.48 (d, J = 6.7 Hz, 2H), 4.95 (t, J = 8.0 Hz, 1H), 4.57 (q, J = 9.7 Hz, 1H), 4.32 – 4.28 (m, 1H), 4.11 (s, 1H), 3.80 (s, 3H), 3.43 (d, J = 15.9 Hz, 1H), 3.29 – 3.16 (m, 1H), 3.10 (d, J = 12.1 Hz, 1H), 2.67 – 2.63 (m, 1H), 2.06 – 2.03 (m, 1H), 1.62 – 1.57 (m, 1H), 1.50 (s, 9H), 1.43 – 1.22 (m, 3H), 1.02 (d, J = 6.1 Hz, 3H), 0.95 (d, J = 6.1 Hz, 3H), 0.90 – 0.77 (m, 7H).

^{13}C NMR (151 MHz, $CDCl_3$): δ 172.57, 171.33, 170.83, 170.51, 155.29, 136.79, 132.83, 128.64, 128.00, 126.81, 124.25, 123.99, 122.94, 120.29, 119.92, 118.91, 118.43, 118.26, 111.77, 111.04, 110.69, 80.06, 59.74, 55.40, 52.69, 52.32, 51.54, 42.14, 31.67, 31.09, 29.03, 28.49, 27.06, 24.48, 22.91, 22.74, 22.00, 19.23, 18.11, 14.21.

HRMS (ESI-TOF): calculated for $C_{39}H_{50}N_6NaO_7^+$ [M+Na]$^+$: 737.3633, found: 737.3636.

[α]^{25}D: +6.7 (c = 0.5, $CHCl_3$)

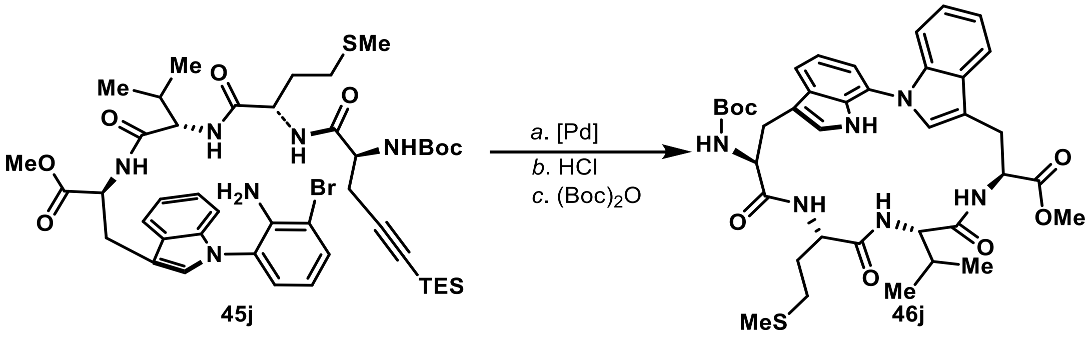

On 0.2 mmol scale, **General Procedure O** was followed with compound **45j** via Larock macrocyclization. Purification by silica gel column chromatography gave the title compound **46j** (67.4 mg, 46% yield).

Compound 46j

Physical State: amorphous solid

^{1}H NMR (600 MHz, $CDCl_3$): δ 8.27 (s, 1H), 7.86 (d, J = 7.9 Hz, 1H), 7.71 (s, 1H), 7.59 (d, J = 7.8 Hz, 1H), 7.48 (d, J = 8.1 Hz, 1H), 7.32 (d, J = 7.4 Hz, 1H), 7.24 (dt, J = 7.7, 3.7 Hz, 2H), 7.20 (t, J = 7.1 Hz, 1H), 6.76 (s, 1H), 6.64 – 6.56 (m, 1H), 6.37 (d, J = 5.4 Hz, 1H), 5.60 (d, J = 7.4 Hz, 1H), 5.40 – 5.29 (m, 1H), 4.92 – 4.88 (m, 1H), 4.62 (q, J = 7.8 Hz, 1H), 4.48 – 4.43 (m, 1H), 3.80 (s, 3H), 3.69 – 3.66 (m, 1H), 3.56 – 3.47 (m, 1H), 3.40 (d, J = 10.2 Hz, 1H), 3.20 (dd, J = 16.2, 11.9 Hz, 1H), 2.78 (t, J = 12.8 Hz, 1H), 2.65 – 2.54 (m, 1H), 2.50 – 2.45 (m, 1H), 2.04 (s, 3H), 2.03 – 1.99 (m, 1H), 1.69 – 1.62 (m, 1H), 1.60 – 1.55 (m, 1H), 1.50 (s, 9H), 1.08 (d, J = 6.8 Hz, 3H), 1.05 (d, J = 6.7 Hz, 3H).

^{13}C NMR (151 MHz, CDCl$_3$): δ 172.60, 170.57, 170.47, 169.74, 155.24, 136.78, 133.42, 128.17, 127.28, 124.27, 122.95, 120.24, 120.12, 119.51, 118.70, 118.44, 111.58, 111.46, 110.78, 79.97, 61.15, 54.99, 52.71, 52.33, 50.57, 31.70, 30.47, 29.80, 28.53, 27.20, 22.74, 19.39, 18.82, 14.80, 14.21.
HRMS (ESI-TOF): calculated for $C_{38}H_{48}N_6NaO_7S^+$ [M+Na]$^+$: 755.3197, found: 755.3199.
[α]^{25}$_D$: +28.2 (c = 0.5, CHCl$_3$)

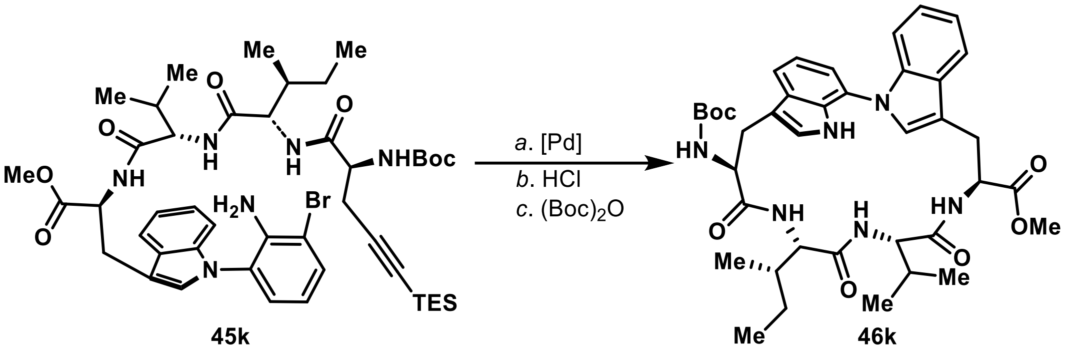

45k **46k**

On 0.2 mmol scale, **General Procedure O** was followed with compound **45k** via Larock macrocyclization. Purification by silica gel column chromatography gave the title compound **46k** (98.6 mg, 62% yield).
Compound 46k
Physical State: amorphous solid
^{1}H NMR (600 MHz, CDCl$_3$): δ 8.39 (s, 1H), 7.76 (d, J = 7.9 Hz, 1H), 7.65 (s, 1H), 7.58 (d, J = 7.7 Hz, 1H), 7.46 (d, J = 8.0 Hz, 1H), 7.31 (d, J = 7.3 Hz, 1H), 7.23 – 7.16 (m, 3H), 6.77 (s, 1H), 6.60 (s, 1H), 6.48 (s, 1H), 5.61 (d, J = 6.5 Hz, 1H), 5.49 (s, 1H), 5.00 – 4.83 (m, 1H), 4.47 – 4.43 (m, 1H), 4.38 – 4.26 (m, 1H), 3.92 (s, 1H), 3.78 (s, 3H), 3.47 (d, J = 15.5 Hz, 1H), 3.29 – 3.14 (m, 2H), 2.73 (t, J = 12.5 Hz, 1H), 2.09 – 2.02 (m, 1H), 1.98 – 1.90 (m, 1H), 1.49 (s, 9H), 1.37 – 1.30 (m, 1H), 0.98 – 0.92 (m, 7H), 0.78 – 0.74 (m, 6H).
^{13}C NMR (151 MHz, CDCl$_3$): δ 172.69, 171.33, 170.42, 170.10, 155.38, 136.66, 133.02, 128.45, 128.08, 126.95, 124.12, 123.97, 122.94, 120.30, 120.05, 119.03, 118.47, 118.33, 111.78, 111.31, 110.66, 80.04, 60.40, 57.33, 55.25, 52.68, 52.42, 38.21, 30.74, 29.33, 28.49, 27.09, 24.72, 19.42, 18.30, 15.03, 11.36.
HRMS (ESI-TOF): calculated for $C_{39}H_{50}N_6NaO_7^+$ [M+Na]$^+$: 737.3633, found: 737.3639.
[α]^{25}$_D$: +46.3 (c = 0.5, CHCl$_3$)

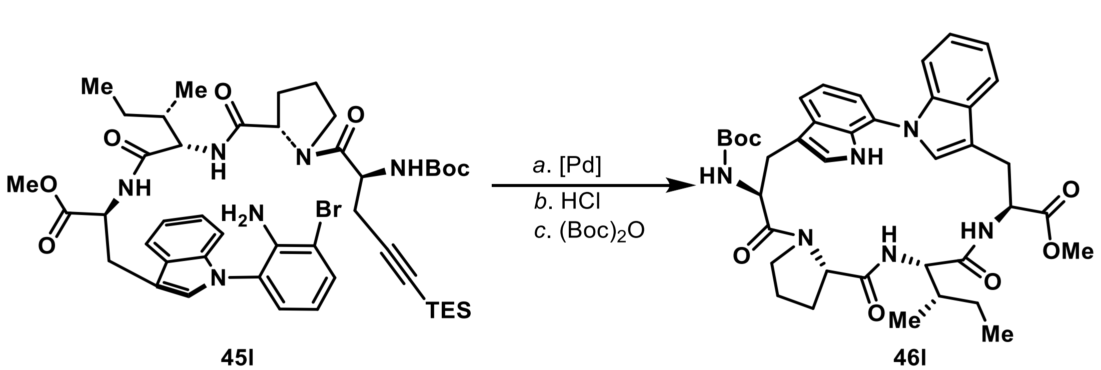

45l **46l**

On 0.2 mmol scale, **General Procedure O** was followed with compound **45l** via Larock macrocyclization. Purification by silica gel column chromatography gave the title compound **46l** (82.7 mg, 58% yield).
Compound 46l
Physical State: amorphous solid
^{1}H NMR (600 MHz, CDCl$_3$): δ 8.33 (s, 1H), 7.73 (d, J = 7.8 Hz, 1H), 7.63 (s, 1H), 7.55 (d, J = 7.8 Hz, 1H), 7.49 (d, J = 8.1 Hz, 1H), 7.26 – 7.24 (m, 1H), 7.24 – 7.21 (m, 1H), 7.20 – 7.15 (m, 2H), 6.84 – 6.76 (m, 1H), 6.51 – 6.50 (m, 1H), 6.12 (s, 1H), 5.26 (d, J = 9.1 Hz, 1H), 4.94 – 4.80 (m, 2H), 4.48 – 4.37 (m, 1H), 3.77 (s, 3H), 3.73 (dd, J = 7.5, 5.1 Hz, 1H), 3.48 (dd, J = 16.5, 2.6 Hz, 1H), 3.38 (q, J = 8.4 Hz, 1H), 3.30 – 3.23 (m,

2H), 2.95 (t, J = 12.3 Hz, 1H), 2.40 – 2.36 (m, 1H), 2.06 – 2.03 (m, 2H), 1.77 – 1.74 (m, 2H), 1.65 – 1.60 (m, 1H), 1.58 – 1.52 (m, 1H), 1.47 (s, 9H), 1.31 – 1.24 (m, 1H), 1.00 (d, J = 6.8 Hz, 3H), 0.92 (t, J = 7.4 Hz, 3H).
^{13}C NMR (151 MHz, CDCl$_3$): δ 172.68, 171.65, 171.25, 171.07, 155.26, 136.74, 133.56, 129.22, 128.17, 127.88, 126.12, 124.14, 122.75, 120.07, 119.50, 118.49, 118.39, 117.90, 111.22, 110.71, 110.17, 79.95, 60.00, 58.55, 52.65, 52.53, 51.92, 47.07, 36.24, 29.84, 29.18, 28.51, 26.86, 26.03, 23.93, 15.30, 11.26.
HRMS (ESI-TOF): calculated for $C_{39}H_{48}N_6NaO_7^+$ [M+Na]$^+$: 735.3477, found: 735.3480.
[α]$^{25}_D$: +72.4 (c = 0.5, CHCl$_3$)

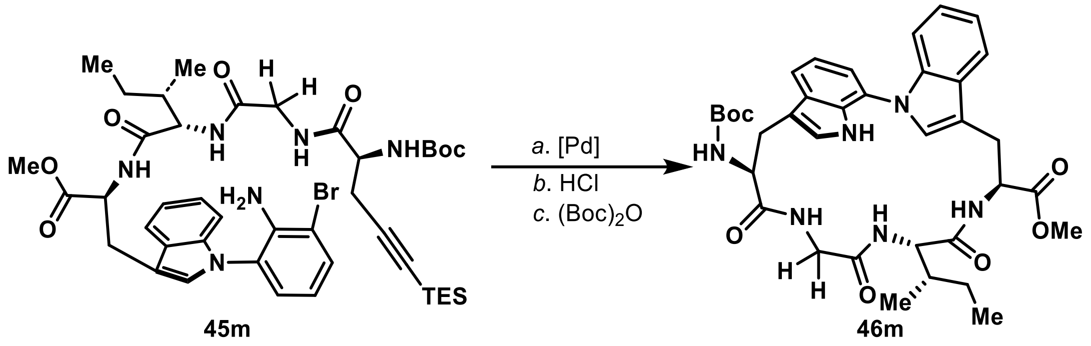

On 0.2 mmol scale, **General Procedure O** was followed with compound **45m** via Larock macrocyclization. Purification by silica gel column chromatography gave the title compound **46m** (80.7 mg, 60% yield).

Compound 46m

Physical State: amorphous solid
^{1}H NMR (600 MHz, DMSO-d_6): δ 10.30 (s, 1H), 8.47 (d, J = 5.0 Hz, 1H), 7.89 (d, J = 4.7 Hz, 1H), 7.61 (d, J = 7.8 Hz, 1H), 7.54 – 7.50 (m, 2H), 7.43 (d, J = 8.0 Hz, 1H), 7.18 – 7.07 (m, 4H), 7.02 – 7.00 (m, 1H), 6.95 (s, 1H), 6.89 – 6.86 (m, 1H), 4.56 – 4.53 (m, 1H), 4.07 – 3.90 (m, 2H), 3.77 (dd, J = 15.4, 7.2 Hz, 1H), 3.67 (s, 3H), 3.27 (d, J = 15.8 Hz, 1H), 3.14 – 2.99 (m, 2H), 2.93 – 2.87 (m, 2H), 1.63 – 1.55 (m, 1H), 1.50 – 1.45 (m, 1H), 1.36 (s, 9H), 1.20 – 1.15 (m, 1H), 0.87 (d, J = 6.6 Hz, 3H), 0.81 (t, J = 7.3 Hz, 3H).
^{13}C NMR (151 MHz, DMSO-d_6): δ 172.83, 172.03, 171.33, 168.08, 155.42, 136.05, 131.92, 130.12, 128.63, 127.72, 126.50, 124.07, 122.85, 120.27, 119.12, 118.89, 117.74, 116.99, 112.21, 110.50, 109.87, 78.68, 57.60, 56.31, 52.52, 52.46, 41.43, 36.46, 28.75, 28.66, 28.34, 26.61, 25.51, 15.41, 11.11.
HRMS (ESI-TOF): calculated for $C_{36}H_{44}N_6NaO_7^+$ [M+Na]$^+$: 695.3164, found: 695.3172.
[α]$^{25}_D$: +7.2 (c = 0.5, CHCl$_3$)

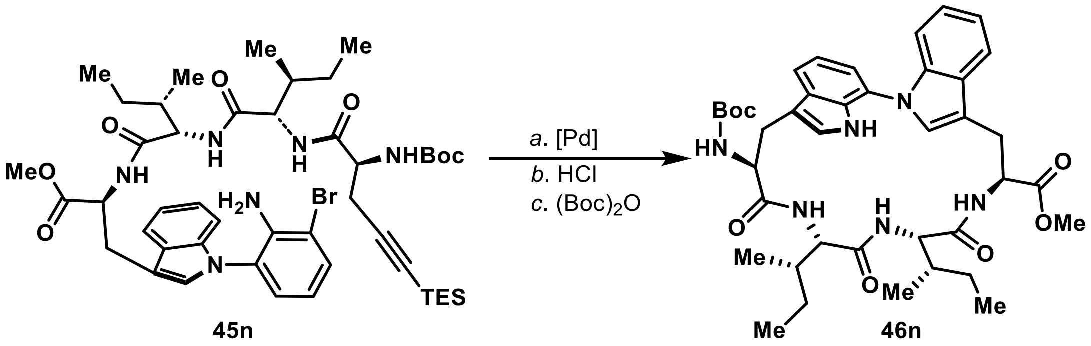

On 0.2 mmol scale, **General Procedure O** was followed with compound **45n** via Larock macrocyclization. Purification by silica gel column chromatography gave the title compound **46n** (87.5 mg, 60% yield).

Compound 46n

Physical State: amorphous solid
^{1}H NMR (600 MHz, CDCl$_3$): δ 8.35 (s, 1H), 7.81 (d, J = 7.9 Hz, 1H), 7.67 (s, 1H), 7.58 (d, J = 7.8 Hz, 1H), 7.47 (d, J = 8.1 Hz, 1H), 7.33 (d, J = 7.4 Hz, 1H), 7.23 (q, J = 7.4 Hz, 2H), 7.20 – 7.16 (m, 1H), 6.64 (s, 1H),

6.58 – 6.52 (m, 1H), 6.09 (s, 1H), 5.58 (d, J = 7.3 Hz, 1H), 5.33 (s, 1H), 4.97 – 4.84 (m, 1H), 4.50 – 4.46 (m, 1H), 4.25 (t, J = 7.7 Hz, 1H), 3.86 (s, 1H), 3.78 (s, 3H), 3.48 (d, J = 15.9 Hz, 1H), 3.31 (d, J = 13.1 Hz, 1H), 3.20 (dd, J = 16.2, 11.6 Hz, 1H), 2.78 (t, J = 12.6 Hz, 1H), 1.89 – 1.86 (m, 1H), 1.75 – 1.65 (m, 1H), 1.62 – 1.56 (m, 1H), 1.50 (s, 9H), 1.28 – 1.23 (m, 2H), 0.99 – 0.87 (m, 7H), 0.77 – 0.73 (m, 6H).
^{13}C NMR (151 MHz, CDCl$_3$): δ 172.61, 171.10, 170.37, 170.06, 155.38, 136.71, 133.21, 128.31, 128.12, 127.22, 124.18, 124.03, 122.91, 120.28, 120.07, 119.13, 118.47, 111.67, 111.43, 110.68, 80.02, 59.54, 57.20, 55.16, 52.67, 52.40, 38.27, 37.08, 29.71, 28.50, 27.17, 26.01, 24.63, 15.04, 14.92, 11.55, 11.36.
HRMS (ESI-TOF): calculated for $C_{40}H_{52}N_6NaO_7^+$ [M+Na]$^+$: 751.3790, found: 751.3796.
[α]$^{25}_D$: +61.4 (c = 0.5, CHCl$_3$)

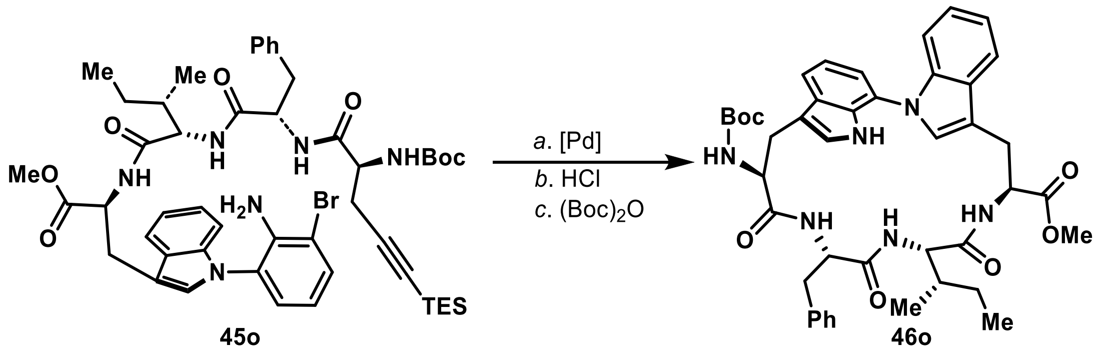

On 0.2 mmol scale, **General Procedure O** was followed with compound **45o** via Larock macrocyclization. Purification by silica gel column chromatography gave the title compound **46o** (99.2 mg, 65% yield).
Compound 46o
Physical State: amorphous solid
^{1}H NMR (600 MHz, CDCl$_3$): δ 8.26 (s, 1H), 7.87 (d, J = 7.5 Hz, 1H), 7.58 (d, J = 7.7 Hz, 1H), 7.45 (d, J = 8.0 Hz, 1H), 7.37 (s, 1H), 7.27 (d, J = 7.4 Hz, 1H), 7.25 – 7.20 (m, 4H), 7.20 – 7.13 (m, 4H), 6.79 (s, 1H), 6.39 (d, J = 6.0 Hz, 1H), 5.70 (d, J = 6.9 Hz, 1H), 5.55 (s, 1H), 5.46 (s, 1H), 4.92 – 4.88 (m, 1H), 4.68 – 4.59 (m, 1H), 4.55 – 4.50 (m, 1H), 3.80 (s, 3H), 3.72 – 3.63 (m, 1H), 3.50 – 3.46 (m, 2H), 3.16 (dd, J = 15.9, 11.5 Hz, 1H), 2.95 – 2.87 (m, 1H), 2.83 (t, J = 12.6 Hz, 1H), 2.63 (dd, J = 13.4, 8.8 Hz, 1H), 1.60 – 1.55 (m, 1H), 1.52 (s, 9H), 1.33 – 1.29 (m, 1H), 0.96 – 0.90 (m, 1H), 0.81 (d, J = 6.7 Hz, 3H), 0.79 (t, J = 7.3 Hz, 3H).
^{13}C NMR (151 MHz, CDCl$_3$): δ 172.55, 170.82, 170.43, 169.38, 155.28, 136.79, 136.19, 133.34, 129.65, 128.81, 128.17, 127.30, 127.20, 124.70, 124.09, 122.86, 120.16, 120.11, 119.56, 118.84, 118.41, 111.61, 111.35, 110.82, 79.89, 59.61, 54.76, 53.59, 52.71, 52.58, 39.18, 36.73, 30.34, 28.57, 27.35, 25.55, 15.04, 11.17.
HRMS (ESI-TOF): calculated for $C_{43}H_{50}N_6NaO_7^+$ [M+Na]$^+$: 785.3633, found: 785.3636.
[α]$^{25}_D$: +12.1 (c = 0.5, CHCl$_3$)

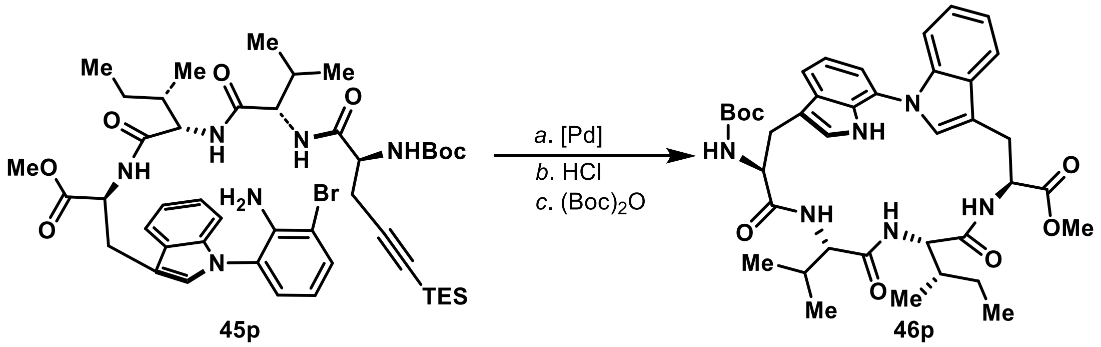

On 0.2 mmol scale, **General Procedure O** was followed with compound **45p** via Larock macrocyclization. Purification by silica gel column chromatography gave the title compound **46p** (85.8 mg, 60% yield).
Compound 46p

Physical State: amorphous solid

^{1}H NMR (600 MHz, CDCl$_3$): δ 8.43 (s, 1H), 7.78 (d, *J* = 7.9 Hz, 1H), 7.65 (s, 1H), 7.58 (d, *J* = 7.8 Hz, 1H), 7.48 (d, *J* = 8.1 Hz, 1H), 7.32 (d, *J* = 7.4 Hz, 1H), 7.25 – 7.12 (m, 3H), 6.72 – 6.56 (m, 2H), 6.42 (s, 1H), 5.75 – 5.62 (m, 1H), 5.50 (s, 1H), 4.98 – 4.84 (m, 1H), 4.53 – 4.49 (m, 1H), 4.30 (dd, *J* = 9.3, 6.8 Hz, 1H), 3.93 (s, 1H), 3.77 (s, 3H), 3.47 (d, *J* = 14.8 Hz, 1H), 3.32 – 3.23 (m, 1H), 3.18 (dd, *J* = 16.1, 11.6 Hz, 1H), 2.82 (t, *J* = 12.6 Hz, 1H), 2.01 (s, 1H), 1.82 – 1.71 (m, 1H), 1.68 – 1.62 (m, 1H), 1.49 (s, 9H), 1.21 – 1.15 (m, 1H), 0.91 (d, *J* = 6.7 Hz, 3H), 0.87 – 0.77 (m, 6H), 0.70 (d, *J* = 6.7 Hz, 3H).

^{13}C NMR (151 MHz, CDCl$_3$): δ 172.60, 171.37, 170.65, 170.19, 155.43, 136.63, 133.08, 128.44, 128.12, 127.05, 124.15, 122.91, 120.29, 120.04, 119.04, 118.46, 118.37, 111.82, 111.32, 110.68, 80.02, 59.32, 57.63, 55.21, 52.66, 52.53, 37.18, 31.93, 29.43, 28.49, 27.14, 25.94, 18.91, 17.67, 14.95, 11.54.

HRMS (ESI-TOF): calculated for $C_{39}H_{50}N_6NaO_7^+$ [M+Na]$^+$: 737.3633, found: 737.3638.

[α]$^{25}_D$: +61.6 (*c* = 0.5, CHCl$_3$)

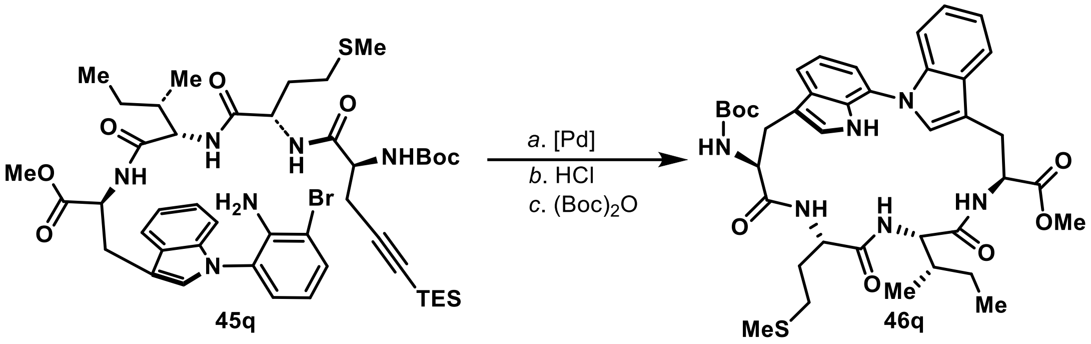

On 0.2 mmol scale, **General Procedure O** was followed with compound **45q** via Larock macrocyclization. Purification by silica gel column chromatography gave the title compound **46q** (71.7 mg, 48% yield).

Compound 46q

Physical State: amorphous solid

^{1}H NMR (600 MHz, CDCl$_3$): δ 8.29 (s, 1H), 7.87 (d, *J* = 7.8 Hz, 1H), 7.72 (s, 1H), 7.59 (d, *J* = 7.8 Hz, 1H), 7.49 (d, *J* = 8.1 Hz, 1H), 7.32 (d, *J* = 7.4 Hz, 1H), 7.25 – 7.22 (m, 2H), 7.20 (t, *J* = 7.4 Hz, 1H), 6.76 (s, 1H), 6.56 (s, 1H), 6.36 (d, *J* = 6.4 Hz, 1H), 5.60 (d, *J* = 7.4 Hz, 1H), 5.34 (s, 1H), 4.91 – 4.87 (m, 1H), 4.60 (q, *J* = 7.9 Hz, 1H), 4.49 – 4.45 (m, 1H), 3.79 (s, 3H), 3.75 – 3.72 (m, 1H), 3.50 (d, *J* = 16.9, 2.3 Hz, 1H), 3.43 (d, *J* = 10.4 Hz, 1H), 3.20 (dd, *J* = 16.2, 11.9 Hz, 1H), 2.79 (t, *J* = 12.6 Hz, 1H), 2.61 – 2.56 (m, 1H), 2.50 – 2.45 (m, 1H), 2.04 (s, 3H), 1.78 – 1.73 (m, 3H), 1.66 – 1.63 (m, 1H), 1.50 (s, 9H), 1.33 – 1.27 (m, 1H), 1.03 (d, *J* = 6.7 Hz, 3H), 0.94 (t, *J* = 7.4 Hz, 3H).

^{13}C NMR (151 MHz, CDCl$_3$): δ 172.56, 170.61, 170.43, 169.71, 155.25, 136.79, 133.46, 128.20, 127.35, 124.26, 122.92, 120.22, 120.12, 119.51, 118.72, 118.44, 111.57, 111.49, 110.77, 79.94, 59.92, 54.98, 52.68, 52.33, 50.54, 36.78, 31.67, 30.39, 29.80, 28.53, 27.22, 25.88, 15.22, 14.78, 11.35.

HRMS (ESI-TOF): calculated for $C_{39}H_{50}N_6NaO_7S^+$ [M+Na]$^+$: 769.3354, found: 769.3362.

[α]$^{25}_D$: +37.5 (*c* = 0.5, CHCl$_3$)

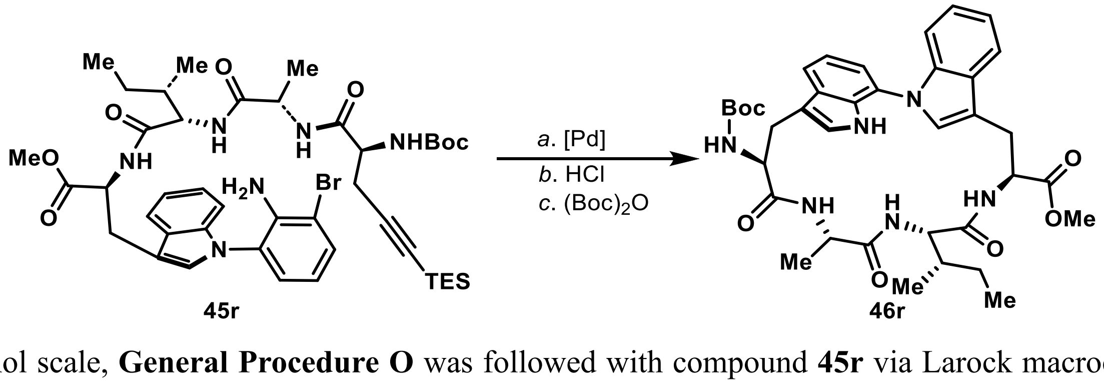

On 0.2 mmol scale, **General Procedure O** was followed with compound **45r** via Larock macrocyclization.

Purification by silica gel column chromatography gave the title compound **46r** (82.4 mg, 60% yield).

Compound 46r

Physical State: amorphous solid

^{1}H NMR (600 MHz, CDCl$_3$): δ 8.55 (s, 1H), 7.75 (d, *J* = 7.4 Hz, 1H), 7.62 – 7.55 (m, 1H), 7.48 (s, 1H), 7.44 – 7.37 (m, 1H), 7.29 (d, *J* = 7.2 Hz, 1H), 7.23 – 7.16 (m, 3H), 6.94 (s, 1H), 6.71 – 6.65 (m, 2H), 5.64 – 5.60 (m, 1H), 5.03 – 4.88 (m, 1H), 4.57 – 4.54 (m, 1H), 4.33 – 4.29 (m, 1H), 4.04 (s, 1H), 3.71 (s, 3H), 3.40 (d, *J* = 16.0 Hz, 1H), 3.33 – 3.14 (m, 2H), 2.66 – 2.59 (m, 1H), 2.30 – 1.93 (m, 1H), 1.88 – 1.78 (m, 1H), 1.49 (s, 9H), 1.21 – 1.19 (m, 1H), 1.11 (d, *J* = 6.8 Hz, 3H), 0.93 (d, *J* = 6.3 Hz, 3H), 0.86 (t, *J* = 6.7 Hz, 3H).

^{13}C NMR (151 MHz, CDCl$_3$): δ 172.53, 171.22, 171.11, 170.95, 155.25, 136.83, 132.95, 128.58, 128.09, 127.10, 124.64, 124.05, 122.92, 120.29, 119.95, 119.02, 118.44, 111.68, 110.93, 110.79, 80.00, 59.49, 55.42, 52.63, 52.37, 48.36, 36.93, 29.51, 28.50, 27.08, 25.77, 19.15, 14.89, 11.33.

HRMS (ESI-TOF): calculated for $C_{37}H_{46}N_6NaO_7^+$ [M+Na]$^+$: 709.3320, found: 709.3325.

[α]$^{25}_D$: +30.0 (*c* = 0.5, CHCl$_3$)

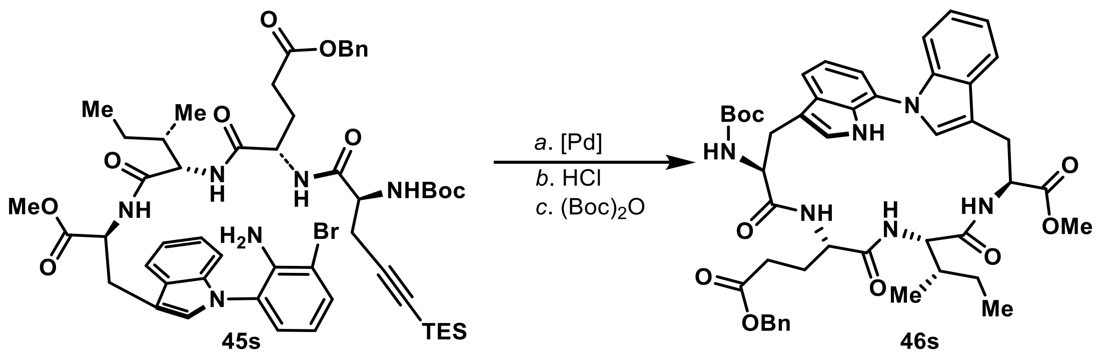

On 0.2 mmol scale, **General Procedure O** was followed with compound **45s** via Larock macrocyclization. Purification by silica gel column chromatography gave the title compound **46s** (103.5 mg, 62% yield).

Compound 46s

Physical State: amorphous solid

^{1}H NMR (600 MHz, CDCl$_3$): δ 8.34 (s, 1H), 7.85 (d, *J* = 7.7 Hz, 1H), 7.69 (s, 1H), 7.58 (d, *J* = 7.7 Hz, 1H), 7.47 (d, *J* = 8.0 Hz, 1H), 7.34 – 7.27 (m, 6H), 7.22 (q, *J* = 7.4 Hz, 2H), 7.18 (t, *J* = 7.4 Hz, 1H), 6.97 – 6.83 (m, 1H), 6.74 (s, 1H), 6.48 (t, *J* = 7.9 Hz, 1H), 5.60 (d, *J* = 7.0 Hz, 1H), 5.40 (s, 1H), 5.05 (q, *J* = 12.3 Hz, 2H), 4.95 – 4.84 (m, 1H), 4.54 (q, *J* = 7.4 Hz, 1H), 4.45 – 4.41 (m, 1H), 3.82 – 3.79 (m, 1H), 3.76 (s, 3H), 3.47 (d, *J* = 16.1 Hz, 1H), 3.39 (d, *J* = 13.3 Hz, 1H), 3.20 (dd, *J* = 16.1, 11.9 Hz, 1H), 2.75 (t, *J* = 12.5 Hz, 1H), 2.50 – 2.44 (m, 1H), 2.42 – 2.34 (m, 1H), 2.05 – 2.02 (m, 2H), 1.80 – 1.75 (m, 1H), 1.49 (s, 9H), 1.30 – 1.26 (m, 2H), 0.99 (d, *J* = 6.6 Hz, 3H), 0.90 (t, *J* = 7.4 Hz, 3H).

^{13}C NMR (151 MHz, CDCl$_3$): δ 173.90, 172.62, 170.85, 170.81, 169.98, 155.24, 136.77, 135.59, 133.35, 128.66, 128.39, 128.35, 128.22, 127.30, 124.47, 124.21, 122.89, 120.20, 120.07, 119.39, 118.64, 118.44, 111.62, 111.30, 110.79, 79.91, 66.77, 59.95, 55.07, 52.63, 52.30, 51.04, 36.67, 31.68, 30.23, 29.32, 28.52, 27.12, 25.82, 22.74, 15.14, 14.22, 11.38.

HRMS (ESI-TOF): calculated for $C_{46}H_{54}N_6NaO_9^+$ [M+Na]$^+$: 857.3844, found: 857.3853.

[α]$^{25}_D$: +49.6 (*c* = 0.5, CHCl$_3$)

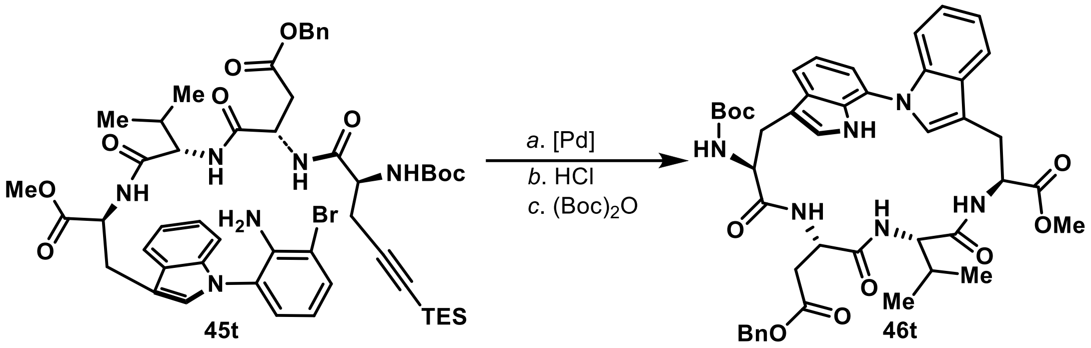

On 0.2 mmol scale, **General Procedure O** was followed with compound **45t** via Larock macrocyclization. Purification by silica gel column chromatography gave the title compound **46t** (103.3 mg, 64% yield).

Compound 46t

Physical State: amorphous solid

^{1}H NMR (600 MHz, CDCl$_3$): δ 8.58 (s, 1H), 7.81 (d, J = 6.6 Hz, 1H), 7.59 (d, J = 7.4 Hz, 1H), 7.42 (d, J = 7.3 Hz, 2H), 7.33 – 7.26 (m, 6H), 7.23 – 7.17 (m, 3H), 6.99 (s, 1H), 6.91 (s, 1H), 6.68 – 6.48 (m, 1H), 5.96 (s, 1H), 5.65 – 5.43 (m, 1H), 5.08 (d, J = 12.2 Hz, 1H), 5.01 (d, J = 12.2 Hz, 1H), 4.91 (t, J = 8.7 Hz, 1H), 4.65 – 4.61 (m, 1H), 4.48 – 4.42 (m, 1H), 3.88 (s, 1H), 3.78 (s, 3H), 3.46 (d, J = 15.7 Hz, 1H), 3.36 (d, J = 11.7 Hz, 1H), 3.20 – 3.03 (m, 1H), 2.91 (t, J = 11.5 Hz, 1H), 2.74 (d, J = 16.6 Hz, 1H), 2.48 (dd, J = 17.3, 7.4 Hz, 1H), 2.14 – 2.18 (m, 1H), 2.03 – 1.98 (m, 1H), 1.48 (s, 9H), 0.97 (t, J = 5.9 Hz, 6H).

^{13}C NMR (151 MHz, CDCl$_3$): δ 172.59, 172.21, 171.09, 170.76, 169.50, 155.35, 136.87, 135.17, 133.08, 128.65, 128.60, 128.49, 128.41, 128.17, 127.15, 124.38, 124.13, 122.85, 120.21, 120.00, 119.34, 118.75, 118.47, 112.06, 111.19, 110.86, 79.99, 67.27, 60.54, 55.28, 52.73, 48.40, 37.06, 31.68, 30.90, 29.40, 28.48, 27.68, 22.74, 18.94, 18.81, 14.22, 7.57.

HRMS (ESI-TOF): calculated for $C_{44}H_{50}N_6NaO_9^+$ [M+Na]$^+$: 829.3531, found: 829.3536.

[α]$^{25}_D$: +67.8 (c = 0.5, CHCl$_3$)

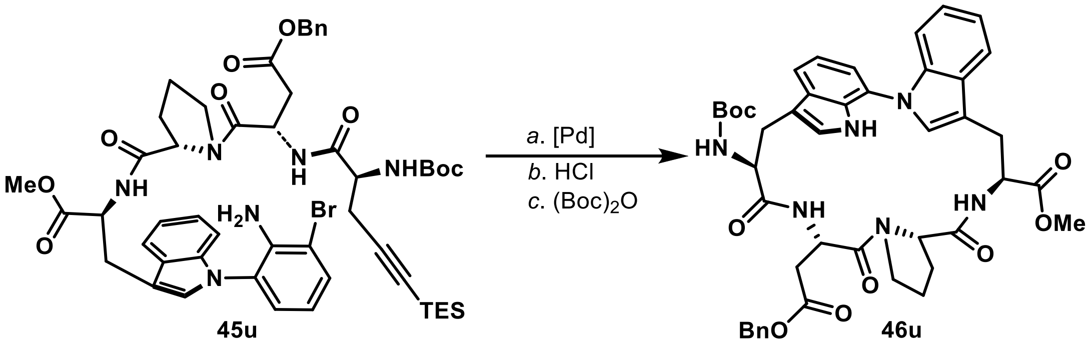

On 0.2 mmol scale, **General Procedure O** was followed with compound **45u** via Larock macrocyclization. Purification by silica gel column chromatography gave the title compound **46u** (101.4 mg, 63% yield).

Compound 46u

Physical State: amorphous solid

^{1}H NMR (600 MHz, CDCl$_3$): δ 8.82 (s, 1H), 7.88 – 7.85 (m, 2H), 7.52 (t, J = 8.0 Hz, 2H), 7.34 – 7.28 (m, 5H), 7.26 – 7.19 (m, 3H), 7.15 (t, J = 7.4 Hz, 1H), 7.01 (s, 1H), 6.73 (s, 1H), 5.58 (d, J = 7.5 Hz, 1H), 5.34 – 5.32 (m, 1H), 5.08 – 5.04 (m, 1H), 5.03 – 4.97 (m, 2H), 4.85 – 4.81 (m, 1H), 4.55 – 4.39 (m, 1H), 4.25 (t, J = 7.3 Hz, 1H), 3.76 (s, 3H), 3.71 – 3.65 (m, 1H), 3.53 – 3.43 (m, 3H), 3.21 (dd, J = 16.3, 10.7 Hz, 1H), 2.76 (t, J = 12.6 Hz, 1H), 2.50 (dd, J = 16.2, 3.5 Hz, 1H), 2.24 – 2.20 (m, 1H), 2.12 – 2.03 (m, 1H), 2.01 – 1.94 (m, 1H), 1.51 (s, 9H), 1.12 – 1.06 (m, 1H), 1.01 (t, J = 7.2 Hz, 1H).

^{13}C NMR (151 MHz, CDCl$_3$): δ 172.90, 171.73, 170.67, 169.77, 167.64, 155.10, 136.31, 135.53, 133.11, 128.57, 128.43, 128.31, 124.62, 124.44, 122.54, 120.00, 119.80, 118.59, 118.43, 118.28, 111.36, 110.78,

110.63, 79.77, 66.98, 60.77, 55.07, 53.67, 53.58, 52.59, 52.22, 47.56, 47.18, 41.74, 37.24, 30.70, 29.61, 28.56, 26.60, 25.47, 18.28, 18.23, 17.05, 11.61.
HRMS (ESI-TOF): calculated for $C_{44}H_{48}N_6NaO_9^+$ [M+Na]$^+$: 827.3375, found: 827.3375.
[α]^{25}D: +57.8 (*c* = 0.5, CHCl$_3$)

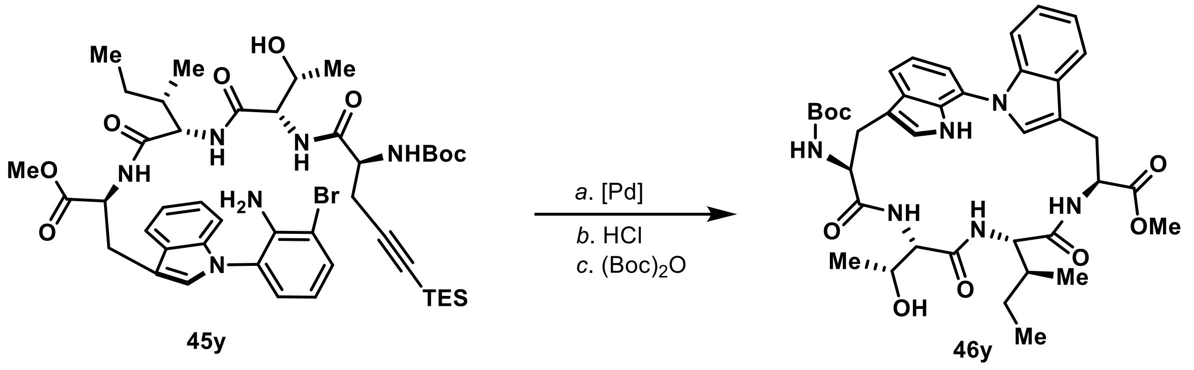

On 0.2 mmol scale, **General Procedure O** was followed with compound **45y** via Larock macrocyclization. Purification by silica gel column chromatography gave the title compound **46y** (88.1 mg, 61% yield).

Compound 46y

Physical State: amorphous solid

^{1}H NMR (600 MHz, CDCl$_3$): δ 8.58 (s, 1H), 7.73 (d, *J* = 7.8 Hz, 1H), 7.55 (d, *J* = 7.6 Hz, 1H), 7.49 (d, *J* = 5.9 Hz, 1H), 7.42 (d, *J* = 7.9 Hz, 1H), 7.37 (s, 1H), 7.25 (d, *J* = 7.5 Hz, 1H), 7.18 (t, *J* = 7.7 Hz, 3H), 6.78 (d, *J* = 24.9 Hz, 2H), 5.69 (d, *J* = 7.7 Hz, 1H), 4.96 (ddd, *J* = 11.0, 7.6, 2.6 Hz, 1H), 4.44 (d, *J* = 7.4 Hz, 1H), 4.34 (d, *J* = 11.5 Hz, 1H), 4.12 (s, 1H), 3.83 (s, 1H), 3.75 (s, 3H), 3.68 – 3.61 (m, 1H), 3.57 (dd, *J* = 5.1, 2.8 Hz, 1H), 3.48 – 3.37 (m, 1H), 3.17 (d, *J* = 13.5 Hz, 2H), 2.71 (t, *J* = 12.9 Hz, 1H), 2.57 – 2.23 (m, 1H), 1.77 (s, 1H), 1.44 (s, 9H), 1.25 – 1.16 (m, 1H), 0.95 – 0.81 (m, 9H).

^{13}C NMR (151 MHz, CDCl$_3$): δ 172.42, 172.04, 171.58, 169.18, 155.34, 136.73, 132.84, 128.65, 128.11, 126.61, 125.16, 123.99, 122.88, 120.23, 119.94, 119.04, 118.41, 118.29, 111.91, 110.78, 80.03, 72.51, 71.35, 70.46, 70.19, 67.73, 61.81, 58.91, 57.13, 55.21, 52.65, 52.19, 37.62, 31.69, 29.37, 28.43, 27.08, 25.55, 19.32, 18.07, 14.98, 13.99, 11.59.

HRMS (ESI-TOF): calculated for $C_{38}H_{48}N_6NaO_8^+$ [M+Na]$^+$: 739.3426, found: 739.3433.
[α]^{25}D: +35.0 (*c* = 0.5, CHCl$_3$)

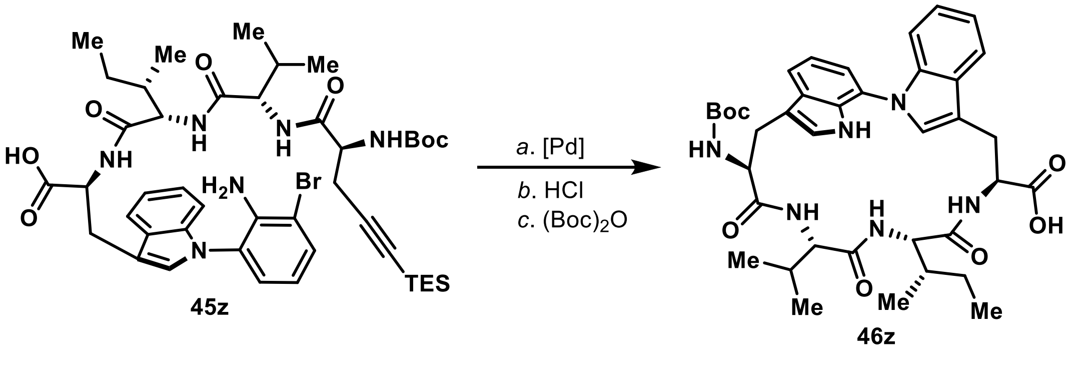

On 0.2 mmol scale, **General Procedure O** was followed with compound **45z** via Larock macrocyclization. Purification by silica gel column chromatography gave the title compound **46z** (59.8 mg, 42% yield).

Compound 46y

Physical State: amorphous solid

^{1}H NMR (600 MHz, CD$_3$OD): δ 9.84 (d, *J* = 2.5 Hz, 1H), 8.07 (s, 1H), 7.81 (d, *J* = 2.9 Hz, 1H), 7.70 (d, *J* = 7.8 Hz, 1H), 7.64 (dd, *J* = 7.6, 1.3 Hz, 1H), 7.55 (d, *J* = 8.1 Hz, 1H), 7.30 (d, *J* = 7.3 Hz, 1H), 7.25 – 7.17 (m, 2H), 7.17 – 7.12 (m, 1H), 6.81 (d, *J* = 2.2 Hz, 1H), 6.72 (d, *J* = 9.2 Hz, 1H), 4.70 (dd, *J* = 11.1, 3.3 Hz, 1H), 4.32 (dd, *J* = 12.2, 4.3 Hz, 1H), 4.06 (t, *J* = 9.1 Hz, 1H), 3.85 (dd, *J* = 6.8, 2.8 Hz, 1H), 3.54 – 3.45 (m, 1H), 3.29 – 3.19 (m, 2H), 2.96 (t, *J* = 12.8 Hz, 1H), 1.84 – 1.73 (m, 2H), 1.65 (dt, *J* = 9.0, 6.7 Hz, 1H), 1.56 (d, *J* =

5.4 Hz, 1H), 1.49 (s, 9H), 1.44 – 1.32 (m, 1H), 1.08 (d, J = 6.7 Hz, 3H), 1.00 (t, J = 7.3 Hz, 3H), 0.90 (d, J = 6.7 Hz, 3H), 0.78 (d, J = 6.6 Hz, 3H).

^{13}C NMR (151 MHz, CD_3OD): δ 173.39, 171.69, 171.08, 170.37, 155.37, 135.56, 131.99, 128.27, 127.93, 126.91, 123.72, 123.47, 123.32, 121.50, 118.95, 118.09, 117.56, 116.45, 116.37, 110.78, 109.40, 108.98, 78.50, 59.29, 57.48, 55.06, 51.66, 35.91, 29.86, 27.88, 26.72, 25.75, 24.99, 17.51, 17.17, 13.26, 10.04.

HRMS (ESI-TOF): calculated for $C_{38}H_{48}N_6NaO_7^+$ $[M+Na]^+$: 723.3477, found: 723.3480.

$[\alpha]^{25}_D$: +63.0 (c = 0.5, $CHCl_3$)

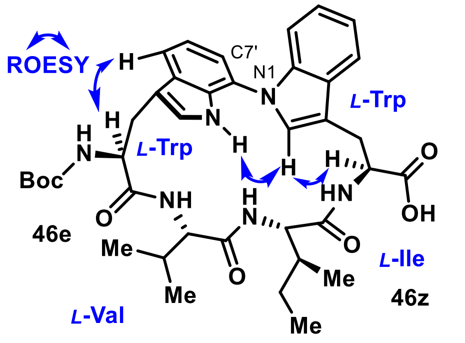

The configuration of the ring system is deduced to be *Sconf*.

Synthesis of extended Cihunamide scaffolds:

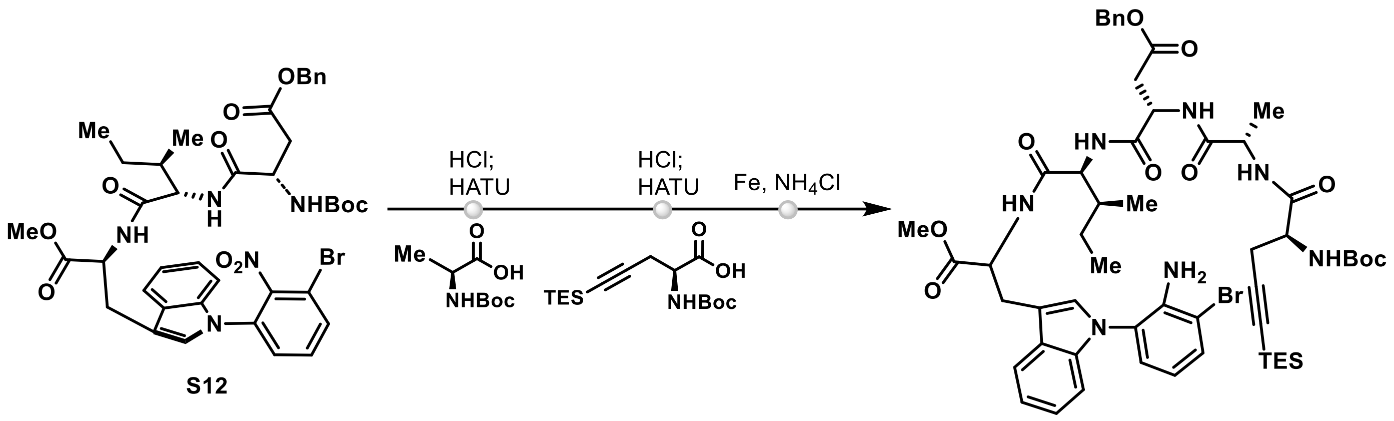

The compound **S12** (770 mg, 0.92 mmol, 1.0 eq.) was dissolved in DCM/4M HCl (3:1, 10 mL) was added portion wise, the reaction was stirred at rt for 2 h. The solvent was removed under reduced pressure to give the crude, the crude was dissolved in DMF (5 mL), Boc-*L*-alanine (191.4 mg, 1.01 mmol, 1.1 eq.), DIPEA (357 mg, 2.76 mmol, 3.0 eq.) and HATU (525 mg, 1.38 mmol, 1.5 eq.) was added sequentially, the reaction was stirred at rt for 4 h, quenched by aq. NH_4Cl and extracted by Hexane/Ethyl acetate = 1:1 for three times, the solvent was removed under reduced pressure to give the residue.

The residue was dissolved in DCM/4M HCl (3:1, 10 mL) was added portion wise, the reaction was stirred at rt for 2 h. The solvent was removed under reduced pressure to give the crude, the crude was dissolved in DMF (5 mL), (*S*)-2-((tert-butoxycarbonyl)amino)-5-(triethylsilyl)pent-4-ynoic acid (332 mg, 1.01 mmol, 1.1 eq.), DIPEA (357 mg, 2.76 mmol, 3.0 eq.) and HATU (525 mg, 1.38 mmol, 1.5 eq.) was added sequentially, the reaction was stirred at rt for 4 h, quenched by aq. NH_4Cl and extracted by Hexane/Ethyl acetate = 1:1 for three times, the solvent was removed under reduced pressure to give the residue.

The residue was dissolved in EtOH/H_2O/EtOAc (3:1:1, 10 mL), Fe (771 mg, 13.8 mmol, 15.0 eq.) and NH_4Cl (197 mg, 3.68 mmol, 4.0 eq.) was added, the reaction was stirred at 60 °C for 4 h, the solvent was removed under reduced pressure and water was added, extracted by EtOAc for three times, the combined organic layers were removed under reduced pressure to give the residue, the residue was purified by silica gel chromatography to give the precursor **S13** (420 mg, 42% yield from **S12**).

Compound S13

Physical State: amorphous solid

^{1}H NMR (600 MHz, CDCl$_3$): δ 7.66 – 7.55 (m, 1H), 7.51 – 7.26 (m, 7H), 7.17 – 6.83 (m, 8H), 6.66 (q, *J* = 7.5 Hz, 1H), 5.41 – 5.26 (m, 1H), 5.06 – 4.89 (m, 3H), 4.71 – 4.60 (m, 1H), 4.43 – 3.98 (m, 4H), 3.72 – 3.66 (m, 3H), 3.45 – 3.22 (m, 2H), 2.93 – 2.59 (m, 4H), 1.94 – 1.90 (m, 1H), 1.43 (s, 9H), 1.35 – 1.26 (m, 5H), 0.96 (t, *J* = 7.9 Hz, 9H), 0.90 – 0.75 (m, 7H), 0.57 (q, *J* = 7.9 Hz, 6H).

^{13}C NMR (151 MHz, CDCl$_3$): δ 172.27, 172.15, 172.01, 171.44, 170.80, 170.72, 170.22, 155.97, 141.96, 136.67, 135.39, 132.43, 128.63, 128.44, 128.38, 127.89, 127.09, 125.09, 122.76, 120.41, 119.05, 118.14, 111.76, 111.55, 110.79, 109.77, 102.40, 86.42, 80.98, 66.99, 58.37, 58.26, 53.44, 53.04, 52.96, 52.48, 49.76, 36.55, 36.26, 35.74, 35.50, 29.77, 28.30, 27.78, 27.65, 24.65, 24.53, 23.19, 17.93, 15.43, 11.53, 11.39, 7.57, 4.40.

HRMS (ESI-TOF): calculated for $C_{54}H_{72}BrN_7NaO_{10}Si^+$ $[M+Na]^+$: 1108.4186, found: 1108.4192

$[\alpha]^{25}_D$: –3.4 (*c* = 0.2, $CHCl_3$)

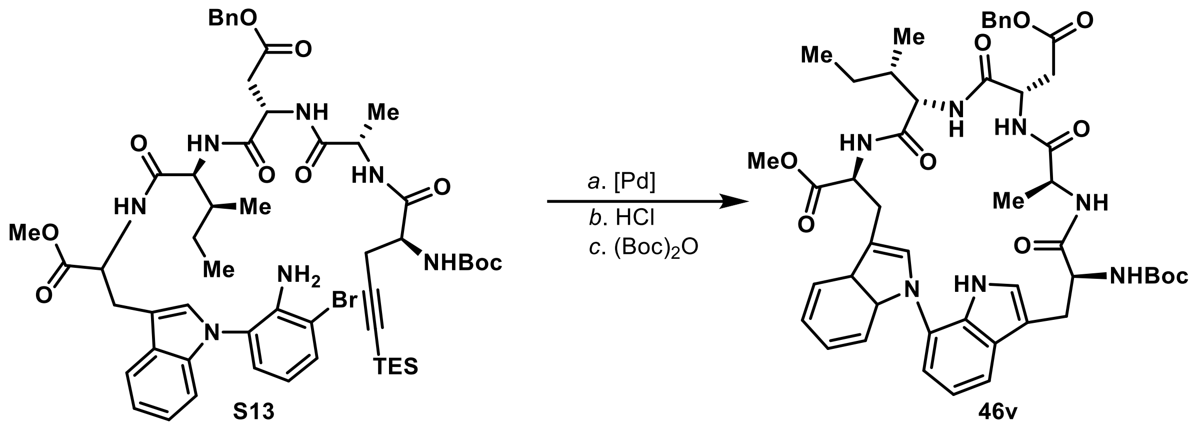

The precursor **S13** (371 mg, 0.34 mmol, 1.0 eq.) was dissolved in toluene (5 mL). Pd(tBu$_3$P)$_2$ (0.034 mmol, 0.1 eq.) and DIPEA (0.85 mmol, 2.5 eq.) were added. The reaction mixture was stirred at 110 °C under nitrogen atmosphere for 4 h. After Celite filtration followed by concentration, the residue was dissolved in DCM/4M HCl = 2 mL /2 mL , the reaction was stirred at rt for 2 h, The solvents was removed under reduced pressure to give the crude, the crude was dissolved in DCM (2 mL), DIPEA (1.0 mmol, 3.0 eq.), (Boc)$_2$O (0.5 mmol, 1.5 eq.) was added, the reaction was stirred at rt for 4 h. The solvents were removed under reduced pressure to give the residue, the residue was purified by silica gel chromatography to give the product **46v** (182 mg, 60% yield).

Compound 46v

Physical State: amorphous solid

^{1}H NMR (600 MHz, CDCl$_3$): δ 9.50 (s, 1H), 7.72 (d, J = 7.6 Hz, 1H), 7.62 – 7.54 (m, 1H), 7.45 (s, 1H), 7.40 – 7.33 (m, 1H), 7.31 – 7.26 (m, 4H), 7.22 (q, J = 7.1 Hz, 1H), 7.18 – 7.10 (m, 5H), 6.84 (d, J = 7.6 Hz, 1H), 6.49 (s, 1H), 5.68 (s, 1H), 5.52 (d, J = 7.6 Hz, 1H), 5.03 (q, J = 5.8 Hz, 1H), 4.91 (d, J = 12.1 Hz, 1H), 4.83 (d, J = 12.1 Hz, 1H), 4.46 – 4.37 (m, 1H), 4.30 – 4.25 (m, 1H), 4.22 (t, J = 8.3 Hz, 1H), 3.75 (s, 3H), 3.36 (d, J = 3.8 Hz, 2H), 3.23 (dd, J = 13.4, 3.4 Hz, 1H), 3.16 (t, J = 12.6 Hz, 1H), 2.26 – 2.06 (m, 3H), 1.98 – 1.87 (m, 1H), 1.49 (s, 9H), 1.43 – 1.39 (m, 1H), 1.26 – 1.21 (m, 1H), 1.08 (d, J = 7.0 Hz, 3H), 1.01 – 0.95 (m, 1H), 0.86 – 0.81 (m, 6H).

^{13}C NMR (151 MHz, CDCl$_3$): δ 172.29, 172.17, 171.74, 170.61, 170.46, 155.45, 136.93, 135.35, 131.54, 129.66, 129.13, 128.74, 128.60, 128.45, 128.32, 127.99, 126.94, 124.76, 123.83, 122.84, 120.40, 119.78, 118.95, 118.56, 117.71, 111.40, 111.09, 110.82, 80.25, 66.98, 58.14, 56.13, 52.75, 51.78, 49.74, 48.56, 35.89, 35.56, 29.78, 29.40, 28.56, 28.45, 28.14, 24.53, 17.96, 15.67, 11.13.

HRMS (ESI-TOF): calculated for $C_{48}H_{57}N_7NaO_{10}^+$ [M+Na]$^+$: 914.4059, found: 914.4066.

[α]$^{25}_D$: –26.2 (c = 0.2, CHCl$_3$)

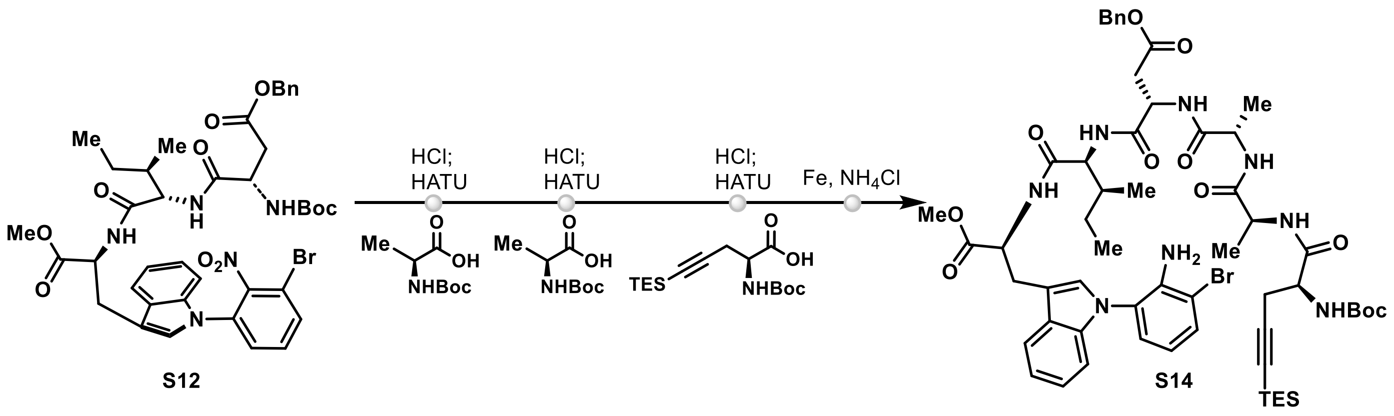

The compound **S12** (1.15 g, 1.38 mmol, 1.0 eq.) was dissolved in DCM/4M HCl (3:1, 15 mL) was added portion wise, the reaction was stirred at rt for 2 h. The solvent was removed under reduced pressure to give the crude, the crude was dissolved in DMF (7 mL), Boc-L-alanine (287.6 mg, 1.52 mmol, 1.1 eq.), DIPEA

(535 mg, 4.14 mmol, 3.0 eq.) and HATU (787 mg, 2.07 mmol, 1.5 eq.) was added sequentially, the reaction was stirred at rt for 4 h, quenched by aq. NH_4Cl and extracted by Hexane/Ethyl acetate = 1:1 for three times, the solvent was removed under reduced pressure to give the residue.

The residue was dissolved in DCM/4M HCl (3:1, 15 mL) was added portion wise, the reaction was stirred at rt for 2 h. The solvent was removed under reduced pressure to give the crude, the crude was dissolved in DMF (7 mL), Boc-*L*-alanine (287.6 mg, 1.52 mmol, 1.1 eq.), DIPEA (535 mg, 4.14 mmol, 3.0 eq.) and HATU (787 mg, 2.07 mmol, 1.5 eq.) was added sequentially, the reaction was stirred at rt for 4 h, quenched by aq. NH_4Cl and extracted by Hexane/Ethyl acetate = 1:1 for three times, the solvent was removed under reduced pressure to give the residue.

The residue was dissolved in DCM/4M HCl (3:1, 15 mL) was added portion wise, the reaction was stirred at rt for 2 h. The solvent was removed under reduced pressure to give the crude, the crude was dissolved in DMF (7 mL), (*S*)-2-((tert-butoxycarbonyl)amino)-5-(triethylsilyl)pent-4-ynoic acid (497 mg, 1.52 mmol, 1.1 eq.), DIPEA (535 mg, 4.14 mmol, 3.0 eq.) and HATU (787 mg, 2.07 mmol, 1.5 eq.) was added sequentially, the reaction was stirred at rt for 4 h, quenched by aq. NH_4Cl and extracted by Hexane/Ethyl acetate = 1:1 for three times, the solvent was removed under reduced pressure to give the residue.

The residue was dissolved in EtOH/H_2O/EtOAc (3:1:1, 15 mL), Fe (1.16 g, 20.7 mmol, 15.0 eq.) and NH_4Cl (295 mg, 5.52 mmol, 4.0 eq.) was added, the reaction was stirred at 60 °C for 4 h, the solvent was removed under reduced pressure and water was added, extracted by EtOAc for three times, the combined organic layers were removed under reduced pressure to give the residue, the residue was purified by silica gel chromatography to give the precursor **S14** (606 mg, 38% yield from **S12**).

Compound S14

Physical State: amorphous solid

^{1}H NMR (600 MHz, DMSO-*d*$_6$): δ 8.39 (d, *J* = 7.1 Hz, 1H), 8.20 (d, *J* = 7.8 Hz, 1H), 8.02 (d, *J* = 7.3 Hz, 1H), 7.91 (d, *J* = 7.1 Hz, 1H), 7.56 (d, *J* = 7.1 Hz, 1H), 7.50 – 7.46 (m, 2H), 7.32 – 7.24 (m, 5H), 7.20 – 7.14 (m, 1H), 7.13 – 7.05 (m, 3H), 6.98 (d, *J* = 8.7 Hz, 1H), 6.93 (d, *J* = 7.7 Hz, 1H), 6.63 (t, *J* = 7.9 Hz, 1H), 4.99 (s, 2H), 4.67 (s, 2H), 4.58 (dt, *J* = 14.6, 7.3 Hz, 2H), 4.22 – 4.16 (m, 3H), 4.11 (td, *J* = 9.4, 4.0 Hz, 1H), 3.53 (d, *J* = 5.6 Hz, 3H), 3.20 – 3.10 (m, 2H), 2.75 – 2.69 (m, 2H), 2.61 – 2.50 (m, 2H), 2.41 (dd, *J* = 17.0, 9.9 Hz, 1H), 1.67 – 1.60 (m, 1H), 1.34 (s, 9H), 1.32 – 1.28 (m, 1H), 1.15 – 1.12 (m, 6H), 1.00 – 0.92 (m, 1H), 0.89 (t, *J* = 7.9 Hz, 9H), 0.74 – 0.70 (m, 6H), 0.47 (q, *J* = 7.9 Hz, 6H).

^{13}C NMR (151 MHz, DMSO-*d*$_6$): δ 172.77, 172.48, 172.20, 171.30, 170.54, 170.20, 155.67, 142.66, 136.63, 136.47, 132.75, 128.90, 128.48, 128.41, 128.24, 124.98, 122.77, 120.29, 119.24, 117.93, 110.93, 109.18, 105.95, 83.09, 78.74, 66.24, 57.02, 53.83, 52.35, 49.78, 48.66, 48.55, 40.58, 37.67, 36.16, 28.64, 27.19, 24.41, 23.75, 18.73, 18.66, 15.57, 11.60, 7.87, 4.48.

HRMS (ESI-TOF): calculated for $C_{57}H_{77}BrN_8NaO_{11}Si^+$ [M+Na]$^+$: 1179.4557, found: 1179.4561.

$[\alpha]^{25}_D$: –36.2 (*c* = 0.2, $CHCl_3$)

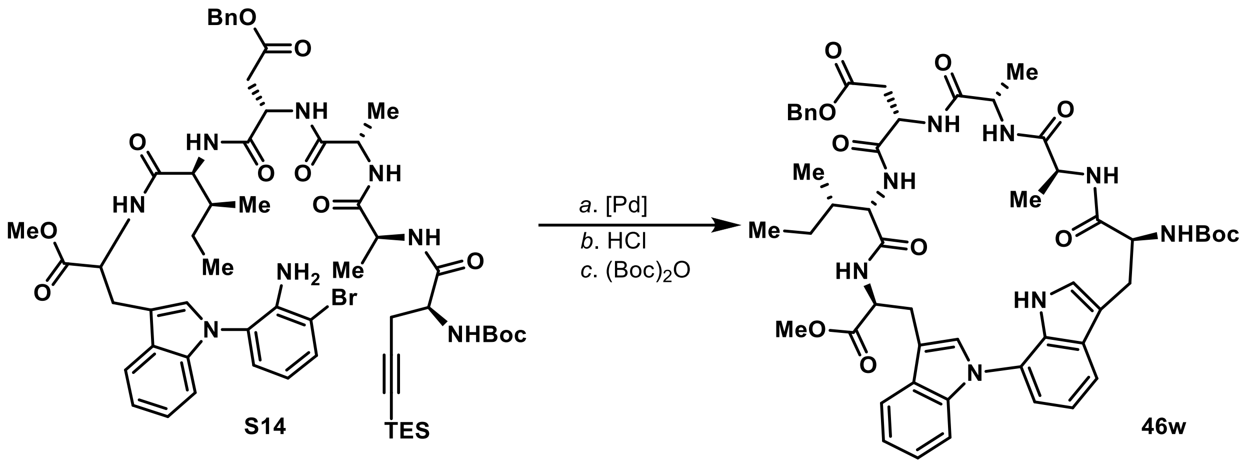

The precursor **S14** (322 mg, 0.278 mmol, 1.0 eq.) was dissolved in toluene (4 mL). Pd(tBu$_3$P)$_2$ (0.028 mmol, 0.1 eq.) and DIPEA (0.69 mmol, 2.5 eq.) were added. The reaction mixture was stirred at 110 °C under nitrogen atmosphere for 4 h. After Celite filtration followed by concentration, the residue was dissolved in DCM/4M HCl = 2 mL /2 mL, the reaction was stirred at rt for 2 h, The solvents was removed under reduced pressure to give the crude, the crude was dissolved in DCM (2 mL), DIPEA (0.83 mmol, 3.0 eq.), (Boc)$_2$O (0.42 mmol, 1.5 eq.) was added, the reaction was stirred at rt for 4 h. The solvents were removed under reduced pressure to give the residue, the residue was purified by silica gel chromatography to give the product **46w** (112 mg, 42% yield).

Compound 46w

Physical State: amorphous solid

^{1}H NMR (600 MHz, CDCl$_3$): δ 9.07 (s, 1H), 7.68 (d, J = 7.3 Hz, 1H), 7.58 (d, J = 7.7 Hz, 1H), 7.46 (s, 1H), 7.33 – 7.28 (m, 5H), 7.26 – 7.20 (m, 4H), 7.17 (q, J = 7.8 Hz, 3H), 7.14 – 7.07 (m, 2H), 6.94 (d, J = 8.7 Hz, 1H), 5.81 – 5.77 (m, 1H), 5.54 (d, J = 7.3 Hz, 1H), 5.10 – 4.93 (m, 2H), 4.89 (t, J = 7.5 Hz, 1H), 4.50 – 4.40 (m, 2H), 4.32 (q, J = 7.4 Hz, 2H), 3.77 (s, 3H), 3.66 – 3.62 (m, 1H), 3.53 (d, J = 12.8 Hz, 1H), 3.32 (dd, J = 13.8, 4.4 Hz, 1H), 3.21 – 3.13 (m, 2H), 3.05 (t, J = 12.9 Hz, 1H), 2.56 (dd, J = 17.2, 5.0 Hz, 1H), 1.97 – 1.84 (m, 2H), 1.48 (s, 9H), 1.21 – 1.17 (m, 6H), 1.06 – 0.95 (m, 1H), 0.77 (d, J = 6.7 Hz, 3H), 0.69 (t, J = 7.3 Hz, 3H).

^{13}C NMR (151 MHz, CDCl$_3$): δ 172.66, 172.29, 172.08, 172.01, 171.72, 171.23, 170.29, 155.39, 136.61, 135.01, 132.50, 129.49, 128.71, 128.64, 128.34, 127.43, 124.32, 123.85, 122.45, 120.23, 119.71, 119.00, 118.90, 117.51, 112.05, 111.20, 110.64, 80.29, 70.62, 67.10, 57.91, 55.76, 53.35, 52.69, 50.92, 50.25, 48.26, 34.81, 34.28, 29.35, 28.48, 28.14, 27.50, 24.32, 17.88, 17.22, 15.51, 11.08.

HRMS (ESI-TOF): calculated for $C_{51}H_{62}N_8NaO_{11}^+$ [M+Na]$^+$: 985.4430, found: 985.4430.

$[\alpha]^{25}_D$: –29.9 (c = 0.2, CHCl$_3$)

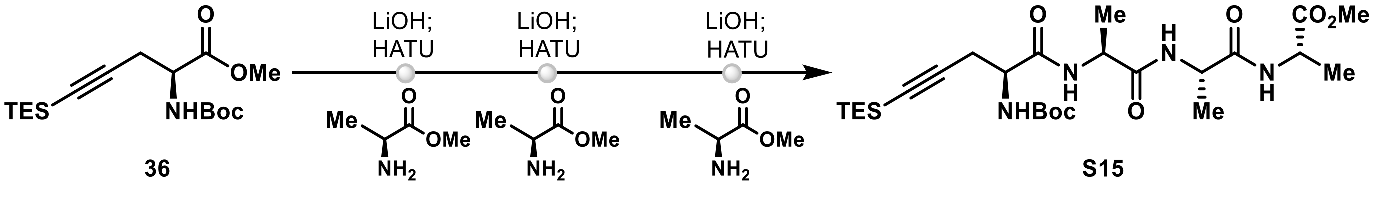

A solution of methyl (S)-2-((tert-butoxycarbonyl)amino)-5-(triethylsilyl)pent-4-ynoate (1.32 g, 3.86 mmol, 1.0 eq.) in MeOH/H$_2$O (1:1, 10 mL) was treated with LiOH·H$_2$O (324 mg, 7.72 mmol, 2.0 eq.). The reaction mixture was stirred at rt for 30 minutes. Upon completion, the reaction was quenched by adjusting the pH to 3 with 0.5 M aq. HCl and then extracted with DCM for three times. The combined organic layers were washed with saturated aq. NaCl, dried over Na$_2$SO$_4$, and concentrated *in vacuo* to give the residue, the residue was used directly in the subsequent step.

To a solution of the residue in DCM (20 mL) was added L-Alanine methyl ester hydrochloride (646 mg, 4.63

mmol, 1.2 eq.), DIPEA (2 g, 15.4 mmol, 4.0 eq.) HATU (1.76 g, 4.63 mmol, 1.2 eq.) sequentially. After stirring at rt for 1 h, the reaction was quenched with 0.5 M HCl and extracted with DCM for three times. The combined organic layers were washed with saturated aq. NaCl, dried over Na_2SO_4, and concentrated *in vacuo* to give the residue, the residue was used directly in the subsequent step.

The residue in MeOH/H_2O (1:1, 10 mL) was treated with LiOH·H_2O (324 mg, 7.72 mmol, 2.0 eq.). The reaction mixture was stirred at rt for 30 minutes. Upon completion, the reaction was quenched by adjusting the pH to 3 with 0.5 M aq. HCl and then extracted with DCM for three times. The combined organic layers were washed with saturated aq. NaCl, dried over Na_2SO_4, and concentrated *in vacuo* to give the residue, the residue was used directly in the subsequent step.

To a solution of the residue in DCM (20 mL) was added *L*-Alanine methyl ester hydrochloride (646 mg, 4.63 mmol, 1.2 eq.), DIPEA (2 g, 15.4 mmol, 4.0 eq.) HATU (1.76 g, 4.63 mmol, 1.2 eq.) sequentially. After stirring at rt for 1 h, the reaction was quenched with 0.5 M HCl and extracted with DCM for three times. The combined organic layers were washed with saturated aq. NaCl, dried over Na_2SO_4, and concentrated *in vacuo* to give the residue, the residue was used directly in the subsequent step.

The residue in MeOH/H_2O (1:1, 10 mL) was treated with LiOH·H_2O (324 mg, 7.72 mmol, 2.0 eq.). The reaction mixture was stirred at rt for 30 minutes. Upon completion, the reaction was quenched by adjusting the pH to 3 with 0.5 M aq. HCl and then extracted with DCM for three times. The combined organic layers were washed with saturated aq. NaCl, dried over Na_2SO_4, and concentrated *in vacuo* to give the residue, the residue was used directly in the subsequent step.

To a solution of the residue in DMF (10 mL) was added *L*-Alanine methyl ester hydrochloride (646 mg, 4.63 mmol, 1.2 eq.), DIPEA (2 g, 15.4 mmol, 4.0 eq.) HATU (1.76 g, 4.63 mmol, 1.2 eq.) sequentially. After stirring at rt for 1 h, the reaction was quenched with 0.5 M HCl and extracted with DCM for three times. The combined organic layers were washed with saturated aq. NaCl, dried over Na_2SO_4, and concentrated *in vacuo* to give the residue, the residue was purified by precipitation in hexane to give the compound **S15** (1.5 g, 70% yield from compound **36**).

Compound S15

Physical State: amorphous solid

^{1}H NMR (600 MHz, DMSO-*d*$_6$): δ 8.21 (d, *J* = 6.4 Hz, 1H), 7.95 (d, *J* = 7.4 Hz, 1H), 7.91 (d, *J* = 7.3 Hz, 1H), 6.96 (d, *J* = 8.3 Hz, 1H), 4.22 – 4.15 (m, 3H), 4.09 (td, *J* = 9.5, 4.0 Hz, 1H), 3.56 (s, 3H), 2.58 (dd, *J* = 17.0, 3.8 Hz, 1H), 2.39 (dd, *J* = 17.0, 10.0 Hz, 1H), 1.32 (s, 9H), 1.22 (d, *J* = 7.3 Hz, 3H), 1.15 (d, *J* = 7.1 Hz, 3H), 1.13 (d, *J* = 7.0 Hz, 3H), 0.87 (t, *J* = 7.9 Hz, 9H), 0.46 (q, *J* = 7.9 Hz, 6H).

^{13}C NMR (151 MHz, DMSO-*d*$_6$): δ 173.48, 172.62, 172.12, 170.59, 155.68, 105.79, 83.19, 78.84, 53.79, 52.39, 48.65, 48.23, 48.05, 28.59, 23.71, 18.68, 18.51, 17.29, 7.83, 4.44.

HRMS (ESI-TOF): calculated for $C_{26}H_{46}N_4NaO_7Si^+$ [M+Na]$^+$: 577.3028, found: 577.3033.

[α]$^{25}_D$: -35.9 (*c* = 0.2, MeOH)

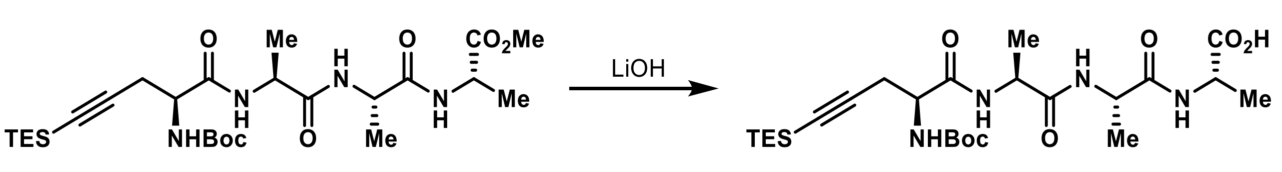

S15 S16

A solution of compound **S15** (1.39 g, 2.5 mmol, 1.0 eq.) in MeOH/H_2O (1:1, 12 mL) was treated with LiOH·H_2O (210 mg, 5.0 mmol, 2.0 eq.). The reaction mixture was stirred at rt for 30 minutes. Upon completion, the reaction was quenched by adjusting the pH to 3 with 0.5 M aq. HCl and then extracted with DCM for three times. The combined organic layers were washed with saturated aq. NaCl, dried over Na_2SO_4, and concentrated *in vacuo* to give the acid crude **S16**, the acid was used directly in the next step.

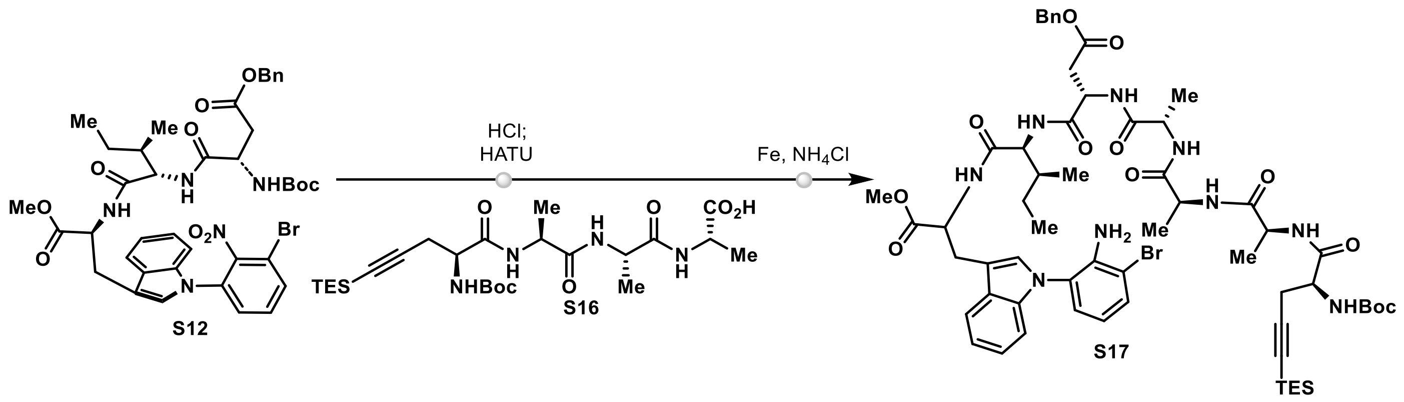

The tripeptide compound **S12** (1.4 g, 1.67 mmol, 1.0 eq.) was dissolved in DCM/4M HCl (3:1, 15 mL) was added portion wise, the reaction was stirred at rt for 2 h. The solvent was removed under reduced pressure to give the crude, the crude was dissolved in DMF (8 mL), the acid crude **S16** (1.35 g, 2.5 mmol, 1.5 eq.), DIPEA (647.5 mg, 5.0 mmol, 3.0 eq.) and HATU (953 mg, 2.5 mmol, 1.5 eq.) was added sequentially, the reaction was stirred at rt for 4 h, quenched by aq. NH_4Cl and extracted by ethyl acetate for three times, the solvent was removed under reduced pressure to give the residue.

The residue was dissolved in EtOH/H_2O/EtOAc (3:1:1, 35 mL), Fe (1.4 g, 25.1 mmol, 15.0 eq.) and NH_4Cl (357.3 mg, 6.7 mmol, 4.0 eq.) was added, the reaction was stirred at 60 ºC for 12 h, the solvent was removed under reduced pressure and water was added, extracted by EtOAc for three times, the combined organic layers were removed under reduced pressure to give the residue, the residue was purified by silica gel chromatography to give the precursor **S17** (617 mg, 30% yield from **S12**).

Compound S17

Physical State: amorphous solid

^{1}H NMR (600 MHz, DMSO-*d*$_6$): δ 8.40 (d, *J* = 7.0 Hz, 1H), 8.21 (d, *J* = 7.8 Hz, 1H), 8.01 (d, *J* = 7.1 Hz, 1H), 7.93 (d, *J* = 7.2 Hz, 1H), 7.88 (d, *J* = 7.3 Hz, 1H), 7.56 (d, *J* = 7.2 Hz, 1H), 7.52 – 7.46 (m, 2H), 7.32 – 7.24 (m, 5H), 7.20 – 7.14 (m, 1H), 7.12 – 7.05 (m, 3H), 6.99 (d, *J* = 8.7 Hz, 1H), 6.93 (d, *J* = 7.7 Hz, 1H), 6.63 (t, *J* = 7.8 Hz, 1H), 4.98 (s, 2H), 4.68 (s, 2H), 4.61 – 4.55 (m, 2H), 4.22 – 4.17 (m, 4H), 4.11 (td, *J* = 9.4, 3.9 Hz, 1H), 3.54 (d, *J* = 6.8 Hz, 3H), 3.20 – 3.10 (m, 2H), 2.76 – 2.70 (m, 1H), 2.65 (s, 1H), 2.61 – 2.53 (m, 2H), 2.41 (dd, *J* = 17.0, 10.0 Hz, 1H), 1.68 – 1.60 (m, 1H), 1.34 (s, 9H), 1.18 – 1.12 (m, 9H), 1.01 – 0.93 (m, 1H), 0.89 (t, *J* = 7.9 Hz, 9H), 0.77 – 0.70 (m, 6H), 0.48 (q, *J* = 7.9 Hz, 6H).

^{13}C NMR (151 MHz, DMSO-*d*$_6$): δ 172.79, 172.48, 172.36, 172.25, 171.30, 170.57, 170.52, 170.20, 155.66, 142.66, 136.63, 136.47, 132.75, 128.90, 128.48, 128.41, 128.24, 124.97, 122.77, 120.29, 119.23, 117.92, 112.15, 110.93, 109.18, 105.96, 83.08, 78.72, 66.24, 57.00, 53.81, 53.21, 52.35, 49.78, 48.61, 48.52, 40.57, 38.77, 37.68, 36.12, 28.64, 27.20, 24.40, 23.78, 18.74, 18.40, 15.56, 11.60, 7.87, 4.47.

HRMS (ESI-TOF): calculated for $C_{60}H_{82}BrN_9NaO_{12}Si^+$ $[M+Na]^+$: 1250.4928, found: 1250.4934.

$[\alpha]^{25}_D$: +2.3 (*c* = 0.2, $CHCl_3$)

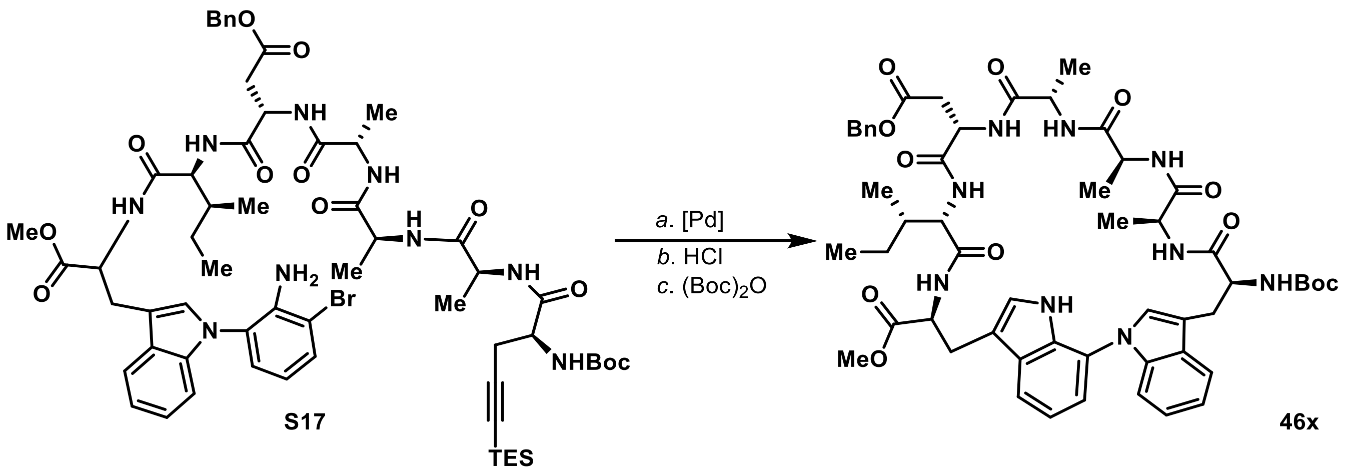

The precursor **S17** (487 mg, 0.4 mmol, 1.0 eq.) was dissolved in DMF (4 mL). Pd(tBu$_3$P)$_2$ (0.04 mmol, 0.1 eq.) and DIPEA (1.0 mmol, 2.5 eq.) were added. The reaction mixture was stirred at 110 °C under nitrogen atmosphere for 4 h. Water was added, extracted by EtOAc for three times, the combined organic layers were washed with saturated aq. NaCl, dried over Na_2SO_4, and concentrated *in vacuo* to give the residue, the residue was then dissolved in DCM/4M HCl = 3 mL /3 mL , the reaction was stirred at rt for 2 h, The solvents was removed under reduced pressure to give the crude, the crude was dissolved in DCM (1 mL), DIPEA (1.2 mmol, 3.0 eq.), (Boc)$_2$O (0.6 mmol, 1.5 eq.) was added, the reaction was stirred at rt for 4 h. The solvents were removed under reduced pressure to give the residue, the residue was purified by silica gel chromatography to give the product **46x** (110 mg, 27% yield).

Compound 46x

Physical State: amorphous solid

^{1}H NMR (600 MHz, DMSO-*d$_6$*): δ 10.60 (s, 1H), 8.23 (d, J = 7.7 Hz, 1H), 8.02 (d, J = 4.3 Hz, 1H), 7.84 (d, J = 6.4 Hz, 1H), 7.75 – 7.67 (m, 2H), 7.60 – 7.51 (m, 3H), 7.47 (s, 1H), 7.36 – 7.15 (m, 8H), 7.11 – 7.06 (m, 4H), 6.82 (d, J = 6.0 Hz, 1H), 4.92 – 4.86 (m, 2H), 4.69 – 4.63 (m, 1H), 4.52 (q, J = 7.5 Hz, 1H), 4.34 – 3.99 (m, 5H), 3.64 (s, 3H), 3.24 – 3.05 (m, 4H), 2.56 (dd, J = 16.1, 5.1 Hz, 1H), 1.63 – 1.58 (m, 1H), 1.37 (s, 9H), 1.32 – 1.24 (m, 2H), 1.10 – 1.03 (m, 8H), 0.97 – 0.92 (m, 1H), 0.65 (q, J = 6.7 Hz, 6H).

^{13}C NMR (151 MHz, DMSO-*d$_6$*): δ 172.61, 172.49, 172.38, 171.91, 171.30, 170.29, 155.79, 136.77, 136.48, 131.25, 130.89, 128.87, 128.41, 128.36, 126.07, 123.83, 122.60, 120.25, 119.04, 118.27, 111.54, 110.97, 79.03, 70.32, 66.10, 57.98, 55.36, 52.56, 49.71, 49.25, 49.05, 36.88, 36.15, 28.72, 27.93, 27.16, 24.61, 18.17, 17.80, 15.59, 11.51, 7.88, 4.47.

HRMS (ESI-TOF): calculated for $C_{54}H_{67}N_9NaO_{12}^+$ [M+Na]$^+$: 1056.4801, found: 1056.4799.

[α]$^{25}_D$: –30.1 (c = 0.2, $CHCl_3$)

General procedure P for the synthesis of S18:

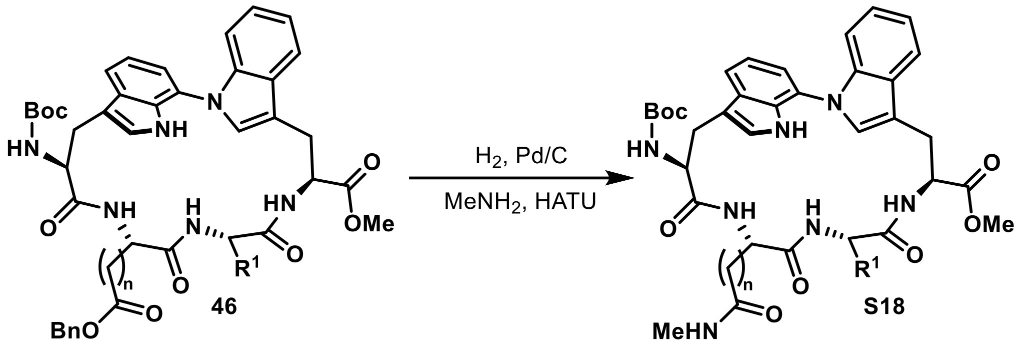

n = 1, 2 n = 1, 2

Compound **46** (0.1 mmol, 1.0 eq.) was dissolved in methanol (1 mL), then 10 wt% Pd/C (0.1 eq.) was added, the reaction was stirred at rt under H_2 for 12 h. Then it was filtered using celite, washed by MeOH, the filtrate was removed under reduced pressure to give the crude, the crude was dissolved DCM (2 mL), $MeNH_2$ (0.3 mmol, 3.0 eq.), DIPEA (0.3 mmol, 3.0 eq.) and HATU (0.15 mmol, 1.5 eq.) were added sequentially, the reaction mixture was stirred at rt for 1 h, quenched by aq. 0.5 M HCl, the organic layers were concentrated under reduced pressure to give the residue, the residue was purified by silica gel chromatography to afford the desired compound **S18**.

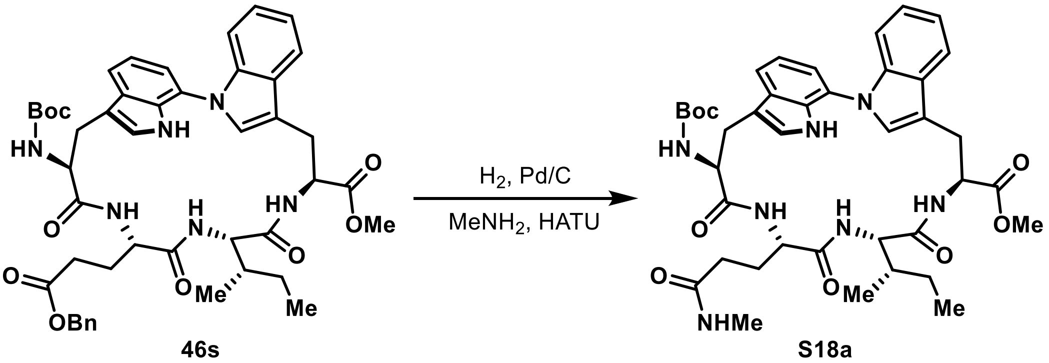

On 0.1 mmol scale, **General Procedure P** was followed with compound **46s** via hydrogenation and amide coupling. Purification by silica gel column chromatography gave the title compound **S18a** (50.0 mg, 66% yield).

Compound S18a

Physical State: amorphous solid

^{1}H NMR (600 MHz, CDCl$_3$): δ 8.96 (s, 1H), 7.89 – 7.73 (m, 2H), 7.54 (d, *J* = 7.8 Hz, 1H), 7.48 (d, *J* = 8.1 Hz, 1H), 7.31 – 7.28 (m, 2H), 7.22 (t, *J* = 7.6 Hz, 1H), 7.18 (t, *J* = 7.6 Hz, 1H), 7.12 (t, *J* = 7.4 Hz, 2H), 6.71 (s, 1H), 6.57 – 6.45 (m, 1H), 5.51 (d, *J* = 5.2 Hz, 1H), 5.31 (s, 1H), 4.81 – 4.73 (m, 1H), 4.37 – 4.32 (m, 2H), 3.85 – 3.79 (m, 1H), 3.72 (s, 3H), 3.41 (d, *J* = 15.4 Hz, 1H), 3.30 – 3.28 (m, 1H), 3.20 – 3.10 (m, 1H), 2.73 (t, *J* = 12.0 Hz, 1H), 2.52 (d, *J* = 4.5 Hz, 3H), 2.42 (s, 1H), 2.10 – 2.00 (m, 1H), 1.99 – 1.88 (m, 1H), 1.69 – 1.66 (m, 1H), 1.49 (s, 9H), 1.31 – 1.23 (m, 2H), 0.94 (d, *J* = 6.5 Hz, 3H), 0.88 (t, *J* = 7.0 Hz, 1H), 0.83 (t, *J* = 7.3 Hz, 3H).

^{13}C NMR (151 MHz, CDCl$_3$): δ 173.94, 172.76, 171.61, 170.87, 169.92, 155.29, 136.60, 133.20, 128.30, 127.55, 124.64, 124.36, 122.69, 120.09, 119.81, 118.88, 118.43, 118.15, 111.41, 110.68, 110.61, 79.94, 59.79, 55.32, 52.51, 52.33, 50.90, 36.45, 31.86, 31.67, 30.02, 29.63, 28.54, 26.91, 26.29, 25.82, 22.74, 15.02, 14.21, 11.30.

HRMS (ESI-TOF): calculated for $C_{40}H_{51}N_7NaO_8^+$ $[M+Na]^+$: 780.3691, found: 780.3693.

$[\alpha]^{25}_D$: +81.4 (*c* = 0.5, $CHCl_3$)

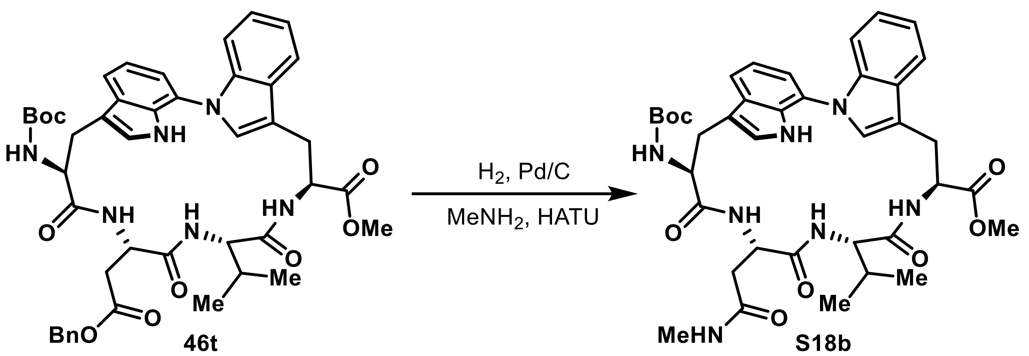

46t S18b

On 0.1 mmol scale, **General Procedure P** was followed with compound **46t** via hydrogenation and amide coupling. Purification by silica gel column chromatography gave the title compound **S18b** (45.3 mg, 62% yield).

Compound S18b

Physical State: amorphous solid

^{1}H NMR (600 MHz, METHANOL-*D*$_4$): δ 9.76 (s, 1H), 7.98 (s, 1H), 7.92 (s, 1H), 7.70 (d, *J* = 7.6 Hz, 1H), 7.61 (d, *J* = 7.8 Hz, 1H), 7.53 (d, *J* = 8.1 Hz, 1H), 7.26 (d, *J* = 7.3 Hz, 1H), 7.21 (t, *J* = 7.6 Hz, 2H), 7.14 (t, *J* = 7.5 Hz, 1H), 6.82 (s, 1H), 4.70 (dd, *J* = 11.7, 2.7 Hz, 2H), 4.24 (dd, *J* = 12.1, 4.1 Hz, 1H), 3.79 (s, 3H), 3.76 (d, *J* = 6.8 Hz, 1H), 3.44 (dd, *J* = 17.4, 3.0 Hz, 1H), 3.23 (dd, *J* = 16.0, 11.8 Hz, 2H), 2.95 (t, *J* = 12.7 Hz, 1H), 2.63 (s, 3H), 2.38 (dd, *J* = 14.7, 5.1 Hz, 1H), 2.24 (dd, *J* = 14.6, 8.4 Hz, 1H), 2.06 – 1.95 (m, 1H), 1.53 – 1.50 (m, 1H), 1.48 (s, 9H), 1.15 (d, *J* = 6.7 Hz, 3H), 1.09 (d, *J* = 6.9 Hz, 3H).

^{13}C NMR (151 MHz, METHANOL-*D*$_4$): δ 172.58, 172.12, 171.35, 170.11, 156.05, 136.32, 132.65, 129.11, 128.51, 127.04, 124.77, 124.61, 124.28, 122.28, 119.66, 118.73, 118.15, 117.18, 117.11, 111.60, 109.79, 79.19, 60.52, 60.43, 55.76, 52.56, 51.48, 49.05, 38.10, 29.94, 28.58, 27.43, 26.21, 25.25, 18.31, 17.89.

HRMS (ESI-TOF): calculated for $C_{38}H_{47}N_7NaO_8^+$ [M+Na]$^+$: 752.3378, found: 752.3358.

[α]$^{25}_{D}$: +9.8 (*c* = 0.5, $CHCl_3$)

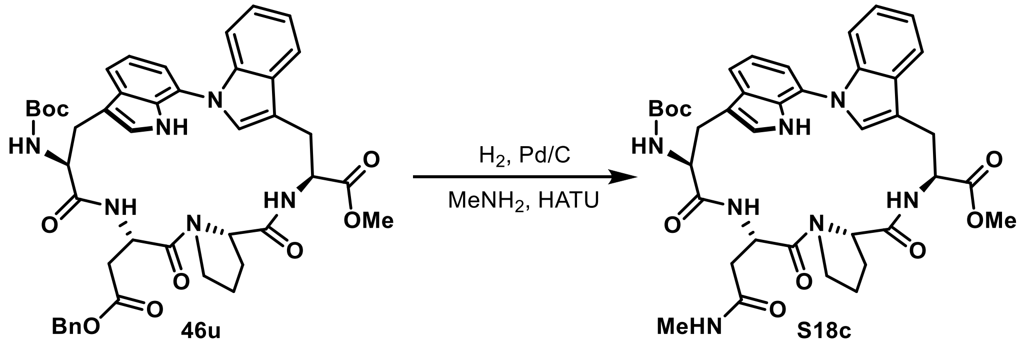

46u S18c

On 0.1 mmol scale, **General Procedure P** was followed with compound **46u** via hydrogenation and amide coupling. Purification by silica gel column chromatography gave the title compound **S18c** (43.7 mg, 60% yield).

Compound S18c

Physical State: amorphous solid

^{1}H NMR (600 MHz, $CDCl_3$): δ 8.80 (s, 1H), 7.89 – 7.71 (m, 2H), 7.49 (t, *J* = 8.1 Hz, 2H), 7.23 (t, *J* = 7.6 Hz, 2H), 7.18 (t, *J* = 7.4 Hz, 1H), 7.12 (t, *J* = 7.3 Hz, 1H), 6.62 (s, 1H), 6.40 (s, 1H), 5.46 – 5.44 (m, 2H), 4.85 – 4.59 (m, 2H), 4.46 – 4.42 (m, 1H), 4.26 – 4.19 (m, 1H), 3.74 (s, 3H), 3.46 (s, 1H), 3.44 – 3.37 (m, 1H), 3.24 (q, *J* = 8.1 Hz, 1H), 3.14 (dd, *J* = 16.1, 11.0 Hz, 1H), 2.72 (t, *J* = 12.3 Hz, 1H), 2.38 – 2.27 (m, 3H), 2.21 – 1.77 (m, 7H), 1.50 (s, 9H), 0.88 (t, *J* = 7.0 Hz, 1H).

^{13}C NMR (151 MHz, $CDCl_3$): δ 172.45, 172.29, 170.62, 169.36, 168.04, 155.15, 136.31, 133.19, 128.36, 128.33, 127.62, 124.49, 124.27, 122.66, 120.08, 119.85, 118.61, 118.47, 118.23, 111.46, 110.78, 110.60, 79.91,

60.82, 54.98, 52.70, 52.17, 47.51, 47.38, 38.97, 31.67, 30.49, 29.59, 28.56, 26.54, 25.52, 22.74, 14.22.
HRMS (ESI-TOF): calculated for $C_{38}H_{45}N_7NaO_8^+$ $[M+Na]^+$: 750.3222, found: 750.3223.
$[\alpha]^{25}_D$: +16.4 (c = 0.5, $CHCl_3$)

General procedure Q for the hydrolysis and deprotection, the synthesis of 46', 48':

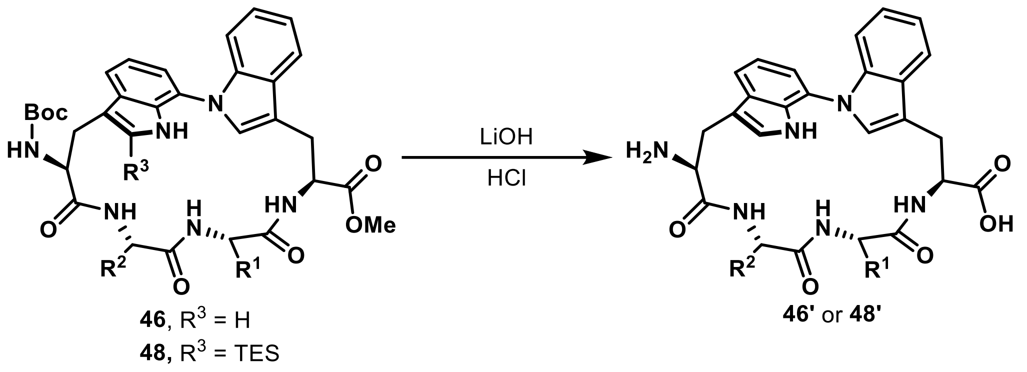

A solution of compound **46** or **48** (0.1 mmol, 1.0 eq.) in MeOH/H_2O (1:1, 0.4 M) was treated with LiOH·H_2O (0.2 mmol, 2.0 eq.). The reaction mixture was stirred at rt for 30 minutes. Upon completion, the reaction was quenched by adjusting the pH to 3 with 0.5 M aq. HCl and then extracted with DCM for three times. The combined organic layers were washed with saturated aq. NaCl, dried over Na_2SO_4, and concentrated *in vacuo* to give the acid, the acid was then dissolved in DCM/4 M HCl (1:1, 0.05 M), The reaction mixture was stirred at rt for 2 h, the solvent was removed under reduced pressure to give the residue, the residue was purified by C18 silica gel (manual reverse-phase silica gel) using H_2O followed by MeOH/H_2O = 1:1 as the mobile phase to give the product **46'** or **48'**.

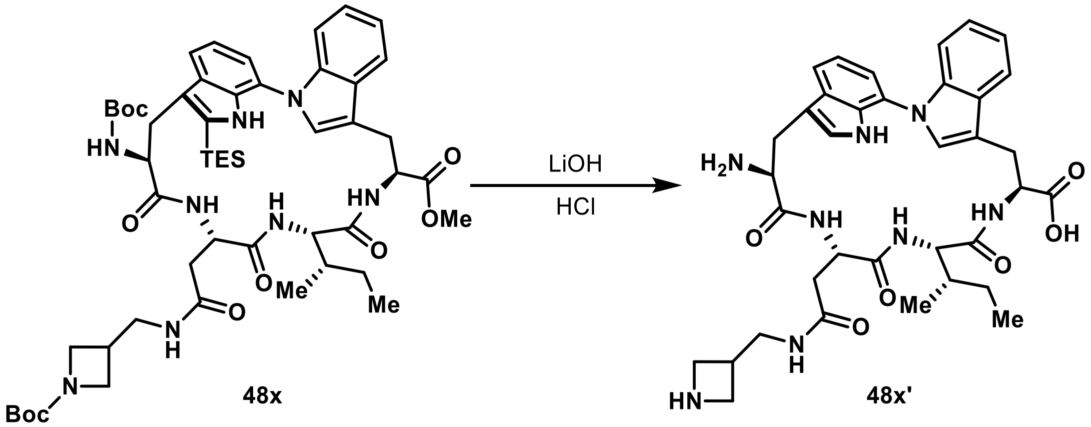

On 0.1 mmol scale, **General Procedure Q** was followed with compound **48x** via hydrolysis and deprotection. Purification by C18 silica gel (manual reverse-phase silica gel) using H_2O followed by MeOH/H_2O = 1:1 as the mobile phase gave the title compound **48x'** (47.9 mg, 70% yield).

Compound 48x'

Physical State: amorphous solid

^{1}H NMR (600 MHz, DMSO-*d*6): δ 10.52 (s, 1H), 8.34 (s, 1H), 7.96 – 7.81 (m, 3H), 7.83 (s, 1H), 7.62 (d, *J* = 7.8 Hz, 1H), 7.56 (d, *J* = 7.8 Hz, 1H), 7.46 (d, *J* = 8.1 Hz, 1H), 7.15 – 7.05 (m, 5H), 6.66 (s, 1H), 4.73 – 4.66 (m, 1H), 4.28 – 4.25 (m, 1H), 4.03 – 3.98 (m, 2H), 3.69 – 3.62 (m, 3H), 3.45 – 3.38 (m, 3H), 3.33 – 3.30 (m, 1H), 3.14 – 3.10 (m, 2H), 3.01 – 2.98 (m, 1H), 2.96 – 2.90 (m, 1H), 2.88 – 2.82 (m, 1H), 2.77 (t, *J* = 12.3 Hz, 1H), 2.21 – 2.11 (m, 2H), 1.64 – 1.55 (m, 2H), 1.33 – 1.26 (m, 1H), 0.91 (d, *J* = 6.7 Hz, 3H), 0.85 (t, *J* = 7.3 Hz, 3H).

^{13}C NMR (151 MHz, DMSO-*d*6): δ 174.94, 172.83, 170.86, 170.39, 170.28, 165.91, 135.99, 132.18, 129.58, 129.34, 129.12, 124.95, 124.60, 122.48, 120.02, 119.47, 118.98, 117.48, 116.90, 112.48, 110.55, 110.07, 58.53, 56.78, 53.34, 49.51, 47.52, 40.56, 39.19, 39.07, 36.28, 32.92, 32.70, 27.40, 25.76, 15.67, 11.42.

HRMS (ESI-TOF): calculated for $C_{36}H_{45}N_8O_6^+$ $[M+Na]^+$: 685.3457, found: 685.3447.
$[\alpha]^{25}_D$: +52.4 (*c* = 0.2, MeOH)

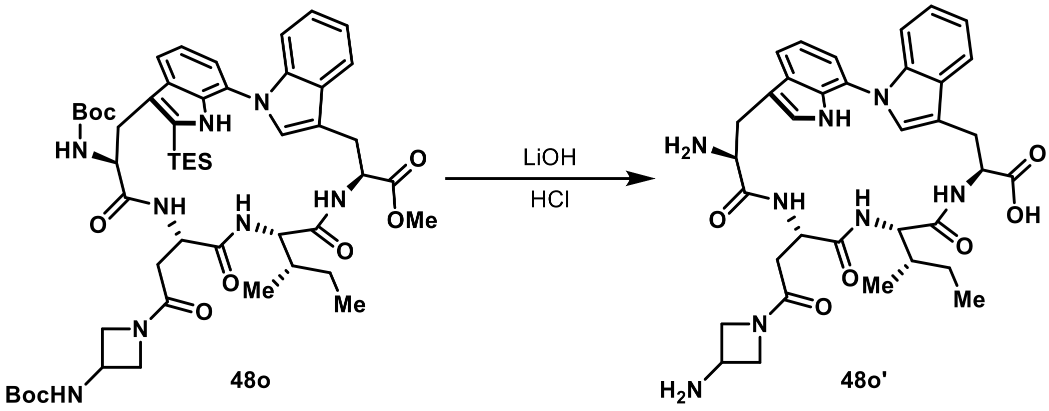

On 0.1 mmol scale, **General Procedure Q** was followed with compound **48o** via hydrolysis and deprotection. Purification by C18 silica gel (manual reverse-phase silica gel) using H_2O followed by MeOH/H_2O = 1:1 as the mobile phase gave the title compound **48o'** (44.2 mg, 66% yield).

Compound 48o'

Physical State: amorphous solid

^{1}H NMR (600 MHz, DMSO-d_6): δ 10.56 (s, 1H), 8.32 – 8.25 (m, 2H), 7.87 – 7.69 (m, 2H), 7.66 – 7.40 (m, 4H), 7.34 – 7.03 (m, 6H), 6.71 – 6.64 (m, 1H), 4.32 – 3.90 (m, 6H), 3.80 – 3.66 (m, 3H), 3.58 – 3.46 (m, 2H), 3.24 – 3.09 (m, 3H), 2.93 – 2.74 (m, 1H), 2.22 – 1.77 (m, 2H), 1.69 – 1.55 (m, 2H), 1.33 – 1.24 (m, 1H), 0.90 – 0.84 (m, 6H).

^{13}C NMR (151 MHz, DMSO-d_6): δ 175.08, 171.12, 170.72, 169.88, 169.16, 168.76, 165.89, 136.09, 135.99, 132.24, 129.68, 129.50, 128.99, 128.61, 125.55, 125.10, 124.55, 122.68, 120.15, 119.37, 119.16, 117.72, 117.10, 112.61, 110.09, 109.46, 58.21, 56.75, 54.96, 54.35, 53.22, 49.06, 41.03, 40.51, 36.43, 36.10, 34.41, 29.68, 27.10, 25.90, 25.76, 15.40, 15.26, 11.80, 11.66.

HRMS (ESI-TOF): calculated for $C_{35}H_{43}N_8O_6^+$ $[M+Na]^+$: 671.3300, found: 671.3309.
$[\alpha]^{25}_D$: +117.2 (*c* = 0.2, MeOH)

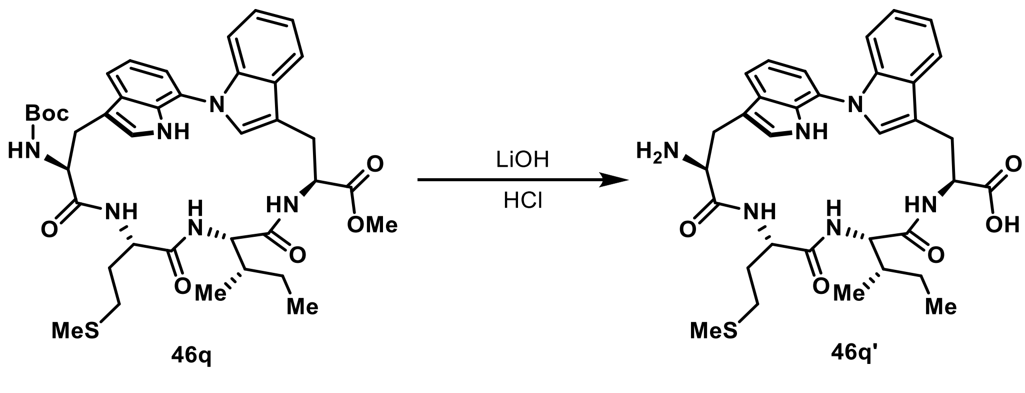

On 0.1 mmol scale, **General Procedure Q** was followed with compound **46q** via hydrolysis and deprotection. Purification by C18 silica gel (manual reverse-phase silica gel) using H_2O followed by MeOH/H_2O = 1:1 as the mobile phase gave the title compound **46q'** (45.5 mg, 72% yield).

Compound 46q'

Physical State: amorphous solid

^{1}H NMR (600 MHz, DMSO-d_6): δ 10.57 (s, 1H), 8.34 (s, 1H), 7.96 (d, *J* = 5.1 Hz, 1H), 7.85 (s, 1H), 7.78 (d, *J* = 2.8 Hz, 1H), 7.62 (d, *J* = 7.9 Hz, 1H), 7.59 (d, *J* = 7.9 Hz, 1H), 7.49 (d, *J* = 8.2 Hz, 2H), 7.20 (d, *J* = 7.3 Hz, 1H), 7.17 – 7.12 (m, 2H), 7.08 (t, *J* = 7.4 Hz, 1H), 6.69 – 6.61 (m, 1H), 4.40 (q, *J* = 8.8 Hz, 1H), 4.33 (dt, *J* = 9.5, 4.9 Hz, 1H), 4.00 (dd, *J* = 6.7, 2.9 Hz, 1H), 3.64 (dd, *J* = 11.7, 3.9 Hz, 1H), 3.26 – 3.17 (m, 2H), 3.13

(dd, J = 16.0, 9.6 Hz, 1H), 2.87 (t, J = 12.5 Hz, 1H), 2.41 – 2.36 (m, 1H), 2.33 – 2.27 (m, 1H), 1.95 (s, 3H), 1.71 – 1.55 (m, 3H), 1.55 – 1.44 (m, 1H), 1.37 – 1.31 (m, 1H), 0.93 (d, J = 6.7 Hz, 3H), 0.88 (t, J = 7.3 Hz, 3H).

^{13}C NMR (151 MHz, DMSO-*d*$_6$): δ 174.54, 170.85, 170.35, 169.78, 165.49, 135.97, 132.22, 129.52, 129.13, 128.99, 125.30, 124.61, 122.62, 120.14, 119.40, 119.12, 117.60, 117.02, 112.18, 110.16, 109.11, 58.40, 54.65, 52.77, 51.41, 40.55, 36.14, 32.62, 29.75, 29.03, 27.25, 25.82, 15.61, 15.13, 11.37.

HRMS (ESI-TOF): calculated for $C_{33}H_{41}N_6O_5S^+$ [M+Na]$^+$: 633.2854, found: 633.2858.

$[\alpha]^{25}_D$: +180.7 (c = 0.2, MeOH)

General procedure R for the synthesis of 48:

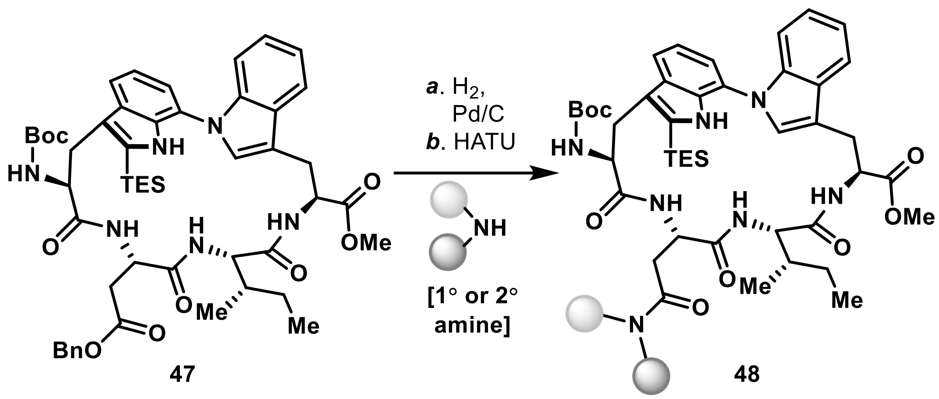

To a solution of compound **47** (0.1 mmol) in MeOH 3 mL was added Pd/C 30 mg, the reaction mixture was allowed to stir at room temperature under hydrogen atmosphere for 4 h. Then the solid was filtered and washed with 2 mL MeOH, the combined organic solvent was removed under reduced pressure to give a residue which was used directly in next step.

The residue (0.1 mmol, 1.0 eq.) was dissolved DCM (2 mL), amine (0.3 mmol, 3.0 eq.), DIPEA (0.3 mmol, 3.0 eq.) and HATU (0.15 mmol, 1.5 eq.) were added sequentially, the reaction mixture was stirred at rt for 1 h, quenched by aq. 0.5 M HCl, the organic layers were concentrated under reduced pressure to give the residue, the residue was purified by silica gel chromatography to give the compound **48a-48x.**

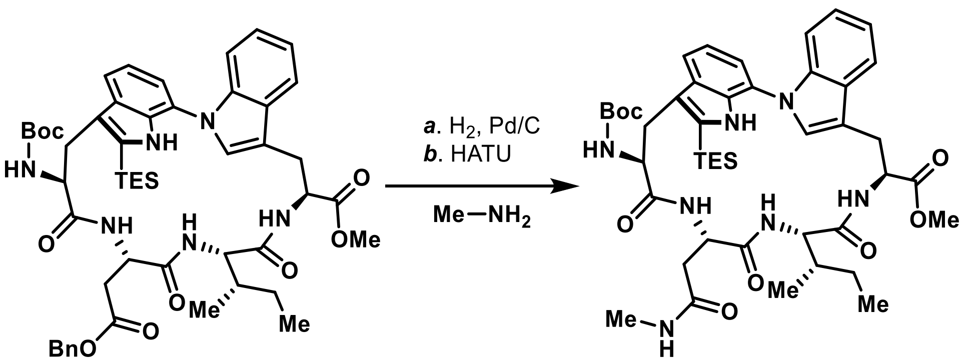

On 0.1 mmol scale, **General Procedure R** was followed with compound **47** via hydrogenation and amide coupling. Purification by silica gel column chromatography gave the title compound **48a** (68.7 mg, 80% yield).

Compound 48a

Physical State: amorphous solid

^{1}H NMR (600 MHz, CDCl$_3$): δ 8.21 (d, *J* = 4.1 Hz, 1H), 8.00 (q, *J* = 4.1 Hz, 1H), 7.96 (s, 1H), 7.73 (s, 1H), 7.62 (d, *J* = 7.8 Hz, 1H), 7.54 (d, *J* = 8.1 Hz, 1H), 7.29 – 7.25 (m, 1H), 7.23 – 7.19 (m, 3H), 6.02 (d, *J* = 7.8 Hz, 1H), 5.93 (q, *J* = 4.8 Hz, 1H), 5.84 (d, *J* = 6.9 Hz, 1H), 5.39 (d, *J* = 6.4 Hz, 1H), 4.90 (ddd, *J* = 12.2, 7.7, 2.3 Hz, 1H), 4.75 (ddd, *J* = 11.7, 6.9, 4.4 Hz, 1H), 4.60 (q, *J* = 6.4 Hz, 1H), 3.74 (s, 3H), 3.69 (dd, *J* = 13.8, 4.4 Hz, 1H), 3.56 – 3.49 (m, 2H), 3.19 – 3.12 (m, 1H), 2.81 (dd, *J* = 13.9, 11.9 Hz, 1H), 2.70 (d, *J* = 4.8 Hz, 3H), 2.30 (d, *J* = 6.4 Hz, 2H), 1.85 – 1.78 (m, 1H), 1.61 – 1.57 (m, 1H), 1.50 (s, 9H), 1.37 – 1.31 (m, 1H), 1.03 (d, *J* = 6.8 Hz, 3H), 0.94 – 0.87 (m, 12H), 0.71 – 0.59 (m, 6H).

^{13}C NMR (151 MHz, CDCl$_3$): δ 172.63, 171.86, 171.18, 169.54, 169.41, 155.30, 136.70, 135.52, 134.69, 129.14, 128.59, 127.31, 124.17, 122.80, 120.29, 120.09, 120.01, 119.94, 119.08, 118.47, 112.42, 111.03, 79.65, 77.39, 77.18, 76.96, 67.16, 60.13, 53.67, 52.62, 52.48, 48.28, 45.14, 39.86, 38.69, 36.38, 31.85, 29.78, 28.58, 28.32, 28.29, 27.14, 26.56, 25.33, 21.27, 20.57, 15.72, 11.19, 7.55, 7.48, 7.43, 3.79.

HRMS (ESI-TOF): calculated for $C_{45}H_{63}N_7NaO_8Si^+$ $[M+Na]^+$: 880.4400, found: 880.4408.

[α]^{25}D: +53.0 (*c* = 0.5, $CHCl_3$)

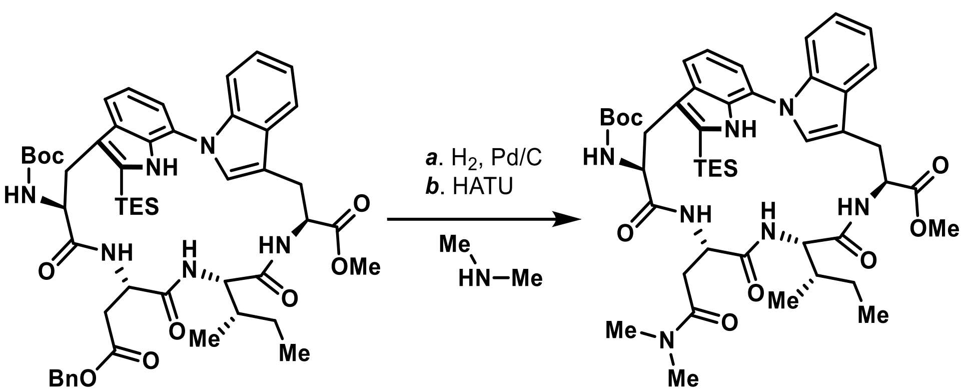

On 0.1 mmol scale, **General Procedure R** was followed with compound **47** via hydrogenation and amide coupling. Purification by silica gel column chromatography gave the title compound **48b** (68.9 mg, 79% yield).

Compound 48b

Physical State: amorphous solid

^{1}H NMR (600 MHz, $CDCl_3$): δ 8.45 (d, *J* = 3.9 Hz, 1H), 8.05 (t, *J* = 4.5 Hz, 1H), 7.96 (s, 1H), 7.79 (s, 1H), 7.65 (d, *J* = 7.8 Hz, 1H), 7.58 (d, *J* = 8.1 Hz, 1H), 7.32 – 7.28 (m, 1H), 7.26 – 7.18 (m, 3H), 5.89 (t, *J* = 7.4 Hz, 2H), 5.44 (d, *J* = 6.5 Hz, 1H), 4.93 (ddd, *J* = 12.1, 7.5, 2.3 Hz, 1H), 4.86 – 4.73 (m, 2H), 3.78 (s, 3H), 3.72 (dd, *J* = 13.9, 4.3 Hz, 1H), 3.58 – 3.49 (m, 2H), 3.23 – 3.13 (m, 1H), 2.91 (s, 3H), 2.84 (s, 3H), 2.58 – 2.48 (m, 1H), 2.39 (dd, *J* = 17.3, 10.6 Hz, 1H), 1.85 – 1.78 (m, 1H), 1.68 (s, 2H), 1.53 (s, 9H), 1.40 – 1.31 (m, 1H), 1.04 (d, *J* = 6.8 Hz, 3H), 0.97 – 0.89 (m, 12H), 0.77 – 0.62 (m, 6H).

^{13}C NMR (151 MHz, $CDCl_3$): δ 172.61, 171.38, 171.03, 169.68, 169.09, 155.33, 136.76, 135.50, 134.71, 129.15, 128.60, 127.34, 124.17, 122.82, 120.42, 120.09, 119.98, 119.16, 118.45, 112.38, 111.07, 79.55, 60.05, 53.59, 52.59, 52.47, 48.15, 38.08, 37.23, 36.40, 35.67, 31.94, 28.59, 27.21, 25.37, 15.71, 11.18, 7.56, 3.80.

HRMS (ESI-TOF): calculated for $C_{46}H_{65}N_7NaO_8Si^+$ $[M+Na]^+$: 894.4556, found: 894.4535.

[α]^{25}D: +21.4 (*c* = 0.5, $CHCl_3$)

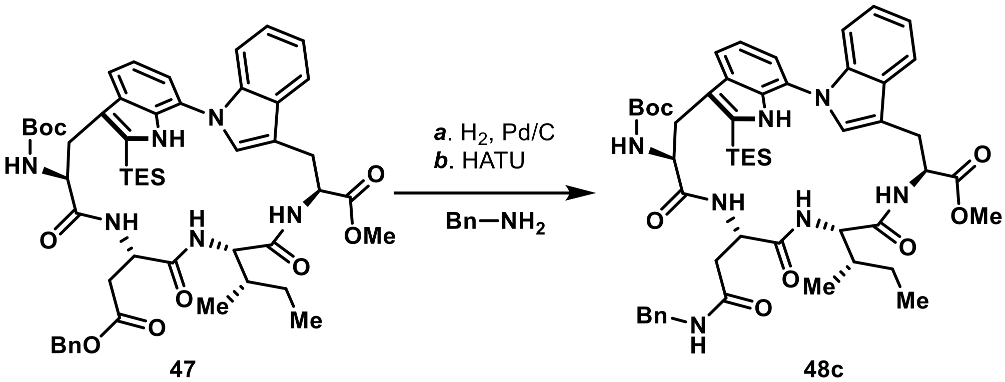

On 0.1 mmol scale, **General Procedure R** was followed with compound **47** via hydrogenation and amide coupling. Purification by silica gel column chromatography gave the title compound **48c** (79.4 mg, 85% yield).

Compound 48c

Physical State: amorphous solid

^{1}H NMR (600 MHz, $CDCl_3$): δ 8.18 (d, *J* = 4.0 Hz, 1H), 8.04 – 8.00 (m, 1H), 7.98 (s, 1H), 7.74 (s, 1H), 7.62 (d, *J* = 7.9 Hz, 1H), 7.54 (d, *J* = 8.1 Hz, 1H), 7.29 – 7.25 (m, 3H), 7.24 – 7.16 (m, 7H), 6.13 (t, *J* = 5.8 Hz, 1H), 6.03 (d, *J* = 7.7 Hz, 1H), 5.85 (d, *J* = 6.9 Hz, 1H), 5.40 (d, *J* = 6.4 Hz, 1H), 4.91 (ddd, *J* = 12.3, 7.7, 2.3 Hz, 1H), 4.76 (ddd, *J* = 11.6, 6.9, 4.4 Hz, 1H), 4.66 (q, *J* = 6.4 Hz, 1H), 4.35 (qd, *J* = 14.8, 5.7 Hz, 2H), 3.75 (s, 3H), 3.70 (dd, *J* = 13.8, 4.4 Hz, 1H), 3.59 – 3.49 (m, 2H), 3.16 (ddd, *J* = 16.7, 12.1, 1.5 Hz, 1H), 2.88 – 2.78 (m, 1H), 2.36 (d, *J* = 6.3 Hz, 2H), 1.85 – 1.78 (m, 1H), 1.61 – 1.58 (m, 1H), 1.51 (s, 9H), 1.03 (d, *J* = 6.8

Hz, 3H), 0.94 – 0.87 (m, 12H), 0.74 – 0.60 (m, 6H).
^{13}C NMR (151 MHz, CDCl$_3$): δ 172.71, 171.29, 171.14, 169.51, 169.40, 162.67, 155.28, 137.54, 136.71, 135.58, 134.72, 129.19, 128.76, 128.63, 128.00, 127.81, 127.59, 127.45, 124.19, 122.76, 120.35, 120.06, 119.98, 119.96, 119.10, 118.46, 112.41, 111.02, 79.62, 77.40, 77.26, 77.19, 77.06, 76.98, 67.14, 60.09, 53.70, 52.56, 52.46, 48.21, 43.86, 39.78, 38.67, 36.61, 36.39, 31.89, 31.50, 29.79, 29.41, 28.59, 28.33, 27.09, 25.99, 25.34, 15.74, 11.20, 7.57, 4.63, 3.82.
HRMS (ESI-TOF): calculated for $C_{51}H_{67}N_7NaO_8Si^+$ [M+Na]$^+$: 956.4713, found: 956.4718.
[α]$^{25}_D$: +12.2 (c = 0.5, CHCl$_3$)

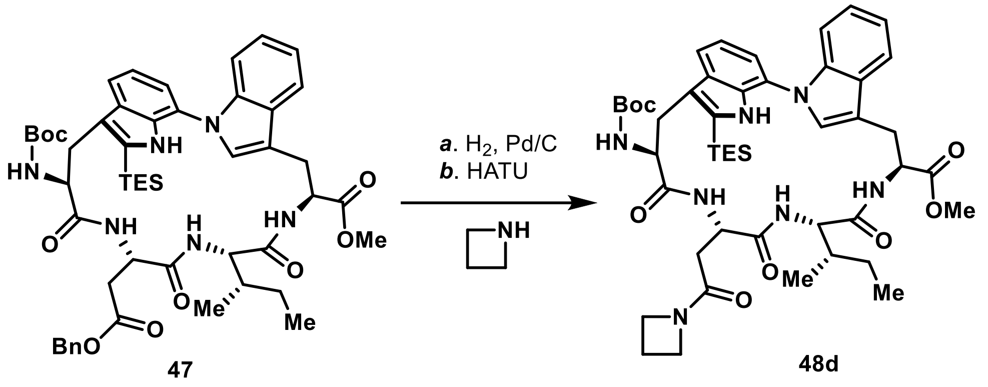

On 0.1 mmol scale, **General Procedure R** was followed with compound **47** via hydrogenation and amide coupling. Purification by silica gel column chromatography gave the title compound **48d** (58.4 mg, 66% yield).
Compound 48d
Physical State: amorphous solid
^{1}H NMR (400 MHz, CDCl$_3$): δ 8.51 (d, J = 3.9 Hz, 1H), 8.03 (dd, J = 5.2, 3.7 Hz, 1H), 7.97 (s, 1H), 7.75 (s, 1H), 7.65 (d, J = 7.7 Hz, 1H), 7.57 (d, J = 8.1 Hz, 1H), 7.32 – 7.27 (m, 1H), 7.25 – 7.19 (m, 3H), 5.95 (d, J = 7.6 Hz, 1H), 5.89 (d, J = 6.8 Hz, 1H), 5.41 (d, J = 6.3 Hz, 1H), 4.94 (ddd, J = 12.1, 7.6, 2.2 Hz, 1H), 4.77 (ddd, J = 11.8, 7.0, 4.5 Hz, 1H), 4.64 (ddd, J = 9.3, 6.3, 2.5 Hz, 1H), 4.06 – 3.96 (m, 4H), 3.78 (s, 3H), 3.76 – 3.72 (m, 1H), 3.59 – 3.51 (m, 2H), 3.18 (ddd, J = 16.7, 12.1, 1.5 Hz, 1H), 2.90 – 2.80 (m, 1H), 2.30 – 2.13 (m, 5H), 1.84 (q, J = 8.3, 7.2 Hz, 1H), 1.62 (ddd, J = 13.8, 7.4, 3.9 Hz, 1H), 1.53 (s, 9H), 1.04 (d, J = 6.8 Hz, 3H), 0.95 – 0.88 (m, 12H), 0.74 – 0.61 (m, 6H).
^{13}C NMR (151 MHz, CDCl$_3$): δ 172.62, 171.17, 170.89, 169.51, 169.26, 155.33, 136.75, 135.51, 134.77, 129.13, 128.61, 127.36, 124.18, 122.81, 120.32, 120.10, 119.99, 119.11, 118.46, 112.38, 111.05, 79.62, 67.17, 60.15, 53.62, 52.60, 52.50, 52.41, 50.40, 48.28, 47.78, 36.28, 35.28, 31.86, 28.59, 28.31, 27.21, 25.34, 15.73, 15.16, 11.06, 7.53, 7.42, 6.67, 5.87, 4.62, 3.79, 3.41.
HRMS (ESI-TOF): calculated for $C_{47}H_{65}N_7NaO_8Si^+$ [M+Na]$^+$: 906.4556, found: 906.4574.
[α]$^{25}_D$: +45.7 (c = 0.5, CHCl$_3$)

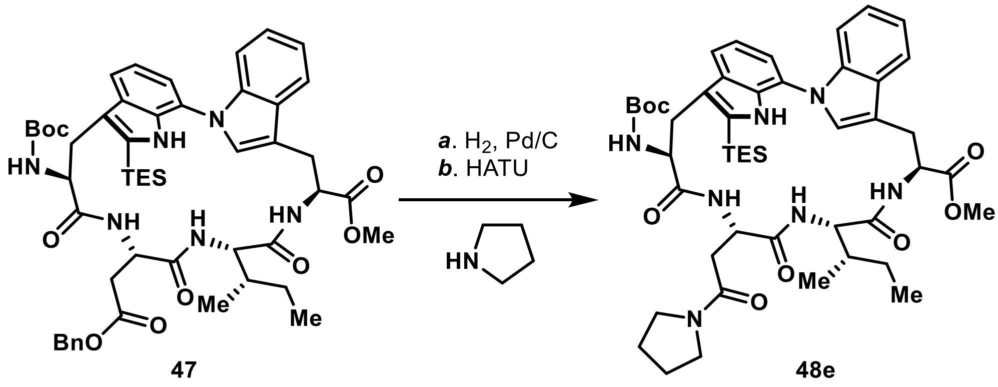

On 0.1 mmol scale, **General Procedure R** was followed with compound **47** via hydrogenation and amide coupling. Purification by silica gel column chromatography gave the title compound **48e** (67.4 mg, 75% yield).

Compound 48e

Physical State: amorphous solid

^{1}H NMR (600 MHz, CDCl$_3$): δ 8.66 (d, *J* = 3.9 Hz, 1H), 8.05 (q, *J* = 4.2 Hz, 1H), 7.96 (s, 1H), 7.78 (s, 1H), 7.65 (d, *J* = 7.7 Hz, 1H), 7.58 (d, *J* = 8.1 Hz, 1H), 7.33 – 7.29 (m, 1H), 7.26 – 7.21 (m, 3H), 5.94 – 5.81 (m, 2H), 5.45 (d, *J* = 6.3 Hz, 1H), 4.93 (ddd, *J* = 12.1, 7.4, 2.3 Hz, 1H), 4.78 (dq, *J* = 12.0, 6.6, 5.6 Hz, 2H), 3.77 (s, 3H), 3.75 – 3.69 (m, 1H), 3.57 – 3.50 (m, 2H), 3.40 (t, *J* = 6.8 Hz, 2H), 3.27 – 3.14 (m, 3H), 2.89 – 2.79 (m, 1H), 2.48 (d, *J* = 17.1 Hz, 1H), 2.35 (dd, *J* = 17.3, 10.6 Hz, 1H), 1.92 – 1.78 (m, 6H), 1.65 – 1.60 (m, 1H), 1.53 (s, 9H), 1.04 (d, *J* = 6.8 Hz, 3H), 0.96 – 0.90 (m, 12H), 0.74 – 0.60 (m, 6H).

^{13}C NMR (151 MHz, CDCl$_3$): δ 172.63, 171.11, 169.75, 169.72, 169.12, 155.32, 136.76, 135.51, 134.73, 129.17, 128.61, 127.37, 124.17, 122.81, 120.42, 120.09, 119.98, 119.15, 118.45, 112.39, 111.07, 79.54, 60.08, 53.61, 52.58, 52.49, 47.90, 46.65, 46.04, 39.04, 36.35, 31.94, 28.60, 28.34, 27.20, 25.94, 25.38, 24.30, 15.73, 11.12, 7.55, 4.63, 3.80.

HRMS (ESI-TOF): calculated for $C_{48}H_{67}N_7NaO_8Si^+$ [M+Na]$^+$: 920.4713, found: 920.4715.

[α]$^{25}_D$: +47.8 (*c* = 0.5, CHCl$_3$)

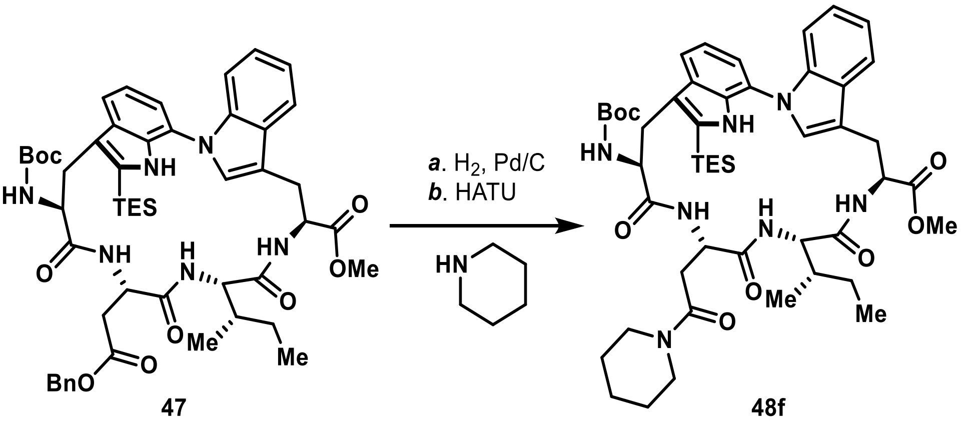

47 48f

On 0.1 mmol scale, **General Procedure R** was followed with compound **47** via hydrogenation and amide coupling. Purification by silica gel column chromatography gave the title compound **48f** (66.6 mg, 73% yield).

Compound 48f

Physical State: amorphous solid

^{1}H NMR (600 MHz, CDCl$_3$): δ 8.41 (t, *J* = 2.7 Hz, 1H), 8.02 (t, *J* = 4.1 Hz, 1H), 7.98 (s, 1H), 7.78 (s, 1H), 7.61 (d, *J* = 7.8 Hz, 1H), 7.54 (d, *J* = 8.1 Hz, 1H), 7.25 (dd, *J* = 5.1, 3.2 Hz, 1H), 7.22 – 7.18 (m, 3H), 6.09 (d, *J* = 7.7 Hz, 1H), 5.89 (dd, *J* = 6.9, 1.8 Hz, 1H), 5.42 (d, *J* = 6.5 Hz, 1H), 4.92 (ddt, *J* = 12.0, 7.7, 2.1 Hz, 1H), 4.85 – 4.81 (m, 1H), 4.78 – 4.71 (m, 1H), 3.74 (d, *J* = 1.8 Hz, 3H), 3.68 (d, *J* = 4.9 Hz, 1H), 3.57 – 3.48 (m, 3H), 3.41 (dt, *J* = 12.4, 5.7 Hz, 1H), 3.23 – 3.11 (m, 3H), 2.87 – 2.78 (m, 1H), 2.51 (d, *J* = 17.0 Hz, 1H), 2.37 (ddd, *J* = 17.3, 10.8, 1.7 Hz, 1H), 1.59 – 1.54 (m, 2H), 1.50 (d, *J* = 1.9 Hz, 9H), 1.48 – 1.40 (m, 6H), 1.01 (dd, *J* = 6.9, 1.8 Hz, 3H), 0.93 – 0.88 (m, 12H), 0.73 – 0.61 (m, 6H).

^{13}C NMR (151 MHz, CDCl$_3$): δ 172.70, 171.02, 169.80, 169.71, 169.39, 169.05, 155.32, 136.71, 135.53, 134.67, 129.17, 128.61, 127.33, 124.17, 122.76, 120.42, 120.05, 119.97, 119.09, 118.45, 112.43, 111.03, 79.52, 67.15, 60.02, 53.62, 52.58, 52.42, 48.23, 46.57, 43.06, 37.87, 36.44, 31.92, 29.77, 28.59, 27.19, 26.14, 26.00, 25.47, 25.19, 24.32, 15.77, 11.26, 7.56, 7.47, 3.79, 1.10.

HRMS (ESI-TOF): calculated for $C_{49}H_{69}N_7NaO_8Si^+$ [M+Na]$^+$: 934.4869, found: 934.4879.

[α]$^{25}_D$: +33.2 (*c* = 0.5, CHCl$_3$)

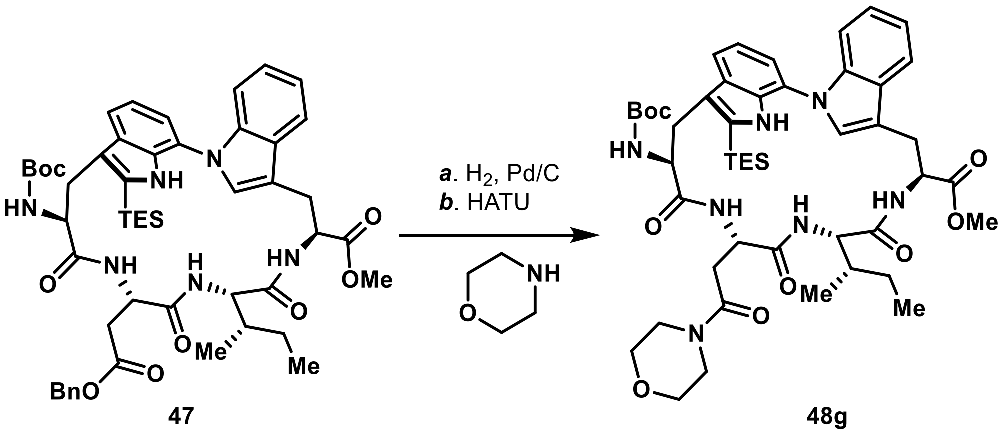

On 0.1 mmol scale, **General Procedure R** was followed with compound **47** via hydrogenation and amide coupling. Purification by silica gel column chromatography gave the title compound **48g** (64.0 mg, 70% yield).

Compound 48g

Physical State: amorphous solid

^{1}H NMR (600 MHz, CDCl$_3$): δ 8.22 (d, *J* = 3.9 Hz, 1H), 8.02 (dd, *J* = 5.7, 3.2 Hz, 1H), 7.95 (s, 1H), 7.76 (s, 1H), 7.62 (d, *J* = 7.9 Hz, 1H), 7.55 (d, *J* = 8.1 Hz, 1H), 7.27 (ddd, *J* = 8.2, 7.0, 1.3 Hz, 1H), 7.23 – 7.19 (m, 3H), 5.96 (d, *J* = 7.7 Hz, 1H), 5.86 (d, *J* = 6.9 Hz, 1H), 5.42 (d, *J* = 6.5 Hz, 1H), 4.91 (ddd, *J* = 12.2, 7.7, 2.4 Hz, 1H), 4.84 – 4.73 (m, 2H), 3.76 (s, 3H), 3.69 (dd, *J* = 13.8, 4.4 Hz, 1H), 3.62 – 3.50 (m, 9H), 3.25 (q, *J* = 4.4 Hz, 2H), 3.15 (ddd, *J* = 16.7, 12.1, 1.5 Hz, 1H), 2.83 (dd, *J* = 13.9, 12.0 Hz, 1H), 2.50 (dd, *J* = 17.2, 2.3 Hz, 1H), 2.37 (dd, *J* = 17.2, 10.7 Hz, 1H), 1.83 – 1.76 (m, 1H), 1.60 (dp, *J* = 11.3, 3.9 Hz, 1H), 1.51 (s, 9H), 1.02 (d, *J* = 6.9 Hz, 3H), 0.93 – 0.89 (m, 12H), 0.72 – 0.61 (m, 6H).

^{13}C NMR (151 MHz, CDCl$_3$): δ 172.67, 171.02, 170.05, 169.46, 169.16, 162.65, 155.32, 136.70, 135.54, 134.71, 129.15, 129.12, 128.61, 127.35, 124.20, 122.77, 120.36, 120.07, 119.97, 119.06, 118.45, 112.44, 111.01, 79.60, 67.15, 66.63, 66.29, 59.98, 53.60, 52.59, 52.44, 48.06, 45.83, 42.18, 42.04, 38.68, 37.62, 36.60, 36.41, 31.91, 31.49, 29.78, 28.58, 27.12, 26.00, 25.25, 15.73, 11.23, 7.56, 3.80, 1.10.

HRMS (ESI-TOF): calculated for $C_{48}H_{67}N_7NaO_9Si^+$ $[M+Na]^+$: 936.4662, found: 936.4669.

$[\alpha]^{25}_D$: +37.9 (*c* = 0.5, CHCl$_3$)

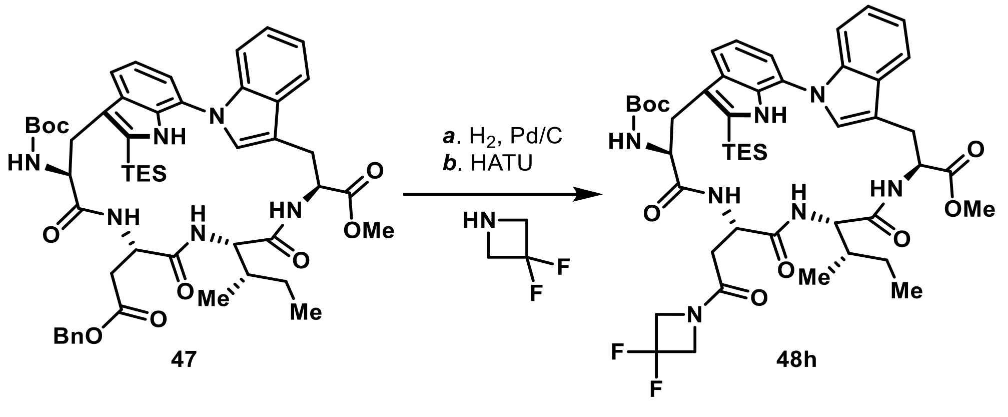

On 0.1 mmol scale, **General Procedure R** was followed with compound **47** via hydrogenation and amide coupling. Purification by silica gel column chromatography gave the title compound **48h** (60.8 mg, 66% yield).

Compound 48h

Physical State: amorphous solid

^{1}H NMR (400 MHz, CDCl$_3$): δ 8.09 (d, *J* = 4.1 Hz, 1H), 8.02 (p, *J* = 3.8 Hz, 1H), 7.98 (s, 1H), 7.71 (s, 1H), 7.61 (d, *J* = 7.8 Hz, 1H), 7.54 (d, *J* = 8.1 Hz, 1H), 7.30 – 7.24 (m, 1H), 7.23 – 7.18 (m, 3H), 6.01 (d, *J* = 7.6 Hz, 1H), 5.83 (d, *J* = 6.9 Hz, 1H), 5.40 (d, *J* = 6.2 Hz, 1H), 4.90 (ddd, *J* = 12.1, 7.6, 2.2 Hz, 1H), 4.77 (ddd, *J* = 11.6, 6.9, 4.4 Hz, 1H), 4.61 (ddd, *J* = 9.5, 6.3, 2.8 Hz, 1H), 4.39 – 4.21 (m, 5H), 3.75 (s, 3H), 3.71 (t, *J* =

6.3 Hz, 1H), 3.55 – 3.48 (m, 2H), 3.23 – 3.10 (m, 1H), 2.89 – 2.77 (m, 1H), 2.32 – 2.22 (m, 2H), 1.84 – 1.78 (m, 1H), 1.62 – 1.57 (m, 1H), 1.52 (s, 9H), 1.03 (d, J = 6.8 Hz, 3H), 0.94 – 0.87 (m, 12H), 0.72 – 0.59 (m, 6H).

^{13}C NMR (151 MHz, $CDCl_3$): δ 172.59, 171.49, 170.96, 169.38, 169.14, 155.32, 136.74, 135.52, 134.73, 129.09, 128.62, 127.31, 124.23, 122.84, 120.30, 120.12, 120.04, 119.12, 118.48, 114.58, 112.46, 111.04, 79.69, 62.15, 61.96, 61.76, 60.54, 60.34, 60.13, 53.58, 52.61, 52.49, 47.63, 36.30, 36.18, 31.85, 29.79, 28.58, 28.35, 28.31, 28.29, 27.17, 25.32, 15.75, 11.09, 7.57, 7.54, 7.44, 4.62, 3.79, 1.11.

^{19}F NMR (565 MHz, $CDCl_3$): δ -100.59.

HRMS (ESI-TOF): calculated for $C_{47}H_{63}F_2N_7NaO_8Si^+$ $[M+Na]^+$: 942.4368, found: 942.4380.

$[\alpha]^{25}_D$: +43.4 (c = 0.5, $CHCl_3$)

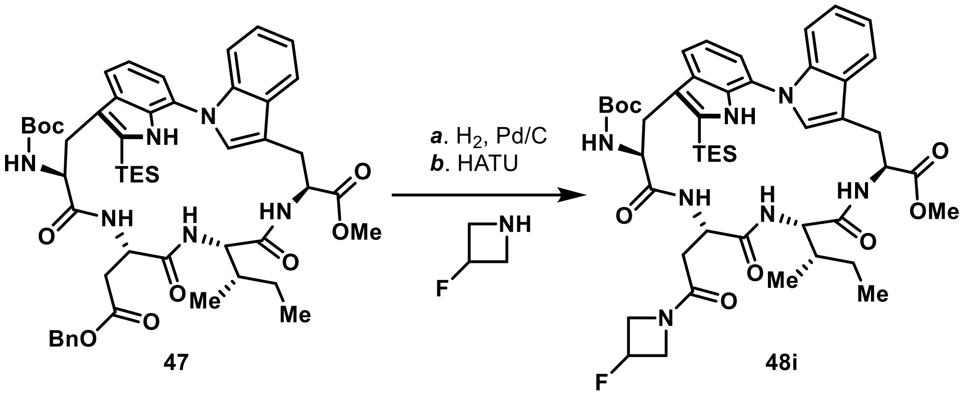

On 0.1 mmol scale, **General Procedure R** was followed with compound **47** via hydrogenation and amide coupling. Purification by silica gel column chromatography gave the title compound **48i** (61.4 mg, 68% yield).

Compound 48i

Physical State: amorphous solid

^{1}H NMR (600 MHz, $CDCl_3$): δ 8.43 – 7.99 (m, 2H), 7.95 (d, J = 6.6 Hz, 1H), 7.72 (d, J = 18.5 Hz, 1H), 7.65 – 7.59 (m, 1H), 7.55 (t, J = 7.5 Hz, 1H), 7.29 – 7.25 (m, 1H), 7.23 – 7.20 (m, 3H), 5.92 (dd, J = 7.7, 5.2 Hz, 1H), 5.84 (dd, J = 22.2, 6.9 Hz, 1H), 5.46 – 5.31 (m, 1H), 5.31 – 5.15 (m, 1H), 4.96 – 4.84 (m, 1H), 4.80 – 4.72 (m, 1H), 4.70 – 4.51 (m, 1H), 4.34 – 4.18 (m, 2H), 4.15 – 4.01 (m, 2H), 3.76 (d, J = 1.2 Hz, 3H), 3.70 (dd, J = 13.9, 4.5 Hz, 1H), 3.56 – 3.47 (m, 2H), 3.15 (ddd, J = 16.7, 12.1, 1.5 Hz, 1H), 2.88 – 2.76 (m, 1H), 2.28 – 2.22 (m, 1H), 2.14 (s, 1H), 1.87 – 1.75 (m, 1H), 1.62 – 1.57 (m, 1H), 1.52 (s, 9H), 1.35 – 1.29 (m, 1H), 1.02 (t, J = 7.0 Hz, 3H), 0.93 – 0.87 (m, 12H), 0.73 – 0.60 (m, 6H).

^{13}C NMR (151 MHz, $CDCl_3$): δ 172.59, 171.49, 170.96, 169.38, 169.14, 155.32, 136.74, 135.52, 134.73, 129.09, 128.62, 127.31, 124.23, 122.84, 120.30, 120.12, 120.04, 119.12, 118.48, 114.58, 112.46, 111.04, 79.69, 62.15, 61.96, 61.76, 60.54, 60.34, 60.13, 53.58, 52.61, 52.49, 47.63, 36.30, 36.18, 31.85, 29.79, 28.58, 28.35, 28.31, 28.29, 27.17, 25.32, 15.75, 11.09, 7.57, 7.54, 7.44, 4.62, 3.79, 1.11.

^{19}F NMR (565 MHz, $CDCl_3$): δ -181.37 (dq, J = 55.9, 21.2 Hz).

HRMS (ESI-TOF): calculated for $C_{47}H_{64}FN_7NaO_8Si^+$ $[M+Na]^+$: 924.4462, found: 924.4468.

$[\alpha]^{25}_D$: +58.8 (c = 0.5, $CHCl_3$)

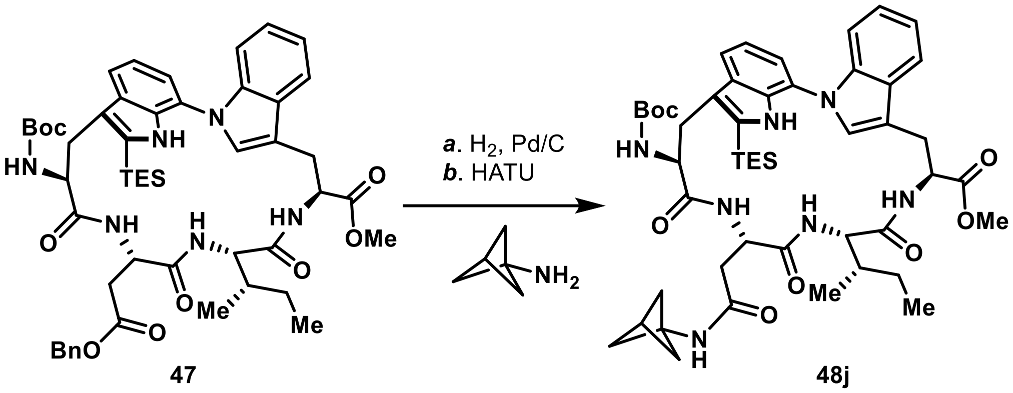

On 0.1 mmol scale, **General Procedure R** was followed with compound **47** via hydrogenation and amide coupling. Purification by silica gel column chromatography gave the title compound **48j** (73.8 mg, 81% yield).

Compound 48j

Physical State: amorphous solid

^{1}H NMR (600 MHz, CDCl$_3$): δ 8.13 (d, *J* = 3.8 Hz, 1H), 8.03 (t, *J* = 4.5 Hz, 1H), 7.95 (s, 1H), 7.75 (s, 1H), 7.64 (d, *J* = 7.7 Hz, 1H), 7.57 (d, *J* = 8.1 Hz, 1H), 7.30 (dd, *J* = 7.2, 1.3 Hz, 1H), 7.26 – 7.21 (m, 3H), 6.03 (s, 1H), 5.94 (d, *J* = 7.8 Hz, 1H), 5.88 (d, *J* = 6.9 Hz, 1H), 5.38 (d, *J* = 6.4 Hz, 1H), 4.95 (ddd, *J* = 12.1, 7.7, 2.3 Hz, 1H), 4.76 (ddd, *J* = 11.5, 6.8, 4.3 Hz, 1H), 4.68 (q, *J* = 6.4 Hz, 1H), 3.78 (s, 3H), 3.71 (dd, *J* = 13.8, 4.4 Hz, 1H), 3.54 (ddd, *J* = 9.2, 4.9, 2.3 Hz, 2H), 3.17 (ddd, *J* = 16.7, 12.1, 1.4 Hz, 1H), 2.83 (dd, *J* = 13.8, 12.0 Hz, 1H), 2.41 (s, 1H), 2.34 – 2.28 (m, 2H), 2.03 (s, 6H), 1.87 – 1.78 (m, 2H), 1.64 – 1.58 (m, 1H), 1.52 (s, 9H), 1.04 (d, *J* = 6.8 Hz, 3H), 0.97 – 0.88 (m, 12H), 0.75 – 0.60 (m, 6H).

^{13}C NMR (151 MHz, CDCl$_3$): δ 172.65, 171.66, 170.81, 169.44, 169.33, 155.29, 136.76, 135.53, 134.65, 129.12, 128.59, 127.35, 124.16, 122.81, 120.37, 120.09, 120.02, 119.15, 118.45, 112.38, 111.06, 79.59, 60.14, 53.64, 52.75, 52.62, 52.40, 48.55, 47.77, 39.68, 36.51, 31.91, 28.59, 27.24, 26.03, 25.26, 24.88, 15.75, 11.38, 7.54, 3.78.

HRMS (ESI-TOF): calculated for $C_{49}H_{67}N_7NaO_9Si^+$ [M+Na]$^+$: 932.4713, found: 932.4711.

$[\alpha]^{25}_D$: +37.4 (*c* = 0.5, CHCl$_3$)

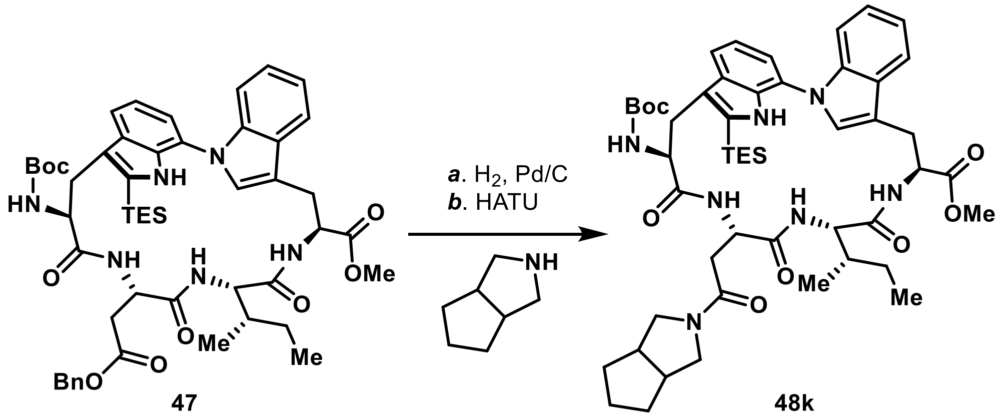

On 0.1 mmol scale, **General Procedure R** was followed with compound **47** via hydrogenation and amide coupling. Purification by silica gel column chromatography gave the title compound **48k** (64.8 mg, 69% yield).

Compound 48k

Physical State: amorphous solid

^{1}H NMR (600 MHz, CDCl$_3$): δ 8.79 – 8.53 (m, 1H), 8.03 (h, *J* = 3.9 Hz, 1H), 7.95 (s, 1H), 7.77 (s, 1H), 7.62 (d, *J* = 7.9 Hz, 1H), 7.55 (dd, *J* = 8.1, 2.9 Hz, 1H), 7.29 – 7.24 (m, 2H), 7.24 – 7.19 (m, 3H), 5.90 (ddd, *J* = 18.2, 7.3, 3.9 Hz, 2H), 5.43 (t, *J* = 6.3 Hz, 1H), 4.92 (dddd, *J* = 12.4, 10.0, 7.5, 2.3 Hz, 1H), 4.83 – 4.69 (m, 2H), 3.78 – 3.73 (m, 3H), 3.73 – 3.67 (m, 1H), 3.62 (dt, *J* = 12.6, 8.8 Hz, 1H), 3.55 – 3.49 (m, 2H), 3.46 (dd, *J* = 11.0, 8.2 Hz, 1H), 3.39 (dd, *J* = 11.0, 8.2 Hz, 1H), 3.19 – 3.12 (m, 2H), 2.96 (ddd, *J* = 15.5, 10.9, 4.8 Hz,

1H), 2.82 (ddd, J = 14.1, 11.9, 2.5 Hz, 1H), 2.66 – 2.53 (m, 2H), 2.48 – 2.41 (m, 1H), 2.37 – 2.27 (m, 1H), 1.85 – 1.66 (m, 5H), 1.62 – 1.56 (m, 2H), 1.51 (s, 9H), 1.39 – 1.33 (m, 2H), 1.02 (dd, J = 6.9, 2.7 Hz, 3H), 0.93 – 0.88 (m, 12H), 0.74 – 0.60 (m, 6H).
^{13}C NMR (151 MHz, CDCl$_3$): δ 172.67, 172.63, 171.09, 171.06, 169.70, 169.67, 169.59, 169.11, 169.08, 155.31, 136.75, 135.52, 135.49, 134.71, 129.19, 129.15, 128.61, 127.34, 124.17, 122.81, 122.79, 120.42, 120.39, 120.09, 119.99, 119.94, 119.14, 118.46, 112.42, 111.06, 79.54, 60.11, 60.07, 53.64, 53.60, 52.69, 52.66, 52.58, 52.48, 51.95, 51.88, 47.98, 47.90, 43.62, 43.60, 41.86, 41.83, 39.02, 38.97, 36.38, 36.34, 31.93, 31.81, 31.79, 31.76, 28.60, 28.37, 27.24, 27.18, 25.40, 25.37, 25.35, 25.31, 15.76, 15.75, 11.17, 11.13, 7.57, 3.80, 3.78.
HRMS (ESI-TOF): calculated for $C_{51}H_{71}N_7NaO_8Si^+$ [M+Na]$^+$: 960.5026, found: 960.5035.
[α]$^{25}_D$: +25.0 (c = 0.5, CHCl$_3$)

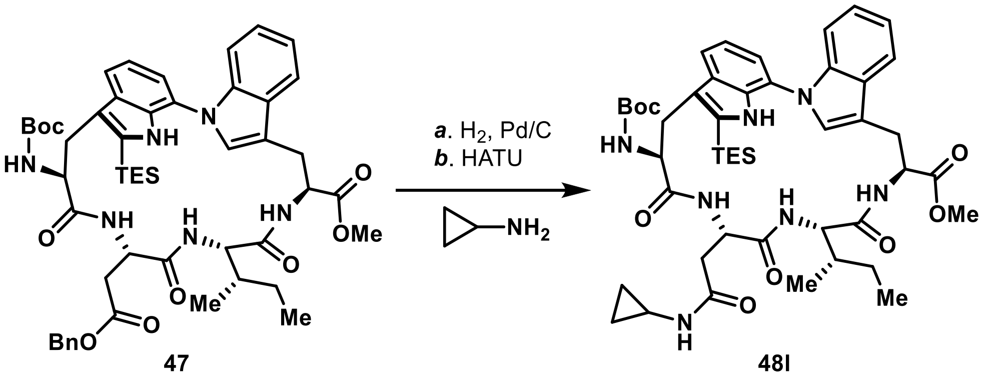

On 0.1 mmol scale, **General Procedure R** was followed with compound **47** via hydrogenation and amide coupling. Purification by silica gel column chromatography gave the title compound **48l** (74.3 mg, 84% yield).
Compound 48l
Physical State: amorphous solid
^{1}H NMR (600 MHz, CDCl$_3$): δ 8.16 (d, J = 4.1 Hz, 1H), 8.06 – 7.98 (m, 1H), 7.95 (d, J = 12.6 Hz, 1H), 7.72 (s, 1H), 7.62 (d, J = 7.8 Hz, 1H), 7.54 (d, J = 8.1 Hz, 1H), 7.27 (d, J = 7.3 Hz, 1H), 7.23 – 7.18 (m, 3H), 6.08 – 5.90 (m, 2H), 5.86 (dd, J = 27.7, 6.8 Hz, 1H), 5.39 (dd, J = 11.5, 6.4 Hz, 1H), 4.91 (ddd, J = 12.2, 7.7, 2.2 Hz, 1H), 4.74 (ddd, J = 11.7, 6.7, 4.3 Hz, 1H), 4.62 (ddd, J = 9.3, 6.4, 3.7 Hz, 1H), 3.75 (d, J = 4.0 Hz, 3H), 3.69 (dd, J = 14.0, 4.4 Hz, 2H), 3.57 – 3.48 (m, 2H), 3.20 – 3.10 (m, 1H), 2.81 (dd, J = 13.8, 11.7 Hz, 1H), 2.64 – 2.57 (m, 1H), 2.33 – 2.22 (m, 2H), 1.87 – 1.80 (m, 1H), 1.62 – 1.57 (m, 1H), 1.50 (s, 9H), 1.29 – 1.22 (m, 4H), 1.04 (d, J = 6.8 Hz, 3H), 0.93 – 0.86 (m, 12H), 0.72 – 0.61 (m, 6H).
^{13}C NMR (151 MHz, CDCl$_3$): δ 176.22, 172.81, 172.62, 172.44, 170.95, 169.57, 169.49, 169.39, 169.10, 155.27, 136.73, 135.51, 134.67, 129.13, 128.60, 127.32, 124.16, 122.80, 120.32, 120.09, 120.02, 119.97, 119.11, 118.46, 112.41, 111.37, 111.05, 79.62, 60.16, 53.63, 52.61, 52.45, 48.09, 39.68, 38.69, 36.37, 31.90, 28.59, 28.37, 27.21, 25.35, 24.44, 23.81, 22.88, 22.65, 15.76, 15.74, 11.23, 11.16, 7.57, 7.54, 6.53, 6.47, 3.80, 3.29.
HRMS (ESI-TOF): calculated for $C_{47}H_{65}N_7NaO_8Si^+$ [M+Na]$^+$: 906.4556, found: 906.4570.
[α]$^{25}_D$: +6.0 (c = 0.5, CHCl$_3$)

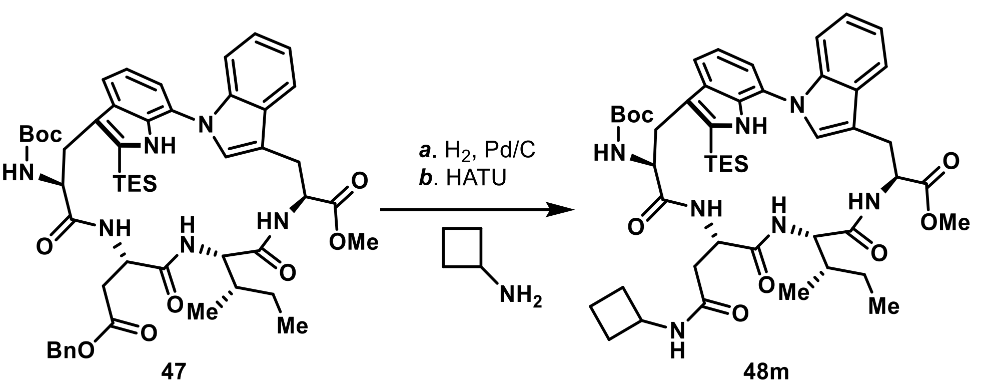

On 0.1 mmol scale, **General Procedure R** was followed with compound **47** via hydrogenation and amide coupling. Purification by silica gel column chromatography gave the title compound **48m** (77.3 mg, 86% yield).

Compound 48m

Physical State: amorphous solid

^{1}H NMR (600 MHz, $CDCl_3$): δ 8.37 – 8.21 (m, 1H), 8.01 (t, J = 4.5 Hz, 1H), 7.96 (s, 1H), 7.72 (s, 1H), 7.61 (d, J = 7.8 Hz, 1H), 7.54 (d, J = 8.1 Hz, 1H), 7.29 – 7.25 (m, 1H), 7.22 – 7.18 (m, 3H), 6.03 – 5.96 (m, 2H), 5.85 (d, J = 6.9 Hz, 1H), 5.39 (d, J = 6.3 Hz, 1H), 4.91 (ddd, J = 12.2, 7.7, 2.4 Hz, 1H), 4.75 (ddd, J = 11.7, 7.0, 4.4 Hz, 1H), 4.59 (ddd, J = 9.4, 6.3, 2.8 Hz, 1H), 4.29 – 4.20 (m, 1H), 3.74 (s, 3H), 3.70 – 3.65 (m, 1H), 3.55 – 3.49 (m, 2H), 3.15 (ddd, J = 16.7, 12.1, 1.5 Hz, 1H), 2.81 (dd, J = 13.7, 12.1 Hz, 1H), 2.31 – 2.20 (m, 4H), 1.84 – 1.75 (m, 4H), 1.60 (ddd, J = 13.8, 8.3, 3.3 Hz, 2H), 1.50 (s, 9H), 1.02 (d, J = 6.9 Hz, 3H), 0.93 – 0.87 (m, 12H), 0.72 – 0.60 (m, 6H).

^{13}C NMR (151 MHz, $CDCl_3$): δ 172.64, 170.97, 170.33, 169.49, 169.40, 155.28, 136.73, 135.52, 134.69, 129.13, 128.60, 127.34, 124.16, 122.80, 122.50, 120.32, 120.09, 120.01, 119.97, 119.12, 118.96, 118.47, 112.41, 111.05, 79.62, 60.15, 53.64, 52.60, 52.45, 52.41, 48.11, 45.02, 39.70, 36.35, 31.91, 31.01, 30.93, 30.90, 28.59, 28.36, 27.21, 25.30, 15.75, 15.24, 15.21, 11.19, 11.16, 7.54, 3.79, 3.29.

HRMS (ESI-TOF): calculated for $C_{48}H_{67}N_7NaO_8Si^+$ $[M+Na]^+$: 920.4713, found: 920.4721.

$[\alpha]^{25}_D$: +62.6 (c = 0.5, $CHCl_3$)

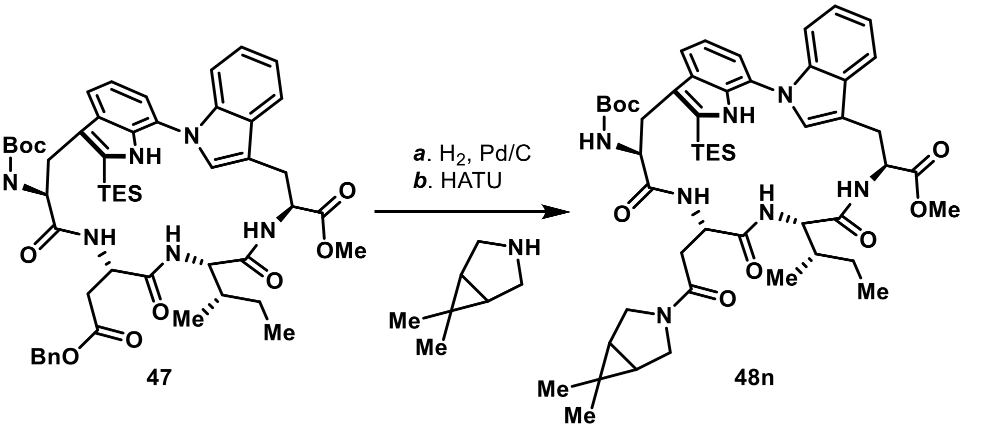

On 0.1 mmol scale, **General Procedure R** was followed with compound **47** via hydrogenation and amide coupling. Purification by silica gel column chromatography gave the title compound **48n** (56.3 mg, 60% yield).

Compound 48n

Physical State: amorphous solid

^{1}H NMR (600 MHz, $CDCl_3$, 25 °C): rotamers (not an impurity). δ 8.64 – 7.94 (m, 1H), 7.89 – 7.02 (m, 8H), 7.02 – 6.70 (m, 1H), 5.99 – 5.83 (m, 1H), 5.07 – 4.82 (m, 1H), 4.82 – 4.50 (m, 1H), 4.36 – 3.85 (m, 1H), 3.83 – 3.59 (m, 4H), 3.57 – 3.11 (m, 5H), 2.94 – 2.65 (m, 1H), 2.55 – 2.22 (m, 1H), 2.18 – 1.88 (m, 1H), 1.85 – 1.69 (m, 1H), 1.67 – 1.55 (m, 1H), 1.54 – 1.39 (m, 9H), 1.36 – 1.18 (m, 4H), 1.10 – 0.58 (m, 26H), 0.57 – 0.34 (m, 4H).

^{13}C NMR (151 MHz, CDCl$_3$, 25 °C): rotamers (not an impurity). δ 172.74, 172.63, 172.37, 170.98, 170.85, 169.63, 169.58, 169.41, 169.26, 169.21, 169.13, 169.06, 155.28, 143.51, 136.84, 136.74, 136.68, 135.49, 134.70, 129.20, 129.10, 128.60, 128.21, 127.32, 127.27, 124.16, 122.81, 122.74, 122.43, 120.42, 120.35, 120.06, 119.98, 119.91, 119.14, 118.89, 118.45, 116.43, 112.42, 111.06, 110.87, 80.26, 79.56, 60.09, 60.04, 58.54, 54.77, 53.67, 53.57, 52.95, 52.60, 52.42, 52.35, 50.04, 47.89, 47.82, 47.15, 46.98, 46.90, 46.82, 46.45, 46.40, 46.17, 39.05, 38.95, 38.69, 36.87, 36.42, 31.90, 28.59, 28.37, 28.01, 27.88, 27.81, 27.70, 27.32, 27.18, 26.75, 26.65, 26.46, 26.20, 26.16, 26.13, 25.32, 25.08, 24.52, 20.18, 19.44, 19.38, 19.29, 15.83, 15.75, 15.66, 15.58, 12.57, 12.53, 12.39, 11.58, 11.31, 11.25, 11.18, 7.56, 7.53, 3.80, 3.76, 3.29.

^{1}H NMR (400 MHz, DMSO-*d$_6$*, 25 °C): rotamers (not an impurity). δ 9.32 – 8.27 (m, 1H), 8.18 – 7.80 (m, 1H), 7.80 – 7.35 (m, 2H), 7.31 – 6.58 (m, 7H), 4.83 – 4.35 (m, 3H), 4.32 – 3.88 (m, 1H), 3.77 – 3.37 (m, 5H), 3.29 – 2.83 (m, 4H), 2.74 – 2.54 (m, 1H), 2.47 – 2.14 (m, 1H), 1.75 – 1.49 (m, 2H), 1.45 – 1.34 (m, 9H), 1.31 – 1.07 (m, 5H), 1.05 – 0.59 (m, 24H), 0.54 – 0.33 (m, 5H).

^{1}H NMR (400 MHz, DMSO-*d$_6$*, 60 °C): rotamers (not an impurity). δ 9.14 – 8.15 (m, 1H), 8.06 – 7.83 (m, 1H), 7.78 – 7.55 (m, 1H), 7.50 – 7.35 (m, 1H), 7.29 – 6.54 (m, 7H), 4.75 – 4.31 (m, 3H), 4.29 – 3.83 (m, 1H), 3.78 – 3.22 (m, 8H), 3.00 – 2.54 (m, 1H), 2.43 – 2.10 (m, 1H), 1.83 – 1.53 (m, 2H), 1.52 – 1.06 (m, 15H), 1.05 – 0.61 (m, 24H), 0.58 – 0.34 (m, 5H).

^{13}C NMR (151 MHz, DMSO-*d$_6$*, 25 °C): rotamers (not an impurity). δ 172.93, 172.51, 171.29, 144.90, 136.70, 129.25, 128.40, 123.79, 122.44, 119.94, 119.08, 116.90, 116.40, 111.67, 111.01, 78.51, 57.14, 52.28, 49.71, 46.56, 46.09, 28.79, 28.68, 27.91, 27.12, 26.42, 24.20, 20.25, 19.19, 15.68, 12.84, 12.76, 11.84, 10.85, 8.03, 7.88, 3.88, 3.35.

HRMS (ESI-TOF): calculated for $C_{51}H_{71}N_7NaO_8Si^+$ [M+Na]$^+$: 960.5026, found: 960.5027.

[α]$^{25}_{D}$: +0.9 (*c* = 0.5, CHCl$_3$)

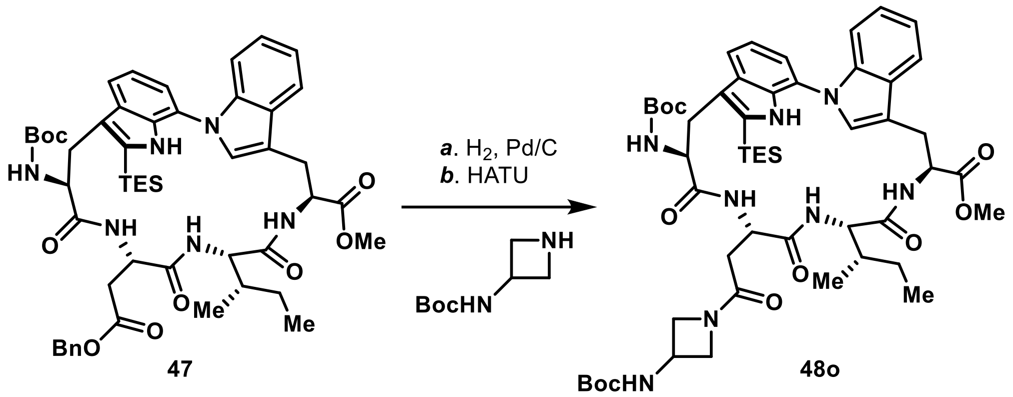

On 0.1 mmol scale, **General Procedure R** was followed with compound **47** via hydrogenation and amide coupling. Purification by silica gel column chromatography gave the title compound **480** (63.0 mg, 63% yield).

Compound 48o

Physical State: amorphous solid

^{1}H NMR (600 MHz, CDCl$_3$, 25 °C): rotamers (not an impurity). δ 8.47 – 7.33 (m, 4H), 7.31 – 6.74 (m, 6H), 6.17 – 4.95 (m, 2H), 4.94 – 4.13 (m, 5H), 3.91 – 3.44 (m, 7H), 3.31 – 2.73 (m, 1H), 2.51 – 1.89 (m, 3H), 1.82 – 1.72 (m, 1H), 1.62 – 1.55 (m, 1H), 1.52 – 1.39 (m, 18H), 1.37 – 1.23 (m, 2H), 1.05 – 0.99 (m, 2H), 0.97 – 0.57 (m, 18H), 0.54 – 0.38 (m, 3H).

^{13}C NMR (151 MHz, CDCl$_3$, 25 °C): rotamers (not an impurity). δ 172.66, 172.37, 171.06, 170.96, 169.45, 169.37, 169.09, 155.32, 154.98, 154.92, 136.73, 136.70, 135.51, 134.74, 134.65, 129.21, 129.17, 129.11, 128.62, 128.59, 128.17, 127.47, 127.36, 124.23, 124.19, 122.89, 122.81, 122.47, 120.45, 120.28, 120.18, 120.11, 120.01, 119.84, 119.14, 119.05, 118.47, 112.43, 112.13, 111.05, 80.12, 79.63, 60.22, 60.12, 58.66, 58.20, 56.13, 53.66, 53.62, 52.67, 52.59, 52.55, 52.46, 48.04, 47.85, 40.19, 39.89, 36.92, 36.39, 36.27, 35.74, 35.63, 31.88, 31.79, 28.60, 28.58, 28.45, 28.41, 28.37, 27.17, 27.10, 25.47, 25.34, 24.72, 24.62, 20.16, 15.85,

15.74, 11.58, 11.18, 11.13, 11.09, 7.56, 7.53, 7.43, 3.84, 3.79, 3.29.
^{1}H NMR (400 MHz, DMSO-d_6, 25 °C): rotamers (not an impurity). δ 9.34 – 7.90 (m, 3H), 7.73 – 7.41 (m, 3H), 7.38 – 6.41 (m, 7H), 5.80 – 5.26 (m, 1H), 4.75 – 4.35 (m, 3H), 4.32 – 4.13 (m, 2H), 3.99 – 3.90 (m, 1H), 3.77 – 3.53 (m, 5H), 3.28 – 3.02 (m, 2H), 2.45 – 2.19 (m, 1H), 2.16 – 1.87 (m, 1H), 1.74 – 1.48 (m, 2H), 1.46 – 1.31 (m, 18H), 1.27 – 1.09 (m, 2H), 0.99 – 0.68 (m, 20H), 0.55 – 0.31 (m, 3H).
^{1}H NMR (400 MHz, DMSO-d_6, 60 °C): rotamers (not an impurity). δ 9.09 – 8.28 (m, 1H), 8.04 – 7.54 (m, 3H), 7.50 – 7.37 (m, 1H), 7.34 – 6.39 (m, 7H), 5.55 – 5.21 (m, 1H), 4.73 – 4.45 (m, 3H), 4.35 – 4.09 (m, 2H), 4.09 – 3.86 (m, 1H), 3.81 – 3.53 (m, 5H), 3.38 – 3.27 (m, 1H), 3.06 – 2.87 (m, 1H), 2.38 (d, *J* = 16.8 Hz, 1H), 2.18 – 1.89 (m, 1H), 1.75 – 1.51 (m, 2H), 1.50 – 1.33 (m, 18H), 1.32 – 1.11 (m, 2H), 1.07 – 0.67 (m, 20H), 0.59 – 0.38 (m, 3H).
^{13}C NMR (151 MHz, DMSO-d_6, 25 °C): rotamers (not an impurity). δ 172.91, 172.51, 171.23, 169.94, 169.31, 155.45, 144.89, 136.70, 136.18, 135.71, 134.94, 130.18, 129.26, 128.40, 128.25, 124.45, 123.80, 122.72, 122.44, 120.19, 119.95, 119.12, 116.92, 116.41, 112.45, 111.63, 110.71, 110.71, 79.72, 78.81, 58.98, 57.52, 54.22, 52.79, 52.49, 52.49, 52.29, 36.37, 28.85, 28.81, 28.66, 26.42, 25.58, 24.28, 20.30, 15.75, 15.66, 11.96, 11.92, 11.79, 10.83, 8.08, 8.07, 7.87, 3.98, 3.94, 3.35.
HRMS (ESI-TOF): calculated for $C_{52}H_{74}N_8NaO_{10}Si^+$ [M+Na]$^+$: 1021.5189 found: 1021.5191.
[α]$^{25}_D$: +3.1 (*c* = 0.5, $CHCl_3$)
HRMS (ESI-TOF): calculated for $C_{52}H_{74}N_8NaO_{10}Si^+$ [M+Na]$^+$: 1021.5189 found: 1021.5191.
[α]$^{25}_D$: +3.1 (*c* = 0.5, $CHCl_3$)

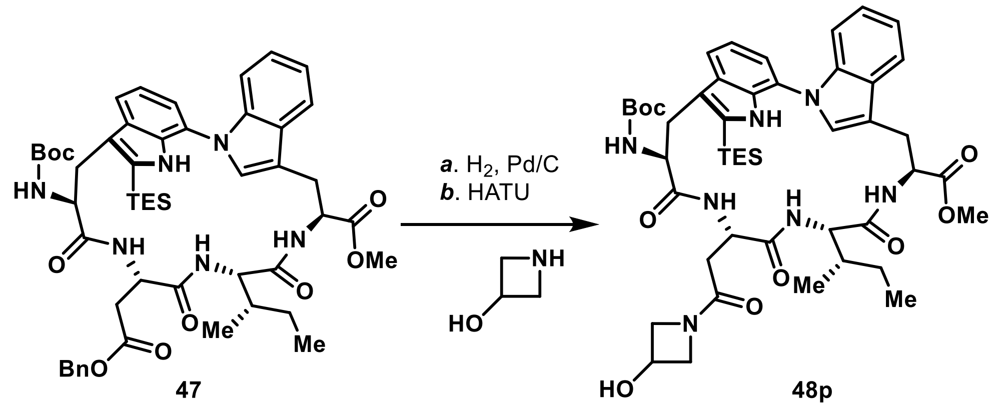

On 0.1 mmol scale, **General Procedure R** was followed with compound **47** via hydrogenation and amide coupling. Purification by silica gel column chromatography gave the title compound **48p** (52.2 mg, 58% yield).
Compound 48p
Physical State: amorphous solid
^{1}H NMR (600 MHz, $CDCl_3$): δ 8.28 – 7.97 (m, 2H), 7.94 – 7.86 (m, 1H), 7.80 – 7.71 (m, 1H), 7.62 (dd, *J* = 8.0, 3.2 Hz, 1H), 7.56 (d, *J* = 8.0 Hz, 1H), 7.29 (t, *J* = 7.7 Hz, 1H), 7.24 – 7.13 (m, 4H), 6.16 – 5.88 (m, 1H), 5.87 – 5.79 (m, 1H), 5.40 – 5.04 (m, 1H), 4.92 – 4.61 (m, 3H), 4.56 – 4.36 (m, 1H), 4.14 (ddd, *J* = 28.7, 9.7, 6.6 Hz, 2H), 3.88 (dd, *J* = 10.9, 4.0 Hz, 1H), 3.78 (s, 3H), 3.69 (dd, *J* = 13.9, 4.7 Hz, 1H), 3.55 – 3.46 (m, 2H), 3.24 – 3.16 (m, 1H), 2.87 – 2.79 (m, 1H), 2.28 (dd, *J* = 15.2, 4.5 Hz, 1H), 1.98 (dd, *J* = 15.3, 11.5 Hz, 1H), 1.90 – 1.77 (m, 2H), 1.76 – 1.71 (m, 1H), 1.61 – 1.56 (m, 1H), 1.51 (d, *J* = 3.3 Hz, 9H), 1.34 – 1.27 (m, 1H), 1.02 (d, *J* = 6.8 Hz, 3H), 0.94 – 0.87 (m, 12H), 0.73 – 0.60 (m, 6H).
^{13}C NMR (151 MHz, $CDCl_3$): δ 172.08, 171.69, 171.17, 169.78, 168.97, 155.32, 136.81, 135.52, 134.54, 129.16, 128.48, 127.34, 124.16, 123.03, 120.51, 120.27, 120.02, 119.94, 119.21, 118.49, 111.90, 111.08, 79.67, 61.22, 60.86, 60.26, 58.75, 53.76, 52.89, 52.77, 52.63, 48.24, 36.35, 35.62, 31.89, 28.58, 26.85, 25.66, 15.72, 15.60, 11.11, 7.60, 7.54, 3.91, 3.79.
HRMS (ESI-TOF): calculated for $C_{47}H_{65}N_7NaO_9Si^+$ [M+Na]$^+$: 922.4505, found: 922.4531.
[α]$^{25}_D$: +38.7 (*c* = 0.5, $CHCl_3$)

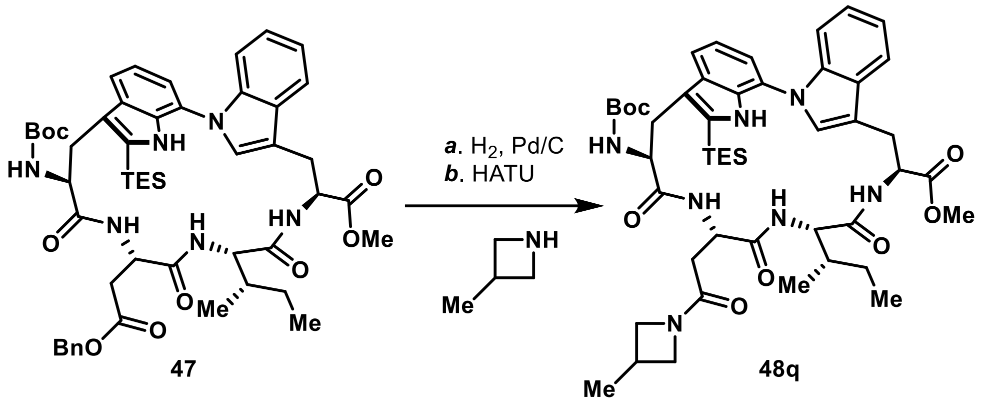

On 0.1 mmol scale, **General Procedure R** was followed with compound **47** via hydrogenation and amide coupling. Purification by silica gel column chromatography gave the title compound **48q** (46.7 mg, 52% yield).

Compound 48q

Physical State: amorphous solid

^{1}H NMR (600 MHz, CDCl$_3$, 25 °C): rotamers (not an impurity). δ 8.79 – 7.98 (m, 1H), 7.97 – 7.06 (m, 9H), 6.98 – 6.72 (m, 1H), 5.98 – 5.68 (m, 1H), 5.45 – 4.25 (m, 3H), 4.18 – 3.94 (m, 2H), 3.86 – 3.57 (m, 4H), 3.56 – 3.46 (m, 2H), 3.43 – 3.22 (m, 1H), 3.20 – 2.74 (m, 1H), 2.71 – 2.50 (m, 1H), 2.37 – 1.95 (m, 2H), 1.87 – 1.69 (m, 1H), 1.63 – 1.54 (m, 1H), 1.53 – 1.39 (m, 9H), 1.38 – 1.27 (m, 2H), 1.22 – 1.16 (m, 2H), 1.14 – 0.97 (m, 3H), 0.95 – 0.75 (m, 14H), 0.74 – 0.58 (m, 3H), 0.56 – 0.34 (m, 4H).

^{13}C NMR (151 MHz, CDCl$_3$, 25 °C): rotamers (not an impurity). δ 172.65, 172.63, 172.45, 172.36, 171.07, 171.01, 170.97, 170.81, 169.55, 169.19, 169.15, 155.75, 155.31, 136.75, 135.51, 134.76, 129.13, 128.61, 128.19, 127.36, 124.18, 122.79, 122.38, 120.38, 120.35, 120.08, 120.04, 119.98, 119.95, 119.14, 118.82, 118.45, 116.48, 112.41, 111.05, 110.95, 80.23, 79.56, 60.14, 60.12, 58.65, 57.01, 56.90, 54.89, 54.86, 54.77, 53.61, 52.80, 52.57, 52.48, 52.37, 47.78, 47.69, 36.87, 36.28, 36.26, 35.45, 35.38, 31.89, 29.78, 28.59, 28.36, 27.21, 25.35, 24.52, 23.74, 23.66, 20.18, 19.55, 19.47, 19.38, 15.75, 11.55, 11.19, 11.07, 11.04, 7.53, 3.79, 3.29.

^{1}H NMR (400 MHz, DMSO-d_6, 25 °C): rotamers (not an impurity). δ 9.38 – 7.33 (m, 6H), 7.27 – 6.37 (m, 6H), 5.95 – 5.21 (m, 1H), 4.81 – 3.98 (m, 4H), 3.97 – 3.83 (m, 1H), 3.77 – 3.36 (m, 5H), 3.31 – 2.87 (m, 3H), 2.65 – 2.51 (m, 1H), 2.47 – 2.21 (m, 1H), 2.06 – 1.97 (m, 1H), 1.74 – 1.51 (m, 2H), 1.49 – 1.33 (m, 9H), 1.30 – 1.22 (m, 1H), 1.17 – 1.08 (m, 3H), 0.99 – 0.63 (m, 20H), 0.54 – 0.35 (m, 3H).

^{1}H NMR (400 MHz, DMSO-d_6, 60 °C): rotamers (not an impurity). δ 9.19 – 7.32 (m, 6H), 7.29 – 6.47 (m, 6H), 5.74 – 5.25 (m, 1H), 4.72 – 4.40 (m, 3H), 4.29 – 3.65 (m, 5H), 3.64 – 3.43 (m, 2H), 3.41 – 3.28 (m, 2H), 3.11 – 2.86 (m, 1H), 2.67 – 2.54 (m, 1H), 2.46 – 2.27 (m, 1H), 2.11 – 2.01 (m, 1H), 1.77 – 1.54 (m, 2H), 1.49 – 1.35 (m, 9H), 1.33 – 1.23 (m, 1H), 1.21 – 1.08 (m, 3H), 0.99 – 0.67 (m, 20H), 0.58 – 0.36 (m, 3H).

^{13}C NMR (151 MHz, DMSO-d_6, 25 °C): rotamers (not an impurity). δ 172.94, 172.53, 171.47, 171.33, 171.20, 170.05, 169.90, 169.30, 144.90, 136.69, 136.18, 135.68, 134.83, 130.15, 129.01, 128.70, 128.40, 128.23, 123.79, 122.73, 122.44, 120.40, 120.19, 119.95, 119.11, 116.91, 116.40, 112.57, 111.62, 110.73, 79.72, 78.71, 78.67, 78.51, 58.92, 56.92, 54.71, 52.84, 52.50, 36.37, 35.40, 28.79, 28.68, 28.68, 26.40, 23.32, 20.28, 19.81, 19.59, 15.80, 15.66, 11.91, 11.80, 8.07, 7.88, 3.93, 3.93, 3.34.

HRMS (ESI-TOF): calculated for $C_{48}H_{67}N_7NaO_8Si^+$ [M+Na]$^+$: 920.4713, found: 920.4713.

$[\alpha]^{25}_{D}$: +2.1 (c = 0.5, $CHCl_3$)

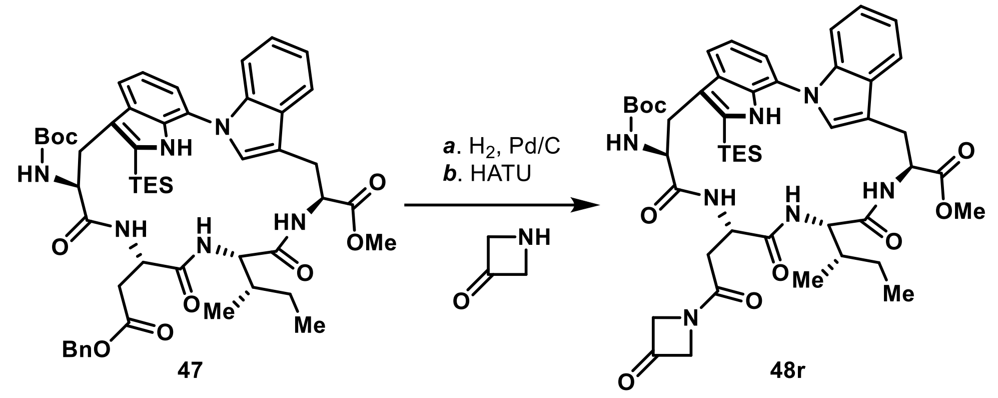

On 0.1 mmol scale, **General Procedure R** was followed with compound **47** via hydrogenation and amide coupling. Purification by silica gel column chromatography gave the title compound **48r** (49.4 mg, 55% yield).

Compound 48r

Physical State: amorphous solid

^{1}H NMR (600 MHz, CDCl$_3$): δ 8.01 (dd, J = 5.3, 3.6 Hz, 1H), 7.95 (s, 1H), 7.93 (d, J = 4.1 Hz, 1H), 7.72 (s, 1H), 7.62 (d, J = 7.8 Hz, 1H), 7.55 (d, J = 8.2 Hz, 1H), 7.30 – 7.26 (m, 1H), 7.24 – 7.20 (m, 3H), 5.90 (d, J = 7.6 Hz, 1H), 5.81 (d, J = 6.9 Hz, 1H), 5.37 (d, J = 6.4 Hz, 1H), 4.88 (ddd, J = 12.2, 7.6, 2.4 Hz, 1H), 4.80 – 4.74 (m, 3H), 4.74 – 4.72 (m, 2H), 4.70 – 4.61 (m, 1H), 3.76 (s, 3H), 3.73 – 3.68 (m, 1H), 3.54 – 3.49 (m, 2H), 3.15 (ddd, J = 16.8, 12.2, 1.5 Hz, 1H), 2.86 – 2.80 (m, 1H), 2.43 (dd, J = 16.6, 2.6 Hz, 1H), 2.33 (dd, J = 16.6, 10.4 Hz, 1H), 1.84 – 1.76 (m, 2H), 1.63 – 1.57 (m, 1H), 1.51 (s, 9H), 1.03 (d, J = 6.8 Hz, 3H), 0.94 – 0.88 (m, 12H), 0.74 – 0.60 (m, 6H).

^{13}C NMR (151 MHz, CDCl$_3$): δ 193.36, 172.54, 171.25, 170.87, 169.39, 169.25, 155.33, 136.76, 135.53, 134.74, 129.09, 128.61, 127.31, 124.24, 122.88, 120.31, 120.16, 120.04, 119.12, 118.48, 112.39, 111.05, 79.72, 71.35, 70.35, 60.17, 53.59, 52.63, 52.47, 47.87, 38.69, 37.13, 36.33, 31.84, 28.58, 27.20, 25.38, 15.74, 11.12, 7.54, 3.82.

HRMS (ESI-TOF): calculated for $C_{47}H_{63}N_7NaO_9Si^+$ $[M+Na]^+$: 920.4349, found: 920.4359.

$[\alpha]^{25}_D$: +24.4 (c = 0.5, $CHCl_3$)

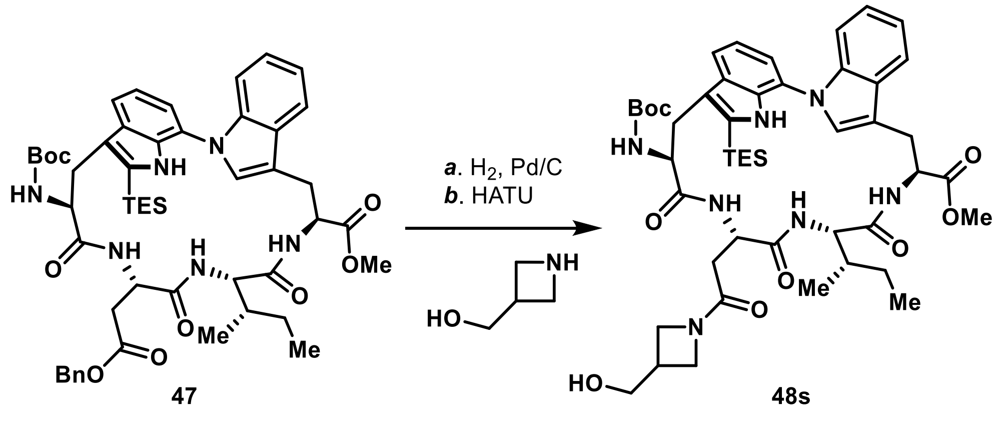

On 0.1 mmol scale, **General Procedure R** was followed with compound **47** via hydrogenation and amide coupling. Purification by silica gel column chromatography gave the title compound **48s** (44.8 mg, 49% yield).

Compound 48s

Physical State: amorphous solid

^{1}H NMR (600 MHz, CDCl$_3$): rotamers (not an impurity). δ 8.23 – 7.97 (m, 1H), 7.75 (s, 1H), 7.64 (m, 1H), 7.58 – 7.54 (m, 1H), 7.30 – 7.26 (m, 1H), 7.25 – 7.10 (m, 5H), 6.08 – 5.80 (m, 1H), 5.52 – 4.47 (m, 3H), 4.08 – 3.94 (m, 2H), 3.80 – 3.75 (m, 3H), 3.74 – 3.63 (m, 4H), 3.55 – 3.29 (m, 2H), 3.22 – 3.08 (m, 1H), 2.88 – 2.56 (m, 3H), 2.28 – 2.21 (m, 1H), 1.52 – 1.43 (m, 9H), 1.36 – 1.25 (m, 4H), 1.05 – 1.01 (m, 2H), 0.94 – 0.87 (m, 16H), 0.71 – 0.64 (m, 3H), 0.48 (q, J = 7.9 Hz, 3H).

^{13}C NMR (151 MHz, CDCl$_3$): rotamers (not an impurity).δ 172.71, 172.62, 172.49, 171.43, 171.11, 169.59, 169.46, 169.31, 169.17, 155.89, 155.32, 136.72, 135.49, 134.75, 129.16, 128.58, 127.33, 124.17, 122.85, 122.42, 120.36, 120.27, 120.13, 119.97, 119.13, 118.85, 118.47, 116.57, 112.43, 111.05, 80.32, 79.65, 63.59, 63.37, 63.11, 60.15, 58.79, 54.76, 53.65, 52.64, 52.49, 52.43, 50.25, 49.86, 47.86, 38.70, 36.76, 36.29, 36.22, 35.42, 35.29, 31.86, 30.30, 30.15, 28.59, 28.36, 27.57, 27.18, 27.10, 25.43, 25.34, 24.52, 20.22, 15.68, 11.57, 11.13, 11.00, 7.54, 4.10, 3.79, 3.28, 3.11.

^{1}H NMR (400 MHz, DMSO-*d$_6$*, rt): rotamers (not an impurity). δ 9.23 – 8.87 (m, 1H), 8.44 – 8.39 (m, 1H), 8.09 – 7.42 (m, 4H), 7.31 – 6.67 (m, 6H), 5.65 – 5.57 (m, 1H), 4.86 – 4.35 (m, 4H), 4.24 – 4.01 (m, 1H), 3.98 – 3.88 (m, 1H), 3.79 – 3.55 (m, 5H), 3.53 – 3.39 (m, 3H), 3.31 – 3.09 (m, 2H), 2.99 – 2.90 (m, 1H), 2.64 – 2.53 (m, 1H), 2.44 – 2.24 (m, 1H), 2.13 – 1.93 (m, 1H), 1.73 – 1.47 (m, 2H), 1.45 – 1.35 (m, 9H), 1.30 – 1.08 (m, 2H), 0.97 – 0.64 (m, 18H), 0.50 -0.40 (m, 3H).

^{1}H NMR (400 MHz, DMSO-*d$_6$*, 60 °C): rotamers (not an impurity). δ 9.03 – 8.95 (m, 1H), 8.32 – 8.22 (m, 1H), 8.04 – 7.38 (m, 4H), 7.31 – 6.45 (m, 6H), 5.48 – 5.40 (m, 1H), 4.64 – 4.42 (m, 4H), 4.25 – 3.86 (m, 2H), 3.82 – 3.55 (m, 5H), 3.53 – 3.43 (m, 2H), 3.35 – 3.19 (m, 2H), 2.98 – 2.89 (m, 1H), 2.73 – 2.67 (m, 1H), 2.63 – 2.55 (m, 1H), 2.40 – 2.28 (m, 1H), 2.09 – 2.00 (m, 1H), 1.79 – 1.54 (m, 2H), 1.47 – 1.37 (m, 9H), 1.32 – 1.11 (m, 2H), 1.06 – 0.59 (m, 18H), 0.53 – 0.41 (m, 3H).

^{13}C NMR (151 MHz, DMSO-*d$_6$*, rt): rotamers (not an impurity). δ 172.93, 172.52, 171.26, 169.99, 169.22, 155.42, 136.69, 136.17, 129.26, 129.01, 124.39, 122.73, 120.18, 119.95, 119.32, 119.09, 116.90, 116.40, 112.52, 110.74, 79.71, 78.68, 63.08, 62.96, 52.82, 52.51, 52.29, 50.21, 40.57, 38.78, 36.38, 30.48, 30.37, 28.80, 28.68, 27.10, 26.41, 25.33, 20.28, 15.81, 15.65, 11.90, 10.82, 8.07, 7.88, 3.92, 3.34.

HRMS (ESI-TOF): calculated for $C_{48}H_{67}N_7NaO_9Si^+$ [M+Na]$^+$: 936.4662, found: 936.4656

[α]$^{25}_{D}$: +0.4 (*c* = 0.5, CHCl$_3$)

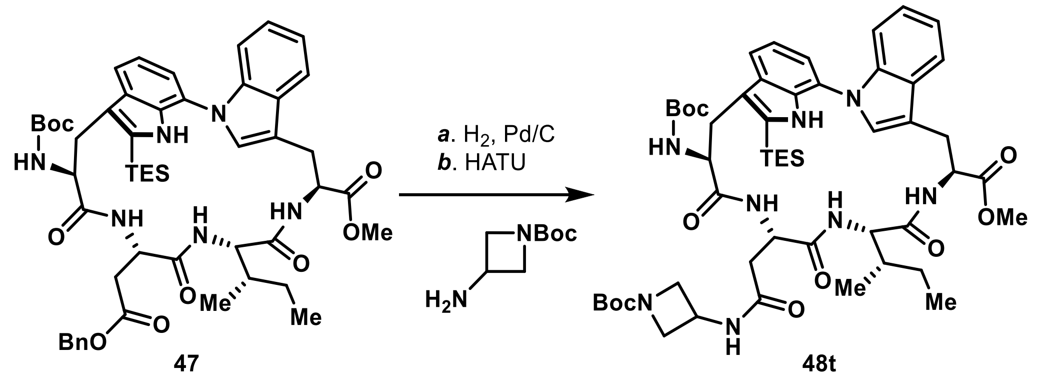

On 0.1 mmol scale, **General Procedure R** was followed with compound **47** via hydrogenation and amide coupling. Purification by silica gel column chromatography gave the title compound **48t** (86.0 mg, 86% yield).

Compound 48t

Physical State: amorphous solid

^{1}H NMR (600 MHz, CDCl$_3$): δ 8.00 (dd, *J* = 6.1, 2.9 Hz, 1H), 7.96 (d, *J* = 4.0 Hz, 1H), 7.93 (s, 1H), 7.71 (s, 1H), 7.61 (d, *J* = 7.9 Hz, 1H), 7.54 (d, *J* = 8.2 Hz, 1H), 7.28 – 7.25 (m, 1H), 7.23 – 7.19 (m, 3H), 5.97 (d, *J* = 7.7 Hz, 1H), 5.84 (d, *J* = 6.9 Hz, 1H), 5.38 (d, *J* = 6.4 Hz, 1H), 4.90 (ddd, *J* = 12.1, 7.8, 2.4 Hz, 1H), 4.76 (ddd, *J* = 11.7, 7.0, 4.4 Hz, 1H), 4.57 (q, *J* = 6.3 Hz, 1H), 4.46 (q, *J* = 6.3 Hz, 1H), 4.08 (t, *J* = 8.5 Hz, 2H), 3.75 (s, 3H), 3.72 – 3.68 (m, 2H), 3.65 – 3.59 (m, 1H), 3.55 – 3.48 (m, 2H), 3.17 – 3.11 (m, 1H), 2.81 (dd, *J* = 13.9, 12.0 Hz, 1H), 2.38 – 2.31 (m, 2H), 2.21 – 1.99 (m, 1H), 1.84 – 1.76 (m, 1H), 1.60 – 1.55 (m, 1H), 1.49 (s, 9H), 1.36 (s, 9H), 1.31 – 1.26 (m, 1H), 1.02 (d, *J* = 6.8 Hz, 3H), 0.94 – 0.85 (m, 12H), 0.73 – 0.58 (m, 6H).

^{13}C NMR (151 MHz, CDCl$_3$): δ 172.56, 171.21, 170.95, 169.50, 169.35, 156.09, 155.26, 136.73, 135.54, 134.61, 129.10, 128.60, 127.29, 124.15, 122.82, 120.29, 120.11, 120.05, 120.03, 119.09, 118.48, 112.38, 111.05, 79.89, 79.70, 60.15, 53.64, 52.63, 52.45, 48.06, 39.68, 39.64, 38.69, 36.39, 31.87, 28.59, 28.36, 27.19, 25.31, 15.75, 11.23, 7.54, 3.80.

HRMS (ESI-TOF): calculated for $C_{52}H_{74}N_8NaO_{10}Si^+$ $[M+Na]^+$: 1021.5189, found: 1021.5196.
$[\alpha]^{25}_D$: +42.2 (*c* = 0.5, $CHCl_3$)

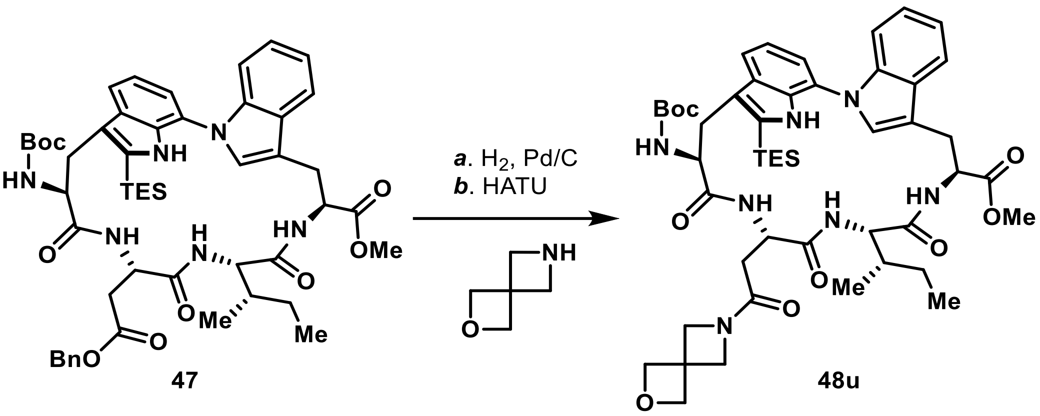

On 0.1 mmol scale, **General Procedure R** was followed with compound **47** via hydrogenation and amide coupling. Purification by silica gel column chromatography gave the title compound **48u** (65.8 mg, 71% yield).
Compound 48u
Physical State: amorphous solid
^{1}H NMR (600 MHz, $CDCl_3$): rotamers (not an impurity). δ 8.13 – 7.92 (m, 2H), 7.75 – 7.72 (m, 1H), 7.66 – 7.61 (m, 1H), 7.60 – 7.54 (m, 1H), 7.33 – 7.27 (m, 1H), 7.24 – 7.14 (m, 4H), 5.94 – 5.83 (m, 1H), 5.37 – 5.34 (m, 1H), 4.94 – 4.84 (m, 1H), 4.79 – 4.69 (m, 4H), 4.66 – 4.60 (m, 1H), 4.17 - 4.06 (m, 4H), 3.82 – 3.68 (m, 5H), 3.54 – 3.48 (m, 1H), 3.33 – 3.09 (m, 1H), 2.87 – 2.76 (m, 1H), 2.28 – 2.05 (m, 2H), 1.50 – 1.39 (m, 9H), 1.05 – 1.00 (m, 2H), 0.94 – 0.85 (m, 18H), 0.74 – 0.60 (m, 3H), 0.51 – 0.45 (m, 3H).
^{13}C NMR (151 MHz, $CDCl_3$): rotamers (not an impurity). δ 172.56, 172.46, 171.22, 171.01, 170.98, 170.72, 169.41, 169.22, 155.72, 155.32, 136.76, 135.49, 134.74, 129.13, 128.59, 128.21, 127.34, 124.21, 122.87, 122.46, 120.34, 120.15, 120.07, 120.00, 119.97, 119.14, 118.72, 118.47, 117.39, 116.45, 112.40, 111.69, 111.07, 80.77, 80.69, 80.63, 80.24, 79.66, 60.09, 59.82, 59.78, 59.21, 58.58, 57.79, 57.69, 54.68, 53.61, 52.91, 52.70, 52.58, 52.53, 52.34, 48.56, 47.72, 37.68, 37.39, 36.87, 36.27, 35.56, 31.87, 29.78, 28.59, 28.45, 28.36, 27.91, 27.13, 26.50, 25.42, 25.07, 24.60, 20.20, 15.71, 11.56, 11.17, 11.07, 7.60, 7.54, 7.50, 3.80, 3.77, 3.29, 0.08.
^{1}H NMR (400 MHz, DMSO-*d_6*, rt): rotamers (not an impurity). δ 9. 16 – 9.14 (m, 1H), 8.40 – 8.37 (m, 1H), 8.07 – 7.82 (m, 1H), 7.73 – 7.43 (m, 2H), 7.27 – 7.01 (m, 4H), 7.00 – 6.63 (m, 2H), 5.50 – 5.47 (m, 1H), 4.74 – 4.43 (m, 6H), 4.41 – 3.82 (m, 5H), 3.74 – 3.54 (m, 3H), 3.32 – 3.09 (m, 3H), 2.45 – 2.23 (m, 1H), 2.01 – 1.96 (m, 1H), 1.76 – 1.50 (m, 2H), 1.44 – 1.35 (m, 9H), 1.31 – 1.11 (m, 2H), 1.02 – 0.56 (m, 20H), 0.51 – 0.40 (m, 3H).
^{1}H NMR (400 MHz, DMSO-*d_6*, 60 °C): rotamers (not an impurity). δ 9.06 – 9.00 (m, 1H), 8.41 – 8.08 (m, 1H), 7.95 – 7.88 (m, 1H), 7.80 – 7.32 (m, 3H), 7.25 – 6.68 (m, 5H), 5.42 – 5.25 (m, 1H), 4.65 – 4.33 (m, 6H), 4.30 – 3.78 (m, 5H), 3.75 – 3.55 (m, 3H), 3.37 – 3.26 (m, 3H), 2.44 – 2.37 (m, 1H), 2.04 – 1.96 (m, 1H), 1.71- 1.60 (m, 2H), 1.48 – 1.33 (m, 9H), 1.33 – 1.12 (m, 2H), 1.16 – 0.55 (m, 20H), 0.53 – 0.37 (m, 3H).
^{13}C NMR (151 MHz, DMSO-*d_6*, rt): rotamers (not an impurity). δ 172.91, 171.31, 171.10, 169.79, 169.58, 169.15, 155.42, 144.89, 136.69, 136.19, 135.69, 134.86, 130.11, 129.27, 128.97, 128.85, 128.41, 124.39, 123.78, 122.73, 122.46, 120.47, 120.20, 119.19, 117.96, 116.91, 116.41, 112.31, 111.02, 110.69, 80.20, 79.72, 78.69, 59.57, 58.82, 57.40, 54.73, 52.86, 52.45, 48.08, 37.45, 37.32, 36.33, 35.63, 28.80, 28.68, 26.39, 25.62, 15.69, 11.99, 11.80, 10.82, 8.09, 8.00, 7.88, 3.98, 3.34.
HRMS (ESI-TOF): calculated for $C_{49}H_{67}N_7NaO_9Si^+$ $[M+Na]^+$: 948.4662, found: 948.4636.
$[\alpha]^{25}_D$: +2.0 (*c* = 0.5, $CHCl_3$)

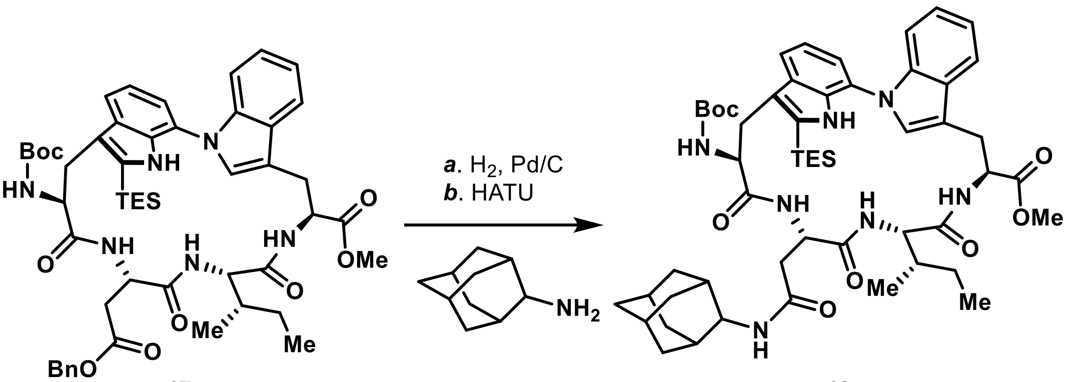

On 0.1 mmol scale, **General Procedure R** was followed with compound **47** via hydrogenation and amide coupling. Purification by silica gel column chromatography gave the title compound **48v** (78.3 mg, 80% yield).

Compound 48v

Physical State: amorphous solid

^{1}H NMR (400 MHz, CDCl$_3$): δ 8.47 – 8.28 (m, 1H), 8.03 (q, *J* = 4.1 Hz, 1H), 7.97 (s, 1H), 7.73 (s, 1H), 7.64 (dd, *J* = 7.6, 1.4 Hz, 1H), 7.56 (d, *J* = 8.0 Hz, 1H), 7.30 (dd, *J* = 7.0, 1.3 Hz, 1H), 7.26 – 7.19 (m, 3H), 5.95 (dd, *J* = 11.5, 7.8 Hz, 2H), 5.88 (d, *J* = 6.8 Hz, 1H), 5.43 (d, *J* = 6.1 Hz, 1H), 4.95 (ddd, *J* = 12.1, 7.7, 2.3 Hz, 1H), 4.77 (ddd, *J* = 11.5, 6.9, 4.2 Hz, 1H), 4.60 (q, *J* = 6.1 Hz, 1H), 4.01 – 3.94 (m, 1H), 3.77 (s, 3H), 3.74 – 3.66 (m, 2H), 3.57 – 3.50 (m, 2H), 3.18 (ddd, *J* = 16.7, 11.9, 1.4 Hz, 1H), 2.84 (dd, *J* = 13.8, 11.9 Hz, 1H), 2.40 (d, *J* = 7.3 Hz, 2H), 1.91 – 1.77 (m, 12H), 1.70 – 1.66 (m, 2H), 1.64 – 1.59 (m, 2H), 1.53 (s, 9H), 1.04 (d, *J* = 6.8 Hz, 3H), 0.95 – 0.88 (m, 12H), 0.76 – 0.61 (m, 6H).

^{13}C NMR (151 MHz, CDCl$_3$): δ 172.68, 170.90, 170.67, 170.21, 169.56, 169.45, 155.27, 136.75, 135.53, 134.70, 129.11, 128.59, 127.38, 124.17, 122.79, 120.34, 120.09, 120.03, 119.15, 118.97, 118.47, 112.36, 111.06, 79.62, 60.20, 53.96, 53.93, 53.66, 52.61, 52.39, 48.46, 40.07, 37.50, 37.17, 37.12, 37.10, 36.31, 31.88, 31.80, 31.76, 31.65, 31.62, 28.60, 28.34, 27.28, 27.11, 25.30, 15.75, 11.17, 7.54, 7.53, 3.78, 3.29.

HRMS (ESI-TOF): calculated for $C_{54}H_{75}N_7NaO_8Si^+$ [M+Na]$^+$: 1000.5339, found: 100.5345.

$[\alpha]^{25}_D$: +37.9 (*c* = 0.5, CHCl$_3$)

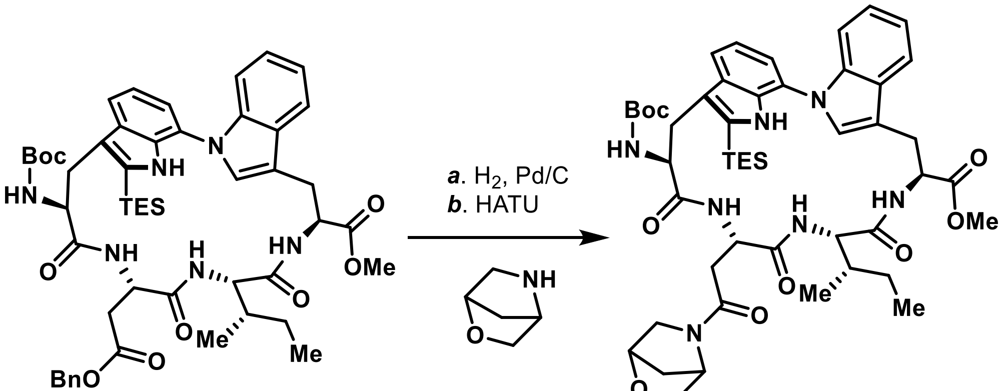

On 0.1 mmol scale, **General Procedure R** was followed with compound **47** via hydrogenation and amide coupling. Purification by silica gel column chromatography gave the title compound **48w** (59.3 mg, 64% yield).

Compound 48w

Physical State: amorphous solid

^{1}H NMR (600 MHz, CDCl$_3$, 25 °C): rotamers (not an impurity). δ 8.61 – 7.69 (m, 3H), 7.67 – 7.47 (m, 2H), 7.41 – 6.69 (m, 6H), 6.15 – 5.31 (m, 2H), 5.24 – 4.71 (m, 3H), 4.69 – 3.88 (m, 3H), 3.87 – 3.58 (m, 6H), 3.55 – 3.49 (m, 1H), 3.44 – 3.07 (m, 3H), 2.98 – 2.78 (m, 1H), 2.57 – 2.08 (m, 2H), 1.94 – 1.77 (m, 2H), 1.76 – 1.68 (m, 1H), 1.64 – 1.55 (m, 1H), 1.55 – 1.41 (m, 9H), 1.39 – 1.13 (m, 4H), 1.05 – 0.97 (m, 2H), 0.96 – 0.80 (m, 15H), 0.79 – 0.59 (m, 5H), 0.56 – 0.38 (m, 3H).

^{13}C NMR (151 MHz, CDCl$_3$, 25 °C): rotamers (not an impurity). δ 172.68, 172.61, 171.10, 171.01, 170.65, 169.49, 169.30, 169.25, 169.16, 168.89, 168.72, 155.86, 155.34, 155.28, 136.71, 135.51, 134.76, 134.68, 129.15, 128.60, 128.50, 128.21, 127.33, 127.26, 124.20, 122.83, 122.79, 122.47, 120.41, 120.32, 120.11, 120.03, 119.98, 119.91, 119.10, 118.47, 118.31, 116.43, 112.54, 112.43, 111.03, 110.87, 79.60, 76.19, 76.15, 75.63, 75.51, 73.95, 73.85, 60.09, 58.32, 55.92, 55.86, 55.16, 55.02, 54.51, 54.40, 53.60, 52.62, 52.59, 52.51, 52.44, 47.94, 47.86, 47.76, 38.68, 38.54, 38.12, 38.04, 37.13, 37.04, 36.44, 36.36, 35.88, 35.78, 31.94, 31.89, 31.67, 28.59, 28.36, 27.30, 27.21, 27.16, 25.29, 25.16, 24.47, 22.74, 20.22, 15.82, 15.77, 15.74, 14.22, 11.60, 11.30, 11.20, 11.16, 11.12, 7.54, 3.80, 3.78, 3.29.
^{1}H NMR (400 MHz, DMSO-*d*$_6$, 25 °C): rotamers (not an impurity). δ 9.36 – 9.07 (m, 1H), 8.51 – 8.35 (m, 1H), 8.19 – 7.86 (m, 1H), 7.81 – 7.40 (m, 3H), 7.26 – 6.67 (m, 6H), 5.78 – 5.36 (m, 1H), 4.73 – 4.58 (m, 2H), 4.56 – 3.90 (m, 3H), 3.77 – 3.48 (m, 6H), 3.27 – 3.10 (m, 3H), 3.04 – 2.59 (m, 1H), 2.38 – 2.08 (m, 1H), 1.79 – 1.69 (m, 1H), 1.63 – 1.53 (m, 1H), 1.46 – 1.34 (m, 9H), 1.31 – 1.08 (m, 2H), 0.99 – 0.64 (m, 22H), 0.60 – 0.33 (m, 3H).
^{1}H NMR (400 MHz, DMSO-*d*$_6$, 60 °C): rotamers (not an impurity). δ 9.14 – 8.74 (m, 1H), 8.40 – 8.20 (m, 1H), 8.01 – 7.71 (m, 2H), 7.69 – 7.57 (m, 1H), 7.54 – 7.37 (m, 1H), 7.28 – 6.54 (m, 6H), 5.54 – 5.35 (m, 1H), 4.75 – 4.60 (m, 2H), 4.59 – 4.48 (m, 3H), 4.46 – 3.86 (m, 1H), 3.77 – 3.50 (m, 6H), 3.39 – 3.29 (m, 2H), 3.06 – 2.63 (m, 1H), 2.36 – 2.12 (m, 1H), 1.76 – 1.70 (m, 1H), 1.63 – 1.54 (m, 1H), 1.50 – 1.36 (m, 9H), 1.34 – 1.10 (m, 2H), 1.07 – 0.67 (m, 22H), 0.61 – 0.38 (m, 3H).
^{13}C NMR (151 MHz, DMSO-*d*$_6$, 25 °C): rotamers (not an impurity). δ 172.93, 172.93, 171.16, 170.98, 169.85, 169.79, 168.44, 168.31, 168.14, 168.10, 155.90, 155.44, 155.35, 144.90, 136.71, 136.19, 135.64, 134.84, 130.15, 130.14, 129.26, 129.06, 128.62, 128.41, 124.56, 123.80, 122.72, 122.44, 120.36, 120.18, 119.40, 119.10, 117.91, 116.91, 116.41, 112.64, 112.45, 111.65, 111.02, 110.76, 79.72, 78.73, 78.53, 76.09, 75.44, 73.62, 58.88, 58.14, 55.65, 55.21, 54.58, 53.28, 52.78, 52.57, 52.51, 52.29, 47.83, 36.92, 36.44, 35.59, 30.89, 28.79, 28.68, 27.14, 26.43, 25.43, 25.31, 20.28, 16.01, 15.88, 15.78, 15.68, 11.93, 11.90, 11.79, 10.84, 8.06, 8.02, 7.87, 3.95, 3.93, 3.91, 3.86, 3.35.
HRMS (ESI-TOF): calculated for $C_{49}H_{67}N_7NaO_9Si^+$ [M+Na]$^+$: 948.4662, found: 948.4651.
[α]$^{25}_{D}$: +5.2 (*c* = 0.5, CHCl$_3$)

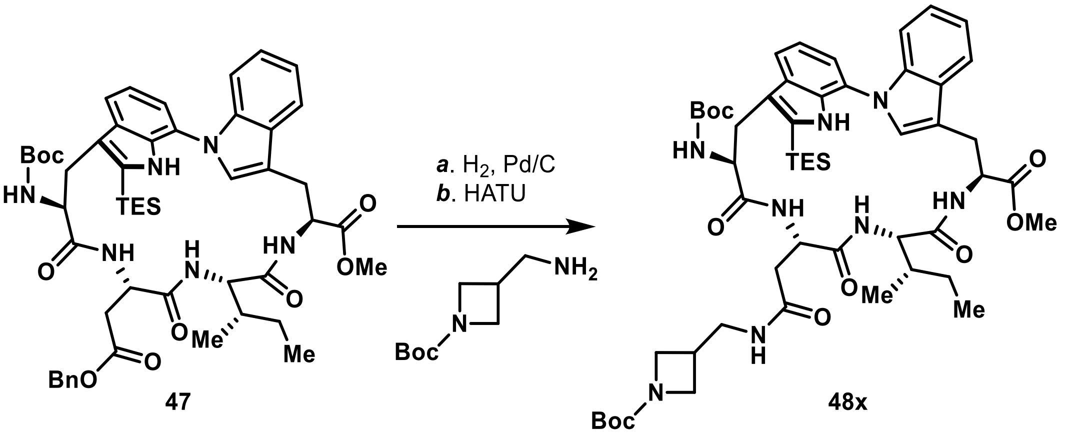

On 0.1 mmol scale, **General Procedure R** was followed with compound **47** via hydrogenation and amide coupling. Purification by silica gel column chromatography gave the title compound **48x** (84.1 mg, 83% yield).
Compound 48x
Physical State: amorphous solid
^{1}H NMR (400 MHz, CDCl$_3$): δ 8.28 – 8.06 (m, 1H), 8.02 (t, *J* = 4.5 Hz, 1H), 7.96 (s, 1H), 7.73 (s, 1H), 7.64 (d, *J* = 7.7 Hz, 1H), 7.56 (d, *J* = 8.1 Hz, 1H), 7.32 – 7.28 (m, 1H), 7.26 – 7.20 (m, 3H), 6.20 (s, 1H), 5.97 (d, *J* = 7.7 Hz, 1H), 5.86 (d, *J* = 6.9 Hz, 1H), 5.41 (d, *J* = 6.4 Hz, 1H), 4.91 (ddd, *J* = 12.1, 7.6, 2.4 Hz, 1H), 4.77

(ddd, J = 11.6, 6.9, 4.3 Hz, 1H), 4.60 (q, J = 6.3 Hz, 1H), 3.93 (t, J = 8.2 Hz, 3H), 3.78 (s, 3H), 3.75 – 3.66 (m, 2H), 3.54 (dt, J = 9.2, 4.4 Hz, 4H), 3.37 (s, 2H), 3.17 (ddd, J = 16.8, 12.1, 1.4 Hz, 1H), 2.84 (dd, J = 13.8, 12.0 Hz, 1H), 2.60 (dd, J = 13.6, 6.0 Hz, 1H), 2.34 (d, J = 6.2 Hz, 1H), 1.81 (d, J = 12.5 Hz, 1H), 1.52 (s, 9H), 1.42 (d, J = 2.8 Hz, 9H), 1.04 (d, J = 6.8 Hz, 3H), 0.96 – 0.88 (m, 12H), 0.75 – 0.60 (m, 6H).
^{13}C NMR (151 MHz, CDCl$_3$): δ 172.59, 171.78, 170.97, 169.49, 169.41, 156.39, 155.29, 136.71, 135.52, 134.69, 129.11, 128.58, 128.40, 127.27, 124.17, 122.84, 120.27, 120.12, 120.04, 119.95, 119.09, 118.47, 112.37, 111.04, 79.68, 79.64, 60.16, 53.66, 52.63, 52.46, 48.23, 43.06, 39.85, 36.37, 31.85, 28.58, 28.47, 28.45, 28.40, 28.37, 27.21, 25.36, 15.76, 11.17, 7.53, 3.80, 3.29.
HRMS (ESI-TOF): calculated for $C_{53}H_{76}N_8NaO_{10}Si^+$ [M+Na]$^+$: 1035.5346, found: 1035.5354.
[α]$^{25}_D$: +21.0 (c = 0.5, CHCl$_3$)

Biological assay of the cihunamide analogs:

Methods

Antibacterial activity assay. The Minimum inhibitory concentration (MIC) was determined by the agar dilution method using Mueller-Hinton (MH) agar (Coolaber, Beijing, China) according to the Clinical and Laboratory Standard Institute (CLSI) guidelines. The tested pathogenic strains included Gram-positive bacteria (*Methicillin-resistant Staphylococcus aureus* ATCC 43300, *Methicillin-resistant Staphylococcus aureus* ATCC 29213, *Staphylococcus aureus* R116, *Staphylococcus aureus* ATCC 25923, *Bacillus subtilis* zk 31, *Bacillus subtilis* BNCC 109047, *Enterococcus faecalis* ATCC 19433, *Enterococcus faecium* ATCC 19434) and Gram-negative bacteria (*Escherichia coli* ATCC 25922, *Pseudomonas aeruginosa* ATCC 27853, *Klebsiella pneumoniae* ATCC 10031, *Salmonella enterica* ATCC 14028). The tested bacterial strains were overcultured and inoculated at a concentration of 1×10^5 CFU/mL in Mueller-Hinton Broth (MHB, Coolaber, Beijing, China). Samples were injected into 96-well plates with different concentrations. Plates were incubated at 37 °C for 18 hours. Vancomycin, Kanamycin, and Netilmicin were used as positive controls.

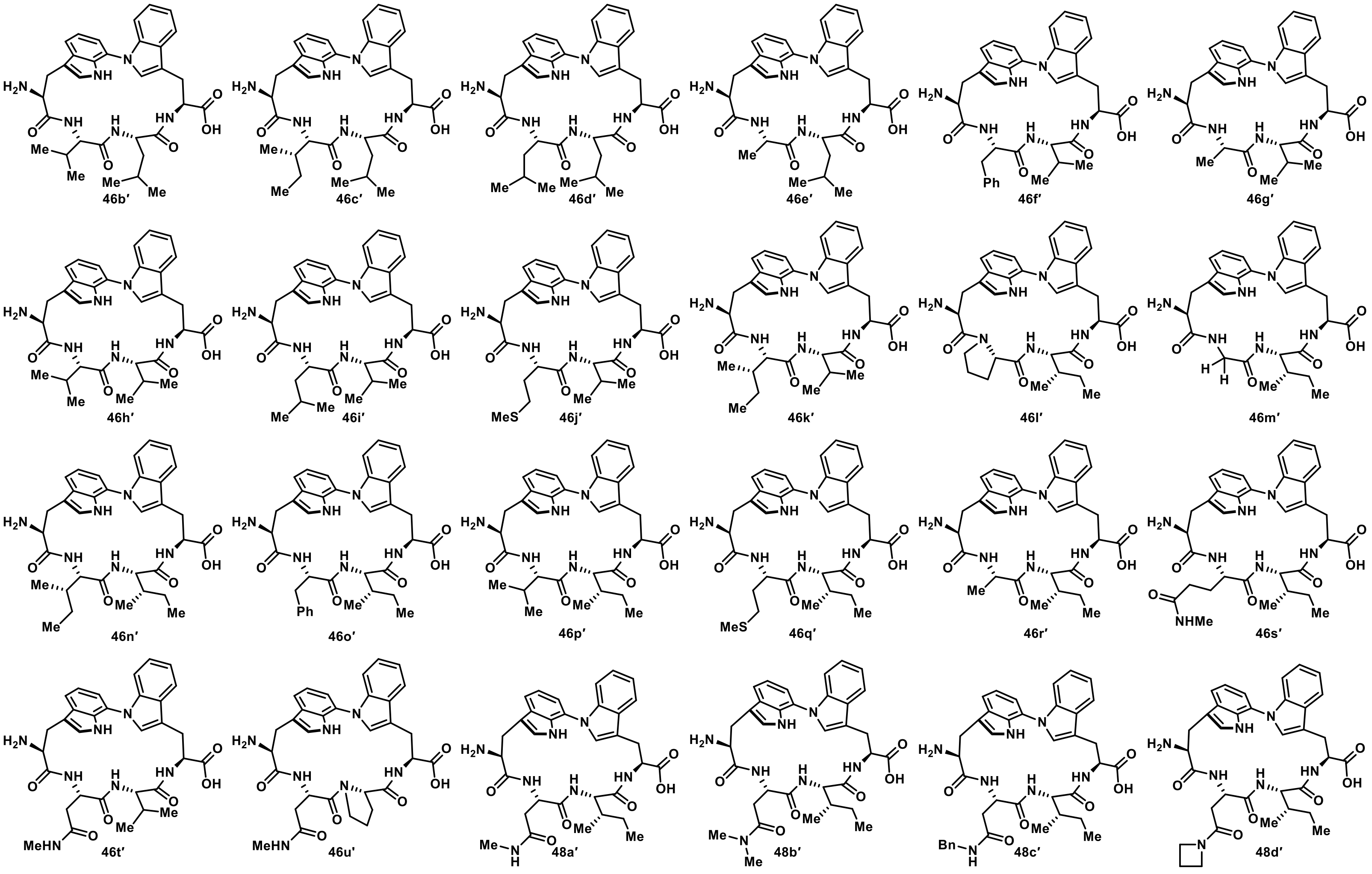

Figure S2. Compounds subjected to the *in-vitro* MIC assay (1)

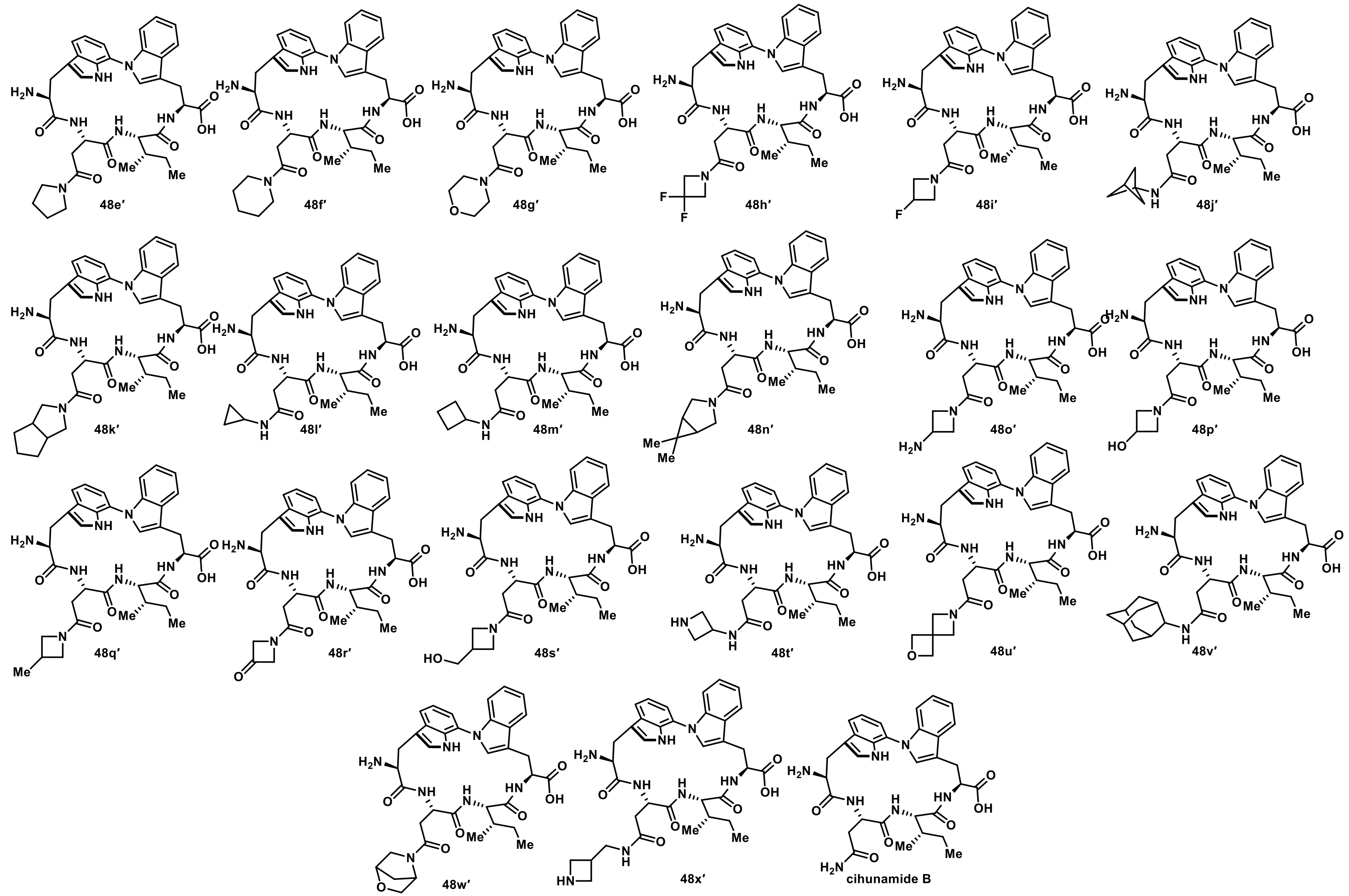

Figure S3. Compounds subjected to the *in-vitro* MIC assay (2)

Table S2. In *vitro* MIC test of Cihunamide Derivatives

Compounds	MIC (μg/mL)											
	Gram-positive								Gram-negative			
	MRSA ATCC 43300	*MRSA* ATCC 29213	*S. aureus* R116	*S. aureus* ATCC 25923	*B. Subtilis* zk31	*B. Subtilis* BNCC 109047	*E. faecalis* ATCC 19433	*E. faecium* ATCC 19434	*E. coli* ATCC 25922	*P. aeruginosa* ATCC 27853	*K. pneumoniae* ATCC 10031	*S. enterica* ATCC 14028
**46b'**	>128	>128	>128	>128	>128	>128	>128	>128	>128	>128	>128	>128
**46c'**	>128	>128	>128	>128	>128	>128	>128	>128	>128	>128	>128	>128
**46d'**	>128	>128	>128	>128	>128	>128	>128	>128	>128	>128	>128	>128
**46e'**	>128	>128	>128	>128	>128	>128	>128	>128	>128	>128	>128	>128
**46f'**	>128	>128	>128	>128	>128	>128	>128	>128	>128	>128	>128	>128
**46g'**	>128	>128	>128	>128	>128	>128	>128	>128	>128	>128	>128	>128
**46h'**	>128	>128	>128	>128	>128	>128	>128	>128	>128	>128	>128	>128
**46i'**	>128	>128	>128	>128	>128	>128	>128	>128	>128	>128	>128	>128
**46j'**	>128	>128	>128	>128	>128	>128	128	>128	>128	>128	>128	>128
**46k'**	>128	>128	>128	>128	>128	>128	>128	>128	>128	>128	>128	>128
**46l'**	>128	>128	>128	>128	>128	>128	>128	>128	>128	>128	>128	>128
**46m'**	>128	>128	>128	>128	>128	>128	>128	>128	>128	>128	>128	>128
**46n'**	>128	>128	>128	>128	>128	>128	>128	>128	>128	>128	>128	>128
**46o'**	>128	>128	>128	>128	>128	>128	>128	>128	>128	>128	>128	>128
**46p'**	>128	>128	>128	>128	>128	>128	>128	>128	>128	>128	>128	>128
**46q'**	16	64	32	64	64	32	32	64	>128	>128	>128	>128
**46r'**	>128	>128	>128	>128	>128	>128	>128	>128	>128	>128	>128	>128
**46s'**	>128	>128	>128	>128	>128	>128	>128	>128	>128	>128	>128	>128

Compounds	MIC (µg/mL)											
	Gram-positive								Gram-negative			
	MRSA ATCC 43300	*MRSA* ATCC 29213	*S. aureus* R116	*S. aureus* ATCC 25923	*B. Subtilis* zk31	*B. Subtilis* BNCC 109047	*E. faecalis* ATCC 19433	*E. faecium* ATCC 19434	*E. coli* ATCC 25922	*P. aeruginosa* ATCC 27853	*K. pneumoniae* ATCC 10031	*S. enterica* ATCC 14028
**46t'**	>128	>128	>128	>128	>128	>128	>128	>128	>128	>128	>128	>128
**46u'**	>128	>128	>128	>128	>128	>128	>128	>128	>128	>128	>128	>128
**48a'**	>128	>128	>128	>128	>128	>128	>128	>128	>128	>128	>128	>128
**48b'**	>128	>128	>128	>128	>128	>128	>128	>128	>128	>128	>128	>128
**48e'**	>128	>128	>128	>128	>128	>128	>128	>128	>128	>128	>128	>128
**48f'**	>128	>128	>128	>128	>128	>128	>128	>128	>128	>128	>128	>128
**48g'**	>128	>128	>128	>128	>128	>128	>128	>128	>128	>128	>128	>128
**48c'**	>128	>128	>128	>128	>128	>128	>128	>128	>128	>128	>128	>128
**48d'**	>128	>128	>128	>128	>128	>128	>128	>128	>128	>128	>128	>128
**48h'**	>128	>128	>128	>128	>128	>128	>128	>128	>128	>128	>128	>128
**48j'**	>128	>128	>128	>128	>128	>128	>128	>128	>128	>128	>128	>128
**48i'**	>128	>128	>128	>128	>128	>128	>128	>128	>128	>128	>128	>128
**48k'**	>128	>128	>128	>128	>128	>128	>128	>128	>128	>128	>128	>128
**48l'**	>128	>128	>128	>128	>128	>128	>128	>128	>128	>128	>128	>128
**48m'**	>128	>128	>128	>128	>128	>128	>128	>128	>128	>128	>128	>128
**48n'**	>128	>128	>128	>128	>128	>128	>128	>128	>128	>128	>128	>128
**48o'**	>128	>128	>128	>128	64	32	>128	128	>128	>128	>128	>128
**48p'**	>128	>128	>128	>128	>128	>128	>128	>128	>128	>128	>128	>128
**48q'**	>128	>128	>128	>128	>128	>128	>128	>128	>128	>128	>128	>128
Compounds	MIC (µg/mL)											

	Gram-positive								Gram-negative			
	MRSA ATCC 43300	*MRSA* ATCC 29213	*S. aureus* R116	*S. aureus* ATCC 25923	*B. Subtilis* zk31	*B. Subtilis* BNCC 109047	*E. faecalis* ATCC 19433	*E. faecium* ATCC 19434	*E. coli* ATCC 25922	*P. aeruginosa* ATCC 27853	*K. pneumoniae* ATCC 10031	*S. enterica* ATCC 14028
**48r'**	>128	>128	>128	>128	>128	>128	>128	>128	>128	>128	>128	>128
**48s'**	>128	>128	>128	>128	>128	>128	>128	>128	>128	>128	>128	>128
**48t'**	>128	>128	>128	>128	>128	>128	>128	>128	>128	>128	>128	>128
**48u'**	>128	>128	>128	>128	>128	>128	>128	>128	>128	>128	>128	>128
**48v'**	>128	>128	>128	>128	>128	>128	>128	>128	>128	>128	>128	>128
**48w'**	>128	>128	>128	>128	>128	>128	>128	>128	>128	>128	>128	>128
**48x'**	128	128	128	128	64	32	128	64	128	>128	>128	>128
Cihunamide B	>128	>128	>128	>128	>128	>128	>128	>128	>128	>128	>128	>128
Vancomycin	1	1	1	1	≤0.25	≤0.25	0.5	1	-	-	-	-
Kanamycin	-	-	-	-	-	-	-	-	8	>256	2	8
Netilmicin	-	-	-	-	-	-	8	256	1	≤0.25	≤0.25	2

References:

[1] Ogawa, H.; Nagata, Y.; Chan, T. K.; Matsuda, Y.; Nakamura, H. Rapid Construction of a Tyr C6-Trp C5' Linkage: Application in the Total Synthesis of Micitide 982, a Noncanonical Cyclic Peptide. *Angew. Chem. Int. Ed.* **2025**, *64* (46), e202516053.

[2] J. Zhang, L. Yu, H. Ogawa, Y. Nagata, H. Nakamura, *Angew. Chem. Int. Ed.* **2024**, e202409987.

[3] a) M. D. Palkowitz, G. Laudadio, S. Kolb, J. Choi, M. S. Oderinde, T. E.-H. Ewing, P. N. Bolduc, T. Chen, H. Zhang, P. T. W. Cheng, B. Zhang, M. D. Mandler, V. D. Blasczak, J. M. Richter, M. R. Collins, R. L. Schioldager, M. Bravo, T. G. M. Dhar, B. Vokits, Y. Zhu, P.-G. Echeverria, M. A. Poss, S. A. Shaw, S. Clementson, N. N. Petersen, P. K. Mykhailiuk, P. S. Baran, *J. Am. Chem. Soc.* **2022**, *144*, 17709–17720; b) G. Laudadio, P. Neigenfind, R. Chebolu, V. D. Blasczak, S. J. Maddirala, M. D. Palkowitz, P. N. Bolduc, M. C. Nicastri, R. K. Puthukanoori, B. R. Paraselli, P. S. Baran, *Org. Lett.* **2024**, *26*, 2276–2281.

[4] L. Yu, Y. Nagata, H. Nakamura, *J. Am. Chem. Soc.* **2024**, *146*, 2549–2555.
